# Draft genome sequencing of *Tilletia caries* inciting common bunt of wheat provides pathogenicity-related genes

**DOI:** 10.3389/fmicb.2023.1283613

**Published:** 2023-11-15

**Authors:** Malkhan Singh Gurjar, Tej Pratap Jitendra Kumar, Mohamad Ayham Shakouka, Mahender Singh Saharan, Laxmi Rawat, Rashmi Aggarwal

**Affiliations:** ^1^Division of Plant Pathology, ICAR-Indian Agricultural Research Institute, New Delhi, India; ^2^College of Hill Agriculture, VCSG Uttarakhand University of Horticulture and Forestry, Ranichauri, Uttarakhand, India

**Keywords:** *Tilletia caries*, common bunt, wheat, genome sequencing, pathogenicity-related genes

## Abstract

Common bunt of wheat caused by *Tilletia caries* is an important disease worldwide. The *T. caries* TC1_MSG genome was sequenced using the Illumina HiSeq 2500 and Nanopore ONT platforms. The Nanopore library was prepared using the ligation sequencing kit SQK-LSK110 to generate approximately 24 GB for sequencing. The assembly size of 38.18 Mb was generated with a GC content of 56.10%. The whole genome shotgun project was deposited at DDBJ/ENA/GenBank under the accession number JALUTQ000000000. Forty-six contigs were obtained with N_50_ of 1,798,756 bp. In total, 10,698 genes were predicted in the assembled genome. Out of 10,698 genes, 10,255 genes were predicted significantly in the genome. The repeat sequences made up approximately 1.57% of the genome. Molecular function, cellular components, and biological processes for predicted genes were mapped into the genome. In addition, repeat elements in the genome were assessed. In all, 0.89% of retroelements were observed, followed by long terminal repeat elements (0.86%) in the genome. In simple sequence repeat (SSR) analysis, 8,582 SSRs were found in the genome assembly. The trinucleotide SSR type (3,703) was the most abundant. Few putative secretory signal peptides and pathogenicity-related genes were predicted. The genomic information of *T. caries* will be valuable in understanding the pathogenesis mechanism as well as developing new methods for the management of the common bunt disease of wheat.

## 1. Introduction

Wheat is an important staple crop around the world. *Tilletia caries*, which causes common bunt disease and belongs to the class basidiomycetes order ustilaginales, is a significant seed-borne wheat disease (Goates, 1996). It occurs in all wheat-growing areas worldwide (Albughobeish and Jorf, 2015). The cereal-infecting *Tilletia* fungi species known as bunt fungi produce teliospores in the ovary of the host plant (Wilcoxon and Saari, 1996; Carris et al., 2006). In India, wheat has an acreage of 31.61 million ha with an average production of 106.84 million metric tons per year, yielding an average of 3.38 metric tons per ha. Common bunt disease, also known as hill bunt or stinking bunt, commonly occurs in Asia, Australia, North and South America, and Europe. *T. caries*, *T. laevis*, and *T. controversa* are designated as three distinct species based on their morphological and physiological characteristics (Goates, 1996).

The spores of *T*. *caries* were found to have similarity to *T. laevis* and *T. controversa*; specifically, *T*. *controversa* is considered a quarantined fungal pathogen (Peterson et al., 2009). *Tilletia* species, namely, *T*. *caries*, *T*. *controversa*, *T*. *laevis*, and *T*. *indica*, are described as infecting wheat and triticale. In India, it occurs in hilly regions, predominantly in Himachal Pradesh (Chamba and Kullu districts), Uttarakhand, and Jammu & Kashmir (Rana et al., 2016). The disease severity ranged from 10 to 15% in the hilly region of India. In organic farming, common and dwarf bunt diseases are an increasing threat to wheat cultivation (Matanguihan et al., 2011). These diseases reduce yields and grain quality with a foul, fishy smell, making it unfit for consumption (Pant et al., 2000; Borgen, 2004; Kochanova et al., 2004). In Romania, 70–80% disease incidence with yield losses of up to 40% has been noticed when untreated wheat seeds were sown.

A typical symptom of common bunt is that wheat kernels turn into millions of teliospores generated by the fungi; the grains are then referred to as “bunt balls” (Mourad et al., 2018). The symptoms appear after ear emergence, when sporulation begins in the young ovary, but infected plants are often slightly stunted. Flag leaves show yellow streaks, and the plants become stunted, with stubby, dark gray-green ears and slightly gaping glumes. After initial infection, the entire kernel is converted into a light to dark brown spherical sorus (bunt ball) containing a dark brown to black mass of teliospores covered by a thin and papery modified periderm. Heavily infested wheat fields give off a rotting fish-like smell. The disease was encountered in Indian conditions and resulted in a 25–50% yield reduction in specific fields (Holton, 1967). *Tilletia* species can infect up to 70% of spikes at very low temperatures. Usually, it is the low temperatures (5–15°C) and the soil moisture that support spore germination and do not require light. The spores are released from infected spikes and settle on healthy seeds and the soil surface. These teliospores survive in seed and soil and serve as a source of inoculum to initiate the disease. A few detection methods have been developed to identify the different *Tilletia* species based on internal transcription spacers (ITSs) and DNA fragments (Kochanova et al., 2004; Eibel et al., 2005; Carris et al., 2006; Gurjar et al., 2017; Gupta et al., 2022). New pathogenic races of common bunt have been detected (Goates, 2012; Albughobeish and Jorf, 2015). The virulence analysis was performed using bunt isolates in India (Aggarwal and Sood, 2006). A few resistant sources are available against *T*. *caries* (Mamluk, 1998). The disease can be managed using fungicide applications (Manninen et al., 1998; Monkiedje and Spiteller, 2002). In fungal genomics, the genome of *T. caries* has been sequenced, but the size of the assembly reported was 29.9 Mb (Nguyen et al., 2019). We have also sequenced the genome of *T. indica* with a size of 33.70 Mb (Gurjar et al., 2019). Genome sequencing of fungal plant pathogens plays an important role in identifying effectors, evolution, lifestyle, and pathogenicity-related genes (Plissonneau et al., 2017). The comparative genome analysis revealed the pathogen variants and host specificity factors. The comparative genome analysis revealed the pathogen variants and host specificity factors.

Therefore, we stated that the genome assembly (38.18 Mb) of *T. caries* was improved and better in quality in comparison to public domain genomes. The genes were predicted based on the model fungus genome of *Ustilago maydis*. For the first time, we have also identified simple sequence repeats (SSRs), or repeat elements, in the genome of *T. caries*. A comparative genome analysis of *Tilletia* species showed the unique and common genes in the assemblies. The putative secretory proteins having a role in virulence and pathogenesis genes were identified in the assembly of *T. caries*. The genomic information of *T. caries* will be useful in understanding the pathogenesis mechanism and resistance as well as developing new methods for treating common bunt disease.

## 2. Materials and methods

### 2.1. Isolation of *Tilletia caries* and DNA extraction

**Figure 1:**
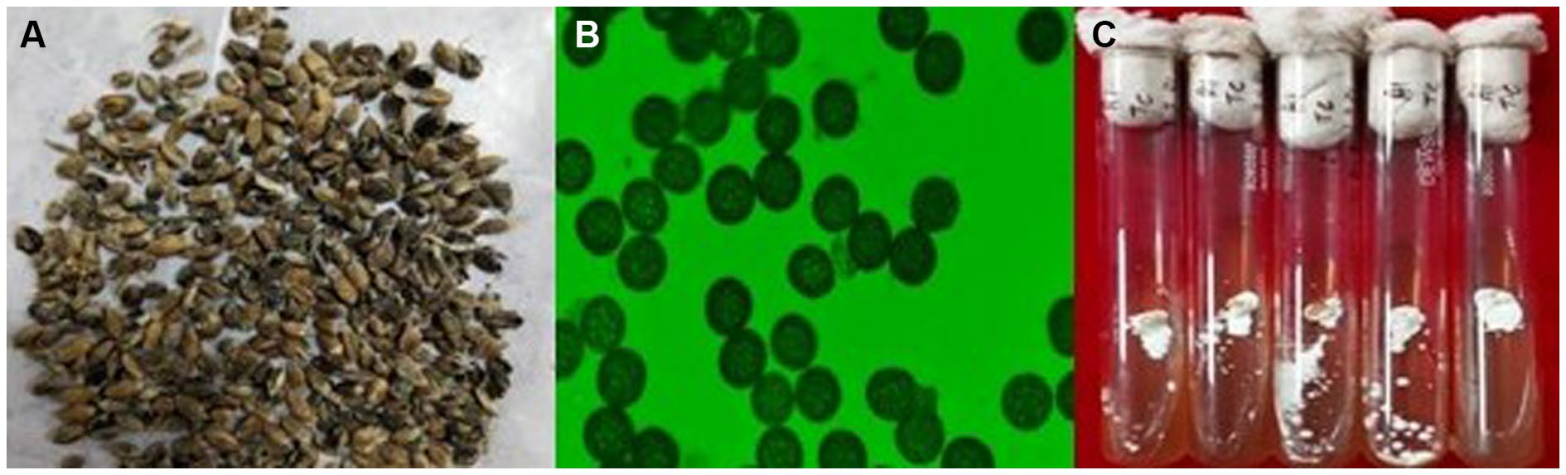
**(A)** Common bunt infected wheat grains **(B)** teliospores of *T. caries* **(C)** mycelial growth of *T. caries*.

The wheat samples showing common bunt symptoms were collected from Ranichauri, Uttarakhand, India, during the year 2019. Infected wheat grains (Figure 1A) were taken to the Fungal Molecular Biology Laboratory, Division of Plant Pathology, ICAR-IARI, Pusa, New Delhi, India. Grains were surface sterilized with 70% ethanol. A pure mycelial culture of the *T. caries* fungus was established from teliospores of *T. caries* (Figures 1B,C). Bunted grains were vortexed in a sterile, capped vial containing sterile distilled water. The tubes were centrifuged at 12,000 rpm for 4 min to pellet down the teliospores, and the supernatant was discarded. The teliospore pellet was treated with 1% NaOCl for 2 min. Again, the tubes were centrifuged, the supernatant discarded, and the pellet was washed two times with sterile distilled water. Finally, the pellet was resuspended in 10 mL of sterile distilled water and kept overnight at 40°C. A volume of 0.4 mL of teliospore suspension was plated on water-agar Petri plates (1.5%). Petri plates were kept at 12 ± 2°C in an incubator for 20 days with exposure to alternate light and dark periods of 12 h. After 20 days, Petri plates were checked microscopically for the germination of teliospores. A single germinating teliospore was transferred to potato-dextrose agar media containing test tubes and incubated at 12 ± 2°C. After 10–15 days, mycelial growth appeared in the test tubes. For DNA isolation, fungal culture was grown in 100 mL potato dextrose broth media in a shaker incubator at 12 ± 2°C for 20 days to obtain the mycelial mass. The mycelial mat was harvested and immediately stored in a deep freezer (−80°C). Furthermore, high-quality DNA was isolated using a NucleoSpin^®^ Tissue Kit following the manufacturer’s instructions. The quality and integrity of DNA were checked on 0.8% agarose gel, pulse field gel electrophoresis, and NanoDrop (Thermo Fisher Scientific).

### 2.2. Genome sequencing and assembly

The genome of *T. caries* TC1_MSG was sequenced using the Illumina HiSeq 2500 and ONT PromethION Flow Cell (R9.4.1) platforms. The paired-end DNA libraries of an average of 496 bp inserts were prepared using the NEB Next Ultra DNA Library Prep Kit and sequenced using 2 × 150 bp chemistry to produce approximately 50 GB of data for the sequencing. The PromethION Flow Cell (R9.4.1) library was prepared using the ligation sequencing kit SQK-LSK110 to produce approximately 24 GB for the sequencing. The reads were obtained from both the Illumina Hiseq 2500 and PromethION Flow Cell platforms. The Illumina reads were filtered using AdapterRemoval v2 version 2.3.1^1^ with an average quality score of less than 30 (Schubert et al., 2016). *De novo* assembly was performed with the ONT data using Flye assembler version 2.9,^2^ and the resulting assembly was polished with Illumina data using the POLCA version 3.4.1^3^ polishing tool. The assembly statistics were generated using QUAST version 4.6^4^ (Gurevich et al., 2013). The levels of conserved genes generated in the sequenced genome using BUSCO version 4.1.4^5^ (Simao et al., 2015).

### 2.3. Repeat elements, masking, and SSR

The repeat sequences from the assembled genome of *T. caries* were identified using RepeatMasker V.4.0.6.^6^ The repeat masking assessed the location of all repeated elements throughout the genome sequence. The RepeatMasker is a program that screens DNA sequences for interspersed repeats and low-complexity DNA sequences. In addition, the MIcroSatellite identification tool (MISA)^7^ was performed to identify the SSR in the assembled genome of *T. caries*.

### 2.4. Gene prediction, annotation, and comparative genome analysis

The AUGUSTUS program was used to predict genes in the eukaryote genome and was the most accurate *ab initio* gene prediction. The genes were predicted in the assembled contigs of *T. caries* using Augustus V.3.3.3 with default parameters (Stanke et al., 2008)^8^ based on *Ustilago maydis* (smut of maize) (Kamper et al., 2006) as a model organism. The predicted genes were compared with the UniProt database using the BLASTx program with an *E*-value cutoff at 10-3. The best BLASTX hit based on query coverage, identity, similarity score, and description of each gene was filtered out using the in-house pipeline. The number of predicted genes with a significant BLASTX match (E-value ≤1e-3 and similarity score ≥ 40%) with the UniProt were identified. The gene ontology (GO), molecular function (MF), cellular component (CC), and biological process (BP) for predicted genes were mapped in the assembled genome using the in-house pipeline. The sequenced genome of *T. caries* TC1_MSG was matched with other *Tilletia* species, viz., *Tilletia caries* AZH3, *Tilletia controversa* OA2, *T. indica* DAOMC236416, *Tilletia* laevis DAOMC238040, and *Tilletia walkeri* AOMC238049, through the OrthoVenn software (Wang et al., 2015).

### 2.5. Secretome prediction and analysis of pathogenicity-related genes

A total of 10,255 predicted proteins from the *T. caries* genome assembly were analyzed in SignalP v5.0^9^ as well as TargetP v2.0^10^ for prediction of the secretory signal peptides. The SignalP v5.0 server increased signal peptide predictions using deep neural networks (Almagro et al., 2019a). The TargetP v2.0 server envisages the presence of N-terminal sequences, signal peptide, mitochondrial transit peptide (mTP), chloroplast transit peptide (cTP), or thylakoid luminal transit peptide (lTP) (Almagro et al., 2019b). Only putative proteins containing signal peptides, which were predicted by both approaches, were annotated as secretomes. The carbohydrate metabolism active enzymes (CAZymes) were assessed using the dbCAN (dbCAN HMMs 5.0) (Yin et al., 2012) based on the CAZy database. Using the pathogen-host interaction database (PHI-base) database (Winnenburg et al., 2006), the putative pathogenicity-related genes were predicted using the Blast analysis with an *E* value of ≤1e-06.

## 3. Results

### 3.1. Genome sequencing, assembly, and annotation of *Tilletia caries*

An isolate of *Tilletia caries* TC1_MSG causing common bunt of wheat was used for whole genome sequencing. The fungus was confirmed using the ITS primers, and the sequence was deposited in the NCBI database (MN871436). The genome of *T. caries* was sequenced using both the Illumina HiSeq 2,500 and ONT PromethION Flow Cell (R9.4.1) platforms. The paired-end DNA libraries of an average of 496 bp inserts were sequenced for the shorter sequences (2 × 150 bp) and the PromethION Flow Cell (R9.4.1) for the longer sequence generation. The assembly was generated with the ONT data using the Flye assembler and further polished with the Illumina data using the POLCA polishing tool. The assembly size of 38.18 Mb was generated with a GC content of 56.10% (Table 1).

**Table 1:** Genomic characteristics of *Tilletia caries* assembly.

Characteristics	*Tilletia caries*
Size (Mb)	38.18
Genome coverage (Illumina)	1,269×
Genome coverage (ONT)	317×
Average genome coverage	109×
Contigs	46
Largest contig	2,689,501
GC (%)	56.10
N_50_	1,798,756
Protein-coding genes	10,698
Significant protein coding genes	10,255

In all, 46 contigs were obtained in the genome with an N_50_ of 1,798,756 bp. Higher coverage of 1,269× and 317× was achieved on paired-end and ONT reads, respectively. The average coverage of the genome was 109×. The gene prediction in the assembled genome was performed using AUGUSTUS v3.3.3 with default parameters based on *Ustilago maydis*. The 10,698 genes were predicted in the genome (Supplementary Table S1). Out of 10,698 genes, the significant BLASTX matches (*E*-value ≤1e-3 and similarity score ≥ 40%) were 10,255 genes.

**Figure 2:**
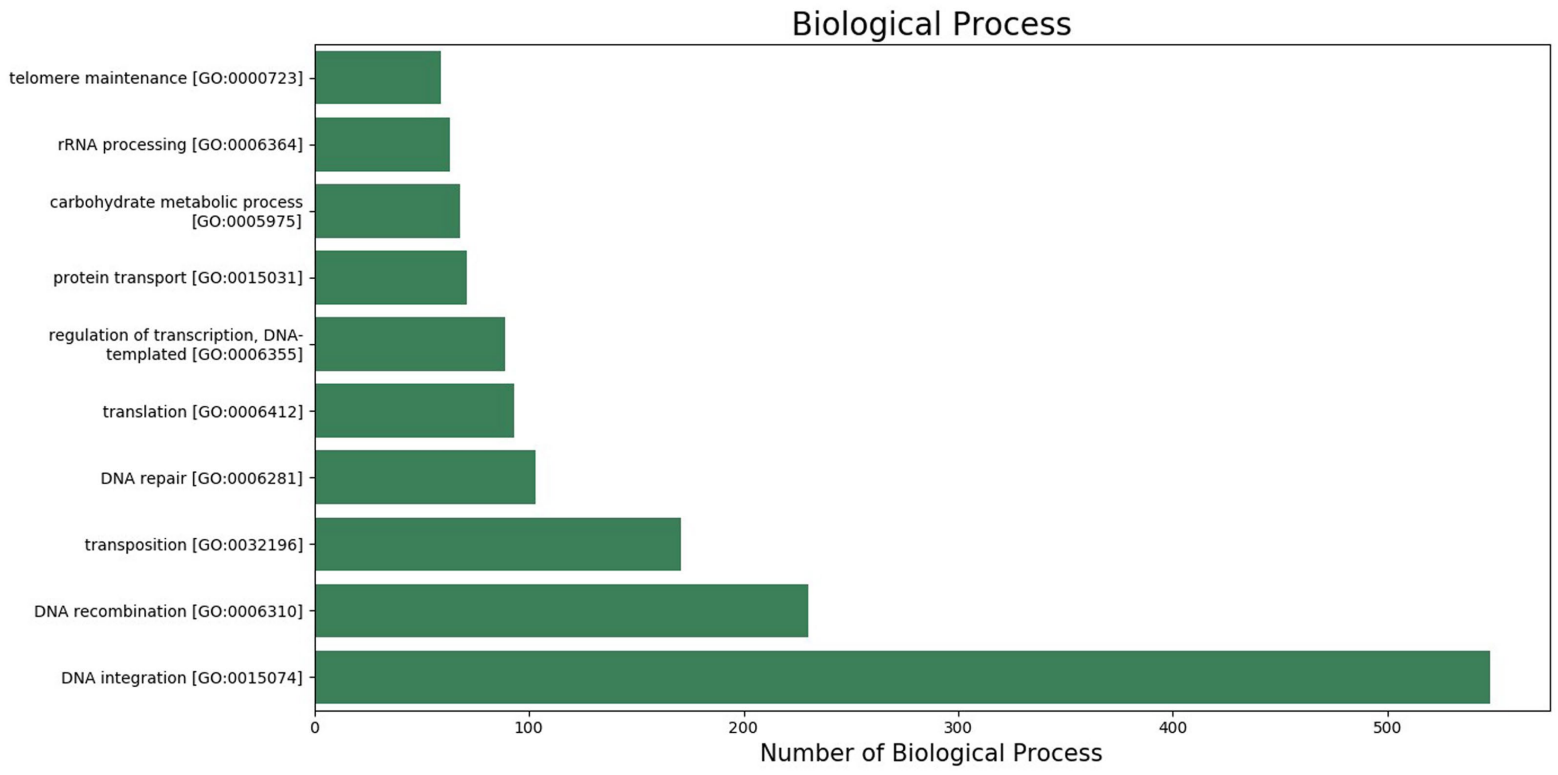
Biological process category of gene ontology in the sequenced genome of *T. caries*.

**Figure 3:**
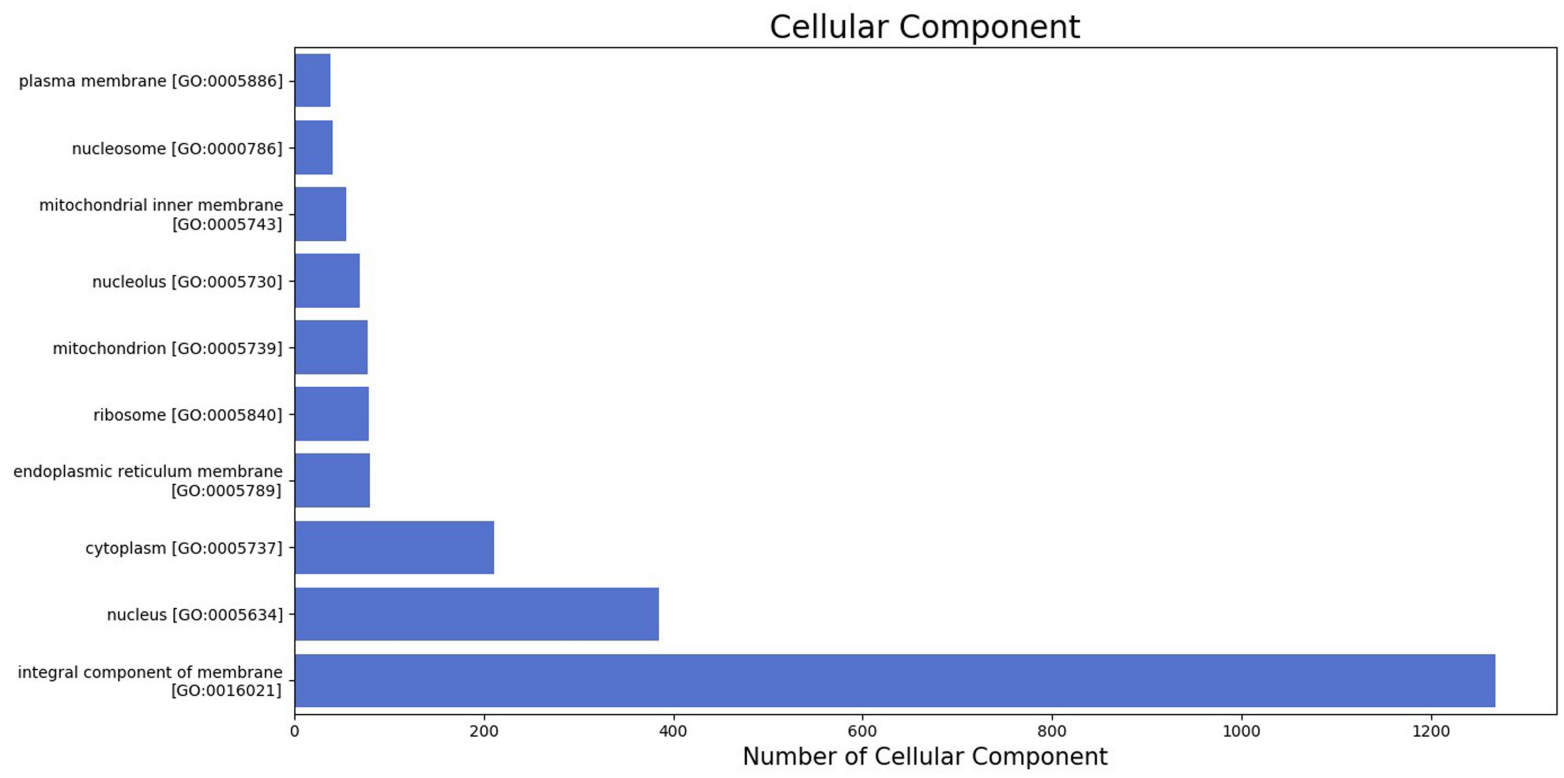
Cellular component category of gene ontology in the sequenced genome of *T. caries*.

**Figure 4:**
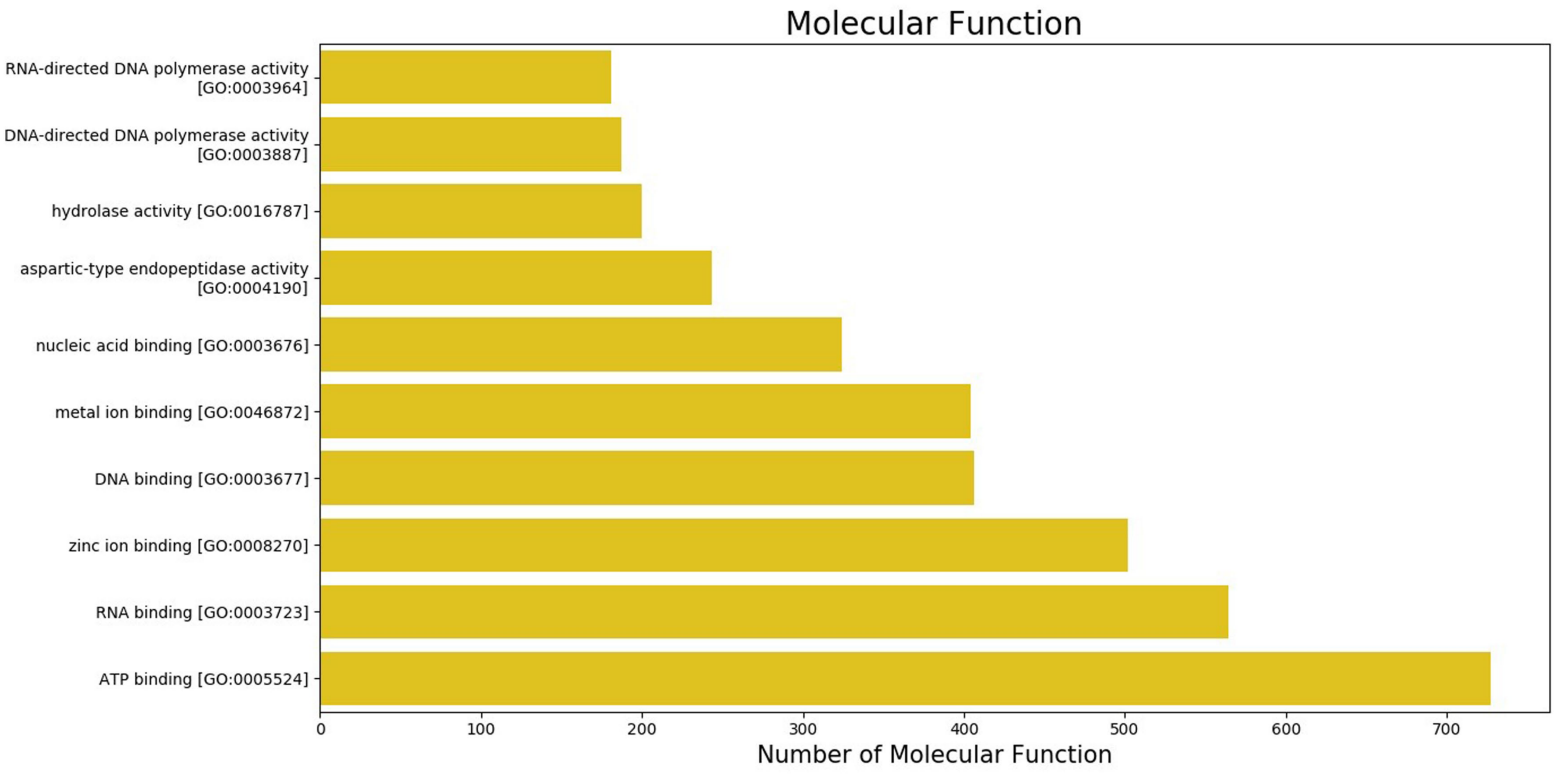
Molecular functions category of gene ontology in the sequenced genome of *T. caries*.

The GO was used to map the BP, CC, and MF of genes in the genome. Notably, 2,388 protein-coding genes were grouped into three categories, namely, BP (960 genes), CC (401 genes), and MF (1,027 genes) (Supplementary Table S2). Maximum GO terms were assigned to DNA integration (548), DNA recombination (230), transposition (171), DNA repair (103), and translation (93) in BP function (Figure 2). GO terms in CCs were grouped into membrane integral components (1268), nucleus (385), cytoplasm (211), endoplasmic reticulum membrane (80), and ribosome (79) (Figure 3). In MF, maximum GO terms were grouped into ATP binding (727), RNA binding (564), followed by zinc ion binding (502), DNA binding (406), and metal ion binding (404) (Figure 4).

### 3.2. Identification of the repeat elements and SSRs

To find out the repeat elements in the assembled genome, we used RepeatMasker v4.0.6. Among the repeat elements, the maximum number of retroelements (895, 0.89%) with an occupied length of 339,809 bp was identified, followed by 788 LTRs (long terminal repeats), elements having a length of 328,735 bp, Gypsy/DIRS1 (424), Ty1/Copia (316), and DNA transposons (283), which were observed in the genome (Table 2). The LINEs types were abundant in retroelements. The gypsy was abundant in LTRs, a type of repeat element, followed by Copia. In addition, 55 small RNAs were predicted to have a length of 84,971 bp.

**Table 2:** Repeat elements in the assembled genome of *T. caries*.

Repeat elements	Number of elements	Length occupied	Percent of sequences
Retroelements	895	339,809 bp	0.89
Penelope	43	4,706 bp	0.01
LINEs	107	11,074 bp	0.03
CRE/SLACS	7	606 bp	0.00
LTR elements	788	328,735 bp	0.86
Ty1/Copia	316	140,302 bp	0.37
Gypsy/DIRS1	424	176,498 bp	0.46
DNA transposons	283	43,263 bp	0.11
hobo-Activator	2	108 bp	0.00
Tc1-IS630-Pogo	121	22,447 bp	0.06
Tourist/Harbinger	52	8,668 bp	0.02
Unclassified	84	22,202 bp	0.06
Total interspersed repeats		405,274 bp	1.06
Small RNA	55	84,971 bp	0.22
Satellites	9	242 bp	0.00
Simple repeats	970	105,235 bp	0.28
Low complexity	27	4,437 bp	0.01

**Figure 5:**
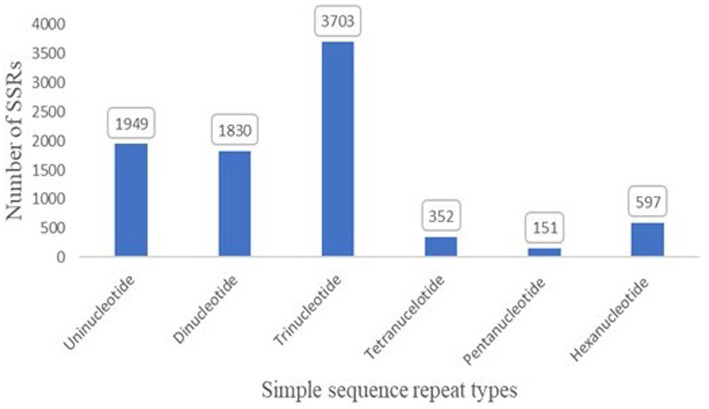
Simple sequence repeats with mono, di, tri, tetra, penta, and hexanucleotide in the assembled genome of *T. caries*.

SSRs play an active role in genome evolution. To examine the evolution, 8,582 SSRs were identified in the genome (Figure 5). In addition, the maximum abundance of SSRs was trinucleotide with 3,703 in the genome, followed by mononucleotide (1,949) (Supplementary Table S3).

### 3.3. Comparative genome analysis with other *Tilletia* species

**Figure 6:**
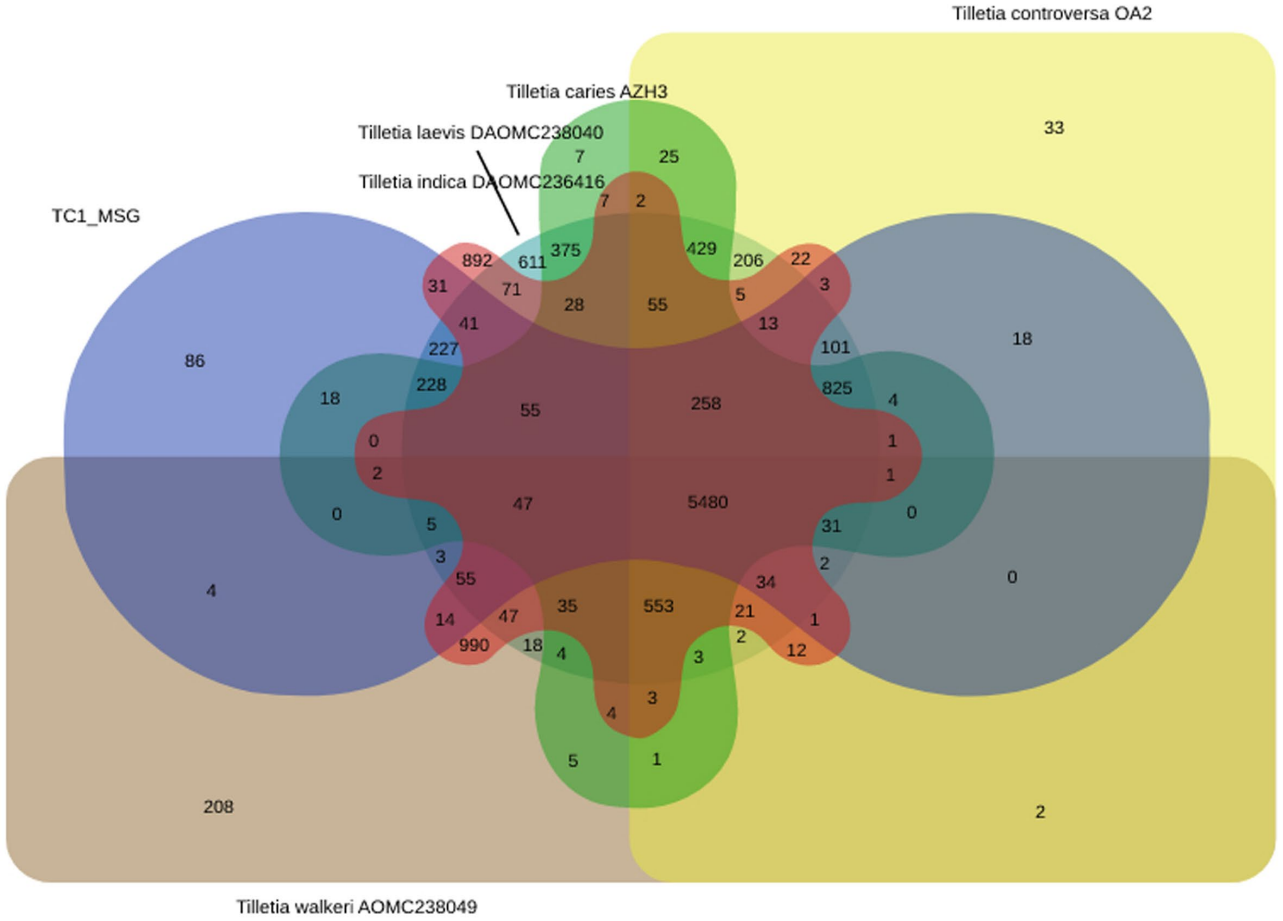
Venn diagram showing the distribution pattern of shared and unique orthologous protein families viz. *Tilletia caries* TC1MSG, *Tilletia caries* AZH3, *Tilletia controversa* OA2, T. indica DAOMC236416, *Tilletia laevis* DAOMC238040, and *Tilletia walkeri* AOMC238049 using OrthoVenn.

*Tilletia caries* TC1_MSG genome was matched with other *Tilletia* species, viz., *Tilletia caries* AZH3, *Tilletia controversa* OA2, *Tilletia indica* DAOMC236416, *Tilletia laevis* DAOMC238040, and *Tilletia walkeri* AOMC238049, to identify the shared and unique orthologous proteins. It revealed that 5,480 protein families of *T. caries* were orthologs in five *Tilletia* species, while 86 protein families were unique to *T. caries* (Figure 6).

### 3.4. Secretome prediction and analysis of *Tilletia caries* assembled genome

**Figure 7:**
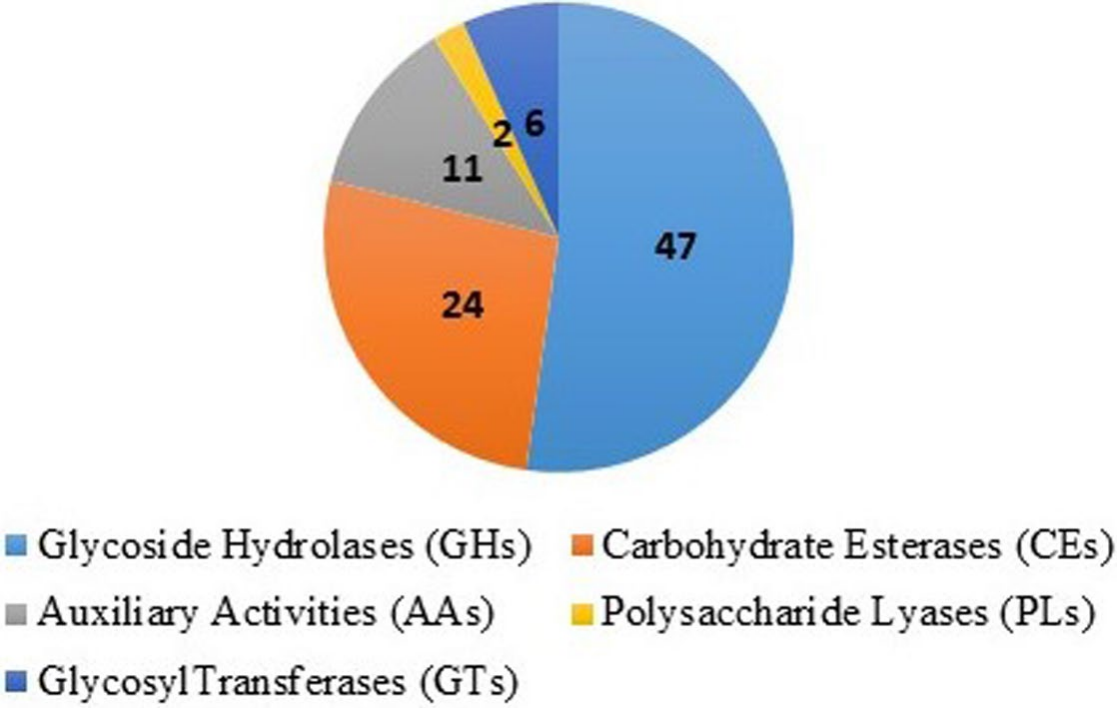
Carbohydrate-active enzymes (CAZymes): 47 glycosyl hydrolase (GH) families, 24 of carbohydrate esterase (CE) families, 11 of auxiliary activity (AA) families, 6 of glycosyl transferase and 2 of polysaccharide lyase (PL) families.

The secreted effector proteins play a major role in infection by plant pathogenic fungi. Using a computational pipeline, the secretory proteins were examined in the genome. In all, 10,255 predicted proteins from the *T. caries* genome assembly were analyzed in SignalP v5.0, as well as TargetP v2.0 for prediction of the secretory signal peptides. Notably, 777 proteins of SignalP and 880 of TargetP proteins were predicted with secretory signals (Supplementary Table S4). The carbohydrate-active enzymes play an important role in the growth and aggressiveness of the pathogens. The carbohydrate-active enzymes (CAZymes) analysis revealed that 47 glycosyl hydrolase (GH) families, 24 carbohydrate esterase (CE) families, 11 auxiliary activity (AA) families, 6 glycosyl transferase families, and 2 polysaccharide lyase (PL) families were predicted (Figure 7). The GH and CE families were highly predominant (Supplementary Table S5).

### 3.5. Pathogenesis-related genes in *Tilletia caries*

**Figure 8:**
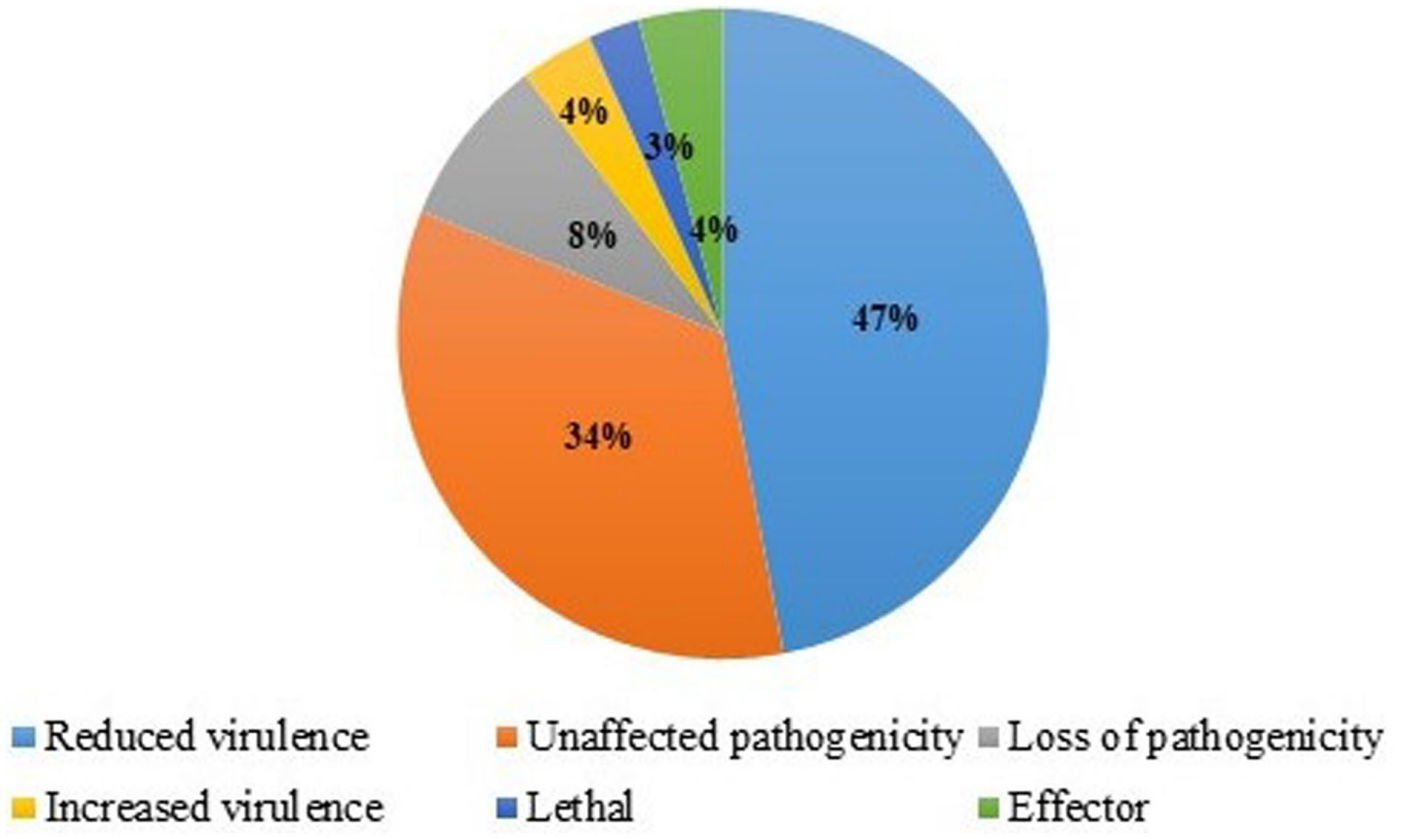
Identification of Pathogenesis‑related genes in *T. caries* using the pathogen–host interaction (PHI) database.

Using the PHI database, 10,255 genes were annotated. Based on the similarity of proteins (Figure 8), 4,922.4 (48%) genes were related to reduced virulence, 3,281.6 (32%) were related to unaffected pathogenicity, 922.95 (9%) were related to loss of pathogenicity, 820.4 (8%) were related to lethal, and 307.65 (3%) genes were related to increased virulence (Supplementary Table S6).

## 4. Discussion

Globally, wheat is an important cereal food crop. The common bunt of wheat is a re-emerging disease in India, causing huge losses. It is distributed worldwide and can be found almost everywhere in wheat-cultivating countries. *Tilletia* species are quarantined wheat pathogens that affect trade (OEPP/EPPO, 2016). The management of bunt diseases is challenging and complex in nature due to complex biology and modes of infection (Gurjar et al., 2021). In the genomic era, genomic data are significant for studying the biology of such pathogens, but to date, no complete genome sequence of *T. caries* is available. In the Indian context, the genome of *T. caries* isolate has not been sequenced to date. The understanding of genomic regions and gene-related virulence is still inadequate, and obtaining such information is crucial for the identification and characterization of virulence factors (Shakouka et al., 2022). The present study attempted to improve the genome assembly and quality of *T. caries* by comparing genome with that of *Tilletia* species and identifying virulence factors causing the common bunt disease of wheat.

In the present investigation, the genome of *T. caries* was sequenced using short (Illumina) and long reads (ONT). The genome assembly was performed with the ONT and the Illumina data using the POLCA polishing tool. Based on the hybrid approach, a high-quality genome assembly of *T. caries* was generated. The genome assembly size was 38.18 Mb with 46 contigs. Higher genome sequencing coverage was achieved compared to other genomes available in the public domain. This genome assembly was larger in size, better quality, and more accurate than other available assemblies of *T. caries*, ranging from 27.14 to 35.80 Mb (Nguyen et al., 2019; Sedaghatjoo et al., 2022). Previously, six *T. caries* genomes were available in the GenBank and NCBI, but these genomes were small in size (USA, Canada) and had a high number of scaffolds (3,606–8,169). Using karyotype analysis, the genome sizes of *T. caries* were estimated in the range of 28–39 Mb (Russell and Mills, 1993). To date, no complete genome of *T. caries* existed in a public database.

In the present study, the gene prediction was performed based on *Ustilago maydis* (Schirawski et al., 2010). In total, 10,698 genes were predicted as protein-coding genes. In eukaryotic genomes, repetitive elements are widespread. Transposition is one of the causes of genomic plasticity and plays an important role in pathogenicity and adaptive evolution (Casacuberta and Gonzalez, 2013; Castanera et al., 2016; Razali et al., 2019). In this present study, 895 retroelements (0.89%) with a length of 339,809 bp were assessed in the assembled genome. Gypsy, followed by Copia, was the most abundant LTR retrotransposons. Publicly available 625 fungal genomes revealed that transposable elements (TEs) have a potential role in genome evolution and correlation with fungal lifestyle (Muszewska et al., 2019).

In earlier studies, Gypsys were the most common repeat element, which was reported in *T. indica* and *T. horrida* (Wang et al., 2018; Gurjar et al., 2019; Mishra et al., 2019). In fungi, the Gypsy group is the most common type of TE (Gorinsek et al., 2004). A total of 1.1% of TEs were in *Ustilago maydis* (Kamper et al., 2006). In earlier studies, TEs may increase the size of fungal genomes (Gurjar et al., 2019). Additionally, 8,582 SSRs were classified throughout the entire genome. The trinucleotide SSRs (3,703) were the most abundant (43.14%).

In addition, a comparative genome investigation with *T. caries* TC1_MSG, *T. caries* AZH3, *T. controversa* OA2, *T. indica* DAOMC236416, *T. laevis* DAOMC238040, and *T. walkeri* AOMC238049 revealed that 5,480 protein families of *T. caries* were orthologs in five *Tilletia* species, and 86 proteins were found to be unique in *T. caries*. Earlier reports revealed that 72 unique proteins belong to *T. caries* (Nguyen et al., 2019). The best method to identify virulence factors and genetic variations was comparative genome-wide analysis (Kamper et al., 2006; Gurjar et al., 2020; Wang et al., 2022).

The fungal secretome is the main factor that enables infection and pathogenesis (Krijger et al., 2014; McCotter et al., 2016; Verma et al., 2016). In all, 10,255 proteins predicted from genome assembly were analyzed in SignalP v5.0 as well as in TargetP v2.0. Notably, 777 proteins of SignalP and 880 proteins of TargetP were predicted to have secretory signals. Genomics and bioinformatics-based analyses of small-secreted proteins provided more phylogenetic and evolutionary interpretations (Feldman et al., 2020). In this study, based on CAZymes analysis, 47 GH families, 24 CE families, 11 AA families, 6 glycosyl transferase families, and 2 PL families were present. The CAZymes are playing an important role in the evolution of fungal carbohydrate-active enzymes and adaptation (Hage and Rosso, 2021). In addition, it is degrading plant biomass and GHs, and CE enzymes were facilitated to cell-wall degradation (Cantarel et al., 2009; Ospina-Giraldo et al., 2010; Zhao et al., 2013). Upon genome-sequencing approaches, the CAZymes were also reported in *T. horrida* and *T. indica* (Wang et al., 2018; Singh et al., 2019). Using the PHI database, 10,255 genes and proteins were categorized. The maximum number of genes related to reduced virulence (48%) was followed by those related to unaffected pathogenicity (32%). Pathogenicity and variation in different environmental conditions are more frequently complex in genes (Sheppard et al., 2018; Lee and Andam, 2019).

## 5. Conclusion

Improved quality of genome of *T. caries* using Illumina and the ONT PromethION and structural and functional annotations were presented. Comparative genomics and the identification of pathogenicity-related genes were successfully performed, which revealed some core conserved genes among *Tilletia* species and some specific genes in *T. caries*. In structural genomics, high numbers of repeat elements and SSRs were identified in the genome. Secretory proteins and pathogenicity-related genes were predicted, which were highly significant findings. Furthermore, these putative virulence genes need to be characterized and validated through functional genomics in order to develop management strategies for common bunt of wheat and other smut pathogens.

## Data availability statement

The data presented are deposited in the NCBI database under accession number PRJNA798867.

## Author contributions

MG: Conceptualization, Funding acquisition, Methodology, Project administration, Writing – original draft, Writing – review & editing, Investigation, Supervision. TK: Investigation, Methodology, Validation, Writing – original draft. MSh: Methodology, Software, Writing – review & editing. MSa: Formal analysis, Writing – review & editing. LR: Methodology, Writing – review & editing. RA: Formal analysis, Writing – review & editing.

## Footnotes

^1^ https://github.com/mikkelschubert/adapterremoval

^2^ https://github.com/fenderglass/Flye/

^3^ https://github.com/alekseyzimin/masurca

^4^ http://bioinf.spbau.ru/quast

^5^ http://busco.ezlab.org/

^6^ https://github.com/rmhubley/RepeatMasker

^7^ http://pgrc.ipk-gatersleben.de/misa/download/misa.pl

^8^ https://github.com/Gaius-Augustus/Augustus

^9^ https://services.healthtech.dtu.dk/service.php?SignalP-5.0

^10^ https://services.healthtech.dtu.dk/service.php?TargetP-2.0

## References

[ref1] Aggarwal P. Sood A. K. (2006). Characterization of the bunt isolates collected from dry temperate zone of Himachal Pradesh. Indian Phytopathol. 59, 318–322.

[ref2] Albughobeish N. Jorf S. A. M. (2015). New races of *Tilletia laevis* and *T. caries*, the causal agents of wheat common bunt in Khuzestan province, Iran. J. Crop Prot. 4, 59–68.

[ref3] Almagro A. J. J. Salvatore M. Emanuelsson O. Winther O. von Heijne G. Elofsson A.. (2019b). Detecting sequence signals in targeting peptides using deep learning. Life Sci. Allian. 2:e201900429. doi: 10.26508/lsa.201900429, PMID: 31570514 PMC6769257

[ref4] Almagro A. J. J. Tsirigos K. D. Sonderby C. K. Petersen T. N. Winther O. Brunak S.. (2019a). SignalP 5.0 improves signal peptide predictions using deep neural networks. Nat. Biotechnol. 37, 420–423. doi: 10.1038/s41587-019-0036-z 30778233

[ref5] Borgen A. (2004). Organic seed treatment to control common bunt (*Tilletia tritici*) in wheat. Seed Test. Int. 128, 8–9.

[ref6] Cantarel B. L. Coutinho P. M. Rancurel C. Bernard T. Lombard V. Henrissat B. (2009). The carbohydrate-active enzymes database (CAZy): an expert resource for glycogenomics. Nucleic Acids Res. 37, D233–D238. doi: 10.1093/nar/gkn663, PMID: 18838391 PMC2686590

[ref7] Carris L. M. Castlebury L. A. Goates B. J. (2006). Nonsystemic bunt fungi-*Tilletia indica* and *T. horrida*: a review of history, systematics, and biology. Annu. Rev. Phytopathol. 44, 113–133. doi: 10.1146/annurev.phyto.44.070505.143402, PMID: 16480336

[ref8] Casacuberta E. Gonzalez J. (2013). The impact of transposable elements in environmental adaptation. Mol. Ecol. 22, 1503–1517. doi: 10.1111/mec.12170 23293987

[ref9] Castanera R. Lopez-Varas L. Borgognone A. LaButti K. Lapidus A. Schmutz J.. (2016). Transposable elements versus the fungal genome: impact on whole-genome architecture and transcriptional profiles. PLoS Genet. 12:e1006108. doi: 10.1371/journal.pgen.1006108, PMID: 27294409 PMC4905642

[ref10] Eibel P. Wolf G. A. Koch E. (2005). Detection of *Tilletia caries*, causal agent of common bunt of wheat, by ELISA and PCR. J. Phytopathol. 153, 297–306. doi: 10.1111/j.1439-0434.2005.00973.x

[ref11] Feldman D. Yarden O. Hadar Y. (2020). Seeking the roles for fungal small-secreted proteins in affecting saprophytic lifestyles. Front. Microbiol. 11:455. doi: 10.3389/fmicb.2020.00455, PMID: 32265881 PMC7105643

[ref12] Goates B. J. (1996). “Common bunt and dwarf bunt” in Bunt and smut diseases of wheat: concepts and methods of disease management. eds. Wilcoxson R. D. Saari E. E. (Mexico City: CIMMYT), 12–25.

[ref13] Goates B. J. (2012). Identification of new pathogenic races of common bunt and dwarf bunt fungi, and evaluation of known races using an expanded set of differential wheat lines. Plant Dis. 96, 361–369. doi: 10.1094/PDIS-04-11-0339, PMID: 30727122

[ref14] Gorinsek B. Gubensek F. Kordis D. (2004). Evolutionary genomics of chromoviruses in eukaryotes. Mol. Boil. Evol. 21, 781–798. doi: 10.1093/molbev/msh057, PMID: 14739248

[ref15] Gupta S. Aggarwal R. Sharma S. Gurjar M. S. Bashyal B. M. Saharan M. S.. (2022). Multiple sequence alignment and phylogenetic analysis of wheat pathogens using conserved genes for identification and development of diagnostic markers. Cereal Res. Commun. 50, 463–472. doi: 10.1007/s42976-021-00193-7

[ref16] Gurevich A. Saveliev V. Vyahhi N. Tesler G. (2013). QUAST: quality assessment tool for genome assemblies. Bioinformatics 29, 1072–1075. doi: 10.1093/bioinformatics/btt086, PMID: 23422339 PMC3624806

[ref17] Gurjar M. S. Aggarwal R. Jain P. Aggarwal S. Gupta S. Saharan M. S. (2020). Comparative genome analysis of *Tilletia indica* inciting Karnal bunt of wheat reveals high genomic variation. Indian Phytopathol. 73, 707–712. doi: 10.1007/s42360-020-00260-9

[ref18] Gurjar M. S. Aggarwal R. Jain S. Sharma S. Singh J. Gupta S.. (2021). Multilocus sequence typing and single nucleotide polymorphism analysis in *Tilletia indica* isolates inciting Karnal bunt of wheat. J. Fungi 7:103. doi: 10.3390/jof7020103, PMID: 33540499 PMC7912946

[ref19] Gurjar M. S. Aggarwal R. Jogawat A. Kulshreshtha D. Sharma S. Solanke A. U.. (2019). De novo genome sequencing and secretome analysis of *Tilletia indica* inciting Karnal bunt of wheat provides pathogenesis-related genes. 3 Biotech 9:219. doi: 10.1007/s13205-019-1743-3, PMID: 31114743 PMC6527731

[ref20] Gurjar M. S. Aggarwal R. Sharma S. Kulshreshtha D. Gupta A. Gogoi R.. (2017). Development of real time PCR assay for the detection and quantification of teliospores of *Tilletia indica* causing wheat Karnal bunt in soil. Indian J. Exp. Biol. 55, 549–554.

[ref21] Hage H. Rosso M. N. (2021). Evolution of fungal carbohydrate-active enzyme portfolios and adaptation to plant cell-wall polymers. J. Fungi 7:185. doi: 10.3390/jof7030185, PMID: 33807546 PMC7998857

[ref22] Holton C. S. (1967). “Smuts” in Wheat and wheat improvement. Agronomy monograph 13. eds. Quisenberry K. S. Reitz L. P. (Madison, WI, USA: American Society of Agronomy), 337–353.

[ref23] Kamper J. Kahmann R. Bolker M. Ma L. J. Brefort T. Saville B. J.. (2006). Insights from the genome of the biotrophic fungal plant pathogen *Ustilago maydis*. Nature 444, 97–101. doi: 10.1038/nature05248, PMID: 17080091

[ref24] Kochanova M. Zouhar M. Prokinova E. Rysanek P. (2004). Detection of *Tilletia controversa* and *Tilletia caries* in wheat by PCR method. Plant Soil Environ. 50, 75–77. doi: 10.17221/3684-pse

[ref25] Krijger J. J. Thon M. R. Deising H. B. Wirsel S. G. (2014). Compositions of fungal secretomes indicate a greater impact of phylogenetic history than lifestyle adaptation. BMC Genomics 15:722. doi: 10.1186/1471-2164-15-722, PMID: 25159997 PMC4161775

[ref26] Lee I. P. A. Andam C. P. (2019). Pan-genome diversification and recombination in *Cronobacter sakazakii*, an opportunistic pathogen in neonates, and insights to its xerotolerant lifestyle. BMC Microbiol. 19:306. doi: 10.1186/s12866-019-1664-7, PMID: 31881843 PMC6935241

[ref27] Mamluk O. F. (1998). Bunts and smuts of wheat in North Africa and the near east. Euphytica 100, 45–50. doi: 10.1023/A:1018343603827

[ref28] Manninen A. M. Laatikainen T. Holopainen T. (1998). Condition of scots pine fine roots and mycorrhiza after fungicide application and low-level ozone exposure in a 2-year field experiment. Trees 12, 347–355. doi: 10.1007/s004680050161

[ref29] Matanguihan J. B. Murphy K. M. Jones S. S. (2011). Control of common bunt in organic wheat. Plant Dis. 95, 92–103. doi: 10.1094/PDIS-09-10-0620, PMID: 30743428

[ref30] McCotter S. W. Horianopoulos L. C. Kronstad J. W. (2016). Regulation of the fungal secretome. Curr. Genet. 62, 533–545. doi: 10.1007/s00294-016-0578-2 26879194

[ref31] Mishra P. Maurya R. Gupta V. K. Ramteke P. W. Marla S. S. Kumar A. (2019). Comparative genomic analysis of monosporidial and monoteliosporic cultures for unravelling the complexity of molecular pathogenesis of *Tilletia indica* pathogen of wheat. Sci. Rep. 9:8185. doi: 10.1038/s41598-019-44464-0, PMID: 31160715 PMC6547692

[ref32] Monkiedje A. Spiteller M. (2002). Effects of the phenylamide fungicides, mefenoxam and metalaxyl, on the microbiological properties of a sandy loam and a sandy clay soil. Biol. Fertil. Soils 35, 393–398. doi: 10.1007/s00374-002-0485-1

[ref33] Mourad A. Mahdy E. Bakheit B. R. Abo-Elwafaa A. Baenziger P. S. (2018). Effect of common bunt infection on agronomic traits in wheat (*Triticum aestivum* L.). J. Plant Genet. Breed. 2:102.

[ref34] Muszewska A. Steczkiewicz K. Stepniewska-Dziubinska M. Ginalski K. (2019). Transposable elements contribute to fungal genes and impact fungal lifestyle. Sci. Rep. 9:4307. doi: 10.1038/s41598-019-40965-0, PMID: 30867521 PMC6416283

[ref35] Nguyen H. D. T. Sultana T. Kesanakurti P. Hambleton S. (2019). Genome sequencing and comparison of five *Tilletia* species to identify candidate genes for the detection of regulated species infecting wheat. IMA Fungus 10:11. doi: 10.1186/s43008-019-0011-9, PMID: 32355611 PMC7184893

[ref36] OEPP/EPPO (2016). PM 3/78 (1) consignment inspection of seed and grain of cereals. Bull *OEPP/EPPO Bull* 46, 49–57. doi: 10.1111/epp.12270

[ref37] Ospina-Giraldo M. D. Griffith J. G. Laird E. W. Mingora C. (2010). The CAZyome of *Phytophthora* spp: a comprehensive analysis of the gene complement coding for carbohydrate-active enzymes in species of the genus *Phytophthora*. BMC Genomics 11, 525–541. doi: 10.1186/1471-2164-11-525, PMID: 20920201 PMC2997016

[ref38] Pant S. K. Kumar P. Chauhan V. S. (2000). Fungicidal efficacy of some bio-extracts against hill bunt disease of wheat. Proc. Int. Conf. Integr. Plant Dis. Manag. Sustain. Agric. 1, 396–398.

[ref39] Peterson G. L. Whitaker T. B. Stefanski R. J. Podleckis E. V. Phillips J. G. Wu J. S.. (2009). A risk assessment model for importation of United States milling wheat containing *Tilletia controversa*. Plant Dis. 93, 560–573. doi: 10.1094/PDIS-93-6-0560, PMID: 30764400

[ref40] Plissonneau C. Benevenuto J. Mohd-Assaad N. Fouche S. Hartmann F. E. Croll D. (2017). Using population and comparative genomics to understand the genetic basis of effector-driven fungal pathogen evolution. Front. Plant Sci. 8:119. doi: 10.3389/fpls.2017.00119 28217138 PMC5289978

[ref41] Rana S. K. Devlash R. K. Jain S. K. Saharan M. S. (2016). Evaluation of wheat genotypes for resistance against common bunt (*Tilletia caries* and *T. foetida*). Plant Dis. Res. 31, 109–111.

[ref42] Razali N. M. Cheah B. H. Nadarajah K. (2019). Transposable elements adaptive role in genome plasticity, pathogenicity and evolution in fungal phytopathogens. Int. J. Mol. Sci. 20:3597. doi: 10.3390/ijms20143597 31340492 PMC6679389

[ref43] Russell B. W. Mills D. (1993). Electrophoretic karyotypes of *Tilletia caries*, *T*. *controversa*, and their F1 progeny: further evidence for conspecifc status. Mol. Plant-Microbe Interact. 6, 66–74. doi: 10.1094/mpmi-6-066, PMID: 8439671

[ref44] Schirawski J. Mannhaupt G. Munch K. Brefort T. Schipper K. Doehlemann G.. (2010). Pathogenicity determinants in smut fungi revealed by genome comparison. Science 12, 1546–1548. doi: 10.1126/science.1195330 21148393

[ref45] Schubert M. Lindgreen S. Orlando L. (2016). Adapter removal v2: rapid adapter trimming, identification, and read merging. BMC. Res. Notes 9:88. doi: 10.1186/s13104-016-1900-2, PMID: 26868221 PMC4751634

[ref46] Sedaghatjoo S. Mishra B. Forster M. K. Becker Y. Keilwagen J. Killermann B.. (2022). Comparative genomics reveals low levels of inter- and intra-species diversity in the causal agents of dwarf and common bunt of wheat and hint at conspecificity of *Tilletia caries* and *T. laevis*. IMA Fungus 13:11. doi: 10.1186/s43008-022-00098-y, PMID: 35672841 PMC9172201

[ref47] Shakouka M. A. Gurjar M. S. Aggarwal R. Saharan M. S. Gogoi R. Bainsla N. K.. (2022). Genome-wide association mapping of virulence genes in wheat Karnal bunt fungus *Tilletia indica* using double digest restriction-site associated DNA-genotyping by sequencing approach. Front. Microbiol. 13:852727. doi: 10.3389/fmicb.2022.852727, PMID: 35633675 PMC9139842

[ref48] Sheppard S. K. Guttman D. S. Fitzgerald J. R. (2018). Population genomics of bacterial host adaptation. Nat. Rev. Genet. 19, 549–565. doi: 10.1038/s41576-018-0032-z 29973680

[ref49] Simao F. A. Waterhouse R. M. Ioannidis P. Kriventseva E. V. Zdobnov E. M. (2015). BUSCO: assessing genome assembly and annotation completeness with single-copy orthologs. Bioinformatics 31, 3210–3212. doi: 10.1093/bioinformatics/btv351 26059717

[ref50] Singh J. Aggarwal R. Gurjar M. S. Sharma S. Saharan M. S. (2019). Identification of carbohydrate active enzymes from whole genome sequence of *Tilletia indica* and sporulation analysis. Indian J. Agric. Sci. 89, 1023–1026. doi: 10.56093/ijas.v89i6.90828

[ref51] Stanke M. Diekhans M. Baertsch R. Haussler D. (2008). Using native and syntenically mapped cDNA alignments to improve *de novo* gene finding. Bioinformatics 24, 637–644. doi: 10.1093/bioinformatics/btn013 18218656

[ref52] Verma S. Gazara R. K. Nizam S. Parween S. Chattopadhyay D. Verma P. K. (2016). Draft genome sequencing and secretome analysis of fungal phytopathogen *Ascochyta rabiei* provides insight into the necrotrophic effector repertoire. Sci. Rep. 6:24638. doi: 10.1038/srep24638, PMID: 27091329 PMC4835772

[ref53] Wang Y. Coleman-Derr D. Chen G. Gu Y. Q. (2015). OrthoVenn: a web server for genome wide comparison and annotation of orthologous clusters across multiple species. Nucleic Acids Res. 43, W78–W84. doi: 10.1093/nar/gkv487, PMID: 25964301 PMC4489293

[ref54] Wang A. Pang L. Wang N. Ai P. Yin D. Li S.. (2018). The pathogenic mechanisms of *Tilletia horrida* as revealed by comparative and functional genomics. Sci. Rep. 8:15413. doi: 10.1038/s41598-018-33752-w 30337609 PMC6194002

[ref55] Wang Y. Wu J. Yan J. Guo M. Xu L. Hou L.. (2022). Comparative genome analysis of plant ascomycete fungal pathogens with different lifestyles reveals distinctive virulence strategies. BMC Genomics 23:34. doi: 10.1186/s12864-021-08165-1, PMID: 34996360 PMC8740420

[ref56] Wilcoxon R. D. Saari E. E. (1996). Bunt and smut diseases of wheat: concepts and methods of disease management. Mexico: CIMMYT.

[ref57] Winnenburg R. Baldwin T. K. Urban M. Rawlings C. Kohler J. Hammond- Kosack K. E. (2006). PHI-base: a new database for pathogen host interactions. Nucleic Acids Res. 34, D459–D464. doi: 10.1093/nar/gkj047, PMID: 16381911 PMC1347410

[ref58] Yin Y. Mao X. Yang J. Chen X. Mao F. Xu Y. (2012). dbCAN: a web resource for automated carbohydrate-active enzyme annotation. Nucleic Acids Res. 40, W445–W451. doi: 10.1093/nar/gks479, PMID: 22645317 PMC3394287

[ref59] Zhao Z. Liu H. Wang C. Xu J. (2013). Comparative analysis of fungal genomes reveals different plant cell wall degrading capacity in fungi. BMC Genomics 14, 274–289. doi: 10.1186/1471-2164-14-274, PMID: 23617724 PMC3652786

