# Supplementary material for: Draft genome sequencing of Tilletia caries inciting common bunt of wheat provides pathogenicity-related genes
Source: Front Microbiol. 2023 Nov 15;14:1283613. doi: 10.3389/fmicb.2023.1283613 (PMC10684912; doi:10.3389/fmicb.2023.1283613)
Supplement: Supplementary file 3 [file Table_3.pdf]

"Draft genome sequencing of Tilletia caries inciting common bunt disease of wheat provides pathogenicity-related genes

Malkhan Singh Gurjar1*, Tej Pratap Jitendra Kumar1, Mohamad Ayham Shakouka1, Mahender Singh Saharan1, Laxmi Rawat2, Rashmi Aggarwal1
1Division of Plant Pathology, ICAR-Indian Agricultural Research Institute, New Delhi-110012
2VCSG Uttarakhand University of Horticulture & Forestry, Ranichouri, Uttarakhand
*Correspondence
Malkhan Singh Gurjar
malkhan_iari@yahoo.com
"

Specifications
==============

"Sequence source file: ""scaffolds.fasta.PolcaCorrected.masked.fa"""

Definement of microsatellites (unit size / minimum number of repeats):
(1/10) (2/6) (3/5) (4/5) (5/5) (6/5)

Maximal number of bases interrupting 2 SSRs in a compound microsatellite: 100

RESULTS OF MICROSATELLITE SEARCH
================================

Total number of sequences examined: 47
Total size of examined sequences (bp): 38181409
Total number of identified SSRs: 8582
Number of SSR containing sequences: 40
Number of sequences containing more than 1 SSR: 37
Number of SSRs present in compound formation: 760

Distribution to different repeat type classes

Unit size	Number of SSRs
1	1949
2	1830
3	3703
4	352
5	151
6	597

Frequency of identified SSR motifs

Repeats	5	6	7	8	9	10	11	12	13	14	15	16	17	18	19	20	21	22	23	24	25	26	27	28	29	30
A	-	-	-	-	-	205	104	60	24	22	17	5	11	8	7	4	1	3	3		2	1			1	
C	-	-	-	-	-	114	89	86	34	45	48	35	17	12	4											
G	-	-	-	-	-	118	95	95	70	52	44	35	14	6	3	1										
T	-	-	-	-	-	206	89	47	33	18	11	12	6	9	10	5	3	4		1						
AC	-	40	21	9	7	7	5	1	2			1				1		2								
AG	-	113	66	44	24	20	12	11	7	1	7			3	1	1	1		3	1		1	1			
AT	-	3	3																							
CA	-	56	24	15	8	3	4	8		1	1	1	3			2			1				1			
CG	-	27	6		1																					
CT	-	106	75	56	26	15	9	7	4	2	3	1	5	2	1	3		1		2	1	2	1		1	
GA	-	141	81	39	27	10	5	5	4	6	2	2		2	3		2			1	2	2	2	1		
GC	-	18	6		1																					
GT	-	37	22	14	8	2	2	4	2	1	3							1								
TA	-	15	2	1																						
TC	-	123	66	43	17	22	11	7	6	2	6		1			2	2	1		2	1					
TG	-	43	24	13	11	6	2	4	1		2	2	4													
AAC	20	8	2	10		1								1												
AAG	16	6	2			2					1															
ACA	26	5	4	5	3			2									1									
ACC	18	16	6	3	2																					

Repeats	31	32	33	34	35	36	37	38	39	40	41	42	43	44	45	46	47	48	49	50	51	52	53	54	55
A																									
C																									
G																									
T																									
AC			1								1														
AG	1			1	1							1													
AT																									
CA						1								1											
CG																									
CT			1	1				1	1	2															
GA					1			1																	
GC																									
GT																									
TA																									
TC			1									1	1	1			1								
TG	1	1																							
AAC																									
AAG																									
ACA																									
ACC																									

Repeats	56	57	58	59	60	61	62	63	64	65	66	67	68	69	70	71	72	73	74	75	76	77	78	79	80	81	total
A																											478
C																											484
G																											533
T																											454
AC																											98
AG																									1		322
AT																											6
CA																											130
CG																											34
CT																											329
GA																											339
GC																											25
GT																											96
TA																											18
TC																		1								1	319
TG																											114
AAC																											42
AAG																											27
ACA																											46
ACC																											

file:///C|/Users/Pathology/Desktop/Tilletia%20caries%20paper/Supplementary%20Table%20S3.txt[07-10-2023 11:15:59]

45

ACG	22	9	5	2	2		1																				41
ACT	2	1																									3
AGA	28	7			3	2					1								1								42
AGC	48	29	8	8	5	2	2	2	1				1														106
AGG	36	15	6	2	2								1			1											63
AGT	2	2																								1	5
ATC	1	9	2	1	5	1					1																20
ATG	5	5	1	1		1																					13
ATT	1																										1
CAA	39	14	7	9	1	1	2	2		1	3																79
CAC	40	15	6	7	5	4		1																			78
CAG	126	78	31	25	27	13	6	6	1	5	1	1	1	1													322
CAT	18	10	5	2	1	1																					37
CCA	31	6	5		4	1																					47
CCG	56	22	16	4	1	1																					100
CCT	74	38	17	10	2	3	1		1	1					2	1											150
CGA	33	13	9	4	4	2			1																		66
CGC	32	8	5	3	2	1																					51
CGG	46	14	3	2	2																						67
CGT	23	8	7	4	3		1		1																		47
CTA	1																										1
CTC	51	21	12	5	6	3	1	2	2	2																	105
CTG	60	29	22	16	8	6	1	2	1		1	1	1	1													149
CTT	36	12	2	2																1							53
GAA	31	12	8	2	1			1	1																		56
GAC	45	22	13	3	1	1					1																86
GAG	90	28	23	5	3	2	3	4	2	1		1							1					1	1		165
GAT	23	9	2	1																							35
GCA	71	24	22	16	6	2		3	2			1															147
GCC	36	13	4	3																							56
GCG	54	17	6	2	1	1																					81
GCT	68	40	25	14	5	2	4	2	2	1	1	1				1											166
GGA	64	32	7	8	5	1	1					1	1		1												121
GGC	27	16	6	4	1	1																					55
GGT	26	18	3	2	1			1																			51
GTC	37	6	6																								49
GTG	24	6	3	3	2			1																			39
GTT	20	12	6	3	1	1					1																44
TAA	1				1																						2
TCA	19	7	4	2	1										1												34
TCC	71	34	17	4	4	4	4	3	1	1					1												144
TCG	52	29	9	3	1	2	2		1																		99
TCT	22	5	2		1																						30

TGA	18	14	6	3													1							42
TGC	87	48	15	16	11	12	7	1	1		1	1												200
TGG	43	10	2	3	2	2	1			1														64
TGT	36	13	9	4	8		3	2	1				1							1				78
TTC	14	5	2	1		2						1												25
TTG	13	9	1	3	2																			28
AAAG	1									1														2
AACC			1																					1
AAGA	1																							1
AAGG	2											1												3
AAGT		1																						1
AATA								1															1	2
ACAA	1																							1
ACAG	1	1																						2
ACAT	2																							2
ACGG		1																						1
ACTC	2		1			1																		4
ACTG				1																				1
AGAA	2																							2
AGCA		1										1												2
AGCC	2		1																					3
AGGA	2		1	1																				4
AGGC	1		1																					2
AGGG		2	3													1								6
AGTC	1	1																						2
AGTG	3	5			1	1	1																	11
ATGG			2				1																	3
CAAA				2																				2
CAAC			1																					1
CAAG						1																		1
CAAT		1																						1
CACC			1																					1
CACG	1																							1
CACT	11	5	2	1	1				1															21
CAGA	1		1																					2
CAGC	3		1			1	1																	6
CAGG	1																							1
CAGT		1			2																			3
CATA	1	2																						3
CATC		1	1																					2
CATT				1																				1
CCAC		1																						1
CCAG	1																							1
CCAT		1																						

1

CCCA	2																																							2
CCCT	1																																							1
CCGG	1																																							1
CCTA	1																																							1
CCTC		1	1		1																																			3
CCTT	1	1					1																																	3
CGAC		1																																						1
CGAG	1																																							1
CGCA		1																																						1
CGCC	1																																							1
CGGC	1																																							1
CGGG	1																																							1
CGGT	1																																							1
CGTC	2																																							2
CGTT		1																																						1
CTAC	1																																							1
CTCA	6	8	4	2						1																														21
CTCC	3	3	2	2							1																													11
CTCG		2																																						2
CTGA	1	1										1																												3
CTGC	1																																							1
CTGG										1																														1
CTGT	1			1		1																																		3
CTTC	9	1	4	1	1			2																															1	19
CTTT	1								1																															2
GAAA	1			1											1																									3
GAAG	1																	1																						2
GAAT	1			1																																				2
GACA		1	1																																					2
GACT	3		2																																					5
GAGC	2																																							2
GAGG	3	2	2		1																																			8
GAGT	9	5	1	3	3			1	1							1																								24
GATC	2																																							2
GATG	1	2																																						3
GCAC	1																																							1
GCCA	1																																							1
GCCT		1																																						1
GCTG	1	1	1																																					3
GGAA	3	3							1																															7
GGAG		1			1																																			2
GGAT	1																																							1
GGCA								1																																1

GGCT	1																																															1
GGGA		1																																														1
GGGC			1																																													1
GGGT	1																																															1
GGTG	1																																															1
GTCA		3																																														3
GTCC		1	1	1																																												3
GTCG		1																																														1
GTGA	2	2	4																																													8
GTGC	1																																															1
GTGG		1																																														1
GTTG	1	1																																														2
TACA										1																																						1
TCAC	4	3	3		1	1				1																																						13
TCAG	2	1			1																																											4
TCCA	1	1	1		1																																											4
TCCC	1	1																																														2
TCCT	2						1																		1																							4
TCGC	1																																															1
TCTG		1	1																																													2
TGAC	2	1																																														3
TGAG	3	6	3	1	2									1																																		16
TGCG	1																																															1
TGGA	1																																															1
TGGC	4	2		1		1																																										8
TGTT	1																																															1
TTCC	1	1		1				1																																								4
TTCT	2																																															2
TTGT		1																																														1
AACGA			1																																													1
AAGAA	1																																															1
ACACG		1																																														1
ACAGA	1																																															1
ACCCA	1																																															1
ACGAA								1																																								1
ACGAG			1																																													1
AGAAC	1																																															1
AGAAG	1																																															1
AGAGA	1																																															1
AGAGG																																															1	1
AGATA	1	1			1					1	1																																					5
AGATT	1																																															1
AGCAC	1																																															1
AGGAC									1																																							

1
AGGCG 1
1
AGGGA 1
1
AGGGC 1
1
ATAAG 2
2
ATCGC 1
1
ATCGT 1
1
ATCTA 1
1
ATCTT 1
1
CAACG 2
2
CAGAA 1
1
CAGCT 1
1
CAGGG 1
1
CAGTC 1
1
CATCA 1
1
CCCAT 1
1
CCCCG 1
1
CCCTC 1 1
2
CCCTG 1
1
CCCTT 1
1
CCGTG 1
1
CCGTT 1
1
CCTCT 1
1
CCTTC 1
1
CCTTT 1 1
2
CGAAT 1
1
CGCAA 1
1
CGCCT 1
1
CGCGT 1
1
CGGAA 1
1
CGTCT 1
1
CTCCC 1
1
CTCCT 1
1
CTCGC 2
2
CTCTC 1 1
2
CTCTT 1
1
CTGAC 9 4 1
14
CTGGG 1
1
CTTCT 2 1
3
CTTTC 1
1
CTTTG 1
1
GAAAA 1 1
2
GACCT 1
1
GACGC 1
1

GAGAC 1
1
GAGAT 1
1
GAGGA 1
1
GAGGC 1
1
GATGG 1
1
GCAAA 1
1
GCAGA 1
1
GCGAT 1 1
2
GCGGC 1
1
GCGGG 1
1
GCTGA 1
1
GCTGT 1 1
2
GGAGA 1 1 1
3
GGAGG 1
1
GGATG 1
1
GGCGA 2
2
GGCGG 1
1
GGGGA 1
1
GGTGG 1
1
GGTTG 1
1
GTCAG 1
1
GTGCG 1
1
GTTGT 1
1
TAATC 1
1
TACTA 1
1
TAGCA 1
1
TCAGG 9 1
10
TCCAG 1 1
2
TCCCA 1
1
TCCTC 1
1
TCCTG 1
1
TCGCC 1
1
TCTCT 1
1
TCTGT 1
1
TCTTA 1
1
TCTTC 1
1
TGAGC 1
1
TGCGA 1
1
TGCGT 1
1
TGGAC 1
1
TGTGC 1
1
TGTTC 1
1
TTCAT 1
1
TTCCC 1

1
TTCCT 1
1
TTCTG 1
1
TTTCC 1
1
TTTGC 1
1
TTTTC 2 1 1
4
AACCCT 1 2 1 1
5
AACGGC 1
1
AACGTG 1
1
AAGCAC 1
1
AAGCAG 1 1
2
AAGGAC 1
1
AAGGGG 1
1
AATCAG 1
1
ACAACG 1
1
ACACCA 2
2
ACACTC 1
1
ACAGAC 1
1
ACAGGC 1
1
ACCACG 1
1
ACCAGC 1 1
2
ACCCGA 1
1
ACCCTA 1 1 2 1
5
ACCTCG 1
1
ACCTTC 1
1
ACGACA 1
1
ACGACT 1
1
ACTCCC 1
1
ACTCGA 1
1
ACTGGC 1
1
ACTTCA 1
1
AGAAGC 1
1
AGAAGG 1 1
2
AGCAAC 1
1
AGCACC 1 1
2
AGCAGG 1 1 1
3
AGCATG 1
1
AGCGAC 1
1
AGCGGC 1 1
2
AGCTCA 1
1
AGGAGC 1 1
2
AGGATG 1
1
AGGGAA 1
1
AGGGAC 1
1

AGGGAG 1
1
AGGGAT 1
1
AGGGGA 1
1
AGGGGC 1
1
AGGGTG 1
1
AGGGTT 2 1 1 1 1
6
AGGTCG 1
1
AGGTGC 1 1
2
AGTGAA 1
1
AGTGGA 1 1
2
ATAAAT 1 1
2
ATAGCC 1
1
ATCAGG 1
1
ATGAGC 1 1
2
ATGAGG 1
1
ATGGTG 1
1
ATGTTA 1
1
CAACAC 1
1
CAACAG 2 1
3
CAACAT 1
1
CAAGGA 1 1
2
CACACC 1
1
CACACG 1
1
CACACT 1
1
CACCAG 1 1
2
CACCAT 1
1
CACCCG 1 1 1 1
4
CACCGA 1 1
2
CACCTC 1 1
2
CACCTT 1
1
CACGAA 1
1
CACGAC 1
1
CACGAG 2 1
3
CAGAAG 1
1
CAGCAA 5 3 1 1
10
CAGCAC 1 1
2
CAGCAT 3 1
4
CAGCGA 1 1
2
CAGCTC 1
1
CAGGAG 1
1
CAGGCA 1 1
2
CAGGCT 1 1 1
3
CAGGGA 1
1
CAGTCG 1

1
CAGTGC 1
1
CATAGT 1
1
CATCAG 1 1
2
CATCCA 1
1
CATCGA 1 1
2
CATCGC 1
1
CATGGG 1
1
CATTGC 1
1
CCAAGC 1
1
CCACAG 1 1 1
3
CCACTC 1
1
CCAGAA 1
1
CCAGCA 3 2
5
CCAGTC 1
1
CCATCG 1
1
CCATGC 1
1
CCATTC 1
1
CCCACT 1
1
CCCAGC 1
1
CCCATG 1 1
2
CCCCGT 1
1
CCCCTC 1
1
CCCCTG 1 2 1
4
CCCGAC 1
1
CCCTAA 3 1 1 1
6
CCCTCG 1 1 1
3
CCCTCT 1
1
CCCTTT 1
1
CCGAAG 1
1
CCGACA 1
1
CCGCAT 1
1
CCGCCT 1
1
CCGGTC 1
1
CCGTCC 1
1
CCGTTC 1
1
CCTAAC 1 2 1 1
5
CCTCAA 1
1
CCTCAG 2
2
CCTCCC 1
1
CCTCCG 1
1
CCTCGA 1 1
2
CCTCGC 1
1
CCTCGT 1
1

CCTCTC	1																						1
CCTCTG													2									1	3
CCTGCT	1	1																					2
CCTGTC														1									1
CCTGTG											1												1
CCTTCG			1																				1
CCTTCT		1																					1
CCTTGA						1																	1
CCTTGC	1	1																	1				3
CGACAC		1	1																				2
CGACAG	2				1	1					1												5
CGACCA	1		1																				2
CGACCC	1																						1
CGACCG	1																						1
CGACCT		1																					1
CGAGGG		1					1																2
CGAGGT	1	1																					2
CGATGG	2	2			1	1																	6
CGATGT	1																						1
CGCACT		1																					1
CGCAGA	1																						1
CGCAGT	1																						1
CGCCCT								1															1
CGCCTG	1																						1
CGCTCC	1																						1
CGCTGG			1																				1
CGCTTT					1																		1
CGGCAG	1																						1
CGGCTG	1																						1
CGGTCG		1																					1
CGGTGG			1																				1
CGGTGT	1	1																					2
CGTCCC		1		1																			2
CGTCCT	1																						1
CGTCGC		1																					1
CGTCGG	1																						1
CGTGAC	1																						1
CGTTCG	1																						1
CTAACC		1				1																	2
CTCACA	1																						1
CTCCCT						1																	1
CTCCGC			1																				1
CTCCTG	1		1																				2
CTCGAC		2	1		1																		

4
CTCGCA 2 1
3
CTCGCC 1 1
2
CTCGCT 1
1
CTCGTC 1
1
CTCGTG 1
1
CTCTCA 1
1
CTCTCC 1
1
CTCTCG 1
1
CTCTTG 1
1
CTGAGA 1
1
CTGAGG 1
1
CTGCCG 1
1
CTGCCT 1
1
CTGCTC 1 2 1 1 1
6
CTGGAC 1
1
CTGGGG 1
1
CTGGTA 1
1
CTGGTT 1
1
CTGTTG 1
1
CTTCCA 1 1
2
CTTCCC 1 1
2
CTTCCT 1 1
2
CTTCGA 1
1
CTTGAG 1
1
CTTGTG 1
1
CTTTCT 1
1
GAAGCA 1
1
GAAGGA 1
1
GAAGGG 1
1
GAAGGT 1
1
GACGAG 1
1
GACGCA 1
1
GACTAT 1
1
GACTCA 1
1
GAGCGT 1 1
2
GAGGAA 1
1
GAGGAC 1
1
GAGGAT 1 1
2
GAGGCA 1
1
GAGGGA 1 1
2
GAGGGC 1
1
GAGGTC 2
2
GAGTGC 2 1 1
4

GAGTGG 1 1
2
GATGCA 1
1
GATGCT 2
2
GATGGA 1
1
GATGGT 1
1
GCAACA 1
1
GCACCA 1
1
GCACGA 1
1
GCAGGA 2
2
GCATCA 1 1
2
GCATGG 1
1
GCCAGA 1
1
GCCAGT 1
1
GCCCAC 1
1
GCCCTT 1
1
GCCGTT 1
1
GCCTGG 1
1
GCCTGT 1
1
GCGAGG 1
1
GCGGAT 1 1
2
GCGGCT 1
1
GCGGGG 1
1
GCGGGT 1
1
GCGGTG 1
1
GCGTGG 1
1
GCTCAC 1
1
GCTCCT 1 1
2
GCTCGT 1
1
GCTGAG 1
1
GCTGGA 1
1
GCTGGG 1
1
GCTGTA 1
1
GCTGTC 1
1
GCTGTG 1 1
2
GCTGTT 2 1 1
4
GCTTAT 1
1
GGAAGG 1 2 1
4
GGACCC 1
1
GGAGTG 2
2
GGATGG 1
1
GGATGT 1
1
GGCAAT 1
1
GGCGAA 1
1
GGCGAC 1

1
GGCGAG 1
1
GGCTGA 1
1
GGCTGT 1 1
2
GGGAAG 1
1
GGGACG 1
1
GGGAGA 1
1
GGGAGT 1
1
GGGATG 1 1
2
GGGCGA 1 1 1
3
GGGCTG 1
1
GGGGAT 1
1
GGGGGA 1
1
GGGTGC 1
1
GGGTTA 3 4 2 2 1 2 2 1 1 2 1
21
GGTCAG 1
1
GGTCCC 1
1
GGTGCG 1
1
GGTGCT 2 1 1
4
GGTGTG 1
1
GGTGTT 1
1
GGTTAG 2
2
GTCCTC 1
1
GTCGAG 1 2 1
4
GTCGAT 1
1
GTCGCG 1
1
GTCGCT 1 1
2
GTCGTG 1
1
GTCGTT 1
1
GTGACT 1
1
GTGAGA 1 1
2
GTGAGT 1
1
GTGATG 1
1
GTGCAG 1
1
GTGCTG 2
2
GTGGAG 1
1
GTGGCT 1
1
GTGGTT 1
1
GTGTGA 1 1
2
GTTAGG 1
1
GTTGAT 1
1
GTTGCC 1
1
GTTGCT 1 1
2
TAACCC 3 2 1 2 2 1 1 1
13

TAGGAG 1
1
TAGGGT 1
1
TCAAGG 1
1
TCACAT 1
1
TCACCT 1 1 1
3
TCAGCA 2 1 1
4
TCAGCT 1
1
TCAGGC 1 1
2
TCAGGG 1
1
TCAGTG 1
1
TCATCC 1
1
TCCAGC 1
1
TCCCAA 1
1
TCCCCT 1
1
TCCCTC 1 1
2
TCCCTT 1 1
2
TCCTCA 2 1
3
TCCTCG 1 1
2
TCCTTC 1
1
TCGACA 1 1
2
TCGACC 1
1
TCGAGG 1
1
TCGATG 1 1
2
TCGCAC 1 1
2
TCGCTG 1 1
2
TCGGCA 1
1
TCGTGG 1
1
TCTGCC 1
1
TCTGCG 1
1
TCTGCT 1
1
TCTGTC 1
1
TCTTGC 1
1
TCTTTT 1
1
TGAAGC 1
1
TGACGC 1
1
TGAGGA 1 1
2
TGAGGC 1
1
TGATGC 1
1
TGATGG 1
1
TGCAAG 1
1
TGCCCA 1
1
TGCCGC 1
1
TGCCGG 1
1
TGCTCA 1

1
TGCTGA 1 1
2
TGCTGG 2 1 1
4
TGCTGT 5 1 1 2
9
TGGCGA 1
1
TGGCTC 1
1
TGGCTG 2
2
TGGGAC 1
1
TGGGAG 1
1
TGGGGC 1
1
TGGTGC 1
1
TGGTGT 1
1
TGGTTG 1
1
TGTAGC 1
1
TGTATG 1
1
TGTCGA 1 1 1
3
TGTCGG 1
1
TGTCGT 1
1
TGTGAG 1
1
TGTGCT 1
1
TGTTGC 1 1 1
3
TTAGGG 1 2 2 2 1
8
TTCAGC 2
2
TTCTTT 1
1
TTGCTC 1 1
2
TTGGGA 1
1
TTGTTC 1
1
TTTTCT 1 1
2

Frequency of classified repeat types (considering sequence complementary)

Repeats 5 6 7 8 9 10 11 12 13 14 15 16 17 18 19 20 21 22 23 24 25 26 27 28 29 30 31 32 33 34 35 36 37 38 39 40 41 42 43 44 45 46 47 48 49 50 51 52 53
54 55 56 57 58 59 60 61 62 63 64 65 66 67 68 69 70 71 72 73 74 75 76 77 78 79 80 81 total
A/T - - - - - 411 193 107 57 40 28 17 17 17 17 9 4 7 3 1 2 1 1
932
C/G - - - - - 232 184 181 104 97 92 70 31 18 7 1
1017
AC/GT - 176 91 51 34 18 13 17 5 2 6 4 7 3 3 1 1 1 1 1 1 1 1
438
AG/CT - 483 288 182 94 67 37 30 21 11 18 3 6 7 5 6 5 2 3 6 4 5 4 1 1 1 2 2 2 2 1 2 2 1 1 1
1 1 1 1309
AT/AT - 18 5 1
24
CG/CG - 45 12 2
59
AAC/GTT 154 61 29 34 15 3 5 6 1 1 4 1 1 1 1
317
AAG/CTT 147 47 16 5 5 6 1 1 2 1 1 1
233
AAT/ATT 2 1
3
ACC/GGT 182 71 25 18 16 7 1 3 1
324
ACG/CGT 212 87 49 16 11 5 4 3 1
388
ACT/AGT 5 3 1
9
AGC/CTG 460 248 123 95 62 37 20 16 8 6 4 5 3 2 1
1090

AGG/CCT	386	168	82	34	22	13	10	9	6	5		2	2		4	2			1					1	1															748
ATC/ATG	84	54	20	10	7	3					1				1		1																							181
CCG/CGG	251	90	40	18	7	4																																		410
AAAC/GTTT	2	1		2																																				5
AAAG/CTTT	8			1					1	1					1																									12
AAAT/ATTT							1															1																		2
AACC/GGTT	1	1	2																																					4
AACG/CGTT		1																																						1
AAGC/CTTG		1				1						1																												3
AAGG/CCTT	21	6	5	3	1		2	3	1			1						1							1														1	46
AAGT/ACTT		1																																						1
AATC/ATTG		1																																						1
AATG/ATTC	1			2																																				3
ACAG/CTGT	3	3	3	1		1																																		11
ACAT/ATGT	3	2								1																														6
ACCC/GGGT	4	2	1																																					7
ACCG/CGGT	1	2																																						3
ACCT/AGGT	2																																							2
ACGC/CGTG	4	1																																						5
ACGG/CCGT	2	2	1	1																																				6
ACTC/AGTG	40	34	18	7	8	3	1	1	2	2				1		1																								118
ACTG/AGTC	9	8	2	1	3							1																												24
AGCC/CTGG	13	3	3	1		2	1			1																														24
AGCG/CGCT	4	2																																						6
AGGC/CCTG	3	1	1					1																																6
AGGG/CCCT	8	11	8	2	3						1					1																								34
ATCC/ATGG	4	5	4		1		1																																	15
ATCG/ATCG	2																																							2
CCCG/CGGG	2		1																																					3
CCGG/CCGG	2																																							2
AAAAG/CTTTT	2	3		1	1																																			7
AAACG/CGTTT			1					1																																2
AAAGC/CTTTG			2																	1																				3
AAAGG/CCTTT	1	1	1									1					1																							5
AAATG/ATTTC		1																																						1
AACAC/GTGTT	1																																							1
AACAG/CTGTT	2	1		1			1				1																													6
AACCC/GGGTT	1	1																																						2
AACGC/CGTTG	4																																							4
AACGG/CCGTT		2																																						2
AAGAG/CTCTT	4			1				1							1	1																								8
AAGAT/ATCTT	1	3		2	1					1	1																													9
AAGGG/CCCTT	2	1				1																																		4
AATCG/ATTCG	1																																							

file:///C|/Users/Pathology/Desktop/Tilletia%20caries%20paper/Supplementary%20Table%20S3.txt[07-10-2023 11:15:59]

																																																1
AATCT/AGATT	3																																															3
ACACG/CGTGT		1																																														1
ACAGC/CTGTG	2		1				1																																									4
ACAGG/CCTGT	1								1																																							2
ACATC/ATGTG	1																																															1
ACCCC/GGGGT	1																																															1
ACCGC/CGGTG	1																																															1
ACCTG/AGGTC	19	5	1	1																																												26
ACGAG/CGTCT		1	1			1																																										3
ACGAT/ATCGT	1																																															1
ACGCG/CGCGT	2																																															2
ACGGC/CCGTG		1																																														1
ACTAT/AGTAT												1																																				1
ACTGG/AGTCC	3			1																																												4
AGAGC/CTCTG				1																																												1
AGAGG/CCTCT	1	1	1		1			1	1					1								1							1																		1	10
AGATG/ATCTC	1																																															1
AGCAT/ATGCT				1																																												1
AGCCC/CTGGG	1																																															1
AGCTC/AGCTG	2	1																																														3
AGGCG/CCTCG	3	4	1																																													8
AGGGC/CCCTG		1	1	1																																												3
AGGGG/CCCCT				1	1					1	2																																					5
ATCCC/ATGGG		2	1	1																																												4
ATCGC/ATGCG	3	1																																														4
CCCCG/CGGGG	3																																															3
CCGCG/CGCGG	1																																															1
AAAAAG/CTTTTT	2						1														1																											4
AAAGAG/CTCTTT	1																																															1
AAAGCG/CGCTTT					1																																											1
AAAGGG/CCCTTT	1							1				1																1																				4
AAAGTG/ACTTTC		1																																														1
AAATAT/ATATTT		1				1																																										2
AACAAG/CTTGTT			1																																													1
AACACC/GGTGTT	3	2																																														5
AACACG/CGTGTT	1																																															1
AACAGC/CTGTTG	16	5	4	1	3	3	1																							1																		34
AACATC/ATGTTG			2																																													2
AACATT/AATGTT	1																																															1
AACCAC/GGTTGT	1	1																																														2
AACCAG/CTGGTT	1	1																																														2
AACCCG/CGGGTT	1																																															1
AACCCT/AGGGTT	14	13	8	8	6	4	3	4	3	3	2	1	2	2		1	1																															75

AACCTC/AGGTTG 1
1
AACGAC/CGTTGT 2 1 1
4
AACGCG/CGCGTT 1
1
AACGGC/CCGTTG 1 1 1
3
AACGGG/CCCGTT 1
1
AACGTG/ACGTTC 1
1
AACTCG/AGTTCG 1
1
AACTTC/AAGTTG 1
1
AAGAGC/CTCTTG 2 1 1
4
AAGAGG/CCTCTT 1 1 1
3
AAGCAC/CTTGTG 2 1
3
AAGCAG/CTGCTT 3 1 1 1
6
AAGCAT/ATGCTT 1
1
AAGCCC/CTTGGG 1
1
AAGCCG/CGGCTT 1
1
AAGCTC/AGCTTG 1 1
2
AAGCTG/AGCTTC 3 1
4
AAGGAC/CCTTGT 1 1 1
3
AAGGAG/CCTTCT 2 1 1 1
5
AAGGCG/CCTTCG 1 1
2
AAGGGC/CCCTTG 2 1 1
4
AAGGGG/CCCCTT 4 2 1 1 1 1 1
11
AAGGTC/ACCTTG 1 1
2
AAGGTG/ACCTTC 3 1 1 1
6
AAGTCG/ACTTCG 1
1
AAGTGC/ACTTGC 1
1
AAGTGG/ACTTCC 1 1 1 1
4
AATCAG/ATTCTG 1
1
AATCCC/ATTGGG 1 1
2
AATGGC/ATTGCC 1 1
2
AATGGG/ATTCCC 1
1
AATGTG/ACATTC 1
1
ACACAG/CTGTGT 1
1
ACACAT/ATGTGT 1
1
ACACCC/GGGTGT 1 1
2
ACACCG/CGGTGT 3 2 2 1
8
ACACGC/CGTGTG 1
1
ACACTC/AGTGTG 5 1 1
7
ACACTG/AGTGTC 1
1
ACAGAG/CTCTGT 1
1
ACAGCC/CTGTGG 3 5 2
10
ACAGCG/CGCTGT 2 4 3 1 1 1 1
13
ACAGCT/AGCTGT 1 1
2
ACAGGC/CCTGTG 1 2 1 1

																									5
ACAGGG/CCCTGT			1										1											1	3
ACAGTC/ACTGTG		1																							1
ACATCC/ATGTGG		1				1																			2
ACATCG/ATGTCG	5	2	3		1																				11
ACCACG/CGTGGT	3	1	1		1																				6
ACCAGC/CTGGTG	7	9	4	4			1																		25
ACCAGT/ACTGGT		1																							1
ACCATC/ATGGTG	3	1				1																			5
ACCCCG/CGGGGT	2																								2
ACCCGC/CGGGTG	2	1			1		2	1																	7
ACCCGG/CCGGGT	1																								1
ACCCTC/AGGGTG	1																								1
ACCCTG/AGGGTC	1	1																							2
ACCGCC/CGGTGG			2																						2
ACCGCG/CGCGGT	2	1																							3
ACCGGC/CCGGTG	1																								1
ACCGGG/CCCGGT	1	1																							2
ACCGTG/ACGGTC	1																								1
ACCTCC/AGGTGG	1	1		1																					3
ACCTCG/AGGTCG	7	7	2	1	1	1																			19
ACCTGC/AGGTGC	1					1					1														3
ACCTGG/AGGTCC	1																								1
ACGACT/AGTCGT	1																								1
ACGAGC/CGTGCT	3	1	2																						6
ACGAGG/CCTCGT	5	1	2																						8
ACGCAG/CGTCTG	1	1			1																				3
ACGCCC/CGTGGG	1	1																							2
ACGCCG/CGGCGT	1																								1
ACGCTC/AGCGTG	2	1																							3
ACGCTG/AGCGTC	1																								1
ACGGCG/CCGTCG	1	1																							2
ACGGGG/CCCCGT	2	1		2																					5
ACTATG/AGTCAT	2																								2
ACTCAG/AGTCTG		1																							1
ACTCCC/AGTGGG	8		1																						9
ACTCCT/AGGAGT	1																								1
ACTCGC/AGTGCG	5	2	2		1																				10
ACTCTC/AGAGTG		2		1																					3
ACTGAG/AGTCTC	1																								1
ACTGCG/AGTCGC	2																								2
ACTGGC/AGTGCC	1		1				1																		3
ACTGGG/AGTCCC	1		1																						2
AGAGCC/CTCTGG	2																								2

AGAGCG/CGCTCT			1	1																											2
AGAGGC/CCTCTG	1								1				1							1		1									6
AGAGGG/CCCTCT	1		1	1	1	2	1				1				2																10
AGATGC/ATCTGC	1																														1
AGATGG/ATCTCC				1																											1
AGCAGG/CCTGCT	5	4	2	2	1		1	1	2			1						1													20
AGCATC/ATGCTG	10	2	4			1																									17
AGCATG/ATGCTC	2		1			1																									4
AGCCAT/ATGGCT	1																														1
AGCCCC/CTGGGG	2	2			1																										5
AGCCGC/CGGCTG	2																														2
AGCCTC/AGGCTG	3	2		1																											6
AGCCTG/AGGCTC	1	1				1											1													1	5
AGCGCC/CGCTGG			1																												1
AGCGGC/CCGCTG	3	1	1																												5
AGCGGG/CCCGCT	1																														1
AGCTCC/AGCTGG	1	2																													3
AGGATC/ATCCTG		1																													1
AGGATG/ATCCTC	2	1	2	1					1		1		1															1			10
AGGCCC/CCTGGG	1																														1
AGGCGC/CCTGCG	1																														1
AGGCGG/CCGCCT	1	1	1																												3
AGGGAT/ATCCCT				1																											1
AGGGCG/CCCTCG	3	4	1		1		3	1	1					1																	15
AGGGGC/CCCCTG	1	2						2																							5
AGGGGG/CCCCCT	3																														3
ATCCCC/ATGGGG	1	1			1	1																									4
ATCCGC/ATGCGG	2			1																											3
ATCGCC/ATGGCG	3	4			1	1																									9
ATCGGC/ATGCCG	1																														1
ATGCCC/ATGGGC	3	1	2																												6
CCCCCG/CGGGGG			1																												1

file:///C|/Users/Pathology/Desktop/Tilletia%20caries%20paper/Supplementary%20Table%20S3.txt[07-10-2023 11:15:59]
