# Supplementary material for: Draft genome sequencing of Tilletia caries inciting common bunt of wheat provides pathogenicity-related genes
Source: Front Microbiol. 2023 Nov 15;14:1283613. doi: 10.3389/fmicb.2023.1283613 (PMC10684912; doi:10.3389/fmicb.2023.1283613)
Supplement: Supplementary file 1 [file Table_1.doc]

Draft genome sequencing of Tilletia caries inciting common bunt disease of wheat provides pathogenicity-related genes

Malkhan Singh Gurjar1*, Tej Pratap Jitendra Kumar1, Mohamad Ayham Shakouka1, Mahender Singh Saharan1, Laxmi Rawat2, Rashmi Aggarwal1

1Division of Plant Pathology, ICAR-Indian Agricultural Research Institute, New Delhi-110012

2VCSG Uttarakhand University of Horticulture & Forestry, Ranichouri, Uttarakhand

*Correspondence

Malkhan Singh Gurjar

malkhan_iari@yahoo.com

>contig_14.contig_14.g1.t1

atgcccttcatactcgatgtcgccacactcgacggcctgagtcctctgtcaacgccccagtcatctcctccacgtcgat

ccttgccgcgcgccagaccacaccctccagctcacctcagcatcaagaatagcataccgtcgccttacctttcgtccgatcgaggactcaactacacc

aagacccccgcgccgcgagccaaccaacgcctagcaacgccgtcctcgagcagtcaagccgtcactatcggcgtcccctccgttctcgtcaagccatt

tcgcacacctctccttcccaagcgtcgtatctgggacacggctgatgatcggcaagagtcgtctggcggttcgcgaggtagcaaggcgcctcgtcgat

tggggacgacgccggctcgagcggtcaagaaaatatcctcggcggacatcttcattccgaagcgtttcatgcgcagtcccagtccggacgaggcctcg

gagaaaggaggacgcatgtatgcgggttcgagtccgaatgctcatgctagctctggtattcaggcgactcttgagtcggctcgggaaggtctcgacgg

ggtgttgaacatacaacatcggcttgacgcgcctttaaacctgctttccgaaaacgatctcaacgccggccagcaagaggggtcgatgtatgcgccct

tacgctcttctccagagtcggcttcttccaacgacgacgctgcctggaagcccacagcgagcggccgcggccacttcgagtttgcgcaggagtttgtg

ctgagtcccagacctcgccccactactgagcacggcatacatgcgcaagtccgaggcaactatgactcgtccagctcgccccaggacggctcaatggt

gcagcagggcacgtacgaccagcgttccgacctccttgtcgaagacggtctcgcgggtcttcccaaggagtacgcactcaaggcacaacgcgatctgg

cgccgtacagcagtccccaagtcaagccgcgctcgtcaagcccgcgtcgtctttataccttttggatcccctacggggacttctctgatttctgttgg

gcatccctgtcaag

>contig_14.contig_14.g2.t1

atgggatcccccgcacgttttcggatctttggatggttcggtagcagtgaccctttcttgagcttctggggcgacctcg

atcttcgccttcatcccttccctcccgctcccgctctccctccccgaacttcgacgatggacagtctcgatctgccgacgtcgcagctcatcctcaac

gcgctcgcgctcaacctcgctcaggaggcaggcgccgggaagctctcccccaacgacgccgccttcctccgcaacaatgccgcgtcctcctcctcagc

agccgccgccgccgccgctgctgcttttgccgcgggcgaggacggcgcggctgctccgtcggccaccaaccacaaccacgcacgctacctgaccgcat

ccgcagtccgcacccacatctccctcgccctcttcgcgtccagcatctcctccgcagccacctccgcaaacgcagaccagcacctcgagcccgtcttg

gaggatctcgtcgctctcaccaaggacctcacaaccgccgagttcgaacactctctcgcatgggcaaactggcccctgcccgaccagctcagttacgc

actcgtctcctctctcctccagattgggaacaacgcggcacaccaccgcaagctcgtccttagcgccgtcgtcgactttgcccagtcggtctcccacg

cactcaccgcggctcagggagacgctcacgtcctcatctcaaagcatgtgcccctcttccacggtctctaccgcgccctcaaggacattaaattcccc

tggtctctctccgagtttgcccggctggccaccagcctcggccctctcaccggcagcatcggcaccacccgcagactcaacgaggccctcctcgtcct

gcccgagcaggacgctgcaaaggaccagggcctgcgcgcaactcgccgcaaggctcgcggacctaccgcagccgccatcgaggaggacgacgagactc

gctccattggctctaccgacacttttgatgccgagggcctcggtcacctcgacaccagcgaagacggcggattcgtatacccggaggagactggtcca

caggaacagctcctccacaattaccgccaggcactactctcccattaccgtcgctccgatcgtccgctctcgggccacttcgtccagtacggcgctct

cgacgccctcgccaacgctctcgctgctgcggtcaaggacgtcaccgaccctgccaaggccggtctcgatactggaagcggcagcaaggcacccgtcg

cagccgccacctccgcactcgcaaacacgggatcccgcattctccagagcatccccgtcgtcggctccgccttggccggtaccgcggacagcagcagc

agtctgctcaacggaagcttcaagggtgtcgacttctcctccctcactggcgtcctctccgtcgtcagctcggtccagcacgccgccagccgggcata

tcacgaccttcagcgcttcgtcgagaacgagggcgagaagcaggccgagctcttcctcgacatttactccgtcgagaccctcgcggcggccctcaagc

ttggcgcatactgctccgtcacacacacccgtctggccggcgagcaactcgacactcatgtcctgctccgcgttcgcactctcctttccgagcagtcc

ttcatcaccgatgagctcttgcaaggttcggctcttcagaccgtctccctcctcgtgcgcgaataccccagcattgccatgcccatgaccacccagct

ccgtcggttcatcaccagccccctccccatatttgagatcgacccggcctcgggcgtcagcaagagtgctatcgtcgacgcgaccgcacgctgtctcg

ccgagtgtgtcaagagtgtgcccggcgacgaccttgtcatatcgacgatgtactcgctcctcaactacctcggccgcgagtcgaccagcgttggcggc

gctggtgcagccgttactggctctgcaggtggtgtggctggttcgggcgcctcggttcgcagtggccactctcgtgcctttactgcccagctcggtgc

tcaggcaggcggcacgacgaccactcaacaaccgtacacgaaccgaacggagaaccagaagaggctcatcgttcccagcaccattcgcgccgtctcgc

tccttgctctcgagtatggcaagcaggacgtcatttcgctcgcgaccagtatgcttctccagcgtcttcgcacggcggatcctctggccgaggctgcc

atcttgcacaacctcgtgcccctggcggtctttggtcccaagagctcctttgtcgatgtcatccgtacctttacccaagtcagccggaccgctaccga

cgaaggcagccacgaggccgagatcgtccaggccgcccagctcaagctcgcccgtgcactgggtgccgtcaaggagaatggggctggcgttgatgagg

tgcacgaggacgacgacctggcgaacaagggtcgcaaggagatctacctctacgagctcctgaacgccttcaccgacaagggcctcgctcttcagact

gctgtcagctctgccaaggccagcagggaggagactgctgagctcaatgccgacttgggtaacatggtccccgcgattgctgccttgctcgcacacga

tgacatcaaccctcagacgggcgcctcgatcgagacgatcgctctcttccggaacatgtggttcctggctgtcctctttggccacgcgacgcctcagc

acgctgtcctgccgcccggtcgcccgctcgtcccgctcgagggaggcgagaaggtcgagcagacgttgccgagcacgttgcccaagcccgaggctcgc

gccgaggtactggccaacgcgttccggatcattgccctgaagacgccgacgctcgtgcccgagaaggcattgaactatctcgagtctgacctcgagta

caactcggtgctccgtcgtgacttctcgcagagcacgctcgaggcgcagcgcaaagctcttgcgggcctgactcctgcgcatgcggccgaggttcgca

ccttcagccctgcccaggtcaccttcctgatgacgacctacattctcgagaacatgcggagtgcgatgggccagccttcgatggtgttgtggtacttt

gtcaacgacgggctcaactcgagtccgctggtgggctcgttggaggacattgctgataaggttatggtgaactttatccagcatctgtctgcccagat

caacgaccatgctctggaccctcgtgtgtcgttcgaggtgaagaacctgcttctgggcagctgccaccgtgtgtccaaggtccggcaggtctcgcgct

cgttcctcgacaagctcgtgggcgcgttgccctcgcttctgtgcgacccggacatcgtgaccaccatgctggagatgctgacgcttttgcgccagtcg

tgcgagtgcgagcaccgggacgagtacgcgcccaagtaccgcttccagtctcgtcgggccagggtcacgtttgacctgagcgacaactacgtcgagcg

ggacgagatcctgacggacttcctccagcggacgaagacgtacctcaacaccctcatgggccgcgcccccgtcgagctcaggggtattctacagcgct

acctcggcacgtttgacgagtcggccttgcctggcaacgctgagctcggcaagtcggtcgctgttgactttgcgcgtctgatgcctgcctcggctcga

caggagaccttcctgcccaagtacggcggctggcgctcggacgcttcgagctcgttcgtcgacgagctttcggcgaagagcacctatttgggcgagat

gacgggtgttcacctcacgttgagcaaagggatgatggagatgcagcgggactcggccaatacgttctcggagaggagcgtggcggagtgcaagcttc

agctgttgcaggtttcggagaacttgattcgccagaagacgttgccgtttgctgagttccggaggttgttgtaccgcacggctgctttggctgttgcc

cttccccacccggactacacgctcctcgcattcgtggtggccattccgatccgcgtcttcacgcctgctgcgatcatgacggcctcccacgtctggac

ctggatcataggcgagcgccccgaggtcgagacaaagatcatggtcgagatcacagtcggctggagctcgactgtcaagctccgtcggggtatcttct

ccgacacgctcacgaacagcaaccccctgctccgcaagacggagatgtcgcccttcaaccgggccgaggtggaggctgagctcgagctggcccacaag

atgttcacgccccatctgacgctgatccagctcatggccagtcgataccaggcgttccggtaccgtgacccgacgaccgtgctggctcttgtccggct

catccagcgctgctctgcggctacggatgagatgagcagccaccccttggcgcgtgaggctcgcttctcgctcatcatctttggtttccgcctgatcc

agggcagccgcctcgacggccttgtggagtacgagctccgatcggcactctaccgtatggcgttctcatggttcgccgttgcgcctcagtggtcgttc

gggagcaactatctgcagatccggacggagatgcagctcatgacggagttccttgaggtgcttaagctcgacaaggtccgggctagctacctgatcac

gagttacccctcgtctttcaccggtgtccgtttccccggtcagatgacggtccagggctccgcaaagctgttcaacgaacgtcgtgccctattgcagc

tcctgctcgagagcgagttgaaccgcttcagcgtgtgggcgaaccctgtccacatgcccggtcgtggggcggactatgttggcgacctcacgaagacg

atgacggacccgctctggtcgcagatggtccacatcgcctggaaggttgacgctcgtctggccgtccagcttcctgcgcgtttcaaggccccggtcat

caggcacgaggtcggcatgcttgtgcgctctcagccgcagaaggcgatcggagctccggctgctatcgagatgctgctcgaagatcatctgcgttcgg

ccgctcgggacgggacagatctcaagtggctccactactgggctccggtcacgcctatcgagtcgattcagctcttccagccgcagtacgagaaccac

ccgatggttcttcagtacgcgatgcgtacgctcgagcagcaccccgtccagctcatcttcttctacgtgcctcaggttgtacaggcactccgagacga

taagtacggctacgtggagcagtttatcttcgagacttccaagatctcgcagctgttctgccatcagataatctggaacatgaaggcgaactcgtaca

aggacggcgacgctgaggttgaggactcgctcaagcctacgcttgaccgtgttcaggacaacatcgtcaagtctctgtctggtgaggctcgtcgcttc

ttcgagcgcgagttcaccttcttcaacgaggtcactggtatctccggcaagctcaagccgtacatcaagaagagcaagctggagaagaaagccaagat

cgacgaggagatggccaagatcaccgtcgacaagggtgtgtacttgccttcgaaccccgatggtgttgtcgtcgaccttgaccgcaagtcgggtcgcc

cgctgcagtcgcacgccaaagcgccttacatggccacgttcaaggtgcaccgtgacgtcgtgaagccccaccctgccgatcagccgggtgagaccata

accgacgagacgggtggctcggtccagaagcaaggtattgatgtctggcagagcgctatcttcaaggtcggagatgactgtcgacaggacgtgctcgc

gcttcaggtgatcgcgatgttcaagaacatcttcacgggagtcggcttggacctgtacctgaacccgtatcgtgtcactgccaccgggccgggctgtg

gtgttatcgacgtcgtgccgaacgcgacgtcgcgtgacgagatgggtcgtgcgaaggtgaacaacttgatggacttcttcgtcgacaagtacggcaac

ccggacagcattgcgttccagaaggcccgtctggccttcatccagtcaatggctgcctactcgctcgcgtgctacatcctccagatccgtgatcgtca

caacggtaacatcatgatcgatgccgatggccacatcgtccacatcgacttcgggttcctgttcgacatcggacccggtggactcaagtttgaggccg

agttcaagctgacgacggagttcatcgaggtcatgggcggcacgacctcgcagggctaccggctgttctgtgacctgttcatcaaggggtacctcgct

ctgcggccgtacgcggatgaccttgttgatgcgtgtcatctgatgctgggtaccgagctgccctcgttcaagggcgagccgacgatcaagcgtcttcg

gtcgcgattccgtctggacctggacgaccgtggagcggccaagtttgcgctgtcgctcatcacgactgcgtctgagaaccctcgttcgattctgtacg

atcagttccagtacttgcaaaaccggattccgtacaacggctaa

>contig_14.contig_14.g3.t1

atggcgcgcggcactggaaacgtcggcccgcgaggcagcggaaagagaacgaacggcggccgcggtggtagcaggaaca

acagcggcagcggcagcaacagcaacagcaacagcaacagcaacagccgcgacgagaatcgctcacaggcagcacagcgtctccagcgcaaggaacag

gcctccgcatcagcaggcggcggcggcggcggtgggcccagcagcgtcctcggcgtttatgattatgtggtcaatgcagatgcagcgggaggcgcagg

cggtgcacctggcaagaagcgcaagcgctccgatcgcgcgctccgacgtgccaccgctggtttcgaggaatctgcaggagcggaggggtccgcgttga

acaagcgacggcgccacaacgacgacgatggcagcgacggcgacggcgacggcgacggagaggatgaggatgatgacgatgacgatctctctgacacc

gagcgcgtacgtgcgcctgctatcttcagcgacgacgaagatggcgatggcgaggctaagccgttcgaaggcgaggatgaggatatcgattcggacga

ggcctttggcgagagtgacgaggagcggtatgacggctgggccttctcggggtccaagtccaagcgcgacgaggacgacgaggatgaagaggatgagg

acgccatgggtctcgatcaagttctcaacgcagtctctgatgatgatgacgacgacgacgaagaagacgacgatgacgacgaagctggcgtcaaaggt

ctcgacgagctcattggctcctcttcatcaggccaacataacaccgacgccaccttcaagcccgccaacttcttcaatttcgaccaagcccaagcagc

agcatcggcctccggacccgtcactctcgaagatcttctcggcccactcgcacaggacgcgtccctaggcggcgccgagctgcgcaaacgcgtctcgg

ccctcgcgtccgaaaataagggaaagggcaacgggaaggaaggcgctggggctgtggattctagaacgggtgcccttgccgctcctctgcctgccatt

ttgcaggaccgcgtggaccgggcggccgcgtacgaagttactcgcgcccaggtcgaggggtggcaaccgacactcaagcgcctccgcgaagcggagca

tctctccttcccgctccagaagcagccggagacgaagtcctcgaactcgactctggtctcctcgatgctctccaccaagcaaggatccgtcctccgct

ccgagctcgagtcacaaacggctgatctgctccgagcggcggagatgaccgattcgcagatcgtcaagaaggaagatcttgcgcttaacaagctcgat

ccggccctggttgaagcgcgtcggcgggagctggcccggatgcgcgagctcatgttccgcgctgagcagaaggctaagcgggtctcgaagatcaagag

taaatcctaccggcggattgcgaggaaggataaggagaagctgaagcagaagatggccgaggcggggctttcgatgggcaatgatgacgacgatgagg

tggactatgaggccgatcgggtggagcgggtgaaggcggagagggagcgggcgctggagagggccacgttgaagcataagaatacgggccagtgggcg

aagacgatgtcaggccggcagggcaataacgcggagacgattgctgcgctggaggaacagcttcggcgcggtcagcaattgcggcagaaggtccaggg

atcggacgacgacgacgacgattccgacgacgagaacgaggacgacaagacgggggcgaacgcgttcgagagcgacctggaggagaatgaggcggaga

tcgaggacgcggctgcgagtggggaggtgtcgaagggatttggcaagttgatgcagatgaagttcatgaaggaggcgcgggcgaaggcggaccagggc

gtccgggaggctgcggacgagttgcgggcgcagttggccgggcttcagagtgacgatgagggggaagatgatgatgaggatggggatggggaggcggc

tgcgcttcggggtcgggtcgcccaggttggtggcactggtggtcgggctgtgttctctgctgcggctggaacggagaagagcgccccgccgaagaaga

cgaagacggcggtggcagcggcggcggcggcagtacccactcagaaatcctcgtcggtgccgctgctgtcgtcttcctcggccgccggtcagaaagac

ggagagaacccgtggctgaacagcgggggtacgaatttgagcgcgatggcgaagaggcaggatcggaatgggacgaagtcgaagcagcgcgcgttgga

acttgcggaggatgcgaaggtggaccttcttgaggatggtctaggcggtggtggcggtggtggtagtgtctcgaaggcgaccccgtcctcgacgacga

agagcggttcgaaggggaaagggaaggcgagcgacgagagcggcccttctacttcgacctctgctgctgctgcaacacgcgggatcgctttcacgcag

cgcgatctcgtcgcggaggcgtttgctggggacgacgtgacggcgcaatttgcggagcagaagcgtgcgcttgtggaagcggatgcgcctcaagagga

ggacaactcgctccccggctggggcagctggtccggcaagggcgtcaaacacagcgcagcgcaaaaggcgcgcctcgccgcgaaccgggcgaaacaca

cacgaacgctaccgggccttgcgccgtcggcacgtaaagacgcggggatggagcacgtggtgatcaacgagcgtgcggataagaaggtggagaagtac

aaggtgaaggatttgccgtatccgtttacgagcgcggcgcagtatgagcttgtgatgaggggtgcgatgggggcggagtggaatacgaggacgcagca

tcagcggatgacgttgccgagggtggttacgaagccgggcaaggcgattgctcctattgagcggaagttttga

>contig_14.contig_14.g4.t1

atgcgtcctctcacagaagcagagaccaagctcctcttcgacaagctcgcccagtacatcggcaacaacatcgtccacc

tcatcgacagaccggacgacccgcacctgttccggctccacaaagaccgcatctactacgtctccgagcccgcctgcaaactcgccgactccgtcgcc

cgtccgcagctgatctccctcggcacctgtctgggcaagttcagcaagagcggcggcaagttcaggctgcacgtcactgcgcttgatttgcttgtgca

gtatgctaagtataaggtctggattaagccgaatggcgagctgccgttcctttatgggaatcacgtcgtcaaggcccatcttggtcgaatcaccgatg

acacgccagagcatgccggtgtcgtcgtgctcaacatgggcggggtcggactgggctttggcgtcacggcacggtcaacggtagacacgcgcaagatt

gaccccacctccatcatcgtcttccaccaggccgatgtcggagagtacctcagagacgaagacaccttgttctga

>contig_14.contig_14.g5.t1

atgtgtgaccacgaccacgccgccactgcagtcccagcgatcgcggcccccaacggagacgtcagccagacacacgtcg

ccgtcgagacccgcgctgcacacgtccccgagggaagacccgcagagcatgccacacagcccatgcgctactccgacttcctctccaacgtgtccaag

ttcaagatcatcgagagcaccctccgtgagggtgaacagttcgccaacgccttcttcgacaccgagaccaagatcaagatcgcaagggctctcgacga

cttcggcgtagactgcatcgagctcacctcccccgccgcctccgagcagagccgaaaggactgcgaggccatctgcaagctcggtctcaaggccaaga

tcctcacccacatcaggtgccacatggacgacgcacgcatcgccgtcgagaccggtgtcgacggtgtcgatgtcgtcatgggcacctccaagttcctc

cgcgagttctcccacggaaaggacatgacctacatcaccaagaaggccatcgaggtcatccagttcgtcaagtccaagggcatcgaaatccgcttctc

caccgaggactccttccggtccgacctcgtcgacctcctctccatctaccaggccgtcgacaaggtcggcgtcaaccgcgtcggagtcgctgacaccg

tcggcgtggccaccccgcgccaggtctacgatctcgtcaggaccctccgcggcgtcgttggctgcgacattgagtgccacttccacaacgacaccggc

tgcgccatcgcgaactcctacaccgccctcgaggccggagcgacccacgtcgacacctccgtgctcggcatcggcgagcgcaacggtatcccttccct

cggtggcttcattgcgcgcatgtacaccgccgatcgtgactacgtcatgagcaagtacaacctcaaggccctccgtgaggtcgagaacatcgtcgccg

atgcggtccaggtccaggtccccttcaacaactacgtgaccggctactgcgcgttcacccacaaggccggcatccacgccaaggccatcctcgccaac

ccgagcacgtacgagatcctgcgcccagaagacttcggcatgtcgcgctacgtctccatcgggcaccgtctcaccggctggaacgcgatcaagtcgcg

cgcccaacagctcgagctcgacatgaccgatgcccagtaccaggaggtgacgacgcggatcaaggcgatggccgacattcgggaacagacgatggagg

atgtcgatgtgattctgcgggtcttccaccggggtgtcaaggctgggcatccggtccatgacgagcaggtgttccatcagctgttggagcagcatcaa

gcggcgaatggggatgccactgctgcgactgcgacttcggctgagcctgccgcggtagcggccgcaccaacagcggcttga

>contig_14.contig_14.g6.t1

atgaagttctccatcttcacctccgtcatggtcgccgtcgtcgtcctcgccatcggggtcatgccgacgtctgcacaga

gagagtcttacgacgacccagccccaacggacggaaaccccgccgcttgcgaggcttggtgcgcgagaaacagtaaggcacctaattgcgctggcgag

tgcgagaggagcggcaatcctccctcctacaatgattaa

>contig_14.contig_14.g7.t1

atggacacagcatcgacagcagcagcagcagcagcagcagcagtgtcggcgagcacgagcacgagcacgagcgcagcag

ggatggccgccctcgacgcagcactcgccaagctcagacaccagaccgcgtctccactcgcctcccagcgcaagccggcacagctcctgctcgccatc

gaagccacgctcgacgagcagcacgagtccaacagcaacagcaacagcaagcgcaagcgactcgccacagaacgctcgcccacagagtatctcatcgc

cctccagtccatgctctcctccaccgtcctcgcaccgacgctctacctcctcgccatcgtcgcaccccacatacagcaacccaccttgctctcccaga

tcaacaccctcctccctcccctcgctaacgtcttctccctctcagcctcctctgacacagcacacgcacacccagacccagacccagacccagagtcc

ctctcactcaccctccgatcctccctcaccatcctcgaggccatcctgctcgcagcctccgccaaccaggcactcaccgcctccctcaggaagaacat

cgtcctcaccggctgctggaactccgccgtacagctctccctcgacgtccgtcccaagatcaggaaacgcgcccatgaactcatcagcaacgtcctca

cctccgcacagccacatccatacgcagattccgccgcagaatgggcagtcggaagactcgacaccatcatcacctcaggcgggctctcctcccgaccg

ggcccgtcttcatcttcatctgcggcagggaagaacaagaacagctcggcgcaaaaacagcagaaggctaccgagttcacctttgacaagaagacggg

acaggcgcgtaatgcaggcgcctttgctgctgttcgtgcccaggccctgcagcagggtctcggacaggctatggtcgaaggctccgtcggaggtgcag

ccgcggcggctgatgcggccgccagtgcaggaatctggctctgccagctgctcaaacttatcgtcaagaccttctcgtcaaagagcgtctcggccctg

gccgccgtgctcctccgtctgccagccctccaaaacccattcctcacctcggccgccttcgaagtcttcgacgccctcttccgctcctcctccccatc

ctcatccacgtcaaccacatcccaaccaacaccaacggtcatcacaaaggataccctctctcgcaccctccgcaccttgctcgacccggcagtcctgc

cgacggagaaccagagtctcagcccggccttgttgtcgacctacctccgcgcactcaccggcgcactcacctcctactcccgcttcctcatccccctc

agcgccggcatgggagaggaggacaaggaggaagagcggaagcagccgccgttggagtggggcaccgtcgtccccgccatctggggcatcgtcctcgc

tcgcgctttggacagcaagacggcaggcacgcaagcgcccaaacaggctcctgtccgacagagcgccgcagaggctctcattgcgtgtgtacgctttg

gtatccctgattctgaagtcgagctcgcgttcgaatatgtcatctcgcatggtcggcatttggcctttcctgccccgccgggcggcgctaaggccgat

gatcgccctttcctcgtcagggtcgtcgctcagcttcaggacgcgctcggcaaacatgcgctccggttcgccagcgtcgttcccgagttgttggacgt

catcaccgccttggtccgtcggctcggtcatcggtcggcctcgacgcttgtcaagaaggacgcaggacggagacaggccgccgtcgagctcgtcctac

ccaccctcatcccgacggtggctgacctacgcacctcgcctgccttccccgcacgcgagaacgcagacgctgtcctcgccgctgcgattcaggtcatt

gggccccggcagattctctcccgtctgccgctcggactgctcggcgagaacccggacggacaggggcgggcctggctcctgcccctcatgcgcaccag

catcagcaacaccgagctctcccacttcgtcgacgagctgatcccgctgagcgagaagctctttgcgcgcaaggaagacgcggcttcccgagcgggca

tggccgaggatcccaagcagaaacagcggttcagcgtcgagtcgaagatgttcgaggcgctcgtggaccaggtctgggctaccttccccgcgtactgc

gacctgccgtacgacctcggacacgtgctgcgggagaagcccgaggtaatcgccctgctgagcagcctgctcttctcgcagcccagtctgcgctcgat

tgtcacgcgcggcttgcagcgccttgtggagcggaacacctcgttggcgcggagtagtgcgccgaaggaggttgcgaaggcggactttgatctcgatc

aggaggacgctaagtcgaatttggagttgcttgctacgcttgcgtaccaggacacccttgcctctgtcggcaatggcggtctcctggcgagtctgatg

aacgtgtacgcccagacggccgcggactcgcgtggctttgtgattgagaccattgcgacgtttgtgggcatcttgccgcaggagcaggtcgacaagac

gtacacccgtgtcgtgaagatgctcaagaaggccctgcctacgttggagaaggataacaataactcgggtaaccacaatgcaccggcacccgtgccgc

acacgatgctcgacctgctcggtgcactcgtgcccttcctcaaagcggccgatgcaaaggatctcttcggcatgctccaccgcgacagcccagatgat

gacgagaaggcccagctgttgaaaagcgccgatgcgggcgtacagaagcggacctacaagcttctcacgcgtcttgtcgagctccggccgaacgaggt

ccttccccgcgccggcggtgtcggcggtgcagccgagctcgtgaagacgctgagcggagcaacgcccactgtgctcgcgggctccaagcgtggacgga

tcgcgctctttgcggccctcgtgccccggatcgggatggacgagctccatctgctgccgagcatcgttcctgaggctgtgctggccaccaaggagacg

aatcagcagtgtcgcgaggagagctatgagcttttggtgcagatgggacggaagatgggggaggctactggtgggattgtcaggcggaagcttgttgg

gcgggagaaaggtgctgcagacgcggacgaggacatggctggcgctggggccgaagtcggcccggctgccaatctgacggagtacttggccatggtct

ccgccgggctggcggcgacgtcgccgcatatgatcagtgcgacgatcacggcgctctcgcgactcgtatatgagttccacgccgagctgccgaacgag

acgcttgacgagctcctgagcacctgcctcgtcttcctcgccagtgcgaaccgggagatcgtcaagtccagtctgggcctggtcaaagtggtgatcgt

gtccttcccgtccgagctcgtcgacggccacttgccgacgctggtcgacttgctcctgaacggcaaatcggcgcagcacaagcagcacttcaagagca

agcttcggcatattatggagcggcttatgcggaagtttgggaatgagcggattgagaagcttgcggatgaggagaatcggaagttgattgttaatatt

cggaagaggaaggagagggctaagaggcggaagacgtccggtggggcggatggtggtgatggggaggaggaggaaggggagcaaggcgccagtgggcg

tgctcaggctggtgttagggcacagaagagtcatgggatggacgcatttgaggaggcgctctatgggagtgactccgatctgtcctctgacggagata

acgacgccgccgaagcaccttcgcaagacaggcgctccagtcgacgaggagcgaccgcggccggaccctccaataataacaacaagaagaaacagcag

cacgaccagacgtacatcctcgaagacggggaggacgaagatgcgatgccgctcgatctgctggaccggactgcgttcgcttcgcgggtggccacgaa

gaatcctgttaaagcggaacagcgacgtcggaagcccggccaggaggcgcgcacgtttgatctcgaagaaggcacgggcaagatgatgatcgacgatg

acgatgacgccggcgaggggaagggactcggtgagcaggccaagttggacgccgaggcgggcgcgggcactgcgttcttgcgcaagcagcgtggcgtg

gatggtttcacgacgggtaaaggcggtgcggtcaagtttaacaagaacaacaaacggacgagggaggacgagatggagcttgatgggcaagcttctgc

ctctgcctctgcctctgcttccgcaggggacgcaccatcgaacgaccctagggcggccgctgcgatctttgcggctgctgcggcgggtgagaaccccg

cttcggccatgggcggtggcggtggtgagaagtcgaggaagaaggcgaagaatgagaaggttgctattggggctgagttccgggcgaagaaggcggga

ggagacgttagcaagaatgggcagagtccttttgcgtatgttccgctcggacaggttgcgaataagaagaaggggaaggggaagggcgaggaaggtgc

tcatgctcatcgtccggtccaatgggcctcgtccgttcgttcgtccatgcccaaggcgtttcaggcatcggacgaacgtggtcgcgtccatggcagac

agcgtatctga

>contig_14.contig_14.g8.t1

atggacgcactggacgcgctgcgctctgcagtggcagcagcacccgccgacgagccgctctcccacatccactaccgcg

cagagggcgaccagcccacagacaacctcgtcctcgcagagaccatcgtctgtctccactctgccttcaacaagtcacacccaacacgcatcctcaag

tccgcaaagacttctcatgatgacccgcccccggacgccttcttgcccctcgaggccctcgtctttgccatccagacccgcgcggagaacttgggcgc

gtatgtcagacaggccacgggacgagccatcccccgtctcgctcccacagaacgggcgccagtgctcgactatctgctgggcaagaccgccgcatggc

caggcgtcgtcgctctccctcccggctcagcctcagcagcgcatcagccagaccacgcgcaatcccaactatcagctgcctccgcatcggacctgacc

accacccgatccaccacaccacccatcgaccctcccgctgccctcaaagcacgcttcgcatcatcatccacagcagcagcatcgacctccgcccgccc

cgtcaaacgagcctacatcccctcccgcgccgacgccgacttcgtcaaacgtctccgcaccaccgcagaacgctccttcctcacccgcgccgacgccc

tccacggcaccaccccctggctcaaacgcgccgacttctccaacttcaggaatcaactccagcccgtcctcctcgtcgcacgcaagggcttggctgct

gtcgctgccgcgggagccaagggcgtccccagtgcagcccctttaaccctcatcccacagtccatgcccgccccttcgggtgggacggccactacgcc

cagcggcctgcgcgtccagagaaaacaacgcgcccaggatcccatcttgatactcagcaacagtcccaccgcgctcatcaacatgttcaacgtcaaga

agctgcttgaggatggcgtctttgtctcccccgatcgggcacggcacgacgcaggcggcttggccgaggccgtcgttcagatcagtcatgtgcccgcc

ctcctcgccgacgctctccatccgtccacatcggccggtggcgcaggcgcaggcgcagggagcggcggcggtgccgctgctgccgcgtcctcaacacg

caagatgaggatcctcgtcgtcgataatgcagatgcgctccagagattgggcggcagtagttcgggagctggggctcgggcgggaggagcgggcgcag

gggccggcaaggacgcgggcgcggatccgagcgaggatgtgtggaatcgggtcgtcgctgtctttaccacaggacagctgtggcagttcaagggctac

aagtataaagagccccgggagctcttcaagcatgccatgggagtgcatgtgcgctactcgaacgaaactctccttcccatcatccgcgactggaacat

tacacaactacagatcgagccatcgaagcggcataccgacaagcagctcgtcgggcacttctggcgcaccctcgaggcatggatcatgcgccggaagc

cgtacttgatgccagtacagcctgtatag

>contig_14.contig_14.g9.t1

atggcccaattcacccccaacaccaccttccaacaaagccccgaatccgactgggcctacctcctcggctccgtaaact

gggccgaccacatcgtcttcaccaaggaatccctcgtcgacttcctcaagactcaaggcatccaggtctcgccgtctttcaaggagctgcagacgctg

cccgggtttgctactgttggcaagttcgatcgggacccgtcggctttgggaggggcggaggcgagggcgcagggggcacagcaggtgccggtgcagcc

accatcacaggctgtgaacgcggcgcaaaatgggcaggagcaggagcagaagcaggcgcaggggcataagaatggttcagctccgccggtggatgtgt

acaggccatcgacacgagttaccgtcatggcgacagaatgctcccccgcgttcccgagaggcacatacacttcctcgatggcgcgcatggccgggggt

tccaactggatcatgcctttccgcacggcggtatttggaaacatcgggtccaacgaccactgtccctcattcgaaaagtcaacgcaatgggacctcct

cggaatggacgagcttgagcttgagcttgggcttgggcttgggcttgatgatgagatcgagacagccgacgacgacgacgacgacgaggacgaggttc

atctcgactctctcgccgatctgcccccttctcgcttgcttgtatttctcctcccgtgtagaacgcagaacccgttccccatcttgtatcctgggctc

ctcgttgccgccgcggttgccttgatccaaaggatccaaggatacgcagtcatcaacgatcaacacggcactgctgcggaagcgatggttgaggcaaa

cgcgttactggccgccgctgaagcggctcgtctgcatgatttggagagtgcttctgcttctcccgcctctcccttaccgcccttcctcgatccgacgc

tgctcgctgcttctcgtcgtgcgaatgagcatgagagcggcggacttcagattcgcacacgacgagacagcgatactgagacggattcggaacatgcg

atgattaacttgaaaaactcgcacaactacgccatgaaatactacgaatatgaaaacaattctcaggcgcgaatgcaagtcgcgaaaaggggaacatg

gaggcttatagagcagggagctaagctatcggcaaggtcaggtctcgctgtcttcatcgcatttgggcctttgaatgaaagtgcgaaggccgaagccc

gagaccatgtctatgtcagtccgaacctctgcgatggcgctcgacctactctgcgcgcaatggcggaacatgctgccaacgatttcaagacaacaatg

catatgtaccgagaagcggcacgagcgaacacagcgcaggcattggaagcaaacaaggttctgattgcggaaaagacagctcttaaagagaagaacga

cgaactcgaagctcttaaagagaagaacgacgaactcgaagctctcgtcgccggtctcgaacgtcgcctagcctccacctcctccgcctctacctcct

ccaacggcctctag

>contig_14.contig_14.g10.t1

atgcagtctcccagcaaaacggcagcccgccgcgggcgagaagtagtagtagccgcacagctacagcaacagctttcat

caagcttgccgtttcctccgcagcaccgcgaaactttgcctcttctgcatcactcatcttcctctactgctgatcctcttccaacttcgtcgtcgtct

ttcctacctcatgaaaatctccccataccatcagcgcccttttctctcttctctgaacaggacattgcgccatcaaagcgacagcgcaaacagcgagc

cagagcacatcgatctggctctgctgctgcgcaccgcgatacctcatcctctcatcacgaacgtccgacaacctcgaatgaagacttcctttcctttg

acgaggggccagcggacaattcaagtgatcgggacgagattcagcactacgcagactcgctgttcattcagcagtctgtgtacgtagcacgtgttgac

cttcggacttgggttgtcgaagactatgtcaatgttatctgccaggaagggaagttctatcatgtgacgctctatgaaggaaccagcccgacaggaag

ggacgaacaacttgcctcatgcgattgctcagctggccgaaatgggttgcaatgctcgcacaagagagtcctgctcgagcacggcgagctgttccaaa

aggaggtcattctttctcagagaatcaatccccttgcaattcagatcgcggcacacctacgtggtgaacttgcttggttctctgttctatctaccaga

agcaccatcaccgcggccagtcaggacggcaagcgttgtgtcgttgcgttgtctggaaacgagcgttggacttgttcggcatcgcattgttcttcctt

tggtcagcctgcaaacgtctgtccacatcgtcaacgcgcccagcagttctggaacgacacaggcaattccatcgagcctgatcttggtggtaatggaa

tgattccatccgcaaagccggccctgctcacatatgccgatctccccgcccccccccattctgccgtctgccctctgagcctattgggaaaccagcca

ctgaaaggggcattcttccaacaacgatcccgcttggagaacacgcccgctgcgactgcggtcgacgcagcagcgttgaagcgccagtacgactccga

ccgtgaatgcagctgccgactcggtcaagagaagaggagcatcggtcccgacctttcggagcacgggcttttcaattggaacaactccatcatcgtgg

cacatgaagtattcaacgcctatacctcccagctctccacctctcctactccttttacagctttctgtctgaccattcaacatgcgtatgaacagcat

gctcctggtggaaagctcaagtttcttcatcgttcgacttttgtcaggttgtactttgcgtacatttccttgcagcgactcgatatcgatttctcatg

ccaactctgcggtcccaatccttctacagtcatagcagatggagtacagcttgccggcttccttctgcagcaggtggcagacaactctgatcagtacc

tcgaattgctgcagcagatcaaggcatctgtcgagacggaatcggtcctggtgatgcgcttgtggtcaggacgctttcagcaggtgctgctggacacg

ttcttggccagggccacactttcgcacaacctcacgaccgcggttcagcaattgatcgaccagttcgctgcacatgagggtgttctgcaactttgccg

ccccccccctctgctgcccactcctgatcgaactcagcaccatcgccttccaaaccgcctccaaggatcgcatcactcgtctctcaaccacactggct

cgtcactgtcccttcctgggcaacgtctttgttcttctggcacatggcatcggcagcatgacgagctcagcagagacgaacctgaggagcagcttgca

atcattgatggcttcgacagcacaagtcgttga

>contig_14.contig_14.g11.t1

atgagctcaaccgatagccaactcaaccggccccggctcatgagggccccgcataccacacctcccctcccagcgacca

cctccctccttctctcccgcggctccacagcctgggacaccgacgacgatggcgacgacgacgggtacgatcaagtaacagcagatagcacccgcgac

gatgatgccaacgcactcatcctcaatgacgggaacaaacgcacaacagcaacaacaacaacagctcctcctcctcccgcccggcgaaatcacctcgt

ccgtctcccgcaagaactcatcctccacatcctcacctacgtccactcagacctgctctacccgccccaagcctcccacccgctctacttccagccgc

tcgaacagcgccagcaagcggcccatctggccttgctcagactgacggctgtcagcgcacagttcgcgagtgcgatcttgagtccgcaggcggggctc

ctttggaggagggtcgatgggagtgctacgttccctgtgccgcgtggtgtgctgccggatgcggaggaggagaagcagcagcagcagcagcagcaata

cggccggccagctcgacatctttccccggccctgctcgtcgcactcgcccgaacggcaggtccggctgtgacttcgctcaatctgcgcggagtccact

tcccttcggccaacggcgaacctggttccatcggcataggatccatattcagcgctgcccaactctttgagcatattcttaaggctcagggtgtgctc

acgaagcagtttgccccgccgagcttctcgtttccgaccaaatcggagcttgggttctctacttcaagtcgctcgaaaaaagtgttggaagagggcag

gatgactacgtccccgacgtcggaggagactccacttcctccccttcccaacaccttgcctgcccctcgcgtcactcagctaacccacctcgacctcc

gctccctccgcatcgactcagtccaacttgcccccgcccgtcgagcactcgccgccctcctctcctcatcgccctacatcaccaccctccagctcgcc

aatgccgatccacgcctggtcgacgacgccctgctcgacaccagtctgtcccatccacacagactgcgagagctcgatctcagtcggtgtgcgggtgt

gagtgcgctggggttgggtcggctcttgcggaaagtggagtcttatcagagtcagatggttcaggctggggatgaggaccgagcgcgggaggcgggga

tgagggtgttgcgtattgttgggctgcgggctacgccgcctgttccgttggacgagggtgctggtagcagtgcggatgtccggagaggtgccggtcgg

cgcggccgcggcagggctgtccgtgcggtcggagattcagagacggacgaggaagaggatccggtggcgcgacagcattccaccgtgtctcaaccgaa

tgggaacggctcttcctccgcccgtgtgcatccgctggaggacatgctggctctgcttcgccagacgagcgcgcattgcctggaagtactcgaggcga

actacgtcctcgagctcaccgacgtgcacatcctcgcgctcacccacgtccgtccgctgagtcctgctgctcctgcctctatggcagggccgcagatt

cagacggcccttgcgcacgagcagatcaagctctcccgcgccctgtcctccgccaagttcttccccctccgtcgctcagccctcgcccgtgcactgga

cgaaagcagcaacacctcaaacgtagtccaccggacgctcttccctcacctccgctccctcgccctctcctcgaacccacggctgacctcggccagct

gcgggttcctcgtcggtgcggtgccgcactgcgagacgcttgagatggctggctggagctcgcgtgcgttcttggaccatgagcgggatgctgatggg

cgacgggtgcggagggcgagtggtgggaggggtacggctgtggtggagttgccgggggatggtccgcttgtggatctgttggagtccgtgccgcgtgt

gaggcggattgatctggaaggcgcaggtgagattacggatgcggtgcttagggcgctcacgcctgttcctcccatccgcgctgggagtcctccgaagg

ccacttcgtcggaggaggaggaggaagaggaggacgtccaaccgggccagcatctgacgcatctcatcctctcgcacgcctatcgcctctcggcccgg

gccacgctggacctcatgcgcggcgcgacgaaactggtccatctcgagttggacgatacgcgggcgggctgcgatctcgtggcgaaggagtgggctat

gttgatgaaggctcgcaagagtgggctgaaggcggccgcttcggcgttggcgttgccgttctgtgcttcttcgagcggcactactgctgctactggag

atggcacggcccaagtcgagcgggctcaggtggcgccgacgccgtacctctccctcgtggactgtcggggcttcagcaaggaggagtatgagcgactg

agtcggcgcgggttcgttcgtgcgcgggaagcgattggtgtccggcctaagcttgcgcatgtggctggtttgaagctagggagtcaggatgaggaggg

agaagttgcaggaggaaggggaagggatgctgttgagctgttggaggcgggtggcgcgggcagctatgagtgggggagagactggtttgggtacgaag

gtcgggcggaggtcatgcctgtcagcactcaccaagctggtaacccgccacagacccaggaacaagctgggccgtcgtccgaggagacgatcgctccg

aatgtgcaggtcggatcgcctagtcgtgcgagtcgattggctcttgcgcttccactcagtttgggtatcgcccttgttcgtagagcgtcgggtcagct

tattggaggcgatgatgaacgtggcgaggcctttggcgggggcgggggtggtggcgagggcggcggtggtgctgcacaactctcgctacccggtcttc

ccttccgcatccgagcccaccttggcggcggtggtggtagcagctcttcttccaacgagcccgagaagaataatggcggccccgatggagacgagacg

aacccggccttgggcgtgttgaagagcttctggggctggcaggctgttgatacgcgagcgaaggcgaggaagaagaggcttgtgaaggaggagaaggt

gcggaaggctgctgctgccgccgccgctgctgcgactgcgagtgcgaatgcttctgcgagcagggcaggacgaccgggtggtggacatggtgcttcgc

gaagtatgtcgtttggcgggctggacgccgctgcgcggaatacggtgggaggcgagtcgggagggagcaataccgggcttcgcaactcgacggctgcg

gcgcttgcgttgctcctccgccgaacgggcgcctcctcggctggggctgcgatgtcagctgcgtttggaccgatgtctccaccgctctctcggagccc

gtcgcctcctgcgactgctggtattccgatccccggtgcgactgctactaccccgacttcctcgccctcgggtggctcgagattgggtctgggtcgct

cgcgtcgctcggattcggcggggttcggatggagcgggtcgccttcgactccgggtgggcaaagtagtatgtcggccttttcgcttatggcactggcg

ggacagcggggcggggcgacgggtgctgcttcgcgctcgggctcaggctcaggaggtggctcttcgtccttctcgttctcgttctcaccctccacttc

gccgacgtgggagcgcggacagcagcatcatgatcggatgcgctcgtcgaccgatggttcgctcgtttcgcatgtctcgtaccgccaggcgttggcat

ttgcggaggcggctgctgaggctgggttgagccctacggaggcggagatgctcgcgatcgctgctacttccgcgcccggttcgcctcttcgtgcgcgt

gctcagtccggctccggttcgagccccggcggcgcgggacccaaacgatcacggatgagctcgcgcgggagtagttctgccttcttgctcttctcgtc

gccttcgagtatgacgtccgagctgagcccggtacggagtcggccttcgagcgtgcggagcttgaaggacagggtccgggagaagggcgatcgggcga

agggtcgaattcggcggttgttgagtcctggctcgagctcgggaagcgggagtggtgcgagtggtctccctcctcggatcagtagctcgcagccgacg

tctggcatgacgacgcctcgccggatgatggcaccggtcgcttcctcgtcggcgattccgttgatggtgaacacgcctgcgtcggagccgagcgttcc

gcagtactacgcccctgtggagcgagagcgtggtggtcgtgggatcggtatggaagggcttgggtgggattccgatgatggtgattatgatatggatg

gccagggatgtgtggtcatgtaa

>contig_14.contig_14.g12.t1

atgctcatcccaacgacctggtttctcttgggcgcgcacagacgggcaagtcgatcccatgcggaaaggcctcgtcttc

gaaaagtacgtgttatcgactcgctctacatcaaaccatgcgatcggactgacagagatgccttgtatacagctcgcctggattccttcccgcgtctg

tccaagtacgagtgtgacgacggaccgcgcaagatcagaaagaggcgatatcaagaggcgtccagggagtttcgcaaggccttcgagctgtatgcacc

tgctatcgctgatttgaccgaatactttgtcgcgctgaatcgatccaagcggacttgggcggacgactcttcgacatctcagtggaagcctgagtccg

aggctatcccggccagcctacccactgcccttctactccattcgacggctcaaagtccagccttctacgatgagctagaccgtaccttttccaagcac

tcgctccgcgtcgtcagattcacaggtcaagagtgcggaacatacaggacgacgatgcaaacgctgacgaagcggatcatcagccaagtcgccaccgc

tctcaccgccggagacgagcaatcactgagcgctgcaccggaggatctgcacgaccttaaacacttctttcaccgccatccttcgaacgggatcctcg

ttcttcactttcctgtcgtggaagaattcccatcgcgtcttctttccgatatcctggtgctcctctcgcaactgcagctgccaattgctgtcttgatg

ggtgtcacttcgcaggatgcctacgagcaggcgttccggtccatggaagcgcagggctcattgcacatctcgcgctttgccatcacttcaggtggacc

ggatgaggtcttcggcagaattgtgtcgcagatcttcttggatcctgtcaagcccccacccttgcatctcgatcatgacacgtacgacttcttgcacc

accaattcttcggcactgacaatgacattgagtccctctttgacgccatcgacctggccatcttgcatcactttcataccgtcccaacatctgccttt

tgtgggcctcccataccagcacacctagcggaacaaacgacaacgtccccggtgccagcgcaagggtggttgccaactttcgtgcagggacttcgaga

ggaggctttaagcccctttgtcgacttttcgcaagacgacgaggaacaagacgacgatgacgacgcacctgtaaaacgtcctcacatccgcttggaac

agctgcaacatgttccgaaccccatcttagtctctggcttcacctttccggggccgaccttcgaagaaacggatggagacaaccgcaagacacacctg

ctcaagatggtacacaacgacagctacttcctgtcccagctgccgtccttgcgtcattgtgtggatctgcatatggcatggcaagctcgcgctatcgc

tgtgctttgctgcattcaaagttttctgcaaaataaggacatcacggagccagcggcgtggggaaggagaagcgcccttcgagaagctcgtgatgcca

tgttcgccatggccgcagactccaataacaacctgttcggtcgcgcccttgagggtgaactcggctccacgctgaaaaagatggaagtgcgaatgctc

gaactcaacgccaagcaatttggaagtctcatcgagctgctcacctgggtcttgtttcgacaaaccggaaagttctacaaggcaactgaagaacccgc

cgacgatgaggggccggagcttgaggagcgccaacaacctgagctcaacgaggacctagcattctatgccgacttcgtctttgcacgagatcaggaag

gaagcgacctttctgacctgcacgagacattacaggactggggccagcagctcgaagactggcgttcggacgaagacaccgcccgtcagggggctgtc

aagcggaagagggaagccgaacgaaaggctcagttggaagtggaggagttcgtccgcgcggccgaaatggaagaggaggagggtcgtcttcccaaaaa

cgtgacggaggatggcaaagtgaccacgtcgatcgacggaccgctgctctttgctgaagagcccatgacgaaggagaagctggaaaagcaatgcaagg

cgagactcgacgctatggacgtagacgagagggatcggcagatcaaatctgatcagcggttctacggtatccgcaaggacatctcgaaggagattcac

tcttttctttcgtccaagtgtcagccacttcgaacgagtgcaagcgtccctggagccacatcggccaacaaggatgcggctgcagcgcccttggcgtg

gatttggcagtgccggtcatcaaagtatcttctagatcatctcgaagcagctccccgacatgacgttatcacacacctcgacaacccgttcacaactc

tggaaggctacggaatggcccaactcatcactggctcacagaggggcgggagcggtcacagtcacagtcaaatcgacgacgcggacgagctgggccca

gcacaaccgggtacgatcaagttcatcaagagcttgaaagacctgagacggtctggtgccttgccggacgtgtgtcgagcatatcagctttatcgaga

cagtccgcggttcttgaactttgcggactggttcgacgccttcgcacaaggagtgcgagcggactgtgcattgctggcagtctccgacaaggatcaga

acgatgttgggggtctagatgacgaagaagttgaagatgacgatctcccggacgccgcagaacttcgcaacgggcgaccgaaacggaaacggaaggac

gacgatatgacgcccaccggcaacagacggaagtctgctcgggctgggcaagctgtggcattgccttcttctgcgacttcaacgaggacggagcacca

agccgaaacagtagccaattcgaaggagcaagaagacatccaggacatccaggcgcgatttgcgcttgctgtcaatgagctggccatcatgggcttcc

tcaagggcacgaggcgcaaagtcgagcatactacgaaggtcatattcgacctgcctggcgtgtag

>contig_14.contig_14.g13.t1

atgccgtcaaagtacgaggaagaagaagaacgagtcgcgtgcgcgttggaagaggcgctcgcggccgaccagcccgact

tttccgctctcgcgaggaagtggaaagtcaaccgcctccgaatctcacaccgatatcgaggcaagaacagcagatcgacgcgcccgccgacgaatcgg

aaactcaacgacgatcaggagagtgcgctgctccgatacattggcattctggacgatctggagctttctgcgcgtcccgagctcatcatttcctcagc

gaaccagctgctctccgcttcccaccaaggatctgatgcgcctcctgttgtgggcgaaaaatggctgccaaatctcgtcaagaggcacgcggagctac

acacggccaagcaaaagtctcgggagctgacgaggatgactcaagaccggacgacgctgagcaggttcttcaagaagctcaaaacaaccatcgaaacg

cagggcatccagccttgcgacatctggaatgtcgacgaggtgggatttagggtcggcatcggaggcaagcagtggatcctcactctgaatccaactaa

ggaggcccaccttgccagcgagacctgtcgagactcggtcacctgcatcgaagcagtcaatacgatcaagagtggcttccgactcactggtatccacc

ccttcaaccccgacatggtcttggtcaagtttcctgagacgaccgaagacctaccggtgaccctgccatccggccctcattcccccgcagatgtcccc

accacgccaaagaccgtgcgcacactgtctcgcgttgtccacgacattgaggagcgacacgggaccgatctctcgccgacagtgctgaaatgcattcg

tggtggtttgattcaggctcagacaggcagtttggcggtgtcggcaatgcgcttccagacggccgcacaaaaggaacggaacaagcgcacagagcgac

aaaggaaggtgactcaatcaggaggggtgttgtatgcggaggaagccagggagattgcggaagagaagttggcaaaggaggaaaaggaggaagcacga

cgagtttgggtgaaagtcaggacagccatcatgaagcggaaggcagatggatggggcaaagtttatgtgcaaatgagaaaagatgtcaagcgccgccg

caggatggcttcggtcgagtcccccgacagtgcggacgaggatttactcgaggaggacgtgttcccatcagcctcttaa

>contig_14.contig_14.g14.t1

atgccgcctcagcacgcaccggcatggcaggcatccggcggcccctggggccaagcctcctccactccttggcgctctc

gtcctccgcaagagtcctccgcagctccgaatgctgtttcgaccactgctacgacgaccgacgagtccggcgtcaccccacttcaagtcttccgcgcg

atgcctcgcgaggacaaggagaccttccgtcgcatcctgggatccatgggcaccacgactcgcgatttccttcggacggttccccttgcccagctgcc

cgaggacgccatccagcgcatagccgtcccactcggcagcaccactccactcaaccctcttcccgacgcttccgacttgggcatcgacgaggccgatc

ctgctgccgaggacaccgctgcgtcagctccggttactgtcgctcctgcatcctcctcgacaccagcactcaactcgtcgcccgaccggtcggaagag

gaaccgctgccaccatccggtccgccgcctactagcgctgccccagctgctccggctccggtcgtcatcactcctccaccaactcggccgctcatctg

ccggccagaggtgctcggagagtacaagggcgacccgtcccggttggaggcgttcctgagccgcgttcgggacatggtccggtcaaacaagaaccccg

gatggaatgcggcggtgatggcgactttgccgatcgttttgaaggacgatgccgcagcatggcacgaaggcatcacgctcgaagaggccgagtcgctg

acgacctacgaggcatgggccgaggcgatgcgcgaggcatttccggtgaacaccaccacacagcgacgcgaggcacgcgccatggcatggaagcctgc

tcaggagacgtccatcggatactacttccgcaagctcttggcgctccgaacctcgtacggccacggtcaatcggatgctgctctcgtgacggacatca

aggacggcttccctacatctttcgtcactctgctgcgcatcccacgaaccgaacccaccctcaagcagctgcgcaaggaactaggcgaggccgagccc

gagtggcggtcgctcactggcatccagattgccgacaccgactctccctcgacggctgtctcgtctactcccacgccctcggccatggcggcccctgc

tgtgcgtcgagtgaccaaccagtccatggttcgctcggcctctgccccggcgttgccggtggcccccgctccatctccgcgaccggctggtgcagttg

atggcactgctgctcgatacgatcctgctcgcattacgcctgctcgaaacggtgagcgacgcaagtacactcgggccaacggccagaccattgagctc

cggcacccgtgtacccgctgccaaggcgaccacttcaactttgagcatgacgcggtcaccggtcctcaagtccgaacgattgaggacagcaccgccgc

ggccggatcggtccacaccgtctcctggtacgatgacccagagaacggcatcgagccgcagcatttcatctcttccggaggtgaacggtgtggaagag

acgtgaaccacggagaaggcccagcgcccaccgtgtacaaatcctctgtgcaatctcacgatgattccgcccatgaacatactgccctgcctgagcct

ccgcagttgttcatcgcgaacaagtccatcttcaacaccgagcgtcgattaaacccgaacgaggtgaatcctccattgtctcaacccacgcgcgcgtt

cgggaacatcgtctctcttccggtgaatcctgcgtccggttcggggcgcggttatcgctctcatgtccctctcaccgtccaggtccgccttaacgata

cggacggccgcgctctttcctcactctttgacacgggcgcctccctctcggttatcgatgcggacctgcttcgccgcttgggcggcgttccctctggc

cagcccatgcgcatacatggccttggtaacgtcatgacattggggtgggtcactatccctgtctttctgcatgcccaagaccctcttggcaaggccac

ccatctcgccttcgcccaggatttccacgttcttccctcgttcgcacccggactctgtctcggtcgcgacttcatcgatgcccaggacctctcggtct

caccggttcggggccggggtcgcatcggccgttataccttccaggtaaacaagcgtctaccgggcccatacgggaagaaacccgagctgacgacgacg

tccgacatcaacctccttgccggcgaacaacgttggattcccgtcggcgctcattgcctcgctccggacgtcgactacgccgtcattccccggctgtc

gactgttcccgacgagtccgtccgtctgctcggtccggctggcttgcttcgccatgcgcctgtgggtcacatcctgatggggaattacgggtcggcgg

tgttttgcctgcagaagggtacggtcgttgcggacgcctatgcggctcgggtcggggacgtcacctccgatgtcggagagctcttcaatttggagcct

cctcgatcttctgccgccacggccggctcacctgcgcctgagtatacgccttccgacgttgagctcgcgctcccgttggacgccttcgatctggctga

tgcctctgggtccagtcttgcggccgacgccgccatatccttggtggacgagcactttcgcgtcggtgtcgatgcgtctgggaccccacactccgacg

tcgtggcgctgcttcggagaacacacgacgctttcgcgttggatggccgtcccggtcgcgttgacgaccatgacatggcgatcaatttgttgcccggc

tccaccttgcgctccgacgctcctcgccgtgccagcccggagaagcgtgcggcgatggatacgaccatcgatcagctcttgtcctgggacgcggtggt

gtacattgacgacgctgtggtggcaacggattccatggctgagcatctccaagccctcgaaactctcctccgctccgctatcagcaccggccttaaat

ttagcccggctaaatgcaccttcgctgttccctccctggtcctgttgggtcggaaggtctccggcgcgggcgtggctgtgtggaaagaccgagccaag

gcggtggaggatctccggcggccgatcacgctccaggaactctaccacaccctggacttgttcggatactaccgctctttcattcacaagttcgccga

gctcgcctccccgctcaccaaactcctgcggggctggcgttacgagtcggccgacggacacacgcgcttggtgaacacggaagggcaggcagtcacag

caagcaaagttccattggaatgggacgtggcccagcagcaagctttcgatgctctacgacacgctgtctcacatccccccatccttgctcaccctgac

ccgtcccggccatacattctgtatacggacgccagcaagaacgctctggccgccattgtacaccaagtccacgacaccgacgcgcccgtcccttcttc

ggctcagctcaaccacctgagcctgtcacagctgcctttctccgtggcgaaggatcattggagtgcgtggttggccgaggatcggttcttcggtccca

tcctttctcggcttcgggactcgccttctgatcccgactgggcgttgaacgatggtgtcctgactcgacggattgacgatcgcgttgccctaccactg

gccgcggtcccgaccatcttgcgcgccctccacgacgagaagggtcactttgggttcttaaagaccttcttgtccgtctctcgccagttctggcggcc

cggcctgtccacgatggtccggtcttgggtgaagcactgcgggacctgtcaagcgacgaagcaggcaccgaagaccggctcgttggacatctccaacg

acccgtcccttcccttcgagtccgtatcgtttgacctcgtctacggtttccctcggtcccaggcaggcaacgacaccgctttggtcattcaggaccaa

ttctctcggatggtcctccttcatccctgtggcaaggacatctctgcggaaggcgtcgcggctatcttcagcgaccgagtcttgcggtacggatggcg

gcctcgtcgcatcgtgaccgattccgaggctcgcgtcagcggggctgtcatgaccgccgtcgcgacgtctctgggcgccgagaccgctccatcgagtc

cctaccatcaacaagcaaacagtgttgagcgcgctgttcagacgatccaacgtgtcttggcgtcgatggctttggactcacgggctcactgggaccgt

cgtgtcttgccggccgttgaattggcaatgaacagttcgccggcgttgacgaccggctatcggccttttgatctcgtcttcttgtcgcaccccgacgt

cgtacatgcggtattcgacgctggcgaacacctgggcgtgggatccttcccggagcgacttgccgcggcccaggaacgcatcgacgatgctcgccgcc

agatcgctgtggctcgcgacgtacagaaacgccgatacgacgggagacgcgcgtccgtcccgcctctcgcgcctggaatgcgggcctggatccgtctg

gcggatcgtcctgtcgccggtgccggagtcgacaagctcgccgccaagaagctggggccattccttgtcgaggaggtcgtcagtccccaccgcgtccg

cctccgtcttccggaccatctccagatcgatccggtcctgaacgtcgagcaactcgacttcgctcctaccgacgtagaccccttcgcggccgaacgat

ggcgtctggctgggccggttcgttctgcggcgacggatgacccgcataggattctcgacccttctgcgttggccggcgcggattcggatcgtgctgcc

ttgccgtcttctccggacgggcttctcgacctccttccggctgagctggcggttccagcagacgggtcgactcttgctgccgagcctcgtgtccgtag

agcgccccggactcttcgcgaatttgatctcggcatggtctacaccgcggggactctggcgaccggtgctgacgtgttggccgatgccctggccgggc

cgatcgatcgtcctcgccgtctcaacgtcgatggccgcgagcgggtgttccaggaacggcccgtcgcctacctgtcccgccttaccactccggcggag

agcaagctggtcgcccccgaacttgagctcgtttgctttacctgggcataccacaagcttgctcatctgttcgagggcgcactggtcacggtcgtcac

cgaccactctccgatggagcgcatgcttcgatccaccaatgatattccctatgggccgacgatttcacgctgcagggccatgatcatgccgcatctcc

cgaacctccgattcatctatcggcctggcgcactgcacaccaatgcggatgccctctctcgactgccgccagcctccggttctgagtgctga

>contig_14.contig_14.g15.t1

atgtccgagacatccgctcacgaggcacaccaaggcgctgcctcctccaccacctcgaccccgggcgtcgacgaactcc

acatctcccttgtgcgcatcactcagactctagatgtcattcttgagcgtcaagacggtttcaaccagcgcctgggtcaggtcgaacattccggcctc

atctctgctcctctcgaccaaggttcgatgactcttccttccgttctcggcccaggtccgacaaccactccttccattctcggcccaggtccgacatc

cactccttctgtcctcggcccaggaccgacaacgactccctctgctctcggccaaggcccgacgccgacttcctctgctctcggccaaggcccgacga

catttccttctgcctcggtacacgatccaccaccccacccattcgtcaacacccgtcgctaa

>contig_14.contig_14.g16.t1

atgtcattcacgggtgagctggacgtggcgtattttgtttcagcggttttaggtgccccgatcccctggaccgaaaaag

aaaactataccctgcctggcgacgggccaatagccattgccctctatcccgacttcatcgctggttttttcaccaatacagagattggacggacacgg

cgaccgctggcgcgagctgatccggaccctgtttcggtcggcagcgtggccgtgacctccagcgcgtcatcgagcgagcagaccatcaagctcggcga

cagcaagaacggcgacgacaagatccaggacttcctctacaccaccttccgcgcctcgcggaaacctttccaagctcaagccttccaagcgccaggct

cgcgaggatggcagacaagaacatccacaacatcctctcgaatctcgtcctcaccgcacgcgacttctggcggtggcggacgtggtggccgcggtggc

ggtggtgagagggggcgtggtcgtggcggcggacgtggaggagcacccgctcctcctcaagctggacatggacagcaggcatctcacccgatcgcgat

cctgtccaacatcggcgttgcggatcgtcaagctttgcgcaagcacctgtatcgtgttcgacttcgcctgactcaccgcccttctcgtcgcttggaga

tgttcgacagcttgacagccacgccggcgacgcagaccaagttcgatcggaacggcgaactagtttccgtcgctgcctacttcaaggacacgtacaat

tacacgatccgcttcccccacaagtttcgcggccagatcggcttcgttctcacttctcctcgagctcagcgccagcaaagcgtggtgcagctccgaaa

cgaggtggttaagtacgagcaggccggcctgctacaggcttgggaagtgcaggtctcaaatgagcccgtgcgtctcgaaggccgtcagctgcccccgc

cccgcgttatgtatggcctcaacaagaccatggaacctgatgggcaaatgtcaacgccggctggagagcagctcatcgagtaccgcgatgcgctcaac

tccgacgctgagaccaccgagtgtctcgatgctctgtcagacctcaacgaggacgtccccgagctcctcgccgacggtgtcagcatcttcgccctcaa

ggccaccggtgttgtcgacggtgtcttcaacgtatggtttccccgccttatcggccagatccagttgatccccctgctccgctgctagcaaatcccag

aaccagcacaggcacacgccttcttcgacgacgccgttgtgagcaagaccgcccccgaggagaagagtgccgaatccaaggcagcacagtcaacagtc

acaagcacaacaacgacaacaacttctgttcagcgcgcacacgatctcagcatctccgccctattcggggacaccatctacacctttggcgagctgga

ccagtccgtgtgcatcgccaacgaagacgtcgatggcccacgcgctttctcccctctctctgagggtcagagcgaaacccagtcacgaccccagtcac

gcaccagccgctccaaggaccaaggactctggcttgccgcccagaacatgaagatcatattcggtctcctcgcaggcagcttttccacagcacagatc

aaccacaccaccgatttcgacaagggtgtcgacaaggagaaccttgccaagcttcgtgacttttacgactcgtgcatggatcatcacgcccaggacaa

ggttggtgacgatcccactctcaagctggtcagcgagtcctggagccgactgaccgtgcgcgaccctactaagaaggacattggcatctcaacctcgc

ccgcccaggaagaccctcgcccctacccgtgtcagggcaaacgccagccgaagcctacccaagccattgactga

>contig_14.contig_14.g17.t1

atgacttcatcctcgggcggccacaacggggcgcattcgcaaattgatggctcgatcggcaccgctgccacgggaatag

ataccaacatgagctcagcccgccgcccgctggatctgccgttcccgcgtgggaccagcacgtggaacaagctggagaagtgggtcaaattcaacccg

gagcgattgaaagcgtgggaagacctcaatcctacactggccgcgcccaatggaagccactcaggcaggatttcgaagttccatcaaaattcaacacc

ttcggtccgccaggagatcgaagactggtggccgatttacctggtcactgcgccgcacgaggaacgaatgacccacttcaatcatgcccacaaggtcc

ttctgaaaatgttattcgacatggaacaagattacggggtgtgttccttcgcagtcctcgcgcatcctgatccagatgtcaaggttggcatagttgcg

tcggagaagagcgaagatcttctggacggggtggtcaaaaatctgaatgcgacgagcaacgcttcgaaattcgccaatctgtttggagcggccaccaa

atcaaaccctccggaatcatggacgcttgcgacacagaggctgaacagcatgacggaagaggacagcactgccgcagtctcgactacgcagaaaattc

gagcaaacatcccgtccaagcttgcagccaaggacctcgtgacagctctttcgggatgggttggctcgggtatggacttcatgagcgtgcagaaagga

gatgcgtggagggaggaaacgaagggcaaaatggtgtataaaggagtcttcaccaagttgagagatgcgggattgtacgtggagggatggccgatcga

agccagacatatgctcgcgcaggatgtcgtagttacagtccagggctcgacgacagcatcgacatatactatcacgtcggggagcctgcacaacactt

cgaaatggaactcgggggatgttcttgcggtccaggacgctctaaaggcgagaacgatgatcttgaaacgcatcatgtaa

>contig_14.contig_14.g18.t1

atgtcccagtccttttccccatcctggaaggacctcggcaagctcagcgaacgactttcgcaggggaccctcgagtcca

aattcgaggacaagaagaacggcattgccgtcacacaggcaattgacgaccgccaacgctctctgtgcccaagcacggcaaggagagaattgattggt

ctcctacgaatctgtagatatgtggtccgcgaccttcaagacgaggatcagaccgagcccctggaagcaatcgtgcccctccccgagaccgtccgaac

ccagccctcacacgactacacctacggctcccaagtgatggcgcttaacccggacacgacgtgcctgtaccgcgcgaccatcgtctccggtggccggt

gtctcgtcatcgtcgaaaccccggccagcctcgcagcccatctgctcgtaaccaacccagatgcaaaggctcagggcgcggtcatcgcatacgacgga

aggcacggctccaagaatgttcgcagattctgctacaggggagcacgcagcacccgcagtccaccacagcagcagcatccaaccaagcaggtatcgac

agcgatgagtgcagcaccgatgatggccagcagcagcaacgacaacagcaacagcgagcaggacatcctcgtccatctcgaggcagttctctcccagt

ggttcatcatcgcattcccatcgcagcactccagctactcatccacaccctccatcagcacccactacatgcatcccatccagcacacagacactctc

tcacgaggcgcgcggctggtcttcgactggatcgtcctcgaggactcctacctcggcaagcaggacgaagccacaggaagcgcaaacatgctctgccg

tctcctcgcactcgcctccccagcactcaacaacgccagcagcccagtcctgctctcctcacccaccctcctcttcctccgaacgacctcgcctgccc

ccgctgacatctgctttgttctcgacctcgtctcgtcctgggtggttcgagacatcgccgacgtgagtcccaatagactccgacaaaaggccctaacc

caggccgtctccgtccttgtccccgatgtgcccggcaccagcgtccccaacggtgctctcgtgagctacgacgtcgacggagccttggtcgaaacgca

tctgcaagaccgtacatcatctccgacgagcctctcgacatcaagaacgctgcctcgctctcagagcagtcgcagcgtgggcaactcggtcttcaaga

acacaaccctcgcctctctggccaagcgcgccaacgtgggtctgcacgtcggctactgccttgggtctgaaatcgtccccttactggcgacactggct

atggccatcaacatggacgtctatgccgtggttggagtcacgctcgttagtgctcatctcggtcaacgagggcgacatcgtcccgctaccacttccga

cgccctccagctcgccttgctccagcatcatcaaatgggcgtcctcgtcgaggccctgccttccttctcgtcgtacacgtgcacgcccagcttgccca

ctggccactgcttctctttccctctccgacacgacacgcccctatccacgcccaagtcgcaggtttgggacgcgatcacggtccgcccgagctcactc

gaggacttcagcatgtacacgcacagcttcgccggttctccctgcctacttctccctgcggccccactcgagaccaagggcgccgaggaggatatcca

gtggtacgtatctgaattctga

>contig_14.contig_14.g19.t1

atgttggatcaatcattggagaccgcgccgcatcaggacctcatgcaaacgaccctcaacgactggttgagcgtcatgt

cgggcgacgtttccttcgattcctggctagcagctgctcccctgtctcagtctgggcgcactgccgtagctggtatcagaaccgccagtcagcagacc

gtgccgcatctccttgactctggatcccacgaggccatggattcggacagtgactcggacaacgatacgccaaacggtgtcctcactacggacgaccc

acttccagctccttcgaccgcagacttacccgcaatcactccctttacctttgcaattgtcccttacctgccgtaccccaagctcagcaagaacgcgc

gacagtccttgaccggaatgtcgaccggctacatgcaattctaccacatgaagaattgggaacctcaagacggatcacagcctcagcgagaggtatgg

cagtgccgaatttgccattcgaccttcaaagtcccatcaacaaaagtttcaaacctgggaacgcacctctatggcaccgccaaacgacctggctgctt

gtacaatcgcggcgacaccccggccgagcaggtgacccttcctcctctggacgccagcggcagaatcttgatcaccacatccatcaacattcacctct

cggaacccaccgacgactgtctttcgagtttcgcgcgctgcgctgctcttgctcctgctcctcgcacctccacgcccgcccgtcctctggctgcttgc

tcgccggcccgtccgcccatcgccttctcgtcctttcggtactcttccgctcgtgtcctcgttcgcgctcgtccttccgttgcctcttggtctcgcgc

gctcgcccgggtgctccttcgttgctggttcgtcgctgttcgtccggctcgttcgtttccggcttctcgtgcgtcggtcggccgcctctcgcttgttc

gtccgttgctgttcggtcgattgcgcccatccggcttgcgtgccttgccggtcgctcgcgctcgttggtcgcacgcgctcggccggctgcttgcgcgt

ccctcgccagtcgcgcctgtcgcgcccgtcgcgttcttatatcggctgctgcttccacgctgcctgccgctctcgcgcgctcttgcgctctcgccctc

ggcacgcgcaagtttgtttacagtacacgcattccaccatggcaccgacgcggaagccgcgcctgtcccgcaaggtgacggaggccccgccccgccca

gcacacaagaaagcctcagcttcggtcccagcagtgaggctgacgattgggtcaattttgacgatgctggtcctgaagaaaaccttctccctttgcct

ttctctgagcaggttgcgcctgggttcaatcccaggggcgacaaaacgacgatcctcgctcgcctcctcaaactacgaaaaacccgccgagcaaacag

aaggaaaacacatgcgttggaggcaactgaggccttccaagaatggtcgagactcctcgacacgctggtcgctccttacatggccgcgacaagacgtg

tgctgccgggacccgctgcttggaacgatctgaagtgcgggtgccacgagctgtattccgaagcgtctcaggcacaagcagctcccggccctgccatc

gccgtgttcgactttgaatcgatacgatatatacttcgcggtcatgcgtccgacacctaccttccgtccttgtccgtgcaggcctctttccagccact

ccctccaaaccacaagcggctttctcgtttggacatggccaggcggcagctgcagtccgcgtccgagtggtttcgcgccttggagactcgaagccgcc

tactctcacagaagctccaagctcctccgacagcactggcgctgccaccggcgggtccaggcttgatttctgcttctcaagctccagcatcgattcag

ttccgacgccctttactcgaacacgacgaccttcaattgacgctgcatgatctttgcgcccggtgtcccgcctgttttggtcgcttaggagacgtaga

tggtcagtctggtcggcgtgcggaggtttcgccgcaagtcattgtgtgtctggacggcaatttccagcacaagcgtcggcgaagaatggacgccttcc

gccgcaatcctcaccctccgacttactttctttcagaccaccaactcgaacaagcgaaagaatacttcgaaagcctctcatcccagcttggacccgcg

acggggtgtggctctcaagttaaagcagcaatcgacaatatggttaaaaccactctggggccattcgatactggaggggttatgggaatgacctgccg

gcacggcgctccccttctctttgccgacgtgaaggagtctggagaagcacactacttcgcctacgcgctcctcgaccatgtcatcacggcatgtggcc

ccactctgcgaaccctcggagtctgctacgatatctcttgcaagatcgacgtgtcccctcgaatgaagaaggctttcgaccagcggccgctccagatc

cactacgcagtgtccttgttccatgtgtacggccacgactacacctgtcagctgaaatacagtcctcggcgcatggcgggtttcggtttgaccgacgg

agaatccctcgaacgcctttggtcggcgttgaccgaccttgtcagccttacccgtggtatgatggctgtccgccgacgggaaacactttgtgcgcgcc

ttgcgaagctttcgacgcagcaccagtccagctcaatcatgctcctggcaaagcgtctgcaaaaggtcgccgcattattccagaaagaagtggataaa

gtcttggaacttcacccctcgatagtcgacatcaagatggccctggaacagatcggaccgctgcagtcaaccacaacatcactcaaccccacacctgc

ggcgtcatcgagctcacaatctgccttcacccccgcggaggtccccaaagattgcgacttctttcggcggatcggactcccggaagagctggttggca

ttgcggtgtatttgcacacacagcctttggcagacgacgccctcgcgaacggcgcatccgggcgagcactgtcgatcacagcagccgcagggtcagac

tggtcctcattcggcggtcagagcacagccacagcgacgttcggtggtgcatcgtcatcgtggagcacagcagcagcagcgtcggacaggttcgcgtc

tggtggtggtccatcttcagtcatcgcagcagcagcagcgtcgggcgggttcgctgagctccgcgctccgcctccggcggagccctccgtcggaggag

tgcggagtccgcaaaaccccagctccgcgtcaactccggggacaaccttagcgtctacagcagcagcagcaacgtcgggcgggtttgcgtctggtggt

ggtcggtcgtcagtcacagcagcagcagcgtcagcggccgaccacgctcttcaacaggcgcggcaacgacagacggctgcatcggatcacctatccga

gcaagctcaggcactacacttaccgctgtcacatatgttttcggacacatcgtcctgtaaatactggagtataataccagtgggatcaaacttcctcg

ccgacttggccacaattgatcccgttcacatcaacctcagcgagctattcaaacgagagacgctaatttaccttgccggacttatagatgcacccgac

ttcacgacagaaccttgggtgtcgaacccagttctggctgatgccatggaccgaacggaactcctactccgcctacacgaagaacgagaacgaatcgg

aatcgaagttgaaaacgctttctcgtggctaaggcgagaacagtcacggctcgtcaaccttcagcagtcagctttggaaacgcattggcagccccact

ccgcggccatctccacgctcctgaaactgcagctcgctgatccaaaggcgagcgcatcaattgctgaagggtccgattctgacagcgaggcggacgat

atctttgcgttggatatcgatgtaacgtctcggactctcaatccacgccgggcagcacggcgtccaccgcaacgtaacgcccacagcacatcaaatca

atgcacagttgaagcggaatcgaggatcgacgacttcttgcgtcaagttgatggtttggaggacgacgaagacgattacggtgacgaatcaacgccgt

gtggatcggatgacgaggagaaggctttttggcaggccagtttgcaagctttactgggctgggttgcgcccatccctctgggccatggctataagaac

aagcttgttcgtgtctcaccaatacgacacatggctcgtacgaagcagactgcaaagaagtctaccggcggtatagcgcctcgagccgcgaccacggc

tgcgcagaaggccgaatccatcctcgcagtgaagcgtccgcaccgctacaagcccgggacggtcgccctaagggagatccgtcgctaccagaagtcaa

cggaacttctcatccgcaagctccccttccagcggctcgtcaaggagatagcccaggactacaagtgggatatacgtttccagtcggcggccattggt

gccctccaggagtcagcggaggcatacctggtctctctcttctccgacatgaacctcgctgccatccacgccaagcgtgtgacggtcatgccgaagga

catgaagctcgcccgtcgtcttcgtggagaacgcaactga

>contig_14.contig_14.g20.t1

atgcacgacggagttgctgcttactcaacgcgctccttctcttcctctggctcctcttcctctggctcctcttcctttg

gctcatcatcctcgcaggctgccttgcctctcgtcttcgtccgtccttcgatcagcgagtggacgaaaccgcttccctttttccaaccccctcggact

ccgagcctgcccctgccccacgcggcgcgtcaaggctttccttcgccgcggagcaaagacaggacgacggagggcggaacgggaggggaagagcaggt

gtcggtcgaggaagcgaggaggagaaggaggaggaggacgggaagggagcacgccagcacggtcagtggatggagtatcttcagcagtagctcctctt

ccggctccacagtcagcaccagctctcgcgaaggtcatggcttgacatcgcttccgccaagtcctcgaagccgagcgcgcacgcaccagccaccaatg

gcgcacatctcacccgtgccgaggccgagcccgacctcgcaactgctgacgattgcgatctag

>contig_14.contig_14.g21.t1

atgcgcctcaagatcaacatcgctgcgctggcgacgttcgatgctggctcgggcttgactcgcaagcagcgtgagcttg

tgatcatgaccatgctcgtcctattctacctttccattggggccttgttttattgtctcttgctcaagatccgcatcctctttgtgcatagggcgtgg

agatatccatccaactactacactactacggctccaaggatcgtcctctttttcctaacccccgttggcattgttcccgtcgccttggtgatcgccac

cacgagagaaacagtactgggacagacggacagttcgaacagagccagccctatcgcgcgtcgctccaatccctctctgagagcatcagcagcgggag

gaggaggtagtggtgggttttgcgccatgacggcgtccatgttcgccgcagcctcactcgcttcgtcctccaatagcagctcggccgcttcctcgccc

tccattctgcgcccgccctctgatctgtcgtccagcgtgggatccaatcggactctcagtctgtcgatcaaaccccctctgttgtctccggcacccgc

gcgacgacctacgctgactcgcagccctgtcgcgcctgttcgaccgccgattgcaacgagctcggctccgcctgcactaaacccgacccccatcatcc

catcgctgctcttcctcgggcccgcgccctcgcatccggcgcactgggccggattgagggcgctgggcgtgaagagggtgttgaatgtggcggaggag

gttgatctcgatgttccgactgtccatcctggaaagggacggcgcggtggtgcaggcacggcaggggaagcggaagcggaagccgaggtcaagtgtag

gaaggttggcattcaggattatgtggaggaacagcctggggaggatatgctccggcagggatctgagttcctcaatgagagcatccacgctcaagagc

cgatctacgtccactgtctcgcaggccggtcgcgtagtccgacggcgatcatcgccagtggcggcgatgagggagagaaggagctcgttgctgtcaaa

gtgaatgaagggctggatcaagaagcaaaggatgtggaaggaggggtggtagggatgcttacgcgctcggcgcctgtctcggcgtcgggtttggagca

cggacatgagacgcacgtgtcaggcacgatggcgtcgtcatcttccacctcgtgggagaccgaccgtactgctcgctccgcagcttccgccgccgtca

acggcagcgtcaatgggaagtag

>contig_14.contig_14.g22.t1

atgtcggcatccacctgcgcactctgcgaatgcgcattcaggatatccaccgttaaccgcgccagtcgcagattctgca

tcacagaccacccaccaccagtaccacccaaaccagcctgcatcacaaccggcgaaaggctcatctcccgttccgccgagaccggctcttcctccgcc

tccgccttgttccccgatgtgcttgaaggaataacggcagaagagacagccaccttccgccccggctcaacagcaatactccgatgcccacgatacgt

cgaagaggtacgtttagcttcatcggtggacggtggacttggaggcgacgaggcaatgtcatctacaacggcgaagaaacgccgtcagttcgcaccag

accagctcccgctgcaacttgcacgcgcagcgatgcatctacactcggacgcttcgcacctgctcgagtcgcaacggcacgtccttgcggagtctctg

agctcggctccttcattgcatcggcgactgcgacggcagttgcgcgtggtggcgtccggtgggaaactcatgcaactctcggcatcaacgccattgcc

gggacatgtcatcatctccttcgacctgcgcccgacgccgaggagcgggcgggatgctacgaagggccagggggaaggccgcgaacagactacaaagc

aaccgccccccgcggccccgccggcctatggtcatgtctgctctatcctcagaacccgctgcgacgcgtttgaaatacgaagggtccgcaccgtgctg

ttgatgcgatgggcgacatggcgaggcgtcgccgaaaatctgacccgacacggcctacccatcaagccgtggaaggttaggcgcattgctcacaagca

tggcatagcgcgctatatcgcccgcgaacgtccagcactgagtgttgctgcggtcaaaaagcgaaaaatctgggcaaataagaaccggattactgact

ggcgccgaattatcttcacggacgagtgttctttggacacttctaaccttaggcgcaagcccttcgtcagccgctttcgaggagccgctgcggttcct

caaaacacttctaggagcttcagaacggctcgcaaaacggttatgatctgggcagcaatcgcctacgggaagaagtctccactggtacgattgtcggg

aacgggctaccgcaaggtcgattcgaaacgctacattgagcacgtcattaatggggccttacgcccctttgtgctgcagtgtgagctcgaggagttca

cgccctacaaggtcgtggaagataacgcttccattcataactcgaaggctaccaacaaggctcggaaggaggcggatattcagcgccattctcacccg

ccactttctccggatctcaacccgatcgaaaaggcttggcgcctgctcaagtcccgtcttcgtgctcgtaacctcagccagaccaagcccgacgagct

ttgggaggcggccaaagaggagtgggcaaagatcgaagtagctcatctcaacaagtacatcgacgatatgccccgacgtgtcaaggtcgtcaggcagc

gccacggtcaattttccgagtaa

>contig_14.contig_14.g23.t1

atgctgaccgcgtcccttccctttccttttcattgtcactttccaccccatctcattccttttcccacactgatcaaga

tcacctcgtgggccttggtgctcggtgcgctcgccagcagcaccgccgcaggcttcgtcaatcgccaacctactgctgtcttcaaccttacgtcgccg

ttgggccgcagtttcctctcggactcgacggctcttcgtgctcggggtgacctccccagcggctccggctgcaacttcgacgcagactgcagcacggg

ctggtgctccttcgacttcgagatcgggaacaacgcatacgtcaactatgcctccaagacgtgtaagccgatgccgtacagcgggccttgctcgtcca

gctctcagtgctcgacagggaactgcgactcgacgtcgtcgacatgcgcatatgccaatgaccccgcccgaacgtgtacgtacaacacggactgcgaa

agtcagttttgcagcaagggcatctgctcgctcaatggaggtgacgcgtgccgcaacgacgttccatgtggtgacaatggctgcaatagctttgctag

ccctcagcccacctgtacccgccagccagtgcgaaattcttgtctccggaatgctcagtacatcaacaacgtctgtggtacgggaacgtcaacataca

attgcggcttcagcttgcaagccgacggatccatcctgcgcagcgaacggtgttggcgctcggattactgccgcttcgcagggatcgggacttcgtgc

gatcccgactactatggcggagactgcgccaatcgattcaacctggactgtgatacggacaccaatatgtgctctttgggactggcaggattccgatg

ctattccggatcggagtgcctgtcgaacgtctgtggtcagagcccctacccgcagctgggatcttccggccacgtcgagcagcacatcatccacggca

ttgtcgagcacatccactacttcctcgcccacaagcacatcgacctcgaccccgacgacttcttccacttccacttccacttccacttccacttccac

ttccacttccacttccacttccacttcgtccacgactacgtcgaagcctgcgaccgcaagctcggtctcttcgacctcaagttcgaccacgactacgt

cgaagccggcgactacaagcaaagcatctacaacatcgagtttgactactgtcacgacgtcgaagccggcgagcacaagtatgagcacgagtgcgagc

gcatcgacgacgaagtcgacaaccagttcctcatcgtcgacagccacgtctgggtcgagcacgacgaccaagcctgctacgacctcggccagtactgc

gacatccaaatctgtgtcgacgagcaagagcagcagcagcaccacgtcaaaatccagcacattgtcgaccacctccaagtcgaccacatcggtgacga

ccagcaagcccacaagcacgtccacttcttccaccacaagcaagtcgacttcgacttcgacaaaaaccactcccgtctcgtccagcacgagccgagcg

acgatgacgacgaccaagtcgagcacgattatctctacccctacgcctaccaagacagtcaccacaaccacgacgaagacaaccacgaaatcaaccac

gacgacgacagcagcgcccttgccgctcggcgctctctgctcgacgagcccgtcctgccaatcgggctactgccgcgccaagctcaacgccgacggca

cgcgggcgtcgcaagcgaattgtga

>contig_14.contig_14.g24.t1

atgccacctcgcccgctggtcaaccagcaaccagtaccgtcctcttcctcctcctccttatcagtatccgcctctctct

ccacagcaccagtcgcaggcgctcacaggaacaccctcaaacaaactccatcgccatcagcagcagcctaccgcaggaccgtcgcacagaactcagca

gcatcgcccgcaacaactgcgcccagttcaagatcgcccagcaccaccagcagtcaaggcgcaaccacggcacatccctcggccatctccgcatccgc

cgctgccttccgagcgagactcgccgagaattcatcacccacatcctcacgtcgctcctcccatggcgcacacagcaacagcagcagcagccaccatg

gctcacgtatccccgtgcgatctcccccaaacacggtaacagcgccctccccgcccgatcccgcaacttcatctcgagtgagatcatcttatctcacc

cagaatggaatcgcgcggtccggccgggcaccatcagcttctcaacatgaatcggggagcgccatttccccatccgggtcggcctcaacttcaacctc

ggcatcaacagcgagaccagcgaatggcaggaagagttcctatttcgagatgtactaccccgcaaagggagcaaaaccagctgggtcacccgcagcag

caggggccgcatcatcatcatcatcctcatcaaccaagacggcatcggcctccaggcacgaatccagcagagccacaacgggtgccaaagcgagtagt

ataccatccaaaacagcagcgataacgaaaatgccgtctgcctcctccgaaggatccgcagtccgcacagccaaggcaaaccgtcgtaaatcaggcgc

cccgtacgctcccacgctcagcaacaacagcagtcggagcagtggcagcatctcacacggcagcgcaggtgggagtaccaatgcgcattcgtcctcgg

gtgggatcccgtcttcctcgtcctccactactaccatgccaccggcatccccggcgtcttcccgactcagcgcggagcctacttcttcgtccggacgc

gatttaatgctcttggctcctgcgcccccgtccccgccccgctcacgactcccgctctccgctcggtcacaggcgccgcatgaggaggtcgccgatct

ggcaccaccaccaccacccaccaccaccaccacttctaccacggcagcagcagccagcccagtcctgctttccccgaacacttcttccaagtgggtct

cctcggcttcctcggccttgtcctcggccaccaacaccaacacctccccagccgcgtttgagccgccggcgaactcgggttggatgcgcgtcgcagat

accactcccgagcgatggcgccagcaacggcaacagcaaccgcaccctcgctccgcatcttcctccgctgctgcgttcagcaacagcagcaacaaggt

acccgaacacgcgctcgacctgtcctcctcctcgtccgactcccaatattcctcctcccggctgacatcgacccggtcccggtccttcgggaacagtt

cctcccgcccttcgcctgcgtccagtccggcgccgttgcccttaccaccggcaccgggcaggaacggttctagtggtggggggagcgccagcacgccg

tctacgagctcgcggtttcagaaggtcgttaaggagcctgttatgtcgattgatgagattatcagggcgcattcggctgcgcttagaccggttgggtt

gtccagaccttcggctggggcgggggcggcttcacaagcgtcctcggggatgggggggaaactttctccgattcctcattcgcctgcgttggttcggt

cgcggacgcctctgccgccgtctgcgagggtggtctcgcagccgacgacgcctatgcctttccccttttcttcgtcttcttcttcttcgttggtagca

gggccgacgagggtagctgatctgtccccggcaggggcagcgtctggcattcctgtacttgcccacggcgcgaaggagagaataccgctcggcgggga

tcgatctcggtccacgaggctggtctcaccgttctcgtcgaggagtggggatgatgcgtatgcgttgatgaagagcgcggcggaggaggaggcggcgg

cggcaggagcatcagtaggagcaggagcaggagcaggagcgagggccgggaagcagtcgttgcccttcttaaatacggcgaaggcgcagcggcattgg

gatcgggatcgggtgggaggagcaggagggagcagcgcggatctgccggcgtccccactcagccccggcgacggaagccggcgtacatcgcgcccccc

atccttcgtcctcagttcgccgaccggtgtcgagttcctcgacgtcgacgtcggcgtagggtctgtcgtactcgacaggcgcaccttggccccgacga

cggcgacgagcgggagtaattgtacgagcgcgagcagtagtcggacggcgtcgttctcggggactgcgttgcctttgtcggtgtctcctgctgctgct

gctgcggcggcggtgatgggaggaggaggtggaggtgggggaggaggagcgcctgcggggtatcctacgcttgatcatctcatgtcgacgacggtctc

gacctcgagctcgagctcgacttcgacttcgatctcggaggatttgagttcgctgtcgaactcggttgaccggccgttgtcgatccggtcggacgtcg

tcggtcggctcaggcacgaacgttcgttcttgacgcgttcgggttcgggttcgggcgctagtgggcggagtgcggctggtagttctaccatggctgtc

gctggtgggctcgggggcgggcctgggtcggaagcacatcggtcctcctccggtgctgatgctcgtgctgctggtgggggtgttcccggttcgatgca

ttggacgaacaatagcagttttgggagtgggacgagcgcgcatacgaatacgagctatggtggcacgagcacggacaccctcggccgctcggtctcgc

cttctacgatctcgtcgtcgttccggcagggcggcggcggcgcgaatggcggccatgcagggggaggtggtggtgtaagggacggcaggtcttatacg

ccctcttcgatcgtggctgcgaatggggggaggagctatacgcctaattcgattgcggggatgtcggaggttggcagctttgtgtctgctagtagtac

gatggggatgggagggcatgggaagagtaatggtgggcggaggaggagtttggttgcgcacgctgcgggcgcgggtagtagtggtcgaaggggcccac

cgacgtcgcccctggagcattcacgtgcgttctcgggctcgtcgacgggcacgcggtccacgtctacgtctacctcggcggcgagcgaggcggaggat

cagttccatgatgcggaagaagggattgggaggtcgatgttggagttggagttgtctgcgccggatgagacggtgccgatgcgggcgcctccgatgcc

gggtttcgtcggcagtacggcgacgactcctgtgccatcttcctcgggccggactgtcggtcggatcgcggatgtgaaacggaaggtgtctgcgcctt

gcttcccatcaggatctgcagcggcggcgcccgtcgttgtgaaaaatgagtcggcggcagagacggacgaagacgaagaaggaggtctgcccggctcg

aactcggaagtggagtcgatctcctcgctcgactcgatcgaccgcgaggtgcgccggacggtggcggcctcgcggcaggcgcaccgtgcgaagcggaa

tgcggagtcgaggcggcgggaggcggaggagaggaaggaggcggaggatgaggaagtgttgaagtgtttgttggggcagccttcgccttcgcttggcg

gtgcgggagggggaggggtgagcgggaacagtggtgggagggaacatatgctcttcccgccgtcttcttccgacggttgggcgtccctctctagcagg

aagggtctgagcaagcggacctcgaaccagagtgtgttgagcggcggcggtgcggggaacaacaacggaggttcgggcttgggttctcccggcagctt

gtcgcggtccggctcggtgttgtctggcacgggctcgtccagtgcgccgcatacgccgcggatcttgcagcatgctgcttcgtttgcggggaaggcga

gtcctgctttcctgaaggggaacaacagcaacagtaacagtaacggtaatggcgcgtcgaagggctcgtcggggtcggatacgctcgggatgggcatg

tggcctgcgccggtgggttcgtggtcgactcctggtacgcgggcggcgtcgccttcgaatcagggcagcgaggatacgcccaatgcgtcccactatgg

tttgagcagcacggtcctcccacccgtgccgcttggcgttggctcctcgtcgtcctcatccaagcattcccagcagcaacaggccgacgcggcttgga

tgagcaaagggaatggaggtgggcagggcaagttttcgaggatccctgttgtgggcatgttggcgcgcatgaaggctggtgcttccgggtcggagggg

ctgaaccactcgaattcgaattcgaatgcgacgagtaacggaggcgggccgagtcaacagtcggctgctatggctgtgctccatcgacgacgagcgga

gtcggctgcgagctcgctctcgggcatctcggcgcgcacggctgcggtcagtctggcgacgacttcggatggcgagtttggtggggatgggtatatga

atgtttcgtatgcgacggcggggtatgaggagaatgggggtgctggtgctggggcgaagaagaatgggcggggtaaaaatgggtttgggaggaataag

tcggcgcccccgcctctggacgtggatgggccagtgatgggccagcctgttgggacgtccccgacgaaagtggtacaaccggcgccgccagtcccgct

gacgccgtcgaacccgatagcgcagtacatccgttctgagcgcctgactcggatcatcacgctcaaacggcatcccttccacggcgtcaaggtgtcgc

tcgccgacgtaggcgccaagaacgggcacccggtcgtcatcttcctcggactgggggcggtgcgatacctggttggactgtacgatgacatcgcgtct

gcgatgggtcttcggctgatctgcatcgaccgatgggggctgggcaagacggatacggtgccggaggaccagaggggggtgctcgaatgggggatcgt

cgttggtgaagtgctggaccaactcggtattgggcgctttgcgattctggcgcacagtgccggcgggccttatgcgatggcgacggtcttgcgtcagc

cgtcaagccggatcgtggggcctgtgcatctgctggcgccctgggtgagcgcgcaggtggagtcgaactggaagtggctcaagtacgttccggacggg

gtgatccggacggcccaggcggcggagtggaagatgcaggcgtggaggctgggcaaacagcctagcttggcgtacgaaggggtcggctttgatgcgcg

tgcggcggcgaacacgtcggttagcagtaccggaagtggggccggtggcttccggaagagcggggaggaggcgtactcgcaggggcactcctctgggc

acggtcagcgagtgtcgaaggacgggacgcgcgcgtcgctcgacagcatttctggcgcctatgctacgtactcccgcgtcttggagctgaacaacggc

ggcggcgatgcgatcagcagtattagcgaggaagacgtgcgcatgagcggcgaaactggtcatcgctcgttggagtcgatcagcgcgtctgtttcgcc

ggtgccgtccagctttggcggggcttctagcggtgggatgggaggtgcgctcagtattgagagcggggtgggacgaagtggtacggctcccgtgtcgc

ctgcttctctttatgcggagagcacgggcagtcggtgggccttcggaggtagtggcggcaacgtgaatgggccgcccttgccgtcgcccaccttgcag

ctgccggagaacaaggctcctcggaaagttgtcaagaagaagagctccaagttgggctctttgttcggcgtgtctggcaccagttcgtcaacttcgtc

tggtggatcagggaagcagcgcgaggggagtggaagtacggctgttcccgtcggcaaaggtgccagcgctgcgtcggatgcgcgacagcggacgacgt

ctctctcgcgactgcgatacaatcccaatcccagttccgaagatgcggcgatggagcatatcgacgaaggagtgccgcagtctggacgagcccggcgt

cagctccggtcggcctctgtcacctccctttcaggatctctttcaggatccctttcagggtcacgccgggacaaggacgtacccgcgttgccagcgga

tgcatcagtgcgctactctgtcgccagtctgcctaccagctcgaccacgagcagcttgcatgaggtgtcgactggtgtgccagaccgacctgctgaag

gcatgcgccgcagtttctcagcgacgatggcccggccgatcgtgccggcctgttcgaacgactccagcttgaagtcgtccaatccgcaatgcggcagc

tcttcgaactcgttgcggacgctggtgcggatgtcaagcgccacagcttccatgaggacattggacgagggcgaccatagctcgatccatactgcccc

gacgcagtccacgccgttctttccgaggatgggccttggcatcggattgggtcttccgttgaacacacctcctgccaccatccatgagccgacaaact

cttcgctcgcctccctgcctgagtacgccacaggcacgcctctctcgtcggtcaacaagagtgctacggccagtagtcgccgatcgaccgcttcgaac

cagtcagcttcggcgtcgtcgaaccaggtgcccgatcttgccacggctctgttgcgagcgtcgcacgcggagtcgttgaaaggcggcacggcggacct

gatggccatcctcggccgcacgtctcggccgtgggggttcacctacactgacgtggagcatccggtgaaagtgtggcatggtgatcgggatgagcgga

tcagtctcagcagtgcgcagtggatgcagcaggagatgaaggattgcgagctgatcgtggtgaagggcgctggacatgggttgatgacaaacgtcctc

gtcgttatggaagcgttggagagtatcgaccgtcatcgacgcgactga

>contig_14.contig_14.g25.t1

atgcagagcggaagcactggcggactggcccagcccacaccgacaccgagagcgagagaagaacggaaggaagtggtcc

cctccggcttccttctggcgatcgacagcgacagcagcagcagcagtggcaatgagtggcagcaagacctccccccacaaggcggcttcccctccgtc

cgctacaagcgcaacctccccacgcgcggaccgggaggaatcgcaatcttcggctccgtcatcgccatctgcgcctacggcttctaccacgtcggccg

ctccaacgtcgaggcccgcgaactcaagcgcgaacatgcctgggcccgcatccatctggtgccgatcttgcttgccgagcaggatcgcgatgtgtatc

ggaggcgcgcggcggcggtcttgagggagaagactattatgcaggacgtccccggctgggaggccggcaagagcgtctaccacacaaaacgatacatt

ccatcaacattcgtcgtcatctag

>contig_14.contig_14.g26.t1

atgccgagcagaagatcaacacgcggcacctcctcctcctccgcagccaaagacctgcccgcccctccctccccacaag

cgtcctcaacatcattacagcacggcgccaacggcaaagcgaagcgtcttctcggcgacgaggatggcagggaggagatggaggctccgcgctcgcct

gctgttagttcttctacggctacgcggagtgctaatttgaaagggcaggagaacgtctttctctttgtgcctaatctgatcggctacgcacgcgtcat

cctcgccgcgctctcgctgtacttcatgaagggcaacccgaaggtgtgcacggtgctgtacggcatcagctgtctgctggacgcgtttgacggtatgg

ctgcgcgcgcgttggggcagtcgactaagtttggtgctgtgctggatatggttacggaccgctcgctgttgaccgggtctaagtcgcacaagattgtg

acttcggacgtgtctcggattctgtggtactactacaacgacagccggatcctgttcctgttctgcgcgggcaacgagctgttcttcgtgtgcctgta

cctgatggcatcctacgacaccccgctcgagctctccctgcccggcatcctaggcccgctaccgtcgtcgctgaccgaacccctgttcgagcagatca

agacggacccgcactcgatcctcgcattcatcgggcgcgaactgccgaagttgacgtggccgcagatcgtgggggcggtgacgttgccgatctgcgcg

gcgaagcaggtgattaatgttgtgcagttttggaaggctgcgaaggtgcttgttggtgttgatcttgtggagcgggagcgggcgaggcagaagaaggc

tgtggaggaggggctatga

>contig_14.contig_14.g27.t1

atgtccgtacaagtaggtccgtactccatctcccccatcggccttggcctcatgagcctcacctggcgctccatctccg

acttcgtcccggacgacgaagccttcaagctgatcaagacagccatcgagaaagccagcccttcgcgcgtgttgctcaacacgggcgcgttctacggc

cccaaggaagacgcgtacgccaacatcaagattttgcgaaggttcttcgctgcctatccggagtacaaggataacgtcgtcgtcaatgtgaagggggg

agctgtgatgtctgaattcatcgagaagggcttcgcaggcggtggattccgagcatcgagcaccgtcgagaatctccgggaggatttgctcgccattc

ggaaggagctcggcagcgacgatggcgggatcgacgtgcacgtgtacgaacctgcccgtcgggatatgaacatcagcgttgaggagatcatcaagaac

ctgcacacgcttcagaaggaggggcttttccagcacatcgctctctctgaggttggggctgacacgattaataccgccgcgaagacagcgaaggaatt

gggcacggagatcgtctctgtcgaagtggagtattcgcccttcttcaccgagatcgaagacaacggagtgctgcaggcctgcaagacgcatcagatcc

cgatccttgcttacagtcccctcggacgaggattccttggaggccagctcaagtcgcgctccgacctcgtatccaacgatcctcggctgcaccaggag

caattctccgaaaagaacttcccaaacaacatcaaagtagcagaaaccttctcccaactcgcagcctccctcaacccaccggtcacgcccgcccagct

cgccctggcctggctcgtccgacaaggaggcgaaggcgcgggtaagacggccatcgtgcccatccccggatcgacgaaggctgctcgggtggaggaga

actttgaggcgatggaggttgggttgggtgatgctgagtttgagaagctgtccagccagatccgttatcgcggtgctcccttcgcacgcgacaccttt

gacctcagcgaatctgatgaaaagggcctcggtggtgactaccgacccgtccatcgctacttcccggacctacgatgtgagttgccgtctgccgagcc

atttctattggaaagagcagtgaaaggtactggcgtatcggattcaccctcattcctgtctttccgtccatactcgcgccaacagtacatcgttgttg

gaggtggtcttgcagggctcgtcgttgccaaccgattgagcgagaacaagaacatccacgttcttgtcattgagtctggcgccgacacccggaatgat

gatcgaattgccagccttgatgcgtacgactccgttttccaagcctccagcacagacattaactggcgtttcaccaccacgaacggcaagacgatcta

tgccggccggggcctcggaggctcgacgagcatcaacggcggcgccatcacccgaggcgacgtcgtccaactggacaacatcggcaaactcggcaatt

cgggctgggactactccagcttgctcacatccatgaagaaggccgaacggttcctgactccgaacgcgggtcaggttcatgcgggtgcacgctacaat

gccagtgcacatggcatggacggttatctcacgatccggtttggggaggtcccgaggtcttcacgacgtgatcttcactccaactcctcttcttcttc

ctctgatgctgaacaggacgcgcggttggagcgaagattctatacgggtccgtatcagcacagctttgtgcagtccatcaagacggccctcggagtag

agctcatcgacgacctctccgccggccaaacgaacgggatcgcatacactcccaactcgatgctgccaggaggagggaatcttcgatcctcatccgcg

accgcatacctgacgcccatcgagaacaagcggcccaatcttgtcgtgctgacgacgtggcgaggctggaaaactacgtggtcttcgaacgcggcggg

taccaccccgactgcgacgggggtcgtcattcagcagtcaagcggcggacagacgtacgatgttgaggcttctcgcgaaatcatcgtggccgcaggcg

cgatccgcagccctgtcttcctcgagcatagtggtattggtgatcccaacattctccgaaagatcgacgtacccgtcaaggtcgatcttcccggcgtc

ggcaggaacttgaatgaacaaacccagtccgtgctcggtggtacccccaagaacggcgtcagtttcggtggagtcggaccgtcgaacttgattgctca

gcccaacatgaggcagctcttctctaatgcttctgccgtgcgctcctacatcgagtcgaattaccagagatgggcccgggatgccgtcagcgcaggtg

cggcggtgagctccgaggggctgattgctcaatggcgcctccaaacttcggctctgttcgactcgaacgtcggtgctgtggagatgttcgtcgacagt

ggctaccccaaccacggcttcggtgtcctgatgtggcctctactcccgtactcccgcggcagcgtacacgcctcctccgcctccaccttctccaaccc

gatcctcgacccacgctacttcaccgttcccgtcgatatggggatgcaagtcgcaggatgccggggcgtgcggcgggtgttgcagacgcgcccggttg

cggatctgttcgctgctgctgctgctgggggtgggggggaggaggtcccgggctttgaggtggcgaagggtggggttcctgacgggagcgaccatggc

gcgtacgcagcttggcagaagtggatctcggacaactatggctctgtgtcccatccgatcggtgcgttcgatctccctcggtacatgtgctcttgctc

ctcgctcgatgggaggggtagtggacccaaccttcaaggtgtacgacacagcgaacatccgggtggtcgatgcgagcacactgccccagcagatctcg

gctcacctgagctcgactttgtatgggatagctga

>contig_14.contig_14.g28.t1

atgcctccaccagcagcagcggcggcggcacttcgccagcagcgcacgtcgggaaggaacgcacacaacccttcaccca

ctgctcatcgccaacagcaccggcaccagcaacagcaccagcaacagccatggctctcacctgccacatcctcatcgccctcttcaccctccgtcacc

gtcacgtcgaccgcaccctccctcagccccttcgacaccatgccaacacacaagactccggcatcccgcccaacaaggccagccgcgacccacaccaa

cagcccaccctcgtccctccccagcaccatgctccccctctcctcaaaacgcacccgcaaaccaagtgtcaaggctcgtgagatggccttcctcaatc

caccacgccctgcctccccagacggcctcgcaagcgctgtcggtgtccgcaaacgcgccttcacctccgctaccagtagggacgacgacgagcttcca

gacgagtcgccctgcattcctcggcggaaacgcgctcgtgacactcaaaatgatgccgggtctagcacagctctcgtgcagagtccgccgttctgggt

cgaaatcccgctccgaaaccagacgaaggaggacgtcaagcatcctccgagcaccgcatccaagaagagcgatatggccgctcccctttccaccatcc

cgcttcgcaggcagcagaaggagcagcaggagaaaggacagcagggcaccccgcgtatcaatcgcgctaccacacctcctagccagatggctcgtaac

cgatcaccgcccccacctcccgcctcagctctgcgtctcgccaacttcggttcctcgtcccgcgagcgtcgtcaacaccagcagcgtcacaatctcgt

ccgcaccgcctcaggctttgagcccactccctcgggcccatccagacttgtcgacttgaacgcgtctcccaccctagacgacgacgatcaagtgaagc

gacccagtccaatcgagttcatctcaccctcccaactgggcgccatccttcggaacccgaactccagctcgtccaaaacgcgaaccaaggatgctacc

agctatatgaaccagatggccggcgagtcctcaggcagcagtgaatcacgatcacgatcgcgtgatatgctcaaactcgccctgctgggcaaggacaa

ggtctctccgccctttgcccgtctttccctaacctcttcgcaggagaagtcgaaggggaaagcaagggccatcgaccctgtctcccctacgtcgtccc

cctttggaccatcctcccagccacgcagctcgcccaccaatccactcggcgcggatgtggaagccgtccggccggtcgagaagcagcaggcaccgaag

ccagtacctgcgtctggcgacgatgtcttggacacttgcagcaagaagtacggccctactttcattactcgctcaggctggcccgtcgagaagtggat

ggagctcgagactcccgcgaggagggaggcacaggcgaaacggcgtcaggtcgaagtcatccttggcttgcgagggaaggacgaagagcccatcgtcg

taagctctccagctcgtactcaaactcaggcagccgaaccaactcagccacaagagtcgaagacgtcgcctgtgcgccaagttgctgagagcagcgcc

attccagctgtctctgcgcccaagcccgagcccaagcccaagcccgaatcagaatcaccaaagccgaagaagaggatcgtccccccgatcaccgacca

agagcgtctcgacttcaactacggctactacggtcacaagtttgccacaaagcaaggctggtccgaggagcgctacctcatcctcaaatctcccgagc

gcaaacgcgcccgggctgcccgtgcggcgatggaggtagccatggccaagggcaccgccatcccactctctcccgtgcgtcgtccccagcgggaacgt

ctcgagttctacgaagaccgaattcgaggtgatgcctttgtcacgagccacggctggaaggaagacgggctggagatcttcccgcgtcctccgaagat

gagcaagcggaacaggaacctggacagccccccacccgggatgagtattcgcgagtggtggagcattcgggagagcctgccgccgcggtacgagacgt

cgtcgaaggatcggatcccgaagggcgcgttggaaatggggcggtcgagggagttgccgccgttgagcttttatgcgcagaataagagggaggaggcc

agtccttcgccgcccagtcggcgggttgtttcggagcagtttgaggatctggtgcggatggctagggagcaggagcgggaggagggagatggggagga

ggaggacgagttggaggatgaggatgcggatgcggatggggatggggaggatgaggatgaggacgaggacgagggtgcggaaggggacgatgagggcg

aggatgaaatggaggtggatgcccaaagcgaggatgaggacggggagggggaggtcgagaacgaggaggatgaagatgaagacacagaggaggaagag

gatgatgatgacgacgaggaggaggaggaggacgaacaagagcacgcggtatggtacgactcccgctccgacctctccgacgtcgaatggcccgacga

tctcgaaatctccgagggagaagacgacggcgatgtccaacggcgtcgacgacggctgcgccgatacaacgccgtgatgggatcgagctactacccgc

ccatgccgcggctgcctgtgaagcggcggagggtgcccggggcgagcccggcgcctacgacgccgtcttcgcagattggcaacaaagtgaagccagcc

agcatccatccgtgccatgcttcagcgggaccatctcgacagcaggaccatctcgaaatgcagcctatcgacaagcgcatgggcgtgggcattcgtta

tttctcggattctgcgcagcgagccagcggaccgacaccggacacatgcaatgccttcctgcttgctgataccctctgcccacttgcgtcgctccagt

cctcctctcacctctgtccctttctcgtcagattgtacggaggcgaccgcaggtatgccgaaggaaacagtgacgggatgcctccagccctcccctcc

tcagatcacgacacagtcaaagattctgtttccgaagcaccgtcgaaacaggtcattcaacggacgggtaaaggcataaaaggagggcctccagacgc

tgattcgtcctcccttttcccttctttcctcacctccaacctccgcaactacgtgtcgagcacggtcgttccccgctcgcttccgaccacaaccacac

cgacaccattcacggccgccgtcatttcctcaaggcaccgccattatcgacgccaatcttccagccaaacgcacaccgtgacccggcaggacgtgtcg

tcgcctactttcggcaggagtcattggcagagcgtcggcctcaccgtagatcgaaacgaggacgagcgatacctttccgcgttcgggcctcaggcgta

cgagttccatggagcttctcatctttcgataccagcacgatccccgagtgcggaacggtacccgctggtcctcgctcagatttccggtcaagccgacg

cgatgccaccgactatctgccgtagtaggcaccgacgcgtcctctcgggtcacggtgcgcgcttcggctggggcttggcgtcgatcgtggcggtgcct

tga

>contig_14.contig_14.g29.t1

atggcaccgcactcgtcgtccatgagcgcgcccgctccagctgcgaacaacagcgcgccagacgcgttgcctcccgccg

cagccgcagccgccgtgcccgccttcgcaccagacgtctccgcaggtccaccgccctacaacccgtaccacaagcatctccagccgcccccgcctgct

ccctcccatcccgctcccagctcgtccatgatggcggggccaggtggcgcagcccaggtcgcggcggcggcagcggcagcagcagcagcggcgtccgt

gtcggccgctgccatgtcaacccactatgcacccaatccagccgcatcattccagcagtcaccaccaccacatctgcagcacgccccgggtccaagcg

cagcagcagcagcagcagcagcacaaccactaccgccgcccccaccaccacccatggcggtccaacaaccaaccatcgcgccagcctccgtctatctc

gccaactactcaggcgtccccgtcttcgagctcaccgtccgaggcatcgctgtcatgcgtcgacgccaagacggcttcctcaatgcgacgcagatcct

caaagtcgctggcgtagacaagggcaagagaacgaagatcctcgagaaggagatacagcaaggagagcatgacaaggtacagggaggctatggaaagt

accagggaacatggattccactatctcgcgcccaagagctctccgcaacacacaacgtatcccacatcctcaagcccctcctcctctttgaccccgca

accgaaacaagcattccaaacgcagccccacgtcgacggcccaaccccaatgccccgcatcaaagcctcggccgcgcagcagcgaacgcaaccgcccc

tgccaaggcaggcgagaacacatccctgcagagcatgacgggttccgcgtccggcatgggcgcttctggcgcagcgtgggattccagcttgggcggct

cggacggtgttcgtggaagtgcacccacacaccaacctcgtttcctcgctcttcgctccccagcgtcttctgctcccggcgctgacttccttcatctc

caggagcccacgtcctccgaccctcgtcctgcccctccccttccgcttcctcttcctttacccgcttctacctctcaacaaatgctctcggcccgaga

atcgaacggggaggacgtctttggacccctcaccgccaccacggccgcagcgagcaagatgctccccggctccggctcaaaacggtccagtgcaactt

ccaacctcggccacgagacgcagtacgatgacgacgatggcggtgggagtgggagtgggagtggggaaccggctgcgaagagggcacgcgggcaggat

ggctccatccctcgctccggtggtagtgatgcgggcatgcttcttggcccttcgcctgtgaaagatttgaatgcgctccatccgtccgcggggcttct

gcaggccgcatctcatccccggcccggaagcgcggcggcgccgttccgctcgccggccgcgtcggcggctatggcagccgccaacgtccgagcgtctg

catggaacgcagcatcgtcctcctcctcctcctccttccaccatgccaatgggaatctcgcaggcgccccgggctcctccgcttcgttgcgcagcgta

ccccgctacgccgaccgaccacgtcttccgcgggctagcgacgaaggcgagcgtcgagcacgcgagcttctcattcatctcttcgtcgatgacggtac

gggcgcaggacacggtctgctccgcggctggaccgctctggatggtacccttgagagcagtggtggtgcgatggggatgggaatggggatcggacccg

gtggttcgaccactgggcatcaactacaagcggcgccggtcaagacgcccgcggagatcgcagcagccgcaagggctcccttcgtgttcggagccgcg

gccgtaggcgccgtgcgagcgaatgcggggccgacgcctggctctggggcggtacttcccccagacgtcgcccagcggaaactggacgagcttcttgc

gctgctcatccgggctgttgggttggatgggttcgaagatgccattgcgagtgccggtgcgggtgggtcgtcctcctcggcaggggcggcggcgggtg

ctatgatgggtgcggtccggaggggcggggcgattgatcttgatattgtcacggatgatcatggacataccgtcttacactgggccgccgcgctcggt

cggctcgagcttgtctctgtcctcctggcccgtccgccggttgggtcggcgggcttacaggctgcgccgttagcgacggacggggacgtattaacctc

ctcctctgagaagggtaaacaacgggaacagcagcaacagcccgcatactcgcactcccacatccacggcatccccgtcggcggcgcgaacccgaacg

caggcaaccacgcgggcgagacggccctgcaacgcaccgtgctcgtcgtgaacgcgtgggaacacggatacttccctcacctgctccggctgctcgcg

cctgcgctgatgacgcgcgattttaggggacgcacggtgttgcatcatgttgcactggttagtgggctccgcggccgcgcggcgggggcgaggtatta

cttggagtgcattttggagtttgctagggaggtttatgggggtttggaggggaggaggacgggaggggcggaggagggagggcagggtgagggtggga

gtgggccgattgggcttgggcttgggttgggacttgggctcggcgtaggtggtgcgagtacaccaaatcagcgtcggcctgtcggtgccggtctcaca

cctggcaccgagcgcgcgaccccggcagggacaggggcaggggcatctacgagctcctcgtccgccctgcgtctgccaacctacgtgcttgagaacct

cgtcgatgcgcaggacgaggacggcgagactgcgctcgggatcgtggcccggctcggcaatgccgggctggtgaagctcctgctggacgccggtgcgc

ggaaggatattccgaacttgctcggcatccgaccttcggactggggtcttgagatggtcggactgggcagcataaatggggcgggagtgggagcggag

acgagtgtggatttgaatggtggggatgcggcgagggttgcggtgggcaacgagcttgctgctacgaggccgcatgatatcatggctgcgcttgggtc

gcgggtgaagcggacgcctatgaagagctcggacattctcgagagcatctcgcacagcctcgaagacctcaactcgacgtacacctccgagctatcgg

agaagaacgcagctctggaagccgtgcagatgttactgaagcaacaaacgcgcgagctggcgaaccggcgtcgggacgtgagcgatgcgcaaggccgc

gcggacgcacgggaggagacggtacatcggctgaacaacctggttggtgtgttgaacagtctgaaggtcgggcctgaggaggagcgggaggtcgtcga

gcactggttatcgtcgtcctcgaccaagaagggcacgaggagaagcggcggtggggacggcgatgtctcgatggaagacggcggcggcagcgcagccc

tggcgccgatcccactcctagacgagctctcctccgcgccgggactgttcacctcggcgcggtcgcagcgggtggagagtgtggtgcggttggagttt

gtgcttccgttgctgcagcgcgaatgtgagacgctcgagcatgcgattggggagattgaaggtgcacagcgggtgaaggatgcgcagtatcggcggct

ggtgtcgatctgtagtggtgtgcctgagcggaacgtggtcgagatgctcgatgaacttgtgctcgctatcgagtcggacggagcggatgtggacctgg

accgactgtcgggattcctggacaagattcagccacggacgcaggggtcggcgctgacaacgacgacgacggctggtggtgtgggtgcagcaggagga

ggctcgtcgatggaggctgatgttgcgacgttggcgcggtctgctgtgcctgttgcgaacggacgaggatga

>contig_14.contig_14.g30.t1

atgtttggcaaggacagcaaggctgcggctgcggcaggcgcgcagaacagcgataacagcaacgacaacagcaactacg

acgcacgcgcagacgaagccgcacgcaggatccagtcctactaccgcgggtactccaccaggcgctccctcaagggatgcaccctctcgcccgacaac

cgctgggacgacctcctcacccgcacccgtctcgaggcagccaacgactccgccgcaaagggggacaacgtcgcgtcctcccgctggaaacgcaccgg

cctcttcgtcaaccagctcgccacctccggccaggacgaccagggcaagggcaaagcggcgggctcggaacagattccctctgggccttcgcttaagt

ctatcggtggggaggatccggcgacgcttaaggtgggcaatgtacctggcgccaatgagggcaagaagatcgataatgtgccttcgattgctcagcct

aagcagcacggactcaggctgatcgagtggtggacgcgctcaggtcaggcacaggcgctctccaaaacgctcgagacacaacattggctcgagatggt

cgactccaagcatcgctacgggtccaacctgaaatactaccaccaagcctggcaagaagatcccaactgctctcaaaacttcttcttctggctcgacc

aaggcgagggaaaagacctctccctcgactcctgttcgcgcgaacaactcgaccgcgaacagatcacctacctctccgccgaacaacgcgcgaactac

ctggtcgacgtcgagccgagcacgggcaagctcttctggagacggaacggcaagtacgtcgacactgccaaggggaaacatcgcgattcggggaacgg

aagagggatcgtcgatttggacgaggacgaacaggccgaagaggccgaagagcgcaaagagcggaaacgacaggtcggcgcgaccgagagcgacctcg

agacgtctgactcgtctgattcgtcctcagactcgagcagcgatgaggaaggggataagtttgatcgtcggttggctaagatggacgcggcgcattat

ggggcggatgatccgacggcgaaacataaggtgtcggctctcaagattaagaattggccagagttgctgctgcggaagacggtgtccgacaacacgtg

gatctttgtcatgactcaaaatcatcagctgttcatgggcctcaagaagacgggcacgttccaacactcaagcttcctttacggcggacgcgtgctcg

ccgccggtatattcaaagccgacaacggcatcctaacctccctgagcccgctctcgggccattaccgcgccggcaccgcccacttccgctacttcgtc

tctacgctccaagcgtccgcggtaaacctggaccgggtgacgctctcgaagtcgttgctcgtcctggccgggatggagcagtacgggaagctggtgaa

gaagaagaagaaggggatcaagcaggcgaagccggcgttagagaggagcgaggtggatgggaagaagaaggggaaggagggggagggggcggggtcga

aacatccgcatttggcgcagatgctcggcaagcttgggatgggcggacactcaaagagcggcgatgggggggaggaggagaagaaggcttga

>contig_14.contig_14.g31.t1

atgtcttcgtccaacgacagcagcgcgggcccagctcccgaaacagcaagcgctgagcggaaaacaacggccgaactta

cagtccctgctcacgatcaaggtccgacgttcgcctttcgcccgctggccgacatcatgcccctcgcgagggccgctgaagaacaagaagaggagtcg

caggacgagtcggagcaagcggcggcggaaagggtggaggaagaagctctgaaggcgaggaggaagcagaggaagatggacaagagaaggcgtggagg

agacgaggatggcaaatcggctgctgcgcaagccaagaagaagcggcggattgatgtcgagagtgccagcgatgaagatcaagacgaagtcgacatca

tggcgctcgcagaagaggcagccctcgcggtggaagacgcgaagaaggccctcgcacgagcgggagcgttggcggatacactcactgcggctcattcg

gcgatggaggccagcaggagagaggttaagacgcggtcgacggtcgagcgatggcgggagaaggtctctgtgccccgctcagcactcacaggtctcct

tccattcctgtcctcccaccttttctccacttcagcgttccttccctttttgcgacctacaggacacatcaagctggccatgcggtactccacgatca

ccctctccgcgatggtgctgtgcgccttcttcattcaagcatctgcggcactgccgacagctgcctcctcaggtctcagcgcgtcgtcggatgccatc

ctcgggcttcgagcagcagctcggccaagtttcgtcgatagcaatcaacggatcgtgatccgccagtcccaagaagtgctgctcgccgatggcctgca

cggcctctcctggtcgtctgcggccgcatggctccacaagccagcctcccgcacatctttcctgtcgccagcgacgtccaagacagtatcggagtttc

aggagacctccatgtcgcgtatcacgaagtggctcgatgaggcggatgccaagaacagtgcgaaggaggctgcgcaggcgcaagggcagtccgcgccg

gccggtgggcgatacacgctcgaggagaagggcaagcaggagatcaacgaaggcagcggcatacccggcagcagtggcagcgacgcattctttgatca

tacggcgaaaggatccgagagcgactcgtggtcaaattccaatgcctggctgcataagccctcgtaccggacttggtttactgggataaggcacaagt

ga

>contig_14.contig_14.g32.t1

atgcccttcacacaagatgtcaccacaatagacggcctgagtcctctgtcgacgccccagtcatctcctccccgtcgag

ccttgccgcgcgccagaccgtaccctccagctcacctcagcatcaaggacgacataccgtcgcctcctctttcgtccgaccgagatctctaccacacc

aagacccccgcgccgcgggccagtcaacgcaaagtgatgccgtcctcgagcagtcaagccgtggctattggcgtcccctccatcctcgtcaagccctt

ccgcacacctctcctccccaaacgtcgtatctgggacacaactgatgatcgacatgaatcgtctggtggctcgcgaggcagcaaggcgcctcgtcggt

tggggacgacaccggctcgagcagccatgaagacgtcttcgtcggcgggtatcttcattccgacgcgcttcatgcgtagtcccagtccggacgaagcc

tcggagaaaggaggacgcgtgtatgcgggttcgagcccgaatacctatactggctcgagcattcaggcggctgttatgcggtctggcgaaggaggcga

cgaggtgaaaaacatgcggcaggggcttgatgcgcccttgggcctgctttccgaaagcgatctcaactttggtcagcaagaggggtcaatgtatgcgc

ccttacgctcttctccggagccggtttcttccaacgacggcgctgcctggaagcccacagcaagcggccgcggccacttcgagtttgcgcaggagttc

gtgctgagtcccagacttcgcccgacaactgatcacggcatacatgcgcaagtccgaggcaactatgacccgtccagctcgccccaggacggctcgat

gctgcagcagggcacgtacaaccagcgttccgattccttcgtagaggacggtcttgcgggccatcccaaggagtacgcactcaaggcgcaacgcgatc

cggagccgtacagcagtccccaagtcaagccgcgttcgtctagcccattcgaacgtgtccctccctcctcccctcctctcctcgaccatcaccacgtc

gcaccctcgcaatggggccagtccaacttccccggctcaggcttcgagaccgagaacggtctactgatgttcagcttgatggacgagctcgagtcgcc

ggaagcgcgggaccgggtccctcttcatgctgtgaacgagctcagcgatgccctcggcgggagtgatggcgcagctgaagatcatggcttgggatacg

gaagagcaggagggggaacgacgacgatacatgcggcagggaacgacggagacgaagaccaccatcccgactccgacggcgaaggggatgcagaaggc

aacgacgacacggcttctcaccaggaatatgtgcccacatgtgcctcggccgaggctgactgcggtctgggggacgatggcgatgtgaatggggaaga

cctaccctcgctcgcggagctgcggtcgagcatgcagatccgcatcgtcacatcttcaaacaggcaggcgagctga

>contig_14.contig_14.g33.t1

atgaccttcccatccgcattcgttacccgctccgcgctctcagcagcgcgctctcccgccgcagccgtctccgcgcgcc

aaacggcagtcgcaagcacagcgcgccgcagcgccgcccgctcctacagctcctcgccctcggtctcagacccgaagatcacctccaaactgccccag

ggcagcgactccatctgggccatcagctccgtcctcgtctttggctccctcttcttctacctcacatcccctcccaaggcgggcgcccacggccacgg

aggaggacacgatgaccacaagcagaacagccatgctaagcctgctgtaagccccgaggaggcggaggagtctgatggagagtcaggggtcgacttcg

tcgtcgttgacgagcctcagctgaccgttcaaacagccggtggtgatgccctcaagcagcgcgaaacggacgaccacggcaagcaagcccaggcgaac

aacaagtcggcaccactgcccaaccggggcgagaccttcgcccagggaatagccgcctccaaggacggcaaccccatcagtgaccccaaggccgtcgt

cgccaaggcgcacgagataaagaaggagaagtacggtctcgcggatgggggcaaggacgtcgctgggaatgcctccaagtcgtcgtcgtcttcttctg

gcggtgatgagaagaaggagaaggaggacaagtaa

>contig_14.contig_14.g34.t1

atgggaggcgacgtttgtttgtttatttgttgggtgtcgatctctgtgcgatgccgcgatttccggggtgggaccgaag

aagcgacgctcggtgaccgactcactcacttcatccagattccagagaatgctgacgctggcgctgacgctgacgtcctccccaccgacagtgaccac

gaccagcgcatgaagaaccggaacgaagtcctcgctgcgcagttcaccctcatcgcctccacgctcggctccttcgtcgcctgtctcctgctcaccca

cttcgtcgcacgcaccacccgcatccccctcctcctcgacctcgccgcagcaaccgacatcatggcctcccaatccgccctcctctccctcgtcatcg

gcttcctcctcagcgtcagcgccctcctctatttcaacgcacagaagcccaaaccggtcctcaacccgagcacctaccagcacttcaagctcgagcac

aaggaccagatctcctccaacaccgctctgtaccgcttcgcgctcggcaagaagaacgccatcctcggcttgcccatcggacagcacatcaccgtcca

ggccaacatcaacggcaaggacgtctcccgctcgtacaccccgacctcgtccgacgacgacgtcggcttcttcgatctcgtcatcaagtcgtacccgc

agggcaacatctccaagcactttgccgagctcaagctgggcgacaccatcgccgtcaagggtcccaagggtcagatgcgctacgcccccggcctggcc

cgcgagatcggcatgatcgccggcggcaccggtctcaccccgtgtctccagatcgtccgcgccgtgctgaagaacccgcaggacaagaccaagctcag

cctcatctacgccaacgtggaggagtccgatatcctgctgaagaaggaactcgacgtcctcgcggccaagaacccagatcgcttcaagattctctact

tcctgaacaagcccccccaggggtggaagggtggcgagggctttgtgacacaggaggccattcaggcgaacctgcctgctcccgcaagtgacatcaag

atccttatgtgcggcccgcccccgatgatcaacgcgatgaagaagcatctcgaggctctcaagtacgatgctccacggacagtcagcaagctgcccga

ccaagtcttctgcttctga

>contig_14.contig_14.g35.t1

atgcggaacagaggaggagcaacggcgcgtcaggcgcgtctcgcagcagcgcgtcaagaggaggagtaccgtcgcgcag

tcgcccgccgcagaaaggcctcctcctcccgctcccagcagtactacaccgaccccctcatctccgaccccttcctgccccacccgcccgagggcacc

tacgtcgaggccggcgacgacggcatcgacccagacatcgacgtcgccctcaacaccgatgtccgcgcgctccgcaagaaacctaagctcgccttcga

tcgcttcgaaattcgctccttgtccgccgcgaggatggacgcggttaggttgccggatatcgcgcaggctggtcggaatatggctgttagtgaggcgc

tgcggagggcgaggatgaagcggagtgcgaagctgttggaggggacgacgtttcttcccagagacagcgtgcgggagatcgtcgggacgcgcatacag

accctgaccgcaggcgcgtcgcagtcccaagcgagtggaagcggtacgtcgggaggatatggggctgctggaagcgcatcaggctcgggtgatcctgg

cacgagtcaggaagctgccatttggaagagtaagagcattgctgctgcgattgcacatcatcgcacgctctggctggcctgggttatggagcacgaaa

atggcacggccgacatcacggtctgcaacgagatcgataatgagccgtctccgccctgggactggtgctttacgaatcaatacatgaccaacaaccta

gccccagatgaggctgccgatcctcccctgcagggatgctcatgcgaagacgagtgcgatccagccacatgcgagtgctttgcccaactcaagctcat

cgaccccatcacctggtcgacggagaccgacttcgcatacaaccgccacggccgtctccgaaacggccatttcgagcagtgcgtcatcctagagtgca

acgacacctgcggctgctccgactcctgtcggaaccggctcgtccagcacgggcgtaccgttccgctggaagtgttcaagacggcgcactgcggctgg

ggtgtgcgcgccaagacgctgattcggaagaacgagtttattgcggcgtatgcgggggagctgattgatacggcgggcatgcatgagcgcgggaagag

gtactcgaagacggcgacgacgtatcagttggatattgactcgttccatatccggtgcctgacccggatcggacctttcaacgagtggagactgcagc

agggactggagcccatggaccctgctctggaggagacgtatgcggctgcggacgactggtgggcgattcatgacccggatcatgttgacccgatcctc

acagtcgatgcgggactgtggggcaacctgacccggttcttcaatcattcctgcgatcccaacatagacatgtatccggtatacacgagctcgcagga

catcatgcgaccttaccttgccttctttgcgcgacgagacattcctgcagggcaggagctgcggttcaactacaatggcgaccttgtcgaagaagtgt

cggttcgacccgtgcctgatgcgatgactccgacgggggccaaccagccgggcacgactcagtcaggcaagacgaacggccgcggcaaggcgaacgag

agctcgtctgcccgaggtcgagaaggtggggccggccggggagctcggggctgggaggacgaagatgaggatgaagacgaagacgacgacggcaatac

gggacagcggtctcgtcagaaagcggtggcgaaggaacgtcgtcgacagacgttgttgatgcagcaggatacggcgtcgaagcacaagaagaaacaga

atcaggaccggctgttggcggggatggacacttcgcggtctcctgaaagaggaggagggggagggcaagaacaatcgatgccggagtcgagctcggcg

atgaatgaggcggcggcggccgctgctgttgccgtggctgagcgggatgggtacgggggcacctcctcctcctcgaacgggccgtcgtcctcttcgac

ggctgcccgttcgctgggtgcgatgatggctgagattgtgccgagcgggagtcgggaggtcgagcttgtcgacggcgtcaaggcgatggtctgtcgct

gtggagcgccgaactgcaagggccaggtcttcacttga

>contig_14.contig_14.g36.t1

atgtccgccaacaatgccgaagatcctcccgctctcgcggcttctttgcgttgtctcgaacgccttcccgatgctcagc

ggcccctcgtcgagcaggccattcgtgcgcagttcgatgcggatcagcaggcctctcaagctgctgccgctcaagaggccgaggaagaggccaatcgt

ctcgacctcgagcgccaggaaatcgaagcagacgagattcgtcgcgctgctgctgccaaagaacaagaggcacagctggctgcggccaaaaatctcat

cgatcttgccaaggcacacgatcttccctccgagggcaaaggcaagggccgacaggtcattcaagacgatgcaatgtccaacgacctcgacattcttg

tggcgaccccctctcaaaaaattcgcgacaggattcgtgcatgcgagtatatcgacctctggcatctcaccgcggaggggatggcagctgccaccaag

tcgaagatttccggggacaccagtttcgagctggggtcggacggtacgttcaagatcaaggactcggtaaccggcttccgaccggatcaagacctctc

catgccctcttgggtcacggccaatggtcactatgtgcggttgatgcaagacgaaggggtgccggagaacatcattcagtcaatgattcggctcaatc

acttgctcacgaaccatccggacttcgagcgccacggtcaagcaattcggatgtggcaccagcatcaacgccgtcagtgggtgatcagtggtcaccta

aacaccgatggtgtccgcttcaacctcgacaaacccaatcaaaatcactacgacgccattcggttgcgactgctggaggagcgcgcggagcgtcggct

ggctccggtcaaacgtacgtcctccgacgatcacgctcccagtgcagacgggggcgggtctcgccccaaggtcgcccgcgtcgagcgcactccgtttc

ggtgtttcgtctgcttctcaagcgcagccggtcatccattcacgacatgcgctgcgaaagagagggtcgacggcaagcatcaacatgtcacccgcggc

gcgtcgggacgaccagtcttcgccgactcgctcaagccgatctgcatcaactatcagatcaaagggtgcacaggcatctgccgcaccggcggagttca

tcgatgtgccaactgcggcattggcgggcatggatgtactaaactagacaagctgtactag

>contig_14.contig_14.g37.t1

atggagcctcgtcctgctctgcctgagcctgttgctgccgatcatcatctcactctgggttccattggctggtccaaga

gaacaccattggatgctgtccacttcgaagccgagcttcggcgtcatggagtactgtctcgctatccttctctttgttccgatcttgccgatggcttc

cgcattggcatccccaccatttcatctaccaccaccttctccaatcacggcagcgctcgctctcatccacacgttctttctgcggcaatcgctcggga

actggacgctggtcggtatcaaggcccattcagcgctgcccaactccagctgctgctcggtcctttccaaaccagtcctttggggctggtcccgaagg

gttctgatgattggcgagtcattcaggatttctcctggccgcgagacggcaacagcatcaattctcatctcagcagcaacgattggcccaccacttgg

ggtgccagccgcgatgttgttcacactactttgggcgcctctgcgctgtgcgctgcgcgtgcgcgcgccctcaatgaggctcggggctgcttgtttcg

gcatggcagctccgttttttcttcagcatcagctcatcgcaatccttgctcgcgcaaatggattgttgttgccttgcgcattgcctcacctgccttgt

gcgtccgggacgtcgccaacgcgtttcgtctggtcccgctgcacccctcgcagtggcctggcattgttgttcgtggtctcgatgatcgcttctacatc

gatcaattcctcggctttggtctggcgcccgccaccggagtatggaccttcccttggtcaatcaacttcgacgtcaagccgccgaatccattgtggag

cggcggacacggggtggcgtcgcttacttcgccgatggcgaaggaaaagaacatgtggaggactaccacttatctcttcaaaacctccgcgcttcgac

ccaccgagctcgtcacctccctgtcaacgatctctgcgatatcagacccgctcggcattccttggaagtctgagaaagatgtggacttctgctcggct

cccgtctacatcgggtttacctggtacatcgccgagcgttgcgttgcgctgccggacagcaaacgcctcaaatacctcgcagctcttgctgattgggg

tcagcagccacggcagaacctgcgcgctgctcaaaagctattcgggcggcttcagcacgccagcttcgtcgttcaacaggggcgcaagcgtttgggct

atcttcggcggttgcgaaagacatggcgtggtggacgtcggcgctttctcgtcccgacttgcgccgtagctttgcgaaagacgatgtttttcgacaaa

cgttttgtcgcctgcgatgcttccaccacccacggcattggcgtggttgtcgacggcgccgaactgttcatcccggtacgtccaggctggaaaagcca

gggtcgcgacattgcctggctcgaagccattgctctcgaagtcacggtacaactgctcgtcgaccaaggcctggcagactgcaggctgcgcgttctca

cagacaactcttccgtcttcttctcggagcgctcaggtcacaataggaacgcgtccgccatggaggcactcgcgcgtatccgtaatctcgaagcggca

tacaacattgaagtggtacccgtcctggttcccagcgcgtcaaaccctgccgatggaccctctcgaggttgttgcgcctcacctgcgcaacttcgtcc

atcggctccacgtcctccccttcctccaccaccagcacgtccacccaccacgccccccgcgtcagcctctgctgctacgcgcacaacgtcaaagcgcc

gtctgcctgtagcagcaggcggatatctcgaggtcccagcagcactacgtctcctggactgggttcctccgggctcatcacttctctcagctcgggac

ccacagcaacgacgacgggtcctcgccgctgcctgggcggagtccacgctagccacgtatggaactggtctcctgcgctggctgctctggtgtgaaca

gcacgctcttcctgcttccgctcgcttccccgcccatgtcggtgacgttgtggacttcattgagtccgttgcgggtcatttctccggcggcagcatca

gatactgggtttcgggtctacgcgcgtggcacgccattcatggctccgcgctagacacagttcacctccgcatcgtcactgcccttcgtggtgccgac

cgtctcacacccgagtcctcaaagaagccccccgagcaccatttcgccttcaccacctctgggcgatccggcgccatgttcaacccgacaaggtggaa

ccccccgagcgcgaagtttgcatgggagaccgctgcgcgtaccaaccctcctgtgatcgccggcgttatttcgctgccatggaccaagacacaggcga

acggagagaagcttgtccttgctccgcagacccaaggtccctgtcccgtccaagcgcttcagcaccatctgcacatcaacaaaccgcctgctgcctct

catctattcgggtactaccaaggcaagaagtttgtctccctgactcgctccgtttttccagcatcggctctcggagctgcttgctgctgcgggtatcg

ccgccgccacactgcaaggccacagtctgcgcatcggaggatgcacagagctgctccttcgtggaacgccaattga

>contig_14.contig_14.g38.t1

atggcgaatacacttggatctgtgagtctgcgcctctgcagatccttgcaagaacatctcccctttgccccagatcaaa

aagggaacgtatggatggtattggacaacgagatggaataccaaacatggatgtcggagttaccgggcgtttggatactcaaaagacgcgacaaggtt

ggcggaagcgacgtagctccggccgcagaagcttcaagagatactccaacaggaagacgtgcggatggagctgttgaagacgatctggatggcattgt

ggatgaggatgacagggttgcgtcggctctgctggcggaagatgctgcgcgtgggcgaggtactgcggggttctctgaagatgaggcggaagaaggcg

cagccgccggaaacgcccggcaaatatctcatctggacgacattggacgaatgcctccgtctcatcttctcatttggaggcatcgacacgattgcaat

catggaggcaagccggaaccatccaagcgcaataccgatctcagcccatccaagaaacgaaagcggctgccttcgatcaaggtgaactgccgcgcaag

attcgtctcgtggcaacgacttggcgacgatcgcatctttgtcaaatggtcacacgctcacagtggtcacaccgtcacctcaatcgccagcatggcgg

aatcgagacttcctgcacgggttaggcattggattcgggatcgtgtgtcagagggaagagattggaaggctattcggaacctgttgaggctggacgag

ggggatttggagaggttggagaagggagtgaatggattggagatgctgtcggaggctcttcgcgtcaaacgaatggacgtctacaacgagctgaggaa

gcagctactcaaagtcgcacggaaggccaccgatcgcaaggagagtctgcaaaagtgggcggtcgaaattgaggaggagggtggtatggcggcagtga

aaacggacattccttgtgcggtgggcgaggccacgtgggcggcttgcttcatgaccagttggcaaaagcaaatgcttctgatgcacggccacgacagc

atcgtctccctggatgcgacccacaacacgtgcttcggcttcgaacataaggagaaggtgtggttgtacagcctggttgtgcgcagctcaatcactgg

gcgaggaattcctgccgcgttcatgctgacgaatggcgagcatgctcagcccattgttttctggctacaagccctccgcagagatcctcgagtgcggt

tcattccgaagcagataatgatcgattgctcttctacggaactcagcgccatacgattggcatatccgccagacaaccttcctacggtcctgttttgc

gactggcatatgttgaaggcaatgtcagatcaaagcaagaagaaggttcgcggacattcgcaacatacccgcggcgctcaaaagattgctgccaacca

gctggctcaaaaaggaggctcgagaatctttcatcgtcttgatgaacgctccggatcaggcagcattcgaaacgcaagcagcggctgcattcaagatc

accgagccagcgagctgagggtctgccttggccttcagtcgtgcgcgctgagtaaagcggatcgcgacgcaaagaagcacgccgacgccgtcaccttc

tcgaacgctcaggcgttggtggtccatgacacagacgattccatcacggtgaagtctttctcgtcatctggtcaagaataccgacttcagcatcgcat

ggacgtccttggggacgaagccgagttggtatcctgctcctgcgaagcgttccgcatgcggacgtttccctgcaagcacatgtggctcgcgcatcgtg

tccttggtttgcctctggcgcagttttccaggaagcccaaagttggcaacctccccgctccgctcagagggcctatcaacgcggcccctccacctgtc

ttagggtcggctgccgatgcagcaccagcacgggtgccaccaccacctcctcccccaggccccccagcaccagcagcgctcccacccccagcgtcggc

accagcagttctcccactccctcctcctcccccagcactggcacaatcagcagcagcgccgccagacccccctgcaccagcaacactcggacccccag

caccagcagcactcggacccccagcaccagcagcactcgggcctccagcgccgcctccaccacgagctgcgctcccactcccagcgctctcagcacca

gcaccactgcccctcccctcatcctcgccgttgtcctctccggaaattggagaggacggttcggcgacattggcctcacccccactctcagcacctgc

acccgggacgcacgcgtcgtccccggaccggtgtcaatcaagctctagccatgcgcccacgcgaaacaacagtcttgaagtgaagtcgctcccgccgc

tgtgtcacgcaggcatctacgctacagccgacaggaaccaagatacgcaggctccataa

>contig_14.contig_14.g39.t1

atgacaaggtccgcgtctgcttctgctgcgtcggcagaagcatcatcatcatcgtcaccggctccctcttcgactagaa

cagcatacaggagatcaccagtcatgcggcggcagcatcggaccttcttctccaccgcactcgcagccgcagcttgcctccttacactcggtcttgcg

gtctccccctccgtcaccgctgccagcgcatctgcgagcaacttccccgctatcgactttgaggccctcggcgctctgggcgtcgttggcaattttat

cggtctggagatctttaatgcgtcggtggaggtgcagccgttgcccacggccaacggatcgtctctcgtcgtcagggcacccaacggtactttgacca

ctattggatccacaaatccaaatgcgtctattaatgcagttactacaggcgggccaggggacggctccgcgccgatggtctacgtcgctggtaacttc

acccacatcggagacatagatgcgagggggatcgcatactatgatcctgcttcgcagaagtgggacgacatgaacggcggtctggacggtactgtcca

tgcactctactattcggcagaacacaacgctgtgctcgccggcggcagcttccgcgcccctatcgacgcaggcgatcagggagatgcctatcgcggct

cggtcgccctctggaacgagcagagtggacaatggcaacccttgaatttcggcggcttgaacggaaccgtgtacagtatcaccgacggctttgaccct

tccttcatccgatttaccggctccttcctcactctctacggaagcggtcagaatgctacttcggacaccatgagttcgccgtcgtccctcactcgggt

cctccagccgtactccctggccatgtcccaggtcagcggtggcccaccgaccgagagcccggactacaacaatgccctccagatcctctgtcctcagg

gcgaagacggtcccggaaactcctaccttttcgcagcaggcatccagggcttgcttaccgtcgaggccttccgagtcctccagatcgccgccattcga

ctcggcaacactcgcattgacggtcgcggtctgaagggctttagcatcgtgtccctgcctgacaaccagattctcgaacttgtctacaaggatcctgc

cacgggcgcaaacgtcacctgctccgacaactgcatactctcgcaccgtggcaacctcacctaccaggacttcttgatctcgcccgctcagggaggca

ccaagaccttgactgggatccagctccgggtcaacagcttctacggtacgggcgcaggtctccacatccttgagctcctttcggacggcgcttggact

tgggcaaacaatgctctcaaccgtggccgatgctcttcgcccgttcccggcgtgaacggcactcgctcgatcacctcccacatcggcgactgggactt

cacttcgtacaaccgcggtctggctcaacagcccatcctcgaattcagcgactcctactcgaacctggctgccgacgtggaggagaacatcaatgtcg

agttccacgctgacatccccgtcgacggcaactacaccggttatctctccatccctggctgtgttcccgacaactcgtgcggacgccggaccctgtcg

ctcgtgactgtataccccacgccggaccagagcgtgtccttccagacgtatgtcaaccagacggtggagcaggacacggtcgtcaagatctttgacgg

cgaagttatcggcacccgagcggatgcgatcgaaggcgtgaccgactttatgcctcgggtcgtgctatcgatcgctcccaatgccaccgcaccggatg

gccagaccgagttcaccattgtggccgacaagctttcgttccagctgcgcaacagcaaccagacgtcgccgatgacgggtcctggctttgcttttggc

ttgatcgagtttgatgtgttcaactcgactgcggcgctgcctcgctatgtgaacactaccttgactcgctccgtcgctactactaatggtacggcgct

ttcgtcgggaggtgccaactcgtcggagagcggctcgtctggcacctcgtctgctctcgcaagcgtcgaccctgctgctcttgaaggcgccgaccctt

ctgtcatcgcaagcgcactcagtgcgattgcggcctccctttctagctccaacggtgcgaaattcgcggcgaagaccaataggcccaaggctcgccag

gtgtccgaaagcggatcggagtctagcacgactcgcttgactcgctctggtcggggctctgctactgcgactgcatcgtcctcttcattgacggcgac

ggattcggccgcattgcctacgggaacgacggccctggagcagtatcagaactacgccctccagctcttcctgccttctttccagaacaccacgcaga

ctccgcttgaccactttgcgactgcgctcttcgaggctggtgttaacggctcgtaccaattcaacagtacggagcttggtcacccgggacaagccact

cgtgccgtcgccgacgttgcgaacttcacctttatcggcggcaacttcacttcgctggcgccccttaccaacagcaccggctttgccaacctggtcgg

gtacaatgtgtccagcggcggtcagaacctcacccgtctgagcggcgggggtttgaatggacgtgtgcttgcgttggctggtgatggtaacatcctct

atgtgggcggtgaattcactggacttgcggactctcccaacacgtcggttccttactttgggaagtacgatgtgctcgcgaacagctggctgcccact

ggatccgccctcgatggtcccgtgtcttcgttgacaatcctgccgaactcgaccctgctgctgacgggagcgttcacagctgccaatggtacccttgg

taacggtggcttcgcgatgtacaacaccacctccggctcttgggtccagcaagatggtctgctgtcgggaaccgtcaacgcagctgcggtggcggtca

acgacgccaatggccggaaccacacgacgacctaccttgttggtccgatcaccgccgtgagcgcctttgctgttcccggagctgccaagattgagcct

gcgcaggcggagaactcgttccctgcaatcaatgcgctcaactttgccttcctgaaggaggcgccggcttctgcgaccaatgagggcagcgccaacgt

tctcaacggcacggtcttgttcaacggcaccgtcgcactccagaacggtactcttgttgccccgaacggaacggttatctactcgcccgctcgcaaca

gcaagtcgccagctggtcccgacagctcgaccagtgccggcagtcgctttgcgaaccgggtcgctcgtcgtgcggctgccaaacaggccgctcagagt

ccgaagatcaagagggatctgcttcacacctaccagcgtcgtgccagcgcgatcgagcccgagattctttcctctgccgaggggatgaatgagatcta

cacgtctgcattctatgcgcgctccgatggcagctacctgcaggttgtcggaggcaacttcaccacgagcaacaatgtggtcaacctgggtgcgtacg

accccgtgacgaagacgttgagcgcgttcccggcgatcccgtccaaccttgccaatttcaccaccgtgcgggccctgcttgtgatcggtgaccaggtc

ttcgtaggcggtgacggcggtctggcgtcgtacaactttgtggcccaggcctgggacaacaccacgactccgctttcgtcggacggtgggaacgcgct

tgtggtgcggtcgattgtgcaccggccggacacgtcggagatctatgttgggggtgcgtttgacaatgcgggctcgttgccttgctcgtcggtgtgca

tgtgggataatagtcagcttcggtggagtccgcttggcacgagcttgaatggtgatattgccgttctggattttgcaggggcgaaggcatctactctc

attgctgctggtaaccttcaagtggcgaacacgcctgctcctctcgctgcgtatgatacgactcaggacagttgggtcgctctcggctctgtcggctc

cgatgccaatcaggcgcccggtgttgcgacggccatgagcgtaaacaatctgcagctgaactcgatcttcgttgccggtcgatctcaagacggtgcgg

cgccttacctggccaagtgggatggtgttcagtacaagcttctcggggacgaggagctccagcctgccacgggcattcaacagttgacccttgtgccc

atcctgaaggcccacgaaggcaacagcatcctggagaacaaccgtctgctggtcgtcagtggtgtcttggagcttgccagctttggcaacgtgtctag

cgccttctttgacggatcgagctggcagcctttcctccttggcgccggaaatgacggcacccacggcattatccgcgccgtcgtcagagcaacggagg

ttctcaagttctcgaacttgcgacgcctggcggttggccttgttatcctgatttcgatcgccatcggcctcggactcgtcttcttgctgacgctgctt

ggcttgctttgggccatggcgcgacggaacaagaataagggagtcgacgccccgctgtcggcttatgatgatgttccgggtgacggcgagcaccaggg

ccggccgtcgagtctgcttgcgactttgaatgctgcgacggagaatgtgatgggagcgaatgccgcaccgggttcggctgcacacggcagcaactatg

ccccgggcgttgcgggcgtcggtgcgggtgccgcggcggctgctgcagctgcgagtgccggacatgctgcgacttcctctggccatggacggcccgac

tctgggccgctcggcacggtcgagaacagtgatgagaatggaggaggtccgtcgacggcgtaccactctgagggtgcacaaacgggccggagcggggc

atcgcagtactacaccgacgatgaaaacctgggtgctgcggaggccgcgggagcaggagcattaggtggaggagcagcggccgctgccgccgccgctg

cagccgcagcgggccattctggcgcgggaggcgtgccggcacgggcacggtatagcttcgaggctacacacgagtcggagctgagcatgcatgcgggc

gaccagatcaccatcctggacgacttggacgagcactggtggctggctcgagactcaagcggacgggaaggcgtggtaccgtcgacgtacgtgctgtg

a

>contig_14.contig_14.g40.t1

atgctgccaggaccggcgacggcgctggcgacactcttccaggacagcaagctcgacattgagctcgacgatgccatcg

agcgcactggtcgagcattcttcgatcagatcctggtgccgacgatcgtgctgcgcttgcccgcgtcgcccaaggtgctgccaccacccaagagctct

tccgaggctcccgcgttcttgctgccaatacggactcccttcccaggcagcagtcccgacgggttctcgacggaacccagcccggcctttccctccga

atacgagcacgacgagcacgacgccagccatgatcacgacgacgagcatgcgccggacggaggcgataggaatcgacccagtgtgccccgtctcttgc

gcacgctgctctccctcatccgcattcaggtagatgggacgtatgctgtttcccgagtggcggccgctcgttctgtccctacttctaccgtgcccgtc

ccgagcgtgcgcgcggaagatcagcagcttttcaaggcgcagagcgcagcagcggaaatgaatgggccgggaggagcggcagagcagcagcagcagca

gcagcagcggccctccgccttggtgccgttccacacccagagctgggcaggcaacaagaatggtcgtcccgcggtcagcagtgctgttcaaggctcaa

gctcgaaagcgaaggattccagggacggtcaagaaaaggagcgcggccagatcacttttgagagtgattctagtgggaagtcgcaagatgggcgatgg

gtcgtgcgatggagctgtgaagtgcctgtctcttttatgcagcttccgtccatgactcccgccctttccctcacggcatctctgtccctcctgctgtc

ctcaactcagcttgccctcctattcgacatccctgcgccgattgacgtctttgtcggcgaccaagacctcctgtccgccctgtccgaaggacccgtct

atcctggcgaatctccctcagatcgagcccagcggaccaagagcagtctagcacgcttgcccctctctcagctctctgcgcaggtcatcggcacgaaa

ctgctcacaccccagaatgcagcaagggaaatcaagggacgctctagccgcgggttgacccttccacctccgccggatgctgtgcaggatgacccaga

agatgcattcgacggcgacctttcctcgagaagaccctctgcgacgccgagccaagccggagccagctccggcgccaaacctgcgccgcctttcaaaa

atgctttctctcagacgacgtccggcacatcggacactggaccctccccagctttctcacgggagcgaagagtgctcaaacgcagttttcttcgtatc

ctggatgtctccagtgctgtcagcgtacgcttacgaacggtgctcagtccggagaggccagctacgttcggatcatcactttctgaacctgatccaca

gctggacgatgctgttgacggcgatgacgatggtatggagccgtacgatgaagacagtgcaggtgcagatgcagctgtggtcacatcagcgccgaggt

tggagcggtacccattcgacgatggcggcgcgttgcttctgtgcgtagaactcgaccatccactctctccagccgcgatgagaaatgcggcaccttca

ttctcaacctcgtccagagcctttggacaactctccttcgagatcatcaatgtcaagattgatgtcctggagcccaactctcagctggcgatgaatgc

caatgccaatggctccgtcgtacatgcggttaagccggctgtgaacgagaccttgcgtgcgacgctgttcgatcctcaacaggccttgcaagacgaca

agacggagtcggggggcagccacaagggatcgtcgttccccttcgtcctgacggagggacaacagcggaatctcgtctactgcgtcgatttcattgat

cccattcccacgatggccgaacctggcagagcggactcgcgtcccccatcggccctcacgttcgccccgggccagccccccgcagacgtcagtccggt

tcggcaggtcaacatcgtagtcgaggcgaggccgctactgaggcggtctcaagtggagccgtacaagatcgatggtgatcaccaagaagctggagacg

tcgggaatgacaaggatgcgacaccggacgcggacatcttgccttcgttttcgtcggcatggaactgcacggtggacattgcgacggcccgtacgaat

ttgcgacgacggaacgaggtccgcttgggcagagaacagcgggagcgcatgctcttggaggagcaagtacctgtatatctagcaacatcagcatga

>contig_14.contig_14.g41.t1

atgatctcagcattcttcatcttcaatcagaagggcgaggtcctcatctcgcgcctcttccggagtgatctcaaacgct

ccatagcggacatcttccgcattcaagtcgtttccaatcccgacgttcgttcgcccatcatcaccctcggaagcacttccttcttccacgtccgacac

gagaacctctacatcgtcgcagtcacaaagtcgaatgcgaatgcggccctcgtcttcgagttctgctaccgcctaattcagatcggccggagctactt

cggcaagctggacgaggaatgcgtcaagaacaactttgtgctcctgtatgagcttctcgacgaggtcctcgacttcggctacccgcaaaatagcgaga

ccgacacgttgaagatgtacatcaccactgagtctgtcaagtccgaacaagcggtccgagaagagagtagcaagatcaccattcaagctacgggggca

acgtcgtggaggagagccgatgtcaagtatcgcaagaatgaggcgttcatggacgtcatcgaaactgtcaatctgctgatgagctcaaaaggaactgt

gctaagggccgacgtcgatgggcagatcatgatgagagcgtatctctcagggacaccagaatgcaagtttggactgaatgacaagctcaagctggaca

aatcgggggtgaacaacccagctgaaggtgctgtcgagttggacgactgccaattccaccaatgcgtcaagctgtccagattcgactcagaccgcagc

atcagcttcatcccgccagatggcgaattcgagctcatgagatatcgatgcacgaccaacgtgaacctaccgttcaaagtacagccgatcgtggagga

ggtgtcgaagtcccgggtcgagtacaacatccatgtccgggccaactttgacgccaagctcagtgcgaacaatgtcgttttgaggataccgacgcctt

tgaacaccaccaatgtcaagtgccaggttgggattgggaaggccaagtacgagccttcggacaacatgattgtgtggaagatcccacgcattcaaggc

atgggagaagccactcttcaagcagaggcggaacggtcagcgacaacgcatgcgaaagcttggagtcgtccgccgatcgaggtcgacttccaagtgct

gatgttcactgcgtccgggctccttgtgcgcttcttaaaagtttgggagaagagcaactatcaaaccgtcaaatgggtccgatacgtctcaagatcaa

acgggtcctacctcgtccgtttctga

>contig_14.contig_14.g42.t1

atgcaggaccacccactcgcagccaacggcagcagtccagacggcagcagcgggacctcactatatgcggacgggaaca

aaccatttctctccttctccgccatatcgggcgcgccagggacagaagacaaagctgtcggcaccaaaaacgggaatcgaagatttacaggcggcggc

ggtgctggccaaggtcgattacgggtcggcgcgtccccttccgacgggccgcgatcatcgtactactggggcccaccagacgccggccgcgccttcgg

gacctcccccgtgggtgtgatcgggcgcgacaagccacgggagatggtccgggtcgagcgcgactacggcgcgggcgagctctgtcagttccaccctg

cgttcccactcgagctcgagggccgactgtcgcccactgtctttgcggagacgatcaacgagatcaatttacaccttatcgacgcgcatgactcggca

tggagctgttttgacaatgccctggccattctcactctctggatcagtccgctctttgtgagcagtcactacataaggtcaatgcggagattagaaga

attcattcaccaagtcaacctgcaaatcttcaacccggccggcctcaacattctgtaccccaagaaatgcgccttcctctttctcgagatcgaatact

actaa

>contig_14.contig_14.g43.t1

atgcatccatcgcagcagcaacaacagcagaggcggagcaaagcacaagcacagcacagcacagcgaaccatacagcag

cagcggcaaggagaagacgcgagcgcaagcagcagcagcagcagcacagaccgagtcagacacgcgcgcgcgccatggaggaagacagggagattgca

cggctgtggaaggtcaaccggaccatccatgagctcgtccgcgacaggggctaccaagtcgcagaagaagaagtcagcatggacctccgcacgttcaa

atccgagtatgctcctcccggcggcggctccggcatcgaccgccaaaagctcaacttcttcaccacccacaccgagcgagcgaacgaccgaatcttcg

tgttctactcgaccgaaaaggccgtcggcgtcaagacgatgcgcacgttcatccagattctggaggagaaagacatccagcgcgggatcatcatctgg

tcggacaagatgacctcagccgcgcggaaggtcatcgatgctatgcggagctcgttcgacctcgaggactttgacgaggcctatctgcttgtcaacat

cacccaccacaccctcgtaccacagcatgcggtgatgacggatggggagaagaaggctcttcttcagcgatatcggctgaaggagtcgcagctgccgc

gcatccaacaggccgatcctgtggctcggtactatgggctgaagcgaggacaggtcgtcaagattaccagagctccgcatccttcctccggtgtactc

gtatcgtctctgcgctcctcctctttgcatcacactcaatcgatctccaatttgcagtatagcccagtttgcgagtcgaggggtattatatga

>contig_14.contig_14.g44.t1

atggtgtttttccgacacaagtcccatcacttccaacgaatcactgacgactcctgtctggacgttttggcattttcgc

ctttcgaaattgggacaagcgcttggaggagcgaaccgaggaactgcacgagaagctgcttccgcctcacctacactcccttcatcgtcgagcttcag

agtcccttcttctccttccttggcaagttcacttgttacgactacgaatctggccacaagatcagcatttccatctggctccgattcttctatcgtcc

caacttcttcattacatacgacgagacttcgtcgcaaaatgtgcacgactatgctgcgaaccacaggcgggaggagacagaggagatcattgcactac

tggtcgttccgacatacctcagcgacatctatgcactccagtatcagtactctctcgaacttgtgggcttctatggcgtctga

>contig_14.contig_14.g45.t1

atgaacatctccttcaacacctcgatcatgctcgccgtcgcgatcatcgctagcatgatgggcacttccgccgcaattc

ctaggtcgaacgtgagtgatgcgtgcctcttcgtttcccccgctggattgcgatcgcaactgacgtcttaccctttcctcgatcggctttccgttcct

ggaccggaagccttcctcgcactcgatctcgatctccgtctcgttcgcttttcctttggctcgttcttgagtgtcctagggattgcgcatctgtctac

cggaagccagcctgccttcccacgatttagttaa

>contig_14.contig_14.g46.t1

atgctgaaacttgctctcttccatctggtcaccctcctgttcttccctgtcaacctcttcctcgccgtccgagatacga

tcaaagcgccgaagggaatcagcttgacgatgcgctatgtcctgctctaccttcgtcttcggcagaacaaggcggcctcggcactgtattgcacctcc

ttccccaccccgccgaaggacgtccacatccgcgaagtcaagttcacctcgccctcgctcttctcctcaaccctcggatcctcccgccacccctggaa

gttcttcctcatcgaacctacccgtgccttcccctcgaagaaacctttcgtcctctacagtcacggcggctcgtttgtgacgacgttccacgacaggc

atttcagctacctgtacgagctctgtcagcagactggtgcgacgatcttcgcgcacgattacccgaggccgccgaacggagcaacccatgaggagacg

tacgccgtcctggtcgagctcgtctctgctctttgctccccttcctcgtcgctgagaaacgcgtccggtctggcggtggacgaaaggcaccttgctga

ggctgcaaggacgaggactaagttggtctttgcgggcgactcggccggtggggcgatcagtctggccctctgcttcgagttgatcgatcgcaagatgg

gcgagctcttgccggaggagaccatcctcatttctcccggtttggatgggcgcttgcaggatgcagagctgtacgatggcaagaatccctggctgtct

ctccctgcctgccacactgtgcttgctgcctggaaaggccccagcgcatctgtcacccatcacctcgtcagtccagctctagcacccaaagatctcct

ccgccagctccttgcctccaagacccgcatcaccaacttgcacggtaccgccgatctactctttccaggctcggtcatcttccaagaacgtgtggtag

aggtggcgaaggagatggggatcgaaaaggttggtgagagggtcaggttcatcgcggcggtggggatgccgcatgactggcttctggttccgctttcg

gcaccggagatcctctctgcccgcacgacgatcatttccgttatcaactcgggagacccctacctccgtgttgtcaacagccgcgtttgccgatgtac

ccatccacaagaggacatcccgacccaccaaccatggcaacctctgcgtcaagatttgctccccgtcgactttcagtggcatcttctcatggtcgctg

catggaccgccagcggagaagcttcccttaagaccctcattccctcgacccatccatgcccgctcgccgacgattggacctctctgggtcgagatggc

cagtcccaacgaagtaccttcccacttcctttcatgtcggcaacgccgcgccgtgtgctgtgctttgcggtggcttcacgggatcttccacggctttt

caaccgcaccatcgcccaaggctctgactctcagacccgccctggcttgtcggatttgaccgtcccgcctccgacccggctaccggcgaccgacgtgg

tggatgtatgcacatgtccctgtcgcttcgccaccgcttcgcaaaacagcgcgcacttgtcctccgcgtcgtccatcgccacgtcgcttctgagttct

ggctctaaggcgtatcacggcgacatcgcccccggcgacactgcgcctgtctga

>contig_14.contig_14.g47.t1

atgtcctcttacgggcaaccagctacaggccaacatgatcagtcccatcaggaaccccgcccttcgacacctcaaagcc

cgaaattctcgacgctggcactcgctctcactcttccgtcttcaagattgctctctttcaccaagggcatgcttttcaatggcctgcttcctgtctcc

tacctcccctcctcccctcctcatctcctcatcgccattcctccatcagatactgcatctgcaagggccaagggccaaggtatcgagctcctaccctc

tcagcaacgtcgtcatgcgcacgctatgacgaccatatttggtttctttcttgatactactccacccatctcatccaagctcaaggttcttcgccatt

cttcgctgatactgcatcatcatcctggagttctcttgcagtacgtcgaatttctctgtcactacctcgctggtgacgatcttcgtgatgttcgcaag

ggtaacggcgaggggctctctgcgatcctatcagaaagatttgaagtgagtgaagtatgcctcgtcagaacatgtctggatgctgtcggtactgactt

ctgtttctacccggacgacgagccagcgggaacgttctgttggacctcagccatctag

>contig_14.contig_14.g48.t1

atgcagttcaccgcctcaatcgctcttcttcttctcgccattgccggctctgcttgcgccgctactgtgcccgcaccca

aagacgcctgcactgtcctgcagcgggcttccctcatcaacctcgacaagcttggctgcgcaaaggtcagcgctgacgtcctcagcaagacgaagccg

atcgccaaggacgagacctgcaaggccctccaggacggtggcctgatcaacctggacgctctcggctgtgccaaggtcgaggccaacgttctcgctga

tggcactgccaagtctgcctcttgctccgccaagaagctctgcacggtcatgcagaagggactcctgaacgtcgacctccttggctgcaccaacgtcg

acctcgacgtcctcggcctcgccgggggtccacttggacggcggccacggcaagcgcaagcacgcccaccttcccattcccgccaagcctgccacctg

tcgcgccgagacgtgccagatcctccagaagggcgttctttccttgcggacgacgattag

>contig_14.contig_14.g49.t1

atgaagttcgccgcccttttggttgctgctgtggctggcctggccacctccgtcgtagccgaggccaacgtcatggctc

aggccaggggggacatccacaacgaattctgcgacaactcatgcaagaaggcctggagcgttaatggacctgatgaccgcagtagcctccccttgaaa

gagttcatgcagtgtggctttcctctgctccccatccttccctcgtcttcctatccactgcagtataaatacggtagcttcctgtacggcaccttagt

gcctcgcccttcgttgcgcctcgactgcaccatgaaattctatccactgtcatcagcccccaggacaagccagttcatccgacgcttgcgatcgcctg

cattgaagtacaagattgctacagcgtcttacgactccacgttactcccaaaccttccgcgataccgagtttcctttgtcgtcctgctcaacaggctg

tggtaa

>contig_14.contig_14.g50.t1

atgacctctggtttcccaaatattggtcagtggcccgacggtccaacaggcagtcaagggttgtcggacgaggctcaat

tcaaggcgggcatcgagcaagctgagtttttagtccgacacgcaggcaatggccatgtccatgttgtcctcacattccgctccgacgaccaagccgag

gtcgagcgagtcaagtccgtggcttcacgcatggtcgcccatgccccgtcttctggggccacagcttcgggcgagcacggggttggggttggcaaacg

tcagtatctggaaaaggagctgggcaagggaagagtttag

>contig_14.contig_14.g51.t1

atggtcttccatcgcctgatcatcctcgatctctaccaatgtacctcaatctccctcgtaccccttccaaacgcccgcg

tcctcgcatctgcatccatacccgccgccacatgcctacgatcgttccactccactcaggccggtcacgtcgagaggctgcggagcatgtcagaccag

ttcgcaaggccaccgaggacgagctcctacgcggctgccaacggacacgggctggtagcctcggcactgtattgcacctccttccccaccccgcccaa

ggacgtccacatccgcgaaatccagttcacatcgccctcgctcttctcctcaaccctcggttcctcctgccacccctggaagttctttctcatcaaat

caacccgcgccctcccctcgaagaaacctttcatcctctacagtcacggtggctcgtttgtatcgacgttcgttccacgtctggcatttcagcttact

tacctgcctcagatgcttctcaactaccggcgcaaatctacgctcgggtggagcatagagaacatcctcctcgatctcacaggcggggtcttctcctt

gttccagctatttatcgattcgtctcaaacaggagactggtcaagcgcagttggcgacttctccaaactcggattgggtcttatcagtatcggattcg

acatcatcttcataatccagcactacgtcttgtacggagccgtcgaggtcgaagaggaaggagggcaggttgaaggtgatcacagcgagcggactggt

cttctcgcaaacggcgcggcggcgcgagagtaa

>contig_14.contig_14.g52.t1

atgactggtccagtgcccaacctctcggagacctccgcggacagcaacagggaatggccgccaggcgccagcagaacaa

ttcaaaaaccgtacagtattactacaaagcctggcacgaagacggctgacacgctggcgtccgacacgctggcgcctggcacgctggcgcctggcacg

aagacagcttctgacgacgacgcgaagggaacacgctccagttgcgaaacgtataaaagacaagactgcgtgccctacaccgagtatctgctcacccc

ttctttcaacaccactttccactcatctgccacacgtcttcatcagcgttcatcgattccttcttcttctacccatctcttaacgtcacctcagcgtc

ccagaatgcgcctcgctactctctcgttcctcgccgccatctttactgcggctgttgcaactgaccgctggccaaaccgtagttccggatgggcggag

tgccgctatggcggcggcattgaaaagctgtgtccgaatgttcaccaactgcccgatgacaggttcgagacaactctcaaggttgtctgcctcaactg

cttgcacaagttttgggcggagagtgaatatggttccaagcacggagaatgtccccatccggaggtcggctgccattgctacaatggctgtgtccaca

acatgtggagaaacgatttggatcaaggcttccacgacgtcggcggagtttgtaaggcctggtgcggcgagggcgcgcagccgctcccgcccaagtgc

tggaagtga

>contig_14.contig_14.g53.t1

atgttttccccttgcctttccctgccctcctttttgttctgtctcctctttatcgccgcctcgagtcagcgtactccta

cgaaagcatcagctgccatgctgaacctcgctctcttccacctggtcaccctcctgttctttcctgtcaacctcctcctcgccgtccaagatgcgatc

aaagcgccgaagggaatcagcttgacgatgcgctatgtcctgctttaccttcgtcttcggcagaataaggcggtgaggtcgatgcacataggcgcagt

cgagtgtctggcgacagtcgtcgggctgacggggatcacgttgccgatctacttcagtgtgctcatattgcccgctgcgtttgggtatgggcgtgtta

agagctttgcggtcggcgcggcgctgtcaagcatcatcatcggacggagacggaaacagcggcgacagcaagaaggaagagggaaaggaactgcagca

gtcgatctgatcaatacgcgcgcgggtacggttctcatcagcgccgcgctcttggacgacatcatcggactcgtgctcgcctccgtcgtcgtcaccct

ccctccagacgactcgtcttcttcttcttcgccacctcgacgaccacaacgacagtggcgccctggcctcttgcttggcctatcgtcgcttctgtcct

catcctcgccctcacggccttccgtgtcgtcgggtccttcgaccgttcatctcctccacacatacctccctcctccgatccttctcccgagtccagca

cttcctcacctaacggacctgaatatacacgtgaatcccactctcgtcgagcgacctcgccctgctcgcgttcgtgaccttgggacgatcgcggaaga

gacgaaatcatcggtcttcatcggcgtcctctgcgcaggcgccatgctagcatactgcgccgaacggatggaactcgccctccgccctccgccccttc

tcctttcccaacctcaactcccacccatggcaccctcacttcgttctcgcatccagactcctcccgatccagaacggcttactcctgcccttcttctt

cgctttgccgctggcgggtgggtcgtcttggctgattggatcgagcggaggagggaagggaaggtgcagggccacctccctactcccgtcgagcaccc

acagattgaagcagcaccgaccgctccatcgcaactacaagagacccgcactcctcaagcaacaccatcctggccccccgccctcttcgtcggcgtct

ccctcatctcttggggcgagatcggatacttgatcctcatcctcgccaggaacgtgttccatcctaccaccacatgcaatcaacctttcacgacggcg

acgtcagcgggcacggtgtccaagctcgtcaacgacgaggcgttcgcagttggttag

>contig_14.contig_14.g54.t1

atgagtcaacgctccacgacttctttcgacgaggttctcggcggggcgcgggactcaacccgagtcattcctgcgccgc

tgcgccacgaacgagcaagtgaaggtcctcgctacggagcatcaagggctgaaggaagtcgaatccgttccttgttgccacccccgctgccaaatccg

cttttgagcgcctccacgacgagggaaacgacagcgcaatttcaatctcagcttgccaccacattcgaccttggcccgactgcccaagaagagaccaa

cttcacccacttcgatgacctcttttttgcctcgggacggtctccctcccctgccaattctgaggcagagcctgtcaatgattatgtggaattgcttt

tccgatacaggacagctctttgcgtgcggctggacgacaggtgctgggtagtggaaggtgtacaagatgacaacatcccagacactggtgtatttcat

catctctttgtgcaggaaggctcagataagctgtccgccagctgcgactgccggaggggcaggcagcacgagccatgcattcacaaagatgcagtgct

agaagctctgcctagcttcaaagcgcagccagtcatctccaggcaagccaatccagcagctattgagattgcctcggctctgcatggtcgacactcct

gggtctcggtcatagcatcgcagaccatgtccggcgcccagcttctgcgtcacgaccaaaagcgcacgatcgtgtcgctcgtgggcccggacaagtgg

tgctgcgatgcctacggatgcagcggtcgcgggtctcccccagcaagctgcgtgcatcgaggtcgtgcgaaagcctatttggagcatgtgctgggtcg

agatgtgaatgatgtagaagagagctcggagagtgagctacaagttgtcatgctcctcagcaccacgcaggctgccgcggttctgtcagcttcctgcg

gagctgaaggaggagccgctgctggtggcggtggacgagcttcccgctgtcctgcgcttcgaaagaattggccggtgcggatgcggaaggagcttcga

tccctcccagccaacctctactcgctcctgtatggtcttccatactcgtggtgtggtgaagagagaggtagaggtgcagcaatgcacatgcagacgac

gcggagagaccagaacgattggaccagatcttgcgtcgtatcagctcttcaattgcttgctgcctcccctaccccactttctgcattccacaccacca

tcacagacgcctacatcgagaaccactgcgatatacacttcgtcgccaaatccacattcatcaaagtctacttcgcgttcgtcaggctgcagaatgtc

aatgtttcctttgcttgcccctcttgtggccccaatccatctgcaatcatcgccgatggagtggtgttggctcatagcacgaagcttcgtcatacgtt

ccttcagcctccaactacgcctattggtcacacaaatacccgagctatgcccaacaaagtactccagccattgccaaccttgcccctgcgaaccgctc

tccgcgaggttgcggacaacctgcttcctttcaccaatgtgcagcgcgccatctctttgtccagcttccgagaatcgatgaaaagtgcgaatctacgc

ggtctcgctcctcagcgtgaagagttcctccgccacctccaaacgttcgtcgattgcagctgtcgaaacgagctccttcgaaatacagacctttcgtt

cgccttacgatgcttgtttcgcgagcttgctgccgaagagggcatttggcaaatgtgtcgtccgatttgcgtggagtcgttgcaacaactttcgagca

gtgcggtcgaattttcctctgcagcgttcgcgaaaaccgccaccacgctttcccgccattccccctctctcggtctcctcgtcaggtcgctgcacgac

tgcggaaagcacgaagtccagctttactcccccgtcgtccactccattcgagaccttctcagctcaatagccaagttggtgggattgcagcacgtcat

gttcggtatccccaaccccaccaccagcccagaccagcccaacgaaccaatacaacctgcattcgattcccttcgtcagccgagcgtcccctacacac

agtctggatccttgtacggcgcagctcgatgtcgtgacaggcctctttaccccagatttggggagaaggccgatctaaaccattcggacgagcagcag

agttctggagatggtcctggtccttgcaggaagttcttctcggactacgtgtcgcagcgacagacgggcggcatcatggcgctctggtgccgtcacct

ggtgtgtgttgggttccacatcattcccaagtgcgagggacggaacgatgtcttctctgcggtgttcacacactggcccaagccacccgacacgatca

tctacgactttgcctgtcaactggcgccgtacagcctcgccagggagcctgcattcttcaagaatgtcctgtttgtggtggaccagatgcaccaacat

ggtcatgctacgtgcacaacgtcttcgtttctatctacttacatgaacacgaatccagagctccagaacatcaacagctctgccgcagaatgtggcaa

tgcaggactgtccaggatcaagaagtccgtctcctactgcagccaagaacatgcgatgctcctgatcaagcagttcctaggtgtatggaacagacgac

gtatgcgtgcaatgttaaaggaatga

>contig_14.contig_14.g55.t1

atgccatcttttgcgcgtacgctcgcgaatgcgattcagggcgcggacgtcgatcctcccagtcccgcacctaccagtc

cagcagcaggaaatggcaacgatacattaaacaatgagacaaatcgcgaatcccacgtgggaaacatcgaaggggaagcacttcccgcggcagatcaa

atatcacccgaagtagcagcgggagcaggagtggaaaacggactgcagccctctgctgaagacaatgcaaacgctgaagacgacgaggaccagactgg

gccgatgaaagattcagagcagcatcacatatggtacaaccttggcaatgcacacgaatatgctgtgaaaaactttaagagggagcctgtcgccaaaa

agagaagcaagattctgaaccggtccctatggagagtgatcaagtccgctgctgacctttccgcacgaacggacacggctatgtttctcgcctgggca

acgttggagcccggaaagcagaagcacaagcaagttgtctgggcgagcgagaacatatgcgatcctgctcgtccggtactgcacagcatgactcgcgc

catgcacgataaattccacgccgacattgccgtctatcgcgagaatcaagtcgcggaagctgcgagacatgcggcagagaaagcaacatggcaggcag

agcggatcgagcttctctcgcgcatcgcggaactccagcataatcgtgccgacggtgagggaggatcaggatcgagctcacagctgtag

>contig_14.contig_14.g56.t1

atgccggtggcctctagccctctgctggattggggggtatacgatgctgatgagttgagggaaaagccatactcgcaaa

ataaaaagcctactgaggttaggaagctacgcgcggtcgcagacgcagggtggcgcagccacccttcgccctgcccgccacttgcgaaagcgggtttc

aacagcagcttgacactgaccgtcacctattcctccttccagcgcaaccacagaccgaataccgatcccctaggactaggacagcaggatggcgacca

aaagggcaaaaagcgcccctcgctgcaacaaaggctgcacagaccccgacgacccaacccagaaggctctgaaggcgcagtgcgcctgcagcaggaag

aggagcagtcagttacaaaaactcagcaggactccagcgcgggcgcaccagcacctgctcccgctccagaccccgctctcgcccctggacccgcgcgc

ggcacctcacagacaggctcagagtccactgctgcgcggcaggggggagaatcttcggcggttttcgatgccgccagcacgattgccgctgtcgatcg

tctcctggccgaaacagcatcccaagcagcttag

>contig_14.contig_14.g57.t1

atgcaattttcagctcatcgatcccactcctatacactcaccgagccccgtcgcgctagtgcgtcagctgcatcctgca

caacctctgcgctccctcgcccgcaacgcatccgcagcgcgccggtcgtacagcgccgccgcggtctcggacattccaagctcgacttcactccatcc

caccaagccttctacatttccttggacgtcacccaggtgaaaattccggctgcatctggcgacatggatatcctcgcagcgcacgtgccctcggtaga

ggagctcaagccggatttggtggcaactcgacacagaagtggctcagccacccaaacagactcgccaacggtcaaatggctcccgcccatcccggccc

catcctctccagcatttcagggctttactaagccgtaccgtaactcagcacttgtccttgtaccggaagctgttgccggtgcttatgcagtcaggtct

agtatcaagtccgtagccaaaaaaaaaagagtcgctcttggattgctcacctcctacatcgccaacggcatgctgctatgccgcatgatcgccgtcac

agcctacaccacgggcaaggcttggggtaccggcgatgccttgtttccagaccgtcaacctggccttgctcgtccagagtggccaattgggacttcga

ttggcagtccaggtcattctacgcacgtaaatgacggcaagtaa

>contig_14.contig_14.g58.t1

atgggtttcgactttccgcgctcgctgtcatccacccttatgctgtcagctcgagttgctctccttcaacctcgtctgg

accaaatcgttcagctggcccttccaatcagctccagggcttcaaatcgtccggaccaggtgttctggccgaggtatcactacgagggtcggtcgatt

gaggttgagctttcggctggggagtggtggatggtgaggagaagcagtctttttacattcttcactccgaaatcagacccggctacaagcacagcatt

cggtccgggcgaagccgtgaagggcatgcggccggtgaatagcagatcttcggtcgacattctgattgtgggtctcgtcttcggcgtgctggctgtgt

tactgtatggctgtgctgatatgggtgccataaatgcttgtacatgcaatcccacctccagctgcgccatgaccgcccccaagggaaaaaccaagcac

ggctcggccagtccgacccgccttggctctcagcaggtcctttcggcttcgaacagcctccggctcttatgcttacacggctacacctcctttcgact

tcaagccgctttttaccgttcgtggcccaccaactcgttgctcgatcctaatctcgatgcaccccccttcttcaccgtccacactatccacttctcga

gcgccaccgatgagagcttgtctgcgctgtcccagccacgttgccctttggatcgacaccgagcgttgaggttgagtgatccctccgcctcgcgccat

gttctctcggtcgcgtatcagctcgccacccttccaacagcgactgaccgtgctccaactagtagcggccgacagtttcagacacgtttcaccggcaa

cagtcagatccccggatcttcctcgcccgcccaatttggggatcaggtgttcgcactacagaggagcacggttcggctcacacagccgatggcgacct

ggcgctgctggctcgtggtcttttcctttcaacagcattggaagatcttcctgccggcccgtctgtctcacaccactgtaccggaaacatcagatgcg

ttcgttgattcgtcgccgtcgaatgcggttgttctttcgtcgccagaaaacgcggccacccaatttgcgtcggcggacgacatcacctcgacgtcgaa

gatgatggtcgctgcctcgctgctgttcgccaatcgtcacgacccaaatacggacggccgacggcgtgcccaactggccggcgtacgatgcgaagagt

ag

>contig_14.contig_14.g59.t1

atgacttccgaagcggacatcctcgctgatgctaatgtactggtcattgccagtggtatcgcactgcatgagcgagaac

tcgacgccgctgcggatctacgaaggggcgagtctcaagaagaacaattgaaggaggccctggacaatttccttgcagcgatcggacgtgccgaacta

ccttggcgcgctctgcaacaatctggactggtgcttgaggctgtttccctcgtcaaattggttcagcaccgcgccaacgagagtaaagatgccgacgt

tgtctctgcgctgcagaatctcctcgacgcggcagggcctcagctcaagcagagcgtcgaggcgttgcaggcggccacaaagcggatcggtgatgtcg

cgcagaccagccgggaccggatcaggcagaatgattcggtgttgtccgtcatcagtgggacagtaggaacgagatcacagggccagaaggacgaaggc

acggcactcctgctcggcgagttcgttgagaacttgctggagaagatggagagctcgctggtggatgcggggagggaactcaagactaaactgggagt

caagtag

>contig_14.contig_14.g60.t1

atgtcggcagctgacaggaaagattctgcttcaggtgcatctcttccagagacggaacgtcaaccggcggatgagctgg

gaatgttgacagctcagaggttactgggcgagtcgcctagtggtgacttggccactgtcatcgagtccgccgcattcttgttatatacgtgtcagcgc

agcaagaagggatatcggctacgcgcgttcctcgtcaagctccttctccagctcggcctcttcaccaaggctactgatcattgggaccatctaaaggc

tgctttcatccaacacgacactcttggccatttgatactcaagagcttcgacgatcttctcttggagtctggcctcctgctcacctttgatcactttc

ctctggcgtataaaaaggcagcgtttgacgaagcttaccgcctttcttcattatctcttccttcccttcccctccttacccctctactctcgccccac

tccctctcccctaattccactctgttcacatcctccggtaccatgtttgcttccaagcccagcactctcgccctctccctcgtactcatcatctttgg

tggatcagtgttgccctcgatcgccagcgcacccaagcccaggtttccgcccctttcttccctcgtccgcgccgatctggaacgtcgggacccgaacg

aataccagctcttccacaagcaggagcacagcggaaatgtctcggcacgcagtgttgtctcgggaggaatctctcagtgcaggactcaagggtgcttt

gctttcacctttgacgacggcccggtgagctcgccctttctcgcaaaaacgcctgctgcttctgccatgttcgaggttctgatactgaagctcgatat

gtgtaaccatcgaaaggtcgctgacgatattgcgtctattggcgcccgcgctacgtttttcgtggttggcaatatgggcaaatgtatctacgatgagg

ccagcgtccaggccctgaggtactcctacaacgctggccatcagatctgctcgcacacctggtctcacccaaacatcgagaccctcagccgccccgcg

atcgaccagcaggtccaactcgtcgaggaggccctgttcaagattctgggtgtggttcctgcttgcatccggcctccttacggaagcgctagcccatc

tacaatccagtacctgaaccagaagcacaaccttaacgttattacttggaacgcctacatccgcgacgctgatggtgctcctgccgacttctcgatca

accaataccgctccctccacgcgccccagaacgtcatcatcttcaaccacgagaccgtccctaccacgagtaaccaggtcattccccaggccctccag

atcgtccggcagaacggctatcatcctagaaacatgggcaccgtcgctcagactgtcggcttcgcgccgtacaaggtcatcggcaggccgggcgttcg

cgattcttcgtggacgtgcgccggtaagccgggtggtggtggctggtag

>contig_14.contig_14.g61.t1

atgcccactgctctgcggccggtctctgccgacgacacctacgcgtcgctagaggacttcaagcatgctgttcgcctct

atgcaatccaaaacaacttcaatcccacatgggaacagtcgaagtcgacgtgggttgtggcagtttgcaagcacaagaaggccagtggctgcactttt

cggattcgggcacatgttgaccaagacgacactgacgtggtgcgcgtctcgaccttcgacgaagggcatgcattttccggcaaagcgccttcgaagcg

caaagcctatgcagatcacggctttcttgtttgctggcctggtgccgtctgctacatgatccagagctgcatgagcatcgatcacaagacgaccgacg

cccaggtgctcgcccagctgaagcagatacacgggattacacttctccagaacacggttaataaagcacgaaatgctgttatcggttctgcgaaggcg

gctcagcaggaggagttccggtacattgaagcgtggctcatccgactcagtgagaaggaccccggcgccgtgacggagcatgtcacagtcgacgggac

gttcacccgcacgttcctcagccccggtcctgctcggcgggcgtggacggcgtgcaagccgttcatggcagtcgacggcaccttcaccaagaacaaat

ttaatatggttttactccttgctgccagcatggacgccgacagcaacctcgtcattctcgcgtgggctttcgttccaactgagagtacggacacgtgg

gaatggtttctcggcaagctacttattgcctttccctctctcgcccagtcgtcaagcacaatcatctcggaccgacagaagggactcatcgcggcagt

ccgagcggtgctccccaatacaaccgagggatattgctgctggaatctggctgagaacgtgaagaagggttacgcgttcccgggtcgacgcttcgacc

acgtgacgtctaatctgagcgagatcgccaactctgctctgcgacagcaccgggaactccctcccctccagttgatggcgggggtcgacacgtctggc

ctcgtggcttgcaacatgggaaagactttcgaagtcactctcgcgacagaccccactaaggcgctgggaaaagacgctaccacgtacgcgtggtatta

ctggacttcggcgacctggcgccgcacatatcagacgccctacaccccgatctctcgtgacgacctgcaggcgaacgaggtggaagcccccgcgattg

ccaacaagcgcggtcggcccgcaacacagcgcaaaaaggctgggcagggtaagggtaagaggaagtcttccaccaatgctttttttgcggattcttct

agctccgcaggtgcaggatcatctacccctgctccccctgtccctacattttgccgaacatgcggtgatggaggacacaccaccaaacagtgccgcaa

cttccatcagtcctcctttctctcctga

>contig_14.contig_14.g62.t1

atgtcggaaggcactgatactgctgagcggactaaccagactccggacgcgacactggccgagatcgcggccagcctgc

gtcgaatggagtcaaggatcggcggcgttgaaaacgcctttgtcgatcatcaagtccgtctatccgccatcgtcaacgcgtatccaatgacggcatca

ggtcctttccatacgccaccgcccatgtcgatgggtccgaactttgggtacatcgtcggccaaacccccggtcatgtacagttccagcaaggccccat

accgacctatggaatgccgtggactgttcgaggttatggaccacctgggtctcctccgggtccagctcataccctgcttcagtcaggatccgttcctc

aagccggcggaccgttcggcctcacgcccccgatggcgcctgtgcccttgcccatgcatcttcggcgtgtccggtttcccgctccccccgaggcaacg

cctattggacctcggctctccaccgccgagacacctcaatcgggaccgagcggacgaccgaacgagccgacattctccttgacggataacgatcccat

tctgttggcagcccgcgctcagctagtcgaggcatattgcttctgctaccgagaacagcatccagattgggattccgatcgcctggagaaggagctga

aggcgaccgtgtctacggagtaccttgccttgcgacgcaagaaaatcgaggaggccgtgcaagctgggaaggtcagcaagaccggcaacggaggttcc

aacgaatcatcccacaagaagaaggaacacttgctcgcctcagttccaggtggaatcggtatcctcgaaggaaccgtgtacggtccccgatacctcag

ccctgacgactcgtcctcccaccccttctacgatcacgcgttggatatcgagctcggacaggccatccttgcgctgatcgaagaagaagttatgggag

acatcatgacgtctcagtcggacggcgaagttcgcggaagcatttactacagagagctacgtaacgctcttcagccatcagccggatatggagccatg

atcctggacgacaacatcgctaaggccacccaaaaggatggcgagacggctcgcaagttcggctcgcgacttcgagggatgttcttcgacatacaagg

cgctggagacccagtggcaccgcacaagaaggtcatgcacttcctcctaggcctgaacccccggttccatacggcaagaaacgagttcatccacaagc

agtcgacaggacaaggaatcgatttcgacactgcccttcggatcgttgcggaggccgagcatgcatatggagctcttatgcaaccacgatcggctccc

agggttccttacctcgcacgaccgacagccaatgccgtggtcgaagaggctatggttgctgcagcaaacccatctgatgaggagtgtcgtccctgcac

ccatgcccatgcacccccaaacgaagccgtggcgagcatggctatggcatttccagcctttggaagcgtggaagcacaggagtgggaggaccagatcg

tttcctggcctgctgcactcaatgtggcgacatcacctggatactcgactccggggcgacacatcacttcgttggcagccacatcacgctcgaccctg

gatcggctcaagctgtcagacgtccttgtggtgccttctttccctttcaatctactttcggttcaacgactcaaccaaacaggagtcagtgtgaactt

ccctcgtgcttccgatatgacaatgctagtcagttcgctcggaacgcatgaggtcaagcgcgatatgaaccgtggcgccactgtcatcagatcgtcgc

agaggggtcatggccattttgctggagcggcagcaatggtgtccaccgaggtggacaagcctgacccaacgggatgctcgcgagcatcacagcctgcc

cccgtatcctgtgtcacgagagactcgtctcaagtgaatgcgctccacaggatgggggcaccctgttccggtacgtgttcggatgtcctacctgctcc

acacatgttcaccgctgaccctttctctcctctccgtttggtgccccacgagcccaagcccccgacaggccagacactgcctagtccgggcgacgagt

gcgtgtgtctgtgcatgcagcacatggctaccctcgtcggagagttggaaaaggtgatgacgcttctccttactgatgcagccaatgcggcacgtgca

ctcgcactcgaactagccaccatccgctggcatcagcgactgggtcacgcaagcttcaagacgattgcacaactggtcggtaatggagtccttggcct

cgaaaagcttggcaagacagcggcccttcgcttggttggacgcgaagacaaatgcgatgcctgtgcgactgccaacctcaaacgagcgccgtacggcc

tgtcggactctagagcagcacagcctggtgacctgttccataatccattgcgatctgatgggaccattgtgatgcagagatggcatcgccttgtgatt

gctcacctgggtcggcgaatcaagatcctccacacggataacggtaccgagttcgacaacaaagccatggaggagtgggctgcatctgaaggaatagg

gtggaaatggacagtgccggactctagcgaacaaaatggccgagtcgaacggcttcaaggagtcatccaggaacgaggacgagccatgatgacagcag

cacgcttgccacatggattctggccattcgcgtggaggtacgcgatgtacatcatcaatcgaacgccccatagtcgcctggactttcagatcccacaa

gagttgtggacaggcgaccgagtcgacttgagcggcctgcttgtcttcgacaaaaccagaacctacgacgagggcaagtcattgaggattcctcaacg

ccacactctccggcaagacgttgaagtcgaaatgaacattgacgaggaacaagccaccgttccaaacgtcatcacgaccgcggaggagctcgaaagct

ttgacaaggacgacgttttgatggaggttgcagggcaggacgcggggcgatcgctggagcaggagggggagaaggagcaggaagagatgacgaaaagg

gtacaggagcaggagggggtaagagagcgtgggccatcggaggaacagcagcagctgggaatcccaagcactgccaatctcgaggagacagtcgaaga

tcaaacgacctcggagatggacgatgcggtccaacgacgcaagagtcctcgactacatggagacctccgcagcaattccggggcctctcagggcattc

cgcattccggttaa

>contig_14.contig_14.g63.t1

atggacgaagacagggcaggggccccgcaggcgcaagccactcccaaagtaagcaccctagatttatctgtatgcgctc

cgactatccaaggcgtgcaatcattgaacggcgcgggatttccctccgcagatgctattgttttcggggagagcactctgtcagagagtgctcagggt

gcagcgtctctgaacgcggcccagagggaaccccatccctcgctgcccccgcaggcaaacaccggcaagacgacaagcacgaccgagagctgggcgga

ccaagttcaggccgccgacgaggccagaaaggctcaagccgatctagaggccagagaggctgctgccggcagggctctcaagcgcaaggctgtgagca

tgtctccgttgaaagagcgctcttctgctctcgacgcgatggaaagcacctcggacagtggagcggacacggacacctctgaagcacgagcctccgtc

cggactaaagcgccgctcgccctcaagcccgcctccgtgttcgcacgacgcgtttcggcgcgaattcaggagaaccacatccagcccgccccggaaaa

gcagacagtcgtcaccggagtcagccgaaacacccgccgcaaaggaagtgcccgctgcgaaggaggcaccagcttcccccgcgaaacgcgctgctccg

gcagcgaagacgagggcaaagtctgctccgcgccctctacaagccctggagcgccgcatccaggctgccctcagtcagcgcgacgagaaggaagcggc

cttgcgtggcttcttcgagacatcggcacgctcctctccctcgccgctggtgtcgcctttgccaagccggggagcactcctatctgccctgagtggca

ttctctcctcgcgagcgccgacctctccaagctccctgcccagtccggcgtcccactcacagccgccgacgcaaccacagccctggacacctctgcct

ggccgctgccttccgcctctggaacgacgaggtcttccaagccctcctcctcccccgcaaacgctgcccgcccggaaaaccgcacattcattcgcctc

ttggacggcaacaaagcccgggacgaagatcccttcgagattcgactgcggctggagaagctcttggcctcactgaaccccttgctgaccattggacg

cgtcaaggcgatcaaatcaggcttctcattcacgcctggtgtgagttgcaacgctcgcgccttcgagccgtacttctcgcaaatccaaaaagcgttcg

atgcgaaggacgttgagggaccctgccagtaccgccaattttgtttccgaggactgccagcaaaaattgtctccggcggtgcgcccaaagcggtgact

ccgcacgacgtccaggtccaggtgtgcgcgcgcttccccgggatacagctggcaatacagccccgtatcctcacccctgcccgccatgagagccccgc

ccccctctggcttctcagcgtcgccgaaggcgaattctcttcggacggccagccctgccccagtcgcgtcgacataatggatcggacatgcaccctcc

gcccataccgcgccaacaacgccagcaaacactgcgcgtgcgtccccgagtgcgcgcattgcaaggcgccacagactaccggccaccccgtctgccga

gcccacccggtatgggacagcaaggtcaatgcggtcattgtgccggaaggagagcggctcgcccgcatccagaacaggggccgatccgagcgcaagct

caccatctcccgactccgcgcacaagccgcacaacgcaccacctcagccgccatcagcgcaacccaaccaccagccaacagcgagtcgtcaactcggc

aagaagacaccacccccaacaccaacacacctatcgacgcatccgggccgcgcagtaacgccctggaacctggcggtttcttttccaactctagaacc

cttcccagaatccttggcaagcattttcaggtctacctgccggacacgcccgacggcatacgaccgcacgtggccacctacgtccgcaaacacggact

cgattgggcaccagcatcgcaaacgacaatgacaaagggcagccgcgacatactcgttctcgagctgaagcatgcgagcgggcagaacattcgggtgg

tcaatgtctacaatgcaccggcgggatgcgaccaccccggagaaggggctaagaagctgatggagctcccggtcacgcgcagtccatgtctggtcgga

ggcgatttcaatctttgccatgacgactgggccgcggagtggtgggcggggagtactacggcgcaagcggcagagctcggagacttcctagggacaga

ggagtgggaattgggcctggagaaaggctccatcacgaggatgtcggaggggggaggatcggccctggacctcgtctttctcaaccggccactcactc

aacggggctggttagccagctgcgaggtccgacctgatctaagcgcaggctctgaccactggccggtgcttaccaagctccagtgcggtcagggccag

gcgctccgccgagagctgcgcttccggttcgttcgcgccgaatgggacaagctccggcagcagcttatgtcccagagagactcccttcttgagccagc

actctccaagctacgagcaggacgggaaccagcatcggtccgacacaccatcgaccggctggccgaggccatgcagcgctggatcttcgctggaatgg

aagcagcaatccctcgcacagccacgaccggacaggggcacccctggtggtcaccagagtgcacgaccgcgacaaaggagctatccaagatggaagcg

aagcttgcccaagttctatccggtggcctcgttgacatggtcctccaagagtcagtggctcaagcaaggtcgacggcgaagcggaaattggcagctgc

gcgccggaacttctacagggagcaggcggggaagatagagggcaatgagatctttgtggtgcggaagtgggctctaggacaacgccagtacgcgtcag

ccgcgatgcgggactccgacggacgtgatttcgtcaccccggccgagaagcgaaccctgctgcgggacactctgcttccccaccacgacgaacaggac

ccactactcgtgctctcacagctctcaccgagtgccgcgcggagcacgcacgcaagtctcacacgcgcggaactgaaggaagcagtagtgctgcgaga

gggatggtcggcgatcgaggaaccgctcttcgtgctcgcgtcagcagccttcaacgagggatggtacccaaccgccttccgtacctccactctatgtg

cgctgaagaaaggaggcaagcgcgatcccgccttggcgcgatcctaccgtctcatcgcattgcttcccgttctcggaaaagccctggagaaggtggcg

gccaaccgactaacgtggttcgcggagcagttcagtgcagtaccgctcgaacagtatggcgcgatgccgaagcgaagtgcgaccgatgcgggggtggc

cctcacacacgacatacacgtgggttgggcacgccacgaccggctcaccacatcggtactcttcttcgatgtggtcggcgcattcgacaacgtcacgc

cgggaagaatggttctgcgcctgtgggagataggtgtcccgctcccacttattgccctcgtcgcctcgtggctggccgccagagttgcagccatcaga

ctggatggtgagatgggcacagcagagccatgtgcaactggactcccacaaggatctccgctaagcatgatcctctttgttctcttcctcagcccgat

gtgggacgccatcccagccggagtccgcctctttgggtttgtggacgacggaggactgcgaactcagagcaccacaatcgagcagaactgccgcacgc

tcgagcaggcatacgacgctgcattgcactgggcgaacgagaacggactgtggtttgacaaagtcaaacgagagctcatccactttccccctccccgc

atcaagcccctcccaaagctgctgcccgtacgactcggtcctgccgaagatgatctggtacaccgcgtcgcgcgagacgcctcgggactggtgaacct

ggcggaagcggttcagaatgtagcattgcgcctaattttgcccgtgtggaggaccacccccatcgtctccctgcagcgcgaggcgagtctgccaccgg

tccgtctggtgctcgactacctacgcgccctttatgcgattcggctccatgcactcccccaacagcaccccgtcgccgttcgcctgactgctgtggtc

ccggcgcaaaaacgcagcagcctccttcaagtgcgcagccgcacgaaccggggccacctcacaataaaaaccacaccattgcttgagatggccaggga

ggtacaggacgtggaacggcatggcccagggccagacgccccttggctccccacgttcgagcaacaggcaggtgtcaccatctctctctttgagggca

gcgacaaggacaccgtggcgaaacagcacaatgctctgatcgaggccaaagcacccaccctgctggtgtactcagacggctcgctatcggcagagggc

gcggcaggagctggatttgtcgtctaccgagcccgcgaaaacacacggaacaaggtccttgacgcgagcatccatctccatcaagacaaggaggtctt

tgatgccgaggtatatggcatgttcacaggtctcaaaactgcgttcagtgccgcagaccggagtggagagcaggacatcatggccttcattgacaacc

aggcagcgctccgggcactcgcacaacgctcaaggacaagtgccagcagcggatctcttctcaccctcacccgccgagaaactgtcaagtggctggaa

tctgacccagctcgctcggttcacttggcatgggtgccgggacacaaagatatcgaggggaacgactacgcagacgaaatggcaaagaagggctcctc

cgcgctgcgagccgacatcagcgtcggcaggcgatggacaagtcttgccaaggctaagcgccgggccaaggaaaggatgcttcaacaatggacgcgcg

agtggagcgacagtgcgcgccatggtcaataccgccgcctccgcacacagtccccatcattgacgccagcgcctcatttgggatctcttccccgtcga

gaactgggcctatggctgcaggccaaaacgggccacggagatttcagcacataccaccaaagacctcatttcaagcaccccgacgcacacatccactg

ccgatgtggccgccataagacacgccttcaccccctcatatgccctacctttcttcaacaccaggatctgctggaccctgcccgacttccgtcaggtc

aactcaacttcgagtacctcttcaacgacaacaagggcatttccctttttgccgagcagacgcgagggcagccctccggcagtagcgaaaaatttgtt

gttactgggtctctggctggacgtcgatctcctcttcccctatgtctcttctccactcttgtatggcccctcttggtgcccgcgaccaacgcttcgcc

cgtacgctccaacttcactgccagttcggtagtggagggtggagctgccgacgcttgtggattgacaaagatcatacatggcaacatcccgcctccac

atttcccacgttacattttcctcgctctcatcctttccgctctcgagctcatccttgtattctttcgacttctctctgtcgcatcacaactcaactca

tacttcactttacttgcaagactagtacttctcacaactagcgtcttgtacatggtgatgagagcgccctcgaccagtccgatttctttctgcatcct

cttttggccccctgagcacctcgacgacttctctcttcccctgcccttctctgtcgacgcgcaggcgcagcttcgccttgaggacggacagccagtca

tttactgtcagcatcgccgctctgacgttctgcacaacgctcttcccgccacgaagactccccgccgcgtgcatacaggggatatccttaccgtcctg

tcgtcggacaacccaaccactgtctccgtccgatatgccgttcaggtggccttcttctccttctcggccgacctgcgtgcagaagtgaacgtcgagct

gcagcggcttcagtccgcgtcggtgctcgcgttacaggctcaaacatcaccccactttcccctcgcgctacccctccacggcaagcttccaactcctg

caccggatggtacgaactgggatggagtgcgggatctcatcaagaccgttcagagtcattccttccagaacgccgatcaagatcgaacgcgtgacggc

gtggtctgcgcatcatcttcttccgccctttccaatctgccgccgccgccgtctttggagtctcgcacgtatgcacagctgatcgccaacgaatcctc

cgctggaccggcatcttcatcatccccgctcgtctcgccctcttcatcatcgtcgagctccggctcaccgttggctgctaccttctcatccgctggac

gcaccccttcggaatctaccggaaacgggacaagttcaaagacctcgttgccggcatccgtgttcatctcgtcgtctggactgcttccatcagcgact

gcgcaggccggctcgatccgcgaggtgtccactccaggctcacccaggccgtgttcaccatccgcacccgtaggagcgtcctcatctgctttcgggcc

atcttcgaatcctgctcggtacgctacagctcaatcggctgcgtcccggatggcatctaccctatctttggtatcggcggaaacggcccttgcacgcg

tccgacaggcgtggttaatgcttcgatatgaggtgctacactctgagctctcggtgaacttggtgcgcgctcctggtcgtgatcagcaccaccacgcg

aacgatgctgtcgatcctccccctgcggtgcagcacagtccttcggatactatcgatgtcgtcctatcacgcgttcgccgggcactgacatcttgcat

tgccgcgcgcactgatgagcattcagcatcgccgatgacggtccctgaaactacttccgccgcgacttctcctaactcctccgtccctctgccgggct

tcgacgctacaagttacggatcaccttccttcccattgcctggtttccacagctatccgccacctccgtttactcccttcatttcgaatttgtggcag

tcattacctctcgccgcgatcgccacgaacatcacccttctgctttctggcatcttcaacccgttcggtcctcatcctcttgcttcgccatgccacgg

tcatctcccggcttcacatcattggtga

>contig_14.contig_14.g64.t1

atgcgaggtcgtcagaaacggaagcgcgtcaacgagagcgaagacttggactacatagacttggagcccgttgcagaca

ccaacacattcaaatgccttccttgcagcgatatgggagtcggaagcggagttggaatagatgcatttgcacacttacgcgacgaggagatcggtggg

agggaagcattggaagacgacgcgcaaggagttgggcttggagatgagtatctacttggagcctctgccagcgaccgtcgagctgaacgcttctggca

ggaggcggaacgtgacgcagatgaagattggctggccctgcggcacgaagaggaggaggaacgcaatcgagagctcgatctgactattcaggcggctg

cagatggtctacgtagacagcataaagcgaacccagatggttggtttccttttttatctaaagaaagctttgcatcaatatgctactggctatctccc

agacttgcggtctccaaggaggagtttcaaactcacatcaagatcgctcgcacgtacggcggaacacctcccattctccatcgtgttgagaccgactt

ggccgcagcacgcaaactctttccggcgcggccacgacgttacctagatacgtccaaacagcccatatatgtcagaagcattgcggacatcattcgaa

acgacttttcctccccctcaacacgctcaaacatgcatctccttccccgtcgggggcctcagctcaaggagtttcgagatggcaaggcgttggcattc

aatgaggacacacggacgtcgcctcccatggtctacctcgagggacactcgttttggacggacgaggtatactgccttgatgatgatgtcgtcgttcg

cccttacatcttctttgaagatgagatcttcccttcagatctgcaagaggaagagctcaggcacggcaatccattacgcgttacggcaggcaacaaga

aggtgtattcggtaccgattggaatcttctgcgacgatctgtctggaaacaaatcgaagaagtggaacaagcacgaggccttgtatttttcgaaccta

ctgttggaccgcgcccttctagacttggacgcacacacacacttcttgtccgtcagtgcatctgtcacggcgactgcgcaactcgaagtggtcgttac

cgctctcatcgatgtctacaacaatccgattacagttttcgatgtcctttgcaacgagctcgttttggttcgacccttcctgctcgcagcattctgtg

acaatccgatggccgcagaactcagcgcatctatcggacggctcagatgcacggtccagcagctatacagacttgacgagcagatcaaggctgcaaag

ggcggcgtgaagaacgggcctgctcgcgacgcagaggaactccggctaaggtcacagccgtggcatgggccactgcttagactgcgcgcggtgacagg

tttcgacgtagcatctcagatgccgatcgaggttctccacacttggctacttgggcccgctaagtatctttggatcgaaacgtgtgcaatgtcggacg

ccaagcgggacgccttgaagaccaggctatcagctgctacaatgaccggtgtgggagatacaggttcgctgcgaggaggatatttggttcagaatgcg

gaccgacctgacgaacgccctcaacaatttcttctgggcggcagcttcgctctttcctgctcgagtggtgtcaaagcggaagttccatcttctggtac

acctcgtcgatag

>contig_14.contig_14.g65.t1

atgcaagcattgatgaacatggccgatgtttccccctgtcttgctgccgacgcagcggcccttgccgacgaagacgatc

ttggcccgatgtttgagaggcatcgaagatggcagggcttgcctaggaatgagaaagaagacgacacgcagtttacggacgagaaaagcgaccttggc

aaggttcctcgccatattcgtacccctcaaaactcccagcgcgcaggaccaagtcagctcaacaggacgcgacgccactcgagcaagaacagcaagcg

agccagtttggatctcaaagacctgctcgcctgctacgacagcgatgaggaagcgagacctgaagtcagggagaactatgaggagatgggtgacgggg

gaaaaccatggtgggaggcggaggatgaagaggaggaagaggaagaggaagaggaagaggaagaggaagaggaagaggaagaggaagaggaagaggaa

gaggaagaggaagaggaagaggaagaggaagaggaagaggaagaggaagaggaagaggaagaggaagaggaagaggaagaggaagaggagggaagggc

agatgaggaggacttcgaaggagaagaggagaaggaggacgaggagatggacgaagaggatggagttgaggaggagaagcgaagtgaggagcgagaag

aggacgacgagaaggaggacaagaacgttcgcaccttccgcaaatcgtacggactaaacgtagccgaagcagtgcaggtacttgacagcattttggta

gtcgacggcaaaggtcagaacgcatctcgagaagtggtatggtctgctgtcgcttccgtcaaggcgatgagcaagatggctatgtctatgcaacagat

caatgcctaccagaagaggaacgagatgttgctcaggcgtgtggaacgaaacaccatacgttttgagcagatgcagaggaacttggagttggtgcatg

caaaactcgaagagacaaacgccaagctcgacgagacgaccgtaaagctggaagagctgcaggagagcctcgcggatatgaagcccacgaagcttcct

aagcccgaacttgtaagcagatactggttcagtggattcaacatcatgcttatcacgttctattgtgctgatatccacctgctggctgctgtgttcgg

gactgatcaggccaaattgaagcagaccgcagcgcagcttttctacagcccaaaggtggagtcctactcggctcaaggcaatcttttcaagaacgtga

aggtgggtggcagtatgaccgctgacccttcatcgttcattgttcaaatctgctaccgaattggaagtactgaagaagcattcaataacgttcctcag

cgctacatcatcagcttcccattggacgtcgggccgatctgcaaaacgatcctagacagcgccgacgaggataccatcaagtccgaaatatgggagcc

tctagcacacaagctcggcacccttagatgcaacttcagggactatctgcatgaatcgatagtgaagcttcaaacactcccggaagtcttccacaaag

cgaccgatagctatgaggttcctatgaccagtgatcgcattcgcaggtttgcacttctgagatgctatgtgaaacaaatcggcgctcgtaatgccgat

gggacaacaaaggcaggcctctggaagaccgttgatgaagctctggaaggcgtcagacagcgcgagaagacggaaccccacttggtgaaagccgacat

gaaggcaatcgtaacccacgacaagaaaaagtacggagaatactcggacaaatcactggagaaggaaccttcgcgcgacgctgagttcgccaaatgct

tcaactag

>contig_14.contig_14.g66.t1

atgaccattgaccagcaagcgggtcctgccatcccctctcccagcggccagcagcggcctccgtcctctcccacccaag

gccccaagtcctcttatcccttccgtcggccccgtctcacctgccacgagcctaaagtcttcggcaacctcctattgacgaaaccttctggcaagacg

ttcgctgcctcattccagagcgcaaccgacagtcagaatcgcgttctgctctccagtagcggcaagaacactaccgaccccgcctcctcattccagct

cacccagtgcgaatccacagtcatgcctgagactggagcagacaattctgatggctcgatcacttactacggccaactgcgatccaacactcatcctg

atcagtgcgttactgccacgactttcggcggttcctctccctctccctcgactctcgtgagccagccttgttcgtggacggacaactctgggcttatc

ggccaatggtttgctctcacggagtacaaggccacaggcaagccgtcgacctatgcactcaactttgtcggaaagacgagcttcagcacgaggaactc

gacctacggctggaccagcactcgcgcaaagaacggcgctcgcgtggtcaagctcatctactcgccgaatggaaacactaccactgctacaggccaac

atgatcagtcccatcaggaaccccgcccttcgacacctcaaagcccgaaattctcgacgctggcactcgctctcactcttccgtcttcaagattgctc

tctttcaccaagggcatgcttttcaatggcctgcttcctgtctcctacctcccctcctcatcttctcatctcctcatcgccattcctccatcagatac

tgcatctgcaagggccaagggccaaggtatcgagctcctaccctctcagcaacgtcgtcatgcgcacgctatgacgaccatatttggtttctttcttg

atactactccacccatctcatccaagctcaaggttcttcgccattcttcgctgatactgcatcatcatcctggagttctcttgcagtacgtcgaattt

ctctgtcactacctcgctggtgacgatcttcgtgatgttcgcaagggtaacggcgaggggctctctgcgatcctatcagaaagatttgaagtgagtga

agtatgcctcgtcagaacatgtctggatgctgtcggtactgacttctgtttctacccggacgacgagccagcgggaacgttctgttggacctcagcca

tctag

>contig_14.contig_14.g67.t1

atgcagttcaccgcctcaatcgctcttcttcttctcgccattgccggctcggctgtcaccgctattgtccccgcatcca

cagacacctgcactgtcctgcagcgggcctccctcatcaacctcgacaaacttggctgcgcaaaggtcagcgctgacgtcctcagcaagacgaagccg

atcgccaaggacgagacctgcaaggccctccaggacggcggcctgatcaacctggacgctctcggctgtgccaaggtcgaggccaacgttctcgctga

tggcactgccaagtctgcctcttgctccgccaagaagctctgcacggtcatgcagaagggactcctgaacgtcgacctccttggctgcaccaacgtcg

acctcgacgtcctcggcctcgccggggtccacttggacggcggccacggcaagcgcaagcacgcccaccttcccattcccgccaagcctgccacctgt

cgcgccgagacgtgccagatcctccagaagggcggtctgggcaacctcgacctccttggctgtgggaacgtgggcgcccacgtcggtccttga

>contig_14.contig_14.g68.t1

atgcctcctcctccttcttcccctcccgctccgtcgtcagcatcagcatcagcatcagcatcaggatcagcctcgccgt

cggcactggtcctgagcgagagtcacatcgcgccactgaggcaggatggatcgacgcccgtcttcctcaccgctctctccactgcgtgtgggtggatc

tacaccgcagcatggtcagcgagcttctacccgcaagtcgtcctcaatgcacgccgcaagtccgtctcgggcctctccctcgacttcgcctttctgaa

caactttgggtttctgtgctatgcagtcttcaacctcgccttcttcgcgtcgccccttgtcagggaacagtatcgagcccggcacggtggacacgaca

acatcgttcgcttgaacgacgtcgcctttgctgtgcatgcgtttgtgagtattaccatgacctatcaccgcgcccccgaccagcggacatcaaaaccg

atccgaatcctgatcgctatctttgccctcatcgtcactgtatcagccttggtctgcctcatcactggcggtaagacaagttctggcaagtcagtccc

actggaatggctcgactttgtccagatcttgtcgagcatcaagctgtgcatcactttcatcaagtacctgcctcagatgcttctcaactaccggcgca

aatctacgctcgggtggagcatagagaacatcctcctcgatctcacaggcggggtcttctccttgttccagctatttatcgattcgtctcaaacagga

gactggtcaagcgcagttggcgacttctccaaactcggattgggtcttatcagtatcggattcgacatcatcttcataatccagcactacgtcttgta

cggagccgtcgaggtcgaagaggaaggagggcaggttgaaggtgatcacagcgagcggactggtcttctcgcaaacggcgcggcggcgcgagagtaa

>contig_14.contig_14.g69.t1

atgtggacagtgatcgtcgctgtctggctggagctcaagatgagatgcctcgatggcaagatgagcatccttgtactcg

cacgacactcgcccgcacaacatccatctggagacgaccacaacgacggcgccgcgcaagaacagcatcgacgagcaccgttgcacccggccatgacc

accagcaagaagagcagcgacgacgacgtggacgagatgctcgcccacgcaaagcagcatttccccgagaacccatttgcggatgcctcagcgcacga

gcctcagcctcaacctcacacagcggggatgagcagtgccagcgccagtaccagtgccaacaccgccgagacgcccgccatcgctgccagcatcggcg

acaagaatcccttccgcgctcacatcgccgcgggggccagcagcggcatcttgtctcctcagcatactgctgcgagtgggagcacagccggtcgtcaa

ggcgggaccggcgcatttaatctcgccggtctgaacgactcgcttcccaggcctcctgcggtcggagaggcgccggcacctgccaccgcggcctcgtc

aggagggccagcgcagactgcagcgcaagcaccctcagagccgaccgtacggagcgagatctcagctgcggaagcatcaagcacggccattccttccc

cacccacgtatgctcccgatgcgccatctcctttgggtgctccatcagcgacagcctcgacgtatgcacctccagtcggaccgccccccgagcaccgg

cctgatgcgcctgaccgcatagattctcatcggcgtgccgcttccaccaattcctcacacccctcacactcgtccgcgcacgtaccacctcctcaggc

tccagctccagttccagccccagcgccgatggcttacgtaccgactaatgtacccactcccggcgcgcccctcctgcgcaagggcaaactcctcgtct

acccacgcggaatggctgaatgccacaaatgccacaacacgggctacaagttcgacgatcctacccatccctgccgcggctgttggtcccgctacggc

gattctgtcaattcaatcttgcaccgatatgcatcctttgatcgtgttccgggcgtcctacagagtccgctgcccatacgctccggtccgtctgttgg

cgtgcatcggccgtatgatggctcgcatcggccctctgcctcggcgggatacccgggcgcgatggccagcacgaatacgtaccgatatatggacaacg

cctacgacggtgggtttgggcacggcattcatacagctgttcttccagcctggctccgagacccgcaaccacaaccgcagcggaggccttcatcgtca

actccggcctttcagcccccgccgggacctccgggaccacagcctccatcgacccgtcctgctcccgctcaggacgaggcgcgtgctgggggacgaca

gcgaacggacgcggaacccacgggcgacgagggcgtctccgccgaaggagaggtgcctccttcgtacgacgaagccgtgtcggccgcagccgacggga

ggaccttgaacccgcagcatgtgcccccacgaccacatggactgcccacgtcgaaccgaccccacgtccaaaccggtccagctccgccaccaccaccg

cggccggccggatatggtgcttacaacgccggtccacagtctccctatggcgcaccacatcatcctcacaacccgcacgcgccctaccaaccacatca

acacgctccttcgaacccacccgtggtcggtccgccggaggcaccctacggctttgcacccgtgtcgaatgtgcattttgtcgactcgaggcgccggc

cgccggctggtgcgctcgttgtcagaccgggcgatcccaggattggcggttcttggactccctctttggtgcgccggacgagcagtgctatgcttgtc

gcggcagtgggcgcgtgtttcgctagcacgagtggccagaaagtcgcccctcgccgacgagcatccaactcttaa

>contig_14.contig_14.g70.t1

atggcaaccaacacaggagcgcgtggcagcgcagtcaagcgcgcaacctatgagaagaagtcactgtacgcgcgtctgc

tcccactcagcatcttcagtccacatcaacagcaggagcagcaacagccgtccttcttcaccgcccgctacgactcgctcctcaataccgtctggctc

gacttcgccgcgtccacctcaactgcgacccacatcgacaacctcggcctctggcacgccggtttcttcgggaaaggaaccctctcccgctccgaacc

cacctggcacactcgtaccctcgcctccgtccagatccaacgcgcacgtcagcaggggcgtctcagcagtgacaaactcacgccggaggaaatgaccg

cttggagacggagggaacgcatgcaggcgaagatcgagagggccaagttggccgtcaaggcggggaccatgctcaaggatggcatcgttgcccttggg

ggtagcctcgaggacgacctcgatgacgatgagctggatgagggagggacagagcagatgggagagactacgactggaggaagcggtcttgccgacga

gggctaccggacctttgtccccggcctggtccatctcagaccaaccgccaagggcaagggaaaagtcgcagacgatgtgatccgacaatcgatgcaag

ccggtccacaagccgtctcccaggcctcactcgaggacatcgtgccagacggggcgaacgacgaggacgactttgacctgctcgagttggagcgtatg

caactctcattgcaggagacgttcttcctcgctgggatgctcggcgtgctggatgtgcgggacgaacaagatcggttattacacctggaagacctata

tcacacccttctccgctccccgctctcgcccacggtacgggctcagccatcagtccaaccgaccacgtatctgcgaccagacaaccctttcctcgtgc

actacatcgcataccaccacttccgctccctcggctgggtcgtcaagacgggcctgaaattctgtgtcgacttgctcatctacaagcgcgggcccgtc

ttctcgcatgccgaatttgccgtcctcgtcgttcccgtgtacgaagatccagaggatgccaagtcgtaccccttcgagactcccctgctcaactgctc

cggtgaactcgactggatcaagttccacacctacaacagagtcaacacacacgtccaaaagactctcatcctggcgcatgtacacatccccgccctct

cacggacgccaacgtcctacctgtccagcccagaggagatggtcaagcaactcaaagctggccagctgtggtccgtccgggaggtggccattcgcaga

tggagtcccgccaggatgcggccgtaa

>contig_14.contig_14.g71.t1

atgaagcccgccgctgtcgagctcggagccttttccacaatcttcctatgtggtgatggtgatgaggcactgaccacct

caacgcctatggaacgccgtttcggccacctcatcaacaaccacgatcaacaaccaacgaccatcgaccatgagcccaagcgagaaggaacagccagc

agggatcagtcccgatcacaagcacctctgcaggctgcaacaggtagcttcctcgtcaaaatgagcaccaggtacagcaacgacaatgaatacgatga

tattgctaatctcatgacgtctggacacagcgacagtgagcgagagatgggaaagtctgtgcgcagtttgtctcctggtggtctcgggtacgccgact

cacgtccgcgcgtggggtcttcctcgtcttacgggcagatgggtgccggatcttcgcgtcttggattcattgcaccacgcccagtttctaccagcgct

gcagctagtttgtctgcccgtctgcataaatctacaggcggcatacgcaccgcagcagccaaagcgtcagcatcaagctctccctcagtgtcatcgtt

caaagacaccgttaacctcactgacggcgtcactctcgtcaaagatcgacgacttctggaccagtcgattgcttctcacactgccaatcgagtgttca

aagatatcacggagcgggagagacttcaggcatttcctgtcatcgggaaaggcttcaatgccgtacaagtgtacaatgccatcatcgaagcctttcgc

aaacagaatcacgacctagtacgcgacggtcagaagggctacgaattctgtcgcctgttgaatcattccgctcgattgcagaccctccggtctgatcc

acccagtattacactcacgggagtcgagctggccacagaatacaaacgaaacaagtgcttcattgtcgtgcgttcagacaagaaattcgacaagtacg

acccactcctccatcagctcctcggtacctctgtggacgacgacagtcctttcgaatcggccgatgatagcgacatcgacttaccagatatcactcaa

agcgaccgcgcctggaagaagtgcgcgttttgcaaggagctcatcggtatcaaagagcacgcggctcacgaatatgaatgtggtatagaatggaagac

tcaaaaggatcttcgcaaagaacttgaagacctccgcaagcaacgtgtcgccgtgaaggaggaagcagagacggcagccgcggctttgaacgaagcaa

actctcttcccaaaggttctgtttccctgcagcctcccgccggtagctgcgcgtgtggcgaggcggatgcgggtgagatggtgggttgtgacggagca

gactgcgaagtctggcgccataagaattgcaccgaagctttccagggttggaccagtcccgagaacgactggtactgctcttttggctgtctcaaaag

agtctcttcagccgcgcataccacgtctgacgttctccccacgtctgctactgatgctgatgtgccgcaggcgacgaagagtcccaccaagcgagcaa

gcagttccaacgcccctgcacagcgatcaagtactcgtgtgcgccgaacctga

>contig_14.contig_14.g72.t1

atggaacgccgtttcggccaccttatcaacaaccacgatcaacaaccaacgaccatcgaccatgagcagaaccgctgcc

atagtcaagcattgagcgacaaatacatacaggtgaacgcctcaacagcaaccgctgcgctcgctgcccttctcgtcgctgcgctcgctgccgccctc

ctcgttgctgcgccgccgccgccactgctctcagtaccttccttcttcgtgctcactccccatcagcctgcactcttgatcacatccattttcgcttg

cgcttctcggcacgtcccaggccctctgcagcccaccgtctcacctttccgtttgcggttcacatcctcgtctttcgcaaatcgcatgtctcctccct

cttcgatggatgccgcagagattgctgagctgcgactggatatctacgccctcctcggccccatcacctcatttatcagcagcgcgatacactcggag

actgcgctcctgacacatatgctgtcagtcgaggagctgttggtgtcattgcatctcatggccaacaaaatccacccacgagtcttcgatgctatcgt

tcagcccatcctctgtctcgaggcacgcttgcacgaaatcaaacgcgcgggacacatagaagaggacgctgccactggctctcgcgaatgggttagga

cggaggccggacagatgaagattcatttgcccgtcgatcttctgatcgagcttctcgagctcggcttctcggacaaagatagcgccctatttctaggg

tgttcggagcggactttgcgcaggcgcagggtcgagctggacctgcaacggtggaatgatcgagacttgtccgatgaggagttgagcagcatcataac

ccagctacagacaatgcacagtgaggacgtcggcgagcgaggcacgtggggtgctctgcgcagtcaaggactgaggatcagccgggcacgtctgcggc

gaatcgtgcaggctacggaccctatctcacagaatccccgatgggcgcggcacctcaaacgtcggcagtactacgtgcctttcgtaaattccttgtgg

cacatggacggccaccacaaacttatcaagtggaagattgttatacatggagcgatcgacgggaagtccaggctcgttactttcttgcgcgcaagtag

caacaatcgagccaacactgtcgcagctctcttcctcgaggccgttcaagctcgcggatcgcccagcagggtacgaggagaccatggcggggagaaca

tgggtgttcttaagttgatggaagaataccgaggtttcggtcgagggtctttcattcaaggtccctctacgcgaaatcagaggattgagcgtctgtgg

gtcgacctgaatggcgccctcgagcacttcgttcgcatgtggaacaatcacaagatgcggacaccgggcctgggtaaccagacacccgaacagatgtt

tctccaaggggcatgggacgctgctcgacgcggctacgatgttgggttactcgatatggcggagagggcagctatgcaggaggttggactcgacattg

agggctgggaggattacggtctagacggatggcgatctgagagacagggcgagcaaccccatgtgcgcgtcgaccgaatcagcgagcgggttcctccg

gtgctgaatgagcccagcacccacgcggctcttcgactcctcctcccttatccgccatttccccctcccgcagattttggcttgaccgcatacctaga

tgtgttggatgctatcaatgaccttatctag

>contig_14.contig_14.g73.t1

atggcagcagcaagcagtccgctcattctccagcgcagtgcgcatgcgcgctcagcagcagggagcagcgcactcggca

gcacgtccactgcaccggccgtagccgcagcaggcacaggcgggcgcacaaccaaccttgccattcacccactgcccatcctcaacatgtcagagcat

cacacccgcttccgctctcaggctggccacgacaatgtccatggtgagactttgtccacatcagtcccaaatcctgtgctgtggtcccccgtggtact

gacttcgcttcgtctccccgcagtgtacggtattctgcttggcactcagcagggtcgcgagatcgagctccataactcgtttgagatcgtgctggtcg

ataacaaggcaaacaaggactcaatatcagcgtcggccccaaaggtggaccacgacatgctcaaggcgcgtcaagaccagttcaagcaagtttttccc

tccttcgaaatcatgggctggtactcaagcgggccggaaccaaatgagcgcgatatgacaatccataaacagttgctcgcatacaatgagacgctcct

cttcctcaagctgtctccatcacatctggacaacgatccatctgcagcatcgtccgactcaagcgccgtgcccaagcgcggagacgatgatctgccac

tctatgcatacgaaaccgtactggaagtcgcagggccgagcaagaaggacgcaacgtccacatcagcaagcagtgcagacgccgggaagcaaacggac

gaagaggcggagtctatgcatgtcctgtgggcgccttgtggctatcggatcgagacgggcgaggctgagcggattgctgtcgaccatgctagcaagcc

tcccgagagcggaagtggcgaagcgacttccttgattgccaatctgacgactcagtacaacgccgtcaagatgctctccgatcgggtccgggcagtct

cgcagtacatcgcccttgttcagagtggcgctttcccacgcgatcacgagacattgcgacaagtgcgcgctttggtcagctgccttcccgtcgtcgga

atgcccgagctcaaagacgagattatcgaggagtacaacgatgtattgctcacttcatacctgtcaaacctgacgcagcaagtcacgaatctcaacga

gctggtcgacaagtttgacgtcacgcaggagccaaacggagcgaacgccgcgggcggactgttcggcggcgggctcggtggacccagacgcatgcccc

gacttgcaccgacatcactgcagcatggaatgggcggcagcagtacactcggcggtggcggcaatctcggtgacctcgacaagcttggctgcgcaaag

gtcagcgctgacgtcctcagcaagacgaagccgatcgccaaggacgagacctgcaaggccctccaggacggtggcctgatcaacctggacgctctcgg

ctgtgccaaggtcgaggccaacgttctcgctgatggcactgccaagtctgcctcttgctccgccaagaagctctgcacggtcatgcagaagggactcc

tgaacgtcgacctccttggctgcaccaacgtcgacctcgacgtcctcggcctcgccggggtccacttggacggcggccacggcaagcgcaagcacgcc

caccttcccattcccgccaagcctgccacctgtcgcgccgagacgtgccagatcctccagaagggcggtctgggcaacctcgacctccttggctgtgg

gaacgtgggcgcccacgtcggtccttga

>contig_14.contig_14.g74.t1

atgaaacggagcgtagctgaggcgggtgtcgcctcgagctacctccgtgatcgcactgccttcgcccgactgacgtccg

acttgggcgcagccggcggaactccattcttccgtaaccggtacaactgcctctccatgtccatgtccgcgatgatttcgcactgcctcatgccctca

ttggtatataaaggtgggccatcagatgtccccagcagtaccctcacttctctctccctcaccgcctcacccactatcaccttcacttcctcatcagc

atctctcagcttgcctcaccacatccctgtcatcattactaccatgaagctgaccgccctttctattgctgtcgttgctggcttagccacatccgtca

tggctcaggtgaaggacaacccattctgcaaggaaacatgtgcgccgtactgggctctctatgtacgttccaagccttcttcctcttttcgatcttag

>contig_14.contig_14.g75.t1

atgggtacggtattgggtatggggtccaccatacccacgaggttagatcgtcagggagaagccatcgacaccggtgaca

cgagcctctacgatttcacctgtttgccatcttgtgacaacgaagcgatcaagcttgctttccaggcgttctctcgcgctcccgacaaagttaccttc

gaccaggagctcatcaagcttcgaaagcctcctgtctcgtcggccccggatcatccgctcgcatctcgttcaaatcctacgctctacctacccggcat

ctccgatcctttgaaagctcccggaccccggttgtcctacctccacctcaaacctgctttcgctcccaaagaacaacgaattggacgaaaacgggttg

cgcccccgccgcagtggccgcactcgaagtcccaacgcgcgccaggcaaacccgactctggcaacgacgactttgacgacgacttctccgaagaggac

gaggtcaaccgcagctccgacctcagagggaaaaggaaggctggttcagcaactcgtcagcagaatgcgccatctaagaagctcaaagcctcttctgc

agaagagcgtgtcttcgatcaggccaggggccgaccacgtaccatcgttccgctcaccagtactccgccgccaaaaggcagagtcccccctgcgacgg

cagatgccgatgaagatgtcgagcctttgaaagatgacgaggacaagagaggagacggtgtgggaacattggagacggttacaattaggcaggccgca

aaggatcgctgggcgcctctgaagtctggagcgaaggagtggcgccacgaatacctcaacaaggtccacagtcagacagtgctgtactacttcgacct

catgtcacgggtcaaaaagaacggaaactggcaactcacctggaagtgtcggtgctgcggcaagccgcgaagcactccctcgacgggcttcagcaatc

tgtcttcgcacattcgtgcaaagccaccgataccacactgtcacatgcgcaagactccctgtgactctgccgttcgcccatggaccccggaagaagac

aacctgatcgacatcgatcattcactccctccctctgccggtccaaagtctgaatcggccgttgtttcacaatcgcgccgcagtatggacaaatggcg

gtccatggttacggatcaggccaatcaaaagcaaactaagctgcagcggcgtgccgtgttggtttgggttgttatgacggcgcagcccttcaccgccc

ccagcaacccctattttcgctcgctttgggccaccgccaatccggaccttgctgccctgaaatcgcctcgcacgcttgtgcgggacatggaggtgctc

tatgattccctgtttggtcaagcaatcaaagccctgcgacgctgcccaggccgcttcactctccaacacgatacgtggacaacaccgagtcggcgcga

agcattcctcgccgtccacgcgacctggatcgacgagaagtggcagcctcgtgcttcctgtatagggttcgagcgcctggggatcgaccatactggcg

cagcttttgccggtcatctcgctgggcttcttgactctcatcagctgtggtctcaatggtctggaatcgtagtttccgacgctgccgagtccaacgtc

cgagcaatccgcttcattgagcgggagattacttccgagactcgatctctccctactgctatccgccagaagctgcttcctttctccattcgggacaa

cgtggttctttgcttcgcacacggtctcaaccgcgcggttgtggatggaacggccgctgctggtgcccacacatccatggtgtcaaaagatacggaca

agattattcgtccagccatctttctggatagtacggaggtaggaggctctattcagagactcgacaaagttgcggaacctgaagctgaaaaccaagac

acccacgccaatcaagccgcccaagccaccgacgggcagatggatgatttagtttctgctgcccgggttgaccgcgaatatgccgagctgcagcaaga

ggttgatgagctcatggaggagctcggaggggggaaagatggtgccagtgggcagtccgattttgatgctgaagtggagggaattgtggacgactata

atgctgatccagaagacgacaacgtcgacagcgccatcacccgccttcacaactgcctcgtcaagatccgttcgtcgacaaagcagtacaagatgttc

aaggaacttgttgccgagctctatcctggcgaaaagaaacggacacttccatcgctgcgtggcgccacacgctggaactcctttcttgttgagctgag

gggttgcgttcggattcaaaaggctttcacatgtctcgtcaacgaggacgacgagggcatctggacgggatttcggttacgaccagcagaatggacgg

caatgaaccaactctgtcaggtcctggagacggccgagctcgtcagcctggacgttcaggcagccgagtccagcatcgctgatgttctctttttccat

cgcatcctacgacgcttcatgattacgcggctcgaagcgcttcacgacgttcacgaggacagtccggcatatggagtcaagaaagctatccttgcaat

tcagatcaagctggaaaaatacgaaaccaaagcgaaggccaacaagatggtggttgcagcctctattttgcaccccaggcgacgccgaaaacctctcg

ccccttctccggacggcgaatcggcatctgcgctcctcggcgttatggctgaccggttcctcggcaaagagtcagttcagccgtcgccaaaaccacga

aacgagccaacgagcgtatcaagatacggcgtcgatatcgacagcgatgacgaggatgacacaaccgtcacggttcccgatccttcgtcaagcgagat

cgacatttacctgtccggccgctatgaatggacagacgactatgggccggatacgacaaagggagccctcacttggcgcaatcttggtgccaacagca

ttaccatgctcacgacgtgcaagctcttgacgcaaagcggattcgacccatacaagatcgacaatcaagaggccgatacaccagctcggaactccacc

tggacaacggcgactactgctgagcctagcatctctcagcgtccatag

>contig_14.contig_14.g76.t1

atgccaccggtatcaaagacattggaggaactgcaagaagaagtgggcgctgctttcacagcagtcggcactgagcctc

ctgcgattgtctgtctggcatgtgaagtcgaggcagacccgcccttcaatcctaaacatctcagacaacacgtcgcatcgaagaagcacaagatcaat

gcaagtttgttcttgcaccctggtcaagcttcgtttccagctatttcgacgggcccaccagcaatgtcgacggcatcggagcctatgctggacgattc

gcatttgttccccgaggacgaagatcgtgctgctctgcgacgcatgcaggacattatggacatggacgacgatgtggctgaatggtctcaggataggc

aagggcaggaagcagaagagagggatacagcggcagagatgggaagagatcggtgcaacagattacaggctatcggaggggatgcttttccgtttctc

tctcgagagacgtttcgatcgctcgcgttcaagctgtcgccaaagcgagccatctcgaacgtcattctcgaagaggcgctgaatctttccacagacct

aggcgctagcagtgttccaaccgtggaggcatgcaaaaagtcgatggaagcagcgagggaggagctgtgcgttaccatggtacgacataagaccaagg

acgggaggatattttactcggacacaattcagcagaagctcagatgggacttcatgaacccaacggttcgatcgagaatgcaccttctcccgtgcaga

ggcgaccagatctccgacatatatgatggttcggctttggcatttgagcaggacacgcgccgaaccccgcctatgatccaacggcgtgatggcgtggc

aatttttacggacgaaaccgtcaagctgctcgacgggcggctcggttggtgtgtcacacttcaagatgccaccctcgaagttcaacgtgaatccatta

ttttcgacccagaacttgtaaacgtctcagcctcagaggagctgagcagctacagagtcgtggacattcttggacgcgagcttcgatccatcaatcct

cttcgcgtcaaagccgacggtcgcctattgatacaggtaccgattgtgctttttatcgacgagacgtccggcacgacaacgaagcgttggaatgaaca

cgtcgccatttacttctcaaacgcagccctgccacgcaaggacatggactctccctccaatgtcaagatgctttctgcgagcgcaaacgtgcgcgatc

acgacctcatggcagctgttgtagaggaactgataagagatgaaaacagcacgagggtaccgaaagctgctaaaggtaatcatgcatggcctccaaaa

tgtctcacaggcttccagttcaaagtcccagccagtctaatcttgattcttcccgtactgcagatcggttcaaaaaagacagccaaagaggtcgtcgt

ttctctcaaggcgcagctctcacttgcagccagcaagggagtcaccgcgcagcttgtggccgaacaaagatcgacttgcatcaaagatgtgtggacga

acgacctctgcaacgagctcatttcccttttcaaagccagcaggaaagccgagagagcagaggtggttgatcgaagtaggcgcgcaattttgaccaca

aaatggcatactcccctgctggcgcttcacgacaagattgggttcgacatctgctcaggtacccctgttgaccttctgcacacatatcagctgggtat

tgtcaagtacgtgtgggcgcagatgctcagcaaggcaacggagacgcagaaggttgagattgccgcacggctgtcggctgcgcctatggatgcagtag

cgaggacgggtgcccttcgcggctcttggctagtgtcaaatgctggaggtttggttgggaaggacctcaaaagtatcgcccaggttgcggcaacagcg

ttctatcccatgatgaaaaatgggacaatggagctcgaagtgtggggcgcgtggagtgctgttgggcaattgggacggctgctgtttgtcgaagagat

tgatcggaaggaccttcataaatacaaggaggaggttaaatgcgctatcatggtctttctcggaacagctgcggttgcattcccagctcaaattctca

gcaagcccaagttccacatgttgctgcatatggccgacaacatcgagcagtacggtcccgcgaaagggttctcatccgagcgatacgaatcattcaac

gccgtactgcgaaaggcttccatttgctcgaatcgatctgcgccttcgaaggacattctgacccgtgtccaaaatcaggaactgattcggcatgtggc

agcttctggatgttggatcaagaatgggagggtcagcacaccgtcaccgctactcaaaacactgttgtctgcgtcccgaaacgaagaatacagcattc

cgaaggtcaacgaggaggccagggactcaaacaagttcttcgtctcgtttggcggggatcggattggtcgcggcaacttcgtcacgacgtttctcgac

gtggaggctggtacacggggtatcgcgcgcgtagacgactttttgtggacggcagacggtctgcttcacatgacggtgacgaatttcgaatacgaaac

cgacgtctctgggctcgagcccgtcatcctgcgagaagtagggcagtcggcgggagtagtggcctccacggtcgacatacaggctacacacgtccgga

tggaacgagaaagcattgaggagtacaagcctgctttggtgcactgtctggcaggcgcgcaaagatacgtgctcaatcattgcctctttcgctcggcc

agttctctggctagtctttatcccaaggttgcaaatcctcccactgtcaaggagatcgccaacatcgcattgttggtgctgggcggagccgatgtaga

cgaggacagcgaggaggacgagctgttctcagaagacgacgatgagtaa

>contig_14.contig_14.g77.t1

atgaacagcagtccagctaaacctgttgattggaacgtagaagacgaggacctctacaatgaccagtaccaggacaact

acgaggacgaactcgatgaggacagcgatgaggacgagtttgacgacaattttggaaacgaggacgaggaagaagacgatgccgacgtcgagcagtcg

cctgcgcaacgaaagcgcaaagatcgggacttcgacgacgagaaggaccgcaatgagcgccgggctccagccagaagcaccagtacgaaggccagagg

cgtgcgcattcctccaatcttgtacaaggccgcaacattgtacaagctcagcagggcgtctgtcaagatctttgaggaagaggttgtcagcagaatgg

agacggacaacgaaatcccctccgcagcagatatttacacaactgcgcgacagatgcgcatctgggagaactcggaagctacgagagagcagctggac

gtgctcaaagaacgtacggatgagcttgcggtcaaagtcaacagcttggccaaggcgaatcagaagctgacaaaggcagaccttacacaacttggcaa

agaagtggcctactgcttctactcagagctcgtcactcgttactgcgccaagggtccccttttcaacaaagttattgcctacttgaagtcggatgccc

ttttggtcagcgagaatttcaagcatcagtttgatgatctcgaggtccgccgggcggagatcaatcccgcggtcaaatccatcttgagtcgtcttcgt

ggagcatgtggcgaccgcatccacgagtctatgggaatagatactggtacgaagaaggaggcgcttccactactctggctgcgactctgcaagggcta

cgacgtaccttttaccacaaagcgagcttatcgcgttgcagtgctgcgatggattgcggccgacgtttcagtgcgccagaaggatggcaagcccacag

gaaagttttggaagactgtggacgcaaccattgagaacctgcaaaagcggcaggatcagcagcccgaagagatcaaaaaacagcttcagcagattttt

gacgccgacaaaagaaagtatggcacctggaacgacgccgaattggttaagggcggctcgaaggagatcgccttgaagtccgcgctgtcctccgtctg

a

>contig_14.contig_14.g78.t1

atggcatccgtcatcaatcatcactcttctgcagctcctccatctcgttactcgctatgcatccttttctcgttgctgt

tctgccgcttcttttcgactttcccagccacccactaccctctctacgtcttcctccgcccgcaaccgaacgtctctatggccttcgcgcctgccgga

gtcaaacgtgcgcgagccggcaagaaccggctccccgctcaggcgtccgggcttaccgccgaaagtcgagagcagaatgaggactggtccatcgatgc

atctcaggcgagttctgtttaa

>contig_14.contig_14.g79.t1

atgcagttcaccgcctccatcgctcttcttcttctcgccattgccggctctgcttgcgccgctttcgtccccgcaccca

caaacacctgcgctgtcctgcagcgggcttccctcatcaacctcgacaagcttggctgcgcaaaggtcagcgctgacgtcctcagcaagacgaagccg

atcgccaaggacgagacctgcaaggccctccaggacggtggcctgatcaacctggacgctctcggctgtgccaaggtcgaggccaacgttctcgctga

tggcactgccaagtctgcttcttgctccgccaagaagctctgcacggtcatgcagaaggggcttctgaacgtcgacctccttggctgcaccaatgtcg

acctcgacgtcctcggtctcgccggggtccacttggacggcggccacggcaagcgcaagcacgcccaccttcccactcccgccaagcctgccacctgt

cgcgcccagacgtgccagatcctccagaagggcggtctgggcaacctcgacctcctcggctgtgggaacgtgggcgcccacgtcggtccttga

>contig_14.contig_14.g80.t1

atgcgaaatcccatcaagaaatggtgggacccagagcccgctcccgcgtccttcggggctggcaagacggtcccagagc

attcagcgtccctcgcctcccgcctgacgttctcatgggtcacccccatgctcagagtggggtggacccgcccactcgagtccgaagatttgtgggaa

ttgccactcgatcgaacagccgaaatcatgggcgaccgcgtcgaagaggccttctaccgtcgagtccgacccagcaaacgacctgcggcggtggcgga

gcactaccaaaccactgttgtcgactcgccgctgacgacacctactgcgagagccgccgcggacttgcaagagaaggtcgttccgggcagagaggacg

atgatgcagcctccaactctctaactgctcactccaccagtgacgccgccccacaaagctcctcccatagcaccgacgaacgtgctgtccccgccacc

ccttccgagaagaaagcggttcaagcaaccccaccgcaagccaggcaaaaaacaagatgggagacaattcacggctacacaattccgttcctgcttcg

aaaaaagaagcccgtctacagcgagagtctcttcggtgctctccacgaagtcttcttctaccgcttctggtccgccgtcgccattcgagccgttgccg

atggcctactcacaacttccccgctcgtcacacgagcgctcctggagtatctcggtcaggcctatgcatattccattgcgccacccggaaccgtgccc

gtcccgccccccgctggcagaggatggggtctggccgttggcctgtgggtcatgcagcaagtggcctctcttcttacgaaccaatactacatcatcgc

gcaaacaactgggttctcttgccggacctcgctcgtaagcttgatcctacgcaaagcacttcgactcgactcccgcgcgcgtctcgagcactccaccg

gcaagatcactaccatgatttccgccgactccacgcgtatcgacatggcgagcggatttgccccaatcattttcattggtcctgctcagattattctc

gcaattggtctcttgattcacaacatgggtgtctctgcgctggtcgggttgggcgtgctcctactgggcacgccagtccaagccatcatcgtcgcgag

gatgatacagacacgccgctctgctgttcgactgaccgatcggcgcgttcgactgatgcaagaggtcttcactggcatccgaatcctggtgctcttcg

aatggcggaacacacccttcgcagagcgcattggcaacatgcgccgagaggaacttgccttcctccgcaaactggctttgcttcgcgccatgaccttc

tctgttgtttacttcctacccgtcctgtctgccgtcctgagtttcattacctacgcactcctcggcaacggcctcaatccggccatcattttctccag

tttacagtacttcaccatcattcggatgccgctgctcttcactccgctcgtcgcgtcctcatgtggtgacgcctacgtcgcgcttggccgaatctcca

aatacatgctctccaaggagctcagaggcgactacgccgaagacaacaaggattacggaatcgaggtgcatggctccttcacgtgggaaaccgttggt

ggccctcctgatgccaatgcgtctacgggtggtcccgctggtacgaagggtcgcaagggacctcccggagcagagggtgtgccgaagggcggagtcaa

gcctgacaaggagaagcgtgccaagctgagcaaggaggacaaggctgctgccaagcagaagaagaaggacgacaaggagaagacaaagaaggacaaaa

tcaagaagaaggctaccaaggctcgatggaatgcgcgccgtgctagggccgaggcaggcgaagatctctcttcatccgaggatgaggctgatgagact

gtcggaaatggcagtagcggcgccaacgcaaaggtcggcagcacccccctcgaagcggacgacgagaaggccttggcgcctgcgcaggggaagccttt

cgcgattcaggatctcaatctcgccatccccaagggcgagctggttgctatcgtcggcagaatcggctcgggcaaatccagtattctgtcagcaatgg

caggcgaaatgcgcaagcaggacggcacggtctcgttcggaggcaccgtggcgtacgtagcccaacacagctggatccaaaacctcacattcaagcag

aatatcctcttcggtcaggaagaggatgaagaacggtaccagcatgtgatcgaaaattgtgcgctcgagcacgatatcgagctcctgcctcacgggga

cgagacggagatcggggagcagggtgttaacttgagtggtggtcagaaggctcgagtttcgctggccagggctgcttattacgatgcggatattgtcc

ttcttgacgacccccttagctcgtcagatcctctcgtcggtcggcatctgatgcacaagtgtatcatgggcttcatgggcggcaagacccggttgttg

gtcacgcatcagctctgggctctgcctttggtggatcggatcatcgtcgtcgataacggcagggtggttgagcagggaacgtactccgacctcctctc

tcgcaaaggtggcgtcttctccaagctgatcgaagagcacggtgtcgaggaggacgaggaacaaaaggtggatcagaaggtagatgagcaagagctca

agaagcccggaaaggaagcggtcaagggcccggcaggcggcggtggtctcatgacggatgaggagagagagatcggggctgtctccttcaaggtgtac

tggaggtacctctcctcggcgggttccgtctggtgggctccactgctgatttcaatcatgacgctgatccaaatttcgcaaatcggtaacaacctgct

gctgcgatactggtccgaagattctattcccggttggagccagggacagtacatcggtctgtacgcaggctttggtgtcgctcaggccgtctttgtct

tctgcggctcattcggtgtctcggtcgcgggcttctacgcctccctgaccttgttcaagcgcagtctgggcggtgtgctcaattcacctatttccttt

cacgagaccacgccgacgggccgtatcgtcaatcggctcagcaaggatgtcgacactctggatatgcagcttccttctaacttgttccaattcttttt

ctccatcggcacaggtaccattggtctagtcatctattcgtacaactggctcggactcatgttccctccactcatgttcatctatacgatcgtgcaag

cttactaccgccggaccagtcgcgaggctaagcgtctcgactccatcctccgatcgaggctttactcttccttcggcgagacactcacgggcctggcg

agtattcgagcattccgcgctcagaagcgcttcattcgccttaacgaggcgaacatcgactacaacaaccgatcctactatctcacgattgctgtgca

acgatggctgtcgattcgcatggatttcctcggcaacatcctggtcctcgctattggtctcgctgccgttggcttcaggaaaagcatctcgcccgcag

tcctgggtgttgcgctgacctacactctccagatcactcaatcactgtcgcaaatggtgcagcaacttgctcaagtcgagcaagacttcaacgttgtc

gagcgtgtgctgcactacgtcgatcttccgccggagggtcccaagcttctgccgaacgatccggaagaggagcagtggccaacagagggtgccattga

attccgcgatgttcagctccgatatcgtgaaagccttccattggtcctcaagggcgtttctttcaagattaaccctggggagcgagtcggcgtcgttg

gtcggacgggtgcaggaaaatcgtcgctgcttgttgcactctggcgcctagcaccccttgccggcggtcagatcctcattgacggcaaggacatctgc

gagattggcttcgaaacactccggagacgcatctgcatcgtgccccaagattcggtcatcttcgacaacacacttcgcttcaatgtggatcctaccgg

tcgcgccacggatgcggagatgaacgctgcgttgcgtcaggtcgggctacttgttgacggtagtgcaagtgcgggtgcgtcgacaccacaagagagtg

aagacaaagacaaggaggtggtttccgtccctaccggagaagtcaggcaacaggctcgcaagttctcgctggacatgccgtgtcgcgacgatagcttc

tccgcaggtcaaaggcagctgatcgcccttgcacgatctatcgtcaaaagcacaaagatcatctgtttggatgaggctacgagctctgctgatgtgga

aagcgacgcgaccatccagcgttggattgccaagcacatggacagaacgctgctgtgcattgcgcatcgcctcaacacaatttgcttctacgatcgcg

ttctcgtgatggacaagggtcaagtggccgagttcgacgctccgctcgttctcttcgatcgccccgactccatcttccgctcgatgtgcaacgctgcc

aagatcacgcgcgaagacatcctcaccatccgggctgctgccgaacgcaagggcagcattgttgaggcgcttgtccagacgtccgctgaggagagggg

cggcattgatccagagtcgtga

>contig_14.contig_14.g81.t1

atggcggacaggaccaatccgaagcgccgcgagcgcaagcgcgagcacgacgagtacgacgaagaagaagaagagagag

ggaagcactcgcggttgcctcactcgccctcctcgtcatcctcctcattcgccgtcgacgcgattcgcacgctcggctcacagctggatgctattgag

cggagcaggatctcgacgagttctaccagcacggctgcggacagcgagacagtgaccaccgcggtgaagcaggagcagggccaggcgaggacggacga

ccaggcgagcggggacaacagcgcaagtagcgtccttgacatctcgcagatcactgccaaccacccgaccacggcggacctgctccagatcctgagct

cgctgcatccgtccatcgtgcttatgccatcgtcaggcgcgggaccgacagcggacaccgtcttcgcactcacacggatcgcactggcgacctacacc

gctttcctcgacaccctcttacaggatgcgcacgccgtcgaagcctccgcctggggctggcatgacattcacgacgacgacaaccacctggcagcgct

gacgtacctggtccagacgctacctgcgcgcacctggtccctttcccaaacggcggtccggatcctgcggtcctcgcaagtgcccgcctcgttctcca

ctcagagcatccagacagttctgcacagtctcaaaaagagtccggccctcctcaccacggccctcttcccgctctctgcctctgggcccgcccctctt

gcttcaggcgaaggcggcgcggcggtcaccacgatcggaatcggacgcgtcaagttcgggttcaatcccctccgcgcaccgcgtgcattcgaccccgt

ccgactcgccgcgcacgaagctagatcaaaagcgcagattctccttgcagaacgggacgcccttgcgacgcagctcggcactcttgcccagcagcttc

ctaccctcgtgtcctcaacaagggacgcggaggggacgacgagccagaagtcggcggtaagcgaatacgaggcggccgttcttgttgcgctccagtcg

gcgggccgagctgttcggcatatccaatcggcactagagcaggagccgatcgctaaatctccttcgccctcggacctctacgacgttcttcacagcct

cctttctacctctatcccacagtacacgtccgcaacccacacgtccctatccgttcccacctcagtctcgaacctctccgccctaggcgagccctctc

tgttcctccgactctggttacccgccatcatcctacctgcctcagcccttgtgctccgctcgacgatcattgcccagtgggaccgtattctggcgaca

gtcaccgatgccaaggagacgatccgagggttctgggtgggctgggtcgtgcagccgggagcacagctcttgcggacactcaggaggggcgaggccga

acgcgggctcatcatcgctaaggaaagcctggcgtcggatctcaagtctctcgagcgtatggtcaccagcttctcggcggagaagtacaatctctcgc

ccgacgagcttgaggatcttgcggcgaaggttcgagacggcgaccttacgagcgtgttacgcgtctacgaggatgagatgcggtcacccttgaaatcg

gcattcacaggctctctgctccgcgctctcctcatccaagtccagaaggcaaaagtcgatctcgaggttgctatgtctggcatcgatcgcctgctgcg

ctcgcaagagctgttgatcggggccgtcgggctcgcgcctgctgtggctatcgtgtgggtgacgacaaagtgggccctcagtggtggtcggttcgggg

gaggcagacgaaggcaattgagagagggcgagtcgttaaggatgagagcctgggaggcgatgcgagaaatcgacaagctcctttcacttcccggagcg

tcttcaggatcctcaacatcatcaacagacaccctaccacctctaacccacggcctcctcctcctccacctgtctttcctccgctcggccgcagatcc

actcaccgccttctacgcccgggcatccccacagaaaaaggcgatagcgaaacggctccgacgggcgttcttgcaggacatacgcgagttggaggcag

cagctgctgcgactggttgggaaggggaggatcagaaggaagccgatgcgcggtcaggttctggatcagatgcggggccagggatgggatcggtcggt

ttgggctggcaagtgcgtcgtgcgacgattcagcggatgtggtctagctggcaaggtctgctcgtgcctcgcggcgtcggaatgtag

>contig_14.contig_14.g82.t1

atgccagcgctcaagcagaagaagcagacggccgagaagcgcaaggctgcggtgccggcctcctccgccgccgcgccct

ccagcaagaagctcaaggcggcgcctcctccacccgcagactcggcctccgacgatgacatggacgaggacgaggacggggatgaggacggggatgag

gacggcgaggacgacgacttcgatgacgacgaggatctcatggccggcgccaggaatggttcaggtggcgatgatgaggatgacgacgacgacgacga

cgatgaggatgaggatgatgaaggcttcgacgaccttgaagcagcggcggaggaggaggagggacaggaggaggaggccgaacctcagccagccccca

ccaagccaacgactccatctgcggcgcagcagaagcgctcaggcacgggtctgtatgcgcttccatctgcagacgagatccggaagctgaaagagaca

acagaggtctacaagaataacgtgctcaggctacagatcgacgagatgctacccgctatcagaccagcatacagcaaatcagcgccgcttgatctcgt

cttgcgtcgtctgaagtcccgactcgactctctcaagcccattgcgtcccagcccctcgctcctgccctcaaagccctgtcgaaagcgtactcgaaaa

ccaagatcaacgtccccttcgcagacccacagccgaagccagacgctccgaatccgttcggcttcgagaaacccacgaacatgcacctcgtcggcagt

tggccgctaagaacggctgttaagcgcggggacggatttgatgttgacatcgcggtagtcatgccctcatcactcttccaggagaaggactacctcaa

ctacagatatgcccacaaacgcgccttctacctcgctgcgttaggagcagcgctctcaagcccagtctctgccgacagcgattcggccctcggcgtca

gtgtccagtttcaatatgcagagggcgaccctcgacgtcccattctcgtcctcgagccgcaaaaggacgcgccgcaggagatcaacttccacaaactc

cgagcgacgatccggatcttcccttccatcgagcctaccctcttcacctacggccgtctggctcccagtcggaatagcgtccgcatcgcaggtgacga

gcaggaggacgaagcgaaacagccgccgacgccgaggtacaatgcgtcggtgctcatggacagtctctttgtgccccatcttgtctatctgcattctg

tcgggaaggagtgtcccgcgttttcagacgcgtgcgccatgcttaagacgtgggccttcaagcgcggctttggctcgggctcgttgcgcaagcatcaa

caggccggtcagaggaggatcatgtacggctccggcagcattcgctttttgctcaccatggttctggcccatcttctccgaggcgaaccgacaccgac

ggaagggccgggtgcgtctagagcggtacgaccgaagcttctgaacaggttcagttccttccagcttttccggggtgtcttggattggcttggatcac

acgactttgcgagcaagccggttcggatgcgggacggccttgactttagtgtcttctctcgcgcggacaagattcccttcgaggagttccagcagcac

tttgatcgcgtccttgtcgatcccacgggcagcatcaacctgctcgctccattcacagccggtgctgttgacactttacaggcggaagcgcgtcggac

cttcgatctgctgaaggtagatgaccgggacgacgccttcgacgtcttgttcatgcaggacttctcggcgcctgtgctctcctgtgacgaggttgcgc

atattgcgattagcacttcggcagaggccgacgcgtccaagcgtgccgactacggaagtgcgttccatgcgacggctgcgtcgctgaacgcgacggtg

cgccaggcactcactgacagggcgcgtcgatcggtcgtcgtactgaacgcggacgaacccggttttgcctcctgggcgctcgacgctcgaccggcttc

ctactcgggcacggtcgaactcggcatcgtcctcgatgcggagcaagctaccaggctcgtcgaccatggcccttcaccggacgatgcggatgcggcca

agcgcttccaggcgttctggggcaagttggctgagctccgtcgattcaaggatgggcggatcatccagagtgtggtgtgggagggcgctggtgcgaag

agtggcattccgcgacggattgtgcgacatgtgctgcagttgcaccatgggatccaggcggcccgggttgcctttttggcggaggcgtttgatgggct

tctggagttgcctgctcagttgccgaaggacgccttcttgccggtacctgtcgtcggtgtcaatcggtttcaacacatccaagggacatttgacggtc

tggtgaagcagcttcgtgccctggaaggcttgcccctatcgctggtgagcgtgcttccatgcgctccagcacttcgtggcacgaacacctttgtgccg

cctgcgatcaacttggcgggacttggaacttcggacgtaccggactgcgcctcgtacctcgcggcacatgacatcgtcctcgtgttcgagagctcgcc

gaagtggccggatgacctggaagcgcttcaagcgatgaagctcgccttctttgagacgcttgcccgactcattcctgccaacatgtctgggacacgga

ccagggtcgctttcgacgaggatgcggctgctagcagtattcaggaccagtgtgctctggagatcatcacgccgacggggtatgcgttccgtgcgcgg

atccaatgcgaacgggagcgccgtctgctcgaagagatcatcgaggagaagcgatacaatactgcgaaggaacagcgacaggccggtctggcgttggc

gcggtactcgcaccgcttcgtcacagccacacggcatcataccgccatggcagcggtctcgcatcgatacccggcattcggggagagtgtccggctcc

tcaagcggtgggtcagcgcccagatgctgtcgtaccacgtagccgaagagctgctggagctcgtcgctgtcgagcttttcgccacggcgggagccggt

gcgcccagttctggcgttgctggctttgcagcgctcctaaaacggctgggttcgtggaggtggaaagacgagccgatcgcggtgcctctcttctcggt

ggccggggcggaggacgagatgaagctccgacagagtggcggcgggcttgcgctcgctacctcggctaatgctgtcaagcgcgacgcggtgattcggt

tcccggccgacaagcttgcgtctgtgcaggctgcgttcacgtcggcgcggcgggcggatccgagcttggagacggctgcgtggtttgtgtgtagtgag

gaggatgtggagagtcgggcttggagtcggacggcgccttttgcgggtgctgcggctgggttgaaggcgttggctgcggcggcgagtgcacgtttgga

ggcgggaggacggctggatgcggttgatgttttgtccattttcacaccctcgctcggccagtacgactttgtgatccatttgaaagctgccgtcctgt

cgcggtacgcatactcgatcaacgcctcacctgtagtgtggcaagacgccgacgagagtacaagtgtgcttggatcggaagctcgaccgggctttgat

gccgccgaggcgtatgtgcaactgcttcagcgcctgtaccaagactccttccgcctcttccacgaccgccagggaggtatcctcatcggaggctcgtg

gaatccacacctccgaaatcaggcacgcaagtaccgagtcgcacttgggttttcggtcctgccggtggtggaaggggagggcgcgggggaggcggtca

agctgaacgttggggccatccttgcggagatggagcggcttgggggaggcttggtggatcggatcgaggttgtgcctgttcgacgcacagcggcgcaa

accaccgcatttccctcgacgccaatgatggatgtagatgttcccaatggcacggtactgtccgccgagctcaacttcttctgtgctggtgtggaaaa

cttttcgggggcggagtcggtcgagcccgccgatgcgtccatccacctcaggtcgaaggaaagctcgcagtggagcccgctcccgcgctcgtgctcgc

gctgggctgattctgagaaatag

>contig_14.contig_14.g83.t1

atgcgctccatcctcactcacagatcagcggcagcgctcaggacggcggcctcgacctcgacctcggcggcggcggcgg

ctgggttcagcagcgcaggtgcagccaggttgacggtgtcattcagctccctcgcgctcaccgccaaccacagaccgtcggcgagcacatcagcaacg

gcggcctcagcaacacagacgcgctcatatgcagtcgttcatccccgtcgcggctctcccaccaacccgcgttacggtccgcccacactcggacagct

caagccttcacagccaacaaaggcgagaagacgtgtcggtcgtggtccgtcgtcagggcgaggtggcacgtcaacacgaggtcacaagggtcagaaag

cccgggctggtaatggcaagcccggaccgggcttcgcaggcggacaaaccccactcatgcgtctcttccccaagcgcggcttcatcagcacccacaag

cgcgagtacatcccgctcaacctcgaccggctccaacactggatcgacgccggtcggctggacgcatctcaacccatcacagcctaccacctcctcca

ctcccgctgcgtgcacaaggtccgcgacggcatcaagatcctcggcgatggcgctcaccaccttcgcacgcccgtccacctcgtcgtctcccgcgcgt

cgcagaaggccattgctgccattgagcgcgcgggagggtcgattgagtgcaggtactacaatgaccttgcgattcggaggatgtgctaccctcacaag

tatggcagtttgaatgtggagggcaagggcaatgcggaggctgtcagcccggatccgatcagcaagaaggacatcttatggtactcatcacctcgcaa

ccgcggctaccttgcgctccgggatgctgcgggcgtgtctccagcaccagctcctgcctcagcccctccggcacggaaaacctcgtcgtag

>contig_14.contig_14.g84.t1

atgccctcacaaccccaggctcaggctcaggctcaggctcaggcccagttgccactgaccttcactcacgccttcacct

tccccgccgacccctcctcaaccctccaaggccagctcatcgccgccctcgtccagaccgtgatcttccaccgcgccttgggaaacgtgaggccaaaa

agcatcccgttcttggagaatagcttcccggcgatcgcggacgagcgggtcgacgaccttgtcgaagagggcgtccaggcttcccgggttgagatcaa

gaggagaggagcggtccagctggtcattggaatcttccctccctcctcacgaacgtcagcaagcgcccctcagaccgcggactccaaagcctcccctt

cgtccggcagcacaagcccttcctcgactaccgcggccccatcgagtcgggagcgactcaagagcctgccgagctcgtggttctcccaagccgtcaat

ctcgccaaccaggcatatacgcacggctatgcgttctccggctatgggctgagcgcgggtgatgtggtacatggtcatggtcatgatcatggtcatgg

tggcgaggtgggcggtcgcatggacagcggcaagtcgacgggtggcgttgcggtggcgggggacgagataccgtttgaggagtgggtgttcagcattc

ggacggtgtcggtcaagacggagcatgaggccactgtggacttcgtcgaccgctgcaaggcgcacctcccccccattccgatccagaacgcagatctg

tttccgtttcccattcgcgtcaaggccatgccctgtcctcctgcctga

>contig_14.contig_14.g85.t1

atgtcgcagccatctgcacccaaaccgccccgtcgctccagcgcaggctcagagctgatacgctactacctcatcgcct

acaactttgccagcttcttcggctggtccctcatcctcttctccctcctcaagcatgtcgccatcggcccacagaccccaacggccgtgcaccgcttc

gccgagttcgccctccgcaacctgaacctccgtcccctccgcatgctcgtcctccccaccttcgcccaaaagctccccgctcccttgctcctgttcct

catgcgcgcagccacagcgcagcagagcatcggcagcctcgtcggcttcgtccagagcttcgccatcctcgaggtcgtccatgctgcgctcggatggg

tcaggtcgcccgtgccgacgacggcgatccaggttggctcgaggttgtttagcgtgtggggcgtggcggagcggtatgcgagcgcgagtgttagtccg

tggtacggtccgatggtgctggcctggtccctcaccgagtgtgtccggtacccgttctacgcccaacagctcctcaagaacgagagcccgttcctgct

ctgggcccggtactccctcttcttcgtgctctacccgctcggagcgatctcagaggccatgctcatcaaaagtaccctgccctccggcttcccttggc

agaagtccgaggcatgggacgcccgcgcctccgtctatgcgggcctcttcctcttctggtggtacggtctgtacgtgatgtacacctacatgatcaag

caacgcaagaaggcgctcgggactggcttcttcggctctaaccctgctggccagcaggctcagcgaaaagtcaaggaggcacctcatcaggcggccgc

gatcgccagcagtgtccagatcaaagcccaggaagccaccaagcggagatga

>contig_14.contig_14.g86.t1

atgcatcagcacacagccgcaacaggcgccgcagagaaggcacgcagtcgctcgcgacaccaccaccagccacgccccc

gcaccagcgcaggatgggcagcctcagcagccatgcccgcaagaaagtcacgcccgcgcactccacgcagcacaccagcaggcccatctccagccatg

gggggaccaccaccagcagaccgcgatgccgacggcgcagggaacccaggcagcgcacccacatcggccctcattgccgtcctgctcgtcgcaagctc

atccggcggctcacacatcgtcttccagtggccaccacgcccacatctcctccgcagactctcacagatacgctacttcaacaacccagaacgacacc

acagcacacatcgaggcagaacaccttcccgcacaggtccacccgaccacaacgaccagcagcagcaacaacaacaacaacacgaacagcacaagcag

cgcccgtcctacgcctacgatcgccagcacaacgctcccaccgaacatccaaaagctgtccacatcgctcccaccttctcacgacgataccgtgcaaa

ccacgacagcagctcctccgcctccttctccgcctccgaaagctccttctcatccacctccgacgtcgccgaattcagctcctcctccgccgcagaag

agaccgagtcctatctctcccgcgacggctacggctccgacgacgaccaaacacgcgaacacagccttgccggcggggacgacaccggcgtagatccc

aatgagtctgccgaatccgatagcgtctccgtccatcccttccaccgctctcgcacaggatctctcgcaccaaactctcgtctccgcaatgagatcaa

cgtctccgctatcacgcccgatctggcaaatcatccacagagacgcacccgaagggcggcctcctccaacctcggacatcgctccctctcccgcggtc

caagtctctatgattccgccggatccatgcggtccaacgagcgcttgccaggcatcgacggcagcgtcgatcggagttatcaacaccatcatccatca

agctcgccccaggccagcatcgtctccgtcctctcgccctccgaggtcgctcacaagctcgttcgttcgccctccgaaatcgctcgcaatgcacgcgc

ctatgacacctatctcggatacgacaacgcctttctcgccgaattgctcaccccagaacgtgacctctgccatcagaagttcgagctcaccgtcgatg

atctcgtctttgtcggacatcccgtctgtgccgatgaccgaggctgggttctcaactctaccaggaaggcgggcgacgacgatcatgatcacgagcaa

gctgccagaggtcgagctcccaccagcaaccggcactcgaagacttccagtttcccaaatcctgccgttgcaagcggtgattgctctcccttcgagtc

gtccacacgctccaactcgttcgcccactatcaaccgcagcaaccaacgaaccagtcgcgttccagcgtccgcggcgaggcatccctccctccccctt

ccgggacagccacgacggggaagactttccccggcctgacctccttccacttcgtcctcatcctcgacaagccagacccggcacccccacacctccca

ccaggcatggcatccatcatcccagcctcccagctgaacaccttcaacatcgaccccaacctcaccgcgcagctctactacgacaacatcgccttcaa

gatgaccgctgcgctctacaacgaacagtgcgtctcgaactacgtcacccatgaggcagcctacctagaccgcaaacgcgaaaaggcgaaaagcaagg

gtaggaaattcacagagttcatgcaacacatgcgcgaacgccccggctccctcgcgaacgccatggcggacctgttcgactcgctcagcaggaacgaa

ggcgcgctgattacgatcaataatcggatcgagacgcatctccagttgccgccgatcgtgcgtgagacgagacggatgctgttcagtgcggatctcga

gacggagcgcgatgcggacgaggcgcatttgaggattgcgccgtttgatgagtatcttgtcggcgcggctggggtcggcgcgggcgcgttggacgtca

gtgccacgagcgcaggcgcaggtgggcccgcgcatcatcatcatcatcatcatcagacgggctttcggatgcaacatgcgcgggatcgccaggtggac

ttgtacgaggagtggaaacggacgacgggcccgcatttggtgccgtggaagacgttgttgatgctgaagccgcagaagaatcatctgtctgggacggg

caagaagtcgaccgcgggggcgggggcgggggctgcttcgggatcgagatcgagatcgggagtgggggcgggggcggggtataagatgggcagggagg

gagagagggatcatcggagagggaactcgtacggctccggctcgcccatggcaggcttacgcggactcagcatgaccaagtccaacacgcgctccgga

tcccgaactcgtcattcgcaggggaacgtcatgttccgcggctggggcgaggcgcacagtcagagccagagccagagccagagcctacattatcagca

gcagcagcatgtcggcggtggggtagttggtcccgagatcagcgagcagagtggaaccctgatggcccgccgtctatcgagcactgcgtccgcctcgc

cctcagcagcggaggacggggacccggacgaggccgaaggcgaaggcgaaggggtctcggctgtcgggctgggccagtctgtacccaacctgttcgac

ggggagatgagtgaggctggtttgttggctgcgtatggggacgatgcggtgcttttggaggcgagggggattgaggcgtgggtgcctaggttcgcgac

ggagttggagccgaagttggatggaatcccgacattgaaagagctcgcggttaatctgaattgggacttgtatcaagatgtgtacccgatggtaaggc

atttgatctattaccgcgaggctcgggtgatcgacgtgccgttcatcagcagcatctattcgatctgcccgctggtcgacgtagcgaacctgccccga

ctgtccaaagagtggtcgaccgccttcccgaccttaccaaaactcccccgcgtcctctcccagttgtcctcctccgccctccccttcgaaaaacaact

agaaacagcggggcgcctctcccacccgccctcgagcgttctccacctgctcgaccgccgggagcgctacctcgacgcgctcttctggctcctgcgcg

cggaactgatcgtgcagatccagattcgctaccggcttgtggccacggaggaggtgaaaaggttggcgaggggcaggtgggaggagagggaggggagg

aggagggaggagagggaggggaggaggagggtgagggaggaggcgcgggcgatgaatttggagcggagggaggtcctgggtccgggtgcgggtgcgga

ttctgagttgggacctgaagccttggaggaggaggaggaggaggatgatgatgatgggtacggatacggcgattcgagttcggaggaggaagcttggg

agtgggaggaccgcgatccccgtccgagcgtgattgcggaaccgggccgtccgagtaaagaggagaatctgtgcttggaggtcatgatggagaatctc

cccagcgacctcgcagctgtctttcgagaagcactgcctttcttcaacggcaagcacagtctggacgagatcgtgtcgcgacgcgatttgtcgctggc

acagcttcgcgctgtcttgaagaggcgggtcgactacatcatcgccttcttccacccatag

>contig_14.contig_14.g87.t1

atgggatccagagcggtaccgacgacagcggcgaggttgtccacagcacacagctctctccacagcgagatggccagca

tgctcgggtccctcacccgcaagcagagcggcgcatcagccaagatgaagaaacaccacgcacaggcccaggctcaggctgctgctgctgctgcacac

gctatgggcaccgcacgcacaggcccactcccaggcacggccgcaaacgggtccgggtccgggtccgtgtccatgtccgctgacggcccactcccact

cgcatacgcatacccagcgtcctcgtccagcggcagtcgccagaacagcccgctcacatcgcccctcaccacagaaccgccgatctccttctcagcag

caggcgcaggtacagcagcagagtcacagccacagtcaccgcacagaccattcctgccgcatctccatcgcacaaagtcagcaagctcagcacgtgcc

atggcaggagcagcaggcggagcaggaccagcaggagcagcagcaacaacagcaacagcaggcaggaacggcgacgatttcttcctcaatccacccgc

catgcccttcccagcagcagcaggatcagcctcctcacacagctcacacacctccagcaccctcgccgcctactccaccacagaaccatccaccgcca

gcagcatatatccaccagccagcgccagttcggtcaccaccgtctccggcacatcctacttccaacagcagcaatctcagcttctcggaaacaacaac

tccgcccacagctccgatcccgtcaacgacggacttgtcaaggatctctgccagaagcgcattcagagcatcgtctacctcaaaaagacgctcgaagg

ccgccagagctggctgaataccgtccgaatgtcaaagcgagatctcgtctccgctttcgagaccgagaagatgaggaagagaacaatccggaatatgt

acctcggtctctccctcgcacccgtcatcgagatgaacagcctcagcgacctctgcaagaccatcaccaacctcttcgcagagctcgaaagctggtca

gagaatgtcgacaaagaccggtccaaggtagtgcgaaacttgtttcgaacgacaagaggctcgaagcgcttgggagccgctagcggaggactcgtaga

attcatgggcgcggaggcacccaacttgctcacaaacgagtatacctacctgctcactcccaatctccccttctcgcccgactacttccacaccttct

tcaccctcgccgacatgctacaagaggtctactacaagatcctcctcagcataccgtcctcctcctccaaatcagccccaccctcggccttcaacccg

ggcacggcaagttcgatctccaccgtcggctccaactcgatctcgatgtcgacctcgcccgtgtcgaagattgggaggggcgcggggggcagtctgtc

cttgtctacttcacagactggctcggcacatgagccggacttgaatgatgactttgggaggttgttgggggtgaccagtgggcaagctggtccgtctg

gcaaggaagtgggcatcagcaatgggctggtcgacttgattcacaaggtcgatggcaagctcaaaaaaatattgatccaaacggcaaaagaggtcgac

gccttggcacggcatttgatgaaggaggagatcacaatcttagagctgcaagtccggaatctgccaaacagcagcggcagcacggctcttgcacccgg

cgccaacctagcgggcgtttcaagtcctctgatgggcggcgatcgaactgcattcgatgggccccgagcgtcccaaataggaggaggcatcggttcat

acccacctggacccggctccgaccgaggttccctcatgacgccctccccagccatgcacggcggaatctga

>contig_14.contig_14.g88.t1

atgcgcttccggaatctcctcggaagagggacggcagcgccctcacatggcgaacatgcgcagaggagaccaagaagat

ggttgtcaggagcgacagcaaccgccctcgccacggccgcggtcatcggcaccctggccgtatgcctcagcgcagcgcaaggcgtagcagcaaccgcg

tccccctcgctctctccccgccaacatgtgacccaagcaggccagcccacccacaagcgccacgagttcccagccacggtcacagactacacctggtt

ccacaactcgaccaacgtcctcgcgcactgcctcgacggctccatctggctctccagcaacgagggcctcacgtggaacgccctcaaggacgtcggcg

gagaccacggggacggacatgcgctcggtgtcattccacatcgctattcgcatgaccgcgcatacctcatcggcaggggccgacgcgtctggtacacc

actgaccgaggctccaactggggcgtcttcgacgcgccggacgtgcccaattcaatgggcctcaacctcctcgaattcaacccgcggaacccagactg

gctcatttggagtgggcagtatgactgcgaacacatctcgtcaccaaattgccgcgcacgagcctggtactcgaccgacggaggcaactattgggaga

agctcgatgagtacgtgaagacatgcgcatggccgcacattgaacacttcaaggagaacccacagacgatcatctgcgagtcctacaagtccaaatcg

ggctcccagcgatccttcacgcaggcaaacaacccgcttgagctcatcgtcggcgacaagttctacaagtccaagaaaaagatcttcgacaatgtcgt

tgattttgcaaccttttcagagtttattctcgtgggagaacttctcgacaaggggactacgctccgcatgcaggtcagcttgaacggtcgagacttca

ccccgccggtcttcccacccagcatgtcgccaaagtcacaggctgataccgtcctggagtcacgaacccatgccctcttcctgcaccacacaacgcac

ccagacaaaaatctggaatggggagccttgatgaagagtaactctaatggttcctactacatcgtctcgcaagacttcgtcaaccgcaacaagcaggg

attcgtagacttcgacaagacccaaagcatggaaggcatcgccttcgtgaacatagtcagcaaccccgaaagcgcagccatcacccgggtcaaacagc

tccagacccgcgtcacccacaacgacggcggacactgggacctcctcgacccccccgcacacgacctcgcaggaaaaccatacggctgcgtagagaag

aactgcagtctacacctgcagagcttcacggagcggccgaacccggcgctctcgtacaccagcccgaccgctcccggcctcatcctcgcggtgggtaa

cgtgggtaccaagctcgacagctgggcacacagtgacaccttcctcagtcgggacggcggtttcctgtgggaagaagtcgggaagggagcgcatatgt

gggaatttggagatcaggggaacatcatcgtcatggcggtcgactccaagccgacgcgtaaggtcagctacacgcttgatcatggcaagaattggctt

gagtacgatttcggggaggagctcgttgtcactgggattgcgacggtgccggaggatacccatcggaagtttatgctgtttggtctgccgcctaagtt

caacgcgcaccgtaccgttggcattcatctcgacttcagccatacggagccgaggaaatgcaatcttgatctcgcccatccggagaacgacgacttcg

agctcttcagcccgagcgaacatcggaaggaagactgtctgttcggacaacagacgtactacctccgccgcaaacgccaagcgaaatgctacgtgggc

caagccctcccgaacccgcacgagatccgcaagaaatgcccctgcaccgacgtcgacttcgaatgcgagtacaaccacgcccggaacgcttcgggcat

ctgcgtccaactgcccaacacgaccccgttaccgtccgacccgtacagccagtgtaaggatgggaagacggagcagtgaaacatccgcctcccaggct

cctcctcctccggcttctcctccggcggacccctctccaccctcgcctccgtcgtgcccttcctccaaggcgtcggcctgatcatcttcagcaagctc

ttcgacctcttcgagagtctgcccgggttgggaaggagcgggagccgctcgcgcgaggcgttcgggtatcgctcgttatcggcggatgcggatgcgga

ggcgttgaatgagtatgatgatgatgaggtcagcgagccaggttcaaagccggacgaggtggcggagaaggagggcgtcttgccgtgtcgagcaggca

aggaagccggtaaaggaggcggcgggaccgacggcggtggcgtaccccctccccctccccctcccattcccgctcctacgctccctatcggcggtaac

ggtcccctcggcggcggaccggccgcttgggcgtacgccgaaggcgggttcgacatcttccccagcctatccgagctcttcctccccagcagcgtccg

tgtggccgtcgtatgggaggaggacgacgacgatgccgaaggaggcgcaggtgcgggagcggggggtagcgggcctggtggtggtggcggcggcggcg

gcggcgggacaggcacagcgggcgccatggaccagccaggcggactccggaacgcagggatgttcggtaatcccggatcaggcggtctcgacggaccc

ggacccggtcccggtcccgcgtga

>contig_14.contig_14.g89.t1

atggctgacccgccgttctcctcgtctgccaaggggaaactgccggcgctcgtcgtccccggtcagcaccgacacccgc

acgaccgcgccgctgacgagtaccagtctcccttcaccctagcctcacgcgacaagaccgacgatgatggagcacaggttatcaagagagagcgtcgc

gctcaggaccacaccgcgccgatgtcgccttacttcgacccctccgagtcccaggaagcctacgactgggctcgcgctcgccgcgccagcaccgatct

ggccacagctgatgagcatgcggtagacctcgattctgaatacggcgtgggacccagcggaccggaagctgtgcccatcaagggacgctctcgccgcg

acagccaggtcggaagctatgacgaccagtccggcagcatcttcgacgggccttccgcgggagctgtacccagttcggtcagcaggcagtgtgctgac

ccgcttatccacattgcaaacagtatgcgtcacttcctgtcccgccctgagctttcttccagagtgtcctccactcgctcgcgccgacgcatctctcg

ggacatcggccgagaacgcaagctcagtagctcgacatcccgcagtcgcacctccctccatgacgacgacgatgccgctgggcgcagcgtgatcagcg

gagacggaatggatcaagatgacgaacaggataccatgctctatggagacggcgcagagtccaccacttctcgtccaggcagcgtccacagctatcgg

cggcggcgcgtctcaacagacgcgacggacgaaccggaagcacgcggaatgttcaagtccctcttctcctcgttccgtggcggcagaggcgacgatga

cgatgatgacgcaaagagcagacactctgcccgcccgtccatcggaggtcgacgtcgcagcacggcctcgtccgtcggtagccgtcgtcgtttgggcg

accggtaccattccgatgaggatgaagtcctggcccaagaacaggacgactttgatggagggaattactccgaccgcagcttgcacgacagcatctca

gacgaagaagctaattccgaccgcggcggcctcttgccttccgtctttggcatgtccagcggagccgtggatccggtctttggcgacgctcgtatcac

caccgagcacgagcctgaagatgatcttgacttggcattggcaggccgggacgatggggtcgacactgccgatctcagagacgaagatggcacacttg

agggcgaagaagggcacactcgagcaggtgctacccaccgtgccgaagctctgtcctctttcccagtatcctcaggtggcgagaacgagatgacccca

gcggcccgtctcaaatacgacgccacctatctggacaagagcgcaaccagccgacagcaagtctacctttcggacgaagacgccctagtccgcttcac

aggctacatcgaagtcaccagtaaacgcatcttgtacctcattgcatgcttcctctccctcggcatcgtctacctgttggccagatggcttccgcgac

ttcggctcaagtgggtatgcaaggagatctcgttccaacaagcggaatttgtccttgtcgaaaaccagtatggggacctccatgtcgaacgactcgga

tctgttccctttgaacgctccttggccactgttttcccgccgtcgtccacagaaccggctgccacgcaggctgagcttcaggcccagctctcgtccag

cgcgagtgttaaccggagcaagaatgcctcacgagctgcctcggttcggagtggaggagtcggatctacacctctggcaccatcggccactgggaatg

gcatcgcattcccgggtggtgatcagcccatcagcaatggcaatggccacagtagcacgaaccactcccaatcagcttcggaccaagtacatgccctt

accttcttcgactatcggtacactcgttttgtcctccatcctccgacgggccgtcttcgcatgatgaaagactggcgcgacccgaactggacgtccgt

catggccgtcgcttccggcgtgaccaacgccggcgaagaggatcgcagcatcatgttcggtctgaacgcggttgacattgcggcccgctcgacatggg

acctgctcgtcgacgaagtgctgcaccccttctacatgttccagatcgtcagcatcatcctctggtccttcgacgactactactactacgctttctgc

atccttatcatctcggctgtcagcatcaccacgacgctcgtggagacgaagcagacggtggcgcggatgcgggagatgagccggttcgaatgcgaggt

tcgcgtttggaggggcggtgtttggaagatgatgagtagtcaggagctcgtccctggggacatctatgatgtcgcagagtcagggctcttgttcttcc

cagcagacagtgtcttgctgagtggagatgccatcgtcaacgagagtatgctcacgggcgagtctgtacctgtgtccaagacgcccatagacgatgag

ggaatccagctcctacagggccgaggatccgacatctccattgacctagcgaagcacttcctcttctctggcacgcgtattatccgaattcgtccctc

gggcggcttggtgcccccgaatgcacccacccacggcggtttggatgcggagattgacgaggtcggggctatggctatggtagtccgcaccgggttcg

acacgacgaagggtgcgctggtccggagtatgctgttccccaagccgatggggttcaagttctatcgcgactctttccgcttcattggcttcttggga

ggaatcgcttgcgttggattcatcagcagttccatcaagtttgccgagctcggaattgcatggcacacaatcgtgatccgtgccttggatctcatcac

cgtcgttgtaccgccagctctgccagcgacgatgagcattggtacgacgttcgccattgcccgacttcggaaggctgggatcttctgcatctcgccca

atcgtgttaacattggtggcaaggtcaatgtgttctgcttcgacaaaactggaaccctcaccgccgacggacttgacgtgctcggagcgagaactatc

gatctgcgcactcagcgcttccgcgaattacacgagaccgtcgatgaaatgccggtcgacggcgccgccacccgaaacgaccccgaagcgcgcaagct

tccccttctgtacgccctcgcgacgtgtcacagtctgaaaattgtagatggcgaggtgattggcgacccgctggacatcaagatgttcgagttcaccg

gatggacgctggacgaaggtcaggaccggtctgcccgagcaacctcaaacacgggtacagccaagtatggcaaaagcaatgtctcggaccggccgccg

gcgctcgttcagactgtggtacgacctcccggcgggcaagccttcggggttgaagacgccattaaatccggtagacatgcgcacttcctggagctcgg

tgtcctgcgaacatttgagttcgtctctgcgcttcgccggatgagcgtgatagtcaagcgtctcaagtcggagtcgatggaagtcttcgtcaagggtg

cacccgaaaccatggccgacatctgtgacaaggaatccttcccggaggactacgacgatctcctgaactactacaccaagcatggataccgcgttatt

gcttgtgcgggcaagacgatggcgggtatgagttggatcaaggcacagcgactgcgccgcgagcaggcggagagcggacttcggttccttggactcat

catattcgagaacaaactcaaagagggcacgacgcctgccatcgcaacgctccggcaagccgatatcatctgcaagatggtgacgggagacaatccgc

agactgcagtgagcgtggctcgggagtgtggcatggtcggacaatctgcccatgtctttatgccgactttcgtacaagggaactcgaggaatcctcgc

gcggttatcgagtggacgagtttggacgacgaccgtgtgcatctggacccgtacagcctcaaaccacgagaggccgatccgcacatcatggacatgac

cgagcttgacttccaagactaccagctcgcgctgaccggggatgtattccactggatggtcgactttgcgcccctggaaacgctgcgacggatgctca

tcaagggcacgatcttcgcgcggatgtctccggacgagaagcacgagctcgtggagcggttgcagatattgggatacactgttggaatgtgtggcgat

ggtgcgaacgattgtggagcgctgaaggctgctgatattgggatttcgctgtcggaggcggaagcttcggtcgctgcgccgttcacgtctcgcatccc

tgacatcacgtgtgtcattgaggtgatcaaagaaggccgagctgccttggtcacttccttttcatgcttcaagtacatggctctttacagtctgatcc

agttcacgagcatcaccattctttacaatctcgcgagcagtctgggcgacttccagttcctgtacatcgatctgttcatcatccttcccgtggcagtg

gcgatggcgcgcacgctgccgtacccggacatccatcccaagcggccgacggcgaacctggtgtcgaagaaggtcttgaccagtatgctcggacaggt

tgtgatttgcgcaagtgtgcagctgtttgcgttcctgtacacccgtgctcagccctggtacgaacagccgatcttgaacccggacgagttgaatgtga

tcaacccggagaactcttccttattcctgatatcctcattccagtacatcctcgtcgcggccgtcttctccgtcggtccgccttaccgcaagccgatg

tacacgaatcccgtcctcacggcgttctctctctacttcctattcgtgccctcaggctggttcttcgacatcctcggcctcgtcagcttcccacgttc

cttccacttcgccctcctcatcatggtcctcgtcaacgtggcgctgtcgatcctcttcgaatgggttgcgatcgagcccgtcactgctttcctcaggg

gtctgcagagattgtggcgaaggcggcacggacggtccgcaacgagtcaagtgcaagatgtcgcggtccttcgattcggcgggtctgtggttcccaac

ttgccatcaaaggttgtcagcggcaaccactctctgagatggaagacccctccccggaacgtccttatcgtcaagaagcggaacgacgtgaaagcctc

ggccgccatgaaagaagtcatcgcccatatccaccagacatatcccgacttcaatgtcatcgttgaaccagacgcttacgaagagttgggcagacaac

aagccgaccttgccgtcgtatcgtcttccacccaggagaagcatcagcttgcggcaaagaccgaccttgtcctgacccttggcggagacgggtcgatc

ttacacgtctccagcttgtttgatctcagtgcggtccctccagtcctgagcttctcaatgggcacccttggcttccttcttccatacgacattacctc

cttccaaccggcctttgctgacttcgtcgcgggggacgtctctctgctgttccgctctcgccttcggatgaacgtacaagggcaaacagaggaggtcc

acttgatgaacgaagtcgcccttcatcggggtgcatcgccacatctgaccatcatcgacgcttttgtcaacggacagcatctgaccgaggccatcgct

gacggactgatagttagcacgccaactgggtcaactgcatactccctctcagccggcggtccgattgttcatccatccgtccagtcgctagttctcac

ccccatctgcccacgctccctgagcttccgaaccgtcatccttccgagcgactcgtgcatccagctcaagatatcccgcgcctctcgatcgcccgcag

agctctccatcgacggccggcattaccaaaccctggctccaggccaacacctccaagtctccctctcgccccacccgatcccatgcgtcaatcggccg

gcaagaccgcagggtaagctcgagtccagcgcgccccaactcgagcacgacaagcccgattggcccgacccgtgctgtcctcccaaggacggctccga

tcgtggcgaggacgactgggtccgggatatcaacaccttgctcaagtttaatgcctccttcgcaggtcgaggcttgctgggcggggaagacgaatcaa

agtga

>contig_14.contig_14.g90.t1

atggaacgccgtttgggtcagcctttcaggtcgcccaacattttttccaggtcctcctcttctccttccgcctgctctg

cgagccactcagcgcgcactacttactctcttccttctcaacctctccccagcaaacagatacgaaacaggatggatccattcataagtttcagcccg

cacagcattgctgcttctggcattcatgctctgcttctgcccggctgccttcttctctcggctaccctggcccttgagtcgttgagtgccatcgagca

caagatgcccatcacgcccgaaaaaagcgaaaaggagggatcttcgctggaggaactgctgacgaagacgacgaacaacgctacgaccggcgccagct

cgcgcgcgggctcgtcgtccgctccgactggcgcatccgcggacagcccgcgcctttctggacgcagcggcttcgccaccagcagcggcaccggcagc

agcaccgggcttcgaggaacgctgtccggacagaggcggcgacgcccgctcaacccgagcagctcctccaaccaggcgcgttctggtctgacctccct

caagcggtcctggtcaggaacatcaaaggacctcggcgcaacgaacaccttcagcaagtcctcccagcaagccagccaaggcggcgccagcgccagcg

gcagtcagggaaagccatcaggaagtcagcgctctgtccgagaatcgagcgcagaagtcttctacgacggttggtcctcctcgcccgacgttcccgag

tccgcaatgagctctggaaagcattcactcgcctccagctcggccgacggtggtccagccaagcggccccgaagcgggcttcagaatcccaccaccgc

tggcgaaagcaagggaagaacgctaccctcgatatttggccagtcgtcctcggacctgctcaagtccaagcgcgccacgtcctcccagtccatattct

ccaaaccgggcaatgtcgcagaaaatggtcccaaaccaacatacgccaaggtcttcttgtccactgaacaacagatggtgctcagtaaggtcgtcgac

aggcaacagaacgtcttctacaccggttcagcaggtaccggcaaatccgtcctgctcagagaaatcattaaggagctccgaacccgccacgcgcgcaa

tccagatgcagtcgccatcactgcttctaccggtatcgctgcgtgcaatgtcggaggtaccaccctccacagttttgctggcattggtctcggagcgg

gaaccgtgcctcagctgatggcgttcctccgcaagaatcgcaaatcagttgctcgctggctcaaaacaaaggtcctcatcattgatgaagtgtccatg

ctggacgccgacctgttcgacaaattggaggagatcgcgaggatgcttcggaagagcaccaagccctttggcggtattcaactcatcgttaccgggga

cttcttccaactgccaccggtgacatacaacaaccagcctaagtttgcattcgaggccaaatgctggaactcagccatcaacttccagtacaatctga

cccaagtcttccgtcagaaagatcagaccttcatcgacatgctgaacgagatgcgattcggcaagcttactccaaagtcaatcgccatcttcaaatcg

ttgaaccgcgttcccaaatacggggaaggcatagaacccaccgaactattcccgcgtcgcgaggacgtcgagaaggccaacaccaacagactggctgc

tctcgattcgtctacccggatatacaacgcaatagacaccggccggctggcgacagaacagcgcgagaagctgctgggcaacttcatcgccccgaaaa

gcttgagcctgaagatcaatgcccaagtcatgctcatcaagaatgtcgacgaaggccttgtgaatggctccatcgggcacattgtcgacttcaagacg

gtcgcaggcgccagtcgcgaagaaggcggcgtccccgatggattgcgagttaaagctgagaagatccccgatggattgcgagttaaagctgagaagat

ggaaggaggcgggtcggggatcgcggctggaggagccgtaggttcctctcgcagtcgcagcatgagtcctgtcaagggtgcggcggaggaaggccccg

agttcccttatgtgttcttcccgcagacgaaccggtacttgctgtgcaagccggacagttggaagaacgaggacaatatgggcgaggttcttgctgag

cgacatcaggtgcccttgatccttgcttgggctatgtccatccacaaagctcaaggtcaaacgatcccatgctgccgcatcgaccttcgcaggatctt

cgagaagggccaggcgtacgtcgcgctgtcccgagccacctcgctcgagggacttcaagtgctcgggtttgatccctccaaggtcatggcccaccctc

gcgtcatcaactggtcccgaaagacgttcgagaacacttcgtag

>contig_14.contig_14.g91.t1

atggcggcagacgcgctcgctctctcatccgctgcggtcccagatctgctgacctctgatgcagacttcgaccagctgc

ccagcacgcccgcgttggcgaacggaaccgaagccgagcccgtcgtgatagacgatgaggacgacgacggggacgagcatcaggacggaatcgatttt

gatcaggaggacgacgacgacgacgacgacgatgatgactacgagatactggaggacgcaaatggcggcgctggccagtccaatgacggtcaggacgg

cgtcggtgcgcgaggtggagcagacgacggcaccgacgctgacgagctcgccggtgaagaagatctgctcggtctcgggatgcattcgacagtcacac

acgtagacacagaggaagaaggcggcggcgaagacgatgcagacgctggcgaaggaccgggcgaagaattcctgccaacggtgctcatcgagtttgaa

gactccctgttcgatctcttcgcacaacaggcaggcgatggcaaacacgacagcgagcagaatgcgattccactgaaggcgccgacagacattcttga

ccaaccgctgtccagtctctttgcaacacttcgcattccagacgcgctgggcgagtttctcgagaaagatttggctctggtgctccgcagcccagagg

tggacctggaaatgacggagaacgacaccaccgctgaacaggtccttttacaagacttccttgatatacatccctccaattccaacggggatgcagtc

cgactacgattctcccaacgtcaatccttcctcactcgcttcaaaatgctcggcggcgtcgtgaatgctgctcaaatgcccgcggcgcctctctctcc

caccacagctgtctcatcgtggatgtcccgtgccaagtcaaactcacgcagagccaacggcggcgacgacgaagctgacgagctcgatgacgaagctg

gtggtgtagatgggggtacacttggtgaacaccacgaacccgatgatgatgatgagggacgcgagccagaatgggatgaagccgagggcgaaggagaa

gtcaacgagcattgggaaggcgaggccgagggtgagggtgaagaggaagacgaggacgaaggggagggagaaggagaaggagacgatgatgtcatctt

gattgaggatgatgacgaccacgaagctgggccgggaccagactacgaagaagggtacgaagaaggatacgaagagggcgaccagatcgtggacacag

aaacaaacgaccatgatctccaagagggcggcccggatgaggacccagaccacagacaaacagacgagcacgcagaggagcaagatcaggacgatgag

gagggcgttgatctcgacgagggtggagaggagggggacgaggtatacgaggataatgagggcgtggaggggccctctaagacatcgggcgaaaaagg

cccctcactgcccgcgacagcagagtccgcgccacccttgtctgcttggaaagagcccgcctcctcggcgacgaacacagtgcctaccgatgggactg

cacaagtagcccccgaacaagttgacgaaggtgaatacgaagacaatgcatccggcgatctcgtcgaccgtgcaggtgctctcggcgcaacatcatcc

ttgacggggaagcgctcctttgacgagactgatgatgatcttcaacaagaagagggagatgagggtgcgaaacggctcaaaggcaccaactccgcaga

ttctctgcttgcacccctcgatcctcagtatcgcatgtgcgctacactacccggcaaccaaacccgaccctccaacaacagagttccgaagcagcttg

acaacccacactga

>contig_14.contig_14.g92.t1

atggccccgccagcggcgctctccctgcttgagtgctgggagcaacgggacctccagcggtttcgtgtcgtccttaaag

gtggcaacctccacgacaggtcgaatcatgcgcagccttcgacttccggcggtggcggtagcggaggaggcggctcaggctcgtatgcgcgctccccg

ccgggcggacatagtgcctcctggggccgagatggtggcagtaacaaccataaccacgtcctcgggcccgccccgccctccgaggtaaaccgacggga

cgagttcgggcgaacgagcctacatctcgtctgtgcctccccggacggcatcgcgatcgacttcctccacgcgctcctctcccacccgagtctgaacg

tgaacctgcaggacttcgaatcaggctatacggcgttgcatctggcgctctatgcggggaacttgcacatcgcgctcacgttgcttcgtaaagcggat

acggatttgcgggtgaaggacttcgaggggttgactcccttcgatctgtacaattcgacgatctcgggtacgaacccgcgctggacgcctgagattgg

cgggagcgagctcttcacctggggcgtgaatcggaatttcacgcttgggttgggggacggggacgatcgggcgctgccgaagtcggtgctgttgaata

aggtatatgatgggtatgcattgaagcagatggaggcgggcgccaggtatgatcgcttgcgcattcgggatgtcgccatgagcaagttgcatactgtc

gtcctcaccaccgagcggagtggcaatgtctgggtctgtgggttcggcgggaatgcacgactcggccgcgcgaaccaatcgcaaacctcactagaacc

cctcaaagacttccacgacagcgccgcaagcgtcgccgtcgcccaagaccacaccgtcatcctctccaccaacggagacgtgtacacctacggccaga

acaagtttgcccagcttgggtacgtgctcgagaagggccagggcatcgtcggctctaccacaggcaactcgggccgcggtgggggggcgcctacggcc

acttttagcgggtcgtcgtcgaatgggcctcagcttgggggcagtctgggcaatagcgcttcggctgggctcgctggtgccattccaggttcacatca

gggtcccctcgagctcgatctgcagatcagtccgcgcaaagtctcgggcgtgctcaagaaggagtacgtcaagggcgctgcggcgagtaagatccaca

cggccgtcttcacctccgacgcgctgtacacctgggggacgaacacgggccagctcgggtacgatcggtccgcgacgcccgtgcaggtcttgccccgc

aaagtgacctcgatcaccgctgcccagcccatcaagcagattgcagcgacggagtttgcgacggctgttgtgttggagtccgacgaggttatcgtctt

ccataacgatggacagttccgcttgcagttccctgcgacgcccaccacccgtttccccgccggcaaggaggtgtacacctccaggcgggtccagcgca

agattcgggtgggcaagatcacaagttcgggcacgacgttcgccgcgctttccgactcgggcgacctgttcacgttctcgctcgagcatccgagcgag

tatgtgaagtcgggcgggaatcatgtccggccggaggtgtctacgatctggagcgtccggcggaagtttactgccgttcgtgatgtggccatcggtgg

ggatgggagtattattcttagtacggagtccgggcatgtttttgtgcgggagcgacgggtgggcggggataagcctgtgggcagcggtcctgccaagt

tttcgaaaacgtcggccgcttcttcttcttcttcggggaagaacgcgttcaagttccatcagattccgtacctgcagcgcgtcgtcaaagtggccacg

aacgagagtggcggatgggcggcgatcaaggccgacgcgccccttcgtgacattcggatccgcggccgttcgatcgaggaggatctacagtcgttgct

gccgcacctcaaagtccgggatatcagccagcacggcttcgaagaagaacgcgagatctccgtcgaggtggttgaggcggaccggaggagggaggagg

ccgtcggcgccgtcggaggagatgaggaggatgtggagagctcggatggggagagcgaaaatgaggagggtgtctcgcatcggcatttgcgcgttggc

aagttgttgggcgaggctgtccggcggtggggcctgtctgagggaatggcgcgagatgcgtatgggcctaacaatctcgtgcccccctttggttgcga

catgttcgtcattgccgagggaaggtacatccctgcgcatcgtatcatttttgccgttcgggtaccggcgctacgagatgcgctggcgtcggcgggca

gcgggagtgtgccggacggtgtgcgggtcgatcacgttacggattcggtggttaccctcactttgcccaattgcggcttccccactgcggtcttcctg

cttcactacctctataccaacgatctgcccgcggtatggacgacgagtatcggcttgcaaattgcggctcagtatcaggccagcaagatggtccccgc

ggtgattcagtcggaactgctggagctggtccagtcgttggaactacaggattccctgcggtcctcgttgacctcgcccaatccaaggtcgcctccgc

ataccctgcggaaagacatggaaacgttgttcgagacacaggttgcggtggaagcgtcctcatcgtcattacgggacaccgagctgatcctctccgat

cgagtcgtgccttgtcactccgtcatcctgagacgttcgccgttctatgccgacttcttccggcctgagtggacgtcgacccggttcgaaaacgggcg

gatccaggtgaacatgcagcatttacgctgggaaatcgcgcggctgctcctgttacatctctacactgatgcgggtgtggagctcttcaaggaggctg

atcaagaccggacaccggaccagtttatcgactttgtgctggaggtgcttgctgctgcggacgagtgggcgcttgcgaagatgaagcaggtctgttcg

ctcctcctgcgtcgacgagtggtgccgacgaacgtgtcggcgatgatgacggacgcggacactttcaacgcgatcgagctcaaggaggcgtgtatgct

ccagacgacgcagacgatggagtccttgcttgagacggggatgttggacgagctcgagggcaagcagcttcgcgacttgacgagctttgtccggcaga

agcaggacgagcgcatccatcgcacgttggcttccgatcggatcttggctgtcatcatggcgaaccaggacgagtacgatcggattgtgcaggaactc

aagagtgccaaacaacgggcacaggcgcggcctcgtaaagccagtgtgtccgtttctgtcgttcagcagcagcagatgccgactagcccggagatgcg

gccacacaaggctcatgcggatgacagtcttatctttgaaatggatgaagatttggatacgctgtcgcaacatggcagtccgagcttgtcggcgctgt

cgaatgtcaagctgagctcgatgccggcttgggcacctcttcccggagctgtccggggtgcatcgcagcaagcgcaccagcaaccggctgctctcttt

ggccgttcaccgcccagtttcgcttctgattcgggcgctcagcgtgcttcgatgacatgggccgccaaatcgaacgtggttgcggaagctccgacaag

tactcccaacggcgtagctgacttccgaagcatcatggccagcgagaaggcgagacggcctggtcctacaaccgtgcagcctgctgcttcgacgccgg

ggacatccactgcgtcccttagcagctggcgccagcccagctctagctctggtagtacgtcgctgacaggctcgggtgcggcgacgccccagcctcca

ccgtcacccagtctcttgccggtcaattttactcccaagttgtcgcagaaggatcggcggaagcaggctatgctgcagagtcagcgacagtcgtcatc

aggtacggccacgccgaccgctatcggtggtgctggtattgctgttgctgctcgtggtggggcaggtgtgccttactctgtcgtgagcggcggaagtc

ctggacttagtacgacgcctacgggaacaccgtggagagtgccttcgtcggctactccgggttcaagtccggctcccggtttggtcaacaaccccgtc

tctccactggctgctcgaacgagggagtcgacgtcgtccgcagcgggcgtgtctgctgccggcacgaccactcccacaaatggctcgccgtggaaatc

tccgccacctagcaagaacatcagtcttccttctcccagtcccagttcattaccaaaagcacgggaagcggtaataccatcctcctcctccttcacca

ctccctccgaaatcccgtccctcatcagcgcctccgcaccccgcaccccctccaagttcagctttgcgcagatacaggagcagcagcagcatgccgtc

gaccaggacctgcgccgggcgagcaatgtgaagaagagctttgctgagattgtggaggaggagcggaggagggaggacgaggagcgggtgcgcagggc

ggaggcggctgagtttgagaggtggtttgagagtgagagtcggagggtgcaggaggctcggggtccggcacaactacaattgcaggcgccgtcggccg

gtggcggtggtcaggctgagggtgagcaggcgaaggagaaggagaagaagggaggccgaggtcgtggtggacggtctgggggcagtcagcgtggggcc

agtcagtcggcttctggcggttctcgacgcggacatgcaaatgcaaatgcaacagcagcagcagcaggaacaggaacagggacaggaacggaagaggc

ggactgcaatgcgcaggaaggtgctgctcagggtgaatctaaggcttcttcgcgcggtcgaagtcggggagggagagggcggggtcgtggcggtcgcg

gtggcggcggcggcggcggaggctcggctccctcagcttcgacttctcatggtacataa

>contig_14.contig_14.g93.t1

atggcagcgatggcagcgatggcagcgatggcagcgatggcagcgaacagcacagcgtcgacatcagcaccaccaccag

caccagcaccgcagcagcccgcactcacaggcaagctcgtcaaggccgtcgacatcgcagcacccttctcccacgaggtacgcgacctcatcaaacac

cgcgtcgagacgctcagcaagcccgctccacgcctcgtcggcatcctcgcaaaaccatacccgccctccatcgcatacgcagaatggacaaagaaggc

atgcaaggatgtcggcgtccagtttgacatctggaagacatgggacgaccagccggagtcctcagcatcatcctcagcatcagcatcagtggggcaga

acggaagtgcactgccgagcgcagacgacatcaaggaggactactccctcgcagccgacgtggaggacctgatcatcgcagccaacgcagacgagact

gtcgacggcatcatggtctactaccccatcttcaccggccgacaggacacctacctccagcagatcgtcgacccgcgcaaagatgtcgagggtctcca

cttctcctactgctggaacatgtaccacaacgtgcgctggatcttgccttcccagctcggcggcgcaccgggaacgatgagcgaacctccgccgagct

acttcgaggacaactcggtccaaaaaccgacctcgcccagtccgatgcttggcaccgaggtcaacgccgccccgggcttcgccaagtccatcttaccc

tgcactcccctcgccatcgtcaagtgtctggagagcatgggcatgtacgacggtcatctgccctatggcgaccgactctcccacaagacggtgactgt

catcaaccggtccgaagtcgtcggtcgtccgctggccgcgctccttgcgaacgacggcgcgcgggtgttctcggttgacatcgacaacattcttgagt

tcagtaagcgcgcggtgcacgagcgcaggcagtcggcggctgtttcacaggaggaatcgggtgcgctgggtgcgccggagggagcgaatattgaggga

gagcggaggaggaggtcgaaagcggtcatcgctgcacaggagaagattgccgagggtcgtctccgatcggctcacaccatcaaggcctgttccaaaac

cttgcaagaatgccttgccatttccgacatcgtcatctccggcgttccctcggcttcgtacaaggtcaagacggaatgggtaaaggaaggagcgacct

gcatcaacttcagctcggacaagaactttgagccggacgtccggtcccgcgccgcacagttcctgcccgccataggcaagacgacgattgtgatggtg

cagcgcaacctgatgagactggtgcaatacaaggaggagattgtccccaaggccgacgtcaaatga

>contig_14.contig_14.g94.t1

atgtcgagcgcacgcacaggtcagaagggcgtcagtcgcggcgcagtatcaagacccgtcgcacgctaccgcccaggaa

gactgcccgagggagccgacctcgccgcagaagctgccctgctcgacgacgacgacgacgacgacgacgccggtgcgaaccaggtcaagaacgaggac

gtcggacagcacgcgttcgtcgtcggcggtggcagcggcggcagtggtgggaatgcacagagcatatcctccttcggtgccgcttcttctggtcgtcg

tcctccggtcaaggtcgagctcagccaggtccagattggcaagcagagccggccacagggcttccaaagcgacgagtatgaaaccgacaccgacgacg

aggccacaccgtccaaaccgacgttccgcaagccaggcgtaggaggaccgggcccatcgaccaccagcgcatccaaggtcaagcaggacgaagagtcg

tcccaatacgagaccgactccgaagaagaggaatccgaagacgaacccgctcccatgctcaaaccagttttcgtccccaagaggttacgggggaccgt

ggcggtgcaacaggtcaaagaggaagtcgatccgaaagaggcggaggcgaaagctgttaaggccgcccagatgcgtaagaaagaaagccacctcctcg

cagcagaaaccataaagcgagaacttgcagaaaaggaattcgaagaaaacctaccagacctatccgacaccgacggcaaagaccccgaagccgagttc

caagcctggcgtctccgcgagctgcaccgtatcaaacgctcgcgtgaactcgatgccgagcgcgaagtggagcgggaggagatcgagcgcaggagggc

catgccggaggaacagcgcttggcggaggatttggcacgggcgaaggagagtcgggatgcgaagtcgaagggacagcaggggtttatgcagaagtatt

atcacaagggcgccttcttccaggacatggacatcctcaaaaagcacgacttcaccgaagccaccgaaggccacgtcgacgtcaacaacctgcccaag

ctcatgcaagtccgcgactacggcaagagatcaagaagcaagtggacacatctcgccaacgaggacacctccaaaccgcaaaaagacgcccgtttcca

aggtctcagcacagcaaccacaggctcgggctctggttccggcccaggcccttcctcctcctctcaagcctgcttcgcctgcggcggaccccatctca

agcgcgactgtccccaagcaggcgctggcggcggcgggtccaaagatggcacgacgacgacaggttcgaatcgagcacccttgggcggtagcagtagc

aacagcagcagcagcgggccgaacggacctcctccctcctcctcctcctcccgctggaacgctccccctcccccctcttcctctcttccccaacgcga

gcgcgaagcagcagaagaacgcgcctacgatccccaaaggcgcgaacgtaggccgagtccgccgcggcgggatgtatttgatgggcccgatcgcgagt

ttgagcgcaggagggaggatcgggtggcggggaggtcgtccggtgctcgtggtgatgggatcgagagcaggaccggcatcgagatcgagacagggaca

gggacagggacggggatagggatagggacagaagatcccgagacgccagagatgtggacaccaaccgcagcgcaaggtgaccagccaaactaa

>contig_14.contig_14.g95.t1

atgagcagcaccctcctcaacgcggccatctccgtcgcaggctttgcctacctcgatgccaagctcggtatctactccg

acaggcgtctcggtggagccgcatacaaggccaccatcatccacaggctccgcagcgtccgcggtcgcgtctcgctctggaactccttcaaggatgcc

gtcgctaagcgtcccaacgacatcgcctacgtctacgagggtcgctcctggacctggaagcaggtcgaccttgagtcgcgtcgggtcgccaacttcct

cctcggcctcggtctgaagcccaactctcgcgtcgccattgtcatggccaactcggtcgcataccccaccatctggctcgcgtgccagtccgctaaca

ttgtccccggtttcatcaactactccctcaccggcaagggtctcgcccactgcatcaatgtcacagaggcagaagtcgtcatctacgaggctgccttc

caggacagcgtcgccgagatccagtccgaccttgccaagacatcccgggtccgcacctacgtctgcttcgaggacggaatccctgaccccaccgagaa

ggtcccctccaccttcaacggcgccgtcgcgaactccatcttcctcggtcccaccgagctcgcccagcagtccaccgccgaagttcccgacagctacc

gctccggcgtcaacgagagctctcccgccgctctcatcttcacctctggtaccaccggtctgcccaaggccgctctctgctcccacggtcgtatgggt

atggccaccatcatctgggcaacttacaacggcttcacccccaaggaccgcatctacacccctatgcctgtgtaccactcttcagccggtttcctctg

tgtcgccgcgtcctggtactctcagtccaccgtcatcatcggccgcaagttctccgcgtcccgttattggcaggaggtgcgcgcctccaaggcaaccg

tcgtccagtacattggtgaaatttgccggtatctcctggcagttcctccccatgcggatgacaagaaccacaacgtccgaatggcatacggtaacggc

atgcgtcccgatgtctggcaaaagttccgcgagcgcttcaacgtccccatcatctccgagttctacgcgtcttcagagggtaacggtgccgtgctcaa

ctacaacaccgggcccttcggtgccggtgccgtcggtcgcttcggtacgcttggcagcaccttccgaccagacttcaaggtggtccaggtcgatgcca

tcaccgaggacatctaccgcgaccccaagacgggcatgtgtgtcgaatgcgcacccgacgagcccggagagttcctgtgccgaatcaacaccagcact

cccatctccaacttccagggttacgcaggcaacaaggcggcaacagaaaagaagattctcggaaacgtcttcaagaagggcgacgcgtggttccgctc

tggtgacctgatgcgcaaggacgccgacgggttcatgtggttcggtgaccgactcggcgacacgttccgctggcgatccgagaacgtctccacggccg

aggtctccgccgcgctcggtgagattgtctccgaggcgaacgtgtacggcgtcctggtgcccgagcacgacggccgcgcgggctgtgctgcgatccct

gaggagtgcgtgaagacgcttgactggaagttgctggcgacgcacgcgcgcaagagtctgcccaagtacgcggtcccgctctttgtgcgggttgtgcc

gaacatggagcagacgggcacggtgaagcagcttaaggtgcagctcaggaacgaggggattgatcattcgaagacgggctcggacttgctttattggc

tgccgcctcgctcggagtcgtacatccccttcgaccgggcggactacgaggccatcgttgctggcaaggtcaagctttga

>contig_14.contig_14.g96.t1

atgcagatcaaaccgactatcgcagccctcgcctgcttgtttgcagccgtgccagccgtcctggctgcttctgccggcc

ctgatggatcgctggacacgaaccggatgacccgggttactcgggcatcgactcaggcgagcgatgtagagatcctccagcgtcgtgggtgcacgatt

gggctgggtgttcatgctggaaacgtcagtatcaacctctcgctgcagctgttgaccgatgtcgtggccagcctcgggttgaacgtgacggacggact

ggcgcagattgcccgcctggacgtatctgctgttccgcagctggggggcatgacggatggggtgacgacgtcgatcaagaccgcgttggctcaattgg

ttacggacactgccagcagcaacagccaggcagcttcttcgaagcgggccgaggacgacgccggcaaggataagctggaagagaacacggcatctctt

ctgcgacaggcttcgcctgcgtttgaccacgtccagggactggtcgctgggggtcatgcggatgctaagtcggttttgggacttcagggcgtgatgga

cggtctggcatcgatgttgatcatctctcgctcgatggactcaagccttttcgacaacctggacgtgaagacgccggcggagtccatcgtccactccg

tgcacctgatgcccgaggccggtctgaactcgacgcttcttaccaaggtgctgtccggcgagaagctagtccggcgggacggggagctcatcaacatt

gggtttgggctcaacttgggtccgaatgtcggtgcgggagcgggcttcaccgtgggacctaaagggatcggtggtgggttcaacatcggggttggtcc

tgttggagctgggggcggcatcaactgcaactga

>contig_14.contig_14.g97.t1

atgcgcgtgtctggtcctgctgctccctcgggcgggctggtggaatacgacctcacctgcatttcggttcactcaaagg

acgcacggacaaagcaccgatcgagggaggtcaaggaggtgatggaggagcatgggtttcgggcagcggtggagaaggtggttgagctacagatggag

gctgcggcggagaagaagaggaagaagtatgcgggcattattaccctggcattccagccattggtgattacgttggatgggaccttgaacaaggagac

tgcggcaactttcaagcactggcgttcgttgagcccttcgtggtcctcggtcagtgctgcgattgcgtgcacactcctccgtacccgcgctgagacct

actgcctttggtga

>contig_14.contig_14.g98.t1

atggcgcagaacggggcaagtggtggtcaagtagtcaatgaatcgagcagctcaaagttcaaacgtcaaaacaccaaca

gctcatcagcatcaccaccaggcatcaagcattcaggatccataacagaccaatctgcaaagacaagcatcatgcgcgctcctcgttgccgccgcggt

tgccttgatccaaaggatccaaggatgtgccgattactcccctgcgagcccggcgcacatagcaaaccgagtaaagatggagaacccgctcgctcacc

agacgcagtcacgacaagcagcccttccgcgcctgctcaggggccagacactcgcgttcctgcccctcgcgtccttgccgcagacgcagtcatcaacg

atcaacacggcactgctgcggaagcaatggttgaggcaaacgcgttactggccgccgctgaagcggctcgtctgcatgatttggagagtgcttctgct

tctcccgcctctcccttaccgcccttcctcaatccgacgctgctcgctgcttctcttcgtgcgaatgagcatgagagcggcggacttcagattcgcac

acgacgagacagcgatactgagacggattcggaacatgcgatgactgcaggtaccactgagatccctaccaatgcgaaaggcaagaaaagacaacgcg

tgaacgtgaaccttcgtccggagcccaacctcaccgaagctcagtatcgtgatatttggcttaacttgaaaaactcgcacaactacgccatgaaatac

tacgaatatgaacacaattctcaggcgcgaatgcaagtcgcgaaaaggggaacatggaggcttatagagcagggagctaagctatcggcaaggtcagg

tctcgctgtcttcatcgcatttgggcctttgaatgaaagtgcgaaggccgaagcccgagaccatgtctatgtcagtccgaacctctgcgatggcgctc

gacctactctgcgcgcaatggcggaacatgctgccaacgatttcaagacaacaatgcatatgtaccgagaagcggcacgagcgaacacagcgcaggca

ttggaagcaaacaaggttctgattgcggaaaaagacagctcttaa

>contig_14.contig_14.g99.t1

atgttgtctgcaggtgcgattgtcacccttcacgcctcacacggcacacctacccggctagtggctacccttcctcact

ccatccacccagccgcaggccttgacattgcgccatcaaagcgacagcgcaaacagcgagccagagcacatcgatctggctctgctgctgcgcaccgc

gatacctcgtcctctcatcacgaacgtccgacaacctcgaatgaagacttcctttcctttggtgctggagtgatagatgactgggcgaaccaatctga

ttttgggatgggagacgaggggccagcggacaattcaagtgatcgggacgagattcagcactacgcagactcgctgttcattcagcagtctgtgtacg

tagcacgtgttgaccttcggacttgggttgtcgaagactatgtcaatggtatctgccaggaagggaagttctatcatgtgacgctctatgaaggaacc

agcccgacaggaagggacgaacaacttgcctcatgcgattgctcagctggccgaaatgggttgcaatgctcgcacaagagagtcctgctcgagcacgg

cgagctgttccaaaaggaggtcattcttcctcagagaatcaatccccttgcaattcagatcgcggcacacctacgtggtgaacttgcttggttctctg

ttctatctaccagaagcaccatcaccgcggccagtcaggacggcaagcgttgtgtcgttgcgttgtctggaaacgagcgttggacttgttcggcatcg

cattgttcttcctttggtcagcctgcaaacgtctgtccacatcgtcaacgcgcccagcagttctggaacgacacaggcaattccatcgagcctgatct

tggtggtaatggaatgatattcaaccgatgtacctgtccactgaaaggggcattcttccaacaacgatcccgcttggagaatacgcccgctgcgactg

cggtcgacgcagcagcgttgaagcgccagtacgactccgaccgtgaatgcagctgccgactcggtcaagagaagaggagcatcggtcccgacctttcg

gagcacgggcttttcaattggaacaactccatcatcgtggcacatgaagtattcaacgcctatacctcccagctctccacctctcctactccttttac

agctttctgtctgaccattcaacatgcgtatgaacagcatgctcctggtggaaagctcaagtttcttcatcgttcgacttttgtcaggttgtactttg

cgtacatttccttgcagcgactcgatatcgatttctcatgccaactctgcggtcccaatccttctacagtcatagcagatggagtagtgcttgcattt

ggtgctccgtttcgcctcgccgacctttgccctcccacctcaaaaggaatagacatcaacgaacgtgcacgaagcaaatcgatcatttcgtttctttc

ccctccttccgcgcgcgcagctgtgaagcagcttgccggcttccttctgcagcaggtggcagacaactctgatcagtacctcgaattgctgcagcaga

tcaaggcatctgtcgagacggaatcggtcctggtgatgcgcttgtggtcaggacgctttcagcaggtgctgctggacacgttcttggccagggccaca

ctttcgcacaacctcacgaccgcggttcagcaattgatcgaccagttcgctgcacatgagggtgttctgcaactttgccgccccccctctgctgccca

ctcctga

>contig_14.contig_14.g100.t1

atgattgatcgatacctgacaggcggcgtcacccctttccgcgcctatctctcaaagcacatcaacgtcgagaacatca

gccccgacaatcttcgtctgcgctacatcccgaacctggtcgcagctcctttcgagaccaccaaggcgtacgccgagctgatattcaatcagacggac

tcctcacgcctcggcaaggccctcttcgcttcactcgtcagatggatcgagacgcaaaacatcatcttctcgcccccttacgacttcgaacgcctgat

cgagttcggtccctcaccgaccctggtcggcatggctcagcgtacacacaagctcaagtacgccaaggcagatctcgcccgtggcaagcgccgtgtaa

tgctgtgccatggcaaggatcaagaagctatatgctattccttcgcagatgcagaggaggacgggtccacaactgccgaacaaggcagcgctcctgag

acgtccgctcctggtcccgccgctgtctcggcatccccagcagctgctgccgccagtgtccccgatgagcccctcaaggctaccgacaccatacgagc

catcttggctcagaagctcaagaagcccatcgccgatattccgctcagcaaggccatcgaggacatggtcagcgacaagtcgacattgcagaacgaac

tcatcggagatctgcagcttgaccaggccatggtcgactacatgcagttctacatcgacagaatcgatccaagcatgggccccaactacgagcttgcc

aaaacgtttggccaacagctaatcgataactgcaaggaggcaatggtcgcatccgccaggtacaaggaggtgcacgatcctacagcgctgaacaccaa

gatcgatgctcgtggcaacatcgtgtataccgaggcgaagcgcatcggcgttcgcaagctcgaagcatacatcaaagagatggcggtcggaactcgta

ttggcccccagatcaatgtcgagaaggcccgcgagaacatcggagagctttggatgctcatcaagaacgagccgagcatgagcaagctcagcaaggcg

actctcaagagtgtgtatatagaggccgtccgcacgacaacggcccggccgacgtctgttgacgtctttcgcatgccggacgatcgtcagccgttctt

gaacatccagcgcaagtttgccggcaagttgcaggccagtcaaaagctgacatttgtcctacttcagtcttctcgaggagatggccagcaatggcata

accttcaggggccaaaatactttggctaccgacaggacctccgcgcacgcgccatgcgccagactgccctcgatggcggtgccatgctcctcttcctg

cccgatactgctcacattctcgagaacgcatattggaaatgccacaacaagaagaagcttcaccttaccaacaatggctggttctgggctgcggaccc

gctctcggactttgcttcgccccagtcgccgtcagcgaggcccagcgcagcacagataacggtcaatctcgcgccctcggtccactcggggcactacg

cagccgcgttgacctcttccgcgggattcgagctgcccgagggcatgaaactctatcgcggcaacaatgcaagcggcagcagcaagcagcgctctcgt

ctcggcgacgatgatccggctctctatctcagcggtcatgacgatgtcatgttctaccacaacaacaactag

>contig_14.contig_14.g101.t1

atgaccaaattggtatgctccctccaaaacctcagcgcattcaaggatgattctgacccatcagccgactggtcagccc

gcgtcgcagcccggcgtgacctcggcgacgccttcggcagcaagaaggcaaaggctaccgtccgggctcaggataggctcaagatcgacgcatcccac

atgaccgccatgctcgaagaggacgctgctactgctgagatctcgcgtgtcatgctctggcactgggtataccatggcacatcgactaacgatggcaa

gccaaccaccgcctccctcattgaccgcatcctcgacgaggaggctaccaagctcacaaagctcagccccaagcgtcttgacctgtccaagcgctacc

tcagccaacagccttccttcatttccctgtgctcattttttctaccaccaatcgtccccacagccgacctcacaacgtcgcctacgtcgtccccggcg

ctgctctcctctggttcggctggaagggatccaacggcggctcgatcctcggtgccaacctccgcgccgtccaagccatcgtgctttgacgggtccgt

acccatcaacggcggcttcatcaaccacaactggatccaactcagataccaactggcagactccgccgccggcttcggctactccttcctcgtcacct

tcatgctcctcttcgccatcaaccgcatctcacactaccacttctgttcctccgagagcgacgaagcgatcggggtcgacctcacccagttcagcgag

gagatctag

>contig_14.contig_14.g102.t1

atgattgaccttgcatccctcagcaagggtgggactgacaactttgtctccctccaccagatgacattgcgagagcggg

acacaacttcgcagcgcatcggctccatcgacctgtgcgagggacgtctgaacttgaccagcgcgtacaaaggcgactggcaagggcggtccgatcca

cgtttggttcacagaggagacaccgaagtcgccagtcgatttaacaccctcgacacctttgtctccatcctcgaacatactggcttcaagttgttgga

cgaggacgactccaacacgcattttaccctgcttcacctacaaacgataaaggacgccgccgtctggagtcacgtcgcgccgctctctgtgcgtatcg

agaccgccggcggtgtttccacctctctcatcaagcgcaacaccactgtcgccaccaagaagtccgagatcttctccacctacgccgacaagcagccc

ggtgcgctgatccaggtctacaagggtgagcatgcacatccaaaggacaacaacctgctcggcaagttcgagctcttcggcatgccgcctgcttcccg

tggtgtcccacagatcgaggtcacctttgacgtcggcgtcaactgtatcctcaacgtatcggccgcggacaaggctaccggaaagtcgcagaagatca

ccatcaccaacgacaaggggcgtctgcccaaggacgagatcgagcgcatggtcagcgaggctgagaagttccgcgctgaggacgaggccgccactacc

cgcatcaccgtcaagaacggcctcgagtcatacgcttataacctccgcaaatcgctcaacgaggaggcctttgcctcaagttggagctga

>contig_14.contig_14.g103.t1

atgtacctcgtacctcaagtcctcaacccttggcagaccagcctcgtccctcgaatattgacagtgtcccttgaggtcg

cctacaacaccacgcgtctctgtctcctcgactccctcggatgcgccatcaaggctctccacttcccccaggccgtctccgtcctcggccccgtcgtg

cccggcatcaccgtcgtcattggtaccaaccatgtccttgaccctatccgtgccgccttcaacaacggtgctttgatccgctggctcgacttcaacga

cacctggctcgctgccgagttacgccacccctccgacaacctcggaaccatcatctccgttggcgactggctctcccgcacagcagtcgccaacggca

agaagcccctcaccgtgcaggacatcctcaccgccgccatccaggcccacgagacccagggcaacctcgccatcgtgaaatcgttcaaccgggtcggt

ctgaaccacgtctctctcgtcaaggtcgcctccgctgccgtcgtttccaagatgatcggcaactcccgcgaccagaccgtcgacgtgatctcccaggc

cgttgttgatggtcagagtcttcgcacctaccgtcacgcgcccaacgcgtcctcgcgtaagtcctgggctactggtgacgcctgctctcgcgctgtca

acctggatctgctcgttcataagggtcagcccggtcttcccagtgtcctcatcgccaaggtctggggcttctatgacgtcctcttcaagggcaaggag

ttcgccttccagcgcccttacatctcctacgtgatggagaacgtcctcttcaagctcgtgcccaccgagttccacgcccacaccgcatgcgagaccgc

catcctcgcccacgccgagatgaccaaggtcggcaagacgtcggacgacattgagaaggtgaagctcctgacgcaagaggctgcctacatggtcgctg

tctcgctcatgaatggcacgttgacgcctgacatgtacaacgatgactacgcagcgaacccgaagattgatgagttgcgggccaagatgaaggttaag

gaggagggccggtacacgaaggaatacatggagccggagaataggagcattggtaactctgttgaggtcgtatttaaggacggtagcaccattgggcg

gattatgcttgattacgaggccattctgctcattgcgaagaagctgcgcaagtacctcgaggaccactttgacgaggcgcgcgagagcaagatcatgt

cccttattattgaggaccacaaggccttggcggcgatgcctgtttcggagttggttgacctgatgaagcatgaagggcgttcgtattctccgtcagca

ctcaccctcgcagatcagattgaagcgttggcatttgattcatcagctgaagacaaggatgtggatgatgaggcgaagggagcgacttgcgagctttg

a

>contig_14.contig_14.g104.t1

atgctcgatcgtttcaactacaagaacctcaagccctcccccagcccacttgaccccaagcagaggctagtcccttatg

agggccaagcctcggaggaggatatcaagctctttcaagcaatgataggatgcctcctttggttggcacaagcatctcgaccagaccttgccttcgca

gtcgccctcctctcgcgccactccaagaaccctggtccagtacacttcggcgtcctcaagcgcgtcatgcgctacgtcgcaggtaccgtggacttcaa

gcttcagatcaccgctcattcagacagccaaatcacagcatactcggacgccgactttggtggtgaccactcggccaagtcgacctctggctggtgca

tttcggttcacggcgcaacggtggggtggggcgccaagctgcagacgttggtatccgattcgacaacgcaagcagaagtcgtcgctgtctggcagaca

actcgggaggtacttgcgattcgaaacctcttcgaggatctcgggctcctcgttcccgcctctggacctccctcactcatttactgcgacaaccaacc

cgccatacatatcatcaacaacccagtcagcagcgggctcactaggcacatggagcgcaaatatctctctacacgcggctacagtgaacgcgggttca

tcaaggtggaatacatcaacacgcagctcaatcccgcagacatgttcaccaaagccctaccgcccatcactatggcccaacatcgcaacaccatcggc

gtcagccacccctcctcgtcggcagcctga

>contig_14.contig_14.g105.t1

atgaccccaaacgcttccggcttctcttcccttcggccagactctacggtcgtcatcacagccagtgggaagcgcctac

gtgcagccggaagagggacggtcacattacagtcagcgaccaacccttcaacgaagctcgtcctctacgacgtcctccatgtgccggcactggagttt

caattgatttcagtgttcaaactgaattgggattacctgcgggtcgggttcacggaagaactggccgcggtaatcacggacccattcgaccggagttt

tgctattgctgctccgtggtcgtcggagtcccaggcgtacctattcccgacggcgggaggtactgagttttccgccctcaccaccactgcaaccggtg

aggagcgaaatggcgcatcggacgacgaggaactggcccttcctcacgtccatggccttccagaatgggtcaagaccctctggattctttggcacgaa

cgattgggacacctcggcagaagctctgtcccaacccttgcgaagaacagcgtcggcctgccggagcagctggtgaagctcatccgtcgcttggttgc

ttcctgcgaggtctgctcgctcaccaacatacgcaagagccccttccctacgtccaccaccaagaccagtcgcgccctcgagttggtccacgctgact

taaccggacgaatcatcgtcaaatccttcggtggcgcggagtacctggccatactagtggacgatttcactaggatggtctgggtcctgccgctcgca

aagaagtcggactttgtggcagcattcatcaagtggagggacgaggttgttcccttcaagggaccaatcgcctgtttgcgcacggacggcggggggga

attcgttaacgccgccctcaatgccgccctctccggtgctcgacacgagatgtcctgcgcctacacctcccagcagaacggcgtcgctgagcgccacg

tcggtctcatcaagcacaccatgcgacctctcctacatgcccgccacttgccggtgcagtactgggctgcggcagccagcactgcagcctacatccga

aaccggtcccccagctccgtcctcgatggcataacacccttcgaagcctggcacggcaccccgcccaaccttgcagacctgcgcgtgttcggttgcct

cgcctttgccatgcttcccgtcaaaactcgggagcatagtctcgctgtgcgtgcgcgtgcgggagtgtttgttgggtacgacaaggagaggaagggat

atcgggtgcattttccagacacaggcgaaatcaccatcaccaagcatgtggagttccacgaagataagggctacgatttcaccacaattcctcgccca

gagccggacgttcactatccacaagaggtgacagagcaggccgctccacaagcacccggccttcgccctcgcattcgggtcattggccctcgtcctcc

tcaaccagtgccatcaatcaccaccattgaggaggtagatgcccctgctgtagcgtcagcgccgctccagcagcagcagcagcaccagctgctcctcc

tgcctcgcgccctcgcacccaccctcctgcgcagactcggcagcacctga

>contig_14.contig_14.g106.t1

atggacccaatctccaatcctcaagtcacagagcaagtcaaaggcctagccggccctgatcccgtctcccttggagacc

ccatccttccctccacctcggttggcgccctcggtatcaacatctgtggaaccgccgactcgcaggccaagctggtggctatgacgaatgctcgtcga

catgcccgcacctccgagctgaaggtcagcaagacggttcccgaccttaccggagccagggacttcgtcgtttggcgaacgatgcttcttggcctctt

cgtgccttacctcggcggacggtactggcatttgctcgaaaaggacccctctaccagtgtggaagcctatcgagttatcttctcggacgatgataagg

ttcttatcgaccgcgaggcagccattgctcatcgcacggccgacctgattgccatacacggtcatctcctccagaccctcgacgcaaacgttcttcaa

agcgtcaatgaggaaacctcctccggcaggttcgacggtgtcaagctttgggatgccttgaacagcaagtacggtggccgagacctaggctccaagcg

cgcggctaaacacgccatcaaggacttcaaactgggagacctctcggtgactgaggcagctcaacagcttcaagccctcttcacgcaagtcttcctcg

ccagtggaggccgccctgttcccgaggaggacaagattgaggccgcactgacggtattcaacacgccgaggtaccagtcccttcgaaccaccattcag

gagtcttggcaaaacggcaatccatacaccttcgatcgcatcatcagtcgattcgtaggtgaggagtctcttacggcagcagcacatgccctctctcg

ccaggaagactctgcggtagcacttcgagcccagacttccggcggcgcaacaggaccaagtggggcaattcgcccgaacccaaagcgtcgccaggagg

gcaaagccaatagcatgacttgctggcactgcacgaagacgggacatcggatgaaggcctgtcggcgccgccttgcgggcgaagcccctcatcccaac

tcgcaccgcgcaaaggagcgcgccggtcaaaaccagactttcggcccgacgagtgctgctgcagaagcattctcccggatcgccgccatcgaatccgc

cctccagcatgcactcggtggcgctggcaatcaccaaatgaggaatgcctcatatggctctggccagcagcagcagcctcatttcggtgtgccctcgc

tcgctcaccgcatcagcgagcagcaccaaacattcacacacaatgggccgcctcaagggacctaccaaaacccctacggctcctcagggtacatgcac

tag

>contig_14.contig_14.g107.t1

atggacgcagcgtctcccaacgcggaaacccccttgtcccccgtgacctccccgtcaacaggcaacgcaacccccgact

cagaaagcagcaacccgatggtctcgacgacgacgacggtcaagaccacaactttggagcagagctcggccggcgatggagtgcaagtggtcgagagc

gaggctcattacacacatctcggcgcgaaaggcgtgaaatcatcggtcggtgaggggggatcggtccatctccaacccgagccggcgagcaaaaaacg

acgagggaagacgcgcactgtcgtctctttccgcgagcgtacttctcacttcgaccgcttcaaccctgcttccggagccgatcaattccgaggattct

acacccttttctggatcggcatgttcctcatgatgctcaacaccatctacacctcctggtcaaatagcggccaattcatctccttgacctttgccgag

ctcttcagcaaagatgccatagtgctcgcgatttcggacggtgtcatggtcggaagcacatttatatgcgtacccttcgcaaaggctcttcatcgtgg

gtggaggtactggccccatttaatctggttccaacatctgtggcaggcattcatgctcggggctgtcatccactggggtcgcttcagagaatggcctt

gggttcagtctggcttcttcgttcttcacacgctttcgatgatgatgaagatacactcctacatggccgtcaatggtgccatgggggacgccttcaac

aagatgtgccgcattgagcgcaagctcgaagagcgcgtctttctggtcgaaggaggcgaaaaggcacaagagcgcgatcccgaaacagcgtccaacga

agagaagcagaaggcttggtcggcagccgtacaagcggcaaactcgcgcactcgttcgctggaaagttcgttaccccctacaacgatcaagctggtct

tcccatcatccaaatccccggctactgcagacgcttctctgctcactcgccttcaggagaaggatgagctctggagcagtatgcatgtacagcgcggt

acgagtgccctgcggcatcgcacgggaggcgcagttgctgcacgtgcccgcaggcattccatcacaaaatctaccccggccgccacagatgcacacct

ggaaagcaagactacaccaaacaaggcctatcctacccttgctgatcgggagaaagaggcggagtcgaaagagagagcgggcgcatcgagtgacgcgc

cagttgcgccggcgggtgcatcgctcaacgtgccggatgtagaagggcggacataccgcgtcccggtcacaccgcacggcacagttctgcgcgatccc

caccctctttccacgcaccccgacaaggaaatttccgaaatggccaaccagattgagctcattcgcgaagacctgatggctgcatcgccagatgacaa

cccgatttcgtcggaatcgagtatacagccccggaccagctggccagcgaacgtgacgtatgccaacttttgggactaccttctcgtgccaacgctcg

tatatgagctcgaatacccgcggacgaaagaaatccgtcccctttatgtgcttgagaagactctcgcgacgtttggcacgttcttcgtcatttacgtc

gtcaccgagcactggatcatgcctttccaaccctcagattcggaaccacttctcaagtccttcctgcaactcgccttgccgatgttgatcaactacct

cctgatcttctacatcatgtttgaatgtatctgcaacgcgtttgcggagctcacacggtttgcggatcgggaattctaccaagattggtggaacgcga

cctcgatggacgtcttctcgcgcaagtggaataagccggtgcactcgtttctccttcggcacgtctacgcgagctcgatcacggcgggcatgccaaag

tcgcaggcgatgttcgtcactttcttgctcagctcgttattgcacgagctcgtgatggctgtggtgacgggcaagatccggggatatctgttcgcgat

gcagatggcgcagatgccgctcatattcatcagccagatccctttcattcggaggaacgaggtgctcgggaacctcatcttctggatcggcctcatgg

tcggatttcccatgttgaatatccttatttcaatgtcttcgcccacgtcgaagtcagacgtggcccaatggccggccggtaggaccatgtacgagctt

ctccggatcaagaagatcgacgagctttgcgatccgggcaaacaaacgtgggtgtacgaaactgcggtgaccggatacaccacctttccccgaccggc

cgtattcggcgggctctcgttatgcgttggtcttcaggctggttcggcatctctattcgaaggaatggacgagaaggacgcctcggaatttcgtccct

attcgtaccaaggctccttcctcgggcctgcactgcccaagttcgccattcgcatcacggtcactacgatccggtccactcggagcttcgtcacccgc

attctgaccctctctcagccggatccggtggactcttcgaaacgacggagcactatggtcgccatttgcgatttcgcccgtcgtggacgacaggccct

tctcgagtttgacacaccggctctcaacccggtgaggggtcaaccctgggaacggccgagcgagataccatccgtgcacgacgtattggacgaaaaga

agaggcgagcgaaagccgcgggcgatcaggacgagctcaagcggctgatgcaagaaggctacttcaacactctctggtatgggtttatcgacaccaag

actccgaaagattcgctcctggcgcagacctattggaccacgcagaatcagctgccgaccacccaggacagcgtacccgtcacagaccgggtgctgtc

cgactggttccggttcgtacctgatctctccgacaagggtgtagcgaagatctccgcggagcgtccggactgtgttcccttccgaccaacgacggtcc

ataccgccttcttcgccttcgcgctcgatctaatcgtgcccaccacatccctcgtgatgaccaagacgccgcgccgaacagccgctcccgaattcagc

ctcgacttttcgatccgcttccatcgcgaggacggcttggacgtgaaccggtggacgctccggcagctcaagacgttgactgctgctgatggtcggac

gtttaaccaggttgcgctgtgggacgaagatggcaggattttggctacgatctctgagcaaggtcacctggtacctctgcctaagcgcgtcgtagagg

tagaggcttcgaaagcaaagctttga

>contig_14.contig_14.g108.t1

atggggcccagcagcactggacggcagcgcactttgtgggtgtggctcacaagcctcaacaccatattgacattcagat

tgcccagatccccaccacctcctggactgttgaagcatatcattgcccgctttggcgccgccctaactttactcttctcttcatcgctcctccgctcc

atcccttccgcctcttctccacttcaccttttctcctcgctcctgttagctttcctctctgtacctcgcgtatcttcgaagcctaccccttgttcgaa

ctcgaaagagtggcagaacttggctacaggtctgctcggacccgcagtacggttgtcgcctcaccctcctcacctcgactcttcggcctctgccccca

gacacgggccgcaaacatctcatcccacggaccgtgtagatctcatcccagtttcgggcatcccagttcctgatctcctggatcgcataaaacgcggc

agcgggcagcctgccatgggtcgctacatgagcccacccccaagtctccacactcctggcgtctcccctgcggagacccacactccgtcctttctcaa

cacctcgacatcacggaagcgacgaagcatagcgctctcggccatgtccgtggactctctcgttggctatgcgcccagcgccaacagccacacgaatc

tcattccgaaaggcggcgtggaggaccgagcgagccttcgggaaggatcaagtctgcggcatgaaaagtccgggatcaagatcagcgccgtcggtcga

cttgacgattcgtcaagttttctgtcacctacgcgaacgtctcttcgtcatcgaagcaacacagacagctgctcggagcaggaggatctcgacacttc

gaacgatacagaaccgccacacgaccttctggacgaaccgcggaagccacgaacacaaaccagtgctctggtagacttccttcgagacacaggccctc

aacggtcgcctcgtcaagtcactcttccgatgtcttcagagtcatcacatgtacggaacgagtccgtgcgacaggcgatcacgccggtatcttcagag

catggacatcttctttgggacgagcctcagacccagtcgaccaacaagctcggtatcttggcgcgagccaagaccgcagttggtttacgccggcagaa

aagcgagcacgccgctgtaatcagtcccaagcgccgagaatctctcagcgaggagaagaagcgggaattcgacgtacaaatccgcacgcagagcctca

ccaaaagaatgcttcccaatggcaagtcctactacactttcaccacgaatggaacggacaacaacgatgaaccaaaatccgatccgcgcctgtcatcg

tcgacgttgcggaagatttcggctccacccattaaactgcatgacgctccttcacgctcgacgagtatgagctctttcgccaatgcatccattcgtcc

tgtcgtcagccaggggagcgtgggtccgatttcgtctaggtcctctcaagacgtgaaggcagatacattccgccaatccatgagccgcaataactctg

tacggtctgtacggtccacgaatgcccgaccgaatcgcgcagtgtcggtgacatcaacagaatatggcccaacgagtatcgcagaatgccacgagccg

gaagaagaggactcacgaatgcaggcacgaagtccagccatgagccacttctccgtacgtaccgctccgaacatgagcactcggaatctggactacga

agccacttctctttccttcggtcgtgcaccgtcgcaaaaggagagtcgaggctccctcaaagcttatctcgctgcgaacgacgagtttcctggcctca

tcgcaagagcgacctctgccatgtatgaccctcagcgggcatccagtgcgagcagggccagtcaccacaccacctcttcgagcctagcatgcccgagc

ccgcttccgccgatgccaacacaggcaggtgatggtgatggtcaagcactttacacaagagcacttgctcgtcccagccagtcatcattgtctcgatc

agcgttggagcgaaacatgtccaatgccatccctgtcacagtggaagataacgacccggcggcgcaatacggtcctcaggcaggacacgtatcctcag

gtcgcagcacggcaccgccgtcgccgaatccaggatcacaagcagggactgcggtggcatctatggagattctccgcccgtcggatttcgctggtccc

agcaatcctgaaagagaacactcccgcgcgacgagtcgtagccagtcatcgtgctctcccgatcattctcgtcggaccgttccacctggtccgttgtc

gccgccaaggtcggttggcaagaaggagggcgcgaagatggtccagtcagagtctacggccgaatctgagcttcgacaagactggacaatgccaagga

atcaggcggcggtgaaagcagctcgttcttcttcgacgtctatcacagttgatcacgacgaacacctactgccccaagttcttcagcgtgctctcagc

ccaccaatccgtaagaaggccagtcgcgctcactctcggactggatctctggatttgtcgggagacaaagctactgcgacccagaaacgtgtctccga

ggacgaaacggacctatctgcgtctctcggtagcgccactcctgtccaacgaagctcgcctctgcgaaacaccgtcgatctcccagcatccgaatctg

acctccagatgtctctggagtccaagcgggccagtacgccgagaaagattcgtcctctgtcgccttcctccaccaagtcaaccaagtcggagcttggc

cctatatcgccttcctccgccaagtcggagcacgagaccgacggcagcggcggcaggagcaggagcaacagtcgtctgctctcccctcaccaggagta

tccctccacctcgcttgaagtggactccagagcctttgggcttggactgatccggtccagctttggtgacaaaacggacttgaacccgagcacggagc

agcgggacccggatgcggcgatcgatcggcccgtgggcaccgcatcgaaccctgcagctctgcgggcagctttggaggcaatgcgagcacaatgcatg

attgctgaacttcgatcggagcaggaagcagcacgtttccgattgttggcgagacgcaatgcggaagccctcgagaaagcccaggaggctcttgaggc

ggcgtggcaatccacctccaaagggcgggcgatttctgggccaacgcgctctcggcagaccaaagcgaagtctctcagcaagcgacaaggacaaggca

tttccacgaagcggacgtcgagggtggagatcggcaatgagctcaccgccgtcatcgcggaccttcgggcgtatgccacgtgcaactcaccacgagaa

tgcggagttgacactcgcgtccgcaactcgtctcttccagcatatccaggtgggaaaacatcgacgactgcgtcgccagcacaaaagggggtgctggc

agtcgccggcgaggacgctgagtccaactctggagccgattgggttgatgttactcaagaagatgacatcaacacggcggcaaaccaatcagtagaat

cgaacaacacgctatcagcgcaggacgacactctgctcggagacaccggtgcttctcgcgtctcatctgcggacgagtatgatgacgacgatatgttc

tctcaccgcaccgatgcttcgaccgagatgtctcgagccacgacgctcgaagatgttcctggatcagcaacggattctccccctccaaagttgcaacg

gggatcgtatggctcgggcagtggtgccagcttacaaggtccttatgctcacgaatgtgattccgaggcgattgaagagatgcaggaccttcctcatc

ccgacatgcgacgaggacgcattcaagacagcagcatgatgccgagtccacgatcggtcggcgccacctcggaggatggcatgttcgacgatgacgat

atgatgaactccgcgtacgacgaggacggaccttttgcgaacggtggacttgtgtacagtcttcggtcgacggggtccacaccatcaaggcatggcgg

cggccgcggctctatgaaccgggcacgacgctgtgagagcatgtccaccatcgtctctgaggatgctccttcatcgagcgaacatggtcaagcacaag

atcctcaggtgctgagccaaggaggatctgctcgggtttggccttattctcctccctctcgctctcagcgacgtccttatgcatcgactgactcggag

ccgaagtcgttttcggtcccatcgagcccttgcttcggcaacgagcgaatagacgcgagcatcgcacgacaggacttggccaacacgaaggtcccaca

acatggtcactcctcgtcagcggtagtagcgggcaggaaacatacgcgatcgagttggtctggaggaccgacggcctcgggatccttcctgaatgggt

tggataaaatgggcagcttctga

>contig_14.contig_14.g109.t1

atgtctgtcaagcagcatctcgacgagtcgcgcgatccctcaatcgcatcgatccggtcggagtcgcagtactcccgct

cgtgttcaaatcccagcacaccggacgctgtcgatgacctgtcgcctcggtcatcacccactgctacctctccggtgacgcctctcatcttccgcacc

catttcatcagtgcgccaggccggattggatccgagctctcgcttggcccggatcaagacgtcgaagcagaaccaaaggtttctcagtccgaacaaga

tgcatcgcttccggcggagggcgctctcggattgactcttgacgcaccggcttttctccgcgcccttgcgggaacacaagatctgcaggtggctttga

acaagaaggaggaggaggaggaggaggatacatcagacgaagagggagatcatgacagtcccgtaggatcttcggatgggcggggtgattcgctgggt

ccggactcgcctgcggccctcgcgctggccgactttttccgcctccgagaacaaatctcgacgacgcctccgcagacgacgctcgcaccagctccact

cccagtcgttccgacattgctactgcctccttccgcctgtgaagacgacagtgctccggccttgctgtctgcggcccacattacggatgggcaacagt

ctgcagctgatcaagatggaaagggcgtggacggtgatggaccatggtggatgctgaacgatagctgggcgtcctggggacctgaagacctgcaggat

ggggaagagcccgctcggaaattatcagccggtcaagtctcgccaaagacgttgccattggcggacgcgcacgtcgaagaaacgccggccagggccac

atcgtcgaaaaagcaagagacagactccagctttgtcactcccatcacgaagggcttcgtcacacccgaacggtcctcagagttctgctcgcccacca

gcatcatcattgcggcccaaagaaaagcctggatcgctcatttctaccctgactccgacatcagcaaaaggccgtaccgcgttcagcttgccaatctc

caactgccttcactcgacgatatcgatgcaccatctgcacctcagcatccaggggatgggtctcctcgtcggatagcgaaggctggtctggaactggg

ttcgcccttcaggggtctgacatcggatctttcgaaggccgaggcgacagtaccagctgcgatgcccgactgtgctgcggacgatgaggttaaggaaa

atcgaactccactgcctcccaaacctaaatacagagcccgggccccctcgttcggcataaatcgcgccagtcgacatcgggccgcttcgacagccgct

ccaccacctccgcccttgattcttgccgattcgaccttcttgccccacgtcaagagaagagctcgagccaactccgatacttccctcccttctgctgc

aaaggcgaactccgaccacgctgccttcgacaacatgtttaagcatgtcggctcggtcaacgtcaaagtgggaaccgacacgtttgttgtagtgaaga

gtatcctggatgagcaagttttcactcctgtctggcctaccactgcgacgagcggcgtgggtatcgctctaatggggtccgttgcttcgcatctacaa

agcttccacaactatcccgtcactggccggccgcggtcccagaccggcgcagaccgagtcacatttgcgcaaggtcccctgcaccctgtacacgctcc

tgcgcttccgacgcgctcgcacagcaccggaagcagcattcggctcagcgctgcagcaaggttcagtcggctccttcgcggtcggcttcaaacccagg

atgccgcgcgggcagtcgtcgcgcaagatccgttcgtgtgcgagccagcagcagcggagacgccgaatccggccgctggaagatatagcactggcaag

ggatggagctcggcacggtgttcgttggatgaatgggaaatgtcgccgtattcgccggagacatctcgacacttgcgcttgcgatcgtgcacggcggg

tgcggacacggaccccgacggagacgcgcgcacagaatgttctaccgcaccttcgacgcccacgatcccggctcatatgctcacgatgtcgccgtggg

aatcctccgaggactcgtgtgctttcctggcatcgccggagtctttcacagctcagttgaggtcgggcaagcctttgccgttgccttgcgtagactcg

acagctgccctacgattacgtccgagcgcgagcgacctccgacagcaggctgattttagtggtcatggagcgttggcctcgccaggacacactgcttt

ggtcaccgacaagtcaggctcgggggcgatctctctgccgttccacgagctcaaggctggttgggaagcccgaacgcgtcatctcgctctcattaccg

atgggggcgctccagtctcgtcatgtcctgctatgtcgcggttcgggtcgcctcgactcggtgccccgttcgggacatga

>contig_14.contig_14.g110.t1

atgcgcgtgctcacctcttctcgtacctgtgctgcctcacttccatctacttccttccatcccgcgtacaatcccgcac

gcgcaattctcatcttctctcccagcaactctctcggttttctgacacccggatacgcgtgcctgatggacaaggaatctgatccctcaggcggatgg

aagttcgcacagtgcttcggcgacaagggcgaggtcgacgatatcaccgaagctgatatcatctccactgtcgagttcgatcacaccggtgattacct

cgcaacaggtgacaagggaggtcgagtcgtcttgttcgaaaggaacgaatcaaaaaagggatgcgaatacaaattccacaccgagttccaatcgcacg

agccagagttcgactatctgaaatcactggaaattgaggagaagatcaacaagatcaagtggtgcagacgtcagaatgctgcacatttcctcctgtcc

acaaatgacaagacgatcaagctatggaaggtgttcgagaagacgctcaaagtggtcgcagagaacaacctctccaacggctggcaatcagcgacccc

tcatttggacccttcgatgttaacgatcccgacgccggtggtgggctcaggcacgagtctgcgcctgccccggatgacccagcacgataacatcgtcg

cggctgtaccgagacggaggtacgcgaacgcgcatgcgtaccacatcaactccatctcggtcaactcggatggagagacgtacatcagcacggacgac

ttgcgcatcaacctctggaacttgaacagcagcgatcgaagcttcaacattgtcgatatcaagcctgtgaacatggaggagctgacagaagtcatcac

tgctgctgagttccacccgcaacactgcaaccactttgtctactcgagctcgaaggggaccatcaagcttgcagatatgcgcgagtcagctctgtgcg

accaacatgcaaagttgtttgaggaagaggaggacccatccaccaagtcgttcttcagcgagatcatcagcagtgtttccgacgtcaagttcagccga

gacgggcggtacatcctgtcgcgcgactacctgaccctcaagatctgggacatcaacatggactcgaagccggtgaagacgatcaacatccatgacca

cctccgatcaaaattgtgcgacctgtacgagaacgacagcatctttgacaagtttgaggcgctctgggggcccgatggcagcaaggtgctcacgggca

gctacaacaattacttccatgtctttgacaaggatgatgcgagtgatgtggtgctgcaggcggataagagcgcttttaaggccaagagaaatgcggcg

gggaagggcaaggggaagggatcgcctggtgtggtgacacaggtgaatacggataatgtggacttcaacaagaagattctgcacgcgagctttcaccc

acgggagcacacgattgcgatcgcggcgacaaacaatctgttcctgttctcacgtggtccatcgcaatga

>contig_14.contig_14.g111.t1

atgctcggctttgacggaatcccgcgcagcgtcgactggtccctctacggcggcgcagaagacgtcgcgccctcccagc

gcaaatacctcgagtccgttcagagacaccgacagggcaccagcaccaccggggacagcagcagcggtgccagtagcagcagcagcatcgccaacacc

gacagcgaaggtcgagggagccaaaccacgagcccgctcacatccgaggcggaggatgatatggacgacgatgaggccaacgtcgaagcggctaccag

ccccaagcacggatcatcaccttccgtcctgcgatcacggcgtacagcccaaacaaaatcttcaacaacagcgaacctcacaccgtcggcccaaaagg

attcttcgaccgccccaccgggtctgcactgggcacccgaagtccttcccgagccacaagacgcgtccgagttcctctggctcctgacggaagagccc

caccgcacccggcgcgccgccatcctgaaggaccacccggagatccgctcgctgatggggcacgagcctctgacgaagtacgtctccctcggcgtgac

gctgctccaactggggcttgcgatcgcgtttgcgcactttggcgtgcacccgttggactggcgcttccttgtgacggcgtacgtggtcggaggcacgg

cgaaccagaacaccttcttggcgatccatgagatcacgcataatttggcgttcaaggggattcgtgcgaacaagctctgggcgatcttggtgaatgcg

ccgattggtgttccgtatgcgatgatgttcaagcgttaccacatcgagcatcacaagcacctcggcgaggacggggtggacaccgatttgccgacgaa

gatcgagctgatgtgcttgaagaatgtgctaggcaaggccttctttgccacgttccaaatcttcttctacgcgctccgaccgggcttcgttcgagccc

aaaagctcacgggctggcacattctcaacatcggattccagctggtactggactacgtgcttgtgcggacctttggagccacggcgctgtggtacctg

attgccagcagtttcttcgcgggaagtctgcacccgtgtgcggcgcacttcattgcggagcactacatgtttggcggcattcagcaggagacgtggag

ttactatggcccgctcaacattcttgcgtacaatgtcgggtaccacaacgagcaccacgacttcccgtcgattccgtggacgcgacttccagcactga

ggaagatggcgcccgagtactacgacgtgctccctcaccacacctcgtggccgttggtgacgctgcagttcatcctgggctcggacagcggtcttttt

gcgcggatcaagcggcagccggcggggacggtgcagattcctttgagcgagcacatcagcatcagcttcagcgtgaagcacgacgacaacgacgacga

cgaccacctcaccactcttcaccgcaccctcccccgtcagcagtag

>contig_14.contig_14.g112.t1

atgagctcttcgggctcgcgcagcacatcatcaagattcaaggtgaacggttatcagcaatcatcgttttcactggacc

tggtgattttgcaactcccgtcgcccgtcgtctccatctttctacaccgactccgtgtctgccaaggcgtcgaggcagccactacgcagctgtcactg

tccctccgccactctcatccacccatccaccaatacaagccttccgagcccgccgctccgctaatggttgccacttgtggccgtccatccacgtcgcc

tgccgtccagcccgccgcctccgccgctgcgtccgccgccgcgcccgccgctgcgctaatggatgccgaattcaccaccctcatcggtccgcccacgt

cgctcagtccgccgctacgctaa

>contig_14.contig_14.g113.t1

atggacgactcgacacgatcctggtgcacgagctgcaaaggataccgtcctattgatgacttccctcaaaccagcagcg

gtcagaggcggaaaacttgccagaaacacctaaagcgagcagtgcccaatgatctctgggttgacgttcagcaagcgttcaagaaccatggccagagc

gcgtcccacagtctccacacggacgtctgcctcagctatgatgaccttcccatccgcgtatcaagagccaacgcacacgaagacgcagacgaggagaa

ggcggctcgaaacgacgcgcacgctatggctcgcgttatatgggagactacaggttttcacttcaagtatcacgataccttggttacagtcgacggat

gcgcaagccgcttccactgctcccaggatgccagtcgtcgctgcgttagcggtcctcatcagccccacgggaacaagggtacccgagcagcagcagat

tccgtggcccagcatcaatgcaatagctcgttgcgcctgacgttcaaacctctagcccgcctgctcaacatacgctttcgtcatacctctcacgatcc

ttactgtgatcgaactctgccacctgtggtcctggaatacattgaaggtcgcctcgattcgacccccggccagatatttcaagacatgcgcgccacct

ttccagatgctgttgtaggccaaatcacgcaaaagcaagtctattattggtggcaggcgctttcctcggctatctggcgacttcaagacgatcccttc

cagtcggctatgcagctcgtcaacgccgaaagtacgcgtggcgatggtccacgctgtagaatgtctgcgaagttgatcgagcgtgatgggcaggtgcg

aggaatgctgctgatagcagaggaactgatcagcatgctggcatcaaccacggtagagatctcaatagactcaacatacggaaccaacaacgccggcc

atgagctcttcggcatcctcgctgagctggacgggacgggcgttccactggcatactgctttctcaacacgaaggacatggaggcagatgggcgcaaa

atcagcattcttacggaggtgttacaggcgcttaagggtcgtgggctcactcctacttttgtcggctgtgacaaggatagcgctgagattgctgctat

cgccaacgtatggcccaccgcaaaggtccagctttgctattggcacgtacgacgagcgaaccttcgccatcactcggaaaggtgcaagtgcgagtgca

agaaggacgcgtcacaatgggatgagcctggcaggctggagtgcaacaaagcagacagcgtcatcatcctcgacttgctgtccaaacatctgaacgag

cacactgcgtttccaacgctacacggtatatacctcacacaagagcagttgtatcgcaggcaggccaaggaggcctacgacttgtgcaaggcaaaggg

atgggcacgcgtgtgggcctatttatgggccaattggtacatgggcaaggaatggaagctgtgggcaaggggagcaaacgagtcaatccccattctca

agtcgacaatggtcaccgaatctcactggcgagtcatcaagcatgactacttacaccgctttgcccgaccacgtatcgaccttgtcaccttcatcttg

cagacgcgtgtactgccgacagccgtcaagcgcgtggtggcattgcgcgcagggaacgaccggattgcaagacctgcctggcgaacaagcatggcggt

agcttggaaaaaagaggcagcaaaggaggtcagcgcggaggctattcaacgctaccgaacagacgtacatgcgtggaaaagtgcatgccctgctttgc

ccatgcatcggttcctgctgtgcaagcatttgaatgctgacgccgctacagctggtgccgacgatgggtctggtatcgacgaaggtgcccgtagcgac

gatgacagcgaagccgatgcacgcagcataaggggcgaatgggacgagggagaagagagggatgagggaggtgaaggcagtttgtcagtgcaggcccg

gttccagcgagcacggcttatgctcacgcgtatgcaggccttgctggaacgcgaggagccgtttgcatcgtcaggattcatcgacaactttttgggac

gcaaactcgaggaatcaacaactgctccgtga

>contig_14.contig_14.g114.t1

atggcaaccatcgaataccagccgaaggtggagaacgctctgctacgggcgtacctgacccgtctccaggcccacccgc

tcctcaccaaagcgcagacgagcgcactcgcaagcgccagcggtgaactcctcgccgcccgcctctccggcgttccccccgccaagcctaccttgcgt

ctcaatggtgtccctggcgagattctcaagaagctcgacagcgctggtatcagcgaacgggtcctgctcatgggcgcgctccagcgtgctttcgcggg

aaaaacttcgacccgggacaagatcctccagatcgtcgctgccaatctgatcacgagccccatcaacaacgccgtctacattgccagcatggcattca

tcaacggagcgagatccgtggaccagatcgttaagaccatcaaggcatctttcgggaaggtcatggtcaccacctggattacgtctcctctcgccatt

gcgtacgcgcagaactacctccagccagagctctgggagcccttcttcgtcttgctcaagttcgtcatgggaacctacttcaacaccattgccaagaa

gaagcagatggagctcgcccggctcgagcgtctccgcaagggcaaggaggccggcaagaggaagtaa

>contig_14.contig_14.g115.t1

atgaataatatccgcgcgacgaagaagctgaacgaggtcgagcttgagaacgcgcttcgctccggcgcgacgggcgcgt

cctggcatgatgagtacaaagattccgcctacatcttcgtcggtggcctgccgaacgagctcaccgagggcgacgtgatcaccattttctcgcaattc

ggcgaaatcatggacatcaacatgccgcgggagaaggactcgggcaagcggaagggcttcggcttccttatgtacgaggatcaacggagcacagtgct

tgcggtcgataatctcaacggggccgacgttctcggtcggacgattcgggtggatcatgtgaaggagtacaagcagaagcgggagcggacggaggaag

gggagtggaaggacgccgaggagcagagcttgaatgctgcgccttctctcgtcgctgcccaagggaatggaggaggacgagggcccggcggcggagat

gagagcgcggacgaatcagacgaggacgaagtcgaccaggaggacccgatggctgcctacttcgcccagcgacgcaaggaggacaaatcgaaagggaa

ggggaaagagagggagcatgggtcggaccgggcggctgagaaggaagctaaacgactcaggaaggaggaacgggcgcgcaaacgggcgagaaaggagg

tcaaatcacgagatcgcggggctgtcgagggggtcaaggacgagggcgagcgggctcagtctcgagatgcggaacggcatcccgaaagcagaagcggt

cacaggacgaaggacgttcgagccggcgagaggatgaccgacatcgcagccgcagtcgctcaccacggcgaagacacgacagatgagcaattcaaaca

aagatttggaagaattgcaagtagtggtgtccaagttccgaaacgcctgtga

>contig_14.contig_14.g116.t1

atgccgtttcccatggtcgcccccgtctacttgcctgggaaggaacccctgcctccaggcttcaccgaagacgaccgcg

ccgccatggcccagatgctccaatcctccaaatacatggcccaagccatggactcctgtcccttcaaatgcggcatggccggcgtcatgggcttcggc

ctcggtgccttcttctccctcctcggctcctccttcgccatggacgacccgctccggagatcgaatgtgcaggcggccataatacggaatggggtcac

gcagcctgtaccggagctcacgactgcgcagcagacgaaggagttctttaaggagacggggagggggatgtataggacgggacgggggttcgggaagg

tcggcgccatctactccgggattgaatgctgtatcgaagggtatcgagcaagacatgacatggccaatcccgtcgcagcaggctttttcacaggtgcc

attctcgcccgcaaccaaggaccccaagcagtattactaggcggagctggctttgcttgtttcagcgctgccatcgactatttaccacgatcacgctc

gaagaaggaggaagggcatcggccatcgagaagcgatgactgcgaagtagtatgtcccaacggccttgaaccaggaaccattgcagctttcttgccac

agaccacttcacctcaccctgactccaccgctgtgcccagaagctattcaagagctgttctcacccaggttctggcttcgatggacgctctcaaagaa

gacgccacgggtttccacagcaacgacacgtatcttatcgttccattcggaaggtggtag

>contig_14.contig_14.g117.t1

atgccggcagctgggctgctggtccccgcttcgatatcgactgccggcgctgctgagtgctgttacggcttgacagcgg

tagatcctcccaattgggcgtggtgcgagaagcgcgtcgcgatgaggtcggcacacgacaatccacatgctttaccccctgcccccgaccacgacgca

cctcccgaccgaaccaacgaaagtgacctggacggcgccataaacgcccttcaaaaggcctttgacgacccctctcgcatctcaaaagacaaggacga

cctctacaaccgcggcttctccgcttggagctatcaccccgccgctcttccttccgtcgtcgtctggcccacctcggttgaggagtgtgtggccatcg

tgaagatcgccaccaaattcggatgtgcaatcgtgcccttcgcaggaggtaccagtcttgagaaccacttctctgcgttaccgcccgaccaggcacgt

cgcgaaggactgccggtccgaccgacgatctcggtcgatttcgagctgatgagttccgttctggaatttcatgaagacgatggcgatgtggtggtcca

gccgggactgaagtgggaggaactgaatcaatggctcagcgatcgaggttcgaatctgtactggccaatcgatccaggaccaggatctgcctttggtg

gtatgttgagcacaggcgggtcaggaacgaatgcggtccgttttggcacgatgaagggagactatgtcttgaatctgaccgtgatcttgcccagtggc

gaagtcattaccacccggcctcgcacgcgcaaattcgccacctcgcctgacatgaccaagctcttcctcggcgcagagggcaccctcggcctcatcgt

cggtgccaccttacgactcgccccacgtcttccctccaccgtcggacgagcgacgttcgagaccgtcgaagatgtatgccgcgcggtgcgcgagatgc

tatcgaatggcgttggggtacagtgtatcgaactactggacgacgtcatgatccgatgtgtgaatcacgccgagtgtcgtggatctgattcgactgca

gtggctgctctaccggagcgaccgacgctgttcatcaagttcatgggcacgccggcacatacgagtgaggatgagcgactggctgaatcgatcgtccg

tcgatttggagccgattcgctcgaagtggcccgagaagcggaggagtgtgagcggctctggcgcgcgcggaaagtagcactgtggtctgcgatggagt

atcgagagagcatctctcgcggctcttcactaccaccatccgactcaagcaaccaacaagctgtgcttgccgagatggtgaagcgggtcaaggcggac

atcgagcaagcgggcattgtcggacctttagtcggacacgcaggcgatggcaatgtccatgctctcctcacattccgctctgacgaccaagccgagtt

cgagcgagtcaagtccgtcgcctcacgcatggtcgcccacgcccaatctttggggggcacagcttcgggcgagcacggggtcgggctaggcaaacggc

agtatctggaaaaggagctgggcaagggcacagttgacctccttcggctgttgaagcgcactattgatccggaaaatatcttcaatcctgggaaacta

tacccgccggaacatgtctctgctcaatga

>contig_14.contig_14.g118.t1

atgtctaccacccccagcacctccacctcattttccaacaacggtccctccatcgaccgatcagctgctctcgccgatc

ttcaatccacctacggtatgtctggccacggctatcgagttcccttctcctttaccgatgcctctccctcctcggacaaagacaacgccgacaagagc

tccatgacttcgtccacttcctcatcgtcatcatcatcgtcatcgtcctcctcatctgcttcctcgctcaaggcgaaagctactgccttcctcaagct

caacggtggccgcaagaccaaagccttgggcaacagcaacgacagtgtgcatactctcgtcgacagacccgtcggcgcggtccaggagaagcccgacc

cgcaactcgtcttggcccacctgtcctcgcaatacggcatcggcgcatccggcgccggctacaagctgcccaactttgaggctgacgagaagaagacc

gacaagaagaaagagcagagaacgaacaagaaaagcaacaacatccctgctccggctcctgattcgcctgaggctgttttggcggcgctcatggatca

gtacggcgctccgggtcgtagccatgctcgagccttcatctga

>contig_14.contig_14.g119.t1

atgacttcaagcaggacaaagttcgaacgcctactcagggtggaaagcgtacgagccgaccgggcgaagctagttcctt

tcaagatcgacgagctcgagaccctggtggaggatcagacggctgttagtggtactgtcaatgaagatgccgacgagagttccggacagaacgccatt

gtagatggaactgcgtctggccgactgagcgatcaacgatcaacagaagctcaggtacgctcggaggtcggacagatggcgggtaacatcgaacctcg

cctcgcctccgtcaataagatcatcgcgggcagactcgttctccagaacggccggctgcagacgccccatcccgattctgacgcctgtgacgatgtct

acgtcgagcttcgcgccggccctcagaccttcaagtggccgaattcgacgctccgcccgttctcttcgatcgtcccgaccgactccgtcttccgctcg

atatgcaacgctgccaagatcacccgcgaagacatcctcaccatccgggctgctgccgaacgcaaggacaacattgttgaggccctttctcagaagtc

cgctgaagggaagggcggcattcgtccgaactcaccagagcaacgcccggcctcacgggcttgtgctgtgctgcggctctag

>contig_14.contig_14.g120.t1

atgcagctctccgcctccgctcttatcctcgttgctatcggcactgcttcagccactttcatccccgcatccgcagacg

cctgtactgtcctgcagcgggcttccctcaccaacctcgacaagtttggctgcgcgaaggctagcgttcacgtccttagcaagatgaagcccatctcc

aagaatgaaacctgccaggccctccaggacggtggcctgatcaaccttaatgctttcggctgtgccaacgctgaggccagctttttcacgtttggaag

ccacaagtctggtccggaaggctctaggtctgcctcttgctccgccaagaagatctgcacagtcatgcagaagggtcttgtgaatgtcaacgtcctcg

gttgcttcagcttcgactttgacttcttccacgccgggggattccacgttggcggcaaccgaagcaagcgtaatgccacccaccatcaccaccacgct

cagaatcccgccccaaccgaccccacttctggcaccgtttacacccctgggacgcctggtactcccgacgtcgctgatcagccaggcacttccggcca

ttcgggccactccagtggcattgctcctgctggagccggtgccgacggtaattctgccagccccggagcccccgctggtgcccccagtcccacccctc

ctgctgagcccaccaattgcgacgcccagacgtgtcagatcctccagaagggtggtctgatcaacctcaacctcctgggctgtgctaaggcgagcgtt

cacatcggcccctga

>contig_14.contig_14.g121.t1

atgcagttcaccgcctccattgctcttcttcttctcgccgctgccggctctgcttccgccgctttcgtccccgcgtcca

cagacacctgcactgtcctgcagcgggcctccctcatcaacctcgacaaacttggctgcgcaaaggttagcgctgacgtcctcagcaagacgaagccg

atcgccaagagcgagacctgcaaggccctccaggacggtggcctgatcaacctggacgctctcggctgtgccaaggtcgaggccaacgttctcgctga

tggcactaccaagtctgcctcttgctccgctaagaagctctgcacggtcatgcagaagggactcctgaacgtcgacctccttggctgcaccaacgtcg

acctcgacgtcctcggtctcgccggggtccacttggacggtggtcatggcaagcgcaagcacgcccaccttcccacccccgccaaccccgccagctgt

cgcgccgagacgtgccagatcctccagaagggcggtctgggcaacctcgacctcctcggctgtgggaacgtgggcgcccacgtcggtccttga

>contig_14.contig_14.g122.t1

atgaaggcgtcgttgctcgaagctggccgtcactacctcgctggtgacgaactttgtgatgctcgcaaggataacggcg

aggggatgtctggcgcgcgatcacgctatccgaaagatgtgagggtgcagccaagctcggtcttgatcatgcttcggtatcgaggccaccaagttatc

caacacaagctcaatgttgccaaagtgacccaacaagggactctcgtacaatcccggtatggttccagcggtgctcagatttgcacatcggagcatcc

gtctgggtcccgtaaacatcaagagccggacgagagcgaggcagttccagctacgctggatgaaacgacaggtcaagtgaccgagttccaggctccgc

ccgttctcttcgattgcctcaactccatcttccgctcgatgcgcaacgctgccaagatcacccgcgaagacatcctcaccatccaagctgctgccgaa

cgcaagggcagcattgttgaggcgctggttcagacgtctgctgagaagaagggcggcattgatccagagtcgtga

>contig_14.contig_14.g123.t1

atgtttgcctctcctgccggccccgctccgctggaataccccatgactaaggaacgcaaacgcggtctgtacatcggcg

ttggcctactcctcttcgtctgcgttgtgccgccctgcgtggtctactacgtgctcagctatgcggtcgagggtgtccaccggagcactgtgtatgga

tatacaagtatttcgatgggagtcctgcagccacttcggtccacccttctgcaaagaaccaaacggacccttttccgggccgatacacttcaatggtt

atcgttgctgatcggtattggcgcctccatacccatgctcgtggcctctcgcaagggcgccttcatccctctcgtcctcaagtaccccatcacgatgg

gtatcttcggattgtcgatcatcctcgtcaccgcgctgtcgtcttctccgggcatcaaacagccttgtaggctgagctcagtcaaggcggggatgccc

ttccgcccggcgttggcgtacctctgggaagaccttacagcggtcgaaggcggtggcgggtatagttcccgccaagccttctcacgccggtacgactc

ttccccaatctttcgagagatgaacaccgtcgtcagcgtcttcctaggaatctccttcctgctctactttgccctcgccctcgttctcaccctcggcc

ttgccaattacagtcgaggccggaacctcgaggcattgaacttcggtgccaactttttcttgtttgtcgtctggaacgcggctacggcggggatagca

ttgcgctgggtacagaggtatattcaggatgaggagaggaggtggaataggaggcagctttatgggatgccggggctctggaggagcagtcatgcggt

tggcgagtttgatggtggttttgaggtgcggcactacggctcggccgacgactggtccatcaattccggtttggagcgggatacgtctgctgaggcga

aggctgtggctcagctggacgcagaatcattggtggatgggcgcagtcgaggatga

>contig_14.contig_14.g124.t1

atgctcaccccgtcatccgccgcgctcaccgaccctttccacgatcacagtaggacgaccacgtcatattctgaatccg

aaagaggtccaccttcgctcaactatatcatgacctcggcaaggaagcgaagtctttttatcactgtcggaacgatcattttcctcaacatcgctctc

ccaaccatcatcttctacgcagccaagtacgggatccatccaagactcaagccagtcgactactacacagccgcgagtgcagcgttgggagcgttaca

gttgccccaagtgccatatcgcttctggtgcctccttaagcaaaacggtcggaggagccctttgctaccagacggcaatcgaaccggcttctggtggg

cacttcgtaggaacgacaccttccaagtaggctttcttattggaacaatccttggcatcgccgtacttgtggttgcaacgagtgtcaacggcgcagat

ggctactttcctctacttgtgctcatgcctgccatcatcttcgggtacatcggcctagcgttactcgccgtcacagccctcagtcaatcagccactat

cgtccagccggcccgattcagcatcgttcccaagggcgaacccttccggccagccctgttctacgtttgggaggacttggtgggttgtgatggtggag

gtgggaacgcatttcggaacgctctggatcgacggtatcgacaatctccaccctttcgacgcatgctcctcaccctcagctggctgatcgcttacagt

ctcaccggactctgcatcgtcgccttcatcatcacctacgccctctacggagacagcagttctcacggaaaatacgaggatgcatcctgggcggccaa

ctttgtcctgctgactctctggctcggtctgaccgcatacgtcggaatcaaacacggcctggacagcatgcgggccgaagagatctggtgggcagaca

agccctccctcgatcgtctgccggagtctcgcgatcatctcgtttcgtag

>contig_14.contig_14.g125.t1

atggcaacagtacacttcatcgttcaagagaaatgtggatacgatatcttgcttggaatgccagccctcaacgcccttc

gaaacagcattgactgtgtgtcaggaaatgctacgatgacgaccgatgacaacaagatcgtggagttgcaggtggtcgaccccgacgaccctgcccac

accgatacacttgagggagcggggcatttacgaacgaaagctgaagctaaccaccttcagttacaggtcaatcagcacaggctcgttagctctggagc

atggaccacgactccgatgactcaccacattaatacgaagcgaaaatccgtggaggataaggtccgacccactgctgtgccgtcctcgaacttcgttc

accaaactttgcgaacgattcaatacgatcgcgatccatacaacacaccattgactgctaatgtccctcccttccgacccgcagaaaagctgaccatg

gaacgaattaacagcctcagttttggccccctagaattcatcaacgacgaagaacgtaatctcctactccatgtgatcacgctctgtcaaaaggcaat

tgccttcgacaaaaaggacaaacgatcccttaacccggcctacactgaccctttcgtcatcaacctctcgaaacacgaaccatggacggacccatccc

ccccatatccaagatcagatcgagaagcagcaatccgactcctcaaagaacaacttcgagacggcgacctagagccatccacgagcccctatgtcact

tcacaattcttcgtcaagaagaagaatggagatctcagaatgatcattgacatgcggacggccaaccaatacaccgtccgggacgccaacattccacc

ctacctacccgattacgtcgacggcttcgtggtcagagtatgctacgggttgggtgatctgctctctttcttcgaccagctacccttggaccccggct

ctcgactactcacagccttcagaactcccctcggcctacttcagaagaccggattaccacagggaggaacaaactctccggcgatggcacagtgcgtc

agcgaccacgtaggaggagaggacgttcccgaccacattgttcccttcatcgacgactttggaatcaaggggccaaagacaaggtacgacgaccaaac

ccttcccaactcaactatccgacgatgggtgttcgaatacgccgtcacattcgaacgattcctgtaccggatagagcatgctcacctggtcgtctccg

gatcaaagctggtcgtgatcaccgatgaactcgacatcaccggaattcgagtcggtttcgagggcaagagcgccggcctggacaagatcgacaggctc

caagaaaggcccaacccctgcccctcccaatcatcactccgaggtttcctcggcatcgcgaatttccttcgccccttcatcaaaaacatggccgtgat

agacgaacccctgcggaagttggtcggacgaacgtttcgctggaaccccgacgcatccgaggctgtgaacagggtcaaagaagcagccgccgcccacc

actatcttggaatcattgattacggatcggaatctccgatagtcgtgtctgtcgactcttcccaaatcgcgatgatcaacaagcccgacgttggcaac

gccaccatgattcggtggatcgccaacctcaaggaatacgactttacgatacgacacatccccggcaaacatcacgttattcccgacggcctttcacg

aacaaacttcaacaacgcagaggaagcggaagcgtggccggaggacggacatgtacggtcaatcgaacacaccgttctcacgcaatcgatcgacacga

acaatgacgtccccgattacaagttcgcgaaggacaagtacgtccaacgcttcggaggactcgcactatggctctccaccggagggacccatcccaac

ctttccaaactgtctcgacaggaacgaaagagaatccgcgggcagctcagcggtttcttcatggaacaaggacgactgtatcgcaggaacccagaaag

gagcccacagctcgtgatcgacaatccagacgacaaggtcaaggtcatggcctatgcacacgaggagctaggccaccgcggacgagatgcgatgacgg

cgctgttactcgaacgtttctggtgggaatccatccgaggcgactgcgccaaacacgctcggtcctgcgctacctgccaacgaagaagccgagagagc

gaggtagaggcccaacgaagcacgccaattccccgtctcttcgaagactttgccctcgacatcgtagacaccggaactggtacgggactacgtcgata

ccttgttatcgcacgagaccttctcacgggatgggtcgaggcccgcgccctcccgaacaagcttgcctcctccgtcgccaagttcatcgaggacgata

tcctcgcacgctacggtcccatggtacggcaaatcctaaccgacaacggcccagagaactcgggagaaacggaggcactattgcgacgcctgggtctg

cgacacgcgcgaatcacaccaaaccatcctcaagggaatgcaattgtggagcgaggacacgccccaatcgtcgaagggctgcttaaggcttcctacga

tgaccgtgaacgatgccatctttacctgccacacatcctcctggcagaccgcatcaccgcccgacgaacgaccggacgatcacctttcgaacttgtct

tcggatacagcccgctcctgcccatcgatcacgacctcacctcgttccagatcgttgactgggcgcagattcacacccgagcggacctcctgggtgcc

cgagcccgccaacttcgaagacgagacgaagaccttcggacggccgctgcactattacaccaagcacgggcgcaaggacgggattatatggaccgacg

acaggaccacaaacgtcgagaacctcttgctcccggcacgatggtactgatcaagtcaacctccaccttcatggccaaggctgaggatcgctggaagg

gaccttacgcgatcatccgacagacccattccggctcgtacgaacttcaagagctcgatggtaccccactcgaacgaaactacggctccgatcgcctg

cgcgaatactttccaaggcctcgtgtgatcaacgaggaacgttttttcctggacgacgcagtcgacggaacgttcaacacaacgcttggaccccagcc

tactctcatccatgtacctcaagcgatgattaacgacttgcccgggcctagggcaactgccacgttcgatgacccgaacccgggcacgctccgagagc

ttcctgatcaccccagctgctacaaaggcgaagaaactgaacgctcgattgactccaacgcacaaacgaacgttccaaaaagcgttccgacaaccgaa

aacgttggccacccaaacgactgctacttttgcacgccgccatgctgccgacctgccctcgacctcgaccccgacctctacccctataccacatgcgg

acaccgaccagccttcgcagtggctcagcaaggctctccggatgcagcgtccgacggccgaaggccaacacccacactcgtcgtcacactcaccctct

cccgctcgccaaccccgtccccgagccctgacgagcctccgcttgcacagcaacgagccaagatcgacgagaccttccgcattgagaagcagtccaca

ccgaacccaaacacactcccagcacccgagtcagcactccccatcgagaagcagtccacacccccggcatccaagtcagcactccccatcgagaagca

gtccacacccccggcacccaagacggcgtctcccaccaagccgcggccctctcccaccatgaagacgcgtcaacacacccttctcttgctcaaggtcc

caaagaccgtcaacacatccttctcttgctcgaggccccaaaagaccgtcaacacattctctcctgcccgacactccaagaccgtcaacacatcctct

ccagctcgatccccaagaccgtcaatctcctctgctcgatcgcccaagaccatcatcctcccctgttcgaccgcattgaggccaccctccatcctgct

caaccacccccccccatcatcctcctctgctcgatcgcccaaggccatcatcctcctctgttcaatcgcccaagaccaccatcctcccctgttcgatc

gcaccgaggccaccctccatcctgctccgtcacccacacgcacacacgctccatag

>contig_14.contig_14.g126.t1

atgatgaccatgaaaaacgatagcatccacctctccttgaaagatgacttgggatacgaacgaagttggatcaaaaccg

aagacgagaccttgggctcgatggccgcaagttacgccaaggctacccgaaccaacgaagctgacttctttctcacgatacgaacagtccccgtcagc

acctacgaacagatccgacaactacctctgatgaatggggacgttttgaccgtagtcatgaacccagcccgagttatccctttccaaaagcccgagtg

gattcgccttctactccaactaagccccgtcggatcggaaggagaagagtatactcactacgatggacctgccacgagaaatgacgaagtacgcctac

aagtcttgctgaccgacctctggcccggaatcccaaccaccgccgtacatctacgatcccgaaaccagctgactctcggcaacgtcaaacgagccttc

gaactcgctctcgaaggaacgtacctccaacattacgaaggcgaaccagacgacgaccttccactctcaaccctcaacaccggaacgatcttgctcca

tttccgccgaagccagctgatcgaacgacctgacgcacgattgaccacccacgaactcttccttggcccaggagttgatggggaccgtgagccaatat

accttcaagtcgacgtcgaatgggaacggcgaacgttctccatgtgcgtaggatcggaagtcaacggggatgacttactcacctggctccggcttcac

ctcgaccaagcgggaatgcccctgtacgcataccatgggctacggttactcaggaacgttcccttgagcggacacggccttcgaagagggcagacgta

cgaaatctcggtcgcaatgggcatcgaaccatcctggatcatccaacctagcacgagaatgacacaacccaaccagactaacaaatcagtttccccgg

tccaccaaagcatctctctcgacagcggaaaccaacgttgcatttttggggacgtttacatcggggctgggcacggaatcattcgaggaaacattgga

caagtccctgcccaaagcaactctgttcgactccaactgcgaggaccggcaaccggattctggacgctcatcctgtcccccagaagccctctgtccac

cgtaacgaactatctggaatacacctatggtcgaacgtttcggtatctaaagaacgaaaaggaaatcaacccgaattaccccagcccacatcttcaag

acggagacactctcgacgcaatctcgtaccagcactacctcgaggaagatacccccaaacgaacgagctcggaccccaatgcgcttaggctcattcag

aaaggagctctcaccacccttccgccgcctccgcctggatacgtaaaactgaggctggagggtgaaaagatcggaacctggaccgctactatcaagac

cacggcctccttctctctcatcgccaaccacctgaatcaggcgtatggagaaacattcaggtacatcatcgacggacagcgaatcttggaacgaatga

caatctatcaaaacgatctcgacaacatggacgagatcgatgtcctgtccgagcaaactggaggaggaacgtcggttgacgagaacgagttcgatcgg

tacagcgaaacccgaggcgaacaagatcaagttccggttaaccagcgagccatgagtccaaccatcctctccagcgcgtcacgaccgcccttgagtgc

aagcccccaagaaacgttgcctgcgcccgcagggcgaaggtacggaatggttccgacgaaaggaacgatcgacgcagtcgggaaattcgagggacgaa

acgcaaaagcctttctgcgaaagttcgaggctctggacatttcggacgaggaacgtttgaaatacctatccttctacgtcgaagacaaggatcccgac

atcttcagcctcatcgaggctcactcctcgtacatagcggggaactggacggcagtacggcacttcataatgacgggattcgaagggcccgagatgga

caagtacactgagcacgatctcagcgccttcgtcctccgaaaccgaaccatcggaacgctcacagaactcaaccattacagcttggccttcaaggaca

tcggagacaagctcgtccaacgaggacaaattggggcgaagcagctgttgcaatactttgtccgaggactgccgattgagctgctcagcacgctccag

aactcatctctgagggacgaggacgcaactttccaacaagtcctagaagaggttcaacgctgtctccaacccgagacgttcttcaagaaggcgacgct

cgagatgcaacgagaacgagctcagatcgatcaccgagctccaacacacgggccggccttaaacttcctcaactttgcccaggccatggagcgacttg

gccctacggttcttcaccacgcaatgcctccccaaggactcgcaggcaacccgaacactgtcccacccccctttccaggaccagtcagaacccagcca

tatgccgggtacaacacgacgctttcacctcagggaaacgtcggaagcgcaccgatgcctacctggaacggcccgaccaacaacaataacaccagcta

tccggccacaggacccaatgcccaacccatccaacgaccgccatcctgtgtatactgcaacgacccacagcatgctcgagaccgatgcccgatactct

tctctcacattcaacaaggaatcgtgcgactggtcaacaactcggtctgctggaatgacggaacccggatttcagctccaaccggccatcttcaagac

ccggtaatggaacgattgcgtcaacagggaccccctccgactcctgccagcaaggtggatgcaatgcaggccaactatctcacgtttgtgttcccccc

agatgataccgaagaccgagggtgccatacccattcgatcaacgtggaggcagcagaaaaacgatcgcccgacaaagatatcgacgatcgaaacgttc

ggcctcgcctcacaaggagccatgccgccggaggacaagggcagggaccacaggttcgattcgatctgcctcccacgccggctgcatccaaaccaaca

acgcctcaggtctcgccaagctcaagcctgtcagaccctcccgacttgcctggacaatccccgagagtccaggatgagatggaggacgaggaccagga

ggacatgatgcaagacccgaagacaccgaagctcccgaatctgtggaaacgagcaagacgaagcaaggttactcggctctcccaacctcaagatgtgg

cccagagtctgatgaacgctccagtgacgctcccctggcagacgatcgtcggtctatcccctgacgtacagaaggccattctcgcccagttacgcgga

gagatgctaccttggcccgtcactgtacggccggtgccttgggacacaagcgaagacacccacccgcagacggtcgcagctcacatatacagccagtt

aatgaacgcgatcccccccaacaaccaggaagaagtgacagaacacctcgtgcaccaaatcaaccacgccaccatccacggggaagaagatggaccat

cttggtacacccgtggactaccgatgggaacagtctacatcggcaaccgaggataccgagccctactcgactccggttcccagatcaatgtgatgtga

>contig_14.contig_14.g127.t1

atgcattcacccaagcctgctcctcatctcctggctcagcaaggctctccggatgcagcgtccgacggccgaaggccaa

cacccacactcgtcgtcacactcaccctctcccgctcgccaaccccgtccccgagccctgacgagcctccgcttgcacagcaacgagccaagatcgac

gagaccttccgcattgagaagcagtccacaccgaacccaaacacactcccagcacccgagtcagcactcctcatcgagaagcagtccacacccccggc

acccaagacggcgtctcccaccaagccgcggccctctcccaccatgaagacgcgctcgatccccaagaccgtcaatctcctctgctcgatcgcccaag

accatcatcctcccctgttcgaccgcattgaggccaccctccatcctgctcaaccacccccatcatcctcctctgctcgatcgcccaaggccatcatc

ctcctctatccctctcaactctacccttcattcacctactcctcatacgaccctcaccctcttcaatcgcctcgcaagtcttatccctcctgcttggc

ccttctccgactggtcgagcccacctcttccggcccgagcgaaccgcacagacccgcactaagcgagaccccatcggcctttcggttcatgaccagcg

caaacaacctcgacacaccaactcgatccatcgaggaagatcccgactactggatccccgaagacaaccagccaaccccctcctcacctcattctgac

tacggagaatacgaggaccaagaccagctagagcaatcggacaccgacgttgacgctaccacgacctccgaaaaggccccttccaacgaactccaaga

catcgtagcgcccctaaaggagctactcaaggacatcgacctcgatcacctccgcgagaccgccgagcgacttcaacgagcgctacaaggaatcgaga

agcacgctccacgcctcgatcgattcaactccgacctcgaatcaagattggagagccttgaggcccaccagcttcgactacggaacgagcgatacacg

ccaacccctcgagcagccagaacgaccgaaaggtgtcagtaccgccggtcaaaccgatccccctcccccccgagcctcgacgaagaccccaagacacc

gccagtactcgatcccgcgactaccgaacaacccaaccctactaccgaccctcggacgtataccgaccctcggaaaaccgacgcaatcgggaccgctc

tcaagaaccaaccaagcgcccttcccgacagaccgacgcccagcagttacagaacgcgatccggaatctag

>contig_14.contig_14.g128.t1

atgtatcgcactgccattctgcgcatctcgtcgtcgcgcgcggcacagacgctgccaacggcggcggtagcaagtgcaa

gtgcagcgagtgcgagtgcgggccagcctcgactcaagtttgacgatgccaccgctccctcagcgacgctgtccccgggcctgctcgaaatggtacac

gcacagcttggtcccaaagcaaagccgactcatgtgcaggaactctgcctccaccacttcttcccgcctccgccgacttccgccaaggggaaacagaa

ggcagcctcacactggctcaaggcagatgcgctaggtccaacggagaccctcatcgcagcagagacaggaagcggaaagacgatggcctaccttctcc

cgcttctccatcatctcaaggccaccgaccgagattttcacccctcgcctgacgtcgacctcgtccaaccacgggccatcatcctcgctcccacccac

gagctcgcacgacaactcgcgggcgcggcaaaaaacctctgccatatcgacaagctcaaagtcctctgtctttcctccggaggctggctcgatactct

tcgtgttggagtagctcaactctcccatgctcacgggcctcagtccgaaagtacgctcgatacgatggaagcagaggaaaatggacaaggaggtgcaa

cggcggggcctacgggacgtttcttacatccagacatcatcgtcagcacgcccagcagattgctatccacactctccaacgctcgcgaagtcaatacc

atcatcgacgatgccgcggctgtggccgagctccgacgaaacggtcggccaagcgacagaaacgcacgagctgcggctgcagctgcgcgaaagaagga

ggatcgtcagccactgattagcctcgtaaacgtacgctccgtcgtcattgacgaagccgatgtcctcctcgctccagactttggacccggtacccgag

cagtcctacatctggcgcgcaaagaacaagccgcagcctcgccggccgaagaggatgcagagtcggaggaagctgctgcttcatcatcatccaaactg

accctggacgacatcactgctccgcatccgaaccccaaaagatcgaagcacgtcgaatctcttgtcagtgacgaatcatcctcatccgattcttcgtc

cttccctttcgacctcatcctcgccaccgccaccatcccaacttcgcttgcaagctatttgcgcaacacccacccaaacatcactaccttggcctcca

atcggctccataagcttccctcgcgtctcaaggctgactttgtcgatccgggcggaaataagatggctgccattgcggggcagattcgcaaggttctc

ggcgagacgccgtctgcgaacgaaaaaggggagggaaacggcaatccggcccctaataaggtgctcatcttcatgaatcggtctacgtccgtagacct

tttgagcaagtacttgaccgagaatgggatcagcaatgtcgctgtgacctcctcagcgggaacgcgaagcaaaaagtcgaacgcgcatatcaagacgt

tcctgcgcgaaagcaagctgtcggaccagcgccaccataccaagtctgcggcagagaacgaagaagcgagcatgaaaaccgaggaggagattgaggct

acgtacatcgaagaagagaagccggacgaggaagtcacgttgccatcagcctcttcgacttcgactccgactgcaactgcaacttcgacttcgactcc

gacctcgtcttcgcccgcttcccctcctccgcgagtgctcatcacgacctccctcctctcccgcggccttgacttcgatccgtccgtgacacacgtat

tcctccccgaccgcatctcctactccacatccggcttcggctcccgtcgtctcgctgtttctgtgcccggcacacctacttcctcttcttcgttggac

acggccgtggcgagcgtggctgtggcgggaatcaacgaactcgagcttctccatcgggctgggcgggccgcgcgcgcgggatcgtcgggtaaactcgt

catcttcgacaagagagccgctgtggctgcttcgcgctctgcgggcggtgcttcggctggtgggaaccggaaggggatggctatgatcaggaataaac

aggggaagtatgttccccgtgctgcggagccgttgcggaaggctgtgaggggcttgcttgttccgaagaagaggacggcgggtccgggggcatggtcg

ccgatgccgaggaccacaacaggtggtgtgtcctcctcgtcagcgcgtggtggcgggccacagggcaggagaggagaagcatttgcaccgagaacggc

gggtggcgacgggtcaaagggccagagaggggatacatctacatcgagaacgtcaggcggcagcggcgggaagaccaggcctgccttgcacttgtcct

catcgtcctcacgctcgccgagcggccggacgccgtcgttcaggcgctga

>contig_14.contig_14.g129.t1

atgacgaccgcattcgacggcgaggacttcatctccttcgatgcggagccagggggaacccgggttggaggagcacaag

ctgagggttccggaacgacgcgcaaccgcattgcagggctgatggcagcggccacagcgcagcggggagcagggaagagcagccagcaatcagcgcct

gcgcagagcaatcctcaggcgtccacctcatccagcgccgccgccgccgccgccacagtggcttccgccttttcttcctcagcaacgcttccatctgg

gccccggctgtcgggaacgaagcggaagcacaatgagattgctgcggacgagaacaggctcagtaagaaggagcgtcaagagctctttgcggcgagca

caccatggtcaatggacgttgactgggatcaatgcagaaatggagcggagatgcttcaccgtgagatcatggctttcgaccaatggctgatgcctacc

actgctgagctcgaatgccgcgatatggtcatcgcattgatacggaaggccatacagtccaagtggccagacgcagatgtccgaccatttgggtcgca

caacactcagctgtacctacccgaaggcgatatcgatttagtcgtgttgtcgtccgcaatgaacacgcagtcccgagaaatggtgctacgaaacatgg

catacattctccggacgaacaacctcacccaaggcaacgtacagatcatagccaaagcaaaggttcccatcataaagttcatctgcgcgtacggcgat

tacaagatcgacatctccgtcaatcaggcaaatggcctgaccgcagccgactacattctcgacaacctcgtccagcaaccggcccttcgaccgctcat

catggtcatcaagcagcttctctcagcccgcaatctgagcgaagtctatcttggcggtctgggaagttacagcgtcattctgactgccatcagcttta

tgcaggtcagtcctactcataaatgctacattgctagcaaaccaagctcaaaagctttcccaccttcagatgcaccctcgcatccaacgatgggatca

ttgaagctatgccttgaagacccgctcgatccgacaaacgacgttgcagccgggtcgttcaacttctacgaagtgaaatcagccctagctggtggctt

cgacctgatctcctctgcgatcggtgagcgatcaatgcagctaggagggccgtcgggaacccgccaatccgccgagcaaatacgaaacgccgccaaga

aaaagaagggcaacagccgatactcggacgacgaagacgagtgggcgcgccagatgctgatggcagagatggacaatgctggtgccaaggacccgatg

agcctgttgggaaagctgttcggaatcaagccgtcgacgatcaaacatcgcaagaagatcaagaaactatgggattccaatgagcttcagcacaaact

gggtcgacccacggcgcggatcacacccgaaccaggaccttcgaccgtgaaaaggatagcagattcacgaacatcatcgcgtacagatcccaaagctg

ctaaatcatcccgcaaagcgctggccagtagtattaaccgcgcggttgtggccagcattcgagcttcgacaaatggccagcaaagaggtgcttctgag

gaggacgtcatcccgatcgatgacgaatcggacggtgaagaggaagagtcccggtacgcgactgtgggctcaagtcgacgcaccgggaaggcagccga

gggccagaaaggcttggctcctggacaggcggtcgagactgccttcgttctggacgactcggcatcttccgcgggcgagagcaacggcgacggcgaag

ctcgtgcgtatacggccgctgctgacgggatcatcgtctcggacgacgaggaccaagatgacgctccggttcctcggaagcgggcacgcgcgtcggat

tcgacttcgccggcgaagcggcgtgcgacgaggagggaggttgatgatttctgggcgagcaagggcaacgccaccggctcgaatgattccagtgacaa

cgacgttgtattcatgggcgcggctgcacagcagtga

>contig_14.contig_14.g130.t1

atggcaccgcatacatcgtcggatgctcatagcagcagcagggacagggacagagacagggacagcagtcgtaagcatc

atcgcacctcttcttctgctgccacatccgcccaccaccactcctcgctctcgcgcagagatcgttcgccaacacgctctcgttctccccgttcccgt

tcccgctctcctccgccacgctcttccgcttcctcaagaccacaagattcctatcgaagacgcgaccaagaccgtgtgagcgaccgggatcgagacag

agactacggcagagatcgtgatcgcgaccgtgattgggaccgggacaggaaccgaggcaaggaccgatctgatggcgggcatcatcgatcgtcttcgc

atcggtacgaggatgatgagcgagatcgatatcgctcttctgatcgctcctcgtatggtcgtgagcgtgaccgcgaccgagaccgcgagcgagatcga

acttatgaccgggaccgaggtgtgagtagcgcagcagcccttgctagagcaaagtcaccgcctcctccaccccctccgtctctaccagctcgaccccc

gccaccgactccagcaggtctaccagcacccccagtgcccgcacccgcatcagcgtcacttcctccccgacccactcctgctcccgccgcagcttttg

gcaatgacgacagtcatgctgccccggcgcccctcaagaagttcgacttgtcccaattaccagacgtcgacatgaacgatgttcagccaccggacgag

gatgaggaggatctggaaacatacgaggaaggctccacaaaacctcgctcaccacgtgcggcaagagcagcctctcccacaattgatgcggcggcggg

agatgcaacagacgaaaatgcggcggcggaggaaaagatggatgaggccaaggatgatgatgaggtcgatgatctcgatgccttcatggtcaacgtcg

agcaagaggtggccaaggtgaacaaggaggacgagaagcgctttggaaagggcaaggcgaaagccacggtcctggagcctgtggacgacgaagcggaa

gaggaggccgctccggacgcggatgatctggatgcgggcctacttcgggcggaagacatccttgcgctcgccgccaagaaggtcaagaagaaggagat

gccgacggtagatcatggcaaagtcaattacgaaacgttccagaaagccttctaccaccctcctgtcgaggtccaagagatgtcagaggatcaagccg

aaaatctgcgcctcgaactcgacgcaatcgcagtccgaggcaaggactgccccaagcccatcacgaaatggtcgcactgtggtctaccagcatcgtgt

ctggacgtcatcaagaagctggaatataagtcgccaactccgatccaagcccaggccatccctgctatcatgtctggtcgcgacataatcggtgtcgc

aaagacaggctcaggcaaaacgatggcattcctccttcccatgttccgtcacataaaagcccagcgtgccgtcgagaacctggagggccccatcggtc

tgatcatgacacccacgcgcgagcttgcggtgcagatattccgagagagcaagcctttcctcaaatcgcttggaatacgggctgtatgcgcgtacgga

ggcgcgccgatttcggagcaaattgcggagatgaagcgctcggcggaaatcgtagtcgcaacacctgggcgaatgatcgatcttttgacggcaaacaa

cggcagagtgaccaatctgcggcgggtcacctatcttgtcctggacgaggctgaccgcatgttcgacatgggttttgaacctcaggttatgaagattg

tcaacaacatccgaccggatcgccagactgtccttttctctgcaaccttccccaagcagatggaaagtctggcccgaaaagtcctgcggaacaagcca

ctggaaatcactgtcggtggaaggagtgttgttgccccggagatcgagcagatcgtcgaagttcgggaagaaggcacgaagttcaaccgactgttgga

ggtcttggggcagatgtacaacgaggaggaagatgctcggacgttggtctttgtggaacgtcaagaagcagcggatgacctcctccgtcaactcatgt

tgaaaggatatcccaccatgagtctgcacggcggcaaggaccaaacggaccgcgactccacgattcaagacttcaagaatgggatcgttcccatcttg

acagctacttcggttgccgcccgcggattggatgtgaagcagctgaagcttgtcatcaattacgacgctcccaatcacctggaggactacatccaccg

agccggtcgcactgggcgtgcggggaacaaaggtacctgtatcactttcatcacccctcagcaagatcgatttgcaagagacctcatcgcagccttga

aagccagcaaggtcgccataccggcagagctcgaggcgctctatgaaaccttcaaggagaagctggcgcaaggcaaagcccacgctgcaggatctggg

ttcggcggcaaaggcctcgagcgactggaaagcgatcgtgagaaggcacttcgggcgcagaagtcggcgtatggcgaaggaggtggcgagggtacagc

cgagggtgcaggcgaaggtgcagccaaggtcaaggaggaagaagcggtggcggcagtgagcagtttggagatcaaggtccagaagggtgctgcgccat

cgacttctacgggcgcaggatcgagcgaggccttcgatcgcgccaatgaactgacgatcgaagtcaagaagggtgctgcgcctgagaacatccggtcg

aacaagacggctggagggcctgcgtctgcgtcgaatcctgctcttgatgctgtgcaggagcggctgaaggcgatcgcgggtgcggacacgaacaagct

tgcgagcgcgctggctgcgatcaatgccatgcagaagaggcctcctactggtgcacaagcagaacaggcgcgcaagccgaaggatccggatgcgacgg

actatcacgccatcgttcccatcaacgacttccctcaacgtgcaagatggcgagtaactaataaggagacgatgagcgagctcatagatacgactggc

gcttcagtgacgaacaagggtcaattctacgagaagggcaaagaacctggaccgggcgagccaccgaagctgcaactcctcatcgagagcaacgacga

gagccgggtggagcatgctgtgcgggagatcaagcggttgctcttggaggctactcaggccgcgctcgaggcggaggcgaggggacctcaacatcatg

cccccgggcggtattctgtccctgatatcgatccgctgaccatgctgagaggagatccactttcatcagacattgcttctcgtaccctttccagcacg

gcgagttgtgagacggcactggccaccggcagacacgggctggtaacaagactcatattgcgcatagcacccattcaaccatacaagaaggtgagtgg

gcgcgtcccttaccgttggagtccgggctgggagctcacgctcgaccccttctcacaactggttatgagcccagatactcgaagtgcgcccatcatgt

cctcaaacgagcaagccaccggatccagtcatcagcgagccgactcgtctgcctcggggacgcctgaagcgcaggccagccagctcgacatcgcgaac

tttggttcctggtttggttcgctctccgtggaggacaaggcacgtgttgccagcatggcaatgagcagtccggaaggaactggtcccgtcgacaatgc

cacgggtcaggcggcgacgactacggcaggttcggctctccctccccgtactcattcgggtctgggtgtgccgcccttgtcggcattcgtagcgccgc

ccccagggccccatggctcggcggtgtcattcaatccgtgggtcaaggcggagcctgttcatcaagacccttatccagcttatacggaacgtcagcac

ggtcccgagacctctagtgacccgcactacgtccgcggaccattcgccaatcagcaccgtgccgtaggcgacattccgaagctgtgcggacgcaagaa

ctggttctcctggaagaccctcttcttcttttcgattcgacccgtcaatggcgcgatcgagcacctcacgagcacgatccccagcgaccagtactcat

actctttcgacattcatctcggagagctgctggttcgcaccatggctctggaccagctcggcccagtcatgatgctcatgagcacaggcgagatgcgt

ggatcaaagctctacgagagcattcgcaagccgttcgagcgcaccgacgctgcgacgaggggacagattgagtccacgctcacggtactcaagcaagg

atcccacactgctgcagagctcaatcagactctgacgcacctgttcgcccaggcactcaacgctggcgtgttcctggatgaagagcgcaaaatccact

tcctctgcaagtcgttgcaccccaagtacggaccattcaacggccagatcaacgctatggctcgtcgcggtttggttccgacttacgaagtggcgatc

gaggacttgtacgtggaagaaggccgactctggcaagaagacgccgagtcacgcaaggtcgacaggagcgcgcaagcgtttgcagtcgccttgtatgg

cggagcccctcagcagaacagcggtggccagcaaggattgaagaagcaagccttcaaaggcaagtgctatcgatgccaaaaggtcgggcacaagcgat

acaactgccctaatcctgcggcagatggcgaagcatcggcgtcaaacagccaatag

>contig_14.contig_14.g131.t1

atggttccgtcgaaggacatcatcacggaattctcgagcgatgcacctgttcaagcagtcaaggtagcagacggtgcat

tcctcgatgtactcggacacggagcggttacattctctgtgggtacgagtggcagaaggacgaccacgcttatgggcgttctttgggtccccgggtta

gcagtgagcctgctctcggtcccaaccttacaacggcagggcggaatcaaggtgacctttgatgagaaaggcgcggtgatcaccgactctgcaggcaa

ccagcttcgtgcatggctggatgcggcagcgggcgtcagcgtcatcaaagcgcggacctcggccgggacctcatcagcgatggtcttggcggcagaag

cattggcagggtcattccctgtaggcggcagcgagcgtgaggccacgatggagtcgctcttcacaggtggcagctcggtcgagccggaggctgcggtg

cagcaagatttgcgaaatgaatctgttggcaccgacgcagatccgacctcggcggcagatcagcagcaactcattgtgcggggtgtgctggcactgat

tcttttgtggcacagacgactaggccacgccggcgtcaaagcaattaggcagttagcgcgggtcgctagctacctgaagattccctacaagcaagtcg

aacgactataccgagcgatgcaagtctgcgatacgtgcgtcgagaccaagatggcacgtttgccattcccacactctttgcaccgatctctcgttgtt

ctcgaactcgtattcgttgacctgctcggcccagtcagcactacgagcccattctcgtatgtgatggtcatagtcgacgacttttctcggaaggcttg

gggggtggtcctttcatccaaatcacaggcgttcgagcgcctgaaggaatggaccaaacagcaggaggatcgcatgcggtcgatgatgctctcagcca

aggcgttagcagagttctgggtcctcgcgttcatggccgccatctatcagtacaacaggacacctagcacggccatcggtggagcccttcctgagacc

aaatggttgggcaagccggcggatctctcgatgctacgggtctggggattaggcagcgaacggaggtttttcggcccggaggagcctgaagaggtgga

ctggcacgaggtcacgggcgaggaccctaaaacgatcaacgttgacacgtacctcggttcaaacgcagcacctccaccagctcacgaatccggcccag

tcgttcatcaaccaccaacgcagcaggacgaccaggtatcggtgggagcacatgggactgtcgacctacaggatgcggtttcggtgggagcaccatcc

gaagctctggatgatctcatgtacgacctccctttgaccgatctgctcgcttctcccattcgcaatcaagaaccaccgatgctgcttgacctcatgcg

tgatacgcctgagctcgaaccgatcagaaacgcagtgcgggcgccaacaccagcaacagccttcttaccgccttctcagcctgcgtttgaagaagaca

ggtcagagccgccagaggcacctttacgctcggcccgctatcttcgggctgaggcacgggagattcggagccaaggcctagcgaagacggttgtaggc

gccgacaagatctttgggcaggcgctgtcctcggtggcaagctcaagaatgcggaccagcgcagacgacgacatcatggtgtctgaaaaggcatactt

gtcaagtcaagtcgacattgtcgatggcggaccggttcgacactttctcggaatcaaaatcaccaagaacaaggaaaccggcgcaattcacctctccc

agaaggcatacatcgaagcaactctcgctcgttttggtctcgatcgcagcacgggcagcaagacccctttgccgctcaagatcccgccacgttcgaag

ccagactcggagaacatggaggaccccctcttcgaccctgatcgcaccaagggatacgctcagcggaccggctgcatcaagtggattgcgttcataac

gcgaccggacttgatttttgcagccaatctgcttggtcgattccagtcagcgccatctgaggaacacgaggagctagtgacccaagtcctacgacgac

caccggaggacgaaacatatcgacatccagtaccacgcggtgcaggaggcggtggagagtggtcggttggaagtcgacagggtggatacggcggagaa

tccggccgacgtatgcacgaaggcatttacagcgatcttagcaagtaccgacgtccacccattggctccgggcactatcacccttcgcgccgcacccg

cccatccgcccagcgagttgtcaagatgcttcaattctggagtgccttcaccgcctcacaaagacgaaacatagccttttatgtcttgggtatcatgt

gctataagttcgcgcttgagtactacaacggtgccttcatcacacttgccaacgagcggttcgccaaagacaaatatcagaagatcggtatcctcaca

ggcatcaactacgccgcccagggtataggatcaatacttatctcccccctcatcaagcggtaccccacccgatcggtcctgtccttggccgttctcgg

tttcgcgatcatctctgccatcctgctgatcaccgatgctgccaccggcgggcagccccgaacgatgcgaaccgatggcaaagacttctacggaaaat

acgacccaaacctgatgttcccgatctttgcgctctccggtatctcctacggcatggttgagcttatccgtcgggtgattccccgtgacatcgtcggt

ggcgacgttcaccgtctgagaaagatggacgccttcgtgcacgtcgtttacgaaattgctggtaccgccggtgctttcacttccaccgccctgatcgg

caagctcggctacaattactcgtttgcgctctcgccacctctcttcgccctcgccgctctcctttggacctttatctccactctcggccacactggtg

cttctcatagggccgctctcaagcagatcgagcactcccagatcgtcgcggctgcacccggctacttcatccagatcgtgtccggtctccgtgccttc

gccaaggccttctacgtcggtgcctacatcatcttctccaggcgcagtctgatctggctggtgccatcctacacactcgctctttacggccaccgtta

cctcgagaacgttctcgctcccatcgtcgccaagcaggtccttggtgtctcatcatactcccagatcatggttggtggctccaatttcggcgaactgt

gcggttccatgtcggtgatgttcttcactaacagcgtaccgacaccgatgccctggcttcgtctcgacgccgtcctgctcatgctcgtctgggtcctg

ccttacatcaaactcgagaccaacaatgttgcatccgcatggcgcctcgccggcatcttcctccccgtctcctacggctgggctgccggtgacgtctc

cctggctgcttacattcagtccacgctcgcaaggatggagtcgaccgaccgcgatgtctctgcccttggtgccgtcatggccttcctctacgttctct

acatcgtcatctacgccctcgtctccaccttcctcggtcgatgggtcgactcacagatcaacggtgccgtgggcgctgcagccgtcggacccgccaag

aaagcgctcgtatacgtcgccggtgttcacttcaccatcctggccgccatcatcattgcctcgaccttcatcccccgcggtgctctcgccttcaaccc

taagcccgaaggcgacctggccactcctaccgacgaagaggtcgaaatggcccggaacggctcggaggcgagcgatgagtacgacctcaaggagggca

tcagcaagcgacatggcgccaaggacgacgtcacagctggcatgtga

>contig_14.contig_14.g132.t1

atgtatcacactgcagcaagaacggttaccctcgtccccaagcttgatcacgacacttatgccacttggcactatcggt

tccgcactcacattcagtcgatccccggtgccttggcacatttcgacggcgatgaatatccgaagggcttccaacgacacggcgacgatttccccgca

ccgatgcaatccgacggctacgaccccgaactcgacgctgcgctcggtaccctgtttgtcaacactgacgagctgactggtgttcggcaacagagcga

atctattcagaagctcggagacaagctgctagggctgttcgagcgcgcagagaatgcgggaatagagttgtcagagcgtcagcaggtagtctatctgg

tgagggctgtcactgagcgctacgcgtcgcgtcggtcggcaatcctggaagcactcgacaggcacgaacctattacattccactcggctctggagaag

ttcaagcaagaagagcagatgttcaacgatcgcaccaaggcggatgtgtcggcgacggcaaaggcagcgaatactcacaagaaagggaacggaaactc

tggcggcggaggtggtggtcgtcgtggcaaagcggaggttccgcccggtctgagagctcacctttggtaccaatggatactcgacacggctgcatcac

accacatgacttggtgcaaggccctgttcgatgagttggacgagggagagttcgaaagcgtcagtctagctgacgatagcgagcttgctatcaaggga

atggggaaactcactctcaaggtagctggggaagatggggagcgcgtgctgcgactgcacgacgtgctctgggtgcccaacctcacgtcgaatctgct

gtccgtcatgcgggctgtggagggagacatcaggtttgagatggcacctggagggatgcggatgggttgtgagggagacatgtcagtattggccccac

atgtcaaggagcggggtgggtgcatactcaccacggaaatcaagggatggcaagcgacggcgcaatacgtgcgcacgggaggcgcgagcctggaagct

cgtctgcttcatcaacggacgggacactcctctcccaaggtcgcgggcgtaatgtgggacaagggctgcgcgaagggaacggcagtcagcaagagtgc

cttaatccaggccagtcacgactgtgacgactgcaagcaagggaaaggcgtgcgcgcaccgttcccccactcagagacggtttcgcatcgtcggctga

gcctggtccatgtggacgtcatcggtcccatggaggagggaaccggtggggatggggagaaatacacattcaccattgtcgatagcgcaagccggaag

gtgtgggcgtatcccatggcggcaaaggggaacgcactgacggtgtttctcgattgggtgcgccgcgcacagaaggagacgggcgagcagctggtggt

ggttcggtcagacaacgggggggagttcatcaaccgacaatcccaagactgggccaaggaactcggggtttggtgggagaccactaacccatacacca

gccagcagaacggagtcgccgagagatggaaccggacactgcaggacagaatgagagccatgctcctgacgtcaggactggacaactcgtactggcca

cacgcaatgcgagcagctgcttacgttctgaaccgtactccacggttgaaggacggagggcgcatacccgaggagatctggacagggcgcaaggtaga

cctctcgaaccttcgcgtagacgacaaagggatggaaggtgtacataccgagcaagtccacccatcggatgagcaccagccgggacgtcatcttcgac

gaagatcgtcggtaccaggaccttccggtcccccccggaagacgggctccctggatgacgtgctcgagtggcacgatgagtggagtctggtggaggac

agggatgaggagcggggagatcagtggtcgggagaaggagttggagcagcaggagcggaagaggcggcaggaccggtggaagtagagggtgaggggag

actcaccaaggacggcgctcaggaggagcggcaggcggatgaccggagggtggtgaagtcgacggagaaggatgcgggagatgtggcggagctggatg

cgaatgcggaaggagtgggggatggagttctgaggaggattggaggtcgaagaggcgatggcgccagcgatcagcgacccaaaccggcaagcaccgag

cgacccacagacgcaacctcaggactcaccaccggcaccgacggcaacagcagcaccacccgaccgaatcccggaggcggagcgcgcgcacatcaagc

gcatccgggagagccgcgaaccgacgcggcgatcaccgcgcttacaccagcaacacggccaagccaagatggtgcgagcgtacgcgatggcggtccgg

gatccccggagcccggactccggggcggcgctccgctcagctccgtctga

>contig_14.contig_14.g133.t1

atggcagacgaactcgctgcgaaagtagagacccagctcagcacggacggcgccggcgtggccgagtccggcagcggca

aggccaacggggcgaacggggacacgaatggccaccagaacggcgccgagaatggcgcagcggacgacgacgatgatgaggatgacgacgacgacgag

gctacagcagagggcggaggaggtgctggcaccgcttcgaaaaagaagaagaagaagaacaagaagagcggagctgcgaagcggagagcgaaggcgaa

caagggcccgaacccggtcatggctcagtcggaccctcctcgggtcggcttgaccaagatctatgtcgatggcatcttccctgttggcgagattgtcg

agtatgaccggagcaagtgggacgacaataccaagcgcgtcacagacgaagagcgccgcgagcgcgagaagatcctgcaagaaggcgagggaaacaac

tacaacgtcctgcgacgagcggccgaggcgcacaggcaagttcgagcctatgcccgtcggtcgatcaagccggggatgacgatgacggagattgccaa

cttgatcgaggacaccaaccgtgcgctggtcgaaggcgatggcatggaggctggtatcggtttcccgactgggttgagtttgaatgaggtcgctgctc

attacacgcccaacgctggcgacaagcgtgtgctgcagaaagaggacgtactcaaggtcgacatcggagtgcatgtgctgggtcggatcgtagacagt

gccttcactctcacctttgaggacaagtggaacccgcttctcgaggctgtgcgggctgcgacgaactccggcatcagggagactggtatcgacgcccg

gttgggagaagttggcgctgccattcaggagacgatggagagccacgagttcgaagttgatggcaagcttcaccacgtgaaatgcatccagaacctgc

aaggccacagcatcagtccgtacgtcatccacggcgggaagtcggtccccatcgttgcacaaccaaacaacaacatcaagatggaggaaggcgagacg

tacgccatcgagaccttcggctccaccggacggggctacgtgcaagactggggcgagtgctcacactacgctcgtcgtcctcaccccccggcagccac

actccgggtgtcgagtgcgcgatcgttgttgatgaccatcaacaagaactttgggtcattgccgttctgccggagatacctcgacaggttaggcgaga

agaattacctgcttgggctgaagcaccttgtccaacaaggcatcgtgacagattaccctccgctggccgatgagcctggatcaatgacggctcaattc

gagcacacgatcatgctccgtccgacttgcaaggagattgtctcccgcggcgacgactactag

>contig_14.contig_14.g134.t1

atgtcgacatcctttgactcccgcctggccctgacctcgtcccgccgctccctgcgtgcgtctggctccctcgagtccg

tcagcagacagcgcagtatgaccgaaaacatcgtcctcgagaagcccacgtcccggaaacgctccctgcccgcctcgcgctccatgcctgtcgtctgg

gccgagtcgcaacagcagggcggcgagggcaacagctccttcctcgccgacatcacatgcgtgggcagcacgccgacgaaggacatgtcctggctcga

gcaccgatccaactcgtcgcagtcgacagtgctgcaacggagcatgcgcaacagccgacgcagcaattccagcagcagtagcagcagcagcagcaccg

gcagcttctctccatgggttggttctcagagctgctcgccctcgcccgtgctcgcagacaccgcaagaatggaacctccggcaccctgggagcagact

ttcgattcgtacgattcgcagcaagaatatctgcaacagcaccagcaccagcaccacgagcgcgcacatagtgaccacatcgtcgaccatctcgccga

cctggaccgctccatgactctctcctccgattccgacgaatacatcgaaacactcgattattcacccgctttttcggccgcgctccttccacccgcat

ctctgcagtcaccctctgcttcatatctttatctgggcgaacctaccgcaccacagccgccgtcggacccggcccatttgataccaggaaaccgaggc

cgtcgggttccgttggctgagatcccactctggtacgccgatgtgcgaggagaggccatcaggaggggaaagcacgagcgatccagtctctcgaactc

gatcgacttttcttcacctcatgttctctctgacaacacatcgcacaatattcctgggagcgattcatactacctatctccatcccaggcgtcctccc

aatcgtcgttctcggtcggagcagaacaaggattcgggaacttgtcctcgattgcagaaaacagcggcatgtacgatggccaccaactctttgtgctt

ccggacgacgagcagcttcaagtcggtggcagtccacgtcggaaggagctgaggtcgctacgacgccaaagcatctcggagcaacacccgtacccgag

caagaagacgatcgccgctcgaggtcggtga

>contig_14.contig_14.g135.t1

atgagcaacggcttccggagcgggatgagccgaagccacagcgacagcgccagcgtcgccagctttaggagcagtccca

gcggcggcggcggcggcagtggcagcttgagcgacagcgacatcttcgcagaccgcgatccacccatgtccatgcccgtgccgccggtctccatgtcg

gcccgctcccggccgctcacgatgggcgagcgcgacctcaacaagatggaggcgcaaatggaagatgtgagttgttgtgctcctcacccctccctggt

gccgcgcagctgtaacaaaactcatgcctatattcccaactttgctcaggctggatatcgagaaggcatcacagccggcaaactcagcaccttccagt

ccggtttcgacgaaggcttcagcaccgtcggcgctccccttggccgggtcgtcggacggctccggggcgaagcgacgagtctgtgcgtctatgtcagt

cgactggctgcggatctgcagccgcagcccgcaggtccaagcgcgcgtgagggctcatcggggcacggcgcggtgaacgacgacgacgacggagaaga

agaagaagaagaggagggttcgaatgaggacgggccacaacagcatatcgagctacagtatgaagacggagatgtgttgcccggtcggcagggcaggg

cggtcaatggacacggcggacagcactcgtcgacagaagctcttcatcccatcttcgccaaagaggacgtccctcccggccccggtcgagcacgagtc

cgcttaaagagccgaggagcgacgattacttcctccacacgaccatcagggaaccaaggcggaagtggaagcggaagcggacgacgaggtgtcggagg

cgcagtcccatcgagccggatgctcgctaacaaccctacgacagcccatctcaagcctgcggacatcttgacactcctgcgcgaagcgcaggcactcc

tcaaagacacacaagagctctcgctcgaaaagctcgccccgccggacgaagaggcgttggcgcacgagcacgagcacgccatgatggccgctgcaggt

gctgcggatggggatgcgcgcgcgggttctgggtcctccgtgcattggacgcgggagagtgagggcgagcgcgcgagacgggaggccatcttgccggg

gctgtatgcgcgcttgcatgcggtcaagacggttttggggctcgtctga

>contig_14.contig_14.g136.t1

atgagcgacgcacagcagtgggaagtccgcttctcaaacacgcacaggctcccgtacttctacaacacggcctccaacc

agtcctcgtgggagcctcctgcaggcctgagcgaggacgacatcagacagctgccgggagcacaccagttccaacagcaggctgccaatgctgcttct

ggcgcaggcggtgctagctccgacaaggtccgcgcatctcacttgctcgtcaagcaccgtggcagccgacggccgtccagctggaaggagcaaaatat

cacgcgatcgcaagaagaagctgaacagacgatccgacagcacatcgatgcattgggccccaacccatcagcagaggaattcggaaaactcgccagcg

tgcacagcgactgctcgtccgcctccaagggcggcgacctcggcttcttcggccgcggacagatgcagaaacccttcgaggaagcgacatatgccctg

cccgtcggcggtttgagcgacatcatcaacaccgagagcgggacgcacgtcatcctccgtacggcatga

>contig_14.contig_14.g137.t1

atggcgaacaagcgcaaggctcacgacagcggacggcggagaggcgcggaggtcgacaagctccagcagcaggaggacg

atctcgttgccagtctcttcggcggcaccccggcgggcaagcgcatcaagaccgctcccagtgttcccacagcaacgacggcgcggaggagcgacgga

atcgagcaggacggacaggatggggacgacgacagcgatgatctctggacggggctcgagcatgtcgacaatggtcaactattcagcgtcgacggtgg

cctagactctcaagaatccgacgaagaagacggagaagagctcgaagatctgagcgagatcgactacgaggccgaaagcgaggaagaggaggacgtcg

acagggagaaggaggaggagacagcaacaagaagaacatcctccgccaagcgcgaaccatcgggtcggaaaaaggcagcatggaccgatacagatgat

gcacaactcgtcgtcgctcttgcgggagccaattcgcgcgccgcagatgggagcttcgtcgggacgaagcggttacgcaagttgcgtcaacacgccga

cgagaaggaggtcagtggactcgaatacgaggcccggctgcgcaagatctttgagaagttgcaccctcggccctcatgggcatcgcgaactcgcaaag

gagatatcgactacaacatggccatcccacccgcttccacggacgcgctccttctctcggaactgctctctcgggatagcggtctggttgctcgcgcg

acgggccagagcaaagtccgcgctaggctgatgccagatggtctcgagatggagcggctgcgcaatgcgaacgagtctcagggacgcgacgacgatgc

tgcggtggagagcgtaggctttcatcctacctcaaatgctaacatcctcatatcggccacccgcaatcgcaaagttcgactgttccagatcgatggca

aagccaaccctctgcttcataccatcaatcttcccgacctcactctccaaaccgcgctcttccacccattgggcaccacagcattcttcgccggcgca

cgtcccttcttctacactcacgacttcatcaccggcaccaccacccgcagtcttccctggagaggaaccgtagacggcgcctatgacggcagcgaagt

cgaacgtgatctcagctttgcgagattccagcctttgcatggcacggggaacctgctggctattggcggtaagaggggcacgatccatctcttggact

gggggaacagcgctaattcggcgtcaggcaccaccggtcgaggctccctcgtcggctcgctgcgcatgaatgcgcctctggttgggatggcctgggac

ccctcggactcggccggtggcaatcgcctcgtcacgctctccaaggaagggcgcgcccacgtatgggatatccggaatatgcgctgcgaggcagtccg

gcttgatctcggcctgtactcccccaagggtctcgaagtcagtccgacttcgatcttgtcgggagattctatcgcgggcaactcgggctcgtcgcatg

cttggctcgtcgggtcggaggctggcattgtcagcgtgtacgacgacaaatttgcggctgtcggcggtgatgtctctgccacgagtgcacgcgggggc

gtgtcggcaagttctgcggtggtggaggtgcccaacttcacgtcgcgagagacgatgtcggctcgtaagacggttgacaacctcaccacggcgattac

gactatgaagtttaatcacgacgggcagatctgtgctgtggccagtcggaataagaaggactcgctcaaggtgctgcatacatcgacgctgcgagtat

ttcccaattggccgacctccgggacaccgttgcaccacgtcactgccatcgacttctcgcgccggggtgaattcgctgcgattggcaatagcaagggt

cgcgtgctctttgataactatatttggcaatcgcacgagatcgcagttcctcaagcggctctacccacagcttcggcagaagacaaggcaccctccag

ctttccgtttctttgccgggacgatggacctgctcacaaagtcacaagcatctcgggccgttctccggagggccgcgatcgtccttcggcccgtcaga

agatggagtaa

>contig_14.contig_14.g138.t1

atgtgggtcggacctggcgcaaattcaggggctccttacctcgagggattcgcgactcaagaaggcaagccagccaaga

aacagacagccgccgacttcattgcagctcacgtcggcgcggtgccgtccaactacatcgtctcctccaccgttcgggccaaactatttgactccctc

tactctcggctgttcgacgaccttccttccccgacagacctcttaccatcgaacgtcgggacgtcagtgagcacagcgctgtcccacctccgcgcatg

cgattggaagctgtccgaggcgcagaagcgcgccgccgagcaggagcggttggccgcagagaagggcaagcaacctctccgtcccctcgatcctaaca

actccgagcccgacccatctctcgagctgggaccagaatatgggccggatcgcaagggaatgccatgcggtcacgtcttcgttaagggagaagccatc

taccgctgccgtgactgtgcacttgatgacacatgtgtgcaatgcgcgccatgcttcaacgcgtctattcacctcaaggaacatcatgatgttgtttt

ctctctctgcgaaaagacgggcgggtgttgcgactgtggtgatgacgaggcttggcaacagcctgtcaagtgcctctatcacgaccacgatcaaaccc

aagcatcggcttcatcttcgcgtcagcctctggacgaactcgaggcatcggccgcagatgaggacatgaaagacgacatcgacgtcgacgtcgacgcc

ggcaacgaggagaccggaacggtgcacagtttctttggcagtctacaaccactgctagatcgatgcccaccagaggtcgctcgcgccatctccaggca

tgtcggcgaagtgctcgacttcatcctagacaccgtcgagcacgcgcctgaagagacgctcggaaatgtatcagccattcgtggcgagcaggttgcgt

cgtcactcccgacactgcgctcaccactcgacgctgaaagtccgcgaatgtacagcttggtgctgtggaacgacgagagtcacactttcaacgaagtg

atcaaccaggtcatgaaggtgactcagaactcgacgcggctcaacaagcagcaggcaagggtgatagcggatggtgtagatgcgcatgggcgacaagt

catcagaacatcggcatcgctcaaggagctcaccgtgatggcgcagaggctgagcaccatcgatctcgccgtctctatcagacccgcatacgacattt

tcgcggaagaccttgcgggactgctactagactacatcttcgatctcgcaaacgcgacagttttcatccccccgacacagcaatcttctccaccggac

gcaggcaaggagaaccggcttgagtccgcggctccaacgctgagagcactgctcactgcgcaattcctgataccctggacgccgcagcggccggtcaa

ccaaggcaaaatgagtcaggaattcttcaatacggcggacttgcgtcgcgtggatgggatcttcttacttgaccacaagatgtggaaggaggcgcgta

tgtgctgccagagcatcctcctggctctgaccacactgaaggagattcgtcgcgaaattgcggtccgcttcgcccaagtctacgccaagttagccgaa

acattcattttcagggaccgcgagccagaccagaccgtatacttcctggcagtgcaactgttcacggtaccatcgattgccctcgagcttgtgtccaa

gcacgactatttgaagaagatgctgcagctcctccaagtgtgctgcacgactggaatgccgacagaagagaagttccggagattggaactaccgccta

caattaccgacactggaccgatcgaaactcggtggtcgctcctaaagggacagcggtgttggcatatcttctgtcatcttcgatatctgatcgcggca

gatggcgtgcagcggctcatcgtggaagaaccccggcacctcgaggactttctcagcttcctgcagatgttcaacgcggtcacgcccgagaaacgggc

ggtcgacttgcacgtcgagtacgagatggacgtttggtggctcgtctttcacgtactgtcccgtcttggcggctttgcccagctcttggcagtcgcgt

atgcgaagggcgatctcctttccctcgtgcgggcgatcaatgtgaccgcgcagagaatcaaggaggcgaccacccaactggcaacaaaggatgccaac

ttggtcagggatgcggaagaacctggccttgagcctgcgttccacactgtcgaggttggaggaaaggagtggcgtgtgcttcgcttccgcgtcgacag

gcagccggtttctttccatcaccctctgcactggctcctgtcaggcctattccggcacagcaagttgctgtcgcaagataatctcgaacagcacggcc

tcaaggacgtgtcggaaattttgacgcggactttcgacaccgatgacatcctgtctatgatggacttcccacttcgtgtctgcgtcaaggttgcgcaa

atccggtcaggactttgggtacgcaacggctacgcgatccggcagcagtcgcatcactaccgcgacgtgaccatgagggacaccatgtacgaccagga

tctgttcatgttgcagtgcggattcgtgctcgtgaaccaggagcagtggctcgcgacgctgatggaccggttcgacgtcgtcaagttcttctccggcg

actttgagcacgacgttctcgatgcacaacagctcatctacatcgttgaggagttcctgctgctcttgatcggtctcctctccgaaccttccagagcg

gccgagtgggaggtcgagcgtcatcttcggcgcgagattattcatgctctggccctcggccaaggcacctactccgaagtgaccaaactcatctcgga

gcgtctgaccgaaagctgcaactttgaccgcgtcctcacacaggtgtccaacttccgggcgcctgatggtaccgcggatctcggcatcttcgagctca

aggacgagttttacgacgaggtccagcctttcttctaccactactcccggaaccagcgggaacaggtggaggaggtactgcagcagcgtcagcggaag

aagggcttgccagactctgagcctgtcgggctgccccagaagatggacgtgccgtacggaccgtttgcagatggccggctgtcgagcacggtggtgac

atcgacatttgtagagatcgtgttcgtcgctctcgccaacgcatactatgcgatggaggacgttcccgatacactggtagaggccactcttcacctga

tcaccatcggtcttgtagagcaggaggctgcatttgctgcatggcttacaggtccatcggcgcggattgccggcacaaacccagcgtcctccaacacc

gaaaaccccgccgatgtcctgtcactcttgtgccggatggaaagtgatgccaacttcaaggcgttcaagcctcggattacctggatcatcgagcgttc

ggcaacactcatcgactgtacgagcaccctgcgaacctatcgcaagcaggacagcgatgtaggcaaggccaagtcggcggaggatgcaaagaaagctg

cggcgaaggcgcgacaggcagcaatcatgctgcagttctctgcccagcagaagagccttctcgaggcgtacggggacgaagacgacgacgacgatgag

gagatggatgaagaaacggatggagcggaggatacgaagccagctgagccgagctacggaagctgcatcatgtgtcaggaagagctcgactcttctca

agcatttggaagcttggtgcatgtgcagacgagtcggaccatgcgactgacgccgtaccacgagagtcgatactttcaacaagttgtcgacactccac

tcactctcgatcgtttcggcgcggatgggaagcgggtgcgtgcgcgtccagaggcgcggccgaatgttgccggccccacaccgacagggatgttccca

acctcagaacatcgcttcggtctgcacgcctcgacgtgtgggcatctgatgcatgtcacctgcttccagagctactgccaatcgacggagcagcgaca

caacagccaggtggcacggaaccaaccggaagacttggagcgattcgagtttgtgtgtccgttgtgcaaaagccttggcaacgtcattcttcccatac

ctcccggtccgaagcaagaagagaatgctgcggctgccaccagttcgcctacgttctctgactggttgcgcaagatcgacatcgatatcctgaaggag

tcgcgtgtatcggccagcgacttccaggacactcagcacggcacaggtacattcctgcagtggtatgctgaagaagcggagaaggcgttccggccgca

tcgctcgatgcggaccgataccggtactcccgagtttcagatgttggatcgacttgtgagcgtggtaggcccgctctcagccgagctcttggcggatc

ggaaggactggcaggcaaggagcattacagcactgccgagtccgaagatgtatctcccggaggaattggttgcgtacacgattggcatgctggaagtc

tctcatcgcggaatggggggagactcgagcatctcagcgaatgccaattctggcctggcgcgtaccgtggctggtgggctgtccgaacagacggtgcg

gctgttgagctcattgctgcactgtctccgcagcttggcagtctcggacaagcacggggagactggcttgatggacatgcgcaggggcttgatcaagc

gacttttgccccattggggccaagaagatgcggtcaaactgccgctgttgcttcgcgacccgctcaccatcctggtcgaaactgcgatcttggctccc

gactgcctcactcacgtcacatcgttgatgtactacgtaaccatcgtccaggttatttacggtctggcgcagtcgatcgtctggtcgcagggccagtc

agcgggcgccaatcgtgtgtcccagtacttgcgcgccagccatgtcgacccagaggatctcaagacagcgcgagaagtgttcccggacgtcaagtggc

tcatcacgaatgtcgtccaagtggtaggctacgttcgcggtaacctctcgattgggatcgaccagtgggatgacgattgcctggcaaaaacgatcagc

agctacacactacctttcctccgccgagcggccatattacactccgtcgcactcggcgagggttctgtcgcagcccttcctgctcacatgcgagacag

gagcgagtacgttcgactgttgaccgccctcaagattccctcgccgcgcgttgccctgcccaagagaggcgagcgccagactcctatgtctagtctga

tagaaggctggattcggcacgcctttgcaccgctcagctctctgttccgacccgctcccatctccatcttcaacccgaccagccgagcaacacagcga

ctgcttgcgttggagcatccacacatctacgagcttgtcgctttgcctgacgacctggcggatttgctccagcaggcgcagaagaaggtctgcaaccg

atgcggaacgataccgacatcgagtgcgatctgtctcatgtgcgcggaggtgcagtgctgccagatgatctgctgccagacggaggaggagggcgatg

aggacgccaagggggagtgtttccagcatatgggagaatgtgccggagcggtaggaatctacttcaagtcgaattccaactgtctgttgctactctat

caccggaacggcagcttcctcgcgtcgccttacctcgacacccacggcgagatggacgtcggactgaggcgaaagctaaccaccaaaggccttctgtg

gaagtag

>contig_14.contig_14.g139.t1

atggcatctgcattctctacgtcggacactgcgatcctcaccgcgccgatcgagctcgacccgctcctgcaggccgcaa

cttcggccgcccagtccatcgatatccgcacgctgtacgacatcgaacgcacagcttctaccattcttcaacatggattccgcagcatagcgcttcag

ttcccagacgaggcgctcatcgatagcgtgcctgtgtactgggcgctgcgcgatgagctttctgcaaagttgaaggtgcagaagcgctccgaccagtc

tgaagagcaacgtgccgggcctagctcacaaggcgcgggcaggaagaacactgcaacagcgatggaggaggatgcgttgccggagctatacattcttg

cggatacaagctatggcaactgctgtgtagacgaagtcgctgccgcccatgtcgatgccgatctcgttgttcactatggacacacctgcctcagccca

accgccaacctccccgccctatacgtgtttccacagctacccgttgacgtcacgcacgcggcgcagtcactggcgagctttgtgcttgacaaagtcaa

atctgaggccagcggatccaagactctcgtcctcacctacgacgtcgcgtacgcgcatgcctcggaggcagtccttggaggacttcggaactctacgg

aactcaaggaggcggggtgggaagtgttttccatggaggttgatacgcgcaggtcctttcaagaccaggttcggaagaggacggttcgtggtgtacaa

aagtcaacttgtgctggtgccactgatggtgcttgttgtaacactgcacccggtgaccggggccagacctgttcgggctgtcaagacggcggatgcat

gcagtcgagcaaaccttctacagcgagtgcttttgagtcaacggaatatactgagagccaagtcgcaagtcttgccctcgacgacagcaacaacaccg

acgggaacacgcctcaggaagccgccaaacaagaattcgacagaataccgcttacttcgggtcgatctgtcaacgtaccgcgtggaaggacgttcgag

gcctcatctagcaccgtgctttatcttggacccgagtcgcgcgctctgaccaatctcctgctccacctcggtccctcatttcccgtggtgtcgtacga

cccgactcggtaccaaccagatgcgccctcttcctcttctgctgcggcgccagtccgacttgagactggcaaaacgaatcgtctgttgatgcagcggt

atgccatggtccaacgcgcccgtgatgcatctgttgtcgggttgctggtcggcacgctcggcgtagcctcatacctgcctctgttggatcgacttcgg

gctcgcctaaccggatctagcaatactggcaacgcgaagacggcaaagaagaacagcacgaagggaagcggtgggcggaaggtgtacacggtcagcat

aggcaagctgaacccggccaagcttgcgaatttccaggagatcgaggtgtgggtgttgattgcctgtccagagaactcgctggtgggatccaagtctg

ggcttgggtcgtccggcgcaggcgggagcggcggcttgagtggggcgaacggacatggaggcgatttctggaggccgatcgtgactccgttcgaaatg

gaattagcactgggaccagagggcgtctggacgggcgaatatgaggtggatctggcaaaattggcggacaagctcgagaagacgagtgatgctcccgt

cggccgctctgatgaggatgccgaaaatggagataccgacgacgaggacgacgaagacgatgatgaggaccggccccactactcattcgtgacgggaa

cctacgtatcccgaacgaaatatcgggatacggctgccgacgacgaggacgatgcaccaggaaagccgggaacgacggttgcgcttcgagcgggccag

gaggtatccgtaatccaatccgcctcgggtctgacgcacatcggcggtcggctgacggaacgagagtggggtgggctgacgcggccggagggtgtgga

tcggtatgtgccgtctgtgctggaggtgggtcggacggggatagcgggtggatacgcgcacgctggtggggacgcggaaggcggtcatcaatag

>contig_14.contig_14.g140.t1

atgtcgggcggaggcggttacaaccatgcagggccttcttctgtaggatcacatccaccagcctggctcggcgtcaccc

gttcaagaaccaatctcttcctctccttccgggagtccgcgccacgcgcatctcgaggctttggggccacctcagcgccgttcataccgaatggcgca

ggcgagtcctcgcgtggacctgggaacgcacgccgcaaggggacctacttcgattcggcgggcgaactctactcggagggtggacaggcggagacagc

tggtctcttggagagcgcggacgacccggctggctacccaccgcggccgaggtcagctgctgtggatatgagcgcgctacctcccaaatgggtagata

tttcggacgaggtggattccatcctggcggccatcaagcccaagatcactcagcttgatcgcttacattccaagcacttgctaccgagcttttcagat

cggactacagaggagcgagagattgagcacttgacccaagggatcacgagggaattccgcaagagctccaggctaataggacaactggcctcgcagac

caacgcgctcgctgcggcaggcaaagcgagctctcatgagacgacgatggcacagaatgtgcaaacagcgttggcgactcgcgtccaagatttgagcg

gcgtgttccgaaagaagcagtcggtctacatgcaacgtatgcgtgggttggagattcggcaagcggacattcaggcggcgagtggactcggtgggaga

aacttgacccgagacagtcagcaagctgtgctggaggatgtcgagctgacgcgtcaagcatcgtcacgaaccaccgactcacagcttctgcttcagga

acaagagctcgcccctcaagattccgacacggcacagttctcaagacgtgaccgtgagatccaacagatcgccaagagcatccaagacctcgcagagc

tgttccaagacctgagcgcgctcgtcatcgaccaggggacacttttagaccggatcgacttcaacgtggagacgatgggtcaagagatgcacaaggcg

gtcgaagagctgaacacggcctcccggacccagcggaggacgggccggttccagtgcatcctgtttttgctactgtgcatcgccctgcttatcacgct

catcttcaccaaaccgctgtggagaagctcgccaaccccgccaagtcctgtccctccacagccgccgacatcggggacgggcggagatcgacctgctt

ga

>contig_14.contig_14.g141.t1

atgaggcctgacgagcggcttcacgagagcgtcagccacacgctgtcctccttggccatccgaggtctgaagggcgagg

ctgccgctccgcgctttctgctttggaagctctggcattcccacctgcccctcgcctcgacctcctcttgggatgtgattctcgacgatgagcgagct

tcctacatcactgctcgaaaggacgcccttcgtgccccggacggtagcctacccccggaactagatgcagactccagcatcactactgtagcaaggcc

gttggggcgaacgcagacgagctcgagtggcaaagtggacaatctgcaagtgaacaacccgctcagccttgaatcttccaacccttggcccgaacatt

ttgcatctttggacgtacttcaaaccatcaagaacgatgtcgagcgagcctttcccgactttgaagcggagaacacggagggagatgctggcgctgat

gatgaatcacattcgggattcttcaaacagggtcgggtagcacaagggttgacgcacatactctacgtctggtcgaagatcaatagcgacattggata

tcgacagggtatgcacgagcttgccggagctatctggctggccagggaacggatgagtgtggcacaatcggcggatgagggtcaggacctgctgtcca

aatttggcgacatccgttacgttgaacacgatacctacagcatctttgacgagctgatgcggacacatcgccaatgcttcgaatggaagcagcccgct

gcgcccgtggctcctcctggtcaggaagcaccaccatcgaagcccacagtctcggctatcattcaaagatgcgagcaggttcaggttctgatccggca

agttgatccgagccttggatccaagcttgacgagctcgacatcgaaccacagctttgggcgatgcggtggatccggatgctgttcctcagagaattcg

atctcctcaccgcattcaggctttgggatggtatcttcatcgcggacgcccactcactcgacttggtcaactttctatgcgttgccatgcttttgcgg

atacgaggcaagctattcggcgcggacaacagcatggccctacaactactgctgcgcttccctacggatcaagctttccctaccgacaagccggaagc

ggttgatttgctcattgcacaggcgaggcagcttcaatctgcacccacaccctcgactggtgtacaggttgtcatgcaaaaccgggacgagcttggga

tccaaatcgagcagccaccatcaggccccattgtttccgatccgccaagattgcggacagtggccgccacaatgggcccctccggtatgcgaccgggc

gaagcacggactgtgtctggcgcccgatcagagcgtcgccgaatcgcgcttccggatcgaagtggcacacaacctggcgttgcgtccccgacttctcc

atcgggtccctttgcgaatttcttgccgacgggagtaagtgaagcagcgaagggcctgtacgagcgctctgaaagtctcggcatcaatcgggcgctgg

cgaatacagtcacgaatgtgcaacgaacggtaaacgcatacgcacaacagggtgttcggagcggaggtgcggttgggagtgatggatttccgagaggg

ttcgacaacttgatacccagtacgcgtgccgagagggagcattccgctaccaccacgatctctccagttgccagtcctatcacttcttctgtcgactc

cgcacctgcggtagctcccagacccgctcccgcaccacggcaaaccagcacacgcgccttccaacgattcgagtcacctcctccggctgcatcctcgt

catcacaacttcagtcgactgcccagtcgcagtcgtcgccatccggacttttgccaaccgctctcggtgggcatgaaaggatggtaccaagttacgcc

acgtacacacggaccaaaggacgctctgcagtagctgcgtccgggacgtacccgcctgatcggaaggtcggcgaggagcagcagcgaagcgcacatga

cgcgacagctggtgtcggccgacaaaccggaccagtcatcactgccgctgggacaggaccgcccgttcgacagcagacggccaagccagtggctccac

cttcacccggcgatcctctcggtgctctctag

>contig_14.contig_14.g142.t1

atggaacgccgtttgggtcagcctttcaggtcgcccaacatttttccagggtcgcctaccaccaaaaccactaccacag

cgaatcaatcctcatccaccgagtatcttcacatacacgaacacacctgcgcacatgcgagcattacgtcctacgcctacaacagctgcgcgcgtgct

gcaccgagcccagaacttgccaaacagatacgaaacaggatggatccattcataagtctcagcccgcccagcattgctgcttctgtcgttcatgctct

gcttctgcccggctgccttcttttctcggctaccctggcccttgagtcgttgagtgcgtcaatactttggccacgtccgtacgtctacaatgccgttc

ctgctccttcgttgcctccattcgcactcatcgttggtgacatgctctcagttctggtattgctcaggccagctagagtgccaggaagcacagagctg

ggtggcgaggttatcagtcagtggagtgaagaggtcgacgcggggtcgcccaaactttccactactaacacgtctcgcgcgcgcttttctgccgactc

tgcggacccttctcctttctccgccgctgtccagctgggtgcttactgctggtaccaatcgctgatcacttcttctctcgccagtcatctccaacctt

gttcaatccttattgttgcagcctcttcggatcttcacgcctgctccttgccggctacagcttgcgctttgcgcgctctcgaatctcgtcatggcggc

aggcttcaagggccatccattttagacctgagccacacgaccgcgcagcaacgaactgcgcacgcgatcccaaaggccaaagcagactcgtccccggg

ttgcatacccccaacctctcacccgcgtcgacctatggcgacacataacgatgcacgggcatga

>contig_14.contig_14.g143.t1

atggtgacccaaacggcgttccataaacgcggaacccgccatgacgccgaacgtccaacaccaatcgccgaacgaccaa

cggccaccagcccaccactctcactactgagttcgtcgacctcatctacagactacagcccccagcgacgtcaagatcactcctcttcgctgctgcct

tccctcgtcctcgcgctcgtccccggctcgtccctgctcgccgcccgctcgcgctcgcgctcgtccccggctcgtccctgctctcggctcgtcctcgc

gctctcgctcgtcccctgctctcggctcttttgctctctctcgactcggctcttttgctcgctctctctcgacccgtcgctctctctcgactcgtctc

tttcgctcgccctctctcggctcgggctcgcgcttgtcttcggtctcctttttgtctttcagcgtacagccgtatacgtggacaatgaatctgatgcg

ctgctgccggcggaggttctgatcattttggaagaggcacgaaccggcctccgcccgtttcggacattcatcgaggataccgagcgcgatataatggt

cattccttcacatctcgtggtcttgcaggagctcattgacgcttttgcgtcactggtcagcgttcttccggctcgagaatacgattccaccatgcgtc

aaatgattttccttcgtgagcagctggacaccctggaacattcaatgccccagagaccgaccgagccactatggcaacgcaccatgagcggacgactg

cgcgtatacatcgacagcgacacactctgcgagctggtcgaaaaggaatccaccgacgatgtcattgccattatgctgggaacctcattgcggacggt

tgaacggcgtcgcgcagagctagggcttcggaaaaggggaggacctgcggttagcgatgatgaactttatgctgccatccgctccacgtacgccttgg

gctttgaggccggtgaacgagggatgcgtggagcccttcggtctatcgggctcagagtcagccgagagcagacggtcactgcctgtttcctcgcggca

actcaacaatggggctggcccagtcgcgtcagagcggatcatggcggagaaaatctgggggttaagggcaagatggaggaagcgcgtggtgtgcgtag

gggatcattcatacaaggtccttccacgcgaaaccaacgcatcgaacgcatgtgggtggacgtgcagcgatggacgacagcgaaatacaaagccatct

tcacgcagctcgaatccgatgggctgctcgatgtcagctcctcagtgcacctctggtgtctgcacctcgtcttccttcctcaattgaacgatgccctt

gcgcactttcaagattcgtggaaccgccacccgatgaggaccggtcagaaaaactggagcccggagcagatgcacaccgcgggcgttctggaagcaga

gaggaagggctttgatttgcggcggaggcctgagatgttggaggaggtggatgcttcggacgatatgagggtgcatgctctacgcgactttgcagatt

atggagcagacgatgggagagaagagcgtgtgcggagagaggacgatcctcatgtcgcggttgacccagtcgaacgtgatcttccaaccccgcttctg

gatccagccgttcggagttggattgagagtcaggttccttcaacctggccccctgaggaagatttgggcttgaacaggtactcacgggcgctgcagct

ggcgtcacaagtactagaggaatcaggatggatgtaa

>contig_14.contig_14.g144.t1

atgaacagcgtgctgccattctcatcaacgtcttccctgtccgcagtgtcaactattccggcgcgtccgggtctcacgc

aggcgtacacagcaagaacggtcgccccagactctgcctctcttctttctgcgcccgcccaccatgcagcgcagcgactccgcggtctcggtctccaa

gccggcccggtgcgcgctgctgctgctgccgccgctgggccctcgcatgactcttctctcacagccatggcggcccgccatcaaccgccggaggccac

gaagatcaagctccacaaggaaagcggcattgatgtggccgacgctggagcgtcgtccgacgcattttatcctgatggaaacgctttgcagtccattt

acgcaaggaagcaggcatacatagtctccatgcgacgagaccctccccagtccatctttccgccgctgccagcaggagcgccaccactacaggcggca

tttgacacggtcaaagtttctcaggtcggccacggcccaagctcaggacgtgccaccaccacgccccgtgcttcttcgggcacatctcagtgcggcac

ctgtgccgccgtcatgcctccacggtttttggccggtcacgaatgtcgtctcgctcgcgtgaagatggaaaatgtgaaggttgagaaaggaaagagca

gcaacactatcgacgatgcaattgaaatcctctccagcgatgaggaaattggggagaccccttctctggtccctaccatgtctcctgaaaaacaggcg

cccaagaagcgtaaggcctccagcgcgaaggagagtacgagtcgcctccggcgtgctcttcctgctgctgtcgcagctggggatgtcgagaacgaagg

agacggcgagaaagaaagggagaaagatgagacgaagcaggtttgccctgtgtgcgggaagaaggagacaccggaaccatggtcggaggacgagaatc

aatacacaagtaaggggaaggctgcagaaattctctgggtctgctgcggcgtcccttcttgcgagcagtggtatcattccgactgccttagcgtcatc

ccaaaggatggtatgaggcaagagatgcctcgtaagggaaaggcttggcgatgcggcgtgtgcaaggaggcaggccttatcgccaagaacaagtag

>contig_14.contig_14.g145.t1

atgtcttcctcttcttctgaaagcgctgctcaagagacgcagactcaaagtcaaggtgaggccctttcgcaggcgccgc

tcgcttccgccacccaggcgggtcagcgtaaggagaactgcttctgggcttcgacagaggacgagaagcttcttgatgtcctcgaagaaggcaaacag

cagggtaaggctagtggaggaggcttcaaacccgagctctgggttagggtccttgaagaaatcgagcctttgcacgagaagggtcgaaaaaaggccct

tgccgatgtgaagaaccattacaggaaccttaaggcccagtacaaggaagtgaaagagctggttgatatgagtgggtttggatgggatgaggataagg

gcgtggccacggccgacgatgcggtgtgggatgacctggtgaaacgtcgccccgagctcaagaagtggagacacaagtcgtttcccttctacacacag

ctggacagcctcaactcgaagtctacagccacaggtgccctttctcgggacgttggtgcagagggagaggccagcagcgactccgaaaacgactctga

gaagggggacaaggaaggcagtgacgatgagaatgatgtgggtagctgtggttatggtgatttcatctttctccttctctcatgggtcaaattcctgc

ctcccgcaagcgcaagcgccccctccggcgttggtgccctggcggagatcgccactgcgctgcgcgggatgagcaaccaggacgagggactgggagag

gccattaccgagcttctggatcgtgacagcgaggcatttgaggtcgacgagcttgcgtctcttggactcgcgctcgccgacaagcctcgccttgcctc

tgtgtacaaatcgtttgcagagagacctgatcttcgtcagggcttcctgcgtaagatcctggactcggacgcttga

>contig_14.contig_14.g146.t1

atggaaatggaaaccgaaatgaaaatgaaaaaggtgaagggaacggcgttccatagcttcttgcatgcacggttcacac

atagctttcagcgatctaaactcgagaagatcatcacttgtatggaacgccgtttgggtcagcctttcagtatggaacgcggaacccgccatgacgcc

gaacgtccaacaccaatcgccgaacgaccaacggccaccagcccaccactctcactactgagttcgtcgacctcatctacagactacagcccccagcg

acgtcaagatcactcctcttcgctgctgccttccctcgtcctcgcgctcgtccccggctcgtccctgctctcggctcgtccccggctcgtccctgctc

tcggctcgtcctcgcgctcgtccctgctctcggctttttcgctctctctcgactcggctcttttgctcgctctctctcgacccgtcgctctctctcga

ctcgtctctttcgctcgccctctctcggctcgggctcgcgcttgtcttcggtctcctccctcattccagcggctcgcgctcacgctcggttctgcgcg

gccactcactgcctcgcactccctctctttgctcgcccttgtctttcgccttgtccaccgttcacggtcgaccgcaccagcagcggaccttgccgatt

ccccgaaagtgctagtttcccgacgcgactatgagttgttgagagaagattatcggagtctgagaacggagaacaccacgctgcgcactcagctgcat

gaacgactgacgcatccagtgccagctccgccgagctccacctacagtgcgcgctccctaccagccccgtctcccacagtcccaccgcccacgcccac

gctcctcctctgccgagggttgcgcccgacgccgcacgcccgcacgcgcttgagtctcgactctacgcttctcgacagttggtcgacctccttccccg

agccccagcccacgaccccacagattctctgcttgtga

>contig_14.contig_14.g147.t1

atgggggacggagagatcgaaatcctcgatgatgccgacgatggtttgtgcatgtttgacggtgagatcgggagcaaag

acttcgcatcaaaacatctggctgccacgaacaccagcctgcgatcgccaatcatatcacgtacgaccgctatgaacttcgcaagcggcgcaggagca

agatggatcccgggctctggccatcataacgggcagggtctggcagcggagcatggacatggtcagcaggcgccgaacgcccaacgtatgggtcagcc

ttcgccgatacaaagtcagcaccagcatcagcgtcagcattctcagcacagctacggatatggaagacactctcacggtccggcgaacatggacgggc

agtacggcggaggccacgttcttcccggatcctccatggacccgcatggcgcgactggattcaacaatcgcttcaatcactacaacccggcccaaccg

cctccgtccgggcaagccccatcgacacctatgatggaacttgcaggcaatcttgcacctcaagcacatgcgacgcaccttttagaccaggatactcg

cgcaagctcgtcttatccgggtactctggattggcagcggaacggaggtcaattcgccgaggaacagatggaattgcggcgtcaggcttcgtctgctg

ccaccaagcgtagacgcgtgactcaggacgaaaatgagacagtggctggcgaaggagaggaggtgggagaggaagagggtcaaggtgacgatgcacgg

gctcggatcgaccacattacgagcactgcaacctcagaggtgggactcagcatggaatcgaaccatcggagcctggacttggacaggcctatcccacg

cggagtcaacacatggaacaagctggagaagtggatcaaattccatcctgagcgtttgacggattgggaggcggccaatccggcggtatcgacgccag

gagcaagtcactccgcacgaatctcaaagttctacgcgaacgcatcggcaatagttcgcaaggagttcgaggactggtggccgctacacctggtaacg

gcgccgcacgaggagcgaatggcaaactacaatcaggcccacaaagtcctaacgaaactaatgtgggacatggagcaggattacggggtctgcggatt

cgctgttctggcacatccagaccccgatgtgaaggccgcggtggtggcctctgagcacagcgaggacctcttcgacggatgcgtcaaaaacttaaacg

ctactgggactgtagccaaattcgccaacctgttcggtgcagcgacaaagaataatcctcctcccgcatgggtcatcgccaccgaacggttgaacgcc

ctgggcgaaggagaggccgtcaaggtggtggagaaggtcacgaaagtaccaacggaaataccctccaaagcggcatcgaaaacgttggtactgaacct

catcaaatggatcaacgatggcatgcgctcgatcagcaaggagaaggaatctgaatggatgaaggacaccaaacaactgaagagtatgatttacaagg

gcgttttcaagaggatcaatgacacaggcctgtgcgtacagggctggccaatcgaggcaaacaagatgttggacggggacgttaaagtgacggtccag

ggtacaacaacatcaagctcgactttcacgattacggcgggaagtcttcggaacacttcaaggtggaaaggcagcgaggtacaggcggttcaagaggc

gctgaacatgaacaccctggtgttaaagcgcgtctaa

>contig_14.contig_14.g148.t1

atggccacgtctccgagcgcgaccacgaccgtgccctcgtacaatctcagccaagacgaccataccatccagccaaact

ctaagcctaacgtggtctccgacggccagatctttgggttcacctgcccgaagatgggccggtctccctactacctcccgctttacttgcccatgccc

gtccactcgcccactctggtccgcgacccggccaggagcgcactgatcgtcaccctccgcaaacctcagcctggcgtgcacatcccggggccctcgct

cctccagccttcactccacccagacctgcccttcatccccgaaataatggacagcggcgcaaaaatccctgtcagctcgccggacaccctcgacaaca

ggcaaagctacctcggaatcgactga

>contig_14.contig_14.g149.t1

atgggtacccactcaggcgcatttgtcgaaagtgcaggaactgaagccagcataacctcatcagcactgtccgcagctg

gcgatgatgacgatgatcagtccagcgccgccgccaaagatgccggggtcagggacgcgatcgcggccgctgctcgcgttgcttcctcgccctctccc

ggcaagatctacccatacggcttctgcggcgcattcactggtcccttccccccttcatcatcaaacatcagcttgggcgctgcctccgtcactggcag

cgcaggccgcacagtggagagcgccagcggaaacgcgtccccccatcccgtccgtagcagggtacacgaacttcagcaagacgtccggcggcttgcgt

ggggacgtcggaggaaggttgctcagctatgtgaagaggagaagtgggacgagggaaggtatctggacggttacgtggatgagtttgaagaggtgtcg

gaaattctcgcttgggtgccagaggaccgacccggcccttcggccgcctacctcgccctcaccaaccagtccgaggcggaggaagccccgtcactcaa

gccgttcgacaagacggcgcgcgaggctcaactagctgcgctccagttcctcgttgctgggagtatcagcacaccggccatcatcacttcatccgacg

acctttctagcggcgccatcccgcgtgctgtcagtcctgcctcgagcataacgggcacatccggttttgcgcaccagccactggacggcgccatgttt

gcgaacacggtgatgtccggtacgcccatcttgcggggtgcgtcccctgatcctgttggtttgtttgccagtaccagcgtgggcgcgaatgctcccat

ttccattgcgggcacggcggggatcggtgcgggtgggttcggatcgagcggcgctatacaaccacacgcgacatctaaccttcccctcgtcacctcgt

cgttcgagtccaccatctttctgacatctctgggcgatttgacgcggccaacatatcagatcagcggtaggcgtcgaaaccatggtaactccgaggag

ttggctgcctggcgccatcattcaacgtctgaggttatcaacgcctcgacaacgctcactcctcaagcatcacatattggtacatcacagatcaatcc

caacattgcggcgatccagacaagtcacggcctggcgtcagaaatctacttcctgcccatctcccccgaatacgtcccgtatgtgcccgaacaggagc

gcccggacggcgtcctgcttaccttcggcggtcagaatgcgctcaacgtcggtgtcaagctggacaagatgggagttttcgagcgtgctcggcagaac

ggcatgttcttccttacagaaatcaacatctccgtcgccaaatcggaggctgtctctactgtgccagaggcactccgggcagcatcagagattgggta

ccccgtcatcgtccgcgctgcctatgccctgggaggtcccggctccggctttgcggaccatgaggatgagctccgggatctttccgcccgctccctga

gtctgtcgccccagatcctggtcgagaagagtctcaagggttggaaatag

>contig_14.contig_14.g150.t1

atggagcactttgatccgctcggtatccacaccggtgtctccatcgtagctgcgccgtcgcagaacctttctgacgaca

actaccacatgctccgcacggctgcattgaaggtcatccgccatttcggcatcgtcggagagtgcaacattcagtacgcgctcagcccagacagccgc

gagtaccgcgtcatcgaggtcaacccgcggttacgtcggtctagtgccatcgaccatgagctgatgctgctcgccaagcagcgcggtttctctgatag

gcagattgctcatctcgtgggcgcgaaggaggtcgatgtccgccgggccagaaaggcctttggcatcacgccttgggtcaagcgcatcgataccgtcg

cagctgaatacccggcccacaccaactacctctactctacaccacctacaacgcacaagcccacgatgtag

>contig_14.contig_14.g151.t1

atggtcctcggatccggtgtataccgaatccgctcttcggtcgagttcccctggtgtgccgttacatgcgctcgctgga

tccgcgggatggggcacaagacaatcatgatcaactacaaccccgagacagtctcgaccgacttcgatgaggctgaccgcctctacttcgaggagctc

ggcttcgagcgtgttatggatatctacgagctcgagcgtgcaacgggtgtggtcgtttcggtgggtggccagctgccccagaacattgcccttcgtct

gaaggacgtgggtgtgaaggttctcggtacggaccctctcatgatcgactcggcagaggacaggcacaagttttcttacgtgctcagtggtgcggcga

tgaacgtcgtttgggacgagtccaccctcgagcaccacttgactgctgccgcgcaagtgaacactgactacccggttgccatcaccaagttcatcgat

ggcgcccaagaaatcgacgttgacgcagtcgcacacaagggcaagctgatcgtccacgctgtgtctgagcacgtcgagaatgcgggtgagcactctgg

tgacgcaacgctcgtcctcccaccatcttctcttaaccagaacgaccttgctcgactgagggtcatacggaaacctccgggcgagggagagacggagg

ccccactcaaggtcatcgagtgcaatctccgtgccagtcgcagtttccccttcgtcatcaaggtgctgggcaagaacttcgttgacattgcaacatgc

gccattgtggaccaagacgttccagaacccatggacttgatgcaagtcgaccgtgactacgtggcagtcaaggcgtctcagttctcgtggacgcgtct

tgcgggcgcagatccttcctcggtgttggtgagtcgatttgatactaatgaaggtgctttatcgtctgctcacccgtccgaatctctcatgtaccgtt

ttcaatcaccagaaatggccagtaccggtgaggtcgcggcgttcggcaaggacatctacgaggcatactgggcctcgatcgctttcacaaccggcttc

cgcgttcctgaggcgaataagggtgtgttgctcggtggcgacgtgacaaagcctcaattcgtagagattgcgaagaagctctgcggcccctcggcccc

accattggcttccacctctggccagtcttcactgcctgagcggccattcaaacttgcgtccgatccttcccgctccacggccccgtcgtccaacgaac

ttcaccgttgcgcatccatcgttcgttcttccactgcccccgttgtttctgctctctcctcttcacttttggcgctcgtccacagtaagcacacacac

cccaccgccgatgagaactgttccacgccgtatcatggagagaaggtcgaatccagcgtcctccagtaa

>contig_14.contig_14.g152.t1

atgacgtctactaatcccatcgtcatcggttcgtcgtctccacccccgccatactcacctcgtcctccaagatacactt

ccccttcaccttttcgtgtcttgcagcccgttctcacgtacgcagctgttgttgcccgacgccctgttggaggttcgatcccggcccccgcttttcct

cgccacccccctctcacggttcagaccccctcctccgtaggttcgatcccgggtcccgattttccccatcatcatcctcccacgggtcagacatcctc

ttctgttcccgtcgttcacgtgatccccgactctcgagctccagtcagaggtacacgccccctttccccgggaccctcgttgagcgacgatgacctcg

ctttcactcctgtgcggcgccgcttgccaacgactcaacgtggtcgggtcagcctggttttggaggacgaggccgagtcggatgattctccgtccagt

gacgaagtgccccccagccaaacttcggaagggccaggtgacgatgtgcctgatgccgaggccatgtcaacggatggagacgaatcgggcgcatctgt

gcaagctatgctcaaggatgacggcgaatcagagtccgaacccgaacctgataccgacgaggatactgctgtatccacgcccccaggagaccagaatg

aagccgaggacgtcgataacgccaaggcggagatcatcaaggcgctccgcaatcctaccgggacatggcgtccctatgcagctgagaattgctccacg

cgcaaggtcggggtcaagtccagcaagactcaaggcaagaagcaaacacaccagtcctcccagagcaaaggcaagggcaaacgccgtcgatctccctc

gcccatccagcaagatttatga

>contig_14.contig_14.g153.t1

atggacgacaacgactcccccgatgtgtcactcgcgtcgtcatcatcatcatcattctcgtcttctaccgcgccttcgt

cggcctcgtcggcatcttcgtcttcaccctcgaagagaatacgattcaacccgtctgaggacaatccggacgtcgtctatgagtggattcacgacgat

cgatccgacgagcctctgttgccctggcgccacgacgacgtcggcaagactagagctgacggcccgggccttttccccgctcgtcgcctgtctaacgg

ggaagttcagattcagtatttccccaagttctggagtccatgttggcgtctctctttgaggcgcgccgctcgcgcgccggctgcaacccagcttctgc

tctcacccctgcccctggcactttcttctgcgaccagcacgtatctcttcaccacgctcgtgcatgtccttatttttgcagataaaggaagccgtgaa

gggaaccgctcgccttccgtttgcgtcggtgccggccccgccgccgttgctgctgctgaggacgatccagccgcgtccgacgcaccaccgctcggatc

cgacgtcgaaggacgagaaggaccctccgtcttatgcgtgtga

>contig_14.contig_14.g154.t1

atgctggttaccgcgtcggttccatgtggcacagagacgcggtactccttcatttttcaaggctttctcctctcactgg

cttcccacatcattgctgacgcgcactcgatacccttcaactgtgtctccatgcccgtagtacgagcgcataagcgtatgtcccggaaggtgaccgaa

gccgcacctcgacgcgccatatggtcccataaggccaccggctcaggcaccaggcccaccttcaccctccagaatcgatcggcacgacccttatccaa

tatttttgcggcagattccgtgtctacgggcactcaagatccttcaaccagcgtaccgagccgggatgacgactgggtggactatggcgattgtaatg

agggttttccgccgtcggccgacctcattcaaaccgacgtacccagagcttctcaacccggctacggacgaaaaggtcgccgccagcagcgcacggtg

cgtcgcaagagtcacaagcgccgggagaacaaggcattcaatgtttgggctgaagccttgcacacgctcgtcatcccatatctggaggcgacctcgcg

caacctccctggacccgcccaatgggcgtctttgcagtgtagctgctactctttgcactctcaaagcttcagagaaacagctccacccagttcccaca

tcgccctttttaacttcgaatcggtcgcaatgcagcctctctggtcgtgttcaaactacatgttagccgtcctggttcgcgccggcctttttccagca

acccccgtccgacctcaagcagcgttttccttcggtctcctgcgattctttcgctcacttcaggacaattctcgcgtcggtgcattcaactttatgaa

tgcgcttgtccagttctttggagacggtacgtctctgatggccgcctgtgcagccatcccttctcaagacgtcgcccggcgtcaactgcgtgcgtcct

ccaactggttccacgcactcgaaacacacagcctcgccaccgttcaacaaccccacccacgaccagtgacgatgctcccaactcaagcagagccaatg

gttgtagcaaaactgcaacggcctttccttcgcgaggacgatctctctccttccctggatgatctctgctctcgctgcccagcgtgctttggccattt

cggctccaatctcgagtcatctactgctgcccgcgccgacaatgaagccgaacctcagctcatcatttgccttgacggcaactttcagcacaaacgga

gacggaagaatgatgctgtcaacaggaatccactccctccttcttacttcctgtccgaccgtcagctggagactgccaagcatcatttcgaaagcctc

tcaagtcagcttgggcctgcaactggatgtggctctcacgtgaaggctgcaatcgaccagatggtgaagacgacgctgggtcctttcgacatcggagg

agtctttggaatgacttgccgccacggagcgcccctcttcctagcagacgtcaaggagtccggtgaggctcactactacgcgtacgcactaattcaac

acctggtcgacacgtgcggtatcaagctgcggacgcttggcatatgctacgacatctcatgcaaaattgatgtttcgccacgcatgaaaaaggctttc

cagcgtcagcccgtcaaagttgccttgacccgaggcatgacgaccgttcagcgcagggatactctttgctctcgcctctccaaactctccacgcagca

tcgaaccaactctgtaaggctcctcgcaaaacgcctccagcgagtcatcagccttttccaacaaggtatccacgccctccgcgaattaagaccgctcg

tatttgacatcaagaccacgttggagaagaccaacaagccatcgttggcagcacaggcttctacgtccacctcatctactctcgatcgtccaccccct

tcaagttcaagtacatccgtgcaacacgcagacacttcaaaggacgaagaatacatccggcgagcaggaatacctcctgatttaacaggcgtatccgt

ctatcttcatgggaaggccgttgcccgccgagaggccgcgtttcaaccagcgtccaaacgcaagaagaatcgagcaaagcgaaaagatatggaacaat

cttccaccgtcttaaacgaggtccaaagggctcgcgagaggttagccgacgccaccgaacgcctgtccgaacaagcacaagctcttcatttgcccttg

gctcaatggcacgctctcacagactcgatacgtcgacgccagtccgaccacaaccatgctctgcaagcgcggttatctgtgtctaaggcaggaacctc

caaaaaagcgaaggcgctccttcaacccttcaatgatgcggtcaagtcatacagcgacgtatcaaaggcaaacggcagacttcttctccctgacgcag

acgagcgtgccactcggtccgaacccaacagtgttctcgccactgtcgacccggtccacatcacggttgaggcgctcttcgacagagaaaccctgata

tacctcgccgggctcgtcacagcaccggacttcacgatagaaccctgggtctctagtccggtgctagctgacgccatgaacaggttagagcttctact

tcgcctgcaagaagagcgccggcgaattgacgtagaagtccgaaatgcctttgcatgggtcgcaagggaacaagcgcgattatcccagctgcatcagc

atacagcttctatagttgtcaaagcacatctcggcagacttcatgccaaactcaaggccttggaaacattctggcggccacatgcagatgtcgtcgcc

gcactactggcttcggactcatacagtcaaacgggagttccctccattcttcacgacaaccctgactcaggcgtcgagcgggatggcatctttactca

agacatcgaccggctatcccgaactcttcacccacgtcggcccagacggcgtcttccccaactgagcgccccggcgccactacacgtacaaagaatga

acgtggacccgggagctattgatcctccatcagacaacactgggactcctatggacgacggcgaagaacgcgatggcgaaggttctagcgcggatgat

aatttcctgaatattatagatctgaacgatctggcggacagcgacgatctgacggacagcggatactggacagaaccagaagatgtgtcgggagcaga

agaggatgaagaaaccgacgcagacaacagcgaggaggaccggtgtggagtcgatagcgaggtggaaacggacaacacgctcactctcgcgctcgccg

cgaatgaatgctcgctcggtctttctcgcgcttgccgcgcatgctcgttctccgccggcctttttctcacacagcttcccttcttcttcatcggagtc

ttgggcggcattgacggtgtctactttaccaaggacctcgcacgccagcttgccgttgtctacggacaagagcgcatcctcctccgtcctcgcgcagg

aaaactcggtctcggaaacagcactgcctacgtacactgccgcacgcatgatgatggcgaagtcaacgccgacgtctcccaccaccctaagttcatcc

cagacatgatcgccctccaattcgaggcatccggcccatcgaccggcgcagtcgtatacagctggatcccttggctgaaacccgtcagtcgaggcgtc

aaccccagacacgtcagaatcactggctccttccggctgtaccagcgacctgtgatcgagcgcgaggtcatgtcggagggcaagagaaagagggacga

ctcccgtacgctccagaccctgacctcggccttacaacccctctctggcgcctcccatatcgcccacaaactccagcacacccactacctcgccctgc

tcgccagtgtaggcgctgcggttgtgctgattgtgttggcgggcatcctggtgcctgtggtgggcgcggggaggacggggagtgcggcgttgggtgtt

gtgtatgctttgtttgtgcttctggcggtcggcgcgggctttacggggaccacgatgctgtatagcgctttcgttggtggggagtgggagaagcgctc

gctgcgcatcatcatgaagaccatcgagctcgagctcacgctaatcaaacagcgtctgaaggaatccgacccacgcacgtccggcaacctagctcaat

catcttcctcctccgaaacgacgactggtgctgctcatagaggctccggctcgggctcggcgcgacaacggaatgggggccagggccaggaccgtgac

gggcaccacacgcctgcaggcatgatcgacccggcgtcgggcaacaagcaacttcacgtccactggaatagtacttgggttgacctcgaggatttcgc

agaccccactctcgccaggcatctctacatacgaatgaaagcctggatgcggggacagcgtcgcattcgacgcgagctaaatctgatacagccgcctg

cccacgaaagggcaccgacgccagtcaaaattgagaagcaatccgcctctccctgcctgcctccagctgaaatgaagccacacccatcagacgaggaa

caagctctcccagacttcgacgcggtcgacgctatggacggcatcgaacggggtgggcggcccaagaagaaggcgcggcgtgggaaccgggccggcca

actggagcggaagaagaaccagaaacggccgtggcctggcgaacgtcttctcgaaagagtcacaatcggccctgcttccgaccaccctcggccccatt

cagtcaggagcagcacctccagccacttcctcctcctctccatcctcgcctcaccacccagcaacagcagcagcaacatcagcacccacgctacgctc

accagccgccacagcactacgctcatcgtcaccctacccaccctccgtacccgtaccactcgccgccaaccagcgcatacccacagcagcagcagcac

cgtcaccagcaccagcagcactacgcctcatcgtcggcctaacaccatccccaccacactccctcccattctctggtccacgtcggcgcccgccaact

ttgacacgggcggctcagaggaggaacaactgcccttccttgggctggcgtcaccgccgacgcgtcaacgtgcctgggacttactcaaggttcttgtc

actgccgatcttgggttggtcaaggcgcatacattgagcatcgtggcccccatcgctctcgtcagcgcctgggaggaacgcgatcagctcgtgcagaa

gaacatgctcgtctcgctcctacctcttctccgcgctctgccagaaacatggcgcgtcgcggactcggcgctaggcggagacgaggatcaggatgatg

atgaggatggagaggagggcagtcagggaggaacacaggatgaaggcgcgggcccatcccagcggaaatcaaaaggcggagtttcttcgagcccctcg

ttctcagctttcgagaagtggatacagaccgggtgtggcggcacgccggaactctgcttctccaccatcgtcctcttcctagtcacgcggcgcatatc

tcggccttttgcgactgcaccgtcttcctcgtgcatcgtttgcttaaagctcccactccctccccctctcaccaaccttcaccaagcaagtccgacgg

gaactcgacctcctcacggaactctacgaccttccgtctttcgccttacactgctccaacccacacccgaccacaccactcttctcgtccacaccctc

tcttttgcctcctccttagccgtggaacagcgtccgcaggcgacgacggtggtgacgcccttactctttacctgccacctcttcgccgttgccaggac

cgggtcacctgtcgctctcccccaggagccaccgcctttgctcgtgtacaagtggtgcaacggcatcaacaatctgcaagatgtgagggagacgagtg

agggcgagtgtggctggcacaaagtacgaacaatgcgcgccatgcgtctcttcggcaagggcgctctcgcccttatcgctgtgtcggccctcctcccc

ggctcagcaaacggcctcgaagtcagcatcacgcagctcacgtgcagcttctgcgtctacactgtaaccctgactgaaggcgaaatcaggcagggttc

acagatccggttcaccatgagcagcggtggcgcgaacggacagacaaagccggctgagacttctctccaatccgcgggcatttcatccaccacacctc

aaacttcgattactacgacctgggccacgcccttcaccagcggtggtgaggcccaagcagtgtacgccgtccttgatgccgggggcaatgtcgtcgtc

gatcctgctaccaactcgtcgtccacgtcggaggtcaccctcactgccaacctcggcacctgcactacgactaccagtacaactacgacgacgaccac

gacgagcacctccacgagcacggccacagctaccacgcctacgacgactaccgcggcccctaccagtaccgcgagtacgatcgcctcatctacgtcgg

gaactttcacctttttcaccgccattgcgacggccactcaagggcagagcactttcatcaccgtcatcacggtaacgccaacgagcaccagctctcct

cccagctcaggttcaggccagagcaacggcggagggtcaaatgccggcgccatcgccggaggcgtggtcggaggtgtccttggcctggctctgctcgg

cttcctgctcctcttctgcctgcgtcggaggaataagcatgatgagcccatggactgggatgggcctagtcagtcgagcgccggagggcccgggcgcg

gcaagctgttcaacccgatcatgcacgacagctctcatgccgagcctactgttcatccgggaagcttggccgccggtggcgcgtttggcggcgcggct

tccgggtatgaatcccacgacgggtatggtggctcctctaccatgggtgccatcgatgacgagtcacgggttcgaaggtga

>contig_14.contig_14.g155.t1

atgtcatctacgcgctcaatacatgaagccgcgtgttcggccacttgtgcgccagcaacaacgggtccaggagcgccca

tcacgtacgaccctagctcaacctcgccaagggccaactcgggagacgattccatggacgattccatgtcagagttgctgcagcagctttcggcggcg

ttggagcctaccgcgtcccaagcctgtattgcgagacttaatccggccaccagagaggtgttatcgtccgcctattatgccctcaaaggattcgtgga

tcagggaatcaatatggattggagtggagtacaggcagccctggatcatatgacggacgaagccgcccaagataccgtcgtcaacgtgctcagacagt

ggttcgacaccgcggcggtctgcaatctcgaagacgcgttcaggaaattcctgccgaatcaaggcggcgtgctcgaagccccacaaccaacgactgaa

agcacagaggctcagaaagtcaacgagactaacggagaccaggatatggatgacgaggaaggcacggatgacggccatgggcaaggcgcggttgtcct

cggcgaatcatacacgcaaaaccagagccattctagccccttcggtaacgagctttaccaaacccagcccgccctggcgaacacggacgccaaccttt

actgcggccctctgtcggcaagtcaagttcgctctctactttcgaagactatcagcgagaatgcaagaacttcgctcacgggacgttcgacggggtat

atgcagttctaccactttaaagggctcgttccggtcgccgaaacgtccgacgaacacggcgggcaagaaatgtggcagtgtcgcatctgccacaaaca

ttacaccaccccgaccagcaaggtttcgaacctaggaacgcatctttacggaactgagaagcgaaccggatgtctctaccgtcggggggaaacttccg

tcgagcgtgttatactcccaacccgcaacagagacgggacatcaaaagacctgacagcagaggatcctctggtccaaactcctcccgaaattgaggag

atgatctcctctttcccacccacccagaccttggcgcaggtccggtcaatgagcagcccaccctccagctattcgcccgcagcgtcagcagaggccta

ccaattccctccccgaagcccccaaccatcagcaatgtcagagcacctgcgcccgctgctgacgctccttcagcccgaatcgccttcgaccccatacc

cgagacggctgcgagtgtccgggcgggcggtgcggacgaggcctcatcggaactcgctcacgatcatcccgtcgcacacgtcaggcaagctgaacttg

aaggcggagtcgtcacccaccgcaagcggcggtattgacgaggagggcaacagtgatgataatgatggtggagagaagacgccgactccttcgatccg

gtgccgacataactcggttgcggagtctacgatgtcctgtgggcctgatgcggtcgagcgattgtcggactggttcggcgatctcatcacgcccgcca

tctacgccttactcgtgatcatcggactcccactctacttcgtgctcaacttctccctgcccctgatcctcggcctgaacctgctcatcttcatcggc

gccatcacggtcgttccccccaagatccgaaggttcgcccacctaatcttggcgacctccatcttgaccgtgctcgtcatttgggcgctggctgcgat

gaaggggaacggcttgaaggagacgctcttgggctcgtaccaggtcggggagaatgcggttccatgctaccgctaccgagccgagctgagggaatcct

tcttcaagctactccgcgtcctcgtgccctgtgctgcgctctccctcttcggctggccgggctttgcccaattgatcaccctggaccgatcacgaagt

ctggcgtttgccgctcgcttcattgcgacccccttggccatcgagttcaacagcacgttgggcggtgacgaaaacaaagtggtcattctcgtcgtcat

cagtgaagttgtcgctgccatcttggaagagcccttcttcaagctgatgcaccgtgcacacgcccctaacttcccgatgccaacgccagagacctcac

ccaaccgtggcaacaacaagatgcaccgacggccaatgtggccctatttctccggcccaagcaccgacggccagccctcaggttccatgcgaggcccc

cacatcatcgaacaacgtggttcaggcgttcaggccgggccggatgccgatatgtag

>contig_14.contig_14.g156.t1

atgagcacctcggctcccgcactgaacgagacccacgagcggcccacggtctggtccaaggctccacctcgcaccgttg

acgaaactctcgtggctaacgtgactacggagctcagggagaaagatgactggcgaaagaaggcgcttgacccagagatcatggccaaatggaaggct

gaagccatcaaggagttcaaattctctgagtcgatggcccagtaccttgaggacgagctcaaatacgaggccgggctctggcacaatgaagagctcgg

catcgaaagcacaggtgtgcttggcgtagttcgtagtgacaagctggtcaaggacgatgtcatcgagaagctccgcctgctttccagcgagctcgagt

cagggccagagaaggattggcatcccgagtccgatgaaactgtgcttgatgtcgtgcatccttcgttgtactgccttgtcgacgaccatacccggctg

cttggtgaagacgaagaagcactgttccccgaagacagtcgagacctgggcaggtggtttggcaaattgcccgatatccacgcaagcgacgagagcct

tcaacgttccgacgaggtccccgttgcacacacctgccaatggcttcctgcagacttccaggtcgacgagcagggcaaggtcaaggcgctttcctaca

tcaacaatctccatccagaccgcagcaagccaacgggaaagctgtacgatgtcattgaagagacactgggagcattcattccaatgttcgaagcgacg

tttgccgaatatcagcttgaaggcttccgaaaagaattccgaaagggctcagacaatgcccactggattgaagaattttggaatccagctgctcctgc

ggaggtaccggtcagcgaattggcgaccacagcagaagatcgcacgtatctcaacaccgatggtgaaggcgcgcacgacaaaagtgcagagtcagtcc

aagcaactttcgaccttgcctcttcggtcactgacgctccactgtccttctacttcccagtcgcatcgagtatgcccgaagagaatattggaccgaac

cgagagtgggtccggattccggtggagaagtttgaacctgtgaagatcaacccaatcagcctgaagaatcgacgattgcaagtaatcgtcaagatggc

gaccattcatctcgacccgaagaagccggaatacgaaggcggagcatggcatgtggaagggacgcatcgagagaagatcagcgctactgggatcctgt

actttgaccaggagaacatcgaagaatctgggctgaagttccgtggctccatcgacacatctcttgcgacagaagaaattccttatgagcagaacgag

tttgacgcgatcatggagctctacggtttcggtaccgactcagagacctcccaggtactcggcaagatcagaaccaagcccggccgttccatcgcatt

cccaaatggtctccaacaccaggttgcacccttcaagcttgctgaccctacaaagcccggcttccggaagatccttgccttcttcctcgtcgatcctt

tggagaaggtcatcagcacggcccgcgtgccaccgcagcaatacgattggatgcggacctactacaaccaagtgttcgcttcccttcctccaacggtg

cctaccgaaatccggcaaattatccttgcgcacgctgcaccagctgcgatcgtgtcacgcccagcggccaatcgggccgaggacggtcagccacagcg

acaacgaccccgtctgaacgcggacaaggacgaatcatgcggcatgtcgattgcccaggcgcgcgagtaccgccttgcgctgatgcgcgagcgggcca

agcaggctgtacacattgaggaggagaatgagcaggggttctcgttttgcgagcactga

>contig_14.contig_14.g157.t1

atgctgttgacgctgctgctcggatcacgtggccgagggcgcgctcgccgtcgcggtccaagccggcgcgatgtttgcc

tcgccctcgccgtcgggctcctctcgctgctggcggtgctcgggctcggccgctccttcgtcgacctggccagctcgctcggtctgagccagcagtgt

cgcatgtcgcgcatgtggcctgcctatgctgtccaccatcccgtctcgccctcgggcctcgcgcgcaagtactcgctgttcctctaccgcgagcgcgg

ctcccacggacacccgcaggacgatcggcccaggggcacacccgcgctcttcatcccaggcaacgcaggacattatgcccagattcgcagcgtcgcaa

gtagctcggcaacccagtactacgaggataacggtgccagcatgcccaaacaggaatgggcccgtgcccgcggtcccgtcgactggtggaccgtccac

ttcaacgaggacttctccgccttccattcgcagacaatgctcgaccaggccacctacgtcaacgaagtcatcgcatacctgctcgagctgtatgcgcc

gatttctagccataacgtcacttccgtccccattctcgctcattccatgggcggcgtcgtcgctcgactcttgctcgaccagcccaactacgtcccag

ggagcatcgacaccatcatcaccctcagcacaccccacgccttcccacccgctccctttgatcgtggcctggagtcaatctacacccgcatcaatcgt

cctgcgcacttgtactctaggcagatgctcgatgcaggcaacaacgctacaaagcaagaagatgtttggcgctcgttcccccactttgccgatgtgct

cctcatcagcatcggttcaggcgctcttgacacccagatcacctccgagagctcatcactcgccctcggatctccctccctgcccgcgctctggccaa

gtgcacaagccatcagcaccttcacatccgccctgcccgctctctggagtagcgtcggacacgtctccatggcttggtgcgaccagctcagggaacgc

atcgctcgcgccagcatgatggacgcactgcttttcgcagatgcacaatcccgtattggtgttcagcatacgcgccagcgcaagaaactttgggagcg

cattcttgggagcaatgcagagtggggactcgatgaacctgatcggcaacttcagcattcgcttccgcttccggaatcgtcccaaaacattcagtcgg

cagatttggaaggtcaagactcgctttctttcgatcttctaggaaagaatgcaggacccgccctcgacatcgaagtcatcacagacctagctgtcggc

aacgatccgactctcgggttaggggtagtttctcagccagagctcgtgatcctcctctgcaaagtccccagcgcacaagctagtccgtctaattgtac

aatggcgtcacctccctcatacgaacttctcccaccgtcctccttccgcccgacgatgtccgcaccaacccagttctcaccttcgatcctcttcccac

acgcagagaccaggtacgagcttcccggccagggactccgacgatggcgtgccagtgtggccgagcttcgcgcgtccggttacagctcggtgaaagtg

tccaagctctcgaacgctcgaggctcgggctggttccgtgctgggtggactccgtctgctcccattgttgtgacacccaaatcggcacaaaatcccgc

tggggcactctcttctgtcaggctcgacattgctgacgctctcagtcagcacccgtcgggctcggacattggccctgtcgtccgtttcctcgtcccct

cgatggactccagtctgctcgcgtatcgccttgtggtcaatatggagtttgccagttccgattctccacctatcagcagtacgcttgccttctcgccc

atgctgcatgctcggcataggggcactggcgacgcagtctggttcccttctcttttggaagctcaggagagaaacggcggcgcgatcagtccatcagc

tcacaatgaccgtctccgaatgagcctgtatgccagctcccctttcatcgcacccgcctcaagcgcccagcaaggcacgttgttctccctgttcttcg

acccgagtgcgctgaaagcgtcgagcgggagggacaagaacgctacccctcagatcggaggacttcacagcatcacggtacagatcgactggatggcg

agtctgggtctcctcgcgatgcgctatcggttcgctgcgctcgtctttcccttcgccatgctctgtctcatcgccggtctgatctgggacgactggaa

ctccccatcggactcggagagcaaggctcggtccgaagacggcgatatcgagacggctttggagaaagtccccttcccggccctgttgtcctcgctct

ggatctacggcccgcgcgtgctcttgatcctgagcatcgcaagcatggtgctcgcgttgctccaggcggccgtcgtgaagatgagcagcgctcaggac

gcgtatgaccagggcatgttgaactggcttttgggctacccaggtccggcttcgttctccttcatcgcccttggacccgtccttcttctgtccacatt

cagcatcctcgtcgtccaggtcgtcttcctggtcggactcgtccgggttctcacgatgctcaccgcaccattcacaggccggtcttctgcgagtgggg

atgcgggtgagggacggaaaggcaatgttgaggttgttggctattcgcgaacgacccttctcgccatcgggacagtcatcgtcgccatcttcttcctc

atcccataccagctcgcgttcctcatcgcgaccatggtccagctcctcaacgccgtccgagcctcggcgacctggagtcgggagagaagacaagccgg

atcgtccgctcacggtaggatcactgcggacctgagcgacagcgcgagccggtatcaccaacagatgctggttttggcgctcatgatgggtgtcctcc

ccattcgggcccccgtgctcgtcgtctgggcacgaaacgtcctcttgggcatcagggcaccggcgacgactccaggcttgggcgatcataatgtcctg

gagattgtgtcgttcatcctgtttgtgcaggtgggcacttcgggacgactccttcaacgagcgccgagcaggatgcttgcgcgtatcacgcagctgct

tttcagctttgtagcgattaagtcgctgacgtggggcttgcggtacacctatctgctatatgacggcctcaacatcatctttgcctggcttctcttcg

tgcagtggagaacgcggaagctcggtgccagttcgtcaacaacagccggtccgtcttcacatgactatacactccttcgtcagcgtggtgactatgac

gggattgaagagaacgctgcacaagctcgcgctgaaaacggtcaggacgctgctgttcattccgccacagcagggccttcagcccttcaagctcggca

ttcgagaccagggccgagtcctgcgctttatgatgagagcgctgctgctcctccccaaataatcaaagcgaacaactcggcacccatcctccgtatgg

acattcccgccgagctctcagcgtccaccgtcctacctccttccctcgcagcctccgccgatctctccaaccccagatcaaagcccaccaccacaatg

cgcactcctcatgagaatctggacgacttgctcaagcgctatctcgacctcgcagcgacgtatcacgccttacgcaccaggagcgccaaacacttcgc

gcagggccatattgatcttgtgcgcgctcggatgcaggtcggtgggtggaacttggcaaagttggggaaagatgggtgggacgcccggttggctgcgc

agactcaggtgcgagcggtgtgggtgaataaggaaagggaagggcaggaggaggagtcgagccggctgcttgggttcgaaacattcactccgcgtccg

tcgacggacgagggggacgaagagaaggagggggtacaaccgtatctcattgacgaggacgacgcgcccgctgtgctgcgacacaggcggaagcccaa

ggcgacctcggacgatgctacggctgcagagacggataaggatggggggaacaagcatcaagaagacgaggacaagaaagagaaggagaaaaaggacg

gcaacggcgatgacaagaaggccaaaagaccgccactacctcccgacccgctctaccaattcgcagccctcccgcctccctcgctacgatcagccaaa

agtcacttcgaaaaggctctgtccctgtctatcggcggcagcggcagcggctcacagacagcggataactcgcaccagccggatatcatctccgtcgt

ccttgagatggcgcagctctccgacgctatagactctgccagacgtacgatagaacagtaa

>contig_14.contig_14.g158.t1

atggcagcacccgcatctactaccgctccagcaccgacggccgcggcagccaatcagcagccggcatgggtcctcccca

cacagcgacagcccacgcccgccctgcacgtctacaactcgctcacacgctccaagactcccttcgtcacccgcacacccggccagctcacatggtac

aactgcggtcccacagtctacgacgcctcccacatgggccacgcgcgcaactacgtctcccaggacatcatccgccgcattctccgtgactactttgc

ctacgacgtacacctcgtcatgaacatcaccgacatcgacgacaagatcatcgttcgcgcacggcatcggtaccttctcgcccgttttaaacaggatc

atgcggccaagccgatatcgaaggaactggttgaaactgtgcgcctcgcttggtccgccttcttcaagagcacccttcagtctctggctcctcccaca

gctcccctcgacgacggcgaagcaccgtccgagcgcgcggttgaggacgcgcaatgggagtccatttcccgtctccggaccactgacccggaatgggt

acgcgtcaattcggaaaagcagcccaagttgagcatgtggttcaccgccctcgacagctcccgtcaggccctcctccccgccgtcgcctctctcgcat

tcagctcatcagcgaacacagccacaagcgattccaacgacagctcagcggaactatctgctaatctcatcaatgcctccgccgatatcattgccccc

tacctcgacgcacaacaaggctctggcatctccaacccctccatcttccgatctctcgccgcccactgggaacgcgccttcttccaagacatggccag

actccgtgtcgaacggccaacgacactcacacgcgtcagcgagtacgtgccggagatcgttacctttgtccagaaaattgtccgacgcgggttcgcgt

acgaggacgaccgcggtaatgtctggttcgacacccgcaagtttgatggcggccaatcgggagcattggaagaggatggcaaggaagtcgatgggcac

gctcgggatggagaagatggcgaatggaagcatacctatgccaagctcgcgccctggagcaagggcaaccgcgaactcatcgaggagggcgaaggctc

tttatctacctcgaccgcgaccatctccggtaagcgacacgcgtccgactttgccctctggaaagcttccaaagcaggagaacccgcttggccatcac

cttggggctccggccgacccggctggcacattgagtgctccgtcatgggctccgacgtcctcggcgcccagatggacatccacagcggcggcgaggat

ctctgcttcccgcaccacgacaacgagctggcccagtccgaggcggccttcggttgtcgacagtgggtcaactacttcttgcacacgggtcatctgca

catcgaagggatgaagatgagcaagagcttgaagaactttatcacgattgaggaggcgctgggcaagtatactgctcgacagcttcggttggccttct

tgaatacgacgtggaatgcgaagatggactttgggaaaaacagcatggcggctgttgcgagtgcggagcagacctttaataactttttcgggaacgtc

aacgccatggttcgagaagccgccgcgcgcgaagtcgaggaggccaagcgtgccgaggctgaagccgctacattagaagccactgccgatttgtccac

ctcgggagccaactcgtcatcacattccccaatcaccgtaggacctcacggctacaacaaagccgaagcggagctcatggcagccctgacctcatctc

aagcggccttccgacgcgccatggccgactccttcgacacgccccagggtctgagcatcctgctcgagctcgtatcgaaagtgaatgtgtatgagcgc

tcgcgtgcggctcgggctgaggtgaatctgggggtgttgcaggctgtgggcagttgggtggggaaaatgttgcggatgtggggactgggtgaggggag

ggttcgggagagagagggtgaggttgggtggggtactgcgcctgaagttggacgggacttggagggtgatgcggccggttcgggcgaggttgaccgag

aggcgctacttgcaccttacctacgcgtgctttcgagcttccgcgacgcgactcgttccctggcacgaaccgcagcttcagctcccgtcgaccccaac

tccaaaccgaaatacccaagcgaactcctcgccctcgccgaccgactgcgagactttgatctggccgagctcggggtgtcactggaggaccaggacga

cggcaaggcgctcgtcaagttcgtctcagccgaccaactccgcgcagcgcgagaagagaaggaacgagcgatccgggaaaaagaggagcggaagcagg

ctgccaaggtagccgctgaggccaaaaggcgcgagatgctgctcaagggcgccacgcctgcctcggacttcttccgctcgaaggagacgtgtggggag

gtctttggtgggtgggacgagagggggatcccgactttggatggggaggggaaggagttgagtaagaagaagaggaagaatgtggagaaggagtttga

gaagcaggttggggcgcatcaggagtatcttaaggcgaaggaggccggcgagatttga

>contig_14.contig_14.g159.t1

atggcagcagtggtaccagcagcagcaggacagggcgaaggcggcgcgcccggggtcgacggacaggacgggcccgttc

ccgacatctacccggagaacctggcgtacaacgcgaagcagatctacttcctccgctcaacagcactctccctcgcaggcgcaagcgcaggcgtcctc

ggtctgcgcaacctctccggcttctacttcttcgccgtcactctcctcctcacgaacctcgttctgcttgcgtacaacgcaagggcccagccgagcaa

gtacgtccctcatctcggcacaatcgcgactcagccgggcgtggtgggattggcggtgcagggcgggcaggagaatctgttcagttacgtgctgtggt

ggactttttggtacggcatcgttcatggtgagtaa

>contig_14.contig_14.g160.t1

atgctcctcagcaccacgcaggctgccgcggttctgtcagcttcctgcggagctgaaggaggagccgctgctggtggcg

gtggacgagcttcccgctgtcctgcgcttcgaaggaattggccggtgcggatgcggaaggagcttcgatccctcccagccaacctctactcgctcctg

tatggtcttccatactcgtggtgtggtgaagagagaggtagaggtgcagcaatgcacatgcagacgacgcggagagaccagaacgattggccagatct

tgcgtcgtatcagctcttcaattggtctgtggatctggtcttcacacacgagctaatgaactcctatacttcccagcttgctgcctcccctaccccac

tttctgcattccacaccaccatcacagacgcctacatcgagaaccactgcgatatacacttcgtcgccaaatccacattcatcaaagtctacttcgcg

ttcgtcaggctgcaggatgtcaatgttgcctttgcttgcccctcttgtggccccaatccatctgcaatcatcgccgatggagtggtgttggctcatag

cacgaagcttcgtcatacgttccttcagcctccaactacgcctattggtcacacaaatacccgagctatgcccaacaaagtactccagccattgccaa

ccttgcccctgcgaaccgctctccgcgaggttgcggacaacctgcttcctttcaccaatgtgcagcgcgccatctctttgtccagcttccgagaatcg

atgaagagtgcgaatctacgcggtctcgctcctcagcgtgaagagttcctccgccacctccaaacgttcgtcgattgcagctgtcgaaacgagctcct

tcgaaatacagacctttcgttcgccttacgatgcttgtttcgcgagcttgctgccgaagagggcatttggcaaatgtgtcgtccgatttgcgtggagt

cgttgcaacaactttcgagcagtgcggtcgaatttccctctgcagcctttactccccccgtcgtccactccattcgagaccttctcagctcaatagcc

aagttggtgggattgcagcacgtcatgttcggtatccccaaccccaccaccagcccagaccagcccaacgaaccaatacaacctgcattcgattccct

tcgtcagccgagcgtcccctacacacagtctggatccttgtacggcgcagctcgatgtcgtgacaggcctctttaccccagatttggggagaaggccg

atctaaaccattcggacgagcagcagagttctggagatggtcctggtccttgcaggaagttcttctccgactacgtgtcgcagcgacagacgggcggc

atcatggcgctctggtgccgtcacctggtgtgtgttgggttccacatcattcccaagtgcgagggacggaacgatgtcttctctgcggtgttcacaca

ctggcccaagccacccgacacgatcatctacgactttgcctgtcaactggcgccgtacagcctcgccagggagcctgcattcttcaagaatgtcctgt

ttgtggtggaccagatgcaccaacatggtcatgctacgtgcacaacgtcttcgtttctatctacttacatgaacacgaatccagagctccagaacatc

aacagctctgccgcagaatgtggcaatgcaggactgtccaggatcaagaagtccgtctcctactgcagccaagaacatgcgatgctcctgatcaagca

gttcctaggtgtatggaacagacgacgtatgcgtgcaatgttaaaggaatga

>contig_14.contig_14.g161.t1

atggcgaccaaaagggcaaagagcggtgagcagcgcgtcagcctgattgaaccatgtcccgatgatgctgatacgagta

tacgaccgtttctcccttccagcccctcgctgcaacaaaggctgcacagaccccgacgacccaacccagaaggctctgaaggcgcagtgcgcctgcag

caggaagaggagcagtcagttacagaaactcagcaggactccagcgcgggcgcaccagcacctgctcccgctccagaccccgctctcgcccctggacc

cgcgcgcggcacctcacagacaggctcagagtccactgctgcgcggcaggggggagaatcttcggcggttttcgatgccgccagcacgattgccgctg

tcgatcgtctcctggccgaaacagcatcccaagcagctcaggcagcagccgagcttgatgtgcagctttcaatgccatcttttgcgcgtacgcccgcg

aatgcgattcagggcgcggacgtcgatcctcccagtcccgcacctaccagtccagcagcaggaaatggcaacgatacattaaacaatgagacaaatcg

cgaatcccacgtgggaaacatcgaaggggaagcacttcccgcggcagatcaaatatcacccgaagtagcagcgggagcaggagtggaaaacggactgc

agccctctgctgaagacaatgcaaacgctgaagacgacgaggaccagactgggccgatgaaagattcagagcagcatcacatatggtacaaccttggc

aatgcacacgattatgttgtgaaaaactttaagagggagcctgtcgccaaaaagagaagcaagattctgaaccggtccctatggagagtgatcaagtc

cgctgctgacctttccgcacgaacggacacggctatgtttctcgcctgggcaacgttggagcccggaaagcagaagcacaagcaagttgtctgggcga

gcgagaacatatgcgatcctgctcgtccggtactgcacagcatgactcgcgccatgcacgataaattccacgccgacattgccgtctatcgcgagaat

caagtcgcggaagctgcgagacatgcggcagagaaagcaacatggcaggcagagcggatcgagcttctctcgcgcatcgcggaactccagcataatcg

tgccgacggtgagggaggatcaggatcgagctcacagctgtag

>contig_14.contig_14.g162.t1

atggcttcactcagcttcccattgcccgacagctcttggcgccttgcctgccctttcctcagccgacctcccagcttca

gcctcagccaaccggaagcacatcgaggcgatccggcatccaacgccgtcaacaggacgccgccatcgagccagcctccaacatcctcggtctgcgcc

gacattattcgggatcttgaggagctactggaggacgaagaggaggaggaagtggtggtcccgagttcggactctctgatggaagatgttgacatcac

tctccagccgtcccccgtcaaggcaatcaagcgcaagcgacgccagtcgatgtcggacagggacgcacgggagttcctccgcttcatgtcgcgcaacc

aactcggcatcggcgcggttctcgagctcttctttcctggcccggattccgatagcaaggccgccatcccaggcgacgatctgctctcttccggatcc

gacttgacggacaagacgtcatactcccacacgatgcactttcttcggaacgggaccacacggtggttcaacaaggttggagcggtctccattgcgcg

caggtacaaggcagcgccacagcccttgctcgacttggtgctggaaatggtcaagaaggagtccaagaacgcctccaggaacgagtacttgtgcggcc

caccagcaggcaagaccaccatctccgatttacgcgacttcgatcccgaggagtccgctcaactcgtcaggcgagacatgcccacggtcagcacagtc

ttgtcggcaatcgcgggagtgcgggacgatgaagcggaggagactgaggtcacccgcgtggaggacaccgagggggcgggggacgaacctatggtcga

acaaatagaccttccgggacgcgtccaggctgtcagaattcttcggcggtacggtcatcggtcacggtccgcagtcgtaacatcggcggtccactccc

tcctcttcgcccgcaaccagcagtgcaataggtggccgttgcatctggcggagcgcgcgtacctcgtcgctcctcgcgtgttcgacagtatggcgaag

gacgaccatcggaggagcctcgagctccttgcgcttccgtcaaccaccttcagcatctcctacgacaatgtcaattggatgcgaggaatacgggacaa

gcgcgtggagaagaaggccgtcatgatggcagctgtggcgggaatggcgtacatcctcaactatcaagcccagtacgaccccgatacacccatctgct

ccgagagactctatgagtcgacattcggctcggggctcttgcggccgacgacgacagtgtgctcacagttacttatcactaagtaccgcaaccaacaa

gtaggcgaagtgagaagtgtgaggattgattga

>contig_14.contig_14.g163.t1

atggtgatgagagtgacctccaccacctccggcgtctcgactgaaagccccagtcccgccacctccgcgtcctccggct

ctggcgcgtccgcgaaggtctacaccgttaagaccgaggacaccggcaccttcgacggcacccccgaaaacctggagcttttcctcgctcgagtccag

gcaatccggttcgccgaaacggacgcgggatgggaccgtgcccgtgctggcctatgccgtatcgattcctggtttgaggccctccgcgagaatttctc

gccgcaatccaccgtcgtccgccaacaagcccgcgaccgaacgtgggatccggaccacgaggatatccttggcttcgtgttcgcgaagatcgccttgc

tcaaggtcggattccggaacatgtccgaggaggatgtcgtccaggagatcgccgatcgtctgcccgtcgacatccagatgcttctccgccagccgcgg

gagcgccaaccatcgctcgtccggcttcgagaggagttgcggatccaggaggtcttctggcgcatccgccacaatcggcccctgcttccctcctcggc

cgtcgagaccgcgagcccgtctctcgtcacgggtggccaacggacctcctcgtttgccaagttgatcgttccggccgcacatccggtctccgcccagg

cttcgtccgaccgtcgccctttccgtggctccgtcgcccagcgccgcgggaaggcatacgccgaggacttcgatccatccctgctgggccgcggaaag

agtcctcgaaccggaaaggagaacactcccaattaccgggtgcccggcaccgatgagacgatctggtgcgagcgaccctgcaaccgctgcggcggcgc

tcatttcgacttcgcccacgaccactcggtctcgaagccacaggctcggttcgctgaggaagcagacgacgatgcaaagagctaccccctcttcccgt

accaccgcatccacctgaacgatgccggctcgcctgccctctcctcgttgcttgacacgggtgcgtcgttgtcctgcattgacgcggcgttgctggca

aagctgggcgggacgccgagcggatcacccatgcgcgtgcatggtctaggggacgtcgagactctcggctgggccaccatcacgttcttcgttccggc

tcgggactcgcacggcttcgccctgatgttggaggtcgagctggacttccatgtcctgcctgctttcgcgcctggtctgctgctcggcctggacttca

tccaaggccacggtgtgaccatcgacgcgccgcgcgggaaggccactgtcatccgttacgcgttcgatgttgccgagcatctgccgagtcccgctgcg

aaagaagctgtcgtgtgttgtgtggagtccgtctgcttgccgggtcgatccatgacgtggatacccgtggatgttgctgcgttgtcgtccgccgttga

ctacacattccatccgcggttgatgaccgacccggatgaaacgatcaaggtagctggtcctgctgcgctcggcaatcgctcgacgacgcatgccctct

tgggaaactacggcacacgccccgttcacctcgaccgccgcacacccgtggctgacgccgttgcagcccagcttggcgacgtgtctgccgagccggat

gtgagccaccgcttcgagctcggctacgctatgcctccggccatggtgcaggccgctgcgaccgaccctggcgcctggacgccctctgccgccactgg

tgatgacgtcgacgacagtgtcgcgtctcctctcgactcgttcgagaacgtcgaggagccggccgcgtctttgactcgtgacgtagagaccgtcctcg

tcgatgaccatttccgggtcggcgtcgatgcatctgggaaaccgcatcctgatgtcgtgtcgctacttcgtcggcatgaggcggcctttgctcttgat

gggcgaccagggttgatcaggggcgaggagatgccgattacgttgcaagaaggggcgcaacttcgtttgactctgctggggcggaaggtgtcaggcgc

gggtgtcgctgtgtggcaagaccgcgccaaggctgtgttggatctgcccgcgccaaccacgctccgggacctgtaccatgtccttggcctgtttggat

attaccgcggctttatccctcggtatgcggagcgggccgagcccttgactcgcttgacccaaggttggcgctgggagtcggtcggcgatcggacacgg

ttggtccggaaggacgggaccgtagcaaatgcagacaaagagcgtctggagtggggcgacgctcaggagcgcagcttcgtcgacctgaaaaatgctat

cgctcgcccgcccgtccttgcgcatcctgacccgacgcgcccgtacgtgctgtacgtcgacgctagcaaggacgcgtttgccgctgtcctgcaccaga

tcttcgaagatccggtggctgatacggccacagctgcgttccctcttgcgcctgctgctttggtgtcggctgagcgctggttggcctggcttcggacc

gaccgtttcttcgggccgattctccgccgtgttgagagcgacgacgccagcgactctgagtggattgtcgatcaaggcttgctgctgcgccgtgtgga

tggtcggttggctttgcccgaggccgctgctccgctggtgtttcgttcagtccacgatgacaacggtcattttggttacacgaagacctgcctagcag

tgatgaagaactactggcggcctcggctggcggagatggtgtccgtgtgggtgcggcactgcgctgtgtgtcagcgcgtgaagctgagtcgtcgcgtc

ggcgagttggatgtggacaaggacgcgcagttcccatttgagacttgctcctttgatctcgtgcttggcctgccacggtcacagtccggaaacgatgc

tgtgctggtgatgcacgatgtgttttcgcgcatggtgttgctcgagccctgtaaagccaggatcgacgctgccggcatcgctgcgctgatcagcaacc

gcatcctgcggttgggctggcggcctcgccgcttggtgtcggattccgaggcgcgggttacgggtcgagtaatgcaggcgttggcggactcgttgaag

gctcggctgaccccatccccgcctcaccaccagcaggcaaacgtcgtggaacgagcggtccagacagtgcagcacgccttgcgcgccctgacggcaaa

gggcgctgctgggtgggatcgtcgcgccgtccctgccgtggaggtcgccatgaatagcaccccaaatgtgaccaccggttacgcgccgttcgaccttg

tattccttgcacatccgggggccgcgcatgccgtcatcgatgccggtaatgcccgacccggggagtcgttcgaggaccggctgttggctgcgaatgag

cggctcgaggatgctcgtcgagccatcggcgtggcgcgcgcgcaacagaaacgtcggtacgacgcgtcgcgtggggcgttaccggtgctcgctgtggg

tgacaaggtgttcgttcgtctgcgtgaccgtcccgttgctggattcggtcagggcaagcttgatgggcgaaagctgggcccgttccgtgtcggcgagg

tgctatcggaccatcgggtgcgtttggacctgccggagtccctggggatcggcaaagagttcgctgtggatcagttagatgttcctcctcgctcgccg

gatccgttcgctgcgagcagagacctacctgatattgttcctcctctggccattgccgacgatgccgcgcccggtcgtgctcttgttgatgatcagag

gcctgcggccgagccggacgcgcgtttcgacccggacaggggaccgggtaccgcagcgggcggccttgctgatgacgaggtagtcgagaatgtcgatc

cgatgctgcggccacgggcgcggggtgcgccgtctgctctgcgcgacttcgttctgtgcgtccaaggcgcggcggttgcggacactgtggtggccacc

gcggaggcggctgacgttcctatggctgtcgggattgcgttggccgctgctgcgaatggcgtaggagaggcgcccgttgttgtgccgactgagctgct

gaggggcccgctgcctgcgccgaaacaagtccagatcggaggccggtcagtgatgttgcgtgaacgacctattgcgtatcaatcacgacttaccaccc

catcggagaaacgcttggtggctcccgagcttgagctttgctgcttcgcgtgggcttttgcccgtatggcccacttgctcgaaggcgcagagatcacg

gtggtaacggaccatcttcctatgggcgcaatgctgcagtccgatgcgggcgcacggtatggaccgacgatttcgcgttgccgagcgctggtgctgcc

gcacttacactcgttccgcttcgtacaccggtccggaaagtcgcacatcaacgtcgacgctctttcacgcttgccagttgctccttga

>contig_14.contig_14.g164.t1

atgcagcgcaaccctcgagaccagcttcgaacaaaggctgctccggccggattcaacccggcttctcttctccctggcg

tggcggacgagaagcagatgctggaggtcatcatttcgcatgctgtgcgttcatgggtcgactcgcatccacggtccggtattcagcagtccgacatc

gcaccgccccctcaagtcctccctttccttccgcgtcgcacacagacgttaggcctacccatactcagcctcgatgaaggctcagtggctggaaacat

tgcggtcatcaaggcctactgcaagttcctcggcatcgacacggaagcgatctctgaagagcggatgctgatcatcgtcgcagacgccttcacgattc

ttcatctgcgctctgggcaggcacgtcggctgtatgatcgctcgaagacaccggcgctcgacaagatgcagggcatccagctttggtctgctctcttt

catctgcagtacacctttcagaagtacttccttgacgcccacagcggtaccgtcgccaatcaaagtcaaatcagcgcaagagttctatgcgacaaagt

cggcatgaagaacctgtgctcggggaacaccgacttccacgaggcagacgcgttcatcaagatcttgttcgcagctttgatcgacgcgatccttaccc

cggcgctgggtgagcaggtctcaatggatgcggatggtggaggggataccaatgcggacgatccggagcaaccattcgtcgacattcccgtcgacaca

gtcgtatccaaggcacgagcgctgctaaagaatctcctgcacggcgatgcggagtccgtggctcttcgacacggggacgaggacaaggccgacttgtt

ttttcttcaaggtcgggagatgctgagggacgcagctatctacctcgagctgaaggactcggtcaagaagggtgacccaggacgccttcttgtggcgg

ccaaatttaccgtccctcggttcgcagcgaccgagcatcatcggtacgtctccgaggttctggaaatgctccaccaggtccgacacgagcttcctcca

gcgttggcgaccaccatgatgtcggcgtcacttgtgaatcactccggtcgttccgacacctggcttcctgcggaccttgacatcgagcatcaagtgaa

tgagctgaagaatgtctttaaggtgacagcatcgtcagacgccgtacgaaacggacacatcggtcaaatcatcaacattctgcgggaacaacgactcc

gatggtaccttgctgtgggtatcagcagccgaaactccaagcactccgagcggtcaacgcacagaagcatccagctactgtcccgccacgtcatcgag

caagccgttttccagtggacttctggacgacggtgccaagcctacgagctcaaccggggcgacgatgttcaccgtcggaagaagatgaacgaacgcag

tccaaagcaagccaaggggctcgcgactgacttccacgtctacggattcgctcggctggttggtgggggaggacaagctggcatgctctcggattgga

tgtcgcgccgaggacgagatatgggaaaggagacagaggccaccagcacaaacgacgcgaccatggctgcgcagcccgatgagcttgaggaggccgat

aacctcgaggatcttcttgacgacagttacttccctgaagtcgacgttgacgctctcatgggtggcttcgtcatgcttcaagatgaagagaacatgac

caccacagccaagccaaggcgcatccaaaacggacaccaccaccaccacaacgatcacgatcacgaggatgacctccccaacggagtcaccaagacga

atgcacaacccaaccaagccgagcacgcagacggagacgagatcacgcccttcccgctacccgcagcccacactacgacccgctcctcatccgctcac

cgtacaacgtctcaccggcgcccaggcactctccctcctcgagcagctcgtcgccagactcgtgggggcctcgaaagatgggcaggaggaggtgcggc

ggcgaacgacgcgctcgccaggggaagctcgacgggtaaagtacggactaccatcgagtggatccgatctgtgctcctcctgcacacctcgtatctca

tggccctcccgcacctcaccacccgactcgcatcgctccatatggcactctcgtcccgcctggcctcccataccaagttgctcggcctccagggcagg

ctcgagttggtcttgagtcagatcgagatcagggctgcctatgctgctgtggcgggcaggggggcacagggggtaaagtcgcgcttggctaatgcggg

cgcgggggctccggcttcgggatcgaagaagaagggtgcgaagggtcgcggtcgcggtcctgctgatgatccgacgcgatggatcgagccgagcgatg

atgcagatgacgatcacatgaagcagcagacacagaacacagaggcacagaacacaatacacatgaaccattacacaatgatttactgctcaacatac

tcatcggcccatccatctggtcgggcatcggagccatttagctga

>contig_14.contig_14.g165.t1

atgagcgcatcaacacaagacacgcaagctgcggcgccagcacggaaacgccctcgcctatctcgttacagcaacgttg

agctggattgggctgcagctgagccaggactcccttacccccccaaacaatggcaaaaggatgcggtggcaggcctggcaagaggtcaggatggactt

gttatcgcgccgactggctctggcaagggctatatcctcaacttactcctccacatgcttcctgataagacattcgttgtcatggaaccgcttcagag

tctgttagaagaacttgcggcccggatgggcaaggaggcagtctacatacatgcggaaaatcgaacgcccaagctattcgaggagatcaaggccggca

atttccgcatggtattcgtctcgcctgagatggccatcggaaacgcatttaaagaggtcctgctggattcgaacttcaaggcatccctgggcggtatt

gtgttcgatgagtgccaggtcctgaaggactgggctgtcgactccacgttccggaaggggatcaaagacgtcagcatcctcagacatttggccgacat

ccccgggctcgcgatgagcgggacccttcctgccaactatcggaaggaggtcctccgccactttgagctcaacgacccttttgtccttgatatcggtg

tcgatcggccaaacatcgagtatgtcgtcgcgccgatccgatacccgcttgcttcctttcgcgacatcctggcgtggctgcccgagctgcatggaacg

aagccggcgaacgacgcggagatgcgggcactaaaggagaggtgcaagccgacgatcatttacaacaataacaaaacgcaacagctggagatgttccg

tcaagtccaggcttggtactcacggctcggactggaggacgtcgttctcctggtgtcggcagacatgagcgattctcatcgcgctcaggtaaaggatc

ttctggaatcgggaaggatcttgtgtgtgatcagcacaagcgcacttggtatgggagcagacctgcaaagaatcctgagggtcattcagtggcggatc

ttggactcacttgcagcaattatgcagcgcctcggtcgcgctggtcggaatccgcgaagccatgccctgggtgtcattcttgccgatccttgggccgt

tcctgcggcgatggccgcacctcaagcccaagctgatgctttgctcgcggccgaggtgggtgaagttgacgaggaggaaccagcgggagccgcagagc

cggaggcagtctcgacgaggctgaatacgacggcagacgaacaaatcgtcaaggtcgttcatcatgcgctcctgcggaaagggtgcatacgtaccatg

atcaatgacttcttgggccagccttcgcccgaaaatgtgccgtcggcagaatacatcttcggtcccaacatcgcaaaagatggaccgtgctgctgcgt

ctgcaatccgaaggagctgccgaaggttcctgacgctctgctgccggagaaggtcgaagaaggtcgagctcggaccgggatcacaaaactgcaggagt

ttatcgagcccaagctccgtcagtggcgacaaacacgatggaaggaacattggtcgaaggtggaaggcgtaccttcgtggctcggtattgcctacttc

ttccgatccgaggatgtggcttcagtcgtgaagaacctcggcaaaatcgacaaggccctcggagctggacaggacgatgctgttggggactacgtgca

gatggaagcgaagtcagttgtgcagcctgagttgacggcatggctgaaggagaacattggaacgtacgatgcggagcgcaaaaaggagcgcgaacagc

agaaggcagaagctgatcgtcgtgccgatgaacgcgctagaagtcgggccgaggagcgtgctgcgaagaaagcacagagggcttcacaggcagcggca

agcggaaatggtatggctggaggcagtgcagtagcgagcggaagtggtacggctgaaggtagcggaacggggagtgggagcgcgcctgcagggattgg

aacgcagcgaacccacaagcagtaccactga

>contig_14.contig_14.g166.t1

atgcccatacattctgctcgtcgccgtcgtctgctgcttgcgctcgtgctcggtgccgctgctgttgcgctctctcctc

aacgtcgcccagtctaccttatccgtcgctcgctcgttcctcccactcagtcggccatcgcccgtgtactgcgaacgaaagacgacatggcatttatt

gctgcgtttggctttgacgtggatattttccgtgatctcctctctcgctactgtccggccgtcgtctctcgtgcaatctgggtcggtctcgaggcaat

gaaagcaatgatgagcgaggacaaggacgcttgggcgatacgctggctcacccctgagatcatgggcgccatggccgctgccgtgcgcgcccgcgagc

cccttctcaagcacgtgttcgggttcgtcgacggtctcaacctgcgcatctaccagccagggtatgtcgaggaacagaacgcctactacaacggctgg

ctaggagacacatactgcagccaggtgataatctttctgaccaatggggagattgcctgggcaagctttaacaacgcgggatcatggcacgaggctaa

gattgcgaacgggctctacgacatcctaagcaaccccgagcgcaccccggcacctttcgccctccttgccgactccgccttccccaacaaccaggagg

tgtccggcaagatcctcagcaagccgaaggagaatgtggcggcaaaggagcaagacgggaacacacaccgcctgtgggaggccatcatcccgcatccg

ccagcgccaagctgcggaatggggaatggaaccctccagggtgcgtttggctttgctacccttcgagctctccgccatgattttccacataacataga

tgatgagcagatggtactccccaaaggtgtaatgtaa

>contig_14.contig_14.g167.t1

atgagcggagtacggagcacgaaaaagcatcccaaaatcagccggaacgccctcgacgacatcgttctggacctcgtca

acctcagcgaggacgaggacgacacggacgctatgggtctcgatgacctcgtcgccaacgtcacgtcgacgccatcgctcaggccgccgagcacgtgg

tcaccgtccaccgagggcgatcaagagccccctcgctaccatgacacggctttctttggccggtccgacgagttcatccagacgatctgcgccctgcg

cgaggaacatccgacaccctcgccctgcggcgcattatgtcccgcctcccgaacgcgacccgacgcttcgacttcggccatcgatcaccctagagcaa

gaacaagttccgccgcaccgactttgaccacatgccttcgggccgggaacacctcaactcgcaagtttgttctatgtcaagcagctcaaaaaccagta

gctttcgcgcctccagcctctcccatctcgctcggcgacgaggaagagctcggagtcccgaagaaggcaggcaataaacgcgctgcctgctggtcaac

cttccagcaactgtctcatgagcagaaggtgcagtgtatcgactgcaaagagaagctcagcttcatctccgtctcccgtaccggtacctcgtcgatga

cgcgccacctcgtgtcctgccccaagaagaaggctcgcttggagccgggacagcagctgctccagctctctcagtccaagatcactgtcgacgtcaag

aagcagctccatgctctcgtgcagcggtggatcgtcagagaccagcagtccctctccgtgctcgatcacccggacttccgggcctttctctctgctct

ccctggcgaccacatgtgccccagccgcttcgtcatgaaggcctcgatcatgtccgactacgccgccgtcaagaacgcggtcaaggcacggcttgctg

ctgcgccaggcaagatcgcgctgaccaccgatgcatggacggccggcaacggggaccagtacccagtgttgaacatactattggagtatggattcctc

tgtctaaccgcgcattggatcgacgagcgctggaagctgggcaatctgcttctggacttcgccccgtttcccgtcccccacacggctgccaacgctgc

cgatctcatcagacgcgctctccaggagctcggcatcgagaacaaggtctccgcgtgcgtcaccgataacactgcctcggcttacaacatctcaaaga

tccttggccagcatatggcccccgccagcttcttccaaggccgctgtgcggcccatctcatcaacctcgtggtcaagcacggcctggacgacgccggg

cgtgacgagatcttcaacaacctgcagcgtctcgagcctgccctccggatcatgttcgccgagcgcgcacacgcagagagcgcctggtctgagcctgt

ctgggtcgctatacgggacatcgtcaagatccttgccgtcttccgggacgtttccgagcacctgcagggccgattgttccctacgatgagcactgcgg

cgcagggatttgtgctgatgacccaagagctgaaggatctgcaggcatcggctacgctttcaacctggggaatcgagatcatcaagttgttccagacg

cgactgactgagtatcgtcatcatatgattgacacaaacgtggttctatgggctacaatactggacccatcgatgaaggcagaggtgatcgttcagga

tcgagtgcaccgcctgtacgacgtcacttcaagcatggccagtcatctcgaggcgcactatcccactcccacccaaggattcaaccaacttctcccca

cgcgtcccggtaatcagccaatctcggggtaccagaagcgcctgcagaagtttgttgcggagaaggaatcaacagcaatccccccaaggaagcagctg

gagatgtacatggccgagcctaccatcgctaccatgtctccttctgatgctctcacgttctgggctgccaacgagggtcgattccccagccttgcagc

aatggcgcgcgacattcttgccatccctgccacgagtgttccttcggaggcagcattttccagaggtggagagctcgttaccaagcgtcgaaatcgtc

tggctgggtctactgtcactgccatcatgtgcttggattcgtag

>contig_14.contig_14.g168.t1

atggaggtgtggactgccgactgcgagagcgacttcctgtactggagcgcaaggtaccgggttgtatattgcgcgatcg

ccgatcgagaagcggttgccgaagcactgagcggcaaggcagaagcggaactgaaagaaatatcggacgctccagaaactcctccgcccctctcgggc

acggtcagcgacaaggaccctctgttcaatgacgtttccgatgaggagtactccacgcctccgccggagtctaactccgatgttgttatcaaagaatg

gcatcttcacaccgtcgcgctcatgctgcgcagcatgagtccagaagccctcgtggccaagctgaagcatacgggtgagtatatagggggtgaaaaca

tgagtgtccggctcgacgccgcgacgctggacgagcgcggccggtttatgacccctgccagcggcaccgagcctgcgcaggagctgcgctggtcatat

gccgccaaagggcgctcggcgtttgggttcgtcaacgggaaaaccggcaacgcattcccgccccagtgggcgaacaattcgacttgtccttctaccgt

cgagctgatcaggtccaactcccacaacaagtcagcaacatgcgaacgaaaagggtacaagggtgcggaagccactcatctcaaggacggattggccg

cagtagccaacagggctaaggacgagtttcgaattgttcctccgccattgcggagtgaggaggacgttcttcaaaatcttcctccgcagtggcggagt

aaggaccagccctacttggagtatcctccggaggagtcacgcgatatcaatggaaggagtctcagctgggcggctctgcgctgtggccacgtgctgag

cgtgcgctgctggtggaggcacggagacgagttggattcggaacgggtcactcagcggaccaccgaccacgacctgttgatgacgctcggggaagacc

agaacctgcgttacaacgctgtcccggacgtaaccgtgcttgacacggacctgaagcagtattactga

>contig_14.contig_14.g169.t1

atgcccaagcatttaagcgcaagcgcggccggccacaagtcgagcgcaaggagaagggacaagggccttcctccaggca

ctgcagcatctgtggactcgaaggccaccgataccctgatgcttctcccgtgcgcacacatttgtgcactagcggcgtctctcaaaaaggctgctgtc

ctctatacgcacagctactggtcaaccaagcactggcgcgcaacctaccagaaggcatacattgtggccgagctcgacaatctcgacgccaacgagct

cgatgcgcctgataccagcacccgccaaaagaaaggacgccctcaaaagcagcgccgcgaagctggaaagggcggaagctctacgctgtcgcctgcac

attag

>contig_14.contig_14.g170.t1

atgagcagccaaaacaacatccccacccttcagcttaagcaggagttcgactccgtcgccgccttcaagctgcaggtgc

gtgcagttgctgccgcagagggttggaacgctaagtatcacaaatccgaggccagctacaccatcatgcgttgtcaccatgacgactgcacctttcgc

gtccggacaaagaccaccaatggcacggtcaaaatcacccgcctcgtacctgctcacacttgtctgggtgtcgtgaaagagggtagtaaggcttccaa

tggtaatcatcaccttgtcgtggctaaggttacagaaaccatggtcgtcactgcccagtcttccggcaaagcaatgcaagcccagctcaagcactctc

tcgggcacgaggccaatgtgcatgcattatacaaggctaaacgaaaaataatgggcgcttccctgggtgacgaggtggcgagcttcgtcgcgtgcccg

cctatgtggacgccttga

>contig_14.contig_14.g171.t1

atgtctggtcgacagtcacctttgcttcctggcgaggtcggatccgtctctgcttcctcttcttccgctgaggccccgc

tcgacagcaccgccaccgctctcgcgaacattctggccgaggtcggcggactgcggcgctcgattcaacgcctcgacgaacggatggacacagtggtg

gacagggtcggccggatggaaacttggatccccaacgacgggggagatacgtcctcggatcgccagctcgacgccggttccatcgacgacaccgaccg

acccaccatcacccgcctcgatccggccctcacacacgcggccgtcacgtcctcaatcgatccgacacgcgatcttccaccccacctcataccccgct

catccatacaaccccctgctgcccgccaagttgattctggcggccccgagtctacacgcgccggatctgagcgcgcggtgtccgagcacgccgcgtcg

ccctctcgtcctcctcgaccggacagagccgctggtcgctccgctactccacacgaactaaagcccccgcaagtccaggcgttccgcgcgcttacctc

aaaggagaaggcggccatccagcgcctctctgcactgctcggccggccggtccgtgactgccttgacggcgacacttcgctagacgatctgctggccg

atcaagttccgggatcgaacgggcagccccagccccgcgcaccatcttccaccaccagtccggctcggcccatccctccgaccaccccacccgactcc

cctccgctccacgtgctcctgccagcctcttcgaccgcaccaagtcctccttggggacgacgaagcgaatcggcaccggcgcgcgtgtcgtcagccga

acttggccaccccgacaccgcctcctcttttggcctcgcctcttactccggtggtcaagaggacccggatgcggagaacgggtacggctgcttgctct

cctcccgcggtgtgttttctgcgctgtccacctggaacggctggccggggataagcctgttccgccacgaccaggatcacatcaaacacacgctccag

gcggcgcatgtatgcacacgcatctggctcgaacagacgatggcggtcgacactgccgtagtttatccaacccgcttcccccaccgttccacagtcca

ctaccatacaggcggcatggccttcgacgagctcaactggctggcctaccgtgaagtcaacctcatggctctggatccttcaactccggccctctcac

tccacccctctcgtcagtcgtcacacacaccggctaaagcaccattccactcgtcagcactcagcagcccgcttgttgttcccgcgactccgccccga

ccgagaccaacttcggtatcaacatcggtactcggttcgggcgcacgcattttcacaccactcttgccttcaattaccttcgattcatacagcttcag

gtccattgccattccagccaccctctcttcgcatgcgcccgtcaccaatcggcctgctcctctcatgcccgctgcccttcccagtttcgatcacgact

cgggacagtcgtcttttccgtggcggcacgtgctggatgaccagcgctctctatcttatctcctttctcttatctcctcttctcccctttctctcacg

cactccgcatag

>contig_14.contig_14.g172.t1

atgaatacccacagcagcgagcgacccatcaattcccacacaatggatcactcaaccaaagatgcccagattgtttcac

atctgaaacaggtccacggcatcagtgtgccgcagcagtcagccaataaagctcgactcgcaattttgggctcgcgtcaagaagaccaactagcggag

tttcggcagatcgaagcgtggctccgtctcgtcaaaaccaacgacgcggacgcgcatgttgagcataaggcagagaacggtcgctcttcctgtgcttt

cgtgtgctccggtgctgctcgcagtgcctggcctcattgcaaacctttcttcgcaatcgatggcacatggaccaaaaatcaacacggaatgattctac

tgctcgctgccgctatggacgccgacgacaaccttgtcattctcgcctggggtttggtccaaatcgagaccgcttccaaattcaaacctctgatgacc

gagcttgcggccctcagcaccggtgctgcagaatacttgcgcgactcagaggatggaggaattccgcacaagttttgggcatcctatgcattcccggg

ccgacgaatcgaccatgttacctcggaagcagatcctagctccacacccccacagcctgtttatcgaggcacttcagcactctcgcaaactcaactgc

accccagccagtga

>contig_14.contig_14.g173.t1

atgggtgatccagacctcaaacaaaaacaaaaaagtcaaagcgccgcacatggccttcccgtggccacctcggagctca

ctctcatacacacccctttttgctacctcttcctcctctcttcttcatcaagatgcctgcagtctcctccctccggcctcatcaagtccgtggcgact

cgccatacccgttctctgcccttgctcaccatcggcacgtcctcaaacccctacaccgtccttgacagcgaggacgacgccgacttccccaccggtcc

caacccaagcaacgacgacctcttcacggtcagcgaccacgagatggaggaagcggttacggacgcaagcactatcgccctcgccgaagccgtcgaag

aggctattgaaggcggcgccaacggcgaccctcccgactacacggagccttcttaccggcagcgtctcgacgacactctcggcgcattctccgtcatg

cccgccgatcgccgacgcgtctggttccaatacttccggcgattcctcactccggagatcgcacaagaggtcgagacacgcacctgcaacttgcgtgc

gaggaacgacacgcttaccagcgacaccgtcgtgctccgccgactgctcgccaaggtcggcgacgggtccactcggctcaacatggacccgcgatccc

aatggaccaagaccaagtttcgcaagtccgacttcaaggagatgcccccacgcaagattcgggtcctcgctcagcaactcctcaaggctgcgaacggt

aacgcctcccaccccatcaatcgacgcatcacggtctgctcggcgtgcatcgaccgcttcaactccctgtga

>contig_14.contig_14.g174.t1

atgtcaactgagcagaggccacagaacggggacgacgggcagtgggacgatgagccggcgttcctcagcgaagaagatc

cggcggaatactcccgccgcaagaaccccactccgaaccaggatagcgtcattaagccgtggaaggtccgaaaccttgaggactggatccgcccgatt

gcgatcggcacctttgttgctacgcagacgacgcagccggactacatacagggtaacaagtttttcgaccgcctcaacgctgcgacgctcgacgacaa

cggtcgtttcatggccccaaacagcgatggtgtgctgcaggagctgcgcttcagctacgcggcgaagggcaaggtcgcatacgccttcaacaacggca

agaccggcaccgcgtttccctctcagtgggcgtccgaggctaccaggccctccagtgtggtgatcgagacttgccacggcaacgccaacatgcgccgg

aagcggaccacggagggtagtcgagagtacaccatcaataccaacaaccagagccctaagacctggtccatgtcgacctcgctgccgcctgcaaactc

tcaggcggacaccgacctccgtcgtgccctggccgcctatgggacccgcatcaaagcggactccgtgctccgcaaaatagctccgcggactccggggt

actccgggctccgcaaatctaaacctagtaaaaagcgctcaaatctcgctccgtgctccgtacccgtgcagagcgtcccccggagtccgcggagctcc

ggggaatgctactccgggacctactccgggaagatccttatggagcgggccatatttgtgaccgcgggcgaggcgctcgagcacggtcgcccctcgga

gatgcgaaaggatcacaacctcgcccgcacgataggagacgaacagggcgttcggtacaatactgtgccggccgttactgtgctcgacacggaggacc

atgacttctggaaagtgtcatggcagctcgcccacgagctcccctccactggcttgtgggagatcggtttccatgtccgggcgtacaagtcccgcgcc

cggaacaagttatacggtcgagagtcagacaaggtgttagagtcatag

>contig_14.contig_14.g175.t1

atggagaagctttctctctccccatccacggcgaccatcttgcgcaccgtagcaagtgtccaggagcgcaagattgaat

tgcttcgagtggtgttcaactcgatgccgatgcaacgtagaggtgaagtgctgagcggcacccagcacaaattcaaggtttataatgtaggtgcagct

actccgacgggaccctcgttcctgcgtcatgcccgttcgaagtcggttcaaggcatcggcgacttttctcgtatcattgccagcaatacaaccttccc

ttcgccatctcgccttctgctgcgcaccggttctcagtcccccgaccgtcagggtataagtagcgcgaccgaaaccggcttcctcaatacgtcccccg

gacaggttcggactctagcacgacaactacttgctgccgctaatagcgacccggaccatccgatcatcaagcgcaacgctgtctacgacgtctgcata

cgccgcttccagactctctga

>contig_14.contig_14.g176.t1

atggacccctcatacgacttctctccccgcccagtcactcccatacataccccgtccaagcatcatggtctgcagcaag

acctctacgaatccccaatgtccaaccagtccccaagcagctcggtggtctcggattcagatcttggcgacactgagaccgtctacagcgagtctgac

tgtcacacggaggacggcgagtgggaaccagactttgttgacattcaagatcattcgaaagacgacgaagattacaatccgcgtaaggatgacgtcga

gatggtcgtcaacattcctgccccggcttctcgggctgtgtcccgcccatctacctcgaccagtgatgatgccgccgtcgcagtcaaggcgttcgcaa

cactcatcatgaagccgactctcgacctccttcacgtcgaccgccaacaccgctttctgtctgcactcaaatctcgatgcgacttccaggcgtacaag

accgacatcgacgacgcttttgatatactttgttgcgatgccaatcggcgcatcgcgtgcgacgggtgggcattccctagctcgaaactgactttcga

tttttggtgcatccagctgttcaggtcaagcttcgctgagcgtcgccgtgccgtggtctgcgttcttggttcggtgctccttgagcccgacttggtca

gggcgggtgacagtgcaggtgcacttgctgaagacgacctcaagaccctcatctaccatcctcgagactga

>contig_14.contig_14.g177.t1

atgcaaaccacttcttgggccgacgtcatctgggtcggccacccaggcaactctcccaaggatctctctgcccgcgcat

ggttcgtccagctcgacaagagcctgaatcagcaagagactcgtgaagtgacctccgacaaagacaaacgagtggtgttgcgagctttctttcggtgt

gcggggcgatgcgcaatgtcaaagcctcctgtacctgcggacgaagaggattcgggagaggagaccgaatctgagggcgtcaaaaagtggagaaggcc

gaagctaacccacccgggagtgcgagggtggggtacgactcatgacctcggtactgccaagatctacagctgcggcaatcatagagacgcaaatgagg

cagatctgcaatattcgcgtcggctccgtttgtacatggaaggtgtggggtcccagcttcgagaaggcacgagcgccgacacagacaccgcggctctg

ccaagagcatacccatttcccgacttcagaatctcgaaggcgaggaagattgacgctgtcatgaagggcatccgttcgtccaagcggttgcattcgga

cccttttgcggcggtcagcatgtttgtcaaacgaaatccgagtctggtgtttgggtacgtaatgtattatgtgggaaacttccctcagtgtttgaccc

tatcgacttctgtgctgtgggtgccgtgcgaaacaattgccattatgcttcgagccttggaagtagaggtggttgcagaggcagaggcgatttgcaat

ggaagcacagatgatctcgcagaaatggaacagaaggagccgctgctactgaagcatgctctgcagatccaggcagtacaagcgtggagccctgcggg

tgtcatgattgacaagtgtctcgcagagctgaacgcaatcaaagaggctgatcatttgtctcgcttccgggaggactcttccgtctccgcacaatctc

acgactttgtcaacgccgacgctcgactggatcaagattccgacgacactttactcccgtattccgctgccatcagtggcaacaaccaccgcacgcag

tctcctccctccggcctcatcaagtccgtggcgactcgccatacccgttctctgcccttgctcaccatcggcacgtcctcaaacccctacaccgtcct

tgacagcgaggacgacgccgacttccccaccggtcccaacccaagcaacgacgacctcttcacggtcagcgaccacgagatggaggaagcggttacgg

acgcaagcactttcgccctcgccgaagccgtcgaagaggctattgaaggcggcgccaacggcgaccctcccgactacacggagccttcttaccggcag

cgtctcgacgacactctcggcgcattctccgtcatgcccgccgatcgccgacgcgtctggttccaatacttccggcgattcctcactccggagatcgc

acaagaggtcgaaacacgcacctgcaacttgcgtgcgaggaacgacacgcttaccagcgacaccgtcgtgctccgccgactgctcgccatagtcggcg

acgggtccactcggctcaacatggacccgcgatcccaatggaccaagaccaagtttcgcaagtccgacttcaaggagatgcccccacgcaagattcgg

atcctcgctcagcaactcctcaaggctgcgaacggtaacgcctcccaccccatcattcgacgcatcacggtctgctcggcgtgcatcgaccgcttcaa

ctccctgtga

>contig_14.contig_14.g178.t1

atgcgacagcaggctcgttctcgtcttcaaatctgcctccaagatcatcaacattcgactcgagcacttgccccaccag

ccatattgcgaccgtacgccgtaccatgccatctgaggtcatcgaatttatcgaggcgcgtctggactcgacagtcgacaccaagacaaatctaccag

gatttgatgtcttcggccgagatgcgatcgaaggccaaggatatcactcagaagcaggtgtggttctggtggcgcgcgggtacctctgcctcctggca

actcagtccggatcctttcgaatccatgaccaagtcgttgaatggagcaatgttggcgcgcgacgatggtcctgatcaagcagctagagccatcgagc

ccttttccagaccctctggctcctcatcaacgtctgtcccgctatcaccatcttctcacgttctagagatctctttggacgcaacctacggaaccaac

agtagcggtcacgagctgttcggcgtacttgcggagctagatggaacaggtgtgccgttaatgtatctgttcatggatacgaaggacatgcttgggga

caggcgcaaaacggaaatccttaccgagagcttagctatgctcaaatatggtaacggcaagaagacgggaacgaacaactatcagcccttcgacgctg

ccgcaacggtcctcggtctcgaggtgtgctggggcgtaccgtcgaagcgtcggcatgacgagcgcgtttgcacctgcggctgtcggtcagacagtcaa

tgggacgatactgggcggctagaagtgacgaaggatgaaagcgaagaccggatcaacttccttttcagacattttaatgtgcatccactgtttcccac

gatcttcggagcggtactcacggctgagcagctacgcgtacaggcggcaacagaggcatatcagtactgcaaagtgcaagcagcgtggctgggcgagg

gtctgggcgtacctgtgaagaaattggtatcggaaggacgaatgggaactgcgggcgagaagtgcaaacgaatcagttccagttctgaagaccaccat

gatctgcgaaagtcatcggcgcaccatcaagcacgactaccttcactgcttcgcccgtccgcgtatccttcatcctccgcacaagactcctgccgcat

gggtccaagcatcaacagatgcattgacagcgtcgaggagatctcaacttttaacagcctcttctctgttttcctcctccatcctgcgcaagagctca

atatactggtggagcaactaatccagcctcatctggacgcggcgggtgcagcagcggaactgctcgaatcgctcaccatcagcatcaaaggacgagac

cctcgacctggcgagctgcagcaagtgctcctgaaggaaggacgactgcggtcgcgagaccagatagcgcaacagcttctgttccggtgccccgttca

agattgtagaggctcatccggctacctcaagagcctcgcccggcactgcgccgtccagcacccacagcctcttccatccattgaagcagacccatcgg

ccctacagatctctggccaacccgtccacaccatcgccccggcgtcctcggccacgatcgcgttcgctcacgagagcgtgtacgtggccgacgcggtc

cgctttggcgcagtcacctggacgcggaccaacctctcactgatcgccgcgctcgcaacttttgcgttgctcctcaacgtcgtcttctgtctcgtgaa

cgccgcgatacagagctcagctggctcggcaactatcttcactatgtaa

>contig_14.contig_14.g179.t1

atgtcgacaccgggcttggcatcgacagcgccgggagtgggagggacgccagacagcaaccagcagcagcagcagcagc

agcagcagcagcaacagccgcagccgcaggagcaggaggcagcactgcgggctgcccttgctgagttccagcgacgaggcctcgctgcgactcggaat

caacaacaccctctcaatgcgggcgcagcggcagcgggagaggccaacccggcagcagcagcaggtaacaatgagaacgaagcgctggctgtccgtcg

aaatctcttccgcgcgtctctcggcgccatcggaagcatgaaccgcgtccgccgcaccttgctcgccatccgtctcctcctctccatcgcacagatcg

cagtcggcatcaccatcgtcagtctgccgtcgtcccatcacgactccttcgctactgcacccaacgacccagagccagagatctgccagccctccgcc

atgctcgtcttcctctccctccacatcgtacgcgtcgccatcaacttgccagccgatgtgtatctcagtatttctccccatcgctccaatcgcgcaag

gagaccgggtgcggacgggcttgaggcacgcgagagaaaccgcgccatcgggaacatctacctcgatcgtcgcatcgccaaagtatcagacctcatcg

ccctcgcacacgtcgtcctcttcatcgtcggcaactacaccttgtactcctcctccgaatgcgcaaagccccccgcacgatccgtgcccctcttctgg

accactttctcctttctcgccatcgactatctcagcatcatcgaagtcgccatcctcgccctcgccgtcatcttcttcctgcccgtcgtcatcttcct

tctccgcctctttggtattgccgatcgactgcatcagcccgaaattcatcccgaacacaagaagatcagccaggagaccgtcgatcagattcccctcg

tattttatcggccagcagatgaagaggaggaacctgggaaggacaagcctctcgaggacgcagcggccgtcccactccctccctcacgagcagagagc

cctcagcccaaggcgccaacttcacctcatcttcgcacccacaccagagatgagagtacagcttcgactgtcaggactcgaaacagcatgacctcggt

ccgaagcgccgcctcgggatcacatccagcctcaacatctgctgccatcccaggcgcagtccatcgacaggggcctctctcccgcctcttcttcgccc

gaaggagcagtcgaagaagcatcggtccaccacaaccatcatcgcctcctgtcgaatcggaggggatctccctcgacaccctcggcccttcatcaaca

gcagcgcacaactccaagctcgaccaggatgacgacgtcattcaaccaacctctccgtctgcgggcgggatgggagcagggtatggccgtacacatcc

actgccacatccactccacccgttaccagcccatcgcgccacctgtccaatctgcctgtgcgacttcgaagagccgccagatatggagcttatcaagt

gggaggccctcgagcgacagcgggccagggagtcgaaggccaaggaggacgaggcggggaagaagactgaggccaataagggggaagaacggcaaggt

gcagggcaggtgggcagaggagggggcgaaggggaggaggaagcagcagcggtagcggcgaatgcacccggtccatcgacgtcacgaacacgcttctt

caagaagccatcaaagtcaaaacaaagcccatcgacaccagcctcgcccaacccgaaagcaacaccaccaacagccgagtccgacgcccgcctgctcg

ccgacgtggatctcgcatcggaggctctcgagccgttaaggctgatgtcctgtaccacgggcctcgtggaatgcggtaagggcacaggcttggaaaag

gctgaggatgccaattga

>contig_14.contig_14.g180.t1

atgtccgtgcatcgctccctcttcacccttccaccctcggctcctcatcacaacgacagcgccgacttcccagcatctc

atctcgccgcgcttcagctacagcccatcactcgcccaaagagtctctcccgtaccatcctcgcccaggacaaacacaagggcggcgtacatgccatc

accggacgcgcactcaaagcacaaggcgtacaagaggccgctcgtcgtttcggattgcgcgtcctagagggcgcagactacatccccggcatcggcaa

gattgacagcagtgttgacgaagaaggcgagagttccgatgaggacgctgccccgcttgatcctcatcgctcagacgatgatgacgacggccacaatc

cgattcttcggcctcgggtgcatagcgacggcgagccggaagaagagatcgacgacgctgcgacgaccaccacagcaacaaccctctccaaagcatcc

tcctccgccatcataaccctccaacagtccctcctctccagccccccgctcaaaacatggacactcctcctcctcggcggcggaaacttcgcagcagc

ggtcatcgccctaaaccccttcgtcgaactctcgctgctgggatcgagcgaacaggcggcgagcttcatcagccagtatgttgctcagcgcttcccca

atgcaccacgcagtcagccacagccacaaccatcgggagcaggagcatcttcttccccgtccagtcaaccaaggcagcagcaagcgaagcagcacggc

aaacagaagctcactactcccaaacagcgacttctccagccagccactgcaaaacgagtggttaaggagataccagaggcattcgggcccgtcggaac

cgtctaccacaaggacaaggacttcgttctcgacggatatcgtcgccccgctgtcattacagcaccgcatggcccggcatctgtccccgcccctccgc

ctaccagcgaaaacgaagccgcttcgacatcgtatcaacagcctactctgatagcagcagcagaacagcaacccaccgcggatgtagccgcacccgaa

ctcgagaccatcgcgcccacagctgccatgagagagctcgatgccctccttgcagagctcgtcgtctccgaagacaaccagggatcagcacctccaga

gctcgtcctgtgcttctgtcaaggacgcgtacatcctctccacccacaacaacccctctgcccaacctgcgccctcccactctgcacctccctcctcc

cctctcccctggcggagggtgcgaagaacgcgtcgaaagagagtgctgccaggaacaacaccgccactccgacagctgcagtcgtcactccagacgaa

gccgatgaaagcgacgacgacgacgcggacgccgacctcggctcgagcgcagtcgaagtcacaatcaccatcctcgtgccagacacagacgacgacgg

ctggcccgcactcgcacaccaagctccgtcctccctctcctccgctgctgacgtcgacgaggcacaacaagcggccatccaacgacagcgcgccatgg

ctcgtaccgtcggaggtcatcggcccctcgccaatccccggctggaggcggggagtagaccgacatatacggcgcgcaagaagcgcgaggcgaggagg

gcggagcaacaccgccgcgcttccactgatactcccactcgcgaacgaaatcccaaactcggccgtatggttcgacgcgttcccctcgcccagcccac

cgccggtcatgagccggtacggatgatgccggagagcgaggagggcgcggaggaggaggaggaggcggaggaggatggtagggaggacgaccgccgcc

gtctgcgccaccgaaggcggatcgcaaaagccaacgccctcaccagtccccgctccgctatggactacgagcacatgtatatcgttcttcgtttccta

actgcaatcaatctgttcacatggaactga

>contig_14.contig_14.g181.t1

atggtggatatggtgcgtaaggggaaggttgacgtccttgttgacttcttgtccaagtacgaggacgagctcgtcaagc

gtggcgggggctggggacctgctcttgccgatggtgatgacagatcgagtgtcacagaacaaaagaacatcgacacgcctttcccgctgtggtggcgt

gctcaggaagctgggatttcgctggacgagctgtcagacgcggggccgggcgagaatggagacacaccgccggcggcggtagaagcgatcccgacctc

ggtccccaacaccctcctccagctcgcagccgaagcaggcgtagaagagatcgtgcaatacctccttatcgagcgccgcgcagacccagccgcaccaa

tcgcacccctccaccgccgcaccacacccccgccctcatcatcatcagacgcaccggcgatcccgcaccggaccgcatacgacctcgcctccgctcgt

gccgtgcgggacgtgtttcgccggctgatggtcgagcagccggactgggccgagtgggcgtag

>contig_14.contig_14.g182.t1

atggcgccgtctcataagatttggaagccttacgcccgtacgcacggcgagcgacctgcgcctgagcggcgacgctgcg

acgcgagcgaagcgcaccaccgactgacgcgtctctcctgcctgcctccatctatccagacccttcgcccaacatacgcacacccgcaacccccaggt

gtgctcgtcacttctcaaacaccctctacgcatcatctccaccctgccgcctcttcttcacaaatagaaggggaaccatggacaatacaatcaacatt

gcttgtctcgcctccatatcaccgtgattatcacaccctctggccgctcccggcggcggtatctggccctgcagcgagcgtgcaactgacccggacat

tggcgaaaacggccaacaagagcgacatccgcaacacttcgtccttcgagaactattccgcttatgtccgcatcgtgatcgccgagggacgcacgcct

gataccgtcttgcgtctcctaatccgtacatatgagagatcaacccgtaagaaattggaatggggaatcgggatccccgcgcaccctgcgtccaggca

tatcgacatcctgggcaccgccttcagcgactgctctcaggagtga

>contig_14.contig_14.g183.t1

atgtcgtcggcatcgcagtccgaccctttctcgcgaccgcactctcccggcggcttcagcattgacgaggaggaggagg

aggaggaagaaccggcctccgaagacgaggaagcgatgcggaggaacgacgagcacaatcaatccattcttcaggctgtgttacaaagccgaggcgcg

tgggcaccaagccagaatcgacttcagggattaacgcagcgcaagtccagcaaacaaaagaaggagaagtggcgcctggttacaacatgcaataccca

gcatctgtaccagtggttcgaatctcagttcaatgattactcctcgcatgacctatcgaccgtaccctgggttaatctcacctggatgggtcacccag

gtgacagtcctcgcgacctgtcagctcgaccatggttcgttcagttcggggccggagtcaacaacaattctaaagacattgctactctttcgaggaag

gagactgctcgcatcgttgttcgcgctcatttccgttgcctgggccgttgcgttccccatccggtcggcggtgacgaagaggaagaggacgagacgga

gacagatgccgatgccgatgagtcggaatcaaaaattcccgagaattccaaagaaatgctgcacggcatcgaatctgcggacctaggcgccctgcctc

gacaattcacccgaccgccgtttcgcataccctcccacaaaaaggtcgaacagttaatgaaaaaccttcggtcttctaagcgcctgcatcaagacccg

ttcgcagcagtcaacatatttgtccagcgaaatccggagcttacgtttggttaccagaagctgcaggttcgggctaagtcgttcaagttcagcgtggg

gatcaaatccctatggtcaatacaaaacctgattcgtcatcacggacgcctcatgcatttggactcgtcgtggcgcaacaaaaacgaaaataggtcgc

ctttgaccttcgttacgattacaaatgaagctgatcatatggttccctgtgctgcctacctcagcgccaatgtgactgctgcctcaatcggctcgttg

ttaacagcgctacagaaagaggtgatggaggaggtcgagcgcatttgcgacctcggagagaaagaattgaatcagctccggaagcgggaccctcagct

ggtcaagcgtgcggcggatatacagaagcgaggggcgtggcagcccgctggcgtcatgattgacaaatgcacggccgagctcaatgcaatcaaaacgg

tgtggcccgaagtccaggtccgcctctgccagttccacgcgatccaggccatcggccgttgggatacggactcaacccaaaatctcaagaacaggaag

tcacccggcatcagtattgcccaaaagctgctgatttgtattgccttccgtgaggctcagcgctgtcgagacggtgctgagtggtcccaatttcagac

cgcattcgaaaaggaggtgaattcgattctggcgcgctgcaccgccgcagtgagaaaacaagtggtgtcgtacttccgggagaattggtggaatgata

cctggagggatattgccacagatattgggctcgaagacacacagacacgggaccatgctaatacaaacaacactatcgagcgggctttcaaaacgttc

gataacatttttttggcgtgcagggtcaacaaaagaatcgatcgcctagtccatatactggcgacggactggctgccctactacgagcagttcagttc

aagcgaaccccgagttaacccggaagtcaagcaaatgatgctggatgctcacaagctgtgggaactgggtacagctatcaggccgggaccgaacaaca

cgttcctggtgtgggatacaattaccacgacaacgaagcagggcgttaccctgaagagggcaaaagagttcactgtcaatctacagggtcaggtcttc

gctatggatgatggtgcagtaaaaactggggttaaacccgcgaaggaagcggctagccagcccgacgaccccaaggccaacaatattccttccggaac

gtcagccggaagaccggcggccgcgcaacctatgcagccgtcacgttcgaaaacagggtcctcgttgagattcagccaaaaacctgggaagcgtccga

aagcaaaagcggacatattacgggaatattattcacagcagaccgtgcaaggcgtgcaaggggccagctcgcctagttgtgatgaatcaacgccccag

agtgccacaactgccgcggaggcctccacagcaaaccctgcccagcccaagaaccctatccccacttacgaggaacagattcaagggataatccgaaa

attggattccgaggccgcagggatccaactgcccggcagcaaaatcagcacctggctggtcgggacagatttcaaggccttcctcagccagcaatggc

tcactggcacactgattgcgttgtggtgcgagctcgttcgacttcactaccacgtcaatccaagaaagcgacctcttgattttcagggcgagccggtg

gaacccactcggacatgcctgacttcggtaacgctgttcacggagacatgggacgaggttgtgatgaaacatcgtatcgaagatatggaacaaggttc

ccttctctcgcaggacaaactagtgtgccctttacacctaggcaccaaccactgggccacagccgtggtagacgtgggcaagaagactgtgacggtca

tcaactcgattcccaacgaagcccaggccaaatctgtcctggcctttttccaggaattcctgccccggcgtgcagagcacgagattgggaaaggtctt

cttgccgtcggtcgcgggtctcaaccagacgactggatcctggcaactgaccctgcagcagagtccgaatacccgcgacagcgggacaattccagttg

cggcgtggtcacctgcgcggtcatactggctgtcgcaaaaggcgaagccccaacgtggcaaaactgcggctacaaagagcagtatatcagcatcgaag

aagccgacaagttccgacgaaagcttctctcccacctcgtccctcacatccggcaggctctcgatggggccgagctcgacaacaaggtccagcctccg

acagcatccatcatcctagtacggcaacatcacattaaccatcggcctctccgtccccaccctctacttcgacgacgaccaatcgcaacgtcggatcg

cgcgtctcgtgcttcggggggaaggggggtagcagtgggatgcaacttcggcaaagcgggtcgtcagtgctggggcggctcggagcggttatggacgc

cgtcgcctccgcactctaccgcgtcaggatggaagagccgtgttcgagcgtctagcagaccgataccggagaggttgctgccatacagcacgcgatct

cagccagtcagcctcgcgccaagcacaagcttctcccacagccatgcatatcgatcaggagcggcaccagcaatgacgcagcaatcaacaggttcgga

tcaagaaaaggtcggtggatcagttggcgaccagattgtcgtcctcaaccctcagcgggatctcgcacaactaggttgcttcaatggagaccgagcca

tcgtcgacctcacagcagcgcgcgtctcccgcctgggccagatctctgccccccttccctcccaggcacaatcagcacaggagcgacctcctctggga

cagcctcgacgacgatgtgttcgccggttcgccctcgtagaggagctcgtcagtatgcacggagcgctcccacgcatgcgaccaactgcagcgaagca

ccgacgtctacgaccctatctcccctctgactattcgcctccatcctccgctattggcagcggaccaaagactactcgggactctgtcagctgtgagc

acgacgggggctggatgcgtcgcgtgctggaggagtcgcttcgtctgcttctcgcctggacctggtggctcggcagtagcagcaaggggcgcgagacc

ctaagggcccggactcctgatcgcactccacgccctgccctccgcacgtcgtctccctcgccggcttaa

>contig_14.contig_14.g184.t1

atgattcaaggatcgaccgcaatctccaacgcagccaatccgtcatggggacagcgggacattgtcccctacagactca

cacccaccttccagcatcagcgcaaactcaacgatctcgtcaacgcacacatcgaccagaccatccagcgaagccccgctaccggtgtagtccaaacc

tccttctctacactcacaccagctccgccgtcgcaaacccccgaacctcatcaatgggccttcaggccggccctcgccaacacgaaaggccatttcta

cttgggcccattggagccaagcctggtcttggccttgctgtcatccgccgggctcagtaagcacgcggaactggcactcaccggcaccgtaggaggct

acctgaaatactaccactttcgtggccattcccaaacaccttcatcacgtcgccgagccgagaaaacgccttctgcagaggaacaggaatggtggcag

tgccgtctctgtcacggagacttccacatccggccgaaccacgttacgaaccttggcgcacacctatacgggaccgcgagatcccggggctgcttgga

ccttcgcgccgacaacccggctgaatggatcccgcctcccgcgcgcgatccgtcgggaaaaattgtccgcattcgccccgacaccccaacaccagccg

cccgcgctcctcgcgggtcacagaacaaatctcgacagccggtcgcaatctaa

>contig_14.contig_14.g185.t1

atgccgcacccgtaccgtcctcaacacccacacacaatcttccccatggcggcggctcgaccgtcctccacatcgccga

cggctatcggcatcctcagtggtgctcctgcttctgtgatcggcgcttctggatcggccctacctgccacgcctggagcagcggctggagcaggcgtc

ctttctcactcggtcgacggacgagtcacgctgccgtctcgctccgccgctgctgccttgacttccacacggcgccagtgtacccgcgactgcgcccg

ctctagcgaaggcttcgtcggccaacaagcgcaggcgcgcctctacgcctgccaagaggttagagcacagtcggccccgccaccacggcacacacaca

tccagacccagacgcaagcgcagaggacggcaagaatggcacacacaggtcagctcccaaatcagcgcccgcctcccgaggacgcaagcagcgcccgt

ctagcgtcgagtcaagttcatccatcaggaccgacccgcaatatcgccggtggtgagctcacttctgacagccgtcgtctcacccttcttcctcccct

ctcccttgcagataaccagatccactgcgccgacccgggcgccgacattggcgaggtccccaaggctgcgcagaccgttctcagactcggttaa

>contig_14.contig_14.g186.t1

atggtcgaacccagtcccttacatgcatccacggctactcacacaggccagacggcaagtacgacccacgttcgatcgg

caagcgcagcgcaccttcgttcccttgaggaacgcgtagcttctcttgaagaacagttgcgggcgagctccggcagtaatgtcgcttcccagtcgacg

cctttctccacgactccgcgtcagcgtaaagagctgagtcgtctgggccgaaacccaaacggagggccttcggcgcctccgatttcgaagaagcaatc

ccacacaaagctgcgccggcattttcgtcggctggtgtacgtcggttacggcagcgtccgaggtggcaaaaacattgttcggctactgtatcctactc

gtggttcctggccaaggcatgcttcttcttcccaaagtatcagcgcgtcagcgtccggcgagactcaaacagctgaagaggagctcaccagtgggaac

caacttcgtttacacttcggccgtccatataacgatcccatcaaccaaacccagctccgttcgtacttcaagtacatgctcgcgcacagacaccgatg

tggagttcctgccgacatgacccttgaggagctcatcgtcgtctgcgccactacgtttgatggctgggctacggcatacacacggtcgctgtcctctg

ctggccggcgaatggcggaagctgcgaaagctcgagcatgccacgcagcgcgtcgagcccggaaagcgacagcacgcgctggagccttgcggttgcac

cggtactacttcttctccgacggcgcaaaggtccccaagacgccaaacgatgctgaagaatcgcgccatgtacgtcgaggagcggttcgagccgatgt

cgagtttgccgctcagccctgtgtcatgagcgacaaggaagaggaggatgaaccagcggatgacgactcttcagataacgacgggatggaaccggaca

gggttcggcgtcgcgtagcagtggagtggcgatctgcttgtctcgtcacaaggctcgaaggtctggacaagaagcgtaaacgtcagccctacaagcgt

attctacctcctacaaagctccgaacgctgccaccaggattcaccttgccttcgagtatccgtcgctggatggttgcacggtcttgggcggaacagaa

ccaggacgcttgcgtcgatgtttccgacaacgctggacccttccaagggccgtattccgtgacgaacggtgcaaaggactggggcgaagacccagatt

ggtcgatgatttccccgcccaaccaacttgacgacgaagagaacggcgatgatgatgttcttgccggaggttcttcgtcttcgtcctcgtcgaggcga

cgtcgtggaagcacctcaagcacgagcagcacaagtagcagcgttcagcgtttgttggatgctgagttcggaggtgaggaagacgggtcgcaatctgg

gatctga

>contig_14.contig_14.g187.t1

atgaccttattcctgccccacggttcgaggttgaaccactacccggagacgactcaccctcatcaacttcaatatttga

cgctggacattgtggttgcggagctacaccgtctacacacagatggccttcaagtccctgaactacgcgaccctcaaactggccagcacctgaggata

cgggttcggcttgcgcgcctgctggcagatacggatgcacgaaccaccactgccggctttcccagccattcctccaaaaagcagttctgtccgtggtg

tactgtggatcaagaggagtgggtggatctgctgttgcgaggagaagaaggaactccgcgaagcgaagctgatcatcgccggaactgccttaaggctc

taccggatgcggtaccaaagctgcgacctttcgaggttgaagctcttcgccgcaaccacggcgcagccatctgcgttctatacaggctccctgggtgg

tctagcttggcgcatgcgcctgtcgacgtcatgcacaatctcgacctcggtctgtgcaaacacttctggatgcagacgttggtggatggcgacctggt

cgactcaaaaaatctgagtctctgtaagtcaattcttcaagaaacaacgtaccccaccggaatcaccaggatcagtcccgatctgggatcgacttcgg

gcggaactccaacagcagctggctggtctatcttgtcacgctacatcatgccagtactcctcgcttcatcttggtccgtccatctgcacaactccgag

cagaagatgttccacagcaccaagaaaaaaatggtcaaacaaggattactcgctgcagccgctagctcagctgccaacccagctatcgcctcaacctc

cgcatcagagcccacgcctacgacgtcagactcctcgcaatttcccacgaatgcgcagtccagtgcgccgctcgacaaatcccgcaaaggaaaaggga

aagcaaagcagaaggacgataaggaaccaaagaaggataaggagccagagtatacgaaagccgtctcacttgtgacgatgctggaggcgtctctggta

ctggcactggcttgtcggcttgcccatgccaggatcattgacgagacgcggatcaatttgcttcacagaagcttggtggcctttgccacactcattgc

ctccgagatcgaatcgacatggattccgtacaactttcacattgcgctacatattgcggaccatatccgccgtcatgggcccgcatgggcgttctgga

gttaccctcaagaacgttcatacgggttgatgaagaggatcagaaccaacaagcaccgaggcggagagctagagcacacgatgcacactcgaagcgag

gatcgccatcggatcaaagcgattctggagtcgacgcccgtcactccattatcgcggattcttcgggacagcatcgggtcggaggagatcaccaagat

ccccgcacccgcacatggagaatcgcagggtcgcgaggagagcgagggtacggacagcgatgaagtcggaagcgacaacgacgaaccccaggaatgca

tggtcaccctcagcattgcagacctgcggcgaatctgcgctttggccaatagggatcgagactcaggccaagctttgttcgttccaatcatcaactac

tctggaacttctccccaactggtcctcgatacccaagcgtacttcgtccccaagctttccgtcggcgagttcaccctacgacccagaagcaagcgcca

tggacccagatcggatccatccgcatgctttgtatctactgcgacgggaccaaggctggcgcagtttcactccgtcctccgacttccatacttcgacg

agcgcatgcgagagcagcacaagcgatatgccgtggttcgtgtccttgacgtcgttgcctggccgaacggacctggattctcatcctccacctggaat

gaactcggatatctcgccgctcaaccaagttcaggcgtgcgaacgatactcgacatcgacagcgtgttgggacccgccatcgtcctgcctgggtccca

catgttcagtggtcgagacggccgacaagtgagaagggcagccaacccctcgactcacccctacatcacctcgactttgactgcagcaccactggacc

accattcgtgtccgacacgtggagtctcgcctgcattccctacgccctcctctcccgccgcttgcctcgctcaagttctcagacccaacattcaggtc

cgcacacctgcgggacggttcattgagatagtcccatgcgatccggttcggataacccttgcgcttggctggatccacacgtccgaagggggtaatcg

cccgatcgtcgttcccttctgtctcctgactcctctaccaccggcaccgccggcggtatcaccgctcttccgcttcagctcgcccgctttcactctgc

gcctcaccctcgatccaagccgagtccgaagcctgcggcgcccacagatcacgcagcatctccctcgcccgcgccaaatcattacgcccttccccagc

tcgggtatgtcccatcaacaacctgacctccgcgcgcagcagcaacgcccgcgacagcgtcaacggcatccccagcgcttgagccgtatcgaccgctt

cgagcgcgtacagctccgcgtcgcgcccgctgccgcgcgtgagagcagcagcctcccgggcgcaggcccaaacggatcctcaaccccaagaatcgccc

ctcccgaacccgaagccgaacccgaagcaactttcgccgacgccgccgccggcaccgcaccacacttcttctctcaagtcgacgtccagccaccctcc

aaatcccccgcgcaagcctcagcctcgcacacgcctgcgcaaccatcgcctgcctctcgagcatctggaactgctcgagttcatgctcctgttggagg

tgaaggcgtttctatcgacaagttccggaggccgaggacgaggccgaggccgaggccgaggccgcccgctcgacagaatgacgagaagggatgggcag

tcatggtccaagatgaatggtgcttcgtcttctcggtacttccgccagagcgagtcgcgtccgatgcagcgatgttgctcttcgcccgtccttctctg

agcgcgacaactccaccgctcgccctcctgtcaagcaaaggcgtgttcaaaccgtggcagggacatcgagatcctcgtcgtctttga

>contig_14.contig_14.g188.t1

atggagcctttctgcgcgccagaccatacggcataccctgcagctcacctcagcacccagaacgacatagcgttgcctc

ccccttcgtccgatcgagatctcaaccacaccaagacccccgcgccgcgagccaggcaacgcccagcaatgccgtcgtcgaacagtcaagccatcgcc

atcgccgtcccctccattattgtcaagccactttgcacacctctcctccccaagcgtcgcgcggctgacgatcccagtccggacggcgtaccccatag

gtcggttggccatagggaggaagaagaacttcctcttgtgctgtgtcgatcggtgcgtctactcggtgcgtcaactgcgtcggccaacaggagcacga

ccaaagtaagtgcgccgcccaacattacgccactgcccgacaccaccatcgttcgcggtaccgttctcatgagaccatcactggtggtgccttccaga

tggacgagatcctctacgagctccgcccagcactcttccggtctcaactgcggccgatgggactacatcttcagcttcatcatcgccaacgcttcatc

accgagggaggcattcgctccaaagtcgccgcccttctcggctactgcacctactggggtggaggatgctgccactgctgatatttcgcgtgtcatgc

tctggcactgggtctaccacggcgcatcgactttcgcctctggtgcagctcgtgaagtaggtttcgaggtcgcgcacggtgtatactgtattgaccga

tcatggcaagacgatcgagagcgcgtccgacagcaacaatatatcctccttccatcgtcaccactcagctccagcgccggcgtagcggagcctagagc

cccaatcccgaccaccatcctgcaagctctcaacagcatctccggccccggcctcatcggcagcggtctcgttgacgacgacgacaacctcatccctg

ggctcagcatggggacccgctcctcatcgtcgcgacggcgagaaggggctcaaatcctgctcctactctcacccattctcaacacacaagagaacacg

tacctcggccgggcagtagtcgacgacccgaacgtccttggtcgcgctacagtttgcttcaagaccctctggatcctcttccacctcgagtacctcat

catgtccaagtcgaacggcgtcgacattcattcgcgagctctggctaattggggccttactgtcgacgacattggagccgtctccatgcacggtacct

ccaccgctgctaacgacaagaacgagagtcacgtttaccacgagatgttcaagccaaggctcggcaactatcttcactatgtagctgctggaagtgac

tatagtggctatttcagcgaaaaagccaacgaaaatttcgcatggctgaaatatggtaactctagtagcttgccgagccttggttcaagcatcggtca

ttcgcccggtcacggcgtccccgccatggcgcagaagtggctacagacctccatcgtcgccggcaaccgtaacgcgtatgacatttctcccgagcttc

ggaacttctcctacctcctctacgcctcgacctccattcagcgtaccgttcaagacctgaatgcggctctcctaacctcattcggcttcggtcaagtc

ggcggcatcttccttgtgctccacccggcgcacgtccttgcccgcttgggcgccgacgagctcaagaactaccgaggcaagacagccaaccaccaggg

gatcacttacacccatatgcactcggcactcacgcacagcgacctcgtacaagtcaaggacgcccctccctaccccaaagatctcaaagatgccgtcc

ttcagaacctcaaagcccgagccggaccgacgctctcgggtacctggaccttcaaggctccactggcggccttccctgcgcttgctgagcgtaagaag

gtggtcaagttgacgacggcgaacgaacaggaggagggaattgcgaagcagatggtgggagttcaggcagttggtgtcgacatccaggacatcggcgg

gctccccgccgacaatgagaccttcatcgagcgaaacttcacgccagccaatatcgcctactgccccgcccaagtcgacgtccgagccttcttctgcg

gccgtttcgtcccctaa

>contig_14.contig_14.g189.t1

atgggatctcccgtcgcgggccgcacatccaccgctgccgacgatcgcaacgggagctcactccgcaagacgctcagaa

gatccataagcagagcatcggtccgcaaatccggagtccctcctccgcctgtgcccgaccttcccccgacgctcacccttcccctcgacatcgacccg

tctgccccgcccaccgcagcaacagcaacaacatcgaccccaaatggattgccccacccctttctcaattcgccctctcagtcccaggcccaatccca

gccacgccgtccaaaactaggcactcatactttcagctccaactccggcaacaacacacgcgtcccaaccggtctcaccgcaccagcatccggcgcca

gtaacaacaacaacaacagcggcagcaacaatggcgcgtcccccgccgcagcactcgcactctccgcatccgcatccttcgcggagtccgatagaaga

tacctcgcccgacaggccgtcgaagagatcacaaatgtccgtctgagcccgcatcatgccaacaccctcctcaccctctgcgcgaccgagatcaagaa

ccgcggtctagagacaccgggaatcttccgccccatgcgtctcgcagagtctcacaaggagctcgatgctctcatccgtctcttcttgctctctgttg

actcagaacgattcgcgcctctctttggtcggaacgggggcgccctcgctgcacagtcctacgcctcatctgcatccgcatccgtcgtcgcagccatt

gctcccgtcggaagtggggccgccgtcggtcttgctgcaggttcctgtctcaacgcgcctggcgaggtagatccactcggtctcgattccaaaacaag

ttcccttctcccctccaaacagtccgacctgcttcatctctttgatgaaaagttggatgccgcatccgcacaggcgcatctccacgaacgcatccagc

acgtctctgtcctcgatgtcgttgccttcttcaaatggggtctccgccatctcaaactggcgccagccgactttggcatcacgagcctcggtgcagac

ccgctctcttggtatgagaccttcaggcgcgatgaaagtgcggcgaactatcccctcaacgcatacagcaagtttctcgtgccccgtctacaggtcca

caccaagcgtctccttgcaactgtcttcgaagtcatgagcagcgtctcggcccaccacctgagcaacttcatgccagcatgcaccctcgtccgcgtgc

tcggcttctggctcctaggtcgtatcggaagtgaccaccccccgccgaacctgactggaatcacaaccagctggtctcgcgcttccatcctcacagag

catcttttcttggcataccttcgtgaccaagtcgcgcactccttgtacgagcttccgctccgtctgaccgagctcgtcgatggctatcccaacctggc

ccagttcagggaaggggatgccgtcattcgcgacgagttcctttccgacgccgaacgcgtctcgcgaaagacgccccagttgccccctacccttcgct

cgcgtcgcgtcgtcgccatcaaggccacccttcgatccgaaaacattatggtctctctcaaacggccccgcaccccctctgacaccctcgccgcagcc

ttgggtgcggagacatgcgatgctgaagaggatgaactcgtggcgtgggatatcatccagagagccgccgcgcgcgcggccggtggacgcgatcggga

gatgagcaatgccagtggtgggcttgtctcagcacatcggatgtccatgtctcaatccgcggtcaatctgggcagtccgactggtgacagcactgttg

aacaagcccctgcggaccctgccgtggcagcggcggagactcaccagcggaagcgggaaacagacgcgaagagagaagcggctgtgctggacgaggaa

catagccggatcttcaacctggtcgcgggggctattgcggtgcgtcgcgctgtgatagaggccatcgatccgcgcgaggcggcagccgctgcagctgc

ggccatgcctacctcgtacggcgcttctcagcagcagcagcgaagcatgacgtccccggtgtatgggtcggtgtacaaccggtacggcggccggaatg

gaaacccgtactcgcagtcgacgtcggaatttgctacattcagtactggttcagccggacggtcttcgatatcgcaaagctccatgaggtcgccgatg

ggatcgcagggtcctgcgcgagggcctacctccacaccgggggacttggtacgcgagcttcgcaagtcgacttccttcagcggcaagccttcgactca

cacgcgggagtcgagcttgacgccgcttccggaggatgttgatatgtcagggaagacgactccgctggggcccgcggctttggtgacagagaagggtc

aggcgcaggcgaatggaagtgcgaagggcacgagcgcgatctctgactccccctcctcgatcctaggctccgactgggccagctttgcctccggcgga

ttcggcgcgtcctcctcgaatgcggaactgacgctcaagagccccacgccattcctcgacaaggagctgtcatccccgacggaagagctggaggagat

gttgactcctgattcgaagagtcggattcgccgcgcgtcgagcagttataactcgatgaggcggaacagtcggcgggcgatgttgaacgcggctgcga

tcaatggggagtacagctctcccaatggcgcaggtagcgccgcggacggatctacgcccgggaccggaggcggtaacggacccgtgcccggcccgacg

cacaagctcgtcgcgctcgagacgattgagatcgacgaaacgctctcggcagcgtggcaagaccaactactcgacttctccctatgcgcccacttccc

acccctcgtcctcgccgcgctgaacagctcgctcatctcgacggtccacgcggccctgccggacgacgccctggcgctcagctggatcctgatcgacg

agacggtgatcccgccccgaccgccgttaccggcctcggcttccggggaccggggcgagtcggcctcgatctcggacaagcgctcgttgtttgcgcct

tcgatccggagcttggcgaccagtttgaggaagttgaggggtgtggggagcttcttgtcgaagcgaccgaagacggagaagtcgtcggcggacgcgat

gatgtcttga

>contig_14.contig_14.g190.t1

atggagtccatacaagaccttgtcatcttgcttcggcagcctgatgtcccccgtccatcggcgcagatcaagcaactca

ccagcgaattcgcgccgcaagactccgagttacgatcccagctggcttcctcatcctccgaagaggggtctgacttggcatcagaattagcgaccggg

ctgtttcgactggcggcaagaagtatccctcggaatgcagagtcttcctcaaggaaaatcgatgctcttccggaggatatcgagatctcgacctttgc

gctgcgactggccagaaatatcgtcgcaggatgtcaagaccttcaggacatcttctggagaagaatcgagattttggatcctgtactgtctttcatca

ccgcctttgcacaagtccatgacgagatctaccgacctctcactcgagcagctgtacagttgctgtccaatctactgatggcaaacgagaaggttcag

gccgctttttgggcccgatacgtggacaaaggatcggcggagctcggagagacgcagttgattcttcgccaacttgcaacctcgtccgtatgtcgccc

agtgctggaaatgctgttatccatcttggagcagtcgttccgggatttcgcggaggccacacatgacgcatcaagcagtccaattgaagagctggacg

agctttcgtccatgacatattctctcttctctcacctctttgcacgacagcttttcggtcccatgctcatcaacgtcacaccagccacatcacaggat

ggcgacgaagagttcgttcctcacaaccccgtcaatgcctcgcagcgaaccttgctcggtcttttcgacgcctacatttacgccggagccgaagacta

tggcagctccttgcagacagaaagctctcaagacttcactgatgcagacgccctgatatcaacatttgttcgacttgggacacatttgcgatctgcta

tgacccagcttcctcgccaagcttcaccaggctctggcagctcagaaggggcggtatcgtcaaatcccagctttgacgcgagaacactgatcggcgta

ccgcaggcaatcgtgctcctcgtccagtgcatgagcgagtggatggtactttcctccaagaggttggacgaggcgaaagaagaaggaatctcgagcac

cgatctgaaagagcccgccgtccttgcaggctgttatctcctggatcgaaaacgccagcttgcgaaagaaggccttcggagcgtggatttggactccg

cagaccctctagccaaccaagatttggacccagatgatcctatcggcgtcgccatctcactgctgcgcgaagccgcggctgtattccctgcagagtca

cccttcaagaattccaatccacagaatgctgcaccatcttcgacaatacccacggaagcaccagaaggtcatgtcctctcccacacaggcacctcctc

tctcctcgctcaatccagtgcagctgcagcctcccagctcgagaacgcaggtgggccagggaagatagctttcgcctacctcaagcgcgaactcgtcc

gattcgtgggcatcgtatcctttgtcgaggctcgtggtgcgagcaacgctgataaggcgggagtcagagatgtgcaagactacgtgagggcaatgggc

ggtctgtatgtcgtacttgggttgacgcagctggacgagctcaatccttacatccgagaacacgccgtcttcgcgatgcgcaatctgctagctggcaa

tcaggccagccaggacatgatcgcccgactgcgcaaagtggacgatccataa

>contig_14.contig_14.g191.t1

atgtcaaccgacctcgcactctcaccagaagacgatgaccaaatgatgacaggaacgccaacccaagccgagcgcgaag

ccaaccagctccgcttctccaccgaactcgaattcgtctcagccctctcctcgcccgcctacctcgtctccttggcccagcgtggattcctccagcaa

ccccgctttgtccggtatttgcactacctcgcccaacattggcggacgcaggcctatgcgcgtttcgtgaggtatccggccgggttgaccatgctgga

gaatctgttggaggaagagttccggggcgctgtgggctcggaggggtgggaggagatggtgaggaacaagatgattgggcattgggcgacatggcgga

ctgcttcggcatcgtag

>contig_14.contig_14.g192.t1

atgattgtgatcccggtccagtcggctgcggaactgctcccgccagagcacacccccgctgctgccgtcatcacccctg

ctccagcagcaaccacttcgcacaggccgtccacatcgcactcaaggagccccagtttgaccccagactacgatgctgctgccaccatcatggtcccg

cctgtctttggaaagctctccaaggacatcgctcgcgactttggtagactctacgaggctgtcgcaggagcacgccgaatcatagtcgtctgcggtgc

tggaatctcggtctcggcacccgccaacattcccgacttccgatcgtcgtctggtctcttccaaaaactaaaagaccaacaccccaatgcaggactca

caagcggaaaggaccttttcgatgctcgccttttcaactcggagaagacttccgacctattttataccatgatcgccgagctcaaagaaatgtccgat

cgagcacagccgacagctttccaccacttcctcaagagactcgacgtagagggaaggctgctcagagtatacacccagaacatcgacggcctcgagga

acgtgccggtctcaccttcggccttaaagccaacagtccagccgaacagtccagcaccgccctcggcaagcgcaagcgggctgaaaacaaagattgct

cgcacctctcggcagaaacagatacctccttatccatggccaaacgagctgcctgggctcgctctcagtcggactcggccgtgctctgggctcagcgt

gcagcaggcaaaccggccatcgacaaatcacagatgcccatgtttcctcgcacaatacctcttcatggctccctcaactcgatgacatgcgggcgatg

cggtcatcaagagatgctcgagaacgccttcaagaccgtcgctgagagcttggcagagtatgaagcctggcgattacaggaagcgcaagacctctcga

ctgatacatccatggacgatgccacggcgcctgcctcatctgcggctacatcccagtctactaccgttacctcgaaacgagattctcgcagggtcgtc

cccgaactcaagcgactcgatatccatgaagtcctcacctccctccgagccggcgaacacatcccctgccctcgctgcgaaactgccgacgaagtccg

gttagcggccggcctccgatcccgcggcgtgggccgcatgaagcccgacgtcgtcctctacggcggccaaaatgacggtgcggagcgggtgggcgagt

gtctgtcgagagacatcctcggtcttcgggacccgaatgagaccctcgtgccggagacgttgtctgagattcgggcccgagagcggaggttaatcaag

gaaggggatgctggcatcagtttggaagtcgggcaagggttgggtcaggaggacaccctcggttcgatgttctttgctgatgatgagccgatggcgtc

gcctggagtggcggaggaggactcggacaagctcgccccccttctcacggaagctttgacatctggtcccgtccctgcccttggggaccgagtcagca

agcttgtcaagacggcctccaagagcaaagcacgcccgaagctaaaacctcttccgccggacttgctcatcgtcgccggcacgagtctgaaagtctct

ggtaccaagcgcatcattcgcgaattcgccaaagcctgtcatgcccgggacgcccgctactaccccggcgatgacgacagtgaggaagcaggttcatc

caccgatgtagactctacccctcgacgacgtcgcggacagcggaaacgcagtgccactgtcgacggtgacgaagaagaggagagggcaggggactcgg

atgaggaggacgatccaaaggggccaatccggacgatcttgctcaactatgagttccccgcaccttcgtcgcagtgggaggacgtctttgacatatgg

atccagggcgacgtccaagcagctgcgcgcggtctatggccgatgcccgacacaaattctgccctcggtgcactgccgttgagtgaggacgacgacca

ggcgaccaaggtggctgcctgtctggcgggcgtgaacccatccgagtcctgggcgcatctgaaggaacatctggaagaacaacgtgcgctggagaaga

agtacaagtccaagtcggccgcttcgatcaagcggcgggcgcgggaggaggaggagacgatgccgccgaagttgatggcattgtcgttgagcacgctg

gcggcgctaccgatgggcgcggcgcagccggtgccgttgccgcagttgggtcggaagaggagcttcgtgaaggcgaagacgttgtcgtcgctgcaatc

gggtgggatctgggatggaggtccgggtcgcttcgcttcggcttcggcttcagcttcgagttcgggctcatcgtcgctttcgcctcctccgtcgcctg

actgctcgttattggagagagtgaccccggccaaggaagtcaagatggcatcgatcagtccgagcaagacccagacgaagcaaaaggacaacgggaag

aaggtgacgccgttgagttcacctttgaagaaggccaaggcgacatcggtcagtcccagcaaaacccagacgaagcaaaaggacgtgaagaaggccaa

ggcggcatcggccagtcccagcaagacccagatgatggaaaaggacaacgggaagaagaaggcgacgtcgtcgagttcacctctgaagaaggccaagg

cggccgtcaaggcgctttctccttccaagccggcagcgaagaaagcggcgagcagggcagtagtctctcccgtggcagctgaggcggcttcttcaccg

tcgaagaaggggaggaaggccaaggcttaa

>contig_14.contig_14.g193.t1

atgcgagccttcagcaccctcctgtctctcgtcgcgttggcttcctcgctcagtctcgcgacagccagcattggaagcg

atcgtcatgcgaatggtggactggagatccgtaaacatcatcatcacaccacgacgaagcatcatcaccgccttccctcggaaccgaccggacggaag

catcacaaaggtcaccacaagcccgacgcagacaatgaagacaagccgaagacgaagaagaagcaccactcaaccaccacgacgaagaagcaccacca

tcacactaccactcccgctcaggccaagcccaccagctcccacaagaccaagacgaccgcgaaggctgctaccactaccgccaaatcgaccactcacg

caacctcgcaccctcaagccactcacaagtatggccttctctatagcgacaaggtacgcggtgtcaatcttggcaactggctcgtggtcgaaaactgg

atggacaaacgtttggacacggctttgaacgccatcgcagtccacgctcctgcctcggttgtcaagaagacaaagggcaaaccgatcgtggacgagta

caccatgggcttgtacgccgacaaggaacaggcttccgccctcgtcatggaacacttcgaaaagtggatgacggagaaggactggaagcggattgccc

agtacggtttgaacagtgtccgaattcccttccctcactatgctttcgccgactgcatcgagaagggcgctccataccttcccctcaatcgtgttgag

aagctcaaggagggtgtcttgctcgcaaagaagtacggcatcaaagtctggatctctctccacacactgcctggctctcagaacggatacgactcatc

aggacgtatcggtccgcccggatgggccaccaaagcggactacgccaccctctccacgcgcgccttcaaccacctcgtctccatcttctccgaagaac

cctacgcatccgccatcctcgccatcgaacccgccaacgagcccaccgcggcacaatcccccgaaatcctcgccaccctcaagaagtactacccgctc

gcatacgacatgatccacaaagccaacacggtcagccggatccagcctgcgagcccgatccgatttgccgcccacgacggctggaagggaatgcggta

ctggtccgaacccgtcgccttcttcgacgagagcgcccggcagcagatgttcctcggcatgcacccgtactacatcttcggggacgacgtcaggaaca

tgacggactcgcagcgcattgccaaggcgtgcacctttggggagaagattgcgacgtacagtagcgtgtacacaattgtggcgagcgagtgcggaatc

aatgctcctcgaggtgacagtgggtggggtcgggatatgcaccaggccgatcatctccagtttgaggatccggaacttgactacccttttagcgacga

gtacatgcgtttcctcgccttgaacttccgtgcacaacaacaggcctacgagcgcggtgcaggctggatgatttggtcatggaagcacttccagcacc

gcgactggtcctacaagagcggtgtccgctacggctggttgcctcgtactcctgctgagctcgaccaccagccctttggcaagctgtgcccttga

>contig_14.contig_14.g194.t1

atggcctcgctgatcagtacaaaggcattcagcgcagtggccgtgtctctccgcgcggctcgctcttcaggagcgcccc

agaacagcattgcggcgatactttgcagccaacaacgctgccgacaaagtgtggcatcgaggggattccactcccgtagcagcggcagcaacaatgac

ggcgccgccccgcgatcaagcgttcgatggagcccctacgtcacaggcacagctctctccctcggcctattcgcagcgttcctgacctacgacgccga

gcaccggaaactgcacttcgacagtccccccggagcgcgcgtcggcatcatccccgttccctcccccggaagcggaccacaacgcaaagacccggcaa

caggcaccgaactccccgaatcgctcccgtccccgctctcctaccagggccccgagctccgcctggtcggtctcggcgtgcggacggtgtccttcttg

aaggtccgggtttatgtggcgggtgtgtatgtggatcagggcgcgctggagcgggtgaggcgggaggctggggtcggggcggaggtgcgtgcgctgga

ggggaagagtacggaggaggttgtgaaggcgcttcttgatgctggtgtgccgttcgtgatccgcatcgtgcctgtccgaagcaccgacttcaaccaca

tgcgcgatggctttacacgctccgttcaagcgcgtctcaaagccgcccgcaaagcaaatatcctccccccagcagcagacgaagacctctccctttcc

ctccaatccctcaagtccctcttcccaccctcggccctgcccaagggatccaccctcgacctcgtcgtccacccgtcctcgtcctcctcctccagagt

agcgggaaaggcacgcatggcgctctcgctggaacatgacgggaaagtgctgggtacgcttgccccgcctgtgcctgggtcggaagcttccaagggag

gctggagcgtcgcgaaagagctcgttttggcgtatgtggcggacaagaacgagatctcgacacctctgaagcagtctgtagcagaggggatgcgcgag

ctcctgggatga

>contig_14.contig_14.g195.t1

atggacgagacattggcattgcgcgcgcagatccaggcgctctcgggaaagatcagcgcacgtcgcgcagatccgcacc

catcagcaccagcagcaccgacaccctctcccgcttgggcggggagaggaagaggaggaggcgccaggggtggataccagccggcatatagcgggaga

ggcagaggcagagggagggggaggggaggtgctacgggaatgcagtggactggcggagctggggtgcggaacaagacactggtattgaatcagccctc

gtcctcggccgtcgacacggcgggaccaggaccggctgcgccctcttcagcatcaagcgcgtcgcggactccggagaatgcagtcgcgtcctcgtctg

cgccatcgggtgcgatcaacccggctgtcccctccggattcgtgaaacgtcacgaccgcggaagtatgagtctcgtcacgtccgacacctacaagaag

ctagaagctgcgcgtgctgcgcatgtagctgcgaagccagcgacaaaggcgcgcaaatcacgtcaagagatcttggagaagaggaagagggaccgaga

gaatcaagcaaaagtggcgcgtcttcaacaacacagcaaagtactcgcgcagttgcagtacttccggacgtgcaagcacatggacaccaccggcacca

gcaccgacaagaagtgtgtctacacgcactcggacaagatcggccctgatacgaagccttgtgagcagttcagcaagctgggctactgcgagaatggc

aaggagtgcgacgagcggcatacctttgagtgcccggagtttgctgcttcgggcaagtgctcaaacccgacgtgcagattgatgcatatcgtttccgc

aggcggtatggaggaagggggtgtaggggagctcgtcttcgagcgggatgatgctgctgccgagggcaacggagatgaggatgaggagatggtcgagt

ctccgaagaagaagggcaagcgaaagcgcgacgatggcgaagatgtcggtgatggggatctcggggatgaggctgaagacgacgtcgaggacttctca

tatcaacctctgaagaagcatcacggcaagtcgttcattacccagcaagacttcatctccttcggcgacgtcagcgaagaagaagaagacgaggacca

tgacgaccgggacgatgacgaagaggaagaccttggcgaggacgaggattctgctgccgatgatgacgacgacgacgacgacgtcgaaggctcggtcg

atggcgaatcgatcgcagcagaggagttcagcatgaatgctggggacgagactgaggacgcgttcgaggatgccgaggagggatcggaagccgaagca

gctctcggcgtataa

>contig_14.contig_14.g196.t1

atgtcgtgggccggcctcaagaagtccgtcaaccgggcgggcacgctcatgctccagaagacctcccaggttgacaaga

ccgtcgacgccgagttcgccgaggaggaggcacgctacaggacactcgagaaggagaccatgatcctccagaaagaggccaaggcatacctcgactcc

gtccgtggcttgtcctccgcccaggctcgcatcggagagaccgttgccagcttctacacagactcgtccgagtcggccatggcggcgaccgcatacaa

gcgcgcaacggaggaactcgacggcaagtgccagcgcgaactcgacgcccccttccgcgccaccattttggaacctatcggacgactatgcgcaatgt

tcccggaaatcaacaagacgatcgaaaagaggaatcgcaagctgatcgactacgacgctgcaagaacaaagcaccgcaagctgatcgagaagccgagt

gacgatccatcaaagttgccaagagcggaacgcgagatggaagacgccaagattctcttcgagcaactgaaccaccagctcctcgaggaattaccaca

gctgatcgacctccgcattccgtacctcgacccttcctttgaggccatggtgcggatgcaggcgcgctttgccgcggaagggtacgagaagatgggtg

gtgtgcaacgcttctttgcggagggtgttcgggaagaatacgcggagggacaacttgacatgcaggttgagggtgtcttgcaagagatgcgagagctg

tcgatctgtggcatgggcaattaa

>contig_14.contig_14.g197.t1

atgtcagcaccatcgggcagcacagcgccgcgaaagcgcgacgacgacgagctcagtgatatcattccaggcaccgccg

acctcagcaccgatccttcacgggctcattcaccctccgctgcagcatccaaagtcgattcagacggcgatgtcgagatggacaccaccactgggccc

gactccccctccaaaacgcaacgacagctcgaaaagcagcgcgagaagcaagatcaagcggcacagcgctcggcggcgaaaagcaagagggccgtcca

gaaccagaatttggaccagaccagagatcaacttgctcagtccaagctcgcagatagtatgaagcgattcggatacctccttggtcagacggaactct

tccagcatttcatcgacatcaagaaggagcgcgatccccagtttgccgccctcctcgctgagtcggaggaacagcagaagaagggcaagcgtaaggca

aaagcgggcactgatggagggaatcgacgtcgcaagacggagaaggaggaggacgaggagctgcttaacgaggacgacgaggaagctgaccaagcctt

ccggttcgagtccagtccttcctacgtcaaagggggagaaatgcgtgattaccaggttcagggtttgaactggatgatctcgctattccacaatggta

tcaacggtatccttgctgacgagatgggcctcggcaagacacttcagacaatctctttccttggctatctccgtgactactgtgacactcccggcttt

caccttgtcgtcgtaccgaagtctacgctcgacaattggcggcgcgagttcgacagatgggtgcccggcttccgcgtcataatgctgcaaggtgccaa

ggaagagcgcactgccgtcattcaaaacaagctgctcacacaggactttgacgtcctgatcaccacatacgagatgtgcttgcgcgaggcttcagccc

tcaaaaagctctcgtgggagtacatagtcatcgacgaggcccaccgtatcaagaatgtcgactccatgctgtctcagattgtgcgccagttcaactcg

cgatcacggttgctcattaccggtactccgctgcaaaacaacctcatggagctgtggtccctgcttaacttcctcttgcccgacgtgttctcgaattc

aggagatttcgagtcgtggttcaagggcaagggcgatgagaaccaggacatggtcatccaacaacttcataaggtgttgcggccgttcttgcttcgcc

gagtcaaggccgatgtcgagaagagcttgctgccaaagaaggagatcaacatctttgtcggcttgaccgagatgcaacgcaggtggtacaagtcaatc

ttggagaaggacattgacgctgtcaacgggatggtcggagcttccggaaagaaagagggaaagacaagactgctgaacattgtcatgcagctgcgcaa

gtgctgcaatcacccctacctcttcgatggtgcagagccgggtccacctttcaccacggacgagcatcttgtctacaactcgggcaagatggtcatcc

tggacaagcttttgaagaacatgaaggggaagggcagccgtgtgctcatcttctcgcagatgagccgagtgttggacatcatggaagattactgtatt

ttccgcgattacaagtactgccggattgacggcagttcggctcatgaggaccgtatcgctgccatcgacgagtacaacaagccagggagcgagaagtt

tgtcttccttttgaccactcgcgccggtggtctaggtatcaacttgacaaccgccgacattgtggtcttgttcgactcggactggaacccgcaggccg

acttgcaagccatggaccgtgcccatcgtatcggtcagaccaaacaagtctacgtcttccgcttcgtcaccgactcctcggttgaggagcgcatcctt

gagcgtgccgcacagaagcttcgcctcgaccagatcgtcatccagcaaggccgggcacaacaggctgcaaagggtcaacagtcgaaagatgagctcgt

ggacatgattcagcacggcgcggagaagatcatcaacaacaaggacccgatgatcattgacgcggatattgatgagatcattaggcaaggcgaggagc

gtacgcaggagatccagcagaagtaccagggcttgaacttggatgacctgaacaatttcaagtcggagtcggcgcttcagtgggaaggcaaggatttc

aagggtcagcgaatcgggtcgctttggattgagccatccaagcgtgagcggaaggccaactactccgttgacagctattaccgcgatgccatgcgggt

gacctccaagccgaccgctcccaaggcaccacgtgcgcccaagcaggtcgccaactacgattggcaattcctcccgaagcgactgatcgagttgcaag

accgtgagactgctgcgtaccagcggggcgtgagctacaaagtgcccagtagggagcccaaggacggggagacggaagcagagattgaagcgcagcga

cagcaggagcaggaactcatcgacacagctgaggggctgacggaagaggaggaaaaagagaaggatcagctgtcaacggaaggattccaagattggac

ccgacgtgacttccaaggctttgtcaggggatgtgaaaagcatggccgaaacaaccttgctgccattgctgcagagattgcggatcccaacaagacgg

aggcggacgtcaaggcatatgccaaggtattctgggcccgtgtctcggagctgtccgactcggatcgactcgtggcccgaattgaggagggcgagtcg

aagctccagaagcagcacgcatatgaacgaactcttcgagaatttgtgggcagcttccgatcgccgctccagcagatgaagctcccgtacacgcagaa

caagggccgctcatactcggaggacgaggaccggttcctgctggtccagctggccaagtacggtctcggtcgggacgacgtgttcgagcagatcaagc

aggacatcagccggttctccggcttccggttcgactggttcatcaagagcaggacgccgttggagctgcaccgacgttgtcagacgttgctgttgctc

atcacgaaggaggctggtgatgattcgacgatggcggatacgaaggcggagggcaaggcgaagacgtcgaagaagcgcaaggtggacgctgtagcatc

acagacagctcgcaagagcaccggtgggcgagcgccgaggaaacaacgcacctcggccgcaagtgcagccgccgagacctccaatgcaggcgccaaca

catccgcctcgacaacagcaggcgcttctaccgcaggccgtcgaaaaccccgtttccgcccgggcaccgtggccctgcgggagatccgcaagtaccaa

aaatcgacggatctgctcatcgcaaagcttccctttgcgcgagtcatacgcgaggtcgcgttagaccatgtgacagacaggcctaatgtggttggact

gaggtggcagagtagtgccattcttgcgctgcaggaggcgacggaggcgtatctggttcatctcttcgaagacgcgtga

>contig_14.contig_14.g198.t1

atgagcggtgcgaggggagatccttcggctcttgtcgaattcatcgctgggactgtcggtggggccgcgagtctcttgg

caggacatcccttcgacacgatcaagacccgtctacagagtcaatctctggctacctcctcagcctctacgtcaaatggagcgcctcccgtctaccga

aatgccatcgacgcatttcggatcatagtcaaggaagaaaagttcatcgggctatacaaaggcatcgcaggcccactgctcggcgtggccgtcatgaa

cgcttccgtgttcgggtgctattctctcgcactcagagctcaattaggtgccaacacctccatcgaagatgcctctctcggtcaaatcatgctggcag

gtatggcgtcaggcgtcatgaccgccctgattacaacgcctatcgacctcctgaagattcgacaacagaacgacacccgcgcagccacttcttcctca

cccattgccattcttcgagacatcgtgcgacgacaaggcatcactggactcttccgtggctacggcgcgacctgcattcgtgatacctacgaattgat

aaaccgcactttaatcagctttcatcatactacaccctatccttccttttcctccacgacagccgacccctctccgggtcccttgcccagcacgccgc

cccgcctttcatctcttgaactcgccctctccggcggtctcgcaggaatggtcggttgggcgtctacattctgggtggacactatcaagactcgcata

caggccacggaaaagcctaccagcggtagcctgtcgactaatgggccgacgcattcccttccgcgaagtgctcaggcgcactcgcgatccgtacagcc

tagcttggccgtcactgggactgctgcagcggcgcgaaggccgaatggaacagtcagcgccgagtcggaccgcttgttaggcaacgcagccccaacga

gtgtcagaacgtcgacccaacaacagtcgtctctcagcacaatgccagaacggaggcgtggacttcgaggatggatcacacgacgcaacgagatcgtc

cgagtctcgcaagatctgtatcggaatggaggcctgcgctcgttttgggtcggtattggacctaccatgctaagagcaattccgaccaacgccgtcct

gttcattgtatacgagtcaacgaagtctaccttcatagattggggatattga

>contig_14.contig_14.g199.t1

atgctgtcaggggaacgattcagctcactggaagctgccctcggtgccctcctcgccgtttgggaccttctcgatgtcc

ttccgtcggatttacaagaccgctttcccaactcgaaatggacccgtctcaccggcttagaggccaccgacgctgcgaaggcttacgtgctcagctcg

ggctggctggtcgatgctcagcgcttcttcttggaatgcatcgtagtggactgggttgacgacctcgcagagcctttgtcgacctcggacgatggcac

aaaatccaccttgcgcgacaaggcgatacaggcggctttcgctccccatgctgtagtcggcgtcaagaacacccaacaggctgggaagaaatctctca

ttgcggtcattgaggatgtcgaggacatcggagccaaatacgaggccaagctcgcggctgtgggacaaatcatcgcctccggcctgtccgccacctcg

caagtcctgacacgcatgtccaaagctcgatcccagtcgtcctcttcctcctccccgcaccatcagctcacgcaggaagcggccttctccgctttgct

tctgctggcggtgcaagacgatggagcggacagggaacgacattcgccactgagttatctcctctgcgccgcggctcggatgcgaaacctggcccaac

gggacaagttctggcaagatgtcgtcagggagctctgtgctgtacctgttcgtgtatcgaacgttctcggagcggccgcgggtccggaagatctggag

gagggccctttcttcgcagcgttcgcaactcagctcgaaggaatcgtgtaccattctacgacagcgagcgatagtgcgcggaaccaggtcagaagcga

gcatctcaaaatcttgttgcagaaattggtccggagcggtgtcaccggtcgagctgtcgaggtagccggcgccacggatggatccaaggcagacttct

ttcaagctctcctcggtagtgctcttcgaagagttcaggactccgttggatcggattcacaagggtggtcaccctcctacctgactagttggcatcga

ctcatccgatcactggagccgaccgagctctctgcgcaatttcttcaacatctagtcgcctacctcgacttcgtctgtttcgtttcccagaagagagg

cgacctggtgattgatgcagagaaggggcggatcggtgctgaaggcaggtcgtttctttccacagagactaccaggtccagtcttctcgtggcccgtc

tcctgaaggtcttcatgccagctccacaaggtcggcaggttcaagacaaggacacgaccaatgcaagatttgtggagctggctgcggatgctgacgat

gcagaggagatgggagaagaagacatgaggagatggcttcggattgtcttcggtgatagccagatcgctgctggtggatcgacctgctttagtgcggc

aatggctcgatgtctggccattcacgtcgcgccaacatcgggcaaaggtgacggcagcgacgaggctcttgcggctgtaattgcgcagaccgtcagcc

gatgggcttccgatgagcgagtgaggcgggcaagcgtggcggaacagcaatacctcacaacgcttctgctcagcctgcttgcccaatgtccaactcgg

agtagcattccagcacagacactgcaaggtccgacctttatccacggtgttagcgcccaccttagtcaccaggatccagcattccgacgactaggcat

ggtcgtcgcggagattctcgatgctgcagtcaaacaaggtgatccggctgcggctggtacgaccaagcgtctggattttgggcaatcgatgtggagcg

gaagaggggatgggaaggaggcgagtcgcgttcttcgtacgatggtggttggctgggacgtgtccagtccacttcgacaacagctacccctcgacggg

ccgaagcttctggaaatggtcgggctcgcacagccagctccaatagccgaaactcccagcgaggagcaagacgacttgtcgcttcctgtcattggctc

catgtttgcaacagaacggcaaccacccagtactcgcgcgctgcccgagcggagacctgcggcgaagacgactcaaggccaaggtccgatagagagtc

tgatcgaggagacctctactctttcctttcttgggggcggtaaggcgcgccgttcgcggcctctgatcgccgatattacagattcaggcgcttctgca

aatgacgccacggtcagtcggccccttcgagcctacgatctcccgaacgagtcggacctcgaggaatcctcctcctccgaagatgactccgacgacga

gcctggggaggaagcccttcctaaatcgagcctcgccaacgaagatgatggacctgaattggtcgacgtgacggtgccgaagaagcgacagcgaagac

gaccggtgtacgttggcgagcttgtcccgctgctgagatcgagcgagcgaagcgagagtcgtaaagggttgaaaaacagcgaggagctcatcaggagg

aagactggatggggcatggaaatcgaggaaacagctgtggacttggcgttggccctggctgcgcttcagaacaactggtcaaccaaacacttcgaaga

gcgtaggaccggcgcgctttcagcgctgattgtcgctgcgcctcaggcggcgggaggatgcatcatcgagcaatacttctccggccactactccattg

ctcaacgcatggccatgctgaactctctagtcgaagccgctcgcgacctcgctgaacgggccgggcagcaaacttcgctcacggccaagcagacagca

ggcaagaatgcagacgtgctgattaggcatgccgcaggagaggcgcgtcaatacggagaacaaaaatacccagcggtcagacaggcgaacaacctcaa

catcctacgtgctccgagaggcggcacgctgatcacggagacgtcttcgtcctcaagtgtcgctgcaaggcgggacggcagacctatcaatgttgcaa

gtctacccggagcttcgatctccaggctgccgcgatatcttgacatcgcctcgagcgtcttcatcttcccgctcatgaaccgcttttgggcggtgatc

aacgacgcaggagtgcgacagagtcggtcgttgcaaggtgcccggggcagtcgacgtgcccagtccgtctggcaaggcgcggggacggggtccgttct

ttcggccccggccatcggctgtctgcttgatgcactggctgtgatggccagcttggcgcgcaacgctctggacttccagcgagtcatcgtaccagagc

ttcttgagctgagcctggctacccctcgagctatgcttcctctgatcggccgcgacgtcgactcggacgacatgggccggatccacgagggcgctacg

acacttgccgtcgttctcgtcgatacggccagagaagctgattcgggacggttcttgcgccgagaaaagggcatgttgttgagggagctggacatgta

cgcggccgaggtcttcaaggtggagaatgaaaaggagaggggtacgagttcggccatgagccgggcgggtcgagccagtgctacgcttctgctccgtc

tgcaagagattcagtcgcaatag

>contig_14.contig_14.g200.t1

atgtccggcttcgactccggccgcatcttctccgcccaggtgctcgccggctcagaagagtcgcgcgcaccggacgcac

ccgtccagaccgaggccaccttcttcgacttcatccagcgcttccgacagggtaacagcttcatctaccgtgaccggctgcggtccaacttgctcgcc

aggcagtatgtgctcgaggtgcagcttgagcatgttcagctctggtcggctcagctcgcacaggacttgcgcgatacacctgccgaggtccttcctct

tttcgagtcggccgttcgacgagccgctcgcctgattctatacccagtcgcagagggcggtgtccggccagaagcaccagaatgccaagtcaccctcc

tctccagcgtgaacctgctcgccatgcgcgacctgcacgccgactccatctcccacctcgtccgtattcccggcatcgtcatcggcgccaccacgctc

gcgtcccgcgccacgtctctgctcatccggtgccgcgactgcggctcgacccgtcaactgcccatttacggtggctttggtggtttcacgttgcctcg

atactgcgacgcttctcggaatgactcgggcgcggggggtgcgatggctaagtgcagtacggatccctttgtcgtcgttcacgacaagtgcaactttg

tcgacacgcagacgatcaagttgcaggaggcgccggatatggttcccgtcggagaactgccgcgccatatgcttctcagtgcggaccgtgctctctgc

ggccgtgtcgttcctggctcgcggatcatcgcgacaggcatctactcgaccttttcgagctcaaagggagggagttctggtggggctgtcgcgattcg

gacgccttaccttcgcgtcattgggttggaaattgactcggagggcgcgggaggccgaggagggcagcggatgttcagcgcggcggaggaggaagaat

ttcagcgtttggcaaggacacctgatctctacaagaagttcgccaacagtatcgcaccaagtgtctttggcggagaggacatcaagaaggctatcaca

tgtctgctgttcggaggcagcaaaaagatacttcccgacggtatgcggcttcgtggagacatcaatgtactgcttctcggtgaccccggtaccgccaa

gagtcaattgctcaaattcgtcgaaaaggtctctcccatttctgtgtacacctcgggcaaaggaagtagtgccgccggtttgacagcatcggttcagc

gtgactcggcctcgcgcgagttttacctcgaaggcggcgcgatggtccttgccgatggcggtgtggtctgcatcgatgagttcgacaagatgcgtgac

gaggaccgcgttgcaatccacgaggcaatggagcaacaaacaatctcaatcgcaaaggctggcattacgacggtactcaacagcagaacatccgtgct

ggccgcggccaaccctgtattcggtcgctacgatgacatgaagagtccaggcgagaacattgacttccagacgacgatcctgagtcgatttgacatga

ttttcatcgtcaaggacgaacacgacgaggcgcgggaccggacgattgcgaagcacgtcatgaacatccacatgaaccgggcgacggacaatgcgaat

gcggtgggcgagctggacatcgagcagatgaagcggttcatcgcctacgccaagtgccggtgcgctccccgactgagtcccgaggctgcggagaagct

gtcgagccacttcgtctcgctgcggaagcacatagcccaggtggagcgcgacaacgacgagcgttcctcgatcccgatcaccgtgcgtcagctggagg

ccattgtgcggatgagcgagagcgtggcgaaagtgacgctgagtccgacggtgaaagaggagcatgtggacgaggcgattcggctgttcaagttttcg

acgatggatgctgtgcaggcgggcagtgtggaggggatgacgaggggcgagctgcaggaggaggtgcagaagttggagagggagatcaagaggcgctt

gccgattgggtggacgacgagctatgcgaagttgcggtctgagtttgttgaggggcagggattcacacatcatgccttggagcgcaccctgttcatct

tggacaagagggatatcatccgattctcggcgcaacggcgagccatcacaagaacgggcgtttga

>contig_14.contig_14.g201.t1

atggatctcgaaggagacgcatcaacaagcacggccagctcgtcgtcagctgcagccatgctgggtggcatcgcggtcg

gtggtatcacagccgctgccaccgccacagccgacgaccaagactccaacagctccccaccttcgcccgagctccctcccctccccgccggcgcactc

gcgctcgccccagccccaccactcgagaccctgcccatcgcgaacatctcccgcatcatgcggcgctcccttcccgaatcggccaagatcggcaagga

cgcgaaagaggcggtgcaaattgtgctgagcgagtttatcagctttgtcacgtcggaggcgaatgagcgctgcttggcggatcgacggaagacgatca

gtggggatgatctcttgactgcttttcggctgttggggctggatgagtaccatgaggttttgcgcatcttcttgacgcggtatcgtatggcgcaagag

gcaaatccacgaccgaagcggagacgtgcatcagcaaagcgaaaagctacgactgaagaggaggggcagggtcacgacgcaggtcaggaagaggagga

cgaggatgacgacgatgggggtgatgaggacgactga

>contig_14.contig_14.g202.t1

atggcatcaacatcagcagcgagcgggagtgcacccgagatcgagatcacccagagcggcatccccaaggcccccttcg

tcgtcgatgtcgagcaacacataggcggcgcagacaagccggtagagcctgcattgcgcaagttccaagaagcgactgccaagtacaagttcatggag

cagaacacggcacagcggcggagagggctggaggagaagataccggatatccggaaaacgctgcagatggtcgagcatctcaagtcgcgcaaggacga

ggaggaggatatggagaccatgtttgagctgaatgacaccttgttcgccaaggccaggatcccgcccgtggacacggtcaacctctggctcggggcca

acgtaatgctgtcgtacccactggacgaagcgatcgacctactccgctccaagctgtcaggcgccgaatcctccttggagggagccaaggccgacctc

gacttcttgcgggaacagatcaccgtgatggaggtcaacacggcgcggtgtcacaactgggacgtggggcggcgacgaagaatacgcgacgagcaaac

caagcaggcgaaggcggagaaggagtaa

>contig_14.contig_14.g203.t1

atggtccaagtctccaacgagctcgagtacctgctcacccgcaccacctcctcctacatcgtcaagcagaacggcctcc

cccgctacttctcccgcgagcccaggaacctggcccaggtccacgccttcaagtactccggcaccaccaacaacaagaccatcggcatcgaggccgtc

cccggcggcatcgtcgtctccacccgcaagagcaaggccgcccctaacaccctcaagggcgcatacagcaccactaccattaagaagggtggtagccg

acgtgccgctggtgttgctagcaacgccgcctctaagaccggataccgacgtgacctgacccaggtcgccgtcgcccgtgccaccaagatcctccgcg

cccagaactcccgcaaggtccgccccaccgcaatcaggccccgcggcaagaaggccatcgccgccgccaacgccaagctcaacaaggcctccgctgag

gagtccgccgccgttgaggagactgcatga

>contig_14.contig_14.g204.t1

atggcagcagcagcagcagcagcaggagcaggaggagggggaggaggaagcgcgggtgggagcacactgcctggggatt

ccttcaacagcagcagcagaggcgatgtcggtgacgaagacgatctgctcccgcccttcctggtcgggggacagcccttcctggacacgaccccgttg

cgcgatctcagcaggcgcaccttcttccacgccctcgcagcagcaacaccgccaatcccaccaccagcagcagcagcctcgtcatccacatccacacc

cagaccacccacagacatcacccccaagaccgtcatcctcgaccccgcactcgcaggtccactcggcctcaccatcgatgtagcatccctcaaggccc

atggcgtcgacaagatgttctggctcgagctcgacgacaaccccaccgagcatgacgagcttgacgacccagcaccaccgccgtcatcagccccaggt

ccgtcttcgtcctcactaccacaacagcagcagcaacagcaacatcaaccagcagatgtactcggcatacgcgcacgctggcctcccaagagactacg

tgtccccgtaaatgcacccacgcgcagcgtcgtctacatctgcagaccagaaatagcatgccacatcatcgccgacctggctgcaaacagtcaggtcc

ccatcaacccctccggacccctgcccccaagctcctccctctcccacacatactccctcctcctctccccacgccgcacggagctctccttactctac

ctctcctccctccgtctcctacccgccccaactcccgtcgacgcatcttcctccaacccagcagccgcatcaagctcagccagctcaaccaccacgcc

gtcgtcctacctgaccaccgtgctcgatctgccgctcgagttcttgccccttgacggatcacccgccccagctccgcctgcacctcaatcatctgggg

ttgctaagggtggtccatcatcttctagcacgattccaccggccatcacctccagctcgctactctccctcgagtggactcatcacttcgccttcacc

cggctctatcgcgacggcgatcatgcatgtatatacctcgccgcacaggctttgatgaccgtacagcacgtatacggcctcatccctcgcatcttggg

caagggtgatctcgcaaagcgcctcgcggacctactcattcgacagcgcagagaacatctctcagcagatcccaccaacccggccttagtcgcccctc

ccacttttatgcccatggctcccgctcctccagcgtccacacaagcgggcggtgcagcatcagcaggggcacccggttcagctaccaccgccagcgca

gggtggagtaatccgaaccgcatcgacgcactcatcatcatcgacaggagcgtggattgggccagtgtgctttgcacccagttgacgtacgagggtct

catcaaggaagtcgttggcattcggaatgcccacatcgacgtggacgcgtccttggtcggaagcgggaatacgcccggccccgccggcacacccagtt

cctcttccgccgtcctagagagccgctcaccaggcgggccccggaaacccaacgcggccacctcaacacccagcacaggcgccaagcgaaaatacctc

ctctcctccaacacggacccgctctacacccgcttgcgcgacctgaacttctccgccgtcggcgagcgtctgcacctgatggccacccgtctgaacgc

gtcctacgcggaccggcacagcgcgaaaaccatggcgcagattaaagcgttcgtggggaagttggggagcttgcaggcgatgcatcaggcgcttgggg

tgcatacggacttgacggagcggatcacgcggaggacgagcgcggatgaggtcttccatcggcttttggagattcagcagaatattgtggcgggcttg

gacacgcctggtcagttgggggcgattgaggatcttatttatgcgagtacgggcgggccgggggggttgctttctggtctttccgtgcgggatgctcg

gccgtctggcgccggtgctgaaggctctgcagccggaggaggagcgggaacggggagtggacaaccatcgacggcgtcgctccttccgatcctccgac

tgctctgtctgatgagtgtggtcaatggcggtctgaaggcgaagaacttggagtatgtgaagcgggagctttgtcagacgttcgggtacgaggttttg

cccctccttttgcagctttccaaggttggcctcttgacgaaggaaggcgcgggcccgggggctggtttcagttcgactgcttccgggatggaggggtc

tggcggtaccgggcccgggcaactcggcgcatcgacgaacatggcagcagggaactcccaaggcggcggtggaagtggagcgggcggtgcgaaccttc

ccacatccctctcggcgaccggttcgacctcctactccggcttctcagccgtgcgtcggacgctcaagttgatcaatgacgacgtggacgagcggaac

ccgcgggacatcagttatgtctttagtgggtatgcgccgttgagtgttcggctggtgcagattgttgcgcagcgggaggcgttgatttctgattctgg

tgtgggtggtggtggggaagcgggggcgggggcgggggccaggagtgcggcggggacgggggggtatggtggggtacgaccgaatcgtctgcgaccaa

gggcacatccgatcgcggggtggaaggggtatgaggacgtgctctctatcctgcccggcgcgacgttcgacgaagtccaacggcccgtgcaaaacttc

cgtccagccgccgcggccgccggtgccactacctcctccatccccgccgccaacaagcggtccacgacgagcaccgcgagcacggggacgcccctcgt

gctccagccgcagctgatcgaccccgacagtccgcggacgacgcttgtgttcttcctgggcggggcgacgtatgcggaggtttcggcgttgcggttca

tgtctgcgcagacgcggaatcggcggtttattgcgatgactacgcatatggtttctggggatgagatccttggtgggctgtga

>contig_14.contig_14.g205.t1

atggcagccacaatgccaggctcgatgatggccaccactcacaacgacagcggcagcggcagaataccaaacagacggt

gggagaagcgcaggaggaaggccaggaggatcgactgcccacgccccatcgctgccatcctcatcgccgccgtcgccatcgccatcgccaccatgcca

gcgacagcagcagcagcagcagcacgaccacaaggaagaagattagcagaacagacaatcgccaaggcacacgcatacacagacaaacaccacgcaat

ggcctccctcgcatccgcagcaaccgcctcctgggcaccccgcgaacgctccgtctggttccagcgcaaagcaatcatcatcacctccgtcttcctcg

ccatcttcatcgtcctcatcatcggatgcgccgtcttcctccgcgaccgcaagtacgacatctccgatcacgacctacacgccgaggaggaggaggag

atcaggtgggaggagatgggcgtgcctgagcatgagagggagcgtatgagggatgatagggagcagatgtggatccgcagggagatggctcgcaagga

ggctgccagggatgccaaggcggagaagcgcagggtaaaacgggaggcaaaacgcagacgcaaggcactcaaactgggcatcccatatgaggagcaca

accacaacgacgacgacgacgatggtgatcatgcagatgaggctcatcctgctgctggtgctgctactgccgaaagtacagcactccagactcgcttc

atcgcaggtcgctgggcaagaaatcgcttacagaccgccataagggtcatccgccccaaccacacctcacgctccacatcctcctccacatccttcaa

caagaacaagaacaagaaggacgctcacttcaacttggccgatctcggccctcccaccaccaccaccatcaccacggccagcctgggctcggcctcgg

attcgataccaccccggcccagaccacactcggacgaaccgacgtcgctgtcgtcttcgttgacttcgcattccctcgtatccacttcatcagcaact

aaccctgcaccggccgtcgttgcaccagacggatcatctctactgccctccttgatggtcggtggcgccatcgggcccggaagacctccttccttctt

cggcggcgacgacgacgacgatgggcgccaatctgatgccgatctttcgcctgccgaggaaggtggtcgggtggatacaactcaaggcaggagaagcg

ctggcaggacacgggctagattacggagtagggacggcaccacccccaccccaacagtcctcgaaccggcgttcctcttcccaccaccagccacgccc

ccacctcgtccatcacgcaccctcgaggacccaccacctccgcatacacccctcggcaccgcaccccgctcgcccgccttcctcaacgcatcggacct

ctccgacgcggaagcgtatgaggagcgcgaactgatacatcgacagcgcagtcggactgcctcgactgccccgcacccgccctcgtctgaggggatgg

gtggtgcgctgccggcgtcggcttcgagtgcgacgctgtcgagttcgtcgatgcgtccgggtgggattggggcgggtacgccttcttcgtcggcgaac

aatgctggaagcggtgctcctgacgtaggggacgaagaggaggagatgttcccgccggcgtatatcgctcctgcaccttcctcccgcgccgcggggcc

ttcttcgagaagaagagccgacgagaagcggagagtggttgatgacgatgatgatgatgatgggcaggaggatctggggccggaggaggaggagagga

ggagggcagcggcgattgaggagcggttgtatgctgccggtctgggacgggtcgaaggggaggatgggacgggctcgtcgaatctaatcccaccgccg

cccattgcgccccatgccgctgcggccgcggcggcgggatcagttcccacgtccacttctgctggagcagcaggaagcggtgcacatgggagaaggac

gagttcggtacggcataccgtccacgttgcgactgatgagaagtccgcgttgcgtgccctggaacaggcgaggggtgcgcccacttcttcttcctccg

cacggcgcccgccatcttcgtcttctgcggcggagcgacctagtgcgccgaccttggctctggacgctgatggcttcgagacgttcgtggtaccttcc

tctgcgcctcgggtggagaaggaaggaggagggtcgtcgtcgtcgttggcggcggcggcggcatcgggtctgcctgctccgccacggccgcatgtccc

tggctggtcggagtttgatgagccttatcgtgcggttgcaggggcaggtcctacattaccatcggcgccgtccgctcctcctccatccagctcgaaac

cgacgcccaagtcgacgtcggcgcaagcaaaagcgcgcgaagctggctttgcgggagaggcagcgccggatgacgacgaagtcgtggctgggatcgtg

cccagcaggcctgggctggtgtcgtcattaccgccatcacatccccaggccgccggagcgggattgggaggggcggggatggggatggggaacttctt

gccgcagtacgaggcttcgcgtggtgcagggtcgagaggacctgaggaaggggaggagattgtcgcacctagcgcgccaccgatgtgggaggaggagg

aggaggaggaggaggaggaggaggaggaggaggaggaggaagaacagggtggaaatgggaacgggcggcatgcggcggtatga

>contig_14.contig_14.g206.t1

atgaacggcaacggcaacagcggcagcggtgtggacggcgatggacatggctccctctctgtcaatcacccagagagtc

tccgacaacaggcaaagatcgacatcgaccagcgcttcagcttcaagagctacgtgtctgcggccgatagtttactcgaaaaggccgaggcagcggaa

cgacagggacatctggaggaagccttcgttaactatctcaaagccatcgcggtgacgcagaagctgcgtggccacaaggagcatcctgtagaggagag

cacaaaatcccacaactggtcagcgttcaaggctctcgtccagcgctcgtaccacaccgtggagcgaaccaatcgaatcgagagtgtgctgctggaca

gagagaagcgcatcagtgagctgcttcgcgaacagcagaagaaaacttcaacatcttcatcatctcgtaccgctcagccggcgacgctaccgaccatg

ataggatcgcccacaatcgagggccccaccgatcagggcgtcgacgccgagctcagctccttgtcagagcgactgaactccctccatcacagcggcat

ggccggaagcctcgtcaatgccagacgcatgagcgatgccatcggtggaggtatacagaacattaacctacgttcaacaccagaggagagaggaaacg

gcgcagcacgagatgagctcaatggatcctcatatgcacccgctcgagggcctcttccacggcctccggcacaatacgctgcggacgcgttcccatca

tcagccaaaggggacttgccaagcattgaagacgtggaacattcctatccgagcctagacgagttcgagcgagacgcgcgctttccacaggccccaaa

gcatttcgtcacagacgaccctgcagctgctgctctagcagcctcggttcccatccgccctttgccagtaccgccttcagcaccagactcgtcgtctc

cacgcggcagcgtgccccaacccttcccagatcgaccgcgcccgcaatcaatctcacaacagcaggatctccatgcacgcgcgaggcaagggtccgac

ccgcgccgaggcccacttccgccacctgccggcccagcagtcgcgagagtggcgcccgcttcctccagcggcggtgtagggccaaaaccttctctgcc

gctgtccaacggggtgaccgtagagcaactgtggacatacctcaatccaggattcgagtccgtcaccgaccctaagacgggcaagcaccggatagaaa

agcggcaaggcttacagattctcctgcttgatgtgcggagtcgggacgagtttgaagctgggcggattcaggggcctcagggcgaaggcgtctgtctg

gacccaatcaccttgcgcgaaggcatgtcatccgaggatctcgaggagaagctggtgctttcacccaccgctgagcaacaggccttcgcacatcgcca

ctcgtacgacctcatcgtgctctacgatcggagctctcggagctttggcgcgccgtcgaacggccccagcaagtttgcctcgatgccgacacccacca

cgatgctgtcgcaacaatccaaggcggcggcagacaaacttgcgatcgtgatgagggccatctatgagaacgagttcctcaaacctttgaagcattca

cctgtgctgttagtgggcggctacgacgcgtggtcgaaggagatcactaggagtgcgccagagtctcggtcgtcttccaatgcgcgggaggcaaagat

ggacgatgtcaaaaggtcacgaagggatgcgcgagtcgtgcctggggagaatgctgtctctggtagggacacgccattgagcccgcaaagctcagcag

ccaccccgggcgatgcaaatggcgcaggcgcttctggtgcgaacgggttcgttcccccctccatgccagcaagagcgtatcatcacgtctcttcgccc

tcccacccacaatcgccggcgtcgatctacccgagtcctcctcagaacacgctcagcagaacacgatcggacgattacttcttcgcggctggtcagac

cggtgtgccattagctggtccgcatggccgcgcgctggcccacatggccgcgggcacacgaagctcgtttgactaccctcagattcatgcgaatcatc

caagcccgcaattaccgccggcagctgcttctgcgtccggcgcaccatcgcgaatgccggcgccgatcacacatccttcctcgtcctcctcttcttcc

acctacagcaacggcgtccggtcctcgttctccaatgactcggcggggaggagaaacgggctgttaccgcatgagactgcgcatgggtaccctgttat

gcctggggcgggggcgatgccggtcgtgcggtccatggtggatgataagcggatcgggctgacggggttgaagaatttgggaaattcgtgttatatga

atgcgacgctgcagtgtttgagtgcgacggtacctctggcagactttttcttggatggccgatacaagaaggcgatcaatcggtccaaccctcttggc

actcagggtgtcctggccgaggcgtttgcccagctcattcgggtcatgtggtccgagctctacgacttcgtctcacccgtcacgttccgcgaagcgat

cacacgcttcgcaccggcattcagaggctacgagcagcacgacagtcaagagttcctcatcttcctgctcgacggtttgcatgaggatctgaactatg

tgattcagaagccacctgcggtggagatgtcgcctgcgcgggaggccgagctcgagtcgcttccgcagcagattgcgagtgcaaaggagtgggcgatt

tacaagcagaggaacgatagtttgatcgtgaactggttccaaggccagttccggaatgtgatgacgtgtatgacatgcgggaagacatcaacgacgta

caacgcgttcatgtacctttcgcttccgctaccgtccgggcggaactactcgcgggtctccctgtacgactgtctagacgccttcgtccgacaagaag

tgctggaaaaagccgacgcgtggttctgccctcaatgcaaaaaaccacgcaaagcgacgaagaagctcagcatctcgcgcctgcccgcgatcctgctc

atccacctcaagcggttctccttcaagggcccttttaccgacaagattgagacgcaggtgactttcccgcttacggagcttgatttgacgcggtacat

gccgccgccgttgccgccgggtgcgggtttggagggagggccggtgagcaagtcgcagcagccgccttatgtgtatgacttgcatggggtcacgcatc

actttggcagtttgaacgcgggtcattacactgcgagcgtccggaattgcggccaatggtggtacttcgatgacagtcgattgacgaagagcgacgaa

cgccagatcaactccaattcgccgtatgtgctctggttccgccgacggacccacacgcgggcgggatcacaaccaccgaacggcggcggtgacggatc

atga

>contig_14.contig_14.g207.t1

atgtcgagctctcctgaccagccgagcatttctatcgctacccgactgtttcgacgtgaacaggagaaggaagatcgca

agctcagggtctctaccactcttttcttcaaggacgtcttccgtgtccgagcgtccgtcttgccacgtgtgtggccatcggtcctcggagtgactgct

tgggccacggccgtcgccaccgcagacttgctctacggtcgtagctggcaaactacaagtgccgtcgtcgctccgctcagtattgttgttggtctgct

catcgctttccgcaacagctccagcattgtcgatcgctggcaggccggtctcatcctctggctcaatacaatgtcagacgctcgcaacctgatccgct

acctttggctcaatatcgacatcgacgctcccgctccagagggtgtcaaggttcggtcacgagaggagcgtctcgaacgcaagaaggccgccattaga

atggtcctcggtttcgtagtcgcggccaagcacttccttcgctccgaacccggcatcggtcacgccgagctacaagatcttctgcccgaggacctcaa

agtccgctggcttgtctccatcaatcgcagcatcgagggccgatcccctcaacagccctcaacctatcaagaagactcacccttgctcgaagcttccg

tcgaggaggacaaggacgacccagctctctgtctgcccctcttcatcctacacgagctcgccgtctatgtcgtcggtgtccgagctgcaaagttaatg

gagccggcaggtcccgccgggttttccgcgatcaatggactcctcaactctctgaccaacaatttggccaatctcgagaggatcgctactattcagct

tcccacgattcttagcatacatctcaagaccgtggtcctgctctaccttttctccctacccttgacccttgtcggtgaactctcatggagaatggtac

ccttcgtcaccatcgtcgccttcatcttcatcggcatagagggaatcagctcggagatcgaaatgcccttcggcgacgatccctccgaccacaacctc

aacctgtacacggcccagctccgacacgaggcagagcacatgatggccttcttcagccagccgccgtcgtacgaataa

>contig_14.contig_14.g208.t1

atgcagatcaagtccctcatccttgcgaccttcgccatcttcgtcggcgccaccgcagtagccgcgtctcttgaacccg

cctcctcagcccaggccgccgctttccggaagcaccagcacagccgctactccgccgacctcgctgctcgggacgattttgttgaccctgggcccgat

gatgcggaccctgctcctcaggttcctgaagccgctacggaccctaacaaggacctcactgacatcgccagcaagctcgaccagcaagtcacagcccc

gctcctcgagatcgccgccgccgccgacctgaagaccatcgacctctccaccctcctccaatccaccaagaccggcctcacacagtcaacggcaatcc

tcggcaacttgatcgagaacctcatgagcgcgcccaccagccccgtccctcaccaacgtcgggcagatgtcgacgtgactgcagctgctgccgccgtt

ctgaacggtgctagcaaatcgctcgtgtctggtgcggctcgcaagatgtttggggctgctaaagatgcggcgcagcatttggtcgaattgaagctcga

tctgagcgcgggtggcgaagttggtgaactcgttgataccgtctttgagctctttgttcaattcctccagacgctggccgaggtcgttcccagcatcc

gcgacctcgaatccggtcctctcgagtcccttcagagcaccgtcggcgagctcttctag

>contig_14.contig_14.g209.t1

atggcagacgcaaaggacatcagcaccagcctcagcgacgtatcggaccgcattgccaacctcatgggcaagggcaccg

cagacgcagcgcccgcctcggccgagggcaagagcgccgatgccgacaagatggcagacgaacccgtcgccgaaggggaatcggtcgcgggaaggaag

ccgacggagctgcgcgagtctagctaccaggtcatggtgacgctcgccgaccagcaagcggaccccaactctcctctgttctccgccaagactttcga

acagctcggactgcacgaggatttgctgaagggtatctacgctatgaagtatcaaaagccttccaagatccaagagcgcgctctgcccctcctccttc

aaaaccctcctcgtaacatgatcggacagtcccagtcgggtaccggcaagactgcggctttcgtcctcaccatgctgtccagaatcgactacagcgtc

aagaaaccccaggcgattgccctcgctccttctcgtgagctcgcccggcaaacgatggacgtcgtacacgagatgggcaagttcactcctgtcacgac

cgcatacgccattcccggtgccatcgagcgcggcgacaaggtcgatgctcagctcattgtcggcacgcccggaaagctgaccgagctgatcaagtaca

agactcttgacacgaaccatgttaaggtcttcgttctggacgaggccgataacatgttggaccagcagggtcttggggagcagagtatccgcgttaag

aacatgctgcccaagtcatgccagctcgtcctcttctccgccaccttccctgatcacgtccgtgacttcgcagcccgcattgccccttcagccaacga

aattcgtctgcggcaggaggagctcagcgtggagggaataaagcagttctacatggactgcaagaacgaagagcacaagtacgatgttctcgtggagc

tgtacagcctgttgaccatcggacaaagtatcatcttctgcgggaaacgcgagacggcggaccagatcgccaagcggatgaccgccgagggccacaag

acggactcgctacacggcaagctggaagcctcggcgcgtgaccggacgattgacgacttccgagacgggaagatcaaggtccttattgcgaccaatgt

gatctctcggggtattgatatccagcaggtgacgcttgtgatcaactatgatatgccgctcacccaggcgaatcagcctgatgcggagacgtacttgc

accgcattggccgtaccggtcgattcggccgcaagggtgtctccatcaacttcgtccacgataagcagagctgggagcacatgaatgcgattgagact

ctcctcaagtgcaacattacccgggtcagcacggatgatctggaggctatggagaagactatcaaggatgcgatcgactgggggacgaagatgtcgca

agcagatgggaagggcaagggcaaggcggagggcccaagctctgggcagacttcctcccagaatccccccagcacatcctctacttccagctacaatc

agctcagcaactctacaacgacaacgtcgtcctcctcggccaaagcacaccatgcgaatcccacaagtttccccagcgcgggcaacgccggcgtccga

gcgggtgggatcgcagctgaattggcggccatgatgcgatcgagcgggaaggctggatccacagcctcagcttcggcagcgggctcgagtgcgcagct

gcttgctgattctgcgctgagtgatgctctgcaaggcgcgggcgcgggtgcgggtggtggcgcagcagtgggtggaccgggcgctgggtttcggagag

cggcggcgcgtgaggaggagtcggcttcgttatcgtccgcggctgggatggagtttgatcagttcgcgtctgggtcggcatcagcagcgggcgtggga

gctggcggggcgacatatgctcaacagcagcatcatccgatgctccgtcctgggcaaccgctctcacctcgactggcggcgtacagcgaggcgctcag

aggaccttggtcctcagcttcggatctcgacggcgagcttcatgcgcaggcgcggcgcgagagtctgatctcgcccatcggggctgattacgaacagc

cccaatcatctgcctgggtcaacgaattcgcggaaggggctgcttctgggtacggacctggggtggacgaagctgcgatggaggaagcttggcgcgcg

tatacgcgagaagtagaagtgggggttgatgatttcaaggtgggaaatggggaggaacaggctaggatgttggatcctgactttcatgcggaatgggc

tcggacggtgcctgcgcccacttttggagctgcggaatgggctacggccggaggtcaggaggtctctggcccatcaggatcgacaggagatgctggag

aggcggacttcttcgctacgctcgaagctgaagaacgggccgaagaggctctttcacgacagcgcggtccgcctgttgtagctcgacccgtgccagcg

gccgtgaacggtgtcttcagcgcagactcacaagcggccgacctggcccagctcggtatcgacgcgagccgactaccctcgggaatcacgacggagtg

gcgaccaccttccccctccggtccgtcgagcgtgagcatcgagcaactacttctccaccaagccctcgccgcgaagcaagaagcgagcgagagtgacg

atgcggaccgagcgagggagcggatccgacctgccgacgacgacgagcgggaagggcagggcgtgtttgcttctacgcctgaggatgcgcttcggagt

gtgtgggatgggaaggcggaggcggagcgggtgaggagggataaggagaagcggcttgttgatctggctgagcggatacggaggggagagtcgcttga

ggagacggaacgggatgtggatcggctgatcgctgatgttaagcggtggttcccgcgcgggacgtatgcagagaaggtatggggccagctccaccact

tcgcctccttcccgcagacaggcatcagtctccgccagatggtccagttcggcaggaatccaacaccgggcaccctcttccaggcaggcagcttcctc

gccgaagagctccccgtccgactcgcacaccgcgtcaaggagctcgacgagcttcccaacggcctcaacgagcagccttccatcgttaaggtcaagaa

ctggtatgctcagtcgtttgaggaactcgtcatgttccccaaaccgcgtctgccagacaccattcagaagcagcttgctgctgctgctgccaagacaa

cctccgagctaccggaatcaacaccgaacccgtccttgtacgagatggactccctcctttccgatccctcttccagcacaggtcttatgagcaagaac

gggtccaagcgaaggatgcccttggcaaatcgatactacacagatgtggatgagattgactggccggtagaggtgtctgagtacaacgagaacttcac

aaagtgcttggagcagatcaagaagaggcacgacgccgtcgtcacaactgttgcccaaggtgtgctcgaatacaagcgatcaacacgtcgtgaaacca

tccaagccgacgttcaacgcttcctcgaccgcttctacctctcccgcatcggcatccgcttcctcatcggccaacacatcgccctctcccactcctcc

aagcgaggcctcggccccggcaaacggggtgttatcgaccacgagctcctccgcggctcgtcctcacagaagtctccagacggggagcagtacgtcgg

catcatatgcgtcaacaccaacgtgggcgctatggcgcatgaggctattgagaacgcgcgctttgtttgcgaggagcattatggactgttcaagggtc

cgcctgtgcagcttgtatgtcctcgggatctgacgttcatgtacgtccccagccatttgaaccacatgctcttcgagcttctcaaaaactctctccgc

gccgtcgtcgagcgatacggcgtcgagaacgaggacaacttcccgccgatcaaggtcatagtcgtcgaggggaaggaggacatcacgatcaagattag

tgatgagggtggaggaatcccacgaagtgaaatgccgctcgtatggacgtacatgtacaccaccgcacagtcggaagacctcgatccagagttcagcg

cgtctgacttcaaagcaccgatggccggctttggctacggtctccccctggcgcgactgtacgcaagatacttcggaggcgacctgaagttgatctcg

atggagggatacggtaccgacgtctacctgcatctcaaccgactctcgtccagcagcgaaccgctaccatga

>contig_14.contig_14.g210.t1

atgtcggagtccctggacgacttcctcgctggctggatcctgcacgatgccaagctcatcaccttccgcctgctcagtc

gcgcacgaggtctgcacgtccaggacgccaaagctgcgctgcagaacttctacgagcagaagagcaaaaagtcgcccttggctctgagcgccatatac

gtgctcaagggcacactgaagcaggacgtccgaggaatccacgggtacaagcgcgaggcggccgacaacgatggcgacatcgccatgaccgacgaagc

agcaggcacctcggcctcgcaaggtcaaaacgcacccgaatcaattccgagaaaacagttcctcctcgtgtccgccgataaactggaagagaccaagg

aacgtttttcctcccttactaccgcgcacatctactctctcgctcccggtcccgacccgcaagcttcctcttcctcctcttcctctgcgaaggctgaa

actacatcgcttgcggaggcggcgcgcaagcagcttcccctgctcgctacggttcaacatgacttacattcgaatcgggagtatgtgatggcgtggga

gaaggcgaggagggggagagcgcttggtgtcatttggaatgcggacgctgtggaggacttctccccgagcgcggctccagcagctcgggcgtcagtcg

caggaccatcagtcaagaaaccattacagaaagaaaaagcatcagaaaagcctgtcaaggtagagccagccaaagtggagaagaaatcgtcctcaagc

aagctggacagcaaagcttcttcgagcagcaagacggacagcaaagcttcgtccaagcaggccttggagaacatgctcttccccaagtccgaacccgt

tcccaaggccgaaacgacgacaacctccgcggcagcgaagaagacatcagaaccatcgagcaaggcgacgacaaccaagacgagcgctacgacgagca

agcggacgacgagcggggcgcagaagcgatccgtgccggagcctgaggaagatgaagatggggaggacgaagataggaagaaagcggacggcggtgct

tcgggggtgaagagacgggtgaagaagacgaggacggtgactaaaaccgagcgctcgaaggacgagagaggatacacaggtacgttccctatccttcc

ccatcccaacccagcttgtatcattttccctaaccccttcctttccaaccaaatcgtagtgcggaagaacgtcgaggtggaagaggagtatacgacgg

atgagtcttcggatgaggtgcaggcgcggccgaaaccgtcgaagatggcgaagaagaccgcggcggcagcgactttgtcctcttcatcttcatcctcc

tcgaggaaagcttcaaacgccaaagcaacacgcgcaccctccccctcaccctcgcctcccccaccaccaccaaaaccagcgccaaagccaagctcaaa

accaggcccagctcctcctccccccgcgcccgcaaaagcaaaggccagcagcagcaccttcggcgccgctcgatcagggagcggctctggctctggct

ctgggtccggtgcggcgaagaagccggtggataagcgacagcagacgttgggctcgttctttaagaagaagacgtga

>contig_14.contig_14.g211.t1

atggcaagaccacagctcgcactcgcaagaacagcactcgcagctgccataagagcaggcgcccgctcatccccagcag

cagcggcggcggcggcggcggcggcggccagtctctcacagcccagctcacaaagaagaccaacagcactcccctcacgatcgctctcgacatcccca

ccgctcaccaacgcaggcctcacagccctccaccagcgcaagatcacaatcgagtccagcgacccaacaaccaagtatcagctcatggtcaaccaggg

cctcatccgcgacgacccacaccagcgcagcatcgtcaacgtcttacagcgtctccacgaggagctcaagacctacaagcagatcccgcccaaggagc

tcacacccgaccaaataaagcacgacctcaacaagaaaaagaagggcttcttccaatcactcttcggcggaggcccggacaaggacaacgcagccgac

gatgaatacaaggtcccagaagacgtcccaaaaggtctctacctctacggagacgtcggcacaggcaagagcatgctcatggacctcttctacgagac

cctccccgccaacatcacccgcgcccgccggatccacttccaccagttcatgatcgacgcccacaagcgcgcacacgccatcaagtccctcacccacg

tcccgagcgggatggtcatgagcgctgtcaccgcggctaccagcactgtgcgctcggctggaccggccggtgggggcacggagcggagtttgcttaat

agggccggagggagtggggaggcggatccgattgggcctacggcaagggagcttgcgagggagtatgaggttttgtgctttgatgagttccaggtcac

cgacattgcagatgccatgatcatgcggaaactactcacaaccatgatggcccacggtgttgtcttcgtcatgacttccaaccgacatccagacaatc

tctacaagaacggaattcaacgaagcagcttcgtaccctgcatcgaagccctcaaatcccaactgcgcgtcaccgacctgaactcaggaacagactac

cgcaagatgccccgcgccctctccaacgtctacttctccccacccacagccgaagccacgcgcgagtacgaaaagatcttcgacgcattcacctccga

cccttccgaccccctgctcgaaaatcggaaagtgcaggtatggggtcgggacctcctcgtcccgcgctccacgtcctccgtcgcgcgcttcaccttcc

aagaactgtgcggtcgtccccgggcggcggcggactacatcgagctctgcaacacgttctcgacggtcttcgtggacgggataccgcagatggggatc

catcagcgcgatgtggcaaggaggttgattacctttatcgatgcggcgtatgaggccaagacgaagttgttgatcacgagcgaggtgccggtgttgca

gatttttagtgggaatccggagaagggtcccacgagggatcagatgcgggcgcttatggatgatctgggcctcaccatggacgacatcggcggctccc

caatattcacaggcgaggaagagctcttcgccttcgcccgcgtcatctcccgcctgacggagatgagcagccagcgctgggccgagttctcaggcggg

agggagacggcggggaaactgaacgtcggttcgaaggatgaagctcaatag

>contig_14.contig_14.g212.t1

atggcacccacagcaacagcaggggcagcaggggcagcaggggcagcaggggcagccccctcagcagaagcagcagccg

tcctcaacgccatcgcagcaaggaagcgccggctcgatcacgaggacagcaaacccatatactcatacgacgacgacgacgacgatcctgatgatgtc

cccagcagcagcgcacgccccagcacctatcttcccctcaagcaaagaaaactgcaggagatacagtccatcaccgcaaagagatcatcatcacgacc

aacaggtgacctaccaccaccagcaaacggcggaaacgacgacgacgacgacgaacacgagcacgacaagggcgtcgcacagggaaccaagtccctcc

tcgccgaggcacgagctctccgagaagcccagccacaagacagcaaaagtctcgcagaaaaagaagcagaagaagaacgcaagatcctcgaagcccac

gcagcccgcacaaaactagccagcgcagccgagctcgcaaagggcgtcatctacaccgaatccctaaccaccacctggaccgctccctccttcatccg

cagacggacccaggaggagaacgagtcgatccggaagaagttggagatcttgtgcgatggggtcgacgtcccgcctgtgatcaccaatttcagagata

tgaagatcccacagtccatccttgactacctggccaccaagaacatcaagaaaccgtcgttgattcagatgcagggattacctaccatcttctccggt

agggacatgatcggcatcgcttttactggttcggggaagacccttgctttcagtctgcccatcgtcatgttcgccctcgaggaggaggctcgtatgcc

gtacatgcaaggcgaaggtcctacaggcctcatcatttgtccatctcgcgagctcgcccgccaaacatacgaaggcatcctcgccctcgcagaagccc

tcctcaagggaggctacccagagatccgcgtcctcctctgcatcggcggcatctccatggccgaacaatcatctgtcctccagcgcggcttccacatc

gtcgttgccacacccgggcgactgcaggatttgctcgagaagaagaagttcacgctcgagagctgcaagtatctgtgtatggacgaggcggatcggat

gattgatcagggcttcgaagatgatatcaggaatatcatgagtttcttcacaaaacaacgtcaaactctcctcttctccgccaccatgccgaagaaga

tccaagacttcgccgagcaatccctcatccgccccgtcgtcgtcaacgtcggccgagcaggcgcagcgaacctagacgtgatccaagaagtcgagtac

gtgaaacaagaagccaagatggtctacctactcgaatgtctccaaaaaacagccccgcccgtgatcatcttcagcaacgacaagaacgaggtcgacga

catacaggagtacctgctcctcaagggcgtcgaggccgtcgccatccacggcagcaagtcgcaagacgagcgagaatacgccatccgctcgttcaagt

ccggtcagaaggacgtcatggttgcttccggcgtggcctccaagggcttggacttcaacgagatccagcacgtgattaattatacgatgccgaaggag

attgaggattatgtccatcagatcggacgtacgggacggagtgggaagacggggattgcgactacgttcgtcaatatgaatacgccggagcagacgtt

gcttgatttgaagtatttgttgatggaggcgaagcagagaataccaccgttcctggcagccatcgacgacccgcgcgcctcggccgccgggggcaaac

cgctcacgggatgcccgatctgcggtgggttgggacacggcatcaaggactgtccgaagttggaggacaaccagcgtcggcagacggctgcgttctcg

aggggcgatgctggtgggggggactaa

>contig_14.contig_14.g213.t1

atggatgctcggtgggtggctcgagcgagggagggaaaggtgttggacttttctcgcgacagggtgtggatgcgatacc

gctgcagtactgcgaccaccttcccactggccaactggggacctcgcccggacgaccctggcttcggggtggacgtggatcgtgagctggacggtcat

ctcgagtacgagcgcagtgtcactcgctcgctcacgccttgcaagaagcctcggatcagatcgctcttcccttcactttccgccacgccaaaagggat

cccgatcgatctcgacacggccatgtcgcgcacagcctcgggctcagctccagccgcgaataccacctccaacagcggcggcggcggcggtggtgcgc

gcggagctgctgctacagccgcaggtgcaggcgcaggcgcaggcgcaggcgcaagtggtacagccacccctcccacctcagccgcatctaccactgcc

ccagccacagctcctaccaccgccccacccgccctctccgacctcacacccctcctaggcacaaccctcaaactcacactaacaacccccggcgcacc

ccccgccacgggcaaactcttcgccttcgaccccaacctcggcaccgtcgcgctcgagacagagctcggccgcgtgccggcgtacctcgtggggagca

tcgccggcggcgcgggtggtttgaagagggctactacgaccacggagaaaggtggagaggacggcatgcttccacggacgggctttcggcttatcaag

attagggaggtgaagaaggtcgaggtcgtacccaccagcagcgtaagcgcaggtaccaatggtgctcccacgccgaacgatggcgctcgagcaggagc

agggccagaccggttgacggctttacatgccctcaacgtcgccacgttggacgccagggcgaacgcggccattcgggacgctcatacgcgcgcgagtc

gacggggcgtgggtgttagtacacaggcgcagggaatatttgatgctttgagcaagacgttaccttgcagatggcacggagcccacatcatcgtcctc

gacgaagtcgtcatatcgggcccgaagtacgacggctcctcaacgaacgtgccctccttcacacaagaccaactccgcgccgcactcgacggcgcacc

cctcccgccctccgcagacggatcaactctaccgccacaggcgcaaaacaccgtccgcgcgaaagcggcgagctggcagcgtgtggtcaaagtactcg

agggagagagggctaagatcttgaggcaagagggtgtcgtggcggtatga

>contig_14.contig_14.g214.t1

atgttcaagaagagcgcgcagctgaagacgagcaacccgctcaagtcctccgaccgccgcaaactactggcccacctcc

agctcgcataccccgtcctcgcacacgcgccaccccaaatcctcgcacgactcgtccccgacggcctacgagtcgcatcagcccttacctccgcagac

gacaaatgctgcttctatactgatgacgatggcgtgccgctctggtttgagctgggcgcgcaggttggcgttgcgcttagtgccgctaaggggaagaa

gtccaccaaggccacggcgccggggacgaagaatggcgcggggcaacctcctcgagtcgagggcgaggtcatcccgacgctgtatgcgctttggatcc

atcccttcatcctgccgcagctgaccacatgggccgctgtcatcgatgatcatctgctcgcgggctcagcactcatggtgcccggtctctgcccccct

ccacataccttccggccctgcccactgcagctccctccagcttcttccgcgtccacatcagctgcagcctccttagctgcgcccacaacggccgagct

tccacgcgcacactccctcgtggccatcacccaaagcgactccttcatccccctcgtcgtcgcccgcctcgagctgtccggcgagaacctgatcgact

tacgatctcaaccgggcgggaaaggcaaggctgctcgcgttgtgcattcgttccgagataccatctgggaaatgggcgggaaggtcaagcctcctgct

gaagctgacttcgagcttgccgagctcgtgcagcagccggaaggaagtggggttgtcgctggtggggagcaagggcaggctggcgcgagcgccgaaac

gaatggagagccaaaagatgcagcggacgctgagggcgaaggtgctggggtcaccaacgaaggagagaccaaggacgaggaagaggaggaggaagacc

tcgcagctgcactggaagcagtcttaatgggcgcaaacaatcaagacgaggacgaaggaacagcaccgacgcaaaccagtgccgagcacacacgaaaa

gcaaaaggcaagggcaagaaggccaaacacacccatgacccacctccatccgacagcatcaacggcgacgaccaccaccaagctgaacaacacgctcc

cgaccacaacaatcacgctccctctctgagcgccacgcaagtcgacgtcgtactcagattggccctcctcaacgccctctcctccctcacccccgaat

cagcctccgctttactccccctgccagcctcgtccctctacagcgcccacgtcctccccggccgaccgtcccatctgcctccccgggttgggaagggg

aaagggaaagctaaggcaacctggttggggtgggaggactggaaggtgagaacgagcgtggctgttggtgatgatgtggaggatggtgatggggaagg

tggggaggttagggttttggcggaggaggcggatgtgaagcggagtagtgcgaagcagttgaagaagtggatcaaagctgcggagaaggacggtttgc

tgaaggcgaaggacgtgcgaggagagctcaccgttgtcgaactcaacgtggcccacgccgaactctccaagtggacccagctatggaccctggcggac

gagtctgccaaagcggctgccgaggaagacgctgctcttggtgaaggtgctcctgcttctgctgctgcgaagaagaaggacaagaactcgccggatcg

gatgggcatcctggtgcagtcgttctatggggtcaagtcggaagcgactcggcagctgtttgaagtctttggccttgaacacgattcgatccagtccg

cttcgtcgctgcggatggccttgtcggactacatcaccaagcgtgcgcttgcccatccgaccaatcaagctctggtgcggccgggggatgacccggcg

ctcgcgatggcgctgaatgcatcagagggaagcgggacgaaggcgaagaagcagaagaaagtggcagcagcaggcagtgaggcagcggcgacggcgac

ggagtccgcgccgcccaaggagatcatgccggagatcaagccgatgcgacgggacgatctcgcacgtaaattgcaggacgtcctacaagagtatcacc

gactctccttcgtccgaacatccaccgccaacgcgatccttgccgcccagacccggcccgacccgctctcctcgtctctactcgcctcggaaacgttc

caggcctcgctgaaggaggacgagcgggtagggatgctaactaaaggtccctcaacacctatcagcgtaagcatcaaacagcgccagggtcgcaagac

agtaacgctcatctctgggttggagccctacggcgtcgaccccaaggtgctggcgggcgagttggcgagggaggtgggtgcgagtacgagtaaccagc

atgctcaacgacatggttcatggttgggggattgcaagacaggctgggctctaaagatggtgtag

>contig_14.contig_14.g215.t1

atgtcgtccttccttttgtacgtatcaagctggcctgagcctgagcgcgactggctcagtggccaagcttacggccgca

tccgacaagaactcgacaagaccagccagcgaagaagccagaaccagaaccagaaccagaaccgagaagaccttcttcacttcttcttcaccaccacc

accaccaccaccaccaacaagatccacccgcgcgcacctgctgaccgctctctcccactcatcgcctccggtcctcccacaggtctcccatcggcaac

ctctctctctttccctctcctccccatccccccagcacatgcgcatgctcagcgcaccacgatcccaccgagtttggtcgccccagcctttcgcagtt

cacgcatcacgagctaccccgccggccacgcccacatcgcccagctgtgtggacgtgttgagattggcaacgcggcaacgagtcaacgaaaggatgct

tccgcgtcccctgtctgtggctcgctcttgaccaagtccatgaaggcatcaggcggtacccgtccctggaccgctctgcgtcgtgcgtgcatcatgga

gatcatagcaacagttcagtcacaagcgcgttccgaccagaggcaggacccgaccatcacatacatcctgcttattattacactgccaacaaagactt

tgcctgcgatgcctgcgaagaaggtgattacgatcgccctgctcgtggcagacacgcccgtgccagccgtcgtcgagacatggggagaatatcccaag

atcttctccactttcctcaaggacgccctagcctccatcccgcgacaccactggcaagaatccatcgaactcgacatcagaccgttcgacgtcgtcaa

agcgatgaaatacccagacgacggccagctcgctgatggactgtacgacgccgtcctcgtcactggctcagcctcgtctgcttatctcgacctcgaat

ggacgaacaagctatcggatttcatccggcatctcgcagaagatcacccactcgtgcgcatcatcggtatctgctacggacatcagattatcgccaga

gctttcggcggtaaggtcttcttgaacgaggccggctgggaggtcgggacgtatcaatgcaccctgaccgaggacggacaagaatacctcggctacgg

tcccgaactggagatggccgtccaacaattccacagagacatcatctccctccccccacccttcgaaggcgcccccttcctaaacctagcccacaccc

ccaaaaccccctcccaagccctcgccctcccgtacccaaccgacgccccacccctgccctcaacagtgtccacctccgacttcgtagccttcgacatg

gacgacagcggcacggcctcctcgggcccgcacccggcgcgcaagttgcatgtgttgagcttgcagggtcatccggagtttgagggggggattgtgag

ggccattttgggggtgaggacggagatgggcgttattacggagggggagaaggaggaggcgatcgagagggctgggaggccgcatgatggtcgacggc

ttggagtgttgattttggctatgctcggagtggaggcggcgaaggacgagagtggtggggctgcgattccggtttga

>contig_14.contig_14.g216.t1

atggccgcccctcaggaccagcatggtaacatcgacgagggcctctacagccgacagctctacgtgctcggccacgatg

ccatgaagcgcatggctgccagcaacgtcctcgttgtcggcatgaagggtctcggtgtcgaagtcgccaagaacatcgctcttgccggagtcaagagt

ctgaccatctacgacccctcgcccatcgaggtggccgacctcggaacccaattcttccttcgaccttcggatctgaatgcaacacctcctctcacgcg

tgcccaggcaacggcgccgcgcttggccgagctcaacacatatgtgcccgttcgcgttctggatgcgcccacgttagatcagcagaccctgacccagt

accaggtcgtcgtcctcaccgacgttccgctatcacagcagctcgccattaacgatatcacccatcgctccggcacgcacttcatcgcagcggacgtc

cgcggtctcttcggcagcgtcttcacagactttggtgacgagtttgtctgcatcgacccctctggcgagcaacctctgagcggcatggtcgtctccgt

cgacaaggacaaggacgccctcgtcaccaccgtcgacgagacccgtcacggccttgaggatggtgactacgtgaccttctccgaggtgcaaggcatga

ctgagctcaacggcaccgcaccccgcaagatttccgtcaagggcccatacacgttcaccatcggcgacacgtccggattctcagactacaagcgcgac

gggatcttcacccaggtcaagatgcccaagaagatcaatttcaagcccctgcgcgagagcttgaagcaacctgaacacctcatttcagactttgccaa

atttgatcgtcccaactcgctccattttggatggcaggcactgtccgcgtactgggagaagaacggcagcttgcctgctcctcgaaatgcggcggcgg

ctgaggagatcctttccctcgccaagtcgttcgctcagcaggttggcagtgaggaggagcttaatgagaaggttatcaaggagcttgccttccaggcc

agtggcgaggtctcccccatggtggcctttgtcggtggctttgtcgcgcaggaggcgttgaaggcctccagcggcaagttccaccccctcgtccagca

cctctacgtagactcgctcgaatcgctccccgagggcacggagtcgctgccagagtccgaatttgcgcctgcgggttcgcggtacgaccggcagattg

cagtctttggacggagcttccaggacaagctggccaacgtgcggcagttccttgtcggctctggcgccatcgggtgtgagatgctcaagaactggtcc

atgatgggtgtcgcttccggtcccaacggtgccattcacgttaccgacatggacacgatcgagaagtcgaacttgaaccgacagttcctcttccgcac

caaggatgtcggcaacttcaaggctgacaccgcagcggcggctgtgattgagatgaacccggacctgaagggtcacatcgtcagccaccagaaccgag

tgggtgcggagacggaagacgtctatggcgatgacttctttgcgcagctcaccggcgtcacgaatgcgcttgacaatgttgccgcgaggcagtacatg

gaccggcggtgtgtgtactaccaaaagccgctgctagagtcgggcacgctcggcaccaaggcaaacacccaagtcgtgatcccgttcatcaccgaatc

gtactcgtcatcccaagatcccccggagaaggccatcccggtctgcacgctcaagaacttccccaaccagatcgagcacacgatccagtgggctcggg

aacagtttgatgaacagtttgtgaagcctgcggagagtgtgaacttgtacctcaccacgcccaacttcctcgaggctgcggcgggcgcgggcagtcct

cgcgaacagctcgtccagatcaaggagtatctggtcgacaatcggccgttgagctttgaggcctgcgtcgaatgggcccgcctgaagttcgaggagca

ctacaacaacaatatccaacagctgctcttcagtcttcccgccgacagcgtgacctcgtccggccaaccgttctggtcaggtcccaagcgggcgccca

agccgctcgtcttcgactcgaacaacccggcccatctggactacgtgatctcagccgcgaacctgcacgcgttcaactacggtctgaagggcagtgcg

gacccggctcagattgtccagattttgaacaacgtcgtcgtacccgagttcaagccgaggtcgaatgtaaagatccagacgacggaggcggaggctgc

gcagcctgcgcagccttccgctggggatgaagacccgtccgagctggctgctgcgctgcccgcacccgccacactggccggcttccgactgcaaccga

tcgagttcgagaaggacgacgacacgaatttccatattgacttcattgcggccgcctccaacctgcgtgcgatgaactacagcatcgcgcctgctgat

cggcataagacgaagggcattgcggggaagattatcccggcgattgctactacgactgcgttggctactgggttggtttgcttggagttgtacaagat

tattgcggggaagagtaaggttgaggattataagaatgcattcgtgaacattgcacttcctttcatggcgttcagtgagccggtggctgcccagaagt

acaagtacaacggtgtcgagtggaacctttggggccgcttcgacgtaccaaacaacccgaccctggcggagttcattgagttcttcaaggagaagaat

ttggaagttacgatgctctcgtcgggccagtccatgctgttctcgggcttcttggcggctaagaagcgcgaggagcggttgaagatgaagatgagcga

gttgattgagacggtgtccaagaagccgatcccacctcatgcaagatatgtgattgtcgagctgatggccgacggcgaggatggcgaagatgttgagg

tgccttacgtccgcgtcgaggttcgcccgtga

>contig_14.contig_14.g217.t1

atgctactcgccccgtcgctcgtgcggctcagtatccaaaagaataaactcgctgccagccccgtcgcacgagctcgtc

gagtcgcccgcgccgtcatggctggcccccgacctccgatcaatcacgctgcagcggccgcagcagcagccaaagctccctttgttagcagtccgatc

cctccgccagctcacaagggcgccaagatggaccaacgcgatggagcgctagtctggatcgactgcgaaatgacaggcctgacggaccaggaccgtat

tattgagattgcgtgtattgtgaccgacggagacttgcaaccggtggacgcaggtatccagtatgtcatccagacaccgaaggaagtgctcgatgcca

tgggcgattggtgtaccgagcatcatggcaaaaatgctttccatcaaggcctcactgcgctatgtctagacccgaccgtagccaaacctcacgaccac

gtccgcgcagccgtgttcgcgtacgtgctggaccgcatcccagtcaagagccaagcatgcctcgcagggaactcggtccatgctgataaggtcttctt

ggaccgcgagatgcctgagctcacggagcacctttcctaccggatcgtcgacgtatcctcgatcaaagagctcgtttcaagatggtacgggaagcaag

ccacgcttaataaagcatcagagcatcgagcgttgagcgacatcaagggatccattgaaggtcagtatcccacctggtacgtatgccaaggtcccgac

tga

>contig_14.contig_14.g218.t1

atgctcaactcgagcctgggcgagccgcgacttccgctgacgatggcaggcgaggtggtcaaggcgctcgcgcacgaag

ctgtccaggacgtcgatgagctggacagaagccctgcggatatcgcctacctcatgtcggatcaattcaagctgtccgtacagctccagggtcacaca

ggcgacgtgcgctccatcgcaactctcgaagctgccggtcgatctctgatcctctccggctcgcgagacgccacggcgcgtctgtggacatcggaagg

ccctcgctctcgtgcttatgaggaaactgctgtgctctctactgaggggggctatgtcaacgcgacccatttcctcgaggacgcagacggaaatctcg

ctgcgctcagtgccggaaaagatacggtcatcaatgcattcgaggtgaagctggacgcgtctggagctgccaagattcacccgcgaccttcgatgaca

ctcatcggccacagcgacaatgtatgcgcgttggacactcgtgatggcacaattgctagcggcagctgggacgccacagcccgggtgtggaaggactg

ggcctgcgtagccgtgctgaagggccatgaaaatgcggtctgggcggtcttgttgatcgatgacgatcgtgtcctgaccgcaggcgcagacaggacaa

tccgactgtggtcgatctcaaaccatgcgaagccaatagccactttctccggccacactgacgctgtccgaggcttgacgcttctcagcggcggtcaa

tcgttcgctagttgcggcaatgatgggaccatcaatctctactctctgatagacattgacggcaacaagccgattgcacctgtcaagacgctctccgg

acacacaagctttgtgtattcacttgctgtcctcccagggccagagggagtgctggtcagctccggagaggatcgcactgtcagaacgatcacgattc

cggccatctctgtctggtgcgtcgcagcgttggagaacggcgatatcgtatgcggcagcagtgatcatgtcatccgagtgttcaccctggacccaaag

cgcgttgcagatgcagcagagattcagacatatgacgagactgtcgcatcccagacgctgaacaagtctcaagtcggagatgtgaagaaggatgacct

tcccggtgtagaggctttgtctgagccagggagcaaggaaggtcagaccaaaatggtccgaaacggtgaaatcgtcgaagctcaccagtgggacgccg

cagggaatcgatggaacaagataggagaggtcgtaggcggtgttggaaacggccagaagaaactgtacgatgggcgcgagtacgactacgtgttcgat

gtggacatcaaagacggcgtgccgcccctcaagctcccttacaacctcagcgagaacccatatacggctgcggctcgtttcttggagaagaacgaatt

gccggccacgtaccttgacgaggtcgtcaagttcattgagaagaacacggaagcggtcagcatcggggcgtcggattacgtcgatccctacaccggca

ccaatcggtatactagctccgcggcaccaccttcctctcgcacaaatgctccagccaccttgaatacgagcacaggcggccagtacaccggcggcaac

aacgtcgaccctttcactcaatcgtcccgtcctgctccagtcacgaatacgtcgtcggtcattcctgtgaaatcacctctcacgtttgtgcagatcaa

ctacgccccgctcaaagcaaagctggcgcagctagaaggacagctgagctctgcaggagtcggcaacgccgcagacgacatcaaggcgatacagggcg

tgatcgccaggctcgaatcggcttctgagtcagggcccgttgacgtcagcggcctcgaggcagctctgcaatcctggcccgcgccaaaccggttcccg

cttctggacctgcttcgagtggcagctcttcaaggaacgtcgacgcctgcggaacgagtggcgagcgctgctcttactgcggcagagtgggccagcga

atggccggaaggcgatgctacgagctccaagactagggacacgaactctatgcttgccctgcgcactgtggccaatctgttgggcggcaagggcaaga

aagatggactagaagagcaagcggatgagatactttcccatcttcgatccgcaaactatgtgcacttgagcaagaacgcacgcgttgctctggccacg

gttgcactgaacttctcggttctagccgtcagcaagagtcagcaagttccctttgcaccatccttgctggacctcatcatagatatcctgagcaagga

gagcggagacccggaggtcgtatatcgaaccaaggtggcgttgggcaatatttcagcgaagcttttgccagccgacgaggttgggattgcgaaggagt

tgctgtcccactggtcagaaatgctggaggaggcccgctttgccgagctcagcaaggagatattggccaaggcctcgatttga

>contig_14.contig_14.g219.t1

atggctttggcacgacgatccttgactgtccggctcgcagagacggcatcgcagtactggtccctcggattgacctcct

tcggggggccgggggttcacgtcgtgatcctgaagaagcgcttcgtcgagaagcttcagtggatcgacatgacgacctatctcgatctgttcacgctg

ggtaacgcgttgcctggaccagggtcgacgcagttggccttctccattgcattggtccgcaatggcaccttggcggccctgctggccttcctgttctg

gtcacttccgggtgcagttggaatgggcgcgctcggtgtgggagtccgtcgtatgccggatgagctcccggaaatcgttcttgccctgcttagcgggc

tcaacgctgcggccgtcggtctgatcgcccttgccgcatatcagctttctctcaactgtatcacggaccacttgacacgtcttgtgcttgtggcaagc

gccagctttggtatctgctatcatgcgccttggatgtaccccattctcatcgtcactggtggtttactcaccctatcctttgacaaccggcacctcgc

attgacgccgatcaagaaggccttccacaaaaggtcggagcgtgcgaagcggaatgcccagagatcttctgctcccagcacgacgccgggcgcggatg

caatcactccagatatcgaactcactgcacttcctcagcacaccaactcaaacggcctgcgaccggaatcgcctgggaacatgtcgactaagagcgga

gccagtacgcggcaacgccacgtcggtgccaatgcttcccttgcgacgcccacagcggaggagagcacccagtctccaaccggagcaatttccggtcc

gctctctcctcctggctctccgatcatcagaccggcagcagacgcccgcgtcggacaagatcaggaggaagccgagacggaggccaaattcatggtcc

tctctcacaaggccgcctttgggctcttcttcttcttcctcgtgtttgtcgtcacgattctcgtcgtccgggccaacctggacctggttcctcgatcg

tttgacttctttacgaacatggtcatcgctggcctgatcatcttcggaggcggccctgtggtgattcctttgcttcgttcgtacgtcgtacagacagg

ctggatctccgatcgcgacttcttgattggatttgccatcctgcaggccttcccaggtcccaacttcaactttgccgtgttctgcggtgttctcgctg

tcaacaccaatcccgccctcggtgccttcctgggctggcttggcatcttctcaccgggcatcatcctcaagctggctctgctccctttgtactccaag

ttccgcaagcacccgtctgtccggagcactctgcgcggcctcaacgctgcggcgtccggtcttgtcttttcggccacgtatcagcttttcatcgtcgg

atacatctaccagtctgccgcaggctcgcgcgatcagaagaccatgagcgggtctctgacttcggatccattctggacggtggtggtggccggcagtt

tcgttgcctctcaattcttctcgtcgcctccatggctcagcatcgccggaggtgccgttgccggtctggcgtggtatggagtccagaagtcgtga

>contig_14.contig_14.g220.t1

atgaacacttcgttctcccgagcgtatcgcagtctgctctccctccgccctaaagttgcacctgtcttcgcccccaccc

tcgcccagcgcacctattcgctctcccatatcaacatggcccctcaacgcgtcaaagtagcttccaagtcggacgtccccaccgggaccatgaagcaa

gtcgtcttcgccggcgaaggagatgatgccgttcacgtcctggtctccaacgtgaagggcactttgcatgccacgtcggccaagtgcacgcattacgg

cgccccgctcgcgaacggcgcgctgtcttccgacggttccgtgatctgcccctggcacggagctcgcttcagttgcaagactggcgacattgaggacg

cgccggcgctcgactcgttgctcactttccccatcgaggttgatggcgaagatgtctttgtgacagcggacgcggagcttttgaagggaaagcccggt

gtggcgcctgggtgcgccggtggaattgagaagatcaagtcaaacgagaagggcactgtcatcgtcggcggcggctcagctgcgattcatttaatcga

aagcgcgcgaaaggaaggatacctgggcaagcttaccgtcttgaccgccgaaccatatggcccgatcgaccgcccaaagctgagcaaggggttgatgg

aggatccaaatgccgttctttggcgttccaagtcgcaccttcagcacaagctcggtgtcgacctccaggagaacgcagtcgtcaccagcgttgacgtg

cagtcgaagagcgtcacccttgagaacggcaccaaggtcgaatacgacactctcattctggcgtccggagggacccctgtgcgattgcccttgcccgg

cgctgaggcgagcaatgttctcactctgcgcggcatcaaggatgtggctgctatcaacgcggcgctcggtaaggatggagataaggatctggtcgttg

tgggttcgagcttcatcggcatggagctcgcgatcgctgtggcttcgaacaagaaggcaaagagtgtgtctgtagtcgggatggagagcgtgcccctc

gaacgcgtgctcggcaaggaggtgggccaaggacttatgaagtcccaacaggacaagaacgacatcaagttctacatggactccaagacggaaagcat

cgagctcggatcagacggcgacaagaaggcgcaagctgttcttctcaaggacaaagacggcaaggacgtcaagcttcccgcggccgtggtcgttcttg

gtgtgggagtcaagcctgcaacacagtaccttcgcgaatcgaagggcttcccagagctgctgaaggatggctctgtcgatgttgactcgacactccgc

gtcaaaggtgtggaggacgtttacgcgctcggagatattgccacggcaccgactcgaagcggcaaagacacggtccggattgagcactggaacgtcgc

tggtgcgcacggtcgactcgtcgggcaagtgttggcgggcaagaagacggcaggtcaagcttctttgcaggcacctcccttcttttggtcagcgatgg

gtgcgcagttgcgctacgtcggctcgggcgagcggcctgggttcgacaacgttcacgtcgatggtaagccggaagagctcaactttgcggcttactac

gcgagaggagacgatgtcgttgcccttgccacaatgggccgcgaccctctggctgtggtggcctccgagctgttccgcgtgaacaagttccctagctt

tgcagagatcaaggccggcaagacgaactgtgccatcttccgttacctcgatagccaggcgattctgaccgacatggccgatgaaaagatcaacaccg

acgaaaacgtccaagtcaacgatgccaacgataccaacgcatctgataaaagtcgacacgaggaaaggcagcaaaacaaaggtggcctcagccacgtc

ggtatgatcttcgcctgcggtgcagccctcttttcggatggatacgtcaacgctatctccggccccgtaggcagtatattgaagcggctttatcaggc

tgaagacgagaaacggttcatccacttcaagaagctcttctcgtccctcggctttgctgggaccgttatcggaatgctcgtgtttggctacctcaccg

atcgtgttggtcgcaagtttggcatgctgtttgcttcgatctggttgtcgatattctgcatcctgagcgcccttgcgtggggtgccgggggttcgatc

gatggcttgttctctgcgttgatcgccttccgattcatccagggtattgctatcggcgcagaataccccgccggctctgtcgcttgttcagagagcac

agagagtgctggcgtcaataagaagcgtcagcaaatgtatttttctctcgcgacgcacacgatgctcgacttaggctttctcgtcgctcatttcgtgc

cgcttgtcctcgtttggatctttggtatgaaccatcttaaggtcgtctggcgtctctccctcggtcttggtgccgtccctccgctctcactgctctac

gcccgcgccaagatgcaggagccgatccattaccgcaagtcttcgatccggtttgctcagatgccttggtggctcatcgtcaagcgctactggttgag

attggcagctgtgtcgatcgcgtggttcatctatgattggatttcctatccagccagtgtgttcgccgactacattatcatacaggtccttccgacag

atgccagcttccaggctaaccttggctggggtgtcgtgatcaacctcttcaatcttcctgggagtctcgtaggcgcgcttgtcaacgaccgtctcggg

cccaagtacactatgatcctcggtcttattgtccaagctgtcattggcttcagtctcgccggcggctatgccctcctcaaggctcatatcggcgcctt

cgtggttctgtacgggctgttcttgtctgctggagaattcgcaacaatctcggtatgtacgtggatgctgtctacatcttctgaaacatctacacctc

agggcccttttaacattggatccgctctcgctctgctttcggccgcaacgatcttcctgttcgttcccaatgtcggggaggacagtatgctcattgag

gacatcgaattcaagcggtatctcgaggcaaatggcttcgacacgagcaccatgggcatcgaagcttcgaagagcagcgtagagacgggcgttccgca

gagcccggcctcggaggatcagaattactccaacaagatcatgtcgcagttcgccccgaacgaaggcgctgcgtccccggcggactcgcctcagaagg

agtttgacgctgtcacgccaccagcggactcccctcagcacgagatccacgccgccacagctccggagcctgtaaagcagcaaggattgcaaggcttt

gccctaatctttgcctgcggtgctgccttgttcagtgatggatacgttaacgcgatcagtgggtcagtaggcacaatcctgagacgcttataccggga

taccaaccctcccgcattcgaccgcttcaagcaactgttcagctctctggcctttgcaggtaccgtcgtgggcatgctcacgttcggagtcttgtccg

ataaaataggacgcaagttcggaatgatctttgcttcgtgctggttggcactgttctccgttctgtcggcgggtgcctatggagccggaggaagcttg

gacgggctctacgcagcacttattgcgtaccgctttattatcggcattgcaattggagcggagtatcccgcaggcagtgtggcggcctcagagaacac

cgagggcgatggtgttaacaaaaagcgccagcagatgtacttctctctcgccactaacacgcctctacccttccagatgatcgactttggcttcgtca

ttgcggctttcattcccctcctcctcatctggatctgtggcatggaccacctccggctcgtctggcgactctcccttggtttcggcgctgttcctcct

ttgatcctgctttacttcagaaccaagatggaggagtcgaagcactaccagaagtcgtccatttccaagttcgggcagatgccttggtggcttatttt

caagcgctactgggtcaggctcgctgccgtgtcgctggcgtggtggatttatgattgggtcacctaccctgccggactgtattccgactacatccttg

ccaccgttcttcctgacgacgccagcttccaatccaaccttggttggggtgtcgtgatcaacctgttctacattccaggtactctacttggcgcactt

gttaacgaccgtctcggccccaagctgttgactctcgatatcttgatctcggatgtcgccacctcgatgttctggaacatcttcacacagtacacgat

gatcctcggtcttctcatgcaggctgtctttggcttcgctctgtcgggtggatactcatctttgaagaaccacatcgggggctttgtgatcctgtacg

gactgttcctctccgccggcgaatttggcccgggcaacaatctgggtctgctggcgtccaaagccactggtcccacggctgttcgaggaaccttctat

ggatgcgcagcggccgtcggaaagattggcgcattcgtgggctcgtatgtcttcgaccctatctccatcagcctttccaaaggaaaccctgatgccga

catctacaccactggaccattctatatcggatccgcactcgccctcgtttcggccaccgtcgtcttcctgttcatccccaacattggccaggacggga

tgctgcgggaggatgaggagttccggaagtatctcgaggagaacggctatgacacgagcaacatgggcacccttaagggcgacgtcgacccctcaccc

tacgaatctgcgtccgccaacgattcgggcagctcagtgcaggaggaaaaaccgaaggcagccgatccttga

>contig_14.contig_14.g221.t1

atggcctctccatcgtacctgctcgatcagcggaacagctcccgcctcagcaacgactgggcctcctacgtccaccttc

actcccagctcgaagaatcagaaccggacccctctatcatcgtcgtcaagaagccgtcagtgacagtctcagcctcagcctcagcgttggcgccacct

gcaccagcgtcccgaggcgcagaatccacatccgtcgagtacgaatccgtcgattccgcctactccacctacagctcgcattccacagtcgacgccag

catgcccgttcccgccgccgtggcgagacagcccgcctcggccctcgacgcacgcctggccagagatcctgatgcgggcgaccgcagaagtgcagtca

ccacacactctgcactcacagatgacatgagcgagtacggcgaagacgacgtcgtcctctacaacgctactcgcgtcggtctgcacagatctccttcc

tcaggcgctgctgtcgtcttcaataggtctacccaatcctccgaaggtcgcgaccgagaggtggctgtgaaacgatcatcatcccatcggtcaaacca

ggatcattcggtcgacgcacccactcacccaaacgccggtactgccgacggccctgcttccaccaggacatccatcggcgaggacaggaaccggaatt

ccattcccagccgggtcagcattggcacctctggatggcagacgccctccgaatctcaatcccagcacagtccccgctcctcgcgctctgttcgtccc

ctgacccttccgccagccgagcctttgccccctctgccgtcgccgaattcgaacactcctcagctacctttcgagggcggcggcgccatgagcagtcc

tgagcatcccacccatgttttgcatcactcctcttccgttggcagattacgccaaggcacaaattccggcggtcatcttccgtcgtcgacatcgactc

cagcaatcgcaccacgcaccacgtctcgggatctcggcttcgaaaccagatcgccgccccaaacaggcgcagcgatcagcccggctaatcgcgaccgg

ccaggcaccgggacctccagtggtagctacatcaacgctgaaatcagctctactgagcaaaggttcatggaccggatcgacaggcctgcacagtcggt

cacaagctcgtcgtcgatttcgtctgctgcgacgccagcacatctccgtcccggctccaacgctgcaaaacgtcgcatgaatggcgacagcagcagca

ctgtgacctctcccgtaaccccgaccgcagcgaccatggcgccgatgtcaccgtcgacctctgctgcattaccgacaacgaacaagccgaccgaccga

gcccctccgtccgccgcaggtaaagcagctgttgtcaaggcgaaaaagtccggccctgtgacggccagccctgcgacattgcgcgcgaatccgctggc

gggagcgcgagctgcagccagcgagcaactccatgaaaaccgagcaccattaatcattggcccggggggaaggaccatgactcggcgagcgagcgaaa

tcaacattcagagtcgcaggacggagatggaggaggacaagtccatcgtcaaagttgcaccacaggagcgctccgtgattttgaagaaggcagagaca

cggtttgccggcgcattcactgaggtcgcgcaagccttcaagcagttacaatccgagaaacgaacccttgagaacatcattcgcgctacaacgccgct

ggacggcttgggcgatcagggccaactagccgaatatctcaagtcgatgacggcaaaaatagccagcagtgaggaggagatccgaaagctgctcgagc

tgctggaccagcagcgcgcggtcatggactacatgctcgagacgcacgaacgagaatcggacgcgcactttgatcagatcgacgagctgcacgcgcag

cttgacagtctggccgaggagtcagaagttcaccgagccaactgcgtgcacctgacggcggagctggaccgagcgcacaacgaagctgtggccgcccg

gtcagacgtcctgaagctccgcgctgcggtacagcaggagtctagcaaatgcgaacaggcgatgactcttctccgtgctgttcagcatcagatgaagg

atcgagaccagatgagcaaagcgctgcgcaccgaggtcgacactttgcgagcccgagaagccgatcagaagcacgcacgcggtgcagacgaggatatg

caggccaacctgtccggccacaacgcgaaagagctttcgtcgatgagcgatgaattgcggcggacgttgagttcccagcacgaggcggatttgttgtc

ccgatccgagcaaattcgaagcgagttggcaaccaagcacgcccgggaattcgccgattataagcgacagctgcgcacagagctggcgacgcaacaca

agagtgaaatcaaggcactgaagcagaagttggagtcttccttaatactttccgaggccgaggccgagcaacgactttcgtcgacaaacatcacccac

gacacggaggtcaagatgctgcaggcgcgtcttgccaagcttgaacgggagggcgacagcaaggcgacgcttgaagctgagttgcgcgaacggaaaac

tcaacatgtgacgctggtcggtcaattggcggagcggaccaacgcgatgaacgagatggagcaaaagagtcgcgctttggagaaggaggttgacactc

ttcgcactgctcctgctcctgctcctccacctctgtctgaaaaggacatgggactaagcggtagtgcgctgcaagcgcggctgcaggctgcggaagcg

cgtgcaaatgcagcggagagggccaaggagcaactgaactcaaaactcacggcactcgaggagggaggttcgaagagcgaacttgcatcattgcgtgc

ccaacttgtagaccagcgttcccgcgaggtccaaattcgaaacgcgtacaagaacttacagtacgagttgcgcaaagcacagcagactaccaaggccg

acgagcggagacggacgttcctcactggcaatgtcggcggaggatcgacgagcgccagtgtttctggtcacggcggcagcggtggtccggcttcaccc

actgatggagttcccggtcttcatcgttcgaactccaattcgagccaaacgagccgacagctgaagcgactcagtcttcccattgtctccaagggcga

tcgtatcctctcagcaaactggatggacacgtatcaacgtgagaacgggaacggaggaggaggatcgggatcgggaacggcttcgcggcccacaagtt

ctcccagttttcctccgtcttcgttccggcagccactctctgcgtctgcgaaccggacttcgttctcgacgtccccttctccatccgatcgccgacaa

tcacagatgactgacggtggagccggggacgaggatggtcggaattctgcgacgggtacggccagcctcgccggacacggcagcagtacgggcggtgt

gctcggttacggctcgtctgcgatttcgaatgtgatggggatgtccggtatgaacacgagcacgggttcttccatgggcgggaattactcgatcatgg

ccaatcagcatggaggtcgtccgttgagtgaggcgtcggcgtacagcatcgaggagggggaggaggaggacgatcctctggagttggtgacgagtgac

agcaattactcgcaaatgtag

>contig_14.contig_14.g222.t1

atgcccgaggaataccagccttcagacaccgacatcatcaccctgagccgacatgtgctccaagaggggttccgtctcc

gtgaggagagcgcggcgagcggtgaccttaccctgttgttgaacgctctgcagacgacctgcaagttcattgagagcaaggtgcgaaaggcacgcctg

atcaacctgatcggcgcagcgggtaacaccaacgttcagggcgaggaccagaagaagctcgatgtgctctcgaacgagatcatgatcaacgcgctcaa

ggcgtcaggtaagactgctgtgcttgtgtctgaggagaacgacgaggccatcatcatcagcgacgacaagaacgcgcgtcacggcaagtactgtgttg

tgtttgaccctctggacggatcttccaacatcgacgccggtgtgaacatcggcaccatcttcggcatttacctcgtcaaggacggctcgaagggctcg

atcgaagacgtgctccgaccgggaagtgagatggtcgcggccggatactgcatgtacggctcttcagccaacatggtattgacgaccggtgacggagt

gaacggctacacgctggacaacgagattggggagttcatcctgacgcacccgaacatccgaatcccgagccggggtaagatctactcgatcaacgaag

gaaacagcgcgtactaccacgagcctgtgctcaagtacctcgacagcatcaagttccccaagggcgacaagaagccgtactcggctcgatacattgga

tccatggtcgccgatgtgcaccgcacgctgttgtacggcggcattttcggttacccggacgacacgaagagcaccaagggcaagcttcgtctcctgta

cgaggctttccccatggccatgctgatggagcaggctggcggactgggaactacgggcaagggccgcatcttggatgtccagcccacctcgatccacg

agcgctgccctgtgtttatgggttcaaaggaggacgttgaggacctcctcaagttctaccagtag

>contig_14.contig_14.g223.t1

atggctccctctcgcaagaacttcgcgcccacaagtgagggcaccgaggccaccgagctcggcaagggcactacgaagg

tagtgtcagaggagaaggctgccaagattgaggcatccgagaagaaggctgacaaggagttcatcgccggcgacgacgatgcggctgacctggcggac

gaggatgatgatgagtacgatgaggaggacgatgaagacttccaggaaggcgaagatgacgacgatgaggaagaaggagacgacgacgacgaagccgg

tgtagaaggcggtaaggccaagagcggcgagaacggtgaggatgaagaaggtgatgaggaggagggcgatgaggaggagggtgatgaggaggaaggag

atgaggaggaaggcgacgacgacaaagccgctgccggccagaagcgcaaagctgtcggagacgcaaagaagaccaacggggagaagaaggccaagaag

gacgacgagtaa

>contig_14.contig_14.g224.t1

atgaagatcacatccagacatatcgagcgtgacggctctgggcgggtgactctggtacccgaagaagatgaggacatgt

accacctctacaacctcatcgaaccgggcgaccttgtccgagctgcggcggtacggcgtgtccagtccgagtcgaatactggctcgattgagtcgcat

cgggtcaagctcaccttgacgattgaggtcaccaagacgagctttgactccagtgcttcgtccgccccaccggacccaaccgcagtcggcgcagaggg

gggcgacgccaacggaggaggcgcgggtgcgggtgggtcagtaggaaacccgcttaatgcgctagggggtggagggggcggcgatggcgtcacgctgc

aagtgacaggacgcgtagcggaggaaaacaacttcgtaaagatgggcgcgtatcataccctcgacttggaatcgcaacggagtctgacgatcaccaaa

gcgtcgtgggactcggtccatctggagcgtctgggtgaggcgagcgatgtcggtcagcgcgcagaggtaggagctgtcgtgctcggtgacgtcatcct

cgcctcaccagggttcatcaaggagaccgtgtacgagtacctctttgcagaaggtcagaagcaaggcgagaagatgctcatcggaaacgaggccaagc

ggaagtttctgaagatacattgcaacagcccccatcttcactctcttatggaggtcctccgatcccccgaagtcagcgcccagctgaaagacaccaaa

tttgcccgcgaagggttgcttctggacaaatttctgcggatgctcaacacggacgaactgcgagcttggtacggcgagaagcacgtcgcactcgccgc

ggagcgaggcgcaatcggttctctgctcatctccgacggtctattccggaacgcagacccaaaccggcgacgcaaatttgtccagctggtggaggacg

tacgaaagacgggaggcgaagctgcaattttcagctcgatgcatgagagtgggaggcagttgaacggcttgacgggcattgctgccatcttgacctac

ccactcgatgtcgagatcgtggaggaagaggaacgggaggtggcagcggaggaggccgcatccgaagccaaggagggcacatag

>contig_14.contig_14.g225.t1

atgaactcacagaacctatccgtcttgtccgaacgtaccaacttcagcaaacagcccgtccagcccgtcgataccgaga

atcctaccaccagcagaatgggtacgcacaagctcatggaagcgctagagcatgacacagacctcgtccaaatgggagcagcagctgatggaagcgac

aagcgctctcacatcactctgtcgtcgaaagcgcatcaaggcgcgggctccaccttgaagatcaatctccatgttgaggcgcgagcgaaggcgcagtc

atctatgcggcaggatagtttgcgcaaagccatcctcacttacatcgagaacagccagacgtaccttcaagcagagtgcgaactcagcggctggaaga

acgtgcctttgttggcaggcaatctcgacgctctgcggatagcggaaaactcttctggacaagagttcgtacccgtcggacaggctcagcttcaaatt

cacatctatcagccggcgctggaagacgcgattcacgagtttgcctcgggcgaggatggggctgaaggcatggatgaagaacaagtcatggcggcgac

tgttgccgagttgcctaatcgagcattagacggcgtgtggaacagtctgatatacgccgatgatgtcaaagtacggctgctcaactacatctacacga

cgctgctcttctccgacgccagtgtggacttcaacattgtgtcctggaatagagttgtactgctgcatggtccgcccggaactggaaagacaagcttg

tgccgagcactcgcgcagaagctggccatcagattatcggacagatactctcacgggaagctggtggagatcaattctcactccctgttctcgaaatg

gttttccgaatccggcaagctcgtccagcgactgttctcaatggtcacagagctcgtcgaggatgaggacggctttgtcgtcgttctgatagacgagg

tcgagtctctgacagcatcaagaaccgcttcgatgtccggttcggagccctcagacgcattgcgcgttgtcaatgctctcctcactcagctggacaag

ctaaagcaccgcaagaactgcttgatcatgaccacatcaaatatctcagaagctatcgacaacgcattcatcgatcgcgcagacatcaagcaatacat

tggtctgccaccgccagaggcagtgttctggatcctgaagacatgtctctccgaactgatgcgcgttgggctcgtcgagcatgtgggtatcatggact

ggcgcgcaaccatgctggccaggtcggacgtaccttccaatctaccagaatgcgatccgaccagcaaggcggctagcatagctctcagtcaactggcc

cagcagtgctccggcatgtccggtcgctcgctccgacgtcttcctgtacttgcccatgcgcggtacatcggcgtcgggtcagctggcggaggcccagt

acagatcagtacttggatggaagcgatgcggcgggccatcttggatgcaggcaacgagatgacacaagcccagcaaggatcctctatgaataacagcg

cagcagactcggcgggcagaacctaa

>contig_14.contig_14.g226.t1

atggccgcattattcgtcggcccaatcacctatacgataagaagcgtactctacgtgttgggcttccgggtgataggtc

ctgcagcgtactccattgcctcgttgatccaggcaaccgtctacggcgcagctgtgaacgcgggcagtctcttcgcagtgctgcaggccatcgctatg

ggcgcggctatcggaccggtgacgcttgcggtagtgggtgctgccctgggtgctacgatcatatacgtgatcgttaccttcgtgtgccccttcctggc

tccctgctttggctgggttcgcgacgtgctcctggtcgatggcatcgagaccttgcgcgaatacttcgactggttccgcaacgtgctcctggtcgatg

gcatcgagaccttgtgcgaattcttcaactgggtccgagacgtgctcctggtcgatgtcatcaagatcttgcgccacctcctgcataatatagccggc

tggtactga

>contig_14.contig_14.g227.t1

atggcatcgaatcccgtcacagtggtcacccgcgtccggcccgtgtccgtgcgcttcacagacgcctccatacaagact

tggacgctgagctgcgcggcaagccaaccatcctcgacaatgacaaccttgcggacgatggagttgaccgggctggagccgcacttggccagcgttca

tcaactctcttcgatgcgcatctggaggaagaggaggggcaacctcgttcgatgggagtcgaggacgaggaagagcttctacagcatcttgcagagga

ggaggacatcgtccgcattttcaagcaaaagatccgtgccgcgcggccgtctctcgctcatcgccgtctccgcttgatccacgccggccgaatcctca

aggacggaatcaagctcgtatcctggctcgacagcctcgactcccaccgctccgcccaagaacgagccgcgaaccgatcgaccggtctcatcatcgga

gagggggcccgtgccgcacttggtggactcagagaggcagccgaacgtggcgccgcgctggatctggacagcctgacgcggagggggaggcaaggcct

gggcgatctctggaggcgattcgaggatgatgagcggaaggagggccggacggatgatgtggaagctgccgcgggtggggaacgctcggcgaaagcga

aaggcaaggcgagacttatcgacgatgacgaggagagagcgtcaggtccgggttgggagtctgctgaggcggcgccaccgcggcgagccggagtcaag

gtcgtcgaagatcggtcagcggatcgggtctacattcaatgctccattggagataatatgacgccagaggaagaagcggaagccaatgctcctcgagc

atccgtaatatcttcagcattgaaaggaagactcaacccatgggcgccttcttcgtcaccaggcactgcgcagtattcgtctccagcaacgggacaat

ctgatccagcaattcccgctgagcattctccctgggccgacatcgatgacgaccccttcagcgacccaacatcaagctggtccaatgcgaatcgatcg

caagcaggcccattgtcctcagaagcagacgacgggaccgctgtccgcgacccagactcgaacgagatcacagacgctcccagagggtttgaccgtct

tgcagccacagccggtctgagcgcgaccgagatcgaggagatgcgttcccagttccgacaggcagcggcacgtggatggggagatggagtgggacagg

gagctgcaggtccgagtgggctgggccggagtggagatgtgctcagggaggctgaggaggaggagcacgcacgggctttggaggaacagtggattgat

tcgatgggcgaagggatcgaaggtgatgccaacaggacagggagtggactactccaagcccgcttcgagggcctattcgtcggcttcttcttcccctt

cctccccctcttcttctggcgggacaagtcaggtcacccagcccggctgccccggagcgagttcgtgtacgaccctcaacggccaggcgaggtcattc

gcgccaacgagatcgactacgactctgaggacgacgacgacgtgattggggccaacgcgcgaccgggaccggggggtaccaacccggccattgcgaat

caggaggcacgaaatgtggtgtttacgcctacgatggcgcaggcaattgttgtgggccttgtgtgtaacgtggcatacggagcgctggcaagcctgtg

ctaa

>contig_14.contig_14.g228.t1

atggcatccacagacccatcctcctccgcagcaggcgccggcgccttcgacaccaacacagaggacctttccctcctcc

cgccagacgatgccctcgcagccatgaaggcgcgcatggcagccatggaggccgaggcggacaagcttcgcgagatgaccgccggtgctgcatcggga

tccgcatcgtctccaggtggtgcgggcgcgaagaacggctcgggtggggatatcaccatgggagagggcggatcacagttcccgaccgaggaggagaa

ggaggaggtggactcgagaagcgtctacgtcgggaacgtcgactacagcaccacaccagaagacctgcactcgcacttttccagctgcggcaacatca

accgagtgaccattctctgcgacaagttcacagggcatcccaaaggatacgcatacgtagaattcgcggatgcatcattagtgacgaacgcgctgctc

ctgaacgagtctctgttcaagggtcgattgatcaaggttacggcgaaacgcacaaatctgccaggtcactcagcgcgaggaaggggaaggggaggacg

cggcggtcgaggcttcccgcggggaggattccgcggtcggggccgtggaggccgcgggcgaggagcattctactga

>contig_14.contig_14.g229.t1

atgactgacaaggacaacgccaacccaatcatccacgcccccaccgtcgtctcctcccgtgcgcgcaacggcgaaagta

gcaccgccggtcgaagcgcgcaatggtggcgcgaccctgcggctgccgaactgaacggcggcctgccctcgatggggagtggcaagaatgtcggtgga

ctcgctgggtccgggtcgacgtcgctttggctgagccagcaagatgatgatgctgaggatctggaggatgatgaggatgataggatggtggaggatgg

tgcgggtgtcaatggatcgagtcgtgcgggggggaaggaccgcgaggagattgactcgcaggaagttttcgacctggtacggtcgatctccgatcccg

agcatcctctctccctggaacaacttgcagtcgtaaattctgcacatatcactgtcgacgaaggagatccggacgggggacgacagccgcgtgtcaag

ctggagttcacacctaccattccacactgcagtatggccactctcattggtctctctctgagagtgcgtctgttgcgggcccttcctccgcgttacaa

ggtggacatctcgatccgccaaggcacgcaccaatctgagacgcaagtcaacaagcaactgaacgacaaggaacgcgtcgcggctgcgatggagaacg

ggcacttgcttaccgtcgttcagaactgtctcagcactgcccatcgtcgaggtatgacagaggcgcaggctcttgagcaggcggatcggatggcggct

gagttgggtctggttgcatga

>contig_14.contig_14.g230.t1

atgtccgatctggcgttaatgctcgaacgcatggagctcaaggtcgacggtctccaagagacgcaggccgctgccgacg

cgcgcattgcgggattggcgtcgaggatctcgaccgtcgagcgtagccgcgaccagcgcgcaccgagcccggacaccctccgtcgcgaccaggtcgag

tcgagcacggagtttgttgccggccgacagcttccggcaccgattgcccgcgtgctgccgccgcacctcacgtcggtactgcagccgattgccgagga

cagctttgccgatcagagcctgtcgcagcggctcatcgaccccggtgttcccgacagcgaccggatgcgcatcctcgaggaagacttcgaggaacagg

ctgctcgtggcggccgcaaggtcgagaacgacgctctttggcgcgcagcctacgagatcgtccgtcgcgacaacccgggcgtcaaccgagcggagctc

ttccgcctcgttaaggccatggtcgacgagcagtctctcaagttcgcggccacagcttcgccacccccacgcaagttcggcgcgggcagcgacaagga

agatcggctcatcagtctctccggcctccagcgcatcacacagctggccgaggacacctacgaggcatggtcggacgacctctggagttacctcagct

ccaaccccaaggcacgcgcgatggtcttcaacaactgcagccttggttacgatcgtcgccttgacgacgagctcggaggtctgctcggcctcacggtc

gacaactactacaagaagacgacaatcaccgtcctccgcaagcaaggggagactcgtggcaccgtcatcttcgcgcagctcaaggccgatgtcaatcg

ggacagcgtgtcgcgtcgcagtctccttcgacagaagatgctcgccacggttcagaagtcggccgaaggcatccaggagtacgccgagcggcaccgga

ctctcttcgcgcagctcggcaacatcgacgacacgctcaacgagcccgagaaggtcgaggcctttctcaaggggctcaccccggcgttcttcggcctg

aaggaggctctctacacttcgcagttcacctctggacgagcgctcacgctcaacgagacggtcaacttcatgatgacgatggaggagaagcgcacctt

gggcgtcatgaccgcgcctcctcaggtccttccccgattcggagcgcgtcaagctgctgtggatgtggataaggaaaccccgaccactctccagctcg

agcatctcaccgaggaagaagcacacgctttcgttgcccgtttcaacagcaagttccgtcccggttcacgccctggattcggtactcgactcggcccc

ccatccggtcacccacaggaggccgattggtttgcgggggaatgcaacttctgcctaaaccccgggcaccgcgaagcgcgatgccgcatgcgcgcacg

tcttctcgacggcgccgggcccggctccaagtacgacaaagatgccaagccggccgctcatactgcttccggcgaggacgtcatggaggaggttgacg

ctcgcctcgcgcagctatttccccaaaaactatgggacgcgtccctcgctgctggctccagcaacgcagagcacgttggccgggaaaccggacagggc

cgcgctactatcgggcagcacagcattcaagagagggagtgcacggtggaagagcggtggattctcgacagtggcgcaacgcaccatatgactgcttc

ggcgtcattcatgcgtggcactaagaggtccgccgctggggtcatggtgcgggttgcggatggaaagagcgttccagctacgcatgtagcagacgtgt

ccgccgtcctccgaggccaggatcgagctcagcactgcgtcgctcttctgaacaacgttctgctcgtccccagcctcgacgccaacctgttgtccacc

caccagctcgcacgccaagggttcatcaccatcatcctccgcaacagcaccgtgcttgtctcgccgtccggtctcgtcatccaggcgcagatcgatcc

ttccacgcgagcagcgtacttcctcatccagcctgaccacttccaaggttcagaggaggtacatgcgaacacggcaagtgtttcgcagcagtcaggag

acggtgtgcctgatcaggcaactatggacccaagggcggagctttggcataggcggactggtcatgcaggatgggctaagctgaggaagtgggtggag

ctcggcctcctggtcggcaaggatgcaccgaccatggcgagcatcggtagtcacctcaagtgcggacgagtctgcgacgtgtgcgtaacggcaaacca

gaagcatgcgccgtacccagatcgtgcaccgtccagagccgatacaccgggcaagctcgtgcattgcgactacatgggcccgttcaccggccacccca

gctttgcatggatcctgacgctcgtcgatgactgtacgcgcaagacttgggcattctacctaccggaccgttctgccgagactctcacggcaaccctc

gacgcctgggcacagcaagtcgaggttaccatcagccagcccgtcgtgaccataaagaccgacaacgggaaggagttcgtgtcggatgagcttcaagc

atgggcaaaggagcgtggcacgacgtggacgtggacggatccgtactcctcgtcgcagaatggggtggcggagaggcgcaacggtctcctcgaggaac

ggatgcgtgcaatgctccgtggtgcacgcctccctttcgggttctggccttatgcagtccagtacgcggcgtacgtgatcaatcgcacccccagcacc

agtctagaaggtcgaatcccacaggcgctctggtccggcaagcccgtcgacatcacggcacttcggactttcgggtgcctctgctggacgctcaatta

tccagagcagcgccaggaagggaagctttcggcgcggggccttcgcgggatcttctcaggagtaggtgaagagcggcgcgcttggttgatcttttgcc

cagcatctccaagcaactcgcttcgatggtcacgctcggttgtatttgatgagagccggacgtatgatcagtcattggcgctagaagaacccaagcca

gtacagccgcgcaatgacgatcatgtggagatgcagctcatacctccaaaacagcgtcaacgcggtaaacggcaagtaccatcgtcgcagccatcaga

tgccgtgctacctactggaacccccagcgatgaggacttgcagctgcaggatacgtctatgagctcgaccccagtcgcgcccgcccccaatgcggcag

atgggatccttccgctctgggaaccgtttgcgacggatccgggcggaccacagtccgagatcgacctcgccacgcgcattcgccaattggagcgcgta

tgggatggagactcggcaacgccgtcaacgatcatgaacctcgatgaccttggtgccctgctcgatgaacaggaacaaggacctccgcgcgcatccac

gcctccaccatcgcagcagctccgccggagctcgcgcatccgtgcaggggcacgcatggcgcatggactctcggagacattcgatcgagacgaggcgg

catacgacgagcggctggcggtaactcagctggttcacgcaaggtccgccatgtccgtgctcggtatggctttgcattgggcttgcaccgccgcagct

catgggactagtgcagacggatcacctcttgagcccgtcaacctcaagcaggcaaagaaccgaccgtctgctgaatgggctcaatggaaacaggcgat

ggacgaggaggttgcgtcgctgacggagatgggcacatgggtgcagagtgagccctgcctgttcgttcggttcggcgccggcagaccagtcatcctcg

cggtgtatgtggacgatatcgtcatcgctgcgcgaggagcggaaggagtcgcgagggtcaaggcagaattcggctcgtggtacaagatcacagacaac

ggggatctctccttcattcttggcgtcaagattgactacgaccccgttcgccgcacggctcacctcagccagggcgcatacatcgacgggctgcttcg

caagtatggaatggagaaggaggatccggtgccaacgccgatgacggaagcgtcacggcatctaggacctaatctggcaggccaggcgagtcggtcag

tgctgggagacctcagcatcaagctcagccgcgccacggacatccttaccgactcaaagggagcggaagcgattgcgcgcgacccggcggatcattct

aagaccaagcacatcgagacgcatcaccatctagttcggcaaatgcaggaacgcaaggaggtctgcgttggacgggttcacacggactctaaccctgc

agacatgctgacgaaggcagttgcgcgggagaagcttggaagcttgagtgggatcatcaagctgggaagaggaggatctattcgggagggagtgcagt

tcaagagttga

>contig_14.contig_14.g231.t1

atgctcctccgtgctccgttgagctccgcgctccgcctccggcggagccctccgtcggaggagtacggagtccgcaaaa

ccccagctccgcgtcaactccggggacaaccttaccccagtccttcacgcacacactcagcttcgcaatcgcacccgtcgtttctctctcctacacag

actcaaacccaggctgaagaaagcagagttcccgtggcccacaacaggtcacaacgccaccacaactttcgcaccccctctgctttgtttccagctcc

gcaacgccacgagcttaccgaccaggctgctcagagccgacagcacgcgctgacgatcatcgggtccctatccgtggccgacacctcttctggttctg

cgctcactcctgcggaggtctggagctctctgtcggcagacaacactccgacttgccacctctccggcaagacggccgacgacctcgatcgagaacac

gtcctggttgagctctccccggctgagcatctcgtctccgctatacacggctctcttcccccacacacgatcaagcagctgcgtcagttccgatcaca

ccgcgtcaagctggacacccgcaagagctacggtgggcacgtcgtggccttcctccgctgggccgacgacttggacattccgcaacacatgcgcttcc

ccatccccccaacgatcgtcctcctctacttttcgtccgcagcccccttctacgcggccaacaccttgtccaagatggtgtcgggtctgcaggcgtgg

catgcggttcacgctttgccttgggcgctagaccgggtcgaagcccgcacgctccgacaggccctggccaatctcgcgcttcctcccctcgaccttcg

tcgcccggttcgtatggccgatttcgaggccatgcaacgccacatggacgttgccgatcgcgcacatactgcggtatacacctgcgccctcttctccc

tctggtcgatggccagacacggagaactcactgttgacgttgtgagtcggcctcacgattcgcgcccccgccggtcggatatccgtatggctacgggt

gagcaaggacagatcaaatcggtcattctgtacctaccggaggacaagacacacgggaataccggtttcaatcggcaaagtcctgtcccttccagctg

cggagtctttgcatacatcgacaagcatggccaaccgcgcgagcttggtgcgtccttcttcatgaagaccatcaatcgatggctggctgccgataatg

ccgagcacatcaccggccactctttccgtattggaggagccactctcctccactccaacgggctctccatcgagtccatcaagcaccttggtggatgg

gcctccgatgccgccctccgatacatccaagacattcacgttcaacacgccgaggtctgttccgacctcgacctttctgctcttcgccgtgccatccc

ggcctccacctaa

>contig_14.contig_14.g232.t1

atgaatgaccggcggaaaggaggtctccgcggagattggctcgtcttcgcgagcttctccgaggctgacggcggagaaa

aagtcgcggagatttgctccgcggagacgcggagacgcggagctccgcgaaaaccgcggagatggtcgacatccttatctcagcatgctcacattccc

gactccgtcgtcaacggattctccatgggcgctctcgcctcccctacagctacgtgtcttcatcccaatcacttccgccacgatcagatcggcatcat

tgacgattggatcgccgactccctcaagtccgacttcatcgctgggccttggtctcttgccgaggtcgaggcgaccgtcggtccaatccatgcttccc

ccttacacatcgtaaccaagacagacgaaaacggagtcgttctcaagcatcgggtggtctatgatgcgtcatacccccgtacacgaccccaagatcca

cctcctccaactccatccatcaactctcagctcgataaggctgactttccttgtgaatggatgacggccaacgacaccaaaatgatggtatgtgttcg

cgcattgaaactccctttcccctctcctccccttcctcgctcctcggagcatacagtcctggctggtcttgcttcggcatacatgctccggcattctg

ctccggcaaccatgctccggcaacctgctctgctctggcacgctcacagtccttacgctcgcgtcgctggtttcgagctagcttgggcattccaacag

ctcagaaacaagcctgagcaacgccctctcttctgcatcaacgtccgcgatcagatttacgctctatttccagacatcattctcaagcattacgtcga

tgattttctgcccgtcgacgtcgcccccccttcttcttctcatcacggtcgcccgttcgagctgcttcttcgcgtggtcaagcagtttggctggacca

tccacccgaccaagcagttcagctggacccgtcgtttcactttcctcggcatcgactgggacctcgacgcaaagacagcctccctcactactcagaag

cagcgcaagttcatgcgcaagcttgagcacgcatacagtccctcctccaccttgtccgagaaggaagtcgcctccattatcggttcatgtcaacacac

ttgcgtcctggcaaccactcgtcggtccaagttgaacgccctctactcgcttcgcaacaccttccgaggacaacacaaggctgccaagaagcacctcc

cgaccgctgctcgcgctgaggttaggtcttggctcgagttctttcaacagggtcccgtcacctgctctctttcctttcccacccatcttcaccctact

cccctctacacagatgccagtgacttcgggctcggcattgtctttggcgatcacgccattacgattccccttcctgccgactatacctctcgccccgg

gatcaacatcggggtgggagaggcatgggccttcgaacttggggtgtatgctgctatccagcatggcgcggtcaactgcattctcaccgcgtacgtgg

acaacctcggtgtcgttcatgccgcacgtcgtggccgctcgcgcaacactctggcgaacgaggcgatcaatcgcgctgctgatgctgccttggccgcc

agcgttgatttgtccatcatctacgttccttcagccgagaacctgtcagatgcaccgtcacgtggcgattcttctagttaccagttcctctcattttc

tcacaactctccctggcttccacacttgttcccatag

>contig_14.contig_14.g233.t1

atgcctcctaaaagcgaagcgtcgacttcaaaggcatcgaagggcaaaggggccatcacgggcaaggcatcaatccgaa

gcactgccaagacgatcataaagcggaaactacccggatctctcgacactgctacctccgaagctgcgaagactgagccgctcgctaagggcaagacc

gcccgtaccaaagcgatgaacaagatcaacatcaaagaagacaaagacaaagatgacgatgacgatgacgatgacggtgacgatgaagaagaacagca

gatgagcgtcgaggctcacaaaaatggtacacaggtggacgcagatgaagacgtttgccttaccttcggaatcatcgtgatgagtgggccttacgaaa

actccggcaaagtcgtcaaggcaggacttcagcgcatcctgtcaattcaaaagcatttcacgtacaacacgttcaagaaacgaatcgaaaacatagtc

caggacgatctttcgaataaggatgtcgacggagatctccgcggagattttctccgccgtcacgtctccgcggcgtctccgtgccactttcccggagc

tccgtctcctctccgtgtttgctgcggagattgcacggagctccggcctatctccgccattttctggggcttctga

>contig_14.contig_14.g234.t1

atggtagctattacagaaagatacataggagggttcgagaccgatcatcgctacgcggaagctcaggacagctccgctt

cccttcgtctgcttcgcaatgagttgcagaccccctccatatgctacaatggcgacgcctacagacgccaggccaacggagaacaccttgcgctgtgt

aaagtaggctgtcggtcaggcacaactcccggtacgtctgggaaatggtctgaactcccctcgttcgcttcgatgccatcggacaagctatggtatgc

ctccgccttggaccgagaactcagctgccagtccagattcttggctatcaggctgaaccaggggtcgaaagggtacttctctctccaaacgaggagga

gcaggtggcagggtagtaagatcgagaagagggagtgtcccagccatctggaaaagaagaagcagatcccctgggtcagctctgggttgcagatccac

tgtcgcaattcgcacacgcagacagaggcgcgcaagagggaagcttcccttcggtcctccgggcagagcaaaaaagcccttttgcacctcgatgtgct

gcgagcatctatggacgagacgggacgtgtgtcgttcgtcggagagcgtcgtgatgaggaagtcggcgtctgcggacgcggaagagaagggtcaacgc

agcgcaccactaccagctcgccggccggagacttgctcaccaaggcgcccgcgtcgaccggcccaccatcatccttccgcgcgcacgcccacgtcaac

agctccatccccaaccccatcgagactcgcgggcccacgccgaccgctccaccaccgcatccctcgtacctgaccgtgcccacgccaaccgcacccct

cgcggccaaccgcccccacaccaaccgccacgccaactgcactccttgcgcccgactgcgcgtacaccgaccgccacgccaaccgcacccctcgcgca

cgaccgcgcgcgcgcgcacgccaaccgccccaccgtcatccccgcgcgaacgcgtgcgcacgccaaccgctcgccccaccgtcatccccacgcgaccg

cccgcgcccgaccgcgcgcgcgcgcacgctaaccgcctcaccgtcatccccgtgcgaccgcccgcgcccgcatcaaccgccgcaccgtcatctcatca

tcgccaccgtcgtcccaccccaaccacccccttatcgccccaccatctccccctcgctcccgaccgcgctaaccccaaccgcttcgctgtcgctccct

cccgcccaatcgcgcccacaccaaccgccccgctgttgtccccgtctccacaccctcagcaatcaaatcaatcgtgtcaagccaatctcacttcctcc

ttcttgtcttcaatgacaggttccattactcctaaggccttgccctcatccttcgacctactccctcgacccaaggcacgaccagccggcgtcgaatc

acagctgctcgagctcgagttcggcttgttccgccctatcaagggaggaggcgtcttcaagaacctccccctctcgggcggagccatcctgttcggac

ccccggggaccggcaagacgtctatcgtctccttcgtcgccgccgccgccgcccgcgtccccgtcgtcgtcctcagggccaactcagtccacagcaaa

ttctatggcgaatcagagaagatcctcgccaacatcttcaaggaggcgagggataagtccccttcagccatcctgatcgacgagctggagtgcatctt

cccctctcgcgtcgcgtccacggccacggaagcgcacaaggccatgctctcgcaacttctcacggagctcgacgacgccaagaacgcgcgtgtggtcg

tcatcggaaccactaactacctttcacagattgatgatgcccttctccgccgtctccctcagcacattgaagtgcctctacctaccgtaggggctcgg

ctttcaatcctcaagcaccacctcactctcggcggccgcaaacaccaaatttccgacgatcaacttcaacttctcgcccaccaactgcaaggatgcag

cggcagcggaattgccaacgcggtcgcctgttccctccaacacctcgcccgtcgcgtttatgagaacgccgagcgggtcaaggacgccgagcgagtca

aagaccggaagcgggtcaaggaccagaagaaggacaagctccagacggatcaaattccaaccaaacccgctcgaacccccaggcggcccctcctcaag

cttccagaactacgatatcgggacttcattcttcctgtccacagccaaagcaggattttctcccatcgcaagaggggtcgaggtaaacgtaactccct

ccatgcttctgtcctccccagtgcctaa

>contig_14.contig_14.g235.t1

atgcaccgccgtccaacagcagagtcccgcactcccacctcagcactcgggcctcagcggatgatgcatcgacgtgagc

aggtcgcagactcgcagatcttgccgatcctgtcttactttcgtgggccgcttcagcacccgggcagcaggttccagctccgtcggcgtcgagccgtc

gaggacaaggaacaggtgaacaaggaagcgggttccaatcgatttgaacgattcttcgaatctccagccagcatgcagcgcccttactacggccttct

ccttccgaagctcaagaacgtctttttctcggtccttgccctcggcacggtgtgctctgccgccatcgcccacccgaaccacgtcagtctctcccgca

aggcggttgattccaagtctgctctcgtcgaccccgtcgtcaacctgcacctaaccctgacagtccccatcaaggagatcgtcggcaccgtcgaggcc

actctcgccaccgtgggcagcgcagtggatctcaccgagagcgcagtgcaccaactctccgactccctgaaaccctacatcgacgatatcaccgacgc

cgtcgagtccacggccgagtacctcgtctccgccctccagaagggcaccctcatcctgacggacgacctcaaggccgccgcccaagcggtgggcgacg

cgttggcgctcgtgctgaagaagctcgagatgacgggcgagctggttatgaagacgttgaaggaggaggtgtatgggcttgcgcaggcggggatgttc

ctctttggccagctggagcaccttgtggaggggttggcgaaggcgatccaggggaccgtggaggagttggtcgtcagggccaacaaggtcggggcgga

tgttgatgggcttttgttcgctattgggaagtcgttgggtcagatccttcacgaccttctctttggatga

>contig_14.contig_14.g236.t1

atgtccagcgacggacagtcagcaaacacagccgccaacaagaacaacaacatctcttcgcttcccccacacccgctcg

cttcctcccgcgcagcagtagacgcagccatggcttccgtcgcttccaaggtacagcagcagcagaaggacgcaggcagtgatgccgccgcctcgtca

tcaacacaacagcaaacagctggcatgtcctccgccccctccgactcatcaataacagacaactcggtcgaccaatccaacgcctccttcgcttccac

cggcaccgacgccgatctcgacgtcatcaccactgttttccaggaccagaaccacttcaacgtcaagcacccgctctggaacacctggaccctctggt

tcgacaacccgtccaccaagggcggcggaggaggaggcggtcacggtgggggtaaaggtgcaccgggcggtaaagatgcctggggtgaggaccaggtc

aaggtcgtcaccattgagtctgttgaggagttctggggtctctacaacaacatcatcccgccctcggatctccccgcccgggccaactactacctctt

taaggatggcatccggcctgcgtgggaggatcctgccaatgctaacggcggcaagtggtccatccagctcccccgcgacaagtccagagagtccatca

acaccctctggctccacactatgctccaagccattggagagactctcgaagcctcctacccagaaaacgtccccaaccaagcaggcagtttcgaagac

gaactcgtcaccggcgtaatcatgtccgcccgtccgaacttctaccgaatcgtgatctggacccgcagagcggacgacgacctcgaggcccacgcctc

caatcccaacgccgacgcccccgagaccaagctggccacgaggctcatcgacatcggcaagcagttcaaggtgaatgtgttgggttataagttggagg

gtaaagctggtgcggggtcgagtgggttcgcgaccgaggtcgagttccagtcgcacagggagagtgagaagaagaaggggaagaagatgatgctctga

>contig_14.contig_14.g237.t1

atgcgcgccctcctcgctccttccacgtacgcttttcgtctctcccccccgtctccatcctcatcctcctccgtcctcc

gagcagccctcgtccgacgcgtatacagcaccaacaacaacaccaacaccaaaaaggacaggccaacagctcccaccctcctcaacccagcacccccg

gcacccctccctccctcacgacgaggcccgcccacgacccgcctcgtacgctggcagagaccagaagacgaagaagatcagtatctctcaaataacaa

cccatcagctcccctcggtcgctccttctctcccccccacagcagcgcatccaccttctccttccaccagccccaacactacgcctccaagtcgacag

ccatcaggatcagacagccttccagttcggccctcttcgctttctacccggccacgactgcaaccgctgtctcaaggcaggcttcgatcgacaatcca

ccctctcctgtcatcaagaacaacaacagcagcgaaacgatcgctcctaccatgtccgcacagccagaactcacacagcgcaagcctgattcggcagc

agttgccaaggaaaccaacggtgtcgcgacgcatgaccaccacgaccattcccactcgcacgcgcattcgcactcgcactcgcactccctgaacccct

tcgcagctcactcccatgcccacggccacgaccacggctccgccgccgaagccgacgcgctcctcgccgccctcaaaggccgcggcgaccgcggctcc

aacatcaccctcctcggcctcttcgccaacatcctgctctccgcctcgaaaggcctcgcaggcgtctacctcaactccgccgccctcctcgcggacgc

agcgcactccctctcggacatgttctcggacctggtcacgctgttctgctggaagatgagccagttgccggcgagcgagacgcacccgctggggtatg

ggaagtacgagactatgggtgggctgggggttagtcttgtgcttgttgcgggcagcttggggattggctttcattcgtatgggctgttgttggaggcg

ttgcagccgacgttggcgcatgcgccgcatgctattcaggtcttgagtagctggacgggctcgttggctagtgtcggtgcgcatttgcatgatcatgg

gggaggggctgaggcgggacattcgcattcgcatcatggcgccgcggaagggggggtgttggatccgaatgcgatgtggttcgctctgatgagtattc

ttgtcaaggaatggctgtaccatgccaccctcaagattgcgcgcgaggagaatagctccgtccttgaggccaacgcaatccaccaccgctccgactcc

ctctcctccggcgtcaccttcctcgccatcggcggctcctggctcgggttccccatcctcgaccccctcggcggtctcctcgtcgcagccctgatcgg

caaacagggtgccgaactcctactctccgccctggccgagctgagcgaccgcggcgtggacggggagacgttggttggctttcgggccgtgttggatg

atgtccaggctgagttctcgctatcgcaaacggggtcagtgacatcagggaagggtgcccatttgcaagggtggcacaccctccgcgcgatcaagagc

ggggtgagcatctttatcgacgtgacgctcgtcatgccgccggagacgacgctggcggaggcccgcgcggtcgagggtgttgttgtggaccggttgag

gcgcagtcggaagggggtcaaggaggttagggtgcagttgtctactgcctttcccccttcttcttcttcttcttgtgggacggagcctggtgggaggg

ggaaggtggggaataaggcggagtga

>contig_14.contig_14.g238.t1

atggctcagcagcagcagcagcagcagcaacacggcgtcgacagcgcaggcgcagactaccccttccagtccccatact

cacaggcatcgtacgagaaccagccatcagcaacagcacagtcagcaccacctcccaacgagcacggcgccttcaacgacgaccatggccagccacac

gcccccgcgtccaacgagctagccccgaccaaggaacgcccagagctatactcaaacactttcaacaacaccggcgctgatccatcccacgactaccc

aacccgcaacgcagccccgacatcctccagaaagggagcctatacagacgcacccgatggggcagcagcctacggccgatccggcatctggacccacg

cccaccgctccgccttccaacgtcgttccacacccatcaaaatcctccgctccctctgctgcgtcctcctcctcggcctcatcgtagccctctccgcc

gtgttgctcatcatcaccttcgcccggccccccaacgtggccatcggagcgatctcgtcgaaccagaccatcccgacgttgggcaagacgtcgctctc

cttcaacatgtccgtcgatattacggttgcgaacccgaacgcgatctcggctacgttgaccgagctcgaggcggtgggctatgacgccgtccgaccaa

ccgtgcccatcggacgcgggaaggtcacgaatgtccccattgggaagaacgcaaatacgaccttcagtctgccgttcgtcgtcgcatatgacgcctcc

accgacgacgaccgcagtctgatcaccgacattgccaccaagtgcggctggctctcgggcagctcggcatcccagctcgacttcaccttcaagatgaa

cgctcatctctccgtactcttcctcgccatcccgttctccttctccgtctcgcccaaattcacctgtccgatacccgccagctccatcacgagtttgc

tcggtcaggcgggcgggtcgctcgatgatatcctcaaggctctcggtgtcggtggctcgggacggaggagatcggaggaggcggaggatgtggcgcct

cggttacagctcatagagtcgggcgcgacgtccctctctctacgggatgtccagttcgcagactttctctccgactggcgcgagcgcgcagcttctct

caccgaggccagccaggtgccaagcgacctatga

>contig_14.contig_14.g239.t1

atggccccacccccgggccccgagcctcgtgcctggtcagccagcccacgcccctcccaagcttcccacccttgccagt

tcccaccctcgctctgtccctcatccccacgcagacacgacccccccaagcctgagcagagcagagcgatggcgcaccgcgtatccgagcgatccccg

ctgacaggcaaccccgtcccgccgcacttcctccacgcctccgacgcctacttccgcgacaccgccggacgcgccgtcctcctccgcggcgtcaatct

ctccggctcctccaaggctcccgtcgacaaaccatcccaggagagagaaggattctgggaagatgctgagcatggcaccgagctctcctttgtcggac

gcccgcttgatatcacggacggctcggctgatgtccatctggcccggttgaagagttggggctttaactcctttcggtatgtctttacctgggaggca

ctcgaacatgacggcccgggaaagtacgaccatgagttcattcagtacaccgtcgatgtccttcgcaagatcaagcagtacggcttccgcgtatacat

ggacccacatcaggatctcttctcacgcttcaccggcgggtccggcgcaccgttctgggtcctccccgcctgcggcatgaacccgcgcaacttcaccc

cgacccaagccgccctcatccactccgaatggccgaaccaggaagacccgtcgccgatcgacttcccagacatgatctgggcaaccaactacacccgt

ctcgcagcggccactctctccgtcctcttctttgccggccgcgactatgcgcccaagtgcatcatcgacggcgtcaatatccaggactggctccagtc

tcattttatcaaggctgtcactcagctcgcacaagccatcgcggctgccgacgatggtgatctgctcgattcgtgtgtcgttggttgggactccatga

acgagccgaacccgacgtacatcggtctgacggcgctcaacaagcttcccgcccactggagactccgcaaggggcccatgcccacccctctgcaaagc

ttcatcacaggcgtggggaaaccacaggaaatcgacaagtatatattcggacctgtcggacctaaaaagcaaggttctctcgtcgtcgattcgaaggg

gaggagcatctggatgaccgccgctgaggacgagcagaagggcgggtccaagtggggctggagccgacacccggattggcccctcggccagtgtctct

gggctgcccatggcgtctggaacgagtccacgaacgagctgctcgactcgacctacttcagcaagtacggcggaccgagcggcacccgatccgtcgag

tttgtcgaagactactggctgcctcactggcgggcgtactcatctgccatccggcccttccaccctgaggcgatcatgttcatgcagccccccgtctt

tgagccgccgccgaagggtctgacggaggacgaccttcgccaccgggcctgtgtgtccagtcatttctacgacggcctcacgcttatcaccaagcact

ggaactggttcaatgccgatgctgttggcttgctccggggtaaatatctcggtgtgccctttgcgctgaagttcgggtacaaggccatccggcagtgc

atgcgcgaccagcttggctacctccgctccgatacactcgacgtcgtgggacagtatccgaccatcattggcgagattgggatccctttcgatctcga

caagcgcaaggcgtactatggcgatgagaaaggtaaagggaagggagacttctcggcacagttggctgcgctggatgcgagcatcaacgcatgcgatg

gaacgaacatgctctcgtactcgctctggacgtactgcccggacaacacgcacaagtggggcgacggatggaatggcgaggatctcagtctgtggggg

ttggacgatgtgaagacggtttcgtctgcggctgtcaacgctggtggtaggggtggtcatcagatggcgatgagcgcaccgggttcgagttcgaatgt

ggacttgctttcggccatgaccacccagacggcgggcgactcggtatcctccctagaccccgcctcgtccacctttgagctgaacctcggcgacggca

gcagcagttcgcaagccttgccagccctatcgaacggcgcgcgctcggctgctgctttcatccggccattcccacgggcgtgtaacggggtgccgatc

gagatcaactttgacatcaagacgtccgagtttgagtttacgcttgaggtggagccggaggatttggcctctcagccggtttctgctgataagcttgc

gtccttgtcgttgttgtcgggttcgactacggcagcacgggcggcgggggtgttgccgacggagatcttcttgccgttcatacattatgcgcgggatg

ggaatggggcgagtgcgatggggaactttgatgtgaagggggagggatttgcgagtgcgggtgggaagcggaataagcggcgggccgcagcggcggcg

gcgaaagatctggccatgtctgcttctgcatcgtcctctgcctctcccatcgcctctggctcggcgtcgacatcgacggacccgagcggctcggtcac

gccgaatcagctcggactgccaccgctggactcggtcgagtttgatgttgaggtggaggtgtctgcggggacgtgggagatccgggatcagtacctgt

actggagcctgcccgcttcggctggagacgtggatgcggtgaaggtggtgctggaggctgcagcggcggctgtagcggccgcggaggaggaagggggc

agtagttcggggcgacagtcttccagggctgccaggcgggagaagcggaatgccaggtcgaatgcagcgtcagcggcggcatcggcagcatcggatac

ggcggctggagagggacctacttcgtacccgttgagtcggacggggtcgagctcgggccagcctaagacgacgccggagggcgatgtggtggttgaag

ttcgagaggctgacgggtcattgtgggaagagcgtgtggacagcaacggcaacggaagcgaggaagaagcggagatccccacgatcctaccgacgatc

acggccgagggcaaggttcggcatcatatccgcatcatcgacttcggttcggcacagtcggcgtggacgacgatggtgggcaatctactcggcatggc

ccgcaccaccgcaccgaactcgtcctccgcgcccgacgccggcagcaccaccagcgtcactggcgcaagcctggcatcgctcaagcgcggcccacccc

acttcggatatgcgttcgacttcatgcgctgggtcggcaccacctccatccaccgcttcttcgaccgcatccaagtcgtcggcggagaggacgtaccc

gcgaagggccctttgatcatcgtcgcttcccatccagccacgattgtggacaccatgatcatcaccacctacatgccccaccgccgcaagatgcactt

ctgggcaaagcgcggactcttcaagcatcccatcgcttcctggcttctccacaactctggcaacttgcccgtcgatcggaagacgaggaacaaccaga

agctctttgcttccacttttctcgctttgaggaagtctcacgcgatctgcctctttcccgagggcggctccttcaccgagcctcatctccactccatc

aaggcaggtgccgcatgggcagctctcgagtatgcccgctacctcgccatacacaatgcccggcacccagaagttaccaaggttcaagtcgttcccat

cggcatctcctacacagacaagtcccgcctgggatcgtctgcagtcatccaattcgggcgtagcttctccatcgatgcctacaccaaagacttcctcc

gcgcacaggacgccacggacccgatcgcacgagagaccgtcaaagcgctcacagaccggatcgagaacgatctgcgttcgctcacgatcaacgcaccc

gattgggaaacgttccacgcggcgcgtatggcgcgggaattgctgtggcctaagaagaagagcttgccactggagcagttcctgaatgcctccaatgc

cattctcgagctggtctcagatgaccggtacccggctcaggaagagtacctccgcaagcgagcgtccaaagcacgcgctgcgctctgccgtctgcaat

gtctctgcatcgcagccaacaccgacctgtggacgctgaacgacttcgcagccgggctgtccaagcgccgtcgtccgacggacagcgtgcccaccccg

cacgggcctctacgcgcgcttacccgccagtcgattagtctgagctggcactcgctctacctcctcccgctcctggctttgcacgcgcctgtccttct

cctcactggatacttgcaggctaccaaggccagccatgaggaggagagcatggcgagtgcgaagacgctcgtggcttggacccttaccttgttcctct

acctcgccctctccttcctcgtcgccgttcccttcctgctcaccccgcccggatgggtgctggccgtcgtcggactgttcgtgatcaaccaggcgctc

cggttgaccatgctgggcgcatatgagcgcgtgaagcgctggacgggctatgcgcgtctcttggctgctagcattgcggagccgctcgagggtttcgc

actgacggattccagcccttcggtccggttggcagttgagaaagtctcggttgggcccgcggtgcaggatacgatcaacaaggccctcgcgcggaagg

gggagcggaggggtttggccagctggaggagacccatgcgggcccggttcgtgggtgtcttgcttcgcacgcgggccgaggcgcgtcaggctttgcaa

ctcctcttggacgcgcttgaggatcgtgcgcatggaggcgatgggaacggcgtctcggctgtgagcacgagtgcttccgaggctggtggggagacgga

gaccgaggcggccgttgctgctgttgccggtgcggagcgggtcacggatgacttttctggcaagtcgttccgtgttggtggattggcgtcgcagttgg

atgcggttgaccagatccggagtctggggggccggctgcggctcgagtatggcaagcagtacgcttctcagtggagctaa

>contig_14.contig_14.g240.t1

atgtgcgatgctggcggccgactcctgcagcagacctgcttcccatccaccgaagatcaccaccatcccgcacgtccca

cctcctcacactcccactcccacgtagcaaggaccatggacagcagcgccacccagccagccctcttccactccctctcccaccacgccgcatccgtc

ctcgcgctcgctgtcgacagcaacgcgaacatcgcctactccgcctcccagtccgaatgcatcgtcgtctgggacctggctacgctgcaggagcgtac

acgtctgaaagggcataccgcctcggttcttgcgctcgaacttgcgcttgataaggggtggctcttctcttcgtcgggtgataacacggtcaggattt

gggacattcactctcatttgcctgtcgctgtcctttacccggccgaggacaatgtgggcgacatcttctccctccaatggagtcagcatcttcagaca

ctctacgtcggctgccaaaacacttccatacagtggatatcgctcgagtccgtcccagcatcggcagcactacccgtcccctccaaaccacacaaatt

cttcgactccgtccccgtcgctctccagcgcagacgacagcgcgaagaagcgcactccatctacgcagcctcgagcagccaactcggctcgccaagct

cgcaggacgcgaacaataatgtgaaggagaaggacagctccctctcctccaccgtcgtagccgattcctcctcctcccgagctgtcatgctctcttcc

agcgcggacagcgagatctcccatatccatcctcccagctccgtctctttctcatcatccaaatcaaagtcgattccggaggaggacgaagatgagga

tgaggacgaggacgaggaggaaggggagaatgtggtccatcttcaattctcaagcaattgtatcgtccccttcgcccacttcggatacgtctacgccc

tcgtcctcttctacccagaccatccccccggccaaccacactggccccacctccgaacgccgagcaacgagcacaaccaatcaaactcgcccggatca

aataacaacaaacaactcccgatcctcgcctcggcttctggagacgaacatatcaaactctggaactgtctaccagacggctccctcgtcctccgtgc

cactttacacaacaaggactcggacgcaggtgggatcctgaccctcgcgcaccagcctaccacgttgcttgcgggcacgcaggcggggacgattgata

tttgggacctcgagacgctcaccacggtgcgcacgctggacgcccatggtgggcatgatgttctcagtttgagcacgttgaggatcgggaatccggag

ggcgcgggagggggcgcgggggagtatgtctgttttagtagtggggccgatggaggggtgcggaggtgggatgctaaatttggatgtacggcgggttg

gagagcgcatgaagggttgagtttggcttgtgtgccgctcgtgttgggacgggagggatgcgtgcgcgttattacgggtggctcggacgcgcttgtga

aggtgtgggatttcccgccgatgaatcttggttcgctcttgcccgtcgcgggtggtggggcgtcgtcttcgtttggtgctgtggggttgggatcgccc

gctgggtctccgggcacggcgttcttgcgcaggtcggcttcgtccgcttctatcaccgcgggactctcgacgcaattctcgtactcgaatttacgccc

ttcgctcctgtcctcctccttctcttcgtccacttccatcatccagcagcaacagcaagaactagacatcctcctacccgccctcgcccgcttcgtct

ctttccgcacggtgtcctcggacgagggatgtcgggaggagagtcggcagtgcgcgcattggcttaaaggctgtcttacccagatgggcgcggagacg

atgttggtcccgggcgcgaagggacggaatccgcttgtgttggctacgttccgcgcgaatgcgacggaaagccaggacaaagtgaagggggccaggag

gaagaagaggtgtttgttttatggacattatgatgttgttgatgatcattcgggggcggagggggctggaaatcatcagcatgagcatggacacggcc

agggccagggccaggggcacgatggcttgttgaggaactcccggagtggcgcttcgacgcttggtgcccatccaaatggctccccaacttcacccgcc

tccccaccaaccccaacaaacccttctacgcccagctccgaaccctggatcctaaccggtcgggacagctacctctacggccgcggggcgagcgacaa

caagggccctacgctcgcggtggcgtgtgcggctgcggagttgttggccaagagggcgctggagatggatgtggtgctgcttgtggagggggaggagg

aggtgggtagtgtggggcttgaggaggctgttaggagggcgaggggccagattggggatattgacgtgatcctgctctcgaactcgtactggctcggt

gaggagacgccgtgtatcaccatgggtctgcgcggggtcatccatgcgacgattgagatgtgcgggccggataaggatgtgcattctggtgttgaggg

cggggccttgcgtgagccgatgatcgatttctacgacgccgtccggcccgtgtcctccgaagagcgttcggccttcgcgacgattgccaagatggagc

gtcgatacggccgccagcccaccccgaccaccaacacaagcaccaattcaaacacaaacacaagtacggaatccctcatcgcgcgctggtgtcgcccc

tcgctctcgatccaccgtctgggcgtttcgggctcggggaactcgacggtgatcccgagtaaagtggaggcggcggtgtcgttaaggattgtgcctga

tatggatgcggagagtgtggggagggcgtttgaagcgcatgttttggggcagtttgctacgttccggtcgagtaatcgggcgagggtccggattgatc

atcgcgccgattggtggttgggctcggagaaggatgcgtactcgcaggcgctggcagactcgattgaggccgaatggggcgaggcgcccgtgagcata

cgcgaaggaggttcgatcccggcgatggcgctgctggagaaggagctcggcgcgccggctgtgcatcttcccatgggacagagttcggacaatgcgca

tttgccgggggagaggattcggttgattaatttgcagcgggggagggcggttgttaggcggtttttggaggcgttgcctgggatctga

>contig_14.contig_14.g241.t1

atgccctgcgacaactgctcatgcgcacccagcaccacagcgtcgacagctgtgggagcgtcatctgacgcagtgcccc

tccccaagtcagcatcccctacctatggcgcaaccgcaagctcagagtcaaccccactcctctggcacacaaccccagcaacagcaacagcaacagca

acagcagccacttccacgcccaccccaacccaatgcgaccgctcccacctcgcaggccgatgctgccaagacatcaacaaatccatcgcacaatcctc

ctcccacccgaccttcctcgacccggacctgatccgggacgtcatcatcggtctctccgacggcctctccgtccccttcgccctcgcagcagccctct

ccgccctgggctcaacacacctcgtcgtcatcggtggctttgccgaactcgtctccggcgccatctccatgggcgtcggtggcttcttgtccgcacgc

gccgagttggatcattataataatcaggcgcgctccactcatgagcgcgtcacgcgctcgtgtctggccgagatgaagagggaggtcgccgccattct

ggagccctttggacttggctccgacgtagccgagaaggtcgccgaacgattggcacacgtcgaacagcagcattatcaacatccatcatcagacagtc

tgcccgttcccgccgtcgcatccccttccgcctctgggatctcaacagccgtggcgcccgaacacggtctcacccctttcctcctcaaactcggacac

ggcctcgaacccatctctccctcgcgcgcctacatctcagcagccaccatcggactctcctacttcctcggcggccttgtccccctgatcccatacat

gatctggcctcgcgtcggagaggctctgatcgcgtctatcatcatcaccggcatcgtcctcctcgtctttggcgcggtcaagcacagattcaccggcg

gcgccccctccgctcgcgcttacacctgggccgctgtcagcaccctcagcgtcggcggcattgccgcagccgcgtcctggagcatcgtacgcgccatc

gagggacaccagtga

>contig_14.contig_14.g242.t1

atggagatcagcatcacgccagcaacaccagcacacgaccagctgccattcccatccaccacgttcaccttgaccccag

tcagcccagcaccaccaccatcaccaccgcagcatgaatcaccaccgtcaccaggttcaataacatcagcatcgacaaccacgatcgcacacgaccca

gcagcaatcaacacagccttcaacgacatcgcagcagccatcgacaacgtacgcagcctgctcttccacatacaagaacagcggcatctgcactcctt

ctcaaccgtaaccacaacatcatcagcaacagcaccagcaacagcaccagcaacagcaacagcaacagcaaccgacacccgttcaccacccgcatcag

taacatcatcatcacgcaccccacagcagccatcctcagagctggacaggtccatcatccgtctcgactcacagctcgaacacatcagcacaaacatc

ctcaacgcagaatcacaactcaacgctctcctcaccagcgatgatcaacagccagaccagctcagagcactcacacgcgcacgcagggatagcaggac

atccctctcctccctcgcctccttcaacgatgcactcctacacaccggcacatccgcaacctcattcgactccttctccacacacactcccatacagc

tccaacagcgcttccacgaaatacaacaggactacgtcgccgtcaccacagacgcagacatgctcaagcgcgaactctccgaggacaagtacctcctc

gtcttccgctccgtctccgaccaggcagacaacatgatgacctccctccacaaggcaatcaccctctcacaagacttcatcaatgcctatcgcgcatc

gcagcaacagcaacaacagcagcatcagcagatagccacctccgcatcccgcctcagcttggacggcaacgacggccagtcccccgaatcacagctcg

aagatctacgcgcactcaagaagaccttcgacgtaaagacgagctactacggccccgcatgcgaacaagtcttccacgtcctcgaacgcggaatcaag

gacaaggcaacctcaaacggcaccatcctgcatcggtacgcggacctcaagatgaggtggaaatccacacgcgaggccatcctacgcaccgatcgcga

tctcaccaagatggaacgacatctgtgcaaggtaatgggtatggccgagttgcctgcctattcggctgaggtgcagcagcatttccgatccgccggca

cttcttcttcttctggactcgcttcatcgcccgcacgaggcaggcaggattcagctgcctcaggagcaggagtagcaggtacaccgccccggctccat

cctaagagctcacagcgcacattgcgacatgtcacctcgcatcaggccagcagtgccgatggcagtttctcctcctcgatcaatgcctcgccaggtcc

taagagtccacccgcacctgccaagccgccgaggagcgagttgaggagggcacccagttcgcagcgcatggccacggccggagctgccaatggagcag

gggcaggaagtgcggccgtgcccattccgggtcggtacactccgtccagcggcggcggagggggatacaaccgcaaccaaccatcatcacactacaac

cagcagcccagcctgagctcgtcggttgggaacgtcgctcggaacgggggaccacatcggcgctcgttctctgcttcgacaaatgagcccatggctga

cgttggctttcgacgggctaatgtcgtgcagggacagggacaggggcagggacagcatccgacgatccccgtccgcgcgggcagtcgggcgggctcga

gcacacccctcggaacgtcctcgtcgttctcctcgaaccccttcaaacacacgggcgccgagtactcgtacaccgccgtcacctccggccggaatctc

ggcgccggcggctttccgagtccgaggagggcgcacagtccgacgcatccgccgagcagttatcggttgacggggtcgaccgctggaagtgtgggttc

agggccggggacggcgccggcgccgaggagcagtaagacgccgcagccgcagtcgcagtcgcagcagtctggctctactttgatgagggcgagttcga

ggaatggggagaggggtgcgtcggagcctcctgagaatgcgcggccgggcagtcgggcgagtgcgagtgggcggatggggggatattcgtccggtgcg

ggtgcgggtgtgttccgaggtggtagtgctggtgctcccgctccggcacctgctcaccagggccgcggccgggcggatagtcgggcctttgatgacgc

gatggaagatctgagtatcggatcgtacgaggctccccccgctccgcgctcggccagtcggaccgggtggggtgcgccttccggcggtggtgcaggga

tgacgaggccgcgtccggcgtcgagtatggcagggtactactaccacccgcccgctgcgcatggacaccaggcgagcgatagccatattccgcgcttg

gctgtcggtgcggagtcgtctacgaatggctcgtcggggacgacgacggggacgtcgaatgggcgcagtcctgcgaattcgttctcgagcttgccggg

taggcctgggtcgtcgatgagtcagacgagcagctcgacgacgggcttcgtgggtgctggtcctgctcctggacctggtccttcgcgtctgggcgggg

gcagtcggactggcttcccgccctctcggaatggaggaggaggaggaggagggggagggccgagtcggttgacgatgcagacgcccgaaccggtcctt

gcggcgcaggtgaagaggctgagtatgttcgcgcgtccttcgacgatgtcgcaacaacaacaacaccagcagatccagcaggcgtatgcgaatgcgaa

tgcgaattcgggtgcaggtgggagtggggtgaggaaatcgagtcgtcccccgccggcgcggttgaataatccgagtcggacggggaggacgacaccgt

tgtcagctgctgcgctcgcagctgtaccccaagcgaaccctgcgctcgcatggatgtcctctggcagcggtggtgggcccgcagacgtgcactcgagc

ccgagccagcagtcctcgtcgagcgtggctgcgtatcgggcgcagcagcgggcgaatgtcggccacggcgggcacaactatacctcgtcttcgcttaa

caataaccggccgggggcggagacgccgttgagcgagagctcgtttggcgggcagagcgtgggtgggtggggcgttcggccttcgacggctactgcgg

cccgtgcgcctgtgggtaccatgtccagcgctacgggcggagcacgcggtggggcgggccagtttgacgtgtacgttcccaatgcgcgggatgcgctg

gacgttgctgttgcggaggttgttaatgcgttgggtgttgcgattgagcgtgttgatgaggggctgccgaggggagttcggacggaggttgcgccggg

gaaggatttgcaggcgaggtattcgattgcggggaagacggttgcctgcaagttgttgatgctgcatcgacccgctggcggggctgggatgcgcgcag

gcacgaatgtgaagaagctcctcgtccgtgtcgggggcggatggcaagacttcgagggctggatcagcgccagtatcttgggccatctcgattactga

>contig_14.contig_14.g243.t1

atgaccaccgcgaccattcccacgaccactcccactcgcactccctcaacgccttcgcagaagccgaagacaacgccct

cctcgccgccctcaaaggccgcggccaccgcggttccaacatcaccgtcctcggcctcctcgccaacatcctcccgctccacgtccaagggcttcaca

ggccctcaacactacgcctccaagtcgacagccatccgacatcctgtcgcggctttcttttgctcgtccccagacagcacgcctctccagcgctcgag

cccgcgtctcttttttctttacctcactcagccagcatccaacaacgggagagcgcaggctccaatacttgctaccacccatcgtacagcacacagcg

acagttctctctctctcgatcagctcttcgtctccttcccatgtccagcgacggacagtcagcaaacacagccgccaacaacaacaacaacaacaaca

acaacaacaacaacatctcttcgctttcccccgcacccgctcgcatccttccgcgcagcagtagacgcagccatggccttcgtcgcctccaaggtaca

gcagcagcagaaggacgcgtgcagcgatgccgctgcctcatcatccacacaacagcaaacagctggcatgtcctccgccccctccgattcatcaataa

cagacaactcggccgaccaatccaacgcctccttcgcttccacctgcaccgacgccgatctcgacgtcatcaccaccgtcttccaggaccagaaccac

ttcaacgtgaagcgcccgctctggaacacctggaccctctggttcgacaacccgtccaccaagggcggcggaggaggaggcggtcacggtgggggtaa

aggtgcaccgggcggtaaagatgcctggggtgaggaccaggtcaaggtcgtcacaattgagtctgttgaggagttctggggtctctacaacaacaaca

tcccgccctcggatctccccgcccgggccaactactacctcgtcaaggatggcatctggcccgcatgggaggatcccgccaatgccaacggcggaaag

cggtccatccagccccccgcgacaagtccagagagtcgatcaacaccctctggctccacactatgctccaagccatcggagagaccctcgaagcctgc

tatccagaaaatgtccccaagcaagcaggcagcttcgaagacgagactcgtcaccggcgtaatcacgtccgcccgtccgaacttctaccgaatcgtga

tctggacccgcagcgcggacgacgacctcgaggcccacgcctccaatcccaacgccgacgcccccgagaccaagctggccgcgaggctcattgacatc

ggcaagcagttcaaggtgaatgtgttgggttataagttggagggtaaagctggtgcggggtcgagtgggttcgcgaccgaggtcgagttccagatggg

tcaacagctcccaccctcctcaacccagcacccccggcaccgctccctgcctccagacgagacccgcccacaatccgtctcgtacgctggcagagacc

agaagacgaagaagatcaggagttctccaacaacccatcagctcccctcggtcgctccttctcgcccctccacagagcagcgcatccaccttctcctt

ccaccagccccaacactacgcctccaagtcgacagccatcaggatcagacagccttccagttcggccctctttgctttctcaaggcaggcttctatcg

acaatcatcatttctggtcatcaagaaaaacagcgaaacaatctctcccaccatgtcgtcgcagccagaactctcacagcgcaagcctggttcggcag

cacttcatcttcaagcccacggacacgaccacggctccgccgccgaagccgacgccctcctcgccgccctcaaaggccgcggcgaccgcggctccaac

atcaccctcctcggcctcctccccaacatccccctctccacctccaaggacctcacaggcgtcttacctcaactccgccgcgactttcaaaccatcac

cagttcaaccatcaccatcataacccttcacatcgcaccgcaatctctctcccagtcctcggctctcggcatcctttcgtccttcctctctgtgatcc

ggcctccctttccgctcccgctctcgactttgctctatcctctggtgcttctggattcgttcgtaatctctccgttctggttcccggtccccggttct

cgcatccttgcttcgttccgctcctgctctctcgactttcctccggtgtcctcttccagcgaggacgaggacgaggacgaggacggcgacgtggacgg

ggccaacgacggggacggatgtgcacaacagacatgtttctattga

>contig_14.contig_14.g244.t1

atggccttcgtcgcctccaaggtacagcagcagcagaaggacgcgtgcagcgatgccgctgcctcatcatccacacaac

agcaaacagctggcatgtcctccgccccctccgattcatcaataacagacaactcggccgaccaatccaacgcctccttcgcttccacctgcaccgac

gccgatctcgacgtcatcaccaccgtcttccaggaccagaaccacttcaacgtgaagcgcccgctctggaacacctggaccctctggttcgacaaccc

gtccaccaagggcggcggaggaggaggcggtcacggtgggggtaaaggtgcaccgggcggtaaagatgcctggggtgaggaccaggtcaaggtcgtca

caattgagtctgttgaggagttctggggtctctacaacaacaacatcccgccctcggatctccccgcccgggccaactactacctcgtcaaggatggc

atctggcccgcatgggaggatcccgccaatgccaacggcggaaagcggtccatccagccccccgcgacaagtccagagagtcgatcaacaccctctgg

ctccacactatgctccaagccatcggagagaccctcgaagcctgctatccagaaaatgtccccaagcaagcaggcagcttcgaagacgagactcgtca

ccggcgtaatcacgtccgcccgtccgaacttctaccgaatcgtgatctggacccgcagcgcggacgacgacctcgaggcccacgcctccaatcccaac

gccgacgcccccgagaccaagctggccgcgaggctcattgacatcggcaagcagttcaaggtgaatgtgttgggttataagttggagggtaaagctgg

tgcggggtcgagtgggttcgcgaccgaggtcgagttccagatgggtcaacagctcccaccctcctcaacccagcacccccggcaccgctccctgcctc

cagacgagacccgcccacaatccgtctcgtacgctggcagagaccagaagacgaagaagatcaggagttctccaacaacccatcagctcccctcggtc

gctccttctcgcccctccacagagcagcgcatccaccttctccttccaccagccccaacactacgcctccaagtcgacagccatcaggatcagacagc

cttccagttcggccctctttgctttctatccggccacgactgcaaccgctgtctcaaggcagtcttctatcgacaatcatcatttcttgtcatcaaga

aagacagcgaaacaatctctcccaccatgttgtcgcagccagaactctcacagcgcaagcctggttcggcagcagtcaacaaggagacgaacggtgtc

acgaccccattccatgccaagggtcacgaatttggctccactgccgaagccgacgccgacgctcttctcgccgccctcaaaggccgcggcgaccgctg

ctccaacatcaccctcctcggcctcctcgccaacatccccctctccacctctgagggcctcgcaggcgactccgccttccgccaggcaccccttcgag

cgcccttctcatccgtcgctcgtcgccctcgccgctgccgtcactcttccggctacttccatctcccgtcatccactgcccaaacaggggcggacgct

cgctattcgccagcgccagcgccaacgccatggtttatcccgcgctctaatcgccatttccatgctcaggatcacgtctccgccgctggagccgaagc

cgacgtcttcctcgccgcgctcaaagaccgcagcgatcgcggctccaaatcaccctcctcggtcacagctcattcccatgcccaggggaattcggcag

cagtcaacaaggagacaaacggtgtaacgacgtacgaccactacgaccgttcacacgagcatttgcattcgcagccgcacacccgctcgcacttgcac

ttgctcaaccccttcgcagtccattcccatgccaagggtcacgaattcggctccactgccgaagccgacgccgacgccgacgcccttctcgccgccct

caaaggccgcggccaccgcggtttcaacatcacactcctcggcctcctcgccaacatccccctctccacctccaaggacctcacaggcgtctacctca

actccgccgcgactgtgactgcgcctggtggagaagacgggcacgggggggggggcaagacacctttctctcagcgtagatgtcctcttgtctaccac

gggaacatcctcacgcccgctccgattttctcgcctcggccctgcccccgggacacatggaggagaatggtccctgcctcacaagaaggtttcttgcc

catcccgcccctgtcttctgccccagttgtagtcgcagtggcggtggggctcgctcgctattcgccagcgccaatgccgtggattctcctgcgctctc

atgcccattcccatgccaagggtcacgaattcggctccaccgccgaagccgacgccgacgccgacgccgacgcccttctcgccgccctcctcgccgca

gcgatcgtggctccaacatcaccttcctcggccacgacctttcccacgaccatgagcacccgcactcccactcgcattcacacgcgctcgtggacttc

ggggctgaatggcatttgcctggttccctcatctttcatcacccccggcaccgctccctgcctcaagacgaggcccgcccacaatccgtctcgtacgc

tggcagagaccagaagacgaagaagatctctcgaccaagacccggtcagctcccctcggtcgctccttctcgcccctccacagagcagcgcatccacc

ttctccttccaccagccccaacactacgcctccaagtcgacagccatcaggatcagacagccttccggttcggccctctttgctttctatccggccac

gactgcaaccgctgtctcaaggcaggcttctatcgacaatccaccctctcctgtcatcaagaacaacaacagccgaacgattactcccaccatgtcgt

cgcagccagaactcacatag

>contig_14.contig_14.g245.t1

atgccgtttgcactcacgctcgcatccatcctggccaccgatccacattccacgctcgcagcatcgacacggatggaat

ccaggatgcttcccagtggcctcgctggcgttcaaggcctggcaagtggcagccgttctcgcgttcgtggagtttgcattcccaaggcaggcacgcat

gatttttgctggcaggccggtacgattcgatgctcattgctcgttaatcgtctatggagcagaggcgctgttcaaggccttggtccttcattccctcc

ttctgttggcaccgtcgcacctcagcagtgtggacccatgggaactccggccctcaagggctcaataggcaacgaaggtatactgtggccagcgcagc

cagaccgaacacgctccaacagcaacagcaacagcaacagcaacagcaacagcaacagcaacagcagcaacaacagcaacagcaacagcaacagcaat

tcgcctctcacactccatcactctatccctccatcactctctcactctctcactctctcacattcccacacttccttcctttccaacaacaacatcaa

gaacagcttcccgatggacaccagacagttccagcagctccagcaggtcaaccaggccgccaaccattgcgagttccttcagaagctcgccgagctca

gaaacgtgatatacgagccctactcgctcgctccctccgcctcctcctcctccttcacctcctccaccagggactgccacgacgactaccacgtcggg

tccaagcgccgccggacaagggatggctcctcgagcagccatcacggccccggagcgatcagcctcgaccgactccgggagttggctggcgctccgga

ggccatcagtgcagcaggaggcagcaagcaccaggcctcacgactccagccaggcgagctcgagaacattatgcagaacgtcgagtttggacgacagc

tcttcgccgagctcattggctcattctcaacgctccagggaatgaaggacgaggcggaggacgacaaggagagggccgaggagcagctgtccgaggct

aacgagcggattcgggagctccagagccgggtgatttcttcgcccctgcccagctcctggatgtccggcaatgtcggtgccgccacctcggacacccg

cctcgtccggcgcttgcgccaggtgccacgcccacctggacggccctcgaggggacgcaccagctccgcctcggtgtctttcggttcagcagacatca

ccaacgcgagtgcgtccactggcacgactctcgttttcggctccgacgctaccttcaacagccgcatcctcgagcttgagaacgccctccagatgcgt

gacgacaccatagaggagctcaaggcaggcctcgaaacccaggcgaaggagcaccggcaggagatcaaagggcatcaggcctcgatcatgtcgttgcg

acagacctgcgacagggtgaggaaggatgcggatgagggctggggaagatgtggtaaggagatcaagctcggggagaagaaggacaaggagatccaaa

agcttcaaagggacaaccagtctctacaagccgagaacgagcggctcactcgcgatgtggagtcgtgggagacgggggcgaacgagctcgaggagaag

aaggctgaggcgaacagacgggttctggtattgaagaggctcttggagagacagaacgagaaggtagccgccctccacaaggaaaccaaggccctaaa

ccgagacaaccacgaacttagggggacgatgtcttgcttggaagtagaagaccacctctccaacaagctcaagacgcagctccaagaggagaaggcca

agctttcggcgaccgactccgagcttcggactgcgaagagccaaacctatgagctcgggagcgaggtggcccggctcaaggccgaactcgcagctaag

gagagcgagctggcggaggctcaacagacggatcgactcgcattgggaactcgttcttcgcgctcgttgggggccaccgacggatgggcgagccatgg

gaagtcggtcggcagtcagcttgctgctgctgcgcggaagagagcggaagcggagatgcgggacaggctgctacgtcctgatgctggtgatgtctcgc

aggacatgatggatcggtcagatgaagaggttgctaccacggtcgcgaagaagcgcggtccttgtcgcaaagacaagacgtcgtcgagtcctcttatc

gacctcttcaaggacgctgaacacccaagcgtcctggagcacggcatcgacgaggatgtgagggacgctcctgtccttccgagcacgttctcctggtg

tgggacggtggacattctccgcggcgtcccgggtcaggataagcctgcacaccaccgacgtgcacgctaccacaagtgcttcgtgacgggaatctgcg

ccctgctaatcggtattttgctcggtattttcgtcttccaaagtccaacgtccgcccgctcgcttcgatccatgaccaccaggaccattctcacaacc

atcggtacacacactggtattcgcactcccccaacaccgtcgcagcccatttacatgcccaggggcacgatcacttctgcgccatcgaagcggaagcc

cacgccctcctcaccgccctcaaaggccgcggcgatcgcgcctccgacatcaccctcgttggccacgacctttctcgcaaccactcgcgcttggtcaa

cgtcttcacagtcgattgtgcccagggtcaggaccacggctccaccgccgaagccgacgcccttctcgccgccctcaaaggccgcggccaccgcagtt

ccaacatcaccctcttctgccttctcgcccacatcctcctgctccacgtccaagggcttcacaggcgtccacctcaacttcgtcgccctcctcgcgct

ggggcttatcgtggctgccctcctatga

>contig_14.contig_14.g246.t1

atgccgtccgaatccagctcaaagtcgaagaagtcatcgtcctcctcctcctcatccaaaaagaagtcatccacagaac

ccacagcgtctgcgagctcctcctcctcctccaagaagagttctaagaagaggaagctcacgccgactgaggctaagctttaccagttcatcacctcc

aatggtgttgcgacacagctagccatcgactcgggtttccaggatgtctctctcgatgacagaatccaggccatcaatggtctcgctcggctgtccct

catccgccttcagacggttggcggagaactgcagtatgaagcagcttcacggcaggaggcatcagtaatgtccgatctcgagggcaacgacctgcttg

tatacactcacatccgagactctggcaacgaaggtatctggacaaagaccatcaaagtccgcgccaatctccaccagacagtagtcaaccgctgcctc

aagtcgctcgaacagaaacatctcatcaagacggtaaaaaatgtcaagttcccgactcgaaagatcgtcatgctctacaacctcgagccttccagtga

gctctcgggtgggccatggtacagtgacaccgagcttgacgaggccttcatcgaggccatttccaacgtctgtctccgcttcattcaagagaggacct

ggccggacggaggacggaagcgactgctctacccagcctcacacgtcttcaacctgcccaccgcccagcagatcctcgaccacgcccactccaccaag

gccttcggggtgacactctctatcgacaacatgaaccagctcctcgacacgctcatctacgacgagaagatcgagaaggtgccgatcctgcccggaca

gcggccctcggcctcgacatccgcatccgcgtcggccagccggaaatcgtcatcgtccaagagcaagaagtcgagcaagaagaagagtagcagtagca

gtagcagcaagaagtcgtctcgccatcacagcgagagcgagagtgagagtgggagcgaaagtgggagtgagagtggggaggaggacagtgatcgagag

aactcgatgtcggaggacgaggatggggatggggacggtcttgatggagtggggaagaaggataaagggaagggaaaatcgaaggaccgcgaccggaa

acgcaaacgcaagcatcgagcctcttcacgctcctcgtcccgatcccgctcaagcagcggcagcaaacgcaagcggtcccgccggagctacgacacgg

attcggacgacggctccggatcagatggctccgacgatagcgagggtagtgagagcgggagtagtagtgcgtcggactcgcatcggcggagcagtagc

aagaaacgcaagcggggcaagtcatcctcctcctcccgctccaagcgctcctcctctgtctcctcgtcacggagcaagaagaagaagcgcaaacgacg

tcaccgcagttcgagcgagagcgggtcctcggacgacgacggttccggctcggagagtgactcctcctcctcctcggagtctagcaagaatagcgggc

ccattgtatcggacgacgacgacgaagatgcagacccatcctacggcggttcggccctgaccgacgcctttcccttcgtcttccgcgccctgcgaccg

atgcacgtcttcagtcccttccagacggacctcctcctcgtccgctccggcgagttcggcacgggcacggcggactcgagctggatgcagacgccctg

tgggcagtgtcctgttttccggttctgcgagccgggtgggccggtcaacgcgagtggccatccatccatccacgccagaaggaagctcgcgttcgcag

ccgccaaagcacagcaagaagacaacgcaacgcaagagacacacagagcgagaagaacgcgcgctgcagagagagagcaagagaagagaagagaagag

aagatgtcgcacctgctacccccaaacctcctccgcctcttcgcaccacgcccaccgctcccctacgcgcccccaccgccgcacaacaccgacccaaa

ccacctcacccacccatcctcccacccaaaacgcaaacgacacgcctacaccggcatcgcgtccttcctcgagtccacccgccaggaagccgtctccc

gcggggaggctacggtcgaggaggatacggaggaggagcgcaagaagttcacgcttgctgaggttacgaagcaggagattcggagggaggaggcgaag

aggaatcgggagagtgagagggagaaggctgttcaatcttataatccgcaagaggacgccaatgcggtcggggatcccttcaagacgctcttcctcgc

cagattaccatacgaaatgacagagaaagacatcagccgggaattcgacaagtttgggcccatcgagagtatccggctcgtacgcgatcacgagggaa

aaagtcgggggtatgcgtttgtgctgtatgagcgggaacgcgatatgcgcgcggcgtacaaagaagccgacggactcaagtttgacgggaggaagatc

ctcgtcgacgtcgaacgcggccggacggtcaaagattggaaaccgatgcgtctcggtggtggactgggtggagcgacgcggaagaagaaggagcctca

acaacaagccatggcgccgcctcctatgacaggtggtttccgcggtggcttccgcggcggtggttttggaggaggaggtgggcgcggtggctttggtg

gacggggtggctttggcggtgggggtggagggttcggaggtggaggtggacctccaggaggagggttcggaggtggaggtggacctccaggaggaggg

tatggaggcggtggagggtttggtggtggaggcggagggggatatggtggtggcggaggattcagtggtggaggtggaccaccaggaggaggaggggg

tgggtatggaggaggaggaggcttcggtggtgggggtgggccaccaggaggaggtggtgggtatggaggcggcggagctggcggttacggagggggag

gtggcggtggcggcggcgggtatggcgggccacctggtggaggattcaacagtgattacggtccaccaccagccaaacgcgggcgatattga

>contig_14.contig_14.g247.t1

atggacgcgctgccaaaatctggacgcgtcccgaagctgatcatgagagaggagcgagctggcaacattctcactaccc

accaccatcgacctcgacctcgacctctccgtgccacaagacaagatctcagcaacatgatggccgacagtgagagcgacagcgagctcgatgtcttc

gacgcagacgatcgcaggccgccgtccactttcacgtacggcagctcacagaggaaggcaaccctccccaacaacacacagcaaacgagctctcaaag

caaggcaacacgaaacgaggaggatcaaaacagcgatgatgaggaagaggagattctcgaggcgccacgatcgagcggcactgtacgcgaagtccacg

acgaagaggacgaggttgagcttcccaagtcaagcgatgctcctgtccattccagtgacgactttgcctccatgccgccagcatgtcaagcaccatca

atgcggcagaagtctgtcatcttcaacgacatcgaggagcaagagcaagaaacgcaagcgcactccgacgcggcgcccgcctcggaatcccagcccga

cgtctttggcgccttcttcgaagacacacagccttttgacgcgcagcagcacgggaccaaggcggatattgacatattcggcaaccccgcttccacat

acgtcccgcgcacgctggaggaggacggcgacgagactcgggtcaatgacgatcaaggattcgatcagcttcggcgggcccaggctttattacgcaat

gccagcccagactcgttcgacgcgctcccttctttaccggaagcttccatggaacaagacaactcggacgctatggacgatgccgaactgcgcgcgat

ggagaagcaacagctcattctgcaggtcgaacgacaacgggcaaacgcgcaagcggacaataagccgcgcgtcatgcacctcaaccgggatggcatgt

tcactcagacagaaccttcacagttcctgttcgacgacacacagtctcagctgttcactcagttctcccaacccgaccctgattttcaacaacaagaa

caaaaccatatgcctagtccagccgtagtgcagaacgtcgacgacatctttagtgaagattccgatcgggacgagccagccgctcccgtcgttcgttc

atcgccaccaacaacccagacccggaagaggaagatcgaagctctcacctcttcatctcctcttactcctctggcatcagaggccaccaccgacagtc

agccaatcaggcacaaacgcaagatcgcattccccgtcccggaaagcaacgacgaagatgaacaacccgcgtcgtcaagtccaagccctcggaaaggc

atcagtcgcatcgtggacagtgaggatgaagatgaagaggccaacgtcacgaaggcggcgtctcctcgtcaacgcaaaatcccactggttgacgcgag

cgatgacgaggaagaggatgcggatatgtcagtgcgcgcgccagaagacgacgacgccgatgacgatgcagatgatgaaggggacgagcttgatgtta

tcaagaagggcaaggccaccaaggcgtgggcgcgggcggacgaggcagacgacgagttcaccgatgaagaggccgaagaaagtcaacggaccaatttc

atgactgctgccggagcaaacaatcgtgttcctaactttcaaccaccgctgtccaactacttcgatgtcccgcgaccaaagtcggccaaggacgacca

acagacggagccgacgaaggcgaagaagagcgcgtacattgctggcgaagccgaggagtctgaagatgacgagcggatcccgggcaagaagagccgga

agggcggcttggggggcgtctttagtgacgatgaagacgcggctgacgaagaagatgaggatgcggaagacgacggcaaagatctcgaaggactcgtc

gacgacgtccaagacgaggaacatctggagaaggaccagctcgttgcggagaagtttaagcaggaccgggcggagcaggataaggcggatgaggacct

gcacatgcgagcgacaaagggtcagctgcgcaagtcgaggccggggaatgggctggacaatgagttggatgatgatgcgcaggaggatctggatcgga

ggaggattacgcagggtccgcagaagcgtaaggttgctggcgagaaggatggtctggatgagattcaaaacacggcgctggcggaagtgtacaaggag

ggcgtccggaacgacgtcgccgacgatgctgacttcctcgaacccatggactccggatccgactcagacatcgtcgacagcgacaacgaggggggaaa

gcccggcatcgtgtccgcgtcttctgtccgatccgagatagttgccagggctcgagccaggaagcgacagcaggtccctgacgacgaggacgacggtg

ctggggaggtggctgacccgactcagattggttctggaagcgacgatgaagatgggtttgggagggtggtgcagatcaagaattatatgcagagtaga

ccgattctgaacatgcgcgaggaggacgatgaaaagcacgccatggacgccctcctcaatccacgaaagcgggacgaggccgagaaggccaagctgga

gcgtaacgctgccgaattcgagagcaaagcggccaaccggaaaaacttcctcgggtccggtggcggcggcggtgggagacagaagaacagctcagtca

atttcaaacaggatcagaatcggcgtgcgggtggcggcagtggtcagtctggctcggctggaagtagtagtagcggggctgctgcggttatgaagtct

gggctgggacacccttctacccgtcgggctgggacgtcttctgcggcacggtcgacatcgaagttggcgacgttgctggagaggaaccggatgctgaa

gttcgaagagaatgggtag

>contig_14.contig_14.g248.t1

atgccacccaagggcacgggaaagggcgcgacacaatcgctgcccgagctcagcgatgtgttccgataccgatccaagt

acaaggagcttaagcgcaagatcagagaaatggaaacggacaatgagcgactgcatttgaagacgctccgggtgaagcgcaacatccaaagaatgcgg

ttagaaagagctatcttatacgagcgcttggagagtgatctagctgctcgtcaaggtcaggccgcctccaccggatcgctcggactctctaacctcgc

aactcttcgccgtcccagcaggccgagaatcttgtcacatcgtgctagccacaaagggcggagcaagacgctcaatctacggggctacggacttcgac

catccaacattccaccagtacgtcaacgcatccggtgcgctctcgacgctcctgacagtccatcgcgggcactccgaaggggccgctcgctcccggcc

atgtcgtcagcccgccgccgcccttcgtccacggagcgccaacaacagcaacaactccaacagcaacagctcaggaagccgcgcaactacctcgaggc

aggcctggtctttggctgcgctgccatcgtcatctggcgcatagcgaacctccctgcaccctttgcaaccgctctagcattagcgctggcgctttttc

tcttctttgtgagaagaccgggagagatggccgtcatttgggccaccgatgcccgcgctttccgaccgtccctggaagacggcgcagtctttggccta

ctcatgggtcccctcgtcactgctgtcctgcttttaatcgcaagcgatcaagtcttgcccaactctttccgatacccaggtggcgagcccatctacaa

ccccgctttccttgtcgagggccctcctcagatcctgaagcattcgaacccattgccaaagggaacctcgacgcgaatactctctagatgcggtctgc

tcaacatgcagaatttgatctcgaccatcttcttcgcacacgtcgcagccgccagatatctgcgcaaacccgatacgctaccaagatcgacatcagca

aaggtacagagctttgcgcttttcgctgtctctgttgccagtctgctctctgggctacatatcgcagcccgtaattacaacttcccgatttgggcaga

acttgaggactgggagttcatcgtcattgcatgcatgtaccaggtcgacctgttcattgtgacaagattagcgcggagaaactttacaatcggcgagc

tcggcattgtggcccaacttggagccacgatcgctctggaaacagtgcacctcacagtctcaaaatcatatcggcaccccactccactaattattttc

caactcgcgctcacggccggcgcttttctggtcggattcctgttatcgcccatcctctacctctcaagacacctcgctcaaaagccaacacataggct

tcgctggcctgagaagcgggatctgcaccggaaactcctctccacctccttctacgtgctcgctgccatctacatccttggcgtgctgggcatgtggg

tccggtggctcctcgtcaagcgggatccgtggttgtggacttttggattcctgttccaaggaacccatcccttctctcgattcgccttgatcgcatac

tggacgtccttgatcggcgcctctgtcttcagctggcagatcatcgtcagcagcatcaagagaccaccgcagccgatcagcaatagaacgacggcgag

cttgagcaggcctgtcgcatattcgacgatgggttcggaaagcaaccccgctctgcgcagcaaaactgtgcacttgagcttgaatgctcgtcgtaaat

tcttccacttccttgctgtcctcctttttctgcccgggatcctttgggaccctgccttcatgcatcttgccttctctcttgcattctctctcttcatc

ttcatggaatatgtccgatacttcgccttacttcccttcggagcatctttgcacattttcctcgcccagttcctcgactcaaaggacgccggtcctgt

catcctcagtcatttctacctacttgccggctgtgcggcttccctctggcttgaaggaccttcggccatctacgacgtgatgggtgttctcgtcctgg

gcatcggggatgctctggcttcgattctgggtcggaagtatggacgacacaagtggcctggctcaccgaagacattggagggctcggctaccttcgtt

gggagtatcgtactgtgcggatggggtttgcaagccgtaggactcattccacctttcaaccttctgcgatttaccatcgcgtgcgtggcgacgggctt

gatggaaggcgcgtctgagcaaaatgacaacctcatcttgccgctcttcgcgttcgtcgtcatgtatattgtggtgttcactggtcagaatggcgttc

aattttggcctcaattcacactgcaaaatgtcaacggccaagtcccacatatccctcgtacctcgacgtcgagctttgagcgtttagcaggcacaatg

cccagagacatcagcgagaaacatagacgcgcatctcgccgcaagaagctcttggctatcttgatgggtgtcaccgcaactgtggcagcaatacatgc

gcgcacctacgttctcaagaaatccaaagtagggtccaggactagcggcttaaagtttctacggcagtacgccggcaaggatgctcatcctcagacgt

gtcaggatttccttggcttaaagcccaactcttttcgacgtttgctccaggttctcaaggacaagggcaagctccgcgacggcactcgatcacgggct

ctgtctgctgaagagaagctcggtatcttcttgcacatggctcgcagtggatcagcctgtagaatgacagccagtatcttcggtagaggcttaggcac

tgttgcaaagtgcttcaaagaagtggtggctgcgcttacgagccgggaaatatacactatgtttgtcaaggcaccaggctccgatgcggaagtacccg

agcgactgcgaggcgacgatcgactgcggcacttcaaggctgttctcggggcagtagatggggtgcatgtggctgcacatccctcggcggtcgacgcc

actcggttccgcaaccgtaaggctggtttaacattcaatgtgcttgccgcttgcggtttcgatctcagcttccattacattctcactggatgggaagg

cagcgccgccgacagtcacgtcttccacgcggcacgcacgtctacctggctcattccgaaaggtcgaacgtatctcgcagatgctggctttcctctct

gtgtcgagctcttgactccgtatcggaacactcggtaccacttgaatgagtggggagatggatctgaaacggtcacaaacaaggaagagctctataac

cgccgacactccggcctccgcagcgtgatagagcgcgccttcggcatcatgaaggcacggtttaaagtgcttcgcacggggtctcagtttgacctcaa

ggtccaagcagcagtttttccggcattggcagtggtacacaacatgctcatcagcgacgatcccgacgacccttaccttctggacgatttcgctctgg

cggagatggagatggacggaacaacagacagcactctgagagcagcagcaggccgcatcagtgatgcagagaggaagagtgggcaggatcagagagac

aagattgctcggaaaaatgtggcggtctttcaagtagaggtggtgatacgtaataacatgcaggctgcagccatcattctcagtgcatcttga

>contig_14.contig_14.g249.t1

atggtcagacgatccgcttctccgccagacagtccctccgctgtcgagatcgatgtggccgctgaagccgccaacgacg

cagctttgattgctcgtctcagcgcaactaacagcgctgcgattgctactactccttccgcacctacttcttcatctgctactggtgcgagccgtggc

acttcggctgccagcgcttcttcggccgtctcccctaccaaaaagaaaattaaagtcgacatgcagatggatggcgctaacttcaaggaaaaacacta

tctgcaggcggccaatgaggtcaatgcgaagcacaagggcagtgctaggacctcgaagcaggctcagacccgctggaagacagttagactgacgctca

ggttgcccaacatgggctactcgccttcctttcgtgaccagctcaagtccactttcgggccttttcgtcgtgtggttcgccgatcaggcttctcgtgg

gactcggaaaataacatgatcgatgcttcagacgacttgtgggagactctcgcagaggaagacgcggctgcggccgaactcagggacaaggtgttcga

gttctacgaggaggtggatgccatcgtcgaggggggcatcgccaacggcgagtttgctctcgaccctggtgcgaaggcttcgaagaatgaaaaagagg

aggattctggtaccgacgaggattcttctgattctccggcctccgacggtgaggaagtcgaaggaacgcacgaggacagtgacggacagacgggtggt

gagggaaagaacaagaagcaaggcaagcgtcgcgctgatgacgaagagtggctgggaaagcccaagcgggtccgaaagacgcaggccggcgagcttac

taagctcaacggtgtaatgcaagatctggccgacaactttaagcagtcagagggcgctagtgccgaggataacgaactccagaacgcaattaagttct

tcaaccttaacgacaaggcccacttcacgaccatggaacagctcgacatcatcgactacttcaacgacaacaagaggggccccttcacctacatggcc

atgtccaaggcggacaacgctgatcttcgcgtggactggcttcgtaagaagatcttctcgacgtga

>contig_14.contig_14.g250.t1

atggtcgacgactcgtcgaccgatttgcactcaaaagccgccgctcttggatgtacagtcctgctgacttgttctcatc

gttctcttcctcttcttcttgctcttgctcctgcatctgctcctgctcccgcccctgcctttgctcttgctcctgcttctgcccctgcttctgcttcg

cccctgctcttgctcctgcccttgctcctgctcttgctccactctgctcttccctttgctccagcagccgagatgacactaccaaggcagcatgtccg

aagaagcctgagtttgcaccagctgcctccgcgccgagcaccaccgagcgtaccgatcccacccgcacccttcccctaccacgacaggaaccgcagtc

acagctttgcgaaagacgattgcaacttctcgcccccctacggcacaaatgaccagttcacgatgtcgtcagactcaactatggttgacccttttgtg

gatgagatgctggaagcccatcaaagtcccttgaacgcctaccccgacatctcagcaatgccggtccatcgacaacaacacccttctccacttcaaag

aatcgttgctgtcagccctttcgtcgctgacgaggccgagccgacggagaacagcgagacgaggaacaaaatcaaattttcggccctgctcgcatcga

ttcccaagagcctgagggcacgagctacatcaaggagcagtgctggcgacaaacgacctatgccgcgctcaagcctttcaggcgaggacacggtcgta

tccagaggacataagcgatctttcacgcaggttgagtctgccaacgtcgagtccgtgcacttggtcgattctcaaccggcctttgcatcaaaatcctc

gagtccgcgcttcaaaaccgcgaacttagatctgcagccagattatccagttgcaggagccgacagggtcaaaaataggtcctccgtcaaggtgttgc

aagacccgaacctcagcaagatatacgacgacgcgacccaatcgccctatcgagagtctgatgctgtcctacccgatcctctcgagctccttcgagcg

aatctcaaaacgatgcttccgtcgtttccaacccccccgcccagacctcacgcgctgaggtcgttcgaatcgtcgccttcgctgcttctttcatttgc

gcaacaagatccgtcgtcagtgccggcttctcagttgttgtcgccgacgtcctgtcattgccgggactcgatcacacgctcgaggaccatgcggaagg

ctgcggacgcctcaaatctgcagcgagacagccagatcgatggcgaaaacgaaatgcccgggcggaaagggcgatccgtgcttgatcacacctctact

gaaatgaaggacgtcgaagacacagaaggaggaacacgcacaagaaagaacacggccaaaagtatctgttcgacgacccagcagtttgcagccctccg

ccaacacgtcctctatcacaaagcggcttccctcctccgccagacacgctccttttgcctaccaaacaaaccacctcctcttcccccaaagcaacgac

gggcgacgctcaatgagtactgcagcatcaagtcagcttataccgcgaacggccgtgcggctaccgagaatcgaagctcgttgttcgaacgttcaggt

catgtaggtcttccgaggattggtggtcctggatgtaagtatagagggaccaagttcccgttggaagagaccactctcgatgagacacaagaggaagg

tgcgcaaatgaccaggactggtactgatgatgctattgagtaa

>contig_14.contig_14.g251.t1

atggtcagaattcttaccagcctcacctccattgctctcgtagccgcggttgcttctgctattcctctcgaggagcgca

gcgtcaaggtcatccacgacaacgacggtgtcgacctcaagaacgccaagattggcctcaacctgctcaacaacctgcacctgctcgacgatggccac

atcctcaagcgcaaggctgcaaccatcgttcacgacaacgatctcgttgacgccaagggcctcaagctcggtctcaacctcctcaacgacatcgatct

tctcaagcgcaaggccaccagcttcgtcgatgatgacgatcttattgacttgaagaacaccaagatcaacctcgacctcttgagcaaccttcacatcc

tcaagcgcggctctgacgccctcgacacaatcattgcccggagcgaagacacgaccgtggaagcacgcgggtgctcgacaaaagtcgtccacgacaac

gacctcatcgacttgaagaacttgaaggcctacctcaacatcctctcccgatccactcccgagaagcgtggcagcaagtatgtttcatacagcgacaa

tgatctcttggacctgaaggacctcggagtcaacgtcaacatcctctcgggccggccgaagtactgcgttcctgttcctcacactcccaagcccaccc

agaagcctacccgcactcccaagcccactcccaagcccactcgcactcctaagccgactactacccgcaagcccaccaccaccaaacacactcccaag

cctactaccaccaagcacacgcccaagcctactaccaccaagcacacgcccaagcccactcagaagcccccccccgacttcttgccccacgacaacga

cctgatcgacctcaaggatctgaccatcaacgtgtgcatccttgacttcagcggcaagtgcggcaaggacagcggcaagaagagccccaactggacct

gccagactgctggcggctgctgcgtgcagaagggcaccaagtcgactaccgtcgtggacgacgacgaccttctggacctgaagaacatcggcatcaac

ctgggcctgctcaacttctga

>contig_14.contig_14.g252.t1

atggtcagaattcttaccagcctcacctccattgctctcgtagccgcggttgcttctgctattcctctcgaggagcgca

gcgtcaaggtcatccacgacaacgacggtgtcgacctcaagaacgccaagattggcctcaacctgctcaacaacctgcacctgctcgacgatggccac

atcctcaagcgcaaggctgcaaccatcgttcacgacaacgatctcgttgacgccaagggcctcaagctcggtctcaacctcctcaacgacatcgatct

tctcaagcgcaaggccaccagcttcgtcgatgatgacgatcttattgacttgaagaacaccaagatcaacctcgacctcttgagcaaccttcacatcc

tcaagcgcggctctgacgccctcgacacaatcattgcccggagcgaagacacgaccgtggaagcacgcgggtgctcgacaaaagtcgtccacgacaac

gacctcatcgacttgaagaacttgaaggcctacctcaacatcctctcccgatccactccccgagaagcgtggcagcaacccacccagaagcctacccg

cactcccaagcccactcccaagcccactcgcactcctaagccgactactacccgcaagcccaccaccaccaaacacactcccaagcctactaccacca

agcacacgcccaagcctactaccaccaagcacacgcccaagcccactcagaagcccccgacttcttgccccagtggcttcgaccacgtgtcccacggc

tccacctgcggaagcgtttacgagagcgacaacgacctgatcgacctcaaggatctgaccatcaacgtgtgcatccttgacttcagcggcaagtgcgg

caaggacagcggcaagaagagccccaactggacctgccagactgctggcggctgctgcgtgcagaagggcaccaagtcgactaccgtcgtggacgacg

acgaccttctggacctgaagaacatcggcatcaacctgggcctgctcaacttctga

>contig_14.contig_14.g253.t1

atgcatgcagaaaggcttcggtcggatcggtgccctgcatgccgagctgcaggtacggcggagcctcctgccaccaacc

agctggctggccccatgtttctctcgctcacagtggcagacctgcgttcttctaccacagcaggggatctcacaacaaagccttcgtcgaccgccgtt

tgtccgaccgaagaaatgcgtgtcgaacttctggtttcctgcctcctctccgcgctggtatccggcctgcctggcaagctcagttattcccaccccga

gcctctcggacgcgtcgctcctgccgaacaaccctacctaacctcgtcaaatctgacgcttcgttcgtcctcgcttcttcgtcgctatgcgtacggag

aattctcgaacggcgatacgtgctcatccccagaccaatgcatcacccaagcctgtatcggctcagtctgcgcaccggccgtgattggtcggggctgc

gaggtcgacggtgactgcttggagggaagctgccaagccggtctctgtctctatcttcaaggaggatccccctgtaccagaaacagtaactgcgcctc

cagcatctgctcatcctctccgggcggcggctcgatgacgtgtctccccatcgctgaaagcggcggcggcgcgtgttcggaaaatgaagactgcgcca

gcttgcggtgcaacagcggcgtgtgcgacgcttcatccgaaggcgaaatatgcggtaccgattccgactgccttgaggatcaggggccatgcacaaat

taccgctgtcgacccccgcacgcatcctgcacggatcgagcccaatgtcaaggtgtctgccgaggcaatccgggcaacatgcactgctttggccgacg

agatgatccatgcaattacgacagtgaatgcgtaagcaactattgcgaccaggacctcggaatgtgcggttctgctcgcgcaggttccatctgctcat

cgtatcgagactgctcgaacaaattcacctgcaccactggcccacaatgtcagaatccctttgcgccttgctgcaccggtcgacagctggatgaggtt

tgctccgactcgacggactgctacagtcattactgcaacagaggcaagtgcgagctagctcctctcgatacttggtgccagttggacgttcactgtca

gtccggccagtgtggtaccgcttcgaaatcgacgattggcgtcaagaaatgtcagcctgccacctcgtcgagctctagcgccacctctgcatccacgt

cttcctccagctccgcttctactccatttgcttctgctccaactcctcctccgacttctactccaacttcggctccaacttctactactccaatctct

gatccgacttccacttccacttccactacttccacttccacttcatcgtcatcttcatcttcatcctcgagtacgatttcgtcagcgacctcaacgtc

caccaccagcacagctttatctacatctaccagcacctcgacaaccacgtcggccatctcctctagccagtcctcaagctcgacaacgacgacctcga

gcacacccactacgccaacgtcaacatcgaaatcatctgtgcctacgacaacatctaagcctacgacgtctacgacttcgagtagggtgaccagcacc

agtaacacgagcaccaagacaacgtctgccacgtccacaacctccgcactgccaacaggtgccgcatgcacagcaaactctgtctgcgggacgggtta

ctgccggaagccgctgctgagcgatggtgtgacacgagcatcgacaggcacttgcgacgtcaagaaagctaacggatcgaaatgctaccagatgcatg

tgcgtcgcagctaccacatcctccaccacctccaagtcgagttctgttacctcgaccaagccgacttcgacttcgacagccacatcgagctcgacgaa

gacgacgacatcctcaacatctacgctgctaccctctgccgcaagttgcacgtcgaacgctctctgtag

>contig_14.contig_14.g254.t1

atgctcgcgtgggcagtcgagcagaccgacgacgacagcggcaaggatcagggatcagcgatcagcacgtggcagtcgc

ccagtcacagcatggactcggtagagaagggcgagggatcctcctcatcctcatcccactacactcgctcacaccccagcgcctcggcctcgcccgcg

atccacctcacccggtcctcaagcaccaatggacacccccatccaaacacacggccagcggtaccaagagcactgtcgtcctcagcagcagcagcagc

aggggcagcaggagcaccaccgctctcgccgctcccagcaatagacggcatcggcatcggcatcggtctcggccccgccagcgctaccaccagtggga

ccaacaccgcgtccacatcagcagggccagcagcagcagcagcagcacccccgccatactcctaccgacatctcccccacacaccctccgtatcaaga

ccaagctcgcccccgctgcccctcaacacctcatcctcaagtcatcaccacaaccagcaccaccaccactccaagacggcctcaggaagcggcgcagg

cttcaccctcggcccagaaccaggcatgttcatggtcaacctcggacgcagtggcgggattcacaccttccgcatcccaatcctcctccagcgcgtcc

tcgccatcgtcattcgcaaatggaggtcactcctcttcctcgccatcgtaggcagtctcaccacctttggcctctacggcgccttctggaccgagtgg

cgcgagatgacctacctccgagtcgacccctttccgctggtacccgagggcgcacagtcgccataccatgcagccgcaccactaccaccggtaggcgc

acccggcgcagtcatcgaccccaacgcccccgccatcggtcccggctcggcgacaaccaagccgcaacgacccatcattcctcccgatatccagggtc

gatgcccaggtccaaagggcacccccatcccgctctctcccgcaaagacatacaagaccccgccgaaccaccctctgcccatcgtcggatcacacctc

ctatccgagctcaacccagaacagtgcatcacctaccgggaccggtacggcatgtactccgacctggccctcggcatcacctaccgcgtcgagccact

caacagcgaggccgccattcggaaaaagaaggaagctctcttcgaccccgaggaccccgagaatctcgtgcccaagttacccctcgacgcagcagccc

aggcagccgctcaagccgaatccgagctcgagctaggcgtagcaggtccagccgacccagacgcagcaaaattcggcaccatcccaccgggaagctcc

tcctcctccaccacttccaacaacgcctcctccatccccgacttcgaccccctcatgggcccacccaaaccacacggcctgccagaccacgactggga

aaaccacctgcgcgaccagatgtggtccatcaacgaacgcgccgaacgcgcgctcgcagacggcgtacagaccttcccagactggcgcgctctcatgg

acgcatgctttgaacagcgcgaacgggaacggggacgcggtgggggggtcgacctctttgatgctgctgccatgcccatcacgcaggagcagggtggg

ggaccgggcgcgggcgtgcatctcccgcctgcgggcggggcgggtgcggagaaggatcctgtgaaagcggcggttgctggatctgcgtcggcgggtgg

ggcgggtggcgtcagtggaggacacggtgggacgggtgcgggcaatggtgctggcggtagtggggcgggtaaggaggacttcaaggcgaagacgaagt

ttagcacgacgccgcagacgaagcgtacggcgcttgtgatgcggagttacgagggatacccatggcgagaggacgacatcttgaacctgcgcgcgatc

atctccgagctctcgctgaacaacccgaacacgccgtacgacatccggatcctggtcgaggtcaagggaaagcacctcgccgtcttcacctcggagtg

ggaccggctgaaggtgctccggtcctcggtcccgcgtgagttctggggccttgtggagctttggacgcaggatgagatgtgtgcgttgtaccccgggt

tggagggcaagttcttgaatgggatgttcccgcagcacacgtaccgcgcttgcttgatggctttgcagaagttctacttggaccaccctgagtatgac

tatgtttacaattgggagatggatgtgcgatacatcggaaactaccttgacttcctagagggtatcgaagggtactctcgtaaggagccactgcacgt

cggcatggacaagtacgacacttggttcgtccgcaacgtcactgtctcggagaacaactgggcggacaacacgacgatgcacacgggccggggcgagg

aggccgacctaatcacgctcggccccatcttcgacccgcgcgcctcgggctggttctgggagtacgatatacagaattaccccctgggccaggcgacg

gaccgtcgggcgagtatcgggacgaacatgcggatgagccgggcgttgctggaggcgatgaacgtcgtcaccgccgaagcgaagaagtcgacccactg

cgaagcgtggccaaccacactcgtgctacactcccaactcgagatcagccgcgaccacagcttctaccccgaggccgggttcacgtacagcctgcccc

taccctttaaaggcgtcttcgcgccccatccggtgtacttccggcacgagtgggatccggacgtgctgcatgcgcggatcaaccggcaggatttttac

aagaagatgaacgagaaggtcatgcgggatacgagtttttactatgatgggaaacactcgaaggagatttacatggggtggaaacagcgggataataa

ctga

>contig_14.contig_14.g255.t1

atggcagccccagccctggcacgcactctgatcgctcgctcagcagcagcacacgcagcctctgcctctgcctcctccc

tcaccgcagcagtcgcagtcgtagtcgctccatcctcagccgcaaggctctccagacacgctccaccaacaccattccaccagcaccactcctgcaga

tgggcatcgtcctcatcttccaccaacagcagcggagacccatctgcagctgactccaaagcgcagcagccatcgcagccaacctcagacacagtaga

cccaaagcaagagccagtctacgcagcccagtcagcagctaccgacggtcagcaatccaacacacccagcacggcaccttcaaaagaggccagcctgg

atgacatcctcggcgcagcactcgcgccctcattaccatcaacatcaacatccgaacccgacaccacagcaccagcaccagcccaggcccggaacaaa

cagccagcctcgcgctacgtactcgcaagcggcatgcagccgcccccgtaccgtctccacatcatgacctcccgcaacaacaccatcctcaccttcac

cacgcccgaggggaacccactcgcctcggcctcggggggcaccgccggcttcaagaaatcacagaggagcgggtacgaggccggttatcgcgctgcct

tccagatgttcgggcacattgccgagtaccgtcgtgctcgcggcttcggcgtcggtccagcccagaactcggcggccgcacgtacctttggtgggttg

gtcactgcgcctacccatggggaggaggacttgcgcattgagacgatttggaatggctttggacagggtcgtgaggctgtctttcgtgcgctgatgac

gaatgagggaaccgacgttcgtaatctggtcaagcgggtcaccgatgggacgcccctcaagattggtggtgtgcgacccaagaagcgcaggagtgagt

gtctgctactgctccgtctctcgtttgctcttgttgaatgcacgctgaccctctctctctcccgtcccgaccgcgcttctgtagtgctttga

>contig_14.contig_14.g256.t1

atgtcgcgcacacccacccagctccgcccgctctccgcgcaaaacgccgtgctagcccgggcagacggcagcgcctcct

tcgccttcggcaccctctccgcctacgcctccaccaccggccccctcgagtcccgcatccgggacgagctcatcgaccgcgccgccctccagatcaac

atcacacccctcaccggcctgcccaccatctcctcccgcggactgggccagggtttgagggaactcttcgaaagtgttgtgttattgcatctgcatcc

gaggtcggtcgtgcaggtgaatgtgcagttggttacgggtccgcccgggttcgtgcccccgtcgaggacgtcggttacgtcggatgctatcctgattg

gcgacgacgacgacgacgaagagcaagagggaggaggaggaggagggaagagtacggccattcgcaaactcagaccatccgctccccacccgaatagc

accttttccgcctcgcaacgggcgacgatcatcaatgctgccatgctcgccctgctggatagtggtctggccatgcaggcgacggtggcggcgtgtgc

gtgtgcggttttgcctgcgaaggaggcgtctcggttgcgcaaacggagacgaagatccgacgacggcggcgacgtagatacgcagatggatgctgagg

gcagtcatgcagagcacgtactggtgctggacccctcgcccgaggaagagtactacgcgacaagcacgcacgtcttcgccttcggcttcgcaggcaaa

tcaacgcctcccgcccgcgagtggtcccatgacgaggagagctacgacatcgcggacctcgctccagctgcgggcgacggacgtcgaaatgcggacga

ggaagaggagatcttgtacgcggagtctcatggccctgttgggttggagacgtatgctgatgctgcggatcagtgccgacgagcggcgcgtggattga

tcgtacctttctacaggagatggctttcacaacatcatgctaagcaatga

>contig_14.contig_14.g257.t1

atgcatctcctcccccgcgagcgcgacaagctcctcctccaccaagccggctctctcgcccaaaagcgactcgcccgag

gcgtccgtctgaaccaggcagaagcgacggccctcatcgcttccgtcctccaagagcgcattcgtgatggacagcattcggtcgctcagcttatgcag

catggcaagacgatgctcggccggactcatgtcatgccgggcgtggcagagctactgcacgaagtcatggtcgagggtgcatttccggacggcacctt

ccttgtgacggtgcacgacccaatatgcaccgagcacggcgacctctccgccgccctgtacggctccttcttccccatcccacccgctgagaagttca

cgcctcgaccgggtatcgacgacgtcgctctcgtcctgccgggggcgaccgtcgttcgcaagtccgtacccgacatcgtactttccgcgggtcggagt

cgggtccgggtcaaggttaccaatacgggtgatcggccgatccaagtgggctctcactacctcttcacggagaccaatccggccctgtccttcccgcg

cctcctcgccctaggacgaagactcgacatacccgcaggcacagccgtccgttttgagcccggcgactccaaaacggtctccctcgtcacaatcggcg

gccagaagatcctctccggcggaaacagtcttttccccggcccgctgtccagcatggacctatccgatgccgaggtacgctcggcgatgagggaacgt

cttcggttggctggcttcaaggatgaggatgtcgggccggatgaaggggtcattgtggatgcgttcttggggacgccacagggtcctgaggttcttcc

gtgtgcgatctcgagggaggcgtatgctgcgctttatggacccactgttggtgatcgtgttcggctggcagactctagtctctggatcgaggtcgagc

gtgaccttacgcactatggcgatgaatgcaagtttggcggagggaaagtactgcgcgatggcatgggccaagcctcgggacgcaaagacgcagaaacg

ttggactgcgtcatcctgaatgccctcattgtggatcactggggcatcgtgaaggccgatatcgggatcaaggagggtcacatcgctgccatcggcaa

agccggcaaccctgacacgatggacggcgtcacggagggcatgatcgtgggctcatgtacagaagtcatcgccggtgaaaagctgatccttactgctg

gagctattgacgctcacgtccactacatctgcccagacctgtgcgaggaggcacttgccaccggaatcacgacgttgattggcggaggcacaggtccg

acttcaggcacatctgcgacaacatgcacgcccgggaaggacaacctgaaagccatgatgcgggcaacggacgacatcccgctcaacttctgcttcac

cggcaagggcaacgactccggcgctgtgggtctggaagagcaggtcaaagctggatgcgccgggctcaagttgcacgaggactggggctcgacgcccg

ctgcgattgatacgtgtctgacggtgtgcgacaagtacgacgtccagtgcaatatccacaccgacactttgaacgagtcgtcctttgtagaagggacg

ctggccgccatcaagggtcggaccatccacacctaccactccgaaggcgccggtggcggtcacgcaccggacatcatcagcgtctgcggcgaaccgaa

cgtgctcccatcgtcgacgaacccgacccggccgtacacgatgaacacgctggacgagcatctcgacatgctcatggtgtgccaccatctctcgcgcg

acatcgtcgaggacgtggcctttgccgattctcggatcagggcggagaccgtcgcggcggaggatgtgctgcaggatgaaggggcgatcagtatgatt

agtagtgattcgcaggcgatgggtcggattggggaggttgttagtcggacctggaggacggcggcgaagatgagggatttgaaagggccgttgcaggg

caaaggggagggagagcaggacgcggaagggagagataacttcagggtgaagcgatacgtgtctaagtacacgatcaacccagccatcgtacacggca

tgtcccactacatcggcagcgtcgagcccggcaaatgggccgacctcgtcctctaccgccccgagaacttcggcgtcaagccggagatcgtgatcaag

ggcggccagatcgcctgggcgcagatgggcgatgccaatgctagcatacccaccgtgcagccgatctatggacgaccgatgtacgcgacgaaaccgca

ggctgcgcatgcgaatagtattctgtgggttagcgacgccagcattaagaatggcaacatcgcctcgtacaccatctcgaagcgcgcgatgccggtca

agaagtgccgcgagatctcgaagcgggacatgaagcacaacaacgccatgccgaagattactgttgatccggagacgtacaacgtcatggccgacggg

aagctgtgcacggtggagccggcgactaagttgccgatgacacagttccatcacttcttctga

>contig_14.contig_14.g258.t1

atgagcccaaggtacacaaagacgtccgggagctccgagaggcatgcgcaggcagcagcggcgccagcaggtgatgcgg

cgctgtcgaccatgctcgcctcggccgagcgcaaggcaacccacttccgctccttccgagctcagattcgttccacacatcagcacggacgaggagaa

gctgcagcagcgcaaggcagtagaagtggtagcagtacacaacgtcctcctcgcgcggggccgagccagcgaacactgccgcgcgttcgcaccgatgc

gacctggacccgcttcttcggcgagaactcggacgcggaggagctcgaggccagtcccgtcacgctcgtcgctacccggccacgcaacacagccgtgc

cctccctatcggcgtccgcgaagggaaagcagcccgtgcggacagacgactccatactggcaagcaccgctgccgcgcaatcgcctatcgcctcgacg

ttatcccccgaggagaaggcccagcatttcgggcccaactatctcgcgcaggaagattcgctccggaacgacttctcgcagtggtacgtcgactcagg

agcgggcgagctcccgggcaacgcggttcggaaccctttcctggcgtctcgcttcgacgagtatcccaagttgaaacgactcgtccagatcaaagagg

agctcgtctcggcaaaagcgcatcccccaacctacctgtatgcggatctgagagcgtccctcgtaccggcatcgcagtaccatcaacagaggaggaat

cttcaaaaggtagaagcggtagggtcttctacatcggaagctggtgttgcaggagtcgcaacccgggctgagacggacggacaccaagcgggagatgt

gggcggcgatgctgcagcgggtccgagcgagcctggaagctccaagggcagtcgaacgagcagcagcgccacgtctctctccaacatgacctcgttga

cgtccgcttcagcttcaacagcggtagcgacagcagctactgcaccagttgaaccaacaacaagcatcgatcctcaaataccagctcctcaacctttc

cacctctccgcgctcatgccgatcaagtacgacgtcgtcatcattgatcctccgcttgaggtatacgactgggaaggcgtcccaactcggaagtcagg

tgcttccggtttggacgggccgcagatccccttcgctcagacatcagcggcagctcctgatcctaagcagacgtggacatgggatcaaattgcgaacc

tgcctatcccacaattgacagccaaggaaagctttgtgtttctctgggtgggcagtggggcgggtgatgggctcgagaggggaagggagttgcttgcc

aaatggggatatcggaggtgcgaggacatcgtgtgggtccgcacgaatcgaggggcggcaaagactcaacatggcgacggcgaggattctctgcggca

gtcatctacatcttcggtcttgcaacggtcggtccagcactgcctcatgggcattcgggggaccgtgaggcgatcttccgacaccaggtttgtgcact

gcaatgtggacatcgacgtggtcatttgggagggcgaggacgaccatgcgggacacgattcgtcgtcgtcgacgcgtgagagtggtggcgggtcatac

ccgaaggctcaggaccacgtccgggccaagccgcaagagatgtactcgctcatcgaaaacttctgctcgggcacgcgtcgcttggagctctttggtac

caatcgcaatctgaggcggggatggctcactgtcggactcgaggttggtccgcatgcagctggcttcccagaagccctgccagcttcctactcgactc

cgcaacagcagaactctcacgagcaaacccagcagcccccggcagagctacagaaggaaggagacgatgccggcccggctccaatcaagatggtcgag

ccagtcgcgtatcagaaacagatgtacgattcctacttcggcgtcgaccgaccggggtgcgacttgcgcgaacgcaagaacctcctgccgttctcccc

ggacatcgacgcccttcgaccgaagagcccagctccgcgcaatgcccactcgcgagtggacagtgataacccgctgactccgcctccgtcgggcgctt

tgagcctgcatggtccacatggctctggctcgccgagcagtggtctaggctgggcctcctctcccgctgcgcatggtaatggccagcagggacccacg

gcgtcagctgtacccccctccgtgctgtcaagtcctcgcgcgagtccccgggcctctatgacagcacgaacgctgcagcataagatgtccatgtcgcc

caccaccttcgtgacagggatgaaccttcccgcgctgccgatgcaacagcaactagcctttggaccaggctcgccgcagggacccggctcgcccactc

gatctcgacacggggcgcctgtgcacaatcaccaccatcatcatcaccactcgcaccatccaggaggagcgggtctgtcgggcttgggggccgggggg

ccgaaggtgtcgtag

>contig_14.contig_14.g259.t1

atggcggcagtactgaccccaaccagcccgggcgctgctcctgcctacgatgtggtcttctcccatctctctgtccgac

tgatcaagaccctggtcaactctgcccccgatgccccgccgctccatcacattcacgggcccatgcagctcgtcgctgtcaccccagacgctcccatg

ctccccaccctcatcctctccctgccaggcttcaccctccccttccgcactttcaacccaggtaccgtgggcggctcgggaattcacgatgccgaacc

tccccgtcccacgtacgccagcttcggcgctggcgagcacggtctcgaggggccctccatctggttcaaggaacacgtgactgaggaaggcgcctggg

ctcgcaagtccctcggtcagcccgcggcgatctggaccctctacctcgtcggcgccccgtccccacttgcaacgcatctgcaagacgtcctcgccggc

tggcctcgggagatgcctcccgctctgccccgatggcttaccgaccctgccatgtacggctggccgtggaaggaccaattcatccagatgccacttcc

gatcgcgctggcccagccagtgcccgttgctgtgtcggctccccgtccgctgccaacccctccgatggacaactcgacaagtgcagcacgaagcgtac

cgaatgctcgtccatcgattccgatcaagccttcgaaactgaggtcgacgacggtggcggcgcagatggagccgccgctcacccctccggtcgaggaa

acccaaacactccagaacgtggtttccgaaaccgagactgtcagcgagagcgcgagtgccgaaccggagcccaagaagaaaaccagctcgcctccgat

accaccaaaaatcttgcgtcagtcggtccagtcgtccggtcgcggcattccccgctccgtcgatgcgccccttgtgcccagccgtccaattgagctcc

tgccttcgcttcctcactccaccatcttgcccagcgctcagtacaacgtcgaacacaatgcgtgggcagaagagagtcaggcagttcccgctgctgaa

gagaaaagggatctcgccagcccagcccgcgatgtggaaccggaaccggcatccttccccgatccgtaccggcacagtctcgtggctgtcgacaacga

aacaggtgaagtcttgggcatcctcgcctcgaacatcatcctgtcgaccgaacaggaagacgaagccgatactaccgtcacgaagaccaaggacgatg

acgtggaagacgatgccgccacccctcacgcgtcaaggttcgtcgagtccaaggccctcccccccgccaaggtagagcccgaacctctgcgagcgttc

accgcatatcgcgatccagacatctactcgccatacgcgccatctaccgatcccgacaaggccgctcgaccgccctcaacacttggcgtgcacacgtt

tgcgcctcctcccctccctgacaaggcggacgacgaggcaggttgggcgggtaccggtgcgagggacagtatggccagtgctgccttcttctctgcgg

ctgaggagctcgactcgggaactgatggatcggagggacacgcaggaactgatgcgggtgcgcccggcttcaagctgggcagtgatcccgtgttgaag

gaggtggaggggtacagagaaggtctgttggtggatgaagtggcagaggaggacgagggtgcggagactgaccggagcggacggaccgtgggtggtct

cctgaagcgcgctttcaagaagaagaacaagaaaggcagcaagaaggatgagaaggtcaaggaagaggtggctgtagaggagctcaaggagcaggcta

cggagcaggctgacaagggcctggcagcagaactaagtcaacaaccagcggaagaggtcacacaatctcacccgctcagtgcgaacgcatcgcaaagc

ccgacggccctcacaagctcgggttctatgacactcgaggctcaagagatcgaggcatccaatactcttcccgttctgcgtccaaagaccgaccccaa

ccggttggcaaatcaccttcctcgggcgctactgcggacgtccgccctcatcgacgtcgaacaccaagtctgggccgacgcactcgctcagaatgaga

tccctgcttcggcaccgtatacacacctcgctgccaaagccacggcggcggacgaagattggacgggtgggagcagggacgaggtggccgtcacgaag

gcaggcggctcgaatagtcctgtcgaccctgacgaatcggtgaatgcttcttcttcgagggcggcggtcgtcgctgcggctgcgggcgagtacatcca

aggtggaacggtgctgctcaagttcttgcaaggcaccatcactgccagcatggtcaaccctgcccagctggctcgatacatgggtggaggacctacgg

aacatgctgaagaacctgcttctcggacgcctgacgctcaagctcgtccagccgccgcggaagagcctgaggaccttcgaacgggcgctatggcttat

atccccgtcttgccggtccatctgctctactacatgggaatcgtgacggagaagccggcagcaagcgagtcgtccttgccctcggccgccaagggtgc

gctcgacagcgttgctcgagctgctggggtcggctttggcgtcggacggctgtggaacacattcatcgagagtgcggtcggacagagctcgtccaacg

ccaccacggcgctttcgtcgatgacgcctgcgaacgtgagcttggacatgctcgggatgagtcgcagcggagttgccacatgctggaactgggtttcg

agtgcggcatcgggagcgctacagagcgtaggcactgccgtgtcaactgccacatccgcccctctcgtccggtcatcgtcgcgggacctggccgtggc

ccggaatgggaacccacacctggactacgacgacgacgatgactgggaagtgacgatcccggactttgacccgaactcgctcggctcgacgccgcggc

cgatctaccgacgcaaggcgaagagtatcctgccttcgtcgacgactgggtttggactggcggatcagcagagacctgcgaagaacagcaacggcaat

aacatcaacggcaacagcatcgattggaagcgggtcagcatcgtgcacttcgatgccaagggggttgggaggagggccgtgatgggcggtgatggctt

cggtgtcctttga

>contig_14.contig_14.g260.t1

atgcgagtcaaagggaagcagccagaacacgagccggacgtcgacgactacgaggaggaagaggacgaggaggacgagt

tcgaggaggaggaggaggatgcgccaaagccacggtacgctgcatatcatggagagagcgacctcgacgacagtgacgaggacgaggaggacgaagct

ggccagtcggaacagtcggaggaggacgaggaggatgatgttgacaaggagaaaatgctgcggcaagaacttggctccctgccgctagctgcattgct

gaaagcacgcgcagcccttgatgcggacgagcctgcagacaataccaaatcaaggtcgcggaggacgaaaggcgcaggggaagaagtcggtagcgacg

actctgacgacgaggatgacgctgccgaagacgacgaagagaaaaatttcaagaaggaaagacgcgagatcagtaagcgcgccgataaatctgcgccg

atggagatgagctccaagagggcagtaacgcggaaacgacaggtcatcgaaacgccaaaaactcacgtccgcgacccccgcttcgactctctctccac

ctccgcattcgacagcaccatcttctcccgctcctactccttcgtaaactcagcccaatcgtccgaactctcctccttacgaaaacaatactcggacc

tccgacgcgccgtcgcggcgcgagctccctccgacgagcgcgccgccaaaggtttgtacagcagtgaggcgatggccctccgtactgagaaggagcgg

gtcggtcagcagattaagcggttggagagtgtggcggctgagaaggagaggcgggagcgggaggcggaggtgaagaggaatttgcgcaaggtcaatga

tgagcgtcagaagcagggacaagagcccgtcttcttcaaacgagcacaaatgaagggagtcatcctacaggacaagttcgagcgtctctcctctgaca

gaccagggaagggcagagccaccggctccaatcggacccaagcctccccatccaccgcctcaaacatgagcgtcgaccgtctacgcgccatgatctcc

tccggctccaacaccgtccccgcttcctcatcttcccgtgggggcagcggcggtgctccggacgacccgtcttccgccaacggtcaaccatcctcctc

ttcctcctcggccattaagaaactcatcgtgcggaaggccaagaaggacgctaagaaggatcggaaagatctgcccttcttggtgcgggatcggggtg

ccggaacaggtggggaaggacagggtcagcgagcctcggctggcaaggacgaagggtcaaagaagcggcatcgtggcagtcgcggcagtggtgggacg

agcaaaaagccgaggacgcaatga

>contig_14.contig_14.g261.t1

atgcagcgaccaggccaacaacgcaacgaagacagcatcatgtccggatcagtgcgctcaggacacatcccgccgcact

ccaggcgcgagtccaccgactcctatgcctcctcagtcggctccaaccgcgccctccgtccgccgaagatcgagatggccgcaacctcgcccttgagc

aagatcggcatcaagggcgacggcctcatgctcttcatcacctgcttcgccagcatcggtgtcttcctcttcggatacgaccaaggcgtcatgtctgg

aattatcaccgggcctcatttcagagcctacttccgcgagccaacctcctacgagatcggtactatggtcgccatcctcgagattggtgccttgtgca

cctccctcgcatgcggaactcttgcagacatcttcggtcgcaaaaaggtcctcttctggggcgccattctcttctccatcggcggtgcgattcagacg

ttcacaacaggttttactagtatggtcattggtcgggtcatcgccggctttggtgtcggaacgctctcgatgattgtaccaacctaccagtctgaaat

ctccccggctgagaaccgaggtatgctggcatgcatcgagttcacgggcaacatcgctggttacgcggcctcggtctggatcgactacttctgctcct

atatcaattccgacctctcctggcgactcccgctctccatccaagtgctgattggtctcactctcgccttcggctcccttctgcttcccgagtcgccc

cgctggctactcgatcacgatcaggacgaggagggtatgcgtgtcctcgcagatctgcacggcggcgggaacccgcgcgagccgcgcgcacgcctcga

atatcgcgaaatcaaggaaaacgtacttacgctcaggaaagagcccgatcgtagttacacggccatgttcaggagataccgctgccgaacttggatcg

cttgctccagtcagatgtttgctcagctcaacggcatcaatgtcatctcgtactatgctcccctcgtgttcgaaagtgcaggttggctgggacgcgac

gcgcttttgatgacaggaataaacggcatcatctacattctctcgacggttcctacatggttcctcgtcgacacatggggtcgccgccccatccttct

ctccggttccgtactcatggccatctcgctcgccctgtgcggctacttcctctacctcgacgcgccttttaccgccaaggccgtcgtgggctgtgtca

ttgttttcaacgcagcctttggcttcagctggggtcccattccttggctgcttccctctgagatcatgccgcttgccttccgtgcaaagggcgcgtct

ctttcgactgcgactaactgggccttcaacttcatcattggcgaaggcactccaatcctgcaggagaaaatcaagtggagactgtacccgatgcatgc

attcttctgcacgtgctcgttcatcctggtcttcttcatgtaccctgaaaccatgggtgttccacttgaggaaatggaccaattattcggcgacgaac

cgtctgctgtcccacaaggagacgactcagacgacgaggacgacgaagaagccgctcggccggtccgacccatccgtacctcgatctcctcccaccct

cgcttcatgaacccagaagagcgtgcagctcgtgcggcagcggcccgcgttgctgctgaggatcgtgcacgagcggagagcgcagggttgttcggtat

caaacggttgattggtcggctcttgggcgcgaatgcgagtgagccggatcggagattgtaccagagcggattcggcccctcttccattccactcgaag

acatcacatcatcaaacgcagcacggcaccgaaagaaggacgggacaaagaaaggtggaaagagaaagggcaagaccccgagcggtatagagacaagc

cgacctacgaccgatcttccgacgaatgacatcccggcggaaacggactatgcggacgaagagacgcagttcaaactcggagactcggacgacgacga

aggggagggaagcggtgcaaagggtcagacgggaccgggtgtaaatgggcgtgatgatagcgatgaggacgatctggagtgggagagcgggcgaagat

ga

>contig_14.contig_14.g262.t1

atggcatcgaatgtaagcagcgcagcgcgcgtggggaagctggcgcaggtgcggctcgacggcagcctgcgctcgcagc

tctgccatctgccggtcgacctccatgcgccgctcgtcgaccgcaagatcgcacctcagaacattctcatcgagctcaccagggaagacgatccacag

cagccgtcgccagcgtcgggatcgtcgcaggtgcccgccaagcgcatcgtactgggatggaccggattcagctccgcgccttcctcgccctttcccgc

aagctcgtcgtccaggtcatcggccccgacaggatccatgcctaccatcgccatcgctccaacagtagcggccctctacagcccgccattggcggagg

gaaccctcgttcggatcactctcctgcgctctcctcccatacccacagcgtctcggatcgatctggtgcccttgacgcccgacgactgggagattctc

tccgcacatgcacagcaggtcgaggacgatatgctggcccaggttcgcggcgcagctgtgggcatgaatcttaccgcccatgtcggctcaggcggtcg

caccgtatgcaagttccgcgtcgaggcgaccgaacccaagacagtccccatcaactcgtcgccctcttccgcagcaccgagcaattctgccgagaagc

tcttcagagctgtccggcttaccaccaatacagaggtcgtcattgctcccaaggtccgatccagtagcaatggtaccaaaacgccctccgcagcgtca

gcgagtcaaattagaacggaaattcagtctacctcgtcgaaacagccatcatctcaacagcgcgcctcgagctccgcatcaacatccactctcaagcc

aggaagaccagacgaggatggccagaatgcacacgctctggctcaggacgaggctgacgatgaaccctcatccgattccgactcactcgcaggagacg

atcctgaagagcacccccatctggcaggcatcaatgacctgttggaaaagtgcatgtcacacattcaaggcaccctacgcgcccggcaactcagaagg

attcaagcgcaacaatcgtcttcaggcacgcaacagaactcttggctccaatctggctcctgtggcatgctcttgaccggcggctcaggctcaggaaa

aacggtcgttagcagagaattggcctaccgcctcgctgcctcgtcgtctgccttcggactcggcgccatctctaccagtcgtgctgcactctcttcgg

aaactcccaacggatcagattcagaaagacaggctgagagcaaaagggtcgcggttccttccggccatatagccacaaaatggatcgactgtacctcc

tttgcagacgaacgtctctccacccttcgagctcggatgggatcatggcttactgatgccgctgctctgacacttccccgtgtgcagaagcaggctcc

atctgatggtgtgggtagtacaacagaagcgtcacttcccaatggagcgggctcgaagaaggtaccagtccctccagcggcagcaaccctcctcatcc

tggacaacttcgatcgcctcctacctgctccttccgagcacactgccggcgacgcgtctgctatgagagttctcgctctcggagagtgcttcgctcag

caagtcagggatgtccagtcgtcctttgacgtctttgtcctcgcgaccgcgcagaatcagaccagtctgcatccgagcgtgggcagtgggtccggcag

taatcgcgtatttggagaggtcataccggtcaaaccgccagcaaaagaggcgaggaaggagatcttggcacatcttatcagcctcaagacggcttcgc

cgaactcagatcactctgaactagccaacggggacgccagccatcacgatcctcattcagaacgggctgaagaggatcatgagaagggggcccgcaaa

acacttctggcaccatctcccgatctcaatccgacaacattggcaactcagacggacgggttcaatgctgctgatctgaaggatcttgtcgaacgcgc

tgtgcatcaggctgccatgcgcgtcggtgatgaactcggtcgggctggccgtcaatcgaagaaggacagcagtgagaccttgctcgaactgactatgg

cagactttgtaaccgcacaagacggcttcgtccccctctccctccgtgacgtcaagctcgaaaagtccacagtggcctggtccgacatcggcggcttg

cacgccacccggcaaactctgcgcgaaacactcgagtggccaaccaagtacgctgccatcttcgcctcctgcccactccgtctccgatcgggtctgct

cctgtacggatttcccgggtgtggcaagacgttgctggcgagtgcggtcgctaaggagtgcgggcttaactttatcagtgtcaagggacctgagatct

tgaataagtatattggtgcgagtgagaagagtgtacgggatctttttgaccgtgctcaagctgcgcgaccttgtgtgctcttcttcgatgagtttgac

tccattgcgccgaagagaggccatgacagcacaggtgtgaccgaccgggtggtcaaccagctcctcactcaaatggatggtgcagagggtctcgaagg

cgtctacgttctcgcagccacgtcccggcctgatctgatcgactccgcgctgctccgacccggccgtctggataagagcttgctctgcgatatgcccg

acgaagaagagcgagtcgacatcctgcgcgccatcgcaggtgttcccacaaaggacgccagtggtcagatcaaggagcccaagattgccctggcggac

gaggtcgatttggaaatgtgggcacagcggacgaagggctactctggtgccgatctacaggccctgctgtacaatgcccatctcgagacgatccacga

gtccatctctgccgcacaggcagacggatcttcccccgagtcctcatcgaaggaagcggccaacggcaacgatgtgcccctgaagtttatatcgctca

agtctcgcagccaggcgcaatctggtacggacggcggtaaaacggtcttgagcggggcagagcaggcagcacttcgacgtcgtatcgaactcatcgcc

tcagatggaactcagacatcctcatcgacttctcaagccaagaacaatcttcccgacgcggacagcaaacccaagagcaaaccaaaaccgcgcgtcac

ggacgcgcacctggaacgttcgctatcttcgacgcgcccttccgtgcctgcggatgagcaactccgactcagaaggatctaccgcgcgtttgccggag

agcgggagggcaactatccggacggagaagccagccacgagatcggagcgcgggagagcctcatgtga

>contig_14.contig_14.g263.t1

atggctatagttcgctcttcccaggcacgcggaggcaccagccgtgcagcagcactcactactcactggagcagcaccc

actactctcgtccctggatgctgcggctgccagctggcgcgttgctgggtgaggcgcatcctgctctcgacgacgacgacgtgctccccctggactgg

ccccgccgtattccagcagcgttaacgcgtcaacctccagcctattctcgatttccctgcgtgtgtgttcgggaggacacagaacgcatcactcggaa

caagtctggcgcacactcgcaggcaaggagatcatcaaccccagcgcagcacgcggtcatcgacctggtggtgatgtcgacagcccttctcgaccgtg

cgtcggtcaccgtgaccgctgcggagaatcggcctcgcggcgcgtcctcagctgcgttcgccgaacggttcaagtacatcctctgcacctccttcctc

cttaccagctccctgtccatctccttctactcggaaacgcacgcaaacaaccatatcgaccatctggaagacgatgaccagaatgacctccttgggtc

cttgcagcagcagcagcgtcccttgtcgtcgtcatcatccctatacccggccaccgcaatctcggccgtcctccccttcgtcagagtcgcatccgagc

gcttacatgctgagcgcttacggttcttggctgaagatgcagacgacatctcggtcagcctaccaaaaaccacggtgcggagtccagccgagtcattc

gacacaccttcgaacgcgattcaggtcatcctaccacctcatctgcatcagcagcgcgatttttcttcgtcagccaaatctgtcgagtcgccctacac

caaaatattcccgccgctttccccagaaggcttaatcctccgtaacacgacagtcgcttcccttcttctgctgttccccatctcgagttcttcggcgg

tgtggtttcgatcgggtctggtgctcgtctcccttgcatgggcaacagtcctcgtctttggatggcacgttggaagcgcatcctcgtacctctccatc

cagctcgattatcctccctccgatattcgtaaggggattatggacaacagccaacacatacagtcacagtccaaggctgtcgaggccactgcgaacag

cacaggagcaattgggccaccagcatcagctgcgactgtactgccacgccttgacatgctgctgccgccgcccatctcggcagtcggtcagctagtta

tccgacgggatgctttccactcagcactcaggttagtgcgtgccgctcagcgactcgacattgagctcaatcgagcgatcgcagccattcaagaggtc

gaattggtcagaagaggctacaaattgacataccctcttgcgccgatctcgcgcattgaaaacatgccttccgcttgcccattgcatccttctggact

gcaaggaaacgccctgtcgatgactcagaacgggtcattgggacaggaaactgctctgctgagaagaatatcgagcgacacaaacggttctgttgcgg

cgggcgctcagcgaacgactgcgacaacatccggatcatccgcagtggatcgccgcatctcgacggtatcgtcgtccacgaatgcatcttcgtcttcc

ccgacttccttgctgagccgagattcgcagttttcgccagggaggaggggatcgggtcaaggtttccaccgcgggttcggtggagccgttcgcacggg

cggagcgactcacgagaaccttatgagcccacacacaagcctaagcagccaggcagaagagctcgaagttctggctgcccgcgatcagcagaacgcag

cgaagtacgaccacgatcagctggacgcgaagactttgatggactggggtcgttcgggtagccaggcacgatccagtatcgcagaaggccaagcagag

ccgaaccagtcgctgttgcggagaatgggctcatacgatcaggcgttcaactcgaatgcggctccctccggtgcctggtcgcgcacagagaccggtgc

tgcatgcacatgtcacagcagacagctcctcgtcttgcgtcataccctggtggaggtgctcaagagcgtcggaaacgaggccggtgtcgtttcggcac

gccttaatcctctggtggacgagatggaattaggcttgctgcttggtatggagggtggcagcgacaaggactccaattcgggtgcggctgagattcca

gctggcacggtgccaaattcaggccaatctacgattgattacgaatcagcatcagttgtggacggcgggatggaacccattctcgacgatggcccacc

cgttgacattgctccaggcccgggtcaagtcagcctgaaggaggagctcttgattcccaactctggccgaccaggcagctccgctcgggcctttgggt

caacatcgacctcttctgaagatgaatccagcccgagcggtgggagtggagccaattcactgcaacgggcgagcgaaatggctgagcgtagacgggct

aggcgtgttgggctccgtactgccgcgcccccatcgtccttctacaaccatgctggcggcagtgctgccagtaccgcaacaccctccgcaccaccggt

gtcttacaaccccttgaactcggcacccgcatctgggagtaccacacctgaccagcattatcgttcaaagtccggtcggcctggtactgccgaatcag

agcccggattctcagtaagcggttcagccccggcgaactccagacacgttcggcgtgggagccgactgcgttacatctcggaccgtcctggaagcgcg

atggaccagtactcggctagcccagcaaacacgatggccggacaacgtcccggacgtcgttcagagtcaccgttgctcggctcgacggcgggtgctcg

ttttagctacctgtcaaatcactctggtggtctgaacgggcacgctggttacggagcggcaggctctgaagaggcttacggtggctactcacctgctg

cgataacatctgcttgggccatggcgagctcttccacttcgacggctgctgcggcaactcgagctgtgaagcgcaatagtttgtcagcctccagcgtg

ggttcgaccgcggggcggaacttgctggcccaactacgagacccattgaacgggaccgaaactactgggtctggagcgggtctggctcctggtgcacg

ctccgccagagattcctggacgagcggacgcgaccacggcttgcaggcacatgatgtccagacgtgggggccgtcgacatctgcatctggtgctgaca

caaagaacaatcgcgaaacgatgctagctctcgagaaactggagggtctcttcactcctcctacaccagcaaaagctctccagcctgtgcaaccggga

gatgccttcggtcgaagcccagctgcactctctggtccaggccttcattcgaacggaagcgacgacctagaccaccttacgctcatttcaattcgggc

gggcttccaagacatgcaccgacgccgtcgcgacgtcctgtaccgactgcttgcgttggaattcgaagcgaacggtcgatttgtcaccgttcgaaggt

taggtgccaccgctggcgcaggtgggcagtcggctcagaaatccaagtctgctgttgcagacatggcaacccacgatgttacccttccaacggtcgat

tactggagcaccgtctgccggtctatggatgctctcagttccaagatggagacgcatgcatcggacattatcgcccaggttgcgcgggagatgggcat

ggattcttccttggaggatcttcccttggccacgtcgacgaagcgacagtctgaattggatcggtcacgcgagcgtgtcttgacggacattttcgggc

ctcagaccaaggatgcgcttgccttggcgcggcaggacggtgtcgacaagttggccccatcctcatcactggttgaaaccatgtcttctcttgggctc

gcgcttcgaaccatccaagtcaagctgcgtgcctgtggtgaagacctgggcatccatcagggccattcgctccatggaatggaaggcggggttgggtc

tggcggtgtcgctcgcttggcgaggaagtgcgtcctttcggcggacgaggtcgaacgagggatcgttgagcaaatgcgtgccgaaagggtctttgctg

gcattggcgacgatctcgcaaccgcatcaaaggagtgggagaacggactcaagttgctcaagcagcagactgtcaggtggcaacgcgctggagctgcc

gtgaatggtagtgccactccggtcagcaatacggagaagacgccagatgagatcgtggccctcagccagcgcctttcgcggttcatcgaagcaaacgg

caggatggatggtcggtcatcctcaggccattcgtacaggtccggtccgacgtctgcaaccgcccatgcttctggtctgcatcaccatctccgggtat

cacaggccatgacttctaactcgatttcgtcggactactcgccagcagcacagtctcaggcagatgaccgggacattgacacggacctgtcacagttg

gtgatgcagcagacctcagcggctcatcttcctgctcctggtccggaacatctctacgaagctgagcctatgcctgctgggtttggcagtgtatcaca

gccgcgtcggtggtcggctcgatcagtcggaaatgcttcatctgagcgccctttcgacccctcgatcggtgctggcgttgtcaacgagctgaaggacg

tcattctgcagcgacaaagccaaaggatgttggtccagggcggactgcctgcagtcctgactcttggttcgccaatgcaagacgaccagctggcaccg

gatggcagctttacgtcgaccatccgaccacagtactctccggtgggacacacaaggcctgccacctctcccgtgacccagtcatcgctttctcctcc

catgttcggcacgacggccaagagaaagtcgcgacgcgtgccggatgacccttcggccgttttcgagatgcacgagagcccgaattcggcgagggaca

cgaagatcacacatgccgacgtggctgcactgcgcagtcggaacagcatgagcagcgaggattcgggcagcttgccgactcgtccttcgagcatgcac

gtttcgggggtcggcagctcgaacctgagccatatccactcggtcatctttgatgatgatggtcaagctagcagggagacgaaagtcgggtcaaacgg

acctgtgggcgggcccactgtagtcgtgactcctggcaagtcgcagggaatgccggccctgaagccgatcgaggggcgtccgctcagcgtcgtcacgg

aggatccatggccgatcgagcatgacgagatgccacgggatgcagcagctgctgcagccgctgcggcggcggcggcgcgtcacaggtgggctcgaagc

ggcgaagtcgagctggaacatagctacgtctcgtcgggcagttctccgaagtcctactctcctaatcctcaggctggccaccaaacaccgacgtcaca

gcaatgggtcgccctctga

>contig_14.contig_14.g264.t1

atggcatcagacgcgggctcccctcccccatccgggtccagcagcatctggaccgagcacaagactgcggacggccgtc

aatactggtatcacaagttcaacaagaccagcgtgtgggagaagccagaggatctctggacaagccgcgagtcttacaaggctgccgatgggcgaacc

tactatgttcactctgagactcaagagtccaagtggacggtaccaccagagattcaaacggaggagaacgacgcgccaacgccggggtcctcggctgc

ggcacccgctcaggccactgcttcttccgcagcttcccccattccagcgtcagtaccacagagatccagacccgacactcaaaccccagatgcggatt

caaagccgatgtatacagggcctccacctgggttcgcgtcagccggctcgcctgctccagcctccgcgacgccatctgcgatagctccgggacctgct

cctggtccagctcccgggccacccccacagatgcccatgtttggaacaggctcaccggcacctggggcaccacctggcccaccgccaggcgcgccacc

tatccgacttggtgttccagccggtcttcctgctttaccctcagctgcagcggtagcggctgctgcatcagcttcagcaaccgacgttggctttgcct

ccaaggaagctgcggaacgcgcgttcatcgatcttctgcgctccaaaggtgtggacatcaagtggaaatgggaagatacgattcggcacatcgttacg

gagccgatgtacaaggctctcaagacgttggcagagcgaaaagctgctttcggcaagtacatacaggagttgcaggaggcagaggaaaaggagaggca

ggagcggaaggacaagttgctgccgttgtggcgcgacttgatccaaggatatggaaaggcgcccgatgggacaggagagatgaaggtcaaggtataca

ccagcttcgacacagcggagaagtggttcagtgggaaaaaggagtggaatgcggcagcaagtcgggaggaagcaaaagagctctacggaatcattcaa

agagagtcgcgcgagaaggaagaggcgtttaaccgcgaaattcgccatcggaatatggacatgctcatgtcgctattcaaaaccttcgaagcagacgt

cttcacaacctgggcggatgcccaacggaccgccttgtcgtcagaagaattccaatccgacgagcatctgggatccatggacccgaccgacatgctca

tcgtctgggaggagcacatgaaaagcgtagagaaggacgagtcggatcgattgaaaaaggtcacagatgcggaacaaacgaaagcgcgcaagaatcgc

aaggcgttcgttgaactactgcacgaactcaaggcccaaggcagcatcaaagccggtactacctggactaccgtgtacaaggccattcacgacgacga

gcgactgattcaaatgctcgggcaaccaggaagtacacccctcgagctcttctttgacatggtggacgaaatggacgccgaacttgatgagcgagtcg

cgagcttggaggaaaccctcaagaagaagggcttccaactctccgatgttttgccttccccaggtgatgcagaggcgaaagaagtggccaacttagct

ggattcgtcaaggtggtcgaaggcgagcacaagctcagtagcgacgagctcgagcagtgctacgaagagatgaagcgacgcgttgagcatgagcaggc

tcgagcccgggcacgcgcggagcgacggcttcggcacgccatcgacgagcttcggtacgagatgaagcgcgttgaatttacagcggaggagctcgagc

tgccatacgatggcattattgccaagctagatgcggaatgccccgagttcaaggcagagaaggacgaggcggtgcgcaagggtgcttgggagaagttt

gtgagaagacacaaggagaaagccgaagagcgaagggaccatcgagacaagaatcgtgatgggcccgccgccgaccatggtgccgacggttccaggac

gaacagcgggaggaagctgggcgccgcgcgcggggctgctggtggaacgaacggtaaccgacgcacagaccgggatcgaaaaatggcgtcacccctcc

gcgagttcagcaagtctggccagaagcttcccgcaatcggcctcggcaccatgttgatggcggctgtctacggccccgcttcgacccaagagcgcgtg

aacgaagtcttgaaccgtgccctcgagaagggatgtctcttctgggacacggccagcgtgtactcggcgccaggaacgctcagcgacaatgagcgtca

gatcgggtcctttctgcgttcccacccgggcagtcgggagaaggagacgatgggagccctgaaggaactgaaagactcgggcaagatcaagctcgagc

gggcggagaagatcgtccacattggtgagtcggtgtcgatcatcgcagcagctccgtctttctcgctcacgtacgccgctttgcttgaccttgttctt

cttgtcacagatgcactccagatcgagataagcctgctttcgactccagacggaactaccatagtggtgtactcgccgctcggaaggggcttcctcac

cgacaacttcaagtcgcccaccgatctcgaggaaggccatttccggaagagcaaccctcgattccaaggcgagaactttacgaagaacctcgagctta

tcacgctggcatgggtgctcagcaagtcggcccagatgtatgtcatcccgggaacaacgtcgccggatcgtctcgtggagaacatcgatgcgggcaag

gccgagctctctgcagaggaggtcgaggagattgacggggtcatcaacagcttcaaggcgagcggcgagcgatacccgcctggcatgaagaaggcttt

ctga

>contig_14.contig_14.g265.t1

atgccggtgaccccggccgcaagcccagcccgtcgtccacttcttcccctcaacgtatccccttctcactcccttcagc

ttactccgggtgcttccgctgccaaacaagacgatgcattcagtgaagacagcccgtctgcccgcgatgtgaacccgaaggaagcccttgggacgtcg

aagggcaagactgctgcgaagcccaagacgcgcacagcatctggttccaaagctcaagcttcaaagtctgctacgaaagaggacaattcagactttca

ggtcagcctcacgatgacgggaacagctcaagctttgaagctgccatcttcgtcgcaaaaacaaggtcgaggccgtcccaaggcgaaggagaccgaca

aattgatcatcatgctgcacctaaaggacactgtcgccttcgaaggcttcaaggacgagattgtctccgcagccactgaagatggcttcaatcctccc

gcatacgacaagctcgatgttaaaggcaagatcagcgctggcggtccgtgggtcaactggttggatattcctgacgagaagacgtttggctctttcac

cgccaagatgcgattcaagaagcgagaaggcttcttgaagctgaaccgctcacccgctgctgcatccgcgatcgtcgaggatacgcactcaagcgaag

acgttgagtccgaggacgaagacattcagatcattggtttctcaaaccgaggcaagagctcgtcgtcgaagggaaccaagcggaaaaagacagaagac

gacgtttccgatgctggagaagaggagccgaacacatccaagaagaccacgaagaaggtcaaagtcaaagacgaggcagaggaggagagaaccaagat

cgctgctgacatctctcgcgtgcatgaatgcacggacggcacgtgctctagcttcacttgctggattcttcgcggcggcatccacctccccatcacca

acatgcttcttgacaactgggtcgctggaatcgttcacgacagccgcaatcacagcattcatcgcattccgaacaatatcacgttcgaacgagcagcc

gatatcgctgtgaaaagggctcgcaccaggcgtcggagctcgggatccacgcgtgcaggagccgaagacgacaacgagctcgaagaccagctgaacga

taatgtgtccgaggacgagctcccctcctctgcgcagcttgctttcctccgccgacccggtgcttccacgtctgcttccggacgctcatactcagcgc

ctcatgcttcaaacggtgtacgacgcgctaatccggaacctggcacctctcggcggggtgccgctcctcctccacctcctatcccatcatccggaccg

gacatcacattcacggctttggcgtctcgcataaagctgggtgaagggctggtgaacaagatggttgcagaaggggttgctaaggcatcatcgtttgc

tcgtctcaacgaggagaagcttcgtgcaatggggtgcttgcagggagagattgaagaggctcttgacgcgcatgatcgatggcgcaggatgactgacg

aaggcatccggcctgaggagctcgtcacttctttggttgtttag

>contig_14.contig_14.g266.t1

atggtgtatcgaaagattagccacgaggtcaaagaaatgatcgtcgagctgctcctcgccggtatcctcacgaaggaag

atgtcctggtcgcaaacatcttctctgtttccactctcgatcgagctttacgacgatggacacccaacggtctttctgctcctcgaaaacccactggg

cgcccacgaaagcttggagacgccgatatcgggttcatcttggctcgggttcagcagaagtcggacatcatgctggatgaattggcccgtgatcttca

agtagttggcggccccgtggcatctcttaccctcctctcccgaacgcttcgacgccttaacattacccgcaagaaggtcacacgtcgagcacgggagg

catcccgccttgcgcaggcagagtttcgattgaggatggcccagtacagtcccaagcaattggtctttaccgacgagaccgggttcgactcaagacag

gtgcaacggaagtacgggtgggcacctgagggggaaaccgcaatcagaatcgtcagcggcaagcgcacgaagcgcgcgaccttgatccctggcctgtg

tgtcgacggaatcatcgccccgaagatttttcaccacaacatcaacactgatgtcttctacgactacctggtacgctatctgctgccagccatgacgc

cgtatcctggcccccgctccgttctggttatggacaacgtgccctttcatcgctccctcaaggttcgacagctgaccgcagactttggtgagttcatt

catctcttcacagctccctctggctacgtggatactgaataccgacatactgctgccttcggctttcaccacaggatgtcgcatagaatacacctctc

cgtggtccccaatgctcaatcctatcgaggagagcttcagcaaaatcaagtccgttcttcgtcggcaaggacgatgcgacgactactcagtcctccta

gcatgcgaaacaataacggctaa

>contig_14.contig_14.g267.t1

atgataagcagcttacgctcacaggatggcaatcggcaagaagacaaagggtcgtgccctctcatcgagcccggcagag

gaggaggttgtcgacctccagcggcgactcctggcaagccagctaccgatggcctctgccccgacatccacttcggcttcggcctcgctcaaggctcc

agctcaggctcctcagcagccttcgacaagcaatcttcgaccgcggggcccattccttcttcaggtgtcgaccacgactcggtggtcgctcggcaaca

ggtcatcgcgcaaatgcttcaagagtccgaggaacatcgtcccggacagagcaaggcgacctccaagggcaaggcccgtcgagtctcgccgcgccccg

agcaggctccagcagagcaccggcctgtcaccccacccagcggcctccatcaggcacacgtggacctcatcggacatttcgagaagctctctcctcgc

tccaaagtggagtggctcatgcagggcggcccggatgacaacacggggagcaagggtgatcctcaatctcaagctgcggccgggcccaacccataccg

gcaacgtcgttctcaagaccattccccagtacgcccacagtctcaggagcagccgccggtcctcaagagacccttctacgtctctccggatggtcttc

ctgttgcccacaagagggcgaaggtcgcggacgacccgctgccggctaggttcgatcaccaacgccaggtcgccagcaacccgctgagcatagcggcc

caccgcaatcggccggcatgctcgatggccaagtccgcggcgctcccggagccaaagcgtcaggacgctgatctctcgctgccagagatgtgggcggc

cgccaacaacatgttgatgtatatggcgatcatagctaacgatgctgtcgacccggacaagaagagcctcctcactcaagaacgagacggctggcgca

aggctttcaacgaaatcttcaaccaccagctcgtgatggaggatccgatttgcggagctactccgcaaaggtatggcttcaacactccggagcacgga

ctggccagatctcagacggagctgagcggagcgccgccccggagtccgggctccggggatctactccgggaagatccttactggccgaccctggctga

gtacgtcgacaacatccggaagaagatatatcttcgaccggtcggctcccggatcaagcccttcggcatccgggacaagctcttccgccagatcagcg

acactgtccagaagcggactgccgcggcggagctctcggccaccattcagtcggcatcggcagccgctgttgctgcggcccaagcggccatacacgct

caactcgcctccagcaacaagcagtcgtcctcaggagctgcgtctggctcgggtggcggtggttcctcatccatgaagtcgaagccctttcgtacaag

tcctgtactggagctccaaaagggcacgatgcccacgccaaacgagacaaggacggcaacatcgtccggcgctctgacggttccaccatctgccacca

gttcaaccttaccggctgcaagcaatccaactgcggcaagggacgtcacgagtgcagtgtctgcggcgacaccgggcaccaagcctccgcttgctccc

gtctcgagccctctccgcagtgaggggttcaagacgctgctcgagcaccacaacctgctgtcggccttcctgcagttggtggaaggcctggagaatgg

attcaacttcgacattcccccgatcacggtcacaaggacgcctccgaaccacaagtcggcgacctcggacctcaagactcttaactcggcgattgaga

aggagatcggattggggagggcctgcgggccttatacgaaggaggatgtcgaggacctcgtcgggccgttccagacatcgccgctgggattggttccg

aaaccgaacggcaagtggaggatggtacaggacttcagctatccgcggaagggggagaacccggcgatcaacaactacatcgcgtccgatgagttcgt

ttgcgcctggcatggctactgggactttgtagacctggcgcgcttcatgccgccgggctcgctggccgcgatggcggatgcgagtgaggcgttccggg

ctgtgggggcggccaaggatcagtggccaggcctcgtgatcctggcgccctctggatggttcattatcgacacgaggcttctgttcgggttggcctcg

gcgacaggggtgtggggctcggtggccgatgccaccctcgtcatcatccacgcccacttcgacacggaggttggcctacgggccgtgaaatgggtgga

cgatttcgtctttatgaagcccccgtcctctgacctgctgctcaacgacgtgctcgcggtgacggagcccctggggttcccatggcacccggagaaaa

gatccgagttcgaaaacgagcccccgtccaaccgttcctcgatgacgtacggggggctcaaccaagtgctcaatgagcatcgggcggtggacaagagg

aggagggcttcgtcgtcggtcgcgggggcgccaatatcgacgaggccaccagctccgactcctttcttttccccatcaatccgaggcgctactgacga

gctcccatcgcacgttgccatctgcgaggagcggttcatcacggcggccaatcggatctttcggtggaccccttcgccgggcccagcttcttctctct

caactacttccggtttcgatttcgacccttcgggcctcatgcggggctcggttaacgagatgctggagttctccctcgaggagtccacatgcacctcg

tacagtaccggcatcatcaagttctgttccttctgtgactcggccggcatccctattcaggatcggtgtccaccgtcgagtggacttctcctcgcgtt

cgtttcttccttcgtcgcaaaggtacgggccgacacggccaagaagtaccttggggccgtggctacttggcatcggctgtgggggcatgactgggtaa

tctttccccaggtcacctatgccctccgtggactcaagcacttccaacctccggccaacgacgtacggcctccgatccttgcggcgcatctacatgct

gtcaaagcacatctcaatatcgattccgatccattgcacgccgccgtgtgggcgtgttcccttttctcttggtgggcggtgtgtcgactcggagagac

aacgtctccttcaacaccggccttcgctccttcacacaacgcgacgcgggcttctattcaggcgcatcgacaagtcctaaacgacaaggagatcgtgc

gggtcaatcttccatcgacgaagacgaccaaggagaagggccagcagaagcacatcgtgaaggtcggtggggccctctgccctgtcgaggccttcgat

aatcatctccgcctcaaccctgtcccggtcaacgaactctcgacgactgctctcttcgcctttcgtgcttcttcaatgcccggctccacacttgtgct

cccgctttcgaagaccaagttcctcgccacgttcaacgcggctctcgtccggtccggccaggaggccttccagggacattccttccgcatcggcggtg

ccacgatgtactggcatacaggaacggacatcaagtcgatcaagctgattggcggatggacggggaactcggtcctcaaataccttcgggaatatgcg

cgcgggcttctaccggcgcacgagcgcgcagcggcggccattgctgaggccgccccgacgtga

>contig_14.contig_14.g268.t1

atgccgtccagcagcttgccttctccccagctcgctgctggcgaaacagtgctgcgatcaagcagaagacgtcctctcg

cggagattcaggcttcccagcctgacgacccgactcagcactcacctacgtccacggcttcgtccccgcctccaaagcgtcagcgacagagtgcacga

catgctggtcatcattcgaagtatggttctccatcactatctcactcctcctccagatctccctctaattctccctctggttctcctcctgaccctct

gcgtgctgccatcccctcccgcgcgactcgatcccgcaacaaatccacacaggctgctcatgttaacatgattcagcttctcagggacttccacgagg

atgcaggcttcattccagagcagtggcagctcgaagtcatctgctgtctagctgctgggtgggatgttgtgctctcagctggcactggaaggggcaag

agcctgatctgggagattgccgcgcttcttcaccctgacaagatcttcttggtgatcgttcccctgcaggccatcgagattgatcaggtctccaagta

caccaaaacgtccatctctgccatcgccctcagtcaaagcgtcatcaccggcaagcttcaagatcagacggactctgtggctcaggcctcaaccacaa

actctgtttcgtctgctcgaaacgaccataccgcgcgagccgcgcggaagaaacttcaaagagatctccgagacggggtgtatcagctgattttcgcc

tcccccgagatgctgctccagaatccaaacatcagtctccttctctccgagcccagcttcaaaaaccgagtcggaggagttttcgtggacgaggccca

cgtcgtcgaagactggggctaccgtgtttcccctggcgacaaacgcgcgttcagacccgagtatgcgaagattcggaccgtcagagcgcgtatcggaa

tggacgttccctgtgcagcagtttctgcaacgcttcctcacgcgactctcgagtccgtcatacaatcgcttgatctcggtcgaaggaatctgcttgca

atcgatgccggcacagaccgctcgaacatcacgtacgaggtgcatcctatccgcaatagcatcactcaaatgacggacctcttgcgcctctttcaacc

atcatatgctcgcccttccgacatcccgaaaacgatcatatacgttcgtacgcgcatggaggcgtatcgcgcctctgcccttctttacgacttccttg

gacgcccgcccggtgtcgttcgtcccttcacatcactctcctccgacgcttacaaaaaatccgtcatgcaggagggcttccgcacaggttcggtgaga

gttgtggtcgcgactgaagctggcggtatgggaatagacatttccgacattgctcgaattgttcaattttctttgcctccagatctgaaggggttggc

ccaacacttcggtagggctatgcgggatccaagtcgggcaggcagagccattcttctggcccctcaatgggcctttgatccacaggcatcggtcacgg

tgaagctggctctcaaggtccccaaggacaaggacgctgctgcgcgtcgagccaatatggacgccgggctcaggaagttcatcaaccactgcgactgc

cgtcgccgagtgctcctggacacgatgcgcttggaccctcaagtcatcgaggctcatctccctggtcgtcgcgtgcgcctgcacgatgtctgcgcatc

ctccgcttcttgcacagaccgttgcgttcgcatcttgcccaatgcagaatcagaatcaggaccagaatcccaaccaacgtcttcctcccatatccacc

cagcggcatcccgaactcgtccgcgttgggatacagtcataatgcggacggaaccaccagccgcacgcacagggagctcgatcttgtgctgcgatgtg

tgcgctgccagtagcgctggtagctcagaatccacgaaccctttccagatcggtgttcccccgccagtcccacagaacattctagccacagtcggaac

gtcaacgaaggccgcatacattccacggccacccccatcagctttcgagacggcgcgtcgcgagcttgcaaacagtctcaggtcgtggcggtctgcgc

ggttcaaagccaaacaactaccgccttttctggactcctcgtgcctgctgtccgaccaggcaatagtcaatctcgtagctcagtgtgtgcggttgatg

gtgcaaaacatgcacggcggctcgcttgaccctccctaccttcgatccctactccaaaccgagcacgtgctgatggacgaggccagtgtcatcgcatt

gcagagtcatcttcaagtttggctccacgctgccgtcaaccgccacggtcatctcatccaccaataccggctgacctcggtgggcgaactgccaagag

atcgagtaagcctctcctcaagtcagcgatag

>contig_14.contig_14.g269.t1

atgtcgacgtcatcttcaacccagcagaggcacgcggacaagattgatcatgtctttcaggggctcaaatcgcagaacc

tgagttttggacggttcgtccaaggtgtcttgaactccaagaacagtgagataaaggcagtcgttacaaaatggctccggctctccaacagcatcgaa

gagagcatcggcggcgtcgaggactcagcaaggaagttcggccctgaggacgttctggacgccctcgtcgccgaagtcaacaatagaagcgatagtgc

tggcgtcaagcgcctcaaccaagcactcatggcccacgcccgccctctcatggttcaggaggtggaggacctctgcgccaacgatggtcagtggctcc

gggtcccttccaccaacgttgtcgagtgctctcggttgcaccagtccttgtcgcctctagccgacttttatcgcaacaaacttccttctctttttcaa

ttcctctccatgctcatcggaaagacacaagataaacaggaaggggacgccgttcatcaagacgacgttgctcagcaagaggaggcgcctggtgcgct

gagtgaagaggagaggatcattttgccgtgtatctcgtcaatcctgtttgcccgaaatcgacttctcaaccgtttccaaatgctggttggcgtcctgc

tcgccgtgtcagacacgccggatatggtgaaagggttcctgcatcattgcgggatgaccgtatcacagccaacgatacgctcgacgttggagtctata

tcggatatggctgtcccaaacgcgaaggaacttttcgcagacaagaacaggatcaaggtcttcctattcgacaatatcaacatatacatgcggcgcgc

tgcactcaggcttacagacttcaacaccgctgccgcattgacgatgcgcacgatctacaccctttcctcctcctgtttgccgtccgactttactgagg

gtcacatccgtcagctgcgggacgctgatcgagcgctccttaccgtcgaagacatcgttccagacggagacttcttggagcaggccgggatcattcac

attgcgcagagtcttatgggatgtgctcaattccagccccgtcaacaaagcgccgttcttcgacgcttggaggaactcaggcggttgcatacgataga

cattctggaaccggaggtgaccacgtttgtgtctctgaaacttcttcgagaggatgaagggacattagaaggcattaagaccgtgtttacagacacat

tgcaggagctgggattggacgctacgatggagccactgctagtagcaggcgatttactcagcgtgcgcaatacgttggcggtacaagatgcgggaaag

ttcgaggacgaaggctctgtgcatcgactggacaaggtgtggccgattagtggaccgtggcacatgttacagagctggctatatctgatctttaagac

tcactttgcggcatcgaagagcgggaaagacgggtgcttggatcgtgtgcgggagatcctccggagagggcgaacggcactccgggaagacaagccgc

tgttcaacgaagcctgggagtttggacaggaggtgtgggaggggaggttgcaggccatgctagagtcagtatacattgttttgcagctgctagacctt

gaacacgagctcacgaaattcttcaccgcgcaggcgaccgagagggcgcgcgatgctggggacgaggtgcatgctgtggtatgctgcttcatgcgaga

ctgggtaattggggtggaatttgaagcagcaaccaaagcaggcgatattgggagactgagccctgagcgtgcggacaccagccgatccagtcatcatg

tgcttgaccggctggaagatcggttgtccgcggttgcagagcaggctcgtgtggtgaagcagtctattgcaaagggctggaacgttccgttgtttcac

cgcaacaaaacaacaaaagctgatcagatcgacatcgcacagatcaaaagggtctcggtggacacacaccttttcagcatcgttgcaggcaggaccat

aggcccttcctctcgcgccgaggaaggaggagcgacggccagaaaggaagcggcaaaggcagcagcgagaaaagacgccgaacacgaggtcggatcgt

tccaactggccgatccgctcatgcgaggctacataaagctgaggaagggagccctgggaaagtggcaggggaggaacagtgggatggagattcgtcag

ttgctgctcgagactctggccgaggaagacgaagctgactacgatgcactcacgagcggggctgacgatggacaagccgcggctgctggttcgcgcgt

ggttgagaggtatcgtcgagatcgaggtgcagtgcgcatggaggagctgtgggaggaatcgaatggtgtacacgacgaatga

>contig_14.contig_14.g270.t1

atggagaccaacaccaccgacgcgcaggacgcgccggtacccaacacttccgacaataccatcgctgcgacttccgcta

ccgtcaatgccctcctcaacggcatcgtcaacctcacccagactctcgcggatcgcgacaccagccgtgatcagcaacttaccgccctgcttcagttc

attcgggagtccaaggccgacaatgacgtccccaaacccgaccattatcaaggccacatgaaacagacgaccaccttccttttccagatcaatacggc

catcaattccaacccacagcgatacgatactgttcaaaagaagattcggttcatgagcagtcggttcaaagatactgccttgatgtggtacatgggcc

tgatccaacgaaacgctcaagcctacgtcgccctcaagaacgcagaacgcgccgccatgattcccgccctgccgccaatgatcgtccagagccacctc

gccttcgactttgaccagtatccaccagtcatcgaccctctcacgacttgggaaggctttacctcccacttcaagtcccattttggcgatcccaacgc

ttgcgcaacggcggagcatactctcgagactctccggcaggtcacttctgtcgccgagtacgcctcaaagtaccagactcactgctatgagctggacg

acaccgaacaacgccgagccaacggcttctacaaacacctcaagcccggagtaaaggacgagatcatgcgactcggaaagcccgacaatcttcccgct

atgatctcgatggccatagacatcgacactcgtgtcatggaacgcgtatcggagcgcaagctagaatcctcgtcggctcttgccgctcccgcccccca

gaccatgttgccgccttcatttgcatccatcagctatccaagctcccaccagcctcctgtcaacactaattctggcccagttccgatgcaagttgacg

caatccaggccagcacagctccttcggcttctcgtcctcgcggcccactcacaccctcggagcggcagcgccgtatggatctgaacctttgcctgtat

tgcggcatgccgaatcacagagcggggcagtgtccaaacagaccttccaacacacgccgacccccgtctcatttcgctcagactagcagtgcgaccgc

atacaccacctacgaccctcatcagtaccaccaagttgtcatgccccaggatgcgttccctgccaccacgactgcctactccggcaacccctcgcccg

cttccaacgatgccagcgagcagggaaaagccctgccgcagcagtga

>contig_14.contig_14.g271.t1

atgtctgagcctttcctttcaccctcctctgcctccctgtctcagatctcagcagcaaccgcaacagcatcggctacca

gcaccacgccctttgcccgtactatccccctcgtcgcgtttgcgcaccccccgcgtcgagacatgatcaccctcgccccgctgctcagcagctccgca

cacccgtctctcctggtcgacatcaaggaggcgcagcagtccgacgacgccgcccagactgcccttgtgtcggacgaccctcactacgcgcaacaaga

cgacctcatcatgtacgacgggcgcattatggacgttcggaaaattggaaatcgctttgattacttgctccgatggcatggcctcgattccagggctc

cggcaactcgccccgccgttccgcaagctcccgcaccccctcccacaacggtgccctcttccaccaccaacctcacaaccactcaccccaccaccacc

cctctcgctaccccttccccctccgccacgctctcgcgccacgctgttcctcgcgcttctccctccgcttttcttccgcccgcaggttcctccgcttc

tgctttccctccttcgcctcttcgcgctcctccggcttcttcctctccttctcttccttccttccctgcagcctcgccgttcctcgtctcggcccctg

cgctctcctcggtcccgattcctcctcccttgccggtgccggcctcggatccctcctcgctcgctgcgccgccatcttcggtgcctcctcctccagct

tcccctgttcctccgcctacagcgcccaccgcccattatcgtcttcgtcgcgtccctcgcctcaactaccgggagtag

>contig_14.contig_14.g272.t1

atggccggtccagaaccagagtgtgtagaatgtcggacagcagaagacgacgtcaattcaacgcgagctgaggcggcca

gcacccccgtcactcctgccgtggacgaagattcgaaacaagatcccaaagagaaggagaagaaggcggacacagaaactgctcccctgccatcagag

cctccagctctgccgacgcctgggatcatcatcgaatactgcgaccggtgccggtggcaaagtcgagcccaatggatccaaaccgaactcctgctgac

tttcccttgtccacgcccgcttcccgtcgacacgcctgcggaaggacaggcactcacgccaggtcttaggagtgtcatcatcctcccacggacggccc

acgacgaaggcggaatctttagggtttggttgcagactcgggaaggggacattttcgatcctgcacacattcctgaggatctcaagtcaagacggacc

gagacgacgtccacgttgctgtgggatcggaagacgcagggaagattccctgagcccaaggagctcaagcagaagattcgcgatatcatcgcgcctga

gcaggatcttgggcactctgacaggaagaaaacttcatcatga

>contig_14.contig_14.g273.t1

atgtcggtcgcccgcccaaaactcgctcctcagcacggtgccgacgttattatggcggaagagtcgtcggcgagatggt

ggaccgacgaggaggataggagagtccgccgcaaggtggacttcaacgttttgccccttatgttcatactctgtgggtaccgatctcctcgatctttg

ctgtggagcgaggcgctgcgaactgacaggtccctgcacctgatagacatgctcgccttcctggatcgctcgaacatcggaaacgcaaagtcgaccat

ggttcctgacctgggcatgtccgactcgcagttccagtggctactcaccatcttctacatcgcctacattctcttccagtggtgcatgatcttctacg

caatctttcccccgagcaaattcgtcgcgatctgcgtccttttctggggaatcgcaagtgctcttcaatccacggcccagagctggagtgccatgatg

gccgccagattcttcatgggaatcgccgaggcggcgtggggaaccggggtagccttcttcttcacgaccttatttccacgaagagaagtcggcttccg

ctttgcagtgttcgtctccggttctgcggtcgcctctgcgttctcgggcgctctcgcatacggtttgctacaggccgacacatccatcgcgccctggc

gtctgctcttcgccgtggagggaataccaacaatcctcatggcacctgtcacatggtatttcctgccggacgaccttgccaccgctcgcttcttgaca

ccgagggagcgaaggatcgccgtagcgcgtctcgtggaacaagaaccggagtccgctccagaattgcgggagaatcggcccaaagacgtcagcgaagg

aggggccatggagtcgacaaaccaacctctgagcgcacgggaacgagcgattcaggacatcaagtccaagtttgatcctaccgcgggcttggaggcat

tcaaagagccactggcatacgtcaccgccatcctctacttcttgctaaacacatcgtacagcagcctgcctgtgtacctgccttcgatcttacagggc

ctgggctacagttcgatcgatgcccaaggatattcggcgcctccctacgtgtgtgctttcgtggtcgcacttaccgcttcgtacatcagcgatcgaac

cggatatagagggacgccttgtgtgctcatggcgttgctcggaggtgtaggctacatcatcctcgcagtgtccaccagcaaccatgcacgatacgctg

cgtgtttcatcgttgcgatcgggctgttctccttcattcctttggtcctgtactggctcctcagcaaccagcacgggaagtccaaacgcggagtagcg

ctggccatcttggcaacgaccggacaagtcggcacggtattggggacgcgactgttccctcaaggcaaataccaaagaggattcttcgtgtgcggctc

gctgctcttcttcgccgcgctgctcatctgcgtctcgctgggctgtatgatcaggtcgaatgcgaagaaggagaggcgatgtccgatcaagggcggaa

tgtcggaagagcgcaagaaggagcgagaccatgacattgcgctcaagggagagaagagtgacttctgtgagtcttga

>contig_14.contig_14.g274.t1

atgtccagctcgcgctccaagcttccggtgccccacaacatcgtgagcacagctgcaaacgtcccgcacccgcagcgac

agcagcttgccagctaccttgtctccttcgcctcatcttcagaaggcggcagagagtataccggtcatatccatcccggcctgctgcctgctgcctcg

gcgacatcacccatccggctcggcgtggccaaggcaggctctggcgacccaacatacggacgagtcaagataccttacacagcacttgagcgacgatg

gatctacgagaacccccgtgtgaaagttaccgaacgacgcgtttcctcgggcctgatcaagaagggcactacgaactcggcgatgcgagcggaggccg

cacagaacgctggccaggcactcgatgcagatacgtggtacaacacgtgggagatacctgaggcaattcagaaggcagatctcaaatcggatgtggtc

atagtccatgggatgaacgactatggcggcaagcttggcccttttgcgatggcatacctcgaggcaggattccgcgtcattgtctacgactttacagg

ccacggtagatcagcaggtgtgcacggctacatccctgacatgcgtgccctccctcaagccttgcacgccgtcatgcgcgacgttttgaagcgcgacg

gagaagcagctcagggccgcaagatgttcctcacgggcacttccatgggcggcttcacgtgcctgtactacgcatccctatacccttcgcagccggaa

accgagaccgaagcgagccaaacagatggacaggcgaacggtacccagcatgtcgaaatcagaatggaagcggaccattaccctaacctggccggcgt

tgcagtcactgcgccgatgatcacaattgcccccgagactaagccctcgccgaccatccacaaaatagcaaaggtcctgcgtctcatcgcccctcgat

tgccgctcgtcgatgctgtcaagggaaacgtatcggacgacccgcgggtggacgaggaattcttcaaggacccgctgacctacaagggcaaggtccgt

ataggaaccggtcttgccatctgggatgggataaacgatctgcagcggaaggcgcatcttatcaaggtaccggtcgcactccaccacggcagcaagga

ccgcgccaccagcgcgcaaggaacgatcgacttcttcccctgtataggaagtgagaagaagacgctcaagatctgggaaggactcgaacatatcatgc

agaagtatgtggagggcatggacgataaggacacagctcaaacggacgcagtcattcatgagctgcgggactttttcctcgagatcgcgaagggttct

ggacgtggctcgcatgaacagagcacactccgcttcctctgcattccgtcgcggttcttgggctag

>contig_14.contig_14.g275.t1

atggatcccaaagctgcaggattgacgaaagccagaaagcgctggtggacacacgaagaagacgtacgagtccgccgga

agctcgacatccacgtcacgttttcttttctcgatcggaccaacatcggcaatgcgaagacgagcatggtcaagtccctgggcctgtccgaccatcag

tttgtctggcttcttaccattttcttcatcgcagatctccgcagcttcactacctttcagtttcttctaatctcgtacgcgatcttcccgccgcggaa

gtacgttgcgacttgcatcttcgtatggggcctctgcggcacgttgcaagcagtcggccagaactgggcgtcctacatggccttccgatttttcctcg

gtgtcgctgaggccggattttcttcaggcgccgccctcttcttttcgactctttaccctcgaaacgaagccggccttcgtttcacagtcttcatttca

gcgggtgctttcgcttccacttggtccggcgctcttgcttatggtgtccttcatctcaacggccgtctagaagggtggagagtattgttcctgatcga

agggctgctcaccatcgcgttctcgcccgtcgtctggttcttgctacccaacaacacggctacggcgtggttccttacggagcgggaacggcagattg

caggagcacgcctgctcaacgacgatctgcagcgcctggacacacctcaagaagtggaagatggtccagaggaaaaagaggaacagcgcgaggccagt

cccatcaagacgaacctcaattcgtatcgggacctgttcgactttaatgttgccgtccgggctttcaagcaccccttggccttcatatccgctgcgct

gttcttctgtgtcagtcttgcaggaagttccttccccatctatttgccgacgattctcgagacatttggccacacgacggtagactctcaacgctgga

cggtgcccccgtatctcgctgcgctggcggtcagcttgtccgcagcctacttttgcgatcgttacggcggtcgaggtctggtcagcggtacaatgatg

ctgctgggtgcgaatggctacctaatcctcgcgctggctgagaacaacaggttgaaatacttcgcatgcgtgatcattgccatcgcggttttcaccag

cgctccgctcatttacttctggatgagcaaccaaatgcatcactccaggagagggcttgcactcgtcatcttaggtaccatcggacaaggcggcccgt

tcgtcggcaccagactcttccccaaacgagaaggaccgagttacaggcgtggcttgctctcctgtgccggtgtgttattcctcggcttcaccatcgcg

atggcaacgacggcctggatgtacttcgataatcgcaagcgtcagaagcttcatccgatcagcaagcccaagacacaggaggaggctgacaagcaaga

gcgcgatatcggccggaacgcggcggagagcctttactcaaagttcggtggcaattctagggagtaccgaatggtgccacgacgtacaggcagaacag

gtgtccgcgacggctgcgccgtacctcaggctcagcaaatgtgctgtacgtag

>contig_14.contig_14.g276.t1

atgccggaccaacacgcattcccctatatcgccaaggcgacgctcaaataccgaagtccgcatgagcaggacttgactt

ttgcgaaaggcgagtcgattcgcgttctctcggcagcacccaaacgcgaagaggatgatgccgacgatgatgatgacgacgacgacgattggcttgtc

ggcgaaagcttggacggctcgcgcaaaggaacgttccccgctgggttcgttgcctatgaaagattggacgagcagggagatgccgcgaacgctggtcc

aacctcgactgcgacaacttcggaccagctgcagcagcagcccgaagcgggatcgcaacctggcgcggagggattaaagacagagagcattacagtcc

ccaaaacttctgtggcgactcaggacgaaagtggggaaagttctgccgctgtcgccaaggaagatgccaggcaccttgccgatgaggacgatgacctt

cccgttcctgtccacgcctcggagccggtccccgccccggaagcgatccctaccagcattccatcgatggaatttgctccccctcccccccttccaga

gccgctcagagcaaccataggacagctgagcctcccgacagacgacaaacccaagtcacaagtcgccgaaactgcagacgcaccaccaacgccgatcg

acatcgagccgacgaccgtagtggccacacctccggttgccattacaagtcaacctctgacgaccacaccgaaacccagcggtccgagctcgtctttt

cggaatcgtattgcagcattcaataagccggtagaaccgggcggagctgcccctccgccagtccctcgagggaagcccggcggttggaagcgaccggt

tctaccgactgagggctcggctcctgcgcccctcttgcctggcaaaccaccggctctggccaaccggcccgtcgtcaagccagcgccgccaccatcgc

aagacgccgacaaggcgagcgagccaaccgccggacacacagcagaaggagcacaggcgacaacaacagcggcagaaactgcaacgagcgaggagcgg

aaagggctgtcggcggccgatgcgaagagcagtattacaatgagtctcaaagaacgcatggctgctctccaacgcggtgctctctccccggagagcgc

aagtgacgatcccacttctgctcctactgcagggagtcagtctctcggagccagtgggggtgcagcacatcctcgtcctcctattccgggcaaattga

gcctggaaaagaggagcattgcaatgctcgacccgggccttggccccactccgccttctgcggcactgtctgcaatttcgcctaccactgagcctatc

agcgcggactcagcagccgattctgggaaggcagatgcggcaggtgcgaccgatcaagcgagtgtggcagggcaagccccggcaaaacagcaggaggg

tccagccgagcccgtaggtgccttggagaccacggcaggaggtgatgacggtgacgcggcggccgagcctgctgcctctacacccgaagaggaggagg

cgcagcgccgcgcggccattgctcaacgtatggcgcgccttggtggacgcaaagttggagctcccggaatgggcatgttcggtcagcccatgccagct

ggtggggtccctcctgtctctccttccgacagcaagccatcggcagcatcggcagataatgcggttaccgaggacgccggcctgatcgcttcagacgg

cgggaccgctgtcgaggcgggagcaaagacctcggaagggactgcgcaagatgaatcagccctcgccaaggcactgaataacgagcgttcagctgtcg

aggcgaaggctgacattcctgccaaagatgagactgccaagagcccgacctctgatcaagttgagggcgcacagaccctgctcgctgtcccacggaga

gcgggtccgccgcgtcgcaagaaggggccggcgcccacacctcccacggctgccgctgtggctgctcccagcgtggataacgtttcggaggaagaagc

accgccttccagaaagaccacggccgaaaatgacgaatcgcccacacctgctttgcctgaggaaagccaagttgatcgaccggtcgcaacgccacttg

aggaaaacctaactgagccagtcactcgagaagtcaaaggaacatcactggaggaacaggctcctgaatccgcagaaacggaactcggtggagaagtt

caacccgaagaagcagtcccaccggccatcaaagaggctcatcgtaccacggacatcggtgacgaggaacaggaagcgacctctcaggaccaggaaga

tgcgccttcagctacgacgccagtcgctgatcaacttgaggaagcggccgaagcacaggacgatcccgaaccagaatcagagtctttcggagatggac

atcgcgcctcgatcgtgccacctcgaccaatgtcgattcctccaccgcctccggtgcgcccttcatctgtgacggtcccagtcgcgtcccctcctccg

cttggaagtcgtcctccgctccctcccatgagtcccgcacctcctgtgcccgttgtgtacagtgccgacgtgtctaaagaagaccaggacatcggcaa

tgacgcaccctctgagcctgagaaggaccagcaagaatcaggtatcgccgctgcgccaccaagtggtctcgttcgaccccaagccaagctaggcacca

cggttcccctcagcgatgcggaagagaaagaagttgcggccatcgagagcatccaggagcctgagcaaccggttgccacttcgaccgaggctgaagcc

cacccagagacagcggggtcagaattgacagaggaggaagaagaggcagatcgtcgtgctcggatcgctcgtcgaatggctgcactgggtggtcagcg

catgggagccctgcctgggatgatgcccgcctttggcgcacctatgccgcggcgaacaacccccaagccaccggggcaagatgatcaggctgccagag

gcggaggcgaaatgggacgttcagagtctgaggccgccgagtctggccctgcgctgcgaatggttgcggaaccgcacgcaagtgagggcggtgctgga

gagcatgagcaagtctctcgcctagcgagtcctcctgctttgccaacaagcccgccgccacgacttcctcgagccgctagcaggtcgtccactcaaat

aagtacgggtgaaagtggctcaatcggacggccaccttctacacgtccggtgatccctgcaggcatgcctccgcctccgcccccagctgccgctgcac

ctccacttcctcctagcactccttcccgccttgatcgcgaggaacaaacaaacgaggacgaggacgaagcggaaacctatgaggctgaaaaggacgca

gaagtcccgcctcccatccgacgcgacagtgtacctgcgctgcccatgagtcctccaccgccgcctcctccggggaagacgcctcctccgccggcgcg

tagtcccttcgtggcgccgatgagtcctccacccccagccccctcctcgggtggaggtgcctcttttgcggcatcagcagcagcagcgccatcagctc

agttccctcctcgccaagaaggccgcatcgtcagcgccaggcagaatgctcgtgatctggatcttatgccagacagtcgatggtggcgccgcgggttg

aagaaccttccaccgacgctctctgatcgcgttgacacgatcacacgtgtaaatgagcaggtcatctcagaggcgaacggagaggtcaagcatatcgt

tgccattcatgtgctcttccaggactactcgcagactgtcgttgacctggactacgtagatggcgatgctgaggagcgctacaccaccttgagtcagc

gtcatctcttcccgccggtgccaccggacggctcaacgcttgtaaagtggtatcgagacattggagcgttcataggtgcccaagcggatcaactggtc

tccaccaaggagactcgggtcgtcggtgatggctcaggctatgccttgccagcttctttgatcggtgcatcgccctctcgtgcactcggaccagtggg

agccagctttggcgctatgattctccacaaagccggtccgacggtgctggaagcagggcccgatgacgtccgagcaggggacatcgtcgtcctctacg

gaaccgagttcaagggcaagaaggcggccttgagcagctaccacaacacctatgggtcggcacgcgaaccggcttttggcgtcgtggtggagaacgag

accaagaagaacaagctccggatcgcgattcagtctgcaggaagcgggggcaagcgcggcgcagccggtgtggaggaaatgtcgctgcgaatggatga

ccttcgggctggtgtgctcaaggccttccgcgtcgctcctgcgcaggggtggttggaggattgcccgagcagcatggaccccagcgacgacgggaatc

gcctctgctttcgcactccaatctccagctggtgcacggacgagcaggacaggaaggtcgctcgcaagatcctcatgatcccgataaagttttccagc

acgatccccagcgtcgccgccgccagaagcgcaagtccaaaactttgccagctgattgacgcacctgactga

>contig_14.contig_14.g277.t1

atgatcaacctcggcaagacagctgccttcctcaagaagacggcatgggacgtatggggcgagcccgcccttaccgctg

tcggcctctacgcacccgcagaccgcagctacgggcgatgcgatatccccttctctcccgccgaggagcgcatgcacgccatgccaaacgtcacagtc

aagttccgtcgggtcatgatggccaacgggaaggactggatgtggtaccaaatctgggaggacaaggaggccgtcctccgcaccggtcgcaaggccga

cttgctcttctgtcacggcaccggtgttcactccggcacttttgcttcacacgctcgacgatacctggactctggcttccgatgcatcgtccctgact

tgctctcacacggatactcatctggcctgcacgtctaccaacggtacatcaactcatactgcgagggtctccacctcgtccttcacgatgtagcacgg

gaggacgacttaatgaacaacaacggcgtctttgtcaccaaggcagagcgtcaccacaccttcatccttggcctctccttcggtggccttctcggctt

ctactacgctctcatgttccccgactcctaccgcgatgaggctgagggccaggacgagattcccatcgatggcatcattggtgtaggacccatgatcg

actacggacacaagattccatcgatcattcaagttttcggctggatggtcattgccttgggtggtctgcgtctcgagttgacagtaccccacaagaca

gtgttggacaaagatcctaaggtgtacaagacattggtaatggaggacccacggtcacaccagggtgccttccggggaggtcacatcttgtgcattcg

cgatgcgatgtgggacattcaacaccgtgctcgtgagatcaggcaccccatgtactgccagcagggtggccgtgaccgagtcgtatcggtagaccaca

ttttcgactggatccgagtcacaggagccgaagaccgcaaaatgacggtctaccccatctgccaacatgtcatctaccgcaaggcgaagacggagatg

gaggaccaagcagggcgcatctgtgtgcttgaggacaacttggcctggatgtgcgaacggtcaccagcctacgggcgtctgatgcgacaaagttccgt

gtcatcagaaatgacccttgtcaacgacctcggcacaccaaagagcaaccccacaacaccttccatttctctcactccttccactccgttcttcaact

cgttcggcgagaatccgatcatcggcatgccacacctccacttcggacccggcggcacgccagcaatcgaggtcaccattgagtcgctccagtcgcag

cttcctacaccagccaccgaggacgcgcctcgttccgctgccaacgaggagaccaccagctcgaagccactcactgactctgacaaggctgccagaac

ataccgcatgcactggaacaagggtctggaggagcgttaccggccgtacgacctcgacgtcaccgccgacaggtga

>contig_14.contig_14.g278.t1

atgcttccgaacggtgctttgcgaaggcctccagtctttgcccctcgtacagggtctgctgtactcggccgtgcggcag

cttccagtcaaccaatcggtggcgcccacatccgtacaccctcctctgccgcccgagccttcgtctcccacactcgcacgctgtctgcctcatcttcc

actcagcctgcgacccaccacagtcgccagggtaccgagaccgtcctccgccgccgcaccttgtccgtctccggctccggccctcttcctggcgatag

tggcgctcacgacgtttctgctgcgggttctcagtcccgcaatgtcgtcgctgctcgtgtctcggcatcaacgcctcagcagactggccgtgtcgcat

ccgcgtccgacttgcgcgctattgaggcgcgggttacacggctggaggagaacagtcagtccagctccggtggcggcgcgtcgcaagagtccgccttc

tcggcctccgagagtcagcgttctgaactcaccctactcggtcgcaatgccagcggtggcccatctgcccctccggaacctaaaggccatgaggcatc

caccattaagctgcacttccgtcgtcttgtgtaccttgggtatggcggagtcaagacaggtgcagagcttgtccgtcagccttatcccgatcgcggat

cctggcccaagcatcccccttcgaggatcgacgacgaagaagaagccgacgatgccggactcacaagtggtaaccaaattcgcctccacctcattcgt

ccctacagggatcccgtcaatcaaggacagctccgcaaaacgtttcggtttatgctctcgcatcggaaacagtgcggcgtcccgcccgaaatgtccct

cgaagaacttgtcgccagctgcgcatccactttcaagggttgggttcgagagtacacgcaatccctaacggcgtcaggtcaaaagaagaagcaagagt

cccagtctcgagctcgtctttcagcaagccgcaagcgtaaagcggggggtcgatccaaggccttgcgacttcatcgatactgttacttcgccgatggc

tccaaggtagccaagccgtccaagaaacaagggcccgtcgaggctgccaacgttcggcgtggcgcggttcgggcggatcttgagttcgccgtccaact

ttacgctatgagcgacgaggaagacgaacagacagcgcccgacgtccccgacgaagccggctggtccgatgatggtctcagcgatggtgagcgaacgg

gagccgcggcacgtcgtcgtatcggtgttccttggcgatcgaagagcctcgtgaagaagctcgaacaacttgagaagcagcgcgctcgcgtccggaag

cctgccaagcctatcctcccgccaactcgccaggccacggtccctctcaacttcaccttgccgtcaacaatccgccgttggatggttgccaagtcttg

ggccgaagccaacaaagacgcgtgcttggacgtgtcggacaatgctggtcctttccagggcaagtactccgttacgacaggtcccaaggaatggggac

ccgatccagattgggaactcaaagaggagactggtgccaatcgcaacgaccgtgacgatggcggcgaagacgaggacgaagatcttgaccttcttggg

aatggcgacgaggacgaggacgaagattttgaccttcccggcttcggcaacgatgctgaaaagggcgacgaggatgacgagcaggacaatgaggacga

tgaggaggacgacgaactccttccagacctttag

>contig_14.contig_14.g279.t1

atggagctcgttacggacaagaaccagaagctgtgcttctgcaacaaatgcggtccagaggggagatcggtcaatcgaa

tcacttatcagaaccacctctttgcgtttgacgaaggatactcttcgggaccggacgcagggtttggtgagcagggccacgacgaggccccagacggg

atggacggagcataccaaggttacatcgactcaggggccggattcgacggcggtgaattcgacggcggtcaactggcgcgcgcgactgcagacctgct

cccaacggaagaagacttgttaaccggtgccttcgagttggagatcgacagcgacgatagcgactcggacccgcagttcgcttcgatccgcgattcca

gctcgcagccggaatggctgcatctgtcggagctgctcctcgcatggctcaacgttgaccacagcgtccccgatcgtacttgcgagctgctcctcgtg

ttcatgcaggcggtcgcgtacaccatttcggcgggaactctgggatcgggcaagcgtaaaggactgagcgctcgaatggcgcgcgtacgcaagactct

gggtctcaacggattaggcatcacgtacgccttgtgtccggatcgatcgtgctggacaccgcacctactcaaggtcgccatcgagcaacgggtcttga

catgtcggaactgcggcaaggacctcttccaaataccacagcattcggttcaaagcctccgacagggtcctcaacccctcatgctgcggcaagtcaag

gtccggtttcgatatgagccgataacgacctggatccagcaactcctccccaggcccaacgttctcgcctactgtcgggagcacacacagcgacaaaa

aaccgcaggagtttacagagatctgtacgacgggtccaattggtcctcccgggacggagacgattccttcgacttggtgctccgtctatctctgatga

ttgactgggtgcagccgttctacaggctagggacggcttcgtatagctgctgcgtcgtctcgttgcgcgtggacgacctcccctttcgactccggaac

tcctcttcatacacacatgtctgctgcattctgccaggcccgcagaaaccgaccgcggaaggcctccacgcagcgctggacatcatggtggccgagct

ctgtcgtcttcaggaggaaggcttacatgtgcccgagctactcaatcatgatacgggccacccaatcaaggttcggatccgcctagagcgtttgctgg

cggatacggacgcacgcaccatcactgccgggttcccaagtcactcatccaaaggccagttctgcccctggtgcacgatcgactacgatgaatgggtc

ggtgcgatgctcgaaggcgaggaggcaccttctcgggatgcagatcgacatcggaggaaatcgctcagtgctttgccggaagcggtacccagacttcg

tccatcagagatcgaatcgcttcggcgcgatgaggccgcctgcctgtctgccctctacaagattccgggctggtccagcctacacaacgcgccagtgg

acgtgatgcacaatctcgatttgggcctgtgcaaacacttctgggtacaaactttggtggacggcaagctactcgaccgggccggcttacacgtctgc

aaggaagtactccaggcaacgacgtatccaatcggggtcactcgaatcagtcctgacctagggcacacgtcaggcggcactccgacagcagctgggtg

gtcggtgttgtcccagtatatcatgcctgttttgattgcttcggcttggacaggtcttctcctcacatccgcgcacaagacgtttcacactgcaaagg

ttcgcatggtcaagcagggcttagccgatggagtggctacggagcctacgccggcctcgacttcatatccacggcagcaagtccgaatccagaacgag

cccggaaaaagaaagcaaaaggataagggaaaagagaaggagaaggagcccgagttcatcaaatcggtatcgctcgtggtgatgctggaggccgctct

cgtcctcgcctcagcatgccgcattgcccacagccgagtcatagacgagccacagatggaggttctccagaccagcctcaagacgtttgtgaccatca

acgcgactgagataggcccgttgtgggttccgtacaatttccacatcgcactgcatattgcagatcaggttcgacgtcacggcccggcttggacgtac

tggagctatccacaggagcggtcatacggacttatgaagaggatcaaaaccaatcggcgtcgaggaggagagttggagcataccatgcaaacgcggag

tgaggacaagcatcgcatcatggcgctactggagtcgacgccgattacacctctgtcccgagtcttgagggaatcgatactaaccggtacagacacag

atcggctcggacaacacccacaccgctcgccagagggtaggtactgggtatacccaagatgtacccatacccacgccgacctctgccgctcgccagga

gacgacgacgaggaccacgaccgttggcaagacgatgaatctgggtctgcggcgggtagcgacaactccaccgacgataggcctgaccgcccgggaaa

cagcgtaaacctcaacaactcggatctccaacagatttgcgtcctcgccaacaagcaccgtagcccaggccaagccagatttgtcccatgcttcaact

acaacaactccgattccgaattcattgttgattccgctgccagctctactcctcatctaaagaccgccgagtacgtacttcgcaaacgaaccaagcaa

cacggggcaaggtctgacccgtcggcatgcatcatctcgacgccaagaggacaacggctagcccacttccaatccgttatcaaggttcggtacaacga

tcggcgctctcaggaacggacgaaacagtacgtagtcgtacacgtactggatcccctatcatggcctgaaggtcccgggctctcatctcccacttgga

accagctcggatacttggctgcctgtcccacgtctctgacccgtgcgattgtggatgtcgaagatgtgctgggtccagctatcgtgtttccaggcgga

cgaatactgggcagacacaacagtgtcattggaacactggtgaattcgataagaagaagctag

>contig_14.contig_14.g280.t1

atggacgaccacgctcctcccgtcgcggacccatcgccgcccgcatcgccgtccgaatcctctacactggcgaatccga

ctacgcctgtgtcgccgtcctcagccgcgttggaactttccgagcgcaattcggtatcacccgcctcggcggcgctcaacctgaacgaggagcccgct

gctcctggcccgcagccactcacatctaccttcgcatcgtcaatcatgcggcgacgtgaagcgccatcgcccgaccctccctcggcagccgagttcga

cgccatgatcgaacgggctttgcgccgccctcctcgtatcaagcacctccgccatcggctcactcccgccggctgggccgagaacgaagccttcaagg

aggcccggagcgaggccgagttacgccgctctaaccccaactataggcccggtgtgaacctcttgcctggaccccgaggttcagacgcacctcattgg

accgacttcgccgcgctccacgactacgagctttggaagcagcagcatcaagacacggaacacggtcattcggaaacgtcgtcctcagaggccgcttc

gagctctcgcgcaccctcctccacatcaaaagagaaagcgacgccatccaaaaagagggtttcctcctccgccccgacagcgaagccgtcttttgctt

ctgcccctggtcttccaactccgtcgcgccgagcactggcgccgttcaccggtgcttcgggctctgaggctgggccttcaacttcggcttcctccgtc

tcgcacccttcgaccctttatcataacggtcggcgcgtactcccgactcgtccgtacggcccagccagtcaggggatcggccgtgtgcttcgtccata

cgaggctatccctggccagacgcctcgccgatgccccatgcccatcctcgttgccggcggcaaccggatggagtttggcaaagaagcggactccaagt

ccgacgccgagccggacacctga

>contig_14.contig_14.g281.t1

atgaccgtcttgcctttgcctgcagcgttggaggtcgagcgctccatggatggcgctgcaagcgttgccttggcagcgc

tgacgagcgctgaggtgctcgactacgacagctacctggcggagtacgagaagtactgcaacgaccgacagccgcccatagagccctaccccatcacc

gcgtccaaagctgcgctcttcatcgatatgacaatgaagcgcgagcattatgtcagcggtcgaggagctggatcgagctctgaggcaaagaagacgat

tgcaggaactcagttggggtactcggccatcaagcagcgtatcaacgcgttggaatggtttcgaaaggtcaatgaggacgattggcgagagcatcccg

actttactgctacacggacaaagctccgtgacaatgcgaccatcaactcgctggagactacctccaagaacgacaaacacaacagagcgggcgcaggg

caagagcaaaaaggccctcgggtcttcctctgccgagggcgacctacctttgcgcccgacttcaagagaaagacccacggcaagtacggttacaggga

atggtggggttgggttgtcttccgtggacgacattctgcatcaagcaaagagatgagctacaagactcatgacggcgcggtgcgaacgatgtttcgcc

tcaacaatgttgatgtgggcaagaccacacacgcagggcgtaccgcgggagccatgcacgctcgcgagaacggggccagtgtttctggaacgaaggcg

ctcggagggtggtccgacggtggagcgttccggtcctgctacgatcgttcgttcccccttgatgcgatttgggcggtcgctggattcaatggacagga

cctggactcgtactatgttgccagggcccacacaaagcctcccgacgtcctcctctcccagctctttccgtggatcgaagacgagcgagagaagctcc

aggagcgacggacggccaacgaaaatgcgtccgactttgctctctctgccttcttggattgtctcgaatggttccgagaggtactgcttcaggatgcg

gcgattctcagccagcatcctgcttggtcgaccttcaagcccttcgcagcgtgtccgaccttctccagtccgcagtttcacgcgtttgcagcagctct

caacacagcagtcaaggcggaggactcagaggcagagaggcagttctcgcaacttccacagcagctcggagccggggtcaagctggcgctcgctgata

tcaagaccggctttgagcgcagggacatggcgttgaatcaaaagctcgacctttgtctgcagcttcttgtctttcaaaacctcccgagtgatgcacga

tatcatcagcagcctgccccccctctctccaccatcaccgcttctgctgctcgtcctctcctttcggtttccaaccagagtctctccctttcggccgg

tgtcggcacctccaactccaaggatgtgagcagcgcgtcagctgcacgtgcgccgtcttccttcagcagcggcaccctcagcagcagcgcagctcctt

tctccttcttcggctccgcagctctcttctcctcccttgccgtcttcgatttcggctccttcttcccccccggaaccgtcctgcgcaacgccgcaccc

gccatcaccggtccaacccctcccatttccccacccgggccgatccacccgaccatcttcgaccagtcctgcttcagccctcagtctgtcgctctcct

ggccgacttcataacggcctcggcctcctccatcgctgccatctccagcgcctctccaccctcagctccggctcctcagtcctctagcggcactgcca

tctcaggcgcacagcaggagaggattcaggagttgaagttgaaggaggaggagcgatggggaaagcagaaaatggatgcgaacgagccatggcaatac

gacgtcaacagggacacggtcttcccctcgtaccaatttcgtccaagtccttcggtgcgagacgtgtgggtggagtacagcataggcgtgaatgggaa

gttcggaacgcggtgcatggaggagatgtgggggacggtgtggagatctgcgccgggcatgaagcaggagtggagtcgtcggcgaaaggttatcgaat

tgatcgaggagatcactcggtctcgcccggcgtggaagcaagaacacgcgctcagattctttgaggcgtgctacggcggctacagcgcgagaagtctc

tccgactggctctcgaagacatctggcttcaaacgcaagcgcgccgacgtcgaccacgggcgacatcaacaagacgcgacgtcagctcgggatgtgat

catgggacggcttcgtacatggttgccctag

>contig_14.contig_14.g282.t1

atgaaggccaaggacacaattaccgaccccaggcccgagcaagtccaggaccttgctgtcgagcggcaagaagcagccg

aggaggatgaggaggaggacttcatgagcgacgctttgctcgcaaagatgctcgctgcaaccgctggattggacaagaatcattcgcgggagacatat

accgagaagcggagaagacaacagaacgagcgtgatcgcaatgcggcggcgaccaagcggaaacagtcgcaggtggagcgagaacgcgaggctcggcg

ggaagccatgggcaagaatctgatcgaacgggagatggcaaataccagaaggataggcgcggattcgacgcccgaccggtcccctgttgcgtctcgtc

tgcatggaccagatacagagctcgatagcgaagacgagggcggcgtaagggccaaaccaaggccacgtacagaccagcaagcgggatcgacctccaat

gccggcccatcgtcttcaggcccaagcgccgccctcaagatgatgttgctcatgggctatcaacacggaaacgccctcggggtcgacccgtccgacgg

gccatccacaacgtcgcttacagatccaactgctggcctctcgtcactttcaccgtcgtcgtctgcgggtgtgaaggaggatcgaacgcagcccctgg

ccgtcgatgatcgctggtcgaaggccagatatggactgggcaacgccgccgtccttcgtacgaaggttgacgactgccctcgagcggatccggacacg

agcgtgaacgcttcggctgcgtctcaggggcaaagctcaagtctgaccgacttccggagtcgtcgagcaaaggaggaggagacacgacgtgcggaaat

gctcctacgcaacgctaggagaacgtgcgaggatcttgatcgcaagatgggactggacttctccgtgttgtggctcgatcccaatctgctgaggcccg

acgcgcgtccgaccgaggaggacgtggagcttctcgatcgcgcctttcctccagtccataccgagaacgagcacgactcagccgaggagctccaagct

caatgccctggcgaggacgaggacgatcattaa

>contig_14.contig_14.g283.t1

atgccctcctggctccccggtttctcttcttcctccaccaccgcagattccggcgcggcagccagcggcggctcaggca

ctgcgttcggctcggcagccggcagcggcggcgccggtggttctcgatggctcaatctcgatacgaacgtgacgaagaacatgggcggggacttgacg

ggcgagaactcagcgtttactgggctgggctttgagatgacgaggcagcagcggctgatgggctttgtgggatgtttgatcggaggcttcgtagtgtc

gctgctcggcacactgctgctcatcacagggtcgctggcgtcgttcgccatcttgtacagcatcggtatcatcatctccctcgtcgggacgggtttct

tgatcgggttcgcaaagcagttgaagtcgatgttcgcgccggtgcgagtagtcgctacgctcatattcttaggagcgtttgcgatggtgtgggtttcc

gcattcgccctcgattcaacaatcctcgccgtcgtctttgtcgtcgtgctatacctggcatatatctggtattctttgtcgtacattccaggtgcacg

ggcgttcttcaagagcatcttcaacaaaatcttctga

>contig_14.contig_14.g284.t1

atgtcaatctcagcagctacttcggctctgtccgccatcgacctcaacggctccaagtccgccaaggctgactcaaccg

acttctccgagcccaacttcctcagcaaggccgcacccagtgctgtccagcaggagaaggttctcgaggccggcgccaaggctgcggccgttcaccct

ctcgataccattgagcgcaagtacgtcggcgacgtcgagctcgccgagcaagatgagcccctactccaggagagcaagcgtcgcttcgttctcttccc

tattcgctaccacgagatctggcaaatgtacaagaaggcagaggcatcgttctggactgctgaggagatcgacctttccaaggacatgcacgactggg

acaacaagttgaacgacaatgagcgtcactttatcagccacgtcctcgccttcttcgcagcatcagacggtattgtcaacgagaacctcgtcgagcgg

tttagttcggaggtgcaagcagcagaggctcgatgcttctacggcttccagatcatgatggagaacattcactcggagacatactctctgctcatcga

cacatacattcgcgaccctcagcaacgcgactacctctttgatgccatggagaccatccctgtcgtcaagcaaaaggccgagtgggccatgcggtgga

tcaacgacaccgaatcgacctttgctgagcgattgatcgccttcgccgccgtggagggcatcttcttctctgggtcctttgcctccatcttctggctc

aagaagcgcggtctcatgcccggcctcacgttctccaacgagctcatcagccgagacgagggtctgcacaccgactttgcgtgcctactcttcagtca

tctcaagaagcgcccacaccctgaggtcgtcttgcgcatcattggcgaagctgtcaccatcgagcaagccttcctcactgatgcgctgccatgttcat

tgatcggcatgaacgcccgtctgatgagccaatacatcgagtttgtcgccgaccgactgctggtcgccctcggcaacgagaagcactacaatgcgaca

aatcccttcgacttcatggagaacatctctctgcaaggaaagactaactttttcgagaagaaggtcgccgagtacgcaaaggctggtgttgcacgaaa

ggacgagggagacgacaagcagggcaccggtatccagacaaacacacattcgttcagcatggaggaggagttctga

>contig_14.contig_14.g285.t1

atgatcctcatcgcccctatcctggtcttgtttgcactggcagcgacggtcgtacaagcggacgctgcggaatatcgag

tcgctccttcgatcaagaactcgtccaaacgcgacgcccggaacagcacactcgccactcgccgtgtgcaccccgggccctttaaaggcgggcacggg

acctggtataacgtggaaaggaacgaagtcgactgtggcggattctacaagcacacagacttcgtcgttgctctgaaccaagttcagtacggcgacct

ggattctcgttcaccatactgcgacaagcagattcgggtcaccgccaacgggaagacaacaattgctaaagtcgttgacgcgtgccctacgagtgcca

atgcgtgtcattatggctcgttggacatgtcgattgggcttttcaaacactttgatgatcttaatacgggagtcatcgacatcgtgtggaagttgatc

gacgccgatggcaacgacgagggcgagtcacacgaacaacacgtcaatcacgacgaccacaaggtacaggtcctttctccttcatcaccttggtttcg

tcttcttgctaatcctcgtcccacactgttgtcactttcagtcaaaacaaggccatcacaaacaccaccaccaccaccaccacaactgacatggccaa

gctcagaacaaggctccagctcttccgttgcagtcccacagattgaagacgcccaagggagcagacagccgcgcagcagctcgcacgagacaatggtc

ctcacaacgggcaccgccgtggcccacatcacgcccgtcgtgctcaaggtgtgttcgcgtgaccgccggctacagtatgcgcctgagaccttcttctc

gcacttctacaagcgcgctcgtcgcggcggcaaggatcgcggtgtcacggctaccgacgagctgatatatgacgaggcgttccacatcgtacagaaat

tctgcgagctcgcaacgcatgacacggtcgaggccattcagaacttcgccaatacacagatcccaactccaccatgggcgctctcgactcccgtcatg

attccgctctcctcgtgtcaccaggctgccgaagcgctcgtcaagtactttgggccggaagaaatcaactcaattgtcggtggtgaaaagtggtggca

gattcgacctcaagacggcgtgcccgcggagtggatcgcgcagcgggatgattgggcgatgatcgagcagcagaagcacagacatcatcccaaagtga

actccgacggcgagcacgtcttcgaggacgacgatgaacaggatgtcaatgagaatgggtcgctggggacgcagaacagtcggaggaagcctaccaag

cagcagaaacggcatcagcgcgccaaagcggcggatgagcagccagatccgtccgaagatcttggccgcctaaaacgcgtcatgctgtatattcatgg

aggcgcctactattggggatccatcaacacccacagacaccagatcatccgtttcgctcgaaagttcggcgggcgtgccttggcggtcaactacagga

aagcgccggactatccatggccagcaccactgcaagattgtttggccgcgtacatgtatctcattcgtcccccgccaggagcttcgcatcctccgatc

gatcccaaaaagctcgtcattgcgggcgactccgcgggaggtgggctgtgcctcgcccttctcggtgtgctgcgggacaccggtatccccatgccagc

gggtgccgtcctgatcagtccatggtgtgacatgacccactcctttccttccattctccaaaacactgcaacagacatcgtgccgccgtacggcttca

tccacaagccttctccgctgtggcccgtccctgcaacagtatccaagcagcgaaatctggatgcggatgttcgtaatggacctgcccagaaggagaat

gacgggcccgagctcaaggctcctcaaggtgtcgactccggtcaatccacagacagttcgcccactgttcccgctgtagccgaaattgagcctggtca

atcgtcaaccgatactcaagcccagccacaaaatgccactctgaaggccccggtcattaagaaacagggtagtcgcctattccgcagtctgcgccggc

gcggtcggaaagacgacgaggaagaggaccatgatattcccattcctcccgggccggagtggcttcactctccaccgatcaaagtccgggtgaccgag

gcagcgatcaagctcgctacccaatcgaagaagaatgacccttccggcaaaattgacttcgcggaggaagtcgagctccgcgaggagggggagaatgg

gcaacgagttggcatctttgagatgaaaagacaaattcaacaatacgcggcgaataagcaggtctttcatccgtacgtctcatcgaaacagcgtcgat

cttccattgcatgttctgacaactttctgcccctcagtctgtgctccccagtaatgcaaggatcgctcggagggttccctcccctgtacatcctggct

ggtgatgcagaagttctgcgagacgaaatcgtctatcttgcgcaccgtgccgcgcatccgaaggaatacccgctgggagagcatctcatgactccccg

tgctcgggaagcggcagccaaatatgacggaattcctacgaaagtgcacttccagctgtacgatgaccaatgtcacgttctcaccctcttcgctttca

caactcaggctaaatacgcattccgagccattgctagtttcatcaagcatgttaccggtgccaagacatctgtacttgacatgccattcccacgagtc

ctcgatggcgacgtggtcaactcgccgcgcggaacgggggccgaggggagcagtgttttaaccctcgacagcaacaacccgccctcaggagttggaca

agaccccgattctgacattgcggaggaggatggagagaccttccatgaggcctcgcaagacgcagactctttggaatctttggaacaaggccagtcaa

gtgcaagcaacgacaccggcgacctcaattcagctggcaatctccagaagcaggagaagcctgcattgtccgtcatcagcgcggacaagaaggcggag

gagaatgtacaggaagcatcgaatggaagcacaggttcgacgaaggacttgcgtcagttcccgggtatgccgtataccgcttctgccgaggaaatgcg

gaaggacgaacagccgaagacaaccgtggacgctctgactccacggaagcaacggctcaaggaagcgagggagcgaaaacacacgctgcgcgcacgca

agcagaagctcatcaacacctctcaacgcaacgagtacgatgggcaagttccattgatgcgtcctgaatatcgcggattcatgattcgtgaacgggtc

gacattcggggccagatccgaggcatggaggacgaaggtcagatgcaagcgcttcagctgacacccggcgaaattggcgtcataaaagaaggtccttg

cttgcggtacatggccggccaaacgttgtgggatcgcaaattcaagcgcacggtgaagaaggtggagcgccagcggatggagaacgagcagaaggttg

ttgagatcttgcggaaggcgtacaagcagggactgctgagcagcagtgctttgactgagcaggatggctttaatggcaagttgtcggagtgggaaggg

tggttggcgaccaatggtcccacaacgatccttaaggagacaccccctcccagtgcaatcgtcgggcgccgagatactgcggagtccatccaacttct

gctgttatgtctgcgagcgcgcggcaaagcgttggacttgcagaacgttcacctgtccgagcgcacgtctcgcccgacgaagagccaccgaaggtctc

ggagtcatcaccggtcctcgtccaagggaaaacgttcgagctcgcatcagtcccgacattccggcagcgggccgtctggagtcccagcggtcgagggt

gaaggtgaaggcgggcaagacgatgagtcggacggtgccagctcggtcactgaagatgaggaatcacgaccgattcaggagggaggcaggacgcacgg

cttgtccgcatggaacactctcatgtcgacgatgtaccgcgctcggacgacgcccaaggaacgccatgaagtcaagctcaaagagcggaggtcgccag

cgccgtcgaggccggtcagcatggcttatgaggaccaaggggaggagaatggcggtggatcacgagttgcgaacagcgagaagactcgtcaagattga

>contig_14.contig_14.g286.t1

atgccccatcggacagctctctccctcgacaaagccgctgccaaggttgctcccaaagcacaccacctcgtcatctcca

accccgtcaactccaccgtgcccatcgtcgccgaggtcttcaagtctttacgaccccaagcgtctgttctgtgtcacgggtcaccacctttgacatca

cacgcgcctccaccttccacttcgacattgccaacgtcaagcccgccgaggtcaatgtgtccgtcgtcggtggccaccttcggcgtcaccattgtgct

tctcctctctcaagtcgcccagggttccatactccaatag

>contig_14.contig_14.g287.t1

atgccccatcggacagctctctccctcgacaaagccgctgccaaggttgctcccaaagcacaccacctcgtcatctcca

accccgtcaactccaccgtgcccatcgtcgccgaggtcttcaagtctttacgaccccaagcgtctgttctgtgtcacgggtcaccacctttgacatca

cacgcgcctccaccttccacttcgacattgccaacgtcaagcccgccgaggtcaatgtgtccgtcgtcggtggccaccttcggcgtcaccattgtgct

tctcctctctcaagtcgcccagggttccatactccaatag

>contig_14.contig_14.g288.t1

atgccccatcggacagctctctccctcgacaaagccgctgccaaggttgctcccaaagcacaccacctcgtcatctcca

accccgtcaactccaccgtgcccatcgtcgccgaggtcttcaagtctttacgaccccaagcgtctgttctgtgtcacgggtcaccacctttgacatca

cacgcgcctccaccttccacttcgacattgccaacgtcaagcccgccgaggtcaatgtgtccgtcgtcggtggccaccttcggcgtcaccattgtgct

tctcctctctcaagtcgcccagggttccatactccaatag

>contig_14.contig_14.g289.t1

atgccccatcggacagctctctccctcgacaaagccgctgccaaggttgctcccaaagcacaccacctcgtcatctcca

accccgtcaactccaccgtgcccatcgtcgccgaggtcttcaagtctttacgaccccaagcggtggtctgttctgtgtcacgggtcaccacctttgac

atcacacgcgcctccaccttccacttcgacattgccaacgtcaagcccgccgaggtcaatgtgtccgtcgtcgatgctcgtagctacgatcgcaagtc

gcagccgcatggcgaagcagacgaaggccgacagccgggtcactatggttccaaggccgacctgttattcaacaaggcgaaaaagttaggggaacact

ctggtaaggcttacagcttacatctcacggctcacacccatgaggacatgatccaggtgccagcactcgctgcaattccgtctccctcagtcctcagt

cctcacagtaaggatcttcccggagtaggccccggagtagcattccccggagctccgcggactccgggggacgctctgcacgggtatggctgcaactc

cccggagcacggatgctccatgtccagccatcagcaaccagccaggcctaagtctcgcaagcatattccagatgcggtcaaagatgttatggctcgcg

tcagttttcagcctggcgttcgtgtcgcggacattgtggcagcaggacaaggcagcagctcagcactgtatcgatcacgggctgagatcaaggtcgat

gataaggtcaaaactcgatccatgtcaagcggtcgaccatctccattccctcctatcgtgatggaatatgcgctcgccttattccactttcgacccgc

cttctatttggacgaagtggttgggcttatcaagcgcttccatggttatacggtaaacaagtcgacagtctgccgctggttgaaaaacaacaacgtga

cgcggaagcgtctgactcggccggcgcgagagcaggacggccatcgcgtgcttcgatggacgttaatgatgggccagtttgacgcctctcaactctgc

tttgcggacgaaacacatgcggaccgcagaacgggcgtgcggaagaacggatactctcctgaaggtaccagggcggtctcgtccacgttcttcgtacg

gggcaaacgtttcacccttcttccggccatgtctcaagacggaatcttcgctccatgggttgtggagggctccgtcaacggagagcgattcgagtggt

ggctagagcatcaatgccttcccttgatgaatccttacccgggcccccaatcggtgctggttatggacaacgccagcacgcataaaggagcccgagtt

cgtgagctcgtcgaagaagctggatgtattttgatctaccttcctccatatagcccagaactaaaccccatcgagctggccttctccgttgacaagcg

ttctctcatgcgaactgctgttatcgaagagtcggatctcatgcatgccgccaaaaactccatcacatctgagggcgccaaaaatatgttcgctcatt

gcgggttcatttga

>contig_14.contig_14.g290.t1

atgcctcctaaaagcgaagcgtcgacttcaaaggcattgaagggcaaaggggccatcacgggcaaggcatcaatccgaa

gcactgccaagacgatcataaagcggaaactacccggatctctcgacactgctacctccgaagctgcgaagactgagccgctcgctaagggcaagacc

gcccgtaccaaagcgatgaacaagatcaacatcaaagaagacaaagacaaagatgacgatgacgatgacggtgacgatgaagaagaacagcagatgag

cgtcgaggctcacgaaaatggtaaacaggtggacgcagatgaagacgtttgccttaccttcggaatcatcgtgatgagtgggccttacgaaaactccg

gcagagtcgtcaaggcaggacttcagcgcatcctgtcaattcaaaagcatttcacgtacaacacgttcaagaaacgaatcgaaaacatagtccaggaa

gatctttcgaaccctttcggcggcagcgtcacggtcaccgtcctcattccaggaaccaaaggagtctttgcgaacgagacgcaactcgatggcgagga

actgtacgctcggctccttcacgacttggaggcggccactctcgcaaacgatagggcgtccgcgaagctccgcctatacgcgtcctcctccgcctcga

aactcaaggtcgaaaatgtcaatgccgtcgttacggccacatcttcgaagacagtgtttgcacaggaagaagagctgctcgccagtgagccagagctc

attccaatcgatcgtgagctccgtgctacatgggcatgtggacgtcaagactgcaagcacaggaattcctcggtcttttgctgcgtgattgatccttc

caaccccagcggcaagcacatccctctacccttcaaacagaggctcgaatgggtcagagctattcgtaacaagagtaaggacgtgagcctcacctctc

caccaacctggttccctttcccaccgtccagcttggcacctacgcagcattcagacgacaaaaagcaagcctccggctcgaaagcaggacttcaaaaa

cgtcacagcagccccggcgtcatcgaagtcatttctctgatctcggacagcgaggatgaagttgccaagaaggagcttccggcggccagtcacaacaa

gaatgagaagaccaaggatcagcctcgtgaccatgttgatcaaaacaatgtggaatcgggtgcgggacaaggctcatcgaccaacctttacggctcat

ggatgacgctggacgactggggcgtgaagtacaatgtttcccaatcaattttgtccgttctcaaaggcgatgggtttccggacccgtgcgcagttgca

cggtgggtcgcaaaccccaacaccgacttgagcaaggaaggattctcgaagaagggcgatcggtactccctcgaggcggctttggatcgatggcgcag

agacgtttctgactcagcccatgtcggtacacctcggcagactcagcagagtgcggaacttgcctcaggtcatcacgatcatcaagccgcagcaatcc

ttacttccctctccagcgccacgacgggaggcggaccaagtactatttga

>contig_14.contig_14.g291.t1

atgacaggttccattactcctaaggccttgccctcatccttcgacctcctccctcgacccaaggcacgaccagccggcg

tcgaatcacagctgctcgagctcgagttcggcctgttccgccctatcaagggaggaggcgtcttcaagaacctccccctctcgggcggagccatcctg

ttcggacccccggggaccggcaagacgtctatcgtctccttcgtcgccgccgccgcccgcgtccccgtcgtcgtcctcagggccaactcagtccacag

caaattctatggcgaatcagagaagatcctcgccaacatcttcaaggaggcgagggataagtccccttcagccatcctgatcgacgagctggagtgca

tcttcccctctcgcgtcgcgtccacggccacggaagcgcacaaggccatgctctcgcaacttctcacggagctcgacgacgccaagaacgcgcgtgtg

gtcgtcatcggaaccactaactacctttcacagattgatgatgcccttctccgccgtctccctcagcacattgaagtgcctctacctaccgtaggggc

tcggctttcaatcctcaagcaccacctcactctcggcggccgcaaacaccaaatttccgacgaccaacttcaacttctcgcccaccaactgcaaggat

gcagcggcagcggaattgccaacgcggtcgcctgttccctccaacacctcgcccgtcgcgtttatgagaacgccgagcgggtcaaggacgccgagcga

gtcaaagaccggaagcgggtcaaggaccagaagaaggacaagctccagatggatcaaattccaaccaaacccgctcgaacccccaggcggcccctcct

caagcttccagaactacgatatcgggatttcattcttcctgtccacagccaaggcaggattttctcccatcgcaagaggggtcgaggtaaacgtaact

ccctccatgcttctgtcctccccagtgcctga

>contig_14.contig_14.g292.t1

atggtctggactcccctcgttcgcttcgatgccatcggacaagcggtggtatgcctccgccttggaccgagaactcagc

tgccagtccagattcttggctatcaggctgaaccaggggtcgaaagggtacttctctctccaaacgaggaggagcaggtggcagggtactcggagccg

gcatggctgagcgtccgctcgcgagaacctggcatcgtaactgttgtggtctgttccgtccaggagagcatctatggacgagacgggacggaagtgtc

gttcgtcggagagcgtcgtgatgaggaagtcggcgtctgcggacgcggaagagaagggtcaacgcagcgcaccactaccagctcgccggccggagact

tgctcaccaaggcgcccgcgtcgaccgccccaccatcatccttccgcgcgcacgcccacgtcaacagctccatccccaaccccatcgagactcgcggg

cccacgccgaccgctccaccaccgcatccctcgcacctgaccgcgcccacgccaaccgcacccctcgcggccaaccgcccccacaccaaccgcccgcc

aactgcactccttgcgcccgactgcgcgtacaccgaccgccacgccaaccgcacccctcgcgcccgaccgcgcgcgcgcgcacgccaaccgccccacc

ggcatccccgtgcgaccgcccgcgcccgcaccaaccgccgcaccgtcatctcatcatcgccaccgtcgtcccaccccaaccaccccattatcgcccca

ccatctccccctcgctcccgaccgcgctaa

>contig_14.contig_14.g293.t1

atggccaagtacatctgtcgggatacatcttgcccgtggaagatagacggtgacaagttctgctggcttccgtactgga

aaccaagcactcacctgcagcttggctttagctctctcaaggtctgggcggctgcactggacaagaacgagcccaatgtcaagcttcacattccgccc

aagatacacccattccttccgccttccagtgcgaccgctccagccaccaatgcatcctcttcatcagcgcgcggcgatgacgcgccggcgcgtgttaa

gcccgtcgtgcaatcggagttcatctatgacgaagacgacgattccgactttgaaatacttggcgcgatacgaggtacggaggttcttcgcaagacac

aggtcaagaccgaacccggtacttcgacttcagccgcttcagttgccgtcaagtctgaaccgcagcccaagtcagtcccagcctcagcacccttggca

gcaccagaagagaagccaaagaagcctttctaccacgacgatcccacgctgccgaagtatggaccaaagatcgaccttgacgagtttggacgcatgga

ggaaactatcaagacgctgaaggaccatttctatcgaattccgcacgcagtggttgcgatgaatcaagaggaggttgagcacgcgcagcttggtgtag

gagaacaacgagcgctgaggacggccgccagactgtggcgagacgatggagaccccagggttggaggcaatgcggtacaatcgaaccaggagcatcgt

gctctgcagcaactcgccgaggcagctgcggtcgttaaccaggaacggccggttcgcagtcggcgctttcgacacagacgactcacaacacgctcacc

gactgcctctcaagcctcgacgccggacaaacaaggaacgctatccgatcccaccggcctggcttttgagccagagtgttccccagactttttgaggc

catga

>contig_14.contig_14.g294.t1

atgtccagccccctcaaacgcaaggcatcgggcgagcctgaagcacagccaggcacggcaaaacccgaacaaccctcgc

tgaagatcaaccttcggctgccaaagcacgcaaggagaggcaatcaggctacaacggcaacagacaaggaaaaccagtccttgaacactttgattcga

gatcactgcgacgcgtccgtgtcggcaaagaagtcccccaccacgcctcgatgctacgagatcaagctcctcttcttccaatccggaaacccgaacgc

caaagtggtggcggaacgaaagctgcgccaggttacggccgaagaacacctcttcaaggtcttcgccaacgacactctctggacgttcatggagcgac

ttgaacgcaaggctaagccaatcattggtggccttcctatcaactggaagatctctgctcaagccgagagctcaaccgctgtatacttcaagaacctc

accctcatcgacattcatgcggacgattaa

>contig_14.contig_14.g295.t1

atggttcggcactggtcctcggggacccatcccaccgagcacagcagcagcaacagtaggacctcgtccccagcaaacg

tcgtcagatttgcctcatccgcgtcctcacagccgccgccgccgccgccgcagcagcagccatcttcgctcctgcactcatccccggccttgacctcc

agcatgagctccggctggggtccccctccgtctctgcagcgcatggcatcctctccagcatcatcggcgctctatccacagcatatcggcccctcccc

aacgtccatgccgccctcctcttccgcatcggctctcacaacacagtccggcggtccacatccgccgcccttccctcccatccgcgcactcaccggat

atgagggcgcagatgacgaccacgacgaggacggagaagaggatgatgaagaggatgacaatgaggatgaagacgaggatgaggagggagaggacgat

gctagtctgccagaggacagcatggcggcctccaagaagcgcaaacgaaaggatagcggcacgacagacgcgcagaatccagcagcatcatcggcagc

tggtccatctacttccacctcccactctcaaaacgctggccaagtatcctcatcctccacctccctcctggcggaagatgaacctcgcgagaagaagc

caaaagtgagccgcggctccaaagcatgcacaaactgtcgccgtctcaaaatgcgctgtgtgccttccacaaacgaaggcggacacccctgcaagcgt

tgtctcaactccggtggaagctgcgtctttgaagtttcccagcgcgggaaacgcacctccaagggcaaacgcacagaggctatggccgcctccctcaa

gaagatggaggaaactctcaactcggtccttcgtagcattcgcgatccagctggttctggcggggggaacgtcgacctcgccccgtccaactcgatgg

gttccgactttgcgaatgcaattggagctacgaacgacttgcctcgttcgcccttcgaccagtttgccccgtccacatcgcgcgttacctcggcacct

gtggcatcaggcaggttggccccgggcgcaagtcctgggggtcagcacatcaatccggcttccatcatcacagcagccgcggcggcagcagcaggagc

aggagcagcagtaggagtagcccctggcgcgcatagctcatcccgcagttctcatggcggctcccaagcagccggcctctcacccgaccaggaacgca

ccgcaggcggtgatagcggctcgggaaatgcttcagccacctatgaccggtcacggcccgcagacgcgtcctcaacctcgggctcctcgcagcaccac

gattcccatccccatcaccacaatcaccatcctcagcagcgcttgcctagtaagagctcgccacggctccatagtctgcccgacaactccctcaaccc

gcttggtctgcttgccgaggccagtctgcacaacaccgagagggcctcccggagggcggccgcggcgatacatcacagaaggggcacttctttcacaa

gctcgcgtgagggcttgttggatgacgatgccggagaggtgggaatcagtccagcggccatctctgaacgaagcgcgcctggtggggagagttctcag

gctggcgcgacggcgggggcggcgggagaggatggcacgcctcggggccgatcgggtacgggcggtgcggcagatagtggtgggctgggaaagaaagc

agatggcaaggcgcgggctatgagctcggcgggtgtgggcgggggtagtacagcaactgctacgtcagccgctgcggctcatgccgacaagggtgaca

acgccgtccacggagatgaagacacaaccaacctgccccgcttcgtccgaccggagggcgatctgtcccacgcgctcggcgtagcctccagctcgtac

ttcaaaccggggcccttgagcaacctgcccttacgccgggtcatcatcgagcgcgaggtcccgccccagctgctgacccagggcatcatctcctccga

agagattctggatctgttccagatctacttccactactgcgcgacgcatgtcctcttgcttgacccggacatgcatacgcctgcgttgatatgcgccc

ggtcgcccttcttattttcagcggtctgctgtgtggcttcgaggtattactcgcataagcggccggacttgtatgccaagtgtttggcggaggcgaag

cgctgtgcttttgacatcatgcaccgcggctacaagtcggtcgagatcgtgcagggcttcttactactttcgatgtggaatcagccttcggagcggta

cgagacagacaagacctggctcttcagcggcgtggcgatgcgcatggcgaccgacctgaacctgcaccggaaatcagtactaacgctgcccccggaca

tgtcacccaacgaccccatcgtgctcgagcgggagcgcgaaattttgaatcgggagcggacatggtacgtatgcttctcgatcgaccgcgcgctgtcg

gctcagatgggcaaaccatacacgatccgggaggacttcctcacgcggaattgcaaatactggtgcgagcagcgggtcagtcagccctgggacctggc

cctctcggccctggtggacctgctccgactgacgtcccgaatgctcgacatgttatactcctccacccggtcggtgaacgggctgaacatggagctcg

actacagcacgatgcttcggatatggaacgagcagctggatgagcttcgggatcagctctcgtacaagggcgtttttcctgcctcgttccggccgcat

tcggacccgttccgggagattaagacgcggattgcggcgcagcgggctcatatttcgcttgggtcgccgtctgttaggtcggcgggagggggacgtgc

tgggtcggcggcgcagggcgctggtggtgccgatgcagcgccggcagcagcaggagcagcccgggagaacggcactcggcgaccgtctgagaagggct

ctgcttctcgggacgaggagggtgatactgatgatgatgctggcggtgctggtgacaagcagcagcagcaaaagcaaccacagccaggcgcgagcgcg

agcgaagggatgcagcaccaccagcgcatactccacttctgtgtccaacaggccccgctccgctggcacaacgccgtcctgatcatcaactcgttcgg

cctccaacgggcgctcgatctgggctcggacgacaaaaactacttctttatcaagtgtctcaccgctgcgaaggggatgttggaggctgcgatgacgg

ggctgagacctgtgttgcgatatgcgccagatgcgcagttcgttatgatctcatatgcgtgtgtcttccttctcaagcttctgcgaaaggaattccgc

ggctggatcgacgaggaagaagtgctctccctcatcagcggcgtgatcgacctgctagacgaagtcgcggtgaacgagacccacacacccgcgctgaa

cgcgacgttcctccgtcggctcttgaacgcgcgggcggagacgaggccggggagtccgtatgcgagtgtgtatccaagtcgggctgggacgccgcctg

cgtctgctgctgctgctgctggtgctggaggtgcggcgggcggaggtcatcggcctgatgcggcgctcgccatgactggcttggacagcggtggttcg

aacgctcgcgctgggacggggacgaaggcgggcacgggaagggacgtgtctgcgggacctgcgtccgggctgaccgatggcgtagcacatgggacctt

ctcgaccaacaacggcggcgggaacgctggtcttgcgggaggtggaggagtagcctcaggcaccgccgactctgggcagacgttttcgaacctgagct

cgttgctgggcggaggagggcctgcactgactggcccagcggcgtcttccactggccccggcggcagcagcagcgggcaacagcagtatgcgcgtttc

gctgctggtttcagtcctaacatcgtctctgcgtcctcgttcggggacgagcacggacatggacatggacatggacagttgaacacgggcatgggcgg

catgaacatgctgagcggcgaagtgtcggcctcgttgaacgtgcccttctcatccgccgacggcggcgggcttggtgcggggctgttctcgtgggccg

gaggactcggaggcggcggtggtggtggtattggtggtggtgacgcgaatcagcaactgctcgggttggacaatgcgcctgctgctgcgtttggagtg

tccagtctggcccagcttttcccgagcaatatgggcttttcgggcacgggggcgccggcggatagcagtcatgcggctgcgatgcatgtgtcgtctgc

tcctagtggggccggctctgggtcggcgggggttagtggctcgatgtcgggcacacagggtggcgcgagcggtagtcaccacaacggtggtggtcttc

agcagcatcatcatcatcacggtgcggtgaacgcgggacttcataacggccaggcaacgacgatgacgacgaacaacaaccacagcctgacggagagc

gtgtttgatgataatttctggagcacgctcttaccgcccggctttgggacctcgagctcgttgagtgggtccgggtgggactttgctggaggaggagg

aggtagtactgggctgaatatgggattcgggatggggatgggtatgggcatgggtatgggtggctggccctcgggaccaggaggaggtggatcaggtg

gatctggcggactggggatgaacgtgccgtctcctttgaatggcggtggtgaggatggtgctcctgctggagcgggggctggttcggcttccaaggcc

ggcggatcgagggcgtcggctggcgcgggcgcgagtgggactgggaacgggaatgagagtgcgggtgggacggggcagcggcaagagactccgagcgc

ggtgggcgccttcaacttctaa

>contig_14.contig_14.g296.t1

atgcgctcatcgctcccacgtcccacttcccaaataattcccaacgctgctgaccatctcgccttgcccatctcgacga

cgagcagctcaaagtcgacagcgagcatcacagcgattcggactcagcgagggatgagttcaacaaccgcaacaagcgcaagcagcagcagcagcaac

aatggcaacagcagcagcaatggtcaacggccagctgccatcttcactccgctgctctcaggaaagctccctagccaggacgatatcgacatctgggt

acagcagcaccgcaccgtagtcactgcagggacagcctccctcgtgtccacaatcgcgtccttcccctttgactccgtcaagtcccgcctgcaggtca

aatactacccctccatctgggcatgcggacgtgctgtcctcgaggaggagggcctggctggcttcttcaggggagtcaccattccactcatcacaatc

acttttgtccgcacctcctccttcagcatctactacaacaccaaaagcgcgctccatacgcgtggttatgccgccgactcttcaaagctctcccatat

cgctctcagcggtctcgcaggcggtttcactagcggagtcattatcagctgtggatctgcacccttcgagctcgtcaaggtgcagagacagctcgaat

atcttatcgccactcagaagggagcagccaagaaaaatgctcagcaacgtgaatcagcagcgcgctcaaggacagctccagcgagcggtggggacgcg

tcgcgctcctactccacctcagcgagaacagcaacgcgtctcccaccacaactccctcttcacccaccggcatcgctcctggcatcctatcaatcaca

tccctcaagaacattcgcatcctcctcattattatcagcagcagcatcgtcatcatcgtcatcaacaacaacaacaacaacgcgctacgtaccccagt

caggcttccaggccgcgcgcgaaatcatgaccaatcacggcgggattcgtggattttacctcggtttccggttgcacttccttcgcgatacctctggc

accgccctctactttggtctatatgacaccctcagggaaatggtcaggaggcacaccggtgagaaggccgccgatgtaggcggctcgtcttcaagaag

gataggcaacgatagcagcgccaatacaggcccaccacctcggatcttcggcatgccaacccctcccatgcccgtcctccagttcctcacgggctcca

cggccggcatcctctcctggctggccgtgtactggatcgacttcctcaagaccaatgtccaacagcgccgattggcagaggccgattctcgcatcact

gcctggcaggtcctcaacgaaaacccagatcagagcgtgttgcagcggatcacaaggttgtaccgcggaattgggatcagtgcggtcaggagtttcat

atcacatggtctcacatggaccttgatcgagcagatctcggaacgcatcgacgcccgcgaacggttacgatccggggatggcagcgcttcctcctcct

cggcctcctcttccccgtctcatctcgcgaacgacacgtcctcgtccccggacgcacctccatcttcagcaacctcgcgctggagataa

>contig_14.contig_14.g297.t1

atggcctcgatcactcagttcagcaagcgcacaccggagtcggctcctggtccgagcgtagcacactcggaatcggaag

caggcacggcttcgggatcggccgatgagagcaagcttaccccgacttccccatctcataatgccaacagcaccactgccagtacaggctggctgtcc

tccatctcgacctacctcggatcctccatcgcgttggtggcctcgcccaacacctccctcgacgaaccagaggatgagatcatcctgcccaaggacga

agagctcgcagacatccaggaggcagacgaggaccaggagaacgaacccgcacccgcaccatcgacagcaccgcccaaaccgacccctgcttacgaaa

ccgatgaccgcggccgcaaactcctcgtcccaaagcctggcgcaaaaccaaccatagacggcctcgaggggaccgggccccgacaggccatcgtcgga

aacgcaggcaccgacgaccccatcgacccactcgccctcgctcaaggctacatcgatcccaagacattccccaacggatccagtctgaactccagcaa

cgaacccttgaacgtcatcatctcagccaactcctcgcccgaagtcctcactcgcaagggtctccagtcataccttcgctccattgacttcgacttcg

agtgtctcaacctacactccggagggcctcagtacgcaaccgtcgatgccacaggaagccaggcacaggtattcctctacagagagaccaagaccagt

ctcgatcacatcttcggaacatgcttggagagtctcgcaggtggtaatcacatccgaggatacctccaaagcggaaccaacgcgtggttcttggccgt

cagcacagaagagaacgtcactaagaagcacaagatcatccccaacggctacgacattggacgcgacgacatggttcaacgctcccagtccgaacccg

acggcatcacagacttcttcttccagaaatggaagacaaccgtcacctacaccaccgatctcctgccggccaaccacatctccaatcacgacattgcc

ttggatggcaagaccgccatcttgactgtcacgcgcttgcccggcttcagctttgggtcgaagacggccaacggttcgagcacgacgacgcccacgac

tgcagacgttgtcgctactcccgctccagttccggctccggtcgctgtccctgctcctgtctctacgaatggcgatgctgtctccgctcccgtccctg

cccctgtgcttatcaacggagatctccccgtcgcgcctgctcctgctcctgcgcctgaagctactcccgtggtagccgcgcccgcgtccgcgtctgcc

cctacggtttcggagaaggaggccccagtcgttgctactgccgcctcagctgtcatcaccaccgccgctgtcgttcccgcagccggccccgcgtccgc

ctccgcgtcagtcaccgctccaaccgaggtcgacaccgccgacgccgccgctgccacaacagcatcccagccctcctcccgcaaatcaaccgatcgca

cccgtcccatgaaacgcttcagtttgggcctgtgtcgccgactgagtcaacaggtcactaaggccgagacggacaaactgctaggagacgttgcggct

gccgaggcggagaaggaggaggcggctgctatcgctgctgccacgggcactaccgccggtgcggctgctgggcctgggctggcgcagagattgaggac

gagggtgaactcccttgctgctgatacgcctgcggagcgcggagtgctcccgcccgtccaagcgcccgttgtgcccgtgcctgcgatccttctccctc

ctggagacgaaccagctgcaggagcaacggagacggaggtggggcacgatgagcagcagcagcagcatgaagagcaggaggagggtcagaaggtgttg

ccgaagttgaagcggaaggagtcggcgttgaggaagttcttgcatctgcagccgcggaagccttcttcttcgtcttctcctgctgctgctcctgcagc

tgcggcggcggaatag

>contig_14.contig_14.g298.t1

atgattccagtgtccaagccagtggtacgcccagtgcggcgggttcccgttgaagatgctcctccgtcacaaccgagtc

cttcatcacccgctgcagctgagcaggccagtccacctcgtccttcccggccgcaaagattaggagcgacgacgcaggcgattgcctcccccgggtcg

aaaaagacgatctggcagtcatggctagctttgagacccagtgtgcgaatggcgatgggcatttctgtcgctgccttcgccgtcctcggcatggtcac

cgcggacaaactgcaagcgtcctacccagccgagaacaccgacaacccatcccagactcaaggctcgtccgcggatgacacaggcgcggacggccgac

ctcggaagtttggtatccgggttttggatcgcgtacccccagcaacaacagcatga

>contig_14.contig_14.g299.t1

atgtccgctcttcacgctccaggctcgctcggctctctccgcgcttcggagaggactgcagcgaccagcgcgcccgctt

tggaggaacgtctccccgatccaaagttgagctcacgtttttcgccgttcttccccaccccatccctccatcgatctgctccgcctccgaacgctcaa

tgtttcgttgactcggagtgcgaatctggcaagtacattccggcggaacagaatccgctctgcgtcggcaaggacggaagcgctcagttctactgtgg

cgcgcactgtggtcgattctcccttggtcaccaatgccgaaacaacggtgaatgctccaagggcctgtgtgtggatcagaagtgcgttcccggtaaga

caggcgacagctgcgtctcggacttccagtgtaccgcacccggtgagctttgtggctcggcaggcaagtgtatctcccccggaaacaacacacttttc

cctcaggagcactgcttgaaggatgctcagtgctacagcggcaagtgtgtcgacgacctgccccttaccaacaagtacgacgagcataaggaacttcc

gatcggctcacctcaggacccttcccgttgcgactatctcaaaccaggtcaagagggcaagtgccgttcctttgtggactgcaacggcggcatctgca

agaaccaggcatgcgtcctcggtaccgatggcgacgcctgtctttacaaccagtactgcaagaacgtctgcggtctcgatggcaaatgctactcgcca

ccctccaaccattcgcttggtgccggtcaaccctgcacgaacagcatacaatgcgcaacgtcagacggctgcaaagccggtagtgcgactcggccagt

tcccgggacttcgggcacgatgcgcaaggtcaccgaaaccatctgcaccggatccaaccccaacggtgcctgcgttcaggacagcgattgcgcagcag

gaggttgccgaaactctacctgcaaggctcttcccgtcggcacttcttgctccacggacacacagtgcacgagtgctactcccgacaagtacaccgca

agcgtctgtgatggtcacggctgtctcgaaaatcggaactgcgaggccgtcctggtcggcagctcgtgccgcacctcggaagactgcgacaccagcgc

tcgctgcacctctggcaagcagtgcaagctcaacaacggcgaagactgccgatcgaactcggactgtgtctcgggagcgtgcaaggatgatgtctgca

tgaagtcatctacttcgacgaagacgacggctaccagcagcaagagcacgactagcaccaccaccaagaagacgacctcgcctacgaccacgtcgaag

accactgcgacctcgaagcgtaccacgtccacgatggtgtaa

>contig_14.contig_14.g300.t1

atggcgcgcacaaagggcaaggccgccaggaaggcggacctcgatctgagaaaggacgctctcaagaagggaccggcaa

gcaacccgccaatcgacgctgccactggtccttctgcgcccggaccgtctgctgcctcgtcatccttcagtgggcccagaggacatgaaatgggccac

gatcctaacggcgagcatcaagggagcggcgaaggtagtgaggatcacagtcaagcagccaacacatctacttcgtctgcaccgggcccacgcgtctg

ctcggtgtgcggcacccaaacccgtccaagactttgcaagacgcctcgcggcaagatgctcttttgcaagacacacgatgtgcgctacactatggtcg

aaaaaaaggccatgagtcatttctcccattaccgaggttttggtggaaacctgactcctgtcgaccttgctaaggccgaaatcgaaggcaagctcacc

attcaataccaagaccaaactttcgccggtggctctccaacgacaaagatatacttcgttaaggagctctacgacctctcgcactccaagcaacgcaa

ggcaacagaagaccctcaggccgacagagcgcgtaaacaggaagcggcccgcaacaagcgtgcccagcgactggcggataggaacaagcgcaaggagg

agaggaaggctcagaaggctcagaaccaggaggagaggaaggctcagaaggcggctgcggctgccgcctcgcaacaggcccaacacgcacctcatcca

gagaactcggctgatctcaccttgccctcctcctcacccatcaacgaggccggcccaagctcttcggtcacggtgtcatcagcttacatcatggacgt

atccgatgacgttggcgacgtccacaatgccgcaactctccaactttctccttcgagcattgcttctgccgatcctcggcccgtccctcaagacttcc

gaatcaaagctgagcctggcttggagccgcctatgctctcttcgagcgccggcatgtcagcattgagcgggacagcggtacagtctaagcgggaagcc

gatcaatctctcttcctccctgacgacttctga

>contig_14.contig_14.g301.t1

atggacgtggatctgatccaagtggtgaaccggctgcaggagaccttcagcgccatcgggggcgactccgtcgatctgc

cccagtgcgtcgtcgtcggctcacagagcagcggcaagtccagtgtgctcgagaccatcgtcggcaaggacttcctcccgcgcggcgcaggcatcgtc

acgcgcagacccctcgtcctccagctcgtgcacactcccaacaccccctcgggtacccccaccgagtacgccgagttcctccacctcgacaagcgctt

tgccgacttcaatgagatcaggcgcgagatcgagaacgagacctaccgcgtcgcgggacagaacaagggcgtctccaagctgcccatccatctcaagg

tctacagcccgaacgtgctcaacctcaccctggtcgatctgcccggcctcaccaagatccccgtcggcgatcagccatccgacatcgagcgccagatc

cgcagtctcgtcacagactacatctccaagcccaactgcatcatcctcgcggtcagcccggccaatgtcgaccttgccaactcagactccctcaagct

cgcacgctcggtcgacccacagggcagacgcaccattggtgtcctcaccaagctcgacctcatggactcaggcacacacgccctcgacatcctcaccg

gtcgcgtctacccgctcaaactcggcttcatcggcgtcgtcaatcgctcacagcaggacatcaacggcaatgtccccatggacgtcgcaaggagggcc

gaggaggagttcttccgcaaccatctcgcttatcggaatatcgctcatcgatgcggcacaaagtatctcgcgaagacactcaaccaggtgagccatct

ccttttctacccaacaggtggcgaatttactagaaagatactgatcattctacaactcccgcaggttttgatgaaccacatccgagacaagctgccag

acatgaaggcccgtctcaacaccctcatgggccaaacccaacaagagctcgccgccctcggcgacaccaccttcctcggcgacgcccaccgcggcagt

ctcgtcctcaaactcatgacccaattcgcacgcgacttcgtctcctccatcgacggcacctccttcgacatctccaccaaggaactctgtggcggcgc

gcgcgtctattatatcttcaatgacgtctttgggaatgcgcttaccagtattgacccgacttccaacttgaccgcgcaggacattcggacggcgattc

ggaactccacggggcctaggccgagtttgttcgttcctgaggcggcgtttgagttgttgatcaagcctcagattaagttgttggagcccccgagtttg

cgctgtgtcgagcttgtgtatgaggagctcatgaagatatgccacaactgcaccagcatggaactccaaaggttcccccgtctacacgcccagctcat

cgaagtcgtctccgagcttctccgggagcgcctcggcccgacgtccgaatacgtccagagcctgatctccatccaggcagcctatatcaacacgaacc

acccgtcgttcgtgcaggactcggcgaatattgcgcgacagacgcgcgaggggcagaatcggaagcatcagttgttgttgcagcagcagagtcagcaa

tcggcacacgtcgaaccggcgctggacgaagacgttggttcggacgaggacgacgtccgaccgcacagcggccgacagtccgtctccaacggcggcac

cgtccgaagcggcagagactccgtccacggccgcgacgcgatcggtggcggtggtggtggtgggtccggcacaggaggagggggcagtaatcgggcac

gcgcctcgatgccgttcacctcggcttcgtcgtcctctgggggggcgtataactcccaacagcagcagcagcagcaaacaccatatcaacagcagcaa

cagcgcgtatcggctgggggaggaggggggtatggagatagtgccggagccggagccggggccgggcgggagaacttcatgacgtacttcttcggcgg

cgtcggcggtggacccatggccggtggtctgtccggtgggggcaacggtgggaatgcggggccgaggggcgcggcgatgggcacggggggttacattc

atgagtttggggtgcgggagatgcgggcgccgaatccgatggcgggcaggggtgggttggaggggagtagtgctgcttatgatatgaagagtttggat

aaacatcttgaggcgacaccaatctcgacagacgaatacgcgctctccgaacgcgaagagctcgaaacgtccctcatccgctccctgatcgcgtccta

cttcaacatcgtgcgccagtcgatccaagacctcgtgcccaaggcagtgatgcacctcctggtgaacttcagccgggagagcgtgcagaatcggctgg

tggcgggcctgtataaggagagtctgtttgatgagctgctgtttgaggatgaggggttgacgagtgagcggaaacgggtgaaggcgcttttggatgct

tatcgggaggcttttggtgtgctctcctcggtcaacctgaaggcgacctag

>contig_14.contig_14.g302.t1

atggaacagcagcgccgcatccaccagcacgcacacaagctcgcacgcccagcaaacaggagaataccatcagcacacc

acagcagggatctcgttgcacactctccagcatcgccccttaacccccgtctcatcttcggtccaatcgacaacggcgactcatcaacaggatcagga

ggcggcggaaagggcggcggcggcggcggcggatcaggctctaactctggcactggcacaggcggaaacagcccatcccacccaggaagcagttccgg

aaagccagaccctcactctggtgacgaccccacaactggctcaggcgacgactcaggcgacaactccggcacaggaaaggaccccgtcaccacatcaa

agtccactcccaagccaacacagacgtccacttccaccccaacacagacaccaacacccaccgcaacaaagaccactcccacagccaccactacaaag

cccacatccaccgcaaaggccaccactgcctcactcaccacttccacagcacagagcgggcctgcattcacattcgtcgtaccctcccaatccaccgc

atacgtgcaggtacagcccaccacatccacagcaaccctcgacaacccatccggcaccggtgagaatgcagacgcttccacaggttcatcccgcgcgg

gacccatcatcggcatcgtcgcaggtgtcctcgtcggcatcgcactcatctccgtcatcgcaagctttgtcttccgtaaagtcaaggctgccaacgaa

gacccctacgaatcagaccctttcgacggatcggagtaccagcgtcacaacgaaacccttcctgccgataacttctcggaggatgaccatcgtgctgc

tgccgctggtatgggcggcatgcagtcccctgacatgaccgagcgaacacttgccaacgtcggaggtcagggtggcatcggaagcccctacctcgacc

agggtcccgccatgatggggatgggaatgggcattggcgccgctgcaggtgcctacgacaatgcatccatcacctctgcctcccgcaaccagagcggc

ggtccccgtccacccaccatgttcgcccgtcacctcgaggcccactccggctcagcagcagcagctgccccaccccaggtcggcggcgtctatgctcc

cggagagtacacccacgccgtcgtgcctgccatgccacccaccgctgccacttacaacggggccggctcggacccgagcatgtcccccttcatgggtc

cgggagtcgtcatgggccacgccaacgtctccagcccgtacgcccatctcgaccgggcccgctcgacctcgtcccagtacgtcgacctggaccgttcg

gcctcgggctcgtcggccggaggaggtgctcccccgcctgggggcgagtacaaccgtcggtctcgacagggtgtgcttgacagcatggctgaggaggg

cagcgggcaggagcagtattcgcagcagcaggctgcgtatgctcagcagcagcagcagcagcaggcgtattataatgccgggcaggcccagcagcatg

atccttaccagcagcagcagtatcagcagcagcagtaccagggccagcaccaacagcaccctgctcagcgggctccttcgcccgatgatgacccctac

ggcggcgtgtag

>contig_14.contig_14.g303.t1

atgtgcacgacccaggctgaggcagcccttcctggttcgctctacctgctgcaccatccccccgtccagtcctcctaca

ccgacttcaagggcacctacgagaccatcgaaggtctgaacacctacgtgaccggggacgcgtcttcgaagaaggctatcgtggctacgcaggatatc

ttcgggcaggcgccgcagactaagcaggcggctgatattgttgctgctgctacgggggcgagggtttatttgcctgattacttccacggcaagcccgt

ccctcagaacgcctaccccccggacaacaaagagaaggaagagctcatccagaacttcttcagcgggatcgcctccccgccgaagaacaaggagatcc

tgatcaacttcggcaagacgctcaagagccagggcgccacttccgtcggcgcgtacggctactgctggggcggcaaagtcaccgtcctcgccgccggc

ccggacacacctttcacagccgtgtcccaaatccaccccgccatgctctccgcggacgacgcctccgccctaaccgtgcccatcgcatcctacccctc

gggggacgaagcgcaggacgacatcgacgccttccagaaagccgtggagggcaactcgaacaagggcgtcaaggaagggtcggtgtttcaccattata

aggagatgttccatgggtgggcggcggcgcgggcggatttggagaagagcgagaacagggaggcttttgatgcggtttacaaggagctggctgccttc

ttcaacaagcatctgtga

>contig_14.contig_14.g304.t1

atgtcacagcacaatctcgacgacgatctgctcgccctcctggaaaacggatcggaccaggacgacgctccttcttccg

ggcgggcgcgagccaaggcagctggtggcggcggcggcggtggtagtggaggtgcgggcggtggtgggggaggctccggatcgggtgcgaatgggagc

ggtgcgggagggaatggtgcatccgctcgcaacggctcatcgggtgcaggcggcggcgcggggatgggttccgggaacgacagcgctcgcggcgccat

ggaggtcgacgaagaggacgaagacgacgaaggtgctggggttcagcagcaacagcgtcggtcttccagcagttcgaagaagcggtctagttcgagct

tgagcaagcggagtggagggggtgggagtagcagcaacaagaagaagcgtcgtcgtcttcactctttcgcggctgaggaagatgaggaggatgacgat

gatgacgatgacgagggggatgggaacccgtatcgactcgacggtctcttccgcgatgaggacgaccgggacagactcatgtccatgaacgaagtcca

gcgcgaagccgagctcgaaagacgtcgagaagagacgaagcgttacaccgatcggctcgcgctcaaacagatgctcgagaagcaacaaggaggagccg

gcggcgcgtcgtccagccgtcggtcaaccagtgcccccaaatcatccagccgcgccaaagcccgccgcagtcgccgcgacgcagacagcgacgacgag

gatgcggagggatcggacgaagacatggccgtagagcaggacgaggatgacgaagacgacgaagaagatcatcggcgatcctcgcgccgcggggtgac

cagcagcagtattgcgactgctgccaagtcgcgcaagcggtctgctgtaggagccagtgcgaagaaggacggcgctctgaacgagctcaagcagcgtc

gggaagcccgcaagaagggtcttcgggaggaagtggacgaggaagacttccagacggcgaccaaggtcgtcgatagctccgacggcgagtcggatgag

tcggacggataccaagattccgatgcgggtcgggccagggctcgattctacccacgctccggcggcaaggacgcgggccattcgggcaaaggcgaaaa

gggcgagaagaagaaggacagaaagccctggtcggggagtgacgatccgccgtcgatgcgcgatctgactctgtgccagctgacgcgggatgacatcg

agaagatgctgcacaagccggactggctcgaacaccttgtcgggaactacatccgattccagtgggagggcgagcgtgaccagaagggcaacaaggtc

ttcccggtgcggatccacgagatcttagaggccaaagcagattcttcgtcctattacgagcttgcgccggggaagccttgtaacctcaggatgaagat

tgagatgggtcccaagccggcatgggtgcaggcgatcgcgatcagcaacaagtctttcacccaggcggagtatgagcgttgggttcttcgtctgcggg

gcaacaagctcaagttcccctcccggtccgagctggacgaccaacagttgaagatggacaagttccactcgcgaatgctcagcgaagatgcgctcagt

aagatggtggcggtgaagaagagtgccaaggacgcgttcaagaagaaacaagacgaggagaaggctgcggcggctgagaatggacctgcaccagggtc

ggcccctcccggtcccccaccccctcgtccacccggactgctggactcggcggtcggatcgggagggatggctggtactgctcctgctgctgttggcg

ctggagcgaaggtgtacaaccagacgacgatggcagagttgaatgagcgtaatcgtcggatggaccgggagcggcgtcaaggtgtgcagcgacgggag

acggcgaagatacaggaggcatggggggtgaagagcagcagcagcagcagtggtaatcaggcgaatcctgctttgggagcggcgggggcgggggcggc

gattcgggcatcgggtgcacctctcgtgggcagcgggacgcctgcgtccttgaaggcttctgggaacgtgtctggtgctgggacgccgttgccttcga

cgcaagggagcgccgatgggtctgcggcggcgtcgtcctcgctgacaaagggcaagcaaggcgcgtcgagtgtggtgctcagtctggacattgacctg

ggcgacttttga

>contig_14.contig_14.g305.t1

atgtcaccctcctccagcaggacaacatcgcgcagcgcttctgacgcgagctcacaaccagcagcttccggtgcagcag

cagcagcagcgcattcaagtcccgcgcctgaggatggagctgcgctcgaaaagcgcctgcgtgacgacctcgcccggtatgcctcccgcccgcacgaa

gacccagatctcgaacggacagacggcgcgccccccgatcgcgctctcgaaaaggaagaagccaggctcgaatcggccggggacgtcatctgggtcga

tttcgaagaaggcgacacggacgatcccttccaatggagcacgcggaaaaagtggttcaacgcgtgcatgacgtgcatcttcactgtcgaggtcgcct

cggctgcgtcgacctatgtcagcgggatcgcgcagatggagcgcgatttgggtgtggagaaacacgtcgtttcgatattgggtatatccctgtatgcg

ctcggattcagcttgccgccgcttgtgcttgcgccgttctcggaggcgctggggcggaagacgatctatgtcataacacactttctttgggggatgct

cttccttggtgtcgggtttgcacagaacattgagaccgttttgatactacgattccttcaaggcgcgttcgggtcaactggaagcacgatggtcggcg

ggaccatttctgatctcttcagaacggcggagaggggcatccccatggcgctctttgctgttgcgggtatcggcgcaaccggacttggacccgcctgg

gccggctggacagctcaatacttgagttggagatgggtacaatggatcagcgcggcagcttcgaaagtagaggaagtgtcctccttgcgagcaaaact

tcgcaagcagactggcgatccgagatatcgcgcgcgagcagaagaagaacgggcgtccatggctgtcctcatcaagacctctctcacaagacctctct

ggcttcttgtcagtgaaccagtcgtggctgctttcagtatctggatagcgttcgcgtgggggatcatgtatgcgctgctcgagtccattggtctcgtc

accgccctgcatgacttcagtctcggtcaaacaggactcgtcttcctcaccctgactgttgcctcggtcctgggcttcttgaccaacttttaccaaga

ggcgctgtatcgacgacacttcccagaaaagggcccggaagcgcgcttgtatgccgcctgcgcgggagcagttctgttccctgtgggctgtctcatct

acgcctggacttcgtacgcccacgtctccatggctgggccaatcgtcggcatcatcgtgttgatgctcggtgtctaccatatatatctcagcgtcttt

tcatacctagccgactgctatctcatctacgccagttctgccctcgccgcacaatcgtttgcaaggaatgtcttcgcgttcgctttcccacttttcgt

cacgcagatgtaccacaatctggggtatcagtgggcctcaacgctcgttgccctgttgggtgccgtgctcggggtggtccccttcgttctgttccggt

ggggccaccggattcgcgcacgatctcggttctcccaatccctacagaaagaggaggacgcgcggcgagagaaagcagctgcgctcgagaagaagggc

gacgtgtag

>contig_14.contig_14.g306.t1

atgccgccctcgcgatttgctacctcgccatggcggaacttcctcgcgaccagtgttcctgacgagctccgcaaacagt

ccggcatcggcgactctgctctccttcaggcgtccggacgagcgttgtacggcctcgactcggcggggaccacgctcacacgggtcgcgctcggcggc

ggaggcggtggtgttggtagcgatgcctcgttctcgcacaaggtcggcgcgtccggtggggctggccaaatcaccgcattggcactcagtcccttcga

ggtcgacggggccggcaatgatctggtcgcggtcggcactcagcaaggcgcggtcaaagtcgtcctcgccggagaggacggagggagtatcgtcgcag

aatggaagtccgatcccgctctgggcacagcgtccatcagcgccgtcgatttccacccacatgcaagcgacctgctcctcgtcggtcatgccaaagga

gcgtctctggcccgcatacaggccaacgaggctcacaccgcactcagcatcgcccttgtcgacaaggtctccttgtacgcggcgagctggagtgtcga

tggcatcaccatcggcacgacgtcctccgatcaacgactctccctctgggatcctcgcgcgtccgtgtcccagccatcagcatcggccgacagcattc

acggcgccaagctccgcccaaccaagctcgcctttacccaaaatcacatcctcacctctggcttcgacctcgggaggcaacgcgaatgggccgtctgg

gacccgcgcgctttgtccagaccggtccaccgagcctccatcccgtcatcttcaaccgccgttcttgcccccattgtcgacgttgatcgctctctcat

ctatctacacggcaaaggcgacacatcgatgcacgtcgtcgatttgtccctggccccatacactgccaaagctacgccgctaccttacccagcatctg

caatcgcactcgcgcccctcttgcggccatcagaaagtgcaatgaagggcgagatcggcaggatcttcctaacgacacccgcaaatgatgccgtgatc

ccagtgtccgtcactattcccagaaggcagtatctcgacttccacgaggacctttacccgcatacattcgcccctttcccagctcacaccgccgacag

ctggctggaaggtaataacctcgagcgagtgcggatctcccaggatcctaccagacggcagagtcagctggatagcgctcggcagaaggcgggatcat

cggttccaaaagtttcgtctgtccctgcagcgccggtaccgaaaatcgaagcggagcagcctaccgcggctcgagccgtggctcccgaatcggctgtt

tttgtgactgcgccctccgaactgtcgaagcagtcgccaaacaaggccgaaccattttcttccggtacaagcacagcttcttctcttcctgctgcatc

agcaccctctccgcaactgtcagagtccaagccgactacacctccggccgtccggcaaagcaacaaccagccgatcccgaatagcgaagccaaagtta

cttcaacaaattcaccggtatcatcaactcccgtacaaccgtctgttggatcacgcccctcccactcttcaggaaatgctggacgtccagtcggcgtt

gcagcgacttcagtaccgcctcagcccgcagccgattggagtcgtcgattcctcgcaggatccaccccgctcatccccgagttcaacaacgtcggcgg

gatcgccagcacgatctctgcagaatcgcgcctgctcggactaaccgtcatcgcctccccctcgggcaccaaccctaagccgggctacctcgtcttcc

cactggccggaccgggcggacgcattggtgttcacccgctctcacagcccggtcggttcccgctcctcgctgacctacccgccctcgacggcgggtca

ggcacagctgtggtcgactttgtctgctcgcccttcctgacggccgtaagccctacgacgggcggcggggaggtgtacgtcctcaatgcggattcgaa

agttcggaagtgggccctgccgctgcctcccccgcctcttcccgctgactgcctgccgctcggtcccggagcgaagcgtcagccgacaaaggacgagc

tggagcagagcgctgttcacgttccgcagcgacctgagttggtcctggacgcgacggcagcagccaaaggcggacgcgtcattcaagtcgaaccgcat

ccgcatgtcaagggtctcgtcgctgctctcgtcgctggaccacccgctcgtgtactcgtatggcgcgaggatgcggctcgggaggctgaggagatcga

gccggagctgagcgtcgatgtcggaggtgccacttctggagcgcttggatttgcgtggagtcccagtggcagcttgatcgctgttgctggcaaggaca

agaagttgcgtgttggcgatgtgcgcgcgatcaaagcgggtcagacaacttgggcggaagtagcagcgcatgatagtccacgagcattcaagctgatc

tggttgtcggaaaggcacatcgctaccaccggtcacggcctcggatcgatccgccaactcaagctctacgaggtagaacgctcctccgcttcctcgga

gcgcactctcacgctcaaggacatcgaaacgctctctctcgactccagtcctgcggtcttgtggccctggttcgatcccgatacaggcatcctccttc

tgtacgcaaggggtgagcgagcggtgaatgcgttcgaagtgcggcttcccggtgcgactccttcaaccgcggcgcccgcggcaagcggccctcgaaag

tacggctccgcggcggctgcttcgggcggtaataagagtgccttcttcgccctgcctgccttcggacatgccaaccctcaacaggcgctcgccttcct

caccaagcgacagctcgacgtcgtcaacgtcgaggtcgcagcggcgtacttgctctcggcgaagggcgagatccagcgcgttccgtggagcatcacgc

gagcccgcccagatttcttccaggacgatatcttcgtcgagacggaagttgtggaagagccaagctcggcagacccggctgcctggctcgacgctccc

acatcacacgaagcggagcagatgtctcgaaggaaggtatcgctctgtccagaaggaatgcagccactctcgctcgcgcctgctgtgcagcacacgag

caagctagccaaaggtccgacggcgaaggagctcacggataaggagaaggaggaggtattcttgcaggccgcgttcaagaaggtcaaagatgaggatg

tgcgaggagctgatgttggtacggaggcggcgaagcgtacacccgccaatgatgattggtcggacgatgactag

>contig_14.contig_14.g307.t1

atgcggagtctgttcacgctggcgcagcgcatcagtgcgccgcttgatggtgccagtatcgagcaactcgcggtcgatc

ccgagaacggaacggtgtttacagccgcagcctcgcccgctgaagacggcctgacaaccatcagcatacatgcacaattcgcacaagatacggaccca

gacgacgccccgctctccagaatcgcacagatcgagactcccgctgcgctacgatctcagccagtgctgtatcctcgaacaaaatcgtctaccacgac

cgacgaagcgcggcgtcaggtcgtctccctccactacctccccgacggcggcgagctctcggcgtatgcgcccgccttgtgtctcgtctgcgcaggag

gcgacattgtcttcctcccgcttccggagtctatgcaggagctccgcggtatcaatggctccgacggcgtacttcgacccgaaatcgttggctcgatc

gagcaaggtatcgccgccgccgcatggagtccggatgacgagctgctgtcaatcgtgacggaggaaacggtggggagcgatgattccgctgcgtcctc

accgaaagtactgctcatgacgaaggtgttcgacgtgctctcagagcggcccatctataccacggacttcggcgaggatcagcctgtcgatgtcggct

ggggctctcgcgcaacgcaattccacggctcagccggtaaagcagctgccgtcgctgccgcagcagaagcggaggcagccgccaaagtggacgcacag

acagatccacgaggacctgccactctcgacgatgatggaaaatccagggtctgctggcgaggagacggcgccttcttcatggtctccgccattgagcc

ctttccgtctgcctcagctctgacatcgtcgcaccgcgcactcggccaccatcgtgtcattcgtgtgttcgctcgtgacggaaatctgtccgccacgt

ccgagcctaccgtccgaggcttgactcaggtgctgtccgtcaagccgactgggaacatcatcgcaagcagtcagcggtttggcgcactggacaaggct

gaaatcggtctcgaaatggagacgagcgaggatcaactgcgcttggccaaggggagagaagggcggcatgatattgtcttctttgagcgcaatggctt

gcggcatggagaattctctttgcgggaggatggcgagtctcagccagaggcaagctatccggatcacagcgtcagcaagctctcctggacgcgcgatc

acaccatcagggacattgcctggaactcagacgggtccgcgctggctgtcctccttgctcggcatcagaccgacaatgctgcggaagatgtcatacag

atctggaccatgggcaactatcattggtatctcaagcaggaagtcgacgcatcggccctgggtctcactccctcccagactcgcgtctccagttttcg

gtggcatccagaggcaccgcatgacctcttcgtcacctggaccacgcaaacggcagggtcgcagcagcacggcagcatcctcgagcatcgcaccttct

tcctcgaaactgtcgcctcgtcctcaactaccccaccgcacgatgcagcgtgtgtgagcgtgaccgacggtgctgcgcttctcctgacgagtttccgt

ctccagaacgtccctccacccatgtcgtcactcacgctcctccccactcaagaggctcaagcacggctcgacacggtgccagtgcatgtggcttgggc

cgacgctgtctcgtcttcgtccacgtcggcgacagcgaagggcagtatcagtatcctggccgtcctctacccgcgcgcggtagtctgtctctatgcgc

tctgctggggtcagatgccgcgtgcggcaccacagggagcgcggacgttccccaccccgatccggcttggaagtgtggaccttacatcggaagggctg

agtacggcctaccagatcgcggcgagcgtacagattcgtgagcgaggattgtcgcagggggacgaggaggcgctggatgtcgatctggctgttttggg

aagggacggggcgcggaaggtggtcggcgtccggagaatacaggtttcgctttacaaggatcgaggctcgttgtccgcacaatcttcgtacgatgact

ccccagtggacacggaccaagacgatctcacacccatcgatgttcggatacccgtgttttgtacctacctcaccattctgagcaagtcgccgctacaa

gccttcggactttcccctaacggacatctctacgccatcccgcatcaagcgcggttcctcccgctccacgctgttttccgggccgacgatggcgcttc

aggttcgattcaggacgatctggacgctgctggaacggcgacgggggcagctaatggcgcttccatggcagagactatggcgctgggtcgaagtgtgg

agcggggaagtcggatcgtcgtggcggtgccgagctcgatgagtctcatcttgcaaatgcctcgcgggaatctggagacggtgtgtccgcgaccgatg

gtgctggaagtcgttcgtgctgctcttgaccgacgacggtacgccatggcattccggatttgtcgcacgcatcgactggacatgaatatcttgcacga

tcacgaccctactgcgtttttggagaacctgtcgctgttcatcgagcagatcaaggatgtcgattacctcaacctgtttttgacttcgttgagggacg

acgacgtcacggaggacctgtacaaaccactcattgaaacggagaagcggtcgggatctctgagcaaagatggtaaagtgaaccgcattgctacaaga

atacgagaagatctacaagcgcgggaccagcgaggctacatcaactcgatcctgaccgcattcgcgtcgatgaaaccaccagcgtacgagcccgcgct

ggagctcctcggccagcttagagaggaggacagcaacctggccgacgaagcggtgcgctatgtgattttcctggccgatgcggacaagctgtttgaca

ttgctctgggcacgtatgactttgcgctcgttttgatggtggcgcaacatgcgaagcgcaaggacccacgggagtacctgccctttttgcgagagctg

agggcggtggagccgattgagtatcaacggttccgtattgatgagtatctcggtcggaggacgaaggctttgaagtggttgcttgcttctggtccgag

tcatcatgaggaagcgatggaatacgtacggaagcacaagctccatgctgaggcgattcagatgttgtcggggcaacctaaaaatttggcggaggttc

aaggcctgtacgcggagtacctcgagggccgtaacaagccgcaggaagctgctctagcttatatggtcagcggtagcagacgtaaggctgttgagtgc

ttccgccaggcggcgaattggaaagatgccttggggttggcgttcgacgaacggcttcctgccagtgaagtcaagtctctggcgcgtgaggcggcaga

tgacctcgagggtaaggggagatactttgaggcgggccggatatatctcgaatacgagcgagacgttgagcaagcggttttgctgttctgcaaggcca

acgacctatcggaggctcgaagagtgtgtgcgactgctggtcgtagggatctgttggagacgacgatcaagccaggcgcctttgaagcacaagaacgg

ctgctggaggagcttgaggagatgcgagagcagatgacgaaacaggtcgctcgtcttgccgaactaaggagcaagaagacggagaatcctgctgcttt

ctacatggagaacgatcccgcgttggacaatgtggatgtgatgacggatacgtcgacgcagatcacccaatacactcggtacacctctgtcccgtctc

tggccagcatgtcgacctcgtccggaatgaccggcagcacgaacaagtcgaggaagacgaagcggaaggaggagcggaagaagaatagcggcaagaag

gggagcatctacgaggaggactacctctttgagtccttgcagaagcttctgaaggagaagctccaaggcttgcaggtggaagtcgcccgtcttctgcc

ccatcttgcggtcctttcgaacgctcaccggaatgccgggaagacactccgggacgccatcgcccgcttcgaaagctcagcccagggtgccgcggatg

agctctgggcggctgaggtggagcaggagcgtgggagggcggccgagttggaccagctcgtctcggctgggccggaggcttggatggctacgtatcga

cggttgaaccagagcccgcttgggcggagggttggggcgagggcgcgggttgaggttgcggcgacgacgtggcggtccgtccgacgctccaccatgga

tcaagcacaccacaagatgcgaaggctcgtccgcacccccagcgagcaccaagcagccccgctcaggaggcagagcacatcccacttcctaggcgcag

acctcgtccgccgccaggacgcgggccaggtcacatccgaccaatgccgtggaggcgcgctctacaccgctccattacaaggcgccgttgtcgactcc

gctcagatgctcaagatcgagtggaacaaggcctgcctgctcgatcagggcaacatcgacatctacgtcgtcacctcggccggtgtcaagcctttggc

tgcatacaagggaatcccctccgaccgtgggttctatcaggcacagctccacccatactggtggggcggcaccgcatctgccaatctaaccctcaaca

tcgtcaagagcggcaatgaggtctgggacagtcccaatcccgttggtccacaatgggtggccacctatgccgtgcccggccctggccagcccgttccc

gattatgccgacggctccacctccaaggccgaccagatcgtcagctacttcttcgccggaggcggcctcacccctggcggcaaagcagccgccatcat

ctgcccgctcgtcgtggttctctgcggaatcgggctctacctgcgcaagcaccacatcaaccgcttcaacaagaccgccgactgggccaacaatatgg

acaagcgcatgtctcgcatctccgtcgactgggaccgcggtggagatggctccaagggccccatgcccggaacccggcctgccagtttctacccgcga

ccctcccaagatcctagcgtgatgggtcaccgcaaccgcgccagtcagtacaagcccgacgagcaggagatggacgagatcgatcccgagatcagaga

cgcgtacggcggtcttgctggactcggcgcatccgccagcagcctcgggcacggccttggcgccggaggggaccactctggcagccggatctcctttg

cagcatcgggcgctggcgatcgtcgctcccgcatctccttcgccccgccccgcgagtccaacgacggtcaatcgtcaggcgtcaaccgtcatcgggcc

agtgcttctattcctcgcgtcggacagaacaactggcgcagaagtgtgggtggcagcagtgcgttccgtgactcggtggcagacagtgacgtgccgga

ggtgcccaagctggccctcaacctgaggagcagtcccctggctggcggatcgcacgaaccggacgaccttctcatgagcccgacccagcatgacggac

ctcgcgccctggccaactccgacgtcgacctcatgcgcccatcctttgacagcttcggcacaaacgcaagcgggccgtccccagccgccggctttatg

aatcgccagaccatggactccacgctctcacacggctactttagcgcaaatggcgcaggagtcggcgcaggcgtcggcaccggcggtggtggcctcgg

ccgactgggaatgggcaccccaggcgagaacgagaactcggacgcgtactccgtctcgacgggtgcccatctcccctacacgagctcgtcgcccccac

cgcaactgcccagcatgcccggcctcggctcctcggcctccgggtttggatcgcaggtgcgcacgggcatggcgccgccggcgaatacgtccagtccg

gacgatgcgatgcgtgcgtatgcggctgcgagggctgctggtacggtgggtgcgggtgtcggtgcgggcggcgcgggtgttggcgcgggtgtggggtc

tggtgcgggtacgggccccggtggtatgcggacgctctttgcgggtgggctggatgacgattcggcccatcgtgcctctccgagcatgggtggcagca

tgggcggcagctcgctgaacgaggatgatgtagttggttataacgaggctgctgctgctggacactga

>contig_14.contig_14.g308.t1

atgagcaaggacacagacaagacagcgaagaagcgcaaggcccaggccgaggtcgtcgccccagtggctgctgaggccg

cggtcactgagcacgagtcggcgccggacaatggcgagaagagcaagaagaagaccaagaacgacgacaacaacgaaggcaaagcggcggcggcaaag

aaggacaagaagaagaacaagcgcaaggcagaggacgacgcgcttccttctcctgttgctcctgctcctgctgtcgttgaagcggaccagcccgagtc

caagaaggcgcgcaaggagcaagccaagcccaagagcaagcccagcaagaaggagaaggagaacagcacacgatccagccagaccgacttcctctcca

acaacaaaggcgcggccgacttcgacaaggagctcgacgccctctttgcagctgtcccaccaccacctccaccgccagctgtcaaccccgctgtggct

ccgtcaagcaagaaggcaggctcctctgcttccgcttctgcacctgcagccacgaagaaaggcgcacgtaccaacgatgccgatgccgaggctgaggc

tgacgaggatgtggatatggaagatgctgatgaggacgacgacgaggaggaggaggacgctcaggccggtgacgatgaggaggcggaggaagatgacg

aagagctcagcgagcttggatcagaggactacaatgtcctatacgatgacggggacgaggaggaggaagacgcgaaaacttcattgaaggctaccacg

aaggcgacgcagggcgaggatgacgattcggacgttgctgatgaggaggaagagggagcagaagacgacgacgacgaagaagaggaggaagaagaaga

agccgagtctgccgacgaagagccggaagagggcaagacagagcttgttcacgagacgcttcgcaaacctcaggacaaggacaagttgagcgcgcgta

gggagcgcgagggcgatgaggagcctcgcgagctcaaggatcgtcgcacggtcttccttgggaacgtcgtcatcgacgcagtcaagtccaaatctctc

caaaaagcgcttcatcggcacatccagtcgttctcgcccgttcctggtttgctcaaggtaatgacggtccgcttccgctcggtacccttcagtgtgcc

gacggactcgatggacaccgaagggaccggggcgaagaagctgaacgagcgcacacaggcgtacaaggaagcatcgcgcctcctggacaacgaggaag

ggcgcgcacagggcaaggtctttttgaacgcgaaccagaagcgcaaagtcgcgtacatcactcaggaattgaactccaaggcggcagcagtgaacgcg

tacgtgaccgtccagcacccggaccccaaggtcgttcgcgagacgtttggcgacgctgctgtcgagagcggcgcggtgactgcacccgtcctggctgc

gctgctggcagtctgtctcaacggatccaccttcgagggccgtcatctgcgcgcagacgtcgtgacacccatggagccctcggacgtggtcaccgcgg

gactggacaagctcaaatcgccagtcgacgggagcctgctcgtcgttggcaatctgcacacctcgcgcgggacgaactcgaaggcgactgtgttcgtg

ggcaacctcgacttcgagacggaggaggaggagctccgtggcttctttgagaagatggttagggaggaacgtgggcggccgccggcgtctcagcagat

caagatcgtgccggcttcttcttcgtcttcgaagagcgatggtggtgcgctgcttttcggcgcggtgacgtccgagcaagagggcgaatgggtacagg

atgtccggctcgtgcgggacaaggcgacacagatgggtaaagggttcgggtatgtgcgctttgtggactcggcgtgcgtggaggaggtgttggcgcta

tacgaggcggaggacgcgtacatgcaagcgctgaaatccgggcgagcagcgagggccccgccggctcctaaggggagtcagaagaagaaggctgctga

tgatgatggagaggatgagctggtatcggcagcgagcagggatacgtaccgccgtcggttcaagttcaagtcgcggcccttgcgagtctcgccttgca

agaacgtgaccgcgtccgccttacccccgaacgccggcggcggacgtgcaggcgcaggagcatcgtcgtcgcaaaacggcagagacggaggagatcgg

aaccggtcgacgaacgcccgaggcggcagctcgaaccgaggctcttttcagaaaggatcatcatcagcagcaccacccagaccgacaaagcatctcga

gcccaacgcagccatcgccgcgcccgagttcttcctgctctcgaaggaggaacgcggcgcagtcaagcgtgcggaccctgctcgaaatgcgcggcgga

tggagaagaagctcgcttcgaagacgaaggggaagggagatggggacgagggtgggaaggcgagggttaagttggcgcaggggaagagtatgaagaag

gccggtggggggcagcatcggaagggggctggatctggatcgggcaagaagacgaagtcgagcacgagcgcgggtgttcgtcgtcggccttga

>contig_14.contig_14.g309.t1

atgttttccttctcccaacctcagcagcagcaacagcagcccgcgcagactgcgggcgccttcagcttcggtcagcctc

aagctcagcagcagcagcagcagcctcaacagcaacagcagccaggcctgttcggtgctgccccagccacgcctggtcctctatcggtcaatgacctc

aaccggcagcagcagcaacagcagcagcctggctttggcttcggatttggccaacagcagcaacaagccggcttccagcctcagcagcagcactccag

ctctctctatggcgcacgcgccatgggcggcgcgggccatcccggcctgcatcactcctcgagctcggggtcgctgtacggcagtgtgggtgcgggtg

tccagggcgccggtcagacgaacccggtgtacgtgcctggttatctgtccaagatccgttcgaacaagccctacgcatccaatcggcgggtgagcgga

catcattcgccgacgcatgacgacgacgcgaacacgagccgggacgccgacggacggggtacagccacgtccccgacgcacgccttccaatcgtcctt

cttcacctcccgaagtccgacgatggacgaccgcggcatgtcggcccggtcggagagcatctttgggcccggcggtctccgtggcaaccgacgctctg

ttgggcctggtggtggtggtggggatgatgccagtctgaggggttctgttggacctggtggattgcgacggagtgcgagtggtgtgttcgttgcgggt

ggtggtggtggtggtggtcgggatgcttctgtggtcgctatggatgaagaggatgacttgccgcctgtgcaagggttgagggagggaggtggggctgg

tggtggggatggatcgtctccctttgggttttctgccagtgttcgatctgggttggatggagggtcgttgtacgcgagtcggttgggcgcttcgacag

cctcacgtgggtctcctgccgtcggactgggtgctagcaccggtggtctcggctcgtcgacagtcggcaacaacggtagttcgccaggctcgaactcg

tcttcggatccgacgcggacgctgctcgtctttgggttccctgctgcgtccaagacggccgtgctgcacttcttcgagtcgatcggcgagattgtcgc

cctgacaccggtatcttcactggacaacggtgcgagccatggtgctggctttgggtccgggctggacggagccccgctcggcggtttgcagtacgacg

ctcttcagatcacctatgccgagccgtggtgtgctctgcgggcgttgcgacggaacggggatgtgatcgctggcgccggttgtcggatcgggatccgc

ttcgtgaacgagaacttgcatcgggaggtcgtgcagaatgggatcaacagtgcggtgctggcctcggcgaacgcggcggtgcttgcgaatggcgggga

cgtgggcgatcttggacccggtcggtcgatgactgcttctatggcgacggcggcgccctcgcctgctccggaggccagtgcgagtgcgagcagtggca

gtcttgcgcctgctcataacccgcacgcacgtcagaagcgagagtcaacgccggcattcggtcgaccgatcagccagatcaacactcccagcctggtg

ctgcgtacgcctggttcgggaggctcgggcgtttcgtcgacgaatgcgagtccgtttgcgaaagcggctgccggtattttcgggtcttcgactggcgg

cgcgtcgtcaagcagcagcggcagggcggacttctcggcttcggtgcacgcgtcgcctggtgggctgggacatggtcggaatgcgagtgcggtgccac

cgagctcgctgttccggacggcgttggggaacagtccggcgcctgctcagggtgcgggtaatggtgcgttggtggctgttcagcagggagggggcaaa

ggtgggaatgaagctggtgctggggcgggcggtgctgggttgggacagcggctgagtgatgccatctttggctggtag

>contig_14.contig_14.g310.t1

atggctccaggattcacccgaccggaaacggtcctcaagcgcgctgaagagctgatcggaatcggccagtcgcaggccg

ctctccaggccctccatgagatcttctctcagcgccgcttcaagcagacgcccctcccgtcgctccagcccatcatgatccgcttcgtcgacctctgt

gttgacctccgccgcgggcgcgtcgccaaagacggcctcatgcagtacaagaacatggcacagaacacagacccaacctcggtcgagcacgtcatcaa

gcacttcatccagctctccgacgccaaagtcgtcgaggcacagggacgcgccgacgccgctgtcgcagagatcgatgtcgatgacctcgaggagggcg

atacgcccgagaacatgctccttggctccgtcagtcaggagcagaccagggaccgcaccgaccgcgctctcgtcacgccctggctcaagttcctctgg

gaggcataccgcaccgctctcgagatcctgcgcaacaacgcccggctggaggttcagtaccaggccatcgccactcaggcgcttaacttctgtcttcg

ctaccagcgcaagacggagttccgccgtctgtgcgacctgctccgcaaccatctctccaacgtcgctcgctttgcgcaccacacccactccatcaacc

ttgcggatgcggacaccctccagcgacaccttgacactcgtttctctcagcttaatgccgccgttgagctcgagctttggcaggaggccttccgcagc

gtggaggacattcacaacctgctcaccatggccaagaagggccccaagccacagatgatggccaactactacgagaagctggcccgtatcttccttgt

ctccgacaacagtctcttccatgctgccgcctggaaccgatactacgctctggcccgggcttcgatcaagaccgaggaggagcatgcacgcatggctt

ctctcgtcttgctcagtgcactggccgttcccgtcatctcatctagcgcccccggaaccggtaacatgaaccggagcaagtcggacttcctccacggc

gaccaggaaactcgcagccgcactggccgtttgactgctctgctcggtctcgcgcggactcccacccgctcgggtctgcttaaggaggctctcaaccg

caacatcctcaagcgtgtcaagcctgagctccgcaacctctacaacatcctcgaggccgagttccaccctctgtcaatctgcgccaagattgagccca

tcatctctcagatcgcacaggatgaggagatggcaaagtacgtcaagccgctgcactctgtcatcctcactcgcctcttccaacagctcagccaggtc

tatgacaccgtcaacctcgacaaggtcatgaagcttgtgtccgccttcaaggcgccatacaactacaccgccgctgacattgagaagttctgcctcaa

cgcctgcaagaagggccacctcgacatccgcatcgaccacaacactcaggccatcaccttccgtgatgacgtgtttgctgccgatgtgcacccatctg

cgtccgtgtctcatgacactgacgttgtcaagctccaggccactccttccgagctggtccgcacgcaactgacccgtcttgcgtctgccctcgacaac

actgcattcattatcgaccccaccctccgcgaacgagccgaggaggcccggcgatctgcgtttgctcgggccgtggcagcagcggaggaagagcacaa

ggctgcagtcgtccgcaggtccatcttggctcgtcgtaaggagcttctcgaggagatggctgcgaagcgtgcctcggaccaggcggccgtccgtgctg

agcaggctcgtctggccgctgaaactgaacagcgtcgtgttgccgaggagaccaagcgccgcgaggccgagcgggtcaagaaggagcttgaggcggtt

cgggtggaggaggcgaagaagcttgcccagagtctcaaggagaagggtggtctcaagcttaccgaggaggagtacgccaacctggacaccgagaagct

ggttcagcttcaggttgagcagttggagaaggagaagcgcgagctgaacgagcgtctgcgtgtggtgttccgccgcatggaccacctcgagcgtgcgt

accgtaaggaagagattcctctcatcgagaaggactacgagcgccaacaaaccgaggacctcaaggcgcacgaggcggcacgtgtggctctcatcgag

acctccaagcagaagcatgctgaggacatggacaacaagggacggctgcagcgtatccttggcgactaccacaagacccgagaggtcattgaagccaa

gcggaaagtcgagttcgaggctcgtcgtgagcgggccaaggccaacattgagcgggagaaagagaagcgcaaggcggacgtattgcgtcaacgagagg

aggatcgccagcgtgcggagcgggaggaggccgaggccgaggagcgtgcacggcaagaggagctgcgcaaggagcaggcggagcgggatgccgaggct

gcgcgggtgcaggcggagaaggatgcggagttggaggcgcagaagcgtgccgagcgggaggcgcgcacggccgagctggaccggactgtggagattca

gcgaaagcgggaggcagaggcggaagagcgtcggatgctacgacagagtggggctgctcttggcactgcaagctcgcctgcccgaccggcggcgggag

caggagcaggagcggcggcgggcgcggggacgggaggagacaatgtttggcggagaggtggggctgcttcgggctcgaatgcggaggtcgctcttctt

tccagtaccgggtaccgaccgggacagggcagattgttgcctcgtacgcgagcggcggaggatggtggcgccgcagcagcgccctcgtctcccgctgt

gaatggatcgcctgcgcctagttcgcctgcaccggcggcggcgacggcggctgctgcgccactcgcttcgtcggggtcgtaccggcccggtatgttgc

gtgcggctaaggaggcgcgcgagagagaggctggtggggcggctagtcctgctcccgcttcccccgctcctgctcctgctgccgctcctgcggctgcg

gctgcaagttcgccggcggcttcgcttcctgcttcgagtggaggcgcgtaccggcctggtgcgtttgccgcgagacggggtccacctggtggtgatgc

cgctcctggctcgccggcgcctgctcgggcggcggcggccccggctccggtagcggaggatggggctgcggttgatgatgatgggttccagcctgtca

aaaaggcggctggaggtgcttacaggccgcctggtcggcgctag

>contig_14.contig_14.g311.t1

atgtggtcaaagaaacaggaagtcgatgggctgggtctcatcgcgcccccgagtcctgcctctcccagctcgaaggtga

ttcgatccgcatctcgcctcgttctgatactcaagatcgccctcatctacactcttatcgcgacctacttctctccttccctccgttctctatttcac

accccattctctcattccacttcttcatcctactcctccagctccgtcgatcagttcctcagatgctcaatcgacaatttagacaagtcctcgctgga

ccaacctctggtcgaacccatccctttcgaagagtttgtcgagcgtcgggatcgaatcgcacaggcactcgttgaggaaggcttggacgcgtatatca

tcgagcccgggttcgatttcctctttctcgctaacctaagcggatgggaggattggggtcctacggaacgacccttcctgctcgtcatcgagcccctg

cccgcaacctccaagtcctccaccaaagtccgctcccgcagcacaattctcacccccaacttcgaactcggccgcgccaaacagctcgtcctcccctt

cgcaaagggagacgccaaccatctgacctggcgcgaagaagtcagcccgtacgacgtcctcgccaccttcctctccaaatccacaggcaagaacgctc

gcagggacatcagagttaaagtcgggccggagacgaggacgttcatcagtcaagggctcgggctggccaatctctctgtcatcccgtacagcgagacc

gagccgacagccgcggtccgacagagtaaatccgctcgcgaagtcgccattatgagggccgcgaacacgctcaccgtcgaaggcatccgcgccgtccg

gaggtgtgcatacgacggtgtctccgagctcgagctgtacgacgtcttcaagaagacgatcctcagcgcaggacacgggatcaacgacgtcgaaggtc

tggtcctcttcggcgaacgcgcggcatgtccgcattgcggcccctcgcagactcgcaaattgatcgctgcagaggacttcatccttatggacgtcggc

gctcgtttgtacggctacatctcagactgcacccgtaccttcctcttgcccaacataacgtcaccagaacgagggtccttcgacccggaaaagctcac

gatctggtataccgtccacgaagctcagtccgcggcgatccatgccatccgggaaggcacacccttgagtgaagtcgaccatgccgcacgtcaagtca

tcgaaaaggccggctacggccccgccttcattcatcgactcggccacagcatcggcttgcagatgcacgagcctccgtatgccactggcaactcggcc

accacactcaagtaccacacgcccctgactgccgaacctggcatttatctcgaggggtatgtgggagttcgattggaagatgtcattctgaccaacgg

ggcaggcaaagaggctgagacgctcacgaaccatcgggccactagcccatgggatccctga

>contig_14.contig_14.g312.t1

atggacttctacctgcgtacagcgcaagttctggaccgtgccagtctggcccagtcgtctctcaagtctcacgtcgcag

ccctgtcccaaacccaaggcgatgccaaacgtctccttgcgctctccgtcagttgtctctcatacaaaccgaccctcgacggactggtcgccttcgca

aagaaggatctcctgagctcgcccagggactggaaagtcttcgaatctgccgtcgctcgctccgctttggcacgaacggaggataccaaagcggcgaa

gggtaaagcgggaagcctcctcctcgtccttcttcacgacttactcctctcgcccaaaaaagcgatccaggcctcggcagcatggcccccgcacgccc

tcctcctcaagtacaagactcgtctccgtgctgaactcacccgggtccaaatacgggcgggcaagagtcgggtggaagagttgcgcttgggtgggcag

gatcgtgaacgggccgaacgcattccgaggtgggcgagggtgaatgagggcaagacgagtgtggctgcggtgatcgtccggctggagcaagagggctg

gaaacaggtcgaagggccacagctttctaaagggcgcaacttcatgctctcgccacacatcccctctctactcgcctttcacccttcgtccacctcgg

ctttgctgtcccacgagctctacagctccggccagctgattcttcaggatctggcctcgtgctttcccgctcagatcctcgcctcggactatcagcgt

agaggtcggtcgctggaggctatcgatgccaccgctgcaccaggaaacaagaccagtcatctcagtgctctcctcggctcaactgcttctgtcaccgc

gttcgagatgtccccgcagaggtatcaaactctgaccagccagctcacgaaagcgggctgcctcactacaagcacaaacaagcaggaagccgcctcgt

cgacagccaacaagaagcggaagcgggctgatctgccagtatccgcaacgggaacactcaatgtgacagctctgcgcgctgatttcttggaaacggaa

ccggctgatgccaggtggcataatgtcgagctcatgctgctcgatccgagctgtagtggtacgggcattgtcaatcggctagactatctgtctggtgc

aggccccagtacggaagaacaagaggaagacgacaccgtcccaccgacgtccacgaaatctgatcctcaatccaaaccttccaccgccgccgcctcat

catcatcatcatcttcatcatcagccctaacagcccgcctcacagccctctccgctttccaactaaccatggtcaagcacgccatgcgcttcccacac

ctccaacggatcgtctacagcacatgcagcattcatgcggaggaggacgagcatgtggtggttgctgccctggagtcggcggaggcgcaggagggagg

gtggaggttgatggggagggagaaggttttgcctgcttggccggttcgaggtagggtggaggactgcaagggcgacacagtcattgcagaaagtgtga

ttcgatgcgttccaggcggcgagctatcgcgggtacgaggcgtggagctgggtacggtgcacatcgaggcgtccaacggcttttttgtcgcagctttc

gaacgaacactcgctgaagacgagaaagaatcgccgtcgactgtctccaatcaggctgcacaacagcccgaaggtcctgaggccaaaaagcgcaagca

gaagaaaaagaagaacaagaagacggttgcgaagagcgttgaggagcagtag

>contig_14.contig_14.g313.t1

atggacgcaagcctcgctgctgagctgtccaagctcatcgccagcctcgataccgacaccgcagcactcatccctgtca

cagatgcagtcgaagaacgcggaagtcaggatggcattccgctactctcactcaagaatgacgttctcctctcctacctccaccacctcgtccttctc

accgcccaccgtctccgcgggaactcccttaccagccaaccgggctcaacactcgtcgagaatctggttcgtctccgactcgtcctagagaagactcg

gccgttggagagcaagatgcggtaccagatcgacaaggccatcaaggcggccgatgaggaggagcgaggagtctcgagctcgagaccgaggagagtca

agcaaggcggcgcggacgaggacgacgaggatgaggacgacgaggaggaagatgaggacgatgatgatgatgaagtggaccccctggcgttccgtccc

aatccctccgccctgatggcagcctccaaacgagcgtccagttcctcccagccctctgcccagcgcaaaccagatcgcaacaacaaagcctctaagac

gtcacgcgcacaagagaccaacaacaacaacgacgacgaagacgaagaagatgtccggggaggcgtgtaccgacctcccaaactggctcccgtcccat

acgacccggactccctctcccgcaaacgcagcgaagccggctccgaagaccgtctaccccccggccggcgcaacgccgccctcctcgcagacctctcc

ctcggcctctcctccaacccgtacgagctctcctcagccggactcggcggcgcaggtcggaactcagcctcctcggcccaggcatccgtacgcgcgaa

ggccctgcgccggatggaggagtacgaggaggagaatttcaccaggttggtgatgaagaagaaggacgcgaataagaggaggagggacgaggctgctg

ttgcgctcggtggggccgggctcgatagtggacggggtggtgggcgacagcgtttgggagctggcttggatgaggagtttggcgatttgctgagaggt

ggtggtgggggtgggggtgctggtgggaagaaggggaagaaacgggcgggtggggatgcgtatgaggctctgagggggacggcagctaagaggcctaa

ggtgtcgagcgggacgctcgcgggttcttcgtcgggctcagcgtcgacgcctaaggatctcttccgctccggtggcggacgagcatcgcagagccagt

tcagcaaggatgtggcgcgatcgcggaagaaggcgcgtacataa

>contig_14.contig_14.g314.t1

atgtgtggtcgattttccaacgcgctcgccctcgaggagttccgtcaagctgtccaggatcagctgcccaagggctgcc

atgcggatctggccccggacgcagagcagtacaaccgctctttcaacgtcgcccctcaaacccggtcccttgtcctccgacgcgctgaagacggtggc

gccggcgacaagcatggcctcgtccttcagagcatgaagtggggtctcgtcccgcgctcgacaaagacgctccccaaggggcccgacgcgtacaagac

tatcaatgcccgagatgataccatcctctccgggtcgggcctctgggcgcccctcttcaagcgggccgaacatcgatgcgtcgtcatggtccaaggct

tctacgagtggcaaaagaaaggctcgacagagtcctccaagcctgtcgaccgagtggcccacttcgttgggatgaacgagccgggaaagggacggaag

aactcgagtgaagggcgggagtatcgattgatgccgctcgctggactttgggaacgcgtccggttcgaaggcgagagcgaagacttgtatacgtttac

catcatcacgaccgagtcgaacaagcagctcggatttctgcacgatcgtatgccggtgattctgccggatgccgagtccatcgcgctctggctcggtc

tgccacagcagcctgtgagcaagcacgaagaggaggcacgcatggaggaagaggaagaggtgaagaatgcccaaggtccatcctcgtcctcggtaccg

ctggcccgagtggccaagcttctgaagcccttcgacaacggagagcttcaatgctaccgcgtcccacctgaggtcggcaaagttgggaacaatgaccc

caagttcatcctgcccgtctccgatcgaaaggacggcatcatggcagccttcggacgggcgaaagccaagaacgaagcttttccttctgcctcaagcc

agaagcctgccgccgtgtcgtctcgcgccaagaaggaggaggaggaggaggaggaggaggagaaggaaggcgaggagaagaagacgtcaacttctcat

gacatcaactccgagacgcatgctcctatgcccgctgacaccccgaagaggaagacggcgggaggacaagatgaaggatccgctggtcagcctgcgaa

gagggtcaagacggagggcgcccaggacgacagcgtgaagtcgtcccccgggtcgtccaaggccaagaaaggcaaacatccgccctcctcatcgccgc

agtcctcctcctccaaggggaataaaagtgccaaatcctcaggcaatcgcgacatccgttccttcttcgcttga

>contig_14.contig_14.g315.t1

atgcagacacgcggatacctcttcgcgctggtggcagcgcttgctgtcagcataatgcaactctgggcgatgcgccagg

caggctggatcatctggaccaaggagtccaagatcgtccaatcctcgatccagtacggccccctgcggacatgctccaagctcgtcgtccacttcggc

gcagaggggacgcaaagtacgcacgagctcggcacggcatcggcggtcctggccaatggacccgcttctacctccgaggtgttggcgggcatcaatgc

gtccgcgtacccaacctttacaggtgacctcctgtccacgctctccgacagggtcgcacacccactcgataaggacgacggctgggtctgccgaccct

tcccacagacggaccaggactgcaactccgacggcgatcgcgccttctgcaacgccttcctcaccgccggttactcgctccaattctcgggcgtgctt

ctcatcgggagcatgatctcgagtctgcttacgattttctctacctcggctatgaacaaggtcgaggggtatcgcgtcgttggtggtatgctaggtgc

ggcttcgctgctgggcattgtggcctttgccattgtgcatgatgcgtacaagaatgactggccatacctgttcggcgcatcgttgtcactcggagcgg

cattctgggtccagctcgttgcttgggtggtcggcgtcctctccacgattggcgtcttgaccgtcggcttccgcgacacaacccaggcaggatcttta

ccggatttggacacgattttcgaccccacgtctcttaccgacgctggccagacgcttacatactcaggatatcgcaaacctcgcaaggttgtctcctt

agtggaaggaaagatgggtaaggagagttcagcgttcagccagggagcggaacctgcgcaaacaaccgtgtccgggcccggcgcacagtccgccgtcc

tgccccggatgacggaccaccaaccgcaagcctcgcccaagggtccccccgagcagtcccagtcacagctccgcttcgccgccctcagcgtccaatcc

ttcggcctcgactcgccccccttcacgggagcggcaggcagcagcagcggcagcggcgaggacggcgccgaaagtgggggtctcagcgatgccgatga

tgctgacttcagcttggagcccgaactcggtccccgctccagggacgccatgcaggatgccggcgccgctgaggatgatgatgccatacccacccaat

cagcagcacggccgacgacgacgacgacggtctcgtcagcagcctcgatggcagatcacgatggggaggacgaagtggccgggttggcggcgtcttcg

tatgcgtccagttcgggcacccacatgcttgcgcatcgcaatcctttcacctctgggtcggaggggagggcctcgcttgagagcgctcggacatcgtc

cgcggcacatgctcctcatccgggcatacagcgaaggagctcacgcgcggcggagaaagccccccgacagggtctgacaccggatacctaccatttcg

cgagacatcgtcttccgcgcgagatgcgcgatgcctccaagatcccgctcataatcgtcgcatgcgggtccttcagcccaccgacctacctccatctc

cgaatcttcgaaatggccaaggacagcatcgtcgagtctggaaagtacgagctgctggcgggatactattcgcccgtatcagacagctacaagaaggc

cggtcttgcgaaagctgaacatcgcgtgcggatgtgcgagctggcagtagagaagacgtctacttggctcatggtagattcttgggaggcagtgcaag

gcgaataccaaaggactgcggtcgtcctcgaccacttcgacaagatgctgaacggacccgacccagtgaccggcgcgaagggtggcatcctcctaccc

agcggcgagcgcaaacggatcaaagtgatgctcctcgcgggaggcgacctcatccagagcttcggcgagccgggcgtctgggccgccgcggatctgca

tcacatcctgggcaagtacgggtgtttcatcattgagcgaacgggggcggatgtgttcaacttcttgttgagccatgacttgctgtggaagtatcgca

gcaatctcaagattgtgaaacagacgatctacaacgacatctcgtcgtccaagatacgcttgttcgtcaagcgcgggatgagcataaagtatctccta

ccaaactcggtcatcaactacatcgaacatcacaaattgtaccgactggaagaagacacggacgcgatggtccacgacggaaagtgggaagagaacgt

ccgccaggacgacgagccgggattctttctctcggcctctgtacaaaagaaaaccttccccacgtctctgtaa

>contig_14.contig_14.g316.t1

atggccgacctagagaaatctaatcacgccggcttcgactctgccggcatcctcgagaacggcggcaagaacatgagcc

ggaccaacacccagagccgggtggagctcgaggactcggatgagaagcaggcggaacggaagcgtcatcaccaccatatccggaaggtgcacctgcat

aagcagaagaaggagaagaagcagagggaacacaggcagattgcgaagaagcttcctgccttcatgcacactgatgacaacaccgatgttgagaagat

gcgaaagcgtctagtcatcaagaacgagatcatcgccggcgtcgctgagttctgcggcaccttcctcttcctgctcttcagtttcggcatcgctacca

tcgccggtcaacaacagctcacggccgacagcacatcgcaagtccaaggcgcggccgactcctccccctcgctcaacacgaaccagctcctgttcagt

agtctcggcttcggcttctcgctcgccgtgaacgcgtggatcttcttccgcgtcagcggtgggctcttcaacccggctgtcacactcggtctgttcct

cgcagatgctattacgtggtaccgcgccatcatcttgggcacgatccagttcgcgtctgctattgcggctgcgggtgtcgcgcagttgattacgcccg

gtggtatcaatgtgcggacgaagcttggtggtgcgacgacgatcgcgcaagggttcttcattgagttcttctgcactgcgttgcttatgttctccatc

ttcatgctggctggcgagaagcacaaggcaacgttcttggcccccgtcggtattggactttctctcttccttgcggaattgtgggcaacaggtcttac

gggaggttcgctcaacccggcacgttcgctcggccccgatgttattgctggtgaattcgacggcaccgcctggatctactacgtcggccccttcagcg

gtgcctttgtcgccttcatgctctactggctgctcaagtacttcaactaccagtccgctgtcgagggtcaagacggtgacgacatccacctcctcatg

cgcgacaaggacggcaacctcaccggcttcgtcgaccaagttgctgccgccgacgcccccgcaatcgtcaagctggcccccgaggagcggcctaccat

gcccacgccgatcgcgacgcaatttgacccgaatgagaagttggacgacacgaacgatgttcctatgtcgcctggtggactcgacgttgacaccgatg

cgggcgagctgcatattgacgacgaccgcacgacgacgacgaagggcgtctccccgctagaaggcaactcggcgtcctctggcaaggagcacagcttt

atctga

>contig_14.contig_14.g317.t1

atgaacagcagaaagttctatgcagcacatcactggactaacgagcccctgccttccaatctccgtcgagatccgagcg

tccctatcgcgactttcccaggcccagcagcagcaccagcagcgtgctgcgcgagcgcacgaccgggagccgatgagcgtcacccaccagtcaccacc

accaccaagtgtgaacgcatctttgaatccgagcctctgtgctttcaacttctcgaatccgagctggatgcgtttctggcagaggaaggcaggtactg

cacattcaagacgcccagtgatcctaacaggacgcggaagtcgcgccacaaagacggtgctcgtagatacactccccgaccgtcaaacgcatccacta

cctatgaggcaaagtacgtttctcgctgcgggtgtagcggcacgtttcaatcaggcagtaaggactcgatctcgcccaagaagcgtatgatcacaggc

ggtgccgcgtcatcggtcaagctcgtcaagatagtctttttggatatcgagtaccattggcggcacgaggggcatgcggttggaacgatatccgactt

ggccagccaacgcaattcaagagaggttaaggagtggatcgaaacccaagtaaagagcggtagaacagccaaggagatcatggcttctgtgcgcatgg

atatggatgagcttcttgccatacactcaatgcacaagctttgggccgaaagtcttcgcgaggaagggtgggaggccagcttcctgaatgacgcaacc

ctcagtgccgcgacggagccagtctgggcaatctttgtcatgcccaggtgggctaaaaaggaatttttccgcgcccagcagttcggtgtgtggcttgt

gcagaaggaaattccagctccgaccgacgtgatgatcgactgctcagaaacagagcgtgctgccatcatcaacgcgttcgcaggcttcacaccacgac

cccaagttcttctctgcctttggcatctgcttcgagcatgggatgataacgtgaagacgaaggtcaaagcgattggagacagccgtgaccccggagtc

agagaagccaatgcatttctccagtccaggtgcagagaagacatgcgcagcatgcagcgagcggaaacagaggagcagttcgaagcggctcgcagcgc

ctttactctcagatggggcgaggaacagcccctcatcacgcgctactttgagaaagaatggctgaacaagaggagcgcacagaactgggcgaagtgtt

acagaaagcatgcacacttcggaatagataccaacaacatggttgaaggttggcacggtaatctgaaagaatacaaaaaccgcatctatgtggacaag

agagaaagtctctacgaagtgacgagctcggacggggtacaggtacactttgtagacgacaagctgtcccgccaatcgcccggttggaaaaagccagg

ggcatccaccagcacgaataacagcatacctggaacggcgaggacatcggaagaggcagaggcggcaggagcgggaggcgatactcgtgcggctgaag

gattggagctaggaggggtcagccatcagggagaaaggggagcggaggaagaagaaaacagaacggggatttcggggcaaagtctaaatgcgcagaag

gccgcggaactggaggcgaaacatcagcaacagaaggaagacttggttcaaacgagcatcgacgaggcactacgactcatgaaggcgcttaatcgcct

acaagagcacaacaactacgccgggctcagcagagctgctctggaacagctagacaacgaaatcagagcagcgcgccgaactgctgagatgttcgtac

gcagagaattcatttga

>contig_14.contig_14.g318.t1

atggcggtcttcggcgacgtaattcactttgagagctcgtcgacgggcagaaacgcttcacagaagctcgacttgtgtg

gtcagtacacactaccgctctatccggctgaaatagacattacgggctctgtccaccatctttatgggctctgtccaccatctttatggggccaatcg

accatctttatgggttcaatgtaccaatcttacgggaccgcagaggcagtcctcgacggcatcggaccctccaccatagtccgcaacgctctcggtca

gtccgtcgtctcccccctccagagcagcgtagagccagagccctcgatccacaaccgcgtcaagatcatcgacacccgcgcagacctctcctccctca

cacacatccccgccctcgaggtcagcgtctctctccttctaggcttctacacgactacacctctcgacctctgcccgccacgctgcatcttctaccac

agcagcagcaggacccgccacccgcgcagcagcagcagcagcattctcctcatcggcacccgcatcagacactctcccacgggccacgacgatcaacc

ttcaatgggacagggcttgggaacaactggtggcatcagcacacgcggggccatcaagctaggcttcttcctgcacactcccttccctagcagtgaga

tctatcggatcttgcctgttcgccgcgaaatcctgctcggcataatgcactgcgacctcatcggcttccacacctacgactacgcccgccacttcctc

tcctcctgcacccgcatcctcggcctccctaccatgcgcaacggtctcgagttcgaaggcagatacgtacatgtgggcacatatcccatcggcatcca

acccgagctgttcgaagagggcctcaggaagcaggccgtccaggaacggatccgggtcttggagaggcgcttcgagggcgtcaagatcatcgtaggcg

tcgatcggttggattacattaagggtgtcccgcagaagctatatgcgctcgaggctttcctccaggatcacccagagtgggttggaaaagtcgtccta

gtccaagtagcagtcccatcccgccaagacgtggaagagtaccagaatctgcggtcgaaagtgaacgaggcggtgggcgcgatcaacggccgatttgg

taccttcgagagcatgccgatccactttctgcaccggagcgtcaacctcgacgagctctgcgcgctgtatgctgtcagcgatgcctgtctcgtcacga

gtacgcgcgacgggatgaacttggtgtcgtacgagtacatcgcttgtcagcaggagcgcaacggagtcatgatcctgtccgaattcgccggcgcagca

cagtaa

>contig_14.contig_14.g319.t1

atgatccttctacctcccattgtgggggggtttggtatcggtccatgggagcccgccgcagatgaggcacaagccaacc

cctccaatggcaccgctgaacctgccagctcaacaacagaaggcatagcctcgacccagggagcatcgacggcaagcgcgccacaggacgaagcagcc

ctactctctctgcacggcaacacaacgatgatgcaccatccttcagaagcatgtctttctccgttgtcaaatacaagcaagcacggacatgtcggcag

tccacaacccctgctgaacgacgatcagcattcaagcatgagtctgacgcagcatagatgtatgctggagcttctacatcgggaagagaggtctgcag

tggcaatggccgtggacatcgccttctggcagctggctgctgccggtttcgaccatggtcctttcaccctacgcgttcacctcaagagcatcacgttt

ggcgttccgacaaatggcatggtttcgaaggacatcagggacgaccgcatcttctcccacggaatcagcgcatgtcctgatccaaattcgagtataaa

tcaagcatggcgcggtggcctgtctgaggatgttggatgtttgtttttcctattgagcgcgaagtatcaactgcgacgcgatactcacccgcatcaac

gaagctcactgtgttgcttctcggacgatgatgagcacgagaatgtctccgatttcagtgcccacatcctccaacccatcatgccgctaccaacttct

cacctgaaggcacgaaggcctatcactcagcttcagcctcactacctgggcgccatggtctatcgatgtggccaccgccattgtctagcacggcactg

gctcgacgaaaaggtcggcggttctcgcactgcacctgtcttctcttcgtgttgtgcacatggaaaagtgaagctgcctcacatcttcgacgcccccg

cacggatcgaacagctctacaaagcaacagatgtcctttctgaacgatttctggcgtcgactcgggactttaacaacatgttcgccttcgtttctatg

ggcggtggttccagagacatgagcgtcgccggtcagctcggggtctacactttcaagattcaagggcaagtctatcaccaggctggctctttggcgtt

ttcgtcccccaacggtcgtccctgctatgcgcagacgtacttcgtccagacagacccggactccaagatcgggactcgcattgacggcgctgcccgtc

aacaggagatcgatcgcgtcacggttgaacgcgtgcaggaggagctgtacaacaacaatccatacgcatcgtttctccgcacggcagcaggaacactt

tctccagcagaagacgaaggtgcagacaccgctatccgtatcatcgatccgggaagaatcggcggagctgatcctcgcacgtacaatcgacctcgagc

ttcggagatcgccgcactcatattacatgatgctcacaccgcaccaaggggtcgcgatatcatcttctactacaaggatgataccccacgagccatct

tcgatgccgatcaagcagtggcccgagctgctttggctcttcccgaggagtacgttgtggacgcttggaccatgatcgaacaagaccgagccgactgg

cagcgccataatcaggacaagctccgtgcagaagtctactccggtcttcaggacgcactcatcaccggtcaagatctgaactctatcgggagacgaac

cacggatatcccgaataccgtcgacggccgaacggtgctaccgctcaaaaacgtacctctggcggccaaccttgcggtaaagtacctgtacaagtaca

ttttcaaaggcccagatcaggtgaccattaccaccgacgctgaaggcaacgctaccgtcgacgagatcaccgattacctcaaagcacgatatgtgtct

ccctgcgaagcggttggtcgtatcctcaacttccagctccacggcctccatcctagcgttcatgccctagatatccacctcgagaaccagcatcaagt

cgtgtttgatcccgacgatgaagacaacgcagcagaacgcctgcaagcagccgaacaaaagtccaaactccttgccttcttcgcgctgtgcgccagtg

accccgcaggaactgccgacttgacatacgcagcagtaccagggcaactcggacgcatctactacgcccccttctccagcggtgaaaagtactacctt

cgacgactgcttcacatcgtcctctgccctaagtcctttgccgacctgcgtactttcaacgggaccgagtaccctacctttcgggacgcatgcgcagc

ccggggcctcattgacgacgatcaagagtggcaccactgcctccaagaggcatcgcaaattcagaccggagcagcacttcgacgtttgttctgcacca

tcatccttaaccacgaggtgcaaagtattgaggatctctatgagcaaaacaaggtcgctcttgccgacgactgcaggaggcgactactccgtcgagga

gtccctaacgcctctgcagaccgggtcctctccctctctcgagcccttcttcgacagacgctgctgaggatgcaaccagactacgacctcgcccactt

tcccattcccggtcctgacgctgccttcgcggatgacatcaacgacctaccagctgttctcgaggacgagttgtcgtatgatcgagtcgcacaactcg

cattcgcccagcaggcatacgcatcgctcaatgaccagcaacggacggtgtttgacgacgtcacacgcgcggtacagcaaagggcctactcctctttc

tttctggacggcccaggtggttccggtaagacctacactctacccatcgtgcgcggaggatccgaagccgatgttctctcagcttgcctccacaagtc

tgcagtgtggaactacatcaccaccctgaggctaactgcgaatgagcgacttcgagcacaaccatcctcaacgtccgagcaagtccgagccttcctga

ctgcgtataacacctgggtcatgaacgtcggtaacggaacacagcaggtcgatcagtacaacaacatcagcatcccgaattggatgcgcttcgaagat

gacgacccgactccgctgatcacctccatctacggggatattcccaatcacatttacaatctcgaatcgtactggagcaggcgagccattctacatgc

tcgaaacgtggatacggagatcaacatccagggtacccaccagagtccctag

>contig_14.contig_14.g320.t1

atggcggtgtcgacgtatagcacctgggcggacgtgtttcaccagtttcccgtggacgaaatcggcaacgtggatacga

aggtcggatttgtttggacgaagtccgtcggcctgttggattccggggacattggaaaccgagtggagctcgtgtggaaccgtgctgaggtggaggtg

gcgcctggagatccggcggatgaggaggagtacgtgggcaggcacttctccagccttcgaatgcaggtccacgcgtttgggctggtgacggcgagcgg

gagtggttcgtttacgattacagggaagacgtacatggtggaggataagataccgttcgagatcatgtttacgacggagagcgaggatacgctgccga

aggttggtacgtttgcaggaggccttggagcgattacctccctggacggtggcgtcacggctcggctccagttcattgaggatggtattcctcaccaa

cccaagctcggtatggcgcgtaagggaccctctgcgtcttcgaagtggtcgagtgcctggaacacaaagaagaaacccgcgtcgacagcgtctactgg

ccccgggacgaatccgagtgcgtcggcgtcggcgtcgacaacaggcgctatggccccgaaggcaacgtcatcgatgttgacgacaaataacagctact

gtgtgtcttcttaccggtttctggtcttcgacgtgtgtgaggagggcggcacaactacggtcacgggccgcacgatcacgaccaaagcggactcgttg

gatttcagaatgcgctggacggtagaggaggctccgatcgtcggcggaatctacagcatgtgcgtctgggtcggcttcgacaaagatgggctcgtgtt

cgagatgattgatggccgggtcgaactgcgtccgggagaggtggagacggaggagtacatggagaatcagctgttcaagcaggcgtggagggtgcgat

ctgcggggaaggtgatcgagtcaaaggcacgatcttttactttggagagtcgtgtcaataccgcaaatgtggagactttgcagacggtggagggggag

cctcgactcgtggttggccttgaagagctgcggaacggggtgcgagaggtcgatacgccgacgcaagcggaaggaggcaagcaagggaccacggcgaa

gaatgcgtggacggcacgctcgaaagcggtctccgacggaaagaaggtggcggtcactgccagagcggatggcgcgaccgcaaacgggaacgaagctg

gtccgagcggctccaaggtcttgacggaggaagcgtttcagctgatgatgaggaagcgcaatcgtgacgacaatgccgctggggacgaaacagagaag

cgtgcacgggttgaggactgcggctga

>contig_14.contig_14.g321.t1

atgtcacacttcgcgcccgacacccgcaaggtgatcctcatcaccggcacctcctccggcatcggacgcgcctttgtcg

acgaactcagcaaatcaccacaagcagcaaacaagtaccgcgtcttcgcatccgcacgcaacatcgacaacctcgctcactttccccccaacgtcgct

cgcgttgccttggatgtgaacgatgagggcagtgtcaaggccgctattgatgctgtccttcgccaggccggccgcatagatgtgctcgtcaacaatgc

gggagcgagcaacaccatcggcgcagccatcgaggtgcccatcgcaaagtacaaggcatgcttcgagaccaacttcttcggactgatccgcgtcactc

aaaccgtcgctccccacatgatcaagcaaggctcaggaaccatcgtcaacatcggcagcacagtcggaatcacctcaactccttgggctgcaggctac

tctgcttccaaggctgccgtccattggtggtcagacgccctccgcattgaactggctcccttcaacgtcaaggtagtcatcgtcgcccccggcgcaat

catgtccgaattcggcgaagcagcaacacaaagcgccacagtcccttccccagacagcccctacgcccccgcgtccgacgtgatcaaggcccgcgcga

tgttctcccaaggcagctacgccactccgacggccatctttgccaagcgcgtcatggcagccgcgctcaagaagaacccgagggcgtatattgtggca

ggggcgaagagcttctcggcgttgatggcgtggtatctgccggtttggatcgtggattcgttcaaggccagggcgttctcgttgggcgaaaacccgcc

ctcagaagagatgcggacaccggatttacgacagctatcacatacccagctattctcattcttttcgatgcttccaccatcctcggccctcactgcga

ctgcctgctcgtcggatcggatcgacaacccccacgctcgcgatcttcgtcggcgccttagctttgtcttccttatcgccaggcaggagaagggggat

cgacccggctctcatcctccagaccggccttcctcctctctcgacattctctaa

>contig_14.contig_14.g322.t1

atgaaatgccaacgacagcgacagcgacagcaacacgagcacgagcacgagcacgagcacgagcagaccgacccacgcc

accacagagatcgagaacgcgaccgcgaccgggatcgcgcgcacagagacagagacagagacagagaccgctaccgggatcgagcatcctacggcgct

ggcgccggtggcagcagcagcagtagcagcaggcgcgatcgcgattgggacaggcacagggaacgagacagggatagggataccagaccgggaggatc

aggatgggaacgagatcgcccagatcgggatgcacatcgcagcgaacgacgtcgcggcgaatgggatgctgatcgcgagcgagagagggacaggcccc

gggaccgcgaccgcgaccgtgagagggaacgagaacgagaacgatccccgcgcaggaggagatcacgctcgcctcccagcactcgcgctgagccacaa

gcggccgcagcaggagcaacaccccgcgcaccggccactgaagcgaataccaacactgccattgctgctgctcccactgcctcagcaagcgcaggctc

gccctccaaagccgctgctccgtcctctactccaatatcatcggccgcccaagaacaacaacaacaacaacaacaacaagaagcccctccgcctccac

cgcccacatcaacagcggacatccccccacccccaccacctcctcctccctccacctcctccgtcccaccccctcctccaccaaacccagcacaagca

caagcgcaagcacaaataccagcaacggcacctacagcgccccgacgcgatcgggagcgcgcagctgcgctaggagggcctgctcctgctcctgcgga

acaacagaagcagaagcagaagcagaagcagaagcagaagcagaaaccgctgccagtgcccgtattgcctgtgccgtaccggtacgaccgtgcgttgg

agcgggaacagacgaaggagcttgttgcgtttcatgaggagcataagcggcttagtgacgaggtcagtcatctcgacaaagcgcatcaggcccgccta

tcactctccttccagacgtacttcgacctcgccccctccccaagctccaacccgaacccagaccccacaccaaccccgacaacacaaacaacactcca

ccaagccctgctctccctctccaccacatcccgcgaactcgagaccgcccgccgccgcgccgaacagacacacaccgccctcgaaggcgcagtcatgg

ccagcgagcggtacgcgaaggccgccgagggtggtgaggaggcgagggtgggcagtccggcggggacaacgccgccaccgccgccgccgccgccgcct

tcttctgctccggctgggtga

>contig_14.contig_14.g323.t1

atgaacagcgcacgcacgaacagcaacagcaacagcaacagcaacagcaacagcaacagcggacgccccgcccttcccg

gcgcaggcactggcactggctccggctccgcgcccgcctccagcccgtccccctcgctcgagccagccggggtcgacgacgacgacgagcaccaccac

cacctcagccaggctgcccacgactctaccatcgctgctgccgccgccgccgccgctgttgccgcccaccacgacagcatcagcacccccgctcacaa

ccccaccgctgcctccctcggcctcaccgcacagcacgacaatggcagcgctactccacagcctcaacaccaccaccaccagcagcagcagcaggtcc

aggtccaacagcaccaacaacagcaaagtcccaatgtgaacaacctctccgtaaactcggtgttcacccagtccgcagtctccgccgcagcagcagcc

gccgtcgcagcacaacaagccgccgccctcggcgcgagcacccaacgcaagtccaccaaggcaacctggacatcctccgacgacctcgagctcatccg

ccaactccgcgtccagcgtgaatcccacgacatcgcagccaaagccaacggcggcaccccttccatcttgggcaatcgcggcatcacctggtccgccg

tcgaggccggcatggagaagtaccggatcgagaagggctcgagtgaggggcgctatgattcccttgcgtgtcggaatcgctggcagttgctcttgctc

cagtatcgcgtcgtcgctgatattcgctgtcagcccgggttcatctggtccgatgccgagggcgcccggatggtgcccagtcatatgtgggacgcatg

ggtcaactcgctcgataccgcccataagatccgcatcgccaagatattccgactcaaggggttcccgctcttcaacgaggtcgcagcgttggcgggcg

acccggccttcctcacctcgaactcatccggctcggcgtcggcttccggctcgcaccataacccccactcctcgggcgggtacggccagcaacagcag

cagcagcaggcgaaccagggcaaggtctcgacacccgctgcgaaccgggcgagggcgttgttgaacaatgggaacaactcgcagtacaactcgcctgc

gggcggcaaccactggtcggtgaacaagcgtcggaacacgggcaaccactcgcacgacctcagccaggacgcctccttcggccttgggggcgacacct

cgtacggcaacccggccggatcgagctccatggccgccgcagccgcagcggcctctctggacccgaacgagcaactgatccagcaggctcaacgacgg

gtccggaacgaagccagcggactctcgatcacccagaaggtcacccttaccaacctcttccggcaggaccttaccgcggtgtggacgtatatggcaat

tggggataacgaggatgagttccgcttggcctggttgaaggaggaggtcgagagggtgaactccagaaaggcggccgacggtggggctgtgggtgctg

gtgctggtgctggtgctggtgctggtggtgcgagtgggggaatggtgcctccgccgcctgctccgggcgtgtcggtctcggccggggacgtctag

>contig_14.contig_14.g324.t1

atgcccggcccgcgctccttccggtcccacggcggcggcggcggcggcggcggtgcacccccgtccccgaccctgtccg

ccacaacacgcgcctccctcgacttcccctcccgcccctcggtcctcgtaacccgctccgacctccgcgcctccctctccgccttcgaaggcctcctc

tccgccgcaaaaggctacacaaacgtcatgctcaccatggcacaggcctccgccgagctcgccggcgcattggagacctgcgccagggtcaagggcgc

acatgagagtggcgccggcttgcatgccagtagtgccttgcatttcttgatgagtaactaccagcaggtcctcgccgacagtctctggaaggacttct

ccatcccgctcctctcccacttcgacacctaccgatccggcgtcgccgagcgtcaggtcggacacgagcaggccgtcgcagagaggagcaaactcctc

aaggagaccgaggcccgcaatatgcgcacgggacggaggaaggagcgcgatttgggatccttccgacgcgcactggccgagttgcaggcccaggtcga

cggcattgacgagatcaaggcgcagtactaccacgaggtcatcgagaatgaggaggaggtctgggagtacatcctctctaaggtgtctctcatggtgc

gatcccagctcgaagtcgccgaacgcgtcgcctccaaggggtcctccgatcccatcttggagcctatgctcgcttccatcccggatccctttgactcc

tatggcccgcctaagcaggacgaccagatcttctccatcctgccaccgacgagcttactctcctcttcgactgtcatgccgagtcccggcattggatc

gggcaatagcaacaaccatcatcatcatcataaacagcaacagcaaatgacgccctcgggctcctcgtctaccgtcgcagcgggcgattcctccaacc

cagctgtgctcggactccgatctcccgccatcgggaatggatccttccaaccgtctggatacaactctcccgcatccacctcccgcagcggaggaggt

gggacaggtggaaacagcggcgccttccccgagaacggcacagccacagtaggctccctactcggcttacattccaccctcgcctccgcgtcctcctc

ctcttcctcctcgtcatccggtggcaatccaggaccgggcgcaggacggctgttcggcgatgacacgctcgacgaagatgatgtgctcctcggcgggg

gaggggttgggggtgggggtagcagtgcgccccctcaaagggacggaggccgactcaagaaggccctcagtatcatcaatgaggacgtccacatcgtg

cccctcgaccagaagcaacagcgacaactcaagcagtccaccacgacggcagcagcagcagcagcagcaggagcagggacagcatcgcagcgaagaat

acaggatgagaaggacgcggagggggaggcggacgaggtcgcggatgtgattacagaatccacgcccacgcgaaccgccaagaagaagaagtcgttgt

ccgcggctgagaaggccgccgctgtgcccgctgtcgtccccgcctccgcggaggatgaggacgtcgaagacggatctcgttcgcagacgaagacgaag

aaggggaaggatccctctaggcgtgggggtagcagaggaagtgcgggcgtagttgacgatgatgaggaggccgtggtggtggtggaggaggaggagga

ggagcgggtcccagggtctgccaatgccagtcagggcgtcgaagctgaagctgagcccgaggttgaagccgaggaggaggaggaggacgagaacgacg

tctcagtcgtaacgccagacggatccccacctcggagaaggaagaacgctgccgcaacgacgacaaggtcagcgtcagcatcatcatcatctcaccgg

gcttcggcagcgacagcgacagcggcaacgtcgacggactcgaaagtcagcgcgaaagcgacgcacccgacggttttgagcgatgaggaggagggaga

ggaggaggacgacgagctgattgagaatgagtggagggacagatag

>contig_14.contig_14.g325.t1

atgtcaacagcagcactgtcatcctcgcccatgtcgccatcgtcatcagcatcaggcgaggcaggccccagcagcagcc

agcccagcgcagaccagcaaatcgcagccgtccagaacaagctctacaagatcaacaccatcctccaagacgcagacccggaggagatcatcgagtgg

gccatcgacaacctgcccggcctctaccagaccaccgccttcggcctcaccggctgcgtcaccctcgaccagatcaacaaagtcagcaaacggcgcgg

acaggcccacctcgtacccctcatcttcctcgacaccctctaccacttcccacagacggtcgacctcgcccaacgcgctgcgagccattaccacgccc

ccatgcacgtcttcaagccgaaggactgcgacaccgtctctgagttcgagaacagacatggccgagaactctggaaccgggacgaagactcgtacgac

tacctcgtcaaagtcgaacccgccgcacgcgcgtacgcacacctccacgtccgcgcggtattcaccggccgtcgtcgctcacaaggcgcagaacgcgc

ctccctcaacgcagtcgagatcgacgaaacgggcctgatcaaggtgaacgcgttcctcaattggtccttcacccaggtggacgagtacgcgaaggcgc

acaaggtgccgtacaacgagctgctcgatttcggatatcgctccgtgggcgattggcactcgacggagccggcgggggagggggatgcgggtgagcgc

gggggtcggtggaaggggagggcaaagcaggagtgtgggctgcatcggatggcggacacgatgcgggcggccaaagttctccgggaggctacgctggg

aaggaacgacgaggaacgcggggacaagatcgacgctgccgccaccgctgtcgttgcttcgtcctga

>contig_14.contig_14.g326.t1

atggccaccttgaatccatacgaagaagcccggctctacacgacctcctccgagcgcgagcagtgggagaacttcgcaa

ccctcttctccctcatcgtcaccctcgactacctcgaactcgcatacgtacgcggcggcgtcaccgaggccgcatacactcccgcatgcacgcgccta

ctagcccagtacaagaccgccctaaaactcgtgcttgggtcgccatcgtcggcagcgcttcagggcaggtcaagacccccgcctgttgagggcttgga

cgagttcatgaggatgtacaagatggacatgccagcagcgcaccaccgtctcctcataggcgtacccgcaaccatcgaacacgggaacaacaacccct

ccgacagtcgcgcacccaaccaacacaacaacaacggcacaaaccacaaccacaacggcggacctaacggcgggcccatgccctccggccgcgaacgc

gccaaacaggtcgcagagaccacccaggccttcatcacgttcaccgacgcgctcaagctcaagctgagggcgaaggacagtttgcatccgctcttgag

tgagctggttgtttgttatacgcgcagtggggagggcgcggggtcggtcaatgaggaaggcagggcgaagatgttgcattggctcatcaagatgaatc

agatgcgagcgagcgaagagatcgatgaagaacaagctagacagcttctgttcgagatcgaaggagcctacaacgattggttccgttccttacaagat

gtgtaa

>contig_14.contig_14.g327.t1

atggccgactctgctgaccgcttcaatatggaagctgacagctccatcatctcactcccaccagcctcatcttctgcct

caccttcatccctatccccatctccatcctcagcagacccctcccagctacaactcgtcctcagaacatcagaccacgcagccttctacgatcgcacc

gcccgtctcgtcactctccactcctcaaccgacctcatcgcacctcctcctcctcctcaggaacagtcacacagcttggcactcctgcctcatcagcc

agccgggcccctcaccaccatcgcctcatcctcaccatcgcccactccagctctcgacaccgcaatagcagcacagcacagcacatgtccaacatgtc

tccgacccctcctgcctacgtaccacaagccacaatccgacatacgccccacctcggcaccgcactggcgtctgcccccgcccaatcactccaacaat

accaaatccgatctcaccgacgacgacgattctcaccaacaaggagcgcagcagacttatgtagcgcccaactacttccgtctgctcgcctcggctag

cgtccctccctcacgagcgagcagtcgaccggggacgcccatcgtcgccctcgttgccgacaacgatgatgatgacgaggaggaagagcaagaggagg

tgattcagtatgctgcgctagactcagatcaggagcgagagcgggtcgatggtgattcgagtgtgctgtcgatccagagggagatgatgcccttccag

cagtctcagcctcaggctcaggccccgtcccaccctcgcacacgaggtctcgtccccagcagtcgcagctctacctctacggccattccgagggtaat

atccgacattcaaccctccaggaatgggaccgacacggcccgaagaacgacaacgacacgaaccagaacaagggagaaggagagagaacgtctgcaga

gctctcagttctcgcagggctactttgaccgcttctttgtgcgtgttcgtaagctgggcagaggtgcgcggggcactgtctggctgtgtcagtatatc

ttgggtgggcagaagttgggcgagtacgctgtcaagcaagatatgttgcgtttgccctcttcttcattttcatgggtatag

>contig_14.contig_14.g328.t1

atgaaggggacgatcgtccgctccgagcagggtctagatgcctgcttgacaactacgacaaggagaaggctcgggatcg

ctatcgccgcggcttggggcggctttggcgcgcagaaggtgaagaatgaacgtttgcttgacgaaggctgtttgttggagcagcttagtggtagcgtg

ggaggggagggtggagctgaggaaggtggcgatgtcgcatcaacgtcggcaccgacacccatcttgcaagagacaaagacgagccgcacgcccagcat

ctactcggcgagggggagctcttgtgcaggttacatacggaagcatggagttccgtcggctgcgcccaagtcggacgtcagttgggcgaaggcatatt

tgcctcgagtagtgcccacccacccaggtcggcccactctcgagctcggcccactctcgagctcggcccatcaatggcccgcgcccaccaacctgcgc

aagaagactgcgggtgcgcagctcagctcgctcagggatgtggcttcgggtctgagctggctgcatgagcacggaagctcgcatcccgcccaccatct

cgatgcttctccaccgcatgctagaccgcaacccagcccagcgaccgagctgtga

>contig_14.contig_14.g329.t1

atggccgacgttcctgaatcattgctacccaggtaccgcgatcatctcggctgctcaggaccgcccttcgttcctagtg

actcggtcgacatcgaatgtctaagtcgcgtaaaggagctacaaaagaacatcgaggagctcagggacgacgtcaagaatttccgtgaggccatcacc

agactcccacaaagcgggacggctgccattggcctggaatacgtgtctgcaggcttcctgtacctcgaggccgtcctgattaagcaacttgagagccc

agcctacgttcaggcgcaatccgcgatcctatggatcctctctcacggtctggaagatgcactcacgacgtccaaggctaccgggctcggcgagcacg

gtgaatgcatgctcaagcatctgagagaataccttccggatttcagcggagatgcattgtcccagctgagacgggacgttcgcgaacgctacgatccg

catgtacggccccccttcgatgagcagataattgtcgaccttttcgactcttatgtcgggctctcacagttcaagcgcaccttcgagaccatgccgtt

catcagtcgtcaacccaggttcaggggactctgcgaagccgtcgatctggtctgcgcatacatcaaggatggttcctacagattcaacaagcatccaa

atcaccagccggacaacctagccgccgtccttgcacaattttgcgtccagatcgatgtgcttcggtcgatgttccaggacttttccaaggagggtcct

atcacagaggaactacgaagtgtggaggagaagaccaagcggatcacccaagacggcatcatgatcactcttgtggctgcaatcgcagcacagtcagt

ggccttcgttctacccacaccaagcaccaaccccatactcgacacggccgcggccatgctactactgtccgccatcatctttgcatacatcactgaga

ttgcacacagaggccaatcctttggattgagctataaccttgcggtcatcctcgtcatcttcttcacgatcgtatgcatcccagcagctgccgcggtg

cttgcgttcgtcatccgagtctattctcggtactcgcgggaaatctatgcctgtgggtccggcgctgagctcgtactcgccgatatcgttggtagtga

agctggagccattgtacctggtgcagccgcctctgcgggagccaagactgtgggcaatggcatcgctgaggaaaccgatgacaccggtgccgatgtgg

ctggttccacagctcctcccgaggccaaagtcggtgaacttggacctaatgctgcctgggccggggccgtaccacctgaaattgactga

>contig_14.contig_14.g330.t1

atggccggatttgtccagcatgatcgccacacggcggccttggaaccgacagcaacgccgattctcgcgctgaaggaca

acgatcgcgacttcttaggtgatgaactggtcaagaacttcaacgacctcaagacgtgcagtcccctggtcaggtacatcgtcaaccccgtcctcttc

aacatcagagcaatcctcaacgacatctactccaatcatcgggtccatttcgaaaaggtccaaaaactggccagcctcatcactgggctgctataccg

actgatacacccctttgaggacactgagtttgtccaggtgccaaacacccagctgctcaatatggtactccacctggacagaaccatgaggagaatca

tcagaaaggccaccgcagacgtcaagaacaagaagtatctggatgaggtacgtgtctatcagttgcgtttgcgaaacgctcagtag

>contig_14.contig_14.g331.t1

atggtcacccgccgggaggaggcgctcggcgccgtcaagttgtaccccggcgagccatacgttggagacgcgtcagtcg

cagcgtcaccttcccttcagtggacggggtccgactcggagttcgtcgacagcgacctcctcgtgttgttccgaaatgtggccagccttgtgccagtc

gcaggccccatcctcgcaccggcgcttgcctacgtcaaggacatcgtgatctgcgtcaagacggtcaaggacaatcaacgccgggcagagtccctcgc

caccgacgtactgagtcacgcgcaaggactccttcagactgcaaaggacgcagacatccgccttttgcccggcactcccgttgcgaacatagtcatcc

gactcgaagttcggctcaaggtcatcctgcaggacattcgcatctgccaggacccatttgcgaacaaattcagcaaggccttcgagcagtttgtcaag

cgcgatgatatcgctgccatcttgtccgttggacgcgagcaactgaaactctctatggcagccttcagcgttcgtatcggtcggtctaaagacccttc

tcacgtatcccatggtcaactgacggattacattcatcctttgaataatccgcagaatgagcccctcttatcgatggccgtccctgcccagtctcatc

tcagcaggacaaagcgcttgccattcttcgagaagcaggctaaagaggaagcggcgctgcccttccgcatcctcgaagccgagctcgagcaccacagc

actgtcggagcagcacacgcgccggacccaaacacacgcgacaaggtggcggccgacgtcgacatactccacctactccagcagatggctggcaagct

caaattagagatggtaaccttgaacgacgatgacagcgcgcggacgacggctctcgagctgagcaagatggtgatcgagtcccagcaaacgggcgagg

acgcccaggacaggttcgagtctatcaaacagctctttgccctgggcacgtgcctaatgcagctcgcaatgtacagcgaggctttgcccgtgtggacc

ctggctgcgtccaactcgcgccaatcgagcgccaaccaggcaagcccccagctcctgcttggattcatcctgttcgccctttccaagtcatactacat

ggtcagccgcatcgacgaggcgctcgacgccgtcgaggagtgcgtcgacatccttggcaaggcagacgttagggacgcagtagtcgcagcgtcactca

acacggcaagggacttcaaggagacgctcgagaaagcctcaaacgacgcagcacaaggtataccgggcccgacgaggcacagcgctccctcgggattc

tttacctaccagccgggcgattctgcgcgcgctgtgctgagcagtgagaagagggtcgaattgtacgagagattgcacgggttggacgcctcacgatg

gcgaccggactatgccaaggcgctctcggatcatgccggatatctgttggagtgggccgagcggtgcgattccgcgacagcagcgcaggtccgcgaga

agagggtcggggtgtacgagagactgcacgggttggacgccccacgatggctattgaactatgtcgcggagctctcggattttgccaagtatctgtca

aaatcggaccagccgggcgactatgaacgcgcaatgcagctccacgacaagagggtcaagctattcaagccgctggtcgcgttgggcaaatttcgatg

gaactatgtcgaggcgctcttggatcacgtaaagtatctggtgaagtcggaccgaccgggcgattccgcgcgtgctgtgcagatcagcgagggcgcca

tcgaactgttcaagcaactgtacgaggaggaagcctcacgatggcgaccgtactacgctgaggcgctctcgaatcatgctgggtatctctcgaagtgg

ggcgatccgggcgactccgcgcgcgcggtgcagttcagcgagaagggcatcgaactgtacaagcaactgcacacggacgacgcttcaaagtggcagtc

ggactatgccgaggcgctctcggatcatgccggatatctgttggagtgggccgagcggtgcgattccgcgacagcagcgcaggtccgcgagaagaggg

tcggagtgtacgagagattgcacgggttggacgcctcacgatggcgaccggactacgctaaggcgctctcgaatcatgccgggtatctgtcgaagtcg

ggccagccgggaggctttgcgcgtgcagtccaggccagccagaggagggttgagctgttcaaggaggacgtcgtacagtggaaaaagccttacgaaga

cgcacaggagagtcatgccagttacctacgtatcttcgaggagctcaaatctccctatccagccattcgatacattcttagtcccgtttccatcagtt

tcaagcacctcgttgtcactttgaagcggacgaacgaacagactcgacaactggccgaggaactggaacagaccatccgcaaggtcatcgtagacctg

gtcgccaacgtagaggtttccgacaatgtgcattccgtggactctgagttcaatgtaatcgcacagaagctggacagattcccagcgatggcgattcc

tggctccatggaaaacgaccaagactgttcttgcccgagtgcaaaggaagagttcgccgtcctctactcaaatctgtgtgctcggcccaacgtgcccg

gccacgtccagcccactctggatctgaatcggttcagccattacccggacctcggtgatttgttgaaggcatgcaacagctccattcctttcgtcgca

gccactatccgtcccgtcctgatcagtttggatggtatcctcatcagcgtacaggacagcaaggacgacgatgtccgccggcagggtgcggccctggc

tgtagagatcctcaaaccggtgaaggggtttgttgacctctttaatggcggcgatttcattcggagtccggatttcgctttcgctgaggctatgcgca

agcttggagatgacctcttcgagcttcggtccgacatgtgcactcgtcaacgcgcctcgggtcattggtggcgtatctttgtcgctcgcaagagcgtc

gagagccttcttgtctctgggcacaaagctttcatggcgaacacaaagcaggcggagatggatgtactctatgaacgtgtacccaaaatcctcaccaa

cacacatcgtcccaccagcggcgcccgctctgctctacaccagcagctcgcgatgttttga

>contig_14.contig_14.g332.t1

atggccgactctgctgaccgcttcaatatggacgctgacagctccatcatttcactcccaccagcctcatcttcatcct

catcctcattcccatccccatccccatcctcagcagacccctcccagctacaactcgtcctcagaacatcagaccacgcagccttctacgatcgcacc

gcccgtctcgtcactctccactcctcaaccgatctcatcgcacctcctcctcctcctcagcaacaatcaaacagcttggcactcctgcctcatcagcc

agccgggcccctcaccaccatcgcctcatcctcaccctcgtccactccagctctcgacacagcaatatcagcacagcacagcacatgtccgacatgtc

tccgccctctcctgcctacgtaccacaaaccgcaatccgacatacgccccacctccgcaccgccctggcgtctgcccccgcccaaacacacaaataat

accatatccgatctcaccgacgacgacgattctcaccaacaaggagtgcagcagacttatgtagcgcccaactacttccgtctgctcgcctcggctag

cgtcccaccctcacgagcgagcagtcgaccgggtacgcccatcgtcgcccttgttgctgacgacgatgatgatgacgaggaggaagaggaggtgattc

agtatgctgcgatagactcagatcatgagcgggagcgggagcggatcgatggtgattcgagtgtgctgtcgatccagagggagatgcccttccagcag

tctcagcctcaggctcaggccccgtcccaccctcgcacacgaggtctcctccccagcagtcgcagctctacctctacggctattccgagggtaatagc

cgacattccaccacccaggaatgggaccgacacggcccgaagaacgtcgacgacgagaaacagaacaagggagagggaaagggagcgtctgcagagct

ctcagttctcgcagggttactttgaccgcttctttgtgcgtgttcgcaagttgggaagaggcgcgcgtggtaccgtctggctgtgtcagcatatcttg

ggcgggcagaagttgggcgagtacgctgtcaagcaagtccctgtgggtgactatgcggacaacttgctcgcgtctctgaaggaagtccagatgctcga

gacgttacggcatcccaacatcatccactaccagcatgcatggatcgaagaggcgcagatcagtccctttgcaccgagagtgcctaccctgcacgtct

taatgatggcagccaacggcggttctctcgctgattggatctccgcccgagccggcaatcccgtctctacatccgcacgcaactccaagtccagccag

gaacctgacgaagaggaggaagagcagcgtcgctcgcccgacgaggacgaggatcgtccgctccgagcagggttcaaacacgtgcttgccgacgatga

tcatgctccgccatcgccgcgtctggaggcgcagaagttgaagaatgaacgtttgaagaaggcggttcggttgcggaggattcaacggcggaatacgg

tggagcagcagcagcagcgtgaagcagggggagagagggaaggtggagctggggaaagtgacggtgtcttatcgtcatcggcaccgggaccgtccggc

tgcgcttctaccctacccgccgctgctgctggaaacaacgacgacgacgagccaccgcacgctcaacacctactcggcgagggggagctcttcagttt

gctcagggatatggcttcgggtctgggctggctgcacgagcgcggagtgctgcatctggatgtgaagcctgagaatgtgctgttgcattgggagcagg

ctgatgatttgctgcctcgagctatgttgagcgattttgggagtagtacgttccttcagggcaactggagacgtatccgatctggacatacgggaacg

atggacttcatggcacccgaagcgatctttcccgaccgcctgacgggcgagctccaggagctcacgagcaagtcggacgtctggagcttgggcatgat

cctccatctgtgtgtcttcttccggctgccgtaccgccattcggacgatattgatcggctgagggaggagatggagcgttatcgggggtattctgttc

gcgacgaacgaaatatgaaccgagcccgcgtcccgcccaccctctcgatgctcctccaccgcatgctagaccgcaacccagcccaccgaccgagctgt

gaggagatcgtgggcgtgcttgcgtctgcatctgcatctgcggacgatgcaagtgctggtgggcgctgggtgctcgtgttggcgcttgcggtgctgag

gagtgtgttgatggaggctgcgttggggatgggtctggttggtggtgcggctgtttggggatgtggagtcgttgggcttgtcgaggttgcgttggcct

ctcgacgtccgcagattgcctcgggcttgttcctcgtctcgctgagcgtccaggtggccatcttctccgtgtccttcgcacgccggtcttctcccata

acaacttaa

>contig_14.contig_14.g333.t1

atggcagcacaacccttcgaccccaacaccgcgcagaacctgccagaaatcgagaagcagatggcagtcaagtgtgtcg

agcatgcacaagtctactggaacctgctcgagaaggtcaagcccagcgctctcaaactcaccaagctcgacgacgagatctacgccgacttccaggcc

accttccccgagtacgcagagccagacaacaagaaggccgtgcgcaagattgacgaggactccctcaagtcaccagagggcaaggagcgctggcgcag

ctttatgaccaagtacgagggaagagtcatgcggttgcagttctacgtgtttgagatcgcacggaaccgacgcgcctacaacgactgggtatgggaga

aggcacgcaaggaggagactaaggaggcagcaaagaaggcaaagggcaagaagtga

>contig_14.contig_14.g334.t1

atgccccttcaaaatcgtgccctgattggccaactgccgatgcgcgtcctcccggatcttatcaagattcgaaggatgc

gtcagcatgtcgacggccagatacaccccgaacgcagcggcgcccagtccgaggccggggaacatgttccggatgtagaaccggttggtgaagagcgg

gtgcttgcgccaggcttcgcgcgcggcccagggatcgcggtagccggagggcattcgaggaaggtactcacccttggcgatgtggcggtcctctgcgc

tgcgaagggcttgagaaccccatccgaagctcgccggccttgtcctcctttgccgcgtggggaagtgccggagggtctggaggattggggtgtggggg

aattggttaatgtgaacgatggtgagctgcacgacgagttctctgacgacgccatacggaaggcgatcaggcgttacccgaagcacaaaaccggcgga

gaagacggagaccacgccgtgctgctcgccgcattgactccctcaagcactagcgatgcggccgatgcggcctccttccctctccctcagcacctctc

tcgtttgctccggctcgtggccgcctgcgaggtcattccggcacgctggggccgctgcctggtgcatctgctgccaaaacgcaaagaaggagatccca

cagcggaaacgagccggccaatagggatgctgccgatgtttcgccgcatcttcgagtcaattctggtccgccgcctgagccccgatcacgactggtct

cagctgcaccctggccaggccggcttccgacgtggctggtcgtgcgcctcggcgcagatgtacaaccacgaatcagcccacacccgtcgaaacatcgc

tgtctttctcgatttcgccaatgccttcccgtcagtcactgccagtctcgtcgcctttgtccttgagactcggggtgcgccgcagtctacgcaacggc

tggtctggtcccttctcactcgcgcagcggctgcggtgctcgtcgtcaacggcagacgctgcagggagctggcgtttcagcgaggccttacccagggt

gccatctgctcgcccgcgctcttcaatctcatcatcgacgagctcgtccgcctgctcaacggccgcgctgggcccgcgcacctgcgcgctattttctt

tgcggacgatggcagcctactggcctcctcagtggcggaggcgcaggagcttctgaacttggccaaggcctgggcggatgaacgtggatttcggttca

acgtgggtaaatgcggtgtggttgcgcggcgtagggtgactctctgtcttggtcccgagcctattccacaggtcgactcgtacaaatacctcggtatt

ccccgcactgccgacgggctggactgcgtcgcgtggctcacgcgagtcaccacgagcatgaacaacaccctccgtttgctctgtgcggtcggcggtgc

gtggtccagcatggtccgcctccggctgtaccaaagtgtgtgccgctccctgtcggattttggtgctggcattgcggctctcactctggcatccctgg

cacctcacgagaaggcatctatgcaatcggccttcgaagcagctgaggagcatcaccgcgcggcggtaaaatggatctgtggctcgggaacacatcat

ggggttggcgaatcgatgacttctcttctgcctcctcaatacaggtttccccttctcgctgcctccctgctgcaccagctgcatacctcgtccagctg

caatcccacgcgctttctccttctctgcccacagcttccgtcttcgtccctccttcagcaagctcgcacgcacacgcgctacgcagagtactgcacag

cagctgcggagcgcgcgcgcgtacagcccctggctgctcctctctcggtccacgcgtggatggcgtcaatgcggcgcaattacatctactcgcacacg

ggccgtttaggcatggtcattgggtcccatcaacgtagccgcggaggcgcagaccgcgttctgtcggtgtctgaacccgcctcgcggagattggctgt

tgcgtggcgaatcggtgtctttggccttggtcggcggtgcccatgcaagccctccccaccgtatcagccgttcaatcgcggccaccacgactgtctcg

cttggcgcattccctggcgcaacgacgatggctcgatgtcaaacgcgcaactcgaccctctactcgaggctggtaccgaatatgccattcgccaacgg

agtctggtgggcgtcccggtcgtcctccaagtctacttcatggatttcctactaggaagcgacatcgacgagtaccacaccatcgccatgaatgctct

aagcgactggtctcttgctctggaaccaatgcctagggctccccgagcatctctctcctcaaccactaccgacccctcgctatccactgctcattcct

cttag

>contig_14.contig_14.g335.t1

atgagctcaattactctggccacggagcgacaacggagctacgcggacaagatcatcgtcaaggttcccttccctcttc

tcccctgcgctgcgcttgcggcgcgctgcgcctcagcgctcagccccggcaagtggccgttcttccatccggcggagtccagcactcggactgccagc

aagagccaaggcatcatcaggggaacgcgactcggtcccgtcgttactgacgcagccaccgaggatcaagctcggcgcgcgcgcgaagaacagcgcgc

ttggttcgctaggcgccttcggaatcggcggcgtcggaggacggaaggggaagctcaacggagtgcggacgccgacagctcagtggtgagctcggcga

ggattgcggtcgcatcgggccaggcgatatcggctaggacactggcgctggcgccacgactcggtcggtgcctaatgatgcgacatacgaggcgcacg

catcgctgcgtggtctcggacgagcgcaagaaggtcaacgtcggcggcgacctaacgccaactcgcggcagtcgttggcagacgcaagacagcggctc

ggaacgtacgatcggcggcccatgcgaccaaatgggcgcgcgcattgatgcggaggcgaagggcagctctgcttacgcgcaggctgaccacggttcac

gtctccggtacggcgcgaggagtgactccctcgttgcggtacacctgaacagcttcggagagccaggctacggcaccggtctagtcacccacggcaga

tcagcaacagcttctgtgctgggcgtgttggcactgatggttcaaggcacccgtggtaagacgcaagtgtaccacggatgggaaggggagaggttcga

gaaggatcatgccaactcgaagtcttctgcttccggacacgacgcccactggtccgggcggggaaggaaccgacacacgacgggaagcaatggcgacc

aaaagcattgcttggggatgcaccgctacctggtccccacaccctgcctgcgagcttcggttcgtaccgcttggcaggatagggacattgcccccagg

ctcgtgcgtgacctggtcagcagggctgaagatggcggagttggaagcggcggtgatctcatcaagcggcggacgacgaccgtcaaggcgtcaaccct

tgcgcgcagcggtgcggcggctttcgatgccgctacacgacggctacaagggttgggcggcgttgtgggacactcggttggacgcagaggctcgcggg

cggcgggggaagtgtcgatcggcattcggcgcaagctctgctccgacgatgcccagcttcctctttcgtcgaacatcgtacgtcacgagcagatgcac

gcgggaggtggagctaccagctttctacccccgagcgcgtgctctgcgatgacggacgataacgaggatcacgccctcacgaacggcccacacagccg

acgtcagaatcaggatctggagttcactgcgggttcggcacgggaacgaggtgattacggcgagcatgcagcgcggtcgacggttatacggccgtaca

ggtacacggctggcagtcaagtgtcaacaccaagttggggttccacgtcggccaccggtcgaacccaccaccacatgacagcacgcagtctcaagatc

acgttcacggttcaccaatgtcagcgacccggcacgacagcatttttccctggtgattctttctctttgggctcgcatgcacggcgcgaagactcctc

gttgcccggccgctacgtctcaccagacgagtatccgagcactgcggagcacggtcgcgataggttcgcggagcaaggaagcaagaacacatcgtcgg

gcacagaccaagtcgctgcggttcatcttcatcagtcaagtgcttcgagagacacaccttcaagacatcgaggcgcggctcccacgatcggtgggagc

gaggagcgaagtgacgacctggcctcgagcaagcgcgatggtcccgggcgtcaagcaaatcaagcttcacctcctcctctgtcgggtcttgcggcgca

gcagcttgctgcgcagccggcgcccacaatacagaatctgcgtatcggtgggagcattggagatgggtacagttacgcgttgggatccgggatcggga

cggtctgccagtctgggcgctgggcgctgggctcagcggcatag

>contig_14.contig_14.g336.t1

atgatcggccgtcaaatcacatccacagtcgctcgcgccggcgcccgcagcgcctccacctccgcgtccgcctcctcct

ccaccctccccatcccccccaagatcgcgaccccgcagagcgtcggtggggccgggggcaactccgcccggatggagtccgtcgtttcgttctacaag

gcgttgccgaagggcgaggctcctgccaagcgtggtggtgggatcaaggcgcggttctttgatgggaagaacgcgagtggggctccgcttgtggctac

tattggcgcgatgcttctgttgggttacaccattgactacaacatgcacttgagtacgttgccgtatctcaccgctttcctgataggtgaaaagaagt

ag

>contig_14.contig_14.g337.t1

atgacttccgcaccagacacagtacagcaagctccctccagcagcaccaaccgcgccgcacccccttccacctccacct

cctccgccgccgtccccgctccagcccaagccccaacaaaaccaacatcccggtacatcgaaacctccgcgaagaacatcatcgcccgcgaagccatc

atctccggctcgcacttcatcgtcctcggtggcaaatgtctcatccacggcggcgcgatcttgagggctgatttgagacggtcacaggtcggcgccgc

tgctgttgttgctgctgctgttgatggggcgggagcggttgcgggagggaagggggcggcgggtgggagggcggagaggggggcggggaatacgccgc

atattgtgatctcgatggggaggtattgtagtgtgggcgagggcagtgtggttaggccgccgtacaagatctaccaagggatcttctcgtactacccg

accaagctcggggaccatgtgaccgtcggaccgaatacggtgctcgaggcgcagacggtgggcaactacgtcgacatcggtgccaactgtgtcattgg

ccggttcgtcaatatcaaggactgcgcccggatactcgacgggactgtccttgctccaaataccgtcgtgcccagtttgacgatatgggctggatctc

ctggtgcgtcccgtccctgtcccgcggaaacaagtggccgagctgcacgaatccttccaggacgaatgcgaaacccggaatcgaagaaacctacggga

gttcagccgagctcttag

>contig_14.contig_14.g338.t1

atgctgagcacgggctctagctctacctccgcctcaggctcaggctcgaaatcgcgaacaacaacaacagagtccgccg

agcatgtcccaggacccgaaaagtcaagtacgaggctttatgtcggcaacttgcacccaagtgtagacgaatatactctgatccaggtcttctccaag

tacggcaagattgccaagctggacttcctcttccacaagacgggcccgatgaagggcaaaccgcgcgggtatgccttcgtagagtacgccttgaaaga

ggaagccttgcaagccctcgtagcgacgaacgacaagatgctccgcggccggaaagtctcggtaacatttgccaacaagtccgagtacgcggacgtag

ccctcggaggcggacagggcggactgggtccgcaccgacatcggcatagcgacaacaaacccacgacgctcagcctgatcaagaacgctcagcaacca

aagagcacgaacgctaagattgcggcgatggaggctaagttgaagttgttgaaggaacagcggagtcagaaacaggctggtggggcagcaggaggggg

cagtggtggcgaagctgcgagcggagaggaccgaatactgcccttaccgcccgccagcgcgggtctgccgtccaagccggcatcggcatcggcgtcga

aacccgcccatcgtagcaatcctacaagatag

>contig_14.contig_14.g339.t1

atgcccgccgccaacgcactcacttcctccctgcctgcctgggaacagctccagcagctgtacgagtccaagggcaaag

ccttcaacctcacacaggccttcgcctccgacccaaagcgcttcgacaccttctcccgcacctacaaggcacacgacgtctccctcctcctcgacttc

tccaagaacctcatcgaccaagacatcttcaatgccctcctcgacctcgcaaagcaggctcaggtcgaggagaagcgcgatgccatgtacaacggcga

acacatcaacacctccgaagagcgcgccgtgcttcacatcgccctccgtgaccccgagaacacatggaaactcaccgagccgggagtcgagggcgtca

agcccgtgctcgagcacatgcgcgagttctcagactccgttcggtccggcaagtggctcggatacaccggaaagcccatctccgccatcgtgaacatc

ggcatcggcggcagcgacctcggccccgtcatggtcatagaagcactcaagtcctacgccaagcgcgacctcgacgcccacttcgtctccaacatcga

cggaacccacctcgcggagaccctgcgcgtgtgcgacccggagacgaccctcttcatcatcgcctccaagactttcaccacccaggagaccatgacca

acgcgagctcggcccgggactggttcctcaagcacgccggcaacaaggagcacgtcgcaaagcactttgtcgccctcagcaccaacgagaaggccgtg

accgaattcggcattgcgaaggataacatgttcggtttctgggactgggtcggcgggcgatactccctctggtccgccatcggtctctcaattgctct

tgtcatcggttacgagaactttgccgagctgctccgcggcgcgcacgagatggacaagcactacaagacggctcctctcgaccagaacctgcccgtta

ttctcgctgtcctcggtgtctggtacaacgacttcttcggctcccagacccacgctcttctcccatacgaccagtacatgcacaagttcgcagactac

ttccaacagggcgacatggagtccaacggaaagttcatcaccaaggacggcgagcgcgtgaactaccagaccggtcccatcatctggggccagtccgg

cacgaacggccagcacgcgttctaccagctcattcaccagggtaccaagatcatacccgcggacttcctcgcgcctgtggagagccaaaacccgctcg

gagagcaccacgagattttgctttccaacttctttgcacagccagaagccctcgcctttggcaagagcgaggacgaagtaaagaaggagctcggcgcg

tctgcggagtcgaactcgttcctggtgaagagcaaagtgttcgagggcaaccgtccgacgaacagcttgttcttccagaagcttacgcctgcgacgct

tggtgcgctgttggcgatgtacgagcataagattaatgtccagggttcgatttggagcattaactcgtatgatcagatgggtgtcgagcttggcaagg

tcttggcgaagaagattctgcccatccttcaatcgcgcgacgcttccaaggtcgacagcgccggcaaggactcgtccacgagcggtctgctcaagttc

ttcctcaacaaccggaagtga

>contig_14.contig_14.g340.t1

atgtccatgccctccgccgccagctcctcctatccccaccacccagctgcaggcccagcaccggcaccagtcgaacaca

cagcccccatcccaatcaccatcccgcaagcccatcaacaggccatgcggacttgcttcccctcccggctccgcgcaggcacctccggcctcatgcag

gccctcccgcacgcaccggacctcgcagacggcatctccgtccccggcctcagcggcagcggtctcatcgacgacgacgacgacctcatccccgggtt

cggcatgggcgccggctcttcctcctcctcctcgctgttgttgggtgggagggggaagatggggctcacgcctgtgggcgcggttccgaagcttgcga

gtgggaccgcgacggcgggaagggggtcgcctgccgttggtgagatggagggcagtttgaggaagtatgtcgggatgcccgtgccgccccatcggctg

gtggccaaggacgccaatcggtctctgcatcagtactactcggactacgatctggaacaggcggcggcgcagggcgagtgtctgatccctgttcgcat

cgagctcgagacggagagccatcggatcagagacgtcttcgtgtggaatatgcgcgagaaactgatcacacccagccagttcgcaagattgcttctgt

ctgacatggagctgccctatgaaccgttcgcggctcagatcgaagctgcgatcaatcaggccatcgaggatgcgaatgcggccgggattgggcattcg

gactttgagactgggaatgaggatgatatccgggtcatcgtagagtacacggtccaaatcctgcgatcgacagtcaaagaccgcttcgaatgggacct

ctgctcgaacctcaccccggaagccttcaccgcccaactatgcgccgacctcggtctttccggcgaagcaagcaccatcatctcccacgcgatccgcg

aacagctcctgatccaccgccgcgcggcgaacgagctcgtgcgggcgaacgagctcgagagccgcggggcgcgggcgttggaggatatttggagggat

ttggaacaggcgagagagtttgggccgttgatggagccgttggctgaggaggagttggagaagattgagatggatgcaatgcggaatagcaggagaaa

tcgtcgcgccgcgcgagcggaagggggaaggaggcggcgatga

>contig_14.contig_14.g341.t1

atggcagactgggacaacgtcactgttatcggctccaaagctcgccccgccggcgccggtggagcaaaagtcggtccta

ccgcgtacgagcgcgcaaaggaggtcggcgccatcacagacaatgaccgcaaggtcgccgccggtacaaacaaaggccacgcccccgtcgaccaccaa

cgcatcgccaagctagaccgggagaacgaagtggcccccccaccaaaagtcgcgccctccgttgggcgcgcaatccaacaggcccgcatggccaagga

gctcacccagaaggatctgggcacgaagatcaacgagaagccgcaggtgattgcggagtacgagagcgggaaagctgtgccgaatgcgcagattttga

gtaagatggaacggatgttgggcgtgaagctgcgtgggaaggacattggtgctcccttcggtccgccaagcaagaagtaa

>contig_14.contig_14.g342.t1

atgcaggatcactacccgcctcccgccagcagcagcttcttccaccacgcagccatcaacaatatcaacgccagcagtc

ccggtggcggaagcgggaatggcagcgcgaacggcagctcgcccgtccccaatccagcaggcggtctctccagagcgaactccctcgcacgcacaaga

gccgtagcaacactcaaacgagccgtctcagcacgagaactcaagcgggaggcatcccagaaccgacacgatcactccacctcccccatccctccacc

caacctcgacatctccaataggagtgagcagcagcaccatggcaatccacccagaccgcacgatcctcggtcggagacagccacagggcccgagctgc

ggaacggagctgagtcagaggcagcggctgggacagctactgatggcggagcaagtgcaatcgcagcatactccggcgcattcgcagccaatcttcca

gctcccattcctcaggcgccaagtagcgatgtcgcagcagcggtctccgtcctggcagcagccgcagcagctcgcaacggtctcgcgataggaggctc

agcagccaccagcggcggacccagcgcgtttgcccccaccctcgcttaccccgaacagggcctcgtgcctagctctatgccggccacgccgcccgttg

cgcgcacacctccgcatctcgcgatgccaggtcccagcatgacgtcccaggtcgcgcgcacctacgccatggccaaacttctcggagctgcgggcctg

cagagggaggcggtggaggaaccgatggtcacgccggaccccatcagtcgaccgacgccggaacctactattccttcttcgggcctgcaggcggaggc

ggatgagcctgtgtttgtgccagatccgatcagcccttccacgccagagcgcaccagtcttccttcggggtcacagatacaaacgtcggagcaaatcg

ttactccagaccaacggatcagccctgtttcggagcgacggatccaaacgtccgagcgagtggtcactccggatgaacggatcagccctacctcagag

ctaggtatgcaggcgtacgagtctccccttacgccagaccgttccagccctgctccggccctgcggatggaggcaaatgagcccatggtcaacccaga

tcctgctccgctttctccagcgtcgttcccgccttatgccacaagcgagcaggactcggtacatctggcggacccggacgagtccgtgaatgtgatga

cctcggtcacctccctcgactcgccgggtttactcctctcggccacggtgaaccagatcatcagtccgagcaagttcgaagagaagtttctcctcctg

ttcaaagacaccctcgtcctggcgaaaccgctcgcgccgccggaggggttggagggtgcgccccgtcttcgggccgaagcgttgatggagaagccaga

cttggattggagtttctccgtcgagagaatcatccatttacatcgggttgggttgatcgtccatcgggaccggctgccggtgtcgaaaccacacgccc

tgatgccttcctttgtccgccatttcagctccaatgccgaaggcgcgatcgagcagctcatctctcttacaggccttggcagggacgcgaagacgatt

gccaagttgctgtaccagactcccgacctcgaccctcagcagctgtgtgagtacatctgctcggcgcgccggaaggacgttctggatgcgtatatcgg

cctacatgcggtcacgggggtctctatcgagtccggtctgcggtcgttcctgctgggcatccggttcccggccgaccagcaggccttcaagacactgc

tcatctccttctctaaacactgggtttcctccaaccggtcactcatcaaaccggccttcagcgccggtctggccgccaacctcgtcttctacatcatg

gccctcaacgacgccctgcacaccaccgagtcctcgtcccggcagatgttcagcggacctcggccgaattacaacgaggaagacttcttgctggagtt

tcgcaagttggacgcctttgccgttctctccgatcggacgctgggacggatcttctcgagtgtccgggcggagcctatccagcaggcgcttcctgcgc

gggagggccccaggttgtcggtccgagtcgccaaaggtctcccgacgaaactgtccccgcacgtgagctcgggtcccatcacggtcgtgatcgaccgg

gccgacgctgactttgcggtgcgggtatacggtaaagacttcgtcttcgaacctcccgtgctcagttttgccaagtcgaactcgtgcagtttcacgat

tacgccgcgctcgttggatgcgaggacgatcacgtttgtgaagattggtcggcgggcgccgcattatgtcgccgtgccctcgagtgagagcgcggcgg

aggtggccctgccgaagagcttcaatgtgacgattgagccgcgcgagctgaggcatacgttctcgcttactatcgccaagggggactcggtaaagcac

aagatcacacttagcgcggagaacggccatcggaaacgggtgatcaccaatgcgctcaaggagcggatcgacatctgcatttcgaagaagagggagcg

gaatcgggggcaggcagccgttcaggctgctgcttctgctgctgcttcctcgagtaacccggacggacgaagcgggagggagggaccgagtccgatcc

cgcctctgaacccttcgggcgcggagaaattctccttctgcgagccggctgcgggatcgttcttgcggcgggtgtacaacaactgcacgccttttgtt

gttgggtcggcctcgtcgagtctggcgccttcgccactcatcggcggtgaaagcagcggcagtggcacgggtggtggttcggcggaggtgttgaagcc

gtgtgtgacgctgacgtttgcgcagtcgttggatgctaagattgcgggtgtaggtgggaagcagctcattttgagtggggaggagagcatgctcatga

cgcacagtctgaggacgttgcatgatgggattctggttggcgctggtacggcggtgaatgataacccacagctaaatgcacgactgctaccagacatt

cctccggtcgaacggctgccgcggccaatcgtgctagacacgcatctgcgcacaccaccgaactgcaagctgctgctgaactaccagaaggggacggg

tcgacagcctctgctggtctgtgcgcggacggcttcgggtatccgtgcggcggagctggaacaggctggtgcggagcttgttgtggccaattgcgagt

ttgatgacttgctctcctggcgctccgtactagccgccctagcgcggaaaggcatcacccgcctcatggtcgaaggcggcgcgaccgtaatcagcggc

ctactcgcctctcaattcgtcgacacgctcattgtgacggtggcgccgaagctgattgggccgcaggggatcagccataatgcgacgttgccgcttgc

ggggccgagtgcgacgggggagcacggccaccttgccatgaggggggcactgaatgccatgctcggcgctggctgtgcgcgagagccgtctgcgaccc

tgcgttcggcggtcgtgatgaggagcgaggtggacatggtcttctcggccgagagactgcagggtataagaagaggtcaaggtcgcgtcgccgtgctg

agaagaaagcatccagcaccagcaccagcaccagcaaaccccagcacgcgctcctcagcactccaacacccagcgaacgacagcaaggcaaaggcgag

gaaacaaaggcaagcaaggatgttctgcgcgatctcaggggagccgccacaggagccggtggtcaacaagaactcggggcagctgtacgaacgcagac

tgatcctcaagtacatcgcagacaaccagtccgatcccatcacgggccagccactcgcagaggacgacctcgtagtgctcaaagcaagtcccaagaca

gccatcccacgcccaccgaccctctcctccgtacccgcattactggtctccctccagaacgagtacgacgccgtgatcctcgaggcattcaccctcaa

gaagcagtacgatgccatccgccaggaactcacccatcttctataccagaacgatgctgcgaatcgtgtcattgctcggctgatgaatgagcgtgatc

aggcgcgagatgcgctgtccaatatccaggcttccctcggcacaggtgccgtcgcgtctctgccccccactggcgactcagatatgactgacgcagcc

ggcgcgtcctcctcccaaggggtctctgaagaggtcggcgctattctcacacagacggctgaacgcctgtccacacaacgtcgtgccaaatcgaagag

gaaagcaccagaaggctacaccaccgcagccgatcttcagcaaacctttgagcaaatcatctcgatcccatccatgcacagcaccaagcctcccggaa

tcacctccctggacctgtcccccgccacatcagagcttgccctcaccggaggcaacgacaaagtcgtgcaaatctataaccgccaaagtgggaaagtc

agcagtacactcaagggacacaccaagaagatcaatgccgtcgcgtggacccacaatggcgagatcaagcttggcgaggcggccgcaccggccgaggg

ggaagattccaatccagcacacgccgtcagtggctccgaggacggaagtgtccgagtctgggcacacaagggcgacgggacctacgccctctcccaca

ccctcgtcgtcagtcccgcatcgggcaagaccaagtcacccgtcgtcggcgtcgcggtccacccatgcggcaccatcgtcggcgcagccacacgggac

gggcattgggccctcttcgacatgctctccggcgacctcctcctccgcgttggtgcacccgaggcaggctccgagcccgaggacgaagccgaaggcgg

gtacgaatatgcgtcgtttgcgttccacccggacggcggtctgctcgccgcgggcacccggggcggggtcgtgcgcgtgtggaacgtgagcagcggca

cgaaggcgagcacgttccgggcccagttgagcggcgagttgcattcgctctcgtttagcgagaatgggtactacctcgccgtcgcagctgcgtcgtcg

gccgatgtggaggtgtgggatctccgcaaggtgacgatggccgcctcgattgcacacccggctggttcgtcgtcgggtgcgggtgcgatccaggcggt

cgcgtttgatccctcggcgcagttccttgctgtggtcgggcctgatgtgcgggtgtatgcgaacaagagctgggctttgcttgcgagctgggaggaga

atgcggcggaggtgacttcggtccggtgggatctgcgtgatgggagcttggttgttggtggcctggatcggacattgagggtgctgggcacgaagaag

gcgtag

>contig_14.contig_14.g343.t1

atgacacagcagcaacagcaacagcaacagcaacagcagcgcgatggcggcggcagcagcctcccacagcaggggcccc

gtcgtcgctccctcttccggccctgcatcgacatccacagcggcgtcgtgaagcagatcgtcggcggaaccctgtccgacagcgaggccaccacaatg

acgaaccacgtctcaacctacccgccctcccacttcgctgccatctataaatcgctcgacatgcgtggggggcacgtcatcaagctcgggcccggggc

ggctaatgacggcgcggcgagggaggcgattgctgcgtggccggatcggttgcaggttggcggtgggatcaatgcggacaatgcgcagagctggatcg

aggctggcgcggagaaggttatcgtcacctcgtacctcttttcggactgcaagtttagcgaggagcgcctgaagacgctctctcagctcgttgggaag

gagcgcctcgtcgtcgatgtcagctgccgacgtcgcggagacaagtggatcgtcgcgatgaaccgatggcaaagcctgacggatctcgaagtcaatca

ggagtccttgcagagattagaacagtactgcagcgaattcctgattcacgcagccgatgtcgaaggtctctgccaaggcatcgacgaagacctcgtgc

aagcactcgggaagtgggtgaccatccagaccacgtacgcagggggcgcacgacatctccaagatctcgaactggtcgaccgcctgtccgagggcaag

gttgatctcaccttcggaagcgcactcgacattttcggcggctcaggggtttctctggagcagttgtcccagtggaacacagaagcacttgccgcgtc

gaaatag

>contig_14.contig_14.g344.t1

atgtccacttctcctgtttctgctgaggcctggcagatcaccaagccaggaccgatccccgacactctgcagcttcgtt

ctatcacgcttcccaagctcaagcctagccaggtgctcgtcaaagtcagccacgctgccctgaacccggtcgactacaaattggccgcgctcttgccc

tctttcatccagaaagttccccgaacggcagccgtcgatttctctggcaccgtcgtcaaactcggctctgactccctctccaagaagtacgaatggct

cggtgaagagaatgtagccgtctggggcctcatttacaacctgccacccacctcagcccttggagcgatgagcacatacacggttgcggaagtggagg

acatccatcccttgcccaccaatacagcggccattgaggctggtatcactgctgagagcgccgcgggattgggtatcgtatccatgaccgcttacatt

cagtctcagtacgtcgagaagggcgacagggtcttcatcaacggtggcacaacggctgtgggtcttcttgcggccgacctcgcgattgcgaagggtgc

gtcgttgatcgtggccactgccagtggctccaaggccgacttcatcaagaaacgcggcgtgaatgaggtcattgatcgaattgctgacgaaaagttcc

acattcttgatccagaccgcgccggtgacccaaagcaagagctcgtaagccgctactcctccgcgccattcgatgtcgtcctcgattgcatcggtact

cccgaactgcacttggcctcgcctgcgtacctcaaagcgggcggaaagtgggtcaacatcggcgcatcgttcatcaagccgaacagcttcatattcag

ctcagaggctctcagcttcatacggtggaatattcgagcaaagctgcccaaattccttggcggacatcagcgcaccctcatcattgccggaccgtcga

agaacgatctggtcgaggtcaacaagcatctggcaaaaggaacgatccaccccgtcgtggacagggtcttcgaatggaaggacggggttgaggcgtac

aaatacttgattgagggccgagcgctgggcaaagtcgtcgtcgcggtcccctga

>contig_14.contig_14.g345.t1

atggagatagcatcatcttgctgcgtgcatcgcggagataacactgtcgacccggacctcacctcctcctcggtccgcg

catcgaaacgcaaagatccgccggctgcgtcaaccgacgcgagcttgccacccacccgtgccgctaagaccgctgcgacggaacgcatccgccagcaa

gccttcccgcgcgcacccaacagcaacgagctttacgccgttcaagccgacctcttcgagaagcgccgcgccgccgacaaaaaggtggcggcctttat

cgaccgctttttgcgcgaccttggtgaccttgagtcatctggtgcgctggacccgaactctgcccgcctcttcgccgccggcgcgcaagcagcattta

ggcaggcgctcactcctggagcgcacgcgccaccggaggtgctcaaagcactcaacgcctgggaccccgctgacaaggcctctgcggatcgcatccgc

cttcacgactcgtacgctgctgcagcagcacccaaccaccggaaactcaccttcccgcgacctcaaaaagggacatccgtcaaagcccgcccggaccc

accatcccttcgacctcttcaaggcttccgtctccgcggtcgcggtgccggcagcgccgaggaagcgctcgagagggccagtggcacggacgacacgg

tttacgtacgcctgtccagccagcactctttgcgttctaaacatgcctacttcctcaagaagaccatcttggacgcttttgccgccgccaatgctccc

tctcacataggcatcgacaccatcaagcacgttccgtctggcctggccttgcaacctctcggttcttgcgccgtctccgagctcttctctcttcacaa

cctcatcaaggcgacattctctgccgacaaggtcgagatcggcaaccaatgggaccgctttgtcctcatgaatgttccgactttcattgatggcgagc

aagttcctgacgaaatcctttccgacgagcttaccgccgccgtcggatgccccctaccagagcccccgcgtcgcctcggccgaccggaggcgctcagc

actcaacggagctcttccgtgctcttttctctgcccggtggacggatgccggcagacacgacacggctttcgatcctcggccaacgtttttccgtccg

cccgtacaaggagcagcgggcctcggaacattgcgaccactgcctctcccagcaccactcaacggcacggtgcaaggaactgaccccccgttgcaagg

attgcgcggctgagggccattcggcagggagcggggcctgcctcatgtctaaggcggctgaggactcggtgcaggcaatcccacgctgctatcattgc

cacggtccgcactcggctttctcacccgggtgcttcgcggcagcacgctactgcaaggaagtgcatgcagtcactattcctaccggatcacgtctcgc

tcgcgcccgccaacttggcgaacgcaagcgcaagcacgaagtcgcccgtcgcatcgtcgcgggcgcagcacgagcagcaaggcagaccggttcggccg

cggcgcagccgaccagtggtgagggagacggggcagcaacggcggctgctatggaggagggttag

>contig_14.contig_14.g346.t1

atgaaccggacaactcctatcgacctggagcagagcccctccgtgctcgatgcggcggggacatcggccaccaactccg

aagaggaggaacgcaaactccctcccatcgcccagctcgccatcatcgtttccatgaacctgttcttgaacgtgtccttcttcatcatcgtgccctct

gcccccgcgtatgcaacctccttgggcgcctcaacctggttcagtggcctctgcatcggtatctccaccgctgcagcaggtctcgtcctctacccagt

ctcgagacgatacagcctagcctacaagactccgatgtccgccagctgtgtttgcttcgtggtcgggaatatcatctatgccttggctggcgttgcca

acacagcatggctgatggccgtaggacgtgttattggcggactggggaacataagctgtcaggtgctcggaatggcaattggcccattctctggtggt

ctcctcaccaagatcggcggcgttcgacaagccggcaaagtttggaacggctttacttctgcaagctggatcatggccatcgcatgggccacgttctt

ggtcgtcttgactcttgtgttcaaagaccctcccggatctgacccatctacaacgctgctggaagaccttcaccgccttgccgggcgaatttcgaaac

taccaccgaacagcatggacatcgaattgaaagtggacgtaaaagctgtggggaaaagccgaacagcggtctcggagcacatttacagggccacatca

tcaggccaagacgataggataacggcacttgcgccgagcaccccaccagccctgccgtcggacgacccaaagggccaggagaacctgaatgctgctca

gtgggcatcgctcggcatgatgtgctggtttgccatggtgaccttcttcaccctcggtgcctgggaggccaacatccccgtcttcagctcgatctttc

cagggcttaactggaccgaggagaaagcgggtaatatcctcgcgctcgctgccatcgtcatcttacccatcctcttcgctctcgtcttcctctccaaa

tacattcaggaccgcgtcatcctcgccacgggcatcgctatcggcttgtccggcttggtcattcacatggtcacactcactccgggcctggaacatag

catcaaccttccgtcctttttcgcctcgtggcttctcgtagccttgggatacaacatgatgagcacagtcaccctctcgctgctctccaagcaagtac

cgcctcacttcaataactacacaagtctcatcatccagctcagcaactacagcggccgtttgggaggtgctttttggggcaccgcagtagaacaagtc

ggacagcgagccatcctgtcgctcgagctcgccctggtcttgttcggaactgtcggcacggcctacatcgagtccttcgtcgcacccgagcgcatgga

cgagatcttcgcgcacttcgaccacaactctcagatcacctaccacgccgtgaacgcgcagaatgacatgcgaacgaaggccaatccgacgcgcggaa

cggctgagcccggcgactatatcccttccggaacggacgcagttgatacattggtcgttctactggcctacgcctccaagggtgccgaaatgacggac

aaaagctgcatcgccgactcagggacgtga

>contig_14.contig_14.g347.t1

atgtccactttccgaaacctgtccaacaacaagtccattcgccagcccactgtcccggccacggtcaagtccaccatca

aggccgccgtcaagcccaacaacaagactatcgttccggccaccatcaagcccaacaacaagaccactggcccggccaccggcaagtccattcgccag

cccactgtcctggccacggtcaagtccaacgacaaggccaagtccattcgccagcccactgtcctggccaccgtcatcgtctgcaacgaagccaccaa

cattattaccgaccagtccattgactctctcggcacttctgagaagaatctcgaccgcattgccgctgatgctgccaccaagaagccatcggagtcgt

atggcgagtccaagtcctccctcgtcgccatcgacaacgatgccatctcgctcctcatgatgcgcaccgaacaggagaagatcgccatggatgtctct

gccgtcggcgacgctgttcgaagcatcgcgagcagcgacgactccgacgacgacgttcctccgactccgactccgacgacgactcacaaagaggaaga

cgaacttcacatcaccaaggaggtcccgttgacgtggccgaatggccccattccttcgatcctcgtcactggacctaccgacacttcctcaacctcgg

atggttaccacgccaacatgccggaattgacctcgcctgatttcctccaagttcctggaaccgagctttgtttctacaatgttccttcgctctcgacc

cgacggagtccgcgcaagcccaagcccttccgagccaacctcggagacgctgagctcgcagatgcttctctcgtcgacgacgaatccgagctcctgtt

tatcacttcgctcgaagagatgcggcggaacttcgacgttctcccagcctgggagaaggtcgaagtctcgaacaccgccgcttccgtcgaagaaaagt

ccaccccgcctcgccaaagctcccccccaccaacttctactgatagccttgcccccaccgccaccatgaccggtcctttcacctcgggctggagacca

atcttgtcaactcgtacggccaaggcgacgctcgtagacttgtcttcaatcgcctccaagaaaacgtcgattgggagcagcaaggcgaagttcgggcg

caaatccttgtcttcgtcccccttccagaccaagcccattggaagcggcaaggcggcgatcgagcgcaaacccttgtcttcggccgcctccaagatca

agtcaattgggagcagcaagacaatcacgactgagggcaccgattcgaatcgacgtgatgacggacaacagcacctcggttcctatcggcctccactt

tcaccaatacagaaggggttctgggtaattgctatctttgagatagatgttgatggaacatcctcccccctccccttcccttcctccctcaaagcggc

atatcatcctcaaagtaagtccgattcccccatcctcacccaagtggccagtccacatgcatgcaactcagatctgatcaagacttccattcctccca

ccgatgcttccccaccaccagaaagcacagatgagtatcctctcgccgtccgcgttcctgattaccgttactttggaagaagcgcagctgactggagg

aaccagtgtcgacgtccttccctttccagtttaccctaa

>contig_14.contig_14.g348.t1

atgacggccaacgccagcttccaagattggacaaaaactctgccgtctgtcttcgacgtctccggcaaggagagagaga

cgattgtgctctacggctttgtcctggatggcgggctcgacgtcgaagtcttgaagcagtcctggaagaagctctgcctcaaatggcctgttctcgct

ggccgactgcggcaaggcgaaggatcattcaaaaagtcgggtgcggcggcatgggaatatcggattccacgcgcacagtctctcgaagcggtcatcga

agcggacatggccccttctcgtcctgaaagcctgcgttcggtcctggtcaacgaggtggaaggcaaggtccgggactcgtacgcctttgtgcaggggt

gtgatctgcctcgggatcgacccactgcccactttggcacccggcccgccgacaccaaggccaagacgcgatcgaagacgatgctcctcttcgcctca

aacgctccgctctctctcgaccacttgttcaaagaggatcggccgatggtcaccacgaagatcactcgctttgaagatgctacgacgattggaatcgc

tctgccgcacgttctgtgcgatgcggcaggagcgtcggaggttatgaatgcatggtcggccatcgttaacggggaggagaacgagatccatgcattgc

ctcagttcggtcaggacttctttgcgcccttggcaccaggcggccaggaggccaagaaggaaagctccgcacgagagttacaggctcaaaagggcttc

cagactcccgccggctggtacgcctacaccttgtgggatacaatccaatttggcatgggctttgcctgggacttgctctggacccgacccgaatccaa

gatggaaggtcgggacatctttcttccgtcggcatacctcgagaagctcaagaaggaagtgaatgaggagatcgccaagacaacgtctgcatcgaagg

gcgaggcacagtgggtcagcacgtcggacatcgtcactgcgatcgcagtcaggactatgtgccgtcctgacgcccgaggtccgaatgacgaacgaccg

gtcagcttcctgtacccatcgaacttccgctggcttcccacaccagccggctgcgatcctttcccaacgccctacctccacaacggcgccttgaccat

cagtctccccgagcagtccatcggctccatcgtgcacaccctaaccttggccgaactcgcccttcagatccgacgcgccttggtcgaacagacccagc

ccgaagccatccgccgcagcctgatctggcgtctcgcgaaccgacaaaagctgctcacttttttccggccttacaacttttggctggtagggacgaac

tggagagctatgaagctgtacgacatctctttccatggcgcgctggagtcttctgaggctgctgctacatccccgtcatcggggcccaccggtcgtgt

gctcaaggtgtggtcccatgtcatcatggacattcccttgcggaacagcttcgggatcgtagccgacgatcctgctggaggcgtttgggtgggcatgc

tcttgagcaaggaccagtggagtcgagtcgacctggagcgaggaagcgcatag

>contig_14.contig_14.g349.t1

atgcagtacgtcggccgcctcgtgtccaccgtgtacaactcgatcaccccgaacatcaacccagccaccctcagcggcg

ccatcgacatcatcgtcgtccagcgccaggtcgagaagcagaatccagcagccgacggcgacggcgacggcgacggcgacggcgacggcgacgatgcc

aaggatgccgctcctcagcaggccgagctcaccaccgagctcgcatcgacgccgttccatgtccgcttcgggaaaatgtccgtcctgcgcccggccga

gcgcaaggtcaccctccatctcaacggctcgcccgaacccttgcccttccacatgaaagtcggcgacaccggcgaggccttcttcgtcctcgaagtcg

acgaggaggactcgtccgacatcccggacgcactcgtcaccagtcccctcctcagcgcaaccggcagccccgtccccggttccgatctcgacagtctc

gaaggggacgatgacgctcgctctggcgcgttcgacgtcgagcctctcgatctcggcgcatcggtcgtcactcccgcggatacaatcgatccgacccg

cgcagctagtcaggcggcctttgttcaagacagcaaccaaggagagagcgccgccgatgccattgcccgtctcagcctcgacgatcatgatctcgact

ccgaggtccgcagcgcagcagcatccagtcagcgcaccgctgttgaaaccgccattcctgtctcgactaaggaactcgacaccatgcttgacatggac

gggtacaaggtcacaggagaggccggccaggatcacaggcatcatcacttcagctccctcttgcgtcgcgccgcacaggaggaagatccgggagagga

cgtcgaggaggagctccgtcgcgaggccgaaaacttctttggtggcactcatcaacgcacactctctaacccgcaagacatgacacatcgacttctcg

cccgcgccaagtcagaccctgccctcgatgtgactgcggcctcagcggccgagattgacggacacagtgccgcggagaagattatggaaaaggtcgtg

cgcggaccggcagctgcaccgcgcactcgcgcagacatcacagccagcacgtccgcaggcacaaaggacaagaaattcgtcaagaccctccgtctctc

gtccgatcagctcaagaccctcaatctcaagaagggcctgaacacaatctccttctccgtcacgtccagctactctggtgtcgcaacctgcacggccc

gcatcttcctctgggacagcagcttccaggtcgtggtatctgacatcgacggcaccatcaccaagtctgacgctctcggacacgtcttcaccatgatc

gggcgcgactggacacatctcggcgtcgccaagctctacactgacatcgcgcgtaacgggtatcacatcatgtacctgaccagtcgcgccgtcggtca

ggccgatgttacgcgcgactatctgaagaacatcaaccagaacgggtaccgcttaccggagggccccgtcatcatgagtcccgatcggctcatgacca

gtctgcatcgagaagtcatcctgcgcaagccagaggtgttcaagatggcctgtctccgagatattgcgcgtctctttgggtgtgatttcaggggtggg

ccgccggcggcccctcatgcgggaggtcactccaccggcgcactggttcctccaacatcggcacagaccgtcggagcggaacttcagaagcaggggct

ggagtccgtcgctgccgcgggggctcatgcgaaccactcttcaacgccgacgcccttctttgccggattcgggaatcgtatcacagacgcgctcagct

accggtcggtgaatgtgccaagttcgcgtatctttaccattgactggaatggcgaggtcaagatggagctgctcgagctggcgggctataacacctca

tatgtgaagatgaacgagctggtcgatcaattcttccccccaatgcgagcagcggaagtctcgaatcctcggcagcgcgaatacaacgatgtgacctt

ctggcgggccccgatcgacgacatcgagctcccactcgacgagctctcgccacctgtatcaccagccctctcggcacgatccggcctcagcatcagaa

gctcgatcagcatccggagctcggccagcaagcggagtgcgacgacgagcgtgctggacaacaacaacgaggagcgttcaccgggcggcggggctggc

ggcgagcgtgggaattccagtggaggacggctcagtcggttcgggctttcgagcttgggcttgagtcggagagagtcgacgagcgtgacgccggaagc

gtcgaggaacgcgacgtgggcgccagcgtcgccggcgtcgccgcagaagtcgaaaacgcaggcgccgtcgggagcaacagcaggaggagaaggagcgg

cggcggggacggcggctcggcttgggattccctttcccgagagcgtgtcgtcgcctgacgtacttatggacgatgatgatccattactggcgcagggc

gaggtgcggttcgattggaaatga

>contig_14.contig_14.g350.t1

atggcaggacaacagcccgtcttcgtcgtgaaccaggcgcccgagcggcagcagggccgcaaggctcagatctccaaca

tcaccgccgcaaagaccgtcgccgatgtcattcgttcctgtctcggtcccaaggcgatgctcaagatgctcctcgaccccatgggtggcattttactc

acaaacgacggacatgccatcttgcgtgagatcgaggtcgcccatcccgcggcaaagagcatgatcgagctcagccgcactcaagatgaggaagtcgg

tgacggcaccaccagtgtcattatcctggctggtgaaatccttgcgcagtcgcttgcctcgctggaagccggcattcaccccgtcgtgatcatcgcag

cgtacaagcgcgcccttgccgaggcactttccatcatcgacgagatcagcgtgcccgtcaatgtggacaacgacgaggagatgcttgctctcatcaag

acgagcatcaacaccaagtttgtcagcagatggtccgatctcatgtgcaacctcgccctcaaggccgtccgaaccgtcgcagtcatggacgcctcggg

accagggggcccgggcaccatggcgtccagcccctcctccggatctctcaacctcagctcccgaccctcttctccctcaggtgcgaacaacgccgccg

gcagcgcgccatcacatccgtcgtcagcctccgctggcgcgtcgggctcgctccacaccgtcgatatcaagcggtacgcgcgcgtggagaaggtggcc

ggaggcgagatcgaggcctcgcgcgtcctcgacggtgtcatgctggccaaggatgtgactcacccaaaaatgcgccgtcgtatcgagaacccgcggat

tattctgctcgactgcccgctcgagtacaagaagggagagtctcagaccaacattgagatcaccaaggaggaagactggaaccgcattttggagattg

aggaggagcagatcaagaacttgtgtgctcgactgatcgagttcaagcccgacttgatcttcaccgagaagggtgtctctgacctcgcccagcactac

ctgctgaaggctgacattaccgcgattcgacgtgtgcgaaagtccgacaacaaccgaattgctcgggtcacaggtgccacaattgtcaaccgcgtcga

ggatcttcgggacgaggacgtcggagagaagtgcggactgttccacattgagaagctgggtgacgagtacttcaccttcctagagaagtgcaaggatc

ccaaggcgtgcaccatcttgctccgtggtccatcgaaggacatcatcaacgagattgagcgaaatctcgcagatgcgatgtcggtggcccgcaatgtc

gtcttccaccccttcctcgcgcccggcggtggtgcgaccgagatggcgatcgccgtcggcttgagcgaatttgcacgatcgtcggccgggagtgcggg

tcagaagggcggcgtccacgcaggtccgatccgcgccgttgctgacgcgatggaggtcattccgcgcacgctgattcaaaattgcggaggcaatgcca

tccggacgctcactacgctgcgagccaagcacgccaatggggagcacagctggggcgtcgatggagacaatggcaaggttgttgatatgaaggagtat

ggcttgtacgagagtgcggcggtaaaggtgcaaacgctgaagactgcaatcgaggtaagtattttccttgtcccaggtctcgtcaacctcgttccaca

gctgacaacgctttgtctctttggccagtctgcatcgcttttgttgcgcgtagacgatgttgtttccgcacggcgtgctcgtcaacaagggggtggtg

gtggtggcccgcaagtgcaaaacatgggtgctccgggcggagaggacgggggtgatggcgagatgcccggcatgtga

>contig_14.contig_14.g351.t1

atgttgtccgtttctcacacgctaccagcgaccgccttgcgaaccatcatcatcctggccattcgccagctcaacctcg

acgtgcacttctgccaggcagcatcgctggcggaagcagccgccgccgcagcaagagcggaggcagaagcagcggaggcagccaggagcgcgtacatc

ggccaggcaggaccatcaacgctgccaggaacctcaggcgcgacatcgctgccatcgacactcgactacgacgctggcgcggagagcggcttcgaggc

agagtga

>contig_14.contig_14.g352.t1

atgccccatcggacagctctctccctcgacaaagccgctgccaaggttgctcccaaagcacaccacctcgtcatctcca

atcccgtcaactccaccgtgcccatcgtcgccgaggtcttcaagtctttacgaccccaagcgtctgttctgtgtcacgggtcaccacctttgacatca

cacgcgcctccaccttccacttcgacattgccaacgtcaagcccgccgaggtcaatgtgtccgtcgtcggtggccaccttcggcgtcaccattgtgct

tctcctctctcaagtcgcccagggttccatactccaatag

>contig_14.contig_14.g353.t1

atggagcgcgaggtcgtcacacagagcaaatccgcccttgtccacaacgtccaggacccacaccaacaatatcttctca

accattgcctttttcgatcggctttgtcattggcaagtttctaccctccacttgcggaagctccgagtacgggcgagattgcccggtttgcgaccgat

atggaggtcgatgaagatgacgacgaagacgacgacgtattttccttggatgacggcttcagtagcgactctaccgagtccgacaaacaagggctcgc

atag

>contig_14.contig_14.g354.t1

atgccggcccactgcaagtcattgcagcagcttcaagacgaggttggcccagatttcaaggttatcagcgcccatccac

ctgttcttgcttgtcgggtctgtagatgggacgccgtccgacggttctcacccaagcatcttcagaggcacattgcgacgcagaagcatcgcgaagca

aatgcggagcgagagaaagagcatctgagcttcacagccgctagttctacagccacgacatcgacagcagcagcagttgcaagagactgggagcagtt

ggacgactctttcttgttcccggacgacgaagcacggcttcaagccgaacagtaccacgacataatgcagatggaggacgatgtcgaggcgtgggcga

gggaggctgcagatatgaaggcggcagaggatcatgacgttgaggcaatgggtcgggacagaagcgatatgctccaggcgctgggaggtgatgcgttt

ccgttcaaaaacagggagacattcttggctctcgcgctcaagttgtcgccaaaacatgccattttaaacacggttctcgcccttgctctcgacttctc

cggacagctgaacgccaacagggtgccctccgtgaatgcatgcgtcgcatctatggcggcagcacgagcagaggttgggatgtcaatgaatcgccatc

agtcggttgatggaaagatattctggtcgagcaccattcagcaaaagctgacgttcgacttttccaacccttccatacggcccaagcttcaggtcctc

cctcgccaagatgaagagtttgcagaattctgggatgggtcggctttatctgcccagcgcgatactcgcaggacccctcctatgtggctccgagcaga

tcaagcgcaaatatttacagatgagacagtgcggcttcgagacaaacggctcgtacggccgctcaatttctttcgcgcagcagcagcatcagaaggcg

ttctctctgaggggaggctggtctatgccagagacgacagtcttcccctggagctattggactctgtcctcacgttcgaccctgtcgacattgacgtc

aatttctcggatgcgctgcgtcagcgtcgaattgtggatagagacggcaaagaactgccctctgtcaacccactgcgcaccatggctaaagggcgtct

gattttccaggtcccggtcatcctctttgtggatgaaacttcaggcaccacaacgaagcggtggaatgaacacgtcgcagtgtacatgtccaacgcgg

gacttgagcggaaggatatggactcgccgagcaatatcaagttactgtccgtcagtaccaacgtgggcgccgaggatctcttgtcggtatttgccgat

gagctggtccagctccaagaacaacctttctcagtattcgacgcctggctggaaacagaggttcttctcggagaggtgaaaactgccaagcaggtaat

cgcctccctgaagtcccagctttccctggcagagaagaagggcgtcacgtctcggcttgtggaaaagcaaagagcgacaggcatcaaagatgcgatca

ccaatcgctactgcgagcagctcatcaattcgttcaaagcgctcagcaaagcggtacgagtagaagcagtcaaagctgtcaaacgggagaaggatgag

attctgtcgaagcgttggcatcattgcttgctctctttgcatgacaaacacggtttcgatgtttgcgaaggcaccccagttgacctgctccacacata

ccagctcggactggttaaaggtccttggctggtttcgaattcaggtggccttgctggtaaggacttgaagtacattgcccaggttgctgccaacgcat

tttatcccatgatgcaggacggcacgatggatcttgggatgtgggcagcttggagtgcggtcggaatcttgggacgactcctcttcatcgaaaatgta

gccaggaaggacctggagacatacaaggacaatctcctaagtgcaatcatggcgttctacagctcggcagcagcctcattcccgtctcaggttgcagc

caaaccgaaatacaatatcatgctccacatgatcgaaaacatcgatcaatttggcccgcccaaaggcttctctgcggaacgatatgaagccttcaacg

ctcgcgttcaagaccaagagcttattcgtcatgttgtgtcggctggctgctggatgaagggaggagaagttcgcaagccctcgccccttctccaccag

ttgccgtcagacgctctgaaggcggagtttagcattctagacccaccttcaatctcgtcccaaacgtcggtcaagttcctgctgtcggcacaagggga

tcgtatcggcgtcggagcatttgctacaatcaatcacgatgtttccagcgagaaacgtagcatagttcgcatggacgagttgcggaggttggaggatg

ggacgatgatgacaacggttactcatatgaactgcgacgtggacgccagcggcttggagcccattgttctcaaagtaggaaggcagcaaagtggaacg

ttcgtgggatctctcttccctttcagatgttcgaccgcctccttaatgtcagccacgactgccatcatcatggctgcaaagtcgacattgctggtgct

gcagtccgaattaaggaagagcacggagtag

>contig_14.contig_14.g355.t1

atggcagggcacgacgagtcgccgatcaaacccgctcggttccgacctgctgcaggtcgaggtcgaggcgctgttcacg

atccaacgtccgacgccccaggtctcgaatctcggttcggagactgggaacctgctggagacgaggaggacacctatggtgaagaagacgatgagctc

gacgattatcttgaaaacggcgaagactccgaggaagaggaggataacgagttcggctcaccttcagaggaagacgagcacgaagaagagcgcgccgg

ccttggcaagcgccagcgaaaccaagaaggaccgaggaagagagcaaggcttcctcccctcctctaccaagccgcaggcaggtacaaattgagcaagg

actgtgtcaagatgttcgaggaggagatcgccgagtacatcagcttcgagaacaagatcccctcggccacaatgctgtacaagacagcccgagagatg

aagacgtaccagaattccgagaccatccaggctgaagtccaagtgctgtgtagcaagactgaactggccaaggctatcgcctactgcttctactcaga

gcatgcgtttctgaaagcgaacccgcgtgctgtcagtgacgacttcaaggagcatttcgacaatgcagaagtcagacgaaaggaagttcagcctgcta

ttcatgcgtcaatgggtatcgactcgggaacaaagaagatgacattgtacgaactctggtcctacctctgcaaaggttatggcgtccctttcaccaag

caaagagcataccgtatcgcactaatgcgatttgtcgcgtcggagaagcccgttcgccacaaagacggcagaccgacgggaaagttctggaggactct

ggacgctaccatcgccgacatgcagcagcgtcagggcgaggatgcggccggagtcaaagaagatttacagaacatctacgaggcggacatggagacat

acggcactcgggacgatgtggaattttccaagggcggatccaaggaagatgccttgaagacagcgatgacggcgaggtctgattga

>contig_14.contig_14.g356.t1

atgaactccgagtttccatcaggcgctcctccggcggaggaggactatgacaaaatgtccattgaggatcgcctcgcat

ccaaggtgagcatgtctgatctccttgacgtaaatgctggccagatctcagtgctcaacttttctttcccccgcaaacagatctggaaagcgcgcgta

tcagcatataccagcctcagctccgcgttcgcgcgcacgccctccgataccgaccccgctttcagaccatacacccgagatcccacgctgctcaaagg

catggcgtcggactcgaatgctgttgcgcaggaaaagggcgtagaagcggtgcggtctttcgtagagtacggtggtgctcaagccgggaaaacaagag

aggtagtcttacctgcccttgtggacaaatgctacggctcggcaagagcagggacgaagaaggcggctatcgagtgcactcttcgctatgtggagatg

gaggacgtcatggggagtgaaggggctgtcacagacgtctgcaacggcttgaacgcgaaacagcccaagctggtcgcaacctgcgtcacggccttgaa

ggagatgattctcaactatggccccaagcaagtctcgccaaagccgattctgaaagtactgccaaatatcttttctcacgccgacaagacggtccgcg

cggaaggatcaacactcgcagtcgagctacatcgctggatcggagcggcacttgaacctaccatcgacaagctcaaagatatccaagccaaggagctg

cgcgctctcttcgaggaagccgacaaggccgatagcggaaagccaaagccagcccgctacctgacttcggagcgcgcggcggcagaagcagcgagtgc

ggccgcagcgacagacggtgctggaggagatggcagcgcgggcgacgacaaagcaaccgaagaagcggacgcattcgatttcgcagagcccttggacg

catttagccacaaggactggcccggagactttgatgagaaggtcgccagctctaaatggcaggagcggaaggaaacactggagaaagcccgtacggta

ctctcgtcgaatccaaagctgaagccagacaacagatacgacaactttgtcgaggctgttgttcttcgagtaactaaggacgtcaacatcaacgttgt

attggaggcatgcggatgcatcgaggcggcggccaaaggtctgcgggccaaaggaggtttcggcaagtaccgcgaaaaggtgatgtccccgctcttgg

agaagtgcaaggagaagaaggccaccgcggttgaggtgttggctcggacattggatgcggttttccagacggtgacaatatcggagatcctcgaggac

gtcttcactttctccaagcacaagaatccaaacgtgaagacagagtcaatccggttccttgttcgatgtttgcggaccacccgaacacccccgggcaa

agcggagatcaagccaatcgcagaagcacttgtagctgcttcagccgacggaagcgcagatgttcgcgaggctggttttcaaggtctaggaacgctaa

tgaagctgatcggcgagcgggcgatgacaccgtacatggaatcgctggacgacttgaagaagccgaaggtgatggaggagtttgccaaggcggaggtc

aaagcgaagatgggcagcgctgctccggctcgggctccgcctgcggcacctgcatcgaacggtccaccagcgcgacttgctgctaaaaaggcgccgac

catcggcaaggagaacgtgaagccgtcttccgcccagcgtggcgcagattctccgcctccagcacttcctcccaaaccgcctgcccgttcgatgccaa

cgaaaaagcccgtcgccgccccagccgcgatcaagaagcctgcagccgcagcagccgcagtgcctaagggcggcaaaggtgcagcggctgcgaccgaa

ccggtcaaataccggttccctccggaggctgcggaggagcgcgctgcggagctgattcctgcttctgtgagggaggagttggcaagttcagtctggaa

ggaacggcttcagggtgcgcaaaattttgcgacttggctcgaggccgaaaaggaatccatcgagagcgagatcatcgtccgcgccctcgcaaaatccc

ccggctggaaggagagcaacttccaggtgctttcggaggtctacagaattttacggatcctcgcagacggatgcccgacatttggacgcgcttccatt

gcactcagtatacaacctctatgcgacaaactcggcgacatcaagctcaagggtcccgccggtgagactctcaccttatacgcggagaagaccagttt

cggattcgttctcgcacaagccttcgcaccgctgtccgctctcaaagcgccaaagccgattgcggacagcctggtgtggatcgaggccaacctcctcg

aatttggcacaggtggagtcgacctgcgaagcttgatcgagcacctgctcgcatgcctcaagaattcgaatgctggtgttcggaccaacgctaccaag

gccttcggcacacttgcccgcttcgttggcggtgcagtcacaacatttctcacagatatcaatccccagcttcgcacgattctagattcggagattga

gaaggctgccggcaatccggcaccgcctccgactcgtttcggtgcagaaaaccaaggcgcagcacctactggtggagcaggaggagccagcaaaggtg

gcggagatgatgcgcttgacgatctcattccccgggtcgacctcgataggctggtctcttctgccatggtcgccaagctcggcgatgcgaactggaag

gagcgtaaagaggcgtgcgaggacattcatgcgattctgcaagcccaccagagattgaagcccaatctgtccgatctggccgtgcctttgaagcaacg

atgtgcggacagcaatatctcggtgcgaacgctttccctcgacatcatcgccaagattgccaccggcatgaacaaaggattcgacacgcacgcgcgag

gattcgcagcaccggtcgtccaggtgttagcagatgccaaggctcccatacgtgcagctgcgacggcaactctcacggccatggcggaacaagtcggt

attgcgcctctcgtcgcgggcatgtcttcggttctcgacagcaaggccgcgaatccgatgctgaaacaggatctcttcacatggctcggagagtggtt

gaacggacatccaccggagaagatcgatttcgcgccccttgcgcaaggtgcggtgctgtgtctggacgacaagctcgcggcggtaaagaaggcgtccc

aatctgtgttaccgttcatcatctcatctgtgggatacaaagcggtagtggcacagacctctcagctgaaagctgcctcgcgcaacacagtcatcccc

ttaatcgatgcagcgaatgctcaggccactccatctgcgccttcgtccgcacctgcgcctaaagcgacaaagcccggtgcaccgactgccatagcgcg

agccacgacaccgagcgcatccaggacacagtctcctgcgcctatggcatcgccccgcacaagcattgcacgtccagggactctcaaggccccaggta

ctttggcaggcaggttacttgccaagccttcagccggccttatcagaccgccttcccaactcgcatccgctgagactccttctgcggtgcccactttc

aagtctcggctcggaggtggtctgaaacgacctacgtctgtagccccaccgtcaatccccccgtcggcttcgcaatccagctcaggatcgaaagctcc

gcctttcctcagtgccgacccgaagttcaaggcagctcgggagaaagcgttcggtcggggcttgaactggattggtgccgaagggtcgcctcgtccgg

agctggtggcggcattatcccaacaatgcgaccaccattgcgactctggattcatggccatgctcttcagcaaggaccacaacaacgaccgcgacttc

atggcggctctttccctttcgctcgactacatctcgaatcccgcctttgcggaggaggaatacgacatgtcgcctcaagaaactgtcggacgggtggt

agccaacagcgaccttatcttcaaatacatcgctgttcgaatgaccgacaacaacacgagcatatcgctgaaatgcctcgatgtgttggctgcgcttg

tcgagctattgcagacggagcagtatcacatgtccgagtatgaggctaactgcctattgccttgcatgatctcacgggccattctctacctgcagttt

ggtgatcccaagttccgtgaccgcattcgggagatcttccgcagcttgacattcgttttcccgcccagcaagctcctgcagcacttcctcgacgatgg

tctcccatcaaagaacgccaaagtgcggacggagtgcctcaccgagctcgggtacttgttctccaagaacggtctgcaagtgtgtactccgggaaagg

tgctgcctgtcatcgccaagcaaatctcggatcgggacgcttcggtgcggacggcggcgcttctttgcctcggggaggcctacaagatctgcggggaa

gacatttggaagctcgtcggaacgctggcgcccaaagaaatgtctcttttggaggagcgtttgaagaggacctcggtgccagccaagcccaacggcgc

tgctccttctacaccgccacgaagtcgacacagtatgctccctcaatccccgaccgcggctgctccgggtgctcgctccaccattgcgacaggatcca

aattggctcggccaggatcgatgatcgggggaccatccgggttgccgtctgcggcggctcgaagccgccttcctggtgcaggaactgcgagtgtacgg

cgcgaatctggtatagcggcccctggcgccatcgctcggcggtccatcagcggggaaagcggcatttcagggtcgaatattgcgatgccgccaccgaa

gagccgcgcaccaccgcccatggaagagactgaagaagttcaggaggacgacgaagacgaggaaacgctattcggtgatgacgacgcggttggacgga

tgtctctggagcaagtcatcaacgaggtgctcagccaagattcggaacggagtgtcgccgcgctgaagcagatcgagcgggatatcaaggagcaagcc

tcacagctgcccgatgttgtggatgcgcttgttgtggttattgggaagcaagtacatgggtccttctcgcaagacaaggataacgactcactggagcg

tctgcgcaaacatctgctggtaaccggaacgtccatcttcgatacgagccggctctgggacgaccgcacgctcggcagctttgtcaaccggactgcac

tgatcccgctgttgacggagctgctgcaacggatgattgacctgtcacctagcaaggaagatacggcacagatgtattgcaagtatttgaacatcatc

gtcttgcggtctttctcggcctgcaacctcaacacactcttaggcgcgtgcttggagatgcttgctcaggcgaccgaggatatgcgggagctgaacga

agagctgctggcgaagagggccaagtttgctgagctggtcatcaagtgcctgtggaagatcacgaagcggctgccggatgcgctgaaccaagatctga

tcggaccccaacagctcctggtcgacgtagaggcgttcctgcaagcggtgcctccggccgagtggaaacgacgagccgctgaccagattgcgctgcag

gatcttccgctccggacggtgaaggtgattttgacgcactttgcacagaacctcaaggaccaagcgctcggttacgtcgaagcacttccgaatgcgga

gagctcgttcgtgtactcgtacctgatgcggatgctgagcattgaagaccagcagggagaccgggccgcgaacgacgacattatcgatgatgcaggtg

taggcgaggacgcctcaatccagcgtcctgcagcgaacgcgggaggcgcccgccacggccggcacagttctcgagccagtatgagcgctgctggtggt

ggcggaggggagaacgaggaggcgataaatgtggagctgcgggcgatctttgagcggatatcgaacaagaacgagtcgcgacagggtattcgggatct

ttatgagttctcgaagcggtatcctcaacggcaggcgagcattgatcggtcactggaggcgactggatccattttccagcgatacattcgacgcgcgt

tggccaaccacgcggcggaagatggagatggtggcggcgacttaacccgagatcctgctcgggactcagcgctgagcggccagcgtgactcgcgtatg

accgcaacttcaggtcggtcgagtgtattcgggggcatggatatagcggcacctgctgctgcggccttcgtcgcaacgacaagaaacagtgcgtttgg

cgacagcgatcccgtgtcgatggcgaacggcgccggcgctgatacagagtcggtggcatctcctgctacttcgccgcctcgagcgtctgtggctcgag

accggatggaggagctgagggcgcggttccggcagccatcttcgaacgggacgcgctctcgagcgggatcaagtgattatggcggcaatcagggcgaa

catcttccatga

>contig_14.contig_14.g357.t1

atgttcgctgcagcagcagttggagatgccgctagtcgcggtggtcgcggtggtcgtggcggtcgtggcggtcgtggtg

gcggtcgtggtggctcaagtggcgccgggtccatcaacatgccagacgttcctggcggcggcaacttcaacgagctgaacgctcagttacaggagtct

atggaacagaataacacagctatgcgacagtgggtgaaggacgaggtcaaagcagatcttcacaagtaccaccaagctgctgccagtttcgctactac

tcacagtatcgcaaacctggaagacgtgcaggtctccggagtcacagacgggcgcatcgtcatcgtactcaatgctgtggagttcgatggactgcaag

atgttctgaggggccaacgagaaatcaagaagacgcttgaggatacccaaaaaacgcttcaagagacccaagaccagctcaccgatgcccaagacgag

attcggatcttgaagcagcaaggtcaaacccaaaggacgttgccgcgaggagtcacccgcgccagggaccaagaggatgaggacgaatcgacagatga

ggacgagtccgggactcacaaacggacaaggactggccatggacgcaaggacaaccgcatacagcgagcgtttgagcgtcttctgcgcctctgcatgg

gacttgcagcgaagggggcctggccatcgttcccaagtgttgacaagtcgtcgccagattggccgcgggaggtaccggaagtggatatggaggatcca

gtcgatccagacgatccagacgccaacatcgatccacggcttcgccaccatgtaccactgggaaagccactgctgcgactcgactgggtcaatggacg

acttgacgacaaggaattttctgccgttcttgcggacttgtcagaggagctggcagagaacgctgatgatcatgacttaccgagcgacccagaaaaaa

aacgctctgttgaatatattcgcagcgcgtgcaaacgcacgctggtcaacatcaaacgcagcgcacgcgagcttcaggtcaaaggtgcggaggtggtg

gagcaagaaaaggctcacaaactgcgcaacgaccgacgcggagggcgacacaacacgagactcaacgcacgacttgccattgccgaacagaattctct

ggaattcggacctgctgccgtcgcgatggttattcctgcgtgtgtagaaaaggacgaatcagaagatgagcttcatgaggagcaaatgcagggcgaca

atcctgaagatgaccgttcggcgacgcgcaaaagggactccatccgagcgatcatcccagagtggtggtcgcaagagaacaaggacttacagactcgg

ctcgacaaagaggtgccgggatcactgtcgtcgtacaggatcaaagcggccacacatctcaagccccacactacaccactccacaagtcaattcctcg

catcgccatcagctcaacgtgggccaatgctcacccagatctcgtccgccgcgtgaagaagaacgccgctccgttcaagccggagcgtggggcactcg

ctgccagcgcagaggagtttggctcgcccatcacagggcttactcttcttcaagcaggttctctgtaa

>contig_14.contig_14.g358.t1

atgacgctgttgaacctaccggagcgtttgaggtatcatccatccaccacctacctcgcgggctgcacgccgggaccga

aggaacctgatgccgcccgcctcccctgctatctccgtcccctgctcactgagctgctcgctttggacgaaggtatgtccagaaacaaagtctgcggc

tttccacctcactcggtgaggattgggaagttctgtggactttgcgacgtcactatacccaccgtcatctccgccttcagtcaatgcgccgaggatct

tgagcgagatgcctcagatcatagggtcgcgtcgcttcgcatggaacagcccttccggagcaagaaagctaagacggccttcgagcgaagcacaggtg

cgcgcgcatctgcgttgaaccgccttccttattggcattcggtcaatcgcgctccgatagaccacatgcacaacctcgagctcggtgttgtgaagcgt

cttttccatcgcacgctcatcgaaggcaagtcaatttctcctctacagcttaaacgattccaatctggcctgttgacggcactggttcctccttctga

gcaagcaccagatgcaaggctcggagatccaggcggaggatcagccacagcagcgcattggtccacactcggcagccgtctcctcgttttgcttctct

ttgtcgcatggcagccgctagtagaggcagacggctctctacccttcgagccccccatttcgaagactgcagcggcagctgtcgcagcagcaacagtt

gcagcagcagctaatggagatgccgatgtcgaagcagaggacgaagacgaggcagcagcagcagcagcagcagcagcaccagcggcagcagtagcagt

ggcagcagcagcagcagcagcgacaacaaccgctgatgtcgcagcagaggacgatgtcgagacagctgcagctgctgcagcgatagcagtcactggag

gaacaacagccgacaacgatcatccgatgagcgcgctcgatacagcagcagacgaacagactcaggatcagcaacagcagagcagcagaacagaaaac

gccggcacggacgtcagatcgaaacgcatacaagcgcgagacgccttgcggaatgcagccctgcttgcgggagcagctactctcgctgctcgccgacg

tatagcatctcacgagatcgacgaccttgaccgtctgttgcgcgagtttggtcgtaccacggctaccttatacggtgagaagtgggttgtgtacaata

cacacatgatgacccatttaccacagcatgttcgacgcttcgggcccgcctaccatttttccgcgtatcacttcgaacgcatgaacggtcagctcggc

agcgtgaacaccaaccgccatcgcaatggcgaaattgaggccacgtacaccagagccttcctcaccaccgcacgatcaggactactcctcgccagtag

caaagacgacatcaatgctgccttacaagctcgtgcgccggtttttgttcctcctgctcccgtgttcttgagtccagtctcggtcttgggcgcttccg

gcgtcgtcgctgtcgagctttccgatggaagacccttcgagcttgcgcgtgagctgcacggacagctgttcgagcacgtgaggaggacgtggagcgac

cagtctccgcctttgctgccaccaacatcatcacagcgtactgggcttcgagtactgcctttcttccaacaacatcggaccatgcgctttggtcacct

ttctttcagcagtatcggcagcagggccaatgcaaaccgtaagcgaacatacgctgcggtcgaaggatcaggcggctccttggacctctttcgcatcg

aggtaattttgtcgcacaccatccacgtcaagtcgcgtgtgcatacgacgttatacctactcggccatcgagggcaacgtgaagttgtgccggcgttc

gacagagttctcggcgaggagctcctacaaaccacactcagatacttttggactcgcagagatgagtggaataccttggagcttcttccggcgcatct

cctcagctccgacgttgccctcatccacaccaaagatctgacaggtgtatctccaggagaagatcaacggcgcctttcatga

>contig_14.contig_14.g359.t1

atgccccatcggacagctctctccctcgacaaagccgctgccaaggttgctcccaaagcacaccacctcgtcatctcca

atcccgtcaactccaccgtgcccatcgtcgccgaggtcttcaagtctttacgaccccaagcgtctgttctgtgtcacgggtcaccacctttgacatca

cacgcgcctccaccttccacttcgacattgccaacgtcaagcccgccgaggtcaatgtgtccgtcgtcggtggccaccttcggcgtcaccattgtgct

tctcctctctcaagtcgcccagggttccatactccaatag

>contig_14.contig_14.g360.t1

atgccccatcggacagctctctccctcgacaaagccgctgccaaggttgctcccaaagcacaccacctcgtcatctcca

atcccgtcaactccaccgtgcccatcgtcgccgaggtcttcaagtctttacgaccccaagcggtggggtttcccccgaccccccctctccttcttcta

acggctctgacgcttcatgagttaaaaatggtgctcatcactgcatatcttcgtacagtctgttctgtgtcacgggtcaccacctttgacatcacacg

cgcctccaccttccacttcgacattgccaacgtcaagcccgccgaggtcaatgtgtccgtcgtcgatgctcgtagctacgatcgcaagtcgcagccgc

atggcgaagcagacgaaggccaggtcactatggttccaaggcctctctccctcgacaaagccgctgccaaggttgctcccaaagcacaccacctcgtc

atctccaatcccgtcaactccaccgtgcccatcgtcgccgaggtcttcaacccgccgaggtcaatgtgtccgtcgtcggtggccaccttcggcgtcac

cattgtgcttctcctctctcaagtcgcccagggttccatactccaatag

>contig_14.contig_14.g361.t1

atgccccatcggacagctctctccctcgacaaagccgctgccaaggttgctcccaaagcacaccacctcgtcatctcca

atcccgtcaactccaccgtgcccatcgtcgccgaggtcttcaacccgccgaggtcaatgtgtccgtcgtcggtggccaccttcggcgtcaccattgtg

cttctcctctctcaagtcgcccagggttccatactccaatag

>contig_14.contig_14.g362.t1

atgccccatcggacagctctctccctcgacaaagccgctgccaaggttgctcccaaagcacaccacctcgtcatctcca

atcccgtcaactccaccgtgcccatcgtcgccgaggtcttcaacccgccgaggtcaatgtgtccgtcgtcggtggccaccttcggcgtcaccattgtg

cttctcctctctcaagtcgcccagggttccatactccaatag

>contig_14.contig_14.g363.t1

atgcctcccgctcgcccgcatcttagccgcccggggctagacgcatccaccggcaagttctggtgcgactgcactgtcc

aaggatgccggacggaatctcgccacttgaacaaagtcgagcgacgcacctattatgctcaccaaaaacagcaaagaagtgctgcgcttcgagcacaa

aacgcaagcgcatttgatttcccgtccatacatgcccggaaagagacgaccgcgcaagatgccagctcttctgagccatccgccgaagtcgatgacat

ccagaacgacaacgacgtctggttccccgacgtcaaagacgctcttggattcgctgctcctcaagataccaccagaggcggcgacaccagcggtgatg

tctccgacgacgacgagttggaggcgagctccgaatttggagacggcattgagcgggagagggtagtcgactcggagcgtccggtgttcggctgtgag

gaggatgaaatggctgaagaggctctctccctcgacaaagccgctgccaaggttgctcccaaagcacaccacctcgtcatctccaatcccgtcaactc

caccgtgcccatcgtcgccgaggtcttcaagtctttacgaccccaagcggtggggtttcccccgaccccctctccttcttctaacggctctgacgctt

catga

>contig_14.contig_14.g364.t1

atggtcaacttcaccatcgccgagatccgtgggcttatggacaagcccacgaacatccgtaacatgtccgtcattgccc

acgtcgaccacggcaagtcaacccttactgactctctcgtctccaaggccggtatcattgcctccgccaaggctggtgacatgcgattcaccgacact

cgtgatgacgagaaggagcgtggtatcaccatcaagtcgactgccatctccatgtacttcgagatggagaaggctgccctcgacgccgtcacccagaa

gactgacggtccccacttcttgatcaacttgatcgactctcccggtcacgtcgacttctcttccgaggtcaccgccgctctccgtgtcaccgacggtg

cccttgtcgttgtcgactgcattgacggtgtctgtgtacaaaccgagaccgttctccgacagtcgctcgccgagcgtgtcaagcccgtctgctgtgtg

aacaaggttgaccgtgccctgctcgagctccaggtcggaaaggaggacctctaccagtccctgtcccgaaccatcgagtccgttaatgtcgttatcgc

cacctacactgacgaggttctcggcgagtgccaggtctaccctgagaagggtaccatcgccttcggttccggtctccacggctgggccttcactctcc

gtcagttcgccatccggtacgccaagaagttcggtgtcgaccagcagaagatgatggacaagctctggggcgacaacttcttcaaccccaagactaag

aagtggaccaagaacgacaaggacgccgatggacagcacctcgagcgtgctttcaacatgttcgttctcgaccccatcttccgtatcttcgactccgt

catgaacttcaagaaggacgagaccaccaagatcctcgagaagcttgaggtcaacctgacctcagacgagagggacctcgagggcaaggccctcctca

aggtcgtcatgcgcaagttcctgcccgctggtgacgctctcctggagatgattgtcattaacctcccctcgcccgtcactgctcaacggtaccgtgtc

gagaccctgtacgagggtcctctcgacgacgagtctgccattggtatccgtgactgtgaccccaagggtcccctcatgctctacgtctccaagatggt

tcccacctccgacaagggtcgcttctacgccttcggccgtatcttctcaggtaccgtccgctctggccccaagatccgcattcagggtcctaactacg

ttcccggcaagaaggaggacctgttcatcaagtctgtccagcgcaccgttctcatgatgggccgaaccgtcgagcccatcgaggactgccccgccggt

aacattatcggtctcgtcggtgtcgaccagttcttgctcaagaacggtaccctgacctcgtcggagactgcccacaacatgaaggtcatgaagttctc

cgtctcccccgtcgtccaggtcgctgttgaggtcaagaacgccaacgacttgcccaagcttgtcgaaggtctcaagcgtctgtcgaagtcggacccct

gtgtgcaggcatggattgccgagaccggtgagcacattgttgccggtgccggtgagctccatctcgagatctgcctcaaggatctcgaggaggaccac

gcccaggtcccgctcaagatctcggaccccgtcgtcggctaccgtgagactgttcaggcatcttcttcgatgactgcgctgtccaagtcccagaacaa

gcacaaccgtctgtatgtggaggcccacaagatggaggaggaggttgcctccgcgattgagtctggcaagattggtgctcgtgacgacttcaaggcgc

gtgccaagatccttgcggacgagttcgggtgggacgtgcaggaggcccgtaagatttggtgcttcggtcccgacacgactggtcccaacgtgtttgtc

gatgtgtcgaagggtgtccagttcatgaacgagatcaaggactcgtgtgttgctgcgttccagtgggcgaccaaggagggtgtatgctgcgaggagaa

catgcgggcaatccggttcaacgtgctcgatgtcaccctgcacacggacgccatccaccgtggtggtggtcagctgatccctacatgccgtcgtgtgt

gctacgccgctgcgttgttggcgcaacccggtatccaggagccggtgtaccaggtcgagatccaatgcccggagacaggtctgggtggtatctactcg

acattgaacaagcgtcgtggccacgtcttctctgaggagcagcgaccgggcacacccatgtacaccgtcaaggcgtacctgcccgtttccgagtcctt

tggcttcaacggtgacctgcgtcaagcgaccggtggccaggccttcccccaatccgtgttcgaccactgggagaccatgaacggtgtggcaaacgaca

agactgccaagctgtacgagcttgtgcaaaaaatccgcgtccggaagggactcaaggaggatgttcccccgcttgagcaatcaaatctgattcgcgcc

caactggttgaccggtccgagctcgttgccagtttctttgcagggttgcccgtcgatgatgagttgaagttattcaggtctcccgagccgacgccgcc

caaggtcaagcccatccccggccatatatag

>contig_14.contig_14.g365.t1

atgatcgacgtcatgatcagcgccagtcccgactccgccatggctctggctcaggacggcggtctgcacagctcaggcc

agcaccgtcagcagcgtccacaaatcagcacgcctgcctcgccagttcagggaagtcccagcccattcaactcccatggcacccgctcccgtggtcac

accgtcacaggagggaacaagttcacggccctcatgggttccatccgcgggtctgcgtccaagtccaatctgaaagacgcgcacgacgcagctggctc

ccacgcatttacttctatcggcgatgactcctcgggcacggcgagaggagggcatagcagtagcgcttcgacgagcaagttttccgctcttggtcagg

gcatccgcgaatttggagcaaagccaccggggaacaggggcagagggggttcccttggaggtcaccagtcgtacgcggacgatggctatgtgccctac

aacgggcccatcgaagctccaccacaacctgcctccaggttgggaaatgagctccttccgccgatccaggcctctttcggtccgacgatcagtcagca

gcttcggggttcagcagccttctcagcctccgctactgctgctgccaatgactctgatagtcgccttcgctccctgtccccgtcagcacaaaatgcta

agcagggaactccgctcaggaacagtcctcagccaggccccccaccgtcctctccgcttcccagtctccccacatctgcggcctttgtctttccacgc

tcgacctccaggcagccgcagatcaccttgtcaacaagtcagacccacagcacttcgatgcttcccactgatgagacaccccacaggtcggaggccgt

ttcaatggcacagcgatattctcagccctacagccggcccgtcgaggccccctctcgaactgccgcttcatgggcttctccgcctcccgccatttccc

accagtcggcgagcccaaagctacaggagggattgctggtggactttgactcggtcgcaaatctccatcgcagaccatccctcggcaccggcttgccc

accgcatacacacatgcctccgctgaccgtcccaccaatatagacgccgcgccgtatttgccggcagccctctcgtttgatagaccgatgcattacca

tcaatacccggctcccgatacctccagtctgaatccggccgccttccgacctacctttgtcggatggacgaaaccagcgacgacttcatacccaatcg

gcagccagcgcgatggcgatcttgactccaagttgggcccacgtagtcgggcccccagtagcattaactctggccacagctcggaagcgccggatgcc

tcttatgtctacaacaaagatcacccctacgaacgctcaggcgttggcagtcactctgcacagacaagtatgacttcggttcggacgttcccgagcat

aggcgctgctcaaaactccagcaacaaagcacggtcccacgtgaacaccccgtccagccaacgtcatcgtcgcggagagaacagctccggtcatggca

gtgctggaacggccttgtcagattgggcagggtccagcagcctggcccacgtaggcaccggcaacaccagacagggagagcacgcttcgccgaacttg

ggtagtatcgactcctttgcgcagatcgagaactccattcgaggaccacacgaagcgcttggactcggtcccgccagcaggtctgtcaacgctgtttt

ggctaggggccggcagccacgcaacgtggcgagcgttccagacttgcgtgcgctacccgtggtcatggtggacgcttccgccgtcgagccagaggcgg

ctgatggtgagctgtcggacgaccgacaatccgatcctgagccagtcgaagtcaccacatcacctcggacggcagatttgcttcgactggaacagagt

actcctgagcaacttcggttggaaacgccaagtggaccgatcagcatcatctcagacttggcgtctcaccgaccgcaggctccgctctcgcctatacc

cagctccatggcctctccccgtaccgaacgcaaccttgacggatcttctccgagcacaagagacatcagcaacagcggagagaactcgacgccactcc

agtccagtcgttcaaacgggtccaggtccgacagcaatgccaccaactcggcgtctcctggtcgacgtccagatcaggtacgaaggcgtggccacagc

gacgtggttttccgcaccccaagcccacaaggtccggagacgtggtcgatgcgggagcgcaaggtgacccaagccaagttccacctgctacaacaact

tcacttggaagagcttattcagcattctgtcgaattttctggtgtgcttgaaccggagagcgggtgcgatgctgtctttcgaccccgtccggcgctta

agaaccgtggtgcggatacatctcgacgctaccagatggctggccttcccgaatgggcatcggcttcttcttctgggcatggtagtcacagcaccatc

gtcccgttggaggccggattcgaacggcaccgtgctcttcggggccaagcccggaacccaacgcaggcgcaccgacgcacgcagactgaaggagcgag

cctcgactcccattttgaactcatgtccagtcatgatcgtcagcccgctgtgctccaggcaggtcgtcagcccttctttcatcaccgcatgccgccag

agccgccccgcgatactacggcggacaagactcagcctgggccgatccgctcggctgccgctgccgttgctgcaccggtagcttggtgtaccaacaca

ggcgatgtgtcttatgagagcgtcgatgagcctgttaagactgcgccagagtacgctacgttgcccatcgagaagcgagcatcgcaggctggaggcga

agtcgagctggtttacagaatgcgctcgattccagtcggagcacagaatccggtcacaccagagggtcgccgggagcgaagcttcagcatcccactgc

gcaaacagccgcctacaccagatctgatccgcactggtgactcatcccccgagatcgatgagctcgatactcccgatctctcgactgtcatagcggat

ggtatctggcttgatgcacagcgagccaagtggcgagagcagtatcgtaaaggcttggactcgagccctgctgttgtatcatggcgtcgccgaagcct

cagccgcagtcgcagcatgagtcgaacgctcaatgagctgccccgaggtgttgatccgacattttacgatcaataccggaagactcggaacgatggtg

cgtcgtcacatgatgacgctgctctggatccccgtggtggtgttctcgacaagctgcagaagaagtacaagggtgatgatgaccagccgcgtggacgg

gtcgcccgtcaagctatcgatgacgatcgcaaatatcgcacccaccagcgtaacaaatccttgcgtcactcgcgaagcagcccgcagttacgcaaggg

tgcattgtttgccgatgaagccggagagaaacccttgccgtccatgccgccgacaacgaggactcgaactcatgctggtctaggactcaaccaagaag

cagagctcaacggtcctcgcagcacgagtccggagattccgacttcgttcgaacccttcgcccgtcgccgtgtggcgtctcttggtaaagaagcggct

cgaaaggcagctgcggctgcagcccgcgagaaagcgcaccggcgcaatcgcagtgcgagcctgagtccggaacgactggctaccaggcaacgtgtcga

tagaaagcattccaagcagaattctggtcttagcgccatcttcggtcgtaagagccgctcgcccgggcccttcgcggtcccctccgcacagccacagt

cgggtcatcggtaccgtaaatcagcgacatccatgtcccgcatgcgcgtgcacagaaacagcgcttcctgggaccgggccgtcgagacagatagccct

gtccttctgatcggcgaggagcgcctcagtgcgttgcaggctgaggcaggagcttgggagcccaaatcggttgtcacggatgtccctgttgtgatagc

aacttctccgacgggccagcggcgctcagacattgagttgtaccacggcagtcccgtcaccgggcatggagctttccagttccctgtgccacccggcg

agggcggtgtcgcggtcgtgtccggtgtggcgatcggatctccgccagcttcgcaactcaacgacagcatcactgattcgtacagctcgaaggctcct

gcattgaccactgctggtcgtcatgcgacaacaccaagctatcatcgtcagacagattccagtgacctgcgtgcgatgcttcggaatgcccatcgttc

ggagagcgagatgtcaaatgaatacaatacgggactggcatctcttcccgcaccatcgcggcgacggggtcggaatcccagcgacgaaggtcgacatg

agagccaaggaagctcagagcttcggctcggtccaggacggctccctcggtcgcctcttcttgagcacccattcaatacccctcaatttgcgatgccg

tcagttcttcaggcaggtgcagcaaacaacatccgagatcgctccgggtccgctctttccggctcggatccacgatcaacgccgaacagtcttggcgc

cggttcccaatctggttcgggaagtaaccgaagagaactgaatgggaaccggtccgctagctcggctgaatcgtgcgatacccgaccaggcagcgtca

cccgcgaagtatggatgatggacagtggatcgagggaagacgacgaagaggcgcagttccagggcctgttcttcatgcctccgctgagtgatgggtcg

gtgccaagaagcccgcacattcacgacggtcgccccatggactcggtggccggcggcagcagagtgatgcttccgctccctcgaggctctcgtgagaa

gattgacgacgagcttgaggagtcttcggaggagatgcagtccatctcgataaaaccgaccaatcaactgagtgcttctcgcgccagctcggacatct

cccccaaggtgcccgccggatcctcgcagagcaggccccgcgaagagaaggtgaaggacgactcgccagttctgggagcctcgactgccaccgttgct

cctgtcgctgggtcgacgtctctcgcggcccgacaactgagccctctgcctgatagtctcgcttcgactacgttcttcctgcgaccagctacagataa

cgcggcatcgacgaaggatgtggccgcgccgagcgtatcgtccaagcgtggcttccgccttggtcatgtggccggtgctgagcaaaccccacagatcg

tggagcggtcgtccatgagccgatcctcgagctcgacgggtttacaacgtacattccgccggggacctacctcgacgtcgagcgacacctacagcatc

cttgccgctaccactctcatggatgagttggcggagagcatgatggtccatccgaacacgtctgccgtcagcgacgatgatctggatcacgatcgtga

agggggtcagccccggaacgctcggctcctcagctttgccagccaagttggtgaggttggcatgtatgaggatgatctagacacaccatag

>contig_14.contig_14.g366.t1

atgtcgctcaagtctggcatctctctcgcgagcttcgtcaagaactcgcccgctctctcccgcttcgtcactcctgtcg

caaatgcatacgcgcaagcagcaggccaccgcaaggtcgggctcaagtacgacgatctcatcatcgaggaggacgagggtgtgcagaaggccatcggt

cgtctcaccgagcgggaatcctatgaccgcgcctaccgtctccggcgggccattcacctcagcgttctccaccgtgagctccccaaggaggagtggtt

gacttcctcagaggaccaacgctacctggcacctctcgtcgaagaggtacgaaaggaggacgacgagcgcgccgcatgggacaccgtcaagatcgaga

agaagcactag

>contig_14.contig_14.g367.t1

atggtgctgatgatcaggtcgacagcctcgtcaagagccgccccggtccgtcaaggcttcaacgtgcttctttcatctc

gagtttggggctcaagacggacgatcagcaacgacacttccgctcagctggatcgcacgacaatcgcagtgctaggcggaggcatcaccgggctcacc

tccgctctcgtcctggccagacgtctgcccaaagatcgattccggattgtattagtcgagaaggaaaagcgactcggaggatgggtgcagagcgatgt

gttgccactcacatcagatgctgcggagaagtctgcgcaagaggccaaagtggccgtgattcgatcactcggtctcgctccaagacttttgtcggtgt

ccactactgctccagccgcaaagaatcgctttatctactacccagatcgtcttcaccgtctcgtctcctcggtcgtcggtgttcccaaggccctgctg

acatcgccacctctacaaggtatcgtccccagccttgccgccgatttcttcacaccagctcgcgcgtgggacccacagtcggatgagtccgtcgacga

attcctcactcgacgcttcggaggtcgaacagagcttccaagtaatctcgtctccgccgtcctgcacgggatttatgcaggagattcgcggaagctct

ccgttcgagccttgatgccgttcttggttcggaccgagcaagagcatggctcactcgttcgcgccatcctgcccaaatggctcaactcgcggtaccag

ggcccctctcaggcggaggttttgagaaggaaaaacgaagccaaggagttggaggaggcgaagcgacggcttggacctgcttgggaacagtggctcaa

gacgacgagcgtctactcgtttccacgaggcatccaagagatcacggatgccatggtgaaggagttaagctcccagccacacgtggaagtacgcacag

cgtccgagttggaaaccctgtcctttcccgccggctcatctgatgttcagatttctttaggaaacggcgaccagctcagagccaagcgcgtcatctcg

acagttccagcctcggcgcttgctgtggcacttcgcgcggatcccagttcgacatcgtcccagcttcgtaatctgctccaaagcatcacatcggtgac

ggtcgcggtgatcaacgtcatcatcccagcgtccgtagtgaaggaggcgggagcaaaaggacgacttcttccagtcgaaggcttcggattcctcattc

ctcgcagtacgccaaacaaccaagatggcatcttgggcgtcgttttcgacagcgacacttttcctgagcaagacagcgtagccagcattgatgtctcg

tccgaggagtcacacgatgccaacgacgcacctccgacttcaacaccgagctcatctttcgaggcgaataacacaaagctgactgtcatgcttggggg

ccactggttcgacaacgccaacaagaatcgctcagcagtaacatccaactccgcggcctcctatccagactcgtccgagctgcaagaacgggccttgc

gtaccttggagcatcaccttggcatcccgccaagcgtcctacgccatcccaatacccgcatccttgcgcggctccaaaaagattgcattccgcagtat

acggtcggccatgtccaacggatgcaggccatccacaacctgctcgcttcgtcggagcacgacgtcacaatggcaggtaggctcatccttaccggtgc

gccttacacggccgcgcccggcgaggacctcgactttggccagaggacgtcttctgcggcggatctgcccatcgatggagctggtgctccgaaacgac

tggttgcgacggcaccagtggcatccatagcccccgctgcgggttccgagaccaataccagcgcaccagtgatcgacacttccgtctcctcatcctcc

gccgccgcaaatgccggcgtgcccgaccacctcctctcaggcccaccctccgagtcagccgaggacatgctcatccagttgaaaaacgagttcgagcg

ccaaaggctcggagaagttggtcgcggaggcgggggaggcgaaggacagggcggctcaggcggaggaggagcgggaagtgttgaggagggcttttga

>contig_14.contig_14.g368.t1

atgtcagaccagaacgaagaaggcgaggacaacttgaacgagtctcgttcccgttcgctccccacggactcggctcgcc

gagagactccgattcggccgactactttcgttgcctccgcttccaacaataatgcgtccaaccccatcgcccgtctcttcggattcgtccactcctcc

ggaccaagtccctcgacgaagctgccgacgaacagccagcatgctgagcggtacatcgacttgtggccgatccagatcgccagtcctgacctcgacgc

cagccatgcttgggatacgtacaccatggtgcttcagttgggcacgatcttgagctacactgggacatggaagggccacaagcttcgtgtatctgtct

ttgtcgagtcggagaagggtgtcgagggagaacgccggcccgtttggtctctcttggacaagcttcggatcccggctgctggcctccgagtcttgacg

ctgtcgtctggatctgtcgctagttacgaggctatcgccggagatccatggtgggaagccttgcgtcaactgcggaaagaagatgagcgacgagcaaa

ggcagcagccaagcgggcctctcaggtcaattcggatacgccaggtccaagcggtgccattgccacgggcagcgagggcacgcagcgacagagcaagc

gcgaccagaagatcttcggcgtcagtctcccacccgaacacatcgcgttcttccagcacagcatgcgaatcggtctcgcccacccacgttcccggaag

gccaaccggacgtggacgggggccggagaggaaagcgagagcaaggacgagctttcggacagtgaagacggaggatctgatttcagtgacgagctggc

caatttgggggatgaagatgagtggttcggtgcgggcagtcagggtcttggattgcgaaggtcctcgacgatcacgtacacgccccgcggcgagcaaa

tgggcaagtcaggccggcagcgggcgtag

>contig_14.contig_14.g369.t1

atggccttcctcttctctcgccgaggtccgaaggattaccccatcgcggaatgcgcaacgctcacgactgcaaagccag

acacgcccgcctcaggcgcgtccgtaccaccacaagacggtgtgcctgcgtgcgactatgtcatcatcggcgcaggaacggcaggtagtgttctggcc

aatcgtctctcccaagatggatccaaacaggtgctcgtcctcgagcgcggaacgtatatgtccgatacccttgcgaagatcccattactgagcgctgc

tcgctctctccaagacttttacacaacgaccatcaagtcagaacctcaacctgggtttgagggcaggaccagcgctgtgcaaattggaagcgcgatca

acgcgaccatatactcgcgcggagcccactgcgaatacgacgcttgggactgtgagggctggcgaggcgaagacctcgacaagtacttcgtcaagtca

gaaaagctgcacaccgaatcggaccagactccgcctctgccaagccgtggtacttctggcccgtggcagacgcgcatcaccaaaccatgcttcgggtt

caatgagcccttccttacagcttgcaacgacaccggtattacacgaactcacgatcctcatgcgtacgaaggcaatatcggacccatggcgacggcga

gctacagcattcagactgcccagacccccgacggggcgcgccaccacgccggctttgcgtacctgccagaggatgtgcttcgagaccgctctaacctg

aaggccgggaagctcgtcgtcgaaggcgtttgggtcgttcagaaaggcaaggtgtacttcgctcgcgccaagcacgttatcgtctcgtcgggcgccat

cttttcaccctgcatccttcaacgctccggtctcggcccagcccccttgctcaaaagcctctccatccccgtcctcgccgatcttcctggcgtcggat

cgaatctcaccgaccacttcctggtgccgctccacttcagcggaccttcgcaggacagtgcttatcacatgttgcgaggcgtgtgggctctgcctacc

ttgatccgcaatgctttgacctactggcgcactggccgaggtcttttctccactgcgttcacagtggagtcaatgagcttcttctccacccaggatgt

tgagatcttgccagtacagagcggagcatcgagcccggaccgtatccgtgtccgctgcaaggacgctcgggagcacgtacccgacatcgaaatcagcg

tttccggtatctgggccgaccagaacatgccccagtattacggcctcggcaagcgtggggtgatctgctttctcgtctggcttgtcaagccgcgctcg

gagggcaccgtcgagatcaagtcaaccaaggaggcagatttccctcgggtggacccgcgctacatgcaagtcgaggccgacacacacgcgctcaggac

ggcagttcgtgtcggtctgagcatcgcagcacacatgcgcaacgcccttggttatgacattggtcctgcgtgtgtcccaggctgggcgccgatggaag

gaggctgggatgcagagtgtggcccgactctgcaaagaagtaaggaggatgccaatctgagtgcagagttcgactttgttgatcctgtcaagattcca

gatgcggctgcttttgtttcggtcactctccagacggtggactctttcatccacagtcatgcgatcccagcacagcacatggcctcaacgtgtcggat

gggcccagcggcaaccggagccgtcctcgacccggcgacgctggaggtctatgggaacaaaagcctcagcgtttgcgatggcagtatcttcccgcgcg

tcctgagcgtccatccttctgcggccatcgtggccgttgcggagaagcatgccgatgacctgatcagatctacttga

>contig_14.contig_14.g370.t1

atgagcaacagtacaggcgacggcacgagcagcagctcagaaagcagcagcagcaacagcgactcgtctgcgccgctgg

gcggtgtccgcccaggcttggtcaccggtggacgtaagcgcaagcgcaagcacaaacacctaccgcgcaagtcgggactcacccctgcaggtcacgac

gaggtgaaagacctgaaggaacgaattcgatctgaaatcgaacaactctccgtcaagcacggcgtgacacctgcgtctattcgccatcggcttgggtt

ttccgtcgaggttggtcgcgaaccccacgatcaccgcaagtggaccgagtggtacgcccacacgcaacccaatccatgtcctgacaatctacaacaac

gcaacaaagagatgtccaccaagtggaccgctctgaaggccaacaaagtccagtacgaccgggttatcgcagatgtcaaagagtggcacctaaagcga

gcagagcacaccgatgttcccgactctaaagagctacttcatgcgttcgctaagcgcgccttcaagctttgcaaagccgccggagtcaacgatgacat

atcagccgtctgcatcgctgcggtacccaacatggcggtggaccctgcaatcattggcctggaaccgatcctggtccagttctccgccagccttcatc

tagcttccgatctctcccacgtccgcgacaacttccaggctcgagtctaccgcaatcccattacctcttcgatgctccctccacaaaccattgaggac

gatccccgattcaaccaactcccccgcggcaccgtcgaggaacgcaaaggcgaccgtgccaaagatatccagttatacatgccccttcacttcatctt

cctcatacgcctcgccctggcccgcgccggcactcaaagtcttcgctgggagcaacgctcgaggaagaacacggtcatggtttatgaggacgtcttcg

atattctcgcggaagtgtctctgtttgttgacggctggcccacgtctctcgaacacctcctcaaggactccgacacctccagcggttcggccaccggc

acccttagcatctacgcgggttcactcatccactggaacacatggagcatccctacgagcccaccgcagaaggcgaaaacgcacaaccgactcccact

gcaattaccgcgaccgccacaccatgacctttcttcttctacaccttcacattaccacttccgtcccttaaactcgcattcatctccgccgccgtcgt

cctggccaactccacacttgccgccatcgacgtttagcccaacgacgaagttcgtcgttcgctccgccgccggtctccacgccgcacacggtgtcgcc

gcccccgagctctttgcccgtggctccaaggctcacttcgcggccaagcccgccgctgctgccaaccattccgacaagaagcacctcctcgacttcgg

catgatgttccagaagaccgccgccgagcacaagggtaacaaggttctctccctcgagcagcgtcaggccatcctccagcccgccatcacgaacctct

cacctctcccatcgaagctctcgtcggccacaccggcccgcaaggtctcggctggaagtcccaagagtccgtcctacgaaactttcgccgggggggac

actcacctgctcatcgccaccagcgtagttgaggaaggactcgacgtccccgcgtgcaacttcgtcatccgcttcgacctgcccgagacggcgatccc

gacgatccacagtcgcgggagggcaagggagcgggggagtacgatttttatgatgtgtgagggcggatcggtgagggatgctaagcttattgagcatt

gtagggggatggagcaggatatgaaggattggctgcagggcttgccggaggatcggctggtttctgatgttgggagcgatgggcaggcggagttcgtg

ccgaactggacggagaccatcaagtcggaagaaactggtgcgaagatcgagcataaagatgccatgtcgctgctcaactggaacgtgcaagcgttgcc

gaaggacgaatacaccccagcaagcaccttcatcagcctggaggaagacgagacgggacccaattctgtctttcggtgcacgctacagatgcccgcta

aatcgaagctgcgcgagcctgagcggatcgcctcggaagtgatcgaggtcaaggacgacgaaggcgaggttcgtctgctgaagagacacaaccttcca

cgtccaggcgcgaagcttttcgctaccgagcaaacagcaatcgagacgcgcacgttccgcatcacgacaatccctttcgactccatcgatacggctgc

tatccaggggtcggtcgcgccgttgctgctactcacctccgccgaggcgcctgacgagcttgcggttgagctcaaccttcccaatggtaaagtcaagc

ttctgtgtcgaccgggacacgccggcactctcacgctcacggaggatcaacatcgcttggcgcaggtgtacacgttgcgcctgctgggtatggtcggt

agattaggaagcgtccccggacccacgcacggctcgtggatgatcctcccgctcgaccagcaggtgaagaacggcggggaggatgaggttgtcgtaca

gtgggaacaagtcgcgatactggcggcacagtcttcccccgtcttccgaagtgcgtcccatgcggagtcgctaggggacgacgtttcgctatgcgcga

tccccgatttctcaaagccgccgtcggcgctgtatcgcattcgggacgaccctactccgtcgagcttgggtccgaggggtcgatggtggtgggcctat

gctggagacgcgggcgatgccgcggttgcagaacttcactacggctggggcgatgcgtgccttgagcaagtcgttgacctttttgatcccggctgtgt

cgttagtccacccggtgccttcgagcgtttatcgcgctgcaatcctgctaccttcgttctgggagagcagaaggccctacatgcctcaatcgtcgccc

tcgccacgccgcccgtgaacaagtcgacctcgctcgaccagcagcccaacaccaataccaccgcggcggggtcagcggcggcgctgaatatcaacgag

aacttccgccgcacactggacatcgaaaccaaagcccgcacactcggtctcaatctgggtgggttcagcgagcagttccatgcgcagatgcaggttgc

tagcacgaagcagcggcaacagcagtaa

>contig_14.contig_14.g371.t1

atggagcaggctcccgggtattgccgccacagcgcaggaggcagatttgaggagaccagcactcacgccttcgccttgc

cacagttgggaccctcagccgacttgcacacctttgacgcgctggacggtgagctcgcctacagtggtgagctcacgggagcattgctggcaaagagg

ctggcccacccgctcgtcgttggcgtcgacattggcagggctgttgtcgttttcgtggaggaaaaagtgcaagaggaggcggcgatagaatcagtcga

gcgcggcggaaacaacttaggcacggcagcaggcggcggcggtccgccctccccacccggtccgccctccccacctgcactcgcagcagcagcagcgg

cggcggcggcaccggtggaacaccaatcaacaacaactccgccccaaccttctccggatcctcacccgccccaacacccttcctcttcccccaaagaa

atgcagccccactcttcctcaccaccaacaacttcgcaagctggcaacagatcttctcgttctggtgctgcagctccacctgcgcggggcacgacgct

gctgttcgaaggaggagctctcctgttcctccgggatgtcctcgtccttgtctttgccgagcgagttgtccgaccgcctcgttctgcccctgaccctg

attcgacggcctcatatgtccttgcccctgccgctgtccctgcgcaagcacaagcgcagcctgttgttgttgttgctgcttctgcttctgcataa

>contig_14.contig_14.g372.t1

atggctgctgccactcaagctaggcgcgctcagctcctcctgctgcggctgccggtggagcgaccgacgctcgcttccc

tgcccatgacattcagcaaccccaagcaagaagaggcggtcatcgactactggcgcagcatcaacgcgttccagacatccctcgaacagtccaagggc

agacctccgttctccttctacgacggcccgcccttcgccaccggcctcccccactgcggccatctcaacgcaggcaccatcaaggacatcgtcacccg

ccacgcccatgccacgggtcaccacgtcgaacgcaagttctga

>contig_14.contig_14.g373.t1

atgtcgcgccatcgtcatgcgatacagaacgagaggcgcaaggccgtcgagcgcatgggacgctggatcgacttcgaca

acggctacaggaccaggaacacccccttcatggagtccttctggtgggtcttcaagaccatgttcgagaagaacctcgtctaccgtggactgcgcgtc

atgccctactcctccggctgcaccaccccgctctccaacttcgaggccgggctcgactaccgcgacgtacaggaccccgccgggacagtctccttccc

gctgctcgacgacccagagatcagtctgctcgcatggacaaccaccccctggacactccccagcaatctcggactctgcgtacacccgcatttcacat

acatcaagattcacgacgaggagcgaaatcagaactttatcatcttggagaagctccttaccaccttgtacaaggaccccaagaaggccaagttcaag

aagatcagcaccatcaagggctccgacatggccggctggaagttcaagcccatctttccctacttctacgaccagtacaaggacactgccttccgagt

cgtcgtcgacaagtacgtgaccgacagctccggcacaggaatcgtccaccaagctcccgccttcggagaggacgaccaccgcatcgccgtcgcgaaca

acatcatcacccgcgaaagcgcccctccctgcccgctcgacgacgcctcccgcttcacatccgacgtacccgagtaccaaggccagttcatcaaggac

gccgacaagaacatcatcaagcacctcaaggctgcggggagactcatcgtgcagagtcagcttatgcactcataccccttctgctggcggtccaagca

gcccctcatctacacggccatcccggcctggttcgtccgcgtcgagcccgtggtggcccagttggtgaagaacaaccaggagacgcactgggcgcccg

gtgcggttcgtgagggccgattcggcagttggcttgccaatgcgcgcgattggaacgtctccaggaaccgctactggggcacacccatgccgctctgg

gtctccgacgactacgaggaggtcgtctgcattggctccattgaagagttggagcggctcagcggcgagaagaacatcaccgacttgcatcgcgagta

g

>contig_14.contig_14.g374.t1

atgtccaggcgcactacccctttgagaacatggaggagttcgagaagagtttttccggcgaactttctctcggggggga

ttgaccagacccgcggttggttctacacgttgctcatcggtcgggacgcacctgttcggcaaggcgccctggaagaacgtcatctgctctggtctcgt

acaagcagcgaaatgaacaacaactgcaacgacgaagtcaccactgtggacctcaaccaggtcggcctgtccgcaggcaacgagctcaacctgtacga

gtacatcgacaaggaccacgtatggggcctcaatctggctatacctgaatcggcacgacaggtgatcaagccgtggaacgaacggaactcgatggtgg

tctatgttgagagtcttgttgatgatcagttcatcatcaacgtgcctttcacctgtccctgtcggattaaatccatccttctcaagcccggccgaggc

gacctggcaccccagagatgtcgagcgtacgtaaatcgtttcgacgaggtagaagcgacgtccgcgcccgaggccccctccttaccaccgccaccgcg

gaccagcctagccggaaacctatcccgactgaccgtctccgcctccgcattcgccccttcctcctccaccgagcaaagaccagcaccagaacagcaac

actacgtctcctcctcctccagctccaatcgcgcgggaagcgtcggtgtaccggggacggggaagccgcaggctgacttcgcgctgttggacgggggg

tcagacacacccgcccttgagaccggccagctcttctacctcggtttccgtggcaccccactcgtgctgaagaagacggagcaggccgatttcgacgt

atccgccgcgttcgagctcacgaattggtacatccgcttcaagcggcgacggctgaagggcgagggtgggcaggaggattggttgacgtctctggact

gtctctttgagacgctgttcaccctgtgccggacgacgaacatctaccaaagcctgcgtcccttcctgccggcgcaggacgcgtcggacaagaacgat

taccggtcgctgcacttcctttcgttcccgagtgtgcgggaggactacattgatgaggttatcttgcgtcggttctcggcgctgcagagtgttactga

gcttgtacggacgttgcgggagaggaatgtgcttgcgcttcgggtcccgctcaaggaactcgtcgggttccacagcgaccaggagtccctggacgacg

tgcagagcctgtcgacgtacatcaccgaggagccgaacgtgcacgacctcgtgctgagcacggacgagaagcgctgcggcgtcggcttcaagatctcg

gcggactggcccaccctcggccggaaactgcggaaagacctggccgggcgggagaaagtgagcttggaggacgcgaaggcctacctcgggacgctgtg

ggaattcagatatctcttcatgtatttgcgatcgtcagggcgtgaggttgggcaagcccaataa

>contig_14.contig_14.g375.t1

atgtcgtccgaatcgccatcacatttcaacttactggcggatgctcgctatatggtcgcctttggcctggtatgcgctc

tggtccttcttggcagtttcagacaaacgctacagcgaagggcgtcgaccgacggacagaaggcggttccgatttccaatgtggcgtcccggagctgg

tacgagccatcggctttcgccttcgccaaacacccctatgtctggctattcgatgcccaattgcgatacggtgatgtcttcgggatgggcttgggatt

tttccgagtcatctggctcactggccccgaagctcccgaatttatcttcggagcggacgaggatacactctctttccacaaagcgatcgagcccaata

tctctaagctctttcccgttcgccaccttaagcagccttggatcaactacctcgttggcggcatgacacaccgaaagcacggaaagcactttgcgcag

tgcgttcttgacatcacggagagcgagacggccaggtgggcgcgcgacacggaaaagactggctcgacggatctgttccagggcagctttgacgtgat

gactttgatcatctccaagtacgcttttggtgacgagtttaccgacaagcatggtgccgagatctgctacctgatctttcaattcgatgccctattgg

gcgacatcggagtcatggccacgcctgactgggcattctggacgaagggcaatcggaagctcaaagcgcaccggcagcgcttctacgagatcaccaat

accgagctccagcgcaggctgagacatccggaactgtacaaagactgcgaggactaccttcaatactatcaagaaggacaatctggagcggcggcaca

gatggcacaatcgaagcgtgtccaggccaagttgcgttccgagctggcttcgctcggagtcacagctcagtctttcacggatgccaagctcaatggca

ccgaagaggagttgctgagcacactggagaactgctcctacctacacggtatcacacacgaatcgatgcggctacgtccctcgccgttcctggttcaa

aaggcgatgaaagacatccgatacggagaacacgtgttcgaaaagggcgcgctgttgacactcgcgcttccagtgatcaaccggaacccgatcgtctt

cccgtgtcccgacgaatttgttccggagcgcttcatggatgatgccccagcggatgtccgaacaccagcggacttcaacctccactcagtcttcgtgc

cctggggtttgggccggaggcggtgtaagggcgagaagttagcggcgatgacgatccacaccaccttggccaggatggtgctctgcaacgaggtcagt

atcgaaggcgagacgacggaacctccgatcaacccattgacgggccgggagaacattgttccaatcgcgcctcaattggacaagatcttcggaccacc

tcacagcgaggagcgatgtgggctacgattggtaccgcgagctgatcaggagagcgactaa

>contig_14.contig_14.g376.t1

atgttttcgcctcttaaactcaacattaggctcttggtcgcgacggccgctttcaatcagctagtaccgttggtggcag

actgcgtattgctcatccgtgtcgcagcggtatttccaaagcggactgtgtcgaatctggtgcgatataccctgctaggaatccccatcctgctcaag

atcggacgactagtctgcattggcctgtttgccagctacttcgtcagaaagggccaagagctcggccccctgctcgtactcaaggccctcattccgag

tccatacatgtacgcggaagccatcatgcagacgtgcgataatgggttctgctccatcttgttcttgtacaagctcaaccaacagcagaacctacgca

agacgcgagcgggattggccaagcgaccgtcagcagagtccacaatctcgcgagtccgacggctgttcattgctgcactcagtagtttcctgcttcct

ttcctgacgaacatcgcgatcattatcctcagcccactcaagccacaagacgagccgatcaccatcatggtcgcgatcaacgcctacgtgaccatcta

ctcgctcctctttgcaacggtgtgggttcccacaggagatccactaggcaccgccctgacagctcagaccggtagccacgcatggtcgtcaggagatg

ccgtcttgcgcgatcgatgcacctactgctcctcccgccgtctctcgaccaagattaacccctccgacttcgaagcgctcgccaactactccggcata

aagtcgcccacgtattccacagccggaagtcgagtcgcaaaccctcccatcggatctgccattactcccgtcggcttgggccacagttcgttggagat

gcgccggataccgcaaaaatccgtcaacagcattcgggtcgacgagaagaccgagagaaatagcagggagcttggcggacgacttcagcagagtctca

ggccaacacatgtcctccaagaggaagaagaggaggaaaccaccacctggtcgttgagttccaccgcagcggacgatgtcgtgctcagcagcccgagc

ggacatagtacgccgttacgatccatagaagggagtcccggcttacgcttgaatgggttcgggccgcgccgaggtccacctgcagacggcgtgcgcgt

acacatcgaacggcaatcgatttccagtgcttcgacaccgccttga

>contig_14.contig_14.g377.t1

atgggaaacaccctagcctctgaactcggcatcctctcccgctccaaacccgtgctggttctggaccctttcagacagg

tccctcccggaaccaatggcggtatctcgctcttcgggacactcatgtctctcaccggcggtctgttcatgggagcgacagccgctctgacgacgtac

ttgtccaatcctgcctgctcggtgtccgccaagtccgcatcgtggctcacgctctggtcgactggcgtcgacggcgcagaccgtttgctctggctgaa

catcattttcctcgggggactgggtggcctgctcgggtccatgcaacggcatctcctctttgcacagattgactctgtactcggcgcgacgttccaac

ggacgtactactcccgcgaaacaaagcaagttctcctcggtcgcttgcctcccaaggagcagtccaagtcgggcagcgatgactcagagtgggtggtc

atcaccggctacgatatcctcagcaacaaccaggtcaacttgatatcgtccgttgccacctcagctctcgttgcctacctgggaacggcagtgctcgt

ctga

>contig_14.contig_14.g378.t1

atgccactgcatagcttcttgccgccttatccagcgacacggggcgaacgagtcttcaactatagccgttcccacacgc

tgagccagcagccttccgcaaacagccatgctcgacctgcctctgccacacaaatacctcttcgccttcaacaccttcgcaaggtcttcgacattctt

catctcagtctattgcgcggcgatggtcctcgggcagcgagagcgctgagacttctggtccgatcgaaagaatggcgacatgccgaattgtggcggta

tgggctggcagtggcgggtctactggcacgcgatgcttccctggaaggaacaacgacgacaaatgggggcagagatgactcaagcgaacatgcggaat

cctctacttctcgtcaagtacagctgaagcaggcggaagaggctgcaacgaatcgtcggctggcctaccttcgtggcctatatcgggccaaacctgct

cttcgatcggaagttctagtcgacattgtctacgaactcatcgagctcaagcggtatgatgaagccatgcgggagatcgaatacgtgctggactccca

ccctttccgaaataatccgtctctacatctgttggccgggattctgaccttggagtcaggctcacaggctcatggcttccctcgcgcgcacgcctcct

cgccctcggcgcttgccgagcttccctctgccgtgcgacgggcggccgagcaccatttccgacgaacgctcacagccacacgcaaggatgaggccgac

aagcaatacgctgcccaagctcgcatggtagtcaagaagcgaagagacgagacggttcgaactcaacgaaaagcgggtctcgaaattggtctggagaa

gcaacggatcagtcgtgcaaagcgtgaagaaaccctcctcggacaagagtatacagccacttcaaccgaaaagccgcccagcagtcgaagtgtctcgg

aaaacgcgcgacaaaaacgcatcaggcggatggagaggaagtatgcgggccctcacaagtgggcgatgaccctcgctcagacggacgagattccggat

agtgacgttgatgacgatgaggagcgagcctctcaacattcggccgtctcggatgtcgaggacaacgatggatcaggggagaatgggcctggagccgg

aactacccatggcgacgatgctgatgaagacgtcgacatggtggatatcgaccgggttccgcaggacgtgaatcccgacgaagatgacgatggggacg

gagaaactccgcgacggctgccctccgcttttgctgccgctgcccacctcttagaaggctggacgaagtcggaggcttcccggcggccatcgacgtgg

gaggcacaagctgcccaatcctacctcgacgttatccaggcagatacctccgacaacgagcacgacgacgacggcgacggcagaatcacaaagaaaca

tccaacaactcaggcctcgacaggaagatggaacaagtccggtatgaagacccgccccggcgtcctgaacggcacccgggtcgactacttcaagggct

cctcagcagtcaaggccatcctcagccccgcatacaagaagctgaagggcgtgccgcaagccgagaatgaagaggaggcggaaaaggtgctgcacggt

atcattccgtatgccttctttctccgcgtcgaccgcggacccacggctggtgcgggcaaggacgcgcctaaggtggttcagatcaatcaacagcagat

gttcaagggtgaattgcactacgtctggctgctccaaggctcgcagctcggtctccgtctcggcgggttcggtgtggtcgcagtcatgcttgcaggtg

tcatgttcccgctctggccgccgttcatgcgtctaggcgtctggtacctctccatcggcattctcggcgtcatcggtctcttcttcgccatggccatc

ttccgtctcatcttctacgtgatcaccatcgtcgtggcgcgaccgggcatctggatcttccccaacctgttcgaggacgtcggctttgtcgattcctt

tattccgttctgggcttgggacgtgactgcgccgaagaaggtgaagaagagcaagaagagcgtcaaggcggaggaagcgggtgttccagatgcgcctc

ttccagtcgccgcgccccctcgtcctcaacctagcgctccggcccctcgtgcccaacccagcgctgcgccttctcaggcgatggacgacttgaattag

>contig_14.contig_14.g379.t1

atgagcgccgaggcagcgacgacgacagcgacgacggcggcgatgagctcggcggcgttcccgctgagtgggcggctgc

gaagtgtgctgctcgatccgcccaatcccgatgcgctgcaggatgccctcgctgctctcgcccatctgtacccagctccaccggactccaactccgag

gcagccgagcagacagtcgacatcgcacgcgcacgccgcaacgtcctcgacgatctcgatgcgcaccttctcgccgccgcagacgaactcgaacaggc

gtacgctcccgccgtcagcgcaatccaagaactctgtaccgtcgtcgatgaggtcgtggccgagacgaaggagacccacgcacgcttcgaagacgtct

acgcgcgcacggccacactcctcgcagagtccgacagtctccagaagcagcgcgtcgctctcacctggcagtccgagcagacatcgctcttcctgcgc

aagttctccctcaacgacgcagagctgctcattctcgacaacccagcagccgcagccgcagaagcgcctgcctcgtcctcgtcgtcttcaagctccca

tggcatcggggaaccattcttccaggtcatcgaccgtctcgagcagattcgagaagattgcgcagagctcctcggtctcgtcgaagaggatgaggatg

acatcgaggaggaagccgcgccgccgaccgcgcccgagtctgtcaacgacgcacaggccttaaccatggccactcctcttccttcgaccagagatcag

tcagagagaaaggacatgatgcgtgcaggcatgcacatcctccgaatcacttcagagcagctcaacacagcgcattcccgcctcgctcgctggtgcac

cttcgaattccgccagccgttccgagaaggtgttgaagtcagtcccgtcatgcgcgaggccgtctgccgactggccgaaggcggacgcgaagacttgc

tcggccccgctctgtccactctcacccagacgcgctccaccgccatcacccgtgaattcaacgcagcactcacacaaggaggtccacccccgactcac

cttccccggcccattgagctctccgcgcacgatccaacacgctacatcggtgacatgcttgcatgggtccatcaggccctagcctccgaacgcgagct

tctcggcagccttgtagggatcggagacgacgacgtggctacgcaccaccgcggcgccgcttctccgtcatcatcgggcacggccgagggcagcgaaa

ctcagatgatgaccgggcgtagaataggacaacgcaggcgctggagtgtcaatccggcagaggtcatcgacatgatggagggcagaaaccaaactggt

gcgccaagcgacaatgaggccttggctggctcactcgcaatggcatccgctgccaccgcacaaaagggagtcattcggcctagggagcgattacgaga

cttgctcgaccgcgacatggagggatgttgtcgaccactcaaagtacggattactcaaactctacactcccaagaaggctcgatcaccgccttcaaac

tctccagtctgatcagcttctacagcagtaccatggaacagaccatcggcaggaaggctgcactgagccaggtcttgagagaactgaccgagactgca

tacagtgcattctttgcgactctggaccgccaagcggcaggcctgatgcgctttgcagcccctcccgaatccgatctgagtccgccacctccattact

cggcgcatgctcgaccctgaaagagcttctcgcgctccatcacagcgcatccgaagaaggcgacctctatcagcccccacccaagcttccctcatcag

gcaaccaagccatcccagaagagccggctctgagtgatttcagccacgtcgtgaagaagctcgtcgatccgatcctcacgatgtgcgatcagatggcg

gactcgcttcgggcgagcattgcgcgacgcagtgtgggcagtgtcagtcgcttgggtgtctcctttaccggtcgcggagcggcggcgagtaagaccaa

ggcggacacggagggcggcgagcagtgggaagtcacggtcttcctggccaactgttggagttatgttcttggtttcttggaagcatatgacttcgttg

gagagaaggttgacgaaatgcgcaagcggattgatgagaaggctgaggcactgacgaagctttattaccggtacatgctgagccaaagtggactcgcc

ccgttgacgacaagcgaagacggagcacttctaccgccagacgaggtcgacaaacacctctcgaaccttgacaaatttctcgcctcgcccagcgcgat

tctcatttcccactcgtccctgagcaagctccagcctgcgcatctgcggaacggcatcaacgcccgggctctttccttgctcgcggacgcgtatgaga

ctgtgtatgagaagtatggacagtttgtcgttgatggagatggtggtggtgggtcaggccgcaaggtagtcaggcggacagttgatgaggtgcggttg

ctgttgggcgcggagatcgcgcagaactga

>contig_14.contig_14.g380.t1

atgtcagcctcgtcgtcttcctctaccgctgcctcccgtgttctgtcccaagtgaggtcgatcatcccgcctctctccg

gcgagctccacaagggacaggccggtcgagtcggtatcgtcgggggcagcaaggactacacaggagctccctacttcgcttcgatggcttcaatgaag

ctcggcgcagacatgagccacaccatctgcgagcccagcgcgggcaatgtcatcaagacctactcgcccgacctcatcgtgcatccgatcatggacca

gaacaagtcgttccccagatcgctcgattccattcggtcggaaatgaaggagatctttgcgcgtctgcacagtgttgtcgtaggcccaggactggggc

gggatcaacacatgcaagactgcggacgaatagccatagagctcgcgcgcgaggcaaacctgtacattgtcgtcgatgccgacgggctctggctcctc

cagaacgagccagaaatcatcagaggctaccgtcgtgcagtcctcactcccaacgtcgtcgagttcggacgattgtgcaacaaactcggaatcgatgc

caagaagaagccagaagaggctgccaaagagctcagcagagcattgcaaggccctaccattctcgaaaagggcaagatagatcgcatcgccaacgcag

acgaagtccttctgtctgaagaaccaggcggtctcaagcgctgcggggggcagggagacatcctctcaggctgtcttgccaccttcctcgcctgggca

cacgtctactcccgaggcgaccaaactactccaacgggccctccgtccgagcacgagcggattccggaggagagactgcccctcctagcagcgtacgg

ggcgagtgtcattgcgaggacgtgctcgagggaaggatttaggaggaatggaagggcattcttggcgaatgacatgattgaggagatcggacccgatc

ctgaacctatggaggcagacgacctcgacgacaacaacgacaacgatcacgaaggccgcgaaaccagctga

>contig_14.contig_14.g381.t1

atgtcaagcaccacaaccaccgactttagcgcctacccctccgaccagcttacgccaagcgaaatggatcacctcgagg

ttctcgtggacaaactcatcttggggacgaagaagcgcgacgaggacggcaggcgtgtcgacgatcatcgctgggatcccacttcgttcgttggcggg

gtcgacatcatgacgatccggggcgtccctaagttgctggaccagcacaaggcccaatttgggatcaacaacaacgcaatctgcgtccttgccaaggc

catggcgaagtacgcggggcattgcggggctcgggatcgcttggagagtcggattgagtccctgatcaaggctgggctcacggagtcgtcgtcatctt

catctgacgcctccgacgccgacgatgatgctgctcccaacgatgccgcttccgacgacgatgcttctgacgagggagagggcgagggccacgagcat

gacggcgatgcttctgacgatgcttccgaccaggacgcttctggcgaggatgcttccgacgacgacgccgaggacgatgacaatgcttccaactccgg

ctccggctctgactcttgcacctcaacttccgtacactcaaaagcgaacctgatcacggatgccaactcggacacggactcggactcggacactgatg

gctcgtctgcgatgtcgttcacccaatgctacaatacagtcaacaccccgctacctacgtacactgcactcgatgccgcggctatcgaagcagacgag

tcctgctcgacatcctctggctcgtcatccgcctattcggacttgatgtcgatgtcgtttacgcctgtccacgtcacatcccgaccgatgcctgaaga

gcgctcggatgtcggcgatactgacggcgaacgtacggactctccctgctcgacatcttcctactcgtcgtccgcctcttcggactcgatcccaatgt

cgttcacccctgtctacgttgcatctcgaccgatcgccggcgaaccctccacgatcaacggtatcaacgacgtcgacgatgacaacgattctgactcg

tgctacgacgaatccacgtcctcgtcgatcgtttcagagcagtactctccgtgtctgtacgcgacgggggtacaagccaccgccccacggcgctttcg

gtaccgcggattactgcccaccatcatcggcaggcccgccagggacttctcgatgaggcaacttgacgcgcagacaccgtccaggatcccagtccgga

cgttgcaggtcaagactactcatcagactgccatgagccccgctggtcgttcacaaggtcgggaatccttcccgtctttcgtgattggcgggatcgaa

cctcgcccggccggcagcatcaaggcccggcatttcaaggccatcaagcccatcgtggccgcgccgcatatcccttggttcatgaggagtcctccccg

ggtcaacgacatcaagcttcggatcgctccgcgagcttccaccaacgcttctacccaaaagaaaattgtgttcccggatcccatcaccagcatcgttt

cctgtcacggcttcgacactgcaacggaacctcgtcccgggctcttcttcgtcctgtcggcttccaaagtaccgggcaccaacctgtaccaacgtccc

aaacctacaccgcagaacccaaccatcaatatcgtactctccgctttgctctccgtacccgcatctctccgcctgctcgtgccttggactctcgccgc

cttcagtctgcaatttatacctatcattgttgttgttttctgtctccttgccatcaccatctag

>contig_14.contig_14.g382.t1

atgggacgcggcggatcacgaagaggcggtagaggaggaggaggaggaggcggcggcggcggcggcggtggaggtggac

gcggtcgcggtcgaggtcgcggaggaggaggaggacgcggccgaggtggtggtggtggaggtggacgcggaggacgcggcggcgcacacactggcgat

caaaacggcgaggaaggcggacgcgactttggcgacagctggaagaaccggccagacgaatacttcaacagcgccttcgagtcgtactacctcgaaca

gcgcctgctcgacacgcccgaggagtggaagcagctcatgcacgccttccgacagcccctgcccaccaccttccgcatcactgccggtctcccctccg

ccgtcttgctcgagacagccatgaacaacgagtgggtgcccttcctcagcaatgtcgaactcccgcgcatcggtccagcagcagcatcgctccagcca

gcagcagcttcaacatcagcagtggatgatcagatagtccaggcagaggacgcggagcaagacgcagaacagacagctgaagacttgggaccagaagc

agccgcagcagacgacgaagcaggagaagaggaagagcccaccttcgtccctccgcccacagtgctccccttctaccatcccgtcggcctcggctggc

aactcgacgtcaagaagaacgtcatccgcaaacacccagccttcaaggccttccagcagtggctcgtacacgaaaccgacacaggctgcatcagcaga

caagaggccgtcagcatggttcccccgctcttcctcgacgtgcgctccgaccacctcgtcctcgacctctgcgccgcccccggaagcaaaacagctca

gctcgtcgaaatgctccacacccgcgggccctcgtcatccgagccccttcctcgcggcaagtacgacgccaacccagaaggcctcgtcctcgcaaacg

actccgacactcgtcgcgctcatatgctcgtccaccaggcctcccgcatcccctccccgaacctcatggtcagcaacctagacgcatccatgttgccc

cgcatcgacgtaccctggctccctcccacagccgacctgctcaacacaaagccaaccacacgcgaactcaagtttgaccgtatcctcgcagacgtccc

ttgctccggcgatggcaccctccgcaagaacatccccatctggaaggactggaactccctcaacggcctcggtctacacggtcttcaggtacgcatcc

tgctccgaggtctcgccttgctccgagacggcggccgcctcgtattctccacatgctccctcaaccctgtcgagaacgaggccgtcgtcgcagccgct

ctcgtcgaatttggcgctctcaagaaggacggcggcgaggttcacctggttgacgtttcccacgagatcccagaactcatccgtcgcgtcggcaagcg

gtcctggaaggtcgctccaggtcgcggtaagcatctcttcaatcatgcatctgcctctgcagcccaaccaactgcaaaggacgagccgcctcaggaag

gtgctgctgtatctgatgctgtcgctcctgccgaagagtcccaactgaacacacctcctgacttcgtcgagtacgagcacttcatcaataccatcatg

gcaggcaagagcccacaagacgtaaagccagacgcagtccgcgaacacgacggtgtcaagctcacctgcaagcgcggcgaatggaaagaccaaccctc

cgccaccccctggctgcccaatttcccctacgtagactcctgggccgagctcaagcaacgcgatccacaactggccggacgcagccccaagacgctct

ggccaccgaccgacgacgcggcggataagctccagctggagaagtgtatgcgcttctacccgcacgaccagaacacgggcggcttcttcgttgccgtg

ctggagaaacgcgcgtcgaaggaggggtatccccatggcgaagagggattggcagcgggtatgcgacgcgccatggagcgagctgatctgctctcgga

cgtgaagggagaggaggagcaggtttctgttgtgaagacggaacagcaacctccccaggtggaagctgcggccgggaagaagcgggagctgagtccgg

ataacgatcgatcggaggcgaagcgcgtcaagaccgaaacgtcggcagcgaacgcgtctgaactggcccttgtcctgcctgaaaacggaaaggaagcc

gaaccagcaccgtcagaccccaacactcatatcaaagccgacaaccacgctgtagaccgggcggtcaaggctgggaaaccaggagtcggcgtgaccga

cttcggccacggacagccaaacggcatgccctaccgcgaagaccccatgacgttcctcagcctggacgacgccgaagtccgggcctgtgtcgacttct

tcaaactgaacgtcgacgacgcgtccgcccaggccaacaacactctccgcttcgtcccgcgcaacctcctctcccgcaacgcagcaggcaagatggtc

cgctccgtgaacctagtctcctactcggcccgcgccatcatttccggcggcggcaaaccgggcctggtgcccaagaaggacgacgatgggaatgtgga

gttcgatggtgatggggaccctgtcatggtgccctcgctgcacccgtccctgaacccggtccgactccggctgctcgcggcgggcatcaagctcttcc

agcgccaagactcctccaagtccgcgtccgcgtcgacgtcggcggcggtggggggcgacatgaacacggccgcgtgcaagtggcgcgtcacctccgac

ggcctgctcgtcctcctcccctatctgaacgcggataaagtggtcaagatcagcatgaacgagctcgcgttcttgatgagcgtcagttatccgtcgtt

gtacgatattgaggagcatgcgccggcgttggcgccgaaggtgcgcaggatggcgttggggagtcatgtggtgcaggttggcgggccggacgcggtcg

atgagcccgagctgggggacgcgccgtcgtcggctttgctgcctgaactgtccgcccggaacgagaacaagggggaaggaggggacggggatggggcg

ccggagggtctgaaggaggaggagaagacgcggttgcaattgaagggagaggccttgttcaagacgaggcaggagtgggtcggtcgaaaacttccggt

ggagattgcactgccgatctggaaggcggctgcgagtgtgaacctgatgattgacaagcaggtcaagagtgctctcagtcaccgtctcttcggccggg

acctaagctccgccacaggcgagcggcacttcaacccagccccgccgccgggcaagaagaagctcaacaagaacaagggagggaaaccgacgcgtgcg

gaggtcgcgaaggatcaggagttggcggatgaggaggatgcggcgatggcggcggcggctgctgctgctgatgctgtcgagggggactcgtag

>contig_14.contig_14.g383.t1

atgctgtcccgctctgctgctggtgccgccgtcgccggtctcgcccgcgcatccatcgcccccgtcgctgcccgccgcg

tgtccgtggctgcatccgccgtccgcacatacgctaccgcaaagcccgccgcatccgaggtcagctccatcctcgagcagcgcatctccggtgccggc

gcagggggtgatgtcgaagagaccggccgtgtcctcaccatcggtgacggtatcgcccgtgtctacggtctccgcaacgtccaggccgaggagatggt

tgagttctcctccggcgtccgcggcatgtgcctcaacctcgaggccgacaacgtcggtgtctccatcttcggttccgatcgtctcatccgtgagggtg

acaccgtcaagcgcactggtcagatcgtagacgtccccgttggacccaacttgctcggccgtgtcgtcgacgctctcggaaaccccatcgacggtaag

ggtcccatcgaggctgccgagcgtcgtcgtgcctccgtcaaggcccccggtatccttccccgtcgctccgtcaaccagcccatgcagaccggtctcaa

gcccatcgacgccatggtccccatcggccgtggtcagcgtgagctcatcatcggtgaccgtcagaccggtaagaccgccgtcgccatcgacaccatcc

tcaaccagaagcgatggaacgacggacaggacgagagcaagaagctctactgcatctacgtcgccgtcggccagaagcgctccaccgtcgcacagctc

gtccaggtcctcgagaagaacgacgcaatgaagtacacctgcatcgtcgccgccaccgcttccgaggccgctcccctccagtacctcgcccccttctc

cggatgcgccatcggagagtggttccgtgacaacggaaagcacgctctcatcatctacgatgacttgtccaagcaggccgtcgcataccgtcagatgt

cccttctcctccgtcgccctcccggtcgtgaggcttacccgggagacgtcttctacctccactcccgtctcctcgagcgcgctgccaagatgcacgag

tcccacggcggcggatccctcaccgccctccccatcatcgagacccagggtggtgatgtctccgctttcattcccaccaacgtcatttcggcggactc

ccgctcttgcgctgcattaccgacgggcaagtcttcctcgaggccgagttgttcttcagcaagtatctccactcttcaacactccagcaccattgaca

gtgagggtatccgtcccgccgtcaacgtcggtctttccgtgtcccgtgtcggctccgccgcccagaccaaggcctacaagtccgtcgctggttcgctc

aagctctacctcgcccagtaccgtgagctggccgccttcgcccaattcggttccgacttggacgcctccacccgtgccgtccttgagcgtggtgcccg

attgactgagctcctcaagcagtcacagttcaaccccatcgccaccgaggtccagattccgctcgtctacgccggtgtcaacggtctcctcgacaaca

tccccaccgacaagattgtcgactgggaggcacaattccgttccaagctcgagagcagccaccaggacatcatcaccgagctggccaagggccaaatg

accaaggacctcgagcagaagatcaccgcggccgtcaaggaggacgtcgcgtcgttcctcagcgcctaa

>contig_14.contig_14.g384.t1

atgctctttgttccagggccaaggccaaggccccaacgcgaatttgttgcctggcccaccgcgacgaagaagaagacgg

cgagcgcgatgggaatcctggggcgtctggtgacttcggtcgggcgtctggttgtgtatctgcaggtgctgtgcttcctcccgctcgccctcgacctc

ggcggacgcgactgcttcctcgcgtacggcgccagtctggcctcctactacgccctgctctccaccttccgcctcgtggtcaagcgcaccagactcga

accactcggtaacttcctcgccgccttccaggtcatcgtcgtccccctctgtctcctcgtctgcttcaatgtctactccccgcccgcagagtcctact

tcgccgaggtcaggcgcacggccgcagaggctgccgcgggatccatcgccggcacagcagcagcgggcgcattgcccgccgactcagcaggcggcgtc

gcagtcagtctcggatcagcagccatcgcagccgcatcacgagctgccaacaagctccccttcgccggatccagcagtatcagcaccggcaccggcac

tggcactgcccgaggtatcgccccagcaccgccgtccccgccggaggacatcaccagcttcgtcgagtggctcctgactccgccgaccagtgaggagg

cccagcgcgcattcgcagcggcggtgtcgaccttcttctggctcgcaaaacgcgtcccgccgtcctggtccgcgctcctccgcttcagctcgcccgcc

ttctccttgctcgaggctgtctccactctccttgtcatccagtccgtcggcaccctcccacgctctctcgcgtctctttcccggcctcgctcctcgtc

aaagcgctcatctaccacactcatcggcagactcatgcccaaatcaggtctcggagtagctgagctctggcagatcctctttctattgctctctgctc

tcacctacgtcgtttcgtctatcatcctcttcctctccttcgaaggcgccacccgcggtcgacccggcgcagctgcagccgtcggcgcatgcgtatcc

tccaccctctggctcaccgccatcagcttcgcacttcacagggggaacgtcgtcgagacctccctgatcgcagcctacgtcaccttcaacgtcgtcca

attgccagacagtctcgccttcaccgcagatcccctcgcgctgattcgctccttcaaatatgccgcagcgggcggaggggctggtgcggatgccaaac

agagcgtcgcaggtacgctcctcactatgctcggcctccttcccgcttcttcgccctcggcgactacgaacgggatcttcagctctccatccctcgat

ccagcccgatacctacctcgcatcgtgatcgagtccgcagaggccctctggaacaccatcggctacgccctcgcccagtccatcgagttcatcgcagc

agcagcctccgcccttcccgcgagcgtcatcatcagtctcgtctaccggctcgtcgtcctctacgcggccatccgcatcttgcccagactcaaggcgg

acaccacggcgaatgcggccgctgctagacggactgcggacgctgatcagcggtgggcgaaagcgaatgcggctgtgatctcacccggtcggaccagt

cgcatgctcggcgttccggctgggccagattcctccactgggcgctcacggcatacctactcccgaagttcgccgcgcaactcaggcgtgcgcgctcc

gatggagaacgactccaccacaccctccttatcccccacggggcccggaagaccgtcggacatggcagacaaatccatagggacctcctccacctcct

cctcctccgccgtgtcgtctctccccagcgtcgaccggctaggcaccgagtccgaaggcatgcgcgaaccgtcgtcctacctgctcgactccgacccg

gccggcgtgctcgatatgagcctgggcgacgagaccgaggccgagacggacgcggaggtgtcgtcggtggtgccgtcgatgattcggagaacgtcggg

agggagggcgaatggggggagtacgttgtcgtcgtccgggtccgggaggaggattaatggggaggcgatagcggaggaggaggatgaggatgaggggg

agctaacagcggcgggggagagacgtctgcgagaccaggagccgtttggcgcatttatttccatcctgatctcctactcaagattgatcctgatcgcc

gtgtacagccacctgctcctgctcgaccagagtcatcagatctactggcgcttcttgaccattgcaagttgtctactattgtggacctttgagattct

acttgggcacgaagaccaaatatacaagcttgactga

>contig_14.contig_14.g385.t1

atgccccatcggacagctctctccctcgacaaagccgctgccaaggttgctcccaaagcacaccacctcgtcatctcca

accccgtcaactccaccgtgcccatcgtcgccgaggtcttcaagtctttacgaccccaagcggtgggttttcccccgaccccccctctccctcttcta

acggctctgacgcttcatgagttaaaaatggtgctcatcactgcatatcttcgtacagtctgttctgtgtcacgggtcaccacctttgacatcacacg

cgcctccaccttccacttcgacattgccaacgtcaagcccgccgaggtcaatgtgtccgtcgtcgatgctcgtagctacgatcgcaagtcgcagccgc

atggcgaagcagacgaaggccgacagccaggtcactatggttccaaggcggacggccccggagcgccccggagtttcggcgaaactccgcactccgct

tgcgctccgtactcctgcggaattcaatcagcgggagtaaatcctcgaaaatgcactccgtactccgtactcccccggacactccagcgaccgtttac

atgctgcgcagcgttcaagcagcagcctagcttctaccaagtgcagcgggacgatacggtcgagaatcaaacttttacaggggacagctccctcaagt

gcggcgtcattcccgaactccccctccggaaagtggattggtgggatgctcatcaatgtgccaacggcgtggcagaacctgaccttgcagcccaaaag

caggtcatgctgaacgtgtcaagagctaccgacgcattatatggagcggaggctggtggggatgcatgctttgcgaaagagggaggtccatcttcgag

tcaacgcagcaacagtccagacgaagacgaagctgacgtttcaatgtcggctgcccctcttgcgtctggctcgttctcttcccccgacaatgccaatt

cacccgcctcccccgcaaatgacgacggcgatcaaggcaagacggagtcatctctgcgatggtacatggatcttccttccctacaaagacccccggta

cctcactctgctttggaagaaccggaggacgcactccaggccctcttaacgacgggaaggacattcaggatccaagagcgtttacccgcatccgcttg

gagcctacgcacctctttccctcagcgacggggtatggtcagtcctcccagacaagtccatttctactggaaacaagacatcatgagggcggctgacc

tgctcaacaagaagcacgcggtgtttcactttggtctcgacgaggtggactcgccttcataccatgtcaagacaccgaacggttggcaacgcctgaac

gatgacacgctgcccccttccgctcagcagctgtttcaatggatttgtcaacgagttcgtctgccttcttccttccctgccttcatgtcaagcgccag

tgaccacagtgaccacagtgccgcgctgtcgcgctcggacttggaggttgaccacagctaccagcccgagattgttttcacatcaccgctaccggaaa

gctacgcacaggtgatggccagcaagcttcacaccagcaatcttcacaaacgctcctttggccgcagctcgatgggagttgactcaatcacgtacagg

gtcaagcaaggtggtgatatcaaaacgatgcgagtgtccaaaggggacacggtctgggttgcccctggcccttcccgtcaaggccaagccattcgtgc

gacggtccgcgaccttttccttttgggccgtgatgacgaggacagacgcacttcaggccgggctttgctggagggatacgaatctcgcctgcaagggc

ggcaagggcggggggagcagactgcctggagcgtctacggcttgcgcccgtgcagtgcagcgggcaatgatgtgaagatcgacgctgaggaagaagcc

gagtttcaacgacttatgcgcgcgccaatcagcgtcgaaactgtgcggtctccgacgttgagcttgcaagaatttgcagctcaacacaagctcaacat

tgcagatgtggaatcgtacccaagctggtcgccaggagcagtccttgccatgaagttcgaagaatcttcggaatgggtcggcatggaggacgtcagcg

agtcaattgctgtcaatggtactgaagtctgggcctgtcgggtgctcagtgccgttggcctccgagccgatcagcagatggtggcacgcgagttccaa

ttgggggacaacatcgtcttggtcccttccaccattggcgcgcaggacgagtccaaacaggaggttttttacggcaagatcatcacgctgaatcttct

caagagataccctttgaagtcatcgaactacaacattcaggttggccccatcatacacgctgcttatcagcgcctccaagagaacggcgatgactaca

agcccgactactacggaaagaagcccgtacctatcaagcaccggtccccttggtgggtcgagaacatcttcaatgtattgaagcgacccgacgccgtg

gatctggaacaaggaggcaacgtagcagacgacacggctcccagcggggttgcttctggctcttcaactcaggccgtgggtagcgcacagagtgcgtc

tgctcctcgcgagagtcaagaagtcgagaaggggacagacggtccaaacgagacggaggctggctccaaagacgtcatggcctcagcccgtgtttttc

ctgctaccaacgatccggccagcaacctcaagatctatttctcgcctcaggcgttccgtcgtccgatcaagtaa

>contig_14.contig_14.g386.t1

atgatacgcgtgcggaccgtcttcgacttggtggagaaggtctgcccggggcgtcctttttaccgccagctgggctctc

tgtccccgttagccctggctagaggattgcagaacattttgcaagcacgctacacggcactgggtggtcgaatagcacaagattgggaaactgctcgc

caaggtgtggactatgcgggagaggcggtcagtctctggttgggaagacggttcggagagactgctctcgacttcaagcgcgtgctctcccttcagct

ggccgagcctttgcggcgatatcacgaggaagcctccaagaacccccagaaaacagcaactctgcacgcctggctctgcccaaaggcatggcagggac

tgcatgacggtcaaggggccacagcacagctattcatggccatctacgaagcccaagtgtcgttacacagtcagcttcggattcggcgcccgcaggaa

gaacagtggacggaccaactccctctgccgttcgggatggacatagtcgcggcttgcctgaatggtccagagctcgcaggaccgatgcttcacacagt

cggcattttcttcagcttcctcgttgagctcgccaagctctgcggcatacgattctcggcccaagctgtcgctcacggtaccatagccgacgatgtgc

tccggaagagcttgggcaaagacacaattccggtccgtcatactgcatactggactcgtcaagaaatcacacccgtgacgtcggcaatgatcatcgcc

atcgagctcgccaaatacatcctggcatctacaaaggcgccgatcctcttgcatgggcggagggactttgtccgcttctacggtttcatacggaacaa

cctcctcgcctctgctccttgccgacgatctcggctctggcgagacatcagtccatttcaaatgattgatgacctcttcttcggtgcgatatgcgtct

cctgcagggcggacatcgacccgcgcgaagcccagctgcaagcgctagacgcgctggaacgagatctcgcaaatctggcgacacgttcgtcttatctc

agacgagaggttacaacgctgatgaacagcaaagcccatctcgaggacaacttggacgcagtacaagcagatctcaaaaaggctcaagaggagctgac

tcagcatcggcgagccactgctgtgctgacagggatggtagggctgactggaagggttatacccgaggatattgctgctgcggtcagcaagcactttg

aaatacgtgctgtgcgcgcgccagaggggaaccctacctcgaccgacccgcctgctgatgcctcagtgcgagcgttggctagcacttttcctgagcgg

atcttgggagaaagagatgcagaggcagtcattcgcgtcgtcccaggatttcaaaacaaagaccgcgagccgacgattcccacgctcgcgctcgcacg

tttgttcatccttgcttcagaccaactactcagcaagaaggagcttaaggatgggcgcggagggccccggagtgctccgtactccggggcccctactc

cggctgactccggctga

>contig_14.contig_14.g387.t1

atggcaacatacaccacgcgcggttctcccggctcgcccttcccagccggaggatcagaatcacaagaggatggcgtgc

gcatgatcctcatcggccctcccggtgcaggaaagggaacacaggcacccaagatcctcgagcgcttcatgaaccgcgtctgccatctcgcaaccggt

gacatgctccgcgaacaggtctccaagaagaccgagctcggcgtcgctgcaaagaagatcatggacgccggcggactcgtctcagacgacatcatggt

caacatgatcaaggaccagctcgagcacaacccttcatgcctaaaaggcttcatcctcgacggcttccctcgcactgtcccccaggcagagaagctcg

acggcatgctccaagccaagggtcagaagatcgaacacgccatcgagctcaagatccaggaccagcttctcatctcccgcatcaccggccgactcatc

cacccggccagtgggcgatcctaccacaaagagttccacccgccaaagaagaccatgaccgacgacatcaccggcgagcccctcatccagcgctccga

cgacaacgcagagaccctcaagaagcgtctcgccacctaccaccagcagaccggtcccgtgaccgactactaccggaaacagggcgtctgggcccccg

tcgacgcagcccaatccgcacaggtcgtctggcagagcatcagcgccatctttgacggaaagcgatcgtag

>contig_14.contig_14.g388.t1

atgacagacgcgacgcccccaaaacgtgcgagcttcaccatcggcggcggcaatgccaatgacgatgccgatgcccccc

gacgacgcgatcccggactccgcctcacccagctcgacggtgacgctgacgctgatggcgacgaggaggaagattcgtggaccaagtatgcgtcgggc

gagcagacgcccaacatctccgaggcggccgagaagatccgtcgcagtctctccgttgcgtccctgcacgacctcgcacacatgaagccgaaggagat

ccgcaagcaggttgcgtctaaggtctggcgcgcccaggatgaacaggtccgcatcccgaccgattgggaacgtctcgccgtccacgtcgtccgcggtg

ccatccgagctggcaatcttgcgttcagtctgcgcgccactatgacgttggtcctcggtcttatcaaggcccttcgcacgcgcaagttccagggccag

acgctggttaagagcatgtttggcctcgacgcctggcgcttcgcgggcatgttcggtctctgggcggccctgtacaaggctactcacaactccctgcg

tctcctcactccgttcccgaccaagcgaccccgctcgctctcccagaagaacatcgcgcacgacaccccaggcacgggtagctcaactgccctctccg

cttcgagcaccgatgtccgtagcacccccaacgcccgcagcatctcgaattctcgcagtaactcggatccccgcagcaaaatctggcatgcctatgtt

gcaggcgctgtcagtgccctcgccgtgttggccgaacgtaaggagacgagagtcacgctggcacagcagctcttcgtgcgtggcttggaggggtcgta

caacgtgatgcacgcgaagaactggatcagtatccctcacggcgccgtcttggtcttcggtatcgcttgcggtcagatcatgtacgcctggttgcacg

ccccggacaccctcccgcgatcctacgtcgcctggatcacccaagcctcccacgtagcgcccacctcccgaaccgtcagtctggcaacgtacagcacg

ggctcagtccacgaggacaccatgctcgactacttccccgaccggaaatggccggagctcatcccgggcacgaaccggtacccgaacatcgtggccac

caaggccagtcgtcgtggtatctccggcaagaatgtgaaggcgatgatcgagttccgtgatcggcttcggagtgggttgaaagaggcccgcccgccgt

gtgcggttgttcatccttgggagaactcgcacttctggagtccgatcgataggttcatcgaagtgactcgctggattctcccactccgtgtcttcctg

aggtcgatctttggagcggtccgatcgagctccttcttgggcgtgttcgtcatcatcttccaaacatactactgctttatgaaggacttgcatggatt

tattaagcaccgaccggcgttgaactctcgcatcccgtcggcgttgacgaatgcgctgattcacccagccacccactggtttggcggcttcttgacgg

cgggctcgctgtttgtcgatgactcccgacgacgagcggagttggctgcctacgtcttgcccaagggtctcgagagcgcgtggttcgtggcacggaag

cgctcgtacgccccagctgtgcctggtggcgacctgctgctcacctcgatcggaatgtcgatggtgatgggcacgtatgcgcagaaccctgaccacct

ctccgggcttgtacgacgcgttgtttaccagttcgtcggtcggaactga

>contig_14.contig_14.g389.t1

atggccggaatctgcagaacaagactggccgaagagcgcaagcaatggcgcaaggatcatcccttcggcttttacgcac

gacccgagaaggccgcagatggcggattgaacttgttgagttgggacgcaggcattccaggaaaggcagggacaccatgggaaggtggattgtacaag

ctcaagctggtattccctgaggagtacccatccaagccgcccaaggtcaagtttactccaccgctttttcatcccaacgtatacccaagcggaacagt

ctgtctgagcattcttgacgaagacaagtcctggaagccgtctatctcaatcaaacaaatcctgaccggcatccagacactgctcatagagccaaatg

catccgatccagcacaagaagaggcgatcaaggtcttccacaaggacaaggaggagtacgaacgacgggtgcgagcacaggcgcgagacatggcaggc

tcagcctag

>contig_14.contig_14.g390.t1

atgtatggcgaagacacgcagtccgacgagcgccaatctcgcactgcccaccttgggcccagcatcatgaccaccagca

tgagcgaagcagacctccagaatatcctcggctccttctccgccggcaatagtaatccagctggctggcctgcgaacgccaccttcttggaccacacg

gccaaccagcagcagcaaagcgcatcagcccagggctccggcggtcagaacatgatgttcacctctgctccaggacccttcgctcgcgcggtacctca

cccggacgccatgtcccaccctttcgcccccgccccccatatggggggaatgcagtcccacatcccgccagactaccggtatcactcctccgactttg

gccagcagctcgcaaccagcattggcgtcggtatcggcgctggcagcagcggtggcggcagcagtacgggcggacagatgatgatgacccctgccatg

atggatacactcttccagcagcagcatcaacaacatcagcagcaacagcaacaacagcagcaccagcaccagcaacaacagcagcagttacaacagca

acaccagcaacaacaacaacagctgcaacagcaacagcaaatgcaaatgcaagaacagaagcagcagcagcagcaagagcaacaacaacagcagcagc

aacagcacgagcaacagcagcagcaacaacatgaacaggagcagcagcaaggacagcatcaacaacagcagccagaccaacagcaggacggcttctca

attccgctgcctccctttttcggtggcgaagaagaaggttctgccgcagcggttggtgctggcgccggtgcaagtaacagtgccactgcggccgctgc

gggtgccatcgtgtccgacttcaccaaacggaaaggatggtctacccggatcgtcgaagagcttctcgacttcgtacacgtgctcgacttcagcggcc

ggatcttattcgccgcccccggcgtggtgagcatcacgggatggaagccggaggaactcgtcggcaaggaggttgtcgagttcattcacccgaatgac

cggatgccgttcgtgcgcgagtttgcgaagctcgtcgatgcctttttggcttcccagcagacgccgctagagcaacaaggcggcttctcgcagatgca

gaccgacggaccgaatccttcgccttcttccatgggcaagagaaaagcctcggccataagcaagggcaagggtaagggtagagcgaacgacagcggcg

ccgatttttccggcatagaggccaatgtcgggctcttctctgacccttccacagcaagtgcggcagcatcaacctcgaccagaccaaccaccgtcttc

ggcgctcctttagccccgtcgacaactaacgccactccaccggcagatcttttattctattaccgcttcgcgaagaagcctctgccgcgacgcgccga

tttattacgaaccaagtcactctctcgcttaacatttgccgacgcgctcgcatcagggaacgtcaagatgggcactcctgggccttctattcttcggc

cgggcatgggcggacgactaccgagtcatgcggctgctttgagtagatccgcttcccgcttcaccgggtctgacgccggcagtgggttattcgatgac

ggatcagggatcgacagtaacacggggatgtccctcgacggctttgacgggatgggcatgatgatgggagggacaggcatgggcagaggatcgagcac

cattgccatgtcgactccgggattgatgaagcataccactccctccaggcctggcggaggggttgtggagggctcggatgacgaaggtggcgctcatg

atccctacgtagaccgggaatgggtcgtcttcgaggtggcaggacacgtgtacgttcctccgcccgacgctttcaccgcggcggcatacggacccgac

ggagatgacaacagggacctcccggccggccgaacctccgctgacctaggggaaaggcggaggagcacagaagcaaaccgtcgagatcacgcggacga

gatcggtccatcgtcgatcaaaccaaacgaatggccacccgaagccgtggacaacgtccgatgcgtcttctgctcatgtcgagtctatccgtcgaaga

acgtgacgatgttcgactccttcttggagctcaaggtcgagaatgagcgcttacgaaacctactcggcgaggctgatgccgcagctccgggccagggg

cagggcgaggattggcgcaatgaggatgggagtctgggccctggtgtgggcgacaccagcctggacgctggttttgtcggcgacatccttgcctctct

cgacgacgacttcgacgacaaatcacatcgacaacggccctccatcacggcgctctcgcgagggccatctgtcatggggcacggcagtggcatgggaa

gtccgaccagtcctgtgagacctttgggctcgacggccatcttggacgatgaggatgagttgtccggatatcaacagggatccggtatggatggcgga

aatgcatgcggtcttcggtgggcgaagaagataaaacgcaaaggtggcgaccccaacgcgacggccaactttcggtacgtcgctcttgtcaccctggc

ttccggaccccgtcaaacatgctttgggtacgacatttatgccgcattctatcttcccacacagacattccgggccgtcaccgtcaggagcttggtgc

cagtcccgagacagctcttcatcgcgtatgacctagcgctgccgcccgcgcgcactgatcccccggcatcggccacatccgttccgtcagaggaagga

gcggaaggaggagggaagaagtggccagtcattccgtggaaggaagacgaatttgctttggagcacaagctcgttacccttctccatgaattcgacgc

gtatcgcaaggtctggttctgccacaagaacgaagcacactcgaacgagaacaagtcttctgccggcaagggccttgcggtgcagcttctgaaggata

ccgtgctcaagaagcacgttgcagaccctaaaagcgcgagtctgaagcactatggatctcgtattgtgcagaaaattgatcagaagaccggccgtggc

cttctgactctcttttatcaggctcgaacggagctcaagatcactggtaacgttcaagtccgtaagagggtgccctggtacttcgagctggcctctct

cgtcggttcacgactagatatcggcactgccgccgtcacgaacagctcttcggacggcgcgagcacagtcttggatgcccgcaataaggcaggtaaga

agcagaagaagaagaaggggacgaaggaaggagaggagaagagcgacggagaggaatcaggagaggagagcagcgaggacgaggaggacagtgaggga

gatgatgaggaagaggaagaggaaggcctggatggagagggtggtgcgattcagaaaggagatggtagtggtggtggtggagaatcaagcagtgatgg

gtcgggagcgaagggcaaaggcaaagttcctgcgactcctgatcgccatggagctaagaggtctctccctgagggaagcagccctggcggaccgaacc

cccccaagaaacgcaagccagttgattcgatgaaggagatggtcatggctcgaatggaggccaaggagaaggagagggcagatctcatcgcccacgag

gctgccaagcaggagcgggagcaggagatggagcgactcaagcaccagaccgaggttcagaagcagaagacggcagagctcgagatcgaacggctcca

gcttcagctgcgtctcaggcagatgcagcgtgacatctag

>contig_14.contig_14.g391.t1

atggaaaaacgcgtcacggacaccggcacgcgtcgacctatgctgctggcggtcaccacttaccttcgccatccttctg

cagcaattcaattcattgcccgtttctttttcgcgcctctgtgtgacctggaagtgttgtttagcagtctctggttgctggttgaatcatcggcgtca

gatatcagggcgaggactgtccaccagacggcatacgatgacctccgggaggcattctatgctgccgaggtcatggacgcttttttttccagcgacca

agaggactctctttccctgcctcgctcggttccggttcttcgtcttccttctctcagagctgcgtcagacaatacgattacggaggccatgagggacg

agctcgtggatgaatatatttacacggctacgcatcggtatctcgagcctcgtcaacctgtgcctcgaacaggtgatctgatagatatcatttttgat

cataagaaaaaccgtcccagtgcttttcgtgaggagctgcgcgtctcgggtcccgcattcgacttcatcctcgatcaaatcaaaggggacgatgtttt

ttatacggcgcccggaaagacgcaaatctcagtcgaggttcagcttgccgtgcttctctacagactggggcactatgggaatgcggctactgagggtt

caattgcgtcgcggtttggattaggcagggggactgtggtcaaatgcacgaatagggttctacaggcggtactacggtcggagacactgaggaacagt

gtacgatggccagcgaaaggggagccacgtgaggcctctaagaacgaggccgcggcacgatctacacctgaatggagaggaggttggtgtggggtgga

cggtacgacggtgcccctggccgagaagccgtacctgtacggacctgcttggttcgatcgcaagggaaggtatagcatgaacgtccaaatgaatataa

tcgatttctctgtgggcaggacgggcagtcgctgtgactctgttgcctttgaggacacgcgactggcccaacgtcattcggagatgctggaagctgga

gaatggtgctgggcagacgctggctataagagacaggactggttggacgatgtacaatacgcttgcttgtggcttcgtgctgcggtactgctccacca

gacggccaagtgggtggagcagggtgatgcctcgatggacccgttctatgcggcgggtacggcctgggaagcggcggaccggggggaccaggccgtag

gggctcgcaatgagaatgctgaagaacagggaggcaccggcaacgctgcaacaggagagtctcgagcgcgacagctcgctcgagcgcacaagttccga

gacgggctcatggctatactctgcaaagccatccccccctcgagagacatatcgtga

>contig_14.contig_14.g392.t1

atgcgcacatcggtcagcttcaaacttcggcagcctccagccttcggtcaagaacggcgcggcaaggcttcgatacggc

ggtcggtaccagggcagatggcccacggacaccacataggaggaatgggacggattgcggcgacggagcccactacgtgtggaagcggtgtctcggcg

gcgagcaccgcgcggagacctacggtccagcgcctgagcagcaaggaggagctcgatgccgtcactggcaagctcctagggctcagcgcaaggcaagg

tcgggatccattcacctccggatcccaggtcaaggcgcgcgagcggcaatcaaggcaccagcagtttgcggtgaccagcagcgctcggcgactcaagg

atctaccgacggcggcccaagaatgcgtttgggcgcgcgcgcaaggtcggagatcattcgggagcggaacgaggcagagacgacttcaggtctcgagc

ttcgacactccaacactttccggtttccgctcatag

>contig_14.contig_14.g393.t1

atgccattcactgctcccaccgatcctttgccttccgttctcagaagccgtgccgtactggccgatcaatgcgccgtga

tgatgcgcttgattccacgcgaaggttccgcagcttctcgattgctgtggaccacttgtgatctctccgcacgtcaccccaatgtgggcgacgctccc

gcagccgacccacgtcgcactgcctctcctgctcgtcgtccaccgaccaccaataccgaaaccgatcccctcctctgcccacagtcgtccaatgcccg

catacacgtgctgatgtgtcaggcacgtatgccgtcgacagggacaggattctgggacagaaagagtgtgcttgacgcccgaaacatgggtagtgact

cggagcgcgcgcaaagggaggtaaggcatggtgtcagcaacttgcgtagacccgtcgatccacgctggttcggcgccgagttgacgccagcgatgctg

ttgtgcatgcccccttcacgcgaggccatatggcccgcgttcattgccaacccgtccagcaacaacggcggtcccgaggccgtcttcggtcgccaggt

cccgacccaggtgtcctgtgtcggtcgcccgagggagtggtggtggcgatcctctactccttcccagactacatgtgtgatggaaccaggacaggcgc

gtaccagccgcgctgtcccccatttgtactactcttgtcgaccgagtcgtagtgcctgtgccgagcatgatcttgtcggcgaggacttccatttcccg

tgcgcctacgcgcgcatcgagagcgatccgagccccggtgcgttcttgaagttgatgcacggctcggtgtcgcgtcgcagaaggtgcttgccggcttc

agtcttggcgggtgttcgacctgcattggcgttcgccagacacccgagtgcatcttccgccttgaatttgaggtccgccgatgtgctggtacctcgcg

cgtcttccgtgctcagcctcatattcttgcgccaagccgaagaccgcaaaccggctccgtcacgtcgcgagagagcttgcgccgctccctatcttccg

tag

>contig_14.contig_14.g394.t1

atggcgattgtggacccaaaacagatgaagaacaagagaaagtatcatctcttgacccatctggaggcggggatcgcgc

ttttcggcccggccaagggatttgctacggaaaggtacgagtcatttaacacggtcgctcgaaatgcgagcatgtgttcaaatcgggcagcgctgtct

cgagatatcgcactgagactttacggtcaagaactcgtgcggcagctcatcctcgacgaaagaagcaacgacgctgcgtctttggtccggtttgcgta

cggctttcaggaaggcgcgctgctgaagttgtacggggtggaagtaaagaagaagaagttggttccaggtgcctatcagaagctgcaagtccagcggg

tgaaggtgttcagccagtttgccgacgctgctcaaatcttgctcggcgaagaaggctttgccggttcttccgaggtcgagtaccactccctgcgattc

accatctcaaacgattcggaacgcatttatcccgggtccttcgtgcgagcggcagttcggatcccctcagacgcagatgatgatggtcctctccaatg

gacgtgcgtggcaatactcaacaaaatcaacatcgttctatactaa

>contig_14.contig_14.g395.t1

atgcttgaagaggacactcactcggtactcatccctgtcttgcctcggcacctcttcgaccagaatcagacgcgtgatt

accccagcccatctacacgcctgccacagactctcttcaatctctccctcattgcgcaagggaaggagcaccagacacagtacttgtgcaagaacgcg

catgtttttgccggtgaggccacctgcgttgttgagcaggtaatagccgtcgagcgtgatcgattcaccaatgccgtcgacgtcgtcggtcccgcctt

tgctgagcgatacaagaccggagaagtgcatttcctggctcttgtctcctccgtcatcaagaagaaggtcaacaacctgcgctctcacatacgcgaca

agatccattggtctctcggactggacgcgaacaggtcgaaacaacacctcttcgacctctacatgaagctaacaaagagctgcggtgtgccggtgacg

aaagagcgcgtgctgcgcatagcatacatcaacaaccctcgaaaggtggacggggagccggtagatggctggtgggagaagttggacgaatacatcac

taagctgctctccactacccgcagagactcggagaaaggcagacgtgtgctcaatgctacatacgatgcggacaagacgaagtttggcgatttcccgc

gacacgggaagggcatctccaaggcaggcagcgaggacaacgatattcgctccgctttcaaggccgctgcgacaggcaacgccaacgacctctga

>contig_14.contig_14.g396.t1

atggtctgctgtgcccatacagcatacctcggacctctctttgatctttcgctggcgcggcaagtgatccttggcaatc

tcaccgtcgaccaagccgtcgcaacaggacacatcagccgcgctacactctttcgcgagctgaagatctatcgcgagaccggacacatcacagagcgg

aagaaatcgtttggtaccaaagccaaagcgtctccagaagctcgcgcctatgtgttggctttgttccagcatcgcccatccctgtacctccgcgaagc

agtcgtaatgctacaccgacacttcggaattcaggtcgataagagcacgattgggagctggctcaaacaagagggtatctcgaacaagaaactaagca

aggtcgccacgcaacaggacggaaccgataggatcgagtataccctactcatggggcagttcctccctcatcagctggtttttgcagacgagacgcat

ttcgattggcgaaacgcaaacagatccatgggctgggcagcgataggacaacgtgccgaggaacgacaagactttcgacgctcaggtgggctcagcat

acttccagccatggacgaaaacgggattctcacggcgtggtgtgtggagggtgccgttgatggcgagaagtttgattactgggtggaacactttctgc

tgccgaaaatgaacccttttccggggtcacggtcagttcttgtcctcgacaactgcaacatccacaaggacacacgcgtccgccagaaaattgaggac

aaaggatgcatcttgcaattccttcctccatactcgccggatctcaacccaattgagaccgcattcaattcgattaagcgatacgtccagtcccatgg

cttgaagacgcgagcggacatcattcaggcgacgtatgctgccgtcaagcccgagcatgccaaagcatactttcgcaacagcggatatgtttga

>contig_14.contig_14.g397.t1

atgattcaattctgggacgcgattctgatcattcaagagccagacaacagcgagcggtggacactcgactccgtggcgt

cgaccttccatctgatgaaggctatctttagccaatgcctttacgtacgatatctccagttttggtcgcatccttcggttcgaacgtcgcaagtagtc

ccggacggggtgcctttcgaagatctccgcgagatcgttctgaccagcaggtaccatgcggactacggcagtatgacagcttccattatgcaatttcc

ttctatctgcataacctccgtccccaaccttcgccgactctccatcgagcatcccgacacagtcttcccaccagacatcgttgacctgctcccttact

ggcgccgatacaaggtccggcgaaacgagtttctctactattcccatcttccatcctcctctttcctctctttctgctgtccccagagcccgcagttc

tcaatcctcattcttacgatcaacaccgccgcactggagtctggacagtatacagcaatgtcccaagctattgcagaagaacaacaacaagatccgac

atccctcacaaacaagaagagacgattcaaaccaacgctttctggccgggaagttatcgggagcttgcaggctcagcagcccggcgatgctgaagaag

aagaggagcgattctccgaggactctgaagaccttcctccagcgaaggaagaagctccaatcccaaaaagagggagatcgactagaacgacggctaac

aaagcattgcgcaccggtgacgcgccgccgctggattggcttccaactgatctcggaatcaagatttttgctgcaccaaatcctaaagccaaggtgga

taaggagaagaagcaccaacaacttggagcgcagcgcatgctgaaattggaaacgcgtaccacatttgcgatgttcaaaggaatgctgctggcagtcg

tacaggaggacaaaaaggagaccatcagtggagattgggacaactggctcgtagaggtttcgctccacagcgccggtcaccttttctcggagaagacg

aagctcaagaacgaggaagttttcgacgactttatcgaggccgctctgcaatcttcaaaacgggccgctacggtgctcgtcaaggagatagaaccgct

gttcactcctgctgtcgcatctggtgcctctgcctcgtcgaagaaggacgactcgtgtatgaagaatatacaggatcttcagcacgtcagcgatcaac

tcaggcaatacgatcgacaggtcgtcaagagatggatttgcaatgcttccgactgccataatcgttccagggcgcatccttcgtgctacattcaccct

cgaaatcctcaacagcacgttaacctcaagtgtgctcaccggtttgcctgggcggcggcgttggcagatagcgctgctggggtgacggtggatttccc

acctgcaacagagatcttcatgccgaaggcaggcatcatcaaagaagaagtcgctaacgaagatcaggcggaggtttccggagcggatgaaggaggca

agcgcggttcagttcggcggaagcccttgcagccgatgaccggaccgggatcaagcttcgagaatagcatcgacctggacgactacttaaaagtcaag

gaggagccggggttgatgaaggagtgttccgcttcaccaaaggttgcgacgatcctgcgcgaggcaggctatatggaggcggatgagctcctgcatct

ggagaaagatgaggaggccctgaaggacctaaagatcgaaaagacgctcgctcatcagttcgcggcggcaatgactcgatggcgccggaagtcaatcg

ctgcaacaagttttgcaagtactgccggatcctcggtctcatcggcatcaacctcgacggatgtcgtcggagcttccaatggcaaacgaaaagctacc

tccgacttggatgattctggagcaaataccagggctagggttgactag

>contig_14.contig_14.g398.t1

atgaaccatgcagaacggtccgacggaagcgttgcttctccagtctttgggtcttgctgctcaagaggcaagatcgtct

tgcccaacatctttgatgctcccgcccaggtccgaggtctgtacacaggagaaagagagctcgcaaccctggctcttcaaccggagttcgatcaggtc

aacccctacgcacgtcttctacatcacgcaagacccattctcgagcaactgacgtcttcgaccgatgatgtcgcaatcaaactactgcaacctggacg

agacaccggcgccgatccacgaacgtacaaccgtccctccgtttcggaaattgcagcccttatcgtcactccctccgggtccgacttcgatgttgcac

caagggacattgtaattcgataccgctcgggggacatacgccgcgtttcacacctgcatgctgctttcctgccactccgctttccggtcctcataccg

tacggccatttcggttggcacgatgccattctgctcggacaattcccacgagaactgttttctgcagatcctgccgttgctgctgcagcactggagga

tccaaacgagtggttggtcgacgcatgggttgtcgtcgagcaaaacaggatcagctttctcaacttcaaccaagatctactgcgcagggaggtctaca

gtggcttgcaagacgcccttatcagcggagccgacttgcatggcattggaaccagaacaattctgccaacgtgtatgaacgaccctcgctatcccggt

cgatgcagtcgcggctatccgatgccattccgcgaagtcacctgcatggatagcgacggctatccagactatcgtcgacgagaccagggcatcgtggt

ggagcgtcggggaagacgcaattcgtacctctacaagtacgtcttcaaaggcccggaccatatctccatgcaagccggttcagtttcggacagcgaga

ttgacaactatctcaatgctcgctacgtgtcccttgcgaagtcggttactttcacccctgacgatcgacgttcggcacgaaacagtctcgccgaatcc

aacgatcactccaagttgctcgcattctttgacctatgcaagcgcctgcccgaagagaccgcaagtctgacctacgcgaacgttccggaccgattcgt

gtggaacgccaaatttcacatgtgggctcctcgacagcgaggtatgggacaggttggtcgcatatactacgtcccccacacggctcaagagaaatact

atcttcgacgcctgttgcacgaattcaactgtcccaaaagcttcgacgacctttacaaatttgaggagcgaacatacaccacgtttcgagaagcatgc

tcggcgcggggtttattatcggacgacgcagaatggcaccgatgccttaacgaggccgcactgttccaaactggagccgctctgcgtcacctgttctg

cctcatcattttccacaacgacgttgaagacatccctagcctctacgatggacacaaatttgaccttgccgatgactttgcgtttcagctacgccgca

gaggcattcaagatcccgttcaagaacgagctctctcgttttcaaggcatcttttgaacgttgagctcagccaggtcgccaacgaacggacgcttgcc

gacttcggaactcctccacctgatgacgcctttgctgccgatacctctgccatcccgtctccagttgctcgagaacagcagtggaacgcggaggagca

gtcggccctctctgtccaacgatacgagcagatgaacgaggatcaacgttcagtattttcggtggttacggcagcgacacttccggttcaacgcaagg

catctgaagcgcagatcatctccatctgcctacacaaatcatacctgtggactcccatgacccaattgcagttgaacatcaacgtatgccttcttcag

tcgaccgctaccgaccaggcagcccaacaagcatacgcagactggattctcagcgttggaactggacaaatcagtacagacgatgggagactggaagt

acttcctcatcttcgaataggactggaagactag

>contig_14.contig_14.g399.t1

atgagcatcacaactgcttccaacttcaaactcagcttgaaaggaggtattccagagggcttgagggaggcgaggcgcc

gatatgacgcgaagcgagcgtctgctccggatctccaggtcggaatgcaggcttggattcgtctccgcgaccggcccgttccgggcaccttgaccgac

aagttggacgccaagaagcttgggccatttgccgtcatcgaggttctgtcacctcatcgcgttcgtctggaccttccctctgatctcgagatcgatcc

gatcttcaacatcgaacagttcgacttcatgcctacggcggtcgacccttacgctgcggtccggtcgccatcctcttccacgattccggagtcatcgg

ccactgtcgatctccccatcgtcccggccatcgtgttggactcggtcgcgagtgacgatgcgttgcctctcgcgcctccgacggacaaccaagacggc

cctccaccgctcgaagacgtgctctcttccgcctctgaggttgaggatgacctccctcccgcaaccatggccgtaaatggcgtttcggttcgtccgcg

tgctcgaaagccgccgtcgacattgcgcgagttccagatgggtctggtccggccggacatgtcccctgcactcaaagatgccctgcgtggtccgctgg

cgcggccgcgactcttctcggaagacgggcaggagttgctgttgaccgaacgaccgatcgcatacctgtcccggttgacgtccccggccgagaccaag

ttggttgccgccgagttggaactcgtttgtttcgcgtgggctttccacaagtttgctcacttgctggaaggcgcagaggtcaccatcatcaccgacca

ctcgcccatggaaacgatgcttcgatccaacgccgcgaccatgtacggcccgaccatcactcgctgccgggctctgattttgccacatctggccaatc

tgcgtttcatttaccgacccggccctcggcacaccaacgtggacgcgctgtcccgactcattcctgaccagggacggtcagcttcccgcggtggggat

gtgctggcaataggccaggtagtacaatga

>contig_14.contig_14.g400.t1

atgatccacaattcctacattgacggagaagctgtagaatgtgattgtcccccttccgaacttcagtgtcctcatattc

aggcaacggcagggggtccacatcaaacgcacaccgaacagaactacgaacgcgacggattcgttgtcgacgataattttgtcgactcgccaacatct

ccagttgcatccgagccatccgccgttctgacggaagacgaggaagcttcgcagacccatacattgcaagagccggcccaaacgacaactcaagtact

atcgctctttcgtttccaacctcgccgcagcgttcggatacgtcacttgatggatgaacaagaaaaacgactcaatgatctgtcagaccttaaacaaa

aggccattgctcttcgaaagcacattgacgatgttgccgccgaatacgaggcagacgaagatggctcggcacaactaccagcaaggcgcacacgaccg

tcggcgcgaaagatcatcaagtcacaatctccctcatcgtcaacgacctcatcatcttcgctatcgtctccgccgtcttctctcagtgtatcgcacgc

tcctttgacagagtatcaaacttctgatgatgcatgctgttatttccccagcggaaagatgaacacggccgtatctgggcgcggatgcccttcttcga

gacgtgggtaccagctcgagagaaggaacatcacaggagcaagaatcaaggagctcagcgactttgagcgtcgtcctttgaagaccgtcctttcgact

tcaccacgcagtaccaacaaatctcctgtcgcctctcctacaaagccaacgtccaagaacatcgctcaatcccataccacaacgagccagtcgtcttc

ggtgccatccatggcagtgcctgcaatcctatcgttggtcgactacgatagcgattag

>contig_14.contig_14.g401.t1

atggtaccatcatccgaaaaggcgagtccacgatggatcagttttcggtctccgggagtaggcctcttcagcagcggtc

gcgggttcatcgagcacgtcgagtacggcgtagagtcgcgattcgtcgtgacactggagagcgcgacgaaccccatcctttcgagctcgctcattcca

atcagtagcgttacagccggaggagaagactggctacaggtctggcaaacccctccccctagtggattcgaagctatcgcgcaggcaggcgttcaacc

cggagccacggcatctatcacctcaaatacgccaagtcagggcagtacgcaagccgagtcggtactttcaagcatctcgtctatcacgccgagccagt

cctcgtctagtatctacaacccgcaccccgagattctgttggcatctccagcgtcgacgactggaactttggtgccggcagcatctggctgggacgtc

agtcccaaggcgatggagaagcgtcctgcttctgacgatgacatcgatcaatcccctacaaagcgttcgacccgtcccgcgactcgtgcttcacacgc

agcgtga

>contig_14.contig_14.g402.t1

atggcggagccgacccagtacaatacgtactgtgagtcgttcctacagctgcctgtcatcgaaattttgccgataacga

actcgaggggtcgttccggatatgtctccattcgcacgctggtcaaaactggggacggacagatcagccgcgctaaggcctctgcctattgggtagag

agtcgacctcccatcaacggacttcttgtcggtccagcaatgttcgccttcacgacgtcgggtgcagaaatgtcgatagaacgaacctcagtggagct

acgacccggggatcccaaggattcatcatacatcaagtcgcatctcggccatgaaccctggagagtccgggtcttcgggaaggtcgtatcgtcctctt

ctacgtctttcacagtgaccggagcaggaaagttcacagtggaacttgtttcttcaaacaactctccgattcctcgtgctccagcaatcggagaattc

gctggaggtagcggcgtggtccaatccatctccaagaacccaccttatatcacggtcatcctcgagagcgtacacatcggcgtcgcgtcccaagccac

tgccgcagaagtcccgcaacagcctcaagcttccgcctggatttcggcactccagggcgaagcgaacgtcgaaccggctgccgtaccccccacagaag

acgtggacggtactgtcgagatcacgcctgtcaacagcttctgtgaaacgacgtttcgtttccctctgttccaaagcgaaatcgccccttcccactgg

ctccgagtccagagcaagacactcacgtaa

>contig_14.contig_14.g403.t1

atgctctccaccgttacctcacaattctaccaatcagacctcgaagccggcctctcctttcgcgctattgcgcgtacac

gaggcgtcagcaagtcttgggtcggcaacctgcgttcgcagatcgagaatctacctcctccgcctacagcggccgcactcccgtgctctccccgcaag

atcgccaacggattacgcgtcttattgtctcaggtcgcctcgaaaatgctgtcgaggttcaccgagagtttactgtttactcgctctgacggacgcaa

gtgctcagtaacaacggccagaagggtactcaagaatgccgacttcaaggcacgggtcaagaaaaagaagcctgctatcagttag

>contig_14.contig_14.g404.t1

atggagaccaatatcactgctgcaagcatacccggccttacaggcgtggagaactttttcaaatggcgtcaagcaatgg

agccgatcctggacgaattttgtgcccttctcatcgtccaaggctacgactgctgtccgacgatgcctgtcatcccttcctcgaccggcctcaaactc

atcgaggcttggaagttggaaggatcgggacgcccgagcccggtcacttatacaaagaaccatcagcggtccgatgtcaacacatacatcgagatggt

ggccgaagcagaagcagcaggtttaccctggggcaagaacgacgacaagaaggctctctgctttctcgacaccctcgaccacgcgttccgtccgataa

agagcgagtggcgtgaattaccaaaagaagaccggtcgtttttcgccctcatcaagatctacaaagaagaggacgttgcccgtaaacgttcgtccgac

cgacaaatcgaagcggcagcaaaggcagctgtccacaaccccaagcagtccgtcaagatcaaggggatatcacaaaacaagaagtctgtcagaccgtc

aggcagcgacaatggaccactatgctatggctgtggctccagggacgggcacgttcgtgccgactgtcccatcaccaagcatcttggtcctggggaga

tcatatgctggaactgtcacgagaagggaagtggaatcgaatatggactggaaacggaaacggatcggaacggaacggaatggcagagaggagtgaga

gcgagaatgagaacgcagcagaattcagaaagggagagccgagcagagtccataccagcacaggaagagcacacgaggctcgatggggagtccgaacc

caccaccgtcaccttcatggtagactccggagcatctcgacatatcgtcgagcacgaatggatgctcagcaattctcgaaacattcccgagattgcat

tcaagaccatgggctgtaagctcaaggccgacaaggctggcgacatcatggggtccctgcctggcggccgcggcatcaccttcaaggacgccgtacat

attccgggagtaggagtcaacgtactgtccgaggaactcctcatggaacgaggctggaggaaggtcattcgcgacaacggtgacgctatcctccagca

taacatcaagcccgaatggcggctgcccctacataaaattggaagggcccgctacctggtgctcaaactcaagtccgtttcaccagtcaagtccgagg

caagcaagaacgtccttgctgccgctcaaccgaccgccgaccgtatcaagtccgaccgtgcttgtccgaaagcatgggaacccagtccgcgagcagag

gagaccgatccttacgaattcattcgcgtcgaagacttccttgagcccgataacgacgacgagcttccggctggagaagggcagagggagcaaccacg

caaggtgatgtgcacgggagaccgacccatccgcgaacctccttgtcgtgccgaccgcgatcagcattcagaaggcgagcgtcaaaacttcgtcatgt

tcattcaagagtcagaaggtcccttgcttccaatcagtccgacggacaaggtcccttccacatataggaggctcttgcacgacctgagcctgatcgac

aagaacgcgtcaatcacatggacagggatttcaattcccctatcaagaccggcaccctcgaagaagtcaccgttccagagggagcaaacgttgtgccc

acaaatgcacgtattcaagaccgtgaccgacatcgacaaggtcagctccgtccttggtatcgccgtccgacgcatgaaggatgcctactacatcaacc

agtatgtctacagtccgaccgcagacatccttaccaagaccgtgtccggttccacactctgcaagcaccgcgagtctatgggactacagcctccgccc

gattcaacatctcattga

>contig_14.contig_14.g405.t1

atgctcactaccactatcgtccgcacccacaagcaattgagcatcacatcactggcacatcatcatcgtccgcagaccg

catcacacatcaccgcaccgattgtcgccttgtgctcagtctcttctgtcgccctcaccaccaccgaccgcgtcaccatcgacatccataccagctcg

tccgcagggacagccaaacggaaccagcacacatcagcgccttgtgcctggcattccgacatcaccgccaacaactaccgcaggagtagttcggcatc

gccctcgcgcgcgcaccagtgccttgtgcttggctgcaccaggtccgtccgtgggactcatcagcagcaccagcggcagcagcagcagcacagggttt

tctttatgaagatatcaatttgctgttcacatgccgtccttccgccgttctatgcccgtttctgtatctcttccacctccactttttccacgccttcg

atagccatggtcaaagctcaaagccctccgaggaagctgatatctcctctgggatcggggcttatatgctcagtgctcgactactcaccttgggagga

ccaacaaggtagaggagagaggccgaagtcgcttgacgagtcgcaggagcaatcccagtatctcaggttcggtgtcggacgatacggaagggatgcaa

cctttgacggggaagtcaaggatgagggcgagaaggtaaaagatgaggaggacgaggtcaaagtcttcgcctacctcaccatcatctatgcaccaatt

accatgccggaccaccacagtccacacgtctctatcccagtcccgcaacttcacaatcgctgcaccgggcttgctgctgcgtctacggtcgtaatcgc

catcgccgctcaccgccgcaactacatcactgcacgccgtccctcgtgcgtcgtcaccgctgccgctgcgccaacggttgtggtgttcatcaccgctc

atcgtcctatcgccattgttactcccggtccctcagtcaccggtcaccgctgcgctaacggttaa

>contig_14.contig_14.g406.t1

atgccccggaccaatgcccacgtcacggcgtacaacgacctcattgacaccttgttcacggtcgagctggatgagatct

tgtgcgaggacctcaacgacgatgaaactcagggcactcgaaacgactcactggagcttccctcactcccatcgatttcagaaccggtttccctgcca

tccctttcgcctatctccgatgtggatgacccggttgtggatgttcaagacgtgatcattcaggagctcgtgcgtacgaccacgacgcgggttatgga

gccgagtcaacctgtactcaggaccggggaatttgtcgaaattttgttcgacaacaaaacgtatcgagaatccgcttttcgggcagaagtccgagtgt

cagcatccgctttcgacttcatcttggattcaatcaaggatgacgacgtttttgacacagctccgggagcaggccacacacagattctggtctcggtt

cagctggctgtgcttctatacagattggaacactacggaaactctgcgtcggtgtcttccgtggctggaaattttggactgggcaagggaaccgtagt

caagcgtcaagcggaggacgaccccttttatgcagcaggatcagcatgggagcacggtgaacgggaaggaggagagggagcagatgaggaggagcaag

ccaggacgagaagcggtgccagagccagagcagctgagattgagcgggcagacgagttcagaaactcgcccaaagggttcttgttcaaggtcaagccg

ccccgaccatcccgcaaagcagcttga

>contig_14.contig_14.g407.t1

atggatatatgcatcactgtcgatccgtgccccgccaagatcgaagagcacgacaagcgacaccacaacggtgagatcg

aggctggacttcgcttcctaccggtcgcgcagcttgacgcgcccctgagagcggtgccgggtctcagaatcgttgatgggttcggatgtggcgaggcg

gactgcctgttcgcggcgcgggctgccgatctggtgtgggaacatcaactggccagtcacggagccgaggagacacaggttgcggtcaaggttcaagg

ctggacagtcgcctccgctttcgtatggcaacttcaagacaatgccgagtcgcagatcgcatcggcttcaagggcgcgcgccaagacggcaaacatgc

tccgcctcgaagacaaagcagttctgatgcgcccgccgggttcgatccaagtcccgatctcaagttttaacgagctcgacgacctcaagctcgctcgc

gatcgaacccgcgagtttcacgccgatgcacttcaaggcttcatcgaggaaatttcgcaagagccgcatcgcttccaaaactacgacataggcccgga

cacagcttttcacatcaactgtgccatctccatcctttcggcactctccttcggcatctacttggtatggagggcacggcaacttcgggcgaggattc

ctgacgccctgcggacagaggccgtcgtccatctcggcacgctcggcgacgcgttcgacctgaacggccaggctcgtaacgcctgccaccgcctagat

caagccctcggaggagtaccggtggaagaacaactccctgccgagcgtactcaaggccccgaggctgtccgggatcttgttgcggccgccgtcgaaga

ccagcggctcggcgaggtgtacgcccatgttctccgggcggtgaccccctccaaccagaggcatcggctgctactcaatgccgcgcacagtatgtggg

cggtgcgcgccttgctcgtgctcagagttcggagacaaaaccacggcgcatcggaccgcaagatgcaggagcggtacagctcagccgcccaggcgccg

ggcttcctcaaggccctggatctcgagatccacgagcacctcgacgacgcaaatcgagaggggttgcctcgcttaagcctactcctagttgacgctct

gattgccgagactggtacgcggcacggcggcgtcctcctctctggcgctccgcgcaacgacgacgggcgcgagctggccaagtatttcaaccatgcac

tcctcgaccttcgaaccgcagacgccgacttggcctacaactttccggccgttgacggcttgacgcagtatgcgggctcttag

>contig_14.contig_14.g408.t1

atggcggatgcgggtgcggatgggagtgggtgggctagggaagaagctggtgctgctgtgggcgagcgcagggaggaca

ctgatgctcgggtgctgatacgcatgcgcatgctatcgcccgggtgcatgggaaggggggataagaatggtgagcagaggtctgagggtgtggaggga

ggtcagggtgctctcggaggagtctcttcggaaatcctgggtcaggataccccaggagatccacaggagactcctggacatgagaggatttcagtcag

gaggatcccgggtcctcttaaaattttgggggtagatctttcgcaacatgcggcaaaacgctgcgtcgatgcaaaagtggttcgttccgaacggccga

caatacctcacggtaaggaagagcacggagtagaccccggaatggatttgcaggaattccgcggagctccgggcagaggtccggcgtcggagagcgga

atgctttcccaacaagtgattatctatcagaatcccgccggagttcggcaacctccgggtaaggatgggcgcggagtaagcccaggaactacgaggtc

aaagacgaggaagatgaatctggaccatccgggctacgtgggcggcgacatcaacagctaccaggacgacaagcgcaaaggttgctttcaacttggct

catcaatcagcttatcggaaacgtcggcgaaggacccctcgagccattattggctacgatgcgctatgaggcaaacgaacggaattcgcgccgtagga

caggctttttccgactccgcctatcctcccaattccgttgccgcatttctcccggctctgacctctgtcccggctccctaa

>contig_14.contig_14.g409.t1

atgttcagaaaggccccgaaggctacgcggctatggcgtctcaaatctcaagcctcaacgcctcaacagcgcgggacat

tacgcatcaagcaatcccgcaccccgcacgcgcaccgacccagtgtctctctctctcccctctctgcggacctcttctcattcggcaacatgtcactg

gagcaggtgtgtgcatccttcgcaaattcagacacaccaacaggagctggccctcttcattcccctcacaagcccgcatggggctgccatgccgacgt

tctacaaaccgagtgcgacttcagcaaggaccaggccaccgcaaaccccgcctacacccccaaagtctga

>contig_14.contig_14.g410.t1

atggacttcattaattgggtctgggaatcgatgattcgtgtcggtttggcgattgtcggcgtcttgcacatcctcgccc

cggttgcggcggttattcttgatcagaggtggaagtgggcacgcgcaattctcgccggcgtcgacaccgtttggagtgtctgggccctcaagctcaag

accgcgcaggacgactacaccgctaattccctcaaagacggaacaagtgccctggactcgcggatggagcgcctgctgctccacgatctcaatctcat

ccacgaggccgaatatcagctcaggtgcaccgagattaccaagcaaggccccggagcgccccagagtttcggggaaactccgcactccgcttgtgctc

cgtactccttcagaatccaatcagcaggagtaaatcctcgaaaatgcactccgtactccgtcctcccccggagacttctcgatatgaacaggtgctcg

actatttcgttcccgaagagggcgatcggatatgcagatggtacgaggctcagggacttggcagacatcatcggtgtcctggagatcttttgcaggca

tcgagatccaccgcgcatcgccgcatgtctacctaacccggttgcggcggtgattcttgatcagagctggaagtgggcacgcgcaattctcgccggcg

tcgacaccgtttggagtgtctgggccctcaagctcaagaccgcgcaggacgactacaccgctaattccctcaaagacggaacaagtgccctggactcg

cggatggagtgcctgctgctccacgatctcaatctaatccacgaggccgaatatcagctcaggtgcaccgagattaccaatgtctgcgacgagcacct

gtttgagcctgcaagtccgtttgcacgcatccaaacccgagtatcaccaagggccagctatcattacgacttggttaaaaactggctcatcgatatca

gctccagaccagagttctggacgttggcggactcctggagacggaacgaagccaccgctggtatctacaacgacatatatgatggaagcatctggaac

caaaaggatcccacaaatcttcggccctgggtctcacaagactggcacctcaatctcgtcctgctgattgactgggtccagcccttctaccgaaacgg

gatggcctcgtatacggatcctccgagtcctcgtcaacgaatgaacgaacttctccgcactggtgttcctgtcacctttctgactacaaacggcaagc

atcgcgacgtcgttctgcgccctcgtatccgccgtcttcaagccgacaccgaggcacgcacttctatcgccggttttcctcagcacgcctcaaaggcc

atcgcaaggagaaagcctatcgaccacgcgaagaacagcattcggacccttcccgacacggacgatcccctctccgcgcctgagatcaaagagatccg

ccagaagcatactgccgggccgtgtgtgctgtatcggatgccaggctggtcgagtttgcaaatggcacccgttgatgtaatgcacgccattgacctcg

ggcccacgaagcacacgtggatggaaacgctgatcaaaggcggtctactcgacgccggggatctaaaggtaattcgcacgcgctcaacgaaacgcttc

cgcaccaccaagcaaaccctccggaacgcgaatgcggtggcagaaactgaaagaatcatcttcccagcgggtgaggacagtgatgatgatgcagacga

gccgccgatgatctttggccggggagaggacctggctccggctcaaccacctcctgaaccggaagctcctcagcgtcgacgtcgcggaccgcctccgg

attcgtactcttgcccagtctctgtctccgggatgtgcaaggcggcattgcacttggcagcggcttcccgcctagcgcacgcctccactctcgacgac

gagctcgtggaggacttacatacccatctcagcgcgtttgtccggctaattgcgacgatgatcaacactgcatgggtgacatacaactattacatcgt

tctccatctgaagcagcacatcaagcagcacgggccgccccgcgggtatcggtgctacccacaagagcgactgtacggcctcatgaagaggctcaaga

ccaatcagcgccgcggtggtgggctcgaactcatcatgcagactcgaacggacgatcgacaccgacttatggcagccatcgccaatctcaccccatca

ccgttggcacgagtcgtgcaagacatagtgcaacctcctgcagcgccgcccagggtccgccatacctctgccgctgccttagacggaagttacccgcg

cagtcgtccaaatctgtccttgagcgacctggaatcggtgtgcgccgcctgcaacacgatgcgagaaggagacgacgagccagtgaccacggtggcac

gataccgttccggcagctcctacactatcatcgatagccccatcacggacgtgtcgagctttgatgtcggaggcgtgatgctcaagccccgcaacgat

cgcaacgggcccaattcggatccttcggcctgtttcgtgacctataaggacgagtttcggttggccttcattacctcaattttctctcactcttatcg

caaggcgaagtcgcaacacgaagtacgacgcatgtatgcgatcgtcgacatcgctgagcaactcccttggcccacagaagaggctttcaactcacgac

tgtggcggaaattagggtacacctctggccttccagcacagagcgtgcgagccgtcatcccctgttctgccatcaccgcacccgccattgtcatgtca

ggctcctacgtttgggcgttgaaggcagcgccatcgcagtccccctatcctcataggtcattgccgttcggcatcaagttcaaggcaaaagccaagcg

caaggtgctcgacattcgccctgcaccctccatcacgatcgccgacgagaacggccacgtcgtcgaagcccccgaacctgaagacggggacagagaag

acgaggttgaggaacccaccggcgctggcgttggcgtcattgcaaggactcgcgcagcacaagggagttatgaggatgaagtttctggttcggataac

cacgcggaggatgaggccgacactgaagcggctgcgaagggagacgatgacgctcttgttgacggagaagatgaggacaacgcgtacaattcggacat

ggagccttctccatcggaagtgggatcggacgacaggttcgggagctatctgctcaccgaggcacaatggcgacaattcgagcgtctatag

>contig_14.contig_14.g411.t1

atgctcatttccaaggtggacaacgagatcatggatctggacaccatcatagcgacagagctcagggagttctgtgaag

caatgtccgacaagctgaagaaatatcgggacatcgccctcgacagccgactcactcttacagccgcccttcttcatccgctcaacaggatggagctc

ttcgacacagcctacccggattacacgaagcgcgcagaaaaggccctccgatctcttctgtttgagctcttcggcaaccccaccacttcattgtcctc

cacggccaaaaagagcatcggcttccccggaagggctgcgagctccatttctcctctttctgcagctagagcacgccgtagacaactgaattcgccgc

caaaaggcagccgcgacaagtcctcaacgaccgacaagacgcctgacgaagtggactga

>contig_14.contig_14.g412.t1

atgtctcccgcctacggctcgctcactggcttccacgatccattttcgctttcttcagctgccgaacgcgcacggtaca

caccagctggagaggatggtgcggctcatagagttggactggatcctgaacacacacacaacacaccagccggcgagggcgacacgggccatagagat

gatgcgaatgctggcagcaaacgaaaggcgtgggtcccttccgtgatctggcggcctcacggatggctccgacgtgtcaaggctgccccgctgcttgg

tcgtggtgcgttcgccagcgctcatcgtgctgtcgatctcctcggcgggccggagcgagcggtgaagcgccaagagtacacggaccgaccatcctcgc

gcctcgtctccaaaatccgcgctctctccatcctaaaacctcatcccggaattgtcgagctattggaactatgctactcggctcgcgtgatcgatctc

gtcatgccaatatattgggggacccttcaggatctgctcgataccacgcaaggacgaaaactgcgtctctgcaccgccaagaatctcactcatcaaat

cttggtcgcggtgactcacatccacaagaacggcgtcgtagacttggacatcatgccagacaacatactgttgtcgaacagcggcgccgtcaagatca

gcgacttcggactggcggtcgaggcgggcacgggtcaagacatgctaacggctggcactgttgattacacggcacccgaatatctgatggggctcagc

gatcatcttcgcgcagcgccggacatcgttgtccagaccggccagcttgtcgcctccgttcttgagccagtgcttggtggtaaccagtcgcgagatcg

attcagcgttgagacgagaacgacggttggtgctgtgtag

>contig_14.contig_14.g413.t1

atggatgaagccatcggcaacggcacggtactgtccgccgagttcaattccttccgtggtggtatagaggccggcctgg

gagaggaatctgtcgagcccgccaatgcatccatccacttcggttgcggccgcatcgaagcgcgcgaaaaagagatgctggggcagaagattgctcga

gaatggtacgagggcgagaggggcgcatggatgctcgccatcggtaccgaacttcttctggagcttacggaacttcgcttcacggagaaggaaacagg

cgtctgtcgcaaatcgcaattgttccagaagcatcattcagcgccggcaagcgctgctcggttacagaaaacgtggtcaggtgtagcaagtactcggt

tgagccaaccggacgatgcgacaaaagacgagcgtcgagctcacgacagcttacgaacgatacgcagcgcaccaccttcttttgccgacaccgacatg

agcaacacacctccttcgttccagctcggatcaggcgctagcagccgacgcgctcctgtggccagatcaggacttcgaagcacaagcgcgagcagcag

ggccccgacttcctccaccccttttgccgccattagttcaagcacggtccaatcactccaacctttcggcacgactcctgcacgcacgtttgcagcgc

tcttgaccgcagagcttccccgcggcgtcaagcgcagcgagcccgacgacggtcaaggcaccgaaaagaccttccaggacggtgacgactcgaggcag

cagcatctcctcggccactcagctcagggacacgaggaagatgcccgagtttcaccttccgaccggatggaggagaggatggaggcgatcagcgcccg

actggcccagcaaccgaccacgcacgcaaccaaccggctgaaacagtcgcgacgatcgaacgcagctcgtgttttccttggcgctgaagatcaaaccg

cctacgactgcacacatcgccctcaaccagatcaggcggctgcgactacacactttgcgcctcgattagacattgtgaatcccctgacgacaccaaga

ggcgcgcatctaacggcaggaccaggtacctccaaagggcttcgcccacgacgcttcacatccgctaagccaagcgacatcggcctgtacgagccgga

caaaggtagcgacatattcagctggtggcgcggcgtcgtgcagtaccaagcatactcgggagccacagaggcggaggttttctcaggtctgcctgatt

gcttctccaagtggcccaggcaatggctggacgagctgattccccgcccagcaacgctgaaggagttccgcaatcgtgcgttccaagccttcatgcgt

gacgctaccctcgtgcaggatgatgtcgaccaccgtaagttcaagcctcaagaggagtcccttgaaacgtatctcttcgacaaatacaagctggtttc

ggagcttcaagtctccaaggcaatggcggcaggacacacggacttcgacaatccgaatccagagtcggcacggatcttgatgacgatcaaggacgcca

ttcagacggtacacaacggtctacccgcagactggcgcacgctgctgaacgtgtatcgtcaagaagaagactggccggcctaccgatcccagcttctc

ggcaacgaacaacagacgagggaggccatcgccgtgttttcagggaacagccagtcaaatcagtcgggtcaggcaaagcagaataatccacctatcgt

caagcacaatactggatggaagccggaacacatcgcgcgttcctatgggcagcccacgctcacgaattcggacagctcggcgcgaaagaaggaccgag

agaagagcctttgttatcattgccacgaacctggctacttcaagcacaagtgtccacaacgcgaagaggacatccggcgcgtgcgatcggtgctggca

acgatggaggatatggaccaccgaaatgaggaccctccggtcgttaggcaggtgcagatgacattggagaatggcccgcgttcagcaggatctggagt

caacaatgtgacgcgagtgattcacgcaaaacgagtccgggtatcaaagtacgaagatgagagcgaaccacagcgggtggtatgcgcccgagcaacga

aaatctcaatggaagacgactcgccagccctttcaccactcgaatgggaacgcggcggaccgactatggcggacgcctactacaccgaggacaacagc

gacagaggctcggcgtccgactactag

>contig_14.contig_14.g414.t1

atgcctggtcgtttccagcagttccgagttaataatacgatccacacccgtgggacgatgaaggcccagacgctcatgt

ggtggaacaaaatgctgatcagcggagtcatgatggagctcggcacggtggaaagggttggcgagattttgcggggtcgaatggcatcacgccaggac

gggctgtacttcaaacttcggatgttctgctcgacattaatcaccagcttgaagctcgatcgacacaaacagaaaagcaagctctatggacggccgga

gctgaccatactcctcctggttaacgaagagcgcatgaaccgatctgcgtacgcggttcaatccggccttcagctgcaagctttgctcctgatctcgt

tttacacgggtctacggccgtcgtctctcgtccagtacgagcgggactcagcatatgctaaggatcaagacataaccatcgtcaagcgcggtcagttc

gcgtttacgatcgagctagaaatcaattctctgaagggatggaatcagagtaacaatgccccgttcgatctcaccgcctgcttgatccctctgctcgc

gttcagaggcggcgttgtcgaggcggagaccggtcgttctatcaggacaatcgacgacttcctgaacagcaaggcttctgtctttcgcgtgttgggcg

acgggcccctctttttgtcggcagggcagggcctcagtgggttgctgcccaataagccgcttcaaggaagcaacatggcctcggtgatcgggtcattg

tgcgaggaggctggcctccctgggtccgggtcctacgccttccgacacgaggccggtaatcggatggcgctgactcagggcgcggaggcggccaagca

gctgctgggccaggacctgcacagaggtcttgtgcggaaacattactcacacgacacggcgaacgtgaatgtcacggccttggcgttggaggaggaca

tcggtccggaggcgcgggcgaccatcgaccgctccaactccttttatctccagtggagtggatatgacgtgcgcgccatggaggtgttgcgcagagag

caaaagcattaa

>contig_14.contig_14.g415.t1

atgagcggactacggagtgccgaaaagcatctcaaaatcagccggagttgcacctctaaccctgcgctccttaattatc

cggaagccttcgcccaaggccaccatcgcctggggagctgctacagcggtatggatgccgcctgcaaagagttggtcgccgtggttcaaaaggacatt

gcccatgagcaccgcgttctgatccgactgccagggctcgcctggaccatcgataccgcgccaatggatcaactacaagaacgggaggccgctttcga

aaagctcggccgagatcaagacgacagggtcacagggacgccaagcaaaaatgagctcgcccgactggaggcggagagtgtcccgcggtggaaggctg

cctatgccgagctcgaagcagcccagcgcgaagcattcgtcgatctcgactcttccagggactccgacgtgcaggaaatcccccacccagcaccccaa

gcaggcaagcacgccttccccaacgcgcagctgcgccacccccacccttcgcactctctgcgaaggcggaagacacggttgacacaggcccaactgca

tggcgtcatttacatgcgtttctccaaaatttccagatctattgtgcgaaggcataaccgacagtaa

>contig_14.contig_14.g416.t1

atgacggacgctccttctgaaggccgcagtctgcgcgatgctgccgcgcgaaagctccctgcgcgttacgcagaatcag

aattggacgacacgcattccagaaactccggcgtagattcttcctctacggtcaagacctcccttcctccctcaacagcctccggcggagttccgccc

atccacagtcgaggtgactctcctgttgcgcctgggcctcctcccggctacattcctccttccggaaccgtcaagcgcggcgccagcaaggcgactgc

tgctatcaacatgcttgcctcctcaagcaaaggtccttcatctcctctccctctccctgctcaaacgccgtcttcacccccagttcccgttgcaccag

gccctcctcgttctcgtccggccacagcagcagcagccctcagcaccgaaccagtcgcaggaacgcctgactcggacggagagcagcccaactccagc

tccgcagaggataccgaaccacgcaagcagcccacagttgcttccaagaacaaggccgagatagcggagaagagcaaggagatacagagcgacgagga

gccgagcgacagtgagctggcgccagcggtcggccttcccaagacatccatcatcatccccaagcccggtgtacccttccgaccagctccccgtcctt

ttgtcccggaaacagaaatcgagggacgcgttcgagcggatcgcgacgtcgacttcttcactcgccgtaaggtcgctcttcgcatagaagcacatcca

ccagaacgccaaactgtgccgctcgaagacgatactgacgacgaagacgcgttcgagcctgcttctacctccaagacaaagtcctcggcatcccagcg

tttctccgccatagcacctctttcggacgacgatctcgtggagaaggaccaagaccaatcagaggaagaggtcattgtcgatctcaccggctcaagtc

cctcccctgccacagctccgccgaaggccgtcaaaggaaagaccaaagccgtcaactccaacaagcaggccaactccatctcgaaggctgtctcctcg

aactcgaagaagcggaaaggcaaagcttcatcgaaggcctcgacggcctcacggagctccgcgcctcaggctcgaacagctgccaacactcccaccac

tcccgttgcttcttcttcgcgcgtccgtctttcagctcgccagtctccggagaaggcaacggagaaagcaacgtcgacgtctccggagaagtcagccg

ctgtcggctccgcgactttgaccccggaggaggcattgaagcgagctcgaactgcctggaccaaggcacacgctgaggcaaacgcgaagtccaagtgc

gaatctgcaggtctggcctacgtagctccctccaacccggcatctccgttttacctctactataacgagccctatctcaagcagaaccctgatccccg

ccacgacaacgtaggcatagctctcgactgtcgttgctgcgccgaacatccgtacacggcgtggcgtgtcctcgaaggaacttcgaccagccttctca

agtcacacgtcaacaccaagaaggtccagggcaacttggaagccgccaaggccaagagtggaatcaccgggcccatagagaggtacctggtcagcacc

aagaaggaatccacctccgctactccgcctctctccggcatgcaagctcgtcaaattgcagtagcctgggtcaccgcgagggcccgaccaatcagcat

tatggaggacgaagagttcatcgaatggctcccggaggatcgacgcggtctggtcccacatcgtaacaccatcacgaaagacatagccaaaacctttc

tcggcacgcaggatgtggtgcgcaagcagctcgcggaggtcgagggggcaatccatctagccatggacatatggacgtcacccaacggccactcgtac

attggcgtggtgggttgctggcaggagataggcaagccggtcagacacgtactggagatgatccctatcacgaaccggcacaccgcacagaatatcgc

cacggagatcactcgaatgatgaagcgactgggcatcgagcgaaaggtgtggttcatcgcaagcgacagtgcatccgtcaacactgctatgatgaaga

ttcttggagacgacgagagcttgccgctgatagagggcgaagcaacacaaatccgctgtatggcacacgtcctcaacctgatctccgaggtgggtgtc

ccgaacattgtctccgtagctccgtccgtcctcccgtcctccgcagtcattcgtccgttcaaccaagctgtgcgagagctcaaaaaaaaagagaaggg

cgtaacggaggaagaagactgggccacagacgaagagatccgagaagacttcgacgtagaggacgtcgacgaactagccggggaagatgatcgagctt

tctccaccaatctgtacccatccgatgcttacgacgaggaagatgacaagctcattcgccacgcacttcaagaaaaaggagtcgcctacgtcggcagc

ggagatcaggcccggccatcttcttcatcctcagggacctctcctcctgttctcacggcggagttacgcgccgacagcggagcggtgggacttcagat

ccgtcagattgcgtgtacgttcgaaatgatcgagagggctcttgcgctatgggacgccatcgtaacctggcaggaacacaactacaagatcgttccct

tgaagttccgcatcaagaggtcccacaagcccgccttcgagatgcttgtcaagcttcttcagccgctcaaagatgccacatcccaattttccgccgat

tccaagccaacgatcgccgacatcgtcggcacgtacgagaacctcgatgagcactaccgcaagatcgaggaggacgaaggttgctccgaggcttggcg

agaagcggcaaagagagcaggagcggtgtgttcgacatactatggcctggcggacaactcctccgtcttttatcttgccgtcatcttgcacccgaatc

tccgcgtctccggcatgcaaagtctgtgctgggaggaagtgtggatcgagaaggccgaagatactcttcgtaggatcttcgaggaccgctatcgatgc

gacgaggttgaaccggaatcgcaatcacgcagccaggatccggaatcggccaagacagctcacgcatcaaagacttccctcgttcaacgacttgagca

aaagcaggcggccgcgcaggatgcgcctgatccaatcacgcaatggatctcgggtgagaatgacgcagaaacaccggtgaagaacaagctggtgaatc

ctctgcaatggtggtggaatcaaaagcagataggaatgatgtggagcggacttactgctctcgcgctcgatgtgttctcggctccggcgacctccgtc

gacgtggaacgactcttctccaaggccggacgtcacgtaactcctctccgacatcgactcaaggcggtcaagctcggccagatggtgactcttggtgg

ctggttccgcgagggctgggtacctaccgactgcctcggtacgtacctgaacgacgaaagagacaagaagcttatggagaaggcgaagggtaagaagc

gcgcggaagtcgaagacccggtcgatgaacaaggcgacaagacaaagcgcagacgtatcgcgggcgacgcagatgatgaagacgatgcttag

>contig_14.contig_14.g417.t1

atgactttcccggacctgttcatgctgttctcctgtatctccccttctggccgacaaatgtcatgaggatctgagaagc

gcagcactcagcaggtcagcagctcaccgcaccgagactcaatatcatcggcagcaccagcaacaacgcgcaagacggcacccaagcagaaggcatcc

acagcatgggtgataacgggtcacccacgcggggttctgcgcacacaacaccggccgcgtcgtcgcccaggggcacgccctacgcttcatcagaccac

ggcggcggccacgaagatccgcccgcggttcagaacccgccctggtctcctctcctggccaccaacaacaacatgcttcaagaccaatatgcccgcac

gggaaacgggtcagccacgtatttcgccttcggctccgcaccttcccacctcctcccctctccccaatccggcttcggtgcttctcctcgctcctaag

tcgccctccccgcatgccgttggctcgtcgtcgcagacgcgtgtatcggctcccccggcgacccaccctccctcacccgcacgcgcaccacgggcggc

gtctcccttccacgcgcgcgcatccacttcgggatccagtcagaagcgacccaggcaatcgcatacgggcgtaccggcttctccggcgacccagcctc

ccacacccgcaccacaggcgacctccccctcccacgcgcgcgcaccatcctcgggacccaaccaggagcgacccaggaaagtcagcctttcgcccagt

cgccatcacccgcgtttcaacgcgaaagggcaaaggcagtgccagaactgcaaggagacaaccccgggcccttcatggcggaagtggaagaaggatgc

gactttccagatctgccatcgctgtcacagccgatacacgcagcggcagaagtcgaaggcggcggcacgcctcgagcaggaagatgaccagcaggagc

aagacgatcagcagaagcacgacgaccaagagccggacgaccagcaagagcaggacgaagaggatgaggctggatcttcatccaacgccgcacgcaca

tgcaacggcgagggtcaggcccaggagcacgaaccagctagcaaaggcaagggcaaggcccgagccccgaactccccaatcatcacccgggtggctcg

ccaccgatcttcacagcgccacaagatccatcgccacctcaagaacaccgcgcggccgtccaggagggaggatggggtgtccgaatcggaagagcggg

agctggatcagtacatggaggaagagctcggtgatgaagacggctattga

>contig_14.contig_14.g418.t1

atggaataccggccacgacttctctctccccgcgccgtcgcttcctttggcgaaacgcgcaacgagttcattctggcgg

tggccagacactacgacgctgccaacgccatcggggagaccgtggacgtcatggacatcctccgacacgcgcaattgttggcggccgaagactgccac

cacgagggcgcggagcagcagcagcaacagcagccggcggacgggcacgggggcgagatgctcccgcagcagccagcggacggagacgggggcgagga

gctccagcagcagccggcggacggggacggagatgcaggtgccgagccccaccatcagccggtcgtcatggccgaagacgaggagcatcaagcaatgg

cgcccggcccggtggcggagggggaggacgaaccggaggatgaggaatgggagggggcacgacgacggatggggacagcacggaaagggaacaaagca

aatgtgacggaggatctggagcaggcacaggaggacatgaaggcggcgaaggcgactttggcggcggcggcggacaaggcaaagggcctgacaggagc

cttgaaggcagcggagaagcggctgaaggaaatggcgagtgattcggttcccggaactgaccatatggcccgtttcaaccagcagcagcgtagcagca

gcgcgcaagcatcaccctcggcgtctcgggctggagcccggggtgcgcccgttgccggacccccagcggacgtgatcaccatcgaggagtcgagcgat

gacgagaaccccgtaccctcgcccaggaagcgacttcaaggcgacgaccggcgcagtcccagcaagcgggccaagaagtcgttcggagcgggggtcgc

caacgtctatgactccgacgccggcaactag

>contig_14.contig_14.g419.t1

atggaagactctatagccccctccgaagacatcaagctacaggacgtcttcgattccaaaggcttgtgggcgaccaccg

ctttcgcaagcatcggactcacagtcgcacagtcgcaccaccatccaaaattcaagcccgcccacgcgggccacatcagcgccggcgcagagagaata

cgcttcgccgaagagccggagccggcctgtcgcagcctaccatggcgagcatcgaagctcaacgagccaaatgcagggcccaaggagccgatgctccg

actcttttccggacttcaagcgatgtggcgagcacaatcatggagtctcaaggtcgaagagcagccgtcacgtaattacaccggcctcgccaagcgac

ttagtttcgcatcatacgcgcccttcagtacaggccacaaatgcctatcgttccacaaccagaatgatgccgacggccgacgcggcgctgttgtctgc

aagccggtatctggaggaacaccgtcaagaaaagaatcaggcttgcactggtcgggaacagagctacgacgatctgaagcaagacgatcgcaagtact

gcgacggattggtttattggctgctgaacgagaaggatcggtctattccgtgcctcagcaagaacagcagcaccctaaccgaccagagctccgcccgc

acgtcgaccggtcgcaaatgctttccgtgcctggtggcacacctggtgactgggaggcgagtatgcgagctgggcagctggcagtgtccgttcgagcg

gatgggattgcaaaagccgacatccgcaaggcaatacaagcccaaaagcgtcaggtacagcagggcaacgtcgctgcaacgtactacctctcgccagc

cgtagcaacggcccagcagctcgaaaactcgcccagctcgggctggccgatgcgggacaacgacaagggcaagaacgaggcggcatttaaagcgcttt

ggtcgattactgccgtcgatgtcgtccggggctcttcgccgcacccagcgcccaggatcgacatgctgcgcggcgtctttcgcctgctcacgacctcc

ttcgatctcgcgcttcaggttcaacatcaacagcttccagatgggaccgtggacaagtcacacctcaccacgccttcgacaacggtaagcacgtcgtc

aaaattccctaccgcgctgaatttgctcaagcggaacctgaatttgctcaagggaggaggttccgcttga

>contig_14.contig_14.g420.t1

atgttgcacgttgcaccactggccccagcagcttcgcccggcttctgcatcgcttcaagacgttccgagcaaagaagac

tcggacatagtctcccggttaagcgttccgggctgctcgtcaacttggacttcgacctcgaacttcctcgctacctacgaaaagcccgcagtaatagc

caagcaagagcgaaggaatggctttcaggcgaagggctagcggtgctgacaaaatggctgccacgcatgggattcgaatcggcagagcaagtagtcgc

ttacgccgtccaacaatgtgagcgtgttgtattatgcgacccgctttcacaagacgaagatgacttttccgacgtcggtgacgagctcgatcaggaat

acaacgaagacgaggacaatgaagacgaggacgaggacaaggacgaggacgaggaacatgagtcgcccacacgtcctctgcggcaggccgccggccct

gaaaatgaggaactgaccatttgcgcgccttatgttggcctaaagagcttgtcctcaatcttcgaagtgggccagctagaaactgaacaggtcctcag

cgggggagtattaccctcccctgatgctttgtcagctacagctgctaaccaagcatctttcccccctccccctcctcgcgcagcgtcggaagctactc

cacagcttcactttgccgaccgtcctcaattttggacttcgcgcatcatcaacacagacgctgccggtcgcctcttggctcagcccactccgtggtgc

gccccagtctcctcactccttgacacgggcgcgtccctatcctccatcgatgccggcttgctcgcacgtctcggtggctcgccctcgggggctcctat

gcgcgtccatggcttgggtgacgttgagaccctcggctgggcgaccttgacgttcttcgtccccgccaaggcagaccatggaaccgatgtcaatcttg

aatgcaccttggacttccatgtcctccccagcttcgcgcctgggctctgtctcggcctggacttcatcgagggcctcggagtgtccatcgacgcagcc

actagcaaggcccgcgtgcgccgttacaccttcgatgtcgtcgagcaccttccccaacccttcgccaaggaagctcagctctgctcgacggcggcagt

gtgcctgcctgctcgatcgatggcttggatacctgtcgatactggagccctggcgtcgggagtcgactacttgatccatcctcggctcatgaccagtc

acgacgagtccgtcacgattgctggccccgctgctctgggaggccgcgacaccaagcatatcctcatcgggaatcacagctcccgcaccgtccacctg

gatcgtcgcactcccatcgcagacgccgtcttggcccatctcggagatcgcgcctcgtcatcgccctccgtcgatcgattcgaactcgggtctcccct

gcctcaggccgatctatggacggcagctgccgatccgcacgcatggaagccagagacgggccgggatgacaccactgctgagcctcttgactttttcg

agggcgttgaggaccctgctcacgcgctctgccgggatgctgagaccgtcctggttgatggccacttccgcgttggtgttgactcgaccggcgcccct

catcccgccgtcgtcgaactgcttcgccgtcacaccgaagcgtttgcgttggatggacgccccgggctgatccgcgcggcgacggcgacattggagga

acacctctccacgttggagaccatcctgaccagtgcatcgcagatcggcctgaagttttccccctccaagtgcaccttcgctgttccaagcctcaccc

ttcttgggcgcaaggtgtccggagccggcgttgccgtgtgggaggagagagccaaggccgttcaggagctgccccgaccacgaaccctgcgagacgtg

taccatgcgcttgggttgttcggatactatcgcgcgtttgttcctgggtatgccgagattgccgcgccgttgacgagcttgacaaaggggtggcggta

cgaatcggtcgaagatcggtctcgtctggtccgcgttgatggaacccccacgaacgccgatcgcgagctcgtgagctgggatgatgcccaagagcgca

gcttcagtcaactcaagcgcgccatcgccaccccaccagtgctcgctcatccagaccctacgcgcccctacgtcttgtatgtagacgccagcaagtcc

gctttcgccgccgccctccatcaggtgtttgtcgacgaggagctgctgtctccctccgcttacccggctgcgccttcccctcttccgaatcccgttcc

cgtatcccgagagcgctggcggtcctggctgctcgccgatcgctacttcggccctcttctccgccgcatggaggcggctggtgctgatgaatcggacg

agtgggtggtggaggatggcgtcctggttcgccgtgttgatggacgatgggcgctaccggacggcgcactgcctttggctctgcgactcgcgcacgag

agcggcggtcactttggctattccaaaacgctgttggccgtgtcgaagtcattctggcgtccacgattggccgagcacgtccgcgcgtgggtggcgca

ctgcctgctctgcctgaagacgaagcctcgtcctcgcgtcggagacttggatgtcgagcacgacccccggctgccgtttgaagacgtgtcgctcgacg

tcgtttcggggttcccctcgttccacggccgcgacgcagttttgttcatcgctgacggctttttcgcgtttgatcatgctggaaccttgcaagtcctc

gatcgatgctgctgggatcgcggccattctgagcaaccgcgtcctgcgtttgggttggcgcccccccgacgtttggtgtcggatagcgaggcaaagct

gacgggccaagtgatgcaagcccttgcgcgttcgttgggtgcagagctgaagccttcgacgccgtaccaccagcaagcgaacttcgtggagaggaccg

tgcaaactgtccagtccgctcttcgcgtcttgactgcggctgggcacgcgccttgtgatcgggttgtcgttcctgccgtggagctcgctttcaacagc

agcccgaacgttaccactgcccatcgtccctttgacctggtgttcgtcgctcatcccgacttggcgcacgcggtctttgacgccgatgccggacgggg

agcggagagctttgatgatcgccttcttgctgcctccgaacgcctctccgatgcgtaccgggcgattgcttccgctcgcgcacagcagaagcgtcgct

acgatgcgcgtcgcggtcctcttcccgtcctacatgcgggtgaccaggtgttcgtgcgactcggaaatcgaccgatcccaggactgggcgaggggaag

ctcgacgcgcgaaaggccggtccatatactgtgcgggaggtcctgtctccgcatcgcgtccgccttgtgctaccagacgacttgcgtatcagtgacga

gttcagcattgatcaactggatcctctgcctggctcacctgaccctttcgcgctgcagcgctgctctcctccacctgtgccgttaccggctcctgctg

ccgaggtcgacgagcccggcgtgattgatgaagtcgccgtgggtgatgatgcggaggacgagctcgacgtcgctccaccactgggtcctcgcgagcgt

cgcccgccgttgagcctgcgtgacttcgatgtcggcgtggtgcatgctggtgctgttccgtcatgggaggaagtgatgcacggtccgatcactggatg

ccgagaggtgatgaaagacggcagatcagtaacggcccgcgaacgtcccatcgcatttcagtcccgacttacctcgccttcggagcagaagctcgtgg

ccgctgagctcgagttgtgctgcctggcatggtccttcgctcgaatggcgcacctgttggaaggcgtacccgtgactgttgtcaccgaccatctcccc

atgtcaaagatgttgacatcaacgggtgctacgcagtacggcccggtcatttcgcgctgccgagccctcttacttccccatctgcacttgctgcgatt

cgagtaccgaacaggccggaaacactccaatgtcgacgccctttcccgcttggttggctga

>contig_14.contig_14.g421.t1

atgccccatcggacagctctctccctcgacaaagccgctgccaaggttgctcccaaagcacaccacctcgtcatctcca

accccgtcaactccaccgtgcccatcgtcgccgaggtcttcaagtctttacgaccccaagcgtctgttctgtgtcacgggtcaccacctttgacatca

cacgcgcctccaccttccacttcgacattgccaacgtcaagcccgccgaggtcaatgtgtccgtcgtcggtggccaccttcggcgtcaccattgtgct

tctcctctctcaagtcgcccagggttccatactccaatag

>contig_14.contig_14.g422.t1

atggcacggggcgaaaacatgcgacaggcccagctcgccgatctgtttgtccttccccttccaaacgagggagcgcaag

atgcgtccgccgtagtgttgatcatggacaacgggaagactaacgcctacgccctcctcaacaagatctggccggatgtcgaaggcgcactcctcgga

atcgagcaaggccgcctggagaaggacctggcggcacagggcttcctccgccttctcgtctatctgcgcacggttctcgtccaggatgctgttgtcat

gcgcaagcagttcccccaacatccaatcttccagcacaccatctttgccgatccactcttcatcgactatgcaaagaacctcgaagtagcgctggaca

agaacgagcgacctctttccgtttccgtacaggagatcttgcccgagcttgctggtctcgtccaaggcgcctttacctccatcctcaacgaggtcgac

tctcttgccaacagggaagtcaaagtgaccgtccatatcgagcagcatcagcagcgcaccggctcttctcctgtcgctgtcgcagccctggctcaatc

agtcgctgccccttcttctacagtgccgcgacagctcctgggccttcccacaacagccgccatcgcctcctcgtcttctcctttggttccgctctcgc

cccctgtctcgcccttctcgtcagctgcgccgttcccgtcagctgctccgctctcggcttcagctttcacgtcttcttctgctctcgttctcgcctcc

tctacatctgcctctacatctgcggtcatcacgcctccttctccttctccacctgctcgcgccctcaccgttgcgcctcacgccctgaccgttgcgcc

tcattcgtcagccgctgtctctgaggattcatctgctgctgtgcgcgatgaccgtctctccatcaccagcaacgaggacgagaacatcgaacaccagt

tggagtcagaacagcagcagcagcgctcagagcagccccctctacttctcccctccccctcgtcctcgttgccatctcagtctccagcatttgctctg

cctctgcgaggaggagcagcagcagcagcagcggaggaggagacggtgccgatatttcagcagagccgcgcgttgacaagcatagttcacgtgtggca

agagtacgagaagggcatcaaccacggaccggcggtgcgagacttggaggctcgatggcagcacaagtggaggacagacacgaagagtcggaaatttt

tctctcagcgtaacaagatatatgccaaggtcatgcgcctcgcacacgacaagcgcatcacagaggttcaagcggcacaggcactggagaacgcacgg

gtggctttgaagcggggagaggagagaaggagaaagatgtgccggggctcgggtgcgagcttgattcgtgccggagccgagttccccacgcaccccga

gtacccaacccgacccgacagcaaagcgcaccgagacgcgcatttcctcgccgagcgcgagagggaaatctga

>contig_14.contig_14.g423.t1

atggcaagcactgctgctggtttcgcagtccacggctcggtcatcgcctatgtatcctcattcgcggtcgacctggaca

cgtccttcatgacaggcgccgctgtcggccttgctattggctcttccgtcgcgctgctccgccggtcctgtggacttgaagccgcgatcagcacgtca

gaaggaggctattccaccgtatcgcgcgcccagcgcatgtgggctgcacagtatctgcgcgcgtccatgcaccgcaatagtcccaggaaaggcgagtc

ctccctcccacccccttctcaccattccaaatccaccgcccaatccctcaagcttggcgtcctcggctgcgccggttggggactcgtcccacatccgt

ctctcccctctctctttcctcccaacaagccttaccacgttgtgccgagatttaccgccatgtgcatcggccgtggctga

>contig_14.contig_14.g424.t1

atgatgccgacggccgacgcggcgctgttgtctgcaagccggtatctggaggaacaccgtcaagaaaaagaattggcga

gtctgcagcaggcttgcactggtcgggaacagagctacgacgatctgaagcaagacgatcgcaagtactgcgacggattggtttattggctgctgaac

gagaaggatcggtctattccgtgcctcagcaagaacagcagcaccctaaccgaccagagctccgcccgcacgtcgaccggtcgcaaatgctttccgtg

cctggtggcacacctggtgactgggaggcgagtatgcgagctgggcagctggcagtgtccgttcgagcggatgggattgcaaaagccgacatccgcaa

ggcaatacaagcccaaaaagcgtcaggtacagcagggcaacgtcgctgcaacgtactacctctcgccagccgtagcaacggcccagcagctcgaaaaa

ctcgcccagctcgggctggccgatgcgggacaacgacaagggcaagaacgaggcggcatttaa

>contig_14.contig_14.g425.t1

atggttccgcccagaatcacaactcgacgtggggacgtcgttgcgaaggtcaccgacaaagttatgcacggcaaaaaca

agcagcgtgactacgaggacgacgaacagcttccccgaaaacgcgtgcgcgcgccgctcccgggctctttcatcgtatggggcacaatgatccagccg

agatccgctagcgtccctcccctcacgttcaccttcctgaacaagccatccaacgcacagcttgaatttcgagttgtggtgctcgatccggaacgtaa

gccgatcaccgtatcgagcagctttaaacacacacggttcgaactttcacctccagagattgtcgcaaaagtcaaaacttgtggcggtaccacatggc

tgcgccacgtgcttccttatcttatctattatcttcgctccttccatttctcatcacctctcttcctctcggccgagagccttccctttgttcctcgt

caccttttcagctctcgctgctctcccttgtactga

>contig_14.contig_14.g426.t1

atgttgcccgacgcgaatgtgagtttgcgtctctttagttcccttcagaacattcttccttattgtcctggagtatctg

ggaccgtctgggtcgttgcggagaacgaagaggggttcaaagactggatggcagctcttcctggaaaatgggtcaggcacaggaaagatcgggttgga

aaccgacttgctgacgagctcggcctcggggaagctaggggcctgggggatgagcaggtaggaggcatgcaaggagatggacgcggagctcagggtgc

caggggtgtacaggacggactggatgcacacgatgcacaacagagagcagctgctgctgctgtgctagcggatggagaggatgaggatggtgaagcag

atgagggagacgggcgggatgctggtcgattgaacgccgctggcagagcagctgccgccgcgttactattgacagcagattctctttcttggcggcat

cgttatagatgtgatcatgcagggaaaccagagaacaaacccaagaacgccgatatcagccctcgaaagcgtcgcaaacgcaatacatccatcaaagt

taactgtcgggcgaaatttgttgcgtgccagcccattggggacgatcgagtctacatccaatggacctttcagcacaccggccacgacgaatgtacgc

tccgcagcgtggccgagtctcgtctccctcaacgcgttcgccagtggattcgagcccgcgtcgaggagggacgcgactggaaagcgtttcgaaatctc

cttcgtctcgacgaggctcagcaggcgatggaacaacatgctcgccacctgcagcaagtaccagaagcgcttcgaattcgatatatggacgtctacaa

cgagctccgaaggcagcttctggcgatggccaggagagacaacaaccggctggagagtctcacgaagtgggcggaggagacacgaggcgaaggaggta

tggcagcggtgaatcttaacattccttgcgctgacagcgagaaaacatgggcagccttctttctttcttcttggcagatcgaaatgatgctcgcctac

ggccagggcagcgttgtttgcctagattcgacgcacaacacgtgttttgggttcgagaaggcagaaaaggtgtggctctacaccttggttgtgcgcag

caccaccaccggccgtgggatccctgccggtttcatgtttaccaacgcagagcacgcccagcctatcatctactggctcgagtgccttcgcagagacc

cgcggttgcgattcaagcccagccacttcatgatcgactgcttcaccaccgaacttctcgccatacgaaccgccttcactgctccctcaactccctcc

gtcctcttctgtgactggcacatgctcaaggcgatgacggccaacagcaagctcaaggtcaaggggcacgaaggacacccacaaggagctctacgaaa

tcaagataaccaactcgctcaaaaggaggctcgttccggcttcctcaagctcgtgaatgcctcggatccacaggcggttgctgaagagttggcgaggt

atttggagcggtggaaatgttgcccggcttggaccaagtacgttgaggagcagtggctgccgtacaaggagctgtggggtcgggcgtggaggcgagag

gcacatcggggcatcgacacaaacaacttcatcgagacgtggcacaaccaactcaagactgcctacctcggcatgatgcggaaacagggcgtcgatgt

tctcctctgcatcctcctcaagcagtgcgtccccgactaccgaagcaacgagcttcgagtttcgctcggcctcgactcatgtgcgatgaccaaggcag

accgagaggcaaggcggaaggcccaagatcttccgtaccttgaggcgctggcgctggtggaggaagcagagggcgggtcaatcacggtggagtccttc

tcggtttccggtcagcggtaccgcttgcgcacgcggctcgatgcgctcggcgatgaggtggagctggttggctgctcttgcttctactttcagcagca

agccagcgctgcgaatcaagccatgcgcgcagagcgcgaccagattctggcggcagcgttgccagagatcggcaagatcttcgccatgggggataata

tccggaggcaggtggacacggacgcattttcttgcagtcgacaagctgcaacggatttggtctcccgactggaggtcgttcaacatataatggtagat

gttctgcaaggccgacaactccaccaccgccagcagtctacagcacgcagcagccgtcttggactctcaactctttcttccagcccacgcgacccgtc

gccccagcaacacatcaccaccatcacttgcagcaatccttcaacactgctcattgtcatttgtcccgtaatctgcacttgctcgattggcccaggca

ttatcccagcaagcagcatcatcttccatcacccacgcggccacacggccacacgccagcttccatgcgcttggcctgtttatatccttttcccagga

tcctccagggcaacacagcagcacagtaggcacaacaagaccatcaagagcgccgaccctgatttgcgctctcaacttggcgcacaagagaactcaac

agcaacccgtgcgcttcttagcgcggcgcgcacaagcttggcgccgtgtttcgctctccacattcagcagcctcattcggcactggaagaaggcgagt

ggcgacagccccactcatccaacaccgtcagagcgtcagcaggtgcagcaatccgcagtccgctctacttcacgttccaccacaagctcgacgtcctc

tga

>contig_14.contig_14.g427.t1

atggaatacacttcgatccaagagcagcgtcttctcgtctcgctccgacggctggaagccaacgaagccaaactcaacc

tgcgcgaggccaaactggcgctgttcgagatgcagctgccggcagcggacaggcttccggagctggcccagcaaccatcatccggcagcgatgcaaaa

tggctcgttcgcagatcgcttacgcccgagcagaacaagtcctactccaagctcaagaaagagggggagcaggggcagttttatgaggaagaggagca

ggagcagttttatgaggaggaggaggaggagcagctggagaaagagcagctggaggaggagcaagagcagcaggagcggttggatgagcaggaggagc

agcgcctcgacgagacactcaagcgagaaaatttcccctcgaccccatcggctagagttcagcagcgcctcgacgcctctgtctcgacgccgcaacaa

ggacgcagcaatatgttcaccgtcggttacctcagcccatcgccgaacaaacgaagcaggactagcaaacagtga

>contig_14.contig_14.g428.t1

atggacccgtacccaggccgacttccgtatatcatactggcgagcagtaaggaggggcgcggagtaagccccggaattc

tgcaggagtacggagcgcaagcctgtcatgtccccggagtacggaacatcacaggttttgaggtctgcatcgggacaccaatcgaggcactgcataca

tggctgcttggtccggtgaaggatctttttgcaagtcttgctcgacaggtcaaaaagaacacacagcactttcaggaggttgctacgcgtctgagtga

cgcgaatctaggaggaacagatctgtcgtcaaagctggacggagtctacttgttgaacaatgctggcggtcttacgggaaaagacatgcgcgctctgg

cgcaggtggtatggctgtcgatacttcccatcaaggaggcaggagtgcttccccagagcacatgggacgcgtggagatgggcaggcacagacgaagac

ggcaatggacaacttttatgcagcaatgctggtcggctctccgaacgaggtcaaggccaagagaaagtttcacttactggtacatcttgcggccagcg

tccagcagtacggtacggtccggcaaagaactttgcaacagaacggtacgaatctttcaactctgtggctcgaaatgctagcatgtgcagcaaccgag

ctgccccctcccgagacatagcactccggatgtacggacagaacctcatccgccaacacacggcaaacgttcgccctgatcgtaatggtattgtttct

gagattgcctgcttcgcaaatgcctttcgcgatggcaagattctctctctctttatggaatggaggcccgaaagaaaagtctcgttccaggcagctgg

cagcgagacgctcaggcagcagcagtcataa

>contig_14.contig_14.g429.t1

atgagaggattcatcgacgtcggcacggatcggccgaacctacacctccgcgtactctccatgcaacatccgatcgaag

gcttcgtggacgtcctgtcggtactgccagagctgtggcaatctgccgccgaaggagcctctactgtggaaaggatgaagaagtttctggtcacgctc

atatacatcaacagcaaagacgcggcaacgaacctgaagcacgtactggacaagtggtgcttgcgcgccggcttcaaggccatcgtccagctctacca

cgccgacctcaccgacgaacaccgctccaagatcctcatgctcatcaataccggaaaaattctgatcgtcatctgtaccgacgcgtttggtatggggg

ccaactgtcgagccatcttgcgagtgatccagttgaagttattcggcggggtgttggcctggtggcagcgtatgggccgagcagggcgcgactttgcg

gatgaggccgaggcgatcttgtttgtcgagcctacgcatatgaccgccgaaatgcgaaagcacatcgccgccacaggagagttgccgacgcctccaga

gatcgatgaagttgatgaggacggccccattaccctcgagaacgacgtggcgttgatttggagcggaacgcacagttgggcggtcgcaaagaagatcg

tatatgacgaactcttgatgcttgctcggcacggtctcgaacgtgatgcgtgcattcttcgattggttctcgatcaccttgcacaacccaaggccgag

acgctgccaagacacgaggagctgtaccattccagccgaccgatccctccaggcagggagatgccctgttgctcaacgtgcgattatcagcctcttcc

gtgcgtgcccgaggagttcgctctgtccaaagagacgatctccgctctgccgggtatcacgagtacccagagcatcttacgtggtcggctggaggcgt

ggcgatcgaaacagtggccggagaaatggaagggattgaatcgagctggacggatgggaattgatgcgtttatggccgagcaggacatcgatgcagtc

gtcagcaacttaaaggcaattgcgcacaacctcagcaaaggaaactcacgatttagtctcttcgtcaacatgcggccgaaggatgtcgttctcccgga

tctgcagaaggagatcgttgcagtgctgcgggaggagcacgagcagggaacgtag

>contig_14.contig_14.g430.t1

atggagctgaagggggaagaggatggcgatggagaaaaggatgaggatgtggctccagaagagaaaaatgataaggaga

ggacgtcccgcaaaagcaagaaacgcggacgaagatcgcagggcgagcgtcaaatgcaagagcgctcggagcaggtcatcgacgcgttcagcacaatg

ggcaagcagcagcaggaatcgatggtcgagcgtctcgagaaggaggagaagatgcatcaagacgacctggccgagcagaaggcattccgaattgcgca

ggagcagagtcacgacgcggatcggattgctcaggaccgtcaacacgaggccttcctgctgttggagcgggagaagatgcaggcggagaccaagcgaa

cccaggagaaaaatcgtcgcttccaacagatggaacagagacttcagacgtgccaggacaccaccattgcgcagcagcaagagctgcagctcgaagtg

cggcaaggacagacggagaacagacagcggcaggacaacattgagcgcatgctctccatcgttgttgctcgacggtttggtaatccaaccaatgatcc

cggcacttcagctcctacggtcgctactcccggcacttcggcacttcggcacttcagcttctacggccgctga

>contig_14.contig_14.g431.t1

atgaatgttgtaaatgctaggtgctcagtgctcgactactcaccttgggaggaccaacaaggtggaggagagaggccga

agtcgcttgacgagttgcaggagcaatcccagtatctcagatacggaagggatgcaacctttgacggggaagtcaaggatgagggcgagaaggtaaaa

gatgaggaggacgaggtcaaagatgaggaggacgaggtcaaaggtgaggaggtgacgagggagagtggaagcaggaacgtgtgtggtgaacttcaata

g

>contig_14.contig_14.g432.t1

atggatcaaaagtcaatatcaattcatctctatgatccgggtcccaaacacaagaacctgaacctgaccgacactctgt

cgtcttttaccaggcacgattacacctcgatcacagggcgcatcctttccggagacatcggagcccgttggagacttgaacgcatgtatcaggatcag

gggccgtctgtccttgtcgatgacgttcaacagcagctcgccaacatcggcccattggcctttccccttctcaaagacgcaggtatttcacattactg

ccgtgtaatggaggtagtggctctttacttggaaccggagctgtga

>contig_14.contig_14.g433.t1

atgagcctctgcagctactcgattgtgttgatgaacagcggcagtgctatcggtgataaactgaagccgcacttcctcc

atgcaacagactacgtcgagcgcgccaaaagttctggaaccgtggatccattaacagatcgagaggacgaggagctttcacagacgaacgtcgatcgc

tacatctccaacggcaaacgtctctacagtgttcccgtcgtgctcttcatagatgatgtctccagagcccgatcaaagagatggaacaaacacgaggc

gatctacttttccaatgcgacgcttgatcgctctcagctcgatctagacgcccacgttaggcacacgtcaatcagcaaagacgtcagcggtgaagttc

aactgtccgccgtggtggacgagctgatgggaaccagttctgtcgactctgccacgtcgatggatctgctcaagcccttcgcgatccagaaggaatgg

ctacatatattacagctggaaatccgcgttcttacacggttgtaa

>contig_14.contig_14.g434.t1

atggaccccaagcttcccgttgccgctatcgacaagattgtttctggtggagtgccgcccgagaccaacccacgcttgc

gcaactgggtcatcaagtacatgcctctctccgacctcacctacggcgactccaacacccaccgagtcacctgtcgccgtccacaggagcaagaccgc

atgattgcccaatactgtcctgccttgctgctgctatgggaagatccctgccccatcgacttagctatgtcggccgacacgtttttaagtgcatcagc

aagggaccatactacgctcactaccggattcgaccagaccgcgagaacaatctcaataggctacatggcgcgtttttggactccatcttacctccaaa

acccttacggtcagctcggcgtacacgagagccaacgcaatagtgcacgcttacaaggccagcatggtccaggatcgaacgcaagctccctcctccgc

tatttcctccgtccctatcacgacacttctgacacctcactcggtgtggccatcacccccagcagctgctgggccatgcacacgccggtctgtgcgcc

tcgtcgactgcacctcgatgcgcggttctgggttcacccagactccctggattcggcataa

>contig_14.contig_14.g435.t1

atggatcagcttcagcacctccacaagctggatgcgcagctgctgctccgccggaatctacaacaccaaccgcgccctc

tccagcgctttctcccctcggaaggcctggatgacgcttggacactcgtcgacagcgcgcttcaccactctctgcgcctactccgagatggggcagct

cagccacgctgcgggatgcgcgactcgatcctcttccagcttccgttgcacctcggcgggcaatcgtccgccaagtttctccaggagatcatgccctc

cctcttcttggatcggtctttccgtttggacggcgccgacgtcccgctcatctctcaggcgaaacgcatggaggccgaatacgcgcggctgaaccact

ccatgatgtcgaaaccgtcgaaaccttcattggaggaggcttag

>contig_14.contig_14.g436.t1

atgacaggcttgcgctccgtactccgcagaattcaatcagtgggagtaaatgctcgaaaatgcgctccgtactcccccg

gattaatactttccatcgctgtcgccgacgacgatcccgtcgtcaatgcccctgttccaaagactcttcccaagcgttcatcagcctgggatgacttc

gagcatctcgtagaccagcggaaggtccagtgcaccaggtgtgggcacaagctgtcctttgtctccgtcagcaagactggtacctcctcaatgacccg

tcatcaggacgcgtgccgcaagaagcgcaagccagagggagggcagcagctgctttccttcgcccccgagatgcccagcacccagaagctcaagatcc

acgccctcctccagcgatggattgtcagggaccagcagtccctcagcgtcgtcgcgcaccccgacttccgtgccttcctctccgccgccgccggtggc

tacgacccaccctcccgatacgtcctgaccaacaagatccgcaacgactacgaggacattaaggtcgttatcaagggatacatgcggtccctccccgg

caaggttgccctgacgaccgacggttggacggctgccaatggcgaccagttcctgtctctgacggcacactgcattggcaaggactggacgatcggct

cactgctcctggacttccctcccttccccgtcccccacacggccgagaacggcgccaacctcatcacacgcgcccttctcgagttcggcatcgacaag

aagatcactgcttgcgtgaccgacaacaccccttccgccttcaacatcacgacgatcctgaagcagcggttccgcccctcgtcgatgctccctggtcg

ctgtgctgctcatctaatcaacctggtggtgaagcatgggctcaatgacgcggacagggacgtgatctttgcgccctgccgctccttcgtcaatctga

ttcaaggatcgaagccaacgaagaacgcgttgagggaggcatgcaaggtgcggggtatcacattcaagatgctctctacggtgatggacaccggaagc

atgccagtctggcttggggtcaacctgtgtggcatgcaaattcaagacctgacgaagatccttgaggtcttttccgatgtctccgagcacatccaggg

acgcaaataccccaccatcagcgctgcagcccaaggctacgttctcatctccaccgagctcgacgccctcggtacgtcccccagtcccacagcatggg

gtatcgaggtcgtcaagctctttcagaaccgccttgtctcgtacagggagcatctcattgacgcgcctctggttcagtgggctacgcttctggatcca

tcaatgaagacaaaggtgattgaatgcgatggggttcatcttgtgggggacgttgtgggctcgttgatggagcatctggtagctttctatcccgagaa

cagtccgacctcttccacgtttgtcccatccgccgatgccgccacttcatccttccagcaacgatgggagaagatggactcggctcccactatctcgc

acctgctcaatcctcgcaagcagctgcacatctaccttcaagaaccagctatcaaaagcaatgttgcctccgactcgcttcggtactgggcggtcaat

gagggtcaatttccaagcttgtcagcgatggcccgagatctactggtcattcaagcaacaagtgtcccttctgaggcaaccttttctcgtggcggcga

agtcatcaccaagcgcaggaaccgactcatcggtaccactgtgaccgccatcatgtgtctcaattcttggatggaggggggatttgatagcatcaggg

cctttgatctcgaggacatcgtcgagcccatcgaggtcgaggagtag

>contig_14.contig_14.g437.t1

atgttcgcagacctttaccttcccgtcatcagcacactcctcaagacactgtgcccagagactatcgccatcctcgccc

gatcagatcttcaccatcctccgaagcccagtcaaattgtggttgccgacagcgtgcgaatgggccgcgatacgatctacatttctccgacgtccacc

ggcaaaggacaagcattcgagcatctggatcacctgctcccagacggggttatcgtaatcatcgagcccctgcagtcgttgacctcagagatggcaga

aagattcaaggaccgtgcaacctacgcggacagcaccaacaagagcccagcccacctgcaatccatccgggacggcgagtatcgcctcgtcttcacga

ccgccgagaccgcgactgaaggcgacttcctcgacatcgtcttgcggaatgatcgtttccgtcaacgagccgtcgcattcatcttcaacgaggcacac

acgctcgaccagtggggtcgcgacttccgtccgaagttcctccagcttggacaagtacgcagacacttgtcggtccctgtcctgatcatgtcagcaac

attgacaccagaagcacgggaggcctgccaatcgacactcaacctgcgtaatccatctgtcatcgatgtgggcaccgatcgaccgaacctgttcttgc

gggtcttgccgatgcgctatccggccgcgtccttcctcgatatcctatccgtcatgccggaactgtggcacggcgtcgatgcagctgcatctgtagag

gaacgaacgcgtcaatttcctgtcacgttgatctatatcaacaacaagaacttagctacatcaatgcaccgcgtcatcaacgagtggtaa

>contig_14.contig_14.g438.t1

atgccgtcagaagccccagaaaatggcggagataggcaggagctccgtgcaatctccgcagcaaacacggagaggagac

ggagctccgggaaagtgacacggagacgccgcggagacgtgacggcggagaaaatctccgcggagatctccgtcgacatccttaacgagtggtgtgtg

cgcgccggtttcgacgatgccgttgatgtctaccacgccgatatgtcggaagagcacctcgaagtggttcattctcgaatcttgacgggaaagaccct

gatcgcgatctgtaccgattgtaccgatgccttcggtatgggcgccgattgtcgcccggtcaagagggtgagccagcccaagcttttcggcagcgttt

cggcatggtggcagcgtgcgggtcgagccggtagggactttgcggatgaggcggaagagatctgctttgtcgagccgacgcatatcgatccggaaacg

aagaaggccctcgcgacaaccggacagcttctccccattcaagatccgcccgtccctcagcacctggacgaccttccttccttggccaaagacgcagc

agtgatgcgagccggcgagaatgaggcgccagcggcgcgcaagatggtagatgacgagctcctcgcactgtccgctcacgggcttgcccgtaccgcat

gcatccgacagctcgttctcgattatctgcagcaaccggagaaagagctgttacccacgcacagcgagctatactcttccagtcgttacgtacctgat

gatcgaaagatgcgttgctgcacaacctgcaaccctgaacccttgcccgaccttcccaacgaacgaaagcctccaccatctccagtgatccccaccct

ccccggcgtaatggctcttcaaaaggccttacaggtccgccttgaggagtggcgggcagagcagtggcgaacgaaatggaaaggaaccaaccgagcag

gcagaatgggcatcgatgccttcctgagcatgcaagccattgaatccgtcatcagtaacctcaagacgattgcccacaatatcgagcaaggcgactca

cgtttgagttcgttcgtcaacatgcgcttcaagtcggaagtgttgccggagcttcaagtcgtagttgaggatctgctgcgggaagaccgggagaggag

ggagcaggagcgggaagaagttcagcgagcggctcagcagagggcggaggagattcgcaagaagagagaagcagagcggatgctgcgtgcggagagag

atcgtcagttgcgggtgcagcaagctgttcagcaagctcagcaggcagaagcgtcaggatccaacacaaccgtctccgagtgcacgatctgcaaggag

atcaaccgcagaactggtgctggtacggtggaaggaagaggccatcgaagtgagtatggcgctgtgtgctttcacactacctcgttcatgatactcac

cttggggttctacaggtga

>contig_14.contig_14.g439.t1

atgctcctccgtgctccgttgggcccaggattgggctccgcgctccgcgtaaattccggcggagtcctccaggcggagt

ccctgcttttttatgatcgaaaattccccaaaagtgagctccgcgctccgccggaggggtacggagtccgcaaaaccccagctccgctcctccgaata

gtcaacgacctgtcacgaaaagcacagaagcaggcacggtcgtcactcctcgacaaaacgaaaatccacgtctttggatacgacaacattaactggca

gcaaaaggtaaacaacgtcggcaccggccacaccacttcgatgaaggcagcgactcacggcacaatctacgaggtggatccatcgaccaagcttcacg

aaggtccgacgcacccggtatgtcatcctcaagtcttcaaagacttgttcggtgaggacatgccgcatccaaccacaactccgacaagtctggtggct

cccgaggatccttccctcatgctcgaactgcgaaggaagagggatcagatgcgcaaacaagctctgcccgcccagcttcagcccgccgacatcttgct

cggagaggaagacgacgagcatattgtgctagcgatcctcagtcacgtccggaaggagtttctgcagcggagctcctcgattgaattcgattcagaga

aacagcccactcctgagcccccacaggtgtggccgcagatgctccgcaagaccaaggtgatccccttaccagtactcgacgtcggcgagggcacgacg

aaagggaaccttgaggttttcaagcagtacttccggttccaactccaaattcccgactcgttctggcgagagaacgtgttgttcacctccgcagatgt

ctactcagtcgagaagctcaagacggggcaaaaaggcatcgtcagtcattatgcatttttgaccacgctctctcccttccagtatgcatacatgcgct

gcctcttctcgacatatggaggctcgaaggagaacgccagtttcatcagctttcgacatctttcagaacgaaatggcttccggcacctgctttccatc

ccacacaacttccacgacggcaatcgcttcctgcacttctgggtctctgctgcgagtgtatctgttgtcgccaacgctctccgcaagcaccggcagcc

tccaaaagagaacgacggccagcaagacgaacctgcccggcgcgacgagcaggacgagtttgacgacctcgactacgacacatccatgcgaatcagcg

aggaggccgtacggaacatgattgggacaggatcttcggacatggtggacgagcatgagcaccaagacgactttgccttgcaaagcaggttgatgctt

ctcgacattggcctttgcatcgagctccagcacgcgatcaagaatggcgatccaggacggatgctggtggccatcaagcaactggttccacgatttca

ggcatccggccaacacaactacgtcgccgaactgctggatatccttgtgacgattcggtacgagctacctccccctctgcgtgccgtcatgctcgcaa

gtttcctcgtcaatcctaagggcaagccggacacattcactctcatcgaccacgttcaagaaaacttcgtcttcgagctcaagaattcgtggcctgtg

ggaggaacaacgaagtcactccagtacaagagcgttatcggctcgctgctggaggtccttgcgaacttgaagatgggcttctggaaggacttggctat

cagtggccagaaccaagagcattcggacaagaagaggaacgctaccatcgactctctccggatcgacttcgatcattatgcggtcttcgaatgggatg

aagcgggacggacctgtgacaccttcgaggtactgcgcggcgatactgtcaaggaaaataaggaccgactgaggaagcagggcaggccagtcggacgg

gttcggcagctcaagaacgtggcctgcgacatgttcaagaccggtatgctcgcactcgaaggcaagacaagtgccaccggcacgtttcagcgatgggt

caggcgtcgagcagcagcgatgaacaaggaaggagctgcttccatcacgattgacgatcaagacgctgtcgacattcctgacgacgatgacgacgacg

ttgtcgatggagaacctttcggtctcagcgttgtgtcgggtaacgtcgacgagttcgaggagctgggaaatgacaatggaatctag

>contig_14.contig_14.g440.t1

atggaccctgtcctgtgctggacgaggtccacgtggctcagttatcttatctcgatctcttcctctccacttccttctt

ttcccacacgcatctcgcatagcaacaccagtacctccacatttcccacgctatcctctctcctcctcatcttttcccttcccaagctcatcccttct

agagacactctagtcttgtacatgcttcaaggcttcaagatgttcgttctcattcgcctcacgcctctcgatggtacgctccctccgtccgatatctt

cctacatccccttctcgaaccagagcacctcgacgacttctccttgcccatttccttccctgtcaacgcgcaggcccagttacacatccacgaagggc

aaccggtcatctactgccagtacagcagctgcgacgttctgcaaaacggaattcccgcttccaagaagcctcggcgcgtgtataccggagactcgttc

acagtcctctcttcggatgctccgacttccatctccgccacatacaccatcgaggtggcttacttctcattttcttccgacctgcgccgagaagtttc

tgtggaattgaagcgcctacagtcttcggcgacgctcgctcttcaagccgacacgagctccggctttccacttcttcttccccttgagggcgatcttc

cagctcctgcgccccgcggcacgaattggaacggcgtgcaagatctgatcaacgggctgatcggcgcggatccttcttctaaaccagccccgaccctt

acattccagccgccctgcatccagacccacgagctccgctcccccacatcggtatccacttcggcactgcggtctccagctccttcgtcagatgacct

ttccgtacagcccccgtctacatcggtactaacctcggtactgtcggctcccgctccttcaccctcgacttttccgtccccaacttcaacatccacat

caatctcggtattggctccgcgtctaactcctttccaagaactggtcccgtccgccaaagcagttcccccttcattgtcgaccgcttcaaacgttggt

tcggctgcctcgacgtccgccgtctctcccggtgactcgttcactgatcatgctcgcctaacatcgtcacgggactcatccatccggttcgatggttt

tgcacttgcgcttgacaggtttcggcagggatggattactgcgagacggatccttctcgcctcaggacgggcgtctgcttccccgtggccgtcgtacc

ctcctgcggtcacgccaaccataatggtcagctctcggattgcttcgatcgagcacgttcaggcgcagctactcgctattcgttcggatattcaagca

tcgataccatctcagaacgcgttacccccggtccatggccgtgcacgcacacccgctttttcgcatcccttgccgccttcatctgcgccattgcggca

gtcctcttctgcatggacgaaggcgtgctgcttgcccgacccacacctcgatgtcctaccggtctccgaccctgaaatgctccttttcccagagcgat

cctttccgactcattcaacggttccagcttctcgcttggctgcatcgcccagcatgggtgccggctcactgtcgtcctcctattatattcgtccagat

tgcttgcactacacggtacacgggtacaccttcttctcactccagcttcccccccgccgcgaccgcaacatacccgtgtctcgaggcccttctgtgct

cctgtcctttcccttccttgcgctccttcttccgctctaccacttcctcgccttcccttacacgttcttcatttcccatcatgactcgggacagtcgt

cttccgtcggtggggatgtgctggacgaggtccacgtggctcagttatcttatctcgatctcttcctctccacttccttcttttcccacacgcatctc

gcatag

>contig_14.contig_14.g441.t1

atgaggtcttattcgtctgaagacttccagggtctgctacttccttcggccgtgaggtttatcacagaatggtacgacg

catcctccaaggtcctttcctggcaagaatcccaagcatcgtcacagcagcaacttgaggatcccaaccatcttcgcccagatgcggttcagcaagaa

attcactgggcttttgacgacgacgacccacctgcattcgcggagatggatgtcgacacggccacttctaacggatcatcaagcggctcagggtcttc

gtcctcatctgattctttcctcgaccaagacgacaacggaaaccttgtcgcagcggagcttccgcttttctttaacggtcaagacgacgacgacgaaa

atagcagcgacgaggacaggagcggaagcgaacacagcgggggtaaccatgaagggagcagcaacggagatagtgacagcgatcgaacggatgccgat

gctattccagacgagctcgacgggaacgaagaagacccagatcctccacctcgatcacagcacggacagcagctccttgacgagctgattggagacta

cacaccgggacctcgtcctcaaagagctcctcgagctgcggatctcctcatctctctcgatgatctctcgcccagcgaaaaggcttcgctcgccaaga

acgtcgaggatgcggccaagacggacgggatcaagatcatgtccaagtacaaggccgccaatttcgtcaagcgcatcaccaaacttgacgagcagcga

ggtcagctccgtaatccggggaatttgtacaatgctcaaaagtcagctccgtactccggcaacggtgagacggctgaggaagaggcttacatgctgag

cgtagacggtgctcaaatggtcgaaaggaaggaatcgaatgggtgggtgatcctgctcacctgcctcaacacgcctcgtcaagctcgctttcgccgaa

cagaatccttcgttgcctcggtcatcccgggcccccaaaaccctgtcgatgtggattcatttctatggccgataatgcaggagtttgctagggccgcg

cgaggccactggctgtgggatggcaaagagcgagaatggtttttgtggaaggcttacttggtcttcgctgccgcagatcagcctggctcacagaagat

caatcacatgaccggaacctcgggctactctggctgtcgtatatgccacattgtcgtcaactacatgccaggtgaagtcaccggttacttccctctcg

agtccactggtcaagatgacgagcctccaatcaactcagatcgcccacagagctacgacccctacgacctcccgcttcgtaccgatcgttcatacgag

gacgctctccttgaacttgatggtctcttggcagcgtcgcctgctttcagcatgccctctttcttccccgtcgacatcttccacctcttcggcatgaa

cattccatcccagctctgggaggtcttcatcaacccgaagccaggagatcctttctctctttcggaagcgcagcagaagcgcttcgctcgcttgctct

ctgaatctggaaagtaccttccaggttcttttgcttctggcttgccgcgcgatccgacggagttctcgaagtcccactataagatgttcgagtcgtcg

ctcgtgtttcataccttcttcccaccttttctgcacgaggtcggcgctccggaacaggtgattgagatgatttcacagcttgaaggagggagtatgac

tctgacagtctccttcaacctcatgtacttagtcattctcctgttgttggaacttctggatgatgtcgagggttatctctattcactacctcctgcac

gtttccgactacatctgctggcacggaattgtcatcatcagctcacaggcgcggacggcgaggaggctgagcagcttgatcgatttcggctcacggtc

aaacttcagaactctcaccgagttctgactgcggacgaacgagaacagcagaacgactgtattcaagcactcgtagctgaagacagcatcgaccgcgc

tccacagaagctcgtcatcagaggtagactcaaccttccatcgggacacatgcttcgtgggagtcgaaacgagtcggacaaggcgcgtatcgcatgtc

ggtttatggccagagaaggtgatggtctcatgtatgcggaggccttgcactttctgtgtcttgggggacaacgcatctcgaacggaggacaggccgag

attctcaccgacagagtttatgtggtggctcacccactcttggatcagctgcttgcacctgttccgtttgagctcagcaccatcgatccggccttgct

tctccaacacccagcgtcagcgtcttcagactctgctgcgacccctgagtatctcgcagccaaggctctggacccgactcggtccgtctggacgcgtg

caattgtacaggctcaagctcttgcccatcgaaaggcgaacgcgaaagcgaaaggcgaagacgaggatgtcaaagaagacgaacgttactcgaaggag

gacgtcatgcgggaccaccacggcaaccccttaaacaaggagcgcatcaaagagatcaacacggtcaccgtctttgcttgtgcggctatcgatgaaca

cgagttcaacccaccacttgcgcctgaggaacaaacgttccggaaggtcaagaccagggccttagcccttcttgtcaggatctgcaaggacatggagg

cgcaaatcgaagaattgaagttttgcgacgatcacttcaaggcaatgctgtgcatcgagctccacctgcgccacaaagttgaaggccggcgcaaaatc

ctgaagacgaccaaaaaaggcaggatctcggcgaccaaagaagacttgtcgaaggcatccagccatgctacgagttccaagacgaagctttcggaggt

cgatttcagcgaggattccgacggaggtgaagatcagcctctcaaaaaaaagacaaagatcctgactcccgttgagaagctgaacgctatggccaaac

ctgatgatgagagtgctacaaccgctgcgagatcgggcaaggtcacacaaagcggcctccaaaactttgagagcaagacgaaggcttcgctcaactcc

actccgcactccgcacaaaatctctccatctcattgcgccagctccgtactccggctacatttggtaaaatgccaaagtccactccgtactccgggac

tgccaagaatctcaccagcagcccaataagctctgccagcactgctaacacgcatacagctgtctttgccactagttccgccaggactcacatatcgc

aacagggagcgaagcaaagtcctccaaagcgacccaaccccaccgtcgcggcgattcaggaatatctcaacctcagctccatatctttcccgtacgac

gctcaaatgcgcgctgctctcaaggtgctgcaagactcggtaaactttggtccggactcgactggacctggagaagaaacgttcatgacgtggttggt

ctctctcgagacgctggaggagacgccggacgacgaggacaaccacggccccagcttcgggcacgatgctgttggacgatacaaatacgcgcaagagc

ttatgaagatcgaagactttggcagcgtccgaaatgcttgccgcctccttgcggccttttttcaaatgtggagggtttcgactaatcagatcgcagca

tctgaacacagccgacacggccctacgcgcaccccctacagacaccgccttcaagatcggcaagtcgtcacgctcttccctgctgcgctccatatgcc

agctccgccgctagcaacggtcactctcactcgccaatatgtcgcccacatcgacgtcacctcaagcacctggctcgctgcgctctggttgccagctc

gttttatccagcatcaatgtcgcttcccttccgctccagatgcgcattcattccgtcccatccctgctgtgctccaaatgccagcttcatcaactccc

gcgctccagacgcgcatttccggtccctcttcctttggctcgccagacggattgagcagctcctctttatttccgacccccaccaccggcattgcgcg

tcgaggtcgagagctttccctccgcgttctggacacaattcagagactttttgcgctcaagacgtgcattccctctgcctgccgactcctcgcgttcg

attggtactgcttcctagcctcacctcgcaagcttcgcagccggcatgaacaccgcgatcctggacttcgcgttaccatcgtcatctttgccaccgca

cagacacgccgcgtcatgcatgcaagatcttcttgttctacacctccttccaccgtcgacaaacccatctacaccgccataggagtctcgatcggctt

cgagcttcccactcctcgtcctcacctccccccagcactctctcttcgcctgcggtga

>contig_14.contig_14.g442.t1

atgtcccaagcaagtcacacagatcaacgcgctgggatccaaaaccctccctccccaagtttggagctgatcggatcac

aggatggcgggatatggacttctgactccagcggcactgggagcagcagcagcagcagcagcagcagctcaagcgaatgcgaccaagtgcaggacggc

aattggctcgattcggacgaggagatcgccgacgatcccgaccagctttcctcgcggcgaacatcagctgcggaacgctcatccggaacctgttcgac

gtggacgcatcaaggtattgccctgagccctgaggttcggaatctgctgcaagtacacgtcaagcctttcttccactcgcagtcagcctacgcgttcg

tcgccgcgtccaaaggcgcaaagaaggtcgcgctggggtctgttgtcgacgagatcaagcgcgaagggaagcgtgcggcccgtatgcccgagattcgc

cgacctccttcaatggatcgagcagaagacatcaggacgtaccgattcgccgacgccatggacgacatcgctcgcaccatgccattcaccaaagcggt

catcgccgccattgcgacgtcaagcgaggacgaggaagaaccaccgagcgcagacctcgtagctgtcgcgcagtccttggccgatctacctaggtctt

ccactccgccgccggtcgagcacaaggcggacgtcgacagttctgcaacagcactcgatcacatgcgcgaagcgtgggaggatgtggaggatctcgaa

gaggagtccaaccagaatgcggaggacggtcgggatgatctctcacaacaggagcccgatataccgaaagcggtgaagggagccacagtcttgcggcg

aggcagtcgatctccggcataatcagccgaggcgactctaaaaattccaaccttctacagtagaacctcgaaaagtcgatcactcgattgggcgatca

cctcgatctctgatcacttttgcccttcccccgagctcgggggtagcgcaacacggggttgcagtcgccgggctccgaatgcgcacgctgctaccttt

gcgagcgcgtcttctgcgtccctcccgctcatccgctccgccttccctttttctcgggcgtctttgtctcctgctccgcacgctcagaacgctggccg

actttcgcagcctctagaatcgttctttgcccccctagacaccggggcggtcgcaagcatcttcggagcgtctggcgttacgggtgtctctactgacg

tcccaactcccagcaacctcattgtctttcttgccgacttgttcgctgctctggaacctctttccatgacgcgctctccgctcttcgttcccttatcc

acagactcctggacctgtgttctcttctcgcagctcgcctcggcactgcaactctgtcagcctcccgtccggccctgcttctttcccgctccgacggt

tccaagcttcacctacgggccgaggcgctgcgatattgcgtctttgcgagaggactccggggcccgggcacattga

>contig_14.contig_14.g443.t1

atgtccggccgcagccatctttgctctgttgactccgagaacgacgatgcgcgcgggccttcacctccgttcgtccgtt

cttcctcaagatcgacattcgacgacaatggtgacgagacgttgaaaatcacggcaaacctgctgccgagcgccggactggagaatcgatcgctttct

ttgccctccgcgctcgttgtccgtttcgccgtcgcttccttcaacctcctcaagatcgccactgtgcggctcggcctccagctctcattccggccagt

cagcccactggtcacgcgagcgccgttcggtgttcacgccgtgcagcacgcggctcacgcagctacggagactgctctttttcacttatgggatactg

tgcaacggttcacggtgggtctcgcgcgtcgcgtcgcgcctgaaatcgacttcggcaccgacgtgccttcctatccgccatcacgatcgagagcaccg

accctcgtcttgtcgcactgtcaacatcgccctcagcccacccatgttctcaattccgatccaaaccagattctcatggtcgacaagggtaatggcga

ccgcgggcacacgggcatacggcggcgcatgaagctgtcgggcgtcgtgggcgcatacaccacagcgcctccatgccatcaagctgagttcagggtat

gcacggctccaagggcccgacggatcaacgttcaaactgctgccggttcgaaaccaccgcgggcgcgtaacgcgttgacgggtctttctgcctccata

tacatcggcttccggggtgtatcaacccaccacgagctcaacatcgccagaaccactccagaccagaccatcgtcgtcgatgtcgacgcgcccaccga

acgagtcgacctcggtaccgacttcggtactcgcggtttgcgtgtcgcacatcctccacccttctcgtgctgtctcctcgcgtgcgctatcctgtga

>contig_14.contig_14.g444.t1

atgtcgtctaccacacctcggggtccctgccgttctgaccattctgttatgacgaccgaggatcagacctttgtttgcc

tgcatccaagcacaacatgtcgtcctcgcaagcagattacccttctcgcaggatcagagccgctcatcctcaccctatcctcccgcaacgtcacccca

ccctctgcacaacgcagcaagggcaatgctatcgccgcgagccactccctagctcagcttcgatctccctcgccatccgacaccgcccagtcactgcc

tctgtctgctcaagtcccagctgtggtccacgtttgtcccgcccgtgtcaagctgcaaattgaaaagctacagaatgcacacagcatcggtgcagact

cggacatgaccgacgtttgggcgtggctggatgtcgatcttctcgcgcttgaagacacgaacagggccaagaagcaagaactggaggtactgcgctgc

gagacggacaagctcgtcgctaccatcggcgatcagaccaaagagtcaccgagcgagcttcgcgctcaacgtactgcaggtctagagatgatcaaaga

gacgaaggaccgtctgcagcaggctgtgttcgacttggctgagtgcctactcatcttcagtacctcactgacgctcatgagtcatgcccatctgctga

cgcttattcacagtagtactgatcgatcaccgatcaccgctcactttcacattttttatacactgtccagcctcctcatcaatgacggtgctatgttg

acagctgaacttcttgcaccccgccgactgagtcattctcctttacccttccgggcgttgcagcttacttatctcaacccccagcacgcctaccctgg

gtttctccgactcttcccctcctttcccaacatcagtcctttctttcttttgaatcttgatgagtccgccgacgaactggtccttcatctctctcatc

tctgcgaggtcttgtgctcatcgcctttgcatatcaatcagcacaggcatcagcgacaatcccgccatcatcaccaggatcaggatcaggatcaggat

cagcatcgcagctcatcctcaattcctcatcacaagctcgtcgcagcaaaacgagctggcatccatcgcactcgcaagattatcacaacacccgacaa

gttctcatccaacatcgctctccctaccacaagtcacaaccacaaccacaaccatgaccgactcctctccaccatcgcctctaccgcaagttgtcttc

ctcgcatcccgccccctcttcttctcgaccctcttcgaaaattcattcgtggtacaggcggtggaggacgaaaggatactcgagagctatctcaaggt

accgtcagtgcacagccagccacatag

>contig_14.contig_14.g445.t1

atgaatgtatgcggaccgaggtgttgctctccttccttccttctcaagtggtccaagcgggcgactaccatcgacatca

gccagcatcaagatcaagaaggacagcatacacgcagcttcgacgacagtgccagagaaagaatggcgcaggtcgaagtcgatcccacacaggacacc

gagttccacgacgctctgcgcaggcatggcatcatcccgcccaagcctccctcgcgctcgccctcgcctgatctgccgtcctcgtccgagctgcgaga

gggcgccctccatcgcgtctcccaaagccagatcgacgctctcctcgatggagagaccgtccgacgcggagatggcgcccggcggaatggaaatcatg

atgacgacgacgatgaagacgatctcaagctggacgaggacgatgatcaggagcgcaagttgatgttgcgactgcgcgaacagcgcatggcggagatg

aggataaaggagaggagagcacgctttggccgcgtcttccccatctcccgggtcgactacacccgcgaagtcacagaggcgtcgaagggggaccccga

tcggcctcaaggatcagatactgcggacaggtcagacggtacaggatccaagtccgagagcaaaggaacggcggttgtatgcttcctttataaggacg

gcttggaactttgcaagctactagggagctacttggacacgatcgcaagcaagaaccccgcaacgaagttcgtgtccatcgttggcgacaagtgcatt

cccaactaccccgaccgaaacctgcctacactcctcatctaccggaacggcgacttgcatcggcaaatcgtcggccttcgtccagagataggactgga

cggaatgaacacgaagcttgctgacgtcgagctcctcctcaccgcagtaggcgccatcgagcgaccggatcaaagagcagccagcaagaacgcaggac

aggatgctgccgaagacgacgaggaggatgaaccccgctcgagatcgtctggcatacggaacggcggcagtggcgctgggacgggctcccgaacgtca

actgtgcgaacacaagaggaggaggacgcggatctggattgggatctgtaa

>contig_14.contig_14.g446.t1

atggacgatctgctggacctcgactggggcggcagcgcaaaggccaagcagccgccgctcaccctcgcacagcagcagg

cacagaccgcagcagcaggaaccacaagcccgaccggaagcatcacccacagcgctgggaacgggtcctcatccacctacagcttcgacgctctcacc

cgctccatgcccgccgccgcaggctcgctctatgtgtcctcgcgctcaggcacccccaacatcgcaggcccactcaagacgaacccaacccctatcaa

cggcagctccaactcagctggcaccgcgagctttgccgaccttgtcaacttcggaactaatgccaggaacggatcaggaagcatggtgacggctcagc

aagcgtctggcggcggcggtggagggtcgaagctgtctggcatgagcatgctggaccgagccaaggcaggagcagatcgaggagcacctgcgcaaacg

gccgacgcagcactctgggactttgacgcctttgacacagcaagcgctccttcgcctgcttctccgattccagcgcgcacggctccggcggtcgcgtc

tccgccagctgctcgcgcgcatgtgggaaaggcttccacgtcacctaccagtaagagtcaaagtgccgggcagaaacctgagatcttctctctccttg

atgatgaagctcaacaggaggagcatcaaccgcctccgctgccccaacgggcgggagctgccaaagatccattcgatttcgactccttcgacgacatg

cccgcctccaacacttcccgcgctgatgtgccaaatgtgtccgtcggtcatgttggaatcgatgacgacgaagacgatttcttgggtgccttaggccg

gcctgtcaccctcaagccagcccgagagccatcgcctccaccaccgacgcacgacaggcttcgtgcggaagcctcctcaccaggtctcggcgaaggta

gctcatcgagttcccgagcgcccagcccagccgacggaggtaggaggcaatatcaggaccgctccaccagggaacccccgcgtcgtcccgtcaaattt

caagatgaggagcgcgccgctcgggaacgagacgaggcgattttgggtggaagtccgcccgcctccagtcgtcgccagcgcgacggtgcggctccgtc

tgtggcagcaagcctctttgggaccagcacggaagaaaacttgctcggagccttaagacggagtctcggatttagtgggactaagagcaatccaagca

agtcggccactcacactccgcctccttcagccagtcttccgtcagaggagaagagacgggttcgaccgcagccacaacttgagacggacctcttcagt

gatactgcaggctcaagcagcaagacccgtgaagtgtcatctccaaccgcacgaccatcgcgacgcccacaaccaactactcagaatgccgctcccag

tcgacccgagccgatccgtgtgccacggtcgatcgtgacggactcaagcagtgcgatctcggcgtctgctgcactcaagttgcaaggcaacgacgcgt

tcaagcgcggcgcgtacgccgaagcggagacgtactacaccgaagccctcgaccagctcgaggaaggctcgctacggaagatccccctgctgaacaac

cgagcgcagtcgcgtctcaagaacggcgaagcttcgggcgcgtcgcacgactgcgagacactgctcggcattatggggcccccctcgaaggcgaatgc

tgacgcgaatacgacgacgctgtaccggccccgagaggaagctgcactacccgaaccactccgatcggagatcaacctgcgggacgcgtgggctaaag

ctgtgctgcgacgtgctcaggcgtcggagatgctggagcaatgggtgtctgcgcgccgggattgggatgcgctcttacgatttgagaaggaggaaggg

agtgggaaggctgctgtttcgaatatgcggagtgcgaaggaggggttgatgcggtgtgaggagatgattagtgggaagatgaagacggcgccgcctca

gcagcagcagcagcagcagccaccacaacgacagtctggtcagtcccgcgccacggagcctcgcgcggaggggagcggagacagggcagtccgacagt

cgagcagcacagcagccgccatcacccgcgccggcgaagcaggcatcgaacgcgtccgtcaagaggcggccttacaagccgccgaagacgcggcgaag

ctctcgctcaaagatcaagttgacgccagcgtgaatggctggaaggagggcaaggagagtaatatccgcggccttttggccagtgtggagagtgttgt

tgtttggcctgagtttgggtggaagaagattgggttgcatgagttgcttacggatgtgcaggttaagaaggcgtatacgcgtgctattgcgaagttgc

atcccgacaaggcgtga

>contig_14.contig_14.g447.t1

atggccaagggctggaaaacgcgctccaactacgttcccagaggacccagtcccgacatacaacccgccccgggcccgt

cgggagatgctgctgctcctgttgctggacgcgttggtcacgtacgatccgcctcaaccgcgtttggggaaccagatgaagtgctaaacgtttcgatt

gtgccggaggttctggctattgcaaagcgagcaaggctgtccaatagcgccacaaacaagcttcatgaactcagcgaagcggtcgtagctgagcagga

cctcgacgacgagtccaacttttcccttcatgcaccttcttctttcctactcgcacagtctctggtattggccaactgggatcttctccggacgttga

acaggaaggcatcatcaactgcgcccgcagaggcatcgaacggtcctcgtcctaagactcagccagctgcagcagccctcgatcccaatatgctgagt

agcgacgcaaaggaaaacgaagaagaggctcaagacatacgcacattttcagatgctgttgcggacaagtggggtctcgatatatcctatgacttcat

cgtccggatggcatggcttcgcaagatgggatcagaaacgatccccattcaaagcgctggagcagcctggtgggacaacgtccaggccgagctcgaca

tcctcgtctctttgtgcaaccatcagggcattgcacatcaacagaaagggcaaaggtgtttcgagcgtgctctccagtgggaattcgacactcatgga

cgctgcgacgtcttggaaaatgagcagaggcccgccgaagaagctgctcttgccgatgactttcttcacgcgggtgaagaattgatcgacgcggaaga

agtcgtggattaa

>contig_14.contig_14.g448.t1

atgctcgatgcgtccaatgtctttgccaaagccgttctttgcccttcggtcttcctcttcgtgcgcggtcccgtcttgc

cggcttgttgcttcgatctcgactcgcgtctcgctccaccgcttcttggcgtccttgctgcctcgcctcgctcctctggccgctgccgctctgccgct

gctgataagagtgcgcgcccggtatatcctcctactttcgacttgtgctggaaggaggcaggcgactcgctcaatgctgcgtctgcatcgaacgacgg

ggaaagcccactgagcttcacaagcatgctgccgcgaccctttgcggttcccatcctctctcttccagtcccaaatcgcgtggatgaagcgcttctcc

ttcacggtcgcatgttgcgtcaggcccccgtcatgtggactgcgcatcatctttcggatagctctctcggtgatgatgaggagcgactcattcagcta

ttggcggatgagatctcggtcgtcaatctcactgcttccgagcatgaagactgggcgcccttccaacagatggtattatctttcgccaaggccgcggt

ccattcctataccaagggaaccaaagatcacggcgttccctctcagaaggctttggatcgatgtcggagacagcttcaggtgacgctcgatgctcccg

cgctgcgagaggcccgcagcgacgctggaaacacgttcttctgctcttccatcgcggcagcaatagcacgcgacttttccaatccatctgtccgttca

tcaatgaccttataccctcgcgagactgctacaattcagaacgttcaagacgcgagtttctttcacagcttgccaggatctctttccgggccatgcgt

cgtgggtatcgatggatcgacatacttcgtcggcgaaaccttgattctcacagacggtcgcgaggttattgccgacatcaagaagtggaatccccaca

tccagctcagcttccagaacgctgcgctggcgtcacatgaactctctcgcgagtgcaatttgcacctttttgcggtcacggacattgccaagtcattg

gagctaatggaagaatttgtcaagcaactcaaggatggtcttcgaacgccggtgcaggtctgggattcccttcatcgcaagatggtgctcgtcagacc

cttcccgctcctcgtacttgccgacaaccccatggcatctgagctatgctctcacgtcggcagtggcaacaacgcccacccctgtaggatctgcaagt

atggaggcaccaatgctttcaaaagctctgttgagggattgaaatcaatgttcaaaacgggcgaagagcgaacatctggaggcacgattgaaaatctc

acaacgctgatcgatcttgctatccagggaaatgcgtcgaagtactccgagcaccagagtgcttccggcgcaaaggatgttattgttgaggacatcac

cgaaattttgttcgagtcgcgtcgtgttctctccggaaaggctccagaccatcctaggactccaacgcatccgcagacgaagaaggccatcctcaacg

agctgctccatgaagctcgtgcattgcagtcgggagcctggcataacgccctacttcagctgtcagaacactgcggcttctcagtccacagccagagt

cctatcgaatgtttgcacactctctggcttggaccggtcaagtacttggcaattgcgacagtgaatatgatcgacaaagacctgctacgcacaaggct

cgagggcttggacacggacggcctggactgcggtgcggtgttgagagcaaagtatcatttgaatcatgttgcttcactgacgggaaaggaattcaagc

tccttacacagtccatgcccgcagcactaccaccccttgtacaccaaaagaaagtctcgcggcatgtgcaagaagcatgggctgccgtagggcgattc

gcgaagatcatatgcgccccatggatcgatcgaacgtgtctagacggccatgttaaagaaattcaagccgcgtcaattggcctataccgagctcttgg

actcatctgcccgctctcactgccagcaaggcccaaattccacatgcttgcccattttccagacaacgtcgagcgttttggtccttctttcagctaca

acaccgagcgcttcgaatccttcaatagtccaatccgagaggcatccgtccattccaatcggcaagctccttctacggatatcctgacccggctatac

gatcaggagcttgttcggcatgtgatggacggtggcgagtggatcgagaagggcaaggtacaaaaggcatcatctcgagtccttgcgttcgcacagag

cgccgagagcaaagtactgatgcagaagtggggcattcagtcggtgaagaagcgtgttactcctgctccgccaagggcaatacttcaagacgagcaaa

tacgagcccaacttttcgaggaagtcctgcagccagtggacgatatgtatgaggagggtctcgaaatatcttctttgagtcaagacctgtgcaaagtc

aagagtcatgtcctggttgaggtggaacaaggcgatctaaatgcaactgtcggcagaatcacagcgatttggaaaggagaaaagcgccaagaagccgc

tctcgttcaactctgtcttctggacgacgctggatggtcaaagaaatgggagatgcggcgactggttgatcgaggacggatggtcattgtgcgcgcat

catgtcagtacaatccggagggcaaagcgaccgtccaagagcggcgcgagacgggtctgacggcaccggcagtcagccacatcactgatggccgagga

aatcaacattatctgctcaaccatcatctcctgcgctatcctccagcacagctctctggacattttccacctcaccgtcactccgactttgacgcgga

ctccgtggccgaagatgtggcgaaggaggtcagacaggcgaggaacgcgaagacaaagaaaaaggccaagcacaagcagagggccgaaccgacaggcg

acgagacggacagcgaagtcgaacgcgagatgggcgacgaaatgaggagagggcctccgggctcaatgcacactacaagctcaatgcacaaacacgtc

gcgtccttccccgtccctttatctctgaccatcacatcgaatcaagcagacatcatgtcttcaacatacaaggaaacagaggagcagctcgagtgcgc

actggaagaggtgcgcgcctgcaaaggaaaacccaacttcgcaaagatagcgaagaagtggaaggttcatcgccagcgcctgtctcgtcgctttcaag

gaaagacccagagcaagtcgacgcgtccacccacaaatcgtaaactcaacagcgcacaagaacaggctctgatggcctacgttgcagcactggaccgc

ttggagctatctgcgcgcccagcattactcgttgcgtcggcaaatcagctgctggcagcagctcatcaaggctctggtccccctcctgttgtcggcaa

gaactggctgggaagctttgtcaagaggcatccacagcttcatcgcgtcaagcaaaagtctcgggagctgtcgcgcatgcttcaagatcgtcccaccc

tcaagaggttcttcggaaagctcaagaagacgatggacgagcacggaattacccaggacgacatatggaatgttgacgaggtcgggttccggattggc

gttggtggaaagcaatggattgtcaccttgaatccctcgaaggatgcgcatctcgccagcgagacctgtcgtgactccgtgacatgcatcgaagctgt

cagtgccgctggtcaacacattgcgcctttgatcatttttgcagcggcacagcattcggagtcctggagcaataacgatctctcggacgataccctcc

tcgcccccatgaaacactggcaccgcgaggcgattgacaaggcgaccagaagcggctgtacgaatttcaacaaaatcgagttcctggcagcgttgacg

agcatcagaaaggcaacgatgcagccaaacacgatcaaagccggattccgactcactggcctgttcccgtttaatcctgagatgattttggtcaaatt

tccggagcacagcaacgattctggcccagcgactggctcatcatctctgccaactgcgacaatttcgacgccaaagacgttgcgctcactgtcccgga

ctgtgaacgtcctcgagcgcaggaacgacctttctcccacagtcatcaagtgcattcgaggtgctcacattcaggcgcaggcgggatcattggcggtc

acagcattggaagttcaaacagccgctcagcaagaacgaaacgcacgcgcagagcgacagcgaaaagtcgtccaatctggtggagttctaaccgcgaa

caagtccaaggccgaagggtggagacaagtctttgcgcagatgcgcaaggtagtcaaggcacgcgcaaaggccaaaagcggagtctcagcagccacta

cggacactttggaggacttggataacgatgcagatgaaggggacaatgatctggacctgttgttgcttccttaa

>contig_14.contig_14.g449.t1

atgggccctcctcctaccagcccgtaccaactgaccagtcaaaaaggtcctccatctcactacgaacgacgaccttaca

atcccgccggaggagcatcggccgcttccgctgctgcttctgtcgctgccgccgccgcttctgctgctgctgctgccgcagctcaggctcaggctcaa

gcgcaggcccaggcccaggcccaagctcaggctcaggctcaggctcaggctcaagctcaggctgcccaggctcaagcgcaagcacaagcgcaggctca

ggcccaggcccaggcgcaagccgaggccgaagctcaagcccatgtacttgctcaagctcaggctcaggcgcgagcccaggcccaggcccaagcgcaag

cgcaagccgaggccgaagctcaagcccatgcactcgctcaggctcaggcagaagcgcaggctcaagctcaagctcaagctcaagcacaggcgcaagcc

caggcagccgctgctgctgccacagcctccagctttcagcgtgatcccgaacgcgaccgcagttggtcaggacctcgggtcgagagcgaggttgtctg

ggagtatctcgaccgctgcgagcggaaagaggctctcctgcggaagatggccatgctggacgatgaagcgcctcccaagggcaagaagcgactcggag

accaagacgatgagctcacagcaaaggacctcaacctcgacccgaactttttcgaacttcctccttcgctctcggctgttgcggacgacaaagcgacg

gtcagggccaggttggaagctgctgcactcgcactggatgcagacctcgactctccggatgcgctcgcactcgccatggcgctcgaaccccccggtgg

ctacggtgtcggcgaagatcgaggacggccggtcaagcatctgggcacctggctctacgactctcgtatcgacccgctgcttcctggcgagctgctca

gcgcgaatgtgggagcgacgttggaagtgcggattcctggcgcgcagctgggacggggtcccagcgctgatttgttccgagcgcttggattggaaaat

gcgcttccgggcatcgagctcgcacccagcgaacggaagcatctgggctcgaacaccatcaccccgactcgaggcaaaggtggcagggctggggccag

gggagttgtgtcggatgatggtgtggccagcgcgacgcccggtccggccggcacgattggtgtgaacgagaacaggggacgatggacgcttgggtggc

gtggacgtgagcatgcgaagctgagggctcagcttgcagcggagagtggaagtgctgtggaggagatgcggaggttcaaacgaaggcggccgctcgtg

tccgttcctgctgaggatgccactacgtccgaagcgacgcaggccacgtctccagaagcggccaaattgggaggtcggaaggatcggttcagcgacca

ggacgcgattgacgctgcgtggtccttttggagcgcgcctgctcttcgggaacggcggtgctggggaacggacgtgtatacggatgactcggatgtgc

tggcgatgtgcatccacgctggctggatcgagccccctgctttggaactggagcatgagcgcgagagggagacgatttcctatgaggaaggacgcgtt

cgggttcggaagggccccggcttgcctggatgggtcccgagtgggaaggcgatgtgggcgtggaagtcgatgagcgccaaggagctggcttctcggat

cccgcctcggtcggaaggacggaggccggggttgggaggtcaacaaccgaatggcgcggttacgccagaggagggtggcgagcgggatgcagcgacgg

tcgacgtaccgttgacatcggtcgccgacgaaatcaagcggcgaacagcggcaatcacgtcgggcacgaccgccacctcgcctgcgaatctgttacgg

cctgccgacccgcgcccgccggatctctctgtgatgctgcgtatcgccccgcggctgatcgcgtaccgtggctgcctgcgcgccgggttcaagagtcg

aagctgggggaatacacacgatggtgtcagcctcgtcgtcgagagtgtagagctcaaagagcctggctatgcgacggcgaggggtcgacgtgcggcga

aggtgcggctgcacgagatgacgacgctgcggcgtgcgggtctcaaagacttccgaaggtctctcaacaaggatcgcgcatcgcgtggagcagcgccc

acaacatacacgggcgtccgcagcgcaacctccgcgacgaaacagccgccatccaaagtcatccgcgcgactcgcgactaccgcgcacgccagcccaa

cgagctcagcttctccaagggcgacttcttccacgtcctcaacgacagcacccatccagaatggttcgaggccgtgaatccgataacgaacgcgaggg

gacacgtccccgccgcttacttcgacgtgctcactaagaccgcacggccgggaggaccatcgatgtccggtcccgggtctgcatcttctgatccaatg

gccggtcccacgtcgcctggcgtaggaaccgattccggcgattccggcctgtccgcccaccttggctccgcaaaaggcgccggtgggctgtatgccgt

tgtcaagtacgacttcgaggcggagcgaccggacgagctcgactctaaagcgggggattccatcatcgtcatcgcgcagagcaatttcgagtggttcg

tcgccagaccaatcggcaagctgggcgggccgggtcttatccccgtcgcgtttgtcgagattcgcgacatggtcacgggacaatcagtggagaatgtg

caggagctcattcggaacgggaccattcccaaggtggaggagtggaagaagatgaaggcagattataccaagaataccatcccgctcgggcgcttcga

cttcgcgggacaacaggttcctccatcacttcccggttctccattcgatggcacgccaggaggcgacggggccggactgggtccaaatgggatgggac

caggagggatgggaggcatgttcatgggtggggcaggaccaagcggacagttcaatcaaggtccatcagacatgatgatcccaccagggatggaacca

tatcaacgaggcgaccagccgctgccatccctcccaggcggcgaagaagtcgagctaccaccgggcatggcgcccggcgaacctcttccctgcggcat

catcgagtctgcccgcgtagactccttccacttcgaacagggcgactactggttccgcgtacaggccacacacatcgcaggtccttctcccgcaccac

cagacgtgttgcgcgcgccaacgggagaagtcagggaattgattctctatcggctctatgaagatttctacgagttccaacttgccttgctcgagcac

ttcccagccgaagcagggcgcgagacgggtccgaacggccagccaaacgaacgcatcttacccctcatgcccggtccaatcgacgaagtggacgacgt

gatcacatcacaacgacgcgccgacttggacacctacatccaagaactgtgcgcgttgcctgaacacattcgacgatctgaactcttacgcgtcttct

tcgagcctcggccgggcgatcattgtacgcgcttgccgctgcagccgggcagtgagggcgccaatgggagcaccatcaccagaaccagcgccggcacc

ggcaccggcgaccggctggccgccgccgctggcggcgatgtagtctcgctcgcgccctccgacggattcatcaagatcaagatcttcaaccgggcaag

tgacgatctggtcgcgattcgggtgcctcccgatgtcaccttctccggcctgatgaccaaggtccgcgaacggctcgggcaggagatctcagtgatgc

ggtatcgggacgagagcgggatgatgcggctgagcgatgacgatgacttgagcgagtgggtcgcgtcggggcagaagttgacactctgggccgatgtg

agatga

>contig_14.contig_14.g450.t1

atggtccaaccttcccttatatcttctcaacaatctgccaccgcggccaacaccacgcctttgacgcgtgactcaagca

tcacatccatcttgacttcctcctacaccactgattcgcaagagctcgagaatccatccacctcttcatcagctgccacatcctgcactcatctcgac

ctctctccgttcgactctggcacacctcaagcatcgtcgattgaagcttctgatggatccggcatcggcacgcctgatattctgccagaactccacaa

cgcacttgatcaaagttcgaagaatgcagcggagcgcttggagaatcctaccgcggcgcaacggtcatctgcctcaatgcgcgttcttcttgtggatg

acaacccgctcaaccttgccctgctcctccgacttctccatcgccgcctcgccgacaagctcgatccagcgcagccccccattgcgctcacggacgct

actgtcgctctcgacctgttgaggggtaccctcccactcgtcgacgcagtctccatcgccacaccgccggcagtttcacgctccgtgcccaactctgc

tcgtgcggtcgccgccgccaacgcactcaccaaccagaccccgacgttcacccacatcatgctggacatccatatgcctgcgatctcagggctcagct

tggcccgtcggattcgcagcctgccgctgtcctcgctgaaccgggaggccaaattgcttgcttgcaccaccgccgttcgggcgcaggaacggcgattc

taccgctctggtggctttgatggcctgattgagaagccagtccgcgaggacacattgcgcgctttcttcgaccatttggctgacgagggcttggtaca

gtcgcgcttagcttcttccgtgctcgaagacgttcgcccatctggaagttcgagccagctcaccggtcggtcgaatatcggaaacgcaagcccgtcag

cgtcaagtccggctcaagatgtttccgaagggttcggaagtctcagcctcggctctcgcacctctcgtcacaagaccgaagtcaacgttggcggaaag

aaggaaatcgtagccctttcctcctcagcgatcttcaagaatcactcgaggggcctcggacctgcggcaactgaggatcgtggtgacttgtcgcgtgt

ccgaggactgacgcggagcgtgagtgtaggatcaacccaggtccagcacaaatcattctttctcgctcttcgcgacagcgagcccgtgatcttgccac

cctgcggccgagatcgatatcaagggcccagttcttccaaccagattagctgtgccagcatcaaggaccatgtcgaggacgaactgatacggtcgcct

cactacctgactcagggcttcttcctgctgggagatgacgagggtgggtgcgccgtcagtggccaagccaccgggttggacaacgccgatgctatatc

tgagccacacctcggattcgaatcccagggccctgcgtgcattgtgcgtcagctggaccaagtttctgctgcgccgatcgacatccgcgacctttatg

agatcaagcgcgaggtcgatttggacagctttcggctggaaggaacgtactttgacgctgcgaaccacgtcgtacgccatgaagaggggtcggtcgct

caggaagcgaaaggcttgtgggatgtcgggagttccaatgggcctgttcacgacggcacggtcgctacgtccaggttgtccgtgacgtcgctcgcgca

ggaactgatgcactcatcgccatcgatcgcatctccggatgctggcaagacgccctcgccgggtccggcaacccacttcatctatgcggccccgagag

ctgcccaggatggtacgatggttctcgatctggagcaagagcttgaaggcgttgttgctggcaatgcagtccaacagccacggacggacgaggcgacc

tgtgctccgtcggcttcgatgcacgcttgcagtccggcttgtctctcggttgttggagtggaatgttcgctgccgctcttgaactttggtatgttttt

ctggcaagacctttcgagcgaaaccgcctcccatggcttcggagctgtcccttacggcatactaccgcatcgactcttgcacgacacccgctctgcgc

cgggaacgactggaaagtacgacttggatctcgtcttaaacctatctgtactctcctcgccatcgccatcgacattctgggccatggcctcatag

>contig_14.contig_14.g451.t1

atgagtcgcaactggaggaatcccaaagccgacgacggggaggccgggtccgatggcgaaggcgaaggcgaaggcggag

gggcaagcaagaccgagctcatggactgggaacagatcgcacactcctccaccaaggacatcgtcctcttctgtatcgacgctggcccgcacatgcat

aagatcaacccagacaccaacgtctccttcctccactccgccctgctcgccgcctgcaaattccaagagaccaagctcatcgcctcgccgaacgatca

ggtcgggatcctcctcttcggaactcagtataccgaaatgcagggcttcatcaacaagggtcccgtgtactcccattgcaccgtgctccagaagattg

cccaagtcgacgtcgagcctcttgtggatatcttcgacttgcttgccgacgacgaacggggcacccagcatctggaccgcatcagtctcacggctccc

tacgaaaagggaaagctcttccgcatcgagcatgcgctcggaaatgccgtgcattacatgatagcacagggcaagaccggctacaagcgcatattctt

catgaccaacaatgacgatcccgctgtcctcaagaacgacgacacggccaaacctattcgatcgtcttcagagcgcacagatgcgacacggaagtgtc

tcaagttcgtcaacgaggccggcctgcgcaatatcgagctggagcctctgtttatcagcagttctgaacacacgttcgaagtcgacaaattctacggt

aacgtctttgcggcctacgacgacccgctatcgtccgacccggaagtgtcgtccgactccgacgatagcgagaccgaggacgatgaggacgagtacga

cttactagctgatctcctcccgaacgagaggcagccatcgagtgccctcaagacaaaggcccaacgtcgagccgagcgtatcgcccggcagtccaagc

gcgaacaacgcgaagctcgcaatcggagcttgtacgactgcgcagtcaagatggaggagatcagtcagacccttgaggagaggcagatgccgaagcgc

gttgttttcaatactgtgatggagattggggaggggattcaactgggagtcaaagggtattctatgtttgcaaaggcgacacggggcacacctgtccg

catggtagaagaagcaagcggataccgcgaagtcgcagccaaagtcgtacccgtctgtgaagacacgggccagatcttgcaaccgaagaaggacatcg

atacggcgttctcctttggcgcaggggctgctgcggccgggtacaatgaggccatgcttgcggaccaagagagacaggctggcgctggcgctggtgcc

ggtccgagcactgcgcgtgaggcggaagcgaacggggatctgggaggcctacccgatctaggaagcatccctggttccgccggccgtggtcttgccaa

gttcacgaaggaggagatgcgcgagatgcgggagatgggtctgccacatggcatccggattctcggattcaagccgcaatccaagctggagttctgga

tgaataccaagcattcggtctttctctatcccactgatgagacgtatgctggaagtaagcgcttctttgcatcgctgctgcagtcgatggcgaaaaag

aaggtatttggacttgcggtgtgcttgccgcgtgctggcagtacaccgtccttcgctgcgatctggccgagagaggaggaggtcagcgcggaaggagt

gcagttgactgcgccggggatgttcatgattccccttccctatgcggacgacattcgcgacaatccggtggagatgtcgctaacgccaaaacgagagg

aggtcgaggcggcagcggcgatcgtaaagaagtactcatacagtcgaccattctacccggatacgtacaacaatcctgcgctggctctgcactatgcg

acccttcgagcggccgctttcgatcgtgagcggccgatgacgagcaagattgcacaggcacgacaggcagctgctgcagctagtgccgctggcgagca

ggatgggatggagattgatggcggcggcggtgctgctgctgtaccttcgtcctcgatggggacgttgggtacgatgctgagctcggtgaggaaggcgg

aggttgtgaaggacgacacgctgcctgagtatgagaacatcgatgcgaccgaggaggaggtacatctcattcgcaatttcaatgagctgatcctgaag

gcggggtacggcagcgtgagaggggcgccgccgaagttcaagaagagggacgaggctacgctgaagtcgaagtgggcatcccaaacgctgagcacgta

caaggtggatgagctcaaggcggcattgttgttctacaagctggcgcagcaacccaaccagcaactaattcccaacttctcgaggactactacaccca

cggctgccatcttcacggtcgacccgaaattccattgccaacatcccattgccatgcttcggactgcttcagccaacatcactgcggtagcgaccggt

cgccactcgctccgccgatctgcaaaagctcttctacacacctccccagcagtcgcagctcacgaaacgcccatcttcccatccgtcgctcgaatcct

ggccgagcattccgaggtcgatgccagcaaaatcaaaggaacgggcatgcgcggaatgatcacgaagggcgatgtgctcgcccacatcggcgaggtcg

gcagtccgctgggcagcgcgaagggcaaggacaagttcctggcaggcggcggaggtgtcagctcgctcagtgatgggaccaggatgaaggcaaatacg

gccggcggctcggaaaccggttctcacagcgctcccgaaaaggtgcaggtgagatcgatcccagagagtgtatactgtatcccggccgctgacgatgc

tacgatctgcttttttcagcctcttgacgcggccgcactgcgctctttgatcctctctggtctggcatcaggacatccaagcgctgtactcggcgaga

ctgcgtcgtcggcgtcgtcctcccgcgccaagacgccatcattcgagaccatcgtgtctggctactcgttctcgccatcgccatctcgtggcccgcat

gaacgcaccaacagtcgccgacactcgattggtcaccaaccttctttcaataaggcaagtttgcctctgtctatcgggcacccagcagcgagcaacaa

tcacgaagcgccaaacagccctggacctcaacaaagcagatcagccgcgagcttcccacaggccggcaaggaaaaggagcacggccgacgccaacgcc

gtggcgggcaagccgtggctgactctggccttggacttatccccgttcggaaagtttggcaccttcgccaccaccacgacccacaggccacagtgtcg

cgacagcctacgaccctgcttcctggtctcttcaagaattgcgatatctccggagcgagggtccagctcttacgaccctggggtggcacagccatcct

cccactctcgtacggccgcgtttactaa

>contig_14.contig_14.g452.t1

atgcagtctccagaagagcacgacctcgccccgctgcagctacagcaggagcagcaccaaccacaaccacaaccacaac

agcaaccacagcaacaaccccgcggcaagagcaagaaggacccagcagacgggtccacctcctcaccaggcaagcagaagagcacgcccaagaagagc

aaggcggccagtcgctcagcaagcacatccatcccagctcccgctccgagtgagggcccgttggctgctacgggcaccagtgtgttccccaccgcccg

catgcaacgcatcatcaagtctgacgaagacttgacgaattgctcagcggaggcagccttcctgatcgccatcgcgactgaatgcttcatcaaatact

tcgtcgaagaaagctatgcgtccgcccgcatggagaaacgcaaggcaatctcatacaaggactgtcaacgggccgtacagcgcgaatgggccctcggc

ttcctccaagaggccgtccctgctaccatgccactctcggtagcattgaagaaggcagaagccctcgaagcgtccgccctgcgcggattcgccacctc

cggaggcacctcggacatggcaggcgggaagccgtcccatgtgaatggggacgaggagatgttctccgatggccctcaaggcgggcatgagaacgagg

gcgacgacgacggcggtgacggtgacggcgatgatgacgacgatgatgatgacgacggggaagacttgccttatccggtcctgccttcaaactcacat

gctggcggcggcggagcaagggaggcagacgaggcccgtgcccgacgaatagcggaggagaatgcgaagcaggaccagaggcgatacgaggaggagac

cttctcggattatgaggatgacttttga

>contig_14.contig_14.g453.t1

atgagcgggagtcgggcggatgggaacgcgatggccgtgagagcgacgagtgaagcgccgctaggctcccagctgcgcg

ccgcgctcgacgcatacgaggaaagcacgctgtccctcttcgccgccatcgagaacgggtcggccttcgaaagcagcaacagcagcaacaacaagaac

gacggggcagaacagcagcagccgctccacgcgctcctctccaccatccacagactggacgagcacctccagtcgagcctgatccatagactcctgcc

cacgcataccgcgcagcagcataccatcgatgccctcgttcggctccagcgcgcgagggatcaggaccggaagacgcagattgagcggcttagtcgga

tgaaggccgacttggatctgctggttaggaggggcagggaggagaagatcgcggcggagaaggcagagactgaaccactctcctacaagcacatcctc

aactacgcctcctacctctcccgaaccacctccgccccaccaggccatcgaccacccggcttcggcctcggcgcagccgtcaaagacgagcagctctc

cgaccaacaacaacaacagcagatggactggcccttcccctccgaggcacagatgcggcgtggggccctcgcagcagcggcagtggcagtggcccgga

cggacgagctccatcggccttcggaagcaggaccgacgggagaaggggcagcagcagcagcagcagcagcagcagcgacgggggggacggcagttgct

gcagctgccgggttggacgtccagcatgctccccagaacttcccactgcctggccaacctgaccatccgtcgcagcaacatcaccatcatcatcacca

gcagcaggagcaggtcgagatggaagaggatgcctttgacctcgacttaaattga

>contig_14.contig_14.g454.t1

atggcgtcttcatcagcatcagcatcagcatctgcatcggtaggaccgctgtacgcattcggtctgccagtcccgctcc

tgctctcggtcaacacgctccagtcggtgatcaacgaaccatatccagagccacagcacatcatccacagcagtaaccaccaccagcagtctcaccac

cagtctcagcctcagcctcagcctcagcctcagcctcagcctcagtcgcacgcagcagcagccaccttcagcatccgattcccaaagccaagaccaca

agatcgagcacctcaaaccacagcccaaaagaaggacaacggcggggagaacgatgaggatggccaggacggggactcagacgacagcgacgatcagg

cacaggcgtacgcttccatcggagcagggccaaacgctcccttcatctggctcaactccacagacccagccctgcacacccagcacttcggcctgcat

cgctccctcttcaccctcccaccctcggctcctcatcacaacgacagcgccgacttcccagcatctcatctcgccgcgcttcagctacagcccatcac

tcgcccaaagggtctctcccgtaccatcctcgcccagaacaaacacaagggcggcgtacatgccatcaccggacgcgcactcaaagcacagggcgtac

aagaggccgctcgtcgtctcggattccgcgtcctagagggcgcagactacatccccggcatcggcaacattgacagcagtgttgacgaagaaggcgag

agctcagacgaggacgctgccccgcttgatcctcatcgctcagacgatgatgacgacggccacaatccgattcttcggcctcgggtgcatagcgacgg

cgagccggaagaagagatcgacgacgctgcgacgaccaccacagcaacaaccctctccaaagcatcctcctccgccatcacacccctccaacagtccc

tcctctccagccccccgctcaaaacatggacactcctcctcctcggcggcggaaacttcgcagcagcggtcatcgccctaaaccccttcgtcgaactc

tcgcccaaactcttccccaacggcccaccgaccccagaacaactcatcaccacagcctccctccggccggcctcagaccgcgccatcctcctcctagc

acataagaccatccaccgctacaccacccgacgcaaacagggcggctcccagtccgcccaggacgcatcgggtaaattcgctaaatcagcaggtgcac

aacttagacggtacggcgagcagtccctgcgcgatgacatccgcgccaccctcggttcacccggttggcgcactttactcgcacagacggatcgcgta

tgggtacgcgcaggtgcacgcgctgccagcggcgtcctctggaactggccctcggcctcgggatccggcgggcccagtccactcgatggacatcgcgc

cagcgacaccgtcgtctccatccctgtgcagacgcgcaagccgacgctgggcgagtgtgtacgggtgtgggctgagttgagccgggtgagggttcgct

cttggaatccggatgagttggctgtgcttgaggaggatgtgagggtggagagggagaagggacgcgctgcggctgagcgcaggaatcgcggtggtgct

ccttccaacgcggcggcagcggcggcgggagaagctgagtctagcattacgaagcgcaaggcggtcgttctctcagatcgagaacgcgcgggacggga

tcggttcctacgcatggtggatatggtgcgtaaggggaaggtcgacgtccttgttgacttcttgtccaagtacgaggacgagctcgtcaagcgtggcg

ggggctggggacctgctcttgccgatggtgatgacaaatcgaatgtcacagaacaaaagaacatcgacacgcctttcccgctgtggtggcgtgctcag

gaagctgggattccgctggacgagctgtcagacgcggggccgggcgagaatggagacacaccggcggcggcggtagaagcgatcccgacctcggtccc

caacaccctcctccagctcgcagccgaagcaggcgtagaagagatcgtgcaatacctcctcatcgagcgccgcgcagacccagccgcaccaatcgcac

ccctacaccgccgcaccacaaccccgccctcatcatcatcagacgcaccggcgatcccgcaccggaccgcatacgacctcgcctccgcccgtgccgtg

cgggacgtgttccggcggctgatggccgagcagccggactgggccgagtgggcgcagatgggtcctgggggtgcgagggtgcctagtgcgctcacggg

ggagatggaggagaagcgcggggggaaggtgaaggagcgccggaatttgttgcgggagaaggcgagggcgagggagacggcgattactgcgaagggcg

gacccgtacaggacccgactcctgctccagctccagctccagctaccatccccaagccgtcgagccagatcgcgaatcggttgggtggagggtcgggc

gggacgaatgcgccgagtgcgctgcggcaggctgtggatcagcaggcggggatcacgccggaggtgcgtgcgcggattgagcgggagaaacgggcgag

ggctgcggaggcgcggatgaaggcgcttcagggtgggaaggagtga

>contig_14.contig_14.g455.t1

atgccactactcgctcactcactgctccaaccttcgagttccagcacagatacacacacagcaaataggcaaggatcca

agatggtcaacacaacagtaacgcgtctggctgagctgacaggtctcgacaacgagtctgtggagtctcagctgctccccttcctcagcaccttcacc

agccccgcctcgctccgggtgcatctcgtcgacctgctgggatcgagcgaacaggcggcgagcttcatcagccagtatgttgctcagcgcttccccaa

tgcaccacgcagtcagccacagccatcgtccagtcaaccaaggcagcagcaagcgaagcagcacggcaaacagaagctcactcccaaacagcgacttc

tccagccagcaaccgcaaaacgagtcgtcaaggagacaccagaggcattcgggcccatcggaaccgtctaccacaaggacaaggacttcgttctcgac

ggatatcgtcgccccgctgtcatctcagcaccgcatggcccggcatccgtccccacccctccgcccaccagcgaaaacgaagccgcttcgacgtcgta

tcagcagcctactctgatagcagcagcagaacagcaacccaccgcggatatacccgcacccgaactcgagaccatcgcgcccacagctgccatgagag

agctcgatgccctccttgcagagctcgtcgtctccgaagacaatcagggatcagcacctccagagctcgtcctgtgcttctgccaaggacgcgtacat

cctctccacccacaacaacccctctgcccaacctgcgccctcccactctgcacctccctcctcccctccccgctccacccgctctcccgctgcccctc

ctgcgcacgctcgccaatcctaccaccaacctcccccctacgcacccatctcatcgcctccctccaatcccagcggctcgcccttgagatcgaggaac

gacagcggatcgagcgcgtgaggagggagaggcgcgccagggatgagaattggaaggaacgctcggcggccgcggagaatgcgtttcctgctcttgat

ggccattcaggctcttcgggcacgtcgtcccctttcatgacctcttcccactctcatcctcccacggggacgtacggtatggccggatatggtccacg

gtcgaatgccgtcgccatcgccctcggcagacgcgcacccacggagaacgagcgtgcggcactacagcagcgcacccatcgtgttcttcgaattccaa

ccaagggcgcgccaccggctaaaagggcgtccaagaaaagcaagaaggtcaaggcggagggtgcgaagaacgcgtcgaaagagagtgctgccaggaac

aacaccgccactccgacagctgcagtcgtcactccagacgaagccgatgaaagcgacgacgacgacgacgcggacgccgacctcggctcgagcgcagt

cgaagtcaccatcaccatcctcgtgccagacacagacgacgacggctggcccgcactcgcacaccaagctccctcctccctctcctccgctgctgacg

tcgacgacgcacaacaggcggccatccaacgacagcgcgccatggctcgtaccgtcggaggtcatcggctcctcgccaatcctcggctggaggcgggg

agtagaccgacatatacggcgcgcaaggagcgcgaggcgaggagggcggcatggctggctgcaggggaggacggaggtgatcatgatgaatag

>contig_14.contig_14.g456.t1

atggctgcgaccgcatacaacgtgctggaggaacagtacgagctctactcggccaaccagcacgcgcccagctcgtcgc

gcgtcaccctcgacacctaccacactcgccaccagcgagcatcaccatcaaatgacaccaccaacaacaacgacgacgacgacgacgacgtcgacgtg

cgcgggccgaacgtcagccagaaagccatcgcctccctcaccggcgccatcgtgacctccgtcttcatgacccccttcgacgtcatcaagactcgatt

acaggtccagggcgcgttcgtccccccgccgctctccttcgatgcacatcatacgcatttactccggccgcagactgccatgggatcagcagcgtccg

ccgcgtcttcttcttccgctacatcgttacctactcggccgggctatcagcatctcaccggcttgttcgatggtgtctcgaaggtgtggagggcggag

ggcgccggagggctctggcgcggtctgagtccgacgctcgtcatgactgtgccgtcccaggtgacgtacatgacctgctacgactacctgcgcaccta

cttcctctccctcaaatcggactccacttcggcgacatatgacaggcggaacggaccgataccgggcacggctgctgtgacggcacattcgctcctgt

cgtcgctcgcagcgggggcgctcgcgcgctctgtgtctgccacactcgtcaccccgctcgagctcgtccgcactcgaacacaagcgtcgtcctcccta

ccatcctcctcctccgcctcctcctcctcctcctcctcctccgcgccctcctcgctctccggcatcatccgtaccctagcacaagaaacggcgcgcgg

cggccccaccgtcctctggcgcggcctaggctcaaccctctggcgcgacgtccccttctctgctgtgtacttcgccggctacgagggtctcaaacggc

tcatgaccggcggtgggttgggcgaggggaacgcgtcgaatcatacggccgagtttgggatttcgttcgcgagtgggagtatcagtgggagcgcggcg

gcgttgcttacccagccttttgatgtcatcaagacgcggttacagaccgaggcgcgcgtcgggggtcggtcttcggagtccccttcggcgtcagggac

gacatcatcaataagaacgtcccgggcgagcgcgcaatcgaggtccacattcgtcgtcgcgagggagattttcgcaaaggaaggcagcgcaggtctct

tcaagggtctctcgccccgcatcgccaaggtcgcgccggcgtgcggggtcatgattgccagtttcgaggtcgtcggacgggcgctcgcctcgcgcgag

tga

>contig_14.contig_14.g457.t1

atgcgcgccatgcgtctcttcggcaagggcgctctcgccctcatcgctgtgtcggccctccttcccggctcagcaaacg

gcctcgaagtcagcatcacgcagctcacgtgcagcttctgcgtctacactgtcaccctgaccgaaggcgaaatcagtcagggttcacagatccggttc

accatgagcagcggcggcgcgaacggacagacaaagccggctgagacttctctccaatccgcgggcatttcatccaccacacctcaaacttcgattac

tacgacctgggccacgcccttcaccagcggtggtgaggcccaagcagtgtacgccgtccttgatgctgggggcaatgtcgtcgtcgatcctgctacca

actcgtcgtccacgtcggaggtcaccctcactgccaacctcggcacctgcactacgactaccagtacaactacgacgacgaccacgacgagcacctcc

acgagcacggccacagctaccacgcctacgacgactaccgcggcccctaccagtaccgcaagtacgatcgcctcatctacgtcgggaactatcaccta

tttcactgccattgcgacggccacccaagggcagagcactttcatcaccgtcatcacggtgacgccaacgagcaccagctctcctccgagctcaggtt

caggccagagcaacggcggagggtcaaatgccggcgccatcgccggaggcgtggtcggaggtgtccttggcctggctctgctcggcttcctgctcctc

ttctgcctgcgtcgcaggaataagcatgatgagcccatggactgggatgggcctagtcagtcgagcgccggagggcccgggcgcggcaagctgttcaa

cccgatcatgcacgacagctctcatgccgagcctactgttcatccgggaagcttggccgccggtggcgcgtttggcggcgcggcttccgggtatgaat

cccacgacgggtatggtggctcctctaccatgggtgcctacccggtgaatggaaacaactacgacccgcaggcgaacttgcaatcgtactacaacaac

ggcccgccgatggcgaaccaggggagaccgatctcgcctccgatgtcggtcgccagtggccagccctcgtatccacctttcaacgcagcgagcccgga

gatgtcgtattacgcgccttcacaccaacaagctccgtacgcgggcttccccatcggcatgacggctgctgccatgggtgccggtgccggtgcgggcg

caggagcgttgggcgtaggaggtcgttcgccgcactcggccaaccaagctctggtgggcagcgggaacacctcgcccggcgcgggcgcgggcccatcg

atgacgagtcacgggttcgaaggtgaactgccgcctgcgtacccggcgggaatggatagcgtcgttcctgggccgacggacgctaagacgggccttca

cacgcacaagacggagggtccttctcgtccttcgacgtcggatccgagcggcggtgcgtcggacgcggctggatcgtcctcagcagcagcaacggcgg

cggggccggcaccgacgcaaacggaaggcgacatctga

>contig_14.contig_14.g458.t1

atgcgcgccacgcgtctcttcggcaagggcggcgttctcgctatcgtcgctgcatcggccctccttcccagctcagcaa

atggcctcggagtccaacttttgtcgctctcgtgcgccgcctgcacatataccgtcttcctgactgaagccgagatcaatctaggttcactaattcag

ttcaggttgaccaccggcggccccaacggagagtcaaaggcggtttcggttggaattacatccagcaacactttcgctacgatcaccaccacctgggc

cacgcctttcatcagcagtggtcaggctcaagcagtgttctccattcttgacgttcccggcgatctcgtcgttgatcccgctctcggctcgtcgtcct

cgtctgaggtcacccaagatttcaacatcggcacctgccctacgactaccacgacgaccaccacgacaaccacgaccacgactacgaccacgacgacc

acgagcaggaccacacctaccagcactaggagaaccatcacctcatctacgtcgggaacgctcatcactatcatcacggtagcgccgactagtacaat

ctctcccagctcggactcagaccagagcaacggcggagggtccaacgtcggcgccattgctggaggcgcggtcggaggtatccttgtcctggctttgc

ttggcttcctgctcctcttctgcttgcgtcgcaagaagaagaaccaggagcctatgatctgggagggacccagccagtcaagcggcggagaggacggc

aagtttttcaacccgatcatgcacgacagctctcatgtcccgccgactgtccctccggcaagcttggccgccggtgccgcctttggcggcgcagctcc

cgggtatgtctcttctacaatggatgcccactcggctaacggaaacaactacgacccgcaggccaacccgcaatcgtactacaacaacggcccggcga

tgtcgaaccaggcgagaccgatctcgcctccgatgtcgattgccagtggccagccctcgtacccgcctctcagcgcagcgagcccggagacgtcgtac

tacgccccttcacaccagcaagctccgtatgcgggcttcgccggagcgttgggcgtaggagggccttcgcaccaagcttcgcccggcgcgggaccatc

gatgacgagccacgagttcgaaggcgaaatgccgcctgcgtacccggcgggaccggacagcgtcgttcctggccctacggacgccaagacgggccttc

acacccatacgagcgaggatcctgccggaccttcgacctcgaagccgagcggtagcgcatcggacacggtcggatcgtcctcgaaagcagcgatggcg

ggcgcggcacccacgcaaacgcaaacggaaagcgagatctga

>contig_14.contig_14.g459.t1

atgcctgaagcgggaagcctggacctcgtcgaccaagtgcatggtggcgataagcaaccgacagaccacgatcgagagg

cgcgtgtgggaagcgggacgcattggggcatgtcgcagccatctgaagctcatgttttgacgtctgcaggcgcagcggaacaagctgccacgcgagaa

actttctcatttcatcgtctcaagatggccgagggcggtgatggcgcggcttcttccggcccttccacttccactggtggaggtggggttctcgctcc

ccccaatctccctcccacttcttcttcttcctcctccaccttctcttctacccagcagatcgtgttcagttctgaacagttccagcagcttttctcgt

ccgcgctctgcgcatacaacaaggacgccgacaaggacgaaggcccctccttcccttccttcaagatcgaccactcctcatcgatcggtgcatacaag

ctctggttcggctgcccgctcacgatccccgacgacctcgtccgtctcttcaacgctggctcgaatcttcctctctcactcctcacagtcgccgcaat

tcgcgacttcggtatcaaccaacgtcgctcctccttccactccaagatgtccggcaagctcctggacgagtacaactcctggcatctccgcgacgagt

ttcttcccttccccgagtggtcgcaggcgatcctcaccctcatccacctcttccgttcggtccgaggccctccgtcagaggacgaggacatcgagcaa

gacccgatccaccagctgacggaacacgtcctccacatctcgacccgggtcaacagctacaactggccgatcttccgggaatacgacaagcgcatccg

gcagcttctctgggcccctcgtccgaagggcgtcggtatgccgttcagtatctcgacgatccacgcgcagactctggacgacgccgaggttcttctac

aaggtcgttccggtgccgccgccgattacggcgcggttctcaactctgcctgggctgcggtcgtgtcagctcctccctcggacattatccgagttggc

aagagactcgccgacctcactcttccatcagctgccaaggtctctgcgaaggacgactcctcatcctccaagacctcaacttctcactctcacacacc

cagctcctccaccaacaacaactacggcagcaatgccaatggcaagcgcaaggcagaagatgacgggcgatctcaatcgccccagtcgtcctttcgtg

gcccgccaaccgctccacccctccctcgcagcccaatgttctgcatcatctgccgtattgttacggatcggcacatctggtacctctgccccggcccg

tacccgcaagacctctaccccaatcctcccggtcaacctcgaggttggacccgtcggggtcagtccaaggacctttgtgccaagtacaatgccggtgt

cccagagcccggtatgctgtgcctccacggccactactgctcccagtgtggccgcgacacttgccgagccatcgaccatggccctcgcgagaacaccg

gtgctcccagctcctcggccacagtcacagttgcgcccactcccaacaccacttccaaccctgtcggattctccggcgccgatcacttccttcatgcc

atcgactccatgccccctgctctcagggatgaatatgcatacattcccaactctatcaccaatggcttctcaatgggccctctctctctcccttcctc

aactcacttccacaagaaccattacaaggaggaccgccaatcatccatcgacaactggttgtcgaaatcgcttgatgccgggttcattgctggacctt

tcgacccggcccaggtggaggcggagattggcccgatccacgcgtcaccgctccagatcgtcgacaagcacgatgccaacggtcggattatcaaggaa

aggatcgtctacgacgcttcctaccccaaagcccgacccggccgaccaccaccccccatcccttccatcaactcccagattcggaaagaggactaccc

ttgcgagtggtttactgcggccgagacaaagatcttgatccgctccgctcctcctcacgcacgctttgctggtttcgacctcaaagacgctttccaac

agctcgggaacttgccgtctcaacgacgcctgttctgcatcaacgtcctcgaccgcatttacgtctggctcgtcggcatcttcggattgacctcgatc

tgcggtctctttggggcgtgctgcgacgtaacttgctccttcctcgaactcctcttcccccttctcctcaccaagcactacgtcgacgactttctcgt

cgccgacttcgcccctccatcttctcctcacgccgatcggcccttcgatctcatcctacacgcggtccgcgagttcggctgggaggtccatccggaga

agttcttctcctggactcggcagttcaccttccttggcttcgagtgggacgccgactctcgctccgccactctcactcagaagaagcaaaccaagttc

cttggcaagctaatcgctctcaacaactctcacgacgtgtccgagaaggaggtggcatctgtgatcggctcctgccaatacgtttgcgttatcgccac

tcatcgacgctccaagctcaacgtcctctatcgcctacgcaactcgttcatcggctctcactctgctactcgtcgatatctcgacactgccagcaaac

gcgaagttgtggactggatccacttcttccaagccggcccagtcacttgctcgctcgatacttcctcttcgttcttccacactccggtcttctccgac

gccagcgacttcgctctcggtgtcgtcctcggttccaaggccctctccatccctctaccccctgactattgcgaccgtcccgacttcaacatcggtgt

cggcgaggcctgggctttcgaactcgctgtccacgccgcgatcgctgctggcgctcgctcctgcgttcttctcgtccacgtcgacaaccagggcgtgg

tctacgccctgcgtcgaggaagatctcgcaaccagcttgtcaacgaggtcattgatcgcacctctgagtacgctctctccttcgacgtcgagctttcg

gttctttacgtcaggtcagaagacaaccctgcagacgctccgtcgaggggggactcttcaggatacgagcctctcatcttcccattcgatcgcccttg

ggagtcgatcctgaacattcgctctttttctcttcgtcggtcctttgagcgcacgcaaccttcttcttcctctactcccctctcttcgtcaacttcgg

ttctcgcttccacttccagatcctcaatctcttcttcattccaggcctctacttcaactgttctccacccttccctcaatcaatccacaacgctttgt

acctcttcatcttcccctgcgctccgtcgcgtgcgtggctccggcaacgctactcgtgctcgggagcacgccgttgctacctttggctctctcgactc

cttctccatcggctcatccgacatggtcgccaatatctgggccaccctctctgaggacgacgttccaactgccgatctctctggcgaggaagccgaca

ggatgcctcgttcggaagcccttccccgtctctctcctgctcggcacgtcgttcgcgcggtcttcggctctctggacgacgacgccatcgatcgactg

agggagttccagctccaacgggtcaagaagttgacgcaagcccaatacgcaggcttcgtcgtcaacttcctgctctgggctgacgacttggacatccc

tctccacctccgtttcccaactccagacaacctcctccttctcttcttctcctcgatctccccctactacgccgaggcgaccctcaacaagttcgtgt

ccggactcaagctgtggcacgcgattcatggtctcccttggagccttgatcgcgtccagggtcgaactatccgccaggccttcaagaacatctccctc

gaccccctcaacctctgtcgacctatccgtatcgacgacttcaaggctatgcgcgcccacatggatcctcatgatccgggcgacgccgctatctacgc

ctgcgtcctgttcgcgatgtggtcgatgtcccgacatggcgagcttactgtccgcacaggatcagaccctccttctcgccgcgctcgacatggtgact

tcaagcccatccaggacactcctgatggccccatcaactcatatctactccacatcccctccgacaagactcatgctcgtgccggttttgaccgggtc

gcttctgcccaggccaacaaccttcccatctgccccgttactgccgtccgctggcatcttcacgtcaacagctctgtccctaaggacgctggtgcctt

cgcctatcgaggaaagcttgggaaactctacgagttgaccagctcccatttcggcaagactatcaacgactggctccgcaaaggtggtcgccccccat

ccaaggacactccttccgcatcggtggtgcgactcttctccatgccaacggcatctccatcgctgacatcaagcaccttggaggatggaaatccgatg

tcgccatccagtacatccgggacattcatctcaagcacgccgagttaa

>contig_14.contig_14.g460.t1

atgcgtcttttcggcaagagcggtctcgccctcatcgctgcatcggccttccttcctggctcagcaaatggcctcgaag

ccagcattaagcaggtctcgtgcggctcctgcgaatacgtcgtcacgttgaccccaaccgaagcccaattgggtgcacagatcgggatctcgatcagc

accagtggggccttcggagagtataaggcggatcagacttctttcgcatccgcgggcatttcatccacccaactttcaactaggctcaccgccacctg

gaccgtgccattcaccaccaatgctgacgctacagcactgttcgccgtacttgacgctggcggcactttcttcgttgatcctaccaccggctcgtcgt

ccacgtcgcaggtcagcgaagctgtcgacctcagcgcctgcactacgactaccacgacgagctcgacgagctcgacgaccacgaccaccaacacgaac

acgagcaggaccacacctaccagcactaggagtaccatcgcctcatctacgtcgggaacgttcatcaccgtcaccacgggagcgccggcgagcaccag

ctttcttcccagctcagaccagagcaacggcggaagatccaacgtcggcgccattgccggaggcgcggtcggaggtatccttgtctcggctttgtccg

gatttctgcttctcttctgctag

>contig_14.contig_14.g461.t1

atgcaagacatgacaggtacatgcctggagtctatctggatggacctcgttgacgaagtgcatggtggcgatcgaggta

agagctcagcatttatcgatcatgtacatagctctacctctgagcattgcacatctttaccactaccatcaccaccgttgccaccgtcacgcactcac

tgctccttgacgcttccgttcaaggcaggcgtcgacaatcccagcccagacaatccgtacacgatcctgggtcttgaccaggaacaagcgagcgatcc

gcggacagtccgacaggcgtactaccgcgaggctctgcactggcacccggaccgcgccctccccgagcgcaagcaagaatgcgaatctcgcttcaagg

aagtcgcccagtcgttcgagctcctctccgaccctgcctcccggcgcgcctatgacgaccgcgtggcacaggaggccgccactgccgagtacgaacgt

ggtatggctgcggacagagctcaatggggtcgtcgccagcagcagcagcagcaatcacaaccgcagcagcagcatgatccttggggtgggtggcaaca

tcagcaccagcaacaacaacaccctttcttccgcccgcacttccaccatcccgcctccgcctccgcctccgcctccgccttcttcggccagcacgccc

aaggtccagcagcatggaaccccttcggcttcccgccctcacacggctttgccgcacgcccgtctgccgacccattccagctgttcgacgagatggcc

ggaggccgcggcgccaactccatctttgctcatcatgcgcgcatgatgaatgacatgcacgcgcaagcatcggcaagggagcgcagcgcagcttctga

cacgcgctcgtctcccatggacgactttttccgcgacttcgagcaggagacgtcccgggagagggagccgtcgcgcgaggacttttcctcccgtcgca

ctcctcgaccccgaaaccagcggggagatgaagactggggtcgggcgcccgcggcaaatcggacttacagtgctcgggtggaagtggacgacgaagta

cccgacatcggacgtctgttcgcccacatcggcagctcgctcctctctgctctgcctgcccttgtcgatcacgccctcggaagctacgtcactcacga

cggcgcttacgacgaacaacagcgcaacaacaacaacaacaactacaacaacaacaacaacaacaatcgattccagtcctcgttctcttctttccaga

gcagcagcagctcgaattacggtgctggcgggccggcgggcctgcgtcggcgaggctcctcgtaccgggcctcttgggaatccaatatccatgctgcg

aaacaacttggactcacctttctctctcccatccgtctgcagggcaacgagacggtcgaaatcctgcacaacgacggacgccggacggtgactgtcaa

tggcttgcccgacagcagtgacggatcgtctcgtcgcgaacgaatcatcgaactctga

>contig_14.contig_14.g462.t1

atggcgatggcgccgaccacgacgttggcgacagcgacagcgacaaggaaagcggaaaggatgagcgtgctgctggaga

cgagcgtgggcgacatcgtgctcgacctcgagaccgatctctgtccgcgcgcaacgaagaacttcctcaagctctgcaagtcctactactacaactat

accaccttcttctccgtccaatcccgctttcttgcacagtctggcgacccaaccaactcgggacagggcggagaatccgtctggagtcatatcgcgtc

gtcgtcgtccgctaccggagccgaccgcttcttcagccccgagatgcccgcctcgctcaaacatcggacgcgcggaaccctgagcctcgcgctggtcg

aacggcctcatcctacgcatacaggccagacgcagctggtagccggaagccagttctttatcaccctcgcagataacatcgactacctcgacggcaag

catgcggtcatcggacacgtcgtcgaaggagccgaacccggcgcagcgcttgacaagctcgacgctgcctttaccgacgatcagaaccgaccccttac

cgacattcgcattcggggtgtggtcgtacttgatgatcccttcccagacccgccgggcatgcgggtaccctctcggacgccctcgccgacccccgaac

agctcgcctccgtccgtctctcagaagccgaggcagcccagacggaagacaccgacttagcacccgaagagcgcgaagcaaacgaacgagccaagaac

agcaaagcggccgccctcacgctcgagatggtcggagatttgccctttgccgagatcagaccacccgagaacatcctgttcatctgcaagctcaaccc

tgtcacccggagcgaggatctcgagctcatcttcagtcgctttggccagatcttgacctgtgaggtcatcaaggataagaaggcctacttcaagatgc

aaaacacgctcatcgatgaccggcgcatctgggtcgacttctcccaatcagtcagcaagcttcacggcgattggctcagcgggcagcggaagggtggg

aggaaagcggctacagagggcagtgctccgggtatgaacgaccagaagcgtccggtcgggatgatgggcggagacgaacagacggcgggaacaggaga

agggaagatggttatcgccgcgacgacgaccgggatcgcgatcggaatcgccttggagaacggcgtcgggaggccagtccgccgccccgaaggcgcgg

ttgtctccttcttcatctccagcgttcccttccccccccccccccccaaattactcactcctaggtcacaggacgacatcctctacggactctactcc

ctctccaacgacctcaccactccccaacacgaacaccattcaatcctttctcagatagcccaaaaacccacaatcacaatggccgacctagagaaatc

taaccacgccggcttcgactccgccggcatcctcgagaacggcggcaagaacatgagccggaccaacaccaagaaccgggtggagctcgaggactcgg

atgagaagcaggcggaacggaggcgtcatcaccaccatatccggaaggtgcacctgcataagcagaagaaggagaagaagcagagggaacacaggcag

attgcgaagaagcttcctgccttcatgcacactgatgacaacaccgatgtcgagaagatgcgaaagtgtctagtcatcaagaacgagattatcgccgg

cgtcgccgaattctgcggcaccttcctcttcttgctcttcagtttcggtatcgctaccatcgccggtcaacaacagctcacggcctcagcacatcgca

atctcggcttcggcttctcgctcgccgtgaacgcgtggatcttcttccgcgtcagcggtgggctcttcaacccggctgtcacactcggtctgttgctc

gcggacgctattacgtggtaccgcgccatcatcttgggcacgatccagttcgcgtctgctattgcggctgcgggtgtggcgcagttgattacgcccgg

tggtatcaatgtgcggacgaagcttggtggtgcgacgacgatcgcgcaagggttcttcattgagttcttctgcactgcgttgcttatgttctccatct

tcatgttggctggcgagaagcacaaggcaacattcttggcccccgtcggtattggactttctctcttccttgcggaattgtgggcaacaggtcttacg

ggaggttcgctcaacccggcacgttcgctcggccccgatgttattgctggtgaattcgacggcaccgcctggatctactacgtcggccccttcagcgg

tgcctttgtcgccttcatgctctaccacgcatccctcacgcatctctcacgcactccccacgcatccctcacgcatctcccacgcatccctgctcttc

tcctcctctcgtcgccgccttcgttgttgtcgtccgccttgtcatcgttcctcatctgcttccttccatctcgctggcaatgccattga

>contig_14.contig_14.g463.t1

atgcgctgcgccggtacttccctcgccctcctcgccaccgcggcaggcctcgtttccgcgcgtctccagccgcttcctc

accagtcccacctcgaccgccgcggcgctccgtccgacgacgtcctcgacatcaacaactacctcgcaaagtactacaacaacggagacaaccccatc

ggacacaaggacggcgacgcgggcggacaggtcgtcgagatcaccagtgctacgggcgccgatgataactcgcagtcgcctcagtgcgcgtcaaccat

cttcgtcggaggagcggcggtcaacggcgcattcaataccggatggcaggccttgcctcagcttactggctttgatctcctccgagacatcgcagtca

ctgacggggacgtcgtgcagccgtactaccagctcagcggacgcaccgcttcccaggttaagcgtatcatctgggtccagcctggtctgccccgtgat

agctggaagtatgtcaacctgatccgaaacgccatgctctgcgctgccgcgaactcgtccaacaacatcaagctctccgaaatcatgatcatggcccc

tgcttggctcaacacggacgacgccagtgctggagcagcccagtccaaagacgtcgtcttcagcaagggaagctggaacatcggctcgctctcccagg

gcccgggttccgccagcgtctcctcctacaccgtcatggacaaattgatcgaggcctccctccagcgcttcccaaacacgaaccaagtctggcttgcg

ggtcactctctcggagcgagctttgtgcaacgatatgccctggtcaggaagcctgacgaccaagatgctaacatcaactactggagtggtaacgctgg

ctcgtacgcttggcccaccaacaacaggcccatcagtccctcgggaagctgtgcaagcacctttgacaactgggcatacggcatcagctcgggtgacg

ctcccatctaccgcaagtcggacgtccaggccaacgtaactgaagtccgccaggaatactacaaccgtcgtgtcgtaattgcccttggcctggccgat

gatggagcaggagactctgcgtgcgcggctcaattcgagggaaccactcacttgtcgcgcggacagaatctgcagaaggagttgcagagtcagcctgg

tggccaacccgccaaccatgcattcgactacgttgaaggcgtcgcacatgaggactacctgatgttttcctctcccgcggcgctgaaccacctgttcg

tcgagaactacaatgtgaagagaacgagctcgaactctggcagcaactctggcagcaacactggcagcaacagtggaaacggcgggaccaagaacggc

aacggaagtgggtcttccagcaacggcaacaacaacagcaacagcaacggcaacggcaacaacaatggcagcagcaacaccggtggcagtggcagcgg

tgcaggtctccgtgctgaacttgtcgctggtgctccccttgcgatggccggcgccgtcgttctggccgtgctcggcagcggactgtgcggtgacagcg

tgagggacagcttcgtcgctagacatcgcgatggtgctccgctccacatgctgcatcgccaggacgatgatcaaccgcgccccagccaagatctggga

ggagagaagccaaggcccaagatgctgcgtgctggctgctgccacttgccagccaattgccaggcaccttccacttcaacgtcaacgccagcacatgc

ttggcggtttttgcactcactctatatctggaacggacgaagagcgccaagccgccataaggcaatgtgcatccgatccgccctccgagactcgcgcc

tcggggcgtgtcactcacttgtcaagccagccagcaaggatcggcaatggggatcgtccagtgttccactgaccgctgacaagggaagaactccacgc

acaggccgcacgccggaaggaccgaccgaagacatgctggctgcggccagccgacaccgcgcctcacggccggccctgattaggaccagatttcccgc

aggcctcgatcgcagactgactatctttgacgctccgtaccatcatcgtttcttttcgatcatcccaggcgacaagttttcccgcctttcccgaagag

ctacccagttacgcttcaacaccatgaaggtcagcgccgccatccccgtcctcgccctcgcagggtctgcatctgtaatggccatgcaactcgaatct

tcgtcacctcaccaaaaccgcctggtccaccgcacactccatccccgcggtcctccctccgacgacgtcctcgacatcaacaacttcttggcccagca

ctacagcagcggtgacaagcctgtcggatacaaagatggagacgctggcaatcaagttgtgaaggccacctcgaaaaagggcgatgacgactcgcaga

caccgcagtgtaaggacactatttttatcggcagtgcggccaataacggtaccaaggagaccggatggacttccttgcctcaggtcactggtttcgag

ctgaagcgggatcttgtcgtcgataagaagagcggggcggtacagccttactatgagctcgcaggccgagacccgtccaaggtcaaacgtgtgatttt

ggtccagccgggactgccgcgggacagttggaagtacaccaacctgttccgcaacgcgctggtctgtgcagcggccaacaccactatgggcgtcaagc

tcgaggacgtcatcatcgcaggtgccgcctggctcaacgaggaggatgtgacagcaggtgcctcccgcaaggacgacgtaatctttaacaagggccaa

tgggcctttggcgccctctccaagggtcccggtgacttgacggtctcctcatacagcgtcctggacgctctcaccgaaaagtactttgatcgcaaggc

gtacccggccctcaaccagatatatatcgcgggtcattcgctcgggggtctgatgacccaacattacgctatgactcggaagccgacgagggacgaac

ccaacatcaatttctggttgggcaaccccgggagctacgtctggcctgtggatggacgaccggccacacctgagaacagcacgtgcgaggacagctac

aacacatggccctatggtatgcaaaacaaggatgagaaggacgtcatcacctacaggagatcagatacattggaagacgtcgaatccgtcaaagagca

atacttttctcgcaatgtcgtctacgctctgggcttggaggacttcggcaacggtgacgaccactgtgaagcacactaccagggagcatcccacctgg

agcgagggcagaactaccgcgaagcactgcgcagcttgcccggtggcgcacccgagagtcactcctttgacttagttcccgacacctcccatcaagac

tacaagatgatctcctcccccgacgccctctggcatctgttcggcgaggatctgaacgtcaggcggaccacttcacgcagcaaatcgggctcgggctc

ggactcggggaagtcgagcaatcgggtgaatgggagcgggaacagcgggaacggtgggacgagtgctgggcagcctcggttctcgaggctgtcgttcg

gaagccttgctggagtcgccctcgcgggtgcgctattcgtcggcatggcatga

>contig_14.contig_14.g464.t1

atggcaagctccggatacgtgtcccgcgttgtaaccaaggccgtcccggccatcgagacctcggaaggagtcggcgccc

gagtgcgacggtcgatcggcacaccaggactgcagaacctctcgcccttcctgatgctcgaccacttccgaatccctcccaaggctggttttgccgac

catcctcacagaggtcaagttacagtgacccttatgctcgagggctacgtaaaccacgaagacttcgcgggacacgccggcactattggccctggcga

cttgcagtggatgacggcaggcagaggcatcgtgcatgccgagatgcccaagcactacgacgaagctggcaaccggttgcctgatccgaccggtcttc

aattgtggattgacctgccgcgggaccacaagtttgacgagccagactaccaagagttgctcggatcatcgctccctacggtgacgccacgcgagtcg

gagcctgaagagacggaggggcaaggatggaagatcaaggtcatcgctggtgaatcgcacggggccgtatcgcctatccgctccccagagaccggcgg

atgcgagtactacgacatcactctccaaccgggcggacgggtcttccagaagctccccaagggctggaacgccttcatctactcgacggccggttcca

tcaaggtcggcgaaaaggagaaggcaaaggtcttcgagaagtatcataccctcgtcctctccaacgctggaccggactcgacggaggacggcgtctgg

ttcgaacacgcgtcttcgggagcggaagccaccgaagaggcgcacttcttcctggtggcgggcaagccacttgatcaacctacggtgcagtacgggcc

cttcgtgctgtgcaccaaagaggatgtcatgcagactcttcaggactaccagctggggcagaatggcttcgagcgggcacccggttggcaatcgaaga

ttgcagccaactttggtcactga

>contig_14.contig_14.g465.t1

atgacgtccgccgcaaaaagatgggtccgacgcaccggccggggcagctcgaaggataacgaggtagatgacgacattt

gggatagaaatgactttgccttcttcgaggaggtatcgcggagccggaccgcctccctggcctctgccttcgatacacacgattcatcgcgagcctct

tactcgccgtcacccctagcagtgcatcgtgatgtgtacaccgacctcgacagccccgggattcgaacgccaagcggcgagacggttacatatacagg

tctcccgcaaggtcaggttcgaaatcaaacatcatcacctccgacgttccgaatgccatttcgaagccctgccgcccttcgcagggcggatgctcaac

ctctcgggtctcccggcgtgcaagatctccgccgaccctcgatgacatcgactcgagccggcagttactcacctcccaatggtgaggtctcaacgcct

tcccgctcgggtgtgcttgcaggcttgaaacgcatcatgagacggagaggatctaagaagtcggacacaacagggtcaccttctttgcagaacagccc

tgttttagatccacatcagagcaggcgatcctatgctgcccatcaggcagagcatcccgactctcggttctttggcctcccaccgagtgcgctggacc

ccacgatcgccaaccagcgcgtgcgcagccccgacgacttcgcactctccacaggtctcggcgtcgcgatcgaggatcccaatgctcagccatctcag

cggagctatcgagcatctcgcgatcgacagggttccgctcccaccctccaagttccgctccgcaccacagcaacgtcacccccgctttctccgcggtc

acgcttcgctgtgtggcaagagcgagttccgttgagcggtccggccttgcgggaccgctcaagtgttgatatctccccagctcgaaacatctcggagt

ggaccagcacaccaccggcttggacgattccgtccccgcctgtaccccgacggcgatcattggcctaccttcagtctatgatggcaacacagtcagcg

cctctgcaggcggacgatgcaacattccccgagcagcaacagctgaagcatcatcagcctcactctccggatgacctgcgctttgaagagtctcgacc

cgtgcgtcctcaacgctcggtcgatctttactttgccagctcaaatacagaccaggaagtcatggacgcattcaatgctgcacacgaccagaaatgga

gcggtttgccatctcgcgaggacagccacgatggccatgccctgcatcattctcggcggaagagtgaggcatcgccccactccatgcttttcgccaat

cttggcacccctggtccgggggtgtctcccgtgccagttgatggccacagaatcgcccggtcacgaccttccacggctcaagatgcaggcgaatccat

ctcacaatcccgtcggcgttctttcacaccttacgatacaagaagaccctccgtagtcttcccatccacttctacacattctgccgccatccatggct

tggtgcatcacggcaagagcagcccagtgaccccaagcagtatctcgcggccactctcggtgtgctcctccacttttgacacgatgtcaatgaacacc

gccaactccaattcgtacgcccctacgttcgcgacatcgttcgcagaacacagtctaccctcgcctcgcacgatggacagcggtcatgggtcaaggag

atatccctcttcttctctctgttcggtctcagacgcgcaggaagagctggagccattgggcgtgcaaggatccaagtcgtaccatggtagtcagcact

accatcagcctcaaccctcaacttacgttttgaagcgaccctcgtcgagcggtacgaacactgtcgatctgtcacatccttcccatgcccacggatat

ggatgcgcagcagacactggttctgctcggcggccgtaccaatcccaggctcctctccatcaaactcctacggcacggccgaagagcgcaacgatgac

ctctcggcggcttggatcggcgggctcgacgctgcaagggatgagcactggccattttcaaggcacagggtcttactctacttgctcccatcctcgac

gcagaccgtcgtttggactggacgtcgcgcctacggcagctgcggcctttgacagcattccgtccccaaacatgatatcggtccatcttccgcgttta

tcgcagtcccaaagccagggcaaacatgcgccgaacatgaacccgtcatccgcgggccagtcggaaccgctctccgtcaagcattgcctggacaacgt

tctgtcgagcgagacaaacgctttctctatgtcaggctcccccaacgccgacgcgtcagaggatgcctccatggctgacattccactcgccgaaccat

tcgagatgagatccccagtcagcgatacgccaatgactccagatctaaatggtgacctagaggagtttgacttcaggctcagtgccctcgtcgagacc

tctagcccagataccctgactgctcgaagcggtgacgtgcaggtcagcaaggattaccgtggacacgaagatgagggtcaactcgagctccttgcgga

cgatttggaaggggacaagatgcaaagaactaggggtggggtcagcgccgaggttgaccaggatgacagtgcggccgatgcggagatggccgtcaaag

ctcccagagcactcctcatccatgcgttgtctaaggaccaccagcgcacacgagagcgacaatggtcgatggacggaggcgggcgtggaacttgtggc

actcctctcgctcatcggacgagcccttccccgcgacagtctttgagccgtcgacgtagcgcacacatcacgcacggttacgcggcgatgaagccttc

gtctacgaacagcccgtcggcgttggcccgcaccaccgacgacttcttcacgtcgctccctacgcccacctactcatacatttga

>contig_14.contig_14.g466.t1

atgggaaagtccgttgcgttcagagacgtggtctacgtcattcctgaacggccagagaagccagtctcggccgtcgccg

ctgcttcagcttggaccaagaactttgccgccactacaaacttgttcagtcgcgtcagacccacgtctgcccacaacacatctggagaaggtgcccat

gagcgcgagagtgcagttgcacagggacggttcgtcgtctcgtcggaacacgcgtccgcaaatggagctgccactcatggggcgtccttggctgaaaa

gtcgggtatctcgtcctcgggatcgagtcctaccggaactcctgtcacagaggaagtgacagcaccgactgcccgtgacttccacgcccgagccaaaa

agttccaaagagcggcatcttccatcagcatcccccgcctgggtcctcgttggctgagcggtagccatagctccagtagcgtacccaccctgggagga

gcagcggcatcaggaacagaaccccacatggcgggcggcatttctgcaccacaactggcctccttacctgcagcagctgcgtcacctcccatcagtcc

gcccgctacgccagaacagacgatgattgccgcagcaccatccgccgttccgaaacggttccagaccggtggggtacatgccgggcagtcgtctggtc

accgccatttggcttcacttcccacccttggctttcccgtaactccgcccctgggtgaaggaagctcctcttcatctaacccgtttgcctcaccagct

gctggaatcctggatcgggcggcgagctcacaagaaatatccgatgtcaccgcgggaagtcacgcccgttctggcaagtcgcctctcaggtcgtgtct

caaacccccaacctctatgttcttcgccgaactcggggtgtttgtccgtatggaccaagccgcgcccgaagagacctccggtgcgcaggatgatgcgt

acttcggtacagctaacctttcagcgccggccactcaagttgggtcaggttcgccaggagccaatgctatcagcgcccaagcacccctcatccgcggc

ttggcgactgacagaccctggcaccgaacggagggacctcccgttttgtctaggcaggccgcgttcaccctagctgctagtagaacgcccgagttgga

gcattcctctcctgatggcttgagctgggccgcctcgatgccgatgccaactcccaccccaggacagcaccacctcgtaccgaatctcctcgctcctc

agcgcacaccttctcctgccgcacagagttcagatgggggccacagtcacgcctcagcggccgctctggaaacgtcttcttccgccggtcccttacgc

gacccagctcaggcagctgacccgcccggaacgtggattcagctcaattcagaatgtgcctgtcagaactgcattgaacgcatttcgaacggcatttc

agcctcgtacacccctacctggtcgcagagtgcgagaaacaagtatctcgccgaccgtgcacaggagcgaaaggtacgcgagcagggcatggatgcag

acatccgactcgcactgaggggcctccaggagacgacggcgaagtggtatgaaggcaggaatattgaagtggtcacagatgcagagctggcccagctc

aatcctgacgacctcctcaacggtcttctcgacgtgcatatggaggaggatgaaggggaggatgaagaggacgaacagtacgtctcgccaccgcccta

cgtgcgtgaagctgtgccggcacaggtcgcgacaggcttgtccccttccacggactcggcgccttatggcaacgtgccggcgcgtggttcatccatct

ctaggaatccttcgttctccttcccggccatttcggactcgccaacgttgagacagtggccgagcaaatctttcgatgcgccttctaccagttctagc

ggttcctgcagtccccatctcggacccgtacaggtctcataccctgtgcggggtgcggcgatcttgcatcatcgcgcttcgagtctagacttcccgcc

tccaagcccgtccgctgtcgcctcggcgatcatcaatacttctctcccagccggacgcaccacgagtgcgaacgaagatccggagctcaaggcagcac

tggccaccccgcccttgtcacgacggaccagcttcggctctgcagcgttccgggcactcaatcgacgggcctcgctcgagtcattacgtgatgcaatt

cgtaggaactcgatcgcaaccctgcggccgcaggctgaggacgagaacagtaacaatacggaactagccgctgaggcggagcaggacgaccagggctc

gggagatgaagactatgggtcagcaaggcagagcatggcaatcgagatcgggcagcctggcgcgatcccactgttgagcgctctggctgcatcagaat

catcatcaacgagggaccacggcaagcaaagcgacgcgaccgagacaggggtgccagcctctctaatctctattccgccgctcgggtgggctttggat

accaccagtacgccaacggtggcgcgccccaatcccatgtttgaagtcctcaagctcgatctcacgcttgcggtgccagggagcgacagtcatgaaga

agaagaaaacatctcaactccagttgccacgaagttcggagcagaggacagagacttccgacaggatcagagcagctccgcgctccaacgcgcttctt

gggctcccactttcgtgatgcgcaatctaatcaaggagcacaaccgccgtgggagcgatgccggcatcctgggcaaacccacaggtgttgtcgctacg

gacaacggcatgcctccagcaagctgggaccgtcggccaagcgatagccaaaacagtgaaactagcatttatgtggacgcacgtcagagctcttttgg

acacgcgacggatgaaaaggtcgaagggttgcgtatcttggcgaacgggatgactctggcggtcgaccggccatcacgtagcgagggtatgcatggca

atgcattctttgcagctgggagcgtcccccctggttccgcagcagcacgagggagtgttgccccgtccgcgccaacgtcaaactcagcatccccgagc

gcgagccggcctcagtctccgcaaaactcaaagttgactctcgcggatcgaagacacaatcgaagagacaccttgccatcggacgtcgaagcggaaat

gagcccatcccttgccaaaactgggctcacgatcgatctgggtccagtggcgaatcaggcggcaaagctgaacaagaaatatgcgcttcacaacccag

cggacgatagggacgcctacgaccagtcagccacacccatgtttgcgcagacgtcagatacgatgcagcagagaaaagtgtcgggcacctatgacaac

caccaccgttccactcggcacaacagtctctcctcgatcgaccaacaggcaatctgtcctgctgcgatcgcccagtacgtgcgccagtcgggcccctc

tatggacggtcctggaaaccggggaattggactagcggccaccgaggctggttggagtatggagagcctgccgatcccgcccagtgtgaacagtcacg

gttcggcctcagcctcggtggagggtctgcacgccaatccatctgttgacgtgggtggccaggcacgtccaggcggaggtgcgacaacagcagcgcag

aacgacatggagacactgatgtcccctgttgcaaagcccaatccgactcagagggattccgagtcaggaaacggcagaaacagtcgaactcattctcc

agattcaggtcgaaatgtcggcctcgggttgggcaaacgggtcaaagatctctttggaaactcgcgaacgcattccaggagctttttcccttccaata

tcttgtccagcccactcagcagcgggttcggaggatccggcggaggcgatggaagcccgcttgcctcggaagtcgactctgaaacttcgctgcctagc

ccagcaacgccgggcggaagcagacccggccgcttcgacaagtttgcttcagctgtcaggcgcggccgtcagaacccttcggatcgacccaagaagag

tctgagcccattggcgccccaaatcaattactctcctcagtgtgatgcaggagccgcaggcaacggcggaagcagtggctcggacggattcttcccga

tgaacaaggtccgatctgcggagccgctgtctgccatgtctaacccgaagtcgaggtccttcagtgggtccgctgttcctaccttaccgaccaaggag

aaagaacctgctgcgccaagctccaaggaaagcggaggatggaaaaagcccaaacttggtcggagctcctccggcaatgcgctccgcgaagctctcaa

tggcgtcggaggcaacagggacagccatgaagccaaaggaagcgcctcccacgtcaacagtatggcatcgggcgacaggcctggtacaagcggtgcgg

ggaacaacaacgtcaagaagtcatccacctcctttgctggcgcgctcttggccacgctctcccaaacgggaggtggtggcgtgccactcccctcggca

tccgcagcgtcgtatcagcgatcgtctgctcctgcatcgaggcaggcgcggcctggcacgtcggacggcttcagcggcgggttcggtgctggtggtgt

tttaccacgatccaagtctggggagcggtactcgcacaggccggcacaaagccagggcgccttctactga

>contig_14.contig_14.g467.t1

atggaggtcaatgatcggtgcggtagtcgggccagacccagaccagatcagcatacagataagataagcacagcaaaat

ttctgccaagtgaccaaggcgcaccaggcccctttccgtccacaaccaccagcagcttcccatccttacttgaatcaatcatcctccgtgcctggctg

ctcctgccctgcccttgctggtcgactgtccctgctcggagtcctcagtcccgtgcctgtgtctcagacctcctctcgcgacgtggtaacctcagaga

ttgcgctctgatcttgctgggctttctcggcggtgctcgcttgccaactctctcccgcgcgtttggacgatcggaatcggcatgctcgcgcgtctaca

ctgctattgcaaacgaaatacatacaacatggtctcatctcctggacattcgccgcgcccatggctctcttctgcgtccgcgtcagctccgtcgctac

gcgaactcgatcgcagctcagcagtgtccgctcgataatctatgggggttcatcgacggcactgtcctggccactgctcgccccatacgaaatcaaaa

ggaatattatagcgggcacaagcgtcaccatggtcacaaataccaggtggtcatcacgcctgatgggatgatgtggatctttgggtcagtggcagcgc

gaaggaacgattctttcgtgctacaagacagcgagctgcaccagtggctagatgagcactcaagggacgagaacgaaaaggatctcctcatttttggc

gataagggctatgccgcgcgcggacacctggtggtcccacacaagggactgttcctcacgcccgcccaagaccaattcaacctgcggctctccagagt

ccgcattgccgtcgagtgggctatcggcggcgtgaccaccctcttcccgcgtctcgacgtcaagcgcgcgcagcgcatgctccacagcgatctaccat

cgcagtaccgcatcgcggtgctgctgcggaacgccctgtcttgcgtctcttcgaaccagacgagtcagacctttcaatgtccaactccaactctggaa

gaatactttgtacctcagtag

>contig_14.contig_14.g468.t1

atgtcgatcccatcgatgtcccctgacgacagcgccaatccgctaggaattccgcctcctgcgcctcctgcgcctttct

tctcggcctcagcagctgcgcaatcaaactcccctcttcctcattcgcagtctcccggagcctcgcgctctaccacgcctgacagccagctttcggtt

cgcagcattgctcggcggcgtgacgaagaagacttggttgctgctgcagaagcaaacgctggcaatacaacgatcgaggatgaggaagaagaagttcc

aacgcaggccagcatcgtggacaaggtcttcacagatgcagacaaggccgcggagaatgaagaaaagaaggccggtcgtcaacagggctcgctgcagt

ggaatggaccggaactgcgagcgctgctggtcgccatgttctcccacgacgttttcgacccgacacggactgcggaccagcgcagtcaatcgtggctg

gacgcggtcaaggagctcaacaaatggaacgaactcaatccccgcaagaacgggcgtttttccgcgggtcagagcgcgtccgagatcgcgagtggccc

gaaggaggacgagcctcagataatgaaagcgtgtcaacatttgaaggagcagtgggaacagagcgtgcgagatgaggcggtgaagcgggaagcgaaaa

agaaggccaagaaccccgacaaggccaaggcactcgacgcacgggacaaggccaccgccaagttggacgagccctccaacgtcgagggagccggagac

gtcgcccgcgagcgcgacacgagcacggaggacgacccgctcacggccaagtccgacggccgcaagacgaccaagcgtctccgcacgagccgcgccga

cggcgccagctcggagctcaccagcgccgtcgagagcatgggctcccggcagctggaggcggcggcggagaccctcaagcagcagcagcaacaccacg

aggaagcgctccgccaggccgagcgcgcacgcgagctcgagcagcggaaacacaaggacctgctctctcgcgaggaccggaggctggatctgcaagag

gcgcaggcaggccaggaggcacggagggatgcgcgggtggctgctgtggagagtaagattgacgatctcttcgcgttcctgaaggagaggctcgagta

g

>contig_14.contig_14.g469.t1

atggccgccttcgcgaacccgcaacaatcgctcggcccagcccagccaggacattccaattcaatcccccacaacaaca

atggcgctgggatggtcagtctttccccaccgaccgaccgcgagctcgtcagcttcagcacaaaatcggcagagaccttcgtcagtgcgtactacgcc

gccacagactcgcccagccgggtacgactcctgcccaacttatacctgcctgcggcctcggtcgtttggaacgggacacccgttcctcccgctgcggc

ctccccggcggatggaacccttcaacttaacggcggcggcgaggtggtcaagactgttggtggagcaggtggcgggttggccgctttgctggatgcga

tgcctgggtcaaaacacgaagtggcgacctttgatgcacatcccatcggatgggggataggagccgatggtatacccgcgcttcgatccatcatcatc

aacgtgtcaggaaccgtacaacatcacacaccggccgcggcgctcccaccgtcgaacgcccccaagtcctcctccacaacgacgaccgtgagcgcgga

ggcgatgatggccctacctcgcatgttcagccagtccttcgtgctcgtcgacggagccggaatgggcaccgagggcggggtccgagtcgtggaggaac

ggatggccagggtggattcgggcgccgggccttcgtctggtgctggtggagcgggctcgacgcagaaggtaaccaggacggcgaggtggctcgttcaa

tcggattcgttccgggcggcccaaagggcttctcagctcaatcagatcggcaatgatgtgcactcttcctgtgccatgcctatgcgatgttcacaagc

gcttgcagtggaatggaaaggctag

>contig_14.contig_14.g470.t1

atgctttctgcaccagcaccgtatccgacggtgtcctcctccatacccgcgccagatgatgctcagatcgtgccctccc

atcggatgccgtccatctgcgtcgactacctgtcccacaactgggctcgcgacgaagatgtctggaacagctggaaggcaatgaccaagtccaaaaac

gaaatcgcaaacggagtacggctggagaacgcttcctggcgcacatggatgaagcagatgcgcaatctcaagactgtcagcccagagacattgaactg

gctcaaagacagtgacgtgacatggctatatggtcctctgcatcagggtcaaatcgaggccgtgcccccaccaaaggtcgcttccacgaccgaacgtc

tcgaccttgtcgagaacccgcgtcaccatacactcaaaaacgcaaactcagcctcggccgcctctcttcctcgcgtaaagctgtccgacctcaggccg

gacagcagcgcgggcaagaagccgtcctttaccggcgatgccccctcgccatccctaccgcagaagcctcacagtgccaacaacaccatcaacagcaa

gatgatgggcaacggtatttcgaaagccttttcccccccgcaccgaggtccgaatgacccctccctctctccgcccgccaccaagtccatccttaagc

atcgcaccatccaagatctccttagcaacccccaggcaaacagcgccgcggggctgggatccgctcttggatttaagagcgcgtcccaacctagtctg

cccgagctcgaagctgccgccgctgctaccagcctcgcccttcgcgagggacaaggcgaagctgtgctctcgcctgaccaggcacgagctcttggcct

tatgggcgttgggaacagcaacaacagcaacagcagtagtaccaccgcttctcccgccactagccatgtcggtctgagtgctattacagatcatgtgg

tcgccacatcacctcccaccatgtctgcctccctagccgacggtgcgatcccaccgccacacgcagaccagcagtccggatcctcgatctccctcgcc

agcaccagtgacaccgacgcggagacggcttccattctcaaatcacccaccgcatcagccgcttcccttgtttctctggcgtcgtcgacgggaagcgg

gactcctaacacggctcgctcgtccgcagtcggactgaacaatgccgccaaaaagtctgctgcccctacgcgtgaacgcagacatatcagcttcaatc

agcgggtggaacagtgcatttccctcgatatggacaatgcgccagagtctctatcctatcgtgacgatgagtatgctcatgacgatgatggcgtcggt

ggcagcggtcgcagaggtatgcatgcaggagattatggatatggcgacgacgacgatgatgaagatggatcgatcatgtggcgtagtgggcccggcca

gcgagttcaacggcgaccccgattcgacgatgacgaagaggaagaaatgctcaccattaacgtccgctcccgaccgagctcgagttcgggagggagca

gcacctcagccacttccctatcgactcgctctcacaggtcgacgcacagcatcagccgacgaaccattgccaaattggcaccgacgatgctccgtggt

ggctccgtcctgcccgacacgggcgagtggatcgatcatgaggagtcagatgcgcacgacgtggatctgaatgcgggagtggggtcggacatgatctc

tgccggtgtcggcgtgggggaaaacgcgcatgtaggggcttacgctcacaaccctgtcgtcaatgttgatcgggaacggcatggtgggtcatcattgt

ctggcggtggcgggaatagcgctaccactggatcaggcgatagggacgctcacgatgcgcccgcggaagcgttcgatggagtccggtctgggtcaaat

gatgatgtagatgaggaagatgaacgcggcagggcgccagagcccacacggacattttcgtacggacgcgtccatcaatctgggcggcatacaagccc

tggctccggacctcaacagaatcactcacatcacgcatatagtggaggcgacgatcatcacgacgactcgtacgaaaatggttcggatggcgggtctg

gttgggacaacaaccgtgcaaatctcccttcttcaggccaggaagaggatgaagaagaggaggattggcagagggaaaacgagcaggagggtgggcga

tatatccgaggaggggtgcctgcggatgatgtcgcgttacggaacaggaacaaatctcgacagggcggtggatccgcaggaagggaatcgaagagcaa

gcagggctcttcgggtggcagtcggtcgcgaggagatggctcggctggggagtctggctctgagtcctgggctgatggagatggggatggagacggtc

ttgatggctcgtcgcatcaagccaatgctaagtcacggcgcgcgcgcgctgaaaacgacgcgccctcgtcgcccgcttccacgtcttcgtccttctcc

tcgtcctcctacaaggacaagtcgtccccacgtggtcctggcgaacgcagacggcagtcttcgaccgaggtggcgttggaggatagagctgtgcatgg

ggtgcttagcatcgatgtggataatgtgccgtcgaacgatccgaacagtccggtcaaccctgctcatggccccacgcctttgaacacacctacgtttc

tgctgggtggcaagccggggaaaggcgcttcgccgtcagcgaagtcggcgcataagtcgcgtcgaggcggtgggagtcgaccgacgccttcctcctca

gcttcggcccacgacaacaaacatggtcaccggtcgacaacttctgcatccacatcagcgccctttgatgacgacgatgacgaggacgactacgaccc

tgcgggcgcggtcgtgccgcaccgacggacgtcgacgggtgcgctggtcgcgccgaagaatcggcaggtgcaggtaccccttgcgcacgactatgtcg

atgagggtgatgagggcggcttgatcaaccgggcggtggagatgatcaacacggctagagatctgatcggtgccctgtggggtgccagtgacagcgac

cgtggtcgatcatggcgggactcgacttacgaatag

>contig_14.contig_14.g471.t1

atggcttcttcttctgcaggaccaagccggccgacggagacggagagcatcgcttcggaggctcagcagcatggagatg

aacccagcgtgtcctcaccgggggattctcaagctgccgtcatccccggcaccgccaaaccggaaaccccgctgcccaagctacatcttgccatcgtc

ttcctcatccgaatcgcggaacctatcaactttgtcttcattctacccttcatcaactcctatgtctgggacctcggcgtcacccgcgatcgatcccg

ggtcggtatttacgcaggcatcatcgagtccctctttgctgcctcgcagttattcactgttctgcattgggccagtctgtcggaccggatcggcagga

ggccagtcttgatcgtgggaacgctcgggttgacgctctcggctgcgctctttggttttgcggattcctttgcgatggctatctttgcgcggtgtctg

acgggactgctgaacgggaacgtggccctccttcgatccatcatcggcgaagtatgcgacgagaccaacgtggccaagggattcgtgctccttcctct

cgcctggaatgcaggtgcctccatcggtcccctcctcggcggcttcctcgtgtacccggcctcccgcttcccggcactcttcggcaacagcgacgcac

cattctactgggacgggttctggctacacaaaccatacctattaccctgcctcgccgtcgcctgcctcagctttgcgagttttctggtcacgctcttc

tttctgccggagacgttaccttccaaagtggccgaactgaaagagcgggaacggaaacgaatggaaagcgagctggaacgggaagaggaagagacgcg

cgtggcggtggagccagaattgaggaggcgaaggcagagggctggtggtgatcgcgaagagggcgacgacgagcggacaccactccttggtgatcgcg

ggctcgacacctcggcatcgcacgcatctcctgataacaggaacgctgacatttcgactacaaatggcgctaccgccgccgagccaatagaggacgac

tccgataaagacgcaccatcatcatcatcatcatggtcaatcctacggatcccacacgtccgcaacgtcctcttctccttcggaattctctccttcct

ctccgtcggcatcgaggccgttctggtcctcttcctctacgaacccctccatctcggcggcttgaacttcacccccgcgcaaacgggcgtctatctcg

gcttttgggccatcttcgcgatggtcgtgcagatgctgtcgacagcatacctcgtccggcgattggggaccaacaggctctattcatattgcttgctg

tgctttcccgtcatggctcttctgctcccgctcgcatctgccgttgctcgatggggcagtcaggggcacgtcggaggggaggccgatccgctgcctac

ctcaacaatggtcatgatctacatcatcctgagcatcttcgcaatactgcgatgctgcagtgcatttggctacgtgtgcaacctcctcctggtgaacg

aatgtgcccgtctgatgcccgggtcgcgtctgggagcagtaaatggcttctccaccatgtcaagctgcatcgcccgcgccctctcgccgtcgttctgc

accttcctcttctccctcacggtctcccccgacaggtacccgctcttccgcggctggctcgtatggggggtgctattcgccattggcgcggcggcttc

gaccaagaccctcaagccgatcgaagttgttgtcgcggaggaggaggcgaggcgaaggaagaggaggagtgaggttagatcttag

>contig_14.contig_14.g472.t1

atgcctctcttcgtgctctcccctgcttcgctcgcccactcggccctcttcgccggctactactcctacctcgccgcca

acgtaatcgccctccgattcaagaccggcatcacccttggccagggcgagagctcgaccgacatccgtaagacggacaagaacaaggtacccaacccg

gctgccgaggttaagctccgccaagctgttcgcgcctacggtaacttcatcgaaggcaccccgctggcgttcctcctcctcttcattgctgagctcaa

cggcgcccccactgcgctcgtccacggcgccttcacaacgctctttgctggccgtgttctgcacagcgtcggcatccgggcagaggactccgtcggct

tcggtcgacccgtcggcatgatcaccacaatcaccataatcattggcacaggtctgtacaacctgcgtctcggctacgagcctctcaagtccttcctc

ggggccaagtaa

>contig_14.contig_14.g473.t1

atggcttcggtcgttgctgtcttgcggaacgtcttcagtcgtccgctcttcctcgaactcgaaaataagggaagcgtgg

cgagagaccatctcgctctggagaggacgtttttggcctggctgaggacatctctcggactcgtcagtcttggaattgccgtcgcccagctcttcaag

atcccagatctcttcgacagcgatcaaataggagccaagacggacgcctctcttctttcctcgcttctcaaagcacccaccacgcctgcggaggaagc

cgccctctcccagctcctggcttcgtctcagccccgccatcaacatctggccagactttctaagccactgggtgcatcgttcatcggactcgggatca

tcgtcctcattctgggatcatatcgatacttcaccgtacaagcaatcctcactcgcggaaaattccttcctagccgagtggagattaccctcaccgcc

ctggtagcaggggcgctgatcacagcatgcctggccgttgccatcggaggtcggattacgggcgcatga

>contig_14.contig_14.g474.t1

atggccatacacgctgcttacttcccaaattgggcaatctacgggcgcaaatacttccccaaggacatcgatgcgtcca

acttgagtcacatcctctacgccttcgccgacgttcgcgcggagacgggcgaagtcttcctcaccgacaaatggtccgacgaggatattcactacgac

ggggactcatggaatgacccgccaggtgggctgtacggcaacttcaagcaattccaaattttgaaagctcaacatcgccacctcaaactcctcctgag

catcgggggctggacctacagcgcgcacttcgccttcgcatccgatccccagaaacgaaccgcgttcgtccaatcggccgtccgcctgctcgaggact

atgggctggacggtctcgatctcgattgggaatatcccaagactggcgacgaggctattcagctcgtcgaattgctccgcgagctgaggtctgcattg

gacgcggcggagaaaatgcggagtggaggcgccaagttttggctcacggttgccgcgccgtgtggtccgcagaatgttcaagtgctggatatcaaagg

aatggagccttacctcgatgccttcatgctgatggcatacgactactctgggtcatgggagagcatagctgggcatcaggcgaaggtccgtggaccgg

ctccatcttcagaagacgccgtctcgcgctacgtatccgcaggcgtgccaccatccaagctcgtccttggtcttccgctctacggccgtgccttcacc

aacaccaatggacccggccaaagcttccaaggcgtcggcgaaggctcattcgaacaaggcgtgtgggactggaaagcgcttcctcatacaggtgcagg

cggcgagagcctccctgtgcaagaggatatggatctgcttgcgtcctggacgcatgatgcgcagatgcagcgaatggtctcgtttgacacggcacgcg

tgctcgaaggcaagatggactgggcagcgagcaaagggctgggaggtgcgatgtggtgggagctcagtggtgatcggataggcgaggagagcgcgatt

cggacggtggcgagaaagctcgggaccattgaccagacaccgaactgcctcacctatcccgaaagcaagttcaccaacctccgcgacggattcgcata

g

>contig_14.contig_14.g475.t1

atgcgtaccacagccatcctcccagcggcgtgcctgctcctgctgagtctggcgacctcgactctcgcctcggccgcag

agacagacccggagctcaacatcctgaccacgtttgcgaacgacttcaacattgtccggaatggggagcgtaaccgggtcgtgctgtccatcacgaac

cctgccgaccgcgcgctctcgctggaaggtatcacgggtgctttcctgaacaagaacaagaaggacggccagaaggggcgtgtgctccggaatatgac

gacgcagcccttcaagtcgctacctctccctcccaagcggagcaagccgcttcaagtgcccttcgacatgtaccccgaattcaagcccggagatgtcg

agattgagcttcgcgtgcttgtcacggacaacggctcgtcgcgcaagtacaacttgaatgcctaccagggcaaggtcaaggtcgaggagcccccaaag

aactggtttgacctccagctcctgtccctatatgcgatgctcatcgccggattctcgtatgccgggtacgtggcactgtccacctacttcacaccgcc

gcaatcgaagcgggaggccgcccgcgctgcgtccgcgaagaaggcagaggcacctgttgccgccccggctccggtcgcttcgagctctgccaccagtg

cagccggctaccaggaggagtggattccggagtctcacctcaagacgccgcgaccgacctcgcccacacggagaaagggcggcaagaacttgacctcg

acggcgcgacacacgcaaagtcgttctttgtcctcgtccttctctccctcgccctcgccctcgccctcgccctcgccctcgccctcgccctcgccctc

gccgccctcgccttcgtcacgccgggacggagacctcggagcattggactcgacggaaggcaagtcacaaggcacgttcccactcaggagccagccag

ccagcaccgacagccgcgggcagaactatcgtgacccgcgacgtcgcctcatctcgtatctgaagaaggttgccgtctaccatcagcccccgccccat

ccccagggcccgaatttcccaggcttctactcgccaaagggcaatctgatctcggcgctcgactcgctcatccttccctccagcgcgcatcgagccgg

cgcgacctcgaagaaacgcaggcccttcccccgacataagcagatcagcgtgagccgtggcggagggtctatcggcaccgcagcgcgcgggggccgta

acggtgccgcgggcggtagtactctcggtgcgaactcgcgctggtcgagtggacctccccgccagctcaaccccaacaccagcgtcaacagtaacact

actgccgcccccaacaacaacagcaacgccagggcagcagccgtacctccgccaaatgctaacgaccagcagcagcagcaacagcaacagcagcagcc

aacgcccagtcgcgcaagtcctgctgctcatgctcctacgagctcccaaacgcctagctcggcttttcccacccttggcgccgcgcaaggcgagaata

cgcaggctaagatgcacgagcgcatcgtctttctgctagcttctctcgttggatccatggtggtcgtgacggcccgcggcggagccaagtacgtcggt

ctcctctcggccgcctctaccgagcaaggtgagctgggcatatccctcgcctccgcccagcagatcgtacctggtgccagtgccgatgtcgaacctga

gctcggaccagcgaagggtgcggtgatcgtgctcggcagggacctggacgagatcgaagcatatgatgtcaagtttggagacccgctcaaggagaccc

gctccacggccgaacgagacagcttccgcaccgacacagatatctccaagacccagttcgacgctctgggcggtggccgtgtgctacagaagtggtcc

gacgatcctggcatggatgttgccgaagcggcccgtgcggcatctatgggtctgaacggaaacccccgcaacagcaccaacagctttggcgggctgga

ggaagcgcccggtcccggcaagtcgggtgcgggtggcgcatgggaccagtttgcggtcaacgagcgacaatttggtgtcaagtcgaattacgatgagg

acatctatacgaccaagttggatcgtagcgggaaagacttcagagaacgcgaacggaacgcggagcggcttgcgcgcgagattctcaacgctacctcg

aacaatccgcacctcgccgaggagcgtaatcaggctacggagggtgctgccggagcggatgaggaagccaaatacggggctgtgctacgcggtccgaa

cgcttatgttcctccagcagctcgcagggccggcggagtcggtgcgctgagcgcaggactcaagcccacgtcgcctttggctacgcatagtgctgcgg

ctgctgcgaagccgacagctgcggcgacgcctgctaccgcaattccaccgccagttgcgtcgggttcaggtggcattaagccacctaccgttgttttg

caagccagttcgaaggatcacactcccgagctggctccgaaaaagattgcacccggcgacgtgtccaaggagttctctaaatttgtcagctcagagcg

cgaacggatgcacgcgaaacgacagcagatccagatgcaggtgcagaaagttcagaaggaggagatgaagaacgagctgcaggcgctcaagtcctggc

acgtcaacttccagctcaaggcgcccctccccgaaaacttggaaagccctctgcggaacaagaacaaggccgacgtctccgacaagccgcgtgacccg

aacctgcagaagagcctcagccctaacgtcacgcacgcagacgctcccgggcccggcgcacacgtatcgcttgccggtgccactgccgaggcgaacgg

ctcccgactgccacctttcaaccgcaacactgcaaatgcatcgagctcgagtggcatgcgtagaccacctggttctaagatccagattgcggcgatcc

cgccctttgacccggagaagttcaagtctcgtcaggctgcgtcgaaaccgggcgttcccggcagcagctctggtggacctacgtcagcttcgagctct

gctgcggccaaaccgacagccactgcagcagcgccggcctcgaattcaactggcgcaaaggctgagaatgctgcattgagctctgcaaagttgagcgc

caaggccagctccttcaagccgaacttcaatccgaatgcgactgctttcacaccaggtgccccggcgaagaagacagagaccccagctcccgctggtc

ccgccgccgccaccacagcgccaccgcctgcgaatcccttctttggcaatcgtgtcctcaaacgccctccttcgcacactccgctgcatgttcgggaa

gaattcaatcctttcaaagtcagcaaggtgccggaggctgtctctatctctcccgaatggcgattcactggcaagaactaccgtcagttgttcacagc

ggtgccgcctcccgccacccaagtccctgttccgatggcgccgcacgtagaggagaactttgtcatgcctccaccgcattcgatgcagcaaccgcagc

cggggatggtgccgatggtaccaggtgcgcaccctcctcacggtccacctcccgcgatgcaacatcatccaggaccacaggtaccccactccggccca

ccgacccacggtggtgggcccccgcagcagatgggcatgcccttccccggtcaaccccatttctacccccgattcccacagcagccccaacacttccc

tcagcagatgcccatgcccgctggccaaggtatcccctacggtacgatgcctcctcaatttgtcggtcagatgcaattctctcctcccatggccccgc

acggcgcacatcccgtatacagtccgcagatggccaatatgcctcctcctggtcagcacggacaatttgtcccgcagcaagggcccatgcaacccatg

ccaggtggacctcatgggcctggtggaggacccccacgcggcggacccggcggacccccgctacatatgcagaagggacctccgcagatgtatttcca

gcagtcaggacctgtgcacgccatgccatacggccacccttctcacttcattcctggcggacctggtggccggccggggatgcctccgatggacgggt

cctaccctggtgtgggagggccgcagatgggcgacccggggaaccacatgtga

>contig_14.contig_14.g476.t1

atgtcggcattccgaccgagcttcgttgccatgggcggcttgctatggaagagcccatggcgcctttcctcaacacgca

agatgcgcgttcgtgagcgtctcaggaacgtcgacgaggtgatcgatactctccgaaggtcacaaatctcactcgctgctctcgaccgggcgctgctc

ctgccgaaggagtccgagatggagcccaaggacaagtacacgacattcagtgccgaagtggacaagattaacactgcgcgagaacccaagaggattct

agggtacatggatcatggatag

>contig_14.contig_14.g477.t1

atgtccggatccatcgatcctggcacgctcgccttcgcagctgccaataatgcggcccatcagcaacagcaacagcggc

agcagcagcaagatcagatccatgcccagctccagtcgcagcatcaacatatggacattccgctcccaacaccgccctcctacgcccagtcacagccc

actcccatcaattccgacacgcagcaattcctggaggcccagcgtcagcaccagcaactgcagcatcaacatcagcagcatgagcgacagcagcagca

gcagcagcagcagcagcagcagcaacagcaacaacaacagcaacaacaacaacaccaacagcacttgctacagcagcaacagcaacagcatttattac

aaagtaccgcccatcagtactcgaccccctgctatcccggtgtgatggtccctcactccatgccgatgtctgcgcagttgcaacatgttatcgcacca

tcagctcaatcacagctgcctggcgcgtcattaccgccccaataccaaccacctcattcagcgcccgcgcacagtcaggctcatcacacccaacattc

gcagccgcgaacaccgccatcacagcagcaccattcacaacagcagcagcaagggttgcagcatagtccgcctttgaatcagcccaatccgtcggctg

cccagcttcaggttgcattcaacggtatcatcgcgtcgccgccacggccgagtccagcagtcgaacagctgcagcagcagcaacaacaacaacaacaa

catgttcacgcagagcagcgccaaatcgctcctctccctcagcatcgtggccatagtgcgtccaggcgaaacaccaacacggaacaaacacgtcccgc

actgcctccgatcgacattacacagaccagtgcggcagtgtttcaagatagcagcaatcttgcttccctctcccaaccgccgtctgccatcgttccca

gtcaaccactgccgttttctgatcaaggcgttcacccggcttcgtcgcgtccgatcaacaccgggcccggtctggagtcgtacatgctcccgaccagt

ggcgtagcattttcgcagcatgtcatgacgatgccgttgacaagtgcaccacaaagttcaggctggatgcagggtgcgggaatcattccttcgcatac

agtgatgtcatccgaagcacagccgatcctcgctccgtcgcttctcactccaagcacagttgaaggcagcataactccttctggactttctgacattg

acagaacaagcttgcgcctcgatgttaccccaaaagcggagccatccggggcgggcattcctacggaggcaatttctcaaatggcctctgggatgttg

cccgcttcgagtgcaacactgggcattatctctgccgatgccaatgtgggatcgctcccaccttccgcgaacggctacttgaccaacacgatccagcc

gcccgtacaaagcgccatagcacccgctacccaaccgatcatgaccactcagctcccgcatgtcgcgcacgacaccccgcagtccattgatgcgtctc

atccaggcgcgtcacaacaccggaggcagctcagtcagcctctggctagtcagatgatgaatggcacctttccgccctttacgcctgcccagaccccg

acacgtctgcagatgacacaggctcatatgactggcgccactggtacccatcttggcacgctcctcaaccagatggcgcagatggctattgctgcagg

caataatcgctcgggaaatcagctcccgttcggttgtgtggaagggttggaacaacttctttcgcagatcaagcaaaatcagttgccatcactgccca

gtacgatctcttcggacagctcgatgcgaagtcgttcgggctctgcatacacgaaacacagtcataacgagtccacttctactctcctctcgagcgaa

ggcagcccccagcgtccgttcgatgaggaggctttcgagcccatgaagtcgttgcgctccgaaaatggcactccgattcatccacctcctccacctcc

tgcttccgctccgcccacggccctgcagagccccgatcagggcactacgccggtatctgctctaccgcccaatctcagctcgcagccaccgagtttat

tgcacaccatgtcggaatcgcaggtgccgcagttgcataccttacagctgggaaatggtgaccgcggtgggcacacactgcctggtggcggggtggga

ctgggaagtcggcccatggcttcgcacatcagccatcggttagcgacaacgaaccagatgagcgcgtcgacggcctctctgggctcaatcatgccgga

gatgcaccagagctcacatcaacttggactggctgtacaatccgttcctcagagcccagtggagcagcagcaacatcaacatcgtcaagatcgatccc

ttgacatggctgcgatggcctttcagaacagttccgcacagcacatgggagtcggtgcagacggccaagctcagcccgtgccttccaatggcggacca

ggatcgggcgtgtactctgatgtacactctggggtgcattcacccattggccccagcttacgacatgtgctcagccaaaactcgctcaacatgggctt

catgcggcaggcgcagatggagcggagcggcgcaggcagcgactcgcgccctttctcgctcccggtcacgcccgcccatccgatgcagcaatccagcg

aagtgcaacatgcgatgcggccgcctcaggtcaaacatgagactgcggctctttctacacccctgcagcaatccttcacgttgccgccgttgggcgac

gaggaggagggacactacgacgagagcatggtcgacgattacgctccaagcagtgcggcgtcctcggtgatcttcaagccgaacccaggagcttcgtt

cgtgggaagcgctgaggacaaacaggcgactctacgagcggcgaaacaggaggaggcgagtcagggcagtctggctggcgttgatttctccatggggg

acgacacgggtgacagcggggagaagaagaatggtggcgcgctcatcccgatcgagccggagcttcaagacaagctggaagtcttctttcgggacttc

ttgtcaaagatctgctgcaacttggatgcaaaggacaaccgaggggaactgatacatcaaacgctcatgccgaaaaagatggcacgcctcgacgaatc

gctcgacttccgacctttcaagtttcgcattcaggcatttacgaatgcgttctatgccgatctacaggctcgcgggatcacggacgaggagtgcaact

tcaagaagatgaagcagttcctctgggctaatctgcttatctcgcgatacaatgaggatggacgcaaggcgaagagcaagggcaaccacgtctggatg

gtctcggctcgcaagatcctgaacgagaatggcggtctccgcggctgggaattctgcaagttcgagcgcaagatcgccggagcttcggacatgcacgc

ttatctgggcgtgccctgggtgtgggcgatgcgtgtctgggatccccaagcatcctccgaaagcatcaaggctacctacactgtagaggaacatccca

gctggcttcgctttgacggagtttctactctgagtgggacgcctcaggatggcgacctgggaggacgtgtcttggtcacggcgcactttccaccggat

gagcaaacgccgccgcttcaggagctggtcagcctcgatgtgcgcaagctgggtgaagcgccgagggcgtcgtcgtcggaagccggcgacgattcggt

tacggacgacatttctggtcgtacgggcgtcccgagctctggcaatgccagcgaacttgaagatggcgaaggggagcactcgtccgtcgcttcttcga

cctctgtcgacggtggatccggctttttcggtgtcgcgggctatggggacggcgacgacagcgctatgagcatgccttacaaacagcagttcttgccg

caggaaatgttctcacagaacatggcggccatgcagttcccgcttacgtctcccatcctggaaggcttccagcacgggtttcaattcgaccggggaca

ggttgccgcttccagtgccagtgggcctaataccgatcttggacgcaaggagactgcgctgcagccgtcggcgttccagtcggccatccagaccctcc

atcaacagcaatctgacttcgtgcttgacaccccgcctatgtctggcgggatgatgcctgcctcaatggattcgctacagcaacagcgtcaacagcat

ccacatctgcaattgcagcaaccgcagcagcagcagcagcagcagcagcaagtgccgcagcagattccgccgcagggtccatcgtcgatgatggatgg

tatggcgcaaattcaagaacctttcccaaacctcccctaa

>contig_14.contig_14.g478.t1

atggtcgaatttgatgcttctcagttgaatctgtccttgctctcgctcggcgcatacattatcttcttcggcttgttct

cctttgtactcaaggaaaaggtcaacatcggtgaatcacccctcgccatcttgctcggcattgccctcggacctcatggcgtcttcaacctgatcaaa

tgggcctccaacggcgacgactccaaagcagatgctctcatgctcggcctcagtcggatcgtcctcggtatccagctcaccctcatcggtgtccagct

gccatttcggttcctgaggacggaacttaagtcgttgctgatcctcatcggacctgtcatgatcatgatgtggctagccaccacgctctgtattcgcg

ccgctatccctggcatcccattccttgtctccctcatcgtcgccgcttgcaccaccagcaccgatcccgtgctctccaacagcatcgtcaagggccaa

ttcgcagacgccttcgttagcgttcgtctccgcaacctcatctccgcagagagtgggctcaatgatggtatgggatatcccttcctttacctggccgt

atactccctccgtcacgccaccttcggtgaagtcctcactcgctggacacttgacacactcttgtggcaaattctcggctcttgtatttacggcactg

tcgtcggttacgtctgtctttggctccttcgccttgccacccgacgcggcaccatcgacaaggagagcttcctgctatatggagtaggcatggccatc

ttcatcgtcggctccgcaggcgtactcggcactgatgacttgctagccgccttcatcagcggcaatgtcttaacttgggacgaccgctaccgagtcga

cacagaggatgacgaagttacgaatgttatcgacctcctgctcaaccagacattcttcctcttcgtagggctcatgatccccttcgacctcttcaacc

aacccgaatacggcatcaccccagcccgactcgtcggcctctccatgctcgtcctcatcttccgccggcttcccgccctactgctccactaccgcttc

atccctagcgtgcgcgatccgagcgaagcggcctttgctggttacctcggacccatcggcgcgggagcgtgtttctatgcgtccctggtgctcgagga

gttcaagccggatgaccccaaccctatgcatcagctggtcaggatctacgtcaagcctttgacgtacgcactcgtcttagcgagcatcgcaggacact

ccctcatggtccccgtcctcaaagcgttcctgatctacaacgacatcggccgcatcaagctcgacgacagcgcctccgactttgacggctcggataca

gagaccatcggagcgaacgatgatcccgccgagaccacgacgcaaagagctcccagcatcgccggcgtctctgtgacctcgagccacctccgcccccc

gctctctcagacgctctcccaaccgctctctcagatcgtcagcccgctcgaccactcctcggtccatcggcccgtctccttcatcgacggaacagcgg

cgcacgtcctcccctcccgccacttcgaggcctccaacgcgcacaaccgctcctcctggcggatgtcttctggacacaagctccactctggcatcgcg

cacgacgaacatggtcgggaggtgtactatctcgcgcgggacgcgccgagtgcgtggacgcgcgcggctcgggacgtcagcagtagctccggtggtac

cggcggcgatggagtgccgggtcagggtacctcgttgcgctcgcgctcccgatcccgctcccgatccagtcgtcggcccatctcggccatctcgggag

gtggtgacgatcttgagcagggagatgtcacgcctccaagtatttctcacgacggtcttcccgtccagtcaactgcggatcggtacactgcggacgac

taa

>contig_14.contig_14.g479.t1

atgctggcgatggaatcatcagactctctccgtacatccgccgcgcccagcccgcgtgtgccgagcatgacgatgcacg

gcaacggcaacggcaacggcaacggcaacggcaacggcaacagcagacagacgtacactcccattgtctggtcggccgatcctgacggccccaccgtg

ctcaccaccccatcccccatcaccctccggccgcagttcttcacacccctcccgcagtcgacgaaccagcacgatgcggcggatccgcagtcggtttt

ccgatcggctggcaatttgggcccacagcagggtccacctctgaccatctaccatcatggcgagatgccgcccccgccagcgcgggttaagaaggagg

agtcgtcttttgctaatgcgatggggcaagggcaggcacagatccagaacgctacccccgtcccatcacccatcttcgatcctggcgcattcgcacgg

ttaccatcacctggcgatagcgtggctagcatgggaatggggttttcttccgcgcccgcagtcgccttctccttggagccaaacgcccagaatgggtt

cgacctggcacaacaacagccgcgagattggagcagcatgatgtccaactcgaactcggcctcgggcggcgtcgaaccagcagcgatgggcgtcatga

cgcccgttacgagtatctctgtcgctgcgcagaacttgtctctgcccaatacactcgaaaatgcgatggcagccgcggtcgcggctgcggccgatggc

agaagcatgtcttctcatgggcatttcaatcagatggggcctggctcgggtggagtggctcactttggtggcatgggcagcgaatatcatcaccatgt

cgcgggaaatgccaccggcgacgtccactcgccgtttgcatataatacggatcgaccacagacgggcggccagcatacatcgtaccttgcccacccgg

gcttgacgagctcgtcttcgtcctcgtcccacgatctcgacaaccgactacgcacgagtccgagttctaacacaatgatcggaatggggatgggagtt

gggatggggatgggcaacggcgccagcggagatggtagtgggcatcattccctatctacgtttgatcggttacggaggtcacacacggctgctccgca

ggggagcgatcggaagaacctgttcaagaacgggcagttgttcttccaaaagaatgcgctggtgggcagcactatcggtagcggtggcgatggcggtg

agatggattgcagtggagacaaggacaaggacaaagagaagaaaggctcccggtttgtacaaaaattgtacaccatggtgcatgatccggagtgccaa

catttgatatgctggaacccaaaaggtaccagcgtcgtgatagtcaactttgacgaattcgcgaaggacgtccttggcaagcatttcaaacactccaa

cttcagctcctttgttcggcagctcaatatgtacgggttttacaaggtcaacaaggtcccgcgtggaagccgatcctcgtcatctgcgggccttaatg

ctgccgatgggctgatttgggagttctcgcatcccaagttccagcgtaatcggccggatttactcgacgagatcaagcgacgtgccatcgagcacgat

tcgagtcgtaacgagtcgaagggtgagatgccgctctctgcgcatgctcaggcgaacgcgtttttcagcgcacaagtcaacgagctcactgtgaaggt

cgacgaactcaatatcgggttgggcgaggctggagagcggaacgcgcatctacgggcgttgaatgggaagttgcgggaggcacttcagtctacgctcg

gggtcttgcgtcagcagtttggaggaacgttgccggatgttttggcgcagcatgaaggacttctcacggaagcccctgcaccggacaggaaccaggcc

gcgttcttgcagtcgccgacatcccgagctgaccagtcgccggagcggtattcacgtgcccggacgaacacagacagttctcaaatgtcgactgggcc

atccaatcaagaagcgacatcgacggggcagaactcgttgcccctcccaccgatcccgctcaccacgttgtcaactcggggatctcggggaacgataa

tgcccctcccggctccgagcgtactgggaccctgtccggaccgacctgtctcccccatcaaggaggatgcaagcctgctttccagcagaggcgactca

cttacccgttcagcgtccagagcaacgttgactgtcgacacgtccgatgtcggcatgagctctaccgctgggtcgaccgatggatactccatatgcga

atctgccacgaatgtgtttgggaacccgcctgcgtccctcctatcggcctcttccatcccctttgcgccgccttccttcaactcgtctgcgagctcgg

agaacggctccgcgccgaggtcggagcggatgcgcgcccatacgttcatcacggccaatgctagcgtggactccctgcagtcatctcagccgagcaag

cagcagcgcacgatgccgtcgacggtcagtgggatgctcccacctcggagttcgtcgcttatggcggcgtttgaatcggctgtcaaggaggccgcagc

ggctggcgcggctgcaactggaagttctgcctcagcctcggcatgcacctcctctaccgattacaatttcgccaagttctcgctggaccacggtgcct

cctcgccacatctcggtggcagcggtacgatgtcctcctcgcacgcgtatcatcagccgcagcagcagcaacaacagagcctgatgtcgtcctcagcc

tcgtcgtcctccatgatgccgagccagggcagcatcttcagcgggttcaccggcccagaatcgtcgacgagcgcaagcacgcggagctctcttatgct

ctatggtgctgggccgggtacgtttggagaccatgggatggtgagcagcaggatgccttcgtccccgaaccccggcgggggtgggaacaagttcgggc

cggcttttacctttggtgaagggcagaaccaggctccctaccaggctcttcagctaccgcagccgggtcagtctggcttctcggtgctgcagcagctt

catcagcaacaacagcaacaccagcagctgcaacagcagcagcatatccaggcaccgcctgctgcccatctctcgtcgatatccatgccgacaaagcg

gaagatcccttcttga

>contig_14.contig_14.g480.t1

atggcgtccaccgctgtagcacccgcggcctccgtcccaacatcgcgcgccgcacccggcaagaaggctgcctctgcca

agcccgtctcctcaaaactcgcacaggcagaccccaacactaggtactcctctagacaaccacggccatacaatgtcgacgagatccaccagttcaag

gtgcccaacctcaccatcaaggacctcctctccgccatcccagcacactgcttcgagcggtcgaccctcaggagcttcctctccctcagcatcgacct

catcaaggtctccgcactcgcatacgcaggcacatacatcgaccctctcctcaagtccgtagacttcacctctagccctctcaactccttcatcagcc

ccgccacccagttctcactcgcccgcttcgccgcctgggccttctggtccttctggcaaggactcgtcttcaccggcatctgggtcgtcgcacacgag

tgtggccaccaggctttcagccctagcaagaccatcaacaacgctgtcggctgggtcttgcactctgccctgatggtgccttaccactcatggcgcat

ctcccacgcacgtcaccacgccggcaccggtcacctcacccgcgacgaggtctttgtcccccgcacacgagcccagcgcggtctcaagaacctccgcc

cgcttgctactgatgaggacgaggacgtcaaggctgccgagcaggtcgctggcaaggtcattcgtcaggacaatgagacttggggagagtggttccac

gagctccttgaggacgctcctctctacagtcttatctacattgtcgtccagcagacactcggatggcctctgtacctgctcaagaactcttctggcca

gcttcactaccctaagggcaccaaccacttcaaccccgacgcgataatcttcgacgcgcgccaccgcaaccagataatcgtctccgacattggtatcg

ctctttccgtctttgcgcttgccgcctggggtctcacctcgcccggtggcttcggcgaggtcttccgcttctacgttgtcccttacctgtggtgcaac

aactggctggtcatgatcacctacctccagcacaccgaccccctgctgccccactacaaggccgaggcgtggaccttcccccgtggcgctctctgcac

cattgaccgtacctggctcggccccatcggagcctggttcctgcacggcatctcggagacccacgtcgcgcaccacatctcgtccaaaatcccgcact

acaacgcctgggaggccaccgatgctctcaaggagcgtctgggcgagcactacgtttgcagcaaggagaacatactgatctccttgtggaagagcatc

cgcacgtgccgctttgtcgacgagaacgacgagatcgcgttctaccggaccattgatggtgtaccccacgccgtcgttgcgcccgacagctcgaacaa

ctcggactccggtgtcggcatgtag

>contig_14.contig_14.g481.t1

atgagcgacttgacttctacaaaatcggcttcgggctcgaactccgacgacgcagcctcctcgtcctcgccggttccgt

ctcccatgacctcgcccgccctctcctcgaactcgtccctggtcgaagcgtgctctccctccctccccaccgccaccgcagccgacggcgaagctccc

ggtctgtccgccgatgaagtttcagccctatccaccgaggaacgcaagaagcgcgccatcgagctgaaggacaagggaaactccaagttcctggcagg

cctgttctcggaggcgaaggacttttacgcgcaggctcttgtgctggacgacagtgtgccgcagttctggagcaatcgtgcggcgtgcgagctcaagt

tggagcagcacgggttggcgatcgaagatgcatccaaggctatcgagctcgatcccacgttttccaaagcctacttccgtcgtgcaacaggttacctc

gccatcttgaaacccaagaacgccttgccggacctgaggagagtgactcagctcgagccaggaaacaaaagcgtacgcgtccagctcgactccacgct

taagctcatccggaggctcgaattcgaaaaggcgatcaaggtcgagtccggccccaagaagtggcaagaagccctcgagcagattcgggatcgagccg

gacaggccgcactcgcacccgcgtcgtactcaggtccacggctcggcgaactctctgccgaagagaaagcgaagctcgacgaacaggatgaggaggat

ggcttcaggggggcggaactgggcaagattgatgggaagtttgtggaggagcttgtcgagttttacaaggagggcggtaaactgccaacgcggctcgc

gtggcagatcatgttgggcgcgttccggttgttcaaccgggagcctacccttgtggagtacacggtagcggaggggaccacaattgatgtgatcggag

acacccacggccagctgtacgaattcgcacacctgctcaccctcaccggcccgccttcggacacccacgcgcttctgttcaacggcgactttgtcgac

cgaggctcgtggtccgtcgagattgcactgatcatcatggcctacaagtgtgcgttccccaagacgacgctggtgaaccgcggcaaccacgaggcgga

gcagatgaacaaggtgtacggcttcgagggcgaggtcaaggccaaatttgggggcgacttcacattcaagctgtttacggaactgttcaacgcgctgc

cgctcgccacgctcattacggcgacgaaggagcctctgcagggtgctgacgtgcccgcgaaggcaccgctcgctcaaaagtcgcccatcctgtctgcg

ccgtccgtcgaggccggcggtcggaaacggtactttgtcgttcacggcggtctcttcagccgggatggcgtcactctcgacgagattcgccagatcaa

tcgataccagcagccgggtcaggaggggctcatgtcggaagcgctgtgggcggacccgcaggttgccaatggccgcggaccgagcaaacgtggtgttg

ggcttgggttcggaccggacattactcgccagtggtgcgagctgaacggaatcactgctgtgattcgctcgcacgaggtgcgacagggcggatacgca

gaggagcatgatggacgctgttgtacagtgttctcggctgcagattattgcggttccactggaaatcaaggagccttcggcagaattgacgatcgcgg

ctcgatcgactgggttactttcaatgcggtcaaccacccgaagaagacattgagagcgatgcattacgcttcgggggccatgggtgggggaatgtaa

>contig_14.contig_14.g482.t1

atgaggggttcgagaacttcaagatgcctgagcatttctcagttcctccttccgtccatcgtccatcactgtctgacgg

aagatctcgatcctggcttcccttgcatgctgaggtcctctgtccctattctcagcaggctcgacaatcgaattgtcatctttgacaggttgaacgac

tttcgaacgatgaagcggagctcaccatcggcctccccggtgttgcacgcatcgtccttggccagcacgagcagcagtaataaaaggtctcgagggga

caatgaagctcaactccatcctcctgccagcacctccgatgaagcgtctacctcgtctggactgcctagcctgcacgcgccctccctcactgctccgc

tcaaaactcccgctttccaaaaaccagcctgcattgccaccttctcctacgacgagaaccggggcctgcaccatgacaaccagtcgctaggggtctac

catcctcctcccccgcctcaattcaaccgcgatgccgatcgaggcagaggccgagtctgtggaccggatctcaagtacggctacgagaggtggaagcg

gagggacgagacggtcgacgagcatctcgactcgctcctgattgcactccaacacaaggcgaacttggccgatcctgagggggaccaggatcgcgatc

gtgctcgggcggacgtgattacctggcggggcatcgccaccaagctctgcacgaccatcttcgcagagaatgactacgactcctggcagctgaacgtt

ttgcagctcggcccaacgctctatttggaagagttcgtcccccccaagacgcggagccagaaggccgaatcgacagaccagcgccagcttggttttat

gtattcgggctacgctttcgagtcgtggtgcgtctcgcctgcgattcccgatacggacgccaaagcggccgccatggacgacagaggcgagcgcgagc

caccaccaccaccaggttggggaggcaccatcaacaacaacgtccagtggtgcgcaatctgcaagacaacgctcggctccaatcgtcttatcattggc

ggagaagtcgactgtatccaaccctcggccctatccaagttccccgcttcctcagctctgctcaacgcctcaacgcttaaccctgacgattttgttga

gctcaagacttcggccgagatcagaaacgaaagagatgcagagcgcttcgaaggaaccaagatgcttcgcttctactttcagagcttcctcctcggcg

tccccaccatcgtcacagggtttcgcgatcgagcaggcttcctcgccgacgtccaatcaattcccacccttaacctacctcggatcgtccgagggaga

aatccggatctcgaagcaggtaaagggctcgcctttgctgatcaaacgctgcagtggatccgagattgtatcagtcgggatacacagaccacaacctc

cgcgcctcccgatgtggaagaagccgagcgatcctttcccgtctttcgcatctcgttcagcccaagtgcttctggagcgccgcgcggagtatcaaacc

acaacagccgcggtggaagtctttccatccggagactgactgatgccgaagttcgtgaggacatcaaaggcgggacagatcaaggtcgcgtgggattt

ttgccatcctcattttacacctttgcacgggagaagctcggtacttcagctggcatctga

>contig_14.contig_14.g483.t1

atgcatttcgcagccccctcatgcgtaggaccactgccaactctctcgaacatacatactgcggaggaggaagaggcca

tgggcctggatcttgatgacgtggtcgccttcctgcgcgttacgcttttccttcccaccttacacgttatcatcctaggtgtagccttgcttggaggt

gaatggctgaaagtgcaatcgtccttccagatccggatactggtcctcctctcgagccttgtgctgttttctcatgcgatctctttcgctggccggaa

gcatcggaacggctggacgcggacaagagtcctcggtgcggaggaatggaagaaggaagtcgtcttgattacgggaggagcgggtggggtcggtagcg

agctttctgctctgctctgtcaggtacatcctctggctgacctatttctttcatccgcagtattcaaacgtcgaggcagaaccacctatttccagtgc

gatgtctctgatgccgatcaggtgaagtcgatcgcagctcggatcaaagacgaggttggccatgtatcgatgctggtcaacaatgccggcgtgttgaa

tggcaagctcttgacggacctcgaggacagtgagatacagaggatcgtcggggtcaatctcatctcccacttttacacgatcaaggcattcctgcctg

atatggtacatcgcagccgtggtcacgtagtcacagtcagttccgtgatgggccatatcggcatcgcgcaagtcggcgactacgttgcgacgaaacac

gcactcgtcggcctacacgagtcgcttcgctacgaattggacaagaaacacaaggccccgaaggttcggacgacactgatttgtcctggtcacatcaa

gacggccctctttgcgcaggtcgaagtccccgccttggcacgcttcctcgctcctctacttgacccgcgcgatgttgcgatggccatcttgcgcgcct

tggaaaggcaagagagtacagacatatatttgccgtggtacgcgcagtgggctcccgccctgcgcatgctgccttccttccttcgcgacttggcccag

actgtatcgggagcagacgaagccattgagggcatgtacaagcgccgtgcagaactggagcgtggcaaatctgaacaatga

>contig_14.contig_14.g484.t1

atgatctcgggacgaccggcatctccgctctctcactttctcagcctgtcgacagtccgcgctcgtcgacttcaacatc

ttcgccacttcaccgcccctagacatcccaacggcctgcctgaccatcaaccattagcagctgaaccgcgactatcatgcattgtgcacatcgaaagc

cggactggtcttgcaggtatcttacctattcctcttgcagtatcgggcggctttcaatccttcgagcaactgggtactccacttcttccttctttccc

ttccgaggccggccgtgagtacaagatcttccgctccacagcttccttgaggtacgttgctgacacgttgaatccctttgcagaatcctccgccatga

acattctcaccactcgcttcgttcacttagctctgctcctcctcagcctcggacccgacagcccactgcagaccggtgtccaagcccttccgttcacc

accagccctcatggccatcggcgctaccatggcagctccgatgttcacatcagatcgttcaaagccaccaggcctcgtatcggtattgtcgtcaagaa

ccaagatgcgccgccccaattcgggggtagcgcattcaacatggaaccgaggggtatcgatcatggagccagcgccgacttgaactacaccctcactg

gcaacaatcccagacatgcgggagaatcgcgtggcttcggaagtgcgttcgattccggcccagggtcgaaagtgctcacgctgccactgcaaaaggtt

ggaggcgtgcggcttaaacaggggaagaatgagcaccccatgatggaattgaaacgtcatctcaacaccgcaaatcgacgactcgccaacatccgtaa

aaggcatctagacaccgtattgttggaaagaagcaaccctccagacctcggtgggacagccatcttgctcgatccgaccctcagcctggggtcgacca

atgggtcacagcagacccaacgtccctctaaaccgtcgaagtcgaaaacccctggtgtctggcctggcaacgacagtccttctttagggcatggtccg

gccatcatccaccaagccaaacagaagcgccttctgggaggtctcgttggggcccttggaggagacactgcggggtcgccctctggatttggcagcgg

acctgcgaacgcaacagggccgacagccggatcgggtctggatggcatactcggaggctggacggaaccttctagcagtagtaaggccagggcaagcc

catcgcctaacgccacaggagcgggctacccggtcaaggatctcgacgctgccaccagcgccgctttgacgggcgctcagcccaccactgctgagcaa

agtttgggtcttgacattgaagccaatgatgttggctacatcgccacaatccagatcggcagcaaccaaaagccattccgaatgcttgtcgactcggg

ttccgctgatacttgggtcgccagctcgacgtgcacagcgtgcgggactaagcatcaaaggctcggagcagatgcgtctacgagttttaaaccgagca

gcaacaaatttacgatcacctatggcactggctctgtctccggcatgctcgcccacgacgatgtttacatcgcgggtttgaagcttgcgaaccacact

ctgggcgtagctcaacaacaaactcgcgacttcaccgatccctcggtgcccttcgacggattgatgggtctcgctcgatccgagttgagcaacgcggg

tacaccgacgccaatcgatgccctattcgctgcaggtcaagtctcagcgcctgtcatgggttaccgcctcggccgcgtcgctgacggccgcaatgacg

gcgaggtgacgttcggaggggtcgacaaactccgattctcgggcaacctgattgaggtggataacatctccaaacagggcttctgggaggctgcgatg

ggcgcggtcacctttggcggacgcgacctgggtctctcgggacgtacggcgattctcgatacgggcacgaccttgattgtggccccgaagaaggatgc

cgatgccgtgcacgccgccatccccggttccaagagcgacggacagggcggatactacattccttgcacaacgacgcagagtctcgctctgacctttg

gcggcgtcaccttcccgatcgatcccagggacatgctcttcctgcctgtggaccagacgaacctgcagggagactgcatcagcgccatttcgagcggg

caagtcggtcaggaggatgagtggctggtgggtgctgtgtggctcaagaacctctactttgcgaccaactcgaggacgaacactatcggcctgggcaa

attgaagtag

>contig_14.contig_14.g485.t1

atgtcccgactcgacgattctgacgacagcctcagcacggctgccagcccaaccttcgatcaactcaacttcctcctta

tctcccgcggctacaccaacaaacctcttctgataccctcgggtacaagcgaggctacaatccaggctcttagcaacacaatccatgcgcttctctct

caacgcgaagaggatatcgaggttcgtgaacaactcggcgctcaaaatcggactctcacggctactgtggagagactcaaacgctctgtagacgagga

gatgcgcaaatcggaagatctcgaacgtaagctggagagcataaaagtcaaggcagactcggctcttgctgctcacgccacagtcgttgccgcccaca

aacagacccaggccgtcctcatccgggcgcgaacagatcttcaatgtgtcaaagttgctgcgcatcagtaccgactttcactaaaccgtggatcggag

cgtctcaagagcaagctcgccgaaacgagtctgactgctcttcgtagtgtcgttcctgcgctccgggttgccacaggagcattttcggactacaaggg

tgtccgtggtgctttgatgcaagcacaacttcgcagtacagcatcttcggggactgtgtctgacggtcacggagcgggcgggagtggggtggtattga

aggaacagcttacagagctcgatcgaagtctgtcttctcgatcgaatatgcctccgcggaaacggaaggcacccgcggaggcacggtcggaggaatca

tctaagagcccgccaaaacggaagccacgcttggaggaggcatccaaaagggcgagtgggtcgctgcggctgagtcgaatcggcgcgctccgcgacac

gagtacgagcggtggaatatcctcaagatcatcgtaa

>contig_14.contig_14.g486.t1

atggccgaccctcgctctcagcacggagcatttctgccccaacatatctcctctgggatgcttccttatcacactgaag

ctccggttcggccgccgatcccaacctattctttctcgccggatactgcatggacgcaagcgtcgtccgcacatttgatgccggccatcgaaatgtcc

tccgagcacagcacactgccgcagttgacacccatccacgatccgtcatctgcaatgttcctccccacagctagcgccggcatgcctgctaccccttt

gccgacctcaggcaatgcgacagatttcggctcaggacaggctgatgcatactcgcccaacgagactgcacctgtttcccccgtcgaatcactctcct

ggaatgtgttcccaccgcctgtcgtcttccctcaatccggctccaactttgcatcacaaggtccatttccctttcctcctttccggcagcaggaggct

gtaggcatggctgatccatacttgcatcatcttccgcaccagcagctccatgctcagccccattcccatccgcagggccccgctctcgccatggggtg

ggcccacgcagcttcacagatggtcaaggctgagaccctgcctccagactcgcccgagtcccaagaggaaaatgtcccagtcctgtacagacatgtgt

ctgcgccgtctacgccgattgcaccgctagaaaacggccgagccttcccagtcgtggatttcagacacggacccacgatcccacagcatatgcatcca

tctacatttttcacaagctcgcctcacgtggattcaacacagcaaatggccggccctagcaacatgcccatgttccagactcctcagagggtcgatta

ttggccttccaactctgccgccccattgagcgacactgcacccattccctatctggaccgatctttcagcaccgagcagtcaaagtacgaggccgtcc

tggctcggtcgcacgccgccgatcgcgctttcttttgcggtgcaaccaacactcgtatctactgccggccctcttgcgcatctaaacgaccggatcgt

tcaaggctccaattcttctgcaatccgaacggtgctgacaaagctgaacaggcgggatatcgcccttgcaagcggtgcaagccgcagatgccgggaac

ggtcgatcagtgcgtgcgttcggtgggcgaatgtgcccggcacatggtcacggccgcacggaacccatccgtaggctcaccgaatgacgacgatgacg

atgaggttcgcaggggtactctgaaggattacagcgcgcgagccaatctctctgcgttccattttcatcgcaccttcaaggcggtcacgagtgtcaca

cttggtgagctcggcaaggccctcaacactctggccttgcaggatgcgctaggagaatggaaaccgtctcctcccaatcagctggcgggcccaagctc

catgtcaatgcgcctatctccacctccatctgaggacatagctcatctccttgttggatggtcggcacggcgggctcgacgagcgctaggcggacttg

ccccgagcgtctatgcggctggttgcccacacgttgtgatacgacatgtgaccacggactcagcgtttggtcccattgccatcttgttcatggtcccg

ccttcagcccgcgatacaaaacgcgcctcagtgctgagccaaatgcaactgggatcctcccccatgccagtgacatctgaggaaggaccgtacttcgg

gcgacagggctccgtttcgcaccaattccagctacctatcgacgcgctgccttctttccctcccgccaacgcgattctgttgggctgcatcctcggcc

aaaatgcggagacggttgtgcaaagacgattcccttcctccgtggcccaacctcaccttgcatcctggatcaggtctaccgtggaggaactgcatcgg

atcgggagccgcgaagtccagctacctcctgaagttgttggcgccgttcgacgggcgcgcgtgtacatcgaggtgatgcatcagatgggatccaaacg

accctcgagcgacgtcggaatcaccacggaggcttcgacttcatctacctcactggaccaaatttga

>contig_14.contig_14.g487.t1

atgagcgatgagcagcgtccgcgcgacacgtcgaatccttcactctcatctctcactctgtccatactcatactacatc

tttccctcatcctccccatcatcctctcgattaccccttcaacgcgcaagcgcgcggttaccccatcccaccccccagccgccggcgcttctgcctcg

gaacccatcattctggatgatcctgctggtataccagccaaagcaggagtcaagcgcgcttcttgttgggacgagttcacacatgaaccagagatcaa

gaaggttcgatgcgtgcattgcaaggggggttctggattacatatccagctccaagaccggtactacttctatgggaagacacctggagacgtgccca

tgtcgtccgaagagcctcgagcgcggtcagcagctgctgcagtttgcgcagacgaagctccccgccgcatccaagacacgcctgcacaacctcctcaa

gcgatggctcgttcgggaccagcagtctctgagcgttctcgaccacccggatttccgagctttcctggacgcgttgccgggggggactatatgtgccc

gtcgaggaagacgatgacgcggtcgatcctcaccgacttcagcagttccaaagtcgccatcaagacataccttgccaccctcccaggacgcatcgctc

tcacgacggacggatggactgcaggcaacggtgatcaattccagtccctgactgggcacttcatcgacagcaactggaccctgcatgcaatcctcctc

gactttgccccgttcccagtcccccacaccgctgccaatgccgccgctctcatatggagaaccttgcaggagctggacatcgacgagaaggtggtcgc

atgcgtcacggacaacaccgcatcagcatacaacatctccgtggatctgcgcaaccgtgtagctcccgaacacttcttcggaggtcgctgtgcggccc

atctcgtcaatctcgtcgtcaagcactctctcgatgatgcagaccggaccctcatcttcgcgaagtgccgggcgttcgtttctttggtccataggtcg

aaacctacggagcaggctctgatcaaggcatgcgagactgctggtatcaaatacaagatgccgaagacggtcatggacgtgcgctggaactcgacgct

gggacagctgctcagtcttctgcaccttgagcctgctctgcgtctgctattcggcgaacgggcgtatcgagatcatgcgtggggagagccagtgtggg

tggccatacgagacattacgaggatactggccatcttcaaggacgtttccgagcatctccaaggccgcaagtttccaacgatgagcgtcgccgcggaa

ggcttcgttctcatgacatacgagctcaacgatctcgctgcgtcagctgttctgtctccgtggggatccgaggttctcaagctctttcaaacgcgcct

ggtagagtaccgtccccatctcatcgactcggacgttactctttgggccaccgtactcgatccaacgatgaagaaagatgtgatcgaagcagacggca

tacacaatgtgcgggacgtggtcagttcattgggattccatcttgaggctcgctatcccaactccgcagatgttcaacaagctttcagtacggcagag

ccaacttccaactacgagaagcgcctgaagaagatcaattcctcaagcagaacgacaataatggtcactcctcgcaaacaactcgagatgtacctggc

ggagaacgtcatcatcaccagcaaaccaaccgatgttttgacgtactgggcatccaacgagagcaggttccccagtcttgcagcaatggctcgggata

ttctcgcgatccctgctaccacagtgccttccgaagcagctttctcccgcggaggggagttgatcaccaagcgccgtaatcggcttggaggcgatact

gtcactgctatcatgtgtcttgattcttggtttgaaggaggacttggtgctattccctccaatgtagtggatgatgagtag

>contig_14.contig_14.g488.t1

atgtccgaacacgagtctttcactcttccggccggtcaattcgacccccccacccacatcgatcccgtcatccagcccg

gccctgccacgccccacagcgttctcatccatgagatccggtccgtttcggcacgactcgacgggttcaatatgcggctaggcgatatggagcgatat

caacgactccaaccaagtcagcccgcttccccatcccctgggcctcgtctcgatcaatccggatcggccaacgcttcgtctggtcaagacctcggtcc

cgacgaccaagacatcgccttgcccgactcggtcaccatccgtcgtggacgcgcccgtcctgacgcacccattccgccccacatggaacgggccgcag

cagcactcgctgcctccaccatctcgcccccacaacgagcgagcgccacaccagcaacaccggactcccccaagccgctcgaccgctacaggtccatg

agcaagggggagaagtccaacgttcgccgcgcattggcaggtcttggactttcagtcccgaccctcatgtcgatgttctccgagaccgcagaggacgc

cgaccttgcttcaaccacggagtctggcgaggataccgctccagccaacacctcggcatcaagcaacgaaagtcctctccgtcccgcgccgtcggctt

ccagcaacgagagtcctctccgtgccgtgccggctgctgcccccgacaccagcgcctcaccagcacctgaggcccgagcatcgtcgagcactccccct

gctgccgcgcccacaccggccatagatccagcacccaacatatcggtcgctgcgaacgcccccactcccgttgtcgtctcgaaccgaccgatgacttg

caagacggagtggatcggcgacttcaacggcgatccctcccagctcggggactttctcactcggctccgcgatctcattcgctcggagactcaacagg

aacttatcccgccgtggatcaaagctgtgcttcgcaccctgccccgcaccttgaccgggaacgctgccgtctggcaccagggcctgtcggacaccgag

gctgccgatctcacctccgtcgatgcttggtccgcggccatgcgtgctgctttccccatcaaccgaggccagcttcggcgggacgctcgagctcggaa

gtgggccccatcggacgaaacgtccattgcctactacttccacaaagtccgactcctccgtcaagcattcggcaaagatcaacccgacgatgcacttg

tcacggacatcaaagatggtctgccggagtccattatcggactcctgcgtcttcctcgggtgggggctacccttgccgacttgcgtgccgagctggga

gattgggaaccaaattggcgtgtccaatacaaagtcccccttcgcacctccacgcttcccgagcagggtacggtctcctccggtgtacctcatacttc

gatgagtactcgcactggtacagttggcaccccggccgcaagccgcgtgcttcagacgaccatgggacgctccgcctcggcgccagccactccagctc

ctaccatgggcttgtcgtctggtgccgcccctcccatctcaaccatcccacgctcgatctcgaagttcgccgccgcatacgatcccacccgggtcatc

caagccagcaacggacagccgcgacgctaccgtccgcccggcaaagacaccgtgatggatctcaaggcaccatgctcttactgtggcggcgaccactt

taatttcgagcacgactacctcaaagttcaggtccgaacgcttattgcggacgacgatgactacgaggagtacttgtgggatgggacacctgaggagg

acggagaaggagaggatgaagatgacccctccctatccttggagcctggccttgcgccctccatgttcccttttgaagttctcaaatcaaatccgact

ctcgagtctgttgacgcccccaaacgttcgtctgatccaatcgcgttgcacttcgcggatcgaagtgtcttcgccatcacacgaacgctaaggtcgaa

cacatccgcccctcctctggcccaggagaagcgcgcgtttgggaaggtggtggccctgcctcggcactcatcaaccggtacggggcaggggtatcgca

accatgtgccgttgtctacacatgtccgagtcaacgacacggatggccgcgtcatgtccaccctcctggaaactggcgcttccctctcgtgcattaat

gctgcgttattgaccaagatgggcggcgctccaaccggcaagccgatgacggtccacggcgtcgggtcgtcgacaacgctcggttggatcactcttcc

cctgtttatcgccgctcaggaccctcaaggcaggcacgtgcacttggaggcgtctcaggactttcacgtcctgccgagcttcccgcctggcatgtgtc

tcggccttgatttcattgatgctcacagcatcagcatctcacccgtgcgcggtcgtgctcgcttaggccggtacacgttctccgtccacgagcggctc

gacaaacccttctccaccgatgtcgagcttcgcaccagttcggccgttaccattgcgcctcggacccaagcctggattccggttaacgcggcttgcct

tgctcccggggtggactacaccgtcgtccctcgtttaagcgtgtcccaggaccatggcgtttgcttgacgggcccgaacggcctcctgactcacggcc

cggtccgccacatcctgctaggcaattacggggatgaatcgttcaccatcgagcaggacacctccgtcgcggatgcatctgccgctcgcgttggcgac

gtcactaccaaggtcaatccctcggtattcacccttcaccgtggcgccgactcgcccgatgtcgaccctccacctctccggccttcggcctcaaatga

tgcgggtatgcccttcgatccttttgacgatgacagctgcgatgtatcgtccctcaccaaggacgctgccacggtcatggtcgacggggtttacaaag

tcggagtggatcctcatggcgacgttcacgccccgattgtcgatcttcttcggcggcattcgtcggcatttgcgttggacggtcggccgggtcaggtc

gcggacgccgatatggagatccgtctcaatcctgacgcgaccctacaatcggaagctccccgacgtgccagccccgacaaacgcgccgccatagattc

cgccatcgaccagctgttagactgggacgcggttgcgactctgccgcgacctaccacgctccaagaactgtaccatacgcttggtctgttcggatatt

accgagctttcgttcgcgggtttgccgaacgtgctgcgcctctcactcgaatgctaaagggatggcgttatgaattcgcagacgggcagtctcgattg

gtgaacactgagggcaaaacggttacagctagtcgggtacccatcgaatgggggccggatcaacaatccagcttcgatgctttgaagcattcgatcgc

cgatccccctgtcttggcccacccggatccatcgaagccatacgtcttctactgcgacgccagtcaacacggtcttgctgccattttgcaccaggttc

acgctgattccgctccgtcgtccgtcgagccggtcgtcgcttcgatgcaccacctgtcgttgccacaactgcctgctgacatcgcgcgcgatcagtgg

gagcgatggttggccgacgatcctttcttcggcccggttcttcgtcgagttcgcgcgtccacgtcaccagatgacgaatgggagttgtccggcggcgt

cttgatccgtcgactggacgatcgccttgccctgcctgctgctgctgtccccttcatcatgcgggcggttcatgatgacctggggcactttggtttta

caaagacgttcctggcaaccaagcgacggttttggcgccctcatctgtcgtccatggttcggtcctgggttaagcattgcgcgacctgtcagggcacc

aagtcgcttccaaaggttggaagtctgaacatcttgaacgacccgacccttccgttcgaaaccatttcgttcgacttgctttacgggtttccccgatc

tcaatccggaaacgacgccgcccttgccattcaggacctcttttcccgcatgatcttgctgacaccatgccacaaagagatcacggccgagggcgttg

ctgcaattatcgcggatcgtatacttcgtttgggttggcgcccgaaacgcctcgtcgccgattccgagtctcgagtgtccggagccgtagcgacctcc

ttggcggcttcgttgggcgctaccgtcaccccgtcctctccatatcatcagcaggccaacgctgtcgaacgggcgattcagaccacgcagcaggttct

tcgcgtcatgacggtcgattcgaagagccattgggatcgacgtatcctacccagagctcggcaagtgaagataaccgcggtggtccctgggtttgtct

catcagagctctaccacgtcactagtacgcccgcattgtcgaccggcttccgaccgttcgacttggtcttcgttgcccatccggacgtggtccacgct

gtctttgatggccatgaacatagtggggtgtcctcctttgaggagcggctgtcggcggctgccgagcgactggacgaggcgcgtcgtgccattttgga

ggctcgatccgaccagaagcgacgatacgatgcgcgccggtccgccatccctgcccttcacaccggcatgatggtctgggttcgccttcgcgatcgtc

ctttgccgggtctcgttcgcgacaaactcgatgctcggaaacatggcccgtttgaggtcgtcgaggtgttgtcggcgcaccgtgtccgattgcgtttg

ccggcggacctggacgtcgatcccgtgttcaatgtcgaacaactggacttcgctcctgttgaggtcgacccgttcgcggacgctcgcgtcttgccgac

tgtggatacagctgctttgccttcggggccagcgttcgttcccgactcggtcgacgcgctgccgacgacgaccgacgctgtagccccggatcaagacg

tggtagagaccgagagtcttgatctgctgctccggggacccctctttcgtcctcgacgattgacgatcgctggagcggaagtcgtgctcaccgagcga

ccggtggcgttcctgtcgaagttgacgggcgtcgcggaacgcaaactggttgccgctgaactggaactggtctgcctcgcgtgggcgttccacaagtg

ggctcatctactcgagggagcgtcagtcaccgtggtaaccgaccatgcgcccatggaacgtatgctcacctcgtcgaacaatatcgcctacggaccca

ccatcactcggtgccgcgccctcattcttccacacctgcccaaccttcggttcgtttatcggcagggctcgcgccacacgaacgtcggcgccttgtcc

cgccttcccatcgaccagggacggtcttctttctccgggggggatgtgctagcagtggtatga

>contig_14.contig_14.g489.t1

atgtcaagtcagtccagcggcagccagaatcggacccagaatcccttcggactcaccggcgagactatgtcgatggcat

cgttggcccacgagatggctaacatgcgcaaggaccaacagcggctgatggagcaaatgcggaccgtcgtcagcgaccagaccgttaacacagcgagc

tcatcgtcaaccgcacctacccatattgccttcggaccccgggacggtcctcgggctcctcctcgtgcacgtaagcatcagctcgcccgcatccgtcg

gaactggcgtcgactgctgcaggacaagatgggtgatgcgcgcaagaagccttacgagtacgactggccctcctccttgtccagttggcccgttcgta

tcgtccgtggtcgccctggcgctccggaccgtcgagtcaggttacttcgcctcaattaccatcggtcctacatgcacgaagacaatcagagtcagctt

cgccctctgttcgatcatatgctcacttaccgcaaccagaatggagtccctgcggccatgaccctcgaggagttgattgggctcatgtccaacaccta

tacctacctgcgtcggcaataccatctgcgctcctcttcggccggtcgacaacgagttgcgcgggagaaggccaggagtcagcttcacgctcgcaagc

gagcgaagagaacgggacgttcttccaccggcggcttcacggagtacgcgcggtttgccgacgggacggagctctggcgcatactcggcaaccgcgtt

gagatcaacaatcggcgattcgaaatgcgtgccgatatcgagttcggcttccagacgttggtgcattctcccgagatcaccgagtccgagccgaccat

tgacgggttgttccggaccgtcacacgccctctctcccttccatggaggtcccgacggctggagaacatgctccatgggtttgacacccatgtttcaa

gggtccgtccctaccaggcccgtccgagcctggccttcttccggttgtccagcacgttcacgttgccgtcctcggtgcggcgctggatggtgtcgtcg

gactgggcagaggagtaccccgaaacctgtgtcaatgtgtcccgcaacgccggcccattcggcgtcgagaaccagcgtcgggagggatttgctgtcgc

cgattcggcgtcggagtggggtagtcccatccccctcgacgagcgcgcagcgggatttggcgacggcgaaggcgaggatgatgggaccgactactttt

ccctacgaggccgacgccgtgcgcctcagtcttctggtggagatcaaccaggtgaggtcgaggaaggtggtggcggggcccagtacggtgatgtgttc

gatgacgagtacggtgatggtggcgacgtgtacggtggcgagttcgtcgatgatgttgaggatgactttgagactggtgttgaggaggacgatgacga

tgatgacgacgatgacgatgatgacgacgagtga

>contig_14.contig_14.g490.t1

atgtctgcgtctcgcacacgcatcgtctgccttccctttggttactccagcgagccgagccagcgcaaacattcctttc

cccgcccgcggtatcctcaaggcaatgtcgagatcgtcggagacaacagcaaccggtaccggggggtcgccgcccaggcaggactcgagaaccagggt

tactacggctccatcgaggatgtgctcaagcctttccacctctacgagcggtgcatgaccgacgcgggggacggctccaaccccaagcacaagcacat

cgcgcacgatatcgaggtcggcgacggtatcccctacatgcgctccatctcgccgccgcccgagacgccattcttaccaccgtctgtgtacgtctgtc

tgctccctcagcttcagactactctcgtgcggctccgcccccctccaccggcgcctacgacaccgggccgcggcggtgatggtgcgcggatgtttcac

gagccgtgcggtgcaggacagtcgactggaggcaaggctcctcgcgcgcagctcgccaagaaggccgagaagctcgctcgtgccgctggtggtgtcaa

gaagccgcatcgatacaagcccgggacggtcgccctgcgcgagatccgcaagtatcagaagtcgactgagttatgcctccgcaagttacccttccagc

gcctcgtacgtgagatcgctcaggatgtcatcaggacggatctgcgcttccagactggggctctcgctgccctgcaggaggcctccgaggcttatctg

gtttccatcctcgcagacacgaaaggcgcggaggaactccgcgcctccgcgcctccgcccgcctacctccgccaaagctccgcgctcatttttgggcg

gatcattccgtccgcccaaggcagtggtgtcggcaaggccggtatcattccgtccgccgtagcagggtggtcctcaccgaggccgcctcctccgtccg

cgtctcagagcggagtaagcgcagaggcgcggagttccgccggaactccgccgaagttggggctgtcgacacctccgtccgcgctcaagtccgggaac

ctcaaatccctccgccctccgtccgcgttcaagtcgttggcggtacttctccgtccgcccctccgctgtcagcctccgcggtacggaggacgtcctcc

ggcggaggaaaatcggcggagttttgcgcacacctactacttatcgaccaccgccaccttctatcggccagctcctgatcgatctcctaacaccccag

tcgccacacgcattcacaaagaccccgccagcgctgccggagagggtggaaccctcgacatggccaggacgaatcgcctcgttctctgcctctgtaca

acacggtgtggtccttcgggacagatggtggctcgggttacccgagatcaacacgaactggacgacctagatccgttcgtggatgtcagccaatcctc

tcacgtcctaggggatccaagtctgtctcgagctgacgaagagtctcgagctgacgaggagaaccatccttttcttttcgacgattccggcgttgact

tcgattgggttgatacacaagacgcgcggttgaccgggacgttggaccagacaacagaacaggatctggcggcagtgctggaggtcgacgaagggtgg

gttcccaccttactccagttccggaagggctgggtggcaatcgacgagtcgggaccggcgtctcgacctgaagggctttggctcttcgagctgctcct

agtatggctcaacgcaacattcggtctgtcggagcaaggctcggtctcggtactgtacgctctctgcccagacagcgcgtgttggactccacatctct

tcgatggcgaaacaccagcgcagtgttcccggtgtcaagagcctttgctgcaaggcaacgacgcttccgacgaggacgaggacgaagatgtacagcat

gttggcaacagccatcggacagagacatcgaagaagcggccgagactctctctccattatgagcctctctcttcttggttgacggaactcgctcgacg

ggaggagttcttcgagctatgcgaccaatggcgcacaagagttcgtcaagagggcgtcttcgaggatatctacgacggggccggctggcagaacgacg

ctctcctccaggatcggtcggtcttccacttgtccctgactctcatggtggactcagtgcagcctttcagtcggaacggcactgcaccgtacagctgc

tgcgtgatatcgctgcggatcgacaacctcccccggcagcatcgcaactccgccgattcgacgcacgtatgctgtatcctgccaggaccaaagaagcc

gaaactcgaagccctgcagtccgtcatgcaaatcatcatcgccgagcagacacagaggcgcgaaccactgtggcaggctttccacattattcgtcgaa

ggaccagttctgcccctggtgcgacgccgacggaaacactcgacgtgttactcctccgtcgagcagcagcagcagcaccttgcgcgttatacaaactg

gacggctggtcgagcctcactatggcaccggttgacgtgatgcacgctctcgatttaggattatgcaagcacttttggaccgagaccttgacggctgg

aggacttctaaaaggtgcagacctcaaacaatgtcagcatctgttgacagcgaccacctatccagcggggatgaccaaaatttcggtgacactaggcg

atacgggtgggggctccccgacagcccatggttggtccatattatcaagataccttcttcccatcctactagccttctcatggtcttcgctctctgaa

accgagaagaccttcgcctcaagcaaacgcactctcaccagccgcgacaaatcgaaggctaagaagcccgtacgccggaagagtcgccgcaatgtgac

ccccagcgacgacggtagttcgatgacgtccgagactacagacggggagaaggaggaggaggcgggcccgtcatcacgtatcggcaagcggaagaggg

tcacctcaaagcgcaagactgcaaagaccttcagcacgcccgtatcgctaactcgactagtcgaggcaagtcttcacctagctcacgcagcacgactt

gcgcatgcttacattctggttgaggcacaactcatcaacctggacgcgcatctatcagacttcgtcaagatcagacctccaccccaaatggctaacgt

acaactatcacgtcgtactacacattaccgaccagattcgtcttcacgggacccctcgaggatactgggcttatccgtccgagcgcctatatggccta

atgaaacaagtgagcccgatatgaaagagtccaaggagggacgcgctgacatctatgtcacttctccatcagtggtcaacagtctacccccaatcccg

ctcgctatgaccctcaagaacatcatcgacgggcatcgccaacaaggggggaaagggatggctgacgacgactttctctttacagatacaaggagatc

agacaagcccttcacgctcacatccacacaactagaagccgtttgtgccatcgccaacaagaggcgaccggcggggaacgaccctctcgtccctctga

cctcgagcatccccaacacacgcgagactatcgtcagcgacgcggcagtacgacgagaccgcgttaccatcaagaactcaactctggccccaaagtcg

acatcgtcttcctcaacgtccgacccatcgttttgcattgtgcaagttgggccccgtcgtcaggccgctcggttccagtacggatttagccacacctt

tttcgagaaatctgcaggcagaatgataacccaacactacgcggaggtccaagccctcgattccgtttcttggcctacgcatggtctattcctacaca

gcatgtggaaagatctaggttatctgttcgcaacgagcctcaagcaccagacactcgtggtaagtatcgaggacattgtttgtgcagcactttcagtc

gacaccgcctcgatcgcatgggccgaagaaggaaccctagtggctgttccaacgtaa

>contig_14.contig_14.g491.t1

atggatcagttcaacgactggcaacaccatcctgcgattacctacgacccgaatggcttcatagccaatctcgatcccg

catacatctcagccgctgagcaggtaccaaccaacaccacgaatccgactcccaaccttccgacgtcttcacagcgctgtctccagctactcccggag

gacatccgagaagcctacaagatcgcttacgaagctgtcaagcgcagcataggacagcatcagcgacctgactgggacttgatctggctggaagcggt

cctgagcccacctgagctcgtcaaggacgcactgaaggctcatttcgaccaactcttcagaagccccgccaactcaactccctcgactacgccatcga

ccttcaatgcccagggcccggtcccaacatcagcgtcctctcctactggccattccgacacccacccaacatccagtcacacggcttcaacacaaccc

tctaccacccccctcggcccgtcaaagatagccaacgaccagcccgagcagacccagccgagctcaacggtttccatgtctaaggcggctcttcaagc

aatttcttcgaagggaacatggaagggctggcattactgcggggtggttaggtgggtacaagggacatgtgacgccgagcccctatcggacaacgaga

tcttgcagcatagccgaaaggtggacaagaaattgcgcggatcaagagaaaacggcacagcgaagggcaagaaggaacgaaagcagacgacgcgcgcg

gaggattacccggacatccgagaacgatggcggtgcgcgacgtgcgggattatccgacacgagaggccctcgccatcgaccccctcaccactatcacc

accatcggacctctcatcgcccagatcatctgacctctcaccctcaccgttggatctcacctacacccttcaaccctcaccctctcaccatttcacct

ctacccctcgtccgacaaccctcgtcggacctctcatcgatccggtcagacctttcatcgccctaatcggatctcctcatcgaacctctcccgacccc

catcgtctcgtcgtcctcaccctcatcctaccctccaacacgctctaa

>contig_14.contig_14.g492.t1

atgacccctgctctgttacgactcctcaatctccgcccatcccacttcgcgaatggaacatgggacccctccctcgccg

ccctcgccgtcgcaggcatcatccccgccagcctgacctacttcacctatatcctccccaaaattgccacccagcgctccgctcctgctgtcgctgag

cggaacggtaacggtaacatcacctatggctcgacatccgattctacgccgccgaagcatagcgctgcctcttccgcttcagcacccttgctcagcgc

cgctgccccgctctggcgtgtgcccgtctcggaatggtccagcattggtccagctttgccgccactcgcatcgatgcgcgactcatcgaaggaaccgc

tctctttggtgtcggctggggtctttctggattggtacgttctcacaccactccttttctgccgagaaagtctacgagaaaggtgtctaagatggaag

ctcatgcctcttttgtttctcctcctctgcccacagcgtccaggcccggtccttgtctctttgggcgccaacatcgcagcttacctcctcctccagtc

cgcgggcaacgccgcgcccaccaccgacgacatcaccaaggtcctcgccgccgccgacatcgagcccgacaccgagcgtctcaacaagctcctctcca

agctccagggcaaggacatctgcgagatcatcgccgaggatgagcagaagctcgcatccgtcccctccggtggcggagatggtggcgctgccgcacct

gctgcagctgctggcggtgccgctcccgcggaggccaagaaggaggaggctaaggaggagcagaaggaggagtccgacgacgacgtgggcttcggtct

ctcgtctcgccagctccgtccttgtcgtggtcgggtcacttcacttctggcgcaagcgctcagccgggcacggcagtctcctccagacctctcctctc

ctgaccttcgtcatcatacggacctctcgtcgctctcggacctcttctcagttctctcttaa

>contig_14.contig_14.g493.t1

atgccgttcgaaccgcactctagtgatgaagattctacgtgggacggcggcgctcaagacactgcggagcctcaagata

ctgccgcggaacactggatcatcgagaaggagtacttgaccggccagttggatctggcggagaggaagaaccgagagagttcctcggagttctggcga

ttaacggtcaaggttcgaggtctgaacggcaagatccggtcgatggacgcgcatcgggaagccctcctccaggatcgacttaccttgcgtgccgacac

tgccgaacttcaagccctcaagtccagcacgtcgaaggagctggagcgggctctggccgtattgcaggaggagaagaaggaatccctctcattgcggg

tccgattggcctgtttggggggagagagagatggactggcgtcaaggctccgcgctgcacgagaggccggcattgaggctacagaaggactcgatcat

ctctcccctcgcgcgatgcgcacccggctgaacgaagcgggcgaggccaattctgcccttcgatcggaggtcaccacgctccgagctcggattctggc

gctggaggcgatcaacactgaccacgagcgcgccaacgaatctctccgtcaacttcagtcggaggccaacttgcgcgtcgccgccttttccggcatct

ttcagcggacgatagagacccctcctcctcaagacgagagctcgggcgcgggtacaccactctccctccagcaggcgcctcgacagagcagcccggtc

accaccactcccttgcctttctactgctattctacgcagagcgctacacagagcgaagactcgctccaggatcaacttctggcgtcgggagaaccggc

cactctggccaagattaacgcgactccgaacgaccaccctcttcccctggactccccgacggcaaaacgggtgcgcacgagacgatgggctagtgccc

aagcgcccacgatctaa

>contig_14.contig_14.g494.t1

atgggaaagggttggtttcgccggttccggcagcgacaccctgagctcgacttcaagggtatcgccaccctgtcgcgcg

caaggtcatctggcatgactgtgtcccgagctcagggattttttaatctgatcgacgagctcaatgaaaagcaccagggtctgcttgccacgggaata

ttcacgatggacgagacgggcgtgcagatcaacacgccgtctgtcggtcagcggtacgcggttccggcgggtgctcagaaagccctgcgacctgcagt

gatcgatggctctcaggagctcaccactgtcctcgagtgcatcgccgccgacggtagtgttttgccccctctttacgtgttcaagggtcgcatgctcg

atctttcgggcatcgtagatgagcgtcacgatggcaaggtcactgccacagagaccggttggacgaacacggctatcacagcgaagtggttcgatgag

gtcttcctgccccgcagcgaggaaattgcgggcaaggggaagcatcggctcttgataatggacggccacaagtcacacttccctctttccattctgga

gaaggcggtttccaacggggtcgacgtcatttgtctccctgcccacaccacccaggcgctctcccctctggacgtctcgtgcttccgcccgctcaaag

agttctgggcggaggagcttcgaaaggagatgatgctcaccggctgcgtgaagcgttctgatgtggtccgactctacgggaacgcacgagaacggggg

atgacctctgccaatgcaattagcggctttgcaagcacaggcatctggccgctgacgcgcctcagggcgattccccagtccaaattcattccggacaa

tccaagcgaagcggagctagaggagcttatcgaggatcaggctgaggcggacgacacagtcgacatgctgtggacgttggtggacagtcagcggacta

aggatgggcgcggagtaagccccggagagtacggagcgcaagcctgtcatgtccccggaccggagtacggagctagcgcaatgagatggggagatttt

gtgcggagcgcggagtggagttgggacgactgcctaaaaatgagcggacggacgaataaggctaaggctgcacttacgcaagccgccaaggactgggc

ccgcatgagggccctcgaaactctattccaccacctgttcgagcgccagcgcaagcacttggaggtcctcaagaagaagccggcaacctaccgtgtcc

atctcgacggcgccgccaagctttacacagccgagtcagccttgcgggaaattcgggaggcagaagcgaggcgggaacgtgaagaagctgacaaggtg

gcacgggcggaggaaagggccaggggcagggaagagagggaggtggctaagcaccgagaccaggcggagaagcagatgcgtgcggaagagagagctcg

gaagaaggcggagaaggagaaagcagcggcggagaagcgtaggcttgcagcggagaggaaggagataaagaggcaggagcgagccgccaccgaggcgc

agaaggcagctgctaaggcgcagaagcgtggtgggtccaacactgacaattctgacggccagcactccaagcgccagaggcgggagggatga

>contig_14.contig_14.g495.t1

atgtcgcccaaatccacatctgctacccgtatcctctactggttccggacagaccttcggctgcatgactctccagcgc

tcctgaaagctctcgacctcaaaccggccagctttttcccggtgtggtgctgggatgtcaactacgtgctctcgcatagggtcggggtgaaccgcttt

cgttttctgctggagtcgatggaggatgtctctcagtccatcacgcgaacgaatcccaactctcagctactcgtactccgcggcgatccaacgacgct

catccccaccttgtgcaagaagtgggggatcacgcatctctgcttcgagcaggatctcagtggatacgcccgcaagcgtgacgagtccatccgtcagc

tcgtcaaggaccagtgcaaaggggtacagatcatcgaggagcagggtcattatctctattcgattcaagacattttggagaagaacgggaagaagcct

acgatgagtatgagcgcgctgcagaaggcagtctcaaaactacccgtccctccccgcccgcgcgatgcacccaaaacgctgccagatccactactccc

agaccaaagcagtgtcaaggaccgtattggcgccctcaaggctctcgcagagaccttgtccgatattccacactacaatatggacaaggtgtccaagc

cggatctaaacgctgcctcagcaggaggcgaccggctgcccgatgcggagaagggtcgcgtgtcctgctacgacacggtgtacggtcctgaccagaag

tccaagaccgagaaggaaatggacggcttgtttggcgtccccacactggcctcgctcggtatggatcccgtcgcggccggcgctttacccgaagctgt

catcaaaggcggcgagacggaggcgcttcgtcgactcgacgctcttttgaagaggaagcattacctggccacatttgcgaaaccgaagacgtcgcctg

ctcaacctgcggatgatccggcgacgacggtcatgtcaccttacctcaagtttggagccatctcggtgcggcggctgtggtacgacgtccaagacgtg

atcaaggagtacaaggggaaggacaagacttcgattccggagaactacgagggtcagcttttgttccgcgagatgtacgccgtgggagaacttgcgat

cggagacgagtaccagcagatcaaggataacaagatatcacgctacatggactggtatctgccaaacgtgtacgacgccgacggcaagcggattgagc

cccggccgcgcggggacgaagcttcggaacgacgacttgaactcttcgactgggacccatgcgcgaatcccggcaatttgtggctttcttgctctgca

tacttttctcagtacttccgggtgtatggcgttgcgagcttcccggccaagtacgacaagtcgggaagtctcgtcagacgatacaagtacatctacgc

acctcacacggcacccatcgcggtgcaaaagcaggccaagtgcatcatcggcaaggattacccgcatcctatgctggacgaaaaggaggagaaggcca

agtgcctggcgaggtgtaaagctgcgttcgcggccgggtattacggaacgagcaaggaagtgctggaaggcaaggcggaggcgatcttgcgcaaggct

catggtcaacccgaaccggagataccaaatacggacaaggcagtgtccaaggggagtaaaatccctgactgggccaagccgagcgtcggcgtcaagga

tggtagtgcgaagcccaaaagccaaacgaaatcggaccaggatgacgacgagaaggaggaagacaacgatcagcaccccgacacagcagatcacgatg

acgatcatgatgttgaagggaagggcaggggtcagaagaagcgagccgtgaaagacgccgacgagcccgtggacgaggacgagagggaggaggaccag

acgagacggagcaagcgcccgcggacctcagcgacttcaagcaaggagtcggcgacggcgacagctccaaagaagaagaagtga

>contig_14.contig_14.g496.t1

atgtgcacacccagcatcatctcgtcgtctccgcatcgcagcccgcagcccgcagcccagcatcgtcgcacagtcggtc

ggcacagaccagaccttctgaaaccatccgggatctacaaaaaagtgtctccggcccaggcagcaataaggcagcaagtaaacattgaaggtgaccag

tcctcgcctttcccctgcagccctgcagcccaatccccaatccccaaaccccgcgcaccctccgatccggacttgacaccaacaagcctgcgcccaca

agcctcaaccctcaagcatcaaagactgccgctcactctctccggccctccgcgcttcccggttgcagcgatgtcattggcacatatatcagcctcgt

ccgccccctcgtccggacccagcggcaacaacaacgcgggaaacaccgcaaacaacagcagcagcagcggtggtcgcgccgacagcggaacaggatcg

tcggcagcagcagcagcagcagcagcagcagcagcgaccacacccatcgctgcctcctacctcggcgtcgggcacagcatcacacccgtcgcagacga

ctcccgctaccagttggatctcattccgggtgtcgcacctagcaacataggcccatccacctctttccacgcgcatctcgcgtcaaacccccacgatt

cctctttcgcacctaccaatcctgccttcacgcttggccctggtgggacatcagggaactcggcatctgcggctatcactggcctcgacccttctttt

ctcaaggttggcgggaacaccacttttgacttcccctctttgccagacgggcttctcgacgaggcgcccgagtacgaatactctgcgcatacaaaagc

gaacgtcttcgaagctgcacaagaataccttgccgacttcgacggagcacgcgtattcgacctcgacagcttcgaccctaccttggccgggctcggcg

gcgacgaatccctctggtccttcgccgaaggtcttgatctcagcctaacacatcttcacggtcatcctaaccagcattcgcacatcgcaggaccgtcg

acgacctcccactttggagcatccgatcctcttctgatggcaagctcggcgttagcttctgcctctgcgactccccacctgcataccaccatgcccga

tgataattcaatgggatacggtgctcaagaagatctgcatcatcctggcacagactcatcatcaaatacgccctcagcacaatacgctctcaactatc

ttccccagcataggatgtccggctcacagacggctcctgcttacccggccctgttccacccttcccaggcgaaccagatgaatgcatcagcatcatcg

atggaacttggcggtgtgggtgacgacagtatgcctgcgtccaagcgaaggcgagggttgtcacctcgcacggcgcctgtttatgacgagaacttcgg

caaggcctcgcctttccagcaacaacggcagcaacatcaacatcaacagcaatcgcaataccatcagcaccagcagcagcaccagcagcagtcgcact

cgtccttcttaccaggttcggattttcagcaacatcgaggctcaaatgcatctcccatgccgaatcaggcccatcctccttactcttctggctatgct

cccttcaatcacgagggaaagtcggtgatgtatccgccgcctcctcctttgccccaccacgaaggcaacgggacttcctcctcgaatgcgcatttgcc

tccgtactcgcgagcccaccctccgtacccgacgacagccacccccgccaccatgacttcgcgttcgaggatgacgagctggcatggatcgccgtacc

ctagcataagtggacccaacggaccggcgacggcgctgcctggtggtccaaacggaggagcggagtggggcatggattccaatgcccctgcccatcct

ggcatgagtagcagtgcgacgtacccgtccatcgtgggcccaagtccggacctgaaccacggccattctggctatcccggccatcgcttgtctgaagt

tagcgttgcgtcgtcatcgatcagtgcggctgctgctgctcctcctggcgttaccggcggcagtaagtccacctttggcaattcggttccgagcggtg

ggggataccacagcggcggccgggctggactgggcattggttccgcaggagccgccggcatgggtctcgtccccgtcgtcggtgtccccgtcgggccc

ggcagcacgatctccatagggccctcacccaaccaacaagcggagatcatccacccactgggtcaggcgctggacgcgacgtcggatgcggatggggt

ggcgcggtgtccgtacccgaattgcaacaagacgtttgcgaagaaccggacgtataacctcaaggcgcatttgaggagtcattcgcagctcaagcctt

ttgctctggcgacaaaccgtacatctgtgaggtttgcggacgaggattcccgcggagcgatgctttgcgaaggcattggtacgcgcgtcgagaaggag

tgtggccacaaagtcgccgagatggaggctcagcagaccggcagtgggatgggcggagccgggacgagcagcatccacggcgaagccgggggcagtgt

ccactcggacgaagacgagacgtcctcgaaccaccacgccggtggggccgctgggaacagcagcgatgggggcagtaatgcgtcgtcgtcgcatcacc

agcatcaggcgtatggcatcaagtccagcatcaacagcaacatgggtactccggcagcggccctagcggtagcggcgacgtag

>contig_14.contig_14.g497.t1

atgtctggctcagggatcactgtcgctgcccgagctgctaccaccccgtcacgaaacaacgcctcctcgacaccttctc

cgtacgtgtggccatccgccatgacgttcgctaccccgtgtgacaaaaagctcaatctcaatgtgccacagataccagaaggaattcaccccgacgct

atcgaagccacgacagagggactccaagtcacatggccagccaagcccctcctcgaatccacctcggcttcagcccaggagcaacaaccgcgcaacac

agaccaagcgcaggagcatgacaaccaccaaccgcaacagcaacagagccagaaccagcacatctccctctacccctggcgctggctcatgaacaact

cctacgcacccgtcctctcccgcacatcctctcctctgccacagggcctcggcgcaggcggggagcatgagcgcatcctctggggtcgcgggattgcg

tctgcgccgcctacggtatcttatgaggacgtcatggggagcgacgaaggggtgcttaagtggtgtaacaagattgctcaatacggcttctccttcgt

ctccggcgtaccagcgacaccggaagcgacccaatccctcatcgaacgcatcgctttcatccgcccaacccactacggcggcttctgggacttcacct

ccaacctcgcccacggcgacacggcctacaccacccttgccctcggcgcccacaccgacaccacctacttcaccgaccctgccgggctgcagctgttt

catctcctttctcatggggtcgcagagggcgccagggagggtgcgcccgtggaggaagttgggccgaaggggggtgagagtctccttgttgatggctt

cttggctgccaatgtgcttagggaggttcatccggaggcgtatcggaccttgagtcgggtcagggtgcgcacccattcggcgggggacgaggctacgc

ttgtgagacctttgcttggggaggggtatccgatccttcagcatgcgcctcgtccggccctccgacgcgtcgacgagctcgccctcgatcctccttct

cctccttcttcgacagacgtagcagcagccccagtcgaagccgcatcaacgacgacgacgacgaacccggtcgagctcgaggacgaattcggcgagct

cgtcatggtccggtacaacaacgatgaccgctcgacgttgcgcgtgccggacgaggatgtggagccgttctacgatgcgctgaggaagtggcatgcca

tcctgaccagcgtccagggcgagtactggtcccagctcgttcccggcacggctttgaccttcgtcggtgcacgacgcctgtgcggcgcttatatctcg

cacgacgacttccgctcgcgcctcagcgtcctccgggcgcagtatccctccgccgagttgggtgtttccacatcttcatcagcgagtccggcggaagg

gaacggcggcagcaatgcggcgggagatcgtggttcgcgctaccatcgcggtgtttgggacgacgcgctctga

>contig_14.contig_14.g498.t1

atgccgatatggagctccattattcatcatcatcgaccttcagccaaggcatttgaaggtcagagccgtcgccgcctgc

aactgggcacgtccgttctccatgctcttgccatcagaacagcgggagtggtgctgactgctgcatcgtcggaaatcggagctgctggctctgcagat

ctcgacatcgacatcgacatcgacatcgacatcaaccaacacctccaggaaatcacagcggaattcgatcccacaaagtaccccttttctctctacag

tcattaccagttgcgcatactcctccctatagaaagccatctctccaagatgcagatctctactgccttctcacggctctccgtcaccctcctcatcg

cactcgcaacagtcttcctcgcctcaacacaacaggcacaagcagtacagcccggtctcccatggggcgcatccccacaactcgcacggaaactcgtc

aagtcgggctcaaacttccaatggtacatgaactggggcaacaaccgccccgttagcacccttggctctctcaactacgtgcccaccttctggggtca

gtccaagattgacgcatggcacagtctcgaggggtggtacaaggctcacgggaagcggcccaagtacatcctcgctcagaacgagcccgacgttccct

ctcaatcaaacacctcccccaaagccgctgccaagcagtggatgaaggaactcaagccctggcaacgccgcggtgtcaaggtcggttccccacaactc

tgctgggacaccaccaactggctcgaacctttcctccgcaacctccgcaacatgggcggcaagcccgacttctgtgccgcccactactacggatctgc

aactgacctcggccgctttaagcaatatgtccgccgtgttcgcagcacatgccgcaagtacggtgtcaagtccatctggctcaccgagatcggtgtga

tggcgtcctcccaccccgcccagggcgaggtcaaccacttcgcacaggaagtcttccagtggctccagacccagccttacgtcaagcgggccgcctgg

atcggtgtcttcgagttcgacacaccttacgacggcttcatgagccagaagaacgcttacttcaactcggacggaaccctgcgctcgctcggccgcat

catggctttcggaaacctcttcggccgtcgctccctcgtccgcgcctccgcccacgccgccattgccgagcgtcaggcagaagtcaacggtaccctca

tcgacgccaaggccgcccttgacgacccccggggcaactgggaccacgcctttgcgggcgatgaccagcacgaccctaacgacgatgcatactgggat

gccaaggtcgcccacgaggacgaggctggcccggccgaccacgacgatgacgtccgtcttcacgaacttgcggacgacgtcgaccactaccaggacgg

cttcttgggcgatctgctcaaggacgtcgtcaaagttgtcgaaggcatcgtccctgctcaagctcaagcttga

>contig_14.contig_14.g499.t1

atgaagacacccgcacaaacacttcttcgctccctactgctcctctccgctaccgccctatgcgtctcagcaagtcctt

caatcctccttcctgaagccgccaatctcgccgtcgcacccctttcgcacgacgtccgctcgcacgagaacacaccgctccatcgtctgaagagtcga

cagttggaagctcggttgccttcatcatcatcatcctcctcctctttttctaagaggcgggtagacggcagtcggctcgtggttcgggctcagtcagg

gacggaccctgtggctgcgaagcctgcgggcgagagcgtcgatgctgatccgagggtagctttccttggtgcgctgcggggagcccaattcagtgcgt

tcgccgatctccttcagaaccagaccgacttcatcttggctgatggtagtgagaaggtggttcttgccatgacaaacgaggccgtccaaaagattgct

ccttacgtgcgacgcgacgcttccctgctacaagccaccttactgcaacacgtcctcgtcggcaccttcccagacctgcccagcaacctgacgtcgtc

gcaaaactcccgcatccacacggtcggctacagcatgctcagccaggggtcgttcgccaaccttccgggtgggaacgggcagccggtcgcgttgagtg

gttcgcgcggggctactaatggatcgtcggaagcttcgaaggaagacgcggcgactatgagcggtaacggtaaaggctttatcaacgaggctctgaat

gatgtcaagttgcgcggggacagcttcaaagtcggcctaatcaccatcgtgcccatactccgtcccatcacggtccccggcaccctccaatacaccct

cgaccacgtcgtcggtggggccttgcaccacagctcaccgctcttcacccctgcgttgctaaaagccagcaagaagaatgggacggggatcgagaagc

tcccggcggcgtatccggctgctgtctccgctcgtgtgacagcactgtcggagacgacgggtttgacgatgttcgtccccgccacagcagccgtgatg

gcgctcctgtcgtccgaagccggccgggcgctcttgaacacttcttcctccagcaagaagggcgggccggacgacgccgtcgggggcttactcaccat

cctcggtcaacacgccgtcgagggtcgaacgttgtacagcccgctcatgcgagatctatgcgacgacactcaagcgccacccgtaaccacctcctccg

gccaaattctcaccttcaaatgctcctggtccgggtccgggtccggtcggggggtcaaataccccaccgcaatccgactcttccgctcgacagacaag

gagcgcaaaaacgtcctcgtgtccgcgaacctggtccagacggacatcatgttcgcaaacggcgtcatccacctcatcgacgctgtcctcttctcgct

agatgcggaccgggacggcgcggtgggtgcgcggtccggcgtggcagcagcgttcgtaaccttggcggctgccatcctcgtcctcatctag

>contig_14.contig_14.g500.t1

atggagaccctcaccagaacattctccaacatcagcgacggtatgcccgtctctgtcatcaccgacgtcagttcatggc

ctatgctcacagccttgctgaccgtcgtcgccaccctggccttctaccagctcgtgctggcgcctccgagctccctccgacccaagggcagcaggatc

aaacccatcttttcctcgttcccgttcctcggatcggtgggcttctattcagacagggtcggcttccttcgcaagcactttggcgaccccgagagctc

cagcttccatggagacgctcgtcgaatcaccgtccgggacgctaccgtcgttctcctggccggcaacaaggacgacgccaaaatgttctattactctc

gcgctctcaagttcacccaagcatacatgaagatcaccacagctgccatcatgaaccaggacaaggctgaccagatgttcaacgaaaacctcaagcag

gccattcgcggtgagcgcctggccaagctggcacccttgttgtgcgaggacacggttcgcgccctggacgctgttccggactggaagaagggcaaggg

cagcatggaccctaaggctttcacgttccccatcgtggaagaattggctggctactactgggcgatgaaagacgcggcatctttttggtcgaccttct

tcaccaagtatccgttccgtacatcaagtttgaaggaagagagtggaaaaaagctgttcgctgcactaggcaaagctgcatacagccgattcgtaaaa

gatctcgtcgaagatgtcactgtcggcatgctgttggacaacggtatggacgcggattacgttgttcgatatgtggtcaccggcttgtttatagccat

catcaactcgacgtcgatgtcgatgtggattttgctgtacctcggcgcccactctgagcttcgacagcgagcgagcaaggaactttctgcggtattgt

tctttcatcctcagaaatcgcgcgttgaacaactccaggcgctgtcccttgcggaatgggagaacggattccctacaattgaggctgcgctgaaggag

acaattcggctcaatgctgatggtactcagttccgtctcaacgctggcgatgccacggcgggcacaagcgcgcccaaggttcaaggcgaggaggtcca

caagggcgagttcctgggatactgtgaccctgaccgctgggcgcgtggggagggcagcggcgacatggaatacttgggctggggcacgggcatgcacc

cttgcactggaatgcgatttgcgaagctggagatcaagaacttcgccaccacgattctcgcgctaatggactgggagtcgtacaatccgcgaagcgga

gaagtctacacaatcggcacgctaccagcaccccagctcaactacggtcatcggttgcctctaggagagaaggttgccgtgaaggaagaggagaagga

ggacacgaaggaggaggaagaagagggggatgaggatgaggatgaggatgaggatgaggatgaggatgaggaggaagagaccaaggagactgccgcga

aggagaaggcagaggaggaggaggaggaggaggccgtcaagaccagtggcaaggcggataacgacaacgacgagtag

>contig_14.contig_14.g501.t1

atgatcggtaagcgaaagctttctggtcagcaggtggcagcttttttgtgcggctttccaaatcgctacacgggtttgt

cctatgaaaagatatattggggctcgcttcttcgatctgtactaccggagttctacaactcggtacagcctacacaaggagattcagttgcggatgcg

gaacatggcaccgagaacgctgctttgattgcggttagggaaggaggaagtattcctcgtccgtccccattctgctcggcgatagcggaggactacgc

ttggagagactcagcgcttgcctcagtctctttttgggactttataacacagtacgtgaagcgaaaacgaggaaaggaacaggagagaatggaagcgc

ttgtagaagaaggattagaagccagagagctgcgcgtgcatgatacggacgacgtcgaagatgaggagccatcaggaggagcagcggatatgaacgac

gtagaggacgcgtctggaacgtttttgccgtttcgatccgggcatgtaagtgccgctacacacggtgtgagacggcgtaaaaggcaggttatcccggt

tcttctaggcccaaacgtccctcgcagagatcgggaagatgatcgagataagtatgctgcgaccatgatgttgttgttcaaaccgtgggacttgtccg

agaccattgcatttaaagctgacgatgaaacgtggtcggctgctttcgataagttcaaagtttctgtaaacgaagaagtctcgtccttgatggataac

atgcagcagctacatgagtgtcgtgacgccaaagatgatcacagcgcgcaccgcaaagctcgggaagccctgctgcgtggctcgggaggaagtgcttt

cgatgatactgctctggataattatgaaggggacgacgaaatctggctggctgctatgacgggcgacgttgcagaggaagcggaacagagtggtcggg

aagcaatgacggcggctctcacgcagcgcgcgatagatcatgccatggcatcgggattttatgaaactagcagcacctgtgttcccatcgacacgaat

ggtgccgtgacggaaggtggagacgatgagatcgcggacgccgatcgacagttggccaagttattgaaggaaaaagaaaatgtcatagccctgcgaaa

aactccgcagagtaatcgggctcccaggggagtcggagcgcaacatcaatcgatgtctaacacatctctggaaccgactctgaccacctgggcggcgg

aagataagaccttgctggatgaatacgtgctaggaacgggacaggacgcagatgagtcaaacataggcatacatcattggcttgcgataggaggagac

gcgtctccacttatctcctaccgcaatggggacaagatcggcaaaggtcagaaccaacgggtgctgcggcatggtgttgctgaagctgcttggtctgt

ctcaccgaagaacgctggagatctgtatgaaaagctgcctctcttggtgcggatgccggtattctgcacggagaacattgcgacagagctcgggatct

ccaatggatctgagggcaggattgcgcgggtttccttctttgagaaggaaggaaggcggtatgcgatctcggcggaagtggatttcccatcgacgctc

gctggtaagagctacgatagatctaaccagcgctga

>contig_14.contig_14.g502.t1

atgggcaatgaactcgcaagcttcaaaaccgtcgctatgacgttcatggttcccccgtcccagcaggcaggctacgtgc

ccgacccgacgacccccactgatgccaacacgggcgcagacgagcccgattcgatccgtccccccgacagtcctctcactgcctctcaactcagtttt

gtgttggctcacctgcgggtgctgaagccgtcggagttccacgccagggttgcggcgactgacgactacgttggctcaacaaccgcgctcggccgtat

ggaccagtatacggtcgacgcccgtggacggtttgtccgcacggacgagaacggcgaggtcgaggagatgcggttctcttacgccgcgaaaggccgat

cggcgtttgcgttcgtgaacggaaagactggcaacgccttccccgtgcagtgggcgtcggaggcaacgcggccgacgacgatcgtgatcgaaacgacc

gccaatgatcccagcatgcgccgcaaacgtgcgacggaggagggtcgagagtatacgtgtaatgtcatctactcgatgcggcgctcaacaccacagtc

cacactgctcacgcctcatgaaggcaacacgaagcgacatgtgtgggcccagccctcgctcggcggcggctcctactttctgcgcgttgagcgcgctc

tcacggtgaagggcggtgatgagcgcgagcctggccgcatgtcggatcgggacggtgatcacgacctcctccgcaccatcggcgagcaggcggcgctc

aaatacaacaccgttcccgacgttgccctgctcgacacggaagaacaagcgtactggaagatcgactggcaccttgttcacgagctccccgcctccac

cctctgggaggtgggtttctttttccgtcagtacatctccccgacgacgaaaaaaatcggctttacgctcgtcttagagtccctggtcgcccacggaa

aattgccggggaacgacctgaccagccccattccacggaagcgcaaggtggactacttctttgctacctattggcccttgccagtacactacttcgca

ggggcggcggacaagcccaacaagcggggcgcggccatgtag

>contig_14.contig_14.g503.t1

atggcgcctgctcctaagaacaagaacaacgcacaacccgctgcctcgaccgctgctgcccccgcggctcctgctgctc

ccacctccaatggcgccgcagacgacgcagcgggctcgccatcgccatcgccatcgtcctcgtcggctatcgacatcgtcaagaccggcggaggcaag

ccagaccagacccatcactacgcacagctcgacgctctcaaggaagacatcgcgaagacacaggctgagctggacaatgtacgctccatcttgaacgg

caccggaccctccaaggacacccccgagggaaagcgcagggacgctctcaaaaccgagctcgacgagatccgcaacaaacaggctggctccaagggaa

gccgcggaaaggtactcgaccaggtcaaggctcttcaggactcgaccacaaagcggatcaaagacctcaacgcagccaaggcgaagctccagttcaag

tcagctgccgacgtcgatgcacagatcagccgtctcaatggccagatcgaatcaggaaccatgaagctcgtcgacgaacgacgcgccatcaacgagat

ctccaacctccgcaaggcaaagaaaacctacgaccagctcgaagtccaacagacctccatcgacaacgaccgcaagcagatcgacgaactccgcaaga

ccctcgacgacccagaatcacgcggcctctccaaacggtacgacgacatcaaggccgaactcgacagcatcgcaaaggaaaccgaaaaacaggtcgga

tcacgctccaagctcctcgaccaacgcaccgccctctccaagaagctcaacgaactcaaccagacccgtcgggaccgccaggcagcctaccgggccga

gaacgagaagttccatgcaaaggtacaggccgagcgcgagaagcggaacgaacgcttccgcgccgagcgagaggccagtgcggatgcgaagcgtaaag

aacatgagcagcagttgcgcgaggatgctgctttacccgcttttgcgaaggagatcgaggactgcgacgtcctcctcaactacttcaacaccaaggct

ggtctgtcggccccatcagccgcaccttcttcatctgcggggccctccggcagtagtagcagcggcgccggattcggcaagcctctcgaggctcgaca

ggtccagtcctcagacgataccctcaagggcatgcgcgtcgccaagaagaagggcgaggaagatgagagtgactacttcttctccggcgccggtaaga

agggtaaaggtgggaagggcggtgccaaggggtccaagcagcccgctcctgctgcagtatcatcggcgaacggtacagccgccgccgccgatagctca

gcatcagcgtcgacaggcacggggagcaccttgcacgtccccctcggccacctctccgccctgctcgcgctctcgatcccgccaccgacatcgacggc

ggacctgccccgggtgatcgacaacctcaagctgaagaagagttactttgtaggcaactcggagcgggtcacgaaggagaacgttgcaaaggtggaga

agatgctggggggtaagggtaagggtgggaaggggggtgaagctgttgccgaggtaggaacggaggaggacacgaaggaggagaaggcggtggtggag

gtggcggagaaggaggaggaggaggaggaggagaaggacacgaaggaggaagaagaagaggcggatgagggcaaagacgaggaggcggagaaggagga

ggaggagaccaaggagactgccgtggaggaggaggagaccgaggcggtcgccgcacccgcggaggctgacgaggtcgctgaggaggacaaggaggaca

aggaggccgtcaagaccaacggcaaggcggacgacgacgacgacgacgagtag

>contig_14.contig_14.g504.t1

atgaagagctcactcgcagcgcagctcgccctcctcaccgacgtcgctcccgtagacaccaacgcagatcgcagcgacg

atctcctccgcgctttccccgtttccagcgctcagcatgccgtccacgacatccacgaccacgatcagcacgaggaacgagaagatggcaacgaccac

gaccacgacgaagatggagagcaagtggaagacgcagatcagtcagatgctcactcagacatgagcgatatccagaatggagatgacgaaggcgacga

gttctcagctcctgacgataaccccaacagcactcatgcgctcgcaaaccaagcagccgagtcagaagcactcatcaaatccctcaaagcagacacag

ccgccgccgctcagcgaggtcgcgccatcaagcgacagcagcgcgactgggacgcagcccttgagctccgcatccgagctcaaaaggtcgtccgcgca

ggaatgaggatcgagacgctcgctatcgacccatttctcaaccaccccaccgcttccaaaaggggcgcaccggagcaccacgcgtccctactccgcaa

cctcgacgccctctccgcctccctcttcgtcctccgcgcccgtctgctcgaacagtcccacccctccgacgtccgtctgcccgccagtctcagcgtcc

agcacttcgaagcaacgcgtgagcgcgcgaggaagcggatcaagttggactcctccgatcaggacgcagacgaggatgtggctgtggctgtggatgtg

gatgggaaagagcacacgttcgcgcttgagaagctcttcgctctcgaatcccgcatcttggaaccactggcgtcttccatcctgaacagcgcccattc

caagctcaacgctgccaacacgtccgcctcctctaaagacaacaaagcggccacagccaagttcaagacggcctctcagacgcccaccgagcagatcg

aggcttccctctctaacgccgagtcgaagcgcagacttctggagcgtacacaggtctggaggggggatgagactcggttgtgtctccccgccactccc

actcccgcagcagtagcagagaaaacaacaacgtcagactccgtagcagaacctacacccccttccgtctccgcccgcgccctcgacctagacacctt

cgacgactccgacatgtactcccacctcctgcgcgagctcatcgagagtaaaggaggccgctcttcttcttcgacgaacacgcctgctgctggcggtg

gtggtctggccggcgatgggggtgctcagtttaatgctgatttgacgctgttggcgcctgggctgcttaacggggggaagaaggcgaaacgggcggtg

gatactcgggcgtcgaagggacggaagatcaggtacgaggtcaacgagaagatcgccaacttcatgccgcccgtgccccgtctgctctgggacgaaga

ccagatcgaccggctcttctccaagctcaatgcccgtcttggtgaccaggacgtcatcaagcgaacccggacggcggcgacggtggaggaagagggtg

atgaggccgatgaggaggagggacagggtgcggcttcgacgaacattattgatggcttgcagttgttcggttaa

>contig_14.contig_14.g505.t1

atggctgctcccacgcgctccgtcgagatcgaggtcgacggcgtcctcttcgacatggacggcaccttgatcgactcta

ccccagcagtgaacgcaacatgggaagagacagcgaagcgattcaaccttgacctcgagcatgtcatggccacctgccacggctggcgcaccatcgag

aatctccgccgcttcgtgccctccctcaccgaggacgagctgccggcggaagtgatcaagttcgagagtcgcatcggggatattgctgccgaggctgt

caagtccggcaagccgggcgctattgtttctctcccgggtgccaaggagcttctcgcgcagatcaatgcgggccgggacgaggatcctacgcgcaggt

caggctgggccatcgtcacaagtgccaccaaggcatacgcgcacaaagccttcccttcctcaggcgtcgcacccgccttcccagaaacattcatcaca

ggcgacctctgcaccgccggcaaacccgacccacaaccctacctcctcggcgcccgcggttcccgccaagcctcctccccctccccttcctcagaaaa

agacgagggcaacgcaaaaggctacctcgtcgtcgaagatgccccaccgggcgtcctctccggcaagagggccggtgcgtcgattaacaatctattcg

agttcaaaacgctgttcggcgccgtccttatgcgtgatatgttgaaagccatgatggagggaaacgtacgcctgccactcatccgcccagacctcctc

ccgatacccgcgcgcacggaggtcccactcgatcgccgcgcggactttgggggagaggttgttttggaagagggaggtgctggcgtgggcaccgatgt

accgcgacccggcggcggcgccctcgtagaaagtctcgatacgggagagggcgtcccgttgggtttgtttggaggagaagaggaggacgaggaggggg

ccttcgagtttgtgggtgtatatacctccaaacaccacccagagagttccgtccccggcgcgcggaacaaggtcgaccgcaccggagagcgcccacag

gcggtgcaaggcagacaacagcaagagcgaagagccagcaagcagtctgaccgcggacctctgctctttcgggaatttttcttcctcgctcgcggact

tccaacttttttgacttggggcaggccactttctctctcagctctctcattctctaccgatttgccaggctcttcgtcttccgcgcaacccacctccc

gaccccgagacaggcccgttcaacggagcctgcgagaaaaaatgggtccaccacctccactccaaccccgaggcctcgagcgtgagcgcgagcgcgag

cgcgacaggaagggcggcgcagtggatgatgtttctgggaagggcaaggcacgcgaccgcgaccgtgatcgtgagcgcgatttgccgccgcggtggag

acagcctgaaccggacgcaggcggcggcggcggagggagaatgagggattcaggctggggcagtcgtggcaaagggccggtgggtgggtcaactaatg

gtcctggacctgtctctgctggattggcagcatcgtctacatcctctgatcgtcttctggaccgagatcggacgaaggataaggagaaggacaaggat

agggatagggatagggagtcggatcgaggtggggctcgggatgatcctagggatcgaaggcggagcggtctttttgacgcggaccgcgagcgcgagcg

tgaacgagaccgaggaggtcccagggacagcagggtcagcgtcagcgaccgggatcgagatcgcgaacgggacagggaacgcgagcgagacagacgcg

atcgagaacgagagcgagaccgagaccgacgcgaccgtgagctcgaacgagatcgtgaacgcgagcgggatcgagagcgcgggccgacggcggcggga

ccgacaactcagcgtccaggtgcaggccccaagcctttacaccacaacaataacaacaacaagaacaagaatggtattccacaaggcccacgcggggg

cgggaatggcgaactccgtcgtcgatcgcccctcccaccccgtcccaccggtcccaaaaatgcaggcggtaactcagccagcggtgggcctccgtccg

gtcctggaccaggtccgggtgggggtcgtctactcgcgcgcaaagcggacgcgggaccaatattggcgcccgtcgtctcccgcccgaacccgcctgcg

ccatcgtcatctacggctagcgcttcgaaccaccggacgcccatctcgcggaatagtaaaggtagcggcgggcccggcgacgaaccggagctcagagt

catctccgctgctgctgctcatgggcgggagagttcggcgagtggtggcgggtggaagaagatcagtggggatcggacgaatgcgatgggtgctgcta

cgacgggtggtagtagtagcagcagtgcgatcccgtcgggcctgcctacggagccgggacagtcgggccggagaagttcgtcgtcgagtggggaggag

ggggaggtgaggagtaggagggatcgtgagcgggatagcgatcgggagctggatagggatcgcgagcgggatcgagatcgagctggcgatcgggatcg

gattcgtaacccgcgagatcgagaacgggagcggcaacgggagcgagaccgaggccgggatcgcgaccgggcgagagagaggcagcgggaacgggaca

gggacagggacagggacagggagacggaccgagatcgcgttcgagagcctcgtcgggccgaacgtgagcgtgaacgcgatcaagaccgaggaatgcga

gatcgaggaggaggaggaggaggagggcgccattcgaacgtccccgacaagccgcgcagtcaccatgaccgctccaccgacaataggccggaccgtgc

cagaggcggcggcggcggcggcatgccggacatcgacggccgaccgatcgacgtcgacttgaacgagaatgaggacatgatggacgtcgacggcgcac

cgctcgacgatgctgtcctgaacggctccgaaccatcccctcccgaacctcctcctccctcctcctccatcgtctacgaccgcgccgtccccggggag

ggctacgagcgtattgcccaagttggggaggggacgtatgggcaggtgttcaaagcgcggggcgagcggacgggtgcgcttgttgcgcttaagaagat

ccggatggagtcggagaaggatggtttcccgatcacggcgatgcgggagatcaagattttgcaggcgttgtatcattcgaatatcgtcaggttgttgg

agatgattgttaccaaagggtcgtactacatggtcttcgaatacatgcagcacgacctaaacggagtcctctcccaaccctccatcgacttcaccccc

gcccacctcaaatccctcgcccaacagctcctctccggcctcgaatacctacaccgcaaatcggtcctgcaccgggacctcaaaggctcgaacttact

cctgaacaacgcgggccagctgaaggtcgctgattttggactggcgaggttgtatgcgaagcggaggaaggggaattatacgaatcgggttgttacgc

tttggtatcgctcgccggagctgttgttgggggcgacgcagtatggggcggaggtggatatgtggggagctggctgcatcttcctcgagctattcacc

cgcacccccgtcttccaagggaacgacgagctgcaccaactacaggtcatctcggacctcatcggcccaccctgtaaagcgcggtggcccgatgtcga

cgcgttgccgtggttcgagctcgtgcggccgtcggctggggtcgagatggacgctggcggaggggctgatgagccgggtcggaagtggactgaactct

tccagaggaattttggccaatacctccctacgcccgaggcgtccaagttgggccaggctctgctcgagtatgatcccaaacatcgcgcgtcggcggcg

gaggcgctttcgatggcatacttcacgacggaagaacccgcacctgagattccagctggcctcctctcgagcgtcgagggcgagtggcacgagtacga

atctcgtaaagctcgtaagacgcggcgggtccagatcgtaccctcctctcaacaacaacacccacaacaacaacaaggccaggctcaaggggcaggcc

ctccccttcctcccccgccagcaggcgcggagggtatggtaagctcggtgccatcgacggtccgtgccgatggcgggtctacagttcaggctgtctcg

gtctcggcggatgggacgcctctgatggctgtcggtgtcggtgctggcagtgctggagtcgtcgtcagtaggaaggagccggctgctgcgcgggtccc

gcatcggaatgaggcgcaggcggatctgggcggacgggggaacgaggtggtgctgttgtctccggggagtgcggcgctgtttgggccttag

>contig_14.contig_14.g506.t1

atgaaatccaaagcaggcggcagttgcgcagttggtaaggcgtactgtatcgttgcggtgccccaccacgtccatccca

tctcgctctcgcactcgctctcgcactcgctctcgcaggcggatccccttcacagtcagcccaagaggagcccgcctttcacttcaatcctccaactc

aaagacgatcagcgaagaacagagcgaccagcccacagctcttccttcccctccgtcggaaatcacattcgctcttttccctctcttccctgccctaa

cccttctcctctgtccccaatccatccctatccgactacttccaccatgctcggtctcgcatcgccacgtgccctgctcaccctcgcattcgcactca

cctccgtctatgcgcaggccaacgacgacgcacaatgtgtctcccttcgcggatcatcaagctgcccggcattccaggacgcttacatcaaccccaca

aacctctcacaggcatggccatggttcacagcagtcaacgatgtcgctaccttcgatcagcagtttgagctctacttcacagacccaaatcgatacca

ccagaccaaattcgagcgccaactgcaatgcaacagcaccgcagcacaaaacacaaccctccaataccagcgcaccatcctctgcggccaattctcac

agatctcatactcagtatcatgcaacctcaagaaccgcgcaaaccccatcatggtctgccaagacacctgtctccaatacgcacaaacagagaacgtc

ctcgtcgcaaacccgcagatctgccctcccgcaaaccaactcaccctcgtccagaacaccacccgcgagttcaacttgaacaaggactacgtcacctg

caccgactggtccactctggtctcctctgacaacgcaacctgtgtcgagggtatcatgaacgaggggaactgcggatggggacctcaggttaacaacc

agctttgtgcccactgcgaccctacgggaaaccagaccatccccgcctgctgtaccgacgcaaagaccgacctctctcaatgctctctgtttggattc

ccaggtgcggcccgcgttcgtcccaccaccagcgtagggacgaccatggtcacctccaccggcacctccgccaacccgacctccacttcatctcccac

aaacaacaccaacaacggtcaacagcagagggggaaacggtacaccggcggccaggtcgccggcatcgtcgtcggctgcatcatcggtgcgctcctgc

tcggcctcctcctcggtctgctctgcttccgtcgtaagaacaacaacaacaacaacaacaacagcaacaacggaagccaagtcgacgctgcttctgca

cggcaggacgatctcgagaagacccggatgttgaacaacaacgcttcgtcggatcctcgctcgggtgcggctgctggtgcgggtgcgggcttggctgg

cgttgggctgggcgccgctgctgctgctggacgtagccgtggggacgagaaggacggtggctggtccagccccttcagagatgagaagacgggcagcc

cggcgttcggtgagaaggaagtcgctcggccgggttcgtcggccctgggcagcatcctgaacaacggtgcccctggtaccaacgccgctgctccgtct

gctatggccgctgctgctcctggcgcacaacaaccacactccaacgcagcgtctgtcaccggcggtcggttctccccttcgtcgagcagatcgggtgt

aggtgcaggtgtgggtgcggcggctgctgggttgggagcgggtgcactcctgggagcaggagcggcccgggcctcttctggcacgccgacgacgaact

cgaactcgaactcgaacccggcctcgaaccgggcagcctcgtcgaccccgacccagatggccggcctcaccgcgggatcgtcgtccgagaaccgtcca

atgagtgcgctctcgaactcgacgacggacgggagggggacgacggtccccgctgttcgggaccagtactccggtctggacattcaaccgaacgacga

ggtcgtggcaatctacccgtactcggccaccctgaccgacgagatcaacctggaagtcgacgacgtggtcaatgtggtccggctgtacgacgatggct

gggcccttggacgccggaccgcagggcctgggatcgtcgctgctgaaggggcgttcccgcttgtgtgtgtgacgcatgcttcggcggtggatgggcct

gcgtcgagggggatgggcggcagtttaccgggtgcgacgagctcggatgatgatcggttgactagtggcgatggcgggatttcggatagtgttgatgg

ggctgttacggctgatgaaggggcgcttactgcggatgaggactttgagccttcaagaaggcgttga

>contig_14.contig_14.g507.t1

atgctcgtcgctaccctcgtcaacactctcctcaccggcttcaccctggtagcagcctctccctcgcccctgctcttcg

gaggcagctccgacctgcccacgtacgcccctggcatcggaaatgctacgtacgacggcaagagcaagtgcaccttccctgctccccagaagggcttc

aacccttccaagtacgtcggaagcctcgacaagcccgctgtctggtaccaactggcatcccaattccaattcttcgaggttggctgcacctgtgtcta

cgccaagtatggcctctactccaacggaagcgtctccgtcgagaacttctgcctccgaaatggctctcccggcggtgtgaacggcacagctaccccac

gccctgacttcggtaccggctcctacaccgtcagcttcggccaacccgccggctcgtgcggggctgaccgacccaactacatcgtcagcaagtactac

accgactcagccggcaagtacaccgctgccgttgttggctccgacagctttgacggctggttcttgctcagcaagcagcgtcttgtaccccgggccac

gattgacagctacctgaaggacatcgccgcgcagggatacgatctgtcgaagaagtacaccaagaagatgtacttcgacaccctcgccaaacacatct

tcagccgcttcttgtccttgaccggctctcccgcagcctcgctcaccggccgtaccatggacccactgcctgcctacccgccatcaatcggtcgagcc

ctgttcgacgcctcgacgaactgcaccttcccgctccccgtccccaacttcgacccagccaaatacgtcggcgccagcccctcctcccccgcaatctg

gtaccaactcgcgtcctcctcaaaccaccacttcgaagaaggttgcaggtgcatgtacgccaagtacgcccctgcgccgtctaagaacgggtcgatcg

tgctcgagaacgtgtgtacccggaccaacgccaccacgggtgaggtgaagacgagcagcgtgcatgggatcgcggaatgcacggcggcgggtgggttt

gggttgggtagctatcaggttcgcttggggccgaggccgggtggggtctgtggtgagcactcgcccaattatgtggttagcaaggtatactatgacga

tggggagctggaggggcaagggctgtatgaagctgtcatcgtaggcggcggacgggacttcgagggctggtacctcctctcccgacagcgcgatgttc

ctcgctccaaaattgatacctacctcaaggacgtcgccagcctgggatacgacttgaggaagccgtacagcctcgccgaacaagggccgtcctgccca

gagccgaccgtctga

>contig_14.contig_14.g508.t1

atggcatcggcactcgcgtcgcagctggcgggaatccgctccgtcaacgccgcccgcatcgccaccctcggatcctcgg

ccggcgctcagcacacagcctcctacctcttcccgcccaagaccgccgcagcacaggacctcaacaccgtacacgccctcgcacgcactgggctcgag

gaactcgcagcccatgaccgctggttcgagtccagcagcgcgtgggcttcggacgatgtcactcgctccggccgcgagcttctttttggagagagtag

caagtcgagggacaggagtgtcctgaccaaggaggacaatgccctcattgacgaagccctcgcgctcttcctcgcccgagtcgcccacgttattaccg

ggagaagtgcggcgaagtgtctcgagtggctcgttcggcgctttcgcatcaacgagttcaacgtacctgtccttctcggcgcattcatgcctttccac

accacacctcaattcgcgcgcatgctcaagatctgccgcatcgagcatcacccgttcctcgctttcctccaaccatgcaaggagaaggagacacctct

acccgctcctctcctcatctccgcgctcctctcctcgccaactctcagcacaagcaacgagctcctgcgctgggtgacaagtctcatcatcgtccgcc

gcgcagcagcctctcagccacatcggaccctgatctccttctgggccgcgactctcattcaactcagtctgcgctgggccgatctggatccagggtct

cgcaccgagctcggtgcagctggtctgtcctccgcatctcacaatcgacgcaacaaacggcggggcggggaaaatcgtcttgacgatgcccagatcca

tctcaatctcatcttgcctgtcgccgttcgatgcgccgccttggtcccatcacaggacgacataagcgagctccgaacggcatcactcatggtcatcg

cagccttgggggcttgcttccccttcagctcggaggcggtgcgcgctaccatcgggtctctcgtctctccggcccgagcagatccccaagctgccgac

gagcaggccatcatgcctctgatctccgcttgtattgctctattcagtcacgccctctcggacgaggatgctctctcccctctgctttccctcaacca

ggcggaacaagaagcggcccacctcgtctctgcagaggctgcacaggttttgctcggtctgcctcgattcgcagagagtgtagcggaagccagcggac

gcgggacagaggtcacacctttggcacaacagattcttgcagctgtcacacgacccaggactcccttggcttcactagatgagaccgcggagtcgttg

agcaagatcattctttccccatcgctcagtgacgatctccgactcgaagccgcccggtacctgctgacgctcaagtacggcgcagaagaacctgctga

aaagcagcgtctccgtttgcgccttctcagcgatcttcgtgagcagcaacctcagatcttcgacgaggctgtcaaagttgtcaattcgggcgtgggtg

aagacaaggctgccgaagaggcattgtgggccgtgctcgaggctgtgatcaccaagcaaacctcgctcaggattcctcagactggcaagggcggtcaa

gtcgagactaccactctttggcttacggtacactcgccctcccccacccagcgcgtcctggcaatccggagcttatacgaggccttgtcagcgggtac

gttgccagcttcggattcgttcgcggtgaatgcccttcaatctcgaatgtccggcgtgtcggaggaggattccgatgtcctcggagcagtttatgccg

atcccagtcaagtcatcgccgtcctcggcgcgaagcgagccactgagctcgttttgagtgcgctgaatccggatgggcaaggggcgaatctgagtcga

caggcgatggaggcccatctcaagttcttcctgggccatctgcttccgcgcgacgcggccgggcttgtgccacgcttgatcaaggaggtgttgtgggg

tcgtttgttgcagacgaaagcggctcacaagacggcggatgcggtggctgatgcgttgcgcaagatatctctccctgcgattcccaaggacggccatg

aggcggtcaagccgtggttcagcgtgctccatgcgctcgtgggagagttgggccggttgagcaaagtccgggacgaccatttgaagtacaacgagggc

gtggcgcaggcgatcagctcccagcttgtcaaggtggtcgcagcatccgtgcactttgatgatctcctcgacttcctcgtggcgaaaacgagggtcac

ctccaactcgccgtccgcaacgcaggcgtcggcacgggcgctcgcgctcctcgtactcgtccgcctcatcccgtcggtcaacaagggacagctcgcac

aacttgcccagcgcgttgctttcggactgcgtctctcgaactcgactgccgatgcgtcgactgaggatcatgtggaagatctggacgtcgtgggtgcg

gatgcatccgtccttagctccggtctaagtgatgccctgtacagcaagccgggtcaggggcgcacgatgcggaggctggtcgcagttctgttcggcgc

tgtgttgaacaaattgcccattaatgggaacggtgcggagctcttgccaatcggagacacttcgaacaatgtcctcgttggcatccggctcttcgtcg

tcctgacctcgtctcagttggcccctcgtacgagccaggcgttgatgcagcgactgcgggacaatctcggtcagtcttatctcgccttcttggcgacg

atctggacggtttcttcggcttcccttgcgcaagctctccacggcgtcaagaagggcgctgccgttcccaccaccgatgatgggcgggaatctgagct

cctgactcgcaagagtgctctgcaggaagcgcagcgattcctgagcaccgctgtgagctcaccaggcgctggggctaccaagatccaggacttccaga

ccgtcgttcctgctttactcctcgctctccaaaatgagtacaaggagattcggcaagctgcgctcaaaccgctcgagaccatcgcatcggcacaggca

attgcttccaagccgaacgtcgagttcaccgagtacggttacgatagcatctacggtatctcatccaaggacctcgtatacctgcactacgtggacgt

ctgcaagtatttgcggagcctgctcgtggagcgcgactcgttcgtcaatgacgagcagaacttgtcgatctggcatgctactgctttgaacatcacca

aggcggacaagcggaaggaagctgaatacaagcaggccgttgtggcgttccttctggcccacgttgtcgcatggccctcgacgctggctggcatcggt

cttctcgaatcgcttcgggacgtgcccaatccggccaagctgcgcacgttgagtcctgtactccgccgattggtcgggagcggagacgagctagtgtc

cttggaggatgctgacgagcaacggcgattgatccaactcatgttcactgctaacgacggtacggcggcgtctgttgtcgaggacaagacgaacggga

cgtggtctctgcttttgcaagcaatgtcttcctcttcagcattcatccagaagtgcgcgtgcagcgctgtttccggtcctttctttaggactttgcgg

ccggacatggcccaggagctgcttcatcacatcgtcgcgacactgaacgaggcgcccgtaacacagactgcccaactgcggcagtgcctgaaggacct

tcccgcgccgtcctctatcttcgcgcccatcctggcccaaacacgtgagaagctcgcaggtgccagcggcggcgaacgacgcgaagcgaagcgggcgc

ggaactctgtcacgtccgaagacgtgcaggctcgggcttcgtctacgcttgctgtcttgctcgagactgtcggtgcgcgggatatcaagggtattcct

tcccttgttgccgagctattcgatgtcctccgaacaacggtcgacgtcagtggcattccggttaccaacccggatcacctgatgcagctcacgatggc

ggcgctgttgtcagcgctgtcgaactcgtcggagaagtcggcacaggtcatccaggcgatccgggctgatactgtcatcagcacgatcaaggcatcgc

aaaacccgcagacgttccagcaggccctcgtgcttttgaaccggatcgccacgatcagccaagaactggtgctgcacaacgtcatgccgatcttcacc

tttgtcggatcgtcgatgctccagcgagacgactcgtacagctttgagatcgtgcagcagaccttgcagagcattatccctgcctatgtgcagtctct

ccgtgcgagcagtcagagtggggatcacttcgagctgctgcggcacggaaggactttcatgcgtatctttacggacgccgctactcatatcccgcggc

atcggcgacagacgttcttcaagtcactgctggacgttctgggaccgtcggacttccttgcggccatctgcatgctcttgacggaccggtcggcccac

aaggtcgcgaagcaatccgccgaggcgttctccgagtccatctcgcttcctctcaacctgttgatgtcgttcaacgtgggcacccaagtgggcgctat

tgagcaaatctttggcgaggttatgaggctgtgggaacaccgggccgtgaacacggtcgaggcgcgggagtcggtcttcctggatcgcatggatcgtg

ccatgcgcgagcattccgatcgtcgtgcgggccctgtccagcagttgtgtgccctcatgagactggtcattgcggccacgggctcgtcgagctttagg

tcccggttgaacaagatgaagaagcaggaggcgagggctgtgattgacgagcagctcgagtcttgtgtgcatcggacgctcaagctggcgttggttga

tgatggagaggtccgggagatctcgaatcaagtccttgagacgctcctgggtcacatctctgttgacggcttcatgagtctggttggcgtccttgtca

cggatcaatcgaatgtggtgagtaagatgtccgactgttcgcgctggcgtagttctgctctcctgaccacaccgcgatcttcctttactcagacgatg

cgcacaggtctgtcattatattcgacacgcttcgctgtcatgtcagccgacgagaaagctgcctcggaagccaaggtgctcccggtcctcaacgtcgc

tgtcaagatcgttgctgctgcacaaacagctgcggagcaacccgtcgaacttgcggaagccttggcaagcatcaaggtgtcgacggtagcaaagctca

cgactttgcaccctgttctggtctctgccgttcccagcctgctcaagctgagcacctcgggcggtctgactccgtccctgcaagtcgaagttctcacg

accatcagccggatcgtcagggtgatcggtcctcgtctcatcccgcatcttggcagcatcgtcactgctatcctttccgtcatcggctcgagttctac

gcacaacagcggctccgtactggcaggcgcgtttggcgtgctgagcagtcttctggcgacattgcccactttcatggtctcgcacgtgcacgtcatcc

ttgcggcgctcgtcaagggagacctattggacaccctcgaacgcgacgaattcgagggcgcagagcggccgctcaatacgctgtatgactctcttgtc

aagtcggtcgctgtcgagaagatgctggaatccctccagaagctgtgggagatccattcgcaggcaggagagctcagctcgcttattagtgttgtgga

gtgcctgcgccgggtcatcattcgtctcgatcgacctgccattgcggccatctacaagaacctcttccgcttcttcctcaagatcttcgacacgcgcc

ggttgcactccaccaagctggacaacgagggcgtggatcaggtcgaggaagctgctttacaggcgttcattgacatggcactgcagctgaacgagtct

gccttccgaccgttgttctaccacatcttcgactgggccgtcttggaactggctgaggatgatcggccggtatccgatcggggactctgcgctcgtcg

gacgctgctgttcaagttggtcaacacgctgctcagtcaactgcgtggactcttcaccacgtactacgccacagttctcgaccataccattgagcttc

tcaacgccttcgcggccggcgagctggaggacgccgtcttgtggcacgaagtgctcagcagtgttgaactcacctgccagttcgatgagggcaacttc

tggaacccgacccggctttccaagatttcggcatcgctcatcgggcaggtgcaagtatgtgccagcggcgagcacaccgacgtcgacgatcaagaaac

gaaacaggctctgctcgcgtccgctgtcgcagcgctggcacagacggtctctgacgaagatagcctgcaagagctgaacgacggcctgctccgcaaga

caagatccggcacatcgacggagaaggcggcagctctgtcggtgctcgacgagctctggagcacacaggccgaggcgatgcagccgtttgtcgcgcaa

acgacgcctttcctggctgagctgcttgatgcggacgacgcgacggtcctgcagcggacgaaccagttggtacaaacgattgagggcattatgggaga

gccactggacagctaccttcaatga

>contig_14.contig_14.g509.t1

atggacaacgaagagcaagcggctgcggctgcgactgtcgacgcacttgcccctcaaagcgctgctcagcctgcgcctg

tcgatccgagcgacccaccgccagctccgcccaagtcaaccgatgacatggatgtagacgaagacgacagcgcgaacaaagcagagggtacaaagaaa

gctgcggctgatgatggtaacggcgacgttgatagcgaagacgacgatgatgatgggcctgtctcagcaaggcggaaggccagcaagccagacgatct

caacctcttcagcgacgatgaagacgaaggcgtcgtcaaagcagcaacctcgggacgctcaacctcacgcaagcttcagacgttgcgcgacgacgacg

acgaagacgatgatgacgatgatgacgatgatgatggagacaaggatgtcgatggccccgctgctgagcgcggcgggggagcagctgagctgtctcgt

cgcaagaaggcagcagccgagcgaatgcgcaaggagaaagcactggccgatctgattcccgacgacccggaggaggacgaggccggcccaggcgacga

cgacgacgacgacgacgacgacggcgcgggcaacaaggatgccaatggcgacgataccgcgcggctcgagcatctcgaatatcgcgagacggacgagc

cctacgtcgaagaagcacagcgtcagacacgtatagccgaattgcacctgcccaactaccccgtccggcggacgaaggaacactggctggcccggctg

cccaatttcctcgactaccgcttgcagccgttcgacgaggacacctttgaccccgagacggacgacaatgcgggccagggcacggcggagctgagtgt

aaaggagcgtttggcggacccggagttgcggatgctcatgaagacgaagaatacgatgcgctggcgctggagcgggaaggtggatgaggatggtgtta

ggattccagagagcaacgcgcgcatcctccgctggagtgacggtaccatgtccttgcagcttggcgacgagtacttcgatgttcccatgactcgcgaa

ggaggagctgccaatgcgaacctcggacccgcacaagtcaatggcatcgtcccacaaggctcgcagggtctggtcaacggagggagtcagcctcaatc

gagcagtcaagctggcaagccaagcgctgcaagcacacagccaatgtccttcatagtcgtccctcacaaagatgctcaagtgttgcaggcggagatgc

cagtcgcaggcacaatgatcttccggccctacaatctccaatccgagacgcatcgcaagcttgcaaaggcggtcaagtaccacaaggtcctcagagtc

ggggcccccacagagctgcccagcgtcgaccctgaggccgagaagctcagccgggagaagaaggctcgcgacgaggaacgcaagatggtcaagaagat

ggacaagtcccgacggacgatcgagaaggaggaggcggacgatgttgatgaccagctccgcggcctttccaggacgggagaagaacgtcgtgttcgca

gccgtcgttcgggtcctggcggtggaggcggaggcggaggtgcttccggtggtagatcggcagccggacgtgctggccgaattcgtgcacgggacgaa

ggggaagactacgaagcagacgacttcatcgtggccgacgactcggacgaggacggcagtggcgatgacgacgcggagggcgaggacgaagacgagga

ggcggaggatgaggacgatgatgatggacaggggtctccgcggaagaagaagaaggataagcggaggaagtcggaggctatggaggtggatgacgagc

ctgatgagatggaacttgctgaacggcgcattgaggagaacgaacgagcagaacgactggcacgcgaaaaagaggcagccgcccccaaaacacaacaa

cagccagagggcaagagcgccgccgccccgtcgagatcgaaagtcctcgctgacagcgacgacgaagacggcgatgcgcaaggtcaggctggtggagg

ttcgtccgctgcggctgctgctactgctggaggagagggtgctagtgctggcgatcaggatgaggccaggaggaagaagcgcgtcattgtggatgagg

acgatgatgatgagtag

>contig_14.contig_14.g510.t1

atgagctccctccagcgcttacgcatcgcaacctcctccgtccttggaatgaccaagggaagtaccaccttccgccaac

gactgggctggcagtcgcctgcgagtgggaccccgacggacgaggaaggccaggcgcggggtttgagcaccggagtgggcgtgcagcctggggctggg

actgggactggggatgaagacgctgcaggtcgtaatgaggagggtagcgcctcgagtgggagcgggacaccgaacacaagcgcgaacaaacgttccca

agccgccaaagaaaaggaaaaagccaaggccaaggctcaagcaaaagcggccgggatcccgcactgcgccgcgtacccgggcgcgccagaagatctta

tggaccgactgggctattcgagcaataaggacacgaacccggacgtctatccgacgctcgtcagtcccgagacgttgacccgcacgggcgtgtttgag

gttcggcctcataagcactcgcttcatcgggatccttttggactctggtatgagcagcatgggaaaggggatacgaagatcttgtttgtgatgggtct

acagtcttcttgcttctcctggctcgactcggtggtatactacggcaaggacccgcgctactcgtgcgtcgtactcgacaatcgcggtttcgcaaact

caggcgcgccttatggaagatatcgcacctcatgcatggcctgggactgcatcctcctcctcgacgagctcggctggacagagcagaggagcgtgcac

gtcgtcggcgtcagtatgggcggcatgatcaccctcgagctggccaagattgcgccggaacggtttgcgagtatcatgcttgtcagcacgacgagcgg

acagggtggaggagagaagagtctgttgactgctttgccgccggcccgaggcgtcgcaacgatgggcaagctcatgggcggcggaggcttcgggatgg

gtggcgaggccgcgcgagcaaggggcgtcgtggagatgcttttccctcagaaatggctttcggagaagcacaacagtcatccagaaggtcggacgaat

cgcgaggtcatgcaagaggtcgtccaatggcgcgcggcattctcccggcaggcacctttccacggcagcgcgtctcagatcggagcagtgctcaccca

tcgcgtcacaaacaaagaattggcgaagatcaacgagaccatccctatcatcaccatcatgaccggcgacgaggacaatcttgtgaacccgctcaact

cggagcatcttgctcagaacatgccacgagcgaggctgatcaagctcgaaattacgggacatgctatcacgatacaacgacccgaggagatccacaaa

gcactagacgaggacattgagaagtcacaagaacggatcaaggaagagagcgaccagtggatcaagagtgaggctcaacttcagcgggacgaggcgga

gcgggaagccaaggcagcgaaggaggaggctgaggaagcggacaagctggcggaagctgcggctgatgcttctattggagcgacagaggaagaattgg

aaatcctgggacggggccagcttccttga

>contig_14.contig_14.g511.t1

atgcagttcaagtccctcttcttcggtgccttcgccctcgccgccgccgtctcggccgtcgctgtcccccagcaggtcg

acaccctcgccgtccgcgctttcaacgttgaggaccccaccatcaccctgtccggctccctgaccatcccgatcaaggagatcgaggccatcctcgtc

gagggtgcccaggagatcgcccaggtcggtggtgccgttaccaaggccggcaaggccctcgttgaccagatcgtcaaggagatctccccctacgtcgc

cgagttcgagaagattctcgttgacggtgccgccgctatccagaagggcatcaagggtgccgccattgccatcacccagggcctcatcgacgcttcca

aggcccttgccgagactttcacctacttcttcaacctcttcgtcaagtacggcaagatcttcgagcaggtcctcatcaacgagctccagactatcgcc

aaggtcggtgttcaactcttcaaggctttcgaggctgccgccaagggtctcttcaaggctctcgacctcgtcggcgaggccgtcatcgtccgcttcaa

caaggtcggtgccgatgtcaccgccctcctgtccttcgtcggcaagtccctcatccaggtcctccacgacgtcatctatggccgctaa

>contig_14.contig_14.g512.t1

atggagactgtcaagcgtcgcgcaggattcaaggccgaagatgctctcgaccaggacagcccgtttgccgtgctcgacg

agcatggtgaagcagagccccttgatgacgcagagcaagcgaagattgttcgagagttgaacgagctgaatgagaaggcgaattacatctaccgggcg

ctcctcctcttcatgctttccctcgtcctcatcgtatacatgacccccatccccgcctacctagccggaacccacccagagaaccacctaatcatgtt

cttcagcccgcaccaccacgagcacacgaacgaagacctcatctacctccccgccgcccccttctacatcttcttcttcctcatccaaggcggcctca

tcttcgcagtcgcctacgaaaccgccgaacggatgggcttcgtcaaattgcccgctaagccatacccgcaacaaccacatcggttcggcaccgcgcct

gattggctcgtgttgatcttggaggatattcggttgcagccttcggaggtggagaagaagaggggggaggacaagggacagcaggccgcacagcgttt

cggcggtcgactcgtctacgtcatcttcctcactgtgcttgcgacccctttgcccttgctcacctttggcgcgggctccttcaccaatgcgggctggt

gggccatcacgcctgtcgtgttggccgtcgtctcggtggtggagtggatgatgggcaaagtcaccgcggacacccaagggctggccacgatgaagtac

aactaccggggtgcctga

>contig_14.contig_14.g513.t1

atgaacatccagaatctttcggcgtacgaccccttcgccgacactggtgaagcagcgctcgacacttctgccagtgcaa

ccgaacagaagagtgcctccgcccagaaggacaaatacgtccacatccgcatccaacagcgcaacggccgcaagacagtcaccacactccagggattg

cccaaggagtatgacgcaaagaagctcctgaaggccttcaagaaagagtttgcctgcaacggtactgttgtcgaagaccctgagctcggtgacgttat

tcagcttcaaggtgaccagcgtcagaagatcgcgcaattcctcgtcgaagaggctattcccaaggagaccatcaaggtccacggagaatcgttccacc

tttccaccgcctcgctgctcaaggtcaacctcgcccacacacgacaacgacaaggagcgcatcagaacacaatgcgcttgctcccacgctcatcagct

gcccaaggcgagcaggagcggacctcaaagcgccggccaacgtccctagcgaagaacatgcttctcaccctcctcgccatcgccagcatcggcctcgc

caacgcacaacaggaccaaggagaagactgcccgccctcgtacccagacattctgggccagccgtgcagcctggggagcaaccggcttgcgctggaca

cgaaccagttcctctccgactgcggatacactgcgttctgtgatccggggacgaatacgtgtcggccgaaccagtgcaggagggatgagtatcctttt

gggtacaagcggaaatgcttctgtccggacgagagcagcggttgtctccctgtgcgaaatgtcggctcgtcatgccagctgaacagagacgacacgat

ctgcctgcacaacacctgccaggttgtcaatgctaccgctggtcaagcgtgcatctccgacaacgtcatttacaccgtctatacctcgtactcatcct

cctacggcgagatcgtgtcacgtgacaactgcgtgtcgggctactactgcgatggcttccgaaatgtctgcctgcaagccaagaatgtcggcgatcaa

tgcagtgccaacaaagagtgccgatcgtacaactgcctcgattcgtccgtatccgtcgacatcccactgaacaattcgccccgtggacgctgcggccc

ggctccctccatcccgacccgaccgggcatctgggtctacatcgtgatcaccgcggccatcatcatcggcgcgatgggactctgctgtgcgctgatcc

agatccacgccaggggccgacgaaggacgaggcaggagcgcaagaagtactttgagaaacaggcgctgctgcgtgatgatgtgtacgaggcgtactac

cgggcctcggtcaacaaggagatgtcggcgtctggtgacgggggcgaaaatttgggcaatggacggatgtatggctccatatcggccatgtcgcagcg

gagtgcggcgaatccggcgacgacgcagcctctcacccttcgcgacgagaaggacacggcacatgggaccgccttgtag

>contig_14.contig_14.g514.t1

atgccgctcaagctgctctccgtcgtcctcttccgcagccccgtgccgtcctcccagggcacagacccctggcacgacg

ccttcggtcccttctgcctcccctccttccccgtctccgccctcgagtcaggaacatcaacaccgctcgcacccgtcgctctcacatcaagcggcctc

ccctccaggaagccaagaccaccaccctccaaccccatgctcaccacccaccacctccacatcctcacccacaacaacagcaacctcgaagacgacgc

actcagcatagccgactccgcagatagccagtccagcgctcccccgtcccccaccaccacagcagcagcctcctcctcctcctcctcccgctccagag

catcgtccgctgtgcctccccggagaccgacgatcgagactaccgagttcacagtgagctccatccccgtcctcgcctccgtcagctgcaaccagtcc

ccgctcgtcggcgccttcctcaatcgccagtgggcaggcgtcgtatgtacctcacaacgcgccgtacaggcctgggcagaggcgctcgtctctatcac

ccatcagaccgtgtgcgagtacgagcgcctggacttcgagcgcagaccgaacgagttgcgcttgcccaggactaccaccgtatggagtcggatcccct

tcttcgctgtcggtccggctacttccaccgcgcttaagaagatcgtcatcggtccgccgctcgggtcgaaagtttcgtctgcttccgtcgccgctgcg

tccaatcccatcaagcctaaagtcattctcggcggcaaaagcacagggaccgcagacatgctcggccgattcatcctcaacttcttcgattgccagcc

aggcgatgatgacggcgaagaaaacactgacgggacaaaaggagaatcgacttcctcgctccgaccacaggcctacgcaatctccaacccactcctcg

tcctacagggcgacaaagccctgccgaacctgccccacatcctctccacagcgaaaccgcacccgatcccattcgagaccatgcgcgtgtacgagacc

ggcgtagacccgcacttcagcgccaatgtggactcgctgttctccctcctggcgagggcacgcggtattgccctcagcaggaggagcagtagtcgttc

cagtcggcgacctagtgggtcatgggcttcgacttccgcttcagcttcagcctcggcttcgggctcgggctcgagctcgctcacagcttcgacctcag

cgagcacagtcgtcggggatgcacccgcgcccgcatcggagacgcgggacggctgggccatgggcaccgctaccctctccgccgaggagattgcgagg

ttacaggggcgacagcaggtacgcgccattgcggcggcgcgcagggcggcaaggaatgcgagggagtggcagttggaggctgatcgggctgctgctgc

gcagcagcagcagcagatgggccagcttgcggtgggggatgatgccagtcgggaatcgtcggcgtcggcgtcggcgtcagtgggggacgggtcggtcg

ttcgtgatcgggcttcgtcgttcgctggtggtagtggtggggagcgagagagtgggcccggcgagacgtcggcgtcggtacattcttccgacgtaagg

gaacgcgcgtcctcaacaggttggtcctcccgccccgaaccgctcaacctctccatgagcaacattatcgcctcaccgagcgccttcacccccagtcc

cgccggcagcgcgatgccctccccgcgcttggacctgcggagactgagcatcggggccagcggcggcagtactctgagcgggagcacgctgcaacagc

atgcgccttctttggctggtgtgaggcctgattggcttgtcttcttttcgccgagtggggtgaagtatgcgctggagacgttgaggaggcaggcgtgg

ttgccgccggcgggagggaatgaggacacggtccgtgttggagatggggttgctcggggtggggagtcgcgagctgagagcccagttgcgtcaacgaa

cggtctccgaatcacaacaacaacaacaacaacaacaacaacaacgacaccgggcaactcagccccaccaccgccaccaccgcctctctacccgcgca

tcgccaccctcggcccaacgacagcccgctggatcatcgagaacttgggccacgaaggcagcccactccctgcctgtgcccccaagatgacggctagc

tctactgccgtaggagtagagccccgtctgtaccagcaacaactcttcgaacgagccaagacggagaatgtgattgcgtgcgcggatacggggactgg

caagacgctcgtggctgtccttctcctgcgctggatgctggaggaggaaagtcggcgtcgggcgttgggggagcggaagcggatctcggtcatggtcg

ttcatcatgtcccgctcgttcatcaacaaggcgactatatcaatgtgaacagccatttgagagtcgagaagctgtacggggaactcaacatcgacaaa

tggaacgaggaagaatggtcgaagctgctctccgaggccgacgtgctagtctgtacggcgcagatcgtcaaggacgccctcgcgcacagttttttgag

cttcgatgacttgaatttgctgatctttgacgaggtccaccatgctacgaaggacgatgtgtatgcgcgaattatgacaagccatccgatttcgtcca

aaagcccgcggttcgccgaagcggcccagaagctcgagatcaccatggacgcccgaatcttcacggtccccatggaaggttggagctctctcgccaaa

gcgaccttcaaggccaagacggagcttgtcaagtactcccctcccgaagctcccttctacagtccgatgtgtcaagagatcattgacgagcttcggaa

gggtaagaagcaggacggcctcattcaacgactggaacgactcggtccttctctcggtccggcactgtgcgaccatattctcgaaggagccgtcagcg

acggcgttccggacgtagccactctccaaacgctccaaggcctcagcaaggaggaacgagagcacctgaagcggtcgtggaatgcggcagttgagaag

acgagctcgctggagtggaagattaaaaagttgaagaggagtacgacggagcatgcggagctgaaggggatgttgaagacggcgagggagcgacatgc

ggaagtagtggaggagcgcgttagtccgaaagtggcgaggctggtccgactgcttctggacatcttcgctgcgtcagaggacagaagaggaatcatct

ttgccaaggagaaggccacagtccacgctctcgccgcatacatctcctccaaccccagcctgacccacatctcagtcgaagcactcgtcggccacacc

ggcccgcaaggtctcggctggaagtcccaagagtccgtcctacgaaacttccgccggggggacacgcacctgctaatcgccaccagcgtagtcgagga

aggactcgacgtccccgcgtgcaacttcgtcatccgcttcgacctgcccgagacggcgatccagacgatccagagtcgcgggagggcaagggagcggg

ggatggcttaccagctgctcaactggtacgtgcaagcgttgccgaaggacgaatacaccccagcaagcattgccatcagcctggaggaagacgagacg

ggacccaattctgtccttcggtgcacgctacagatgcccgctaaatcgaagctgcgcgaggtacgttcggagtggcagctgtcgaagaaggcagctcg

tcaggaggctgcattcaatgcttgcaaacagctattcaaaatccgagagcttgacaatcgcctgcttccacggaagcctgaacggatcgcctcggagg

tgatcgaggtcaaggacgacgaaggcgaggttcgtctgctgaagagacacaaccttccacgtccagtcgcgaagcttttcgctaccgagcaaacagcg

atcgagacgcgcacgttccgaatcacgatgatccctttcgactccatcgatacggctgctatccaggggtcggtcgcgccgttgctgctactcacctc

cgcggaggcgcctgacgacgagctttccgttgagctcaaccttcccaatggtaaagtcaagcttctgtgtcgaccgggacacgccggcactctcacgc

tcacggaggatcaacatcgcttggcgaaggtgtacacgttgcgcctgctgggtatggtcggtagattagaaagcgtccccggacccacgcacggctcg

tggatgatcctcccgctcgaccagcaggtgaagaacggcggggaggaggaggaggttgtcgtacagtgggaacaagtcgcgatgctggcggcacagtc

ttcgcccgtcttccgaagtgcgtcccatgcggagtcgctaggggacgacgtttcgctatgcgagatccccgatttctcaaagccgccgtcggcgctgt

atcgcattcgggacgaccttactccgtcgagcttgggtccgaggggtacgaggcagggtcggcttggtgagacgttccagcagttttatgagcgcgtg

tcggggaatagggtcgatggtggtgggcctatgctggagacgcggacgatgccgcggttgcagaatttcactacggctggggcgttgcgtgctttgag

caagtcgttgacctttttgatcccggctgtgtcgtacgtccacccggtgccgtcgagcgtctatcgcgctgcaatcctgctaccttcggtcctcgaca

gactgaatgcgatgtgcctggcgagggagtgtcagttggaggtgctaaaggatataaaagtaccgcacgaagcactccttgctgctctgactgctccc

tcagctggcctgtcattcagctatcagaagtttgaatttattggagactctgttttgaagctcgttgcgacgtgccatgctttcgtgagccggattga

agcagatgagggcgagcttacctcgtactacatgacgctggtgagcaaccatccagaaactcacctagacaacatcccgccaggctcggacgccttct

ccacgaagacatgggcccctcccggctacttcgtgacagacgaggaagacggcttatccaaggctcccagtcttccatggtctgcaaaacgactggcc

gatattgtcgaggcgattctgggctctgctacgacgtacacgatcgaccagaagctgcaagttgcgttgcagctgggtttgctgcctgaggagatttc

gagctttgctgcgtttggcaccgctttccagggcaagcttgaaggctacatgcctacggcagacatgcttggcttgtcggaatttactaagcggctca

tggagaggatccagtacaagtttgagcatccgctgcttgcgatcaatgctgtcacccattccagctgcatgggcttcgagctgccttcgtacgaacgt

cttgagacacttggagatgctttgctcgacttcctcgtggtggagatgctgcagaagaagtacgagttctttgaggagggcgagctgacgattgtgaa

ggcgaactgtgtcagcaacaagacgcttgcggctttggcggtgtcactcggattgcacaagcacatgaaccaccacagctcgtccttaagcggcgcga

tcgcagcgtacgtcgacgaagtcacagtggagcgagaaagggaactagaactcggacgcccaattcctcaccaatactggtggagcctgttgcctccg

aaggcgttggcagacattgtggagagcttgctcggtgcggtcctcgtcgatgcgcgtttcaacctcgatgtcgcgagggcgtactttgaccggctcta

tcgtccgttcctggagaacttttgtcggagggagaacgcgattctgtcttctgctgagagtcttttccagctgttgctgcaacgccgctgtatggact

ggtgcatcaccggagcagatacggcatccaaagaggagaatggcgatgctgctgctgcttcaggaacggtcgcaatggaggaggatgagcaagttgag

gagaatcgactggttcgtgtcatggtccatggttcagtctgtggcgacatcaagcgcggtgacaagcgattcctagaaaggattgaggaggcaatgaa

aatgctcgatgactggagatcggacaaaccggaagcaccaggcgagggcgagcttctcgacgtgctacgggaactctggccggggccgctccggacga

agaagatgtgcatgccgcgacttgtgctcgaggcgcaatgccattgccgtccttcgcagagggaggaggacgactggtga

>contig_14.contig_14.g515.t1

atggaacgccgtttcggccacctcatcaacaaccacgatcaacaaccaacgaccatcgaccatgagcccaagcgagaag

gaacagccagcagggatcagtctcgatcacaagcacctctgcaggctgcaacaggtagcttcctcgtcaaaatgagcaccaggtacagcaacgacaat

gaatacgatgatattgctaatctcatgacgtctggacacagcgacagtgagcgagagatgggaaagtctgtgcgcagtttgtctcctggtggtctcgg

gtacgccgactcacgtccgcgcgtggggtcttcctcgtcttacgggcagatgggtgccggatcttcgcgtcttggattcattgcaccacgcccagttt

ctaccagcgctgcagctagtttgtctgcccgtctgcataaatctacaggcggcatacgcaccgcagcagccaaagcgtcagcatcaagctctccctca

gtgccatcgttcaaagacaccgttaacctcactgacggcgtcactctcgtcaaagatcgacgacttctggaccagtcgattgcttctcacactgccaa

tcgagtgttcaaagatatcacggagcgggagagacttcaggcatttcctgtcatcgggaaaggcttcaatgccgtacaagtgtacaatgccatcatcg

aagcctttcgcaaacagaatcacgacctagtacgcgacggtcagaagggctacgaattctgtcgcctgttgaatcattccgctcgattgcagaccctc

cggtctgatccacccagtattgcactcacgggagtcgagctggccacagaatacaaacgaaacaagtgcttcattgtcgtgcgttcagacaagaaatt

cgacaagtacgacccactcctccatcagctcctcggtacctctgtggacgacgacagtcctttcgaatcggccgatgatagcgacatcgacttaccag

atatcactcaaagcgaccgcgcctggaagaagatcttcgcaaagaacttgaagacctccgcaagcaacgtgtcgccgtga

>contig_14.contig_14.g516.t1

atggaacgccgtttcggccaccttatcaacaaccacgatcaacaaccaacgaccatcgaccatgagcagaaccgctgcc

atagtcaagcattgagcgacaaatacatacaggtgaacgcctcaacagcaaccgctgcgctcgctgcccttctcgtcgctgcgctcgctgccgccctc

ctcgttgctgcgccgccgccgccactgctctcagtaccttccttcttcgtgctcactccccatcagcctgcactcttgatcacatccattttcgcttg

cgcttctcggcacgtcccaggccctctgcagcccaccgtctcacctttacgtttgcggttcacatcctcgtctttcgcaaatcgcatgtctcctccct

cttcgatggatgccgcagagattgctgagctgcgactggatatctacgccctcctcggccccatcacctcatttatcggcagcgcgatacactcggag

actgcgctcctgacacatatgctgtcagtcgaggagctgttggtgtcattgcatctcatggccaacaaaatccacccacgagtctttgatgctatcgt

tcagcccatcctctgtctcgaggcacgcttgcacgaaatcaaacgcgcgggacacatagaagaggacgctgccactgcctctcgcgaatgggttagga

cggaggccggacagatgaagattcatttgcccgtcgatcttctgatcgagcttctcgagctcggcttctcggacaaagatagcgccctatttctaggg

tgttcggagcggactttgcgcaggcgcagggtcgagctggacctgcaacggtggaatgatcgagacttgtccgatgaggagttgagcagcatcataac

ccagctacagacaatgcacagtgaggacgtcggcgagcgaggcacgtggggtgctctgcgcagtcaaggactgaggatcagccgggcacgtctgcggc

gaatcgtgcaggctacggaccctatctcacagaatccccgatgggcgcggcacctcaaacgtcggcagtactacgtgcctttcgtaaattccttgtgg

cacgtggacggccaccacaaacttatgaagtggaatattgttatacatggagcgatcgacgggaagtccaggctcgttactttcttgcgcgcaagtag

caacaatcgagccaacactgtcgcagctctcttcctcgaggccgttcaagctcgcggatggcccagcagggtacgaggagaccatggcggggagaaca

tgggtgttcttaagttgatggaagaataccgaggtttcggtcgagggtctttcattcaaggtccctctacgcgaaatcagaggattgagcgtctgtgg

gtcgacctgaatggcgccctcgagcacttcgttcgcatgtggaacaatcacaagatgcggacaccgggcctgggtaatcagacacccgaacagatgtt

tctccaaggggcatgggacgctgctcgacgcggctacgatgttgggttactcgatatggcggagagggcagctatgcaggaggttggactcgacattg

agggctgggaggattacggtctagacggatggcgatctgagagacagggcgagcaaccccatgtgcgcgtcgaccgaatcagcgagtgggttcctccg

gtgctgaatgagcccagcacccacgcggctcttcgactcctcctcccttatccgccgtttccccctcccgcagattttggcttgaccgcatacctaga

tgtgttggatgctatcaatgaccttatctag

>contig_14.contig_14.g517.t1

atgttcggcgaagatcggtcacagagcccgcgcatgccctccccatggacatcccgcctctccacctccctcgacccct

ctccgctcgccgttccagagactcccagacgagcccagcgcacccaagcccaccactcgccctccctcgccccactcaacccggaagaagacaccatc

gggaacatcgagtacaagctcaaactcctcccgcccacacgcgaccgcttcaaccggctcgtcacacagctcaaatggcgcctcctacagggtggcgg

catggccatctacgaagtcggcgttctcgacgacggcgccctcgtcggcttgtcccgccgcgagatgcgcgcctcgcttgataccctcgagcgcatgg

ccgcacatctcggagccagggtcgaagttagacgcgtcattgtcgtacagcgatatccggggcatgatcctgctaccaccactactactactaccacc

accaccaccaccaccacttcgtcctcatcttcatcctccaccccgccaacccaacccgcgtcccggccgagctccagcctgccagacttccctcctca

tctctcccttccatccacaacgatcaacggcgtccatacaggcgccctcccgccatcctcctcctcctcctcctcctcctcctcctcccaatcccatt

ccccatccaactccgccgccccacaagtcatctccgaccgaataccccgcatacaacaaaccccaacagatacacctccctccccctcccaccaaccc

tcgtcctccctgccacccttccgcaccatggcccaaatccacgccgcgaacaaagtcctgaacgaagcctacctcctgcgcgagctcggtgccaaaag

acgcgggtcgcgcgcggggcctgcggacattgttcatctgggcctgttggacgcgggacaggcgaggaagtggatcggtggggggaaacgcagtttca

gtgcggatatgcgcttcgatctcgcttctccttccttggcccgcaatgatgtgggggaggactctctcgcggcggtgtcgctcccgaccatgctcgag

ctttccacgggctcatcgcaccctcacgcccaggactcgcataggaaactagcagcggagatggcgctccctctcgtcgtcctacccgcagacgtcct

ggcgcgggagatcgagcatgatcagatgctcgagcagtgggaggacgatctcaaacggcgcagggccgaggcggctgttctgagccttgctgctgttg

ctgccaatggttccggtggaggagggggagcggtgacgcagcgtcagcgtcggaaacggagcacgaatcggatcgacaacggatcgccgacgttgagt

gggatggccttcttctcgcacggcgggaagaaaccgtggcttcttgctgagcctgggcttgctttaggcggtgtgggcgtgggcgtgggtgtggagga

ggaggatccggattcagacgcggcggtgcttcagtccgtcgtgggccttgctaaggaagggagcgggcctgttcggatagcgggtgctggatcggacg

ggttggacaccgagggcggtaaacgacacgctggccgagaaggcggagatcaagatggggcgggagtatcggatgaggacgaggatgggagtgaaggc

tcgaccgagaagaacgacgacgacgacgacgacgaggatgaagatggagatgaacagacggacgagcgggactccacttccgcttccgcttccgacga

tgacgaagatgaggatgaagacgaaggcttcttctccttcagcctgtcgctgagcgacgacgaagacggccagctcaaaggcggccgtcgtaagaagg

accccaagccgaaaaaggcgcggatagccaagccgcctctaccccagcacgacgccaactcagttcccaacgcaccaccgcctcctctcggcgtactg

gtcaggaggccgaagagttcgggctcgtcacagcggaaggcggaccagcgggcggctgtgcaggagagtgcgcttgtcagggctgatgcggaggcgcg

gagtagtcagcggcaggggctgtatgttccatctgcatctgcgtctgcgtctgcgtctgcgtctgcgtctgcggctggctcgtcgggcgtggaggttc

ctgctgtgctgtttgagttgggttcgggtggcggtgaggtccgggctggagctggggccggggtggatgatggaggaggggaggtggaggtggagatg

gaccgagcagcgagtgcgggagcgggagcgggggggatgagggacacggccacggccacggcgatccggttcatcgtggaggcgaaggtgttgcggaa

gctgaagaagggggatgtgttcattgattatgcgggcatctga

>contig_14.contig_14.g518.t1

atggtgagcgagaagcagctccgcaagcgcaagctcgtcgcgcactccacctcctccggccaacaaggacgcgtacgaa

gcagcaccagcaccagcaccagcggcccttcctcccgcggcggcggcagcagcagcagagggcgtggatccaacaccaacagcagaggacgaggacgc

gggggcttccacgtcggcccgacccacgcacccgcaggctcctacctcggccgcgcgaagaagatcaaggaggagctgatcgcgaaggcgaagacgaa

gaaggcgtattatgcgctgttgaaacgggatgggatgtctgttcctgagcggcggtcgaggggggcagaaggggctggggaagggaagggtgtgggtg

ggaaggatgatttttttgatcatgggtcgcaggatgggggtgatgatgatgacgggactgatgccggggatggggacgaggacgaggacgaagaccaa

gaagatgtcacagacgaacaacaaagacaccgtcccccgacaagtcgcttcacactctcccacgacaccgcagaagaagaccaagacaagccgagcct

cgaccctctccgcatgctcgaagagcaacgcgcggcgcggaccgctgcggctcagcgtaaatctaagaaggtcctcgctgcggaagcgaagcagaccg

gcgatgcggatggggacgacctgagccggaagaggcggagggatgtccaggaggatggagcacggggaagtgcgaagcggagggcggaggaggaggtt

gtcatgcatcgacacttcaacaacaacgccgacgccacctccacctccgaatcgtcaaaacggacgaatcaccccaagcaacaagcagaccatcaacc

agaacgttccaaaccggaagaacgacaacgacagcgcgaagcatggaacaaactctccaattcggcccgcgggcgtgcacgtggccagccggatctgg

gggcgagggtgggtgtgatgctggagaggattgaggcgaaggcgcttggtgttggggctggtgctcccgccggtcgggatggaggaggaggaggggag

atggggggagggcttgaggaggggatctccgaacggaacgcttcgtccttctcccctacgacgatgttccgtctgccttccctcgcccggtcatcctt

ctctcgcatctacccagcaacaacaacgaccaccaccaccaccaccacgagaatcagaatgagctccacctgggtaccccccggcctcgacgccatcc

ccgccccgcccgcgcccgtccggaaggacatgcactccctctccaacatcgcccagatccacccgaaacacatccacctcgactgggaaatcgactgg

acgcgcaaagtcatcttcggcagcgtcacccacaagatcgggatcgacgaggacgggctccaggagctcgtgcttgactcgagttatttggacttggg

gaagatcactgttgatgggctgaaggtggaggccagggttgcggagaggcaggggagtttggggtccgctcttaggatcccgctgagtgcgcctgcga

agaagggggacgcggttgaggtcaagatcgagtactccaccacgcagcagagcactgccctcggctggctcaccaaggaacagacactgagcaagaaa

ggcccattcctatactcccaatgccaggcaatccacgcccgctccctcctgccctgcatcgactccccctcgcaccgcacgtcctacacagcaacagt

caaaagcgcctacccggtcctaatgtcggccctgaacgacaacgaattccacaaagaatcagcttcgaagcacgacccaaacacctaccacttcaaac

agccccaacgcatcccgtcctacctcatcgccatcatctccgccgtgctcgagttccgctcgttgggcagcagggtcggcgtttgggccgaaccgagt

gtggcggatcaggcgcagtgggagttcgaggcggatgcggaacgcttcttgaaggcggcggaggagactaaccccaacgcagtcacactgacacccgc

agtcatcgtaggcaaccgaatgcaagtcgacgtcctcctgcacgagctcatgcacagctggagcgggaacttgaccaccaacgccgactggcgctcct

tcttcctcaacgagtccttctgcgtctacctcgagcgtctggtcctccaggtcgtccatgggaaggaggaggggcctgcgtaccgtggtttctcgtac

atcatgggcgccaaggctttgcgcgattctcgactgggtttcaaggatactccgcgcttccagagactggtgcccgtctatgagcatggggaggatcc

tgatgatgccttctcgagtgttccgtacgagggtggttccaacctgctcctctacatcgagaacctcgtcggaggattggacaacttcctcccgtacg

tccgcgcctacttccacaccttctacgaccgctccgtcaccgtcgaagaatggaagtcccacctgctcgcctatttcgcctcctcccccgagctcacc

caaaaaatcaccgacaacgtcgacttcgacgcctggctgcatggcgaaggtgtcgacttgcccgtcgacatgacgccgtattatgatgatacgttggc

gagggctgcgtgggcgcttgctgcgcggtgggttgcgtttgagggtcagggtgggaagaaggaggagtttgggaagcatgacatagaggcttttaatg

ctagtcagattgtcgtcttcctggagaagctccattcgggaccggacgtgcccccagctgtggtcaagaagctcgacgaagcctacgacttctccaac

tcgaacgacggcgagatcctcctgcgcttctacgaagtcgcgctcgaagtcgaggccggcacctttgcgcccaaagcggccaagtgggtccagaccgt

gggccggatgaagtacgtccgtccggtgtaccgggcgctgaggcgggttgatgaagaactggcggtgaagacgttcaatgaggcgaaggagttctatc

atccgatttgtcgggctcttcttgtgaaggatttgggactggcgtga

>contig_14.contig_14.g519.t1

atgcgacaacagcttctcactcaagcgctcagcgcaccgcatgccctcgtgctgggtcgcagcgtatgcgtgccaggtg

caacagtgcgagcatctgcgcaaggaagacggcaaacgcaagcttcctcttcgccttctcgaccctttgcttctgctgcgacacgatggcagagcatg

agcgcgagcgcgagcgcgggcgccattacctcggacgcgtcctctcctctcaacgcggatcttccagagcgcagcggccgcagcactccaacccccac

cgaagcccttcgacctcccccgtcctcaagcagtcccgccctccgagatcgccacggccgtgaccactcctacctgcgcatctccctcaccgagcgct

gcaacctgcgctgtctgtactgcatgccggaggaagggatcccgctgactccctcggagaagctgctcagccaggctgagattggcaggttggctcgg

ttgtttgtgcgggagggggttactaaggttcggttgacgggtggggagccgacgttgaggagtgatttggttggcattgttgaatccctgaatgacct

ccgacccctcggcctccgccagatcggtctcacctccaacgggatctcgctgcaccgcaagctgcccgccctcgtctcctcaggactgacccacctca

acctgtccctggacaccctcgaccccttcaagtttgaattcatgacgagaagaagagggttcgaggctgtcattcgttcgctcgataaggcgctcgag

ttggggatggagagcgttaagctgaatgtggtcgttcttaaggggttgaatgatgggcaggacgttttggactttgtgcagtttacgagggataagcc

tgtcactgttcgcttcatcgagtacatgccattcgacgggaataagtggcagatgggcaagctcgtcccatatcgagatcttttggatcgtattcagc

acaagttcggtcctctggagagattacgggacgacgacaatgacacgagcaaaggatggcgcgtgcctgggtttgctggcagtgtcggcttcatcacc

agattgcgaatcacagctgacggaaacttgaaggtctgcctgttcggcaacgccgaaatctccctccgcgacgcgatgcgcggtgtcgcttctacctc

ctcaacctcgtcgtcgacgtcggcctcggatatcttgccacgcgtcacacctgcgaccgacgatcagcttctggacgtcatctccgcggcggtcgggc

gcaaacatgcacgtcatgccggtatgtcctcgctcgatgcccttgcccagggcgtcaatcggcccatggtggctattgtaagatcgtccgttcagtgg

agacagccaagcactcaacgtcgagcgatccctacgcatctactcgggtcgtcctcatcccccgcgcttgatgctccctcctccatccggcatttcag

ctcgacggcctcgccaaagcgagggggaaagggaggccggccaggagacgatgcaggggaggatgacgatctctccgatctggacttttcgtacgagc

ctcattttgagcgtctgggtggtgcacccgggacggcctccacgtccaaccctacatcatcatcatcatcctcactcaacacgtcctcccaacccgaa

cccgcccatgaagcatctcacggcacagcagccgtctcccagcccgtcgtcactcgcccacgactcacacacgtcgacccctcggaccagttccgtgc

ccggatggtagacgtctcgggcaaagcgtcgacagtacggaccggtgtggccgtaggccgggtgtacttgtcccaattagcgatggcgctcatccgag

cgagcgaagatggcacaggtggggggcataagggccctgtgctgagaacggctcagttggcggggatcatgggtgctaaacggacggcggaactcatt

ccgctttgtcatccgctttcgttgagccatgttgatgtcgtgttggagctggtcgacctggatgcggatgaggatgaggggaaggagagggaagcgga

ggagctggctgcgttggcgtcctcggaggcgttcgtccggcaggcgactgcgggcgggcaaaacgaggaggcggaggcgggcggtgggcgggctgcat

ttgcccctggaggtcaacaggactttggcttccggattgaggatctgatggatggggatcgatggcgggggaaggagtttgacatgaatgatgctggt

gtgggaggcggaggtgaatacggaggtggaaacggcacattctcgaacgagttgcgaacatccatccaagccagagcacccttcgccacgtcctcgca

accgcatgcagaacggaaagtcaaccaagccaccactgcttccacagctgccagcaccgacgccgccggctacgtccaagtgacctgcacggcacgta

cgacgggtccaacgggtgtggagatggaggccctggtcggggcgaacgtggcgtgtctcacgatgtactggatgggctggatgcgtccgagtcccgct

gcgctgtactggccgcttccgccgccgccacctccgccgctgttcatcgcttgtcagattggcgcggtttccgaagggaagaagaggacgagcgcgat

gagttctttaccccttgtggtggcatgggtaaaattcgggactctcaggaacaaggccttctccatggaaattttggaagcacgaaccacgacatcga

catcgacatcgacatcgacactgctgacctcgacaccgacaccgacaccgacaccgacaccgctcccatatatctcgcacacagccagccagccagcc

agccagccagccagccagccagccatgccgaacccgcactacgactacctcatcaagctcctcctcatcggcgacagcggagtcggcaagtcctgtct

cctcctccgcttctgcgacgacgcctggacgccctccttcatcaccaccatcggcatcgacttcaagattcgcacaatcgagctcgacggcaagcgca

tcaagctccagatctgggacaccgcaggacaggagcgtttccgcaccatcacaaccgcctactaccgcggcgccatgggtatcctccttgtctacgac

gtcaccgacgagaagagcttcggcaatgtacgaacatggtactctaacattgagcaacatgccagcgaaggtgtcagcaagattctagtaggaaacaa

gtcggattgggaggatcaccgggtcgtcaccgaagaacaaggccgcgcactagcccaagaactcggcctcaagtacatcgagacctcggccaagacga

acgcaaacgtggaagaggcattcttctcccttgcccgcgacgtcaaagcaaagctgctcgacagtgccgcggccgctgccgccgcttcaccgagtggt

gggggtgctggggccgatgggaagggggctgtcaaggttggctcggggagtggtgggaatccagcggcgggctgctgcacttga

>contig_14.contig_14.g520.t1

atggcggccagcaggtcctcgcgctccaaaagccggacccggtcaggcgtccaccttcttcaacaacaacaacaacctc

aacctcaatctcaatctcagcctcaacctcagctccagcctcggccctcgctgctcaccggtctcaacttgtcgataaccccacccgtcaatgctgaa

cagatcgcaagcgagcttcgagcgcgtatcgagttcgcatccttgaagcctgccaatggactcaccctccacagccttcatgagatcgagcaagcgac

actggaagccgaacgcatccacgcaggcgtcctcgcgcaacagaatcacagacaacatagcgcagcactcccagctacacatgttacagagtctgctc

ctgtgcagtcgaccagaagatccaatctatcagagtccgtctcgccgcatgctcggccttcgaatcctaatcctaacatttggaactcgccttctact

gccatcaatctcgcggttcaatccgaatcacaaccaccccaacaacaggttgctgaacaaggaaggaatcagcctcggacaatttttgatgccatcgc

agctcgtgcatccacacatccaccacagccgaccagcacttcatacctgcttttccccaacgtgcctctgacatcgaaaagcatccaacagccgcagt

catcgatatctggacagtctcctcaatctgtcaaggccgcatcttcgccgccttctgcacggaccgccgtctctgcagtgacgcttctgcatcgagct

gccagcatccctcaacttggttcacccagacgccttgtgaactctcggactgagctcagcgatccgcataatgaaccatcgttggaaacgaaggaaca

agagcgcaaactagcggtcgaattcaagcaacagcaacgtcgggctgctgagcgggagagagccattctggcacaaagacgagtccagagctcagcga

cgggttccctcaagcgtagtcatgcttcttcgcttccttccatcaaggagcacgaaatacttcctcccccttcaaaacgggctcgattctcggacgcc

agtctttcgtcacagcatggtcaatcgtcatggctctccgcacattcaccggcgggtcaccttgctttgctttcggcccgcagtccgtccaccaagct

cgcctcgtcatatgctcgtggtgtagctgcctcgtctgccatcggtaccggtcttgcccgttcacctgatacacggccttctcgatccatcaacgcaa

caactcacgccagttccctcgcttttcagtctcctcgcggcttaactctgggacagggactcggcatcacctctcgaacatccctgaattctgttccg

ggttctccactgaacccgaatcccaggagacgagaggaatcgtttgagcggagtgcacctagcccggcagctccttccaccaagtcttccgaattgat

gcgtacgtatcaaggatcacacgcatctgcgagtatcagggttggccagcacgtcccttcgatagcatctgagcagcgcacacgcccgcttctttcgc

ctccgccttcgagcccagcgatccgggacgaatccaggatgcacagacttgtcggggctgcaccgcatctcagacctcaggaccagcacgtaccaagg

aaatacgcccatttcagccctcaccatgcaggagcggccttacggcggcgccaagatggatcggcgacgagtgcagcattgacctctgcagcgcccgt

ggcagaaactcgtagtccagcacgaatgcaacggctcagtccgaagcaagcagatggagcagggactgacctgaaagtaaatgcgccttcttccaccg

tctcagacgttggccaagccgatgaatcgactcggtctgctggtacttcggcctcacaaacgaatctgggactcggattcgacttcgagcatggcgct

cccgccgatatgccagcgcccgctacgcccaagaaatcagccgcacgaacgcttgtagccgatgagatgcacgataagggccaacggacgcaagatgt

cgacatggacggctccgaggctgcccagctcatgctctttctggctggctcaccttctgcaccaccatctacccgtttcactgcgggcgttacaccca

gtatcaccagtttggcgtttgagtctactcggaagggggatgaaccgtcgtcggtggacggtcaagagatccagaactctggaccgatgaggtcggtt

ggaagacttcaacggtctggcgatgcttccaacacgattttgaggggcggacgtggtctgggatcgcctggacctgagggcaagacgatcgctcggat

gctaagctatggcacctctggttctcatcaaaaagatggacccgcctcccaaagtcctcggtcaaattcgacagcgtttgccccgacgttgggatccg

gctctgccgagatttccagcactgcctcgtccagcacgttgcgcagttatcagtccgacataattcaggataatggcggtcagccggcgacgagcaag

agccctgcgaccccaccagagccatgtccgattgtcgtcacagtcagccaggacgagtcggacagcagatatgcgcgaactcagactcccccgccgca

ttcttcgaacacggcccagaccgtgactcccccgcgccaacctccgtcaactcccaaagcgcccggctcgtccaacttcagctacgccgagtacctga

atgtgagcccgtcgcctcagcctcggctgcgcaacacgcctcgactcggcggcggcggcaagaaggtcggtcggtcttccgctggcgacaggccttat

agcgctgatccgtcccggacgcccagtcggatggcccgcggtcggttcttggactatgatcaggagcttggtcttcagagtgctacgattgagcatcg

gaagcggtttgcgcagatgatgcagattggggaggaaggtgctggtggtgctgggtctggccatgtcgtcagtgggccagggcacggtgacgtcctcg

tccccggtccgctggaaagcgcagcgtcagagccgcttctaggcgccgtcgtgtgcgacgcctcgcctgctgcgctgaaggagaagtcgcacaaccat

ccttcgggcgtgctggaagcgagcgcgatgatgatccctcggacgacgggcgcgggcgcggcggcggtggcgatcgtcaggtcgatcgggggaggact

gggtgcgagtcctgtgttgaaggcgcagatggctcgtccgcttgcgctgttggcgccaccacctcgagacgttgcggcggcgctcatcgtcaaggacg

gccctactgagatcacgagctcggggacctga

>contig_14.contig_14.g521.t1

atggagcaagctgggagagctcctttcggcgtggaggcacacgtggagaggcgcgagagggtgatgagatcggcgaggt

tggacacggtgtgtatgctgacagcaagagagacgcgcgtcgagctcgagatggacgtcgctgtcgagcacggtgttgttcttggcggcgatgcgatg

gatggcgatggcaacaacgaggagaggagggcgggcgcgtcgcgtctcagtttctgttgtgtggaagcaagaaaaaaagttgagcaggtcaaagacgg

cgtggatgagatcgacgcgcttcatcttgacagcgagaccaacagggaaaacagaatggaacgcgtgcagtactcactcgagcggacgctgccgtcgc

tccacaccctcgactccaccaagaccttctcgcgcgacgagctccgtgcgctcaccgcacagcgcgccagcctcgagtccgccctcgtccgtcgcgtc

gccaaccgggacgggtccgactacatcgcctacgtcgccttcgaggacaagatcgagaagctcagactcgtccgcaccgctaaagctgaggcacagac

gcaggccgatcgcaagggcaagggcagggcaaaggcggcacagcagcagcagcagcaggatgacgaggcagtgccggtgggaggcacgaaggagcagc

aggctcggctgagggcttcgtacctcgcacgctgcatctccatcttcgaacgcggcgtcaccaagctcaaggacagcctggccctgtggcaagcctac

ctcgcctgggcccgcgcccgcaacatgcgcatcgtcgtcggtcgcgtctttgcccgcgtgctcaacctccacaccgcccatatccccctctggatcca

ggcagccggatacgagctcggaaccaactttgcagcgaatacagctcgtctgctcttgcagcgcgccatcagtctcaacacgttgccgttcaccgaag

tcgcccttcccgcaacagccctcccaaaccaagcggaggaagccgacgaagccgaaacgacgcctcccaagaaaaagagacgtcagagcaaacagacc

gcatcggctattgccacaagcatcaactctctgctcgatgacaaacatcgtcctatcgcattcaccctcaacctcacccctgccgagcgaaacatgct

ccgtctctgggtagaatacgcccgcatggagctcgtcttcctcgagcgtctccgaaggcggtggtcaatcttgggtctcggccaagcttccaacgcga

ccgctctcacatctcgtcctgccgaaggctcctcgtctcaagccaagggtatctcccagcgctttgtcgatgcggccgcgcaatcaacaggtgaggaa

acgatcgatacctccaaactggccaatctgcccggcgatggcgcggtggggatcgatgagaaggcgaaggaggatgtcgagcagcgggacgaggcaga

agctctcgaaaatacagacgtgcccacgtcggagctcgcccttccctctcctcctcccactggcgagtcctcactccaaacacccgcccgggccgcac

agcatgccatcatgtccggcggtcttctcgcgcccctcatcagcacagccttcacctccgtcgcgcccaagctgcgtctccccctcgcactcggcctc

atcaagcttctccgaagtttccccttcctcgacgctgagccttcgtcgtcgtcggagatcgagagtggactgcagtttaacattcgagctgagcttgg

cggtcgggtcctggacgcgtttacgaggtcgactacagttgtgcagacgtcccacccggactcggcctccaacggcgcaacgcgagcgatcctcctcg

ccgcccggcccatcttcctcgacgcgcctgctgcctcctctcgctggtcctcagcaacacgggaaaaggaaggcaaatctcacggcaagcttcgggcg

caggggaggctctgtcccgtcgcggacgacgaggtcgatggtcttctgaacatcgctaaggaggatctgaaggttcgacaacagagtcaggctgggcg

aagtgctgctgaacttctggtcagtatgcctggtccgcgctctggctctgttggcgatgacgacgctcccacgcacttggtctcgctccagatcatcg

atgccctcactccgctggcgtcagccgccactcggaccctcctccccatcctgctcggtcaattcgtcgggaacctgttcacgtgtgcccagaaatct

tctgctggggccgacgctggcgtgacaccgggaaggaaggcgggcagcagcattagtgctgcggcgtacggtttgtccttgtcacccgcgggcgaaga

ggcccgctttggagctctggctgctcaaaccctcaagacggtcctggaggcgcggcttccggttaagaaggtgcggaaagtcgaggagatacgcggtg

acgacgaggaggagcaggagcaggagcaagacgatgatgaggcggaggttgaggcggaggcggagaagcaacgtggcgaggttgccggccagctactg

gttcaggaagccaacttgcggacgttgctggagacggcgatcaagcgcttcttgcaagtcaagtag

>contig_14.contig_14.g522.t1

atgaagacactcttccacatgatcaaggacagggccgcaaaatcctcgaatgcgcattcccactatggaactgacgatg

actccaacccatcctcgtctacctcctcgcacacttcagatgcatcctttcgagctgcgcgcaggtcccgatccaatacccagccgacgaagcagaag

aaggagtgcttttcttcgcatcctctgcctctgccaaaaggacccgcagccaacgggttcatcgttccacatccatctcaatctacaccttctctcgc

cattccaaggtctagccctcagttgccaaatttacccggcaagttcaggaacaaccagaacgctgacaaaagacagggtcgcgacgagggtcgatcca

acactgttacttcccacacagaacaagtgaatgaactctgtccaccggcgtccccatcacttgcgcagttcccagggaacagagaaccggcatccgca

gcaaacacgatccgtccatgtcggtccttccgagatagcccactcggctcactggtcgtcctcaaggatcggctcgcagtgtcgtcctcctcgttcct

tcacccagacgattccatcgaccctacatcggacgacgtgcatggcgaagcaggcatgggcagtgctttcagcgatatgaccgttgtctcaggtgtcg

accacgcctctgcccttcgttacatttctagcaagagatcgatggatacctccgctaaggacgccgtctcgacctcgtccggctcggcaagctcgatc

cacaatacacgacgaagcatcgaagacacgatatcgcaatacagacgtctgcaagccgaaactcctactccccctccccgggcgccatcccgttcgcg

ggcccgtactgtcttgcaacgtcttcggagaccactcacagctggttcacgcagaccagaggaacctttcgacctggctgcaatgaaaccgcacggat

ttggcttcaatcagactgctgcgccgataaccggcaacccgaatctttccggtgaggacggattctcatcgggagtgacgcccagattaaacgctggc

cacccgtcctctctcgaatttgattcgtctgtgcgaggcgtcccgagcgacgtatcatctccaaattccgcgacattcactacgtcgcttcaccgacc

ctccaacgtcagcgacgagatttcgccgatggaattgtcgatactggtcaacgagctgactggacctgaaaccaatcccgaagacgtgtctgctcagg

catctcggatcatcagatgggcagaacacatcgcggcaagagaaatttcgcacggcacggtcgtgggcggctcccgctcgctcggacctcaaggactg

ggcattctttcttccagccttctgagaagcattgattacctcgaaatgatggtgcaggggcacctcagcaagggtgaacatagtgtgggagaatgccg

gaacatcttggctgcgttccaccatgcgaaagatctttgcaccccaatcgcaactccgaacacacctcttgcatcggagctgcctagcggaactttcc

acacaggtcttgcggtcgccatggtacccaaccgtcggcacggatcaatctcggcggaattatcgtcagagccacttccacccccaggtgcacttcct

ctaacaccacccaacatcctgaacctcttcgacccacaccacaagggctctcctgctcaacgtttcacggacctccatggaggagcaacggcaccgct

ttcccctacgtccatgaacagcacgcccaacccagccgcaacgacggagatgaacttggccacgctcgtgactccgccagagccacggaagctgatcg

ataacgtatcaccttttgaatcgcaacttgccgaaacctctggaaagggagcgacagtctcgctcacgcctgcaccaagacggagaaagcgacccacc

acggcgccggagacgagtggaaatgggggtctgcatgcgtttggagcggcgttgagcctaactgcggcagctgcgcgagaagggctgggaaccgttat

ggacaagagtcctcggctgccatcgccaaggattccagatggcggtcgtgagactcaaaaccgcagcgccagcaacaccgagcctctagcgagcgctg

tgggcagcccaccgaaccgtcgaacgatgttgcatcagccttcattctcgtatcctggcagggacgccgatgctgagaatgaactggacggagggcag

ctggagcttttgcggagtaattctcccttcgatccggcatatacataccagcaccatcattcttctccctcgcaccacgctctgtcacaacaggatcg

gaacgggggcgagatgatgggtcgtggtggtgctggtcgtcggataggtggcccgaaccaatctcggcccaacacggctacgtcagtgttgacggtga

cgggatcgaggagcgggaccaatacgccgcaggagatcttcacgcctgagtcgttccgtgcggggcctagaccgtccagtcgatcgacttcgtcgaac

aactcgaccccgtacctccgctcggacgctagcgtttcgcagaggagtctggcaaagtcgggcgcggccacaccacttacgccgccgtctttcggggg

cgtcgccgcactgggagcacccatcgtgttgacgaacgggtccagctccggtctgggcatccggctcggggagagtggcgacttcgcgcagcacgcgg

accacccctccatccgctcgaagcagtcccagtcctcgatcagcgacattgatccgcagttcaatggtcgccaggctcctgagtctgtggccgggctg

cggttcggcggggcgaatccgaatgagctgcgtgcgttcactgctgggaaggtgttccggatggcaccgggtttccgtaaggcgtccatgggtgctac

ttcgacgactacggccgctgcagtttaa

>contig_14.contig_14.g523.t1

atgttgctttttccctccgaaacggcgacggtagcggcagcgaacagtcggggcccagacggcgggatgatctatcttc

tgctcaatattcaatttgctcatcccaatccttccaggagctcgaccgcaagttacggcttagtgcggcagggcaagtcggcgtcgaacccattccgt

gactgcagcctgcaggtcaccggtaaccggtgctcgataccgtcctcagaactttcgccgccagtattcgcctcgcggagaccgtacggagcgcgggg

gctgttgttgttcgtcagcgattcggatcaagcccttttccccctccaatcttcagtctccgatctccagccgccttcccccccgtgtccaaagacac

ctgcactgttctgcagcgagcttccctcaccattcaccaaactcgacacgcttggctgcacaaaggtcagcattgacgtcctcagcaagatgaagccc

aattccacaaacaaggcctgccaggacctccagcacgatgagatcgcttcgtgcccgtccatctcctgcacccggtcagcccacatcgccccctcctc

aacaacctgccccgacccaagccgcgccagtacgacctccccgtacacgcagagacctcagaccgactcgctccggaacgagatcaatgagttctact

cctacgtcgagatcaagacgtgccccatggccaagaagggaggctcacccgaaaccccacctcctccctctccttttcctctctttcctgagtcctga

>contig_14.contig_14.g524.t1

atgcccttcaggcttcagcagccaaacgaagagcagccaattcgatcgcgaagttctccattgacgatggccatctttg

ggcagatgccctggtggcttagtttcaagcgctactgggtcagactcgctgccgtatcgctggtatggaacgccgtttggctgggtgcttactgctgg

taccaatcgctgatcacttcttctctcgccagtcatctccaaccttgttcaatccttattgttgcagcctcttcggatcttcacgcctgctccttgcc

ggctacagcttgcgctttgcgcgctctcgaatctcgtcatggcggcaggcttcaagggccatccatttttgacctgagctactcgcacgctatcccaa

aggccaaagcagctctgtgcttcctggcactctatcacgcatccatcatgtcatcatcgaacaaggaacgatatgctcaaccgcttctttttgctcgc

tcacgtagggtcacctatcttgacggactgtattccgactacatccttgccaccatccttcctgacgacgccagcttccaatccaaccttggctgggg

tgtcgtgaatcagaactacttcaacagattatgtcgcagttcgccccgaacaaaggcgctgcgtccccggcggactcgcctcagaagggagtccgctg

tcacaccaccagcggactcccttcagcacgagatccacgccaccacagctccagagcccgtcaagcagcacggattacaaggctttgcccacatcttt

gcttgcggcgctgccttgttcagcgatggatacgttaacgcgatcagtggatcagtaggcaccatcctgagacgcttctaccgggacaccaaccctcc

cgcattcgaccgcttcacagctccctggcctttgcaggacgcaagttcggaatga

>contig_14.contig_14.g525.t1

atggcgaagcccatgacctgtctgggcgacgcggtcgatcaacctttccttgcgccgacttctttcttttactcctcat

tcatcttccttcagtccaatcacaggatcgattctcacctgcggcaggccggacaccagcaccgtgtgaggctaccggcgtcggcgctttccatgacg

agcttaacgcgacgagaagccgatttgggcaacagcagtattctcggggtcgagtcgatcgaaaacgaagacggtggatttaatgatagcctcgagca

tccgcccgctacccgcagccaaaatttggtcttggctcctccttctaaccctgaagccattgcggccagcgccgcggcaatgcccacgggctacagat

ctcaatttcagcgcatgtgcgaaggactgcgtaacgttccatcagatgccaaacacccgcccgacttgtggacggcggacgcggctgactctcaggaa

gagggcatcgatgaagaggcactagaggccttactggaatcgttcaagtcggcctgcacaaatagcgacgatccagaagactgggcaccgttcccaga

caaagaaacattcctagttgcagttgcagctctgaaccctcgagcgccaatgagcgtgggtggcgtgcaaatgatgctgacatttgcgcgtgcagttg

gaggcaagaatgtgccctcgtatgatcggtatctcaaagccctcaccgcgctacggactatgtcaaccgggttgaagcagagacaaggagcggccgga

aacatcttctttactgtcgacctgcacagcatcgtcgcccgccagttctgcaatcctgacgtacgccgacgtttacatctctaccctcgaaggggtga

tgttattcagcacttctgcgacggagacatcttcagcgcgaaaaatgtggacaacaatcttccatcaaatcatgtccagccaatggtcacgctgctca

gtggccagtacgcctacctccatgagcccgtcgttctttcggatcagcgcgtgatggtgccatttgtgtggtgggaggatgacgaaggtcgaatactt

ggcactgccaacgcgttttccctccccattgatccagaatgcctcgcgttcgtggaaggaacgttcaccctcgacccagaactcatcgatcacaaggc

gaccaggacgttgtggaagcagattgcggatcaaaatgtctacaacgaacaaggcgatgacattagccatatgatctgcatgccaaacgatctgcgtc

tcgagtggaaggggcaactttgcgtgacgctgcccctaatcatggcgattgatgactggggaggggggcgctcaaaaaaatgggatcctcatcacagc

ctcttgatcatgcttgcggggttggaacgttccgatctggatcaagagaagaacatccacttgtatggtgcctccaaccatgcaacccctgctgagct

agtcgcagcattcacccaggaccttcaagatgctgccggggagttttgggactgccaaacaaatacaacgatatggatccgtccctacctgctcgttg

tttctgcggacaacccaatggctgcggagctggcggcatcaaccggtatgaaggccaacttcccctgtagggtgtgtagctggggaggcaccaatgca

gtgaagtacacgagcaaaggagtaaaggcgatggctgagggcggaaagattcggacccctgccagcacaataaaagagctggataagcagctgcagct

cgcagccatggactgcagcgatgccgagatcaggtctcgttgggcgacgactggtatcaaagatcgacagacaacagagtgttgcacaatcctcttcc

agcggaatcaagagctttccggcaaggccaagaaatcgcctcacaatcgcagtaacccactctcaccgtcacagatccaaaaactgatggctgaggaa

aaggccagcctcatggccacggattggaaatcgtctctcttggacttacatgaatcagaacagctgcaattcgacgttcaccaacaaatgcctccaga

ggttttgcacaccattatactcggacccctcaaatacttggcggcgattaccgactctacactgaacgatgacgcgcgcgatcaactacaacttaagg

cgctcgcacaagtgatttcttccgcgatggccccgctggtagaggctggctctatctccaaacatctccagcatgcgtggcttcggacagccgagctg

gcgaagcttgtgtgggtagactctatcgatcgagatgcgatggacgaatatcaaaatgaattgcaaaatcgagtttgggcgatgatggaggcgcatgc

gcggattgccccgaagtccatcttgtcgaggccgaagtttcacctgctaatccatctaggcgatgcgatacgaagatacggccctgcgtcaaactttg

cgacggagaggacagagagttttgtcgccgtccaacgcgcagcggtcgctcacaccaacaggatggcaaactccagagatgttgcccaacgcctcgtt

gatcagcagctccttcggcatctcatgtcggggggcatgcggaagtctctcaacaccggggagccttctactccatgcgcagccatcgttcaggtgac

acagagcccaaaaaacaccgtgttcaggcagagatggggattgcaggctgcggagaaggaaaaaccaaaagggcaagctgagatcatgtcaaccggcc

gggctagtgcgacatggagcttgtcaggagaacgatgtgaggtaggcacatttgtgctatatgatggggctgatgacggaggagacgaagatgaggat

gatacggtcccgttgagcgtgggccagattcaagaagtagtcatgcaagacaggtcgactgccagcgttatcattcgacactttgaacagcaccagct

caacaaaaaatataacatgatcagcattcgctcaactgagcgacaagttgccatcaaaccatcggaagttattgggatcgtcaatgctccacacgact

gcatctattctcggtgcaaaatcgacgttgaaggtcgtgaggtggaacaagaacgactccgtacgggcgtcagcaagcctagtatcattcacttggag

agaggtccacagcgccgccatctaatcaatcacacgttgatcaggaacatccctctttccttcagacctcgtcaaccgcagcctgctgccaacccttc

gagctcaaaggatatcgtgaaggcctacgaactcggcatgcaagaaacgatcgaagcaggcgcaggcagcggcgggggtcggaggaagcgaaagcaaa

agcgaaagcgaaagcgcacgcaagaagatatgggtgatgaggaggaggaggaggaggttgtgaatgaaggtgcggaaaaccatgacgtcgacgtcagt

gaagagggaggtgaggatgaggaggatgaggaggatctagccgaatcggaaccggaagaagacgagtttgacgatatggacgagggctattga

>contig_14.contig_14.g526.t1

atggaaaaacgcgtcacggacaccggcacgcgtcgacctatgctgctggcggtcaccacttaccttcgccatccttctg

cagcaattcaattcattgcccgtttctttttcgcgcctctgtgtgacctggaagtgttgtttagcagtctctggttgctggttgaatcatcggcgtca

gatatcagggcgaggactgtccaccagacggcatacgatgacctccgggaggcattctatgctgccgaggtcatggacgctttttccagcgaccaaga

ggactctctttccctgcctcgctcggttccggttcttcgtcttccttctctcagagctgcgtcagacaatacgattacggaggccatgagggacgagc

tcgtggatgaatatatttacacggctacgcatcggtatctcgagcctcgtcaacctgtgcctcgaacagtgtacgatggccagcgaaagggggagcca

tgtgaggcctctaagaacgaggccgcggcacgatctacacctgaatggagaggaggttggtgtggggtggacggtacgacggtgcctctggccgagaa

gccgtacctgtacggacctgcttggttcgatcgcaagggaaggtatagcatgaacgtccaaatgaatataatcgatttctctgtgggcaggacgggca

gtcgctgtgactctgttgcctttgaggacacgcgactggcccaacgtcattcggagatgctggaagctggagaatggtgctgggcagacgctggctat

aagagacaggactggttggacgatgtacaatacgcttgcttgtggcttcgtgctgcggtactgctccaccagacggccaagtgggtggagcagggtga

tgcctcgatggacccgttctatgcggcgggtacggcctgggaagcggcggaccggggggaccaggccgtaggggctcgcaatgagaatgctgaagaac

agggaggcaccggcaacgctgcaacaggagagtctcgagcgcgacagctcgctcgagcgcacaagttccgagacgggctcatggctatactctgcaaa

gccatcccccctggtcagtctcgaagaacgttctga

>contig_14.contig_14.g527.t1

atgtctacccaaccgtctcccgcagcgctgccgcccgcgcgcactgatcccccggcatcggccacatccgttccgtcag

aggaaggagcggaaggaggagggaagaagtggccagtcattccgtggaaggaagacgaatttgctttggagcacaagctcgttacccttctccatgaa

ttcgacgcgtatcgcaaggtctggttctgccacaagaacgaagcacactcgaacgagaacaagtcttctgccggcaagggccttgcggtgcagcttct

gaaggataccgtgctcaagaagcacgttgcagaccctaaaagcgcgagtctgaagcactatggatctcctgctccctgcacctgcatgtggatcgtgg

tgctgacactgctgagcgctgacggtgctcgaacggagctcaagatcactggtaacggtgtgaaatctgaacatgacttgagtgacgagtctagcaac

ctttgggtccgtaagagggtgccctggtacttcgagctggcctctctcgtcggttcacgactagatatcggcactgccgccgtcacgaacagctcttc

ggacggcgcgagcacagtcttggatgcccgcaataaggcaggtaagaagcagaagaagaagaaggggacgaaggaaggagaggagaagagcgacggag

aggaatcaggagaggagagcagcgaggacgaggagggcagtgagggagatgatgaggaagaggaagaggaaggcctggatggagagggtggtgcgatt

cagaaaggagatggtagtggtggtggtggagaatcaagcagtgatgggtcgggagcgaagggcaaaggcaaagttcctgcgactcctgatcgccatgg

agctaagaggtctctccctgagggaagcagccctggcggaccgaacccccccaagaaacgcaagccagttgattcgatgaaggagatggtcatggctc

gaatggaggccaaggagaaggagagggcagatctcatcgcccacgaggctgccaagcaggagcgggagcaggagatggagcgactcaagcaccagacc

gaggttcagaagcagaagacggcagagctcgagatcgaacggctccagcttcagctgcgtctcaggcagatgcagcgtgacatctag

>contig_14.contig_14.g528.t1

atggttcggattcacccttcgcgtcaaaacctcgtagacgaagcggctgcattggccctcttccaacgctggcgagaaa

acaactcgggtgatagcaacgaataccctgttcgcccctctctctttcattgggctcatgtgagcgacctgctcacacgtcgatatctggtagagcga

atagaagttccaaagtcaatcgcttggatactaaacattcttcccgagctcgacgacgaacgatttcgcaactgggttcgaatggatcggagcagctt

tgcgttcgtcctttctttgatcgaggacgaccctgtcttccaagggtcgtctcgctcgccacaggctccggtggccaatcaacttgcagttgcccttc

atcgcatgggtacaaatggagtaggaggtgcaatcagaatggaagcaggccactgggggacgtcggagggtcacgtcttcaactgtgttcgtcgcgtg

gtctcggcacttgcttctctcaaagactcttggatcgtttggccagatgcagcggcgcgcatcctggaatccgccgactccaagaagcgagcaggcct

caagggggtcattgggaagctcgacggcacggatatcgtcctgcgaagccgcggagagctcaatggggatgaattcttcaatcgaaaacgccgttacg

caatcaatctgacagcggtgtgcgatcgacggttgaggttcatctacacgctggttggctggcctggatccgtccacgacagccgcgcgtggtcagtg

tcatccattcagagggagcccgccagcttctttgagacgggtcagtttctcgtagcggacagcgcctataccaactccgacattgtcgttacacccta

caagaagcccctcgccaaccgccggatgggttcccgctcgtccagatccgctcgcgttggacttcccttggcacctcctcaccatttctggccaacgc

ctcgccctcgtgtcaacggcatctgtccgtcgctccctccaaggcgctcccggcccgagctgcccccaatggcctacaatttctttttcctcccccct

gcaggcgtcttccttctgtactcgcccgcctcgctcttcatggcgcctcaggttcccgcctctcttcgcgctcgctttgcgctctggagatcgtgtgt

cctacggaccgtgtacgtctgtcgccacgctgctggagtctccggtcgcgaagctggtatcccccccgatttcttcttcctcacggcctcagacgttc

tctcctccaccgccaccctactggccgatctcctcgctgcggcgtggctcagggtgctgcagctacccgacgagactcgccctgccgcggtcacagct

ttcgggaagaaatgggtctccggcggctcgttcgccctgctgatcgacaaccgtctcacattcgccgccgtgtccgtcgatcccgcttcgctttcgaa

cccatga

>contig_14.contig_14.g529.t1

atgccgcctcaactctacagtccgagcaccatctttcatcttcttttcgccttttctttgcctattttgcctgcctcga

actgttttgtctgggcgcagtgcaagaactacagggacttcacggacctcaagcggcgcggccttgcgcctcctccctcgcttcaaggaatcacgcca

gacgtggtgcgtgcgaccatcaagacgctcaagaatgacaccagcggtggggccggaggatggacagttccccttctccagttgtcggcgaagagcga

tcggttctgtatgttcattgctacgcttacccagcaaatggcaaacgagacggccccagcacgtcagcaactcacctccgccatcctcatcccctttg

cgccagcagcagcaccagccccagcggcagcattacccgtcgcccaggcaaacggtgaccaagaccagcaacaacaacaggaccgtcaggcaccagcg

gcccaagcacaggcggcaggccccgcagcagacgctgatcgtcaacctcgccaagaaggtgaagaggacggccaaatcaacgaccctaacctcaacga

cgtcccggagggtgaacagcaacgacacgaaggacctgacattaccccggaacagctggccgaagctgaagtcctgatcgccaaccgcaaggcatggt

tccaagcgctggcctcagagctcgacaagaaggcctggaaggaccttaccgagctagaccggcgcagaagaccgcgtctgaccagtgaccgacagaca

caccgacctgcccaccaacgtcaagaccatgcaagtccagcccatggcccatgggcccaccagctcgcgttttcctttgctcctcctctcgccatttc

ctcttcagacacgatgccactcaagaagagtcgcacgaaccctagcacggcggtacttgccttttcctccgacgatgaaaacgtagacggcttgaatc

gagcacagccccgcggcagagacgtcctgtccccagcaccttcagcgtgcgaatctttctactctgaacttgcggagcatccatcagtgatgacagcc

gccagaaagcgcaaactgaacaacaagacaacgcatgagctcaatgccttcgcaggcttgagccgtgaggctatgactcctgacgatcagcttggtga

tgacgtcgaagacggtggtcacgtttccgcccaaaggccagctcccagcatcagcatggcaatcgagcagcacagtattctgctgagtatcatggaat

ccgtgcgagctctggaagccaagatgactcgcatgcaggcacagatcgacgacatcaattcgaagcttcctgtgttggtggccttcttggttgtcgac

aacactacgaacatcgacgccgctgtaaaggctgcattctggtctgccaactgcccggcgtactctcatttttcgaatgctatagaagaggtcgtctt

tgagctcatattgaagaagaacgaaattttgggtgcaaatggtgcccagctcatggacgaaaacaacaaggccggtcgcgatgccgtgcgccgcgcaa

tcaaagtttctatcgacaatacacgattcagtgtcaaagactcactccatgctgcgggtggtggcgatgatggaggggacaaggtcttgctcaaagaa

ttcgtggaagacgtcctcattgcgaaccggaacttaaagctgacgtggggcttgatataccgtgccgcgatgctattggcttgcgctatcagatttcc

tcccaaggtcaaaggtcaatggaacaaggctttttgggaaaagattgatcccaccattgccgatctggtcgacaaagctcaaggcagtgcatcacaaa

aggatgaggcaagaagcattctcgacaaggcgatgctcacctgcaatcgacattatggccggtgcgagttcgtcaacgtggtttcggacggcgaaacc

gagaagatcttcaaaaagcttttcgaaacgggcggactctga

>contig_14.contig_14.g530.t1

atggcatgccgttccgctgagcccgccgagggccccggcgcagagctttcgcttcctcgcgaagacccttatcgccttc

ctccccaattccactactatcaccaccatcttatcagcggctcaaacgacatggcaagtgcaaatctgaacgtcaatagcggccctaatgtcttcaag

aaccttggacagcgactgttcgataatcacccagaagttttcaccatgctcgagtccgaggtcgacgcgttcctcgagcaggagagacagtacgcagt

cttcttgcgaccctcacacaacaacaaggagcgaaagacgcgtaaaaaagaggagaccatgaccattgtggtccgtggagatgcacgccgttactctc

ataaggcggccacgcacttcgaggctacgtttgtcagcgcctgtagctgctccgggcagtaccgagatcgtcgcgacgaggatgaggagttgtccccc

aagaagcgtcggattacgggccagggttcatcttcctacaaagtcgggtgtaaggcaaagatctgcatcaaggtcaacgcgccgttgtccacagcagc

cattagcagcagcagcacagattccgcatctttgccttccactggaccttccatcgcggtcggtatcgattcgagtgcgggcggcagcacgtcgcatg

cgcgctctaccactggcgattctaccttctctctccctggcccccctcgagaagacagcatcgtcacgatcagctactactggaagcacacgaaccat

gatcccggttcgtggattgacgctgcccagcaacgtacctgtcgagaggtgcgagagtggattgagaggcaggtatctgaaggtcgcagcatgaagga

gatcatgcgctcggtgcgtcttagtctggatgatctgcagaaggttcgaacttccacaacagccattgatgcgagcatacgagtccgttatcaagacg

tctacaacgtgatccgacgattgaaggtcgagcaggcgcgaaaagctgctgacgggtgggagtcctgtcgtctgtgggcagcggagttggctgcggag

gggtggagcagcttgttctgcgactccacagtgctcagcgcgaccgcggagtcaaaacacattctccctgctgcccccgtcaactgtcggcgtcgtac

aggtacggacgccatagcccaggggcacatccatcacacaggcgatgggatccctggcacagacgcgcaaatcgcgaagcgcaagctcatggtctggg

ttccaccaccgagcgacatcgtggtcgattgctccacgacagagcaggcagcaatcaatagagccttcaaccatcttcccactccaccccgcatcctc

ttgtgtcaatggcacgtcttacgagcctgggaggataacatcaaggacaagatccgcgttctcatctctctgctgctgatccctgcagtgcgcactgc

agcagctggaactgtagtcctcagttactgcagcctgcatctggattctggcttggaggatgacgcgcgggaaaaatcacaacagctgcagtcgcgat

gccgtgcggacctacgacttctggtctatgcggagacgcccgcgcgattcgacgagctgatgacggcatttgaagcggaatggagccctgcattttcg

gtcttcgtcgcatatttcaagcgcgaatgggttacgaagcgaccaccatcactgtggtcgatggcattcagacagcacgctcactacggaattgatac

caacaattatatcgagagttggcatggttctctaaaggtgaagttgtggcgatcattgtggtcaactacctgggccttctgcggaaacagcgagtgga

ccatgtggtcttcgttcttgcgcgagaggtcatacctgactatgcacgacgattattgcaatgtcaacttgaaaaagaatttgtgctcccaggagaag

tctgcgcgaaagcgcgcttttggattgagctggacggagctgtttgaaagagttgatggagatgtgaaaggttcactggcaatccgaggcagcgatgg

tgtcgcgggcaatgcgtacagggtcagctttgacctgtcgcattgctcctgtcctgcattcaaggccaccagtctcgcatgtaagcacctttttatgg

gcagtcgcttcttgggtctgcctgtcttgcgctggaagccgtccactatccctctctcatcgacagcagacggaacagcatcgaccctgcctactctg

cccgccaattccactcatggaaacgcggagatcgagcagagacagcatcagcacaaggaggacctactgagtcaagcgttggacgaggttcgtcgcct

ggccgagtctctgggtcgcaatcaacgatctacgaccacgcttcgaaacactgcgaggagcaagctggaggagatactggcagatattgaaggcgata

agggcgtccttcctcgccctccatctctagacaacttctcgctctcgttgtacttagctctctctctctctccatcattcaacaccactcttattcat

cgcttctggtcagcaccgtctgaaccgaatagaaacctggctttcctcacccacaatggctccgcccaaagcacccccgacacacccactcgagacga

gttcgcggcgctcgacgccgcccgcgccaaactcgacggtgatgtgtcggcgctgcgcaactccatcgactcgaacaactccgacattggctccctca

aggctgaagtcggctcacttcactccaagttcgatgagcttctgaaccgcctcgcccatccggtgacaaccaagaccgagccagcctccacccacact

ctcgcgtcatcaaccaccggcgcatccaccgcgtcgtccaccttcgacgccgcatccaccccgccttcgcccgtcacacccaaatggaacagcgccga

ggctgctttccgaggtgggatgactccctcttccgtctccgtcggcaagaacatacggattcagctccctgacgccgaggagtccggccgctccttca

acatcaaacccgaggagctcggcttcttccacggcgtaccagaagacacggcgctgtttctctcaaacattgaggcaatgcgcgtctcggagaccgac

tccgggtgggacaaggccatcttgcgggcgcttccacgcactctgcgtggccctgcccgactctggttcgccgcgctacctcccgagcagcgcaagtc

ttcgctcaccagcctagacgccttcatcgcgacgatccggaccaacttccgcccccattgccacgtcgtacactatacgttcgtcaaggccgcgatgc

tcaagaccggctgggagaacatgggggaggacgagctcgtcagcgaagtgatggagggcatcgacccatctattgccaagctcgttcagactcccttc

cgagccgaccccacactctctgcactgcgaagcgagctgcgcatgcaggagacgtactggcgcgcggagtacggtcggcctctgtctcgtccggcgaa

cgcatcttctgctgctggcgttccggcatacgacagtctggtggcacctttgtccgccccgaccgtgagcgcatttattgctcaagccgacactcagt

caggttcaccttcggccgacgctttccctcaagctcctcggcccaacaccgccagctcagacccgcgtcgcggtcgttcgattcggtcggacttcacc

cccgcgaacctcgccatccgcatgcatcccgaactcaagaggatgatgatggcgtacaagattccggacaccactcgccttatgtggtgtgtccgtcc

ctgcgaccactgtcggggagaccacttcggtttcgcccacgagcactgttctaagaacacggacaagccggcagagcctcccccgccccccgccttct

tggctcagccagtgctgggtgccgggagtgctggagaagaaaaacctggcggctcggactacacttccctgagctgctgcaacccccctcggcaggag

atggaagccgggggatgcaagagcgtcgtcgagagggttgcgacctccttgggcccaagggaggtccgcgacgacgggtacagcacgatcccgaccct

gggacctggcggaggacatgggccctcatccgaccagacaacccggggtcacccccaaggcgagtatcacacttgccttcccaacgttgcgtctgacc

agagttctactgatgatacccctttgccttccactcaggctagggactatcgtcagtctcccgttctctacctagcggatcaatggcttcgacaaccc

acgacctcgccaacaatcgcggtgtccccaaagcgcccgcgaggtgaggttcggggcgtcaagctatcccgcctggtagagacgggctctggtctcgg

tcacctacgccatgctccgacgtcctgtcaggtccagttccctcacactgacctggcacctgtcccttgtctcgtcgataccggtgcttcgctgtcca

cgattgacgcaagcctggctgagagactcggacatgctccctcgggagggacactgagcatcaatggcattgggaccgagcgcacgttggggttcgtc

acgttgtctttcagtattgaaggcacggacgacaccgacggagccgtccgactggatttcgaccacgacttccatgtcgttccatcctttgcgccggg

cattcttcttggacaggattggatttcaggtcacggtctggtcgtcgacccctccagcaacaaagcttcgatgcaggggcatcacttccgagtgcgca

gtgacaagcctccagcacataccttcctgggcaagatctgcactcgaaaagcggttgtactcgcccccggttatcatacctgggtgccggtcgactgc

gctgcgctcattgccgacgtcgactacactctcgatccgacttggcacttggacccagcacaacaacaaatggcagtcgccattccctgcatcatgga

ccgccagacctctcgcatcctcgtcaccaacttcagcgatgaggcactcgaggttccgagccgcactctgcttggcgaggccacggcctgcggtccag

gctccgtctccactctccaggcatacgcctttccactatccgacaccacccccatccctgcaccgttctcgacacccccaggctccccgctcgatcag

ttcgagttcgacgaacaacctctcgctgctcgcgcacaagagaacgcgacgatcatcattgatgacgtctttcgggtcggggtggacgacggcggcat

tccacatcgcgaagtcgtggcgctgcttcctcctcttacggccctcacaaaaggtctcgcgtatcagcgtgttggcagtgcttggagactgctacggt

ccgacggtacacctacatcgaaacaagcccagctcctcgcatggtcagacaaagaggaatcagcattcaatgatctgaagtctgctctcatcagcccg

cccactctggcgcatcccgactacgaccgaccctttctgttgtatacggacgcgtgcaagagtggcttcgccgccgctattcatcagatccacgtcca

tccggtcccttctgttccgacagtcgttgctcccgcatggcccaacctaacgcctccgttggacccactcgcttggtccgatgctcttcttcgtgatc

caacgtttgctccaacgatgcgacggttcgctgcatcaccaacgaccgaggacgacgacggtgtctacgccgtgaaggatgggatcttgattcggcgc

gacgacgggagggtctgcgtccctcgctcgatgctgctcgccgtgcttcgtcacgcgcacgacgacggtggccatttcggcttcggcaagacatacgc

cctggtcgccacgcagttctggcatccccgtctgtcgagtctcgtcgaggcgatcgtgcgcctcggttggcgtccagagcggctgacatccgatcacg

acatgcggatcatcggcgaggcgggccagcagctcgccggattcttgggtgctcgcatcaccgcgacaccgcctcaccaccaccaggcgaatccagtc

gaacggcacgtccagatcgttcagcgcgttcttcgggcgatgagtaccgctcgttccggcctctgggatgacgaagttcttccggctgtcgagctagc

aatcaatagctcggtcagcacggcgacggggttgacgcctttcgatgcgatcttcatcgactcaccacggttgctagacgctcttctccgcgacggtg

gacatgacggcaaggaagggtacgacgaacgacacgccgccatgcccgcgctagctcccggctcatccgtctggatccggctgcgcgatcgaccggtc

cgatccgccccatcagggaagatggatccggtccgattgggtccgttcgttgtgcgtcgtgtgctgtcaccgcaccgtgtcttggtcgacgtaccgcc

cgagcttaatatcgacgacgagttcgacatatcccagctggagccacatcccactggagacgatccgttcgaggacgagcgcgacttggacccgacag

caccttccacatcagcgtctgcatcgagccctcagcccgcagtcgctgaagtggatccgccagccgcctcttccgagatattccctcgtcgcggccag

cgacaacgtcagccggcgctcatcgtccgtgacccgacgtacgaagtaaatgcgcttcgcccagacctttccaccccgatcctctcccagtctgccga

cgcgttcgcagacccgatcgcaagggcgaagatcgttgattgggagggcacccgcatggaaatggtcgagcggccggttgccttcatgtcgcgaacga

cgaccttcgcagaatcgaagctggctggcgctgagctcgagctcgcgtgcatttgttgggcgtttacgcgatcgcagcacatgctcgagggtgcgaag

gtcactgttgtgactgatcacgctccggtgcatggaatgctccattctcgtcctggagctctgccctacgggcatgcagtggagcgcgcacgtacgat

cctccgccctcacctcgacaacctccgcatccaccgtctgaaccgaatagaaacctggctttcctcacccacaatggctccgcccaaagcaccccccg

acacacccactcgagacgagttcgcggcgctcgacgccgcccgcgccaaactcgacggtgatgtgtcggcgctgcgcaactccatcgactcgaacaac

tccgacattggctccctcaaggctgaagtcggctcacttcactccaagttcgatgagcttctgaaccgcctcgcccatccggtgacaaccaagaccga

gccagcctccacccacactctcgcgtcatcaaccaccggcgcatccaccgcgtcgtccaccttcgacgccgcatccaccccgccttcgcccgtcacac

ccaaatggaacagcgccgaggctgctttccgaggtgggatgactccctcttccgtctccgtcggcaagaacatacggattcagctccctgacgccgag

gagtccggccgctccttcaacatcaaacccgaggagctcggcttcttccacggcgtaccagaagacacggcgctgtttctctcaaacattgaggcaat

gcgcgtctcggagaccgactccgggtgggacaaggccatcttgcgggcgcttccacgcactctgcgtggccctgcccgactctggttcgccgcgctac

ctcccgagcagcgcaagtcttcgctcaccagcctagacgccttcatcgcgacgatccggaccaacttccgcccccccattgccgtcatccgtcaacaa

gcacgagctcgtcgctggctccccgactcagaagacgtcgtacactatacgttcgtcaaggccgcgatgctcaagaccggctgggagaacatggggga

ggacgagctcgtcagcgaagtgatggagggcatcgacccatctattgccaagctcgttcagactcccttccgagccgaccccacactctctgcactgc

gaagcgagctgcgcatgcaggagacgtactggcgcgcggagtacggtcggcctctgtctcgtccggcgaacgcatcttctgctgctggcgttccggca

tacgacagtctggtggcacctttgtccgccccgaccgtgagcgcatttattgctcaagccgacactcagtcaggttcaccttcggccgacgctttccc

tcaagctcctcggcccaacaccgccagctcagacccgcgtcgcggtcgttcgattcggtcggacttcacccccgcgaacctcgccatccgcatgcatc

ccgaactcaagaggatgatgatggcgtacaagattccggacaccactcgccttatgtggtgtgtccgtccctgcgaccactgtcggggagaccacttc

ggtttcgcccacgagcactgttctaagaacacggacaagccggcagagcctcccccgccccccgccttcttggctcagccagtgctgggtgccgggag

tgctggagaagaaaaacctggcggctcggactacacttccctgagctgctgcaaccccccccctcggcaggagatggaagccgggggatgcaagagcg

tcgtcgagagggttgcgacctccttgggcccaagggaggtccgcgacgacgggtacagcacgatcccgaccctgggacctggcggaggacatgggccc

tcatccgaccagacaacccggggtcacccccaaggcgagtatcacacttgccttcccaacgttgcgtctgaccagagttctactgatgataccccttt

gccttccactcaggctagggactatcgtcagtctcccgttctctacctagcggatcaatggcttcgacaacccacgacctcgccaacaatcgcggtgt

ccccaaagcgcccgcgaggtgaggttcggggcgtcaagctatcccgcctggtagagacgggctctggtctcggtcacctacgccatgctccgacgtcc

tgtcaggtccagttccctcacactgacctggcacctgtcccttgtctcgtcgataccggtgcttcgctgtccacgattgacgcaagcctggctgagag

actcggacatgctccctcgggagggacactgagcatcaatggcattgggaccgagcgcacgttggggttcgtcacgttgtctttcagtattgaaggca

cggacgacaccgacggagccgtccgactggatttcgaccacgacttccatgtcgttccatcctttgcgccgggcattcttcttggacaggattggatt

tcaggtcacggtctggtcgtcgacccctccagcaacaaagcttcgatgcagggggcatcacttccgacggttgtactcgcccccggttatcatacctg

ggtgccggtcgactgcgctgcgctcattgccgacgtcgactacactctcgatccgacttggcacttggacccagcacaacaacaaatggcagtcgcca

ttccctgcatcatggaccgccagacctctcgcatcctcgtcaccaacttcagcgatgaggcactcgaggttccgagccgcactctgcttggcgaggcc

acggcctgcggtccaggctccgtctccactctccaggcatacgcctttccactatccgacaccacccccatccctgcaccgttctcgacacccccagg

ctccccgctcgatcagttcgagttcgacgaacaacctctcgctgctcgcgcacaagagaacgcgacgatcatcgttgatgacgtctttcgggtcgggg

tggacgacggcggcattccacatcgcgaagtcgtggcgctgcttcctcctcttacggccctcacaaaaggtctcgcgtatcagcgtgttggcagtgct

tggagactgctacggtccgacggtacacctacatcgaaacaagcccagctcctcgcatggtcagacaaagaggaatcagcattcaatgatctgaagtc

tgctctcatcagcccgcccactctggcgcatcccgactacgaccgaccctttctgttgtatacggacgcgtgcaagagtggcttcgccgccgctattc

atcagatccacgtccatccggtcccttctgttccgacagtcgttgctcccgcatggcccaacctaacgcctccgttggacccactcgcttggtccgat

gctcttcttcgtgatccaacgtttgctccaacgatgcgacggttcgctgcatcaccaacgaccgaggacgacgacggtgtctacgccgtgaaggatgg

gatcttgattcggcgcgacgacgggagggtctgcgtccctcgctcgatgctgctcgccgtgcttcgtcacgcgcacgacgacggtggccatttcggct

tcggcaagacatacgccctggtcgccacgcagttctggcatccccgtctgtcgagtctcgtcgaggcgtacgttcggtattgcggggtctgcttgcga

actaagtgcgggaggaagacgggctctctggacatcgatcacgatgctgttcgtcctttccagcatgtgtccatcgacatcgtgcttggcctcccgtc

ctctcgacgccgaaacgacgcgtacttggtggcggtggacaccttctcgaaaatggtactcctcgagccttgcacgtccgcgttcacggcgacggatg

tcactaagttcgtcatgaacaggatcgtgcgcctcggttggcgtccagagcggctgacatccgatcacgacatgcggatcatcggcgaggcgggccag

cagctcgccggattcttgggtgctcgcatcaccgcgacaccgcctcaccaccaccaggcgaatccagtcgaacggcacgtccagatcgttcagcgcgt

tcttcgggcgatgagtaccgctcgttccggcctctgggatgacgaagttcttccggctgtcgagctagcaatcaatagctcggtcagcacggcgacgg

ggttgacgcctttcgatgcgatcttcatcgactcaccacggttgctagacgctcttctccgcgacggtggacatgacggcaaggaagggtacgacgaa

cgacacgccgccatgcccgcgctagctcccggctcatccgtctggatccggctgcgcgatcgaccggtccgatccgccccatcagggaagatggatcc

ggtccgattgggtccgttcgttgtgcgtcgtgtgctgtcaccgcaccgtgtcttggtcgacgtaccgcccgagcttaatatcgacgacgagttcgaca

tatcccagctggagccacatcccactggagacgatccgttcgaggacgagcgcgacttggacccgacagcaccttccacatcagcgtctgcatcgagc

cctcagcccgcagtcgctgaagtggatccgccagccgcctcttccgagatattccctcgtcgcggccagcgacaacgtcagccggcgctcatcgtccg

tgacccgacgtacgaagtaaatgcgcttcgcccagacctttccaccccgatcctctcccagtctgccgacgcgttcgcagacccgatcgcaagggcga

agatcgttgattgggagggcacccgcatggaaatggtcgagcggccggttgccttcatgtcgcgaacgacgaccttcgcagaatcgaagctggctggc

gctgagctcgagctcgcgtgcatttgttgggcgtttacgcgatcgcagcacatgctcgagggtgcgaaggtcactgttgtgactgatcacgctccggt

gcatggaatgctccattctcgtcctggagctctgccctacgggcatgcagtggagcgcgcacgtacgatcctccgccctcacctcgacaacctccgca

tcgtgtatcgcccaggccgcttacacgtcaatgtggacgcgctgtctcggctcgtccgtgatccaccggctcgcccgggacggcgagcttctttcggg

ggggatgtgatgggctctctatga

>contig_14.contig_14.g531.t1

atgcgatcccacagctggaagccccccttgtgcgcagttcctcagaatcccgcacggcgagcgcattcaggcgctagta

gctccatcctaggcaagcggagagcgcgcgacgagccagaggaagacacccgctggtcgaagcgtgccaagatcctcgagaagaccaagctacagttc

cagccccgtccgcatcaagtggtggcgtcggatgcgatccaccgaggatgggatgtcgtcttcaagtcccccacgtctaccgggaagagtcaggttat

cgactacctcgactgtctccttgatgacggggttatcatcaccattggacccctgcgagttctgctgaaagaatacgttcaaagatttggggatcgag

cgacgcatgtggacgccaaccacaaagacccagcgctgctggcttcaattcgcgacgggaagttccggtttgtcttcacgactgcggagtccgccacg

gaaggcgccttcctcaaccaggtcctaatgcacgagaagttcaggaagaaggtcaaggcatttgtctttgacgaggcgcatacactcgatcaatgggg

aagagacttccgcccaaagttccttcagctcggtcatgtgcgcaagcatctctcagcgcccgtactcatcatgtcggcaacactcaccaaggttgcgc

agcagctctgcgagacgacccttcagctcttcaagcccctcgtcgtcgacgttggaaccgacagaccgaacatcttcctccgagtccagccaatcatg

tacgcggagagcagcttcctcgacatcttgacggcgttgcctcagctgtgggagcctgccggggatgaacagaccatggaccaacgcagacaggaatt

ccgaccgaccgtcatcttcgtcaacaacaaggaaacggcgacctcgatggtatatcggctgcaacagtggtgcaagagagccgggttcggagacgtcg

tcgacgtattccacgcagacatgtcggagaagcacaagggtatcgtgcgcgagcgccttgataacgggtcgacgctcattgcagtctgcaccgatgcg

tttggaatgggcgccaactgtcgtgttctcctcgacatgttccagtggaagatgtacggaggggtctcggcttactggcaacggacaggaaggtgcgg

acggaagttcaacgatcgcgctgtggccactctcttcgtcgacagtactcacatcgactcggaactcaagaagaccttgacgaccacgggccaactcc

caggcgacgatgaggcggaggatcgagctcgagagtcgacatcgctcgaagaggacgttcgaatgatgcgaggcgagaagaaggcaggggatcgacaa

gttgcagacgacgagatgattgctcttgctcggcatggtcttgctcgggatgcatgtctgcgcaagcttgtccttgaacaccttggtcagcccagcat

tgacaagttgcccacgcttgctgagctgtttggcgctgggcctaacgacgacggatcggacttaccttgctgctcaacttgtgatccatacgatcttt

cggttttgccgaaggatctacatgcgccaaaagagaacgttccgcttttaaaggacctcgttgccacacagaaaatccttcggcctcgcctggaggag

tggcggcgcaaacaatacaaggagtcgtggagacgcgctggtggagctggacggttgggaatcaacgcttatatgagcttgatggatatcgatgcggt

tgtctgcaacttaaaaatcattgcgaacaacatcagcaacggagactcacgcttaagcgacatcgtcaacatgcccttcaaggaaacggtgcttggcc

cactggaggtcgaaatcaaaaacgtcctgcgggaggcagaggacgaacgagaagctgcaaggaaagcacaagaggagcaacgtcgtcagcgggctgag

gacacaaggaagcgtcaggcggacagaagggatcagcgaactagagcagaaatgcaacagagggttcagcaggcggagagctcgagctcttcatcgtc

gacacgaactcgagcatgcacaacgtgcacggacatcaatagtcatctaccgccgaccgacgagaagctcgaggcacgaggacacaatagtaaggctt

ctgactga

>contig_14.contig_14.g532.t1

atggacagccgcagtccttctccggaatggagcacttcctcgctctcttcgctctcctccgtccccgccgaaagctctg

gccctcaaaatgcgtcttctcggcggcgcagaaacctacaagatgccgacgcggaatcgttctttgattggctcggaggacggaacatctcctgcagc

gccctactcagggcattgttcgaccccgcgcaatccagcttctccaaggacacactccgacaggtccgatcccgggtcaatcctttcttcggtacgat

agcggccaagtcgttcatcgacaactgccccagcgcgcaagaatcggcagtcaactacatactgcggcaggttcgcaaagaggcttccaaggcttccg

cgcttccagagatctctcgaccacaaggccacagccgcgctgaagacattaggaccttccgactcaagtccgctgtcgacatactcgaaagcaatttg

cctatcaccttcgcggtcacacaagccatcgcaggagtagggaaagacgacgatgcgccggtcgaaaacacatccactgcagtcgaagaggccatgtc

gacgctggctccgctcgcatcaaccgctgacgacgaggaggccttaaacgaaggagcctcggacagtgaactgcaacctttggaagactcagggtcgg

acttggccgctgccgaggaagatgagctccgagatgtatgggacgatgtcggggaggatattagggacgatgttgaagaggggcaacctgccactgcg

gatggcaatgacgggacggaagcggaaccgaaagagacagctccggtgtcacgacgacgcaaagggaagaggaaccccaagatcgtggcagccgccaa

cacactgtgcctgctcatgggccgcaacaatcggaacggcagatttggcctgaacctcggccttttcttgtttgcctcaagaacgggacggcggacga

ttgaagctctgggtaggatggggctttcagcatcgtacctgaccattcatcgggcggtcaaggcactgtctcgatccgcgcgccgccagctcatcgcc

aagatcaaggaccccttctccgttctcgcgcttggctacgacaatatcaactggttgcagcgagcacggaacaagggtcttacggtgaccaacacgat

gcgagctgcggtccacggcgtagtttacgaggtagacgcgtcggttcagctacaagaaggaccccagcatcccgtctgcgatcctcttgtggtcgacg

agatcttcggatccgacatgccaaagccatcaacaacgccgaccaacctggcggatcgagcacacaacccggacggtctgtggccaaagattcgagag

cagcgggatcagatgaggaagcaagcaacgccggacatccaccctttttcacttctcagcagcgagacagacgacgagcaattcgtactgtctgtcgt

cagtcacgcgcgcaaggctttcctgacgcagcatcaatacctcgactacgaccatccttttcccgagccccctcagatctggcccttgacgcctcgga

agaccgtcgtccacacgcttccagtccttgatgtcgacgaaggatccgtagaaggcaacatcgaggtgttcaagcaatacctcgacaagcacgtcaag

gttccgaattccttctggagagacagagttctctcgatcatcgccgatgccttcacgacagagaagatcaagcaagcccagcggacgcgaaacctgga

ccgttctccggactcctttgacaagtttgacgcccagcagccatgggctgcgccctggcatctcatgtatgcgtacatccgatgcttgttcctcaatc

acggcggggctaaagatcagcccagtgccatcagcttccggcatctctccgagcgggtcggctttcgaaacctcctcacccagccctacaacttccac

gactcatctcgattccttcacttttggttcgccgctgcgtgcaactctgtcatcgaagaggcagttcgaaagcacctggatccactgtcaaccgcatc

ttctgccgacaagcctgcggacaatccagctcatgccgcccaaccagctcgaggaaccgcagagcgacgagcacggcatccgggaggcgagaatgacg

cggaacatcccgcagaggagttcaagggtcggctcgaccatgagacatttctgcggatcagcaacgcagccatccgcgatctcctcaagtacagcgca

caagaccttcttgacgagcaggaggaggacggaaagaagcgggacgacatcgccctgcacagccgaaccatgtttgccgatgtcgcattgttcatcga

gctccaggctgcgatcaaggtgggaggtgcaggacgtctgatcctcgccatgaagatgctcgtcccacgctttcagtcgactggacagcacaactatg

tgtcggaattgctggagatcatcgtctcgcttcgattcgagatgcctcctgcgctgcgtgcaatcatggtcgcatccctcttcgtcaacacggacggc

ggccgaaacacattcgtgccgacagatttgctccaagagaacttcgtcttcgacctgaaacatacctggccggtgggaggaacgaccaagtctctgga

gtacaagcaggtcatcggctcgctgctccaggtcctcgctggaatgaagatgggcctgtgggaggacttgggaatcgcgtcgcaatcacagaatcatt

cggacaagaagcgtgccattaccatcacggctctgcagcgagacttcgaccgactcaagatcttcgagtgggacgaccagggacgagcaggctccgag

tttgaggtcatgcgaggcgacgcagttcgtcaagaacgatccagtttacaggcagcaggaaaaagcaccaagcacatccgccaaagcaagacaatggc

atgcgacgcgtgggcaagcggaatgcttcagctatccggaacgtcaaccggaagtggctcatacgaccgatggaaggcaagacaagccctctcattgg

gcaaggagggcgcggcagcgacacatgccgacgctgatcaaccagtattggaaggtgccgctgcagaagaggggtttgcatatataggcttatcatca

tccacagctggcttcgaggaactagaggacgatgtttgctga

>contig_14.contig_14.g533.t1

atggcgggttcaacaaacgttacaaatgctagcgaccaagcatcgtcgtcctcatcaccaaccagcttctggctcagga

agcccggcatcctgaagatccggtcccagcgccgagcaagtgctcccagtattgaagaggatccggcgggtcagcacgcggaagagcacatcgacgac

gacgcaggcgccgcgcctccccgtcaactccgccccgcaagcaccctaaacggctcacgaagtggaagtgcaaccccactcgatcctcgggactctcc

cgctcttcaagctcaagaccaatcgctcggtctcgatggtgggaaagatgagatcgaggaattggacgaagtcgttccggaccgtatcgagtttttcg

cgccgcaccaaccgtacttctggctgagcaatgccgctccatacgagattgttctggacggggtcaggtatccgacggctgagcattgcttccagagc

ctcaaattccttccgcatcgaccggagatcgcccgcaaagtacgcaaggcatcaacacctgtcgaagctgtgcgaacagcgcgaaagcacacagcgga

cgttcagcgtggttggcgaagggacggtttgaatctggtcaacatccgacgggtaatgctcctcaagttctctcagcacacagcgctccgcaaagccc

tgctccagacggggaatgcccagctggtgaatgccgtaccgaccgacgtgttctggggaaacggcggcgggaggggacggaacgagtttgggaaagca

ctggctagtgtgcgtgaggtgcttcggaccatcgatctacttggggcgggaagcggagccattacggtctaa

>contig_14.contig_14.g534.t1

atgcctgcgcagagcatctacgacgagatcgagctggaggacatgagctacgacgacgtcaagcgcgtcttccactacc

cgtgtccctgcggcgaccgcttcgaaatctcacgccgccagctcaaggacgccgaagacatcgcacgctgtccctcctgctccctcatcattcgcgtc

gtctatgaccccctcgactttgaggacgacgaggatgaggatgacgttcccgtcgaggctggtgtagaggaagccgagccggtcaaacgccacatcga

tgagcggatcggcgcggtctcgtctctcgtgatattccagaagtcgcaggacaagcgcgatgactcgccctcctcgtcaaccagctccgcaagcagct

acgtgctgaagaagcagaagcacgcatcccacaaccacagccacagacacggagacagatccccgaagagcaccctgctcaacatcaccaccagtatc

aagcaggacgtctgcagatggagcggtgttcgtatcggagcctacatgttcggacgctcaatggcaagacgtctggatcttgcaggagcacgctcatc

gggtcggcactctcgtcctctccacacgagtttttcatctgcacgggttacctttgactaccttagcgaaggtcgatggggaggaaggagctccagcc

tccagggcgtcagtaccgcggccacctcggacactgtctctccgtcttccgacagtactccctgcatcggtctctctggtccccacctcctcgtatcc

aactcttgccctgtctccattggtcgccccaaatcaagccacgttttacaatgcacgaatgagggccagacgtgccgtgccactctcttttctgcgcg

gatgaaacaatga

>contig_14.contig_14.g535.t1

atgcagcagcattcaacacaccggctccccttcgccgatatcttggcgcactcgcacgacgaccccgacccgtcttcgt

cggcggtgccactcgcgcgcaaggctggagaagacgatcagcagaacggaagaggtcttctgctcgaattcgttgcgaaaaccagcatgtactgccct

ccccgccaatccccgctgccgctctccagcaagacgacacccgaatgccgtcacccagctcgggcgctcccgcgcgcacgacgctctcgactggcatt

ttcgctgcttctctccgttggagcaatcttcggacttgtatgcggcaccctaccggtcggtgccatactcgctccgggcgatgaccctaatcctgtga

tcggcacggcggaagccatcgcctcagccctaccaacacttccggtcgccataccatctggggacgctgtgcaaacggataccggtccgtccgcgcac

gacgcgcaggaggcgtcgggccaagttctagagcccgtcaaggtcgctttgcctccgcgtgttgcaaatccgccactgtcaagaacgacgatccccat

cgaggtacaaggaactacattactgtcatctttctcgtcctattcccagtctctcgtcgcaaaagcttcagatcctcctcaggccgttttccaagcat

cagcggcctcatcgccgtccgcaagtcactcccctcccgtcgacgtcacgatcgcagcatcttcctcacacgcctcggaatcgactccaactgctaca

tcagcaggctccattgctacgacacagcaaacagcagcgacaccagatatacccctcccaccagtcccaactgccgaaaatgtaaccccgtcaccgtc

agcaggagcagcgggaacacaaagcgcgactgtaccctcttcggagccgtctcttgcgagcacgagcccggaagtcactgcaacaactgagcatatca

ctgaccctacatctgctgtgcccccgccttccacgacaaatgatgtgccggtagtgactgagccagtcagccctgagaccgcttcatctactcctgct

cttccaaattctcggccggcagtcggcaatccaggacctctgctcgcccagctcaagcaccgatggaacttcgcctccatggactgtgcagccgtcgt

gcaccgcaccaacccgggcgccaagttcgcgtcttccatcctgagcgagaagaaggatcgatacatgctctcaccctgtccagccgatgggaagggcg

gcggacagttcgtcatcgtcgagctgtgcgacgatatcatgatcgacaccgtcgtccttgggaactacgagttcttctcccggcagttcaagcggttc

aggctgagggctgccgccaagctccatgcgcgggaagaggactggttcgactttggcaccttccgggccagaaattcccgtggacttcaagtcttcaa

tgtcgaaccgagggacaacgaggtggaagctctggctggagccgaggctggtgtgatcccgggctacgatggcgaagatggcagtggcgaccagcctc

tacctcccccaccatcgagcaagaaagatgcacgattcttccgctacgtgcgcatcgacttcctcgagcactatggatcagagttttactgtcccgtc

agcgtcttgcgtatctacggactcacagcgctggacgagttttatcaagaccaggaggaaggcggtgacggcggtgcagcggcggccgttgcggccgt

ggccaatcccattgtggcgaatgtggtagagttggcggaccagctcgactttgcttatcagtatgctgcgaacggatggggatacatgccctatgaaa

gcaaaggcccggacgaatctcgactcattggggacggtacaaagcaggctgagggacatgtcgctggatcgggtactcaggggcgtcctgcgctgggt

gcttttgactcccattgggagaacatgctcgatgctgccgtcgccgccgtgaagtcttcgtcctccgctttcaattcgaacacagctacgtttgcgga

cacaagcggacaatggaagaagggacctgcctcctcctcgcaggcggcagcagctgcagctgcagcttcggaggcggcggtacgtagcaccgaggggg

tggtggagacataccggatcgacgttgatgccggtcagccgcatgtacatgtcaacataccaggtgcaggctcggcgctcggtgcggaccctatcgtt

ctcgccagagataggttgacctgctcacctgaagatgcgccgacgtgtggtacatcggagacaccatcttcctctcctgttccgggttcttcagcatc

gaaggttgcaagtccggctgcgagcgaaatgcctcagccggggagctcgatccaagcgggggtaccttctgcaacgagtacgcagcaaacaactcggc

accacactccgtcgtcatctgctcaaagtatggctgggccggctcagggtaacacggaggcacagcacgtggttcctgtcagcgtaagctcggaggca

gctcagagcagtcattcaaatgcttctgctgcggcgaacgcgacacgttcgaccaccgttcaagcaccagcagccggcgcgaagagcactgaggacag

agatacgaccgacacagctaatgcttcaggccctcctgcggggcagcagccggtgccggcttcgtcaccggtggcgtctgggaagtctacgaatgcaa

gtgctggaacgggtacagctatcccttcaaacaccaccgcgacccgccctgttgcctcgaatgcatcggtgacggcaggcggggcgtctggcgagagc

cctcgtgcacccgtgcctccgccaccctctccttcccagggcagtggaggcggggcaggttctgaatcgatctatcgggccatcaacaaacgactggc

agcgttagagtacaactcgacgttgaatctggctttcatcgagcatagtcagcgacttcttcgagacagctttatgcggatggctgatcggcatcaga

ctcggatcgacgatatggtccgggcactgaatgcgagtaattggaggcagtttgacatgctgaggcgacggacccaggttgatttgcagagggcgctg

tttgaggctgatattcggaggcagcaggcggattcggagcggtcggccatgatggcgcagattcatctgttgacggaagagaagaagatcagcctggc

tcagttgttcctaattctaggactgttcgtctttgtggcgttgacccgcggcatgcgtgctccacaattcatccaaacgggttttgggaacaaagtcg

ggatgagcagtcggccttcgacaccagtcgctatcagtgcggccggcagccctaacctcgacgctcccagcacggggcccgtctcgatattcccaaca

cccgcgttcatccgccagcgactgaatcgttcccgccgagtctcggctccggctcccagggcgtcgtctgctgcggctgggaatgcttcgaatccagc

tcagcacaacaatcagaacggctatcaccccgggctcgcacaccaacctaccgaccgcagtcgactgtcgatcgtcgttcctgccctttcgcatcctt

cgaacgtgtatcggttgaacagcggtgctagcagtccgaatggctccatactggaactcaacgagctactgccccatcaacatcaccacccgcagccg

atccgtggggaacactggcgcgagcgaatccgctcgaacgggacgggatccatcgtttccttcctcgatgaggagttcgatgaggaagacatggaaga

agaggaagggctgagcgatacgcgtcgacggtcgacgtcctcggttgccttccgtccgcggcggcgatcgacctcctcgacgacaaccaggagcgtga

ggtcggcttcgatccggtctggctcggatgggaagagcatgacgggaagtgtccggagtgggaatggtcgcggaggccccacggcctcctccccatca

ccaccgccgccgatacagaaggacgcagtcgttatggtctccgtttctgacgagtctgacatgacggcggtgattcagcacagagcggacggcgagat

gatccctgctgagcaggcggccaatgggagtgccagtgtcgaggtcggcaaccgtgatggctcgtccgacaccggcacgaagctgaagaggaggcaaa

gcgcagcgggcccctccccggagaagaacaaggccgggaaacgtgctggtggcttctccttcaccttttcctctcctgcttcgaaatga

>contig_14.contig_14.g536.t1

atgagcatgttccgctccctcggcgccgtcgcccgcatcctctccggcgtcctcgtcctcggggtagcctcgaccgcat

gtctcctatggcgctaccagggtctcctcgtctacccggccagcttccccagcggctcccgcaccgtcgtcgacacgcccgaaagcttcggcatgccg

tacgaggaggttacgcttaccaccccggacggcgagcaggtccggtgttttgtgattttgcagcggactccgtcggacaaggagcgcgcggcggggct

gagtggcgtcggcgatgatactgcgcgcagacggccgacgattgtctttttccatgcgaatgctggcaatgttggccatcgcctacctatcgccgccg

tcttctaccgccgcttcggcgccaacgtcgtcatgctttcctaccgcggctacggtctttcgacaggtacaccttccgagcgcggcattcggatcgat

gcgcagaccctcatggactggattcacggccatcctgtgctgcgctcaactcatctggtggtgtacggtcagagcttgggaggtgcggttgcggtaga

tatagctagtcggccaagtaataagatcgctgctgtcgtccttgagaacaccttcctttccatccccgagctcatccctcacgtcctcccaccagcga

gacttttcacattcctttgtcgtgaaatctggcctacaggggctttgattcgcacgcttcgaatgcctgtgctcttcttgtccggtcgccaagatcag

ctggtaccaccttcccatatggatatcctgtacgagcgatgcccaagcaagaacaaggtcttcaaggcgttcggtgacgggtcacataatgacacatg

tgttaagcccggctatttcgaagagatctcgaacttcctccttcgttacatcgtacctctaccaccccgcccgtcctctcccacccttccattatctc

cactcgacgcacctgactcgtcacattccgaactcagaagacgaaaccaacaacaacaacagcagcgcgaacgcgagccatcctcaacgggttctgtc

gtcgcttcttcctcggcatcgatcatgtccggtgcgacaagtccaagtctgatcgatgcagccgagatctcacctgccagtccccctcatggtgcagc

agcagcagcagcaggagacgagtcggatggagagggaacgggtagtgactgggtcgagatgagcatgacggatgctgtaccgcctgaatcagccaagg

gaagagagcccgggacggctgcgagtgcgggtgaagtcaaggcgggagcgggcggcttggcgccgaaagacacggtgaagaagatgttggaggaggag

gcggagcgagcgaagggcaagacgacggggggcaaactctga

>contig_14.contig_14.g537.t1

atgggcagggctatggtgctcgacgtcgagatgaaggcaaccgcgcgcttgcctcaccttcgacgcacaaggaccactc

ctcaatcaacatcctctgatcgcctgccatggactgctgcgatgcagcgcatcatcgagcaacggcagatgaaggacttcatgaagctttacgtcggc

ctggtcgagcgctgcttcgatgcgtgctgcaacgacttcacaagcaaggccctcagcacaaaggaggagacatgcgtgaacaactgcacaacaaagtg

gctcaagcactcggagcgtgtcggtgcaagattccaggaggagaacgcaaagcgtgcctggtttcgctctaacggcggtgtgaaacgtgtgacattga

gaggtaggattggggaggcgggtctccctaagggcagcgggtcgggtccctcccctcccctgtctttgtggctgatgacggggaggaggaggaccgcg

tggacgtcgttgggtcgttctgaagctgacgctgaagtgagcgtgtgcacgaacgacgagcaggaggaggaggcggtgaaccgaacatccggcatccc

acacacagcatcagcatcagcaacgatccggcgtacaccccccctgtgtgccagatctctcagcatggcatcctctgccgccgtcgacagtcgcagcg

cagcagccggggcttcagcggccatcacccggcccttgttcgcatcaagagaagcatcgaccgcctcggacgcctcctcatccacctcagacgcatac

aacaacgagcacgacctcccactctccaacagcacttccaccctcgcagacaacaacggcaacgccctcgtcccacgccagcccctcgatcacgcttc

ctatatgagcaacccagctctcgatctgcccaacgatcaggagcacgatcacacaatcaccatgagctcaacctcctactcacaacagcagaaacaac

gcagcttctcctcctcttcctcttcgacagggacagcatccccaagcaccaccatcctgcccccgacctccttatcgtccaacaaaacctccggcgga

ggcagcgccgatcaaggaggcctcggcacaggcactggccaagtcgcccgaggcggtcgactcccctcctcctcctcctacccgcccatcagcgagga

agacgacgaggctcgcgaggtcgaacgaaacctcgagcgatgggccgccgaggaaaaacagcgtcggaaagccctgcgcgcctcccggaccaactcct

tactcgggcccccggccgcctcggctcttgctcggcgcttgagctccctccgtctgtctggcacacctcagggccggcccttgagtcagatcagtacg

aacgtcggcggtggtggtggtggtggtagtggaaggggaggagatgcgatttctccgagcgagcaagttcccttggaaacgctcgctgatgaggaagg

cggaagcagtagcacacactcaggatccttcggcagacgcgctggtcaaaccggctcacgcaggagtagcgggtacggcccacctggcccgctccgca

aactgaacggccgggagagcgtaagcagtctagactcctacaccagctccgacgcgctcactcgctccgtctcctcgaaccaagccgcgctcgcggct

gcagtggccatccatcctgcccagcccgtctcgccggatgcgacgcctaccatcaagcaggccggctttttccagagcgacgggtcggaacatgtctc

cgagggcgaccctgcatcgaacgggacgcccaggccgctcaagaacacgacggctgcgcaaaggggtgctaatggcggcagcggcgacgccatcaaca

gactgaagggtaaaggtcgtgcgctcgactacgagtctgatcaagccgacaatgcgaacccgaaccctttcgcagacgacgccgatgaacaattgaac

gcagagggcgacgtctccagcgtatccatgtcactcgagcacgacgtggagaacccgaccaatcgacagcaaacgccccaactgagaatcaccccaga

ccattcctcatcatcgtcaccaccaaatcagcaacaaaaacgtctctcccgatccgcctcctcgtcatacgcgagcaccaccttacccgcgtacgcga

aaccgaaaccgtcggcgagtaggccgatcgtgaccgtagggcgggaagcacctgccacgctcgagcgccagcggcgcgacgctgatcgggggttccct

tctattgtggctacggacgcggatgcggaagcggctgaggaggaggaggcagcgaggatggctaacaagcgcggcgggaaagcgggggacggaggtgc

gtatagcatgaacgccggtgggggttcgtatggccaacaacaaccggctatgaagcagccacggtcgtcgaggaggctgggctcgcggaggagtcatc

ggacgattgccgagggggaggaagatgaggaggacttcgaagatgacgagggtgggagtgacgaacttgatcggagagcgggtgtgggaactcattct

gccccgaaacgccgctcgacgcacgactcgcgctttaacgaggtcggccttgaagacgatgacgacgacgacgagaacgatggggagcgaggagccga

agcggagtgggtcgctgcgcagcgtcgtcgtcgtcgtcaagcggcccaagtgcagaggcgggaggaggaggaggagttgaggaacggacggaagatgt

ggtggaccgagtggctttgtggatgtgggcggcatgcgatggatgaggatgaacagcaacatggcaagacgaatcctatgggatga

>contig_14.contig_14.g538.t1

atgagtgcacctgcctcagcaccacacgacggcatcaagctctacatctcccccgatgtctacgcgtttgagcccgcag

gccagagcgccgagaaggccggcgccgtgctcaccatcaaccgcaagacgggcgacgtcgccaccaagcccaacatggacggcctcctcaccgctcgc

gagcgcaccatggacatctacggcatcctcggcctcctcaagctgtcgaccaccgagttcctcgtcgtgatcacctcccgctcgaaagtcgcctccct

cttcggccagcccatctacctcgcaaccgacttccgcgtcctgcccatctcctcctcggtgaagtccaacctgctctcgaacccagtcgaggccgccc

tgctcgccatgctcaagacacacctctactcggccccgttctacttctcctacggcgtagacttgaccaactccttccagcgtcagagcacacacccc

gtcgcttctgacctgtccaagccgctctgggaacgcgcggacgagcgcttcttctggaaccggcatctgaacgcccggctcatcgaggcagccgggtc

caatgcgcaagtgagccctttcatcctgccttgcatgtacggtttcctcgccatcaagtcggccaccatcaacgggaaggactttgtcttcggtctcc

tcgcccgacgatcccgacaccgcgccggaactcgctacttcagccgcggtatcgataaggacggccacgtctccaacttcaacgagagtgagcagttt

gtcgtctacgacggcgaaggtggcgctgtcacgggatccgccttcgagaaggagaagacctccatcggcggacgcgtcgagggcaagattcggatgag

ctacgtccagacgcgcggaagcgtgcccgtcttctgggccgaggtcaacaatctgcgctacaagcccgacctgctcatcatggagaagccggagaccg

tcgaggcgacgcggaagcatttcgaggagcagacgcagatctacgggacgaactatttggccaatctggtgaatcagaaggggtatgagaagccggtt

aaggaggcgtatgagcaggctttgcagacgttggagaatgataaggtcgtgtatacgtactatgacttccaccacgagtgcaagggtatgaagttcga

acgcgtcatgggcctcgtcgagacgctggagaagaagggcctcactcaggacaacttcttccactacgacgacagcaggtcagggtcgaaggtggtcc

gcgagcaagacgccgtcctccgcacgaactgcatggactgtctcgaccggaccaatgtcgtacaatccacgatgggtcgctgggtgctgaataagcaa

cttgtcttgctcggcatcttgaaggagggccagggagtagagcgcgagacggagtttatgcggatcttccggaacatttgggcggaccatgctgatgt

tgtgtcgaaggcgtactctggcacgggtgcactgaagaccgatttcacccgcacgggcaagcgctcgaaggaaggcgcgctccaagacgggatcaact

cgatcacgcgctatgtgaagaacaactactttgacgggcttaggcaggacgcgtatgacctcttcacgggcgcgtgggcgccggagtcggataaggtg

ccgcatgtggatgcgcggacggtcgttgtgaaagggatgccttgggtcttcttgttctcgttgatcatgttcatcctgaccctcgtactgccacgcac

caagaatatggtgtccggcaccaagctcgccacgttctggtttgtcgtcgcctctggctcgctttggtacatcgtcgaccgtggtctggagtacgtcg

cctggccgacgctgaaccgaccaaacgacatcatgttctacgatggacccggctactcgagcggtcgctacggccgcggcgcggtcacgggacactgg

ggcggcgcgtcgaacaagcctacgcggtcgccgtcgacgccccggatcacggagaacaacaagggccggagaggagctgcgtatcgggctgcaggtgg

ggagagcaacgggacgtcttaa

>contig_14.contig_14.g539.t1

atgatcgcagccagcctccgcaccgccgccctccccgcgctcacccgctgcgccttcgcagcctcgcgcttctcctcct

ccgccgcccgtcgcgccttcacaagctcatcctaccagctcgcacggcgatacagcaaagaccacgagtgggccgacctggaggacggacagagtctg

gctaccgtcgggatcaccaagcatgccgcgaatcagcttggcgatgtcgtctttgttgagctcaagtcgaaggggacctcggttgaggagggagctga

attgggcaccatcgaatccgtcaaggccgtctccgagctctacgctcccctcagcggcatcattgaggcggtcaacgagcaggtcgtcgaagacccca

gcatgatcaacacgtcacccgaatcggaagggtggatcatcaagctcaatgtcacctcgcccgccgagttcaagaacttgctcagtgaggaggcatac

aaggcctacgttgccgagcaggagtaa

>contig_14.contig_14.g540.t1

atggacgagcgcgagacggaaggcgtcgatcgcccacccagcccttcgcaggcggaggcaggagccgatcctgcagcga

cctcgacctcgacgtcaacgacagtcccgctctccaaacgagcgcagaaacgactggcgcgcgagcgagaatgggaagaacgcaagccgctcatcaaa

gctcaacgtaaagagaaagaccgactggcaaaggaagcaaggaaagcggccaggagcgctgcggaagatcatgcatacaccagcggagaagatcagca

ccaagctggacgaccctcagcctctaaaaagattcgaatcgatcatcatgaaggagaagaaaaacaaccgcttccaccacgcagcgagttcaacgcta

cgctcgtcattgacctcggcttcgacgacctgatgaatacgaaggagatcaagtcgcttgcccatcaactcacctttgtatatgcggccaaccggaat

tcccaggcgccctttcgggacctcgtctttgtcggctcaggaggtggcgaaaatgataattccaacaatcggaaactgttctccgaggacggctgtca

acttcctcctcccgctgcaggaccttcttccattcagcaaggtggggcacgcaaggctctttccttcgcagagtcacccactggatcccaattggacg

ctcgaactcaaggcagttggcagcggtggcgaggtctacaagtcatcgacagtggagaaggcttgccggcactctgggctgagtcaacgacgccaggt

gggctgactcgggcacggcaggatgtcgtatacctcactgccgactcggagaatgtcattcatgaattggaagaaggcaaggcgtatgtgatcggtgg

gattgtggataagaacaggtcgaagttttccagtctgacactgatcttctcttcccctacaacccagaacttatgcttcgcaaaagcccaagccttag

gcatccaaaccgcacaactacccatctccgaagcgctcctcgatccctcaccggacgacgacgacgacgacgacgacagcgacagcgaagcggaagta

gaatcttccgccaacgccaacaataaggaaaagggatcggtcaccaccaagcttcgcacacgaaaggtcttgaccgtcaaccaggttgtcgacatctt

gcttgggtggacggagcaacgggcgtccaacgtgcacgttcgagagagcacccaaagcggagcagctccctcggaaccgaaagacgctgacgttccca

tgccgacgcaggaccacgagcaggagcagagcaagtcgtcttccctgccccttcccgatcccccagcaaacgcagacagcagtgaaccctggtcaacg

gcccaactggacggatggtggcgcgctgccgtccgaaaagcattcccgcagcgcaagctcaaggaacggacctctcgacgcagaccgcggaatgaaga

gctgcttgctgccgctgctgctcaggcccgagatattagatctgccaatgaggatcaagacgacgggcggggtgaggtcatggaagcgatggccgacg

tagatgaggaccagttgtacaatgatgcgcaattacacgagaagtga

>contig_14.contig_14.g541.t1

atgaaggcagacatccaaccacaggccaagcggtacacggccacggaccgaccgaagcggtcgatgaaggaaacatggt

accgtgagaagcaatggtcacaatggaaccttgcacagcgactcctgttcctcgtcagactcatgccacccaagaatgcgcttaagccacctcataaa

gaagccaccgaacccgtcggagttttcaccgagctcgctcaatgggcattttggtttcctctcgcggcgacgacaccgacgcttcaatacatctacat

gaagaccaccggcgagacgtggtcgcccttcgctgcgtactgcatctacctgttttccttcatcgtcttctcgatccttacggttcagcaactcaatc

gcctgggcctgaagcacggttacttcgacagtaaacacagccgagacggagttccggaccagcaaagccacacggtgctttggtctttgaccgccacc

gtcgcatttcgtccaattgtcggcttctacttttcgtacgaccgcaacgagctgccctggatctcaccttggttcccacttcaagcctttgcctacgc

taccacactggacctcttcttctactgctaccaccgcgtcatgcacgaggtccctttcctgtggaaattccaccaacgccaccacgagactcaccacc

ccaacgcgctgctcagccctttcgcggatttagagcaggatgtgtttgacatcgtcgtcgttccctacctcacattcgccactctgcctatgaacttt

cacacattctggatgatctcgctcggcatcctgtacacagaagccgcgggtcactctggtgtccgattgtatctgcccgcgcccctcaccggctacat

tctccgcccacttggcatggaacttgcactcgaggaccatgacatccaccaccgacacggatggaaggaccgtggaggaaactacggcaagcagacac

gagtatgggacttcatgttcggcaccatgcggccccggatcgagactcaatggaagcaggtcgactggaacaacaagcactccttcaacgccggcctt

gcgctcgcacttttacccaacggtgaacagacgaacatgggcgcagacggtggctccataccccgccgcgacgagctcgtccgcacacgaggccgcga

cgcctcctccgccgcccaagacccggccctcctcctccgcgccctctggaccctctgcgccctctcccgaacgagcctctccaaaccgatcgtgagcg

atgcgatgggtcggctgtacaacaaggaggcggtcgtgcgtttcttgctgggcagggcggatccgggtgcggagagggatccggttgatgaggctgtt

gctggtcatttgaagggtcttaaggaccttcgtgagcttaccctcgaacccaacccggcataccgtcgacgaggctcgcccagctcatcctcatcatc

agctgacgcagcagcagcggcagcagcagcagacgaagccacaggtcccttccccttcgtctgcgccctcactcaaaaggagctcaacggcaaaatgc

gattcgtctacttacgctcctgcggatgcgtgctcgcagaatctggtttgcgcacggtggcggcggctggatcgtcgtctgcccctatctctgcctct

gcgcttactgccgtcaaacgggaggaaggagtgaagctcaagcaacacgactgtccgttgtgcagcaagcccttccgcgcatcgccggcattcttctc

taacaagatcgcatcgtcctcatcacatctcggcgcagcggcggcggttgaagaagactctacttccacgagtgcggaggtggaagctgcactgctca

ggtacgacccatctggagatatcgtacccctcaaccctccccccgccgaacaagaagccttacgagcggccttcctcgcgaatcgacccactacttca

tccaagaagcgcaaagcggcagcagcaactgcaacaggagttattgagaacgggaatgggaacagcagtgtagcggcaggagaggaagtgaatgcaaa

gggaaaacaaaaagcagaagctaggtcggcgcgcaaagcagccatagctcaactctcagccgaactagcctccgatccaccggactcggcaacatcat

cagcatcatcaccagccgtcaagcgggcacgcacgtccgggcccgctgctgcttcgcattctacgacggcgccgcaggagaagaaggagatgagcgcg

gcggttcggagcatttatgggttggatcggcctaaggatcctaaaggcgagagttggatggtgcgtggtactttcaatcggtatgcctga

>contig_14.contig_14.g542.t1

atggttggccaatcttcgtcttcggtcgatcccgatcttcaagaggaccgcgaaaacttgagggccacggacgaagatc

tcggcaagcctggttggtctccccggtcgatgttcccattcatcgactcttcacgttcgcatctagtttccgcgaatccaactcatcgagaggtggag

gaagcccttggatggaagcgtcaccacagtccgggatctccgccctccccaactatgcaaggacgattttggaatgacttccttgtctctccacccaa

ggactccaatttatcgtctggacaggccaacaacggcacgtgtccgtccgcttccgctcctcctgttaccgatgcaaggggaaaagcaaggatgcctc

ttgacgatgtgacgggtcagtctcaagccggtctacccgcggtctcgacgtatcctcgttcgccagcatcagcagccctcggttacggtgatcctgcc

tccgggtccgaagatgacaggtctgttctgtctccagcagcaacgcgctcatcacgatcatcgacggtcaccactgctgtctctgatacccctgctgg

tacatcctcccaagctagctcgcggccacctcagcgaaacagaattcgacacgtcccttggctcaagatacctattggttggacggacaggaaggcct

atcgccggttgccatcaccgcctagggtgccctatgcctattcatctgaagtgaacacactccctcgcgctcgcggcagctctccgcattcccctgat

tctgtgtccgacgaagaagatcatcctccgcggaaggtgccaaagttgggtcctgtgctcgagtcccccacacccgtcgaagacgacgatacagaagc

tgtgggcggaaggatggcatcacagaccgaatcggaaacggcatcagagacggcatcggagactgcggacgcgccagctccctccgttacgaatggat

ga

>contig_14.contig_14.g543.t1

atgagcgttgctgaccactcccgtgattctggttccgggatgcccgggcagaaactgtgccagtgcaggtcaagatgtg

gcaccaccggccgctgggtcgatcggcagacgtacgagaatcacctgctcgacttcgacgatgggtgctcgtctgggtccgatgacggtggtgctagc

ggaggaatgggggagcttgtagatgatttcggccaggtgagtacttccttgtccccggtctctgcggtaccgatggcgtattcactcgcaatgctctc

cacttcactgcaggatccaatggactacatcaatcaggaggaagatatcgagctcgacatacgtcctgatgctggcttgctgccggcagaggacgacc

tcttggctggtgggttctctttggacatcgacgatgacggcagcgactcgggtaccggaccagacacatcggacccacagccggaatggttgcgactc

tttgagataatgctcgtttggctcaatgtggagcacagcgttcccgaccgtacgtgcgagctgctcctcgcctacctgcaatccgtctactacacgat

ctcgtccgcatcgtcaagctcagcgcgccgcccattgccaagcgctcgaatggcgcgcatcaggaagtctcttggtctcgaaggatacggaatcacct

atgccctgtgtcccgaccgatcatgcttgacgcctcatttgctgaaggaagtcatcgagcggcaaacggggtcatgccgccgttgcggactagctctc

gttcacgatcctcagccgcaggcgcctctcgtactttctgttcctctcaagccgcatcaagtgaaggtccggctccgatacgagcccattacaaactg

gcttcagcaactcctacccaggcccgacatactcgcgctgtgtagacaccacaaacgaagacagcatacccccggggtgtatcgagatctgtacgacg

gttcgaattggactgcacacgaaggagacagtcgattcgacttggttctcagtctttcgctcatgattgattggattcagccattctatcgacttgga

accgcgtcgtacagctgtggcgttgtatcggttcgcgtggacgacctaccgtttcatatgcggaacgcatcagcgtacacccatgtctgttgcattct

gccaggacctcagaagcccacagcggaaggtttacatgcaacgctagacattgtagtcgcggaacttcaccgtctctacgaggatggcctacagatca

gcgagttggtcgacccagtcacaggccggcctgtatccgtccgcgttcgtctcgctcgtcttctggcggacaccgacgctcgtacctctaccgctgga

tttcccagccattcatcgaagggccaattctgcccttggtgcaccgtcgatcacgaggattgggtccggcttctactccaagggcaagaagggcaggc

cagaaacccagcagaccatcgtcgaaactctctgcgcgctcttcccaatgcagtaccacgtctgcggccagccgagctcgattcgcttcggcgcgatc

acgccgcttgtctatctgtgctctacaagatacccacacggtcgagtttggaccacgcgcctgtggacgtgatgcacaacttggacctgggcctgtgc

aagcatttctggacccaaaccttggtcgacggaacgcttctggatcaatcagacttgcgcgtttgcaaagaggttctgcaaaggaccacgtaccctac

aggagtcacaaggatcagtcccgacctcgggctgacctccggcggtacgccaacagcggctggctggtcagtgttgtctcaatacatcatgcctgtcc

tacttgcttcgtcgtggtccagcctccttcaagcatcagcacagaagactttccaaagcttgaagccgacaatggtcaagaaaggattggtcaatcca

gccgcagctccggcttcagcgccaattcttcaggacaatgtacccggacctagctcgaggccagaagcgacgagagcgactcgccctcccaaaagagg

gcaaaaaaaaggagccagtgcccaaattcacaaaaacagtctctcttaccaccatcctggaggcgtctcttgttttggcattggcatgtcgcctagcg

cacgccagggcgataggcgaagtccaattgcagcagctgcatag

>contig_14.contig_14.g544.t1

atgggccagcttgggcgttttggagttatccacaagaacgatcatacgatcaggaccaacaagcatcgaggaggggagc

tggagcatactatgcacacccgaagcgaggatcggcatcggattatggccctcctagaagcgatgccagtaacaccactctcgcgcatactgcgagac

agtatcaggtccgagaaggcaacaggtccctcctcctccacccgtcccatacgatcggaaagagaaggtcaagacggagatactggagacaacgccga

agacgacgtcgacggcggtgaattagacacttccgaaactgtcgtatctctcagcaacgcagatatgcaacgcgtctgtgctctggcaaacaaggatc

ggaacgcggggcaggcacgatttgtcccgtgtttcgactataacggccgcattccggaactggttatcgactcgaccgcaacgtccgttgctcgattg

tcggtcggcgagttcatactgcggccaaggaaccagcgccatggaatcagatccgatccatccgcgtgcttcgtctcaacaccaggaggtccacgact

tgcccgcttctgttcggtcgtacagatcccatatgtcgacttacgcttacaggatcggttcaaagactacgctgtggttgagatccttgatgctctgg

actggcaggacggactcggtctctcttcgtctacttggaaggaactcggatacctcgccgccagatccgcgtctgctatccggaccatcgttgacatc

gacgatgttttgggaccagctattgttctcccaggaacgcgcatcttcggcctgccagacagcatcattgcaacaatgattcatcctgctggcaggaa

ctaa

>contig_14.contig_14.g545.t1

atgattgaccgcggtcctttgcgtacgtcgccgactggccctggtcgcatggtatcgcactctcgatctctctcgaaca

gccaaccgacagcccatgagcgcacgccttcgcgctccagcgctttcccggccacccagccaaccgctcatgagcgcacgtcttcaagctctcgttct

ggggctcccccgaacagccaacagagtcagcactctggccgaaccgccagtgcggccgattatcgagctcttgaagagcgtgtcgccatccttgaaga

atccacgaggtccgccgcgaggtccgaagtcgctgaggcgcaggattctgagttcgctccatccgtacgccagcgtaaggagctgaaccagctctctc

gaaacgcaagcggcggtccgtctgcgcctccggtcgcgaaggtccatgactgcaagatttaccgtcacttccgccgacttgtgcacatcgggtacggc

ggtgtccgaggcgggcgaagtatcgtcaagctgccgtaccccagacgtggtgcctggcccaagcatcctccatcccaaagtatcagcacaacgggagc

agaacaagatgggatcgccgaagacgaactcaccagtggggatcagattcgcttggactatgctcgatcgtataccgatcctgtgaaccaaaggcagc

tccgctcgaccttccgatacatgcttgctcaccgccaacgatgcggtgtcccggtagacatgtcgcttgaagacttgatcgccaagtgcgccacgaca

ttcaacggctgggttatcgagtatactcggtcgctatcttctgcaggccggcagaagaagaaggtcgccctgaagaggtcctgtcactctgcgcgccg

aaaacgtaaggcgggctcgagagccaaggcgttgcgtctgcaccggtactttttcctttctgatggggctaaggtcgccaaaacaaatcaggccgacg

aagcgcgccgcgtgcgactgggcgcagtccgcgcggatgttgagtttaccgtacagccttgcgccatgagtgacgaagaggacgagtgggaggtcatc

gaaaacggcaaggctccagtccgtccatcaaacgattcatccgatgctgaaggcgatggcttgagcgatggggggcggcccggccgacacgttcgcag

gcgcgttgggatcgaatggcgttcgtcctgtctcatctccaagctcgaagaactggacaagaagcgaagtcgtcagccatcgaagcccatcctaccgc

ccaaccgtcttcggcccttacctcaaggatttacgcttccttccagcgttcgtcggtggatggttgcccgctcttgggccgacgcgaacgaggatagc

tgcgagcatgtctcggacaacgcaggaccatttcgagggccgtattccgtgacgacgggtgccaaggattggggacaggatccagactggacgatcta

tgatcgtgatgaagacgacgagggaactgaatctggcggcgcaaatcatggcgagaacgggactggtggccgccgggatggtcctttgcgccgtgacg

aggattcatccgacgaaggggcaactcgggagactcaacgactgttggatgccgagactggatggggtggatcaggggacgaagatgaggaggaggag

gatgaagataatgtcgaagaggaggatgagctgttcagctaa

>contig_14.contig_14.g546.t1

atggccaacaggaagaaggccaacaagaggcaacagcagcaacagcaacaggagcggccgaagtccttcccatcgatca

gcgcgaaagcatcggccgccgcgcctgcgacgacttcgacggtctccagcgagtcgcttctctctgccgaccgctatgatgtctcgcaaggaacgcag

ttcccagcctctgcatcagcatcgtcaatcgcgagcaacgtccatgtcccatcgtcactctcccacttgttcgcacaggatcgagagtcacgcactcg

gaccccctccaatgcgtcgtcctcccatcgcccatcgatcgccatacacgcctcgccggctaggaacctcctttctaattctccccgcccgacttcct

cgcctttctctaccgctctttccgtctctccgccttctccacgcaagcactcctacgacccgaactccaccttcgcatccgcatctctcgaccaggac

atgacgtccgattcgatccctgctcccggtctcgctgtaccagcattggtcagctcgtcctcttcggcaggtgccggcggtgctctgaagtcgcttca

gcgtgtcgcttcgcgagacaagctcaagcaggagcaggctgaacaggctgctgcacgtaaagacaccaaaaagaagaagaaacagtcgaagcatagtg

accaagctcacgatgattcggataggcaggctggacaatctaccgctactgcgaacccggacactgatcgccaatcaatcaagtcggaagcaggtgtg

gtccagcctgcgccttctgctgcccaccaggaatccatcttgcaccagtcctccgctcacgcagaggctgaccgtctcatcgaagccgatcgcgttga

ggccgagcaactggaagctcatgtggtcaaacacgaaggcgatcagacagccgagattgagaacgagtcccgacagaccccacaggaactctcggccg

ccttccatccttcgtaccagcctcgaaagcgcgacctcgagcgcatacagcctgcttccagtcccattcccagtcagcctgtgaagcatacccagtct

ggatcgccaacgaccgagcagattcctgaggacgatccaccgaccgcaatccagcatgacgagcctgatcccgagggcgcccagcgttctcatgacga

ttggcatgctcagctcgagcaagatcgagcacgcatcggagctgcggttggggacatggcgaagatctccggctcgtcgcgtgagaaggagcgcgaat

cttcaggctggcgacagtggaaacggaccgaccggagcgccgaggtcggatccttgctccacgctggcctttcgggtccacagctcggactcgtctgg

cacgggcccgggctgagcatcccgggcaaggtgaatgcactcgcaaactcggccaagtcggttcttcgacgcatcaccacgaataggggcgaagcggg

gcgcgaaagcagtgaggacactcatcagaggggacagcctggccctgtcgagcttgtccgtgtgcgagctccgctcccgcttctcgtgcctcactttg

ccctcgaatggacgacggctgcgttgggcgctgagggaagcaaggttcggagctcgaaagtcgagaaggacgtcgatgaggaaggcgacccgaacgct

gaaatacagaagatgctgctgcccaacgacatggaggtcgtcatgctcgacgacaggctcaggccgatgaaggagtgggctctcgtgaacacagccgc

aggagaatggcaagcttacatcgtcgccgaaccaggcaggtcgttcgcgatccagctcggagtgaaggaccggagtaaagaggacctcactgcggata

tctggctgaacgggggcgcgaagaagagtcatacggtcaagtttcccgcgggcgcgccccggcagaacatcgttcgcgggattgagaaggagattgca

gacgcgtgcgaggacacgaaggtgttaggttcgatgggggaaatatgcgtgacttcgtgtccgtacgatacgaggccgagaaagaatacgtggaaggg

cggagagggcgggggacgagatccgagccttccgaatgctgacctcaagttctacgagaagaacaaaaagctgatgacttctaaccttcgcgtaggct

ttggccaagctgaacagctaggaggtcgcggaaagggagtacgcggtttccctgcgcacgtctacgtccgaaaagaccccgtctacaagatccggttc

ctgccacggacggagtatgcactggagcgatccggcatcatcgaagatccggatgaaggcgtagaagtaacggggagcagccgtcgtccaagcggtgc

taaagcagaaggttcgtcagggtcggggccaaatggatcgccgaaagtgttgcggcggaaggaacccggcatgcaaaactcggcgtctttttcaagct

ctgcatcgtcgacggcccatgctgaatcaagtcgcagtagcttgggatcgcgggtacgtgagcggtccaagtcgcccgagtatgaggtggtcgacgtc

aagcgggagaagcaggatcagaagcggagtcgacgcgtatttgctggtcctggtcgctcggctgaggaaccgcttgagatcccggacaacagcgagga

cgagtgtcccttctga

>contig_14.contig_14.g547.t1

atgagcgcaaacggttcgcggtcgcggatccaaggcctcattctcgaggtccttaacgcgcatccttcggcgagggaca

gcaaagcgtacctccaatcattcggcatctccatgcctcccctccctcccgcgaacaacagccagcaacaacagcagtccgcacagaccaaccagcct

ccgtcgcccatccccatcggcagcagcacctcgcccgccttccacttcacccagcacaccgcgctcgtcaaggtacagggtcccttctccgaccgcca

gctgggcagcatcgccgatggcctcgtctacctcaagcgtctcggtctcatgtccatcctggtcctcgactcggagcggtggcctagcgcggcggcgc

ggacgaatcagcaaggtcaacttctcgtcccgcgcgcctttcttaatccagatacggccgcggcggcttcgggcgatccgaggagtgcgcatgcggct

acgatggcagctgccgctgctgctgctgccgtgtccgcgggttcgtcagctgcggcgcgtacgcctgctgcgctcgtcggtaaagcgcaagaaggctt

gaggaagcgcatgattcatcaggttctgcgtctctcgcacttgctcgaggcacgcggggcgcccaccatgccgttgacccagcccgtctttcggatcg

acccggacgcggtcgcacagctgcactcgcgaataccagccccagctcctgcgcgacacgggaacgggaacgggtcatcctcgcacgcgtcatcgagt

gcgtcagcggcgaatggggttaccaccgcctctgcgaatcggtcgcgctcccccttcatggccacctcgcggaacccagacgaactctcgatatacca

atccccactgcacaaccgcgtccctctagtctctgccgacggactcgacgcgatccgcgcagccctgctgagcgaccaactgcccatcctcgcgcccc

tcgcgttctacgaggacccgcatgaccaaggcgtcgagcgcgcgctggcggtcggggcggatgatgcgctcgtcgcgctggcgcgggagatgagtgat

gcggctattcaggcggctgagaggaggaagtcgctctcgaggcaggcgctcaggcagggggagcaggatgccgcggaggctatcgtctcgggcgcgat

gacggaacaggatgatttggatctcatgccgttgaggcttatggttatcaatcgcgaaggtggtattccatcgcatgcacgaggtggcagtccacatc

tttcgatcaacctcgcctctgagttctcccagatccaccggacgttcatctggaaccgatcgcattcatcggccattcgaaatctccgcactgtcaag

gactgtctcgcctacatgccccacccgacctccagcggcgtcgtcgtctcgcaccgctcgcccaagagtctgatcgcgaacctgatcacgaacaaggc

tgcgcactcgccgagtctgccgcacagcctgctcacaggccggaacgacgtccgacacacgcccacgattgtgcgctggggcctgcccgtccgggtcc

tggagcggatcgaggatgtcgagctggacaagctgggcgacttgctggcgatgagttttggtaaaacgttggatcgggtcaattactttgagcggttg

aggaaacggctcgattttgttattgtggcgggggactatcagggtgcggcgattgtcacgaaggagtgggcgccgggggaggaggagcaggctgaggc

gttgcgggcggagttggctgctggtagggccgacgcgtctcaatcgtcgtcactggcggaagcgggtgggagtctgcttccggacgaggcggtggacg

cgaggaccacgtcggaatggatcgacgctgcttccgcggccgcttctggcgatgcggcctctcgccctactccgctcgaacccatcgcgtatctggac

aagttcgcggtgctgccgtcgctgaggggcagtggtgtggtcgatttcttatggggagccttgcgggacgaggtacacggattgggtctgctcgatgc

gctcaatgagaacgggggtaaaggcggctttgggcaagggcgggatcttgtgtggaagagtcggggggagaacacggtgaatcggtggtactttgagc

ggagcaatgggttcgttcgaatcccagctggcggcgcgccaataccccaaacgaagaagaacggcaacattgcggccgcagcagcaggagaggatgac

gacgacgacgaccaggaccggtatccgtgggtgatgttctggtgcgacgctgaggaacgactggcgaacatgagcggagagaagcggttgggtgcgga

ggctacgtatgaggatgtgttggatctggcggtggagagggagaagcgacggctggaggaagaggaggaagaggccttggagcggaatcaggatgggg

aggagaggatgatgagacggatgtgggatgaagaggatggacgaggcttgttgtcgtcgtcgtcgaatggcggtcgttcgtcgttgtcggaacgagga

catgggccgtacgagggcgatctgtggtcgacaatcggcggcggcggtgcgaacggcggctctggcagctcatcgtcgacctcctcctcggcacgatt

ctcgactggctcctccgacccgttctcgttcgggtcgtccccatcgtcccaaatgtcgacatcagcctcaagctcagaatcggcggcgacggagatgg

gctcgatggggttgtcagggggcagcctgggcaatctgtttacgcgtctgccgcatatcgctcctgaggagcgtggtcggcttgatcggtgggcgaga

tgtatggcgagcatcccgagcgcgtggatctga

>contig_14.contig_14.g548.t1

atgcctctctgtacctcaaaaaatcagaatcagcatgccgccctttctacgacccccccgcccgacgatgcctttgaca

gcaaagaggcagggctgaagttcgcacagacgtgggccagagccgaaggatacgcaatcgtgatctatgcatcttcaaagaagggaaggtacaactat

ctccgctgcgacaggggcagcaagtaccgaaacacgcgcaagataacggacgaagatcggcagcgcaagacatccacaaccaagatcgactgcaagtt

cagggctcggctcgtttgcaattcggaagggggttgggagctgagcgtgcaggagggagcccacaccgaccacggccccacggagtcctgcctgtcac

actccattcagaggcgattcgatgcgctcacgactgagcgcgttctggagctcgcgcgaaccggatctcgtgtccgtcaggtcgtttcattgcttcaa

gagcaggttccgccggtgctcgtcacgtcgcgggacgtatccaatctcatctccaagtatcgacgtcagctcctcgaaggtcggccaccggctgaagc

ccttctcgactggctgaaagagcagagctggccgttcatcgagaagcgagtgccggtttcggagaacgcagatcgcctggaggggttgctttttgccc

atccgcgcggaatcgagctgctttgccgctttggtggtgtcgtagtcatcgatgctacgtacagcacaaatcagcaccgccttcccctacttcacatt

gtcggatttaccagctccaaccgcacatttactgccgccctcgcctttcttcccaatgagacgacagtcacgtacacatgggcgctacaagcacttca

gctcatcgctcctacgtgggaatcggacgttctcgttacggatgaagacgcggctttgagcgcggctctaatcaatagtttccctccttcggtctcgc

aggtgctgtgccagtggcacattcggcagaatatgcagaagcacgcgcgaaaacgactggtcgacgaggatcaatacgagcttctgatgagcgatttc

gacgaaattcgcgacgcacggacgttggagcagcttaccgaggccagagacgtgttcttcgaccactggcaagactctcatcctgccctttgcgacta

cctcgacgaaaagctgtctgcggagcatctttgggtatcggtctacatcaatcggcatccgcatatggggacaaaagtgacgtctcgcgccgaaggcg

ctcatgccaccttgaagaattggcttcactcccgacacggcagccttttcaacgttacgcaggccctgagggaccacttcgtcgttcagcacaatcag

atcgcaagtgacctccagaatgacatgagcaaaatccccatcaccatcctcggcgaccctttcttcgtacaggtctcgaggacggtgtctcgatatgc

gctgaatcttataaagcaacaggacgaagtcagcagacgcccccgccacaaagaggatggctatgctgctcctgttcccgtagatctgcagcccaaca

agtcgcgtctggtcgtctaa

>contig_14.contig_14.g549.t1

atggccaccagcgcaaccttctggaatagccgcgacagcgcgcgcccaggaggcacccgaagctcatccgtccctccca

tcgaaccaaccgccaacctggaggcagcatcggacatgaagaacctcgccgacctcacgaggaccaaccccgccgcggcagcagcagtccgcgccctg

atcaaggcagaccacaccatcggcgccgccctcgcgatgatgcagacggccatggccactgcagcacctcccaaggctcaagaccgaccatccacccc

caagcgagaccacgcacgccgccccggttgcagcaactcggcacccctctcgccctctccgctgccgaaccgcaagcgggtcgcgccccacgaaaacg

acgccccgcgcaagaagcctaccaagggggcccgcgctaccacccccatcatcgtgggaagcgaggaagaagacgacggcctcgacatagactacgga

gcgcgcaaccacaacagcttcgaggaacactggccgtcgaacaagatctgtcgcctcgttaagagcctcgacttcgtcgacatgtggttcttcacccc

tgccggttgcagggcagcagcaaaggaccgccgcgacggcacggagcaagcgatgctgctacaggcggatggaacgctcaagcccgcgggaccgcctc

agaccctagtcaaagactgggatctcgacgcccacgatttccgttcggctgccgacaagtggctgaaggtcgccatgaagatgggcgtctccacgcgg

atgcagaactccatcgcccttctcaacaacgagatctgcggacacgagagctggagcacggagccaacacccctcattctctggcaccaccatcaacg

cgagacctgggcccgggaccggtttactccaggctgttacgctctccacgccctcgacccggccacatacgccaacatccggctgcgcggggcggtag

ccgcgagcgacgccgcaaacacgcgatccgcaatcgcagaggccgcggtggccacaatgcgccagcagcagcaaggctccaccagttcccgccaggcc

aatcaccaaccgcagggcgacaattttcgtcacgccgggggagccggcgaccgaacgacgacgcagcacagctcgtccacgccctctggcagcggcat

tgcctgcctgcgttgcgggtcaagccgctcccaccactttccgcgctgcaacagctccacccgctcggacggatcgtcccttcgggtggatcacgacc

agcacaaaggaggcctccaattcatcgccacgagggaaccagcatgcgtccacagtgtcgacaggcccctcaccgccgctagaggcgccctcggcccc

acccagcttcgcgcctcgggatggtcggccgccctgcacaaagcaggactcgcccagcgctatccaacgatcgtcgcaggcatccagcagggtttcca

tgttggattgccatgcatcacttcgaccttcgtcccacccaaccacaagtccgccacgaaagatcccgcaaccgtacgcgccaagatcgacaaggagc

tcgccgcgggtcgatacatcggccctctgtcacgacccgcctgcgaggcagcagttggtgggccgttccagtgttctcccatctctctcgcccccaaa

cccccggacggatggcgcttcattcaggacgcttctttccctcgctccgatccttcccaccagtccatcaattctcagattcgttccgacgactggcc

atgcacctacgccggcattgcaacagtcatatggcaatccccaccgtccccgtttcccccttctggtccccagattctacaccttcccccctccgccc

aagccgccgcacgagatgtgcgctcggcctaccgcgccatcgtccttcacccctcccagtggccggccgccgtcgttcattgggagggggagttcttc

atcgatacatgcttggctttcggcatggcctcctcggccggcgcgtggggaatcgttggagacgctctggcggatgtgctccgccatgctggcatcgg

tcccatccttaaatgggtggacgactacctgttctttcgcgttccccgccgcgaactccacagcatcaacaccgaacgcgtcctgctcgccgctcgcg

tccagcgagaacatcgcggtggcgtcatcctctggcgcgatggggatcgcgagctcaccgaggacttcagcacagccctcctcgccctttcccccgag

cccgacgagtgggcgtacgacttttcctcggtcgacaccgtctgcgcgcctcttggtgtcccttggtccgcggagaaggaacaggccttcgggcctag

gatcacatactacggggtggaatttgatatccccggccgcatcgtctctctcccggagcgaaagcgcgccggactcttactgctgcttcgggaatggc

tcactcgggctacatttcggcgggacgagactgaatccattttgggcaagctccagttcgccacccacgtggtccccaatggccgacagtacctcacc

ggcctcgtcaccttcctcggattatactag

>contig_14.contig_14.g550.t1

atgccaagttctcctacacgacgtcggaccaccgagctacatcaatcagcctcgacatggcagtggcccaacgacacaa

caccagcgcccatggtcccttcaccccggaaccctcagaagtcaatcattccgcagcgcaatacaccgagcaacgaaacagatcggcccacccctccc

acacctcaaaggccaccagaagatcgccgatccctcaaacaccttcgcgacaacctggccgctatcgaaaaggacgccgacaaccttgacctcgcaaa

ccctacagacgcggcacgcctccctcgcctcctctctcgaatccaaactgctggcgatttactcacagacagagtatcgatcggcagcatcaaaccat

cttcaacacacgcttttcgcccatcgacctggatgtccggcgccctccagcgtacgggcgggactgcacacatcaaacgacttcgagacaccaccggc

atcatcacggcggacgagtctcgaatgctcaacatcacccagacattcttcaccaacctatacagtcctcctgtctctccggccaactacgacacagc

tcaagcactcctactgtcagcggcccgtacctccttcagcgcagaagacgtcaccatgctagcctccccttacttactcagcgacctcaaggcagcac

tacgtaccacaaactcatactcggctcctggacctctcggcctaacatacccccttctccggctgacctccgaaacaactggccctcacatcttatcg

ctcctagaaggcctcgccgcgggccactcgctcccggtcctcctgcaaacaacattactctacaagaaaggcgacaaggccgacctggcaaactaccg

acccatctcagtgtcggacaccgctctgcgcctggttacccgaatggtagcttttcgccttcaggtggccacagggaacgctctcccctggaaccagg

ccgctttcctacccggaagacgcacatcaagcgtcgcaggtgcccttcagggcatcatcgatcatgtcgggacgggcagacctggcatgccaacctcg

atcttcatcctacttctcgaccagcaaaaggcctacgaccgagtcggccacccatggctctggtctgtcttacaggccgctggaatccccagcacgtt

cctcctgatcttacaatctttctacagcgatcctcacattcaagtcatgatcaatggcctactcacagacctgatctccctacgcgctggcctcctcc

aaggagacccgctctcctgcgttctatacaacctggctctgcagcctttcctggaccttctcgacaccttccaagttggaatcgacgtccccggccta

ggccgcttagcgagcctggccttcgcagacgacgtctctctcctcctcccaggcacccctgagggcctcacacaatggccctctgtcctccacgccat

tgcggcatacgaaacagcgagcggagctcgtctgaaccgggcaaaatgtggtttcatccaagtcactcacccctcacacgatccgcaacatagcgccc

cactgcgcgctgccctcattcaagccggtttcaaccccctaccgacacaacacggagaaatcgtccatctgggtcaccccatccacactcgcggagcc

ggcagtccatgcattatcggattcaacgaccgcatcgatgctatcggagcacgcatccccctcattcgcaagttcggatcggaccttattactcgcat

tcgactaagcaacagcatcctaacccccaaactctggcacgcaaccgccataggtggcctccccatcaccgcacgcaccgatgtgtctcttgcactcc

gcaagttcctctacctcggtgacacaccgtggtttgagacggcgaacatcagcgcccctctccaccttggcggcctgggctttatccaccccgaccac

atgttcacagcgcagtccatcacctacctagcacacaacctcctccgccctgacgagtacggaacctggcttcaagagggcctcgcctggcacctcca

ccacgtctaccattgcagccctgctgcacttctcatcccaaacggcgtgcaccgggcccgcctcacagcggacactaccagagccgctggcttctggg

gccgtctcctccacgcattggcatcggtcgacctctcactcgacccgtcgtggccggacctcgggggccctgcattgcttgagctcccgtggtatttc

gacagtgccatcgctcgcacacccaagccctggcccctttcccgctaccgttccgcagcaacccgcggctggatcacctggggagacatcctctggaa

gtccaccctcgccacccgcgaccgaacacacgcatcatcttggccccttggcccccccagcccccgcgcagcggccgctaatctgatccctcgcgcca

acgacatcagggaccccaaagggccggaacttggaacgatcttcgccccatactgggcgtcactcccaccggacttccgccaaaaacttatccaaacg

acggacatgccattggcgaccggcgtggacccctccctgccggtaccacgcatccgcgacccatcggcgcgcgccttcccctggcacctgctccaggt

aaacaccaaaccactgcagcaggcgactacccgctccacccgcctggccctcggccgaaccacaacaatcaccgtgcgctggccttcggcaatgctcc

acctcccaaacaacggaccaccagacccgctccttccccccaacatggagtcctcctctgagcccctccctccggcacccgacaccgccgcctggggg

acagcctggaccgagctccaccgcacccccctgccgagcccggcagtggcaaattgcttcctatcgatgcaccgacgggcttggcttgctctcgatgg

caagaagactcaccaatgcactctccccggctgtgacaaacacgacagtcaggaacacgcctacctcacttgctctgcgatcacccctctatggtcag

cagctctgcctgtccttcgaatgatgggcatcactgcacccatcccgctcgcgccacacctcgtcgccttggcctggccgacaattcgtcgcttccgg

cctcgtcttgtgctatggcggaccatcatcatacacctcatcacggaagcccgtcatcccgccctcgctcacctcaaacgtaccggtacctttgtacc

caccgccccaaacccggagctattccgcctcaaggtcgaacggttgcttgcggagagcataaccacggcccgggcccgctgcagggcccgccaacgtt

ccaccaactccaagaacacacctaccgacacattcacaagcacatggatttccggctcaaccctcgtctacgccgacgagaccggcggagacccaacc

tcacggcatttccttctgtttccggccaggtccccatccaaagatcctcaaggacgatcaaggtttacagaacgagagcccttcgaggcggtgtccat

tgatcttgctctgggtttgccgcggacccgcaacggcatcgacgcagtgctgttcatgcaggacgtgttttctcggatggttctccttcatccatgct

cgtccgagatcactgctgaagggatcgccgcgatcgtatccgatcggatcttgcgcatgggttggcgcccgcgacgactggtgtcggattccgagtct

cgcatgacgggagccgtgatgcaggcgttggccgtatcgttggatgcgtcgatcaccccgtcccctccgcaccatcagcaggcaaactccgtggaacg

tttcgttcagacggttcaaggcgccctgcgcgccctgtgtcagacctcgtccgctgcttgggaccgacgagcggttcccgcggccgagctcgcgctga

attcgacccctagtgtctcgactggacagcgacctttcgatctcgtgttcgttagtcaccctcgtattgtccatgcagtgttcgacgcttccgagcat

gacggtgtggggtcatttccggagcgtcttgcagcggcgaaagagcggttggaggaggcgcgacgcgtggtggagcttgcgcgcgcggaacagaagcg

ccgttatgatcaacgtcgtcgtgatctgccggttctggccgcaggggataggatattcgtgcgcctgacggatcggccgattcccggtgccatcacgt

cgaagctcagcccccggaaattgggcccgtacgcagtggcggaggtcttgtcccctcatcgggttcgcattgccctgccctcgccgttgcggattagc

gacgtgttcgctgttgagcaactcgacgccttgcccaccgagatcgatcctttcgcgacggtccgtgatgctgatgctgtgcgcgctgtggaagagtc

aacggtttcgtcagttcctcgctcgctgtcggagccagacgcacacgacgaggagaacgtaccatccacgcgcccttctcgagctgctcgtcttcctc

gtgctctacgagattacgagcttgagggccttgctgttgatgcctcggctgccggcgaagcacacgtgtcaatgtcccccgccgtattcgagctcctg

cggacaccttcgtcaaagcctcgccttgccgagatcgaaggccggccggtcgtcttggaagagaggccagtcgcatatctgtcgcgcttgacgactat

ttctgaaaagaaactcgtcgcgccggaactggagctgagctgccttgcttgggcattcgggcgcctcatgcatctccttgaaggggttccggtgaccg

tggttacggaccacgcgccgatgggtcctatgctaaaggcaaaagcggcatcagagtacggtcccacgatcaccaagtgccgtgcgatgctaatgcct

caccttccgaacgtcgagtttgtgtataaacaaggccgcaagcactccaatgtggatgcttttgtactggatagctctaaggagaggctaccagcaag

cacagctttgtcgcgcttggtctcgaccggaggtgacgacgagaaggacgatccgggtcgatcgtcttctgaggcgggggatgtgctggatgagtag

>contig_14.contig_14.g551.t1

atggcctctcttcgccagcgcatcacggtctcgactgccaaggcaggaacgggactcaacagtcccatatgcatcagca

gctcctcccaggagtcgtccggcacagactcgaccaccccttccagccacctccaggactctcaagaccctgacgaggactacgagcccgccctcggc

acccagaccatcggctcggaaggagccgctatccaagaagagctcgaggatatcctcaacgctcctggcgccgtcgacaccgacatgctccaacatga

gcatgacaagacaaccccacccacgagtgccaccgctggatctaacacggccacatacaccatcatcctccgcaatttcaacccggaagaccctaccc

accaatgcaggctcgtcgctgacaccgagaaacacggtctcaagaagggcttcaagggtgactttgacatcaaaatacaccccctcgccggacgaaac

aagatcgtggagattgtctgggaagacatcgaggatttccgcctggccaaacgcatcaacttcaagatctactggaagtcgatcccgattacgaccag

caactggggcccagccctcgactgccgtgctcgcgccatcaaggtccatcttccccctggccagaaaccggaggactacctcaacgccttcgtacaat

cgtgccaagacaacatcaccatcacccatgcctgggtggtcggaaccggccccaaagacgcttcaaaagccacgaaatacggaggaatcctcgtggcg

gtcgtccgcctcgcaggcactggtcacctcactctcgaggaactacactcctccctccctgcatggacgctatttgctggcagaagttacgaaaacca

cttcgctggcgccatccagcgctgccgacactgtcgccataaatccctcacaatccactcaagtgaagaatgtccagtccaatactgcacagcatgcc

gcaaacgccacctcccagacgaatgcgacaagtggggctaccgaggaaccgcaccacaaggaaaaacctcctcaacctcttcggatgaccaacctcga

gctcaaccaaaagcctaccccacgattcacaaccgcgagtcccacaaggacgaccgtactcaagctcccccggcgcctccttcagctcatgatcaagc

tggaccgagccgccttagggaagacaccacagtcgctaccctcccgaagactcccccgcgaggatcatacggagccatcattgcgaacctcaacgcaa

aagccatggacccgccgaagacatccggaaaagggaaaggcaaagctactcaatcatccgcctccgtcacccagccatccattgtcgccttcttcagc

cccaagaggaagccggtcgtcgatcccagcggctctccccctcctcgcaagatggcccgccaagatcccgagtga

>contig_14.contig_14.g552.t1

atgccgaatgccttcgttgtagccgctcacgtctggacgaaacgcgtgcacaaaattactgctcctgccgagttggtgg

cccgccgtattccgaaggatatcctggagggcgaatacggcttcttgatccagaccaaaccggttgaggaaggcgggaacggcgaggcgattggcttg

gaggtgctcggcgcatacgccatggcccgcggtttcatgcgacagggtcaaggtcagcccgacgagactcgctcagcccgatacgttctcaaggataa

aaaggagtggaaagcgcctgtgtgtgctcccaaggcccagcacgcacgccttccccttgccccctccccttctgcactcccgctctcacgcgctcctg

tagccatccacagtcactcaacctcatcagcatgccctctctcgcaattctctggaatgctccagagcaatactcccgagtatcatgggagtgatctg

gacagcatgacatggagcaaattgggaattcccagcgtcgcagaaaggtgctgcgaggtgggcgatgagattccagcgataaaatga

>contig_14.contig_14.g553.t1

atggaccccgacaccgcatacgtcgacggtatcgcaccggccaagcttatggaggtggaccatgtgaaagaagtacttg

ggagcggaaaggatccgtataagctcaagaggcagctcacatccttccacgcactcggatcactactcgtgcacctgtccggcctggcgtttccagac

cagagtctcgggcacggccaggacgtcaggacgtgtaaacatctcaaggctctacttggagaaaagtacgaaaatgaacgagtagcagcagcaagagg

ggtcagcttgatgctcgccgaaacgtttgcgatcgatggtaacgtcgacccgaccggctggtggctgagcgagaagcttgatggagttcgcgcgtact

gggacggaagctcaatctggtcccgagcctctgtcctgtacgacgcgccgcaagacttcaagtataaacttccgaccgacatgtctctcgatggagag

ctctggatggagcgagatgcgttcgacaggacttcggctcacctcatacgcttcctggtaaaggtctttgacgtcgtgggtgacagtaacccattcga

acaacgcctggaagcactcaagcagcgattcggcgaacccctgacgccgaccgaagccctatctcaaaagggggttccgggaggcaggatcgtcgtcc

tcaagcacgagaagtgtacctcccgcgaccacctgctcgaagagctcgccaaagtcgaagccgtgggcggcgaaggtctgatgcttcgcaaggccggc

agccagtacgaccaccgacggtcgcgctcgctcctcaaggtgaagacgttctacgatgccgaggcgatcgtcattagtattgaacaaggcgagggcaa

gaactcgggtcgtatgggtgcccttggatgtcagatggagaacggagcgcggttcaaggtcggaacgggttttacggactcgcaacgcaacaatccgc

ctccggtcggatctatcatcacctacaagtttcaggaactgtcccaggacggaattcgtaggttcccggtcttcaagggcattgccgccgacaagacc

aagccgaaagacgccgtcgtccgctcgacccggctgcgccctgataaagcagccgccgtcgctttgactgccgctacagaggcgtctacggaggcgag

ctcttccggcactcagaagacggcgccggcgaagaggcagaagaagtag

>contig_14.contig_14.g554.t1

atgagcacacgtctttccacaagcaccatcaccaaccggccggacttgagggtgaagggtccgccgaacttgagcacct

gggagcgcagggagagctcgatgtacgccttctggagggcctcgaggggagcgtcgctcgttacggccgagagacaaaaattctgggacccggcgaac

gttcttggcatcgacgaggtcggcggtagcgaggacggtgttgaaggtgtcctgcgagtgagcttcaaccgcccgcctgtcaacgcctggcacgatgc

gatgtggagagagcttcacaagctcttcgacaccatcagcacagacaacaacgtcgccgtcgtcgtcttgagtggcgagggccgagccttcaccgccg

gtcttgacccgacaggcaaagaaggcagccaaggacgaggtcgccgagaaggcaaagctgaagaagaggccctcgcacaaggaaataccgaaggcgcc

cgtatctacgcgtccaatgcgatccggaagaagaacgagtcgctgaacctgcttcgcctgggaagtcggatcgacgccatcgcgctggtcatggacaa

gttcgaaaaccaatttgaagacatggacgcccaaacatcctacatggaaggcgcgatctcccaacaatccgcccaaacaacacccttggaccaagtcg

acctcctcatgcaacaagtcgcagacgagaacgggatcgagatcaaacagcgcgtcggggaggtggacctggtggtcgggaaggtggccgcgctgagt

cccgcggcgcagcaggagcaggcggagaaggagagggaggagaggaagttgattgcggaggggtcggaggtgggaccgagctcgggtgtgggcgcggg

cgcgggttctggggctgcgcctgctgcggccgctgcggcggggccatcttcgactgcgcggtctggggacgaagactcccggcttgcgggcaggtcat

tgaattctggcccctcccgaaccctgctggcacgcccttctttcgaggctgaggctgtcgatccggtagacatcggtctcgcagcggacatcggcacc

ctccaacgattccccaagacggtaggtaacgactccctcgcccgcgaactggcatactcagcccgaacattcgacgcccgcgaggcgctcgccatcgg

cttcgtcagcaaagtgaccgagcaaggtcaaaccgctgtcgtcgcggcggcactctccttggccaaggacatcgcctccaaatcgcccatcgccgtcc

aaagcacgaaggcgatcctgctccacgcccgggaccccccccgtcgacgcaggcctggccttcacggccgcttggaacgcggccatgctgcagacgga

ggaatcgcaactcaggctgtgatggcgaagcagaagcctgccttctccaagttgtag

>contig_14.contig_14.g555.t1

atgcacgctcccctgagctcacatcggtcgaatccacacattggcatcctcctcctcaagcaccccaccgccagtcttc

ttcaaccagtcgatcaacaaccagctcacgagtttgcaggcgtggtccacaacggtatcgagtccggagacatgcagctcatcggcgaggcatattat

caccggctatttttctttgtgctggaacgcgtcgcgcctgaggagaagacgcgcgagaagaacggcgagaacgggttcgcggagtggggcgatatcct

gaccacaacaaccaaagcataccatccagacatacgtgctcaccctttttaccgaggcattcgaccctacaaagcaggctcggaacttggcgccgccc

ctccgactcggcgccttcaacctcgccccacactctcctctgcccgccaggtcgaagtcgaccttgtcatgcatccccagacacctctctggctgtcg

cacgacatctcgcaggacctccaagaccgcatcgaggacctgctcatggtcgagcgtgctttcatccacgtcgaccacgagacggatcaccggcctga

acatcgcaagaacgtctga

>contig_14.contig_14.g556.t1

atggtctgcatgctcggctctcaggcgcccaacgtcctcagactcgttctcgttcggcttaccaagctcaatcaggatt

caggacgcctacctaaaggtagaattcgtatggtgacggctgggaccgagatgatcatgatcaaagtaaagacgccctatacgccggtcaccaccgct

tggtcaacaaagcagcacatcaccatgattggatag

>contig_14.contig_14.g557.t1

atggtctgcatgctcggctctcaggcgcccaacgtcctcagactcgttctcgttcggcttaccaagctcaatcaggatt

caggacgcctacctaaaggtagaattcgtatggtgacggctgggaccgagatgatcatgatcaaagtaaagacgccctatacgccggtcaccaccgct

tggtcaacaaagcagcacatcaccatgattggatag

>contig_14.contig_14.g558.t1

atggtctgcatgctcggctctcaggcgcccaacgtcctcagactcgttctcgttcggcttaccaagctcaatcaggatt

caggacgcctacctaaaggtagaattcgtatggtgacggctgggaccgagatgatcatgatcaaagtaaagacgccctatacgccggtcaccaccgct

tggtcaacaaagcagcacatcaccatgattggatag

>contig_14.contig_14.g559.t1

atgaccgtcttgcctttgcctgcagcgttggaggtcgagcgctccatggatggcgctgcaagcgttgccttggcagcgc

tgacgagcgctgagccgcccatagagccctaccccatcaccgcgtccaaagctgcgctcttcatcgatatgacaatgaagcgcgagcattatgtcagc

ggtcgaggagctggatcgagctctgaggcaaagaagacgattgcaggaactcagttggggtactcggccatcaagcagcgtatcaacgcgttggaatg

gtttcgaaaggtcaatgaggacgattggcgagagcatcccgactttactgctacacggacaaagctccgtgacaatgcgaccatcaactcgctggaga

ctacctccaagaacgacaaacacaacagagcgggcgcagggcaagagcaaaaggccctcgggtcttcctctgctacgcttagaaaagaagaggtcttg

aagttgagtgagtgggcgctgggacaaggtcgtacaccagccaagttgggcaccgcggcgctgtggtcgatgcaacttggaagccgagggcgacctac

ctttgcgcccgacttcaagagaaagacccacggcaagtacggttacagggaatggtggggttgggttgtcttccgtggacgacattctgcatcaagca

aagagatgagctacaagactcatgacggcgcggtgcgaacgatgtttcgcctcaacaatgttgatgtgggcaagaccacacacgcagggcgtaccgcg

ggagccatgcacgctcgcgagaacggggccagtgtttctggaacgaaggcgctcggagggtggtccgacggtggagcgttccggtcctgctacgatcg

ttcgttcccccttgatgcgatttgggcggtcgctggattcaatggacaggacctggactcgtactatgttgccagggcccacacaaacgagcgagaga

agctcaaggagcgacggacggccaatgaaaatgcgtccgactttgctctctctgccttcttggattgtctcgaatggttccgagaggtgctgcttcag

gatgcggcgattctcagccagcatcctgcttggtcgaccttcaagcccttcgcagcgtgtccgaccttctccagtccgcagtttcacgcgtttgcagc

agctctcaacacagcagtcaaggcggaggactcggaggcagagaggcagttctcgcaacttccacagcagctcggagccggggtcaagctggcgctcg

ctgatatcaagaccggttttgagcgcagggacatggcgttgaatcaaaagctcgacctttcagcctgcccccctctctccaccatcaccgcttctgct

gctcgtcctctcctttcggtttccaaccagagtctctccctttcggccggtgtcggcacctccaactccaaggatgtgagcagcgcgtcagctgcacg

tgcgccgtcttccttcagcagcggcaccctcagcagcagcgcagctcctttctccttcttcggctccgcagctctcttctcctcccttgccgtcttcg

atttcggctccttcttcccccccggaaccgtcctgcgcaacgccgcacccgccatcaccggtccaacccctcccatttccccacccgggccgatccac

ccgaccatcttcgaccagtcctgcttcagccctcagtctgtcgctctcctggccgacttcataacggcctcggcctcctccatcgctgccatctccag

cgcctctccaccctcagctccggctcctcagtcctctagcggcactgccatctcaggcgcacagcaggagaggattcaggagttgaagttgaaggagg

aggagcgatggggaaagcagaaaatggatgcgaacgagccatggcaatacgacgtcaacagggacacggtcttcccctcgtaccaatttcgtccaagt

ccttcggtgcgagacgtgtgggtggagtacagcataggcgtgaatgggaagttcggaacgcggtgcatggaggagatgtgggggacggtgtggagatc

tgcgccgggcatgaagcaggagtggagtcgtcggcgaaaggttatcgaattgatcgaggagatcactcggtctcgcccggcgtggaagcaagaacacg

cgctcagattctttgaggcgtgctacggcggctacagcgcgagaggtctctccgactggctctcgaagacatctggcttcaaacgcaagcgcgccgac

gtcgaccacgggcgacatcaacaagacgcgacgtcagctcgggatgtgatcatgggacggcttcgtacatggttgccgtag

>contig_14.contig_14.g560.t1

atggccgaccctccgccagcgccctggattgggtcagtagcaccagaatcggtgctgtggcctggcgtagaaggcacac

aacaggcggcggagtggcataatgcgcccatcgatacttggtttctaccaaaatacgcccacctgccgcatccagtcgtgccggatgaagatatcggc

ggaatgctcacggagctgagcgctacacttcggcagcgcctcgacgctgtgcctccaccaccgccgtctcgtaggaccaggcctggagagcgcccaga

caattcgcgcttgaagacctatcgcacccggcttcggccgaatacacagcaaaaagaggcacttcgaaaaacggctggaatattcaggtggacgtaca

accgggcggtggcgtacctcaaggctcatccaaacgcctctttaaacgacgtccgggaacattcagtcacagaggatggccttcccctcatgaacctg

tcatgggtgactccggtgccatacagcatcagggagcaggcgtgccttgaggcccgtacagcgcttcagaatggcttggcggccgttcgggacggcaa

aattccgaacttcgagctgaagttccggaaaaagcgggcgctcttctcctcgttccggattcggacccgtgactgggttggaggatgcctcaagatcc

caaatttcggtctgggtccaggtcagcaccagaccaagattcggacggggatcgacgttccatcgctggagcatgatggggcatgcatcaagcaggag

aacgggtcatggttcctgctttggcagaaggacatgggggaagagaagatgcgggttcctgcagcacgtgggccacaaatgtgtgccattgaccccgg

cgttcggacatttgccacgatttacgaccctttgcggggaacggcggcaaaatttggcgatggagaacacaagcgtcttttccgcctcggtacgcact

tggatgacttgatctcgcgatcaacccaagctaaagcaaaaaagcgttaccgaatgcgccgcgccgcaaatcgactgcgtgcaagaatccggcatctt

gtcgacaagctccattggaagctgcgtggctacctcaagcgaaatttcgacactgtgctgctgccaacattcaacgcgcaccagatgtctcggcgagg

tcaaaggcggatcgggcgcaagacagtcagagccatgatgacgtatattagcgagcctttgtag

>contig_14.contig_14.g561.t1

atgtgctggacggccacgtggctccctctactggctccctacaaaggctcgctagtatatggtgatgagaggattctct

tgctgaccatcgacccgacgatcctcagcagcccacgacgacacaacccccagttccctcgtcgagacaccctttgctccagccccggctccggcatc

ccagaccgccccaggctcaagacatcaagatcatccccatcggcaggcacgggcggcggcacccccacacatattcctcgcatacttccgactatccc

ttttccagcgatcatctccgacacggggcgtgcaaggcggtctagacttctgctctttggcactgccggccccccctctctcgagtctcgcccatgtt

ttcgattggcatcctcaatccatccgtcatcgccgtcggccgtcacaccgctctgtatgatcgccacagtcggccatcagcagcgcgcgcaaggaccg

gctttctcctcatgcaccacttcggtaccgacatcgggatctcaaccaccttcggcatctctgccgccaccgttggctccgcgcacacccacctctgc

gtcccagcatcccaccggtgaacggcgcgacgtgaccgtcgtgtaccgacgatacgagatggactgtgctgccacaggaatcaaccggcaagagaggt

ccgagctgatacactctgctggacgacgccatagttcgaaccagctcagcagcaccggaatcctctgcctgccgtcccatccagacgtccccaacaaa

gtgcctcccgagccgcctgccgcgacctcgggacccacttcggtactgggcttgccgctcgccaacaccgcgcaccgactctcaccgtgtctcaacag

gagtggggagactggagacaggcgacggctccggagcgtggcaaagacgaatctgggcggaaacgtgtggagtcgtggtgggcggtggctcagtatcg

gccgagctggcaacgcactccgcaaaaagcacagcatgagggcacgcaaactcgccaaggtcgttgctgttggccaatggttcctagacgactgggtt

cccacaacactgctgacggacattctggagcgcgagcaagaggtcaaggaggcggccaagcaactgcggagtactccgcactttgccaaaaaccttag

cgtccacggccttatttcgcgcacgcgacgcatggctccaggctcgggctcttgttgtcgcggcctccttgtcgaccgatactgccgcatcgtctccg

agaacgcctattcttccgtcttcgtgcagctacacggtacaccgcaggcaacaacagcggctgtgccacgcacgtcttcggctttcacgatcgcgact

tccgcaccggcgatatcagcgcccttacccgcgtacgccactcttcgacggctccttctccctcatccgttgtcctgctgcccgcttcacttcactct

ccgttccggcaccgctctcattccacgcgcgcatggtcactcgcttcccttcccctatgcggccatgcccgctgcagttcccacaacaccctacctcc

acttttcccacgtcacacttttccctcgctctcatcgttacccttctcgagctcatcattgtacttag

>contig_14.contig_14.g562.t1

atggatccctccgacttcctgttcgggctcaaccaccagctctcgcccgatagcaacggacgtcgaccgccctcagcgg

cagataccccgaccgccctccacaacggtctcgacgtcgtcttcaccaacgagctccaacacgcagccggtgctggccagggtcccaccgccgacgac

gagttgatgcgagcaggcgttcccaacttggccgaacagctcgcactctggacaaacgcaaacttcagcttcgacggccccaccggccacgcactcct

tgccgacgacgaggaaaaagagaaggagaaggacgagggagacggttcgaaagataagggtcccaagtccttcggcttcaacaacccaaatcaccgtc

tggaggaagctactggtcggcctttaggtggacctgcacagcccttacccggaccttcacgacagtacgccggcgccgctggtcccggtggctcgggc

aacaacaacgcgtctggcggtgggctcgatttaggccgcggccgcaataattcccaccaaaaccccggtttgccgaacggttcacagccgagccccgc

tcagtcgatccctacttcctcggctcaacagggcgggccgggctcggcatcagtagccacgtcgcaacctccgcccggcgggcttgcaggattcggcg

gtctgaactttggtggtctggcgggcttgggagcacccgtgccccagaccccgagttcggccgcccttgggtccctgcccttcctgtacaatgccgat

cctgccgtcgctgccagactgggagcggctcaagctgggttgggaacgtcgccgcctgcgttggacatgacttcgatcctggcgctccagcatttgct

gacgagcaatccacttgcgcttgcccagctgagctcgagcttggcgagcatcaatccgcttctcgcccagatgcagcttgcacaggctcagcaacaac

aacaacaacaacaactgcagcagcagcagcagcagcagcagcagcagcagcaacaacaacagcagcaacagcagcaacagcaacaacagcaacagcaa

cagcagcagcagcagcaacatcaacagcagcagcagcagcagcagcacaatcttcatcatcaacagcaacatcctcaaaatacacctcactcctctgg

gatatcttcgcctcccaacggttcccttctcccctttggagcaagctggaatctccccggaccaggcgcaccgccgcctccaaccccgcccgtagggt

cagcaccaccacatgtacaagcagtcgtagcaccacaaccaccaccagcagcgggagcagagcacggacatacaggcgcgagcggaggtggcagcaac

acgacttcgccgccatcccaggcgttctggtcccatcccagtctgcaatcggacgcccgctttagctcattttcgggtccgagcgccggtggagcgga

agggctgtctcatcatcccggcgctggggcggcgtctgcgggcccgcatgcacctcctcatatcaacggtggaatcggtggacatggcggagcgggga

cggggttgcccttcttccccagtgctggtgatatgcaaggtgcctccgcgcacatcaacggactgggagctgattccaatctgggcgatgacgaagat

gactaccctctacctccgaagcgaaaacgcggttcacggcatacggccacggctgcctcgtcatctgctgctgcgatggctgcggccatggctgctgc

gaacgacggtagcgcgacggaggggtttggtggatattcgaatgcgggcgattcggattcggcggggccgtcgggtcgggagagcgggaggcagtccg

ggctggagcggagactgtcgttggatgtgccgccgcttaagttgattgatactggcaatccggaggcggacgcggaggcgaatcgggtgttaattgag

gaggacaagcggaggaggaatacggctgctagtgctcgattccggatcaagaagaagcagagggaggcagcgctcgagacgtctgcgaaggagatgtc

tggccgggttcgcgagctcgagcaggagaatgcccgcttgaggacggagaatgggtggttgaagagtctgatccaggtccggcccaatggcagcggcg

ggtccggagatgcgaacggcggaggtggaggtgggggtgcagcggttgcgggaccttcgacctcgtcgtcttcttcctcattgccgacgcagcatctg

ccaggaggaggaggaggagggtcgagccgcgatacggacctcgggccgcgcgatcggtcccatgcttcgtcgtcgagcacggctatcaacggtggaag

tcgatatggctctgggccgtcgggtgggaagggggccgcgtcgggaagtggggcgaacgcgtcgcaagggcctcggtcgtctagatga

>contig_14.contig_14.g563.t1

atgtcggttgccaacacgaccccatcgcacgaaaaggacttgctcgccagcgacctggccaacgcccagatggaaccac

ccggactggaagcaggcggtcctttcaagaagcacaatagcaagaagagctgggttcggaccaagtacgattcgcctacctttcaggtctttttggtc

gccgtcgtctgcttcttgactccgggcctatacaatgcgctctctggtattggaggcggaggtcagctcgacaacagtatctccgagaaagcaagcat

cgcgctctactcaacctttgccaccgtctccttcttcgccggctccatccacaacaagctcggaacccggctcaccctctggatcggctccctcggct

acgccctgtacgcggcctccttcctctcctacaacctgaaccagaacgccgcctttgtcgtcgctgcgggggccatcttgggcgtttgtgccagtttg

ctctggtgctctcagggcgcgattatgatgacctacccgaccgagcaggagaaggggaagatgatcgccctcttctgggccgtcttcaatctcggcgc

agtcattggatccgtcatcgaactggggctcgcccagaacaacgaggcgaatacggtcaacaacggaacctatgcggccttcttcgtgttgatgtcga

ttggctcatgcatcacctggcttctcaagaagccagcggacgtcgttcgtaatgacggctcacgtgtcatggtgccgccgcaggcgagttggaagcac

gagattcaagatccgtggatcatcctcctcttccccatgttcttcgcctccaactttttctacacatggcagttcaacagtttcaacggctccctctt

caccctccgcacccgcgccctcaacagcatgctctactggctcgcccagatggtcggcgccttcctcctcggcatcctcctcgactcgacccgtctct

cccgccgccgacgggcctgcatcggctgggccgtctgtttcgggctcgtctgggccgtgtggggcgggagttatgccaagcagcgcacgtatacccgc

gagagtgtgcatgagcagggcctgggcggctttgttcggctggactaccgtgatggcggccggtatgcgggtctctgcattctgtacatcatgagcgg

ggttgtggacgcggtttggcaatgcctagtctactacctgctcggcgctgtatccaacgatctcggcaaactcgcagcgctggcaggcttctacaaag

cccttcagtccgcaggcgccgcgtcagcattcggggtcgacgtcagcgggagcccatacatgactctcctcgccgtcacatggtctctgtgcgtcgct

ggccttctcttcgccatgcctgtcatctggctccggatcggagatgtgacgaaggaggagcgaactgtgctggtttcgggggcggcagccgagtcgaa

gatcagatcctcggctgatgaggaggacggcgagggtatgaagggcgagggggctgtcgtcctgggaccgccgagacctctcctctga

>contig_14.contig_14.g564.t1

atggcatccgaaagctcgcccctgctgggtgaccgcacccgccgatcctcggccctcgcctcgtcgacccgccccgact

tcctcgaggaaggcggcggatcggtgatcgagcaccagtcccagctcgaggacgccatcgcacaggagtccgccttcggtgcgatccgcgatgccgca

gccggtgtcgaccccaacgcatccgccttcgtgccccagccggacgcctcccacttctccgtccagcctctggcagcccagccaggcgcaagtgtcat

ctcgattgccgcacccccccagcgcgacgatgtgctcctcatcatgtctgccatgtggatcggcaccttcctcgccgctttggacggtactatagtcg

cgaccatcatgtccaccgtcggatccgagttccgcgtctctcgcgagatcggctggctcggaacctcgtacctgctcacccagacagccttccagccg

ctctacggccgggcgtctgacatcttcggtcgcaaggccgctactctcttcgcgtccgccatcttcctcatcggatctctcttctgtggtctggctac

caccttcccgcagttgtgcgcggcgcgtgccattgctggtatcggaggcggcggtctgactaccatgtccactattgttacttcggaccttgtctcca

tcaaggcgcgtggcacatggcaaggtctcggtaacctcgtctacgcggccggtgctgccttcggtggtcccctcggtggtgctctcgctgagagccgt

ttcggctggcgcatggccttccttgtccaggtccctttgtgcgccatccacttcggtgtcgtgtcctacaagatagacatccccgcaggtcccggaag

catggtcgagaagctcaagcgcatcgattaccttggctctctctccctcgtcatctccgtcaccctgctccttaccggtctcagcttgggaggcaacg

aacgtgcatggaacgactctatggtcatcggtaccattgctggcggtcttggtggtctcgtcatcttcactcttatcgagaagtacgtcgcgcgtgag

cctttgatgcccatgagcgttctcttcacccgcacgcccggcttcgtcgccattgcttgttggttcatatcgatgtctcaattctcgatcatattcca

agttccgctctacttcaccgcagtcgagcagaccaaggcgtcctacgccggcttgcacctcatccccaacgctatcgttgcctcttcgtgctcgctcg

gctctggtcttattatggcccgcacaggcaagtaccgccgcatgctcctcaccgccggtgtcatggcgttcctcggtcccctcctcatgtgcttctgg

aagcgcggcgagacacacgagtggttctactggatctcgatgctgcccggtggtgccggttacggtggtatcttgaccatcagcttggtcgcgctcat

tgcctcgatcgatcccaaggacatggctgccgccacaggcgtcacctacctcttccgtgccaccggttccgtattgggtatctcgctttcaaacgcca

tcctgcagaactacttgcagagcaacctcgagaagtcgggacttcctcaaaagaccatcgacatcattcgtcaggacgtctcttcgatccgcagcttg

cccaaggcgactcgcgaactcgcgatcggtgcttaccagagctcgatgcacattgtcttcatcgccacggccgctgccggtctgttcgcgctctttgc

cttgcttcccattgcggagcacgctctgcctggacacaagtccaacccggcgagccagcccgcgcgcgaggagactatattggaagaggaggagcctc

tcgagccagaaaacggtcactga

>contig_14.contig_14.g565.t1

atgggtatcaaaggtttgacaagtctcatccaagatgaggcccctgatgccataaaggaggccgacatcaagacctact

tcgggcgcaaggtagcgatcgacgccagcatgagtctgtaccagttcctgatcgccgtgcggcagcaggacgggcagcagctcatgtccgacagcgga

gagacgacgtcgcatctgatgggcttcttctaccgcacactgcgcatggtcgagtacggcatcaagccgatgtacgtctttgatggcaagccgccgga

tctcaagaaggctgtgcttgcgagtcggtatgggaagcgcgaggaggcacgggaggagcaagaggaggagaaggatgttgctgacaatgagcgaatgg

accagctggcacggcgccaagtgcggccgaccaagtcacacaatgacgaggttcggcaactgctgaagctgatgggaataccatgcgtgacagctccc

tcagaggcagaggcacagtgcgctgagctatgtcgggcgggcaaggtgtatgctgcgggctcagaggatatggacacccttacctttggctctcccat

cttgctgaagcatctgaccttcagcgagcagaagaagatgccggtacatgaggtcagtctcgccaaagtaagagagggcttcggtatggacatggacc

agttcatcgatctgtgcattctattggggtgcgactacctggaccccataaggggcatcggtcccaagacggccctcaagctgatacgggaacatggc

acgattgatgctatattggagcatctgcgaagcgagccgaaaaagagcattgtgatcccagaggtgtggccgtatgaggaggcacgcaagctgttcaa

gaatccagacacgcagaagggcgacgatcttgaattgaagtgggaggctcctgacgtcgaggggctggtgaagttcctgtgccaggacaagggattca

gcgaggaccgagtacggaagggtgcggacaagttgacaagatcgctgtcggtcaagcaacaaggccggctggatggcttcttcacagtactgccatcg

agcaagccaaagccagaggcaggttcgagcgggaagggcggcaagggcaagggcaaagacaacaagggcaaggacgacaagaagaagagcggatcgtc

ctcctccagcaagccgaaagcggtcctgacggcgaagaagcggtga

>contig_14.contig_14.g566.t1

atggttctcactgacgatcttgtgtatctcgagaagggcgattggccgcttcatcgcgagctcaaagcccgcgcaaatc

gagcgggtcgcagccacgtatcggagcatcagaggaatacatgcggtgtccaccccgtcaagcttctcggctgggtcttcctcgctgccagtctcggt

ctgctgtacgtaggcatcttccactcccactccctccagagtctgcactcggtcgcgggcgacggtcttgcggactactgggatggggtcttcggcga

tgctgtcatgcacgacgtcttctctgagtcttcaaaggggaccgacgatctgtgccctcagcaacccgcattcaagctcaaggacaccgcattcacgt

tcaccgagcccagtgctgcgcttcaggcgagtcgcttgagtggtgcgatccaagtcgatacatcggtccaggacgaatacccgcctttcaatgacaat

cctgagctctggaagggtattttcgacccactcgctgattacctcgaacggactttcccactcatccatgctccccagggacccgtcacccgcgaaaa

ggtcggcggcgtcggtctactctacacctggcccggttccgatccatccctgaaacctcttatcctggccgcacatcaagacgtagtccccgtcgaca

gtaagacggtcgaccagtggacatacccaccctttagcggcttctacgacgagaagacgggactggtctggggtcgtggcgccagcgacgacaagtcg

agcttggtcgcccttctcaccgcgtttgagtccctgctcgaggcacgggacacggagggaaacccatgggttcccaaacgcacagtggtgctctcctt

cggtgccgatgaagaggcaagtggacggatcgccttcgaattggcgaaacatcttgtcggtgtctacggcgagaagggcgcatacatgatggtcgacg

aaggcggtcccgtcgttccagcctcagagtcgcctttcaaccgcactctcgctgtggtcgcaacagccgaaaaaggctatttggacgctcggatcacc

gtacatgcgcccggaggtcactcctccgagccgcctgcgcacactgccatcggtcatctggcaagtctcatcacacttatcgaaggacgaccctacaa

agccactttgggtgtcgacaatcctgccttcaccatgctcggctgcctcacgacatccgacagcgttgcccctgggctcaagaaggcaatgaaggagc

tccgtcatgcattgaagaagaagaattcgaaatcgcgcagagagcaaaagcacctcgagaagctcaaacagaaagtccttgacctcgccggagaagat

gtcggtttgagcttcgtcacctcccaagctgtcgaccttatctcagccggtgtcaaagtcaacgcacttcccgaggaagcgacggcggtcatcaacca

ccgtatcgagatctcctcctctgtggccgacatccgggagcacatcgccacaacgctccgtgattacgcggaacagaatgacatcgagctcgacggct

ggggcaacgcgacgctttcgtacacgcccaagaacaagaactctatcatggacaacgaaaacgacgagacgttcctgcagcaagtcagcgcacagacg

accatgcacaacaagaggtcgtggtcccacctaaggaacacgacgatgacgagtgtccacacagctgcaaagccaatgcttcggatcgtgctatccga

cgcgttcgagtccgccctcgagcccgcgcctcaaacgccgctggaaggcgaaggatccgagccatggaagctcctgcagggcgtcatccgcaccagct

actcggatcaagacatcgtgatcgtcccggacctgatgggtggcaacacggatacgaagagctactggaacctgacggacgccatcttccggttctcg

cctggctcgccgaacccgacgccgggagggctgtcggatgggattcacactgtgaacgagttcgcaccggctgacgcgctggtctatggatggcgggt

gtggatctcgctcatccgggctgttgcagactcgtcccttcctattgacggccacatggagggcagcatgatggcaggctattccggcctggaggagg

gacaagcatcaacagatgctcagagggacctcacccagtacatactcttgctcctgagcgattccaatctgcccactggtggcttcatcgcctcggcc

ggcctggagtcatacgtggtccacggtctcctctcgcaatgtgcccaagaattgcatggtgcccctcctggaactgggcaaggtcaggatcaaagtca

aggcaggccaccgagcgtcgctcagacatgctcggcgttcctcagcttcctcggctcctcgctcgacaactacgcacgctcgtcgatgcccttcctgg

ccgatgcacacgtcctcacagctcgactggctgctgcttccgctccgcagaatcaggacgagacctgcgaccatgggcagaatgaacaattggagaca

attcgaaaattcgtcgagagtttcgcccgacttgacgaagcgtatcattccatggtcctgaatcacgtcgcgcgtcgagcatcttgtgcccaaggcgt

agctttcctcgccctcttctcgaaagccttcgcccgggctcccgaactcgccacttcgatcgatgtttcgatacggagattaccaccacggccgtcag

caaggagggcccagcaggcgaacgcaagtcaggaccacacattagccaagacgaagacgaatggggatgcggatacggatacgaaggcgaagaacgct

ctcgtctcagcagcaattgacgctctcaagctcgccatccgccgtggctccacacccggccatctgtccctctgcttcggcgtcttcactgccgcctt

aggactcgaactgcgctcggcagcacatctgcatcttttccttcaggctcgggcgctcctgagtactgcgatccgattgaacatctttggaccgtatt

tggcacatcagatgctcctattccatgtaaggacgctggtagattcgacgttggcgggcatggatgcattcgacccctcgagcgaaggccaggaatcg

caaccttccactggtttgctcatgacaacggcgacgatgcatggaagaactagcaagcagggtctccgaccgcacgaccagcctgtgaggaacgacga

tcccgatcaaggctgggcatgggactggccggacgagggagcatggggtgcgcatgtctctctcacagacgcgccaacaacgacctggcctctcggcg

agctcgcccaggcgcgccatgaccagctccagtcaagactattcaacagctga

>contig_14.contig_14.g567.t1

atggcttgtcccaaggcttctttgctgcttggcgataatgaccttgatgagctgcagccacccggcaactacttcttat

cttcttcttgtgctcctgctactgctcgaaccgtgcgcacgacacccgtctcggtcccaaccaccaccgccgttgtcccagctgcgaacccctgccca

gctatttataatcccgcgcgccgtccaggactccaatcccccggccctcttcattccccgtctgcttcatcctccttctcctcttcggccattcaaca

ccatcaaacccacaagatgtcgtccaagctcgccctcgccgagatgctcaagcacaacgagcactggtgctccgaaacccacaccctcaagcccggtt

tcttcaagacgtgctccgagggacaggcgcccaaggtgctctggctcggatgctccgacagccgcgtacctgagagcgttgtcaccaaggcggacccg

ggtcagatcttcgtggcgaggaacattgcgaaccagttcaacccggacgatgacaatgtcgtcagtgtcctcacatacggcgtccaggccctgggtgt

cgagcacatcatcgtcgtcggccacacatcgtgcggcggtatgattgctgccacgaacgtagccacgaagggcgaagctcctggaggagccctgggtc

gatacctcactcctctcgtcgagctcgccaagagcctgaaggagaacgaggaggcagctaagctcgaggatgctgacttcgtccgcctgctcactgag

gaatcagtcaagcggagcatgcggctcatcgcggcatctgatatcatccaggacaactgggccggaaaggtgtcaccgctgagcggccaagaagctat

cttggcacgggcgctgacgtcgccttacccccccccccccctctcgcgcgatgcgcatccagtcatgtccaagatcagcattcacggctggatttccg

acatcggcacgggcaagctcaaagacctcggggtctccatcactgccccgggcgaagatccgacagcagtttcgttgtcgggatga

>contig_14.contig_14.g568.t1

atggctcacgatgacaacgaggacatcccctccttttccagccccgaggacgaagtcagatactggaagagcaaggtca

atgacctgcacgacaccctcaatgaaacggaagcgacgctgcaagacttcatggaggggtccaaggagcttgaggcagagatggaacgagaaatctca

gcttcgaataagcggctcaccgacctccagaacaagaacgaggcgatgcgggcagacgtagacaattggaagagcaagtaccagagttcgctgtcgga

gcacaacaagacccttgcggatctgaacaaggaggtcgagaccctgcgggacgcgcataacaactacaagaacaaagttcgcgacatggagctgacga

acgatgaactagaaagcgcggagcgaatggtccagtcctcgctgatggatatggaagggcgctacaacgtagcgatggagaaaacagctctgctggag

gaggagctcttcacaaaagccaccctggaagaagagaatcagcggctgaaggatgagatcagggagctcaacgaagagttgtcagtggtcagaaacat

gacgccctcgctcagccgcgctcagagccggtccgatgcgcccggtcccgccgccgaagacatcgtcagcgattccctcgagaccgacttgaacctct

ccgacctggtcgtcaaccgacccatgtcttcggcgtcttcggtgtcgactggacaacggagttctggatctgcagctgtggctgagcgtgcattgagt

ccaaatacaacggtgacgagacaacggaccatgcctgcgacagcttccacagcacgaccttcgcacctcggccactcacgacgcgggagtcgcgactt

gcgcggcttgagcatgcaggattcggctgtatcgcgtcctccgatcagcggctcgtccagaccagcacaatctgcccgaatttcccgtccgatgggct

catcttcgtcccggcggtcgggtgtgcctgccactccgagtggtggggctcagcggatggttcaggatatgatggcgcggatgaagacgctcgagtcg

cgaatcaactcggcgcgcaatctcagtcggctgcctgtgagcacactggcgtcgtcgacgagctcgcgttacggcatgagcgatgcgcctagtgcgat

cccacgcccgagctcccggctgggcaatagtgcagggccaggtgcggcgggggggaacagctatacaccacgtgcttcgttagcgagacgggacagct

tagatgcgcgaccgggcggaagtgcgattcctattccttcgggtgggttggcgcggtccacgctgggtccacggccgtcctcgcggctgtcgagtgcg

ggtcgggatacgccacctctgccgaacaatggtgctgtgtccccctcggatgagctcttctttgcgacaggggccaacggacagtcggttgctggggg

gaacactgtcatcggtggcgagagccgactgaatcggaaacggagcgggacggcgggctcatcgctcggtgcgaagcctcgggggagtgggagtgccc

tgcctctgccgagcagtgctttgctgaaagcgtccactggtgcgggagtggcacggatgagcacgggcatgatggaacgggaggggtccgacggagga

ggtcaccatccccgcacagttggtccgccgtcgagctggatgacgcggatcgggggcgcgccaggtacaccgtcggttccgggaagcggggccagcac

tcctactacgacgatggcgagtagtggcacttcggcgcgaccgcggtcgaacagcgggacgcgacagagtggagacgagggcgaatccctttcgacga

gcttctcctgccgcgtcgtcatcgcaaggaagtcgagagactctgtatacatcatgcctaagggatacgccattcacgactcgtccaaatggaccgac

ttcaaggtcactgacttcgagctcaagaccgccgaggccgacgacgtcaccctcaagatcacccactgcggtgtctgcggatccgacgtccacaccat

ctccggcggctggggccccttcgcaaccaagtttgtcatccccggccacgagatcgtcggcatcgtcaccgaggtcggcgacaatgtgaaggatatca

aggtcggcgaccgcgtcggtgttggcgcacaggtcggctcatgcctgaactgcccgccttgcaaccagaagcaggagcagtactgcattggtgacgga

gagaagggtctcgttgacacgtacaacgctatctactctcacaccaaggacgaggcgcaaggtggttacagcactgcgatccgtgctcaccaacaatt

cgttttccctatccccgaaggtctcgagtccacagacgccgccagcatgctctgcggtggtgccactgtgtactcgccccttctccgcaacggtgctg

gccccggcaagcgcgtcggtgttgtcggtcttggtggcctcggtcactacgctgtgctccttggcgcagcgatgggtgctgagattgttgtgatttcc

cacagctccagcaagaaggacgacgcgctgaagatgggtgctaaggagttctactcgaccgctgaagacgacaagtgggcggagaagctgcagcagaa

gcctctcgacttcattgtctccactgcctcttccaacgcagtcgacttggcccagattttgagcacacttgtcgtgcacggcaagcttgtatacgtcg

ggatgccagaagatgccttcaagctgaagatccaggagatggccggcaacggtgcctttatcggctcgtcgcacattgccagcaagatcgagatcctt

gagatgctcaagctcgccgcggagaagcaggtcaagccgtggattgaagtaatggacatgaaggagtgctccaaagccgttgcgaacattgaggcagg

caaggtccgctaccgccaggtccttgtccaggacattgagccttga

>contig_14.contig_14.g569.t1

atgcaccgcccactatccacaggacagcagaacagcgcaacgcatcgcattcattcaaaatggccacctctaacctctt

ccattgaatcaggcggaagagaaggacacgaagcaggcatggagcatgcgcagaggatggaggcagcgaatatcttcgcccaacgattggcatccgac

gatcccaacctcgtcctggccgaattcctcactgaagatgcaagcgttcaacccgtgctgaccgggcagattgtttcaaggttgtccacgctctcgca

tgcggccgatttcgactccctctcaagactatgccgagccctcttgggaaatctaagagcgctcgatgtcatcgtcggccatgtcggatgccaaagat

taatcgaacctgtctccgtcttcttgcgggacgaaaggcaggcggaagaagtcgatgatgcgtcgatcctaacgtcccatctgttctttgctcaggct

ctggtccagagacaacagtcatcacacatcaaagagccacccacccccattcccatgctggaagagtaccttcgcgtgagatctctcagctaccagct

gaatcagcttagcgagaatgagcgggaactcatcgggcgatgggtcaccgcgctcttcgactctgaagtcgacctcgacacgctcagaggcgccttga

cttacttcctccaagacttgctgagctacacactaccgggtccaatcatctggctcctccgccaattgacacactacccgcctccctcgccggactcg

tctcttaccttgggatcctcccatgctttcggcgcagaggccaagatgagatggtgtctctacctcgatgtcctcgccatgctcctcttggcagatac

ctgcccggagagtgtcattgtcgtcactgcccccgctctgcgtgcgcttttcagccctcagatacggcttcgggcgagaactgccttcccacaccctg

agcgaggacttgaagacgttgtctacgctgtctctgccttctcaccacgtccccttcagctgagctcggacaagatgtcgctcctcgacgtgctgtac

ggctctcaaacggcccagcagaacggctggcaagcgtcccttctcgaccttcgcacaggttcgagtatctccaaggcccgtctacacctcgacgcgct

gagggtggcgcgaagtcttcacgatggcggcctcctgcgcagccggattgccgagaatacggtccttctccatctgcccaattgtctggcatttgtac

cccttctgctcggagcgctgacagctggcgctacggtcgtcttggcggatcctcatctctcaccggaggatctggccgacttgttgcgaaggagtcaa

ccgcaagtgatcgtgacctcgaccggaacttcaggagaagacgtgatcggccagtctctcgacctgattctgtcctccgccagcgacgacccgtccaa

gcgattcgcagatgacctggtccagtcacatcgcctcgccatcgcgactcgactacacccttccgagttggcaccgtacaagaatcgcagaatctgga

cggtcaaccccgaggccgattactacggcacggccttcatccttcgcacgcccacggcgcctgcgactgtataccgagccgactcccaagactggacg

gtccttctccttccaccgcccggacacaagcatgcaggtaatcaggagttcgaccaagttggttcgatgaccatccagggtcaacctcggttgccctt

caagctttctcagatgaacaaaccagcctcggactctgctgccttcgtcacgtttgccagcggagaatttaaggaatggactcatgatgccgcagtcg

agctcgtacgtgcgatcatcacccattcggccttgaaaccatctacaggccaatcaacaccctggctcagcagcctctcctggtcaactgccgaggga

ttattcggccaagtcctcgcatcgctcgcgggtggcgccagcctgctggtcttgcctcgtcaattgcaggcacagggccaaaaccagcaggtacagca

cctcttcggcacccagtcagcgtcagtcgcccatgtccacactgtcaaagaagcagagaacgtcgcctcggcccaacttcccttcctccgcgccgtcg

ccgttgccgaagacacgtctcagacaacagcgggctcattgccgctgctgagcatcggccggcttcttgcctccaagaatgtgtcgagcccgaacaag

tcgaagctttga

>contig_14.contig_14.g570.t1

atggccaaagcaacagtacagacaaggagtgtcagcctctttgcctgcaatgtctttgtcacggctctcgcggtcggat

tcggtatctggatcggcacatggtcccttcttccaccaccgcagccgaccacaagccgccaattgaaatacacggaccaccaaggacgagtcgtcgga

gtcgttgcgctgggcgtcgtcacgttgctatggaccctcatagaaactctggcgctcattctatatgccgtccagatgttctactggaagcgaccatt

cgacaagctgcccttggctttggagcgaattttctctggctttgcactcatcaccttgggtatcttcatccctttgcgtctcgcgtctcgtgtgcaca

tcttatgggcgaaccactgccagaagacaacgtcgtacgcatttgtacagcaacagaacgagtttgcgatggcagaggagagtgcagatcagcgagcg

tacgaccggctggctgcgatccgcctccagtactcgcaagctcaggacgatcaatcaagaggcaacgcaaacatcgcgcgatag

>contig_14.contig_14.g571.t1

atgcacggacaggccacatactcgcgcgaaatctacgtctcaaacctgcgcggggtcgagacctttccgaccacgctca

aagtcgtcgaggagttgggcaagaagaatgtgcccaaatcagcgtgggactatgtcgcgggctccgcctcgaccgagcagacgtacgactccaacagg

cgtgctctcgacaactggcagctcgtacctcgcatgctcactgcggctgcgacggacgactttgatatgacgttgcgcttgccggcacttggtcccgc

gacggacgacaaggcgacagaagctggcgaaggtcaaggcgcagcattgggcaaactgttccctacgccgcttgttctctgcccaattggcgtgcttc

gccagctacacgaggagaaggatctcgccgtcgcacgagcggcgagtgagctcaacattccttataccatgagcagtgctgcctcagcaccatgtgag

gaaatcgctgaagcgggggagtgggatccgaaggaaacgcccggaaaagaagcttggttccagctctactggccttccgacgacgagttaacagcttc

catcctgtcgcgagcgaagaagtgcggtttcaccactctcgtgatcacattggacacctgggccctgggttggcggcctcgggacttggataacaagg

gttacaatcctttccttcacggcgagggcgtagccaacatcttctcggatcccgtctttgtccagaagtactgcgagggcaaatcgccgctgtccaag

gatgccacgcaggaagatatcggacaagcggccatccaggcgatcggtctcatcacgcctggcttttctcggggttgggaagacctcaagattgcgcg

taagctctggggcgatgggccgattgtgctcaaggggattcagacgccatcggatgcactccgggcagtccagcatgggatggacgggatttgggtct

caaatcatgggggcagacaggtggacggtgctattggatcgttgcaggcccttcggtccatcgtacaggtcgcccatgcgagtgccagcactagtggg

aaggcagacggtcgaaagcggcctgctgtcatcttcgactctgggatccggactggtgcagatatcatgaaagcactagctcttggcgcagacctcgt

cggaattgggcgaccttacgcatatggcctggccgcgcacggccaagtgggcgtcgacgccgtgctgcgaagtctcctcgccgatctcgagctcaacg

cggctctggcgggctgcaagagcgttgaagagattcgtcagcgtgggacaggcgcgacaggtctggagcctgtggtcgttcgcacggggtcagaactc

aagctgtga

>contig_14.contig_14.g572.t1

atgctgctctgtctttcctttctcttccagttatggatgctgatagccagcgtcgttggtgtacctgcttcccacgacc

atctcaaacgcagctgcggtggaacttcgagtcttccggatcctcgcatcgacatcaagcagggccccatcgttggctacacggggtgcgtagacgac

gtcccgatcagttacttcctcggtattccatacgcccagccacccacaggttcaaggcggtttgccaagcctaaacctatatcggagaaaaagccggt

cattgacgcgaaagcgttcggacatatctgctggcaaatacccagttcgacctacccacgggaaaggatggacgaaaactgcttgacgatcaacatca

tggcccctgccgatgcacaacatcgagacaagaagctcccggtcatggtctggcatcatggtggcggcttcaccacgggagccagcacctatcacaat

ggaagccgattggttgctgaatcagtcaagctcgaagctcctgctatctgggtgaacttcaactacaggatcaatgcgtacgttctctctggctcgac

gcacgcttcgaatcctactctagcatctcacgaagcttctatgttcaatagcttcggcttcctgtcgtcacgtcaggtgctcaaagccgctcaggcag

gcgaagctgcgctgaatgtcggtctatacgatgcgcgagaagctttacgatggtgtgtctgtgtacacctgtctcgttccgagcaggatggctcacaa

tcattggtgtctccatccagggttcagcagaacatcgacagatttgggggcgatcctcaacgatcggctggcgcgtatgcagttggcgctcagctgct

cgccaacggaggcgattccgagggtctcttccagtctgccatcatgcattcaggctctcccgcaggagggaccgccttgacaccggcccatccgcgtc

tacaggctacatttgcaaatctgactgcaatggtcaactgtccgaacgacgaaacatcggtccaatgcctgcgacagattgaccaggttatccttgca

aatgccagtgcaatcatcgctcagaattacagcagcaatccttatttgggctactggccttggacgcccgtgcaagacgccgctgtagacggcttctg

gttccgcgaacaaccctcgaagttggtgaacaagggccagtacgctgttgttcccatgatcacgggcgactgcgctgatgaagggaccatcttcgcag

cccacgatctcgaaaacgcaacatccttcgatcagtggttccgtcacatcgctgtcgccgatgtcagcacggatcccgaccagcagagtcgcgtgact

gggctcctgcaacgtctctacgagctcgtccctgacgatgtcacccagggttccccatactttaaccccccgaacgccgtcagcaagggcgtgaccaa

cgtgtccgatccaatcttccagccggcaaccaatcagtacaagcgcgccgctaggatgttctcaacatggagatacgaagctccgcgccgaaaattcc

tgcgtctcttcgcgaatcaaacgaagtcaggcgcttggacttatcgtttccatcagcacgacaactcaaatgcaattgtcggaacatcgcatggctct

gaaatcgtctatgtaagattactggcacccggacacctcgttagattagagctgaccaacagaacgctacacactactatacagatcctcggaaatac

ctcgaccatcaactcgacaggcctttatcctcccttgtcgaagttgatgcagagagcttggatatcattcgccaacttcaacgatccgactgcactcg

gccacctcgattggccgcgatactcggaggaagagcgctccatgatccagttcaagggcctgaacgttacgctcattcgcgacgactaccaagagggg

gcgctcgcgtacatgaggagcgacgagatgtctgccatcctcagctcctag

>contig_14.contig_14.g573.t1

atgctgctccgtctttcccttctcttccagttatgggtgtcggtgcctaatatctttggtgcacatgcccttcaacccc

gtcagacttgcggctgcaatggaacttcgactctttcggatcctcgcatcgacatcaagcagggccccattgttggctacacggggtgcgtagacgac

gtcccgatcagttacttcctcggtattccatacgcccagccacccacaggttcaaggcggtttgccaagcccgtgcctatgtcggaggacaacccagt

cattcacgctaaagcgttcggacatatctgctggcaaatcgccagttcgacatatccgctggaaaggatggacgaaaactgcttgacaatcaacatca

tggcccctgccgatgcacaacatcgagacaagaagcttccggtcatgttctggcatcatggtggcggcttcaccacgggagccagcacctatcacaat

ggaagccgattggttgctgaatcagtcaagctcggagctcctgctatctgggtgaacttcaactacaggatcaatgcgtacgttctctctggctcgac

gcacgcttcgaatcctactatatcgtctgacaatggttctgtgtcaaatagcttcggcttcctgtcgtcacgtcagatgctcgaagccgctcaggcag

gtgaagctgcgctgaatgtcggtctatacgatgcgcgagaagctttgcgatgggttcagcagaacatccacagatttgggggcgatcctaaacgatcg

gctggcgcgtatgcagttggcgctcaattgctcgccaacggaggcgattctgagggtctcttccagtccgccatcatgcattcaggctctcccgcagg

agggaccgccttgacaccggcccatccgcgtctacaggctacatttgcaaatctgactgcaatggtcaactgtccgaacgacgaaacatcggtccaat

gcctgcgacagattgaccaggttatccttgcaaatgccagtgcaatcatcgctcagaattacagcagcaatccttatttgggctactggccttggacg

cccgtgcaagacgccgctgtagacggcttctggttccgcgaacaaccctcgaagttggtgaacaagggccagtacgctgttgttcccatgatcacggg

cgactgcgctgatgaagggaccatcttcgcagcccacgatctcgaaaacgcaacatccttcgatcagtggttccgtcacatcgctgtcgccgatgtca

gcacggatcccgaccagcagagtcgcgtgactgggctcctgcaacgtctctacgagctcgtccctgacgatgtcacccagggttccccatactttaac

ccccccccgaacgccgtcagcaagggcgtgaccaacgtgtccgatccaatcttccagccggcaaccaatcagtacaagcgcgccgctaggatgttctc

aacatggagatacgaagctccgcgccgaaaattcctgcgtctcttcgcgaatcaaacgaagtcaggcgcttggacttatcgtttccatcagcacgaca

actcaaatgcaattgtcggaacatcgcatggctctgaaatcgtctatgtaagattactggcacccggacacctcgttagattagagctgaccaacaga

acgctacacactactatacagatcctcggaaatacctcgaccatcaactcgacaggcctttatcctcccttgtcgaagttgatgcagagagcttggat

atcattcgccaacttcaacgatccgactgcactcggccacctcgattggccgcgatactcggaggaagagcgctccatgatccagttcaagggcctga

acgttacgctcattcgcgacgactaccaagagggggcgctcgcgtacatgaggagcgacgagatgtctgccatcctcagctcctag

>contig_14.contig_14.g574.t1

atgccaccgcgtaagaacatacgcggcagagctcgaccaggtcttgctagtggatcatccagcgggagtcccttggctt

tggccggacacaaggcaggaacgcgcgcagccacggcagcagccgcatccacacccaccggcaggagagcccccgttgcacggacagccgccgcaacg

atgtcgtcggcagcctcgcccaagacaacaccaacagcagcaacaacatcagctgtagccggtccttcgtcaggcgttgttgcgtcgcctgcgcagcc

agcacttgccgcgccggcatctgaagggtccgcaccagcaacagccccagcgacgtcgtctgcagtagcaatctctacacccggcgatgcaaccccaa

acacttctacgacacgcatttctgcgggtacgttcgccatcgcaaatgccgccgcagaagccgccgctcgtcgcgattccagcccctccacatcagcg

aacatctcctcgccctttggcgccatccggggcagtccgctccagcacgcaacagccctcagccgcgccacggccgcttccgagctcaccagtatgca

ggcacgcacaaaggagaccattgagcgcatcctcgactacaagatcgacagcatgctgccccagatgcaagagcgtatccgcaagggaataatgcccg

gcctcatgcagaaagtcacggacgtagtcaccgatcgattcgagcaattcgctcaagagtatgcgcgcgtgaagagctccctcacgcgtgtgagtgca

accccggctcctcccgcaagattgccggcaccaactgcagcaactggtactcctgttgcccaggcatcgacgtcgacacttcaagccggagctgtgcc

tgcgcccactccttcttcggcctcagtgcctgcgcagagcactgctctcactgtacctactggcaagacattcgcccaggcctcgcccgctgcagctc

aaaaggcctcccttgcgagctccagtactgccatcaccactccagtccagaatcgtattcaagctccagtttcagcagcaatccccgatcaggcttct

gcaagcgcttcagcaacgacgagtgttctttctgcgcttccacccatgccagcaaagtctgccccggtcgagcaacggatcgaagctctcgagcagcg

tgacgcatcgctggtacatgcactgaaaacgctcgacgctcaacttcgcggcgtcagcgcccaagtcgaacatcttgtccgatggaagaagctcatcg

aacaacatgctctccgtgctgaacaggctcctaagaaggcgccggtccctgcgcccgcagtctatcgccctgtgcttccgcaaacacagcagcttcag

caacaggcagcggtagggccttcatcgtctactcccgttcctgctcgcggacgcaagcgcaagtcgaccatcgctgagccccctgctgatcttagtgc

ggcaccggataccactgcgggttcgccaaccaagaaagctaagaccactgctgacagttcgacggacgccagccgcgtcaatcaaccggatcgggatg

tgcaatcagctttcctcgagctgcttcgttccgagatgggcctgagcgaccgtaagtggcctcccttcccaggccacggtgttcccgaagaacgctgg

ccgcgtttcgacccgacagagcaacagctcgctgcgtgggaaacccttccggccgacaaacgaccgccgcgggaaaccgttggcacccgcaaagtccg

tctggactggaacttgccgcctaccaaccctacgatcgcaggatatctggtcagactgcgtgaccggatcttggcggatccggacaagcatggtattc

cgaatgaccctgttcggcggaacccggacgtggccctgaatgctatcaagcgatcgtacttcgaggtgcgacggcacgggaagcaggccaatgcgctg

gctaaggactctgaaccggctgccgggactacgtctaccgttgcggctccggcttcggcttccgctactgctactgcttctgctacaaatgcagctcc

tgcgacagccgagacttccgctgcgggtgcggctgcgcagggagatgcgaatggcaagggcaaaggaaaggagaaggcggacggaactgttgctacca

agcggaagtacacgaagagagctgtgaagagcgatgcagcggcggatgcatctgctgcttcgacctctgctgctccggcagccggggctactggagag

aaggcggcgaaggaccctgagaaggaaaggtcccgaactgtctacaatattcggcaacgtcgcgttgcacgtcgtaagagtcgtcgaacgcaacggga

ggtcgagctcaagaagcggaagggctgggcaggcacggacattgaaaccgccactgcgctggaagctgtcagcgaagatgagacggacggagagcaag

aggctccggaggatgaagctgacaaggacgatggcgaaggtgggaagaagcgcaagccacagagcccacccgcccgcgccgtcgttcccgagtggcga

tcgaaggaacttgcgctggtcatcgccgatctggagaagacgatcaaacgctcgcgctcgattctgcctccgctcggcctgacgccggttacggtgcc

gctgccggccaccttgcgccgatggcaagtcagcgagaagtttgcaaaggagcatccggagcttgtcaagagtgtgacgctgaatgcggggccgttca

tgggccagccgtcgaatggctcgccgacggtggcgaagagcgcgacgtgctggggtacgcctttggtgctgaacttctcgggagcgggggcgccgacg

attgtgatgggtggcggagcgccgggatctgggacgggaggtggcgcaagtggtagtggtactgatgggcagagtggcagtggtgctggtggtgacag

tgcagcggctcctcgatcaaagagtacaggagaagcggagggcgggacttcttcagctggcggtgctgtggctggacaaggcgcctcgcggcttgcca

ccgcagttccgactgcacatgctgagtcggggtcaccttatgcctctgccgccaatactgcttccaccctcgagggcggcgatacgggacacaacaac

aatcaatccggcggaggatatacggcgtcgttgcgggccgcttcggcctggcctaccatgggccagtctgtctctaacaaccactcctctctggctga

tgggttcggcacggcagcgtacggggctggctctgggggtggtgacacgcccgatccgacggcgcgaaacgatctcccttcgcgcgcttcaagcgctg

cggacacgggatctgcgtatcagggtcaccagcaagctgcgactcacggtggctacgcaagcacacagcagggccatgatgggataagctggtatcag

ccttcgggctcgacagctgccgcttctcagacgcagacgcagcagcagcaagaacagcacgcttcacagcggactggctacttgtccctgacgccctc

accgcaagcaggagcacgtggtacaactgcgtcaacggggagcggagtggatgaatacgctgcggcagcaggcacgcactcgaggggcagcagtaaca

tgcggcccactaccgtcgctgccaaccacgctcatacccacgctcacggcggcggcagcatcgccggttttgggacgcaagcggccgtacgatcggga

agcggtaatcatgactacgcttcttcgacctcgtcttcgagttcgacgcatcggcctagctactctgccagctcgactgctggaatccatgcgctttc

agggtatggtgcctctgctgcgaacgctgctcgaacggtggctgcggcacaggagcaacagcgtcaataccagcagcaacagcagcaacagcaccaac

agcagcatcagcatcagcagcagcaagctgcttcgaacacgggtgcgagctcgtactggaatgcttttgggccttctggctacagcggcactgccgcg

gcacaggcgcagcaaccgcagcaacatcagcagcagccacagcagcagcaacatcaacaacaacactcgcagcactcgacgtccaactcgaatcttcg

atcaacctttggctggggattctcttcccagggctga

>contig_14.contig_14.g575.t1

atgcgctcttccccttttcaaatgctctcgaaggcagcagcagcactcctctgcctcgtcgcttccacttccgccttct

cgcacccggatcctgccctggaaaggcggcagggcctttcagccgctacttccgatcctttgggaaccatccaggtcaatagcccagtccgcccgctc

cctacgtgcgcatcagctgatatcgaatggcagtggactggcgacctctccgtctccaatacggtcgcaatctacattcagaatctcgccgagtacag

cttctcgcgccgagacattggcggtggtggttcagcagcccgctcgtctcgcatgcacaaggtccgtcgcggacccggcggcgatacggcatcgtctt

cgctccacatgctccatgctcgcgcaggcagtcggctccttgcgcaggacatcagtgttgtcgatcagaactgggtttggcccagcgtcgatgttaaa

ccgggcctgtaccgcttctatatcgttctccagggaacaactcagcaggcaacctttggcagcacctccgccttctccatcttgcccggttcagacac

tacctgtttgggtggaaatcccgccgagacaactaccaccgccgatccgacatgggccttggctcctgtcgcctcgtcaactacatcatcacccacag

tcgtatcgacgccatcgcagtcagctgcagtcagttcgactccctccgttgctgcgaccaccaccaccgcatcgacgcccgtcgtcgcacctgccaca

accagcaatggctcgaccgcaaattccaccttatccggcaacagctcatcatctcagcccgcatctggcctccacggtcccatcattgcggccgtcat

tatccttcctctgttagccgtggtcgccctcgcaatgggtgcggtgcacatccttaggaaacgccaagaagagagctcttcgccttacattcgtagtg

tcccctcctcggacgctctcgcaggctcaggtgctggaggagcgggtgcttgggccggacgcttcctgaccagatccgcaagtcggggcatggacacg

gatgtcgcggaggtacatgcagagaaacgcgaggacaaccgagacagtgtcgcctcggacgtctatggagggatgggacaggttgagcaccagcgtac

accttcgcacgactccatgggactcggtcgtcccagcatggccgaagtatgcctcgacgattcgccctatctccactgccagtcggtctcgccgggac

ccacgatgtcagcctcgggccgaccgagcatggcttctctccgcagcatgcccatgtcctcgcctatgcctctgatcattgaggaccgtcccacgggc

cgcagctacgacctgtcgcgcggtacggcagcaacaaccccgcaagccgtgttctctcccacagaaagcgaacaggccttttccactggcggttggac

gcagtatgcaaatgtgggcacggtcgatgtcgagggtgcccatgccctgagcaaagatgaggtcgacgttatcggtagcactccttctttcgccagtc

agcaacaacagccccctctcacccgtgccctgccaattccaacccatctcgagaccatccctggctccaagggtagcaaaggcaccgaaaccgaggga

acggcggaccagctcccacaaaccctcctcaccgagctcaacagcgaaccaagcccagagatgccgtacccagacacgcctcggccgtatgacaccat

gaggcatgtcgacgaaggcgcctcccaccctccccgcagtgcgtcgagcatggctagcaggaacatcggcggaggaagcggtgcatatgcgactgtcc

aacgatcagcctcgaacgggagcacattcagcgtccgacggaagcctgttcccacgacacccctgaccgactcggccactgcgaccaacggcccattc

tcggatgtgaacgccatcagcagcagcccgacttcatcgtcgacggagttcccatctccagcggcgggagcgcagcgccctcggacgccgaagaagga

cgagttcccggagacgcctaaccccgctgctgatgaggaggagcaggaggagtccattgcggccccaaatgcggacgcggatgcggacgccgatgaga

tcatgggcaagaagcagtatcagctcagcgtagacctcagcatggacccgcttcggtttagcgggttctga

>contig_14.contig_14.g576.t1

atgcagattcgaggccttgtcgcagccgtcttcggactgtctgcgctcgccagcgcccttccagccacccctactcttg

aagcacgccaaacacaaacggtcactgtcaccgtcttgccctgccctacaggtcggcccaactgccactgccctaccacacgcccctactgtgtcacc

ggccctccaccatccggcggtgggacgggaacaggcggtggcggcacaggtggcggaggtacgggcggtggaggtacaggcggcggcacaggtggcgg

tcaaggaggctatggtggccaaggcgggtacgggggtggatacggtggtggatatggcggtggacagggtggctacggcggaggaggatacagtggcg

gaggtgatggaggaggtggctacggtggaggcggctacggtggaggtggctacggtggcggaggatatggcggtggaggctacggaggaggaggagga

tacggaggaggaggcggcggatacggcggtggtgatacggaggcggtggatatggtggtggcggatacggaggcggcggatacggaggctaccgccgc

actgtcgcaaagcccattgaggctcgcgcaacgacctccgtttgaagctctgttcccgaccctgcccaccctgctcccaagttcagctcaatgcctac

actacgtgaccaaaatggcgaaggctcccaaagcaaacggaaaggcggctgtggccaaggctcccgccggcaaggctgcagtcagcaagcccgcgccc

agccccaaggcggctcccaacgcaaagccggtagctgcgacgaagaagaacggcaaggcagcacccgctgctcctccagcaaaggctgccgaggccct

cgatcaggagggagacgacgacgatgacgatgacgacggctttgaggacgtcgaagacagcagcagtgacgaggaggacggtctcgatgtctcacgga

agggcatggagcgtcttctagccgcgctcggcgacgacagtctggacgaaatgtcaaaggcggccctagaggagctcggtcaaggagcggacgatgag

gacgaggaggaggatgaagacaaggaggagggagacgatgacgaggaggaagatgatgaggaattcgaggatgttgacgaggaggacgatgatgaaga

cctcgaagacgaagatatggacgaagaagcgaccctggaggaggatgccgaagaggaagaggaggaagacattgaactcgacgccctggacggtgcga

ctgcttctgccctccccgaagagatccgcggcatgcgcatcaacaaggtcaagcaaaacaacaccgaagcactccgacgcgtcctcgaggaagttcga

gcaggagcagcttccagcggtggtgcagacgggaagggcaaaggaaaggggaaattgccctggatcgagacgatgagcatcgcatggaacaagagcgt

tgaggaggaagtcggcgaggacggaaaggacgacgatctcagaagagaactctcgttctaccaacaagccctccacgccgccgtcgaaggtcgaaagc

tcgtcgaagcctcaggagtcgtcttctcccggccaacggactactttgccgagatggttaagacggacgtccatatggaacgcgtccgccagcgtctc

ctggacgagcgcgcgggtatcaaggcgagcgaagacgcgaagcggcagcgtgagctcaagaagttcggcaagaaagttcaggttgaaaagctgcttga

gcgtcagcggtctaagcggcagatgaaggagaagatcaagggcttgaagcgcaagcactctggtctcgaagaagacggttccggtggcgctggccagt

cagcctttgccggcaacgacgacttcgacgtccgtctcgaggaagctattgaaaacccgggtggccccaacaagaaggcccgcaccagctccggcgcc

agctcacgaggaaacgccgccggcagggccaagatgccccgtccggcccgcgacgacaagttcggctatggcggcaagaaacgtcatgccaaagagaa

cacggcggagagctcggctgatctgtatggcagtggtgatggtggacgaggtggccggggccggggccgtggaggtgcgcgaggcggacgaggtggac

gtggaggcggaggaggtgggcgaggagccgcgggtcggggtcgtggtggggcacggggaggatttggcggagcttcgaggggtggctccaggggtggc

tccaggggtggttcgaggggcggttcgcggggcggtggacggggaggaaatcggccgggcaaggacagccggtcggactcgcgcaagtga

>contig_14.contig_14.g577.t1

atgcgatacacctcatacctcggtctattggccgctcttgcggccagtggagcctttgcactcgactctactcaacagg

gccatgctaacaccgtcgtcaacaccgctctgaactacggcaccactgcgcctgatcaggcttgtgtaactgcagtggcgacagccattactgaatcg

gccctctacaactatgccaacagcggggtgcccgcctcgctcaactaccctcacgacagggttggctccgatcatgattcggtcggtctgttccagca

acgagttagcatctacaagaacttggctgccgatatggatccggctggttccacccgccaattcttagctgagatggtccgaatcagcggatggcaga

ccatggaccccggcacgttggctcagaaggttcagaggagcgcctaccccacgcgatacgccggcaacgtcgcccaggctcgtagcctctgcggctcc

ggcaacggcggtggaggaggaggaggaggaggatcctatggcgggggaggcagcggcggtggtgacggtagctattacggtggtggcagtggtgatgg

tagctactacggtggcggtggtgacggtagctactacggcggtggtgggggatcctacggtggtggtggaggatcctacggtggtggtggaggatcct

acggaggtgggggatcctacggaggtggaggcggaggtagcgatggaggaggatactatggtgggggcgggggtgatggtggaggaggatactacgga

ggcggcggtgacggaggcggtgggtactatggtggcggcggcgacgggggtggtgggtactacggaggcggtggcggaggttactctgcttccatctt

tggctcgggtgggttcgcctggccctcgggccccgactccagaatgcacacaggggacgaactgttctgggattcttcctttcttccgctgacctcta

ctttcatccgcttcccctcttcttcccctttccacaagatgaggctcacgtcccaccttgttctgctggccgctcttgctggggcctctgcactcgat

accactcaacatgccaacactactgtcaaagatgccgccaaagcccccgtcaaagctaccgccgaccccactaaaaacaccatttcgagatacagcct

cgactcggtccaacagggacacgccaataccatcgcatatactgctctgaactacggctcctcctatgcgtctcatcacgcctgtgtcactgcactca

cagcggccattggggcatcgtccctctatgtttacgccaacgcccgggtgccgctctcgttcatatactcttacgatggtgttgtggtcaccgatatc

gatgcggtcggtctgtatttgcagcaggtgtccatctaccctaatgtcggtgccgatatgaatcccactgtttccacccgccaattcctggacaagat

ggtcagcatcaaaggatggcagaacatggaccccgccacactcgcccagaaggtccagaagagcgcttcctctgcacgttacgccggtcacgttggcc

aggctcacagtctctgcgatcgcggcagaggcggtggtggtggcggcggcggatactatggtggtggaggaggggaaggggatggcgatggcgatgga

ggataccacggtggtggtggagggggaggcggatggggatggggaggcggtggtgacggaggatgttcttcttattgtaagatcagccgtcacgctca

acatacctttgggcttatcgaaggctga

>contig_14.contig_14.g578.t1

atgagggggcgcagccaagtgattgtgacatacggcccaaaccctcccaaggtcgccctcctgctcgagtggctcaacc

tgtcatacaaggtcgaacccctggtcttcggggatggcccgaaaggaatcaagaatcctgaattcctcgccattaacccgaacggcagagtgcccgcc

ttggtcgaccactccaacaacgacttcacggtatgggagagcggcgcctgcttgatctaccttgctgacaagtacgaccaggagggacgcttctacgg

tgtgtctaaagatcgagggcttggtaggaacgcggaggagagagcactgacggcccagtggctcacacaccagctcagcggtctcggcccatctcaag

gccaagtaggctggttgactaactattatgagcaaaatacaggcatcaaggtccagcccgagatgattcagcgctaccgtgaggagacagatcggctg

tacaaggtcctcgatggccagcttgctaggcaagccgaggctggctcgcagtacatcgtgcacgatcgcatcacgattgcagactttgccttctacgg

ttgggttcacatcgtcagcttcgccaaattcgacttgtccgactacccgaacgtgcagaagtgggcaggaaacatcgcgaacgacgagcgcatgaaga

aggcatacgcaaagatggacgcttaa

>contig_14.contig_14.g579.t1

atgtttgctcttaaatccagcacgctcgccctcgtcctcgccgtcctcggtggatcggccttggcctcggccgccagcg

cacccggccccgacttccccccgctctctcccctcgttcgcgccgacctcgaacgccgggacccgagcgaatacgagctcttccacaacgagcagcgc

agcggcaacgtcaccacacgcgctgtcattcctggtggcattacccgctgcaccaccaccaagtgcttctcgttcaccttcgacgatgggccgtacaa

ctaccaccaaaagatcgccgacgagatcgcctccatcggcacccgcgctaccttcttcgtcaacggaaacaactaccagtgcatctacgacgacgccg

cggtcgcagcactcaagtactcttacaacgctggacatcagatttgctcgcacacatggtcccacccagacatcgagaccatcagccgcgcccgactt

gacaaggaggtccagttcatcgaggatgccctgtacaagatccttggtgtcgtgcccgcatgcatccgacctccctacggaagcgccagcgatgccac

gatccagtacctcaacaacaagtggggctacaacgtggtgctttggaacgccgatactaaggacgcggatggcgcagcagtcagcttctccctcaacc

aataccgagcactcaaggcccccaagcacgtcatcatcctcaaccacgagacggttcctaccaccagcagcaaggtgattcctcaggctctccagatc

gtccaggacaacggatacgaacccaagtacatgggcaccgtcgctcagaccatcgggtttgcaccttacaaggtcatcggcaagcccggcaagcgcga

ctccacatggacctgcgctggaaagcccctgcctggctcatga

>contig_14.contig_14.g580.t1

atggcagtctcagccggtgtccgaccgcgggcaacgctggccagcctgccgacagagctccatgcgctgatcgcccgcc

tgatctacctcgactccatcccctccggcagcctctgcaccctcgaccccctcaccttgccaaactcgaacccactcgcagacggctccgaccccgtc

agcgcccggccccttgcctccaaccagaacgagacccgcaagaccctgtatgcgctctgtctgaccagcaggtccttctacgtcgccgcacgccctct

cctcttccgccgagtcgtcgtcaccctgccacacagcttcgtcctcgtgctccgtaccctcggcgcagcccatctcgcgcaggtctatgacaaatatg

ctgccagtggcaaccaggacttcctcctgcgtgaccctgccacgaccctcgcctcaggggcccttggcagcgagctccagggtcagctcgtcaaacgg

aagcgcgaacggactgttaccttcgaggagatggtcgctgctgctggctttgcccacgctgccggtggcacgctcgtcgtcaaggcccgcagcaaggc

tgccctcgaacgacctcaggggcgcttgcagtcacagccagccattgtccaagagcccgggtctacgcagccttctggctcggcttacacggcggagg

cggacaatgcgagagagatggatctcgtctggatcgaggacccaacttccaagtccaagtccaagcgctaccacgtccatctcgctccattcaagccc

acagcactcacgctttccaacgaagagctcttcgcagcccatcgcttcgtgcgtatgatcgactttagccggtatacctcccagggtatgcgccgctc

cgtcggagactcctccaagaatcgcttcgtcaccccttccaagctcttggccctcatcaccgctgtctccgagttcctagaatcatttggcgcgtcag

agactatggatagcgcattgacgtgcgagatattggaggctttgctgttccgagatggagagtgggcaaagccatcccacctccgaggcgtctcaatc

gagcgtcgtcgctccgtctcccgacaccgcggcacctccatgagccgagttggctcagccaaccagcgtgactcggatcacaagatgctccagagctt

ggatctctgcaactgcgtcagtcctcgcttcggcgaagcattcacacgctttgttcagctgtacttgcgcccccgcgccgtaggtataacggccgacg

atgtgccggacattgagccgccatcgggcccgctggagatacatgcggaggaggaagacgcgacaacgtctacagagacagagacggagactgaggtc

gagacggaaacggacggagactctgaagtcgacggcggccgtcctgaggcggcccaacgtcgtcggactgtgacgattgatgaagaagaggaagatga

ggagatgatggagcgtggtcgtgacactcgctcgcgacggatcttcggctcccaggctgggtcaaactcccgctctcgatcgcgaactccgttcgcgg

gtgcctcggccgcatcgtcttcgtcatcagcgacggcaacgacaatatcaacgcccagaaccccttcacaatcgcgcctgagcggcgtcgcgggaggt

tctgcccgacttggcagtccttcggtccgcacgacgagtctgacgcggaacgcctcccagacgcgcagtcggtcgactgccttcacacccgcgacgtt

ccccagcttgcgacggctcgggatgaacgggatcacgttcccgatggacattgtgacacccttcgtgctggcgttcccgcacttgacccacctcgatc

tgtcaaggtcgaagacggacggggcgctgcttcgcgcgttggctgcgagcgacacacttgctttacaatcgctcagtctggcgcgctgtcggtccatc

gatagcgagagtattctcgagctcgtggtccgctcggacgtctgtgccagtctggtggagctcaacttggagggcacgctcatcttcccgacgtcgat

cacccgcccggacgcgatgacgctgatcaaggaggctcctgcgatcaagtccggcgcgatgcggtacctcgacctgagcgggtgcaacttgacggacg

cgcacctggctgtgtttgcagctcagccgagtatgctcgatctggggctgggctgcaacccgcgcattacgctacagggggtggctacgcttctgaag

gagaaggctccgaatgtgcaggtgttggatttggcggatagttgcacggccacgtctgatgacacgggtggagaatatgatgatgatttcgacatggc

cgacgggccgccgatatttgggagtgcaggtgggagcgctgtaactgctgtacagctgcatcaacagctgctcgctccgtgctgtctggccccgcccg

tgccgctctctcaacagctggctgaaatgggcttcaaccatctggcgcaagatccaagctcgtccagcggtgggacggccccttcgaacctgactcca

ccgcgtccgccgacgaatctgcgggtgatcgagctgggcgcctcgagtctgcgttcgatccgaagtgggattgagacgtggaaggtggtatggggtgc

gggtcagcgtgggtggattgtggatatggctgcgggcccgaatccgcaggctgtcttccagaaggatacggatgtgttggggaagggagccgaggctg

aggctgatggtcgtggacgtggtcggacgggtcagccgcggcgtgcgtctcgcgccggtggcggtggactgggaggcgacctgggtgagacgggtcgt

cgtgattcgccacggcgtcggtcgtcactccggcaatcgagtgcaccctcgaactcccgctcgcgatcacgcgcgcggtcgacgtcccgtgtccgggc

gcctggtcggatgatcagtctcgacacggacgaggatgatgatgatgatggaggagaagagcgcgatgaggatgatggggttgacgatcggagacgtg

gtgctgggatgccttcttcgttgcgagggtcggcgagtcggtttggacgggtggataggacggtcgttagcgatgacgaggacgatggtgcggccgca

tcagcacgagcgggtgatacgacactccattctgcgcccgcgccagaaccggcaccgaccgacagctcagctcacagtggaagcacgtctgcggctcc

caccaagacgaccgtcctcaccgcgcggaacgaggtcgtccggggtcttccgccgacccatccgcgccgggaggcactggaggctcttggtcgggcga

aaggtcacgttcccggggatacgggctggcattcgcggaagatggagatcttgcttgggtttggattcatggggcgggaggcggggtcgtatgctatg

gttgcgtatcagacctaa

>contig_14.contig_14.g581.t1

atggcgtaccagcagcagcaaggatactaccctccccagggccaacagcagggatactaccctccccaacaaggtggtc

aataccctccccaaggtggtcaatatccaccccaaggccaatacccaggtccccaaccctacatgcaacaaccacccatgatgcagcagggcccccca

ccgggtatgagcatgcgccaacaccgcatgcagaaggcacaagataagcacggatcatgctgcgggccttgtgccgcttgctgcgtcggtgcgatggc

ctgttgctgtctcgaggacatttgctgtctcgagcctcgcgtttcgcgctcgcggtacaacccctacttttcggcggatagaggaaccaagccgttcc

ttctggcacgcttcttttcctcggcacaccgacgtaacgatggacccatcccagatcatgggccaaccgtcaaacgcgttcgattcgatgcaaacgcg

cccctccagcaccttatcacggactatacgaaccttttcttgaccccctgcctgctgtgcacttcgcactggaatgatacacgatatacactcacgcc

aaaggagcgtcatccaggctgctgttga

>contig_14.contig_14.g582.t1

atggctgaaggcacaacctccgctgcctcaaacgcccccaccggggccccgcaacctgccccagcttctactgcgagtg

ggccgaccctccttcccactcacggagcgctttcagagaagcagccgcagctcgagccaacaacaataccatcgaacatgcctccactcgaccaaatc

gagctcgtcggcgcagaagatccgaagaactggtcccctcaagtcaaatggacctacgtcgccgtcatctccctcgccggcttcatctctcccatggg

atccagcatcgtcgtccccggcgcgcactctctcgacaacgacttcaacctggacttgccccttgaagagctcctgccggtctccgtctacgtgctag

gcctgggaatcggtcccttcctccttgcgcctatttcggagctccggggtcggaagaacgtctacctcgtcagctcctggatctttgtcgtcctcaac

ctcgggtgcatcttcactcaccacagcttcgtcgctctcaacatagtccgcttcttcgccggagccgcgggcagtgctgggccctccttaggcgcagg

aagtatagccgatatgttcctaccgtcagagcgcggaaaggcacagagtttatatgctctcggcccgctcctcggacctgtcataggcaatgtcgtcg

gtggatgggtcgctcaggaagccaagacctggaggtggcttctggccgtgctgacaatctgctcctttgtcatcgccgtcatcttcacattcaccctg

aaggagacctaccagcccgtccttctccgacagaagcgccaagcctacgcaaaaaaatgggcccgtaaccacagaatccctctgacctccctcaccct

caccgacgccgaaaaggagaccgcagctcgagttcccaaatggcgtcgccacctcactctctcccccgacgcacgtctgaaacttaaccaagccttct

cccggcccttccgcctcctcttcaccaaccccgtttgcgccatcttcagtctctacctcggcttctgctacggtatcatcttcctcttcctcacccaa

cacccgcttctctttgagcgacgagagagggacgagtatcgcgatttgctccgggccgaacttgccgggtgggggattgactacgacgatgtcagggg

taactcgaccttctttaaggagcatggcttggactggagtcgtcggcaggatgacgacgatctgaacctgccagggcccggtggcagtttcaacatgt

ctgacattccgccggattacgggtcggggtcgggatctggctccttcatggatggctccggttcaggcgatcccctcccgcaacttcccaacttcgga

tggtcgagcggcgtggctggactgacctacctcggtctgggcgccggctttctcgtgtccgccctgatcaacaccttcatgcaagacgcgatctacca

gcgcttggccgcgagtgatggccagctcgggtggttccttctcaaaagcccggaagagatcaggcagattatggaggagagggagctcaggcgagcgg

agcggagggtgaggagagctgctgtggatgcgaatgcgctggacgatcgggaggcggacacagatgtggagaacgacgcggcggatctggacggcctg

accttgccagcgacttctctccccgtaaatgaacacacctggccgggtcgtcgagggagtgcggcatctacagcgcacgaaggatccgtggcagatgt

tgctggccgatccagttccgatctcggctcgactcggaatcaccgtctccgtgtttcggctggcccggtaataccgaccagtctgccttccgctgact

cgcaggagggcgttgcttctactaccatcgcttcatcaagtctcacagcagcacataagcccaaggcacagagcagcgggctcggccttgatgctgag

agggacgggaagaaggaggtacgggggcaggatgaggaggaggaagacgacgacgacgaagacgacgatcagaatcactcgcggagaaagaacggagg

agggggcagcgggtcgggactgggcagcaagagcgaggacaagctcacgccatttgcatcaatatcaaaagtcccatcagcctccgagaagcccctag

gaacgaacgcgaccatcacagaccctacatcaacgactgcagcaacaacagcgctccccgcctcctccaaggccgacagcacaaagaagaaaggccgg

cccgaataccggctgccactctgcctgctcggcatgtgtctcctccccatcggcctcctctgcttcggctggtccgcccaatccaaaacgcactggct

cctccccatgctcggctccttcctcacagggatggccaccatcctgtgcttccagaccatcctcgtcttcctgatcgacgcctttgtgccctttagcg

cgagcgcggcggcgtgtgcggttctggtgaggagtctgttggccgctgcttttccgctgtttgcggggaggatgtatgcgaggattgggtttgggtgg

gggagctcgttgcttgcgcttacggctgcgctggggattccggtgcctattgttttgtttcggtatggcgagcagttgcggtga

>contig_14.contig_14.g583.t1

atgtcgtctaccacacctcggggtccctgccgttctgaccattctgttatgacgaccgaggatcagacctttgtttgcc

tgcatccaagcacaacatgtcgtcctcgcaagcagattacccttctcgcaggatcagagccgctcatcctcaccctatcctcccgcaacgtcacccca

ccctctgcacaacgcagcaagggcaatgctatcgccattcgccgcgagccagttgactcagcccaacctcagcttcgatctccctcgccatccgacac

cgcccagtcactgcctctgtctgctcaagtcccagctgtggtccacgtttgtcccgcccgtgtcaagctgcaaattgaaaagctacagaatgcacaca

gcatcggtgcagactcggacatgaccgacgtttgggcgtggctggatgtcgatcttctcgcgcttgaagacacgaacagggccaagaagcaagagctg

gaggtactgcgctgcgagacggacaagctcgtcgctaccatcggcgatcagaccaaagagtcaccgagcgagcttcgcgctcaacgtactgcaggtct

agagatgatcaaagagacgaaggaccgtctgcagcaggctgtgttcgacttggctgagtgcctactcatcttcagtacctcactgacgctcatgagtc

atgcccatctgctgacgcttattcacagtagtactgatcgatcaccgatcaccgctcactttcacattttttatacactgtccagcctcctcatcaat

gacggtgctatgttgacagctgaacttcttgcaccccgccgactgagtcattctcctttacccttccgggcgttgcagcttacttatctcaaccccca

gcacgcctaccctgggtttctccgactcttcccctcctttcccaacatcagtcctttctttcttttgaatcttgatgagtccgccgacgaactggtag

tctgcaacaagctctcctcacctggtatctttggcagcttcttactgactctcatccagcgaggtcttgtgctcatcgcctttgcatatcaatcagca

caggcatcagcgacaatcccaccatcatcaccaggatcaggatcaggatcaggatcagcatcgcagctcatcctcaattcctcatcacaagctcgtcg

cagcaaaacgagctggcatccatcgcactcgcaagattatcacaacacccgacaagttctcatccaacatcgctctccctaccacaagtcacaaccac

aaccacaaccatga

>contig_14.contig_14.g584.t1

atgcgcttcgtctgtcacgatattcgacattggcatacgagtagcagcggtcgaagcgaccgcctcacggacacccaag

tcgccagcatcagctacgtatcttgccccgcctgccaaaactactaccgtcgcgccgccgacggcacgcccaatgaccacaactgcacatgcgcctcg

tgcggcgtggatcttggacgtggacgagcggtcgctcacgattgcagcttgcccgacagtgcccaccccggtactacacaagacgctctcgacgaggc

cgacgtggccgctttcctcggcggagaagttcctgcaccggcggcagcatcaccagcagctcctggtccggcgtttcctccggctgaagatctgtcgg

acgaggccgcccttgaggagtttgtcctgctcctcggcatggccaacaccaccctcccaaccggcctcaccaaggattggcgcgcgctttgcggaaaa

ctggcccaacggtatacgaacaaccccagcacccgcgccttgctcgacatcctggcggcaccaaagattgccctcgcccgccttcgctctcctcgccc

ccaacacaaaattaccgcccggatgcacggttacccggacgttccccgccctcctccccgcaaacgctctctcggccctagttccgaccgccaagtct

ccgagcttgtgcatcgccaactgaagaagggttacgcgcggcgtgcggcgagggtcctccgtgccgagtcgtccatcgcgcctctcacggaagatgtc

ctcgctcaactccgcaataagcaccccgagggtccggtgaacgcgtttcaggcagaggggaagaatccgcggcagcaccacttggcggcagagaagat

ggatgtcctgggcgttttcaagtcattcaaccgcgaggtcagcggcggctgctcagggtggactcagcctctcctcaaaatcgcgctcgaagaagacc

ttttcgtccagttcattcagctcctctgccgccagatggcgcttggatcggcacccggacgagtactgctgggggcggccagcctcacgcctctcaac

aagactggcggcggggtgcgccccattgccgctggtgagcttctctacaggctctgcgcaaagatactcctcaaggtcctcccgccatcggacgcttt

gttgccatctcagctcggtgttggatctcctggcggcgttgagccgattgttctgcgagatctggcggcggagctgcaagacttcctcggcgagaatg

cgcgcctcgtctgctacttcgacgattggaccatcctcgccaaggatcccggtgtgctcgagaaggtcaaagctttctttgcccgcaaggcgtcagcc

ggacagtcgtgtggtctcgacctcagcagcaccaagtgtcgggagttgagcagggacgaggtccagcgggacggagtgcaggtgttggggacgatgat

cggcggtgtggaggcgcgcgcgcggttcctgcgggagaaggtcgcggatgaagtcgaagtcttgggtcgcttgcgctcactgggacgacaggatgcgc

tgctccttctcaccaaatcaattcagcacacccttcgccacctactccgcacactcaacacggacggcatcacaaatccctggcacaatctcgaccat

gcccttcacgacgcgctcgtctttgttcgtggctccagtgcccatgacgaggaggttgatcggattctgtttttcgctaccggcacgactgggcggct

gcgcggtccccctgagaggggccctcgtcaacgagatcggctgaagccggtgtgggccggcatgctcgctttcttggccgttggcccaagtgaggatt

cgcctccccgctccatcgaccagctcgtcaccgtcctcggccacagctccccactcggccggcgttggctctctgtcttccctttcgacatcaactca

caactttctgatcgagaagtacaggtcggcctgcagctccgaactctgcagccgatgccacacgctcgctgcttggactgtggacagcgcgcatcgca

tggacacgacgacatatgtgccatggtcacacatcgacgggtcaagcggcataatacagctgctcgtttcatggacgatggtctgcagcgtgtgcccg

ggacgatggtgatccgtgaacctcatttgatcggtgacaaccatcgcaccgacttgcgtgtctcgggtccggggggctcctcgcggggtcctcaccga

gttcgacctcaccttcatctccaccacctcaaaggaggcacaggcgattgcccaccgaatcgagcgccagctgctttccacaggacaggaccggtggg

cgatcgctaa

>contig_14.contig_14.g585.t1

atgccccatcggacagctctctccctcgacaaagccgctgccaaggttgctcccaaagcacaccacctcgtcatctcca

accccgtcaactccaccgtgcccatcgtcgccgaggtcttcaagtctttacgaccccaagcgtctgttctgtgtcacgggtcaccacctttgacatca

cacgcgcctccaccttctacttcgacattgccaacgtcaagcccgccgaggtcaatgtgtccgtcgtcggtggccaccttcggcgtcaccattgtgct

tctcctctctcaagtcgcccagggttccatactccaatag

>contig_14.contig_14.g586.t1

atgaatgagaagagagcaccgtccaccatcctactttcatcattcctcgattctcccctagccaacagtacagttctgt

cacccagatcctcctctcgacccgccatcccgcccagcatgtcttcgggacaggcgcgcatcgatctcgaagtggactctcgcatatccggcgcttcg

ccatcgcgtgacgatgctgcaatgccatccggagcatccggacagaataggtggagtcaaataggggggagaggtacaaagcgaagatctgatggggt

agtctacactcccaacaagaaacgctgcgcagtgtggtatgctttcggtgatcccacaacgcgatctgtgtctgccaaggaccgcgccaaagctctcg

gggtgggagcaccaaccctctatgactggaggaagacatttccggaagcatggagtgggatcggctccctcgatactagctctcctgtagcacagcag

atgcgcgtcataactggacgatctgcagctcagaaccttgccttctttcgtacaccggacgatcctgccacgtggaatcccagcggtagcggaccaca

aacagcctccgcttctgcctcccggccgtctctcaatgatcctgatgccttcgctcgtcctgacggtctgggtcccgaggcggagcctcaagagacat

ttggaggccgtgggcctggaattcttcctcctgaactggttgagcagctagcaagggaaggagctcttgctacagacgaggaagtggccgctgttcat

gctaccacactcgacagcgacacaggcactggagcggcgccctacatcaatgagacgatcgacctcacactgcacctcaagcaaagcacactggcgcc

taaggtcaaggagaattgggaccgctgcaagcggaacttcttggtcttttggaggtgtctcaatcgtatcagagctggacaagaactacaagaaggcc

agcagggcggaagcgcacagaggcctcgctcggcagagactggagagggtgaagatgagttggaggaggtcctggtgtcggaacagaagttggtcacg

tatttgaactgctttgtgcttcacaagaaccctcgaatcggagatgagggtgttgaaacacacatcaaggcaatggtcaacttgtgggagacacagac

tctgcaaggtcgcaacagcttccctcacccgcgtgatggggcgctgatcaaggcattcacgaaggcattgcgtactggaaggagtgcattggccgcag

cgaagctggacgacgcgtggaagcactcgctgcgggacggctacgatccggacgagcacaacaagatcagcaaatggtattttgaacaagccgtggtg

gacccacagggctcttgggctcgcgcacggttcgatttcctgatgcagcacgccattatggggcgttcggaagacctgcggaatgccaagctctcctc

gctttaccttcactcatcccgaccgcaggcctctaaggcagtcgttgtgagcatcaagcacagcaagaccaacaccgaagcccgtcgggagttcggca

ttgcagcacgtcacaagaatgaagacgtctgtccaataggcgcgatggcgttctacttctttgaccgctttcacgtcaagaatgagcccttcccggac

ttctcgtctagggcggcgtggtacaatcttcaattgctggtggatgaagacgaacgtgggggagtgacctggagtgaccaagcgaagatcttgcgcaa

ggcgttcgaggaactcaacatttcttcctccaagctcactcacgccatgcgcggagcagcagctcgccacgcgcacgaagccggatgttcggagtcaa

gcattcgcattcatggtcgatggactgcttcaggggatcagttgattgagcgctacctcacaggcattgcggtgcagcccgtacgcgcgctttcgggc

tttagcatcagtggaggcgaccattacctgcctcggactcttctcgaccccccaaccgacctcgctcgcaagctctggccgcaggtcgacgaggctga

gaccgctgtgcgagctagacaggcagcgggtggagagaaggatgaggccgcactagcattcctcgagatgatgcggtggttgcgtttagttctgcttc

aggatgcagcattgttgtcgacaacctacccctctctgccgatctggtccatggaaccattcacgtcgacggagttccacgcttttcgctctgccctc

accaccacgatcgctggcacaccgcaccctctagaggtcacagtcacggagctcataccacgcctgggccatgcgctttcagacttgcgaagtagtgt

cgccgagacgacggaggcgttcaagcaggcgttctccacgcaggaggcggagaacgaccgtctccgcctcagcttcgaaactgccatcgccaccttat

ccagttctatcgagcggcgtgaccgagcagacgaggctcgagtcatgacacagcaagtcatcataagcagcatcaccgctcttgcaagcgcatcttcc

agtctcgagtcaccccacaccagtcaacacgagcagcagcagcaaaccatccagcttcttgttcgccttctgtccaacagcaccgtttcccctgctct

ttccggccctccagtgtcggctgcaaccgtcccagctgcgtctgccatttcttctccgctttctggatcccagagtctcgagctcacaggaaccacac

ctacttcatcttcgccttcatcacctccggtgctttcctcctcagctgccgcacttaccggacctgcaccaacatcatcccccgtcccgcctacaaca

cccctcgtcccgcctgccaccgcgccctgtccctctctgcctgctgcaccagttcttctcccgcgtaccatcctttgccttgcagcgacggtcaagac

agtcaatgaacttgcaagggagtggtacgagggcagagatggatacatgggtatttgcgctcgtctgctcgcaaaggattaa

>contig_14.contig_14.g587.t1

atgtatcaggactacagaccagctttggcgttccccaaggccgcagacccggatggtcctccaccattggcatggtacg

aaccgcggatggccaacggcatgcaaatcattcagccgcatgctcatagctatggtgggatccgcaaggtcggtgacccatacgacccctgggattac

cgtgacgaggacgggacgggtgaccttttcgaagagcccagctacccggacgacctatttcaagatccttatggcgaattcgacaagcttgggtggct

ggcaagccgcaacgccgacctgcccttatcatctgccgtggagaagggggatgtcgatgcagtccgcgccttgttggacgacagcacgtcggacagca

agaaagcccttctcagcgcatcgcatcccgacacgggcgaggccgccgtgcacatcgccgcagcaacatgccaagccggtattctcgaattgctgttc

aaggctggtgctcaggtggacatgctcgaccgtcaaggttggagtccgatcatgtacctcgccttccactccggggagcccgttcgcactggtcgggt

ttcagctactgcccttcaagaatgcgtcaaggtcctgctggataatggtgcggatccactcctcatatcggctgccaatcctcgaagggacatatcgg

actggcatggcaacagcatcgactgggcttcccactcatggaatcacaccttcttacgggccgtgatcccccgcttgtctggagaagctccattgcga

gaagaaccgattccaccatcttctgacccaaggattacatggggccttccccagccaccgctcggctcacggttcgtcacggatagcattcacccgtt

ccggcgcagcgtcggccatccacgcagaatcatcatcagcagtgggcggtcccccttcgataaaccatcttctcctggcgactactcgtgggatttca

ccggatcgcgtctggtcaaggcagtcgtgtctgagaatgccaccttgccgatacatccgaccaacgacgacgttgcgtacgggaacatttccgagtgg

gaaaagaccctggacgcgctgctcgatgggggtgccgaggtcaagctgctgccagagctttggtacacaggccatttcagcgtaccagcccgcgttgc

tctgaggttgttcgatgcgagcaagatcgatgtcacccaagacgttcccttccatccttctcaaaactcaaacggaggctctcttgccgcacgcatcg

tcgaatccgagctgtgctccgcgttctctgaggcagttcccctcgtccaaggactgaaagatcgcggcgtgaatatcgtcaacgccaaactgctgccg

aagccatatgcggacgtccctcgccttcaggctccgctcacgcatctgacccaagcagtggctcgacgcacggcgcaaaagtacgaggagcaagcacg

ggagaacggcgaaagtacggaaaccattgagagagaaagcttggaaacgttcaagcgtgtgcatgggcagccggtacccatcctcgctctcgcagtgg

cgacgttggacccggacctcatcgactacctcatcgggctcggcgtatcgacagaacctccaaaggacttccgatggagcgacttcctgccttcgccc

aagctcgaaacgatttggccaactaccaacaccttctgccgacatctgcttcccggccggccgtgtggcgacgacttgttcgctgcacacctcctccg

ccggaatgccacctgtgccaagctcgactccttgggcctgtcatga

>contig_14.contig_14.g588.t1

atgtcggtgttgacgctcaacacgccgcagcagcagcacacgcgtctcgctggcacgacgcgcagtggacgcacgtgcc

gcctcccagctcggtttcggcgcagtgaccacgcggacgacgaggagcatgtggaggacgaggatgaggacggggacggggacggggatgaggaggac

gaggttgaagctccgacacgtgctcgaagcagatcagtatctgcaccgagatcaagagcgtcgtctgtaacgccgcccggagcaggaagtggaggagc

gtcagcggacgacgagcggccctttcagtgcgaacatccaggatgcggcaaggctttcaagaagcggaacaagttgaacagacacatgctctcccata

cagacgagcgaaagtacgtctgccagcatccaggctgcgacaagaagtttctccggtcccagcatcttgctgcccatcagcagacacattccggccca

tcatcatcaccaatgttatcagccatggagctcgacggcgcagcgatcagtgaggcgcaaaaacaacacgtttgcacccacccgatgccgaccactgc

cgactcggctcaaagccctgtcccagtctgcggcaaacgcttctggaccaaacagaaactcgacagacacctccacaccgtacacgaggtctgtccac

ccggcgaggaactcagcctcagcgctctcgaggacgaagagggagcctcaggatcaccactcggcacgagcacgaaaaagtcggctaaactttatcct

tgcgatcaggacgagtgtagcaaaattttccgcaaacgcaagcatctgcgcgcacatatctgggacacccattcggacgcgccccacctttctccata

taccacaccctctcgagccattcgtgcctctgttgagcctggcacccccgccgccgccgccgccgccaacaaccaccctgagcgtgatgttcgacagc

ggcccttcccatgcaactggccaggctgcaccatgcgcttccccacaaactcacacagacgcatacacgagcaaaggcatgaagaaaagaagcaaaaa

tcatacacatgcgccttgccgcatccgccctcttcttcctcggaaggaagtgcgggaggaatgctcaccttccccacgtggtccctcctccgtgcaca

catgcgcgaagtccatcctccgcgatgtcctcgggccgagtgtgcagtcgatgccgcgtcaggcttaggaaggagcttcaggagccaggagggtctca

aggctcatttgaaacggcacgaggagagggacgcgctattggaggcggaagaagaggcggaggaagctgaagaagccgatggtgatgacgaagacgag

gacgaggatgaggacgaagaggagcacgacgacggggatggagcccagaatgcgtccgggcccttctacggcgacccaacaaagacgggctaccggtt

cgcatgcacccattcatctccccaaccatcacaacagcagccgacctgtcctaaacgcttcaagactgcccgagcactcgacgtccacacccgcgtcg

tgcatctgggcgaacgcagattccagtgctcgagaggctgtggacgacggtttggatacaagtgtacgaaggagcggcatgaggctgctgagaagtgc

gtccttgctctcgctgacgatagagaggaggaggaggaggagatggaggacgattactttcgagaagaaggaggggcggtacctgagcgtgactcgga

gcgggttgggccgtcgacaagcagggctcaacctgcaccagcgattccggctgagctttccatcctctccccacctcctcgcgctctcgtggaggcag

cgaagcagagcacgaatgcagagaaccaggatcagaagaaggcgaaggagaagaagaaggtagcgcataccggtcgagcgcgtggattgcaggcgtac

ggagtcggctcgtcttcgaccggccagtcgtcctcctcaaggtcgggacagggacggtcgttgtacgatctcttcacgggcgaaggctacgcgcccgc

gtctgctacggtatctacggcccactcctcatccgagagcgcggagaagaaacgcaaacgaccgtccaaatctggtgaggaggacggagaggatgcgg

atggggaagaggacggggaaggagaaggagaaggaagaggaggaggcgatgaaatgcgtggcagtgcctcatcaccaccaccaagcaagaaacgcaaa

atacgcgatcggatcttcccctgcccctggcacaggatcgtcgaactacaacgcaaagcagacgccgaggccgaggccgacggtgggagcgacgatga

ttatggtgttgaggaggagggggagcggcaagagccgttgggcccgcttccggattgcggattccggttctcgcggatgtacgacttgcgtcggcatg

tgcgctctcggcatggagtggacctgaccaagtttgacaagggtgcgatggaggttatcatgcagcagcagtag

>contig_14.contig_14.g589.t1

atgccagcgcccacagtgaacggaagcagcaagaagcgcaaggcagacaacgcggtaggtgatcacggcagcaacggaa

agaagcagaagaagaagaaggcgcgcaacagcaagtacaagaacgcgcctggcatccttccccagccccagccgcagtcgcagcgcaatgtcgaccca

gacatcctcaaagcgcgcgctgccctccccgtcgcggcaggaaaagacgccatcgtggccgctctccgccagcacgacaccatcatcgtcctcggaga

gaccgggtccggcaagacgacgcagatcccacagttcctcttcgaggcaggattcgcaaaccacaaggacggcggtacaaatgcgaacaagaatcgac

agaaactcatcggcgtcacacagcccagacgggtggccgcgacgtcgctcgccagtcgcgtcgctgccgagatgggatgtacggatccagccaagctg

cccctccgatctcacgccatggcagccaaggcaaaaggacagaataacaaggctcccgccctggttggctactctatccgcttcgacgatcgttcggg

tccggaaacaagaatcaagttcatgactgacggctggattctgcgcgaaatggtcggtccttcgctccttgccagcaaacgggcggagaaacaggctc

tcattactgccaatgggaagaacaaggaaaaaggaaaggacaaggaagaaggagccgtcgatacgtccttcagcgagatgtcctccctgctcctttcc

tacgccgttatcattatcgatgaagcccacgaacggacactcgatacggacctcatcctcggcctcgccaaacggatccaagcaagacgcaaagtcct

tcgctctcagtggttgaaagatcaccaggccgatcctaaccacccactggcccgcgttaccgagctcaaacttgtgattatgagcgcaacattggatg

cccgtgccttttccaacttcttcgcttccactccacctgtcctttctgcagcctcctcatcttcatcacagtcttcttcgtcaccagcgccgattcta

tacgtcgaaggtcgtcaacatccagtcaccctctaccatacccccgccccactccaggactggaccgatgccgccctccgcaccatcttgcagatcca

cacctccaaacctgccccgggcgacatcctcgtcttcctcacaggacaagaggacattgagaagatggcggcgtctctcaggacggtggccgagtgga

ttccggattggctggagacgcgagggaggctggcggggacgaatgggatagaaaatggggcgagtttggaagcggagaaggatgggcagtcttcttcg

gaagaggaaagcagtgagagtgaggaagagtcgtcggaagagactgcggaacagtccaacatcaatcccatcgatcgtatcatctccaacggtctctc

caagaaacacctgctcaacggatcttcctcctcgcgtccctctacaaaactcgcctctcatcacgtcgctctcaacccttccggcgtcccgaccgaac

tcctcatcgcacccatctacgccgccctcggctcctccgccaccatccccgtcttcgccccgaccccacccacagcccgcaaagtcgtcctagcaacc

aacatcgccgagacgtccgtgaccatccccggcatcgtgtacgtcatcgactgcggcttcgccaaggagaagacgtactcccccgaatcgggcatcga

gatccttcgtgctgtccccatcagtcagggtgcggcgagacaacggatgggacgagcggggcgactgaagcctggggagtgttatcggttgtatactt

cggacgcgttcgagagtctcgacgaggtcgctacgccggagatcctcaagacggatctgagcagtgcggcactgcagttatacgccgcccagctcgac

ccgttctccttcgactggatggacgcgccggagatgggctccttgcaggctgcgttgcttgcccttgttcagctgggggcggtcagcatacctgatac

ggcgaggggaacggagaacggcctggtggcgcggatcacgccgctcgggcggaggatggcagccttgcccttatcgccagtttacgcccgaacactga

tcgcagcctcggagcggggtggacccgccgtcgctcgacaggctcgcgacctcgtcgcgctcctcagtgtggatcggagcgtgtttgtggatgcgtct

gggatggcgcatggtggggataagaagaaggcgggagtagcgtcgacgatggacgtggaggaggcgcgcgaggaagcgctcgctcggcggatgcgggc

cgggttgatccatcggagcggagaccacgcgacggcgcttaacgtgctgtatgcgtacctcgaagtgctggagtccggcggcggcagtggtagcggtg

gtcggttcaagggagcacagcatgcggcggcggcggctagtgtacgggcgtggtgccatgccaactacgtgcatgagaagacggcgaatggagcggag

gtggatgacaccacgagcgacagcagtgcctcacccgaccacctagcgcaatccgacgaggaggacgacgacgataccaccacgggtagctcttcacg

gggcctacagacgggagctacaagcggatcggaaaagggcaggcgagttgcttcccacaccttcaagattcatccgagctcggcactgcatggtcgag

gggcgagtgcgatcttctttgaggaactcgtccatacgacgcagcatttcgccaggacggtcagtgctatcgagcctgtatgggctcagaccgctggc

gggggtggtggggagaagtaa

>contig_14.contig_14.g590.t1

atggcatttccaagcggcagcagtaacagcaacggggccagcgggaacggcggcggcggcggtgggagcttccttggga

ccagcaatgcaggcagtagcagtctgctcaacctcagcggtctccgaagccagagcatctccggatcggcggaacaggccgaggactcctccttctcg

ccgcgatccaagctggtcacaagctaccgcgcgctcctctccggcgagcagccgtggataacgtcgtcagcgtcgtcggcagcatctggcaaacggag

agccgctccgcgagatacctacctctccgacttgctcaccgtgccccttcccagctccgcccagctcgcccagctcttccactctctcttgccgcccg

cttcgtccaacacggcgtacgcgctgatcgatgacgatccgaacagcgcgggtgctcgtattcgggataccatctcgggactctgggctcatgcggta

gcgctatggctcgcctcgcccttgcaggacgacgactcgtcgtccatgggccgtcgcgcgggacgcagcggcagcgggagggatgatgacaatatggc

cgcactgagctcgcctttacgactgaatgcggtccagacgctcctggcgctgtcgtctgtcatcctttcccagcgctttcccaaccacaccctcgatc

tgatcaacgtcttcgcaggaagtatctccgaggcggatgatctgttcatccagctcgtcgtcggaatcgatgatgtcttatccgaagactttccgcgc

gtcgagcatgacagtcctgagctcgatctggacgactcgtctctgtctggcgcaaagcgcggaatgcgaccagcgcagcatctgcatttgcaacacat

gacagtccagctcgttctcctctggctgtcttacacctcccacacctctctgggcacctacttcctccgccgagacctcttcctctccctcacctctt

tcctcgaacgaagccctctcacctcccgcttcggattcgagatcgggcttgcgattggtctcctggccggtgtgggccagcagggtgggagtctgggt

atgagcacgcactccaagcgcgcctcgctcagtatcgcacatggcgccggtccgaaccctctcagcagttcacatccgtacatccggcatttggccct

ctggtatgcctccgcctcgtccgggggcagcagatccgcacgctctggaaactacgtgccccgtcccgggatcgacaccctcattctcgtcggagctg

ttgccttcgagaagcaaggatggcgagcatacaccgctgtccagcctgacggcgaggaatcgctcctggagggagaaggaggcgacagtgtaacgtat

atggaagcgggtacgaatgcgatgagagccattgttggagctcctatcaaaggcgtgcaggtgattggagatgaagtcatgcagctctcggaaacgtt

ggttggtagtctgcggtggatggtaggtggtagcggtagcacggccgcgggttcaagtggatcaggaggggtcaccgctgatccattcgttgtcgcac

acagtcccaccgcgccggccgcgtacctccttccgctctacctattttcccgatcaaatccgcacttcatacactccaccttctacacgtctgagcag

agctccagcgcttcccgtcgtccctccaaaccaatcaactcaacctcaccaacaaatccagacgatgcctccccagcaccaattccaccacctcagcc

agaccttcccgtctctatcctctctttcgcatcctatctcctcacgcacgcctcggttgcgccgccttcgtcttccgcagctggggctgaggcgtact

ccgaagtcggtgagaaagccttactgcgggctgaaccggtcgggacgtccggcccttctgcttctgccgagggaactgacaaggaaatggccgcgacg

agttctcgtgtaggacgggtgcggagatgcaagcagcgcgagcatgtgggcggaggtgggacgggctctgcagcttcgagttcgagggtggcaaggtc

ctcttccaacgcgagcgcgggatcgtcattcctgccttcatgggcgacgtttggtcgtaccaaggtcgaggcgtctgcaactacggatgctgatggag

gtgcgactctggccggtcccggtgatgggccgcgattgattggatgcttgctggatgtttgtggcgcttatgtgcgcaacaacgtcagtctgcgcctt

gacgtccggtctttctccctggcgttgcgaatcattcggaggtcgatgctcctgttgtcagccgctcggattgagctgaagaccacgactctcggtcc

atcggcgttggctgcgctgaccgacgctcgcgatatgccgccgtaccaatttacagcggtaattctgcgccccctcctctccctcgctcgcttccttg

cgacgcgatctgcgaacgagctccgtctcagtgcagcagccgtctccctgcctggctccacctccgcctcgaattcttccggcgccaacgcgggtccc

gtctccctctcctcaaacgcagcactggcgtccctgattcgcctgctccttctcacactctcgacgtacctcgtcaatgcggatcgtctgctctcgtc

tgcagctgaggtgcatgaggtgatatacgagcttgtacgagaaggtgatgcattgcgcaagctggcggatggtcttgtgcgggacgtgttgggacagg

gattggacagtcttccgctgcgacctggtcacaggaatgcgaatgcttcaacgcggacgggcgcctcaacaggccctgttggcaatggcaacatatcc

gcctccctagccgccgacgaaacagtccgctcggtgggctggtcgctggcgctcgagcccgtcctggacgcagtagactccaagttggcctcctcgcg

gacagtccttcgcgactcagcaagtgtgatgaagctcatcgcggagctggacctggaggcgttgctgggtgctgcttctcttgtgggggcgggctcgg

ggtccggcttaaacgcgacgatggtcgggcctgggctggttccactgtcatcgccgcctgctgctgctgtcggcccgggtgcggcgccggcgtccttc

tcgaccttccgcgatgggttctcgtcgtcggcgaatgattccgctgcaagctcgggcgttggggacggatcgaccacgggattggagacgacattggc

accgagagtctcggcgctcttggatcgaggcgagctggatgaggcctggtttgaggaggtcgaggaaggcgcactcgcggaagtcgcaagggtggttg

ctgtcgatgtcggggagatgctgcctattcaatga

>contig_14.contig_14.g591.t1

atgcactcatccgcgcaccaagctctcaccgaacctcacaacgagctaccacatctaacctgggatagatcgttcctga

cgctcatgacccacgccaagctctccaaagaactttccagcctcgactcatcagctcaccctctctctccgttctgcttctgttttcacttcttcaca

gcttctgtcactcccgcgtttgaaccgccgcgatcgctttaagccaacaccacccctgagaagttcgtcaagaccaccggcaaccctatcgccgggac

caccatgaggtaccgcctcgacgatgcccaacacaggaacagcaacaccatcgtcgagaccgcgctgaactacaataccaacgcgcctgaccaggctt

gtgtagctgcattgaccgccgcgattactgagacgtccatctacatctacgccaacaaccaggtgcaggcctcgatgaactaccgtcacgacaaggtt

gcatccgagcaagactatgtcggtctattccagtatccagccagcagattcagggacatcggggccgatatggatcccgctaaagccacccagatatt

cctgtaccagatggtccgcatcaggggatggcagaacatggaccccggtgtgctcgcacagcaggttcagcgccgtggaactgctgcgcgttaccagc

agtgggtttctgcggctcgcaatctttgccagttcggcgagggagataatggaggaaacagtggaggatataatggaggcaacaatggaggatataat

ggaggatataatggaggaaacaactacaactactag

>contig_14.contig_14.g592.t1

atggtgcgaaggccgtgcaatgatccgtacaagtcaaaacagctcaccatgtctagccctcagtttaccctctattcgc

acaagggccctggcccgaaccctcccaaggttgccctcctgctcgagtggctcaacctgtcatacgagatcgtgccccgcgtcttcggggacggcccg

aacggagtcaaggcccctgacttcctcgccctcaacccgaacggcagagtgcccgccttggtcgaccacggtaacaacgacttcacggtgtgggagag

cggagcctgcttgatctaccttgctgacaagtatgaccaggagggacgcttctacggcaagaccgccgaggagcgagccctgacggcgcagtggctca

cataccagctcagcggtctcggcccatctcaaggccaggtgaactggttgattcactactttgagagcgcgtcaggcgtcaaggcccagcctgagatg

cttaagcgattccgtgacgagaccgatcggctctacaagattctcgatggccagcttgccaggcaagccaaggctggctcgcagtacatcgtgcacga

tcgtatcacgattgcggacttcgctttctacggttgggtacgtatcatccacatggccaagttcgagttgtccgactaccccaacgtgcagaagtggg

cagacaacctcgcgaacgatgagcgtacgaagaaggcgtacgcgaagttggagctgaagttgcatagcagagatgccatgccttcagactactacttg

cactga

>contig_14.contig_14.g593.t1

atggcagcccccatcttgccagtcatcaacattcccgtcgattacgcggaggagaaagaaaaaattaccgacttcctgc

gtcacttcaaaggagccgtcccagcactgcccaagccaagctcaagcacagggtcaagcaacgccatcaatggaaaccgcagcgccgctcacactgac

gacgatgatgaggacgacctcgacgctgagtttgatcttgaattcgaagatgacggtatggacgatgtcgtccagggcgtggaccgaatggccgcagg

tgttcagactcggggcgcggcgcgcaataagcttaagtacatggagcaaatgcaacgcatcgcaaacagagaacaagacctcctcgttatcgacctca

aagacgtctctaagcaccgcaacaccgtcaataacgagtcttacgcacctttcgtcgccgccataagcgccaatgcccgccgctatgttgacctcttt

tacgagtgcatcgacgccgagatgccggacccgaccagggacatcagtcacaaggacgatgtattggatgttattatgcatcaacgtcgagagcggaa

tgctcgagccgctgccgacgctggtggtggggcgcagggtgatggcgctgagggagcagcaggcgggcaagatgaagaagcgcccttccctcccatgc

ttctgagaagatatacactttacattaagcctaacacaaccaaggcggatgctccgctagccgtccgaaccgttcgaggcggccatctgggcaagctc

attactgtccgcggcatcgtcacccgtgtctccgaagtgaagcccttcctgctcgttgacgcatacgcatgcgactcctgcggtgccgaaatcttcca

ggaggtcaccagtcggagctacatgcccttgacgcagtgcagcagtctgcggtgcaagacgaatcagacgaagggacaactgttcagccagacccggg

cgagcaagtttgtacccttccaggaggtcaaggtacaagagatggctgatcaagtcccaatcggtcacattccgcgttctatgactgttcacgtatac

ggtcccctcgtgcgctcgatgagcccgggcgacattgcccatctgggcggcatcttccttccgatgccttactccggcttccgagccatccgagcagg

gctgctgaccgacacctacctggaggttcagcacgtccaccaggtcaagaagcagtacatcgacatggagctcactcccgagatcgaggagcgcatag

cccatctgaagtccgatccggctctttacgctaaactggccgcatcgattgctcccgagatttacggccacgaggacgtgaaaaaggtgctcttactt

ctgctcgtcggcggagtaactaaagtaatgggtgacggcatgaggatccgcggtgacctcaatgtgtgcctgatgggtgacccaggtgtggccaagtc

gcagttgctcaagtacatctccaaggtcgcaccccgcggtgtttacacgacgggccgcggttcctccggtgtcggtctgacggctgccgtcatgcgag

acccggtaacggaggagatggtgttggaaggaggagcactggtgctggcggacaacggcattgcgtgcatcgacgagtttgacaagatggaggaaggc

gaccgtacggcgattcacgaagtgatggagcagcagacaatcagtatcagcaaggccgggatcacgacgacgctcaatgcgcggacatccatcttggc

agctgcgaacccgctatacggacgctacaacccccgaatctcgcccgtggacaacatcaacctgcccgctgccctgttgagtcgattcgacattcttt

tcctcatcctcgacacgccctcgcggctggacgacgagcgtctggctcagcacgtcacgtatgtgcacatgcacaatgcccacccgcctctgcagggc

gacggggaggacacgctggacccgaccctgatgcgacactacattgcgctcgctcggcaaaagcggccgacagtacccaaggcggtatcggactacgt

cgtgggtgcgtacgtgcagctgcgagcacagcacaaggaggacgaggcgcgcgatatgggttacacgtacatgtccgctcgtgcgctcttggcgatcc

tgcgtctgtcgcaagcgcttgctcggctgcgatttgcggacatggtggaagtgccggatgttgacgaggcactgcggctgatggacgtgtccaaggcg

tcgctccacgcggacaaccgtgctggtggaagggatcagcaacatggcggagtgggcatcggggatcagagcttcgtcagtaaaatttatcggatcgt

tcgggagatggcgatggctgcgggagcggctcgggacgaggcacgtcggcatcgacgagctggtggcgctggctcgagtcgcctgggccgcggaccgg

agggcgagcgtggtgggaatgcgatggaggtcgacgaggacgttgacgacggacttttggatgccgagggcgaagacgaggacgagactgtgggccgg

cctgcggaactgccgcttcgggaggtgcgcgatcgaatcattgcgagtggatgggtagaagaccagcttcaggactgtctcaacgcatacgaggaact

cggggtctggcagatctcggagacgggggctcggctggtcttcctgtga

>contig_14.contig_14.g594.t1

atgtcgatgccagccgtcaaagggctcgatgcagccctcgacctgatcgagtccgagttcgatctggagaaggaagacc

tgtatgccatcgtgcgcaagttccaccagcttttcgactacgggctcaactatgacaatgcggatatggccatgatcccttccttcgttactggcctg

ccgaatggctcggagaaggggacctatctcgcgctcgatctgggcgggaccaaccttcgcgtctgtaagatagagctgctcggcgacggcaagtttga

gcttgatcagcagaagttcaaagtatcgaatgagctcaagacgggccctgtggccgatctgtttggttacatggccgaatcggtgggcgtcttcctca

agagcacgggtgctttgccggggcagagcgacgagcctctctctatgggcttcacattctcattccccgtccaacaaagctcgctcgactccggaacg

ctccttcagtggaccaagggcttctcctgcccagacgccgtcggccacgacgtcgtcaaactcctccaaaactccctcgactcgcaaaaaaggcgtgt

ccgcgtccgagccctggtgaacgacaccgtcggcgcgctcctcacctcgtcctaccagacgggcggtaagacgcttatgggtgccatatttgggacag

gcacgaatgcggcgattgtcagtgagattgagcgggttggtaaaatctcgatcgggaaggatgggaaggtcggaggagttggcaaggatcatccaaca

catatgctcatcaataccgagtggggcgcatttgaccagaagggatcgtcactaccttctaccatgttcgataactttgtagatcggctagcgatccg

acaacggcatcacaggttcgagaagatgatcagcggaatgtatctgggagaaatctgccgagccgtcttcctctacctgatcgacaacttcgtcctgt

tccaaggcttttcctccccaaagatgaacacccagtacggcttcgacacagagtacatgtccacagtcgtggccgacaaggccgacccgttcgaacgc

gactgcgcgacgcggaaagtcatcgttgagacgatgggcatctcggatcagtacgtgcaggaggaggacgtattggcggtgaggagagtatgtacggc

tgttggaaggcgggcggcgaggttgagctcggtagctgtcgcttcggtcctcctccacacagggttcgcagactcgcccaagatcccttccgcccttc

gctgcgaacgcgccggctggcctccacaaggcggcggcgtcaccgtcggtccaaaaggcgaagtccacatcggcatggacgggagtgtggtagagcac

tatcctgatttcgtcttgatgctccggcaagggttgatcgacatcgtgggcgaagagacggagaagaaggtcgtgatcagtatggcaaaggatgggag

tggagtaggcgcggcgttgggggcgctgcaggcggagaagcaggagaaggcggggcatgttgtcgttgccgtaccgtcacctgcacctacgcaggtga

tccacatgttcaggattgttcgacggtcggtcgtccgcgggaacgcttcgacgagcaccacgctgcaaacactcttgatccaacgcgacgatacgctg

tacgaccagacgcgttcggccgaggacacaacaacagccggcagatctttgccaaggcaaagggaccgtggccgtgaccctcgctgcaatagtgccag

ccaaaagacatcggtgtcgtctcctggagcctggacgcagccactgactcgacagacaaagcgggacaagaaggatggccccacagcacccgaggtcc

acggcaactggtccgttgctctaggtgccgatggaaagatggagcacacccgtcaccaggatgacgacctcatctccctcaagcgcaccatccaccgc

gaccgggcccacttacccgaaacccgcgagcgtacgatcgttttcaacatcggctgcatgctatctcaactcctgaagcatgcacatatcaagaagct

catgaacggtactacgactcttctgatcaaagtatcgaaagccgacggattgatagcatcagtgcctcatcctatcctgctcagatggaacgttccag

gcgccagccttttgaacggtggtatgtggtgggacgatatcagcaatacatag

>contig_14.contig_14.g595.t1

atgactgcttcggcgtcattcatgcgtggcactacgaggtccgccgctggggtcatggtgcgggttgcggatggaaaga

gcgttccggctacgcatgtagcagacgtgtccgccgtcctccgaggccaggatcgagctcagcactgcgtcgcccttctacacaacgttctgctcgtc

cccagcctcgacgccaacctgttgtccacccaccagctcgcacgacaagggttcatcaccatcatcctccgccacagcaccgtgcttgtctcgccgtc

cggtctcgtcattcaggcgcagattgacccctccacgcgggcagcgtacttcctcatccagcctaaccacttccaaggttcagaggaggtacatgcga

acacggcaagtgtctcgcagaagtcaggagacggtgtgcctgataaggcaattgtggacccaagggcggagctttggcataggcggactggccatgca

ggatgggctaagttgaagaagtgggtggagctcggcctcctagtcggcaaagatgcaccaaccatggctagcatcggtcattacctcaagggcggtcg

agtctgcgacgtatgtgtcacggcaaaccagaaacacgcgccgtacccagatcgtgcaccgtccagagccgatacaccgggcaagcttgtgcattgcg

attacatgggcccgttcaccggccaccccagctttgcgtggatcctgacactcgtcgatgactgtacgcgcaagacttgggcattctgtctaccagac

cgttctgccgagactctcacggcaacgctcgacgcctgggcacagcaagtcgaggtcaccatcagccagccggtcgtgaccatgaagaccgacaacgg

gaaggagtttgtgtcggacgagcttcaagcatgggcaaaggagcgtggtacgacatggacgtggacggctccctactcctcgtcgcagaatggggtag

cggagaggcgcaacggtctcctcgaggaacggatgcgcgcaatgctccgtggtgcacgcctccctttcgggttctggccttacgcagtccagtacgcg

gcgtatgtgatcaatcgcacccccagcaccactctagaagagcagcgccaagaagggaagctctcggcgcggggccttcgcgggatcttcttaggatc

attggcgctagaggaacccaagccggtacagccccgaaatgacgaccatgtggagatgcagctcatacctccaaagcagcgtcaacgcggtacacggc

aggtaccatcgtcgcagccctcagatgccgtgctacctactggaacccccagcgatgaggatttacagctgcaggagtataaattggcgggcgagact

caaaccctcgaacctcccagttctgtgctcccacgaccggaaatcgaagagcacgtctatgagctcgaccccggtcgtgtccccaatgcggcagatgg

gatcctttcgctgtgggagccgtttgggacggatccgagcggaccacagtccgaaatcgaccttgccacgcgcattcgtcaattggagcgcgtgtggg

agggagactcggccacgccgtctacgatcatgagtctcgatgatctgggtgccctgctggatgaacaggaacagggacctccgcgcgcatccacacct

ccgccatcaaagcagcttcgccggagctcgcgcatccgtgcgggggcacgcatggcgcatggactctcggagacattcgatcgagacgaggcggcaga

cgacgagcgggtggcggtgacccagttggttcatgcaaggtccgccatgtccgtgctcggtatggctttgcattgggcttgcaccgccgcagctcatg

ggactagtgcagacggatcacctcttgagcctgtcaatctcaagcaggcaaagaaccgacctgagccctgcctgttcgttcggttcggtaccggcgga

ccagtaatcctcgccgtgtatgtggacgatatcgtcatcgctgcacgtggagcggagggagtcgctaaggtcaaggcagaattcgcctcttggtacaa

gatcacagacaacggggatctctccttcatcctcggcgtcaagattgactacgatcccgttcgccgcacggctcacctcagccagggtgcatacatcg

acgggctacttcgcaagtatggcatggagaaggaggatccagtgccaacgccaatgacggaagcatcacggcatctagggcctaatctggcaggccag

gcgagtcgtggcggtgtcttcgaccgaagcggaactccattacatatactcctcgctcgttcggctactccgcagaccatgccttaccaacctcgcaa

ggtgtacatcgtcggcgtcggctcgaccaagttcagcaagcccggagaggctgactacccggacttgggtctggaagccgctgtgaaggccctgctcg

acgctggtctcacgtacgacgacatccagagcgcggccgctgggtacgtgtacggcgactccacctcgggccaacgcgtgctctaccagctcggccag

acgggcatcgagatcaacaatgtgaacaacaactgcagcaccggttcgtctgcgttcgctctggctgcgcaggcggtggagtgcggtcgcgtcgaatg

cgcgatggcgatcggcttcgagaagatggcgtccggctcgctgggctctacttttaacgaccgcgccaacccaatggagggcaccatcagccacctgt

tcgagctcgagtcggagcgcgaatacgaagaggccgagtatggacctttcgcggcgcgcatctttggcgcggccggactggagtactgcgagaagtac

ggcgccaagccggaacacatccacgagatctcgtccaagaaccaccgtcactcgaagaacaacccctacgcccagttccggatcacacccagcgcagc

cgaggttgccaaggcccgcaagatcacgcgcgagatgacgttgccgatgtgcagtcctacgagcgacggcgcggcgtgcgcgatcatcgcctcggagg

agttcgtcaaggcgcacaagctggaagaccgggcgatcgagttggccggactaggtatctcgactgacacgcctgcgctgcatgccaaggacagccgg

attgagctggcgggtgcggatatgacgcgcaaggcggcggccaaggcgtacaagcaggcgggcattggagcgaaggatgtgcaggtgattgagttgca

tgactgctttgcggcgaatgagcttgttacctacccagctttgggcatgtgtgcggagaacgaggcgcacaagattgtcgagcgtggtgacaacactt

tcggcggcaagtgggtggtcaacgtctccggtggactcgagagcaagggtcacccgctcggcgcgacaggcctcggcatgatcttctacatcgtgtcc

cagctccgcggcgaggcaggtccgatgcaggtcgacaacgtcaagtacggcctgacgcacaacattggcctgggcggcagctgcgtggtcacgatcct

gaagcgggccgacttctacaatggcaagccgggcgggttcaaggcgcgcttgggatacaatgctgggtttgagtgcaagacgattacttccgatgact

tcgagaaggtcaagagcaagtcgttcaccgactacctttaccccagcaagctctga

>contig_14.contig_14.g596.t1

atggtagctccgccttccgtgcactcaccacagatcctcaagatgcccccccaagcaaaccaccgagggagaacccatt

accggcgcagccgcgtccggctcccccccacgcgcgcctcgacccagccgacgatgtccgatctggcactcatgctcgaacgcatggagctcaaggtc

gacggtctccaagagacacaggccgccgctgacgcgcgcatcgcgggattgacgtcgaggatctcgaccgtcgagcgcagccgcgaccagcgcgcacc

gagcccggacaccgttcgtcgcgatcaggtcgagtccagcacggagttcgtcgccgaccgacagcttccagcaccgatcgcccgcgttctgccgccgc

acatcacgtcggtgctgcagccgattgccgaagacagcttcgccgaccagagtctgtcccagcggctcatcgaccccggtgttcccgacagcgaccga

atgcgcatcctcgaggaggatttcgaggagcaggctgctcgtggcggccgcaaggtcgagaacaacgcgctttggcgcgcagcctacgagattgtccg

tcgcgacaacccgggcgtcaatcgagcggagcgcttccgtctcgccaaggccatggtcgacgaacagtccctcaagttcgcggccacggctccgccac

ccccacgcaagttcggcgcgggcagcgacaaggatgattggctcatcagtctctccggcctccagcgcatcacacagctggctgaggacacctacgag

gcatggtcggacgacctttggagttacctcagctccaaccccaaggcacgcgcgatgatcttcaacaactgcagtctcggttacgatcgtcgccttga

cgacgagctcggaggtctgctcggcctcacggtcgacaactactacaagaagacgacgatcaccgttctgcgcaagcaaggggagactcgtggcacgg

tcatcttcgcgcagctcaaggccgatgtcaatcgggacagcgtgtcgcgccgcagtctccttcgacagaagatgctcgctacggttcagaagtcggcc

gaaggcatccaggagtacgccgaacggcaccggactctcttcgcccagctcggcaacatcgacgacacgctcaacgagcccgagaaggtcgaggcctt

cctcaaagggctcaccccggggttctccggcctcaaggaggccctctacacttcgcaattcacctccggacgagcgctcacgctcaacgagacggtca

atttcatgatgacgatggaggagaagcgcaccttgggcaccatgaccgcgcctcctcaggtcctcccccgattcggagcgcgtcaagctgctgtggat

gtggatgaagaagccccgaacactctccagctcgagcatctcaccgaggaagaggcacacgccttcgtcgcccacttcaacagcaagttccgtcccgg

ttcgcgccctggattcggtacccgatccagccctccatccggtcatccacaagaggccgattggttcgcgggggaatgtaacttctgcctaaaccccg

ggcaccgcgaagcgcgatgccgcatgcgcgcacgtcttctcgacggcgccggacccggctccaagtacgacaaagatgtcaagccggccgctcgtgct

gtttccggcgaggacgtcatggaggaggttgacgctcgcctcgcgcagctatttcctcaaacctatgggagtgacagtaaggttgaagccagggttgg

gcaagttctctcgggtaatctggaatag

>contig_14.contig_14.g597.t1

atgacacgcagcactctcctctccgacccgcggctctacctcttcctcgccgtcctggtgctcgcctccctgcccgccg

tatccgcgttcggagcaggaaacgtccccaactggtccttctcagagggctccagtttccgccatggtgacctcgaggacatactcgtcgatctcctc

aagaagacgggtggaggactgctttcgcgcggttccaagttcagcggattggatgtcaagcgcgtctactttggtaactggctgagggattatagtca

ggctatggacgtcgccgcgctccaaaagctcaagccagtctctatccttagcatcgtcatggtcctcggcttcatggcgcacggctacgtcaccgaag

aattccaggtcaccgacgagcgtctgggagtctaccttcccactgagcacattgacaacccaaagggctacgccgataacaaggacgcgcgcgagtac

gaccgtcgtcttcgcggacccgtcgacccccgtgagctggaaattgacccgcgctcaggaatgaagaactacattgccaatgagtccggtaactggga

cacctcctccgccctcgtccgccgcaccctaacccaatgcatcgaaatgggccgccgagcccgctccaccggccgcgtcgaagacctctacgaagcct

accgtctcctcggccaagccttgcacacgctcgaggacttgcccgcgcactccaactggtgcgagctcgccttgctcaagctgggcttccgtgacgtg

ttctgtcatgtgggcgacaatgtgcgcgttcaggcgccgggcggggagagggttcctccgcttgttacgggcacgtttggaagtgctgatttcatgca

ctcccttcttggagaggctcaagatcatttgagcgaagcttccgtttcggacctttccaaggccgtcgagggagcccgctcccagcagggtggcagcc

gcggtatcggagaaggcggctcgggtagtgctttcagcaacctcatgggactgctcaacagcgttcctggaggtcccgatggctctgtcagtcgggat

atcgaggatcttagtcgggcgcctgctggtggtgtggaaaacatgagccccgaggagatgtaccagaacctttggcgcattctgtcccttcgcgaccg

gatcatgaagagcattgagcagacgattgagaagattccgggcctgggctccttgctcgagtctgtcagcaacgcgatcagtgtcttcatcttcacca

cgctcgagccgtacctgaagccgctcgtcactcaggcgatgaccggactctcgacgggttcggcccaggtcatcaagaaggaagaccaattcgaggtt

ttcaacgacccaaacgcgtccgaccctacgcacagtatgctggcgaaggatcactttgccaagatcctgaacgaggtcgcgggcaatgttgccaagat

tatcgtcaagcacactgtgaacaatgtcgtcaaggcatgggatgatcagcgtgcgagtgtcgaccagattgtggatgagagtcttgcgtgcatgttcc

atccctactggcaccaccctcaggcgatcgtccagcgcgagatgctcgagtacatcgaaagctggtcgcgcacccaccacaacgagatccgccgtctg

gatcgccagaactgccggaaccacaccgacacgcgcaccggcaaggccgaggagcactcgcatggccacggcggcggcgggaacaacaacaatcaggt

ccctggtggagtgggcggtaaatttggcgcccaagctgcgggagcgttctcgaattacatggggaacaaggtgcagggcatgatgccgcagcagtttg

ggtcgagagaggctggtgagggtggaggagagagtgcgagctactatggtggtgggggtggagatggaggcgagggtcgtggacatggtcagagtggt

agtggtgttggtggcggcggggactatggtcggaacgagtcggaggggtacggaggtcatcgcgcgcagggcagtgggggaggcggaggatacggtgg

aaatgagggcggataccgtcctcagcagccggagcctcagcgatatggcggtggtggggatggagggtaccgtcctcaagagtcggagcctcagtatg

gctctggcggaggtgggggtgggtacggtgctcccccttcgatgccgaatgagaggcactaccttggtggtggtggtggtggtggcaacgatggcgga

tatcctgatgctggtggatatggcggacaacagcaacagggccctcctgatttccctgctcccagctttggaggcggcaattcgtacgacggtggtaa

caaccagtcgatgtacccgggccagcagcagcagcagcagcaaggccagtaccctgggcagcagcagcaggggtggaacccgccgcagaaccagggat

acgggggcgggggcggtaactaccagcagtactga

>contig_14.contig_14.g598.t1

atggcaagtagcaatgcgaagaacacgaacagccgcctctgcgcgcgctgccagaccgcccgcgccgtgctaaagcgtc

ccaaaacaggcgacaccgtctgccagccctgcttctaccacatcttcgaaaccgagatccacaacacaatcacctcctcccgtctcttcacacccgga

gaacgagtggccatcggagccagcggtggcaaagattccactgtactcgcgtacgtgctcaagctgctcaacgaacggtacggctatggactcgattt

gttccttttgagcatcgatgaggggattactggctatcgggatgactcgctcgagacggttaagaggaatcagatgcagtatgggctgccgctcaaga

tcttggggtatgatgagctctatggatggagcatggatgatattgtccggcaggtgggccgtaagaacaattgcacgtactgcggtgtcttccgacga

caggccctcgaccgaggagcagccacgctcggtatcgaccatatcgtcaccggccacaacgcagacgacatcgccgaaaccgtcctgatgaacatcct

ccgcggagacatcgcccgcctcgagcgctgcaccgaaatcatcaccaaaggcccagacaccgaaagcgacatccccgactccaccaacccctgttccg

ccggcagcggcggtgggctcgaccacatccgccgcagcaagcccttcaagtacgcctacgagaaggagatcgtcatgtacgcctacttcaagaagctc

gactacttctcgacggagtgcatctactcgccgaatgcgtatcgcgggtatgcgcgggcgttcttgaaggatttggaggcgattcggcctagtgctat

cattgacattattcattcgggggagagcttgaaggtgaagggggaggtgaagagggcggtgcagagaacgtgctctcgctgcggatacatctcgtcga

acgaactatgcaaagcctgtgtccttctcgaaggcctgaaccgcggcaatccagccataggcatccgctccgagcgctctcgagcggtcaaggacgcc

ctctcacaggaagccccaccttctcaagctccagctcccagctccatcatcgacagaatacagagcgcacgagaggccgctgcagccttcgcccaata

g

>contig_14.contig_14.g599.t1

atgttgcgcactaccttccaacacgccgctcgatgcggcagccgaatgcaggctcagcagcagcagcagcagcagcacg

ccctcaagcgcacctacgcgtccgccccaccttcctcaggcagcaacagcagcgtcccactctacctcgccgcaggaggcctcgccggtctcggagca

tggtacgggctcggaggctttggtcccgccaacaaggagaaggcacaggccgctgtcgacaaggtacagtccgctgtcggacaggcgaaggaggctgc

cttgtccaaggacgcctttgttgactttgtcctcaaggaggtcaagccttacaaccacgactcttccaccttcgtgttcgagctgcccggcgacaaga

gctcaggtctgaccacctgctcagccgtcgtcgtcaaggccgccggtgacggtctcaaggatgacaaaggcaagcccgtcatccggccatacactccc

atcaccagccctgaaacctccgggcacatcgacttcctcgtcaagaactacgagaagggcaccatgagcaagcacatccacaacctcaagccaggaga

caagctcgccatcaagggtcccatcgagaagttccagttcgaccgaccgaacatcttcgaggaggtcggcctaatcgctggcggcaccggcatcaccc

cgcactggcaactcatccacaagctgctcaacgaccccgaggacaagaccaagatcaccctcatctacgccaacaagagccaggatgacattctgttg

agggaagagtttgaccgctgggcacaacagaagcctgaccgcttcaaggttgtctacggtatcgaccagggagaggcgcccaaggggggcttcaaggg

gtacttgagcaacgaggtcctgagggcgaacctgcctttgcccggtaaggcggacaagatcaagctatttgtgtgcggaccgcccggtcaagttgagg

ttgtttccggcggcaagggtcccaagggctcgcaaggagaactcaagggtattctcgcggacctcggctactcgcctgaccaagtatacaagttctag

>contig_14.contig_14.g600.t1

atggcatcgagctcagcaacaacacacctcacaaggagaatagcagcgggagcaggaacgacggcgcgcggggtgcatc

gctcgtttgcctcctcatcgcgctcagcagacagcgaaccaagacgagctccaccaaggatactcccgcccctgctctcgccctcgacctcaagatta

tcaggctcaggctcaggatcaggatcaggcggaggaggattagcagaaagatcttcactgctggcatcgccacctccaccaacaacagcatcgcgcta

cgtcctcatctccaacttacccatcaactcaagaccatcagacatctaccgtctcaccgacccgagctccctccaccgcctgcgcgacctccactccc

tcaccttcctccgcacgccgaccctcaatccgacaggagaggccatcgcagacttcataaccgatacatcggcacgccgattccagcgcggcatcgac

ggtagccgactcagtggacttcatgttcgcgccacgcagctggctccacaaaaggccaagcagatgctcacctctcatctatcggccaacctctccat

tcagctgatacatctgcttctcataagccagacctcaggattcaccgtcctcctccgcggcttaccaccctccctctacgtctcccgtctgcacgagg

tcctccacaagggatactctctcgctccgcctaccaacatcagacagagtcgcaaaacgattcatctccgctcccttttcgacacaagctcgatcatc

acccagcgcacaaaagctgtcttgtcaaaacggaacgacgagctctatcgcgtcttggtccgcaagcgcaaaattggtgtgaatgctctgcgcgcgtc

cttggaagaagaggcacaagaacgagccggcaacattgccaacaataattcaaacaatcaccaccgcgagtcagacgaagctgacaacgcagctcttc

agcatgaagaacaagagccatcggacacagacaacgacctagagtcctaccgccctgcctactcctcccccttcggcccttcccaaatcgacggcatc

gaaggacccgacctccgtctcgactacccacacgaggacgccgaaaacccatatgcatacaccagcttcggcaaggcgtactgggacgtcatctggca

agccgccaagacgggctactcgatccagcgtctcaaagatctccaacagccgcatgcccaagagcagatgcagggcatgactctcggcgatcatgatg

atgatgacgatggcgatcatagcagcagtagtctggcttcggcaccgggctttgtgccctttttgcctgtcaccaagctgccgtacccgtctgcgcgg

ggagaccataaccagacccgggcatggttcctcgtccgcatgactgatgctgctgatgcccacaggcttgttcgtaggtggcataacatgtattatgc

tgagagcatatttggacagcgctatcatgtcgaggcgcaggtgttgcattga

>contig_14.contig_14.g601.t1

atgcagctcaactccaagctctccctcgtcctctgcgccctcctggtcgcccagcccatcgcgtccgctgccattactg

gcgacaccaacgcagtcgagctccgcccccgctctgacgacggtggcaagaagggattcgacctcggcgatgtccttcgtgccggcggtgtcttcgga

ggagacaacaaggacttcggccagggttctggtggtggccacttcggcaacgacaagagcaacaaggacaatgacgacaagaagggtggagacaaggg

tggcgacgatgatgacaaggacaagggcaagggccacggcggcggacccaaaaagagcttccgcgagaagaagctcaaggctctcaaagccgctgcga

acgaggacaagcacaaggacgctgcggctgtcaaagcagccgccaagttcaacgctgcggctgccaaggaggaatctcaccacaaggaggcggacttg

aagaagttcaagaaggagaagaagcaggactccggcaaggacgactttggcttcgatcgccgtgatggctttgggatcggcggtggactcgacttcgg

aggcgacaagggcaagggccacggaggtgacgacaagggtggcaaggacaagggcaagggcaagggtttcggcggtggatccaacaagaacaagaagg

agaaggtcgagaagatctcgaagcacctcgacaagtccgacaagcacaaggctgcggccaaggtcaatgcggccgccaagttcaaggctgcggctgct

aaggaggatttcaagcataacaaggccatcgcgaagaagctcgcgaaggagaagaagaagagcggctttggcaagggcttcttctag

>contig_14.contig_14.g602.t1

atgcagctcaactccaagctttccctcgtcctctgcgccctgctggtggcccagcccatcacgtccactcccgtcgcca

gcaacaacaacgccgcccagctgactcctcgctctggcaccatcggttctggcgagaacaagggcgtcgacgttggcaaggtgtttggcaacttcttc

gaggacaacaagaagggaggcaacgaccacggcgatgaccacggcaaaggccacggcggaaaccacaagcttgccgagaagaagctcaaagcgctcaa

ggccgccaagaaggaggacaagcacaaggctgctgtggctgtcaaggcagctacccacttcgatgctgccgcctttaaggagaactctcagcacaaga

aggccgacctgaagaagttcaagaaggagaagaagcaaggctccggcaaggacgaattcggtggcttcgaccgtcgtgacggcttcggtttccgtgag

ggactcgactttgaagaccttggcactggccccggtggctttgagggcggccgctacggtggatttgaaggcagcgtcggcaccttcagtggcagcag

cagcacgtccggcttccaaggcggtagcttcctcggtgctcgcagcttcgacggcggaaagggcgggaagggcggctctcgccacaagaaggagaagg

tcgagaagatcgaagcgcatctcaagaaagacaagaagcacaaggctgcggcggaggtcaatgtggccgccaagttcaaggctgctgctgctaaggag

gatttcaagcacaacaaggccatcgcgaagaagctcgcggagaagaagaagaagaacggcttcggagtgggcttcatctag

>contig_14.contig_14.g603.t1

atgctgctgccgcctataactgaagcagaagcggaagcagaagcggaagcagaagcggaagcgaaagcagaagcagaag

cagaagcagaagcagacgcacaagcacgagaagaaaacgacgaggacgacgacggagagaacgacgaggaggagggcgagcaagacgacgagcaagac

gacgaagacgacgacgaagacgacgacgaagatgacgagtga

>contig_14.contig_14.g604.t1

atggagagagggagtaaacagcatcgtgccgcgacgcacacgcgtcacgcgcgccgacacgtgcactttttgaccagat

cacccaggaacgccaacccagcaacaccaacgaccgtgctgctccgccagaagccccacaatggctcgaacaaccaggtcttccggaacgaccttctc

cgtgataaccttgtcccccccgcgcccgaagatacggatacggatgagaatggcgtaacccagcaggggcgccgcatgcgcacccccgtcccggatgc

tccagatctcctcactagcgccgaagccgagcacgcgagcgtcttgcccgccgccaaacgctacaagctctcgaatgccgctgttcatggcttgaaca

cggcagcaaagatgatggtcgatgctttcgaggctgaagatggccatctcgaagatcgcatcgagatggaagtggacgggcgtgctcaggccttgtcc

acgacagacatgatcgtggagctgctgaaggaggtgaagctcttgcggaaggaggtggaggtaatcaacagcaagttgccgacactcctcccgctgtt

ggaagtcagcaacacagttcgagaggaagggagagccaatatcgaagcagccgtaaagaaggcctggtggaaccccaactgccccggctactctcatc

actgccgcgccatcgaggagcttgtcgtccgtatgacagagaagaaacctgagatgctgggtacgaacggaggacagctcatggacatcgacaatgcc

gacggccatgatgcagtcctcagcgtcatcagggcatgcatggatagcatgcgctttgcttggaagcagaagatctacgacgcagctggcggaggaag

aggaattgacaagatgaatctgaaggattttgttgaagatgttcttctaagctgctctcagctcaagctcacctggggtctgatttatcgggtggctt

tgcttcgagcccttgctctcaaagtcgacattaaggacaccaagggtcgttggaaccgcgacttctgggccgagctggacccggcggtcgcggaagtg

gtggatcaagcctctggcacggaagaagagatcgcaacgtcgctggagtcggcactcagcaagtgccagtcgaagttcggaaggtgcaccttcatcaa

cgtccctcgccaatccgaggaagagcaaagattcatgaagatgtttagcaccggcaaagccttgtag

>contig_14.contig_14.g605.t1

atgtctgatctaccggaggaatttgaggagcttcccgcctcgtccaaagctcgctgcaagatctgtcctggtccacggg

ttctgaaaagaagcaaggtcctggaccacgctcgttcctctggtcaccagcaccgtttgcgactccccccttgtgctcttccctccaccagtacccgc

cggcaacttcaagatctcggcgcggcagtgtctgctgcagaagacgaaggacctccgtgcgatgacgacgtgcagcatgatctcgacggagtcgagta

cttcagtcattggacgtttgcgccagacgtcaacgagcagcatgcatctgcccccaacatcgatgcagcgctgccaactggctatcgatctacctacg

aaggactgctgcatcagcagccctcggaggacgacattcctgcaggcaacaacagtcttggtcgagatgtcccagaggacgggaacaatgagaacagt

tcggaaagggcaatcgatgcgttcttgcaggcctttaagctgatcggccaatctgacgtgcgagactgggcaccatttccggacagagagaccttcct

tgtggcggtcgcctcgctcaatccacgttctcccctgagcgtctctgagatcaagacgatgctatccttcgcccacgcggtaggaggcgaacgagtgc

caagctacgacacgtacttgaaagcactgaagtctttgcttcgcgagctgcgggacggcgaagaattcggaaagcagcctggactcggcaaggctcaa

cctatggtcactctgcgggacggcacaagcgcttatattggggttccgatagctatcgttggtcaagatagggccattcttccgctggaatgtcaagt

ttgcgatgtggctgttgacacgctcacaacttcgtccagttttgctcggggtatccgccattaccgcctcgccacggaccttcccggctatgagttcg

tcaagagcctcaggaggcgattcccgggcaagatggtgtattccctacccatcattacagcgatcgatgattggggcggaggaaaatcacgcaagtgg

gatcctcaccatagcctcctcctcatgcttgctggcctcgaccgaaaagaactcgataaagagcagagcattcatttgtttgccgcctcgaaccacgc

caatccctcggagctgatggaagcgtttgttgcggagctgagccacggtccgctcgacgtatgggactgccagtctcagaacaatgtcatagtcgtcc

cgtaccttctggctgtgtgcgcggacaaccccatggctgcagagttgtcggcatcttccggtatgcaggccaattttccctgccgcatctgttcctgg

ggaggaaaattcgttgaaaaggcaacagaagatggggtacgccaaatgattgaaggtggcagtctgcgctctcctgaaaagacggtcaaagaattagg

caaacagctgcgattggcctccacgaattgcaccgagcagacactgctaccactcgccttcgacgttcacgcccaaactccccccgaatgtcttcaca

cacttaccctcggtcctctcaagtatctggcagcaataaccggttccacattgaacgatgatgctcgcgatcatcttctgcttgccctggaggactgc

gccaccgagggcatcgactgcggcaagacgtttcgagcctcgtatcatctgcaacatctcaattcactggtgggacgggagcttaaggtgctgagcca

ggtgatggtcgcgtctatggctcccttggtcaagaaggaactggtttctgtggacctacaggaggcgtggttacgtgcagcggagctgtccaagctgt

tgtgggtggacgagattcccaggaacgaattgcaggaatacacggtgagcaaggttgacatacagatttcagagttcgatgaaatgtttacacttcag

attcaattgtactgtcataaggaccgattaggacatgccacgagggcgttgatggctgccgttgcaaagatagcacctaagtcaatcgtctctcgacc

gaagtaccacctcctcacccacgtccctgcagccatcaggtccttcggagtggccgctaactttgccacggagcgaacggaacggttcgtcgccgtgc

aacgtacagcggtagcccactccaaccgcatggcgaattcgagagatgtggcccagcgactgctggaccaacagcttttgcgtcacctgatgacgggc

ggataccgcctgaatcatcagcaagcaagggtcgaacaaccctgccacagcatggtagagatcatctctcaccgcaagaatcgaatcttccgggagaa

atggggattgactagcatggataacctcccactctttttcaagtctcagggtcgtattcatcacattcgctcgaagggagaagaaaactgtgctgtgg

gtaacttcgtctactacgatggggatcccgatgcgagagcccagctagaggtcgggcgtatatcgggattccgactttccgatgaggatgaatgcctc

gtcacggtggaacgatactcactccatgccatcaatcccaactacggcatgcgaagcattcgatcaaccggcatctctatcacggtggctgtcgacgg

acggcatcaacggtatcttctcaaccacacgatgctctgccacccacttcccttgttcaggagccgaatcagccagcaattcagcgtctccaccatcg

acgacgtcacgacagcgtttcagaccgggatggagaaccacgttcagcaggagcaaaagaaacgacggaagcgcaatgatggagctgcttcaacattc

gaccaaacacaacatgaagacgacgaattctctgaggacaacgacgatgtttag

>contig_14.contig_14.g606.t1

atgcgattctcctccctcattgtccttttgcccgctcttgctagtgcctttgcactcgactcggccacaaaagccaaag

tcacccctgagaaccctaccaccggcaccaccatgagggtccagctcaccgaggcccaagagaggaacaccagaaccatcacccagaccgcgttgaac

tacaacaccaacgcgccttaccaggcttgtcttgctgcaatgaccgccgcaattactgagacggaaatctacatctacgccaatagcagggtgccggc

ctcgatgaactaccattacgacggggttacaaccgagcaagactatgtcggtatattccagtatccagccagcaggttcaggaacatcggggccgata

tggatcccgcttatgccacccagatattcttgtaccagatggtccgcatcagcggatggcagaacatggaccccggtacgctcgcgcagcaggtccag

caccgtggatatcctgaacgttaccaacaacgggttgtttcggctcgcaatctttgctcgtggggcaaggaaataaatggagggggaggatactaccg

taaggcgtag

>contig_14.contig_14.g607.t1

atgactgactttgtggtattgaccagcgatgagggacaggaggatgtcaaacagacgctcctcagcccgctttacttgt

caagaacgatgtgcaagaccgacatcatccacatcatttgtccgcactgcttcgagcccaccaacaaggagcatcgcagcttcaccgcctgtaactgt

gatgactgcgacgatgacgaagagtacaacagcaccaagtgctcactcgtctcattccgactcccaccaggctccagcaatcctcgtcaatacttccc

gtctctcctctccggctctggttcaactttgtcaccgaccctcgcatcaactttcgcattcttacccaacagcaagctttcgcattcgcaatttattg

gtcaccagtcctccagccttcagttcttccagtgtaaacgtcacctcaaggctcaaggtatcgcatcgaacaagtcatcaaattctctcatgatttca

tccaaacccccccccaccgcgggcgtcgctactggggctactggagctgcaggcagccgagataccgagaccgaaccctttctcagtgtgatcccttc

ccgtgatggggccaagctacctttcgtaactgatgatggcgttctcatctctctcagccacagcgcatcgctcaatcgatccacagccctgatgtacc

ccctttccctctctgctggtcagtctggatccccctcgatcttagcgtggatgtcttcaccaaggttgtag

>contig_14.contig_14.g608.t1

atgcagctcaactccaagctttccctcgtcctctgcgccctgctggtggcccagcccatcacgtccactcccgtcgcca

gcaacaacaacgccgcccagctgactcctcgctctggcaccatcggttctggcgagaacaagggcgtcgacgttggcaaggtgtttggcaacttcttc

gaggacaacaagaagggaggcaacgaccacggcgatgaccacggcaaaggccacggcggaaaccacaagcttgccgagaagaagctcaaagcgctcaa

ggccgccaagaaggaggacaagcacaaggctgctgtggctgtcaaggcagctacccacttcgatgctgccgcctttaaggagaactctcagcacaaga

aggccgacctgaagaagttcaagaaggagaagaagcaaggctccggcaaggacgaattcggtggcttcgaccgtcgtgacggcttcggtttccgtgag

ggactcgactttggagacttcggcactggccccggtggctttgagggcggccgctacggtggattcgaaggcagcgtcggcactttcagtggcagcag

cagcacgtccggcttccaaggcggtagcttcctcggtgctcgcagcttcgacggcggaaagggcgggaagggcggctctcgccacaagaaggagaagg

tcgagaagatcgaagcgcatctcaagaaagacaagaagcacaaggctgcggcggaggtcaatgcggccgccaagttcaaggctgctgctgctaaggag

gatttcaagcacaacaaggccatcgcgaagaagctcgcggagaagaagaagaagaacggcttcggagtgggcttcatctag

>contig_14.contig_14.g609.t1

atgcccagaacgaacggttctgcgcagtacggcaagcttgagctcagctggctcttccactacgtcgaaaaacatcgtc

cccatggcagcaactactgggagaaggtagcgaaggactacgtccttcgggctacgagcaagggaaagccgccccggaccgctgccagcctcaagaag

aaattcgacaacttgtgcaagagtgtcaaaccaaccggcgatcctacttgtcctcctgaagttcggaaagcaaagcgacttaggcgcgatatcgagtc

cgagcacgcggtggagagtacggaggacgacgaagacgacgcagatttgggtgaagatcagcctcaagaagacgacgacgaggccctggaagattcgg

acgacgacgaccccgacaacttcgacgtcacggattcggaggacgacgacgaggcctcaccacccgtcgtcaccgcttcctcgtcgtctgccgcccta

aagcccccctccaccaccaccagcattccctccatcagctcggacccgaccatcaccaccaccaccagcatcccttccccaaccgccgacgccaccac

ctctcttggccccgccgcgtcgcttgctccctcctcgcttcccgcgacccccagggccgcgaccagcaagaagcgggcccggcctgccctcgtcgccc

cctcctcatccgcctcctccactcccggtcccgcttcgacctctgccacccctctcccagacccctcagccgcccctgcccccgtcttcacccccaac

ccaaagcgcgtcaagatctcggccaaggcgggggtcggggcgctgagcgcttccccggcgcctgcggcctctcagcgccgactcctgcagtcgctcac

gagcgctgcggagtcgttgcagctgacccagttggccttctcagtgaccactctgggcagtcagctcgctgcgctcacctctcaggtggcgcagctgg

agatgcaggtgatgcggatgagcttccaggtgtccaaccctctccctgtccctaaccccctctctgaccctgcccccagcactgccctctttgcctct

tcgtcctcttcctccctcccaaacacagtccccttcaccttctctcagtcacgcgacgctctgcgctcttccccctcccttggatccttcctccctcc

tcctcctcctgctccagcatcttctgcgtcttccatctcgttttcctcttccatgccatatcattga

>contig_14.contig_14.g610.t1

atgatcttccagattttccacagcacttcctggatcaaaaccttcctcctctgccaggtcaagttttcctcatctcctc

cacacctcacctgctcggctgctcaagatttccctggccgtgggcgtcgccgacgccaggactcgaatcatcgcctgctcgacggtcgacctctcttt

tcctcccccggcctgtcgtcctcctctcgctcggcgctctccggctctccgtcttccaccttcctcgccggccgcccacctcaccatctctctctgcc

ccggcctcctgctgccgcctctccgttctccgctctcgtccctgcgcctcatattcctcgcatcgtggacactgtcggtgcctctttctggcgcgcgt

ggctccaacagcaccgacggcgcgtgttgatgggtctgcttctgggcgctgctgcggtcatcctcaggccaaagcgccgccagccctgcctcactcga

gcctcccttctctctccatccctctcggctgcagcgcatctcctacacgcgcgcaaggacagcgggtacatctccgtcttcggcctagacgtcaagac

cttcgatctcctgctcgccaaagcgtcttctttcttcccgcccccacctctcaaagtccgtcgtcctcgtctgctctcagtccgcgcacatctcgctc

tcactctgtactatctcacaactccacaacgacagaaatcgatctcaatcgtctttggtattgttccgagcgcggtctcaccggcaatctgggttggg

cttcgcgcgctacgggtcatgatggaagcagacccagactattgggccatccggtggccttccccagacacgatgaaatccatggcagcagctgtcag

agctcgggagccgctgctcaagcacgtcttcggatttgtggacggcctcaacttgcgcatatatcagcccggcgacgttgacgaacaaaatgcatact

ataacggctggctcgcggatacctattgcacggcagggctgtacaagctccttaccaaccccacccgcacgccaggatccttcgccgtccttgccgac

tccgcctttccctacaccgcggagattgccgggcgcatcctgagcaggcccaaggacaagatcgtcggcaaggagaccgacgtcagcaagctgcggct

gtgggaggccgtaactcgccaacgtcaatcggcggagtgggggatgagggctctgcaaggcgccttcggccgccttgacctccgtctccccgtccaca

agaccaagcgagcgctgctcctctacgtagtcttccgccttcacaacttcaggacgaggatagttgggttgaatcagataaaagaggtgtatttccca

gaatggcaggcacgtcctaattga

>contig_14.contig_14.g611.t1

atgcgattctcctccctcattgtccttttgcccgctcttgctagtgcctttgcactcgactcggccacaaaagccaaag

tcacccctgagaagttcgtcaagatcaccggcaaacctatccccggccacgactcggccacagaagccaacaccacccctgagaagttcgtcaagacc

accgacagccctaccaccggcaccaccatgagggtccagctcaccgaggcccaagagaggaacaccagaaccatcacccagaccgcgttgaactacaa

caccaacgcgccttaccaggcttgtcttgctgcaatgaccgccgcaattactgagacggaaatctacatctacgccaatagcagggtgccggcctcga

tgaactaccattacgacggggttacaaccgagcaagactatgtcggtatattccagtatccagccagcaggttcaggaacatcggggccgatatggat

cccgcttatgccacccagatattcttgtaccagatggtccgcatcagcggatggcagaacatggaccccggtacgctcgcgcagcaggtccagcaccg

tggatacttccctctctttcacaacgacttctcgaattcatccaccatggtccagagtcaaagcggcaaaaagcgcccgtgtcatggagggaaaacca

tagtgctcatcaatcttaatggggcccattcagcgatcaacaatggcaagaacgtgatcggtcctgtcctccgtgcggtggatacatatcggcatatc

ttcgggaatgtgattcggccagctccaacctggttcgtcaatcgatgtctcatcgctcgtcaatcacagagacctccaggatcagactggcatgcttc

atcaaccaatcccgccgtcgactataaactctggcgagaagcaaaagcagaagatgacgaggaagacgaaatcaaaacacaagcagaagcagaagacg

acgaggacgaggacgacgatgaagaagacgctgaagcagacgacaaggacgacaaggacgacggcaaggaagacggcaaggaagacgacggcgataac

gacgaagatgaccagtga

>contig_14.contig_14.g612.t1

atgaagcaaaagttctcggtgtgcgaactcatcgactggtcgccgagcagcatctccagctcggcaagattggcatcag

cagctatctgccgtctaacacagtgctccaaggagaccaaaaagccaccgccatcgccgatctttcccggcctccatttcaacaacaagccagacact

gttgaggactgtgcaggaggacccgacatcgcccacaatctcacttcttcgacctattctagccgagtggtcgagctgagtatgagtgaccgacccaa

atatcctgccaccagtagactcaagacaaacgcgagcaggaacccacgatctggagcatggtttggcaacctcaacgtgatcttcacgcccctcttcg

accgcaatttgatcggtcgtactgccaagatcatgcaacccgctgatactgtctaa

>contig_14.contig_14.g613.t1

atgcgctccgcttccttcgccgccctcttcaccctcgtcgccgccttctccagcgccaacgctgccattctccgggccc

cgtgccgcaacctcgaccttgacaacatcaacaccgagaaccagtgcagctacggcgtcggcgcaaagcacgacgtcctctcatgtgcaagcttgccc

gacagcccgggcggcggtccactcgactgctgcgtctactcgactgtcgacaactggcggcccgaggttctcaaggagatcgtcggcaagatcctcga

cggctgcggcgctcctcagaacggcgcccagcccggcggcacggtcaacggacgctactacgatgaggcaggccagcgcgtctgcatcggtaacggag

acggctgtccaatatcacatcaatcgtctatgtcctatgaattgcgagcttccttctctgcatcattcgagtccggaccagactccagagatgaagag

aacaaggaacagcctggtattagcgaatgcgacctgaagcggtttgttgtgggctcggaactgggagatcgacgatttgtttgttcaggagttggaga

taattcggcttcggacttgtgcccagtcgacctggtagaactcggggccagcaacgatgtggttgttgagcagggtgaaaatttcgagacgtccctgt

tcatgagagctatgacgcggctcctgtggtag

>contig_14.contig_14.g614.t1

atggtttctgcgctgcagcctgcgatcatcaccgcgtcatctgcacgcatcgaggccaggcgccagctgccaggattca

ggaacagtgcagagggcgtcgttccgctcacggagtcatggcccttcactgaccactacagccgatcggcgcgggcgacagcactgcgaaccgagact

gctgctgaagagatcagcggtcactgtcagcgtttcggtggcgtgggtaaatctgcaattgcgccccgcacttctgtggcgcttggcaggcatgttct

tcgggatgccaaggcctactatactgctagcttccgaaaagctatgtaccgacgccttgggcgcttcggcctgcctgtgacacgtgaaagcgcaaagg

tttcgaacgatatgtccgatgcgatcgcagctgccgttctcagctgggcgccgggcgctgacgcactgatgctgacggctgatggctggtag

>contig_14.contig_14.g615.t1

atggctcgcctcgctttcgccgctctcgccgcccttgccttcgccgccatcaccaatgcaagcccactgatcgcgtccc

gacagaaccgcaaccctacctgcggtaccgccggtgatgccacgctctcggactgccaggcgctcgtcgacaactggcccaacttcggcaactacgag

cgcacctgtaccttcagtgtgagccaaaccgcctacaacccggcgtgcttcggcaactgctgcgtgtacgtcaccactaaccaggcccgctgggacga

ccccgacgccgagatcggtgacgtccgcaaggctgcccaggccattctcggatgcggcagtgccgagaagaactcggtcaacggtgtcatcgacgtcg

aacaggatggcgtccagggccgggtctgcctctccgacggagctggctgcggtgactgcttctctgactga

>contig_14.contig_14.g616.t1

atgaagttctcagcatctgccattctgtccctcgccctcgtcgggctctccgccgccgccgccgttcctcagcagctcg

tccagcgtggcacagtctctgacctcgccgacgtcggagccaagatcgcccaaatcatcctcaaggctcaggctgccgataacgacgtccgtgcccgc

ttcgcgcagaacatggtcgatggcttccgccgcgaatacggcgatgctaccaacatcgttgtgatccacaccgagcacgactacacatggaacggcgc

acagggagacgcctgggagcactggcactacgagcttgacgtccagatcggcggcaccatcggctacgagatgtacgcctccaaggtcggcggctacc

tcaagcgcaccggtgacggcggctctctgaactgggcatggtacggcatcctcgccaaggaccctgaggaggatggctctcgcctcacctttgccgac

acgggcgatctttga

>contig_14.contig_14.g617.t1

atgcagctcaactccaaactttccctcgtcctttgcgccctcctggtcgcccagcccatcgcgtccgctgccatcacca

gcgacgcccacgtagtcgaactcagccctcgctctgacgaaggcaacaacaaaggattcgatctcagcgacgtccttcgtgccggcggtgtctttgga

ggagacaacaaggtcttcggccagggttctggtggtggccacttcggcaacgacaagagcaacaaggacaatgacgacaagaagggtggagacaaggg

tggcgacgatgatgacaaggacaagggcaagggccacggcggcggacccaaaaagagcttccgcgagaagaagctcaaggctctcaaagccgctgcga

acgaggacaagcacaaggacgctgcggctgtcaaagcagccgccaagttcaacgctgcggctgccaaggaggaatctcaccacaaggaggcggacttg

aagaagttcaagaaggagaagaagcaggactccggcaaggacgactttggcttcgatcgccgtgatggctttgggatcggcggtggactcgacttcgg

aggcgacaagggcaagggccacggaggtgacgacaagggtggcaaggacaagggcaagggcaagggtttcggcggtggatccaacaagaacaagaagg

agaaggtcgagaagatctcgaagcacctggacaagtccgacaagcacaaggctgcggccaaggtcaatgcggccgccaagttcaaggctgcggctgct

aaggaggatttcaagcacaataaggccatcgcgaagaagctcgcggagaagaagaagaagagcggctctggcaagggcctcttctag

>contig_14.contig_14.g618.t1

atgttccagactctcagccgcgccttctcgccctccctcttctctcgctctgcagccgccttcggaggcagtgctcgag

tacagtaccggccccgagttggagcggtgccttcctaccccacgacgacgcaggaacaaacttccatggctaccaaggacactgatatcccttcagga

gccgcactggaggatatcgtcgtctccatcggcacgctgtccgcacctggcgtgcgcgtcgaagtctacaatgacgcctag

>contig_14.contig_14.g619.t1

atgaatacgacgggtcgcgacctggaagtctggaacgcgtgcgcactggccagcacatcaaacggaatccacctcgacc

tcgacctcgccgacgacagcagcgacgacgacagcgacgacagcgacgatccaagtgtcgcgccgcaggaaaaggcccgacgtccgtatctcatctgc

atcgccagcggaacgccgcttcccatccatactcagaccgaccaaggtggtcctgtccaggtcgcagtccccatcgctgatcccaaacgcgtggctgc

cgaactcgcaaacgactcccacgccgtctccttcaaagaagacgcttcgcccgaggagaaggcacgcgccgccctcgctcaaagctccaacgtccccg

gcggctccaagaagcagcccgaagaaacgtcgggagcgggtatggtctctgatatcggaggacatcgcgtcaaggcgaccaccaacctcgacgagatt

caacggatcaacgagctcgagaagggcgaggccaccgctaaaggcaccgcggcccaagctgcgggcgtacagctcaaagacgacgtctctgtcggatg

gaccggtcaccgcgacagcatcctgaagggagcagccgcgcccggtccggacgaggagaaactcgtgcccgaccacgagacaaagttggtccagaagt

acgtcggcgataactacatcggctacttctggtggaacggagttgccatggtcgtcaccatactctccacgtactttgcgactcgctttggcggcgga

ctcttcagcctgctcgtcatcggagccttctgtactacgtactacaatgcgtcgatgcgccgcactcgccagcgtgcccgcgacgacatttcccgcga

gctccagaagcgacacatggacacagagaacgaaagtgtgcactggatgaactacttcctcgaccgcttttgggccatctacgagcccattctttgcg

cgactatcattcaggttgtcgacggcatcctcgttgcgcaatgcccaaccttcttggactccatacgcttgaccacgttccgtctcggcactaaggct

cctcacatcgagcacgttcgctccttctccaagaccgaagacgacatagtccaaatggactggaagttcgctttcacccccaatgcgagtccggacgt

ccgtcagaacgcgatgcgcgcgaaccccaaaatcgtattgacagcacgcctcggacgcggcaaaatcggggctggtctgcccatcctcgtcgaggata

tgatgttcaagggcagcatccggctcaagatgaaactcatcagcaactaccctcacgtccaagtcgtcgacttctgtttcatggagcctccggagttt

gactatgtgctgaagccgattggtggaaacttgctcgggttcgacgtcggagttgttcctgagcaggttcacgccaacttgggaccgatgatgtacta

ccccaacatgttcaccatcgatctcgagcagttgctctctggcactcccctcgacactgcctgcggtgttcttcaggttactgtcttctccgctcgtc

agctcaaggccaccaagtttggtggtggacaacctgacccttacgtcgccatttccatcgatgacaaggatgtccttgcgaagacgaagtggaagcac

agcaccatcaacccgcagtggaaggagacaaagttcatcctgctcaacaacctcaatggcatgcttacattcgtgctcaacgacttcaaccagcaccg

cgcagacactcgcttgggtgttgccagcttcgacctcaaagagcttgagcaggatcccgagcacgagctcatcagtacgcctgtcgtctttgatgcca

aggaccgcggtgagatccaatattcgctctcgtactaccccgtcatcaagcccgaaaaggccgaagatggaaaggagaagccattgcccaacaccact

tctggtgtcgtccgcctctctcttttccaggccaaggagctcgacaagcgcacgggcttgatcaacgagatcaatccctttgcccgtatccgactcaa

tggccaacggatcaaagactccaagacagtcaagcggagccacaaccctgcgtttgacatcaacgcggagttcctcgtcaccgaccgtaagcacgccg

tcattggcgtcgagatcatcgacgaccgtgagctcaccggcgacgctgtcgttggccagatcagcatcaagttggacgacctgctcgccgccaaggac

aagcagcaagactggttccccctggccaagtcgaaagcaggtcgtctccgtatgcaggcccagtggaagcccgtccagatgtccggtagcaagaatgg

aggaaacgggtaccagcctgcgatcggtgttgtcaagttctggcttgacaaggcgatcgatgtcaagaacgttgaagcgctcacgggcgggaagtcgg

atccctacgtggcgatcaagctgcgaggacaggtcatggctgcgtccaccattgtcaacaacaacttgaaccccatcttcaacgaaatcttgtacgcc

tccgtgcacaaccttcgcgagaagatcaccgtcgaggtcctggactaccaaaactctggcaaggaccgctcgatcggaaccatcgaggtcgagcttgc

aactctcgccaagaagagcgagcctggctccacgttgccattcgagagcaccggccgccaaactatccgcgctcgtctaaacctgggccgaggaattt

acaagggcgagctcctcttcgactgccaattccttcccggtgtccagctcaagaacgccgagtttgaaggcgccggtaacgaggtcgcccaaaagacg

atgatgggcactgacgatggcggagaccctgatgcgccctcttcggacgatgaggccgaggaggaggccaagcatgtgcgcgagatgagccatgcgtc

ggtcaagctgggacaggatggggaggtgacggacaagaaggtgaacggtgctaggcgccagggcagcattgcttccatcgcctctgtcgacaccgtcg

ctaccgtttccaagaatgagggcgttttgatgagcaaccaggagctccttgctgcgccggccggcgtgttcgtcttcaacatcctcggcggaaacctt

gcacgcaagaacgctcgacttgaaatagccttcgacgacgcctactggccgacgtacactactgaacccgcccgcacggcgcactgcacgtgggacga

ggtcggcgaggaggtcattaaggagctggactggtcgcgcatctggctgaagctgcgttccggcagtggcgatgcggagagtgatgcatttgcagagt

tccagggcaacacgaaggacaacaagcccgcggagttcactctgcagaacatcaatggaaacggaaagagtacaatcacgatggcgtgcaagtacatt

cctgttgatatccatatcgagcccgtggagagtgtgaacaaccaaggatacctgaccgtcaccattctgtcggccaagaaccttcgagcagcggaccg

aaacggaaaatctgacccgtacgtgaagtaccgacttaacggcaaggaggtcggtaagacaaagaccatcaagaagacgctcaatcctgagtggaacg

agaagctcgaggaggtcactgtgccatcacgagtggctgccgagtcaatcatcgaagtgtacgactttgaccaggttggcgccgcggacccgctcggc

actgcggtatgcaacctcgccgagctggaaccattccagtccgtcgagaagacacttccgctcaccggaagcggtgccaacacggagccgagcttcgt

gactgtccgtcttgtgttccgacccgagttcgtcacgggtggtcgcgaacgcaagggtacctccatcgggcgcacgttcacccaagttggtggcgtgg

cgggtggcgttggcaagcttggtgtgaagggcgtcactggtgtcgcgggcggcggattggccgtcggcaaagctggcttcggattgggcaaaggcgcc

gtaggactcgtcaccggtcgcggacaccgcaaggcctcatcgtcgggcatgcccgaagacatccctgccgtgcctgtgattccggattccgtcgacgg

gatgccggccattatgcccgacggattgggcggatctgctgtggcaggtatgcctctggcgagcggtagtaacatcgctgcgctgcaagcctcgcctg

gccgtccgaatggcgaggatggtgacgacgcgtctttcgctgagtcctcgcacacggggacaccgtccaagaagaagggaagtcggttccacaaccct

ttcaagaagcacgacaagtag

>contig_14.contig_14.g620.t1

atgaacacaattcagacacactactgggcggtgcgcgactatctcgctcccgtcctccgagaatccaaattcaaggagc

acggccgtatcacaccagaggaattcgtcatcgcaggcgacttcctcacctacaagttcccgacctggtcatggaccgccggagaagcttcaaaaacg

cgtgatttcttgcccgcagataggcagtatctcatcagtcgaggcgtaccctgtctgcgacgcgtctcccagatggagagcgcggccattggcagagg

cgcaggagctgacaagagtggggatcagattctggactttggagatggcgacggcggcaaagacgatgaggggtggctcgcgacacatttcgattcgt

caaattcaaataccgaacaacgcgaaattgccgacattcctgactcggagccacctgctgccgacaatctttccgcctctcaagaggacgcgcttgcc

tcgcgcgtggcagggatatccgtagcggaccacagtgccgctggagcatcagcaccggacgcgcgcgcgtcttcgattgatgacgatattggtgacat

accagatatggacgaggacgatctggctggcatgggaggcggcgtcgaagaggaggaagacgctgcgacggcaccacaacctgctgcagcagctgcaa

cagcctcgtcatcgacagctcctggcacaggcgctgcgggatcacaagcagtcacaaccacgtccccgtccgacaacctcctcagcgtccgcacatac

gactgcctgatcacctacgacaagttctaccagacgccacgcatgtggctcgtcggatacgacgagcacggagcgcccctcaaacccgctcaaatctt

cgaagacgtctcgtcggactatgcccagaagacggtcacgattgagcccttcccgcacgcggccagcatgagcacagcaagcgtgcacccctgtcgtc

acgccagcgtgatgaagaagatcatcgaacgcatgaacggaagtgtcgtcgaggagcagaggaaggcaaaagcggctgcagtaccagcaggatcgggt

ggaggcgagggcaccgccagtggagagaagggagccaagaagaagaagacgtgggcaagtctgggcagtgcggtgcggaaagtgaccggtggttcttc

ctccaaggactcgaacgcgagtgggtcgagcacgcctggatccgctgctgcggccaatgctgatggagcggtcggggacgtgagcgccgaggacgcgg

acgagctcgaggggatccgggtcgaccattacatgatcatcttcctcaagttcatgagctccgtgctaccggctgtggagctggacgcaacgagcgga

tgggccgtcaccaagtga

>contig_14.contig_14.g621.t1

atgaacgaaaatctcgcgcccaagatccgacgcggtccaggatcaatcatggtgtggggcgctatcgcctacggaaaaa

aatggcccctctttcgctttccgttgtcgcctggtcgggtcgtgaacggggagcgagtgagggcggaaactatcactgcggtcaaatactcagattgg

atactcaaagaacgtctgggaggttacagcgacgaactagtcgaagcagtgggccctgtcgtgcgcgtggtcgaagacggagcgtccgtacacaccgc

tgcggtctctcgtcgcgtccgaaacgaagtgttcattattcctcaatctcatcccgccaacagcccggacctcaaccccatagaaaacatctgggcca

tcctaaagcaacgggtccggcgctggaggcccgtgccccgcacaacagacgcgctttgggacgttatccagaaggtgtgggacgacattgatcaagat

gttatcgacaggcgcgtggactccatgacagagcgagtcacggcggtgcgacgcgtcggtggtagcatgaccatgtattaa

>contig_14.contig_14.g622.t1

atgaacaacgatcaagaacgccgagacgagaagcgcatctgcttgctcgatggcgagaactggcccgagtgggagttcc

agatcgaagaagtgctgcgcggcaagaagctctggaaggcggtcgaacccgagaccgcaccagcagcaaacgcgagcaccgagaagaaggaggaatgg

gacgagaagtgcgacgccgccttctctaccatgtccctcaactgcgccgccgatatccaaggagacatcgccggcaagaacaaggagcgacgaaccgc

tggcgaaccgcgcctgacaccgaaggagctgttcgactgccttcgcgaggagtacgctcctcagtcccttcaagccgtccttcgcatccgccgaaagc

tcgcagccgccctcgccgagcccccggcatccatgcgcgatcacatctcagcgctgtccggcttctacacccagctcgccgagatcggccgcaccacg

tccgaggatgagcgctgtattgcgctgctcaacagcctcccgacgccctaccgacactgggtcaagagccacaagagcatcaccagctgggcggaact

ccgagtcggcatcctcgaggaagaagacgacatcaagacagaggacgccaaacacgcgccaagcaagaacgagacggtattcttcgtcggaccctcaa

cgacagggggcacacaaggttccagcagcagcggaaacagcaacaggggcagcggacagagccgaggtggttcccgcggccgaggaggcagaggaggt

tacaggtttccctacaggtgccatatctgcaagcaagagggccatcagatgaacgaatgcgtccacaagcccaagcacgaccagttcctgcagtcact

gcacttcttcatcctcgacactgccagcacccgtcacatcgtcaagagcaagggggacctcacggaataccgatttgccccgcctggaagcggcatcg

agtgcgccaacggggacatgatggccgtggagggctatggaaccctcaaggtcatcacagaggaaggtaacatcatcaccctgcaagacgtcgcgcac

tgtccctccacgataggcaacctcatcggcggcaagaggctcacgaaagccgggtgccggatcaccttcgacgacgcgtctgccacggtcgtgactgc

agagggaggccatccggtcatgcgagcagaggttcgaggcggcagctgggtcgttcggctgcacaccgttcaagacatcgctgcgctcgtcaagggag

agcccgacatcgatcccgtggccgttcacgcccaccgttgcttgggacacgtcaacgagaaggtcctcaaggcggccgctgcttccggcctcatccct

ggcctccccaagaccctgggtgacatcggcttttgttcggcctgcgcgcaaggtaagctggccaggactccccatcatcgcctctctccactcgagcg

agcaacacgagcaatggaactgatccatgttgacaccttcggccccgaacgcatgcaggactcgaaatcgttgggaggatctaagtacggatcggtat

tggtcgatgacttcacaaaacacgtctgggtcaagcttctccttcgcaagtccgacgcgatcaagtcgatcaaagcgttctgtctccaagcgtcgacg

agactcccgagtctcccgatcagccgtgtacgaacggatggcgccttggaattcaaaagtgcggatctgcatgaattttggctgaatcgaggtgtgac

acatgaggtgacggcgcggtactcgccgcagagctacggggtggcggagaggaacatcaggactgttggatatatgcatcatccatga

>contig_14.contig_14.g623.t1

atgctgtttgggaagcctcccgcgccggaagacttcaggccgttcggatgcgttgcttgggccaagaaggcgagcacag

agcgacacggcaagttcgacccgaaggcaaggccctgcgtatacctcggcccagcattcaggggtgcgtctcgcctttgggatcctgcgacggagaag

gagatcgttgagcactctgtgacattcgatgaccatcgagacgccagcgcgctcgtgtaccccggacagcagcggctcacggatgcgccgggcgtcac

cagcgcctcagcccgtcccgcaccagcccctgcagtaccaccaacaacagcaccaccagctcctccctcgggacagcccgctctagcccccgctcccg

ttctcatccgtgccatgcagacggtgctcgacaaggacatcgtgcccgacgacgtcgtcgactcggcgaggacgatcgcgggagggaacccggtctcg

ccccggaccctctcaaccttgacgacccggctgagggaacggacaaaccagattgcgcggggagcagcaggggaacagccttgcaaggtcgacccgac

gatgtttgtgcgagagggagacagtccggccatgctcctggtttatgtcgatgatattctcgttgcggcaccaaagggcggcggaattgaaggcgtca

agaaagagctcctaagtctcttcaaggggacggacttgggcccggcacatcactgtctcggtattcgcatcagtcggaattgggagaacgggacgatc

actctggaccaggaacggtacgccaaggaggtcctctcgcgctttggcatgacggaatgcaatccagccaaaacaccaatggatcagaaagccagtct

ccgaaaggcaacagacgacgaggctcgggcggacaagagagagttccaggccatcgttggctgcctggcttacctagcgcaggggacgcggcccgacc

tagcctatgctgtcagcaccctcggcagattcagctccgatcccaccgaaacccaccgcatcgccgccaatcgcgtccttcggtacttgaagcggaca

tcaggcgcaaagttgaccttcggaggacagaaggacggcaacgttagcctcgaaggcttcgttgattcggattgggcggcggaccgggacgacaggag

gtcgacctccggcttcgtgtttactgtgggcggagtgttgattttctccagctgcgagcgaccagcaatggtgctccagcatatgctgtga

>contig_14.contig_14.g624.t1

atgctcactaccaccatcgtccgcacccacaagcaattgagcatcacatcactggcacatcatcatcgtccgcagaccg

catcacacatcaccgaaccgattggcgccttgtgcccagtctcttccatcgcgcttaccaccaccgaccgcgtcgccatcgacatcgacacaagctcg

tccgcagggacagctttacggaaccagcacatatcagcgccttgtgcctggcattccgacatcaccgccaacaactaccgcaggagtagtccggcatc

gccctcgcgcgcgcgccagtgccttgtgcttggctgcaccaagtccgtccgtgggactcatcagcagcaccagcagcagcagcatcacagggctcttc

ccacacccctttcacccgtccgcgccacctgtccgtccgtcgacaccgtccgtaaccccgtcgacaccgtccgtccgtgcctcatccgcacttgtccg

tctgcaccgtccgtccgtgcatcgtccgtttaccccgtccgcatcgtccgcgcccgcgtcgacgtcgactccaccgtcgtcgacctcatcctcctccc

tgccgcgctcgtccgtctgcgcttgtccgtccgtcctccgagtctgatcgtccgcaagtccgagcctgggttagaggttgagcacgtccgtacgcccc

tcctcatcaccctcgccatcgtcgatcccatctttagggctctcgacctcatccacaagtccgtcgacctcatcaccgatccctcatttactcggccg

aaattttacccgatcccggtcacgtgcccgcccgaaaagcataccaccgaaacgcccgtctcaagctctgatttatcatcttgttttcaagtccgtac

ctttcgtcctcgccgagaaagaaccctccgaactcatccttcactttcggggtcgatcaaattccggttcacccactag

>contig_14.contig_14.g625.t1

atggagaccaatatcacttctgcaagcatacccggccttacaggcgtggagaactttttcaagtggcgtcaagcaatgg

agccgatcctggacggattccgtgcccttctcatcgtccaaggctacgactgctgtccgacgatgcccgtcatcccttcctcgaccgacctcaaactc

atcgaggcttggaaggatcgggacgcccgagcccggtcacttatacaaagaaccatcagcggtccgatggttgctatgaccagagccctcaagacttc

ttcggagatgtggcttctcctccacgatctcaacgacctcaacacgtccgattaccgtgccacaatcaatcgaagactcgcaaacctctttctggagg

agtccggcgacatgaccgaacatctcaacacatacattgagatggtggccaaagcagaagcagcaggcttaccctggggcaagaacgacgacgagaag

gctctctgctttctcgacaccctcgaccacgcgttccgtccgataaagagcgagtggcgtggattaccaaaagaagaccggtcgtttttcgccctcat

caagatctacaaagaagaggacgttgcccgtaaacgttcgtccgaccgacagatcgaagcggcagcaatggcagctgtccaaaaccccaagcagtccg

tcaagatcaaggggatatcacaaaacaagaagtctgcccgaccgtcaggcagcgacaatggaccactatgctatggctgtggctccagggacgggcac

gttcgtgccgactgtcccatcaccaagcatcttggtcctggggggatcatatgctggaactgtcacgagaagggacacgcacagaccgagtgtcccat

cgaccatgcgaaggcggtgcgcacggaggacgaggacatcctcggtgcactagtggaagagcatacgaggctcgctggggagtccgaccccaccaccg

tcgccttcatggtagactccggagcatcccgacacatcgtcgagcacgaatggatgctcagcaattctcgaaacattcccgagattgcattcaagacc

gtgggctgtaagctcaagtccaacaaggctggcgacatcatggggtccctgcctgacggccgcggcatcaccttcaaggacgccgtacatattccggg

aggaggagttaatctactgtccgaggaactcctcatggaacgaggctggaggaaggtcatttgcgacaacggtgacgctatcctccaacataacatca

agtccgactggcggttgcccctacagaaacttggaagggcccgctacctggtgttcaaactcaagtccgtcccaccggtcgagtccgaggcaagcaaa

aacgtccttgctgccgctcaaccgaccgtcgaccgtatcaagtccgaccctgcttgtccgaaagcatgggaacccagtccgcaagcagaggagaccga

tcctcacgaagtcattcgtgtcgaagacttccttgagcccgataacgacgacgagctcccggctgcagaagggcagagggagcaaccacgcgaggtgg

tatgcacgggagaccgacccgtccgcgaacctccttgtcgttccgaccgcaatcagcgtccagaaggcgaacgtcagaatttcatcatggtcattcaa

gagctcgaaggtcacttgcttccaatcagtccgacggacaaggaccctttcacatacgaggaggctcttgcacgacctgatcctgatcgaacagaacg

cgtccatcacatggacagggatttcaattcccttatcaagaccggcaccctcgaagaagtcaccgttccagagggagcaaacgttgtgcccacaaggc

acgtattcaagaccgtgaccgacatcgacaaggtcagccccgtcctcggtatcaccgtccgacgcatgaaggatgcctactacatcaaccagtctgtc

tacagtgcgaccgcagacatcctcaccaagcccgtgtccggttccacactctgcaagcaccgcgagtctatgggactacagcctccgcccgattcaac

atctcattga

>contig_14.contig_14.g626.t1

atgctcactaccaccatcgtccgcacccacaagcaattgagcatcacatcactggcacatcatcatcgtccgcagaccg

catcacacatcaccgaaccgattggcgccttgtgcccagtctcttccatcgcgctcaccaccaccgaccgcgtcgccatcgacatcgacacaagctcg

tccgcagggacagctttacggaaccagcacatatcagcgccttgtgcctggcattccgacatcaccgccaacaactaccgcaggagtagtccggcatc

gccctcgcgcgcgcgccagtgccttgtgcttggctgcaccaagtccgtccgtgggactcatcagcagcaccagcagcagcagcatcacagggaatacg

tggcggcaggggttgccgggcgcgagtcactgatgctcagcggcgttctcaaggcagccggtcaggacatgggtaccgacaaatcaagcattatcagc

acgcccaagcaatcgggatggcattggcaacaggagcatggaggcctggtctag

>contig_14.contig_14.g627.t1

atgagcccctcgcgagcaagcagagctcgccacgacgcgccgaacgcgcccaagaacgacccatttgggctacccacgg

ccaagattggctcaagacaccactgcctcgactttttcgagcacacctttcctgggcgcgaagcgccctcccacaatatcccagcacgcatgcgagcg

tactcactcccagcaagccattgcacgcgtacggcggcacgcatgtcgctcaccgccgctccacctgcgacccctgcgcctgccaaacacaaccttcc

cgcgcaggccgggttttcttcgaaccgtttgggcgactcgcacacggcaccatcagcacgaacaaggagctctctctcgcagcgcttgcttgatcagg

aactcccacccatcgaaccatacccttcgcacgaccacggccggacgggaccccctcctagccgcgtcacccctcttcggcacccactggccgatcaa

tgcacggccctgcccgcgttcctcccttcagctcatggagtccgagagcatccgccaggcctgtacccgagtccgagccaagcgcccaagccggtctt

cacggacggaaactcccaacaccggttcgaggaccagtga

>contig_14.contig_14.g628.t1

atgagcccacagtccagactatcggacaaacacggcggcggcggcggtggggaggccacaatggacggcaagctcaagg

aggaggacggggaagcggcagctcatcggcgagcgccgcgcggtaccccatcggatcggcacgaaggtcacgacgggtctcgtccgcgagcccgcatt

attccagcaccttcggcgatcaggacggtcgggtcgctcaatccggacccgacgaccatcgagcaagctccaagagcacatgcggcggcgccgacggc

catcggccctcgcaggcactcaagatgttcgagcattggccgacagcagccccaggatgcgatctgggcgctggctacacaggtcggcaatcgaacga

gacgagagcggcacttcccccacaagaagagcgagtcggatacgcaagcgcttggcgagctagccactcaagaggcgcactgcgtacgggtacgaggt

accagctctcttggcggacctcaccctcattcgcgcatgacggcaaccgaacggtcatcggtcttcaacactcgaaaggagaacgacagcaccaaatc

gccggccgcagcgcaaccgacataa

>contig_14.contig_14.g629.t1

atgacgccgtggtggtccctcgacggcgacgacagcgagatggatcggatatgtgacgacggccaagcacagcactatc

accaccaccaccaccatctggcttcgcgacgtgccgggttgacgatggggacatctcctaccggtgcgtatacacagcgcacccctacaaccagtacg

tcgaccaccgtcaaccttctccatctcccttctctctcctccctcagcctcgggaccgtttgcggtcggggcagctcctcgcgcacgcctgcggagag

gagcagtgcagctgcctgcacgcagcacgagcgacacgctctcggcaagacatggacgaggagcagcgactactccaacgctcaaggctcggcaagca

tacaggcgttcgaagccgacatggggcggcggcagcggcgctatatgcgcacggtcagggtccagggactaggtcccaggacgtatgccatcatcggc

atcgaacacagactcgagccgacctcaagaccaaccttccctcacgtcgactacaccgatcgttcgatcagcttcttgttcgtcaggtgcagcttcag

catacgcgaacagccgactcaaaacgccgagcgacgtccagaactccagcagcagcagcagcacacctaccagctctccgcgtcacagcgagagcccg

cgcaccagtcagtccagctctggcgacaggacctcaccgtctcgccaagaccatcagcatacgacttcaacaggacgagccccaagtccactctcacc

gagcggggcaggtcagcaacactagtacctgtgtggaggcaacgtcaagtccgtgcggctacggcaagcggaacgagcgagaccatgagcaacaccga

ctcatggagcgcaaccccggcgctagctgccagcagatcagcactcagcagcgtcggcagcccaccacactttgtaggcacagagccaaggacgcctg

catgcgcatcagcagcgtccgccctccatcggcgaccgattggttcggcaccagcttccagccttccacagcgatcggctcgcagctcgcagtacgcc

agcaagtcggagcggcacggtgcggcaaagacatgcctttggggcagtcttgtgacaaaagggttggatgagtttggcactttggcaggatcgcagat

tggcacgggatcgcagcagctcagtcctagtacgggtggctcggagcatcgggccgcgatcggtatcgacgccggcggcagcaccagcagcggcagca

cgcgcaatccgcgtccgctctgtcctctcgaccaccacatcaagaacgaccagcaggaggcgattccatcgccatcgacgacgacatctcttcctcgc

gttcacacgcactcaaggcgcgtactagggtcgcatacggcatcagacacttccacccttctcctgctcggcatctggcgccacgagatcacgggcgc

cgacttatcggcatatgcctacagtctgacggggcagaccaggaccggacgatgggaatttggtatgggatttggaccagtgggtgactggattgggc

ggttcggcagtgtgcgggagtggagggaattggatgagagggcagtgaatatggagcatttggtcagtcgacttacggtcaatatcgatcatcatcat

cgaccatcaacatcgaacatcatcatcaccttcgtcatcatcacctttcaccatcatcaccgcaacctcatgttcaggattccggtggccgtcaggca

ggagcaccatcatttcagcatcgagcgtcagaacagggatactcgagcttcacttcctccttgttaa

>contig_14.contig_14.g630.t1

atgtttgcttccaagtccagcactctcgccctctccctcgtacttatcatccttggtggatcagtgttgccctcgatcg

ccagcgcacccaagcccaggtttccgcccctttctcccctcgtccgcgccgatctggaacgtcgggacccgaacgaataccagctcttccacaagcag

gagcacagcggcaatatctcggcacgcagtgttgtctcgggaggaatctctcagtgcaggactcaagggtgctttgctttcacctttgacgacggtcc

gtccatcaaccatcgaaaggtcgctgacgagattgcgtctattggcgcccgcgctacgtttttcgtggttggcaatatgggcaaatgtatctacgatg

aggacagcgtccaggccctgaggtactcctacaacgctggccatcagatctgctcgcacacctggtctcacccaaacatcgagaccctcagccgcccc

gcgatcgaccagcaggtccaactcgtcgaggaggccatgttcaagattctgggtgtggttcctgcttgcatccggcctccttacggaagcgccagccc

atctacaatccagtacctgaaccagaagcataaccttaacgttattacttggaacgcctacatccgcgacgccgatggtgctcctgccgacttctcga

tcaaccaataccgctccctccacgcgcctcagaacgtcatcatcctcaaccacgagaccgtccctaccacgagtaaccgggtcatcccccaggccctc

cagatcgtccggcagaacggctatcgacctagaaacatgggcaccgtcgctcagactgtcggcttcgcgccgtacaaggtcatcggcaggccgggcgt

tcgcgattcttcgtggacgtgtgccggtaagccgggtggtcgtggctggtag

>contig_14.contig_14.g631.t1

atgtgggcgcgttcgcttggtcgtcttgctgcggcctccaatcggaggtcgtccggagtttctctttctggccgtacgg

acagcggactggctcgacctgttcatggtgaagcttcccgtgccgccaacttggactcggccacagctcaaccacgacgcgacttccacggatcctcg

cagtcgagggctcaggatcatgccggccaacattcccgcgcggggtcgaagtctgggcctgatggggaaggcaaatcggcaccctttgcattcttcaa

ttcaacaggattcgaaacggccatgggagcctcggtcggcgtacttcttctaggctgcggatcgctggtgtacatgtcctggtataagaagcgtgtga

taaagaagatgattcaggctttcgatccggattttgatcccgtcagctacctggcccaacaaggtgctgaagtagccaagattcatcctgctgaccgg

acggagtacgaggttatccggaacataatcggcggaagggaagctggccattactacctggtcctcggggccaagggaagcggcaagacgggtatgat

catcgacgccatgggagagatacaagccgatggatgtgcctacttcgatgcccactccgacgcagccattacggtcgaccgcttcagcgagagcatca

acttcgccatgaaccgcgattacctcggcaacctcctcggcctcaccgatctcagcgggatgaactcgtttcaaatgttggagagaagtctccataag

ctcgagtcggccttgatccagcgcaaacagaagcaaggcaaaccggcggtcatcatcatgaacaatgctcatctgttgcgcgacgaggacggagagag

attgctgactgtgttccaacagcgtgctgagaaatgggcaaagcactacttcccgaaccagcctccactctccgatgacgtgctcgaggaggtttaca

aagtcgcgggtggacgagtgaatctgctcaacaagctagcacgccgcgccgacatcttgaaatcgaccgatcaacttgttgaggatgatatgcaatgg

ctactgagcaagactggtatcattgtggaccacgacgacgatgttatggacgagcaaaaatgggctacctgctcatggctgctcttcaccgagctggc

tcaaagacatgcggcatttgaagaagcagcggttgacctggctaaatcgaagaacgtgtcgatagagtccatcgaagagcaagaggaggagccagtag

aggaagatacgaccgacatagtggaagcttcggctgaccctgcgagggacctgattcacaaggaaggcgaggacatcctccccgctgtgagcctgacc

tggggcgaggccagacaagtaatgactcgtccagactacattctcgagctggatcgactcaatatgatccacatcgatcgacaccatcgcatctatgc

cgacaacatggcccttctccgtgcgatgcgacgtgtcaccgaaacggaaggcttcgcagaaaagcttgagtccgtgatggaccgcgtcgcagccatcg

aatccctcggccgcacgagggaactggtctggaaggagcaggtcgacggcggcaagttcatgatccggaaagatgggaaagggcgggagatggagggc

tggtaccttctagggggtgacgagcgactgggccgggaggcggacgaagctcaggacgactcatcttaa

>contig_14.contig_14.g632.t1

atggagccctcaggtcggtcaggttctgcttccaacgggagcgcggtgccgtccgcgtcgccgtcgagccgcaacccct

tctcttaccaggccaagtacctcgagcggtccgcgtcccggagcacgaccgccaacttctccctcgcaggcgccggcagcgtgccatcgtcatctaca

gggctcaacaatgccatgtcctcctccacagactcgacaggaagtagcggcggtggtggcccacctcgggtcggagtccttcccaccgcgggcgccgg

catggcacgctcaggctcggtgcgcaaatatccaatgcctcatcggcatgcagcctctctcgacccagcacgcacggccgagttgcgctccggcagcc

tgcgcaacggacagtcatctctgaccggctcgccgactacacctcaaggctcgttcgcgcctctgccagaaagctcgccgccttcgagctcgccgtat

tctccgcttgcgccgagagcggctcatttctcaccaggctcgacatcgacgtcttcaacaggtcggccgatctctatgccaccagcgccgcgcgacaa

tggagtgcctgcttcagcaaagcgggcaatgtctccgacaaagggaatgtttgagtcctcctactcgcccagctcgcggccgatgtcgccattcaagg

cgggacaggtcctcccaccggctggttcccccacgaaagtcgacgattcttggatcaacggctcaccgcgcaacgcccagggaagcttttggtcttcg

tcatcacccgcccctgccgctactccctcagcggtccatttaggtggcgtgagcgatttgaagagatcaagtgtcggcgctctcaagatgatgcgaca

gtacacaactcgggctgacagtgtggtcggcttaggcggtccggacgcaggacatactttgctcacagttggtcggaaaaacagcacgcgtgcgaatg

ccgacattcaagaggacgacggctgggagaagccgccaaggcctgggcgaatggagagtgaagatcaggcaggggagatcattctgcctggtatcacc

accaacgccgaagacgtgacgggtctaacaggtcgcattcgtttggcccgtcaagatacggctccgacccagctcggcatgtcggcttccttgccata

ctcgaccaacagtcggtggatggacacccagcgccatctactccaggcgtatgagtacctctgccactgtggtgaggccaaggaatggatggagcact

gtgtcgaggaagagctgggtgccatcgtagacatggagaacgaaatgcgcgacggcgtctttctcgccaaacttgcgcgtaaattcgaaccggaatgc

gtgccgcggatctttgtcaatcccaaactccaatatcgacacaccgacaacaccaactactttttcgccttcgttaacaaggtcggcctgcctgtttt

cttccagttcgagctcaccgatttgtacgagaagaaaaacttccccaagatcgtctactgcattcacgcgctcagtcacttactcgcgcgccaaggtc

gagccttaaaggtgggcaaccttctcggaaaactcgagttcacagatgctcagttgcagcagactcagcggggcctggatgctgcgggtgtggcgatg

cccagcttcggagcgatcggcaaggcgctggcgaaagagatgaatgtggaacctgaaccagagccggagacggagcaagaacgagtcgaccgcgagct

gtccgaaaacgtcgacgcggtggtcgctatccaaactgcgaccagagcattccttggtcgcatcacacgagaaaggcttgcagaggaagctgcatacc

ggcggaaacgtgaggaagaggcgcggaggaaacgcgaagaggaggagcgtcgtcggcgcgaggaggaagaacggttacgtctggaagaggaggaacgg

gtgcgacgcgccgaagaggagcgcagaaggatagaagaggagcgtcggcggaaggaggaggaagagcgccagcgccgagaggaagaagagcgtcagcg

actagcggaggaggagcgcttgcggcgtgaggaggaggagcgacgaagaaaagaggcggagcggctgcgtcttctggaggaggcgcgtatcgccgaag

agaagcgcctggctgaggtggcgcgcattgccgaagagaagcgcctagctgagttggcgcgcattgccgaagaggagcgcctggctgaggtggcgcgc

attgccgaagaacgacgtctagcggaggaggccaggaagaaggccgaggaagaacgcatcgtagcggcctgggctccaaaggtccaagctggtattcg

cggtttcctggcgcgacagagtcatcaggacaagctttataccttgcagcagcaccagtccagcatcaccgccctgcaagcacaggtacgcggagctt

tggcacgcaagcgtcatgaagccctcattttcggactggccgatgcgatggaggacgtcatcaagatgcagagtgcctgtcgtgccgttcttgcacga

cgcgctttgctcaacaagatccgggcgatgcgggcgacgacggactccacggtcatggttcaggcgcaaattcgcggtgtattggcgcgcaagtcata

tcttgcgatgagctcggcactgcagcagatcgaggttgtccgttcggtcggtggtctgcaatcctttgctagggctgcactggcgcgcaggaagcacc

aggagcagatcaagcagttggagttcgttgctccggatgtcgtcggactgcaagctcagctccgcggtatgctggtgcggcaggagtacttctggtgg

cgcgaccacctccacaggagcgaagctgtggttgtacaccttcagtcattgattcgaggtgtgctgcagcgtcgaaagtacatggccaagatgcggca

ctacaatgagcacatggacaaggtcgtcaagattcagagcttgttccgcggcaaacagcagggcgagcaataccgaagtctcactgtcgggaaaaacg

ttcccgtcgcgaccatcaagaattttggccatttgctcaacgactcggatcacgacttcgaggatgagatcgaggtggagcgcctgcgaaagctcgtg

gtgcgcaacatccgcgagaatcaaactctagatgtggacgtgacggagctggacaccaagattgctctgcttgtcaagaacaagcttggcatcgaaga

actcatcaaggccaagaacgagcgcggtctgcttcgacaacgggaagcggctgcgatggtgcagcgaagaaattcgttgcttgtcgctgctggtgatc

cattcgctgatcagaatctggaccgaggggcacggcgcaagctcgagctgtaccaggagctcttccacacgctccagacgcgccccgagtacctcgcc

aagctgttcgtgcgcgtcagcagggtcgagatgtctgacaggcagaagaagcagatcgagaagattgtgttgacgctcttcggctatgcgcaaaattc

tcgcgaagagtatctcctcttgaagctattccagcgttgcgtagtggaggaacttacgggcgtatctacggtgcaggagttcatccgcggcaatgccc

agttcatcaagctggtcgtacagtacaatcgcggcgcgaaggagcgtaaatacctccgcgacttgctcagcccactcgtccgcggggtgatggacgac

gacactatcgacctcgaaacagacccttgcgcgatctaccggaacacgattaaccaggaggagatgcagacgggcatgcccagtcggagaccacttga

cgtcaacttccacgaagcgctcgctgaaccgactgcgcgggtgatcttcattcgtcacttgcaggcgctgcgagggactacagaggcgttcttgtctg

cgatgatcgggtcgacaaagaagatgccgtacggaatccgatacatcgcgcgcgaggtcttccgtgcgcttcagaccaagttcccgaatgagcccgag

acggctttgatcaaggtggtcggccacctggtctactaccgctacctcaacccagccatcgtcgcgcccgaaggattcgatgttgtcgagacgctcgt

cgggcccgtccaacgcaagaacttggccgagatctcgaagatgctgacgcagatcgctgtgggcaagctgttttcggaggacaacccgtacctgcagc

cgttgaacgagtatgtgtcgcaggcctcgcagcgatttacgaagtggcttttcgctgtggtagacgttcaggatgcagagatgcagttctcagcgagc

gagttcttggatcacgcggcgaatcagcggccggtcatttacatctcgcctaatgagatctactcgatgcacggtttggtcttgggtgagcttgacca

cttggctcctcatcatgatgacccgcttcgcgctgtgctgctcgagctgggagacgctccgatgtcgaacaaccaggatcttaactcggctcgcgact

ctgagatcacgcttgagttgaagagtaatgtgcgaacgctgaaagatcccgaagcggagaccaaggcgctattcgtcgagaccaagcgactcgtgctt

gctattctgaaggttcaaacgggcaagacgcttttggatgtcttcgtcgcgcccgtgtcgccccaggacgagatgctctgggaagagatcgtgtctct

ggaggtagctgccgagcgacagcgccatcgcggtcaacgtccccctgcgccgccccagctcggaccaaagctggaagacgtgcgccgcttgacgttcc

cggagctcaaagcgcgcacactggagaacatgctccgactcgagaagctgggcaaggtgtcccgggcgaacaactaccaggacatgctcaatgcgatt

gcgattgatatccggaacaagcatcggaagcgggtacaaaggcacaacgagaaggcagccatgttgacgacgctggacaacttggcggagaagaagaa

gtatctggaagaccagatctcatcttaccactcgtacattgatgcgagcatgcagacgatgcagaagaagggacgcaagaagtttgtgctgccgttca

cccagcagtacttccacctgcggagtctcaaagccactggcaaggtgcccaaattcggctcgtacaaatactcggctaaggtgttacacgacaaggga

atcctgatctcgatcgaccagttctcaaacaagcaatttgatcagatcaccctgacgatcagcagcgacgaggccggactgttcacgatcgaggcgag

tgtgctgggcgtttccggcggctcgtgcgatatccggatcgaagacttgctagaggcgcagttcaacaacacgaaccagctcactctgatggacggca

tggccaaggtgcacgtcaacatgttcattcatttgatcaacaagaagttctacgcgtag

>contig_14.contig_14.g633.t1

atgccttctccttctgtttacgacacccactgcgaaggcccaggtctctcttccagcaccaggacgtggtcgcaatggt

actgccaataccggcgctttgcgggtgatatcaacaccactcaggaggaaccgcccaagatcgacctcactggcaagaacgtgatcatctctggcgcc

aacagcggtatcggctacgaagccgccctgctctccgcgcgctggggagctaacgtgattctcgcgtgcagagacccgcctgcacacgagccccaccc

agagaagtgcatcgagcagatcatcgaggcgacggggtgctccagggatcggctggagtggtgggaggtcgactacaccaagctgaagacggtgcatg

cgctcggaaagcgatggcagcagtccggcaggacgtgcgattacttgtttaacaatgccggtctcagcattgctcactccctaacggaggatggattt

gatcttacgcatcaagtcatgttcctcgcgcacttcctcctcaccttctatgtgctgcccaccatgaaagacgccaaagccccacgcatcgtcaacac

tgcctcatgcttccattacggtgggcaactcgacttcgccaacttcaactttgagcggaaggtcaacgaaggcccacgcggagtgacgctctactgca

ccaacaagctcctcgtcatgatgggcctcaccaaggagctccaggcgcgtctatcgcgcagtgaacactaccgacacgttatcgtgcagggtgttcac

cccggcttcgtcgcatcgaacatttggcacgcgactaagacgcttccatggttcgtgcaggccatacttcaatttctgatcagaaatctgtcgatcaa

cacccaacaaggcagcatcgctctcacctacggcggctaccacccggccttcggtttcccccagggcgagctcgagcaagggactccagccccaggtc

gctccgcggagatcggaggcaagtacatcaaccgcattagcgcgcaggagcctcgtccagagatcttcgacgtgcttgcgcgctcgcggctctggcag

cgcgtgttggaggatttgaaggtcaaggatcatggcttggcaaaggacttgccgggacaccttcccgagctcgtctttcatgtgcgctccttttcttc

ggctttcaagtttcatgtcccatcgcctgctctcatgaatgtcgctctgccttcgcgcgctgtgttgctggccaatgttgtaccaaaggaagagtcac

cgagtcacctttttgtcagtgttggtctcggacgcgacgcgtctggaggcgtgttggaaaagtcgcgaacgtcagcgcttctcagcatggcctccact

ccttcgacaaggcccaagagggtagcaagctcttcgcccacaaagtcagcggccccgagcgtcgcgaagaaggcgaagctggatccttcccccaagaa

gaccaatggcagcaacaccgacccacccgtaaaggacgatgacatcgaggacgaattcgatgacggggacgcactggacaatgagcttctgatggaag

tggcggaacaggccgaacgagaagctgcgcgcaagctggcgcagaagggcgccgccaaagcgaagacgtcagcttcatcgacggcggctgtctcagcg

cagaagccagccagcgatagccgatcagcgccggcgcgagccactaccgccgctgctgccactagcagtgtagccgcttccagtgcggggacagcgaa

tgctgcttccaaagattctgacgatgaggttcataccatggaggccgaatggcggggcaggctgcaagacgagcttaagaaggagtattatgtcaaac

tgaagcaatttctggcgagcgaacggaaagcgggcaaggctatatatcctcctacacatctcgtacataattggtcccgtctgacaccgttgagcaga

gtgaaagttgtcgttgtcggcatatcacggcgcgggtcaagcttgcgcttttcagttcccaaaggtatcgcggtcccgggcagtttgaagaacatata

caaggaactccaaaatgaatacacggactttgtgcctcctaagcatgggtgtctggacgaatgggccaagcaaggcgtcctgctcctcaacgcatgtc

tgaccgtatacgcaggtcaagcgggctcccactctggcaaaggctgggaaccattcacgcgcgtcattctgaagacgatcgcagacgaagccgccaaa

ggatcaggcggcagtggaggtgccgaaaagcgtgtcgagaagtcggcggccaatccgttcgggtggagcacggtgggaacctcgaagagcattgctgc

agcgggtgggaaaacgtctgaagagaaggcacccgaagcatcagctgtccccaagacggccgcttcgaagaacgatggtgatggtgatggtgctgggt

gcaaaggtgtcgtattccttgtctggggccaacctgctgcaaagaccgttgctgcggccgggatcaccgagaagtctcccaatgtgctgatcctgcgc

tcgccgcacccgtcaccgctctctgcgcatcgcggctttctcggaaacggacacttcaagaaggccaacgagtggctggagacgatctatggtcctgg

cgcaaagcacagtgtccctgcacacacccgcgatccctttgcactggccgtgcccaagatgggcataaaagcggcgatcccaaccatcactttccctc

ctctgccatcgaccccctctccccctccgatcgctgcaaagatgactcgtctcaccgagttcgtcacgaaggctaacgaccgagtcgccaactctgcg

gtgggctcgtacttccgtctcgatggctcgacccacccgctcgcccgcaaaggctcggtcttcactaccgagatccgcgccggactcgtgaccgcggc

cgccatgctttacattatttcggtcaacgcgtccatcctgtctgcgtctggcggaccttgcgagtgcccgcgcacggccgaggacttcgcgtgcgata

acgacgtagcatacctcgcgtgtgtcgatgtggtccgtcgagactacgtgacggcgacctcggccatctcgacgattgcgacggggttgatgggtctg

ctggcgaatttgccgcttgcgttggctcctggactcggcgcgaatgccttctttgcattctcgatcgtcggttatcacggcacgggtgggactacgta

cggctctgcccttggcgctgtctttctcgaaggcatcatcttcttcatcctctccgtctttgggctccgacagtggctcggccgcgttcttccccggt

ccgtctcgctcgcaacgggtgtggggatcggcctcttcttagccatcatcggattgggtccgaatggtttgggagttatcggcgggaactattcgaac

ctcgtcgggcttggagggtgccctgcggcattcaaggatgagaacgggtactgcacgggacatgtgcttcaggatccacgcgtctggctcggagtctt

cctcggtggtgtgctcacgggactgctgctcctgtatcgcgtccgaggtgccttcctgcttccgatcttgctcgtcgccatcgccagttggccgcgga

gcagcgccatcacgttgtttccctatacggacgcaggggactccaacttcgcgttcttcaagaaggtcgccacgttccatcagttcgagctgctcgga

ccgagcaacatcaactttgactacgccaacggcaaagtgtggttggcgctgatctccttcttgtacatcgatgtcatggacacgacgggaacgatgta

ctcgatggcgcgttatgcaggccttctggatgaccgcacgggcaacttcgaaggctcctccgtcgccttcctggtcgatgctggctccatcagtgtcg

gtgcgctcatgggactcagtccctcgaccgccttcatcgagagtgcggccgggatcggagagggcggcaagactggcttgactgcgatcacgacggcg

gtcgtcttcttcttctcgctcttctttgcgccaatcttcgcgagtattcctagttgggctacaggaagtacactcttcctcgtcggctctctcatgtg

caagaacgctgtctccatcaactgggatttcccgggcgacgcgattccggccttcttgatcatcgccgttatcccgttcactttcaacatcgcatacg

gactgatcggtggcatcattgcctggatgctcctccacaacatcccactgctcatctcctacgcgactggcggccgtgtcctgcctcccgggtgggag

accgagaaggagccttacgcccttgtccgacgggcggacgacaagagcccggcggccccactgttcccgccgtggcttcgcaaggtgctgaagggcaa

caagaagttctggaaattgaccccggaggagattgaagaactcaagcagggccgcatcatcaccgccatcaggacaaggagagcagcagcacagcgcc

aacgggagcgcgacgagatgcgcaacccgctcggtgcatcggagcaggtcgaattcgaggacgacggggacatccgggaagctcgagatgagatggcc

gaggacggaagcatgggggatgtgagttcgcgtaaagagggccggctgggacggacggggagcgtgaaggatcttcggctggagggtgggcttgctgc

gttgagcgatcgacaggctcatttggatgtgatgcatggggcgcggaggagtctgtctcaggtgcggatggagagggagacgaagctggagtga

>contig_14.contig_14.g634.t1

atgctctctgtcttccacccaccgcgcggcggacccagcacccagagcgcagagaggcaacgaggccagaacgcgtttc

ttgaagcaacccacactcggaacggcggacagatcccagcgtcaggagtcaggacagcacgtccatcccttcgctccttccgccgcatcgacgcagcc

gaaacgaggtacgaaagaggtggtgacctgggatacataagcaaggcacagggcagccgcatttctcatctcatctcgtcctccggcgttcgtcagga

ctcaagagtatctcatcttcctcccgactcgtcgcccctcgaggctcgaagtactccgaacccatatcttatcagggattcgtccggaaccatgccga

aggctccatccaactcgcatggccgtcgcacggtgtcctccccgtatgagaacgtccaaaacacccctgcctcttcccaacactcggcggggtttgga

acggtaggcgactcccccgcctcagcagcagacacatctgcatctgccgccggcgctcgctccgtcacctccgccgcctgtctctcgtgcagatccgc

taaacggaaatgcgacggagagcgccccgtctgcacacagtgcacccagcgaggtatcactcctgccaccgcttccggcgtaggtggatgccactacg

tcgccagcaagcgcggtggccccaggtacaagggagtccgcggcgctgccaccatcgccaagaccgaacagaagaagtgggagaagcgggccttggcg

gcatctaaagcgagtggtgacgtccgcactgcgccgccaccggtggcatcgtccatgcatggacagcgtagcctcagcagggacggcacatcccagtc

tgcatacagtcattcccctccgctgccgcagccgcagcctctaccgacgacagctcctactttaccgtcgtccttgttagcgcgccgcatggtgggtg

ttgccccgatgcatctgcctcccccccatgcccttcttcaaacgcaaaccgatcaagaaaggatgcatgtcggcggtggtggagctcctctcgcttac

catccgcatgcccttccaccgcccatctccactggcatggttggacctccaccctccttgggctccacaggaacaggacaacaggatagcatttctcc

tgccctctcccttctccagatgtctgcctcggttccctcgacctcgagtcagccatcctccgaacggggctcgccggtgacaatgcccctcgaaggag

gtctcgggatcggataccctacccacggcagtaatcaactcccggccatccctggtggtcgccaccctcaaggatggcggacttcgaccttcccgacg

aatccttctggacatcatgttcgaacgagctcgtactcaaactcgagcaatcaaagccatcttcagcagatgcatccgtcggtcgccatggctgcacc

taccggtttggcccccatgcggaacacaggagatggcggtagtatgataacgcatcgcagtgcttcttttgccagttcgtcgggacacactgcgacat

cgtacccttctgtcggcgggtcgacggcctcgctcacctctggtgaggccatgatgaacacaggcagcggacccatcggcgggacccagatgccagtc

tcgcacagtatggcggcacccacttgggcagcctcgtccatgaacgccccggggccacaattcaacactacacgtcactcggtcggacagcacggtac

tggtcacgggtatcacaacactggcagcagtggtccttcacccaacacgacgatcatctcaatgccgccccagtcggtcaatcttcccattccggacg

tgtatgccgacagtatgactctcgtcggcgatctctcattcgagaagttggagatgtggtcgaaattgcagcaatatcagcttccctcatcgacatcg

ggtgtccagactactgccattatgccctcctcaaagtcgacgacgcagacggccatgtcactgaacaacatcgccaccaacgctcctggcgcccatct

cggcgggacacttcagcacggcggtcccctggatacggctctgtcaccgagttccatcaccaaccaggcccacctcgccttcagcgacttccttcaga

aactcgaggcacttcccaaagctggagtcgtcggtggtgcacacgatggaggggaacatggagatgctcatcatcacccgagtgctggcttcagcggt

gtgatgggaatggacgaaactgaagacggcaacaaccgcagcggcggtggtggtggcccgtgcgggaatcgcgagtgtggcgatgatgtaggagaggg

cacggatggcgtggagggtgcaggtcgtctgcggggcacattcgataccgaacgacgtgtcaaggcgctattgaccgactactatcagctcgtgtacc

cggcgagtccggtcatgcttcccccaatgcatctctcctcgctcaccttccactactgcgagagcgggccaaacgcgctgcacgcggccatctccgcc

tcgatcgcccagcatctcaatgattccgaggcgcaaaagacgctgcagggggatgcggcttggtcccttgctctgagcttgggcaaacggggtgggcc

ggtggagctgacgaggttggaggtcgcggcgcaccatgcggcttcggcggagatgctgttgctcagtgcggaggagggactggcgagtgctggttgtg

gtccttcgggcgtggcgtccccgcatgggttgggcgggagtcagcaacagcagcaacagcaacatcagtcgatgacggccagtcccggctgcagctcg

tccgcgggaggtatctttgcctcttcgaacccgttccgatcgtcgccaccagatctcgagctcgtgcggatcgagtgtgtcgcggcgcgggtgctgtt

gtcgcagtactactacggccaatgcgggcatcgcgggcacaagttgggtcacgcacatgccttgcgggcctgggagaacgcacagtcccttgtgctga

ctcttgctggcgaggagggtgggccgagatcggaggctgtgacggtgggtggtggtggtggaggagatggtgggacgacgatgggagggtcgcatttg

atgtcgcctggtgggttcagtccggcacagaaggtggagtggggacggagggtgtatcactcttgcttctcggctgtggtcgtcctggcgacaaccgg

tggatttgaacccgtccaagcggcccaagacgtccttaccgcgctccaaacacggccttcgctcggtaatgacgagaacgcgtgggggacgcttattc

gtggctcgcactacgtctgcgggacgtaccaggcgctgtacgaccttgagtcgctgcgtcggggggacattgcgagctcgggtgcggtggtgcagtcg

caggcggaggcgtatgcggtgcggaacgagatctttgcgcggatgagccatttggacatcgatatggccaactactgtgtgtatgatccggagtgggc

gactgcacagcggagtgcggccgcgggtggtgctggtaggatggggcggtcgaggggtgagaatgactttgatgcggatgaaggggcgccggttttcg

ttgttgaggttgagaaggagttggcgcgttcgctcaagattgcgggtaagctgatgacggcaggatccatcatcattttgcaccgggcgcaagcattc

ggcaatgctcgactgtttatggacaagccgcagtgtgggttgcctgctttcaacacgaaggagcttgcaccggctccgtccaaggactcgcatgagtc

gagccaggacgacgacgatgcgctctggcaccgatcgaaagaccgggaggcgagccctacggagtcgaatgcgacgtcgacgttcactggctccggtt

cggcttttacgaatacggatgggggcacgggttcgccgctgtttgtgaggggccaggcgccttcgtctgtggctacgagtgcgccgtcaacttctggg

tcggtgacgtcgtcgtcgaaggcgaaagcgagttcgttgttcaaggctgtggctacgagtcaagccggaggctcgagcagtgggccaggtggtgggag

tgtgcctgcgaaggcgttcttccctccggacgatcggtacatgggcggaccgtttgacgctcgggtgtcattacatcggtgtaagcttgctgctgcgg

tgatgcacgagacgttcctgaaccaggagcgggagaaggagcgggagatggcgagcggtggggatcgacggcggcggttgagcctgacgcctgttttg

aattcggacggttttggtggtggtggtgaagggacacgacggcaatcggcgtatatggaccagtcgggcgacggcggcgacggcccatcatcaggcgc

aagaacgggggaagcgacccctcgtctacccccatactcggcgtgctcgtacgtactctcggcgtacgtgtggctgatgctgtccctgctcgccctgc

tgggaacggacgaccgcgaacaggctgcgcagcagattgagatgcttcggagtcgtgcgcggagtatacattcgatcttggatcggatgtcggccagt

tggcggaatgcgagggagtatcgggctgaggtggagacgcttttgttggcgaatgagaagctggctgggtga

>contig_14.contig_14.g635.t1

atgttgtatgctccgatcgcaaagacacagctcgagtggctcgcctccctcagcccactcgagcactccgtcgagaact

caaacaagttcggacgcaagacgtcgatcatctccaccatcggtcccaagaccaactcgcctgagatgctccagaagctccgcaaggctggcatgaac

atcgtccgcctcaacgcgtcccacggctcccacgactacttcctctccgtcgtcaaaaacgcgcgtcaagctgtcaagggtgggtccgcaacacgcat

ccgatctggccaaattacacacactcccggccgatcactcgccattgcgctcgacacgaaaggtcccgagatgcgcaccggtctcatggtcgacggcg

aggatgtcaagattccgctcggacacgagttctacgtcactaccgacgacaagtacgcagaggcgtgcacggaccagcatctttacatcgactacaag

aacttgcccaagaaggtcgagcctgggcggacgatcttcatcgatgatggaattctctcgctccaggtcctcgccatcgagagcgacaccatggtccg

tgtacgcgccgtcaacaatggtgttctttccaacaagaagggtgtcaacttgcccctgaccgaggtcgacctgcccgccatctccgagaaggaccggg

gcgacatccagttcgcagtcgacaacgacctcgactacatctttgcctccttcatccgccgcggctccgacgtccgcaccatccgcgagatcctcggc

gagaagggagcccacgtccgcatcatctccaagatcgagaaccaccagggcgtcctgaacttcgacgagatcctcaaggaatccgacggcatcatggt

cgcccgtggtgatctcggaattgagatccccgccccgcaggtgttcatggcccagaagatgatgattgccaagtgcaacatcgcgggcaagccctcga

tctgcgccacccagatgctggagagcatgattgtcaacaaccgaccgacgcgggcggaagtgtccgacgtggctaatgccgtgctcgacggcgcggac

tgtgtgatgctctccggcgagacggccaagggagcgtacccgatcgaggcggtaacgatgatggccgagacggcgtacctggccgaacagtcgatctc

gtacatacccctgttcaacgagatgcgcagcctgacccaggttcccactgagacttcggagacggttgccatggcggcggtgagtgcgtcgttggagc

agcacgcgggtgcgatcctgctgatgagcaccagcgggaacacggcccggctggtgagcaagtatcgtccctcatgcccgatcctggtgatcacccgg

aacgagcagacggcgcgtggcgtgcacctgtaccggggctgctacccgttcctgtacccgcacgagcgcccggacacgcttgcgaagtggcaggagga

cgtggataaccggatcaagtttgggttgggggaggcgttgcatttggggattttggagaagggggatacggttattgcgttgcaggggtggagggggg

gtaaagggcatacgaacacgatgcgggtgctttcggtgcctgatgcttcgaggagctttgcgctggatgggactaagttggatggatga

>contig_14.contig_14.g636.t1

atggttgacacgatcaagaccgccgtcgactccctcgcccagggcgtcgcgtccatcaccggcgtcggtgctccgtccc

agcaggccaccagccccgaggaggccaagaagcaggtcttcgtcggaaacctcgccttcgccaccacagaggcagacctcaagaccctcttcgcagac

gcaggcaccgtcgcatccgcacagatcatcacccgcggcacacgctccctcggatacgcattcgtcaccttcgactccgaggccgccgctcagaacgc

cgtgcagaccctcaacaagcgcgaggtcgccggtcgcgagatcaacgtggagggcgcaaagccacagtccgccaacacgaacaagaccaacggtgcac

gcgctccgggtgccgaggacggccctaacaagggcggccgtggtgccggtgccggtggcgcacgaggcgctccaggcggcgctcgcaccgggagggga

ggtcgcggcgccgcttcttcctccgcccgcacacccggtggtgcccccggtgccacaggcacatcgtccggtgccgcacgccggttaccacgcgccgc

cgccggcgccgacggctcagcaccctccggcgagccctcccaaaccctcatcttcgtcgccaacctgcccttcagcaccgacgacgaaggactgaagg

ccgcctttgccgactacaagatcacctccgcctttgtggccaagcgcttcggtcgcaagtccaagggctacggctttgtcgatgccgagtcccacgag

gagcagacccgcatcatcgagaacggcaagggcctcaccattggcgaccgcgaggttcagctccgcgtcgctctcaagaaggcagactccaccgacaa

cgctgctgccggtgccggtgccggtgccgacgcagagggtgtcgagggtgccgctgcccccgccactgacgccgctcccactgagaccgcttaa

>contig_14.contig_14.g637.t1

atgagtgcggcggacggagacgcagcccacagtgtcgcacgcgcactgagcgggaacagcgacgacaccacacagcaac

tgctcgccatcgtcgacagcagcagcagcagcagcagcagcagcagcgaggacgggctcggactgggagcagcactcgcacgcacgctgcatgcaatc

atcagacaccacatcctcttccccctcgcactcaccacacccgcactcctcgccacagtgacccacatcgcagcaagactcgccccaccccagctcca

gtccgcaccccgtccaccggccggcatcgacacccagaacacagagatcgcgcctccaccagcatcgcccttccacgacttcctcatcgatgccgtct

ggactgtcgacgcagagctagaactgctctccgacgcagcactcggcatcgcatcacagacacagctccccctcaacaagcagatcgaacgcagggca

gaacagatccgcatcccacatccatcaagcgacaaccagatcaccttccacgacgcacaggaaagactcgcagcactcgtcaaatcactcgtccacgc

ccgtcatctcaccagagaactcgtcgcagaacggctcgagctgcccctcctacaccgcctcaacctcttcccagaaccgctcaacaccatcaatgcac

gcgtcgcaaaagtcaacacgtccatcaactacaagcaggacaagtacaatctcctcagggaagagaacgagggctataccgctctcatcaagctcctg

ctcgaatccataggacccgcgctcaaaccggtatatgttctcgatcaaagcaattctcattcctcgtccactgctccaacgggcgcgaactacacctc

ccctcccatcgccatcatagagaccgagtccgcacacgaacgcacaacacgcgccaccatcgtcctcaagaagatccgcgccctcgtaggttacttca

atttggactcctcccgcgtgcttgatgtcctcctcgacgtcatgggtcttcatctcacacagcactggcccttcttctgtaccctgctcgagctctcg

gcatggggcgagcatctgcgctcggggagaagtaaagctgatgatcatcatcatgagatggacatcgacagcgacgctgaatcccaatcccaatccca

atcccaaaagggcaaaggaaaggcaaaacagacaggtaccgcccatacaagcgcacaacgtcgtccccgcccactaggcgacctcgaatccgaaatca

acaacctgccagactacctcgcctccgaaacaggcaactccacctgcgcacagctcatcggcttcaaattcagagactaccaacgccccgaatccgcc

gacgtcctacccccggaactctacctcctcaccgccctcctcgtccatcgcggctttgtccgcctgctggatctcgtgccccatctgacaccggatgc

ggctcagatgcgcgagttggagctcgagcatcgcaagaggatgcgggcgggcaatcggaaggttgagcttaataatgcacttgccatggctgctccct

tggaagatgatggcgccggagggggagggcgtcggcctgcttcctcttctagcggcgcattttcatccaaccctactgcggacacaggcaccaaccca

cccgcctccggcaccaacacagacgcggggaagaaaagggagaaccaaatcgtcggtctgacccgggccttcctcgcgctcggctccctccgacatgc

ctggtttttcctcggccgctcgccctggttgctcaccgcccacccggacctggcagacgcgtactgtcgcctgttgaaggtcgtgctcgcgccgacgt

atcggcatgtggcgtcgtcgcagttggagccggtgttgcgctctacgccgcctttgagccgcttggctgtgaagccgtcggcgatcgcggcggggagt

accgacggcggaaaggcagcgtcgacatcgccacagagcttaccatcaataggccccttaccgacccatgcgctcacgctcaccctacacgcgcccga

gccgctctggacagccgagaagcggttcgtcttcttctaccccttctggacggccgagcttccgcactgcgccaaaccggccgatttcttcactgtct

ttgtgccgatgctcaagtttctcgggccgttcttctatcgggatttggagctgtactcggctatttgtcggttggggaaggtggggttgaggatgtgt

gtcgggaaggcgggggttgatgggcaggtaggggatggggagggcgcaggcgcaggcgcggagctttcggcagtggcggtggagcagaaggaacgttg

gttggacgttgtaaggacatacctcctcccggcgcatatcctgaccgacggcctatcctacgtcggcacggagctgtggcggatgctgtcctgtctcg

agtacaaggagcgctacggcctgtatggggagtggaagtaccgcctctacctgcgggacgacctacaaggtcgccgggccgacgccgagcgggacgca

cgaagcgtgctgcggcggatcagcaaagactcggacaaagccaaggcgatggcgatgggcaagaagcttgcaaaagtggcgcattcgaacccgattgt

gtttttcagcgtggcgttggcgcaggtgcaggcgtatgacaactttgcgttctcgctggtggagtgtctccggttcgcctcgctcaccccgctcgagt

cggacgtgttcatgttcgtgctgctggacgcgctctccgacgacaagaaggaccggctgaagagcgacgggctgaatacgagtctctggcttgcgtcg

cttgcgacgttctcgggcatcttgctgaggcggtgcgagaaggtcgacccggtgcccctgctgcagtacgtggtgaactcgctggtgagccagaacac

gaacgacatcgtgattgttcgggagctggtgaagcagatgagcgggctggttgtgctggagacgttggatgaccgacaggtggatgcgctctttggcg

ggcgactgctccgggtcgaggcgatgatggcgcctgagcataagcctgattccgaagggcgggaggcgatgttgaggtctggggagcggcttttgggc

agtttgagacggagtcggatgggggtgccgttgcttgtgctgttggcgcagatgagggggagttgtgtttttgcggaggagatggggcttgtggagga

gggttcgtcaggtgcgggaggtgggcggaaggcggttgctgctatgtttgactcgtgtcaaatggtcctcttccagttcatcgagttcctccagtact

tcgccgacgcgcggacgtacgccgacctggttccctcgctcgaggcgctcgtgttgcgctttgggctctcgcctgcgattgctttccatattgcgcgg

ccgaacttgaagcgggagatcgagttgggcgcggaggcggagaaacgggcggcggcggagaaggagaaggagagggcgagggagaagaagaggttgga

ggaggaggaggagaagttgaagaaggaggctgaggctgaggcggaagcaggggcgaaggaagtggaggagaagggggagggaaaggaaaaggaaaagg

gaggggaggatgtggagatggagaatggcgagaaggctaaagaggaggatgtgaacggcaaggacgcttctgacgcgtcgggtgccaatggtgaagcg

aagacggcacctgtcgcaaccgagccagccgccgccgtagctacgacagcagcagctgataccgacaccgacatggtcgaacaagcctccaacaaagc

cgccgaaccctggctacccgcactcgcccccgccctctcagccgcacacaagttcctacccgccgaagtccaagacagcctcggcgcgttcttctacg

tgaccttctggcagctgagctacgcagatctggacgtgccctcgagccggtacacgattgagacgagtctgagtggccggagtaatcggagtattgag

gcgctgagggcggagaatatgaagcagatgcaggcgcagaatgggacgcggttgaggttgttgaaggagaaggttgcttggtttccggacgaggtgcg

acaggtgcagcttgctgatcaggtgattcagtactgcctccatcctcgggcgctgatctcgactgtggatgcgatctttgcggccaagttcttgcgtc

gtcttcactcgttgggcacggctaacttcccttcgctgaggatctatgataagctgttcacgcaacatgtcacgttgactatttttagctgcacggag

gcagaggctaagtactacggtcgattcttgctcaccattctcgaggatgtgacgacgtgggccaagtcggaggaacgctacgagaaggaggtgctcaa

gaccaaagacggcgttgaccttcctggatttcgacagtccgtcaagggctcgccgttgtcgtgggccgacttcctgcagatatccgataagtggcatg

atgcgctgcgatcgggcctgcaaaagtgcctagagagcaaggactacctctgtcgccggaatgcgatcgcgatcgccaacctgatcacgccatacttc

cctcgcaaggccctgtacggcgacagcatgatgacctacatccagaatgtcatctccgaagagacgcgcgaggatgtccgtctctctgccaatggtct

gctagcgcgctacaaggcagccagtaagacgtggctccgcccaagtgcgaaggccttcgaaccgccgacgccggcccgctccgaggtggcttcgtcca

cggctgccaaagatgggtcaagcgccaacaagaagacggtggagccagctgctacgagaccagcttcaaataccgcgccggcgagcaagacgagtgca

ccatctccagcgttgcctacgggacctaaagccgatggacgctcgacgttgccggatcgaccgggtgctgtacggactgtgtcgtctacccagatggc

cccgccgacgggtcctaccgggggccggcatggcttgccacctcggcctacgacgggccgaacgagttcgacgacagttccgcctactggacctgctt

ctacttctgttaatcgggagcgtggacctctgaatggtcctggtcctggtcctggtcctgtgtctgggcgaggatcggatgtgtcggccgttggggag

aaggacggtgctgctcagaaggagaaggaacgccctgagaagccggttgctgcgattgcgagccgggcggtcgctgccgaaatttctgctcctgcacc

gccgagtgggaacggcaacttaacgcctgtatctcactccgctatcgcatccccacgccctgctcgctcgcgcgatgtgtctccctcgacgaatccgc

gacgaagccctgagccgggctctgcatcgggatcagtccgaggcacgaaacgtgatcgggatcgtgaaggtgctgaggagcgccctgcacttggtgtc

gatgcaccgtcgcgtcgtgctgatcgtagcccgcgggattcagaccgggatcgagaccgggatcgcgaccgtggcgatcgcgaccgggagcatcgaaa

ccgggatcgagaccgggaccgtgagcgagatcgggatcgtgaccgcagcgacagacatcgggatggcgaacgggaccgggagcgcgaccgcgaccgtg

atcgggatcgtgagaaagatcgacgggaccgggatgatcgcagggatcctcctcgcggaccacgaggagctgtggatgagaaggacaaggttaaagat

aaggtcactgctgatcgtagtgacgaggctgctgccaataaacgccgacgcgtggagcgtgaggaggcctcgggtcggaatggatccggatcggggaa

cgggtgggacgctccgctgccgccgactactacggctgctgcacccagccgtgctccgacgcatccctcaagctcaaacgctacagccatgggcccac

ctcgtcgtcccgaaggaactgatacgtgggtgaaagaaacccctgcgcgtcgttccgagacgggaacagatacgcgggtcaaagacacccctgcgaga

ggggagcccgctcatacgtcgagctcgaacgcgacagcgatgggcccgcctcgtcgtcccgaagtggctgagacgtggggcaaagagactcctgcgcg

tcggtccgagtcggggacggatacgtgggtgaaagatgctcctgcgcgggttgaacgggacgaacctgagcgggaacgagagcgtgcgtggggaccta

atgccgtcggtggcaggaatgggcgggatggagttccgactggtccgaggacaccggtcgtcagtggggcttccgctgtcgtggccgatgcgccttca

tcctcggctcgggcttctcatgatcgggatcggggtagtgaaagggagcgggagcgagaacgggagcgtgacggtcctcgcgcccgcggacgggaggc

tgagcgagaccgtgagcgcgatcgcgatcgtgaacgtgatcgtgatcgtgacaaaggacgcgatcgagacgaccgtggcaaaggcggtcgtgcagagg

cccctgtcgcctcgcaagctgaagcacctccagcgactccgagcaaggtcgaccctgcgcctgctaatatcactccggcagagaaggacaagggcaaa

caagaagcaacaagtcggccccgcgaaagcgacaacaaagatgtctcggcagacacaagcatttcggttcctcagaagcgcagtcacgacgagcaggt

tgaaagtgcggacggtcctcaagatgcatctgctgatagtgtgagtggagagttctgcgactctgcgtttcgaaagctgtgtataagaagctcatcgg

aacataacttcactcctgatcctcagaagcgcgccaagcgtacacctggtgcacgagcggaaggtcaaggactgcggttgtctggcgttgccgctgca

gcggccgcgtcagcagcctcagttggaacgggccgtgatgctggcaaaccccgtctgacaggcgggaatcgggtggacggatctcgagctggccgcgc

tggtcagtcgatattcggcgcggcggcgtctgcgtccgcaccgactgcgagtgctccggtgacggccgcggcttcgaactcgggttcactcatggctc

ggctgggtgctgagcctagcccgtccaagggttcgcctgcgtctgcaggggagagtacgaccaatgtgaagtctccttctggtgacggcagcgttcct

ggtggccggatcgtgaccatccagggaatggcaagtcgacgggagaaggatcgcgacgtcagtgtaccaggtgcggccgtggaatcgcctgcccggcc

tggacctgtcgaaggctccagcaaggatcgcgaacggtctaatgaccccggcccgaccaacaacggcggagaggcagatcgggagagggagcacagca

accgcagtcggcgaaacagtcgtgacggaggacaagtagcggcccccgccatcgcagatcgggatcgcgagcgcgagcaacgggatcgagaccgtgaa

cgcgatcgggatcatcctagtcatgcccgcggaagcaacagccacggccgaaatgaccgtgaccgggagagggagagggaacgcgagcgagatcgagt

caaggacagggacagggagcgtgatcgcgatcgtgacccgcagaaggtttggtcgccgcagtctgtcacggatggtctcatcgtgcttggttttctgt

cctcctcggcccagaaggccgggcgcattgtagctgctgaaccgtggcacaagcatacgcgcaacatcttctgctcgtatagcgtcgttgcagcgttc

aagtccaaatcgacagcccgtcctggtcggcattttggacagctgccttcgaaacaaccgcgtccgccgtggttcgtaaccgtggccgcgcccttcat

atcttctcgcgaagtgaccgcccccgacgacatcgccgagatcccgggacgattactgatccaaagcttgcagcacgaggacaccccttccacaggcc

ctcaaacgtacaaatctcgacggcggtcagcaccacaagattcagccatgttccagactcacagccgcgccttctcgccctccctcttctctcgctct

gcagccgccttcggaggcagtgctcgagtacagtaccggccccgagttggagcggtgccttcctaccccacgacgacgcaggaacaaacttccatggc

taccaaggacactgatatcccttcaggagccgcactggaggatatcgtcgtgtacgtcggcgtgcctttccttgcgaacggaaaactcgcagccgagc

ctcactttcacgatctgctcagctccatcggcacgctgtccgcacctggcgtgcgcgtcgaagtctacaatgacgcctag

>contig_14.contig_14.g638.t1

atgcagctcaactccaaactttccctcgtcctttgtgccctcctcgtggcccagcccatcgcgtccgctgccatcgcca

gccacgccaacgcagtcgaactcagccctcgctctgacgacggtaacaacaaaggcttcgacctcggcgacgtccttcgtgccggcggtgtcttcgga

ggagacaacaaggaccttggccagggttctggaggtggccacttcggcaacaacaacaacattaagaaggacgacgacgacaagaagggaggaggcaa

gggtggcgacgatgatgacaagggcaagggcaagggccacggcggcggacccaaaaagagcttccgcgagaagaagctcaaggctctcaaagccgctg

cgaacgaggacaagcacaaggacgctgcggctgtcaaagcagccgccaagttcaacgctgcggctgccaaggaggaatctcaccacaaggaggcggac

ttgaagaagttcaagaaggagaagaagcaggactccggcaaggacgactttggcttcgatcgccgtgatggctttgggatcggcggtggactcgactt

cggaggcgacaagggcaagggccacggaggtgacgacaagggtggcaaggacaagggcaagggcaagggtttcggcggtggatccaacaagaacaaga

aggagaaggtcgagaagatctcgaagcacctcgacaagtccgacaagcacaaggctgcggccaaggtcaatgcggccgccaagttcaaggctgcggct

gctaaggaggatctcaagcacaacaaggctatcgcgaagaagctcgcgaaggagaagaagaagagcggctctggaaagggtctcttctag

>contig_14.contig_14.g639.t1

atgaagttctcagcatctgccattctgtccctcgccctcgtcgggctctccgccgccgccgccgttcctcagcagctcg

tccagcgtggcacagtctctgacctcgccgacgtcggagccaagatcgcccaaatcatcctcaaggctcaggctgccgataacgacgtccgtgcccgc

ttcgcgcagaacatggtcgatggcttccgccgcgaatacggcgatgctaccaacatcgttgtgatccacaccgagcacgactacacatggaacggcgc

acagggagacgcctgggagcactggcactacgagcttgacgtccagatcggcggcaccatcggctacgagatgtacgcctccaaggtcggcggctacc

tcaagcgcaccggtgacggcggctctctgaactgggcatggtacggcatcctcgccaaggaccctgaggaggatggctctcgcctcacctttgccgac

acgggcgatctttga

>contig_14.contig_14.g640.t1

atggctcgcctcgctttcgccgctctcgccgcccttgccttcgccgccatcaccaatgcaagcccactgatcgcgtccc

gacagaaccgcaaccctacctgcggtaccgccggtgatgccacgctctcggactgccaggcgctcgtcgacaactggcccaacttcggcaactacgag

cgcacctgtaccttcagtgtgagccaaaccgcctacaacccggcgtgcttcggcaactgctgcgtgtacgtcaccactaaccaggcccgctgggacga

ccccgacgccgagatcggtgacatccgcaaggctgcccaggccattctcggatgcggcagtgccgagaagaactcggtcaacggtgtcatcgacgtcg

aacaggatggcgtccagggccgggtctgcctctccgacggagctggctgcggtgactgcttctctgactga

>contig_14.contig_14.g641.t1

atggtttctgcgctgcagcctgcgatcatcaccgcgtcatctgcacgcatcgaggccaggcgccagctgccaggattca

ggaacagtgcagagggcgtcgttccgctcacggagtcatggcccttcactgaccactacagccgatcggcgcgggcgacagcactgcgaaccgagact

gctgctgaagagatcagcggtcactgtcagcgtttcggtggcgtgggtaaatctgcaattgcgccccgcacttctgtggcgcttggcaggcatgttct

tcgggatgccaaggcctactatactgctagcttccgaaaagctatgtaccgacgccttgggcgcttcggcctgcctgtgacacgtgaaagcgcaaagg

tttcgaacgatatgtccgatgcgatcgcagctgccgttctcagctgggcgccgggcgctgacgcactgatgctgacggctgatggctggtag

>contig_14.contig_14.g642.t1

atgcgctccgcttccttcgccgccctcttcaccctcgtcgccgccttctccagcgccaacgctgccattctccgggccc

gtgacgagcccaagctcgagtgcggtgtcaccggcgacgcccccctcgcagcgtgccgcaacctcgaccttgacaacatcaacaccgagaaccagtgc

agctacggcgtcggcgcaaagcacgacgtcctctcatgtgcaagcttgcccgacagcccgggcggcggtccactcgactgctgcgtctactcgactgt

cgacaactggcggcccgaggttctcaaggagatcgtcggcaagatcctcgacggctgcggcgctcctcagaacggcgcccagcccggcggcacggtca

acggacgctactacgatgaggcaggccagcgcgtctgcatcggtaacggagacggctgtccaatatcacatcaatcgtctatgtcctatgaattgcga

gcttccttctctgcatcattcgagtccggaccagactccagagatgaagagaacaagtccctcaccgcccagtga

>contig_14.contig_14.g643.t1

atgctcctcaaggctgtcaacgtgctcgctctgacgctgctggccactgtctgctccgcagaacccagtccagacttcc

ctttcgcaaatctgccacctcggccacgcgcgacagcagctccggccacgtacaaagacttgcagctgcgcaacaacgaggactttgtcgatcatgtg

gtccagccgcacgagcggcagctcactctcacccccggtgctaccccaacgcccatctttgtcccattagcagatccgaaggacttctacagcgcctg

cttctggggcatgggaaatgccgggaagagcgtttacaagatctttgaatcggcgccgtcgggcgaagagtggcaggcattcatggttggagggccca

cggaagtatcagtctaccaggacaggctgaccacgctgaggaacggaaccttggagaccgtcatcgccagcgccacctgcaagtatgatggtgaagcc

gtgggcaaccgcgtcaaggccaaatgcgactatatggcagtgatcgatccaccccacacaagttggtacaaaaaaaccgacgaagtcccagtaaccta

cacccccggcatctgcgacctgaacctcgtcaaacccagaccgtcatgggacattggcagcgggaacggcagctttgaccccaacccagaatccgttc

cgggatcttgggccgtgcaccgcttcgagtactgggccatgacctccatctggggcatcggcgtgatcggcctgctggccatcgttggtctgggcgta

ctttga

>contig_14.contig_14.g644.t1

atgttcctgccggagtttgcggatgcggtggtgcattcggtggagggctgtgcagcgctgcagctcgcgggcctgctgg

cgatccagtctgcgctcgcccatgccctcatcgccaaggtccccgaccggtcgccgcggtacatcgtcgtcggcctccgcagctgtgctgccttcctc

gccgcgccctgggacgacatctgtctccgccatgtcgagtgcctgcatcacctgtctaccatcccgccccgggtgtccgggcgcagcctccagcagct

cgcagtcgaggcagaggcactcggcaacgcaaccaaggcacacgcagaggtcatcagtctcttcctccgcttcttcacctcgcttcagtccgggcgct

gggcgctgcctactctgcgcgtcctcatcatcgaccaccgaagactcagccaggacgcagatcgcgcatccgcagcagtagccgcggccgccctctcc

ttctcagggaacgccccatccgccgatggctcagccggtacagccgcagcatcccaggctgtcattgcagccgcaaatagcagccacaaacccaatgc

ccacaccgaggagtttgcgcgtcagctgaacaaggcatttgccgcatgcgtggccgaccgcgcatctcctatcgaacacagtcggaagtggggaacct

acgaggtcattggattcatcttcagagtctacttccgactgaaaacatctcatctctgcaagaacatcattcgggcactcaatgctgcagatatacca

ccgctctcttctttcccccgggcgcatcaggtcaccttccgatactacatcggagtcctcgctttccagggcgaagactacgcaaaggccgaggaaga

gctatcatgggcacttaacaactgcctcactctggcaacacaaaatcaacaaatgatcctcacctatttgatcccaacacgtctactacgaggctacc

tcccttcaccttacctcctctccctcttcccccacctacagaaagtctactcaccactcagcacagccatgcgcgcccccaaccctttcctctacaca

tccaccctcctctccgacccatacatcgaacgagcgctcgtcaggagtcggacctatctcgcctgcgaacgcgctcgcgaagtaggcgttcgcgccct

ttttgagaaatgctggaaggcaagtggaagtaagagtcggttgcctattgccgtcctggggagtgtgctcaagctggcggcggagcgcagtcggagtg

cgacacaggatgccgagagtgccgaggcgaaggaggacggtctgagtccggaggaagtcgagtggcaggtcgctgtcatgattgcaaagggctacgtc

aagggttacatctcccactcgcatcagatggtcgttctcagtcagcaagatcccttccccaaactatccgctgtaccactcgcggccacctag

>contig_14.contig_14.g645.t1

atgagcattataccgacccagacgacctcatctgtccgcatcgggctgctccactcgtacagccaggttcagcgcgacg

acgaggaacaacagctgctcgacgcctacgtcaagaatgcgggcgagtcgaagcccatcagcacgcgcaagctgactcagccgcgtctccgggctgcc

gctctggtcgatgccgagcatggcaactcggcggcgagaacagctcgacctcccagcctggctgcgctcgcgcttgatgtcacagcgcgtaatttccc

aaggctcctgcgcgttgctcaaaccgaagatggcgatactggggaagcgacgcgctcaggaaacacggccgttcccagccctgctcgtggtccagctt

caacaccggcacggggaatgcgcaagagtcgccgcgtcgcggagcttgcgaacgagggtgctagcggagacggcaaccgtcgagcgcatctcatgtcg

ctcatgggacctgatggcagaccatcggcagagctcagtcaaagaacgcacgatcggctgcggctcctcccggctcaacatctcgccagcctacaact

tctgctgcaaaagtaccatcgccgaaccctcacgatgctcactctccgccgctacttttttcctccgactgcttcgcgtctgcatttgtccccagctg

tccccctcatcaccgagaatccagacaccggtccgattcgactggtctctgccgttacctcccacgctcagcacttgacacatgcctcacttatcgca

ctcaatcgtctagacgcaaaattcttctccaagatcatgctccaagccacgagacttgagtatttggacctttccgcatgtctcaatgtcaacgcgag

cgtcatccaagccctagtgacggggtgccgggatgaaggcaaaccgagtcggctcagatacctgaatctggatgcgactggcatcggtgcccatgggc

ttgccgcagcaatttcttcctttcacagcttggaggccctgaaagcagcaaatgtccaaggcctggacgatgtaggcttttatcaggcgacattgaat

gcgatctctgtggaggcagacgtcagcgaagcagggtcgggtcttgcgtcggcatcccgtccacttggctgccttcgatccctcaaacttcgtcgcac

cgcggttggcgacggctccttgtcgcttctcctgggcatgtgtgatccgtctctgctccagactctggacatcggatttacgcgctgctccctcgaag

gcctgttggaagctatgcaggcgccagcgcagcactcggatccagatgattcggaattcagtgcccgagtcggagaagcggacgcattcacagatcgt

cttgaccatccacgccaagtttcgccaggtgtgggtagccaaggaggaggaggaggtatgacgacgtccctgtctcagccagcaactcgtaattcgga

cagcattgagcccagactgtcacagcaactcgcacaaggatcccgacctgccgatctcggaccttcaggttcacaacaaccgctccctcaagcggaat

acaggtccaagttttcgctacgaaagctatcactttccgggttgtacctacattcctcacgaggtccaacagaaaatcgtcttcaacctctcaagaac

ttgctcaaaactcagactgagcttcggagcctgtaccttcaacgcgttccgcatttcagtcgcgagtgcgaccttgtccaggaccttgtcggcgtcct

gcgcgaaggcctgagaaatatgcttgagatgcgagccggcccagatggacttccggacatagaaggcgcggacagttcgagaactccctccagtgttc

cgctcttcaagaagttggttctgtcggacaaccctgcgctcggcttccgcgtcatcgatggtaggacgggtcgaggctgggtattcttacatctcctt

caggaagaggaggagatgcaggccggagaattggccaaattgatgcacagcgttggcgtgttttgcgaggagatccgactgtccaatgttaccctcct

cgccccgcatctggccttttccccgtatcaacggatcctggaactcgaccacaccgccgtggagaacttcatgattctcgcagccctcgaacgcgcga

acatgtatcggcccgatgtggttgagctcgaaccgccgctgattgctgccacgcctctagagactgcaatgcaaatcggtcggacgcgaagcggacgt

gccggcgcccctgctacgtctaagcggactgcttctgggaaaggcaaagcacgtgaggtagacgtgaaggaagacatccttaccttcgatgtctccct

caagccacttctcgacgcgaacccatttctgcaggagattgacctgacttcgtgtcgaagtgtgccggtcgtacaacggcgcaactactttgagcatg

ttctcggggaggacgagatcggagatgacgacgaagacgcggacaacacgcggtccgacaccgatagctatgaggtacgacaacgaagttcgcgactg

aacccgcaccgaggcagttcggaccgtcgcgctgctatcgcccgccgccaagccgcatccggcacgacgctcacgaggcaagagctccacgacgatga

ttttatcatggacagcgacagcgactatgatgaaggtcgcccgtctcgtgcacgcagacggtctaatccgtctagcccccggaagcgatccggttcaa

cctcgccttctaagcgctcggttgcgtcctcttcttcccaagctgcgcagcggcggaacttggaaggtgcgatggaggcggtcaagtcggccaagacg

gcccgattggcggcggcggcgcgccttcatgcatctgcccattttccagagtcgtcgacttctgcgtcgtcgtcgttcccgcgaagtctgccttcggg

tcctaggccctcgggaagccctgcgaagagacgggctgtcgagcctgaagctcaggacacgtcgagcgaggagagcgagatgacggagctgagctctg

acagtgacgtgggctcggattcggacggggacgaggaggacgaccgtccactctctcaacgccgactgaaacgccgtcgactgaacaccgacggatca

acgtcagtcgctggccgtcgacgacggcctacgcgctcggcggcgcgcaatctttcccttgtggatcagtttccggtcgaagataacgaggacgagga

tcctgacttttccgttccttcttcgcgcggtggcggcggtgggagcagacaatctcctaacaagagcggtcggcaaggaccgatcggcggtagcgctc

gttcggcgtcgagctctgggcaggccatcggttcgagcagtacagctcacgggccgacgccttttacgtcgacatcctcccgaactgttgatgttggc

ggcaataatagcaacggcaacagcagtagcagccgacgtcgaagcgcgagccccacccgctcgccttcgagccgagtccgcgaccacgctgcgcgcgc

cgatgaggtagcgcgagaagcacgcctccgagctcgggagctggctgcggaggaggagcaggcggctcgggctgctcggttggcgaagcggacgaagt

ga

>contig_14.contig_14.g646.t1

atgcagctcaactacaagctctccctcgtcctctgcgtcctcctggctgtccagcccattgcatctgctacgatcacca

gcgacaatgacgcagccgaactcagccctcgctcaggcgacgttggctctggcaacaacaagcccttcgacttcaacggcttgctcggcatcttcgga

ggagacaacaaggacgacggcaagggctctggaggtggccacttcggaagtgacgacaacaagggggacaagaagggtggcgacaaccacggcaacgg

cggacgcaaccgcttcgccgagaagaagaacaaggctctcaaggccgctgcgaacgaggaaaaaaagaagaaggctgcggctgtcaaggcggcggcca

agttcaatgctgcggctgccaaggaggactctcaccacaaggaggccgacttgaaaaagttcaagaaggagaagaagcaggactcgggcaaggacgac

ttcggcggtttcgatcgtcgagatggctttggcttccgcgagggactcgatttcggaaatgacttcagcggcggccgcggtggcttcgacggtggccg

gtacagtggatttgaaggcagcgtcggcagcttcagtggcagcagcagcacttccggcttccaaggcggcaatttcttcggtgctcgcagcttcaacg

gcgggaagggcggctctcgccacaagaaagagaaggtcgagaaggtcgaagcacacctcaaaaaggacaagaagcacaaggctgcggccgaggtcaat

gcggacgccaagttcaaagcggcagctgttcacgaggatctcaagcacaacaaggccatcgccaagaagcttgcggctgagaagaagaagaagaacgg

cttcggcgcgggctttttctag

>contig_14.contig_14.g647.t1

atgaagtcgatcagctccttcatgctcatcggcatgctgtttgccatcagcggcgtcaatgccaactctcctgttcaag

atatctgtatcaaactctcgcagcattgcgagcctgcaaacagttatatggatgcaattaagagctgcctgtgccaggtctggactgctaaaggcaac

ggcgactgctacaagggatgcacggacgaccatcctcataataaaaaaggcaaagaatgggaggactgtcgttaccggtgtgacaaatttcgagaaga

agccaacggcggatgcccctccgtaccgaagagctgccagtcgggctag

>contig_14.contig_14.g648.t1

atgtgcaagaccgacatcatccatatcatttgtccgcactgctttgagcccaccaacaaggagcatcgcagcttcaccg

cctgcaactgcgatgactgcgacgaggacgaagagtacaacagcaccaagattcctggtgtaagtatgtactcactcgtctctttccgactcgaacca

ggctccagcagtcctcttcaatacttcccgtctctcctctggttcaacttcgtcaccgaccctcgcatcgattttcgcattacttacccagcagcaag

cttccgcattcacaatttattggtcaccagtccttcagtcttcatttcttccagcaccaacgtcatctcaaggctcaagggatcgcatcgaacctatc

atcgacttctctcgcaactttatctaaccccccgtgcgtcgctcccggagccactcaaactgcaggcagctgtgtcgacgtccagcttcccacgcgtc

tgtcctcccactccccggaaccctcttctgaggacaattttacatctctattccagagccgatgcatcctaa

>contig_14.contig_14.g649.t1

atgcgcctcacatctcccttcatcgccttactcggcctccttcctctcgcacaggctctgccttcgtcatcgcccttcg

tcggcgtcgacatgatcctcaagcggtacggcatcgcctctcgctctccggaggactatgcgagccagcttcccgctctgcgcagatccaagtcgcag

cctcggccgcccacgagctcgcgtctggacccgacgccagactaccgtgcgaggacgccgttcgactgggccagtattgctcttgctttgcaccaaga

gtacatcgagctggatgcgttcaactacgcaatcacccgcttcactcctgctgagtgggaggaatggggaatcagccccaactacgtccacctgatcc

gtttcttcgctcagcaagaggaagggcattcgactgcattctccaacatgcttggccctcgtgcgccgctgccctgcgagtacaactacccgtgggcg

aatcagacaaacgcatcggtgccgggcttcatcgactggtctcagaaaagcacccagcttggcgagagtggcaccattggctggatcccaattatgga

tgagccggcaccggcatccatcgttcagcaagccatcgtcaccgagtcacgtcagcagtttgctttccgccagctggagggcttgttccccgtcaccg

tttggttcattaccggtctgacgcaggccatgcactggacgatcctctctcgctggattacccagtgccccaaggagaacaagccactgccgtggccg

atcttcccccgcctctacgtagacaaccagccagacgccattgctgccggtgcaagcgctggtggacccaacatcgcccacaacctcacctcgctgac

ctaccctggccgagtggttgagctaagttgggatcgacctggcgagattcaaggaccctaccaccagctgactcagacaaacacgagcggggtgccac

gctttgcagcatggttcggcaacctcaacgtgaccttcacgcccctcttcgagcgtaatctgaccagtcgtacagccaagaccacgcaacccggtgac

actatctaccctgacgtaggccagcgcgtcatcaatggaactgcgtttatcgctctcgtggacagggacgtctttgtcactcccgaaaatctcacgct

gctcaacgaccacatcgtcgctggccccgagttctaccaggctgattga

>contig_14.contig_14.g650.t1

atgcagctcaactccaagctttccctcttcctctgcgccctcctcgtggcccagcccatcgcgtccgcgcccgtcacca

gcgacaacaacgccgccgaactggctacgcgctctggcaacatcggctctggccagaacaagggcatcgacgtcggcagcatttttggcaacttcttc

gaaggtgacaagaagggaggcgaggaccacggcaagggccacggcggacaccaccgctttgccgagaagaagctcaaggctctcaaagccgctgagaa

tgcgaagaagcacaaggacgctgcggctgtcaaggcggcggcgaagttcgatgctgcggctgccaaggaggactctcaccacaaggaggccgacctga

agaagttcaagaaggagaagaagcagaaatccggcaaggacggctttggcggcggctttgacttcgatcgtcgggacggctttgggctcggcagtggg

cttgactttggaggcaaagacgactctggccacggcaaggacaactccggtcacggaaacaagggcaagggacacggcaagggcaagggcttcggcgg

atccaacaagaacaagaaggagaaggtcgagaaggtcgaagcgcacatcaagaatgacaagaagcacaaggccgcggccgaggtcaatgcggacgccg

agttcaaagcggcagctgttcacgaggatctcaagcacaacaaggccatcgcgaagaagcttgcagctgagaagaagaagaagaacggcttcggcctg

ggcatcttctag

>contig_14.contig_14.g651.t1

atgagcgagttactcagcgcgtcctggttgaagatcagatacaagcagttcatgagcaacgagaaggtcgcgaggagtg

gtgatgagtcgttggatctcatgatgcaggaggacagtagcggccatggcggagctcgcaggcctggcttggggaatcatgctaccagctcgagcttt

cacggcagtctcggcggcttctcctcgtcctcgccttcgccctcgctctctcccgcgccaggcggatcgatgtcaccgccactacaggcgacaactat

gatgatactcacgaaggatacgatacctgctcccaaagccatcgggccgatctcccctgcgccgtcgcgctacaccgttccgagctcgagcgaggcca

gtctgccgatccgaccggtgatggcagccgtgcacccggcctatcttgtcgatgcgattgagaatgcgggcggggggcgatgtcgcaagctgggaacg

tgtacacacgctcgggtaccgtcccgcttcccggcggtgtgtcccgtcgaccttccggcgagctatactacgacacagagggaggcgatggagaggga

gcagaagcggtggcgacaaaagcggcagcagcggctgtacaggcgcaggcacaggcacaggctcgagatcagacgcaggtgcaacgcaagcacaggcg

gcgcaggcccaggcggcaagcgaggcggcgaacgagacgacgaaggcaaacgggttgggggtgaggcgccctcgttttcctcgtcggtaccccgagct

tcggttcatgtgccggcgtctgcggcggatgagattccggcttatgctgcttcgcctgcaccagcgcctcattacggagttggagcggcttgttgcgt

tcggctcatcacaagtctcaagggcaagaatgagaatgacgacagtgagggcgactcgtccggggaggatgatgatgaagaagacgacgatgaggcat

ga

>contig_14.contig_14.g652.t1

atgaagtcgatcagttccttcatgctcattggcatgctgttcgccatcagcagcgtcaatgcagcctgcacatgcgcga

tctggataatgaacggcaaaggcgactgcaacaagggatgcatggcagactatcccaatcaagatgcaaaggactattgtagcgagcactggccgaac

ccccacatctcatcaggcccgcaacgcaagatcattcttgacgcttttggtacgcccctcttcagattgaccgagagatggtgccgcgggtctcctct

cacgcgagaggagcgtccctaccttcctctcactctcttatccagcatcagccctcgctctgatgagggcggctccggcaaaaacaacggcttcgact

tcggcgatgcgctcggcaccttcagaggagacgataaaggcttccacaagggctctggaggtgaccacttcagaaatgacgacaacaaggagggaggc

ggtggagagcgagcagaaggcggtggtggcgaaagcggcagcggcagctgtacaggcgcgggcgcaggcacaggctcgagctcaagcgcaggtgcaag

cgcaagcacaggcggcgcagactcaagcggcgaacgaggcggcaaaggcgacgagggacttgacttcggaaatcacttcatcagcggcggccgcggtg

gcttcgacggtggccggtacagtggatttgaaggcagcgtaggcagcgttggcagcttaactggcagcagcagcacttccggcttccaaggcggcagc

ttcctcggtgctcgtagcttcgacggcgaaaagtgcggctctggtctccatcaaaagaaggagaaggtcaagaaggttgcagcgcatctcaagaatga

atga

>contig_14.contig_14.g653.t1

atgggctcgcccatcatcaatgaggagcattactgcaacgatccggtttgcgcggagatgaacacgaagtacaccgaaa

cagagtccgaagattggtgccacgactgtacccaacgaagtgaagaagcggtagcacaggcagacgacggcgaggaaggcaacgaggaagacgacgaa

gaatccggcaaagaagacgacgaggaagacgccaaggaagacggcaaggaagaagacaaggaagacgccaaggacgaccaagaaggcgacggagatga

cgacgaaaatgacgagtga

>contig_14.contig_14.g654.t1

atgcatccccagtcgagacgaggcccggggccagcgatgggcatgaacagcggaagcggcatcaatggcagcacggctt

acacaggcgtcggcccatcctcctcctccacagcatcagccgccaacatgtccgcacggcagaaggccgccctcgcaagcgcatacgaagaactcgga

aaggagctccacgcctcctcagcactcaagaccgtcgggaactacaccctcggaaggccgatcgcagagggcaccttcggcaaagtcagcctggccac

acatcggcttaccaacacccgcgtcgccatcaagcagatccccaagtcacactcctcccacgcagccgccgcgctcaccagggagatccaccatcacc

gcaagctgcaccacccacacgtcacccagctctacgaggtcctcgcgaccgagtcttccatctggatggtgtccgaactctgcgcggggggcgagttg

tacgactacctcgtcgagcggggaaccttacccgacgccgaggcacgccgtctcttcggacagctctgtctcgcagtcgcatacatccacgagcgagg

catcgtccatcgggatctcaagctcgaaaacgtcctccttgacgagatgtgtaacgtcaaacttggcgattttggatttactcgcgagtttgagcggc

ggaagttgatggacacgttctgtgggacaaccgggtatgcggctccagagatgctggcggggaaaaagtactttggacaagaggttgacatctggtcc

ctcggcatcatcctgtatgcgctcctcacaggcgctcttcccttcgacgatgacgatgaggacatcatgcggcttaaaatattacagggcgattacga

cattcccgaagatctctccgacgacgctcgtgatctgatccgctccatcctcaagcaaaatccagtcgagagactgggaatccgcgccatcctcagcc

atccctggttcactcgtcacccaatgcccccgctgccctcactatcttccttcggcacctcaggcgccgagtcggcagaccataaccaccctccctcg

tcctatcgacatccacacctgtcgaatagtgccccgcccagcgacggcggatccgaagttctccgtgagcgcgagagtgagaacagggagagggaacg

aagtcgggcggccagtcggacaggcggtggtcccgtctcggacctgccgccgaccacggaggcagacaatgagagcaacaattcggtctcccattctg

caggtgatgaggcggacggggataggacattccggcaagagcaggcgacaggggacgaaaccgagatcaaggagaaggattcagacgagccggtcgtc

ggcgtacacgcagccgagggtgagaatgcacaagcagacgcggtaacgacagatgtcacgaaccggtctcccgaaatcgttgcctcttcctcctcctc

ctcttcactttccgcgcccgtaggactccatcagaccacggcctcgacaatgtctcagttgtccgatctcagcttcgtctccgctgtctctgagagcg

agtcggcgtcgatggaggagagcacgccgcctcgctctgaatcgacaagtccgacgacggaggacgacgacgaggaactggaagggggagcagcgggg

agtagtgcgagtgctctcgccaaacctgctggggaagttgcgcccggaggggaagacgcggacaagaccaaatcgtcgtcttccgatgagattgatcg

tcagcgattaaaaagcatgcggaacgagagtcagacgacgatacggcgcaaagactcgatgggatcggaccagtctcgtaaggcctcaggtcctacga

gcgtgggtgccaatgcatccgcgccgttgcccacccatcacgagtcgcctacttcgtccatcgatgatccccgggacacgacggtgaggtcaaatagt

gggccgtctcaaagtagtgttatgacgacgacgacgacggcgactgcgcagtcggtggcctccacctccgccccgacgctgacgggtccagagggatc

gaacttgaccaaattccactcgcagaattcgtctaggtcccttgctacggccggaggtggcggaggaggacccgcgggtggaccgggcgctggtcact

tccgcacaccatcccgcaccaaacgtcggtccgcctcttccatcgctctctccgtcgagggtatcctcccaccccattccgcctcagccgcctctgcg

actcgtttcgtggactacgtctccctgctgacccagctccaaccgacgctcttctcgacgctggtggagcagcggcttctgtttgcgctctcgatggt

cggcgtcgaggtgggccagattgtgcatagtgtcacgacggacgcgtgtgatgcgagtggggcgctttggtggatcttgaagaagaaggcggaggaga

gggagcgggagaaggagagtgaggctgctcttgctgctggggtggtgccgggcgcttcttcgaaggacgcggcggaggtggtggcgagtggtgtgggt

gtcctgggcggcggcggagctgttcctccgagcggcgtccaaagaccggtctcgccgcccctcccaccgacccctcagacagatccgatgagcccacc

gccgatcccgccaaaggacccggcccgacataggacggacagcgaccacgcccgagcggctgccgtcgctgctcttgtatcgatcggctcgaacgagg

gcctgccgaagtcggcccaggactcgttccgggagcatctgaatcggttgccgggcgcgagtgggacggcggccatggctgctctgcggcagttggag

cgggatcgcagtttggcggcggtttcgggggtgtcgtcggcgtcggtggtgggtagtcagtcggactcggtgcttgccctccagcgggctgcgagtcg

gagatcgaatgcgagttcggaagcggcggcggctactgctactggagctgcccgtggtggtggtggtggtggtgtcagtggtgatacaatagctggtc

ggacatcctacagcgcggcggaagtgccgaggcctggtgctctctcgccatctgttacggctgcgcagagctcgacttcgccattacgccagcaggtc

ctgcccgagacgccgagccataccacaccttcctctccagtttcggcctcggccgctctcgcgacggttgctctgaacatcacgccaaacaaggggac

gcgtccgaagcgagcggatcggccacgtacgaactcgttgagcatccggacgtttgcgaatgcgctctcgggaaggagtggagatgggtcgtcgccct

cgaatggcgctggcggtaattcgagcggacaccaggatggggagtcgaatcatcccgaacgcgccaagagtcccgtctcggcgctcttctcccgtcgt

ggtgcgggcagtttgattgccaaggcgggtgggggtcgagggaaggaagctgagcgtacgcccacgattggggttaccggcccgggctcgggaggcgt

cgctgggtcgtcgattggtcccagggcggatccgttccagcccggcacgccggcttccattctttccaagacgtccggcgtctcgccaggctcgccgt

cgaagagcgttgatgtaagcagccataccccgacttctgttggcgcctcctcaaaggcggcccccggtctgactcccaaaaagagcgtcaactcggtg

aagcaagggccgctcggtgcgtccatgtcccaagataccttcagcacggtccaatctcaaggcgagacggacgagacgaagaagcggagtgagaaggc

ccgacgtccgaagagctcgttcctttctacagttcggacctggctgaatgcggatgagcgacacgcgaagaagcggaagcagaaacagagccaggcgg

ctgcgttgtcgcttgcccatggctttgcaggcagccccgcctcgtcggcacttaggggaggtgggacgttgggcggcagtgcggcgcattcacgcaat

aactcggcgagtctgtctgggagtgtggctcggaacacgggctcggtacggtcccgtcagaacccgtatccgcccgtgagccctaccgtacgccggtc

gttggtgggtcccttcgccgtcgccagtggctcgaacaggggcagcatgagtcgtcgcagctcgaccgggagcaccgtcttgcctggaattgcagcgt

ctgcgtctgccatccctacgagcgcgagcgcgggtgcggcaggtacgagcacgaacatggtcacgaatgctcatgatggcttgaaccggcctacgaat

ttccggcggcagagcgcggggagcatcacgcctacgggcacggcgtttggcgactacgcgcacgaaatgccgggcttgttcgctgcgccgttacgggg

tagtagaccctcgagcatgcattcgttctctcagcagcagctccatcctcgtcggttgcctgccaagacgggttcggtcgggtccgcgagttcattcc

gccgaccgtacgatgccggcatgacgaagagcggctcgaactcgggatcgttcaatgccggctcgttcaacgggaacagtttgaagtcgatccataat

cgtcggccgagtgcggatggcggtacggtggtgaagagacataagagcgggtacggcttggcgttgggtgtgaatggtcctcatcagcggcatcacca

tcatcggacgggctcggggcattcacgcgccagctcgcctggaccgagcatgctgagcgaaatagctcgtggggaagggcccaatgaggccgagggtg

gaggaccgacagccccgggccaaattcctacgttcacgcgtggtggggggagcggcgatgtcccttctaaccgcagctcagcatacgagggcgacctg

ctcgccgtcccttcggccatcacggatgaaccgagcgagattctcagatctgcgagtggctcactgaatggattgccaagctcggcagcgcattcgac

cctgttcgtggcgcacaagtctcggtcgccgtataagcctccttcggccaatcatgcgctgcagggtgcgatacatcgggcggctgcggtcagtcaag

cgcacggtcaacaagggcatggcggcgcgaatctgactcagccctcgagcgacctgcgctctgtcccgctctgcacctggcatcgaagctggggtcgc

ccacctcctggctggactgggcccgtcgatgactcgcctgcgcctcccaaacggacgagtccgatccagcggggcgttcgagatgtctttgcgcgacg

gaagaagagcgggtatgccgatgccgatgacgaagatgattgggtggacgaggaggacgagccggcgtatgtgggtgggctgggccagttggactcgt

cgaaggtcaatgggctgtgggtgtcctcttcgtctggccttcggacatcccatgcagcgtctcaggggttcgcttcgctgcccggcttccaccccttc

agctcgacaccgtccgggctgccgcagagctcgtcttcggccctgcatcctgctctgagctcgcctatccctggccgtgggtttgggcaatttgatgg

ctttatgccccgtggcggtagcagtggcagcggcagcggcggcgcggctgctcctggaagcagtcagcgcggcgcatatccatcgtatctttcgcgcc

acactccttcgtcatccaacaatggacccttctcttcggcctccgcctccggtcaggggcttcgcggcctgttcgctccaccttcgttggggagcgac

gtatacccacgccagacggtcatatcggaatcgtcgagcgtcccggccgcgagcccggctgtgtctactgccaaccctgctggtgctgagggcagtgg

gagtcaggtagctgagggaggaggagcggatgttgctgcagtcggagtcgggtcagggtcagcgtcagggtcagggtcagggacggcgggaggtaccg

aggcgggcgggtccactactggcacaggtgtcggtgctgtcggaccgcgtcgcgcagctgcggctgctgcctcggccttcgtctccatcgaggagggg

gaggaggaggaagattga

>contig_14.contig_14.g655.t1

atgcccaaccttcgccttggatccgtcgcccccgacttcactgccgagaccacccacggcactctctcactccacgagt

acctcggtgactcatggggcattctcttctcccaccccgatgacttcacccccgtgtgcaccaccgagctcggcgaggtcgcacgcaagagccccgag

ttcaagaagcgcggcgtgaaggtgatcggcctgtccgccaacgatgtcgcgtcccacgaccggtggatcgccgacattgaggagattgccaagtccaa

agtcgatttccctatcattggcgacaaggaccgcaaaatcgcaactgaatacgacatgctggacgctctcgacgcgaccaacgtcgatgcgaagggca

tcccattcactgtccgcgacgtctttgttatcgaccccaagaaggtcatccgcctgaagatctcctacccagcctccaccggccgtcactttgacgag

atcatccgtgtcatcgactcgctgcagatcggcgacaagcacaagatcaccacccccgtcaactggcagaagggcgacaaggtcattgtccacccctc

cgtccaaggcgaggacgtgaagaagctgttccccgacgtcgaggtcgtgaagccctacctgcgcttcaccaaggaccccactgcttag

>contig_14.contig_14.g656.t1

atgaagctcagaactcagaaggccagaagcgggccaagaaggaaaggcttacgaccgtgcagcgtaccagccacgcctt

ccggcttcccaatcacgccatccacgccgcgcaccgctgaggcaccgagcgtcacctcatacggccggaacatgctcgatgatcgcggccatgctctc

gacgatggcgagctgctcactcacgctgcacccacacgaacgaacggaacgagtgcacgaacgagcgagaagatgtccacgcgcaacgccgccgccgg

gcccagcacggtgcgcaacaccccggccacctcccgcaacgccgcacagggagcaggcctctacaacacccgcggcaccaagcgcaaagagcggccct

catcaggcgcagcagacgcgcccctcccgcgcgacaagagcctggggaactacatcgagttcgacctcagcaaggtccgcaactccaagggcggcttc

ttggtcgaggatgacgacgcgcccggtggcaacgctgccaagaccgtcgaggagttgaagaaggagcgtgagaggcagatgcagcgcatggagcagga

gcaggatccgggcatcatcctcgactcgtccaagcagccccactgccacgtctgcaactccctcgaggttgatgagcagtttcgccgcatcttcagta

ttctcgtctgcaagacatgcatgaaggccgacccggacaagttcagcttgctcaccaagaccgaagtcaaggaggattatctgctcacagactctgaa

ctgcgcgatcccgaactcctcccccacctcctccgtccaaatccacacaaagcaacctactccaacatgatgctcttcctccgctgccaagtcgaatc

ctacgccttcggcccctcccgctggggctcccccgaagaactcgacgccgagttcgcccgtcgacaagaagccaaagcaaaacggcgcggcaagaaat

tcgcagaggacatccaggcactccggaaacggaccagggacgagacgattgcgaagaggaacgaggtcgcggcacatgagcatacgtgggttgaggtg

ccgggacaagtcggcaagcaggagtgcgaaacgtgtgggcaggagatcgaggttgaagagttctga

>contig_14.contig_14.g657.t1

atgaatataccgcccttcaacgagtctgagtccgcccataaccacttccccaacacgaaccatggtggcgccaacgccg

caaccaacgctcccaatggagccctcctcgccgcagaggacggcggagcagcgccggacgaacccccctgtagtgggcactcccccgtcaaccatacc

ctcaaacatgaccgccgccagctagaagctattgaagtcggtgcacagtcacccccttctccctcggccgagtccacaacctcgtcggcctcgtccat

caccatcgcacagggtgtcgccgcgacgggaacgccggccgcagtccaggagatcagcaacgctcacttgctccccgtcgatgccgtgtgtgctcagt

tgggcacccaccccaaggcaggtctcgcatccgccgcagttgaggatcgtcagcgtcagttcggactcaaccagttgacggaccccagccgcgtctcg

gccctcaacatcctcgcacgccaggtcttcaatgccctcaccttggtcctgatcggcgccctcgtcctcagctttgccgtcaaggactgggtcgaggg

tgccgttgtatgcgcggtcattgtcatcaacgtcggcctggctttcgttcaggaatggaaggccgaacgcaccatggcctcccttcgcaagattgcct

cccccacggcccaagtcttccgcagtggacacgacggaagcatcaccattgccgctgtggacctggttcccggtgacatcgtcgagctctccaccggc

gacatcgtccctgccgaccttcgactcatcagcgtcaacggcctcgagatcgaagagtccgctttgacgggtgagagtgtgcctgtcagcaaactcgt

ccaggctctcgtcttccccagtcaggttgtcagcaacagcaagtcggaaggcgaagcagctgatgctgccatgacgcacgatgtgcccctcgctgacc

gagtcaactgcgcctttgcctctaccatggtcaccacgggccgcgcaacaggtgtggtcatcgccaccgccaacgacacccaggtcggccagattgcc

gcccagatgcgtcttgggtccgccgccactcgcaaagcggccgatggcgacgataatgccaacgggaacgcctttgtgggccgtctccgccgtattgg

tgccaagatcctccatggacttggcaagtttttcggtctcaatgtcggtactcccctccagcgccggatgaacaagttcgccttcctcctccttggac

tggcgcttgtctgtgccattattgtctttggtgtggccaagtttgatatcgatgatcaggtggttctctacgccattgctcttggcatcggagtcatc

cccgaaagtttgacggccgtcttgaccatcaccttcagcgtcggtgccaaacgcatggctgaaaagaatgtcatcgtccgtcgtctcgaagcgcttga

agcacttggatcatgcagcatgatcgctagtgacaagaccggtaccttgaccgaaggaaagatggtcgtccgcgagtgctggctccctggcgcagtcg

acgaggacggccaatctctcgagctcactctttccgcagacggaacgggaagcatctccaacactaccaccggtaacgcttctcccaccacacaagca

atcggacccgatgttctctcggttggtctccgtgcgatggctactgtcgcctcgctctgcaatgtcgcccagatcgccgtcaaggcagagaccactac

tcctccagcgcaagggcagaaggacagccctgctccggccacccgcggcaaccctaccgagatcgcactcgccttgttcgctacggcactgggaatga

gcaaggagaagctcatcggcaagtcatccggctcgtcgacagaacccgccttcggattgccggctgatttcagccgcacttcgagcccgatgaaggag

aagaacgacgttaggattcgctccgtcgaggtgcccgtcagcacacctgctgcctacgtcgaactcggcgaatatcctttcgacggaacgatcaagcg

gatgacgagtgtctaccgtcccacgaccttctcccctcacaacaccgatgatgtcagccaagtctacttcatgaagggtgcggtggaacgagtgctcc

tcgcctgttccaacaccgtcgtcaacgatggtgacgccaagggccgcgatttcgccttgttaccgactcgagaactcgacgagcgcacgaaggaggct

atcctcgctcgcatggaaacgatggctagtaaaggtctccgtgtccttgctctcgcccaacgcttcgacacggcatccgtctcgagctcgttccctga

tcagaaggccgacaaggtcatcgacgaaggcatccctaccaaggccctccagctgtccaccggcgtcgagagtgtggccgaggcatacaagttgcccg

cccgagaggaagtcgagcagggtttcaccttcctcggcctcgttgccctacgtgacccaccccgtcccgagactcgcccgagtgtggaagcctgtcgt

cgcgccggcatcaccgtctgcatggtaaccggcgaccatccctccacggctcgtcagatcgcgctcgacgtcgccatcctgcgcggcaacgaaggtcc

cggggcggtcatggaggcccgtcagttcgacgccttgagcgatgccgaggttgacaagttggccgagttgccgttggtcattgctcgatgctcgccca

acaccaaggtccgacttgtcgaggcggcggcgaggcgcggcaagttcctggcgatgactggagatggtatcaatgatgcacctgcgctcaagcgaagc

ccgatcggtattggaatgggccagaacggcacggctgtggctcgggacagctcggacattgtgctccaggacgataacttcagcagcatcgtggctgc

catcaaggccggacggaccatctttgacaacatccaacgcttcctgatctcgctcctcgtcgccaacgtcggcgaggtcatcctcctgctcggcgggc

ttggcttccgcgatgcacagggcgagtccgtcttccccctctcgccgctccagatcttgttcacgaacgccgtcgtggcgtcgttgcctgccgtcgga

ctcggtctcgagcccgcggagcgtggcagcatgcgtcgtcacccggaccaattcgcctccggcgtcttctcccgcgcggtcatcgtcgacatgctcgt

gtacggatgcgtgatgggcgtcacctgtctgctcaccttcatcgcgatgatgtacggcgtcggacaggcccagtttgggattggctgtaacgagaatg

cgagtccggagtgcgatgctgtcttccaggctcgctcggcgacgtttgtggccctcatcatgcagaacctcttcctcgcgtgggagctgatcagcatg

caccggtccttcttcgccatgaagccctggcgccatctggctcggaaccctctcctgctcggctcggtcatcttcggcctcgtgctgatcccgatctg

tctgtacgtgcccggcttcaacgaccgggtcttccgccatggtccgttgaggggcgaggggtggggtatcgcgctggctgcggttgtcgtgtttgtgc

ttgcgctggaggggtggaagtgggctgccagggagggcggctggccttggttgaccgaggtgtccggtgggcttcagatgaggatccccccggctgtt

gatggcgaggacgacgaggaggaggaggaggatgaggggcgcagcgttgaggagaaggagagcggacgaaggcagtcggtcgagaggatggcgtag

>contig_14.contig_14.g658.t1

atggaagaaccaggcgaaggaaagggagaaggaagcagcccatggacactctcagacctcacaacacgctccctcgatc

ccacgctgacacgatcacaagcccagcagatccagcagattatcgcagctcaagttcaaataccagaagatgggtcagcaccacacccatggcccttc

aattcttccaaaacgacactccgcatcgcagccatggacatcagctaccccgtagccccggcccaggcctcaacacacacagccgcctcgctcgtcct

cgtccagattccatggcctctacccgctcttcttggagatgcggtagtcatcacccacctccgaacactcaccatccgctcaccaccgccttcctttc

cttacatccccggcctactcgcttttcgggaactaggtcccctcctgcatcttctctccctgctagaggacgaagagagaccggacgtacttctatgc

gacggacagggtatcgcccacccggctcggtgcggactcgcctcccacctgggcgtactcaccggcttgcccagtatcgggtctgccaagtcttggtt

cttcggacgactttccgaagacgaggacgacgaccgtcaggaaggaggacatcctcatcatcatcatcgaccgacgtttccgaaccttggccatcatc

gcggctcacgtctgcccctttattactcaaactcaacattcacagctacggcgactcatcaacaacaccagcgaagagaacccatctcccatgtcctc

cgctcccagccaggcatcaaccccatcttcgtctcgcccggccacagattcaccctcaaccaagcatcccagctcgttctcgcgctttgcacgaagta

tcgactgccggatcccattcggatggcggatcacagtagtcgggaagcgttgcgggaatgggcggcggaggcgcagcggaccgagacggaagacaaga

caaaggacgcacaaggagagatgtcagctctattatcttga

>contig_14.contig_14.g659.t1

atgtctgcgtggcgcgacaccctcaccatcgagacgatggagaaggagcgcgcgaaccccggcttcgacgtacgcgccc

tctccgtcgccatgcacggctccgaactcagcctgacacagaaggagcgcttcatgctcgaactcagccgagaccccgtcttctacatgggcgactgg

caggacctctccaaggaccaggtccgcgaacgcaccatgcagaagatcgccgctcttgtgcactacgtcatgaacgagaccgtcgacgtcttcaccaa

gcgcatggagctcatcggcatgcacgaccccggcttctggactcgtttcggtgtccactacggcctcttccttggtgccatccgtggtagcgccacct

ccaaccagttctcatactggatggagaagggctgcatctcctgccaaggcatgtacggctgcttcggcatgaccgagcttgcccacggctccaacgtc

gccggtctcgagacaaccgccaccttcgacgagaggaccgaccagtttgtggtccacacccctaacctcggcgccacaaagtggtggatcggaggtgc

cgcccagtccgccacacactgctccgtctttgcccagctcgtcgtcaacggcaagcgctacggtaccaagaccttcatcgtaccgctccgtgacccca

agagcttccagcttctgcccggtgtcaacgtcggtgacatcggaaagaagatgggacgtgacggtatcgacaacggcttcattcaattcacaaacgtc

cgcattcctcgcgcatacatgctcatgaagcacacacaggtcacacgcgagggtgaagtacgagagcccccgctgcagcaactcacctacggtgccct

gctccaaggccgcacagccatggtcgccgacgccgccaacactgccaagaaggcactcaccattgccgtccgatacgccgccgtacgaagacagttca

agtcctccccgcaagctgcgctcgaaacccagattctcgactaccccatccaccaacgtcgtctcatgcccctcctcgcccaggccaccgccttcggc

ttcaccgcgctcgagatgacccgcctgttcgaggagacctcaaccgctcttgaggccctcgagcccggagaccctaaccttgagaagacgattcagat

gcttaaggagacccacgccacgtccgccggtcttaaggcgttctgcacctggggaactctggaggccattgacaagtgccgacaggcatgtggtggtc

acggttactcgtcatacaacgggttcccttccatgtacgccgacttcgcggtacactgcacatgggagggtgacaacaccattcttgccctgcaatcc

ggtcgctccctcatcgcctcgtacttggaagccaaggacaacgggaaaaagttctctgacgccaccgcatacatgaacgacattgagaccgtcctgaa

ggccaagtgcgccggcaacgaggcgctcaacaccctcgagggcatcgaggaaggttgggccactgtcgccgcccacgccgtcaagaaggctgccgagg

actacgtcgccctcgtcaaggctggcaagaacaaggacgaggctttcgaggcgtgctcccaggtccgcttcgtcgccgcctcgatccactcgtccggc

tacatctaccggcagttcaagcaggccgtcgagcgtctgcccgccgacgccaaggacggcgtccggcagaagcttgaggtcgttgcgaagctgtacgg

actctggcagatggaggagaaggctgccttcttcctccgctcgggctggctctcggcccaacagttggatctcgtccaggcgcgtgtcagcgagttct

gcgctgcgctccggaagtttgcgatcccgctcatcgacgcctttgcgctctcggaccacattgtgaactcgccgatcggggtgaagagcggggacatc

taccgcgagtacttccgcgctgttcgtcgggcgaacccgcaaaataaggaacacccgtacttcgagcgcgtcattaagccgttcctgttcaggaaccc

gccggagatggacgatgcggatgatatgggggttgatgatgacattgaggagatccgggaggagctggaggaggctgagaaggatcggttgagtgcgg

agaaggagcgggcggaggcggagaagaaggctacggtcgagtag

>contig_14.contig_14.g660.t1

atgccgtctgaagtcgagcgcgacgcagcaacgacggcggccctcctccccttcggcgccgcctacgcaggcgcacccg

cagacctcctcaagcgcaacggaaccttccaattcagcaagaacaggcgccggacgaggaccaacgacgagcacgacgacccctcggtcttcaacccg

gcgacctgcacccgcaagggcaagcatctcgtacccctccacccgaaatgctcacactccaagccgttcaacatctactatgagcaacacggtacggg

acctatcaagctcgtcttcctcatgggtctagccacgtcttgcgccggatggctaccccaggttgaccacttcgccaacgcgcggaatgggaatgttg

ataggtactcctgtctcgtgtacgaccagcgcgggttcgggagtagcgatgtgccaaagggtcgctatcgcacctcggacatggcgcacgacttgctc

agcttgttggcagagttgcgatggatcgatggggatgagtacaggaacgtccatcttgtcggcgtcagtatgggcgggatggtcacgctcgagttggc

gaagatggtgcctcaccattggggtagcattacccttgtctctaccaccagcggacagaacctgggctccaagtccgtgatgaccggtatgccaccgt

tccgcggcatctcaaccttcgccaacatcatcatcaccaccgtgctcggcaccaagacaccgcgccaacatctgaacgccatggtcgagctcctcttc

ccatcctcctacctctcggaaccacacccagacagcccgcagggcctctcccgccgggaatgtttccagcagatgctcctctggcgatttcagtacag

cagacacgccccgcccgacggcgtcctccgccagatctccgccgtcttcacacacggcgtcacgcagtcccagctggaccggatcaacagagaggttc

cctccatctccatcgtgacgggggacgatgaccacctcgtcaacccggccaactcggattatctggccaagtatctcgaccgggctaggttggtcaag

atgagggagacgggacatgcgttgcccatccagcgcacggaggagttgaatctgcttattgagcagactgttgacttgggcgtgcagaggtcgaagga

ggcatactggcgcgatcgcacggcgcgggctgtgctttga

>contig_14.contig_14.g661.t1

atgtcgctcgcagccacaagcgccttccgcgccctcgcacgccccaccatgctccgctcaccagcagcagcagcagcag

cagcagcagcagcagcctccccagcgctggctgcccggaccgcgttccacaccaccgcactcgcacacatgcgctccacccaccgcaagaacgccgtc

gcaggcaacccaaccaaatcatacatcgacggcacggtgaacgagccgacgaccttcccgcccccgtccccctcgcacggctcctaccactggaccct

cgagcgttccctctccatcgccctcgtgcccctcatcgccgccggtgccgccaagcacggcgcctcgggaatcctcgacggcgccattgccctcagct

tggtcgtgcactcccacattgggttcgatgtgatcgtgcaggattatctgcacccgcgcaagtaccccgttgcgggacctattgcggcgtggaccttg

cgggccgctactgttggtaccctggttggcatttatgagttccagacaaacgacgttggcttcaccgagctcatcgccaaggtgtggaccgcatga

>contig_14.contig_14.g662.t1

atggcatccgcgcgcatcagcaaccagcaccagaacgcatccacctccggctccagcacatcctccgcctccgcctctg

cctccgcatcctccgcctccctcctcgccgcacactcccaccagcaacatcgcttccgcgcgtctcccacaggcggaatgagcggtgcacagccacca

tcaccatcatcatcacagctgccacagggcgccggtggggggtatgcgggtcttcagcttaatgggagtggaaatggaaatggaaatggcaatggtcc

ctcacggccaccgccagatcggtcagatatcgtcggccgcggcaccgtcctcgatccgtacgggcttgactttaatggtgcggccgcaggcgggggac

tgccgatgccagctaccgcgggcacacaatcaggctggggagcaagtttcgcaaggccagcccggtcgcactacttcctctcccgctccgaaatcgtc

aagggcgtggccaaccggttcgtccattcgactgcgtatctctacttctacggaggcatggccgtcgcctccctcctcaccgtcgccatcagtcttat

tcaagattgccccggcacgctcttctacagtctagagctggccattaacgtccttctcattgccgaggtgggcatcaggctatttgcgtttggaaagc

aattctggaagtcgacattcaacatcatcgacctctgtctcgtcgtcttctgcgccctcacccttctcgtcatcttcttctctcacgactgctctccc

aaccacggcggcgacggcggcggtgactacggctccagcttcgacggcgaccaggaccgagacgcacgaagaggaggccggggcggcagatccgaaga

actcctcgactccatcctcctcatcatccggaacgcaatgcagtgcttccgcctcgtctccgtcgtccgcagatcgaggcagaacgtaaactcgcgcg

tgaccaatatcgaccttgcctctgcgcaggctggccgcggcgctgctgatgccgatctctatcctggctttgagtccgctgcctccggtggggggatg

cacgatggcgataatccctatggggatacagggataggaggcggagacgggggcgggggcggtggtttgggaagaggtgcgggcgggcccctcggcgc

cctcggccgagcagcgcgcttctccctcgacatcgacttacaagatgagggggccctcgcacgggagcgcatggccgacgggggcgatccggccgagc

ggaaacggagggccgagcgggaacgatggcggacaagcagcgggggtgcgggagtgggagggggtttcttggctgggatgtacaaggatggcgcggag

gagcagcgggcgctgtttgatgcggatgatgatgatgatgaggagctttga

>contig_14.contig_14.g663.t1

atgagcaacggccgaccatcccaaccgcccatcgcgcaagtctacaaccccatcgagttccaagccgaagtaaacatcg

ccagttccgccttcgaggacctcctaagcccgacgacagaatggaaggactacggcgaacaagacggcgtccgaatctaccggggcgacccccccgtc

cttcccggtcgcgaccccagcccgggaaggtcggtcttgccgctctgcaagagcgaggtcgagatcgaagacgcgaaaccggtcgaacttctctcatg

cgtccaccaggcggcctaccgatgtctcttcgatccccgcctccggacgtcgtactacctccgccgcttcagcatgttccacaaccaattctacgccg

tgctctccttaggccgcgagtgtcccacacgggaacttgtcggtgtccaatttgcgcgcttctttgaggaggacgggaacgaggtcgcgcatcctacg

atgaagttgggcaggattgatctggtgtttgcgagtgtggatgacgtgggggcgccgccggtggaggggaagtatatctcgcaggttgattatgggca

acctataccatcatttatccacaaggtcatgtggctcgaagaaccactcaccgtcgggcggttaaaggagaccttccgactgctcggttttccgccgt

acgtcctcgatccgacgcataccatcgtcatgcagctccagagcttcgacgtcgagactcgggcggtgacgatctcggccctggtggtacgtgcgggt

acttttactgttgtacttgacagcaaacggatgtacaaggaaggcgttttcgtcagtgatcgttcaggacctgcgatcaagtcgatcggcatcgagga

gatggacggcaacatccaaataaagacgatcgaaagtgcagtcgggcatgcctttgagctggtcgccgagctggtcctcgaatctcaaaatgtatcgt

tatcgcataccgaaggcatccctgtcttcgccctaaccccttttgacgatcccgcgtctttccgccatccggcggccctcaagcttgaggtcgagttc

atcagtttccacgctcgcagcaacctgtgtcctgtacccgaccgccgaagggaggccgacgaggcgatgagcgcgacggatttgattcgccgatttga

taattggctcctccttgccagacagctccgaacgctgctcaatggtccactgcattcatgccgagccaaagatcgctcattttcctcttctctgagcc

actcggcggatcgaaaggagctcgcggctgacctcttttgcccagtgctgctgaccactttcttccccgttgttgcctccaagagtctttctgctcgc

ttgctgctgctgctgctatgcctagctcacactttcgtctttgcgttttga

>contig_14.contig_14.g664.t1

atggtcgctctcctgaacctcgtcggagctctcgctctgggctcgacccttgtgcacgcactccccccgctcgagcgta

acctcgcttacctctccccctcagcgcgtctcacccaccttggccacgacgtcgcctcgattgcgagccgcattgttgacaatcatgtcgagctcgtg

aagcgatcgactcaggctgatggattggttgccaactggaccggcgactttggacccgatggccagatggcctacaagggcaatgtcaccttccccta

cggtgttgcctccggtgacccttacgacacatcggtcatcctctggactcaccccgtccctaccgacagcaacgtctcccagccgatctgcctgacct

accaggtctccaccgaccaagccggcaagaacgtcgtcgtgtccaactacgcctggacgacttccgacgtcggttactcgtacaaggtcgaggctacc

ggtctccaggccaagacgaactactactaccgcttcgcggcttgcgccgaccactccgttcagtcggaaactggacacttccgtacgctgcccagctc

caacgatcttttcgtgaccagcctcaagttcgcggtgttctcgtgctcgaacttcccctttggacacttcaactcgtacgcagcggctgccttgaagg

acttggacatttggttgcacgtcggagacttcatctacgaatatgcaggcgatggctccgcggactcgtacggtgacggccgtaacatctctcgcgtc

cctgagccgaaccgcgaaattgtcactctggacgactaccgcacgcggtacgccacgtacaagacggacgaaggtctccgtgctttgagcgctgctca

tgcactcttcgctgtgcccgacgaccatgaggtcgccgacaacacgtggaagggtggatctgccgacagcaacaacaccgctgctggaacgatctcgg

gcgtcactttcactcagcggaaactggctgccgtccaggcttaccacgagtggatgcccattcgccaggttgacaccaccgatggcctgcgcttctgg

cgtcgcttccagttcggcagacttgcttctttctctatgctggacacgcgtcaatacgagcgcgatgttaccgatctctactacaacaccgccgaggt

cgctgctatgagcaacgacaccaaccgatctctgatgggtggcaagcaggagcagtggttgtacaagaacctcgcgaatgaccagaagagcggcatcc

agtggaagatcgtcggtcagcagatcatcctggccgtcacccacgaaggcgcgaccgccgactcatacgactacgatgcctgggacggctacaacgcc

aaccgtcgtcgtctcttcaagaccatccgcgacaacagcgtgcagaacaccatcttcctctccggtgactcgcacgccgcttggtcgtccgacttgtt

cgaggctgagcttcttaacacgacggcgtatgaccctaccacgggcaagggctccattgctgtcgagttcgccggcacgggtgttacgtctccaccct

cgtacggacaaggaaagacctcccagcagtacgtcgaccgcgccaagtatctcacgcagatcaaccgtgggctgcaattcgcagagggagagctccgc

ggctactttaccgtcgagatcaaccccttcaaggcgaccgccaagttctggggcactcccgatatcaagaccgcgaacacgaacgagactctcatcgt

ccaactcgacgttcaggccggtcagaaccgcctctcgcgtccgatcaacggtggcaagaacgtcacctacggcgcgctgcagtccgacgtggtcaagt

acaacaccacgagtgccaaccggggttggaacggaacggcgtaccagctttga

>contig_14.contig_14.g665.t1

atggaagccttcaacagaagcggcggacgtggaagcaacggcactcatgctcgcaatgctcagccaggcccttccagaa

accccgtcatgagcacacccagccctgaaagctccagttacatgcagatgtccagcacgtccagaccagactccgcaaagcatgcttcccccttcagc

aacgcttctgcctccgcttcaagacttataccctttgacaccaacagcagtgacttctacgcgcgcgcacaggctatgggactccgcctctccagaga

agatctcgagcgcacacgcgcaggcgtcgcagccatcgtcaagcgcaatcgaaccagcccggcctcctccgtccttgcacatgctcccacgtcatctt

caccctgcccacccaccatgggcggtccggtattgagaaaagtcactacccctcactcagggtccaacagcagcagcagcagcagacgatccatggca

ctctccagcgggggcagccaaagcgccggcctcaagcccacattagagcaaattagcatccgtaaagagcaagacggagacgatcggcgccgaaagag

gcaacggcgcatatcagagagcactccaaatcagcctgtcctacaaccaggtctactccatacccttcatcagcaccagcactaccaccaccatcaac

acaacctccttagctcgtcctcatcacaattcatcgactcctcctcccccggcagccatttcgatcctttcaccgtatcggccccacgctggaaatgg

atggaccgatcaggccggcctcggacccggcctcgtatgacactcgacgaagttggcgtccgggccgaggaaggcagtgacgagaagcgcaagtccag

acgtcgccgacgcaccgccgaggagtccggggccgaactcaccgacaacgattcagtccggcccgacgaggacgacatgctgcctctccttcgatctc

tccaccccacacatccaccctcatcgctctatacggaccgcagacggaacgtcgccgaagattctcatgctgaccttactgatacggattcccttcgg

ccagaggaagaagatatgctgcctcttcttctctcgcgccaacctcctgatcttccgtcttcgtcccttcctgtacttcctccttctgctccttcgaa

caagaccgacgagtatctccaaacgctgctcgcctaccccaccgtcccaatcgtcgcacaggaagagccggtcgccgacgagtcgatcgcaggcgagc

ctccaccctcaagcgcggcagacttgtccaagtcccgttccgacatggaggacgagggcgatacgtcggcatcccagcttatgctggccacagacgag

tttgtcgaggcgtgggattacaacgatggaactcgggtcgtcaagcttgtgtcggaagagttggcgtacgctctgcgtgcgggaacactctccgagtc

cgacattgaaccgcgctactacacccttcccgtgcattggggtaaagaaaagatgaagccctcccacgcatcgtacgcaggcttgatcggcctcgcta

tcctgtcctcgtccgacggcaagctcggcttggcagagatctatcagtggatcaccgccacgttcccacactacgagatgcgcgaccgcggctggcaa

aactcgattcggcacaacctcagtctgaacaagagttttatcaaggtggagcgaagcctgctcgagaaggggaaaggggccctgtggagtattgcacc

cggacacgaatgtcgcttcgagaatgggacgtacaatgcgaagaaggcgatcgtgtcccagcctgcggggccagggcagggtggtgcgcggcatgatg

tgacggaggacgaagctgatgacgagggtgatatggacgacagtcatgcacgcgccggttcgagctccggtgcatctcggaatgcctctacctcaaaa

ggtgggatgtcgtcgtctacggcagatgcgcttgcgctgatgaagaagcatggcggatcgcaggtactgtgctacaccctgcagcacggcacgtcgca

caggtaccaccggctcaagcgcgatcccgtccaggacgtggccaagggcctggctgttgctgttacgctttcgatacccgccgacaggactggcggcg

aaacgagcacccctaggattggtagttcgaccagcttaccagccctcgtccctgcttctcaagaaagcgtgcggacggagaccagtcttccgaccggt

tcgttctcctcgaccccagcccgtcctcttgctccgccaccacagcccaaacgcggcaggcgctccggaggagctgtgccgtcgaaagctgcgcaggt

ggaggcactcagccaggcggtgtccaaagctgaaacagcggcggctcgcgcggcggccaaagttggcgttgtcgcctctcaagatcctgcgtctgcgt

ctgcatctgctcctgtggtgacccggtctcggaagaggataccgctgaaggacaagacgaatgtcggggtgggcatcaaagctgtgccggcggtgcag

aaggacattttctctgataatactatggttgccagtacggcggctgcgaaggggaagggcaaggcggaagaccctgttaaagtcacgacgactgcata

ccctcagatctcgatgggcgcaccgttccactcccccaaggctccctcgggaccgacgatgatgggcgccttcacgcccggaccggcgaactcgtcga

ataacaacactacgacgaggtggccgatgcgctcgcctatatcgtccctgcggagttcgatgggtggggccttgaagagcgggagtgggagcgtcatg

cttcccattgcaggtcttacgcccgctcgtcggatcttgggcggccatacggggctgggatcccaattcagtccgctgttcaatacgcccggcgggct

gggttccgggatgggcatggagcttgggctgggcgtcggtggtgtggggggcggcaataacgggatgggcctgagcagtctgcggagcgcggcgatgg

gcgctggcacgggtttgggcagcgtgatgagtcctgtccatccgctcggcggggtgcttcagacccctgcgagggggatgttgagtggcagcgcgatg

gggggcgacgagatgcatcatgctcagatgtccgggggcggtggtgctgggcggcatcggaggcgggagagcacagggcctggagcggggatggacgg

accgagcctcggtgggaacagcagtcagtggatcttctcctcgacgcccatccgtcgtctgatgagcggggcggccgggtcggagagcgcggggaggg

ggatgagcaatttagggaatatcatgtcgtccccgatctcgtcctcgatccacgaacggacggggagcccgtatgcgcgttcgaaccggagtcatgcg

ggcagcggcggcggccacccttccagttcgatcggctcaacctcgacgccctcggccgccgtacctggttctcacaaccgatcgacaggtctgacgcc

tgcggggggcgacgcgcactggctcggcgatccctttggctaccagggcaacttctcgcacgagctcgcgttcacgactgagatgggcgggttaggga

cggggattgattcgagtccggcgaaggatctctggcggtcgtga

>contig_14.contig_14.g666.t1

atgtgccgccgcaggaaggacggcgagccctcgtcagcaactcccgtcgtgatcctgttctccctcgtcctgctcacca

cctccatcgccttcctccaggccttcgagtcctacgtccgccgcacacgcaagctcaacaacaagcgcgtacgcaccctcctcaagcagatctacacc

gaggaagaggagaaacatctcgcacagaaggaaggcgggcagaaggagtacgaatacctgaataagcgccgcgaggaaatcaccatcgtcggcttcct

ccaccccttctgcaacgctggcggcggcggcgagcgcgttcttttccaggctctggcgcaccaccaggatcgcgaccctaacaccctctgcgtcgtct

acaccggcgatctctcggaggaggtcaccaaggaggacatcttggcccgagccaaacagcgcttcggcatctccctccgcccagcccgcgtccacttc

ctcccgctcacaaggagattcctggtcgaagatgcctactggaagcatttcactctgctcggtcaaagcttcggagctgccattctcggagcgcaggc

tatggcccttctcgtgccagacatcttcgtcgtcgtgcgcttcttcgtccctggcattcccatcggcgcatatgtccactaccccaccatcagcacgg

acatgctcgaacgagtcaagaatcgtacagcaggacataccaacgacgccagtgtagcagggagcaagtggaagacagtcgcaaagctcatatactac

cacatcttcgcatacgcatactcctactccctctcccgcgcagacgccgtcgtagccaactcaagctggaccaaagcccatctcgactcgctcctctt

cggcaagccgcgcaggcaggctcgtcccaaacccgtcctcgcttcgtcctcaacctcaacctcaacctcagacagcaccggtcttgcgaagacaaaca

agaaatcagcggtgcagtggaagtcgaagaaggcggttgagattgtgtatccgccttgtgatacgactgccttttcggccttccctcttgaaggcccc

gctgagggcgaaagcagagaggccggtctgatcgtctccctagcccagttcagacctgaaaaggagcacgccacccaaatccgcctcctccgcgccct

cttcgatctcgacccagcctatcgcaccggcggcaccctgaagaccgactctgccgtcgtcgtcgttcccaaggttcggttgaccatgatgggcagct

gtcggaacgagggcgacgaagaccggattgaagatttgagggagcttgcacatcagcttggtgtacaggattccgtctgtttcgtggtgaacgcgccg

ttcactgacatcctgcacaccctctcacgggcctccatcggactgagcacaatggtcgacgagcacttcggcatcaacgtcgtcgagttcatggctgc

gggtctgatcaccctctcccacgcctccgccggaccctacctcgacatcgccctccccatcctcccctcccctacctcctccacaaccctccccaccg

gcttccacgcaacaacaccagcctccttcgcctccaaactcgccgagatcctcgcactggacccggaggaaacgctggccatccgcggacgcgcgcgg

gcgagagcgatcgaatgttttggaagtgaagggttcgaggagacgtggagatttaatgtgtgggatgtgcttgtgcggaggtgggaggcggagcggct

gaagaaggagaagtctgagtcggactga

>contig_14.contig_14.g667.t1

atgtcttccacccagacctcagcatcggaagcgcagccgcaggcacagaccgacgcgcaatcgtccgcgcccgcgcccc

gggaatccaactcggcctcaacatcctcagaaccgagcacgtggacgagcaacctgtcttcctatctatggcccttcggaagtgggaccagcacctcc

gaagcttccccaccttccgcccgcagcccctcctcctcctcatcgcccccgccgccagtgtggagaccgagctacgaagagttccgccgccacgagcc

cccacaggcgcgcctcatccgtctccgtgggctcttcgataaattgctcgataagagcggggaatctgcgttggctaatggacgcgcgaaggacgctt

ggacgaagagttcgattcgggctcggatgggcgcgggtgttaaggtggaggagaagggcgggatgtgtgtatcgtcggatttgacggggaagtcgtcc

cattcgagcgcgggcggggcagctgctgctgcttcttccgcggccgcggcgccgtcgagtgggaaaacgtcgcaaggacctgaactggccgcgtcgga

gttaccacagccgtcgtatgcgactgagctacttgcgaagtgcaagcgctcgcacgatgtggacgacagtagcgaacggaaacgcgcgacgcaggtcc

ggaaggaagttgctaaagtcgaatcttcctcgtcctcctcgtctccctcctcttctaccccggctccatcttcgagctccgcttctcccctctctggt

cgcgagcgcgccgccgacgcaagccgtgacggcgaagccggctgggtaggttccggcgtctggggcctcaccgccgtcagtctagaccatctcgaaga

cgctgctgctaccagtcagagcgaggaggccagactgggcggggcgtccgctgaggaggtgcgggagttggaggagagggagaaggtggaacgggagg

aggtggaggggttgaggcagcggaggatagagtgggaggggtttttgaagtatgctgaggagaaggagcgtgaactctacaccatctttcaagacatt

gacaagaatggcgacgggatcgtcgacattggcgagattcgcgccgccctcgagcgcgccggcatcgatttggggtcggcgcctctcgaagacttcac

agcctgtctggcgtcggcgcggtattacccttccccacccgtgtcgggcccgtcggagggaagcctgtatgtcacgttccccgagttccgcgactatc

tgctccttctgcccagaaaagcaaccatgccagagatcttccgtttctaccaagtgagaaaagcgtttggtctctttgtggagggtggagtcttcgag

gaactcggacggggatggggtcgaaccaaacgtggtgcggccgtggtcaactttgatggggatgtcagccttgccggcgaagagaagatgaagggaag

ctcgagttcgagctcgatcgcccccgctccttcctcgtccggagcggcgacagccgcagcggtcacggcgacggctgtccaagctcagattccgacaa

gcgatagcaagagttctcgttcgcaggcacacacttcgcacccccagcaccaccacggcgccggccattcgtccgaaggagacgaccacatcgaatca

accagcggcgcctcctcctctgcttccagcagcgcagcccacgcccactcaccagcccaggaaggagaagaagaagaagaagacgaagaagaggacga

cagcgacgcgctgcatcccgagatcgcggtcaagttcctcctcgccggcggcatcgcgggtgcggtgtcccgaacggcgacggcgccctttgaccgat

tgaaggtgtacctcataacgtccgcgaagcagacggggacgggcgatgttgcgagtggggtacaggctgttgcgcatgctgcggcggcgggtacgagg

gctgctggtggcggggttttgggtgcggcgagtgggggggttactgctgctgtggggcaggcggctgctgttggtggggttgcggcgaataagggggc

tgggctgttggggcaggctattgtgacgttgtatcgggatggaggtggggttaaggcgttctgggtcgggaatgggttgaatgtggtcaagatctttc

cggagtcggctatcaagttcatgtcgtatgagatgtcgaagcgtgcgttcgcgaagtatgtcgatggtgtatcagattcgagagatatcagcggaacg

agtcgatttataagcggaggtatcggtggtattacgagtcaactagccatctatcccatcgagacgctcaaaacccgactaatggcctcccaagaacc

aggcaagagccagaagatcctgctcaagaccgccaaggacatgtggaacaacggcggaatccggctgtactaccgcggcctgaccgcgggcctcatcg

gcgtctttccctattcggcgatcgacatgtctgcgtttgagggcatcaagctgttctatatcaagtatacggggaaggaggagccgggtgtgttggcg

cttttggcgtttgggagtattagtgggagtgtgggggccacgacggtgtacccgctcaacttgatccgcacaagattacaagcggctggtacgcctgc

gcatccaacgagatacgatgggtttaaggacgcggtccggcagacatacaggaatgagggcgtcggtgggttctatcgcggtttggtccctacgcttg

cgaaagttgtcccggccgtgtcgatctcgtacgtggtatatgagcacaactttaccttggacttcccttctgccgtctctgggttcgagttgatgttg

actgatctgcgcgaaggggatgggatgggaagcacttcgacttctttcttcagtcccagcgtttcccgctgtctaccaccacgactcgatacagaccc

tgctctacgccctgctgcctgctggccttacataggaaaggaacgaatcatgccagaggccgaccgatgcacaatcccgactccacgacgcggaaaag

ggcctcaaaaaacccagcttgaagccgaagcgccctccctctccctacccgcagccctgatccattcgaaacacccaagagccctcctccacgaattg

cacttgctcaaccaactcctgtacaagtcgcacgcccaactgcggagcgcgaagtgggtccagagcgttcgggccgtacgcaaatgcgggaagcggat

ctttggggatgcgggtcgtgaggatccggatggggagcaaggaggagaaagaaggagcaagaggaggaggcttatggagcaccgcggcggtggcgaaa

gcaaggtcggaaatgcgggcacgacgagcaagtcggagatcctctcctcttcttcgtcgtcaaacacggcaggcggagtcttcggcaaggtccaaacc

cgcgcaactgcattgggcatcgctggctcggacaagaacgttgcagcgtccgcggcagcgactgcggcggactcttcaaatcattcttcttcatcagc

gtcggcgggtgtcattcggttacggtctgctgagatggatggcgagactgctgcggcctggcaacgaccacgagaacgagctcgacaaggccgacgtg

ggcgtggcggtggcggtggcggtggcagtggcgacctccgagcatctgtgacggatctgatcagccttcttcgccatctggtcgagcgatgtaatgca

tcatacgcgatcatctctgcccatctgcgaacgcctcccgccccgtttttggccccgctcgccactgctctgctcggggtgtgtgcacagtgcggtac

gacggcttctgcatgggctgatgacttagagcggatcaactgggagcgggatcgtgatccgaatgcggctgggagcgtttga

>contig_14.contig_14.g668.t1

atgtcgtcttcaacccccgttcagaaggacgcagccgccacggccgccctgctccccatcggcgcagcctacatcggcg

caccagacgacctcctcaacaccgacggcgtcggctccaagcccaacggcttcccctccgtcttcaacccggccacccggaccgccggcggggatcgg

cacgtcgtccaaccggacatctcgggaaagacgctcaagaaggagccctttgggatctactacgaggtccacggaacgggacccatcaagattgtgtt

catcatgggccttgcgaactcctgtgccggctggctgccccagattgaccacttctcgaaccctaaaaacggaaatacggagaagtactcgacgctgg

tgtatgataaccgcgggtatgggtgcagcgaggtcccctcggggcggtatcgcacctcggacatgggacatgatctgctgagtctgctcaaggagctc

aagtggactgagaagccgagggcggttcatctggtcggcgtcagcatgggtgggatgatcacgcttgaattggcgaagattgcgccggagctttgggg

cagtatcacgctggtttctacgacgagcggacaggggattggcgagaaggcgcttgcggtcggtctgcctccgatccgcggcatcagtgtgatcgcgc

aagtcgtcggaacggccatgttcggcataggtgacgagaaatcgcggataaacaacgttcttgagctgctcttccccgacagctggctgtcagaagta

aacccagacgacccacaaggccgaacgcggcgcgaagtcctcttcgccgtcttcgcctggcgcttccaattcagccgtcgcgcacccccacaaggcat

cctgagccaaatcgccgccgtggtctcccaccgggtgacgaacggccaactggcgaagatcaacatcggcatccctgcgatttcgatcgtgacgggcg

acgtggaccatctggtgaacccggggaactcggtgcatttggcgaagaatatgccgaaggcgcggttggtgcagatgaaggagacgggtcatgcgttg

ccgatgcagcggacggaggagttgaatgcgttgattgaggagacgattcgggctgggatggagaggagtggggtgcaggccaccctggccaccatgac

ccgaagtcgctccaccctgatgaccgccgttgcgaaaggtcagatgatggtcagcctgccggtgcccaggtatgcacagtttaccgcttccgatcagg

tgttgcgatgccacacccgcgcacacccacaccttgctcactctcaatccgatcacttcctacttccaggtaacgcctgctgggagctctacctgaac

gagcacggcctgtcccacgacggccgcatcatcgccgaggacggtatttccgacgacggtttctccaccttcttcagcgagaccggctccaagaagta

cgtcccccgctccatctacgtcgacctcgagcccaacgtgatcgacgagatcaagaccggtccttaccgccagctcttccacccggagaccctcgtca

ccggcaaggaggacgccgcgaacaactacgcccgtggccactacaccgtcggcaaggagctcatcgaccagaccatggacaaggtccgtcgtctgtcg

gaggcctgcaccggtctccagggcttcttcgtcttccactccttcggcggtggaacgggttccggtttcggttcgctcctgctcgagcgtctccagca

ggactacggcaagaaggccaagctcgagttcagcgtgtacccggccccccaggtcagctcctcggttgttgagccttacaacgcggtgttgaccaccc

acaccacgctcgagtcctcggactgctccttcatggtcgacaacgaggccatctacgacatctgccagaagaagctcggcatctccagcccctcgttc

gccaacctgaaccggctcattgcccaggttgtgtcctcggtcactgcctccctccgtttctctggctcgctcaacgtcgacttgaacgagttccagac

gaacttggttccttacccccgcatccacttcccgctcgccacctacgctcccatcatgtccgccaagtcggtcacccacgagcagaacacggttgccg

acatgaccaacgcctccttcaccgttggcaaccagatggtcaagtgcgacccccgtgacggcaagttcatggcgtgctgcctgttgtaccgtggtgac

gtcgtgcccaaggacgccaacgctgcggttgcagccatcaagacgaagcgcaccatccagttcgtcgactggtgccccaccggtttcaagctgggcat

ctgcaacgagcccccgagctacatccccggtggcgacctcgccaaggtgtcccgctcgctctgcatgctcgccaacaccactgccattggtgctgctt

gggagcgtcttgaccgcaagtttgatctgctgtactcgaagcgtgcgttcgtgcactggtacgttggtgagggtatggaggagggcgagttctctgag

gctcgtgaggacttggctgcgcttgagcgtgactacgccgaggttggccgtgactctcccgacgaggaccaggaggtcgctgacgagtactaa

>contig_14.contig_14.g669.t1

atgtcatccgacgcctcctcctcctcctcctcctcaggcctaacccgccctccccgccccaccctctgcggccccccaa

caggccccaccccaccctacccactctacctcgaaggcacaatccaacgcggcttcggccgcggcggcaaagaactcggctgtcccactgcgaactta

ccctcgactgtcttccttgacgcgtctacgggccggacgcgcgaggggcttgattcgacgggggtgtattttgggtatgcgagggtgtatggtccttc

gtatgtgcctccttcgaagcagaaggagggcgtggagggggatggggatgggaaggatgtgaatgcgtcggagttacccccggcgccgagcgagttgg

gggaggaggagagcgcggtcctgccgcaggtcatgagtgtcggctacaaccctttctacaagaacaccacgaagacagcggaggtccacatcatgcat

cccttcaaacacgacttctacgggaactacatgaaggtcgtcatcctcggctacatcagaccggagctcaactatgtctccaaagaggccctcatcga

ggacatcgagacagataagcgcgtaggactggcctcgttggaaaggccagcgtatcaagcatacagggacgacccgttcttcaagtcctaa

>contig_14.contig_14.g670.t1

atgaagtcggctgccttgctcctcgtcgcgctcacagcgggacagcacgcagccagcgtcgccgccgcatccctgccgc

tcaccacaacaacaacaccagcagcggaagcagtcgggctggacgagctgcaactgcagacacagcgcgagaagcagcagcacgtccccggccgcttt

ggaaagttcttgcacatcaccgacttacatcctgattcccactacacagccggagccaagatctcaaaggcgtgccacatcagccacaagaaggagaa

gaagaaccacggcggcaagttcccgcccatctccagcgcacgagctcttctcgacctggtatatggcgccatcgacaccgtcctcacatccgaactcg

tctcccagtctcaaaaggaccgcaaggcgtccaagaacaaggcaggccattggggctcgcccgacagtatctgcgacagtccgccggcgctaatcaca

ggctccctccgctggatcaccgagaactggctcaccgtcccatcaaaagggaaggcggcccagcaccaccccggcgtccaaaaggaacacgacgtcgg

cttcgactttgtcctcgtcaccggcgatctggcgcggcacgacgtcgatgcgcagcagtaccctcgcacgttacctgagatttaccgcgcgaaccggt

ggacgatggagaagatcgagcaggcgtttccggacatccctattattccctgtgttggcaataatgatatatggcctcataatatcatgaactctgga

ccctccgcagtcacccgcgagctgtcaagcatttggtccaaacacatccccgaattcgagctgcacaacttccagcgcggggcttacttcgccaagga

ggtcatcccaaacgagctcgccgtcctcagtctgaacaccatctatctgtatgactcgaacaaggtcgtggatggatgtgtgaagagtaagaagggca

agccggagaaggatcgggatgcggggactgtcatgctggattggatggaggtacagctctccctcttccgctcacgacacatgcaggtccatcttatg

ggacacgtcccgcctaccgcgggcaactacttcccgctctgctacgaacgctacaccgacatcgtgcttcggtatcaagacaccgtcgtaggacagca

ttatggacacatgaatgtcgatgccttcttcgtccaagaggacgaggaggccgttatggacgatccaactggagacaatacaacgacctcggacacct

cgatccacatctcttccctcgctgacgacctgcaaaacgattatgccacccttcccggacggaaacggacgaatctggattactaccataccttcttt

gccacgccttcgatcgtgccgacgttcctacctacgattcgcgtttacacgtataacaacacgcgcagtgcgaaacgctcctttttcgatctctcgga

ggcgaaggatgctggactgcaggacgtgtcgagcggcagctttggcgatgacgatgacgacgacggtgacgatgaccaacgcgtcatattcccgacaa

gccgctcccatcgcagaagacatcgcaagcacaagcgcaagaaacaccgcaagaaaaagctgccgcggcacgcctcgccggactctcctgcgcggacg

aacacgtacctgagcatgctcgggtatagccagtgggtgcttgatctggaccgggcgaatgcggagtgggagcggagacatggggatgagcatcagca

ggatgatgatgatgatgatgtcttctcttcacgtgagaaggggaagaaggaggaggaggagctgaagctggagtatgagctggagtatgtgacgtacg

agcctgagacgctgtggcgggcttatttggaggaggaggaggaggggaagaggtcatacaggcaggcgacggacgggcatataccggttccgaaggcc

ttgttggatcggctattggaacgcgcgggcgtgtcgccgccgcactacgtcagcgcggaggacgaggatgcgctgtgccttcgcccgggctctcacct

ggagattctcttcttccagcgccatcccgacgacatgctcagccccaccaacctcctcctcctccagcgacgccgccttgccaccctcccgagcgggg

acgcacaagctgcggttgccgaaggaggtgaagcatttgacggagtatgggatggcgtcgttgactgttga

>contig_14.contig_14.g671.t1

atgagtgcgccaacccccgccctgccattccgcgaggagacgcagcgctcagccaggcccccgtccgtacccgcgcccg

cacccatcctcgtcccggccagtcacgccctcccgtctacgattaccagccgtatcaaccatttacagacttccctcgcccagcttagtgccctcgac

ctcgacctcgaccgatccacgctcagagaagagaggaaatcgatcatgcggcccgaatcatcctcctcctcctccgcatccgcctcctcctcctcctc

cggtctgcccatgcgaccaccaaagctcccgcgccgctacgactcgacccaccacatgccctcgcttcgcgggacccatacctacgaaactcagcaat

ccggcacatccgatggcgatgggatggtcacttcaccggacgcatacagcagcggccgtccggagcctagtgagggtgctgcttctgtctcgctcagg

agcttgcagtcgcgtgcggatctctctgttgagagtgcgaaactcgatttgcgcgagatggctatcggattcgtatgcgcttcttcccttctcgtccc

cgaccttcagtcgaagatattgaatcctcccgccctaagtcggctcaagatggcccacgagctcatcatccggagctgcaacatgactctctgcgcca

cgatcttctgcacggagcagcgcctcatcgtcgccaacgagaccgccaatcgcgacaagcgcagcataacggaagtggaggaaaagctactgagagca

ctgctcgagcatgttgcagaaattgaccaggacgtgatctatctctccaatctcaggacttttgcggaggagcatgactgcacagaaccgctcttcca

aactttcgtcgcttctcccgtccggatccctccctccgcttcagaagcccccatcgtcgtaggcatgctcgccgcattcgatgtcgtccagcgcgacg

cactctccgacgaagacgccgcatacatgcgccatctcgccgacgcagccactttcatcgtcgaagacctcttcgccgcagaccgtagacgcggcgaa

ctcgtcgtacagcgcgccatcgtcggtctcaatcgactcctcgccggtgaacgggtgcccgaagataataacaccgaaggaggtcccgcggggggagc

ggggagaagaccgtcgagtggcacggaggacgctaagacgaatgtctcgctcttcgccaatgccgcccaggccttgtgcgacgctttgaacgccgaag

cggctgtcattatcgacgtctcctccttccgtctccggcccggcggtcgggctcagcagagcacctccaccctcttcggctccgacagcgatacggcc

tcgctcgacgccagtacctccgacgaaggcctcaccacaccgggccctgtgcatccgcccttcaagcgcgaacagtcgacgatgcacgaacgtctggt

ggagcgcgaaccgacgcgggccatcttgaacaccgagacgtcctgcttctacggcctgtcggcgaacccgctgcccatcgtaggcgcgtccggcgaac

ccgcggtcctcgagcatttctccaaacttcccgcgaccttcaagaaccctccgtatcaatgggaggagccggacttccttctcgtcgagcagatggga

gcggttcttctaagcgctgtgcaccgcgctcgagcgagggcggtcgatgttgctcaaacgtattttatgaggaaagtttctcacgagctacggacacc

tctgcatgccatcttgggcgtcacagaaatgacccgcttggcactgccgcctcaaaacgaggaactgatctcactggtcgactccatcagtctcgccg

cggactcactcagtaatacgatcgacgacctggtcgacttcaacgctctgaatggaacgatcttgcatggtgcgggcgtgaaggtcaccgattcgacg

atgacggcgactgtgcttgatatctttgggactatttcggacgctgctgttgcgcaatatacacaccgccgacgcgtactacggcaatcggacctcgc

ggagatccaaaagccaatcaacctagatctcccaccaccagagctcttcatcaagctctccccgatcagcaatgcggcgctgaaaaaggtcgacatcg

acgtcgaagcgctgcggcgcatcgtcagcaaggtggtcagcaacgcacttcgggcgacagaaacgggcatcatcacggtcgctgctgcgatctctgcg

gtcgatgcggactcgccgacgccggaaatggggccgttccgtcgaaaatctcgcaagaaggcttcggagcttgagatcgtcatttctgatacgggtaa

aggtatgaccgacaagtttctcaaggcacggctgttcgagcccttttcgcaggaggaccccttctggtcggggactgggctcagtttgacgctctgtc

ggaatatgattgatgagttgggagggcgggtgcaggtctcgtctgttgttggcgaaggcacggactttttcatccatgtgccgttgccgggtgggatc

gctgagacgccttccaagcgctcattatcctcgcctcggtccgccacgccggcgtacttcctgagattcgacgatgaggagcgtttcgggcataggac

ctaccggtcccatctgatccggcagttcgacctccggccggtcgaggaggctcagatcgggcaggccagggtcatcttcgtcatctccgaggcgctcg

aggacggctcggctcttgctgtactcgagggcaaacttggccgtctgaagaataaggcgtcgacgagcagtcagggcccactcctcgttgttctttcg

gccttcgggttcacggacgaccccgagcaggggaacagcctggcgagattggcggaggcggagcagtgtgaactgttgatcctggagcgtcccttcgg

ggtgaagggcatcctccttcttgatgacaagttgcggacaataggtgatgcggagcgggagggacgtggggagttgatgcagaaggcgggcatgctgg

tcactggtgctctggatgttaacgggaggcggtcgcctaatatgcgcacatcggccgagttcagagtgctcgttgtggaagataaccctctcaatgct

cggatcttgacgacgatgctgcggaagattggggtcggcttccatgaggcgaccaacggacgggaagcggtggatatgtatcgcaagtacagtcacga

tgtggtgctgatggatatctctatgccggagatggatggatttgaggcgtgcgagaagataaaagctatggatggatcagaaggaagccgaatcatcg

cgatcacggccctgagctccgaactggatcgaattcgaggtcgcgaggtcgggttttccgaatggatcgtcaaaccggtccgattaggcccacttgcc

caggacgtgaagaagtggaaggaggatgccgatgccagtcgcatcttggagtggaaggagaataccgaagccagtcttatcgtcggggtggatcagga

gatggccgtttacgcctag

>contig_14.contig_14.g672.t1

atgaacagcgtaattccacgacgctctgcgcaagcatggcatcatcccgcccaagccgccctcgcgcttgccctcgccc

gatctgccgtcctcgtccgagctgcgaaaggacgccctccaccgcgtctcccaaagccagatcgacgctctcctcgacggagagaccgtccgacgcgg

agatgcgcccggcggaatggcaatgatgccgacgacgacgacgacgatgaagacgaactcaagctggacgaggacgatgatcaggagcgcaaattgat

gttgcgactgcgcgaacagcgcatggcagagatgaggacaaaggagaggagagcacgatttggacgcgtcttccccatctcccgggtcgactacaccc

gcgaagtgacagaggcgtggaaggaggaccccgatcggcctcaaggatcagataccggggacaggtctgacggtgcaggatccaagtccgagagcaaa

ggaacggcggtcgtatgcttcctttataaggacggcttggaacttttactgattatccccatcctcgcatcctctctgctcctcttttgctgggaagc

cgacgacgagaggaacactgacgctcccaagtcaaacacttgcagtgcattcccaactacgccgaccgaaacctacctacactgctcatcgctcatct

accgagatggcgacttgcatcggcaaatcgtcggccttcgtccagagataggactggacggaatgaacacgaagcttgcagacgtcgagctcctcctc

acagcagtaggtgccatcgagcgaccgaaccaaagagcagccagcaaaaacgcaggacaggatgctgccgaagacgaggaggatgatgaaccccgctc

gagatcgtctggcatccggaacggcggcagtggcgctgggacgggctcccgaacgtcgactgtgcgaacacaagaggaggaggacgtggatttggatt

gggatctgtaa

>contig_14.contig_14.g673.t1

atgaggtcgatcagttccttcatgctcatcggcatgctgttcgctatcagcgccgtcaatgccgaatacagcgagaagc

gttgcttgaacaagggtattaaaaacccatcgtttgattacatcctcgacgtacgttgcgtgtatatcatttcaaacactcaagctgccccactgaca

tcgcactctccgtcttgcttcctgctctatggagctgcctgtgcacgtggtggactgctaacagagataacggatgcctcaacgatgattctcacctc

tcacgcgcactcgacaatcctatgttctgtgacgacacctgtgatgtcttccgccgtcgattcggcaacggatgcggcaacatgccgaagagctgcta

gtcgggctagccatccggtgccgcaggacgagcggtaa

>contig_14.contig_14.g674.t1

atgagcgagttactcagcgcgtcctggttgaagatcagatacaagcagttcatgagcaacgagaaggtcgcgaggagtg

gtgatgagtcgttggatctcatgatgcaggaggacagtagcggccatggcggagctcgcaggcctggcttggggaatcatgctaccagctcgagcttt

cacggcagtctcggcggcttctcctcgtcctcgccttcgccctcgctctctcccgcgccaggcggatcgatgtcaccgccactacaggcgacaactat

gatgatactcacgaaggatacgatacctgctcccaaagccatcgggccgatctcccctgcgccgtcgcgctacaccgttccgagctcgagcgaggcca

gtctgccgatccgaccggtgatggcagccgtgcacccggcctatcttgtcgatgcgattgagaatgcgggcggggggtcaggtggggctgattcacgt

tcgtcgggaggaaacggcgcgagtgagagcggattgaacggcgtgccgaatggagcgacgacactggccatgctcggcctgcatctttctcgcgcgga

acccactcccatcagcgatgccgcaagctgggaacgtgtacacacgctcgggtaccgtcccgcttcccggcggtgtgtcccgtcgactgccgcttccg

gcgagctatacgacgacacagagggaggcgatggagagggagcagaaggcggtggcggagaaagctgcagcagcggctgtgcaggcgcaggcacaggc

acaggctcgggatcaggcgcaggtgcaagcgcaagcacaggcggcgcaggctcaggcggcgaacgaggcggcgaaggcaaacgggtcgggggtgggcc

gccctcgttttcctcgtcgttccccctgcttcggttcatggatgtccagctgcgaaggaagaagaagaagaaggtgatgatgatggatgaggaggacg

atgacaattcggaggaggaaggagttgtcaagggacctggtactgctctcgctctttcgaagtcggtgcccacgtctggcgtgaataccacgaacgcg

acgggtgcacgtagctccaggccagcggcacttcctcctccttctcactcgaagatgtcattcaacgcaacaccagtgacaaccgcctcatcagcgca

tcagccttcactccagcggctcatcacaagtctcaagggcaagaatgagaatgacggcagtgagggcgactcgtccggggaggatgatgatgaagaat

acgacgatgaggcatga

>contig_14.contig_14.g675.t1

atgcagctcaactccaagctttccctcgtcctctgcgccctcctcgtggcccagccaattgcttctgcccccttcgcca

gcgaaaacaactctgccgagctggctgctcgctctggcaacatcggttctggccagaacaaaggcctcgacgtcggcagcatttttggcaacttcttc

gaaggcgacaagaagggaggcgaggaccacggcaagggccacggcggacaccaccgcttggccgagaagaagctcaaggctctcaaagccgctgagaa

tgcgaagaagcacaaggacgctgcggctgtcaaggcggcggcgaagttcgatgctgcggctgccaaggaggactctcaccacaaggaggccgacctga

agaagttcaagaaggagaagaagcagaaatccggcaaggacggctttggcggcggctttgacttcgatcgtcgggacggctttgggctcggcagtggg

cttgactttggaggcaaagacgactctggccacggcaaggacaactccggtcacggaggcaagggcaagggacacggcaagggcaagggcttcggcgg

atccaacaagaacaagaaggagaaggtcgagaaggtcgaagcgcacatcaagaatgacaagaagcacaaggccgcggccgaggtcaatgcggacgccg

agttcaaagcggcagctgttcacgaggatctcaagcacaacaaggccatcgcgaagaagcttgcagctgagaagaagaagaagaacggcttcggcctg

ggcatcttctag

>contig_14.contig_14.g676.t1

atgcgcctcacatctcccttcatcgccttactcggcctccttcctctcgcacaggctctgccttcgtcgtcgcccttcg

tcggcgtcgacatgatcctcaagcggtacggcatcgcctctcgctctccggaggactatgcgagccagcttcccgctctgcgcagatccaagtcgcag

cctcggccccccacgagctctcgtctggacccgacgccagactatcgtgcgaggacgccgttcgactgggccagcattgctcttgctttgcaccaaga

gtacatcgagctggatgcgttcaactacgcgatcacccgcttcactcctgctgagtgggaggaatggggaatcaaccccaactacgtccacctgatcc

gtttcttcgctcagcaagaggaagggcattcgactgcattctccaacatgcttggccctcgtgcgccgctgccctgcgacacccagcttggcgagagt

ggcaccattggctggatcccaattatggatgagccggcaccggcatccatcgttcagcaagccatcgtcaccgagtcacgtcagcagtttgctttccg

ccagctggagggcttgttccccgtcaccgtttggttcattaccggccatgcactggacgatcctctctcgctggattacccagtgccccaaggagaac

aagccactgccgtggccgatcttcccccccgcctctacgtagacaaccagccagacgccattgctgccggtgcaagcgctggtggacccgacatcgcc

cataacctcacctcgctgacctaccctggccgagtggttgagctaagttgggatcgacctggcgagattcaaggaccctaccaccagctgactcagac

aaacacgagcggggtgccacgctttgcagcatggttcggcaacctcaacgtgaccttcacgcccctcttcgagcgtaatctgaccagtcgtacagcca

agaccacgcaacccggtgacactatctaccctgacgtaggccagcgcgtcatcaatgggacgtctttgtcactcccgaaaaatctcacgctgctcaac

gagcacatcgtcgctggccccgagttctaccaggctgattga

>contig_14.contig_14.g677.t1

atgaagtcgatcagttccttcatgctcatcggcatgctgttcgccatcagcggcgtcaatacagccaaagctgcacatg

cacgatttggactcttagcggcaaaggcgactgctacaagggatgcatggcagaccatcatgatcccggactcgatgttcctcggttctgtaaagaca

actgtgacaactttcgccaacgattgccccgtggatgcggctccattccgcaggaatgccaccaggactagccattcgtcgccgcatgaccagcgata

a

>contig_14.contig_14.g678.t1

atgagcgctcttcaggctccccacattcaggacttccacgatggctccaaggtcgagccccattggggatacgcggaca

gagtcattccctgtaccgtagatgcaggcagttgcgagtatctcgatgtcgtgtactcggctcatgaccgcggcatgatttacgtcgccgtcatctgg

gccaccatcttcgccatccttgcgctctatgggaccggccgactcatcgctcctaaattcacttccaagattgacgcctgcggccccgcctcttccga

tgaggatccctctcaccagcgagcgccgtcagtattgaaccgccacatcgcggccgccggtgcaacactccgacactacctgcttccagatatcggcc

ttcgacgcatcttcggccgagtgacccgcttccaagtcctcattctggccatttttgctggctatatcatcatatggtcctttcttggaatgtcctac

aagcgctggatcacgcctgtcaagaaggctccgggtaaatacaacagtcgaagcaccttaggcccattcgcggaccgagtgggcgtcctggccttcgc

cctcactccactctcgatcctgcttagcagtcgcgaatcggtcctgtcattgattacgggagtgccgtaccaccacttcaacttcctccatcgctggc

tgggccacatcatcttcattcaaagctcggtccacaccatcggctggtgcatcgtcgagatccgactctaccaaccacaaccatccgtaggaatcaac

tggatcaagcaaccctacatgatctggggcgtggttgcgatgttcttcctcacctcgctctgggtgttgagcatgccgttcgcaatccgagcctttgg

gtacgaattcttccgaaaggcgcactatgtcctcgccatggtatacgtcggcgcatgctggggtcactgggagcatctcaaggttttcctcatccctt

ccctcgtcctgtggggtatcgatcgtgccgtgcgcattgggcgttctgccttgcttcactacaagtgtctgcccgggtcgatgtctgcgagtcgattc

ggattctccgctgccccggcccaggtcacctacttccccgacgagaagaacggtgacattgtccgactcgactttcagcatccgcagcggccgtggaa

catcggtcaatacttctacctctgctttactgagggaagcatctggcagtcccacccactcacaccgctctcccttccgattcaggactcagccaagc

tcgtcacgcactcgtacatcttccgggccaagaagggggagacgaagcgcatcgcgatgctggctgcggagaagtgcgcaatgatcgatggcccaagt

tcagaggcaggcaagcccacgacgggcgtcattctcacaggtccgtacggcgcttcgattatgaatggcgtcggacacgatacgaatatcttctgcct

ggcaggtggcacgggcgtcacctttgtcctgccagtactcctccacctcgccaacactcttgcccacgacaacaagacagctcgcaagatagagctct

gctgggttgtgcgccaggcgaaggacgtggaatgggtcggagcggagcttgatgtgcttcgtgcccatcccttgatcactgtgcggacctttgtgacc

agggaccgggttggacatgaggccaacacggagaagaacgaggtggtcgacgaggcgatcggtaaagaacatcactctgcacctggctcaaactcggg

ctcgggctcagcggaagtctcggtcgtcaacctgccccggatcacatcttcgtcccctcccaccgaccatcaccgaccggatgtgagcgaattgatac

gcgacttcgtcggcaccaccgtctctactggcgagacaacggttttcgtgagcgggccgggcgagatggtctcggaggctcggagggcagcggcggcc

tgcaactggggtcgggcggtgttgaaggaacggacgggcgactacctgcgctcgagcgttcgctttgtcgatgataaccggctggagttttga

>contig_14.contig_14.g679.t1

atgaagtgtgatggcgccgaggtgaaggtctggaacgcatcccatctcttcgtgccccgctcgcagggaggttcacggc

ccccgtacgcgatcatcctgctcaacgcgcccatctccggctcccagaaggaaatattcctcggcctctggagcggagccaggcgcagactctgtgct

gatggcggggcaaatcggctgatcgaggcgttcggcgagcaagcccttctggactctacggttcctttgcctgacgaggtccgcggcgatctcgactc

catgcgggaagacgcccgagccttctttgaaacgcagagggttcccatcaccaagcgcgagtcgcagtatgcgaccgatctgcagaagtgcatcaagg

ccgtcgaagtcttcgaacaagacgaacttgaaaaagggcaaatctcacagccgctcgagctcgtgatctacggcggcctgtccggtcgattcgatcag

acggtacatactctgcacgtcctttggacgctcggtgcaccgctcccagaaaccggtccgggaccagaactgctttcagagcgggacgaggatgctac

ccttgcgcggagagcaaagacatgggtcgtcggtgaacaatgcttggtttggatcctcgaaccgggtcgacatgtcattcatgtatcgaatccgccgc

taggtcctacatgcggtctccttccccttgccacaagtagtggagctcgcgtgatttccaaggacggtaccattcctagctcactcccttccttcgtc

tcgacctcaaatcaaatccctttcactggcggaaaggcgaccatcgaagtcgagacggacaagacaatttactggagcatagagctgcaggaatcgga

atag

>contig_14.contig_14.g680.t1

atgcagctcagctccaagcttgccctcgtactttgcgccatcctggcagcagctcccacgaggtccgccccgacggcga

ctggagaatccgacattgccccgcgtagtcccgacaaccatgctctcggctatggtggccacttcgatcaaggtagcgaggatctcgccgatgaaggc

caaagccttgccgacatcgtctccttccgccgctcaaccggagacttaccgggcgatggaggaaacagcttccatctcgtccgccgcgacggtatcga

tgacaacctcggcgttgacgagtacgagagcccggacaagaaggagaccgagaagaagaagctcgatctgatcaagaagatccacgacaaggagatca

aggcggccgaggtactgctggtccagaagatcagcttcttctacgaggacaaggacgaccacctcttgcacaagttcgttgacaaggagaaactcaag

aaggctcacggcggcggcaagaaggactaa

>contig_14.contig_14.g681.t1

atgaagtcgctcagtgccctgatgctcatcggcatgctgttcgccatcagcgccgtcaatgcgaactggggctattgtc

aagagacgtacgcgggccattgcaagaagaacacaatttgtgacgatccgaaggacccgggctattgcgatgtgacagacgatagcgacctcgcaatc

ccgagctgcatgtgcacgatttggactcttatcggcaaaggtgacgagcgatactacgtcagctccgcctacccggccgaagccttcttgcaaagctt

ccacttcggacaggaaccgacaccccacttctcatcagacccgcaacgcaagacgtttttgtacgcccctcattggattgaccgggcgatggtgctgc

tgtcctattcccacccgcagtacgtcttgacttcggtcctatatctatcagcgacagcgacgcagtcgagctgcaccctcgctctggtgacgtcggct

ccggcaacaacaacggcttcgacttcggcgatgcgctcggcatcttcggaggagacaactaaggcttcgacaagggctctggacatggccgcttcgga

aaagaccggcctgaagaggttcaagaaggagcataagcaaggctctggcaaggacgactttggcttccgcgagggactcgacttcggaaacgacttcg

gcggtggctgcggtggtttggaggcggccgctacggaaagagcagctccggttcccgccacaaggaggagaagatcgagaaaaatcaaaacggacaag

aagcacaaggccgcggccgaggtcaatgcggacgccaagttcaaagctgcggccgctcacgagacgttccaccgcaacgctcttctcgccaagaagat

tgcggctgagaaaaagaagggcggcttcagctggttcttctag

>contig_14.contig_14.g682.t1

atgcagctcaactccaagctttccctcgtcctctgcgccctcctcgtggcccagccaattgcttctgcccccttcgcca

gcgaaaacaactctgccgagctggctgctcgctctggcaacatcggttctggccagaacaaaggcctcgacgtcggcagcatttttggcaacttcttc

gaaggcgacaagaagggaggcgaggaccacggcaagggccacggcggacaccaccgcttggccgagaagaagctcaaggctctcaaagccgctgagaa

tgcgaagaagcacaaggacgctgcggctgtcaaggcggcggcgaagttcgatgctgcggctgccaaggaggactctcaccacaaggaggccgacctga

agaagttcaagaaggagaagaagcagaaatccggcaaggacggctttggcggcggctttgacttcgatcgtcgggacggctttgggctcggcagtggg

cttgactttggaggcaaagacgactctggccacggcaaggacaactccggtcacggaaacaagggcaagggacacggcaagggcaagggcttcggcgg

atccaacaagaacaagaaggagaaggtcgagaaggtcgaagcgcacatcaagaatgacaagaagcacaaggccgcggctgaggtcaatgcggacgcca

agttcaaagctgcagctgttcacgaggatctcaagcacaacaaggccatcgccaagaagcttgcagctgagaagaagaagaacggcttcgctgaaagt

ggttggaacttgatagtgctggagctaagcaagagcgaaacttctgggaggtttgtagccatcatgggaaagccgtcgtgtaccagcgcgtacgactc

aggcgaggcacaaaagcaagcagatctgaccggctggcctcatatttctctcaaagagtcaaccgacttcaatcgaacccgcctcaaattgcggcagt

tacatctcgaagaattggtctgccttgaccatgaagcgacgccgctagccatgctcgacctgcaccattgccgcgcgaaaccactcccatcagcgatg

ttgcaggctgcgaacgcgtacacgcgctcgggcaccgtcccgcttcctggcggtgtgtcccgtcgaccgccgcttccggcgagctatacagctgaaca

gagggaggcggtggagagcgagcagaaggcggtggtggcgaaagcggcagcggcggctgtgcaggcgcaggcgcaggcgcaggcacaggcgcaggcac

aggctcgggatcaagcgaaggtgcaagcgcaagcacaggcggcgcagactcaagcggcgaacgaggcggcgaaggcgaagcggattgagggtggggcg

ccctcgtattcgtctcagaatgctgcacaggtgcagacggagttggagcggcttattgcgttgttcaaggcgaagggagggccggctgcggaggtaga

tgaggacgacgaggacgacgatgaggaggaggacgatgacgattcggaggaggaaggggttgtcaacggaccgggtactgctctcgctctttcgaagt

cggtgcccacgtctggcgtgaatacgacgaacgcgacgggtgtgcctacctccctgccagcggaacttcctccttctgctccctcgacgatgtcatca

aacacaacaccaggggcacccaccttatcagccgttgctgtcaagacgtcgaccgacgccgctccatataatgcgatccagagcgagctccagcggct

catcgcaagtctcaagggcaagaatggggatgggaatgaagatgatgaggacgaatcgtccggggaggatgatgatgatgttgatgacgacgacgatg

aggacgagtag

>contig_14.contig_14.g683.t1

atgtcgccccacatcacatcagcaacacccacctcagcaagcgtcaggcacagcgcactcaccaacaccacaaaccagc

acgaccgccgctcctccctcccaggcatcaacctcagcccagggccactctcacccgcattcgcaggcacatcagcaatcagcaagcgcgactccgta

cactcgcccctctccgcaacccaccaccaccacaacccgccctggctgcacgacaccccccgcttcgtcactcacatcagcgacggcgatgaccacga

cgacgacgacgacgacgaggctggtaaaggtcatggcggcaggatgtccgttcccccgggaagaccagactccagaccgctcttcccactcactctca

gtccccagcctcatatcaccatctcatcctcatcctcatcctcagctgcgcccgtattactcgcacccagcgcacctctcatcaccaccaccggtctc

tcgccgcaggtttatggacccctctcggcggacacctcctccaaacttctcaccgccagtcccatcatcgtaccacaggcgcgcaggggggccatcaa

tctcaagtcaaaggcctcattaggatcccacgcagaccgcaagagacgccgacggagaagggcgaggaggaaggcaagacaggccgcagaagccgccg

ctcttgctgcaccagggacttccacggacggcctgctcgacatggccgccgtggctgaacgacaccaccatcgcagactcggctccaacgagacggag

cattcctcccagtcaggccggtacgggccgtactacacctcccaccaccgccgcagccacaccaacgaagcagcctcctacccgctctcccaccggcc

cgccttatcgtccatgcgctccatcgcctccatcctcttcttcaacaccgtcgccgccttcctccacccgcagacgacggccaggcggatcgggtgct

ggttcatgcgcacctgtcgcgaagtcgacgatgccttccgggacccgcgcacgagggagagggtgtggaagcccgagtggttggacgcatatgtgccc

ttcttgatttggttggctgtcagtttgggaagtacggggactgtgattgtttttcatacgaaggtgttcaccgcgctggatgagctgtcgcagacgct

gcagaggttgggcctggggggccagttcgtcatgggcgggctgatcttccttaccaccttccctccgatgccactctactcgaccctcatcgtcctct

gcggcttctccttcggcatgatcgaaggcttcatcatctcctacatcgccgccctcagcggggccatcaccgtcttcatcctctcccgcaccatgctc

aaaggctggatgcagggcctcctcaacaagagcggcgggttgaagaaagtcgtacgcgccattgagaaacagcccaagttgctgttcttgattcggct

cgcgccctatccgtacaacttgatgaatacgctgttggcgagcagtccgacgttgacgtttaggacgtatacgacgtgtacggcgttggcgttgccca

aacttctcgtacataccggcctgggcacgagcataaagaactttgcggcgtatcatggggctgagaatgggtccggcactggctctggtccgggggag

ggagggggggaggagagcgaggaggagaaggcacggagcgcgacggccgagcgcgtcaagcggattgcggggcttgtgggtgtggtgctctgcatagg

catcttcttctacctcatgcacgtcgcgcggaaggcggtggatgggctggatgatgagaatctggcggacgatgggggggcagatgatggggagaagg

aggcgagggtgggacgcggcgcgtcgtctaggaggagaaggaggagacgggcgaatgcacggtcgtcggcgtcggctggttcgtcgttctcggtccgt

ggcggagcgggaccctcggtcgaggcggagcgccagcggctgtttatcggcgatgatcttgatttcgacgaggatgagcaggatgatggcgattctct

ctatagcgatgattcgctgtactccgacgacggggatgacttggacgactccgaggacgaggatgactttgactctgaagaggacgaggacgaggatg

acctgcctgcgctcgacggtggacgtgtaggcgaggacggcatccccggctcctcgtcgttgtcctccccgttctcgcatcatcaccagcggccggtc

tacggacgcgcacccagcaccgacgcggcgatcgacttgcgccagactgggggttaccatggtcgacatcagtggggcacgggcaccagtggcgggcc

gtcagtgcagcagtcgggaggtgttggtcttgtggggcggtttaaggctgggcttgggttgggtgggccgacgtcgtcggcaggtgcgggcgcgggct

cgtcgaagacgcggtatgtggccttgccgctttcggacggtcttggtggatcttcctcctcggaccggtctcgttccggtcgggcaggagggggagca

tcgacccacgcgcatgccgccgcaggagcgtcctcatcgtcttcggcagcgggtgcgggaatgggcgcggtgcagatgtccgtcgtgtcctcggtatc

gacgccgatgtccagcctgcctgcgtccgtggcgaacgggagcgtgcccggggggcggagggccgtcgtcctgcccggcggtgtcggtagtggtggtg

gagcgggtatcctgggagctcggccgcgtgtgtctgggctgggcgagccgagctcgtacgcagcgggtccgcggagaccgacgtctacgccgatggct

gctgctgctgcttcgtctacggccggcgcgggtgtgggtatggcgaatcggacgcgtgcgttcgtgccgccgaggttggggcctgaggctgttgggcc

tgctgcgggcgggtatggagggatgagtatgctcagtccgattgtgggaacgccgggtgtggtagtccccgcctcgacctcctcaataccggcctcgg

gcccagctccggcttcgtacctgccgccttcgacgctctcggccgctcccggcgggacgaaccctgctgctgtacctactccggacgctgcagcatca

gatggagggggaccgagggaatccggtgagctgctcgggatgagtatggaggatcggattgcggagatggagaagagtgcggaggagtacttctcctc

cggtcggcggtggaacgcttcttcgtcgtctaccacagattcggatggagtcggcggcacattaggaggaggaggaggaggaggaggaggaggaggag

gagtaacggcagcagggtcgccggtggtggtggtggttggggacggcgacgacgacgtcatcccaaccacgacgacgacgacggagatggatctggtt

gagatgcagagattggcccagcagcagcgacggggcggtccgcgctcggcgagcgagatgggagtcgaagaggcgagctcgcaggtcggcgaaagtag

caccagcatctatcgtccagcatctatcgtccagcaggccatcgtcaccgaggctagtcaacctttccgccagctggagggcttcttccccattaccg

tttggttcattactggtctgacaaagtgccccaaggagaacaagccactgccgtggccgatcttcccccgcctctacgtagacaaccagccagacgcc

attgctgccggtgcaagcgctggtggacccgacatcgcccacaacctcacctcgctgacctaccctggccgagtggttgagctaagttgggatcgacc

tggcgagattcaaggaccctaccaccagctgactcagacaaacacgagcggggtgccacgctttgcagcatggttcggcaacctcaacgtgaccttca

cgcccctcttcgagcgtaatctgaccagtcgtacagccaagaccacgcaacccggtgacactatctaccctgacgtaggccagcgcgtcatcaatgga

actgcgtttatcgctctcgtggacagggacgtctttgtcactcccgaaaatctcacgctgctcaacgagcacatcgtcgctggccccgagttctacca

ggctgattga

>contig_14.contig_14.g684.t1

atgctgcaagtggacggcgaccgtgctcccgttgtcgctgtcgtgctcgcccttgtgctcttccttgctcactgcttct

tgctcgctgctcactgcttcgctgctcactgcgtcttccctgtgcctccccccgtgtgtctaggtggtcgcgttgcttggggcgagagcgtgcagcgc

aatgccgtctccactgtctgggtctgggtcggcaactggctcgccgctccaggagcagcagggccagctccaccaccgccgcgccaactctcccctgc

cgccctatctccagcacctccatga

>contig_14.contig_14.g685.t1

atgtctccatcttctgctctgacctctcctcttgatcatggagactgggacgagaccatcacagcacacgacaccctca

gcctcatcccaccccaacacacaacaccacccttcgcagacctcggcagcgcatacatgcaggacctcgagtccttccactccgaattccagcagaag

aagaacagggacaccacacctcaggacctcgtatctatctacggactccaccctggttctgctccgcatctcacgccggaactggccgctgagttgga

atacttgcatgcgcttgatcaggttgaactcaccagtgtcaacaggaccgaacggagcgggatcttcgtcggctgggatcctgttcacgctcaagaac

gggcaaagtctagggagttcgtccagatcaggacagggtggcgggatgtcgtcacttggttggtcgagcaggacagtcgggaggttgagatgcatgtc

atcagcgtgagctggtcagcaactttcatcctcgccggcctccgatcccacccagactccctcctccccccagcctccacctacccaacgacactaaa

cgcgaacgacccagtcctaaacaccaacaacctcggcgccggagccctctccaaatcctcctcaaaatcagaatcaggagtaaaagcaggcatccgaa

caggcaagcacaaactcgacgtcctccgctccctcgtccatccagcctctgggacgacgacggcgacgacgattacaatctacgtaggcgatagcagt

accgacctgccctgcctcctacacgcccacatcggcatcctcatcggaaacaagcactccatgctcacgaagctcgacggattgggcgtcgttgttga

gaatgtggacgccttcttggagaggtggcaagagatgggcaggcgggcgttgggtggcgggattgggtctacgcaggaacccaggctggtcggagtgg

cagattgggtcgacgtcctccgcatcttccgcgctctcagctga

>contig_14.contig_14.g686.t1

atgtccagcggcgcgcagcagtacccgccttcataccgaacactacaccacaaaggcgcgaaggacgaagaatcagaag

ggagaatcgacgagatgccgctccttggacataccacgtcctcgcgccggcccaagcgatcaggcgacgaatgggacctaggcacaaccaaagctgga

aatcgcagttccacatcgaccatacggacctacctcctcccgcgctcgcccctccatcgtctcctcctcctcgtcctcctcctcgccgtcttcagctg

gacggcatggaccggcgtctccagctcctcctcgcgccgcaacaagctcggcatccaactcgcccgtccgaaagaccgggtgccgcatcctgcccatc

cagcagcggacgcccccgacgccctcatccatcgcaggttggtggccgtggcggacttacacggggattatgagcatgcgctgaacgtgctcgagatg

gccaacctgattgatgcctcgcccgaagcgagactggaagatggcacgccgcgctgggtcggcgggcatgatgtgctcgtcagtacgggggatatcgt

cgatcggggtacggacacgatcaagttataccggatgttcgcgtccttgagacggcaggcttccgggctggaccccccggggaaggtggtgaatctgc

tggggaatcacgaggtcatgaacgcattgaaggactggcggtacgtcacctcggccgacttggactccttcgggggcgcgagggagcggaggaaggcg

atctcgtccaccggctggatcgggatggagtggttggatcactacaacatcactgcgtcggtcgacttactgccccgcgagttcttgccggaggagta

cgagacgccccgggcgaacttcgtgcatgggggcatcacgcccgagtgggctgcgagtggcggggacggtagggatgcgggcatcgacaggatcaaca

cgctcggcacgagtttcctacggaaggcactgtccaacccgaacccggacggacgttcaccaccagacacgacacccaccgaagcatcgctgtactcc

gccgacggtccgctctggtaccgcggctacgccctcgacgcctccgactccctcgtctgctcccgcgccgagaacgcgtcccacctcctgctcgtcga

ttttctcatcatgggccacacgcccaacttcgacggggtggtggtgcggtgtccgcaggcgaagatcatggtcattgatacggggatcagtagggcgt

atggcggtgagcagagtgctgttgtttttgataccgagttgcagagggcgccgacgccggatgtggggcggcagttgggtctgggctcccctgctgct

gtggtgggtgggatgaagaagaccgtgcaggcggggaaggaggtggaggtggaggaggaggaggaggaggttacgccgatggagcattcgaaggggat

gcatcgctgggtggagcggcggagtgttacggcgttgtatgtggggcgggtgccgaagacgttgcacatgggcgagcgggacgtgtggctgtag

>contig_14.contig_14.g687.t1

atgcttcccgtcgtactcatcagttctctcatcgggctccctcgttgtgaccagaatttacagtatgacagcctcgcgt

tccctcttcgccagctgatcgctgatctcttgcgttcttcttatcttggtgttcgtggtgttgctcttctctcggccgaatggaatcgaggttcggtg

acctttggctttgcaacagcagctgggatggtggcccgttcgcggagctcgctcttgactgtcacgcacggagcacgccagctgtcgacgttctcgct

ctcgctctccatcagcaccagcaccagcacgcgcacgaggaacacgcacccagcttcgtgcctctctcttcgatcccgtaccagcactcccactccca

ctcgcagcttcaccgcttcatctacagccatgggtgtcactgtcgaatctctcgctcccggcgacggcaccaacttccccaagcccggcgaccgcgtt

cacatgcactacgtcggcaccctccccgacggcactgtgttcgacagctcccgtgaccgcaacaaggccttcatcactgagattggcgtcggtcgcgt

catcaagggctgggacgagggtgtcccccagctcagcctcggccagaaggcaaagctgacccccgactacgcttacggcgctcgcggtttccccccgg

tcatccccccgaacgcaacgctctgcttcgaggtcgagctcctcaagatcgagtaa

>contig_14.contig_14.g688.t1

atggtggaagtcgagttcgagtcagtcgggaacgggttccaactttccaagggttcacctgcagcaaagtcagacctca

tcccgcctcctttcacttcttcatactccctccgtcgtcttccctcgtccgctcgccctacgttccgctacttactgctctcgacttcgacaagattt

tcgaagcgcgtccttcgcttgttcccaccagtctacagcaaagctatggagcacgagaagggaccgtggaccggaccggacgtccggcctttcgtcag

cgacggacctcccttccaatgcttcgagcatatcttccacccgccgtggtatcgaccgcaccatgagcccatcctcctcccgatcgacacgctcaaaa

ccctcaagaactacctgcagcggcagtgcaagcggcttgcggatttgggcatacatgtgccaaagcagctcaagaacgccaaatacgagtacgaactt

gcttggagacgtcggaaggggcttcccatcaacagcaggatgcagcactgggagcaagctgtgctgatcccactgcgtcagaatctttctatcctttc

agctgcacaagatttccaatccacgcaagcaggaggcagtcgagacctgtccagcggaggctttcgttgcccgcgcctcagtccgctgccagacgcgg

caacaacgccgcacggttcggtggttgcgaagggcggcaggaagcaagttggagacacgttccgacctcgtggctcctcatctcccgacacgtcctcc

cgttctcctttcgtttcatctacatacgtggcgccgtcgtccaggaaactcgcccgcagtatctgcatctga

>contig_14.contig_14.g689.t1

atgggtgtcactgttgaatccatctctgccggcgacggcaccaacttccccaagcccggccagaccgtcatcatgcact

acgtcggcaccctcgagaacggcagcaagttcgatagctcccgtgaccgcaacgagcccttcgagactcagatcggcgtcggtcaggtcatcaggggc

tgggacgagggtgtcccccagctcagccttggccagaaggcaaagctgatcatcacccccgactacggctacggtgctcgcggtgctcccccggtcat

ccccccgaacgcaacgctccacttcgaggttgagctcctcaagatcgtctaa

>contig_14.contig_14.g690.t1

atggacaccgcagcagcagcagcgacgatgcagccgccaacacagcaagcacagccagcagcaccagcggcatcggtag

cagcaacaggggcagcatcatcaagcacagctccagctccatcccaggcagccccagcgtacgcgcccatcaagagggaaatcgcctccgtgcctcag

aagcgcagcatatcagccgtctctgccggtccgtcctcggcgggaacagcgccgccgcctcagaagaagattgtcatcgagaccatcgcagggccgcc

gatcgccatcaagcaggaacacagccagcgagctctcccaccaccaacagcaccaccagcaccaccagcaccacggataccagcacccgcacaaccca

ccacaaatccacatccacacccgcacccgcctccaagaccagcaccacctccagcacccacgcccgctccaggcacttcttcaacggcagcatcttct

acctccgcccctcggcctgcctcccaacagccacagcagcctgatccacgctccaatcccgcatatgcgcatctcctcgcagccagtgcgaacaaccc

agcactcgctgccatcctcaacaagatcttgcccaaggtcacaggcccaagtcccgtctcaaatgtagcaccagcagctccagcaccggttccagcag

cagcaccagcacaggcgccagcatctccagcacctcctgtaccaaagcctacagctgccagctcagtgccggcttctgcgccggctcctgtagcaaga

ccaccggctggatctgtcagtagcttctattcggcggcggtgcctcccaaagccccagctccagtagcaaaaccaccaccaatagcagcaacaccagc

atcagcagcggtggccgcacgagtagcaccagtgccagctacagtggcagcacctccaccacggcaacaacaattgccgcctgcgacacagccagctc

gaacaatagctcctgcgccagctcctactccagcgagagtcaatgtagcgccagcgcctacttccgcgcgtcctgcaccgccacccttaacttcagcg

ccagcgtcagcgccggtagcgccgccgccaagaccaataccgtccccttcactagcttcagtcgcggcccccactacaccagcacctgcacctgcgcc

cgcaccaagacctgcgcccattgcaccatcaccagcgtcagtaccagcgccagcgccagcaccagcaacgcccatgccccgactcaccacaacaacag

ccccttctcccacccacagaccagcagttatccccagaacgaattccgccatgcccggggcactcccacgaccgcctcacttgctcaacgcgacgaat

ctccccacacgaagtcccctcttaccagcacagtggctcgcatcgcgcgcagccctcaccagaccaccccccttaccgcccggcgccttgcctccgcc

cccgcctccaccaggcgccgtactgccgccttcgatgatcatggccaatcgggtgggagggagcggacgcgtatatgcgcgtccgtcgggcgggatgg

catcacctgcggtgcgcaatacgggcgtgggaatgaatggaaagccgaacgcgatgggcgggatgaacgggcctgcgtcgtctcggttgactgggtta

aatgcgccaggaggcatctcaaggatcgggggtccttcttcttcccggtctgcgccagcggcaccttcgaatgatccgcatgcggcgggcacgacctt

accttcgtatgaggacatgctcgtagaagctatcactgcgctcgaagctgctggactcgcgcatcccgctccgacgtccaaacatgcgggtcagcaag

gtgtcccgccccgcgtgctcttccactggatggcagaccgctacccgctcatgaagaacttccgtccgtcggcgtcgcaggctttacagaagtcactc

aagcgcggccgctttctcaagattggcagcttatacaggttgaatccagactttgacgagtcgttggatctcatgatgcaggaggacagcagtggcgc

agttcgcaggcctggattgggtgatcatcctgcccgcccgagttttcacggcagtctcggcggcttctcctcgtcctcgccttcgccttctccctctc

ccgcaccaggcggatcaatgtcaccggcactgcaggcggcgactatgatgatacccaagaaggacaccagacctgctcccaaagccatcgggtcgatc

tcccctgcaccgtcgcgctacaccgtcccgagctcgagcggggccagtctgccgatccggccggtgatggcagccgtgcacccggcccatctcgctgg

tgcgattgggaacgtgggcgggggatcgggtggcggcggaggcgccggtggggctgattcgcgttcgtcgggaggaaacggcgcaagtgggagcggac

tgaacggcgtgccgaatggagcgacaacactggccatgctcggcctgcaccattcacgcgcgaacccactcccgtcagcgatgttgcaagctgcgaac

gcatatacgcgctcgggcaccgttccgcttcctggcggtgtaccccgtcgaccgccgcttccggcgagctacacggccgaacagagggaggcggtgga

gagggagcagaaggcggtggcggcgaaagcggcagcggcggctgtgcaggcgcaggcacaggcgcaggctcgagctcaagcgcaggtgcaagcgcaag

cacaggcggcgcaggctcaggcggcgaacgaggcggcgaaggcgaagcgggttgggggtgggacgccctcgtattcgtcgtcagtactccgagctccg

gctcctgcggcggcagcggctgtgcctccggcctatgctgcttcgcgtgcaccggcgcctgcgagcagtcagaatgctgcacaggtgcagacggagtt

ggagcggcttattgcgttgttcaaggcgaagggagggccggctgcggaggtagatgaggacgacgaggacgacgatgaggaggaggacgatgacgatt

cggaggaggaaggggttgtcaacggaccgggtactgctctcgctctttcgaagtcggtgcccacgtctggcgtgaatacgacgaacgcgacgggtgta

cgtacctccttgccagcggatcttccaccttctgctccctcgacgatgtcatccaacacaacaccaggggcacccaccttatcggccgttgctgtcaa

gacgtcgaccgacgccgctccatataatgcgatccagagcgagctccagcggctcatcgcaagtctcaagggcaagaatggggatgggaatgaagatg

atgaggacgaatcgtccggggaggatgatgatgatgttgatgacgacgacgatgaggacgagtag

>contig_14.contig_14.g691.t1

atgaagtcgctcagtgccctgatgctcatcggcatgctgttcgccatcagcgccgtcaatgcgaactggggctattctc

gtgttgccgaaaagtgtcaagagacgtacgcgggccattgcaagaagaacacaatttgtgacgatccgaaggacccgggctattgcgatgtgacagac

gatagcgacctcgcaatcccgagctgcatgtgcacgatttggactcttatcggcaaaggtgacgagcgatactacgtcagctccgcctacccggccga

agccttcttgcaaagcttccacttcggacaggtccacaccgcatcctcaagtcgcgcagaaaatacaccatcgcaccacgaacgtgacttctcaagac

cggaggagctacctccccaagcaccactcttctcaacatctcagtccttcttcagtcctttggcccttcccaagcctcgaccagtccctagtctccac

ggccaagcttcatagaaccgacaccccacttctcatcagacccgcaacgcaagacgttttttgtacgcccctcattggattgaccgggcgatggtgct

gctgtcctattcccacccgcagcgcggagatgaaaggtttaaaaagggtagtcgaccttcgcccactggcaacatacagcttactttcaagctcccct

tcgtcctctgtgccctcctggtcgcccatcccatggcatccgccgtagctatcagcgacagcgacgcagtcgagctgcaccctcgctctggtggcgtc

ggctccggcaacaacaacggcttcgacttcggcgatgcactcggcatcttcgaaggagacaactaa

>contig_14.contig_14.g692.t1

atggccgcttcggaaaagaccggcctgaagaggttcaagaaggagcataagcaaggctctggcaaggacgactttggct

tccgcgagggactcgacttcggaaacgacttcggcggtggctgcggtggtttggaggcggccgctacggtgggtttgaaagtagcgtcggaagcttca

gtggcagcagcagcaacttccggtttttgtggtggcaaattcttcggtgctcgcagcttcgacggaggaaagagcagctccggttcccgccacaagga

ggagaagatcgagaaaatcaaaacggacaagaagcacaaggccgcggccgaggtcaatgcggacgccaagttcaaagctgcggccgctcacgagacgt

tccaccgcaacgctcttctcgccaagaagattgcggctgagaaaaagaagggcggcttcaactggctcttctag

>contig_14.contig_14.g693.t1

atggcagacctgccgttcaccgagaaagtccagggcagtctctcgcaggcggtccagctcgcaaaggactacgcacatc

cacaggtcacacccacacacatcgcactcgccctcttcaccgacacagcatcgcgcggcccagtcacagacacacaagcagactccctcttcaggtca

acgctctccaaggcaggcgcagacacggccaagttcgacaccctcctccgcacggccctcaagaagatcccctcccaatcacccgttcccgacgacgt

cagcctcagctcagccgcggtcaagctgctcaaaggggctgacagcctccgcaagactcagcacgacaccttcatcgcgcaggaccacgtcctcctcg

ccctcatagaagacagtcaaataggcacgctcctcacacaggctggtctggccaatcccaacctcctcaagacggccctcgaccaggcacgcggcggg

cgcaggatcgacagcaagaacgccgaggccggttttgatgcgctgaccaagtactgctccgacttgaccgctctcgccgcggagggcaagctcgaccc

cgtcattggtcgcgatgacgagatcagacgtgtcatccgagtcctctcccgacgcaccaagaacaacgccgtcctaatcggcgaaccaggcgtaggaa

agaccgccgtcgtcgaaggccttgcgcagcgcatcgtcgaccgtgacgtgcccgccagcctgatcgggcgactcttttccctcgacatgggcgccctc

atggcaggagccaaataccaaggcgaatacgaagaacgcgtcaaggccgtcctcaacgagatcgagaaggcatccgacgaaggcacctccatcatcct

cttctgcgacgagctgcatctcctcgtcgcgggacagggatctagctcgggcacgatggacgctgcgaacttgttcaagccgatgctcgcccgcggca

aactccgctgtatcggcgcgacgacgttcgcagagtaccgccagcacatcgagaaggacagtgcgctcgaacgccgcttccagcaggtattggtcaag

gagcccagcgtgaatgacacggttgccatcttgcgtgggatccgagacaagtacgagcttcaccatggcgttcggattcttgattccgctgttgtgca

ggcggccattcttgctaagcggtaccttacctcgcgtcgcttgccggattcggccatcgatctcgtcgacgaggcttgtgcagatgtacgcgtcagtc

gcgacaccgtgcccgaagacatcgaccagctcgaacgcaagcgtatccagcaacaagtcgccatccacgctctcgaacgcgagaaggatgcggtctcg

aaggagcgactgaccgacgcccgcaaggaactcgctcaaatcgaagaagaacttgcccccaagaaggccgcatacgaagccaacaaggctaaaggcga

cgaggtcaaccagctccgcaagcgcatcgacgaactcaaagccaaggccgacgacgcagagcgcaagtacgacctccagaccgccgccgacattcgat

actacgcccttccggacctgcaggagaagctcaagaccgtccaggccgaagagcgtcgacgcgaagaagccagtctgaacggcgaaggccccagcgca

aatgccgtgacgcccgagtcgatcgcaggcatcgtagcccgctggacagggatccccgtctcccgcatgctcgagacggagaagacgaagctgctccg

gatggagagcgtgctgaaccgggagatcgtggggcagccggaggcggtcaaggcggttgcgcaggcgattcggatgaaccgggcgggactgagtgcgc

agaatcggccgattgggagttttttgatgtgtgggccgagtggggtggggaagacgcagctttgtaaggcgttggcgcagttcttgtttgatgatgct

tcagctatgcttcgcatcgatgcgtccgagttctccgagaagcactcgatcagccgtcttatcggtgccccgccgggctacgtcggctacgaccaagg

cggagtgctgaccgagtgggtccgccgtcgaccctacagcctcatactgatcgacgaagtcgagaaggccgctcgcgaattcgtcaccctcttcctcc

aagtcctcgacgacggtcggctctcggacagccaaggccgggtggtcaacttcaacaacacgctcgtggtgttcacgtccaacctgggctcggcctac

ctgaacgcggacatgtcggaggaggtcacacccgcgacgcgcgagctcgtgaatggtgccatctttggccacttccctcccgagtttatcaaccggat

cgacgcgacggtcatcttccgcaagctgggccgcaacgatatccggaacattgtggacatccgtatccgggaagtgcagcaacggctgaacgcgaaca

acaagcggatcaagctcgtccttgacgatggcgcgaaggacttcctcggctcgatcggctacagccccacgctcggtgctcgtccgctgaaccgggcg

atccagaacgagttgctcggtccgctctcgatctcgatcttgacgggtcagatacgggagggggagactgcgaatgtcgtttttgacgggccggcgaa

tcggattcgggttgaggctaaccatgaggttgaggaggggttgatggatgatatggatgagttgcgggaggaggcggatatgatggaccaggactatg

tggaggctcaggtcgaggagttggactga

>contig_14.contig_14.g694.t1

atggctgatgacgacgacgacatccatctctttctcgttcccattcaattctctcccactcgctgccatcctctgccca

atgccaacgcctgtgcaccgcgtgactcggccggcgtcagtgcgaccacacacacgcctcgcatccgagtacgtcgctcacacgctcctcgtactcct

acaccgcctgcgctgcttatcgctgactcgctccttccctttctctccctcgctcgcagggtcaacccgttcgagcgctccttctccgacaaccccaa

ggactcgcctagcaaggcagacaagaacagcagcagcaacagcaacaatgagcccaacaacagcgtcatcccagcagccaccactccctccaagggca

tcgcagcagaggcaccctccacaagacccgagtacaaaccgacactgccccccgtcgcttccataacatccccctccggtgactccgtcgccccgtcc

tccttcccatggggctccagtctcgcaggctccctccgcgcagggcccctcagcccagccatgctcgccggcccacagaaccaaggcaactcaaatca

caaccacaacggacagggtgccaccaactcatcctcagggccgaatccatccagttcgagtatgagcaacaccaacagcagcaacaatcacaataacc

accactccagtatcggcttcgatccacagtccttccgtactggtttcacccctgacctcagcaatttccgaacgggcctgactccccttgcaggtggg

cccacttccttccctccaccctccccaaacacggccgccttcttggccatggtcaccaatgggaccaacggcgtcgcccaaccaaacaacggccccac

catcactcccaacaccctcgctgctctcaccggagcagccaatgcaagccagcaaggaggagcgaacagtcatgcccacgggtcttacggcaacaaca

gtcatcacaaccacaaccatcaccacggccatcaccaccactccgaggggaacaatgccagcgactttgatattgcctttggtcgctccttccacggc

catgatggtacaggaaagcaacagccgtcctcccgactgcgcacgtccacccgcgcagacgacgaagcatccgcctccgtcagtcccgaactcgtcag

catgcccctcggtcccggcggccggaaccagtccaaccagccgaatggcgacccggtacccggccacggacccaacatggccgccaatggcctcttcc

tgctctcccaggctcaccagatggagatggccaagcaggacgatcccgaggcggccgcggccgctgcggctctccagggtgtcagtcagatcttcacc

catccaggacagcagcctcctgcctcgggcgctccgggcgatccctcttggcccggtgcgatccctggtgtcccgcctggacagcagcaacagcaaca

acaacaacaacccagcacgaccggggtagacgatctgtccgggatgaatgcggccgctccgcaacaaccgaacggcaaggctgctcctgctggtggtg

ggaagcgggcggcgaagcgcaagaacagctctgcgaatgcaaaggccggagcggcgaacaaaaaggatgccggtgggggttctgctctgcctgttgcc

aagcgggccaagacgagtgcgtcaacaaagggagctggagcagcggcggcggcgggaggcgctgacaagccgggccacaaccgccgcaccagcgcagc

ggcgtccctcaccggcggatccgaggacaacaacaacatgaacttcgacatcaactccttccgtccgggaggggagatggactttggcgactttgacg

atgaggatgatgatgatgatggtggggaggacggggaggaggaggaggatgggatgggtgggaagaagaagaagagtcggaggccgaataagagtggg

tcgcatatcgatgatgatgagaagcggaagaactttttggagcggaatcggcaggccgcactcaagtgccgacaaaggaagaaggcatggctggcgtc

gctgcaggcccgggtggagtacctgaccggggacaacgagcaactccaaacgacagtcgcccgccaagcgcaggagatcatgtacctcaaatccgccc

tgggcaaggcgcagagcgtagcggtctcgcacggtatcccggccacggagctgcaggtgcctccgatcaacatgaacgcgatgatccatgcgcaccat

cagcaccatcagcagcagcagcagcagcatatggtgatggtttcgcaagggccgatgatcccgcctggcgtaccgatcccgagcgcgatgcataccaa

cccgcattcagcagcgccgaaccctgccgccgtggcagggctgggctcggcgaatgtatcgcctgcgaactcggtgcggaccttgcctgttgttactt

ctaccacgcaggcgatgggcggcggcggtggagcggcgggtgtgtattctgcgcattcggtgccgaggccgcagcaggggcagtttggaggaggagga

ggaggaggggggggtgctccgatgcagcctccgccgcctgcgcctggtgctggtgctggtggggctggacggtccgggtcgagtgaggttgggggtgg

gggtgggagtcggtctgctacgcctggtcatttggttgggcaggctgcgtaa

>contig_14.contig_14.g695.t1

atgttctccatagaccccttcgcacacgccgttcccaagtcggtcgatgcctccatctcaaatgcaaacgcagcctgca

tcgcattcaaccacgcctccgccgcacccgcgctgatcccacccggtcccaatggcaatgctcagactgcctcagagacgcaagaccacagacggcta

cactccctcttcgcagggcactacctcgccgcaggaagactcgacggcatcgtagccatctgggacatcgaaacccgctctcgtctccgctggttcga

cgcccacatcaaacaggtaacctcgctcgcctggtccccgcatggacgctatctcgccagcagtagcctcgactggaatgtgatcgtatgggatctcg

gtctcaagacgggggcgacacagcccgcacgcaagcgcaccattcgcttcgacgcgcctgtcctcgaggtccgcttctcgcccgtcagcagcttgatc

ctgctcgctgtcctcgagacccagcaggcctacattatcgacctccgaaggccaaagtatacccttgaacaggttcccttgcatactccccaagacga

gcccaatcaggcaggtccctcagaaaccactccaaatagcacactcccgacaacgaaagccgaagagagtagtccggtccctccctatccgctgcctc

ccgatcctctgcctgccatctccctcgagccctcagcatacagtaccgcagtattcaccccagacggcaagttcatcttcgcggggacgtccaaaggt

catctgcacgtcgtcgacccacagactggcaagactatttccaagctgagtgcgaccgctccatcaggcatacgagagctcgccttcgaccggaccgg

aagcaaactgctcatcaacgccaccgatcgcaccatcaggactttcctcgtcgaatacgactacagcaccctctcccctgatggccaatccacctggc

ccttcccagcgccaatctcaagcgatccgcagcacgacaacaaggcaatcgacaacgcgccagaccacaacatggacgtcgccaaccccgcctccgaa

ccggagatcaagcacgacatccagtccatcaccacgaccgcagcagcaacaccagtcgtcctcatgcccgtgcacaagctcatcgacctcgtcaaccg

cacgccctggaacggcatcggctggagcgggcacgggggcgagtatgtgtacgcaggtgcggcgcataaggcctcgcataatatctacatctgggata

tggctacgggtacgctggagaaggtgctgcagggacctaaggacccgctcgtggatgttgattggcatcctactcggcccgtgatcgcgtcggtgtgc

tcgacgggcgcggtccacttctggttcagcaagtcggaggaggcttggagtgcgtatgctccccagttccaagagctcgaagagaacatccagtacga

agagcgcgaggaggagtttgatctcgaagatcaagacgagctcagccgacgcaagcaggacgaagaagaggcccttgtcgacatctccggcaccttcc

cgcccagcttgctgccccccggcatgcccctcctcaccgggctaccgcacagcaagatgcaagacgtcgacgacgatgtgaaggtccgccgcacgaag

tcggacctgctcggggctggagagggcacgggacgagcgaacgacgggaatggggtgcaagttgcggctcgtgctgcgtttacgtggcgctgggtacg

gactggtgggagtgggagcttcgaagcggacgacgatatcgatccgcacttcgtcatacccgtccagctcgaggacgggaactcgtcctcggatggga

gcagtcggtctggttga

>contig_14.contig_14.g696.t1

atggacactcgaggaggcttcgtgttgccgcgggaaggcgagccgcagctgctgccttcactccatgcgcacagtcacg

agggggaagcgccgccgctgccgccccgcccgctgacagatcggcaacagactcgtctgagcgcgtacttggacgaagcgttcggccggatcagccgg

ggcttcctccgccgacacgagcccagacccgagcgcgccgacggagaggcgaagacgaaggcggaggaggacgcggactggctgcccacgctgcactc

gctcctcgctgcctgggcatctgtgcttgggttgatcgcgcgcgtcccaccgctcggctcgtccgcgccccttctcgtctccttgctgctcagggcca

cgtccgagcttacggagaacatcagcggctacaacctcggtgagcatggcgcgctgcacgacctcgacacgcagctgcatgccgcgcttgctcttgtc

gatgcgctggacagtatctgggcggatcttctgcgtggacaggctatcgatcaacaccaggctcgtctgcagcttctcacaacctcgccagaagctcc

tccatcttccgacacgcagtacggtcttagcacggatccgagtggacgctttcaccactcgcaggtctcgcggcgctccggcccatcatcacttgtac

actccttcccacccccgccttccactcccttcgcagatgcctccaccagcagcagcacagccacctccgtctcggccctctcccaaacagagcgcatc

cggctgcgcaaccttgccctcctcgcccgcgaccgtctcttccgctggatgagggacgtcctcgacgcaccgccccctcccgagacggacgacgccca

ggatgaaggcggcgacagtcatgcggcgatggcgattctgggcaatggaccgcgcgttgcggctttgcttgcgtttgacgagcggatgcagcgacacg

acgatgacgaggctgcgcgtcggccggggaagcggagacgattggaaggggagggaggagggctgggaggatcgagcgctgtggctcgtgaggacgat

gatgcgctcgaagagcaggaacgggagttcgcggagctagtgggcgctcagggcgatcttgactcgggcttaccgcctgtgcgcgacgagcccgtgga

tgagccggacgacgacgacgacgacggggtggaggagctggaaccaccgacgacgaggacgaggacgacagcagacggcgaggaactcgagcttgaga

tcgaggtcgttattgatgacgacgacgacgcgcctagagacgagggcggcggaggcggggatggtgatgcgttgcctggcgaggacgagggaccgctc

gagcaggagcaccagcactacgcagatctcttctctcgaaagttggatccggacgcgtccgacgacgacgacgagccccatgatacggacgaccatcg

caagaggaccaacgcgaatctttcctccgacccaccatcctccaaggacaaacggcaacggtccggctcaattgacagcgacgacgcgcgtaccgtcg

acccatcagcgggggcaggggaagagagcgcctcccgagcttcgcgggccgtgcttgggcagtgggatcttgagttctcccgctgttttcggcggacg

ctgcggttgcttgctgaacaggctgatcgtgcgcaggagcgggaaagggacagggagcgcgatggagattga

>contig_67.contig_67.g697.t1

aagagcgtcgagaatgcgaagaagtggtaccgcaaggcggccagccacagaaacgccgacgcacaggaacgcatcgacg

acctctccaaaccacagccgcacgagcttagtcggacggaccaccaggcccatatggatgccaagctcgtgcgcaaacgcacccatgcccagatcaag

tcggatcatcaaaaagcgggctcgagcagcggcggcggcggcggacatgggcctgctggacacccggctgccggtgcgcagcacaagcaggcagtcga

cgtctcccgtcgtcagaccatgaagatggtcgatgcgtatacggggtatcggaggcagggtcgacaggaagcttcgatgcccaacatgcccgcgcatc

acgtcctcacccggcctgtgtcctcctccgcgtcctcgacggtgtcttcctcggtctccgtcagtcagggtcaacatggggcgggctatagtctgagc

gagtcgtaccaccacaaccaccagcccgtccctactccagcttcggcaggggcaggcgtaggggtaggggggatggtagggacgagtcctggtcggaa

ggtgcttgcgaaggtgtcgccttcgattccggttcatgctacctcggccgcaggtgcccggcctgggttcccactgtcggcatccggtgcgcttccgg

cactgccgcagccgcaagggcagcagggaacaagaccgaaccaccaccaccaccagcagcagcagcctgagaggaacaaggtaccacagccacagcag

cagcagcagcagcagcagggcggtcctccccgtccgggtgagcctgcgtatagcacgtttgctgagatgggctttgcgcctaaaccgagtggggccga

gaaggactgtgtgattatgtga

>contig_67.contig_67.g698.t1

atgaagatcttcaattccgctgctttggtcctgctggcgctcgtatcggtgcgctccgttcaaggagtaccaataccgg

agtctccgcactccccgccagttgaacgagccgctgctcgcatctacccggaagggcatcgtgattccggtacttcaccgacgaacggtcgtcgtgcg

gttgtgactaccactccggcggcggaaacgcatcgagatcccgtacgacacgaggcagccgattccatcaactctgcgaagccgatcgcgctcaacgt

cgagtatcatcaggtagtgagcaaagagcccgagaagaacgccagagcattggcgacgggagcgatgagccctgaggcctaa

>contig_67.contig_67.g699.t1

atggctcgggctaggagatgcaagaaaagcaccaagatcgacgatggcggctcggagatcgactattcgggggaacatg

aaaaagatccaggcatcggcgagacggaaggacctctgttcgacctcgggagggcactcgccgcggaagacagcgagcctagcgagttcagcttcggc

ggtagtgcagacttccttcctgctgcgcccggtttgttcatcgaaggtgttggcaaagtcgtgctcccactcatcgacgagcaacaggcggataagat

cgtcaagatatgcgagccgacgccatccgaaccagaactcgataccatcgtcgacacatccatgcacagctcctggcagctggattcctccaaggtca

agctccagaaccccggctggacctccggcatccataaaacactgccactcatcgccaagaagttcggcgtcactggcacgcctattaacctgcaccta

cacaagctcctactctacaaggagggcggccaccatgcgaagcaccgcgacacggagagggaggaccgctcgttcgcgacgatggtcgtccaacttcc

ctccgcgcacaaaggtggtcaacttcaagtcttcaaggacagctcggaggatcccatcacccacaacttcggcgcggaagctggcacggccgagtacc

agtgcaactacgctgttcactacgccgacgtcgagtatgcagtccagcccatcacggaaggataccggatcgcgctggtgtactcggtctgttggcct

gcagacagcacgcagccagcaccatccctttctggcgacagccaggcgcccatgctcaaagctcttgctgagctggccgatgccaaccgcgacttcca

ctactacttcgagcacgtttacacgcccaaaagcatcagagatctcggtgttggctgcctcaatggtcaagatcgtgctcgcgttgacaagctgtgcg

tgttaaacgctatgttgcctcctcatcaacgcttcactttctacttggtggaaggagtgcgcagggcacattactaccgcttcgattacaaggtaaac

cacgccgactggaagcaactgagtccgccgtcctacagattcctaccgcttctttccttggagggaaagtacgtggctgtagaatccgtcgacatcga

aaacaacgatgttctcaatccagatggcaagacacgaatgcagaggtggcgcggtcaccgcgtcacgacgttcgaagaggatcacagcagcgacgacc

tcgacaaggacacaacgttcgaaaagtacctgctgctcgcctggccggccaagctgacggaagacaagatgttcgtgctcaccggagtcgaggcacac

ttcaccggcatgctctcagctggggttgacccagatgccgtacgggagtttgtgcagcgtgtcacgagggtgaacgcaataggcctactcaaggctga

aagggagatcaagttcggccaggctctgtaccagcacatcatctcggagccgcagctctacgaccttttccccatctacttcgacatcttcccaaggg

gaacccacggcccggaatatcctactcggttgtcttacggagaatctccagtctcgacgacgcctttcattgatatcatccagatgacccagcagcct

cgctactgggagaccttggaagggaaagtcatcgaagccttcgcaggcaaggtgcttccaatcctggacttcatggaggaggtcgggaaatcaaaggt

cctaaagacggagatgaaggaccagctcaccaagaagttgctggctctttttcgccaaccctaa

>contig_67.contig_67.g700.t1

atgtgcattgagcgttcgctgcgacgcacggtgtgggcgccgtttgtcgacattgcggtgaagttctgcgaggcgccgg

gaagtcttatgcaaacaacatcgcactcgagcacgaccacgcccagaccccttttgttgaaggagttgtgggacacggcgatcctgatcgacagcaag

gcggaggacggcgaatcggtctcggatgctgctgtaaaattgctcacacgctacatcacggaggcaccgatcggccttgctgcggcaatcgctcaaga

tttcgaacacggagttgaagaagacttcgacgacctcattcacctggcgcacaagtttcccagtgtatgggaggcgtcgaagtccaaagtcttgagca

tcatgcgcggagcggacctgaagcgctcgctcgtgttcgtccagcagtgtcggctggccaatctgccggaggcagtatgggccccaagtgtagaactc

tgcttgaaaacaggcgggattcccacatacgatgagctcaccgacgagaggttccaagcggcgctctggggcgtcgcagttgatctgccaaatctgac

tttgtgtcagctgtcggtgaagcgctaccttgtgctaccccttttgttcgcggaaatcgcgcgctcagcattgattgctgcctgcaaggcgaattcgc

aggacttcaagagcaaatgccacctgctggagcctttgcttagagactggtcccacccagtacggcgggattttcacgagctacagagaaagttcaca

gaagcgaacagctggagcacagctacggccacaattaccggccacccttcactggcaaccttcgcggagagcaccgaaacatcgacgaggattggcgg

atttgatagccttgctgccgcacggaagtgcgcccaaagtatctacttcaagaattgcaagggcactgccgaggctggcggtcgtggcaaagatgcgt

acgtcctagtcaccaaggacctcgactatgtgaaacagctggagagcgagtatcagcgggccaagcagcagtacgatgatcttcgggacttactgtcg

caatccgaagcagagaaggtcggaccggcggtcaagaagcggagagcgaagtag

>contig_67.contig_67.g701.t1

atggctgagaaccagatcgagcaaggacgcaatagagacgacggaactttggatcgaggcatcggtacgagcaatggcg

gcgcccaggtggagaatggggatggacggcgcgacgagtctggcggaacagagaaggaaggccctctcttcgatctcgggaaggcactcgctgcaacg

aagtgcgagccggacgaattcagcttcggaggcagcgcagacttccttcctgctgcgcccggtttgttcatcgaaggtgttggcaaagtcgtgctccc

actcatcgacgagcaacaggcggataagatcgtcaagatatgcgagccgacgccatccgaaccagaactcgataccatcgtcgacacatccatgcaca

gctcctggcagctggattcctccaaggtcaagctccagaaccccggctggacctccggcatccataaaacactgccactcatcgccaagaagttcggc

gtcactggcacgcctattaacctgcacctacacaagctcctactctacaaggagggcggccaccatgcgaagcaccgcgacacggagagggaggaccg

ctcgttcgcgacgatggtcgtccaacttccctccgcgcacaaaggtggtcaacttcaagtcttcaaggacagctcggaggatcccatcacccacaact

tcggcgcggaagctggcacggccgagtaccagtgcaactacgctgttcactacgccgacgtcgagtatgcagtccagcccatcacggaaggataccgg

atcgcgctggtgtactcggtctgttggcctgcagacagcacgcagccagcaccatccctttctggcgacagccaggcgcccatgctcaaagctcttgc

tgagctggccgatgccaaccgcgacttccactactacttcgagcacgtttacacgcccaaaagcatcagagatctcggtgttggctgcctcaatggtc

aagatcgtgctcgcgttgacaagctgtgcgtgttaaacgctatgttgcctcctcatcaacgcttcactttctacttggtggaaggagtgcgcagggca

cattactaccgcttcgattacaaggtaaaccacgccgactggaagcaactgagtccgccgtcctacagattcctaccgcttctttccttggagggaaa

gtacgtggctgtagaatccgtcgacatcgaaaacaacgatgttctcaatccagatggcaagacacgaatgcagaggtggcgcggtcaccgcgtcacga

cgttcgaagaggatcacagcagcgacgacctcgacaaggacacaacgttcgaaaagtacctgctgctcgcctggccggccaagctgacggaagacaag

atgttcgtgctcaccggagtcgaggcacacttcaccggcatgctctcagctggggttgacccagatgccgtacgggagtttgtgcagcgtgtcacgag

ggtgaacgcaataggcctactcaaggctgaaagggagatcaagttcggccaggctctgtaccagcacatcatctcggagccgcagctctacgaccttt

ttccccatctacttcgacatcttcccaaggggaacccacggcccggaatatcctactcggttatgacccagcagcctcgctactgggagaccttggaa

gggaaagtcatcgaagccttcgcaggcaaggtgcttccaatcctggacttcatggaggaggtcgggaaatcaaaggtcctaaagacggagatgaagga

ccagctcaccaagaagttgctggctctttttcgccaacctaacgttctcacctcggacgcgaatgctcgaaagaacgagaagctactgcagccaaagc

tttggtcggctgtacttgcgaacgaaggatcagagttcgtcggcaatttggcccgcacgtacgtcgccctttttcctcaaggggcttatgcgacgact

cagccagaaggcaatcagcgattcgaggatctcctacacttggcaacgtcagtcagtgcctgggactcgatcagtcccactatcctcgacgcgttcga

gggcgacttgtacggcacgctcattttcatcagaatgtgcattgagcgttcgctgcgacgcacggtgtgggcgccgtttgtcgacattgcggtgaagt

tctgcgaggcgccgggaagtcttatgcaaacaacatcgcactcgagcacgaccacgcccagaccccttttgttgaaggagttgtgggacacggcgatc

ctgatcgacagcaaggcggaggacggcgaatcggtctcggatgctgctgtaaaattgctcacacgctacatcacggaggcaccgatcggccttgctgc

ggcaatcgctcaagatttcgaacacggagttgaagaagacttcgacgacctcattcacctggcgcacaagtttcccagtgtatgggaggcgtcgaagt

ccaaagtcttgagcatcatgcgcggagcggacctgaagcgctcgctcgtgttcgtccagcagtgtcggctggccaatctgccggaggcactgtcggtg

aagcgctaccttgtgctaccccttttgttcgcggaaatcgcgcgctcagcattgattgctgcctgcaaggcgaattcgcaggacttcaagagcaaatg

ccacctgctggagcctttgcttagagactggtcccacccagtacggcgggattttcacgagctacagagaaagttcacagaagcgaacagctggagca

cagctacggccacaattaccggccacccttcactggcaaccttcgcggagagcaccgaaacatcgacgaggattggcggatttgatagccttgctgcc

gcacggaagtgcgcccaaagtatctacttcaagaattgcaagggcactgccgaggctggcggtcgtggcaaagatgcgtacgtcctagtcaccaagga

cctcgactatgtgaaacagctggagagcgagtatcagcgggccaagcagcagtacgatgatcttcgggacttactgtcgcaatccgaagcagagaagg

tcggaccggcggtcaagaagcggagagcgaagtag

>contig_67.contig_67.g702.t1

atggctgagaaccagatcgagcaaggacgcaatagagacgacggaactttggatcgaggcatcggtacgagcaatggcg

gcgcccaggtggagaatggggatggacggcgcgacgagtctggcggaacagagaaggaaggccctctcttcgatctcgggaaggcactcgctgcaacg

aagtgcgagccggacgaattcagcttcggaggcagcgcagacttccttcctgctgcgcccggtttgttcatcgaaggtgttggcaaagtcgtgctccc

actcatcgacgagcaacaggcggataagatcgtcaagatatgcgagccgacgccatccgaaccagaactcgataccatcgtcgacacatccatgcaca

gctcctggcagctggattcctccaaggtcaagctccagaaccccggctggacctccggcatccataaaacactgccactcatcgccaagaagttcggc

gtcactggcacgcctattaacctgcacctacacaagctcctactctacaaggagggcggccaccatgcgaagcaccgcgacacggagagggaggaccg

ctcgttcgcgacgatggtcgtccaacttccctccgcgcacaaaggtggtcaacttcaagtcttcaaggacagctcggaggatcccatcacccacaact

tcggcgcggaagctggcacggccgagtaccagtgcaactacgctgttcactacgccgacgtcgagtatgcagtccagcccatcacggaaggataccgg

atcgcgctggtgtactcggtctgttggcctgcagacagcacgcagccagcaccatccctttctggcgacagccaggcgcccatgctcaaagctcttgc

tgagctggccgatgccaaccgcgacttccactactacttcgagcacgtttacacgcccaaaagcatcagagatctcggtgttggctgcctcaatggtc

aagatcgtgctcgcgttgacaagctgtgcgtgttaaacgctatgttgcctcctcatcaacgcttcactttctacttggtggaaggagtgcgcagggca

cattactaccgcttcgattacaaggtaaaccacgccgactggaagcaactgagtccgccgtcctacagattcctaccgcttctttccttggagggaaa

gtacgtggctgtagaatccgtcgacatcgaaaacaacgatgttctcaatccagatggcaagacacgaatgcagaggtggcgcggtcaccgcgtcacga

cgttcgaagaggatcacagcagcgacgacctcgacaaggacacaacgttcgaaaagtacctgctgctcgcctggccggccaagctgacggaagacaag

atgttcgtgctcaccggagtcgaggcacacttcaccggcatgctctcagctggggttgacccagatgccgtacgggagtttgtgcagcgtgtcacgag

ggtgaacgcaataggcctactcaaggctgaaagggagatcaagttcggccaggctctgtaccagcacatcatctcggagccgcagctctacgaccttt

tccccatctacttcgacatcttcccaaggggaacccacggcccggaatatcctactcggttgtcttacggagaatctccagtctcgacgacgcctttc

attgatatcatccagatgacccagcagcctcgctactgggagaccttggaagggaaagtcatcgaagccttcgcaggcaaggtgcttccaatcctgga

cttcatggaggaggtcgggaaatcaaaggtcctaaagacggagatgaaggaccagctcaccaagaagttgctggctctttttcgccaacctaacgttc

tcacctcggacgcgaatgctcgaaagaacgagaagctactgcagccaaagctttggtcggctgtacttgcgaacgaaggatcagagttcgtcggcaat

ttggcccgcacaatgtgcattgagcgttcgctgcgacgcacggtgtgggcgccgtttgtcgacattgcggtgaagttctgcgaggcgccgggaagtct

tatgcaaacaacatcgcactcgagcacgaccacgcccagaccccttttgttgaaggagttgtgggacacggcgatcctgatcgacagcaaggcggagg

acggcgaatcggtctcggatgctgctgtaaaattgctcacacgctacatcacggaggcaccgatcggccttgctgcggcaatcgctcaagatttcgaa

cacggagttgaagaagacttcgacgacctcattcacctggcgcacaagtttcccagtgtatgggaggcgtcgaagtccaaagtcttgagcatcatgcg

cggagcggacctgaagcgctcgctcgtgttcgtccagcagtgtcggctggccaatctgccggaggcagtatgggccccaagtgtagaactctgcttga

aaacaggcgggattcccacatacgatgagctcaccgacgagaggttccaagcggcgctctggggcgtcgcagttgatctgccaaatctgactttgtgt

cagctgtcggtgaagcgctaccttgtgctaccccttttgttcgcggaaatcgcgcgctcagcattgattgctgcctgcaaggcgaattcgcaggactt

caagagcaaatgccacctgctggagcctttgcttagagactggtcccacccagtacggcgggattttcacgagctacagagaaagttcacagaagcga

acagctggagcacagctacggccacaattaccggccacccttcactggcaaccttcgcggagagcaccgaaacatcgacgaggattggcggatttgat

agccttgctgccgcacggaagtgcgcccaaagtatctacttcaagaattgcaagggcactgccgaggctggcggtcgtggcaaagatgcgtacgtcct

agtcaccaaggacctcgactatgtgaaacagctggagagcgagtatcagcgggccaagcagcagtacgatgatcttcgggacttactgtcgcaatccg

aagcagagaaggtcggaccggcggtcaagaagcggagagcgaagtag

>contig_21.contig_21.g703.t1

atgtacaccctgtacgcacctcgtccgcggtcgcatcggtccaatgaccggagtgaggagttgcagaacgcttcccacg

ctatcatcttttccttcttcttcgacttcttctcctaccttcatcttcgcatatcactgactctcatcttctgcgacgcgctcccccagtctctccaa

cacccgtcgtctcatgagctgcttcctcacggacagtacgatgccaatgcaagccgaatcatcctcaccatcgactctacacactccaccaccagata

tcccgtgtcccttctgcaaacttctcactcacctcccatcgctccttcttcctgcatttgcccttacatttccttcatcttctcatttatcctcatca

gcagcgttgctctcgccctatcttttgcaggagacgccctcgcgcgaacaatgtcgggattatctgaggtgctcgaagctcaaggccgtgactctgtg

cggcacatctttgcttttgatctttcggacctcggcgcccatctgcgcgatccacgggaatggttcgacggaaggaagagctcgcaggccgtcggacg

gcgtgacagctgtaggcttgttttctcagtttccacgcttaccgggacggatcttgttgtcgaagtggacggtgctcgcaagtttgagcaccgggatc

cagcctcgagcatcgacatcatcatcaactattcgaaggatacctcaacaaagtacttccaggaattgctgagcctcgtcgtcctcgagttcggccaa

gatcccaacgaaatacttgtcctcggtcgcgactcttttggcgaaggatcagctcagccatcccaatttacatcatcgccgacggctgtttttcaact

ctctcccgtcctcggtctgcccgtctccgcggctccgcatgtcgtcttcgtgcagtacctcgccgcactcgccgtcgtccttgcgctccagagtggga

tcgtcttgcctccgggactcgcttcatcggaggaggagaagtcaaagatggtgcgcggcatatctgcatctgtcgagccctctgtggctctcgcgcaa

gacaagcaagctctctacgagaagcaagctgaaggcgacaaactggccaacttcatcatcgaccaatgcgagcctggcactctcgaagaattcctcgc

gcttcgaggcactggccacactgcggaggaagcgatcgctgaactgggactcaacttcgaagacttcgaagacttcgaagagcctgatgtctcggaac

ctgctctggatactgaagacaagcaagccgacactggtcgcactactcgagctcgatcggcatctgtcccgtccaagaagtctgctggcaagaagaag

gctacgcctcaagcacaggttgaggagtcctctccccgtgtgcagccaactgcctcgacttctaagacctctggcatcaagaggatcagggtcgtatc

gtcgagctccgagggcaacgagcgcggcgtccccaaacctcgcgtcgaatcagagactgcggaagcggaatcggtctcggtcactcgcgacatcccag

ctccagctctgggtcgacctgctcaaccggtactgtccaaggttcttgctcgacctacaaccattactcgctcgtcaacgcctcctacgtcggctctg

tctctcgcatcggcttttgagcagcttagccctccttcaaagaaggcgatcaacgaaattctcagccggtccaagcgaattcctcttgaggccgaagc

tgttggttccgactcgatcctccagcctcctgcgcctaccacgggttctgggcttggcctgtttggtccctctgagtcgaccttctcgttcaaccaag

cacctgctcaagactcagggccttctctctcgccctcgatcggtcttgccattggctctcctgctcggcaccctatcggtgcaactcgcttctcgggt

gctgctgctcctccgccgctcactcgaaacaagaccattgctgaccctctggggcatgtcggatacaaccccaacggggtagtcggctcgttcttcaa

gatgccagccacatcggtgatcggaaaggtggcggcttcgatatatgtcccgctgtggcatttcacgaccgaaggaatcgccgaaggctatcaccaca

tgtccaactggactgaagtcggtaaacgtctagccaacgtcctgcaatctgaggtggaactgccaccgataaagatccccgacgagttcatgggattc

gagatcttcttccaagctgccgactaccaagtcaaagtcctgcgcgagatcgccaaaggcgagatcgatcctctcaagcagatggtgctgtctgagga

agcagctatatggacggctgcatacaacgagtgcctcaagcgcagactcgccaacaagaacctcgactgggcgctcatcatgcgctacatcttttact

tgcgctcgcagtactactcgataccggcgggcccagctcggatcaacccttctctgtggcaaccagccatctgggacacggttctgtctgagcggcaa

ggttcgcagctgtccggctcaactgcttacttcccatctggacaagttcagcagccacagcacttggctttccaataccagtcccagcccttcgctca

gcagcagcagcaaccggttcagcagcagcagcaacagcaaggtcagggccaaggatcatcgtctcgtaagaaggcggccaagcaacagcagccctttc

agactggctcccgaggtggtacccaggccgccccgggagcatgtgtcatctgcggaaagaccaccattccacacaatttcaaagagtgcaagtctcca

ccaaccggaaagtctgctccctttgcttcccgaaacgaagccggtcatctcgttcgcgtctcggacggagcagctctctgcatgcgatggaaccgcac

ctcttgcgccttgtgggctcgactgttcccggacagagcgcctgaggatcggcccttcaaccctgatgccttcgagaaggctctcaaagagctcgacc

tcattcatgctttccccaatcttgccggacacatgcgagaaggatttggtttcggcatcccaccaatccgcaagactcaaacccctgacaatcacttc

tatctggagatagagagcatcctccacagccctttccaaacctcgccactcaagttccttgtcaaaccgaatggtgatctgcgtcctcttcaaaactt

ctcgttcccatacgacgattcgtacccgagtatcaactcgtacatcgactccaaccatttcctgacagaatgggacggggtacggatcgttcaacaac

agattcgggatctcaagtctcggtactcctatctcgacgctgctactgctgacgccaaggaggcattcagagcctgccttgcgcgatttgaccaactt

cccgggacggtcatcaagtttggtccagaccagttttatctggatctcttcatgggttttggccttgcctccgccaccggagtctggggaacggttgc

ggatgctctgaaggcaatcctgaaagtcaggttgtcagggaaactggagttgtccaagtgggtggacgacttctttttgttccgtcttgacccagcct

tgtccattaatcaagtcatggaggcggttcaagatcttcggttcccttggaacccggagaagactacggacttctcaaagctgcctcgttatatcggc

tgggactgggacctcgacaatcttaggatgatcttgcctcggaacaagaaggacaagtacgtcgacaaagcaaagggctttcaggaatcgactcctcg

atcgctgaaggaagtcgaagagctcgtcggttcgctgttgcatgtctcaatcgttgcgagagacctctgcccctaccttgcggaactgttcaagatgt

ctgcgctctttgatccctcgttgcctctcaagaagcactacgttccggtccaagtgcgacatgacatggcgcagtggataacggctctgtcagccact

cccttcgtcaggtccttcaaggtctcggagagtcagttcggagatgtggtctatgtagacgcaccaactgaatgggggattggcatcgttgtcggtga

acgatgggctgcttggaagtggttgcccggttgggatgctgagaacccgggcatcaactggggagaagcggtagctctggagctgggtctgcgtgcgg

ctgtggcgtacggcgcggccaagaaggtcgtcaccttctggtctgacaaccagggtgtcatcggctcgtacaagaagggattctctcattcagcgcac

atcaactcggtgctgaagagagtggtccagctcgagatggagcacaggctccacgtcaagatcgactatgtcgaatcggctctgaaccctgctgatgc

tccctctcgtggagaagttctgcccgagaaccggcggctcaatgccaacaagaagctcgagttcagcgccttcggggggaggcaagatctggattcct

cgtttccgtcggcattcccgcctcccaaaggcacgacagcctttccagcttctgaagaagctttacgacaagatctgttcccgaacgttgcgactcac

gaacgtctgctctcttggaggccgggtctaacagcggccgctcctctcacactcgcgtctggtaaactcttccctgcctcggtcgtacacaatcaagt

tcgctctgctctacgcgcctctctcgagccttcgactcgggcaaactacggttccgctattggcgtcttcctcaagttctgcttctctgctggtctcc

tgatacaagacatcttcccgattactcaaggcctccttctcgctttcgtttcgtcggcatacggctccaaatcggacagaacgtttgccgactcggcg

aacttacggttccttcccctctcgtcttcaatcctgcgagacacgtttgtcgatcacatgtcggtcaagctcgttctgtcggtcaagatctggaggct

ctacaaatcgatataccttggaccaaaaccaccggcaccgatgtggcatcttcatctcaactcttcgactactgccgatctcgaatcaacaccactct

tctcgtacaaagtttcggctggttctggcttccgtctcgttcccctcacgaaaggcaagatgctggaggtgttcttcgaagcactcctcagagctcga

cgaccgaccttttccggacactccttccgaatcggaggtgcaacacactactggcatcaaggagctacggttgatcaaatcaaactgttgggtggttg

ggcgtcggactcctttcgggtatacctgcgtgatcccatcgctggcattgctccattgcaacaacagctgggaggataa

>contig_21.contig_21.g704.t1

atggatccgattctgcagaaggcgctgctcgaccgcgcatacgagaagcgcaagcaggccaccctcgacctcgagcgcc

aggtccgcgagtgtctcgcacgcaacgagctcgcacgcgtcgacctgatcgtgcaccagctctgcgcactcctcgcatcctcctcgccccacaacctc

aacgcacgcaatggcggcctcatcggcctcgccggcatcgcaatcgccctcggcgtacgcatcgcaccctacctcgagcagatcgtgccccccgtcct

cgcatgctttgccgacccggacaacaagatccgctacttcgcctgcgagagcttctacaacatcgcaaaggtctgcaagggcgagatcctcatcttct

tcaacgacatcttcgacgcactctccaagctcgccgccgactccgaactgtccgtcaagaacggagccgagctcctcgaccgcctcatgaaggacatc

gtctgcgaggcagcaccgcactacgtcagccagcatcaagacatcgccctcattcgcgcacgtcaggaccaggccctcggacgcggcaacctcggcgg

cgaagcagagatggacatcgcacgtgagaagaccagggaggcagcctggttgctctcacacccttacagcgcagagagcgtcattcgcagagggcagg

gcctcatgcctgcctctatggagaacagagcaggcaccagcgcaggtatgtcctccaaggaccgaggtctcaatggcgggcagctcaccgccggcgag

atcgaccgcgaattggaggcgcacacccagaccatgacctcacaacacgccattgccgccgactccctctccacctccctcaaccgagccttctccct

cgcacgcttcatccccctcttggccgaacgcctctacgtcctctcgcctttcactcgcaacttcctcgtcggctggatcacaacgctcgactccgtcc

cggacctcgaactcgtcagctacttgcctcgcatcttcgaccccctcctcaagtacctctccgatcccaacatggacgtccgcgtcgccaccaagggc

gttctcggcaacttcctccaggaggccaaagaggccgccagctcagctgcagcgagggaagccgaggagaagcagcgagagtcgcgtcgcaggaaaag

ggaacaggacgaggccgatgcagaagcagacgccctcgcagcttccgtctccgcttccagcctcacagacgagctcgatcctcgaggaggcactcgcc

tgccccgcctcactccagacacatccggcgccaagaagtccaccaagtcagcaacctcctcaaagggtactgccactcctgtcgatcccgaagcatca

ccagcaaatcgctcattagcagcttccagcaaggacgacaacgacgacggccggttgacacctgtcgctgggggcccgtcgatggccgtctcagcgtc

aaaagaagagctcgacaggggcgaggaagacgacgaggatgaggtggacgatgtggatgtggatcatcaatctgcaaatgccgtggcaggagcctacg

gcgttgatggggaacgaagtgcagcgcgaggagagaacgggaggaggaaggagaaggaacaatggattccaaaacaagagatccgtatcgactacgcc

gcggtgatggatatcctcatcggccacatcgctgatcctgacgaggagatccaggcaacgacactgcaatggatagcagagttcctgctcgtgatcaa

gtcggttgtcctgcctttcactcctcgcctgatacctgcgatcctaccaaacctggcacatcacagcccagcaattcagcatgccgctcaacgcacca

acgacgtactgtaccggaccatcctcgagctgcccaacccagatgcagaagacggcggtaaggtcggagatgttgcagggagagtgcctgatgggaat

acaccgcgacccgccggtgctcgagaacgcgaacgcagtgccacgatcagctcaccgccgctcagtccacgattgagaatgcagcctttgctcggacc

ctcgatacgggcggcctcgggaccgggaccgagcccagcagcagcagcagcagcagcagcgggacgcaaccttccttgtgattcttcctcgagttctc

tgctgcctgagccattcgactatcacagcacggtcaacgcgctcactctgcagcttctggacgagcatgaggagacgagggtcacggcgctgcagtgg

ctgctcatgctgcacaccaatgccccgcgcaagattctggcgatggacgacggcacgttccctgcgctcctgaagacgctgtccgacccctccgaaga

ggtgatccgccaagacctccgactgctggcgcagatctcgagtgcgagcgaggacgagtacttctttgcgttcatgcggaacttgctcaacttgttcg

ccaccgaccgacgtctgctcgaaactcggggcagcctgatcatccgacagctgtgtgcgagtctgaatacggaccggatcatgcgcacgcttgcggag

attctggagcgggacgaggatgctgagtttgcgagtattatggttacgaatctgatgattatcatcatcaccgcgccggaactggcggacttccgacg

gaggcttaagagtctggacacgcgggacggccagctgcttttcacgagcatctacaagagctggtgccacaatgccgtggcgacgttcacgctgtgcc

tgctcgccggcgcatacgagcacgcgttcaacctgctccagatcttcgcctccgaactcgagatcactgtcccgctcctggtccagatcgacaagctg

gtccagttgctcgagtcgcccatcttcaccagtctgcggttacaactgctcgaacccgaaaaacacccgttcctgttcaagtgtctgtacggcctgct

catgctgctcccgcagagctcggcgtttgtcacgctgaggaatcgactgaacgcggtgagcacgatggggttcgtgcatgtcgtgccaagggccgtcg

cttcggcaggtggaccgggaaacgcggtccagcgcggtggttcctcctcggctctcggccccgggggtggatccggctcgggcgcgggggtgggaagc

tcgctcatgagcagcagcaatgctagcggcggcggcggcggtggcgtaggctccagcacgggcaagatgttcacaggcggccactccctcgtccgatg

gaacgagctcctgagccacttccgagcggtgcagatcaagcatgagcgactgcgacgacagaatttggggatggggatgggtggtcaggactctgctg

gaaatgtgctgacgggacaacaggactggaaggagcagcgaggtgggggtgggacgacgggttggtcgagaggcgcgatcaacgagtcgacgacgacg

acgaatacgcgggaacaggattccagtggtggtggtgcgaaaggcggccggatgatgaaagggccgagcgcgaagggcagatga

>contig_21.contig_21.g705.t1

atgccgccctttctacgacccccgcccgacgatgcctttgacagcaaagaggcagggctgaagttcgcacagacgtggg

ccagagccgaaggatacgcaatcgtgatctatgcatcttcaaagaagggaaggtacaactatctccgctgcgacaggggcagcaagtaccgaaacgcg

cgcaagataacggacgaagatcggcagcgcaagacatccacaaccaagatcgactgcaagttcagggctcggctccggcgattcgatgcgctcacgac

tgagcgcgttctggagctcgcgcgaaccggatctcgtgtccgtcaggtcgtttccttgcttcaagagcaggttccgccggtgctcgtcacgtcgcggg

acgtatccaacctcatctccaagtatcgacgtcagctcctcgaaggtcggccaccggctgaagcccttctcgactggctgaaagagcagagctggccg

ttcatcgagaagcgagtgccggtttcggagaacgcagatcgcctggaggggttgctttttgcccatccgcgcggaattgagctgcttagccgctttgg

tggtgtcgtagtcatcgatgctacgtacagcacaaatcagcaccgccttcccctacttcacattgtcggatttaccagctccaaccgcacatttactg

ccgccctcgcctttcttcccaatgagacgacagtcacgtacacatgggcgcgccaagcacttcagctcatcgctcctacgtgggaaccggacgttctc

gttacggatgaagacgcggctttgagcgcggctctaatcaatagtttccctccttcggtctcgcaggatcaatacgagcttctgatgagcgatttcga

cgaaattcgcgacgcacggacgttggagcagcttaccgaggccagagacgtgttcttcgaccactggcaagactctcatcctgccctttgcgactacc

tcgacgaaaaagctgtctgcggagcatctttgggtatcggtctacatcaatcggcatccgcatatggggacaaaagtgacgccctgaggaaccacttc

gtcgttcagcacaatcagatcgcaagtgacctccagaatgacatgagcaaaatccccatcaccatcctcggcgaccctttcttcgtacaggtctcgag

gacggtgtctcgatatgcgctgaatcttataaagcaacaggacgaagtcagcagacgccctcgccacaaagaggatggctatgctgctcctgttcccg

tagatctgcagcccaacaagtcgcgtctggtcgtctaa

>contig_21.contig_21.g706.t1

atgctcatgcgctcatgctcgggtctcatctcggttcccagcaccaccttctcgacatccagcatctcagcgtcgacat

cggggggacaagcacagacacagacgcaaggcatggcagacagcgcccgttcacgctcaccctcaccctcaccctcacgctcgcccgggaggaggaga

gacagcgcagagagagacaggcgaaggtcacgctcgcccacacatccccgtccaaactcgccaacgggccgctcagcaacatattcacctcgatccca

cagctcatcgcctccacgctcccgctctcgctctccatcacactcacgatctcgatctcgatctcgcagccgctctccgccctccgccggccgcgctg

tgcctgagccaggagatgacgcccctcccttcatgctgcgcgtcgtgctcagcgtaggccagttccggcccttgcgcgacttcgatcagcccgatagt

gcacgtaacgatcggtatcactcccgctcggccggcggcggcggcggcgcaggcgcaggcgcaggcctggcaagggacgagttcaagctgtacctctg

gcgcaactcaacactgcgcgatgtggtccgcctcgtgcacgcgtcggcgcacttggcgaagcaccccgtcactcgcgacccagccgcatgcagtgcac

aggccctgcacgagattcggctcgtctactacgatccacgtacgagaagctgcattgcgcgtgagatgggtcgcgatattgcccgagtctccgtcgcc

gagtacacatccgcactgctcgggatcggcgtcggggcgggcgtcggggctggggccagtggcgcgaccgcggcagcagcagcagcagcagcaacaac

aacaacaacagagctcgaatcccaactgctggtcgaggagaagcgggccttggagagactcaagacgagggatcgggcaagcgacgcaaccaggacac

tgcgcaagtgtcagctgcgcgacggagacgtgctcgagtgtgtcgttttccccgacaaagctttcgtgggtgcagccgctcctgctgctgctgctgct

gctgctgccacagcaccgccacgagccggactcggaccaggcacaagcttcggtcgcgacggcccaccgatgcacaacagcagaggcggtggccacaa

cgccagcggtagcaggatgggcttctcgtcatcgcgaggcggcagatggaactga

>contig_21.contig_21.g707.t1

atgtactcctcgccgcagtacaccttcctcgcccccagcggagatcaggatgcgacccctgcgcatccgacctacccgc

cccagccagaccgcgtccgaggcccaagacagccaagcgaccatcgccatgcgccccgtctgtccgatcggagccaggcctccaatcaatcctaccag

cactcgcactcatactcgaccgaggcccgaccacctgcggtaaaccgggattccttccagagacagcctctgcatcaccaagcgcagccttaccaccc

acctgcgcaccgagatgctgggcaccagccccaacctgcgcgtcgggacgagcgccagcatttaactggggcttcctaccccccttttgaccctatga

gctaccacggtcccagtgcatacaaccacttgtccgcctcgcatccatctcacgacgatcgaccaatgaagccaggaggcggcggataccgccccctc

cacatttccgtaccagacacccgcttcccgcagcagcagcagcagcagcagcttcactccgcccagcccggccggcggctgccctggttcctccgacc

ctggcagacagccttcgaagcgccgtgcaagtccaacatcgccctcctcctcatcagctgtgcggcggcgtacaccctcatctggctcgtccccaccc

atatcaccaagctcatcgagatgaatctctactgggcccgtgccatcgtcagcgcggccaccgtcatctcaggtctgatggtttccgtgcccctgacc

gagatctcccaccgcatgctctactcggccatctggaccgccgtcatcctggaggaagacatgacactccgcgacctcgaccagatcgcagatcggat

tggcgtcatatcaggtttccgcttgctgtggataaagtttcgtgcgggctcgaaacgcgtctttgggacgacgccgcgcgctgatcagagcgataagg

cgagtatccatggatctaagtcgcgcattcgtaaagcgatttggggcgtcgaagttccggctagcttgggcctgcttctcctcgccagcgccttcagt

ttcgccgcggacagagctatcaagatcaacctaactctcaggcctcaaactcaactctacgataccatatcggtcggaggcgacctttctgatgccga

catctccgcagccgcatccctccagcctttcttcgacaacttcgtccgcacctggaccctacagtccacagcagcactcaagctcccctcaaccgtca

agctgctcctgccaaatcagacggaccagtacgcctacttcaccgaggtccttcccgataacttcctgccccagtttgagggatacggcagcttcacc

aatgatactcatacgacgtcgaacaacgcgtcgtggcaagacccgagtcgggaaatgcagaagcaggacgattcggacgagtggacgactatcgttgg

tggatcggatgggaaggaggtcaccgtgctcaggaatgtgaggtggccccgatgggggattcagaacctaaaccaatacctcgtaccagacatctccc

agaccccagatgcgaccgtcgccaaccgactctccgtcctgttcctcacccgccaagtcctgagctcggtcctatcaacactcgacgtctccctaccc

tcgaccctccaaatggctctggacctgaacatgacctacggcgatctcaacaaggtcttacctgccgggacgaatgcggccgatgtcgccatagccca

cccgttttacgcgaacggagtcgcgcagtcgttcttctcttcgcctattatggctgaggatgagattcagtttgatggctcgcttgcggacgcgtaca

agaatacgccgggaagcgcgggacgggggtgggtctcgctggaggtcgtcctgattcgcctgaatccggaattggcaggacccggtgcgcagtttggc

atggtggccaatgttaccgacaacaatggcaactttgggaacgtcggctacgacgtcggtgtttgcgtggagcggtttgacccatatatcgtggaagt

ctatcaggggcgaagcgtccaaagcatatccatcctcagccaagcagccgactccagctccgaaagcggcctcctccgctcctccctagtctcccccg

gatcatcgacgaacctgagctccctaggcaagaacgcagcctacaccgtggcgcacgagaatgcgcggaacgcgctcctaaaggacaatgggcgggat

tcgtcttgggttcctaatccgacgctgacgagcatgtccagtgggggaggtggggggccggcggggtatggcgttctggatccggaacgtacacgcct

gttgctgccctacctcgtgggacgcggcaagatcgaagctcgcagctacgagttgaagcagatcgcacgcgtatcctgcatactccccttgcttaaca

tcgtgttcggagccatcctcgggtgcggtttgctgggcatcttcttcgtgccgcgcctgcctgcaggcctaccacgacgaagcacgagtccaatcagt

tggctcgtggccttcagcggcgcaggatccttgcgaatcggcagcacaccagccgaagccatcttctccggcaagtccaccacaggaagtcccggaga

ccgcaagagcgcctcaggatccttcgcatcggtccaccatctctcccacgacccatctcgtttcagctcatacgacgacgacgagcagaaattgatgc

gaggaggaggcgaaggcttccgtccgagatcgtcgtccgatgggcggatgttggttgggggcgcgtgggtcacgctcgatgaggttgagaagcggctg

gggaagaagccggttagctatgcggcgcctgctgctcctggcgatggcggggactcgatggcgggcacaccgtgggattcgcagccggttttggcata

ccgaacggacttttga

>contig_21.contig_21.g708.t1

atgaactcgtcgtcgcagtacaccttcctcggccccagtggagaccaagacgcgacgcctgggcagccgacctacccgc

cccaacgagactgcgtccgaggccaaagacagccaagcgaccatcgccatccgccccgcccgtccgatcggagccaggcctccgatgcctcctaccag

cactcccactcatactccaacgacggacgaccaccggcggtaaaccgggattccttccagaaacagcctcctcatcaccaggagcagccttacaaccc

acccccgcaccctgatgcgcatcagcctcaacctgccgagcaccagcgcccaacctcggcctactccaactttgatcctatgagctaccacggtccaa

gtgcatacaaccacttgtccgcctcacagccatctcaacaggatcgaccaatgaagccaggaggcggcggattcgacccacaccttcactacgccctg

cccggacggcgtctgccctggttcctccgaccctggcagacatctttcgaagcgccgtgcaagtcaaatatcgccctcctcctcatcagctgtgccgc

agcatacaccctcatctggctcgtccccacccacgtcaccaaactcgtcgagatgaatctctactgggcccgtgccatcgtcagcgccgtcaccatcg

tcgcaggtctgatggtatccattcccctcaccgagatctcccaccggatgctctactcagccatctggaccgccgtcatcctcgaggaagacatgaca

ctccgcgacctcgaccagaccgcagatcggattggcgtcatatcaggtgtccgcttgctgtggatcaagtttcgtgcgggctcgaaacgcgtctttgg

gacgacgccgcgcgctgatcagagcgataaggcgagtatccatggatctaagtcgcgcattcgtaaagcgatttggggcgtcgaagttccggctagct

tgggcctgcttctcctcgccagcgccttcagttttgccgcggacagagccttcaagatcaacctaactctcaggcctcaggctcaactctacgattcc

atatatgtcggaggtgatctttctcttgctgacctgtccgcagccgcatccatatggcctttctacaacgactacgtccgcacctggactctgcagtc

cacagcagcaatcaagcttcctccgaccgtcaagctgctcctgccgaacgaaggcaaggtctctgggatgattcaagcaaatggttggatcgggcgat

cgatcgcgagtcccttctgggtgaggtttgtgagcgcaaaaccgctcactgcctctcccaaaaagagccccgccaaaacttcaaacacctctgtactc

cagcgcatccagctcctcaaccagctaaccgaacacgctgaatacacctgctacgtcttcatcttgaggcactga

>contig_21.contig_21.g709.t1

atgagcggtgaaaaaacaaggaagtatgtactcaccgttgatcatcagatttggggacatctgacgtcgcttgagcgca

acgagagcctgaaacgagacgcaagagggcgggaatggtgcccggaggcggttccatcgcgtccaccggtgcgcctggacgacctctacgtgattcgc

aatgcattgccgattgagcgagaccgggcatcggcggcgatctgggcgtgcctctcctttgctttcttcgcccttgctcgcctgggcgagttaacagt

cgacgtcctctccgctcctcgagaccctcgtgaccgcagctctcggtgtcattggtcactgcggaatctagaaggttcggtggcaccgacggcgatcc

tccgtctccccacggagaaagttcagggcccgaatggcgtggatctcattgcagcggaacaacgcacgtccccttccatctgtcccgttgcagcagtg

cggtggcatttgcacgtaaactccgaggtcgctccagacgcaggtgctttcgctttcattgacaaaaatggtttggatcacgagcttcgtacacagtt

ctgcgtcgataccgcaaatgcagctcttaccgcagcccgacaggcacccatcacggcacactgccttcgcatcggcggcaccaccttctacatggctg

cggggatctctgaagcagacattcggcgacacggccgatggaagtccgatgcgatgctcgtctacctccgttggtttgcctggtgcctggtcttcgtt

tatccgcccactgcgccttcccaatatcatttccttcaactccagtcgttcctctcgctcactcggggtctagcttcgtcgctcatttggggtccggc

gtgtccaccgcagcaggccggttgggctgcttgggagaccgcacgcagcggcggacacggcgttcgagctccaagctcgacccccaattga

>contig_21.contig_21.g710.t1

atgttgcttgctaaggcaatcatcttagcattgtgcctttacgagtacagtggcgagcggaaatggccgcataaatcgt

ggcatcccactaagtggtgctgcaaagtcaaaggaacatgcggccatattgcacccctccccgtctcccctcactactacgacctcgccgaggtcatg

tgcgatcgcatctcgacaaaccccaccggcagctacagcaccacatcgaacgttgatgctaccggtgacgctctccgccgctccgtcatcggtttgga

tgatgacaccgatgaggacgaggaggcggagagcgatgaaggggagatcgaggacggagagaaagcgaaggaggggtcgaagaagggaaaggagaagg

aggggagcgagaagggaaaggagaaggaagagggaccggtcaagaagaagatgaaggtgaagaaggagggaactggtaggccatcggatacgggccgc

aagttcggaaagctggacgcgtccagcagcgagttctctccccgctatcatcggactgcgtcagaaacgcatgatgctgacactttttctccacctct

ccactctgctgcagctcccaagcgcaaaaccaccggcgtcgaggctgcgctggatcagcgcagcgcggagaagacaaagcaggacgcacagctcaacg

agcccaaggtcaaggcagagaagatgggaaccttgaggcttgatggccttggcccgcgagctccgggaggctga

>contig_21.contig_21.g711.t1

atgccgctcaaattctttgccagtggcatgttcgacagaatgtcgaaaagttcgcgcggccacggcttccaaatcaggc

cgaatacgctatacgagcagttcctgtccgactttgacgagcttcgagatgcccgtacattggaccgtctccaagatcaatcggacgcactgcgtcat

gaatggcaagatgcgcatccagccctggtcgactacatagacgagaaatttttacccgccacgatgggaactccgtgccaccacacgcttcgtcttct

gcacgatctcggtctgcccatccctctccagtccatccatccacagtggagaaccattgcggagtgttccgagcgcgtttcccgtgtgctcgaccctc

tccgacctcggaagaagggtcaaacgcttgccacgaccgtcaatggacggataatgaccggaagtgagaaaacaccaggagaccatattgaccaggat

cctcctcaactcattttggaccggttcctcgacatgtccaggagaatacatgcgcagcagctaccgagcaacccagcacttggtccagcgcaccactc

ccactctcagctcagctctgccgccaccgccacatcgtcgctcgcagtaccggcgtaccgccatcagccgccatcatctgtcgcggtctatcggccgc

ccaccggcagtcggcacatctcgcagcttaccctctttaccctctgcggtaataccgcgcatctcagacggcagacatttctcctacgtacggttgcc

gctgcgcgtgcgctcactcggcctcagcgcaccgcgcagaattctcacgacagcccaccagcgccccgtccacgccaaccacagcccatcatgcccca

ccaagacctcgtccaggcccaccaggcccaccagcgcctcatccggtcttaccagtcttccgcgcacaccaagaataagggaagtcgttggtag

>contig_21.contig_21.g712.t1

atggcagcctcatgcgcatccaatgcgatgcttcctcctcctcaagagtcttttgaaaacagcaaagcagcgcaggaat

acttgcaagtctgggccagggcacatggctacgcggttgtcctctcgcgttcggccaaggcaggacgatacctttatttcatctgcgacaaggacggc

gagtatcgaaacacgcgcaagatacaggacgaggatcgtcaacgcaagacctcaacgcggaaggcgggctgcacattccgtgcaaacatcagcggtag

caccgacttttggaccattacggttacctgcccagcgcatacaggacacgatgcatcgaacacgactcttgcacaccctgtgcaccgtcgcgttgacg

tggcgtcgcgagagcaagttctcgagctcgttcgacacggttccactgcgcgacaaattgtccctctcctcgcccaacgagaaccgcctctgttggtc

acgtccaaagatatcgccaacctgatccaaagctatcgtcgagaggtcctcaaaggtcgaccacacgcagaagccctcttggagtggctcgaggagca

gcattggccacatcgtactcttcttggagcggtgagcgaaaaggtccagcggctcgagggtcttttcttcgcccatccacaagagctcaagttgcttc

aacgctttggatcggtgatcacagtggacgcgacgtacaacacaaactcgcacaggatgcctcttcttcattttgtggcctcacgagctcgaatcggt

ctaaggaaaggcgtcacctccgcgctctcggcggagttccgcgtcctccgcgcctccgtaccgcaaaactccgccgattttcctccgccggaggacgt

cctccgcaccgcggaggctgacagcggaggggcggacggagaagtaccgccaacgacttga

>contig_21.contig_21.g713.t1

atgtactcctcgctgcagtacaccttcctcacccccggtggagatcaagacgcgacccctgcgcatccgacttacctgc

cccgaccagaccgcgtccgaggcccaagacagccaagcgaccatcgccatccgccccgcctgtccgatcggagccaggccttcaatcaatcctaccag

cactcgcactcatactcgaccgaggcgcgaccacctgcggtaaaccgagattccttccagagacagccttctcatcaccagggacagccttaccaccc

agccgcgcaccgagataggcaccatcctcaacctgcgcttcaagatgagcgccagcagcatccaacttcggcctcctaccccacttttgatcctttga

gctatcacggtccaagtgcacataaccacttgtccgcctcacagccatctcacgagaatcgaccaatgaagccaggaggcggcggacaccgccccctc

cacatttcagcaccagacacccgcttcccacagcagcagcttcaccacgcccagcccggacggtgtctgccctggttcctcagaccctggcagacagc

cttcgaagcgccgtgcatatcaaacatcgccctcctcctcatcagctgtgccgcagcatacaccctcatctggctcgtccccacccacgtcaccaaac

tcgtcgagatgaatctctactgggcccgtgccatcgtcagcgccgtcaccatcgtcgcaggtctgatggtatccattcccctcaccgagatctcccac

cggatgctctactcggccatctggaccgccgtcatcctcgaggaagacatgacgctgcgcgacctggaccagatcgcagatcggatcggggtcctctc

aggcgttcgcttgctgtggataaagtttcgcgcgggctcgaagcgtgtcttcggcgcgacaccacacgctgatcagagcgataaggcgagcattcatg

gatccaagtcacgcattcgcaaagcaatctggggcgtcgaagttccggctagcttgggccttcttctcctcgccagtgccttcagtttcgccgcggac

agagccatcaagatcaacctaactctcaggcctcagactcagctctacgataccatatcggtcggaggggacctttctcttgccgacatctccgcagc

cgcctccctccagccttttttcgacaacttcgtccgcacctggactctgcagtccacagcagcactcaagcttccttcgaccgtcaagctgctcctgc

caaatcagacggaacagtacgcctacttcaccgaggtccttcccgacaacttcctgccagagtttgagggctacggtagcttcaccaatgatactcat

acggcgtccaacaacgcgtcgcagccagacccaagtcgggaaatgcaaaagcaggacgattcggacgagtggacgaccatcgtcggtggatcggacgg

gaaggaggtcactgtgctcaggaacgtcaggtggccccgatgggggattcagaatctagaccaatacctcgtacctgacatctgccagaccccagatg

cgaccgtcgccgaccgactctccgtcctgttcctgacccgccaggtcctcaacacgatcctatcaaaactcgacgtctcgctaccgtcgagcctcgag

aaggctgtggacctcggcgatctcaacaacgtcgtccctgctggaacgaatcctgccgatatcgccatagcccaacgtttctacgcgaacggagtcgc

gcagtcgttcttctctacgcccattatggcagaggatgagattcagcctgacggttcggttccggacgcgtacaagaatacgccgggaagcgcgggac

gggggtgggtctcgctggaggtcgttctgattcgcctgaatccggaattggcaggtcccggcgcgaaattcggcatggtggccaatgttactgacaac

aatggcaactttgggaacgtcggctacgacgtcggtgtatgcgtggagcggtttgacccctatatcgtggaggtctatcaggggcgaagcgtccaaag

catatccatcctcagccaagcagccgactccagctccgagaccggcctcctccgctcctccctagtctcccctggatcatcgacgaacctgagctccc

taggcaagaacgcagcctacaccgtggcgcacgagaatgcgcggaacgcgctcctgaaggataacggccgggattcgtcttgggttcctaatccgacg

ctgacgagcatgtctagtgggggaggcggtgggccggcggggtatggagttctggatccggaacggttggcggatatgcttgctagtgctgagtacgt

ttcaatccccagtcccgccacacctgactacgtacagcctgctgatagtgtaaacctttcccccccccccccctctctcagtacccgcctgttgctgc

cctacctcgtaggacgcggcaagatcgaagctcgcagttacgaattgaagcagatcgcgcgtatatcctgcatactccccttgcttaacatcgtgttc

ggagccatcgtcgggtgcgggctgctgggtattttcttcgtgccgcgcctgcctgcaggtctaccacgacgaagcacgagtccaatcagttggctcgt

ggcattcagcggcgcaggatccttgcgaatcggtagcacacccgccgaagccatcttcttcggcaagtccaccacaggaagtcccgaccgcaagagcg

cctcaggacccttcgcaacggtccaccaccaccaccacctctcccacgacccatctcgtctctgttcatacgacgacgacaagcagaaactgatgcga

ggaggcgaaggtttgttggtcgggggcgcgtgggtcacgctcgatgaggtcgagaagcggctggggaagaagccggttagctatgcggcgcctgctgc

tggggatgtcggggactcgatggcggggacaccctgggattcgcagccggttttggcataccgaacggacatttga

>contig_21.contig_21.g714.t1

atgaactcgtcgtcgcagtacaccttcctcggccccagtggagaccaagacgcgacgcctgggcagccgacctacccgc

cccaacgagactgcgtccgaggccaaagacagccaagcgaccatcgccatccgccccgcccgtccgatcggagccaggcctccgatgcctcctaccag

cactcccactcatactccaacgacggacgaccaccggcggtaaaccgggattccttccagaaacagcctcctcatcaccaggagcagccttacaaccc

acccccgcaccctgatgcgcatcagcctcaacctgccgagcaccagcgcccaacctcggcctactccaactttgatcctatgagctaccacggtccaa

gtgcatacaaccacttgtccgcctcacagccatctcaacaggatcgaccaatgaagccaggaggcggcggattcgacccacaccttcactacgccctg

cccggacggcgtctgccctggttcctccgaccctggcagacatctttcgaagcgccgtgcaagtcaaatatcgccctcctcctcatcagctgtgcggc

agcatacaccctcatctggctcgtccccacccacgtcaccaagctcgttgagattaatctctactgggcccgtgccatcgtcagcgccgtcaccaccg

tcgcaggtctgatggtctccatgcccctcaccgagatctcccaccggatgctctactcggccatctggaccgccgtcatcctcgaggaagacatgaca

ctccgcgacctcgaccagaccgcagatcggattggcgtcatatcaggtgtccgcttgctgtggatcaagtttcgtgcgggctcgaaacgcgtctttgg

gacgacgccgcgcgctgatcagagcgataaggcgagtatccatggatctaagtcgcgcattcgtaaagcgatttggggcgtcgaagttccggctagct

tgggcctgcttctcctcgccagcgccttcagttttgccgcggacagagctatcaagatcaacctaactctcaggcctcaggctcaactctacgattcc

atatctgtcggaggtgatctttctcttgctgacctgtccgcagccgcatccatatggcctttctacaacgactacgtccgcacctggactctgcagtc

cacagcagcactcaagcttccttcgaccgtcaagctgctcctgccgaaccagacggaccagtacgcctacttcactgaggtccttcccgacaacttcc

tgccccagttcgaaggatatggcagcttcaccaatgatactcatacgacgtcgaacaacgcgtcgtggcaagacccgagtcgggaaatgcagaagcag

gacgattcggacaagtggacgactatcgttggtggatcggatgggaaggaggtcaccgtgctcaggaatgtgaggtggccccgatgggggatccaggt

cctcagctcgatcctatcaacgctcgacgtctcgctaccgtcgaaccttcagacagttgtgaacctgacgacgaactacggagatcttcacaacgtcg

tccctcttggaatggatcctgccgatatcgccatagccgttcctttctccccaaactcagtcgcgcagtcgttcttctctacgcccattatggcagag

gacgacatcgagcctgacggcgtgaacaatgccaagttcaaaaatgtcccgggaggtgcgggacgagggtgggtccagctggaggtcgtcctgatccg

cctgaatacgacattggcaggtccccgcgcagaattcggcatgaccgccaatgtgtatggccacgattgggaatttggagaggtcggcttcgacgtcg

gtatatgcgtggagcggtttgacccctatatggtggaggtctatcagggacgaagcgtccaaagtcttcgaatcatcagccaaacggctgactccagc

tccgggacgggcctactccgctcctcgctggtctctgccggatcagccatgaacctgagctctcagggaaagaatgcagcctttaccgtggcgcacga

gaacgctcggaacgcgcttttgaaggacaacggccgcgatcatccttgggttcccaatccgactctgatcagcatgtccagtgggggaggcggtgggc

cggcaggctatggggtcctggatccggaacggttggcggatatgcttgctagtgttgagtacgtctttaaccccttgttcccaggtcccgccacagcc

tga

>contig_21.contig_21.g715.t1

atgtcctgcattcgacccatacttaacatcatattcggagccatcgtcgggtgcggtctgctgggcatctttttcgtgc

cgcgcctgcctgcaggcctaccacgacgaagcacgagtccaatcagttggctcgtggccttcagcggcgcaggatccttgcgaatcggtagcacaccc

gccgaagccatcttctccggcaagtccaccacaggaagccccgaccgcaagagcacctcagaatccttcgcatcggtccacgccccatctcgtctcag

ttcatacgacgacgacaagcagaaactgatgcgaggaggcgaaggtttgttggtcggaggggcgtgggtcacgctcgatgaggtcgagaagttgctgg

ggaagaagccggtgagctatgcggcgcctgctgctgctggcgatggcggggactcgatggcgggcacaccgtgggattcgcagccggttctggcatac

cgaacggacatttga

>contig_21.contig_21.g716.t1

atgttcgcgtcgcgcccaccgaatccgttctccatgccgtcgccgaacgccgcgtcgtcagcagcgatgatcctgcccg

cacagccgcccgccgcagccgcagccgcagctctcaacgggagcggcgcagagggtcacgccgagacaccagccacagcgccatcgccgtacgacgca

gcactcgccatcgaacgcgcctcccagctacaggcctacctcgccgagcccgtccgcttcgtccgccaggaagacccctcagcgtcgtcgtcgtccaa

aggtctcagcgcgtccgcctcaaaaaccgaagctcagcgcctgggctacgtcccctgcaaccggcccggactcaacctcgctcccgacgcagcacagc

ccgcctcgtcgcccgctgcagagctcctgccttccccgaacaagctccccaacacgcccaccagctcgcttcctgcgctcgatggaagccagaaggcc

gtctccagctccggtggctcatcgtcccgtacggtccctctcacccgccagaccagtcccacaccttcaagttctacagagcaggcctcgcccttgac

accaaggaccaatctccagaacaacgccgcacccgctcccgctcccactctcgccgccgcaccatcagcatcgacctcaaaagcaaccgccacgccag

cagcaccagcagcttccaccctccgagaccttttcctctttcccatccccatggcattcgcacgcgacaccgtctctcccacaaccgcacccgtcgga

ctccgcaactacggaaacacatgctaccagaactcagtcatgcaggccctcctccacaccgcccctctcgcaaacatactcgtcacacgctctccaga

cgagctccgaggtcgcggcggtagcctgcagaacaagggattttgcaccgtcgaggccacacagaactttgctcggcgcgccctcgttgagcgcaaag

ccacagcgcccatcgagctcaatcggaatctcagtcaattcgcgagaccattgcgaatcgggcgtcaggaggacgcgcatgagttcctgcgtttcctc

ctagagggcatgcaaaaggcctgtctgaccaaatcacccaaagtcttaaaaccctccaaccctctgacgagcaccacctttgtcaacaagatcttcgg

cggccgtctccgctcccgggtacactgccttacatgcggacacgactccgacacctttgatcccttcctcgatctcagccttgacattcgctccgggc

ccgtcgtcgggggcttttccaatggtggtgggggaggaggcgctctcgagagtgtaaatgctagtttggctgcatttacacgcctcgagaagctggac

gggaagaacaagtacaagtgcgagaagtgcaagaagctcactgctgcccacaagcagttcacgctcgaccaagtacctcccatcttgaccatacagct

caagcgcttcaccctcacaggcgccaagatcggacgcgccatcaacttccccgaggttctcaacctccgcaactacatgtccgcaaactcacaggccg

gcagcagtgcgaagaagtccccacacggtggtgctccggacgacaccaacccttcctaccggctgtatgcggtcgtccaccacttcggcggcggtcct

aattcgggacactatgtcgccacagtccgtcgaggcaaccagtggtaccgctgcgacgactcgtcgacgtacccgacccagagccccacgggcgcaca

aggcggaggaggaggcggagatcgttcgagcgcatatgtgctgttttaccagcgcgagggtctgagcgttattgatttgaagaacgggaacgcggtgc

ggagggccagtaatgctggggcgacgcctgcttcgtcctctgctgtgaatgggccttcgccagttaagcggagggactcgatgccgccgccgccgcgg

ccagcaagcgccacgctcgagggctcacccaccaagattcgcagacagtcgatgtctgccaacgggtctggcccacgattggagtcggctgcgggaaa

ctcggagttcaagcctcgatatgtgtcgaatggcgcagcgggagagggtgctggattgctgctgaagaagaagaagaagagtgcgacgatgggggctt

tactggctcgggcgctggacgcgaagcctgaggtcgctgctgtcaatggcacgaacgcgaaggcgaacgcgatggatactgagggcgatggcgatgat

gttgggcagctcgttgaggacgaccagactgtgagctcggtcacggagcaagtcaatggcgcatcggcgcagaagcggaagcggatcttgctcgatga

cggagaagacgacgctgccgacgatacctccgcaagcgttgtgccggcgcgaggtgctgacgaggaaggacttcgccgagccgtcatgcagaacaaga

agaagcagaagcagcgcaacaagcagctcggcatcgtccagcgctcgcccgatcgcgatcgcccgcagcaggaggtatcgaacagcaacggcgtcgct

cagaagaagaacaagaagccacgcaggcgggcctcggctgaattgggtgggcctgggggtagggtatcggtcgggagtccgtttgttgctggtggtgg

gtttggcggtgctggtgcgcaagggaggggtccgctcttggggcagcgtcctggagccaagatgggtggcggtgctgggggcggtaactttgcgtcga

agatggcgccaaggacgacaccgcggctttga

>contig_21.contig_21.g717.t1

atgtcgttcgacttcggcctctcctctccacagcagtccgaccccaccgccgacttcctccagcgcgagcgggacgccc

tcggcgcagacgacgctgatctcttcggcggcggcaacagcaccgcccaaccaggcaccctcgccgccggcgcagcagcagcagcagccagcggcccc

ggcgccggcgctgacctcgacttcgataccgacaagttccccgatctcgacggcgacgcacccacctcgtccacagcggcgccccccaaggatgactt

ctcctccaagttccccaacctcgacgacgctgacgacgacgacaacgatgatgatgatggactaaatggcttccagtcacggcaagcaccagcagcgg

ccgctcccttatcatcctccttcacacccgccgccgtccctccatcagcatccgcaggcgcagctcaactaggatcctccaaccgcttcgcacccaac

gacgacgacgacgatagcgcagagtccgacgccatccgggaatggcgtgacaagcgcgccgacgacatcgccgcacgcgacgcagccgccgagcgcaa

gaaggcagaaaccatctccaaggcagagcgcgacatcgacgacttctacgcagactacaacaagaagaaggagaagaacatctccgccaacaaggaga

ccgaagcacgcagcgtagccgagcgccaagccgaactcgccgaaggaaccacctggtcccgaatcacaaagctcatcgacctccaatcccccgcgtcc

aagacgatcctcaacacctccaccgccaagaacccggtcacgggacagcccgtcggcgatctgacccgcatgaaggacttgctcttgggtctcaggag

ggagggcgacaaggcccctgccgctggttattga

>contig_21.contig_21.g718.t1

atgtccttcacacacagcaccaccacttcaatctccaacaacggtccctccgtcgaccgatctgctgccctcgccgagc

ttcagtccacctacggcatgtctggccaaggctatcgagttccctcctgcttcgccggtacctctccctcctcggacaaagaaaacgacgacaagagc

ttcatgacttcgtccacttcctcatcctcatcgtcggcctcttcggcttcctcctcctcatctgccttctcgctcaaggcgaaagctactgccttcct

caaattcaacggcggccgcaagaccaaggtcacggtcaaaaacaacgacagtacgtccaccgtcgtcgaccaacccgtcagcgcagtccagtcgaagc

ccgacccgcaactcgtcttcgcccacctctcgtcccaatacggcatcggtgcctccggcggcggctacaagctgcacaatttcgaggctggcgagaag

aagagcaagaagagcaacggcggcatcgctgctccggctcccgattcgcctgaggctattcttgcggcgctcatggatcagtacggcgctcctggtcg

tggccaagtccgagccttcatctga

>contig_21.contig_21.g719.t1

atggcgatcggcaactgtagcgacaccgagtccaaggacggcagtatttcagggaccgtaccaccagcacccgccgctt

catcccttcaaaccaggtccaaatccccaccccacctcgacaacctccccacagagctcctccgacaaatcatagcccacctcgaccccccatcccta

ctcaccctcgccgccctctccacccacctccgctccgtccttcttcacggcgcagaggacttactcgagttgacatgcaggcggtatgtcgggtggtg

gtggaggaagagcgcgcttagctttcgaccgcatgaggcttttgtgcagcttgttgggctggtacgggagcgggcttgtcccgtctgtcgactgatgc

cggagcctgagcaaaaagccgttggccccggggacggaggcgagggcgagggcaagggcgagagggaggggcaggagaaggataaccccggcgcatcc

gacctggcgtgctcagcagcaaccccatccacctcctcgcagaagcaccccaccaacaagaaaaagatgacaaaccccgcaaaagcgcaccagatcga

aacccacattctcctcccacacatccgcgcctgccgaacctgtctgcgcacccacccctccctcgcgctcatcaccgccacgaccgcactccagaaat

acggcatcgatcagtcgaccctcgaccgagtcgcacgctcgtgctcggcgtacacgcagtccattcggtatccccacggcaagcctcgacagaagacg

ctctatctgctacaggacatcgtggcctacgtcgccaatcgacaggcagaggacgtaccagctgcgaaggagggctgcggaaccgtaccgcctacaaa

gtacaatccactcccgcgcagtgaaggcgaacaggaaggggctactgcacgtcaagtgaacggaggcgcggacgattcggtacgtcaggctgggaaag

cgaaggatggattgagataa

>contig_21.contig_21.g720.t1

atggcttcagcagcagcagacaacccctacctcgcacacatgaaccctcgctccggtggaggaggcagcggcagcggca

gcgatccgctctccggcctcatcccgcgcaaagtcactggcgaccagatccgcgccatcatggacgcaggcctgaatccgtggggcggcagaggcgcg

accttctcgaaccggtacaaggacatcctcaagaagcgtcgcgagctgcctgtgtatggacagatggacgagttcttccagaagtacgcagagaacca

gatcatggtcctcgtcggcgagaccgggtcgggaaagaccacgcaaatcccgcagttcgcattgtacgcggatctcccctcaacacagcgcgcaagaa

acccagacggatcgctcgagccaccgcgcatgatcgcatgcacacagccgcgcagggtcgccgccatgagtgtcgcaaagcgtgtcgcagaagagctc

gacgtcgaactcggcaagcaagtcggctacaccatccgtttcgaagacgccaccgagcgcggcaccaccgtgctcaagtacatgaccgacggtatgct

gctccgcgaagctatgaacgaccgtaatctctcgcgctactcgtgcatcattctcgacgaagcacacgagcgcacccttgcaaccgacatcctcatgg

gcctcctcaagggcgaggtcatcccgcgccgacccgatctcaagctcatcgtcatgtctgccaccctggacgcactcaagttccaaaagtacttcaac

gacgcgcccctcatcaaagttcccggccggaccttccccgtggagaaactgtacaccgaaaagccagaggaggactacgttgaggcttccatccgcac

cgtcctcatggtccaccaagccgaagacgaaggcgacgtcctcctcttcctcaccggtgaggaagaaatcgaagacgcctgtcggcagatccgcgccg

aggcagagcagatcgcacacgaatcgccggacttggccggaccgttactcgtcgtgcctctgtactcctcccttccacctcaacagcaacagcgcatc

ttcgacccggccccaccaccagccttccccggcgggccaccgggtcggaagatcgtcgtcagcacaaacatcgcagagacctcgctcaccatcgacgg

catagtgtacgtcatcgaccccggcttctccaagcagaaggtgtacaacccgcggatccgggtcgagtccctgctcgtgagccccatctccaaggcgt

ccgcccagcagcgcgccggtcgggccggtcggacgaggccggggaaatgcttccggctgtatacggagaaggacttcgtggctgagctcgtggagcag

acgtatccggagatcttgaagagtaatctggccaatactgtgctggagttgctcaagcttggggtggagaatatcgtcaagtttgactatatggaccc

gccagctccggagacgatgatgcgagcgctggaactgctgaactatctcgccgcactcgacgacaacggcaagctcaccccactcggcgagctgatgg

ccgaattgcccttggatccacagctggccaagtgtctgctcgtcagcccggaattcaaatgctcgaacgaggtcctgaccatagccgccatgctcagc

attccgaatgtgttccttcggccgagctcgcagcggacggctgcggatgctgctcatgcggagttcgcacaccccttgggcgaccatctcaccctgtt

gaacgtctatcatgcctggaagtcgagctgtcccgacccccgagcaggtggaaactggtgctggaacaactacctctcccatcgatcgctgctccagg

ccgacaatgtgcggttccagctgcaacggtcgctggagcggctcgacatcgaactggtctcgacggcgtttgaggatcggaagtactacaccaacatc

caacgggcgattacatgcggattcttcatgcaagtcgcgcacaaggaaggcgcgaaaggcgcgtatgtgacgatcaaggacaaccagccggtcagctt

gcatccgtcgacgacgttggagagttacccggactttgtgctctaccacgagttcgtgctgacgacgcgcaactacatccgaacggtgatggaagtca

aagcagactggctgatcgaactcgcccccgcctatttcgaaccgaaagaattccccgagggggagatgaagcgcaagttgttcggggccacccgggcg

cctggcggtgcgagtgggagtcgggctggtgcggaaggagcggagcggggaccgaagaagaagaagcagaggcattga

>contig_21.contig_21.g721.t1

atgccacgcacattcaccgccgaacacgacttcccgtacccctgggcgcagacagcactggccatctaccacaagtatc

cgaacccacagacagcccacgtcgtcagtctcgacgtgctctcccagcactacgaccccatcgcaaagcagctccgtctcgagcgcatcttgggcgtc

aagcagggcgcgcccaagtgggccatgcgcgcccttggcggcgacaacctcacctacgtacgcgaggtcatggtcgtcgatcccctccgcaagcgcat

cgagatgaccagcacgaacatgagcctcagcaagtacctcatggtcaaggagtacatcacctacgaacccagcacgctggtctcaccaccaacatcaa

catccatatcgccatcaccagaatcgagctcagtattactaccaacacagtcatcagacagcacagccttccgtcaggtggccgccatgtcgtccgac

tccttcctgggcgcctcggccatcgaagactggtccttcagccggttcggggacaacgctgcgaagggcaggaacggcgcgtatggcgtcaagcgtca

aggctcgagcaagcaggaagtcgccggaaaggtaaagaggatgagctggtactcgccgcgcatgacaagcgcgtcgccgtcgtcgtcgggcgtggcac

gagggagcagcagcagccatgcgagcgacggggcagctacagcatcagcgccacctccgcagacaccttccaccgtcccagacttgcacccggcgctc

tgtcgcgacctgatccagtttcgtcacttcctggccaagtaccgcgccctagacgaccgaatcacctccctcctgaaccgcaccctcgccacctcccg

cgaatcaggccggaaccagcccccgtccctgctcctcccgctcccgctttccaacccagactccccgctcctcctgcacgccaggaataatacacccg

cccacttcgctgacgcgggtgtctctacgtatgcacgtgcggacccgacgtcgtgcgctaccctctgggaccagctcgtccgagtatggaccggccgg

gaggacagcgttcgatactgtctcgaggtcacccaggcccaagccggtaaccgtctaacctacactaccggtccagcttccccacccggaagaggaag

aggagcaggggaaccggacgagctcgcgagcttggacgcggacaggagccggccttctgacgtgagtgccgcggaggaagggggcgggaggagggcca

ggaagacgaaggatcgggatgcggagcgggcgccggagcgctggaggggggatgaggatgaggacgcgtggaggggtaaacgggaggtggaggaggat

gccttggcccgacaactgcacaacgagctcaggatcgaccaaattcttcgtcgacggtcaatagaagtctcgcaatacagccctcctcctcctccttc

gccgccttcttcgccgtctgcgccttcttcctctcctcctcctcccgtctcggcgcgtcgacgcgacagggtttgcgcagcatgcccgacttccgcta

gcctttcccttcccgtcctgcgaggggcaaaaggtttggatcatgcgcgcgcgggaggcggagtgcagaacctgtgttcgaaatgcatgctgggtcgc

ttgccaagcatgatccagtcatcagcgcatcagcagtcgcatcgtctggagttccttatccgtatcgctattcgctgcacgccgtcacagttactcga

cctgctccccaccttgtgtatccccagcccgcctattcccctggccctcgcctcaacctcgacgtctctgctctttccttgtccaatcacccgtcctt

ccgtcctccaggcctcagtgttctcgttcctggcgacacgcgcacggcggccggcgcagacgacggactacggcagatctcgtcctctgagccaagtg

agctactaccctgcccattctcccccttgcgcatcatgcatcagcagcgcgtaa

>contig_21.contig_21.g722.t1

atgccccatcggacagctctctccctcgacaaagccgctgccaaggttgctcccaaagcacaccacctcgtcatctcca

accccgtcaactccaccgtgcccatcgtcgccgaggtcttcaagtctttacgaccccaagcgtctgttctgtgtcacgggtcaccacctttgacatca

cacgcgcctccaccttccacttcgacattgccaacgtcaagcccgccgaggtcaatgtgtccgtcgtcggtggccaccttcggcgtcaccattgtgct

tctcctctctcaagtcgcccagggttccatactccaatag

>contig_21.contig_21.g723.t1

atgaggttccccgatgagcgagtcagtgggtcgggcgagggaagggaagctgctgttgctatgcagccagccaacagca

cgacctcgacgaattcaattcgcgaattcgctgatgacgaggacgatgacgaggacgagccagcaaacagaaccgcctcgcctggtcactggtcggct

gggacaaaggcgtccgtcgtagaaggccgctgtctcaccttcggtgatacgacctactacgccgcaaacagaatcgctcccagccgtcctcacacggc

ttcaaatcttcgcttcgcatcagccgtccagagcccccgtgataagcacagtttccggccgcgcccacgcttcgactcggcacttccctcgcttcctc

cctctcgtcccacgcgcaaactatactcgccaccagcctcgaccatgtctcccatccactcctccgcatctccctcatcagcagaatgtgcagcggcg

gaagaaggacaggccagtgaggattcgcccccggaatacgaagaaagtccggccgagcccagtgaaaaaccgacaggaatgggctcgcggggcgatca

agagctcaagctgcaaacgcagtggcgatgggacgatcgttacccccgaccggaatccgaacccaatagtcccacgactgcgccctccagtccggttc

gggaaacattctcagctgacgatgcgactaggcccagctcgccttgggtgttctcaagtccgattcgggaaaagtcaccagctgacgatgaggaggta

aacgatgaggaggagggccagtcttcacgtcccacatcgcctgcggcgagcgattcgtccggcagaagcccgtcgcccgaggtcgacacttcgactga

ggaggacgcgtaccgcttgatgggctcgaggacgttgctgctgccaatgtcgggcgatgaacgaaggcagcgatatcctgctgggtcacgtcgatcaa

gattcaaggacaaggacaaggaaagcccaggccgagttgggatccttgcttcccaatcgtggacgtttcaatcaattggacctacgggcttgacgctt

gacgaaggcacagagacgcctaatcatggacgcagtcgacacaccccgctctctaccaccgtagaggcatcgaaatacggtcgcagcgggcagtcggc

tcagttcagcaggaccatcacaaccgataaggactccagagacgaagaggggaaggccactcgcgtcgaagtctcggatgctcacacgacaccctcct

tcaacccttccctaccctctgaagctctgatctcttatcctctcttgccgcaaagtcattcccagcccgccttcctccaccctccatatccccaaatc

tacagaatgtgcgaacatacctccgtgaccatcctgtgcgccatctgccaagtcaacgtcgtcgatcgccgactggaacagcaaccatgcggcgaggt

ccagcagggccggatctgtcccaagcaggcctccatgaccgcaggtctcatactataccgtcaagacgtcggatcgaagctctgcgactcgtgcactg

cgcaagcgatccaagaacgaggggaggaaggtcggagcagtgcggtttcgcctctgccttccgacgcgaattcggccgcggccagtcgggcccgctca

gagaggatatcatggggcgaccaagaactaaagctgaagacgcagtggcgctgggacgaccgttttgaccgtcccgaatcaagcatcggcagtcctac

gaccgatctcagctcgccttcgacgcccgccgacggacgttcgcagacgacgagcgagacgggacatgctgatacgggcaccggcagtcggtcctcga

gccagtcttcggacttggggaactcgactgagttggcggggcagggcttgaacgaccttatctacggtctcagcttcaccttcaagtctacagaagtc

agcaggcggacacaggacggcctggaatccacaagggaggatgaaatctcggacctcctggtcgaaaagctgaaaggggacagaggcatcaagcaaca

cgcgagcatcttcttccgctcgctgctcggtttcccctccgttttcgttgctctatcggccaagcgcgcgctttccaccgcgatcacttgggctggtc

tgggaaggacgagctcgagcgtcaaatgtcaaagcatgatgcatgatggttggaaggaaggaaggaaggaaggaaggaaggaaggaaggaaggaagga

acgaaagcgctgcgctcccccgctccaccttcgtcggaccgacttcccgacttccccactttcccaggaacgcaggaacgcgggatcgccgagtttat

ccgcccgaaacacatgctgttttccattgaccagtccaatgcagtttctaataacgcccataacagcgccgcgcagcggagctttacaaatttcaaac

ctctcagatggaacttcctaatgaaactgtacacttcgattgtagctgaacagaagcgggctgcttacacaaagagagagaaaaagagtgcgaacacg

atgccttcacctcccatcagaccatctccagtttctatcgtctttgtcacacaacaatggcaggcagcaagcgcaaggcaaaaagatacctcggccgt

gatgacagccgcgagcactagcaacgccgtggctgtgattcctgcggctggtatcgctccaaagcctctcactccggccgagctagagatgtccatgc

aggcgtcgatgctccggacggtcacacgggcaattgatgtgcgaccccctaacacatcgcgtacctacaatccaaagcagcaggagtggaggaagtgg

tgcgaagagaaaggtttcgaggcgatgacacactcgcggagtcaactcggccgctcatccacgccctggtccggtccggatgatgctcggtactcgga

cgctcgaggaacgggatcgcaagaagcgcgaggctgcgagaccgactggctttccttatctctcattacgcccttctacgaggagacaacgcgcggaa

acttgagctcgccgaccttcaccatctcgagctggaaggggaaggccccctcaagttgctttgccgtggtactgttgctggccaatggaaaaacgaat

caagcaggtcgagtggagtacgggtcgtttatgcgcaacagggatgtccagctgtgtccagttggtgcactggctctctacctgttttcgagattcca

tcacagcgctgaagcattccccgacttgagccaatcatctctgtggtacgatatgaaggtgatcaaggctccaaagagcagcaccgacccgattggcg

acgagactcatggcaaggcgctggacgcggcgttcaaagcgtgcggggtcttgaccaccgccaaaacccatgcagctcgaaagtccggcgctcaactc

gctgaggctaagggtgcgcctgaggcttcggtcaggagagcgggtcggtggaaccagacgacaatggagacgtgctacttgacccgcctaccgcgagc

ggtgcttcgcgttcatgccgggttccatgcggacggcgggtcttacttcctccgtcgcgacgttccccctcctcaggagctccttgagcgcgtcttcc

ctcaagctgatgcatgggatactgccatcgctagcagacaggctgcggatggttcaccggtcgaaatcaacatcgcatcgcgtgctttcgtgcgcctc

ctcattcgccttcgtcccgtcctgctgcaggatgctgtgctgctgaagaaagagtttccgcagctctatctctggagccatgccatgttccactccga

cgcattcatcagctacgagaacgagctcctcaggacactcgagaagcctgacacatcgggcgacgaccagctgcgcgcggttctgcctttgctcgccg

agcagatccaggcacactcttcggccacgcagcagcacgtcgatgaaggtctcaaggagctgcgggaggacgttgaacgcgaccttgaaatggtggtc

gagctgctgcacgggatgagagagcagcaacagcagcagacaaacgagctgcacgcaaccttgcgtggtattctggggttccttcttgctgcaaatgg

tggctcgtctctttcatctccctcttctcttgctggcccttcgtcctcttctgtgacatcaccctcgtctttgatctcagcagcatcagcagcagcaa

ccacctccgcagtttcacgtatcggtcagcgctctagacttcacggcctctcggccacatcctcttcgtctccctctctcgtcgccaacaaggtcatg

ccggccatggagcaggacagaggaatcagctcttgcgctgagctctggcgggagtacaaggatggtctgaagggaatgttgtcggtggaaagaatgga

cgaggagtggggcgcggcttggcgatcatccgagaaggaccgcaagcactacgagcgtcgaatgcgcataatcagcaaaataaaggacctcgccatcg

agaagaaggtgtcggagagggtggcattggttatgctggagcatgctaggaatcagctcccgaacaagtcattggcaaagttgtcggatcatatacga

cagggcaaggagccgacgtatcttgatctgtctcttaacgctgtatag

>contig_21.contig_21.g724.t1

atgagcagctcgacgatgagcagcggcagcaaacacgccgggcccagcatcaccatgctcgcccatgcagaggctcagc

aggcagcagcgggccccagcagcagcagactcgacagcaacagcaacagcaacatcaacatcaacgaccatggcgctcatcctcctctccctgctccg

ggcacagctgccaagcgcagagcagcagggccaggaaagcgcgtctcgtactactatgactccgacgtcggcgcgttctcatacggctaccagcatcc

aatgaagccgcatcggatgcgcatggcacataatctcatacaggcatacggtctcgacagacacatggatgtccacagaccgaagcgcgcaaccccga

acgagcttacacgcttccataccgatgaatacgtcgacttcctccgcatggtcaccccagagactgtcgccatactcacaggagactcggccagacac

ttggccggcgctggcgaagattgtccagccttcgaagggctctgggacttctgctccatcagcgcaggaggctcgatcggcgccgctcatcagatcac

cacaggaaactcagacatcagcgtcaattgggccggtggactccatcacgccaagaagcgcgaggcctcgggcttctgctacgtcaatgacatcgtcc

tctgcatccttgagctcctccgcgtccacacccgcgtcctctacatagacatcgacgtccaccacggcgacggggtcgaggaggcgttttactccacc

gatcgcgtcttaaccgcaagcttccacaagtttggtgacttcttccccggaaccggcgatgtcaaggataccggcatgaagggcggcaagggttacag

cgtcaatgtgcctctcaaagatggcatcaccgaagaggcgtacatggacgtgttcaagccggtcatgtcgcacattatggactggtacagaccagacg

cagttgtactgcagtgcggcgcagacagtctggcaggcgacaagcttggctgcttcaatctcagcatgcgcggtgagtctcttatcgacttccatgtg

gtccctgccctctcactcacttccgatctcgacattcctcctttctgtatcgcaggtcatgccgcatgtgtagattatatgatgacgtacaacgtacc

tctcatcatactcggcggtggtggatacaccattcgcaatgttgccagaacatggacctacgaaacaggcgcggtgctcggcatggagctctctcctg

acctaccatacaacgcgtatatgagctactttggccctgactacaagctcgacgtgcccaagaatagcatggaggactacaactcgaagacatatctc

gaggacataaaatcccaaataatcgacacactacgcactttgcctggcgctcccagtgcacaaatgcaagaagtacctcggggacccctctttggcgt

aggacgaagagaagaggatctgtcgaatgacgaagactccgacatggaccaacgcatcactgaaagaataaaggacgcgcatatcgagcggtatggcg

acgagctttcagacgacggtgaagaagatggtgaattctatggcgtcgggccaaatccgcccaaccggatgagattgaggccagaagggtacgaagat

gagagcgaggacgaggtcatgtacgacggccggatacaggatcgacaacaacaacaacagcaacaacaacagcaagcaggctcaaggagcagtcgccg

cgggagctcgtcctcggccctagtagcagcgaacggagcgagcagacggagccaagacgacgcgcgtgcccaagcgctcttaccgcccgtctggcgcg

aaggaaccgctggcccacctgcatcatcatccacctcttcgcacccaaacgcatggaacgcagatccgggcgccatcgtgcgcgcctttcccttccac

aacccgtactcccgccaccggcccaaacggagctttttctcctccggctcgcgtccgtcggcgagtctggacgagatacgcgccgcgctggcccagca

ggggtataccggctacggcggctattacggtgctgggatggggggttatggagctgggatggaggaggatgacgaagatgaggatgaagaggaggaga

gtggtggaggtgggaatggcgggaatgggacttcgtactacgacgatcgaaaacgcgagagggacggtcaatcggattcggaccgggatcgggcacga

agaagaggtgtcggtatgcctgctgccggcggcggccgtggtggctggttggacgagatgggcctgagcaccggcggggtcggtggccgtggtttcac

ccgagacgggcgcaaagtggacgcgagtgcgaatcattatttgtcgtcctcgtcgtcctcgatggctggaggaggtggcggtgggaaagcaggacgtg

atcgggccaaggagcgggagcgcaaagctgcgcgggaacgcgagtgggaacaggcgcgggagtggagagtcgctgcgtag

>contig_21.contig_21.g725.t1

atgcgtctctcaatcaccacagatgctacacaggttcttctgagcccgcaccgccgcagcacggagaccacgcccctcc

tcgtccaggtccaccttcagcttggccctcatgaacggccaccctcccgcttgacatcgctcacgatatctctccagcgattcgagaatctggaattt

aacaacaggccagagtccaccgtcttcgaacccatcaaggcgtcctttgacgtctccgaccgggttctgcttccaggcacagtccacacgtggcaggc

caggctcgagatccctcacactgctgcggcttacaacgtcatccagaacaacaagtcgcaacagtacctcctcgccgccgctcactaccagtcgcgtc

tccgcctcagtcgaatgatcacagcgaagaaggagctctacctgatcccccaaccggctgtcgcagccgactcggacccgttctgctacacccacgtc

cagactggtgcacatgacggtatcggacccgtgctcatccaagtacaggcccagcatctcacgattggggggatgattcgggtacgagcacagttccc

tgctccttcccccttattccggctgagctccgtcgagtttgccatcgatcagaaagtcacgctgcggtcgcggaagataaaaggcgtcgagcaggagt

tgccatcaagacggctggtcctcttacatcagtccacgtcgaagttagagacagatggctggttgctgcgcatgccaagttgtgcagtgatgaggcca

agcagcgagtcgaagaggctgggcatcgacgtcgaacacctattcaccgctcggttcaccttctacatgctcagcgagaacgagtatcaacaggaaca

gcaggaacagcacgcggaccacacccgatcccaggacggcgcagacacacagctcacgaagggggagaagaaacagcggaaagcgacgagcagcagca

gcagcatccctcagcgacacgtcgcctacaccctagcctggtccatccatgtccccagctgcgccctccgatgggaatccatactgctgcctgcctac

tcggagacggacccgagtccagtgccggaggcggatcggacggagatcgcgtccaggaagagtgggcgacgactctgcgtgtgtgggaaggagcagga

agagctgctcgcactcgacgagctcatggcgaagatacacgacatccccgacgaccgcaaccacttcgacagaccggagtctcagctcgcggacaagc

ttcgatccaactacgcctgctgcagcaaagacggcggctggactaccggcgccagcaatagcggcagcagcggcggtgcttgttgtgatggagaggct

gctcagcacggccatcagcgccgatgttccgcgctctcctccttctcgcacgcgtcgtcgtgctcgtcgggcccggtccaccacagctag

>contig_21.contig_21.g726.t1

atggctcgcagcggagcatccctgacgaccttcttcgtcgtcggcccggaggagtatgtcgagtttgccgaatcaatgg

gatccaaggcgttcctccaacacatctgggacaactcaatcacacccgtcgcggccaaggacttcgacggtaactctcgcctgcacgctacgggcgcg

cgcatcgtccgccccaaggacggatcaccccggatcgagttccccaccgtaaagcgtatccgagggaacaagtacaagccgacgggcaagtggatttc

gtggcaggccctcaagcgtctcaatccactcgctgtcaccgacccgccggacttggaagtctacatggcccgcggcggtcaggtcggcacaggctcgc

gtagggcaccaggcgcatatctcgagcaaacctcaagcaagaacatgatccccttcgccgtggccgaggagcccgacccgacagagaatggcgaattg

atggagtctcaatcacatccatctacaaaccgagacgatccgtcgcacacttccaccgccccgcccagccgagccacgaccccgtcgctcagtaaaga

ctcccaaggatggatcggcatggggtccgcgaatagcattcacgcgcctgcgcgggccgtggtcgtccgcagggcccgacagtcctcctcaccagctg

cactcgctgccaaagctgccatggcggcaggacgtccaggtccgagtccactggcaaattccattgcggcgcccgccggtagcaatgctactgtaaca

ccttccaacgcgcatctcattcgcgcctcgggtgtcgcggcgaattatccagcttctcctgcgcgttcctcctccccgtcaacatcatcgtctatcac

gcccacctcgacgcctcccgcgcggaactctcccagattctctcacgcgccctcggcatctgtcggtaccaactttccctctccagtttcctccatgg

ggacccctccgtccttggtgaatgcatcccttcatcagcagtcgccgaccgggtctgctggctctagcggctcgcgcgcaagtcggaaaccgaatctc

aggatcccttccatgcctgggccacctcaacacctgtcctcctcctttcgagccggtccgccagcggcaggaggatcgatctcgggatccgacctgac

acctggctcgctgacgcctggtcaggcaccgggcgcgaggacaccgctgagcgcgacccatccaccgcccgagacacctctgaagcatcagagacgga

gggcgaaggacatggccgaggcagctgacgcggcggaagcactggcagctcgaggcggtggtggtggagatggcgccaacgatccaacaacaacagca

acaacaggagaaggaacagcggccttggagacgacccccaaaaaagtgagcgcgggtggtgttactgtagatgtggaggacgaggagaacccgatgga

cttcacagccaaaacgtccctcctcaccacaggtattgggatccaaatgagtccttccggccacacccccggagcggcgggagcaggagcaggagcag

gagtaggagtaggagcaggagcaggagcgggcaccggtctgcgcacgccggacctgcgcggattggccggcttgaatcgggcggatatggaacgggcg

cggttggcctttgagcggacggccgcaggacaggacccgctgttgtctgcctctgcgaatgtgcggacgactcctggggcgactggtgttccgcttgg

tgggccggggactgggtacggatttggattcggagcgacgggggctggatggggtgggctgggcgaatgggcgggcgggatgcaaggttcttatggag

gaggaggaggtgcaggaggcgggttaggaggtcttccgggtcctgcgatggccgcttcgattgcggacgcgcgaaggtcgagcttgcctccgctcacg

cctgctacggaaggtccaccggcgggtacttggggcgccccagcaccgccgccgtcattatcgcgcggtggatctgctactacctacagagaaggcga

ggacgacggtgtgttggggcacgggcttgggctggagccgggcgaagcgacgggtcagcgaagtccgtatacgatggttccggcaaggcaaggatctg

accagggagggaggtggatgatgggaccaggatccggagctggagctggaagtggagccggagccggagctggatatggcccacctcgcccctcaccc

ccacccgaggaagcggacgcggaagcggaaggcggtcggacccccaacatgaaccgcgcccgtgctcccaggtggatcatcaaggaggaggacgtcac

gtcatcgtcactggcatcctcgatgcaatctgttcgtctcggcgcagggcccggtacggcctccctcgagcaaggccgcgtttaa

>contig_21.contig_21.g727.t1

atgcaactttcccagctggctatcgccatcgtcctcccggccctcgtcgccgccgttcccaccgacaagcgctccacca

ttgccacaggcctcgtcttcgcctcttcttccgatggaccgttgtcccagagcgccacgggaggcgggatgaacaatggcaccctccagaacgggccc

gtcgtcgctggcaaagcctttgaccgaattacccacatctggctcgagaacaccaatgcggctgtggccttttccactccggccttcatgaacctcgc

ctcgaaaggtcttaccctcacttcctcgtacgcggtcacacatccgtcggagcctaactacatctcgtccgtggtaggcgactactttggattggcgg

acgactcgtttaatcgcatccccacgaacgtgacttcagtctttgacttgctcgacgagaaggcgatctcgtgggcctgctaccaagaacagctgccc

acgacgggttacaccggcttcaactacacccagaaggcttacatcgaaggatctgcaacgtggacgtactacgttcgcaagcacaatccgtgcgctat

ccttgaccgggtcagtgggaacgccactctccgcgaacgaaaccggaacttcaacgacttcgctgccgacgcttccgccgacgccctgccacaatgga

tgttcatgacacccaacatggtgaacgacggccacgacacttcgccggccttcttcagcaactggacagacttcttcctcctcccgctcctgaacaac

accgcattcaacaacaaccgctcgctgatcgtgctcagctttgacgagaacgaggactacacagtgaacaaccaggtggccaccatcattcttggtaa

cggtctgccccagaacctcgtcggcactgaggatgacacgtactacactcactactccttcctgagcacggtcgaggccaactggggactcaagactc

tcggccgaggggacgtcaacaagacgatgtccaacgtcctcagcttcgtggcgaacgtaactggctaccagaacgtaaacgtccccgtccaggagcga

cccttgacgaacctgaccggcgtcgctcccggtccgctctcggctgatctcttcaccgagttctacgctcctccgcaaccgaacgtcatcggtgccgg

tggacaggccgcgctgatccttcccggtctgaacgtgaaccaagtccttacggaatcgacgagtacaacgaacttgacggcgacgggtggtaagcggc

catacggtacagacccattcgcgtcctcgtctgctgtttctgcgacgtcgaccagcgcggctgttgcccagcttcgcaccggcgcgaatgtacaccct

gtcatggtggctgctgccctggcagttttcggtggagctcttgccactctgctttga

>contig_21.contig_21.g728.t1

atgatgctcggagctcggaccatggataatggatgcgctgctgctggttggggccagcgtggagagtggagaggagagg

tggcagcggcacagcgagaggtttggatttggaattcccacgaccgacccacgtcgagtcccacaccgccgggaaaagcttcgcagcgtccaaaacaa

ggagcgttttcgcttgcggcgaccaaccatgccgacggcagcagggcttcctcgcccgccggttccggcttccgctggttctttactccgcctctccg

ccactcgcccactctctcgctgttcctcgcctgcctccaacagccctcgggtcctcgtctgtccctctccccgctgcctcctgcgggatgcctcatgc

tcggatga

>contig_21.contig_21.g729.t1

atgactcacgcaccaggagcgtcctcttcctcgacaccttccgtcaccgggactctcgtgggagggcccgatccccgcg

ccatcgagaagcagcaagcaaatgtacagcaatcttcaaagccaaagaggaagctcaccgtcatcctagcctccctcgtcatcctcctcgtcatcctc

ggactcgccctcggtctaggcctcggactgggtctccggaatcggaaatcaacgagcaaatatgcccctgccacaggcgcagtgtcctcctggaccgt

cgggagtaaactgcccttcgtcccaatcgaggaactcgtcgacccttcgcaattcatcctctcgcctacattcgacactcatgccgagcctcagactc

gagagtatacgtgggacttaaccgaagtcatcgctgcgcccggaggcgtcactcgccggatgctcgtcgtcaacggtaaatttcctggtcctacgatc

gaggctaacttgggggatcagatcgtcgttcatgtcaacaatcaactgtcgaatctctctacaattcactggcacggacagtatcagaacgggtccaa

ctacatggacgggtcgtactcaatcacagactgcggtattgccccaggctctagctttacgtataactggaccgttcagaacaccggcacatactggt

ggcatgctcacgccggtgcccagtacaacgacggtctgatcggtgccctgattcttcacgggcccaacgatacatacggcttccgaagttctcctacc

gacacttcgcggcccgccaacgtctcctacgacggcgactttatcatgatcgtcaacgacatgtaccacctcttctcgaccgactggctctctctctt

cctgaacctccaggttggtggtggggagggcgacgagcctacccctgactacggcgtaatcaacggtataggacaggcgaactgcggtgggacaccag

accgacagtcctgcgttaacaaaggaaacgcggggctctaccacaaccttactgtgcagtccaatcagcgctaccgtatgcgggtcatcaacgccggc

gcacttgcgtcctttgacttttccgtcgacgggcacccgctcagcgtgattgaggttgactctacccccgtagcacccacgcccgcggcgagcgtcag

cgtcctcgtggcccaaaggacaagcttcatcatcgagacgaatcaaacggcaggagcatactggatacgtgcgaccatctccgaggacatgttggcat

atgacaccccgaccatcgtcaaggatcaacgggcggtgatgcggtatacaggtgttgctgagacgaccttgccgtccaacgaagacattccttcgctt

gggaacgtaccgagtgacttcaatgcgagtgcgttggcacccctcgaccccatcaacccaccaaaccccacgatgcagaagatagtctacatgaactt

tggtaattcagcgaatggcgcctaccacgccttcttcaacaacacagccttcggcgccccctacgctggctatagcacggtgacgaacgtccagaatg

ccacactagcaggcaaagcggttgactatggaggagcattcgtcgtcaatgtggacaacgtcgaggttctggacatcgtgatcaacaatcaggacgac

ggaggtcacccactgcacggatatgcgcccttcctgctagggaccggcgaaggcagcttcatcccaggaaagagcatggacacgatgcaaacgttcgt

gaatccgatgcgacgggacaccttcgtcgtacccgcgtactcgtggctcgtcattcgagtcgtggctgacaacccgggcgcgtggcctttccactgcc

atctttcgagccacatggctgcgggcctgatgatgcagttcaccatgctgccgtcgaagattgcgcagttcgatctgccagctgactaccggcagcag

tgcgctgccatcaaggccaagggcctttcggacggagcatga

>contig_21.contig_21.g730.t1

atgacttcgaacaccaacacggccgacggcgggagctaccacagcgcggcccaccaccagaacaccgcgccccaggact

gggataccgcaccagcagaggcacacgcgctcgaggtcgtcgccgccgcagctgctgccgccgccgcaggccttcaggtcgacaactcccacgccggc

ttctacgcctcccatcacgcacaacatgcaccagccccacccggggtccacgagggagtgcccgcccagccctcagccgcggagcaggccctgatcga

tgtcaccgccgaaatgtatgcggctgcttcccacctctccgctcacgaccccgcctcgatgtcgggcgggtccgaagccctcttcgactgggggcccc

ttgctctcgccaacacttcggcacctccgccccttgctcccggcacacacctggacagcatgattcctgctccagaagtgcatgacaccatcacaagt

tcacaaagtgatggccttcccacaggccgcgccagtcgcgcgcgaggcggacgcaagacaacacgctccagcggcaccgttgcagaagggccatcctc

gtcgcaggcaggacccagctcatcccgctcaaagccagcaagatcgaagaaagctcgcgtcccgccgaccgccgttgtctctgccactggtaagggtg

gtgacgatgatgatgatgacaacgacgacgatgaggaggacgatgatgacgatgatgatgacggtggtgctatgaatgcgggcacatcagccgctcgc

ggaaagcgtaaggcagggtccggcaaggacatcccgtctactgtcgggggcccggagctgcaacaggtgtggagaagcgcgatcgaagcacgcatcgc

ggccatgaaccaagattcgcgccatgtccatactacccaaaagcgattcctcactgccgatgctcagctcagcgagccagcctcgccctgcgattctt

tcggtctcgtccggcactcccgcaagcgcaaagccctttccgaacgcgacaccaccctccgccacgcccggcagggatacatccagaagtggcgcaca

ctgatgcgatccatcgtcacggaatctcggtcacgcggagcggaagacattgccaagctggtcgaagatcggctgggggcttgcatcaacgagatggc

ggaggatttggagtcgtgggtgcccaaggaccccattctgcacgtcaagcccggcaagctgcaagcgtag

>contig_21.contig_21.g731.t1

atggcggcgctggatagcagcataaactcgactgccggatcctcgactacgtcgctgaatgctgagtgccacgagctcg

aaaagcagcacgcaacaaatgttcgccccaccccgttctaccgcagaccgctcatcctggctttggccttcatcgccgctgccgcattgttcggcact

gctctcggtctgggcgtgctgagcgaccaattcgccagccatcggttgacgacgttactggcaagggcggatgccaggccgcctcccccctcacctac

tcgaacgttgacagaaggaacagtatcgtcttataccgtcggagcgccctacccagtcatcccaatccaacagctcgtcgacccttcccagttcatcc

tctcgccgaccttcgacaccaaagcttcccctcaaacgcgtgaatattcctggactgtctctgaagtcatcgctgcacccggtgggatcaatcgacgg

atgctcgtcgtcaatggcaaatacccaggcccgacgatcgaggctaacttgggtgatcgcatcgttgtgcatgtgacgaacaaaatgagcaatatcac

caccattcattggcacggtcagctgcagaacggtacgaattggcaggacggggtcttcgccactaccgagtgcggcatcgcgccaaacactacttata

catatagctgggttgttcagctgacaggcacctattggtggcacgcgcacactggagccctatacaacgacggtctgatcggtgccttgattctccac

ggccgggacgatgtctacggattccgctccaggccctctgacaccacccgacccgccaacgtctcgtacgacggagacatcatcgtcatggtaaacga

tctttacaatctcttctcgacggactgggtgtccgactacctgacccagaaggactcccccgtcttcggggcggtgccccttccggactacggtgtga

tgaacgggatcggtcaggcgaattgttctgcggttcctgccggccagacatgcgaacggaatggaaatgctggcctgtatagcagtgtcaccgtcgct

cccaggcgtcggtatcgacttcgcgtgatcaactccggagcgctcgtggcatggaacttctcggttgatgctcacaccttgaatgtgatcgaggtgga

cggaactgaggtgttctcccggccggcccaaagtgtggccattgacgtagcgcagcgtacgagtgttatcatcgaaacggatcagcgtcccggtgctt

atttcatgcgcagccatatgctcgaggaagatattcccttcacacttgcctcacaagtgaaggaccagaaaatggtcatgcgatattctggcatatct

tcgagcatcacgcccaacctcacggcttcacctcctactcttccagggtcgccgccccgtaacctcaacaccgacactctgactccgatcgtgtcgat

caatcctccgccggcgaccaagcagatcaccctcgacatcgactttgcgaaggatgctgaggagaataacgagctgcgcgcgtacttcaacggctcga

cattcggccggccttaccctggatacagcacgatcgacgctgtcaaaggctcatacttaaacaacacaattccggtcacgaacgggctttttgtcacc

acggacaagtacgaggtgatcgacttgatcgtcaacaaccacgatcccggatcacatcctctgcacggctttgtcccgtacatcctcggttccgggcc

cggcgactatccagcgggcagcctcgacctgacaaagacgtttgtgaacccgatgcgtcgtgatgtgtacaccatgcagtcgtctgcgtgggctgtca

tccggttcgtggcagacaacccgggcgaatgggtctaccactgccacttgtcgagtcacatggctgcaggtctgataatgaccttctccatccaaccg

gcgaaggttgctcagttcacgctccctgccgaccatactgcggagtgcaacacgatccgttcgagcggcatcactgctgaagcctaa

>contig_21.contig_21.g732.t1

atgtcgactattagcggtgtcaacaactcgatgtccgcttcaactgcgaaggtcgtcgacgttctggaggttccggaca

agcccgagctcggccggttcgaaagtgagagcaggaaggggacgactctcgaggccggggccgacgccctttccgccaaacctgcattcgcccttcta

gcggaagagatccagggttccttaaggaaaacggaaggcttcgatgcggaattgacttggacaagacgggaggagcgatgggttacggtcaagacgga

tctctgcgtgctcattcccgtcttcctcgtcttcgtgttcttggcattggatcgatcgaatgttgcgaatgccctcacggacaactttctgaaggact

tgcacatcacccaaaacgagctcaacaacgctgtcttactcggcaatgccgccatcggattgttcgacataccgagcaacatcctcgcaaaaaggttt

ggtcccaatcgctacttgccgcttctcgtccttgcgtggggcgctgtgactgtcggccaggctttcatcacgaataaggcccaactgtacgtctgtcg

attcctagtgggcgctttcgaagccggcgcgatcccgggatacgcctactacctccttcgattctaccgagatcgtgagatcgcatcgcgctacgcct

acttctggtcggccaacatctttgcgtcggctatcgggggactgctctcactaggcattttgaagcttcggggaacccatggacgcgctggttggtcc

tatctgttcatcatctacggtaccatcacaatcgccgttgccttgtttgccttggcgtggctgccggacagcgccgtttcggcctcgcgtgggacttt

cgctcgactgtggtatacacctcgacaagcctccatcctgacgacacgcgttcttctggatgatccagagaagtcggaggagattcacgccgcacccc

tcagcagagctgaaatcttcgtcactctgaccgattggagattgtggacactcgttctttgcgctgccgcgacggcagtgctatacagtcctatcaac

acatacagcttactcctcatcaagtcgctcgggttccgagggtacactgccaatggcctcgccgttcctggcttcgtcctatcgatcgtctggtccat

tgtgatgggtatcctcatcaaccgacacggtcgacacgccttctatgtcgccatcaccaacattggcgcggtgatcggcctgctgtggatctctctcg

tgccgcggaataaggctgctcaatacatcggtattctgtggttgctcaccttcaacatcagtgctgtgggcgtcattggcgcttggacagcacatgtc

gttccagcaagacaaagagccgttgctcttgctttgacggtcagcttcaacaatctagccagtcttggagcggacgcaccgcgatacaagcatggtct

gtgggctctgtccggaacctgtaccgcaggagccattttggccggcgtggcccatctcgcacttcgacatcgacgaagaggtcgataa

>contig_21.contig_21.g733.t1

atgaccggaatgctcccctacattctcccctgcgtcgagctctcatcagctctcttcccggacgaagcatcgccgccag

ccacaggaagagcgacaccgacaaagcccgtccaaagctccgacctgtcagtacctggcaacatggagcggtccgccgagcatacgtgcttcttcacg

cccctcaacatccacatcgctgcccagcccttgcccgactcggcgctggctcatactgcgctcttccttgcgcccgtacacccgcgcaacaccgtgat

cgcgaccgacctccgcaagagtacctccctcccatccgacgccgtctatcggccggtacctacccctactgtgtacagcgtcttcaccacctccgctg

cctcctccgctgggccgcaggtgagcactggccccgacttccagaccctcgccgctcttcaaggcttgctcgaggctatccgcttcgtccgcgagaac

tatgcccggtcgtccctctgcgtccatctgcgcactgataatgagattgcggtccgcgcatggaaccactggattcctcgctgggatggtacaggggc

tgccagccagagcgacacttttacctccagaggatggccgcaactgcccggtcggcccgataccgccccatcgcgatcgacgttgaagtccttctcat

caacacccattccttcatcagctcaaggtcttgtcatcgcttctgggcgcagctcagacagcagcgaaggcgtcgccgacagggagatcatgttcacc

gcgctctcaacctactcactgcagtccaccgagcgcgccaactctgatgacgtaagaagaaatacatttcgcgacttcgaccacgacaacctgtccaa

tgcgaatgttcaggcgccagcacctaaagcactaccttcagtatctgcgacgggcatgaatgcgggaagaaacgaactcaatgaaccgaaattgatgc

ctatgggcccacctccgatcccgcgccagatcccagcctcaacctatgcagctgcatatccgctcctccgagctttagctgtgttacatcggttcttc

gcccatgccgacgacgacgccttcacaccaaaccacaagtggaacggccgcatgcggatgacggtatcgagacttacgccgggcaagctcaatacagt

gaccgcttatcttgacgaagccctccgggcgaagcagatgagcgcatctccctcatcatcgcctactaccacgaccctcgaggtgacccggagaaact

cgtcgatctcccagccatttgcggtcacgacggtcgcatggggacaaaagctctccgatacggagaagccgggggagacgatggaggaacaacgtaag

ctccgagctcgagaatcgtcggcggagtcgtcgtcgagttcacaccgtcctggtcgattaccagccgtgcctggccttttctggaagcgtagaccaaa

tacggccagcgcggatacggtggattctgtcttcgctcgaagtcaacatttgggtaatgaggagatgcctaatggagttgatcttgctcgacggggca

gccggacaaaggccatcttcttccggagatcatctctcagcagctccgctatgcttgcgactggccacggcggcgaaaatggaacagattctatttct

tatgttgcctcaacaccattgatgacaccaccctcgacgaacccaacatcgcccaatgtcgccattacctcttcgcaaagagcggcaaagtatccgac

gattcctcctgccgtcgatgccgactcgactgacagccagcttgtctgccctccttcgcctggcaatccgctgggcagcatcagatgcaagccggtac

cggacttgttgccgcttccttcctccttccgatccaatcatggcctgagtacggattcgaacgcggcggcagtggcggccaggagacgattgaccatg

ctcggattagacttggacctgcccggaaattcgcttaattgggaacgcctcgagatcggcagccgcaacgcgagtcgcagcgcatcgaggaacaagga

gcacctaactggcagcgaagaaagggcagatgattttctacccgcgcttatggcgggtctgcgtggtcgaaatgatactcccatagtcgcgcgcgatt

cggtcgacagcaactcccggacgatcagcagtccacatcgccggacgaactcggtgaagattgactcgctgacggagagggagacgagctcgagaaaa

cgggcgagcacggtgacggtgacgccgacgaacctgaggtcgggcgaacatgaggaaagtgctcccaacatgggccaccaaaactcggtcgcgttccc

gaaggtggagagcaaggtactggaagcgtccccagcgccggcgccggcgcagaagcctacttggcgctgggctgacccgaagcacagtgagagcatca

agcttgcgcccatctctggccttgtactcgacccttcgagcgggctctacaaggtggctgcatcgtcttcctcgtcttggccggcttggaagtgggcc

actccgggtcagtcgagcacggttgcgactcccggccatcattccggccaggcacactcggcagcggtgtccgctaccacatcccccgccctggcttc

ttcctctgcttctcccgccaatacgcgcccgccatcgatttcagaggcggtgcgtcgtgctcgtttgacggagcaacggccggacgcatcccgcgctg

ctagtccttctgcggcctttgtgatcgatgttcagcaacatgagacgtccagtccgcatttggcgagcctcacccggacgtcgagcatcgtcagcggt

gaatcgggagtgggaggtgtgcatcagggcagcggagcggtcactgtggacaatcgtgccaatgctgggtggccgcaagccccagcttcggcgagctc

gagtgggcgcagtggattggcgggcaactggaaatggtcgaacgattcgcaaactcgtctggagcggcaaaaggcacgagcggaggagttggccgatt

tggctgcttcgaaacaagacgtgaatggaggttcgagaaggagtagcgaggctagcgggaatctgcctccctcgagcctgttcagtgcatcgccatat

ggcagtgcgggaccgggcacccagacggcgctcaccctttctgcggccgggtcgagcaaccacattgcgccgccttcacctggcaagccaccggcact

gaatctgcacgaggcaatgagcttgcagcagacaatgaccgcgactccacggtataccatgcggccacagcgaagcgttcccagactttcgaatgtgc

ctgacgccgagcattccgttgtgcaagccccgacggcggcttcggcggaggagacccagacagccccggccagagctgccagggccgagttcgaggac

gacgatgttctggtggacgaacatggatcgccaatcgctgttactgacatggtgctctcgcgagagcgtcagctcgccactgcggcctggacggcctc

gatcgcgcctctcctcagtcagcagcgcgacgatctggcatccgaaccgaggttctccggcgacagcaacaggcaacacagtggtgctgctcggcatg

gccaggctcctcgcacggtggtgcctgcaacagcagcagacatggccatgactccattgacggagaaggccccgctggcgactacgagagtcgctaga

tctggtacgcgatcgcgcatcttgaattccatccagcgacctaccgttcctgcggatgcgtcgacggcgatgggcagcaagcttaccgcccggacgac

cgagttcccctga

>contig_21.contig_21.g734.t1

atgctaaagttccgatcagcagcaagctgcagtttgcctggcgagtgccagcgtgccagcggcacagaattctttgcgc

gaataatggcgaagcgggacgagggacgaacccgctcgggacgtccaagccacgtctcgaaagaacaatggaaggccaccaccgccccgaacggcccg

ctcactcgcttatcgctcatcacgcgcttcctccagatcctccacctcgcggcatctccacgacctccacccacgacctctatatcttctcctcgccc

ctgcctcgatgcggacagcggacgacacaggtttccttccagtatccacggcgtcctggcacttctggtcttccttccctcacagatcacacgcagct

tgcgaatcgatccttcggcactcggcagtcccgaaagggtttccacccttcggcgcgttcaagcgttcaagcgttcaagcgttcaagcttcaaggccg

ttgctgtcgagcatgctgctcttcttcttcctcctggatcaggcctcgagcgtgaggaagaccttgcatggcctcatggcgccttttccttacggtcc

ttcagcagccagagccaggcgccagtcgcgtcggtacaaagttgtggtcgacatggcgtggatgatgggcatgatccaccgcctgaatggcaagctca

aggaacggtaa

>contig_21.contig_21.g735.t1

atggaagagctcagacgccaggttatcgagcaggaggccgcgggtggacacgcgctccagcctccccgccgcgatcacc

acgcccccgagcccaagatccacctccagtccgccgtgtccgcaggcaccgccccaacgaaccacagcgttctcgccaacgtgagcaatacccacaac

cgatcctccaccggcagctttgacaatgccatagtcattgacgactcccccattgctgtccgcaccaagccctcatcgccgatcgccagtcccgattc

catccccatcccctatgccggcccagcgcttccttcgggcgacagcatcatcagcgcggccgaccgcgagaaacagctgatgaacatgatgacctcta

tggtcgctgtcgtcgaccacgtcaaggaatccgataccctcgtccccggtctcacctgcgctctcatgccccaccaggttcagggagtaaagtggatg

atgagcagggaatcgggagtcgagaaggggggtattcttgcggatgatatgggtctcggcaagactgtccaaaccatcgctctcatcctccagaatcg

gcccggtgcacgcaagaacaccatcgagtggctgcgcacgccagacgcggacgagcaggccaagctcaacggaataacgcgccgagttccagcgaaga

agctcgccaagaagcccgcccccgcgcccaagaagagccgggcgatcattatcgacagcgacgacgaggtcgtcccggaagcggaggaggaggcggag

gaggaagtcgatgacgaagaggaggacgaggaggaagaggacgagctggagcggacggatatcaagtccaccaccaccctcatcgttgcacccgtggc

cgtcgtccggcagtgggagaaggagctcgccacaaagagtacgaaggtaccggctttgcccttgcgcgagcccaagatggtgcctcaggacgacgagc

ccatcgacctggacgccgacgaggacgaggagtcggcggcctatcatcgacagctcaagctcattcaacacaaggacgcgctcaagagggcggccagg

gacgtaccgcacagcaagcccttctcccgtcttcgcgtcctcgtccaccacggtgctaccaaggtcaagtccgcaagacagctgcgcgagtacgacgt

cgtgattactacctaccctaccatggtgtcggagtacaagagcaccactgcctacgcgaagaatgagtccaagtccggcaagggcaaggcggcttcga

gcgacgacgacgacgacaagggcgacgtcctcgccctctcctccgacgagtccgatgacgatgagcaggacagcgacgacgaccgcttcatcaatgac

gatagcgaccttgacgagttctcgaactcggacaacgagatcgagagtcacttccgcccgatgtcgaagccgaaggcgtaccggccgccgaaggtagg

cgcgtcgagctccaagaagaaggcaaaggcgaggagtgtggggcggaagaagagtagcgcgaacgggaagccgacgccgctatttgatatggactggt

tgaggattgttttggacgaggcccagaatgtcaagaaccacacaacacaggcagcgaaggccgccttccagctgtccagccgcgcgaccgcgcgctgg

tgtctcaccggcacacccgtccagaacacaatctatgagctctggtcgctcattcacttcctccggattaagcccttcgacgattacgcacactttaa

ggacaagattggcgagcccctcaaccccaaaaagaagaacgtccatcaaatgacggtcaactgggcgatgaagcgtctccatgtggttctccaagcaa

tcctgctgcggaggaccaaagccacaatgcacgagggcaagaagttgattgacctaccgccgaagcacatcagcctggtcgaaaccgactttgacgac

cccatggagcgtaccatgtatacgctcctcgagacgaggattcggaatcgcgtgcaggaggcggaggaggtcgggggcagcaagagcttcggtcagat

tgaaatcttgacgatgttgacgcggttgcgtcaagcatgcagccatccggcgctgctgaagggcaaggtcgatctcgacgtcggcacggctgcgtctg

ccgccaagcccagctccggcgctggcgcgagcgcgaacgcgaatgacgacgtcgacggacttgcagccatgctaggcggagtcgacatcgccggcccc

gccaagtgcgaacaatgtctcctgcccctccaacaacaggacaccctcgccatcgtgccccccaccgcacccgacgacagcgccgacgactcgatcgt

ggagatgcagacgcttacgagggaaggccggaagctttgtgttggatgcgcggagattgttgatcagcagagggcggcggggacggattggagtgaga

aggcttctactaagttcacgacgtttcttgacctcctcgagcctcatctgcggaagagcaagttcaagtacgagcgatacgacggccagatggacatg

agggaccgcgacctcgcgctaaaggcggtcgagacccggcccgacaagaccgtcctgctgatctccttcaaagccggctcgacaggcctgaacctgac

cttctgcaaccacgtcatcctctcggagtcctggtggaacccagctttggaggaccaagcgttcgaccgggctcaccggctggggcagaagcgggaga

cgttcatctacaagctgtgtgtgagcaatacggtggatgggcggattttggagatgcaggataagaagcggaagttggcggagaggaaagctgctgaa

ggaatccttcaggggtccggcttctcgttcaaggtccagcgtcctgaggtcgtcggtcccgtctgggcgttcaggttccgcagaacggcatcctcgag

ttcgccagggtatggatggacgtcctaa

>contig_21.contig_21.g736.t1

atgcctcctagagcccgcgggagcacctcgcgtgcccccacgaacgcttcgtcttcgtccactggtgcgcgacgggcgg

acaacgagcccgacgccccacaatctccacctcatgcggctgccacgtcagcaccctcaaatgacgaccctctcaactcgtccactgcgtcctctatt

atacaggacgccctcgctatcatcagctcgacggccagcgacccagataccggtaaacggtatcgacagccgaaggacgccgagctccacaacgtcaa

gcgagcgctcgagagactccaggagcatctctcttcctcagcagctcccgctcccggcccttccagcacaccgaatgccgcggatgcctctccgccca

tctccaggtcggatttcgagatactcctcggccgcttcggtgcgctcgagcggacggtgctggaaacagctgctccttcccccccgacaaccgactcg

acccccaaccgacgaaatcctcctcccggcacgattgccgcacggtctgcggcaacggtgtatgcgcacaggcacacatccacgccgcgcgtgcgctt

tcgcgtcgtcctcaatacgagagagctgcctccggaccaccccgcgcgcaacctcggcgcaccacagatcttcgacgctctcaaggacccaatttttc

gatcagagatggtcacaccactctcggtcgagaagttgaggagcggcgacatcggcatcaactttgccaaccaactggaggtcgagcaagtcctcgca

tctgaaggcgatcgatggcttgcggaagccttccccgaccaagaagcactccccactctgcgcgaactaacgttcgggctccttcaaacaatcgtggt

tcacggaatacctttcgccgacaaggatggtgagggcatggcagctgctctcacctccaccaacaacatcaacttcatcaacacgcggtttctcgcct

ctccagcgaagcgggaggcccacccaaacggtttcggccccgcatcgtctcggaatatcggcccaatcttcgactccagcaatgcgggcactgtcaac

agttcggccacgtcgcgcgtcactgtcgccgatctcctgtctgtcggcttcacccttcacgcctcacacggcacacctacccggctagtggctaccct

tcctcactccatccacccagccgcaggccttgacattgcgccatcaaagcgacagcgcaaacagcgagccagagcacatcgatctggctctgctgctg

cgcaccgcgatacctcgtcctctcatcacgaacgtccgacaacctcgaatgaagacttcctttcctttggtgctggagtgatagatgactgggcgaac

caatctgattttgggatgggagacgaggggccagcggacaattcaagtgatcgggacgagattcagcactacgcagactcgctgttcattcagcagtc

tgtgtacgtagcacgtgttgaccttcggacttgggttgtcgaagactatgtcaatggtatctgccaggaagggaagttctatcatgtgacgctctatg

aaggaaccagcccgacaggaagggacgaacaacttgcctcatgcgattgctcagctggccgaaatgggttgcaatgctcgcacaagagagtcctgctc

gagcacggcgagctgttccaaaaggaggtcattctttctcagagaatcaatccccttgcaattcagatcgcggcacacctacgtggtgaacttgcttg

gttctctgttctatctaccggaagcaccatcaccgcggccagtcaggacggcaagcgttgtgtcgttgcgttgtctggaaacgagcgttggacttgtt

cggcatcgcattgttcttcctttggtcagcctgcaaacgtctgtccacatcgtcaacgcgcccagcagttctggaacgacacaggcaattccatcgag

cctgatcttggtggtaatggaatgatggcattcttccaacaactatcccgcttggagaacacgcccgctgcgactgcggtcgacgcagcagcgttgaa

gcgccagtacgactccgaccgtcatgctcctggtggaaagctcaagtttcttcatcgttcgacttttgtcaggttgtactttgcgtacatttccttgc

agcgactcgatatcgatttctcatgccaactctgcggtcccaatccttctacagtcatagcatatggagtagtgcttgcatttggtgctccgtttcgc

ctcgccgacctttgccctcccgcctcaaaaggaatggacatcaacgaacgtgcacgaagcaaatcgatcatttcgtttctttcccctccttccgcgcg

cgcagctgtgaagcagcttgccggcttccttctgcagcaggtggcagacaactctgatcagtacctcgagttgctgcagcagatcaaggcatctgtcg

agacggaatcggtcctggtgatgcgcttgtggtcaggacgctttcagcaggtgctgctggacacgttcttggccagggccacactttcgcacaacctc

acgaccgcggttcagcaattgatcgaccagttcgctgcacatgagggtgttctgcaactttgccgccccctctgctgcccactcctgatcgaactcag

caccatcgccttccaaaccgcctccaaggatcgcatcactcgtctctcaaccacactggttcgtcactgtcccttcctgggcaacgtctttgttcttc

tggcacatggcatcggcagcatgacgagctcagcagagacgaacctgaggagcagcttgcaatcattgatggcttcgacagcacaagtcgttgaccat

caagttaccttgctcaatcctcgaccaccacctctgtccctgcccaacaatgcaacgatcagctatacgaagtcgggaactttgtacggtgcatccaa

atgtcgaacgaggcccaactatccgcacttgaatgagggattggaagggagaagcggtgagaagcaggcaacggcggagggcggagagacggggcacc

agtgccgcaagttctatgacgaatacaccaagcgccagcggacgggaggcatcatggcgttgtggtgtacgcattgtatatgcgttggatttcacgtc

attccgcacgcagaaggtaggaacgatgtcttctcagcgatattttcatactggccacgccctccctctgtgatcgtatacgattttgcatgccaact

cggaccatactccttgagacgagaaccaggattcttcaaggacacgctctttgtagtggaccagatgcacgagaaaggtcattcaaattgctcggcag

ccagtagactctcgacatacatgcgaaacgatccggcactccaacatctgtactcgtccgcggcggaatgcggaaactcgggtctggcgaggatcaag

aagacagtgtccttctgcaatcaggagcacgcggttgcactcgtgcgggtgtttttgtgcgtctggaaccggcaaaagacgctgaggatgagctag

>contig_21.contig_21.g737.t1

atgcgcgacgcagtcatcaacgatcaacacggcactgctgcggaagcgatggttgaggcaaacgcgttactggccgccg

ctgaagcggctcgtctgcatgatttggagagtgcttctgcttctcccgcctctcccttaccgcccttcctcgatccgacgctgctcgctgcttctcgt

cgtgcgaatgagcatgagagcggcggacttcagattcgcacacgacgagacagcgatactgagacggattcggaacatgcgatgactgcaggtacaac

tgagatccctaccaatgcgaaaggcaagaaaagacaacgcgtgaacgtgaaccttcgtccggagcccaacctcaccgaagctcagtatcgtgatattt

ggcttaacttgaaaaactcgcacaactacgccatgaaatactacgaatatgaacacaattctcaggcgcgaatgcaagtcgcgaaaaaggggaacatg

gaggcttatagagcagctaagctatcggcaaggtcaggtctcgctgtcttcatcgcatttgggcctttgaatgaaagtgcgaaggccgaagcccgaga

ccatgtctatgtcagtccgaacctctgcgatggcgctcgacctactctgcgcgcaatggcggaacatgctgccaacgatttcaagacaacaatgcata

tgtaccgagaagcggcacgagcgaacacagcgcaggcattggaagcaaacaaggttctgattgcggaaaaagacagctcttaa

>contig_21.contig_21.g738.t1

atggcaggatttctacgcggaggcggcggcggcgcaccacccgctgatcgcagcccatactctcgcggcggcaccggcg

cacccaacccatacgcatcggccggcaatgctccctacgcctccagcaacagcatggcaggagcaggcgccggagcaggtgctggtgctggtggcgct

ggcttccgctcagcatacggcgcccaaccagcagccgcacacgcagcatacggcggcgcaggcggagctggtgggggcagtgccggatacgagaaggc

agcatacggcggcggcgcaggaggaggaggcgcagctaacggaggcttcaccgtcgcaccatgcccgtccgcattgaccctcgccaactgcatcatca

catgcgcgcaggactggtcgccaaacactcagtacctcctcgtcaaccagaactacgtcctcaccgtgctcccggacaccacccgcagcattatgccc

ggatctctcggaatgggctccatcatgcgcgcttggctcggtctctccgctggtgcggtggtccaggccactccatacgaccctcatgcgaacaacgc

gccaggaacattcgtcgcctcaatggacatcgagctcaactggctgtccgcagcacaagcaaccaacgacactttcgactccgacaagatgcaagaag

cattctgtcaggctttcaacaaccaaatcttcacaatcggccagagactcgtgttcgagttccgcggcacaaaccttcatgcttccgttcgcggcatc

acggccatcgaaggcggcggcggaaagggtgctcctccagcccgctccgacaagggcatcctcatgaaccaaaccgaggtcaccttcctcaaagaagg

cggcagcacgatcaagatcaagggcagcaagaaccgcgccgcgcctaatgccattcttcggcccgacttcaagttcgaagacatgggcatcggcggcc

tcgacaccgaattcagcaacattttccgtcgtgccttcgcatcccgcatcttccctccgtccctcgtcgacaaacttggcatccaacacgtgaaaggt

atcctcctcttcggccctccaggtaccggtaagaccctcatggcgcggcagatcggcaagatgctcaatgcgaaggaacccaagatcgtcaatggacc

ggagattctgaacaagtacgtcggtgctagtgaggagaacattcgtaaactcttcgccgatgccgagaaagaatacaaagcacgcggcgacgagtcag

atctacacatcatcatctttgatgagcttgatgcgatctgcaagcagcgtggctcgaccggcggcgggaccggtgtcggagactctgtagtcaaccag

ctgctgtccaagatggatggtgtggaccaactcaacaacatcctcatcatcggcatgaccaatcgtctggacatgatcgacgaggccctcctccgacc

cggccgtctcgaagtccacatggagatcaatctccctgacgaaccaggccgtcgacagatcatctccatccaaacggcgaaaatgcgcgccaacaagg

tgatggacgccgacgtgaacatggacgagcttgcgtccctgacgaagaacttcagcggggcggagatcgcgggtctggtgaagagtgcgactagtttt

gcgttcaatcggcatgttaaggttggcacggtcgccggtgtgggggaggatgtggagaagatgcgggtgaattggggggacttcttgaatgcgttgaa

tgaagtcacgccggcctttggtgtttcggaggaggagcttgcgcaggtcgtgcagaatggggttatgaagtttgcacctcacattgacggtatcctac

gcgatggttcgctccgcgtcgagcaagtacgaacgtccacccgcaccccgctcgtcaccgtcctcctccacggccccccgagctccggtaagacggcg

ctggcagcgacgatcgcgacgatgtccggcttccccttcatcaagctcatctcaccggagaacatggtgggcatgaacgaggcctcgaagattgcgta

tatcaacaaggtcttcaacgactcgtacaagagtccgatgagcatcattgttgtggatgcgattgagaagatcgtggagtgggttccgattggaccga

ggttctcgaacccggttttgcaggcgctgtctgtgctgcttggcaagcgtccgcccaaggaccgtcgtctcctcatcctcgccacgacctccaacaaa

gccatgctgcaggacatggacgtagcagacgccttcctagcggacatccggataccctcgatctcctcgatcgcctccatcgagcacgtcctgcgcga

ggttgagctcttcagctcggaggaggagctggcgcggtgtgtgcagtacctgcagagcacgggattggaggagagtggtcggttgcagattggaatca

agaagcttctttctgagattgagatggcgaggctggatcaagaccctgcggacaagctcgtcgctgccctcaccgctctttga

>contig_21.contig_21.g739.t1

atgacactacaactcaaatcgactcctgtcatcactatcactttacacaacgtttacgtcgttccagcgttccctcata

accttctctcggtccaacgactaaaccaggcaggatacgatgtcaactttccacatgcgacgtccgagaccggacggggcacccccctcagcttccga

agcatcgcagtcctcatcggtttgggggtcgaaggattgcgccatctcaacaagagcgcggctcttcgactcgtcgatcgagaagatcgatgcgatgc

atgcgcagtgtcgaacatcaagaaagcaccgttctcatcatcaagctcgagagctgaagacagagctgctaaggtacaatgcgacctttcaggcccat

atactggaaatgaagagtacagctactcggtcaccacgcaagacgactattctcgaaagggatggaccgtgccgactgtcgacaaatcaagctcgacg

gtgaccgaggtacttcagcgttggcatcgacaggtcaaaacctttacgggtcggaacgtggtgacgatgcacacggataatgggacggaatttgataa

cgctgcgatggacaaatgggctgcgagtgaaggtctcaattggatttggacagttccaggctccagtgagcagaacggtcgggtggagcgtttacaag

gcctgattcaagaacgcggacgagcaatgttgacagcaggccagcagcacgactcccgatcggtttttggcccttcgcatggcgatacgccattacaa

gacgctcgtcaccggccacacgggaagctatcggcccggggtgttcgtggcaccttcctcggcatctctgaagacaggaaaggatggctcatctacgt

cccttcggataagtccaaccctcttcggtatagccgatcagttctgttcgacgaaaccagaacttacgacgaaggcttgtcattggagattctacaag

gccaccaaccacgtcaagatgatcacgtccaaatggtacttcccttgccccgaagacgtcgtcgccgagtcggagagggggagaaagaggcactcaag

gtaccaaccgacctacgcgacgatttggacaacctgggggggtacacgaaagacgacgaagtggccatcgatatttcatctctcccaatcccaaacac

agcatcaatggaccagcccgacgagacacaagagcaggagacagaggtagagcagctggaggatgagcaagcggtgggagaagcggaaaggttggttc

aggacacagcagagcaagaaggggcgttggagaatgtgcaagagggaacggaggtagcagacgaggaacagagacaagagggggtcaatgtggggcag

ggacgtcggcggactaaggaacgcattgcaggaaaacgacgaagccctcgattacacgatgtccaagcgcttagctcgactctgtgcacggaaccaat

cgatcaagactggaatcgagtcgagctcatgggcatcactgaagcttcagagctggaagcaatggccttactggccaacggcggaacggaaccgagga

cccttgacgggcaactcctcgaaccggcaaccttcgccgaggctcttgcacgtcgacgtgcggccgagtggcgaccatga

>contig_21.contig_21.g740.t1

atgcctgcatcctcggagaccggttcccaaggtgcttcggctcaaccgtccctgtccgacttgatgtccaagttggact

ctattagggacgatcttgcgacgcagaagcaagagctcgccgcccagaagattgccctctcgggcgttacttcttccttcgcggctcgggaaggctac

ctccagacccccatcatgggaggtcttggacaagacaatccccgacaactcgagaccgagtccgccctgggtcttacagcaggccgaccagcccttca

acttggccaacaaggcgggccctatggtctttccccggtcttccccgcacgggatgaccgacggggtcacggacgacccagcatgggagtgtccggct

tcccgctcccacctcaagcaagtccggcggtcaacctcaatcgcggaccgaaccctaccgcccaacaggctcgtcaaggtccagcaattcgcaagaca

gttcctcgcgattcggatgaggcgatggtgcagaccagggccgacttgatgaccacgaatcagttctacttccggatgttgcatccggaatggactga

tgatcaggtcaatcgagaagcaaacaagagaaccgcggaggactacttgtccctgcgaaagaaggccattgaggaagccctcaacgacggaccagctc

agacgagctcatcgcagatctacgatgtcggtcgtcaagataatggccgtcaggactccaaccgactgtactttcggttcaattggggaatgatccgc

tatatcaagaagctgaactccttcaactggtctgaatggaggagctccatatacagtctgctcggaaccgttcccggcgcaatcggcatactcgaagg

gatcatcttcggccctcggtatctcaacccaaatgatccctcggcccaccctgaatacgacgaggcattggatctcgaactcggccaggtgattcaat

catgcatcgacgaggacacacagtccctactcctcaagccgatctctgatggggaactgcgtggcagcttcttttatcgggaacttcgggtttggttg

gtccctaatcaaggatatgcggccctgaagctcatcgctaagatgggacgccatcgccagcgggacgacgaatcggctcgtcagttcggggagcgact

ccgaaagttctaccttgaacttcagtcagctggagagacactggaccagacaaagcaagtcggtttcctgctcgccggactccgacatcgcttccaga

cggcaagagacagtgttgtttcgcgccaggcggcaggcgaagtgttcaccttcgagaaggtgctccacattctcgcagagactgaagaatcgttcggt

gcgttcgaggatcgtcgtcgtcaagttccacctcgctcagtacctccattgtcgcggacatcggctcatgtggccgaggaagatctggacgatacgga

aacggacattgatgttgaagaggttcaagcgttagccgcccagatcgctcagcaaatgctcgctcgccgaagggcacaagggtacaatagacccttca

gtggagcatgcttccaatgcaagaaggttggccaccgggcagccgaatgtccggacgtcgttgaggcgtcaaagaaccctcagacaacatcccgagca

ctcgctgctgatggctcggatggatcaactcctcaagtccagcatacatgtggcaaggacgacgccagagcgtcaatggcaacggcggttccttccct

cggtgcaatggaggcccaaatttgggaagacgaactcgacgcattccctgcagcgttgaacgtcatgacatcggaatag

>contig_21.contig_21.g741.t1

atgccatcctctcgctcctcatcgtcgttcctcgatgctcatcaggaggtactaccaccccttcgacgtcctgacctgc

tcgcgccagcctgcttcatgccgtccgaggaggacttcatgctgatcaaaggcgccatgtcgccatctggctgggctctgaaaggcctacatgtcttg

gtagccgccaagtcatcaattgattattggaagacagcgcaagaacagggatccaacgaggatcgagtgagcatccggacgagggccatcaatgtgac

ccgcaatcatgtcggtcttctgcctcttccggaaccacccttcccttcggagtgttgtaattcttgtccctggtacagcagtggcaccctggacgcag

aagagtacgaagagattcgactggtccaaaatcaggtcgagaactggcggagcacagacgagccagatttccagatgacggaacccaggatacgtcgg

tcactgtctccgtcacccggtgacggtcacccaattgccaaacgagcgaggctcgatggcctccaatccaaggatacagcagcgcaagacgatgcggc

cgtgcctctgcatgcgtctgctggtccgtcggcactcccagcatctcggggtctcggagacgtggatcaaccggcggcgaaagcctttttcggccttc

ctgagctcctatccttgctttgcacccacctcgcatacgacaaagtcgacctcgtcgtgctatcccgagtgtcgagacgagttcgggcggtggtgctt

ccgaccctctatcagactctgaatgtccgccttaccaaggttgccctattgagccgggttttcaaagccaatcctggactgattgagcaggtccgata

cctgcgagtcgtggatgacgcggctcggtaccacgctcgctgcaacacaataggcaacagcaccctacccgcagccttccacagtcaagacgagcctc

gactcagcgcagctacatggacttcgctcggagacttccttctcgccgtacaaaatcggagaaatttttccaagcctcgagtcgagctatcgtttggt

cagatgagccttatcgagctctacaatgagctgcgacgcgctccacgcgtgcttgagaccctcaccgccctattcatcatggacgactttgggatggc

tcacttcaagtatctggaccgtaaagcggtggagaacacgtaccacgatcatggcacagcgatctccgaggagcttacggcggtcttgcgtctcatct

gtgacgtccaggacgacgcagacgtggacaactttcagaccttcgagttcaaggctcttaggctcaagacggaggaacggggatcgcttctacccatg

ctgccaccgcgtcttctaaagcggcttggtggtcgaattcgaagcttattcgttagcatcggcgagggtattcgatcagacgcagagagcttcaaagt

gctcaccgaggcgaattggtggaaacttcgcaacttccgagtacactgtggttgctgcagcgaagaagtctacgcgagcattcaatcggtcgtgacca

actttctccggcgacacatgcatttggtcgaaaccgacatcagaatcgaagaggacaggtggggtgaaggtgggccgcactgggtcaatgtctccctg

ccccggctcgtggcgtgccaccttcagcccacgcatgtgagcacggagctgcaactcgacttcgccaatgcgcaccaagacattcggaagctctgggt

cacgggcttcaatggcgcattcgctcttgctagccaccgagcgatcataaaatcacttcgtttcctccgagcagagcccggcgttgcggaagaatttt

tagatgcaggtgcggccatcagccatcttcacgtggatttaatcgcggacgatgagggatattgccgcttccccgagtgcttatacccaggcggccag

ccaacgacctccatcaccagtttcgttcttcaatttgaggagattgctttcactcacatcatccagaagccggagacgtatctaccaattcatcgcct

gcccaatctcgtcgagttttcgctgaagtggacaagcggcgcggtgagcaaggttcgcgactcgccggagcagtctgcccaatgccttgcggcgctgt

tgaacgagctcgcgaagcacggcgccaagctccgtgctgtacgcatcgactacgaggccgctgccgacctaccttccgacaataatcttgctcaagca

gtcaaaacatttccgcccaagctcgaatatctggtgtggcacattccgtgcatcaatcgaacggatcactaccgtgtcctgaggccacaagcgaattc

ccctccatcctccgcccaagttgcccagtcttcccgatcacgaccgggccaaatcacatcgtccacgcagacgtccgagacgcagcccaaggtccgtc

tgcagcgcttgcccgccatcttccgacccaaggtcgacccgaagacgggaatgtgggaagaccttgacgacgagggctgcttgagtctcttcgatcat

atgggccgcgaacctcggttgaagttcatccccttcgatccccagacgtcgagggtcgagcgggacgagggcgagggtagcacagcagcgatatcgat

cgacaatgccgaagagtcctctgcatcgctcccctgctcgggacccgcccttcctgatcctgatccgaaccgcgagcgtcggcagcccccttcccagc

cagtggctccttcctcccatccgtcctcgaatccgccgacgctggatgcgcccccgaatctctcacccgcaagtggcgaactgtcgaggcgtcctgtt

gatcgagctgcacgtcctccctggcagcaccgacagccggccggggaagtgctctcggtcagggattggtcgatgaacgtgacgatgggcgatccgga

tggaaaggggaaggatgccgaggtttga

>contig_21.contig_21.g742.t1

atgggttacagaagcacttcgagcagagagatgggagatttggagaggaaccacgagctcgaccctgcgcaaggtgctc

cctctcaagactccgcttccgggtggctcaccccccatcttggcctggagccgcatcgcatcaccaaagtcaccttcgccagcaggatgcccgccacg

caaagcgcaggtgtaggcgcaggcgcaagctcggcctcagcttcggacatgggcccaccaccttccacgcccaagcgattagcaggcggtacgcattc

ctcccagccttcctcagcactcactcaagagcaccacagcggctatcccttaccaaccccacccacctcccatctcgaagatttccacagcccgacac

atacagcaactccggccgcagagggtcgctctttcgcagcggcaggagcgagcacaggagccggcgcaggatcagcggacgacagaactcctacattc

accgccggagctcgatcccgtggcggtttactaacttcacccagagatcatcaggggcacagcttgcggcaacagcagacgcccaccgccagatcaat

cgctttcgccaggtcaaatccaggacagagcgacaacgggacctacacctaccaagacgcatatacccgctctcgaactccttctcccattttaccac

gtctccaacctgtaccagaagcagcttcttcgaccggcgacggacaccttaccttcccgccctttggcgtgtcgcaggcgcagatcgattccatctta

catgacccgaccacggacagattcgagccgtccctcatgcccctgccggacatcgaggcactggctatgtcaatacggcaattaacagttgatatggc

caatgcggaggatgccaagctacagcaactggtccacaatgcaaagacgctcggtgaacaggctctgggatatgcacagcacaatgccagcctcaggc

aatcactcaacaatacccgcatgcatgcagtctccgccatcaacacccttaatgcctccgcttcccattcactttccgcagagcgcgatcttcgccaa

cgtctacaggtagagctagacggtgagcgggcccagtctcgcatgctcgccggacggatgggacgtggcagtcgtaagcatgaggaggagcattatgc

aggcgatgggtcgggttcagaagcgactgagattacaagtccttcggagctcctcaaggaacgcagtaaattaattgccgacaaacggttcctcaaga

gtagggtcaaggaagcagaagcgcaagcactccgcctcgagtcagagctgaaggctctccgaccacacttcgtccgcgctggttacggtaactcagct

tctatccgcggccacggttacagtcgcggaactaatcacagtcacagccacagtcacggtcagagccacggctacattgcggatccctttgcactcgg

aagcagccagagcagcagtcaacgtcgtcgtcgtgcggcgacattgggcgatgccgggtcggaacatctcatgctggccgcccgtcgctatcgcgaaa

acgatcgcgaagtgcatcggcgtgatcagacggccgcccaggagatgatagatcacgctgccagtctgtacaactcgtccatgccggtctatcacccg

tatcccggctacgtaccaccggagatgccgcatacagtcccgtcctcgcctgggcctggtgccagggctgcgatgggtggatatgcgcttcatgcctc

gggttcggctccttatccccatcagtacgggggctaccctgtcccggacgcacgggaaggagcggctgcccacggccaacagccaaacactcaagtgt

cgtttgcgctcggagggggaggaggtcccaatccctttgatcccaaccagttcggcaccaacccgaaccactacgccggccaactccccagtcaaaga

cccatgtcgagctcggcatcatcaacgggtgtcttcaggggaaacccgtacggaagtccgactgggttcggtaatgtcggccccacgccgcgtacacc

tccgccgatgcacgaccgtgtgcctcggggcgctcgcacggacggctccatctcacgaatccgcagtatcccgtccagtcctcggggcagtccgtcgt

cggccgcgttgcgcggggcggagtggtaccgtgccggctccggggctgggctcggcactacgtctggacctggaacagcaccgggcgcgggagctggt

ggatcgcaacaaggtccaggaacacctggatctggttcgctcgaagacctgttgcatgccgcccaatctgcgttccggcctggacaagagggacgagc

tctcgtgcagagtcggattcaacagcattcgcacggcggtcgtcgatccgatcttcgtgggttggcttcgataccctctgtcggtggtcgcaccagtg

ggggcaagcgcgtgagcatcggaggggatgatataccctttgagcacgaagaggacgataacgacagcttgccgtactccgaggatgaatcgggggac

gacgaaggtggccggcgtcatcatgccgatgatgatgatatcggggacgagtcctttgcgaagaagatgccgggaagcccgaaacgtcgtcggacctc

ggctgcgctcaactcgccctcgaagcggatggcgaaggggcggagtaaagcttcgacgagtgggtcggttcctggaagcccatccaaggatcggacac

cgcggccgccgaaggggaagagcggagggggaggaacaacccccaaggcttcggatggcagtggtacaagtgcgggtgcgggtgtcggaggtctgacg

gcgctcgacttgcttgcggatcaggctgcagcgagctcgacccaaagccagtacagcggcgacagtggatccgaacatcaatcccagccggccgggtt

tggcgaggcaccgatcgtactcggtgggggcagcagtagcagtagtagtagcagcgcccgacgatctggtgcggatggcggcagtggcggtccggagg

aagagggcatggacgttgagcctacttcgcctccgttgagtggggtggctgctgcgaacgcgtcgcatgagaagcggtcgacgtatactcgttggtca

gcggaggaagacacgaagttacgggctgcgatcaagcagcatgggcagagatgggaacttgtttctcgagctgtggagactcgctcgtaccaccaatg

tcgtcaacgctatctcctgttgcgccgcaaagaagcagccaagtcggacgggactggcgggagcggggtcaagccaggcgcgtccaaggttgctggcg

acggcaacggcaatggaggcgcattcgcgatgccgggctcgccgtcgaaggctggcagtagcagcggtggcaatggcggcagcggaagcggtagtcga

tccggtcggtcaaactcagaaggagaagaggttgatgagatcgaggaagaggaggaggtcgagaccgagcgagacggaccggccgtggaacttgggcc

ggacgagcttctcactagtcaggccggttga

>contig_21.contig_21.g743.t1

atgacctccgccgactcagcactcgctctcgaattgagattgcgtctgctcgaaaccattctcagcggaccctccgaac

aggcttacgatccaacagagtcaagattatcttcctcctcccatcatcaacaacagcaagtaccatcctctcggaccctgaccgttcggaccgcacga

gcgacaaacgtcttacattccaccgtcaaggaaaatgcaagcctcgccgccttcgtcagagcgtacgacaagaatctacccttcttgaccccggcctt

tgcactcgcacaaccaggctccaaccctgtcgaagaggatctacgactgagtggggtcgacccttcttcatcaacaccatcggcacccgagtcgaacg

gcaagaagagcgaatccgaggcagaacaatccgaaacggaaaacatacccctctccgccctcgccctccaacctcaagctcaagcagccctcctactc

gaagttgagcccgaactccaagcactcgaacggcagctcgccgaatgcaaagtccttgatgaacgtgacaccgcaggggcgggcacactcgctgagac

cgagacgctcgcgccaaagctcaaggctgcatgggaacagcacgcgaagaggaaggcggaggtggagtcgctcgagcgacagctgagcaccgtattcg

attcatacacagactacacggacgcagcttcccaaatcttcatcgcttgggacgaattactcacatccatggagcttcgcgtctcgaaagcggagaag

gatagagtgcagcaagtgtag

>contig_21.contig_21.g744.t1

atgtccgcagcaccatccaaggaccagacctcgcctgccgcggctgtcaaacccgaccccagcattgaaccggcatctg

tccgtacgacagtgcgtatcgacggtttatttgctattcctgcgcccgccggcgtcaatactggcggcttcgaccttcaaatgtcagccgagcactgg

gcgctgattggcaatctccaggaggttcgtatcgaggatagttacgtcccggctaccgtcgtactgggttggatcccaaaagagcactggatggcact

gtcgaaggactgggcctaccctacaacgcccgcggccgacgtcggtagtcgtgtcacgggaacaagcttctctcttcgagatctggcccaagtgtatg

ccctcaacaagacacggtgccccgactcgacgtatgcggcccaatatgctctcctccgaatggaagccgtcgtagccggctggatcgacgttccgaat

cagaccgaagtgattctcgtggatcctttggatgacgccacggcgatacaaacgctggtatctgacctgtcgaccatggaaaagtacaagattcagca

ggccaaaaatcttggatacgtaattcccctcatcagtgaatacgccttccggactacgggatactactggcgcagggaccaagcagagttgcagatgc

acgttctgaaggctcaacttgactacgacaggcttcctaaaccgctcaagctcagggcctctcatggccctgcaagtaccgagctgatcgaggcatgc

gtacgagttatcaacgttctggaggacctgaaaatcgatttcgacctcgcttttggtcaaggctacgatttcgagctgctgaagacagtcttcgaggt

gatcacccaagatcccttcaagtatcaccacgcgagtcgattctatggcgtggcttctcctacgccagccgagtggtccatactcgacaaagccaggg

acatggccatcgagtgtgcggcaatgggtcaagcctgcatcacaatgggcgtcggacgtggcctactctccgatattacggcccttgaagagtatgcc

gagctcaatcctgcggagcagcgccgctgcgtgatgatcctctccgaatgtaaggatttcggaagattctga

>contig_21.contig_21.g745.t1

atgagttcccagcacaccgcccccgctgtcgagtccgcctacgtgcccaacggcactcttacccgcagcgacgcggaac

aggccctcgctgccgccgccggacagcagcacggcagcatcaacggcagtgccagcggccctctcggcggaggctcctccatctccgccgacgatgag

gacaatcccctcaacgcgcgaggccgtgatggaacctggcccgtcacagccgataacctcggtcgcatcccacaggcagatcagctcgcgcccggcat

ccgactccccaacaacacccccgcccaggatggacacgatgacgacgacgagtatgaacatggacatggcgttgcgccccccgatgggatcacgccca

gacggaaaataggcccgtcacacgtcggctttttcggagaagccggcgaacctatcccgggtgtacctccggagctggatccgcgagcggccaaattc

aggcgtcccacatcgcctggtgccgcttccgtcttctcgctcgcggtacaacccccggcctctcccaaggcggcacccatcgacggagacgcagccga

cgcgggcacgtacatctccacacccggcatgacttcctcagagcttccctctctgtcttcgcgtatgagcttcacgctgccttcacccagcgtcaaca

gctcgtcggcttctgtcttctcgggcccgcccagtgcttctctcacaggtgggctcggcacgcctctaggtacaggccccggcatgagcacgtacagt

ccgacgatgacgggaatgcctggcatgggcgttcaggctcccggtgttgcagccagcattgctgccacggctgggtcttccagttccgcctcgagcgt

ggggaactcacgtccgaactccattcgcgctccttcgcctcccatgagcgtcgtcagcgcgccaggaagcgacgtcagcatgggtcctccctctgctg

cagcgtcttccgtcgtgcctcctccaccacactcggcttcctcaggcagcttctatcggacatccgggtaccaggccggtccaagtatgctctctcga

caggctgcccttgtcagtcaagccccttccgaggtgggcagtgtcgagacaacctcttcctttgacgctcccctttccagcagggacggtggcatgga

cacggacaatgatgcgggccgccattacgatgatgaagagggtgaaccagacggaggggacgtcatctcgcgacgggccaacaacattgaacgtcaac

gcatgcgggaacgtgagcagttgggtggtgaggctggtgtcggtggactttcttcgcgtcctcctaccattccctccctcgcaactctcatgcgccaa

gatcgaacagcgggaggcttcggatccgggacgtccctgctctcgccctctcggccgggcagcacggctgcgacgattggcacagcctctccgcctca

gtccacggtatcgagctcagatgatggacacgagatgtcatcggcgaggagagacagtggcatgggaggtagtcacgatgatgccggatcgcttctgt

acggccattcacagatgcagaccccgccacttcggccttcgcccgccttcgcgtttggagccggtgcaagcaccgtcccacctccagcaccatcatcg

acgtccgcatccagagccggctcgagtgtcgcagcggttgcgttctcctcagccgcgaggggccccggcgagctggacgtgccctcctcctcatcgat

cggcgacttcgcgcaccgatcaagacggtcctcgatcgagagcacagagacgattgcgagtagctcgcaccagcctgtttcggcgagtagcggcgcga

gcgatcgggatcgggatgaacgggagtactggcccagtggcggggctggtgctggtgccggtgccggtgctggcgcggggcctggtggggcggaggat

gagacgatgggaggtacgacgggggctgatctgagggggatgcgactggaggagacggtcttgctggacggtccgcagatcggatttggcaagccggg

cacgccgacgggtgaggagcggatgcagttctga

>contig_21.contig_21.g746.t1

atgggactaatcatcgtccttctccgatggacctatctcgtcttctcggtccgagccgcccacaaggccatcaaccaat

ccgggtccagaagaaggccgggtgttcaaccgtcgtctgctgcaaacggacgccgcctgcataattcgaattcgaatcctcgcagaggcagaccagcg

cgaagaaagcagcaggatcggtctgacgtcttgcgaacgatactgacccgcttcttcgtatggatcctctatcgacgcgcggagggcattctcgacgg

catcggaaagtggattctgttctacgacacagtcaagactctcttccttttctggcttctggtcagccatgacttcgctgcctcacaactattccata

cggtactggaaccgataatccagccgtatgaacctctcatcgacttagtagccctgcgcgtcattcgcggcttccacgcatttaacttcctctccctg

ctctttgtcacacatctctcccatttgattccccccactctgagcaagggagcgcaaaccgctttcgaccgaatagccgactcattcgcgtggcttga

agccaagcttacagagcgacgtttgcgagctgtttcagtccatgacttggacgatgcgatcgaaggagacgcgccccggcggtcacgatcgggctctc

cgcctcccttcctcatcccaccatcccgtgcagctctccatagcgcgcacgcaactttaccaggctcatggcggccccttcattcgccagagaaggct

tcccgtcccggcctccccgacgcatggggagcgtccacatctcgagcgggtcccagcactgcgctcccaacccgccgcatcgtttctaacggaaacgg

gtccatgcccgcttccacatcggcaagcaacagcaggacaaacgggtactcgcacgcaacatactctgctcgagccctctccaaacttcgtcaacctc

cggcaaacgacttctcgccttctgtgccaagggtccgactcatgccggactggcccgattcgccgaacgcgtcctcgtccgacctcactgcgagcatc

gagaatggagctcagcttggacggccgccgtcgcctccatatcagctcgcacgggctgccggtctcggttctcactcaagtcaaagcggactgccccg

gccgtcagacgccacgaggatagattcttcctcagccaacgccacaccgcgcaaacggaagaagcgcgagtctctagacgagaaaccgtacgacgagg

attcgtcagcgggaaatgcgaaggaaaaggactccattgcatccaagtccaagccgagaactccgaaaagggccggtaaagctagcgcctccactcgg

gtggcgaagagtaacataccggtgacagcaaaaggaaaggaacggcagaggccctcagtcaaagaagaagaggccgaagatccggacagagcggagat

ggtcgagccggcccgaaagcggacgagaacggtcaagaaggaagcagcgactcaagacgagatggaaatcatggaactcgcaccatcctactcaaccc

gaacactccgagctcggcccaatcgcggaagctcaagcaacacacccagcatcgcctccccacctcctcccgaaaccaacgcacgcccgacgacaagc

acgagcacgagcacgaagacgccaaggacggcggtagctcggaagacgaaggacaagccggcttcacctgccaagacgggcgctgaggggaagaagaa

gttgagctcgacaagtgcggggacgcgtacgcggggattgaaccggcctgatggtgcgggtgggagtggtagtgcggctacgacggaggcgaaagggt

caggctcgtcaggtgatggtgttgtttcgcgggcgaatgggaccggcacggtacggcagagtcgacgcacttga

>contig_21.contig_21.g747.t1

atgtccgccaacaatgccgaagatcctcccgctctcgcggcttctttgcgttggctcgaacgccttcccgatgctcagc

ggcccctcgtcgagcaggccattcgtgcgcagttcgatgcggatcagcaggcctctcaagctgctgccgctcaagaggccgaggaagaggccaatcgt

ctcgacctcgagcgccaggaaatcgaagcagacgagattcgtcgcgctgctgctgccaaagaacaagaggcacagctggctgcggccaaaaatctcat

cgatcttgccaaggcacacgatcttccctccgagggcaaaggcaagggccgacaggtcattcaagacgatgcaatgtccaacgacctcgacattcttg

tggcgaccccctctcaaaaaattcgcgacaggattcgtgcatgcgagtatatcgacctctggcatctcactgcggaggggatggcagctgccaccaag

tcgaagatttccggggacaccagtttcgagctggggtcggacggtacgttcaagatcaaggactcggtaaccggcttccgaccggatcaagacctctc

catgccctcttgggtcacggccaatggtcactatgtgcggttgatgcaagacgaaggggtgccggagaacatcattcagtcaatgattcggctcaatc

acttgctcacgaaccatccggacttcgagcgccacggtcaagcaattcggatgtggcaccagcatcaacgccgtcagtgggtgatcagtggtcaccta

aacaccgatggtgtccgcttcaacctcggcaaacccaatcaaaatcactacgacgccattcggttgcgactgctggaggagcgcgcggagcgtcggct

ggctccggtcaaaccgcagccggtcatccattcacgacatgcgctgcgaaagagagggtcgacggcaagcatcaacatgtcacccgcggcgcgtcggg

acgaccagtcttcgccgactcgctcaagccgatctgcatcaactatcagatcaaagggtgcacaggcacctgccgcaccggcggagttcatcgatgtg

ccaactgcggcattggcgggcatggagcctcgtcctgctctgcctgagcctgttgctgtcgatcatcatctcactctgggttccattggctggtccaa

gcgaacaccattggatgctgtccacttcgaagccgagcttcggcgtcatggactactgtctcgctatccttctctttgttccgatcttgccgatggct

tccgcattggcatccccaccatttcatctaccaccaccttctccaatcacggcagcgctcgctctcatccacacgttctttctgcggcaatcgctcgg

gaactggacgctggtcggtatcaaggcccattcagcgctgcccaactccagctgctgctcggtcctttccaaaccagtcctttggggctggtcccgaa

gggttctgatgattggcgagtcattcaggatttctcctggccgcgagacggcaacagcatcaattctcatctcagcagcaacgattggcccaccactt

ggggtgccagccgcgatgttgttcacactcttttgtctctccctgtctctgcacaaggtgcagtccgggacgtcgccaacgcgtttcgtctggtcccg

ctgcacccctcgcagtggcctggcattgttgttcgtggtctcgatgatcgcttctacatcgatcaattcctcggctttggtctggcgcccgccaccgg

agtatggggtcgggtggcagatgcactcgccgacatccttcgcgcacacgaccttcccttggtcaatcaacttcgacgtcaagccgccgaatccattg

tggagcggcggacacggggtggcgtcgcttacttcgccgatggcgaaggaaaagaacatgtggaggactaccacttccctcttcaaaacctctccgcg

cttcgacccaccgagctcgtcacctccctgtcaacgatctctgcgatatcagacccgctcggcattccttggaagtctgagaaagatgtggacttctg

ctcggctcccgtctacatcgggtttacctggtacatcgccgagcgttgcgttgcgctgccggacagcaaacgcctcaaatacctcgcagctcttgctg

attggggtcagcagccacggcagaacctgcgcgctgctcaaaagctattcgggcggcttcagcacgccagcttcgtcgttcaacaggggcgcaagcgt

ttgggctatcttcggcggttgcgaaagacatggcgtggtggacgtcggcgctttctcgtcccgacttgcgccgtagctttgcgaaagacgatgttttc

gacaaacgtttttgtcgcctgcgatgcttccaccacccacggcattggcgtggttgtcgacggcgccgaactgttcatcccggtacgtccaggctgga

agagccagggtcgcgacattgcctggctcgaagccattgctctcgaagtcacggtacaactgctcgtcgaccaaggcctggcagactgcaggctgcgc

gttctcacagacaactcttccgtcttcttctcggagcgctcaggtcacaataggaacgcgtccgtcatggaggcactcgcgcatatccgtgatctcga

agcggcatacaacattgaagtggtacccgtcctggttcccagcgcgtcaaaccctgccgatggaccctctcgaggttgttgcacctcacctgcgcaac

ttcgtccatcggctccacgtcctccccttcctccaccaccagcacgtccacccaccacgccccccgcgtcagcctctgctgctacgcgcacaacgtca

aagcgccgtctgcctgtagcagcagccggatatctcgaggtcccagcagcactacgtctcctggactgggttcctccgggctcatcacttctctcagc

tcgggacccacagcaacgacgacgggtcctcgccgctgcctgggcggagtccacgctagccacgtatggaactggtctcctgcgctggctgctctggt

gtgaacagcacgctcttcctgcttccgctcgcttccccgcccatgtcggtgacgttgtggacttcattgagtccgttgcgggtcatttctccggcggc

agcatcagaaactgggtttcgggtctacgcgcgtggcacgccattcatggctccgcgctagacacagttcacctccgcatcgtcactgcccttcgtgg

tgccgaccgtctcacacccgagtcctcaaagaagccccccccgagcaccatttcgccttcaccacctctgggcgatccggcgccatgttcaacccgac

agtccgcttga

>contig_21.contig_21.g748.t1

atggcgggctggtatcgtcgattcattcccgactttgcccgccgcgctcgacctctggccgaccttagtcgtcaagacg

atccatcgactcctttcctctggttggcggcggcggcggctgcctttgacgatctgaagggggcgctcgtagccgcaccggttctccgccggccggac

tatggccgtccgttcattttgtcagtcgatgcctcgtcggcaggatttggggccgtcctcgcacagacggatgacgacggtcacgagcacccggtcat

cttcttgtccaggcaaacgaaggaaggcgagagcaagctgccggccacgcatctggagctggtggcgctgcattggtgtatcgggaagctgaagcagt

acctggacgtatcagagctcgaggttaggacagaccacagggcactactgtggttgtggtcgatcaagtcgaacgagcttacaggtcgtttggacaaa

cttcgtcttgctctggcgccactggaggggaaggtacaggtggtgcataaagcagggggggtcaatgttgtggctgatgctctttcccgtgctcccat

ctcagccgtcccagaagcagcagatcaagaaacatcggtggagatcgagttccaagcgcccgagtctccggaccgctcggctgtgcgtggtctcgacg

ccaagacggagagcgtggctcacgatacggatcccagtgcctcatgggacgaggactacggacaggacccgcattggcggacaatctggctgcgcttg

tcgcacgaacagccctccctgccgtttgtcctcgaccagggacgaatctggttgccgaagccgggtggggacgagggcaagcggctttgcgttccgca

ggcacgggtattcgaggtgttgcgagagtaccactccagcattcgagcggcgcacccgggcgtgcacgggacggtcgaactgatccagcggcacatgt

tcttccatgacatgcgcgcgcaggtgaagctgttcattctggagtgctacgaatgccaaaccaacaagaaccgtcgtcaacggaagcacgggctcctg

gaacccatcatggagccccacgaggtttgggcgtgtctcgggatggacttcgttaccgggcaacctcgtcttttcggcctgcacgaggacgcggcggt

atccacgggatcgcccggtcactctgcttgggttgacgacctgcgagcggcacgtgtggaggctgggctggtgatcgagatgtcctccgcccgacaga

aggcgtggttcgacgttcatcgtgacgacctggtactccaggagggggaccatgtgtgggtcgacctccgtagttatcgggcgccgctggattcgggc

gcggatgcgcgacgcaagctttcagcgaagtatgccggtccctttccggtcgtcgaggtggtgggaaagcgggcgtaccgacttcgtcttccaggctg

gtttcgcgcccatgacgtcttcccaatcgatgcccttgttcggttctacggcgatccttccaaagtacgccctcgcccggtgcatggtctcgacagca

gcgagggagagaacgagcatcgagtctttggatatcttggccgccgacccaccctgttcgacgatggcaagacgttcgattaccttgtggagtggtct

cacgagcctccctcctggcagcaagactctcggctgcctcatctcgcctgggcgacgcgagatttcgacaaggcggcgcgggaacatctacatgtcac

ccgcctgcctcgctcaactaccatcatcctcccgggacatgaggaggacgcgcgcagagccgatgcagaggaggatcaggctttggtggatgcggacc

acgtcagccctcgcggacgagggcagcactcggtcgcgacgtatatccctcatcacaaccgtaccgcccacctcctctgcgccgacctcgcgcccggc

ttcagcgtgcgtaacatcggcttcggcctcggcatcgtcgacacctgcttccaatcctaccggcccggacggcacgtcattgtcgagcctcacccgga

cgcgcttgcctatcttcgtcagaagggatgggcggatcgggaaggggtcgagattgtggagaagaggtgggaggaggccttgttggatcctgacttgg

ggagctttgatgtcgtctatttcgacacgtacagtcaggactacaaggatttgaagaagtttttcgatgaggtgcccgcactgttaaatggaccggac

gcacgcttctccttcttccacggcctggcagggacaaacgacttcctctacgatgtctacacccggctctccgagctcgacctgcaatccatcggcct

ctccacccaatggcacgccgtacacccacaactgaccgaggaagtctggaggggcatccgacgcgagtactggagcttgcctagatttctgatccctg

tctccaaaatgaacctatag

>contig_21.contig_21.g749.t1

atggacaccccgcgtgcgtacgtcgcagacgagtcacaggagcagtcaaggatgcttcctgatatcgccggtgccggag

aacacagtccggcccagccatccgacgaccccaatgatcagcgcctccaacacatcgaagccctcgaagccgaagacttggcggcagcagaggacctg

caacgaagggaggtagcgctccaagcagccaggcgagcgcacgaggagcaaaggagggcccgtcgaaggaccataaatcagctgcggacggacgagga

agggccgctagactctgattcgcaagtgggctcgaccactaatggggttctgaatcagatccagtcccagctgggaacgctcacggagctcatctcag

ccaatcgtcgcgagatgttggaacgtttccgtggtctggagaacgcaggagacgcttcgctggcacagctgaggccgactatggcgggcgcaaaccca

gcgcctcaggtccagttcgcccctcaaccgccgccggcacagtttccccagtttcagccacccgcgacgggaccggctcccttcctcctcggcagcgc

cggtccgaggctcaacgatccgcaccagagggtacccagaccttccgccatcaccccgttcacgttcggtcagtctgctccattccaacagcaacagc

ctccggcgggtgcaggatttgtcccacatgtcggtccctttcctccgcaatggcaagacggaggatttgcggcaggacaggttgccaacccccagccc

ggccggaactggcctcctttcccgcctcagtacggtcaggctcggcagtctgcagcgggcgcagcaaacgccgcagcatcgtcggacatggctcagtc

cttccgacggaacgattatccaaggtcgaaggacattggacacttcgacccggacgatgagaagacgaaggcttacctatggtggcagaagctcgagc

acttcaccgtcgtttccagatacggaaaggcggagacccttccagtcctcatgagctgcatgcagggcagagcggaaacctggatctcgtccatacag

cccatccccaccacatgtgcgcagtggagaaaggcgttcctgcaggagttcattccttcggcaacgaaggcccacatggatctggatgtgcgcaaatt

tgagccggataaggagaccatgtcggcctaccttcatgagaagtacagtctggttgggcagtggcaggtggctaacgctctggaacgaggtctgttgg

acgaggatcgtgatgcgattcgggacatagttggacgtatcagtatcatggacctcattttctatgtccatcagggtctcccggcggaatggaagctt

gagaccgacaatgtcaaggcttccgcccagtcttggtccgactatcgatctgcgatggtcgaaaaagaggacaccgtccggaatgcgcacgctgagag

gcaggctttgatgcgcagctcaatctcacgacagacgcccagagcggacgccacttccaagttcaccacccgggccgttccaacggtcgctcgaccac

agccagacaacgagaagaaaaaggcgagagcggaggatcgggctctgggacggtgttacctgtgtcagacggtcggccactttgctcgcgactgcccc

tccaatccagattcggcacgcggtgttcgaagggacgagcgcctcgcagcacgacgtgccatggcgggcgaggccatcccgcacagcagcggcgagga

ggacagcggcgaaagcgacgaggaggaccgtgttggcaccgtggcgatcgcacgtcgtatcggctcgctcgtcgaagaggtcggtctctcagctctcc

ccgacgacgaccaggctttcgttaggggactcaaatcggatcttccaggttctcgtttccggatcactcaggccggaacgctggcggtttgggtcgac

ggctccccgagacgctacgacatctgccccgactcgggagccgatctgtccaccatctcttcgtcgacactcgcagccgtctcgccgggagcgaagat

cctggccccagcaaaggtggagatccaggggtacgcaggaggtatggcgcagaagccaacaggagtagtgcgtttgcctctgcacttccaggctgtca

acggaaaacgagtgtcgagggaggcagaattccatgtggtgtcagagtgcgccgacggatggttattgggggcggacaacctagcgcattttggattt

gttgtcaacttgcgagatcgggtggtcacgttggcggacgataataccatcaagatccccctccggaacggaaagggtgtcgacccggccgctcgtct

tctcgtagtgtcccggtccatcaccatcccggcacacgcaagcgcgctcgtcccggttcgccctccgcggcaagaagcgactcttcagccgtaccttt

tccagaatgacgatctttccgaccagtggatctccgcgggaaagggtctggtaggccctcaaacccttggagtcctcttcctgaacgcgagcgatcga

accctagcccggagcagaggaaggccatcgacgagaccctga

>contig_21.contig_21.g750.t1

atgtcggaggcacccacagacacgctcgacgcgcggctcgcccttaaccgcactctcctccgtgtcgtccaggccggcg

acctggccgctgctaaggaatgtctcaaggcgggcgccgatgcatggttcgaatcggacgaacaggacggtctgggctggagtgctctccatctggct

gtcgttgccaattctcccgaacttctgcagttcttgcttcaacgaggggccatttggaacgcagttgacccgagagggatcactgccgctcaattggc

ttggtcgctcaactatgtggaatgctatcagcttctcttcgaagagggcgtcagacattcgatgctcatgaacatgctagctggggacgacgacgaag

ccgaagatgacgctgaagacgaagttgacgcagagtccggggcgatcaccctccggcccgaaggcaacgaactcgccaattccaacgccaagtttctc

cagagccgactcatctttgttaccgataaagatgggaaggaaagagtcctcgacggggatagcaacttggtcatggcagcttgggaaagcgacatcat

ggaccgttgtaaggagctgcctgagggccctgcattggttacgttcattccctga

>contig_21.contig_21.g751.t1

atggacgtagatgccattcaaccctcagccgcgggccgtactattgggactcccatcgtctttgacgtcgacgacgaca

attcccctccccgtgccactgtcaagcgagctgctgcttggagtgagtttgaggagctgccggatgaagagaaggtgcagtgcgcgcactgtggagag

aagctcgtgtacaagggaggcaagactgggacttcttccatgggacgtcatctggaaatctgcaagaagaaggtcaggctggaggcgggacagcagac

cctctcgttcggcctcatcaagcttccccacgctgccaagctcaagatacacggcatgatcgagcgttggatgctccgcgaccagcagtccctcagcg

tcttggatcacccggacttcaaggccctactcaagactcttcagcctgactacgattgcccgagccgcaacgtcatgacgcgcaagattagcgtcgat

tacggcctcctcaaaactcaggttggcgcttacctccgctccctccccggcagagtcgctctcaccacggacgggtggacggcaagaaatggggacca

gttcctgtcactcacggcccacttcatcacggcagactgggagctacagacgatgctcctagacttcgcccctttccccgtccctcactctgcagaga

atgccgccaatctcataaccagcgcgcttcaagactatggaatcgagaagaagatctcggcgtgtgtcacagacaacaccgcctccgcctacaacatt

tctaccatcctcaagcgtcgcgctgcgccggctcccttcttcccgtctcgttgcgccgctcacctcatcaacctcatcgtcaagcacggtcttcagga

tgccgggagagacgagatctttgccgcgccccgggccttcgtctcccttctccaccgctcaaagccgacggaaaaggcgctgatcgctgcatgcgagt

cagtgggcatcaagtacgctgtccctccgaccgctatggaggtcaggtggaactctacgctggcacagctgcttgggctgcagcggctcgagcctgcc

ctgcgtcatctcttcgagagcggccacagcacttctgcgtggtctgagcaggtctggcttgccatgagggatacgatcaagatcctcaacggattcaa

ggacgtcagcgaccacctgcagggccgcagcttcccaactatcagcgctgcggccgaaggatacatcatcatgaacaagggtctggaagagcttcgag

cggcttcgactcttactccttgggggatggaggttctggcgctctttgatacccggcttaccgagtaccgagagcatctgattgatacgtttgtggtt

cagtgggccaccattctcgatccctccatgaaggctgaagtcattcgtgccgacggagggaacgatcttcgatacatcatctcggaactcaagttcgc

tgctgcggcgcccgcccccatcactatcgacgccgggaagcagcttgagctctacctcgctgagccccccatccccacccctgctccctcagattccc

ttcactactggtccgtcaatcagtctcgttacccaagcctggccgctttggctcgcgatcttttggccattcctgccaccagtgtcccttcggaggct

gcattttctcgtggtggagaggtcatcaccaagcgcaggaaccgactttcgggaacgacagtcacccacatcatgtgtctcgactcctggatgaacgc

tggcttcaccaccatcaaagactttgtcctcaacgatctggtcgaggacgagtag

>contig_21.contig_21.g752.t1

atggccgacgccccgaaccccaacccagctcctgctgctgctgctgcggccgctgcacaacagaccggcctcgctcgcg

cctttgtggaggaactgcggctattccgcgaagggaacgcagcgggcgacaagcaacaaggtctcccgtccttccaagtcgaccccgaccatgcagac

acggtcgtcgagccttggccaggcgcatccttcaacgtcccggccgacatcgtcgagctcgtcaggacgggacgccgccccccactgatctggttgac

ggtcgaggcgctagtcagttctgaggacggggagacgcgcaagccgctcaccttccccctcaacaacaagaaccaggagcgcctcaacgacgcttacc

gcaaagactccttccttccccgcggcgccactcaccaagccatccaggtgctggccagcctgtgtcgcgccctggggtctcccaccgaagacgccgac

aagaaccatgccgtcctcctcgaggagatgcacctcaccgtcatcacccgcgcgaccgacgagcactgggccgtctggcgcctctacatcaagcgctg

tctcgaggcaatgtgggcgaagcgagagtcaggtgtgggaatggcgtttgacataggaaaaataaacgaatcactgctccggaaggccgagcgtcagt

gcaacaagccgccaggatacgtcgacgacttcacaagaacgacatccacactcgtcgccgatctcttgcgcgccgagggcgacaccatcggcaaggtg

ggagcggccatggaggcgctccgcgaccagagcgtgtacggaccgacagtagctccgctgccctcggctgctgcccctgctccatcctcaaagttcca

gcccacccagccccaagcctcatcatcctctggcgggccttccgtaccggccctcaaacgccgagagcgcgaatgggacgacatgccctttcagcggg

cgcctccctcctactctccccgggacggaggcgcaatccctccaaaggggccccgtgccggcagcgaccggcccgaccgccgctctactggacggcct

gctgccgctggtgcgaccacgacttccgagactgccgacaggcgttctcccccgacctcgagaatctcggaaagctgggatggcgttggaagggcaag

tctgtcgctttctgcgaccgattcaacaggtctatcgattgcctggcagcaggaaactgcagctggggccactactgctcgaagtgcggcgctgacgg

tcacccagcactcaagtgtcacggctccatctagttctggaagcgcgtggcacgctttccgggtagcgcgactctctcctttagttgcgcagacgttc

gagaccgcgcttcgagtacttccagcttcacatcaagttcggctagcccacattgtcgagggattgaagacaggcttctcggttggtcctctgtccct

cccgaacaagacagtcattgctcccaaccacttcaagaacggtcagatcgacatcgtgcgggcgtggtgcgacaaggccttggaggacgggtacgcac

acggtccatttgcccagtccgacatagagagggaggtgggacccttcatttcgatccctctcctcattgtccacacgccagcaacgtcaaccaaacca

gaaaagaaccgagtgtgttttaacgcctcgtggccgagactcgagcacacggacgccaacgcacaagcaggagaagggatcaagtctatcaatgagga

ggttaaggatgagggatgggaatgcgaatggtttctattggaggaagttaagaccttgctttccacggctactacgacggcgacggttatgggtttcg

atctttccgacgcctaccagcaagtcccaaacctcccagcccagcgccagcggtttgtcttctcggtggagggtcaattcttcgtgtgggtagtggga

atgttcggcatcgcgacgatgtccgcaatcttcggccaactctgcgacgtcttgtgcgcgtggttggagcaccgctttccctctgtgcgagcgcgaca

cttcgccgacgatcacatggtgatgcgggacgcgccggtactgacggaggacgaggtgtacgaagaggtcaaatcttttggctggaaggttcacccaa

caaagcgctttgggtggacgcggcagtttgcctcgctggggtttgtgtgggatctagatgcggggacggttgctataggagaggagaagagggacaag

tatctgcggaagctgacgagcttccagaacaaggagaaggtgacatatcgggacgtttcaagcatgttgggcactctcgttcacacctgcgcggtctt

cggtgagaggcgctcccacctcaacgcactctacgcactacgagcccgtttccatcgatctaatcctcaccacagcctcgagctttcgactgctgcaa

ggcgagaggcaaaggagtggactgcctttctccagctcccaaacctctccagatcgttccacatccccgcccggtcatttgagcacaccctgcgctcc

gacgcctccgacatgggatgcggcgtggtgattgacgggcgggcaagattctggccgctgccaggatggatcgaacgtcccgaagtcgacatcggcgt

catggaggcatgggcgctccaccttgcccttccagccagcattgcggagggagcgagggattgcgtggtgaagttcgaggtggacaaccttggtgtgg

tttttgcggtgcgcaaagggaggtcacggagccggtggaccaaccggtgccttatgggaatgtcagagattgcctcacaacacggcatctcgatggcg

gtgtcgtacatcgcgtcggcggcgaacccagccgacgcgccttctcggggtgactgcacgcagttcacctcactcaccttcgattgggtggcaccctg

gcaggagctccaggtttccggggcgccagcacgtactcccacgagttcaccttacttccactgcccgtctctggtatgtcgggcgcccagcgtcgcga

tcgtcgtctacgatccgacaatccgacctcgaacccgtcgtctcccgctgcgttcgagctcagctctcagcgatcagcgtctgctctttcctctctca

ctctcccagccactcaactctctccgaccataccctcctcgtcccgccccactctgcctattccagtcccttccagctccgcacctcgccgctctcga

tttcggcacatcccaccctcatcccttacgactcggtcccggtccaccctcgcagcagcgtgcgttggagccggcggctcccgcagacaacccctccc

agtccttgacccagatcgtcccagcaagaagcctcctcacagcggacgtgtggtccactttgtccgtcgacaatgtggccacctccgtcctcacggcc

gacgctgcacacgcgctgaagcctcgagaggagcttgcgctccggctgactccggcctcacacttggtccagatggcgcacgggtccgcggatctggc

ggttgctcgcgctgcgtccctgcttctgtggtcgtccgtgaagacaaagacgcgtgtaaagtatgcgggccatatcattgcatttctctgctggtgtg

acgagttgggcgtacccctccacttccggtttcctacagcttccaatattcttctcctctacttgcacaaagacatgacgagcctgcgagcgtccaca

atcgagcaccgcacgcacgcacttgcctggtggcacaaggttcagcgcatgccgtggtcactggagcgcaaggagcagaaggcgttccagaaagccgt

caagatcgagtcgcttccccccctcgagaagcgtcgccctgtccgcctgaacgacattgtcgtcatctcccagtatcccaagcccaacgatcgcgccc

atgtggctatcgtagccgcggctctctttgcgttctacgccatgtgtcgcccgggcgagttcacggtccggaacggcacgtcaccccatgaggatcgt

cctagatggacccacctcatcgagcaacctccagtatccccaggtggtccggcttcgataattctcgcgcttccatctgagaaggcgcgcggaaacgc

aggcttcgaccgaatcgtttctgaacagcgcaatctcccgaatctttgtccggtgaaggcggtgcaacgtcaccgtgctctcaatgcaccgcgtgctg

acgagtcagcagacgctctgggagcgttctcgtacgtgggagagaacgggaaccggtacgagctcaccgagggtctcttctcgcggaccatcaatgct

tggctttcggcagcggggcgggaaccggtgacgggacactgcttccgtatcggcggagcaactctcttctttgcagcaaataaaccaatggaggatat

tcggaccagaggagggtggcagtccgatgcctacctcacttatatccgcgacaactacgtacgccatgcagcatcctttggcgaccttgcgcccgacg

gcctgttctacggctga

>contig_21.contig_21.g753.t1

atgtcgcagcgctctgctccctccatctccagctcgacaggtcgccagatagcggctcttcaagacgaggcacggctca

tccagtcggatgctgccggtgcgggcgaggatgttggcggagatgccttcatggctgcgggagaaggcgatcacaaggaggacgacaaggacgacgac

gccgaccaagctgcttcggcaaccgctcatcaagaccatcatccactcaactcctacatccgtggaggcgaacgtcccgcgcgtgatgttcagcgcga

catcgagcaaggcatcatggactcgctcacggacccttaccttccctccaacgccggagatcagtccgtccaccgatacccaccctctctcaaccttc

cgaaggtgtactcggtcaagggcaaggccaagaaggctcggtcgtcatctcactcggccgattcggatgaggaggttcgacgcatcgtcgcgaaggac

ctggaagaagggcgtccgataacgcctgagcctaccaagatagaggacctgttctccctcttcgagcagctcaccccgacatctcgcaagaccgccct

aggtcggttgttgcctgtcgcgaaggggcccaatcatgcggaatcggacagcgagaccccccaggcccaggacaacccctatcgacgtcctgtggcta

aggctcctcctccgacatcgacaccatcatcaagcaaacacaagctgtcgccgcaaggccttccatctctcaaaaagattcgacatcaagctcctctg

ctcggccgctctgacagcgagccttcactcatcaggggccgacccttccagccaaaggcgaagagcacggccacggtcgacctcccctccctcgtcgt

catcaacaagctgcgtaactcgatgtacgtaccgctgtggcatttcacccaagaaggcatcgtggagggaagagcgcgcgatgcgcacgtcgtacaca

cgacaactccgaccatcgctcaactcctggactcgtccggtccaaacgctcagcttggtcagcacaagaaacaagacccggcgatgtcttacgctgag

atgtcgtcggcattcaaggccttcgacaagtccatggagaaggtcgtcgcggaagccaaaggagaggtcaaggaatggctccaaatcgagaaggacgc

ctgggtggacatgtggtcgtccgttcgcgatcaccctctgatagggaagagtggaggatgggcgatccttgctcgctacgtcgacctgctccgacacg

actactacgatgccccatacggctatcgactcgaccctgccacttggcagtctgatatcatggcgcgggtcaagatgttcttggacttcaactccatc

atcgaggcgtcgtcttcttcctcgggtgctcatcaagctggacacggcggcaatcaacaggcctcctcctcatcgggcaagtccgctcagaagaagaa

caaggcccaagggggcaagcagcccttttcatcgggcgcctcgactagtcccgatggtcttggcgcctgcttcgtctgcgggtcccgggttcggcaca

acatcaacgactgcaaccgagtccccgaaggtcgagagaccgcgcacgccaagcgtgacgccagccgcaagcttgtccgggcctcggatggcacaact

atcatctgccaccgattcaatctctccaacggatgcatgtcgactgctccaagctgctcggccggaaagcacgagtgctccatctgcggcaagaccga

gcatgggtgccaaggttgccctgccgtcaccaagccctctcaagcctcagggctttga

>contig_21.contig_21.g754.t1

atgtcggacgcgcaggaggcgttccgggcgatcaaggcgcacccctctcagcttccggggttggtcattgcgttgccaa

aaggaggatttggcattgacacccgtctgccctttgggctggcttcggccacgggggtctgggggtcggtggcggacctcgtcaagatgctgatcatc

agcacaatgcccgcactccgggtcatcaaatgggttgacgatttcgtcttcatcaaacccccggactcggcaatcacgctcaaggacatccatcaagc

gaccgaggtgctgggtttcccctggcacccggtgaagcagtcggacttcgactcgaaggttacttacctgggattcatctgggaccttcatgcgatga

cggtcaagcttccggattcgaaacgggaagcgttcagcttacgggcctcggctttcctgtcgtcttcacctcggtccctcaaggaagtgcgcgagacg

tgcggtgcacttcagcatgtggcctccatggcccgggacctggctcctttccttgcggagttcttcggtttcatgaaggcttgggagtccaaggagcc

ttatcatcgacttcacgtccctgctcaggtacaagacgaagccaaggtttgggtgcgtgccctcaggcacgaaatggtcaggtcaattgccatgcctt

cgaaggagtttcctcacgtcttgtacgtcgacgcgtctacaacctggggcgtcggggtgacgtgtgacgatcgatgggcggcttggatgttgctctcc

ggttgggattctgatggaaaagggattggctgggccgaagcggctgccttggagcttggcgtccggcaggcggttgcgatgggcgcctcggactatgt

ccttcggatcttcagcgataacaaaggtgttatcggggctttcaggaaaggtcggagtcgaggacgatcggccaactcgatcatgcgctcgctcattg

cttttgcgatgcagcatcatctcgacgttcgggtcacctacgtggctaccgacccccccttcgacccttccttcatgccacacggagacgttaagtgg

taccaaagacaacttcgggaggagcagaaggctcgatatcctcgcacaaggcggcagggggctgcgtccttggcaggaaccaaatctggccggcttcc

aactcccttcacccatcgagccccgcgacggggctcatcttcatcatcatcatcatcctcctcttcacaaccagcagctttgcatcaatcttccatca

ccaaatcactctctccatccgccctgacgagcctggctctcgttgggcgccccaagcctctcaagacaatcaagacatcgaaaccctcgtccttcggc

aagaagcccctggctcgtgatcgaatcatgtccatcgcccccatcagtggggacaatcctggactcaagacgacatcggggttcgacttcgacccgca

aggctgccactggcaggctgttcaccagatcctcgcgtcctcgctcgaggcttcaacgcgtgccaattatggcgctggcattgaccatttcatcgctt

tctgcaacgagagcggcatcgctcatcgacatcgataccctgtgtcccagggcttgctcctcgcctttgtggctcatatgagcgacaaggtcagggct

tcaacactcagcaactacatcaccggcatcgccacctggcaccggacctggaatcaacctttcgagaggggcgaggctgtcggcctggccctcagggc

tctatcgaaatcaggacctcccaagctgccccctcgcccgccggtcactcctgaggagctcatggccgtattccgcgttctggatgttgccgggaatg

ctcatcatgcagcggtatgggcttgtgcgttggtctcatggtggtccatgtgtcggttgggggaaacaaccacggccaacctctctcctcaggctgat

cgcaacgtgatgcggtcccactttcgaggtttctcgacgggagattcgggccacaggatggcggccttctggctaccatggacgaagacgacgaaggt

ccaaggtctggacaagttcgtggccgactgcggtggggctctcaacccggtcttcgccctccgccatcattttcgactctcgcctcacgttccggagc

tcgccggcatcacttcactcttcgctttcagggagcgggctgacaagcccctccttcctctcacaaagaagtcttttctcgagaccttcaactcggcc

ctttctgctgccggtcgtggttcgcatgtggggcactcgttccggattgggggcgcgaccatccactggcgtgctggtgtccccctggataccatcaa

gctcatggggggttggaagtccgactccgtacttcaatacctccgtcgcctggatatcggattggccagcgcgcatgcgctcacggcagcattcatcg

ggaatcccttgtga

>contig_21.contig_21.g755.t1

atgaccgagtccgtaagcgccatgaaccaggaagccgtgcgcgcggccatgcaggctgaattggatggcgtcgacgcca

tcgctcgattccggttcgacgacgagttcaacatctactctttcggtcaagcaatcgacgccattcccaatgcatcgccggggctgacggcgtctctg

cgcaaggttgcatacgcattcgatgagccggacacccacgccgaggcttttcaatccacaccatcgctctcgttcccacagtctcaagaacggtacaa

ggcacttcaggagcggctcaactacgacgagtacctacaatttacatcgagccagtcacagcagattggtacgcaagcagaatcccagtcgcagtcga

caacacaagctgcgtcgacgccatctgctttccgcgctcctccctgcgctcaaccttccaagctacagatcccgaatcgcgaacctgaagcacccaat

cccagacgatctcctcgcggcgtcacccagtctcgtcgtctctcgggatacgctgttggagaagagtcagaggagcgtagtcgcggcacccatcgttc

accagtaccaggctcgtcctcgaagccccaaggccaaaaaggttccgcgtcctccaccaagatgctctcactgtcgagcagcgactctgaggcggcgc

aaagcgagaaagaagacttcgactcaggccgccctcggcctcgcccacgacgcgctcgtccggcctccaagaggtccgactttgtcgttccggacagc

gactctgaggaatcggatgtgcctgttaccggatccaagcgaacctcgggtccagtccatgaagatgctcgcgagccagtttccaagaaggccgacat

cggctcttccaagaacacgtcccgacagcctcctgtcccgcctacacgtcaagagcgcgagcagcatgacgctgcggccaagatgaacgccccttcga

agaggacacgcactgagtccgctcaatcgggtgtagtcaccatcgaggacgacgatgatgatgacacaccccgcgctgggagagtgttcaacgggaag

aacgctcacaagcgcttcatggagaacgacatcgccatctccgctgccgccaagaacagcattatcggcgctgtgcaagactcggatgcctacgatgg

ctacaaatacctcagcagcgaatggacggacggtacgaagacgtgcgtgaaggaaaagtggtggtgcctcgtctgcaagtcctccaagacggcatcga

aggctggcaagcccacgaatcttcggacgcaccgaaaactttgtcccattcctccggagggtattgccgacgaggaagagatgggagaggccaatccg

gatttggaaaagcgtcgattcaagctcaaatgcaagccagcctcttttgccgcttctaccaccctggtcggcaactcctaccacggacctagccttca

cggatggctgaatgggcagcagcccatgcacgtaaagctgacgcgacgcctcggattggtcatggtaatccagaacgcccttccctttcaacatctcg

cctcccaagccatgcgcaaaatggtcaagtccatcgacgcaaaggcgacgcttgcgctcatgtctggcgccaccatccgacgcgaccttgccgtcttc

cacggcaatctggagtcggacatgaagaaacggctgcaaggtatcgacacactcttttccgtacagcacgacgcatggacgaccaagggcttccagta

ttcattcgtagccatgctggcaacaaacgtcgatcgagactgggagttcagcgagacgctactgtccttcgatgtcctcaaggagaagcacacgggag

ccacgttcagtgggcacttgacgaggactctactagccaacgacctcggtgacaagtgggctggatccgttaccagcgattccaccggtaccaaccac

aggatgatggacgtgctggagtacgctcccgagctcgacaaactccaaccgaagggacgtggacaagtcacgctggatcgcaagtcggctgctcaagt

gaaggcatcggtctcagctctgcctcgcgccagtatgcgccagtcgttccagtggaaggcaaacgagaacaagattctatgcatgaaccaccacatca

acttagcagtgcgagcgggctttgcgagcctgggcgtggcgatcaagaccaagacgcagcggaaggttctcgacatcaatccgatcccggccatcgtg

gtccaagacgaggagggtcgcactgtggaggtcgacttcgaacggtggtgccctgacgacccaccgctctcggaggcaccggcatctaaggcggtctc

tcttggagcggctcctctccctgcttcggaaggaggagggaagggaaaagcacgagcaatcgaccccgaagtggacgagggtgtcgacctcgacactt

catctgcctccattgtcgatgccgcggccaatctggcagactcacagcccgagccagacagcaaagacgatgcatcggatgaagacgagagcccaggc

gacgatgaagacgactatgaatcctcgggcgaagaggacatgggtccagaggcaggcgagctgctctccgacgacggcggcggcccagaggtagatgg

gccctccaagacgcggatctccaagaccaaggtcaacgccgtcaccaagctggaggcgttcacgacgtctatccacagaggcagtgaaaggagggacg

actttcgcaccaccatggccaagaagtaccacaggaggcccaagctggccaaagcgccctttcccccgaagccaaatgctacacgctggaactcccac

ttccggatgatcagggcggcgctcaagaatcgcgaggcgattgactcccactgtcgcgccaatatcggaaagaagagcgacaagctgggctcctacct

gctcaccgaatcggaatggacgatgttggagtttctgatgccgatcctagagctggcatcggaggcaaccaaggagctcgagaaggctcgtggaacgc

tatacatggtcctcgatcaccacgccagtctccgtgacaagatcaaggccaccgtcgccgaactggacacggcagatctcggaaaggagacgacggaa

tcgctgaagtccttcttggcggccatgagctcaaagctcttgaagtatcgcacccttgcgcttcacaatcgatacacgctcgtcgcagcgttgctgga

tcccgccaatcgtatggagctcttcgaattggcctacccaacgtatggaaaggaagctgagaaggcgctgcgggacatcatgagcgaatggctagggc

aggaagccaaacgacttcgacagcaacagcttgcggaggaaagacgcaagaagacggacaccgattccgatgaggtggcacggtatctcaacatcaag

aactgtccttggcgagactccgacgaaacaccgtacaagtggtggcgcgacaacgagaacgtcttccccaatattgcaaagattgctcgagtaattct

tggtatcccaggaagttcatcctccgtcgaacgggtcttctcgcaggcagcgctcttcagcaccagcaagcgccaaagcctgtcttcgaaggcgctct

cggaactggtctcgaccaaacactggctcatccacggcgcggacgagctctctggacttcctgccgaggtcaagcagattgcggagacgataaacaag

ctaccggacctttga

>contig_21.contig_21.g756.t1

atgtcttcgccgccgcagcgcagctctcctgtggatatcgagaagcagcctcgagacgtcccatctgcccctgctgaag

ctgacggtgtgcgacagccaagcaatcctcgcctgtcacttcagctcgacgcagagcaccgggccttcaagcccgtacaacccgtccgctcccactct

tatccgaacgatggccgacctcctccacacactcgaaccagccaagttcgctcaatcaaggcaagcgagttcgaatcagaccatacagctgcaacagg

cacaccccctccgttccaccaactccagctgtcagagcagcagcaagaacatgtgcaagatggccagtccagccggccatatgctctcgtccggattg

tcccctctgcgatcactcacaatgtcgagctgaaactctactggacacgtgccatcgtcactgcgactaccaccatcgcaggtatgatcgtttgcggg

ccgatcggaaacatcgcgagccgcatcatgtactcggccatctggacggctgtcatcctcgaggaagacatgacgatgggcgacctcgatgcgatcgg

aggtaatgttgggatcatctcaggtatccatctgctccatctgcgactccgtgccggtgcaaagcgcgtcgcgggtgaaaaacgatccgccgatcaga

gcgaccgcgcaggcattctcagacgcatgtcacgcttccgcagggcaacctggggcacccctgaaattctagccggtttgggtcttctgttactcgct

gttgccttcggtttcgccgcggacagaaccatcacgatcagcgctcagttccagcctcagaaccgattcttcaatgcagtatctgtcggaggtgacct

ttccgatgcagatgtttccgcagccgaagcattacgacctttctacaaagatttcattcgctcgtggactctgcagtccacagctgcgctcaagcttc

cttcgaccgtcaagctgcccttaccagatcaacgagaccaatacgtatacttcaccgaagttctccacgaccacttcatggccaactatgagggctac

ggcagctttacaaacgagacgcgtaccgcatcaaagaacgagaccacgcaagcgcaggacgatgacgacaagtcgaaggatggtgatcagtcgatgac

cctcattaccattgacggaatacaattcagcttacgaagggcgaacgtcaggtggccccgatggggaatccggaatgttagccgttacttggcgccag

acattgctgaatcccggaatgcctccgtcgatgaccgcaagtccgtcttcttcctgacccacgaagtcctcggctcagtgctttcgacacttaacgtc

tcgttcccatccagctacaagaagccggcggatctaaacacgacctacggcgatctccacaatgtcctgcctgcgggcttgaaggcagctgatgtcgc

agcgaccaaacctttcgcggcgaacggggttgcgcagaccttctggtccagaccactgtgggcagaggacgagccacccgagagcaaaccagggagcg

caggacgagggtgggtgtcgctggaggtcatcctggtccggctgaatcagacactggcaggttcaggcgctcagttcggcatggttgcaaacgtgtct

gatgaccacggcaatttcgggaatgtcggcttcgacatgggcgtatgtgtcgagcggtttgatccttacgtggtggaggtctatcaagggctgactag

tgttcgaagcacgtcaatcattcgccaagcggatgatttgaccgtggagagcggccagcgccgctccttgctagtctctcccggaacgacgagccagc

tgagctccgagggaaagtacgcagcgtacgaggtggcacacgaaaatgctcgaaacgctcttattaaggacaatggccgcgatcatgcttgggttcct

aatccaacgctgactagcatgtccagcggtgctggaggtgggccggcgggctatggggttctggatccggtgcggttggcggacatgcttgcgagtgc

cgatactcggctgctgctgccgtaccttgtggggcgcgggaagattgaagctcgcagctggctggcagcgtataaattcttgcgaatcggtggcgcac

ctgttgaagacatactgttcagaacatctacaacgcagctgccaatggaaaacgcaacgagtccggatcgcaagactgctgcacgattcctagcaaac

tttcaccagcatccccaccatcgttctcgcctcagtacagcaagaagcggaggcgatgcgggtgaagtcgacattgagcggagcgcaccaggagcgta

tgaacttcatcctcggccgtccgatgagcgaacgcgggccgaaggtcccttgatcacgctgaacgagatcgagaaacggctgacgaagcagccggctt

cctcatcctatacagcagactgcgagatgtgctccgtgtcacagaacaactctaccgttgatgtcgagaggccatctcgggacatcccatctcctggc

cctcaaggcgattgcgattcaagcttgcaacagccaagtgacctccacctgtcattgcagctcgatgcagagcaccaagcacaacccgtccgttccca

atctcatccgaacgatggccgacctcctgcgcacactggactcagccaagctcggtcaaccgaggcaagcgacttcgcttcagatcacacagctgcaa

cacgcccaccccctccgctccaccaaccccactgctcagagcagcagcagaagaatgtgcaagacgaccagtccagctggcgtggatcccgcttcctg

cgaccttggaagacaacattcgagtccccatccatacacgaccttgttctcctcctcattcactgttccatagcatatgctctcatccggatcgtccc

ctctgctatcaccaacaacgtcgacatggatctctactgggcacgtgccatcgtcactgcaaccaacagtgtcgcaggtgccatcgtctgcgtgccta

tccataacatcgcacaccgcatcgtccactcggcaatctggacggccgtcatcctggaggaagacatgacaatgggcgacctcgatgcgatcgcagat

aatattgggatgctttctagttttcgtttgctctttctgcgactgcgggccggtgccagccgcctcgctggtgcagatcgacgcgctgatcagagcga

ccgggcaggtattctcggacggatgtcacgcttctacaaggcaactcggggtatccctgagatcatagccgctttgggtcttttattcctcgcgcttg

ccttcggcttcgtctcagaccgtaccatcgacattcgcgcccagctccagcctcagtaccagcacttcgatgccctgtctgttgcaggtgatctcacg

ggtgcagatatctccgcagccgaagccttacagcctttctacgacgattacatccgctcctggactctgcagtccacgtctccaatcgaccgcccttc

gaccgtcaagctgcccttgccagatcaacgagaccaatacgtgtacttcaccgaagttctccacgaccacttcatggccaactatgagggctacggca

ctttcaccagcgacacgcataccgcatcaaagaacgagaccatgcaagcgcaagacgattctgataagtcgaagaatggcgatcagtcgacaactctc

attctcaatgacggaaccgaggttcccctgcgacgaaacacccattgtcaacgccttgaaaatctcagccgttacttggtgccagacatatctcaatc

cgcaaatgcatccgtcgatgaccgcaaggccgtcttctttctgacccacgaggtactcggctcagtgctttcgacacttaacgtctccttaccatcca

gctacaagaagccggcgaatctgagcacgacctacggcgatctccacaatgtcctgcctgcaggcttgaaggcagctgatgtcgcagtggccaaattc

ttcgcagcggatggagttgcacaggccttctggtcccagccgataaggtcagatgaggaggtcctgctggatgaagcgtcgtccaggtacagccctgg

gagcgcggggcgagggtgggtgtcgctggaggtcatcctgatccgcctgaatcagacactggcaggtcctggcgctcagttcggcatggttgcaaatg

tgtctgaccaggagggcaggttcgggaacatcggcttcgacatcgccgtatgtgtcgagcggcttgacccttacgtagtggaggtcgatcaagggctg

aacagcgttcgaagcgcgtccatcgttcgccaagcggatgatctgaccctagatgacggtcagcgccgctcctcgctagcctctgctgtaacgacgag

caatctgagctccaagggcaagtacgcagcgtacatcctagcgcacgaaagtgcacgaaatgctctgatcaaggacaacggccgggatcattcgtggg

tccccaatcccacgctgatgagcgtgtccagcggtgctggaggtgggccgacgggctacggggttctggatccgctgcggttggcggatatgcttgcg

aatgttgatggcaaggtcgaagctcgcagctatgatttgatacagattgcccgcgcatcttgcgtcgttcatatgctgaacatcgttgttggcgtcgt

catcggttgctgtctgctgggcgtcctcttggtcccgcgcctgccttctggtctacgaagacgaactacgagcccaatcagctggctcacagtgtaca

gatctttgcgcatcggcggcctccctgtcgaagacgcattgttcgacgagtcttcaccttctacgactccggatcgcaagaatcccgcgcgattcttc

gcatccttccaccaccctcctcacgattcttctcgtctcagcgcaacaacaagcagcggcggtgcgggccaagctgacatcgagcagacagcacagcg

gaatcatcctcatcctcctcggtcggtagataggcagacgctggtcgtagggggcacaatatcgctgaacgatgtcgagaaacgggtggcgaaggagc

tggtcagctatgcggctaaagcaaacaagagccgggcgagacagaacacaagtatctttggtttcatacctcctgatacggaacgtaatgtcacgtct

tttcttccaggtcgtctcaatgtcatctacgttttcaacctgaaccgatatgaacctgcccctcggccttgctccgtgcggaaaaacgctccgaagct

cgggatctctaagatggctcccaacatgtcgcgtagcaagcggcgcacgccttcttctcttccctacgtcttacccagcagcaccactcgcgcccgcg

cctccagcagcatcacccgcgcccgcgcctccaacaactgccccttgctcccacgacgcgttcatggtcgcgacattgggactgttgtcagcttcttt

tgcttttcatcatcctcaagctcagaaggtcgtgccctgcaagtgttccacagctcaatccaggctcctgatcgtcgccatgcgcctgttcagcgtgc

gtctcattctgctgaaccagcactctcgtctgccaacctcctcgaagggcgcactctcatctccagcccgacgtcgaccggtgacctaagctctggtc

accctctggaccctcaagcgagcagcgccatccacgtcgcgcctccttcagatgacatctccttcagcaatgggtcaagtcggcttgcccagttgtcg

ctgtcgccctttcgtcccagcgaggcctcgtctccttcagcaccacctccttcaccaacacgtccggatgccgtcaagaccgaacccgaacccacatc

ggactccgcttggatcgccccggctacatggcgacctccagggcgctacgtgcgcgtccgccacgctccgctgatatctcgtggcggcagtgtcgagc

ttgctcgagttgtggacgtccttctcggccgcacggtgtgcgcgcgcaagcgcattcggttcctcaacgagccaccgcgctttttgctcttcgaggtc

gaggccctgacgcctctgcagggccacaaaggcatcacccagctcctcgatgtgtgctatacaacacacaaggtggacatcatcatgccactttactg

gggtacgctgcaagatctttttgaccaggcaaacggccgcgaactggagacgagtctggcgaagaacttgacgctgcagcttctggtcgcggtctctt

tcatccaccgcaaggaggtcattcatctcgacatcaagcccgccaacctcatgctgactcgagactttacgatcaaggtcggggacttcggcatctct

gcgctcgccggacagaatcaagacgcgcgcacgtacggcactctcggatatacggctccggaatgcctcctcggatccatcaagcccacgtttcaagt

cgacgtctggtcgacgggctgcgtaatcgcgcatctctttcttggccggcccctcttcaccaactcgtacgatgcggccagctccgtggaagacatac

tgcaattcaccggccatgcaggaggacccgtatttgcacgagctcggtttccgccgtctgcgttcgacttaaccatggactggaggccgttcgaatct

caggcatcggctatatttgaggacgtggacggggatgcggcagacatcatcgagaagatgcttttctcttcaccaccgaccctcgtccaaaccgaatc

aatcccacgcttcctcgcagtaacgcgtcaattcgatgagaccaccgacgaggggtag

>contig_21.contig_21.g757.t1

atggatgccctctgtctaggacccgtcgctcaagtccacccctggcgtaattcctcaatcccattgatcttccactttg

actttcatttcgtgtctgctgatcgcttccttcgtctcttgaccctgcccaccccctcccccgtgcccttcgttcagacccagacgacggagcaatct

gaacacccaggcttcttcggcggctctgaggctcatgtcgattccgagcgcagagcggtgcggaccttccactacgaccttcataccatgtctgctga

ctgctttcctccgtctctcgcccttccgctgatcaagcagcgtttcgaggtctcagctaccggcctgcgtgctcccagcattctggcaggcaaggccc

aggtgcaggacgtagcatgccagatgcgggtgcgagagggctcccagcgaagggccccaacaatcttgagcggacctaaggctcaggtcgatcccgag

ctcattgctgatctcggatcttcaacttcgcgatga

>contig_21.contig_21.g758.t1

atgtccgctgaccgtgtctcttacctgcccaacccgttctctctgccctctgccctttttccagacccggacgatgaaa

cccagctgatcgaatatgtcttcgatccagattacattgctctgcttcagcctggtgagattgtcagggtctgcaaaaccaatagcaaggccaagtgc

aagagtacccagctgactcgatcgatcttcgcagccgcctcggcgcaacgctcactgttgatattcttcccaatccctctctttttgacctgcgaccg

tgacttgatctccttcacgtcgaactgcgagatgccttcgccgccgcagcgcagctctcctgtcgatatcgagaagaagcctcgagacgtcccatttg

cccctcctgaagctgacggtgtgcgacagccaagcaatcctcgcctgtctctgcagctcgaagcagatcaccaagtcgtcaagcccatacaactcttc

cgttcccattcctatccgaaagatggccgatttcttccacacactcgaaccggccaagttcgctcaatcaaggcaagcgagttcgaaccagaccatac

agctgcaacaggcccaccctcttcgttccaccaaccccacctgccagagcagcagcagcatgtacaaggtggccaggccagccagccatatgctctcg

tccggattgtcccctctgcgatcactcacaatgtcgagctgaaactctactggacacgtgccatcgtcactgcgaccaccaccatcgcaggtatgatt

atttgcgggcctatccatcacattgcaaaccgcatcatctactcggcgatgtggacggccgtcatcctggaggagaacgtcacgatgggcaacctaga

ctcgatcggaaatgatgttggaatgctttctggtcttcgtttgctctttctgcgactgcgggccggtgcctatcgcgtcgctggtgaaaatcgatccg

ccgatcagaacgaccgagcagacattccaggacgtatatcacgcttctacagggcaacccgaggcacccctgagatcatagctgctttaggtcttcta

ctcctcgcacaaacctttgggtttgtctcagaccgtaccatcagcattcgcgctcaaacccagcctcagtaccaacacttcgatgccatatctgtcgg

aggtgacctttctgatgcagatgcctccgctgccacagtcctactaccattctatgaggattacatccgctcctggactctccagtccacagctgcgc

tgaagcttccctcgaccgtcaagctgcccattccaaatcagccagaccaatacgcgtacttcaccgaggtcctccacgaccactttttcgccaactat

gagggctatggcagcttcaccaacgagacgtacaccgcgtcaaagaacgagactgagggctcgagcgaacccacgcaggagcaagacgattctgacaa

gtcgaagaaaggtgatcactcgtcgacgactctcatttccaatgacggaacagagatcaccctgcgatggaacacccattgccaacgccttgaaaatc

tcagccgttacttggtgccagacatatctcaatcccggaatgcctccgtcgatgaccgcgaggccgtcttctttctgacccacgaggtgctcagctca

gtcctgtcaagactcgacgtctcgttaccatccaattgcaaagtgccccaaaatcttaccacgcccgatggaacaattctcaacaacgacatcctacc

tgcggacttgaaggcagctgaagtcgttttggccaaacctttcgcagcggatggagttgcacagtccttctggtccctgccgatgaggtcagatgagg

agatcctgctggatgaagcgtcgtccaggtacagcccagggagcgcggggcgagggtgggtgtcgctggaggtcatcctgatccgcctgaatcagaca

ctggcaggtccaggcgctcgtttcggcatgattgcaaatgtgtctgaccacgagggcaacttcgggaacgtcggcttcgacctcggtgtatgtgtcga

gcggttagacccttatgtggtagaggtcttccgagggctgagcgttcgaagtacgtccatcattcgccaagcggatgatttgaccctggagggtggcc

agcgtcgctctttgctagtttctgccgaaacgacgaacaaactgagctctaagggaaagtacgcagcgtacaatgtagcgcacaaaaatgctcgaaat

gcgctcttcaaggacaacggccgcgacagtgcttgggttcccaatccgacgctgaccagcatgtccagtggtgctgtaggtgggccggcgggctacgg

ggttctggatccgatgcggctggctgacatgctggcgagtgccgatactcggctgttgctgccataccttgtggggcacggaaaactcgaagctcgca

gttacgagttcatacagattgctcgtgcatcctgcgttgtttactggctgaacatcgtcattggcgtcgtcatcggttgctgtctgctgggaatcctc

ttggtgccgcgcctacctgccggcctaccaagacgaagtacgagcccgatcagctggctcgcagtttatggatcattgcgcatcggcggcctccctgt

cgaagacgcattgttcggcgagtcttcaccttcaacgactccggatcgcaagattcccgcgcgattcttcgcatccttccaccaccctcctcacgatt

cttctcgtctcagtgcaacaacaagcagaggcggtgcgggccaagctgacatcgagcagacagcacagcgggatcatcctcatcctcatcggtcggta

gatgggcagacgctgcccgaaggggccaggatatcgctgaacgatatcgagaaacgggtggcgaaggagctggtcagctatgcgtgcaaagtcaaagt

tcgaaagatgtcagaggcacccacagacacgctcgacgcgcggctcgcccttaaccgcactctcctccgtgtcgtccaggccggcgacttggccgctg

ctaaggaatgtctcaaggcgggcgccgatgcatggttcgaatcggacgaacaggacggtctgggctggagtgcgctccatctcgctgtcgttgccaat

tcgcccgaacttctgcagttcttgcttcaacgaggggccatttggaacgcagttgacccgagagggatcactgccgctcaattggcttggtcgctcaa

ctatgtggaatgctatcagcttctcttcgaagagggcgtcagacattcgatgctcatgaacatgctagctggggacgacgacgaagccgaagatgacg

ctgaagacgaagttgacgcagagtccggggcgatcaccctccggcccgaaggcaacgaactcgccaattccaacgccaagtttctccagagccgactc

atctttgttaccgataaagatgggaaggaaagagtcctcgacggggatagcaacttggtcatggcagcttgggaaagcgacatcatggaccgtaccgc

ccacctcctctgcaccgacctcgcgcccagcttcagcgtgctcaacatcggcttcggcctcggcatcgtcgacacctacttccaatcctaccggcccg

gacggcacgtcattgtcgagcctcacccggacgcgcttgcctttctccgtcagaaggaatgggcggatcgggaaggggtcgagattgtggagaagagg

tgggaggaggccttgttggatcctgacttggggagctttgatgtcgtctatttcgacacgtacagtcaggactacaaggatttgaagaagtttttcga

tgaggtgcccgcactgttaaatgggccgaatgcacgcttttcgttcttccacggcctggcagggacaaacgacttcctctacgatgtctacacccggc

tctccgagctcgacctgcaatccatcggcctctccacccaatggcacgccgtacacccacaactgaccgaggaagtctggaggggcatccgacgcgag

tactggagcttgcctagatttctgatccctgtctccaaaatgaacctatag

>contig_21.contig_21.g759.t1

atggccatccgtccgggatctatacgccctgccctggggatgcctcctgacgacgtttgggcctccctgaacgccacga

attcaccaacatccgacatctcccataccggcgcgtgcgccatctccccgcccagccatgctgcccttctattgaccccagcgctacaccttctcacc

gccgcgcatggcacggcagacctcaccgtcgcaaagagtcagtcggagtcattttggaactccgtcaaagagaagactaaggtcgattactcccgcaa

gatcatcgcctacctcgcgtggtgcgacgacctgggcatccccgagcattttcgctttccgatcgctgcgaacatcctggtcctttacttgcagaagg

atatcactacactacgacccggatccgtcgacaaacgatcccacgccctgctatattggcacagggttcagcgactaccatgggctctccacaaggcg

gattcgcgggccttatcaaaggccgcaaagattcaagggttgccggcactggagaagcgcagaccggtgcgtctgttggacctcaaggcaatggttga

ccatcacgaccgagacgaccgcgcacatgttgcaattctggctgctgccctactggccttctttgccatgtgccgcccaggggaagtgacggttcgtt

cggcttccgacccgcccacagaccgggcacgatgggcacacatcgtagaacagccctcacatgcagtaggtggacccgcctctgtcgtccttcgcctt

ccgaccgagaaggtacatggcaacgacggcttggaccgcattgcggccgagcagcgtcaaatgccggagctctgtccagtcgcggcgatgcgccgaca

tctgctcatcaacgcgttacgcccaggggaggagggcttggctcatggcgccttctcgtacgtgtcccgctcgggctcccggaacgagctgacggagg

gtctgttcgctcggtcgataaacacatggcttgcgaaggggaaccgggaacgagtcacgggccactgttttcgcattggcggagccacgcttctgttt

gcggcgggcaagccgctggacgacatcaagctacgcggaggttggggctcggacgccttcctgctgtacttgcgggatctctacgtacgacaggcgac

tatgttcagcgacctggacctccagacgttatgctacggctga

>contig_21.contig_21.g760.t1

atggctctcaccgaagaggagatcgtgctcctggttgcgcgtactcggcgcgcagagcgagaggaagaggctcagcgtc

gagcacaggaagaagctgctggcgctggcgaaaacgaagccggaggacgcgacagagccattgcggctaccgagcgatccaagcacatgtcgctgctg

cacgacaagacgatcgctgagcgggcgatacacccgtggccgaactcctccattcggattccgaaggacatcgtcaacttggctcggcacggtgtaca

gattccgttgatttggctcacggtggagggtttgttggatacgtctggccagcaccgcaagttcgtcaccgtccctccgacaccgaaggtacaagagt

acctggcggcggcatacgagaaggaccgtttcttgccgcgcggcgcattcagccaggcacttcaggctttcatagcgctatggaacgccgtcggtccc

caggccccagccggcacggcaagtcaagcctccctgctggaggacatgcaccttgcgatcctctcgcacgccgtcgacgagcattggccgatctggcg

agcgtacgccaagaaggtgttggactggatctttgaggagcgagcccctggcgagggtattggcctgaacatcgccgagatcgatcaacgcctcgtcg

cagaggcagagcgcgaatgcaaatacccaccggggcgcgctatcgacttcaccaactcggacaacacgatggtcggcaagctgctgcgggcggagggg

agcgaggtcgcacgcattggcgcagagatcgatcaactccacagggcggccacgtttggcgatatccgcacgggggacacagctgtttccactgtacc

tccctctggtccgcgcgctgagcgagctccgcgtggtaacctcccgtccgctggcccttccagctggcagcgacgcgactacgaccgaggcccacagc

cctttcaacgcgcgcccccgtcatacccctcgcgcgacggcggcgcatggccacagcgagcataccgcgccgaaagggactactacccggcccgaact

ccccaattttgcaccagctgcctggagatggtcagcgatcacgacttccgctcatgcagacgaccgtacgcgaagaacctcgaggaatacaacggagg

atggcatttccccggcaaggacttccattctgcaaccgatacaactgtggaggagagtgcacacgcgggtgcacgtacggtcactactgttcctgctg

cggtgacagcaggtgtcatgcggccaagcaccttcgcaccagcgcaaactccaagtagtccaactccatcagtccagtccgccttctccttcttccgc

cgatcggctctccaagcgtcagcttttgctcatgcgctggaacagctaccagtatcattccgggcccgactcgcgcatgtggtcccgggagtcaacaa

cggcttctcgatgggcgaattggcaatgccatctcgcacgatcctcgccccgcaacatttctcaaaggagcaggcaccgctcatcacggcatggaagg

acaaggccctggaggatgggtttgctgtgggaccattttccggacgcgagattgaggactctgtgggaccgtttgcgtgcgttccgctgacagtggta

cacacccccgcgaccgagaccaaagcagaaaagaaccgcgtctgcttcaacgcctcatggcctaccaatgtcgagagtgggagcatcgggattaggtc

catcaacgacgagctcaaagaggaagagtgggaatgcgaatggctactgatcaacggggtaaagctcctgctgtcgcaggcgcactccacggcatgcg

caatgggcttcgacttggccgacgcgtatcagcaactgccgaatgctccatggcagcgtcggcgcttcgtgtttgcggtggacggcgaattcttcgtt

tggatcgtgggcatgttcgggattgcaaccatgtcggcactgttcggacagctgtgcgatgtcctatgctggtggatcgagcgctgctttccttcggt

ccgagcgcgacacttcgcggacgaccgtctggtgctgcacgacggcacggtcccttgcccgtcgacggaggaggtctatgcagaggtcgcccgctttg

gatggaaggtccacgccaccaagcacttctcgtggtcacgtcgcttcgtcttgctgggtttcgactgggacatggacgcgcagacggtcgcgctgacg

gaggagaaaagaagcaagtaccttcggaagttgagcgcggtcctgtctcggccagcagtacggtacaaggagacctccagcgtagccggcactctgat

tcacgtctgttccatctttccggaacggcgcagccacttgaacgccatctatgcgctccggaacaggttcgatcgcaggcgccgcttccacagcctgg

acatcccgactcccgctgccgccgaactgcgagactggctcaagttcctcaagaccccggtgctcgtccgatctttccggccccccactgcctcgttt

ccgcacctggtgtactcagacgcctcgaatctcggctgcggcgttgtgatcgacgggaaggcgcagggatgggcgttgcctggcatgatcgaccacga

cgaggtggacattggcgtcatggaggcttgggcgcttcagctggcgttggaagcgtgcatcagtatgggagccggggagtggggactgcgtggtacgc

ttccagaggtggcgattgcggcgaacatcacaatgtcgatggcatacgttgcctcggctgacaacttggcagatgccccatcgcggggcgactgctcg

cggttctcaccgctcaatctcattttggccgccccctggcaggagttcatgggggtcgccacccattag

>contig_21.contig_21.g761.t1

atggcctcaaaaagctggtggtccgaccacaacctgggcgacgatgctcgtggcagagagcccgcagaagagggcaatg

gttttgatgttcatccttgtatgaagaagaaggagatcggggatgtgaaagcggggagaagtatctggggtgtgtgtacgccggacggaggagctggt

aatcatgaggccgaacgtgcgctggcgtatgtaatttgctggctgtctccgcagacgcctatctcttccatatgtatcgcctcatccgaagaacagct

ggacctggccgacaagcccaaacaaggaggagaagcgccgggcaggggagagagctgggcggccggcgacgacgagggatgccctgccgaggtggagg

acatcggaggtgctcgagactttgtgagattgcgcggtggttgtcaatcttctgctccactgaagcagccttgctcggaacatgaccttcgcaaatgc

cccttttccatgggcaaggtggaggcggtggaggggcagaagcaacgagagcaaggcaagcaagcaagtgggacgtgggagaccgagcgctacagcgt

tccgcactcgcccgcacctccctcccttgttcgaagcacgaagcacgaagcacggtcaccttttcccgtctcctcacggacgccagtcaatcagttgc

acacttgcacctcgccttcgccaggtacacatacatctcctctttactacctcgacgacctcaacacccagcctcgccgtatcgaatcacacacccag

accacagcatctcaattcaacatggcgtcgaaccaagacaccgaagaacagccacagccacagcagcagcacgcccatggccaggcacaggcacagcc

aaccctcttcaacgtaggcagcgagtacaaaatcgtcgatgtcgtcggagaaggagcctacggtgtcgtctgctcggctattcacgtgcccaccaact

cccgcgttgccatcaagcgcatcacgccctttgaccactccatgttctgtctgcgcaccctccgagagatcaagttgctccgtcacttcaaccacgag

aacatcatctccatcttggatatcgtcaagccgacctcatacgatgcctttaccgaggtcttccttgtccaggaactcatggagaccgacatgcaccg

cgtcatccgcacccaagaactgagtgatgaccactgccaatacttcatctaccagactttgcgaggtctcaaggccctgcactccgccgaggttctcc

atcgggatctcaaaccgtcaaacttgctcctcaacgccaactgcgatctcaagatttgcgatttcggattggccaggagcgcgaatcagccggaggcg

gagggaaccgggttcatgacggagtatgtcgccacgcggtggtaccgcgcgcccgagatcatgttgaccttccgagagtactcaaaagccattgatgt

ctggtcggtcggctgcattctggcggagatgctgagcggcaagccactcttcccgggacgcgattaccaccaccaactctcgatcacgcttgagatcc

tgggcaccccttccctcgacgacttctacgcgatcacctccgcccgctcccgggactacatccgcgcccttcccttccgcaaacgccgcaactttgcc

caaatgtacccaaacgcaaacccgctcgcggtcgacctcatggagcgctgcctgaccttctcccccaagaagcgcatcaccgtcgaggaggcgctcag

ccatccgtacctggagccgtatcatgacccggacgacgagccgacggcgttgccgctcgatccggcgttctttgcgtttgactacgccaaggagcagt

tgagtcgggcgcagctgaaggagctcatctatgccgaggttgtgcgcgacaactttcccgaatgcgcgttccctcacatcaagacctttttacaactc

gacatttccacataccctttggcggatttgggcccgctgttcagtgttcaccgctcttcgggtcctgcttctccctccgctgtcgagatcgatgtggc

cgctgaagccgccaacgacgcagctttgattgctcgtctcagcgcaactaacagcgctgcgattgctactactccttccgcacctacttcttcatctg

ctactggtgcgagccgtggcacttcggctgccagcgcttcttcggccgtctcccctaccaaaaagaaagtcgaaaaagctcaggccaataagggaaaa

agcaaagctggctcctaa

>contig_21.contig_21.g762.t1

atggatggcgctaacttcaaggaaaaacactatctgcaggcggccaatgaggtcaatgcgaagcacaagggcagtgcta

ggacctcgaagcaggctcagacccgctggaagacagttagactgacgctcaggttgcccaacatgggctactcgccttcctttcgtgaccagctcaag

tccactttcgggccttttcgtcgtgtggttcgccgatcaggcttctcgtgggactcggaaaataacatgatcgatgcttcagacgacttgtgggagac

tctcgcagaggaagacgcggctgcggccgaactcagggacaaggtgttcgagttctacgaggaggtggatgccatcgtcgaggggggcatcgccaacg

gcgagtttgctctcgaccctggtgcgaaggcttcgaagaatgaaaaagaggaggattctggtaccgacgaggattcttctgattctccggcctccgac

ggtgaggaagtcgaaggaacgcacgaggacagtgacggacagacgggtggtgagggaaagaacaagaagcaaggcaagcgtcgcgctgatgacgaaga

gtggctgggaaagcccaagcgggtccgaaagacgcaggccggcgagcttactaagctcaacggtgtaatgcaagatctggccgacaactttaagcagt

cagagggcgctagtgccgaggataacgaactccagaacgcaattaagttcttcaaccttaacgacaaggcccacttcacgaccatggaacagctcgac

atcatcgactacttcaacgacaacaagaggggccccttcacctacatggccatgtccaaggcggacaacgctgatcttcgcgtggactggcttcgtaa

gaagatcttctcgacgtga

>contig_21.contig_21.g763.t1

atgtacgagtatgcgcttcgaggagcagacctcagacctggccgagcgtcgcctcaggtggtgttcactggtcagaatg

gcgttcaattttggcctcaattcacactgcaaaatgtcaacggccaagtcccacatatccctcgtacctcgacgtcgagctttgagcgtttagcaggc

acaatgcccagagacatcagcgagaaacatagacgcgcatctcgccgcaagaagctcttggctatcttgatgggtgtcaccgcaactgtggcagcaat

acatgcgcgcacctacgttctcaagaaatccaaagtagggtccaggactagcggcttaaagtttctacggcagtacgccggcaaggatgctcatcctc

agacgtgtcaggatttccttggcttaaagcccaactcttttcgacgtttgctccaggttctcaaggacaagggcaagctccgcgacggcactcgatca

cgggctctgtctgctgaagagaagctcggtatcttcttgcacatggctcgcagtggatcagcctgtagaatgacagccagtatcttcggtagaggctt

aggcactgttgcaaagtgcttcaaagaagtggtggctgcgcttacgagccgggaaatatacactatgtttgtcaaggcaccaggctccgatgcggaag

tacccgagcgactgcgaggcgacgatcgactgcggcacttcaaggctgttctcggggcagtagatggggtgcatgtggctgcacatccctcggcggtc

gacgccactcggttccgcaaccgtaaggctggtttaacattcaatgtgcttgccgcttgcggtttcgatctcagcttccattacattctcactggatg

ggaaggcagcgccgccgacagtcacgtcttccacgcggcacgcacgtctacctggctcattccgaaaggtcgaacgtatctcgcagatgctggctttc

ctctctgtgtcgagctcttgactccgtatcggaacactcggtaccacttgaatgagtggggagatggatctgaaacggtcacaaacaaggaagagctc

tataaccgccgacactccggcctccgcagcgtgatagagcgcgccttcggcatcatgaaggcacggtttaaagtgcttcgcacggggtctcagtttga

cctcaaggtccaagcagcagtttttccggcattggcagtggtacacaacatgctcatcagcgacgatcccgacgacccttaccttctggacgatttcg

ctctggcggagatggagatggacggaacaacagacagcactctgagagcagcagcaggccgcatcagtgatgcagagaggaagagtgggcaggatcag

agagacaagattgctcggaaaaatgtggcggtctttcaagtagaggtggtgatacgtaataacatgcaggctgcagccatcattctcagtgcatcttg

a

>contig_21.contig_21.g764.t1

atgccgcaaatgccaaagaacatcatcaagggcgggaaggaagagctcaagctccaagaagcctccctgctcaagaacc

aggcctacatcaacggcaagtgggtctccggctccaagacgttcaccgtctacaacaaggcgacgaaccaggagatcggaaacgtgccggacctgggc

gccttcgagacccagtccgccatcgaggccgcctccgccgcgttcaagacgtggtccaagacctccgccaagcaccgccacgacctcctcctcgccct

cttcgcccagctcgagcagcacaccaccgacctggcccacatcgtggtggccgagaacgggaaggcgctcgcggaggccaagggcgagatcacgtact

cgaactcgttcatctcttggtttgccgaggaggcgttgaggacgtatggacataccgtccccgctcccacgcctggggtcaggaacgtcatcatcagg

gagcccgttggcgtggcaggtatcatcacaccgtggaatttccccgcagccatgatcacacgcaagctcggacccgccctcgcagcaggctgcacagc

tgtgatcaaagcccccgccgaaacgcccttctcggccctggccatcgcccacctggccgagaaagtcggcttcccgcccggggtgatcaacattgtca

cctgcgccaagggggagaacgagatcgcggtggggaaggagctgtgcgagaacaagaaggtgaagaagttgagcttcaccgggtcgacgagggtgggc

aaaattctgatgagccagtgcagtgggacgctcaagaagttgagcatggagctgggtggaaacgcgcctttcatcgtcttcgaagacgcagacatcga

tgcagcagtaaagggcgccatcgcctgcaagttccgcggttccggccagacctgcgtctgtgcaaaccgaatcttcgtccacgccaacgtctacgacc

agttctccaaagcgctcgcggccgaagtcgagaagtttgtcgtcggatacggaatggaggaaggtgtcacccacggtccgctcgtgtccgaagctgga

cagaagaaggtcgaggaccacgtgaatgagtcggtcgagaagggtgccaaggtgctcgtgggtgggaagaggggcgagggtctgttcttcgagcctac

tgtgctggtggatctgcccagaggattgcccatggagaccgacgaaactttcggtcccttggcggccctgttcaaattcacctccgaagaagacgtcc

tcgagctcgcgaacgactccgacgtcggtctggcgggctacttcttctcgcgtgattacgcccggtgttggcgggtggccgaggcccttcaagtcggc

atggtcgggatcaactgtccgatactctcgcagaactgcgtgccgtttggtggagttaaggagtccgggttcgggcgcgagggcggacgaggcggcat

ctacgagtacatgaacgagaagcttctcgcatggggtggtatcaactga

>contig_21.contig_21.g765.t1

atgtcgggctctcaacccgacccaagggaaacagaccccgtcattttggacatcttcggacagcacacctttggagatc

tcggagttggtcccttccagctgcagttagaagagcttggccttcctccgaaccagagccagcgatggtcggaattctcacaagaatgtcagcagtcc

ctgaaccgggacgaccccgaatccccgcaggtcccgggcgaaatcctcaagcgactcgtgagcagacacacccttcccctcctccgctcccagcagta

cgtcgttctaactcctttgtcacacgtaatccagcccacagtccaggatccgacaacgcgtctgtgctgggaagagctcatcaacgacccgacggccc

acacgtggaaagcgctcgtcgaaggcaagcacgcatctcagcaatccactggccaagctgccgttcgcagctccgctccgacttccacgaatgtttcc

gccgctctcggaccttccaagcgtcccacggcacggtcgctgcaggctacctccgcatccgtcacaacacgagcaccgacacaggaagtcctcgacga

cttctttgatcggtccgctgcacgagttgccgccgccggtcgccagtccgcgcgcaggcgctcggttccgtcacatctttcggactacgagtacgaaa

ccgacgatccaactgcccctccgagcaaaggccccggcaaggacatggcatccagctccaaggcccccagagcctccacttccagcaaaaaaggagcg

gcatccagctccaaggcccccagtgcctccacttccagcaagaaaggagcggcgagcacttcagcgtcgaagtccaagaagggcgccgcgaagaagcc

gaaaaagcccactccgatgtccgcccttgagcggcgggaaaaaaccgcagcgatgcgccgccgggctccggaaagcgacgagtcggagtccgaagcag

aagttgaagtagtcgagatcgatgatgaatccgacattgatgagttccaggtggaggaagaccagcacagcagcatgtcctcttccggagcttccgaa

gaggaaagcgacgaggaaggtccgagcagctccccgacctcacggcggcgcaagcagggcaagaagggtcaggcggccaagtccaagaagaagtccaa

ggccaagtccaagctccatctggtgatggactactgcagcaccgatctgcagcaagttctcaacgaggagggtcagcgcagaaggctaactccaagag

tggtgtgtggtatagctaaacaactggcgtctggattgcgggcggttcatcagatgtattacattcccctaccagtgcaacgtcgctcatttcgacgt

caagccttcaaatatactgatttcgctagacggcgacgtgcggttgtcggacttcagatgctccaaccgaatggtcagccgctctggtacgcggggcg

cccggacaaactgttgtgtcaacaggtgagagatctgggacggatgggcgtggaactctggccaggctcggactcttttcctggcgctttaccggacg

atcagatgctgtacaagtctctctggcggggtaaacatcgattcttcaactccgacggcagtaccacttcaagctctttggcagaacgtcttgccttg

cacaccttcaattatgcgggactcatccgctttctgggattcgtgctggttctggatccggatcggcgaccaactatggatcaggtcttgaatgatcg

attctatggcctcgacaggaaagatgcggaccccccctacccttgtttcccgagcatcacagtgatgagaagcctcctgcttagtagaaacacgtag

>contig_21.contig_21.g766.t1

atgaacctcctcctcactcaggacgaggacgaatgggacgtctccgcagccgctacctgcatcggtcgaatggcagcct

gtgtcggcgacgccatcgacatgcccgccatccacttcatcgaggccaggaccgtccatctgaccgagatcgcccaccatctgcatcctttccttcaa

ggtctcaaggatgcccatctacgggtaaaatgtgcgcggttgaagggtggctgcgccaccctgataccgcgtggcgcgtacgaaggtacatatacata

a

>contig_21.contig_21.g767.t1

atgcactccgtactccgtactcccccggatgcatttatgcgcctatatcaccatcaccttaccatcaccatcactacga

tcctcacccgcatcatctctccgctcttgtcagtagcattgatgataatttacgtggacgacgccctgcgtcccccggccggccctggcattggcaaa

tccacagcacaggttgcgtccagcgccgagatggacagattcttcgatgggcttgacgagctcagtacagagctcgatacccacaaaccaaccaagaa

gctcgcaacctccacagagcgcctccacgaaggcgccgagaatttgctccgcggtttcgtcaaccgctacttcgccaagaagggacttccaggacgcg

atcaagatgtcctgaagcctcaaggtttacagctgctcgccccagcgaggggtatgcggaaggacgcctcgaagtttcgtcgaagcgtcctgtattct

catccagatgctgctccgggcctggacccgacagtgccaatcctcaaaattctgcttgaggatcaagccttcgttgggatcgacacccaagacgacct

gctgaacctttctccagcacacctcgccaccttggaggacgggcagctcgagctgcccctccgctccgaatactccgaaacgtgctccgacgggccgc

tgctcactgtgccgatttcaatgaccaggagatggtcttttacactctacgaagaatggtggtcagtgcgaccaaccgcaccgacgtcacgtcagaga

gcgcccaccttgtcgtcgggcacaagctcggcactagcacccgattctaaggatgggcgcggagtaagccccggagagtgcggagcgcaagcctgtca

tgtccccggaccggagtacggagctggcgcaatgagatggggagattctgtgcggagcgcggagtggagttggggcgactgcctgaaaatgagcggac

cggagtcagccggagtaggggccccggagtacctccgcgcccatccttacccgattcacgtggacgtgcaggggatcatcctctttgcaggagagaga

aaggagtggaattcagcaccggcaatgctgacggcgccctgggagcctctgacgccgaaggaggtcacagaggcggagggaaaggcgcccatcttgaa

ggagatggaagcagctttcaaagcggcatcgggtgccgaacgccagcgcctctaccttatatggatccggaagtgcgcttctattcggcgcgaggccg

gcagagccaaagctcaatgccgccaactgctcaagcagcactctgctgtgacagcggctctttccaacgtcaccttccttcaggatctcatcagcagc

aaccacgagcaggaaaacgacgatgcgggcacagaggctgccgtcgtctcaccggagcgatacgacctgctggacagcaaggttaagagcaccgccca

gctcgccgatctcaccactctgatcaggttgggcgtgctaagcgacaacatgtcctaccggcatcctcgcgagacggacggtaggcacatcggctggg

ctaaagcggttgcgttggagctaggtttcctggctgcgttggcgtgcggtgcgtcgcggtctctgatccgcttttggtcggacaaccacggggtcatt

ggcgcctacaagaaggggcggagctttggcaagcaagccaacacggtgctgaagagagtcctggacaaggagcggcaagaagtcgaaaccctcctcaa

cacagtgatgcggtcacttcttctggatgatgcgtctcattcgtcgacaaccggtcgtgacctgggggggtccaatgcggtcggtgctagcacggaaa

cagcatacatccccactccggggtccaaactggacacccctgctgagctgatgctcaggcggtctctcttcccgaacgtcccaacagtggaaaggctc

ctaacttggaggcctggcgtcaatctctctttcgcgctgctcacgagttccggcaatcatctcccgaattccaagactcatcaagcacaagtgctccg

ggcgttgcggtcctctatcgaggagaacacgcgaaaaaactacggctcgggcatcggcattttcctcgcgttcgctttcgatcatggtcttgctgtca

cagacatcttcccggtgtctgaaggggttctgcttgcgtttgtgggcagctgcggtggtgttcgctcggcccggacggtcaagaagtacctgtcggcc

ctatcgttctggcatcggatgtggggccagcagtggcggatctttccgatggtcatgcaagcactcaaagagaccacctctccttcggcgaagttcgt

ggatccctctcggcacgttacgagggaccggatcgaacctttcagggaggttgcgggcgtccttacggtggaagtacgaattccttggacaaagacga

cgcatacggatggcgggtccaaggtgctctcgacggcagatggcctactcgaccccattcacgcgctgcgatggcatttgaccttcaacacggttgct

tctgcgattcctcccaccactacccttttcgcatacagggtcaaggcgggtagggactggcgattggtaccccttaccaagaagacctttttggacgc

cttctctggcctctgcggcgttgcgggcggcaggaagaccgacttttccgggtcattcctttcggatcggcggggcctgtttctactggcaccgcggg

gcaacagttccagagctcaagctcttgggtaa

>contig_21.contig_21.g768.t1

atgcacaagcttgcgggggcaaatccaagtccgcttagcgcggacgggtacgagaagatgctcaaggacctcgacctct

tccacgcctacagtgaggtcgtcgagggccttcgctccggtttcgacttcggaattcctcccgtttcctctttccgttcccctcctaatcacggctcc

gctaccaacgatttcgacacgctcaacgcctccatcgacaaggaggtctctctcggtcgttcccttggccctttctctcaggatcaagtggaagaaat

gctcggcccgttccagacttcgccactcggcctcgtccctaaaccaaacgggaagtggcgcatggtgcaagatttctcctaccccaagaagggggctt

acgcttcagtcaactcttacatcgagtcggacgagttcgtctgcgcttgggacggtttcctcgctctcgttgacttgattcgttcgtttcccagcggc

tctgatgccgcaatggccgatgcaggggaagccttccgggctatccctgctcgtccggaccagctgcccggtctcgtctacaagacgccggataaccg

tttcattgtcgaccttcgtctcccctttggcttggcctcagccactggcgtgtggggtctcgtcgcggacgccacgagggcgattatcgagcgccttt

tcgagaaggagggtgtccgggtggtcaaatgggttgacgacttcgtgttcgtccggcccccaacctgcccggtgtccctggacgacatcatggctgcg

actgctccgcttggcttcccgtggcatgccacgaaacgatccgcttttgctccaaccatacgattcatcggcttccatttcgacctacaccactacac

cgtatggctgcctgacgacaaggcggcgaagttccttgatcgcataaaggtgttcacgacacgcggaccgatatcgctcaagcagactcaagaggtca

tcggctccctccagcacgtggccgtcatggcgcgcgacctcgctccctacctctcggagatggtcgcctccctttacgcgttcgaagacaagaacccg

ttcgccaagttgttcgttggcgacaaggttaggagcgacgcaaaggagtgggtctcagcactctcctccaacttccgtcggtccttcgctgctcccgg

tcctccattcgagttgccgatttacgttgatgcctccacggagtggggcgtgggcatcctggttggcgagcgttgggctgcgtggcggcttcaacccg

gttgggaagccgatgctagcgggatcctctgggccgaagccatcgctcttgagttcggaatcctcgctgctctcgcgctcggtgctgaaggtcactcg

gtactcgttcacggcgacaaccaaggcgtgattggttcgttccgacgcggccgtagccgcggtcgccaagcgaactcggtgctcaagaggatcctcaa

gctggaacggacctggcgcgtagagttcctcgcggaacgaaagcggcgggttcgggcgaaggcgatgatcttgccggtcgcggggaccagcaaggcaa

gttcatcaacgcctccttccattccctctccgctcccatcccctggcgtccccctgtccacgaaggggggtccacctggtcatgcagatggtaaacgt

atcaagagcatcgatctcatgcttcgcactgccgctctcgctgcccctccgggcccgctccgttcgcgctccggttctagtttcgaccctgacggctc

tctttcggcaaacgtctcttccgttctttcggcttcgcttgaaccctccacccgtagcaagtatggcacggccgcacgggcatcaggaagttccgagc

tttctgcgacgctctcgacatcccgcttctccgaacgttacccaatctctcgagatcttcttctcgccttcgtctgttctttcgctggcaaggttcga

cgcaagaccgtcagcgactacttgacgggcttggctagctggcacgccatgtggaatcggccttggattcggcataactccattaccgtcgcccttca

aggtatcgctcgcctcgctccccctcccaacgacgaacgtctccccatcctcacggaggatatgcttgcggtccgaggcgagctcgatatcgacaacg

atcctcgtcatgctgccgtgtgggcctgcgctatggtgtcctggaccgcaggcatcgtcaaagccgtcgtatctgtcggtggtcgactggacccgctt

gcggcgctccttcatcacctctcgctctccccggttgatcacgctctcgcttcctcgactccgctcttcgcctttcgctctaatgactctcctcccgg

ctcgcttcttcgtccgctcaccaagattgaattcctggcggagtttaacgctgctctcgttcgcgccggtcgtgtcgccttcctggggcactcgttcc

gcattggcggcgccactgtccattggcatgctggcgccgcggtcgcagacatcaagctgcttgatggttggaccagcaactcggtaaatcggtatctt

cgagattacggtcgcggcctcctcgccgtccatcagcgtaccgctgcctcgattgccaacgtctcctcggctcaacccgttcccctcgctcccgtttc

gcttcccgctcccgctccacgtccctga

>contig_21.contig_21.g769.t1

atgcctccgcctcaagactggtgcttctcggttttcgcgttcgacaagccatcgacgcgcttctctggtccagcgcgca

gctgtgatcggccttcgttctgctctgcgtttgatacgccatcgaatcgcgacattcacatttgcgggttgtccgcctcggggtctgccactctgccc

cttcgacgcgcagccgtcttctccttctttacacccggcctcattattttcgattctcgacacgccatcgatcatcgtccatcgcacgcttttcctcc

cggtctccgtcctcagaccgtgtcgaggtcgagcaatccctccggccccgacagctgtttcttttcgcgagacttctccagaacacatcacggagtcg

gcgtcgccacatcacaacccggcgtcgttccctttcggtcctcgcttgctgctgcagcgattcgtcaaaaccaacatcgtccccttgccattatgcac

gcagaccacagtaccgtgatctccaccgccgcacgccatctcgtcgacaatgtcgtagaggacaaccagctcaacgccctcctcgagaagatcaagca

gtcccccgtcctcaaggaaggcaccttcgaccgcacttccgccctctccgaccagagcgctcgcgtcgtagctgacctcaagcgcgtcatcgagcaga

tcatcaagctcggcgacagcaagaacggcgacgacaagatccaagacttcttctacaactccattgccggtccggagtcggcggcgtgtggaaatcgc

ttacttcatccactatccatctcgcactggcaacagcgttttccgcgatccgtggcccccgcgcatgtgttgaggtcgagaaatccctccatccagca

gagcactcagactgctcgcacctccagtcactctccgtcctctcatacccgtccgcgtcgagaattcgcacattccgctccgagatggcttccctcgc

tcgctccgctcgccccttggtgggtgccgctgccccctggctgctgctcgctccttctccgcctccgcagcacaacaagccacttagccaccctccgt

gagcttgagcaacgagtcaagtcggtccggaacatcgagaagatcaccaagtccatgaagatgattgcaaccaccaagctcaacaaggccaccactgc

catgcaggctgccaaggtctacggcaaggccaacggtgatatcttcactaaatccgaggcccttgcaccatccggtggccgagagctcttcatcgtcg

tctcctccgacaagggtctctgcggcggtatccactcctccgtctccaagcggacccgtgccgagctcgccaagatcagccccaccgccgccaacccg

gactcgcccgagggtccagccatcgtcgtcctcggcgagaagtccaaggctcagctacagcgttcgttcaagaagaaccttgccctctccttctccca

ggtcggccgtgatgtgcctacgtttgcggatgcggctgccattgccgacatgatcttcaagtcgaacctcaagtttggcaaggtgaacatcgtctaca

acaagttcctttctgcgctctccttcgagtccgacatcctcgaggctttcagcgagaaggctctccgagacgcaccctttcctccatacccctctttc

caccttccttctctcgttcgtccgcctcactcctcatcgtttactcttcatgccgactcgggacagtcgtcttctaagggtcagtaccctatcggcac

gctaccgatacaaaaacgagcgcgatag

>contig_21.contig_21.g770.t1

atgacatcgaaatacaaagaggaagaagaagccgtcgagtgcgcggttgaagaggcacaggctatcgttcggaatggag

gcaaaccagatttttcagctcttggccgcaagcacaaggtggatcgccagcgcatctcaaggcgatatcataatggattaagcagatctacacgtcct

gccaacaatcggctgctctcagacagtcaggaggccgcgcttgttgcttggattcgcgtgttggacgatctcgaagccacaaatgaggcgaatgcgcc

caccgaagtgggcgatcactgggtccataacttcgtcaaaaggcacgaggagcttcatttcctcaagcaaaaatcgagggagctctcgagaatgacta

tggattggacaacaattaactggttcttcgtcaagctggataaagtcatcaaacagcatggaatattgcctgaagacatctggaacgttgatgaggtc

ggcttccgtgtaggcataggagggcgacaatggatcattacgctggttccatcaaaagacgcacaccttgcgtcggagacctgccgggatagcatcac

cgctatcgaggccgtcaatgcaacaggacagcatattgaaccgatgatcattatgtctgcaagcgtgcactctgaggcgtgggtggacaacaaactcg

aggacgagactctcctcgccgtttctcccaaaggatacaccgacgatatcattgcgttaaggcgcacgggaatctggccgtacaatccaaatgaggtc

ctgtccaagtaccccaacccaaagtactccggtacttcatctcaccaggccacctcatctcagcctaatgttccttcctcgccgagtcagcccgagcc

tccagtcacgccgcgcactgtccggtccatcaggcgctctgctcatgagatagagaagcgatacgatctctcgccgactttagagaaatatatcagag

gcaccgtggaaaatgcgcaaacagggagcctcgcaatttcggcactcgatcaacacacagcagcacaggtggaacgaaacaagcgtaacgagcggcag

cgcaaggttacgcagtcgggaggggtactgtacgcgcgggaggccagagagatagctcaggagaagttgcaacaggaggaaaaggaagaggcaaagcg

ggaatgggtgaagacgaaaaaggagatcaagaagcgcaaagatgagggatgggctaaggtttttgtcgaggtgcgcaagaaaccaccaggagaccata

ttgaccaggatcctcctcaactcattttggaccggttcctcgacatgtccaggagaacacatgtcagggaggatcctcagaccgaaatggaagcatcg

gaatttccctcagcgctgctcatgtcctcttctttcttgtcagactga

>contig_21.contig_21.g771.t1

atgtttcgcatccctctaaccgacttcactgtgcgcatcaacaccctaccaacagttattgatacgctcagctgcgtta

ggagaactgagtactactggacgcagcgaggattgatgctgttgctactgttcgatggagaccccttctcctatcgcacggattactaccgtgttctg

aggccgcaagcgactccccctccatcctccacccaagtcgcccagtcttcccaatcacgactgggccaaagcacatcgtccacgcagacgtcagagac

gcagtcgaaggtccgtctgcagcgcttgcccgtcatgttccgacccaaggtcatgaagacggcaatatgggaagaccctgacgacgaggagtgcttca

atctcttcgatcatatgggccgcggacctcggctgaagttcatgtaa

>contig_21.contig_21.g772.t1

atgcaactcaccgtcgccgccgtcctctccctcatcggcgctgccgccgctgccaccacgggtcccttctccgtcggtg

ccgccacctccggctacgagatcggtgttctcaactccaccctcctctgcaacgtcacctccaccggtctcaacctgaagaaccagcagatcctcttc

ggcatcaaggctgccctccccaaccgtgtcagctccacccagagcttctacatcactgccggcacccgtctcatcgtgcccgcgtccatcaacaactt

ggcctacggcttcggtgcccgcacctacgccggtaacgctaccaaggtcattgtcaacgcccagggcgccaccccctccaccattgacgccgccgtta

cccccatcgccatcccctcggcaccggtcgtctccggcggtgtctccgtcctgaacgttcccggttccggcaagaccctcaaggttggccccttcaag

ggcgccgctgccaacagcaaggtcattgtctccattggccagatcgaggccaccgtcaagacctacaacgacgctggtgccgccaccttcctgactgc

cagcatcgtctgccccgctcaggcccgccctgcctcgctcgcctacatcaacgttggcacctccggctccaccaccacacttactcccgccaacaacg

gcattctgcctacgattcccgtgaactccaccgccggcgccactggttacacttacacctgcactttcaccgacctcggctcgactgccgtccgtgtc

tccctcggtggtgcccgtgccaacaacgctgccgttgctcccggtggcgccatctccgtgaccaagggccagggtaacatttactcctccgctgccct

cgtcagcctgattcagggcaagtacgccaacgccgccaccttctccgtcggcatctctaccctcaacttcaacgctgccaacgccagcccctccacca

agaacggcatcccctccaccggttacacctccgccaagatcgccgtctcctccaccgctgttgccaccatcccctccggtgcccccaccacgacgctc

cccgccgtcaccttcaccgctggttccacctccggcaccaccgccaccgtctccctcggctcggctgccggtaacctgaacctgttcgacagcgccgg

tgccagcatcgccactgtccccttctcgtgccccgccctttcccccgatgtcccgatcttccccttcgacatccagtaa

>contig_21.contig_21.g773.t1

atgacctccacagcgtcaacatcggcaagcgcggcggaagcggacccatacgcaaccatcaggagctcacactccaacc

gcaaggctccctcctcacgcactcccatcttacccgcaacctcagcctccccgccctccttctcggcacccaggacatcaacccccaacacctcctcg

tcctcctgctccccgcttataaggccctcctccccgaccccgtccaccttctcagcctcgtcctcctcccactcctccaccacttcctctttcaaacg

gcgaaggactagcgtggatagcgttaccacagactggacaggctctctcccgcggaccaacttcagagccaaaacaggctcacgattactccgtcacg

gccttcattccaacagggactccctcggcagcagcaccagcgccccaccagccgaagccgaaccagatgcagatgcagatgtagacgcggaaggagaa

ggagacggcctcaagggctccgccgaaagcaccacctcctccacagcaaccacccaatcagcccaagccgccttcgaggagctcttccacgccacagc

cttcgccctcgaacgctccaacgagctcctcctctcgaccctcaactcccgctcccagctcgcatcgctacgggcacagcagagcgccatcgaacaga

acatgaactcgcgcgagttggagcttcgcagacgactggaggccaatcgcgatatggccgagtgggttcggcagagcgctgaacagctcgagacgctc

gtcagagagttggatgcggaaaggttgcgcgcggctggtgggaaacctgttcttaactcgactgccggcgccggtggtggcggagcagggtccgcttg

gggtttcgctcgtccggcccactggcgcgttccttccctcggcggactaggctccgtcttcggcgcagcgggggagaacgaggcagcaggctctgtcc

cgaattctgctgccgctgctgctgctgctgccaccacgtcctcgttatcatccgaagcgaacgtggggaacaccgctgaggacgggacgttgacgatt

ggtaaaacggcggcgaagcgacttgaacgggtgcttatcaagcataagagggggcagagctctctcagccttgcgagctctatgcagggccagcaggg

ccacggtaggagtgcatccgtggcttcctcccggttggagacgcccatcttcctcaacggcggcggcggcggtggtaatggggcaggggtcggcgaca

tgaagctaagctcgccctcccattctcccctcgcgaaaatggtcgacttccaagacgaagctcgcgatactggggccgacaatgacaaggcagcgccg

ccgtccaaggaagactccgagcttcatgcggccaacacgagctcgagcgtcgtgtctaccctggcgccttcttcttcgtccaacatcccggcgacgcc

caaatcgactattgtgcggagtcggtcgtcgaaactgatcaggccgggcaagcctgggctgggacttcgacttgggccctcggcgccaacgacgacga

cgacgcctactgcggccttgagcagtcccacagttgtagtttctcgcagcgccgagacgtcatcctcccccgaagtaggcggagtcgcggctcggaac

tcgttcgcgagtcgacggacggagtctcatacgagtctgagctcgattaacgaagatgagggcaactcgttatctaccacgggagggcgtactgggcg

actgttctcctttggtgctggggatggtggtggaggtgctggtcctgcgacgtcatcagcagcacccgcctcgcccacagtagatcggtgcgtctcgc

ctgcgctatcgatccaattcactcccggcgcagcaggcggaggaggaggaggtgcgcgtcggaccccttcccagcggggcagtatcagttcgttgaac

agcgtgaattcggtccggagcgagcggtatttgaacatgaagacttcgggcgggatgagttctcctgggtttgcgaaacctcgggcgttggttgctgt

tgctggttcgtcggcgcccgcgtcccggttggaggtgccgactactgtgctttcgggatcggcgccgtttcccggttggtcctctgggcctgctgatg

gcttgcctgacatgacggcggcggcgggtgcgggcgggatgggctcgttgatgttgatgaatgcgggaggtggaggtgggggaggtgcgtctggtacg

ctctctgctcttctgagtcggagcccgtccagggctgcttctgccacctctgcgcagcgcagtaccgccggtagcgagatgggcgaaggagagggcga

aggagaggaggatgacagtgcgcaattcagcgcctacctccgtaacagcgagaacaaggactcctctacctcttcggaccagcagcagcagcagcagc

agcagcagccaaaaatcccacccgcaagcccgctcaaagtcgacgctcgccgactctcgtcccaatcgaacgcgagcaggctgagcaatctctccatg

aacctgcgctcgccccgtctggcccggttggactcgatgaacaacgcggggagtgcgctggctgcgcaggctagttcggaccttgcgtccgggtggac

cggcgagggtgggcataggcgggcggataagatgatggggaacggtgagggggctggtgggaggggggctggggctggtggtgcggctggtggtggtg

gtggagggaagggagccttggcggcgctcaagaggttgaatgagcagaagggcgtgttgcccggtgggccggaggtggatggccggttcctttctgag

acgggcgccggtgccggggctggggctggtgctggcgcgtgggcgtcgatcacgagttgggttgggttgggcggtctggctggtggtggtgcggcgcc

ggctgctggtgctggggaggatacgtcacaggaagaggcgatggagaacggcgctgttgtggccaaggactctgagcctgcgtcggtggtgggcacgg

ctacggtacaggaggtctga

>contig_21.contig_21.g774.t1

atgagtgctgcctcgaacgcacccgtgctgcaatcccagcagccgccctggacggaaatcaaaccagcgtccacagctg

acaaggagagtttcgcatatacctcctgcctctcgcgctggccgacgatcctgacgggcgtgatcgacagcctcgtccacgcctccgccgccctcgcc

tacgcacgcaacgcacagctcgcacaggaacgcatcgacgaagccaaggcgctcatcgaatccatctccaagctcaaacacgacctcgcacgcgaccg

gccactcgagcccatcgtccattcttctactgcctcctccgacgggctcgattgccgtcattacaatgccgtggtcgaggctgagcatccgacctggt

tccgcgcgtcctggctgttcgcagagtgccatctgtacgccaccctacgagccatcttcgcaaactcaatccactggcagaacttcgaccccttcgct

cttcagaaactacagaccctcaagagctccggtaaggccatccgcaccctcgccaacaccgtgaacaacatcatcaccgccgccgccagctctaagga

catcgagagcgtgaccaacagcgcagagggtcaaaaggccctcttcctcgaaatggccaccatgagtctctggggaaacgcaaccgacctctcgctgc

tcacctccctctcctacgcagacatacaggccctccaggcatcctccgcagagcaacagcaggccaaggctcgcttcatcctctccaacgacctcgaa

cgcgtctgggacgacgtctttgcgcccctgcctaccttctcctccgtcgaagaacgcaaggagaagggcgtccgtgtcgatgttgtgcttgataattc

cgggttcgagctgtacacggacttgctctttgcagactggctcttgagtactggcaaagttcgagaagtcgtcttccaccccaagaacatgccatggt

tcgtcagcgacgtcaacccttcgggtacgtatcccaccgttaccgatcccgctcctcttctgcataggtcactaacacgctcaagaacccccttagac

ttccgacacatcatcaccgccctcctctctcccgaagacttcttcctcggcgaagacggcaccgccgctacgaagtctagcagtggtgccgtcgtaga

gacccccgagggcatcgacccagcttactttgcagacgcacgcggccgaccctgctcgccctccgcatcctacatcatcgactccacctccgtcaccc

cgccctccgtctccgacatcgcccctgcccctccctcctcctccggagacgacgagatggaagcactcaagtccaccgccgctcgctgggtcgagcac

ctcgactcgggccggttcaggcttagcatgccgcttgatgctgagctcggtcgacgggcgggtgctggcgatttctggacggagccgatgggctattc

cgagatggaaaagtacgcaccggacctgctcgcagacttgcaagaatctgcgctcgtcatcttcaaaggcgacctcaactaccgcaaactaacctccg

acctcaactggccctccacaacccccttcaacaccgccctcggacccctggcaggacaagccaacatcctcgcgctccggacgtgcaaggcggatgtg

tgtgtcggcttgggggagggcgtggaggagaaggtcaaggcggaggatgggaagtggagggtgaatgggaagtgggctgtcgttcagctttcccttcg

tacttga

>contig_21.contig_21.g775.t1

atgaacggcagcagcagcagcagcagcagtattcccatggggggcagcaacttcgatcaccacgaacacttcccgcgcg

gccgcctcacgcacacccacagcagcaccagcaccagcaccagcaccagccacggtggcgacggcggatcgcccttctccgcacactcccgcgagagc

tcgctcggaaaccagccgcagactcagcagcaccgcccacgcgaccactcgccgttccaagactccgccgccatcaacatgcagcatccacagcgcgc

cataggcttcgaagtcggccgcgccacggggaacggacaccatccggcttcctcctcctcacccggcggcggaggcgacttctcctcacgcgccagct

ccctcggccccgtcgcacgaccgcagccagctggaccagccgggaccagcatgccgcgcacaacatccgccatcgggcccagcggatcctcacaacac

atcagcagcagtgcatacggatcctcctcttcctcctcgtcgtcctcctccctcgcctcttccggctttggcgggtttggtggcagcggctcatcctc

cgccctcggctcgctcgccataacagacgaagacctcctaggcgatctcgagtccctcaacattggcagcaatggcgggccttatggccacggctctg

catccacctcctccgtccttgcccgtgtccagtacgacaccgcacagatccagcgacacccttcggtccgcgtgccatcgaaccacgcagcacagaca

ggcgtccatgcagcatccgtcccctccggcttcggcctcggacagctctttgccaaccaccaccagcaatctcagcaccaccatcaccaccaccacca

ccacagctcatcatcagctctcggcggtggtgggggcaacggatccgtctatggctccagcgcaggctccaattcgagttccagctacggcggcgctc

atgccatccccgccgagatgagcgcgagtaccaacagccatctctttcatcggagtaaccagcagcaccagtacagcccgcaactctcccatcatcac

cacgtgtccagtaccgggtccaactcgagcgctgctgctgccgctgcagccgagtatctgcgcggtgggactccttatggcagtggctcccatccgac

tcaacagcagcagtcaaatctcttctcacagcatcagcaccagctctatggacacagtcacgctcatgcccatcatccccacgcccatcccctctccg

ccagctggaacgactccggcgcagtcacgccgggaacatccgcccctcccgctcctggcgcgcgcatgcacgaactcgccgaacgactgcaacggcac

cagcaggtacagggacaacagtcctacctgcctctcaaatctaaacacgtcccgatgccttcaagagatctcggcggcgcttcctggggtacctcagg

atctcccactgcgctcggtgggttgggatccagtcctggggcggctgcagcagccgctgcggccatggcggctgcgttgggacgcagtcccgcatcta

ccagtggcctcggacgccgcgtcgtccctcgctctggatcacagacgtctccctcccgacgttctctcggcggcggtggcggtggcggcagtgacttt

gatgcccagagctcgcatagcgagagcgattgggtcgacgatgatgggcctgaggatgggcctgagtattcgggcccgcatgggaatggcgggctggg

tcactatggccatcatcatcatcaccagcacgcacaccagcagtaccagaatgggatgggtctgcaggggtatcagcatcagcagcaggctgagggcg

gacgcggacatgggcatggcgaggagtatgaggagggcgaggaagggatgggcggggcgggggctcggactccgactgcgcggacgtctagacatccc

ttcaatggattcacgagtatgagtgggcgcgcgtcacagcagcaacaacatcagcaacaagctccgccaccactaccactgcactcgctcgacgccct

cgccgcgcacggtctggacgaattgacgatagccaccctggccgcgctgggcgcgagcggagcggcgggcgcggcggatcttatgggtgcctttggtg

gccaaggaggcggacacggcgggcaaggaggaggcatgattccaggcgggtcgtcgtcgttcgggatgcatcctggtgggccctttggtggaccacag

tccttcgggcccggtggtggcggcggacatccgcaccaacaaggacccggcgggcctacaggcttcgcaggacccggcgtggccgagctcgggaaagg

cgtgccgctgcagacgttgttgaaggagacgcctttgtacattgtggagtttaagcaggggcggaaggaggtctttttcgcaccggtggacctgcaga

tgcagtgggagagggagcgggagcggattgtgaatggggatttggtgattgtggaggcggatcgggggaaggatttgggtacagtcgttacggactcg

ctcactgtcgagcaggtccaggcgtatctgctgcaccactccgaagtgctacaggcccaagtgaacgctcagcagcgcgcggaaggaggcgccggcgc

gggaggaccactatcaccaggcctcgacggcaccaacggcaccagcagtagcaacggaggcggaccccacccaccaggcagcaccaacccgaacagca

gcagcagcagcagcaacgagcttccgatgtacgcccgtcttgcgcgctcgatccacccgaagcgtctgtatggcaaggcgagcacgaacgataccagt

ctgctgttgacgaaggcgcaggatgaggagcgggcgttgcagttgtgccggacgaaggtgtcgcagcgcgggttgccgatgagtgttgttgctgcgga

gatgcagtgggatcgccgcaagttgactttctactatactgcatccgcgcgcgtcgatttccgcgatcttgtgaaagaattgttccggctgtacaaga

cgcgcatctggatgtgccacctggctcgatga

>contig_21.contig_21.g776.t1

atgtccttctacgaccttccactgtctcgaggctcggccattgcgccttctcatttccatgtcttcctgtcttcgcccc

tcttccagacgaccaaccactctatttcctcggcgagaatcctaccctcgactgggttctcgactacaatcatcaagaacgaagtctatgacacgctc

gcaacctgctcgccccaacgcctcaactcgctcgcattagctgcccagttggcgcaggaacgtcgcctggcgcccgacgcaagaagcttccatgcaga

agtggatacgcaaacgtattgggacagtgctggaccgttttcttcgctcgacaaccacctctcaactccttccagtcaacccgtatccggtgtccatc

tacagattggagtagattctacgcaaactcggattctggtgtcgttgaacaggaccttcagcttgaggagtgcacctttcatcggtctggacaccatc

caacacatcaccctagcggaacgacgaggtttcggtgccctgcctactgtaacagccttgccagagatgttcactcacgagcaccaagctcagcttca

ccattctcatccccatgctcaccaagctcaccatcaccacggcatgcccatgcaaatgggtgcaccgatgagtgcgccaccaaccatcacaggaatgt

caacgccaaccggcgtgcagactcccaaagtcgaatcgagtgctcctgtcgctcactggacccaggaggacgacagaagattacgagctgcggtagag

aatgtctcatgcggtcggaatggttcggctgggtcttcggcgaacgctgccgcccttagcgggacggctggaaagtcatggcaacgcatcgccctcgt

tgctttccccaacggcccgcacgacaaggatgcgtgtgcggctcgctggaaggcattaaaccaacccagcagcatcaagggcccctggtcgaaacaag

aggacaagcaacttatccgccttgtcgatcagtacgggccggagcactgggtgaccattgctgaaaagctcggcacccggactggcaaacagtgtcgc

gagcggtggcacaaccatgttgatcctacgatcaacaagacatcgttcacgcccgaggaggatgccatcatcatgaacatgtacgacaagattggaag

taaatgggcagagatgagcaaggttctcaaagggcggccggacaacgccatcaagaaccactacaacactacccttgtccgccggcggaagctcatgg

aggcggtcggaaagcagcagcagcaacaacatcagcaacaacaacaacaacagttccatcatgtacaacatcaaattcttccaccaactcccatgtcc

gccccacccatggtccatgcccaagatcccatgttcgtcaacgccatgatggaacaccggagatacctgctgcagcagcagcagaacgagaacgcaaa

cgcaggcatggtcgttcccgacgggaccatgtcaagcccctacatcacggaaggttgctttccgatgatgccgttccgcctggaggatcaagagaacg

aagctatgccccggttcgcccccggtgccgtccatcagttccaggatcgattcggcatgggtggcggtggtgggtccatgatggctcgctcggcctcg

attcctgcccagtcgcacgtccgactgggccttccgatccctctccagcgatcgacctcgtacacgaatggattcgtgccacccccacctgcgccggg

aacgtttatcaacccggacgagttcaacttcatgggtgccccaggggagaacgcttccagcagctttgcggatccgagctcgattcagttcggagggt

tcgggaacttgccgcatatggtgatctcgagccaggagtcgtacaacaacttgacgccaccgccgccgccgcctggtgtatcgctggacatggtgccg

ccttcgcatcttcatcagatgcacgagtcgctgtcggagggcgtctcgcttccgagctcgcatcaggactggcagctcgaacatatgatgcaggcggc

cgcatcgcagcagtcgttacatcacggctcgcaagcatcggacgcaagtgggctctatgcgacatatgggaacatgctcggcgtcacaggcatgccgg

tgacgatggtcctgtcagaccactcgcctcaggatgcgttcgcgcacgccaacctgcccgagctgtccagtgccggcaccagtccgttcttcggcagc

attccgctgggcatgcctgggtcggcaacgacgacccacgtccaggcggggacggtgatgcagcatcagcattcgcaacaacttgcactggaggctcg

gcaacagctccagcaggagcaagaaattgctgcgatccgggttcgggaggctcagctgcatgcgatggcgatgcaacagcagcagcagcaacagcagc

ccagtcagacatcgtcgacgacggcctcctcttcttctgctgcgcacgagtccaacggtcgctcgaccttgtcggtggccccaagcaagagtgcgacg

aacgctcgcggtggagtgtcacctaagacgagcaccatggagctatcggacaagccgagcaagaagaggaaacactctgccggtctgacgacttctga

ctctcgggatgcgaagaacgaggggacttcgagcagtaaagccgacgcttctgctgctgcctcctcttcctcatcatcgtcatcaatccaccgcgact

ccatcgcaggccactccgtcaagctacccagcatgcccgaatcccccttcagcccgcgccgtccccgcgaacgtctcccctccctcgcagccgtagct

ggagccgaggcactaaacgcctcccccaccacaatcaacaacgcagcagcggtgatcgcgatccggaaccggtcccgcacctcgagtctgctgggcat

cgacgagacgcataaagcgctgctgcccgcgttcaagcagcagatgtcggcttcggcgcataaggcggcggctgcggcggctgtggctaatgtgcttg

ctgccaacgccggttccggttctggtggcggtgggaaggacagggacggcggcttggcttccgggcggtcgagtcctggtgggacgggtctatcggcc

atcgtcctcggcggcggcacaccatcctcggccagtcgtcgattgcgctccggcagtcagctgagcgaaggaggtaatggagactcgccctcgaggcg

gatggcagcgcccccgttgaaacggtcgcggttgagcagtctctcgacggatatcttgccttcgacgatcgcttcgagtaactttacgttcagtgtgc

cgtcgtcggctcctggtgagggtaaggctgatgcagcagcgggggcgggagtgggtgacacgtcgacctcgagcgctcggtcgtccacgagcagtgcg

agcggtagtagtagcagcagcagcagccacctcgccatgcttcccgccctctccgccgactcctccatgaactcgaactcgaccaggagcatgacgtt

gctcatgagcccggacgacatcgagccgtcggaggattacacgagtgcgaaggtgctccctccaccatcggtatcgtccgcaacgacgtcggcgggta

tactgggttcgagcatgctgggtaacttcgctagcttcgtgctcccgccttcgttggacgggtatggcttctccttcgggaactcgctctcgccttcg

ggaccctcgatgacttcttcgtcctcgtcctcgtcctctgcgctcaaggagaagaacggtggcaataacggcaacggcaatggcactacgtcgcacat

ggtcgccaacctgctcggccttcatcatgcgcatcatcagcaggggaacgggaacgggaacgatgagaacgacccctctggtgggaatggacatcact

cgaactcgaactcgtcccggatgacgttcgggaacagccatctgcggattgggtcgaatagcactggcaccgtcggttccagtcggcctgggtcgagt

tga

>contig_21.contig_21.g777.t1

atgagcgcagacacagcagcagcagcgacggacaaggcagacaaggcagcgcgcaaggcagcaaaggctgcaaagaagg

aggagaagaagcgaaagagagagcagcagcagaaggaggagcacgagcagggcgatgcgcaggaccgcggcaacgacggcgaccaggagcgagaggac

cagcagaagcccaagaagaagaagcagaagaagagcgcagacggaagcacagcagaagtagccaacgacgacagcaagaagcagaagaagaaggacaa

gacaatcgcacccacagacccagctgcagcacaggacttcctcaccgcaaacaagatcaccatcgaagtgccagacaacgacgacgactcttcggcca

ccccgccgctccccatgctctccttcgcagaactcaagccacatctccagccagacctcttcaccgccctccagtcacagggcttcgcacagcccacc

cccatccaggcatgctgctggcccatcctcctcgcaggctcagacgtcgtaggcatcgcagagacaggctcaggaaagaccttcgccttcggtctccc

cgcactccaactcatcgcatcaaccgacgcacaacagcagaagaagggcgcaaaggcgaaggggaaggggaatgcctccgccgcagctgtgtccatgc

tcgtcgtcgcacccacacgcgaactcgccatacagacacacgaaaacttagagctcctcgccaaacccttgggcatcggatccatctgtctttacggc

ggcgtcagcaagcacgagcaagtggccgccctccgctcctcacagcctcccatccgcatcgtcgtcggcacaccaggtcgcgtcctcgacctcgcacg

cggtagttcctcaggaggcgattccgctctcgacctcagtcaggtccagtacctcgtcctcgacgaggctgatcgcatgctcgaccgcggcttcgaac

ccgacattcgcgccatcatcgggctctgtcgctcccacacccatccggagggcgggagacatacctccatgttctcagccacctggcctcccgccgta

cgcggtcttgccgagtccttcatgcgcaaccccacacgcgtcaccgtcggctctgacgagttgagcgccaatcgacgcgtctcgcaggaggtcgaggt

ccttgacgacggacgcgcgaaggaacgcagactgaatgagatccttagacggattggggccggtgggaacggcgctgctagcaaggacaagatcctca

tctttgccctctacaagaaggaggcacagcgtgtggagcagaccctccgaaggaacggctatgctgtctcgggcatccatggcgatctgtcacagcat

gatcgtatggcttccctcgatgcattcaagacggctaagacgcccttgttggtcgccaccgatgtcgctgcgcgcggattggacattcccaatgtgga

gtatgtcattaactataccttccctctgaccgtcgaagactacgtccaccgaatcggccgcacaggccggggcggcaagaccggcaaatccatcacct

tcttcaccgaagaagacaaagcacacgccggcgagctcatgcgggtcctcagggacgccgaccaacccgtgcccgaatcgatgaaccggttcccctcg

acgatcaagaagaagacgcataaggtgcaatgcattccagccatccactccgagatcaaaccatgcatcaccatcactccgggcgcgggtctgcatgc

gaagaagacgatccgatttgctacgctacataagtga

>contig_21.contig_21.g778.t1

atggtgtcttcatacgcagcagcagcatcgagctcagccaagccacaggcatcctcgctcggggccaaagctgcaaaac

caacacagacagtcaaacagtctctcctcggcaacacaacagcaccagctaccaaggacagcaacgacgacgacgacccactcccaccacgcaagcca

ttcggacaggaagacagcgacgacgacaatcctgatctcgtcttcgaagatgcaagagcagacaccgaaaacgacaacgacgacgacctcatgatcac

agacacaatcgaactcgtatccacaacctcaaccacagcacccacaacaagatcagcatcagcatcagcatcagcatcgaacaaaccctccttccccg

ccctcgcatccggcgctgcctcctcagccatcacctcctccaccgtatcagcaggagcaacggccatcgtcgaaaagcaggaacgcaagatccccatc

ccaccacaccgtctctccccgctcaaacgcgactggcccaagatctacaccccgctggtcgagatggcaaacctccaagtacgcatgaacgtcaagcg

ccacaccgtcgagctccgttcctccaaacacaccctcgccagcgcagccgctggcctcggcgtctccggtggactcggcatgctccagcgcggagccg

atttcgtgaaagcgtatgcgctgggtttcgatgtcgatgatgctattgccctcctcaggatggacgacctctttgtggacacctttgagatcaaggac

gtcaagacgcttcatggggaccatcttagtcgcgccattggtcgcatcgccggtaaggacggaaggacccggtatgccatcgaaaatgccagtcggac

acgcgtcgtcctcgcagataccaaaattcacatcctcggctcgttccaaaacatccgtatcgcacgcgatgccgtcgtctctctcatcatgggctcgc

cccccggaaaggtctatgcaaaccttaagacgatcggtgcaagacagcgacagcgcgcctga

>contig_21.contig_21.g779.t1

atgcaacgcgccggccacagacccatcacctccgagcgcgccgccaccgccggcagccccgccacgcccggcggcccca

actccctcttcacacgcgcaacacccggcggcggcggcggcagcaagagcccaggtgtcgcgctccggaagggcaacgagcgatacccgacccgactg

tcgttctacaacgtgccgccgacgcaggagatcaccctgtggcagttcgagacctgggccatcgacagactgcgtgtactggccgacatcgagtcctc

ccaagcgcgaaaccggacctacgaagaaacccgggatctagtaaaaacaacatcccggacctacctccccctcaacgcccacacagccaaagagaaac

cgctccaacacgagcgcgagcgcgaccacgtctcgcactttgtcctccggctcgccttcagtcggagcgaggagcttaggcgcaggttcgctaaggcg

gagacgatgctttttaggctgaggtgggaggaggatgattcgatcgagagggcggagttcgtggctagtttgaacatcgagtgggagcacgtctctga

cgaggaaaagcaaagattggcgccccagctgaaagccgtcaactcatgggccgcgacaaacttcaaggacgagcatttttacaaggtccactggacca

aagtcctcgacctcgtcgaaaagcgcaaagtcctcctcctcaacggccaagcctacgtcccctcccgcgaacaatcagccctagtcgtgtccgagttc

cacaacaacctgctcgccgcgctcgaacagaccgcgcgcgcactgccccggctgaacgaggatgaccgcctcgttcccgtgctggagcacctttcgat

ggggttcatggcgggtatatcttcggattatcagggtcaggctgggagtgggatcggggcggagttgggtgaggctttgcgagcggagatggtcgaga

atctcgtgcggcaacacgcgccgatgtgcatgcgtcatctgcaagagacgctcacggagaagaagcatctcaagcatttcggccggttgcagtataat

ctcttcttgaaggagatggggttgccgattgaggaggcctgcgcccggatcctgacgcaggaccaaccaggcccacaagactcccacggctgtccctt

ccgccacttctccccgcaaaacctctccgccggcctgtcctcgctgtacaacatcccggcctcggagcaagccgagatcctcaccgcggtcaaggcag

gacactaccacgtcggctgtacgcgcctctttgagctcacccacaagaaggtcggggtgaagaggggcgggggtgtggatgggaagggggagagtgtt

agtcatccgaatcggtactttgagcggagctttgggctgtcgagggaggggatggaggtggatgaggagcaggaggttaaggaggaaggggagaagag

ggacccgggcgagaactatgtcccttccgttccgaaatgggggaagacgtctgtgctgggagaggatgggaaggtggagggggagattggggatgagg

agttggatgctcttgttggtgagcagcagggcggaggggacaagatggaggtggatgatgggattgatgatgatgcggaggcggaggcgcggctgttg

gacgcgatgtacgccgtgacggcttga

>contig_21.contig_21.g780.t1

atggcgaatatctacgtgaaagagcctccgacagcggggaagatccgcctcgtcacgtccagcggcgacatcgacatcg

agctctggtcgaacgaggctcccaaggcatgccgcaacatcatcggcctcgcgctcgaaggctactacgacaactgcgtcttccaccgcatcgtcaag

aacttcatcatccaaaccggcgatcccacgggaacaggcatgggcggggagagcttctacggcgaacccttcgcagacgagatccaccaacgactccg

cttcacccgccgcggtctcgtcgcctgcgcgaattccaacgaaccaggcacgaacgagtcccagttcttcatcacactcgaccagacgcccgagctcc

agaacaaacacacgatctggggacggatcgagggtccgacgatttataacgtcctcgcgcttgcggatgtggagatgcggcaggacgagcctgatcgg

ccggtatatgcgcccaagttgctcacggttcaggtgctggagaatccttttgacgatatcgtgccgcgcatcacgcgcgaggagcggaaggagcagga

tcgggctaagaaggaggctctccgcgcgaagagggaaggcagagccggaccaaagaccaagaaaaattcgacactgctttcctttggcgaagcggaag

aggtcgaagcgaccgccctgagcaagggtgttagcaagtccagccacgaccttctcaacgacaagcgtctcaagcgcgaggcagccgtcgtcataccc

gcattcgacaaccccgcaccagcttccacctcaaacaaagcgaaccaacaacgcgccgaggagaatggagacgaaccgagcgcaaagaaacggaagac

ggaaccagcggcgggggagaccaaaatggaaagcagcgacctcgcgtccttacgcgccacacacgcgtccaaatccgccaaatccggcctctcctccc

aaatctccgagctcgagtcctccctgcgcgacatgagcggcgccaaagccaaagcgaaagcagcggccgaggaggaggagcggaagcggaagaaggcg

aaccgggggagggagctgctcgaagaggcgcggcgggagtacgtgctgaagggaggtgatgtcaaagatgggaaggatggacatggacagaagaaggg

gaaggctagggaggtatcgggggaagatgcgacgttggctgctatgaagcggttccaggcggagattcgttccaagggggctgctgctggcgatggcg

atggcggtaaggaagctcagcgggatccaatagcgtctgtggaggctgtggcggaggaagtctcggtcttggatagggaatatggtgcgagcgatgat

gacgatgatgattggcggaatcaccgcctcgattcccgcggcaagtcgatgtttacctccagcaccaaaggagcaggcggggacagactggacgacta

cgaagttctcgatccgcgtcaaccctcggctgccgctgctcgtcttggattcggaccggctgataatgtggccaaagctagggaggaggaaaagctgc

gggtcgaaggtcggcgcgggcgggattgggtggatgagagggattggaagggatggagtggtcgagaggaaagaggtggtggtggtggtggtggtagt

ggcggaagtaggccgtctcgagacgatgatcgacctggacacggtcaaggtcatggtcaacgcgacggtggaggagggcgagggaggggaagggggag

ttggggcgagcgtggagaggccgataagcggccgccaaatcacagcggggggtgggatggccgatcagggtcgaaaagggacgagttgggcgaccggt

cgggtgcgtcgcgggatcgacgatga

>contig_21.contig_21.g781.t1

atgtcagcgaagggccaagggtcggacgcggtgggcgaacaggctgtggatttcctgccctcccgggacctccaacacc

tcgccgttcggcggactccatcgctcagcaaagtcgaagctgcccttaaaaacacggcaagagccttaggagtgagcgaaaagggcaaggacgacgaa

cgcgagattctgttcgacgggaacctccaggatccggatgaagacgaagatgatcgaagcggcgaagaattcgagatgcgtgtcgtccgtaattggtt

gactggcatcgttgcttccgacttggactggctcgccaaggacggcgacggcgacgaacaaaaggtcgaagaacggagagaagaagctgtgtctcgcg

ctgcggaactgctcgccgtctgcgctgggaagtcagcgtccggtccatctcgagtacgatacaatttcgcaactccctccggcaccctctcggtccgc

tcccaagaagcgaccctgaccgaagatgcgctcggaggccgaacatggggtgccgccccgctccttctccaacatctcctcaaagacccggacacgag

gcaatccttgtcccttgattctccctgtagggctggtcgcccccttcggattctcgagattggggcaggaacaggtctggtcggtcttggacttgctc

tcgccagacgaaaacgaccgtcgaacgaaatgaacaaagcagaggacaagatcgatctccagctcgttctgacggaccaccatccgaacgtgctcgcc

aatctgacgtccaacgtcgctctaaacggcatcgagactggcagctcgacccaggtacgacggttggactggcaggctgtttatgatgctcaggcggg

cgaacatacatcctcgacatactcttccaaagcccagacgctgcctgtccagtccgagtccagcatggatccaacctggctggagcctcttccgttcg

aggacggaacggtggacatcctcattgctgcagactgcgtctacgacccactccacccgacctggatccgagcagtcgcagaaaagtacctgcgcaga

ccatcgtccagcacgaccaactctcagcacccaactcgcgaggaggaaggaggcctcatgcacatgatctcgcccctccgctccacgcacgccgcgga

gatgacagccatataccatgcgttcccgagcagcaaatcgtcgcaaggtaccgacgggccgcagcgggaagaggaagggaaagacgaggtcaagttgc

tgatggcggtcgaggcggagctgaagggttatgacgacttcgggccaccgcgcttacggcttggaacgcacgcacagggcgcacggaggatggatggc

actcagaccatgtaccgtcgtttcgaaattcgatggaggacataa

>contig_21.contig_21.g782.t1

atgggagtgatcccgagtccacgtggtggtcatgctcccgcacagacccgcttgctcgaagctgagctccttgggccag

catgggatgtagaaccatcgcttccctggagctgcatccctgcacgcttttccctcgcccccttccttgcttgcatctcgagccgccccgcccggtct

gaagtgtacatctcgcaactccgtctccccaaaatgaaagaagtcctgaccgacgccaactttccgcgtgatgcggaggggcatacctatcacgttgc

gaccaagctcggccaggtcgccaaccgtattatcacagtcggagatcatgtcagagcccgccgcatcgctgagctgttcgacggtggcaaagccatct

ttgagaagagctcgcagcgtctctttttgactttgaccggtacctacaagggtacaccgattactgttgtcgctatcggcatgggcttcagcgcggtc

gacttcttccttcgcgaatgccgagccgtcgtccaaggcgagatgattgtagtcagacttggatcatgcggatctattgacgagagccttcggatcgg

aaccgttgtcgtgcccttccaatcagtaggcgtgacccgcaactgggactacttcagcccagagctttcaggcaaggagcgtagcgctctcgagccgt

acctcatcactcagcccatcgcatgtgacaaagaagtgcatgatgcattgctcaagtcactcgaagcatctactgtagctccggccaggtccgacgtc

tttgggggcgagaaagccaaggccgttggcaacaccaccaatgccagtgccgacagcttttacgctgcccaaggtcggaatgacccagctttctccga

tgccaacgataccctgattgatgatctcaagaagcggcgccctggcgtggctacgttcgagatggagacgtatgcgctgagctcgctggctgcgtgcg

ccaatgctgacggatcaggtcggatccggacaggcgcggtgcagatgatctttgccgaccggaactcgtcaggcgtcatcacaccgaccgaggtcgaa

ttgttggagctttgggcgggaaagagtgtatgcgatgcccttatcagcctgcccattcctgaagagctcacgcagaaggagggcgtgtggagccaggc

atcgacgtcgagctag

>contig_21.contig_21.g783.t1

atgggtatcagccgatcatccagacacaagcgcacagcctcgggtgctcgccaggcccactaccgcaagaagagaaagt

tcgagctcggtcgccaggccgccaacacccgtatcggtgccaagcgcattcactccgtccgcgtccgtggtggtaacgtcaagcagcgtgccctccgt

ctcgagagcggtaactttgcatggggctctgagcacatcaccgccaagacccgtatcctcggtgtcgtcttcaacgcttccaacaacgagctggtccg

cacaaacaccctcgtcaagggtgccgtcatccaggtcgatgccacccccttccgtcaggcatacgagaagcactacggtgtccccgtgactatcaaga

agcgcgccggtgcccccgctgaggtcgaggaggtcaagaagagcaagcacattgccgctaccctcgttggccgtcagaaggacgccaagctcgacccc

ctcgttgagccccaattcggcgctggtcgtttgtacgccgtcatctcatctcgtcccggtcagagcgggcgggcagatggttacatcctggagggcgc

tgagctcgagcactacgttcgccggctccgtaccaagggttccgccaagcacggttaa

>contig_21.contig_21.g784.t1

atgatgaatgttgatcgcccaaacgcagcagagcatgacgctcttgccaaagtccagggtaggcactcctctcacgttg

cagttcccccagccagatttccttccaattgcagcattttcttctcttccagcgcagaagaacaggtcgacttagctttgttcgaatgcgtcaaggac

aagctgtcgctcgaacatcagtcgaacaacattgtgcgctcagcactcgcggagctgaccaagacagcgaccggcctccgaggtggaatcgatcagaa

gaccgccgacattcttgacgaacaaggagtccgactgtggaatgcatcgattacgcttcgacacctcagcgaaggggatgccggagcagacaacttta

tagccgatgtccgatcgacggcggtgtctatgctcgatgcaggatcaagtgtcagcctcgacacggatgccgctgtcaggctcatcatggcgtactcc

agcctgctcagtctgttgagctccactaccaaacatgaatttgagggaccgctgtctcgcgccaaggaggacttcgcatcaggtcgctacgaaagtgg

agatagtcatctgtccaagcttcttgagcccatctggaggcggtgtttccagctcggacagcatttcctcctacagctacctgagcaagtccaaaccg

aaggagaaggagctggtctgggcgagacggagattggacaatgtaaaattgcggttaagtggctgcaaaagggcattacgctccaagagcgggccggt

gtggtcggagtgcccgtggtcaaagccctgctccaactggcgtatgcctactttattgctggagcaacggagcgaagtgcactcgcttcggcagaagc

agctttgaacgagtcatcgaaaatgcttcaggctgatggcgggcagaacagcgagctcgtcgagtacatcgcaatcatgcgcttccgagtgacaacgc

gacacggcagcgacgcggatatctctcgagcactggagcagctgtgttcgacactcgccttcaatgaaacaaacactgcccaactgctagacttcgtc

acccacggcccgaagcgtatgctcgctaccgggttccgcatcatcatccgtgccatgctgaagtgcgatcaagaagacgtgcagcagcatgtcacgtc

cgtcgttcttcgagctctgttcttgctcagcgacccatcaaccttgtcgcagctcgactcgctgttggacgacatcgaaagctcagacatcgatctga

gtcaggatgctgtttgtgctgcccaaatgatgatctggcgccgaggggacgttttgtacaaaaaagagatgttcgaacacgctgccgacgtccacatg

atcgccgtgcatcgggtctttgcttccatgcccgacaacattgccaaaagtgcaagacgtatcgcggtctgctacctcgcactgcaccagcccgaggc

tgtgaaggcatgcttggatctctgctccgagccttatcgcaacaatgcgtcctcgcatctctgcatgtaccttgctgccgttcaaatgggcgacgagg

acgaggctttgggagcgttccaggcgttggtgcgagctcccgacatggagcctcaagtccttgttgaggttgttgaagaggcgcagaaggcgaacatg

gaaagagttctccacgtcggcctcgaaacgctgctggacgctatcaaggtcgactctcagatggaggctaaggtcgacattctaacgctgcacaagac

cctcgtttgcattttcctcccgcctgacaacgagcgagcttggaccgagcaccaagccaatcagcttgaggtgtatctgcgtcaagctctgaaagcgt

tgagcactcaaatggactcgaacgaggacgcacgcaagcacgcagaatggctgtggaagcatgtcctggcatcccactctcttgacgcaaacatcacg

gccagtctctttgacgttgctttcgatttgatggaagagcggcatcggttctcgggagaagctcctgatagctccatcgtggttgcgagttggtgtgc

aaagttcgcttcggtcgaagttgcggcgctcagcgttccgagcgtagagaacccctctgaacgggcgagtctgtacaagatgctgcaagagcaagtcg

agcttgcccagaagctacgaaaggcggcacatgctatcaatccggaagctgcaccggcagaggcggagtgggctctgctcatgcttttgctaaaactg

gcagtcgcgacccaagattggtcaggtgtcaaggaacttttgcagagtctcgagggccggctggactccatgcctttcaaggtcgcagaggcgctcgt

tcatgccgtgagcgtggaggccgagtccgaaatcccggtcgaaatcgaggtccagtgcttagagtttctgctgcggagatacaatcttgaagaagatg

agaagacgatcagtcgaacgctgcgataccaccgcttcctgctgatgtgcctccttgaagacggcacgttcgcacaagcagaataccgccgaagagca

ttggcctgcttcgagtcggtacgggacgctgtggtcgaggcgcaaggcaaggagaagacggaatcgcacgtcgacgatatcatgtggtacctggcgca

agcgtggcaaaggggcacggagctggcgatggcggcggtgctagaagaggcgaaaacgttgagcgagctcgctctgagcttcagtcggttcattcctt

ttccggacgctacaagacgcaggatggagattcagtaccagaccttgctacgtcgcatcagcgaagcaccacccctgccgcctttcccgcagaacaag

caagcgtcctga

>contig_21.contig_21.g785.t1

atgccaaaagccagctcatctcagccggtgtctggatcatcgaagtccagctcgtcgaagaagacgtcggaggacaagg

agaatcgcgcggcgggcaagcaggatgcccagcagcactctgttggcgatgcggcaggggaggtcgagcggctgacgagcaagatcaagctccttgaa

ggtcatttagctgatatggaagcggagcgcgaaggagacgtcaagcatttcgaggagctccaaggtttgcggaataaaaagatggagcaacagctgga

gagggtgaaggagaaggcgatggggaagcatcaattacaagaaaagctaatcagcacactcaaggagcgcgtcaaagctctcgaagcaacgattacct

ccctggcagcggcgccctccaatggcgaaggcagcgcggggagcgcatcgaccgtgcggaatgtggggacgtccgcatctgctcaagctagcaatcgg

gacgagggcacgtcggaaatggcagccgagctgaagaaattgcgggaaagcatggcaaggagggagcaagagtgggagctagagcggagcagcttaaa

agatcaagtgcgacaactggagagagagaacaaggcggagagagaagcgtcgaaggcggcgcttcagaagacgaatggcacacatcaagctgtcaagg

agcctgctgggaacccgctcaatgagggtacggacaagattcgagcgttgtacgaggatctcacggggcttactatcagctctgtggagacgtatgac

tctcggaatgggtaccgcaggttccgaggagtcttcgcgaatcagggatatcatcatctccaactgtgcctcgaggagtccgcatcggaagtaccacc

ctcgaccgcggagaaatcatcccggaagccagccatggccccacggaagcgacaggaactcgtgtacattcctcagatccaggacgaccgcgacgccg

agttgctcaaggcggggacgttgcctgccttcttcttgggacagatacgtctccatcccgtcttcctgttctctctctatgtttacaccgaccttatc

accggcgacgagatggcctccgactcgttcgaccttaagctcgtcgacgacatcgtcttcgaggtcgactcccagctgatcaccctcaaagagggcga

agtcgacatcggcggcaacccctcggccgaagaggccgacgaaggcgtcgaagacggctcgaagacggtgaacaacttcggcaacgcgttctcgctcc

aaccaacctcgttcgacaagaagacgtacctgacgtacttgaaggggtacatgaaggagatcaagagcaagctgccggaggacagggtgcccgcgttt

gagaagggggctgcggcttttgcgaagaaggtggtggctaactttaaggactacgagttcttcactggcgagtcgatgaaccctgacggcatggtcgc

catgctcaactaccgggaggacggtgtcactccctactggatcctctggaaggacgggctcaaggtgaccaaggtctaa

>contig_21.contig_21.g786.t1

atgctgtcgcgctttgccgctctcagcatcgccggctcatccacgcgcctcccagcagcagccctcctcgccgcacgcg

catcagcgacagcccgtctcgcttccaccgcagcagcagcagcagcagcagcaactgcccccaagaagaaggcacccgcagcgtccccatccgcaact

accaagaagacggccacatccgcaaaatcaaagcctgctgctaccaagaaggccgccgctgctaagaagaccgcagccgctgcgaagaagaagaaggc

tgcccccaagaagaaggctgctcccaagaagaaagcagcggctcctaagaagaaggccgctcctaagaaggctcccgcaccaaagccatgggtacgac

tcgatgccgatggcaagccggtgcccctcccacaagagacaagacccaggctgctccccgcatacatcctcttcatgaaggaccacttcagcaaccca

gaggtcagggaacaaaccgggagcaaagcgccccaagacctcttgcgcggaatcgccaatcaatggaagtcgcttcctcaagcagacaagcagaaata

cctggatgcagccgcagccgcaaaggagacctaccccgccgagctcgagacctggaaggcctccctgaccccagacgacatccagcgccagaacgagt

acgagcgctacctgaaccgtcgtcgtcaagcggccctcaagaagagcgaagcgggcgatgcctccctcaaggtgcccaccaagcgacgcagtctggcc

ctgcgcgacccgctcaagcccaagcggcccatctcgcccttcttccacttcatgggcgactaccggatcaggcactacgagactgtccttgcggctca

tgacagtgtcaaggcggcccagaaccagctggcgaaggatgctgcgaaggagtggaactcgttcagtgctgagaccaagcagccatacgaggaccggt

tcaagcgcgacaacgagaactaccacgtcgccgtcaaggcgtacgagttgcgaaacaccccccagtaa

>contig_21.contig_21.g787.t1

atggcaccgcggacagtgaagacgtcgcacggagaggcgatcggcccgcttcaggctgccgagctcctccgcgcacgcc

gcatcgcaaccggaaagaccgcactcacccccgaggaagaggcacaccaagcagcgcgccaggcagaacagcccaccgcacaggacgcgctcccactc

ggcaggctcctcgcctccaccgagaagcgcatacgcgacggcgccattcgctccctcgctgcctttgtctcccgccccagcagcagcagcagtagtgc

ccaccaggacgaccctccatcgacctcggctgtcatcttgccagcagagatggccaagctctggaagggcatcttctactgcttctggatgtccgaca

agcctctcgtgcagcaggccctcgcctcagaacttgcccaactcgtcctccgcatcgcaaacccgccgcagggaaagaagcggtccagcactgaagag

cagcaggaacagcacgacgccctcgatcccggcagggtcgactccgccatcgctttcctccaaggcttctgggccgccatggcaagggaatggccttt

gatcgacaagcacagggtcgacaaatacctcctcctcatgcgccggttcacaggcgccggcttccaactctgccagctcaccaactgggacccgtccg

ccctcgcaagactgaacgacgttcttaccacacccacgcccgcaccaggcagcgcttcccaggacgattccaacgaccccttcgcaaggggcggacca

gggcccctcgcagtacacgacatcaagctgcctgacagcatctcctaccacgtcgctgatatctgggtagatgagctctccaacgcactagcaaccgc

agatgagccatcatcatcatcatcatcatcgcgcgccgctccactaccagaagtgttccaaccgatcatggcagccctctcccgcgccgcactctcga

agatgtacgaccgaatcctacagagcagccttcagcccctgcttgacgatctccagaaggctatcgagttcttggacgtcattgcaggggaggacgat

gacgatcttgatgatgaccacgaagcggaaggagaggagcagggcgagggaaaagccaagtctaaaggcgcttcaagaccaaagcggaatgctcagag

aaaacccgacgaaggagcagacggagaggaagacgacgactttgacatcgaggagctatgggaagctctcgattacccctcgctcttgcagtcggccc

tgacgttggacgcgtcgtcgcagccaaccaccatcgacacgcaagccgcccggcctgccaagcgcgcaaagtcgtccaaagcgacacggaaggaagca

gagctcgaaccagaaatgcagacgactccttcctccccaacgccgtcagcaaagactctcctatgccgcgcccgcaaactccgactcgacatctacct

cgccctccgcgcagcagcacgcagtacatcgactaccaccacacccgcgaaccccgcccgggcccgctcgctcgagaaacttgtccgctcccaactct

ccccaacggaagaccccgagccccagtccccctccgacgaaggcgtcctctccgcctccgagcgccgcacactcgaacgcaaggtccggatggagcgt

gcgaaggcgaggatcttgagacggaagaggaacgtggagatgcaggctgctgttatgcggaaggcggcggagaggagggcgaagaaggcgaaggttgc

cagtcaggggagggccgcggctgagaaggctaggttggggatggacgggccggtcgttattgggccttcgctttcgcgtaagctggcgcgacggaaga

agtga

>contig_21.contig_21.g788.t1

atgccgatcaatcaacctgccaatcagatcaaactgacaaatgtgtcggtcgtgcgattgaagaagggcggcaagcgct

tcgagatcgcctgctacaagaacaaggtccgggaatggcggacgggcgctgagaaagacctcgacgaagtcgtccaaatccaaaacgtgttcgtgaac

gtgtcgaaaggccaggtggcgccgaacgaagacctcctcaagatcttcggcacgaccgagctcgagccgatcgtgctcgacattctcaagaagggcga

actccaagtcggcgaaaaggaacgctcgcacgagctctcctcaacctggcgcgacatcgcaacccaagtcgtcgagaaatgcatcgacccgcgctcgc

aacggccctacaccgtggggatgatcgaaaaagcgatggccgaggtgcatttcagcgtgaagccgggcaagagcgcgaagagtcaggcgttggagttg

atcaagatcttgcaggagaagaagacgatcccgattgagagggcgcggatgaaggtgcgggttacgatgccgaataaggacgggaagcggattaagga

gaaggtggtgccgttggtggagaaggtggaggatgaggattggacggatgagtgggaattgatcgcgatgatcagtcctggctcgctgcgactcatca

acgagctgttggaggaggagaccaagggcaggggacgcgtagagacgctcagcttcgcaactgtgctagatggccacgacggcgacgagagaatcgat

tag

>contig_21.contig_21.g789.t1

atggccgcccttgccgctgccgccgccgccgcgtgcccgacttctctccagacactcgcagcctcgagctccctctcgc

ccatcaaagtcttgagaataccccacctcgtgccatacaacctcggcacggccctccaagagcatctctaccagcgtcgattggatgcccgtgccttc

gtacgggagctgccgagcccttcgcctgcgtccaggatcgatgctgcaggacgagggagggggagagaagcgctcgcaagagaggtggcgaacacgga

tcatctgctcttactccagcacgcccccgtctttaccgagggcaagcgccagaaggagatggatcaagaattgtccaacgcttgtcggaagcttgggg

tagactacgtccttgcccgccgcggtggcttgatcacgtaccacggtgttggacagatcacgggatatccatcgcttgacctggcgccgatgcaactc

tcggtcaagtgctacgtctcctacctcgagaccctcttccgcaccctcctcgcctcggaccgatacaacctcccaaccgtcgaaccaccggacggcca

cacgggcgtctgggcagacgactaccacaagatcgtcgccctcggcgtccaagtccgccaccgactcaccctcgacggcttcgggttcaacatcagct

cagccccgctcgatttcttctcgatgatcgtcgcttgtggcattcagggaaggttcatgaccacgctttggacggagcttggcaagaaggccgcggga

gggggaaagttgccttcagctgaggaggcggagaggatatgggcggcgaacccagatctgaggccatcggttgcggacatgatgtccgttgttgccga

gcactttggtctgcagttcggacgaaggatggaagatgttcgggaggaggaggcccagtacgaagtcgagtccacttcgcaatggatggatgagaacg

gaaagccggccttgaagctcaagtccatcacgctgtacggcgagaggatagacctcctcgtacctccgtaa

>contig_21.contig_21.g790.t1

atgccatcgccgtccggcccgaccgcgcccgcgtccgcaacccgcccgccgccgccgcctcgctcgctcaagctggtgc

tcattggcgacggcggtgtcggcaaaacaacgcttcgttcccgtttcctcacaggccacttcgcgcccgcatacagagctaccattggcgccgacttc

ctcaccaagacgctgtcgttgaaccccgatgaccccgccagtcctcgtgtgacggtgtccatctgggacacggcaggacaagaacgcttcgcctccct

cggcgcaagcttctaccgcggcgcagacgctgtcatcatcgccttcgactccaatgcatccctcgctcatatccaggctcggctgacgtactggcaca

acgaatttctcaccaagacgcccatcccgcccaacgaacatgcccgtcgcttctgctgggtctgtgtgggttgcaaaaccgatctcatccccgatagc

gaccaggacactgctctaaccctcaagcgcaaccaggtccaggcggtgctcgacgcgattctgccgcggcctaggtccgacatccaagctgaagacat

ctcgtctgagccgcttctccccccttctcccatccctgcttcttcctcctctaataccctcagcaagaatgatctcacactgaagctgaacgataccc

gcgctgatcgaaggaagtccctgccatacccggcagctatggcaaaacgcgcgtccaaacagagtctgcgttccaccatttccgtcaagccgataccc

acgcctcccccgccgaactcagccggttccgcctctcgctctcgaagccggcgcatctcagctcggaaccccccgcgtcattcgaacgagcaaggctc

accgcttacatctccggctttgggattgacaacagcgagcagctcctccgcttccacccctactcccttcggagggacgcgctccgacccaatagacg

tcacacccaccaaagcgtcgcgtcctctgcccctgaacgacgacgatcttccttccgcgcagggtcacaccgactcatcctcggccatcagcatttcc

tccgcctccgcctcagcctcagcccactccttccctattctcggcccttcctccgctggatcatctgcctcgtcgaccaacaacccgctcctctactc

cggcggaatctacgtgaacgggaagatgcacaaacgggaccggctcgacagttccgtctccaatacgtcgaccatctaccatacgccccggaacagca

ctttcttcccctccagtccggcaacccatcggtcgggtttgactactggctcggcgatctccggttccgatccagctgtacccccttttgtggatgga

cgacggacgcgtacgagtctagagagtacgcggagtaatgggtcccatccgagcgtcgtaagcggtctgcggagacgggcgtcgaatgagagtgtctc

ttctgtccggactgtcaagggggatgatggggccagcagtctgggtcacggatcgaacgggacgagtatccgctttggcggcggtgcagaggcgaagg

aacgcggttcgggaagcgggcggtcgtcgctcgagctggatacacaagtcaagcgcaagtcctcgctcgcgttcagctataagggcgaagatgatcag

gaagacgggaaggaggagagtactgaagaggatgacgcacacgagatgcggaatttggttcgaccgcgggggatgacgcgtcgggagagctcgtccga

tacggtacaagcgccgcaacgtgcagaagcttcggaagaagaattttccgctgtggaagtagacgacgcgtccatttctcggccacagtcaggtgtaa

gcacaagcaccagcatgacggagcgcagttgggatcagcccacgccccgtcctggctcgtgggcgcaaactggggccggcggcggcggtgacagtggc

gaaggagggtctatcatcctaagaaaccaagctgaagtcgatgacattgacgaggaggatcaggaggacgaggacgatgaagaagacgaatacggatt

gcgaaggacaagcactgctttcccactcgagccaatccccttcccctccgccgccatctatcacgaaccgcaccacttcgacgacgacctggacccga

acttcagcctcccgggcgggtcgtccaataccctcagtcgagcgacagcgccgcctgtgctgcccagtctctcgatcaccgatacgagcgatgacccc

ggcggagcgccgagtacgctgtatcagagcaagaaggaggcggcgcaggcggagatggaacgatggcgcgaggcgcggtcgaggaagcggagatcgag

gatgggaacgggggcgacgacggggacatggacgggtacggggacgacgacccgggtggattcgctcaggatggaggcggaagtgagttcgatgagct

cgagcttgacctcggctggaagtgtgagcgcggggaaggcagacggtgcgagtgagccctcgaggagtcggacggtgtcgaacaatttggagtcggag

agagaggagcgggggcgggacgacgcagatggcgaggtggacgaggctgggacgacgtctggccggggtcgtggagagacgggcggctatgttgtagg

ttcgcacgatgcggatctgagtagcagtcggagaagctccctcttccggcctcaggatgatacgaccataaccaataacccgctcaccgctgccacca

acgtcgacccaaccctgacctcgacctcggccccaggcccaacgaacccaccccgcccatcgagtcccgcgaccgacctggaccccacagacccggag

cccgccgcgctcgaagaaggcttccacctcttcctaacctcctgcaagatcggcacgaacgtctcgaatgtcttcacgcacgtcgtcgagcgcgtctc

ggcgcggtgggcgtacgaggagtgggaggagcgggagtaccggcgggctgtgagggagtggaaggttgcccggcggttggagagggagggtggggttg

gtggggcgcagatggggaggaaggggaggtggatggctatttttgggcgggggaggggcgctggttctggtgctggtgctggaggagggaggaatgcg

acttcgggggccgcggaggagtgggagttggatgaggatgaggatgaagaggaggagcggacgagggttgctgtccgacgggcgattcggatcgcggc

tggcaaggactctggggcgggcgcttggtccgtgaagggctgttgcagtggttga

>contig_21.contig_21.g791.t1

atgtctccgttggtagcagccgcagccgcaaccgccgagagcgccagtccgcgctcctccctcgacaaccagcccgccg

acgacagcggcaaggatgacgccaaggatgacgccaagggaaaggcccgcgtcccctccgtgactttcccgtccagcgtcgtcgagcgcactcccagt

cccgaggtgcagatctaccgcacaggatcacagcgccggccctcccactcgatccaccagcacccgctccagctcacaaacatccagcaagacgcagg

gacgagtcgcatgggcgacgcaaatgacgcgcttgagctcggagaccgccgctcttccgtcggcggaatctccttcggaaaccccgtcaccccgcgag

acgagccgacggacccgctcctcctccgcagccgcctcgtagccgacaacgacctccgccggcggggcggcaagggcaagcgtaccgacggccgaatc

aacgaattctacaacacgcaaaactcgcacatcgagaatctgctcaagccgatggcgaagcatgccacggacggcgaggaggagcgggagggcgcggc

gctcaaggtgaagattgccatctatgcgtcgatcattgccaacttcgtactggccgccttgcagctctacgctgccatctcctccctctccctctcgc

tcttcgccacagccgccgactccgtcttcgacccgttcgccaacctggtcctgaactggctccaccgcaagtcaaagagcgtcgacgagaccaagtgg

cccatcggcggcgcccgcttcgaaaccatcggcaacatcgtgtacgccttcctcatggcctccgtctccgccatcctgatcgtccaatcgctggtcga

catcgcaggccactccgggaacgacaagaacgagcttcacatcccctcgctcatcgcagtcggcatcgcgtttgtgaccaagtttgtgctgtttgtgt

attgctatgggatccggtcgcattcgagtcaggtgcaggtcttgtatgaggaccatcggaatgacctcttcatcaatggcttcggtatcttcacctca

gccgcaggtgccaagttcgcctggtggctcgaccctctcggcgccatcctcatctccatcgccatcatcttcagctggacccggacggcctacctcca

attccgcgaactcgcaggcgcaggtgccccgccggagttcctctccctcatcgtgtacaatgccatgttgcaccacgagcatattcagcagatcgatt

cttgcaaggcgtatcattccgggccgaagtacatagtcgaagtcgaccttgtcatgcatccccagacacctctctggctgtcgcacgacgtctcgcag

gacctccaagaccgcatcgaggacctgcccatggtcgagcgtgctttcatccacgtcgaccacgagacggatcaccggcctgaacatcgcaagaacgt

ctga

>contig_21.contig_21.g792.t1

atgacctcgttccctcagccgtacaagacgctcgccaccgaattccacctggtccaattcgaggccgaaggtgtcctgc

gagtgagcttcaaccgcccgcctgtcaatgcctggcacgatgcgatgtggagagagcttcacaagctcttcgacaccatcagcaccgataacgacgtc

gccgtcgtcgtcttgagtggcgagggccgagccttcaccgccggtcttgacctgaaatcaaacgaactcttcaccacgctctccacaatcgaggacgt

cggacgacgggcctttgcgatccatgcccacatcaagcacttccaggctgccatctcctccatcgagaagtgcggtaagccggtgatcgccgctgccc

atgggattgcctatggcttggctctcgacctcatgtcggcctgcgatatccgcatcggcctcgcagcggacatcggcaccctccaacgattccccaag

acggtaggtaacgactccctcgcccgcgaactggcatactcagcccgaacattcgacgcccgcgaggcgctcgccatcggcttcgtcagcaaagtgac

cgagcaaggccaacccgctgtcgtcgcggcggcactctccctggccaaggacattgcctccaaatcgcccatcgccgtccaaagcacgaaggcgatcc

tgctccacgcccgggaccaccccgtcgacgcaggcctggccttcacggccgcctggaacgcggccatgctgcagacggaggatattccgatcgcaact

caggctgtgatggcgaagcagaagcctgccttctccaagttgtag

>contig_21.contig_21.g793.t1

atgagtgcagcgcttcagaaagtccagcacgccctcgagcgcaccaccggcgcacccggccgccgatcgtcccgcaacc

cgtacacccgcgcagcaggagccgccgccgctgtcctcacactcgccggactcggcatcggactgtatgtatattcgcgttggacaccggactctcag

ccgggtcagggcggggatgattgggctgctggcggtcgctcgaggaggagcgggggaggagatgatgagacgcgagcaacaacatcagaggacaaaga

gggagcctcagcacaacaaccaccaaaatcagcagcgccagcaacaggcgcaacgcagcaagcccgtcgtcgccgtctgcctagaccaagtctctccc

tctccctcccacccaccttcgaccgctcctcgccacaagccctccagctcttacacgacctcgtcgccgccctagccgagcacttcctcatccatctc

atcctaccagactccccttcctcctcttccaattccaccagcgagagcccgtctccagcaactgctgctactgctactgggcaaaatacaggcgctct

agcagccgtcttcgccgacgtcctcgacttcgacacccggcgcttattactctacggaacgcccgcagggcgacaggccctcggtctggccctggcat

gcgatgcgcatgtcgagatctccctcgtcggtgtggttgctgatcctaaagaggaggaaggaggatcatcgtcgtcggatgggaacccaccagaggag

agtatcaagaaggtcgagcatctccagcgcacagccggcgtagtcgtcctctgcgtcctgcccgctcccggccgccgacaaggacagggcgaggcgtc

ggtctccccggcgacgagcgcaagcagggcaaacctcgatgccgtgctcgaacatttcaaaaaggcgtcgttcagcaggagcgtccgcatcatcgata

caagcactccctcagcagctgagggtcccgctgccaccggggcacaaagtgacgagacgcagtggaggaccggggcagcgagaatagtagaactcaga

gcaaagatgaagtag

>contig_21.contig_21.g794.t1

atgtcacgcgaagcaaagtgtctcgtcatcgtccatggcggatactcgatccgggccggcatgggcatccatgatctct

tccagcagccatccgtgacacttaacgcccgcgtcggtctctcgcgcgcatggctcagacagcgcaggaaatcagcagcagacccatcatccttccca

ccgccccaagcactcgactacctcgtcggccaagccctcgacgacgccctctccgccaacccgtcggatatcatcatctaccggcccctccagggagc

ccacgtcgccgattggcaacagctcatcgctctctggcgacatctcctcttcgacctcctccgcatcaagcgctcccggaatgattcgcatacgctcc

tttctctgccggcgcctattcatcgggacacgtataccaccgctgcgcaggtcttcttcgagctcttcaatacgcccgcgctgtccattgtggagacg

ccccttgtcagcgcgtacgctgctggcaccctcaccggcgtcaccgttgatatcggctgggcggggtgctccatcacgcccattgtagattgcgaggt

ccggcacgaggccatcgttcgtacacgcgtaggcacccggctatgcaccatctggctcgcccatctcctcgcgcaggactcgagtatcgtgcaggaac

tgggccggatcgcaggatcggctccatcgtccgccgaggcctcctcctcctcgtctacaacacagtcggccgtacacgagctcctggtcgatctcgcc

cagctcttgttggaggaaggtcatatccggatgaccgacgaaggggatcattcctccgcgcgggcccatgagctcgatgatgagggcaatctcgatat

cgccgctgctctagtccaagggcgcgaacgcgacttggtagaggagcaagaacggaaaaaggcggcggaggcagcagaggacgcggctgctaaagggg

acgaagaagcgggcggggcggcagctgctgctaagagggccgaagcgcagaagcaactgcagcagcagggccaagcgggcgggagcaccgatccgaat

gcggtcacggttgatttccgcggacgccggatcacggttggaccgtcccggttccggtttgcggagccgatgttggctgatccgacggtcttgcatgc

tgtcaagctgccgcagctccaccgacatccgttcctctcgcccactgcgggcggcgctgacgaggagggggatgaccaggcaggagaggatgagcgag

ggtttggtgcgggattacatgagggcgagcgtctttctcaggcattactacagtccttcgtcctagccgacgacgaggacgacccttcgatttccaag

aatggcaaggcaataacaacgacgacgttcaacagcgcgatgacgctccccgaagccatcgcgctcgccatccaacgcaccctcagcgtcactctggg

taacgcctacctagacgcctcggtccccttcgccgtatccgaacgccgactgacgctctgggagaacctcgtcatcacaggcgcgccgacgcggatca

agggtttcaccccggccctaacagcagcgtgcggtttacatgtgggcgctgcggcccagggccaaggccacggcggcgggggagcaggagagggaggc

gagggtgctgggttggaaggcgtgggcgttgttgggttgggaccggggacgcaggctcggaatgtgagggcgttgaaggtacctgattactttgctga

gtttaaggatcggacggacttggcgccgttcttgggggcgtcgatctatacgaagcttacccttgggaacccggcagggacgtcgttcatctccaagg

aggcgtacaacgaccggggcccatctgtctttttcacgattctaggtccagcatag

>contig_21.contig_21.g795.t1

atgcggacagcatcatcgtccacaacaacaacctcgacctcctcgcctcccctcgccttgccttcgaacgcatgcatgc

tacctccctccatctggtgcaaccaagagaaggaagagacgggagatctcgctggcaaaaaaaaatggcatcgcagtctggctccgtcgatgccgcgt

ggtttcatggtgaatttcggggagaagacaaagcgacgacccattcctgaatgctggacaactaggacgtatgagcttgtaaggcagattcctgctgg

aaaggtgaccacgtacgattctcgaatggtcggtgcggctctcaagtggctagcagacgataccgtcccatggcaaagagtcatcggtgctggcggta

tcatcagcgaacgaggcgatggaggtcatggtgcatctcggcaagctcaggctcttcaagcgggtgaggaagacagcctccttcctctcgctatcaaa

gctggacccgcgatgttgatcgtcgtatcctattttcgatcccatgcagaaggtgtcgaggtcaacgaagctgctggagctggcattggtacaggcgg

tcggtggcgagtttcgatggcaacctatggctggtgtgagtccctctaccttcagattttgatgctcatcaaacaactgcacgaggaacaccctctcc

ggtctcaaacttcaccttccatcgaggacaactccgtccagccaaccccgtcccagccttccgctgctgcggaccggagactgcaaaaagccttcttc

gcgcagcgacaggctctctcgcgttcctcctctggctcttcttcgattgacagaacaccacaccaacgagacggtcctcatcgcgacgatgttctgac

aagggccagcagtctgcctgtctgcttctccaggggcaagctgtgcaggaaggaggagccaaagtgcgaacgcggtgagggcctgaacactgcacaca

agcggaagtcggcacaaacgtccgacgcgtcctcatcgacgacaacgtcccttcccaccccagtgaccgaatcgtatacttcgaactcagctccgccc

agctcgtccaaacgtcgtcgtcttctcgccagcggctcatggtcgcaaatgcgatcggtgggtccttctcaggcaagagcagggagcgctccttccta

cacacccgtgcaagcacctgcctatgtgctttcaccaagaattccttccctttcgctccgtgaatcggtgccgcccaagatagacgaaaccgcctccg

acacgggacaatcggagccctcagccccgtcccggaccccgccaccgccccagtccagcctgattctcccccttcccgcagcacctcgcactcctaca

gcacctcgcacccaaacatcactctcgcctcctcctcccgctccagcagcccctcccgccgatccttctcaaccaggaccgccttccacctccaacat

caccaccacttccgccgctcaatccagacacaaccctcgcatccttgcgttcgcaatgaaccatcggccgggccagtttgcaaagcgcaagctgctcg

cgaaggcggagacgaagaaaggcaccggatga

>contig_21.contig_21.g796.t1

atggctcacggaactcaagacagctcacaagccgggccctcacgctcccagcaagatccagatgcctcacagccgatgg

acgtcgacggggactccaaatcattcagccagagccaggcacaagcgacagcaaagaatgccttccaagacgcgaacgagaagagagagctccgcaag

aagtatcgaggtttgctcgcggctgccgaagaccagacatcggagagcttgacgtccatgctgaagcagacagatgacttgttcaacaaagtgaaagc

accatcagaaggtatcctcgatgcgcgcctcctcggccagatggtcgacatcggcacacacatggcccgaaccctcaagacgaacgcagacgcctttg

acaccgactccttcctctcccgtgtcgcacgtgtggttggaggacaagtgcgctccggcgcgcggagaggggcggacgatctggatgacgacgaggcc

gtgctcgcggccaatgagtggaattgggacgagcttggacgcttggcggcgaaacattcgcgacgagcggtctcgttggacttcttgcttgggcctct

gcaggcaaagcctaaggagagaaaacagacggagaggacaaagaaggtgaaggagaaggttggcgtggccgtacggccggaagagctcaaagacaccg

atatacaacgatcggaaaacgagacaacagtcatggtccgccaaattgcaaatctgctcgaagaggtcggagaagtacatctcttcaatttcgttgtg

gatccaacgagcttcagtaataccgtcgagaacctcttctacgtcagctttcttgtccgtgacggcaaggtttcggtggattttgatgacaagacgaa

cgagccgatgctgatgcggacggaaccaccaagagacgaagacttccaacagggcctcaccaagcggcaactcgtggtagagttcgacatgaagcttt

acaaggacatcattgagacgtacgacatcaccgagtccatcattccaactcggaaggaggccaagaaggaggccacttcaggaagatggtacggcaaa

atgacctcccaatacaacccagcagtcgatccgcgcgagtccaagctgttgatccccaagacagccaaagcagccgtctcctacaaggacaacggccc

tgtccagctcgagacggtccccgtcatccaggcagagcaactcgagcgcggcacggtgctggtccggatgctcttcagcggagtttgccacacggact

tgcacgcgctgcgcggtgattggccgatgacgaagaaattgcctttgatcggtgggcatgaaggagcgggcgtcgtatgcgccctcggaccggatagt

gaggagtactgcaagctgggcgaccgcgtcggtgtgaagtggcttgcggacgcttgtcttagctgtgcgttctgtcgggctggcgatgaggggaattg

cagctcggtccagcgtctgtcggggtacgtcgtcagctgggcgcgctacgtgacgcctattcctcctcccatcctctgtgccggagtgaccgtctaca

aggctctcatgacggccgagctcaagcctggccaaagcgtcgtcgtcgtcggctccggaggcggtctgggccatctatgctgccagtacgcctcggcg

tcgggataccgcgtgatcggcatcgacaccggcgaggaaaagcgtaggatctcactcgagtccggcgccgagcacttcatcgacttcgccaactcctc

ctcctcctccaacaagctcaccgccgacgtcctcgccgcaacccccgagaaagcaggagcccacgcggccatcgtcgcggcctcctcggcggcgtcgt

acgagcaggcgctggagtatctccgcccgcgcggcgtgcttgtcgctgtgggcatgccggcggatgcgaagatggaggtgggcatctttgggtttgtg

gttggtgagaagggcatcaagggtagttatgtgggcaaccgccaggatgcggtggaggcgctggacatggcggctcgtggtaaagtcaaggtgtccta

caagcttcgcggactctcggagctgaatgccgtcttcgacgagatgcagactggcaagatggctggccgcgtcgtgcttgacctcgacaagtag

>contig_21.contig_21.g797.t1

atgaaccgcctcttcggctcgcgcaatgccgcacccaagcccaatctccaccaggccatcgtgaacaccgagtccaggg

cggacagcaccgaagtcaagatccgcaagctcgatgctgagctcacgcgctatcgcgaccagatgaagaagatgcgcgatgggcctggcaagacggcg

atccaacagcgcgccatacgagtcctcaagcagaaacgcctgtacgagtcccaactgacccaactccaacaacaagccttcaacatggaacaggcaac

gctcaccactgacaacctccggaacacaatggccactgtcgacgccatgcaaaccgccaacaaggagatgcgtcggacgtacggcaagatcgatctcg

ccaagatcgagagggtgcaggatgagatggaggatctgatcgagcaggcgaatgaggtccaggagacgttggggaggacgtatggcgtgccggaggag

attgacgaggcagacttacaggccgagttggatgcgttggaagacgacttcgcagaggaagaatccgcgatgccgtcctacctacaagaggggccgag

ttccctgccagacttcatcgacgagccaccgactgcgacaacagaacctgcagcaggcgaggcgatgcgggcggcatga

>contig_21.contig_21.g798.t1

atggcaacagacacgctcgtcccgctccctagtccacacggagccgggcccgccatcatcacctccacagatgccacta

cgtcctccatgcttatcgacccatcacaaccagcactggcacaggcctgcctctcagcaaacgtccaatcatcatcaacatcgacagcaggaccattg

ccaccagcagtagcggaccctcagcaacaacaacaacagcagcagcagcagcagccacaaccatcgagcaaagcacgatctatggcattcccaacttc

cctctccgacttccggctgcccttatcaacacaacatctccaccacccaggagcaggcagctcagcctcctcctcctccgcttcgtcttcgtcttcat

acgccttccctacaacaccagccatgtccgcttccgacccgaccttctcagaaggaacaccaccacctaccgccttccgccgcggcacgccttccgcc

tcctccgttatggtcggcagattacggaggagcagcatgctcggccgcgaactcacccccgatctcctccccgacgatggaggggatgatgacagtct

cggggattccgacatgcgcactttcgaaggggccgctcgcactccttctccctcaccgctcctacctccacgatcgggaccagcaggagagatcggaa

cagcagcatcggcaacgggaggggagaaaacgatgacacccctgtcattacccaagggcaaagcagcacccgcgagcctcatcacaacacacgaacat

gtaaataccgggttgtttgcgcccgctccctcttcttcttccaccaatgcgtcacccgcctccgccgaaccattctggatcctcaacagacatacagg

acgaatgccttccagaaaatcgtcatcgcatcgcaaatcaggaggaagcgtctccttccgggaccgatccgtgctcagagtcgccgccagtctatgtg

acgaaagcagcccggccgacagcgagatcgcctccgaagccaaactcacccgaaaagtaaaatcagactgctgtgcctctccacgatccatgcatttc

gcaaccatgcccgcaccatcatcagctggcccacaacccaagaactgcgatctgcacgatacggactatgtcgatctcggccatatggactttgagga

ggatttgagctcgttgagtagcgacgaggacgaggacgacgatgccggcggagggtgttgctcgcactcttcggttgtcgtcgggacgagtgaaggag

gaggaggagactcacgccggatcggctcgggagatcatgctgatgccggctcgcgcagtctggatggcggaagcgatggcgaggtggagatgagcttc

gagggcgacggcactaccactaatcgccggacgaccctttcctccgagtcgaacgggggcggatccgaatgcgccgcttctaccaccttgggcggcga

atcatcattagcagtgtcggcgaacgcggcgccacctagcaacaattcgtcgtcctcgctgctgctcatgagcaccagtatgcccacgccgccgtcca

gtctggcgaagaagggggcccagtggaccaacttccgcgctgctgctcgggctcggtccgcagcaaggaccagtccaggtttggaacggatctatcgt

cctcgaacaccgggtccgcccagcaccaccactaccaacaacaacaacaacaacaacaacaatggcacctctgcgtatgcggctccgctctcagcccc

atcttctgcgtcgtcgatggacacgtcggagatgcctcagagcggaccccatgcgccgctgttgatgtccccgagcatgtggacgtttgttcggcatg

gcaaacggaagttgggcatgggagattcttcgcagcagcagcaacagctacaacagcagcagcatcatcaacaacagcagggaggaggagggtgttat

gcgaatgagcggtatgagccttatgctacgtcagctgcgaagcggagggcggtctccccgattgcgtatctggcgtcggtctctgcggggaactcgtc

gggtctgatggggaacggtgcgggcagtgcggttgcgttggggataccgtcctcgttagcttcatcctactgtggaggcggaggaggctatgcaggcg

cgggtgcgattgggtcggctacgagctcacccttgacgggctcggcgctcgcgaacggtctaccgacaccaactccgctctcgatgccgtcgcccacg

aacccgctcgtgcagccgttctgggcgaattcgtcctcgtactccgtcggtaccggcggtggggctcacatttaccacaaccacaacccgcatcatcg

gacagcgtccgggccgtacgggcgcagcgcgagtgcattcgctactgcagctgctgcgtcgtcgtcttctcgctcgggatcacctttgacgagaccgt

cgactccgacgaatttgagtggtgggtttgtcgcgggtggagctggccccgccgttgttgttggcggctcaggtacgcccggctcggctccggcggga

cttccgctatccctgcgcgagattcatcaccaccaccataacatgagcgctggtctgagctcgccctcgttactggccacccagacgccggggagtgc

ggggagtggacctggctatggctcgatgccggctgcgttgggactttcgttggggggcaattcggctgcgaggacgagggatgcggaggaggaggagg

gcaatcgcggcgggatggatgaaggggttcggatgctggggcttgggtga

>contig_21.contig_21.g799.t1

atgaacttgccagcctctccacagccatcaacctatcttggcgaacgcaggagtcgccttcagctgggccacaggctcc

aggctccaggctccaacagggccgaaccggagcactgtgcaggctcatcgctcatcgcctcacgttcttcctttccttcgtcggctttaacgagcacc

cgctccagccttttggagggcaggacggaagatcagatgcatgcgcttgaggttagcttcggccgtctttcgaacttcgagcaaaggactgcttccca

atttcccatgccatgtcctgtggatctccttgcatcccaccatatctccagtttggacaaagatgaaggaaccgtgctcgctcgcaagagccgacatc

caaacggtgaggtcgagagacgagatgccatgctggggcctggagcgggtcgcaagctcctcctcgccagcgtcacagtcaccttcctcctccttgcc

ctcgcagcatctgccaaaggcttacacgtcaccactgcctcccaggtccccttcggccgcgaatccagctctaatgccggtacccagtccgtcattcg

caagacgacgaagaagcatggcacggcaactacagctgcccgggccttgcgaaagtggggcagccgcgttgtcgatgtagaccgcatggatggtctct

tggatgatgcattcaagcgctggcgggcggatgctcagaacgcatcggtcacacagtttgactacgctgggctggtcaatccatttctcggtactgaa

gctaacggcgacccaggcaatgtcttccctggagcttccatccctttcggtatggcaaagatgggcatcgatcttaccgacacatacgccccggcagg

ttaccaggatgacatcaatgctcccgtccgtagcttgggtgccattcacgactctggcactggctccggcgacggcagtggtggcatcttcggcgtca

tgcccgtcgtctgccccggagatgacttctacaagtgccccaccaccgtcgaggctcgcaagattacccgagccaagaacaaagacgctgcctacccc

ggctacttcacgaccaccctcaacaactcgatcacgatggagaccaccacgacgcgtcgggcgggcttgcagcggtacacgttcgaccacaagacgct

tgcctcggcaaaatcgctccctcatctcgtctttgactggacgcgggacaacccgagctcgtgggctggcggcacgattgacttcgactatcagaagg

gtcggatcatgatgaatggatcgtggtacagcagctttggcgtgcgaacgcaggagtatgaggcgaaccggtataagggcttcagctgcatcgatcta

ctgcatggatcacagactattgccaaggccggtctttgggctggcgatcgattcgggcaagacaccaagctgattggacagacgcacgccaacttgag

ccagaacttcatcggctcagaccccatccagagcggcgcgctcgtctccttcgccaacgcggccaaagtaggatgcgacagtcaagtcacgatccgat

acggcgtctcctacgtgtccgccgcccaagcctgtgccaacgccgaggaagagattggaccgaactgggattttgccgccgtggaggccgcctcgcgt

tcgcagtggaacgagaagctcaacaggattgtcctgtcgccgaacacgacggatgaggttgcgaggctgttctacagctcgatgtatcgctcgttctt

gagcccgaacaatgcgaccctggaggcgccgttcccgacgaagacatcgtactttgatggactgtactgtacttgggacaccttccgtaccgagttcc

ctttcctctccctcacatctccggacgacttccgcaacatcgttgacaactacatcgacggtgcctctgcaaccggatttgtccccgagtgccgcgcc

aacatggtcccaggtctcacccaaggcggcgccaatggtctgaacgtcatttccgactacatcgtaaaatacggttactcgtccctggccttcacgaa

ggagcagatccttgcccagctcaccaaggaatcgtacgtcaccccgacggagtggaacagctacggccgtcagattggcgtgtacatgaagtacggct

acgttccctttgccgtcttcgacaccgaatcgaccggtcgacagacgagggaggcgtccaggactctcgagtacgctttcaacgacttcggagtggcg

ttggctgccaaggagctcggagacgacaagttgcacgcggacatgatcaagcgatcgatgaactaccgcaacatcttcgacccgacggtgaagtttcg

tggcttcaaagggtacccgcagaagcgtcgcaccaatggtgagcaaggaacgctcatgaaccctgccgacaatgcgaccgaccactcctgctcgctgc

agcaggagaacattttcgggttcttcgagagttctgccgccgagtacgccttctgggctccgcatgatggcgccggtattgtcaatctgacctcttct

tccacgtccgagttcgtgaagcgtttggacgacttcttcggagacactcaagcatcgctataccaagtcggcaatgagccttccttcgtcttgccaac

catgtaccactacgtcggccgcccgtcgaaatcggtccagcgggttcgcaaggtggttcacgacaactttgactccgccaagctgcctggcaacgatg

acaacggggcgatggctacgctggctgcgttctggctccttggcttcttccccattccgtctacgaaggaacttctgatcatctctcccttcattccc

gaatacaccatcaagaaccctctcctcggcaacattcacatcgtcgcagacggcttcgactcggcctcggtcgccgaaaccatcccgaagggcgtcaa

ggcgtacgtcgagaagatcaccctgaacggcaagccctactcgtcccggtgccggatctcgtttgatgacctcttcccgggagcgcctggcaagacgg

cggaggtggtcttgacgctcacggccgatgcggacgcggtcaacaactgcggcgcgggtggagcggccgacctgccgctcagtctgtcgactggtggc

tggaagtag

>contig_21.contig_21.g800.t1

atgttctgccttcgaggacgtaagctcctcctcgccagcgtcacagccaccttcctcctcgtcgccctcacagcagcag

acaaaggctccgacaacaccaatgtctcccaggtcctcttcgcccgcgaatccacccctgatgtcggcaccgagtccgacattcgcaaggagaagaag

aattccatcattcgcaagacgacgaagaagcatggcacggcaactacagctgcccgggccttgcgaaagtggggcagccgcgttgtcgatgtagaccg

catggatggtctcttggatgatgcattcaagcgctggcgggcggatgctcagaacgcctcggtcacacagtttgactacgctgggctggtcaatccct

tcatcggcaccgaagccaataacaacccaggcaatgtcttccctggagcttccatccctttcggtatggcaaagatgggcatcgatcttaccgacaca

tacgccccggcaggttaccaggatgacatcaatgctcccgtccgtagcttgggtgccattcacgactctggcactggctccggcgacggcagtggtgg

catcttcggcgtcatgcccgtcgtctgccccggagatgacttctacaagtgccccaccaccgtcgaggctcgcaagattacccgagccaagaacaaag

acgctgcctaccccggctacttcacgaccaccctcaacaactcgatcacgatggagaccaccacgacgcgtcgggcgggcctgcagcggtacacgttc

gaccataagacgcttgcctcggccaactcgctccctcatctcgtctttgactggacgcgggacaacccgagctcgtgggctggcggtacgattgattt

cgactaccagaagggtcggatcatgatgaatggatcgtggtacagcagctttggcgtgcgaacgcaggagtatgaggcgaaccggtataagggcttca

gctgcatcgatctactgcatggatcacagactattgccaaggccggtctttgggctggcgatcggttcgggcaagacaccaagctgattggacagacg

catgccaacttgagccagaacttcatcggctcagaccccatccagagcggcgcgctcgtctccttcgccaacgcggccaaagtaggatgcgacagtca

agtcacgatccgatacggcgtctcctacgtgtccgccgcccaagcctgtgccaacgccgaggaagagattggaccgaactgggattttgccgccgtgg

aggccgcctcgcgttcgcagtggaacgagaagctcaacaggattgtcctgtcgccgaacacgacggatgaggttgcgaggctgttctacagctcgatg

tatcgctcgttcttgagcccgaacaatgcgaccctggaggcgccgttcccgacgaagacatcgtactttgatggactgtactgtacttgggacacctt

ccgtaccgagttccctttcctctccctcacatctccggacgacttccgcaacatcgttgacaactacatcgacggcgcttctgcgaccggatggatcc

ctgaatgtcgcgccaacatggttccaggtctcactcaaggcggcgcaggtggcctgagcgtcatttccgactacatcgtcaaatacggctactcgtcc

ctggccttcacgaaggagcagatccttgcccagctcaccaaggaatcgtacgtcaccccgacggagtggaacagctacggccgtcagattggcgtgta

catgaagtacggctacgttccctttgccgtcttcgacaccgagtcaaccggtcggcagacgagggaggcgtccaggactctcgagtacgctttcaacg

acttcggagtggcgttggctgccaaggagctcggagacgacaagttacacgcggacatgctcaagcgatcgatgaactaccgcaacaccttcgacccg

acggtgaagtctcgtggattcaagggattcgtgcagaagcgtcgcaccaacggccagtttgtgtatacggacccgacattctgcagccccgccgacaa

tgctcaagatcattactgctcgcttcagcaggagaatattttcggtacttacgagagctctcccgccgagtactccttctgggccccgcatgacggcg

ccggtattgtgaacctgacctcctcttctaccgacgagttcgtgaagcgtttggacgactttttcggagacacccaagcatcgctataccaagtcggc

aatgagccttccttcgtcttgccaaccatgtaccactacgtcggccgcccgtcgaaatcggtccagcgggttcgcaaggtggttcacgacaactttga

ctccgccaagctgcctggcaacgatgacaacggggcgatggctacgctggctgcgttctggctccttggcttcttccccattccgtctacgaaggaac

ttctgatcatctctcccttcattcccgaatacaccatcaagaaccctctcctcggcaacattcacatcgtcgcagacggcttcgactcggcctcggtc

gccgaaaccatcccgaagggcgtcaaggcgtacgtcgagaagatcaccctgaacggcaagccctactcgtcccggtgccggatctcgtttgatgacct

cttcccgggagcgcctggcaagacgacggaggtggtcttgacgctcacggccgatgcagacgcggtcaacaactgcggcgcgggtggagcggccgacc

tgccgctcagtctgtcgactggtggctggagacagactgagcggctggaatcccaaaactttcgcaacccgctgtacgccttgctcgctcaggtcccc

cttgttccccccgccgccttgtcccgcaaccgcaaccgcaaccgcatccgcatccgcatcccgtccccggtgtccaagaataaggaagcgctcgagac

caacacttcggacgcgaaggcaactccaacagacgcgtctgcttcgctttgggcatccgtatcacgaacgtcgcgtccgtttgtcgcgcccctgcccc

tgttgcttcgcgacatcctaggcggatcttcgcaaccggcttggaaccctaccaccgtcgaggccttcttgctcgagaagacgttcctcgaggatgtc

gtccggcccctcgtccgcgccttggtccactcccgtctccaaacggccggcctggcccccggtacctctcacactccggcgcttcaacgtgatctgat

gggaatccgggactcgctccttcagggtattgactccatgagcggtagcgcacctggtcccagcactcgtgccgctgccgcttctgctccagctcctg

ctcctgcaccttctcctgcgcctgcgccttcttttggcaccccggttcggagcggtggaatgggtcgtcccgatcctgccatacatgcgttgctcaat

ggcattcccagcaacacacatactcgccccgatcctgccatccaagcgttgctgaacgcgcctcccagcagcacacatactcgccccgatcctgccat

acaagcgttgcttaacaattctcctgctccaaattcgacctcaggcgacaacagcggcggcggcggcggcggcaacgactcggacgcgaacatgtcga

tcgatggcggcccttcaggaagtatgtctcagctctcctcctttgacatgggcaatttcagccaaatgcggagatggacaccagctcaacgcaagcgg

accgctacccggcatcgagtctgcgcaggatgcggcatcccccacaacagcaagttccgccgtggtccaagtggacccggaacgctgtgtgacaagtg

cggcaccaaatggaagaagatcacagatcaggccaaactggagaacagctatcttcaggtcggccaaggctccaactctaccggagcagctggtcaaa

accaaggcggcctcggaccggacgggcgctctcactcgggcccttcctccgctgccggcccatctggtcgtcaggaagatcccggagataacgcttgg

catgtaatgccctcgagtcgggataggattcagccccaagacaatctggacgaaccaggcgaaggtcactcatccttcgctcagggtcagcgcggcca

ggatcggcgccactctcctgtcccaagcggcggcggaaacgggcacggaagtggttctggtccaggtcccggctcgaggggatcaccttcctcttctt

cgtctggccacgttcccacttcgggcctttcgatgggcgtaaacttcgctggtggcggtggtggtggtggcggcggcgatgtctcctcagaacgctcc

tcccatcattcgcctcagcaccacggacggcccattcctcgcagcgtaccgggcccatccttcacacccttgctgcagtctcgcggcgacggaggagg

aggcggagggggaggaggcggagaaggcaacggcggcgaaaacaactcgaactctcatcctccgtcagctccactgccgtccgggtccgggtccggtg

caagtccgcagcagggcgaagtttcgcacgcggctgcgcaggcgttcttacgggccatgcatcagcgaaatgtggtgtaccctggatctggatga

>contig_21.contig_21.g801.t1

atgcagtcagtcggcaccctgttgcggtccgcgtctgccgcaaagccagcagtctggacctcggcatcaacagtgatcg

caaggcgcgcattctcgggcgaggcacaaaggtactcgaccattcacgcattcttccgtcgatctgcgcgcgacgcgctcgctgcccaggttcaggca

gcaggtctgggtaggagaagcatacattcctccctttcgcgatcgcaaggagcggacaaacctccaggcacttcgacgtcctcatcgacgtctcagga

ggacgaggctacgaaaacgtccaaatcagagcagacgactggccagccgaagtcggatagcgtccagcatgcatctgcacggcaccatgccgccgcat

tccgaaattggccagcgtcccttcgcgagctcgccttgacggcagtcggggcgggagctgcccagaacgcctcttcatcatcaacatcaccatcatca

tcatcatcgaacagcaaaggcccgtcgcccctcagcacacagagctccccccaccctcccgaccacccacaccacgtcacatgggctcgtccaaccca

ggaagacctcctccgtctcgcccagggcttctggcaacggacccgtatccgcttcaagtggttcaccatccgttccttccgacgcttcaacgccgacg

acatctccgccttcttcaccctcggcggcctcagcaccatcctcctcctcatcctgggaacgacgaccttcgtctccgtcttcgtctttgcgctcaat

gccctcagtttcgaccagtggattgcgcgcaagattgccgattatctcactgcggagacgggcgttgtcgtcgtttttgagagtgcgatcgtgccaaa

gtggaaggacagcaagattgcttttaagaatgtttacttgaccaaacggccccaacctaaggaggccaccccgcagagcaaatacgagcgcgccaaga

ggctccttcgtactcgcaatcgacgtgggacggcgaccaccgccggtcaagggatggcatgggagggcatgcattacgacgaagacttcgcacttcaa

ggcaacagcgtcattgcgcccatcaacggagacgatcgcagagtgctcgaggacgaagacgacgagttggccgatgtcgatacgaacttcaccatgtt

tgatctcaatgtagactcgatcgacatctcactcagcttctcgcgctggtgggatggtaagggcttggtcacgaacgctaccatcaagggcgtacgtg

gcgtcgtcgatcggcggaacgtttggtgggatcctgcgaaaccttatgatcccaaagaacaccgccgcaggcccaaaatcggcgatttccagctcgac

tctctcgtcctcgaagacttcctcgtcacggtctatcaaccctccccaccgggctcgctcccaggcgccgcgcccttccggcccttcaactttagcat

cttccgcgctcacctgccccgcttccgccgacagtggctcttctacgacttgctcaatgctgagtatatcacgggccagcttgatggatgtcttttca

gcttgcataagccgcagacgattgggaggagtattgagcaggaatgggagcggacgaatggggatcatccggatggaaggccgtcgccttggaagacg

atgtcacgcttccgcatcgatggtgtcaacattgaccacatcaagagtcaaaccggcataacaggtcccatgtcatggatcacctctggcaagtacga

cttcgtggccgacatccgcttcccgcgcgaggactcggacaacgtcgacctccaagtcatccttgccgagctcgtagataacttgtctgccgtcgtca

gcggcggtatcggacccgatcgctctaacggccccgaagccgatgcgcaccgtgccatcccaggccaagctcgtctgagcgggccggcaattgagttc

cgcaaggcgcagatccggagtgcggccgagggcactgggaatgcgggtgcttatgggagcgacgaggatgaacgcgtcctgaacgctgctgagctcgg

ggcggaggaaggactgggtctgggtctgactcgaagagaaggcagtcagcgggacgaagcggagttgcgagctgagctggcgaagcatctcccgccgg

caccttcgccggccgtcgtgattgatgtggacatccggttcaaggacatcaaggcggctgttccgctctttgcaaaggatttgagctatcgggataat

gcgtttgtgcggccgattgttgcgttcatgaatgcgaacaggactttgatccccattcgctgccgtattgtgcatgatctttccgagttcgacggctc

cttcgacctttcccaaactggcctgataccaataatgtcggccaaggtcagtgtcctatacgcgtccccttcaacgccggatcccaagtccgccttga

atacgcaacctttgctcaacataggtctgttcttctacccctcacagatacacgaagctctggcacaccacgtcgcctcccaaaacgcaaaccatcaa

cgcctccgaaacgtctccctctggagtctccagatgacagcccacgcagcgctgacgctcgtcaagaatgtgcgtgactcatttgcgcgctcgacgcc

gccgacctattcgggcacggagcctgagcagggcaaagtggcgctggggagtttccgtcggaattctcctgggacactgccgccgacatga

>contig_21.contig_21.g802.t1

atgatggaacatggcatggcatggcatgacatgggaccccttcgatggcagcggtgctcgaatgctcgaatcgcatgtg

catcgccccacattcgatcctcacgcctcgacgctggcctcctcaacgagaccgagtggcacctcctcgtcgtcgcacgagagatcgatgccgaacag

ttgctcaacgctgtctcatctggcagtcctcatagagtgaagagctcatatgcggtcacgattgggattccatcgaaaatctacggtcggtacaggtc

gcattcggacgagctggccagcaggcctgctcccgccctgtcctccttgctcgatcagttgccggcctcgcttcgttccggtgtcgatgggaaaccgc

gcgattcccagcatttagaagtgtcggacgacctccttcggtttgcggatgagctggagcggaaggaggagtttcgccaccgaccagtggtcatgctc

aatctgcttcaattccaagaaggcaagcatgagtcgtacaagcagtacggcaagggcttcgtgaccatcggatcccgacacggcggagtcgccaagct

tgtcggcctcgtcgttgccccgccagccgcctttgcgtccgacagccgcggcgacaaagcccgaccatcgcatgaatggtggaacgaggcagcatatg

cattctacccttccattcgtcactttgtcgacatggctgctaattcggactatcaagaaatcaaccagaggttccggcttccttctcttcgcgacacg

acgatcatctgtacgacggagctcgactggggtcatgctgcagataagtcggcactgtcgtccgcgagccggaccgggacagcgtcggcttcgcgcaa

ggcgaggatttga

>contig_21.contig_21.g803.t1

atgcccgcccaccgggggacacctgcgcactatatcggcctggaacgggcgtcggggcatcccttcttatcggcaccca

tgaattttacccccagacttctaatgcttatgaaacgctccgagctgagtctggagacatccgccgaagattccagagcagctttgccacctccatct

tcgaccgttacctcacaattctaccaacctgtcacagctccttcttcctcctcgttatctccccggtctcaacgcgtcaagtcagatctcgaagccgg

cctctccctttgcgctattgcgcgtacacgaggcgtcagcaagtcttgggccggcaacctgcgttcgcagatcgagaacctgccccctccgccccaca

gcggccgccctcccgtgctctccccgcaagaacgccgacggattacgcgccctattgtctcagtaacgacggtcagaagggtactcaaggatgccggc

ttcaaggcacgggtcaagaaaaagaagcctgctatcaccgacgcgcacgccaaggcccgtctagcatgggcacgtcagcaccgaggatggacggcgag

cgactgggagaaggttatatggtcagacgagaccaagatcaaccgctttggatccgatggtcggaagtgggtatgggtgcccaagggatcggcattga

cgaatagggaggtcacttccacgcacaagtttggaggtggtggagtgatggtttggggatgcattactgcctcttcagtgggaggtatgactttcatc

cgaggtatcatggactcggagaagtatgtggagatcctgcagagccgccttttcccaaccctggacgctctcgacctcgtcggtaaccccaatgagcg

ccagcagcgaattttccaacacgacaacgacccgaaacacacttctcgccacaccaaagcctggctggctcaacacaaccccaccgtcctcgactggc

ctgcgcagagcccggacctcaacccaatcgaacacgtttggcgaaagctgaaagagcagctaaagacatatcccactcccgcagcaaatcttcagcag

ctgaaggatcgcatccaagaacagtgggatctgattccagctgactacatccagaggctagtagcctcaatgccagccagagttgaggccgttatcaa

ggcaaaggggcgcagcaccaagtactga

>contig_21.contig_21.g804.t1

atgtctctctccgcaccttccaacggtccccagatcaaggcagcccgccaacgcggtgcttcccacatcgacttcaaat

cagctaccaccggtgttgctgccaagaccatgcgcaacgagctccagaatctcgtcaatggcgtccctgacggccccgagcgcaaggctttcgacctc

gagatgcagggcttcttctccatcttcaaccggtacctcaacgagcgcgccaagggcgagaagatcgaatgggagaagatcaaggcacccgcggacga

gcaggtcaccccatacgctcaactccgccaggcttccgacccttccatcttgaacaagctcgctgtcctcaagctcaacggtggtctcggcaccacga

tgggctgcactggtcccaagtccgtgatcgaggtccgcgaaggcatgactttcctcgatctctctgtccgacaaatcgagcacctcaacacagaacac

aaggtcaatgtgccgttcatcctcatgaactcattcaacaccgatgacgacacagcccgggtcattcagaagtatgccaaccacaacattgacatcct

caccttcaaccaatcccgatacccgcgtgtcaacaaggagtccctccttccctgcccgcgctctgcaacttccgacaagagcctctggtaccctccgg

gccacggcgacgtcttcgacgccctcaacaactccggtctgctcgaccgcctcatcaacgcgggcaaggagtacatttttgtctccaacgtcgacaac

ttgggtgccgttgttgacttgaacatcatgcagcacatgatcgacactcaggccgagttcatttccgaagttacggacaagaccaaggctgatgtcaa

gggtggtacgcttatcgactacgagggcaacattcgtctgcttgagattgctcaggtaccctctgaccatgtcgaggaattcaagagcatcaagaagt

tcaagatcttcaacactaacaacctctggctcaacctcaaggccatcaagcgcgttttggacaacgatgagcttgacctcgagatcatcgtgaacaac

aagtctctcgactctggcgaggctgtcatccagctcgagaccgctgccggtgcggccatcaagcacttccgcggtgccaagggtgtaaacgtaccccg

ggcacgcttccttcccgtcaagtcctgctccgacttgctcctgatcacctcggatctgtacagcttggagcacggcaagctggtgatgaacccgtccc

gaatgtttgggcaaacaccggtggtcaagctcggcgacacgttcaagaaggtggcgcagttccagaagcggttcaagagcattcctgagattatcgag

ctcgatcatttgacggtctcgggtgatgttgtgtttggacggaatattacgttgagggggactgtcatcattgttgccaacgagggcagccacatctc

tattcccgatggcgcggtgttggagaacaagctcgtatccggtaacctcacgctcatcgaccactga

>contig_21.contig_21.g805.t1

atgtccactccctcgtcgcagtctcaggcagagtcaggcttgctcctcgtttttgccgaaccaggaaagaacgtcactc

tggaggagttccacgactggtacgacaacgagcacatccccctccgcaccgagaagttccacgaattccgctccgcagcgcgataccacgtcccatcc

tccgtcgcctctgcctcccgcccctacacatcctcaacctggtcagcaatctacaccatctcctccctctccctcctcctctccgacccggcatacac

ctccctccgcacgaaccgctcggagcgcgaggctgaactgctgggccggatcgggattctggatcgcaggatttacaagttgacgtatgattcggacg

tggatcccaaattccattcgacggcggcggagagagacggtggcgacaaatcgaaggacgacgatcatcatcatagatggagcacacgtccaaccaag

gcgtccgaaatcagtcccgtcgtgatcgccacaagcgtcacgcccgccgaagggaaccaagaagcctacgacgagtggtatctggacgaacacgtcga

ggcgctctccaaggtgcccggctggatacggactcgtcggtttgagctcgtcgacgccctcatcgtcggaaacgacgctgcgccggaaaatgcggacg

cgaagtctgttcctcgctgtctgggactgcacgaaaacgcatcgccagagttctcctctacgcccgaattcgccgctgcgctggacacgccctggcgc

acacgcatcatgcgtgaggacggctcgggcatcgtccaacaggagcgacgtgtcatgcagctttaccgcgcatgggaccctacggtagccttgcaaca

cccgcagtcaggatga

>contig_21.contig_21.g806.t1

atgcgaccggcgagatacacatcctcatcctcaacggccgtctctctccatgccagcgtcctcagtctgcgcggaaagg

aagtgacgccccaacccattcgctccccggacggccgactggttctggcctggaacgggcagatttttcggcatgaaggtgacgagaataatgatgag

gaaaaccggagttcgttattcctcgacgctgcgaagaatgacggcgaggaactcctcaaggtgctcgaacgggtgctgcagacttctgagtctggcga

agacgtaggcttgaccctcgcaggcgttctcaaggcgatcgaaggtccatatgcctttgtgcttctcgataccgagacgcagtatctttactttggta

gagacccattgggacgacggtcgcttctcatgcggcgctctgttgagacaggggacgaatctttgatgctgtgctcggtggcggatcagggtaccatc

gccgcaggatcgagcttcgtcgaagtggattgtagcaggtcgatctggtgcgtcgacctcaacgcgaatcaaagtccaaaagctgtcgaaataccgcg

cggactcgagccccttgtcctatccgatctacaatccgcaacaccgctccacgagccctgcttctcggaacgacttgcctcagcgcacaccttccgcg

ccgtactcgaggacagcgtccgccgacgagtgaccaacatccgagcctacacgtcctctcacgaggcccatgtcgccgtgctgttctcgggcggactg

gactgcgccaccgtcgccctcctcgcacaccaatatctcgacccagcccaaccgatcgacctactcaacgtagccctagagaacccccgctccctcgc

cgccgccgccgccgccgctgccgctgccgcccgagctcgttccgacacccacaccccctcaccttcttcggaggaggaggtggtcgtggatccgtacg

acgtgcccgatcgccagacggggaaagcgacgtttgtcgagctgtgcaagctgactccggggcggaagtggaatttcgtcagggttgacgtgccttat

gatgagtattgtggggcgagggaggagattagggggacgatgtacccttcgaatagcgtgtccatcgcagcagcgctctacttcgccgcccgcggccg

cggcttcctcctggacacgctggaaccgtacgaaagcccagctcggatcttgctctccggcttgggagctgacgagcttctcggcgggtactctcgtc

atcgggtggcttttcagaggggagggaaggctgcgcttgtccaagagctccaactcgacttggatcgactgccaacgcggaatttaggccgggacgac

cgcatcctatgcacacacgccaaagaagcccgctacccgttcctcgaccgagcggtgatcgcctacctgaactccctgcccgtcgaacacaagatgac

gttctcacccgagcatctctccttggccccatctccccctcctgcttcttctgaagaaggatcatcgacacgaacagcgtacccgggcgacaagcttc

tccttcgtctggtcgcgggtgagattctgggtttggcgggtggggcgcggctgaagaagcgggcgatccagtttgggtctaggagtgcgaagatggag

gttgaggggaggggagggaaggtgaaggggcatgaggagttgggatga

>contig_21.contig_21.g807.t1

atgggattgggattgggattgtgcacgcgctgctgttcgatgcgcgcgctcatggcagctcagcgcgcggctgtcagaa

ctcctgctgcactcgcgcccgtctccatcgccatcgcagcctcctcacagcacagagcagcatcgcaaccgcagcggagaccattcgcgacctcgact

tcacaccgcagcacacacagcacaccaccccgcccatccacaaccacaaccaccgtgctcggacaggagcatcccgcagacgactacaccaacctgcc

caagtccatcctctcccgcgtcggcacaacacccatcctgctcgccaacccatcccacccactcagcctcctccagtcacagatacaaacccacctcg

cctccctcacagacaccgcatccttcacccccatcacaccaccaccacccgtcgtctccgcagacctcaacttctccgacctcggcttcccacaagac

cacccgggacgatcacccaccgacacctactacctcaaccgcaccacctgtctccgcacacatacctccgcgcacgaggttgaagtcttcagacaggg

acataccagctggttgctcgctgccgacgtgtacagacgcgacgagatcgatgccagccattatcccatcttccaccagatggagagcgcacgcatct

tcccgctcgaacactaccagcctgcggagggtggaggaagcgggcggaagagtctcgtcgaactcgaatgcgaagccatcgaagctcgactgtcagcg

tcggacgtacagatcgaggtggaggacaacgtcgatctggaagacgcaggtgggatccagccctcgcatgagaaacccgtcgccctcctcgcggcccg

gcatctcaaggccgtcctgaactcgctcgtcctcgagctcttcggcgacagacatcgcgctgacctctcatcatcatctgcaacagcagcagcacccg

aaccactccgcgtccgctggatcgccgccacgttccccttcaccaccccctcgtacgaggtcgaagtctggttccgcggcaagtggctcgagatcctc

ggctgcggcgtcgtcatggaccgcaccttacagaacgccggcgtacgcaatcatctaggctgggccttcggactcgggctcgaacgcatcgccatggt

tttgtactccatcccggacattcgcctcttctggagtaatgatgaacgcttcttaaggcagtttgctgggccttcttcgctgcgctctgatgggagcg

acacgcacgcgggcgtcagtccagcagggggtctcgtcgactccgcctccaagcccacctccaaacgtcccctcatcacattcaccccatacagccga

caccctccctgctacaaagacgtctccttctggctgcccactgcaaatacggaggcagctccagcgacggccttccacgagaacgacttctgcgaggt

cgtacgcgataccgcagccgacctcgccgaggacgtcagtcgcatcgacgagtttgtccatcccaagacgggcagacgcagtttgtgctatcggatca

attaccgcagcatggacagaaatcttgagaatgaggaagtgaatgcgttgcatgcgcaagtcatcaaacgcctcgtcgacaactttggcatcgagatt

cgatga

>contig_21.contig_21.g808.t1

atggacgtgcacgagtaccaagcacaggagaacgcaacagcagcagcagcaccagcaccagcaccagcagcagcagcac

cacaacagcagcaacacggcgatggtgtgcccgcgcccgcccccgccttcgaggcagagatcaaccagctcacatcctcgctcacctcctggtgggga

tccgtcaccaaacagtcacagcagaccctagaccaggcacgtacccacatccaaaaacaaggcggtatcctcaacgccgcaaagtcagaatggtccaa

gctcgaggaccacctgaacgacgctcaacaacgcgcacgcgatgctgcctttgcacccgcagacgccgacgatcacacccaacatactggtcccggtc

ccggtcccggtcccggtcccgtccctgcgcccactgacgatgatctgctcgaagcatcccagcctcccacgcgaatgagctcagcgaccagctccgtt

ctcgacaagaagggcaagggccgagctcccccgagtcccatcgatcatcaagaacaaggcacccagggagatcgtctcgacaacggcgacgacgacga

ggttccccagatgtactctgccgacggtacccccatcctcgaaccaggcacccgggcccgaggagccgcatcatcacacaaccgaaactcagcctcga

tcgacctgaacgccgtcgcgaaggaggcacagacacaagccagccaactcgccaacaatgcgacctcgttcttctctcggatcggatcgcagatcgcc

accgatctccgcgtggctgatttgaaggcgatcaccaacagtctctcctctggtccaccttcgtcgcgcgcacaaggaggtgttgatgggccgaactc

gactgactcggagaagtctgctgctgctgcgggtcgatatgcgattaccgatccatcatctacagcgtcctcggggacgcagcagaacaataccctgc

ctacctggtcagccacccaagcgctcgctcgcaagtactgggccgaggcagaggccgtcgctcgcgacgtgggcaaggacgtccgcgatctggtgaac

gaagtcgtacaggtcgtcccaccatcagaagaaggcaaggtactaccagcaggcagcagcagcagcagcagcagcggtgtcggtatctcgcgtgagga

gttgcaggatgaggtggacaagattgctcaggagaaggaacgtggtgcgtctgcttcctcgcaacggaagaagaagagttcaacaacgggtggagagg

gggaggacgaggaggacgatgatttcgattgggacgaaggggatgatcatgcgggtgcacaagcgtccaaacagtccaaggctggcggaaaagcagct

gaattatcaccgactgcggagacgccgtcctcagcttcgacttccgctcccgctcccgtaccccaagcttcgacagtactgccagccactgtgagacc

gacgacgacgacgcttcctgcctcgggatcgtccgactcgcacctacagccgacgacgggtccgcagacggatcggttcgaggtgtcgtcgggtcgtg

cgggcgcagacgacgacgaggagggcgactccgactgggaatga

>contig_21.contig_21.g809.t1

atgatctccatcatcgccatcctcgacgtcaagggcaaatccctgctccagcgctcgtaccgggacgacatccctccgt

cgaccatcgagaagttcctcccgctcctgctcgagatggaggaggagtccgggggcagcgccgtcactccttgtttgagtgttgatgggattaactac

atgttcattaggcataataacctctacttgctcggcctgtcgagacggaacagcaacgccgccgaggtcctcctcttcctccacaagctcgcctccgt

gctggaagagtacttcaaggagctcgaggaggagtccatccgggacaactttgtcatcatctacgagctcctggacgagatgatggactttggatacc

cgcagacgacggagagcaagatcttgcaggagtacatcacccaagaatcgcacaagctcgaggtccaagcccggccaccgatggccgtgacgaacgcg

gtctcctggcggtccgaaggcatccgctaccgcaaaaacgaagtcttcctagacgtcgtcgaatccgtgaacctgctcgtaaacgcaaacggggccgt

cgtccgctccgagatcctcggcgccatcaagatgaagtgctacctctccggtatgcccgagcttcggctcggcttgaacgataaggtcatgtttgaga

gtaccgggcgggccagtcgggggaaggcgatcgagatggaggatgttaagtttcatcagtgtgtgaggctttcccggtttgagaacgatcggacgatc

tcgttcatcccgcctgatggcgagtttgagctcatgagctatcggcttagcacgcaggtgaagccgctgatttgggccgaggcggttgttgagcgcca

tgagggcagtcgggtggagtacatggtcaaggtcaaagcacagttcaagcggagatcaacagcgaacaacgtcgagattatcatccccgtcccagacg

acgccgactcgccaaagttccgggccgccatcggctccgtccaatacgcccccgaaaaatccgcaatgatctggaagatcaagcaactaggcggcggc

aaagagttcctcatgcgtgcccacttcggcctgcccagcgtgaagagcgaggacggcagcgtggacaacaagcggacgcccatcagtgtcaagtttga

gatcccgtattttacggtgtccggcattcaggttcggtatctgaaggtggtggagaagtccgggtatcaggctctgccatgggtgcgatacatcaccc

agcatggggagtacgacctccggacggcgagttccaaagcgggcactcgactggcacctttcgggcaatga

>contig_21.contig_21.g810.t1

atgatgcctcaaccacgcgatcccgatctcggtaaacatcacgtgctcattctccagaagaacgatcgatcgatcgctg

gccagctgagcgcacgtgacgccgcctgcttttgcgttggaagtggagtcgacaccttcccgtcgacacccacgccatccaccaaggtccaataccgc

aagatgggtatcgacatcaagcacaagcacgtcaagacgggtcgccgtacggcgcccaagagtcaggacccttacctcctcctcttggtcaagctgta

ccgcttcctcgcccgccgcaccaactccaacttcaacaaggtcgtcctccgccgtctcttcatgtccaagacgaaccgcccgcccgtgagcatctccc

gcgtggcctacctgacccgcaaccaaggcctcacccccaaggccaaggccgccctcgacccctccgtcggccccaagacggtcgtcgtcgttggcacc

atcaccgacgacaaccgggttctcgaggttcccaagctcagcattgccgcccttcggtttaccactactgctcgcgcacgtattactgaggctggtgg

agagtgcttgacgcttgatcagcttgctctgcgcgcgcctactggttccaacacgctcctcatccgtggacctaagcttgcacgtgaggccgtcaagc

acttcggcatgggtccccacaagaacaagaagccctacatccgctccaagggccgcaagttcgagaaggcacgtggtcgcagaaagtcgcgcggcttc

aaggtataa

>contig_21.contig_21.g811.t1

atgtccaacaccttccaatcgctctggaaattcatcaccgaacctccccctcgcccttgccctcatcatcaggactact

tcgacgcagacgcacagccccccgctgcacacgcagacgcagacgcaaacggagccgccccagccggttccaacaataccgcgaacgacgacgacctc

ggtctcccgccaccccgtcgaccctccgacacccgctcccgctcccgctccagctcgcgttcgcgctcccgctcccgctcccccatgcgcggcatcgc

aaagaaagaaggcaacgacgacgacgacgacgacgagcgaagatcgcgctccccgctcccgcgccgctcccgttcgcccctcccgcgacgcgacggag

gagagcgagggtcgcgttcgcgcagtccgccaccgcgtcgattctcgcccccgccccctccgcgcccgcgccatgcgcctgcggtggtggagcctacg

aacatcgtgggtgtttttgggctgagcattcggacgacggagcgggatttggatgaggaatttggccgggttgccccggtggagaaggctcttgttgt

gtatgatgccaggtcggagcgatctcgtgggtttggcttcgtcacgatgcgcgatgtggatggagccgccgctgtgattaaggaattgaacggcatgg

atctccacggccgccgaatccgcgttgactactcggccactcaccggccgcatcagtcgactcccggccgctacatgggccatgtccgacccgatgac

cgctggcgtcgcgacgggggtggtggtggcggtggaggccccagtggcgcccgttcccctcctcccttccgtggtggcggagcaccaggcggctggcg

tgaacgcgaacgcgagcgcgattacggcccgcccccctcccgtggtggaggctggggggccaacgaccgctggggcgggggaggagatcgtggtgggg

accgctacaacgaccgagaccgtggggaccggtacaatgaccgtgaccgcggcgatcggttggattcccgtccgccgccgccgtcgcgcggtcggtac

gatgatgactggcgtcgtccgagggggtcgccgccgccgcccgctcgtctgggtggtggtgggggcagtagtcgcggagggggcagtcggggtggacg

atcgccgagcccgccgccgcggcggtatgggacgaggtcgcggtcgccgttgcctccgcctccgtcggcgggacggcgtgatgatgacttgccgtttg

agacgtga

>contig_21.contig_21.g812.t1

atggcacgcacattctccatcttctccatcgtcgccggcttctgcgcctttgtcctcctcgtgctctgcactgtctccc

tgccgactacgctgaatgacagtacccccttcgactacgtccgcgccagcaacctcgagggtgtcaacgatgtcggaattgctgccaatactgaccga

aacttgagccgtatccatcttggactctggggttactgctcttcctccatcgagaagcccgacaacttcgacttctgcgatcgtcctggacatggtta

ccaagtctccctgaacgcaacgaacggcgaccgccaagccgcgtacgagaacgcgaacatccaagccagctggacccgcggacttgcgatccacgtcg

tggccatggtcgcagctgccattgcggtcattctcagctttgtgccgcacacgctggtgatgctcattgcgttcttcgtgtaccttgtcgccgctatt

ttgactttgatcgcattcatcatcgatatcgtccttttcgtctacgcgcgaaaccgcattcaccgtgtttcgcccgatgcaagcgtcatgcccggtcc

cgccttctacttcgccctcgtctccatccccctcctcctcctcgcctccatcaccgtctgctgcggccgccgccacaagaagcgggacgacgagtacc

cggccaccaccggtgcgagcagtacctacccggccccgacgcactatgcccagggaacgacgggaaccactggtactgcggctacggatagctatggg

atgtcgactacgaagtcgtcgaagcagaaggtgcttgatgctttccatgcgtcgaagaattag

>contig_21.contig_21.g813.t1

atgcacgtgtatctagggatactcgttacttctatgacctctagtaaacagcttcacacgcaggcagactcaagaccca

cactgactggcatcggggcttccaccttcgcaacgcgcgcgacagcaacagcagcagcagcagcaacgcgttcagcgacagcgacagtgagagagatg

acgctggtcacgctcggagtctgcacaggctcgatctacactgccttcctgatctggggcctgctccaggagcggctcaccacaacaccatactccgc

ctcgtcctcatccccgcccgagttcttcaaatcaccgctcttcatcaatgccgtccaggccgccctctccagcatactggccgccctctacctcctct

tccgcagaccgactgccgatcgcaggcccgtcggcaacgtccttggcttgcatctccttaccccggctggggcccagtctgcccgcaagcacggacag

aagagggaacactcgaatgggtccccatcatcaaaagcatcgaacgccgccgccaatgggagaagcgtgtcgcccgtgctggcgcgctacctgctcat

cgcaaccttacaatcaataggctcccagctcggcttcctctccctccgacacatctcctacccaaccatgatcctcggcaaatcatgcaagctcgtcc

ccgtcctcttcatgaacctgctcctctaccgccgcaagatcgcaggatacaagtacgccgtcgtcgcactcgtcactctcggcatcagcatcttcatg

ctcttcggacacgagggaggtaccgctgctgctgctgcaagtgcaggcgcgagcgcagtgaagaagatcaagaaggccagcaagaaacacgctacgcc

cgccagcagctccctcttcggtctcgcgctcctcacagccaacctgctcgcagacggcgcgaccaactccacccaggacgaaatcttcactcattata

ccctctccggaccacagatgatgctcgccatgaacgcgttctccgtcgtgctcctcaccgcatccctcctcgtgccggacccgagtgcgttcgtggcg

tccataccgctcctcagtgggcttgtaggtggggcgaacgcgtcggcgagggcggcggcgggggcgggggcgaccaatgagctcggcaccgcgctggc

gttcattcatcgacacccagaggtgctcagagatatcatggcctatgccattgccggcgcaacaggacagatcgccatcttcgagaccctggagcgct

tcggcagtctcaccctggtctccatcactgtcacccgcaagctcttcaccatgctcctctccgtcgtcgtctacaagcacgccctctcgcccttccag

tggatcggcgtcctcgtcgtctttgcaggcatcggcatcgaagcacgcgagaagcgacgcgaggggctggccaagcgcgtcgtgaatgatcaagcgaa

ggcaaaagtcaaggatgcctaa

>contig_21.contig_21.g814.t1

atgccccatcggacagctctctccctcgacaaagccgctgccaaggttgctcccaaagcacaccacctcgtcatctcca

accccgtcaactccaccgtgcccatcgtcgccgaggtcttcaagtctttacgaccccaagcggtggtctgttctgtgtcacgggtcaccacctttgac

atcacacgcgcctccaccttccacttcgacattgccaacgtcaagcccgccgaggtcaatgtgtccgtcgtcgatgctcgtagctacgatcgcaagtc

gcagccgcatggcgaagcagacgaaggccgacagccaggtcactatggttccaaggccagtcagcactcccgaattgtcatccaagctctctccctcg

acaaagccgctgccaaggttgctcccaaagcacaccacctcgtcatctccaaccccgtcaactccaccgtgcccatcgtcgccgaggtcttcaagtct

ttacgaccccaagcgtctgttctgtgtcacgggtcaccacctttgacatcacacgcgcctccaccttccacttcgacattgccaacgtcaagcccgcc

gaggtcaatgtgtccgtcgtcggtggccaccttcggcgtcaccattgtgcttctcctctctcaagtcgcccagggttccatactccaatag

>contig_21.contig_21.g815.t1

atgccccatcggacagctctctccctcgacaaagccgctgccaaggttgctcccaaagcacaccacctcgtcatctcca

accccgtcaactccaccgtgcccatcgtcgccgaggtctttaacccgccgaggtcaatgtgtccgtcgtcggtggccaccttcggcgtcaccattgtg

cttctcctctctcaagtcgcccagggttccatactccaatag

>contig_21.contig_21.g816.t1

atgccccatcggacagctctctccctcgacaaagccgctgccaaggttgctcccaaagcacaccacctcgtcatctcca

accccgtcaactccaccgtgcccatcgtcgccgaggtcttcaagtctttacgaccccaagcgtctgttctgtgtcacgggtcaccacctttgacatca

cacgcgcctccaccttccacttcgacattgccaacgtcaagcccgccgaggtcaatgtgtccgtcgtcggtggccaccttcggcgtcaccattgtgct

tctcctctctcaagtcgcccagggttccatactccaatag

>contig_21.contig_21.g817.t1

atgccccatcggacagctctctccctcgacaaagccgctgccaaggttgctcccaaagcacaccacctcgtcatctcca

accccgtcaactccaccgtgcccatcgtcgccgaggtcttcaagtctttacgaccccaagcggtggtctgttctgtgtcacgggtcaccacctttgac

atcacacgcgcctccaccttccacttcgacattgccaacgtcaagcccgccgaggtcaatgtgtccgtcgtcgatgctcgtagctacaatcgcaagtc

gcagccgcatggcgaagcagacgaaggccgacagccaggtcactatggttccaaggccagtcagcactcccgaattgtcatccaagctctctccctcg

acaaagccgctgccaaggttgctcccaaagcacaccacctcgtcatctccaaccccgtcaactccaccgtgcccatcgtcgccgaggtcttcaagtct

ttacgaccccaagcgtctgttctgtgtcacgggtcaccacctttgacatcacacgcgcctccaccttccacttcgacattgccaacgtcaagcccgcc

gaggtcaatgtgtccgtcgtcggtggccaccttcggcgtcaccattgtgcttctcctctctcaagtcgcccagggttccatactccaatag

>contig_21.contig_21.g818.t1

atgccccatcggacagctctctccctcgacaaagccgctgccaaggttgctcccaaagcacaccacctcgtcatctcca

accccgtcaactccaccgtgcccatcgtcgccgaggtcttcaagtctttacgaccccaagcggtggtctgttctgtgtcacgggtcaccacctttgac

atcacacgcgcctccaccttccacttcgacattgccaacgtcaagcccgccgaggtcaatgtgtccgtcgtcgatgctcgtagctacgatcgcaagtc

gcagccgcatggcgaagcagacgaaggccgacagccaggtcactatggttccaaggccagtcagcactcccgaattgtcatccaagctctctccctcg

acaaagccgctgccaaggttgctcccaaagcacaccacctcgtcatctccaaccccgtcaactccaccgtgcccatcgtcgccgaggtcttcaagtct

ttacgaccccaagcggtggtctgttctgtgtcacgggtcaccacctttgacatcacacgcgcctccaccttccacttcgacattgccaacgtcaagcc

cgccgaggtcaatgtgtccgtcgtcgatgctcgtagctacgatcgcaagtcgcagccgcatggcgaagcagacgaaggccgacagccaggtcactatg

gttccaaggccagtcagcactcccgaattgtcatccaagctctctccctcgacaaagccgctgccaaggttgctcccaaagcacaccacctcgtcatc

tccaaccccgtcaactccaccgtgcccatcgtcgccgaggtcttcaagtctttacgaccccaagcgtctgttctgtgtcacgggtcaccacctttgac

atcacacgcgcctccaccttccacttcgacattgccaacgtcaagcccgccgaggtcaatgtgtccgtcgtcggtggccaccttcggcgtcaccattg

tgcttctcctctctcaagtcgcccagggttccatactccaatag

>contig_21.contig_21.g819.t1

atgacatccggcaagaagacgaagggtcgtgccctatcatcaagcccagcggaggaggagatcgtcaacctccagcggc

gactcctggcaagccaacctttggcccctgccccgacatccacatcggcctcggcctctctcaaggcttcgacacacacacaggttcctcagcaacct

tcgacaagcagccgcccacagcttcgctctgccttgtccggtccctcgtcctcgacagcggtcaagtcttcgaccgcgggacctattccctcttcggg

ggtcgaccacgactcggtggtcgcccggcagcaggtcatcgcagagatgctgcaagagtcggaggattctcgtcccgtccagaacaaaacgtcctcta

agggcaaagcccgtcgagtatccccacagccggagcaacacccatcggcctcagttccagcagagaaccgaccggtcacgccgcccagcggccttcat

caggcgcacgtagacctgatcggccacttcgagaagctgtctccgcggtcgaaagtcgaatggctcatgcaaggcggtccaaacgacaaggacgagag

caaggaggttcctcctcctcatcctgcggctgcggccaatccttaccgccaacgtcgtgcccaggatcattccccgtcgcagcctcaagctcatcagc

agccgccggtcctcaagagacccttctatgtctcaccggacggcttccctgtcgcacgcaagagggccaaggtcgcggacgacccgctgccggctagg

tttgagaaccaaccccaacaacccgctgcgccgcagagcggcccaccgcaatcggcgtgggtcgacgacgatgacgacgagtggcttgaggagggcat

gctctccggacacgcgcgtggcacaaaggccaaagacctcccggattctctgagcgggatgctcgactcggtcgccaagtccgcggcgctcccggaac

caaagcgtcaagacgcggatctctcgttgccagagatgtgggcggcggcgaacaacatgctcatgtacatggcgatcatagccaatgacgcagttgat

cccgtcaagaagaacctgcttcaccaagagcgagacggctggcgcaaggccttcaacgagatctttaaccacaacttggtaatggaggatccaatcaa

gaactggccaacgctggccgagtacgtcgacaacatgaggaagaagatatatcttcgacctgtcggctcccgcatcaagcccttcggcattcgcgaca

agctcttccgctcgatcagcgacactgtccagaagcggacggctgcggcggagctctcagctacgatccagtcggcatcggcagctgctgttgctgca

gcccaagcggccatacacgctcagctcgcctcgtcttcgtcaaagcagtcgtcctcaggagctgcgtctggctcgggtggcggtggttcgtcgtcgaa

gaatcctgcactggctctccgaaagggcacgatgcccacgccaagcgcgacaaagacggaaacatcgtgcgacgctccgacggcagcaccatctgcca

ccagttcaacctcactggatgtaagcagtcgaactgctcaaagggacgtcacgagtgcagtgtctgcggagacactgggcaccaagcctccgcttgat

cccgtctcgagccctctccgcagtgaggggttcaagacgctactggagcaccacaatctgctgtcggccttcctagagttggtggaaggcctggagaa

tgggtttaacttcggaatccccccaatcacggtcacgaggacaccgcccaaccacaagtcggcgacctcggacctcaagactcttaactcggcgattg

agaaggagatcggattggggagggcctgcgggccttatacgaaggaggaggtcgaggacctcgtcgggccgttccagacatcgccgctgggattggtt

ccgaaaccgaacggcaagtggaggatggtacaggacttcagctatccgcggaagggggagtacccggcgatctacaactacatcgcgtccgatgagtt

cgtttgcgcctggcatggctactgggactttgtaggcctgcggagcgcgcgcatgtcggtcgactgcgcagcggggcatgccggtcggctgccccgtc

ttctccgtctggcgttcgccgacggagggccattgggctttgggggggtctggcgttcgccgaccccggcgcgcttcatgccgccgggctcgctggct

gcgatggcagacgcgagtgaggctttccgggctgtgggggcggccaaggatcagtggccaggcctcgtgatcctggcgccctctggatggttcattat

cgacacgagactcccgttcgggttagcctcggcgacaggggtgtggggctcggtggccgatgccaccctcgccatcatccacgcccacttcgacacgg

aggttggcctccgggctgtgaaatgggtggacgacttcgtctttatgaagcccccgtcctccgacctgctgctcgacaatgtgctcgcggtcacggag

cccctggggttcccgtggcacccggagaagagatcggagttcgcgagcgaggtcagatacctcggcttcgaattcaatgtccacacgttcgtcgttca

actcccagaggacaaggcggcgaagtaccgagagcgggtgcttcccttcaccaagtcgggccacgtacgcctcaaggacgtggagagggtcttcgggt

gccttcagcacatcgtgattatggctagggacctgggcccccacctcgccgacttcgccgagttcctcggggtcttcgatagggacgacctgttccag

cacaggtttatcccagcccgagtccaggacgaagccaaggagtcgtgccagatcctatgggcggaggcggtcgcactcgagtttggcttgctcgtcgc

catcgagatgggcggggctggtcattcgatcttgatccacagtgacaaccagggtgtcattggtgcctttcggttcggacggtgccgaggccgacagg

ccaacacggtcctgaaaaggatcgttaaccttgagcgcgctcacaagtttgagctgcgcatcaagtacgttccgacggccagcaacccggccgacgtg

ctcaatgagcaccgggcggtggagaagaggaggagggcttcgtcgtcggtcgcgggggcgccaacatcgacgaggccgccagctccgactcatttctt

tcccccagcgatccgtggcgcgactgatgagctcccatcgcacgtagccatctgcgaggagcgattcattacggcagccaatcggatctttcggtggg

ccccttcgccgggcccagcttcttctctttcaacttcttccggtttccaattcgacccttctggcctcatgcggggctcggtcaacgaggtgctggag

ttctccctcgaggagtccacctgtgtctcgtacagcaccggcatcatcaagttctgttccttctgcgactgtgccggcatcccaatccaggaccggtg

tccaccgtcaggtggacttctcctcgcgttcgtctcttccttcgtcgcaaagatacggaccgacacggccaagaagtacctaggggctgtggcgactt

ggcatcggctgtggggtcatgactgggtcatctttccccaggtaacctacgcccttcgagggctcaaacacttccaacctcccgccaacgaccttcgg

cctccgatccttgcagcgcatctgcacgccgtcaaggcgcatctcaatatcgactctgatcctcttcacgccgcagtgtgggcgtgttcccttttctc

ttggtgggcggtctgtcgactcggggagacaacgtctccttccacaccagcattcacgccttctcacaacgcaacgcgagcctcgatccagtcacacc

gtcaaatcctgaacggcaaggagatcatacgtgtcaatctgccgtggacgaagacgactaaggagaaaggtcaacagaaacacatcgtcaaggtcggt

ggggccctctgccctgtcgaagccttcgacaaccatctccggctctctcctgttccggtccacgagctcgctaccactgctctcttcgctttccgctc

atcatcgatgcctggttcaacacttgttctcccactttccaagaccaagttcctcgcaacctttaacgctgccctcgtccggtccggtcaggaggcct

tccagggccattccttccgtatcggcggcgcgaccatgtattggcacacgggcacggacatcaagtccatcaagctcatcgggggttggaaggggaac

tcggttctcaagtacctgcgtgaatacgcgcgcggactactaccggcacaagagcgcgcagcggcggccattgctgaggccgccccgccgtga

>contig_21.contig_21.g820.t1

atgatcacagcacagacacatctccatactccaatctcgcaacaccgtgtcgacgagattcccactcacctcgcatcat

gctcgcactcgcctcatcgccgcctgcctgccatcgagcaccccaacccggccaccattcattccgcaacgacctcggcccgccaaatcatgctctcc

tccgccgtcgcgtcctcctcacgctccaccgccgccgccctcgtgcgtgcctccgcctcgtcgcgcaccacccttgcgcccgcatcagcggcaccagt

atcagcagcagcaatcacacggcggtacgcttccggcggctcatcctacaaccaacccaccggccacctcttcggcgagaagcccgcgccaaagggac

agaagcgccaaaaggaggactgggaagatatctggatgtacggcatgttcggcggcatggcattcgccctcgtcgtcctcatgtaccgacctgacaca

accattcaaacttgggccatgggcaaggctaaggagcgcttggccgagtccggcgagatctggcagtacaagccttcgccaaactccggatacccaga

aggcgttgccgagaagtga

>contig_21.contig_21.g821.t1

atggttcgcctgctatccctacagcgcgccctccgcctccgcgccgccctcccttcgtccccgtactcatcctcagcag

gcccaggccccagcccaagcccaagctcccaggttgtccgatgtcgattcgccccgtccccaaccgggttcctccacctcggcggcctccggacggcc

ctcttcaacgtcctcatggtccgaagttcggcgccggggtcgaagttcgtgctgaggatcgatgatactgacaggaatcggtatgtcgagggagcggt

cgagtcgcttacaagaacattgagatgggcaggcctgcattgggacgaaggtcccgagcaaagcgaccaattcgggccttacttccaatccatgcgtt

tgcctctctatcaacgctacgtcgacaagctcctccagacaggcggtgcatatcgagatttccgcccggcgtcatctgaggccgcgacaggccaatcc

gcaggtatccgtctggcgccttacatcccaccgaccgaagcggagtctcaacagatgatcgacgctgggaaaccgttcgtcgtccggctgaagatgcc

agagcgctcgattgactttgacgacatcgtctttggtcacatcagtttgaatgctaaggatttgggtaatgatccgatcctgttgaagagcgacgggt

ggccgacctatcatctcgccagtactgtggacgacatcgagatgcagatcacccatgtcttccgaggagaggaatggctaccatcaataggcaagcac

ctagccctcttcgacgccctcggcgcgaacccaccccacttcgcccacctaccgctcctaatcaacccggacggctccaagttgtcgaaacgaacagg

cgacgcgcacgtgcaggcgtacatcgataaagggtacgagccggaggccgttctgaatttcgtgggtctgatggggtataatcatcagaatgcgcctg

tttcttcttctgcttctggcgcgggtgtgggtgcgggtgcgggtaaaggtggcgcttcgggggaagaggatcattcggaggtgatggatttggaagcg

ttgatacgtggtttcgacatcagccgaatcgcccgttcacgagcatcccccttcttccccaaactcgactttctcaaccgtcagcacatccgacagat

cacgagtgtacctgatgatgccgacgacgactcggctcagcagaagcggaagaacgaggtgattgatcgggcacggagtgagcttgttgctgcttttg

ggccaggggcggaccttttaagggacaagatggtggaagttctcctgacgatcggcgagagggcgactactgtcaaggatttggttgaggaggctcgg

ttcctgttcgagacaccggattggactgccgaaccggctcaaaagctgctgaggagcatcgtcacccagaagaacgttcctgcaggggcggacgtcca

tgctgcgatgcgactcatcttggacagatatacgcacgagcttagcggactgaaagggaaggaagacaatacgtcaagtaaccaatcggaagatccga

tgactcgcgtcgtgcccgctgtcctggagacactcagtgccgggaacgaaggcgccaactggaaggcgaaagtactcagcacacttgccaagaagacg

ctccgatgggcagtatccggcgggaagcctggccctgctgttgcagacgtgatcggcgtgatgggggcggaggccatgcaaagccgactgcagacggc

acgaggtgcgctggatgcatctttgtcgcaggtagatccgccgtcaaatggaagcacagcaaaggcgcagtga

>contig_21.contig_21.g822.t1

atgctcttctcttgttcttcccgacctcgacgcgcacctgagcaagtcactgtccatcgcttcgaggcagccgcaagtg

gagcgcagagcaaggtgggaacgcgcgcgctggaccaaaggtcgacctccaagccaagaccgcgcccgggtcaaggtagaaagctcaacgctggcatt

gctcttcctcaagtatcccgctcgtcgaccgatcctcaaacttccgaagatatcaaaaagctgaaagagagcccttcattacagtacgtgaacagcgt

gttggtctatcgcgccggcgggaccaacaggctgaccggcctcatgctcgccgccggtacgctcggtctcctcctcgtcgggcccggggtgatccagt

acctgcccgtgatggtcgtcgcagccctcatctttgtcctcggcatcgatctcgccaaggaggccctgtgggataccatcggcagggtgtcccgggag

gaacacatcaccatcgtcattatcgtcgtcgtcatgacctactgggactttgtcattggtatcctcgccggcatcgtctttgcctgtctcttcttcgt

cttgcaggccagtcgacggcggaatacgatccgggccatctttgatggaagtatcgcgagaagtacggtccggagacatatcacccagcgacgcttcc

ttgagcaggtcggcacccagacccagatcctcaaactccaaggcttcctcttctttggcacgatcaactcagtcgaggatctcattcgccgcgctctc

gacatagccacctggcagagcaatcccattcgatttctcatcgtcgactttgatcttgtctctgggcttgacttctctgctgccgaggcatttacccg

ccttcaacgcttactcgaggccaaggatgtcgtgctcgtgttctgcggtctacaaccagactctaatgttggaatcgccctccgctccgtcgatctct

gggccgataacggagttcgtctcgaagttttcgcaaacttgaatgaagccctcgaatggtcagagaacgaataccttcgcggcatgtactcaagcggc

tgggcttcagcccgcgcttttggccgcgcaacagcaggtccaggagctagcggcatcgaagtccctcgaaccaacttcggtgcaagcggcagcgacct

gggcccacacaaacctccagcaggaggcggtgctgtgcccggcctgggtctcgaagccgccggcgtagacataccggacagcaagacaagacaacctg

cctttgtcctcgattcaagctttgagaactctccgcggcggtctcatctccaacaagcggcgaggaccgccctcgaacgcgccggtggggatggcatg

atggtcggggcgctcaactcggtgcccgagctcgaggttgaggacggggcttcttcagccgatggggacatggtcatagagtcaatcgagcgggaacg

gccaggagagagaaggacagcatcctcgggcgaacagttgcctccaaatgcgctaccccagcccacggatggccagcagcaccagtcacaaaatcaat

caacagcggtgttgcaaagaaggcggaaacttctccaacctgtgccgctgttgacgcttacttttaaaccgtatgcacaggaggacgtcggtcaagaa

ttcttcgccaacttggcgccatactttaccaaggttctcatcagtaagggccagaagttgtgggcgcgaggagacgaacctgatgcattgtatctcat

cgagaaggggattctgaaagcccgctacgacttccctcaagaagactttgaaattaacgaagccatgctagccggtaccatcgcaggcgagctctcct

tcctctccggccaagtccgcaacacctccgtgcacgccgagctcgactgcgtcctgtggaaactggacggcgcgcaaatggaccggatgcaagtcgag

cgtcctgcgggtgatttccgaaccttcttccgcatgttgctcaggcgtctgcgcagcttgggccatcccgctcccgatccgctgccatcaggatgttc

acccgcggcagtcatctcaagtccttcctgtcgactcttccacccaagatctcggctcgctacgcgcgctgaagacgctgctcagccatgccccgctg

gagagaccgtggcccagcctcactcagtccatgctcatcctggtgaggacgacgaccacaacctcaacgacgacgaggatgagctcgacagcaacgat

ggccgccgccgccgtcaacgccctcgccctcgacctccgtctctagccagtcatcggaatcgggcccttcaccctcggcatccccctccggcacacaa

tcgactacctccgctccaacccagcccacttcccagcgtacacctcgtcctcccctccccctcttcatcctcgaactcgatcctactctccatcttgc

cccatcgtcatctcgtctatagcccgctctctcaggcctcagcgccttcagcttacacgcccttgctcgacagcccggcgagccaggatcgggttcac

tcacgacaccctgcagcgggtcagggtcaacagggtcaggggcaacaacaacaacagcaacaggaggcaaccaccctccttacgcgcactggcatcca

tcagacctttggtcccacctacccaccccgccgcatccccaggcaagactga

>contig_21.contig_21.g823.t1

atggccgacagcagcagcaccagcagcaagcccgtccagcgcgactcccagcccgctccaggcgatgcccgcacggagg

aagtcccagccgggcccgagcgcgtccccaaggatgcaatgagccctcctgctcctgctcccaccgcactccatcagcgactccaacggagcatgggc

ccctcattaggatcctcgcccatgcactcgccgcgcagcgcactccgagctcttgatccagagagtcagacccgcctcggacaccacactcccacttc

atctccaggagcaggatcatcggccctccccatccatctcgaaggtcgttcctaccgcttccagcattcccacaacaccgacagccccggcgccacca

aggcacagctgtctacctctccgcctgtggagtccgatggtgtccagtactcgtcctcctatacggccacgtccaatgcgatcagagacaacaccgcc

atgctcgcaaatatggcactctatgacgacgatcacagtccagatcgcgaagaaggaggggaagaagaagaacaggatcggacgcaggtacagagcgc

gagggtacaatggcgcgacagctcgtcttcttcctccgaactcggttctggcttccaattccaggcggcccagtcaggcgatcgcatacccgatcagg

acctactctccacctcgccccgagctcagccgaaccgaggacccggcatgctcagtctggccctcggtgtaatggacgacggaaaccaaccgcctgcc

attgtcacgtcgcctccaccgatagagtacagtgagccaacaccccgattggctggtgcggtctcccagtccaccttttccgaatttgggcagcgctg

cgaatccaactcaggaacgacggacacaggaagtgacttctcagtccgtgcaccagcgccagtcgtcccagccatcgcaaccgcctccgccggcgctc

ccgaagaaaatgcagaagcgcggcgagcttcggtaccgacccgccacattgccgccgctcagcatggcgaacaacaaggcccggctcgagctatacat

atgcaacggcgaaccagtgtgggtgcggctacgcctcaggatctcttcatcgcgcgagcgacctcgatgtccccggcaggcactggagtcaatgcctt

caagccgagctccttctctggctttgctgccttcggcgcgtctcatcgaggcagtttcggctttggcgccctcgcacaggcgcaagcgcaagccgagg

ctcagactcaacaggcccatcacactggaggaagtggggcaggagggggagggggaggaggcagcggagatcgaacaagcggaacgtctgcaatccaa

ttgccaggtggatttagctttgcaaacgctcgaacgtccggagtgggcgggcggagaagtagcattgcccgcccatcaaacccccctggagcgtcttc

cttctccatacatcggacatcgagcatgggtcggcgatcgcttccccacggcgggggcggcaccaacgactcgaactatggaagcgtcgtgagttccg

acgtgagggacgacaactacgagcgcggacggtacggcggccggagagacacgcacgacagtgatgacacagacgaacgtcaggcactgctgagcgct

gacaggtcctggcggaacgtgcttggtggtcgtcggcaggatagcagccccagaccggcccctacgcgcgcggcgacaatctacaacgcggtcaccta

ccccgtccgcgctgtaagctcccttctcttcagcccacgttctcgctcgcgatcccccgctcggtcatcaagcagtgggacacccgctgttggggcga

gggaaaggatcatgaagaagatgaccaaggcgaacttaaaggctatggtcaccgagccgatcactgtgcttccggccgtcgtcctcgggttgctactc

aacctacttgatggtgtcagttatggcatgattaccttcccgaacagcaatccgatctttgctcactttggtggagacggcgtcgccatgttcttcgt

cacatgcatcatatcgcaactaatatactcactcggcgggagtgttttcaaggcgggaaacggaagcatgatgatcgaagtcgtccccttctaccaca

tactcgtccgcaagatcgtcgaggagattggcgacaacaacccggcggccgtgattgcgacgacatgcatggccttcgcgctctcctccgtcctcacc

ggcctcgtcttcctcctccttggtctcctccgtctcggcgtcctcataggcttcttcccgcgccatatcctcgtgggctgtatcggcggcgtgggcat

cttcttgctcgagactggctttgaggtcgcaggtcggctgcagtcggaggatgggttccagtacaacctcgagacgctcaagttcttcttccagagcc

cgcacgaggtggcgctttggtcgatcccgctcgggttggccatcttgctccgcgtcattacggcccggttcaaccatccgctcgtggtgcccgcgtat

ttcgtcatcatgccggccgtcttcttcgccgtgactcttcccttcggtttctcgctcgcccgactccagggcagtgggtgggtattcgacatcggcag

cgcagccgacgcgcccttctggcgcttctacacctacttcgacctgacgcagacgagctggatggcgctggtggcgacgatgccgacgcagtttgccc

tcgtcttcttgagcgtactgcacccgccgctcaacatccctgcgctggcggtcagtgtgggacaggacgatgtcaatacggaccgggagttgactgcg

catggatggtcgaacatcatcgctggtgctcatgtgctgattccggcgcttggcttcttgattgcgggcaagcatctacaagcatga

>contig_21.contig_21.g824.t1

atgcacgtcgagggcgccgtgtcgccatctggctgggctctgaatggccttctcatccgcaaagtcgctttgcaatcca

tctcgctttgggagaaggcgcaacaagagggatccagcgaggatcgagtcagcgtccggacgaaggctatcaacaaggtccgcaaggctgccaatgcc

atgcccaatccacacgcccccttgccgccgcactgcaagaatccctcccctttcgaattcgagtccgatctgaatgctgaagaggcgcaagagatccc

gctggtccaaagtctgatcgagaactggcccggcacagaggaggcagactttcaaatgatggcgaacccgaagacgcgtcggtcgcagccatccgatt

ccgaatgcaaccaggcaagcgccaagcgagcgcggatcgatccccagtccaacgaaacaggagcgcaaggcgaagtggtcgtttcttggcatacttca

gctggtccctcggcgctcacagcatatcgcggacccggagatgtggatcaaacggcggtacaagcctttttccgcctacctgagctcgtacttctcgt

ttgcacccacctggcgtacgaaaaagccgacctcgttgtcctgtctcaagtctccaaacgagtccgcgtcacggtgcttccgaccctttactcgaccg

tgagggttagattgaccaaggtcggccaactcagccaggtcctaaaggacaatgccggattggtcgagcagattcggtacctgaacatctgggacgac

gtggcccattaccacgctcgctgcaacactgcaaacttcagacccatgtccaagtttcggagtcaagtcatacctcgaaccaccacagccacttggtc

ttcttttggacactttctcctcgccataaacaatcgtaaaagctcgtccacgcctctgtttgagctatcattcggccagatgaatctccttgagctct

acgttcagctccgacgcgcccctcgactgatacaaaccctcaccacgtttcgaatcgtcaacgatcttggtgcgactcacttcaggggtctgacccgc

aaagcagtggagaggacacttcgcaaccacggcacaacaatgtcagaggaccttgcggtgatcttgcgcctcatctgtgaaatacaggatgattcgaa

agtggacaacttccggacgttcgacttcgacgccctgcggctcgacacggaggaacggcgatcggtcctgcccatgctgccgccacgtcttctcaaaa

cgcttgctggtcgtcttcactgtctaggtcttagtgtgggcgacattattcgagcagacgcagaaatcctccaagtactcacagaagcgcattggccg

aaacttcgcggcttccaattttacgctgacggtcaaagtgatgaagcctacgaaatcgtccaatcaagcctcaccagcttccttcatcgtcacatgca

tctggtcgaaagcgacatcaggattcatgatgactgctcacacgaaggtcgaccgcactgggccaacgtcaccttaccctatctcgaggcgtgccata

ttgatcttgagaaggggagcatggagcagctactcgactttgccgacgcgcaccgagaccttcgcaaactcttcgtgactggcgagtccgattcaaat

gccctcgctagtcatcaagcggtatcgaaatcacttcgcttcctccgagcggagccagaagccgcagaggagtttctacgagcgggtgcggcgatcgt

ccatcttgatctggacgtcagtgtcggtgaatacgatgagcaacgaagccgcttcccacaatgcttacacccggatgatcggccaacatcctccatca

ccggcctcaatcttgcatttttcgagatctcttacactgcgattatcgagaagtcagaccggttcctaccgctccatcagctgcccaatctcgtcgag

ctctcgctgcatttgacgctaagcgccgtggacggaactcgcgactcgccggagcagtctgctcaatgcctggcggccctcctgcacaagctcgcaaa

gcatggcaacaagctccgcgctgtagccattcaatacgatgctgccggcgatctgccttgcgaccttaaccttgcgcaagcagtccatacattcccgc

ccaagctcgaatatctgacatggcacattcccttcagcaatcacacagattactaccgcgtcctgaggcctcaagcgatccttcttccgccctcgaag

caacttgtccagtctttacagtcgccaccgagccaaagttcctcgtccacgcagacatcggaggtgcaggccaaggcccttctgcagcgcctacccgc

catcttccgaccaaaggttgattcgaagacgggaatgtgggaggacattgacagcgatacctacctcaacctcttcgatcatatgggccgtgaacctc

ggttgaagtgcatgttaaagtcaggcgctttggccatttgtccccccgccccttgcgtcccgtccgcagcagcagggcaagatgacgccctactcatg

catgacctccttcgactttgtcgccgagatgcagcgtgcgagccgttgttgccgctgctcctcgccgtccgtgaagcccttggttcgctctttcgctg

gctccgagcatggtgtccgccggccgcaagcgctcgtcccgagcaggatactccagtttcgcaggccgaccctctacatctgggccagcttctgcaaa

agggaaaggaggcacgagggcggcaggggcagcacaagaccaacgccatgcttgcactcggtactctaagcccgtcgcagccggcgacgcgactgaat

gtgaaggcgagtgtccccttgacgtcacccgtgctcgatgttgccgacgccggtcttgccgtcgaacatctccatggccagagaagtgaggcctga

>contig_21.contig_21.g825.t1

atggcaacaaacagcttggctgacaccaccctctaccctcgtcaacaacccccgaatggccctcgatcggtcaagagtc

caccggtctacgggttcatgggtgcgacctcctgccgtctgccgccgtctttaggtttcctcccccgcaagcatgtcccgtccgtcctgcccttcgtc

aagcctaccgtttcaaagcccaacgtctcaacacagaacaaccctgacgccaagacaaccaatgcccactcctcacgggtcctcaacaaggtcaaccc

tcaacactctctatacaccgaccttgacctacactcggcgtcatcggtcggcaagggcaccaccaaggacgggcgggaccccttttcctcatcttatc

ctctcactgtgttgactaacgtgaccctctccctcttggctgtcccatctcccatgctctcctcatcgacaccggcaccgtccctgtccccgtctcgc

cctcaccctcaccctctacctcccttggtcgccatcaccgacaccatcaccttcgacctcaccctcgacattgccttcgccttcctcctcaacatcga

ccttgacctcttctga

>contig_21.contig_21.g826.t1

atggcgttgtccccgtcgcagcctgagccatcgccggcaacacccagactgaggttcacgcagaccgtcccagggagta

ctgaggcagaaccgaccccgctctccattcctcctcctccttcttcctcagcgggcagcatagcgtccctcgaagatgaccccttcgctcctcccatt

cccgcctcgaatcactccatggccgctgcctttgccggtcctggcgtggctgcggctagtacggctgtgtctccgccgcagacatgcgcacatacagt

cgtcatctcgggagggtcgggatttaatgacctcgtgtgcgcaactccacaggctacttttgtcatgccaatttctgacgacggagggagttcgtctg

aaatcatcagagtactgggcggaccctccatcggcgacatccgatcccgcctcgtcagactcatcccaacctcccaccaacagtacggcctcctcccc

actgacccccgaccgccgccttcaaacgacgccatccacgccctgcttgcataccgcttgccacctcaaggaagcattcgactcatcaaggaagagtg

gttggccatcttggagggcagacatcgcctttggaaggggattgagccggagaggagggagtgcattcgtggcttcctcgtccatttcgagagtgaga

tcttaagacgcgcgcatcgaaatttcaacttccgcggaggcagcattggcaacttcttcctcgccgccgcccaaaagtttttccgctccgtatcctcc

gccatcttcctcttctccgccatcaccctgacttcaacctaccgcggctcacgcgtcatacccgccatcaacacgaaccataccgcaactatcgccgc

ctctctgctgaatggtgaggtactcgtcgggcagtgcgagatcagtcatccttccaagccgaggacgagggctgtctctcaacatcaacaacaacggg

ggcagccacattttcttgggtcggcggtacggcaggacacgattcgggatgcctctgctgctgcggccgatgcagctgccgcactagcttctctccac

ctccaatccatgacctcgcccagtccgagcacggcagccgcagccgcagcggtcgaacacgagcccatgtcccgcaacaccagcagtcaagggggtca

acacagcgccgattacacttttcccggaacgccgagtagtgcgatctttgaccccttctcggaggaagactgcgaattgcgctcgcggtatagctccg

gtcctgcctcggcgcggttcttcgggcattcacccgcacagtacgcttcgccttcttctcccgcctccatgtcggtcgactcgccaaatccagctaca

gctgctccagcgcgaccattgctgagaggcatgtataacacgcatgatcttcggacgacgtcgacgacttccattgttgggaatgctggggatgggct

tgatccggaggaacggtctgatgatgaagaggaggagggtgagaaacggaaggggaaggggaaggggaaagttgggggccggcggagaaggcgacggc

aacagcagctacaacccagcgggagcgcgggcggtgatgaggtagggtacagtgagggagaaatcgagacggttacggaagagtcgaccattggagct

cacgatgatgatgatctagaggacgacgaggacgaggaagacggggacgactatgagggggatcagggcgatgaccagtttcgggaagccgtggggaa

tatcctctttgcgaaggatcagagtggcgagactgcgaatcagcctctgcctgctccgatcgaacgaatcttctatgtgaacgcgtatcgaagcgaga

tctttccacgaccctcgtccgattatctgtctgtgctgggcacggcgcgtacgcttctctattcatgcggctccctctggaccagtatcatcccctgc

ctcgccctgcggaacgtagcaacagccatcgcgcgctcgccgaagctgacacacaagatcctcctcctcaacacgacccacgaccgcgagtcggatgg

gatggatgcgttgggtttcgtgggcgctatggtggatacgttgaatcggtgcgatgtaccgcctacttcgtcttctggggcgcagggagggaaaaggg

ggaggggcccgtcgagctatggtccgagagactttattacccatattatctacttcccgaccgggagcataaagatcgaacgcgccgccatagaggca

atgggtatcgtgtgtgtgcccgtgccgggaagcgagggagtccacaagccgagattcacagaagccagcgctcatcgattgtggcgcaagcccaagtg

caactataccgaatgcagcgtcgtagagcgaagacatcgttcatcctatgcaagggatatggcgttcgtcgaggacttcttcaagacatctccgggac

cgaccggccaggccgacttcatcctgcgttcgtcgacatcgatactgactgcatccagcctcaaagaatga

>contig_21.contig_21.g827.t1

atggcagcaaccgactcccgctttgatacctccgagtggaaggcagcaggaagcgcatccgccgtgtcagcgcccgtgg

tcgatgcgcccggggagcaagtgcatagcgatggggagcacgatgttgatgaggacgaggtcgatgacgatgagaacgacggcgatgaggacggcgat

gaggacgacggtgatgacgacgaggacggtgacgaagaagaagaggagaaggtcgtgaagccgctctcgaaaagcgaactcgaagcctaccaacgcaa

acaacgcaaggcaggcatcatctacatctccagcatcccacacggcatgaccccgcccaaggtccgccatctgctcagcggattcggcgaactcgaac

gcatctacctccacgacggcacccagggcaagttcggctcctcctcgtccgactccaaacgacgctcgtcggcccactacaccgagggctgggtcgag

ttcgccaggaagagcgttgcgaagcaggtcgcggaggtgctgaatgccaaacctattgggactgccctcatcgcggcgggcgtcaagatgccgagggg

gagtggaggcggtggcagtagttccaagaacgcgggcggggcacggaggtggaaggatcatgtgtggacgatgaagtatctgcctggattcaaatgga

ccatgctcaccgagcagatgggtgagcagcatcgcatcggcgaatttttcgttcttaggcccttttctgactgttttcctttcgtacatcaaccagct

gccgaacgcgcctctcgcactgcccgtctacggaccgaactcgcccagtcggccctggagcaaagggactacctccaaaaagtcgaacgcgctcgcgt

catgaacgacaaggagacccgtcgtcaacgaagacagcaggcctcgtcctcttccagcacgtcacaggccctgccttctcagaaggacgcgatggacg

atgcggccatggccagagatggcgctcggatgcgcacgttcaagcagcgcgcgcctgtcttgaccgacgtacgcgatcaggatcggaaggaacgtgtg

gcgcagcaatcttcctcagaaccgcagcgtcagcagcagaaacggaaggcggactcggacgggatgggagcgaatgcgggtggcaagaagaagaggaa

gcaggagggcggggagggtggtgggagagggagagaggctctggattctgtactgggcagcattttctga

>contig_21.contig_21.g828.t1

atgacgaccgccgcgccgacccgccagcaaatactgcgtctgtacaggcgctacctcagcacatcccagtccttcgcct

cctacaacttccgaacatacttcctccgtcggtcgcgcgacatgttccgctccgctctgttgccggccagcgaggccaccaccgcgagccccttcagc

aaagcagcctccgtgaccgcgcccgtcgtgcccgcctctctcgtccatgcgcctatctctaccccagcaggcgcatctcccttcggcacatccctagc

agcgtccctccagcacaaggaatcgtcgtcagggacgcccgaggagcgtctggctgcgttctacgagagggcgagtgcggagttggatgcgttgaggc

gggctgcggttgtgaatcggctttatcagggggagcggctggttgtggaggagccgaggttgatcattggtggtggagggagtggggcggaggctagt

gttggtggtgggggccagcctgtgtaa

>contig_21.contig_21.g829.t1

atggcatcagcagcaacggccccggctcctacgccaccctctgcgagttccgcggccgcctccagattcagcaggatgg

gcgcagacgccctctctgcgctcttcaacgcaccgtccccaaccgatggcactcagcttgggtcaaatgctagactgacgcctatacatgcgtcttct

cgcgccactacggtaacaaacgatggaactattcgcgcaagttcttcgccggctgcaaccaaggtcggcgccaacacgacgtcgcttcctctatttgc

ggctccggaaggcaccacatcggcagcacattcctccctaccctcggaaccgacgagcgaaagccgcccctctgtatcccgtcccgacccagtctcgc

aacccgacgggaaggccgcaatgtcccgatcatactccaccacatccgccgcttccggttcctcgtcttcgtcttcatggacggccgcagcgcgaaat

gcgagcatggcgccgtatcgcgcattggcggccgccttgcccagcctgctcaacgtctctcctggtctcggagcaagcacagacacctcgccgcccag

ttcagctcctaccagtctcgacagtgatctcactatgacgccggccatagttcacactatgaaagagagcagattgaggaatagtagcggtatcgaag

aagaagaaggaggggaagcggaggacgatggactcgattctggtgcagtgacggcatggcagacgcccgagatgggcgtcatgcttgccatcggacag

gacgcatctgcgagcgggaccgaccatcaaccaagtgtcggtggcgagaggacatcagcggctgctgcgctgatgcgatccctatcgacagctagtcc

ccattcgtcaacttcggcggatgaggaggaggatgagcgggagcgagagtatatcaggaatgtcgcaaaacggccttcgggcgggttggcgtctgtcg

tgcatgttcacgaaacagcgccggcctctgattcggattgggagcctgactcgtcggacgaggacgaggagcggcagcgaaggggcattgacatggtg

atccatccgtacgaggatgaggaacagcaacagaggctcaatgaagtggcggtgcctgtacacgcacacgctaacgaggacgttccttcttcgtcggc

tatggcggtagagaatgcagaggaccctacacaacaacaacagcgcagacggcggagaagaaaagaaggtcatcaacggcagcaatcttcttttttca

gcgtcagctccgtcttcccgccgccttcctctgggcacgacgagcagcggtcgattcggcctacgttcccgccacggttgtcgtccttagaattcgac

attccggtggtggagggacgacaagctgaagaagtgcaagaaccacagcgtgtcttactgccttctcccatcattgccgatggctctctcgccctgac

acctcttcctccgcacgccggtgtcccactctcgcagggagtgatgtcccgcacaccttcctcgtcggtcccttcgtcttcgggcctttcagacgctg

ttggagtctcctcggcgtcagcgatggccggctccgacgcgctcatgctgggacttgggctggtggacatggcatcgtcatcggcatctccttcgtct

tcctcggaaaatgctgacgagtctgtcgacgtgacggaaaagctgggtcatcgtccgtctttgttgtcgctaaacacgaccatgcgccaaacacaacg

agatgctttcttgtctcacgatagaatggaaacgggcgctcccggtgcagtcgaggtccaagatgcacacgtcggaattggggaacaatccatgttcc

cctcaggactggtggcgtcgcccgcctcactcgaaggtccgttcgaagaggcccgtttcggtctgccgcttgcaggtgtcttcacgcctccgagccga

gcgctccctatgggcgcgtcttcggcacattcgacgcctgcgactgtcgttcctggggttgcgacggtcgtgcccaccacgccggtgctcgagtcgga

cgtgcctttctcctcgaccggtgccgatgcgcctgcgaacggcggggtggaggagcaaggcgagactgcacaacaacggctgcaagtggcattggcgc

gcgcggaacatcggcgcgagttgcagcggtatgcgctattagagctcgtccggacggagcgctcgtatgcggacgatctcgcgttactcgtcatggtc

ttcttcgagaacctggtggtgctgccagctttcgcacctgctgtgaacaacggtggcggcagtagtaatgggagcgtcgctggtacgaacggggacat

agaccggccggggtcgcggttatcggagacccaggaaggcagtgatgatccagaggtgctgatggcgagttcggatgaagggagtggctcagctgtcg

tctcggcgaatcccggcgcgaccagttctgctatcggatctggaagcactacgctccaaccccctgcaccgccgcactctcgctcgtcctcgtatcag

ccaactgcaagcggaagtggtgcaggagcatcaccgatgtccgctgccttttctcgcaattcctccgatcctgccttggagcgacttgatctggtcac

gcgcaatgccgaggagttgctcagtctgcatcaatcggcggcatggcgtcttgaggccattgtctctcagtacgggctcgaccaccccgagtctgatc

cgaacgtgaaggtgaaggtatcacctgcggcgcttgaggaaggcacgcgtaaagtggctgaatacctcaccaatacgctggctccagccttacgcgac

ttgtacgcgcgcttctgttcgcgccacatggaggctctctcgatcgtcaaagacgccgagaggaggggtgcggctgaatgggtcgcgtatgagcggat

gtgtgcggagacgttgcaggttgttgcggcgaggatgtcgccgacgcttccttctccggtacaacaagaagcggcgacagcggctgatgtgccaactt

cgacagctgccaatgccaacttggtctcgatctccgcctctgctacgccgggtgctcacccgacctcgagtccgatcgcgccgctgttgaacaagggc

aatcgaccacaccgactcgtcttccacgattactttgtgaagcctatccagcggatcgcactgtaccccctcttgctccagagcttgctcaagtactg

ccatccagtcagtccgcctgccagcggggaggaggatggtagccttgatgacggaccaggcgtggacggcgtgtcgcagatgggtcagagcaggccaa

gtaaaggcaaggaccgggaggcggtgtttggggctgttgccgcgcttgttggtgtcgttgagcatgtcaatgaggccggtcgacttcgccacgaggag

cggctgtcacagacgattgtagacaggattgaaacccactacaatgtgacgccctcgttcctacactcgctcgggcactgtgttgtggccggcaccct

cgatgtgatgtaccaccagcgaaacggtcacccgctcacttcccccctccgactcaagtatcagggctgcgttctctaccaaggctttatgcttatct

tcaaagtccgcaaggcagccgcgtacgagcctaagttctggttcccgctctggctggccaaactgagcgacgtgcccgaggagtccaggttcctgccg

catgcattccgactgagcgtccgcgatcaccatttcgagctggttgcttctaatgcgcgggaacgcaagctctgggtcgacgcgttgggcgagacgat

ctcccaggcgcttattcaccctccaggcacggaggcggcattccctagtagcctgcacgtcgactcggctgggactggtgagagctctagtcggccct

cgagccgagctactcagcgcgcatccttccggctgtcaaattatgcggctcctgccgtacctggcagtctggacgctggaatgatcgaagagatggac

ccagcggccgagagcatgggtcggttcgaagcaagccctcggaaaggacgattcgctacacgtggacaagggcactcgcccgtcggagcgttgtcaac

gccatcgtccccattgacgccgtcatctgcgaacttcaacctgaactcgccagctcaaggtggaaacagcgatgacgtcttgcagcagtacttcgagg

agacagctgagattcaggtgcgacggtcttcaccttaccaacgctcggtgatcgaccgctccatggcgttctcggtctcggtggttggtgcgcgtgct

cctcgcagcgcccatgattacgttggcgggtcgggttccctggcgttgagcaattcgtttgctctcgcgacgcctgctcatgggcccggctgctggca

acagtctctcggcgccctcgtcggcttgggtcggatgggttcgatggagacttcgacgctgaaaatgcaacggcggaagagctacgccgatcctgtcg

aaagcctcttcatcccaccgaactcagacgcgctcatgtcgtcttacgcggccttggtggctgacggctccaacggtaatatcggctcccacacagtc

ccgttcccccagcacactcccaccggaaacacgaacggcatgtcaaaccgtgccatgagaacattccgccagctgtctctgaagacgtcgtcgcgagc

atctgtgcgggcgatgagcgagcttccggccgtggccatgccgacgtctgccccgatggacggaggaacgacgggtgacacgaacgggtctacatcga

caggacagcagagtctgtccgtcccgcagcgtgcttccgcgtcccgtcgcgagtcgtttgcggccagccttcgcgatgcgctgacggtttcctttgga

cgtcgacagcgctctcagtcgtcgcaggaagtcgagtcggagtcacacaccccctcagggacggacgccgcaagctcgggcctgccttcgggcgccaa

cacgccgtatgttccttcgatgcagcagtcgccgtcgcttcatgctggcctagccttgactcccgtgaccggcgaggccgagaatggggcttctaaac

ctggccgaacggactcgatacggcgggtttttggcagtatacgctacaagcgtgaacgcacgttgagtatgccagcgccgcctgaggtcgacgcgagc

acggaacagcagcagtctgcattgggcctgctgaagggttcggctccgacaggactagccccaaaatcggacggagaggccgccacggcttctcgaga

ctcgcttttgcataatgaaggagaagcgtctttgcaagtcaacatttcgcaagaggacgggtcgattccatcgaatcctccaccacggcggtccttat

ctcgcgcgtcgctgcagcgatcccggacggctttgggcaagatctggttcggaggacaacagccggctgagagtggctctggcttcttgtctgtcgct

gggcctcagccccatttgaaccgaaggagcaccgatggcttcgttgctgcgcgcagccagtcggccgtggcgcttcccacttttcccacgccattctc

ctcgtcctactcttccgatggcagcaacacgggaggcgatcccagcccggtagcctcccgttccggatctgtttcggctggcggggctagcgtgggcg

gcagcagtccgcctgagggtgcggaacgtccgatcttcttctcgacgagcaagaagcagggcgcggaacgagtgggtctgtctagctcgggttccctt

tcgagccgacggcctcggatcaacatgaggttgggatcgggctcgcatcggatgacgactccggtaccgccgctgccgacgttgcccgccacgccgtc

ggaagagtcggaggcacatctggacaacggttttactgtagaaggtgctgcggtgacgagctga

>contig_21.contig_21.g830.t1

atggacgcaaataagcccatcggcgacgacctcttcgccccggccatgcactcgaccacaccagccggagcggccaact

tcacccacgacgacgacgatgacggggccgacagtcccgccatgacgcggacgtatcacagcgctgatgcgggtccctccgctggaccatcagctcct

gcttttggcggccggcccgcgccgggatcaagactctcacagatgtcgattccccagttccacctcccgccctcttcttcgtcggcgtctcccattca

gcagcagcagcagtatgcctcaccatcgtcggcagcgtacggctttacgcggtcttccgcgaaagctgcagggtactcgagcaagccgctaccgcctc

cgcctccaccagacagtgatttcaattttggcgcgtcgggatctggtggcccccccactcgatcgcaagacacatctttgcccagctcatccaggaat

acatcgaaccagccaaactgggaaatcaccgcagtatcatcaccatccggaatgatttccagatcgccacagattccgccgttcgcaccgactccgca

gcgacctaaagttgaaatcccggtcccgagtcaccctgccgaattcggccttcccgaaacgcgcagaggcccagccacggtcgtcgagccgccctcat

acgcttccttgccagacacggaaaatttacctcccgacttcgccgatgcttctggggcagcgccagctacgggcggcaggttcccgcgctggcggggt

tggctcgaaaagcgggacttggagcgaaggtacgcggccgtggaggaggctgctctggctgcggcggaaggcaggcctgcacggaagaagagctgggg

agcgggcgttcacgatgctgatgcgctgccgtatgatggcgaagacgatgacgaagacgacgacgagtacgatgaggaaatagacgacgacgatgata

gcgagggtggcagtgacggcgatggcgaagatcgtgagggacacgatcagcagcgccggagagcacatgcgtataatgattcgacgactagtgccgga

ttccatgccgagagtcaagactcgcgcccagcagcacgctcacgcaagcatcccaggacaggcccctctcctcccctccacacgcatcactttgggtc

ccgattcctcccacatctaccctctcagccgctctgtgctgccttccttgacatcccgccgaagcctattaccccaattttagcggtcgcggctgcgc

catctatggtccaggatcccagtccgacagcaggtccgtctggcggagcgcggctttggaatcgggatgcgaaggtaaaggaggcgcggggaaagacg

cgcccaccgcagcggaggactgccatcttgatcggcacggcggaggggctattcgcggtcgagcttgcccgacccgtcaggaggaaaagaagacggaa

cagggacggtgatgtccaacccggcccgaacggcaggcactccacgagtgatggagagatggaaacgccaacctttgcctccgccagatattccaatg

tgggacgaagggcaggttcggatacggaagagggacaaggtgacgacaggctaggggatgagaatgaggatcagaggtctggctggaatggtggaatc

cgcgttgtgcaagtctggagtggactaggtgtcttccagctctccatcttacggtctcgtccaaccccctcctccatctttggtcccgacggggtcac

gaattcttcgctttctcgcaggagcggcaacgcattcatgggcggctttggcgcaggattctcccccatgaacgctgctccggtacaaggaggaggag

acagcgcgccgagtggcgctatcttactcgccttgacggctcccgctcctccttcctacgcaccgtgtccgccgctcactcgcgtcagcccgttcccc

aacgcgttgttggaaaactccgcagctggttcaggtgtggctcatcattatccacaccatgcgcggtcacactctcatgcgcacggtcacgaagatgg

tctcgccgagtcttacaaggctggagtgggaggcggcggcggcaagggactttccgttggtatgaatctggcacggaatgttgcgcctggctcgttcg

gcacggattcgggcggcggaccaggtcgcgctgtacctacgacgctgtcttcgtcgttggcagctcacgtcgcaagcttaccagcggcagcagctgcg

ggaatcaatgcctcagatgctagcgcttccgcagcggcagcggccaccattgccacgctgtcggcagggggcactggaactccaaacggacaggtcag

aatgtggaacttggaagcgttgaggggatgcgtcgcgtgggctctagattctcagcaaccatgtgatcctctcgacctctcgaaccgcgatggattag

gaggaaagaaacctcacaaacatgcccggaataggctttctcgtgcctttaagaaggtgttcggcgtgctgggcgactctggctcccctagcaaaggc

ccagcccgacccggcctctctgaccccggctcgctagacgcagtcgaaacgttggccaagaccagcgcagcgacgcatgggaagggcaagggcagaga

gggtaaagaccgagacgaagagcggaaacgacggctcggccagcgcacagccagcgatggtagcgttgtcagcagcacttggacggttattgatggct

tggacgccgcgtcgccgaccagtattgcgacgcccgccgatctggaaagtgagtccggacatggggctggatacgaagggtcgatccgttctgtgggc

acgtatggccggagctttgaggaggatcgacgagcgatgtcgccgctgagccctgatgctggcgggagcggcatgtttgaggacccggctatggtggc

tcaagaagcatcgaggagggctgccttccgacttgcgctcagctcggtcgtcatccccgcgggctcgggctcggccagtacggctggtacgttcccag

gaccttctcttccaggcttggaagatgatcggaacggtggtcaagggaagggcaaggagcgtgctgggtacgaccgtggaggaacttcatcggctgga

ggcaattcagggttgggcaactcaacgaacacgccttccggtagcgggattggaccggtcaccagcagctccggctcacccaagcctgtgatcttcta

cgccgtgcatgaagctgctcctggggccaaaggtgctggaacttggtaccttgccctggcaactacgaagacgatcacggtctacgaagcgcgaccac

ctaagaacgaccaacgcacgacttcttcatttcttcccatgtcactgcagaggggtggaccgagtacagggcacagctgggcccaagatgggcggact

cccgccggagggatgggtgaggcacgctcttggagcttgctgcgggaactgtacacgccccaggccccgaaggcaatctcctttgcccaagcgagcac

gactgacattccgctcacggccaaggagaagggtggtggcatgcgtagtgatagcgtgcttcagccccaattctccggcgggatggctggtccgcgag

gttggatgggtgccgacttgagtgtactggtcacctttggaaaccgagcggtggtcatccggctgacggatctcaacgtacgcgactttgaccttgct

tccaacttcacccttccgccccaagtttggcaacaacgcacgtctccagacttgcgtggaagtgctctagcggacgacgcgagcaatttcgccctgga

tcctcacagttctgtgggcggtcgaggcgcaacttcgaattctcaacaccgccgcacatctagttcttctcagagccagatggtagagaggcagaact

ggatcggaatgcaaacggtcgatgtcaaaatcatgctcaagcagagtccctcacccaatctcgcccaagatctcagcgccttcgagggtggagtgccg

atggctgcaagtcatagtgcggaaaccacatttggacgatctggaggcgccgaaaacggtcgggggaacctggacatgcgaccaaccccagttttcac

tagtgttctcagtccaaatgcggcttctgagcaacatgcgttcgatttgggcgcacatcggagccctcctcgcccttcgaccatgcaaccctcaagat

cttcttcgtccactggcgagagcgaatcgacgaccaccaacgaagacgacgactccgcggatgggaacaacgaggacctcgatcgtagtgaggtcatt

tacaccgcaacgggccgcagagctgtcgcccttcgggatcgatccgtacatcttacatctggttcgcgatccgaccccaccctgacagttgtcgaccg

agcaggcgggcccactccgcgagaggccgtcgcactgcccaaacgcaacgcagcccttcgccgccgtgaccgggagaagcagggcgagacggacaatc

cgggcaagttggtatcaacgtcgatcgtgctgataacaaaagggcccactacgcagatcttgccacttcccctcccggcggatctgtgcaagcccaag

gctcttgacgtgctcaactggagcggagttcccaatgccgtgagtgcttgggctcgggtagtcgggctcgaacgcgagcggacgactggagcagtgag

cacagcggacatcggaagccgatccaatggtcgtgcgatgccccctcgagggagcggagatgattatggggcgacgagcactatcatacttcacctca

gcgtcacggctcttgctttcatggcgagcaaagtggagtcccaacggacccgagtgcgtgtccacgtctcgctctcgttcccactccttcgagatgcg

gagatcgagctcttgccggtgttggatgcgcttctgccaactccggtcccgacagctgttcgtctgcctgctcaccatcaacgaacgcaaagtggcgg

aggcgccgagaacggatcccatcagccagagatgggcatcaagatgaagatgaacccacgcgcgtatgccttgggcgtcagccgcgccaacgacttgt

gcatctgcgccgatccaccccgagtcgtcggttctaattcgtccgcctctgatgccgctcgacggcgctctctggccgagccacacggacgggccgag

caggagttcgaatacctgtcgggctcgcttgtcggtctgcctcatcggggcggggtgagggctgcgagtatcgccgcggtggatgtgtgtcggccgtg

ggagatgcggggcaatggcggtgtcttgggctgggattggcgaggagggagagattacagactctttttcgtgggcgcagagatctcatcgtctcact

cggacacttctttctgcgacaatggactttttcgtcgaggtacatacacgccctttcgcacccgcaccaccgcatcacaaggttgggaattcgggatc

atcacctggctttacatcctacccacttcgttcaatctcggcggaacatcctcggcagaagccttctaccgctttcctcgggaaccagtgacgacctc

attgagtcctgagaagaaaatttatgtcctccatacccattacccacatccctttcctctgcccccacccttctccacctacccgcgccttccatacg

agacaatgaccaccgcggtgttcaacctactcatggtcctgtccatgctcgcagtagtcgcttcggcagtcaaagtcacgttccagggcgatggcttt

ctgtcctcctccctcaacggaccgctgccgaagtgcgacgacgtgtgcgacgacggcatcgatacccttccgacgtgtgcaatcaagtgcaatttgga

agactggcagcagggtcccaagctcccaagactggaagcatga

>contig_21.contig_21.g831.t1

atggtctcctacacgctttggtactggccgagcatcccggggcgaggcgaatatgtgcgtctggccttcgcagctgccg

aagttgcgttcacggacaattcggaaggcgaggtcggcgcgcttgtcaagcacattgggcctcaaggcgagacagggcacccatatcactttgcgtgc

ccacttctggaggtctcgaatgcatctggcgggagtggcaagacagacggagctcaagacgcgactaaagggtcaactttctacatctctcaaacacc

cgtcattctcgcgtatctggcgcccaaactcaagcttgatggaacgcaggaggaggaagatctcagcatcgaagaagcagagatccgaagagcgcacg

tctctcagttgaccgccactgttctcgacctgagcaacgaaatccatgatacgcatcaccctatagcagctggagcctactacgaagaccagaaggac

gaagctgctcgtcgggcggaggatcttcgcaacaaccggatcccgaagttcctcaagatcttcgaggccaccattgccaagaacccatacaagtcagg

tttcctcatcagcaagcacgtcacgacggccgatgtggtactgttccaggtgcttgaggggctctccttcgcctttcccaagctcagcaagtcccttc

aagatggaggtgactacaagcacttgtatgcgcttcgggaccaggtggccgccattcccggcatcaaggcctacctgaactcggaccggcgcaagcct

ttctcgaacggcatcttccgcttctaccctgagctcgacgctgcgctcaaggcgtga

>contig_21.contig_21.g832.t1

atgccaaaaggatccggcaagtctaaggaagagccgaccacctctcggtccggttctcctttgtccgacccacccgcca

tcaccctcgaagatgttgcctcgactctggcttcgttcatgaccaggatggactcccggatggacgctgtggactacaagctggccacgttggccacc

ccagctcaaccgcccatcgctgcggctgcggttgctcccgacacctccaccaacgtcgcacccgacgtggccaccaaatccccctctgacgacgctcg

atcggtctcgtcccctaccgagcaggcagccgcacgactcgccgcgcaacatccggcgttgtggtcgcgtctgccttccgattcccgaagtgtcgtca

cttccgcgcttggtcgcgcgggatacaccctcaccgatctcgactcgcgccctccgacagcggcccctcccgtgtcgcgtccgctcaagtgccagcgg

gagctcattggctcctacgacggcgagcccacccgcttggagcggttcctcagtcgggttgccgatatggctcgctccaactccgatcccgcgtggga

acctgcggtcgtggtggcgattccccaggcgctggaagggatcgcggctcgctggcatgcaggcctgccaacgacgactgtgcgcggcctccgcacgg

tggaggattacgcgcgtctgatgcgtcgcgcctttgctgtaaaccgggcgcagcttcgtcaggacgcgcatgatcgccggtggcaagagtctcaggag

tctggaatggcttacctgtttgacaaggtccagctcttgcgttacgcgttcggcgattcgtacacggagagcgttctggtgcaagaggtggccgacaa

gttgccgccctccatgtatgcgtacattcgcttgccggaggaaggtgccgtcctcgatgatcttcaggacgccatcatccgttgggagccagtctgga

ggaaggtgcacaacattcctctcctcgactcgagctcgatgtcgtcggacaaggtcgtcaaaaccccgtcagctccggtttctgccccgatccggcag

gctccgacgtccgcgccccctcctcgtcctccccgtgcggtgcatcacaccgttcctcccgcatcggtgacggccccgggcccgcagacctccttgcc

ggcgacatacgatcccgctcggatcgtgccggccgcgaatgggctgccgcgcatgtaccgccggcccaactccaataaggtcgtccgcaacgacggta

aactagtggcgggcaccgacgtcgaacgggcccgcccgcctcctcaccctcccgatgcgcccgtcgtacatgctgcggtcgtggagagcttggaagtg

acggagacggagtctgattcagtctcatttgtccgtgacgacgttaaactatcgaccaatactcactggtccatcccacatccttcctctaaccagac

tgacgcagggccctatgccagccctgtcgaagctgcggtgaggctggatcttggaagggagggaagcgcccctccgctgccaagcagcaaggatggtc

agcacgactgtcctgacgagctcgcgccggagacagctcgtcgtagcccctggactcacactgagaccagattcaagagcggcagggagagggctacg

tccccctctggtccaagacgggacctccctgccgggtaccatgggatcagagatgaagctgtgaacgcacctgaggatggggagggacaaccgtccac

aagtggaaacgcgcccataccggcgcttgctttacgcatcgaacctctgccgcagttcttcactgcgcgggcgttccctgtgaaccgagagaatagac

cattgcgtcagaagacgcgacgcattggaaagatcgtgacaattgctcctggaccggaggtgggcacggggacagcatacagacatcatgtgccactt

accatgcacattcgcattaatgacgcctctggtaaccccctgtcctccctcctggacactggctcatctctctccataatcgatcgccagctgctcga

gacgttgggaggcacggtccaaggccaacccctgcctatccacggcatcggctccaagtcgtccttggggtggagtacgatgacgttcttcgtcgccg

tcgatgatgccgtcggccagtccttgtgcatggagtgcaccgtcgacttccatgtgctcgaggcgttcgcgcccggactgtgtttggggcaggatttc

attagcacacatggaatcgccatccactctcgtcgtggcactgccgtgctggagcacaatgggaatccactcacgtttccagtgcgtgagcacatgcc

ggggccctatgcccgtcaagccgagctttgcacgacgcgcgacatcgttattcccgctcgttcccatgcctgggtccccgtcgacaccgccccattgg

ctcccggtctcgactacaccgcgtttcctcgactgatgtccaacgccgcggagaccgtgcgcttggcggggccactggctgtgatcgactcccggacg

gcgcatgtcctcctgaccaatgtcggtaccctctccacctctctttctcgccgtactccggtcgccgacgctgtggtcgcccaacttggtgaggtggg

ggtgcatgcggcgcacgtcttcgacttgcctgcggccatgccgggagaggcgtatcatgcggccggctcgtccttgtctttctctgtcgaggtcgaca

tcgacccgctggacgtgtgtgaatcggtcgatgggtccgatctcccgtcgccgacggaggaggtgtccgttgcgatggtcgacggtgtgttcaaagtc

gggcttgacgctgagggtcgtccgcatccggacattgttgcggtactgggtcgtcattcggcagcgtttgctcttgacggtcggcctggtcgagttgt

cggccatgagatgtccattgatcttgacgacgatcgcaacgttcaccccgaggccccgcgccgcgcgagtccggagaagcagcgcgccatggatgcaa

cgattgatcagctgttggagtgggagcggctgatggactcgctgcttggggatctgcgatggaaggtggctgtggtttacattgacgatgtcgttgtg

gcgacgaacaccatggacgagcatgtacacgcgttagagaccttgctggctcgcgcggaagacgtcggtcttcgcttcagccaagcgaagtgcacctt

cggcgtgccgtccctgacgctactcgggaggaaggtgtccggcgcgggggtggcggtctggtccgatcgtgctcgtgctgtgttggatttggctcgac

caaagacgttgcggcaactgtaccatgccatggggctctttgggtactaccgctcgttcattcctcgatttgcaaccgttgcggccccattgaccgcc

ttgacacggggatggcggtatgagcggtgtggggatcgcactcggctcgtcgcgaccgatgggtcccctgcctctgcggatagggtcgagattccgtg

gggtgacgagcaggacgccgcgttcgagacgttgaagagggcgattgcttctccgccggtcctcgcgcatccggatcccgctcggccttatgtgcttt

acgtggacgcgagtaaggatggttttggtgcggtattgcaccaggtctttgaggtggatgatccggcaccggctgtgtcgctttccgctgcggcgatt

cccgtgcagcagtcctcgttgcttccatccacggtcgcacgcgagcgttggcatgcctggctatgtcgagaccgccactttggcccaatccttcgcgc

tctggatgctggtgttgaggagcctgagtgggtgatgcaggatggtctcctcattcgccgccaagatgggctgctcgcgctacccgaaggcggtgttc

cggacatccttcgcaccgtccatgatcaacgcggtcacttcggattcaccaagacgtatctggctgttcggcgacatttctggcgtccggcgctgtca

gttgctgttcgagcctgggttaagcattgcccaccttgcgttgcaacgaagttggcacgccggaccggacggttagatgtcgaggatgacgcctccct

cccgttccagtccatctcggtggacttattgctgggtttcccgcggtcgagggctggtaatgatgccgtccttgtggtgttggacctgttctcgcgca

tgatcctgctcgagccgtgcgccgcgtcggtcacggctgagggtgtcgccgctatcctcagcgaccgcgtgctccgttacggatggcgaccgagtcgc

ttgatcccggactcggaagcgcggttgtccggcgtcgttatgtctgccttagcgacgtcgctgggagctgtgttgtcgccgtcggtccctcatcacca

ccaagccaacccggtcgaacggtctatccagaccgtcaagcgcgtcctgcagacgttgtgcttggactcccgtgctcattgggacaagcgcgccgtac

ctgccgcggagttggcgatgaattccaccccgtccgtgtccaccggcgtttgtccgttcgacctggtcttcgttgcccatccggatgtggtacacgcc

gtctttgatgcgcaagagcatgagggtgtcggatcatttgttgagcggttgacggcggcgggagcgcggttggaggatgctcgtgcctcgctggcgga

agctcgtcgcgctcaaaaagcgcgctatgatgcggtccgggcgccgttgccctcgtatgctgtaggggatgccgtctttgtgcgtttgccggatcgac

cgattccgggttcccaggagaataagctggcgccgcagaagatgggtcctttccggatcagtgcggtccttagtgaccaccgggtgcggttggagctg

ccggcggacctgaggattggagacacgtttgcggtcagtcagttggatgctgtccctggggatgaggatccgtacgtcactcaccgtgcgggttcgcc

cgtcgaggacgctgatcgggttgcgcctgcttctcctttcgcaccgtcttctcgtgcttcctctccggttcctcgtcccccgcgggctcggcgcactc

cgggtgtgttgcgagagtatgacgtgtcggcgcgcgtccatgctatgtcgtcctcgttgtacgatgctttgcgtggtccgctccatcgcgctcggcgt

ctcgaggttgatggccggacggtgatcttgacggagcgacctgtcgccttcttgtcccggttgaccatgccatcggagcaaaagcttgtcgcgcctga

gctggagctgtcgtgccttgcgtgggcttttgggcgattcctccacttgttggagggggggggccgacgtaaccgtggtgacggatcacgctccgttg

ggggcgatgctcacgagttcggccggggcgcgctatggaccggtgatttcgcgttgccgggcgcagctgatgccgcatctcgcgcatctgcgcttcgt

tcatcggccaggcaccacacacaccaatgctga

>contig_21.contig_21.g833.t1

atgtccttagccgcgcgaatggcgccaatcctcggcctgcctcttctcaaacccgtagcgcgactagttacccatctga

ttcccgacgcagtcacgcccgcgccccgctccttactcagcacacccctcgttcttccgccgcgctcgcccaagttcccctatgacatcctcgtctca

tccccgtctggcgctgactcccagatcgacgaggaggaccgcagcaggatgcgcatgtgggcccaggattgggctccgcgctccgctgagctccgcgc

tccgcctccggcggagccctccgtcggaggagcatctcccccgcagccttgcctcagctcgacatcggagatacacgcgatctcattctctcccactc

tggtcagccctccactcggcacaccatcacctcgggtccagacgtacccgcgcctgacacttggcgaggtcctcagcgggagaggcattgccgcccat

ttctcctccagccataacggcggcggcgatgacgaggtcgaacccgtcggagtcgcccttctccgcaagcaagcacaagcagcattggcgcgcaccca

ggcagccaaaggtggcgacagcagcggcagtgcaaaggcagaacccgagttgaaagagactcttgcccgtctcgatgcactcgcacagcgcaaacgca

aggcagaaaagttgcacgccgatatcgacatcatgccatggcacaggactaggggtgagaccagtctcgacccaagtgtcatggatgatgcatatatc

caacatacctccgatcctcttctttcttcggccctcggtcttcttccttctctgcctctcttcttcggcatggacgacaccaactcctccgccatctc

cggcattcctgccctcgtcggcctcgaaaacttcttcgcctggcgagaggcaatcgagcccgtcttcatcggcatccgcgcgttcgacattgtccgcg

gcgtcgagactcagccaactctgccgcccaacgccacctccaccgatgtgcgcctgtccgagtcctggaaggacagggacgccaaggccatgtcctac

ctccgcaagacggtcagcggggccctcaaggccatgattcgagacctctcctcgtccgccgacatgtggtccctcctcgccagtcttcacgacttggg

ttcctcggatcatcggagcgccatcaaccgcaagatggcgaacctctttctcgacgagggcggagacatggtccagcacctcaactcctacatggagc

tggtggctgaggcgagcgcggcgggtcttccttggggcacggaggacaaagacaagtgcgaccgtttcctcgacacccttcctcattcgctcaggctc

gtccgggtctacaacgaggagaacgcttctcagaagcacacggcggagcgcagcgccaacgccatggcaatggccgctgccagccaacatctccacat

cgccatcaagggcgtggcaaacaaggcccgctccggcaagtcctcttcgggtggcggcaagaagaagtcttcttcttcgccttcttcttctcaggcga

cctccggcagcagcaagggcaagggcaacagcaacggcaacagcaagcgtctgctctgctacggctgtggtgctcgcgaccacgaacgtcctgactgt

ccagtcgcgagccaccttcttcccggcaatggtccgatctgctggacatgtcacaagcaaggccacgtcaagtccgagtgtcccaagtcgacttcgtc

gtccggaaaggctgccgccgccctcgagtcttatccagacgccgacgagatgctcgcagccatgtttgagccctccgttcttgccgcctccacgacgt

cgaccttgattcccttcatgatcgactctggtgcatcacagcacattgtcgaccgacccgagctgctcctcaactctcggaaagcggacaagaccttg

accttcaagacggttggctccaagcaggcatccgaccacatcggcgatgtcactggacttcttccctccggccgccgcgtcactttcctggacgttgt

gcttctccccggcgccggagtcaacttgctttcggaggaactgctgcgcgagcgtggctggatcaaagtcatcgacgaggatgacaacgcccacctcc

agcacaaggacaacgcctcttggcgcatgtctttgaccaagcgtggtcgagctcgatggctgctgttacacgtcgaccttgcggcctccacatcgtct

ccctcagcaatgctggcaccaatgcagcccgctcagcgcgatcccgctctcgaggttcacattcgcctcggtcacttgggcttcagcaccatcaagaa

gctcgtccgttcgggtcatcttcctcccattcctgggctgaacgatgccacgccttggtgcgatagctgcgaagctaccaaggctacccgtcgaccgt

tcaacgacgcgcctctccacgcgagttctccgctggagatcatcctcacggatgttggcggtcccttgtacacggactacaccaaggtcgacttactc

cgctccaaggccgaggtttttgctcgccttgttgagttcgtcaaggtagccgaacggactttcggcaccaaggtgcaagtcattcgcaccgacggcgg

cggcgagtacgtcaacaagcacatgtccacctggactgcttctcagggcatcctccaccacatcacaacgccccacaccccggaactcaacggtgtgg

ctgagcgcaagaatcgtaccctcaaggagatgaccgccgccatgcttcacagtagcggcctcccggttcagtggtggggacatgcgattttattcgct

gcggtgttgctcatgaaggtaaccttcgtcaaagacggtcggacggtttgggagtgggtacacaagcgcaaaccctcccttggcaagactcgagcatt

cggttctccatgctgggttcatattcccgccgctaaccgcaccaagtcggacctcaccactcccaaggcctggaagggacgtttggtcagctggccga

cacagcgcagcggctggggggtgcacaacgagaccacggggaagattgtcttctcccgcaacgtctctttcggccccaagctctcgtccaaagtcacc

gaggtctccgagccttccaacatcgtccagctgaacgtggagcctggtgatcccggcttcctcgagtacgacgaggaagacatttccgacgatgacgc

ggagccaggtcccgacgccaataatgtcgaacttcttcctcttggccgcaaacgccctgagcccgagggggagtcggaagggtttctttgcaagattg

ccctcgtcgacgacgaccccacaagctacggcgacgccatgtcgagtcctgaacgtcagcagtgggccaaggctttcgaagcggagctggagtctctg

atcaagaacgagacgctcgaggaggtggtcctccctcccggcgccaaggtctcggacgcatga

>contig_21.contig_21.g834.t1

atgccagagtgccagattctcgaagctgactccggtctcttatgcacctctcctaaagcgacagccgtgatgactcgtc

tgccccctgctgggttcgcgtcggctccttcgagcttcgagcgagtcgcgcagtatgggaaagcgtccgcgtcctgggcgagtacatggtccggtacc

tgttccagtgagacacctccgccgaccatgggtccgagtccgggtcttgacagaccatgggcctcttcgccctccagcacctcgtcctcgcgctctcc

ccgctcatcggttcaaacacacgcacggcccggctccactgtcgggagagttggcgagagtggaggcggaggagttgaagaggttgtcggatggaatg

agcgcagtgagggtgaggatgagaagactcagcatgagctgatcgacacgcgtctggtcgtgctcgagcgtctggacttcagcatccatctccgccga

ctggccgacttcccctgccttcctggcgggcacgtctcgggtcccaacctattcgccctgttgagggcctcgtactccgttcagtccctcacctcttt

tcatgatctggaactgaccaactga

>contig_21.contig_21.g835.t1

atgcagattcaagttgaggccttcgttccggaccgagctgcgtccatgtcaagctacgttccgacagacaaatttaagc

tctccggcatcgcgcctgctccccctggtgtcccccagatcgaggtcaccttcgacgtcgacgccaactctgtcttcaacgtatcggccgcggacaag

actcccggcaagtcgcagaagattaccatcaacaacgacaaggcccgactgtccaaagacgacatcgagcgcatggtcaacgaggctgagaagttccg

cgctgaggacgaggccgccactgcccgcatcaccgccaagaacggcctcgagtcatacgcctacaacctccgcagctcgctcaacgaggaggcctttg

cctccaagttcgagctgacgacaagactgtccatcgtccatttccagaatgtcgagatgaccagcgcaactgcaaacttcactgacccatacatcttc

cgcgtcacctttgaatgcatctctccactcacagagcttgagaccaactggctgacatctcttactccacccctacctcaggtctacttcaacgagat

cgagcttctcttcaacacacactggcctcttgtcgagcgcctcatcgttgtcgctgccaagaccctccaagcctctacatcaacattcagcgtcacca

attctcacaggtcgaccagcatcgatattacggtaccagcaagcctcgactccatgatatcggaaacaccccattttgaaccgtctgcgcagcaagac

cagcagcagcagcgtcccatgttgctggcccacgcagaagccaaccaactgaccgcctccagcctcttcaacatccgttcgcgctcaacggaatacac

cccaggtccgcagcttgtctcctctctgctgacgctctcttacacagcctcgtactgttctgaggcatttccgcctacctcactcacctccggcgtcc

ccgaccggcccgacatcaacgaaatcaaccgtcttggctcttttctggcaggccttcactctctcgctacccaacagggcgcgcctcgcggcaccggt

ataggaagtgtggaccgactggcgatcatcgacgatcatttcgacccttggcgccctgcaacccctcactcgggatga

>contig_21.contig_21.g836.t1

atgactgatcactttgtgctccagtgcgcaatcacgctcctgcttgtcgctcacctctttctcactgacccttacattc

ttctcttgactcccttaacctcttctgtgtctcttgaggtcgcctacaacactgcgcgtctgtgtctcctcgactccgtcggatccgccgtcgaggct

ctccacttctcccaggccgtctccgtcctcggcgccgtcgtgcccggcaccaccgtccccaacggtgctcgcgtcattggtaccaaccgcgtccttga

ccctatccgtgccgctttcaacaagggggctttgatccgctggctcaacttcaacgacacctggctcgctgtcgagtggggccacccctccgacaacc

ttggaaccatcatctccgtcgccgactggctctcccgcacagcagtcgccaacggcaagaagccctcaccgtccaggacatcctcaccgcccccatcc

aggcccatgagatccagggcaccctcgccatcgagaactcgctcaaccgtgtcggtctggaccacgtcctactcgtcaagtcctgggctgctcgttca

gaagggtcagcccggtctgcccagtgtcctcaccgccaaggtctggggcttctacgacgtccttttcaagggcaatgagttcgcattccagcgccctt

acacctcctacgtgatggagaacggcctcttcaagctcgtgcccgccgagctccacgcccacaccgcctgcgaggccgccgccctcgcccacgccgag

atgaccaaggtctgcaagacgtcggacgacattgagaaggtgaagctcctgacgcaagaggctgccgcgcgaatcatcagcaaggcgggcacgctgca

cacctacgccgaccgggatcactcgcgccagtacatggtcgctgtctcgctcatgaatggcaagttgacgcctgacatgtacaacgatgactatgcgg

cgaacccgaagattgaagagttgcgggccaagatggaggttaaggaggagggtcggtacacgaaggagtacatggagccggagaagaggagcattggt

aactctgttgagatcgtctttaaggacggtagcaccattgggccgattgcgtttgactaccccgtcggttatgcccgtcgccgtgaggaggccatgcc

gctcattgcgaagaagctgcgcaagtacctcgaggaccactttgacgaggcgcgcgacagcaagatcatgtcccttattgaggaccacaaggcgttgg

cggcgatgcctgtttcggagttcgttgacctctggctcaccgctcaaggcttggttgtccgccttgctcgtccgccgggatctgtttag

>contig_21.contig_21.g837.t1

atgagcaattgcccttcaactcttcaacacccaccagccttctggtcctcgccttactctcacggctcagctgagcttc

atcaagcggctcaatctgcagcatcagcgcgagccaggtactggtttctccctgcctaccaagggtggtacggtcgtgttccgcgctcgcacatctcc

atatcagcccacaactacggcaaggctcgttcagcgactgttgggccgttgattgtcgtgcgcctcttgtttctgccgtcctgccggtacgaacccgg

gcaggcaatccttctcgcatcattaccacagcagcatgcggaacgcattcaaggcgtgtttcagcaactgtctccgcatcacttgagcttcggaaaat

tcgtgcgcggagcgcttcggtctcaggacagcataattcgcagcactgttaccaaatggatgcgaataccagagccaggcccgacaagcaacgccgaa

gaagcttcgacaaatggtggggacgttgtgcataaatatggaccggaagattttttgaacactctcgtcctcgagacatacgccagaggcaacagcga

cggaattaaacgtctcaaacaggccctcctgcagcatgcggtcccgcttttggtggaagaggtcgagctcttttccagcaacgacagtcagtacctac

gcgtctcagcacccgatgtggacacctgctctgccttgcccgactctcttggacccctcgtcgatttatatcgagcgaagcttcccgcccttttccga

ctgttctctctgctcctctccgccagtgcgactatcagcaacagtgacgcagtcacagtattgaccgctgaggagagaatcattgtaccttgcatgtc

ggcgatgctctttgcccgcaatagactcttcaatcgctttcaaatgctcgttggcgtcttcctcgctgtgaccgatactcccgacgtggtgaaagcgt

tccttcatcactgtggcttggcggtatcgcaacccacaactcgtacgacgttggaaagcatctcggaggcagccattcccagcgcccggaagctgttt

gcggacaaggctctcatcaaagtctttctctttgataacatcaatatatacatgcgccgacgccaaacgcggctcacggactacagcaccgccgccgc

gttgactatgcggaccatctttgcccttccctctttctgtgacccctcgcacttcaccaaagagcacatacgacagctccgcgacgcagaccggtcac

ttgtcagcatgtccgacgccctccccgaagagcattttctcgaacgagcggctgaaatccacatcgctcaaagccttctcgacgtggtcgaatgctca

gatcgaacacgttcatccatcaagaaacgtctagaaacgctctgcagtgagcatgcaatggaccttgttgcacctgaagcaaccaagctggtctgtct

gaagcttctccgccagaatgaaggaactctcgaaggcattcagactgttttaacggagactacaggagacgtgaacagcgatgtggatccatatctgg

ttgcgggagacctgttgacggtccgaaatacacaagcagtacaggatgccggtcaatacaaagacgacgatgcgctccaacgtctcgactatgcctgg

gcgataagcggaccatggcacctccttcagagctgggtctaccttatcttcaaagcgcacttttctgcgaccaaggcaggcaaagatgcttgccttga

ccgcgtgcgagaggtccttcgccggggacggaccgcactgcgtgaagacaaacccctcttcaatgaggggtgggagtttgggaaacaagtgtgggctg

gaagattgcgggcaattctagatatggagcgcggagtcgaaccaaacaaggaaacggaaccatggtgccccagcacaaagaagttctttgccatgtgt

aggcatgtgtaccgcaagtacctgaccgcgacggcaacagagcgagctcgagaagcaggtgatgaagtccatgctgctgcctgttgcttcatgcgcga

ctggctcatcgatatggaatatgctgccgcaacgaagaatggagacttagggagaatgagatcaacggagctcttcatggggatggctttctttggag

caggtcaacacaactatgcgcacctgattttggaccgcctacttgctgacaaggtgctgccaagctcgacctcgagaatcctacgtattgcccagctg

gtcaaccgcagagggaaggaaggggcgtttgagggcgccgatcatttccaggagactatgaacaaagagattcagagggcggacaccagcagagcgag

ccatcatgtgttggatcgacttgaggatcgcctctccgccattgcggaacactcacgtcacgtcaaagagacgtttgcatcggcttggaacacaccac

tgtatgatcgacaacgcaagacaaagggagctgagaaagatatcgaacagataaaacgctttgcttcggacacgcacctctttgacaccattccaaac

agaaggatcatggtcacggcatcaaacccaacacacggcaaggacgacaagttgacggcaaaggaagcggccaacagggatgcggagcacggtgcgcc

gccgttccttttgctcgacgtgctcgagcgaggctatgtgaagctgaggaagggaagtctggcaaagtggcaaggcagaaacagtggtatggagatcc

gtcagctcctgctcgagaatttggccgaggaggacgcggaagactacgaagctctcaccgatgaaactgacaatagccgggcccaagcagctggcatc

aaggttgcggcagggtatcgtgaggctcgttccttcatacgctcgaaagaggtgtggaacgaagatgatctgggtgtcgtgggcgaggcggaagagag

agctggcgattgcgcgcacgacgaggccactgcgcgtatgccgaggcctaagaacttgtaa

>contig_21.contig_21.g838.t1

atggggatgcgaaagggaaccggagagagcggaggaaagggaaaggacatcatggtccgcggtgttcactctgcgggag

ccaagattccgaggaaggagggcgcgcttccctcctcgacactctcgtcagtatgtgcttcgctggaactcggcaggcgcgatctgctggccatcgat

gcgggcacggaccgatccaatatcagctacgaggtccattccatgcgatcgaacgtgaccgagatgctcgacctgcttcgcctcttcgagccccgata

caattctctcaatgacattcccaaaaccgtcatctacgtacgcacccgcatggaggcgtatcgtgcctgcaacctcctctggaacttcttcggccgtc

cactcacaggcgttgttcgtcccttcacttccctatcctccgacgcatacaaagccaacatcatgcagaggagttttcggacaggaccggtgagagta

attgtggctaccgaggcaggtggtatgggaatagacatcccagatattgctcgtattgttcaattttcccttcctccagacctcaaagggctagctca

gcactttggccgcgctatgcgggatccattgcgaacgggacgcgccttgcttttggcgccgcagtgggcgttcgatccgcagtccaatgccagccgcg

ccctcggagtcaaggttgcaaaggacaaggacgctgcggcacgccgggccatactggatgctggcctcaacgatttcatcaatcactgtcgatgccgt

cgtcaagttcttgttgactctatgcggctcgacaccaccatccttgcctcgcatcttcctaatcgagcccttcgcatggttgatgttctttcctcacc

acccagtatcgacaactgccttctcgtctctcgcccgcatttggacccatccgaagatccatcctctcgttcgaatcagatgtgggatctggtggttc

tccaatgcgactctccttcctcgcgcagaggtcatgccgatatctgttgtgacttgtgctctgcatcctcggcatcacattcagactctccgcctcat

gccctacgaatcaactctgctgtcccaagcacccagcaccagcagtcaagcactcagcagcagccatctaccgtcagcactgaagcatcagtggtcaa

acatgcatacgttcctcgcatcccccctgatgacttcggcgtcgtgcgccgcgagtttgcagccagcttgcgattgtggagacgtacgaccttcacca

agcgtcctcgcgcctcttatctcaatgcttctacacttttgtcagaacaggccatcatcaatctcgttgcccaagccgtccgcatactcgcgcagcgt

gtacaagggtccatactcaggccggcatacacccgctcgctcctccaagccgaacatgtgctctttgatgacgatgatatcgaagcgctgatggatca

tctgcgcttgtggttggatgaggccgaagtcaaataccggcaccttgtgctagaacaccacctcacgcccatgggggaggtgccacggactcgcgcgt

cccagctcgcatccaccagcacatcatcgacagctcaatga

>contig_21.contig_21.g839.t1

atgatctcaaacttcgatgtcctgcagcacgaggactgttccgccatctccagctttcaagccaccctcgaccagattc

tgagtgagggcgaggtttcctgcacttcgccagttaccgcgcaaaggaacgctcgcaagcgaaagcgtcagccactggcggagcttcccaacggtgcg

caacaaccacaagatcacccaactgccgtgtcttcctcgtcggcccttgtcggcgatgcggagtcgagacccagctcttcccgcaagcgcaagcgcaa

acacaagcgcaagcgtcatcgtcaaggtgcccatatcagtacaggtgacactcaaccttccctcctcccttcttcagctcaggcaccctcttgtccca

cctcctccggcagtgcatcgtctcaagaccaggacgacctaccctcagatggccgccctgcaatgcgtcataaggacatcgttcaaaagttgcgggaa

tttcgcaaagatcctgacttcaccccagaactttggcaggtatcgaagtacgagaagacttccatatccgcagtagcactcagccaaagcgtcatcaa

cggtcgcgctgatgcgcagggccatcaagcacaggctacaaccacatcgtga

>contig_21.contig_21.g840.t1

atggtgtccgaaattctggccaccagcgcggcttctgcctcgccctcctccaattcggaatcaagtgccgtcgaaacac

ctccggtcaacgagaaggatgtcgctctcgccaccagtccacgcaagacaaacctagaaaagctttcccgccaccaaactaaggctccgctttttctc

tacctcttcaccttcggtccacccatctcgctccgctcgccgagcgcctttttcacccacccgagaacattgtacttcatcggctttgtatcagccat

catcgccgcaggaaccattccctccctcgacttgaattacggttactgggtcaacgctctgtcgcgcgtcgacatcaccaatgacgaacggacctcaa

tctcaaatcgaacggctggcgtgtctgccgctctcggtttggccaacgctatcttcactaccatctattttaccgctttctgcctcgcctcggagcgg

ctaagttccgacttgcgagagacgtacgtcgccgccacgctcgctcaggacgtgagcttctttgaccttcacggtcccggtgaggtcgcatcacgggt

cagcaaggatgtcaataatgtccgggtcgctttcggagaaaagacggcttatttgatctacggcgtcagtaatttggtcgttgctctctcttttgcgt

tcgctcgttcacccaaggtagctgggatcatgtttggactcattcccctcaccatcgctgccttcgcaatcctcggcatattcagcgagaagattggg

gcacccgccgctgccatggaaggtcgagtcgccacctacgtcgagcagatgctcagtagtccgcgagtggtccaagcgttctcaatgcaagccgctct

ggttcaacgactcgagaaagccatgctctcgccattgagagttctaggccgtcgccgcgccgctatccgaggcgccgaagtctcaacgctgttcttta

tcattttcggagcgtacgccctgttcttcgcctggggagcattcctcatctctcacgggtatacgagcgtcggcgcctctacgactgcgttctggtca

gctatcaactcgatcttcggtctggctaatgtggtgccgcacgtacctggcatcatcaacggcttcctctccctccggacactccgcaccaccatcga

gcgtcaacccaaaatagatgtgcgcgatgagggcggtgtcacccttccccccggctcctttgagtcctccttcgagctgaaggacgtcaccttcgcct

atgcggctcgcccagcgcacgccagtctacgagacgtcagcctaaagattgaaccggggaagatgactgcgctggtcggaccgagcggatcaggcaag

tcgaccatcgccagtcttctaatccgcgaagccgacccagagacatctaacgtcctctccgaagaagatgccgctctgatggctttcattgcggaaag

caacaagagggagaagaacaaaggagaaaaggatgacaagaaggccgacaagaaagccaagggcaagaacgatagcgcgagcgaaagcgagaagcccg

aattggatgtcgaggtggctgatcgagtcaaaggtcacggcgttgtctgttatgccggtcacgaccttcgcacgctgaacgtcaaatggctccgatcc

caggttgcagttgtccaacagcatcctcaactgttcaccggaacagttttcgccaacgtcgccgcaggtttagtcagcactccttatgaatggatcga

cgatggcaaatccgaaatcgacttgagcaaggaggagaaggaacgctacgacgctactcgtgccaaagttcagtctgcgctggagaaggcacaagcgt

gggaatttgtccagcgcttgccacaaggtgtcgacaccgttatctctggtggtcgaactggcgtcctttcgggtggccaaagacagagagtcgccatc

gcacgcgctctcgtctccgaaccgatgatgcttgccctagacgaagccacttcagcccttgactcgtcgacagaggaacgtatccggctaatgctcga

agaagagcagcgcacacgaggaatgactgtcgtcaccatcgcacatcggctttcgacggtcgagaaggcggaccgaatcctggtcatcgatcaaggac

gggtcgtcgatgatggaacgcatgaggaacttatgcaatctggccggaagaatcggacataccgcaagatggtgctccgacagcgggctgtggcgcaa

ggcgacgagccggacgatgtggaggttgaggctctcaccagtgcaggcaccgagacggagagtggtgacatgctaaccagggtggcttcggcttccta

cggccacgagcgcgacgagaggccaggactccaaactgcgaattcgggcataggaagctcggcccttgccctggccgctgattcacacactgccattc

agaggctgaactcacgatcggcatcgacgccgcacggccatcagaccgcagagccatcctcgacgcctgcccagcacgccgctgctcgtcgacggtat

caagccgctgcgttgagctggggtgggatcgtagctgtccccgcaccgacgtcgtcccagcaccacggtcccacagatgtgccaacgatgaaacggct

cggtcggacgctctgggctcgagctcggccgcagatgtcgttcttcgcgatcggatgcatcgccagtgttttcgtcggtgtggctttcccgctcatct

cctggttgacaggccacggcatcgccgccctcggccttccggatcccgtcgagctccgacgccagtcgtacaagtggtcgctctacctgctcctgata

gcatttgtcgtcttcattgcgtgcgggatgcaaggatggttcctcgaagtcgctagtgatcggacgctgcgtctgtttcagcgggacaccctcgaatc

cttgctgtcgcaggagattggctacttcgatgcagaggaccaggccagcggtgggttgacggctgctttgacctcccacccggaaaaagtcggcaatg

cgtctggtataatcttcggccaattgttgatgagcatcgtcaatgtcttcggctctgttctcctcgctttcctgctcaactggcgtggtgccgtcgtt

gagttgagcccccttatcctggtcgccatctttgcgtacactaacgtcgtcttcctggaaaagtacgagaccatggcgaccaggcccgtgcagcatgc

ttccgcgtacgtctccgagcagatcgacggaattcgtacagtggcggcgctgggacgccaagaagccgtgctgcacacattccggacgaagaacaagc

gactgcggaatcggatcaagtatctcgtcaatggcgcgttcggatactcaatgaacacggcctccatgcttttcatcgccagtctcgtcttttactgg

tccggcgtcctcctcaaccggggccaggcgaccaacgtggaggtttttggaggcatggaggcggtcatcgtcggatcgttcagtttgatgcgcctggc

ctctctcctgccagactatgcgcgcggactgagttcgctccgggtcttgaccgactggctggagcgtgttccgaagattgccaagtatgtgctcgtca

agccttcggagggcgacgacaacaggactgatcagccgaagaaacatgacggatcatccggcgccgggtccgaggactataagaaggacgagggcatg

ttcgacagcgttgctcaggacattgtcttctcggacgtagagatgcggtaccctcaacggccggaccatcccgccttgcgatcgctcgatctgaccat

caaggcgggcgagacgactgcgttcgtaggcacgagtgggagtggcaagtcgtcgtgcctcaatctgctctcgcgcttttacgacccggtggctggat

cgatcacgtgtggagggatcgatattcgggccatgccgctggatgagtggaggggccgggcggcgctggtgtcgcaggatcccgtgctgtacgaaggc

tccgtgcgctggaacttaaagcttggcgccatcgacccggagagtgttacggacgaggagatccggttcgcatgcgagcaagcgtgcatcttggactt

catcctctcgcttccaggcggctttgacgaggacctgggcatgaagggcatgaacctcagtggcggtcagaagcagcgattgtgtatcgctcgcgcgc

tgctgaggaagcctgagattctgctgctggacgaggctacgtcggcgctggatgcggagtcggaggcgacagtgcagcttgcgctggagcgggcatcg

aagggaagaaccacggtcgtgatagcgcaccgcctgtcgaccattcgcaaggcggacacgatccacgtcgtcgaggatggacgtatcgtcgaaagtgg

ctcgcacgcggagttgctggctcggaagggtcgctacctcgagctgatcgaggctcaactttga

>contig_21.contig_21.g841.t1

atggccctctggcgcacgaactcgaacacgtctacagcctcgaaggcctcgaaagcgtctaaatacagcaagaaggaca

aggagaaggataacggaatggatctgtttcgtcaggatgaacatctgcgtggacgaccgggccatctggatccgggacagcagcatcagctgaagaag

ctgcgccagttggtcgaagaagagaacctctacgtgcccgcgcgccacgacgacgcctgcctatgtcggtttctgcgtgcccgcaaatggaacgcgga

cgaagccttgacgatgttcaccgcggccgagaagtggcggaaggagtttggtgtcgacgagctgtatgagaactttgactacccggagcaggcggagg

tggataagtactacccgcagtattaccacaagacggacaaggaggggcgaccgctttacattgagcaactaggcaagcttgacatcaaggctctttac

caagtaacgacggcggagcgacaactgcagaagctcgtggtcgagtacgagaagttccagcgggagcgtctgccggtgtgctcgcgagtgcataatga

gctggtcgagacgagctgcacgattatggacttgaagaacgtgggcattagccagttttggaaggtgtcgacatatgtgcaacaggcgagcaacatcg

gacagcactactacccagaaacgatgggcaagttctacatagtgggctggctcgacccggttacggtggagaagatcaagatcctaggctctggctac

tcggaagagctgctgtcacaaatcccagcggagaacctgccgtcgacgctgggcgggaagtgcaactgccccggcggctgttcgctctcggacgccgg

gccgtggaacacggacgagggccgggcgatcttggaggatgtgaagcgggaggcgagggagcggattgaggctgagatggtgaatgcgaagaaggagg

ggaagacggtcgatgttagtgggacgatcaagggtgcgcctgcggggatgaaggcgtga

>contig_21.contig_21.g842.t1

atggacatcgaccccgccacccccggcgcttccaggcgccagcaacagcaacaacaagcccaaccccgcaatcgaaggc

tcaacatcgccccgccttctcctaccagcacctcccaatctgtcatcgcagacgaagacgccgatcccgaaatcgacgacgacgacgacgaagacgac

cccgaggacgagctcgcagacatcaccaggggcgcagcggtaactcccagtggcgtcttcgatcatcctccagcatcctccggtcccgtaccccccgc

acgcgccgtcgcggcaagtccaaatccaaggagcgccctccgcacacgcaccgacacctcctccaccttgcctggctccggctccctttccgctggcc

tgacccagctccagactcccggggcttctctcgccaccctgtcgtcctcttccgcagctgcgctcgtggtagccagtgggagtggacgtgcgagtagc

agtaccagcagcagcaacaacaacatgggacgcggtggcgcaactcaacagtttgctgccgccattcccggcctctcctcccgtctgcccgccgaact

cctgaactacatcctccgcttcgtcgcaaccgatccccgcgacctcgcacgctgtctccgcgtcaaccaactctggctccgctgtggtgtcgagctgc

tctggcacagacccacgctgcgcaacattggttccttccacagcctcctccgtacactgcagcccgcaggcgagctcgtcggactcaagcatctcccc

gcccgaatcatcgtcgagcatgtcacctcttccaacagccgcggtcgtgaactttcggccgatctggacgatgacgatgatgtggaagaagacgagga

ggctgagcaggctgccgtagctgcggctgaacccccttctcgcggcgtgacccctcagcccgggcccgcctcacccatggacctctccgagccagcgc

cttcgtcctcctcctcctctacctccgaccaagacttctcccggccctcctccgcagctaggaccaatgtcaccgaacctgagcaggactacaaccgc

cgtcagccgacgttccactacgagcagttcattcgaagactcaacttcgccctcttctcccacagcgtcaccgacgactacctccgctgggtcgccgt

ctgtgatcgtctggagcgacttaccatcgccggctgctccgagatcactgatgcaggcctctgcgccatcctgccccactcacccaacttggtcgcca

ttgatctcaccgatgtcagcaagatcaccgatgccactttacgtgctattgcgcagcactgtcctaaagtacagggtatcaacctctctggctgtgtc

cagatcaccggcgcaggactggccgacctggcccaggcatgcaaagccctccgacgcgtcaagctgtgcaaatgccccttaatcaccgacgcaggcgt

caagaccctcgtccgctcctgcccgctcctcctcgagctcgatctcctggacgccgtcgagctcggcgacgaaggcatcgcagagatgtggcgcacgt

cgagacatattcgcgaactgcgctttgcgggcattaggaagctgacggatgctgcgtttccggacccgtccggtcggccgcctgctgcggcgggcacg

acgccgttggcgtttgggtcgaatccgagtgtgagggtgggcaatgcgctggcgatcccgccgccgctgaacctttccgctgcggccatgggcggagg

tgagggcagcggtgctggtggtgggcggatttcgggcacgggtgtcggaggtattttctctcctgcttcggggacgtcgagtccgctcctgggtgggg

aggctcagatgctgttggatcggagatatcggagtgcggagccgtcgccgtcgccgaggggccggagtatgccgatgaaccctgctgtggcgagtctg

ttggcggagtcttcctcgacggcggcggctgcttcgtcatcatcgatgtctgtcgatcggatgggcggggtctcgcctgctcggctgtcgtctactgg

tcctggggatgaggacgacgacgatgagattgagattgatggtggtggtggtgcgatggtcgtcggcagtgcggttgaggccatgcgttcggcgggcc

agttgggcagtgttgacacgacggggttgaatgcgatctcggctgcggacgcggcggcgttgcatcagcagatggtgcaccatcaggcgcggagcttg

aatgcggtccatgcggcccaacagggtggcggtgatggctcacatcattcgcggcatcatcaccaccaccaccatccgcatgggcaccgacatcacca

taatcatggtcatcatggtgggcaccggaatctggatcgggagcaggggcatcagcattcgtcggcgcctggttcgacgtcggcttctcgctcgggct

cgaaccatggtgctggcggcggcggccatcatggaggcggtgcagcttctagcggacacggtggacctgattcaaacaacaacggcggcggcggcggc

ggcgcgaacccggatatcctctcctccgcctccccactctaccgtcccttcgcgacgcggctgttcgagcagctgcggatcattgatctgacgagctg

cagtcatctgacggacgcggctgtggaggggattattgcgaatacgccccggatccggaacttggtgttgaacaagtgtgctgggttgacggatcgga

gtgcgattgctattgcgaggttggggaagaatttgcatcatttgcatttgggacacgtgcagaggatcacggatcgcggcgtgactgcatttgcgcgg

tcgtgcacccgcctgcggtacattgacctcgcgtactgttccgagttgacggatcagagtctgtttgaggttgctgcgaatcttcccaagttgcgtag

aatcggtctggtcaaggtgatcaatctgaccgatgccgctatcatgcgcctggtcgagaagcacacatgcttggagcgcattcatctgagttactgcg

agaacatcacggtggacgccgtcttcgccctgctgaacaagttgcacaagctcacccatctgtcgctcacgggcgtaccttcgttcaagactccggag

ctccagcaattctgtcgggagccgccgagctcgtacaaccaacagcagcgcgagaacttctgcgtctactcgggctcgggtgtgcgcgaactgcgaca

ctacctccaatctctgcatctggagtttgcagatgaaggtatgacgctgcatgaagctgtccgcatggttcagcagcgcaaggaggaggcggcgcgtc

tgcaggaagttgaggacgcccgtgcgcgcgaactcatggagacgcaggagatgctgcagatgcaggccgagatcggagcggagatgagcgcggagcag

attgcgcgtaattcgcagacgtgggcggcgatgcaggctgctcggcatgcacagatgcacactgcccatgcttctcgcagtgcggattttaatcagct

gcgggcttcgcttgctgcctatacgcagggtgtgcccggcttgcctccgtcgcagacgccgttctcgcaccatgcggcccaccagcttcagcaacaga

tcggcggtgcgcatggacctacgttccagtctagtttccctgggccgctgccgagttgggtgagcggcttgcctgcgtctcatcctgcccacctcagc

gcggcgcatgcgcatgcgcacggacagggcttggcgccgtctcctgcttctatggctggtgtcggacagggtatgaactcccccggcggtggcagcag

tagcagcagtgcgacggccgtgccaggctcggcaccgggctcagcttctacgacgacggactatcaccgcgccttggcggcgtctatgcaggaccgtc

acatgcgtgatctgcacgagaatgctctggctgccgctgtccttatagcacgagccagtgggctccacgctgattcgcccgaggcgcgggcacagtca

cgtgcgctgttatctacgttcccgcacatcatggccaactatgcggagagccagaatgctagcgggagcaggagtcccgtgtttaacgtcgcgagctc

cagtgcgggcgctggcgcgaatgccgccggtggtggcagcgaggaggctggaggttcggctcccagctcgacgatggcggtcattgcgggtcggaaca

acaacaacaacaatgcgagcgccgcatcgacgagccgacgtcaaggaggtggcctcctggcgtctgccaatggtatgagggcggaggctgtgatgagc

cagtacgaggagacgatggctcggtaccgagcccatcaggaggcggaggcgcaacgggcgttggtggagcaggctgcggcgcgagtgaatgcgaatgc

tgttcaagctgatgtggcgtcgtcgagtggccgtgggattggtggctcgggctccactggaggaggaggaggatctcgttcggcgactatgcctgatg

agttgagtacagcaagaactggagctggagctggagtcgaggcccgggctggaccttccatggctacaggggcaggttcgtcgacttctaccgcattg

ccgccatcgtcgtcgagaaaccaaccacgcaacaagggcacccgccgagcggtccttcaacgcttcttcggcggagcaaacggtggtaacgccgaggc

ttccggttccgggtcggctcatcacagttcgcctgcaacttcctcggcttcgagtcctgctgggggctcgagcggtgcggctttgatgctgccgggca

ccgctgcgttggaagcgatggaggtggacgagcaacagcagcagcagcaggtgcgatcgtctacccggtccggtggcgttgctcagacttcatcacag

acccaggctcaggttcaggctgggtcttcgtcaacgacgacgagtcccggtgatgaggatgggtcggatgtgatgtcgacccatcaggctattgcggc

gcttgctacgcctcctgctcctgcgcatgcgacgctccatatggagtcgcagcctgcgtgggcgcagtcgagttcggccgcatcggcggctggtacga

caccgacgaacaattcccgcggtacgtctgggtcggcggcggcggcgggacttgggtttggattccgttcgagcgcgaggacgggggcgacttcttcg

gctgctgctgcgtctgcacagacgccctcttcatcttcttccgctgctgctgctgctgctgcccgccgaacgtctggttctcgagcggcttcgggacg

tacgggagctgctggtgctgcctcgagttccgattcgttcttggttcgcgggcagaatcctgtcccattccgctcctttcccacctcgggtcctgcga

acagctctttatcctacctcgccaatctgcatcaaggtcttacgtcttacgccgccatcaccatcaagcatcaagccgcaagttgcaacgctcacaac

acccacacacatcacaacaatcctgctgtatacataacagatttctttccaccccttcaagctcgtccggaggaaacgcatgcaacatcggttgctcg

ttctccaaaggacgcccatacattacgacacaatcattcagacgcgccacttccacaggcgcctccatcattccaatctttcaaccttacatcaagat

cctttcggccatttggccttcgacctcgacctctttacgcggtggaacctcaacctcgtctctattctctcggcccgtcatctcagcaccaactcgtc

gaccatatcctacagcccaccctacccatccagcctccaaggatggcttatgactccctccccttccccccggtgtctctggtttaa

>contig_21.contig_21.g843.t1

atgtcgtaccccaagggaaagcacaagcgggtcgacagcggcggtgacggccgcagcgacggacatgacggtaacccac

gcctgtacgtcgaggactatcatctgccgcacaccacccacctctcctcagggaaggcgtgggtatcctcagccgctcgtctgcgcaccatcaccgga

tcatccgccgactcctcctccgactcgggcctgtcctcagaaccgactcccaccatccctcagcagccgtgctcaccttcgccagccaagacagtcgg

actaggaagaccttccacctcccgcttcgacagtctccatgacttgctccaacaggctggatacaaggaaacccgcattgtcactccccaaacccatt

cgcccgtgtcgcgcacacattctctcacccaatcagcggcgcagcaggccctcgccctcaacatgctctccgactcccagacccagcagcaacgccga

gcatcaaccgcgtcacagtctgccgccaacaactccggctggttctcgtccttgctcacccgaccaccacggcggacgacctcgacttccatcgagcg

cgacccatatcggcctggaatcccgcactcgtcttcagaccccaccatctctacaaactacccttccattccaacactcacaatcgatccttgctccc

caacatcgattccttctccgctgcctacaacacaccatgccgcccgtctcggcaccctgcattctcatcaacataatcaactccgcccggccttgcgc

aaaagagcctcgagaacatcacacgccctctggacgggcagtatcgcctatcgcaactcccatacacttactaatcatctccatcagcatctgcatca

acagccaaaacggtcagcaaccgtcagtgggcccactcacaatcggtctcgacccagtctacacgcagcatttgccgccagtccaaccaagtctctca

agagtgctagtatggctgcccctgctacggtcaaggtcaaaactgcaaccgagggtgacgatatcagcactcgaagggccgaattcgaagctcagtat

gacctccatcggaagctgcaccagagcccaccttcatctcccaaggaagcacaagaacaagcgatgatgcaaccaacagagggcagccgcaggaggca

gaacaagcggtcgcagtccaccacagccctcatggcctatccggctttgcccgtcctcttcgccgcacccgccgtcgcactccggcacgaagcttgtc

tgattccttcctccatcgcgctcatcaccgacgcacgcgccagcgcgtctgacccgctcgcgtcgtcccatgccggtatgggatctgagcagatggaa

gcccagggctccacccaaagtcaacaatccatccgtcggcccggcctgggtcggatgaggagtgtggacctcctccgagacgtacttgacgccatgct

ccgcatccgagagaacgcagccgatccgtcccagagtctggcagccgtcgacgaagttgggtatgccaccgtcactccaacgacggcaacagatccgg

atacgagtggagaaggaggacgacgaacaacgtcctcgtccgggacgctcctctccgttacgaactcgctaggtttgagcggcgagactgatgtggct

gcgagcacggagctcccccaacccgtacctcagctgttcctctcctcgcccagggggatcacagcaccgcagccagttggcttcgagagcatcctgga

ccctccgtgcgacgaagtgacgatggtcgacggtgctgggacagaaacgacgggcctcggttccgcgttcctctccctcttcggacggggtaaaaaca

agaccggcgctgttgttcctccgccgtcgacatctactcccgcacccgcaccttggtcaatcgtggccaactcagctggggcgaatgggatgatcaac

tcggcctcgatcgaagctgagctcgaccggatccttgccggcgcgggcagtgggcgtgtatctgcgtcccctaccaaacccatgggacgacgggggtc

acgcgtgcaaaggccagcgatcgaatatggagaacgccggtcgagctctgactcgctcgaagtcatcaagccgctccgaatccggcgcagccgacgac

gaagtcaaggaggaggcggaggaagtcgtccctcgagcatcacctcagccgacaacgtctcggacaatcctactcggtgcatgtcgccctcgcagctg

gaccggatcgagagtgccctccggggctcggcgggcacgttgagtggggcgcggagggtgagcgcgttgaggagtggtgttgaggtcttgcgtgcttc

gcgggatggtggcgcgagtggagcgactgaggacgagcagttggaacagcagcagcagcagcaggtggacgagagcagcagcgaccagtccggctcgg

tgtcgaagagcgaggaggacccgtccggctcgctttcggagagcgatgcgagccaccgctccatatcgattcggacgcaaaccgcgctggagagcgag

gtaaatccgttccttatcgactcagactcaagcgcggcaaccaacacccttctgcgggatgtcaccccgtcttctggaccactcagcacgacgactga

gcaggatacgacgacgacgtccagcgctgtcagcacaagctcgagcacgacggccgctcctggagcctctgagatcgctgtgcgtaagttcttcgaga

gcctggtcgagctcgagacgccgggagggaaggagatggtgtcgcagcagctgtcggtgtacgaggatgaggaggatgtcctcgggcccgtggtagcg

gcagcggctgtcgatcaggcgggatcggcagcgttgacgaagggggctcagcctgctgctgggacgatactttctgaaaggcctgctcacgccaatac

cgatgctaagaccgaacagcaagtacctcagtccaagcgcgaatcgttgtcggcatcgtcgtcgtcgtccacagggtccgtaacacctgacactcatt

cgatccactcgaaagagcctggtcggggcgatacagcggacattcaagcttcatccctaggctcgagcgctgtcatcaagaagcctaagggcaaacct

cacggtccacgaaagcccatcatcgccatcacccacaaagacatgaaccggctcgtcttggacgccgaatccaaacgaagagcgaaattgggagcaca

acaggcagaaggagaacggccgagaacgccggacgcccacaggagcaacaagacggcaccttcctctccgcgctgctcaggacgattgttgtcatcgc

cagtggcggccctgttcgaccaccgggatttactgcgaccgcatgtcgtcctggtcgccgatgagaactatctggaccagagtccgacgagcacgatg

cttgctcagcggcaattgcaccagcctcaacatcagcgggccgccaagacggggatggcgaacaaggcgttgagggcgacgacttcgcagcctgtgtt

ggggaagagtgatatcgttgggtga

>contig_21.contig_21.g844.t1

atgctgttcgccagctcatcttctctctcccgccgcgtcccagctctgctcctctcccagtccaacccctcgctcgcaa

ccttccagcacgcatccgcctccacccagtccgccccgggctcctcgtccgcaaagtacaacacacagcgcaagcgcgagtgggccgtcgtcgtcacc

gcagcaggtggtctcgccgtctccgggtcttggtactggttccacgtccgtccacgcagacaggcagccgccgttcaacagcacgagttgctcttgca

agaacagcaacagcaacagcaacaacagcaactactacaacaacagcagatccagtcagcatcaaacatggctccacgcggctcacgtcccttctcca

cctccgcaacttcctccttctccatcccctcccgctcccgatcagcctcaggcgaacgccaacagaaagtcatcactctcctcaccgaatcacaggtc

aaccaaaaactacgcgcaaacgaacactcggtaattgtcgatcgtccagcgggcgtatcgctgatcacccgctacgactccaacgtcgtaccctccaa

caatccctgcgaagatcggagagtcgagatcatcgtcgagagcgatcacgtcatcccggaagtcgctaccaagaccaagggcgacctcttcttcggag

ccatcatggacggtcacgctggtttccatacctcggctctcctcactcagaaactcatctctgccgtcgccctcgagctcgacaaggtcttcagtgcg

aagggcgaatatgccgatctcgtaggccactctcgtaagggtggcctgctttcgcaacagaaggggagcgtcactagcaaggtgtggaaatctgtatt

tggcgctgcccccggtccctccgctcagcacaccccaacctttgtccagccaccgaacatcacaggctctcctccttcgtcgccgtctcccgaagcga

aagcggacgatgttacagtctcctccggcctcgagtccgatccggaaatcatcaagcacgcgatccgaaaggcgttcctcaccctcgactccgaaatc

gtcaacacgccggtagaactgttgaaggaatacgagctctctctcgcagccgctgcttcggcttctcagcagaagcagggcacggacgcttcatcaac

gaaccccaaaccgattcggcccgccacagcctcggcacagtcggcaggacccgcacacagtaatgcgacggtgacgaactcgggtgcgctgagccaat

tcgcatcctccctcttcccttcttcttcctcctcctcctcagcagcggcattcacagcgacacagcgaacggtgtacgaagccatgctgccggcactt

tccggcagttgtgccctcctagcatacatcgactccgcacggggtgacctctacgtggcctgcacgggcgacagtcgcgccctggctggctactacga

ctccgcccataacaagtggatcatcgaacccctctcgaccgaccagacgggccggaacccgtccgaggtgaaacggatgagagccgaacatccgctct

ccgaagcgtcgacggttatcatgcgcggacgggtcctgggtggcctcgaaccgacgagggccttcggagacgcgaggtacaagtgggacaagagcgta

caggagcggttgtacgagactttcttgcctggtggacgcgcggcggcgaggggcccgccgcagctgttggagacgccgccgtacgtgaccgcgaggcc

ggaggtggagttccgcaagattgcgcccggtctcgtgggtggggcgacgaagaagaacgggcccgagttgaagtttgtagtgatggccaccgatggac

tgtgggacatgttggagaacgaggaggttggggcgctcgtggcgggccatttggagaatgtgcgcgggacggtgctagcgtcgtccctgcacgaggcc

tgctttaaccctcgttcctcctcgtcggcatcaccagcaccgacgccggcggatcagcagcagcaacggggtggcggggccggtggggaacgggcgca

tccgctttcgcaggcgcctgcgaatttgagggcgtatacgtttgaggacgataatttgagtacgcacctggtgaggaatgcgttgggcggagcgaatc

gggatcgggttgcgggcctgttggcgattccgagtccggagagtcggtggtaccgcgatgatatcacggtcaatgtgattctgttcaatacggcggct

caggagggacaacagcagccggaagggcagcaggggcagaaggagcaggcgggcaagtcggacgatggatcaccgcgggcgaagctctga

>contig_21.contig_21.g845.t1

atggagtcgacgtcaagagaaagtcatgggcgcgagcgtgggagtgcgagcagaaggagccgacggtgcgcgatcacgg

ccaaggcggtcaagctcctcgtccttctcgtcgtcttcatggcagcggcgggttgcggacatgcgcttgcagtggactccgcagcggctgcacgactg

cctccacctactgtccaatccgcagcgccgtcgggcgagtcctcaggcgtgcgcgcagcagacggaacacccgctcccactcccactcctgctccgaa

gaccttccgatcgcacctctcctcctctgtgatctccctcctcaacgtcgtcctaaccgacctagccgagatcttctccctctccgaagcatacgcct

cctcctccggcttcggctgggaacccgaggacgccgtcgaatatccacaagacgatgggagcttagatggaggggggaatcccgtcttcaactgggag

gggagttcccttgtcgtcgtgaggtcgcaggcgtcgtatcttacccggccggcggcgtttgggccccgcgtgcaggcggaggatgggatgaaggggat

gttgttgcctattgggatgttctatcggcctcctgttgggcatggggggaaaaaggaggatggggggaaggagtacaacagggcatgtccttataagg

gcgggccgggatggagagataatacggaggataacgaggatgagacgttgagctcttccttctggagctggaatgcgccggctggggagcacgaggag

gaggaggaggagggtatctccatgagctctgctcccggacaagacagccaggccgccttcacaacagcagcatcagcagacgaggccaccttgtccga

actacgaccgccccgacactggatcgccctgatcgagcgcgggacctgtcccttcgtcgccaaagttcgcgtcgcacaggcgttaggagcgatcgctg

tcgtcgttggcgatgcgcctagcccaggttggactccgcccccaccgatcttgtctcaaaaacgatcccgcgttctcgacgatgttgcggcggcggat

gaaaaggggcagcatggaggcgaaggcgggagagaaggggaggatgggaagaaggagaaggagaaagagtcgttctgggcttggttgaagcgagtcac

ggggtgggacgccctttgtgagctcctttttggtcctccgcgccatggcaaaccagttgcggacgaagacgacggtgcggatccgggccagagcaaca

agcgccttgttaccatgtttggcggcggggacacgtcggacatccacattcctgccacattcgtcacccgaccgtcgtatctcgacctgatccgtctc

atcgacgaggtagagcacgagcagcaagagccgcgacagcggcgtcgtcgccctcacaacggagacgaaggagatcaggatcaggatgaggacgagga

tgaggatgaaggcgagggtgatagcgagcggacgccccgaggactggaggttattcttgaacgcgacgagctcgtctgggagtggcccctcatcgact

ttgccatcttcctcctcctcctaccttcgatcatgaccctcggtacggtgctcattcaccgcgttcgactcgcacgacaacgggcgaaagatcgcgcg

ccggaaattttcgttgccaatctgccctgctacatttggagaggcggtggtgtgccctgggagaaggtggagggtgatccagacgcattcgacggggc

gggcaagaagcggtcgactgctggcggtaccagtaaccgagcaggaatcgaatcggcggaagcagcggaggaagaggcgagtctgttggggaactctt

cgaatggtggcacacttgatcctgagactggcgaggcggactttgatctggaggcagcggggggatccagtcgtggaatctcgtcgagcaagatgcgc

aatagctcctcgtcggatgacgacgttggcccatcatcaggacatgaacctggtctgccttcctcctcctctacggccgccgctcaaaaggacgacaa

caacagcaccacgtcgaggaaagtgtcctcgcgtctttccaaagcggccgcctcgacctccttcgacttcctccccgccggacgtcagtactacggca

cggtcgaatgcgcgatctgcctggacgactttgccgacggcgaccaagtccggatcctgccctgtggacactgcttccatcgatcggaggtggacgag

tggttgacgcggatgaggaagttgtgtccggtgtgtaagcgggactgtacggttgctgtgccggattgggtgcagaggaatgaggtgcccagctcgtc

ggttcggccgagtccgagtgggagtcggagcgcgagtgctggcgtggcagcaggtcgaactttaacttcggaaggggttgatgatgtggggggatccg

gagcaccaccgccggcttcgtctgccgttgacgtcgtctcgctcgccgaagcgctcgaactggatgggcagccttcgtcgcggtcacgagatggatcc

ggatcgacgcgaggcccggcgaattga

>contig_21.contig_21.g846.t1

atgccacccgctcccaactcggtggaagccatgtgcgactatcatgcctggacggccgccaaatccatacagcctcaag

catctccgacgtcctcgatctcggcagcgcatccctcgtcgtcgccggtcgcccagcgcccagctctctccctcctgggcgcttctccttcttgcttc

ggcttgtcggccggctccagctctcagcttcggacgtttccctcatccctgccagatacttctccccgctttcacatccccgatctccttcttcttcg

taccaggatgaacatcagcattattgccctcttctgtgggctctttgccgcgagcatcgtcgcggcgaatgatggagatcgcggctttcatcctcacg

aagaggatcaagaactcctgcttcgcggccgttatccgcatgacgattcatccgctgccatcactaaatgcatggatgttgagtgctactcgaacgat

caccgctgggccatcgacgaatggttccagggtagctgctacgggtaa

>contig_21.contig_21.g847.t1

atggctcccaaccgccccctttccgacgacgaggtcatgacagagatgaagaagatggtctccttcatcaagcaagagg

ctctcgagaaggcgcgtgagattcagatcaaggctgacgaggagttcgccattgagaaggcaaagatcgtccgtcaggaggcgatcaacatcgatgct

tcctacgcgaagaagttcaagcaggccgaagtagcgcagaagattgcccagtcaaatgccacaaataagtcgcgattggccgtcctccaagcccggga

agcaaagctccaagacctcttcacagccgcccgcgaaggtctcgccgatatcacaaaggacgagagcaagtacgagaagctcatgagcgacctcattc

tccagggcctgctccagctcatggaggacaaggtcgagatcactgtgcgtgagcaggatgtcgggcttgctaagaaggccgcggactcggctgctagc

gcatacaaggagaagagcggcaagagcacaagtgtcgaagttggcgcagggttggggaagaacagtgctggaggtgtcgctctctctgggttcggagg

caagatcaagatcaacaacactttggaggagcgtctgcgtctgatggaggagaagatgctgcccgagattcgaatggacctgtacggccgcaacccgt

cacgaaaattcgacaactga

>contig_21.contig_21.g848.t1

atgccaagctaccattcagcatacaacgacgacaccgacagccgccagtgcggcaacatggccctcctgcccatccgca

ccaagctgcgcgggcccgcaccgccgcccgtcgacgccgagcaaccagacatcatcgacgagtccctcgacctcttccgcgccaactccctcttccgc

aacttcgagatcaagggcgccgccgaccgcgtgctgatctacctcatcctcttcatctccgactgtctctccaagatcgcagcgagccgggttcccat

gagccagaacgaggctcataagcagctcagcacgtttgcgattgataacgtgatcgttcccggggatgccagtttccccctcaatgcgctctatcagg

ctcctgcgaaccggaacgagaccgatcagctgcgacagtatctctcacaggcccgacaggagaccgcgttgagactggtcgacaagatttacatcgac

ggaaagccgagcaagtggtggttggccttcacaaagaggaaattcatgggcaagagtctgtcttga

>contig_21.contig_21.g849.t1

atgacggcatgctcgggccaagttcgttccttcccgcccgcgacagacgatcaccagcaaggcatcactcatcagcaac

gacaacaacagcgcacgcgtaacagccaggcctacacgcgcgcgctcatatcagcactcacttcacgcgcccctgtctcgctcccctgcgcttgcgaa

ctgcgaccgacatcgctcggagacttgcctgttgcccagcacaacccccatcgaaaaggaaaccaaatgcaggcaacttcgctccggctcagcgggag

agtgctcgcaggaggaagaggagcagcgcccatctcgcgccaacacgcgtcgcttgctgtcttccgccatgcctccctgcgttctggcgggagagctg

cctcgtctgctcctcggactgccatcggccaaactcagcttcgggtcgccggtcagcgctttgctagcatcaataccccgccgccgccgccaccacca

cctcctgcttccagtgctcagccagctcagtccgcagtcttcacaggtgcgtcgtccggtagcggtaccggcgggggcggcaaacctccacgaccccg

cacgcgtggacttcccctgcctcgcaaccccatcatccgatacaccctctacaccatcggatccgccgtcttcggcctcaccgtcgtcgtcgtcggtc

tgctcggctacgacgccatgacctaccgcgtagctcacatcgacggcatacccaccaagccactcgcgctcaaccctcggccgggaggtccgaagaac

ttgccgatcgtgtcggatcaggtcgatgatttggaggacgatgctcatcagagattgagcgggaaggagcgcttggttattgttggtggaggttgggc

tgctgtagccgtcctacaaggtcttgatcccggaaagtacaacgtcaccattgtttcgccatccaacttcttcctcttcacccccttgctaccttctg

cggtcgtcggaacggtcgaaccacgttctctggtcgagccgctccgcaagatcggcgctcgtgttaaatcgcacttcatcctcggcaccgcagtcgac

gtagtcatgagcgaagaccctaacaagcgcttgctcgaagtgagcgtcatcagccatgccgactgcgtcggtgacgagggcaccggtcaagccttcca

aggtcgtaaggagaccgccggaaagcacgtgtacataccctacgacaagctcatcgtcgccgtcgggtcgatcacctccactcacggcgtccccggtc

tggagaactgcttccacctcaagacgatacaggacggccgtgccgtccggagtcggctcatggacaatctcgagattgcggcacttccgacgactacg

cctgaggagcggaagcggttgctcagctttgttgtttgtggaggtgggcccaccggtgttgagacggcgagtgagattcatgatatgatcaatgagga

cgtgctcgaatacttccccaagcttctacgtgcggaagctcaggtacacgtcatccagtcgcgcgaacacatcctcaacacctacagcgagaagattt

ccaaatacgcagagtccaagttccttcacgacgacgtggacgtgatcacgaatgcgcgcgttaagcgagtggaagctgacaaggtcatctacacgatc

aaggacgagcaaggcaacgtcaccgagaaggagctcccctccggcttcacgctctggtcaacgggcatcgccatgagccccttcacgcgtcggttgac

ggagatcctgccgaaccagagtcacttgaaggcggtgcaggtggactcgcacctgcgcgtcagtggggcaccgctgggcactgtgtacgcgcttggag

atgcgtgcacgattgataatcagctgattgatcatttgtacgagtttgtggacaagtgcgacacggaccgggacaacgccctgacgttcaccgagttc

gaagaactcgctgcgacggtcggcaagaagttcccgctcgccagcaagcacttcgagaagctgcgggatacgtttgaccggtacgataaggacaagga

tggcacgctcaacttgaatgagattgcggatatgtttatggagacgtcgagcaagatgacggctctgcctgcgactgcccaagtcgcctcgcaacaag

gcaagtacctcgcccggaagctaaacaagctcgcccgtgtccgagatgcgcacaaggagatccccgacgagcagaagaaaagcccaggcccacgggac

ctggacgacgctgtctatcggccgttcacgtaccggaatctgggctcgttggcgtacattgggaacgcggctgcgtttgatctgccgctgccggagcc

gattgggagctttgcgggcgggttggcggcgatgtatgcgtggcggtcgttctatctgtctgagcaggtctccatgcggactcgcgctctgctcatga

tggattggatcaagcgcggaatctggggccgcgacttgagccgaatctaa

>contig_21.contig_21.g850.t1

atgctcaccgtcctcaaattcacgccactcgacccgaagaatcacgctttcaccttcctcttgcatccacatctcaagc

gacagaacgtcattgacgacgatcttctccagctcccctttgtctttgcggacgaagtacgaatacagcttcgaggggatcatccgaccatctgttct

tcgcgcgttatactcggtgtttacgtcaaccatcaactcctcggctccaccccttgtattctccgcgacggcgacatcctttcctttccacaacgtcg

tcacaagctcacaactgcggctcgttttctggtcgagatggaggtcttggtgctgagctgcggggtagacaaagagctgcttgcgcttcaggggtttg

cggaggagctgcaagacgagaaggcacgcaagacctctcagagcactttggagaagaagcgcaagacctctccaagcacatcggagacggcacggaag

acctctacaagcacttcttcggccttgtccacggtgtcgcactcaatcggcagcggtaccgggccgccccggatcttctccatcctcgccgcagcatc

ggtcacctcgtccgctgtggcgtcccgaaccctgctcggacacagttcctcgcgttcccttgattctacttcttccgaccctcgtcctgcttctccta

ccccaacttcgatctcagcctcggtacttgaagcagcatcctcgcacgttccaccaagcacatcttctgccgccgcttgcgcgcccattacgtcgtcc

gccgacccgagcagtacttcggtactgacttcgggactggatcactcttgtcttgtctctgcagcgacttcccgcagcctcacctcgactgatgcggg

cgtcgctcaaggcacctccgcgcattcacccacagcatctgactctgcatcagcttccgctcaggtcacagctcctcgtccctcggaatcgtcatcct

cgacggagcgctcctcatcggtgcattgcgaggatggacccgtgcgcgtcatgttgcgttcggcagagagggcgcttcaccgtgttggccacgctttg

ctgcaggctcgtctggccacctctagcatgacgacggcggctgttgcagtcgagcgcatgcccgacaatcccagtccttacccctgcgcttccacctg

caggctgggcgaccgggcccattgtgccaaccatggtgtggcaccggaatcggacgtttccgatggcacccgcgacttcacatcttcgcatcgttcac

cagtgcttgctctcgccgacggtccgtctttttggtcgccccaattccattcccatccagtcgactcgggacagtcgtcttttgtcggtggggatgtg

ctggacagagtccataccacaatgctcaccgtcctcaaattcacgccactcgacccgaagaatcacgctttcaccttcctcttgcatccacatctcaa

gcgacagaacgtcattgacgacgatcttctccagctcccctttgtctttgcggacgaagtacgaatacagcttcgaggggatcatccgaccatctgtt

cttcgcgcgttatactcggtgtttacgtcaaccatcaactcctcggctccaccccttgtattctccgcgacggcgacatcctttcctttccacaacgt

cgtcacaagctcacaactgcggctcgttttctggtcgagatggaggtcttggtgctgagctgcggggtagacaaagagctgcttgcgcttcaggggtt

tgcggaggagctgcaagacgagaaggcacgcaagacctctcagagcactttggagaagaagcgcaagacctctccaagcacatcggagacggcacgga

agacctctacaagcacttcttcggccttgtccacggtgtcgcactcaatcggcagcggtaccgggccgcccccggatcttctcgtacgctgctctagt

tgcgtcccgcgttccaccagctccacaaccttcagcatcctcgccgcagcatcggtcacctcgtccgctgtggcgtcccgaaccctgctcggacacag

ttcctcgcgttcccttgattctacttcttccgaccctcgtcctgcttctcctaccccaacttcgatctcagcctcggtacttgaagcagcatcctcgc

acgttccaccaagcacatcttctgccgccgcttgcgcgcccattacgtcgtccgccgacccgagcagtacttcggtactgacttcgggactggatcac

tcttgtcttgtctctgcagcgacttcccgcagcctcacctcgactgatgcgggcgtcgctcaaggcacctccgcgcattcacccacagcatctgactc

tgcatcagcttccgctcaggtcacagctcctcgtccctcggaatcgtcatcctcgacggagcgctcctcatcggtgcattgcgaggatggacccgtgc

gcgtcatgttgcgttcggcagagagggcgcttcaccgtgttggccacgctttgctgcaggctcgtctggccacctctagcatgacgacggcggctgtt

gcagtcgagcgcatgcgtgcggcttggagggaggcccgtggcgcagtcgattctgttgctgtttcgctggtcttttcagccgacaatcccagtcctta

cccctgcgcttccacctgcaggctgggcgaccgggcccattgtgccaaccatggtgtggcaccggaatcggacgtttccgatggcacccgcgacttca

catcttcgcatcgttcaccagtgcttgctctcgccgacggtccgtcttttggtcgccccaatgtcagcagcttcccttcgtggcccttccctctcacc

tctcctcctcaccttcaacattttcgggctttccaatctgtgcccgtacatcatcatccggctgccgagacgttcatgccttgtgcttcatggccatg

gacatggcctccgtatctcccacatcttccaccatacatccattcccatccagtcgactcgggacagtcgtcttttgtcggtggggatgtgctggaca

gagtccataccacaatgctcaccgtcctcaaattcacgccactcgacccgaagaatcacgctttcaccttcctcttgcatccacatctcaagcgacag

aacgtcattgacgacgatcttctccagctcccctttgtctttgcggacgaagtacgaatacagcttcgaggggatcatccgaccatctgttcttcgcg

cgttatactcggtgtttacgtcaaccatcaactcctcggctccaccccttgtattctccgcgacggcgacatcctttcctttccacaacgtcgtcaca

agctcacaactgcggctcgttttctggtcgagatggaggtcttggtgctgagctgcggggtagacaaagagctgcttgcgcttcaggggtttgcggag

gagctgcaagacgagaaggcacgcaagacctctcagagcactttggagaagaagcgcaagacctctccaagcacatcggagacggcacggaagacctc

tacaagcacttcttcggccttgtccacggtgtcgcactcaatcggcagcggtaccgggccgccccggatcttctccatcctcgccgcagcatcggtca

cctcgtccgctgtggcgtcccgaaccctgctcggacacagttcctcgcgttcccttgattctacttcttccgaccctcgtcctgcttctcctacccca

acttcgatctcagcctcggtacttgaagcagcatcctcgcacgttccaccaagcacatcttctgccgccgcttgcgcgcccattacgtcgtccgccga

cccgagcagtacttcggtactgacttcgggactggatcactcttgtcttgtctctgcagcgacttcccgcagcctcacctcgactgatgcgggcgtcg

ctcaaggcacctccgcgcattcacccacagcatctgactctgcatcagcttccgctcaggtcacagctcctcgtccctcggaatcgtcatcctcgacg

gagcgctcctcatcggtgcattgcgaggatggacccgtgcgcgtcatgttgcgttcggcagagagggcgcttcaccgtgttggccacgctttgctgca

ggctcgtctggccacctctagcatgacgacggcggctgttgcagtcgagcgcatgcccgacaatcccagtccttacccctgcgcttccacctgcaggc

tgggcgaccgggcccattgtgccaaccatggtgtggcaccggaatcggacgtttccgatggcacccgcgacttcacatcttcgcatcgttcaccagtg

cttgctctcgccgacggtccgtctttttggtcgccccaattccattcccatccagtcgactcgggacagtcgtcttttgtcggtggggatgtgctgga

cagagtccataccacaatgctcaccgtcctcaaattcacgccactcgacccgaagaatcacgctttcaccttcctcttgcatccacatctcaagcgac

agaacgtcattgacgacgatcttctccagctcccctttgtctttgcggacgaagtacgaatacagcttcgaggggatcatccgaccatctgttcttcg

cgcgttatactcggtgtttacgtcaaccatcaactcctcggctccaccccttgtattctccgcgacggcgacatcctttcctttccacaacgtcgtca

caagctcacaactgcggctcgttttctggtcgagatggaggtcttggtgctgagctgcggggtagacaaagagctgcttgcgcttcaggggtttgcgg

aggagctgcaagacgagaaggcacgcaagacctctcagagcactttggagaagaagcgcaagacctctccaagcacatcggagacggcacggaagacc

tctacaagcacttcttcggccttgtccacggtgtcgcactcaatcggcagcggtaccgggccgccccggatcttctccatcctcgccgcagcatcggt

cacctcgtccgctgtggcgtcccgaaccctgctcggacacagttcctcgcgttcccttgattctacttcttccgaccctcgtcctgcttctcctaccc

caacttcgatctcagcctcggtacttgaagcagcatcctcgcacgttccaccaagcacatcttctgccgccgcttgcgcgcccattacgtcgtccgcc

gacccgagcagtacttcggtactgacttcgggactggatcactcttgtcttgtctctgcagcgacttcccgcagcctcacctcgactgatgcgggcgt

cgctcaaggcacctccgcgcattcacccacagcatctgactctgcatcagcttccgctcaggtcacagctcctcgtccctcggaatcgtcatcctcga

cggagcgctcctcatcggtgcattgcgaggatggacccgtgcgcgtcatgttgcgttcggcagagagggcgcttcaccgtgttggccacgctttgctg

caggctcgtctggccacctctagcatgacgacggcggctgttgcagtcgagcgcatgcgtgcggcttggagggaggcccgtggcgcagtcgattctgt

tgctgtttcgctggtctttttcagccgacaatcccagtccttacccctgcgcttccacctgcaggctgggcgaccgggcccattgtgccaaccatggt

gtggcaccggaatcggacgtttccgatggcacccgcgacttcacatcttcgcatcgttcaccagtgcttgctctcgccgacggtccgtctttttggtc

gccccaatgtcagcagcttcccttcgtggcccttccctctcacctctcctcctcaccttcaacattttcgggctttccaatctgtgcccgtacatcat

catccggctgccgagacgttcatgccttgtgcttcatggccatggacatggcctccgtatctcccacatcttccaccatacatccattcccatccagt

cgactcgggacagtcgtctttttgtcggtggggatgtgctggacagagtccattgtcccgacaaggctgtcgatctcgacaagactcacaacaacaag

ggcggcggtaacaagaacacctccggcgactctttcaaaagccactggatcctggacactggagcatcgcaccacatgacgtggtgctctgagctgtt

tggcgaattggaaaagggggagcatggcgacgtcagcttggccgatggtagtacactgcccatcaagggcatcggctcggttgcactcactgtgctcg

gcaacgacgggaagagggtgttgcggctgcgggaggtgctgtgggtccctggactcacgagcaacctggtggcggtgatgcaggcgatggcgggggat

gtgaagatggagctggtggcaggaggaatgtgggttggacgggaaggagacatgtcagtttgggcaccacatctggtggagcgcggcggctgcgtact

caccacaggtgagcaccgggcggccacggcgcaggtcgcgcaggcggatgcgggcgagatgggagctatgctgatgcatgagcgtctcgcggatccgg

cgcagaaggcggccggggtgatgtgggacctgggggcatgcaagggaatcaatgtcagcaagaacgcactcatgcgggcaagcgcggcctgcgattcg

tgcaaacgggccaagggcactcgagcgccgtttcctatctccacgcgcacagtcaaaaagcgccttgcgctggtgtgtgtggatgtgatcggcccgct

cgaggaaggcacgggggacaatggggagaagtacacactcaccattgtcgacgctgcgacgagaaaagtgtgggccatacccgtcgccacgaaggcga

acgccttgccggcgttcattgagtgggggaagcgggtgcagaacgaaaccggggagatcctcaccgtcgtccgctccgacaacggcaaggagtttgtg

aaccggcagtccaaggagtgggccaaggagctcggcgtttggtgggagacgacaacgccctacaccagccaacagaatggtgtggtggaacgttggaa

tcgaacactgcaagatcgcatgcgagcaatgttggtcagctcgggcctaggcaacacattctggccgcacgcgatgcgtgctgcggcgtacgtactca

accgcactccgcgcctgaaggaacaagggcgaattccccagcgggactggacggggcagccggtggacttgtcgaacctgcgagtgttcggctgtctg

tgctggccgcttgttcaatctggtcaaagggaggggaagctaagtgagcggcgcgttccagcaatcttcttgggctacggcgaatctgtcaagggctg

gctcgtgtacatacccaccaagtccactcatcggaggaggtatagtcgcgacgtgctcttcgatgagcagcgccggtaccaggacgttccggtgccga

ggcaaactgcgacgctggatgacatcctggattggcatgaggagtggacattggtggaagagaataatggatcagcggatactgcgggatcggtggga

gctggaatcaggaacactgaggcctcaccggagcaggtggctgatggaggaagagacggtggtactgagaaggtggctgtggtgcctgccagtggtcc

gccggaggatggtgatggtggagctgagcaggtggctgtggtgcctgccagtggtctgccggatagtggtggagccgagcaggtggtgcctgctggtg

gtccgccggatggaggagaaaagaaggatgttgctgatgatggggatgaggaggcaatgcagtccggtctgctcgaagaactgctgcttcgcgcacag

atcgacgggttgcctgctgctgcatttgcggaagaggcagtagaggagatgatgagcgagctgcaagcggggacaagtggggatcaacacaccggatc

agcgccagacgttgatcgaccaggagacgcagagcgcgctcacgttgagcgcattcggaatacgcctactcggcgctccgaaaggttgcgagcacagc

ggacaaagggctacacgaagatcgtgcgtgcctttgctatggctgcgcagggtgctctgctggtcgggaaacggacaagcgccgatggtgtgctgctg

gaaccatacaatctcagcgaggccaagacccggccggactgggagaagtggtggaaggcaatgcagtcggaatacgactcacttatgtccaaggagac

gtggaagaagattccgcgcaagctcgcacgacgcttgatccgatgtcgctgggtgttcaagctcaagttggacgagcttggtcgggcactgcggtaca

aggcaagccaagaggaatgcgatcgcgtcaaagcatttctgcgggcggagttcgagatcacggacaatggacccattcatcatttcctaggggtgcaa

gttcagcgcaacaaggagactggggacgtgaggctgaatcagaccgcgtacgccaaagcaacactccagcgctacagaatggaggatgccaaaggatc

cgacttacccatgaacactccgggtactgtcgcggctggcactcccatacagatgggagattacgcagcacgggttgggagcttgatgtgggaggcgc

aatgtacgcggccggacatgagctaccccgtgggagttctcgcacgcaagatggcgaacccggatgccgaatccactgcaaatgtgcagaaggctctt

cgttatctgagcaagacgacggatctggggctgagctactcacctagctcagatatcgtcctcgagggctattcggacgccgatcatgacagaagaca

accacggccacgaccccatcccgatccccaacaacagcatacaaacaagatgatgcgccccgagggttccgagctggacgaggcccccaacccgctcc

agcagttcgtagcccattccaacgtggcccaacagcgcgtccaatcgttcctcgactccaccaccccacacgtctaccaacgctgggccgcaacatcc

ctcctcctcctcctcttcctcctccgcatcgtcctcgcacagggctggtacatcgtctgctacgccctcgccatctacctcctcaacctcttcctcgc

cttcctgcagcccaagttcgacccttcgtacgaacatgacctcgctgagcaggatgtggaggagggcgagcccgggttgccgacgtcggctgctaagt

cctcaagggccaataactttgggttaggatctggtgggaatacgggaggcgggctcatgagcggcgtctttggcggtggggcgcagaacggacagggt

accgcgccgcctgaggaggaattcagaccattcatccgccgtctgcccgagttcaagttctggctatccgcaacgcaagccgtctccgtctccctcgt

ctgcaccctcaccgaactatgcgacgtgccagtctactggcccatcctgctcgtctacttcctcatcctcttcgcactcaccatgcgcagacagatcc

aacacatgatcaagtacagatacgtccccttcgacctcggtcggaaacagcgctacatctaa

>contig_21.contig_21.g851.t1

atgtcgctcctcgtcatcgaggatctcgacggacggctcatctacgcccgcgcaggcgacggtgaggccgtcttcggct

ccgatggcgaattcgagttcgcagacgatagcagcgactggtgggagtccgaggacgaagacatgaacgacagcatccccggcgctctcgaaccagac

gacctcgaagacgaagaaaatcaaggcaacgaagccgacaacgagtccgaggcgcaggcacggctccatgtcctccagaatgccacctcgacctcggc

acacaccctttcccaaccagatccctccaccgcctcttcctcggctctccccaatcaggccgcactagctggtccctcaacttcgtctgccattgacg

gagccaacgaggccaccacttcggcagcaaccggccgcaccaagtcagcacgcacactcgcaactaccagcgcagagtcttccctcgagcccgatgaa

ggcgagaccaccgacgaacttggctacgaagaaatgccatatccccgtctcctcgtcggctccattgctccccgcggaagcggtatcagccgagctcg

tgcccgagcaagtgcccaggccaagcggaacaacatcaacaacaaccacgccggctcgtcctctgcctccctcaacatcaacggcaacagacatccgc

cgtcagcgatgcagacgatgcccggaggcagcatgagcaacagtaccaccaccaccaccaacagcagcagtaacaacagcatatcgtccaacaccgtg

cccactacacccaggatgtgccccccgccatcgccgtcgataccgaacggcggacagcagccagcaatgacagcagacgccgtgccctccacacccgg

acccagctcggccgcagtcgccgtggcggcagcggcggggaccggaggagcaggcccaccgccgctgccgtcgttccagcggccggaaatgggcacgt

tcattccgtcccaggacaaggcggtccatcgggccattatcgacggagctagtaaggcgccgagcccgtaccagcgtgggaaagacgtcatgaccaag

ggtctcgcaccgaagcggaagcatgatgactccgacggacgtagcgcccgacaaaggatcgagaggagagcccaccgcggccgctcgccgatgagcat

gagcagcgcccgaatgggcggcagcggtggcggcggcgggccgagcggcagcagcagcaacgtgacgcacacgaacaaccacaaccacaaccacaacc

acaccaacggcagtatctcgctctcgaacgggatcgtggctgcgcaccggtcgtcggcgcggagcgcgaggaagaagaaggcgcgggtgccggatatg

gacccggggatttcgcttgatgagcttttggatatggagctcctttcggagggcggtcttgttcagcgttga

>contig_21.contig_21.g852.t1

atggcgacagcagcgggcgacggacgggcaccgggcaacggcagcgcgacgatgcggacctcgtccaccttaccgcctt

acgtgcatgggcgcgtctatccagctgccgagcggatcagcccaaatatcatcccgcaaaaactgcccatctgggatgcgtcagaaggctcgggcgac

gtgccggccattgtcatcgacaacgggagctccgagctccgcgcaggcttcgcgcttcccggcgttgatacaccgccgctcgtattcgacagcgttat

aagcagatatcgcgaccggaagcgctcgaccaatctcctcctcgtaggctccgatgcgtatgtcgacacccagagtcgctccggtatccgaagaccgt

tcgacatggatgtggtgtgcaactacgatgtcatggagagcgtcttcgacttcaccttctcccgcctcggcatcgagtccgagacggtccaacatcca

atcttgctcacggagaccttgtgcaatcccgcatacacccgcgcccagatgaacgaaatgctcttcgaatgctacggtgctccctccgtggcatacgg

cctcgatgctctcttcagtgcgtatgcgaacgatgtcgagaattctctcatcgtctcctcgggcagggctagtactctcgttatccctttggtggacc

ggaaagcgctaataggaagctcaaaacgtctgaactggggcgggcagctcgcgactgactacctgttacggctcgtccagctcaagtacccatccttc

cctctcagagtctctcagacccagtcagcatggatgctagagcagttcacgtttcttgccgcgccttctacaacgtccggcttcacctcaacctcagg

agcagactcctatgaagagttcattggctcactacccgccccatacgaaaagaagatggaggcacaaagaccgccagcacggggtgcgatccttccgc

ctgccaagccgaccgtcagaccattggctcacggtcttgccgagttagatcgcgtcgtacagttccctttcacagagtccgagcaagaagcaagaaga

gcggcaggcgaggacgtcgagaatggcgaacaacccaagcgaaatccttcccattttgccaagaagcccaaaccaccaccaggagaagaaccagtagt

ggaggaagagatcaaggtaccgtctttcccgcttgtcgacgtgccagaccatcagttggacgaggagggcgtcaaggacaagcggcgacaacgcctca

tgaaggctgggtacgatgctcggatgcgggtcaaggctgccaaggatgagcaagcgcggacggaggaggaagaacgtcgcaaggacgaagatcagcgg

acgaatgcgtttgagcagtggaaggagagaaaacgaaaggagtacgaggatgccattgagcggatcaaggagcggaaaagaaatcaggagatgctttc

agatcgcaagtctctagttgcgcagcaacgcatgaaggccatcacgactatggcgagtgagcacggtatgggtgctgccggtggtgcgggtggtggtg

ctccttccaagaagagacggcggaatccagacgatgacggtttcggcatggatgactcggactgggccatctaccgagaggtgcacggacaggacgag

gaagaagaggaggaagaggagcaagcggccgttgcgtccatcgaggcgacactgctcaagtacgacacctcgttcacgcaggatgacacttttgccgc

acgggaggagcgcaagacccatctgacgacgacgttcttgcggggtcattggccggcgccgtggaaccctgatgactcggcgcagattcatcagcttc

atctcaatgtggagagaatacgagtgcctgaggttctctttcaaccgagcttggcgggcgtcgatcaggctgggttggacgagctgtgcgcccatgtg

ttgcggaatctcgacgcgcctgtcagggaccggttggcaaagaacatcttcattaccggtcaacacaccctctatcccggctttgacgaccgtctgtt

gtcctccattcgggccacccaaccgatcggtacccagttggaactccgacgggcacgcgatgtccggttcgacccttggcgtggaatggcgcgttggg

ctcgggagtgtcgggaggagctgatggaggcgcaggtgacgcgggctgagtatgaggagaagggctcggagtggttcaaggagttccggttctcgggt

tccgttcagaaggaggaggaccatcaccagcagatcactcccagctcccatccaatcaacgtcgtgcagcaggccgcaggttacccccccgaccacgc

aacctacggttcctttcacaacatgacgggagagggtacgtttgcgcaggatatatcgcttgatgcgttcgcgcgcatcatcatgttcagcgacctgg

aatgcgactacatcaaccctatcgacctttgcaacaagctgaatcagttcacactcccggagatggttgcgcacgcggttttgacggtcttcttcctg

ttgagcgggcaatggattgcattcatattgaatgcaccattggtagcgttcaatgtgaaaaaagtgatggataacaaccacatgctggacgcgacgga

gatcttccggacactatcccagcacaagaaggagtgtttcatcaagctcgggttctatttgatttccttcttcttctacttgtaccgcatgattgttg

cattgatcgcagtccatcccttctcggctgggaatggtaccggcctggggaacgacgaccggcaccgattcgattgggcgtcgatgcatcccttcatg

actggcggcaagacgtacttgacaacaggcgtaaccggatgtctgatgataccacgagcacctcaacgacagtacgccctctttcgaccgggtccagc

tgtgcgactctggcccgtgcagggtcgtccctccccggtcttcttccacaccaaaagcaaggacaggcacctgtccgcggcgcgctcgtcctctgccg

gtacctcgttgcatcccagtctcctccactactccaccatacctgatgttcgtattatcccctgtcaacaatga

>contig_21.contig_21.g853.t1

atggcatcgttcggctccgagcacgagtctgcgatcccgactccgccttccgaggcggcaccatcctcacaaggatcga

gagtgggcgaggcacagacctcattacctcgtccaaacatcgcatcaaggtcgttgaaggggggagatctttatgtccatacctcgggccacgcgcct

agtgggactcaaagccccaacacccccgcaaacactcccgcagccccggggcgcaggttgaggagaccaacagaaatcgggtctggatcggtaccagg

gacacctctcacggcatcagtgccgcgtcggcggagaccaaaagatcgacacctcgcctcactggaagcgattcgtgacttcctccgcgaaaggtcct

catacgatgtcctgccggtctccttccgtgtcgtcgtgctggacacgaaacttgtggtcaagccggcattggatgtgctctggcaagcgggaattatc

agtgccccgctctggcacagtactgtccctccaccgcttgaaagaaacggagagcaagaagcgaatggggaccaggcgccgtcgcaaacaaactcagc

cgagccagtactgcccagacagcagtctgccgaagcgtcaaaggaaggctctcagctgtcttccacagcggaccttccctcatcaaaaatcaagccag

gattcgctggtatgctcactgtaaacgacatcatccacttgatccagtactactacaagtttcacgcctcctacgacatcgccgccaaggacgtagaa

cacttccgactagagtcgttgcgtgacattgaacaggcactaggagtaccgcagcctcctctgctcagcatcgatcctatacggcccttgtatcaagc

ttgtcggttgcttgtcaagacccatgcccgcagattaccccttctcgactacgacgaaactacgggcgcagaaaccgtcgtcagcgtcctgacacaat

acagagtcttgaagttcattgccatgaattgtcgtgagaccgctgggctccatcggtcgctgcaatcatgcaggatcggaacgtacaccgacaatgtt

tccatagcgactgccaccctcgacacaacggtctttgacgttgtgcacatcttctccgaattgggcatcagtgctgtaccgatcgtggacgacgacgg

cttcgttgtcgacatgtacgagacggtcgacgtcataaccctcgtccgcacgggcgcatataccgtcctggatatgaccatccgacaagcactcgatc

gaagaccgaaggatttccccggcgtgtacacatgcagtccacaagacagtctagcaaacatcttcgccctcctgcgcaaggtccgcttccaccgactg

ctcatcctcgaacctgaagacgcgtccctcccaactccgggtgccggagacacaagtgacgaggcgcagcaggctcaagaagactttgccgcctctgc

agagttggcttcggcaacggtcacgactcccttggccgccacgatgaacaagcccctcgagcttccggaggacatcgccaccggaggttcgagccgga

cgagcagcgggagcaaggtatctgagaaggttccgcagggtcggactatcgagcaggagctcgaggccgggttacggacccgcaagagggggcgtctg

gcgggcattgtgtgtttgagtgacatactcagatacatcgtcggagccgacgagaaggatctcggcagcgctcttcggcaggcgaccgcatga

>contig_21.contig_21.g854.t1

atgtcgacgtcctcgccaatacctgcgccgggttctgctgaggcgatgacctcagcaggagccgcaatctccaagacaa

ccaccctcaaggctgtaggcgctgcgcgtcctaccatcgacgccctcactagcgagacggctcctgtcggcacggcggccccgaagctgagtggtgct

gccaatgacgcgcccacggacgaatgcgacaccgctaggaccttgaagcgcgtcctgctctcagcgcgcagcgacactgataatcacgtcagccagga

tctcatcgagcttgtcaccgagtccttacgagtcggcgcgggagagtatgtccttcggctcgggtccgccgctcaagacggaggcgcggccaaggctg

cgcagcatcaacagaagaagacgggcgctggcgggaagggtgccgaagcgcagacgaacgttgccacgcctgatgacttaatagggtacacgaaggac

gaattcgagaaggccatagataacttgaccgatgctgcccgcggcatggactcagtcgtgaccgttcttgagtcgcggatatggactgtcgggggacc

cgacaacgcttctgagcgcgaggcgagtgctgcggatagtaccgctggctccaacgagactgtcgagcccatcacgtacatgtctggatacgtccagg

tccgtcgcaaccccgaatccgtcgtctcgctgattgagtcccgcgttgccgtcattggcaacgtcgacgccggaaagtcgactttgttgggcgtcttg

acgaaaggcggactggatgatggacgcggtcgagctcgtgttcactgtttccgccacaagcatgaggtcgagtctggtcgtaccagttccgtctccat

ggaaatctgcggtttcgacgcgcatggcgagcccgtgtacaattccgctgtggtgaagggtaacagcaacgccaccacgaaggatcacacggacgagc

ctcatcgccagctgacttggcagcagatctgcgagcggtcggccaaggtcgtcagcttcattgatctggccggacacgagaagtacctcaaaagcacc

gtcttcggcttgaccggttgcctgccagactatgtcctgctcatggtcggagccaatgcgggactcattgggatgtccaaggagcatctcggcattgc

gctcgcgctgtcggtccctgtagctgtcgtagtgaccaagattgacatggcaccgccgcaagtgctcgagaccaccatcaagcagttgaccaagatac

tgaagtcccccggatgtcgcaagacacccgttttcatcaccgaccgcatgtccactgtggacgctgccctgcggttgggccagggccgtgaccgcatc

tgtcccatcttcctcgtcagcaacgtcactggggacggcttgggcaatctgcgaagcttcctcaacgtgctgcctcagagcgcattgacgaagtactc

gtccaagggaccgttcgagttccagattagcgacattttcagcgtgcccttcgtcggcaccgtcgtttccggcgtcatcgttagcggctccgtcaagg

ttggagataatctcctgatcggcccggactcgctgggaggcttccttcccacgacagtccgctcgatcgaacgcaagcgcgtcggagtggacgtggcg

tacgccggccagtcggcatcttttgccctgaagcgtatcaagcggaacatggtacggaagggaatggtgatgctgtcgcgtccgctcaatgacgcgga

cccgcctcccctggcgcatacgacgtgcgacgcggaagtcctgtgtctgtatcacagcacaaccctcagcatcgggagctgcatggttttgcacaccc

agagcatccgccagaccgtccgtatcatcggcatagccaagcttggccctggtggtgagctggcagagcaagaggagggctctggcgttgctcagatg

gctgtcccaggaggcaagggcaagaccaaggctccgggacctgtcgaggacaagtccgacacgctgacgggcgcgaatatgcccgttgttcggactgg

agatcgtgcgctcctgcgactgcagttcatccgctttgccgagtttatcaaggtcggacacaagctgatcacccgagagggcaagacgaagctgatcg

gcgtcgtccgggcggtaggggctacaggccctctggttccgcctgctcagaagcagctcgacgctgctcattaa

>contig_21.contig_21.g855.t1

atgtctcgacctcacgaaccgccaactttctatgtgccaggcgaagcggtatctactggagccactccgcatgcaacgc

cggctcgaggtattgtctcctctacaacggcacccgccctgactcgtgtccaatccagccgtgtcctgcatgatgcggatgttggcgcgctcgacgca

gggtatggattctactccagcgcccgctccggccttgacgatgcaagggcgacgccatttcagacaagacctcgctcagcctcacaagcccctgaaac

ggcccgcggacggtgggcaaggggacggatttccgctggtccactactccggcgagctgtcaccaatactgccgattcaccagatcaacgggtccggg

atgtggccacagggaccagacgcggatggttctcgcgcggagcgtcccaagctcatgctgatgggaatgctgagatcaatggggctccttctcacacg

gcggcggcgacggcaggaacgcaagcgcatcaccatgttctactcgaagccattcaaagagattttgaggctgatcgcgtagaagaagcgacagaagc

gcgggaactggcagaagtcagaaagggagggaccgggcaacacgcattcaataaggcacacaaagcgagtcgagagtcagcggtatcagagaaggttg

agaagggtgatgttgaagctcagagtacgacggggctggggacgggaactgacgacacagcagtgggatcggaggcggagggagcagaaaaggggttg

ccggcttcggaaaaggagcaagatcctaatctagtcgggtgggattcgccttcagatccggcaaatccgcgaaactggtcgaacaagaagaagtggct

cgttacgaccatcgtcagctcgtacactcttctcagtccgctcagctcctccatggtcgcgccggccctgccaatcctggatcaggaattcggagtaa

caaacgagattcagtccgctctcatgctctcgaccttcgtgctcgcatatgctatcgggcctcttttattcggcggtctctctgagatgtatgggcgg

cttccggtccttcagattgccaacattctgctcatcatctttaccgtagccggtggtctggcccaaacctcagtccagatgaccgccttcaggttctt

cgctgggctgggtggttccgcaccactcaccataggcggaggcgtgatcgccgatatttatgcggtggaggagagaggaaagggaatgagcatctact

cgctcgccccattgcttggaccatgtataggtcccgtcgtggctggctggattatccaagccggtatttcgtggagatggatcttctgggttgtcctt

atcgcctctggcgttgtggcaggctttggtttcctgacactgccggaaacgtatgcgcccatcattcttgcgaaaaagcggcgacagctggtaaaaga

gactggtaatactcagctccgcactgtcttcgatgtacaacgcaaggagtcctttcccacgcgtctaagacggaacttcttccgacctttcatattca

tgaccactcagcccgcggttcaagcgctgtcgctgtacatgatggtcgtgtatggaacgctctatttgcttctcaccaacgtcgattcgaccttcagc

aggacatacaaccaagggcctggcatcgcctcactgcattacattgccctggggcttggcttcacggccggaggtcagattggcggacgtcttgtgga

caaagtctaccggatcttgaagcgcaagaataatggtgttgggcgaccagagttcaagctgcccttcatggttgtctcttcgccgctagtacctgcag

gcctgctactctacggatggccgattcagtatgcagttcactggatcgtacccgacatcggcttattcctctttggtgtgggcagtgagttgctggtt

ctccgagcgctggctcagacaatagcctatccctcagatgctttgctaaacaaccttcttatccaattccgtgccggtgcacgaacagtgatgggctc

gttcttatcaacccaaaacttccttgtcgacacgtatcagctttacgctgcctctgctgttggcgctgccgtcttcctgcggtctatcgctggatttg

cattgcctctggcagcaccggcgctctacggtacgctgggcagcggttggggcaactcgctgttggccctcattgctgcgctgatcggcatcccagct

ccagtggccctctacctctatgggccaattctacgcaagaagagcaaatacgccagcgaggcatcgtga

>contig_21.contig_21.g856.t1

atgccgaccgaagtcaccagggaagaactcgaagaactcaacgcgcagcgtatcaagcttggccttaagcccctcgagg

tcgaaaccgtcgattccaaaacggatggcggcgctgcagcttctgaagatccagatgccgtcgctgaggcaaattttgcgcgcgtcagagatgaggcg

cgcaaggagcgagaggacaaggctctagacgaacgtattgcaaaggcaaagaataagcgcgaattggcgcgcaagctggcagggaagggacttggcga

agccgctgacgatcggggaggagacagcaagcccgaatcggcattggcatggctcaaagcatccaagaaacgcgccaaggaaaacgcagctcgccgac

tccaacagcagatcgaagagcaagaggcccaagtccaagccgaatacgggcaagatgatctcgcaggtcttcgcattgggcacgactacgacgaattc

gaagaggaaggcgaggaacgtattctcacgctccgagatgcgcgcgtccttgaagatgtagaggatgaacttgtcgaccatgcccttgcgcagaggga

acaggatgcgttgaaggacgagcgcaggaagggagcgaagaagtacaccggcttggacgacgaggagtttggtgaaggcgtcggtccttcctcatctg

tcgtaggcaaaaagcgcggagtcttgtccaagtacgatggcgatcttggcgaagaggacccactgacgaaatctggtggggaagtaggctttcgtatt

ggagggccgaaaaggaacaaggaggagatcaaggcgcaggaagctgaggaggcggcacgattggcgaacaggaagctgttaaatctcgattatgctaa

aaacgtggaggtctctgattatttgaccgaaggagaagtcgggttcaagaaaagcaagggtaaaaagaagaagcgcacggcaacaagggtcatagtcg

atctggacgacgaagccgaacctgatcaagccgcggcagcgacaacgaatggcggcgacgcgatcatggctgaacaagagtctacgtcgatggcttca

ggatcccgacagcggttggttgagaccgacaatttcgttgacgatgacgatcttcaggccagtctcgccaaagcacggaggcaaaaagcaaagaagac

tcttaacaagctcacgcctgagcagatcgcagccaacctggcttctcaacgagccaccgaggacgaagagtctcgcaaggatgttgaggcgaagatgg

aagttgatgattctggggacggggaacagccactgacgttcgatagcaccactgaatttgtgcggaatctggctttcaaggcggctgcggaggagccg

aggcgacagatcaagtcggaaccgtcggacactattgcaaccctgcctcaagaaccgggaccgtccgaagtagttcgtagcagcgtcaaagtagagga

gcagcaagcggcccccccgtctgaggagccttccaagaaaacgcaagatgggcacgatgatgagatggacgtttcggaggacgaggacttcgagaacc

tggaagtctttgctgtgcccgccgaaggagctggcgagggcgagggtgaggatggtgcagtagatcaagcggagtcaagctctgagccgttggcgggt

ggcagtatggctgcgaccgtatccttgctgaggcagcagggtctcttgcaagagtacacgcccgagctcaaacagaaggaggccaagcaacgcgagta

cgatctgtggatggcggagaggcgtcggcgggaacgagtgcgcgaggaagagcttgctgcaagtaaagcgcgaggtagcgcggtcgaccaggctacaa

gggagtatgagaatcagcagagggatctagctgacgccagagaggcgcagaaacggtttgcgaattacaagccggacgttgagctcaagtaccacgat

cagtacggtcgggagttgagtcaaaagaaagcctggaaggttctcagtcatcgtttccacggcaaagctccaggtcacaagaagcaggaattggagct

gaggcgtctggagaacgaacggaagcgcgagaagatggagtcgggcgacactccgacgggtatgagcaaagctttccaagaccgatccgaacgcacag

gacaggcgcatatggttctcagcgtaggagctcgcggaaatgcgccccaggagctgtccatgctgggtccggatatcaccaagtcggcacgaccgcct

cctgcagccgctgtgggcaacaacggcacgagcaaagggaaggggaaagggaaagcgtccagtcgtcatcccgacatgtcatcctccatctccagcat

cgggttacaatctttcgtacctcgccatgttggcggtggtagcggattggcgagtgtcttgccatcgtcgaagcggaacgggagcacagggtctggca

cgccgttgcctccctcgggtgctgctccgtcagccccgcccccgccacctcaatcttcttcggacaccgccggcaacggaggagccatgttgccgcca

ccggccgttcgacctgtgatgaagcctgcctttgcacctatcaaacgagcggccgcggcctctacaccgatcggtcatggtacggatgcaggttctgc

tacagcagcggcgcctgctcctgctggggcggctgatggggcgggccggttcaaattgcagttgggctccaaacggaaggcggaggagcccgcaacgg

gtagtgagccgcaaggaaagcggcgtggttga

>contig_21.contig_21.g857.t1

atgctttcctccagcgctctcaacagccacgaaaccgtcgcgtcccacaccagtcagtttctcaggacgcgcatcagcc

agtggctggccacgtcgggtccaacagcagttgctgagagatggaaaggagcgccgactttcgcgctctctctcgtttgcgaggcgctcgtcaaggaa

gcaaagcgaggaagcaagatcccctatctcgttggcgcggcagcaggcaagacgacaatggcggaactggaggagttcgatccagagacttcggccga

cttgatccagcagaagctgccgaccattcactccgtgctctccgccatcataggtttggacgcagcgcagaagcaaaacgaggacgagcctgaagctt

cccaggaacaggaggacagcgaggacgatcaaccacgcgaggacgacacagctacgcctcacttccagctccacggtcaacgaggacgtccagcggtc

gtgtcctcgtgcgcgcatactttgctctttgcacgcaactctcggtgcaacagatggcccatgcagctcagcgtggatctggtcggtgcgcgcgttcc

tcgacgctcttgcgagttgctctcacgactggggttcatcggcttttggcggtcatcgcagcggttgctcgagaacatggccaaagacgatcacgcac

gggtaaagcgactagtacggcttccatcctcggcaatcagcctctcctacgacaacgtcaactggcaaagaggcgtcagagacaaacgcagcgagaag

aagcagcagatgatggctgcaatatgcggcatggcctacctcctggactacaaggcacagtacgacgagagccagccaatctgctgcgacgagctctt

caagtccatcttccatcccgacgtcccccgcccttcggcatcagagccttcgccgatgaacccactcgacgaacaagggagatctatcgccatgaagc

gatctctgtacgacgatgcccgtgcgaaggtcaagctcgacgacatcaagctctcgcaattttttcctggacgcgcagaggaagaacacttcttggag

gtcgtggtttcgcacgctatacgagcgtgggtcgacctacacgacgacagcggagtggacaccagcaagattcgccaaccaccacaagtactgccctt

cttgccgcagcgcactcagacaatgggcttgcctgttctcaacgaggatgaaggatcggtctccggcaacatcgccgtgatctacgcctacttgaagt

tcctcggcatcgatgacgacgctcgatgcggcactcacaatcttccagtggtcgccgacgcgttgacggttggccatgttcgatctgctttggctcgg

agagtgcatgacaagtcgaccaaccccaagatcgaccagcttcagttcctgcaacccatggcggccttcttccacttgcagtacaacttccagaagta

ctggatcgacgctcatgccggcacagctcagcaccaaagcaagatcagcgcgagagttctctgcgatcgcgtagggatgaagaatttgtgcacaggcg

gaaccgacttccacgacatggacgccttcctcaagatgctgttcgctgctctggtggacgcgaaggtcacgcctgttttacgtgcggcggggcacggt

ggtgaagaggacgacttcgcgaatctcagtgccgaggcccttcaagacacgatccggactgaactggcaccgttgctgaagaaaatgttccgggacac

cgctatctacctggagctccgagacgctgtcaagacgggagatccgggtcgcgtgatggctgcatccaagttcaccgtaccacggttcgcggccaccg

gacatcaccgatacagcagcgaggtactggacatgcttgttcaagtacgctttgagcttccaccggctctcgtgacgacaattctctgcgccacctta

gtcaaccacgccgggaagaaggacagctggtttgcggccgatctcgacattgagcaccaagtcaacgagctcaagaacatcttcaaggtgacgggtgg

cagcgacgcgaagtcgatcgacattctggcgagagacttcgtcaacctcaaggcgttcgattggatcccgcggcgatcaagcaagtgcttcgagctgt

accgaggcgacgaggtgcggcaacgaaagaaggacggggagcgctttgtcaagcagtcgaagggcgttgcgaccgacttccacatctacggcttcgca

agactgtcaggaacggaatcactctccttcaagaactggctggcccgacgtgcgcgtgatgctggtaaggggagagacgctgaagtactgtctctgga

tggagttgacgatgtcgaagccccgcccactacgatcacctatgacgcggagaccgtgatggacgagttcatctctttgattgatgacgaattctga

>contig_21.contig_21.g858.t1

atggagccaactcagccatccgacgcacgaacgtcaacacaggctggtttcccagacaaacggcccacaccggacaagc

aggcgcggtcggaaaagcagccactgcgatgggcagaaattgtcaaggagcttccttattcacccaagcaataccagatcgatgccgtagaggctact

gccagtggaaaggatgcgctagtcatcgccccgactggctctggaaaggaggaactcaaagatcggattggtagcgaagcagtctatgtacatgcggg

caaccgcactccggagctcctcaaggatatcaggaagggaaagtaccggatgatcttcctcagccccgagatggccatcggcaactcgttccgggaag

tcttgatggacaaggtcttcgaggcctccctcggcgccatcatcttcgacgaggctcaagtcctcaaggattgggctgtcgactcacagttccgccgt

gggttcagggaaatcagcgttctccgtcatatggctcgcgttccagggatggccatgagcggcacactcccggcagcatacagggaggaagtccgacg

ccatttcgagctccagaacgaggtggttatcgatgtcggaacggaccgtccgaacatcgagctcgtagtccagccgatccagcacagtttggtttcct

acctcgatgtccttgcctgcctgtccgagcttcaccaggatctgcccgagaccgaggagcagcgtgctgcgctaaaggcgaaatgtaaaccgaccatc

gtctatatcgacaacaaaactcagcagctcgatctgttccacaaggttaggtcgtggtactcacgtttcgggctcgaggatgcagtgctgcttgtctc

cgccgacatgagcgccagccatcgcagccaagtaaaggtcctggtcgagaaaggagaggtcctgtgcatcatcagtacaagcgtgctcggtatgggct

cggatatgcgcaagatcctcagactgggacgggccggtcgagatcctgcgaccaaagccgaagcattcatcctggccgacaagtggtccatcccccgt

gtcacgcaagaggcactcgaccgcgacgggcagctccccactgagcctgttccagaaggagaagaggaaggtggtattgctgttcggtcggaggtgac

caaggcgagggagggggcggacgccgacctgctgctcgtggtgcagaccgccttgctgcgccgtggatgtattcgtcgcctggtaaatgatctccttg

gtcagccttcgcccgaccagctccctgcacctcaggacatctttggaccgacccttgccaaggacttaccgtgctgtgtggtctgctcgaaacgggaa

atcggccaggtaccggacgcgtatcttcctacgacgacagaggaaggcagggctcgggacggaatcaccaagctgcaggagatggtggagcccaagct

tcgtcagtggaggttgtcaatgtggaaggaggagtggagcaagctcgacgcaccttcttggctcggggtttccttctacatgcgagtcgaagacattg

ccgacgtggtgcggaacctcggtaagatcgaccgaggcttgactgcggaggaagacggttttgtcgagcggttcgtcgccatggaggcaaagtccctt

gtcgttccacagctgacggccaagctgaaggagatcatcgccgagtttgatgccgacaaggagaaggctcggcaggaaaagcaagctgccgcggacca

ggctcgacgagagcgagccaaagacaaagccagcaagcatgctgcaaggaaaagtgcggctcagacttcacgcgccgacaatgctggagaggggagca

gtcagtcagtcaggtacacaccagctaggcaagcttacacgtgcggggcttgcaaaaagtggaatcaggagaatcctgacaaggaacctctggatgcg

gtgggacatacagccaagtacaagaactgccccgcaaagatcggaatagcttcaacgagccacgctccggctgcatccagtagtaccagcaacgcgaa

tgagaaatga

>contig_21.contig_21.g859.t1

atgagctctcaccaacccaagctctttactcgtcaagagcttcccatcttgatccccaactatcaccgatggatggcca

aggagcatcccgactatgtgctcaagaaggatgctcttttctcgcgagaacagatgaacagtttcgaattcgacgacgattctcaatggcgcactgtg

acatactcgaaattcgcagagctcattaatgccgcgacattcctactcggaaaggacgacttcgcgggatggccgctcaggactgctaaagatccgat

cgacacaacgccacccaaggttgcagctttcctaggaacggatattgcttcggtcgcgacgctcgaaggcctgatccagcgtggtctctcaaccctga

ctttgtctccgagaaacacgcctgaagttctgatcgcaatgatcgtcgatcaaggcatcgagcacatcgtctacgcaccggcgttcgccgcggtcact

gccacggtcgctgccaagtttgctgagcaacactcttcccgccgcttgtccaccgccgaacttccgtctttcgaaaagctgggcgcttatggtgccga

aactcccttcccgtacgacttggacactaccgccgagtaccaaaagacggtcctttacgtccacacgtcggggtcttcggggaacatgcccactttgt

gcccatactctcacgaaatgctcctcaacatcccgcaatcgtaccagctggagcatcttgtcgggcagtcgcattacggtcccctcccactgttccac

gtcatggcgcacttctttggcatctactacgcgagtgctttccttcggaccatagtcactcagccccccgggccaccgctgacgggttccgaactcgc

agcgttcattatcaagacgaagccgggtagttgcctcctcgttccgcagcagatcgacaccctcactgaagttcccgagggcctgaaagccctcgctt

cggtcgacaccgttctcttcggcggcgcttcccttcggcgatcgacgaaggtaaagctcgatgaagccaaagtcaactacatgtccggatacgggcta

tcagaagtcacggcttgtgccatgtcattcaccaaaatccgaccgtcagggctcgatggcgagtggctaaaggccttggatggcggcgtcaatctgga

gtttgacctttacagcgaggcttccggagccgtcccagcactgtacaccctcatcgcacggaacggcgccctttcatgctgcgtaaccaacatcgaag

gcgggtacgataccggcgatctcgtggagcgacaccccgactaccctgaatggttcaagatctacggccgcaagagttcctttgtggttctttccaat

ggagagaacgctcccgttccgcccatcgaggacgccgtgtcgtcgcacgagtcggtcggcaccgcactattgttcggcaaaggtcagcctcaagtcgg

acttctggtcgagctcaagccggcgtacgttgtccagcctggcgatcatgcggccgcggaggctgcgcgcgacaatctttggccgaccatcgagaaag

tcaacaaaaacctgccagacttcgccaagctctacaagaacatgatcatcttcaccacaccggatcgtccggtgccgatggcggacaagggctcgccc

aagcgtcaggtggccctcaacatctacgacgatgacattgaagctctatatgcatcagtcaccgctgggacagactccgctgtcaacctgggatcgat

tgaagaacccgctctgctcgacatggtaaagagtgttcttgcctccgtctacggcactgagacacctatcaagccggacacctctctcacactcgagc

ttggccaggactcgctgcgcgccaccatgactaggacctcgatcgtgtcctccttgcgtgccgctgctaagtccggtacgatttcccagctggctgct

ttcgatcccgacacgttgccggcaactcttacttatcagtattcgacgccagctgagctagcaaccttcttgcacgaagctatcactggaaatggcaa

aagctcgggcgatctgacagcttctcgaacggcaatgatgaaggagctcgtcgagaagtacagccagcagatcattgcgtcccgccgcagcacagacc

acagtacgaacaaggaaggcgagccgctcaagcccggtctcctcgacaggattttcaaacataacagctcgaagccccgggcgcatactgtgctgctg

accggcagctctggagctttcgggaccacacttctcgagcagctcgtcaatcactccggtgtcgagaaggtcatttgcctgcttcgctcggagaaccc

tgatcgaagtgctctacttgctcgtcaggtacaggcgtacaccagccggaacctcgaccctgcccctgcacactcgccaaaggtggaatacctccatg

gcacccctaccgacccgaacctagtcattcctgaggtcacaagcatcatccatgcggcttggtcagtcaacttcaacctctccctgaccgactttacc

cccgagatcgaaggcgtggttcggctgcttcagcactgccaaaggactggcgctcgtctggtctttgcgagctcagtcagcactgtcatggcagcgcc

acaggatgccggcaaagaatcgaccattcctggaatccggctcattgctgaggaccttgaagtcccgcttgagtggacgtcaattgactacggaagaa

gcaaggccgtggttgagaagctgatcgcaaaagcggttcatgaaggcggcgttgaagcagtttccgtccgatgtggccagatcattggagatgctcgt

actggtgcctggaacaaagcagagtgggcgcccaccgtgttccgttccgccgaggccctcggcgtgctcccggacaacctcggcatcgtagactggat

cacagtcaacgacacgatgaaggtccttgtcgccgcagcagttgaacctgatgtggagagcgcgacgaccgcctcgcgcaccaacgtcgtcaacctcg

tcaatcctcagcgcatgccctggagccaggttgtcgaagcgttccgcacgcacctgcccgcccacgtcaaggtcgtccccagcacggaatggctggcc

catgtggaagaggccagtcggaagctgagcacgtcgaaggacccgaaggcggacctggctcggatccctgctgtcaacttggtgacgacgtatcaggg

cttggtggcgtccaggcatcaagtgctcctcgaggtgaaggcggccaaggacctgacgaagggtgagattgattcggccgtcgtggtggacgaagacc

tggccgggcgctacgtccgcttctggcaggagcaagcctcttcttcctga

>contig_21.contig_21.g860.t1

atgaggttgtgcagcatatcaaaactgttcccatcactattctcaccggctgcagatgcctccttgcgctgcgtcctcc

ccgtctactgcacaaccccagtagtggcgcttcagattgaagcttcggtgccacccatcgagcttgcactcgatcgggccgcgcgccggtttgccaag

cgcctgtggactctctccgacgaccaccctctccttctgcggctggcgggactgcctggacaaccaagccccatcgaccagacgctgctgcccccgga

gcacctcgcaacatcgccgttcttccgcccacccactggtccgaccctctcactggacgactcgccagcagcagaggcggccgcgcgctcttacacca

ctgtcctggtcaaccacgcctcgagggtggacaggccgttagagcgcgctgtctcagtcgcctgggcgccgtggagcacgcccatcaatcagcacccg

cgtgttcaagtcatgcttgggccccgggatcaggcggtgagtgcgcacgagcagctgctgcagcgagccgctcgcgatggggacatggtagcatacag

tgatgcttcattgatgggcatggacagaggtgtgggagtcagtgtgaggcatgggctccgactttgtgtggaaacatattgggcggtcaccgctgagg

ctgaggtttttgacttggaggcttggggattgtttgcggcgttctgtcatgctgtggcggcccaacgtcctttcagtactcacattgtcatcttcaca

gataacctctcggtcgcgcgatcagcactggcccagccacgcggctcgagccaaaggaccttcctgcggctgcagcaacgtgtgcggcactggctcgc

cgttgacgcccggcaccgccttaccatcgcctgggtccccggtcactcggaaattgagggtaacgagcgtgcggaccagctggcaagagctggcgcag

agcgcaaggcggtggacggacgcgcgcctgatgaggcagtgtgggaagagcgcaccttcgcctctttccagcgttgggaccggcagcagctccacgag

gtttggtcattgcggtgggacctcatcaagggcgagcaccaacactacgcgcaatacaggagcaccccctactctgcgagacctacggccccctccag

cacctactccacgacgacaacggtcgaatctctctcccgtggctactcgactctcggcagggctctctcgcctttgcacggtatgctcaggcatctaa

tgccttcaactgcaaccaccagcctcctgtcctccagcaggcatagttag

>contig_21.contig_21.g861.t1

atgagctccctcgtcgcaacccttccatgctccgggacggactccgctgtcaacctgggctcaactctgctcgacatgg

tgcagagtgttctcgcgtccgtctacggcactgagacacctatcaagccggacacctctctcacactcgagctcggccaggacttcctgcgtgcaacc

atgattaggacctcgatcgtgacctcgttgcgtgccgccgctgaatccggtgcgatcccccagacggctgctttcgatcccgaaaagttgccggcact

tctcacttatcagtactcgacgccagccgagctagcaaacttcttgcacgaatctatcactggaaagggcaaaagcttgcgcgatctggcagcctctc

aggcggcattgatgacggagctcgtcgagaagtacagccagcagatcattgcgtcccaccgcagcacagaccgcagtgcggacaaggaaggcgagctg

ctcaagcccggtctcctcaacaggatcttcaaacgcaaacgcagcagcagctcgaagccccgaacacatactgtgttgctcaccggtagctctggagc

tttcgggaccacacttctcgagcagctcgtcaatcactccggtgtcgagaaggtcatttgcctggttcgctcggagaaccctgatcgaagtgctctgc

ttgctcgtcaggtacatgcgtacaccagcaggaacctcgaccctgcccctgcacactcgccaaaggtggagtacctccatggcacccctaccgacccg

aacctagtcattcctgagacggtcacaagcatcatccatgcggcttggtcagtcaacttcaacctctccctgaccgactttacccccgagatcgaagg

cgcggttcggctgcttcagcactgccaaaggactggcgctcgtctggtctttgcaagctcagtcaccactgtcatgggagcgccaccgagtgccggca

aagaatcgaccattcctggaatccggctcattggcgaggacattgaagtaccgtccgagtgggcatacctcggctacgggagaagcaaggccgtggtt

gagaagctgatcgcaaaagcggttcatgaaggcggcgttgaagcagtttccgtccgatgtggccagatcattggagatgctcgtactggtgcctggaa

caaagcagagtgggcgcccaccgtgttccgttccgccgaggccctcggcgtgctcccggacaacctcggcatcgtagactggatcacagtcaacgaca

cgatgaaggtccttgtcgccgcagcagttgaacctgatgtggagagcgcgacgaccgcctcgcgcaccaacgtcgtcaacctcgtcaatcctcagcgc

atgccctggagccaggttgtcgaagcgttccgcacgcacctgcccgcccacgtcaaggtcgtccccagcacggagtggctggctcacattgaagaggc

cagtcggaagctgagcacgtcgaaggacccgaaggcggacctggctcggatccctgctatcaaattgctgtcgacgtaccagggcttggtggcgtcca

ggcaccaagtgctcctcgaggtgaacgcggccaagacgctgacgagggacgagattgattcggctgtcgtggtgaacgaagacctggccggccgatac

gttcgtttctggcagaagcaagtctctcctccccgccagcagcagcagcggacccatcgcccggccagaagtcccgccgtttcgacctcgatcgactt

caaactcgtcgacacgttggtctacggcttccgagcatcgaattcggacggtctcacaagcgttacgtcctttgatcaggagaaatccccctttgtac

ggagcaccctccccgccctccgtcatgttcccacctcacgtcggggtagcgagccccttttccagtctttgccacttgtagtatag

>contig_21.contig_21.g862.t1

atgggggcagagcgaaggcctcagaattggggaggaatgtggactcttttcttgttttttcttgctatctggaggttcg

gtggtggtggtggtggtggtggtggtggtgatgatggtggtttcgactcatctccatcacctcgaagctcgaacggacgaagatctcaaatacataca

ctgaccggaccgaacagaaggcatctgaccatgaacgtgctcgtgtacgacggcccaggcgtctcccactccgccctccaacacaccctccgctccct

ccgtcgtctcctagcagcatacgacgtccagaccatcaacgcaaggcaaatcgccctcgaaccatggcaatcttacaccgccctcttcgtcctccccg

gcggacgcgacctgccatacctgtcctcgctcgcggaacgcttccaacttcaatcccaatcccagtccggtacggccgctgaggagattgcgagattc

gtacatgcgggaggcaagttcctgggcatatgcgcgggggcgtacttcgcgtcgtcgcgttgcgtcttcgaaccgggtacgccgatggaggtcatcgg

tgatcggccggcatttcgattcttcccggccgaatgcgtggggacggtctaccctggcttcgtgtacgagtccgatgcgggagcccgtctcatcgatc

ttgagctcgagctcggcgaagatccgaaaaatcgggatatgttcgccagtatggactctttcaagtgtcaatataacggcggtggggcgttcctacag

gccgatttgtacaaatcgaaaggagtcgacgtcctcgctcgatactcacactccgaccccgtcctcactcaagcctcctcctcctcctctgatcaaaa

acccaaatttgcaggccaagctgcagccgtgctctgtcaagtcggtgccgggaaagcgcttctgtacggcgtccatcccgaattcccattgtcgacaa

tgtctgtgtacttttccgaaagtgtacagcatgatggaacatcaccgagcaaggaggatgcttcgatcatgtcggacctcgaacgcgctgaggtagtc

cgactcgccgtctttggtcgacatttggccctgctcggcctcaaagtggacgctcctcgaagtctggacagcgatcgaccagcgtctgtgcagcagcc

tccagagtcccccatcatgccaccctcgatcacagaccccccacgactgacgccacttctgctagcctcgcccgatctccccaacttgcttgccaagt

tacagcgagcctcgacttcgaccaagggcccgacggtcaaacacttgtcttcggtcgcggctctcttggcgaaggatcaacttggccaaccggctttt

accatcgtcgacggtaatgacaccttacacatcctttccgcccacacgattcgctccggcaccacgcgcgaggacgggagcccgtcggacgcagatgc

actgtggctggcgactttacaggccacatacgacgattacgtcccacacggacccccttcctcctcatcctcagcagcagcagcagctggaacggcga

cagaaagcgtcgaagccgaagccgaacccgacctgcactccgtcccgaaatacatcgtccccatcccagccgaagctccagacgcgtccacacaagag

cagatccggcacctggcgccccatttcgacgtatttgcgtacctcgctgcgctccgccgcggacgggagcgcatcacttccctgctccaacatcagca

gcagcagcagcagcagtcttcgttgctcgctggcattcaggcgggtcctgcgcgctggggcaggggaggcgcgcggttgggggatgaggttatgtatg

ggcaggtcgtgactagcacgcagaccatgcttgacaaaaacttcaaactcctccgcaccgtacccaccggcttcaccagcatcgcaacccatcaagtc

tccggccgcggtcgcggcaagaacgcctggatctcgcccctgggctgtttccagttctccctcgtcctccgacttcccgtctctgcggcgccctacat

cgtcttcgtgcagtaccttgccgcactcgctgtcgtccttgcgcttcggagtggggttgtcttgcctgccggttcatcaggggagggggaggaggagg

aggtgaggaaggaggacgagcaggggttggaggcgtcgatggcggcgttggggctgggcgagaggaaggggacgttcgtgaaggacgggaagcggtat

gccaagctgggcggaatcttggtcaatagccaatttgcagataacgagttcatgctcgtcgttggctgtggtgtcaatgtcctgaaccgtcgcccgac

gacctcgatctcggacctgatcttagcagccaacagcaccgccgtcgagcgcaacgagcccaccttgcccatcctgagccaggaagacttcggcggcg

gcgtcctggccacctttgagcggctgtggagcgtcttcgagcaggcgggtagtttccagcccttcttggaggcgtacagggaggtgtggctacactcg

aaccaactggcaaccctcgccgacaccgaaccgcccacgcccatccggatcgtcggcatctcgtccgactacggcttcctccgctctgttcatgccag

ctcgacgctaacgacggcgagtgcgggagcgtggggagactcggactcgtcgggagccggagccaaggcggagcgggagaagggcaggtggatcgatc

ttcagccggatgggaacagctttgatatgctccacaacctcattcggcggaaggactga

>contig_21.contig_21.g863.t1

atgtcagcagtccctgaaccgggacgaccccgaatccccgcaggtcccgggcgaaatcctcaagcgactcgtgagcaga

aacacccttcccctcctccgctcccagcagtacgtcgttctaactcctttgtcacacgtaatccagcccacagtccaggatccgacaacgcgtctgtg

ctgggaagagctccaccgaatggtcagccgctctggtacgcggggcgcccgcacgaactgttgtgtcaacaggtgagagacctgggacggatgggcgt

ggaactctggccaggctcggactcttttcctggcgctttaccggacgatcagatgccgtacaactctctctggcggggtaaacatcgattcttcaact

ccgacggcagtaccacttcaagctctttggcagaacgtcttgccttgcacaccttcaattatgcgggactcatccgctttctgggattcgtgctggtt

ctggatccggaatccggatcggcgaccaactatggatcaggtcttgaatga

>contig_21.contig_21.g864.t1

atgatcggagacttggggattttaaacagcggtaaggcctcgcggctgaccgaagagcaactcgccagtattggatgcg

tcgagtgtcgcgctggtggacactactacgggcgtggcgcggatggtctccccaacgatcataactgcagctgctcgacctgcggtgccgacttgggt

cgcggccgtcctcttcctcaccagtgttcctccgcatctgcacctatctctcaagacgccatggacgaggcagacgtagcggctttactcggcggcag

tgcacctccttctaccgcagttccgatcccgtccactatctcctcggccccggttgacagcttgtcggatgaggatgcgctcaaggagttcactttgc

tgctcgatatggcgaaaacgacactctcaaccggcctggtcaaagattggagagccttgtgcaagaaactcgctcaggcctacatcctcaacccgtgc

aaccgcaccttgttggacgttctcggcgcccccaagattgcgcttgctcggctccgatcccctcggcctcagcacaagattggcgcccgtattcacag

ctatccggatacaccccgccctccgcctcgaacccgctcgaagggtcctagttccgaccgccaagttcctgaacttctccgcaataagcaccccaagg

gccctgcggatgcgttcgcggcggaaggtcggaatccgcgtgaccgcgaagccgccttcgtcgagtttgttcagctcctctgtcgccagatggcgttg

gggaatgctcccggacaatctctacttggtgcggctgctcttactcctctcaacaaacaagggggtggagtacggcccattgcagccggcgaactctt

gtaccgcctctgcgccaaggtcctgttgaagtcgcttcctccggctaatgcgttgctcccctcgcagctcggtgtgggctctcccggcggtgtggaac

cgattgtgcgcctcctggagcgatttgtggacgaggtagagggagaaggtgaatttgaatttctcattttgctcgacttctccaatgctttcaacgcg

ttgtgtcgagctgcgatgagtaaagccattctcaagcattgcaacaatctccaccgcttcgccaaatggcgctacaacagcccctgtcccctcttctt

caacggttgcgccccggatcaacctctcctctcctcccaaggcctcccccagggtgatccgttcggtcccctgttcttctcggtcacggtgagggacc

tcgcggcggagctgcaagagttcctaggtgaagatgcgctgctcgtttgctacttcgacgactggaccatcctggccaaacgccccggagttctcgaa

cgagtcaaagctttgtttgcagccaaggaggcggaaggtcagacttgtgggcttgcgctgagtagcggcaagtgcaaggagatgagcagggaagaggt

acgcacagaaggagtacaggtgcttgggacgatgattgggggtgtggcgccgcgggcgcggtttctcgcggagaagatccgagaagaagtcgaggttc

tcggtcgtttgcggtcgttgggacgtcaggatgcgctgctccttcttacaaaatcaattcagcacaccctgcgtcatctcctccggacgctcagtacc

gacggcatcgattctcattggaacgaactggacaacgcgctgcacgaggcgctggtgttccttcgcgggtccactgcacacaacaaagaggtcgatcg

aatcttgttctcgctgccggcgcgtctcgggggctgcgcggtccctcagtttgcggatatcgctccccacgccagggactctatgcgcatggaatcgg

atcacaccctggctatccgccagttgctccgtccctacacgtcgcccgcgtttcctaggccttcctccttggatgatgacgcagaagaagagcggggt

cctcggcagcgggaccggctcaagcaggtctggacggggatgctcgtcagcctcgcagtcagctcggatgattatcctcccctccttcccatcgacca

actcatctcggtgctcggccaccgctcgcctcttggccgccgctggctctcagtcttccctttcgacatcaacacagcattatctgatcgggaaattc

aagtgggcctgcagccctgcagcttcgtaccttgcagccgatgcacaatctttgctgcttggattgcggactgcgagccccgcatggccacgaccaag

gctcggcaactatcttcactatcaccgctccaccacatcgtcgcaccgaagaacgtgatggtgtcctaccgcgagctgtccaaggagccatactgtgc

gctctcggctcagaacatcaaagttattcgcgcgtacggacagaaacaatgctggcctgatcaatcctacgatttcgacgacatccaagcccgactgt

tctcgagaccagattcccccgcctcgcggtgtattgcctcactatgggccgcctaccgcgatttcgaccttctccagctcccggtggacgtcagaggg

gctggtccgtacggaacgatgggagaagaatgtctggcgcaggccgcatgtcaagaattcctcaccctcgacagtacggatcccgaacgcgctcggcg

tcttgctcttctcgccgttacggagctagctgagcttctcctatcgtcggacaccggatttcctttggccaccgtccgacggtcgattttcgatggta

tcctgtacggacgagtcatgtttcgcatacttcccttagccgtgctctcccgccgcgactcgccaagacccaactccttttacgacatcatggatcac

gacgcagatccatccgatccggtttccccttcgcaaccggattgcggttacgatttcgacaactcttccgcgccagacgtgttcctaaccccgcctgc

ccccccgaccgtctccccctccgtccgtcgttcctgcctccccgaagccgctggtccgactcgttccgaccaacctcccaaggcaacgttcgaggctg

gcccgtcgaatgcggctgcgacggcaaccccggagattgcggacgacttaactatagtcccaaccctggccaccgcggcgggctgcgatgcatcctcg

gaacgggcgaacatcgacacgtccaccgacctcgttgcgcttcggaagcgggaggcctggatgaaggtcgcgttagccgccgcttttcgtaaagggta

tgttctagctgaagaactttgggatccttccgaaggaggggtgggtcggattatcttccctccgtcatcgacggagctcgctgctatccacgaattga

gtggtcagatggacgtgatggatgcagacacgaccgaggcggcgattggcgagcctggggttaccgagtccacaggttccgacggggcgactctccgc

gtgatggtcgaagttggcgtttctcctccctcgcggatgatagggtccccacctcactgggtcgcgcagccgtcttggcttcccatcagcatctgctc

ccccgctgtggccaataactacggcccactttatcaacccttcgacccaccgccctactaccaaagcttcggtcgatacgagcagggcgatcgcatcg

gcgccggcgtacatgggcaagtgtacagatgccgggatcgcctcactcaacaggttgtcgctgtcaagtggtcgaaatgcgaggaagccagacggcct

ccgaattcgacgctacgggagatcagcgcgctgaaacgactgcaacacaagagcatcatgtacttgcatgcggtagtgtacgacggcatcggaatcgg

tctcgtcttcggattggctcagcaagatctacgggcggtcatcaatctgcgtggccgcggatctctcaacgtccctctcatcaagctgtatatgcgcc

agctcctggaggggttggcatacctccacgaacaaaagtacgtgcatctggacatcaaaccagagaacatccttttgaatgccgatcatcgcctgagg

atcacggactttggtctggcccgccgggcgggtccagcaggtggtcagaggtccgttgtcacgatgtcgtatcgtcctcctgaggtgttctttcgtac

acccaccttcacctcggccgttgacgtattctcggcaggcgttgtactggcagaaattttgggttcagtaccatggagccagtccgcgtggcaacctc

tgccttgcttccaacacatgctggaatacctagggtgtggacctggagaggtatggcccggcgcggataacaaccccggtgtctggcatggcgacgat

gtcgtgggagacgagccgatgctcctcgccaagaaaggccggatacggttccaaccacgtcctcggcgtatcccgcaagacctgcggtgtgtcttcga

ccatgctggagaccttctcaagctcgttgataagtgcctagaagtcgatccagagcttaggccgcgagcgaagcatctgcttcgggcccatcctgcat

tccagcgggaccctaaaccggccgttttcggtttcgccttgaacagtccgcgcaccgcatcgatggctggacgcgttccacgagatcaacgagtcgtc

tgcgttgttcgcaacgaagggggctggacggacaccctcacgcaagggggccgggtttattcgcgagtggaaggaggagaactcggtgtggtcctatc

acaaggtcgacttcgtctggaggtaccttccgacgacgaggatgctgcgggtactcataccgttagcgcaaccggtccgccgagcttgttgagcttgt

ctcttccccccgtcgtcaaccctggcgcgacagcgatcacaggacccgaagaagtgcgttctaacacaggcaaccttaccacgccatacagcggtgaa

ggaggagcagttggaagcgtaccgaacgactgctcggacgaagaggacgtcgccgcacctcctcctgggcagtcatccactacgaccaccgttcccag

cctggacctacctctgcacgccgcccacctcacccttcaagatgcgcggagcccagcggcgcgacgggcgctcaagggtacagccactggtcgaatga

cgcagtggcaccttgaagacgtggtcgtctacgaccccgatacacctccagcggggatcaagaggacgaaaggcagcaaacgggcaaagttggacaag

gacggaaaacagatcaaggagatttggcgcgctcatatcaatgcctcggccatcggttccaccccttccatccttcgtcagacgtcaatcacgtcaga

caaggggcgcattcaagttcaacacaatcaacagagcatggcactttcaaacggtgagaagcggtag

>contig_21.contig_21.g865.t1

atgcctgagggcagtcgtcgccgcatggtcccatctgtccatgcccgtgggaagaagctgacgctcagtgcggtgcgca

atcgcctgggcgactttggaggtggtggagatgaaggtgaggtcgaactcggtaaggaccccacgaggagcgcccggccccgagacgcgcagatctgt

gcgatggttgtccccaatcaaatgaggttcccgcaccaccatcgtcccaggcacccgatccaagccctgctcaacgaaatatcccacagcattatgac

gcttgacacagtccacaagctgccacagcctgccactattccatatcccccctcaccgcaactctccactctatcgcccagcccaaggctggcatccg

acatgacgagcaacgagcgggccttgcccaagttcgtaacccaggccgtcgcgcaacgcgaggccgccgccgaacaagaccagccagacaatgccgcc

gcctccgccgcgatcgcgccagccgagcaagaccggcggtgcttcgacatcgcctgctcttacctattcggcgatctcacgttaatgcaggccggcga

acaactcgacgccctctctctcggacctctgcgccgcaagaactttggcgcattcttaagcggactcgacgaggccaagtccaccgacggcttgacga

ccacgtcagattgctggaccattatgcccgcgtctgccacgcaagaaggggacaagctatgggccgccttccaggcggatcccactgtcggcaacagt

cggcagctgctcaagggttaccaggcgggcgagtcggcactcggcaagcgtcggcgtcgggcgcccagcggcctttccgactatgcgaccgacggcga

cggagaggggcgatcatcaggccggcagggaacggcagcatctgggagcgccacggccatccggcaagcgaagcagcctcgctatgtggctgcgccga

agggcgcccccatcaccggctcagacggcaacgagaccgagcgccccattcctcctgggcgctcgaaggggaagggcaaggcggtcccacccaaggct

cccccgaagaaaactcagtcctcgaccagcaagaccaccaaccctttgctgtccgacaagcgccgcgcccttacggcgtcggagaaacgggtcatgac

aaaggccaggtcggggaagccagggtccgactcaggttcccacgggggcatgacctccgaagaagaggaggagctgcgcggcccgtcggctcgcacac

gcacggccggcgagaccgcatcaacccagtccggcgaggtcgatcaacaggacaagggttcggagggtgatgacgaggatgacgatctccccgagatg

gagaatcggacatccacgcggttcttcaagttcgtccgggcgaccaagaagcccaagactcgccggaccggcgagaagtacaaggtcaacggctcgtt

ctgcagccggctgttcgaccccattgacaagtccttcgccgggactttccagcgcacgaatcaagacgccggcaaggacaaactcgcgtcgcccgcca

tgtcatctttctcgactccatcggttcggccgggacggggtcagcaaggactcgacggttggatgagctcacagaaagctcaggcgacggaggccaag

atcaccgaagtacgccgactggtactcaagtgggttatcgcgtcgaaccagccgttcacagagcctcagaacccccacttcagggagatcatccgagc

aatcaacccttccctctctccggcactcacctcggcccggacggttcgtcgcgacctcgaagccgcccacggagagatcctccgcgaagcggttgatc

atttaaagcggaatcagctcactttcagtgtctcccacgacggctggacgtcgccgtctcggcgatacacgttcctcgccttcgtcatcaactacgtt

gacaccgactggagtttccagcagtttatcctcagcttcaccgtcttacgcggtccacacaccggagctgctctcgctgggcaccttatccgcgctct

ttcagagcacaacctgctcgatctgtggtccggcattctcgtcggcgacgccgcctcggcgaacaggcggatgtgcgacgctctcgaatacgaattcg

ccaaggatagagacgaccctcagaaggcggttgagcatcgacgcggggaccacaacatcttttgctttaaccatgtcctcaatcgaggcatggtcgac

ttctatagcggaatgggcctcaaagccgatgccaatgatggtgttcttcgtgcgtccgcggataacccagaggagtccaaggagtccgttgccgacga

tcccgtcgatgactcggataacgagccgctgtcggacgacgaaggtgccgctgagaccgaaatcacctctgcgggcaacggaatcggaggtgccgaag

gagagaagggaggggctggagacgatgctgcggttgttcaggcgggaggggttcgtgaaggcgaactggagccagacgatccggaagcggaggaggcc

cttgtgatgtttgcggacgttcgctgcaagttagcgacggtggttgaggaagacgaagaagacggagggcctggagagggcggtaacgaaggtaatga

tgatgccgaacagggggataaaggcgagggcaccaaatcgggagcggccgacgtcgagggcgaggacgacgaagatttggccaatcctctgaacaaga

tcgccagcgtcgtcacccagatctacagctcgtccgattcgctgcgagagtttaagaagtggatgtccatggcctaccgcgatcatgacaaccccagg

ctggtcgacaaggttcctcccaagctgaatcaaacgagatggaacagccgttacaagcaggtgcaggccgcactcaagatccgagaaggcctgttgct

ctacagcaagaactccactcgccccaaggtcaccgcgatcggcctgacggagcgcgacttcgatattgctcaacaaatccgctggtatcgtcaactcg

ttcacatcctctcggtcgagatcgaggaggctggcagcaagaacggcgacgttgctgcggaactgaagactgggttgatgcagatgcggaccaagatc

aaacgttaccaggaggcggccgctcagaacaagacgctgcttctcgctggcgtcctccacccacgataccgcctcgaccctttcgaagccgactaccc

ggacaaagtggaacgcgtcaagacattgctcaatgaagaggtgcgagcactctctgacagcccatctggtccgtccgagcccgccgttccaggttcct

ccaagcataagtctgttcttccgaaaaagtggaggcgcgagctgccgaatgccagctcagcggcgaccatccgggccgagcagacggaggtagacgcc

taccttgcgaatcggaacccttatcaactagccgagaaggggtcaaaggtccctggtgatacggtgctgggttggtggaaatcacacgaggctcttta

cccaactctcgccaagcttgctcgtagggtctctctgcacccggatcgacttccgaggttgagcgcgtcttctcgcgtgctggacgttacgtgtcagg

acgccgtccgctgggtccgacagctttggaacaactcgtcgtcaccagtcagcttcttgccagaggcttcaccgcctccattcatgccaagcgcgtga

>contig_21.contig_21.g866.t1

atgattttcctcttatataacctcgccccagctgacattggtcttcccacctccctctcctcaccccacacccccccct

tcgctcccccaccgaccttctactttgccttctctaccatggctactccgccccccttctttttcccgcctcctctcttggacgccaccgccggcttc

accccttcccaggccatctcggtcggcggcacccaacctcagggtgatgagtttcctcgttctcctacgtcttcatctgcacctcttgcgggcacagc

tcaccccgttctcgacgatgacctcgccgatggcagctctggtagcttgccgagccttgctcgcaccactgccgctactcttgaggacgatctcatcg

actacgacgaaacaatgcaccccggtcacccgacaacgaaagtacctctccttgcgagccccgccccaacacaccggagcaactccacgacctcgtca

tcacacctccttggcggaacacaggatagcatcatggatgaggatcaagacttcatcgctccagacacccagcccgtcgtccctaactctcagctccc

cgacgaggctgccaccctctcccaggaagtcgctggccttagtcctcccagctcctcacccgtggatcgatacctcaaccgccccgccgaaggtacct

acgctgaagtcgacgatgacgacgaagattacacggacgacgagcaagacgatgcgacttcttcctcctctgctgagcccgaggccattctccctccc

gctgccgtccctcaagtctctccttccagcgtcaacggcctggaagctatatccttctaccgctcgcgcaggggacgacaccccctctgcgacccttt

caccattcgcgccgccaaggctcggacagttacttacagttgtccattttggtgggcacagttatacagtcgccgggccttgcgcgccgccgccgggg

cctacggaggaatccctccgggcgcctgtcggtatttcctgcctacttggtggaaacacaacaacgacgctcacctccaatgggccgaggacgctttt

gaggaggccctcacccaacgcttcgccaccttcccaatgcctcgcgaattcactaccttcttccacacccagggcagcgaggcacggcagaaggccgt

tgacgtctacacggcccacactgagctccatttggccctcgtcgactcttccctggttcttcccgggaacgattcgttcagtcacgccatcctctccc

cacccaaggctggcctggctatctccccgcgatatataccttgccgggttcgtttcgacgccaagactactggcgatgaggccaaggccgccctcctc

cacaccttctccaacattcacactgcttacgttgtggaggcctgggaactcgtgcggcgccgcccgaacggcctcgtattcaaacacgagatcatatt

cctggtcatggtacccgtccctccccttggacctcgcgctccggacgcgcccccatacacaggcatggccgatccagtctccgaccatcaacgcggtg

ggatccctgcctttgccttccaaatcggcaccgctgacgtacagatcgacttccccttccgaccgaagcactgctggggctgcaaaggtttctccact

gctttccaccgcacggaaatgtgtatcaacgcgtctgtcccctgtgcacccgaggttcttggccccgaccctgcggtccagactcatcaacagcccgc

cccgacgccccctgtcacctcacacacagccggcgatcatccctccggttcgacgaacaacccctctgctttcaactacgcctccttcgtccagggca

cccccggaccacaccctaccctcgccatttcctcctcttccatggatccccctccacccatccagtcggctccacactcctcaacccccacaacggcg

aacactcgtccctccgggtcaaagcctcgcggtagcaagcgaccctcctcctctggcgcgacgccttcgccctccctgccccgtgtcgcatcctccgg

taccgctgcctcttcctcctcgacgggcacgcacccccccccccccccatctcaacacctcgctccccagccaagaaaatccctaagggcaagaagaa

ggcgactgacgggctcatccaaacctcactcaccttcgcccgcccggccacctccactaccttctcattcaccagttccactgtcgcatcctcgtccg

ccgcaaccctggaacccactacccctaccaccacgtccactacagccccctctaccccctccaccacctcatctaagcgctctcgtatcattcgatga

atccctcctgctatctcctcacaatacttctctcctcacctcatctcctgtctcctctgatctgctaccatctgctctggaaagcctgcacgtcgtca

cactcaattgcggccctggtggtcttcgcgcccgccaacgggagttcacccctgccgcccgcaacctcctcgccacggccgacaccatttgcctccaa

gaaagccgcgttcgctcgcccatcctccccgaccaacctgacttccttcgtgcctacttcaacacccctctttcaccgaccacgcgcgccctcctcac

caacgactgcagcatcctcatccgcagcccctccctcgtcatcgaggactgggacttcggaaaacactgggcatatatccgcgtgagtctcccacttc

ccccttccttctccctctcccgaccttttccaccgcctgtctacttcggcattttctccatccacggtcccttcacccacaacgactggatccctatc

cggactgctgttagtcgcctgcatccagacgccaccctgccgtgcatcttcggggccgactggaattccatccccgacgccatcctcgactcctctaa

tgctcgacctaccggtaccccctgctccacccctgctaccttcctctcccctctgcgcctcactcatacttttcgctccctgcaccccacagattcag

gatggacgttctttcgcatcactcgggctgccgacgcccccgtcctcaccagcgcgcgccgcctcgacagcatcttatgctctggatctttcgtcccc

tccctccacacttccacctcggtgttcacgtcctcggaccaccacgcagtctccgcctccttcggccttcccagcacctcctcgacaccctatatcgc

caccaccgacccctcgaccaacaggactctcacctgggccctacaccctggcctctggcagtccatccctttcctccaggatgtccgcgccttcgtgg

ctcgctaccagcccacatacgactccctccccctcacacccctccaagcatggaagcttctctccgtcaccacgcaggaccaattccaacgcctctca

atctctcatggctctacgcaacgactcctcctcgacgacctcgccgccgtatctgcagcgttggtgttgctcgactgtcgctctcccctcgacatgac

ggctcttcccttcctgttggcccgattccgcaaagccttaaccgcgctcgacctcgcactcacactcccttacgcctctcggctggccgcagccgaac

ttcgcctgtcctcctggcctcctggctctccaaggcctcggcttcacctcgcaccacaccactcccaacccttatgttcgatggccatccctactgac

tgtgccacccttttccaccacctgctttcctccctccctcccgcactagccgagcagctctcgtcacctttctcgaccgatgaagtcgccagcgccct

tcgccgagccaatcggagggcctctcccgggcctgacggccttccctatcgcgtctggtcggagctcctgccggttgccggtccactcttggcttccc

tcgccaacgccctcggcgcgggggcctctcttgaggtcagcgcccgcaccatcctactcccgaagaccggggacctgtcaaacctcgccaactatcga

ccgatctccataagcgattcctacgtgcgtactttggcccgcatgctctctgcccgattactcaccgtagcctctttcctcttaccgtggaaccaggc

ggccttcattcccgggcgccgcaccaccctcgtgtccggcctactccaaggcctcatcgacaccacgacggcaaccccgtcccatccggctgcacccg

ccgccttcttcgtcatctcactggaccagcgcaaggcgtacgaccgcgtccgccacgaatggctcttcgcgtgcctccaacgcctggggattcctcgc

atcttgctcctgcttctctccgccctctactcatcggcctcaacccgcgtctctaccgccgatggtatgaccgcccccattcccttcctctcaggcga

cttggcttcctcacctctctcgccttcgccgacgacgtcctttatcttcatcgacgcctccagtcgtggtttccagcaatggcacgcccttcagtccg

ccctcatcctctacgagcgcgcctcaaacgctcagattaacaccaccaaatcctccttctggctcgtcggttcccccaccgatgccaccgccgcctcc

gccctagccgccaccatcgcgtcgtacggcctcgcgaatcaaaaccaccgcgctttcctcacccacctgggtcaccccatcaccctgtggcccaggcc

tcccacggacgctctcgtcgcccgtctcgcagctatccgcactcgcgccctctgtttccccaccattggcgtgagtatcctgagccgcatacaaaaat

ctaagcaatttctgacatcgcgcctctggcactccatctccctgggcgggctacctcacgatttcaacgacctctacttcgaggctctcaacccttac

atcttcgcccccaccaagccatacatctcccgggacgacctcacgcgccctcgctccctcggtggtttcaacctccttcatcccgaccacatggccat

cgccctcagcatctcgttcctgcggaactacctctcggaccccggccccatcggctcctggcttcgcctaaatctgaccgccgagcttcgccaccgcc

actcggcggtcccagccatcctcctcgcacgctccagcccctccttcacagccctccagcatgccgacgcgcgtgccgagggactgttcggccgcctc

ctccacgccctggccagcttcgacctcggtatcgccccggagtggaaggaattgccggctgcagcgatcatcaccctgccctggctcctcctcttccc

ttcgcctgagctcaccgaccagcgccaggtcaaatacctgcgctcgcactgggtcacggtcggtgacatgctctggctcggccctcgtccgcgccctg

ctgcgcccgtccataccttcttgggcctaccgccgagtcgctcagcaacggcccacaaccgcctgtcctcggcgcaaaactcctttggaggtgtcccc

tccctcccgtggtctacaatgtggtcccaactcgagccacgactcgctgccgccttgaccgctcactgcctcggctcgcccaacccctaccggctcct

cacaccagacgccttgagctctcgaacaccgtcggaaacctggatccccctctcggtggatcccgcgtacccctccttcccctggcatctcctcacaa

tcgccggccggcccctcctcgcctcatccccacgctccgtccgccgctccctcaccccacaggagccccggatccctggttggactctcgaccctccc

gtcccttccagcttctggacgacggcgatgggggtctccactcccctctccttcgacccgcggaccatcattggagcctggcaacacctgcctggcct

ccgcggccgcctcgtgctatggaggaacgccgtcctcgtcacccttcacacggctcgcaccattgcgggccgcgacgcccggactagtgcctctccgc

ccaactttcatcactgtgacgccatggacgtcctgtccttcaccaccgcgtcggtggtgtcggcactcacgtccgcctggtccaggctcccgcaagag

tcagacgtggccgtcgcccgtttccgcgcgcgatgggtcctgggatcatccctcttacgcatcgagggcccctccctggccgtatccgccctccccac

cgcaccaccgccatctcctccggcaccgtag

>contig_21.contig_21.g867.t1

atgatatcgaaatacaaagaggaagaagaagccgtcgagtgcgcggttgaagaggcacaggctatcgttcggaatggag

gcaaaccagatttttcagctcttggccgcaagcacaaggtggatcgccagcgcatctcaaggcgatatcataatggattaagtagatctacacgtcct

gcgaacaatcggctgctctcagacagtcaggaggccgcgcttgttgcttggattcgcgtgttggacgatctcgaactggataaagtcatcaaacagca

tcgaatattgcctgaagacatctggaacgttgatgaggtcggcttccgtgtaggcataggagggcgacaatggatcattacgctggttccatcaaaag

acgcacaccttgcgtcggagacctgccgggatagcatcaccgctatcgaggccgtcaatgcaacaggacagcatattgaaccgatgatcattatgtct

gcaagcgtgcactctgaggcgtgggtggacaacaaactcgaggacgagactctcctcgccgtttctcccaaaggatacaccgacgatatcattggcca

ttgcgttaagacgatgaagcccaatacgattaaggcgggattcaggcgcacgggaatctggccgtacaatccaaatgaggtcctgtccaagtacccca

acccaaagtactccggtacttcatctcaccaggccacctcatctcagcctaatgttctttcttcgccgagtcagcccgagcctccagtcacgccgcgc

aatgtccggtccatcaggcgctctgctcatgagatagagaagcgatacgatctctcgccgactttagagaaatatatcagaggcaccgtggaaaatgc

gcaaacagggagcctcgcaatttcggcgctcgatcaacaaacagcagcacaagtggaacgaaacaagcgtaacgagcggcagcgcaaggttacgcagt

cgggaggggtactgtacgcgcgggaggccagagagatagctcaggagaagttgcaacaggaggaaaaggaagaggcaaagcgggaatgggtgaagacg

aaaaaggagattaagaagcgcaaagatgagggatgggctaaggtttttgtcgaggtgcgcaaggtagccgcaaaggaggaggtcaagcgggcggaaaa

acgaaggcgtcttgagtag

>contig_21.contig_21.g868.t1

atgatagactggctccctctactggctccctacaaaggctcgctagtatatggtgatgagaggattctcttgctgacca

tcgacccgacgatcctcagcagcccacgacgatcacaacttccaagttctctcgtcgagacaccaaccacttcagaccgcctcagctcttgctccggc

cctctgctccagaccgccccagctctcgctccggccctctgttctcaccgtcgccaacgccgagcctcatctggccctcgcgcgttcccgacttcttc

tccgtcgtcaccgccgagcctctttcgaccctcgcgcgttcctgaccacccttccctcgcatttctcttcgcatctcttcttcctaattccttcgcac

ttcttctctctcttctcctccgccttatcaggctcaagacatcaagatcatcaccgtcggcctcgaacgcgaccccgactacggccttgatattctct

cgcctttcccgcacctcctttactttccgacgcacaccctatcggcaagcctacccacaagtctttctattgccctttgatcttacagataccgactt

cagggccgggcgcaacgcgctgtcgcgctcccacatcggtaccaacctcgccacattgttccggccatcgcctatcggaccggccctcgtcttttctc

tccggtgcagtcgcaagcgcaacaacatccactccttcccctgctgcttccttgtcggaccgctcccggccgccggcgccacccttgccacgatcctc

actggactcagaagctaccgccgcttgatgtctcgaatggacggccggttggacacggtggtgaatggcttcgccttgccgcgggaagggaacaaagg

gttcgacggttggtttccagcatctcaacaagacaacaagccggagctgggcctcggcgctgcatccgtggcggagatcggcgacgtcatcatccagc

cgctgcccgtcattgcggacgcgttgtatccgacatcagggtttggcgcggacgctgctttcgcggcggcacacacagcacacgacagccggtttaca

atgcatcatgccgagggagagcctgatagcgacggcggatacccggtcgacgcaacattggcgttcttgcccgactctactcccgctcatcacaaccg

tcggactcacttccggacgcggcctcacttccggacgcacctccggaagtccggatgcgcgtcccctgctgcaagagcatccctccctgtgccacgct

tcagttccgacaccgtcacccgttcaccccttccggtcgagtgtcgatacattcgcaaaccggctctctacacccaccttctgcagaactgcaatctc

gcccctgtgcctttcggaccgatcagtatcaacctcgttatcgaccttggtactcgtccacacagcttctctcccctgcccgtcctggcaacgccttc

gtcccatgcgctcgtcgccattcctttctattcccccatgctcgtcgcagttcccagtctcgaccacgactcgggacagtcgtctttttctgggtggg

gatgtgctggacggccacgtggtcacacacgctccctctactggctccctacaaaggctcgctag

>contig_21.contig_21.g869.t1

atgacagaccccaacttcagcaccgccaccttcgaggccgagctccactccctcgcctcccagcacaacctcgactcca

cctcccacgcccagatcgccgccttgctcgacgacgcagacccgctcgccccgcttcggaaggagtacaatctccccagtatgaagggcctgaaggct

gccttggcacgcaaggctgcgcgtgacggcggcggcggtggcggtggggctcccacttcgacttcgacttcgaccgatggagatgcagatggagacgc

agacgcagacgcagacgcgctctacctctgcggcaactccctcggcccgcaagcccggctctcctccaccctcctcaaccgcgaactggccgtctggt

cctccgtcggcgtcctcggccacttcgaccacccgcacgacgagccctggctccgcgccgacgaggcgtgcgccgagctcatggccgatctcgtcggc

gcgaagagggccgaggtggcttgtatgggcaccctgacgggcaacttgcatactttgctcgcgacgttttatcggccgggcgtcgagggtggtgtgag

gttaccgcgggacgggtcgatcaagcgcgatgagagtgcgggtgagaaggggaagaggaggcataagatcgtgtacgaatggaaagccttcccctcgg

accaatacgccctccaatccgtggtccgactggcaggtctagacccttccacctctctgatcgccctccgcccgcgcccaagcgaagacaccctccgc

acctccgacatcctgcacaccctccaaaccctcgccgcagaaggcgagaccgccctcctgatgctctccggcatccagtactacaccggccaattctt

cgacatgcagcaaatcacgcgcgcggcgagggagtgtgggattgtggtcgggtgggatctggcgcatgcgttcgcgaatgtgccgttagcgctgcatg

attgggatgtggactttgcggcttggtgtacgtataaaccggcaggttggttcggacataaccgcgccacccgtttcgccatgccctcccaattcgac

cccatcgacggcgcagcaggcttcataacctcgaacccgtccgccctcgacctcgtcgtcctccggggctcgctcgaaaccttagcccgggcacctca

gctcgcccccgccgccgccaccaaggaggaggtggggcaggggatggtgggattcgggaaaatcatggcccccctaagggcaaaatcactcaagttaa

cagcatacctggcacacctcctcctcctgcccaacttcctcccggacgaatgcgaagcggtcctcgtgaccccgcgtgacccacaccagcggggatcg

cagctttgcgtccggatcgtggatcaccggccttcctcctccccctcgcaagaaagggaggaggaggaggaggatggggaagggatcccacccccact

ctccccgacctccctcctcgcccgcgcacacgcccgcgccgagcgggagcacgggctcatcgtggacgtgcgtgcgccggatgtgatccgcctcgcgc

ctttggcgcagtattcgacattcgaggaggtgtggcgggcggcgcatgcgttgagggatgcgttggttaaggagcttgctgtgattgcttga

>contig_21.contig_21.g870.t1

atgttcgcccaacgcgccttcgcacgcgcactccagcggccactcgcttccacagcagcagcagcattctccacatcag

cacccgcatcccgcatcatcagcacacagcccctccgcgctaaggaggctcccaccccgctcggcgccacagccggctaccccatcatcgaccactac

tacgatgccatcgtcgtcggcgcagggggaagtggcctccgcgccgcattcggtctcgccgaggctggcctctccaccgcctgcatcacaaagctctt

ccccacacgctcacacaccgtcgcagcacagggtggcatcaacgcagcactcggaaacatgaccgaggacgactggaggtggcacatgtacgacacca

tcaagggctccgactggctcggcgaccaggacgccatccactacatgtgtcgcgaggcaccacgcaccgtcatcgagctcgagcacttcggtctcccc

ttctcccgcacaaaggaaggcaagatctaccaacgcgccttcggtggccagtccctcaacttcggcaagggaggacaggcataccgatgcgcagccgc

agccgaccggacgggacacgccatgctccacaccctctacggacagtccctccgccacaacaccaacttcttcatcgagtacttcgccatcgacctca

tcatggaggacggcgagtgcgtcggcgtcgtcgctctcaacatggaggacggcaccatccaccgcttccgcgcccacaagaccgtccttgcctcggga

ggatacggccgtgcgtacttctccgccacctccgcacacacctgcaccggtgacggcatggccatggtctcccgcgctggattgcccctccaggatct

cgagttcgtccagttccaccccacaggcatctacggtgccggctgtctcatcaccgagggatcccgcggtgagggcggctacctcctcaactccgagg

gtgagcgcttcatggagcgatacgctcccaccgccaaagatctcgcatcccgtgacgtcgtctcccgctccatgaccatcgagatccgcgagggacgc

ggtgtcgggcccgagaaggaccacatctacctccagctctcccacctgcccccggaagtcctccacgagcgtctccccggtatctcagagaccgccgc

catcttcgccggtgtcgacgtcaccaaggagcccatccccgtcctgcccaccgtacactacaacatgggcggcatccccaccgactacaagggacacg

tcctccgcaccacctccgacggccgcgacgagatcgtccccggcctctacgctgcgggcgagacggcctgtgtctccgtgcacggcgcgaaccgtctt

ggtgcgaactcgctcctcgacatcgtcgtctttggccgcgcctgtgccaacaccatcaaggaagagctctcccccggcaagccccacaaggaactcac

cggcgacgcgggcgcgatgggcatcaaccgcctcgacgccgtccgtaatgcgaacggccccaccctggtctctcagctccggctggagatgcagaaga

cgatgcaggacgacgccgctgtcttccggacgcagacctcgctcgaccagggcgtggagaagatgaggaaggtgcacgagaaggtgcgcgacttgaaa

gtcgtcgaccggtccatgatctggaactcggacctggtcgaagggctcgagctgcagaacctgctcacgtgcgccgagcagacggtggccagtgcggc

ggcgcgcaaggagtcgcggggcgcgcatgcccgggaggacttcccggacaggaacgataaggagtggatgaagcatacgttgagttggcaggtgtcgg

agcatgagcctgtcaagcttgattatcggacggtgcagatgaacacgctggatgagagtgaggcgaagagtgtgccgccgtttgcgcgtacgtactag

>contig_21.contig_21.g871.t1

atgtcagccgcatcacgtgacgcagccagcatcctcggagggttggcacttggcagccatgatcagtcaccgatgatca

tccgaagatcgacgcgcctcaatccgcagtactcgtactcgtacgcagcacagaaagaacagcgacaacgaaaggaacgatcgatgttcgcagcaggg

agcagtgggagtgccagtgggagtgcgagtgcgggtatgggtatgagtggggctgtgccaggatcgccagcaccgtccctgcacgacagcacacagca

cggcaaggctccaggcgcacagctcttcttcctcccgcccagtccaccaccgacaactccaggcgcagactccatatccacatccacagcatcagcat

cagcatcagcatcagcatcagcatcgaccgcacgcccgcccccaaagaagaagcacaggaagaacgatcagtcgctcatcacaccgcccatcatcaca

ccagcaccctcttcctcaacagcggcagccaactcacaactgcactcgcaccagctgctcctcagacacgcacacgacgaactacgcatcgacaccgc

aacactcacactccagcgcagagaactcaaacacgcatcacacataatcaacacacacatcgcacacctcacaacccgcatccactcgcgtctccagc

gacatgcagaccagcaccgtcccacccgcactgctaaactcatcatgccacaaggaacacacaagcgctgcgtcactcccgctcacatcgcacagacc

tgcttcgatcgtcaaaaagcagcaggaccagaacagcaccaatcatccaaacacgaactcgccctcctcaaccggctcagaccaggaacttccacttc

cacttccacctctgccaacacagacgctccctcccatccggacgacctcctcatacacgaagcactccagcgatatctatacccagcaaatatcgaca

gaggcaagtccttccatccaccaccacgcaaggtacgcgtccggaaaccacagccacgccgcccatcctcctcctcctccaccacccagcagcaacca

caaccgcaaccgcaacagacggaagctcaaatcgctcgcaatatcgtacacgcgtccttagcgtccatcatggaggtctcagaagctcaatcgacgat

gtacgaaccttccgcgcaggctcctcctcctcctcctcctcctcctcctcctactcctactcccatgatgaccacacagcctaatgacggcatggcca

ctgggaccgaggatcgctggcatagcttcatagaagagctcttggccgctgcagctacccaggcccagcccgctgcctctgtagtggctgtggctggg

cacgaccagcagtccggcgcagaggcgcaagggggaatcgaatcatcaacagcggcgctcctcctctcctcaacagcggtatcacagtccgaggcggg

tggagcagcgtcactcccctcttcgaacgcgcccttctcggttgccgatgccgatgcggatgcggatgtgcatgcggagatggacatggcggcatggg

gacacatattcgcgtcggccgctgctcgatcggcggcccgtgatgcagaagacatgggagacgaaggcgacgcgaagggggggatgatcatacctcat

cgcttcctgatcacagctgaaccggctggtggggacgcggcggtggtggagggggaggagggtcagaatggggatgggaatggggatggggacgggga

cggggacggggacggggatgcacagctcgcttcgttactcttggcgtctgcctcgggggacacataccctataagcgaagagagagcagcggtcgatc

atccgagtggtgggatgatcatacctcatggcttcctgatggcggctggaccgactggtgggacggtggcggcggtggtggagcaggaggaagaaaga

gagggagggagagatggggaatgggatggggatggggacgtgagtgggaatgggcaggcacagctcgcttccttattctggggagcagcggtcgatca

tgcgagtggtgggatgatcatacctcctggcttcctgatcacggctggaccgactggtggggcggtggcggtggcggaggtggggaagaaggaaaggg

gggaagatcagaagggggatggggatgtgggtggggaggcacaacttgctatgttactcttggcgtctgcgtcggggcaaggataccctacaaacgga

aacggaggaggaggagtagcggtcgatcacgatcacgatcacgatcagcaggatggatcgtctcattgtcatcctcatcaccatcatccgactggctc

ggacgaggcggaagcagaggctggcatgctcttgctcagcgccagtggaggaggaggaggaggaggaggaggagaagcggggatggggatggggacgg

agatggaaccggagctcacgccttcaaaacggcgcgtcgaggagatcgacatgaatctcgactttgataccctcatggagcgtctggccgggtga

>contig_21.contig_21.g872.t1

atgaacatcaaagttactgctttactctgcgtgctgcttgccagcattgcagccgcaggtgggtttcccaagcctgacc

ctaagccagatcaactgccagagctactgcaagcattgcgtcttcaagaagcccaaggaccccgtctgccgttcttaggcatgcggctctttacgttc

agcgcggaccgacattgccctgctcttgccacaagaacgagttcgcacatcgaccaggtcaccttccggcgtcgccgcggctttggaatcaagtgcgg

atgcgggataaggggctactgcagtcctcagaaaacgctcaaaacgagcggcttgacggttttgggggaccccttgcgcgcggtaccatcggtatccg

tcatccgtgacactcgtcaagcccgccgagcctcgagcacctccgatgtcctcgacctcggcggcgcatccctcgtcgtcgccggtcgcccagctctc

tccctcatcactggtccctggcgcttcgtctccttccttcgcgctgcgtccgtggatctgccctcgctcagccctcgtctgtctctcaactcgccgag

acaccagttacttcctcccccctttgacataccagatccgcaagcttccagtcttcgttcgaggatgaacatcaacatcattgctctcttctgcgcgc

tcgtcctctccgccagcatcgtcgccgcggatgatggagatcgcggctttcatcctcacgacgagaataaagcactcctgcgccgcggccgtgctccg

catcgtatcgatcccctgcatcccgaacctccgtttgtccaccctattccggaccccagcctacacgatctgtgcttgtcgagctgcaacatccatcc

aaacttcaagaaaggcgcagaacagcagtgctcgtcttcgctccgctccctcgccttcatctcaagatcgtcggtcacatcatcacctctccccattc

acacacctgcattgttcacccatagcagcgcccccgaagcacaaggacaaggccagggatacccaacccacaacccatacgcccccaccccgcacgga

tacgactacgcgcccgacgacgtcaacaaccacgcaccaaccagcggcggctggggcctcggcggcggcggcagcggcggcggacgcaggaaaactcg

cgcaggcacaacaaccccaggcctctcccgattcaacgccctcgtccaacgttccccgctccgctccgtcctcatcttcacctgtttcctctcgggcg

tgtacctggccgtgggtgcgtacagccagttccgtgcgctcggtcgggctgcggagccggctaggttgaaggtctttgatggggtgcagggcgggctt

tttggggttgctgctggtattgaggcgtttggggtgtatgctgctgttaaggccgagtacaggctcgcgcgaatctactccctcgcctcctgcgtctc

cctcgcctgtgcactcgccggcgagttactcggcatcatcaaccacttcatctcccacaccctcatcctcgacgcctgcaccaagcgcaacacaggcc

ggatcgacccgagctgggataacggctggtggggctcgtcgaactactacgacgctgacgatttccctgcggtctccgcgatgaatgggacggcgaat

gggacggtggtgtatacggaggcggacgcgagggcgtggtgcgaggcgctgtacaggcggtatacaccctggctcatcatctggctcatcgcgactgc

gttcgtaggtgcgatcttcgtcgtcatctcctttgccttcgtccggcagattgaggacccgtcggccgtgggcaggggacggacgcgtgtggcgccca

gtgcgcagtatagtcggaatgcgggcgcgggcgcgggagggggagggggaggggcccagatgccggagttgaggtatgactcgtacccgatgggcgat

cagcctcactctggtggtgctggagcaggggcgggagggtacggctacggccgacgctcgacggactccgactacgcgtgggactcgcgcgacgccaa

gtcacccacgtacgacccgtactcctatggcaactttgaccagcacgacgatgaacgcgacgcggaggcgacgacgacgaacgcgcgagatggcggcg

gtcagaagaaggggggaggcgctgatgttgatgcgaacacgttggttggggaggaggatcggaaggataacctgcccacctcgccctcactcgactct

ttcgactccactccgctcgcctcgaacgatgcttccacatcctcgccgttcttcccttcgatcccttcgcaaacgaccgcatcaacaccgccagctgc

ctaa

>contig_21.contig_21.g873.t1

atggtcgcgcctcgcaggagagcagcgcgcgccttcagccgggctacagctgccgccgcgtctccgtcatcgtcaccgt

cttttctcagcagactctcagctctggcggctgcggccctcgctcccacaacatcagctactccgcctgcttctccaccagtagctcccgctcccgct

cccgcaagacgcctccctcccaagaagccatacaaagctcctgtcaatccatttaagcatcttggccctccccaaaggaaaacagttgccgatgcttc

acgcccagcgcaatcggcagcagtgaaaccaccttcctcgcaaagcggtagcactcaggtcaccgattctggcaccaacgacgggcagccgaaaagcc

atagctccgcacatgcggaatcaagcgcgcagcctcaggtagcagcagcggaaaaggctcagaaggtggcggaacgtcccgtaccgcgtcccaaaaaa

atatggcagaatctggcgcatctggagggggcggtactgggccgcctcagcgggttagacgtcagtccagagactgtcacgccgctgcgggagatgca

tgtagccacccttgcacatggcctcgatcgcgtattgttcaatctccgcaacattctcgacccggacctgttcgactacgatgccctaccgccatata

tcacctccagtcaggacaaggagctcgaaaccattgccaagaaaaatgaagctagatattgcgggagcacgagcagcatcgggcggaaaaatcctgac

ctctcaggcttttctgaaagcttcaaagctttgggcaacggcttcacgatgggcgcccagctacctgcttctatcaagctgatgcgcaaaccaaatca

tgacggctcaaaggaggtctatgccatcgatgcagacaaggcagccgccggcgaacttgacaacagcaacttcgtcctcgcggcactgggcaagagta

tggagaagatgttgacagtcgacgataacgagttccgcaatttcgaacgaacggagcgctggacaagattgagcaaaggggaagttgacccccctgcc

caacctgaggcgtaccactattcgaaattgtccaagttcatgatgcggtcacagctcgattgctacgattcgcggttacccaacaagacgtttgacct

aaagactcgcgccgtagtggcgatccgtcaagaccgcgccaactgggtggagtcgtctggctatcaaattcgatcggctaaaggtctactggagtcat

ttgagcgcgaatattacgatatggttcgcgctgccttccttaagtacggcttccaggcgcgcatcggaaatatggacggcattttcgtggcgtatcac

aacacgaaggaaatctttggtttccagtacgttccgctggaagagatcttcacccgcgtctttggcacggtcgagatgggcgaacaggcattctcgct

cagcgtgagattgttggagaagatcctggattccgccacggagatatacgaaaagcagtccctgagactgacgcttgtagtaggcgaaggcaaagatc

caagcatgaacgtatttgtggaaccgtacgacgagaaagcggcgtcccaaagccagcccgtttcccagccaagcaacgcggactcgacgtccatacct

gatcccttcggtacaccgatcgagaaagtcatcgactctagcaataagccgtttgtgccattgagccagcggaagattgtgcaactcgacgtcaaagt

ggacaggtggctcgacgggaacatggtgcacggagtgcctgtcgactttacgtctccgcccggtcgcgcggttaacagcttaaaagaggacgagatcg

caagaagatcgcggcagaagttgggtcccctacagtgggatgtcgactatgtcatccagccacggggcgacctgacggaagcggagaccaaccgccac

ctgaatgtggcccgcgcggcccaactctccgtgtcgagtctgacaatgcccaatctggacgctgttgcgacccgtgaggccgagttggaggaacgaat

gcggcaatatggcggagaagaggctgtggagcgctggaggtcggagcgtagggacggcaggatagggcggatgccgagagcgcccgggcagaagcctt

tgagtggactggaggggactgaggaggaggctgtggcggcggtggaaggggaagcagatggagggtcggagggatcggtcgcagctacatcggaagac

ggggagagcgctgctggcgctgcagaggcagcctcgacgagccaggacgatgctgctgctgccgccgctgcgtcccgcgatcttcctcacataagcct

gctggatcgcggggaaggtctgtggacgacgcggttgagcgccttgaggagggttcaacatttgagggagttggcccgcttgggggcgcaggaccgcg

tcttgcaggagaagtcggatcggaaggaacaagtgtga

>contig_21.contig_21.g874.t1

atgttcaaaactcgcaacaccgggcaacagcaacacgacacgcggcacgaagagctcggcgccgcgatgacttcactcc

tgggaggcgcgaaggctgcgcaggctacagtcggtgccatcgacacctccgcctccgcctcaggttccggcacaggcgccggcgcagggttcgaaacc

accacgatccgccctcaggagcgcattcccggctttgacgacgacgacgacccagccaaacactccattcataccgcggcacagcacggagacctcga

gacgatccggcaccttttggactcccagctcgcctcgcctgctgagcgtgatgaggacggaatcaccccgcttcattgggcggccatcaacaatcatg

tgggaatatgcgccttcctgctcgatcgagacgcgcctatagatgcactcggcggagatctggtcgcctctccgctgcagtgggcagcccgctcgggg

catctcaacgtcatgcatcttctcctggcgcgcggggctgacccgctcatcaaagacgcccagggcttcaactcgcttcatctgaccacgcactcgtc

tgccatcatgcccttactataccttctccaacatcctgcgttccagggtgtagctgcagaatatggctcgctggagtctcacacgtccgatactgccg

ccatcgacgtgcccgatgctcagggtcacaccgccctgatgtgggcagcgtatcagggcgacggcatctccatcgacattctcctcaagcttggcgct

cagatcaacagacacgatcatgcaggacttactccgctccactggggcgtcgtcaagggaaatcggctctgtattcgcaagctcgctcaggcgggcgc

cgatttctgggcaaaggagcgcgacggtaaaacggcacgcgacatggccgtggagctgaagagcatcgtcgcctatcgggctgcgctggccgatgcag

ggtttgaagcagacgggaggaggaaacggacggcgtgcatggcgttggcgccaaacaatctgcgcgtctcggaacgactaacgcggttaggagtcctt

cttctcccgcttatcaccctcagtctcgtcttcagcacgcagactttactcccttggttcaccggtcttcccatggccgctgcagaactgtttgcgat

gcaccatgtcatcactcgtgtattgctcaacgacaagcaggacggctccctcaaacgcagtccttacttcctaggcattgtcggaggaagcgtggtac

tggtcggctgggaatgggcgagaaagttggtcaatcacaccccggcgcatgcaaccgcgaacttcatctttgcttttgccatcgcgctttgtgcatac

aatctcttccgtgcggcgacgatggagcctggctatgctccctgtccagccactgacatcgaacgtcgcgagagcatcgaaggcctcttccagcaggg

tcgacttaacggcttgaactgtccgcagccgtgctacgttgatccaggtcgagtgatacgtctgctcatcctgctcgcaaatgtgcacagtcggaact

acgcgcttaatgcccccgcatatgttcccgacctgacttcgtcctgcatcctgccgagctcagtctgcgccgcgacaacgtacgacacctacctgttc

accctggcgtgctggtccggcctccagctcacctggacgatcatccttctcatcgcccaaagctggcagatcatgcgccagctgacgacgctcgaagt

gagcaacttgggccggtatggttacatgggtgggaagagcggtgcgagtgcgaatgcacagacgggaatggtcgagcgggccactgcggctgtccagg

ccagtcaggaggcgagcaaagctgcccaggcgctgaggcaacaacagcaggctgccgccgctgcttcagggactgaggatgaaattggggcatcggcc

gaaggagggggctcgattggagggagagatggaggactcctgccgctgcattcgccagctacgcagccggtgccaccgactcagcagggcctaaatca

acatcgacatgcgcctggcttcttggctcatctcggtggggcggggaactgggtgctgagcattgttgggctagacctgtatacgcgcgggaaagcgg

gcgaggggttgaagcgggcggccaatgccggcaaccctttcgaccggggctttattacgaattgcgaagatttctggacacgcggcaagaagctcggc

gtgcggtacgaggagctttacgatgtgccacccatgggcttcggaaatcagcctgtccagcacgacaataacgacgacggcgatgatgacgatgcagg

ggtggattcaaggggagggtcccgtcggcgatggtcgatgtggggcgcggttcggaatgcgggtcgaggaaggggtggtggggacgagtcgagcaggc

ctgggcaaagcgagtattcgctggtgcctggtgccgtcgtctga

>contig_21.contig_21.g875.t1

atggcagactcgtccttcaaccccgttccctccgacggctcttctgccggcaaaaccccccaggtccagatccgcggag

tctctgcctacgaaagcgctctgaggatgttcaccatcgctcatcgcacgatgaagcagcaagagattcgggtgtccaaggcccatgtcaccttgctc

gaaggcaagaacaactgggaagtttggcgcatgcgtctggccaccatcctccagcccgttgccggtggtcgactctggcatctcatgctcaaccaccc

atccaccacagttgaggcctaccgccacatactcgcaccggaagacgacaagccggtcgacttcgaagaagcgaacgccatccgcggtttcgatctcg

ctgacgttgccaatgcgatcaaccagaccttgacggaccgcatagccgaggacgtcaaccctccaagcgaggacttccttattcctgcagacggacag

gtcatatggaacaagctcgtcgacaagttcgcctctcaagatatcgcctcacttcaggaagccgaagccgaactctcgtcgttcaagctcggcaatcg

gacagctgtagaggtcggtcgcaatctccaaggcatcttctcccgcatcttcaacgccggaggaggtgcagttcaagagcaccgcaagattggcgccg

tgctgaagtgctacacgcaatctcccttgatgaacgacaccagggcggccatccaacagacctggtcgaacaagcacgaatttaccttcgacgaggtt

gtaaagcggttcgctgccgaagaggcgctgttgggtgtgcacaggcagagtgagcctgaaagctcggacggcgctcaagcattgcgcatccagcacgg

ttcctccatgcctcaatcatcgtcgcagaaacagccctcacagcagcaacagaccggagcaaacaggcccaacaatgcgcgtcgcaacaaggctaaaa

acgggctcgaagtgcaattgggatggaccaatcagccactgcatcagctcaatgcaggttacccacagcctcagcttgtccctgctccttacgctgtc

ggagcgcgcgcgatgatggtacaccccggcatgatgcacgccactgccctgcatgcttctcatttcggtggttacgaccaccccggcccctacgtcta

ccctgcatacgcagcgcctgaactcgcggccctgtcttcggcatcgctgcctcggcaaggcccagatataggtgttccgttgaagtggatcctcgatt

cgggtgcctcatctcatatgacgcccaacggagaactgttctcggacattcagccggactctaccacagtcaaaacggccgatggcaagaggctgcgc

gctcgcgggcgtggctccatcaccctcattacgaccaatcctacttggaacgaactcatccttcacgacgttttgcatgtccccgagctggattgcaa

cttggtttcggtcgtcaaactgaaccaagactacttacgagtcgagtttaccgaacaactcgaaactttgatcacggaccccttccagcgagacttca

ccatctcagcgacctggtcgtggcagtcccaagcgtatctcctccccgttgtgggatccgtggagagcgcatacgtttccgccttcgtccttcatgac

gatgagcgcagtgcgtcatctggagcatcggagctagctactccaactccttctgaccaagtgtttcggctttgggtattgtggcacccacgactcgg

ccatctgggcaattcgtcgttgcctatcctagcccagaacagcgtcggacttcccgcgtcgctgatcaagaggagcgccaccggggcgttccagtgcg

aaatttgttctctcacgaacatcaagaggttgccgttctccgcctccaccaccaagtcgacacgacccctcgagctcgtccacgccgacctcaccggg

cgcgtgatctgcaagtcgttgggcggtgcggagtatctggcaattctcgtggacgactacacccgcatgctgtgggtcctccccctcgttcagaaatc

cgacttcgtcctccgcttcatcgagtggcgcgaccagggatatcgaattcactttccggacaccaaggaactggtggtcacccgcaatgtcgatttcc

acgaagacaaaggttacgatttctcctccctcgtagtcgccgagtctgagccaccacttcccccagaggtcgtcgtcagctcggctccacggcctcct

ggcctccggccccgcctagttgtcatcggccccaggcaaccggaccctgctccgtctgcttcggtcgacgtggaactgccagcagcagcagcagcacc

tccaccagcagcacctccaccactgccaccactgccaccggtatcagcaccggctccgggcccggctccagctgctgccccagccgctccgtcagggg

cgccacgttcctctgcctccgtcccttccgcgtcggtgggagctcaggcgccagcagcagcgccaggcgcacggcctcgtcctcctcccacaaggcag

taccagacgcgccagcatacgagcaggatggagcaaagacggcagcagcgtgaaacggagtggtactttaccctccgcgcggcgcttatcgagctcgg

cttcgaacagtcagcagcagacccagctatcttcatttttgggcaaggcacttccgccatcatcgtcgcagtctacgtggacgacgttctcgtcctcg

gaagcaacaccgaggagatcgatgcagtcaagactgcactgggcaaacggttcaagatgaaggatcaggggcaaattgggcacttcctggggatgaag

atcgagcgcagcgaagattctcggtcctttttcatctctcagtcagcctatatccgatcgatgctcgaacgattcaactacaaggacatcaaggcagc

gcccagtccccttgaccccaagcagaggcttcaaccatacgacggcagtgcttcggatgaagatcgtcgccgcttccaagccatgctcggttgcctca

tgtggctcgcccaggcgtcgcggtccgacctttcgtatgcggtctctacgttagccaggcacgcctcgaaccctggtccccaacacttcgggtgcatc

aagcgcacgatgcaatacttggcgggcacagtcgaattcaagctccacaacaccgctaattcagacgaggacatcgtcgcctactccgatgccgactt

tggtggcgatttctctgccaagtcgacttccgggtggtgtgtgtttgtacacggtgccacagtcgcttggggctcgaaattacaaaccctcgtcgcag

attctactgcacaggcagagctcatcgccgtctggcaaactgctcgggaggttctcgcccttcgacacttcttcgaagacctcggcctccgccaaatc

aacgcgggttcccctactgtcgtttattgcgacaatcaacccgtcatccacattctttccaaccctgtcagcaacggcttgaccaggcacatggagcg

caagtacttggctactcgcgagcaccaagaacgaggccttgtgcatgtcacctacatcaacaccaaggtgaatccagcggatatgttcacgaaggccc

tcatcccagcaaccttccaacaacatcgctcagccatcggcctacgataa

>contig_21.contig_21.g876.t1

atgactcgcgccagcagcagtcgccgccgggactcgagcaccagcgcagcgacgatcacgcccacaacaacaactccgg

gctccagcgcgctcaccattcccgcctcaccctccgcgagctcacgcttgaatgccgcaggagctggtgcagggacgcgctcgagcagggacagcatc

tactccctctcgctttcctcgcgccggcatctgacgccccttgcgaacccggcaccctcaacttctaccacctccctcagcccgtcccacgcaaggtc

gagcgggaccgtgaccagcacggcaacgagccgacttcgcaatgtcgctagcaacgcttcgaaccgacttggtcttccaagactggcgacggattctg

gcttttctgtctcaacatcaccgccaaaggccactgccgtatcgccgccaccagcaccgccagcatctgggccaagcggtggaacgtcagtaccggcg

tcttccgcagcggacggcacgaagcgggctggcactgattccgagccaacagcacctgcgcctccttccgtgcagcctcttccagacacgcaagcgca

ggaccgggtagcgaccgcagaagcaggagcagcggcgatgaagcgtaaaatacctcctggtgcgacactttcggcggcggaagcaggcaactcgcgga

ggaggacatggttcggcttcggatctcccatgactgctgagcccacctctatcaaccctattggagagactccgtcgacacctccgcccatggacgcg

gatgaagctgcattccgtctcgcactcgttgctggcgcccctactcaaccggcggcggctgaaggcgctgccgcgcagcccgccgaggggccaaaggc

tgccttaagtccagaaacgaccggcgcgacggcaaagattcaggcagagccgcagacggaggaggtagcagacgggcagagcacggccgcgtcatcga

ttaccgagcaagtgccgaccaatcccagaacgactaattcaagaggctggtttacctggcgagcacaagcccaagcgcctccccctcctcctggatcc

ccacttttgccagccgaggcgagtcagacaattactccaaaggcagttcagcagcttgccccagctcccgcacaagcgccacctcagcctcccacact

cacagcacctgcgcccagcaagtcgagtcagccagcttatgcttctccgttgagacggatgtggaatcgctccgagcaagcggctgatgcgccgacag

agcctgagacgcaaagtgctcccacagcgtcccccgtcaaagccgccatggagcagcaagcacctcctgcaccagctaccgagcaacatgcaccagat

ggttccggtgatcgaagtggacagcccaacgctgctggctcgtcgacgtggcggttctccctatgggcagggcgcgctccagtcgatccccagatgaa

ccagggcccggctgaggatgtcaagccgaatgtggtgtcgtcggctggtaattccgacataccctcgatagtgttgaacaaggaggtgaccaatcatg

cagaacatgagtcacaggccggcgctagcgatcccagtacactcgggcaggccagctacgcatcatacatcacgaactggattccaggctggagttca

gaaattccacaagcttcgtcttcctcgacgctatcgcaggacgcgacggacgggccgggcggcgcctccttcgtgccccgaacaccggcagagcaagt

caaagccgacgcgctggcccgatctgagcgcccccgcgtgttagacactgtcagcgggagtatccagaagctggcaagtccgaccgaggccattctga

acagccaaacacgcaagagctgggtgtcctatttcactacgcggaacccccacgccattcgaagaatccagggtacccccgcctcggacggtgggcca

gaagtgatggatctggacgaggacgaacctgcggagagctcgaccgcaccttctcttgcggagagtgcccctgctgttatgaagggaggtatgggcaa

gggtgctgctgcggccaaggcgattgtgcaaaaggcatcagccgtcacatcggctgcgtcatccgtcgtcctgaaaggcggagcgaagagcgggagca

agagtgctagtggatcggctccaacttcgcccgtcatcgggcccacaagcaaaggcggctctccgacaaaggatggatcgactgtggcgagcactccg

ggtagaagagggagtagcgaccctaagggtaaaggatcagcagcaacgacagtcagaacgcaagagtcgactgtctccttgtctaacaaggcgactgc

aaccgcgcaggaacttacagtacctacgcccgacaacaaagcagcaacaaattcaaagctgtcaaagaagggctccgtggttgttctgaaagcgccaa

accttgtgctacccagctttgaagacacctttgccacggcgccgcgaatctggcctcccaaggtcggaatgctggagcgaacaatacaggcattcaat

acatacctgttcaacaagcctccggacttccaacggctcggagcccctacagcaacctcggcctcggccagtcaagggacgggcaaggcccggagacg

cagctccaccttgttcaccgaagagactgcacagcgcttgccacgtagttgggaagtgatgggcgacaagacacgtgcctcgcagcgtggcgttggct

ccgttcgcaaggtagtcgttctgggcattcgaggttggttcgcccaatttgcgatcttgaagaacgtgatgggcgagccgacggggacgtccttcaag

tttgcgacgatgatgacggaggccattcggaagcacttcaaggaggccggctgggagctcaatccggaggcaattactgtcattgcgctggaagggga

tggtcgagttgcggatcgggtgcatgacttgtcggtcaatctgctgagcaatccggcgtgggtcaaggacttgcacgaggcggatgccatcttctttt

cggcgcacagccaaggctcgatcgttgggacagagttgttagcacgattgatcgagcaaaagcacatccagcccgaacggacgcgagtatgcttgcta

gctatggctggcatacacgagggaccgtttcctcacctccaatctgccatcatgtccgcgtacttcacctacttcgagactgccgcggccaaagagct

cttcgagttcatggcttcgacagcgcacgtctcggagtcatatgcggccgcccttcgcatatgtctgtcaactggcgtgaaggcggtttacgtagctt

ccgtcgacgaccaagttgtacccctccacggagccctacacgcggcaatttcgcatccgagcattctccgtgcactctacatcgatggcactgctttc

ccgcgggccgacttcctgacgaacctcctcgtgctctgcacacaggttcggaatgcgggcttgaacgaccacgaggtgctttcccttttgtctacgac

tgtcgccggcagtctgtacggcggcgtgggccacagcaacgtatatgaagagccacaggtgtacgatttggcagtgcggtacctgttcgagactacgc

acccatgcagtgagccaacgtgcgtgggcgaccatcgcgtttcgatgccggcggtggaccgaaggatatttcaagtacaagcacacaacccatactcg

ctcccctgggccattcgcggcctgctggaagatccagatgtacgggagctcttctcagaacacatggccgacctgctggagcaatttgctcaatggaa

accgacgaccaagtcgcagaaggacgtccatttccgactcagtccgctcaagagcatccagattcctggtgtcaggcaacgcacggcctcgaccgcat

catcttccatgccgccatcacccaagttcccttccagctccaaattgtga

>contig_21.contig_21.g877.t1

atggcgagcaacaatgcgccttcagcatcctccagcaacacaacgggcacggctccggccggaaacccagccggcgttc

ccgccgtggtaccacccgttccgtcggattacaactctctcgatttcaaccctgccgatgctgcttctactgtggttcctttggtctacccgttcaac

aacagcccttaccccattccccaagacctcttcgatctgtacagggcaggtcatccagttcccttgtctcttcttacgctcgtctgcctggtcaacca

ttctcgcggaggacgtcccgaaatcaaactgcctctcgatccgacctcggcacggatcctggacgaatgggtctacaccgacagcatgcatccccctt

ccggccacaaggaagcggcagtctacgcgggtcacatcctctgtgttgctggcgctcatcacggcggaaactgggacattctacgggaatacgatcat

cgtgtccgtgcagcgcatgctgcggcccgcaagacttcctcgaaaattggtttcgacattcgtgtcgtcaaccaacagcttctgggcctctccgcagg

agcagtccgtccagacggaggagtgggcgcgagcatcgacgttgcaggtgcctttgccagtccttgggctcggctcgcaggagctgcctacccggatt

gcatgcgtatcttccagtcttggtccatgccaacagtcactcactcggcgcatgcgacgtcgtatcatgcaccctcttctcctaagagaaagatgcct

cgtacagtctcctcgtccggtggaccctcggcttccacgtctggctcgtcaagtaacccctctcccgtctcagcttggtgcccgtccgctactcgtgc

gctccgggaacggctggagtttttgctgggaaggagcggcaagtttgcctcaacttcaattgcaacctggcttgctccaccggccgttcgcgaagcgg

ccaacattccggttcggcggatgcgtcttcttaccactgtccctacggccattactgctcccgctgcggtggagaccataggtcgtgcacaaacgtct

gcgcggcagccgttgcttcgtcctcagctcctgcgctggtgcagcagcctttttcttggctcctcgggccctttctcagcgtttcagcctacctccgt

catcccttccggctcaggcccatctggtttcaactatcccgacgcctctcttcaacaattctttttccaccctcaacatcaacccttcagcttcctcg

cagcactgaggaggcgcgtcgcacagtggagcttggccatttcgatgctccccgaggctctgcaggctgagtacgctgacattcccctgcagattcag

tttgggttttggctgggtctgcctctcaaaccgcccacaacattcctccccaacaatcgattctcggatgccgaagcccctgtcgttcaagactggat

cacgacggaagttggcctcggtagggcctttggtcctttcaccgacaaagagatggccacggcgggtccgtggagatcagctcctctcagcgtcatcc

atacgccagcgactgcggagaagccggagaagaaccgaatcgtcgaggacctcagccatcctcgcttccaacgtcagcgacctctttccactcagtcc

ctcctggccctcgaacatccctcggtaaactcgctggtttcgaataacaccttcttttgcaggtggtttacggtctccgctcagattgaacgatcggg

cctctcgcgttatcgcttcctcccgcccacggcagacgttatgggcatggacattaaagatgccttctacaacatctgtcctcatccttcccagcgtc

cctatctctgcgtacggttgggaccggaacaaaactacgttcgagcttccagcccgtttggccataagggaatctgcgcaatttttgggcgtaccgtc

gactgctcgtgcgacatagtctcgatgcgcctgcctggaatcctctttgggtcttgggtggatgacattggcgccattcgcccggccggcccgtcttc

gggtatcagcgaacatcaggttcgagcggaattcgagcgtctgggatggccattgcacccttcggatcgaaaggggttttccttttcccgccgtttca

cgctcactggcatcgagtgggacttggacgctcagacgatgaccctccccgaggggaagcgccaaaagtacctcggcaaggtgcaagcgattctcggc

gactcttcccgtcgtgttggccgcggtgccggctgcgccagactctcgttttccgtcgcgctctatttgccccttacgtcttccatcacgcaacttcg

gccctgcggggcgagctccgacactgggccagctgcctcgcgagtgggcccatctcccgttcttttcagctccccacccgcttcacaacgaccttgta

tgcgtctgatgcctgcgacaccggcctcggcatcgtatgcggaccctacgctcatttctggcccctccccgctggctggaaagaccgggacggtctag

>contig_21.contig_21.g878.t1

atgacctccgatgggcggtccttcccttccacttcgccgctgggggaaccgaccgagactcccgttgcgtcagcggcgt

ccacctcacatgctctccctcctctcggcgtctccctttcttctccttctttctccgttcagtcccatctctcctcttctctgtgccccctgacattg

tcacctttccaaccgtcatcaatttcttccatctcgtcttctctgggtgactgcggtttcgcacgtttcggactcccccagtcgctgctcggccgggc

ctctccctgcgaggccggcgtcgttttggtgcaagacctggttcaactcctcgtttccccccgctgtcggtcgccctcgccgcggcggttgcccgtca

agacgtttttgctcctcgttggcccccttctcggccacctcagtccccgttatgccgacctgacggaggttcagacgtggaagattcaggtttccatg

gcgtgctcggtcaagatcaagaccttgaaggactacggcagtgcggttcgcgttttttcttcgctggtgcgagagtaa

>contig_21.contig_21.g879.t1

atggtttcaacctccaaattgacgatgggatgtcgagacgcctcggtcgggcagctcgcctatgtcaacctgaggccat

gccttcccgacctccaatcagaatttttcgatcttggcgtcatacggaattcgttacgggtggagaacgaccgcgccatggcagccgtctgggcatgc

atctcgttcgcctttttcggtttggcgcgtctcggcgagctcacggtcgagactttgtccgagccgcgtgatcgtctggcccggagtctgcgtctaca

ctggtctgttcgccccgcggaaggttcggcggctcctacggtcacgctccgcctgcccacggataaggttcaaggccccaatggatccgacctcattg

cgtctcagcagcagacatccccgtcgctgtgccctgtggcggccgttcggtggcatttacacgtcaaccgtgcggttcctcaggatgctggggctttc

gcctttattgacgcggctggatgcgttcgcgaactgactactgatttttgtatcaagactgcgaatgaggcgctgtcgtcggcaaacctccaacccat

ctctggtcattgtctccgcatcggcggaactaccttctatctttctgcaggcattccggctcgcagacacggacgatggaagtcggatgcaatgttcg

tctatctgcgccgcctttacgtgaccgctggtcgggtattcgcgaacgttgacacatcccacggatga

>contig_21.contig_21.g880.t1

atgtcgagttccagtgtcgacggcttcggggggagccacgatgtcgacctgctaatcccatcggcctttccacccccgc

tagactcgacggctttccctgccgacgaagacatgaccagacgaactctctttccgaacataccacccgaggaccgccttatcaaatggcgcccgggc

agatcgatacgcaacgtcctcaccctctcttcggggaaggagcttcccggtacttcttttctcaacttggctgtcggaagagcactcggctcgtctct

gaagccctcaacccgatcaaactacggctacgcgcttggcacctttctcagattctgcttcgaactcggactctcaacgatcgacatcttccctctct

ctgaagggctccttctcgccttcgtctccacccaggctggctccaaggcgaaaggtacggtctccaactacttctccgccttgtcagcatggcacgac

atctggggcgtcgagtggaagcgtttccctctcgttgaccgcgcgctccaaggtatagcccgacttgcgcctgacaaactcccgcttcgtcctcctgt

ccgactctccgatctcgcagccgctcgatccatgctagacatcgactccaaccctctcgacgctgcggtctgggcctgcgctctcgtcaccttctggg

ccgcgtgccgattgggggaaacgacctgtccgtcacaaaacttcttcaacccggctcgacacgttactcgctcagctgtctcggctatgagtctcctt

cctgacgacgccctggccttgtcctttcaccttccatggacgaaaacgacgaaacgcgccggcatgacaaaagttttttcatcccaacctgatttgct

cgacccgatctcggctctgcatcatcacctgtcgttcaacacgactccctcgacagacaactcttcgacttctatcttcgcttaccgagtcaaggccg

gtcgaggatggaggcttgtcccgctctccaaagacaaaatgctcgacgtcttcggccacgctctcgctagagcaggcctgccctctctccctggccac

tcgttccggatcggcgccgcgactttctactggcaccacggcgccacggtggaggagatcaagctcctcggaggttgggactccgactcgttcaagat

ttatctccgcgacccagtactcggtcttgcgccgctccaacagcggcttggtgcttcgtga

>contig_21.contig_21.g881.t1

atggagagtttgcagaagaaattggagaccgtggcaggtcagattgcacgcgagatggacctgcagcgggcgcaattct

tggagacgatcaagtacgccgacggagtggcgagcaagatcgcccgtcagcgacaggctttggtcagtcgggttcgaatgcaacgggctcaggttgcg

gaacgggcggacgacgtaatgagggacgacgaccaggagcgggctatggtgcttgcttcgatgaacctctggtccaacgtggtcaattcgtggtggtc

cagtgtgctctacccggccacggatgcgcccaggttcaagaagggtaatgatcgctttgatctgtgtagcagtggacgtccgcgccatcgagtgggaa

acaggtctgcaacacgcgaccgtctcccctccatccttgagcaaggcgacagcgtcttcgtcggagcgcacacttcggctggaaaaacggtggtcgct

gagtatgcgattgccttggcgatgaaacacataacaagatgcatttacacctcgccgatcaaggcactgtcgaatcagaagttccgcgacttcaagca

gaccttcggtgcccagaacgtcggcatcctgaccggtgatgtccagatcaacccggaagcgccttgcctcatcatgacaacggagattctcagaagca

tgctctatcgtggtgcggatctgattcgagacatagagtttgtgatcttcgacgaggtgcactatgtcaacgatctagagcgtggtgtcgtctgggaa

gaagtcatcatcctgtgtccgagccacgtcaacttgatccttctctccgccaccgtgccgaacaccaaagaattcgctgattgggtgggccggacgaa

gaagaaggacatctacgtcatctctactcccaagagagctgtcccactcgagcacttcctctacgccgggaaagagcttcacaaaatcgtcgatgcca

acggccaattccagaccggaggcatgcgagaagccggcgaagcactccggcgtcgacaagataaagaacgcgaagctgctgggctccttcccctcaat

catccagctgcacgatgtggagctgctgcgggacaacgcggtggtacacgaggcggaaggggcgggggcgctgctggcgccaactcgcgaggaagagg

ccgcggagcgatgcacgctgcccgtggtgcgatccacaacgcaaaggagctccttcctgtggtcacgttcgtcttcagcaagaagagatgcgaggagt

atgcgggatccgacaagaagttgccgcagattctacgcatgcgggaactcctgagccgaggcatcggtgtccatcacggcggccttcttcccatcgtg

aagggagttgtggagattttgttccagcgcggattggtcaaggtcctcttcgccacagagacgtttgccatgtacacacaaatgtctggtcgagccag

tcgtcgtggactggataccacaggttgtgatcatcaattcatcagataccttgcccgaaaggcactaaaggtcgaagaaatgatcaagcggtccttct

cggaaaacgcatcacagaagatgctgcccgaccagcagaacaaggtcaaggatatcgagcgacagttgaaccagttggccaaggtggccggacccgga

gtgggaagcggaacggcgcttgatcagcagcaggggagcaagaatatggcgtcgggaaggatcgtgcttgtgagggatcatctctttgagaacgacgg

agcggtgattgttaagcagatcaacagtcggctattccttacattggccactgtcacccctgagcggaagtcaaagactctcgacgtcgcggagaatt

cgaagccgccactgtggaacccgacgctcaaggggcgtgtcctggacggcctcgtgtacgatcttgtggaggtaccgctcacaagtatcgtcctggtc

accaaacatgtagtcaagatcgagccatcgatgatcatggcccatcgtatctctgccatgcaaggcgcggtgaacgcgatgatcccttaccttctgga

gtggtcggagcaaggcgtcattccggaggtggagtggtccaagcttcggaaacttgacttccaagaagcattacgggctcgcgacgggtacgtctcga

agatcgcacagcagtcgtatatcctaggaaaggaggacttcgccaaagactacgcaactgtagacaaacgcaagcgtctccatcgtgagatcgcctcg

ctccgaatgagcatctccgatcagaacctcgagctcctgccagactacgaacaacggatccaagtgctccaaacgctccactttgtcgacccgctcaa

cgagagcgtcctcctgaaaggccgagtcgcatgcgagatcaactcggtcaacgagctcgtgctgacggagctcatcgtgaacaatgtctttgcggcct

atcagccggacgaggtcgttgcgctgctgagcgtgtttgtgttccaggagaagacggaggtcaacaacatctccgtcctccccgtcgacgaccaagtc

aagtgcatcgagggcatgattgacgcagccgacgagcgcgtgcgcgcaaagggcatccccactaacttcgacgagtggatcgtgcgcatgatgggtgt

cggtatcgcagatctcttcatgcgaccttacaacttcaaggtctgggccgttcccaccactcagcgtgtcgctgctccctcactcaagctcgtcgtct

ccaacaccctcaacaagaaggtagccggcaactggggtcccaacgccacattcaagttccctgccttcggcggcacgggtgccatctggaaggccgtg

gccaagacgctgccgtcggacaagcttctgttcaagaagcgcgtcgccaagatcgatgccaagggtcacctcgcccaccttgaggacggcagcagtgt

gcagtacaagcacctgatcactacgatggagctcgacttcctcgtgaacaactcggagaacgtcgagccgaagtcgcacggcatcataaaggccgccg

tgcgcgaaggtctggtgtactcctccactcacgtcatcggcattggaatccgtggtgtgctcccgcctcggatcggtgacaaatgctggctgtacttc

cccgaggacgactcgccgttctaccgggcgaccatcttcagcaatggccacgatccgaaaaacttgcccaggcggtcgttgacggcgggatcgcaaaa

tgacgagcaagggaacggcaacccctcgttccgaccttctttccctactggcggcgccgctggtcgttctgcaagctcatcattggacgctgtcatct

gccctggcaccgaggcaaggtctacatcctttctcccactcccaacctggctggaagagctccatctggcactccgctttgacggttcacacccgcgt

gctctggtcagcgcccgcacaaatgccagtgaccaggatgcaacaagtcgttctccggttcgtcttcgctcgcccggcaccgtcttcatttccaatag

catcaacaagaaagagaacgaaacagtcgtcgacatgaccggtgaggcggtgaccctccgcacgcccaagggggagctcgcgaagcagcgacggcagg

acaagaaggacaagtccaagacggcagcggcgaacggcaacagagaacaccattcggtgcactcgcctcgaggaacgcattctgtccgccaaggacca

gcaacatcatctgctacagctctgtgcctctccttcctcggcctcgataggcccacccaccatagcagcttcctctctagtcctcctctcaggcatgt

cagccagtcagctttcgcctttcagacgatcaaaagtcagtgtcagaccctcgacccgaaaacgttgtctacaatgtccgaaggacaggaaggttcct

cggctgaccaggccgccattgcgcgcgctctggcagctgctcaactccgcgcacgacaagctgcttccgacatacaacaacagcagcagcaaggcacc

ctccaagcctacgcgcaagctcgcgacaaagacgtcgctcacggtgaagctcttcaagcagccagctccggcacgtctacgaatccgaacacctcgac

ctcgtccgccactggttcaactccagctggccctagctcggtctcgatactcccagcaacagctacagcctcgactgtgggctcaacacctgcccaga

cttccgcatcgaagaggactcgacaagcctccaagcccggtgacgccacaaagcagccggcgcagaagcaagctcgggtggagccccaagcagctcgc

acagggccgacagatgcccagcaatcctcgagcacagcgagcaccgctcagtcgaccagcacaccagcccgctcgtcgactcctcccccgaacccgtc

catcaaggctctgatcgacgctttcacacagttctcgccaaacagccgctccaacctcatcgcctcgctggcgaacatggacaaggttccgggggacg

ataccagcatacaatccggcctcgaacaagaacgacaacagagcggcaaccccccgactcttggctcgtctcgcgtcgaagctggttcgtcttcacag

tccagtgctcaccctcatcctccaggagcctcgacgacaagccatgagagaggcgctcctcctcatgcgtctcaacatgctccctcgctgcctcgtct

ctcctcgcaacctccggtgatccagcgacctgtcgggtactcaaaatcggcgatactcgacaacttcatcctgctcccgagtccggctgtcgtcacca

agatgacctcgtcccagttggtgcctctgtggcatttcacgaaggagggcatcgcggcgggcttcaccaagtactcctcccagaccgacacaagcaaa

cgactcctcgagaccctcggtgcggacattcctctcgccaaggaacgaatggacgacgagcagctcaattacgagcagttctcgcgcgccaccgacag

ccagatcgacatcctcaaagatctggcgaaggccgaggtcgaccccatcaagcgacagctacttcaagaggaggcggacatatgggagttggcctacc

ttcgctgcgctaaggacgaccgagcgaagcgccgatggccaatgatggccaagtacgtcgcgctgatgcggcataactggtatacaactccagcgggc

gagcgtcgattcgacccctcgaagtggcaggatcccatctgggagcacgtcatgatcctcaaggagtcagacgccttcaccgagcagccgtccacggt

gatgcagcaccaagcctcatcctcctcgcaagcgcactcgtcgacctctcacacgcagcaggcctcgagctcaaagcacaagcagccacctcgacagc

aacagcagccctttccggcaagctcctcggcggaaacccgcacctaccgaggagcgtctgtctgcatcatgtgtgggaagggagcctcaccaggccac

aacttcatctcctgccgtgccaccccgcccggtcgatcatcctgctatgcctatcggaacgatctcggacacctcttccgggaatccgaccaagcctc

gctctgcatgcgcttcaaccgaggcatctgcccccgcgccgagacaggagggtcgtgctggctccgacccgacctagcgggcaaggggcggcccctca

gctctgagggtttcgcgtccgccctcaaacaatacaaccttctcgacgctttccagaccactgtggacggccttcgagatgggttcgacttcggcgtg

ccgcggatcaacaagacgatcacgccgacgaatcatgcctcggctcgagacaatcggcaggcactgatcgacattgctgcaaaaagaggtggagaagg

gccgtccagccctctcggaatcatcccgaaaccaaatgggaaggtccgactcatccaggacttctcgcacccgaagaaggagaaggcgaaaagatccc

tcggctacaacagcatcaacgcatacatcgattccgacgaattcctcaccgggtgggactcggtatcagacgtcttcaagcttctcaactcgctgccg

accacagtcgaaggagcgacgatggatgcttccgaagccttccgcggatgcttggctcgtctatctcagctccccggtctcgtcgtgtgtcttgacga

cgacactttctatatcgacttcttcttgggctttggcctagcgtcggccacaggtgtctggggtctcgtcggcgatgctaccaaggcgattctcaaag

ctcgtctcgccggtcggatcagagtcctcaagtgggtagacgacttctttaccattcgcctggacccaacaatcacgatcaacgacatcctcgctgcg

acaagagagctggacttcccctggaatccagataagacggttgatttcaaggccgacccgaagtatatcggatgggtcttcaacatcccagagaggaa

ggctgtcctgccccgagagaaggcagagaagtacgtcgatcgagctacacctttcgagaaagggctgcaacaagacaagcacacaacacagaagctgc

taggctctctgcaacacgttgcctttgtggctcgtgacctccgaccccacctcggctcgatcgcctcttttctcgccagctggccggaggagaagcag

taccagaagcggttcattcctgtcgaggtccagaaggaagccgggttctgggtcaaggccctgtcgattgtccctttcgtccgatcttatgctctccc

tccgaaacggttcccgaacacgaggggtcacagcaggggaaaacagatcaatactgtcctcaagcgcgttgtcgacatggaacgagacgatcgactac

aactcgatatcaagtacatcgaatccaagctcaaccctgctgacgacccgtcgagaggcgtcttctcttctggccctcgcctgccaactttcaaaatc

cccccgctcgtctccgcctatgtcgtcccggccggggacctatag

>contig_21.contig_21.g882.t1

atggcaccgctgccacgactgcctgctgagatctgggatctcatactcaaccatatctttgccgatccggactcgcagg

ccactctcgcacagttgagtaggacttgctattcatggtaccatctgcttcgcttcaagttgctacagcgagttcgcctttcgtatccgaacggcagc

tctgctttctcccgtcagctttatacggagctggcattaggatggtgcgttcgccgcctcgaattcactgggtcgctgaaggaaacggatctctaccg

cgatcaacaccgtagcgcacggtttcgggtcctgtcgaagcgatgtcctttcctcgaggttttcattcttttccgcggagatttgacggaggatggag

agattgtggcattctctgcccaatttcctttcctccacaccatcgtcgtccagggcttctccaagtgggacctagatacgttctccggcacgatgacg

gaaaacttctgcttccctaccatttggaaatctgtcgcaccgtccctgcggcatgtcatcgtttccgaagactactacgatgatctaccgatatgctc

ttgcatcgacgcgcagtacacgatctttgtcaatcgcgtctctgcaaaatcgtctccacccgatgccgttagatcttgccgggatccagaactcactc

aacaggcatacctcacatga

>contig_21.contig_21.g883.t1

atgctctcatcaaaaagtctcgccaagatcggagacggcgatatgatcagcgtccttctgcacggctggtggcgcgatg

agcgtcctgtggagggttcgatcattctagggacatttatggtcgctagccctcaaaccggagtcgagcttttctttgctcgctcggaagtcgaagtt

cgcccaggcgcggctaccgatccagactacatggcgaggcacatttcgttccttcctatgtgctgcaagcctgcaaagttcggaatgaagcacgcaag

ccctgctgcaaatcccactgttacattgctcaagggatatgttagcagccagatgatggcacactgcgaatacggttag

>contig_21.contig_21.g884.t1

atgtccttggacgtccacgaggaaggcggtcaacgtgtgacgtttgatccggacgatttgaatggaactctcgagacta

tggaaggagaagaacgtcgatcgaagttgctcgcattctttcagctctgcggtcgctctggatcagaaacggcggagctaacctatccagaggttccg

tcccaatacgtatggaatcagaaagaccgcacatggaagcggcgctcccaaggaaggcagatcggccgtgtctatttcgtcccacagacggcgggcga

gaaattctatctcagaatgctgctgcaccatgtccggggaccaacatcgttcgctgacgttcggacgttcgaaggattcgtctatcctaccttccgtg

ctgcttgcctcgctcgtggactccttaccgacgactctgagtgggactactgccttcgagaagcttctgcgatacagcacggatctgctctgcgtcgc

ctattctgcgctatcctgctaaaccaaagcgtggacgatcctgttcgactctacacaaagcacagagaagcccttgcggacgattgtcgtcgactcct

tctccgccgtggagtccaaaacccatcgttcgaacgatgcttatccctttcacgagctctgcttcgtgacgttttggggtcgatgcaagctgatgtgg

atttggctctgtacggtatacccccaccggatgacatatacagcttcgatgaagtacgtattccggtcgcggtactggaggaagtcggctacaactgg

gacacacagcggctcaagtacgaagaaatggtccgcctcatgaacgcagatcaagcgatggctttcgagaccatacaggccgcgatcgacgatcctgc

ttcttcgtcaaccaccttcttcctcgatgggccaggcggatacgacgcctctctacagatggaatacggtcgctggacgatgcacattggtaatggcc

actgccctcaggacaacaagggacgagtcatggttccggatcccattcggttgaatggaaacgacgcttccagtttaattcgttttgtctacaaatcc

atcccggccgccttttctccagtaaccgcacaaaggtattgggctcctcgcgcaattcttcatgcacgaaacgatgctgccgacaaaacgaacgagac

gatattgtcaatgcttccaggcgaagcaattaccttcttcagcgccgacagagcggtcacggccgacggcgacgagtctcctgcgttctctgcagaat

acctagcgacgcaacagccctctggatctcccccgcatgcactttcagcaatcagccctaaaaatccccaagacatcacgaacgcggccacctctgca

tcgcaaatcgatatgaactatgttatcgaagaagctgaagaggacgactcctcaagctctcaatctaccttgacgagtcttttttcagaacctagcgg

agattcgatcggaactgcatcttcccagtcggcaccagacacggaaggagacgaatctgattcatcggcggactcggatacgtcaattgttcagttca

ttgtttccgacaatgaggacgtgacgcagtctaccggatcaccagacgcttcagaaggaactgcttctttcacggacggagtctcagatgactccaac

tcggattcaacgtcatcttccaagctcagcgctcacagcctcaaacgtcgtggtcggacgcgcgtggaccctgaacagcctcggcaggagttggagac

agcggcaggtcagatcaaggacgaatatgaactagagtggatggacggtgctatacgaaagcgtgtgaacagttccggagacatctttgctttcatac

ggcagcgatggatacccgatcaaaggccagagcacatcatttgggacatgacaccagaacatgctctgctgcaatga

>contig_21.contig_21.g885.t1

atgaggattgaagaagctttttgcgagacgtcgcatcggttcgtactcaaatctgcgatcaacaacgaggtggccggtc

gggcctattccctcgacggagaaactcatgacgttcgccttgcctggaactacggccatacacctccagcagagatcatctacggtatcacaggaccg

ctcacgttcgaggagctggcgctccgcatcgacgtgaacgaagggcgctttgaggcgcgacacggagataaaacggaccctgcctaccttataaatca

gagtctttctctgcaatggcgcgtgcgaggagttggtcgagtggttgaaacttcagctgggctttttagcctggagattgtgtccgagacgaacggga

aggtgacggtgtttatgatggacgttcgcattccaatcgacaacaagcgttgggcaaagtatcggacagtcccggtcggaagcttcgtgggcgtgcgc

ggaacggtagagacgatcaagtataacgacatcgccaagatcgtcatcaccctggagcatctctgcagcccaccgcctaacagaagtggaggccctgg

aggtccatctcccccgaactcggcagaaacgtctagcagcattcagacccgatacatgaacacgctcggtatcacgacagcagacccctccgctggat

cttcctccggagctggtacctctatcgctgcgccatctccaccaaagctaaccctcgagtctggacgcagcgtgcctccagaaattgcagcgacttca

gctttcactgcgcctactcagtacatcggcgacgccgcacatcatcccgcatcccggcagaacatcgaattaaacgtcaacgccgaggaaacatctgg

tcagaactttgttcttcttgcccagcagatcacccgacagccagcggaccttggattggcagcaggaagcgcggagcgttcctctgcagcggcagcag

cggaaggaaaggggaagaaacgtagccgagaagatgaaggcaaagaggtcatgggtgcaaaaagggcggtctccgtacgctaa

>contig_21.contig_21.g886.t1

atgatcaacgacgaacttgcgatggagtctcagggagatgaggtgcttgaagaacggcagctcgatgactttttcttgc

aggaaaccgacataccgtattcaacgactgcatccctcgtccttgtcatctatctcggccaagtgttcaattttcaatcatctgcagcagcgaagcca

cgtcctcccgcttcgacactcgacagcagctcgctcgcgcgggaggtatctttccggccttgccagctcgaccaagacgggccggccccaagctcggt

agtcaaacgcgcctccgcttggaccacttcctcctcagctttggggcctacgcctcccgcccgctctgctatcctagcccggttgccgtcgccgcctg

cacctggtctcgtcgcccactggactgcaggccttacggcgccaccacccggagctcgttctggatacctctga

>contig_21.contig_21.g887.t1

atggcgaacaatgggttcttcgctcacccgggagccccgaccgatctgttgcccggggagatttacttcccgaccccca

tgcatctcgatccgccggacacgcccattcaaggcgagggcgtggacgacgaccacgacgaagacgatgccgacgtgacggacgaagacgagatagtg

acactcgatagacacgacgaggcgatggacgaggatgttgctggaccaagtaacgctgttgcaggtcccagcgcgagcgcagtcgtcgccgtcacaga

agcggccgcagctgcgcgacccaagcagaagatgaaggagtttaacgagctgccacaagagcttctcatagaggtccttctcaatctcgagccgatcc

atcttgccgttgcctctcggacgaacaagcggatgtggacgatcacgaaggacctctcgttccgggctactgaccacaactcgccactcccgacctct

tacagagacccctcgaccaaggagcccacttctcccgctacctggcgcaaatggtcagttttgcaggagagcgccatcatctgcaccaccacggctct

aatcgcatgttctccaacatatgcctttccaagctatggcaacgacatggctatcagtcgcgtctccgatgatgccagcatgctgagcaacctcttga

tcacgttcaagcgagattcagactggaagttcatcgaagagctcttcaacaagtacaatttcatgtactactctcggcacgattccaatcggatctat

ttcgtccatcagattgcccaggagcctcggctggcacctttggccaccaagaacggtctcagagtatctacggatgactgcgacacgatcatgagaaa

tgtcttcggagatagcgtgaagacggccgaggagaaggctgcttccctctcgactttcatcgagtccggcctaccgggacttcacgtttccaagacca

tggcgatcaaggccatcttgcatcagactggcgatgtgggaagtcggttgttcgatttcaccgctgctcgctcgacgtcctacagggccctcaaggtc

cttgccgacagcggaaagctgaagctcaatctgagtcgggagacttccctgaagactgaagtcatcccactcttgcctcgcaaagctgctttgatcaa

gttccttgcgcacaccgactatggcactgatactgcgcggaacaagcttcacaaggctcttgccgacgcacgcccccatctgacacagcgcctgattg

tcgatctggttgcgaatgagttcatgactactgcggtcccgctgcaatacgctcgcgacctccttcgcgacgacgaggtgaatgccaaagtagcagct

tacgaggcgttgcggaagtgtttttggtttgatttggaccagctgcaagccgacgatcctgacaacagagaatctgaccacagggaggagcggcccgt

ttctttgcttgcctggaactatgcaggagcgatgatggatgtggacgcctgctccgacgagaccacaaacaatccgtaccagcttcatgacaagggta

aaggcccggcgctcgacaagcccgcggaaacgtcaacgggcaacgatgcaagcactcctggcgcagagcagaaccaaccgcagcagcaagcagctata

tccgctacttcggagtctgagctgaaggaggaaccgcttccggacaagaatccgttcctcagctctaatccatgggccgacgacaacctggcgtacga

cttcgcacaatgtttttcgcctctttccattcctctggctgaagctgtcctggaggtgttcggagcgcattcggaagctgccgacatcatgtatgggc

atgcgttgctcgacggcaatggggccatcatggacctgtaccactctcgcatgttctacccttccctcatgcacctgcgccttctgctccacgtcggt

cggcgacttcccgacaaattccaaactcggctcgagcgagcttcattccgcaagcgtccgtacggctcgatggagaacgaggttcgtggccggacaaa

gctcgaatccccgttctggtgcagcaccgacaagctgccgctcaccgcccgtcctggatctgcggatgcgaccgtcaacctcgagaggtgtgatgact

actttgggttcggcgagaacgaaagcgacgacgaagagttgataagctggcgtcggcacactcgcgggaaaaatggcgtcaagaaggaaggagcggag

tcgccgggtcccagtccgcggaagcgtagttcaaggacgccgcgtactggcaccaagcaggatgcagcgccgtttcatgcccctctatcggcaactgt

aactgcacctgcttctgttcctactcctacacctgcacatgcgactatcattggcgaacttgcaactgcacctgcacctgtcgctacccctgctcctg

ctaccacgattggcgacctggctgctgctgtctttgcacagtccgcttcaatctctaccatcgcaccgccagcttctgtaccttctgtcgcagccacg

gctccagttaccgcggttgcgacgccggcttctgcatctgctcctgctactgctgttgcagcctcgactttaccctctgctgtcgaaggcgccacctc

caatgctggggtcgcgccgccagtcacgacaactatcgcgacggctgagatgcagacgggtacctcggcagcggcggatcgcaaggcagccgcagccg

tgacgtactttgccgatgaaaactcttcggacgactttgaagtgaaccctgtgccagctcagcaacagtctccggtcaaatctcctgtgaagaccaag

gcggcgcccgcccgaggcaaaaagcgcaagattggccaagacgacgatgtggagttcatattgctgggagtgaaagagcacgacgtgaagccggtgat

accgatgccggttttcatcgggcccaaccagctttcgagcttgcaggtgctcagcctttgggagtcgaccgcggagattgaggtgatgtggaagtggg

cgacgtttgatcggcactcgaaagtcctcgcagctctggatgcatga

>contig_21.contig_21.g888.t1

atgttccacgcagcagcaacagtagcatcaccagtgacaccaccaccggcagcaccacaaccagcagcagcgccagcag

cagcatccagcatcagcatctccccgtcgcctactccacatccggcgctctcggctggaactggcgtgcgcgccatctgctcgccggcatggtcctat

gcgcttgtcatctctcgcttcattcttctcgactccgtgctgctctacttcaccttccatggcatggtcaagttccactcctatcagaaccggtgcgt

caatcggcgcagccaccagttcaaatgggtcggcttgtttgccatagccctcattggtctctgcagcatggaggatctgtgggaaaagtttggtggtt

tccgaatcagctcacagatgagcagtctcttcaggctggtctcaagggcaatgagtttgcaaaaaaaaaaccctgttgaggctgcctacggtagcaag

gtcacgttcaagaacatgggccaaggcggcggtctcctgcactctcacctctattacttccaggaggctaatatgagcacagcacccacttacacggc

aagagctttcgttctagtccatggcccacgccccgaacgccccttctcatgctgcagcataaatttcgccatcatgccttcagctacctccaactctc

cccccaccagtcacacgacggacttcgtcgctgtcgagtccggtgccagtacgcccgacactacagtttcccactcgtcctcatacgcggtcacccgt

cctctcaccatcggcgccaacggagtccacgtcaaggtccaagtgccttcctatcaggacggctggctcgcatctcaactcctccgtgagggttttga

gagctcctacaagaaggctcgccgcgggagcaacgacagcgctcggcccgccctcgctcctgcggactcgtccgatggtgacgagactgctgtgggct

cgacgctccctaacggagctgagcctgcgcatgtctcgctcacagcttcgttcctcggcttttccgcctgcaaggtgaacgaggagcccgacggaccc

gtcatcgagtgcgtcaaggccctctggacttttttccacgccgagtacctcaccatctcgaagtctaacagcgtcgatattcattcggtcgctatgaa

tgctgcggcgttcgacgagcagtgtcttttgtgcgattacttcactgcttacgtcacgctggagaagcagggcaagttgccactgctgccaactccga

aactgctcagcctggccgggaagggcgaagctggcattttcgctctcttcggaggacagggtaccaatgaagtctacttcaacgagccccagcttctc

ttcaacacacactatgaccctttcgagagcctcatcgtcaccgctctctacatcaaccaggtcggcatcagcctttacatctacaacggcactcgccg

atacgtcttcactaaccagcttcggaaggacactcttcaagagaccttcaccaacgttgccggggaaggctcggtcgagttcgccagcatgaatgcaa

atggtgtgcaggtgctcatgagcctcgctaccgctctcgagatggactgccccatccaagccatcgtcgcctgcatggaccttcacttccgagtcaac

ctgatcaatgtcgagctgttcaccgatgctgcgacgtcccaggagagccttcacaaagccaatctggccaagtctgcgatcaacggcacggatgtcaa

gttcaagatgctgagcaactctgcgatgaaggccgatgtcgctctgaaatcgttcacggtctcggacacccggtcagtcaaggataccaagttcaggg

agattattccggccgtcaagcacaacgctcgccagttcgtgctgaactacaggatgtcggccggtgccaacgcgtcctctctggcgcttgtgactgtc

gacagtccgaacatcattttctcgctcgacgctgccttcgcccttatgaacttcttcatgagtgcattcccaccagcgcccgcggaggccagtgcctt

ttcgggcaaaggcagtcagcaggcgaagaccagtacgagcaagccccagacgaagcaagactcctctccgccctcagaaaacaccatggcgttccgcg

tgcacgtgctggatccgaccattattctgcttgctgcaccaaagcgtgctgacacggaggccatcgtgctgtcgatcaagcagttcctgctgggcgtc

ctggcgttgaaggtggatcagatggggatgcttatgaggaggatgaaccggcccaaggagtcgtcgcgcttccctgacgacttcgaagtgacaacagt

cgcggtctgtacgggcgtccgactcgtgccatcaatactcgatcgtatctctcgagcttcgcaagaggagaacagctcgactcgtacgaattggagag

cagctggcagaccaaccccgccagcggccagccaggggtcgtcatctttcaaccccgcgatcgcacatcctctttgctgcgcctattcatactctagc

aaagcggctgaaccctcagcacaatggcttgccatcggcgacaacgaaggccgcatcattctcctcaacaccctcgacgactccacagcagacgtatt

cacaggcccaccacaatggcgcgtcaccggtcgagacctcggcataactgcgaccgggcctacccccttggtcttcgaactcgcctggcggcaggacg

acgtccttctcgcttccagcgcgtccaattactcggtggcggtatgggatgtcagcacgcatgcatgtactgacgtctttgctgggcctcagggaagc

gctcgaagtatccagttgtag

>contig_21.contig_21.g889.t1

atgaacatcaacatcattgctctcttctgcgcgctcgtcctctccgccagcatcgtcgccgcggatgatggagatcgcg

gctttcatcctcacgacgagaataaagcactcctgcgccgcggccgtgctccgcgtcgtatcgatcccctggatcccaaacctccgtttgtcgaccct

ttcccggaccccggcctagcagcctcgagcacctccgatgtcctcgacctcggcggcgcatccctagttcgtcgccggtcgcccagctctctccctcc

ctggtccccggcgcttcgtctccttccttcgcgctgcgtccatggatctgccctccctcagccctcttctgtctctcgactcgccgagttgagaaagg

gtacgacaccagacgcttctcccccttttggcaagccgaatccccaagcttccgttcttcattccacgatgaacatcaaggtcatcactctcctctgc

ggtctcgtcctctccgccagcatcgtcgccgcaggtgatggaccaagcggagatcatgcccgcagatacaccgtgactgtgaaacccagacccccgac

catcaaaccgaccccagcgtgcatgttaatgtgccaagagcatcgacacctccagataaagtatggcacctgccaaaagtactgcgactactgcaaga

cggttgagcactctatggaggacgactgctggtattggtcaccgtag

>contig_21.contig_21.g890.t1

atgcagaggttcatcgcatcaagagcagcagcagcagcagcagcgacgggggcagcgacagcagcagcgcgctcccatg

tcgcagccgcgtgtctcgcactcactctcgcgcctcgccctgcgctccctgcatccaggagcgagcggcaggcgtcaacatcagcacataccctcgct

cccattccaccaccagcgtcgtccctgtcatcgtcatcatcgccatcatcaacagcagtagcgacggcatcagacgccagcgcagtagcccagtttgt

cgatctcggtctgccccggcctctcgctcggcagctcatcgcagccttcccacacattcacgcccccaccctagcacagaccgctctgctcgaggcca

tcacccagcccaacgatgtcattctcagagcacatacaggcacaggaaagagctttgccctcctgctcgccctcctcgccaagccgcgcgtcctcttc

tcccagcctgcttctcttagctcgagcagcgaggagcggcaacagcaacatggtatcgcaagcatcatcatagtgccgagcaacgagctcgcattcca

gtactactcctgggcacgcgctctgctgccacaggccggtgccgagcttcatccgatcatgcaagtcctcgtgcgaggacacccgactctctctgccc

aggatcagctcgatctccttcgcaagacaccgccccatctcatcgtcgcaacaccgacccgcctgctcgagattctctccggaaagacaaacgatgga

catgcgctgctcggcctatccaccctccgtaccctcgcacttgacgaagtggacgcactcctcgatctgcccggacgtttcccgactcacaagctcaa

atggaaacacacggtccacccggcacctgctctcgtcttcctcaacgaggtcatgcggctccgtccttcttgctcaggaggccagctcttgccttcag

caggcctcgaacgcgacgagggcagacatttcgacgaacgcagaccacccgatgcgatccgtcgtactgctcacagatcacgagaggctctcatctcc

aaacagcaacaagccccaaaacaggagtcatcggcgcattcgttctactccgccttcaactttgcccttcccaagccccgatatgggcccacgggcag

tccacccattcaactcgtcgtcaccagtgcaacagcgaatgccgttctacgacacttctttggcgcccggactggctggcttcgaaccggtgttaagg

atcagatcggaagaccgcaaggtcaagtctcaaatcacgcatacgcacgccaagaacgcatcacaggcgtctggctcgatctcaccggtcttaccaag

gcctctgccgagctcactgcgtctctggacaaggggaaaaggcagaaagcagaagcggacgatcatgcgtcgacattcccaacaggtgccgagggcag

tctctctgccaagctcaaggctgaaggacgtgtaatcgacgaggctgataggactacgttcccaatgacgatgccagaagagctcacccactactgcc

tcgttgtcgacgagcccagtccaaacgagcgctccccactcgaatcagagggcaccgacagcaatgctaccgtctccgatacaaccctagcgagcgca

ctcccacccatgcgcaacctcgatccacgcgccttcatccgaccctggcgcaaggccctcgatcttgtccggaccaccggccaaccagacctggcgac

gaccatccgatcggcttcgccaccggcacacgaggtcgacccgcacttgctcggagcgctcgcattcgcgttcgcgtcggatgagagctcgcgtgggc

ttgcgcttgtgccggcgcagtggagtttgaggaagacgaaggaaatgttggaagggtggggggttcctgttcgcctcctggatgggggaggcagcagt

gagggcggcgatggagcagagacgacggcggcagcggaaggaaaggattctccggtgctctacctgctccaggccacatccgcacgaggtctcgacct

gcccgtcctcacccacgtcttcattgtaggcatcgaagcggtcggcgatgcagtgcgatacactcatctggcaggccgagcctctcgaattggaccca

acgcagaactggctaccatcccccaacctcaagaccctgacaacagtgaggatggcggtgggaacaggaacatccagacgacactgcgaccgttggca

aaggtgatcaccatcatccgcggcctcccacatgcctccgtcctccgccgacgagccgccctccgagcctggtcttccctcacaagagaggaacgcgc

cgcgcgcgggatgaagaaaccgcaacacgacgccatcatggtcagttctgccgagcgtaagctctggaccgtgtggagaaggacgggggtcagtgggc

ggaaggcggggcaagggaggagtgggggatggagcaagtgggatttgagattggttggtggggagacggttgaggatgaggttgcactggatgggtgg

acacctgaggaggatgaggccgagttcgaggaggggaggcaggagtttgaagaagaggaggaagagatggaagacgaggttgcagatggggaaaggga

aggtcatggcttcaaggctgctgcgagtccgagatgggagcccagagaagaaagaaatggaacccgcgagcgtgcttcgtcccacctgcgcgaacgta

gagaggagaagaatggtaactccaagcgtacctcgtctcctcggtgggaacggagggaggacaagtacgcagcttccaaccgtgcttcacttcctcag

cgcgaccgcagggaggacggaggtggtagcttccgacgtacctcggctcctcggcatggactcaaagcagagagagacgttagtttcagacgcgcctc

ggtccctcagtggaaacgttcgaacgctaaagatggaggcttccagcgcacttcggattctcatcgagagcaatcagacaacacggatggaggattca

agcgttccttcgctcaacgaggagatcgcggggagggaagggacgatggctttgtctccaagcgtggctcagctccccaatcaagacatccaagattc

gatcgcgagggcaggaaaggggctacgaacggtggtgaagatcggctggaatttgggggctcggaacgcagctctggcccagggcgtagacggcatga

ttcaacgggacgaccacagacggcatcagcacggaggtggtaa

>contig_21.contig_21.g891.t1

atggaggtcagcggtcagatactgtatatcttctcatcagcaacgaccttccacagacatacacacgtcaatagcaagt

cgctttctttctcgtacaacgcgcgcagacaggtgtctacttcagtgatggcgcaccaaagaatattcgttgtcggcggctctggtttcctcggctct

gctatcgtcaagcaggccgttgctcgagggtgggaggtcgtcagtatcagcccgtcgggtcggcccttcaccactcccgccggccatcgtccgggctg

gtctacctctccgctcattcgctggcaaagcgctgatgcgctccggccggagacgtacgcagacgagctgcgctcatgcacggctgtggttcatacca

ttggcatcttgctcgagtcggattacaagggcggccggtcacgaactgcgggaggggcccaggagtctggcgctggtggtggcttgcttcaagggctg

ctgaagggatgggggtttgggtcgggcaatcctctgaatgagtcggcgggcaagctatcgtatgaggtcatgaaccgcgattcgggtgcgtacatttc

ccagccgttctcactgccattgccgtcgccgctcaggcatacgcaagcattctatccatcacgacatgctgataattctatccatattacgtatcgaa

tagctttgagcgtagcccgggctttcatcgaatcgcgcactggtacatgcccccttacctcaacacctacttcgacatccgctccgttcgtctacatc

tccgccgaagacatcttcagacccatcgtgtcggaacgatacatcaagacgaagcgggaggccgagtacgagcttgcccgactcgcacttgactcgct

caatgcatcttcctcctcctcttcgacttcgttatcctccctggcgaccgcggcggcgaaggagcctgtcatccgacccttttatgtccggcctgggc

tgatgtatcacccgcaaacgcggccgctctcgacgttccctgcggcactgctcgacctttctgcctccatacatgccagactggagccggcatcatcg

acatctgtccccggaaacgacgaccctctatcatctcaaagacaatcgtcgtcagcgtccagactacccttcggcctacggattcccactccagcctc

tctcatccggtccttgggtccagaaggagtccagccactcgccgacctgctcacgctcccacccctccacatcgatacggtggccaaggcagtttgcg

ccgctattgcgggcgaggcgcaagagcagcggatcgggctttcggtgacggctcaggtggaagcgagggaggcgccgatctctgtaggcgaagttttg

gccacctctgatatccggagactggctggactcgagcatggccgagtcgaccacccccccaccaacaacaagtccgacctggagaaggtcctggccgc

ggtcgaagcctcgaccacgtccaacaatcgccgcttcgaccgattcgaagccgagctcgccgagctcaaggccaaccgcacgatgtcgaccatcggcg

gtacccccccttcggcgagcgttttccaccgccccctccagacgcccgtccaggctacgcccaccgcccgcggtgtttccgggttcctcgtgcccatc

acccccaaggcctcgaggaacaaggacgagaacgtcgtcgctttccgccacctcgatccccacgtctcggccgccgagcgcatcgaggcctcgcgtcg

ggccagtgacgagaccagcgacgaggttcgccaactcaaggccgaactctggacggcgtgcctctccgtcgtcgagaaggagaaccccggcgcctccg

acgtcctgatcgcggagaagaccgaccagatgttgtcggagcagtggctggctctccgtaaggaccgcatcgctcgctcgttcgataccgcccaccgt

ggcgacacgagcaaggacttcggcaagggaaacgctggcaagaaagaatggcagcgcaagatcagccagtcggcaccctcccgcgtcctcaacaaact

cacccacggcaattacatggactgggtgtccgagatcatcagcatcctcgccccgatccaaggcgccaccgagatactcgagggtatcgagcatggtc

gcggatacgtcgaggacgacccggacaaggagaccgaaggctacgacgtccgcctcgacttggagatcggaggactcgtggccggttccgtctccgcc

gaatgccgcggctacgtcgtctcgcgacagtcgacaggcgaataccggggcagtgttctgtggagggacctggtgctcgtgatgcgccgcggcgacgg

cgtcgataaggcgacgatctttgaggacgtgattgccgaaaggcaggaatcgaacgaaggcgtgcgcccatacgcggagaggttgcggacgaacttca

tcaagctgggagcaatcaactcgccgctctcggcagacgaccaggttatgtacctcctgcgtggtcttcagccccgcatggatacccaccgagtcgct

gtccgcgccttccggcagtctggaaccgcctacacgttcgacgcggcactctcgttcctcctccagcaagaggccgccgcctcactcgatcggtccca

ggccccaaagcgactgtcgctcttgtcccggccgaccgctaactgggccgcgggcgagaatcaaggacaagtccgcaagttccatgtcattcggaact

cggccaacaagggacagccaggatacttcaatggggaatgtcgccattgccacaagtggggccacaagcaggaggactgcgctcagaggaagcgcgaa

atcgatgccggtctggtcgatcccaagcacccgagcgccaaggtggcccagtccggagaagtggaggaacaagaggagtccggatccgatgttgaggg

cgacatctacgaagtcatggacgcaaacggggaagtcttcttcgcccggctcgccaccggacccgtcggcgatacggacaacgaagccaacgcggccg

tggccatgatggcccacagctccatcgccatcgcttccaaggctgcagtctcacacccagactggaccgacgaggacctcgacagtttcagcgaggct

cacatccccaagaacgcgatcaagtggatcctggactccggcgctactcatcatatgacgcccaggtccgagctcctcccagtccgcgtcaaggccgc

cacgggggcagtagtccacatcgcggacggaaccaaggccaatatcgagcgccgaggagcctgcatcgcgtcaatgttcggcgacggcggcacggagg

ccaaaaacgtggtcctcaagaccgtcctcgtagtgcccagtttcaccaccaatctcctctcggtccagcgtctcgccgaggacggttggaccgtcaag

ttcgcgcgaactggagcggaactcgtcagcccaaagggagagcgggtggaaatcttcaccgagccaggcacaggagcacccttcgtgatgcttcggac

ccagcctgtgaagtacatcgacgtcaagggctcggcaaggcgtatgggaccagccatggccgcggcccacgtcgccatcacggaccgagacttggacg

aggcagctaagtggcaccagcgaatggggcacttgagctaccatgcgctccgaatcctcgcttccaacccagcgttcgcacgccctagccggcctagc

ccgcaggccttcgccaggatcatcgcggaagaagatctctgcgagccttgcgtggaaaccaagcagccgctactccctttcggcaactccgagaccgt

gccggaggcaaagatgagcgatgtggccttcgatctcgtaggaccattcgaaggcaataaggagttcaaatacgccttcaacctctcggaagcttggg

caaggatgctgtggagttttcctatccccaacaaggaagcagccaccgtcttcgccgtcttcaaggagtgggccgacctcatgaagacacacggccac

ggcagcatcagacgcatccactgcgaccacggcggcgaattcgagaatcgcctcttcgagtcgtgggcggccgagaacggcgtcgagtggctcagcga

ctccttctggccatacgccttccgagctgccgcagaccttatcaaccggtcgccttccaccagcatcggcaaccaagtccccctcgtcctctggagcg

acaagccagtggactacgagagcattcgcgttttcggttgcctctgctgggtcatcaagccgcgccctcaccttcgcatcactcgacacgtcgtcttc

aacgagcgtctcacgtacgatgctcgcttcgcgctcgaccagaagcagcagcacatcgggtcggcagacgacatcatcgacctcgtcctccccaagcg

tgccgttaagctcagcgatgtgcccatcgagcccgaccaagcaccgatcggcgaatggtataacgcggatgaggaggacaagcagctggatcacgagg

actggaccgttacgttggacaacggcaaccgtgatgatcaagtgcagggccagttcggcgagtggccagccctcgaatcactgctcggcgaggacgac

gatgacgaagacttggtcaccaacgagttcacgggcgagctgtctcgggtcaatactcaacagcacctgccaccgtccgtccaaggcacccgggatgc

ctactcgcaagtcgacgacttcgacgagcccatcggcctcgacgacctcgaccggatgttcgaagatactcctacccaggtcacattcgagccgactg

tgggcgccgttcttccagcagcaccacagccccgtcgcagcaagcgcatcgagtcccaattcaaggctctgaccgcgactatcgagttgtcccttctc

gccgcatctggcgatgaagtcctccagcgagcgacagagatcgccgcccgggcattcagcgctgcgatccgatcgacggcggatggcgtcttgatcga

gcccaagtccctcgccgacgccaagaagcgcgacgactgggcaaagtgggaggagggccgggtgtgcaagctccgacgcagcctttacggcttgaaac

agtcgggacgcgagtggttctccgtcgtggcgacgtggctcaaggctcaagggctctggccaactcagtccgagccatgcgtgttcaccaagcgagac

cgatacggcaacgccatcatcatcgtcctctatgtcgacgatgtcctcatcgcctgccagaaccagtccaccatcgacagcattcgcgactccttctc

gcagcgattcaggatgacggataacgggccgctatcgcaagtactgggcctgaaggtcgatatcaaccacgacgcgggaacagccacgatcagccaac

aagcctacatcgacagcatcatccgtcgcttcgggatggatgaggccaacgcagtggactcccccatgacggcggcactgcagggcctcggccgaagg

acggacggccaagcaacagcggcagagatctgcgtcttcgcatccttgatcggttgcgagatgtgggccgctcagggcacgcgaccagacatcgcgct

gggcagggcatag

>contig_21.contig_21.g892.t1

atggacggaattgcatatctccgctccgacaattggagctttgcttccgatcaggtgtccttcgccatccaaatgcaag

acgaggacgatgacccgtctgcgcgacgcgccaatacattcgaattctacaatacgatcctcaccttcgcggtcaccggagcggttctgatttatctg

ctgcaatccgcagttcgcgggctcggacgaaaaatcgaacttcggcatggaagccacggtattagccatgatgcggtagacagcgccgcggccgagac

agttgaaggggacgacatcatggcggcttcagtccacccaattctcagcgaaccggtcatggaagacaagtacaacggcgaaatacgttggagtcgga

atctcctgctgctctgggtcattatctcgctcgtattggccttggccaccttcgtcgagcttcaaagacgcaaagcacccttcttcacgattctttcg

ctcgaagccgactcgctcctttggttctcggttctggcctttcttctcacgcctctacgaacgcagggccgatggccgcatattcttcagctaaatgt

gctgttgactgcatccctcatcacacacggtgtccgagtcgtcacaccacctccgctgtcgtgggatgagcaacacgacgtaccaatacaactgcctg

ccgtctttgagggcaaagtcttgtggcttcaggcaacagcgactatcatcaccttgttggcctggatcacgaatgggctgacgccctccggtcctctt

cgagtcgtgccctcgccaaatccaggaacggttcctacggtcagagttcttaaagaccagagcagcggcgcttcgatgatcggctttcttttcttttc

ttactgctttccggtcatcaaggcatcctacatgaaggcgcagttaggcgtggacgatgtgcctcatgttcccgactcgttccgggcatccgagttga

acacgcaaacacagctggcttttgctcgcttcgccgctagcgacaaaaagaatggcgtggaaaccaatcggaccaaaacctggcgcgcattcaggtta

atacaagtcctagtctggacgaacaggacacttgttgccgccattattgtgtcgaccgccctcgcagctgcgtcctcctacattccttgggtgctcct

ttggctcgtcatctctgccattgagcgttccgaaaagaacaaggagtccaccgccatcgccgtccggcagaacatcgtctatgcggtcatattcgccg

ttggccaaatcgcgtcctcttgggtactcaatcacgcctggggaatgtctgccacgctgttgaagagccgattcagatctcagctttcggtactactt

ctcaacaagtcgttgcgaaggaaagacaccacaactgtcaaggacaaggggacggacggagagggagcagattcgaacgagtcaggtggcaagggaaa

ggagggcgacgaagagtctggcgacgattcccccgcccaatttgccacgaaaagcaagatcatttcaatccaaacggtggacattcagcgcgtcgagg

agctcagtttgcacatcttctccctcataggctgtccaacagagctggttgttggtggcatcctcgcctaccgcatcctcggcatctcagtcgtgatc

ggcctcgccacaagcttgctcacagcccccatcgtgtacctgatcacatctcggatcgagaaggtgcaggaaaagctgatggcagcccgagacagccg

gtcatctgctatgaacgagagcttccaggccatccgaatgatcaagttctcgagctgggaacgccgcgtcacggaacggatcatgaaagctcgtcggg

ccgagttgaagcaacagagatcagagcttcgatgggcaattacgcagcttccagacacgctctcatccgccatccaatcctggacatcgctcaagcgg

atcgcctcgtatttggactgtcctgaggtcgacgtacccgagtcggtcgacctgacctcggcgcatgctcttgccgccgacggcgagatccaactgca

gaatgccacgataggatggcctgttgtcgtgttgtcgcaaagctcacaagccggctctgtggctccagccttccgcctgcgagacgtttcggtccagt

tcacaccgaggcagttaaatctgatttgcggaaagctgggaagcggaaaatcattgatgcttcaaggacttctgggcgaagctgacctactggcgggt

agcctcaaatgccctcgttccatccccaatgcggtcgaacaatcaggcgatccgcggatctacgcacgcggagaatggatttcgccgtcgctcacagc

ttacgtgcctcaaagtgctttcttgacgaacgccagtctccgcgacaatatcctttttggactgcccatgattcgtaagcggtacgaggccgttctag

aagcttgcgcgctgctcccggatcttgcgatgtttgaggacgcagatatggtcgagataggagagggcggaataggactgagtggaggacaaaaggct

cgcgtctcgctggccagggctgtctattcgcgcgcccaaactcttctgctcgacgatatcctaagtgctgtagatgctcataccgcctcccatatcca

taagcaactcctgcaagggcccttgcttaaagaccgtaccgtcctgctggtatcccatcaaattcagctcctagctccttcggctgctaaagtggtgc

tgctggacaatggtgtggtcaagtaccaaggccaggcgtccggcgagtccatgaacaaggttcacggctactttctccattccaaacactaccgcgga

cttcttggtgacgacaaagaggaaaaggacgctcatgacaagagaggggatggtacgtctgaatcggcaggcaaggacaaggaagcgggagccgccgc

tgcgaatggcaaggcagccgctaaggacgtcgctcccaggacaactgcacctcgcaaactggtggaggacgaaggtcggaatgtcggtgcaatcggtc

tccaaacgtggaagcgatacctgcaaatggccggcagatacagggtcatcttcctcgtcatcgccataatcatcccctccatctggacgctcgttagc

tcatggtggctccgcgccttctctgcagatgcgaagaatgacaaggacacttcgcactcgatcgtctactggatggggatctacacggctcttgtcat

gatggagtgcgtgttgcactttgtgaagtggctcgctgcctatactgcatccctgcatgtgagtcaggtcatcttcgaggctgcgctcaacgcggtgt

tccgggcgcctatgcgcttccacgatacggttcctcgtgggcggctgctcatacgattcatgaatgaccaggaggaggtcgactcttcgcttgcgtcc

aacttcgtcgtcttcatggacgaatcgcttacggttgtcttcgccatcgtcgcggtctgcgttggtggcgctccctacttcctggtagttgttgcgat

aatggccccactatactactggactgcgaaagtattcgccggtgctgcacgagacctccgtcgactacagcagaccgcgaagagtccagtcgttcaaa

cgttcgccgatgtcgtcaacggtgttgcggtcgtgcgagctttcggagcatcggctacaactctcagttccttcttcgtccagctggacaacagtctg

cgctacgccttcttcgaccagcaatgcagtcggtggctcagtgtgctgtacgcagtcgcgacggccgtgctgtatttctgtgccacggcactcatcat

gatctcaggcgccgactcggccgtggctgcgtttgcgctgtcctttttacttcaaattgggcctcaactgttctacgccgtttcaagctactcagaga

atgagtccaagggcgttgcaatcgagcgactcgtggagtacgctgatctagaaccagaagctgcgctcatcgtcgagcctcggccgccggcatcgtgg

cctcactccggtcaagttacgttcgagaacctacgcctccgatacgccccggacctgcctgaggtgctcaagggggtttcgttcgacatttcggctgg

tcagaaggttggcatcgtgggaccgacagggtgcgggcttgtggacaatttctcgggcggacgggttctgatcgatggcattggtgagcgtcgatctg

ctggtacgcatgaagatcaagtagctgaacatacactctttttgcgagcaccagacatctccaaaatcggtcttgaggacctgcgatcgcgtctaatg

atcgttccccaggaccccgtcatcctcagcggttctctgcgcgattcgattgacatatttcaagagcacacggacgaggacatcatagcggcgctgaa

gaaggtccacctgatcggatctcccgatcctaactcctcgttactctctcaagtggttgctccagagagcaatgagcagggtgaccagaacgtgaata

tgttcgaagatctcagctacaacatcgcagaaggcgggactagcttgtctaacggtcagcggcagcttctctgtatggcccgagccctacttggccgt

gcaaaagttgtgatctttgacgaggcctcgagctcggtcgacgtggacgccgacgacctcatcacggcgacgctacaagaggagttctccgactgcac

cgtcatcacgattgcgcaccggctgcggacaatcatgcattccgatcgggtggtggtgatggacaagggaacggtctccgagtttgcgagccctgtgc

agctcttggagcagcccgagtcgcacttttacaaactgtgtgcggcgagcgggaaggcggagtttgagaagttgcgggagatggctgctttctcgagt

atatcttcgtcttcgaagacgctttcaacatcacctaaataa

>contig_21.contig_21.g893.t1

atgtcagcccagacagcttcctccgcagactcggcggccctcaaagacaccttgctcaacctgtactccaccattttag

acgcagtcatccccattctgccgcctcgtcccccaagttccgcgccggcaacttccctccaagcatcgcaatatgccgttgcccgctccattgtgact

cgactcgccgccccactggaggactcggatgccgtccgacatgctcctacagcggcatcgccaccgaaagccactcccgcgccaagcttgggagcatc

aacagtagggcttgcctccatcttgccagacatccttcgcctctgctccgcacgagcagctcctgtccgggacgaggagatccaaaagttgcttagac

aggccatggacattgatcgagaggctcttaacaacatgggtagtaacgacatctctctgcaatcccgtctggtggctcgcttggatactctggcaagg

gcgattcagcatctctctgaagagatgatgaccgacatccggacccatcatcggaagcgatcggacgcgtccactgcaagtcacgccacatccaatcg

acctgtctcgcccttcgacgcaggcggtggcaacgccgcggaagcaggagccaatgcgtcaccgaacaacgtctctcaagaaagcctcattctcacag

caccaccgggtacggccgcagatcagtttcgaaggcatgtccagtccgacctcgagttgtccatgcaacgcggagaggtttatgccgtctggggaggc

gaagaacaggtccaagccaattggaaggattgggtcgcgtctacggcttccgcaggcgaaattgacacctcttcgacgctggagaccatgcgatggcg

tgccggacttctcgacgcgatctacagcgaccatcctgtgcggttacctattctaccgacccgcgccaatctggaggcaaagtcgaaggagaaggaag

gagagtcatcgacgaccgcatcttcctccctcaagcaaaccgaaaatacgctgccttacgcccttgttgcaggcgctactaccctatttcagctgcaa

aacaaggtacaagcagtcgtcgtcctagcttgtctcgtcgcagttgtcacatccgcagtcgggagcagggccatcccacagcgaactgccaatcatgc

caacaactcggtcgcacaaggcgattcttcgagtcttcctgacgatccggacctttgggtttcacgattgtggatgcttctggaagacttcatcgctg

aaccggatcatcctgcgcccgtcccggattccctcaccgagccagccagttcatcttctccggctactcgacctgcgccagcagaagcggaacgtagc

aggctcaagaacctcacggccgaatgtatccgggcctatcgcgaactaagaggtctttcgacgcagacggcccgctcagacgacacagctgccgatga

agttaagattgaagcgggcgttcgtggggcgctgagatatgaggatcccgtcttcaagatcctcaggaaacgacttcatgcggccatgctgtgtcttc

tgctcgagtctggtccagcttccattggaagtaaggctacgagtactcctggtgggactactggcacggcagcctcgccggctgcctcgcccaacccc

tcaagccccgtccctcttcctccctcaccacggggtggcgcacccgcccgtctggccacaggacgaagtcatccgactcgacagacagcgtcagtgaa

cgcgacatcggcatcgacgctccgcctaccgcccgtccccggtttcacgcgtcccggatttgtggcagagaaggcaaacgaggtggtgcgggaggtgg

acgcggtctgtacggcgattgtcaaggtctggggcaaggatctgtctttagaatga

>contig_21.contig_21.g894.t1

atgctgaacactactcttactcgcgcctcgacccgtgttgtggctgcctcgcgcgcctctctgacggccggcgccatcc

ggtccaagcacacccttcctgacctcagctatgactacggagcactcgagcccgccatcagtgccgagatcatgcagacccaccaccagaagcaccac

cagacatacgtcaccaacctcaatgccgccgaggagtcgctcaaccaggccctccagaagagcgacgtcaagtcgaccatcgccctccagaaggctat

caacttcaacggcggcggacacatcaaccacaccctcttctgggccaaccttgcccccaagaacaaagacggcggccatcttccctccggcggcgagc

tctccaaggccatcgagcgtgactttggctcgctcgacaagctcaaggagaagttcaacgctcagctcgctggcatccagggctccgggtggggatgg

ctcggttacaacaaggccaccggcacacttgacatcgctaccacggctaaccaagatcctctgctggcccttactcccctgatcggcatcgacgcgtg

ggagcacgcttactacctgcaatacaagaacgtcaaggtcgactacttcaaggccatctgggaggtcatcaactacaaggaggccgagaagcgtctca

tcagcgcgcagaagtaa

>contig_21.contig_21.g895.t1

atgcaagcaggcgcaccccttactcctgccaccaatggctcggctgccatcgccgccagctctactaatggacagcaac

cagcggcgacgtccccagccattgagcaattagctggcggccaacagccaccggcttcacaaccaccagcggcaggcacaccccccggcgggccaaat

gcaaccgctgtctctgccacgactcccacgcaacgtcgtccccacctctacgttggaaacctctctcccagcgtagcagaacacatgctcatcgacat

attctctaccgccgggcccgtcgcttccgtcaagatcattcccgaacgcaaccagcagcaacaaccgagttccccgactcggtatgcctttgtcgaat

tcagagatatcagctccgctgaacaggccctccagaccctcaacggcaggaagctcttcgatcacgaaattcgcgtcaactgggctcatcaggccacc

aacaacggcggcccggccgggaacaacggaacagcacccggcccacaccccagcattcccggctcagggcccaaagctgacgaggcccacgtcttcgt

cggcgatctggctcccgagatcaacgacgaggccctccgcaaagctttcagcgcctttggcagtctctccgaagcccgcgtcatgtgggacatggtct

caggcaagtcccgcggttacggtttcctcactttccgtaccaagggtgatgccgaacaggccattgctaccatgaacggcgagtggctcggcagtcgc

gccattcgcgttaactgggccaaccagaagaatgaccccgcacagggcggctatggaggcatgggaagcggtgcgatgggcggacacggcggcgctcg

aggcgggccgcatcatcacatgcacggcggcggaggcccacacggttacaatgctggcatgggtggtccgcacatgatgggcatgggcatgcctggag

ccccttacggcatgggtggacccggaggtggtgccagtatgccaccgggaatgcccctcacctacgaacaaacactcgcggcctcccacctccacaac

accactatttacgtcggcaacttgctccccttcaccacccagaacgacctcgtaccctatttccagaactacggctacatcactgaagtacgcatgca

ggcggatcggggctttgccttcgtccggttggactcgcatgaaaatgctgccatggccatctcccagctcaacgggaccccgatccacaaccggccca

tcaagtgtggttggggtaaagaccggcatgaaggcgggcgcgctggagcgggcggtgttggcggtgcagcaggcggcgcaggcgccggtggtgcctac

ggtggagcacagggtgccggtatgggcccgcagggctacggtaacatgatgtacggcatgcaggcccagtacccgtacagcaatgcctttggtggcta

cccgggtgcacagggcatccccggcgcacaggggaacatgggcatgggcatgagcggtgcaggagggatgagcatgcctggcgcggctgcgggtctgg

caccgcctggctcggcgacttctgttgctggctccggcggaggccaggatgggtcttcgatgggcatgcctattcctgtgcctcagcagcaacagcag

cagaacggcgggtccaatggtattttgggaacgtcccctgttaccacccagaacgtcgggaacggtggtggtgtgcctggctcggttggctcagcgag

cgcgggtgggttggacgcggcgatgcagcagcagatggcggctgcttttggtggtgctggccaaggtggctatggcggtctgaacggaagtgcgcaag

gcggccaagtgtttgagcatcttgcagagtactacaactcgtatgcggcacaatatcaggcgctcggagcgggcggctacggcggcttcggtgtctct

ggccaaaccggcaacgggacgggcgcccaaggtcgatga

>contig_21.contig_21.g896.t1

atgccccatcggacagctctctccctcgacaaagccgctgccaaagttgctcccaaagcacaccacctcgtcatctcca

accccgtcaactccaccgtgcccatcgtcgccgaggtcttcaagtctttacgaccccaagcggagtacggagcgcaagcctgtcatgtccccggagta

cggagtacggcaaggcctggacctaacttttcgccggaccggagtacggagctggcgcaatga

>contig_21.contig_21.g897.t1

atgcccaagtcacgcaagccggtcgttgcagccgtccgatcgaatacatcgctcggcgagtcattctctgctgccgaag

acgacgatcgagatgctgtccatcttcccactgcagaggatgttttggatgctgctcgaaaccgaagccagaagaactcagatgcgcgccagagttca

gagcctgaggaggaggaggaggattttggatccgctgatgaggatgaagacgatcagccgagcaacaaacgacagcgagctcaagcagacgaggcaat

gcgtccagagcaacaagcagccgttcctccagtactgaggcagtgggctcagcgtttccgactcgggcctcaagcaagagatctacttgaggatcggt

atcagctgcagatgtcgttgccgacattttttttgactgggcccgcgacctttctacctgggaaaaattcgacccaatccggacaaatgttggagcag

gtcatgcgaaagatcgatcagctaaaggatgacgtcgagaagctcaagaaaggaacgggaatactgacgaagggagacttgaacaagttcggccaggc

tgtagccttcgtgttcttcagcagtcaaattacagagtacagcgcggactcaagtattgctacgcgcacaatgctctaa

>contig_21.contig_21.g898.t1

atgcctgcgtaccgcaaaacagcaggggcgattcaggcgcagcttggtccggagttccgtctcttggacggcaccccgt

cgcggatcgtgtttttggcctgcgatgaagatggaagacgacctttcgtgccaaagcacctcggtcagcacgcgtcgagtagccgtcatcaacgtaac

aagtcaacatggcaacatgatcaggagttggagcaggacaggagtgcgacgccagtgaccgatcccttcttcgagtcgggagaggatcacctgctgat

tgataatccaaacgaccatgcaaaggaggacttctttcgtcgcatggaccttgacgacgacatggcgtctttggtggaggagggactggccatgcagg

aggaagatgaagcgttcacgcaggagataagcaaggacaacgatcatcggaggcacaacggaggcagcgacgatgcattcccctttcagtcaaaggag

aactcgcctcaacacgtcctgtccgaggcgtcgctgggcttcgccctcgactttgccaacgcgttggatgcgcgcggaactccgtctgcgtacgcctg

ctctcaagccatgaagctcatcagatcggacgtgtcccctcccctttatcgtcacacaacttcggacaataagatcttcttctcaagcagcgtggagg

ataagatccgtaacgactttgccaatcccattacgcgcacgaagatgatactgctgcctgttcgggatcaatcaatgagggaggtgtttcacgggaac

gagatggcgcatcagacggatacgcgcaagacaccgccctgctttcgcctcagcaacggtgagactgtctttaccgatgaggttgtacaggtcactgg

tggccagcttctgcggccttatacattcttcatcaatgaagagggcgacccgagatgtgaggcatggcaagtgattcggcgcggcgatcactttgagg

catatattcaaggcgtgtctcccgtcgagttcgatcccgagacaatcgagacctgggaacgctcagtacttcacgaagttgatgtctttgatcagcac

gggacaaatttgcctcgaatcaatcacctccgcctgaaggccggcaatcgccttgtctatcaggttcctgttatcctgttcgaagatgagacgtctgg

ttcgacaacaaagcgttggaatgaacacatcggaatctacatgtccaacgcagcattacccagagccgagatggacaagcgcatcaacgtcaagctgt

tgtccgtcagcaccaaggtcagcggacacgacctgatgagtgctgcggttgacgagttgatcgtcgatgtcatcaaggagaatcgtaaacaaatcaac

ttggctgccaaaggcgtggcatcgacgctgtccaatcgacaacgcgatactggaatcaaggacggggtgactgctgtgctgtgtgatgaattgcttgc

actatccaaatcgaaaccagaccgacaccatgacgaagctgtgaaggcacgacgaaaggagatgcttgagggaaaatggcactctccgctgttgcggc

tgtacgacgagacgggattcgacgtctgtgccgctacgccagtggacctgctccacacctttctgctcggcgtcgcaaagtacttgtggattcacaca

gtatcgagcatcggtgggacaaagtttagctccacccagcgagacgctgttgcccggcttcaggctgtgtctctctcgggtgtggtcaatgcaaagtg

cctaccgggaaaatggctgattgacaatgcaggcggtctcatcggcaaggacctcaagaagatcgtacaagttgcggcagttgcattctacccgttga

tgaaagcggagacaatgtccctggaactgtggatggcatggagtgctctgggtgttttgggacgtcttctctttgttgaagagatttcatgggaagag

atgggtcaatacaaagaagatgtcaaggccgccctcatgatcttctatgccgccgcagctgcagttttcccttccaaactcacaggcaaaccaaagtt

tcatattctacttcacatagtcgacaacattgaagcgtatggaccggccaagggatttacatcggaacgatatgaatcctttaactctgcggtccggg

atgcgtccatcaagagcaatcgctctgctccctcgaaagacattctgcagcgcatgatggatcaagaagccattcagcacctggcggctggaggtagc

tgggtcaaagacgacatcgttcacaggccggattccaatctgctttcactaatgccttccagtctgcaagaactttacggcctttctacggaggagga

cgaggcggtctcttcggtgaagaggatcatcacatcagcaggggatcgagtgagggttggcgagttctccaccttctggtttgatcacagaactggtg

acgggaggatcgtccgtgtggatcgaatttttacagtgagcactcagctccaggtagaagtgatggtcatgcactggggcgaagatctgggactgcag

ccaagcattcttcaagaggatagacaagagatcgtaggcattgagggatttgaacgactcgtcaatgtcatgcacgactgccatcaccatggatgcaa

ggtggtcggcgacgccaatcaagtccgcatggagcgagaggagacgaccttcacggatgcaggcgtcatccacagcgaggaaggccgacaacaatacc

tactgaatcattgcctttttcgctcggctctcgagctctcattgtgttatccagcacttccagacgcccccacagaacaagagatcgcagaactagct

attgaaatcatggacgagcaagacgacatagggcagaatgatgcgcgagccgaacgacatgaaaaccaggacgaagacgatgacttttggaccgaatc

tggcagcgacgacgaggaggctcctaatgacagaccggaagcgctggacctttga

>contig_21.contig_21.g899.t1

atgccccatcggacagctctctccctcgacaaagccgctgccaaggttgctcccaaagcacaccacctcgtcatctcca

accccgtcaactccaccgtgcccatcgtcgccgaggtcttcaagtctttacgaccccaagcgtctgttctgtgtcacgggtcaccacctttgacatca

cacgcgcctccaacttccacttcgacattgccaacgtcaagcccgccgaggtcaatgtgtccgtcgtcggtggccaccttcggcgtcaccattgtgct

tctcctctctcaagtcgcccagggttccatactccaatag

>contig_21.contig_21.g900.t1

atgccccatcggacagctctctccctcgacaaagccgctgccaaggttgctcccaaagcacaccacctcgtcatctcca

accccgtcaactccaccgtgcccatcgtcgccgaggtcttcaagtctttacgaccccaagcgtctgttctgtgtcacgggtcaccacctttgacatca

cacgcgcctccaacttccacttcgacattgccaacgtcaagcccgccgaggtcaatgtgtccgtcgtcggtggccaccttcggcgtcaccattgtgct

tctcctctctcaagtcgcccagggttccatactccaatag

>contig_21.contig_21.g901.t1

atgcctgcgtaccgcaaaacagcaggggcgattcaggcgcagcttggtccggagttccgtctcttggacggcaccccgt

cgcggatcgtgtttttggcctgcgatgaagatggaagacgacctttcgtgccaaagcacctcggtcagcacgcgtcgagtagccgtcatcaacgtaac

aagtcaacatggcaacatgatcaggagttggagcaggacaggagtgcgacgccagtgaccgatcccttcttcgagtcgggagaggatcacctgctgat

tgataatccaaacgaccatgcaaaggaggacttctttcgtcgcatggaccttgacgacgacatggcgtctttggtggaggagggactggccatgcagg

aggaagatgaagcgttcacgcaggagataagcaaggacaacgatcatcggaggcacaacggaggcagcgacgatgcattcccctttcagtcaaaggag

aactcgcctcaacacgtcctgtccgaggcgtcgctgggcttcgccctcgactttgccaacgcgttggatgcgcgcggaactccgtctgcgtacgcctg

ctctcaagccatgaagctcatcagatcggacgtgtcccctcccctttatcgtcacacaacttcggacaataagatcttcttctcaagcagcgtggagg

ataagatccgtaacgactttgccaatcccattacgcgcacgaagatgatactgctgcctgttcgggatcaatcaatgagggaggtgtttcacgggaac

gagatggcgcatcagacggatacgcgcaagacaccgccctgctttcgcctcagcaacggtgagactgtctttaccgatgaggttgtacaggtcactgg

tggccagcttctgcggccttatacattcttcatcaatgaagagggcgacccgagatgtgaggcatggcaagtgattcggcgcggcgatcactttgagg

catatattcaaggcgtgtctcccgtcgagttcgatcccgagacaatcgagacctgggaacgctcagtacttcacgaagttgatgtctttgatcagcac

gggacaaatttgcctcgaatcaatcacctccgcctgaaggccggcaatcgccttgtctatcaggttcctgttatcctgttcgaagatgagacgtctgg

ttcgacaacaaagcgttggaatgaacacatcggaatctacatgtccaacgcagcattacccagagccgagatggacaagcgcatcaacgtcaagctgt

tgtccgtcagcaccaaggtcagcggacacgacctgatgagtgctgcggttgacgagttgattgcactacacaacgatcccttcccggtccatgattgt

ttcttaaacgaagaagcactcgtcagaccgattttgatcttttgcgtggccgacaacccgatggcggctatgctttcagcatccatcgggatgtccgg

tcttcatgcgtgccgatgctgtcgagcgggagggacgaggagacagatgaaagaggtgctagggtttgccaacttcttaaagctcggaacgaagaaga

acagcgtcgatgtcatcaaggagaatcgtaaacaaatcaacttggctgccaaaggcgtggcatcgacgctgtccaatcgacaacgcgatactggaatc

aaggacggggtgactgctgtgctgtgtgatgaattgcttgcactatccaaatcgaaaccagaccgacaccatgacgaagctgtgaaggcacgacgaaa

ggagatgcttgagggaaaaatggcactctccgctgttgcggctgtacgtgcctaccgggaaaaatggctgattgacaatgcaggcggtctcatcggca

aggacctcaagaagatcgtacaagttgcggcagttgcattctacccgttgatgaaagcggagacaatgtccctggaactgtggatggcatggagtgct

ctgggtgttttgggacgtcttctctttgttgaagagatttcatgggaagagatgggtcaatacaaagaagatgtcaaggccgccctcatgatcttcta

tgccgccgcagctgcagttttcccttccaaactcacaggcaaaccaaagtttcatattctacttcacatagtcgacaacattgaagcgtatggaccgg

ccaagggatttacatcggaacgatatgaatcctttaactctgcggtccgggatgcgtccatcaagagcaatcgctctgctccctcgaaagacattctg

cagcgcatgatggatcaagaagccattcagcacctggcggctggaggtagctgggtcaaagacgacatcgttcacaggccggattccaatctgctttc

actaatgccttccagtctgcaagaactttacggcctttctacggaggaggacgaggcggtctcttcggtgaagaggatcatcacatcagcaggggatc

gagtgagggttggcgagttctccaccttctggtttgatcacagaactggtgacgggaggatcgtccgtgtggatcgaatttttacagtgagcactcag

ctccaggtagaagtgatggtcatgcactggggcgaagatctgggactgcagccaagcattcttcaagaggatagacaagagatcgtaggcattgaggg

atttgaacgactcgtcaatgtcatgcacgactgccatcaccatggatgcaaggtgcgaggaaggccgacaacaatacctactgaatcattgccttttt

cgctcggctctcgagctctcattgtgttatccagcacttccagacgcccccccacagaacaagagatcgcagaactagctattgaaatcatggacgag

caagacgacatagggcagaatgatgcgcgagccgaacgacatgaaaaccaggacgaagacgatgacttttggaccgaatctggcagcgacgacgagga

ggctcctaatgacagaccggaagcgctggacctttga

>contig_21.contig_21.g902.t1

atgctgtccatcttcccactgcagaggatgtttttggatgctgctcgaaaccgaagccagaagaactcagatgcgcgcc

agagttcagagcctgaggaggaggaggaggattttggatccgctgatgaggatgaagacgatcagccgagcaacaaacgacagcgagctcaagcagac

gaggcaatgcgtccagagcaacaagcagccgttcctccagtactgaggcagtgggctcagcgtttccgactcgggcctcaagcaagagatctacttga

ggatcggtatcagctgcagatgtcgtatggtgatgacccaccaacagttgccgacatttttgactgggcccgcgacctttctacctgggaaaattcga

cccaatccggacaaatgttggagcaggtcatgcgaaagatcgatcagctaaaggatgacgtcgagaagctcaagaaaggaacgggaatactgacgaag

ggagacttgaacaagctcggccaggctgtagccttcgtgttcttcagcagtcaaattacagagtacagcgcggactcaagtattgctacgcgcacaat

gaaattccttaaggcaaatcctcatcaagtcggtcagcatttcaaagagctggtggaggatccggagacgtgcgattgccacgtcgagccaaaagtgc

acaaatcgatgggagtggacgacgatacggaggtacagagcggagatgaattgtgggacaccatttgcaaaggatacaaggtccccttcaccaagaag

cgcagtcgccccaactccactccgcactccgcacaaaatctctccatctcattgcgccagctccgtactccggctaaattcagtaaagtttcaaagtc

cactccgtactccggggagttccgccagcttcaggaccgtgatcctgatgctgccaaaaaggagctcgaagccgtgtgggtgaatgacaaaaagcgtt

tcggaattttcgatgacagggcgatgaccaaggcggacgagaagggagaggaggccctcgccaatgcagtgcgatcaaagatgcagtcgcaggaagag

gactgtgctctgccaacgtcggtcatcacgccatcagtcatgcgcagcacggccaatcacactccaaagctgcgcagctggcaagatcgtcagtttca

agctgcggttgcaggcgaagatcgtaaaggggaagtgggagatgccgggccgtcttcggcgggcacacgagcgcagaacggcggctgcgtcggctatg

ggtgcctcagatccatagctcaggccaacacgcctgcgggacggctcattgagacactcccaaagatcaaggcgctcggctggacccactcctcgcca

ccagctgctcttcgccagatccagcagcatcagcaccggcactgccgacggtatcgccccagcagcgccgacctcgctggaggacatcaccagtctct

tcgagtgggtgctgactccgccaatagtgaggagggacggtgtcgacctacttctggctggcaaaccgcaaaacgcgtcccgccctcctggtccgcgc

tcccccgcttcatcccgcccgccttcttgttgctcgaggctgtctccaccttcgtcatccagtctgtcggcagacagcaccctcccacgttctctcgc

gtctctttcccagcctcgctgctcatcaaggcgcatctaccacacttatcggcatactcttgcgacatgcccaaatcaggactcggagtagctgagct

ctggcagatcctcttcctatccattgctctacatatgctctcacctacgtcgtctcgtctatcgacctttttctctccttcgaaggcgccactcgcgg

ccgacccagcacagctgcagctgtcggcgcatgcttacactcaacactctggctcggcatcactttcgcactccaagatcgggatttttcagctctcc

atgcctctcagctcgtcatctgcctcgcatcgtgatcgatctgaaacaccaacggctacgctcttgcccaatccaacgacctcatctcagccacagcc

tcctccaccctag

>contig_21.contig_21.g903.t1

atggtgtcaggtcgccctcggcgagctgccgagctcaccagcacgctcggctcgttcctcaagaagaagctgagtgcca

gaaggcgctcgacatcgttcagatccctgagcgcatcatcaccttccgacgacaaggggcaaatgccacgtcgatcgcagttgccgttctcagggtac

gcgtctgacgagcagtatccgtcttgtggaaagaaactgaattggctcaatacgctcatcttcattacatccacctcatcacccactcccaaccactg

gctcggcaattga

>contig_21.contig_21.g904.t1

atggacatgaccatcaccccatcggccatcgcacactcgcccagtatggcttcccagggtagacgcagtatgatggtca

atcggtccccgtcggcgctgtcgagcaacctgccctcgtcctccgttccccccggcagctcggtctctcccgcactcaccaagtttgtcggcgccgag

tgggtcgcacaccggtacaccaccctcgggccacgcggaagcgacaaggacatcttcaacgctggtggagaggcgatcatttccaatgacgagtcgca

accgtgcgcaaactcccagctcgtcaagggtgtcgccttcaacaaggcattcgcatacgcccgcttcgagcagaaacccgagagctctcaacgtcgaa

ctcgagcgacagtgcagaggtccgcatcgccgaggtggcagaatacaagatcatcgtcgatgcagagtggatatgaattatga

>contig_21.contig_21.g905.t1

atgcttccgaaccacccgctacgaccaatcaccaccgcccccgaccccgagcccgagcccgacgccgactccgcccgct

ccacacccaccctcgccttcccgccctcaccagatctctcatcaccgcgtggtggtgggctggctgcgtcgctctcggtgccccaacgccccggccag

aatcgcggacggagtagctcgcttttgaagctggagcatattcaggactcgcacgatgtcatgcttgaccagaacgcgggcttcaatgccaatgccga

ctgggtgaaccacaagggcgcctgggtcgtccacatcgtcctcatcctcgccggcaagatcctcctcgacgtcgtcctcggcatcacccaagacgtct

cctggacactcgtaacctggactacaaaatttgcgccttcagctatcagtgttcctcgacgctcgctccaaattccgattgttgacgccttcatctct

tccgcagcgcagtaa

>contig_21.contig_21.g906.t1

atgccccatcggacagctctctccctcgacaaagccgctgccaaggttgctcccaaagcacaccacctcgtcatctcca

accccgtcaactccaccgtgcccatcgtcgccgaggtcttcaagtctttacgaccccaagcggtggtctgttctgtgtcacgggtcaccacctttgac

atcacacgcgcctccaccttccacttcgacattgccaacgtcaagcccgccgaggtcaatgtgtccgtcgtcgatgctcgtagctacgatcgcaagtc

gcagccgcatggcgaagcagacgaaggccaggtcactatggttccaaggcaagatgcacgtgacctcgtctacatccacacacgcaacctcaggccgt

gcgtgcacctcgccttcacctcacagccaagctcgcgacgtcgacatcagcacgaacaacaaacacagcgcgcacgcacatatacacactcacaggga

acagcgcgacaagacatggacgaaatcggaatcagtggcacccgccagcccctcggccagcgcgaagaacccactttcgacgatgcagtcgcagagaa

gacggcgctccaccaggaaatgtcccaggaggaagacatctatctccgcatgaagaagctccaacaacatctcaacttcctcatcaccatggaggagt

atgtcaaagacgaacagcgcaacctcaagcgtgagctcgtccgcgctggtgaggaggtcaagcgcatccaggcagggcccctcgtcatcggccagttc

ctcgagccgatcgatcaacacaccggcatcgtgggctcgaccactggatccaactacgttgttcgcatcctctccactatcgatcgcgagttgctcaa

ggcagggagtagtgtagcccttcatcgtcactccaacgctcttgtcgacgtactaccaccggaggcagactccagtattgccatgcttgggcaagatg

agaagccaacagagacatacgcggacgtaggcgggatggacattcaaaaacaggaaatcagagaagcagtcgagttaccacttacccaattcgatctt

taccggcaaatcggtatcgacccaccacgaggtgtcctgctctacggtcctccaggaacaggcaagaccatgctcgtcaaagccgtcgccaacgccac

aacagcctccttcattcgcgtcgtcggctccgagttcgtacaaaagtacctcggagaaggcccccgcatggtcagagacgtcttccgtctcgcccgcg

agaacgcaccggccatcatcttcatcgacgaagtcgatgctattgccaccaaacgtttcgacgcgcagaccggagcggatcgagaggtccagcgtatc

ttgctcgagttgctcaaccagatggacggcttcgaccagggagctaacgtcaaggtcatcatggctaccaatcgcgcagataccctcgatcccgctct

cctccgtcccggtcggttagatcggaagattgagttccctctgccttccaggagagagaagcgactcatcttccagaccatcaccggtacaatgaacc

tcgcgcccgatgtcgacctcgaagactacgtctccagaccggacaagctcagttcggctgagattgcatccgtctgtcaagcggctggtcttcaagca

gtcagacgcaacagatatgtcatcctacctgcagatttcgaagaagcatggaaacagaccgtcaagcgcagcgatactggcaaactcgagttctacca

gtga

>contig_21.contig_21.g907.t1

atgggggtcccgacaacacgatgcaacgcatctgttctcgaaaggcgaacttttctctcctcaccatcaagcatcaccg

catcacccgccaagttggctcccgatgagctggtactggtatcgatcctgatcgcaccctctcatccagaaccagcaacccagatgcaaggccagcca

gaattctcctcagaaacggctgttatagacacgctccggtgcaacgagttcactgctcaactactcgatagcaccggcgcaccaaagcacttatacgg

catcaaagagaaagatcatttcgtagaggcatatgtcgaggccaaggaaggagaacaagtctccgcaaagctcgactcatgggatcggtcccacatct

acaagcacagcagaggctatggcgcgtcctgcagcttcgaagagaccttcactccaatttatttccaattcaactgcgtcacccgtgctgagctgcag

cgactcggccatgttttgtcggatcatgccaaagaccgcttaatggaggaaggggaggaccaggaccaggaggcgcaggaggagaatgtcagcacacc

gaaacatgccaagaagcgcccaagggaggaagatggcagccatgctaggaaaaagcgcctccagaaggaactcgaagagcttcaggagatggagagac

tcgaggacctcgttcttgagaagcgcagacggcttacgggcggttcgaatagcttgcgcaatgcgaatgcaagcacccaagcgggcgaatcttccatc

agcgtcaaaaaggagccaaccgagtctggtgccggcagcgcaggcactgcagacgagcccttgatcattgccgacctgagtgactaa

>contig_21.contig_21.g908.t1

atggactccagcacgacgagcgcagggggctccgctgccgagcgcccagcagcagcagccgcagcaatcggcgagcttg

ccggcgacgtcagcgtccatcagcaacgtccgctaccgcccagtgcaatccgaaaacggtcggccgcataccacgcttccggcgccagtagcagcgca

cccagccctcgcctggacgacgctttcgaggaggacatctccagggaggatctcgagcgcgcgctcgagggactctctcgtgatgatctcgtcatcgc

acttggaagggcaaaaaaggcattggactcgcttgatgcacagctcgacgcgcggaccggggagttccacgagctgcaggcttttacagaagacctca

tcgccaagtcgaacctcagtgcggcagaccaacaggcgatcaaagacgccgtcgcacagcgtgatgagcggaatgaccatctccttcgcgaacaggaa

cgtcttgaggaggagctctacgctcaaatacaagtcgtcgaacgcttgcgccgtcaattgcaagagtccgagcggtcccaggccgagatcgagcgtcg

gtacaccgatcagaccgcgactgcagacaaggaacgacaggcattttccgacatggaagccctcctgcgctcccaaaaggcatccgaatcagctgcca

acgagaagctgggcgcggaaaatctggcccttcagcgcgagaatgagcgcatggcaaaccacatcgccgctttgcaagcctcgacgggctcaaccctc

gcccttgatgacgaagtcaagggcgatgatggtctgaccccaggcgcacgtcgagcacgcgccaaatctgctgccgatgccgaagccgttgcactccg

atccgaactcgctgctactcagaaggcgcaagggagttatgtcgaagctgtgcagcagcttcagacggaactcagcgagctgaaatcggaaaatgcct

cactgcgagacacaaattattcgctgatcgacatattgcaggagaagtcgttctcaggcgccctcttcgccgagagcgctgtgctctcccgtgggttc

caaggtaaaggcggacgtgcggccgcagcggcatccatgttggcagaaaccgaatccgacacggacgacggctccgacgataccgcaagcaacgggac

gggtgacgtggaggatgacgttcccgatcgacccgaatcgccggtcaagccgcgtagaaggaagggcaagcagaacccggctcggtcgaggagcaact

cgaacgcgttgctggaagtgcctactgatttgggcagcgaattggcgcagagtggagcattcggtcagggtaaagacaagggcaagggcaagggcacg

aagcgtgacaggtccggccatgaagtctcaagcgatccagaagtactccagaaggaactaatcgaactgcgcgagacgaactctgcgctcactgttta

tatctccaaggtgatcgaccgcgttcttgcgcgcgacggattcgaacgtgtcctctccaccgacccagagacaaaacgcgccggaatcggctcgcagc

ggtccaaagcacgctcccgcccatcgatccttcccgtcaacacagaatccggtaacaagaactctcctcctacctctgcagggggaacgccagaaatt

ggtgcaacagcgtcagcaaacaagcgacagagcatgggtattctcggctttggacggagtccgcttgcgctgtcctcgtctcttgtaacatctctctc

ctcatcatcggtacccgaaaatggtatagccaccaacggatcttcgacagcatcgtcttccaataagcctgcgtctgcggcagcggctcacggtcgtt

cccgcatgtccgtcgactggcgcggacttctgagcggcatgggaggcggcagcggcgcccccgccaaagacccgcgcgaggccaatctgcgtccgctc

gtcttgcggaccgcgagcatctccaccgcctcgggcgccgcgcgcaaggtgtccgaatcggaagaaatcgaggatcaggtggatcggatcgagcggga

acggatacgggcggatctgcagatgcagggctattcgcctcctgataatcagctgcgccagcggacgacgagcacgacggggaactctgcgtccggct

ccggaggtggctcgagtatcacggccttcttgtcccgggtcgtctcgggctcgtcgactggtagtggcgggaatagcaccaacacttcgacgggcacg

acgaattctcctcggggaggagctgggtccggccgggatctgttggcggagcctcgggcgagaacgtcggcagcggggacaccggtctcggaggaagg

ggagcctctgggcaagttgcgattcccttcgaccgactcggggccgccggggacgaggacgagctcgatggctggggacgagtcctacctcgtccgag

gaccttga

>contig_21.contig_21.g909.t1

atggcgcagtacgcactcgacacccgggacgcgttccctctcacgctgcgcttcagcaagtgcgcaattgtcgtccccg

tccagccgtcgacctccgtctccgagctcaagacggacattgtcgaagccattcgcgcgctcactgcctctggcaggatcctgcatgaggccaacggg

tgggaacaggtcggaggacgagatcctgcctccgagcttgagaaagagcatatcggcattttccgaggctcggcggcgcctggtgtaaatgccgagga

gcgcggcaacgtagcccctggctgggagccaccgaataccttccagcgcgtcgactcggaaaaagcggccggtccaagatcatcatcgtcctcgggcc

cctccgtttctagcctcggcttcaaggcgggctcggaccagctggcctatcttggctttgctccaaaaggatcaaacacgattgcgcgtccagtatac

gtgactcccgcattcctagacgaggacgaggaagaggaggaaggcgagccagactcacaaatgggcgcaggggccggggatgccgaagctgacaacgg

cgcggacgaccaagaagaagaggacggcggggaagaggatgacgaagatgaggatgcgtggattcgcgcaggggtcacactcggctcagcaagtgcag

gtccttcctaa

>contig_21.contig_21.g910.t1

atggaggccacgttcaccgaacgcatcccgagcaatccccaggaggcagacgagcagcgccgtcgagagaatgcactgc

gcgaagagcgtggcgagcccaaagtcggaggatccgctggccgccagcagattgccaacaaggacccgaaagagttcgacgtccgaggcggtccaggt

ggcgaccccaagccgaagcaagaccctgttatgtga

>contig_21.contig_21.g911.t1

atgccgaccagagccaaaaaaaaatatggccccgttttggaactgtcgctcgagattgtagcatcgttgcctttgaatc

tggcgaatggttgtcaagtcaatgtggccaccttcggtcgtggccttgcggagaagccagagcttgaccgaccttcccagagcaacgtcgcgccccac

gaggtcgctcgaaggagcgaaagcctacggcccggaactcctatctgcccttcactctatcgcaacctctgcgaatttctgcgccgcggtgatatcag

cttcggacgataccttctacgagacgcgcgtccacaccaccgccaaccagaccatggtacatcatcggacctggacagggcctcggccccagcatcga

caattctcagcgggctgcgagagatgctcggtgctcggagcaccgagcgaagtcaattcgtctcgatctcttcgcgcgttccttcgattccctctcga

ccggctaacgccctctgtccactgctccatctcgaccaggtggttcgccacttcacttcggaccgcacgttccaaacttccaacctttccatgctcgt

cggtacccgcgactttccctctcactcaccgatttctcctcagcttcgctatttgcacttccctccctcctaccggccttctgtcatgcacttcaagc

tcctcctgagcctcatagccctgacgagctccgccatcgctgtcacgactgggccatacaacgtcggcagtggccatctgacagaggtgccaagcaac

attttcgctcagcaaggccaggtcaacactgtcccagctccgccttgcccagtcgtgccaacgattccagcatctactatttcggcaggactgaggac

cgatatcaatattggggtacctttggctgtcacctcgtacttctccaaggcgggagcctcgtcgctgacggcaaacaacgacgctattctgggggtca

agttcaccggctaccgcgacagcccggtagttaatgctcgtggaaatgcgacgtcgacagtgcagatcacagggacgccctacacaagtgtttccctc

ggacgtttcaccttcgatacaaacgtaaacttttcagctggccagttggccgcagtccccagtcgccggttcgtctttcagcgtgatcaattcacctt

ctcgctgtcagccgcgggcaagacagcaaacgtcacctgctccgccggagcacggcgacagaattttggtgttgcaaacatcgccagcttgccgatat

ggacccaattagcacgtttcgcgatcgtcgcggtacgcaacgagacattgggcactccgccgctgaactccacctcccttgttagctcgtaccgaccc

ctcgaacctgtcgtagttctagacgaacagaaccagcccatcagcctctcaagcccaggtgtcttcagtctgggccagagcaactttgagatttcgaa

taacctgactgcctttctttggaccaagtatccttccaccactcaagtgagcctcaagctaagcgccttcaacgtttcgatctcaaatgcgacaccgt

cggtcaagaacctcctcccgtccggtggctacgccgcatccgccaagctcggcgctggaagctttgctacattcccatccaatgctccgtcatcgacc

taccccagcccaacgttcacacccattgcctcgagcatcggcaagggacaattggcgttcctctcctacgacaacgtcgccggctccttcgaactctt

cgacacgaactcggcgtccctcggctccgtgacattttcttgcagcgagggtgctggaaagtaa

>contig_21.contig_21.g912.t1

atgagtcaacctgtccgcgcacgaaggatcggtgcgcgtgtcggccaggctaccgccgatgccggaacgagcgcagtcg

cgtcgagcgcagctgctgccgtgtctgctgctatagccactggaggtgctgctgctcctgtcgtcgctgctgctcctgtcgtcgctgctgctcctgtc

gccgctgctgctcctgccgccgccaccgctcctgctaccgctgccgctgctcctgccgctgccgctcgcactcctgctaccaccgccgccctcgctcg

tgctcgtgctcgtgctcctgctctcgccgccgcacgcgcagctgctgctgctgctgccgccgccacccgtggtgctccttctgctgctgcagccgctg

ccgctgctcgtggcactactgctgctgcttctgctgctgcggccgctgccgccggtcgtgggtctgctactgcagctgctgctgggtctgctgcggcc

cctcctgctaccatcactgcagcacgcgcaaccgcagccgccgccgctcgatccactgtctcggccgctatcgcgcgtgctagtggaggggcaggtgc

aggagcaagtggtcgtctccacgagggctcagcgacgcaaaaaaaacagctcgaggacgccttgatggagaatgtcctcaagaggctggaagatcacg

agaagaaggttgcaaaagaatgggatcagcacatcgatcaaaggctcgaatcgtatcatgcagctgccaactctttcagcgacgctcaccagctggtg

gacatcctggatgtggatcccagtcgttctgaaggggacaaggtggtcatcgtctttaacagcattcaactcgacgagatgaagacgatggtgcggtc

attgaggactcaccagaggcaccaagagctcctggagcaaggtgtccgtgatatgcgcgctcaactgtcagagctgcagaggctgcaggcgcgggttg

tgattctggagagaaacgtggatgaacaagatggtcagcactccgaacacccctcgccgcagaggtctcgccgtcgcaacgaatcggactctacggca

gagccgcctcccaagcgcgcccggacgaagcctgtcgcagccgcagaagagggtgagctggtcggagagccgatggatcggttggactggatggaagg

gagacttgacgatcgtcagtttaaagccatcctgtccaagcatacgcggactatcatggagaaggcagacgagttcgacttgcctgatgaagagaatg

ttcgcacgcaaaagtatatctatgccgcgtgtaaggaagagcacggagtagaccccggaatgggtttgcaggaattccggggcactccgggcagagtt

ccggcggtacccggaacccggaatgctaattttcaatttttgtcagtttag

>contig_21.contig_21.g913.t1

atgcggaatgccctgagaggccccggaattgctgcgcaggtcctccccggagtgcggaaattccggggcactccgtgct

cttccttagccgcgtgtcatcgatcgctagaatggttgaaacgcacctgtcgacagaagcgagaggtgggggctgccgccatgaaggagaagaaggac

gcgaaggaccaaaccgaccgccggaccaaacgtcgcaaagcgcgacgagaatcccgccacaaggttgctaggggcggctcagataggatcagcggtgc

gatggccgatatggttgtccttccatgcattgaggcggacgagtctgggatggaggagggtctggaagatgtcagcgatgacgatcttggcttggacg

acattgcgccgggaaaacgaaccaagacgcttcggcctatcatccccgaatggtggtcaaaagaaaacaaggaccttcagcacaaacttgcgcgtcgt

gctccagcttcgaacatcaagtgtagagtcttagaggctactcacctgactttgcatcaagacccactgccagcggaggtaccgcgaatcgccgtcag

ctccacctttgccgaacggtatccgcatcttgttctggacgtgaaggaaaactcacctccgtttaaaccagagcggggagcggtggcggagagcgcag

ggagttggggttcgcccattgccgacttggtgcttctcggagaagcaaacagcgggactggaggtggcggagaagatgagctgggtggaggaggagct

gaagaggacagcgatgaagtggaggtcgatgatgaagacgaggatgacgatgatggtatggatatgagctga

>contig_21.contig_21.g914.t1

atgtcgcccatactgagctcgcacgaacaacccgtgcgcaccgcgacccgcgcaggcctcgcgccgacccgcgcgactg

agggttctgaactgacgtcgccctggcctcgcactgacctgtgcgacgaggcaaaaaccttgcagcactcagcagcgctccctcatgccccccatcgc

gcggggccgccgcgcaaggctggaaaagatcgagcctctgggaagtggtggtgcggatgcagcgtaaacggctgcggctcctctgctcagcagcttcg

tcaagtttccgagcgcacatacttctatcacaaagcgcaacaggagaggcacaacgcagacggtcaattccttcttggcggttctgatcagcttcagt

cgtcaacttccgcatccacctcccagccgtctcatgcagtcaccgacgaggaccaccagcgatctctcaacagtcttggccctcatgacgcagcctct

gacgaggcaggttccccagatttggacctcggcccaggattggagttcgattcatttcctgcagacgctgcagctaggttcagtcctgacttctccct

agacagtggagacagtactgacgatgatactgactcagagtttggcgacggtgtggaagcagatccggttctagatcccgaccgcgccattttctccg

tcctcgatgacatcgataatcctgagcagcaacaaccaccagcaacatcgattgacacaaacagaagggcgcccgcgtccctcacctctccccctctc

cttgctgagcagcgcgcatatagtgcccttgctcgaaagtgtatggtgaccgccctttgtgtctattttggccttggaagggagccggccggcttact

tctaacatttttctcatccaccattcagcctttgctcgatcctgacacttccccagtcccactgcagattgacacagtccgtcgccatttgggcattc

ttcctcagctccagcaccatgcagtatgtcctgagtgcgaacggctagcgcgacggactggtggatcggacagctgcgtctcgtgccagacgccccta

tacgagcgacccggcaaacccctcgtcacgttctcgtaccagtctctcatcgaatggttaacttggctgcttcaacagccagggatcgaaactgcgct

tggggagtggagggatcgagaccctgtctcttccatgcgagacatatacgatggttccgcctggagctcggagatcgactcgcggggtctacctttca

ccgatcacagcctctctatcctcacctccttgggaattgactggttctctcctttccgaagccagtacacgtcgtggcactccactggtgctgtgatg

atgtgtgtgctgaaccttccacctcgtctccgatacagtgtctcatccacatacatcgcaggttgtactcccggacctagcgaaccgaaggctacgcg

agtcggcgagtatctgaagcccatgctcgacgagcttgctcagctggacaaaggcgtgatcattcgcacccattcggcaccaaaggggcgcctcgtgc

ggctccgtgtagcattctatttgatggccttcgaccgcaccatcgagacgcgcgaccctcaccagcatcgatcagacgcggagcggttcaagcagcca

ttcacgaacaaggagcagcgcgaatatcacgaacgaaacacaggcgcacgagacagtccactcctccagctgccctactggcaatcggtctcgagagc

gcccgtggatttcatgcactgcctcgagctggggctcgtcaaacgactcttccatcgcgttctcattcaaggggaggttatcacggccgagcacctga

ggattgttcaacatgggctggggacttgtcgtgttccccattccgagcaagcccctgatcgacggcttggcgatccaggtggcgggtctgcaactgcg

gcacattggtcgacgttagggcgaagattgcttcctctcctcctttttgtggcatggcggccaatcctcacgacgaatggtgatttagagcacgtggt

gacgtcggtggacaaaactgcaggtgtcgcacctcagcccgtttctcagcgacggcagcaaggagccgcattgctaccagaaccaccaccagcaaaga

tcaaaaagccccgcaagacactgcgacacaccacgccgatgaacgcgaggacggtgtggcgcaatgtggtccagctggcggctgtcacaacgttggcc

gcgcgaagggcgattctgcccaacgagatcggcaagctggatgccatcctccgcgactatggtcgcacaactgccgatctttttggagcccgagcgat

catctacaacctacaccttgcctcccatctgcccgaacacattcggcgctttggaccctgctaccacttctcagcctatcattttgagaggatgaatg

gtcagcttggtcgaacctccacaaatcgtcaccgaaatggcgagatcgagacaacgtacacgaccgcattcatcaccaacggtcgatttgaatatcag

ctcgcccgcgaggagatccagcttgaagcctctatcagcaggcgcatgcctcctatcaagcgtcccgtcctcgatcagctcacgcgtcagtctcttct

ctcaaccattggtatcgtcgaggtattgttgtcggccggtgatgcgtacgaccccccctccgaaatttaccagcagctttgcaatcaccttcaccgtg

cgttttctcataccactctctgcctccttcccatctggtccagccggtccgacgggatcaagatcatgcgacgcatgaggcaacatgcgactcttact

tttggccacctcatcttcggtggcattggcgtgcgaaagaacgccagcaccagccgtacatacgcggtcgtggacgccccggacggcagcctggatat

cttccacatccagaatgttctcactcatgagcttcatacaccagaacaaacccataccagcgttttccttctcggtcgtcgcgcagtccgacaatcag

tacccacgttcgacgccatctttgctgcatcttatgccgatatcctacgagttttctggaccgtctcagacagctggcagacttccgagcatcttccg

gcacaccaactcgtttccgacgcagccattgtcttcatggacaacctcgctggagtgtcgccaggcgaagatcaacgatcagcaccctag

>contig_21.contig_21.g915.t1

atgtcttccttacccaccccctcttatcctcctcactccctcactgacctccaatccaccgcactatcagctcaaggct

caccgctcaccagcacgcgccgttcactctcactctccgttcctatcgtctccgccctctcggatagcccactaccaatgagatggacctcgagtctg

cgcgcaataaaaactcccgttgtcgatctcgccgcccgcgccaggcggcgcgcatcaacggtagcagtcgcctttgcttggtctgctttcccccctcg

tccacaggtttccagttcttaa

>contig_21.contig_21.g916.t1

atggacgacaccaactcctccgccatctccggcattccttccctcgtcggcctcgaaaacttcttcgcctggcgagagg

caatcgagcccgtcttcatcggcatccgcgcgttcgacattgtccgcggcgtcgagactcagccaactctgccgcccaacgccacctccaccgatgtg

cgcctgtccgagtcctggaaggacagggacgccaaggccatgtcctacctccgcaagacggtcagcggggccctcaaggccatgattcgagacctctc

ctcgtccgccgacatgtggtccctcctcgccagtcttcacgacttgggttcctcggatcatcggagcgccatcaaccgcaagatggcgaacctctttc

tcgacgagggcggagacatggtccagcacctcaactcctacatggagctggtggctgaggcgagcgcggcgggtcttccttggggcacggaggacaaa

gacaagtgcgaccgtttcctcgacacccttcctcattcgctcaggctcgtccgggtctacaacgaggagaacgcttctcagaagcacacggcggagcg

cagcgccaacgccatggcaatggccgctgccagccaacatctccacatcgccatcaagggcgtggcaaacaaggcccgctccggcaagtcctcttcgg

gtggcggcaagaagaagtcttcttcttcgccttcttcttctcaggcgacctccggcagcagcaagggcaagggcaacagcaacggcaacagcaaacgt

ctgctctgctacggctgtggtgctcgcgaccacgaacgtcctgactgtccagtcgcgagccaccttcttcccggcaatggtccgatctgctggacatg

tcacaagcaaggccacgtcaagtccgagtgtcccaagtcgacttcgtcgtccggaaaggctgccgccgccctcgagtcttatccagacgccgacgaga

tgctcgcagccatgtttgagccctccgttcttgccgcctccacgacgtcgaccttgattcccttcatgatcgactctggtgcatcacagcacattgtc

gaccgacccgagctgctcctcaactctcggaaagcggacaagaccttgaccttcaagacggttggctccaagcaggcatccgaccacatcggcgatgt

cactggacttcttccctccggccgccgcgtcactttcctggacgttgtgcttctccccggcgccggagtcaacttgctttcggaggaactgctgcgcg

agcgtggctggatcaaagtcatcgacgagtatgacaacgcccacctccagcacaaggacaacgcctcttggcgcatgcctttgaccaagcgtggtcga

gctcgatggctgctgttacacgtcgaccttgcggcctccacatcgtctccctcagcgatgctggcaccaatgcagcccgctcagcgcgatcccgctct

cgaggttcacattcgcctcggtcacttgggcttcagcaccatcaagaagctcgtccgttcgggtcatcttcctcccattcctgggctgaacgatgcca

cgccttggtgcgatagctgcgaagctaccaaggctacccgtcgaccgttcaacgacgcgcctctccacgcgagttctccgctggagatcatcctcacg

gatgttggcggtcccttgtacacggactacaccaaggtcgacttactccgctccaaggccgaggtttttgctcgccttgttgagttcgtcaaggtagc

cgaacggactttcggcaccaaggtgcaagtcattcgcaccgacggcggcggcgagtacgtcaacaagcacatgtccacctggactgcttctcagggca

tcctccaccacatcacaacgccccacaccccggaactcaacggtgtggctgagcgcaagaatcgtaccctcaaggagatgaccgccgccatgcttcac

agtagcggcctcccggttcagtggtggggacatgcgattttattcgctgcggtgttgctcatgaaggtaaccttcgtcaaagacggtcggacggtttg

ggagtgggtacacaagcgcaaaccctcccttggcaagactcgagcattcggttctccatgctgggttcatattcccgccgctaaccgcaccaagtcgg

acctcaccactcccaaggcctggaagggacgtttggtcagctggccgacacagcgcagcggctggggggtgcacaacgagaccacggggaagattgtc

ttctcccgcaacgtctctttcggccccaagctctcgtccaaagtcaccgaggtctccgagccttccaacatcgtccagctggacgtggagcctggtga

tcccggcttcctcgagtacgacgaggaagacatttccgacgatgacgcggagccaggtcccgacgccaataatgtcgaacttcttcctcttggccgca

aacgccctgagcccgagggggtgaatcatgcgcccggcaacagtcctgccgtggccgatcgacctcaaggcattcgccggcttcctgcgcgcgctggc

cgcggtcaacgttcgtctgctttttcgggacacgtacgcagcgttcatgcaggagtcggaagggtttctttgcaagattgccctcgtcgacgacgacc

ccacaagctacggcgacgccatgtcgagtcctga

>contig_21.contig_21.g917.t1

atgttgctcctgctttgtgcggctgctctgcatcacatcgagtaccgactatgtagcgcgctttctctctctccgccgc

acctctctctgcagtcacctctaccgagccctcagtcccctaccggctacttcaacgtcctctccagcatatccaacttcaagaccatcgagagcacc

ctccgtggatggacggttcgtcaacgccttcttcgacaccgatcgctctcgacgactttcacctcccccgccacctccgagcagatccgaaaaacagc

ctcacccacatctgtagcagccccgtgtcacgcctcggctccagccgccgcttccgccgctgactgctctccggcacccaactacgcaagcgaaccat

tccgcatcttacccaccccacccccagctacagcaacaacaggatcctga

>contig_21.contig_21.g918.t1

atgagcttccagtccgataatgacctcatccactcagtagtagaggacatggaagcgttctattccgcagaacatccgt

caacaccttcgcagcttccctcaccaccgcatataataccttcacaagcacgtcttccaagtacccatctcatcaacattgcccccgtccctcaacct

gatccaacacccgttctcgcgtcaggtccgccacctcctccagcaccacaaggtctaccacctccatcacctccacccccaccacctccgccctctcc

acctcctcccgacctgggaaacgatgagccgttatcatggtggtcgaggttctcaagaacagcacaacgcctgttacgcaaagaccccgaccctctgc

gatctacatggcggcacgagccctgtaacttctgcgggaccctgctgctcgactccgagccgatgtctttctgctgccagttcggacagaacgccctc

gacccccttcctaaccttcccccgttccttcaatcgatcgagcaccacaacagcgcgggacgagcgtcactccttctcaatcaggccacccacttcgc

cgtgcagggctacactcacagaaggatcaacttcgacaccgggccaggagcgatggccatccagggctccatttaccatcgcctactgccagtcgacg

aagaccagtctccgctcaacatgtttctgtatcagcgagagccaaccggacttcaaggcacacaaaatatgccggaggactggattccgtcctttcga

agagaacttcggctccacaatcccctgagtcaaggctttcttcagctcgccgacgtacacccagacgtcccttcggcgaccttggagctccgacaaga

aggaccagcaaacgaggtagctgctatcatgcactacggagcaccttccaggcgcgacccacgctccatctatgtccaccgacggaccgacgacggcc

ctgtccgaatcacagcaacgaatcccttctacgatgccctcgcgtttcccgtgctctttcctcgtggagaaacgttcgtcgaccaccgccggtggacc

atccgcaagatcgcccggttcttccttttgtctgaacctcggttccgcaacttttggcgcgtcgcaaacctttggatgctcgatgtgatgtgtcggat

tgaggaggatcggttggcatttgtcgcagatggaatcggagcacacacacggcgaaacgagcttccctcccatgcgattgcggagatggatccgaacg

aagacaacgatcccgatcgccgcgtcaacgccgcactgccggcttccttcgtcgggtcgcgtgcgtacagagccgagcacgtcgccgacgctcttgcg

ctgtccacccgcttcggtcctcctcaggggatgatcaccgtgaccacaaatccggaatggtcggagctgaaggaagtgctgaactcccgcgcaggaca

ggcggccactgcggttcctcagatcaccgcccgcgtcttttcccaacggctctccaagttcatggcgaagctgaagacaatcttcgcaccactcctct

acatcatcaaggtcatcgagtttcagaagcgcgggctcccacatgcccacatcgtctttgcagccgatccaaagctccctgtcaccgcgattgactcc

gtcgtcagcggggagattccgccggagaccgagccacgcctgcgggagttggtgctgaaattcatggtgcatccggatgatcacgttttccgtgcgga

cggcagtgtcaatgaacgaatcgcaccaaataggaacaatgaggcatccccagaggaactcgctgccgctgccgctgccgcgaacgactatatctacg

cacgatacctttctgcgtccgaggccgtctggcggatcttcggcttcgatctcacatccaagactccgactgtgatccgcctggccgtgcacgagccg

aatcaaaaccgtgcccagttccgtggcagcagtaatccaggttcatcagcgagctctctggccttcgacgacatgcgatacgtagcatactacgagac

cgtcacttctcgcgctgccacggctttggagatcgagcatcccgagcaactgcaccctttagagcatctggagagaaccgagcgtgcgtccaacttcc

cgccgcgtgtcgtccaccgacgcctccacggaaaggccgcagcccgcatcaagaccgttcgcccgtcggccggagacgccttcttcatgcgtctgatc

ctactccatcggccgatagccaactggatggactttcgacgcactgcagacggtcagatgcatgcttccatccgagacgccgctgcgcacgaaggact

aattgagggcgagaacgaggcacaccaggtgatggtggaggccacgcaaaaccactcggccccaagcgacctccggttccttctcgctctcctcacct

tcgaaggcgcgccttctccaattacactgtggctacaacacaaggaggctctggcgcgggactacctccctctcgcgttcgccagccccgccacggcg

ccttcagcctcgcgccgtctctctgagcaggctgcgctggacgacatcgaccggagtcttgccacgttcggcctatccaacatcgacataggcctccc

tgtcgcgagctccaggccagccctcattgaggaggagctcaactatttcgctccccatcggcatcgcctccgtcaagacgcagcacagagccgagcgc

tgttcacgcagcagcagcatgcaattcgcgccgcaatcctcgacgacatactcgcggagggaggaccgcggcttcacctcattcagggacgcgcatgt

cggggcaaaaccttcctggtcaaagccatcatcgacgagcttcgcgggaacgggcacgtcgtcgcgacctgcggggcaaccggactgtccgcctctgc

gttcacgcgcggcacgactgttcataaacgatttgctattcccgtgattgaagataacgatgaccccgccactcctccgccacgatcccagctctccc

tcacctccgacagggccaattacctgaggcagtcctccgcgctgctgatcgacgagatctgggctctccctcgccctgtgatcgagacggtcgacgcg

ttcctgcgagcgctgatggagtctgacgcaccattcgggggcaagtgtgtcattgcagtcggggaccctagacaaactgctccgatcacgaaagaaaa

caccagacaggctacgatagatgcttcctttctctccacacaactgttccctcgattccagctgcacgaactgcacgcctcccaacgccaggcccacg

acttgcagtttggcgagtgggtcgacaacatcggcgacgactccagctcggccgacgtcgacctctccctgatgtttgcctctgtttcaacaccacag

gcagccttcgacttcctcttcccgcccgctgtcctcaacaacccacaagaggctgtccagcgctgctttctcaccccgttgaacgtcacggtcgacga

cttcaacactcttgcggtcgacagcctcccaggccaggcccagcacaagacagcgcgcgactccatcaaagatgcggacgagcatcccaacgctgccg

ccgacatcgaagcgacgctccagattctatctacagtcacccatcccgggatcccggaccacaccctgcgcctcaaggtcggacaaatctgcagcctc

ttacgcaacatcaacgtgcagaaaggcctggtgaagcatgcgagggtgaccgttacccacatcgatccccacagcatcgcagtccggctcattctgac

cggccggtctttcacgcttcctcgaatcaacttcgagttcacgccgatcggatctccattcaaactcaacagaaaacagtttccgctccgcgcggcat

atgcatcagacacatcatctctcttcttcaccaacttttcatcccccatactccatagcccttcgccttttccttaccttacccacatcagattcacc

atggagcgcatgttgttttccaccctcacaggctcgattcaaattgactcgatcgagcgacagaaggcctggtatcaattcaaaggaatttgcctcat

gggccagtctacctcggccgagtgcgaggtcgtcggtacaatctacgacgccggcacgatcaagctaccgagcgtcgtggaagtcgcagctggactca

ttggcataacaaccaagggcgagctcaacctcactgtgcataggtgggaggttctatgttcggctgaggacgtttcccacacgatcgcgccacccgtc

atgacgggcgtcggacaggtgagcgaggttaaccaagcaaaagcgctcatcaaatacaagagcgccgggtacaaccggcaggacggaagaaccctcat

cacggagtactgtagtgtccgcgacagtctccgatggaaaaacgttccgtacgcacaggtgggaaacttcattgccatcatcggacagcttcatcacg

tcgagtccgtaagcaatgtcctgttcatcaccctcgacgacgctgcgtggacgagcattggcgcgggaacgcatcaatcccccacggcagcccccggc

gcccggaagttcctcggcaaacgcaaacggaacgagcaagaaacgaacggtgagccctgcacttcgacttccgcctctgcctcctccgccgcgacgtc

aacatctgccacccccaacggctcctcctcccactccttcaaccgcacgtga

>contig_21.contig_21.g919.t1

atggacacgatgctcccacacagcaacattcttcaagcctccaagcagcgaagccggaccagcgcgctcgtttccgata

cgctgcgaagccgcttctctccacaaccttgtgctccgtcgtcgcccgccagcctgtcgaccaagcatgctccatgttcgcaccagcgcacgtttgcg

catcctcaagatctcaaggcccaaagcttgccgcgttgcgctttcgctgctttagcctcgagctcgtcagcacattccccatttcccaagaagcgcag

cacccaccacagaaggctgtacaaccgcgcttcaaacgcaaggcgatggcttgcttcgcagatgaggaaccaggaaggcggttatggctcaccctcgt

cagtactgaggttgtacaacgcgaaactgctcgctcaagtccagaacgcatccgtcgtcgccgtcttgatacgcacatcgtcaccattgcaagtttcc

ggcaaggttgcgcagtcctcggccgccgaggcccggagagctccgccgggctctcctcagctgtcacggatggatagcgtgtctcccgctcttgagac

gctgtcggcacgcatcacacgcggtgctgagacgcccagaaccacagcgtctaaacagcgtctcaggagcgtcagcagcgtctcaacatccggtccac

ttttcctgattcaattggacattgcagccattcaccgagcttggaccccattggcaagcttggacctcacgccgttgcctttcttctctctcgccagc

tccggtaagccccacttcccttctgccggttcgcagggtgctcgcttttctcgtgtcggcgcccgtgttgatgatcttgcaccgggtattggaccaaa

tgaggtactcgtttcagccgaactcccagatcccatcttgcaactttccgatggcaaagaaccgatcctcgtcccgaagctcctggcttgggccgccc

aggagaaccctgacggcgtggctcgccgcgatcccttgttcagctgggacgacgtgcagtcgttgaagacgttgccatggcgcgaaacgacatggaag

gatttcaaccagctcgtcgatgccggtgcgtttcttttcagcgaacgcggatggccggtgcgcggcaagaacgagcagcaggccacggagccaatcaa

ggtcgccgtgttcctgtcgacggaactggcttcgtgcatcgctctgtatgcgttgatcaagtgtggtaactctgtgctcctcccctcgccacgcaaca

gcatcaatgttctggtcgacatgctggtccaggaagggattgaacgcatcattttctcgtcggtatacgaagctgttgcaaagtctgtcggggatgag

cttgcgaagcgtcaggtcggtctcgctcccacactcgctccaattccctctcaggaagagcttgacgaccgcaaagagacgtgcggtgtctttcccta

cgagctcgacgacgagctggaatggaagcgaccgatctggtttgtccacacatctggatcgtcgggaatggtcccaacgatctgtccgtacacccatc

aaatcatggtagagacggcccgtgctatgacactgggaaatattcaaggcgaaaatcacatcagcctggcgccactcttccatctgatggtccacatt

cgtggaccgatctattgtgcccttaatcgcgatacggtgacgacggggccgatgggccctccaccatcggcaacgcaactggccgctcttatcagcaa

gtccaactgcgacggaatcaccgtcgttccccagctactcaaagcgctgacggagacgcccgggggactcgacgctctgaagcgactcaagggtgtca

agtacggtgccgctgccctgccgaacaagacaaagcgcgctctggacgacgctggcatcaaacacgtgaactcatacggtctcagtgaggcctcggtt

ctggcagactgcgaaccggatcgctggcctgccaacgtcaaattggacacagactggatgaccagcaccggtcttcacacactgaagtttgagttctt

ctcgaaggccagtgacggcgtgccggatctctacgagctcatcgcactcgcaggcgacattccttgctcggtatcgaatgtacctggagggtatgcca

ccggtgaccttgtagagcgtcaccctgactatccggagtggttccgcatttggggccggaaaaaggctatcgtcgtgctggccaatggtgaaaatgcg

ccgatcagggccctggaagaggccttggaggcacatccttgggtacgaacggccctcgtttacggtacagctcggcctcaggttggcgtcctcgtcga

gctcgatcccgaacacgctgtcggaaaaggcaaccaggaagcggacgcagcggcgcgcaacgaactgtggccgacaatcgagaaagtcaatgcggagc

ttcccgacttcgccaggcttttcaagagcatgatcttgttcgcgtctcctgaggtaccaattccacgaacggacaagggcacacccaaacgtcaggtc

gcgatgaagatgtacgaggacgacatcgacaacctgtacacctctgtcgctgctggtactggcgcagccgtccagttcaactccgtcgatcaggagtc

ccttcagaaaatggtcatcagcatcttgcacacgatctacggtgatgataaggagatcaaccccgacacttcactcacactcgagctcggacaagact

cgcttcgtgcgacgatgactcggacgtccatcgttgcctctctgcgcgcggcttcacagtccggcaacgtgcccgagctcgccggcttcatgccagac

gacgttccgacgcttattgcataccaatactctacccctgctaccttggctcacgcccttcatgagctggtcaaaggtatcaccaggagcccccacga

tactgaggcgtcagaaatcgaactgatgcgtcagctcgtgaagaagtacagcgatgtactgaagtccacgcatcgacccaacggcaacgccctgagca

acggcagccacaactctggcggtcgtgtttcgactcacggcagtcagcaggttgtcctgctcacgggaagcaccggagccttcggaacctgccttctc

gagcaactcgtcgacaacgcgaacgttcaccaggtcatctgcgtgattcgctccgcaagctccgaccaggctgcactcaaagcacgtcaggtcacagc

tttcgtgagcaagaaccttgaccctgcgccggcacactctccgaaagttcagttcctgcacggcaacccttctgaagaccaccttcagattcctgagg

aagtaacgacgatcatccatgcagcgtggtcagtcaacttcaatctttcccttcaagatctcgttccggaaatcgaaggcacggttcggctacttcaa

cactgccagcgcaccggtgcgagactggtctttgccagctccgtcagcaccgtcatgggagcacctcctgaaggtcaagggtctcggttcatcgaaga

ggatgaggatgctcctctggagtgggctgtcatgggctatgggcggagcaaggctgtcgtcgagaagttgatctcctctgcggtccacgaggccggag

tggaggcgatgtcgattcgcttcggtcaaatcactggcgactcgcgatctggtgcttggaacgctcaggaaagggtgccgagcatcttccgctcggcg

actgcactttcagccttgccagacgacatgggtaccgtcgactggctgaccgtcaacgacgcgatgcaggtcacggttgcggctgctatcgggcagga

tgtcaccagcgcgacgtcggcatccaaacaaaacgttgtcaacctggtcaaccccaacaaagttccatggctcactgctgtgaaggccttccaggctc

acatgccgagttcggtcgagcttctacccacgcgcgaatggttccagcgactcttggacgcccagaaacgtacggaggcagcaggcgacttcgaacga

gcatcagtccaaattccggtgctcaagctcgtctcaagctttggtgggcgcgtcggacaagcgatggcgggcactgccgccactcttcttcaggtgaa

agaggcaattcgcttgagccagggccagctcctctccgctacggccatcgacgagcatacagcagggctttacttgcagttttgggagtctcagacga

agcaggaacacgctcgggcttga

>contig_21.contig_21.g920.t1

atgatgctcggtactcggacgctcgaggaacgggatcgcaagaagcgcgagtatgtggatcgtggcattgggactgcac

ttgacggatacagtcagactcaattcggggatgtcagcaactacttcctcttgcgcaacagcgagatagggctgcgagaccgactggctttccttatc

tctcattacgcccttctacgaggagacaacgcgcggaaacttgagctcgccgaccttcaccatctcgagctggaaggggaaggcccctcaagttgctt

tgccgtggtactgttgctggccaatggaaaaacgaatcaagcaggtcgagtggagtacgggtcgtttatgcgcaacagggatgtccagctgtgtccag

ttggtgcactggctctctacctgttttcgagattccatcacagcgctgaagcattccccgacttgagccaatcatctctgtggtacgatatgaaggtg

atcaaggctccaaagagcagcaccgacccgattggcgacgagactcatggcaaggcgctggacgcggcgttcaaagcgtgcggggtcttgaccaccgc

caaaacccatgcagctcgaaagtccggcgctcaactcgctgaggctaagggtgcgcctgaggcttcggtcaggagagcgggtcggtggaaccagacga

cgatggagacgtgctacttgacccgcctaccgcgaccggtgcttcgcgttcatgccgggttccatgcggacggcgggtcttacttcctccgtcgcgac

gttccccctcctcaggagctccttgagcgcgtcttccctcaagctgatgcatgggatactgccatcgctagcggacaggctgcggatggttcaccggt

cgaaatcaacatcgcatcgcgtgctttcgtgcgcatcctcattcgccttcgtcccgtcctgctgcaggatgctgtgctgctgaagaaagagtttccgc

agctctatctctggagccatgccatgttccactccgacgcattcatcagctacgagaacgagctcctcaggacactcgagaagcctgacacatcgggc

gacgaccagctgcgcgcggttctgcctttgctcgccgagcagatccaggcacactcttcggccacgcagcagcacgtcgatgaaggtctcaaggagct

gcgggaggacgttgaacgcgaccttgaaatggtggtcgagctgctgcacgggatgagagagcagcaacagcagcagacaaacgagctgcacgcaacct

tgcgtggtattctggggttccttcttgctgcaaatggtggctcgtctctttcatctccctcttctcttgctggcccttcgtcctcttctgtgacatca

ccctcgtctttgatctcagcagcatcagcagcagcaaccacctccgcagtttcacgtatcggtcagcgctctagacttcacggcctctcggccacatc

ctcttcgtctccctctctcgtcgccaaggtcatgccggccatggagcaggacagaggaatcagctcttgcgctgagctctggcgggagtacaaggatg

gtctgaagggaatgttgtcggtggaaagaatggacgaggagtggggcgcggcttggcgatcatccgagaaggaccgcaagcactacgagcgtcgaatg

cgcataatcagcaaaataaaggacctcgccatcgagaagaaggtgtcggagagggtggcattggttatgctggagcatgctaggaatcagctcccgaa

caagtcattggcaaagttgtcggatcatatacgacagggcaaggagctgacgtatcttgatctgtctcttaacgctgtatag

>contig_21.contig_21.g921.t1

atgtccgcttcttctgtttctgctgaggcctggcagatcaccaagccaggatcgatccccgacacgctcgagcttcgtt

ccatcacacttcccaatctcaagcctaaccaggtgctcatcaaagtcagccacgctgccctcaacccggccgactacaaattggccgcaatcttgccc

actttcatccagaaagttccccgaacggcagcctccgatttctctggcaccgtcgtcaagctcggctctgacgccctcgccaagaagtacgaatggct

ccgtgaagagaatgtagccgtctggggcctcattttcaacctgccacccacctcagcccttggagcgatgagcacatacactgttgcggaagtggagt

gcatccatcccttgcccaccaatacagcggccattgaggctggtgttactgctgagagcgccgcgggactgggtatcgtgtccctaaccgcctacctt

caggcccagtacgtcaagaagggcgacagggtcttcatcaacggtggcaccacggctgtgggtctcattgcggccgacctcgcgattgcgaagggtgc

atcgttgatcgtggccactgccagtggctctaaggccgacttcatcaagaaacgcggcgtgaacgaggtcatcgatcaccgcgccagcgacccaaagc

aagagctcctgagccgctactcctccgcgccattcgatgtcgtcctcgattgcattggtactccagatctgcacttgtcctcgcctgcgtacctcaaa

gccgacggaaagtggatcaacatcggcgcatcgttccatcgcaccctcgtctttgccggactgtcgaagtacgatctggtcgaggtcaacaagcttct

ggtagaaggaacgatccaccccgtcgtggacaaggtcttcgaatggaaggacggggttgaggcgtacaagtacttgattgagggtcgagcgttgaccg

cttgcaccttcggacgtaatatatgtgtccacaccttgaatgcctgtctgcctgtagaccgcaggtgcgaagcccacacggcggacgatgcactgacc

gacgagcagcaggttgggcaaccatggcacagcggcaacagcaacagcagcgcgatggcggcggcgactgtggttgatgccagtgtcgtccagagcgc

gcttcgtctcgtccggcaggcgaggaggtttgcgcggctgcgcggtggctcgtttacatttgtgtag

>contig_21.contig_21.g922.t1

atgctcctcgacgttacgcgcaagctgtacgcgcgcagtgcccgcgtcaaggccgtcgacttccacccgaccgagccat

ggatcctcgccggtctctacaccggccaggtcaacatctggaactacgagaccggcgccctcgtcaagaccttcgacgtctctgaggtccccgtccgc

tgcgtcaagttcatcgcacggaacaactggtttgtcgctgggtccgacgactttttcctccggtgcttcaattacaatacccacgagaaggtcaccgc

cttcgaggctcatcccgactacatccgctccatggccgtgcacccgactgcatccctcctcctcaccggttcggacgacatgaccatcaagctctggg

actgggacaagaactggaggcatgttcagaccttcgaggggcacacccatttcatcatgaacctcgccttcaacccgaaggactccaacacattcgcg

tcatcctgtctcgaccgcacagtcaaagtctggagtctgggctcgtcgcaagcaaactacaccctcgaggcgcacgacaagggcgtaaactacgtcga

atactaccatggctccgacaagccatacctcatcaccaccggcgacgaccgcaccgtcaagatctgggactacctttcgaagtcatgtgtccagaccc

tcgagggtcacacctccaacgtctcctacgccgtctttcacccgtccctgcccctcatcatctccggctccgaggacggcacaatcaagctctggcac

tccaacacataccgcctcgagaacaccctcgactatggcctcgagcgcgcctggtccatcgcctacaagaagacgggtaacgatctcgccctcggctt

cgacgaaggtgccgtcgtgatcaagattggcaaggaggagccaagtgtcagcatggacaactcaggcaagctcgtctgggccaaaaatgccgaggtcc

tcggcaccaacctcggcggactcgtccctgccgagcttccagcggatggtcaacgaatcaatgtgggcgtgcgagagattggcggcagcgaggtatat

gcgaccaacctcgtgcactcgcccaacggccgcttcgtcgccgttgtcggcgatggagaatacatcatttacaccgctctcgtctggcgcaacaaggc

cttcggacccggtttcggcttcgcatgggccagcgatagcaacacctacgccgtcaaggagagcgattcaaaaatcaaggtcttcaagaacttcaagg

agcgtccaaatctcatcccgatcggatacaagattgtcgatgtcaagggaggcaacttgctcggggttatcggcactggattcgtctgcttctacgac

tgggagtcgggagcgctggtccgtcggatcgacgttgacgctcgcaacatatactggtcaacaacgggggagttggcggccatcgtcggcgaagattc

cttttatatcctggcttacaacgcggatgcatacgccgcagcgctggacgcaggcgaggcgatcgaggacgagggtgtggaggacgcattcgaggtgg

tgaccgagatctctgatacagttcggacagcacgatggacgggcgagtgcttcatgtacactacggctgcgaaccgactgcagtatctcatcggcgag

cagacgaacacggttcaccactccgatcgcgagttgtacttgctcggatatctgccacaacatggtcgcatctacatggcggaccaggatatggggat

ctacagctttacgctgtccctgtcggtggtggagtaccaaacggcgatcttgcggggagacctggaggcggcctctacaatgctggagaacgtgcctc

tcgatcagcgcaacaagctcgctcgattccttgagacacaagaccacaaggagctcgctttgcaagtgacacaagatcctgatcacaagttcgacctt

gccgtttctctggacgactttgacactgcgctcgagattgctcagaccggcccacaaactgggtccgagccgcgctggcggacgataggcgacaaggc

catcgggcgatggaacctgacgctcgcccaggaatgcttcgagaaggccaaggatctcaacgcgctcctcctcctcggaacctctaccggcgaccgga

cgctgctgagcagggtagcgaccctcgccctcgagcgcggctcgaccaacatcgccttcgccgcctggctccaactggacgacgcggagagctgtgtg

aagctgctgctggataccggccgggcgcccgaggctgcgttgttcgcgcggacgtatgcgcccagtcttgtttcgagggtcgtcggggcgtggaaggc

ggagctggagagcacgaagaggtcgaagcagagtgcgattgcttcgcggattgcggatcctcagtttgattcggctgcgtttgaggaggggtgggagg

aggcgttggcgcgggagggggagcttcgtcggcccagttcaattggaaaatgcagccattcagcgagtttggaccccaccggcaacttggacctcacc

ccgtactcttcttcccctacgccagctccggggacctctcactttccttctgccggaacgctgagaagcgctgaaggctggcgaaggcccgtgttgat

gatcttgcaccggcctcatccatccctccagcctcaaccctccagcctcaagatgactcaactatcaatgacaactttcaaagaaccgatcctcgtcc

cgaagctcctggcttgggccgcccaggagaaccctgacggcgtggctcgccgcgatcccttgttcagctgggacgacgtgcagtcgttgaagacgttg

ccatggcgcgaaacgacatggaaggatttcaaccagctcgtcgatgccggtgcgtttcttttcagcgaacgcggatggccggtgcgcggcaagaacga

gcagcaggccacggagccaatcaaggtcgccgtgttcctgtcgacggaactggcttcgtgcatcgctctgtatgcgttgatcaagtgtggtaactctg

tgctcctcccctcgccacgcaacagcatcaatgttctggtcgacatgctggtccaggaagggattgaacgcatcattttctcgtcggtatacgaagct

gttgcaaagtctgtcggggatgagcttgcgaagcgtcaggtcggtctcgctcccacactcgctccaattccctctcaggaagagcttgacgaccgcaa

agagacgtgcggtgtctttccctacgagctcgacaacgagctggaatggaagcgaccgatctggtccgtccacacatctggatcgtcgggaacggtcc

cgacgatcttgacgacggggccgatgggccctccaccatcggcaacgcaactggccgctcttatcagcaagtccgactgcgacggaatcaccgtcatt

ccccagctactcaaagcgctgacggagttgcctgggggagtcgaaattctgaaacgactcaagtggatcagatacggtggttctggcctgccgaacga

gacgaagcgcatcctggatggcgctggtatcaactacattaactcatacggtcttagtgaggcctcggctctggcggatggcaacccgaatcgctggc

ctgccaacgtcaaattggacagagaatggatcagcgacaccggtcttcacgaactgaagtttgagttcttctcgaaggccagtgacggcgtgccggat

ctctacgagctcatcgcactcgcaggcgacattccttgctcggtatcgaatgtacctggagggtatgccaccggtgaccttgtagagcgtcacccgga

ctatccggagtggttccgcatttggggccggaaaaaggctattctcggcctcaggttggcgtcctcgtcgagctcgatcccgaacacgctgtcggaaa

aaggcaaccaggaagcggacgcagcggcgcgcaacgaactgtggccgacaatcgagaaagtcaatgcggagcttcccgacttcgccaggcttttcaag

agcatgatcttgttcgcgtctcctgaggtaccaattccacgaacggacaagggcacacccaaacgtcaggtcgcgatgaagatgtacgaggacgacat

cgacaacctgtacacctctgtcgctgctggtactggcgcagccgtccagttcaactccgtcgatcaggagtcccttcagaaaatggtcatcagcatct

tgcacacgatctacggtgatgataaggagatcaaccccgacacttcactcacactcgagctcggacaagactcgcttcgtgcgacgatgactcggacg

tccatcgttgcctctctgcgcgcggcttcacagtccggcaacgtgcccgagctcgccggcttcatgccagacgacgtcccgacgcttattgcatacca

atactctacccctgctaccttggctcacgcccttcatgagctggtcaaaggtatcaccaggagcccccacgatactgaggcgtcagaaatcgaactga

tgcgtcagctcgtgaagaagtacagcgatgtactgaagtccacgcatcgacccaacggcaacgccctgagcaacggcagccacaactctggcggtcgt

gtttcgactcacggcagtcagcaggttgtcctgctcacgggaagcaccggagccttcggaacctgccttctcgagcaactcgtcgacaacgcgaacgt

tcaccaggtcatctgcgtgattcgctccgcaagctccgaccaggctgcactcaaagcacgtcaggtcacagctttcgtgagcaagaaccttgaccctg

cgccggcacactctccgaaagttcagttcctgcacggcaacccttctgaagaccaccttcagattcctgaggaagtaacgacgatcatccatgcagcg

tggtcagtcaacttcaatctttcccttcaagatctcgttccggaaatcgaaggcacggttcggctacttcaacactgccagcgcaccggtgcgagact

ggtctttgccagctccgtcagcaccgtcatgggagcacctcctgaaggtcaagggtctcggttcatcgaagaggatgaggatgctcctctggagtggg

ctgtcatgggctatgggcggagcaaggctgtcgtcgagaagttgatctcctctgcggtccacgaggccggagtggaggcgatgtcgattcgcttcggt

caaatcactggcgactcgcgatctggtgcttggaacgctcaggaaagggtgccgagcatcttccgctcggcgactgcactttcagccttgccagacga

catgggtaccgtcgactggctgaccgtcaacgacgcgatgcaggccttccaggctcacatgccgagttcggtcgagcttctacccacgcgcgaatggt

tccagcgactcttggacgcccagaaacgtacggaggcagcaggcgacttcgaacgagcatcagtccaaattccggtgctcaagctcgtctcaagcttt

ggtgggcgcgtcggacaagcgatggcgggcactgccgccactcttcttcaggtgaaagaggcaattcgcttgagccagggccagctcctctccgctac

ggccatcgacgagcatacagcagggctttacttgcagttttgggagtctcagacgaagcaggaacaggctcgggcttga

>contig_21.contig_21.g923.t1

atgagctcagagaatacccccctcctgccccaacacgcgcagcgcacggctcagcgtgccgcccatgaggtacagcacg

ccgtctccaacccgcgcgacacagcaaagcggctcactccctccaaggatgcccagaagaacctcgcacacgtatacgctgccctccaggctggcaag

atcccctcccagaaccagctcaacgccctcctcgagaagatcaagcagtcccccgtcctcaaggaaggcaccttcgaccgcacttccgccctctccga

ccagagcgctcgcgtcgttgctgacctcaagcgcgtcatcgagcagatcatcaagctcggcgacagcaagaacggcgacgacaagatccaagacttcc

tctacaacacctcccgcgcctctctcgacgcagacgtcggaaacctctccaagctcaagccttccaagcgccaggctcgtgaggacggaaatgctctg

atcgagtctatccgcaccctcggatacttgctcatcaccgacaagaacatacacaacatcctctccgacctagtcctcaccgcacgcgacctctttgc

cgatgccgcctctaccgtcgccgaccaggcctccgaggtcgcccgcaaggcaaagccctccgaggaagagcgcagctccgatctcacccgcaaatacg

ccaccgaccctcgcggcagcgctgacaaggcgaagaagcgcgctgaccagatccgcaaggactaccgcgccggtgtacacaagcagatcttccgcgac

tacgccgaccgtgcaagcagctgggtcgaggagaagactcccgacgatgccaaggatgcctttgttgagcgcgtcaagaagattatcgtcagcgtcca

gagcaacgacgagtaccaccaggccgtcactaccattgtccagctcgtcaagaagtggggctccatagctgtggagaaggccgacgctgccaaagacg

aggtcgatgttgaaggcaacgaacaccttcacgccgcccttgacaacctccgctccattatcgagacattcgccggtggcaagtctctcgaccctctt

ctcgagagcgtccgcaagataatcgacgatgtccgccaggacgagcgtctgaccaactactggaacgagatcgactcttacgtcgagcgtctcctctt

cgacgagggcttcgtcacctcctccaaggccgcccgcaaggctgacgacctctacgagaaggcacaaaagctgttggagagcaacgccggctggaagc

gcgacgcagaggcgctcaacgagcagcttgtcgagtaccgcgacgcgctcagccacgacgctgaggccaccgagttccttgacgccctgtcagacctt

aacgaggacgtctccgagctcctcgtggacggtgtcagcatctttgccctcaaggctaccggtgtcgttgctgatgtcttcgacgtatggcttccccg

cctcatcggccagatcaagtcgatccccctgccccgttgcgagtacaagagtgccgacatcgactttgtgctcgacaacatcagcttcgagtccatca

gcggctccttcatccccgaccgcgtcaagttcaccaaccacaacgagctcgagctcatccagggctacgctgcctttgcgagcacaggcacttctcgc

gtccagctcaccattgagggcgtccgtatcaaggcgaaagatatcgcctactggctccacaagaagaccggcttcggctggaacgactggggctttgt

cgatgtcgagatcggtggccagaagggctgcaccatcaccgtcgagctcgagactgccgaggagaaggacgacgagcacttctacaaggtgctcaacg

tcgacgtcgacacgtccagcttcaccgcccgggtgcacaacacccacaagcccattgccaacacgctcctgttcccctttgcccgtcctgcgctccgc

aagctgctccagcacacccttgaggagacggtccgggaacagatcggcgacatggacggcaagctgcacgacattcaccagcgcagcatcgcccttgc

ccagtactcgtaccaacaaggctcagcgcccagcctggccggcttcgcgaagggtctcttcagctcctcgttcctgccctccgtctccggcggccgcg

gcggcgtctccgtctcgaagcgcgggatcgtcaagaagggcaagcgcggggagtacctgctcgccattggtgtttcggaccacctgcttcccggcgtc

ggcgggccgactgggttctaccaggagcaggcgaagaaggtcgctgaggaggcgcagcggttcgggttggctggcgttagtctgaagcaggggctgag

tgcccaggctaaggacgtcaagcgggaggcgcgacggcttgctcgggcggagtctcgggaggagacatggcgcagtgatgcctttgacttgcttctct

ga

>contig_21.contig_21.g924.t1

atggcagctgctgcgtttgcttcagggagcgcaaggttgctggcgagacgcgccgtccagccgaccaacacactcgctg

cctcaacagcatcatgctggcagcgcacaacctcagccccagccccagccccagccccagccctacacacgcgcaccttctccctctcccacagccgc

cataccacgccgcccaaaaagccagaaatcaaatccatcgcagccttacgccaggccatcccaggcacgtccatgctcaaagcccgcgaagccctcct

ggcaactcgctcccccgaagctccggacacggacaacgtcgaggctgcgctcgcttggcttgaggaggatcggcggaagtctggcgctagcaagagtg

agaaggtcgctgggcgcacggcggcggagggtctggttggcgtatgtgtccttgccgacggtctggtcccctcctcttcttcctcgacgaagcagagc

ggaggagcatcgccattaccacaggccgctattgtcgagcttaactgcgagactgatttcgtaggacggaatgaggtctttggacagctcgtccgcga

tatcacccataccgccgccctcttccccgtcctcgccggcttcgtcccacccacctccgatccaacctcgagctcgcccagcatggtcgaactaccaa

tccaagacctgctccaattccccctcctctcctcctcggcagactcccaaccggtggagagtgagagcgtaaagacgatctcggcggccatcatggat

accgtcgcacggttaggggagaagatcacgctctcccgcgccgccgcgctcgtctcccccacaacccccgcccccaccgcgccccgtcgctccgagtc

cggctcagcagcagggagcgaagtcgcccatctcgcctcggcgtacgcgcacggtggaccttcgttgatcagtggacagcaggcttcttcggcgacgg

cggcggggcaggggtattccctgactagcggacgcgtcgcttccctccttctcacccgtatcgctagcccggacctcccctcccagctcacccaggtt

cagggcactaccacaccgccctcgctccacatcgccgcgcgtgcattggctcgctccctagcgcgtcaagtagcgggtatggagacgcgttccatcca

tcccggtcctgaggacgtcgtctccctcgacggcccgcccaggacggctctctacgcccagccattcgccatgctcctcgccgccgcggctccccagc

cggacagcgcgtctcactcggtagcggacgtgttgcgcgcgtggggcgagagcaagggacttggtcaggcggcggtggaggtgcagagccttcagcgc

ggactatgtacgaggatcccatctaggactcgcacggcagcagcagcagcagcagcagcagggacgatggccgcgatggaggcgacagcgagcgcagc

atcctcagcgacagcagcgtcctcgacaacagcacacacactcacagcacacgacgacaccacggccaccgcggcagcgacagcgacagcgacagcga

cagagaggaggagcaacagcagcggcggggccagcagccatcgcgtggcatcgctcccgctcccgctccccctgcgcaacaagcactgccacttcgtc

ctcctcgccgagttcgacatcgaccgcggatccaccctcgcacatcagtacccgtcccccatcggacacgatgaccacatactcgcagagctcatgct

gcccgacggcgcgcatgcccgctccgaggactggaccatcttcttcctcaagcagacctcgccgcgctcccgccagaagcatgcgtccgtcaatggca

atggcaatgccaatggcgactcagcgccgggcccaaatggcactgccgacgccgagccagccaaggcgatcgacaccgacatcgtctatgtgttgaat

ctggtgcggacaaagcatgacaactccgtcagaagaggcgcaatggtcaaggcaatgtgcatcggcacctcccatccctacgtccagattttcaagcc

caccctgctcctcgctttggacgactactttgccgacccaggcatggactgcctcgcccgcttgtacgaggccatcaacgagatggacctgacgccca

tgcccatcttctccacggccgagaagatgatcctgcgctcgagcgaacggagagatctgttcgaggagcgctttatggctgtgcctcgtagggacgat

aaagatggaccgtcggggcctaaggatacccatttttatgacacccacatcacctttcgacagatttgcatccctatccgcttaccattgaccacttt

ccccgaagaagtaggagagaccttctccggacccaactcgaatccgaccgggccacaacattcacacctacactccaacgggtccctgactcacccca

tcatcatcctcttcaacgccatggcaacccagaaacgagtcatcttcctcggccacggccaaccagcgaacgacgtcgctacctttgttctttctgct

tgtgcgctcggcagcggttgcggcgccgtctttcgcggcttcaccgcgcgggcttttccctatgccaatctcaccagtttggacgagatggagaaggt

gcctggatacatcgctggcgtcaccaatcctcggttcgaagatctgccggcctgggacgtcatctgcaacatcgagaccggcaggatcacgatctcta

aagacattgagcccgccccgccgttgggcaccgtatcctcccctcactcccacgcacacagtcggcagggcggcctggtccgcgatcacttctcgagt

ggcagtctgagctatgcgtccggaagcatgagcaacattgccggtgtgcagggtggagctgccacagatggcgtatcaagcacgggaacggcagcggt

atcaggttctggggctacgccttcccacgggccgagcgagtccgcagttggaagcagcagtcgggctcccagtgaggctgactcccagagcctctcgg

gcgggtatccgggcgggtccactgctgttggtacactctctcggacggggacgacgctgagctcggggaaggagaaggcggtacatatcgagggccgg

ccggatgcgttggacaatctgctgatcgaagatatcgtagctgcgatccaatcccgctacggcgaaacctatatccgtgcccggatcgtcgactacgc

cgccttcttcggccgggtcgtctcccggcacgaggagtacttctggggccacaccaagattggctggccgagtcaaccttttctgaatgggcagctgg

ggagcgggctggtgtatgtggatcgggagatggagatgcgcgagattcagatcaatgcgatgagggcggaggggtggagggcgagtagtgcgtacccg

ctctttcggcaggatgcggtccatcgcgaggtcacgcgggagatccgggccttcgatgtcgttcatcagcttcacagaatacggcgggcgaagcagct

ttcttatggagagtccgacttgatatttgccacgattgcaaaattgatcagaaccccggaacagattgtcgagctcttatcccatctacccgcgcaca

ccggcggcctcaacccactcgcttacgggctctaccaccccgcatccgcagtccggaacgcgatggtcgagttcttctcgaacatgtgcagccacccg

gtcgcgcggaagctcatcaccaacctgagcacgttccaccggctggcgctggcgaggttgttgaatgagcgggagtcgcagattaactcgtcgaggat

ggcgcaggctcttgggcagcaggggcagggtggaatggcgtatgttggtacatcgtctccaccatatccggtgcctccgcacgctccgcctccaccac

catcgaatgtgcaaggcaacagcagcggtagcggcagtggaagtggcagtggcagcaatgcatccccgtccagcaccagcgccgccaactccagtccc

gtcccacccaccaccaccactacccccgctcgtcacagccccactcgcgccacctcttccttcctccgctccggtcgtgccaccgccgccaccaccag

cagcggggtcgtccctcatcgcgaagattacgggtcgggcgcgggcgaattcgtcggtttcgacgcagggatccggcgggtctgggtctggggtgggg

tcggcgcaggcgccaccggttccgagtcgggagtcgcggctctcgcttgcgtcgaaacagcttga

>contig_21.contig_21.g925.t1

atgtccaacctcgccgcgtccgtcaccgccgccgccgccagtcacagcaacggccgcgcctcaggcatggggccatccc

aagcagcagcgacagggacagggactgggacaggaaccgccgtggtcgggagcagcaacggcagcagcagcgcagccctcgcagccggcgccgaccct

ttcttccgtcagctcgatggcgccgcttccgtcgtgcgccagtatctgcaggaggatgtctccaaccctgacctctccgagcatctttctgctaccac

gccgggacactatcgtctcgtcctgaatgagatcagcacgcctgtcgagcggaagcgcaacatactcatccctgacgcggtcctagaagccatcacct

cttccaacgcttatgccaaccaaggccttttccccgagatagagcgcgcatggatcactatcgataacaacctcttcctatgggactacttggatgga

cggaacgatgccatcgaatatcacagacactccgactacgtcatccaagccgtcggtctagtacgcgcccgcaaagatgtactcgtcgataccataaa

atacgtccttgtcttgtccacaaacgactccgtcactctgctcggtctagcgctcgaatctcccaagccaccatcaggatcatcggccggttctgctt

ctggccgcaaggaactcaagatctacggcctcgacatggtcgtgccaacccaaggcgtcaccatggtcgacttttgcagtactgattctggcaggctc

ttctgcagaggcagagaggacaattgcatctatgagatctcataccaggcccaagaatcgtggttcaagcagaaatgcactctcaccaacctcacagc

tccgacgctcagcaatctggtgccgagtttcctgcagagcaagcagcgagaggacctgactacgattattgtggatcgcgagcggagcgtcttgtatg

cactctacaagcgcacggagatcgaggtttggaacattcctgcagatcacagcaagcagccttacaaggtgacacgaacgagcgacctctgtagacag

gcgacgaacatctgcccgagcacaaacatgctgccctctttcgacttctccatcgaatggatgggagcggtgagcccgagcgagtcaaggacacttca

catggtcgcagtcaccagcaagggtgtccgtctgtacttctcacactcccgcagcttttatcgaagcgcgtctggggccgccccgccggtagactgtc

ttgatctccgagccgtgcggccaccaccgcagccgaactctgtcgaggaccccgaggagcgcgccctacacgcccaatcgtccggtaacacgtctgat

atcccccctcaacatcccgcatcgggaaacatccgcactgcatactactcgcaaggcgtcttcctcgccgccagcacgctgacgaaggaccacgacag

cgcgactgataacatcctctatatcgctcgctcgcccgcgcgtgcctcgagagcgggaacaaataccaccctcggtcttcctcagaacgtgggcacgg

cctccgagctcgctgagacggcgacggacttgattcatcgcggtagcaccactgtcatcgcggaggcgccgcaatcagtcggggtgccgattgtggat

gggaacgggatcaggctcagtgtgtttgggcttcagctggtgttgcctccccggcaattccttatctttgggagctcgggtgtggtcatattgtcgga

gagaagaccgattgatgtgcttcgggggatgttggaggcgaaccaagcatatgagcagacgattgtgccgttcttcaaccggtatgggtatcctcagt

gctgtgcgatgcttctagccattgcggctcgtaacaagaacgcgactcaatccaccgcagtcgacggcggggtcgtctcatccaacggcgccgactct

cgcataataagcgacaacgccatctccagtgcttggcgactcttcttcgagttcggcggggcacccacaagccttgcgggtctgcggactactcaggc

actaagcgaaagcaacgtccgcgtcagcaaccggttcgaaggtctcgcattatacctcgctcgtctcctgcgcccgttttggacatcgtcagtcacca

agacatcgcccgtcgctggcaacccgaaccgacaagatgcagctgtgtcggcggctgtactgaccagtgcacaagcggacctcacatcccttgcgcaa

ttcttgcaggcgaacgctcaattctttgtgctcaatgctcaaaatcagcgaagtttcggagctcaggagggcgaagcatatgcctggaaggtagaaca

cgaccgcgtcgagtcaatccgggtactcatctcccgaacgctcgaggcgctcagcttcgtccttctgctgatcgactaccagcttccggccgtggtca

gcgtgctgaaacccgaacgccaaaaacagctcgcaggcctgacctttgttgacctcatcacaacctcacagggccgcgatgtggctcgtgccctggtg

gaggctgtcattgatcggcaaatagcgtcccaggtcagcatcgactcgatcgcggacgtccttcaacagcgatgcggtagcttctgcagcgctgccga

tgtacagctgtacaaggccttggagctggttcggggcgcaaaggaggcgagagaccctgcggagcgggctgcttgcttgagggacagcttgaagctgc

tgtaccgggcgacgccgcagttgccgtttgagaagctccaggcgatatgtgcggacttcagggagcttaagcatgctcggggcgctattgagcttccg

ctgaagtgtgctgcagcttgggacccgtccaatgtggccaacacgatccctccgaacgcctctccggcattgcttcagaatcacccggcgcttccgat

cttggagaagcgcaacgcttgttatgcgcttgtgctggatatgctcaaggacgtcgatgacgccttggacaaggccgagcaattgcccgagtctacct

ccgaccgactgtctgaaccatcggatctgcgtcaggatgcatacgccttggcgctggagcagagcgacaagctgtttcactggaagctgtacgactgg

ctccttgaacgggggatgacggaacagctgctcaaggtgcagtcgtccttcatagaggagtatcttctgcaagagccgaacagcgttgagaagctgca

cctgttgtggcagctttatcgaagagatgaaaagtatcccagcgcagcgcttgtgctctgcggcttggcgaaagtgacagacatcgagaccgtcgacc

tagtcagtcggatcgagtatctatccatggctcttagcttggccaagagctcctacacgcccatgtccgagatggtacacgacatcgaggatacactt

gatgtggcgcgcgttcagctggaggtacagcagtggctgcgacagaactatccggacgatgagcggatcttggatctggacgagcaactgctcgatat

gacaacactttggcttgattatgccaatccctatgcgatgaacgaggtcaagatcatgattttccacatcgctgccttccgggaggtcgacctggtgc

gagccacatggtcgcagctcatcgaggacgctcacaacgaccccaacttcccggagagccagcgcttccaagcagtgaatatcaaggtggtcgacatt

ggtcggcggttcatgccttccgatacggcttgcccaatcccggtgttgctggaaatcctggaacagtatgcttacaatgagcggaacagccaggacat

tcctgtgggatggacgccgatgacacttctccaagctggtggcaacgcgcaagagatcttcgacagccttgactcgatgctgagcagtcgtgtcgagc

catgggacaccaaacaggcgcgagtcttcctgctggcagagatgtcatcatacttgaccatctggctgcacgatgctctaacacacagccggccaggg

gtcggagcgggtcatgttttccctgcgagtcgggtcgacgatgcgatcagttcctacctgtctgcggcggcatcgctcgcgtctgcggttgccaaggg

cgagtttgcaatggaagtccccagttcccgtcccgcacaaggtgaaatgattcagctcagacgaggcaatggaggcctgccgcgcgccgcgacgggga

agacgagggaggaagaggagaaacaaagaaagaaccgccgcacgacccgccgggacacgagctttccgcttcctcatttccgcgtcgttgtcgtcgag

gatcttcttctgacttcctcgacccctctcgtcacttggtgtcctggaccgtcccagctgagggagcaagagttgtttgagagaatgtggaagctact

cgggcagaagttgcctctgatgatgtcggtgagggccgaagctcagcaacagcctgagaccccggagaggacctcgctcgacgggcatggcatcttta

ccatcatcgtctttgccatcgtgtctctcgtcgtcctggtccccgtcaagatcccaatcccgcatcccatcgccgctatctttggcagggacaagaag

gaacgggctgcggtctcagcagggtcgaatgccccgccgtctcaagctcaaggccctgcatcgaagccggcgaacgacttggccccgaccccgtcaac

gcactcgcaggcgaatctgttggcagcccagccgcagccggacgccccgcccaagagacgctacctggtcctgaaccacgtcaatatccccttcatcg

gagtacttgtcctcctcgcgacgcggtcgatagacggtaaacaagtcaaagatggcatcgtcggggcagcaggcgtcgagccatacgatgtcctcctc

ttcttcataagtctcgcctatatcgccatcagtctggacgcgaccggtctccttcgatacatcgcctttaccgtatgtatcaagtccggatcaagggg

tcgacttctctacctcctgctctacttcttcttctggaccgctggcgttctggtcgggaacgatcctgtcattctcagcggtacggctttcttggtgt

acctcacgcgcgtcgctggaattgttcccccaagcgcctggatttgggcccagttcgttgccgcgaatgtgtcctccgccgtgctcgtatcctccaac

ccaaccaacttggtaattgcttccgccttcggcatctcttttccaacatacaccgcctacatggtcttgccctctttcggttccgcgctggcagccct

ggttactctcctgatcttcttccgggatcggacaatcgtcaagaaagaagaatcggagacccaaggcggagcaacattcgccaaattcgcctccgcga

ttcctgctaagatccgacctgggtttttggaacgtcggtcgaccaaaggcgtcaactcgtcacccgcagcgaatggacctccttcagcagagacagaa

gaggccgactattctcctccgacgcagaaaatatcatatatcccttctaccattattcggccggatatcgacccacgggccgccttggtagacaagcc

gggcgccatctttgtctccttcatcatggctgcgaccctcggtattttgatcacaacgagcgtgacaggccacgtcaaagtgtaccaaattgctctgc

ccggcgcagctgtctgtctgatcagggacgccgtcgtcgactacctcgcttggcgtaaagggcgtctaggcgttgccaaggccgaaaatggcagccag

gagccatctgctgctgggaccgtcccgcctaagaatggcaccgccccatcaagtaaagacatcgagctcgcgacgatttccaaaacttcccagccagc

cgaggttgatggagtcgagtcaaagccggcaggtgatgaagcgacgacaccagccgaaactgcctccaaagacgacgcatccaaagagcaccaatcgt

tcttcacgcggttgcgcaacctgcccgccaagcttgaggagatttttcccaccgtatcggtcgtgatgtctcgtcttcccttcccactcctgcccttc

gcgttcggcatgttcatcctcgtccaaggcctggcacacgtcggattcatctggattctggcggacggtgtcgggaagacccgtgctgatgatgctac

atcactacctttcccttcactctctcaggtggctggtaccaacattggagctacaatcattcttacggaagccattcagtcaccatccttcacgcgac

atttgcctcctgcctcggccgctctgatcacgaagacggcactctattctatcgctctcggttcgaacatcggagcccttggtggcactttcgctgcc

agtcttgctggcttgctttggcgggaagggctgcgacaaggtggaatcaaggtgacaacgggtcaatttgcgctctggtgcgcagtggttatcgtacc

tgccaccgccgctggcgtgggtatcctgctggcagaagtcatctattttagagtcggcgaatga

>contig_21.contig_21.g926.t1

atgccacccaagtcgcgggcgaaaggagctcgttcggctagcgggagctttcgagccggcccaagttcgccgctaccct

ccgtcgcatcagacaacagcatcggcagcaccaacagcaacggcggtagcaatggcgttgccaaacggagcaaggccaaggtcgctcatgcaccattg

ggaggacggccgaattcagttctcttacttcccgtcctcgtcgcattcatcgccctgcttggtcacattacgctccgcttgcactacgccttgcctac

tccacttaccatggccgattcggctgcgagcgaagctaaaggtcacggtgcgctcttctccgaggagaatgcgatgactgtcctctcaaagctagtag

atatcggctaccgcatcgtcgggactcccgagcacgtcgaggcggaggactggctcgagaaacaggttcgcaaatacgagggcacgcatgcgactggc

actggatccgggaaccaaacacaagtggaagtctggaagcagatcggcgacggtgcacacagatttgacttcatgtcaagtgtggtctggaagcgata

ccatagcatgagtaacatcatcgttcgcctcagcgatggcaccaacgagggcaaggagcatgctgttctggtcaatgcgcatctggacagtacccttc

cgagtccaggggctgcggatgacggggcaggtatcgctattctccttgagatccttcgaatctacaccaccgcgcctcgaccgaagctccgtcactcc

gtgattctgctgttcaataacggggaggagtcattgcaagacgcctcgcacctctatgctactcagcaccacacgcgtgattccgtacgcgctgtggt

taacctggaggcctgtggcaccacaggaccggaactcttgttccaagctacttcgctcgaaatgatcaaagcgtatagcaaggtaccccatccattcg

gaaccgtactcgccaacgacgtgttttccagtggcatcatcctctctgataccgactttagacaatttgttgagtacggcaatcagtctggactcgac

atggctatcattggaaacagttacctttaccacacacgaaaggacatccctgccaatgtcgagccaggtgttttacagcacttcggcgaaaataccct

cgcgatcgtgaactatctggtcacctcaccggaatctcaactggccacgaacagggctttcactaagtccgagtcgcctgtctacttctctctcatgg

gacaggtgttcatttacttggacaacgttacgttccgcaagctcgttcatggccttgttgccgttctggccgcccagatcctctatgctgttcgggcg

gagcgtcaccttgattcgctcagagcgactgtcatcgcgacgctcggtatttacgccaacttcattgccggcgcgttgctcagcaacgtcgtagcctt

cgtcctttccacagtgctgggagctggtatgagctggtaccgaaacgaatacttctcattcgtagtgttcgggccaccgacggtcactggcatattga

tcgtccaagcgttcatctccagcctctttgacacacctgcgaagaaggcctacctcgagcgcgcaaccctgaacgggatagccgtgttcttcataatc

aactcgctcgttgtgaattaccttgacatcggtagtgcctacctatttgtcttagggcttgtcacatgcatcctttcgattgctgtcaacgactatgt

cttcgtcggcactgatcggatcaacaagcgtcaagtcgcggctaacaaccgcgtcgctgctgcgacctatccgttgctgtccttcctgcccacattgt

tcgcatttgacggcttcacgattctggacttgttcgttccgctcaccggccgtatgggagagatttcccctgtcgatcacattatcggctcgttgacc

gccattctcacggttatgggatttcccgcactgattgtcatcggacacagataccccagtggattcaagtgggctctaatgttcaccgtttccgccac

cgtagcatctgtggccgttttctcggccgcatcactaccggccaccttgggtcttggcccaggacgtacgttcgacgaatggcaccccaagcgcaact

tcgtgcatttgagcacgaatcttaccagcgacaaatgggagctccactttggcactgccgatcccgcccctagattcaaggagttcgtcgacgagatg

cagggcttgatgggtgtgcccggtgaaaaggcagttctcacgcgaagcgatgacaaggccgcttgggccatccttcatccggtcagcgatttcttgac

accgtacagcttccagcttccaacgccggatgtcaagaagtatcctcaacaatttactgccccggctcccgtcaaaaatcacttccgcatccgatctc

tgaacgagcaactcgatttggaggctgggactcgccgcatcaagctgatcatcgaccacccgaacctaatctggactgtcgtgtctttccacggcgaa

atcatcgagtgggacttgcccacgacgccgccgaaaggctggcagaagcattacatcaaggaggtctctcgacagggcgtcagtgtgtgggagatcaa

cctgctcctcaagctcgacaatgcgggcgtggctgcggccaagtcacgaggacagcagaagggcgaatacggccatctggtgcgccaccttcctggcc

aagagccggacgttgccagagatccctccagactccacatcgaatacagcgctttggatgcgtccggcatgtggccgtcggcctacaagcgcttctat

gctggcgacaaggacaccctcaaaaagccaagcgtctcgttcttcgagaaactggacaagtacctgctggaaaagcacccggagatggacagcatgct

cctccctatcattgccgccgtcgctcaagtttag

>contig_21.contig_21.g927.t1

atgccggacatcagcagaactcgttcatcgacacagccaaatgggcgcgctaggagttcaaacaacgacgaagatgctg

tgccgggcggatcgatgccgacagcggctggctcaagccgaaaacgcggcagagacgagatgctcgatctcgacgaggatgaagacgaggacgaagat

ggcgaagatcaggctacccaggcagccaatcgcaagaagcaacgagatctagccatcgagcgtactacgcaggaccgagacgtggacgggtaccttcc

cggatccattgtgcgagtcaagctcaagcacttcgtcacctacgacgcggtcgagttccgtccgggcccatatctcaacatgatcataggtccgaacg

gcacaggcaagtccacaatcgtctgcgccattgcgctcggtctgggatggagaccaaatgtacttggacgcgccaaagatgtggctgccttcgtgaag

cagggctacgacgacggtgccatcgaaatcgagttgaaaggaaggcctggaaccaagaacgtagtcatcagaagagaaatcactcgcgccgacaatcg

tagcgactggttcctggatcgccagaagacgactcagaaagaggtagacgcccgagtggaggcacttgacatcaaaatcggtaatctctgctcattct

tgccccaagacaaggtcgccgatttcgcgcggatggcacctcctcaactgttgaaggaagtccagaaggctgctggcgaggaaggtctcatcgaccag

cacgtcaagcttacagagcttgggaacaacgaagtcaaaatcgcggacaagttggcttcggagcaaatcgaagtcgacaatctcaagcagcgtcagga

agtgctggagcgcgacgttcaaagattcaaagatagacaggcgattgagcgacaggtgaaagcactggagatccgggtgccacaagtaaagtacctca

aggcaagagagcagacaatgaggtacaagcgcatcatcgagaagcggaaggtcaaagtcgaggatgccgaaaagcagattggtccttacacgcaagtc

gaagtagactacgacgatcatgcggagaaactcaagctgcgcaaggacaaggccaaaagtagtcttgacaaggttgcccaggatcttacaaaatgcgg

gactgctctggagaagaaggaaaccgacgcggagaagctgcatcgagacatgtccagtctggacaagcgcgaagacaaccacaggaaaaatgtggaga

gactcaaggctgaggtccgcgaactgcaagaggctgtcgccgaccctcccgaacatgctagcacgcaagtatcggatcagcaaatgaagaagatccga

atcaaagagcgcgggttgaactctgaccgggcggaagtcaaggccggaatcgacgagatcaacctggagaatcgatctctacgcgccacgctagacga

ttcgcgcagaaaactcgaggccttggacaacgtccggcatcaacgcctcatggctttgcagcaagcggacacccctgcctaccgggctgtaatgtggc

tccgcgagaaccaacagctattcagtggcaaagtgtacgagccagtgctactcgaagcttccgtcaaagatcaacgcttcgcagctgctgtggaatcc

tgcatcaactgggccacattcaggaccttcgtctgcgagaaccgttcggactacgatatcttcactagggagctcgtggacaggcaaaagttgcgctt

gaacgtgttcgaggtggaaggcttcgaggacaatctgacgcgcggcatttacccaccgggagtgcttcaacaatttaacttcgacgcaactgccgtcg

acatgatcgacgcgcccgcgcccgttctgaagtttttgtcacagagcagcaatgttcatcgcattcccatctctcttaacgcgaacccgtcgacccaa

gatctcgaacgagttaccagcagtggtcaaattcagcgctttgtttatggtccgatgaactacaatgtcactgtgtccgcctacggcaacaggcgctc

aaccacaacgtcgaagccatataacctgagaccacgaaacttcggcacaagtgtcgatcaaaacaggaaaagggagcttgaacgttcggtcttggagt

gcactactcgccagcgcgaaagtgagagccgcgtcaacgacctctcccgacgtgaccaggctctcaaggcagagcttcaaaagctggaacaagaaaaa

gcgacccttcaagaagagttgaaggctgcgagtgcggcaagaacaaagtacgagaaagacaaactcaaactcaatacctccaagcggcatcttcaaga

ggcgatgaaacggccctcaagtgatgctgaacgagtccacattcgttcgcgacttcaaacgctagcggaggaaagggcaaacctcgcgcaagatatta

agtctcacatgcttctacaaaacaagcttcgcatggattacgacatccatttcttggatatcttacagcacgaagccaatcgcactgcccttgcagaa

ctcgtcaagaacgcctcggagcagctggatcgtgccaaagatgcactttcggaggtgcaagaggaagaaaagggaaagcggaaatcggccaaatcgtt

gctggccaagtatcaggcgctgttagatgatgccgagccggaggttacggaggtactgaagcagtttgaagaacgcgaagggtatcatgaagagacaa

tcgacagtcttgagagtcagctggagtctcaaagaactgctctcgaatttgctcacggtatcagcgacgatgtcattcaagcttacgaagctcgacaa

cgacagattgccaagcacactgatatcatcgagcacctgaagactcaaaagcaagagatcgatgcggagataaaggagatcagagaccactgggagcc

tgctatccgctccttggtgcaggaggtcagcgcgaaattctctgccgcctttgatcgtattggatgtgctggcgaaattcgtgttgccacggaccgtg

attacgacaagtggggcatcgagattctcgtcaaattccgggacaccgaagagctgcagctgctcactggtcaacgccaatccggaggagagcggtct

ctctcaacgatcatgttcctcatgggtctcacagagctcgggaaatctccgttctcgcttgtggacgagatcaaccaaggcatggatcagcgtgctga

gagagcggtgcacaaccagatggttgacgtcacctgtggtcaacatgcccgacaatacttcctcatcacaccgaagctgctgaaccacctcaggttcc

acgaacgcatgagagtgctcatcatcaacaatggcgaatggctgcccccgaaactcagctcggcaaccacgagcctcaaagccatcaagcagcagacc

acccctgaaaatgtcgtcaaggggcacgaagcgactcttgctgatatcttcacggcgactccgctcaccaaggctcagcgctcgaagctgttcaaaga

tgaggtcaagatcatcgaaacgttacgaagttgccacggcgtggaacaaacccacggcgtcgcggagaccttcacagacgacctacaggcatttcagg

ctcgagaaaaccacaactccgccattttcctcaacgatttggacgctacttctaatcttcgctgcaagcagctagacgcctgcatgcttgcgttacag

cctctcaatcacggcaattccagcctcgtttcgcttctgcaaggcactgtcgggcagctctttttccagcgtctgcatttcttgacgcaagagttccg

ggactcctgccagcacgcgcacctcgactgccctccggcgttcatcatccgagccctctttcatgtcatcacccctggggtgcaggaaggcggctatc

ctcgcgaaaagacaggcgccccggcgcgtcgcttcacgggtgtgggcttggacatgggcgcagagcttgactccgactttttgttgatccaacagttc

gtgctggaccatcattatcctataatgaacgtggctctacttggagaggttgcgatgcggctgcaagaacgacctgtcctatgccgcgatctcggcac

gctttggattggcctgttgcaagagctatccaatcgatccaactcctcgaatatgctggccgacgcacaatctggcaagctgtcggctgaggattatg

tcctggcgttcgctgctgcttctctcgatttcacaaacatcgtggaaatcggtaaagtcgagggactgtgtcatgatcacacccatctcttcgatgac

gattcgagctacggtgcgaggaacgacggtctgcgcgaagcggccgaacgacgtatattcgttctcgcgaccaatcgggctctgcaggctctggaacg

tctcggaaccctcacgaacaaagatccacagcaccctgcttgtagaatctatgcgtgccttaagcttcagttgggaacgcttgaaggcaatctcgacc

atattcaacattcgatcgagcgtcttcatctcttggccgcccataatccaagcttgccagatccatctaagttcgagactccgctccaactcctcatc

gaggcttattggaagcacgctgaggcttcgatctcatttatcgcgagcgcaggatcactccgggcgaagaaagagcgagcagtgatgtgctcaggatt

gcattccattcgcaaaagcttgaagcttcttcggattgtgacgaccaaagaccctaccaacattgcaatgcgggagaagtacggccaaatcttacgga

gcgcgtgcacatgctgggttttggcaggcgagtttggcgatccgccggcgggatggagaaacgaggcgattcgggtcctgtccgcggcattcgaagta

ttcctcgccctttacaccgacgcaagtcgcattcgcaacggtacggcgaaggaagacgatgccagtggcttcgatggatctacatctggctctgccga

aggattcgacgttgcgcactcgagaggaggaatcccaaaatatacgagggacagtgccacactgtccgcaacagagtatatgttcgaatctgttcgag

tcggaggcgtcctgatcgaagttctcgaaggcgcggtttgcctggatccaaccgactgcagcattaatgcccgctttacagacctcgcgcacttcctc

tcttcaccggagagctcgccagattcgacccaccaccttgcggcaggcttgacggcgatcaaaaccttcagaaagctgttggcttccatcaagaagat

acattcgaaactactgttgcttgccggacggaacccttcctttcaagatgcagtctcgaactcggcttggattgcttcccagctgctgcgtcgctgcc

accttgaacgtcttcacccttaa

>contig_21.contig_21.g928.t1

atgcaagccagcgaagaaggtgccgtgcttcagccaggcggggacgagatcgccagtggcaagcggaaatcccatgtat

gctcccctcttttggatcgaaccgtggcgatcaggcatacgatatggacgcacttgcgactcgctctacccgcggtcttcgccagcttcttgctgtct

gtccttgtcctcgttcgagccgtctcggactggactggtccgtttgccttcctggtattctttcacctccatctcttcttcttcccgtcacctctgac

gataggagagaacatacagtcgtcgctcctgggtctgctcggatcggcggtcggatgcagcatcggtctgctttgtgtggcgtgtggaatttggatcg

atggggaccagcagttcgaccggagcatcctcgcgttcaccccttatgtctccagtgatgcaagagcattcgccgcatgcgccctcctcgtcttcgcc

ttccttaatggcctgctttcctctgcagttcctagaggatcggccgagcactctatcggctacaaaatcttctccgaattcttttacccaagtctgct

cagcgccggggtcggcatcctcgcaaacctcctaatcttcccgcgaaccggtcatagtgtctttacggatgatctgctcaacaccattggtctactcg

gcaagctcgtcgacgattccgcagtgctgttcttttccaacgagcgcagtcgccaggcaaaacaagacatcgtggctctgagggcccagttcacgacg

gctgtcacacggctggcacctagcttcgaagcggcaacggcggaaattgccttggtgcgaatacccgtgtcagcgtacgggccgtttgtcaaagcgtt

gcaacaaactcgcgggtggattgcgagcggcatcgaggtcgaagtggatcaagagcatacttcagcttctgctggcagcctttcagatcttgagggtc

ccgtacgaacgctagccgttgagacgaagcaatctttgtccctaatccgggtttgcgtcctcctcgcaacgggtcgccaagcgagtttcagcgctgcc

cttgttgccgtcggccaacctcacttactcgagtctattttggagggcgacgtcaaggtcgatcagtcgctggctcatcgagcagtcttgcagcagcg

tcacaatctcaccgctgccgtcgatcgacttaagcacgacctacaaactgtcttagatgttgcaacaccacagcttcaaccaccggcatcaacagtaa

accctcgatctttgcccaacgttgaggtgaacggacactctcggtctgtgtcaacagccgctgaggagtcaagggacaaggatgcgcaacagacaccg

cctcacactttccagaacaaccgcatgtatgcagccgcctgtctcatcctttgcctgatcgagattgcgcaggcgtgtctccatgctctcgtaacgac

tcagaacctacttcggctctgggacggcagcctcaaacgaaagatccagtggccatggacgattgagcatggctcccagctttcgacgagtgcaggga

gctggcgccgttgggccaacagagcaggtccaggcacgctcaatgacatcctggggtcgatgcgggcggagaaatacggtctcgagaacgaagaccct

ttcgacgaagacgaaggcaagtcaaggacagaagtggccgaagcggagctcaacgaaaaagaagcggataaggacaggaaaagaggtcttcgggacgc

cggaaagggactcaaatctgaacgctcaagattcaacatattcgcggaggcggaccacacaatcgcagacacatcacgaagcacctcaccttctgata

cggaggccgctcgtgcgcccgtggtgatatcgaagtggcaaagaatacgaaaagcacccttcatgctactacggctttggagaacgaaaacggccatc

atctatcgggttcgagtgtccaacgcgttgagaaacttcaaagagagtcgtcatgttcggttcgggctgaagctctctatcggctgcctgatactcag

cttgcctgcttggcttcctggcaacaatgctcgcaaatggtggtacggagagcgtggacaatgggcactgatttcgtacgtctggtgtctggagacga

gcacgggcgcgacagtacgagtggcgacatttcgaatgatcggtacggttgcaggtgccgtgtacggttatgtgatctggctcatatgtggacagaat

gcgttcggtcttgctttcttcatcattctcgctgagctcttcgcggcgtatatgatgctcgaaacgacggcacaaggcatcggtgtcgtctttgctct

cacgttcccgatcatagcattgatcccatatttccagcagataccatccagcgtggccatcctcgcctgggaacgtgggtacatggttgtgatcggga

tcatttttgcgctccttatcaacctgttgttatggccgtttcatgccagggttcagctggtacttcacattgcccgagcgacggggcgactccagcga

ctttatctttcgctgagtcgtcaaatgctcaataccgaccaggtcacaacccgtcatactcgcaaagcatttgagcgactggaacgcagtattctcaa

gacgctggcgcagtctcggtcgcttattgctctcatgtcattggaggtgagcttggcgccgaagccgaccgtgctacttggcaagatcatcaatcacc

tcgcaagaattgcggatttgctcgtgggcctacggctttgtcgcgaacacgcgatgaaggggaagcttcacgcgcatctggtgctgaacgtactcgaa

aataggacagagttgatctctgccatccttatttcattctttgccgtcggtgcatccgttcggaatcggactcctttgcctcaatttctaccctccgc

ccgggtggcgctcgaagagctgaccacgtcgctccacgaacaactttctcaacacgactcacgcggcgcagcaaccgcgggtacttcaacgcccgacg

cattccatgcactgcgcagtctctacggtcgtcggccaggctggagaagacaacactcgaattccgaacgagtctcgcgctcgcgttcgccaacaaat

catacgccggagggtgtgtcgacaccagctcgcgcctcgatagggccgacgccatccaacacgcacaatttgctgcaatccgcgatccagcacaacgc

gcggcgctatgcagcgcctccaaagctagcccacttctttgccctggcggagcacagtctattggaacagatcgtctcagagctagagcatgtgctcg

agcttacgcggaggctctgtggaacagcgaccatcctcgaaatggcgtatgatcctccgcctgccgttgattcaccgcattcgcgagctcatagcacg

tggcaatccggccacatcccttag

>contig_21.contig_21.g929.t1

atgccagcagcctcgtcctctcgagcttcgtcttcaacaccctcttctcacagcaaatctttccgcccctcaaagaccg

gcccctccgctcctacacctccctcttggacactcgacgagcatgtcctgcatctcacagcccttttgaacgctcatcccgcggcggaagagcttcat

tcgacttgggaaggatacgcgaccgcgccgatgagtcaggggagcaggccactcgcgctcggtctgaaggattacaaggcggccctggactccggacg

gagaatcagcggcaacaatggcaagaatgattcacgacaaggcacaagcttgaatcgctcgggactcccgctctgggtcgtcgtccgtatgctgaaag

cctcctggcatgccgaaggaagttggccccttatcggcgccgacgcatcggagaaggatacccaattaagccttccaacgaatcctcaacgaagtatt

gggacgtcggtggcaacgtcgacgccgcacccgccgtcgtcgacaacaacgatgaagattcctcgatccgtgcatcccactgctctacttctaaaagg

aaaggccccactcgtgacgacaatgacgccagagccaggccctcccgcccgacctgatccatcgccagatgaagtcgcccaagcctaccgtgcacgag

gtatccttgtcgcagatcctccagtcttgatcccaagcgacgacgagggcgaagaggcgcccacaggaggagaggcaatcgacgacgacagtgcataa

>contig_21.contig_21.g930.t1

atgcgcaccgacttgctgcccaccctctacctcaccctcctcgccagctggctcctcctcgcagccgcgcagtcagccc

ccgcatcgacacgcgacagagacctcgctctccgttccgcgcgccatcacaatgccaacatcaggcggagtggaaagtcacctggtgcaggagacggc

cctcattccacgcatgacggcagctccaagccgggccaggcctcaggagcacaaataccacgaccgaggagtgcggccgacctcgtgctgaccgacat

cgtcctcgtcaccaccgtcgaaggcggcatctacggccttgaccgcgcgacggggagcaccatctgggaggtccccgcgcataccgcgagctcagcca

tccccggcgaagaaccattcgccggcttcggacccctcgtacggtccacctacgggcccaagcggagctcctttgtcgagctcctccagggaggcgcg

gcttcttcctccggctcctacgcgggcggtctggcctcgcctcctactcggcccgcctatgcgtccgatgacgaccagggtccgaacgcagatgctgt

cgcgctgctcgagcaggtcggcatttacgtcgtcgaaccgtcctcggcgggggacatctacatgctcacgcccgcctccccgaaatcgggcgcgagtc

ctggtggggatgagcagcagggcgaaggggcggacccgctctctgtgccccgcacacatctctccaaacttcccctgacgttgccccagctcgtccaa

ctgtcacccttctcattcgcaggcgacgacacgcgcatcttcatcggtcataaatcgaccacgctcgtcgaacttgacatcgcgaccggatccatcgg

cgccgtatggggcggctcgtctcctgactccgcagcgaccaatgcctcctttgacgaccactccacccatgggcatgaccaggcccgtcggccgtgga

cctatattggacgcacggattatacgctcacgattcatgcccgcagccggccggcgctctcgcagaccttgcacttctctacctacgcacctaccgct

gcggacctcgacatccagaccatctgggcacaggcaggacatccggaccagcgcgccgtcatcgggacgggcgaacagcagactgtggtctgcttcaa

cactacttcctcggcatcccatgctggtgctcctgcgactactgtagcggaggatcaggatgaacgtacggtttggaggacgagcattggtgggacgg

ttgcggggatctttgatattgtgcagcccagtagcgtcactgctagggctgagtatcatgggtcggggactacagctacttcgaagtcgcccgcacct

gccgcggctgcctctcctgctgcttcttccgcgcgatatcatgcgggaggagctcggcctgtcatcgtgcctcacccgcccatctctctctcctctct

gttcggatccagtcggcaagtcagtgtagggctaggtatgccctcgtcgcgtcgcgctggcggtatcgtcaatgctgacgacgacgacgatgatgatg

atgatgacgatgacgcagagcgggatcagacggaggacgagaatggtcagagtccgctctttgatctgcagagtatgggaatgtcggactcccagcga

cctgcttatctgggcttctccggcgggtgcctgttcgcgatgggctcgagcaagttcccactcgctgccttcagtccacgcgcagcggccggtctgcc

gggaagtaccacagcggacggcccatcggacctcccctcgggccgaccatcgccatttgaggagttcggcaggagtgagggcgccgatccaaacacgg

gcatgagtgtccgcccctgttccgcctttggctgttggttgggcagctacccggtcgaagtggacgcggagacggatcggaatgcgcaggaggcgctg

ttggggtctgcgaggaggccgttggggattgcggatcatcatgaggcgtatgacttttggggggtgccgagtcggagtgtgcctgcgattgacgcgcc

gcctgctcggtcggaaggtacggggccggagcggggttcgtcatcgtccggcccggggacgaacagctccttggaggaggacgagggagaaggaggga

agcgctctgggtctaagatgcctagcctcactacgctcctcttccaagtgatcggtctgggctgcatcacgctcttctcgtacgccctcattctgggc

gtaaacgagcagcagagacagaacgaagaacggtccaaccagctcgcagaggtcgtcacgctctgtgaccaggacgcgccgtacatgacggaggaaca

ggcggggttgcagatggttatcaacccaaagacgggcttgcctgtccttgtcatgcctgaacctgagactccccctcaagctttcgcctctccttctt

cggtcacagcgacgcctcagctcaaggagaagccgttacctgcgactccgatcaccctggaggagatggagcgagcgagacgaatggcagaggaggac

gaggcggttgcggcggagctttcgaagaagaaggaggagttccagcgattggctcaggcgacggacgttaatacgcttgcggcgtttattgagcagca

acagcagcagcagcagcagcagaacggagcgggctcttcgtccgccaatgggcattcgaacctggcggatagtgggagaccgacgtcttcggctgggg

gtggagccacgcccaatggtaaatcgggaccgggcggaggaggagggaagcggaggagaaggggtaagcgtgctggtgcggcgttggctgcgaaagtg

gcggcgcgggcggcggcggctgggttagatgccggcgagggggaggacgaagaaggtgatgaggacgatacgggagagggaagtccgccgccggctat

gctggtgaatgggagaccgtccccaccgggtagtgcaggcgcaggcgcgagtgggggcggcgggaagaaaggcggtgctcttttgctcaagcaaacct

ccgatcggaaatcgaacggcgttcgcttccttgagcaggacgcgacgggcgtcaagttaccccccaaccggttgactgcgacggggttggcatccgca

tcgacattgggcgcaggcgcggttgcaggcgcggacgtgtttgggacgtacccgggcccgtactcgcccaccaccactgcgctacttgacggcgggtg

gctccaggctgggcctgattcgcagtcggccgaggagcagttggctaagatggcggcgctggcgtcgactcctggcagtatgcgtgggcctggcagtg

gtggtggtggtggaaatggggacgggaggagtgaggagattgggatggggatagggatagggatagggaactcgggatctgctgggcggcaggtggtg

tccaagagttcggggttgacgatctcggacgaggttcttggatacggaagttcgggaacggtcgtgtttaaggggacgttccaaggccgcgctgtcgc

tgtcaagcgactgttgcgggactttgtcaatgtggctagcaaggaggtgtcattactggaaagcgcggacaatcatcccaatgtcattcgatatttct

acaaggaactcaccggctcgttcttgtacatagccctcgagctctgcccggccagcttggcggacgttatcgataagccgaacgagttccgggatttg

tacaatatcctggagccgaagcgggcgttgcagcagatcgcttctggcgtgcgacatctgcattctttgcatacagtgatgcccaacagcaagcggct

caagatgctcctctccgactttggcctgtccaagcgactcgacggcctagcacaatcgagtttcagtcagacggtgaacaatccaggtggtaccgtgg

gatggcgtgcacctgaaatcttgaggggtgatgtccagttggataacggtgggggctcggagagcgagcagagttcgattggggttgcttcagatggg

acgagggagcgtgggccggcggaggagaggaaacgattgacgcgggctgttgacatctttgcgcttggttgcgtggcgtactacgtccttgccaacgg

agagcatccgtttggaagtcggttcgagcgtgagatgaacataatccggaattgcaagaacctatcgcgtctggacggtctcggggaggaaggcgtcg

aagctcaacacctgatcaacaccatgattgccgtcgaacctcgcgaacgaccttcggcgtcagacgtgctgtcccatccgtacttctgggacgcaggc

aagcggctcaacttcttgcaagaggcgagtgatcggtttgagatcatggagcgggacccgcctgcggctccgctcgtggcgcttgaggcgcagtcggt

gacggtgctgggcggggagtggtacaagaaggtcgatcgggcgtttgtggatgacttgggcaagtttcgcaagtacgattcaaggagcgtgcaggatc

tgttacgggccttgagaaataagaaacatcactaccaggacctcagtccgcacctcaagaagctgctcggtccactaccgggcggtttccttgcgtac

ttcacccgtcgcttcccactgctgttcatgcacgtccactccacgctcagctcgcagtcgcttatcccacacgaggctcagttcagagaatactttga

acccaacgagcacgagcattga

>contig_21.contig_21.g931.t1

atggacatcccactcacctccgacgcgctcgacatcgccttccaccccgacccacagacaaacatcatcgcagcaggcc

tcctctccggcaagatccagctcatcgactacaccgccgcctccaccaacaccttcaagaagaccctcaccctacgcccgaccatcaaatccacccgc

gcgctcgccttcgctgactccaccctatatgcagcgtctaaggatcgcgcgctgtttggtgtcgatactcatacggggacgcttgtgtcccattgggc

gaacgtacatgccgccccgccttcgcgcatgatcacactcgactccggcctcatcgtcacaggcgacgacgacggcgctgtcaaactttgggacccga

ggatgcccaccacctccgcctcctcttcctcttcagcgtcaacttcggaagctcccgccgcgaagaagaccaaactctctgcggcggcaggaaacgtc

gttaactctattcagccgacgactgccacgcctgtgaggacgtacgcgcatcatttcgactggatcagcgatttgctctggtgcgaccacctcgccca

accacggacccaaaacctgccccaaaagggtaagaagaaagcaacagcggcggcggcggatcagcagcaacaggaccaaccggtcacgcttcgctccc

ggctggtgtgtaccagcggagacggtacgctcagtgttattgatttccggcagggcgggtcgggaagcgcgggtgcgggtgcgaaggcggacgtgggg

aagaaggcggggaacgtgaagggcgtagaagtcagtgaggatcaggaggacgagcttctggcgatcgcgcctgtcaaaggagggtcgaaatttgtcgt

cggcacccaactcggcaccctctctgtctgggcccccacacgcgccatgcttgaccacgtagaccgcatcccaggccacccggcctccgtagacgcca

tctgcccactctacgacccttcttcttcctcatcagcagagaacgacgtcgtggcccgtttcgcgcaagacgacgacgtcctcctcaccggctcgtcc

gacggcctgatccgcatcttgcatatcggaccaccgcatacgttgctgggcgtgttggctgatcatgggggcagtgggatgccggtggagcggattcg

gaggaaggggcgtgaggatgaggatgaggagggcgagggggagagttctggagttgcggggaagaagaagaagcgggcggtgaaaggggatgacgata

gtgatgatgatgaggacgaggatgaggatgaagaggaggacgaggaggacagcgatgatgatgacgatgcaccggacgcgccactctcaaaagcagct

gacgacgaagacgaggactccgattccgactccgactccgacgcagcacccgcccccacatccggccccaagaccaaagcagcccaactggcgaaagg

gatagcaaaaggcgctctcctcgcccctgtgacctcgacctcgtctggctgggatgacgatgatgatgatgaggagcgttcgggcaagaagggccgga

agggcggtgtgaagaaggagatgaagggtcgggatcaggctgcggcgaagcggaatgagcgggttgcattctttgcagacttataa

>contig_21.contig_21.g932.t1

atggcgaacccgttcgctgtgatcttctctgtcctccgcttcctgatctcgtccctcactgccatcttcgcgtcctcaa

cggcgtcctcagcacacatgacggcccgtctccactcgtcccgcatcctcgtctaccgcggcccagacacctacgtacccatcaccatcctcgcacac

aacacaaacgtagacgctgacagcctcaaggtcaccctcatccagaagggataccgcgccggtctcttcggatgggcactcgctcgctacctcggcga

catcagcgagaaagccatcgatgtcactcccgagtccatcaagaactggaacgacaccgacaccgactcagcatcagcagcatcttctaccagcacaa

tccagaaggaggataacccaaactcgtctctctccactcccaacctcacccagacccaacgctccaagatcgccaacgagctcgagtccctcctgccc

accatcaatcacaaacacccgctcgccaccttcttcgtccgcatccccgtccgcagcggcgacggctacttccgattcaaggtcaaaaccggaaacaa

gaccgtatacactcccaccctccgcatcctctccgtctccctctcctccgcctccatcaagggcgcatccatcctcccaccaaccatcgtccccgagc

tcctcatccgcaccctcagcatcgcattcaccaccttcctctacggcctcttccccatcgccgccttactcgacaaagtcctgcccaagaagtggtca

cgcaagttgctcggatggctctaccgccgactgggcatggagcagcgtcagcagcagtttatggacaagcatggccagaaggtactcgacgccaggaa

gaagaccatggagtcggtcccgtttgcgacgttgggcgtgaggacccactacgagatcgagaaggacgagaagaagggcaggggcgggatcatgtacc

tgcacgcgtaa

>contig_21.contig_21.g933.t1

atgagttcgaacggcagcttccccaagcccaagcgcgcgccacgtgtcctgctggtcaacgatgacggcccgccctcgt

cctcgtcgccccacgtgctgccgctgtacaacgccctggtcgccctcggatgggacgtcaccgtcgtcctcccctccggacagcgctcctggggctcc

atggcattcaccatccgcggaaacctgcccgtctggtactactacccgctccgaaacaacgacgacggctcacatcacgacgacgccgcgtgctggag

taccgagcgcaggcaggtacgcacccagcacggcgagcttggcgaatgggtcctcattgatggatcacccaccaccgctgccaacgtaggcctcttca

acaccgacgtcctcttcgggccccagacgcaccccgtctctcgcaacccacccaccgccgatggcacctctcctccgttctcctcctgggcagacctc

gtcatctccggtcccaactttggcagaaacacgggcactgcatttgcactctcctccggaacactcggggccgccctcgccggtgctctctcgggcgt

caagagcatcgctgtctcctacggccatttccaaagggggtctggtcctcaacggtttgctgtcccctccactacgtcagaatcggaatcatcatcag

cggcatcggtgcagaacacgacggaaccggtaaagaccgaccctggtgcaggccacatcgtccgctccccacctgcaccacagcatgttgaacagctg

gccaccgatctgactgtcaagattgtcaaacagctctgggaggagtgggaagacggcgtgcagtgctacagcgtcaatgtaccgctctcctggacctt

ggaggaaccaaagatatactggacgcgcatgtgggagaatatgtacccaaaacaagtcagtctcgatcaggaagatggagatctaccatcgtcaacag

cggagggaaaacgacccaagcctgcgatgcatctcacttttgctccacccatgggcaccatgctcgcacccgatcagttgctcgaaggaacagacatc

tgggcgctgatgaacggctgggtatccatagtaaaattgtgcccgaagtacacccatgtcgacgaacagtcatcagcaacaacactcgagcaagcatg

gtcggatgccgcacccgctggccaaactgcttccagcagcttgggcggtccagggacaagatggatgctttga

>contig_21.contig_21.g934.t1

atggcatcaaccgcccaaaccacgtccaccacgtccacctcctcatccgccggcatccccctcccaacccgccgccaac

gcatccccccccgttccgaattccgcttcgagctcgaaccaggcgagcggatctccttccgactcgtgccttcctccggtggggacgccgagtgcttt

ggggcgccgctcgtcgcgggggaggggaggtgggtcaagcgagggaagcctgaagcttctgggtcatttgctgaagtgaagggaagcagccaagcgac

cttcgaatccatctccgacgagccctcccccaccttcacaacccacaccaagctccacctcgctctggaacgaagccgactcctcgcccggaccttcc

tccgatcgaacgaggaactgttcgagacgggcatcagtaacgctcctgccgtcgttcatccttcgaccgctccgatttcgttcgaagttggcgcggct

gaggggggcggcgctgttccagcttcttcttttccgacgttcgagagttggagcagtgatgaggcgacggcctatcggagggagggccagggtccgcg

cgtgatggtcgtcggtggcgaaagttcgggcaagtcgagtttggtcaagttcttggccaattacgcgatgcgatcgccggtgttggctagtctttcgt

cgtctgcttcgacgacgaaggggagggatgctgcttctgctggtaacaataaggaggatcagccggaaattactgggtggtggccggtcatcgtcaac

gtagatcctggcgatggcgccgctccgctccccttcaccttctccgccctccccctgaacccaatcccaaccgccttcctcgcatccacctcccccgc

cctaccctacggcctcacaacctcaacaacaggcgcaatccacccaacctccacctccgcccacatcgtctcgccctacaccctctggctcgggcggg

acaacgtccgggacaacgagatgcatgccaaaagggtcgtcgacttcctcgcggaggggttggagcgtcgattggcccgggacgcgagggcgaggatg

agtggggttgtcgttgatactgcgggcgtcattacggctgatgggaggagtcggtatgcgtggattcagtattgcgcgagggcgtttaaggtggatac

gatcgtggtgctgggacatgataaactcaatatcgagcttaccaggctgtatggtcatccgggctcgggggtgagcgtgatcaagctgcccaagtcgg

gaggggtggtcgagctcgacgacatctaccgcgcccgtctacgatcgctccaggtgcgctcctacttctacggcggctcgggacctgcgaagatcacc

atcccggacctttcccagccggatgggaacgcaaccaaggaagtcacgctacctggccacgaagaaagtctggggggcgtctcgacgctcggtgcgtt

ctcgagtacgttgcccctcgacctgatgtggatctggcgcgtcggacaagaggccatggcaccctcctccgccctccccatcggcgcccaacgaacag

taacccaaacccagctagtcaagctcgaaccgacgaactcaaccgcggaccaagcgaggctcctgcactccatcgtcgccctcgtgcagacgcccagg

ggcacgggaccgaaagtcggggcggcgaaagcgaaggcggaggcagacgctgcggcggggattgggattgggattggtgttgaggacgatcaggacgg

tttgacggatgatgagatattgggtgcggatatggacatgaaccgcaagacgatgacggtcctggcacccacgaagggcaagctaccggcgttgactg

gtcttgttgggggcttggagtggaccgatgcctag

>contig_21.contig_21.g935.t1

atgggaggaacaccttgcaccttgctcactcactactcacttcctcgctgctggctcactcactcacccactccctcac

tccctcgcactactctggctgtctccgctgggactccggtcgaactgcaccagcacgcccttgctaatactccagctatctctgccacgtccacgtcc

acgcccagctggtcgttcaccttcctctccggcgcacagatcgagtacccgcccatcaggcacggcatcgatccacgcgcagacgaacacatcccaca

ccacgtcctcaccgcaatgtacgcgtcgtcgtcctcaacagcagcagcagcagctccagccacagcaccagcaccagccatgattcccatgggtccac

ccacctccctcccacccgcaccatcaccagcagcactcttccaacagcagcagcagcagcagcaacagcaacagcaacaacagccatacggctacccg

cagtcctcatcctccgcatcaaccactgccatcttgtcagcatcaccacacacaacagcacatctcaacctcggcccagaccatcaccaccaccagca

gcagcaccaacaccagcagcagcaacagcagcagcatcagaagatgaacgggatacccaacccacccaccacctccatacgaccgcccacaccatcca

catccgcatcatcacccaaccacggtcagcagcagcagcacgtccgcaaacgaaatgcatctctctccctcgaccagggcggatcacccgctccacaa

cagggcaaggagaagagacctcgcaactcagcggcagcagcaggcaccaattcaaaacagggcgccagtccaaggaacaacaacacagccaaggataa

caatggcaatcccacagccacaatcgcgcagcaacccagcgatgtcacatggggcgaagaatcagcaaaactcgacatctacgtttgggactacctca

cccgccgcggcttcaccaacgctgccaaagcactcatctcagacgctaccatgcccggacatccagaaataccattaacaacaccacaaggccttctg

cacgaatattggcgcgtcttttgggacatgttcagtcgcacagagaagaaccagccctactcctcccgcatccacccggacatgcaagatcacaaggc

agacaacatgcacgttcgcaaacacgagggtctcgatccagccttctccgccgatctgagtcaacagcagcagcagtcaggcatgaacccggcctggt

tcctcggtggacaggacggccattctcagggaattatggcaccaggaggtgggggagcaagtggaactctccatcctcagaatggtgcgtcgaccatg

aatcaagtcatgattcgcaacgctgtcgcaggtccagcagcggccgcagcagccatggccgcctcgaaccacttcaacgccaaccaactatcctcgac

ggccaataacaacgcatcatcatcgtcctccttattaccatcctcagcattaggagtgggagcaggggcgggcgtaggcgtagggatgcccctcctca

acccaccctggaacaacctccaaccggtacagcagtaccaactcctctccaccgcggccgtcaaatccggcttctccattgacgacgtcaaactcatg

acacccgccgcgcgcacccagttcctcgctgcgaatctgacgccccagcaacaggcgcactcgttgaaggtgctgcaggcgcagcatttgcagcaggc

tcaacaagcccagcagcaggcgcaggcacagcaacagcaaggtggagggaatggggcgcgcgtgggtgggtcagagtctggtgggcctggcgggaacg

cgttgggccctgctaatcaggccgtccgacagatgcagctccaacagcaacagcaacagcaccaactctcgatgatgcagcaacagcagcaacagggt

cagggtcaaggtggtcctggtggtcagcaggatggatcccagcgcgggctcggaccgggcgtaggcagttcgagcatgcagggccgatcggcttcgtc

catgtcgaatggcggtggggggccggggagtgatcagcaacaacagcagcaacaacagcaaaggcaggcgggtcccggtcccggtcagtcgccgttgg

cgggtatgccgcaggcgatccctgctgttagtggacctggttcacagcagggacagatgcatcagcaggggatgagagggccgcagccgtcacctctg

atacagggtggtcctcagcaacaacaacaacagggacagagggtgccgcccggggcgacggatcctgggcccggtcctggtccgcaacagcagcagca

gcaacattccatgcatcctggtggaggacctcctgggatgcagccggggcaggggcaggggcagggtgcgggtccgagtggacagaggggtgggatgc

agtttgggatgccgccgccgccgcagcagcagcatcagcatggacatccgatggtgccgccgcagcagatgacgcctcaacagcaacagcagatcttt

gggcaaatgcaacaactgcacgaagctcagcaggtcgaataccacaattcccagatgatgcagcaccagcctgaagtctcgaatcaggcgatgggccg

ggcgcgcgagtatcatgcgaagttgcagcagcttgggagtatgtttccgggggtgtatcagcacttctcgcaggcggcgcagttgcagcagcagcagc

aacagcagcaacagcagcagttacaacaacagcaacagcagcagcagcagcaacagcaacaacaacaggcgcagcaacagcaacagcagcaggcgcag

gggcatcttatggggaatgcgggcgggccgcatggattcgggccgcagggtatgtccggccagccaggtgggatggtaccccgaggccccgtcaacgt

cggcgccggcctcggccaaggccagaatcaaggccagggaggaggccaacaacaacaacaacaacaacaggtcccacagggcatgatgaaccccgccc

aagcggcggcccagatgggccagatgatgacgcctcagcagcgggaacagttcgcgcagcggcatcaacagcagcagcagcagcagcaacttgctgtg

gcggcggcggctgcggctgctgctactgctcagcagcagcagaatcagcatgggcatcagcagcaggggcaacaagggcagggacagcagggtggggg

tgggatcccgggtgctccttctcaacagcaggctggtcaacaacagcaacaacagcaacaacagcaacagcagcagcaacaacaacaacaactccgga

tcgcgaatgggtacaacaacatgacgccccaacaacgggctcatttcgcacaacagtcgcctgcggccttggtcaataatccggggttgatggctagt

atgatgcagcctggtcctgggccggggcaggggcctggtgggatggtttcacagcaacagcaacagcagcggatgggtatgatgcctggccaacaaca

acaacagccgctcacgcctggattgggacctggacaacctcttggcggtggacctccctcgcatccgccactacccactggaccggggggcatgaatg

gcaataatggcgccggtcctggacctgggcccactggtctgttcgaggattataacgcacagctggaggctcaacggcgtcagcagctcgcagcgggt

atgcagcccggtggtagtagcaatatcaacggcggcggcggcgggccaccaccccacccgcccttcggcgtacccatccgctccggccccacacccga

tggcgacctctccaaacagcggccaagcatgcaacgcacggcctcgatgaacaacaataatggtggtggcccaccgggctcgatgggcccgccgcaga

gtgtgttgttgagctcgtcgccgagggggttgccgagtgggttgcagttgggtcctgctggggttgtgaatggcgcgggggcgtcgccgggtgctgct

gtgcctagtacgcccaagttggggaatgtgggtggtgcgacgggggacgggagtgggagtagcaatgcgagtgctcccggcgcgggtggggcgacggc

tgtcactgcgagcgggggtaaagcgccgaagcagaagaaggagagtgcgaggacgaggaagaactcgaaggcgactacgacggggaagacgcctgtgt

tggcgcctgctgctgtgccgcagtcgcagtcgcagtcgcagacggccggctcgggttcgggttcaggttcgggcgctgcggcgtcttctgcaaacggc

gcaggggcagcgtcgtcatcttcctccggcggtgctgggaatggtggggcggggacaccagctgcatcggctgctcctacgccgtcgtccaaaccgac

gccgaacgcgatgacgcctgcgatggggaagatggacctccccacgccgcagcaggctccgacgcccgtccccgcttctgcgtctgcgatggcgccac

cgccgccgagctcgggcaagagtctgccgatggcgttgccggatgtggcaggcgcagcgggtgcgggtgcgggtgcgacgccttcgtcgtcttcgtct

cagcagcaggcgaatgggttggcgatgtctgctgcggcaggtggttcaggttcggatccctccggtagcggcagcgggaacaacaacaacaacggcgg

cggcggcttactttctggcagcctaggacagacagccatgctcggctcgtcgtccctggcgccttcgtcgtcgtcgtcctcgctgttgatgtcctcat

catccgatctgaacgggccgccgaaccctgtgttgacgtcgaacccgagcatgagtgggaatgggagtgggaacgggaacgggaatgggaatggcaat

aataatggcggccataatgcgtttagtttcgggacgaactttgggagtgcgggtgggatggacgattattcctctgtgttcccgcaggacttctttag

cagctttgaccttggcggtgggggtgggaacgggaatgggccgacgaacctatttggcggagggggcggcgccggtgggggtgggggcaacggtggtg

ggggaggcgatggctcgggtctgaatggtgcggagggcggtggcttctatagtgggctcacgagcgatgtttgggactcgaatagcttgaatatgctg

tttagtgggtcggacacgcttgctgggactggtgggggtgggggtttatga

>contig_21.contig_21.g936.t1

atggcgcaccacaatctccatctgcatggtaacggaggtcccatgcaccaagccttctcgcccaacaccatgagtccct

ccgctggccctcaatccgcaggcgggcccaactcagctgggccacagcgaagattcggcggcccaggcggcggcggcaacttcggtccaggcggtaat

ctcggacctggcatgcgccaacttcaacatatccaatcccaactgcacgcctcacaacaaggccatggtcatccgcagcaggggtctcccgctggctt

tcagcagcctggtggccgtcgaccaggcgcacccacccgcctcggcctcaacgccatgagcgcacccttcatgcccggcggcgcaggtggcgcaccag

cgcccatgctcagtccaggctttgtcaacgcagccggcgcactctcccccggcagtggcatgcacacgcccggagcacccaacatggcaggtatcgca

accggccccggtccaggcatgattcacgcacccggcaacggcggcatgatggctcccatccctcccggcatgggcatgggtatgggcatcgccggtat

ccccatgagtctcagtcccagcgcactcaccagtgcaggcttgggcccacccacccctctcagcgggatgaatgccttccccggcggcggcggtcata

atggagccggtgggccgcccagcggcgccagtacgccttccatcgctgcctcttctgcaccccagggtcgcatgggcggcatctcccccatccctgga

gcaggaggacccggcggtggtatgtcggcctccgcatcgagcaacaacctcatgggccgtggacaggcaggacgtaaatctcgcggactgccccaaca

tacccctctcaagaccaccggccacaactccaagccctcggtcagcatgagtggaccggctgtgttccagctgctgtccagcgtacagggagccgctc

tcaagaagcgtaacgtcgtcgccctccccaaggagatcgacgacgccgcacacgaggaggccgtcgccaacctggccgctctacaacaggatgcggat

cagagtgcgaacggcaccatgacggagcaggacaagtcggctcggaaagctcggaacgcggctcgggacgccgcggcggtccgttccaaatggcccgc

ccgcgaaccggagccgttcgacccggaactcctccaacccgacgcccatccggacctgatcctctccgacgaacaaacggtctcggcggcgatgtacc

ccgaggcctggccgtacgaccagggtctgccggagtcgatcgatatctacctccctgggaaaggggcgtgggatatctatcgggaacagcggattcgg

gaacggatcgaggcggctgaggtcgaggatcggtcgtatgacgctcttcagcagcagctggatggcgtggctagttcgcagtttgatagtcggagtgc

gagtcggactgggtatcggggtgcgatgcctccgactggtggtgctgaggtcggcgagacgagctggggatacggcgttggcgctggtgatagctcga

cctttggcattgtcaatcatgagaacaacgacaacgacggacacgcagcagacgacccctcccacgcacacgcactgtccatctccagcccggccgac

cacaacatggtctccttcaagctcaaccggtacctccagtcccaagccgatgcggataacccgcaccacgagtccaacaacacgggtccgggtgggcg

gagactttcatcgtactcggactcggcggataaccggtatgagaccgcggttccgcagagcggaacgcacgcgccctccgcttcgctcgccatcgctc

ctacgcagtatcttgagaacttcctgaatgcatccccggctcagaacgcgcttgcgaggtcggcctcggaccggggcacgacgatgggctttgatggg

ctgggtcggccggagatggtcgcgcagcgcggggggatggtcatggccgaacgggtcagttcgtcgtatggacggcttgactcgggtgtgcatactgc

ccctggggatatttcggcttcgcttgaggagctggatgccactgcggacacgacggctgcgagttctgcggctgcgaataagggcgatcggagtggga

cgccgaattggaaggaccttgcgcgtgggtttgggtatgatcttgcggaggttgatgaggagggtgatgaggaggtccggagcgggccgcacagtcgc

aagacgtccaagtcccatcggaacggagttgcccgtcccggcgataatgacgatgatggtggcgacaacgacgacgacgacgacgacgcggacatgcg

tacgaacccatctgaagacgcggacgactcgaacgtcgaggatgaagatgaggatgacggcgaggatgaatcgcgcacccatcggcgtcagacctccc

aggcgagcgagcgacactgggcccgacgaagcacggcgcaactgtccgactttggcacgcgtccccccgctacgacgaccgcggcgggccatgcgcgc

gcctcgatgcgcggctcgaacgagatctccggtctgagcctgagcgagctcgaacttcataaccacaaccggcgccacgaacatggggagggggagct

ggagatggaggaagaggatgcgatctcggaggcgacgacggaaggtgcgagcgaggaggtgtatagtaacccctcggacgaagaggctgcgcagcttc

gacgggaggaacggagggagatgagggagcggaggcgggcgtcgaggggtgggatgggctcgtaccattcgcctggggcgcagcggaatgcccgagga

ggcaatggtggaggtggactcggtcgaccgcgttcgggcacggttaatacgctccctagcagcagctttggtgaaggggacgactatgaccagcgacg

gaacccacgtgctcgtccgcctgggttccccggcgcgtcgatgggccgtcgtccctcggaccggtacgagatcgtcagtaacccgtcggatgaggatg

gggaccagttcagtggtggtgcggagacgtttggtgggcatgggccgttggatcacccgctctctggtggtccgagtgggggccatgtcggtcggatg

agcgaggacttcaggttcccgccttcggttggggcgtatggtgtcggcggccggagtaacgagatcagtccgtaccagacgcaggcgtaccacgagtc

cgggaacaacgtgatgcctcgctttggtaccggtgcgggtacgattgggaacaacagtagtggcacgattggtcggtccgcggggaactcgatgttga

accctgctgcgaaggagttcagctttggcgacatttcggggacgagcagtcgaccctcgctcaatgcggctgcgccttcgttcacccctggcaagttc

accttcaccgcgcctggtgtggcgaaggtcacgatcccagaaccgatcccgctggtgtatcccgcccgtgcgctgccctcgcaggaagtcgcgttgga

gaagtccgcctcgggcgtacgcgatgcgcaggggcgggagaaacgaccgcgctttgggcggaatgcggagatgctcctgttggacgagggcgagttct

cgttccagcagtctgaggacgcgtttgatcctacgatgcacttcacgccgcagcagcggcccatgaactcgacggcgagtatcgaggggcccttgagg

aacttgcaggatgggacttctgtcaggccgttcgggagcttggacagtattcatgcgccggcgagggcgggcgcgggcgcggtgcctgctgcgccgag

tttgccgaacatcatccagcaccatcggccgtcggcgagtggggattactcgttacaggctcatgcgcagccgttccagcctacgtggggccggtcga

cggctgcgggacctgcatcgcgtcctgggattcccagctttgctggcatgccggccgccgcagaacgtccgatgccgctcatctttggcagcagcggg

gtgttgtcgactgtgcccgcgtcggatttcactttcactattgggaacaaggcgatcccgatccgaaggcccgacgaggcgggcatcggtggcgccgc

cgacatttcgaacggatcgcactcgcacgaggcgtcgatccatgccgacggtagtaggacttcgatgcctgatgtgcctagggacatggactcggaac

atgaccaggagaacgacaggcgtggcagcttggcgcaccggccttcgatcggcgatgtgcggcggatcagcgagacgtcgacttcggcgcgggtgcct

ctgtcgtcgatgtatgaccagtactcggacgacgcgacggcagcggcttcggcgctcgtgtctgcctctgcggttcatgcggccaagcagaaacttgc

ccgctcggcgcctgtgccgatcccgatgctccgaccggaccaggccgcacagcaggccgcaggacacgcgagcagcacgggtctggcgcgtagtttcc

gctcgcacggtcggaacgggactgcggagagtctggcgcattcgctcacgtctggtcgggcgatgcgtcatcgtcgggcgagcacggtcgaagttgac

agtctgggcgaaggggatgaaggccggagcggggacgacgatgttgatgaggatgaggacgatgaggatgctgtttcggacattattgaggagcttgg

tgaacgtattgagaaggcgcttgatgggtgggctggcaagattttggacgaggtcacgattatgggacaggtgcggccgatgattggtgccaagcttg

acgctgcggatcgtgatgctcttttggagagctttggcgtccgggtcgaggaacagctgcaggatcactttgctcggttcaaggagacgacggctgct

cagactgctgctgctgcggccgctgctgctgccaaggccgagatggacgccacgaaggacacggattcgactgtgatgcctacccggactgcccaccc

ttcgcgaactggctctggtcatccgcctttggacgcggtgggcgagttggactttgactacgtgtcggacacgctggatcagaagattgccgatctta

agcgggagcttatggctgcgtttgacacggcgttgccgagggctatggagtctgtggtggacaagatccctgcgccggctcccgtagctgctcctgct

ccagcgacgattacggcttccaacgcggcgtccgcgactttcccatccactatggacctttcgctcagctcgtcccaagagctcgctgatcttctcct

tcgacggctgcagagcgctctcgaggccaagcaggactcgacgaacgaggtccttcagaacacgctggagaaggtgctcgagcctctgctcgaagatg

ggttcgcccgtgctcaagtcaagtccgtcgtcgaccggaccgagtctgcccaccagctcactgagctcatggtctccaagtttggcgacctcgagaat

ctcatgggcgagtctcgtcagacaattgaagaccatctgcaagtggcgcttatcaacggcttggtgcctcacatcgagacgctgcgtcaggaaccgat

cgaccctgacctcgtagccgctcgggtgcttgagatactcgtgcccatgcttgaccgcgggtatgagcgcgagagtagctcgaggaagaacagcgagg

agggacggccgcagcctgttgtgcagacggacgacatcgccgacacgacggtggagcgacttcttccgttcctcaatgcgctcaagccggctcatatc

gacatcgaagagctcggggcgatgctcgatgtctcgttggcaaagaagcacatctctgctgagactgatctcgagcccgtcattgctctgttggagcc

actggtctccaaccaggccgatgcgcggactatgtctcggcagacgattgaccgactcaaagaggtcgaaagcactctttcggtcctgcccggcgcta

tcaacgcaaagacggagatcttccttacggccacccagcagacgcatgacacccagggatcgatcttggagcgtatcaacgacctgggccaccagcta

cagaccatgctgcatcgcactgctgagctcgacgagcaacgcgatgagctcattcggcagcttgacgaagtgcgcaaggaggtcaacgaacgcaaagc

tgaggctgacgaggcaaagtctgagctcagtgctgccaacggctacggaaacacgctcaaggacgagctcacgcgactcgaggcacgtctggatcaag

ctgagagggcagctgaagaggtcactgatcagatggacgagctccgcacccgtgctcaacaggcccgtgagcgtgagcacatcgctgagcgcagggcc

gaccagatgacggctcaggctcgggaggcccagcatgccggggacgcggcccaggcccagaacttgctcctgcagaagcgacttgaggaactctcctc

tgagctggcggaggccagggccgagaggaaccgtcagcgcgaagcggcaacgcaatctactgccgatgcgatcgtccgcgcggagcgagcggagatgg

cagccgacgaagcgcgccagcgtgccgatgcgcgtgtggacgaggccgctcgtgtggagcgaaacgcacacgagcaagcctccgccattgctgagcga

gcagcgaaggcggagggcgaagctgccagcctggagaagcgcgtgatcgagcaagactccaagattgcgaatctgcaacagctgactgcgacacagaa

gcagaaggctgcccagtcgcagcagaagctgtccgagtcggacaagaagatgcagcactttgagcaacaggcgcaagaccttgccattgcgaatgcca

gactggcggagatggaggcgcggctggctgacaaggaggctgtggaggatcgtcttgcgcatagccaagatgccgagatgcgtctccgcgaggagcta

tccacgtaccacacccgcttcctggaccttgagaaggacctcatcaccatgaaggaaagcatggtcgggcgggacgagttggaagccgtccaggccga

gcttgcgggaagccgcgaagatgttgcacgtttgaacggccagcttgcggagcgcgaacaacaggctgctgccgcgctggcggctgccaaggagctgc

agcagcaccaacaacaacaacaacaaccactatgggacggctcacacgctcagcatcccggcaacgtcaacacgtggacttccatacaggccccaccc

aaacggcagggcgacttccacaatgacggcgcgcatcagtacccgcgggagatgccgactccaaccactgcggctccgagacacacctatgcttcgaa

cggggccaacaactggggcgactcggactcgtcgacggactcctttgcgcctaccggccagtcggggtacagcagttatgcgcagattgcgccgccca

gcaagaaggcgccgtccttctctggccggagcgagtcctttgggccgactcgggtacctcagcgagatgcaggtggttggtggagctga

>contig_21.contig_21.g937.t1

atgtctcgcactctcgctgctggttgcgctggggaaaactttctgggcgagaacgacgaacgacgtacgagtagacgca

ggactgactcggactcggaaacaatggcaggcggatctcagctctcgcagctgaaggcgcgcatcaagacagaaggcatcagcgaccgcagacagcag

accaagaagtcgtccaacaagaacaagaagaagggaaagccagacgagtcctccgccgtgcgcgcacagcgactcgcagccatcgtcggcgagaaccc

gttcgaatccaagatcaccaagcccaagcacgacgtcgtctccctcaagaagcatcatgccgctggcctcaaggcaaagccaggcgcgtccaagcaga

ctgccatctccctccgccaggcccagctcctgccagagtaccaggcacgcgcaaaggcagggtccttcgtcgacaggcgattcggtgaagcggacccc

accctaacccccgaggaacgcatgctcgagcgtttcacaaaggagaagcaaaggcgctccgctcgcaactcgtcggctttcaatctcaatgatggcga

cgatgcaggcgacggcgccgactccagcttccagctcactcactacggacagtcactcaacggactcgacgaccttgacgacatccccctcgacgatg

acgaagaggagggcggcatgtcgggcaacattgacaagcgaaccaccagtcgatcccatttcggtggattccaagagaaggaccaggacgaggaggaa

gatggagaggaacgcaaaaagagcaaggccgaagtcatggcggaaatcatcgccaaatccaagcttggcaaacacgaacgaaagatcgtcaaggatcg

cgacgatgatctccgtcacgagctggacgccgaactcggcgacattctcagtctgctcttcagtatgcctggtcaggatgctcaagacgaggaggaag

aagcacggaaacgggaggaaaagcgcgctcggttgggcgatcccaacaagtccggctccaatgccgtcccactcggtggcggagagaaggagaagccg

aaggacaaggcgaaggacgcagtcgatccgactccctcgacttctaccatcaccgccaaaactccgaccgactcgtacgacgccttcgtccgcgaact

cgcctttgaccgacgcgcaaaaccgacagatcgcctcaagaccgaggcggaactcgcagccgaacaggcagaagcgctgcagaaacaggaacgcgcac

gactcaagcggatgcgcggggagcgcgatgacgatagtgatgcagaagatggagcacgcaaacgtgggcgcacgggtggggcggcgcaggcggacgat

ctggatgacgactttgtgctcgatggcactaccgctgccgacgtttatggtctgggccgaggtctggctgacatgagcgaagacgaagacgaagatga

tgccggagaagccgtggctgtggaccttgaagatgatgacgacgacgaagatggagaggatgatgatgatgacgacgatgatgacgacgatgaggagg

aagatgaagacgatgacgaagatggggaggaaggtgaggaaggagatgacttcggcgatatggtcgaccctgacgcgctcgctcagcctggcgaggac

gaagacggggatgtcgaaggcgatcatcaggacctcatcggtgctactccatctctgccagtcagcaagacgaagaagaaagcagcagcgacaacggc

agtagcagacccaaagaaggcttctacgcttcccttcacgttcccttgtcctcgcacccatcccgaattcctcgcgttgcttcaacaacacaacgtac

aggctgcggacgttcccacggtcatcaagcggattcggacgctctaccatccgagtttggcggaggagaacaagttcaggctccaagccgttcttggg

atccttctcgatcacgcactctacgtcgcaggtctccagcagagctctcaagcttctgccggcggcccagccagtggtggagtagcgagcttcgacct

cgtgaatgcgctcctgccacccatctttgaactcagcaagacctacccgctcgcctcggcacagcacttcgtcgccaagctcaccctgatgcagcgca

acctccaacgcggtcttgcggcgggtgccctcctgcccacgtcccgcacctggccccgatccggtggcgagctcgccttgctgaggatcgcaggcgcc

gtctggcccacgagtgactggcaacatcccgtctctacgccccgggcgttgttgatggcgcaataccttgcccatgcgcgtgtccgctgcacgggcga

cgttgcctgtgccctctatctcgtcactctcattctcgagggcgagacgctcagcaagcggatcgtacctgaggcgctgaatgcgttgagtcagagta

tggttttgctggccaaggaggctgtggcagctacggaggaagggtggaagagggtgcaggaggcccaggtgaagtggggcattccgatgccggatgtc

ggggcggcgcatactcagcacatcggagtcgcgctggtggaagatggctcgtccgacccggacccgactcagcagttttctctgcccgctctactcgg

cctcggaccgaaggaagacgtcaaagacaattcactctctgaggcagccgcggaccagcttctccgatctacatttgcgctggtcaagcgctttgcca

agctgtaccaaggcagttcagcctacgttgagctgttcgagcccttcctgaccctactctccgccttccccgccgaaggagccggtctgctaccccgc

acgggaaaagccatggtcgagctcaccacctacctaaaccgacaagtccaactggcctcctcctcccgtcgggccctccgcctccaagcacaccgcgc

agtctccatcgcgacctacatccccaagttcgaccaagaaggccgcggcggcgcctcccgtctgcgcggaggtgtcttcgacccggactccgtccgat

cagaggagtccaagctgcggacgcagctgaagagggagaagaagggcgccatccgcgagttgaggcgggacaatgccttcttggccgaggttcgggag

ggcgagaggagggagggggataaggagtataagaggaagattgataagattgttagtgggttgcaggaggagaggagcgaggagaaggcgcttgcgag

ggataaggcgaagatgaagaagagggcggggaagaactcacccctttcgatcctttcctcgccgcttcgctcctctgcctcgaaacgagcaatgagcg

cgacagctgccctctacgcgatctacgatggcatccttgccaacatcgacgcaacacaagacctcacggacgagaccatcacggcgctcctccgcctc

ttcgacgagaccttgatccaagcactctaccttgtcgaccgtggtgccgttccatcgtcgaggtccaatcgcgccacccggaaccgacccgagcggtc

gttatctcgcctcgaggcacgtctgtcaggcaagactggtgcgaatgtgcagagttcacactcgtggctggaggagcgaggggctcgtcgatgggacc

gcacgcgctgccttttcccgtttgcgtccacctgcttgctgcccacctcgctcaccgcacaggtcgaatcatcgtcatgggcgaaggacagacggccc

ggggatcagagaaccaagtgctactcgatcgtctaa

>contig_21.contig_21.g938.t1

atggatgtcagctgtcagctgtcagcgtcagcgccagtgttccatgccgtcgtgcctgcattccttgatccttccgtcg

cacctcctgtcgagacgcactgttccagacagagcaaacaagaattcgatcagcaccgagccacgagccagcggagaggagacaaggtcctggaacgc

cgccatcttggggacggcaacaaaggcttgaccgtcgagcactgtgcctgccaaaaaggtcagccgggggaaggatcatcgttgaaaggaagagaaga

acagaatatggtcactcactgttctgctatcgtcaagcaggccgttgctcgagggtgggaggtcgtcagtatcagcccgtcgggtcggccattcacca

ctcccgccggccatcgtccaggatggtccacctccccgctcattcgctggcaaagtgctgatgcgctccggccggagacgtacgcagacgagctgcgc

tcatgcacggctgtggttcataccattggcatcttgctcgagtcggattacaagggcggccggtcacgagctgcgcgaggggcccaggagtctggcgc

tggtggtggcttgcttcaagggctgctgaagggatgggggtttgggtcgggcaatcctctgaatgagtcggcgggcaagctatcgtatgaggtcatga

accgcgattcgggtgcgtacatttcccagccgttctcactgccattgccgtcgccgctcaggcatgcgcaagcattctatccatcacgacatgctgat

aattctatccatattacgtatcgaaaagctttgagcgtagcccgggctttcatcgaatcgcgcactggtatatgcccccttacctcaacacctacttc

gacacccgctccgttcgtctacatctccgccgaagacatcttcagacccatcgtgtcggaacgatacatcaagacgaagcgggaagccgagtacgagc

tttcccgactcgcacttgactcgctcaatgcatctttctcctcctcttcgatttcgttatcctccctggcgaccgcggcggcgaaggagcctgtcatc

cgacccttttttgtccggcctgggctgatgtatcacccgcaaacgcggccgctctcgacgttccctgcggcactgctcgacctttctgcctccataca

tgccagactggagccggcatcatcgacatctgtccccggaaacgatgaccctcttttatctcaaagacaatcgtcgtcagcgtccagactacccttcg

gcctacggattcccactccagcctctctcatccggtccttgggtccagaaggagtccagccactcgccgacctgctcacgctcccacccctgcatatc

gatacggtagccaaggcagtctgcgccgctattgcgggcgaggcgcaagagaagcggatcgggctttcagtgacggctcaggtggaagctagggaggc

gccgatctctgtaggcgaagttttggccacctctgatatccggagactggctggctgggagcgtcggacgtgggagctccgcttggcacttgggcttc

gaggcacacatgacacagccgctttgttctgtgccttgcatgtggaagtcacgcctgccttggatgtcggctcgtcggcccgcggcccgaggaacgcg

gtcgacaagcgaccggtaaataagaggttcatcgcatcaagagcagcagcggcagcagcgacgggggcagcgacagcagcggtgcgctctcatgtcgc

agccgcgtgtctcgcactcactctcgcccctcgccctgcgctccctgcatccaggagcgagcggcaggcgtcaacatcagcacataccctcgctccca

ttacaccaccagcgtcgtccctgtcatcgtcatcatcgccatcatcaacagcagtagcgacggcatcagacgccagcgcagtagcccagtttgtcgat

ctcggtctgccccggcctctcgctcggcagctcatcgcagccttcccacacattcacgcccccaccctagcacagaccgctctgctcgaggccatcac

ccagcccaacgatgtcattctcagagcacatacaggcacaggaaagagctttgccctcctgctcgccctcctcgccaagccgcgcgtcctcttctccc

agcctgcttctctgagctcgagcagcgaggaacggcaacagcaacatggtatcgcaagcatcatcatagtgccgagcaacgagctcgcattccagtac

tactcctgggcacgcgctctgctgccacaggccggtgccgagcttcatccgatcatgcaagtcctcgtgcgaggacacccgactctctctgcccagga

tcagctcgatctccttcgcaagacaccgccccatctcatcgtcgcaacaccgacccgcctgctcgagattctctccggaaagacaaacgatggacatg

cgctgctcggcctatccaccctccgtaccctcgcacttgacgaagtggacgcactcctcgatctgcccggacgtttcccgacccacaagctcaaatgg

aaacacacggtccacccggcacctgctctcgtcttcctcaacgaggtcatgcggctccgtccttcttgctcaggaggccagctcttgccttcagcagg

cctcgaacgcgacgagggcagacatttcgacgaacgcagaccacccgatgcgatccgtcgtactgctcacagatcacgagaggctctcatctccaaac

agcaacaagcccaaaaacaggagtcatcggcgcattcgttctactccgccttcaactttgcccttcccaagccccgatatgggcccacgggcagtcca

cccattcagctcgtcgtcaccagtgcaacagcgaatgccgttctacgacatttctttggcgcccggactggctggcttcgaaccggtgttaaggatca

gatcggaagaccgcaaggtcaagtcccaaatcacgcatacgcacgccaagaacgcatcacaggcgtctggctcgatctcaccggtcttaccaaggcct

ctgccgagctcactgcgtctctggacaaggggaaaaggcagaaagcagaagcggacgatcatgcgtcgacattcccaacaggtgccgagggcagtctc

tctgccaagctcaaggctgaaggacgtgtaatcgacgaggctgataggactacgttcccaatgacgatgccagaagagctcacccactactgcctcgt

tgtcgacgagcccagtccaaacgagcgctccccactcgaatcagagggcaccgacagcaatgctaccgtctccgatacaaccctagcgagcgcactcc

cacccatgcgcaacctcgatccacgcgccttcatccgaccctggcgcaaggccctcgatcttgtccggaccaccggccaaccagacctggcgacgacc

atccgatcggcttcgccaccggcacacgaggtcgacccgcacttgctcggagcgctcgcattcgccagtgagggcggcgatggagcagagacgacggc

ggcagcggaaggaaaggattctccggtgctctacctgctccaggccacatccgcacgaggtctcgacctgcccgtcctcacccacgtcttcatcgtag

gcatcgaagcggtcggcgatgcagtgcgatacactcatctggcaggccgagcctctcgaattggacccagcgcagaactggccaccatcccccaacct

caagatcctaacaacagtgaggatagcggtgcgaacaagaacatccagacgacgctgcgaccactggcaaaggtggtcaccatcatccgcggcctccc

tcatgcctccgtcctccgccgacgagccgccctccgagcctggtcttccctcacaagagaggaacgcgccgcgcgcgggatgaagaaaccgcaacacg

acgccatcatggtcagttctgccgagcgtaagctctggaccgtgtggagaaggacgggagtcagtggacggaaggcggggcaggggaagagtggggga

tggggcaagtgggatttgagattagttggtggggagacggttgaggatgaggttgcactggatgggtggacacctgaggaggatgaggccgagttcga

ggaggggaggcaggagtttgaagaagaggaggaagagatggaagacgaggttgcagatggggaaagggaaggtcatggcttcaaggctgctgcgagtc

cgagatgggagcccagagaagaaagaaatggaacccgcgagcgtgcttcgtcccacctgcgcgaacgtagagaggagaagaatggtaattccaagcgt

acctcgtctcctcggtgggaacggagggaggacaagtacgcagcttccacccgtgcttcacttcctcagcgcgaccgtagggaggacggaggtggtag

cttccgacgtacctcggctcctcggcatggactcaaagcagacagggacgttagtttcagacgcgcctcggtccctcagtggaaacgttcgaacgcta

aagatggaggcttccagcgcacttcggattctcatcgagagcgatcagacaacacggatggaggattcaagcgttccttcgctcaacgaggagatcgc

ggggagggaagggacgatggctttgtctccaagcgtggctcagctccccaatcaagacatccaagattcgatcgcgagggcgggagaggggctgcaga

cggtggcgaagatcggctggaatttgggggctcggaacgcagctctgggccagggcgtagacggcacgattcagcgggacgaccacagacggcatcag

cacggaggtggtaa

>contig_21.contig_21.g939.t1

atgaacgtcaagatcttcactctcctctgcggtctcgtcctctccgccagcatcgtcgccgcaggtgacggaccacgcg

gaaatcgtgcccgcagaggcattataactgtgaaacccagacccccgaccgttcaaccgttcgacgacgcgtcatctactccctcacatcccgctcgc

cattcgttcaagatcgtcggcatggcggaagctaccaccgtcaaatgtcaagcctatcgcctactgatggtgtttgtgcaggccaacacagcgagtca

agaactcgtcaccccccacaccttcgatttctcgcacaacgacctttcttcattcattcattccctcatcagcagcatcagcccatacttgtacctca

cggtcttcgatcaagagatgtttgaaacccccactacctactatcccttcgccatctgccaaaccgagttgttgatcgtttatgtctatctgtcggcg

cccggtacgcatcgcgttctgcgaacgaacgttctgctggagccgtctcctaagtctgagtcacgcctgagatcgctatccactcatcaatga

>contig_21.contig_21.g940.t1

atgtcgcgcctggtccctaaaccagctctgcgtcgggttccacatagagagcttccacgtccccacgtggccacgtgtt

tgcctgacgcgcaggacggaacgcgcaacctggacctcatctcattgaacaagtctcgcagcatggtcgacacgccgcccacggatgcaggcatgttc

gctgccggaactccaccggtcggtacgccagcagcgcccgcgtcaaccacgagcaaggcgagccggaagcgcaaggcaaccaaccagcagcagcagca

gcagccgaacaacagcagcacgcctcggccgcagcaggatcacagtcagcaccagcaccaacagcatctcctacagcagcaggccgcccaggcgcact

ttgcagctcaatactacgctcgcggcggccaggccttaccacagcagccgcaaccacagcagttcccgccgcatcttcaggcagcccaaggtgggaat

ccctacctacagcaacagccacatctagcagcgcaagcagcagcacaggcccaggcccaagcccaggctcaggctgaagcacaggcacaggcacaggt

tcaagctcaagctcaggcccacgcgcacatgatggctgccgcagcggctcaacaacaacaacagcagcagcagcaacagcaacagctgcaagctcggc

cggctggaaagacaagcatggccctcggcatgcctcagcatgacaatcacggtgactatgtcctggacgactttgagagaagcacgccccgcgacttt

gccatagcgagatatacgcgcaatcatgagctcctcaaccaagtcttcgatgcccgcagaatcgacatgatcaaggtccctcaatcaccatacgcatt

caaggacgcaaacgacctcaaacgccaacgcgacgcgctccttgctgagaacagagacttacaatccacgcatgccaaacggatgcgacacctcaaac

acgtcctcgacagcgctggcgcttcgagctctctccctgcgtcgatcgacgacgatgacgactccctcctaccagacgcgaagaagatcggcgacgcc

gcagaaccggaaggagaagacggtaccgacttcaatgccgactgggctcaacatcctttcgaaggtcctatcgtcggtatcggtctcgtccgcgccat

cacgcccgagccgctccgtattgtcatgcgcgaggcggacgaggcgcgcgccaggcaggacgcactcgacgccgcgcgggaacgggagaggagggaga

ttgctgtgcttgcgcaggcgcaggctcaggaacaggcgcaggaacaggctcacgcgcaggctcaagcccaggcccaggctcaagcgcaggcacaggct

acggctgcggctgcgttgttgcaggctcagcaacactccaattaccagcagcaagagcaagagcaagaacaagaacaagcgcctcaacatcagccgat

gcaagtgcaagagcaagagcgtcagccggtgcatcagcaagagcagcagcaagagcagcagcaagggcagcagcaagagcagcaacaagagcagcagc

aagagcagcagcaagagcagcaacaagagcagcaacaagagcagcaacaagagcagcagcaagagcagcatcaacaacaaggggagggcggaaatcag

gctccgctggagccgcaagatgcggttatgaatctaggttcggtcttggaggcggcgcaacaggctcaatcgatggccgaagcgggcgccttccccac

cagcacgagctcagaggcggaggctgcgcctgctggtgttgctgtggctgtggaaacaggtgccgatgcggtcccggctgtggaagcagctacggtcg

aggaggtggctcctactccttcggatgtagatactgcgccaatgggagcataa

>contig_21.contig_21.g941.t1

atgcgatcatacactctcctcgccgccatcatcatggccttcctggcttcagcttccctcgccatcgcctccagcaacg

tcgaggcatccgaccttctccagcaccgctccgccaccgacaaggccgagcgtcgcgcacgggtccacggtcagaagaaccacaaccgcgttcaccgc

aagcgcaagtcgtgcaaggccaagagcaccaacggcaagaccaaggtcgacggcctcggcgctgtcaacaatgtcaccgttagcgacgacggttccgg

tgtcctccgtggcatctgctggggtatggacgacagctacgcccacaagctcgatggctccaagctcgcctggtaccaccactggcaagatggacccg

tcaatggtcttaactctcagggtgccgaatatgtccccatgttctggggcgctcgccacagcggtctttggaaccagcgcaaggcttccttcggtaac

aacctccccaagcacatcattgcccagaacgaacccgatgtgaagagccaggccaacgtcgacccttacactgctgccgccgactgggcccgtgagct

cggccctttccaaaagaagggtgtcaagatcaccactccccagatcgtctacgatgtcaactggatggccaccttctactctgagctcaagaagaagg

gctacacccccgacatcattgccctccactggtacggcggctctgaccagatcgactccttcaaggcctacctcaacaaggtccactcccgcttcccc

aaccagcccatctggatcaccgagatgggtgtcacccgcaccggtgccggctcccagaccgatgtcctccagttccagcgcgaggtcatgaacttcgt

caaccagtctggctacgtcaagcgtgttgcatggatcggtgcctttgtccagggtcaggcttacgacaacttcatgtccaacctgaacggcttcttca

actcgggcggcatccagggctgggctcgcttcttcctcttcggcagctcctccaagcgggacatccactacgaggaggctgagcgtgacgccgcttcc

gtctctcgcgacgtcgtcgacgtcgatgctgagcgggacaccaccaagctcttcgtcgcttaa

>contig_21.contig_21.g942.t1

atggccttgaatccagtgcagaggccatcagaatggcacccgccagcggcgctaggcataccgccgtctctccgcgatc

acgtctcgaccttatccggcgcctacgtcaactctggccgaatggcttccggagtcgcccagctcaactcggccactgcgatgtggaccgagatgtcc

gggcagaggcaggacacgatggatcgccgaattggtgaggtcgctcaggatctcgggagcgttcggagagatgtgcaagcggacctcgaacgtacgca

tcggaaccttgctgcggaggctgaggtgatgcaacagcagtttgaggtgatgcaacagcaatttcaggcgatgcaacagcgatttcagaatcggttgc

aagacgaactcggccataccaaaactcgggtccatgagcaactcgagcgatgtttcggccaggttcaaagtcggcttgcgaatctgggggcggacgaa

cgtgtccaggttgacaagacgctcgaacacgtcaggacgtgtacttctcggctggaaaacgacatcatggcgacggaggtcagctatatgcaaagtct

ctcccacctcgttgtatacaactcatggaccactgcatggcccgaagctgtacgtaacgcacagcagacttcgcagcggtccgttccgcaagacatcg

tacaaggcccgccttcccattatctgtcaatacgcaacgaaggatcatcagtcgccagtggtcaaacgcaaaatgctctctccaatttcttagacaac

ctcgaaaggtcctctcgtgcccctgctgagcccccggctactcctgctcttgctctcgctcctgcttctgtctccgctcctgctcctcctgcccttgc

ttctgctccggttcgggcttctgcttctgcttccacttctgctcgtactcctactgctaccaccttcgccgccccgactactgctcctgctcgtaccc

ctgctcgtgccggtcctcctgtcacgcgcccacctgtttccacccaatcccgacagccgtcggcatga

>contig_21.contig_21.g943.t1

atgtcggagaccgcagacgtccccgccgccgcccgggcatccaagacgatcttgagcgccgccgcttcaggtgccgcat

cagcccagactggagcggaagtgtccgcttctggcccacctcccaggattcctggtatcgcctccgtcaagcctgagtatgtgatccgacccattggc

gacgctcgaggtccgagccagcgcgaccgcgaccgcgaagcagcaggatcttcagctgcgcctcagtcgggagctgacgtctcggacgcgcgcgactc

gaagcgcactttctctggaaccgacatgggcgatgacgatgcggcagaagctgcaggcgcaggcgatggccagcgcggttccaaacgaggccgaaagg

agcgaggtcagaacaagggccgcaaatttgcgaagcagaacgatgatgtgcgcatgtgcaatatgtacatgcttggtaaatgctcctttggaaagcaa

tgtcgatactctcatgaccttgcggcgtacatctcctccaaggaacgagacttgacattcatgccctctccctctccatccggctctttacgtcctct

cagcgcagaagcccgagaagcttatttgaacgagcactactcgacaagcgcgccctgggtcctgtcttcctcctcaaagcccgatcaatctgcagatc

cgacggcattgcctcacggttccatcgacacctctgtccgttgccccaacttcacaaatacaggcgaatgtcctgcagggtggaagtgccgctttcta

ggaggccatgtccgcattctaccttctgattcggaagcggcggcacaggaagaagaaacagaggttgcaggcacgatccctgctgtggcaccgactcg

gtctggcaatgttgagcttcttggacttaatcttgcccaggcccacggctcttcagcccccactgaggaaggggacgacgacttcaggcgcggggaaa

ccaactttgtttccaagacatcgctgagacttttgaagaacagagagtacttcttgcccaaggcgaagcaagttcttgaaatcctgaatttggagacg

gaggaagcccaggcccatggacatgcacggcctcgtcccatcattctctccgaggacggagcggaaaagccagcgaagaagaagctgaccggagccga

cctagaagatgctatgatgaacggcttcgaacccgagacgtcatcgcacgcgacacagcaggacggcgcttcatcgaccgtcgaccatgcgcgttacc

gaccaacggagaagcgacggctggactggaagggcaagctgtatctcgcccctttgacgacgacgggtaatctacccttccgtcgtgtgtgtgcgtcc

ttcggatccgacattcactgtggcgaaatgggcttggcagaaagctatctccacggcacaggtggcgagtggagtctggtccgacgttgggaaggcga

acggatcttcggcacgcagatttgcggaagcaaaccaaacctactcgtacctgctgtcgaggcattggtgaaggaagtcggatccggactcgatttcg

tcgatctgaactgcggctgccccatcgacctcgtgtacaatcgcggagctggatcagccttgctcgattcggcaggtcgattgggcaagatcttgcgc

gggatcaatgcggtcctgggcgagatccctctgacgatcaagttgcgcacaggcacgacgtcgaagaatactacgcacaaactctttgcccggctcca

gacggaatggggatgcagtgctgctacgctccacggccgcagtcgcaaacagcggtacaagaatctggccgattggaactacatcaaggagtgtaccg

atacgctgcgcgattctgtccgcgtctggaacgaggagagtcggcacgccgacgaaccggagatggtgcctgtgcctatctttggcaacggagatgtg

tacagctgggaagactaccgggagaacatggagcggacaggtgtcgatggcgagatgctggcgcgcggagcgctcatcaagccctggctcttcacgga

gatcaaagagcagcggaattgggacatcagcagtcgggagcggctggacatgtaccgtcagtatgccgagtatgccatctcgcattggggctccgaca

cgcagggcatcaacacggcacgacgatacttctgcgaggcgttgagtttcacacatcggtacgtgcccgtcggcattcttgagcatctgccgccgcga

ttgaacgatcggccgccgatctggcgcgggcgggacgagcttgagacactgttgggaagctccaacgcgaatgactgggtcaaaatctcggacatgtt

ctttggaaaggcgccagacagctggcatttcactccgaaacacaagtctagctcgtacgacgcggacggcgggtctgagcagcaagggtga

>contig_21.contig_21.g944.t1

atggcgacgaaggcagcgggatcgacaagatttgcaccgcgccagcctgttccatcatggagaaagaccagcggaggag

cagcaatctcacgccagcctgtaccatcgtggaaaaggaccagcggaggagcggcatctccaggccagcctgttccgtcctggagaaagaagagcgga

ggagaagcttttccacgccagcctgctccaccatcctggagaaagacgagcggaggagaagcactcactgtcgccgaccatgcaaagctaaggaggga

cccgttgccacgccatgcctcaagtgtacaacgcgacatctgggatcagcaaatcgagatcagtaccaagctcaaaaggctggctgaccgggtcatgg

ctgcggaagccaagatcaaggttggtggacgaatcggaacggaggaatggaacaagtttacctcagacaagaggtcggaggcacttctcggttttgag

aaggcggagtacatcgcgaagatatcgcctccagaagcgatgaagaagtcaaatctgtatccaactgccttgaccctgtctatcatgttcagcgggct

gaactcgttggcccggcggaagtcgttcgaattgggattggctcgggacaagcagttcacggagcaagtcgcaagccttgctcacgacgtccttcaac

ttcacgtccatgcgtccaaactatgggtgactcccaaggatcgaagactgtcaccgaatcaacttgccgaagcgcagaaggaacatgcggaggcagta

cagctgatcaatatgtccagtgcagccctggccgaggctactccggcgatgaggatggcggcctcgcgtcagaaagcggccgcagagctcgaagaaaa

tttcaccaccctgtcgcgtgcttattccagctacatcatcttgctctctcgtaccaccaaaggtcggccggcaatggtctggcaggtgtacgaagcgc

tgagacgaggcgtgggcaggcccgagggcatatccgagttcgacttcgcgcgacctttccgcagcaagacgattttcaccaccatgctcaccggggtc

ttcctccaggtctcaactccgactagaagacaagagactcgccgtgcggtgcaaagggctgcgttatcgcgacaagctctgacgggggcgacgcagga

ggtcgatcaacagacgagcgctacaaacaatgacaacgagaacgactctgacgacgcgatggcacaagaagctcccgaactgccatccacgcctttcg

gccaagcaagacagctgtggctcgactgggaggacaccattgaggaagccgagcaacgaggcatcaatcgcagcgactggcaagaacttgaagtcgac

gagaaagctctcgacctgtttctcgacttcttttccaaatgtcgggaagaaaaggagcgcgtctttgccaagtacatcaaagagagtcacatcaccag

aaaattgcggaaatag

>contig_21.contig_21.g945.t1

atgagcgatctcagcaacagccccgaggccagccccgccaaacgccctctggaggacgccgctgatccagctgcgcgca

agaagagagccaggacttcaacttcaagtgtcaaatgcgacgcgaaaagtgtgctggcgatggacgacgcggccatcggtgagctttcccaagccaac

gctatcacctgcctcatcaacctcaagtctgcatacgcgcagctacaagaagagcacgccgccgccatcaagaaggcagcctcatcgcgtgccgcccc

tgcagcatcatcatctacggctgcccctgtttggacacctgccaaggtggctgaagcggctcagaagattcgcaagaacacggctgctgcaatcaaga

agcagatgaagtggcagccgagctgcaagaaaggcactacgaagtggtcaatctccgttcttgcacctacagtcgaagtcttgaaggccgtcttcggc

ttccctgccactgacaaggaggtcaagctcaagaaattgacgatggaagagtttgagcgcgcgttcggttgcatcgaagtctcgattcgatacgggtc

cctagaaattacgggaacgaccgtcacgttgcgatggtctgccgagaccagtcagttctcgctcagcggaacatacggagtgccagcatga

>contig_21.contig_21.g946.t1

atgaagtttgctcgctatctggaggacgcctcggtacctgaatggcgtcgaagctatgtcaactacagtggcctgaaaa

agctcatcaagcgcgttgctgatcaccggacgcagcgcctgtcgctagatctctccaagacaacgagcaagtcatcaggtcgccccaaactacacgtc

atggagagcgccgcctccatcttgcgcgttcgacgctctagctctgtcgagcacgggagttctggattcttcgctccgcaaagccccacctacggcgc

tgcaggctctgaacgccagcccgtgcagcgctcgggccagggagaccttgaggcaaatcgaagggttgagccaatcagcctggaagggactgggcttc

acatcgatgaagatcataccctagatctgcatggcagggttcgcacaaatgatacggatcagacggcggtccaacaaaatggctctagtcccacccat

cccaaggaagacctcgcgactgagacaagcgatgccaatcgtggcagccttggatcaccaaccgggtctcaagctcgggcggagcaaggatctactca

cctgcgctttaacgtcgccgacacctctcaaggcgcatcaagcgcagtgtcgagaccgcctttagcagagagcgggtccgcgaacgctgtacgcacgc

ggtccactcaagctgcttcacaaaaggctggcaaatctaagacaaaagcctcgtatccggaaaatctgagccaaatcatccaggaaaactttgaccaa

gaggaaaggaaaatttttctggctcttgactccgagattaggcgcgtcattgccttcctggaagcccgggaaaagcaagctctcgatcgcttcgaggt

acttgctctccagctccaagaactggctcaacatcgaattcagttcaagagccataatcaaggctccaccgtaaggaaagtggcctcagacgctgttc

tcaatttggcatccagtcgcatgccgaagatttccagcgtcgtttcggccctgcctgcacgaataacatcggtggacgggaggcgtgggcttgtcggc

gggcaagaagcaacggaacgacgtggctcgggcgcatctgcgactccagttctttcagatccatcccaaccaggctattgggacgaaggcgctgagcg

ccgatccctcgccctgtcccggatcccgcacttcaccaagggtcaactcttcacggcagcagatgaagaaatcaggcgggcgaatgaggctgctgctc

tctctcatgatccggagcgttaccgaagcgccaaaaagaaaatgaaggctgcagttctggaagagtaccgccttctggagctgatcaaggatttccgg

actctgaatcgcacagcactcgccaagattctgaagaagctacagaagcaaacaggtgtccattgctccgacgccttcttcaaggcacgtgtcgcacg

atcgatgctcgtaaaatcggaagagatcgagcgactcttgaaaagcacagaggagctgtacacgttctttttcgaacacggtgattccaagaaggctc

gcgagcgtttacgatcaggaaacgcctttgcgacactgagcagctcccttcctcaccagtcccaccacatttcagtcttcaaggccggtattttcctt

ggcgtagcgttatgtgcgacggtcgctggtcttgtcaaagctgttgagcctgcgacgaagctcgacatcccccagtggccagcgctgatgcgtgttta

cggcgcgctgttcctccctgcgctctttgcctttctattcgggctgaatttggcagcttggcgcaggacgagggtcaactacgtgttcatctttgaat

tcgacgtgcggacaatggcagattatcaccagtactttgagcttccagcattgttcctcttgatgctgtcgctctgcttctgggtaaccttcctcaat

ccgttcccggacaagatagctccgacaacatggccgcttgtgagtctctttagctcggatcattctgttcaaagacactgtaagaagctgatggctca

cctgtcgtccgcaccaggtgtggatcgtgaccgtgttggtggtgctcctgatgccggtcccattcctttcattgccgagcagaagatggctgacttct

tcctcggagacgagctgaattctctcgtatggtcgtttgcgacattatggttcatcggatgccagtatcaacgccagtggcctgcgattgacacttgc

gagcctaatggcaccttctggaccccggtacttggtgcgctgccccccttcatacgtttcatacagtgttggcgccgttggatcgacagcgatatgaa

ggccaggatacacctgatcaatgccgggaagtacacgaccgcatgggaccttaaaatcgattggggtctgttggaccctcgggcgcgctggccgtacc

tgcgcaatgatctctactacgaatggcccttcctctactacttcgcaattgtttcgaacgtgatcatccggtttgcttgggtcatctacctcataccc

gggccggcatcttctctcatgaagatcttcatcattgccctgctcgaagcactgcggagatggcaatggaacatgtatcgtctcgagaacgaacatct

cggtaacgccgatgacttccgagtcgtcaaggacgtaccgcttccctacacgcacggaattcgggtcgccgaggaagatctggacgaggacgaaggac

cttcgagcaagaagcaacggcgcaacatattcaaactgtccgagaccttcaagcggaaagactctgaccagcactcggatccattcgcggaggagcga

atggcaccaggtgcggagggcgagggtatgtcggatcgcaattcagacgcgctcggaccggcaccagatgaagctcctggtctatcttcagctccaaa

gaatgacgaccgcgacggtgggaagaaagagtgctcttgtttcctcttctcgttcggatacccacccggaatacacagtggcacgcttcaggcgtcct

cgaacacggaacaacagaaccacgtttcgttcgctgcaggcagcagtaggaagatgtcgtccggagtctctgccctcgcgctcgccgcttcctcgcag

gaccccgacgagctcgcctttgcgtctgcgtctgacccgaccgggtctcggtccgatgcctcgctgaaggcgtacttccgtgagctcaagaaggtcat

ctccctctccgacgtcctcctccaggttctcgacgcacgcgacccactcggatctcgatctatcgccactgagagactcatcctctctcagaacaaac

gcatcgtgtttatcctgaacaagattgacctcgttcctcaagacaacgtactcgcctggctaaagtatctccggaatgactgccccaccctcgccttc

aagtcgagcacacagtctcagcgcgacaacctcggtcagaagcactccgcctccggatcaggcgcgctcgctccgcatcagaagactcagacagctct

cggcgcgcagtctctgcttcagctcctcaagaactactctcgcaacctcaacctcaagacatccctcaccgtcggcatctttggcgctcccaatgtag

gcaaatcaagtctcatcaactccctcaagcgcgcgcgcgtgtgttcggtcgcttccactcccggtcacaccaaggtcatgcaaggagtcatgctcgac

aagcacgtccgactgctcgactgtccaggaatcgtcttttccgatgcgggcgataatgaggaccagagcgtcgtccagcttaggaacgtcatcaaggt

cgagcaggttcaagatccggttcttcccgtccaggcgattctcactcgcgtatcggctcacatgctgaacaagctgtacaacgtcgacgtcaggtcaa

cttctgctgatgtctttgacccagcctttcccgctccccaaccagacacacaagacttccttcttcgtctcgccctgcagcgcggtcgtcttggacgg

ggcggtatcccggacacagacgccatggcgcgctcgttgctccatgactggaacacaggaaagataccattctacacggtcccgccgaagctccacac

ctctgctctactctccaaggacaagaagaaggacaatgaggacaagatggatatcggaactaattctattgcggacgttgccgctgccgctgctgctt

cttcatctggcgagcgggcgatcgcagagccgcagcagaccgatctcgaacaatcggcagccatcgtcaactccttctctgaagcattcgacctcgct

ggtctgctcggccaagcagacgcagaattattcgccggaacgggtgtatcgtcttctaaccctactccttctgacggcaagggcaagggcaaagcgtc

aagcaaggcggaaccaacagcctcagactcaacacagccaccgctgtcgcccgccagcaacggcaagtcagccctcggcaaacgtcgtcgcggaaaca

acgcagactcactagcgccagaagacgaagtcgcagatgagccagcgtggaaggcacgccaacggcatcgcgcccaacaatctacccggcaccaacag

gtcggcggatcgattccatctatagtagagcatgatggtgccatggcaaacagcacactttcgcgcgagaatctcaagaaggccaagaagaaggcgaa

gaggaaggacgagatcgagcggaaggaaggtctggctgggcctggcgggttgctggatcagatggagctggtctttactcttgacgggactgaggggg

atcgcgagggggacgagtctcgggattcggaaatgatgacgagtgcagaagctgttggcgtcaaggtgcaacagcaggtcggagaagaggcggaaatg

gcaatggagtcttcagcagcgggcacgggaccagcttcttcgaccaagacgaagaatgatgaagacgatgaggagtggtaa

>contig_21.contig_21.g947.t1

atggcgcgcacaacaccaggaggacaggctgcgcagcgtgcggctcctcgctgtcgcaaaggatgcaaggatcctgaca

acccaatcttaggcgcaatcaaggcaaactgcagctgcagtcgtccaaagggacgtcttcgtgccgatgtgcctaccattacttccgcagcaactgct

cccgccgctgccgcaaattctactcccgccgctgcctcacctccttcgcctgctgccgcagacaccgtggcggaagccttgcgagtggcagacgaagc

tctgagacttgaagccgcacgatctgccgtaccggcgtcggcgtccgcgcaggatcggctctcctctcctccatcgctccgcattcttccgttgtttg

cgcgcacagcacccgtggcagaaggcgatgcctctccttctggttctggcgtcggcgatgaacaaatgctaggaggcgttaattcccctcccggcttt

cccctatcgcgacaggcacacggcacaattcacaataataattctgcgcatggtgatgatggggacgatagggaaacacgagctgcagacatggacat

tgatctcgatgaagaggcagatgctggaaatgctgctgatggagtctcgtcgtcttctgagtctgcagcaggcaataaaaagaagacaaaggaaatca

gtgacgctgaaggcgcaagtatctggctggacatagacaatgcacacgctcagctgctgaaggataaggtcaagtaccagaccaatccagaagcgcga

gccaggtacttgagacgctgtgcgcgaacaggaacggccgtgttcttcggatttgcccagctggagcccggcaagcacaggctcccccattacgtcta

cgccgatcccgttctatgtgatccttctcggggctcgcttcatgacatggcagcggccatgttcaaaactttcgtgaaggagaccgacttctacaggg

agagtcagcgcgttaacatcgtcaatcagctgcgagagcgtcacgagtggcagcagaaggagatggaatatcaggacaagatccgtcagctccaggct

cgcgtcgctgctcaggacatcgctcaagcatcaacagttcaagcccaagatctgcttgccaatgctgcgtccacgtccaacctcgcatcgcttgcaat

ggtatcctccgacacccactcaggcgtgcgagagaggcaggaagggaacgagtga

>contig_21.contig_21.g948.t1

atgcgtaatgcgatagcctcttggggtctcgacccacaatacatctctcttgttctcttccttggtctaccaccactct

cgatatcgattcccggtccggcacgacaagaaacagaccaggtcaagcgcgggtgcaagagggatctggcatcgcttaatctgttcaactggaataat

cagatgatcttctcccatgagcttttgcactcttacagctcgcagctctgtgcctcttctaccccgctctcagccttccacaccaccaccagcaatgc

ctacatcgagcacggctgcacacttccttttgtcgcgcgttcaaccttcaataggatctttttcgccttcctgcaactgcaacaaaaagattcgccgc

tttcctgcccaagctgtggtcctaccccttctattgtcattgctgatggtgtcgttttggcgcacgacacgaagctgctacatgccaatttgcgacct

ccaactactccaggtcctgtggacgatcgacaagctcgcactgggcgtatgctcccatttcttccaacagcagcactacgaaatcaactccgatcaat

ggtggatattgttctgcccctttcccagcactaccccgtccacgtggagtccttccgctcagcaatagacctggccaaactcgattccatcagcccgg

ctcggtcggagtggattcaagcacttgtggatgctctacaggctgccttcaacgactgtgcacgatcctcggaggtagcgatgcgttggcggaacctt

atccagcagctggcggcagaggagggcgttctacagctgtgcaggcctctgtcggtgcccttgctgcgatcccttggctcggacacggtcacgctggc

ttcgaccaccttctcacagcaggcgacctctctggcccgtcattgtcctgcgattggtcttctcttgctggcataccaggatcaattcgctcatccgt

cgctcgcaatcgtcaagatacgctttctgctcgtcaagactgctgctctcgtcgtccaccagcttctcgtgttgggcgtagtcgaatcgaacgcagtg

gaaactcgtcgtccaagcaatccagctgtgacggtagacgcggatcaagctgaggaggttcctttcttccagagcggcgttctctatggagccaagcc

tgtgcgcgagcgcaagtggtattcgaagattgaggataagggtgggatccatgtggatgacagcatacagcaggacaacgaggccatgggttcggatc

actgtcgcaagtactttacggcctatgtcaccgccaaacgaacgggcggactgatggcattgtggtgcaggcacctaatctgcgtggggctccatgct

ataccaaaggcagaaggtaggaatgatgtgttctctgccatctatcggttctggccagtcgccccagaagtgatcgtgtacgactttgcttgccagct

tgcaccctactctcttcatcgtgaaccagagttcttccaaaacacactgtttgtcgtcgaccagatgcatcagcatggtcataccaagttcagtactt

cctctttcctcagtacatatatgggtgtcaatcaagatctccatcgcctcaacagctccgccgccgagtccggtaacgcaggattggccaggatcaag

aaatcggtttcgtattgctctcaggaacatgcacttgcgctggtggaacacttcctgtcgatatggaatcgcaagcgggcgctggagatgggaaacat

acttgtcagctag

>contig_21.contig_21.g949.t1

atgggaaaagcacagcaatgcgagcgggtggtcgggggtcctttccaatcttcaccgatcgcactcgcacccaagcccc

ctgacgggtggcgattcattcaagacgcctccttccctcgccacgaccactcaaccaagtccatcaacgcgtccatcaactctgacgactggccgtgc

acgtactctggcatcgccatggctatatggcagattctgtctcttcccccgttcgcgcaagcggcggcacgcgacgtgcgttcggcatacagagcgat

cgctcttcggcggtgccagtggcccgcggcggtcgtacagtgggagaacgacttctatgtcgacaaggcgctggcgttcgggatgtcgtcgtcggcgg

gcgcgtggggcgtggtcggcgacgcactggccgacatcatgcgcgcggaaggcatcggacccatactcaagtgggtggacgactacctcttcttccgt

ttccccccctccgccctctctaccgtcaacgctcatcgggccgcactggctgcgcgcgtcactgcctcgggccgaaacgggtgcctcttctggcgcga

cggtgagcacgagctttccgaggacttcactcgtccgcttcgccctctcgacggcccggccgcgcagtggaccttctccttcgcccacgtcgacgctg

tctgtgccccactgggcatcccgtggtcagaggagaaggagcaaggtttcggcttcgaggttacttactacggctgcgtcttcaaccttcccgcccga

accattgcgctcccggagcggaagcgcgccaagatgcgggccgacattcaagcatggcttggcgagtcctcccgcactcacactctcgccagcgcaga

agccctcctcggcctcctccagttcggcacacacgtcgtgccaaacggccgaccttacctctcgggtttggttcagttcctcggaatgcacgccgcag

ccgcggcgcggcggcgcgccgcaggacaacgcgcagcattcgagtccgcgggcctccatcctccccgccgcgtgcgccaagatctcctatggtgggac

agcatcctggcccagccagtcgtttcccgctccttcgccaacgacctcgacctgctcgaccccggtctctacaccgacgcaagcgggagcggcgctgg

agtggtgctcgacgaccgcgtcgccgcctatgcttttgacccagcctggcgggacggtggtcgaaacatcatgtgggcggaggcagtgggagctgagc

tcggcgtacgacatctcattgcgaacggggttcgagaccgacgtgttcaaacgaggcagccaaccactgcctcgaacggcttttccggtttgcctctg

accacaacatcaccttcgtttctactcgcgtcgcttctgccgataaccccgccgacggcccctcccgaggcgtttcttctgccctcgctcggctcccc

gttctacctctccccctcgagctcgccacagtcctcacccgcaagtggtgataggacgacggccatcgcggccgccctcttgaccgcggagtaccgca

gcagcagcagcagcagaagagcgatcctctcctcccccacctctcctcccacgcgcagagcctcctcgtcgactgcggcgctcaatcgccgtcagttc

cgaccgcgcccagcacccgccccagcgtctctggctgcgcttcccgccggctctcgcctggtggcctaccggccgtcggcagcggcgcagcagtttgc

gggtctcgaagacgcgcgcctggcccgggtgatggagatggcgttcgcgccatctacccgcgtcggctacggcaccggcctcagccacttcttggcgt

ggtgcgaagatcgcggagttgacgaggagctgcgctgcccggcttccgaggagctcctgactcgtttcgtagccgctcaggccggattctactcaggc

gactacatcgccaagatgatatcagcagtcggcgcatggcacaagatgttcggtgtcccctggtgctttgtttcgggctccagtctctctcttgccct

gcgcggtgccgccgccatgacgcccgcatcttccaaacgcccgccgcgggacccttacacgccggcgtacttacgcgccatcaaacctcacttcgatc

ttcgagacacaaaggatctggcggtgtgggcttgcgcgctctgcgcgttctggggattggcgcgaattggtgaggtcacagtgccaacccagaagact

tacgaccccgccgttcacatcagtcgcgcgggatgggcgctgcgccctccctctcccggttccacggcagccggcaccctgcgccttccatggaccaa

gacgacacgatctgcgggcgcggtggttgttctctcggtccaagacgttgatctttgcccggtgtcagctttgtccgcccatctcaccacttcggcgc

tcccggactcttcggcgcttttcgcatactcccagggcaacaagcgggtcccgctatctcgctccgtctttctttctcgacttcgccgagcagccgag

gcagcggcgctgcccaccctacacgggcattctttccgaattggggggtgtacggaacttcttttgcggggggcagccattgaggacgtcaaggcgca

cggccgttggcgttcggacgcctggaccgtgtacgtccgcgaccacgccggcgtgttctcggatcgtttgtcggtggccccactggtgcatgcccacc

tgggtgcctga

>contig_21.contig_21.g950.t1

atgttcgtccttgttcgcctttcacctcccgacagcgccctcgaccagtccgatttctttctgcatcctcttttggccc

ctgagcacctcgacgacttctctcttcccctgcccttctctgtcgacgcgcaggcgcagcttcgccttgaggacggacagccagtcatttactgtcag

catcgccgctctgacgttctgcacaacgctcttcccgccacgaagactccccgccgcgtgcatacaggggatatccttaccgtcctgtcgtcggacaa

cccaaccactgtctccgtccgatatgccgttcaggtggccttcttctccttctcggccgacctgcgtgcagaagtgaacgtcgagctgcagcggcttc

agtccgcgtcggtgctcgcgttacaggctcaaacatcaccccactttcccctcgcgctacccctccacggcaagcttccaactcctgcaccggatggt

acgaactgggatggagtgcgggatctcatcaagaccgttcagagtcattccttccagaacgccgatcaagatcgaacgcgtgacggcgtggtctgcgc

atcatcttcttccgccctttccaatctgcagccgccgccgtctttggagtctcccacgtatgcacagctgatcgccaacgaatcctccgctggaccgg

catcttcatcatccccgctcgtctcgccctcttcatcatcgtcgagctccggctcaccgttggctgctaccttctcatccgctggacgcaccccttcg

gaatctaccggaagttcccccccttctccactcacttcggtactcccgtcttccgggctcgtagacgggacaagttcaaagacctcgttgccggcatc

cgtgttcatctcgtcgtctggactgcttccatcagcgactgcgcaggccggctcgatccgcgaggtgtccactccaggctcacccaggccgtgttcac

catccgcacccgtaggagcgtcctcatctgctttcgcgccatcttcgaatcctgctcggtacgctacagctcaatcggctgcgtcccggatggcatct

accctatctttggtatcggcggaaactgcccttgcacgcgtccgacaggcgtggttaatgcttcgatatgaggtgctacactctgagctctcggtgaa

cttggtgcgcgctcctggtcgtgatcagcaccaccacgcgaacgatgctgtcgatcctccccctgcggtgcagcacagtccttcggatactatcgatg

tcgtcctatcacgcgttcgccgggcactgacatcttgcattgccgcgcgcactgatgagcattcagcatcgccgatgacggtccctgaaactacttcc

gccgcgacttctcctaactcctccgtccctctgccgggcttcgacgctacaagttacggatcaccttccttcccattgcctggtttccacagctatcc

gccacctccgtttactcccttcatttcgaatttgtggcagtcattacctctcgccgcgatcgccacgaacatcacccttctgctttctggcatcttca

acccgttcggtcctcatcctcttgcttcgccatgccacggtcatctcccggcttcacatcattggtga

>contig_21.contig_21.g951.t1

atggacgataactctgctcctggggctgcaccccagctccaatcacgcgccggctctgcaccccctgacggtgacgcag

agaacgcgcgccgtctcgaggagctggccgagtcgaaccccccggcggctaacctaccccgggcgctcatcacagcaggccactccatcccagccgcc

atccaaatggtgatggcagccgtcgcgccggcagcacctcccgtggtgggccagggaactcccaacggaggtagcgcacccggctcaccagaccgggc

ccgcatcgtcgccgtcggcacgccccgacgccaagagggcgacgtcgcaaacatccgaggcggctcggttcctccaccctccccctctccggcccgcc

agcgtcgcgttcgggaggagctctctccaagcccctccgccaaacgacagaaagcggccactggcgacgacgccgacgccaaagccctcgcagacttc

gagcccaacccgacgtacctcaacccacacgaggtcgacgaggtgtggccggcggaacgcatctgcaagcaggtcaagcgcctcgagttcatctggat

atggcacttcaccccagccgggtgtcgccaggcggaggagcagcgcgcggacggcacagacgaggcgatggtcatgcagtccgatggctctttcaagc

cggctgttagcacccgggtgacgaaagacgagctgctcccagcccaccagtttcaggcagcggctgctctctggctccgggtcgcgaccagaatgaag

gtacccagcgatattctctgcgcaatgactctgctcaacaccaagatcttcggccatccgcactggcaaaccgacccggcaacccttcaagtttggcg

tgcccatcagcgacgttcgtgggccacctatatggccaaccccgaagtcgagaagcccttcagtctccacgccctcaatccgtcaacatacagcagct

tgcagctccgcggagcggtccacgcggcgcaatcagcggagcgacgcgctcagcaggccgaggccctggtcgctgccctgaccagcagcatcaagagc

cagtcgtcgaaggtgcgtgacccccccgcaacgttttttcgccaagccgggggagccggcccatcaacgttcaacatccgaggctcagcctcgtccgg

acggcactcgtactcaaaagcatgtctccgatgcgggggagcagccacggccgaccagcaagtcaacaagtgcatgccagcgcagcgcaccgatggct

ctcttcccatcctcgagacagacccgggctggagaaatggaatccgtttccaggacacccgcgagcgagcgttcaccaggactgcagccaagccccac

accggcgctgaagagccgctcggcccaacccccctactcgccgacggctggcagtcagccctacagcgatggagtatggggacccggtacccaaccat

cgtggacgggatttggaacgggttcgacgtcggtctccccaccatcggcacaacctttgttccccccaaccacaaatcagccaccaaggaccccgcca

ccgtcgccgccaagatccaaaaagagcgcgacgccgggcgatacgcgggcccgttctcgcagcagcaattgtacaagacgctagttgtgagaagagtc

atgggaaaaggtaagaggaaagataaggagaacgaaaggagattagatatggatgccatgtggcagagccacgtggtcgtccagcacatccccaccga

caaaaagacgactgtccctagtcgtgaaggaaagtag

>contig_21.contig_21.g952.t1

atggcatccgatgttactgcagctgacatccgtttaccatcggatactccttcggcacttcatttgtgccatcctgctc

cttacttccgcttcttctcatcaccaacgtacggttccatacaccctgtcagcatcatgacatcccagaccgaacagaaggccattctcaacagaggc

ctcacagaatattggccttctcctagtgtcgtgcagcaactcgaccttcacaagcttctctttggtcgaccagacgctcccggctcgaccctcgtgcc

gctcttggcgaaatatggaccaaagcctgagaagctcttcgtcagtggcgacacgatcacatggacggacacccgcggagccggccctctcggtcctg

cgatggagttacggctgctccttctctctttccagcagctcgagggacaagcaccggatcttgacatcccacatgtcgcgcagcagctggtgccctgt

ttggcctctcatgtcctcactcatctgaccggcgcagccttggagcagagcgaggcgttgcttcaggatcctcaggcgaacgaatatggccggatggt

ttttccaatgggcccagcgttgttgcaccttcaggttcagcaagcaatttcgaccaaagaggttcacatgctaaggcagtttgtggagactcagacat

cggatgcatccaacagtcaacagcagaaggacgagctcgacgagatacgggcagagctgcggaaagcagagcaggcgattacagcggcggaggaactg

gaaacgctgctcgagaaggctcgttctgacgtcaaagaactggaggcgcaggcggtgcgaaaagagacggagatcgtccacgcgaaggatcaacttcg

ggcctctgcagagcaaagcagccaggagatcgccaatctacaagaccgccttcgagaggccgtacgaaagggcgaaaatgcatctgccgacagccatc

tgcgcctcaccacagctgaggaccatctcaaggcctccgaagaccgtctcaaagtctccgaagaccaactgaaggccgccctgcaacaggaaaaatcc

ctcagaactcttcttcgcgaggctgatgcgcgcaaggcctcggacgacgaccgcttgcaagcagcgcgcaactatggcaaagctttggaagaacgact

gcaacaggcggaagaccgggtgaatccactggaggctcagctcaataccttcgagcagcggagccgaacagcacaggttaacacggataagctttcca

aacagaagcaagatctggaggataacgtgcggagcttgacgtccaagtgcgacgaagcgacggagaagctgcacttttccgagatcgaatgcgccaag

ctcagggaggagctgcggacaaagatcgaagcagagaaacggtcggagaacacgaggagatcaaaccgacacgccgggagcgacactgcgtttgctac

gacgatcgcgctggatgccttgaaaaagaacttgaaatcggcagaccaaaaggcgcagcgtttgcaagctgagctggacggcatgcgagcgcagatta

cggagaccaatcgtacagccgaggcttctctgcaagctcctcaagaagtacgtctccttcccacgaaccccatttggcgcgacgcagcccgttattcg

atcgaccctcaaactcgtaccgtgggtgccggtgcgcacgggagggtgcgtgtggtccgcgatgaccttactcgcaatcgtgtggtggtgaaggtgaa

cgagattcctcctcctggggttcttccgcaacatatagggcgcgagatcgactttctcgcagaatcgagctgcgactacgtcgttccactcctggacg

tggttcagttcatggagggacagcatcactttcttggactggtcatgccgttctatcctcgcgatctggaaggggttatctacgacttcgcaaggaac

ctcagcgtcaaagacatcacccactacgccagtcaaatcttgtccggactttctttcctggcgtcgcgcgacatcatccattgcgacttgaagcctgc

taatttgctggtcggaaagaatcaccaactggtcctcgccgacttcggcctggcggagatcagagtccgcccgcagcagagacgggttatcgcatcga

tgaattaccgctcgcccgagtgcttcttccggactgaacaacgcgggcagtcgctggacatttgggcgtttggggttattttggcggagatgatcggt

cgagccttgccctgggatggatccacgcccctggacatttttgaacagatccttcgtcacaccggtgttccttatcaagtggagctgttcccgggtgc

aggcaaacaccccggtgtgttccacggacaagacatgcacagcgaaaggtcaatcccaccgactgggcaagtcaagttcgatccacgccctcggcttc

tgcccatgtcgatcgacacctgtggaacctctcgagatcatttccgtgatctccttgcgcttgtggaccggacactggtcctcgatccggcacgccga

ccgtcggcatcgcgactgctggagtcgaccgcctttcgggattcagatccacggggcgataggaagatgtag

>contig_21.contig_21.g953.t1

atgatcggacgagatctcgacaagatgcattcttcgctgttcacccaggctatcgaagcgatcaagacggcaaacacca

agttttccatccaacacgatggctggacgacacgtaacagacgtgcggcatttctcgcagtgcacgctagctggatcgacggcaacttcgttcgacgg

gaggcatgcatcgcgttcaatcagctcggcgccgaccataccggagctacttttgctgcgcacatcgtctccattctcaaagagaacaagctgtggga

ccattggtcgggagtcattgtctccgacgctgccgagtccaacatccgcgcatcgcgcttcattcaactggaagtcgacagcgaaatcggctccgtcg

tcccaatgcggcacaagatcgtcgactcccttgtcctttgctttgctcacggactcaaccgcgcggttctggacggtacggctgcagcaggagccgag

cttgctatggtcgagaaggacaacgacaaagttcttgcccctacgattatgatcgacgagaccgaggtcgggggcgggctgcgtcgatacactgggct

ggctgccgagcaggagctcagcgtcgaggagaaggaactacaacaggaagaggagcagatgcgcgcggagatggccgagctgatggaagagcctgttc

cggatggcgacggcgaagaaggggacggagaagatgcagatgcacttagtcccgatgatgccgaaatcgtccaagaatacgaggcctcggacgtggac

gaaacttcctcagcacttacccgaattcaccaatgcttggtgaagattcgatcctccccgaaattactgacgcagttcaccgaactcatcaagagctc

atacccaggagagaagaagaaggtggttccgatgcttcgcaatgcgacgcgatggaactcagctctggtcgagatgcgaggctgcgttaacgtccaac

gggcttttacagcgctggtcaattcggacgaaactggcaagtggggacctttctcaatttcgagaccggaatggttggcgatggagaagctcgtcgac

gttctcgagtgcgctgaagtgctcagtctcgatgcccagtcgcgggaagcttcgatcggcgacgtcctgttcttccatcacgtccttcgaacaaatct

acagcttcagctcgactctctcaaggagatcagagaaggcacagctgcatacggcaggaccaagaagaacgagttgatcatcgtagccacgctcatgc

atcccggataccggctcgaatggctcgaagcgcactttccgtcacaggctcacggtgcgcacgatctgctacgcgaatacctcgacacattcgttggc

cgggaatcaagagccgaggagaataccattcagacgaagtacgaccgcttcatcggaagccagcgttgcgctccaacatcttccagctcgcaggccga

cgaagtcgagaaatacctgtcgaagctatacagctggggcgcaaacgacttgcagacaacggacggagcagtcgcgtggtggaaaaagacatgcgatg

ctgcttccgcgactctccattctcgcaagagtggtgttgtcgacccctgcaagtacagcggtagcggaacgcgcattcagaagctgacgatgtcaaag

ctcctgatgctgagcggttttgatggctacggtgaacccgacatcgtcgatgagcttccgcagctcacgcccaagtcagtggcatctgaacaatccga

agttggcaacccttcaacagcaatggcgactcctaactcggcgcaggaggctcagcggaccttgtag

>contig_21.contig_21.g954.t1

atggatttgtcctcttccaaacatccgctagcccgccgcaagtctcaggccgctgcagctgcaaacagaagcaatgacc

ttcacagctatttcgtcgataccggacggattcaatcactgccttccatttcgccttcagcgctcttgccttggtccagtgcccctgaatctcgtacg

ttgggtgcggtagacgcggatgttgctagaaggcaagcccgaagcacaaaatccggtcacgctcttcaagaccggcgccctgcctcccggcagccgcc

ttcgcagcacgctcatcctctttccgggcctggtccatctctgatctcctcctcgaacccctcctcaggagtggaagcgtatagcggatccccgaata

gctcaccacaagttcaggaacagcccaccttcaatttcgacctcggagacagtcatgctgtggacttcgggccgagcggggacgaatcgatggatcac

tgcttcggtctcaagaatcgcgacaaagacggcgaagacctcgatgttgtagcgacatacggtagtctgctattcgacgaacgatactgtcagtggtc

ggcatggatcccggtcagatcggatgattgtctgcttttggttagaatgatcatcggtccatcacctgatctcatccatgcgcagcacgctgaaaaga

cttttgaactcaagctcttggatatcaccgggcaatggttttgccatggccttgagaccgaggccatgggcgaggcgctttcttcgctctctctgttc

cctcaggccaccgcaagctggaaggccttcctggaatacaacggcgagttcatcgacaagaaggagcccgaggaatacgacttctctaaacgacctcg

catcgacaactccatcgtggaggcagcgtttaagcagtttgagagcgatcccaccccggggaactttgacaagatactcatggacatcaagaaaaaag

gtcagccttcctctcggcagcccacctctaccgccggaacgtcatcgtcgctcggagtaggttcaaagctccatgtcccgactcgaaaaggatcggtc

tcccccgtacgcgcaagcacacgagcgcgtacgcagaaccgcagatacagcggcaactttgaactctcgggtgacgaagggtctcgcggacgccaagg

gaaggccgcttctccgtcggaccgatcagatcctgccgatgacgatggagatatcacggttgtcggtcgtaaacaccgcaagaccatccttgacgaca

gcgacgacgaagatgaagacgctcttccggcctcccccgtgagaaagaagcgtaagaagggcacggatcgatccagcgcagaaactgctccaaagaag

aagtttcgcccgacgcaacaggaacaagaagtctacaccaataccagagcatcttctgcggtgcaatcccatcatcgaggatctgcacctcgattgtc

gggtcaggctttgaatgacgcgcggatcgaacaggacggcgatgggcccgacaatgaccaggcagcacaagacgaatgcgagcgccgagggtttgggg

ctgaagaaacgcgcaaagcgcgagccgaagccaaggatcgacagaaggacgtcgttagggcaaagaacgggcgaatccaagactggcgataccctgct

ctcaacgaagtccagagcatcacagtcctgaaatacttcgacccggtttccaagctcgaaccgagtcctggattcatcaagtccatcacttggaaatg

tcgttgctgccaccgcacgtttactgcggctccagggattaccacaaacctcctcggacacctgaacgcgaaaccaagcagagggccatgccccgacc

gggacaatcctttgacaaaaggtgtcgaaccctggatcccgtcatgggcaaaggacgacgagtctgcgcgtgtgcccgaaccagcgaagggacgtgat

ctagatgcaatgtcgcagcggtcattcatgtcgaagtggacgaagtag

>contig_21.contig_21.g955.t1

atgtccaccttccgcacccaactcttcccagacgcacccaaggaccgcttcacgtccttctccttcggccacatcatcc

cgccttccagcctgcacgccgtcttcctgacaaccgcaccccggactaaccgcgtcttcgagttcaaatgggacgtccggccggataccgccttgctt

aacgatctgggcacgggtgccgtcgttcccgggggccttgccgtctttgtgccgtcgtatgggttcctggaggccggccaacccagctcagacacctt

caacatctcgctccgccgccacaacacgggggcactcatgtttgccgtcgtcggtgccaagctgcccaagcacatcaacttatcggacgacctagccc

gtacggccgtcaaggtcgacatgccccatacaaatgcggcgaggccgaagctggcagagcggatgaggtatgtgagggaatttgcgaatacttctgga

ggattggagaagggcgcgtctaaaccagatgcggctgccctgatcctcctcgagcgacactacgcccgtcccgacatcaggaaccgtcttcctggctg

gatccgcaacgaagtcaagacggttgagcaattcgggcctgccatggccggcttgggcgccttctttcgcgggaagaagcagcaacaggatgtcattg

tggaccgaccttgtcctccgccttctgccgcgcgcctgggactattaatcgagcgctgcaacagtcctccaggcctccataagttgctccgctccgtt

tccctccgggcgcccatcctaccgccctcgtggttccacacagcacggtgtgctgatggtgcagtcgtccagatggccggagacgggagcgctattct

gagcaagaggcactga

>contig_21.contig_21.g956.t1

atgctgcacagacatactgacttggctctgcctttacttccccttgcagacgtggacgagctcgagctcaccttcctcc

ccaagcagatccaggacgtcagctccggcatggtcgataaccagaccctcgacaacttcctcggccgcatccatctcttcctcctcaagatcggtatc

gaccccgcccgtccccgcttccgacagcaaatgggcaaagaggtggcgcacaacgcctccgactgctggaatgccgagatccacacctcctacggctg

gatcgaatgcgtcggcggtgtcaatcgtgcggatttgaagtacgacgaggatgaccggaggagaagccggatcggctgcggctcgttgctaaggatga

gaagggaggagcgaatgcagattctcgaatacgaagcgaagcgatcgcggcgcgagaaaaaggcggaggctgatgcccagggtgacggtggcaatcgc

tcgtcctcaccctcatcatcatcgtcgtcttcgtcaagcaacggtaacggcagggcggtgacggataagacggtggcggctaaagctgtggagaagga

gacggtggccgaatcaacgacggcgaacgaacaggaggaaggaagtgcgaagctgctggtcggatgtcagggtgctggcgtcgacgtcgaggacatcg

gtgggttccctgcagacaatgagaccttcaacgagcgcagcttcacgccggccgagatcgcctactgccgcgcccaagcctccttctgcggccgtttc

gccgccaaggagaccgtcttcaaagcgatgggcgtcccctcaaagggagcggccgctcccatgcgggaagccggtacccatcctcgccctcgcagtgg

tgacagccgcaaaaggacttcgatggagcgacttcctgcgttcgccgaagctcgaaacaatttggcgaactaccagtaccttttgccgccatctgctt

cccggccggccgcccggccctgtggcgatgaacttttcgctgcatacctcctccgccggagcatcacctgtgccaagatcgactcctcgggcctgcaa

tga

>contig_21.contig_21.g957.t1

atggcgggcagggcgaagggtggctgcgccaccctgagggcgcgaccgcgcgtacctattcaatcctcagtggctattt

tggttcagctcatcaatcccactcctatacactcatctcgagttctcgacgcctcccggcccgtcgcgccagtgcgtcagacgcgtcctgcacaacct

ctgcgctccctcgcccgcaccgcatccgcagcgcgccggtcgtacagcgccgccgcggtctcggacatcccaagctcgacttcaccatattccaccag

gcccgctctctacatttccttggacgttaccggcgacatggatatcctcgcagtgcacgtgccttcggttgaggagctcaagacggagccacccagtg

acgacttcctcaccaaatacataaaccgtaccccacccaacctggttatatgtgcgcagccttcgcccttcaacgcatctgctcctggtcaacgcaac

actttctgcttctcttttcctgcaccgtgcacgactccgctctgctatcgttcacgcttccttttgctatttgcttacgcttcgtttagcggcatcca

aggtcagaagagtaactttcgtaaacgtctcgcaaatcatccatggaagtcgagctcaactggttgtccgcagcacaaacagccaatgacgcttttga

ctcctatcggctacaagcagcatttttccaacatcaccgccatagaaggcggcggcggcgagaaggcttcttccaggattcagcccgcttcgacaagg

tcgccttcctcaaggagggcggcagcacaatcgatattaagggtatcgggaaccgtaaagtgtgctaggcgggaaggtggatccaagggagagaagct

tgcagcagggactatggccaccgtcttctgggccggcgacatcttcggctacaagatccgcgtcccacgctcttgctctgccggccacaagatgacct

ctgcagagcaggagcacaacgacgagggcaaggacgggaaggaaatggtcgggttgcagaccttcttgcaggatgcgtatcttagggcgttcggggag

ttggccgatgcggttggagggttaaagagcgtgttgggcttcgaggtcatgaatgagccccatcgcggcttcgtcaatctgcactcctttgacgcgtg

ggactacgataccgacttgcacatcggctaccttcctttgttcctccaggccgtcgctcttggcgaagcctccgccattctcgtcatcgtcacttcct

ccaacccacccctctggcgacccgacggaccctcccgcggacggtgcatcaaggaagctgtctcgggcaagccggatgcgctgcgggccgactacttc

gagtacgatcctcgcttgctgacaacggcactcgacactcgtactcatgttgacccttctgtcacctgctccactcctactagagccagcgcatcagt

cggcgcagtcaccagtgcaaactgcaaatgggtcggcttgtttgccatgaccctcgttggtctccacggcatggaggatctgtgggagaagtttggtg

atttccgaattccagtgggaacatatgcaagacattggtttgtcagagcaatcgctctcatcgtaatgcccgccatcatctacatggtctcgttcaag

atccacttcgtccttcccagccactccagtccaggcaactcacagatgagcagtctcttccaggctggtctcaagggcgatgacttcgcaaagaacca

tgttgaggctgcctacggtagcaaggtcacttccaagaacatgcggacctcggcggtggcttttaccgcgtccccctcgtactacatcgtcggctcag

ccctctccaaacctttccgcgcctatttttccgctgcttctctcggtctcgccggtcccctgtacgggagacaggaggttctcggctggaatgtatac

atgctcgaggacgtcggtgcgaacccttctcaagagaagatcaaggagtacctctcgtcaactatctggctgagtcattcctggcccgaactccgctt

cgccatcctgacccgatgctacgactcgcgcccggagctggaaacttcgcctctcgtgcagctcgcgaagctggttttcgaggtcgcgcacggtgtat

tgatcgagcatggcaagacacaccatcatattccccctccatcgtcaccacccagccccagcaccagcgtagccgagccaacagccccaatcccgaac

accatcgtacaagctctcaacaggccatacaaacctgctttccctcccgtctccgccatctcagcccccagccgcagcggcagcggtctcgttgacga

cgacaagctcattcctgaaatctga

>contig_21.contig_21.g958.t1

atggtctacaccaacagacaacgccgcggcagcacaggcagacgatgggcggcctggcttacgatcttggtcacgctct

ccaatgtcgccgcagacattgaagttcagcggagactgtcgtcacaacttcctcagctcggccatgttggtctaccatattcctggacatttgcagat

gacacattcttggcgatgggggacgatcaagacagttcatcaggttcctccacatccccttccccgtcctcctcggactcgatctcattcgaggtcga

gggactgccctcttgggcctctttcgacgcaccatcacggacgttcacaggagcaccacagtctcacgatcaaggccggtcatcagtaaaattgactg

caaccggctccgacggctcatcgacgcaagatagcttcagcctcattgtcgtcgagggtccaccgcccaccctcaaaatgcctttggcacctcaacta

cctacagtgaacggcctcggttctcagtacatccttcccgatggctcacaatttgtgccactcggatggtcctttagcctgggattctcggggtacag

cttccaaatgccaaattgggagcacatctattacaccgctacccgcgacgacggctctccactcccgtcatggctcacgttcagtgccgaaagcttca

cctttgacggagtggcacctgcgtcgcccagccctgccgacagcgcctttaccgtcgtgatcaccgcctcaaacatgcgcggcttcggcggtacgtcg

gactcctttcgaatcgtcctgaccaagcagccactgaccctcgcacacccactgcgaccggtcaacgccactgctggttcatctttccactatgcagt

accgacaagcggggtccagctcaatggaaagcaggcaccacaagacgattcgatcactgtcgatgcgatactcagcccttcatacccgtggctctcct

tcgactctgcgacatcaaccttgtcgggaactccacctttcaacttttccacttcgaactcgaactcgctggctcctctgccggtagaagtgccagtt

ctcttcaacgaccggaccagcaatttcaccacgaacacaaccctgacgttgaacgtgttcccgtctatcttcaccctcgaaacaataccaaacatgaa

tatcgacactgggatgccattcaatatttcctttcaacagttcctgcgcgcaccattggatgcttccaccgtacagctcaacctgacttacgatccag

cggaagcgagcagttggttcgtattcaacacatcgacattcttcctatcgggccggacacctgcaaatgcagtgctagacaaggtcaaggtcagggtg

gacgccattagcgatgtttccgacgacttcacacgggccggttcaaccactttcttcgttggcctgaacgggaatgaacctgatcctacgagctcagc

gactgatggccaccatcaggactcacacgggatgacggacagaacgcgcatggccctgggagcatcgttggggacggtcggcggcgtgctgctcctat

tcctgctcattttggggtgccgacgatgggtcgcggacgaggcgaacgaccacgataatgatgtttacattcataaggacctcaactcctttgaaacg

gatgataagggcaaatctccggccatgagcggtctgtacgtgccgcactctcaactccggatgtcgaagggatacaatgcgtcgcaggtcaagatcaa

gacgagcccgtatctagagcctgccagcatcttggtcgcaccggtcctgtcggcgaaccccaagacgaaggatggggttgtagcctgggcagatagcg

atcaacattcggcggggttggagcaggttgtggtggacgatggcagaggcagtccgacgttgaatgaggcggtcgcagcatacagacaagcggaggct

gtggcgatgtcgaatgcggctcatcgcgagagaccacggccgaacggtttgctcgctgcaatcttcacgtccaagaagccaagaacgtcgcagagagg

cgacggtggtctgatcacacatgggccgtacgttgcttctccttcggcagggctcgggttgtccggtctcgggatcggcgaggatgtcgacgatcacc

cagataacggtagcggtcgagttacacattcgcgcagtcgacacacgattcagtcccggaagagcagctgggaagaagggatctggcacgatgatgat

cctcgggcggcaggtggtaacaccaacaacctacgagtacgagacgatgtgctcgcaagtcccgagagcggacagtcacgggcaagcactgctccgag

catacctcaacggcgtggcggggccaatttactgtttggaaacaagagtgggctgggacctatgcgacaccggaatagccacatcaacgacagccccg

ccttccagacgacgggcacgtttgacaccacggcgagcagcggcgagagtggaatggcgttgtccgagtcaaggcagcggatgtttgacgaggcacgg

accgggttggaacagcgggtgatgagcaccggccttggactcagcagcgccgctgatggaaacgcaagagatggacagaatatcatgaacgtgcgttc

actaggcagtcggcatccgatctcgatcgaggaaagcaagttacggaacgtcagccatgcggtttcctccgaaggtgccttcgaggatgcggacgagg

acattgccagcgctggaatgcaccaagcgcaccaacaatatcactatcataacgacctggatgacggcgaagatgacgatgtgctccagcaggaactc

agcacggtcgagattgtggcagcgggtcgagcgctgacaaccgagtcagcgacggccgccccggcagtacaacggcatagcgtgctcagctacacgac

aaatcacagcagtgacgaggccgtccaaggtgcggcgatcttcgctgaggcagctctgaagacaatcccgaccttctcccttcggccggaggagacta

tgcgtgctgtgggcgatgcgaagatgttccggtactag

>contig_21.contig_21.g959.t1

atgtcgcttcagcgcgttccggccaaatccatatatccgggtcttttctccgagcttctgcaacttccatactacaggc

gcctgtcaagaaacttccctctcaccaacaacaccaagcaatcctgtgttctgctctcctctcgccgatcatccacttggtggatcagcacgtcactc

ataggcttgctgttgcaccttcaatttctctcgtcttgcttttatgcaacattcgacgatcccgccaaccgcaagcttcctcgatggcaagttacagc

aaatcaacctcaagttctctgcccaacccaattttctcaagttctggtcgcgcttggcattcgctcttgtctttcttcgtcccgtcgcctcaacaaca

tcctttgccctgcttcggtctcgccttcctcgggcttcaagtgcttccgggttcggtctgatccacgagtgaccaacgttctactctcgatggtgtcg

tctccgtccggtcggcttcgcacctccgcctctatcaacaagttcgtactggccaacgcagaacgtcaggcagctggcgaacatagctcgctcggctc

ggccgaaatgcaggaaggcgctgggaaccacaagcatcaacgcatcggccttttctttttcttttctccttctcgagcttcgggccctcaattcgatt

catctcccatggttgcggacgagcgtgagcgcgtccggaccttcgacttgccgacccccaagaccgtttctacacgtcacacagacacctacgctctc

cacgtacatttgcaaagcacgctgccctccccacatgcctccaacaagaatcccatcgctctaccgctttga

>contig_21.contig_21.g960.t1

atgcagcgaggcccgcacgaaagccaagtcggaccaaatcaagaaggcagcatcggccagggacgcgagctcgagcaac

aacagaatgactcccacgatagcggaggtgcgcatagcattgcgtcaacagacggtcctcgcaaggaacgtatgaacagtgcgcagcccgtcgcatcc

gctttgcaggatcactttgctgccaaccgcgcaggcatgcaacactggaggtctgtcacggacaacgccaggccactgcgctccgtcgcgcaccccag

tcctcctcatggcatgggtgactctggtgcgggtgcgggagggcagccgcatcctggccaaggtcctccttttggtggtccccttgacgtacccttca

ccctttctccttctctccgtttcgatcgatctcgttcttcagcatccgaccgagcttcggcagcgtctggagttaacgactcggcatcatcattcggt

ctcacaagtgattacacttcatcccggacgttcatcagtccgccctccacgctttccactttcagcgaaatcgtgccgcacggcaagcatccgcagga

gggcaatagtggcattgacaaccggatgccatggcagatgtcagtcgctcagcctatgtacgagttccttctcccacccgctcattcggaacgcggca

tgatcgctggtccccgtccgccagccaacaacgtcgccgataagaacatccccaatcgattcccgggtcacatggcaggagcacaaatgcccaagcac

tttttgccgggtggctaccccgcagcggaaggggaacggaaagccgaacctggacgtccgccttcagtggctggtggatggacggatccacagatggc

tcgcatgagtcctcccgagtatctgccgccagactactttcaagttccgggtcttcctcttcccccgcccaatatggcaaggcaggcctctggtgcgc

agggtggtggtgcgcctggaggggcacaggaattcagtatcttcgtgggtgacttgagcctggatttacgcgagaatgatctagtcaaccagttcctg

cagcctccaccttggccgccttcgcatccgtttgccatcgtgcacgcacatgcgcagcaagcgcagggcaactacaatccaccggcacgcatcggtcc

cgctccattcttgtccacaaaatctgctaagattatgaccgacccggtcacgggcatgtcgcgcggcttcggtttcgtccgctttacgcaagaggccg

actgcgctcgcgccttggttgagatgcaaggcgttgttgtcagtcctgtgcacgggttttctcccggccggcctttgcgtgtgtgcaccgccactccc

aagaatcgtaatcccaattctggcccgcagttcccggagcaagagcctaaccacgctttctatggacctggaccaacgcctggacactttggacctaa

cttcaatcgacctccccagcaccagcctgctggcaaccagcctggcatgccgggtatgtcgccgttgcctccgatgcagatggcttcctttggaggcc

ccggtatgatgccgcgccctggcggtccctatcccgggcccgagcaaatgcgagacttgcgagcgatgtctcctgccggtggtcagccacctgctggc

atcatgcaggcacaggctctggctcgaaaccagccccatatggaccggccgcccgttgggttcggtcaggccttgagtcaactatccatgccgccgca

cggtaacgtccccgataatggatccaacgggttcccgctgcagatgggttcgccatcgtccgccatcgggccaagtagcgcagcttcaatcctatctt

cttcgagcgccctcgatccgacaaacacgactgtcttcgttggcgggctgtcgtctttgatatctgaggagacactcaagaccttctttgtgcccttc

ggcgagatcacctacgtcaaaattccgccgggtaaaggatgtggcttcgtgcagttcgtgtccaagaccgatgctgagcgcgccatcgagcggatgca

aggtttccccattggcggaggtcgcatccgcctgagttggggtcgtagccagggtgataaagcagcggctgctgcagctcaggcggctacgcatgcgg

cgcagttgggccagttggctggtctggcaggcctgagcggtcttagcgcgacccagttggctcagctcgctgggctcagctcggtcttgagcgcagtc

cagcgtagtgcgtcggcaggcaacggtacaagcaacggtgttgaccccaacacggccttgctgcagcagattgctgccaatctgagcggcgcgagtcc

gccgtccaacctcaatggcgctgccatgcccacatcgcctcagcctcatcagcagggatacggtcagcaagcccatcacatgtcgatgcctcagcagc

agcaacagcagcagcagcaacccaagcatcagtctcctcctggatcgttggaccttaatgcttttgccatgctcagtgattccctgaatcactcacag

ctcagctccttgctcgggaacctcactagacaggcggctacggacagcagtggcaccatcagcaacagcatcagcaacaacaacaacagcaacagtca

caacaacggcaacagtcacaacaacactggcaactccaacgagtacaatcagcatgatattgaccacgacgccttgactgctacattggcaaagatgg

cgattgaccctaccgtgctcaaccgcctgcgggacgcgtccggcggcagcgactttggcaactccaacttctcccagtctggtctgagtgggggtcga

ccctctccattgacgaatttgcttaagtcgtccacgttctcgccctttagtccggccatctctcctgtcgtcggtggctctacgcagcttcctgccga

ggactccaacggtcaagcgggccatgggtacgatcctcgtgaccatgtgggccggcacaggctggatcagagggagtttgaccgcgggcaggatggtg

aagaaaatgggcagcaccgacagcagcagcagcatcaacagcaacaacaacaacagctgcagcaccatctacagcagcaacagcaacaacaacaacaa

caacaacaacaacaacaacagcagcagcagcatcagcagcagcagcaacagcagcagcagcagcatcaacagcaacagctgctgcatcagcaccaaaa

ccagcaacagctggcaaaaagtccgagcctggccgaagcctatcgaaacgggaatcttggttcactggatcacgacgagatgcttcgagtcttgatgt

ggcacgagcaatttggtcctcaatatcacccatcagggccagcccagggctttgcagggcgatcacaggagatggggtccgaagcggcgacgccctcg

tctcggtcgcctcctcagggatcgttggaccaggcttaccggcagaatgacaaagagcgagggccttcgggacactcggctgcgacgccgcttgcacg

ggcggcggaggtgttgaggtctcctccgccgatcatgctccgcggaggcatgtcgcatgatcaaagcacggacggccaggctcatggattttga

>contig_21.contig_21.g961.t1

atgaccatctcagaaattccaagaccaccgtcgctccagcggacctgggttagcgacagtgccgccgagctaccaacct

tgcagaccattggtgaccttttacccgcacaacagaaccgggaacaaggagcagggcccggacggtcacgatcgaaacgcttgccatacctgtccacg

gcgcagatatcgtatgcatccgacacacaacgagccgtctttgaaagtccatttctatgcttccctcgctctcgtagcgctagacctggcaatgccca

ctcatcgtctgactcgaatcgaccgtctccaccgggctccccgaccacagatttcgaagagctctctgcagtcctcaggctgatcccttctacgagcc

ggatgttcccagcctctggtgtcgccagttctccctccctcgttcgaaggaactcgtcgtcaccgagtccgctgtggcagccatttcgaagccaagct

aagtctcgatctcaatcgcgttcccgtgctggaagctcgtccggtcacctgtcagatacaactttacgtcgtactcagacttcggatcctgcgccgag

gagaccatcgctcgcaatctccattacgaacaggtcacacgtatcaaccatcgacttggatcctgcttggtcatcttctgacggtcttcatacaccga

gttcgcaagtcacagaacccacgccagcgataccggccttccaagatggagaacacgaaggagaagaatggcttgcctatcctgatgacgatttaata

ggcagacaaggcgctgaaacatacgtcaatcgtcgttcatggggcaccttcaggagggcaatgtccacaagctccgcatcttcatccatcaaagggtt

tccgatcgacactggctcccatgttccggctgctgcaggtccttctgtgggatcacctcgccgtgcttccactcgcatggagtcatggaattcgggca

ctcacgacgactgggctggcccaccaacagccaactccgataggttgaagcgaagacggagatcgaaggcaacgcacgagggatcgaagagccccagc

acttttttggcttttttctgggacaaggacaaagacaagactcagccacagcgcacagtagcctctgcgcaggttcaacccatattttcggacgatat

caaaggccgtctcgattctgaaggctcgaagagtcaaggtccggatcgccgcggctcgatgaagccatcacacgcctcggagaaatgtgtgcagtcga

tgcctgctgcttacgacgtttcgccctctgcacctcgcagtatagtgtctctgtcggaccgacgaaacgttatgacagcgaggtctgtctcctccgca

tcatcattggcgtcttcgagctatcaaatgtctccgcaatcgagcgccggtacaagctcgtcccagtgcatgtcgtctattcacagcctgaacgtgga

cttcaacgcgacgagcagtttccgtgcaaacagtgttcgcagcaacagcgcaagcgcagagtctcacaacgaccaccggctcagtatcgtcaacagct

ctcagacgaaaactggggacagcagtcgttcaagcgggtcgcactccagaacgaagggaccaaacgcgggtcaaatcaagaagaagtcgcccggcgct

gcggcttcgactgcgattatggaccccagcacaaaggcaatgctctcggccgaccaagcctttgctgccatgttcccttacgacgaaagcagaacact

gcgccggcaacgacagaaggccgacagcatttcggttgcggcagagaagctcgaacgtggctggaatcacgcgccacaaccacactcgtcctcgtttg

gcgttcctgatgcaaccggtcaccaggattacttgacccgaatcatttctccgaaaacgacggtggtgccggacgctaaggcagcccagctgaaggcc

tcgacgcttcgccggcccaagacggaagatggcagcggtggagaaggtacacggaaacagcgtggtggatttggcactcggttttctgcatggttgga

taagatctga

>contig_21.contig_21.g962.t1

atgcagagccccgcacaaatctgcccacccgctgaggaagccccgggacggctggtagccgagagcggtgcgagcagct

caccatactcacgaccaccatcaatcttcgtcttcaactccatctccaaatcctacaaaggccgtgggccacttggcaaacgcatgcagttcttcgga

gcgattggccatgagagcagtgcatgtaatgagccccacacgacggactcgaagcagtgctacggctgcggccagaagggccacatccgctcggactg

ccctaacctgacgcgtcctccgcccaaggtacagacccccaacgcggcccccgtcgccatcgcgggcacggcaaacgccaacgtacagtgccacaact

gcggaggtcgcggtcacatcaaggcgcagtgcaccagcccggccgtggtgccccaggtccaggttcaggcccaggcccaggtccaggtccagtgccac

cggccgcccggcctgtggcagccaaagcgtgctaccgttgcgctcaggtcggccatatcgctcgcgattgcccgtcgtcggcttgagctgtctctcgg

tctcagttctcagagaacaggtcgcatcctttcaaagtgcctcaacctcgaggccatgggcgctccgtttcccgacttcaatgcggaatttcaccttt

tgcccttgctgcggccgttccctgaggccatcgccgccatcgccccgccacaggcagaattggcctacctggaagctcatcgacagcctcgcattgcc

tttcgaagcaacggagtcgactgggtcgtcaccacgcacgtccttcctgccgcttatcctcgtatgcatgacttggcaacgtctcccccttcgaagcc

tctgttccccctcggagcgggcatggacgtaaagccgaatgctactacagacgaggttcgtgaagcgcagtgggctcttatcgaggagtgcaccaggg

acaatccgccgcccacctactacccaccccgaacgaagctggacgctgaaatcgacgctgaaaagcgaagggcaagccggcagcctcagctatggact

gtggtggaacggatcgttcccatgcgaaccgtccaggagcaacgggcaattgatcgagccggtcaggttggactaaccgtcgtcgctgctcatgcaaa

tggaatgcacaaggagacctacgaacctttcttccgagctttgaccgaacagctgaaggccaaggtagaggacgcgactccccggatcgacgagatct

ggagtatcgacactatcgattctggtgacggtggcttgctgaatggtcggatcttgggacgatgcggccattggttagaccaaccccgtgacatcctg

aactttctacagtatcacttgccacaagctaccgcgagcattgcggagcaaggaaagtcgacgtcttcagcaaagaagcagagcggcatccaggctgg

tgccgcgtcacaaccggatgcaggcctccggccgacgtggccaacccgcatcctgccacgcttgaacaagggggaaggccttcgaggcaggagagttg

tcctgatgggtcattcgtacagtggaaatgcagtcgccatgatcagtgccctcgagccgaaacttatggagaggaccatctgcatcgaccctgtcatc

atctctgtcaaatgctgggaggacggcatagtaggcccgtggccatcacaggccttgctgggcagaggagccgcgatcaggcaagaggtgtggccatc

ctcggctgcggcactcgcttccttcaagaagaagcccttctttactccgtgggatccccatgctctcgaactgttcgtccgttttggcctccgaccca

ccaaacccgatccgtctctctctggctctggcgggccacagacgctggctatgaataagtggcgggaagccgaagccttcaaccagaactggttggcg

cgatactgctgggccctgctggaacgaggccgcggaatgtcgtcggagacgcgttctccgcttcatctgttcacaatgtcggcatccacgttggccgg

tatctattgcgaatcaccggacacagcagcccagcttaccaatctcttccgcgcccaaacgagtgaatctacggccaggcagcttgagggaggacatc

tggtcgtccaacaacaacccgccgttttggccgctgcgatcatcgacatcctctctggctcaaggtcttcttcaactgtaccacaggcaaggctgtaa

>contig_21.contig_21.g963.t1

atgtcgtcgtcgtccaaggtcaccaaggagtccgtccttaagcagttccgcaccattaccaatgcaacctctaccgatg

cgcagcgtatcctcaaggcacacagctatcgcctcaccgccgccctggacgcattctacgacgatgcgggcgcggtccagaacgcatcgaaggcaggg

agcggaagcggtagcgcgtcaaagaaggccgagaaggagagcagagatcgcttgaatgcgctgtttgatgagtacaaggacgaagacggagagaacat

caccatcgaaggtgccatggagctctgctccgatctcggcatttcgcccgaagaccccgccatccttccgctctccttctacctgcgcagtccttcct

tggggacattcacccgagaggaatacgtcgagggctggcgcagccttggatcgggcttggacaccaaggagaagcagaaggagtacgtagcgaagacg

ctatcgaaagagctgcggcaggatgcacctgtgcgtggacccttggcgacgcagggtaagggtggactgtatcggcgggtatacgagttcacctacac

ctttgcacgtccagaggggcagaagagtcttcctctcgattccgcggtggctttctgggacttgattatcccttgcgcgcccgtctttgagggcaatc

acgatatgtctgggacaccgggggagttctctcagagacagctcgatctgtggaaggaatacctctccgagacgatgaagggtcgggccatcagcaag

gacacctggtcgcttattaacgaagacttttccaatcatgacttggacggagcctggccgtccgtgattgacgactttgtgacgtgggctcaaaagcg

aagcgcggcgaacgacgaaatggagtcttga

>contig_21.contig_21.g964.t1

atgccaccgtttctggctcctccgccagaagacacgttcgattcgaaggaagcggcaatgaagtatgcgcagaagtggg

cgcgagctgaaggatacgcggtcgtcacacacgcgtctgcaaaaaagggtcgtttcgtctaccttcgctgcgataggggaagcacatacaggaacacg

cgcaagctccaagacgacgacaggcagcggaaagtctcatcaagcaaggtcgactgccaattccgagcgcgcatccatctcaccgacagtggatcatg

ggaactcaacgtccaaaatgaggcccacacagatcatggcccctccgaatcctgcttgtcgcactcaatccaacgccgattcgatgctttgacgacga

acgtgtgctggaacttgctcgatccggatctcgaggacgtttccaacctcatcgctcgataccagcgagaactgctccaaggtcgccctcccgccgag

gcgctgctcgattggctcaaagagcaaggatggccgcacctggagaagactgttccggtctctgaagacgcaaatcgcctggagggactcttgtttgc

gcatccccggggtctggagcttatgcatcgcttcaacacggtgctcgttattgatgcaacatacagcaccaataaaaatcgacttccactcctacact

ttgtaggcttcaccagctccaatcggagcttcacggccgccctcgcgtttttgcccaacgagacgacgatcacatacacctgggcattggctgcattg

cagcttctttgcccgacgtgggagccacacgtcctggtgacagacgaggacgctgcattgagcgctgcaatcctcaacacgtttcctcccacagtttc

gcaggtcctatgtcaatggcacatcagacaaaatatgcagaaacacgcgcgtcgctcccttctcgacgagcaggcgtacgaactcattctgtccgatt

tcgacgaggtacgagacgcgcagacgttggaccaactcaccgaggccaggaacgcgtttatggagcactgggaggactcgcacccgaagctctgcgac

tatctggacgagaaattgtcggccgagcacctttgggttaacgcatacattaaccgccatccacacatggggacgagagtcacttctcgcgcggaggg

agcgcacgctaccctgaagaactggctccattcccgccatggaagtctatttgtcgtcacccaggctctcaaaaatcacttcgttgtccagcaaacag

gcatttcttccgacctcgagaatgatcggtccaaaattccagtcaccctcctcggcgatccattcttcgtgcaagtttcgcgtacagtctcgcgccat

gccctcaatctgatcaaccaattcccctggatttcatccaccctcagtggcgcactgctgcagggcttcttgacgcacgagttccccgacttctggat

ccattgtcgcccagccaacgcagagtcgcaaatgaggctgcggccgcgaccacctcttccggcagaatcctcacaggaaatgaggctgcacagcaggt

gcacactcgcaggtcccgtcggtgcaaaaactgccatcagaccggtcacaattcgagcaagtgtccgttaccatgtgggcgctgtcaaggagatcatc

agcttggccaatgtccccattgatggctga

>contig_21.contig_21.g965.t1

atgatacatcgcaactggtcggtcacaggcttacttcaagccgtttccagctcccaggcctcggatcagcaatcgacca

catcaatgcagtccattgccgaatctccaggaccgcgaaacagatcaaatacattcagcaatgactccgactctgccagcctccagtccaacacttta

ccaagaagaaaaaagctcaatgggctgatgcatcgttccacaagtggctttcacgctcttattcgagggttgctccccgaaaactcggcagacgcatc

cgaagcagggaagggagcgccagacgaccatctgtatccaccctcagaactcgtccacgaccgaccgcgacggaaaagcgacagcgctgatgaattac

tcagagatccgtcaaggccaagaaagccgcgcggtattgttcagcctgcttcctacgaaccacacacgctacgatcgggcagcgatgttccacccaac

tcttacacaaggatccaaccacagcaaatacctggacctcgttcagaggctgagttgagatataggaatggcggagcagaccgaccgcctccgccgcc

acaagtgcggctaccgtcacataccagagaagcttcaaggaagcaatcgtcctcgacgcttcaaatgctcacgccggattgctgcaacgccgctggtg

tgaaaacacccaccttcggaccagttgacgccggtcgtgactctttctttggtgatgaagggcggggcaagcaacgtattcatgatatacaggccatg

ctggacggctaccagccttcggtccagggcggcttgggtcttgctctgacgagggagctactgtaccaacaatcccggaacaacgctagatcgcagtc

tcactacacacctctcggacgggattcaagcacagtccagcgaacattgtcctctaaagaggcacatccacgcactcataaatccctgtcctcaagag

ccgacctcaccaaattaccgcctttgccttcgccgccggctcacgctacaccgtatcagttctcattggctggcaactcaaccttgtcgttgaacagg

gatgttatcaccccatcctccacgccttcaggatgggtggagacgtatcacgaactggggtcatccaaaggctcggcaggattggccggagtacccga

cgaagcgcgttcattgctgcagcgtcgttctcgcaagcctttggaaatcggattgtttcaggggtcggacaagcaagattctgctaacggacagacgg

cggcggtgtcaacaggctggcaatcacaaggttcaacctcgatgtcagcaatggtcacaagcccaaagtctccgggaagcttgcgttcgccgcctcct

ttccctcctccacgcatgcctctcccagcagtcccgaagagtggatcgacacacatcccaatgccccataccccgatgcccaccctcttacaaccgga

cacggctggatcgagcaaaacgttctatggcaagcattcgcctcttatcggctcggcgaagcttgctgcgacggaagtggagggtcggagtcggtctt

cttcgctggacacaccctcggctgctttgtatcgacgtcagggccaagcttcgggcaggggccgatttgattcgggcagcgcgtgtaccttcaccccc

ctcactgccctcccggccccaatccggcctccacgagcacaagacaatgttgcctatagagagcatgcgccacaggtcgagctcgctttcctcgccaa

atcatcggcagcccccttcaatgcaacaggaagtgagctcggtagcctcggcaagttgcggaacgacctgaatgactacggtcgggtatctctcgaaa

attcatctccgcggccctcgagtccaggctatacttctcaacccacgtacagcggaagtgaagatgcggctacatacggggatgatgctatgcgagcc

caagaggctcgcaatctgactaggcgtcgacaactttcgtcaccgcccctggtgctgcatgccgaagccaacaaccctgctctgacacgaagtgattc

gtcccgaggccggcgttcttccatcagcatgctaccgtacaagaacgagccgtcatggtcattggatcattcttctcctccgcaagctcagcgcgagc

tacagcaatccgagaggtctgccctcggacacgctccgtcctccagtctccgccatatggcagctttcgtcgctgcgtccacaccggcttccagacct

ggacctgggagaggacatgcggcgaacagtagtgcaggaagcatgtccagccgcgatacgagtccgctcatcccgatggtccacacgtcctcgcatgc

tggctcttctgcaagctccgccagcttcgatggcccgcccaccccagccgactcaatcatggagcggttcagcacggtagaccatcctccctcggcga

cccggcgatcgatgtcggtgccgagaattctgctgctgggtacgcaagagatccctgccgagtacgaacatttgcttcgcgaccgggcggataccatt

gcgaatgaaaccgacgtgctcaataggccaccaatgggctccactttgtcgcaacttcccgatactggccttcgcggacactctccacaaaagttgag

ccccatccttgcgtccggtccagggtcgtttgagacgcactccagcggaccaaagttgatcagtccttcggctcggaccagcgagattatccattcgg

ccgactttccattcgtcggtcacatcgagcgacgaacccctgagcaattctcagcggacactccaagcgcgcgaacaagtcgaataacttcgtacgcg

gcttctcttggtcacggccaaccacacatgacaaccgactcaacgtcacccaacacacatgcttacacagacaaccttcaccgatcaccgagcggaag

gagctggaaaccgtcgcttcctgcttctcgtaaggccagcaccaacagtcgcggcatgaccggctcgtatcgtcgagacgacgagattgaccaaacca

gcatctatggcatggcaatgtga

>contig_21.contig_21.g966.t1

atgagttcaggcgcaggaggaagtgcaagttccgcaatgacgacacctcggccaccctctggctatgccgcgatcaagg

tcagcccttcggacgtccttctcgagctcacgatatcgaacaaatgcggccaggccttccggtggaggagcatgcaaatatgggagccgcgcgaacct

gttccgaaggaagagttcccacaagtcaagaaggaagacggggaagaagtttccacgggcgaagacaagaagaacggtcccgaaggtatcgaggaaca

gacggagtggtcgctgtgtctgtccgaccgtgtcgttctcgtccgacaggatgtcgaatgcgggtacctgtaccatcggacactcctaccgtctccga

atcgcgcgagtggcgacctcgaaccgatcctggccgagcccaactcgctggccgctcaggagacgactcagtggctcaccgactacctcagcttgcga

gtgccggtcgcagaactgagcgtgcactggtcttcccgcgaccccatcttcgccaaacaaaacaagaaattcacaggctttcgaatgctgcgtcaaga

cccctgggaatgtctttgtgccttcatatgcagctccaacaacaacatcgcccgcatcggccagatggtgtccaagctctgtaccgcgttcactccgc

ctttgctcgaatacacgtggcctgcgccaccacgtcctgaaaatgcaggtctctcatcgaacgaagcggagcatgccacagcagatctgcctgtccgg

ctcgtgtaccatcccttcccgtcggctgcacggcttgcagaaagcgatgtcgatcaacatctacgggagctaggtttcggatatcgggccaaatacct

tgctaacacggccagtatgctgtgtgccgcccattctcgcccggaagatccgaccaagctttcgctagaccagtacgaggcattgcctgcgtcgcggc

tcacgctggatggtatcgtgccacgatcgaacgcagtgccagcctccgctgaagagtcacgtccacgagtaaagagacggcggacagaaccaaaggaa

ctggcctcagcagtggatatcacgccctctggacattcagtatcatcgtatctggcctcgctccgcgacatgtcctactccactgcacgggagaatct

gatacggctaccagggccgtcctctattccggtggatcgacacgttttcaactttgctgagaagctatacggcatgcgtggtgcaagcacgcggggtc

ataaagggtatgaagaagttgccgaaaagctacgcgcaatctggggaggatgggctggctgggcacattctgtcctgttcatggccgagcttcgcgcg

tttgccaaattcgaacagccatcaatcgtcaagaaggaggaggacgtcgacccagcgctaccagtggccaaaaagcccctattacagcatgcaaagtc

ttcaccagcagtggtcaagcttgaggcacagttggcccagcaacccgcgctgaatttctctccaacggtcaattcgagtgtgaaaggaaccatgccgc

gaaccattgtcgttgtcgatggcggggcagctgtcatcgagatgctgctcaatgcgaacttggctgtgcagaaggggacctcacagtcaagcgcgcgc

ggaacgcagccgggccatgctgtgccggttcacctacccgatatcctcccttcggcgcctttccagcgcttcgcggcaccaggagcgacttctctcgg

cgaagcgttccagacgctggaagatttcgaaccccattccctgccaaggcggccgggtcgatcgttgcaggtcagcgagagcgccagctgcgtttcca

ggtcgacggacgatcatttgcgaggtgcatcttctggctcatccccggacacatcgggcaagacgaatcaagatggcacccctccgagttccctgcct

gaagacgagcttgtcatggccgccgccgacgcgcttacgacggccgtcggtcccaatgtgctcagtgctaagcctggatacttcgccagctccttggg

tcaacagcctctgccgtcttcactgtccatgtccaactcggctgcacagccggggacatcagagcttctttccgggttcgagatgccctccgcgacat

cgccctacagcgatttgttcaacgctacggacgtgtcatctgaatatttcaatggtcacgtcccattttcgacccctccaacatcgccacctcagcca

ccccgagttttcagccacgagtcggccgaattctctctgaagtctttgcacgcgacggacgctgaagccaaaagctccacgacgtctcacggttctgt

aagttcaagatccagagagggttcggacatcgaaatgttctcgcctttggccatgtcgtgcatagacgcaccgtcgaagtccaatcgcttcaaaactc

caggaactcctccagtctggacactgcgctccctgatggaaggtagtgccgtcgacacagcaccacagagcacatatggatccaagtgcgtcgagacc

atgaaggtcaagtactcggacccgacctcgatgaggaacgctatggcgggtgttcatggtctctaccttggcctagacgcattttccagtgctcctca

gctcttcgccgaggacaacatcaaaaacatcatatacgcagccgtcagcgcaggtgtccaacacgtcatatttgcgggccatgtgtgggatccagatc

tggacgggcttatcatcagcggaaacccgaaggattccactgatgctgagtatacggcgggtacaaacgcatccacagccacatcggacgaacctggc

ccgcacaacttgagctcgctgcctcttgctgtgcaacgctgcaacgcagtgcacagactcttacgcaggctgcaacaatccggtctgttccgccttac

ggtcttcagcctcccggtttgcttcgaagccggctttgaggccggtctactctgcgaagattcgtggttcgctcttcgtccgaagtccaacacgtatg

tcctagacatgcctgtcgctagctctacactcatgcctttgtgctcactgtcggatgtcgggaacgctgcgctgcaggtgttctcccggcccgagcgc

ttcgatggggttgtactcgatttgacatcggacatcgcctcgccgcgtgagctctgcgtgcagatgacccaggtcatctacgtcagcggaaagagggt

catacattgcgatgatcttgccaactggatttgcgaacacgagcgggctgcttctgcgggcaatcttgactcgcagcagaacatgatgggttcaaaag

tggccaactctcttttgctgcgcgagttcatcctgttcatggcgaccttcacgcgcaagcctcttcctcttctactttcccaggtgtgcaccaacgca

cggcgatacaatctgcggctaactccatgggtgagatcttccacttctgctggaccccaagtcggctttcagcccctgcccgcggcaagccgagccga

ctggagtcactcgcaagaccgacacgggctcaaggacaattccattccactcttttcggtctccataccatctttcatcgacagtacatag

>contig_21.contig_21.g967.t1

atggactcgtcagacggcaaggattggaagtcagtcctgctcccgtacgcagaggcttggggctcgcatgcgaactcgc

cctatccagcatggacactctctggactgtcggcagtcccatccccaactcccatcggtcccgtcggacaaggctcggtaggatccaccttggccaaa

gcggcggcgagcacgtcctcatcagcaactacgcaggcagggcggacctatgctggttcttcgatcgtcgcagccgctagagcgccacttgccgctct

gccgcccttctaccagctcgtcggcttttctgcagtctttgcgggaggaggatacatcgttaagcagggggactctctcaatgggagcggtgtcatgt

cagcttggtccatgctcttcctcttcttccggctcattccgtcccttcgaccccgagcacccgcccagcccctccccttcatcctctcctcatcgacc

ttggccatcggaagcatctacgcagcacactacttcagtcggacctcctggcgcggcgccgcaccttttacgcctccgcctgcgcccaaggccgcggc

ccggaacgcggagaagaagggcaaggcgctcgcggatatgacggagcgagtcggaacggattcgtcatacgccctgggcttgagacgagggacaggca

cgacgacgacttcttctactgcttcgacgtcggcatcgactcggattgtcgtcggcgacgagcgcaacatccacaacgcgctctttccctggttgcaa

tccccctcgcagtacatgaacatcgagctagcctctactgactgccgcgacttgcctcctgttgtgcctcgcattggtgcctggtggacggactgccc

gtttccgtgcggaaacggaccgtactacaagaggcgcaaacttgatcaccgagctcaccccatcggcaatcccatcaccctgcagagacgcactgcgt

ggggccgttgtatgacagctgtcgtaaccgcagcgaagatggcccaacctctcatatcacgccatccaaagccgtcgctcacagtgagcttcttcaac

tacgtcgtcaagcaacaggtccccgtcatagcagcgcagacgctcctggtaggcgagatcatcaggaaggtccatgtcgactaccgcatgggtgtacg

ggcgcgcaaacgtggtcaccttaatgtgagcgagctcctgtatatgaccatcaactggctaggcgctgatatgtcctcatcgttgtccccaggtgctt

cgccatcgttgccgtcatcctcgattgtgttcttcaggaattcagctagcaccttcctttggtacacgtcgtcgactttggttgctgcggccttggcc

tggcgaacgtacatcattttcggtcgaagtagacgcgctctgcgactcctgctcggaggccttgcgcttcaattcaccatgacgattcttgcgatctg

caagacacacaagcttatgcttatcgacgatggctactgcgactacgacgaagagcagttcatggacctcaaacgaccctggtaccacctcgctgcgc

cgtggttcatttttgtgaccttcctatttgacgcgagcgtggtcacggcttccacctataagattgtttcgggcgccaggagcccgctcggcttctcc

gccctggctcgtgtgctgatcacgaacggggtccaatacgctgccttggtctgcacgacgaacttcatcgaatttattctgatcgctaccgtctccca

caagataccgagccttctcaacttgtccatcacgatccagatcatcacgggcctgaacctgatcgcggaagagcaggacgctgtgcacggctacactt

gctcccaagcacacaacggaccgaaacactacgagtacgcatccgacaagagtccggcattgcctcgaaccaacagaacaggagcaagcagggacacg

ggctcggtcagtggatacggcatttctaccacccagcacgactacaaggaaggcatgtacccaagcatgatctgcgaggaccccgacgttgtggagat

gcacacgtgcgtcgatgtcgaggctcgctcggaccggtccgacccgcagagcggacccatctcggctgatagcgcaccgcaccactcgcgcagtccta

gtgtgccgtgggagatcaccatgggcagttcctccgttcgacgcaagccggctcctatcccgactgccggtggcgccaaagttcgacacaccatcgcc

tattcgcccacggaagctgaacagtcccacacttcgagtgtgcaaccgggactgactctcaatggtggtggtggtgatggcgtcgacggcggcgcgga

catcgagatgcaagagcgaccgtcgacgacgacgacgacgagtatgagcaacactctcggtctcgagcgtgccggctcagtcgcacactccatctcga

ggtcttcgagcttccagcgggcagcgtcgtcgagtgccggtggtggcgagggtgcaccgattcctaacactccgatgggcggtgtctccattcgctcg

atccgacaggcgatcgagaggggtactactttggacgttccgatgtacgcccgtccggatagcacgctggaggtcatccctacgagctcgcagtggcc

caacaaccgaacgggggagtag

>contig_21.contig_21.g968.t1

atgtaccaacttccaccttccggggccgtcaacacatgggatgatttcactccggcgtgaagtcagaacaggaaccaca

gcgctggcgaacagtgcctattccctaccttgaccctgaactctttaagatctacgaagcagggtctaaattggaaacgaaccatcgtcgttcgcagc

caaccttcgtccatcagcagcatctcctatcttctgtcccagccgccgtcttctcgcaagttgcaactgcctttgcatttgccaggagtagcgacaag

ggacgaagttcaggacgaagccaaaaccaaagctcattcgcccccgccctttgcctggccaagatcccgagcgccaactcgctcatcgtcatcggagt

tcggacagcattctgcatcttcgccctcctggttctccctgcatcagggccatcccgaggacgtccagcatccagcatccaaaatccagcaccatcac

gcattcgttcgaggtcttctgcctctgcgatcttggcgtgttccttcccaaagcctcgtagccatagcatcggctaggccttctcgttctcgttctcg

ttctggatccaccgacccatctctccttcttcttcccctagccatgtcgtccttccagcttcccacagcacgcggaaacgtcgtctccatcttgaacg

atcaacgcggcggcagcaccagcagcaccgacaccaatctcagcagaccgagcccgacatcgactcaatcacagcctacgatggccacatattctcac

aacagctcgctcaaacgcaaattggtcgacgttgacggtcgcccctcgtttacaggaagcagccgaccatcgctcgaacctagtccgtctcggaggag

aagcgagcttgcggttctcaataagcgacccgactgcgttgctcgatcgtcgctctatgggagaccaccctctagcgtccacagttttcacacacaga

gcggaagcggtagcggtagcggaagtagcagcagacttgcagacgtatccaaatcaccagggccgtctcctctccagagtgcacagggcgggggagtc

atgaatggccaaatccccgctgtgagcgaacacgtacagcggaaggactacagtgtggccgacgagcacgagtatgcccatccactagcgcggacagg

gtcccggacgagcagcgggacagcacgagcgcatggaagtgggcccactcctccacgacggaacccacatcgcagtcgacgatccgagcggagtcttg

aggaggctcaattggctgaccagggtgatgagggtgaatcagatcgggcacgaagtgggaatgaagtcgccccatcaggtgaagccagtcagagttcg

ctgagcggcgtcggctctggcgaacagattacttctgccgaagcgtcgcctccagggaccgccagcgccagcggcagtggcacaggatcgggagcaac

cgacagtgttttgacttcgaccagcgggagcgggaccgcctcttccgccagcatgttcgatcgtgtaggttccaccggcctcggtcggagcgggctgg

acgcatccactgatttgtccacgcctgattcgattctcggctcatttggagaaggcagcggcaaatttgacggggccggagtaggaggagagaagaag

ccaggcgacggtggaaaggccggccatgcgtgcccttcccattactttgcgggagcagtcgaaggagagggacccgaggtcggcactcaagtcgtggc

tgagcaggagaaggccaaagctcggtggcatagcgaatgggcggtggcccatcctggcgaagatgagttgagcgaatcggaggacgaggaagagactg

ccaatcggacctggctatcacagatccgagctggagcgcatggtctgccatatccgctgcgatcgtttgggtccagcgattctgtcacgacgcctctg

aagacgttggcagccgttgcaggagcgcagagggagctgaaggcaaaaggtcttgaggcttatcctcgtcatgagcggcatccggggcctgcggagcg

ggctgcattggatgtcgatgcggcatcgccgcctgcaatggacgacgatcgtgtgcgtcatggcaggctacatcctcaccaccacccacatcagcatg

atgtacatcatccacaacatcaccatcatccacagcagcaacagcagcaggagcaacatcgacctcgtcaccgtcgtcaagattcgtcgcgtgggtcc

gggctaactggacagaaaatgattcgaaaggacaacatggtcagcagtcctgagccgatggacgactcggaggaaaaacggctcgaggatcaggttga

ccatgatggatatctgcagcaggacggggacaaacattcgtcacagccacatcagcagcagcaacatggtcgctccgaacaacaagtccatcacagtg

atgaatcaggggtgccttcccggcccaagcttcctcacctgacggggacaccgaatgggcagcgttcgatcagctctgctgcggctcgactgccgctg

ccgtctctcagcggtgcagggagcaactcgatgaacacctacagcaagacgcttcccaacctcgctcgactcaacctctcgccacacccgggcatggc

ggagcctcagcatcagcattctcaccagcatggcagtgatggcccgattctcgatggtgtcatgccagatcggggatttctgcaacaccgccggctac

cgagtggtcacgtgacggagatgtcatcatcgtcgttgtcaccggggcgggtgaagatgatggagcttgacaaccaggagcttggtagcagtcgacgc

agcagtgcaagtggagtctcaacaagccacaagtacctcccgcttgctgctgctgcttctgccaaacacgcccacccgcttccaatcaatggacacgg

tcatggtaagctgggtgtccctcccttcctgccgcctctgacatcccaacaccacagctacccagcacggcccagccgctcctctgcatccacacaag

acggctccttcggcatggctgcccgaggtccttccatcgcatccccggcattgtacacccatccggcccatcactcggctggctcgcacccaagtcgc

tacggtgccaaattgcatccggccgatccggctgtgctcaacgctgcttccctgcgttcgcggccccttgaagtaaggacggtccagctgccttcgtc

tgcctacctcggctcaaccggcgagtcggccatgcctggaatgatgaacgacggcatgcagcagcccggccctggtaatggtggtaacgctcccgccc

ctcatctggttggacagatgcaattgggacacgctttcaatgcacaggagtactactcagctcttcccggagctccccagaacttcggctttcagcaa

caccagccttcgtctcagatgatgtacgccgagcaaggccctacggtgacgtcttccgcacacggattgcttcctcctcccgacggcgccttcccgag

cacgattccttcctcctcgctcccaccacctggcccatttgcgacacaccttcccggacaactcgatcaaggtcacagactgcctccgctcgattcat

cgatgaatggcagcggtggattcgcaggattcggtcctgtcatgggagggaacgcgataccacctactttcccaggcgcgctacttggttcgtccacc

tatttgtccgctcctcctccgccgccagcaagctactatgacctctcctccaatggtgaacctgatggcatcaaaagtaccgagggacaaggcctgcc

gttcgctagtgatttcaacaagagtgtcatggtaccggcgaacgcgattgcgggcagcagtcacagtaacagcaacaccagcagcactcatcgactcg

gcagcgggcgaaatgagagaacagctcgttcgagtgacccgtacccgacgaacagccgagccggagcaggtgcgctggctggaacaagcatagttctc

gaggtcccaccccggacgcgcatcgtgccccagaaagggccagacgggaagaagcggtacccgtgctcgttcccgggctgtgacaagacattctccac

cagtggacacgctgcacgacacagtcgcatacacacaggtcaaaagccgtaccgatgcacgtttccaggttgcaaggcgaaattttcgcgtcaggaca

acagcttgcaacactaccgcacccatatcatccagcccaaagcacgtccgcgtgtccggcgcgaggacgactcgattggctcccccggtccgtcctcg

agtgacttgcgattcatgccccaacaccccgccgaggatgactcgatgcaagctgaggacgacgacattcgatcgatcaactcggacatgatgactcc

cagtgtggccctgggccgaaaggcgctcgaggaaggcacagccatcgcggtggtacacgacgtgcttgactctaaaggtcgcaagaagggagagacgc

ttgagcgcatggtcggtgctgggggcaaacagggccagtcggctaatcactggaacggcgagggcacaccgtcggggacgacggatgacaattcgagc

ccgcctgattcctcggggaatggcaattctggacagagcagtcagacgcggagcaagtccggcgcacctcattcgcaccatcctccatccaacctggc

cggatcgtcgcacgaatctcggatgtcctccgttggtccgtcgacgtttgatcaccagagcgagcgtacgggccggacgacagagggctcgactccgt

tgaatgacatgcaaggcgtggtgcggtctgaggccgactttgctctggatgcgaatctgatcaacgggcacgatggtacacggaggacgcgtccggtt

gtcggtaatgggcctgccaggagcgcgtacgagtcccagatgtatgatcgctcggcacctgcaccgatgaccggccacggactcgtatatgccacgga

cgggcatcccgggtcgcgaccggctgccttcccacaacagatggctggcaacggcaatggcaatggccctgccaaacgcaatggcgagcccgtgccgc

tggacggtcccaccagtattctggacgacttccgaccgtcgaagacccgacatgcactcatttctgactcgggcccgacgtggatgaccacggccact

tcgaacggctacggtgcctggcccggcgtggcatacgtctcagccggccctgtcatgcccggcggagctaccttgcagcctccgacggagttcatgcc

gaacaaagacccaggcacggcggatttctctcccgcgcacagcgtgcaggcgtcgccgaactcgatgcgtgcttggcaagggttgaacgacgctgatc

atgcatgggaacgagaacgcgaggagcggatggcgctctatgctgaggctggccgagaagctgctcaaacccattcggacagtcgcatgcctgtcgca

aactccggatcggggcgtgtgtcgcagcctttccagccgaatttgcccacggggaaccccaactggcagatgaatcagcgacctgtacattgcggtgc

ccataattggtcgctgagcctgacatcgaatggggctggactggctcttgatggcaatctcactgcggccgttgtcgcaggaaccgcgcccaagttct

cgcctcattggcgttcgaacccggccatgccgtcggacagcaacggcaatggcaacggcgttctggctcggccaatgaccacgaacgtcatcagttcc

gccgtgcctcgaccgacggcagactcgagtatgtcagccccacccgtgcctagtcacgcttctgctgaaacgttgtcgcgtcgacagtcgactgtcac

tgtgcctgcgaccgtgccgagtcctaacttgtccgccagctctcattcgaacgtgaataatggcggtatgggtaactcaaactctgccgcacctcaat

cgagagttgtgctgcctcagaacgccgaccccgtgtcgcccactatgtcaacggcatcgtcagcactcctctcgctctcggtcctttcctcggcggca

ctcactgctacacctgcccactgcggtccaaccgctggctcaacaaacaaggggccttccatcaacgggagtgggagcactgccaggctgcccttctc

gacgggtcgcacgacgtcgcacctcgagcgaaacgccgagtttacacacaacaacaacaacaacaacaacggtccgcctgttacttcttcctcctctc

cgacaggtagccgaggaaattcgaagacgggttcgaacttgatcgtgacagctcggcccttgccgtaa

>contig_21.contig_21.g969.t1

atgatgattaggaagtcggtcagagcatcgagctcgacagctcgcgtggcagcgggactgggactgtcactgtccccgt

actcggcgtcggcgcccgcatgtccccgatgctccctccgccaaaatgcaggccagaacattcccccccatgtttccgttccgaggtccaagatttct

gctcctcagcccttcctccgctccttctcgacctcgacctcgacctcgacctcgacctcgacccggacctggtctctcgcagacggcctcgcgacgcc

gtacctgccggacctgatccggaatatctccattatcgcccatatcgacgcaggcaagacgacgctgacagaaagactgcttctttacgcgcaacacc

aacaggcctcgttgtcctccgcaacttcacccgcaggttcgagctcgtcgccgtccgtctcccaggacaagcatctcagccatgtctcactgcccggt

agcgtcgatactggcaccacggtcaccgacttcctcgacgcggagcgcgaacgcgggatcaccatccaaagcgccgctgtcggaccactgtattggaa

caccacaacatcaccttccaacccgaccccgaccccgaccccgaccccgaccccggccgccacagccatctacctcgtcgacacgcccggtcatatcg

acttcacaatcgaggtagaacgcgccctacgagtcgttgacggctgtgttgtcgtccttgatggcgtggaaggtgtagaggctcagacggagggcgta

tggcgtcaggctgcgagatatgggatccaatctcacatcgtcttcatcaacaagctggatcgcgccggcgcctctgtaccccggtccctgctctcgct

cattcagcgcaatctccactcccgacccgtcctcctccagctgcctgtctacagtagcagccaaggagaggacgaatccctcacaggtctcatcgacc

tcctccacctcgatcacctcacctttaacggcaacgcaggcgagaccatccatcgcgaacctctatcacctacccaccctcttttcaaagaagcgctc

aaagcacgatcaagtctgatcgaaaccctcgcagagcaagatgaggacctgctggagcaatacctcgaagtctctgacctcctcgaagaagcatcgac

agatggaacccagcaagaccaacggcagcgacatctcgatgcgatctcacccgacatccatcttcggacggccctgcgccgacagacaattcgaggaa

gtgtaattcccgtcctatgcggagcggcggcgaaaaacacaggagttcagcccctgctggacgccgtggcgtggtacctaccctcgccaatcgaagcg

gccgctgcagcgagcgccaccgccgcagcgacagaggcacaggaaaccctcgagcctgtgaaaaagcatcagcagggagataaaggtgccaagggcaa

agttgtcaaacagaaggagttggcacctcatgagcgagagctggcagcgttggcctttaaggtggtctgggacaaacggaaaggtcccatcacttttg

ttcgcgtatattccggcaccctctctcgttcctcgaccttgttcaacacgacgactcaggcccgcgagagactctccaaggtcttcctgccctttgcc

gaccaatacgtcgagacgccctcgcttcgggccggccagatcggtgttctgatgggactccgcgacacgcgaacaggcgacaccctgatcgaccctag

gtccgtcggccccactccctccgccagctccgcttcctcctcctcctcgaaacaactccgcctgagaccgatacatgtccccccgcctgtgttcagta

tgagcatcgagccggagagcaagtcggacgaggcgagcgtgagcgaggcgttgagtatgctcgtccggactgatccttcgctccgattggacgaaggg

ggcagtagtagcagctcgtcgtcgtctgcctctgggggcagcttcggagccgcaggcacaggccagacggtgctaagcgggatgggcgagttacacct

cgagatcgcgcgcgagcgactggcgaatgagttcggtgtgcgggcgcggatgggttcggttcgggtgagctaccgagagacgctggatgagaatgtgg

gggaggttgtggttgaggagacgtgggagaaggagttggggggtaagaagttgaaagtcgggtgtacacttaccgtcaggcgtttacgggacgacgag

ggggaggtcggcgatccgaatgcgggtgggaatgtcgtcgtggtgcaactggacgacgatggactcgaggatgatgtatcgtcagcggacaaggacga

cgatccgtcctcgctggaccctgagacggtccgacaagcgttcacgacaggcttgatcgcggcgctctcccgcggcccgctctcctccaacccactga

cgaacttgcacctcaccttgtcgtccccactgctcttcgggcccgacctctccccgccccgcgcactcagcatggcagccggagccgccttgcgaagc

gcgatgaagtcggcagcgccttcttctggcatgaatgcagacctggcaccaccaccagcgacggccgcgaagatgatggagcctgtgatgcgggtccg

gatcgagtgcgatgcggcccatatgggcaaagtcgtcggtgacctcacggccgaccaagcaggcacggtggtcgatgtcgagcagccggggaatcaca

atgacgagggcgcgggcgcaagggaggacgggtcctcgaccacgatcaacgtctacatccccccggacgacctctccagctcgagcgcaggcgagagc

agaggctcgtcagcatcgtcctcgtcgtcatcggttcggacccggaccatcgtccatgcgctggtacccctggcccgacttgtgagctattcgagccg

gttacgggccttgacgggtgggatgggcacgttcacgatggagcttcatggttttgctcagacgagtcgggagcgcgagcgggagatcttgcgcgagt

tggggaaggcggcgtga

>contig_21.contig_21.g970.t1

atgtcgcgcaagcttgcaggccaggtgggcacgagtgtctcgcgtctcctgcgcggcggcttcctcaagcaggagcccg

tctggttccaggccgtgatcgcgcacccgcccgcaccgcccaagccccgctcgcccatcgtccggccggtggaggacctgccgccgagcctcgccgcc

cgacagaagcgtctcgtccgcaccttcctcgggcaggcttcctcacagtcaggcgcggcatcatcctcgccctcgtcctcgtcctcgtcatcgtcatc

ggtaccagactccaagcggccgccgcgaatgtccgccaagcgtctccgagccctcacgcccgaactgcacccgcagccgatcttctacctccaggacc

gcgtccgccggcagttctaccgcgatcatccctgggaagcgtaccgtccccgtacgctggtcgaaatggacaaggtcgtggtcatcgataagtcgtcg

cccttgggtgctcccgaagatgctcgagagcttcgcgcgtggggcaggaatccaggaccagaggacgtcgtatcttgcaccatccacctccacgagaa

gcacggcctgtcactctctcaagcctaccaccacagcatcgcatcctaccacgccctccgcgccgaacacgagaccgcatcccgctacgccgtcctcg

aggcagaagcatacggcgcccagttcggcccgacggagactacgcttgccttccgccgcgagcaccgtcagatcttcggcacggaccggatcgaccag

gcggacgaggccgccccgaccaccgccgagtctacacacgaacgcagcgctagtcgtcaaccgcccaccatgatttctgtcaccccggaccagctgcc

cgttcaaaaagacgcactaggaggctcggaccagactgcccatttgtacgtcgagccgcgaggcaagttcgatcgtgcccggaggatcaagaatgtcg

atgatacgtttactgcgggcgacaagtacctggaggctgcgttctctaccagtcaggctgcgttggcacgactgcgatcatag

>contig_21.contig_21.g971.t1

atggtccgcgtcaagaagcgtatttcaaagcgtgtcggcacaaagcagcgcgtcaaggtcgagaagaaagtccgcgagg

gaaaccggaaggcgaagcgagccgcaaagaagaatgtgcagtggaagagcagagtcaaaaaagacccgggtatccctaactcgttcccgtacaaggag

cagctcctcaacgagatcgaaatgaagaaggagaaggcggaacaagccaagatcgcagcgaaggacgcccgaaaggaagctgcgcgcgccagcaaggc

agctgctcgggtcgcggctgaaggtggcgttgagccaggcacaagcagcagcagctccgaagaggctgccgacgcggatgagctgcagtccgtcgccg

ggccgagcagtcccgtcgagggcgacgagatggatcatgacgacgatgacgagttcgaggatgcggacgaaggcgaggatgacggagaggagggactc

gcccacgtagctcttgtgcccatgtctggcaaggagaggaggagaaccaaggcggcagagcagccggagaaggcgaagcgagaacccaaggctcccaa

ggcggcgaagaaggtggccttccaagcgtcgtctacgaaaacaagagcggcagccagtgcgacaccaaagtcgacagggtccgaccagccgtatgact

tcagtgccctcttctga

>contig_21.contig_21.g972.t1

atgcgcatcagcattcatgacctcagcgacagcgaggacgagcccatcgtcgtcgtccccaacatcaccagcgtcggcg

acaacgagagcgagagcgaccttctcgactcatcacagctagcgggcctcgcccactactcgagcgcagcacgccagtttttccccaaacgcgcgcaa

agcaacccgcccgctcgggcaccattgagcgcccgcgatccgaggcgcacgccgagcgcggagccctcacgaaatatcaagcgcgcttacacgccgta

ttggaccactgcgcagccatcgcccgaggagctatccaagtcgttttcagcgattggcctggaagggacgtctgacttcgaggtcgtgttgagctctc

ccgacagtacagacgccgctgcggtctcctctgcggatgaactggacgactcagcggattcggaggaggaacgatggcctgtgaatcctcgcggatac

cacccgcgaagggctgtcaaggggcgcgcagggaatcagaagggtcggaacgcgcgcaacgccggtgtagatgaggaggtaatgaggagaatacgact

gagcgctctgcccgaatctggtcgcacgtctaatgccaaagaaaatgtctgggccgtcgccacgagcagatatcatgcgcaagcccgagctttcagcc

agaaccgagtcacagctgcaccaggctccggaaatgctaccgcatttggcttgaggaaggcgccattggcatccctcaaacaccgaactcctttcgga

ctgagcgtcgtgtcagaggaagccagtcgtcaaccttcggcagccataaatgaggtccagaatctcctcagagagcttcacatgcgtcgagacgagga

cgacaagcgtctgcaaaatgacttcgaagcgcgtagtaagcgactatgggatagcatagaggcgtcgatccgtgaagcggagcacaagaacgaagccg

aggctgctgcgcaaaccgccgtgctgatcgccaaacgaaaagagcaagaagccaacgaacgagtcgcccgcgaagcaagagaagcagaggagcgcaga

gttgctgaagagaatgagcggcaacggcaggtggaagaggaggcgaagcgcaaggaggaggaggaagctcggagacgggaggcggaagcggaggagga

acggaagagggagaaggcggcggcggaggccgatgctgccagtacgttgggcggggtcgcaggtccatcgctgtggacacaagccagggacgactacg

agaagtggtactctcgcatccaggacatcaagaccaatgtgcttcctcaaatatctgacaaccctgactggcgcaagcagtgcttcatggccaagcgt

cagattacgcccaagatatcgcaactgacaaactctcggagtgagatcattcgaattactacgagcatcgccgacgtcctcgggcaagcgaagaacgc

acatccggcgatttacacctggatcttgaaccatctgtctaaatgtctcatccgccaggccgagcaagaggtagcagcgagatccagcacggccttcc

cgttggcacgtgttgtggtatggctcatcttcagcggtcacgaggagctcggggaggtcctcatggctcgcttggtgaagaaatgctgttatgtcgta

gcatactggcctcaaaagggaaagacacaatccgacggcgagtatcggaaacagctcggccgaccaaggccagacgagacgacgcacaccttcaattc

gcgaatgtctggcattttcgcgctctactgcgccatcctgcagacgcttccgacaccacctccgacccaaactggcggctcgctcaacctggagacga

tgccagcatgcttccgctccaatatgctgtggacgtggcaggctcgagccatgacgcgaccgatgacggatcaccctctgacaccgacactgctcgcg

acatgtttcgaagtggcaggccgtcgggcacagggctattacgggaagcagatgagcaagctgtggcggctgttacttgtcgaaggcctccgggaacg

caaggctggctatgtgcacaggtatctcgaggcgggggccgaggatgggggagcgttggcggcgtcgtctcggttgcagttgcttctggaggagtggg

agaagacgggcgtcgctaaagctgatcctgctgccgatcggatggacccgtga

>contig_21.contig_21.g973.t1

atgagcggcctagagaaatccctgttccagctcaagttcacagccaagtccctacagcgacaggcaaagaaggcagcca

aggacgaggtcgccgagaaggcaaagctgaagaaggccctcgcacaaggaaacaccgaaggcgcccgtatctacgcgtccaatgcgatccggaagaag

aacgagtcgctgaacctgctccgcctgggaagtcggatcgacgccgttagttcccgggtcgagactgctgtcacgatgcggcaggtcaccggcagcat

gaccagcgtcgtcaaagggatggatagggcgatggagagcatgaatctggatcgcatcgcgctggtcatggacaagttcgaaaaccaattcgaagaca

tggacgcccaaacatcctacatggaaggcgcgatctcccaacaatccgcccaaacaacacccttggaccaagtcgacctcctcatgcaacaagtcgca

gacgagaacgggatcgagatcaaacagcgcgtcggggaggtcgacctggtcgggaaggtggccgcgctcagtcccgcggcgcagcaggagcaggcgga

gaaggagagggaggagaggaagttgattgcggaggggtcggaggtgggaccgagctcgggtgtgggcgcgggttctggggccgcgcctgctgcggccg

ctgcggcggggccatcttcgactgcgcggtctggggacgaagactcccggcttgcggaacggctcagagcattaagaccggcaggtcattga

>contig_21.contig_21.g974.t1

atgatcgacaaccacatccagaggctcaaggagggcgaggtcgacctcggaacctccatcatggctgttgcctacgatg

aaggtgtcatcatcggtgcagactcaagaacaacaatgggcagctacatcgccaatcgagtcaccgacaagctcacccacatcaccgaccgaatctac

tgctgccgctccggctccgcagccgacacccaagccgtcgccgacgccgtgacccaccacctctccctcgtcgacgtcctgcacggccccccggaagt

ctccactgccgcccacctgttctccgagatcgtttatcagaacaaggaccggttgagcgcgggtattattgtggccgggtgggataagaggaatgggg

gaactgtgtacaatgttccgttgggaggtggtctgttccagcagccttgggctatcggaggctcgggatcgacatacatctacggctactgcgactcc

acctaccggccaggatggaaccgacaacagacagtcgagtttgtcaccaatgctctcgcactggccatgcgacgcgatggctcgtccggaggcactat

ccgtcttgccgacatctccgagcacggcgtggagaggcatttcatcccaggagacaagcttcctgagttgtcggcaaagctctgctga

>contig_21.contig_21.g975.t1

atgaacgatgaacccagctcgctctccagtctccaccctggccactcactcactcgctcccacaatccttcgccttcgc

ctctctctcgctgccgacccgctctgcctgccatgtatctcctcggcgcacatctgcccgaccacaagctggtccacatcgcgctcaccaacttcctc

ggcatcaaccaacacacagcccgacgcctctgcgcccgtctccaactacacgaaacagcaaaggtgtcctccctctcagaagcacagatcaaccagct

ctccgccttcctctcctcgccgagctccatccccgcccgctcggccgcacctacaacgcccgtacccttcaccttggcccaccggcgcaaacccacag

cagcatcaacatcaccagcatcatcaggagcaggagcgacagaagcctcccctcccgcacccacagaccccaccctgatccccccctcccaacggccc

tccccctccaccgaccccctccgctccctcgtcctcgaagccgacctgcgccgccagatccagtccaacatcgcacaccaccgcatggtaggctccta

cctcggtcgtcgacatgccggcggtctgccgggtggcgatgaagccctcctgctgaagaaaaatcaatcgaatgccaattcgcaagaaagaaggctgc

caacgatgcccgcgacgaggaaggagaaggagacgaagatgaagaagcggcgagtagacggacgtccgtccgaccctgcccccggccaatcgtccaag

aagatcaaaagaacaggccaccctccaccagctccggccacaccccaaaacggcagcacgtccaaatcaataacggagactccctccgggcccgaaga

caaaccagcgaactggccgaccgaggtacaatacctctccgaagcctgtatcccttcccccaacctccccccgcacttcaaaccgaccttctgccacc

ccttccccccctcccacccacccctccccctctcccacctctccatccggcccatcaccgccctcacacccttccgccggcacccactgctgcagaac

acggtccacccggcgctcggccagcacggtctgttcgcggaggtcgatctgccggggcggaggctggtgtgtgcgtatttgggggtggtgcacgggga

ggaggagacggatcggcggtcggagtatgatgctcaggtttgggctaggggtactggggaggtgctcgggttcgagcgggatgttgggttggggattg

atgcgacgtatgcgggtaatttgggtcgattcatcaacgacttccgcggcatcgcccaacgagccaacgtcaccttcgaagactga

>contig_21.contig_21.g976.t1

atggcgctggacatcaccatggacgacctgatcggatccatgcagcactccttccacgctggagaccgaggacgcgacc

tccagaacatcagagacaacctcgcctttgcactcgctccacacgcaatgggcccagccagcaccaccgcagcacagcccttcgccatcgtacgcaca

cagccatcgtcctcatcctcagccgcccactacggcatggtcggctcatccgcttcctccgcttctgctgcctccataccctacgacgcaagcagaat

gccgtccctcaacaacagctttggaagcacggcatcaacatcagcatcaagaccagcaccaatacccacaaacaacacattcgccacctcacagccag

catccctcagcagtagcagcggcagtgcatcatatgcataccctccaccacccccaccaccacaagcacccgccaacactcccatgcagacacccgtc

gaccgcgtagctggcgaacactggccagccccagctaccgaaccagatccaccacgctatcatgccccatcagcatcaaccgaggaccaccatcaaca

acaacaacaacaacaacaacaacaacaacaacaacaacaacaggcgatcaacatggacatcggcggacaacgcacgcctgtcttccaccaatccctca

tgtttggattccaacaagcacaggcacaggcgggtgccccacccaacagtcccgcacagttgggacatgggacgggcgaggcggggatggagtggcct

gcgatttcttcgcatgacgggagtagtagtacccgtcggcttgctcatctgctctga

>contig_21.contig_21.g977.t1

atgctgagccgcaccatcgtcagtgcaatcccccgctcaagcgcgagcgcgagcgcgagcaaggtctcccccacggccg

ccactctccttctcctccgccacgcctcctccaccgcgccgagtgcacttctcggccatcctcctcggacgggcaccaagacgctcccccgtacctgc

gtgaacaacaagaacaacaagaccgcagcgcgcgcgctgcccgtctccgtctccgtctccatccgccgcggcctgtccacgtccacgtccggctcggg

atcgggagcggagaacgatcggtcctgggttgataaaggcgaggttacgtatgcggagctcaagccggagacgcaggctccctctggtaacatcacaa

tcatcgacgtgcgcgaaccagacgaagtagcccaagggatcatcccctctgcagtctccgtgcccctctccgagttcacctcggccttctcacccgcg

aacggacgggacttcgtgaagaagttcgggttcgagcggccggagtttggcgggaaggttgtgttttattgtaggagtgggaagaggagcgcgcaggc

gttggagtttgcgaggtcgcaggggtggtggaactcgcgcaactacaccggctcgtggttggactggactgccaacgaggagaagaagtag

>contig_21.contig_21.g978.t1

atgtcgtcctcattacaagccgcacacgacgagctgctgcacctcttcgcacgcgcaacaacacccgcacagctcgcac

ccgccgcagcacagctcagcaaactcaagatcgaactcgccaaggccggtctcctcgtcccctccgcagcatcactagcatcctcaacgccggcctat

gtgcagcagcttgtcttggcccgcgatgtgctggaggtaggcgccttttacagcgtgcgggtgcaggatgttagggggtttgatcggtatatggatct

tttgaagacgttctactccgactttggatcaaagctaccaccctcggagaaccaccacccgctcctcggcctctccctcctccgcctcctcgcctcca

accaaatctccgccttccacaccgcactcgagaccctgcccccggacgtcgtccagaactcggcctacgtccgccacccggtcaacctcgagcgctgg

cttatggagggctcgtactcgaaggtgtggagtgcaaggcaggaggcgcctagggttgagtatcagttctttgttgatcagcttatgggcacgattcg

aaacgaaatcgcgtcatgcgaagagaaggcatacgaatccctacccctcgccgacgcagccacattactcttcttcgacaacatgaacgacgtcctcg

tcttcgccaaggagcgcggctggaacgtcgaaccctctacgcagaaagtcaactttgccggcaagctggacgcctcgcgcgtcggtgggattggcgcg

agggactcggcggccaagaaggagacgatcaagctgaatttgcactttgcgaaggagcttgagagcattgtttga

>contig_21.contig_21.g979.t1

atgtcgttccgggtgagcacacgtgagtacaagagctacacctacaccgactcccgcaccggcgcatccgtccaaaaac

ccatcagcctcgacgtatacctccccgagggcatcgactgctggcgcaacccgctcctccgcttcagcgagccacacaagaaacagcagtcctccatc

ggcttcggcggcatcgtcgtcgagcgcgagcgagtcgcacaactcgtcgtctggatccatccgggcgggttcttacagggtaaccggaaacagatccc

accgcatttgcaacgcgctgtgcagaatcatagacttgtcgtcgtcatcccggacttcaggctctgtccgcaggtctccatcgtggagagtctgaccg

acatccgcgacgcgtgcgagtacgctctcggcggcgcacttggtgattcagttaataccagcacatgggccctcggcgggaatagcgcaggtggatgg

gccgcactcttgctcggattgggacttacagcttcgacaccgacgggctcggcggtacagctacccaccctttctagattgccgcgcgccgtctttgc

catctatcccgtcacggacatcacacgcgaaggcggcgcgtgcttctacgagcctttgaagccgatcacctggactccctctgtcagcgctggtgtgg

gaggagatgggattatcgatggtgcgctccttgaacccttcttgcggaaaccgggccaggctcccttgggcggaagcacagagcgcccagacgcggcg

ggtgtgaatcaggacgaagaaggggaggaggaggaggaagacgcctcggcggtcgtcacctcttccgatccggagaatgaccccatccgatcacagct

cttcaattacgcccgacaagaaggtatattcccttcgcttgtgcttccctcggacctacctcccgaacgcgtctctataccggcactgctccggggcg

aagctctgacgagcgcaacggcatcagcagcggctgcagcggccgccgcttcatcctcatcctcaagctcaggacccccaaactccctccctctctcc

acctcccaatcctccctctcctcccgctccccgctcctcaacctcccaccaaccttcatcgcctgggccgacgccgacgaccgggtaccgccctccca

gagcatcgagaccgtcaaagcgttgcgggcgtgtgtggggggcgcggaggtacagacgcatgtcgtcaaggggaaggagcatctgtttgattatgagg

aggacgcggagattccggggctttggaggtggcttatgcagaagatgggggggttgacgtga

>contig_21.contig_21.g980.t1

atggaatcagcccacacccacctataccgccgagcaaccttcccccgcaacccaaaagacccagtcccctccgacgaga

tctccgtcaacatctacggctacgagcccagcatcgtcctcggcatcgtcggaagtgtcgtcttcttcgtcgggctggtcctgcatctcgtctggttt

ggacggtataagtcttccaggacgtttcaggcgctgttggcttttgggtgtgcgttggaggttgttggatacggagcgcggacggcagcctcccaaaa

tccattcgtcctcaactccttcgtcctccaatacttcttcatcgtctgcgcccccatcttcctctccgcagccctgtactgggccctatcggtcatca

tccgctcccggcccgaataccgcgcgctgtcgcccatcggaccccggctgctgctgggggtgttcatcttctttgatgtggtgacgattgtggcgcag

gtcgttggtgcggctttcgtgggtgtatcggagtcccagtcgggcaagggtcagaaacctttcatcacacccgaacaatccaataacatcgtcgtcgg

cggtctagccgtccaatcagccgccttcctcgtcttcctcctcctcttcctaatcttcgtctaccgcctccagcggcacaactacctctcctcccaaa

ccacaggcccgaacaagctcatgctcctcaccctcgcaggcaccgccttactcatctacatccgcacactcttccgcctcgccgagtcagccggtggg

ctgttgagctatatcgcgacgaaccagacgctgttcggggtgtttgagacgttgccgattatgctgtctgtgctttggtggggtgttgtgccgttggg

tcgctttacggacgcgccgggtgggttgggagggagggagggccagggccagagggtcggcagcgaggcggtggagaagaacgcgatctga

>contig_21.contig_21.g981.t1

atgcttcgactggcagcccgaacgactctctccctctccttgctgcgtcctgccatcgctactgctaccaccaccacca

ccaccaccaccactgtccgcgctcccacgtctcgagcgatctccttcaagtctatcttcggatccagcaagccagcaacttcggattcaacttcgtct

tctacatcttcccctcgacgacgcatgcacttgcccctcgccttcactttcgccaccgtattcatcgccatcgccgctttcgccacttccaaatccaa

ttcgaattcgccacacaccatgtccttcctctccaacctcttcggccgcaatagttctagcaccggaacacctcattcagccatgtcagacagcttcc

ccaagggaaagtccgattcggagtggagagcacagctcagtcccgagcagttccacatcctccgagagaaaggaacggagcgaccaggaacaggcgaa

tacaacaaagtctacgacaagggcgtctacgagtgcgcaggctgtggcaacccgctctacaagtccgaaaccaagttcgactccggctgcggatggcc

cgcctttttcgagggcatccccggctccctgcgtctggtcgaggatcgcaagttcgggatgcttaggacggagatggccaacagcgtgtcgctcaagt

tcacgcctgccgaagatggcaaggcaaagctcagcatcgttccacccaaggatcttgctccaagatag

>contig_21.contig_21.g982.t1

atgacttccttgagaacgacagcaagacttctcccacgcctgccatgcacaccagcggcccgtccctgcggctgcgcgc

tctctcgctcatcaacagcgtcttcctccgacaaccagtaccgccgcaatccagccccgtccttcgctcccttctcctcatcctcgatctcactccaa

caacaacagaagcccagtaaaagcgcttcttcagacgtttatgagctcttcggacttagtctccaagatctgccacagaacggctggggggtcgacga

gaaggatgtcaaatcgacatggaggaaactcatgggcggtacccatcccgatcgaatggcaggtcgctcagaggaagagcaggcagaggcagcacagc

agagtaccgtcgtcaatcgcgcatacgaaacactcttcaacccgctcctccgagctcaacacctgctggagcggcacggcatcacttcctccgacgag

gccgagggactcgacgacccggacttgctgctccaagtgatggaattccgcgaacggctggaggaagcctcgtccgagtcagaagcggccgaagtccg

ggcagacaatcgagaacatctcgagcacacactgaccgccctcggaaatgccttcggcgcgtcaccaccggacctcgaacaagcccgaaaactgacta

tcgaactcaggtattggctcaacatcgacaaggccgcgcgcgaatgggcgccgggagcacgcgtcgagttgcaacattga

>contig_21.contig_21.g983.t1

atgggtcagcaaggatcacagctcctcaccgagatcgagcaaaacacaaatttcagcgcatcggagattcagcgcttga

aaaagcgattcatgaagctggaccgagacggttccggctccattgaccgcgacgagttcctgcagattccagccatcgcaaacaacccactcgcatta

cgaatgatcgccatctttgatgaagacggaggaggaacggtcgatttccaggagttcgtagcaggtctgagcgccttcagcagccaagggagccggga

ggagaagctgaggttcgcattcaaggtgtacgacatggaccgggacggctacatttccaacggcgagctcttcctcgtcctcaagatgatggtgggca

acaacctgaaggaccagcagctgcagcagattgtggacaagacgatcatggaggctgataaggatggggatggcaagttggactttgaggagtttcag

gatatggtcgcgagtacggatgtggcgaagcagatctcttctgagagcggtgcacccgatggaaacctatactcgattctgctcgtaacaccttccac

cgatcccgtctctccgccctgggcttcagttgcgtcacccgagcccccacagaatgcctga

>contig_21.contig_21.g984.t1

atgacctcgccttcgaatggcactttcaagcggaggaaaatcgactggaagggcaagggcaaggctgtcgatggtgggg

aaagcggtggtgatgatgtggcattgcctcctgtcattgtcgctctcgacgtcgattgcttctatgcgcaggcaagtcgtttgcgcgacccatcactc

atcggcatcccacttggagtccaacaaaagacgattctcgccgccgtctcatacgaagcccgagcgcaaggcgtgcccaagatcgtctctatgaggga

ggcccttcgaatccttccctcgctaacgatcgtcaacggcgaagacttgtccttcttcaggttgctcagcaatcgaagctggaggctcttacgctcac

ttgtatgggatgaacgtgtcgagaagtgcggcatggacgaactctgggcagatgtcacggagacggtcgacgaccatctcctaaggtgccagctcgat

agggtgaatcgagataaagctcttggcatcttcttctctttcgaccaacttccactcgagcacatgcagccggtcgacctcgcgagagggttctggta

tcgcccctccgaccccatcccaggatacttccagcccgaccctcactccttcacccaaggctggccacaggatccctggagacagcgctacgctctcg

gcgcccatatcacctccttcttccgacgcatcatacgcgaacaggtcaacctcgattgttccggtggcgtctccttctccaaacagatggcaaagctg

attaggagcgccaacaagccgaataagcagacttgcttttgcccagcgcagcctttggaaggtgtcaatgtcactcgcaacgattttcatcgcaggtt

tctacaagatgcacaagattacatcgatcctctcgacctcggcaagctgaacgggttcggccacgtatacgtcaagaagctggaagagattcagcagg

gccaacaatctggcttttctaacgccacgaccaacaccactcgcgaagagactcactcccacaacgactggctgaacggccaccacgactccgtcaaa

gccaagcaccgcctcaccgtcagtacagcccgccacctcttcaccgccgcccaattcaactccctcttcggctccaccatcggcccccgtctctggtc

catcctgcacgcaaccgacaccgagcccgtagccatcgcctccgacctcgccgtccagcagatcagcatcgaagatacctaccggaacttacccccgg

gcccggctgcggtggcggagatgcgtaagctcggtgagagcttgatcaaacggcttgaggtggagtgtgtagagatgccggatgaggcctgggttgcg

gctgtgcggggggcgaccaggcagcgggctgtgttggagacggttgtcgtgggaccttctgaagagcatggtcctggcgtcgagaaggaggagaggat

gttgactcaagtgcgcaattatgtggatgtggaagaagaggatgaacagcagacggaggaagtaggtcgtgctatcggcagcgagatgcctgcttcta

ccactgcgtcgaacaaacaacccagcgctcgaaggccaaagtcttggagaagatacccactcagcgtccgcctctcccttcgaatcggctggggcgct

cgcttttccaaaacgggcaagatgcccatcggaatctttgacctaacccaaccacgcaagaagcgcgtcgagatcctgctcagcacagtggtcaatct

cttccgacagatgacggccagccgggacatcagcgaaggcttcaacctaatcaatatcgccgcgctcgagctgagcaagtctccgccggcctcccatg

atatcaagaatttgctcgagaaggcgtcctctatggcgggcggcggcggaggaggagcgggggcgatgaattggataccgcaggttcaggacgaaatt

ggtcatgcagccgcggtggggatgaatgattcggtatatcctcagcctgggcctcaacctgagatcgacctcgatgtgctgaaggacctgcctgcgga

catcgccgccgagatagcgcaggagtatggaattccttggccattgcccacgcgcccgccactactaccatcacaagccgagcccgaacttagatctt

ccccttcctccttggtcgagccagaacgatcgtcgtttccgccagacgcaaggccacggacaccatcaaccccgccctttccatcacgctcgctctcg

cctttaccgcgatcaccatcactatctccgtcttcgtcgtgcctatccttcccaccctcccaactctccgcacaatcgttctctccgcctccgcagcc

tcgctcgatgccaacgacttcatccccatcaccacctcctgccgaagagcctcccgccccgatcgaaccccacgaggacgtacaggtggaccaaaaac

aagaagatgtcgacgaggtgacccaacacgccccatcatcccaacctggagcaggctccgagacttcccgtctgtcgaggcggttcggccatttcgag

ctgagcgggtgcgagatgagcattgatgagctggagaggctgttggctccacctacgccgcctctattaccgtctccctcgccttctccttctcctcc

tgataaggatgacgacgctgtgacgacgctaccgcttccttctaccggcccgccgcgagacccccttcttccgccttcacctgatgagacagaacaac

atcggtctgtagaagtacagggtgctgcaccacaaggctcgtctgtagaagtacagggtgctgcaccacaaggctcgacggaacctgtcagcacccag

gagcagcaggaacaagacaaaggcacgatggacgtcgactgggagcaagaggcgtgccggcactgcgggcgacaaatggtcatctggctccagcacga

ccacgacgtctttggggagagcgggctgcctgctgggcagcaacaaagcagcagcagcagcagcagcagcacagcggccgtgggcgatggacaccccg

ctgacgcagaggaggtcatgcgcacctactccttccacgacgaccccgctaccgcagccgccgcctcttccgccaggagacgcggccgccgctacgca

agccaggacaccggaaccagcatgctcgccgcctccgaacgccagcagctgggccaacaccatcagcatcagcagcacacggcttgtcaacgatggtc

gacctctacgaccaactcgacaaacaggagaagatctgcgaggacgctccgtccatcctccagctcatcgcacaggacggcttcgcgtcgtcgtccaa

taagccatcctctgcagggtgccctcctctgtgcgcacgaaccagaaccatcactgacttcactcaccgcttcgttgacatcgaacccatcgtccaag

tcaatgccggtcccgacctcgacgctcagcaagcccgcgggcgacagtgccggctccagcgcagggaacaaccccgctgctgtcgctggtctcagctc

cccaccaagcaacatcaactccaacatctggctcgcagccggcgagggcgatctcgactcggtccgctacctgctcgacaaccagccggacctgaccc

caacatcccccgacgatttctcctacaccgccgttcacgccgccgcatcttatgcccagctcgcggtgctcgacttcctcctgtcccacccaaaagca

gccctcttcacgccttccgccgagaactcagcggcaccatccggcgtacctgcccccaccggcgccgccaacgtacaagattccgatggagacacacc

cctcttcgtctgcgagacggtcgagcctgccaagttgctcatcgagaagttcggtgccgacccgaaacatcggaatctagatgggaagacggcggcac

aggctgcctacgagaatgaggcgtttgaagtttgcgactatttgcgctctctcactggcgagccggagccgacggatgatgattacgaagagggaggg

tacgagcgcaaggatgacgatgacgacgacgacgacgacgacgaagaggaggaggatgacgatgatgaagcggaggaggccgacgccgtgatccacca

cctctccctcgtcgacgtcctgcacggcccgccggaagtctccaccgccgcccacctgttgtccgagatcgtttatcagaacaaggatcggttgagtg

cgggtattattgtggccgggtgggataagaggaatgggggggccactgtgtgcaatgttcccttgggaggtggtctgtttcagcagccttgggctatc

ggaggatggaaccgacaacagacagtcgagttcgtcaccaatgctctcgcattggccatacgacgcgatcgctcgtccggaggcaccatccgtcttgc

cgacatctccgagcacggcgtggagagtcaattcatcccgggagacaagctcccggagctgccggcgaagctctgctga

>contig_21.contig_21.g985.t1

atggcgacccaccgcgactccaagttcccctcgaagccatacgttgacccgaccccgcttccctcggcgatcccggagg

tgcgcgagatcggcacctcctccgccccgctcaagtcggccagcttcttcatcggcgaggcgtgcaagagctacaacgaggacttcatgctgtgcaag

aatgagagtgcggacccggagcattgcttgaaggaggggagaagggtgacgaggtgtgctcaggatctcatccgcaagctcggtgatagctgtgggaa

gcaatgggaggagcactgggcatgcttagaacacaacaaccaggaattctacaagtgccgacagcccgaacggacgctgaacaaatgtgtctttgaca

aactgaatctggcgaagaagattcctggcactgccgagggcaaggagcaagtgcacgaactcaagcggcccgataccgagtctgtcactccgtcaaca

atgcaagaacatatctttttcggaaccccctggctgcctggctggagtgcctgcctcgacggcgtccgcatgcacgtccgttgttcctgttcaccttc

tgagagagcgagactcgagagagagagcgtcggcccgttcggcccgagatactgggaaatggtcgccgtgagggacagcttagctgagaagaaagtca

agctcgtgtctgcgtcgatctcgcgcgtcagcgctgccgccgccctgcccaatgcagcgcacgcagcagcggccgtgtcggtgtcgtcgatgtcgatg

tccgccagcctcagcggtgttggcagcagctcctcgcgctcggcctcgctccggaccatgagcacgcgcgccaaccgcaggaccagctgcagggactc

agccgcgctcggcactccttccacctccactctcacctccagctcagccgcggcgtccctgactctcacccgctccctcgcaaccgaagtccagcgct

cggccaccttcacaaagctgaacccaacccacatcgcaaacctcgccaaatgtctctccaccccgaccacctcgatcctcaccaccatcgctgcgccc

gaggctggcatcaaggcagtctccaaggacgagctcgatgcgtacaataacgactggatggacaagtaccacggctcctccgcctgcgtcatccggcc

caagacgacgcaggaagtcagcaacgtcatgcgctactgcttccagaaccgcatcgccgtcgtgccccagggcgggaatacgggcttggtcggcggcg

gagtacccgtctttgacgaggtcatcgtcaacctcagcggactccaccagattcgcagctttgacgaggttgcgggcacgctggtttgcgatgctggc

tgtgtactcgaggtgttggacaactttgtgggcgagaaggggtacatgatgccgctcgatttgggcgcgaagggcagctgtcacatcggtggcaatgt

cgctaccaacgcgggtgggctccggttcctgagatacgggtctttgcatgggaatgtgctcgggttggaggtcgttctgccagacggaaccattcttg

acggtctcagcacgctgcggaaggacaacactggttttgacctgaagcaactcttcatcggctcagagggaacgattgggctcatcaccggcgtctcc

atcatcacgccccggaaaccgaacgccgtcaacgtcgccgtcttcgccgttcccagcttcgaggctgtccagaccaccttcaccaccgtgaagcaaca

ctgcggcgagattctctctgcctttgagttcttcgacgcacagagctttgagctcgtccagggatatgccacgcgctctgtcagagatcccttcgagg

aacggcatcccttctatgtgcttattgagacgagtgggagcaagaaggagcatgatgatgagaaactggaaggcctactcgaacacctcctcgaatcc

tccaccatcaccgacggcgtcctagcccaagacacaacccaagtctctagcctgtgggccctgcgggagagcatccccgaggccgcgggcaagatggg

caaagtgttcaagtacgacttgagcatgccggtggagaagatgaacgagctggtggaagtgatgcgggccaggtttgaagggaaggggatgatggggg

aggggaaggggatcaagactgttgttgggtatggacatattggtgatggcaacttgcacatcaacattgtcgcggactcctacagccctgagatcgag

gccgtcatcgagccttacatctacgaatggatcgccgagcagaacggctccatctccgccgagcatggactgggtctgatgaaggctaccaagatcgg

gtactcgaagagtcctacttcgatcgagtacatgaagaagatcaaggcggcctttgatccggagggcatccttaacccttacaagtacatccaagact

gcttcgatcaatgggattga

>contig_21.contig_21.g986.t1

atgaaccgatccatcacagacgcgtgggcagcaggcgcagcagcggggacggcagcgggaggcgcggcatcccagcgca

ggagcggcgcaactcgcatctcgctcaacacccccgtcgctcagctccagcagcagctccagcagcagcagatccaccaccaacagctccctccagga

tcagcagcagcagcggcggtggcagcggcggcggcatcagcagcgcagggcactcacaaggaagcgaatgcggaaagtggtacccctacctcgccagc

taagcccatccctgccctcgacggcccagccctagcagcgaacgggtccgacgtcccaacaccaacaggctcccgcacgccaggcaagcgcaaactcc

gcatctcccaacgcaccgaatccacccaaccctccacctcaacctcaacatcgacatcctccacctccaaacgacagcgctccagtctccaggtaccg

agctcctccaccattgcggagaagtacaccccgcccgatctccggctggccgaccttggcggcgtgtcagctgcgatcgagaaggtgcttgagctcat

tgctactccgctctgtcatcctgagatctacgcgcacacgggtgtcaagccgccgagggggattctgctccatggcccgccgggatgcggcaagacga

tgctggccggagccatcgctggagaactcggcgtgcccttcctctccatctccgccccctccatcgtatccggcacctccggagaatccgaaaagaca

ctccgcgacacattcgaagaggccatccggatcgccccttccatcctctttctcgacgagatcgacgccatcacgcccaagcgggagaccgcacagcg

cgagatggagcgtcggatcgtggcgcagcttcttacttgtctagatgatctgtcgtgggaaaagtcaggagggaaaccggtcatggttattggcgcaa

cgaaccggccagactcgttagatcctgcccttcgacgagcggggcggttcgaccatgaaatcgccatgggcgtgcccgacgaggacggacgcgagcgc

atcctgaaagtcctggctaaaaagctccgtctgtcgggagacttcgacttccgcggactggcaaaagctaccccaggctttgtcggtgccgacttatc

ggccctgaccagtgcggctggcatcgtagctgtcaagcgcatcttccacgagctgggcacgtcactagaagtcaatggtgctccgaagcaaattacag

cgggtgaagacgtcgacatggcggtcgatggtgtgccgcagacagccgcttccactgcagagacaccggatctgagtaacgcagggtgggagatgagc

gcgtccgctcccgttacggaggtcgccgccgtttccaccgaacgctccttcgtccgacccctgcagtccttcttcgactccctccccgtctccctcca

aaaatcctccatcgcgctcttcctccgctcccaccccgacccgctcaccccagcccaactcgagccgctctccatcagcctgatcgacttccaaacag

cgcttccctccatccaaccttcgtcgaagcgggaagggttcgctaccgttccggacgtgacctggtccgacatcggggcgctgcatgcgattcgggag

gagctgagtttggcgattgtacagccgattcgcaggccggagctttttagggcgttggggtacgtcggcgagagtgagcgagcggtccgtcaggtgtt

tgcacgcgctcgtacatcgtcgccgtgtgtgatcttcttcgatgagctggacgcgcttgtcccgcgccgcgaggacggcatgtccgaatcctccgccc

gagtcgtaaacacactcctgaccgaactggacgggctcgaggcgcgcgttcagacgtacgtgatcgcagcgacgaaccgaccagacatgatcgacccc

gcgatgtgtcgtcctggccgactggacaagctcctgtacgtcgacctcccctcgccggcggaacgactggagatcctcaagacgctcacgaagaagtc

gcccctggtccgcggcgatggcaatggtgctgaggttgatttgagtgcgattgcgtttgatgggagggcggatgggtatagtggtgcggacttggctg

cgcttgtgagggaggcggcagttatggcgctcaaggaggcattgggtacctccctgacggccagtgccacccgatccttcgtcgagggcgaaggcgag

gagaccgaccggcagccgttgagctcgttcgacgaggacgtggtggtggaagtcgacggtatcgggcccggggccaggatgaagatcgcacatggaca

cttcgtcgaggcgctcgggaaggtgcagccgagtgtgagtatggcgcagaggaggcggtatgagatcttgaggagtcggttgtctggtacgcctgcgg

gggcgcgggcggggacggagacgaggcaggagaaggagaaggagggtcagcaggggggtggtggtgcgggcgtcgtggaggctgcgtga

>contig_21.contig_21.g987.t1

atggcgcccgcaacatccaagaacaaggtcggcctcggccacgccatcatcaaccgcaaggccaaggacgcaaaggcaa

tgaaccaacgcacaaacacgtcctccattcacactgcagacccgaccgcgccacagcacaaccagccaggatgggtcggcctccgcagtatcaccgca

cagaccagtctcgacgagttcctctccaccgcacagcttgccggacaggacttcaccgcagagcgaagaaacgtcaccgtcgtctccaccccgggcgg

gctgcctggaatgggcgaggacagcacacgctggaacccgtacctcctcaatcccgatgaggagcgtaacttgcttgctcagcagcgcgatcatgccg

ctcgactccgcgtcccgcgcagaccggagtgggaccagagcacaactccacaaggcctcgccaagctcgaggctgaaagcttcctcgattggaggagg

tctctcgctgtcctgcaggacggaccatcgggcagcggtaccaccagcagcatcccacagggcggcttcgtcctcactcccttcgagcggaacatcga

ggtgtggagacagctgtggcgtgtcgtcgaacgaagtcacctcgtcgtccagatcgtagacgcccgcaacccgctccgtttccgatgcgaagatctgg

agaagtacgtcgcggagcttcgcggtggcatgggcggacagggtggcatccagtacgtcgatggggaagagcaggacgagggagatcagtatggcaag

cggagaacattgctcctcatcaacaagtcagacttgctcgactacaagcagcgactcgcctgggcagattactttgatacacagaatatccagtatgc

cttcttcagtgccgccgaggccgcagaaatcttggagcgcgaacagctcgcggaacagcagaagctcgccgctgaggaagcggcgacggccagagcag

cggagaacacggattctgacgacgagggcagcgacgaggaggaggaggaggaagaggaggaggttgaagatgacaacgacgaagactacgaggacgaa

gacgaggacgtagatgaggcctctcaacatctacaaggcacgcatctcgacgctgacgagcacaaagaagacacagccgcagagggtaccacctcgga

cccgcgaactcgaatcctgtccgtatccgagctcgaggagctgttcctctcctgtgctcctccgctcgacaacttccctgaagtcccagcacccatcc

cgccgaagcccaagcatcctcacgccaaggtgccacagcagccggttgaagagcctcaacctcaagcagcgccctcccgtgtgctcactgtcggtctg

gtcgggtacccgaacgtcggaaagtcgtccacgatcaacgctctcctcggatcaaagaaggtcagcgtctccgccacgccgggaaagacgaagcactt

ccagactctgcatctcagtccggacgtcgtcctctgcgattgtcccggtctcgtctttccgcaattcgccaccacgagcgcggagctcatcgtcgacg

gtgtgctgcccattgatcagatgcgcgagttcactgctcctgccgagttggtggcccgccggattccgaaggatatcctggagggcgaatacggcttc

ttgatccagaccaaaccggttgaggaaggcgggaacggcgaggcgactggcttggaggtgcttggcgcatacgccatggcccgcggtttcatgcgaca

gggtcaaggtcagcccgacgagactcgctcagctcgatacgttctcaaggactacgtcaatgcccgactcctatactgccaccccccgccccgtctct

ccccaagcagcggagccgaatcctccgtcggactcgaagaaggcgacgcggacgagttcaacgcgggtatccgtgaccgcgcccgcagacggatcaac

cagcgccgcggccgaggactcgaagtcgacttcgacgagcagacgcacagcacggtccggttcaacccgttcgacatcggacgtgcgaaccatgcgca

gggagcgaagtcccgcgcgctcgattcggctttcttccggccggcaggtggggggatggcggcgaagtttgcggtgaaggggagacgggccactcccg

gggcgaatgtgattgagaatcgggttgcgccggatgggtcggtcatgcctggccgtgcggctggggctggggtcggggcgggggcgggagctcaggcg

ggtgggaaggagtttaagaagcatttcaaggggcgtccccgccgtgctcactgctcacctgccatgccttccagtcacacaccctctgcatctgtccc

tgcgcctcccttcttcggccttcctttcgagcttgtcaaatttattctggagcaatgcgacgactatcgcacccttcacactctccgtgggacctgca

aggctttcaagaacacgatcgacaatgaccccttcttcgacaaggttctctttcgcaatcgacctctcaccaatccaacgctcacgggggccgacatc

gccaagatctggagcctggaaaaagacggggaggaggaggagattggagatgcgatcaacacgttattccttgagcgtggcaacccctacatcgacgt

ccaccctgctctttcccgtgctttctggtccaaagacgcatgcagcagcctgaacgagatcagcctcaccccggagctcaccatcggagacgttcccc

acgtcctcaaggaatgcgcgacctggccgccggtctgtagtgtcggattagggatggaaatcggctacagcggccatctgcgcacgagaataggcggc

tcgtacagcatgggacctcccgagcccgtcctcgtcgaggaggtcctgtcgcatatcctcggatggtcacaaacactgcccaactggaactccgtcga

aaaagaactctcgtacccttggcgcgttgccgaagctccgaagccgaggcctttacacctcagttggtcgctcgccaccatacagaagatacgacata

aaggcctgctgtacctctatttttga

>contig_21.contig_21.g988.t1

atggacgacatgccgtcctcctccttcggcctgccctcctcgccatcagccgcaaccggcctcaaccgcgccctcgccc

cttcttcccaagatgccaacggtgccggggccagtggcagcggtgccaacggccgtgcaagcgccagcggtggcaacagtgccttctacctcaactca

aacagccgcggcagtgcggcgaatcgtggtgggttcgggcaggacccggatgatgaggatgatcccatgtcggacgacgtcggtggtcgggctcctgc

ctcgctacagggaagtcgagcgagtgcaagtgcgaatggcgatgctggtgctggagccggagctggtgctggtgctggagagggcgagggcgctcctg

aagaggaaggacgacaggctcgcggacgtcgacaacctcgtcaacgcgatttgcggaatctcgaggatgtgccgcctgtcacggacgacactggcgag

cgtgttcgcgagagttttgccgagtttctcgagagtttcatcgacgataagattctcaacgaccctgcgcagggcagccatccccgcgagccactcta

catcgaccaaatcaaggcgctgcgggacttccaacggacgacgctctttgtcgatttctcacacgtgctccgatacgaggagatcctcgcccgcgcca

tctcggagcagtactaccgtttcctcccctacctccgacgcgccctgctcgacctcgtctcgacgtacactcccaactatctccacatcaacgcgaac

gcggctagctcgaattcgggtcttgtgcctcgcgacttcaacgtcagcttctacaacctcggcgtcgtccagggcattcgcgccctccgcaccgaccg

gatccgtcggctgtcttgtatcagcggcaccgtgacgcgcacgagcgaggtcaggcccgagttgctctatggcacatttgaatgtcaggagtgcaaga

cggtcgttcgggatgtggagcagcagttcaagtacacggagccgatcatgtgtcgtggtatcgggtgtcagagccggaatatgtggaagcttatggtc

gagcagagtcggttctgcgattggcagaaggttcgcattcaagagaacgcgaacgagattccgaccggaagcatgccgagaagtcttgacgtcgttct

tcgcggagacatcgtcgaacgtgcaaaagctggagacaaatgcgtcttcacaggaacgttcatcgtcgtacccgacgtctcccagctcggtgtgcccg

gagtcaacgcccagatccaacgcgacaatgacggagcaggccgggatgccaactccggtatcacaggtctcaagagtctcggtgtccgcgatctcacg

tacaagacggccttcctggcctgtatggtgcagaatgcggactcgcgtggaggcgccaatgatattcgcgcgtcgactgcgaacgaggagggcgcggt

cgaaggcgaggaggatgcacggacattattggagagtatgacggaggcggagcgggacgagctcgaggttatggtctctgcggatgatatatatgaac

ggcttgtgggcagcattgcgcctgcggtgtatggacatgagatcgtcaagaagggcatcttgctccagctcatgggcggcgtgcacaaggtgacacct

gaaggcatccatctccgtggtgacttgaacatctgcatcgttggcgacccaagtaccagcaaatcacaattcctcaagtatgtgtgcagcttcttgcc

acggagtgtgtacacctccggaaaggcatcttcggctgccggtctgacagccgccgtggtcaaggacgaggagaccggcgagttcacgattgaggctg

gtgctctcatgctcgccgacaatggcatctgcgccattgacgagttcgacaagatggatctcagcgaccaggtcgcgattcacgaagccatggaacag

cagacgatctcgattgcgaaggcgggtatccaggcgacgctcaatgcgcgtacttcgattctcgctgctgccaaccctgtcggtgggcgatacaaccg

caagcagacattgcgggcgaatgtcgcaatgagcgccccgatcatgtcccgtttcgacctgttcttcgtcgttctggacgactgcaacgaggcgacgg

ataagaacattgcgcagcacattatcaatgtgcatcgcttccgggacgcagctatcaatcccgaattcaccacggaggctattcagcgatacatccga

tatgcacggacgtaccagcccaagttgacgcccgaagccagcgacgtgttggttgagaagtaccgtctgctgcgacaggacgatacgggtgcgggcgc

gaactcgtaccggatcactgtccgtcagctggagagcatgattcggttgagcgaggctattgcgcgcgctcactgtcgtcacgagatcacgcctggat

ttgtgcgcgaagcgtacgccctgctgcgacagtcgatcatccacgttgagaaggacgacatcgatctggaggacgacgatgatgatgatggggtcaac

cccaacgctggcggctttggcggcggcggcggcggcaacgatggcggcgacgacaatgatggggacgacgatgacgatgatgctgacctgcctgatat

cgctccgcggagaccgcagggcgatagcagtcaagatccacagtcttcgctcggtccccagacagacagcatggagatggacggccaagcgcctcctt

ccggccaggctggagcggccgagacgcagtcccagtcacaagcgcaaccgaaacgcaagatgcgaatcacatacgaccgatacatggagatcacgaac

ctgatcgtgctccgggtgtccgaggtggagcgtgcgacgatgcgcggtatccccaaggacgagctcatccagtggtacctcgagcaacgggaggaaga

catccagacgattgagcagctcgaggaggagaaggagctgattcggaaggtgctgcacaagcttgtgaaggagcagtacctgattgagctacaggcga

gctctgcggacgaggagggcgaggattcggagatgcgtcagggcggtagtggtgctgggcaaggtggggcggatgcgtccctgtctaccaccggggct

gatcagacctcggctacggttcaagagacgatggcgccctttgaagactcgacgcttgaccagaccaacaccacgacgaccacgacgtcgggagcgaa

caacacgtcctcgggtgctgggggggatgctacgacttcgtcggtcgacaatgcggcgggacggtcggcggggacaccgatgacggttattatgcttc

atccgcgcatcgatgtcgattcgctgggcgatgcgagcaccgcttga

>contig_21.contig_21.g989.t1

atgttcgactaccacgccaccaagacgagtcaaaccctctccgaaccgactgggagcttcccaggccggggttccatcg

cctctgtctccacgccaattcaatctcctccagaagacgattctccgtcaagaagagcgccgtcgccaggctccgctcacctccacagcatccccgac

gccgaaaagaaggagaacgatgcggcaatcataagcgctcaacgcaagtctgttaccgaccatgatgaacaaccggacgaaagctccttacaaacatc

agtggcaaaggcaggaagccgactggcgtcgtcgcagcccggggaacgctcattaatcgggatgagcgatctcggtgcgttcatgtccaaggcgagcg

cggacggggacgcgattaatgctacggcgtactctggcgcagaagagggcgtcatggccgaattcaatacctcgcacgaaatctttgtgcttggaaat

gcactctactctctcacgcctttactgctggctcctctatcagaggtatacggccgaaaccgcatcctgatcggatccgccttgatctacgccatctt

ctacatcccacaagcgctcgctcccaacatggccaccttgctcgtctcgcgctcgattcaaggagcggtggcgtcggtgggcaacagtctggtcgggg

gtgtgatttcggacatgttcgaggctaaggaacgcgggaatgtcatggcggtatacggaattgcaaacttcgtggggcaaggcatcgggcctgcgccg

agcacatacctcgctgtagcgaagggttggccctgggtgttctggtggcagcttatcatcaccagtgcgctgctggtcctgatgatcctcttcgtcca

agagtctcgtggctcggtcctcctcgtccgtcgaacacagcgattgacaaaggagagctttgcccgcgctgccaccgccgctgctgctgtccgtccac

aaggtgtgccaagatatagtacgagttctggtactgatgcagagaaggagaaggagaaggaggggcaggaagggagaggtgatggggattctagaccg

acggtgtacagatgtcgggctgcagaggagcgggcaagtctggcggtgatggcgaagacgagtctgacgagaccgctcgtgtatctcgtcaccgagcc

agtcattttctggatggccctgtgggctgcgacggcttggggcactgtcttcctactcgtcgacgtggccctgttgctggccaagctgatcgcttggg

aatcttcctcagatcctctctacgtctgcatcttctcatatctcgcagatgtgtacgagcggtacgcttcctcggcgttggcagcacagagccttcta

cggaacctcgccgctggcctgattccactagtggccacccagctgtacaacgctctgggaacccaatgggcgtccacgctggtcgggtgcgtagctgc

ggtcttgggcatgtgccccttcatcctgttccgctacggcgagcagattcgggcgcggagtcgggtagcgagggcgatgcggcgggaagaggaggaga

gggagagaaggacggagctggagcgggaggcggtggacgccaaggtgggccgggcggaggagaggagacggaaggcggctggagctggcggtggcggt

ggcggcgacggcgaccagggcatagaagctcggacgctgcgaaactcggatgggtcgatggcgtga

>contig_21.contig_21.g990.t1

atgtcggacgccatggaggcgttccgagcgatctcggccctgccttcgcagtggcctggcctggtgattgccctcccgc

tggggggcttcgccatcgacactcgcctcccatttggactggcgtcggccacgggggtctggggctccatagccgacctcgtcaagatcgccctttcg

cggatctttccaagacttcgagtcatcaaatgggtggatgacttcatctttctcaagccggcagatgagccgctctcgctcgacgaagtccatgaggc

caccaaggagctaggcttcccatggcacccaacaaagcggtcggagttcgccacgaccgtcaagtacctcggtttccattgggaccttgctgcgcaca

ccgtcacccttccggacgacaagcgggtgcatttcgccgagcgggtcaagtcgtttacgacctctgacccgaaatcactcagggacgtacgagagctt

gctggctccgtacaaaacatagccatgatggctcgggatctcgcgccgcacacggcggagatcatcagtttcctgtcggcctggaactctcaaccggc

ttacaagaagctccatgtcccctcggccgtccaatcggaggccaaggcttggctcagggccttggggggtgagctgatccgctccattgccgtcccgc

ccaccacctttccccacgtaatctacgtggacgcctcgacaagttggggcgtcggggtcacctccgatgatcgatgggccgcttggatgctcctgtcc

ggatgggacaaggacaaccgcggcatcggatgggctgaagcggcggctctcgagctcggggtccgccaggcagtcgcgatgggcgcaaggaactgccg

agttgaagtcttcagtgacaacaaaggtgtaattggggccttccggcgtggacgtagccgcggtcggtccgcgaactcgattatgaggtcgctcatcg

ccttcgagatggagcaccatctcgatgtgcgggtcactcgtcagggggctgcggctgcgcggatcgcatatccgaacccgtcggcgacttcatcatcg

gccggaccacatctggtcttcggctctgaagcttccgacattcgtcgccagcactctcttccatcgacatcacaccaactatcttcaacatcgaactc

actcacacatcgttcacacacactcccgggcggtctgcccgttgcaaatcaatccaaccaggtcgtcaagactattcccaactccaagaagaagcccg

tgcttcaggatccgttggccggcttccctctcaacaagcaaggcaagatttatgcgaaggatcgcattctcgcttacccgccattcagcggtggaagc

atgtcccttctcacgtcgtcagggctcgagttcgacccctccggcttccactggtcgaggatcaagcatgtgctggcctcggctctcgaaacatcaac

ccggagcaactacggcgcgggtatccaacacttcctggatttctgcgatgagcagggcatcccccctgagcatcgtcatcccatttctcaatacctcc

ttctggcattcgtggtcagactcagcgacacatgtcgcgcgaacaccatcagcacctacgtctcctcgctcgtcacttggtgttggtaccagacctgg

aacagggtcctcgtccgtggtgacggagtcaaccttgccatcaaggccatcgccaagaacggcccaccacctctccctctccgtcccccggtatcgcc

agatgacctagacgccatctttgcggggctggacgtggccacaacgccgctccacgcagctgtgtgggcgtgtgccacgctctcgtggtggtccatgt

gccgcctcggggaaacaacaatcgcgtcttatcccattcgagcggatcgacacgtcatgcgctcccacttgcggggcacggcgatgtcggacgctggg

ttcgcggtggccaggatctggctgccctggacgaagacaacaaaagctaaaggcctcgacaaatatatcgtcgactgcgggacgcacaccaatgcggt

attcgccctcaagcaccatctctctctctctttacactcactcgcgctggcctcttcaacacctctgttcgcctacaaggcgactccgacatcctcaa

gcctcacaccgctgacgaaacaagtcttcctcgcggtgatcgaccgcgcgctcatcagtgccgggcgggaaacccatgtcggtcactccttccgcatt

gggggagccacagcccattggcgatcgaaggtcccccttgagaccattaagttgatgggggggtggcagacagatgtcgtcactcaataccttcgtga

ccttggcaaaggcctggccgacgcacacttgctcacagccaaccacatggctgcgcggcgcctgtcctga

>contig_21.contig_21.g991.t1

atgccccatcggacagctctctccctcgacaaagccgctgccaaggttgctcccaaagcacaccacctcgtcatctcca

accccgtcaactccaccgtgcccatcgtcgccgaggtcttcaacccgccgaggtcaatgtgtccgtcgtcggtggccaccttcggcgtcaccattgtg

cttctcctctctcaagtcgcccagggttccatactccaatag

>contig_21.contig_21.g992.t1

atgacggacaatccgtcaggcgaacacgcccttccccctccaacttcccagcaagctgccggtgcggcacccccttcca

cagcacaacaagcttccaccgccctcatggcagggttcaagtcctccctcaaggaggcacagcgcattcgcgctgaccaaggaggtccttcggcgtcc

cgacagctcgacatcgatgtcgaggattccgacaacggcttcggtgaagaggaggaggacgaagagcatggagctcaagacaagggcaaacagcggcg

catttcgcctcggccatccgtcctcggagaccaacacgaggcccccgtcaccgcgccctcaacccctccgaagcggttcatggagccgatcgtcaagt

cgcgcccggaacaagtccgcgccatgcaactcaacgagtttacggctctcttcgacgacatgtcgccaaagacgcgtcaggccgccatctcccgggct

ctcgacttcgaggccaacattgccaaggaagaggaggccgacgaggagcgacgggccagcatcaacccttaccgcaaagcagctgctgagtcttcaac

taagactcaatctcgacaagaagtcacggagtccctcaagaggcgccattcgccccgggagatccaagacctcaagcgctcccgcctcgatgcccatt

cccttcaccactcggcgtccgaccctctcatcatgaagggcgcgcccttcaagcccggcgcccagagtggttcgtccgtcgacatgccatcgttggtc

atactcaacaaactcagaaacagcatgtatgtgccattgtggcatttcacccaaaaaggcatcaaggctggccgcgcgcgtgacgccaacgtcgcctc

ggcaacaactgcggccctgtcaaagctcctcgacactttcggcgcttccgcccaactcggcggtcatcgcaaagaggactgttacatgacatatcaag

aaatgacatcggcgttcaagatactgttcaagatatacgacaagctggtccttgagactgtcggggaggcgaaggagtggattcgcaccgagaagcgc

gcctggcaggatggttgggagatggtccgcgaaagtgaatacgcggaccgcgagtcgagctgggaatacctggccctgtacgtggacaagctgcgaca

ggagtactacaactcgccatacggagatcgacctgacccaagtgtgtggcaagatcggatctggcgccggaccaaggacgccatggacatgcgccgtg

cgctccccgccccgtcggccccaactcaggatgcccccaagcaaggcaagacgtcggcatcatcatccaacaagacctttccgacaagcacccccggc

aagaagccacagcagggaggtgcttgcttccaatgtggcagccgcgccaagcacaacctcgaggagtgcaacaaaggcccggacggaaaggacgggct

gagcatggttgccagtcttgccctatcaccaagtccgcttaagcccgagggctttgacaagttcatcaagaagttcgacctttcgcacgcattcccgg

cgatggtcgacaacatcaagctcggcttcgacttcggcatcccaccaattcgggagacgctgattcagccaaaccattgctctgctgatgacgagatg

gagatcctccaagccatcgtggctaaggagatggaggtgggacgtgtggtcggtcccttctccaaggaggaggttgaggccagagttggtgcctttca

gacttcccccctgggcctagttcccaaaccgggcggcaaatggcgcatgatccaggacttctcatcgccacgccgaagccctattgctgcgatcaacg

actacatcgattcggacgaattcgtctgctgctgggacggctacctcgcgatggtggacgaggtcagtgcccgttga

>contig_21.contig_21.g993.t1

atggctgagcctatcagtatccccgcaagggcagtcgtcctgccgctgccgtacccgctcgtcctccacccgggactga

tgctgagcatcccgcagtcgtactcccagtcgctctccctcctcaaggcagccctccaggtggccgatgcgtcgccgaacagctccggcagcaaccgc

tcagccgcagaccggcccatcgtagtcgtctgcgtgcctaccctccgcactcctccgcatgtgcagcccccatcgcccacaagagcgctgccctcgcc

tcccccgggctccagctcggacatcgtacagccggttcggatcaacgacctcttcgactggggcgtcgctgctcgcctgcttcgtctgacccgccacc

cttccacccagacctgcactctgctcgtcacgggccttcagcgagtccgcatcgatcgatggctctccatcaaggcgcccctgacgaccagcgctctc

gacaagaacaacagccttaccgtgcccgatgtgcccgtcccgctcgcagcgattactcccttcaaggatcccatctcgggatggccagcagcatctga

tgtacccactcagcaatcgaacaagaaggaccgcgatcgcgatgttgagacggttcgtcagttccgcgaggctgccctcggcttgctcgacgccatcg

cgcagctcaaccccgtccaaacggcctcgtctgtcgctgaccaagtcgacaagtttaagcctaataccaacccagatcccatcgcgccacccactccc

gcgcttcccatcttgccgcccgcgctcttgaagcgcattcgagcttttgtgaaggacgccacccgacccgatcactctgccttcccggcctctgcatc

agctgcggccgatgtcttgatcggcaccctcggcggcgcatgtccctgggcttctcgcgtttctctcctgtcagagtgggacccgcgccagcgcgttc

gtgccgccacagctctcttgatcgaaggcactcagcgcgtcaagacggcccgtgagctcatcagctcactcagcgctcccctcaataagtctgcaaag

gaaactctggcgcgcagtcagctcgaggccctgctcaaccagctcgctgccctcaatcccaatatctccgccaagattaccactggaaacaccacctt

cagtatcggcagtgctctcgacgacaaggacggtgacaaggacaagggtggctttggtatcatatccatccgtcagcctcagcgcggcgaggacgatg

acgcgaacaacagcaataataacaacggcaagccagccatcatcatccgcggtaacctgcccggctttggagctcgctcccgcggcagctccggcaga

ggagggccctttgacggtccgccaagtggcgcctccctcggtggcaagcaggacggtggcgaggatgaagacgaactcggagaactccgtcggaaact

cgacgaagcctccctctcccccgaagcgcgcaaagtatgcgatcgcgaacttaagcggctcgcgcgcatcccgccgcagtccgtcgagcgcggtgtac

ttgtctcgtacctcgaggtcatgtccgagctgccctgggagcgtgtctcgtccgaggtcgcgccggagaagctgaagatcgggcgtgatgcggaagac

tcgcagcggccgaggaagagcatcaaggccggtgagggtctggtagagcgcgccaggcggatcttggaggaagaccattacggtcttgacaagatcaa

gaagcgactggtcgagtaccttgccgtgctcgagctcaagacgcttcaggctgaagagcagctcgccgaggaggaggctgcggaagtcgctgccgctt

cggcaaaggcaggagagggtggaaacaacgctgcagaccagacctcccgctccgatgggctgcgatcaggggacaacgacgatgatggcttccagcac

ctggaggatatcgctgaggagcaccgggaggccagggtacagaaagatcgggatacgctcgagcatgatcgggcgaagaagcgtaggaaggcagccac

tatcaaggacaagggtcccattttgctacttgtcggtcctccaggcgtcggaaagacgagtattgcacgctctctcgccactgcactccagcggcccc

ttacacgtctctctctcggtggtgtccgcgatgaagccgagatccgcggccaccggcgcacctatgtaggcgccatggctggtgcgattgcaagctcg

ctccgcaaagttggggtcagcgaccctgtcatgctgctcgacgagatcgacaagttgggcagcgggaacgggatgcacggcgatccgatggctgcgat

gcttgaggtgttggaccccgaacaaaatcacgccttcacggaccattacataaactgccccatcgatcttagtagagtcctcttcatcgccacagcca

actcgctcgacacgatctcaccaccccttctcgaccgtaccgaggttatctacgtcagcggatacacgattgacgagaagatcgccatcgcgcgatcc

tacctcgtccccaagcaagtcaaggcccaaggtctcaagccggagcacatcgtcatctccgacgacgtgctcagcaagatctcctcaagctacacgcg

cgaggcgggtgttcggagtctggagcgtgagattggctctgttgcgagggcgaaggcggtcgagtttgccgaggcgaggaagggcgtgaaggacgagc

agggcgaggcgaaggtgtacgaccctgttgttacccttgcggacgtggagaggattttgggtgtggagacgtacgagcctgaggtggcggatatggag

gcacgtcctggtgtcgcaacgggtctcgcataccaaggcagcggcaacggcggtgtcctcttcatcgagacggcactcctccctcccggctctggcaa

cgtgaaactgacgggctcccttggggacgtcattcgcgagagtgccgagctcgcgatctcttggatcaaggccaacgcgttcctcctcggcattacca

gtacgcgcgagtccgagttccccaagaacgacctgcacgttcacttcccctctggcgcggtgtcgaaggatgggccctcggcgggcattgcgattacg

tgtgcgcttgttggactgttcactcggacggctattgatcctaagttggcgatgacgggtgagattacgttgcgtgggcaggttacgcctgtgggcgg

gatcaaggagaaggttctcggagcacatcgtgcgggcatcaccaagatcctacttccctaccggaaccgcaaggacgtcgaggccgacctgccagcca

agatcaaggcggacgtcgagattgtttacatcaagacgacgtggcaagccctcgatgctgcgtttggacatgccctgttcgagcacgtcgtcggccac

cacgaaggtgttgaggaaggtcgcaagcggatgacggagatcgtcgaggagcatcgtctttga

>contig_21.contig_21.g994.t1

atgagtccgtacgcaaagcccgtctctttcgcgttcatcaagccccgaagtccgtacgcaaagcccgtctcttccgcgt

ttaccaagccccgaaggtacgccccgagagtacgccccgagggtacgccccgaagctgccaattcggcgcctctgcccacatcgaactgtccttcctg

cccggctctttctcagccgtccagcctcgcgccggaggagaagagggtgtcagcggggcaaaagaactcctggatggcggtcacggcaaatccgaggt

ggacgagtggagggccgaagtgtcgaagtatcgaagctatggttcgggtctgacggagggaggtcagcagtacgtcgaaggtctggagaagctgatta

aagcagccgaacagcaagaaaaactggaccagtggagggccgaagtggcgaggcttcgaagcctgttccgaagtcagcagcgaccctga

>contig_21.contig_21.g995.t1

atgtataatcgttcccatcgcatccaaaataggggtcgtaccagcaaggtcgatatcatgtcgttttgcaaagtcgcat

tacttcgagctcgtctcgatctcttgaatggaagccaacgcgtttctcctcgacattaccagtacgtgcgagtccgagctcccaggaacggtttcctc

cccggcacggtctcgaaagatgagccctcggcgggcattgcggttacgtgtgcgctcgttgagctgttccgtcggatggctatcgatcccaagttggc

caagttggcgatgatggatgtcgtgcggacatcaccaagatcctggttccgtatcgaaatcacaaggacttcgaggtcggtctgcccggcgaagggaa

agtcggacgtcgagattcttcccgtcgcaagatcacatggcaagctctcaacgttgccttacgtcgtcggacgccacgacggtgacgaggaaggccag

aagcgaatggatggaagtcgtcgaagactaacacgcgtcgaaggtaattgcgatgtgttttga

>contig_21.contig_21.g996.t1

atgagtccgtacgcaaagcccgtctctttcgcgttcatcaagccccgaagtccgtacgcaaagcccgtctcttccgcgt

ttaccaagccccgaaggtacgccccgagagtacgccccgagggtacgccccgaagctgccaattcggcgcctctgcccacatcgaactgtccttcctg

cccggctctttctcagccgtccagcctcgcgccggaggagaagagggtgtcagcggggcaaaagaactcctggatgacggtcacgtcgcatacttgct

caaactgtccgaggcgtcgggatattcgaaccaggcggcgcattatgaaagcttaggtgagaagctgacggatggcggtcggacctacgccgaaggtc

tgaggaagatggcacacgaaaaagaaggtggaagcgtggagggccgaagcggcgaggcttcggaaccttggttcggcgctgacggatgccggtcggaa

gctcgccgaaaaactggagaagcaggtagacgaagccgaccagcaggccaaagactaaagaggaacatctcgcccgagaagccgcccaacgggcagaa

cgagcccggcatcagcagcgaccctgacgatgttctcgctatggatgtgaccgcgtttcatacgccctgcacggcttccgttcgttgccgccgtctat

gccaggaagagacgtttctcacacagctgacagcagaagtcttccagctggaacacatatttggaggagcaaaaggagaagagaagtgggaaagagac

acgttttcgaaagctagagcagagaaacaaggcaatcaggactcgagagagatggagcaccgccgtccgaacgaacagaccaacaagcgcaaacgtga

ccgcaatgaccctcagaaggctcatctttcgagaccgcgccggaaggaaattgttgctggaaactcggaatcgcacgtgctggtcatgccgaggagga

acgcgttgtctcgcgacgaagtgaggaactatacactctcgccgatggagtttttgcacatctgttcggggtttgaccaatcactcctgggtaatccc

ttacattcgtttctgctgacatcttcccctttcctctcctcctcctcctccgccctggtttccgtctgcaatgggtctacacaggctgaacgtattct

ccacaggaagttccagcgtttccaggccacagaactgctatga

>contig_21.contig_21.g997.t1

atgccgagttcgacgacgtacgcgcgcgagtgggccgagtacgacgctgcgcacttcggggtcgggctggagaagcttg

gtcaggcatgtgcgcggtattggagctttaaggcgcggtataccaatttgaacgcgggtgcgtttggggcgtgtccgaatgtggtctgtcaggcgtat

aaggagattcaggatgagattgaggcgtgtcccgattacttcatcgactctgtccttccgaaacgtctgctggcctcccgcgaacgcatcgccccgct

cctcaacgtatccccagacgacatcgccttcgtccccaacaccaccaccgccttccactccgtcatccgggacctgccccacaccatcttcgagccat

ccgacgccatcctgacgctctcgaccatctacggcgcgatcgggctgaatgtgcagtacctactcgactcaaacacagggaatcaggcaggctatcgc

aaggtggaggtcccgcttcggtggccgctccgggatgaggagatcctcaaggcggtggaggaggtcatcttgcgggatcggagcgaagaagcactggc

tcagcggaaggggaaaggcaggattcgcctggcggtgtttgatgcgatttcgagtatgcctggtgtcaggctgccatgggagaagctcgttgctctgg

cgaagaagcatgtgatcatgacctttattgatggagcgcatgcgattggacagattcaacttgacattggcagtgcagatcctgactttttgctgtat

gcgcatcggggatgtgccgtattctacgttgccaaacgaaaccagcacgccgtccaaggtgttcccatctcgtggatgtacatacccgccgcacagag

agcggaagccgacaaggctgccgtggaaggggcattgcccacgaattcttggagggaatcgtccggaactgtagattggtcaagcttgatgacggtcg

acgcagcactcgacttccgacagaagctgggaggcgaacgccggatcatggcatatacgcactgggttgcgctccgaggcggtcaactagccgcaaag

attctgggcactgaaatgctggaagtattggacgatgttaccttggctgcggtgaccaatgatcccgatcagccttcgacgacaaacgaggacaaggg

agagaacgcaagcaccacagctgctcccaccacggaaagcagcagatcgtcatccagcctcccgcagcgccaacgcccgcacacgatgtcgctagtga

acctccgtctgccgctcaccagcgccgtcttctcgccggcccatcccgccgaacggaaagccaagctaaaaagcgtacttcttccgaacgctttggga

cgagttccgtacggcgattccggtgtatga

>contig_21.contig_21.g998.t1

atgagtccgcacgcagagcccgtctctttcgcgttcatcaagccccgagggtacgcctcgagggtaccgcccgagtcaa

gtatcagtccgaatccgaccttcatcatatctgccaattcggcgcctctgcccacatcgaactgtccttcctgcccggctctttctcagccgtccagc

cttgaagctcgcgccggaggagaagagggtgtcagcggggcaaaagaactcctggatgacggtcacgtcgcatacttgctcaaactgtccgaggcgtc

gggatattcgaaccaggcggcgcattatgaaagcttaggtgagaagctgacggatggcggtcggacctacgccgaaggtctgaggaagatggcacacg

aaaagaaggtggaagcgtggagggccgaagcggcgaggcttcggaaccttggttcggcgctgacggatgccggtcggaagctcgccgaaaaactggag

aagcaggtagacgaagccgaccagcaggccaaagactaa

>contig_21.contig_21.g999.t1

atgcagtcgtcctcctcagcaggtgcgactgacccttgggcagaggaagagtcccacgctcctgcgctctctgccatgg

tctcatcctccgacccaactcagcacgacgacgacaacggcgacaacgacgaggataacgataaagacgacgacaaggatgcggacaaagaggaggag

gaggaggatgagccagcctcgacggaggatagcgaccatctcgcgcctcccacgtccgagctccacaccctcaacctctcctccgcgccagcttcgtc

caacccctgggccgaatccctccccgcagacccgctcgccccttcctccaccacccccacctcccgccccaccgaccctctccggaccctctcctcct

ccacctccatcaccggcggcattggtcccgcaacaacagccgagcacgaccctacgccgagttcgacgacgtacgcgcgcgaatgggccgagtacgac

gctgcgcacttcggggtcgggctggagaagcttggtcaggcatgtgcgcggtattggagctttaaggcgcggtataccaatttgaacgcgggtgcgtt

tggggcgtgtccgaatgtggtctgtcaggcgtataaggagattcaggatgagattgaggcgtgtcccgactacttcatcgactctgtccttccgaaac

gtctgctggcctcccgcgaacgcatcgccccgctcctcaacgtatccccagacgacatcgccttcgtccccaacaccaccaccgccttccactccgtc

atccgggacctgccccacaccatcttcgagccatccgacgccatcctgacgctctcgaccatctacggcgcgatcgggctgaatgtgcagtacctact

cgactcgaacacagggaatcaggcaggctatcgcaaggtggaggtcccgcttcggtggccgctccgggatgaggagatcctcaaggcggtggaggagg

tcatcttgcgggatcggagcgaagaagcactggctcagcggaaggggaaaggcaggattcgcctggcggtgtttgatgcgatttcgagtatgcctggt

gtcaggctgccatgggagaagctcgttgctctggcgaagaagcatgggatcatgacctttattgatggagcccatgcgattggacagaatcaacttga

cattggcagtgcagatcctgactttttgctgtatgcgcatcggggatgtgccgtattctacgttgccaaacgaaaccagcacgccgtccaaggtgttc

ccatctcgtggatgtacatacccgccgcacagagagcggaagccgacaaggctgccgtggaaggggcattgcccacgaattcttggagggaatcgtcc

ggaactgtagattggtcaagcttgatgacggtcgacgcagcactcgacttccgacagaagctgggaggcgaacgccggatcatggcatatacgcactg

ggttgcgctccgaggcggtcaactagccgcaaagattctgggcactgaaatgctggaagtattggacgatgttaccttggctgcggtgaccaatgatc

ccgatcagccttcgacgacaaacgaggacaagggagagaacgcaagcaccacagctgctcccaccacggaaagcagcagatcgtcatccagcctcccg

cagcgccaacgcccgcacacgatgtcgctagtgaacctccgtctgccgctcaccagcgccgtcttctcgccggcccatcccgccgaacggaaagccaa

gctaaaagcgtacttcttccgaacgctttgggacgagttcctccatgacgatccgatggctacggtgcttgatgcattgatccgttcgtggaaccacg

ctttcatggaccatatatcaacagacaacaacctagataagtga

>contig_21.contig_21.g1000.t1

atgagcgactcccacacagctgccatccttgctcctactcctcctccccaggacagcccatcaactgcagccaatcgca

ccatgggcacctcctcctcctcgacctcgccctcgccgtggctgccgcaattcgccggatcaaaggcaaagcgcactcgccagatcctctaccttctc

gccatgcaaggactcggcgcagcttttatcagtggaggtatcaattttgcagtctccgtggccatgtatttacgagcgtccgacatcaccatgtggat

cctcgccaagaacacaatagcaggcgacctcggcgtgacaatcctgatccaatgttcctttactggccaggttaccatctctttcattatcacctcct

ccctcattcgaggggatctgcgctcaggcttagccatccctctccagcagccgtggccccctgctcccccgtccacaaacgccgccaaaggatcagcc

aatctaccaccgtcgacccgatacgaggacacacagcatcggtccacattccaactgctcaagcacaatatagaaactttctacatcgtcactcagca

gaaccacatcttccagaacgacctctccgcacgcgagcgcttcttcagactcgcccggactgctctccaaggcttcgtcctcgcatgctgtgtctttc

cctggttctggggcatcgccatcgccatcttggcccccatctactcgaacaagcaaatggcccatacctggatccccatggtcatcaagggcgtctac

ggcgccttgctcggtctggtcaccaacccactcatcgccctcatgaacctcggcgcacacgacgttgctggccagcagaactcggcggctgcactgtc

ctcagcccctgaccgatcagccgagaccgctgatttggaaaagacgggaacgccggccgggcatacttctgctagctcggggactcctcgtcttcccg

acgaagaagaagagcaagaggagcaggagatcgttcctagctcatcgagcggcgccgcatttccagcttcggactctcggatgaccatcgacggtgtc

tccagcaacgcacatgaggacgacttccactcggtatcggacatgcccatcggcgttacaactacacaagctgcatag

>contig_21.contig_21.g1001.t1

atggacagaagaactcgactgccaacgatcctcttccctcggggagccggcacagacgatgcatcccagtcgtccattc

cagctgccacctctcccaacaagacacccacgaagtcgactgccaccaagaacaagaacaacgacgacgacggctcaggcaacgagctcaccccaaac

ccaaagcacaagcgatacatgtcagccagggactacatcacacagcgacatgtgccagactccctcggcttcccagcaagtctcaacgaggccatcaa

ctcagcctttgccgaggagcagtacgaggccggcgttgagaccatcgaccacagccgcgcaaacggcattataccagcagagcaccacctccgcatcc

ttttcgcattctccctccgtcccaccccagacccaatcacctaccgccaaacagcaagctccctcacaggcgcactatccgcaccaccctcactccct

ctcatccaccgagcccgcacgctcctacttgacctcgcccacctacacggcccggaccgaatcctccgcgctctctccccagctaagatcgcgcacgc

aggcggattggacgcacccagcaaaggatcccttcgcgccactgtcaagacgggccgtaatcgctcttatggcactctcggtcgggccaaggataaca

gtcccgagtcggacgaggatgatcaggacggcacctttctcgaaaatgagccggcagccgctgcgcttcagaagaatcctttcctcgttcccttccct

ctcgccgaggcctcttcctcgtcctcctccacctccttcggctcccatctcgctccggccgtaagggacagctgggaactccttcgtacacccccgcc

gggatccctcacagaggcagacctctaccgacgagccctcgctgcggccgaggcggacgagtctagcaacgggaatatgaccctcaacgctgccgcca

ttcgacaaatagctttgcgatattccgcacgaaggcgaggacgcggtcgcggagggaacagcgcagccgctgcatcgtcctcgtcctcacgcgtccca

gccgacggcttcctgacacatgaagatctccttcgacactcgcctgccgccattcagcgctataacgagattgtatacggcaaatccggtgctcggga

caccctccgcacccgagccgtcaacggcgaggcactcgtcttcatggcacccttgtggcaggctgagcgcagacagatcatgtatggctccaagaagg

gtaaagagcctgctcttgatggccaacccgctgagcggctgtacgacgaagtgaacgcgctcaaggcgctctacggcctcagtctcgcctgggacttc

cccatcgatcgcaaattggcccagtcgatgtcgaccgccgcgaggaagtcgaccatgcccaaggaggcacctgcagtgaaagaggacgcgagcgcgag

cgcgaacccgcccccgcctgccaagccggccaagacgcacgccttcttcgcacagcgcttcgcctcctcggccccgtccaaaggttttaaccgcgggt

cgcccaacataggctcgagcccacctgcgtccgcagcaggcggcccaccatcctcgcccgccgggcccgggcccggtcccacctccgcctcgggatct

ccctcggcgtcccgagtcgcgaggacggggccgcaggggtgtgcggaggtcgggcaggcgctggacgtcctttttgaggtgttcgcggggaacgtgct

cctgtccgatcgggacgggctgtgtcggagtgcgttggcgctcgttgcgtcgaatggtgctggggggaatggtctgagtggggatgttgcgacgacga

acgggtcggctgtgtcggcattggggagtggggcgccggcggtgtcggcgaagtggagggagatggttggcgctgcggctactgcgttgtacgaggtg

cttcaattgactcggcaggcggacgagaagagtgcacctgtgctggatgcggacgcgctggagcgtggcatcgcgcagcgacttgcatgggcggacgt

gggtgctgatgctattgctatcactgaacaaaatctgcgcgcaaatgttgaggaagaacaagctcgctcagtccaagcgcggggaacgtcggcttcgc

ttgtcgtcccgctcgagtgggagacggagcctgtcctcagttggatgagggctctggtcaaccactttagacgcgtagctagcgtcctccgtcgagga

tctgccaagcccacagacggcccgaatgccgagacgccctcgtcctctcagcgttccagtcgctcggcggcctcgctggtctcggccacctcgacgaa

gtgggagcaaagtctgggccaactcctccagagtctgtcgcctcacttcttctggatggagatccggcgggagcgcttggcggaggtgcaggatgacc

tcgaagatgatgggcaggcccgctttgtggacattgatgcgaagcgggaacggcgcgtttgggccgatctgatggaggttgaggctgctgagggggag

gctgtgcctgtgccgcagcgggctgcacgaagggcacgggagggctcgcctgagatcatctgggtcttctcgggcgggcctgcgtttgaacctggctc

ttctcgtgctgggccgtcgtcgtcgatggacacctcggacgattcgcccggctcgcccggttacgagattgtcagcgtgacaccgtcgagcaagctca

gggacgtacccatggtttctgccgcgaagagttcctcaggctcaggatcgaagccgggcggatctagtagcaacagtagcgcacaggccgccaaggat

cgaccgaagttgagccggtcggatgcgattcaggtgcaagcgcgcgaatgggtgctcgactggggtctggacgtcttcagcgatctcaagtcgacccg

gcgtccctcggcgtcgcccatccggaccgaggcggccgcagacgctacgttggaagatcacgatgtccggctgaatgcgctcttgagggttgcggagg

tcgtcttgggcgaggttaggcggcgaaggaggggggcgttgatcaagtttgctgtgctgatgtgggctcgacgatgtgtgccgccgctgtcggtagca

gcggcgggcgcgggtgtgggcggcagccagcaggagggggcggtcccgagtcagggtacctcggctatggacgtggacgaggacgagttcagagctcc

tacgtcgtcggcgattgcgtctgcctacctgggcacccagacctcggaaagctcgacgtcgacgtcggcgtcgacttcatcaaccgtcagacaggccc

tgctagacaagctggacagcttctgcacggacgcagccgcgtctgcccagcggtcgttgctgcaggtgcgtgtatgggagggtgcgctgcgatgtgtg

tgggaggaggctattgggttgggcatcgtgcctgcgacgaacgggcgggcgaaggggtttaagtcggagatggggaaagctgtgaatgtcgcgtttac

gatggagatggagcggcttttgcaggcgattggggttgaggtgcagggcttgggcgggacggtgatgggttggagcatgtatgcgcttgagggtggtg

ctctttga

>contig_21.contig_21.g1002.t1

atggcattcggcggacctgtgggattcatggcatcggaaccttcatcgggggtccttcaggcccgaggaactcacactc

tcttcttctatggtacactagtccacccagccattctcgcccgagtcatcgggaaccaaggcgcccacctccgcgtgcggaacgccctcctccccgcc

cacgcaacctggcacgtccaaagagtagactaccccgccctcgtctccctctccgatcggcggccccaacaacaacaacaacaacaacccgatgctcc

cacatcggcttcactcacagcaggtgatgatgcagagaccgtcgtccccggcaccctcatcagcggcttgacggattccgacctacgatttctggact

tgttcgagggagaccagtacgtccgcaaggaagtctccgtccagcctgtcgaagaaggggacggcatctcgaacgccctccgaccgcgggatgcctct

ctcgccgacatcctcgccgacctggatccaacagcaaacgagctgctcaagtcgaagctcaagctcgagaccgtccgggcacagacgtacgtctggtg

cgccccacttagcatgcttgaggatcggagatggatctttgctgagttcgcgagggagaaggcgaagcggtggatcggcgctcaggcggatcatgagt

acactgacgtggatcgcacgcggagcgggactgcataa

>contig_21.contig_21.g1003.t1

atggattccccaccttcaaaggaacagctcgtggagatcttcaagcatctgaagaacgcccacaagaacaacaaagtct

gcttcgactgcggcgcccgcaacccaacctgggcctccgccaccttcgccgtctacatctgtctcgactgctcgagcgtgcaccgcaacctgggcgtg

cacatcaccttcgtgcgctcgacgaacctcgacagctggtcatggcaacagctccggctgatgaagatcggcgggaacggcgcggcgacggagttctt

taataaacatgggggtggaagcttgctccagccgaagacagaggggaaggtgaagtactcgagtagtgtggctggtgcttataaggcggagctggcga

agcgggcgctggaggatgcgggagggttgccgctcagcagtgcggtttccttccccggtatggcgagtgctgcttctgctgctgctgcggctgccgcg

ggcaacggagccaaggcgggcggtgcggggaatgacgatgacttcttcgacgactgggacaagcctgcgaagcctgctgcctctacgtcctcgtcatc

agcagcaccggaggcgcctgtcctgcccgggattggagtggtagcgcgatcctcagctgcgcctgcacccgcgtctccgcaattggacagtccttcct

cgggcacagcatcgccaagacccaaggcacccactgtgacctcctcctcgagcctgcagagctcgtcttcgtctgctactcgttcgggcgcccgttcg

ctcggtgcggtccgatcggctggtggagccgggggcagcaagctgggcgcggtgcgtacagcgggcggcgcgggtggcaagcttggtggcgtagtgaa

gaagggcggagcaggctcgatcaacttcgaagaggcggagcgaagggcccttgaggcggaggagaaacgacgcgtattggaggcggaggcggagcggg

agacggactctcgcacgaaggcggagaaggctgctgttgctgctgtggcggctgcacgggaggagatagctacaaagcaggctgctgctgctgctacg

acgagtgcgcagaaggctgctattgtggccaaggcccaggcgaacccgcttgcggagcgatccgagagggagacggatcggctgggcatgggtttcgc

tcggttgggattcgcccaagctcaggtggccgcgcaggctgcggcggagaagtcggctgctcatggggcgcaagaagacgatccggagagcacgtatg

cgcggaacaagttctcggcgcagaagagtatctcgagcgatcagtacttccagcgtggcgggtatgatccgcaggcgacgtcggaggctcaggctcga

cttgccaatttccagggccagacgagcatttcgagcaaccagtactttggacgggaagaggaggacgacgacgatcttggtggggatggggtgtcgta

ccggggcggcggtggaggcggacaatacggcaatgacttcgggactgagctggaggcaacggcgcgggagtactaccagcggttcatggcgaacgaag

acgtgcagagcggactggacgcgttccgtcaaggggcgatgaagcttacggacatgctggcggacatgagccggaacggagcgtga

>contig_21.contig_21.g1004.t1

atgtcactctcgcaggaacaactgagcgaaggcttcgagccgtcgtcgtcagccaccaacacatccgacatcgtctccg

tcgcgaccgaggacgaggaggatctcaaggactactgcaagggcggataccaccccgtacacgtcggcgacaccttctccgacaaccgctacctcatc

gtccgcaagctcggctggggtcacttctccaccgtgtggctcgcaaaggacttcaagatgaacaggcacgtcgccctcaaggtcgtcaagtccgctca

gcactacacagagaccgccctcgacgaaatcaagctgctccagaaactcgtagccgcaaaccccaaccacgccggccgcagacactgcgtctccctcc

tcgaccacttcaagcaccgcggccccaacggcacacacgtatgcatggtcttcgaggtcctcggggaaaatctgctcggcctcatcaagcgctaccaa

catcgcggcgtgccccccaacatcgtcaagcagatcgcaaaacaggtcctcctcggcctcgactacatgcattccgagtgcggcatcatacacaccga

cctcaaaccggagaacgtcctcatctgcatcgaagacgtagaggccgtcgtacaggccgaactcaggacaaacccagcagccgtacccaccaagatcg

tcggcgtacccccttcccagggacgaggcgggacccaaactccacgcagggagggcatcttcatcactggttcacagccactaccgtccccctccagc

agtctcggatccagtcccatgttcgacaagtgggcctttggcatgagtcgcatcgacaacggtgactccgatcgcgttcgcagtggcgacagtcactc

aggcacaggaacaggcgccacttctaccagcggcctcggaggcgctccctccctctccgccctcgcatcaggatccggcgcaggcagcagcagcaaca

gcaacagcactggcggccctactactgctgctgctgctgtcaccccaggaggttctgcagcagtctctgcgggaacttccaaagacgcagatatcctc

ggtcacgggttctcccagatgagcacaaacgagccgaccccggagccatttgggaccaagaagcaactcaccacggccgtcgtacatcacgggccctc

gcttctcacccaacaggtgcaggcacaacggcaggctcaggaacagcaacggcagcagcaagaagcgtcagggtcaggatccgcatcagggcacggct

cggcagcgccggcggcggcatcagcggcagaaaacgcgacggcatccactacggacaccccgatgagcaccaacggctcatattcgcctgcatctcat

cactctgccatgtccaccgatccaccgacaatacctgtctccgagcccgctgccaccacatccgcttccacagacgcgactccttccggcgcgcagga

tgcatcctcagcgtcatcttcatcacatccacagccaccgccactctcggaaagcacaggcgtagccgtagacagcagcagtgcaactaccaccacca

ccaccagcggcaacgctgcacccggtccctccaacctttcacaatactacgccccagccccagccgcaggcgacccgaacacgctgcccccaccgccc

ccctacgacccgagctcgctcgagcggatcacggtcaagattgccgatctaggcaatgcgtgctggacggaccatcattttacgaatgatattcagac

gaggcagtatcggtgtccggaggtcatcttgggggcgaattggggtacgagtgcggatatgtggagtgcggcgtgtatgttcttcgagctgttgacag

gcgattacctgtttgaccctgcggcgggcacacggtataacaaggacgacgaccacatcgcgcaagtgatcgagctgctaggcgacttccccaagccg

ctcgccttctccggcaagtactcggccgacctgttcaaccggagaggcgagctccgacacattcaacgtctccgcttctggccgctcatctcggtcct

gcaggagaagtacctgatgccgttcgacgaggcgaaccagctgtcgacgttcttgctgcccatgctgcggctgcatccggaacgacgcgcgcctgcgc

gtgatttgacggaccatgagtggattggcgggattaaggtgcagggtgagctcgagctcgagtgggcgcgtgcgaggggtcagcttgcggatgcgagc

atgtcggacgcgcctggtgttggaggaggcggcggtggtcctgctggctctggatcggcgccggtggatatcgcgcggatgatgggtacggcgacggg

cggggcggaggacatgaatgacgcgctcaagccgatctcgagcagttttgggagtacgggcggggttgagatgagtccgaatgtgtcgcagatgccac

ctgggcttgtgctcaaccacgcctcgatggatgaggcgaagcggagggcgctgcaggcgagtacgttgcgcggggcgacgaaggacgaggaaggggag

ggggctgcgcaggggggctcgtcgtcgaggcttggggctggggaggaggcgatgccggagggcgaggcggctatttcgtctttgctgttggcgacgga

atcacgctcggtcccgccgccggcgacgagggatcatgcgcctgcaggtgctggtgctgcatcgcaaccaccggcggtggccgcgccgttacggtctt

cgacgcttgcgacgtctgcgcctgcaactgggacgtcggcgagtggatctctcgacccggcggcggcggtggcgggggcgcctcagcagcagcagaat

ctaacacctgcggcggttgcggctcaatag

>contig_21.contig_21.g1005.t1

atgctcgagatcacgccgctgcagaacttctccctcggcgcccaggtcgccatcctcgtcggcggaagcgccgtcctcg

ccgtcgtcctcaacgtcctcgcacagctggccctgcccaaggaccgctccgcgccgccggtcgtcttccactgggttcccatcgtcggttcggctgtg

acgtacggcatcgacccatacaagttcttcttcgactgtcgcgaaaagtacggcgacgtcttcacctttgtcctcctcggccgcagggtcaccgtcgc

cctcggtgcgaagggaaacaacctcatcctcaacggcaagctctcccaggtgtccgccgaggccgcatacacccacttgaccacgcccgtctttggca

aggaggtcgtgtacgacgtccccaatgctgtcctcatggagcagaagaagtttgtcaaagtcggactctcgaccgataacatgcgggtgtatgtcgac

cagattgtggacgaggtgaacaagtacatcgcgtccgacaaggtctttgctcccttccagggtaagaaggagctcaaggagaccgacgagacccggct

cgacatcttcaagtccatgtcggagatcacgatcctcaccgccagccggacgctgcagggtcgcgaagtgcgcgagaacctcgacgccagtttcgcac

agctctaccacgacctcgacgccggattcacccctctcaactttgtgatcccgaacctgcccttacccggaaacttccgccgggaccgcgcccagctg

gccatgtcccgcttctaccagaccatcatcaagaaccgccgcgatggaaaagcgagtgggattgacgatgacgagcatgacatgatcgctgcgcttat

gcagcagagttacaagaatggccgtcagctgtctgacgttgagattgcgcatatgatgattgcgctgcttatggccggtcagcatacgtccagcgcgt

ctggttcatggattctcttgcgtcttgccagcaggcccgacattgttgaggaactctgggccgagcaaaaccaggtctggggcgatggatccggctct

ctccgcccgctcgactacgagaccctgaagtcatcgactccgctcctggacgcggttatccgcgaaacactccgtctccacccgccgctccactccct

catgcgcaaggtgatgtcccccatcacggtgcccccgacccttgcctctcccggatctcgcaagtcaggaggagcaggagggggcgacgacgcagcgt

acgtgatcccggagggccactacgtcatggccgctcccggcgtgtcgcaagtggacccgcacctgtggccgaacgcagaggagttcgaccccaagcgc

tggttggacgcgtcgagtgtggtcgtccagcaggagcgtgaggacgccgagtcggagaaggttgactttggatggggcgctgtttccagtggggcgaa

cagtccgtatttgccctttggggctggacggcatcggtgcattggagagcagtttgcgtatcttcagctgggcacgattatttctacttttgtgcgcc

ggttccgctggagcttgcaggagggtttcccggacccggactacacttccatggtcgtcctgcccaagcacggcaagatcatcatcacaccacgcgtc

tag

>contig_21.contig_21.g1006.t1

atgacccagacactcaccgacaccgtcgagctcccgccgccgaaagagcccgcaacatggcagcatatcaaacacgtca

tctgccgcgggaacctcgacgacctctaccgcgaccccgtctgtgagctcgcctaccgcgcatggtccaaacccgtcaaggcgcagtggggctccctc

gaacgcttcattcgcgtcgaacggcttcactgggacgatacacactcgtctccctcctccgccagtaatgccttctttgaggatatcgacgacgaatg

gctcgtcaagatgatccccaatcactggccgtacaacatcccgctcaagtgccggcactggtgcgtctggtcccgcaaacccatcacacacatcaccc

agctccccagcgcgcccgacagtcctggtgggcctgtccaacataccccctccacgaacggtcatggtcacgcgcccgctgcggcccaagccaacgga

cgcgtcgctcctagctcccccgagtccctacctagctcctcctcctattcttcctcctcctcctcctcttcccaatccccatcacagccctccgctcc

aaacgcctcctcgtcgtcgtcctcctccaagctcgcccacgcgcctcacgccagacccgtcccctggcccttcccgcacatgagccggaaggagcaag

aagacctctacgcagccgtctcgcacgatgggattcgcgccctcgtcttgcccgacgaagacgcgcgcggcgccgcccctgagcctgtcacaggcgag

tatgtcctcgagttcttccgctcaagaccgcatcatgccgccagactcgttgcgccatcaccatcacagcatgtagctggcaccgagcccaaaacagg

ggcggaggcggaagctatggccgctcgcgcagcagacgcgaccatggcctacttctcccagcactgccacagcttcgtcctcaagaccttcccaccaa

aacaaggctggcgctccgtactcttctgcaacccgccccaccttcgcaccgtccccggactcgatcactggcatatcatgtctctcgccccggacgcg

ccgccgtccgtcgaatag

>contig_21.contig_21.g1007.t1

atgccacacgccgcttctgccaacgcatcgcccggtgagcaccaccaccaccagcagcagcagcagcagcagcagcagc

agctgtcgagcatcctcatccacgcacacacacggtcctcgtacggaagagacgacgccatcgagccacccgcgcccgtcgcactcccactcggccgc

ctcgcctccaactccaccaccactaatgacgatccgctcgcccaagcccaccactatccaccctccgtctcctccagcggacagcatcgccagccggc

cactcctgtatcctctacatcgcgctggctcccaccttggcttcctcccctgccctccttctccggtgcccctccgatcttcaacagcagcaacaggg

gcagagacaagggcaagcgccgcgcctcctcgtcctcacaaccacctcccagcgacccgggaaccgacgcagtagcctctccctcaaagccagcctcc

gttcatcgatctccctcagtcaagaaacgctcttatcggagcacggcccctcgcaccaggggcaaaggcaagagctccaagaagtccaggaagcggag

gaactcgcgtgcttcgaatatcagcaagaccaccaccagcacagactccgacagtctcgtcaccatcgaggatgatccccttccacccttagctgcct

ctgcctctgcctctgcctcggctcccggtcggccctccacaagttctcgccctgagctgcatggtgcagaagcccagccggagaagaaggccagacct

cagctaccgagcgcggcgacctccctcctcttccgtggccgacctcggctcaggaccatcgcaaacggccttactcccgccccatctccctccggttc

cctcgcacgcgcacgccgacatgacgaagccatgtccgctgcctttgccggcctcaataatccttcttccttcagcgcagcccattcccgcgaaggtt

ctgtccgtcatacttctcgctcagcgtcaggggcaagggccactccccccaccgcggaagatgccggcttcactggacgagccacgagggcactcggc

ctcggctctctccttcgctcccaattcaacagcgaggatgtggccaatcgcggtgcttccgcctccatctttcctggcggaaaccatgacgaggaaga

cggtgatgccgatacagaatttgaaatggcttctttccaggacggagctggtccttcaggcaggttcattaagggtaaattcaaggatcccgaaggag

gcgataccaacgaagtttcctttggcgaccatctctccccacccgcctcaccagacagtatgacccgcgccctcgctcaaatcgtgcctccaactgac

ccacacaccggtgcacgcgccctccaatccgatcgggatgcttccgacctcggtttgccatccctcatagcgccagaaccgacagaggccaccgcccg

cgtcctcgaggctgcccgctctactcggcccacaaaggtggtgcgagggtctcgcgcggcggccgctcgcgctgttgctgaagcagcccaaatatcag

cccaggccgcggtacagtctggtcgattgactccttctgggttctttgcataccagtcgcccctctctctgtctcttgctaccggagcatctgcttta

attcccttagcagcacagcaacaggaacagcagcagcagcaacaacaacccgctcactcatccgccgccaacgtggccttctctccacagctcacagt

ttctcctcagcaggacagccccttgcagcgcggtcccttacctttggtgaggctaggtggaataccgcttacctccgtccttcgatcggcgagtggac

ccgcaccaggagtgagccccctttctccctccttcggacccgaagctaccgctgccccaggcggcgcggcaaggctgaccttcgccccagagcaacga

cgtgtgcgacggaaggatctgccgcccggcttcgaggtcggcgatacttaccgggccctggacgcgtacgttctcgagtccagagggcgacggagggc

ggaacgggaggggaagagcaagcgtcgctcgaggaagcgaggaggagggggaggcagcagcattcgcgggaagggcagccggagctcaagttttggcg

agcacgccagcacggccagtggttggagcagctccagcagtagctcctcctccggctccacggacagcaccagctccggcgaaggccatggcttgacg

atcgcgaggctccgagcctttttcggtgacgccgactcgtctagctcgagctcgagcagcagcagtagtagtagcactagttcggattcggacaccgg

cagcagcagagacggccgcgtctccggcaccggcacctcgcgattgacggacacctcgtcctcttcctcaagctcttcctcaagctcgagctcctcgt

ccggtcgttcgagtcggagtgggttctccttcagtctgtcccgcgtccggtccaacatttccggtgtgggcggacggaggcgaggaatgagtgtcact

gcgcgcggaggagcggctgacagtgccagtattctgtctgggggtgggggaggcggccgaggcggcggcggcggcggaggaacgggtgcagacctgac

aacgcgcacaccacgactgaacccgattggagataccacccaagcgtctcgaaatccgcagcagcggaagcaccaacggcggcaccggcagcagcagc

gccggaatcgggaggtccaacgccggcgacgacgagctgctcgcaaacaaatgcagaagttcgagtcgactgcgcgtaaggcgggcttactccctccc

aagcccagcaaatcggcccggaacaaggcgaggctggccaagcagctcgcaggcggcgtgacgcagtggatgctttacacgccccttccgctgcctgt

cagcacgactcggaggggcttgaaaaggagagagggcgacagtcattgggatcacgatgagctggatgctctctctgcgagcgataaaggtggtgacc

tgtcagcttctgctggtccttcggcaccggagcgtccaccgctgaagcgcgctgccaccgcgcaggccgttctcacagcagccgataccccgccagag

cacgcaatccatggttctcggccgctgtcccatcatcgacaccatggatacgccggggcggccgacgcggaaccgaaggagcaggtactcgagacgat

ctccttcctcgcggtcaaggaacgattgtatgctcttcgacaacagatccggcagcctgagcacgttggtggtgatttgggaatgatgagctcgagtg

ctgtgctggatgatccggggggtatgagggggcgggctggagctgcggcttttggagcgggagcgggtcggtttgccaccccgccctcgagtggggac

ggtaccagtagctcgaagccgtctgttggggcttggtggctggacgtcaagtgtcctacctacagtgacatgaaagatttgagcaagctatttccgct

ccatccactcacggtcgaggacatcttgcagcaagacccgcgcgagaaagtcgaagtcttcgagaagctgggctactactttatcaccgttcgagccg

tcgacgaaacctacttccggacgagctcgggcgacgctgatgatgggaaggcccgcatcaatctgctctcgggtgtgcctggcaaggacggtattgaa

ggtgtcagtgtgggtgcgatcaatttgtatattgtcgtcttctcgcatggcttgattacttttcattttgaagatctgtcaaagcatacgtcgcgtgt

tttggaacgcctcatggacctgtcacggtcgctggactacactgctgattggattgcacacgggctggtggactcattggtggatgccttcttcccac

tgttggatctggtcgagtctgaagtcgccgaattcgaagataccatcgaattagcccccgcagccagcccgacgtctggtccgcccgatcagcaccga

cgtcgagcttggaccaaactctttaacggacagcgcgggaactttatcaagctcgggcagtctggacccaaggcagccaagaacaacagcaccaacga

ggagaagcaaggtccgagcgatcagagtgccctgttgaagcggattgcgactgtccggaagattgtgacgggtctgacgcgcctgttggctcccaaga

gcgagagcgtgatcaacctgcggaagcggtacatggagctgagcgcgatgagcataggctccaaacgggccagtcgggcggaagtatcgatccatatg

ggcgacttacacgaccacatcgtcacgcttctgacgcagctccatcaatccgagggccggctgaacgacacgcacgcagaatacctggctatggtgca

gattcggaatcgacagagtcgggaccgatccgcccggtccctggtcggcctcgtcaccattaccttgacggcggtaatgtgcacgttcttcacctcga

tctggagtttcaacgtggtgataccgacgaacgagcacgagtcgccgaaccatctgatcttcttcctcgtcgtggccacgcttggcgtcatcacggtc

tcgttgcaagtctatgtccgatacttgtggatgaaggccaaacagaagcgggtcaaccgagcggcgcatcgataa

>contig_21.contig_21.g1008.t1

atggatgcgacctcatcagctccggcgctcccgtccgcattgggcgccttgcctctggatgcggcgacagtgccagctg

cggatgtacctgcccaatcgacatctgcagcggtggcagtgccggtctttcaacctcagccttcctcagcgcctttgcaagttggaggccgtggagtg

aagcggcgtgcggacggcgtcgtctacacgagggacaagaagttcattgcagtacattacatgtttggcagtagcacgtctcggacacttcccgttgg

agatcgtgtgaaacggatcaaggtgggcaaagctaccttgtacgattggcggaaggagcatcgagaggcttgggaagcgacagcaggcgggccctctt

tccttcgccctgatggcctcggcccacaagcagagccgagtaccaccttcggagacttgggcccgggcagtcttcctccggagctggtggagtccctt

tgtcgggttgagggtcaaagcgatgtgacacgtctggagcaggatcaagttcttgcgatgcatgcggccgtagacgacgacgtctcttccggaggggt

tccttttgtcgaccacacactcagccaggccgcctacctacaacgaggtaacctgcgagcgtctaccaacaagttcgcagcaaacgttacggctgagc

acgattcaatttcccgtctcctgcagatgttctggcgcgcgttggacaagctgagagcgccagccagcgatttcatgccgcctgcagtggcttctgct

acttcttctacttcagccggagtttcatccagcatctcactcgccacatcgaccgtcgctctcacatcgctctcctctcttgatcccgcgacatcgac

catctcccccacatcaacatcttcttctccttccgcgcctgcccaatccattcttccgacgcttgacaacagcacgatcattgtgactgagcagaaac

tagtcacgtttctgaactgtttcgttcttcatcgtaaccctcgcctgagcgacgagagcctgaagacggaggtcaaagcggtgacgaacctgtgggag

acgcagcagctcttgggtctcaacaacaatgccagcccgcggggaaagctgctgaaatcgtacctcaaggccgtcaagaagcaccgagctgcccatgc

tgtcacaatacaggaagactggtggaaacactctcttcgtgacggctacgatgcgcaggagcactacgagatatccaggtggtatctgagacaggctt

cgaacggtaacacccaagttttgagcagctcacttcctgcttcactccctgattctcgtctcccttctagcctcgacagcaactccaacagcgacagc

gacagcgacagcgacagcgacagcgacagcgacagcgacagcgacagcgacagcgacagcgacagcgacagcgacagcgacagcgacagcgacagcga

cagcgacagcgacagcgacagcgacagcgacagcgacagcgacagcgacagcgacagcgacagcgactctagcagcgacagcgatgaagacgacatca

ccgcaggaaacctccccccgccgctctcgcccctctcgacgttcccttggctccgcccttccattccatccaggagtgccaacacagctctctttgcc

tcctcctcagctgctgcgtcgtcgtcacctcttaccgcacagcagcgcgctatgcgcaacaggtgggcgtccatagcgatccggtctcgtttcgagtt

tcttatgcagcacgccctcatgggccgttccgaagatctgcgcaacgccatactagcagcaacctattcgatctctgcccctacaaggtcccagccgc

tcggtagcagtcgttcaccgatcccgcagtggctggaggctgagatgcctcattctcgccctcaggaagcgcgcgccttcgttgttacactccgcagc

agcaaaggaaacacggtcggtcgcaacgagtggggtgttgcaacgcgacattgcgatgtggagtcctgccccgtgggtgccctggggctgtacctgtt

cgagcgctggcaccagcaccagcatcctactcccacgttcgactctcgcgcatcttggtacgacgagaagcctctcgtatcggcagacagcactgcca

ttgaatggtacgaccagtcgtatctgcttcggcacgccttcgacgaggtcggcgttgcctcttccaaactcacacatgcgatgcgctcgggaggcgca

cgctttgcccacgaggcggggtgcacagaggacagtttgcggatacacggtcgctggtgtggggatcggatgatcgaacgctatctgggaggggtctc

aattcagccaatccgtgcgctggccgatttcgacatcaagggtggagacaactggcttccacgcgcgctcctcgagccggacgcgagcctgcagcagc

agatctttccttttgtggaatctgaagaggccaaggtccgccaacgtcacaaggatcgcggcgaggaagactatgcggcggtgaactttcttcagctt

ctcaagtggttgcgggtcgtccttctgcaagacgctgtcctcctccagcccatgttccctgcgcttcctatctggcgtcatgcgcctttcagcaccgc

cgcgttccaagcattcgcccagactctccgcaacacgatcgcgtccacccccactcccttcgatgttactatgtccacgctagtcccggagcttggtc

gtgctctcagtggacttcaggccatgctctcggacatcgcaaacaagtcggccacagccgccatagtacagcatcagggtcagctggtgcgctctgcg

gtttcggatgcagttgtggatctcaaggagtacattggaggtctctttcgcggcttagcgtcagccgaccacgcgcagaggacggcgcaacgggcgtt

ggcgtttgggatgcagcagcttgccactgcctccgcagaggtagcagagctcttcaagaacacgagctcatcatcccatcccaccgaacccggcatct

ctcccgtctccccctccccgaccaccacagaatatcctactcctggccccccctccttcgcgcccaatgccgatctctccggaaatccttctgcgatt

ccggcacttccaactcctccagcacctccacctcctcccccggccagcattctggcgctggccagggaggtccggtcggttgcacagttgatggtcga

atggcgggatggcggaagcgggcgccgtgggttgaagctgcgtctggatgagggggacaagacgttggagaagggagctccgggagcggcgaagcaac

tctcacgctggagaatcgtttttgagctggtggagtacctgtcagaaacccgggcagaagctgtgaaaccgttccccaaggcccaatcttcgtcttcg

cctcgacttcacagtacaatcctcactggcgacgcggaagacatgagcttggctcctgctgaagatgccttgatgctggatggctttactgaagcact

gggagctgagcgaggcctgatctatgatcgacaggacgtcctcttcctcactcctcagtcgctcagtagtcgtctctaccttggcaccttgcccgaag

gcattgcatttgaccaatggattcctggcgtctaccacacgcatacctcggccaaccacatggcgatcaccatggaatccagccataccacagctcac

cgtcgcatatttttccttctctttcgtctcgatatcagccttggaaaggcaagcttccgcgtctgtatgttcctccacttcgacaagcgctttgccga

cttcaatgcgatcaggcgcgagatcgagaacgagatctaccgcgtggctggacagaacaaggctccgctctcaagatcccctccacggtatacctgga

acccaacctctgacaatatgtcacagccatccaaggggaccgcgaaccccaaccccaggcccggtcgcaccggaacgcgcagcgtcagcgctacagca

aggctcccatctgaaggcgtcccgccctcgagtctggctgtccctcgcgcgcccattccagccaccgagacctcatcatccccgcccgatcagcgggg

gcaccagaggaccatctctgaggagcttgtcgacacatccattgctgtcgaccagcctccaccacgatcgtctctccgtccgaccgccgaggatgaag

aggacgaacctcagcagtcggggtccgaagactcctctctgagtgtgggccaagtccagagcatgctgcgcatctctgaagcacgcgcatcgaccgac

ttgcgcaaggccctacaagatgccgaccgtcgacaccagcagctcgcagccgacatcgaacggcggcacgacgacaagatggatcggatcatgacgct

gcttggccagctgcagcaagccccgacagtgccatccaggacacccagtactcccactgctggcgaccatcgtcgagcacctcgcgtctctgccgctt

cgcacatggtctttggcgcagaagacaccccgcgtggtccgatcccgccgcatctccgtttcgctgagccggccccgccagcagatcgagctcctgcc

gtaggcgcagcgattcgcatggaggacgaggaggaagaagcagagccgcgtcacagctcgcgcagcttcttccgcgccaagtccaaagacatcggcac

cttcaacccggagcaaggcgacgacatctacagctggtggtccggactcgtcacctacatggagtgctcccggtacacggaagcagagatgttcgccg

gcctgcccggatgcttcgagggctcgtcaagacactggttcaacacgctcaagccgcgcccgaggactctcgaggagctccgagttcgagctttctcg

gcttacgttcggaacgagtcgcaggtctgggaagagctcgatgatcgccgcttcaagccggagagcgagcgactcgacgtgtaccttgcggacaagct

caagctcatctccgagctgcacgtctctcgagctgtgaaccgtgggagcctcagcctcgaacctttctctctcgactcgatcatgaccacacagacgg

tctccgacgccatccagaccgctcatcaaggtctccctccggattgggccattcttttggactcgtcgcgggaagcagctcaggactggacggactac

cgcaccagactcctcggcaaggagcgcggaacaaggacagcaattgcactcctccagcgtccagcttcctccccggttccctccatcaacccccctcg

agctcggcctgcgagaactcacgcggcagcgccgagagagttcgacaacactcagtcggcacgaccgcgtgctccttctatgagcccggtcgatgagg

agacccgtaggctggatcgggagcaaggacggtgctttgcttgtcacacggccgggcacgctaagagcaactgtcccagtcgcgcttcaacggttcgt

gtcatcaagcgcatcctcgcccgcctggaagacgacaacgacgaggaggacgaacacttctctatggtggtccgacaggtagccgtacttcatgctcg

acaacggcacaccgatgcagattcggcgtcggaaaccgatccagaggacaacgtcgcccgagtacgatacgcccgcagcgtgtctgtactcgccgacg

agcgctcgccgcccagtttcggcatttctcctcccacagagcgcgacgtggatctcaaggggcaccgacatcgagacgttgttgcccacaaagcagcg

gtgtatctggggaacgaaaacgaacgtctcgagctacacgttgatggtggatcccctctgagtatgatcacgccgagggccctaaggaccattgcacc

agacgctaagatcttaccaccggagcctttgaaaatacgaggctacagaggagatgactttcaaaggccgtccggtgttactctcctccccgtctcgt

ttcccacagccaacggtgccccagccgtgatacatcacttcgagtttcacgtggtcgaagagtgtacaggcggttggatcctcggcgtggacaacatg

aaggccgacgggatcgatgccctgtccaaacgggagctgctcgtattcgagagccggcccgacgcagaggttcgtctcctccagacctcctccgtagc

ccgccacgtcggatcgcccagccgcctcgtctgcgcgaagaaggtgcggatcgaaccacatacggttcgcgtcgtggagatcgaccccgtggcgagcg

acctagtggtccgcccatggtggttctcccatgaggagtctccaacaggcttcgtgaaggtgccatgctgcctcgtcggtgtcgacacccggggcgtc

gaggtcaccaacacgtcggaagcagctgtggatttggaggaaggcgacgtattgggtgaggtcgagcctcttcttcagcagctgtgcgacctcagtga

gctcgaagatgaggtcaacaaccgaccgaaaggaagactcggtcatcaaaagcgggacctctctcgctcgctcaagagctggcgtcggttaactgcag

cctcccctccctcggaactcaaaacgtcgcgacgtgtcacgttcactcgcacggaggacgacgggaacgaagtactgcccgagatcgacatatccacg

cctgagaatcgccagcagaccgttctctgctccgcagggctgacagtcgatcaacgttcccgtttgatgaaggtgctggaatcccacgctgtctggcc

aacaccgcttcgtcctctcggcctatacgaatatggagaggtttcgctgcgtctgcgcgagggacaagatcattggacacacacggaaccaccgcgcc

gtacatcccccgcgcagaaggatgtcatcgacgacaccttgagggagcacgacgaccttggcatatcagagccatctcaaggttcctatgcttcgggc

gtggtgttggtgcaacagagggacaagatcagattctgcaacgactatcgaccgctcaacaaagttactgttgacgattactatgccatgccaacggt

tgacgcgatatttgatcagctgggccaatcgaaagtgttcacagtcgtcgacgccaacaaggggtattaccagttcctgctcagtaaggactcgagag

atctgacgggcttcatcactttcagagaacccacgaacgcggccgaggctcgacgtttcttcgcgatgtcagcctggtatcgtcgtttcatcaaggac

tttgcggcgagggcacagcccattaacaagtcctttgtcggcccaacgttcgtgtggcacgcggcggagcgagcggctttcgaggatctcaagaccgc

cattaccaccgctcctgtcctcgcccgtccagacttccgtcgaccgtttgtcttggctgtcgatgcttcgaagcaaggtctcggcggcgtccttctcc

agaccgatgccaacgaggaggagcgcccccttttcgagatgcacgaaacgcgcgtccagaagttcaaaatggcgttggctccgctggagaacaaggtc

aagatctcataccatcgaggaagagataatgtcgttgctgatgcactctcccgagctcccattcccgaccaatctcacgccgacaaggaggccacgct

tagcttcaccgctcgcgagtaccaacggcttcccgaaatcgacatggcggctgcagcggataagatcataaggtcaatttcagtgctgaggtttggag

cagaggagctaggtttgtgggcggcggcatatgcggcagatccgcactggcggcgcatctggaggcggtccgagactagagggacggcagtcgcggct

ctcccggcggacatcgaagatgaagagctcggagaggtagaggatcgaggcgtcgaggtcatagcggacaatgacaaaccctcgacagccgtcgacct

cgaaggtgcagacccgcccgcgaggccaacagctcctccccgtcgcgagcttcgaccgcgtcgcatcttggccgtccaccgcgcagaagagaactcca

tctttttcatcaaggatggcctgctctatgtgcagacggatgagacggtacgcttgtgcgttcccaaagcgagacttgacttattgattcatgagtac

cacagatcgatccgtgcagcccacccctcagctgaacggacccacggcatgatgaaggaccatatctttgcccatggactcgcccagcatatcgccac

acacgttcgaacatgttacgagtgtcaggccaacaaggtgccgcggcacaagccctatggagaattacagcccattgttacacagaacgaagtgttcc

acactctgggcatggacttcatcaccggtgtacccaaaaccgaactgggccatgattccatactcaacgttgtgtgcaacacaacgttttcgccatac

cgtctggtgttcaaccgcgagccgtcggtcttcccgctgcgcgatctggttgaagccacagacaccacctctgccaaggtgaacgacatcttcgccgt

ccatgcagacgctcaagcagccatggacatcgcgagggaacgtcaacggtcggcatacaactcgcgccaccagccccaagagttcgccgtcggagatg

cagtaatgatcagcctcaaaaactacaagtttgatctggatccaacggaaaacgctcgggctaaactggcggcacgccagggcggcccgtacaaagtc

aagtgtcgcatcggtcgccttgcgtacgaactcgacgttccggaatggttccgagctcacccagtgcttcccatatccgcgctcgagcccttccacgg

agacccagaagaatttcaccctcggacacaagcaggtgtggcaggcggtgatggaggagatgagctgagagtcagggattttctgggtcggcggccaa

cgcaactgctggagggaattacgttcgactatctagtcaagtgggccggcccaaagccaacctggcaatgcgacagtcgccttccggtgctcgcgtgg

gccacgaagaaattcgaacgtttggcacgcgagcaacttcatctcaccaggttgtctcgtcacgcaacgatcattttgccggaggcacaagagcgagc

cgatgctctagctgcggaggacaacgacgacgccgtgaggacgctgggctcttcgttggggggggcgaatacttcattcgcgttaatccttacaaagg

gcgtctccaagctgcccatacatctcaaggtctacagccccaacgtgctcaatttcaccctggacgatctgcccggcctcaccaagatccccgtcggc

gatcagccatcggacatcgagcgccagatccgcagtctcgtcacagactacatctccaagcccaactgcaccatcctcgcggtcagctcggccaatga

cgaccttgccaactcagactccctcaagctcgcccgctcggtcgacccacagggcagacgcaccattggtgtcctcaccaagctcgacctcatggaca

caggcacacacgccctcgacatcctcaccggtcgcgtctaccctgtcaaactcggcttcatcggcgtcgtcaatcgctcacagcaggacatcaacggc

aatttccccatggacgtcgcaaggagggccgaggaggaattcttccgcaatcatctcgcttatcggaatctcgctcatcgatgggcacaaagcatctc

gcaaatacactcaaccagaaagctgccagacatgaaggcccgcctcaacaccctcaagggccaaacccaacaaaagctcgccgccctcggtgacacca

ccttcctcggcgacgcccaccgcggcagcctcgtcctcaaactcatgacccaattcgcacgcgacctcgtctcctccatcgacggcacctccttcgac

atctccaccaaggaactctgtggcggggcgcgcgtttattatgtcttcaataactattcggacggcgattcggaactcgacggggctaaggccgacat

ggtcagtcctcacctcacctcgaacatagaagccccctcccctctcaagctgacaaaatcacccattttctccacccaggaactccaaaggttccccc

gtctgcacgcccagctcatcgcagtcgtctctaagcttctccgcgagcgcctcgacccgagctccggatacgtccagagcctgatctccatccaggcc

gcttacatcaacacaaaccacccgttgtccgtcaaatatcgcgagacagacgcgcgagggccagaatcagaagcatcagttgttgttgcagcagcaga

gtcagcggccgacagtccgtttctaacggcggcacagtccgaagcgggagagacttcgtccacggctgcgaagcgatcgctggtggtggtggaggggc

aggccgcaggaggaagcaatcgagcacgcgcctccgtgcgattcacctcggcttcttcttcttctggtggggcgtataactcccaacaaccgccagag

caaaacgcatcaactctgatctttccctcgcccgacgaaagcggcagcggagacggacacgaagaataa

>contig_21.contig_21.g1009.t1

atgtcaggatccggattaagcgacttctccgtcctgcaggacacaccgccgcgccgcaacgcgtcaccggcgcatcgtg

ccagcgcccgcactgcattcgacttgagcgatgaggatgaatctcctccacgccgccagaagcgcaagctggtcgagatggaagaggaggacgatgac

gggaccccgtcgaagatcaaccagctgcctgccgacatgaaggagtgggcaaagagggtgaagttgagcaagctgcacatctctgaccttgtgcaatt

cggccaggattacatgaccgagaccaatgaggactgctgtcctttctacctgtaccgcctgtacggccagtcccttctccagacccagcttgcccgag

ttggcatttccacaacgaatgagctgcgcgaggaggttgtcccagcgatgaagagagctcggtcgatgctcgtccgcgttgagggcgatgtgaaagag

ctgaagaagtcggcagagagtaagctcacggatgctgatgcggtgagagttctgattcatgcgcgtaccaaggactgcagatgctcatcatatattgt

tttcaaggtcgccattcaacgcatgatagccagcgtcttcttcagtgccgacgttcagacatacagcgagaagggagatcttcccagggtgacctcaa

tttacctcgaacgcaaccctgatctcgtcggccccgcttttgccgagcggtacaagaccggggaggtgcacttcctggcactcgtctcatctctgatc

aagaaaaaggtgaacaatctgcgctctcacgtacgagacaagtaccattggtcgcttggagtggacaagaacaagtccaagcaggacctatacgaact

ctacatcaagctcaccaagagctacggcgtcccggtgacgaaagaacgcgtgctccgcgtggcatacatcgtcgacggtgaacctgtggagggatggt

gggagaagcttgacgagtacgtcactaaactgctctccaccacgcggaaggacgcagagaaggggagacgaactaagtttggcgccttccagcgacac

gagaaaggcatctccaaggccggcagtgaggacaacgacgttcgctccgccttcgccgccgccgctgccgccgccaacgcgaacagcctctga

>contig_21.contig_21.g1010.t1

atgccggcttcacgagcagatccagacgcagttcagcgtgaaattggatcccacttccaagtcgtccctgggcggtttc

ctgtgcacgtcaaatgcctggcctgcggtcacgaaggttcgaagccgtttcggtatgcgccctcggcattgaaggagcacatcagctccaagaagcat

gaaaagaacgcgaggaacttccatcctgctcgagatacagtgatttccgcttatgaccttccgctgccattcgatgggtttgcagatgacgacgggga

ggacagtgctcctgcttttgcggacgacgctgctttgccgatctggttcgatggagagagccgagacgccttctggatgatggtcgaccagcgtgaag

atgtcattgcgggtctcgaagtcgagctggccgaattgcgtcgcgaggaacaggctctcgctgatgcgcatgcagcgcacggaaatccaatgcggcat

cttcttgggcaagagcaaggatcatggggcccgttcaaagagaagaggatcttcgcagctttgagcttcctcctcagtcctcgtcaagcccactcgct

caccagtctcgagcacgcccttgggcttgcggaagctctgggcgctcacaacgtccccagcatcaacgtattcgatggagaccaagagctctcgcatg

tcaattctgatcgtgtttgctgcgatggcaaacgtcttttcagcataccaatcgttctgttcatcgacgatctgtctggagcaaggtcaaagcgatgg

aacaagcacgaggccacgtacctttcaaacgccactcttgatcgatcggatttggacctggactccaacatcaaactcgtgtccatcagcaaagcggt

gtcgggggaggggcaactgtcagccattgtggacgagctcatccacctccatcagaatcccttcactgcgtatgactgcgagctcaaggaagacgtga

tggtacgaccgtacctgctgtcggtcatggcagacaatccaatggcagcgagtttgacctcgtcgatcggactgatgggcaacaagtgctgccgcatg

tgcgacgtggacggcagcaaagagactctgcaaaccgccgaaggattcgttcgctatctccgaccagggtcggctcgatcggtgcagacgatcaaagc

agcgctgacagagcaactcgatgctgcagagcgaggacagccacagtcgttcatcaatgagctgcgcaagcagactggcatcaaggacactctcacaa

cgaagttctgtgacaccttgacggagctgcgacgccaactgcgagaacaaggccaaagtcgagacgagattgatcaagctgcgcaccaaagaaggaga

gagatcgagtcaggaaattggtacggcccgctgcttcgtttgcatggattcgaagtttgtgccggcctgcccattgaggcacttcatacgtggctact

tgggccgatgaagtacctagtcgcggaagtgacccggacggtcaagaaagagacagccctcttccaagaagtcgctgcccgactgtcgtcgatggacc

tggacggtatcggagagtcatcttcgttggacggaacgtacctactcaacaactctggcggattgaccggcaaggacatgcgtgcgctttcacaagta

ctctggctggcacttcttccgctgcgggatcaaggcaagatccaagagagtctttgggcggcttgcagatgggctgcagtgatcactcgtctgacaca

gtttaggaccgcgcttgccaacttcttcgctgcgatggttttggttgatccaaagcagatgcgcaacaaacgaaagtatcatctgatgacgcatctcg

aagcaggcatctccttgtacggtccggccaagggattcgccaccgaacgctacgagtcgttcaacactgttgcaaggaacgcgagcatgtgctccaac

cgcgcagctccctcccgcgatatcgccctgcgcttgtacgacggcgcgttgctcaagctctacggagtggaagcacgcaagaggaaactggttccagg

ttcatttcagcgcgagcaggtcgagcggacggtgataaccagatgcttcggagatgctgccttgcatctcttcgacgaggagtacgtcgactcttcgg

agctggaatatcattcccttcgctttgtcgtttcaaatgcccacgagcgcatatacccggactctttcgttcaggtcgacgctcggcttcctcctgaa

acagaggaagagggacattgctcgatgttcggcgacccggtcggactcgaggacgagcatccgaacgccgccaaaggccccctgtttctggtccagcc

aatgtatgccggtcatctcagtactcacggcctccgctga

>contig_21.contig_21.g1011.t1

atgtcgttccgcttcgactggcccgagttcggccccgacttccacgcgcacgcagccgagatgctcaacagcgcactca

acaagggcccacgtcccaaagtcatcgccgacgacatcaaggtcaaggagctcagcatgggcaccgtcccgcccgagctcgacatcctcgagatcggc

gacctcactaccgaccgcttccgcggcatctttcggctcacctacatgggcgatgcgcatcttattctgtctacgaaggtgcaggccaacccgctgtc
[truncated: 19,723,978 more chars]
